# Transcriptional Signature and Memory Retention of Human-Induced Pluripotent Stem Cells

**DOI:** 10.1371/journal.pone.0007076

**Published:** 2009-09-18

**Authors:** Maria C. N. Marchetto, Gene W. Yeo, Osamu Kainohana, Martin Marsala, Fred H. Gage, Alysson R. Muotri

**Affiliations:** 1 The Salk Institute for Biological Studies, Laboratory of Genetics, La Jolla, California, United States of America; 2 University of California San Diego, School of Medicine, Department of Cellular & Molecular Medicine, Stem Cell Program, La Jolla, California, United States of America; 3 University of California, San Diego, School of Medicine, Department of Anesthesiology, La Jolla, California, United States of America; 4 University of California San Diego, School of Medicine, Department of Pediatrics/Rady Children's Hospital San Diego, Department of Cellular & Molecular Medicine, Stem Cell Program, La Jolla, California, United States of America; University of Washington, United States of America

## Abstract

Genetic reprogramming of somatic cells to a pluripotent state (induced pluripotent stem cells or iPSCs) by over-expression of specific genes has been accomplished using mouse and human cells. However, it is still unclear how similar human iPSCs are to human Embryonic Stem Cells (hESCs). Here, we describe the transcriptional profile of human iPSCs generated without viral vectors or genomic insertions, revealing that these cells are in general similar to hESCs but with significant differences. For the generation of human iPSCs without viral vectors or genomic insertions, pluripotent factors Oct4 and Nanog were cloned in episomal vectors and transfected into human fetal neural progenitor cells. The transient expression of these two factors, or from Oct4 alone, resulted in efficient generation of human iPSCs. The reprogramming strategy described here revealed a potential transcriptional signature for human iPSCs yet retaining the gene expression of donor cells in human reprogrammed cells free of viral and transgene interference. Moreover, the episomal reprogramming strategy represents a safe way to generate human iPSCs for clinical purposes and basic research.

## Introduction

Genetic reprogramming to a pluripotent state of mouse somatic cells was first achieved by ectopic expression of four factors (Oct4, Sox2, Klf4 and c-Myc) using retroviruses [1]. Such cells were named induced pluripotent stem cells (iPSCs). Subsequently, this method was applied to human cells using the same factors or a different combination in a lentivirus vector (Oct4, Sox2, Lin28 and Nanog) [2]–[5]. Both mouse and human iPSCs are similar to embryonic stem cells (ESCs) with respect to their morphology, cell behavior, gene expression, epigenetic status and differentiation potential both in culture and *in vivo*. However, to date, a comprehensive transcriptional analysis has not been reported comparing human ESCs and iPSCs. One reason is that the technology used to derived iPSCs is not “footprint-free” and thus, subjected to transcriptional interference.

Viral vectors are known to affect the transcriptional profile from target cells, altering their behavior and sometimes inducing apoptosis [6]. Moreover, the reactivation of the viral transgene was also implicated in tumorigenesis from iPSC-derived chimeric mice [7]. Also, random integration may influence the molecular signatures of iPSCs by interrupting regulatory regions in the human genome. Interestingly, a transcriptional analysis revealed that transgene expression from not completely silenced viral vectors could, in fact, perturb global gene expression in hiPSCs [8].

Several attempts were made to generate a viral-free, integration-free iPSCs. The generation of iPSCs with later excision of reprogramming factors was recently achieved; still, the genome continues to be affected by random solo-LTR insertions from viral vectors [8]. Mouse iPSCs were also generated by multiple transient expression of Oct4, Sox2 and Klf4 from embryonic fibroblasts at very low efficiency [9]. Recently, a two-step seamless factor removal from iPSCs using transposase-stimulated excision was recently reported [10], [11]. Although evidence that the system might work in human cells was presented, it needs further validation in more rigorous pluripotent assays [10], [11]. A “footprint-free” and highly efficient system of generating human iPSCs would help to determine the molecular mechanism of cellular reprogramming and accelerate the search for efficient compounds that will replace the original factors without side effects.

The timing of the reprogramming and the factors required seem to vary depending on cellular context [12]–[17]. The susceptibility of a somatic cell to reprogram may depend on how similar its transcriptional profile is to ESCs. Of note, mouse neural stem cells (NSCs) were reprogrammed using only one (Oct4) or two factors (Oct4 and Klf4), due to the endogenously high expression of pluripotent genes, such as Sox2 and c-Myc, as well as several intermediate reprogramming markers [14], [17], [18]. Fibroblasts that already carry the Oct4 transgene can be reprogrammed with fewer factors, facilitating the study of nuclear reprogramming [19]. Moreover, although reprogramming can be achieved without c-Myc, iPSC generation is more efficient when the gene is present [20], [21]. Furthermore, recent data suggest that c-Myc expression primes cells for iPSC conversion, accelerating the initial steps of reprogramming to achieve high efficiency [22]. Such observations prompted us to use human NSCs expressing c-Myc, as a model to facilitate the generation of iPSCs and to study the reprogramming steps.

## Results

### Oct4 and Nanog can reprogram human neural stem cells

**Figure 1:**
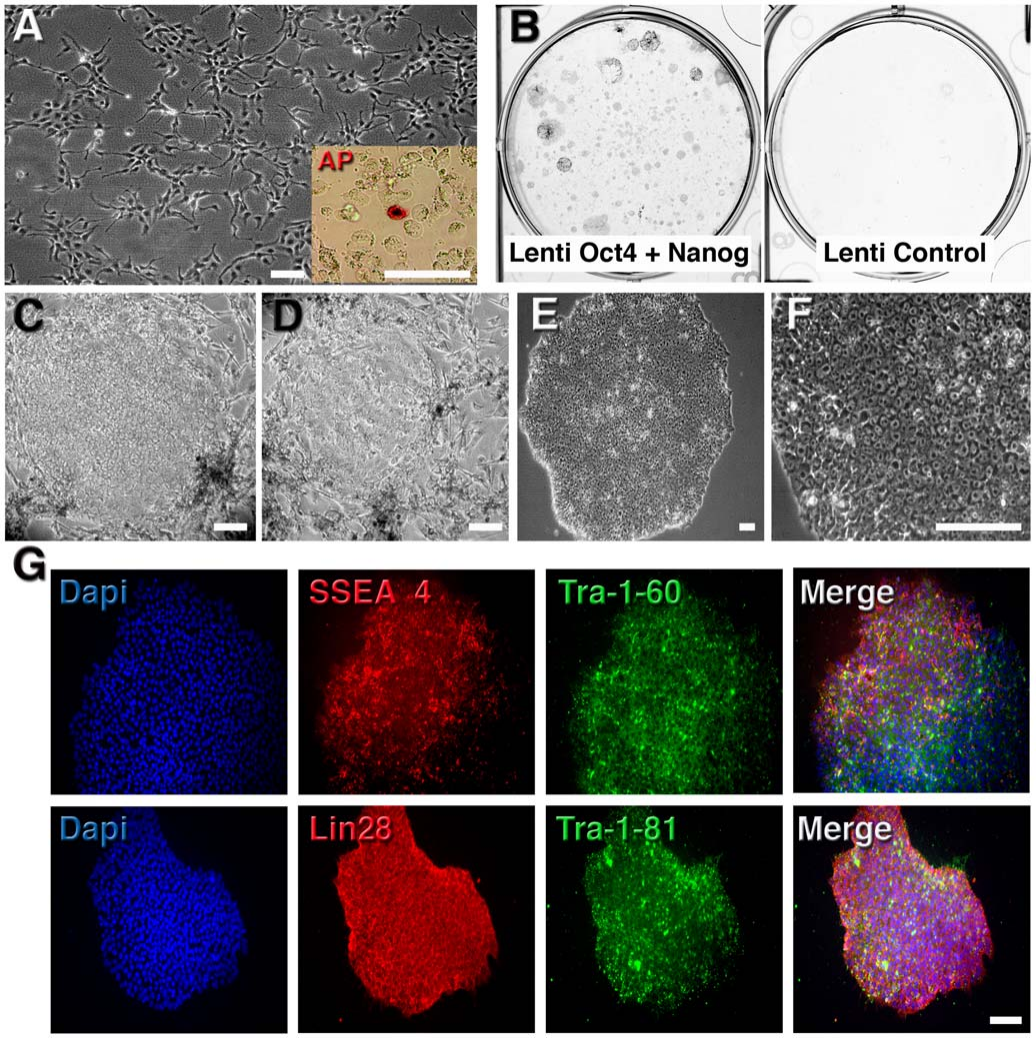
Efficient and rapid generation of iPSCs from human fetal NSCs using two factors. A, Morphology of human fetal NSCs before lentiviral infection. Inset: after 3 days post-infection with Lenti-Oct4 and Lenti-Nanog, individual cells expressed alkaline phosphatase (AP). B, Example of infected plates stained for AP at 14 days post-infection showing several AP-positive colonies. Control infection did not result in any AP-positive colonies. C and D, Aspect of colonies 14 days after infection growing in MEFs. E, Established human iPSC colonies, with well-defined borders and compact cells, are morphologically similar to hESCs. F, Typical image of iPSCs growing in feeder-free conditions. G, Representative immunofluorescence analysis of iPSCs growing on matrigel. Clear expression of pluripotent markers is observed. Bar = 150 µm.

Our starting material was a multipotent, karyotypically normal, c-Myc-immortalized human NSC line derived from a tissue sample of human midbrain (10 weeks of gestation). Our rationale was that the elevated expression of c-Myc and Sox2 in these cells might prompt them to reprogram more easily than reported for other types. First, we examined whether the combination of Oct4 and Nanog would reprogram these cells to a pluripotent state [23], [24]. The human NSCs have a typical, undifferentiated neural stem cell morphology when expanding as monolayers on laminin-coated plates (**Fig. 1A**). NSCs were infected once with lentivirus expressing Oct4 and Nanog (ON) and plated onto a layer of irradiated mouse embryonic fibroblasts (MEFs) in human ESCs (hESC) medium [25]. Individual cells positive for alkaline phosphatase (AP), a marker for pluripotent cells, appear as early as 4 days after infection (**Fig. 1A**, inset). Interestingly, the efficiency was around 1–3%, as measured by the number of AP-positive colonies, at 14 days post-infection. Single infection with an empty control virus or Nanog alone did not produce any colonies (**Fig. 1B**). In the first week after infection, hundreds of small colonies grew rapidly and had hESC morphology (**Fig. 1C, D**). Two weeks after infection, iPSC colonies with a mature morphology similar to hESCs were distinguished from the original NSC population (**Fig. 1E–F**). The NSCs-iPSC(ON) colonies were then manually isolated and propagated under feeder-free growth conditions on matrigel-coated dishes. They expressed markers of undifferentiated ESCs, including Lin28, TRA-1-60 and SSEA-4, confirming the genetic reprogramming by the two factors, Oct4 and Nanog (**Fig. 1G**). Several iPSC lineages were established from independent infections and mechanically expanded for at least 20 passages while maintaining a normal karyotype (data not shown).

### A viral-free, integration-free reprogramming approach

**Figure 2:**
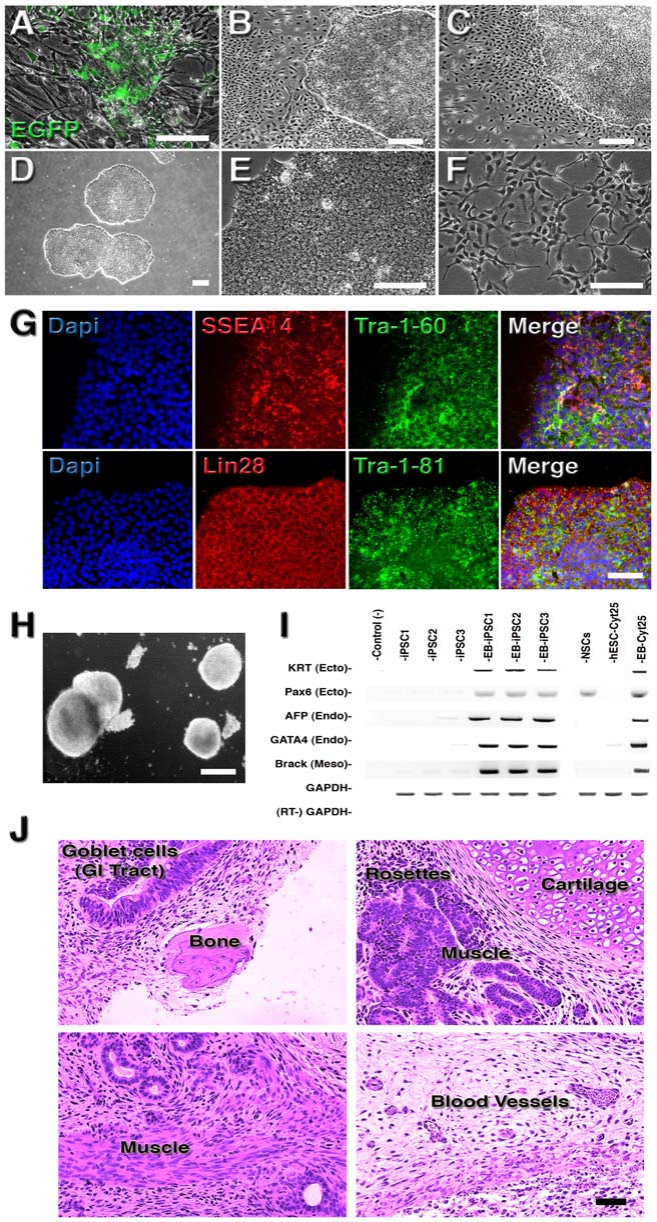
Generation of virus-free, integration-free human iPSCs. A, Aspect of human NSCs after plasmid electroporation and plating on MEFs. B and C, Some selected colonies display a strong differentiation tendency in feeder-free conditions. D, Established iPSC lines are morphologically similar to hESCs. E, iPSCs have a large nucleus-to-cytoplasm ratio and prominent nucleoli when compared to original NSCs (F). G, Immunofluorescence analysis of iPSCs growing on matrigel showed clear expression of typical ESC markers. H, *In vitro* differentiation of iPSCs into EBs. I, RT-PCR from undifferentiated and EB-derived iPSCs showing expression of markers for all three primary germ cell layers. The hESCs Cyt25 was used as a benchmark. J, Hematoxylin and eosin staining of teratoma sections generated from integration-free iPSC lines showing differentiation in three germ layers: goblet cells in gastro-intestinal (GI) tract (endoderm); neural rosettes (ectoderm) and blood vessels, muscle and cartilage/bone (mesoderm). Bar = 150 µm.

To generate human iPSCs without the use of viral delivery vectors or genomic insertions, the Oct4 and Nanog cDNAs were independently cloned under the CMV promoter into a plasmid (pCEP) with the *trans-*acting Epstein-Barr associated nuclear antigen 1 (EBNA-1) gene and the *cis*-DNA element *oriP*. The combination of EBNA-1 and *oriP* elements allows for a transient extra-chromosomal (episomal) state, avoiding genetic integration in human and non-human primate cells [26]–[30]. The constructs also contain a mammalian selection marker (the hygromycin resistant gene). Human NSCs were electroporated with equimolar concentrations of the two episomal plasmids (pCEP-Oct4 and pCEP-Nanog) or the EGFP-reporter plasmid and plated on MEFs under hESC conditions (**Fig. 2A**). Previous data in the literature suggested that reprogramming factors should be maintained for up to 12 days during iPSC generation from mouse cells [31], [32]. Hygromycin selection was maintained for only a week, but transgene expression from the plasmid carrying the EGFP reporter gene suggested that the plasmid remained in the cells for another week before being eliminated (**Figure S1**). After 10–12 days, small iPSC colonies were first noted. Colonies were mechanically isolated and propagated under hESC conditions on matrigel. At this point, some colonies seemed unstable, with a strong tendency to spontaneously differentiate and form a heterogeneous population of cells (**Fig. 2B, C**). Undifferentiated cells were manually selected from differentiated cells according to morphology until a homogeneous population of iPSCs was achieved (**Fig. 2D**). The iPSC colonies were morphologically indistinguishable from hESCs, forming tight colonies of cells with a large nucleus to cytoplasm ratio and prominent nucleoli (**Fig. 2E**), and they did not display the NSCs' original cell morphology (**Fig. 2F**). The efficiency was higher (0.1–1%) when compared to fibroblasts reprogrammed with retroviruses. We established several cell lineages from three independent transfection experiments and chose three lines (iPSC1, iPSC2, iPSC3) for further characterization.

**Figure 3:**
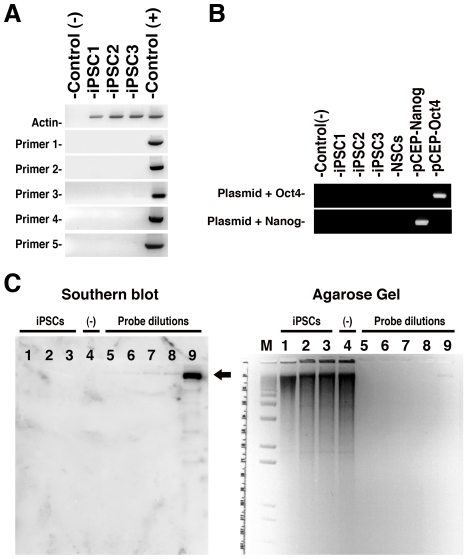
Absence of plasmid integration on virus-free iPSCs. A and B, PCR analyses for plasmid integration in genomic DNA from the iPSC clones. Controls: (−) water; (+) pCEP4 plasmid. Primers were designed to specifically amplify plasmid backbone (A) or transgenes (B) (see Methods). c, Southern blot (left) membrane hybridization of 10 µg of *Bam*HI-digested genomic DNA (see corresponding agarose gel on right) using a DNA probe from the pCEP backbone. Plasmid DNAs of pCEP-Oct4 and PCEP-Nanog, diluted to the equivalent of 0.5 integration per genome, were used as controls for probe dilution. Lanes: M, DNA molecular marker; 1- iPSC1; 2- iPSC2; 3- iPSC3; 4- NSCs (negative control); 5- probe 25 ρg; 6- probe 50 ρg; 7- 100 ρg; 8- 200 ρg and 9- 50 ηg. Arrow indicates expected probe size.

These three iPSC colonies expressed several pluripotent markers and were able to form embryoid bodies (EBs) *in vitro* (**Fig. 2G, H**). They were also able to express markers of the three germ layers, suggesting that they re-established pluripotency at the molecular and cellular levels (**Fig. 2I**). PCR DNA fingerprinting confirmed their derivation from NSCs rather than from a contaminating hESC line (**Figure S2**). All iPSC clones could be successfully propagated for more than 30 passages while maintaining a normal karyotype (data not shown). Plasmid transfection may lead to random integration into the genome at low frequency. To test for genomic integration of plasmid DNA, we designed several sets of PCR primers to amplify various parts of the vector and transgenes (**Fig. 3A, B**). Teratomas containing derivatives from all three embryonic germ layers confirmed that the hiPSCs (but not the original NPCs used) were pluripotent and able to differentiate to complex tissues in two different experimental settings (**Fig. 2J** **and Figure S3**). Additionally, southern blot analyses did not detect integration of plasmids in these clones (**Fig. 3C**). DNA from the transfected plasmids was not detected in any established colony using either method, indicating a lack of genomic insertion and suggesting that the episomal vectors had been diluted from the cells over time.

### Human iPSCs have similar levels of myc when compared to hESCs

We then analyzed if myc levels from these iPSCs derived from NSCs would change after reprogramming. Interestingly, despite the fact that the NSCs were immortalized with ectoptic expression of myc, the transcriptional activity of myc is higher in iPSCs compared to NSCs. Moreover, iPSCs clones have similar myc transcriptional levels to hESCs (**Figure S4**). Together, these observations indicate that the myc expression will likely not interfere with the global transcription profile on the iPSCs.

### A transcriptional signature for human iPSCs

**Figure 4:**
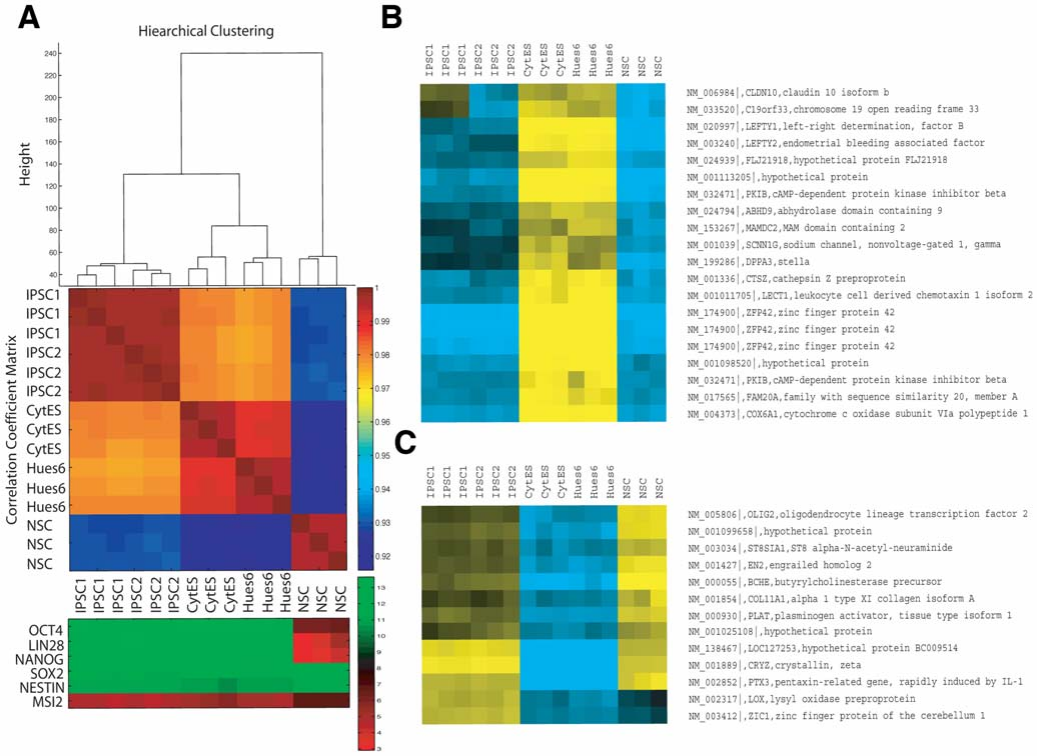
Transcriptional analysis of human integration-free iPSC colonies. A, Hierarchical clustering and correlation coefficients of microarray profiles of triplicate iPSC1, iPSC2, CytES (Cyt25 hESC), Hues6 and NSC. Color bar indicates the level of correlation (from 0 to 1). Panel below illustrates marker genes implicated in pluripotency of NSCs, with color bar reporting log2 normalized expression values (green/red indicates high/low relative expression). B, Refseq-annotated genes that were insufficiently induced in iPSCs relative to hESCs (yellow/blue indicates high normalized log2 expression). C, Refseq-annotated genes that were insufficiently silenced in iPSCs relative to hESCs.

Next, we asked if the global molecular signatures of two plasmid-free iPSC lines (iPSC1, iPSC2) resembled those of available hESC lines, namely HUES6 and Cyt25. Gene expression profiles measured using human genome Affymetrix Gene Chip arrays were grouped by hierarchical clustering, and correlation coefficients were computed for all pair-wise comparisons (**Fig. 4A**). We observed that the two iPSCs lines were almost indistinguishable from each other and that the two hESC lines were also highly similar to each other. Clearly, the iPSC and hESC lines were globally more similar to each other than to the NSC line (**Fig. 4A**), and combined with manual inspection of the gene expression of several known pluripotent (Oct4, LIN28, Sox2 and Nanog) and neural stem cell markers (Sox2, Nestin and Musashi2) as measured on the arrays, we concluded that the reprogramming was successful (**Fig. 4A**).

**Figure 5:**
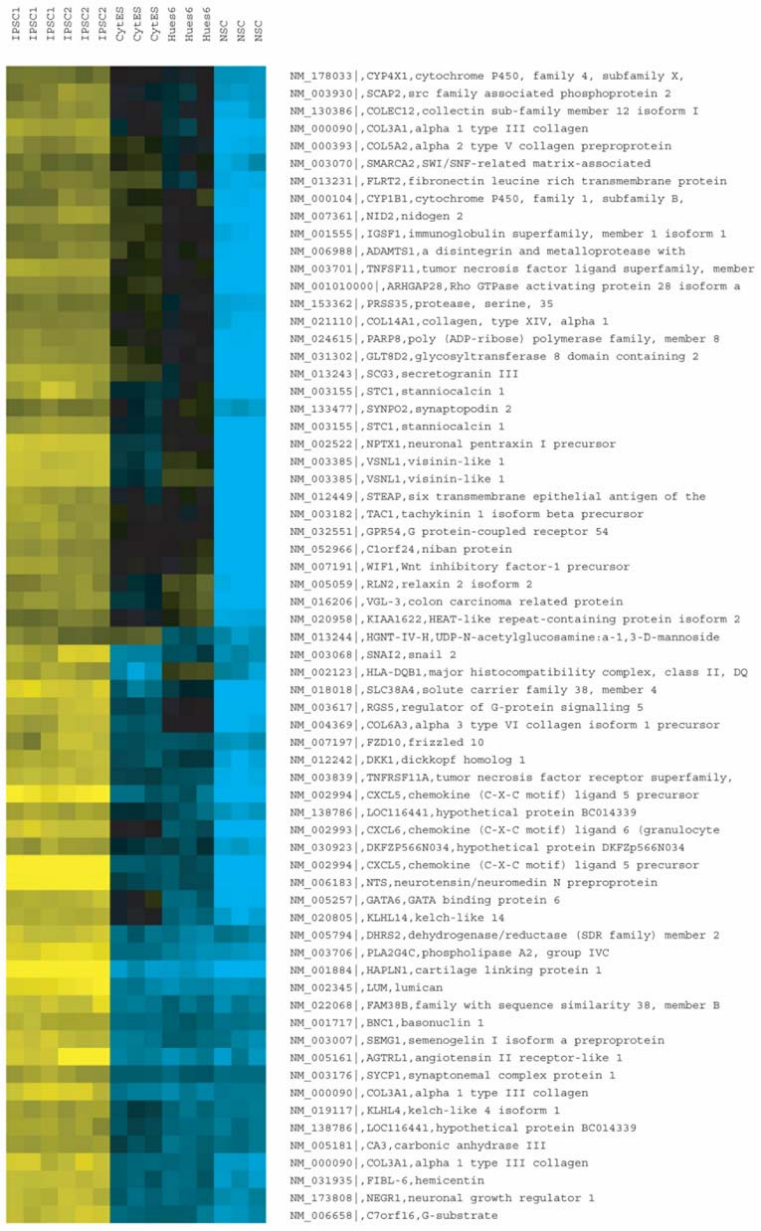
Refseq-annotated genes that were upregulated in iPSCs relative to both hESCs and NSC. Panel illustrates marker genes implicated in pluripotency of NSCs, with color bar reporting log2 normalized expression values (green/red indicates high/low relative expression).

Despite the global similarity between iPSCs and hESCs, the profiles were not completely indistinguishable, which led us to study what the molecular differences were. Four independent (A versus B) group-wise comparisons were performed to identify differentially expressed genes: (i) iPSC versus hESC (1,952 Refseq-annotated genes were significantly enriched in iPSCs versus hESCs; 1,072 genes were enriched in hESCs versus iPSCs at P<0.01 after correcting for multiple hypotheses testing); (ii) iPSC versus NSC (3,347 genes were significantly enriched in iPSCs versus NSCs; 2,959 genes were enriched in NSCs versus iPSCs); (iii) hESC versus NSC (2,376 genes were significantly enriched in hESCs versus NSCs; 2,541 genes were enriched in NSCs versus hESCs); (iv) iPSC and hESC versus NSC (3,730 genes were significantly enriched in iPSCs and hESCs, versus NSCs and 3,638 genes were enriched in NSCs versus iPSCs and hESCs (**Tables S1** to **S8** contain the full list of comparisons). Restricting these differentially expressed genes to ones that changed by at least 4-fold in any comparison, at a stringent p-value cutoff of P<0.0001, we identified three groups of biologically interesting genes. The first group of iPSC-expressed genes was not sufficiently induced to comparable levels as in hESCs and was still at their original levels in NSCs (**Fig. 4B**). This group contained factors that were important in early embryonic fate, such as Stella, ZFP42 (REX1), CLDN10, LEFTY1 and LEFTY2. It is noteworthy that ZFP42 has been shown to be dispensable for pluripotency in mouse ES cells [33], which may explain why the factor need not be induced highly. Lefty1 has been shown to be important for pluripotency as well [34], and it is downstream of Oct4 and Sox2, but perhaps without the use of Klf4 the Lefty1 expression is not sufficiently induced. The second group contained iPSC-expressed genes that were not sufficiently repressed, such as ZIC1, OLIG2, EN2 and PTX3, which were associated with the neuronal lineage (**Fig. 4C**). The third group consisted of genes that were upregulated in iPSCs, which were silenced in both NSCs and hESCs, suggesting that these genes may be downstream factors in the reprogramming step to induce pluripotent cells (**Fig. 5**). Overall, our transcriptome analyses indicated that, whereas the iPSCs are globally similar to hESCs, they are not indistinguishable, primarily due to the insufficient suppression or induction of NSC-specific or early embryonic-specific genes, respectively, as well as a class of genes that was upregulated during the reprogramming step.

### Oct4 alone is able to reprogram human NSCs

**Figure 6:**
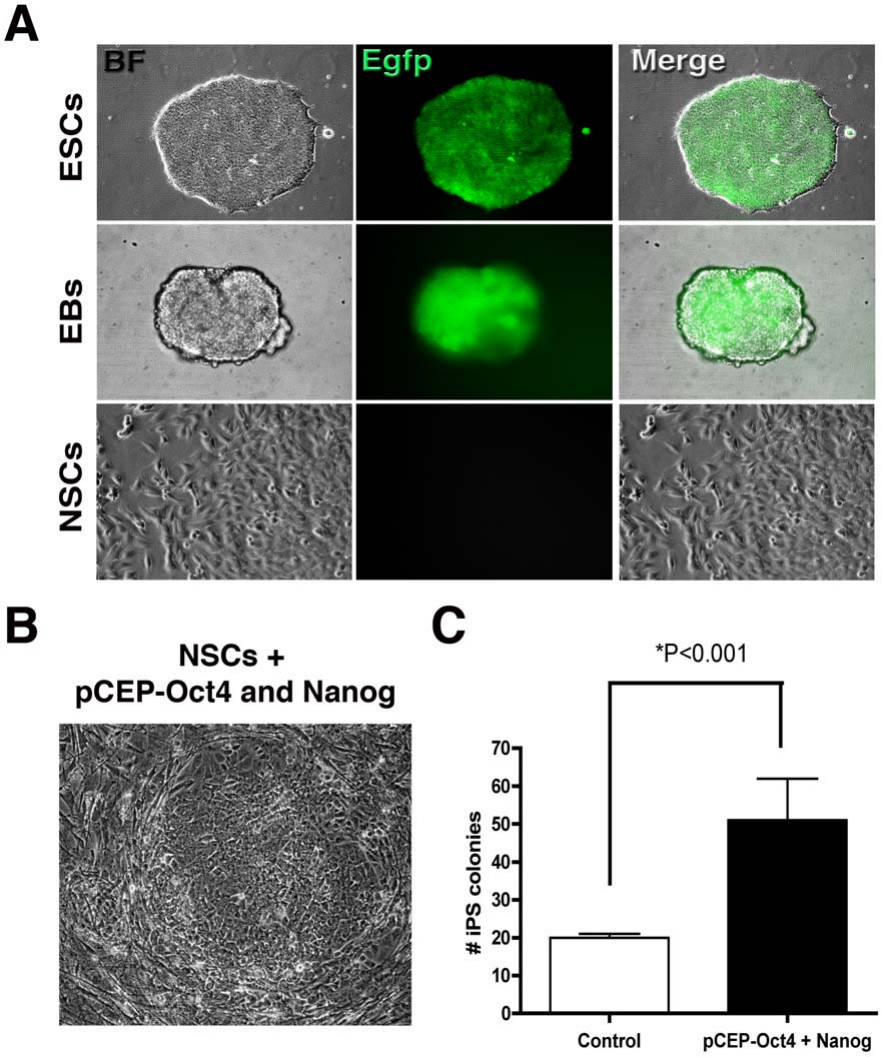
The dynamics of integration-free reprogramming. A, Undifferentiated H1 Oct4-EGFP hESC line expresses the EGFP reporter gene that is gradually turned off during NSC differentiation. NSCs are morphologically distinct from hESCs. B, Small iPSC colonies can be detected 10 days after transfection with pCEP-Oct4 and pCEP-Nanog. C, Typical number of iPSC colonies obtained with electroporation of pCEP-Oct4 and Nanog or with Oct4 alone. Bar = 150 µm.

Next, we repeated the transient transfection using NSCs derived from the H1 hESC line that contains the EGFP reporter cassette knocked in the endogenous Oct4 gene by homologous recombinantion [35]. The H1-Oct4-EGFP cell line expressed EGFP, which turned off during differentiation (**Fig. 6A**). NSCs were generated using our previous established protocol and consisted of a cell population with a genetic profile distinct from both human fetal cells and hESCs [36], [37]. NSCs derived from the H1-EGFP do not express EGFP (**Fig. 6A**). An EGFP-negative population of NSCs, isolated by FACS, was electroporated with both episomal plasmids carrying Oct4 and Nanog. Several iPSC colonies were observed as early as 10 days after transfection, becoming morphologically indistinguishable from the original H1-Oct4-EGFP cell line (**Fig. 6B, C**). As a control, we electroporated the same cell population with Oct4 only. Interestingly, we detected several colonies when cells were transfected with Oct4 alone. These colonies were positive for pluripotent makers, such as Nanog and Lin28, suggesting efficient reprogramming (data not shown). Our findings in human cells recapitulate recent data demonstrating that Oct4 alone is sufficient to reprogram mouse NSCs [**17**]. The observation that we could reprogram using only Oct4 but not Nanog alone, suggests that Oct4 is likely an upstream factor for cellular reprogramming. In fact, a previous study suggests that Nanog may function to stabilize pluripotency rather than being essential for the pluripotent stage [38].

## Discussion

**Figure 7:**
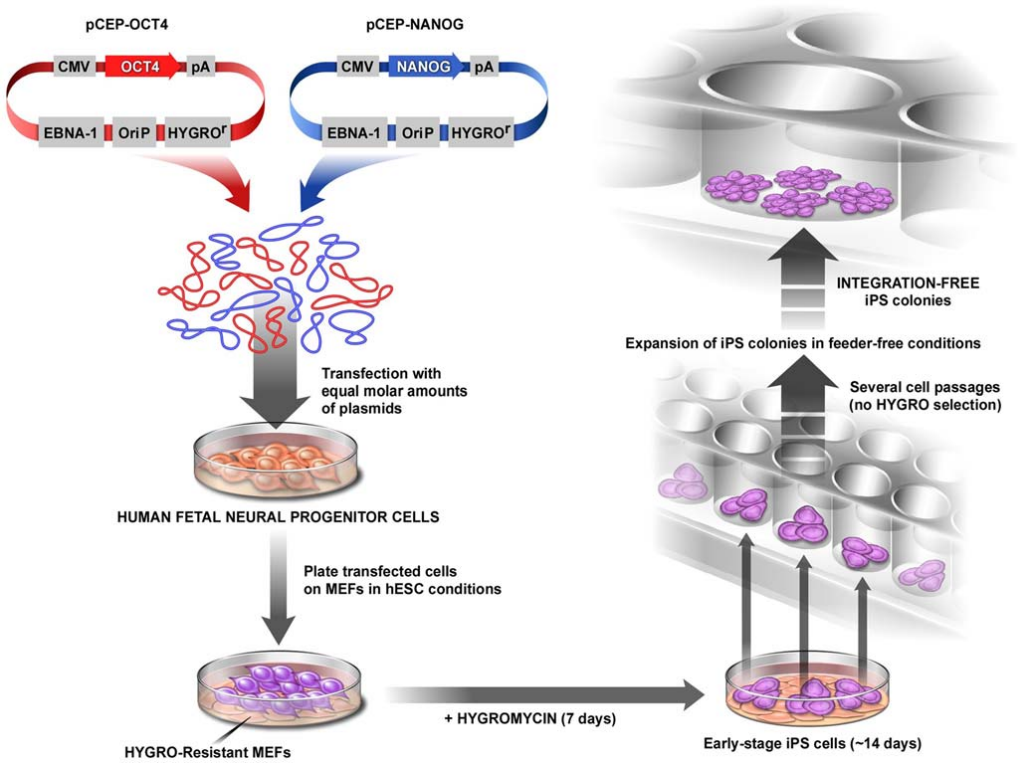
Schematic model of integration-free human iPSC generation from NSCs. Episomal plasmids carrying reprogramming factors are transfected into NSCs and cells are plated on MEFs. On the following day, medium is changed to the hESC condition. Resistant selection is kept for a week. After 14 days, iPSC colonies are visible and can be transferred to a feeder-free condition. Individual colonies are expanded and ready for characterization. At this time, no evidence of plasmid integration is found.

Using a simple methodology (**Fig. 7**), we demonstrated that it is possible to generate human iPSCs at a high frequency without viruses and with no evidence of genomic insertion. Human iPSCs were achieved using transient episomal vectors carrying the cDNAs for Oct4 and Nanog in a cell type that was likely more prone to genetic reprogramming, such as NSCs. Also, we demonstrated here for the first time that a myc-immortalized cell line could be successfully reprogrammed, opening new avenues for the study of several previously characterized immortalized cell lines that are relevant for the biological understanding of several disorders. The Myc-immortalized NSCs represent a reliable, homogenous and commercially available tool to dissect individual factors required for reprogramming. Myc levels after reprogramming is similar to hESCs, thus it may be a better standard model for fundamental reprogramming studies than fibroblasts. Furthermore, our data from hESC-derived NSCs indicate that reprogramming can be achieved without ectopic expression of the tumor-associated genes, c-Myc and Klf4. Future studies will show whether other sources of primary human NSCs can also be efficiently reprogrammed by such non-viral methodology. Human iPSCs generated by episomal vectors were then used to assess whether human iPSCs and ESCs are really equivalent at the molecular and functional levels, avoiding artifacts that may affect their genetic signature, differentiation behavior or developmental potential. Almost all previous studies have shown that the genetic profile of human iPSCs is comparable, but never identical, to that of hESCs. The slight differences between these two cellular populations could be attributed to viral insertions in the genome or incomplete genetic reprogramming. Our data suggest that, although the global transcriptional profiles of hESCs and iPSCs were globally similar, small but significant differences indeed exist and cannot be attributed to random viral insertions in the genome. Although we still do not know how relevant these differences are, we anticipate that they may correspond to a unique differentiation potential of iPSCs. Such a potential may also be dependent on the original cell type and may suggest retention of the gene expression memory of the donor cell in iPSCs. We recognize that the use of the oncogene myc to immortalized NSCs might have caused permanent epigenetic changes in the starting cell line that could be carried over into the iPSC stage. The use of only one NSC line further limits the conclusions of this work. Future experiments using iPSCs reprogrammed by the episomal virus-free, transgene-free strategy, in different original primary cell types is needed to validate such hypothesis.

Our results support earlier observations that viral integration is dispensable for genetic reprogramming [15], [39]. Our data point to the fact that viral integration does not facilitate iPSC generation, and the efficiency is probably due to the duration and level of the transgenes achieved with episomal plasmids. It has been estimated that each cell contains as many as 50 copies of each episomal plasmid in the nucleus [40]. After a critical amount of time, selection is removed and the episomal vectors are eliminated from the cells during duplication. Although we never detected episomal plasmids in iPSC established colonies, eventual leftover plasmid will likely be severely methylated when cells reach a pluripotent state, avoiding excess transgene expression after reprogramming [41]. In such a system, the amount and time of gene expression can be easily controlled. We anticipate that different cell types will require a distinct cocktail of pluripotent factors, under specific timing and expression conditions. While this manuscript was in preparation, a similar episomal strategy was used to reprogram human primary fibroblasts using a distinct pluripotent cocktail of factors, validating our methods, but with significant lower efficiency (only 3 to 6 colonies/10^6^ input cells) [42]. Finally, the strategy described here may be a valuable tool for creating safer patient-specific cells and thus could have major implications for future cell therapy.

## Materials and Methods

### Ethics Statement

All animal work was conducted according to relevant national and international guidelines. Protocols were previously approved by the University of California San Diego Institutional Animal Care and Use Committee, the Institutional Review Board and the Embryonic Stem Cell Research Oversight Committee.

### Cell culture

Human fetal NSCs (ReNCell VM, Chemicon) were cultured on laminin-coated dishes in ReNcell maintenance medium (Chemicon) in the presence of basic fibroblast growth factor 2 (bFGF2), following the manufacturer's instructions. The hESC Cyt25 (Cythera, San Diego) and HUES6 cell lines were cultured as previously described [25]. Two days after infection/transfection, cells were plated on mitotically inactivated MEFs (Chemicon), with hESCs medium, in the presence or not of 50 µg/ml of hygromycin B (Invitrogen). After 2 weeks, iPSC colonies were directly transferred to feeder-free conditions, on matrigel-coated dishes (BD) using mTeSR™1 (StemCell Technologies). Established iPSC colonies were kept in feeder-free conditions indefinitely and passed using mechanical dissociation. EBs were formed by mechanical dissociation of cell clusters and plating into low-adherence dishes in hESC medium without bFGF2 for 7 days.

### Lentiviral and episomal plasmids

Lentiviral vectors containing the Oct4 and Nanog human cDNAs from Yamanaka's group were obtained from Addgene. The cDNAs were then subcloned into the pCEP4β episomal plasmid (Invitrogen). Plasmid transfections were done by electroporation of equimolar amounts of pCEP-Oct4 and pCEP-Nanog (5 µg each) using the nucleofactor for rat NSCs, following the manufacturer's instructions (Lonza/Amaxa Biosystem). Lentiviruses were produced by triple transfection of HEK293T cells followed by ultracentrifugation as previously described elsewhere [25]. Fetal NSCs were infected with both Lenti-Oct4 and Lenti-Nanog at a titer of 0.5×10^10^ gene transfer units/ml overnight, followed by a 2-day recovery period before being plated on mitotically inactive MEFs.

### Immunocytochemistry

Cells were briefly fixed in 4% paraformaldehyde and then permeabilized with 0.5% Triton-X in PBS. Cells were blocked in 0.5% Triton-X with 5% donkey serum for 1 hour before incubation with primary antibody overnight at 4°C. After 3 washes in PBS, cells were incubated with secondary antibodies (Jackson ImmunoResearch) for 2 hours at room temperature. Fluorescent signals were detected using a Zeiss inverted microscope and images were processed with Photoshop CS3 (Adobe Systems). Primary antibodies used in this study are SSEA-4, TRA-1-60, TRA-1-81 (1∶100, Chemicon) and Lin28 (1∶500 R&D Systems). Alkaline phosphatase activity was detected in live cells using the Vector Red Alkaline Phosphatase substrate kit (Vector Laboratories).

### Genomic PCR and Southern blot

Genomic DNA was isolated and prepared using standard molecular techniques. The PCR primers were designed to recognize the pCEP4 episomal vector (Invitrogene). The primers pairs used to amplify the plasmid back bone were: CEP19-F: 5′- tatgatgacacaaaccccgcccag -3′ and CEP19-R: 5′- aaagcacgagattcttcgccctcc -3′; CEP20-F: 5′- gaaaaagcctgaactcaccgc -3′ and CEP20-R: 5′- aaagcacgagattcttcgccctcc -3′; CEP21-F: 5′- ggcgaagaatctcgtgctttc -3′ and CEP21-R: 5′- cggtgtcgtccatcacagtttg -3′; CEP22-F: 5′- cgcaaggaatcggtcaatacactac -3 and CEP22-R: 5′- tccatacaagccaaccacgg -3′; CEP23-F: 5′- ggatttcggctccaacaatgtc -3′ and CEP23-R: 5′- tgaacaaacgacccaacaccc -3′. The primers used to amplify the transgene only were: CEP1-F1: 5′- gcgtggatagcggtttgactc -3′; Oct4R1: 5′- aaatccgaagccaggtgtc -3′; NanogR1: 5′- cagtcggatgcttcaaag -3′. Southern blot with 10 µg of genomic DNA, previously digested with *Bam*HI, was performed using standard molecular techniques. The probe used was a fragment of pCEP4 plasmid cut with *Nru*I and *Sal*I enzymes.

### RNA extraction and RT-PCR

Total cellular RNA was extracted from ∼5×10^6^ cells using the RNeasy Protect Mini kit (Qiagen, Valencia, CA), according to the manufacturer's instructions, and reverse transcribed using the SuperScript III First-Strand Synthesis System RT-PCR from Invitrogen. The cDNA was amplified by PCR using Accuprime Taq DNA polymerase system (Invitrogene). The primer sequences were: hNanog-Fw: 5′ cctatgcctgtgatttgtgg 3′ and hNanog-Rv: 5′ ctgggaccttgtcttccttt 3′; hBRACHYURY-F: 5′ gccctctccctcccctccacgcacag 3′ and hBRACHYURY-R: 5′ cggcgccgttgctcacagaccacagg 3′; hKRT-18-F: tctgtggagaacgacatcca and KRT-18-R: 5′ ctgtacgtctcagctctgtga 3′; h-AFP-F: 5′ aaaagcccactccagcatc 3′ and AFP-R: 5′ cagacaatccagcacatctc 3′; GATA-4-F: 5′ ctccttcaggcagtgagagc 3′ and GATA-4-R: 5′ gagatgcagtgtgctcgtgc 3′; hGAPDH-Fw: 5′ accacagtccatgccatcac 3′, hGAPDH-Rv: 5′ tccaccaccctgttgctgta 3′. PCR products were separated by electrophoresis on a 2% agarose gel, stained with ethidium bromide and visualized by UV illumination.

### Teratoma formation in nude mice

Around 1−3×10^6^ cells were injected subcutaneously into the dorsal flanks of nude mice (CByJ.Cg-Foxn1nu/J) anesthetized with isoflurane. Five to 6 weeks after injection, teratomas were dissected, fixed overnight in 10% buffered formalin phosphate and embedded in paraffin. Sections were stained with haematoxylin and eosin for further analysis.

### *In vivo* spinal iPSCs grafting and identification of teratomas

Adult Sprague-Dawley male rats (320–350 g; n = 6) were anesthetized with isoflurane (1.5–2% maintenance; in room air), placed into a spinal unit apparatus (Stoelting, Wood Dale, IL, USA) and a partial Th12–L1 laminectomy performed using a dental drill (exposing the dorsal surface of L2–L5 segments). Using a glass capillary (tip diameter 80–100 µm) connected to a microinjector (Kopf Instruments, Tujunga, CA), rats were injected with 0.5 µl (10, 000 cells per injection) of the iPS (n = 3) or proliferating H9 cells in DMEF/F12 media. The duration of each injection was 60 s followed by 30 s pause before capillary withdrawal. The center of the injection was targeted into the dorsal horn (distance from the dorsal surface of the spinal cord at L3 level: 0.5–0.7 mm). Ten injections (500–800 µm rostrocaudally apart) were made on each side of the lumbar spinal cord. After injections, the incision was cleaned with penicillin-streptomycin solution and sutured in two layers. Three or four weeks after cell grafting, rats were deeply anesthetized with pentobarbital and phenytoin and transcardially perfused with 200 ml of heparinized saline followed by 250 ml of 4% paraformaldehyde in PBS. The spinal cords were dissected and postfixed in 4% formaldehyde in PBS overnight at 4°C and then cryoprotected in 30% sucrose PBS until transverse sections (30 µm thick) were cut on a cryostat and mounted on Silane-Prep slides (Sigma). Sections were stained with H&E or immunostained overnight at 4°C with primary human specific (h) or non-specific antibodies made in PBS with 0.2% Triton-X100: mouse anti-nuclear matrix protein/h-nuc (hNUMA; 1:100; Millipore, Temecula, CA, USA); goat anti-doublecortin (DCX; 1:1000; Millipore); mouse anti-Nestin (hNestin; Chemicon). After incubation with primary antibodies, sections were washed 3× in PBS and incubated with fluorescent-conjugated secondary donkey anti-mouse, or donkey anti-goat antibodies (Alexa 488, 546; 1:250; Invitrogen Corp., Carlsbad, CA, USA) and DAPI for general nuclear staining. Sections were then dried at room temperature, covered with Prolong anti-fade kit (Invitrogen Corp., Carlsbad, CA, USA) and analyzed with confocal microscopy (Olympus, Fluoview 1000).

### DNA fingerprinting

DNA fingerprinting analysis was performed by Cell Line Genetics (Madison, WI).

### Microarray analysis

The Affymetrix Power Tools (APT) suite of programs and Affymetrix HG-U133 Plus 2.0 library files and annotation were obtained from http://www.affymetrix.com/support. Gene-level signal estimates were derived from the CEL files by RMA-sketch normalization as a method in the apt-probeset-summarize program. Hierarchical clustering of the full dataset of 15 (2 hiPSC lines samples, 2 hESC lines, 1 NSC line in triplicate each) by 54,675 probeset values was performed by complete linkage using Euclidean distance as a similarity metric in Matlab. The t-statistic t_A,B_ = (m_A_−m_B_)/sqrt (((n_A_−1)s^2^_A_+(n_B_−1)s^2^_B_)(n_A_+n_B_))/((n_A_n_B_) (n_A_+n_B_−2))), where n_A_ and n_B_ were the number of replicates, m_A_ and m_B_ were the mean, and s^2^_A_ and s^2^_B_ were the variances of the expression values for the two datasets, was calculated representing the differential enrichment of a gene using gene-level estimates in cell-type(s) A relative to cell-type(s) B. Multiple hypothesis testing was corrected by controlling for the false discovery rate (Benjamini-Hochberg). Four independent (A versus B) comparisons were performed to identify differentially expressed genes: (i) iPSCs versus hESCs; (ii) iPSCs versus NSCs; (iii) hESCs versus NSCs; and (iv) iPSCs and hESCs versus NSCs. A total of 653 probesets were retained at a stringent cutoff of p<0.0001 and fold-change of 4. Probesets were centered by mean expression values, and hierarchical clustering was performed by complete linkage and uncentered correlation as the similarity metric using Cluster 3.0 program. Results were visualized using Java Treeview. Gene ontology analysis was performed as described elsewhere [43].

## Supporting Information

Figure S1 Sustained expression using episomal vectors. A, Human fetal NSCs were electroporated with an episomal plasmid carrying the EGFP reporter gene. Transfection efficiency was around 95%. B, Percentage of cells expressing EGFP in the presence or not of hygromycin. Bar = 150 Î¼m.
(1.00 MB TIF)

Figure S2 Integration-free iPSC colonies are genetically identical to the original human fetal NSCs. DNA fingerprinting analysis at 16 independent loci indicated that both iPSCs generated by lentivirus infection (iPSC colony 19) and by transient transfection with episomal vectors (iPSC colony 1) and the original human fetal NSCs (ReNCell VM) shared all alleles investigated and were different from commonly available hESC lines.
(0.81 MB TIF)

Figure S3 Development of teratomas after spinal injections of iPSCs into lumbar gray matter. Lumbar spinal cord sections were stained with H&E at 3 weeks after grafting (A, B). The presence of rosette-like structures (A, yellow arrow) and ectoderm-derived squamous epithelium was identified (B, yellow arrow). Staining with human-specific nestin (green) and DCX (red) antibody show well organized nestin positive cells in primitive neuronal tube and numerous postmitotic DCX-positive neurons at the periphery of grafts (C, D).
(9.63 MB TIF)

Figure S4 Myc levels in neural stem cells before and after reprogramming. The myc levels in iPSCs are similar to hESCs.
(0.27 MB TIF)

Table S1 IPSC-enriched probes in IPSC versus ES. Probesets enriched in group-wise comparisons: Column headings are probeset identifiers, T-statistic, P-value, Fold-Change (log2), Refseq identifier and Description of the gene. (NA indicates no Refseq annotation).
(5.43 MB DOC)

Table S2 ES-enriched probes in IPSC versus ES. Probesets enriched in group-wise comparisons: Column headings are probeset identifiers, T-statistic, P-value, Fold-Change (log2), Refseq identifier and Description of the gene. (NA indicates no Refseq annotation).
(2.66 MB DOC)

Table S3 IPSC-enriched probes in IPSC versus NSC. Probesets enriched in group-wise comparisons: Column headings are probeset identifiers, T-statistic, P-value, Fold-Change (log2), Refseq identifier and Description of the gene. (NA indicates no Refseq annotation).
(8.67 MB DOC)

Table S4 NSC-enriched probes in IPSC versus NSC. Probesets enriched in group-wise comparisons: Column headings are probeset identifiers, T-statistic, P-value, Fold-Change (log2), Refseq identifier and Description of the gene. (NA indicates no Refseq annotation).
(9.01 MB DOC)

Table S5 ES-enriched probes in ES versus NSC. Probesets enriched in group-wise comparisons: Column headings are probeset identifiers, T-statistic, P-value, Fold-Change (log2), Refseq identifier and Description of the gene. (NA indicates no Refseq annotation).
(6.06 MB DOC)

Table S6 NSC-enriched probes in ES versus NSC. Probesets enriched in group-wise comparisons: Column headings are probeset identifiers, T-statistic, P-value, Fold-Change (log2), Refseq identifier and Description of the gene. (NA indicates no Refseq annotation).
(7.45 MB DOC)

Table S7 IPSC, ES-enriched probes in IPSC, ES versus NSC. Probesets enriched in group-wise comparisons: Column headings are probeset identifiers, T-statistic, P-value, Fold-Change (log2), Refseq identifier and Description of the gene. (NA indicates no Refseq annotation).
(10.18 MB DOC)

Table S8 NSC-enriched probes in IPSC, ES versus NSC. Probesets enriched in group-wise comparisons: Column headings are probeset identifiers, T-statistic, P-value, Fold-Change (log2), Refseq identifier and Description of the gene. (NA indicates no Refseq annotation).
(12.08 MB DOC)
