# Supplementary figures and images for: Transcriptional Signature and Memory Retention of Human-Induced Pluripotent Stem Cells
Source: PLoS One. 2009 Sep 18;4(9):e7076. doi: 10.1371/journal.pone.0007076 (PMC2741600; doi:10.1371/journal.pone.0007076)

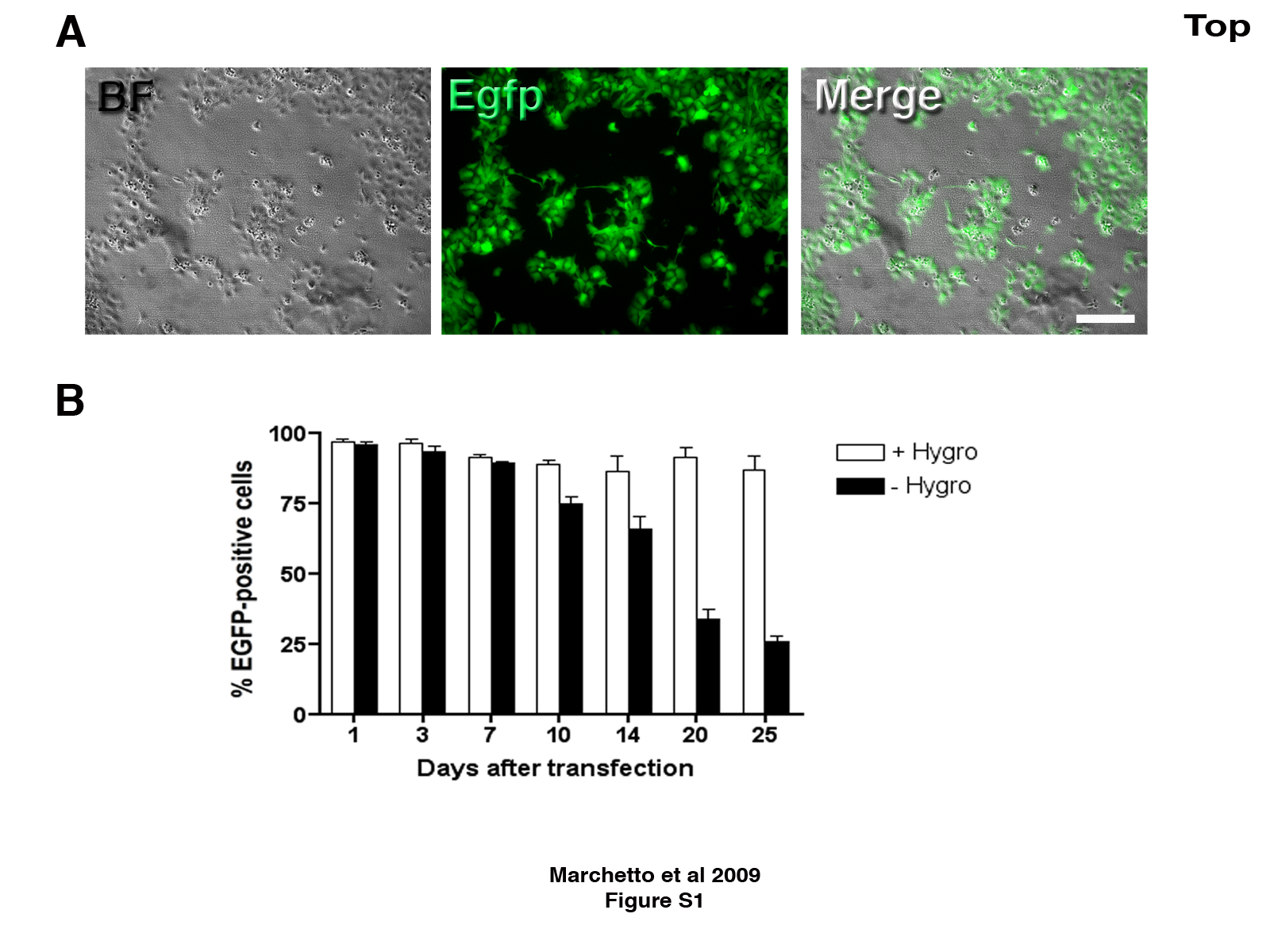

Supplement: Figure S1 — Sustained expression using episomal vectors. A, Human fetal NSCs were electroporated with an episomal plasmid carrying the EGFP reporter gene. Transfection efficiency was around 95%. B, Percentage of cells expressing EGFP in the presence or not of hygromycin. Bar = 150 Î¼m. (1.00 MB TIF) [file pone.0007076.s001.tif]

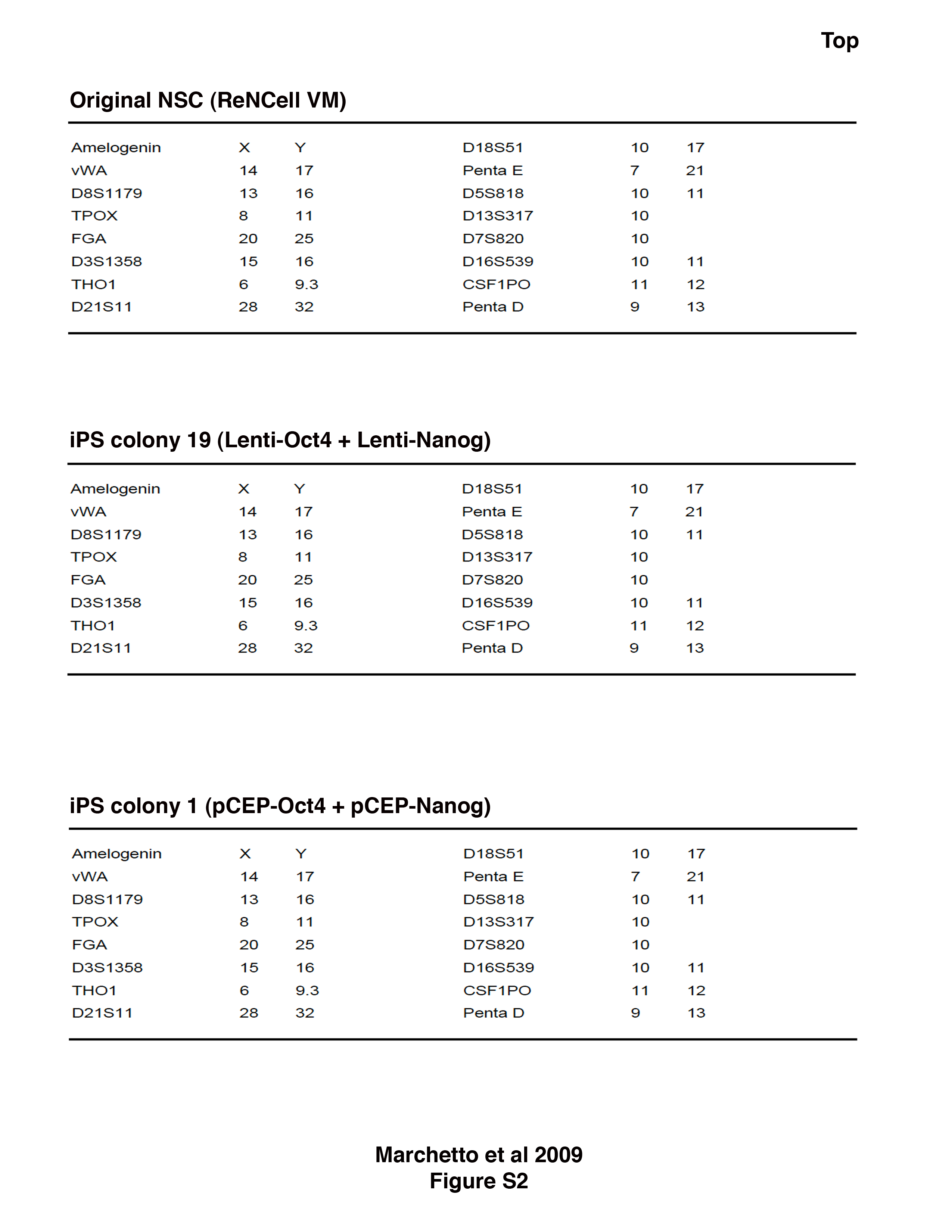

Supplement: Figure S2 — Integration-free iPSC colonies are genetically identical to the original human fetal NSCs. DNA fingerprinting analysis at 16 independent loci indicated that both iPSCs generated by lentivirus infection (iPSC colony 19) and by transient transfection with episomal vectors (iPSC colony 1) and the original human fetal NSCs (ReNCell VM) shared all alleles investigated and were different from commonly available hESC lines. (0.81 MB TIF) [file pone.0007076.s002.tif]

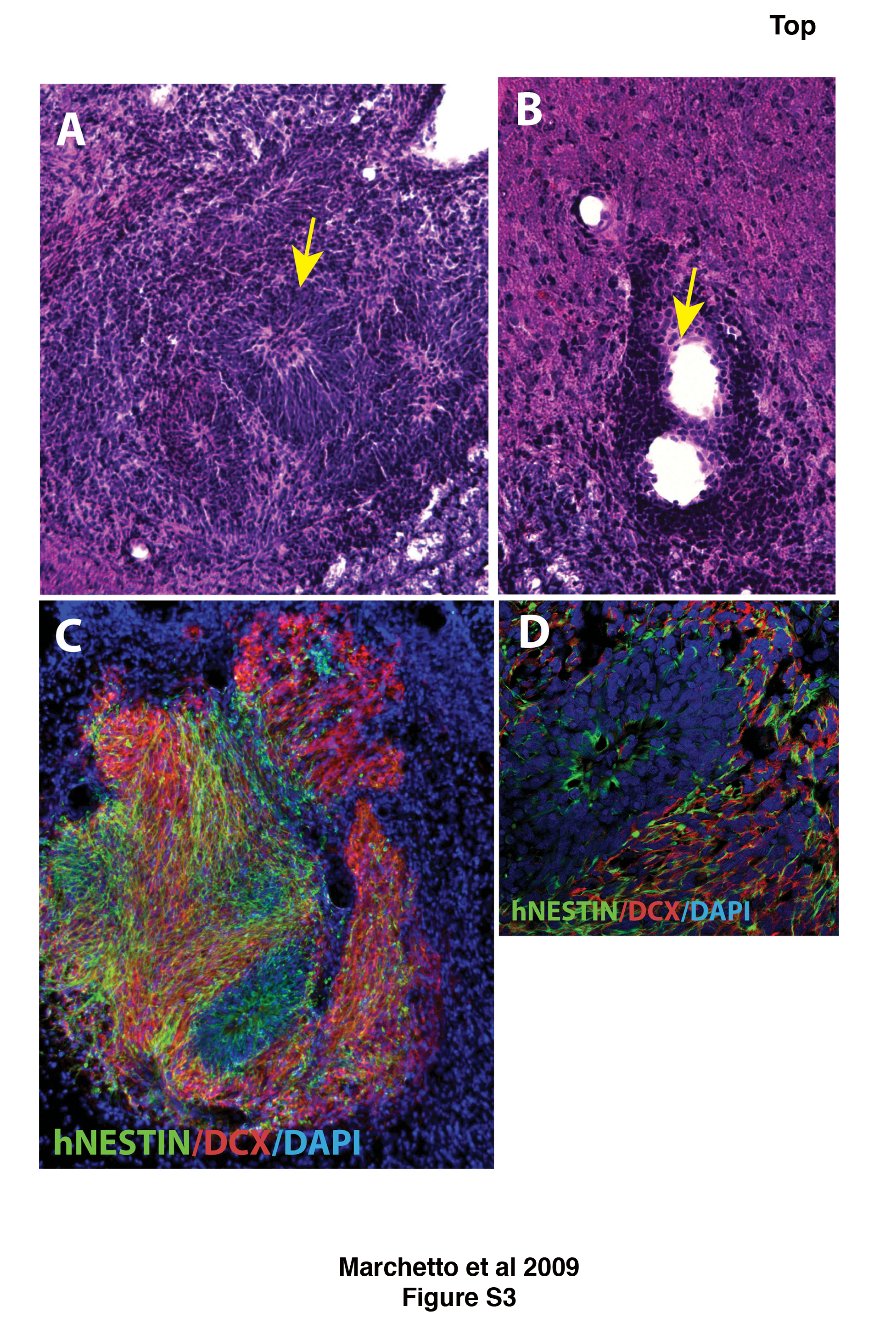

Supplement: Figure S3 — Development of teratomas after spinal injections of iPSCs into lumbar gray matter. Lumbar spinal cord sections were stained with H&E at 3 weeks after grafting (A, B). The presence of rosette-like structures (A, yellow arrow) and ectoderm-derived squamous epithelium was identified (B, yellow arrow). Staining with human-specific nestin (green) and DCX (red) antibody show well organized nestin positive cells in primitive neuronal tube and numerous postmitotic DCX-positive neurons at the periphery of grafts (C, D). (9.63 MB TIF) [file pone.0007076.s003.tif]

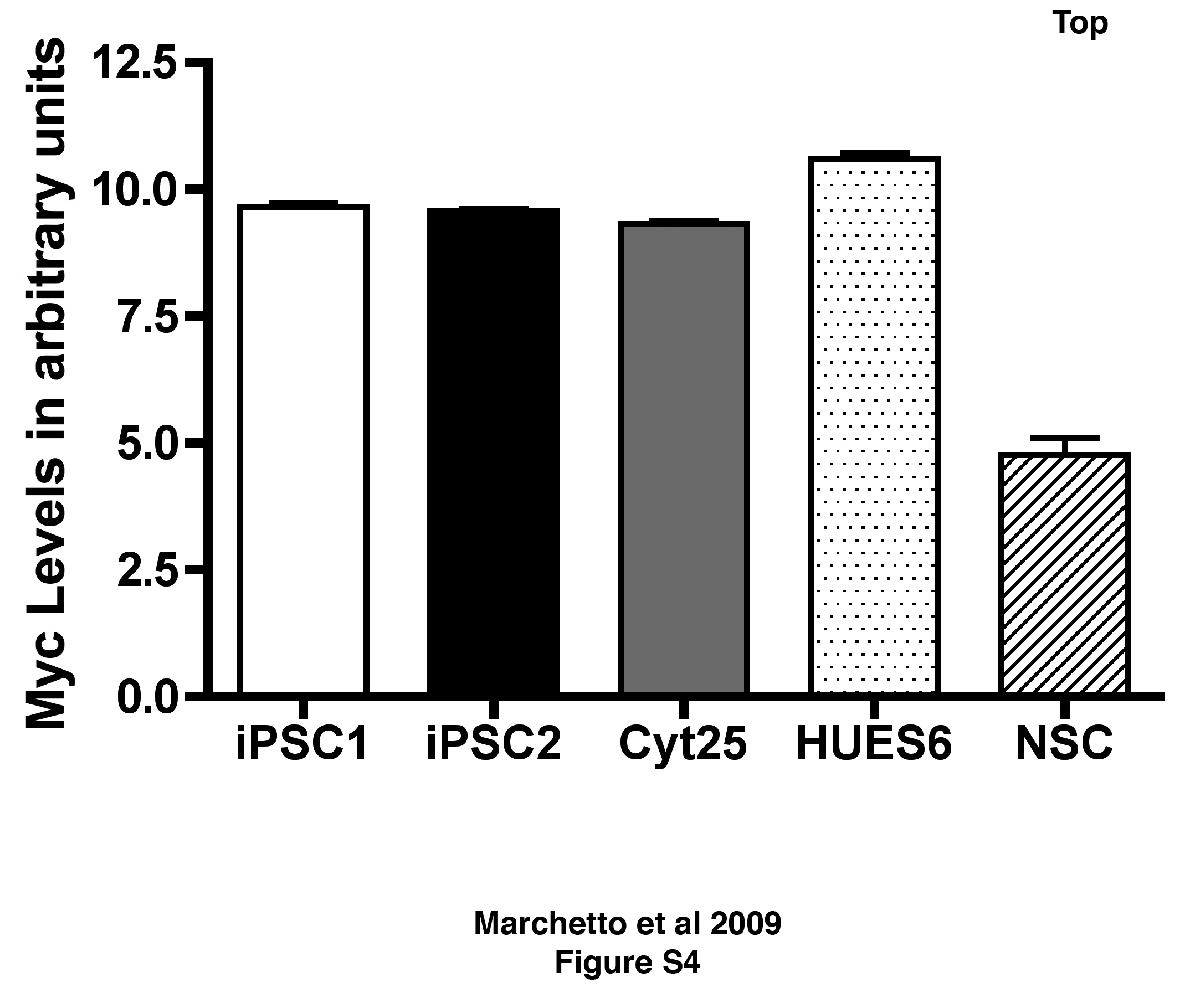

Supplement: Figure S4 — Myc levels in neural stem cells before and after reprogramming. The myc levels in iPSCs are similar to hESCs. (0.27 MB TIF) [file pone.0007076.s004.tif]
