# Supplementary material for: Transcriptional Signature and Memory Retention of Human-Induced Pluripotent Stem Cells
Source: PLoS One. 2009 Sep 18;4(9):e7076. doi: 10.1371/journal.pone.0007076 (PMC2741600; doi:10.1371/journal.pone.0007076)
Supplement: Table S1 — IPSC-enriched probes in IPSC versus ES. Probesets enriched in group-wise comparisons: Column headings are probeset identifiers, T-statistic, P-value, Fold-Change (log2), Refseq identifier and Description of the gene. (NA indicates no Refseq annotation). (5.43 MB DOC) [file pone.0007076.s005.doc]

| Probeset | T-statistic | P-value | Fold-Change (log2) | Refseq | Description |  |  |  |
| --- | --- | --- | --- | --- | --- | --- | --- | --- |
| 1555623_at | 88.5 | 2.27E-11 | 4.017012418 | NA |  |  |  |  |
| 206157_at | 82.34 | 3.11E-11 | 2.282096701 | NM_002852| | PTX3,pentaxin-related gene, rapidly induced by IL-1 | | | |
| 202950_at | 77.19 | 4.44E-11 | 2.876233165 | NM_001889| | CRYZ,crystallin, zeta | |  |  |
| 236896_at | 63.61 | 2.45E-10 | 2.374881112 | NM_003412| | ZIC1,zinc finger protein of the cerebellum 1 | | | |
| 207168_s_at | 60.68 | 3.05E-10 | 1.2027755 | NM_001040158| | NA |  |  |  |
| 214974_x_at | 59.38 | 3.05E-10 | 1.594214987 | NM_002994| | CXCL5,chemokine (C-X-C motif) ligand 5 precursor | | | |
| 242455_at | 58.22 | 3.30E-10 | 1.971076115 | NM_005604| | POU3F2,POU domain, class 3, transcription factor 2 | | | |
| 227141_at | 57.13 | 3.58E-10 | 2.665519378 | NM_138467| | LOC127253,hypothetical protein BC009514 | | | |
| 209465_x_at | 54.92 | 4.10E-10 | 1.274733426 | NM_002825| | PTN,pleiotrophin | |  |  |
| 231040_at | 54.26 | 4.10E-10 | 1.609523673 | NA |  |  |  |  |
| 1559470_at | 54.11 | 4.10E-10 | 2.221935636 | NA |  |  |  |  |
| 212513_s_at | 53.28 | 4.34E-10 | 1.143167935 | NM_015017| | USP33,ubiquitin specific protease 33 isoform 1 | | | |
| 204918_s_at | 53.13 | 4.34E-10 | 1.671237936 | NM_004529| | MLLT3,myeloid/lymphoid or mixed-lineage leukemia | | | |
| 237034_at | 49.8 | 7.83E-10 | 2.09212433 | NA |  |  |  |  |
| 201860_s_at | 49 | 8.41E-10 | 1.908288195 | NM_000930| | PLAT,plasminogen activator, tissue type isoform 1 | | | |
| 224354_at | 46.88 | 1.12E-09 | 3.333887978 | NA |  |  |  |  |
| 200862_at | 46.87 | 1.12E-09 | 1.206624287 | NM_014762| | DHCR24,24-dehydrocholesterol reductase precursor | | | |
| 234623_x_at | 45.04 | 1.45E-09 | 3.407692968 | NA |  |  |  |  |
| 208103_s_at | 44.94 | 1.45E-09 | 1.154704637 | NM_030920| | ANP32E,acidic (leucine-rich) nuclear phosphoprotein 32 | | | |
| 225342_at | 44.46 | 1.56E-09 | 1.267066094 | NM_001002921| | AK3L2,adenylate kinase 3-like 2 | | |  |
| 209267_s_at | 43.9 | 1.70E-09 | 1.471498303 | NM_022154| | SLC39A8,solute carrier family 39 (zinc transporter), | | | |
| 206291_at | 42.58 | 2.03E-09 | 1.537836156 | NM_006183| | NTS,neurotensin/neuromedin N preproprotein | | | |
| 238846_at | 41.94 | 2.29E-09 | 1.553211629 | NM_003839| | TNFRSF11A,tumor necrosis factor receptor superfamily, | | | |
| 238762_at | 41.71 | 2.29E-09 | 1.56059585 | NM_001004346| | MTHFD2L,methylenetetrahydrofolate dehydrogenase (NADP+ | | | |
| 206373_at | 41.02 | 2.63E-09 | 1.576531495 | NM_003412| | ZIC1,zinc finger protein of the cerebellum 1 | | | |
| 219134_at | 39.77 | 3.27E-09 | 2.024392062 | NM_022159| | NA |  |  |  |
| 204288_s_at | 39.74 | 3.27E-09 | 1.388489076 | NM_003603| | ARGBP2,Arg/Abl-interacting protein 2 isoform 1 | | | |
| 226558_at | 39.71 | 3.27E-09 | 2.052069933 | NA |  |  |  |  |
| 229461_x_at | 39.44 | 3.33E-09 | 2.001541379 | NM_173808| | NEGR1,neuronal growth regulator 1 | | |  |
| 201744_s_at | 39.33 | 3.33E-09 | 2.463965631 | NM_002345| | LUM,lumican |  |  |  |
| 206932_at | 39.18 | 3.33E-09 | 1.690970782 | NM_003956| | CH25H,cholesterol 25-hydroxylase | | |  |
| 1559471_s_at | 38.68 | 3.71E-09 | 1.656424844 | NA |  |  |  |  |
| 1552487_a_at | 38.58 | 3.72E-09 | 1.990680444 | NM_001717| | BNC1,basonuclin 1 | |  |  |
| 235944_at | 38.24 | 3.85E-09 | 1.789126768 | NM_031935| | FIBL-6,hemicentin | |  |  |
| 223250_at | 38.22 | 3.85E-09 | 1.108108502 | NM_001031710| | NA |  |  |  |
| 223529_at | 37.71 | 4.23E-09 | 1.497328035 | NM_020783| | SYT4,synaptotagmin IV | |  |  |
| 213227_at | 37.71 | 4.23E-09 | 1.115706474 | NM_006320| | PGRMC2,progesterone membrane binding protein | | | |
| 204348_s_at | 37.39 | 4.50E-09 | 1.247789655 | NM_001002921| | AK3L2,adenylate kinase 3-like 2 | | |  |
| 214844_s_at | 37.09 | 4.68E-09 | 1.128822067 | NM_018431| | DOK5,DOK5 protein isoform a | | |  |
| 211161_s_at | 37.05 | 4.68E-09 | 2.404677996 | NM_000090| | COL3A1,alpha 1 type III collagen | | |  |
| 222557_at | 36.44 | 5.43E-09 | 1.160219595 | NM_015894| | STMN3,SCG10-like-protein | | |  |
| 226452_at | 36.35 | 5.47E-09 | 1.191592868 | NM_002610| | PDK1,pyruvate dehydrogenase kinase, isoenzyme 1 | | | |
| 219885_at | 35.72 | 6.41E-09 | 1.428542988 | NM_018042| | FLJ10260,hypothetical protein FLJ10260 | | | |
| 231925_at | 35.64 | 6.41E-09 | 1.501555941 | NA |  |  |  |  |
| 220780_at | 35.6 | 6.41E-09 | 1.170490546 | NM_015715| | PLA2G3,phospholipase A2, group III precursor | | | |
| 237449_at | 35.39 | 6.57E-09 | 1.328911574 | NM_182700| | SP8,Sp8 transcription factor isoform 1 | | |  |
| 226281_at | 35.18 | 6.86E-09 | 1.672484128 | NM_139072| | DNER,delta-notch-like EGF repeat-containing | | | |
| 201852_x_at | 34.56 | 7.83E-09 | 1.751293889 | NM_000090| | COL3A1,alpha 1 type III collagen | | |  |
| 205029_s_at | 34.43 | 7.94E-09 | 1.704321519 | NM_001446| | FABP7,fatty acid binding protein 7, brain | | | |
| 205523_at | 34.24 | 8.22E-09 | 2.391594874 | NM_001884| | HAPLN1,cartilage linking protein 1 | | |  |
| 227607_at | 33.95 | 8.83E-09 | 1.406189254 | NM_020799| | AMSH-LP,associated molecule with the SH3 domain of STAM | | | |
| 223122_s_at | 33.68 | 9.24E-09 | 1.139881544 | NM_003013| | SFRP2,secreted frizzled-related protein 2 precursor | | | |
| 211959_at | 33.65 | 9.24E-09 | 1.276228584 | NM_000599| | IGFBP5,insulin-like growth factor binding protein 5 | | | |
| 235381_at | 33.3 | 9.62E-09 | 1.256139245 | NA |  |  |  |  |
| 206307_s_at | 33.27 | 9.62E-09 | 1.448934909 | NM_004472| | FOXD1,forkhead box D1 | |  |  |
| 225197_at | 33.26 | 9.62E-09 | 1.109129388 | NA |  |  |  |  |
| 203510_at | 33.2 | 9.68E-09 | 1.232299467 | NM_000245| | MET,met proto-oncogene precursor | | |  |
| 209841_s_at | 33.05 | 1.00E-08 | 1.557884759 | NM_001099658| | NA |  |  |  |
| 205030_at | 33 | 1.00E-08 | 1.502016545 | NM_001446| | FABP7,fatty acid binding protein 7, brain | | | |
| 209466_x_at | 32.92 | 1.01E-08 | 1.262786209 | NM_002825| | PTN,pleiotrophin | |  |  |
| 225978_at | 32.75 | 1.04E-08 | 1.300231745 | NM_020734| | KIAA1238,KIAA1238 protein | | |  |
| 223121_s_at | 32.61 | 1.06E-08 | 1.126585869 | NM_003013| | SFRP2,secreted frizzled-related protein 2 precursor | | | |
| 241998_at | 32.57 | 1.06E-08 | 1.938072607 | NM_001099334| | NA |  |  |  |
| 229498_at | 32.55 | 1.06E-08 | 1.473444244 | NA |  |  |  |  |
| 234432_at | 32.54 | 1.06E-08 | 2.869350479 | NA |  |  |  |  |
| 220231_at | 32.49 | 1.06E-08 | 1.702012986 | NM_006658| | C7orf16,G-substrate | |  |  |
| 205814_at | 32.29 | 1.11E-08 | 1.410891006 | NM_000840| | GRM3,glutamate receptor, metabotropic 3 precursor | | | |
| 216917_s_at | 32.13 | 1.15E-08 | 1.854765652 | NM_003176| | SYCP1,synaptonemal complex protein 1 | | | |
| 208940_at | 32 | 1.19E-08 | 1.142246681 | NM_012247| | SEPHS1,selenophosphate synthetase | | |  |
| 226041_at | 31.73 | 1.25E-08 | 1.294740341 | NM_001122838| | NA |  |  |  |
| 224624_at | 31.44 | 1.32E-08 | 1.052418707 | NM_001127244| | NA |  |  |  |
| 221059_s_at | 31.31 | 1.36E-08 | 1.073221154 | NM_021149| | COTL1,coactosin-like 1 | |  |  |
| 214717_at | 31.29 | 1.36E-08 | 1.398675283 | NA |  |  |  |  |
| 211737_x_at | 31.21 | 1.36E-08 | 1.263610951 | NM_002825| | PTN,pleiotrophin | |  |  |
| 228780_at | 31.2 | 1.36E-08 | 1.834432549 | NA |  |  |  |  |
| 201193_at | 31.17 | 1.36E-08 | 1.068624446 | NM_005896| | IDH1,isocitrate dehydrogenase 1 (NADP+), soluble | | | |
| 207060_at | 31.07 | 1.37E-08 | 1.694513436 | NM_001427| | EN2,engrailed homolog 2 | |  |  |
| 202540_s_at | 31.05 | 1.37E-08 | 1.145714772 | NM_000859| | HMGCR,3-hydroxy-3-methylglutaryl-Coenzyme A reductase | | | |
| 224964_s_at | 31.03 | 1.37E-08 | 1.439569883 | NM_053064| | GNG2,guanine nucleotide binding protein (G protein), | | | |
| 221654_s_at | 30.93 | 1.39E-08 | 1.304129575 | NM_006537| | USP3,ubiquitin specific protease 3 | | |  |
| 209785_s_at | 30.91 | 1.39E-08 | 2.048492052 | NM_003706| | PLA2G4C,phospholipase A2, group IVC | | |  |
| 209988_s_at | 30.86 | 1.40E-08 | 1.763716212 | NM_004316| | ASCL1,achaete-scute complex homolog-like 1 | | | |
| 217902_s_at | 30.79 | 1.42E-08 | 1.091158262 | NM_004667| | HERC2,hect domain and RLD 2 | | |  |
| 203447_at | 30.63 | 1.47E-08 | 1.136542715 | NM_005047| | PSMD5,proteasome 26S non-ATPase subunit 5 | | | |
| 219602_s_at | 30.42 | 1.52E-08 | 2.03443428 | NM_022068| | FAM38B,family with sequence similarity 38, member B | | | |
| 228987_at | 30.38 | 1.52E-08 | 1.224736366 | NA |  |  |  |  |
| 227164_at | 30.36 | 1.52E-08 | 1.198081234 | NM_001078166| | NA |  |  |  |
| 201250_s_at | 30.28 | 1.54E-08 | 1.238126562 | NM_006516| | SLC2A1,solute carrier family 2 (facilitated glucose | | | |
| 226776_at | 30.21 | 1.57E-08 | 1.365908113 | NM_020189| | e(y)2,e(y)2 protein | |  |  |
| 238360_s_at | 30.18 | 1.57E-08 | 1.740324572 | NA |  |  |  |  |
| 218980_at | 30.09 | 1.61E-08 | 1.195833631 | NM_025135| | FHOD3,formin homology 2 domain containing 3 | | | |
| 204697_s_at | 30.02 | 1.63E-08 | 1.346644298 | NM_001275| | CHGA,chromogranin A | |  |  |
| 206442_at | 29.93 | 1.66E-08 | 1.895437928 | NM_003007| | SEMG1,semenogelin I isoform a preproprotein | | | |
| 204743_at | 29.93 | 1.66E-08 | 1.226718662 | NM_001008272| | TAGLN3,transgelin 3 | |  |  |
| 200897_s_at | 29.9 | 1.66E-08 | 1.086723917 | NM_016081| | KIAA0992,palladin | |  |  |
| 1561775_at | 29.79 | 1.69E-08 | 2.465996306 | NA |  |  |  |  |
| 229347_at | 29.71 | 1.73E-08 | 1.675160069 | NA |  |  |  |  |
| 219090_at | 29.68 | 1.74E-08 | 1.23257311 | NM_020689| | SLC24A3,solute carrier family 24 | | |  |
| 213927_at | 29.6 | 1.77E-08 | 1.140494184 | NM_033141| | MAP3K9,mitogen-activated protein kinase kinase kinase | | | |
| 229127_at | 29.42 | 1.86E-08 | 1.3557484 | NM_021219| | JAM2,junctional adhesion molecule 2 precursor | | | |
| 212915_at | 29.3 | 1.92E-08 | 1.210202373 | NM_015009| | PDZRN3,PDZ domain containing RING finger 3 | | | |
| 228146_at | 29.3 | 1.92E-08 | 1.560860017 | NM_001113434| | NA |  |  |  |
| 214633_at | 29.07 | 2.03E-08 | 1.298663036 | NM_005634| | SOX3,SRY (sex determining region Y)-box 3 | | | |
| 219410_at | 29.05 | 2.03E-08 | 1.185894674 | NM_018004| | TMEM45A,transmembrane protein 45A | | |  |
| 204304_s_at | 29.04 | 2.03E-08 | 1.060832552 | NM_006017| | PROM1,prominin 1 | |  |  |
| 242770_at | 28.86 | 2.14E-08 | 1.882919998 | NA |  |  |  |  |
| 202503_s_at | 28.82 | 2.15E-08 | 1.096483609 | NM_001029989| | NA |  |  |  |
| 235919_at | 28.81 | 2.15E-08 | 1.226143802 | NA |  |  |  |  |
| 209625_at | 28.73 | 2.19E-08 | 1.256423635 | NM_004569| | PIGH,phosphatidylinositol glycan, class H | | | |
| 204451_at | 28.49 | 2.33E-08 | 1.355135196 | NM_003505| | FZD1,frizzled 1 | |  |  |
| 218377_s_at | 28.45 | 2.34E-08 | 1.199074606 | NM_016940| | C21orf6,chromosome 21 open reading frame 6 | | | |
| 215446_s_at | 28.43 | 2.35E-08 | 2.003156944 | NM_002317| | LOX,lysyl oxidase preproprotein | | |  |
| 214769_at | 28.4 | 2.35E-08 | 1.195883218 | NM_001830| | CLCN4,chloride channel 4 | |  |  |
| 224773_at | 28.27 | 2.42E-08 | 1.142331293 | NM_020443| | NAV1,neuron navigator 1 | |  |  |
| 228365_at | 28.12 | 2.53E-08 | 1.29831997 | NM_153634| | CPNE8,copine VIII | |  |  |
| 200644_at | 28.07 | 2.54E-08 | 1.073685703 | NM_023009| | MARCKSL1,MARCKS-like 1 | |  |  |
| 226390_at | 27.82 | 2.67E-08 | 1.233003696 | NM_139164| | STARD4,START domain containing 4, sterol regulated | | | |
| 204347_at | 27.78 | 2.67E-08 | 1.231671622 | NM_001002921| | AK3L2,adenylate kinase 3-like 2 | | |  |
| 201204_s_at | 27.77 | 2.67E-08 | 1.074702596 | NM_001042576| | NA |  |  |  |
| 202619_s_at | 27.76 | 2.67E-08 | 1.214666018 | NM_000935| | PLOD2,procollagen-lysine, 2-oxoglutarate 5-dioxygenase | | | |
| 207826_s_at | 27.74 | 2.67E-08 | 1.188031016 | NM_002167| | ID3,inhibitor of DNA binding 3 | | |  |
| 212912_at | 27.7 | 2.70E-08 | 1.238908977 | NM_001006932| | RPS6KA2,ribosomal protein S6 kinase, 90kDa, polypeptide | | | |
| 208065_at | 27.56 | 2.82E-08 | 1.863116034 | NM_015879| | ST8SIA3,ST8 alpha-N-acetyl-neuraminide | | | |
| 226775_at | 27.52 | 2.83E-08 | 1.292634636 | NM_020189| | e(y)2,e(y)2 protein | |  |  |
| 212928_at | 27.52 | 2.83E-08 | 1.13112771 | NM_021648| | TSPYL4,KIAA0721 protein | |  |  |
| 211984_at | 27.43 | 2.90E-08 | 1.07678201 | NM_001743| | CALM2,calmodulin 2 | |  |  |
| 228955_at | 27.42 | 2.90E-08 | 1.422326942 | NA |  |  |  |  |
| 200795_at | 27.29 | 3.00E-08 | 1.800927226 | NM_004684| | SPARCL1,SPARC-like 1 | |  |  |
| 242344_at | 27.15 | 3.09E-08 | 1.749712733 | NM_000813| | GABRB2,gamma-aminobutyric acid (GABA) A receptor, beta | | | |
| 214119_s_at | 27.14 | 3.09E-08 | 1.199317958 | NM_000801| | FKBP1A,FK506-binding protein 1A | | |  |
| 204612_at | 27.09 | 3.12E-08 | 1.205261223 | NM_006823| | PKIA,cAMP-dependent protein kinase inhibitor alpha | | | |
| 226676_at | 27.07 | 3.14E-08 | 1.138322303 | NM_015461| | ZNF521,zinc finger protein 521 | | |  |
| 1552626_a_at | 26.99 | 3.19E-08 | 1.402827708 | NM_030923| | DKFZP566N034,hypothetical protein DKFZp566N034 | | | |
| 211990_at | 26.91 | 3.24E-08 | 1.315144725 | NM_033554| | HLA-DPA1,major histocompatibility complex, class II, DP | | | |
| 225961_at | 26.91 | 3.24E-08 | 1.120925575 | NM_020782| | KLHDC5,kelch domain containing 5 | | |  |
| 209840_s_at | 26.9 | 3.24E-08 | 1.791311087 | NM_001099658| | NA |  |  |  |
| 1554016_a_at | 26.76 | 3.40E-08 | 1.119832005 | NM_024598| | FLJ13154,hypothetical protein FLJ13154 | | | |
| 211713_x_at | 26.75 | 3.40E-08 | 1.256959463 | NM_001029989| | NA |  |  |  |
| 203998_s_at | 26.64 | 3.51E-08 | 1.209542762 | NM_005639| | SYT1,synaptotagmin I | |  |  |
| 204012_s_at | 26.6 | 3.53E-08 | 1.134282224 | NM_014793| | LCMT2,leucine carboxyl methyltransferase 2 | | | |
| 214079_at | 26.58 | 3.53E-08 | 2.051294284 | NM_005794| | DHRS2,dehydrogenase/reductase (SDR family) member 2 | | | |
| 203595_s_at | 26.51 | 3.60E-08 | 1.246309217 | NM_012420| | IFIT5,interferon-induced protein with | | |  |
| 231579_s_at | 26.5 | 3.60E-08 | 1.100214657 | NM_003255| | TIMP2,tissue inhibitor of metalloproteinase 2 | | | |
| 211719_x_at | 26.41 | 3.69E-08 | 1.184840662 | NM_002026| | FN1,fibronectin 1 isoform 3 preproprotein | | | |
| 215101_s_at | 26.38 | 3.71E-08 | 1.716660343 | NM_002994| | CXCL5,chemokine (C-X-C motif) ligand 5 precursor | | | |
| 228381_at | 26.37 | 3.71E-08 | 1.290792519 | NM_024997| | ATF7IP2,activating transcription factor 7 interacting | | | |
| 222877_at | 26.34 | 3.73E-08 | 1.385288562 | NA |  |  |  |  |
| 218888_s_at | 26.13 | 3.96E-08 | 1.185324908 | NM_018092| | NETO2,neuropilin- and tolloid-like protein 2 | | | |
| 202752_x_at | 26.09 | 3.98E-08 | 1.146981144 | NM_012244| | SLC7A8,solute carrier family 7 (cationic amino acid | | | |
| 236824_at | 25.9 | 4.20E-08 | 1.403069363 | NM_052907| | KIAA1906,KIAA1906 protein | | |  |
| 213258_at | 25.87 | 4.24E-08 | 1.322614096 | NM_001032281| | NA |  |  |  |
| 230738_at | 25.82 | 4.30E-08 | 1.346976474 | NA |  |  |  |  |
| 202741_at | 25.76 | 4.37E-08 | 1.077626528 | NM_002731| | PRKACB,cAMP-dependent protein kinase catalytic subunit | | | |
| 213058_at | 25.74 | 4.40E-08 | 1.171959779 | NA |  |  |  |  |
| 227719_at | 25.68 | 4.45E-08 | 1.563834233 | NM_001127217| | NA |  |  |  |
| 226084_at | 25.66 | 4.45E-08 | 1.122785821 | NM_005909| | MAP1B,microtubule-associated protein 1B isoform 1 | | | |
| 216092_s_at | 25.6 | 4.48E-08 | 1.131043081 | NM_012244| | SLC7A8,solute carrier family 7 (cationic amino acid | | | |
| 219355_at | 25.59 | 4.48E-08 | 1.447286681 | NM_018015| | FLJ10178,hypothetical protein FLJ10178 | | | |
| 204424_s_at | 25.59 | 4.48E-08 | 1.538240276 | NM_001001395| | LMO3,LIM domain only 3 | |  |  |
| 226809_at | 25.59 | 4.48E-08 | 1.184210443 | NA |  |  |  |  |
| 221019_s_at | 25.58 | 4.48E-08 | 1.291797798 | NM_130386| | COLEC12,collectin sub-family member 12 isoform I | | | |
| 227568_at | 25.54 | 4.50E-08 | 1.288607542 | NM_173497| | HECTD2,HECT domain containing 2 isoform b | | | |
| 228298_at | 25.47 | 4.61E-08 | 1.260631625 | NM_138371| | MGC16044,hypothetical protein MGC16044 | | | |
| 203625_x_at | 25.42 | 4.67E-08 | 1.063120669 | NM_005983| | SKP2,S-phase kinase-associated protein 2 isoform 1 | | | |
| 203548_s_at | 25.37 | 4.76E-08 | 1.728284603 | NM_000237| | LPL,lipoprotein lipase precursor | | |  |
| 242517_at | 25.34 | 4.79E-08 | 1.238361996 | NM_032551| | GPR54,G protein-coupled receptor 54 | | |  |
| 204595_s_at | 25.29 | 4.84E-08 | 1.223524519 | NM_003155| | STC1,stanniocalcin 1 | |  |  |
| 205381_at | 25.27 | 4.84E-08 | 1.479412104 | NM_001031692| | NA |  |  |  |
| 203428_s_at | 25.18 | 4.97E-08 | 1.076150296 | NM_014034| | ASF1A,ASF1 anti-silencing function 1 homolog A | | | |
| 218404_at | 25.14 | 5.03E-08 | 1.088428838 | NM_013322| | SNX10,sorting nexin 10 | |  |  |
| 203325_s_at | 25.1 | 5.08E-08 | 1.338503093 | NM_000093| | COL5A1,alpha 1 type V collagen preproprotein | | | |
| 227461_at | 25.06 | 5.15E-08 | 1.251511143 | NM_033104| | STN2,stonin 2 | |  |  |
| 202620_s_at | 25.03 | 5.18E-08 | 1.213280037 | NM_000935| | PLOD2,procollagen-lysine, 2-oxoglutarate 5-dioxygenase | | | |
| 209684_at | 24.92 | 5.37E-08 | 1.454207269 | NM_018993| | RIN2,RAB5 interacting protein 2 | | |  |
| 219918_s_at | 24.84 | 5.53E-08 | 1.091581272 | NM_018136| | ASPM,asp (abnormal spindle)-like, microcephaly | | | |
| 228397_at | 24.83 | 5.53E-08 | 1.304427299 | NA |  |  |  |  |
| 213592_at | 24.8 | 5.57E-08 | 1.943718997 | NM_005161| | AGTRL1,angiotensin II receptor-like 1 | | |  |
| 228092_at | 24.76 | 5.63E-08 | 1.327013978 | NM_001881| | CREM,cAMP responsive element modulator isoform b | | | |
| 1557545_s_at | 24.73 | 5.69E-08 | 1.524102134 | NM_152470| | C18orf23,chromosome 18 open reading frame 23 | | | |
| 209566_at | 24.71 | 5.69E-08 | 1.269435193 | NM_016133| | INSIG2,insulin induced protein 2 | | |  |
| 204602_at | 24.68 | 5.72E-08 | 1.447521435 | NM_012242| | DKK1,dickkopf homolog 1 | |  |  |
| 233847_x_at | 24.46 | 6.20E-08 | 2.931312576 | NA |  |  |  |  |
| 220865_s_at | 24.46 | 6.20E-08 | 1.105841382 | NM_014317| | TPRT,trans-prenyltransferase | | |  |
| 214074_s_at | 24.37 | 6.36E-08 | 1.226201381 | NM_005231| | CTTN,cortactin isoform a | |  |  |
| 200777_s_at | 24.33 | 6.43E-08 | 1.065698816 | NM_014670| | BZW1,basic leucine zipper and W2 domains 1 | | | |
| 201241_at | 24.31 | 6.44E-08 | 1.043563514 | NM_004939| | DDX1,DEAD (Asp-Glu-Ala-Asp) box polypeptide 1 | | | |
| 214608_s_at | 24.23 | 6.58E-08 | 1.31715479 | NM_000503| | EYA1,eyes absent 1 isoform b | | |  |
| 210495_x_at | 24.22 | 6.58E-08 | 1.17783138 | NM_002026| | FN1,fibronectin 1 isoform 3 preproprotein | | | |
| 1554863_s_at | 24.1 | 6.88E-08 | 1.164511301 | NM_018431| | DOK5,DOK5 protein isoform a | | |  |
| 238453_at | 24.1 | 6.88E-08 | 1.304337957 | NM_152429| | C10orf13,hypothetical protein MGC39320 | | | |
| 203560_at | 24.08 | 6.88E-08 | 1.210660505 | NM_003878| | GGH,gamma-glutamyl hydrolase precursor | | | |
| 236016_at | 24.07 | 6.88E-08 | 1.331226645 | NA |  |  |  |  |
| 212464_s_at | 24.06 | 6.89E-08 | 1.179904825 | NM_002026| | FN1,fibronectin 1 isoform 3 preproprotein | | | |
| 203474_at | 24.04 | 6.91E-08 | 1.142432597 | NM_006633| | IQGAP2,IQ motif containing GTPase activating protein 2 | | | |
| 202720_at | 24.02 | 6.92E-08 | 1.128551617 | NM_015641| | TES,testin isoform 1 | |  |  |
| 212486_s_at | 23.94 | 7.14E-08 | 1.198956336 | NM_002037| | FYN,protein-tyrosine kinase fyn isoform a | | | |
| 204493_at | 23.92 | 7.14E-08 | 1.157920716 | NM_001196| | BID,BH3 interacting domain death agonist isoform 2 | | | |
| 230630_at | 23.92 | 7.14E-08 | 1.295979081 | NA |  |  |  |  |
| 204694_at | 23.85 | 7.32E-08 | 1.262267232 | NM_001134| | AFP,alpha-fetoprotein precursor | | |  |
| 201564_s_at | 23.8 | 7.44E-08 | 1.123006516 | NM_003088| | FSCN1,fascin 1 | |  |  |
| 218138_at | 23.79 | 7.44E-08 | 1.091550315 | NM_018848| | MKKS,McKusick-Kaufman syndrome protein | | | |
| 220102_at | 23.78 | 7.44E-08 | 1.695380544 | NM_023067| | FOXL2,forkhead box L2 | |  |  |
| 244523_at | 23.77 | 7.46E-08 | 1.178541873 | NM_012329| | MMD,monocyte to macrophage | | |  |
| 218854_at | 23.72 | 7.54E-08 | 1.052954484 | NM_001080976| | NA |  |  |  |
| 228850_s_at | 23.7 | 7.59E-08 | 1.757900841 | NA |  |  |  |  |
| 201890_at | 23.66 | 7.68E-08 | 1.125282318 | NM_001034| | RRM2,ribonucleotide reductase M2 polypeptide | | | |
| 218353_at | 23.63 | 7.73E-08 | 1.895165438 | NM_003617| | RGS5,regulator of G-protein signalling 5 | | | |
| 223154_at | 23.61 | 7.77E-08 | 1.125218703 | NM_020236| | MRPL1,mitochondrial ribosomal protein L1 | | | |
| 225670_at | 23.61 | 7.77E-08 | 1.155124909 | NM_199133| | LOC134145,hypothetical protein LOC134145 | | | |
| 228635_at | 23.59 | 7.78E-08 | 1.454079452 | NM_020815| | PCDH10,protocadherin 10 isoform 2 precursor | | | |
| 231130_at | 23.5 | 8.04E-08 | 1.60063611 | NA |  |  |  |  |
| 229391_s_at | 23.49 | 8.04E-08 | 1.41789521 | NM_001010919| | NA |  |  |  |
| 224516_s_at | 23.46 | 8.09E-08 | 1.093489763 | NM_016463| | CXXC5,CXXC finger 5 | |  |  |
| 212614_at | 23.42 | 8.22E-08 | 1.587310858 | NM_032199| | NA |  |  |  |
| 219151_s_at | 23.39 | 8.27E-08 | 1.200650289 | NM_001003789| | RABL2B,RAB, member of RAS oncogene family-like 2B | | | |
| 213113_s_at | 23.37 | 8.32E-08 | 1.195254972 | NM_014096| | SLC43A3,solute carrier family 43, member 3 | | | |
| 217967_s_at | 23.33 | 8.44E-08 | 1.188703995 | NM_052966| | C1orf24,niban protein | |  |  |
| 216442_x_at | 23.32 | 8.44E-08 | 1.179377854 | NM_002026| | FN1,fibronectin 1 isoform 3 preproprotein | | | |
| 233955_x_at | 23.3 | 8.50E-08 | 1.076635493 | NM_016463| | CXXC5,CXXC finger 5 | |  |  |
| 218625_at | 23.28 | 8.53E-08 | 1.9182502 | NM_016588| | NRN1,neuritin precursor | |  |  |
| 224694_at | 23.27 | 8.53E-08 | 1.086094587 | NM_018153| | ANTXR1,tumor endothelial marker 8 isoform 3 precursor | | | |
| 204284_at | 23.08 | 9.18E-08 | 1.4426062 | NM_005398| | PPP1R3C,protein phosphatase 1, regulatory (inhibitor) | | | |
| 201843_s_at | 23.05 | 9.26E-08 | 1.534616803 | NM_001039348| | NA |  |  |  |
| 230895_at | 23 | 9.42E-08 | 2.27602776 | NA |  |  |  |  |
| 219763_at | 22.97 | 9.53E-08 | 1.128526627 | NM_020946| | KIAA1608,KIAA1608 protein isoform 1 | | |  |
| 230204_at | 22.83 | 9.97E-08 | 2.07222233 | NA |  |  |  |  |
| 201842_s_at | 22.82 | 9.98E-08 | 1.26837629 | NM_001039348| | NA |  |  |  |
| 225163_at | 22.81 | 1.00E-07 | 1.223186593 | NM_018027| | FRMD4A,FERM domain containing 4A | | |  |
| 201163_s_at | 22.77 | 1.01E-07 | 1.670922085 | NM_001553| | IGFBP7,insulin-like growth factor binding protein 7 | | | |
| 225716_at | 22.76 | 1.02E-07 | 1.14979978 | NA |  |  |  |  |
| 212296_at | 22.7 | 1.03E-07 | 1.078959086 | NM_005805| | PSMD14,26S proteasome-associated pad1 homolog | | | |
| 222122_s_at | 22.69 | 1.03E-07 | 1.048079631 | NM_001081550| | NA |  |  |  |
| 238551_at | 22.63 | 1.05E-07 | 1.310085001 | NM_173540| | FUT11,fucosyltransferase 11 (alpha (1,3) | | | |
| 242300_at | 22.6 | 1.06E-07 | 1.384536186 | NA |  |  |  |  |
| 244881_at | 22.59 | 1.06E-07 | 1.159851728 | NM_033029| | LMLN,leishmanolysin-like (metallopeptidase M8 | | | |
| 226360_at | 22.59 | 1.06E-07 | 1.152585242 | NM_032173| | NA |  |  |  |
| 204115_at | 22.56 | 1.07E-07 | 1.442712356 | NM_004126| | GNG11,guanine nucleotide binding protein (G protein), | | | |
| 237472_at | 22.5 | 1.10E-07 | 1.510674871 | NM_005986| | SOX1,SRY (sex determining region Y)-box 1 | | | |
| 223470_at | 22.49 | 1.10E-07 | 1.114060973 | NM_145167| | PIGM,PIG-M mannosyltransferase | | |  |
| 222803_at | 22.46 | 1.11E-07 | 1.218302571 | NM_020200| | PRTFDC1,phosphoribosyl transferase domain containing 1 | | | |
| 205493_s_at | 22.45 | 1.11E-07 | 1.138022555 | NM_006426| | DPYSL4,dihydropyrimidinase-like 4 | | |  |
| 238867_at | 22.38 | 1.13E-07 | 1.281671674 | NM_144632| | FLJ30294,hypothetical protein FLJ30294 | | | |
| 202259_s_at | 22.33 | 1.15E-07 | 1.160717532 | NM_014887| | PFAAP5,phosphonoformate immuno-associated protein 5 | | | |
| 211675_s_at | 22.22 | 1.20E-07 | 1.524003556 | NM_199072| | HIC,I-mfa domain-containing protein isoform p40 | | | |
| 230264_s_at | 22.2 | 1.20E-07 | 1.183752903 | NM_003916| | AP1S2,adaptor-related protein complex 1 sigma 2 | | | |
| 222908_at | 22.2 | 1.20E-07 | 1.551399111 | NM_022068| | FAM38B,family with sequence similarity 38, member B | | | |
| 224733_at | 22.19 | 1.20E-07 | 1.07388572 | NM_001048251| | NA |  |  |  |
| 242486_at | 22.15 | 1.21E-07 | 1.450152786 | NA |  |  |  |  |
| 227547_at | 22.12 | 1.22E-07 | 1.103385248 | NA |  |  |  |  |
| 231867_at | 22.05 | 1.25E-07 | 1.62360198 | NM_001080428| | NA |  |  |  |
| 212195_at | 22.03 | 1.26E-07 | 1.236430351 | NM_002184| | IL6ST,interleukin 6 signal transducer isoform 1 | | | |
| 228095_at | 22.02 | 1.26E-07 | 1.139075903 | NM_001007157| | PHF14,PHD finger protein 14 isoform 1 | | |  |
| 204178_s_at | 22 | 1.26E-07 | 1.105791301 | NM_006328| | RBM14,RNA binding motif protein 14 | | |  |
| 203851_at | 21.99 | 1.27E-07 | 1.170099041 | NM_002178| | IGFBP6,insulin-like growth factor binding protein 6 | | | |
| 228894_at | 21.94 | 1.29E-07 | 1.136920315 | NM_001489| | NR6A1,nuclear receptor subfamily 6, group A, member 1 | | | |
| 203708_at | 21.9 | 1.31E-07 | 1.402526567 | NM_001037339| | NA |  |  |  |
| 204368_at | 21.89 | 1.31E-07 | 1.318156209 | NM_005630| | SLCO2A1,solute carrier organic anion transporter family, | | | |
| 201952_at | 21.88 | 1.32E-07 | 1.112375673 | NM_001627| | ALCAM,activated leukocyte cell adhesion molecule | | | |
| 227484_at | 21.78 | 1.37E-07 | 1.141995696 | NA |  |  |  |  |
| 212080_at | 21.77 | 1.37E-07 | 1.116869246 | NM_005933| | MLL,myeloid/lymphoid or mixed-lineage leukemia | | | |
| 223503_at | 21.76 | 1.37E-07 | 1.435710158 | NM_030923| | DKFZP566N034,hypothetical protein DKFZp566N034 | | | |
| 225655_at | 21.75 | 1.38E-07 | 1.179179612 | NM_001048201| | NA |  |  |  |
| 201069_at | 21.74 | 1.38E-07 | 1.238030454 | NM_004530| | MMP2,matrix metalloproteinase 2 preproprotein | | | |
| 222108_at | 21.74 | 1.38E-07 | 1.334021242 | NM_181847| | AMIGO2,amphoterin induced gene 2 | | |  |
| 223253_at | 21.65 | 1.42E-07 | 1.09472583 | NM_017549| | EPDR1,upregulated in colorectal cancer gene 1 protein | | | |
| 208743_s_at | 21.65 | 1.42E-07 | 1.04605161 | NM_003404| | YWHAB,tyrosine 3-monooxygenase/tryptophan | | | |
| 229390_at | 21.63 | 1.43E-07 | 1.605528149 | NM_001010919| | NA |  |  |  |
| 234973_at | 21.62 | 1.43E-07 | 1.222447203 | NM_033518| | SLC38A5,amino acid transport system N2 | | | |
| 236465_at | 21.58 | 1.44E-07 | 1.130691278 | NM_173662| | RNF175,ring finger protein 175 | | |  |
| 212328_at | 21.54 | 1.46E-07 | 1.19205803 | NM_001112717| | NA |  |  |  |
| 203963_at | 21.49 | 1.49E-07 | 1.34440259 | NM_001218| | CA12,carbonic anhydrase XII isoform 1 precursor | | | |
| 211423_s_at | 21.47 | 1.51E-07 | 1.072758484 | NM_001024956| | NA |  |  |  |
| 242447_at | 21.44 | 1.52E-07 | 1.498161283 | NM_001025266| | NA |  |  |  |
| 201847_at | 21.43 | 1.52E-07 | 1.113303581 | NM_000235| | LIPA,lipase A precursor | |  |  |
| 1558964_at | 21.43 | 1.52E-07 | 1.431002121 | NM_001008781| | NA |  |  |  |
| 222317_at | 21.41 | 1.53E-07 | 1.298824146 | NA |  |  |  |  |
| 208051_s_at | 21.39 | 1.54E-07 | 1.13364045 | NM_006451| | PAIP1,poly(A) binding protein interacting protein 1 | | | |
| 229824_at | 21.38 | 1.54E-07 | 1.854698893 | NA |  |  |  |  |
| 225732_at | 21.33 | 1.57E-07 | 1.100072273 | NM_020782| | KLHDC5,kelch domain containing 5 | | |  |
| 208398_s_at | 21.31 | 1.58E-07 | 1.066443483 | NM_004865| | TBPL1,TBP-like 1 | |  |  |
| 208766_s_at | 21.31 | 1.58E-07 | 1.053155788 | NM_001102397| | NA |  |  |  |
| 218829_s_at | 21.29 | 1.59E-07 | 1.054518278 | NM_017780| | CHD7,chromodomain helicase DNA binding protein 7 | | | |
| 225199_at | 21.26 | 1.60E-07 | 1.050819816 | NA |  |  |  |  |
| 202464_s_at | 21.24 | 1.61E-07 | 1.285336434 | NM_004566| | PFKFB3,6-phosphofructo-2-kinase/fructose-2, | | | |
| 225145_at | 21.24 | 1.61E-07 | 1.237233935 | NM_020967| | NCOA5,nuclear receptor coactivator 5 | | |  |
| 230943_at | 21.21 | 1.62E-07 | 1.396170946 | NM_022454| | SOX17,SRY-box 17 | |  |  |
| 201084_s_at | 21.21 | 1.62E-07 | 1.090391219 | NM_001077440| | NA |  |  |  |
| 210643_at | 21.2 | 1.62E-07 | 1.235941835 | NM_003701| | TNFSF11,tumor necrosis factor ligand superfamily, member | | | |
| 225728_at | 21.19 | 1.62E-07 | 1.405643999 | NM_003603| | ARGBP2,Arg/Abl-interacting protein 2 isoform 1 | | | |
| 214501_s_at | 21.18 | 1.62E-07 | 1.16386596 | NM_001040158| | NA |  |  |  |
| 225820_at | 21.16 | 1.64E-07 | 1.07513274 | NM_024900| | PHF17,Jade1 protein short isoform | | |  |
| 1560258_a_at | 21.08 | 1.67E-07 | 1.342748586 | NA |  |  |  |  |
| 219133_at | 21.08 | 1.67E-07 | 1.227309893 | NM_017897| | FLJ20604,hypothetical protein FLJ20604 | | | |
| 235518_at | 21.08 | 1.67E-07 | 1.59759365 | NM_001112800| | NA |  |  |  |
| 212218_s_at | 21.07 | 1.67E-07 | 1.157763587 | NM_004104| | FASN,fatty acid synthase | |  |  |
| 204320_at | 21.02 | 1.70E-07 | 1.434994937 | NM_001854| | COL11A1,alpha 1 type XI collagen isoform A | | | |
| 205407_at | 21.01 | 1.70E-07 | 1.223835726 | NM_021111| | RECK,RECK protein precursor | | |  |
| 224583_at | 20.99 | 1.71E-07 | 1.104912305 | NM_021149| | COTL1,coactosin-like 1 | |  |  |
| 232856_at | 20.96 | 1.74E-07 | 1.567998841 | NM_001005210| | FLJ45686,FLJ45686 protein | | |  |
| 222654_at | 20.94 | 1.75E-07 | 1.323410249 | NM_017813| | IMPA3,myo-inositol monophosphatase A3 | | | |
| 205830_at | 20.91 | 1.77E-07 | 1.188259528 | NM_004362| | CLGN,calmegin | |  |  |
| 244455_at | 20.88 | 1.79E-07 | 1.385814474 | NM_198503| | SLICK,sodium- and chloride-activated ATP-sensitive | | | |
| 218951_s_at | 20.88 | 1.79E-07 | 1.073013751 | NM_018390| | PLCXD1,phosphatidylinositol-specific phospholipase C, X | | | |
| 230960_at | 20.85 | 1.80E-07 | 1.239603682 | NM_004884| | PUNC,putative neuronal cell adhesion molecule | | | |
| 224963_at | 20.85 | 1.80E-07 | 1.084989444 | NM_000112| | SLC26A2,solute carrier family 26 member 2 | | | |
| 228181_at | 20.79 | 1.84E-07 | 1.148408056 | NM_021194| | SLC30A1,solute carrier family 30 (zinc transporter), | | | |
| 227705_at | 20.75 | 1.87E-07 | 1.132103522 | NM_152278| | TCEAL7,transcription elongation factor A (SII)-like 7 | | | |
| 206039_at | 20.66 | 1.95E-07 | 1.381729467 | NM_004794| | RAB33A,Ras-related protein Rab-33A | | |  |
| 231823_s_at | 20.65 | 1.95E-07 | 1.104332624 | NM_001017995| | NA |  |  |  |
| 204373_s_at | 20.65 | 1.95E-07 | 1.066072174 | NM_014810| | CAP350,centrosome-associated protein 350 | | | |
| 227997_at | 20.61 | 1.97E-07 | 1.11449043 | NM_001080973| | NA |  |  |  |
| 239913_at | 20.55 | 2.02E-07 | 1.496088877 | NM_152679| | SLC10A4,solute carrier family 10 (sodium/bile acid | | | |
| 1555967_at | 20.55 | 2.02E-07 | 1.694668514 | NA |  |  |  |  |
| 200988_s_at | 20.55 | 2.02E-07 | 1.251389005 | NM_005789| | PSME3,proteasome activator subunit 3 isoform 1 | | | |
| 214144_at | 20.55 | 2.02E-07 | 1.159959199 | NM_004805| | POLR2D,DNA directed RNA polymerase II polypeptide D | | | |
| 202809_s_at | 20.53 | 2.02E-07 | 1.107776159 | NM_023015| | FLJ21919,FLJ21919 protein | | |  |
| 204917_s_at | 20.53 | 2.02E-07 | 1.332292539 | NM_004529| | MLLT3,myeloid/lymphoid or mixed-lineage leukemia | | | |
| 201791_s_at | 20.51 | 2.02E-07 | 1.15826932 | NM_001360| | DHCR7,7-dehydrocholesterol reductase | | |  |
| 229885_at | 20.5 | 2.02E-07 | 1.345299065 | NA |  |  |  |  |
| 225597_at | 20.49 | 2.03E-07 | 1.154202578 | NM_001080431| | NA |  |  |  |
| 229271_x_at | 20.47 | 2.04E-07 | 1.792724014 | NM_001854| | COL11A1,alpha 1 type XI collagen isoform A | | | |
| 206197_at | 20.47 | 2.05E-07 | 1.208754217 | NM_003551| | NME5,non-metastatic cells 5, protein expressed in | | | |
| 205436_s_at | 20.44 | 2.06E-07 | 1.085977521 | NM_002105| | H2AFX,H2A histone family, member X | | |  |
| 205433_at | 20.44 | 2.06E-07 | 2.257217952 | NM_000055| | BCHE,butyrylcholinesterase precursor | | |  |
| 225566_at | 20.42 | 2.07E-07 | 1.15389116 | NM_003872| | NRP2,neuropilin 2 isoform 2 precursor | | |  |
| 202351_at | 20.4 | 2.08E-07 | 1.095751073 | NM_002210| | ITGAV,integrin alpha-V precursor | | |  |
| 1560425_s_at | 20.39 | 2.10E-07 | 1.441686648 | NA |  |  |  |  |
| 225585_at | 20.37 | 2.11E-07 | 1.075349786 | NM_021033| | RAP2A,RAP2A, member of RAS oncogene family | | | |
| 223541_at | 20.34 | 2.13E-07 | 1.206229574 | NM_005329| | HAS3,hyaluronan synthase 3 isoform a | | |  |
| 213825_at | 20.22 | 2.26E-07 | 1.634422148 | NM_005806| | OLIG2,oligodendrocyte lineage transcription factor 2 | | | |
| 200853_at | 20.2 | 2.26E-07 | 1.037164898 | NM_002106| | H2AFZ,H2A histone family, member Z | | |  |
| 227288_at | 20.2 | 2.26E-07 | 1.222790231 | NM_173829| | FLJ36754,hypothetical protein FLJ36754 | | | |
| 226348_at | 20.19 | 2.26E-07 | 1.198418835 | NA |  |  |  |  |
| 222984_at | 20.14 | 2.31E-07 | 1.040383242 | NM_001033112| | NA |  |  |  |
| 208920_at | 20.14 | 2.31E-07 | 1.162368782 | NM_003130| | SRI,sorcin isoform a | |  |  |
| 202589_at | 20.12 | 2.33E-07 | 1.083216035 | NM_001071| | TYMS,thymidylate synthetase | | |  |
| 225614_at | 20.09 | 2.36E-07 | 1.077609042 | NM_138421| | LOC113174,hypothetical protein BC012010 | | | |
| 228902_at | 20.08 | 2.36E-07 | 1.186698488 | NM_005085| | NUP214,nucleoporin 214kDa | | |  |
| 218656_s_at | 20.08 | 2.36E-07 | 1.631967348 | NM_005780| | LHFP,lipoma HMGIC fusion partner | | |  |
| 210220_at | 20.07 | 2.37E-07 | 1.105960972 | NM_001466| | FZD2,frizzled 2 | |  |  |
| 202368_s_at | 20.05 | 2.39E-07 | 1.22326698 | NM_012288| | TRAM2,translocation-associated membrane protein 2 | | | |
| 221185_s_at | 20.01 | 2.43E-07 | 1.151257088 | NM_032263| | IQCG,IQ motif containing G | | |  |
| 219814_at | 19.99 | 2.44E-07 | 1.425810478 | NM_018388| | MBNL3,muscleblind-like 3 isoform G | | |  |
| 210102_at | 19.98 | 2.44E-07 | 1.247245234 | NM_014622| | LOH11CR2A,BCSC-1 isoform 1 | | |  |
| 235117_at | 19.97 | 2.45E-07 | 1.202830151 | NM_001008708| | LOC494143,similar to mouse 2510006C20Rik protein | | | |
| 226864_at | 19.94 | 2.47E-07 | 1.293157216 | NM_006823| | PKIA,cAMP-dependent protein kinase inhibitor alpha | | | |
| 210073_at | 19.94 | 2.47E-07 | 1.664244054 | NM_003034| | ST8SIA1,ST8 alpha-N-acetyl-neuraminide | | | |
| 227496_at | 19.91 | 2.50E-07 | 1.179219295 | NM_001489| | NR6A1,nuclear receptor subfamily 6, group A, member 1 | | | |
| 226177_at | 19.87 | 2.55E-07 | 1.240606767 | NM_016433| | GLTP,glycolipid transfer protein | | |  |
| 200680_x_at | 19.84 | 2.57E-07 | 1.018041096 | NM_002128| | HMGB1,high-mobility group box 1 | | |  |
| 201101_s_at | 19.82 | 2.58E-07 | 1.088319654 | NM_001077440| | NA |  |  |  |
| 209933_s_at | 19.81 | 2.58E-07 | 1.452309088 | NM_007261| | CD300A,leukocyte membrane antigen | | |  |
| 219984_s_at | 19.79 | 2.60E-07 | 1.153988563 | NM_020386| | HRASLS,HRAS-like suppressor | | |  |
| 238521_at | 19.76 | 2.64E-07 | 1.30347461 | NA |  |  |  |  |
| 233487_s_at | 19.72 | 2.67E-07 | 1.116403174 | NM_001127244| | NA |  |  |  |
| 211936_at | 19.69 | 2.69E-07 | 1.058825457 | NM_005347| | HSPA5,heat shock 70kDa protein 5 (glucose-regulated | | | |
| 204064_at | 19.68 | 2.70E-07 | 1.060473745 | NM_005131| | THOC1,nuclear matrix protein p84 | | |  |
| 219368_at | 19.63 | 2.75E-07 | 1.089829442 | NM_021963| | NAP1L2,nucleosome assembly protein 1-like 2 | | | |
| 205097_at | 19.63 | 2.75E-07 | 1.122471095 | NM_000112| | SLC26A2,solute carrier family 26 member 2 | | | |
| 1564031_a_at | 19.61 | 2.76E-07 | 1.107861518 | NM_173828| | C5orf16,chromosome 5 open reading frame 16 | | | |
| 225765_at | 19.61 | 2.76E-07 | 1.054247801 | NM_002270| | TNPO1,transportin 1 | |  |  |
| 226556_at | 19.59 | 2.78E-07 | 1.133117051 | NA |  |  |  |  |
| 211784_s_at | 19.58 | 2.79E-07 | 1.057929381 | NM_001078166| | NA |  |  |  |
| 227760_at | 19.58 | 2.79E-07 | 1.099101369 | NM_001007563| | IGFBPL1,insulin-like growth factor binding protein-like | | | |
| 242082_at | 19.55 | 2.81E-07 | 1.121489321 | NM_052845| | MMAB,cob(I)alamin adenosyltransferase | | | |
| 224899_s_at | 19.51 | 2.86E-07 | 1.062318241 | NM_032121| | DKFZp564K142,implantation-associated protein | | | |
| 205861_at | 19.47 | 2.90E-07 | 1.394984822 | NM_003121| | SPIB,Spi-B transcription factor (Spi-1/PU.1 related) | | | |
| 200633_at | 19.47 | 2.90E-07 | 1.024272036 | NM_002954| | RPS27A,ubiquitin and ribosomal protein S27a precursor | | | |
| 228837_at | 19.46 | 2.91E-07 | 1.210552313 | NM_001083962| | NA |  |  |  |
| 218108_at | 19.45 | 2.92E-07 | 1.069066902 | NM_001100417| | NA |  |  |  |
| 214463_x_at | 19.45 | 2.92E-07 | 1.184500035 | NM_021968| | HIST1H4J,H4 histone family, member E | | | |
| 214930_at | 19.42 | 2.96E-07 | 1.391888941 | NM_015567| | SLITRK5,SLIT and NTRK-like family, member 5 | | | |
| 208716_s_at | 19.41 | 2.96E-07 | 1.075975388 | NM_019026| | LOC54499,putative membrane protein | | |  |
| 227494_at | 19.41 | 2.96E-07 | 1.143715811 | NM_001489| | NR6A1,nuclear receptor subfamily 6, group A, member 1 | | | |
| 200880_at | 19.4 | 2.97E-07 | 1.108710231 | NM_001539| | DNAJA1,DnaJ (Hsp40) homolog, subfamily A, member 1 | | | |
| 202364_at | 19.38 | 2.98E-07 | 1.15530063 | NM_001008541| | MXI1,MAX interactor 1 isoform c | | |  |
| 226347_at | 19.37 | 3.00E-07 | 1.181483551 | NA |  |  |  |  |
| 225358_at | 19.35 | 3.02E-07 | 1.120251848 | NM_145261| | TIM14,homolog of yeast TIM14 isoform a | | | |
| 213056_at | 19.31 | 3.08E-07 | 1.301016766 | NM_015123| | NA |  |  |  |
| 228964_at | 19.3 | 3.09E-07 | 1.500919453 | NM_001198| | PRDM1,PR domain containing 1, with ZNF domain isoform | | | |
| 231810_at | 19.3 | 3.08E-07 | 1.16625023 | NM_080626| | BRI3BP,BRI3-binding protein | | |  |
| 230061_at | 19.29 | 3.09E-07 | 1.841262329 | NM_138786| | LOC116441,hypothetical protein BC014339 | | | |
| 236029_at | 19.28 | 3.10E-07 | 1.409214969 | NM_001008781| | NA |  |  |  |
| 228449_at | 19.28 | 3.10E-07 | 1.369557224 | NA |  |  |  |  |
| 206230_at | 19.27 | 3.10E-07 | 1.632460289 | NM_005568| | LHX1,LIM homeobox protein 1 | | |  |
| 204510_at | 19.26 | 3.10E-07 | 1.091627218 | NM_003503| | CDC7,CDC7 cell division cycle 7 | | |  |
| 224463_s_at | 19.25 | 3.12E-07 | 1.235122251 | NM_032930| | MGC13040,hypothetical protein MGC13040 | | | |
| 212492_s_at | 19.22 | 3.16E-07 | 1.088750924 | NM_015015| | JMJD2B,jumonji domain containing 2B | | |  |
| 244075_at | 19.2 | 3.19E-07 | 1.642965694 | NA |  |  |  |  |
| 213435_at | 19.19 | 3.20E-07 | 1.231464101 | NM_015265| | SATB2,SATB family member 2 | | |  |
| 225805_at | 19.18 | 3.20E-07 | 1.060941466 | NM_004501| | HNRPU,heterogeneous nuclear ribonucleoprotein U | | | |
| 203423_at | 19.16 | 3.22E-07 | 1.179839631 | NM_002899| | RBP1,retinol binding protein 1, cellular | | |  |
| 238444_at | 19.16 | 3.22E-07 | 1.108785098 | NM_133374| | NA |  |  |  |
| 202457_s_at | 19.15 | 3.22E-07 | 1.149771557 | NM_000944| | PPP3CA,protein phosphatase 3 (formerly 2B), catalytic | | | |
| 238194_at | 19.15 | 3.22E-07 | 1.367946561 | NA |  |  |  |  |
| 218332_at | 19.15 | 3.22E-07 | 1.085346864 | NM_018476| | BEX1,brain expressed, X-linked 1 | | |  |
| 209608_s_at | 19.14 | 3.22E-07 | 1.149248899 | NM_005891| | ACAT2,acetyl-Coenzyme A acetyltransferase 2 | | | |
| 200881_s_at | 19.14 | 3.22E-07 | 1.07060012 | NM_001539| | DNAJA1,DnaJ (Hsp40) homolog, subfamily A, member 1 | | | |
| 229480_at | 19.14 | 3.22E-07 | 1.327701853 | NA |  |  |  |  |
| 219869_s_at | 19.13 | 3.23E-07 | 1.437145259 | NM_022154| | SLC39A8,solute carrier family 39 (zinc transporter), | | | |
| 204742_s_at | 19.11 | 3.26E-07 | 1.151982253 | NM_015032| | APRIN,androgen-induced prostate proliferative shutoff | | | |
| 200953_s_at | 19.08 | 3.29E-07 | 1.068717316 | NM_001759| | CCND2,cyclin D2 | |  |  |
| 227095_at | 19.07 | 3.31E-07 | 1.225065114 | NA |  |  |  |  |
| 224925_at | 19.06 | 3.32E-07 | 1.17591378 | NM_020820| | PREX1,PREX1 protein | |  |  |
| 202669_s_at | 19.05 | 3.33E-07 | 1.137948533 | NM_004093| | EFNB2,ephrin B2 | |  |  |
| 201280_s_at | 19.04 | 3.33E-07 | 1.445025683 | NM_001343| | DAB2,disabled homolog 2 | |  |  |
| 205588_s_at | 19.04 | 3.33E-07 | 1.121943392 | NM_007045| | FGFR1OP,FGFR1 oncogene partner isoform a | | | |
| 203295_s_at | 19.03 | 3.34E-07 | 1.266917627 | NM_000702| | ATP1A2,Na+/K+ -ATPase alpha 2 subunit proprotein | | | |
| 231397_at | 19.01 | 3.36E-07 | 1.572431702 | NM_001010861| | NA |  |  |  |
| 219033_at | 18.99 | 3.38E-07 | 1.214491838 | NM_024615| | PARP8,poly (ADP-ribose) polymerase family, member 8 | | | |
| 212866_at | 18.98 | 3.39E-07 | 1.150065336 | NA |  |  |  |  |
| 221750_at | 18.96 | 3.43E-07 | 1.176923284 | NM_001098272| | NA |  |  |  |
| 223075_s_at | 18.94 | 3.45E-07 | 1.051115065 | NM_031426| | C9orf58,chromosome 9 open reading frame 58 isoform 1 | | | |
| 203299_s_at | 18.94 | 3.45E-07 | 1.211208829 | NM_003916| | AP1S2,adaptor-related protein complex 1 sigma 2 | | | |
| 204976_s_at | 18.92 | 3.47E-07 | 1.060346468 | NM_001025580| | NA |  |  |  |
| 208146_s_at | 18.89 | 3.50E-07 | 1.208261801 | NM_019029| | CPVL,serine carboxypeptidase vitellogenic-like | | | |
| 210809_s_at | 18.85 | 3.56E-07 | 1.710524799 | NM_006475| | POSTN,periostin, osteoblast specific factor | | | |
| 209765_at | 18.79 | 3.67E-07 | 1.221935962 | NM_023038| | ADAM19,a disintegrin and metalloproteinase domain 19 | | | |
| 1556329_a_at | 18.78 | 3.68E-07 | 1.686023474 | NA |  |  |  |  |
| 217966_s_at | 18.77 | 3.69E-07 | 1.185090609 | NM_052966| | C1orf24,niban protein | |  |  |
| 239911_at | 18.75 | 3.72E-07 | 1.420213828 | NM_004852| | ONECUT2,one cut domain, family member 2 | | | |
| 220227_at | 18.73 | 3.76E-07 | 1.177289014 | NM_001794| | CDH4,cadherin 4, type 1 preproprotein | | |  |
| 242981_at | 18.69 | 3.82E-07 | 1.313974971 | NA |  |  |  |  |
| 218966_at | 18.67 | 3.85E-07 | 1.134164503 | NM_018728| | MYO5C,myosin VC | |  |  |
| 222146_s_at | 18.66 | 3.86E-07 | 1.064802229 | NM_001083962| | NA |  |  |  |
| 228184_at | 18.6 | 3.96E-07 | 1.255500218 | NM_032890| | DISP1,dispatched A | |  |  |
| 212691_at | 18.57 | 4.02E-07 | 1.060430404 | NM_015354| | NA |  |  |  |
| 204865_at | 18.55 | 4.05E-07 | 1.801085474 | NM_005181| | CA3,carbonic anhydrase III | | |  |
| 204444_at | 18.54 | 4.06E-07 | 1.068588074 | NM_004523| | KIF11,kinesin family member 11 | | |  |
| 223673_at | 18.54 | 4.05E-07 | 2.052686217 | NM_002920| | RFX4,regulatory factor X4 isoform b | | |  |
| 222734_at | 18.47 | 4.19E-07 | 1.128917335 | NM_015836| | WARS2,mitochondrial tryptophanyl tRNA synthetase 2 | | | |
| 203282_at | 18.43 | 4.27E-07 | 1.263462285 | NM_000158| | GBE1,glucan (1,4-alpha-), branching enzyme 1 | | | |
| 212489_at | 18.4 | 4.32E-07 | 1.303635913 | NM_000093| | COL5A1,alpha 1 type V collagen preproprotein | | | |
| 202599_s_at | 18.39 | 4.32E-07 | 1.24952598 | NM_003489| | NRIP1,receptor interacting protein 140 | | |  |
| 206554_x_at | 18.39 | 4.32E-07 | 1.217303135 | NM_006515| | SETMAR,SET domain and mariner transposase fusion gene | | | |
| 200935_at | 18.39 | 4.32E-07 | 1.175824486 | NM_004343| | CALR,calreticulin precursor | | |  |
| 206261_at | 18.38 | 4.33E-07 | 1.174105578 | NM_001099282| | NA |  |  |  |
| 206414_s_at | 18.37 | 4.34E-07 | 1.098524609 | NM_003887| | DDEF2,development- and differentiation-enhancing | | | |
| 218950_at | 18.37 | 4.34E-07 | 1.103000815 | NM_022481| | CENTD3,centaurin, delta 3 | |  |  |
| 201855_s_at | 18.36 | 4.34E-07 | 1.11802382 | NM_015251| | KIAA0431,KIAA0431 protein | | |  |
| 214913_at | 18.36 | 4.34E-07 | 1.215178102 | NM_014243| | ADAMTS3,a disintegrin-like and metalloprotease | | | |
| 214452_at | 18.36 | 4.35E-07 | 1.168800904 | NM_005504| | BCAT1,branched chain aminotransferase 1, cytosolic | | | |
| 221933_at | 18.35 | 4.36E-07 | 1.078098576 | NM_020742| | NLGN4X,X-linked neuroligin 4 | | |  |
| 232914_s_at | 18.34 | 4.37E-07 | 1.573097274 | NM_032379| | SYTL2,synaptotagmin-like 2 isoform b | | |  |
| 235168_at | 18.33 | 4.39E-07 | 1.30232595 | NM_145167| | PIGM,PIG-M mannosyltransferase | | |  |
| 206740_x_at | 18.32 | 4.39E-07 | 1.48433505 | NM_003176| | SYCP1,synaptonemal complex protein 1 | | | |
| 229415_at | 18.32 | 4.40E-07 | 1.305636594 | NM_018947| | CYCS,cytochrome c | |  |  |
| 209967_s_at | 18.29 | 4.46E-07 | 1.120225361 | NM_001881| | CREM,cAMP responsive element modulator isoform b | | | |
| 226103_at | 18.28 | 4.47E-07 | 1.297057104 | NM_144573| | NEXN,nexilin (F actin binding protein) | | |  |
| 205281_s_at | 18.27 | 4.48E-07 | 1.166874529 | NM_002641| | PIGA,phosphatidylinositol | |  |  |
| 213484_at | 18.24 | 4.56E-07 | 1.106863663 | NA |  |  |  |  |
| 244033_at | 18.19 | 4.65E-07 | 1.19400155 | NM_152446| | NA |  |  |  |
| 205150_s_at | 18.19 | 4.65E-07 | 1.2215529 | NM_014817| | NA |  |  |  |
| 209897_s_at | 18.18 | 4.67E-07 | 1.393314033 | NM_004787| | SLIT2,slit homolog 2 | |  |  |
| 202766_s_at | 18.14 | 4.75E-07 | 1.182504 | NM_000138| | FBN1,fibrillin 1 | |  |  |
| 226123_at | 18.14 | 4.76E-07 | 1.071264521 | NM_017780| | CHD7,chromodomain helicase DNA binding protein 7 | | | |
| 226077_at | 18.13 | 4.77E-07 | 1.120305497 | NM_144726| | FLJ31951,hypothetical protein FLJ31951 | | | |
| 226117_at | 18.1 | 4.84E-07 | 1.492844629 | NM_052864| | TIFA,TRAF-interacting protein with a | | |  |
| 203487_s_at | 18.1 | 4.84E-07 | 1.119148248 | NM_014154| | ARMC8,armadillo repeat containing 8 | | |  |
| 213051_at | 18.1 | 4.83E-07 | 1.124600299 | NM_020119| | ZC3HAV1,zinc finger antiviral protein isoform 1 | | | |
| 219798_s_at | 18.1 | 4.83E-07 | 1.076957923 | NM_019606| | FLJ20257,hypothetical protein FLJ20257 | | | |
| 1555867_at | 18.07 | 4.89E-07 | 1.307818963 | NM_001098721| | NA |  |  |  |
| 212865_s_at | 18.04 | 4.96E-07 | 1.213228508 | NM_021110| | COL14A1,collagen, type XIV, alpha 1 | | |  |
| 203192_at | 18.02 | 5.00E-07 | 1.120403549 | NM_005689| | ABCB6,ATP-binding cassette, sub-family B, member 6 | | | |
| 226865_at | 18.02 | 4.99E-07 | 1.151662978 | NA |  |  |  |  |
| 213001_at | 18.02 | 4.99E-07 | 1.135858162 | NM_012098| | ANGPTL2,angiopoietin-like 2 precursor | | |  |
| 207196_s_at | 18.01 | 5.02E-07 | 1.113358571 | NM_006058| | TNIP1,Nef-associated factor 1 | | |  |
| 242141_at | 18 | 5.02E-07 | 1.146561174 | NM_001527| | HDAC2,histone deacetylase 2 | | |  |
| 235798_at | 18 | 5.02E-07 | 1.339046909 | NM_001100829| | NA |  |  |  |
| 225031_at | 17.99 | 5.03E-07 | 1.144754874 | NM_032221| | CHD6,chromodomain helicase DNA binding protein 6 | | | |
| 205524_s_at | 17.97 | 5.09E-07 | 1.596727475 | NM_001884| | HAPLN1,cartilage linking protein 1 | | |  |
| 204584_at | 17.96 | 5.10E-07 | 1.181726015 | NM_000425| | L1CAM,L1 cell adhesion molecule isoform 1 precursor | | | |
| 205933_at | 17.95 | 5.10E-07 | 1.236330836 | NM_015559| | SETBP1,SET binding protein 1 | | |  |
| 226790_at | 17.94 | 5.11E-07 | 1.194194906 | NM_194270| | MOPT,protein containing single MORN motif in testis | | | |
| 238529_at | 17.93 | 5.11E-07 | 1.264780132 | NA |  |  |  |  |
| 236217_at | 17.92 | 5.15E-07 | 1.255349937 | NM_001859| | SLC31A1,solute carrier family 31 (copper transporters), | | | |
| 236115_at | 17.91 | 5.17E-07 | 1.397562875 | NA |  |  |  |  |
| 201083_s_at | 17.9 | 5.20E-07 | 1.181268914 | NM_001077440| | NA |  |  |  |
| 209025_s_at | 17.88 | 5.23E-07 | 1.059377434 | NM_006372| | SYNCRIP,synaptotagmin binding, cytoplasmic RNA | | | |
| 219697_at | 17.86 | 5.28E-07 | 1.106531038 | NM_006043| | HS3ST2,heparan sulfate D-glucosaminyl | | | |
| 223024_at | 17.85 | 5.28E-07 | 1.112133976 | NM_032493| | AP1M1,adaptor-related protein complex 1, mu 1 subunit | | | |
| 223392_s_at | 17.85 | 5.28E-07 | 1.154501286 | NM_020856| | ZNF537,zinc finger protein 537 | | |  |
| 226652_at | 17.84 | 5.30E-07 | 1.32486192 | NM_006537| | USP3,ubiquitin specific protease 3 | | |  |
| 209505_at | 17.84 | 5.30E-07 | 1.26718994 | NM_005654| | NR2F1,nuclear receptor subfamily 2, group F, member 1 | | | |
| 230720_at | 17.83 | 5.31E-07 | 1.162480024 | NM_152737| | RNF182,ring finger protein 182 | | |  |
| 230883_at | 17.8 | 5.37E-07 | 1.456818475 | NA |  |  |  |  |
| 214499_s_at | 17.79 | 5.39E-07 | 1.147256552 | NM_001077440| | NA |  |  |  |
| 226338_at | 17.77 | 5.45E-07 | 1.185601916 | NM_018710| | DKFZp762O076,hypothetical protein DKFZp762O076 | | | |
| 209757_s_at | 17.76 | 5.47E-07 | 1.038591531 | NM_005378| | MYCN,v-myc myelocytomatosis viral related oncogene, | | | |
| 210768_x_at | 17.76 | 5.47E-07 | 1.078909045 | NM_019026| | LOC54499,putative membrane protein | | |  |
| 212190_at | 17.76 | 5.47E-07 | 1.050639 | NM_006216| | SERPINE2,plasminogen activator inhibitor type 1, member | | | |
| 206243_at | 17.73 | 5.51E-07 | 1.247844399 | NM_003256| | TIMP4,tissue inhibitor of metalloproteinase 4 | | | |
| 209470_s_at | 17.71 | 5.58E-07 | 1.57555948 | NM_005277| | GPM6A,glycoprotein M6A isoform 1 | | |  |
| 201309_x_at | 17.69 | 5.61E-07 | 1.079166755 | NM_004772| | C5orf13,neuronal protein 3.1 | | |  |
| 222996_s_at | 17.68 | 5.64E-07 | 1.072879709 | NM_016463| | CXXC5,CXXC finger 5 | |  |  |
| 226990_at | 17.65 | 5.73E-07 | 1.076165547 | NM_005898| | M11S1,membrane component, chromosome 11, surface | | | |
| 1559006_at | 17.65 | 5.72E-07 | 1.278054902 | NA |  |  |  |  |
| 202429_s_at | 17.65 | 5.72E-07 | 1.178469391 | NM_000944| | PPP3CA,protein phosphatase 3 (formerly 2B), catalytic | | | |
| 239237_at | 17.64 | 5.73E-07 | 1.31909047 | NA |  |  |  |  |
| 208753_s_at | 17.62 | 5.78E-07 | 1.109024427 | NM_004537| | NAP1L1,nucleosome assembly protein 1-like 1 | | | |
| 223623_at | 17.62 | 5.78E-07 | 1.315205019 | NM_032411| | ECRG4,esophageal cancer related gene 4 protein | | | |
| 239288_at | 17.58 | 5.90E-07 | 1.487651023 | NM_015028| | TNIK,TRAF2 and NCK interacting kinase | | |  |
| 219682_s_at | 17.55 | 5.98E-07 | 1.654210104 | NM_005996| | TBX3,T-box 3 protein isoform 1 | | |  |
| 209859_at | 17.55 | 5.98E-07 | 1.483017086 | NM_015163| | TRIM9,tripartite motif protein 9 isoform 1 | | | |
| 225540_at | 17.54 | 5.98E-07 | 1.46933197 | NM_001039538| | NA |  |  |  |
| 229944_at | 17.54 | 5.98E-07 | 1.415189966 | NM_000912| | OPRK1,opioid receptor, kappa 1 | | |  |
| 201899_s_at | 17.48 | 6.15E-07 | 1.066315936 | NM_003336| | UBE2A,ubiquitin-conjugating enzyme E2A isoform 1 | | | |
| 207455_at | 17.47 | 6.16E-07 | 1.369060077 | NM_002563| | P2RY1,purinergic receptor P2Y1 | | |  |
| 202947_s_at | 17.47 | 6.16E-07 | 1.234099816 | NM_002101| | GYPC,glycophorin C isoform 1 | | |  |
| 213308_at | 17.46 | 6.19E-07 | 1.078585599 | NM_012309| | SHANK2,SH3 and multiple ankyrin repeat domains 2 | | | |
| 205893_at | 17.46 | 6.19E-07 | 1.488754438 | NM_014932| | NLGN1,neuroligin 1 | |  |  |
| 218903_s_at | 17.38 | 6.41E-07 | 1.15831317 | NM_024068| | MGC2731,hypothetical protein MGC2731 | | | |
| 225710_at | 17.38 | 6.41E-07 | 1.084897665 | NM_021629| | GNB4,guanine nucleotide-binding protein, beta-4 | | | |
| 213075_at | 17.38 | 6.41E-07 | 1.090881962 | NM_182487| | OLFML2A,olfactomedin-like 2A | | |  |
| 235470_at | 17.35 | 6.49E-07 | 1.11413057 | NA |  |  |  |  |
| 211964_at | 17.3 | 6.65E-07 | 1.093174671 | NM_001846| | COL4A2,alpha 2 type IV collagen preproprotein | | | |
| 207156_at | 17.29 | 6.66E-07 | 1.452652855 | NM_003509| | HIST1H2AI,H2A histone family, member C | | | |
| 208962_s_at | 17.29 | 6.66E-07 | 1.11183157 | NM_013402| | FADS1,fatty acid desaturase 1 | | |  |
| 206729_at | 17.29 | 6.66E-07 | 1.124372414 | NM_001243| | TNFRSF8,tumor necrosis factor receptor superfamily, | | | |
| 37892_at | 17.28 | 6.68E-07 | 1.418056769 | NM_001854| | COL11A1,alpha 1 type XI collagen isoform A | | | |
| 213007_at | 17.27 | 6.68E-07 | 1.06805924 | NM_001113378| | NA |  |  |  |
| 207956_x_at | 17.27 | 6.68E-07 | 1.068945484 | NM_015032| | APRIN,androgen-induced prostate proliferative shutoff | | | |
| 210868_s_at | 17.25 | 6.76E-07 | 1.114755158 | NM_024090| | ELOVL6,ELOVL family member 6, elongation of long chain | | | |
| 1564520_s_at | 17.24 | 6.78E-07 | 1.055403021 | NM_001039619| | NA |  |  |  |
| 229710_at | 17.24 | 6.77E-07 | 1.142552962 | NA |  |  |  |  |
| 235068_at | 17.22 | 6.81E-07 | 1.419849161 | NM_178566| | ZDHHC21,zinc finger, DHHC domain containing 21 | | | |
| 226353_at | 17.2 | 6.89E-07 | 1.115282906 | NM_032802| | SPPL2A,putative intramembrane cleaving protease | | | |
| 219553_at | 17.16 | 6.99E-07 | 1.097142064 | NM_013330| | NME7,nucleoside-diphosphate kinase 7 isoform a | | | |
| 213365_at | 17.16 | 6.99E-07 | 1.069959553 | NM_080663| | MGC16943,hypothetical protein MGC16943 | | | |
| 230413_s_at | 17.16 | 6.99E-07 | 1.264906093 | NA |  |  |  |  |
| 225851_at | 17.15 | 6.99E-07 | 1.132915759 | NM_002028| | FNTB,farnesyltransferase, CAAX box, beta | | | |
| 204256_at | 17.12 | 7.11E-07 | 1.098817609 | NM_024090| | ELOVL6,ELOVL family member 6, elongation of long chain | | | |
| 211980_at | 17.11 | 7.14E-07 | 1.102748602 | NM_001845| | COL4A1,alpha 1 type IV collagen preproprotein | | | |
| 231776_at | 17.1 | 7.14E-07 | 1.461741082 | NM_005442| | EOMES,eomesodermin | |  |  |
| 218249_at | 17.1 | 7.14E-07 | 1.116927836 | NM_022494| | ZDHHC6,zinc finger, DHHC domain containing 6 | | | |
| 224851_at | 17.1 | 7.14E-07 | 1.192570092 | NM_001259| | CDK6,cyclin-dependent kinase 6 | | |  |
| 221258_s_at | 17.1 | 7.14E-07 | 1.098856533 | NM_031217| | KIF18A,kinesin family member 18A | | |  |
| 201563_at | 17.09 | 7.15E-07 | 1.051597965 | NM_003104| | SORD,sorbitol dehydrogenase | | |  |
| 204767_s_at | 17.07 | 7.23E-07 | 1.040289354 | NM_004111| | FEN1,flap structure-specific endonuclease 1 | | | |
| 203358_s_at | 17.07 | 7.21E-07 | 1.150098576 | NM_004456| | EZH2,enhancer of zeste 2 isoform a | | |  |
| 210609_s_at | 17.06 | 7.25E-07 | 1.108713875 | NM_004881| | TP53I3,tumor protein p53 inducible protein 3 | | | |
| 213541_s_at | 17.06 | 7.25E-07 | 1.635521504 | NM_004449| | ERG,v-ets erythroblastosis virus E26 oncogene like | | | |
| 204112_s_at | 17.05 | 7.26E-07 | 1.428420153 | NM_001024074| | NA |  |  |  |
| 221530_s_at | 17.03 | 7.32E-07 | 1.327462984 | NM_030762| | BHLHB3,basic helix-loop-helix domain containing, class | | | |
| 227019_at | 17.01 | 7.38E-07 | 1.062588052 | NM_001085375| | NA |  |  |  |
| 204142_at | 17 | 7.43E-07 | 1.07646062 | NM_001126123| | NA |  |  |  |
| 212327_at | 16.98 | 7.49E-07 | 1.211976282 | NM_001112717| | NA |  |  |  |
| 206070_s_at | 16.98 | 7.49E-07 | 1.806634014 | NM_005233| | EPHA3,ephrin receptor EphA3 isoform a precursor | | | |
| 202660_at | 16.97 | 7.49E-07 | 1.382034613 | NM_002223| | ITPR2,inositol 1,4,5-triphosphate receptor, type 2 | | | |
| 202245_at | 16.96 | 7.51E-07 | 1.092845054 | NM_001001438| | LSS,lanosterol synthase | |  |  |
| 226891_at | 16.94 | 7.59E-07 | 1.091855943 | NM_152531| | C3orf21,chromosome 3 open reading frame 21 | | | |
| 233536_at | 16.93 | 7.61E-07 | 1.446185738 | NM_030632| | NA |  |  |  |
| 211744_s_at | 16.93 | 7.62E-07 | 1.227860234 | NM_001779| | CD58,CD58 antigen, (lymphocyte function-associated | | | |
| 211708_s_at | 16.92 | 7.64E-07 | 1.236813856 | NM_005063| | SCD,stearoyl-CoA desaturase | | |  |
| 209083_at | 16.9 | 7.70E-07 | 1.240992232 | NM_007074| | CORO1A,coronin, actin binding protein, 1A | | | |
| 228062_at | 16.89 | 7.73E-07 | 1.16012863 | NM_153757| | NAP1L5,nucleosome assembly protein 1-like 5 | | | |
| 206330_s_at | 16.88 | 7.77E-07 | 1.227641379 | NM_016848| | SHC3,src homology 2 domain containing transforming | | | |
| 227033_at | 16.88 | 7.76E-07 | 1.078502692 | NM_005313| | GRP58,glucose regulated protein, 58kDa | | | |
| 223455_at | 16.88 | 7.77E-07 | 1.204842991 | NM_032300| | MGC10854,hypothetical protein MGC10854 | | | |
| 218224_at | 16.87 | 7.77E-07 | 1.073070914 | NM_006029| | PNMA1,paraneoplastic antigen MA1 | | |  |
| 201634_s_at | 16.87 | 7.77E-07 | 1.091391322 | NM_030579| | CYB5-M,cytochrome b5 outer mitochondrial membrane | | | |
| 235241_at | 16.86 | 7.81E-07 | 1.069322849 | NM_173514| | FLJ90709,hypothetical protein FLJ90709 | | | |
| 225484_at | 16.84 | 7.87E-07 | 1.135944315 | NM_018718| | TSGA14,testis specific, 14 | |  |  |
| 218242_s_at | 16.82 | 7.96E-07 | 1.098903933 | NM_016028| | SUV420H1,suppressor of variegation 4-20 homolog 1 isoform | | | |
| 227896_at | 16.8 | 8.04E-07 | 1.139598652 | NM_016567| | BCCIP,BRCA2 and CDKN1A-interacting protein isoform | | | |
| 205070_at | 16.79 | 8.07E-07 | 1.096573776 | NM_019071| | ING3,inhibitor of growth family, member 3 isoform 1 | | | |
| 218382_s_at | 16.78 | 8.10E-07 | 1.112037188 | NM_001012478| | U2AF2,U2 (RNU2) small nuclear RNA auxiliary factor 2 | | | |
| 224605_at | 16.75 | 8.23E-07 | 1.232432273 | NM_001001701| | LOC401152,HCV F-transactivated protein 1 | | | |
| 213454_at | 16.75 | 8.23E-07 | 1.08761263 | NM_198544| | NA |  |  |  |
| 209064_x_at | 16.74 | 8.24E-07 | 1.12186176 | NM_006451| | PAIP1,poly(A) binding protein interacting protein 1 | | | |
| 1559942_at | 16.74 | 8.23E-07 | 1.52668328 | NM_199072| | HIC,I-mfa domain-containing protein isoform p40 | | | |
| 209398_at | 16.73 | 8.24E-07 | 1.258977037 | NM_005319| | HIST1H1C,H1 histone family, member 2 | | | |
| 214804_at | 16.71 | 8.35E-07 | 1.161759206 | NM_006733| | FSHPRH1,follicle-stimulating hormone primary response | | | |
| 228200_at | 16.69 | 8.43E-07 | 1.198719748 | NA |  |  |  |  |
| 214004_s_at | 16.69 | 8.40E-07 | 1.083964982 | NM_014667| | VGLL4,vestigial like 4 | |  |  |
| 1552557_a_at | 16.67 | 8.49E-07 | 1.252296684 | NM_144969| | ZDHHC15,zinc finger, DHHC domain containing 15 | | | |
| 223275_at | 16.65 | 8.56E-07 | 1.123438041 | NM_018137| | HRMT1L6,HMT1 hnRNP methyltransferase-like 6 | | | |
| 201128_s_at | 16.65 | 8.56E-07 | 1.047426693 | NM_001096| | ACLY,ATP citrate lyase isoform 1 | | |  |
| 229225_at | 16.65 | 8.56E-07 | 1.183591957 | NM_003872| | NRP2,neuropilin 2 isoform 2 precursor | | |  |
| 224469_s_at | 16.63 | 8.64E-07 | 1.165030001 | NM_001031714| | NA |  |  |  |
| 234111_at | 16.61 | 8.69E-07 | 1.272180991 | NA |  |  |  |  |
| 1558487_a_at | 16.58 | 8.82E-07 | 1.250854622 | NM_182547| | TMED4,transmembrane emp24 protein transport domain | | | |
| 224560_at | 16.58 | 8.82E-07 | 1.093369095 | NM_003255| | TIMP2,tissue inhibitor of metalloproteinase 2 | | | |
| 206619_at | 16.58 | 8.82E-07 | 1.325384132 | NM_014420| | DKK4,dickkopf homolog 4 | |  |  |
| 235644_at | 16.58 | 8.82E-07 | 1.104973722 | NM_144978| | FLJ32745,hypothetical protein FLJ32745 | | | |
| 219933_at | 16.57 | 8.82E-07 | 1.239313788 | NM_016066| | GLRX2,glutaredoxin 2 isoform 1 | | |  |
| 32836_at | 16.57 | 8.82E-07 | 1.101126191 | NM_006411| | AGPAT1,1-acylglycerol-3-phosphate O-acyltransferase 1 | | | |
| 213094_at | 16.55 | 8.90E-07 | 1.351050408 | NM_001032394| | NA |  |  |  |
| 227413_at | 16.55 | 8.90E-07 | 1.070757539 | NM_145049| | MGC10067,hypothetical protein MGC10067 | | | |
| 226834_at | 16.55 | 8.90E-07 | 1.127982895 | NA |  |  |  |  |
| 228218_at | 16.53 | 8.98E-07 | 1.33175726 | NA |  |  |  |  |
| 236562_at | 16.51 | 9.06E-07 | 1.529714026 | NM_152262| | ZNF439,zinc finger protein 439 | | |  |
| 208939_at | 16.5 | 9.12E-07 | 1.071348249 | NM_012247| | SEPHS1,selenophosphate synthetase | | |  |
| 223215_s_at | 16.5 | 9.10E-07 | 1.068398327 | NM_001098625| | NA |  |  |  |
| 218517_at | 16.49 | 9.12E-07 | 1.066298803 | NM_024900| | PHF17,Jade1 protein short isoform | | |  |
| 222278_at | 16.49 | 9.12E-07 | 1.264154294 | NA |  |  |  |  |
| 1557155_a_at | 16.49 | 9.12E-07 | 1.644798443 | NA |  |  |  |  |
| 1557128_at | 16.49 | 9.12E-07 | 1.252396165 | NM_198947| | CANP,cancer-associated nucleoprotein | | |  |
| 203640_at | 16.47 | 9.16E-07 | 1.46664843 | NM_144778| | MBNL2,muscleblind-like 2 isoform 1 | | |  |
| 203999_at | 16.47 | 9.19E-07 | 1.098486869 | NM_005639| | SYT1,synaptotagmin I | |  |  |
| 204781_s_at | 16.45 | 9.25E-07 | 1.366189686 | NM_000043| | FAS,tumor necrosis factor receptor superfamily, | | | |
| 1555427_s_at | 16.45 | 9.25E-07 | 1.076728636 | NM_006372| | SYNCRIP,synaptotagmin binding, cytoplasmic RNA | | | |
| 226901_at | 16.43 | 9.30E-07 | 1.080129879 | NM_181655| | LOC284018,hypothetical protein LOC284018 isoform a | | | |
| 218689_at | 16.42 | 9.33E-07 | 1.084506102 | NM_022725| | FANCF,Fanconi anemia, complementation group F | | | |
| 236782_at | 16.41 | 9.36E-07 | 1.59878843 | NM_001017373| | NA |  |  |  |
| 204549_at | 16.39 | 9.44E-07 | 1.17162199 | NM_014002| | IKBKE,IKK-related kinase epsilon | | |  |
| 242317_at | 16.39 | 9.46E-07 | 1.237783662 | NM_001099668| | NA |  |  |  |
| 218768_at | 16.39 | 9.44E-07 | 1.029702522 | NM_020401| | NUP107,nucleoporin 107kDa | | |  |
| 204684_at | 16.38 | 9.49E-07 | 1.319514157 | NM_002522| | NPTX1,neuronal pentraxin I precursor | | |  |
| 209817_at | 16.38 | 9.47E-07 | 1.098806386 | NM_021132| | PPP3CB,protein phosphatase 3 (formerly 2B), catalytic | | | |
| 219960_s_at | 16.37 | 9.53E-07 | 1.074400082 | NM_015984| | UCHL5,ubiquitin C-terminal hydrolase UCH37 | | | |
| 204470_at | 16.36 | 9.56E-07 | 1.433409632 | NM_001511| | CXCL1,chemokine (C-X-C motif) ligand 1 | | | |
| 1568678_s_at | 16.34 | 9.66E-07 | 1.116823905 | NM_007045| | FGFR1OP,FGFR1 oncogene partner isoform a | | | |
| 1552330_at | 16.32 | 9.74E-07 | 1.300926814 | NM_145039| | MGC16385,hypothetical protein MGC16385 | | | |
| 204812_at | 16.32 | 9.73E-07 | 1.058823631 | NM_004724| | ZW10,centromere/kinetochore protein zw10 homolog | | | |
| 214591_at | 16.31 | 9.78E-07 | 1.826411269 | NM_019117| | KLHL4,kelch-like 4 isoform 1 | | |  |
| 211945_s_at | 16.31 | 9.78E-07 | 1.043408774 | NM_002211| | ITGB1,integrin beta 1 isoform 1A precursor | | | |
| 209852_x_at | 16.3 | 9.78E-07 | 1.073705684 | NM_005789| | PSME3,proteasome activator subunit 3 isoform 1 | | | |
| 206710_s_at | 16.3 | 9.78E-07 | 1.140718264 | NM_012307| | EPB41L3,erythrocyte membrane protein band 4.1-like 3 | | | |
| 235699_at | 16.28 | 9.94E-07 | 1.214480305 | NM_173527| | FLJ38964,hypothetical protein FLJ38964 | | | |
| 235498_at | 16.22 | 1.02E-06 | 1.492562305 | NM_001105659| | NA |  |  |  |
| 201951_at | 16.21 | 1.03E-06 | 1.144159667 | NM_001627| | ALCAM,activated leukocyte cell adhesion molecule | | | |
| 209170_s_at | 16.21 | 1.03E-06 | 1.143222898 | NM_001001994| | GPM6B,glycoprotein M6B isoform 4 | | |  |
| 205173_x_at | 16.2 | 1.03E-06 | 1.176565037 | NM_001779| | CD58,CD58 antigen, (lymphocyte function-associated | | | |
| 204587_at | 16.19 | 1.03E-06 | 1.136845119 | NM_003951| | SLC25A14,solute carrier family 25, member 14 isoform | | | |
| 218894_s_at | 16.19 | 1.03E-06 | 1.16885632 | NM_018048| | FLJ10292,mago-nashi homolog | | |  |
| 222033_s_at | 16.17 | 1.04E-06 | 1.123889458 | NA |  |  |  |  |
| 203296_s_at | 16.17 | 1.05E-06 | 1.184096288 | NM_000702| | ATP1A2,Na+/K+ -ATPase alpha 2 subunit proprotein | | | |
| 205794_s_at | 16.16 | 1.05E-06 | 1.304012648 | NM_002515| | NOVA1,neuro-oncological ventral antigen 1 isoform 1 | | | |
| 222048_at | 16.15 | 1.05E-06 | 1.226325812 | NA |  |  |  |  |
| 200816_s_at | 16.14 | 1.06E-06 | 1.075665072 | NM_000430| | PAFAH1B1,platelet-activating factor acetylhydrolase, | | | |
| 219474_at | 16.14 | 1.06E-06 | 1.132861421 | NM_024616| | FLJ23186,hypothetical protein FLJ23186 | | | |
| 202094_at | 16.13 | 1.06E-06 | 1.184090749 | NM_001012270| | BIRC5,baculoviral IAP repeat-containing protein 5 | | | |
| 219469_at | 16.13 | 1.06E-06 | 1.128756417 | NM_001080463| | NA |  |  |  |
| 215143_at | 16.13 | 1.06E-06 | 1.346971665 | NA |  |  |  |  |
| 36566_at | 16.12 | 1.06E-06 | 1.111583904 | NM_001031681| | NA |  |  |  |
| 208964_s_at | 16.1 | 1.07E-06 | 1.115188267 | NM_013402| | FADS1,fatty acid desaturase 1 | | |  |
| 206552_s_at | 16.1 | 1.07E-06 | 1.265913064 | NM_003182| | TAC1,tachykinin 1 isoform beta precursor | | | |
| 218763_at | 16.08 | 1.08E-06 | 1.089088053 | NM_016930| | STX18,syntaxin 18 | |  |  |
| 213172_at | 16.05 | 1.10E-06 | 1.232310573 | NM_015351| | NA |  |  |  |
| 202743_at | 16.04 | 1.10E-06 | 1.094791263 | NM_001114172| | NA |  |  |  |
| 205046_at | 16.04 | 1.10E-06 | 1.100303659 | NM_001813| | CENPE,centromere protein E | | |  |
| 209774_x_at | 16.03 | 1.11E-06 | 1.596847631 | NM_002089| | CXCL2,chemokine (C-X-C motif) ligand 2 | | | |
| 219654_at | 16.02 | 1.11E-06 | 1.062340738 | NM_014241| | PTPLA,protein tyrosine phosphatase-like, member a | | | |
| 216942_s_at | 16.02 | 1.11E-06 | 1.227294388 | NM_001779| | CD58,CD58 antigen, (lymphocyte function-associated | | | |
| 227606_s_at | 16 | 1.12E-06 | 1.261988464 | NM_020799| | AMSH-LP,associated molecule with the SH3 domain of STAM | | | |
| 223228_at | 16 | 1.12E-06 | 1.062928584 | NM_032287| | LDOC1L,leucine zipper, down-regulated in cancer 1-like | | | |
| 231358_at | 15.99 | 1.13E-06 | 1.283404053 | NM_001127174| | NA |  |  |  |
| 208891_at | 15.98 | 1.13E-06 | 1.578414101 | NM_001946| | DUSP6,dual specificity phosphatase 6 isoform a | | | |
| 230836_at | 15.98 | 1.13E-06 | 1.457663591 | NM_005668| | ST8SIA4,ST8 alpha-N-acetyl-neuraminide | | | |
| 207850_at | 15.98 | 1.13E-06 | 1.326464927 | NM_002090| | CXCL3,chemokine (C-X-C motif) ligand 3 | | | |
| 208638_at | 15.97 | 1.14E-06 | 1.042644359 | NM_005742| | TXNDC7,protein disulfide isomerase-related protein | | | |
| 219412_at | 15.95 | 1.14E-06 | 1.075081329 | NM_022337| | RAB38,RAB38 | |  |  |
| 217608_at | 15.95 | 1.14E-06 | 1.210109857 | NM_173829| | FLJ36754,hypothetical protein FLJ36754 | | | |
| 213374_x_at | 15.94 | 1.15E-06 | 1.097131058 | NM_014362| | HIBCH,3-hydroxyisobutyryl-Coenzyme A hydrolase isoform | | | |
| 210705_s_at | 15.91 | 1.16E-06 | 1.25451835 | NM_033034| | TRIM5,tripartite motif protein TRIM5 isoform alpha | | | |
| 207030_s_at | 15.9 | 1.17E-06 | 1.074414391 | NM_001321| | CSRP2,cysteine and glycine-rich protein 2 | | | |
| 235147_at | 15.89 | 1.17E-06 | 1.627203287 | NA |  |  |  |  |
| 201916_s_at | 15.88 | 1.18E-06 | 1.076674235 | NM_007214| | SEC63,SEC63-like protein | |  |  |
| 212983_at | 15.87 | 1.18E-06 | 1.109258355 | NM_005343| | HRAS,v-Ha-ras Harvey rat sarcoma viral oncogene | | | |
| 226852_at | 15.87 | 1.18E-06 | 1.137000905 | NM_020744| | MTA3,metastasis associated 1 family, member 3 | | | |
| 209108_at | 15.86 | 1.19E-06 | 1.064906173 | NM_003270| | TM4SF6,transmembrane 4 superfamily member 6 | | | |
| 205862_at | 15.86 | 1.19E-06 | 1.153708934 | NM_014668| | GREB1,GREB1 protein isoform a | | |  |
| 226810_at | 15.86 | 1.19E-06 | 1.18719868 | NM_024576| | OGFRL1,opioid growth factor receptor-like 1 | | | |
| 221903_s_at | 15.85 | 1.19E-06 | 1.211208741 | NM_001042355| | NA |  |  |  |
| 226421_at | 15.83 | 1.21E-06 | 1.062506375 | NM_001025580| | NA |  |  |  |
| 39729_at | 15.81 | 1.22E-06 | 1.038724649 | NM_005809| | PRDX2,peroxiredoxin 2 isoform a | | |  |
| 201240_s_at | 15.8 | 1.22E-06 | 1.072711968 | NM_014752| | SPCS2,signal peptidase complex subunit 2 homolog | | | |
| 1562275_at | 15.8 | 1.22E-06 | 1.320669961 | NA |  |  |  |  |
| 226766_at | 15.8 | 1.22E-06 | 1.319385396 | NM_002942| | NA |  |  |  |
| 225687_at | 15.79 | 1.22E-06 | 1.029227554 | NM_030919| | C20orf129,chromosome 20 open reading frame 129 | | | |
| 206928_at | 15.79 | 1.22E-06 | 1.25421984 | NM_003431| | ZNF124,zinc finger protein 124 | | |  |
| 224604_at | 15.78 | 1.23E-06 | 1.217161618 | NM_001001701| | LOC401152,HCV F-transactivated protein 1 | | | |
| 218309_at | 15.77 | 1.24E-06 | 1.15842793 | NM_018584| | CaMKIINalpha,calcium/calmodulin-dependent protein kinase II | | | |
| 208787_at | 15.77 | 1.24E-06 | 1.022179305 | NM_007208| | MRPL3,mitochondrial ribosomal protein L3 | | | |
| 212822_at | 15.76 | 1.24E-06 | 1.143443904 | NM_020733| | NA |  |  |  |
| 212279_at | 15.76 | 1.24E-06 | 1.109920051 | NM_014573| | MAC30,hypothetical protein MAC30 | | |  |
| 228714_at | 15.75 | 1.25E-06 | 1.189701212 | NA |  |  |  |  |
| 204244_s_at | 15.74 | 1.25E-06 | 1.075180535 | NM_006716| | ASK,activator of S phase kinase | | |  |
| 1553118_at | 15.73 | 1.26E-06 | 1.241670932 | NM_053055| | CTMP,carboxyl-terminal modulator protein isoform a | | | |
| 210793_s_at | 15.73 | 1.26E-06 | 1.345524887 | NM_005387| | NUP98,nucleoporin 98kD isoform 3 | | |  |
| 224848_at | 15.72 | 1.26E-06 | 1.147246766 | NM_001259| | CDK6,cyclin-dependent kinase 6 | | |  |
| 203137_at | 15.71 | 1.26E-06 | 1.111033837 | NM_004906| | WTAP,Wilms' tumour 1-associating protein isoform 1 | | | |
| 209071_s_at | 15.71 | 1.26E-06 | 1.325624855 | NM_003617| | RGS5,regulator of G-protein signalling 5 | | | |
| 223273_at | 15.7 | 1.27E-06 | 1.191779549 | NM_032490| | C14orf142,chromosome 14 open reading frame 142 | | | |
| 41329_at | 15.7 | 1.27E-06 | 1.080672074 | NM_020423| | PACE-1,ezrin-binding partner PACE-1 isoform 1 | | | |
| 217599_s_at | 15.7 | 1.27E-06 | 1.668904944 | NM_199072| | HIC,I-mfa domain-containing protein isoform p40 | | | |
| 221683_s_at | 15.7 | 1.27E-06 | 1.144361416 | NM_025114| | Cep290,centrosome protein cep290 | | |  |
| 209552_at | 15.68 | 1.27E-06 | 1.151912062 | NM_003466| | PAX8,paired box gene 8 isoform PAX8A | | |  |
| 201111_at | 15.68 | 1.28E-06 | 1.054829505 | NM_001316| | CSE1L,CSE1 chromosome segregation 1-like protein | | | |
| 221864_at | 15.67 | 1.28E-06 | 1.098958779 | NM_152288| | MGC13024,hypothetical protein MGC13024 | | | |
| 200790_at | 15.67 | 1.28E-06 | 1.068389032 | NM_002539| | ODC1,ornithine decarboxylase 1 | | |  |
| 224646_x_at | 15.66 | 1.29E-06 | 1.852050628 | NA |  |  |  |  |
| 209789_at | 15.65 | 1.29E-06 | 1.363074335 | NM_006091| | CORO2B,coronin, actin binding protein, 2B | | | |
| 1553646_at | 15.64 | 1.30E-06 | 1.362869792 | NM_144657| | CXorf43,chromosome X open reading frame 43 | | | |
| 228489_at | 15.64 | 1.29E-06 | 1.532356112 | NM_138786| | LOC116441,hypothetical protein BC014339 | | | |
| 227911_at | 15.64 | 1.30E-06 | 1.187671733 | NM_001010000| | ARHGAP28,Rho GTPase activating protein 28 isoform a | | | |
| 232667_at | 15.64 | 1.29E-06 | 1.112111548 | NA |  |  |  |  |
| 235320_at | 15.61 | 1.31E-06 | 1.182084734 | NM_032146| | ARL6,ADP-ribosylation factor-like 6 | | |  |
| 207828_s_at | 15.61 | 1.31E-06 | 1.037420526 | NM_016343| | CENPF,centromere protein F (350/400kD) | | | |
| 228400_at | 15.6 | 1.32E-06 | 1.082323089 | NM_020859| | ShrmL,Shroom-related protein | | |  |
| 227198_at | 15.59 | 1.33E-06 | 1.908807007 | NM_001025108| | NA |  |  |  |
| 220887_at | 15.59 | 1.32E-06 | 1.221339701 | NA |  |  |  |  |
| 223044_at | 15.58 | 1.33E-06 | 1.636121271 | NM_014585| | SLC40A1,solute carrier family 40 (iron-regulated | | | |
| 225171_at | 15.57 | 1.33E-06 | 1.225798225 | NM_033515| | ARHGAP18,Rho GTPase activating protein 18 | | | |
| 210391_at | 15.57 | 1.33E-06 | 1.251523136 | NM_001489| | NR6A1,nuclear receptor subfamily 6, group A, member 1 | | | |
| 207014_at | 15.56 | 1.34E-06 | 1.304871037 | NM_000807| | GABRA2,gamma-aminobutyric acid A receptor, alpha 2 | | | |
| 238520_at | 15.53 | 1.36E-06 | 1.16808944 | NM_033502| | TRERF1,transcriptional regulating factor 1 isoform 1 | | | |
| 204362_at | 15.53 | 1.36E-06 | 1.219526936 | NM_003930| | SCAP2,src family associated phosphoprotein 2 | | | |
| 225967_s_at | 15.52 | 1.37E-06 | 1.074983139 | NM_001086521| | NA |  |  |  |
| 203549_s_at | 15.51 | 1.38E-06 | 1.553135797 | NM_000237| | LPL,lipoprotein lipase precursor | | |  |
| 239562_at | 15.51 | 1.38E-06 | 1.319297839 | NM_001004346| | MTHFD2L,methylenetetrahydrofolate dehydrogenase (NADP+ | | | |
| 201278_at | 15.51 | 1.38E-06 | 1.449908356 | NM_001343| | DAB2,disabled homolog 2 | |  |  |
| 202297_s_at | 15.5 | 1.38E-06 | 1.082760028 | NM_007033| | RER1,RER1 retention in endoplasmic reticulum 1 | | | |
| 221107_at | 15.5 | 1.38E-06 | 1.426093777 | NM_017581| | CHRNA9,cholinergic receptor, nicotinic, alpha | | | |
| 223339_at | 15.49 | 1.39E-06 | 1.332418131 | NM_016311| | ATPIF1,ATPase inhibitory factor 1 isoform 1 precursor | | | |
| 226677_at | 15.49 | 1.38E-06 | 1.15369284 | NM_015461| | ZNF521,zinc finger protein 521 | | |  |
| 226918_at | 15.48 | 1.39E-06 | 1.111563074 | NM_032452| | JPH4,junctophilin 4 | |  |  |
| 236565_s_at | 15.47 | 1.40E-06 | 1.195500646 | NM_018357| | FLJ11196,acheron isoform 1 | | |  |
| 212055_at | 15.46 | 1.41E-06 | 1.032975924 | NM_015476| | C18orf10,chromosome 18 open reading frame 10 | | | |
| 209598_at | 15.45 | 1.41E-06 | 1.140635444 | NM_007257| | NA |  |  |  |
| 226460_at | 15.43 | 1.42E-06 | 1.060665641 | NM_020840| | NA |  |  |  |
| 224819_at | 15.43 | 1.42E-06 | 1.076052351 | NM_001006684| | TCEAL8,transcription elongation factor A (SII)-like 8 | | | |
| 202440_s_at | 15.42 | 1.43E-06 | 1.115458356 | NM_005418| | ST5,suppression of tumorigenicity 5 isoform 1 | | | |
| 230498_at | 15.42 | 1.43E-06 | 1.339801598 | NM_005297| | GPR24,G protein-coupled receptor 24 | | |  |
| 242385_at | 15.41 | 1.44E-06 | 1.398343496 | NM_006914| | RORB,RAR-related orphan receptor B | | |  |
| 1558345_a_at | 15.41 | 1.44E-06 | 1.294292358 | NA |  |  |  |  |
| 238030_at | 15.41 | 1.44E-06 | 1.114710411 | NA |  |  |  |  |
| 57588_at | 15.39 | 1.45E-06 | 1.234951393 | NM_020689| | SLC24A3,solute carrier family 24 | | |  |
| 208892_s_at | 15.39 | 1.45E-06 | 1.688176446 | NM_001946| | DUSP6,dual specificity phosphatase 6 isoform a | | | |
| 238722_x_at | 15.38 | 1.45E-06 | 1.300084458 | NM_001122838| | NA |  |  |  |
| 226301_at | 15.37 | 1.47E-06 | 1.142631866 | NM_052831| | C6orf192,chromosome 6 open reading frame 192 | | | |
| 38158_at | 15.35 | 1.48E-06 | 1.072431828 | NM_012291| | ESPL1,extra spindle poles like 1 | | |  |
| 235850_at | 15.35 | 1.48E-06 | 1.485580865 | NM_019069| | WDR5B,WD repeat domain 5B | | |  |
| 241772_at | 15.34 | 1.48E-06 | 1.225099001 | NA |  |  |  |  |
| 229641_at | 15.32 | 1.50E-06 | 1.298306327 | NA |  |  |  |  |
| 209384_at | 15.32 | 1.50E-06 | 1.099599787 | NM_007198| | PROSC,proline synthetase co-transcribed homolog | | | |
| 39966_at | 15.31 | 1.51E-06 | 1.12982782 | NM_006574| | CSPG5,chondroitin sulfate proteoglycan 5 (neuroglycan | | | |
| 223433_at | 15.3 | 1.51E-06 | 1.155221184 | NM_020192| | C7orf36,chromosome 7 open reading frame 36 | | | |
| 239552_at | 15.3 | 1.52E-06 | 1.118382859 | NA |  |  |  |  |
| 202108_at | 15.28 | 1.53E-06 | 1.073821356 | NM_000285| | PEPD,Xaa-Pro dipeptidase | |  |  |
| 236641_at | 15.27 | 1.53E-06 | 1.086933811 | NM_014875| | NA |  |  |  |
| 230013_s_at | 15.26 | 1.55E-06 | 1.274508964 | NA |  |  |  |  |
| 1557487_at | 15.23 | 1.57E-06 | 1.616067002 | NA |  |  |  |  |
| 201535_at | 15.23 | 1.57E-06 | 1.06990923 | NM_007106| | UBL3,ubiquitin-like 3 | |  |  |
| 223217_s_at | 15.2 | 1.60E-06 | 1.166954509 | NM_001005474| | NFKBIZ,nuclear factor of kappa light polypeptide gene | | | |
| 227617_at | 15.19 | 1.60E-06 | 1.112918519 | NM_001010866| | RP13-15M17.2,novel protein | | |  |
| 205632_s_at | 15.19 | 1.60E-06 | 1.139239207 | NM_003558| | PIP5K1B,phosphatidylinositol-4-phosphate 5-kinase, type | | | |
| 219263_at | 15.19 | 1.60E-06 | 1.463752124 | NM_024539| | RNF128,ring finger protein 128 isoform 2 | | | |
| 217718_s_at | 15.19 | 1.60E-06 | 1.041460932 | NM_003404| | YWHAB,tyrosine 3-monooxygenase/tryptophan | | | |
| 218409_s_at | 15.18 | 1.61E-06 | 1.084717681 | NM_022365| | DNAJC1,DnaJ (Hsp40) homolog, subfamily C, member 1 | | | |
| 214830_at | 15.16 | 1.62E-06 | 1.185687122 | NM_153811| | SLC38A6,N system amino acid transporter NAT-1 | | | |
| 214383_x_at | 15.16 | 1.62E-06 | 1.053228491 | NM_057161| | KLHDC3,testis intracellular mediator protein | | | |
| 222270_at | 15.16 | 1.62E-06 | 1.389735976 | NM_001122964| | NA |  |  |  |
| 219181_at | 15.15 | 1.62E-06 | 1.474290445 | NM_006033| | LIPG,endothelial lipase | |  |  |
| 202710_at | 15.15 | 1.63E-06 | 1.073145802 | NM_005868| | BET1,BET1 homolog | |  |  |
| 218979_at | 15.13 | 1.64E-06 | 1.082449606 | NM_024945| | C9orf76,chromosome 9 open reading frame 76 | | | |
| 212624_s_at | 15.1 | 1.66E-06 | 1.055019105 | NM_001025201| | NA |  |  |  |
| 209277_at | 15.05 | 1.71E-06 | 1.342182722 | NM_006528| | TFPI2,tissue factor pathway inhibitor 2 | | |  |
| 204466_s_at | 15.04 | 1.72E-06 | 1.091661615 | NM_000345| | SNCA,alpha-synuclein isoform NACP140 | | |  |
| 1560916_a_at | 15.03 | 1.72E-06 | 1.202439321 | NM_015283| | NA |  |  |  |
| 220179_at | 15.02 | 1.73E-06 | 1.259314909 | NM_022357| | DPEP3,dipeptidase 3 | |  |  |
| 201672_s_at | 15.02 | 1.73E-06 | 1.033016044 | NM_001037334| | NA |  |  |  |
| 219250_s_at | 15.01 | 1.74E-06 | 1.178059293 | NM_013281| | FLRT3,fibronectin leucine rich transmembrane protein 3 | | | |
| 210951_x_at | 15.01 | 1.74E-06 | 1.125483855 | NM_004580| | RAB27A,Ras-related protein Rab-27A | | |  |
| 225556_at | 15 | 1.75E-06 | 1.065150872 | NM_001017980| | NA |  |  |  |
| 209763_at | 15 | 1.75E-06 | 1.223753429 | NM_145234| | CHRDL1,chordin-like 1 | |  |  |
| 205003_at | 15 | 1.75E-06 | 1.103845331 | NM_014705| | DOCK4,dedicator of cytokinesis 4 | | |  |
| 201361_at | 14.98 | 1.77E-06 | 1.118193843 | NM_024092| | MGC5508,hypothetical protein MGC5508 | | | |
| 213069_at | 14.97 | 1.77E-06 | 1.103988117 | NM_020733| | NA |  |  |  |
| 217834_s_at | 14.97 | 1.77E-06 | 1.086150426 | NM_006372| | SYNCRIP,synaptotagmin binding, cytoplasmic RNA | | | |
| 202814_s_at | 14.97 | 1.77E-06 | 1.087719591 | NM_006460| | HIS1,HMBA-inducible | |  |  |
| 210633_x_at | 14.96 | 1.78E-06 | 1.110558057 | NM_000421| | KRT10,keratin 10 | |  |  |
| 228839_s_at | 14.96 | 1.78E-06 | 1.107628358 | NA |  |  |  |  |
| 212752_at | 14.95 | 1.79E-06 | 1.041808915 | NM_015282| | CLASP1,CLIP-associating protein 1 | | |  |
| 218217_at | 14.95 | 1.79E-06 | 1.04534721 | NM_021626| | SCPEP1,serine carboxypeptidase 1 precursor protein | | | |
| 227702_at | 14.95 | 1.78E-06 | 1.232009457 | NM_178033| | CYP4X1,cytochrome P450, family 4, subfamily X, | | | |
| 213238_at | 14.94 | 1.79E-06 | 1.211612709 | NM_020453| | ATP10D,ATPase, Class V, type 10D | | |  |
| 232235_at | 14.93 | 1.80E-06 | 1.352863419 | NM_032160| | C18orf4,chromosome 18 open reading frame 4 | | | |
| 202936_s_at | 14.92 | 1.81E-06 | 1.187014869 | NM_000346| | SOX9,transcription factor SOX9 | | |  |
| 1553978_at | 14.92 | 1.81E-06 | 1.148521875 | NM_005919| | MEF2B,MADS box transcription enhancer factor 2, | | | |
| 222481_at | 14.91 | 1.82E-06 | 1.181562069 | NM_012192| | FXC1,fracture callus 1 homolog | | |  |
| 1552575_a_at | 14.9 | 1.83E-06 | 1.200797726 | NA |  |  |  |  |
| 225391_at | 14.9 | 1.83E-06 | 1.068624432 | NA |  |  |  |  |
| 205510_s_at | 14.9 | 1.83E-06 | 1.195821929 | NA |  |  |  |  |
| 229302_at | 14.9 | 1.83E-06 | 1.204920106 | NM_152390| | MGC33926,hypothetical protein MGC33926 | | | |
| 220294_at | 14.89 | 1.83E-06 | 1.427814802 | NM_014379| | KCNV1,potassium channel, subfamily V, member 1 | | | |
| 1555833_a_at | 14.88 | 1.83E-06 | 1.061242965 | NA |  |  |  |  |
| 203816_at | 14.88 | 1.84E-06 | 1.093254491 | NM_080916| | DGUOK,deoxyguanosine kinase isoform a precursor | | | |
| 202935_s_at | 14.88 | 1.84E-06 | 1.228670534 | NM_000346| | SOX9,transcription factor SOX9 | | |  |
| 204201_s_at | 14.88 | 1.84E-06 | 1.140691787 | NM_006264| | PTPN13,protein tyrosine phosphatase, non-receptor type | | | |
| 237053_at | 14.88 | 1.84E-06 | 1.155911026 | NM_001105579| | NA |  |  |  |
| 221031_s_at | 14.87 | 1.84E-06 | 1.447409656 | NM_030817| | DKFZP434F0318,hypothetical protein DKFZp434F0318 | | | |
| 203886_s_at | 14.86 | 1.85E-06 | 1.172351933 | NM_001004019| | FBLN2,fibulin 2 isoform a precursor | | |  |
| 227210_at | 14.86 | 1.85E-06 | 1.438615502 | NM_001029880| | NA |  |  |  |
| 215867_x_at | 14.85 | 1.86E-06 | 1.14681671 | NM_001218| | CA12,carbonic anhydrase XII isoform 1 precursor | | | |
| 204717_s_at | 14.83 | 1.88E-06 | 1.153408568 | NM_001532| | SLC29A2,solute carrier family 29 (nucleoside | | | |
| 204597_x_at | 14.83 | 1.88E-06 | 1.255337089 | NM_003155| | STC1,stanniocalcin 1 | |  |  |
| 206110_at | 14.83 | 1.88E-06 | 1.450676205 | NM_003536| | HIST1H3H,H3 histone family, member K | | | |
| 223087_at | 14.82 | 1.89E-06 | 1.107224241 | NM_001002030| | NA |  |  |  |
| 207808_s_at | 14.82 | 1.89E-06 | 1.463533038 | NM_000313| | PROS1,protein S (alpha) | |  |  |
| 228335_at | 14.81 | 1.89E-06 | 1.240602235 | NM_005602| | CLDN11,claudin 11 | |  |  |
| 203729_at | 14.8 | 1.91E-06 | 1.170863447 | NM_001425| | EMP3,epithelial membrane protein 3 | | |  |
| 208343_s_at | 14.79 | 1.92E-06 | 1.567508736 | NM_003822| | NR5A2,nuclear receptor subfamily 5, group A, member 2 | | | |
| 202662_s_at | 14.79 | 1.91E-06 | 1.348324691 | NM_002223| | ITPR2,inositol 1,4,5-triphosphate receptor, type 2 | | | |
| 236856_x_at | 14.79 | 1.91E-06 | 1.356493304 | NA |  |  |  |  |
| 204744_s_at | 14.76 | 1.94E-06 | 1.016459914 | NM_002161| | IARS,isoleucine-tRNA synthetase | | |  |
| 227687_at | 14.76 | 1.94E-06 | 1.112342388 | NM_145014| | FLJ32915,hypothetical protein FLJ32915 | | | |
| 209469_at | 14.76 | 1.94E-06 | 1.467268792 | NM_005277| | GPM6A,glycoprotein M6A isoform 1 | | |  |
| 235497_at | 14.74 | 1.96E-06 | 1.354288564 | NA |  |  |  |  |
| 221773_at | 14.74 | 1.96E-06 | 1.220121335 | NM_005230| | ELK3,ELK3 protein | |  |  |
| 223212_at | 14.74 | 1.96E-06 | 1.070712654 | NM_032327| | ZDHHC16,Abl-philin 2 isoform 1 | | |  |
| 234394_at | 14.73 | 1.97E-06 | 1.5095547 | NM_003431| | ZNF124,zinc finger protein 124 | | |  |
| 205542_at | 14.72 | 1.98E-06 | 1.231035341 | NM_012449| | STEAP,six transmembrane epithelial antigen of the | | | |
| 204026_s_at | 14.71 | 1.99E-06 | 1.049661312 | NM_001005413| | ZWINT,ZW10 interactor isoform b | | |  |
| 238833_at | 14.69 | 2.01E-06 | 1.268994979 | NA |  |  |  |  |
| 213170_at | 14.69 | 2.01E-06 | 1.089188015 | NM_015696| | GPX7,glutathione peroxidase 7 | | |  |
| 242273_at | 14.67 | 2.03E-06 | 1.194084942 | NA |  |  |  |  |
| 221311_x_at | 14.66 | 2.05E-06 | 1.127314015 | NM_020466| | DJ122O8.2,hypothetical protein dJ122O8.2 | | | |
| 226542_at | 14.65 | 2.06E-06 | 1.13838076 | NA |  |  |  |  |
| 202414_at | 14.65 | 2.05E-06 | 1.053175911 | NM_000123| | ERCC5,XPG-complementing protein | | |  |
| 207761_s_at | 14.64 | 2.06E-06 | 1.324681805 | NM_014033| | DKFZP586A0522,DKFZP586A0522 protein | | | |
| 224635_s_at | 14.64 | 2.06E-06 | 1.067788633 | NM_016252| | BIRC6,baculoviral IAP repeat-containing 6 | | | |
| 219993_at | 14.63 | 2.07E-06 | 1.411267796 | NM_022454| | SOX17,SRY-box 17 | |  |  |
| 202223_at | 14.63 | 2.07E-06 | 1.076456215 | NM_152713| | ITM1,integral membrane protein 1 | | |  |
| 227712_at | 14.62 | 2.08E-06 | 1.105684134 | NM_020466| | DJ122O8.2,hypothetical protein dJ122O8.2 | | | |
| 211796_s_at | 14.62 | 2.08E-06 | 1.348157502 | NA |  |  |  |  |
| 219304_s_at | 14.61 | 2.09E-06 | 1.180645923 | NM_025208| | PDGFD,platelet derived growth factor D isoform 1 | | | |
| 242093_at | 14.61 | 2.09E-06 | 1.421096878 | NM_138780| | SYTL5,synaptotagmin-like 5 | | |  |
| 201956_s_at | 14.6 | 2.10E-06 | 1.058122577 | NM_014236| | GNPAT,glyceronephosphate O-acyltransferase | | | |
| 226517_at | 14.59 | 2.10E-06 | 1.071166065 | NM_005504| | BCAT1,branched chain aminotransferase 1, cytosolic | | | |
| 238663_x_at | 14.59 | 2.10E-06 | 1.511355638 | NM_000829| | GRIA4,glutamate receptor, ionotrophic | | |  |
| 236281_x_at | 14.59 | 2.11E-06 | 1.596577918 | NM_000872| | HTR7,5-hydroxytryptamine receptor 7 isoform a | | | |
| 213918_s_at | 14.59 | 2.10E-06 | 1.129655632 | NM_015384| | NIPBL,delangin isoform B | |  |  |
| 225348_at | 14.58 | 2.11E-06 | 1.126283388 | NM_006625| | FUSIP1,FUS interacting protein (serine-arginine rich) 1 | | | |
| 229969_at | 14.57 | 2.12E-06 | 1.15828252 | NA |  |  |  |  |
| 225634_at | 14.57 | 2.12E-06 | 1.093597245 | NM_020119| | ZC3HAV1,zinc finger antiviral protein isoform 1 | | | |
| 1555225_at | 14.57 | 2.12E-06 | 1.262950133 | NM_001098616| | NA |  |  |  |
| 213107_at | 14.57 | 2.12E-06 | 1.562704362 | NM_015028| | TNIK,TRAF2 and NCK interacting kinase | | |  |
| 206314_at | 14.55 | 2.14E-06 | 1.106809995 | NM_018651| | ZNF167,zinc finger protein ZFP isoform 1 | | | |
| 205330_at | 14.55 | 2.15E-06 | 1.246311424 | NM_002430| | MN1,meningioma 1 | |  |  |
| 1560019_at | 14.55 | 2.14E-06 | 1.432572397 | NA |  |  |  |  |
| 202903_at | 14.54 | 2.15E-06 | 1.146431562 | NM_012322| | LSM5,U6 snRNA-associated Sm-like protein 5 | | | |
| 201645_at | 14.5 | 2.21E-06 | 1.284172306 | NM_002160| | TNC,tenascin C (hexabrachion) | | |  |
| 222565_s_at | 14.49 | 2.21E-06 | 1.106709653 | NM_005813| | PRKD3,protein kinase D3 | |  |  |
| 228573_at | 14.49 | 2.22E-06 | 1.289462278 | NA |  |  |  |  |
| 226560_at | 14.48 | 2.23E-06 | 1.227797678 | NA |  |  |  |  |
| 210015_s_at | 14.48 | 2.22E-06 | 1.361072635 | NM_001039538| | NA |  |  |  |
| 207169_x_at | 14.47 | 2.24E-06 | 1.03838148 | NM_001954| | DDR1,discoidin receptor tyrosine kinase isoform b | | | |
| 231202_at | 14.47 | 2.23E-06 | 1.334240495 | NM_001034173| | NA |  |  |  |
| 223614_at | 14.45 | 2.25E-06 | 1.117906837 | NA |  |  |  |  |
| 224727_at | 14.45 | 2.25E-06 | 1.074725015 | NM_175063| | LOC284361,hypothetical protein LOC284361 isoform 1 | | | |
| 235022_at | 14.45 | 2.26E-06 | 1.116688579 | NM_001098801| | NA |  |  |  |
| 214061_at | 14.44 | 2.27E-06 | 1.080690908 | NM_145647| | MGC21654,unknown MGC21654 product | | | |
| 225826_at | 14.42 | 2.29E-06 | 1.171757331 | NM_052845| | MMAB,cob(I)alamin adenosyltransferase | | | |
| 243357_at | 14.42 | 2.29E-06 | 1.437025693 | NM_173808| | NEGR1,neuronal growth regulator 1 | | |  |
| 210105_s_at | 14.41 | 2.29E-06 | 1.050382065 | NM_002037| | FYN,protein-tyrosine kinase fyn isoform a | | | |
| 228280_at | 14.41 | 2.30E-06 | 1.346772439 | NM_080660| | MGC14289,hypothetical protein MGC14289 | | | |
| 209284_s_at | 14.4 | 2.31E-06 | 1.115187347 | NM_001112736| | NA |  |  |  |
| 221787_at | 14.4 | 2.31E-06 | 1.06391374 | NM_001029863| | NA |  |  |  |
| 202560_s_at | 14.39 | 2.31E-06 | 1.039097861 | NM_015607| | DKFZP547E1010,DKFZP547E1010 protein | | | |
| 240069_at | 14.38 | 2.33E-06 | 1.310177685 | NA |  |  |  |  |
| 212623_at | 14.37 | 2.34E-06 | 1.080822001 | NM_015012| | NA |  |  |  |
| 218507_at | 14.37 | 2.33E-06 | 1.184819503 | NM_001098786| | NA |  |  |  |
| 215716_s_at | 14.36 | 2.34E-06 | 1.133425673 | NM_001001323| | ATP2B1,plasma membrane calcium ATPase 1 isoform 1a | | | |
| 205189_s_at | 14.35 | 2.35E-06 | 1.128858793 | NM_000136| | FANCC,Fanconi anemia, complementation group C | | | |
| 203102_s_at | 14.35 | 2.35E-06 | 1.0817172 | NM_002408| | MGAT2,alpha-1,6-mannosyl-glycoprotein | | | |
| 205113_at | 14.35 | 2.35E-06 | 1.379093814 | NM_001105541| | NA |  |  |  |
| 208893_s_at | 14.34 | 2.36E-06 | 1.642238161 | NM_001946| | DUSP6,dual specificity phosphatase 6 isoform a | | | |
| 222244_s_at | 14.33 | 2.38E-06 | 1.056434106 | NA |  |  |  |  |
| 202539_s_at | 14.31 | 2.40E-06 | 1.085106284 | NM_000859| | HMGCR,3-hydroxy-3-methylglutaryl-Coenzyme A reductase | | | |
| 59437_at | 14.31 | 2.40E-06 | 1.128593035 | NM_001048265| | NA |  |  |  |
| 41858_at | 14.3 | 2.41E-06 | 1.06185735 | NM_014489| | FRAG1,FGF receptor activating protein 1 | | |  |
| 226969_at | 14.29 | 2.42E-06 | 1.155723199 | NM_000254| | MTR,5-methyltetrahydrofolate-homocysteine | | | |
| 229333_at | 14.28 | 2.44E-06 | 1.191317701 | NA |  |  |  |  |
| 225187_at | 14.28 | 2.43E-06 | 1.053116086 | NM_021174| | KIAA1967,p30 DBC protein | | |  |
| 215076_s_at | 14.28 | 2.44E-06 | 1.334964308 | NM_000090| | COL3A1,alpha 1 type III collagen | | |  |
| 218820_at | 14.28 | 2.43E-06 | 1.136569103 | NA |  |  |  |  |
| 227680_at | 14.27 | 2.44E-06 | 1.096127755 | NM_182975| | ZNF326,zinc finger protein 326 isoform 3 | | | |
| 204775_at | 14.26 | 2.46E-06 | 1.069429417 | NM_005441| | CHAF1B,chromatin assembly factor 1 subunit B | | | |
| 220138_at | 14.26 | 2.45E-06 | 1.62580333 | NM_004821| | HAND1,basic helix-loop-helix transcription factor | | | |
| 235413_at | 14.25 | 2.46E-06 | 1.16043475 | NM_000821| | GGCX,gamma-glutamyl carboxylase | | |  |
| 226610_at | 14.24 | 2.48E-06 | 1.070920185 | NM_181716| | PRR6,proline rich 6 | |  |  |
| 242794_at | 14.24 | 2.48E-06 | 1.16618592 | NM_018717| | MAML3,mastermind-like 3 | |  |  |
| 201200_at | 14.23 | 2.49E-06 | 1.077176755 | NM_003851| | CREG1,cellular repressor of E1A-stimulated genes | | | |
| 213761_at | 14.23 | 2.49E-06 | 1.125169373 | NM_017440| | MDM1,Mdm4, transformed 3T3 cell double minute 1, p53 | | | |
| 204140_at | 14.22 | 2.51E-06 | 1.074905398 | NM_003596| | TPST1,tyrosylprotein sulfotransferase 1 | | |  |
| 202308_at | 14.21 | 2.51E-06 | 1.194592252 | NM_001005291| | SREBF1,sterol regulatory element binding transcription | | | |
| 206336_at | 14.21 | 2.51E-06 | 1.481421845 | NM_002993| | CXCL6,chemokine (C-X-C motif) ligand 6 (granulocyte | | | |
| 212186_at | 14.2 | 2.53E-06 | 1.059349809 | NM_198834| | ACACA,acetyl-Coenzyme A carboxylase alpha isoform 1 | | | |
| 218434_s_at | 14.2 | 2.53E-06 | 1.080084833 | NM_023928| | AACS,acetoacetyl-CoA synthetase | | |  |
| 227545_at | 14.18 | 2.55E-06 | 1.061517652 | NA |  |  |  |  |
| 201381_x_at | 14.17 | 2.57E-06 | 1.040376515 | NM_001007214| | CACYBP,calcyclin binding protein isoform 2 | | | |
| 209773_s_at | 14.16 | 2.58E-06 | 1.094670962 | NM_001034| | RRM2,ribonucleotide reductase M2 polypeptide | | | |
| 222869_s_at | 14.16 | 2.58E-06 | 1.296300863 | NM_018696| | ELAC1,elaC homolog 1 | |  |  |
| 202390_s_at | 14.15 | 2.59E-06 | 1.090908216 | NM_002111| | HD,huntingtin | |  |  |
| 228170_at | 14.15 | 2.59E-06 | 1.648728736 | NM_138983| | OLIG1,oligodendrocyte transcription factor 1 | | | |
| 222269_at | 14.14 | 2.61E-06 | 1.276123766 | NM_198450| | CXorf33,chromosome X open reading frame 33 | | | |
| 1565595_at | 14.13 | 2.63E-06 | 1.19183046 | NA |  |  |  |  |
| 219079_at | 14.12 | 2.64E-06 | 1.099986111 | NM_016230| | NCB5OR,NADPH cytochrome B5 oxidoreductase | | | |
| 226502_at | 14.11 | 2.65E-06 | 1.067591375 | NM_153702| | ELMOD2,ELMO domain containing 2 | | |  |
| 242348_at | 14.11 | 2.65E-06 | 1.268397784 | NM_001005527| | FAM19A4,family with sequence similarity 19 (chemokine | | | |
| 1558522_at | 14.09 | 2.68E-06 | 1.1370466 | NA |  |  |  |  |
| 213030_s_at | 14.09 | 2.68E-06 | 1.24857031 | NM_025179| | PLXNA2,plexin A2 | |  |  |
| 202557_at | 14.09 | 2.68E-06 | 1.050351766 | NM_006948| | STCH,stress 70 protein chaperone, | | |  |
| 206976_s_at | 14.08 | 2.69E-06 | 1.067344237 | NM_006644| | HSPH1,heat shock 105kD | |  |  |
| 203185_at | 14.06 | 2.71E-06 | 1.158110452 | NM_014737| | RASSF2,Ras association domain family 2 isoform 1 | | | |
| 200907_s_at | 14.06 | 2.72E-06 | 1.087651371 | NM_016081| | KIAA0992,palladin | |  |  |
| 204658_at | 14.06 | 2.71E-06 | 1.07031891 | NM_013293| | TRA2A,transformer-2 alpha | | |  |
| 231325_at | 14.05 | 2.74E-06 | 1.086865572 | NM_080872| | UNC5D,netrin receptor Unc5h4 | | |  |
| 227276_at | 14.04 | 2.75E-06 | 1.071532152 | NM_032812| | PLXDC2,plexin domain containing 2 precursor | | | |
| 219764_at | 14.03 | 2.76E-06 | 1.599673105 | NM_007197| | FZD10,frizzled 10 | |  |  |
| 217814_at | 14.03 | 2.75E-06 | 1.066197591 | NM_020198| | GK001,GK001 protein | |  |  |
| 227732_at | 14.02 | 2.78E-06 | 1.193122459 | NM_020725| | NA |  |  |  |
| 227451_s_at | 14.02 | 2.77E-06 | 1.119672838 | NM_001031713| | NA |  |  |  |
| 244170_at | 14.01 | 2.79E-06 | 1.232084058 | NA |  |  |  |  |
| 206224_at | 14.01 | 2.78E-06 | 1.389290164 | NM_001898| | CST1,cystatin SN precursor | | |  |
| 204750_s_at | 14 | 2.79E-06 | 1.13775681 | NM_004949| | DSC2,desmocollin 2 isoform Dsc2b preproprotein | | | |
| 226763_at | 14 | 2.79E-06 | 1.056373238 | NM_178123| | SESTD1,SEC14 and spectrin domains 1 | | |  |
| 1556061_at | 14 | 2.80E-06 | 1.23038731 | NM_001104546| | NA |  |  |  |
| 225386_s_at | 14 | 2.79E-06 | 1.050777408 | NM_138394| | HNRPLL,heterogeneous nuclear ribonucleoprotein L-like | | | |
| 241727_x_at | 14 | 2.79E-06 | 1.185309498 | NM_176815| | DHFRL1,dihydrofolate reductase-like 1 | | |  |
| 230570_at | 13.99 | 2.80E-06 | 1.147178568 | NA |  |  |  |  |
| 224329_s_at | 13.98 | 2.83E-06 | 1.15586412 | NM_032488| | CNFN,cornifelin | |  |  |
| 228438_at | 13.98 | 2.83E-06 | 1.334374387 | NA |  |  |  |  |
| 211758_x_at | 13.97 | 2.84E-06 | 1.049270469 | NM_005783| | TXNDC9,ATP binding protein associated with cell | | | |
| 219225_at | 13.97 | 2.84E-06 | 1.097786097 | NM_024554| | PGBD5,piggyBac transposable element derived 5 | | | |
| 220786_s_at | 13.96 | 2.84E-06 | 1.685095556 | NM_018018| | SLC38A4,solute carrier family 38, member 4 | | | |
| 208985_s_at | 13.96 | 2.84E-06 | 1.039613723 | NM_003758| | EIF3S1,eukaryotic translation initiation factor 3, | | | |
| 204932_at | 13.96 | 2.84E-06 | 1.337264169 | NM_002546| | TNFRSF11B,osteoprotegerin precursor | | |  |
| 200929_at | 13.96 | 2.84E-06 | 1.037823055 | NM_006827| | TMP21,transmembrane trafficking protein | | | |
| 214636_at | 13.96 | 2.84E-06 | 1.186060584 | NM_000728| | CALCB,calcitonin-related polypeptide, beta | | | |
| 211162_x_at | 13.96 | 2.84E-06 | 1.261567291 | NM_005063| | SCD,stearoyl-CoA desaturase | | |  |
| 203300_x_at | 13.95 | 2.85E-06 | 1.149119247 | NM_003916| | AP1S2,adaptor-related protein complex 1 sigma 2 | | | |
| 37232_at | 13.94 | 2.86E-06 | 1.142118215 | NM_014749| | KIAA0586,KIAA0586 | |  |  |
| 201648_at | 13.94 | 2.86E-06 | 1.087701948 | NM_002227| | JAK1,janus kinase 1 | |  |  |
| 229748_x_at | 13.94 | 2.87E-06 | 1.273296938 | NM_001033515| | NA |  |  |  |
| 202754_at | 13.94 | 2.86E-06 | 1.048741603 | NM_015361| | R3HDM,R3H domain (binds single-stranded nucleic acids) | | | |
| 238536_at | 13.93 | 2.88E-06 | 1.212603424 | NA |  |  |  |  |
| 210017_at | 13.93 | 2.88E-06 | 1.10598807 | NM_006785| | MALT1,mucosa associated lymphoid tissue lymphoma | | | |
| 228628_at | 13.93 | 2.88E-06 | 1.113222709 | NA |  |  |  |  |
| 217879_at | 13.92 | 2.89E-06 | 1.062416577 | NM_001114091| | NA |  |  |  |
| 202409_at | 13.91 | 2.91E-06 | 1.300083368 | NM_000612| | IGF2,insulin-like growth factor 2 (somatomedin A) | | | |
| 201587_s_at | 13.91 | 2.91E-06 | 1.149146108 | NM_001025242| | NA |  |  |  |
| 225558_at | 13.91 | 2.90E-06 | 1.139291647 | NM_014776| | GIT2,G protein-coupled receptor kinase-interactor 2 | | | |
| 225316_at | 13.9 | 2.91E-06 | 1.22890294 | NM_032793| | FLJ14490,hypothetical protein FLJ14490 | | | |
| 214531_s_at | 13.89 | 2.93E-06 | 1.064110472 | NM_003099| | SNX1,sorting nexin 1 isoform a | | |  |
| 222129_at | 13.89 | 2.93E-06 | 1.228591126 | NM_024293| | C2orf17,chromosome 2 open reading frame 17 | | | |
| 235242_at | 13.89 | 2.93E-06 | 1.083416399 | NA |  |  |  |  |
| 201307_at | 13.89 | 2.93E-06 | 1.068357746 | NM_018243| | SEPT11,septin 11 | |  |  |
| 204712_at | 13.87 | 2.95E-06 | 1.242726881 | NM_007191| | WIF1,Wnt inhibitory factor-1 precursor | | |  |
| 225126_at | 13.87 | 2.96E-06 | 1.103669533 | NM_138777| | MRRF,mitochondrial ribosome recycling factor isoform | | | |
| 222867_s_at | 13.86 | 2.98E-06 | 1.171418248 | NM_016060| | MED31,mediator of RNA polymerase II transcription, | | | |
| 213111_at | 13.85 | 2.99E-06 | 1.043468097 | NM_015040| | PIP5K3,phosphatidylinositol-3- | | |  |
| 223213_s_at | 13.84 | 2.99E-06 | 1.205560659 | NM_001017926| | NA |  |  |  |
| 231807_at | 13.83 | 3.01E-06 | 1.126876444 | NM_001098500| | NA |  |  |  |
| 206513_at | 13.83 | 3.02E-06 | 1.310499928 | NM_004833| | AIM2,absent in melanoma 2 | | |  |
| 224413_s_at | 13.83 | 3.02E-06 | 1.050560536 | NM_001024380| | NA |  |  |  |
| 209680_s_at | 13.82 | 3.03E-06 | 1.0268096 | NM_002263| | NA |  |  |  |
| 223151_at | 13.82 | 3.03E-06 | 1.055540787 | NM_032299| | MGC2714,hypothetical protein MGC2714 | | | |
| 226802_s_at | 13.81 | 3.05E-06 | 1.103088433 | NA |  |  |  |  |
| 228925_at | 13.81 | 3.04E-06 | 1.484189723 | NA |  |  |  |  |
| 242293_at | 13.79 | 3.08E-06 | 1.124416897 | NM_019071| | ING3,inhibitor of growth family, member 3 isoform 1 | | | |
| 219987_at | 13.78 | 3.09E-06 | 1.179874819 | NA |  |  |  |  |
| 222011_s_at | 13.78 | 3.09E-06 | 1.128980029 | NM_001008897| | TCP1,T-complex protein 1 isoform b | | |  |
| 219372_at | 13.78 | 3.09E-06 | 1.249988101 | NM_014055| | CDV1,carnitine deficiency-associated, expressed in | | | |
| 220159_at | 13.78 | 3.09E-06 | 1.371829258 | NA |  |  |  |  |
| 223627_at | 13.78 | 3.09E-06 | 1.136680825 | NM_032246| | RKHD3,ring finger and KH domain containing 3 | | | |
| 219855_at | 13.78 | 3.09E-06 | 1.045191025 | NM_018159| | NUDT11,nudix (nucleoside diphosphate linked moiety | | | |
| 213302_at | 13.78 | 3.09E-06 | 1.054088425 | NM_012393| | PFAS,phosphoribosylformylglycinamidine synthase | | | |
| 235113_at | 13.76 | 3.11E-06 | 1.060946294 | NM_152329| | PPIL5,peptidylprolyl isomerase (cyclophilin)-like 5 | | | |
| 203304_at | 13.76 | 3.11E-06 | 1.089276575 | NM_012342| | BAMBI,BMP and activin membrane-bound inhibitor | | | |
| 212994_at | 13.76 | 3.11E-06 | 1.064767638 | NM_001081550| | NA |  |  |  |
| 203396_at | 13.75 | 3.12E-06 | 1.022910057 | NM_001102667| | NA |  |  |  |
| 1552400_a_at | 13.75 | 3.12E-06 | 1.22316537 | NM_152335| | C15orf27,chromosome 15 open reading frame 27 | | | |
| 237116_at | 13.75 | 3.14E-06 | 1.212912117 | NA |  |  |  |  |
| 238623_at | 13.74 | 3.15E-06 | 1.177766686 | NA |  |  |  |  |
| 238604_at | 13.73 | 3.16E-06 | 1.165774972 | NA |  |  |  |  |
| 227335_at | 13.72 | 3.18E-06 | 1.359803352 | NM_022105| | DATF1,death associated transcription factor 1 isoform | | | |
| 228160_at | 13.71 | 3.19E-06 | 1.261772 | NA |  |  |  |  |
| 201287_s_at | 13.71 | 3.20E-06 | 1.101319764 | NM_001006946| | SDC1,syndecan 1 precursor | | |  |
| 224968_at | 13.7 | 3.22E-06 | 1.099929463 | NM_080667| | MGC15407,hypothetical protein MGC15407 | | | |
| 212843_at | 13.69 | 3.22E-06 | 1.523604783 | NM_000615| | NCAM1,neural cell adhesion molecule 1 | | |  |
| 222526_at | 13.69 | 3.23E-06 | 1.090588948 | NM_017660| | p66alpha,p66 alpha | |  |  |
| 35156_at | 13.69 | 3.23E-06 | 1.105470885 | NA |  |  |  |  |
| 226464_at | 13.69 | 3.23E-06 | 1.087671197 | NM_173552| | MGC33365,hypothetical protein MGC33365 | | | |
| 221480_at | 13.66 | 3.27E-06 | 1.1068316 | NM_001003810| | HNRPD,heterogeneous nuclear ribonucleoprotein D | | | |
| 32625_at | 13.65 | 3.30E-06 | 1.19284457 | NM_000906| | NPR1,natriuretic peptide receptor A/guanylate cyclase | | | |
| 225167_at | 13.65 | 3.29E-06 | 1.254293445 | NM_018027| | FRMD4A,FERM domain containing 4A | | |  |
| 202252_at | 13.65 | 3.30E-06 | 1.052488651 | NM_002870| | RAB13,RAB13, member RAS oncogene family | | | |
| 218298_s_at | 13.65 | 3.30E-06 | 1.097394877 | NM_001102366| | NA |  |  |  |
| 200782_at | 13.64 | 3.32E-06 | 1.073673585 | NM_001154| | ANXA5,annexin 5 | |  |  |
| 209103_s_at | 13.64 | 3.31E-06 | 1.072917997 | NM_001035247| | NA |  |  |  |
| 202640_s_at | 13.62 | 3.35E-06 | 1.067423104 | NM_003624| | RANBP3,RAN binding protein 3 isoform RANBP3-a | | | |
| 205292_s_at | 13.62 | 3.36E-06 | 1.029499662 | NM_002137| | HNRPA2B1,heterogeneous nuclear ribonucleoprotein A2/B1 | | | |
| 213869_x_at | 13.6 | 3.40E-06 | 1.130686736 | NM_006288| | THY1,Thy-1 cell surface antigen | | |  |
| 202007_at | 13.59 | 3.41E-06 | 1.104828925 | NM_002508| | NID,nidogen (enactin) | |  |  |
| 209694_at | 13.59 | 3.41E-06 | 1.068346258 | NM_000317| | PTS,6-pyruvoyltetrahydropterin synthase | | | |
| 202596_at | 13.59 | 3.41E-06 | 1.074710793 | NM_004436| | ENSA,endosulfine alpha isoform 3 | | |  |
| 236106_at | 13.59 | 3.40E-06 | 1.339392901 | NA |  |  |  |  |
| 237675_at | 13.58 | 3.42E-06 | 1.295386639 | NA |  |  |  |  |
| 229160_at | 13.58 | 3.42E-06 | 1.475818515 | NM_152423| | MUM1L1,melanoma associated antigen (mutated) 1-like 1 | | | |
| 212248_at | 13.58 | 3.42E-06 | 1.158446462 | NM_178812| | LYRIC,LYRIC/3D3 | |  |  |
| 215091_s_at | 13.57 | 3.43E-06 | 1.03446643 | NM_002097| | GTF3A,general transcription factor IIIA | | |  |
| 206854_s_at | 13.57 | 3.43E-06 | 1.049828439 | NM_003188| | MAP3K7,mitogen-activated protein kinase kinase kinase 7 | | | |
| 231859_at | 13.56 | 3.45E-06 | 1.153634856 | NA |  |  |  |  |
| 219615_s_at | 13.56 | 3.44E-06 | 1.084488893 | NM_003740| | KCNK5,potassium channel, subfamily K, member 5 | | | |
| 210191_s_at | 13.56 | 3.44E-06 | 1.181047498 | NM_006608| | PHTF1,putative homeodomain transcription factor 1 | | | |
| 1555968_a_at | 13.55 | 3.47E-06 | 1.310608708 | NA |  |  |  |  |
| 227143_s_at | 13.55 | 3.47E-06 | 1.101794219 | NM_001196| | BID,BH3 interacting domain death agonist isoform 2 | | | |
| 219493_at | 13.55 | 3.46E-06 | 1.119523873 | NM_024745| | SHCBP1,SHC SH2-domain binding protein 1 | | | |
| 203276_at | 13.52 | 3.52E-06 | 1.055173736 | NM_005573| | LMNB1,lamin B1 | |  |  |
| 225816_at | 13.51 | 3.55E-06 | 1.064004167 | NM_024900| | PHF17,Jade1 protein short isoform | | |  |
| 224870_at | 13.51 | 3.54E-06 | 1.086679551 | NA |  |  |  |  |
| 218542_at | 13.5 | 3.57E-06 | 1.078464428 | NM_001127182| | NA |  |  |  |
| 244463_at | 13.5 | 3.56E-06 | 1.148065279 | NM_003812| | ADAM23,a disintegrin and metalloproteinase domain 23 | | | |
| 203260_at | 13.49 | 3.58E-06 | 1.161160108 | NM_016063| | C6orf74,chromosome 6 open reading frame 74 | | | |
| 205967_at | 13.49 | 3.58E-06 | 1.02696794 | NM_001034077| | NA |  |  |  |
| 203674_at | 13.48 | 3.60E-06 | 1.105825144 | NM_014877| | HELZ,helicase with zinc finger domain | | |  |
| 225522_at | 13.48 | 3.60E-06 | 1.090455924 | NM_014911| | AAK1,AP2 associated kinase 1 | | |  |
| 219292_at | 13.48 | 3.60E-06 | 1.086438463 | NM_018105| | THAP1,THAP domain containing, apoptosis associated | | | |
| 224511_s_at | 13.47 | 3.62E-06 | 1.067548319 | NM_032731| | TXNL5,thioredoxin-like 5 | |  |  |
| 1557129_a_at | 13.46 | 3.64E-06 | 1.360252573 | NM_198947| | CANP,cancer-associated nucleoprotein | | |  |
| 200594_x_at | 13.45 | 3.66E-06 | 1.027911948 | NM_004501| | HNRPU,heterogeneous nuclear ribonucleoprotein U | | | |
| 203405_at | 13.45 | 3.66E-06 | 1.073598037 | NM_003720| | DSCR2,Down syndrome critical region protein 2 isoform | | | |
| 204817_at | 13.45 | 3.66E-06 | 1.065044978 | NM_012291| | ESPL1,extra spindle poles like 1 | | |  |
| 222691_at | 13.45 | 3.66E-06 | 1.090945215 | NM_015948| | SLC35B3,solute carrier family 35, member B3 | | | |
| 237203_at | 13.44 | 3.67E-06 | 1.171493156 | NA |  |  |  |  |
| 219982_s_at | 13.44 | 3.66E-06 | 1.127253608 | NM_021967| | SERF1A,small EDRK-rich factor 1A, telomeric | | | |
| 205345_at | 13.43 | 3.69E-06 | 1.063902182 | NM_000465| | BARD1,BRCA1 associated RING domain 1 | | | |
| 227070_at | 13.43 | 3.69E-06 | 1.211479025 | NM_031302| | GLT8D2,glycosyltransferase 8 domain containing 2 | | | |
| 1559007_s_at | 13.43 | 3.69E-06 | 1.361212638 | NA |  |  |  |  |
| 214290_s_at | 13.43 | 3.69E-06 | 1.180842891 | NM_001040874| | NA |  |  |  |
| 201506_at | 13.39 | 3.78E-06 | 1.233047125 | NM_000358| | TGFBI,transforming growth factor, beta-induced, 68kDa | | | |
| 201266_at | 13.38 | 3.81E-06 | 1.027317485 | NM_001093771| | NA |  |  |  |
| 239043_at | 13.37 | 3.82E-06 | 1.195234533 | NM_001033719| | NA |  |  |  |
| 213346_at | 13.37 | 3.82E-06 | 1.094262367 | NM_138779| | LOC93081,hypothetical protein BC015148 | | | |
| 221896_s_at | 13.36 | 3.82E-06 | 1.049380903 | NM_001099668| | NA |  |  |  |
| 202600_s_at | 13.36 | 3.82E-06 | 1.286320595 | NM_003489| | NRIP1,receptor interacting protein 140 | | |  |
| 235535_x_at | 13.35 | 3.85E-06 | 1.211354795 | NM_004477| | FRG1,FSHD region gene 1 | |  |  |
| 213469_at | 13.34 | 3.87E-06 | 1.093001097 | NM_024989| | PGAP1,GPI deacylase | |  |  |
| 212934_at | 13.33 | 3.89E-06 | 1.095086671 | NM_001077619| | NA |  |  |  |
| 204165_at | 13.33 | 3.89E-06 | 1.081034387 | NM_001024934| | NA |  |  |  |
| 212169_at | 13.33 | 3.89E-06 | 1.106053684 | NM_007270| | FKBP9,FK506 binding protein 9 | | |  |
| 222400_s_at | 13.33 | 3.89E-06 | 1.14034462 | NM_018269| | MTCBP-1,membrane-type 1 matrix metalloproteinase | | | |
| 214130_s_at | 13.32 | 3.91E-06 | 1.295696864 | NM_001002810| | PDE4DIP,phosphodiesterase 4D interacting protein isoform | | | |
| 215983_s_at | 13.31 | 3.94E-06 | 1.156910285 | NM_005671| | D8S2298E,reproduction 8 | |  |  |
| 204467_s_at | 13.31 | 3.93E-06 | 1.210525877 | NM_000345| | SNCA,alpha-synuclein isoform NACP140 | | |  |
| 209987_s_at | 13.31 | 3.93E-06 | 1.419767319 | NM_004316| | ASCL1,achaete-scute complex homolog-like 1 | | | |
| 213307_at | 13.3 | 3.95E-06 | 1.233294809 | NM_012309| | SHANK2,SH3 and multiple ankyrin repeat domains 2 | | | |
| 226621_at | 13.3 | 3.95E-06 | 1.320880776 | NA |  |  |  |  |
| 224722_at | 13.3 | 3.95E-06 | 1.202820609 | NM_020774| | MIB1,mindbomb homolog 1 | | |  |
| 1556123_a_at | 13.29 | 3.96E-06 | 1.159592029 | NA |  |  |  |  |
| 212372_at | 13.29 | 3.96E-06 | 1.031057026 | NM_005964| | MYH10,myosin, heavy polypeptide 10, non-muscle | | | |
| 244052_at | 13.29 | 3.96E-06 | 1.05063077 | NM_032783| | CBR4,carbonic reductase 4 | | |  |
| 210045_at | 13.28 | 3.99E-06 | 1.185667832 | NM_002168| | IDH2,isocitrate dehydrogenase 2 (NADP+), | | | |
| 201544_x_at | 13.27 | 4.00E-06 | 1.024141932 | NM_004643| | PABPN1,poly(A) binding protein, nuclear 1 | | | |
| 226628_at | 13.27 | 4.00E-06 | 1.155469363 | NM_001081550| | NA |  |  |  |
| 221911_at | 13.27 | 4.01E-06 | 1.087752014 | NM_004956| | ETV1,ets variant gene 1 | |  |  |
| 200077_s_at | 13.26 | 4.03E-06 | 1.022371506 | NM_004152| | OAZ1,ornithine decarboxylase antizyme 1 | | | |
| 229025_s_at | 13.26 | 4.02E-06 | 1.204210476 | NM_144981| | FLJ25059,hypothetical protein FLJ25059 | | | |
| 233016_at | 13.25 | 4.05E-06 | 1.205852692 | NA |  |  |  |  |
| 205090_s_at | 13.25 | 4.04E-06 | 1.074127264 | NM_016256| | NAGPA,N-acetylglucosamine-1-phosphodiester | | | |
| 204863_s_at | 13.24 | 4.06E-06 | 1.348514107 | NM_002184| | IL6ST,interleukin 6 signal transducer isoform 1 | | | |
| 208580_x_at | 13.24 | 4.07E-06 | 1.186878707 | NM_001034077| | NA |  |  |  |
| 224352_s_at | 13.22 | 4.11E-06 | 1.097626607 | NM_021914| | CFL2,cofilin 2 | |  |  |
| 203790_s_at | 13.22 | 4.10E-06 | 1.078815467 | NM_005836| | HRSP12,heat-responsive protein 12 | | |  |
| 236756_at | 13.22 | 4.11E-06 | 1.095903694 | NA |  |  |  |  |
| 214512_s_at | 13.22 | 4.11E-06 | 1.033886433 | NM_006713| | PC4,activated RNA polymerase II transcription | | | |
| 222642_s_at | 13.21 | 4.13E-06 | 1.097030516 | NM_018126| | TMEM33,transmembrane protein 33 | | |  |
| 232297_at | 13.2 | 4.15E-06 | 1.291201127 | NA |  |  |  |  |
| 201121_s_at | 13.2 | 4.16E-06 | 1.072349431 | NM_006667| | PGRMC1,progesterone receptor membrane component 1 | | | |
| 202420_s_at | 13.19 | 4.17E-06 | 1.065725327 | NM_001357| | DHX9,DEAH (Asp-Glu-Ala-His) box polypeptide 9 isoform | | | |
| 224822_at | 13.19 | 4.18E-06 | 1.318837418 | NM_006094| | DLC1,deleted in liver cancer 1 isoform 2 | | | |
| 235390_at | 13.19 | 4.18E-06 | 1.057066622 | NM_173829| | FLJ36754,hypothetical protein FLJ36754 | | | |
| 200049_at | 13.18 | 4.19E-06 | 1.055072882 | NM_007067| | MYST2,MYST histone acetyltransferase 2 | | | |
| 212721_at | 13.16 | 4.24E-06 | 1.078119102 | NM_001077199| | NA |  |  |  |
| 227875_at | 13.16 | 4.24E-06 | 1.116267266 | NM_033495| | KLHL13,kelch-like 13 | |  |  |
| 217775_s_at | 13.15 | 4.27E-06 | 1.070824811 | NM_016026| | RDH11,androgen-regulated short-chain | | |  |
| 235334_at | 13.15 | 4.26E-06 | 1.099680708 | NM_152996| | ST6GALNAC3,ST6 | |  |  |
| 223737_x_at | 13.15 | 4.26E-06 | 1.116955115 | NM_031422| | CHST9,GalNAc-4-sulfotransferase 2 | | |  |
| 226649_at | 13.15 | 4.26E-06 | 1.076032902 | NM_138316| | PANK1,pantothenate kinase 1 isoform gamma | | | |
| 242828_at | 13.15 | 4.27E-06 | 1.067700224 | NM_018086| | FIGN,fidgetin | |  |  |
| 1552256_a_at | 13.14 | 4.28E-06 | 1.081607689 | NM_001082959| | NA |  |  |  |
| 230369_at | 13.13 | 4.30E-06 | 1.223667332 | NM_007369| | GPR161,G protein-coupled receptor 161 | | | |
| 238205_at | 13.13 | 4.31E-06 | 1.144602667 | NM_178470| | WDR40B,WD repeat domain 40B | | |  |
| 228692_at | 13.13 | 4.30E-06 | 1.293471193 | NA |  |  |  |  |
| 226892_at | 13.12 | 4.31E-06 | 1.114258084 | NM_015652| | C10orf12,chromosome 10 open reading frame 12 | | | |
| 219951_s_at | 13.11 | 4.34E-06 | 1.141385502 | NM_001099407| | NA |  |  |  |
| 1553613_s_at | 13.11 | 4.34E-06 | 1.628874608 | NM_001453| | FOXC1,forkhead box C1 | |  |  |
| 230496_at | 13.11 | 4.34E-06 | 1.691152763 | NM_152704| | FLJ25477,hypothetical protein FLJ25477 isoform 1 | | | |
| 222742_s_at | 13.11 | 4.34E-06 | 1.117085624 | NM_022777| | RABL5,RAB, member RAS oncogene family-like 5 | | | |
| 218984_at | 13.11 | 4.35E-06 | 1.051386827 | NM_019042| | FLJ20485,hypothetical protein FLJ20485 | | | |
| 201433_s_at | 13.11 | 4.35E-06 | 1.039132753 | NM_014754| | PTDSS1,phosphatidylserine synthase 1 | | |  |
| 227478_at | 13.1 | 4.36E-06 | 1.247016968 | NM_015559| | SETBP1,SET binding protein 1 | | |  |
| 204246_s_at | 13.1 | 4.35E-06 | 1.070622843 | NM_007234| | DCTN3,dynactin 3 isoform 1 | | |  |
| 201585_s_at | 13.09 | 4.38E-06 | 1.086553466 | NM_005066| | SFPQ,splicing factor proline/glutamine rich | | | |
| 1555037_a_at | 13.07 | 4.45E-06 | 1.066585499 | NM_005896| | IDH1,isocitrate dehydrogenase 1 (NADP+), soluble | | | |
| 200967_at | 13.07 | 4.45E-06 | 1.04895623 | NM_000942| | PPIB,peptidylprolyl isomerase B precursor | | | |
| 214894_x_at | 13.06 | 4.46E-06 | 1.057606359 | NM_012090| | MACF1,microfilament and actin filament cross-linker | | | |
| 212907_at | 13.06 | 4.46E-06 | 1.099114808 | NM_021194| | SLC30A1,solute carrier family 30 (zinc transporter), | | | |
| 220840_s_at | 13.03 | 4.55E-06 | 1.073269021 | NM_018186| | FLJ10706,hypothetical protein FLJ10706 | | | |
| 201412_at | 13.01 | 4.60E-06 | 1.123912704 | NM_014045| | LRP10,low density lipoprotein receptor-related protein | | | |
| 212651_at | 13.01 | 4.60E-06 | 1.181434342 | NM_001032380| | NA |  |  |  |
| 209070_s_at | 13.01 | 4.60E-06 | 1.300602682 | NM_003617| | RGS5,regulator of G-protein signalling 5 | | | |
| 227068_at | 13.01 | 4.60E-06 | 1.099349636 | NM_000291| | PGK1,phosphoglycerate kinase 1 | | |  |
| 228389_at | 13 | 4.61E-06 | 1.204880609 | NA |  |  |  |  |
| 212736_at | 13 | 4.60E-06 | 1.116302772 | NM_033201| | C16orf45,chromosome 16 open reading frame 45 | | | |
| 228260_at | 12.99 | 4.65E-06 | 1.127119684 | NM_004432| | ELAVL2,ELAV (embryonic lethal, abnormal vision, | | | |
| 224644_at | 12.99 | 4.63E-06 | 1.042485417 | NA |  |  |  |  |
| 214097_at | 12.98 | 4.69E-06 | 1.109297204 | NM_001024| | RPS21,ribosomal protein S21 | | |  |
| 204512_at | 12.98 | 4.68E-06 | 1.066315048 | NM_002114| | HIVEP1,human immunodeficiency virus type I enhancer | | | |
| 213188_s_at | 12.97 | 4.70E-06 | 1.24837439 | NM_001042533| | NA |  |  |  |
| 210186_s_at | 12.97 | 4.70E-06 | 1.145062456 | NM_000801| | FKBP1A,FK506-binding protein 1A | | |  |
| 212949_at | 12.97 | 4.69E-06 | 1.034250853 | NM_015341| | BRRN1,barren | |  |  |
| 226085_at | 12.97 | 4.70E-06 | 1.148602253 | NA |  |  |  |  |
| 236468_at | 12.96 | 4.70E-06 | 1.420599685 | NA |  |  |  |  |
| 212233_at | 12.96 | 4.71E-06 | 1.065052327 | NM_005909| | MAP1B,microtubule-associated protein 1B isoform 1 | | | |
| 241379_at | 12.96 | 4.71E-06 | 1.303862098 | NM_173545| | C2orf13,chromosome 2 open reading frame 13 | | | |
| 229013_at | 12.95 | 4.73E-06 | 1.183254782 | NA |  |  |  |  |
| 209278_s_at | 12.95 | 4.73E-06 | 1.223932399 | NM_006528| | TFPI2,tissue factor pathway inhibitor 2 | | |  |
| 224367_at | 12.95 | 4.73E-06 | 1.097183456 | NM_032621| | BEX2,brain expressed X-linked 2 | | |  |
| 225346_at | 12.95 | 4.73E-06 | 1.118139068 | NM_001033050| | NA |  |  |  |
| 222853_at | 12.94 | 4.77E-06 | 1.2228809 | NM_013281| | FLRT3,fibronectin leucine rich transmembrane protein 3 | | | |
| 209725_at | 12.93 | 4.78E-06 | 1.067841749 | NM_014503| | DRIM,down-regulated in metastasis | | |  |
| 1569349_at | 12.92 | 4.80E-06 | 1.437041998 | NM_020193| | C11orf30,EMSY protein | |  |  |
| 212985_at | 12.92 | 4.81E-06 | 1.078455468 | NA |  |  |  |  |
| 238554_at | 12.91 | 4.84E-06 | 1.260095304 | NM_030579| | CYB5-M,cytochrome b5 outer mitochondrial membrane | | | |
| 212488_at | 12.9 | 4.85E-06 | 1.161979334 | NM_000093| | COL5A1,alpha 1 type V collagen preproprotein | | | |
| 219837_s_at | 12.9 | 4.87E-06 | 1.334141705 | NM_018659| | CYTL1,cytokine-like 1 | |  |  |
| 228630_at | 12.9 | 4.85E-06 | 1.117623839 | NM_003428| | ZNF84,zinc finger protein 84 (HPF2) | | |  |
| 225422_at | 12.9 | 4.88E-06 | 1.070376761 | NM_139286| | CDC26,CDC26 subunit of anaphase promoting complex | | | |
| 225016_at | 12.86 | 4.98E-06 | 1.351130227 | NM_153000| | APCDD1,adenomatosis polyposis coli down-regulated 1 | | | |
| 201722_s_at | 12.86 | 4.97E-06 | 1.055385047 | NM_020474| | GALNT1,polypeptide N-acetylgalactosaminyltransferase 1 | | | |
| 218604_at | 12.85 | 4.99E-06 | 1.047100371 | NM_014319| | LEMD3,LEM domain containing 3 | | |  |
| 226609_at | 12.84 | 5.03E-06 | 1.17828499 | NM_173674| | DCBLD1,discoidin, CUB and LCCL domain containing 1 | | | |
| 209870_s_at | 12.84 | 5.03E-06 | 1.076474155 | NM_005503| | APBA2,amyloid beta A4 precursor protein-binding, | | | |
| 222661_at | 12.84 | 5.03E-06 | 1.065341051 | NM_018046| | AGGF1,angiogenic factor VG5Q | | |  |
| 215719_x_at | 12.84 | 5.03E-06 | 1.373547892 | NM_000043| | FAS,tumor necrosis factor receptor superfamily, | | | |
| 204703_at | 12.84 | 5.03E-06 | 1.084117434 | NM_006531| | TTC10,Tg737 protein isoform 2 | | |  |
| 208634_s_at | 12.83 | 5.04E-06 | 1.064038169 | NM_012090| | MACF1,microfilament and actin filament cross-linker | | | |
| 227548_at | 12.83 | 5.05E-06 | 1.143469365 | NM_016467| | ORMDL1,ORM1-like 1 | |  |  |
| 231798_at | 12.82 | 5.09E-06 | 1.379623417 | NM_005450| | NOG,noggin precursor | |  |  |
| 212872_s_at | 12.8 | 5.17E-06 | 1.049739478 | NM_004275| | USP49,TRF-proximal protein | | |  |
| 207046_at | 12.79 | 5.18E-06 | 1.19312093 | NM_001034077| | NA |  |  |  |
| 220295_x_at | 12.79 | 5.17E-06 | 1.067638496 | NM_001114120| | NA |  |  |  |
| 243198_at | 12.78 | 5.19E-06 | 1.313818758 | NM_198524| | TEX9,testis expressed gene 9 | | |  |
| 218708_at | 12.78 | 5.21E-06 | 1.062322403 | NM_013248| | NXT1,NTF2-like export factor 1 | | |  |
| 218248_at | 12.78 | 5.20E-06 | 1.125985511 | NM_022074| | FLJ22794,FLJ22794 protein | | |  |
| 223300_s_at | 12.77 | 5.23E-06 | 1.116500984 | NM_024725| | FLJ23518,hypothetical protein FLJ23518 | | | |
| 1557621_at | 12.76 | 5.27E-06 | 1.172402923 | NA |  |  |  |  |
| 235182_at | 12.75 | 5.29E-06 | 1.322314355 | NM_080826| | NA |  |  |  |
| 209227_at | 12.75 | 5.31E-06 | 1.155728497 | NM_006765| | TUSC3,tumor suppressor candidate 3 isoform a | | | |
| 214129_at | 12.74 | 5.31E-06 | 1.231582919 | NM_001002810| | PDE4DIP,phosphodiesterase 4D interacting protein isoform | | | |
| 213757_at | 12.74 | 5.31E-06 | 1.142937342 | NA |  |  |  |  |
| 213287_s_at | 12.73 | 5.34E-06 | 1.13337643 | NM_000421| | KRT10,keratin 10 | |  |  |
| 213243_at | 12.73 | 5.33E-06 | 1.121485247 | NM_015243| | COH1,Cohen syndrome 1 protein isoform 3 | | | |
| 227524_at | 12.72 | 5.35E-06 | 1.096341096 | NA |  |  |  |  |
| 225447_at | 12.71 | 5.39E-06 | 1.062797057 | NM_000408| | GPD2,glycerol-3-phosphate dehydrogenase 2 | | | |
| 207871_s_at | 12.71 | 5.38E-06 | 1.250789781 | NM_018412| | ST7,suppression of tumorigenicity 7 isoform a | | | |
| 231855_at | 12.71 | 5.39E-06 | 1.121146753 | NM_020890| | KIAA1524,KIAA1524 | |  |  |
| 222774_s_at | 12.7 | 5.44E-06 | 1.098097136 | NM_018092| | NETO2,neuropilin- and tolloid-like protein 2 | | | |
| 227449_at | 12.7 | 5.42E-06 | 1.12969616 | NM_004438| | EPHA4,ephrin receptor EphA4 | | |  |
| 205429_s_at | 12.69 | 5.44E-06 | 1.096103826 | NM_016447| | MPP6,membrane protein, palmitoylated 6 | | | |
| 230300_at | 12.69 | 5.45E-06 | 1.395018622 | NA |  |  |  |  |
| 215285_s_at | 12.68 | 5.48E-06 | 1.176612235 | NM_006608| | PHTF1,putative homeodomain transcription factor 1 | | | |
| 203103_s_at | 12.68 | 5.50E-06 | 1.048285171 | NM_014502| | PRP19,PRP19/PSO4 homolog | | |  |
| 214012_at | 12.67 | 5.51E-06 | 1.261261966 | NM_001040458| | NA |  |  |  |
| 1553171_x_at | 12.67 | 5.51E-06 | 1.259386534 | NM_152611| | C20orf75,chromosome 20 open reading frame 75 | | | |
| 219479_at | 12.67 | 5.53E-06 | 1.101096858 | NM_024089| | KDELC1,KDEL (Lys-Asp-Glu-Leu) containing 1 | | | |
| 212330_at | 12.67 | 5.51E-06 | 1.103271425 | NM_007111| | TFDP1,transcription factor Dp-1 | | |  |
| 213328_at | 12.66 | 5.55E-06 | 1.093117542 | NM_012224| | NEK1,NIMA (never in mitosis gene a)-related kinase 1 | | | |
| 238565_at | 12.65 | 5.57E-06 | 1.117947446 | NA |  |  |  |  |
| 244414_at | 12.65 | 5.57E-06 | 1.08684955 | NA |  |  |  |  |
| 228339_at | 12.65 | 5.57E-06 | 1.246533682 | NM_001077693| | NA |  |  |  |
| 217762_s_at | 12.65 | 5.58E-06 | 1.08282616 | NM_006868| | RAB31,RAB31, member RAS oncogene family | | | |
| 225643_at | 12.64 | 5.62E-06 | 1.0587883 | NM_144578| | C14orf32,MAPK-interacting and spindle-stabilizing | | | |
| 213139_at | 12.62 | 5.67E-06 | 1.788345607 | NM_003068| | SNAI2,snail 2 | |  |  |
| 216252_x_at | 12.62 | 5.69E-06 | 1.380072035 | NM_000043| | FAS,tumor necrosis factor receptor superfamily, | | | |
| 208616_s_at | 12.62 | 5.68E-06 | 1.024349694 | NM_080391| | PTP4A2,protein tyrosine phosphatase type IVA, member 2 | | | |
| 223393_s_at | 12.61 | 5.72E-06 | 1.139393217 | NM_020856| | ZNF537,zinc finger protein 537 | | |  |
| 211042_x_at | 12.61 | 5.71E-06 | 1.052364354 | NM_006500| | MCAM,melanoma cell adhesion molecule | | | |
| 223707_at | 12.6 | 5.74E-06 | 1.166169237 | NM_000990| | RPL27A,ribosomal protein L27a | | |  |
| 239069_s_at | 12.57 | 5.84E-06 | 1.293599059 | NA |  |  |  |  |
| 1564521_x_at | 12.57 | 5.85E-06 | 1.041386922 | NM_001039619| | NA |  |  |  |
| 213817_at | 12.55 | 5.94E-06 | 1.516073661 | NA |  |  |  |  |
| 215566_x_at | 12.55 | 5.94E-06 | 1.047264779 | NM_007260| | LYPLA2,lysophospholipase II | | |  |
| 222498_at | 12.55 | 5.91E-06 | 1.185903776 | NM_022461| | AZI2,5-azacytidine induced 2 isoform a | | |  |
| 243816_at | 12.55 | 5.91E-06 | 1.13257038 | NM_021916| | ZNF70,zinc finger protein 70 | | |  |
| 203753_at | 12.54 | 5.95E-06 | 1.047623969 | NM_001083962| | NA |  |  |  |
| 202535_at | 12.54 | 5.95E-06 | 1.057168738 | NM_003824| | FADD,Fas-associated via death domain | | |  |
| 227670_at | 12.53 | 5.99E-06 | 1.117911278 | NM_153028| | ZNF75A,zinc finger protein 75a | | |  |
| 201349_at | 12.53 | 5.97E-06 | 1.096213645 | NM_004252| | SLC9A3R1,solute carrier family 9 (sodium/hydrogen | | | |
| 239888_at | 12.53 | 5.99E-06 | 1.208934955 | NA |  |  |  |  |
| 230048_at | 12.52 | 6.03E-06 | 1.1672003 | NA |  |  |  |  |
| 204891_s_at | 12.52 | 6.03E-06 | 1.120367523 | NM_001042771| | NA |  |  |  |
| 212767_at | 12.52 | 6.01E-06 | 1.112212604 | NM_138384| | GTP,GTP_binding protein | |  |  |
| 219596_at | 12.51 | 6.03E-06 | 1.08300363 | NM_020147| | THAP10,THAP domain containing 10 | | |  |
| 224736_at | 12.51 | 6.05E-06 | 1.057175704 | NM_018237| | CCAR1,cell-cycle and apoptosis regulatory protein 1 | | | |
| 243681_at | 12.49 | 6.13E-06 | 1.309744729 | NM_012309| | SHANK2,SH3 and multiple ankyrin repeat domains 2 | | | |
| 203115_at | 12.49 | 6.13E-06 | 1.083707158 | NM_000140| | FECH,ferrochelatase isoform b precursor | | | |
| 229256_at | 12.49 | 6.14E-06 | 1.053814563 | NM_173582| | PGM2L1,phosphoglucomutase 2-like 1 | | |  |
| 213331_s_at | 12.48 | 6.17E-06 | 1.133417903 | NM_012224| | NEK1,NIMA (never in mitosis gene a)-related kinase 1 | | | |
| 219833_s_at | 12.48 | 6.17E-06 | 1.096969338 | NM_018100| | EFHC1,EF-hand domain (C-terminal) containing 1 | | | |
| 221229_s_at | 12.48 | 6.17E-06 | 1.077641671 | NM_017910| | FLJ20628,hypothetical protein FLJ20628 | | | |
| 213169_at | 12.47 | 6.20E-06 | 1.198963234 | NM_003966| | SEMA5A,semaphorin 5A | |  |  |
| 205466_s_at | 12.47 | 6.18E-06 | 1.36913803 | NM_005114| | HS3ST1,heparan sulfate D-glucosaminyl | | | |
| 238431_at | 12.47 | 6.20E-06 | 1.078614174 | NA |  |  |  |  |
| 1554572_a_at | 12.45 | 6.26E-06 | 1.078517378 | NM_024670| | SUV39H2,suppressor of variegation 3-9 homolog 2 | | | |
| 1557411_s_at | 12.44 | 6.32E-06 | 1.128739786 | NM_145305| | LOC203427,mitochondrial solute carrier protein | | | |
| 219521_at | 12.44 | 6.32E-06 | 1.096903909 | NM_018644| | B3GAT1,beta-1,3-glucuronyltransferase 1 | | | |
| 210445_at | 12.44 | 6.32E-06 | 1.354050117 | NM_001040442| | NA |  |  |  |
| 229007_at | 12.43 | 6.34E-06 | 1.309019748 | NA |  |  |  |  |
| 224937_at | 12.42 | 6.38E-06 | 1.077668746 | NM_020440| | PTGFRN,prostaglandin F2 receptor negative regulator | | | |
| 232037_at | 12.42 | 6.39E-06 | 1.16911867 | NM_004884| | PUNC,putative neuronal cell adhesion molecule | | | |
| 215016_x_at | 12.42 | 6.38E-06 | 1.075461265 | NM_001723| | DST,dystonin isoform 1e precursor | | |  |
| 33494_at | 12.41 | 6.40E-06 | 1.1307727 | NM_004453| | ETFDH,electron-transferring-flavoprotein | | | |
| 243840_at | 12.41 | 6.41E-06 | 1.115329869 | NA |  |  |  |  |
| 1554452_a_at | 12.4 | 6.43E-06 | 1.20049536 | NM_001098786| | NA |  |  |  |
| 214948_s_at | 12.4 | 6.43E-06 | 1.041504415 | NM_007114| | TMF1,TATA element modulatory factor 1 | | | |
| 203233_at | 12.38 | 6.54E-06 | 1.103422584 | NM_000418| | IL4R,interleukin 4 receptor alpha chain isoform a | | | |
| 218035_s_at | 12.38 | 6.54E-06 | 1.05942766 | NM_001098634| | NA |  |  |  |
| 205768_s_at | 12.38 | 6.54E-06 | 1.133787416 | NM_003645| | SLC27A2,solute carrier family 27 (fatty acid | | | |
| 223349_s_at | 12.37 | 6.58E-06 | 1.106498939 | NM_032515| | BOK,BCL2-related ovarian killer | | |  |
| 205822_s_at | 12.37 | 6.55E-06 | 1.223276751 | NM_001098272| | NA |  |  |  |
| 229285_at | 12.37 | 6.55E-06 | 1.397031257 | NM_021133| | RNASEL,ribonuclease L | |  |  |
| 214841_at | 12.36 | 6.61E-06 | 1.350620692 | NM_152495| | CNIH3,cornichon homolog 3 | | |  |
| 227763_at | 12.36 | 6.62E-06 | 1.312245415 | NM_194317| | MGC52057,hypothetical protein MGC52057 | | | |
| 213880_at | 12.36 | 6.58E-06 | 1.825401427 | NM_003667| | LGR5,leucine-rich repeat-containing G protein-coupled | | | |
| 206960_at | 12.35 | 6.65E-06 | 1.134750571 | NM_005296| | GPR23,G protein-coupled receptor 23 | | |  |
| 1552344_s_at | 12.35 | 6.65E-06 | 1.120134097 | NM_013354| | CNOT7,CCR4-NOT transcription complex, subunit 7 | | | |
| 202218_s_at | 12.35 | 6.64E-06 | 1.177414295 | NM_004265| | FADS2,fatty acid desaturase 2 | | |  |
| 204807_at | 12.34 | 6.68E-06 | 1.423921434 | NM_014254| | TMEM5,transmembrane protein 5 | | |  |
| 211126_s_at | 12.34 | 6.66E-06 | 1.080215146 | NM_001321| | CSRP2,cysteine and glycine-rich protein 2 | | | |
| 227928_at | 12.34 | 6.67E-06 | 1.13977426 | NM_017915| | FLJ20641,hypothetical protein FLJ20641 | | | |
| 200600_at | 12.34 | 6.66E-06 | 1.05431137 | NM_002444| | MSN,moesin |  |  |  |
| 223185_s_at | 12.33 | 6.71E-06 | 1.463201626 | NM_030762| | BHLHB3,basic helix-loop-helix domain containing, class | | | |
| 202126_at | 12.33 | 6.70E-06 | 1.068302386 | NM_003913| | PRPF4B,serine/threonine-protein kinase PRP4K | | | |
| 1557181_s_at | 12.32 | 6.72E-06 | 1.345821596 | NM_207645| | LOC399947,similar to expressed sequence AI593442 | | | |
| 235348_at | 12.32 | 6.75E-06 | 1.153389456 | NM_032859| | C13orf6,chromosome 13 open reading frame 6 | | | |
| 224663_s_at | 12.32 | 6.75E-06 | 1.121471035 | NM_021914| | CFL2,cofilin 2 | |  |  |
| 209167_at | 12.31 | 6.76E-06 | 1.158174779 | NM_001001994| | GPM6B,glycoprotein M6B isoform 4 | | |  |
| 203868_s_at | 12.31 | 6.75E-06 | 1.318000052 | NM_001078| | VCAM1,vascular cell adhesion molecule 1 isoform a | | | |
| 236584_at | 12.31 | 6.75E-06 | 1.131001635 | NA |  |  |  |  |
| 218639_s_at | 12.3 | 6.80E-06 | 1.117015451 | NM_001040653| | NA |  |  |  |
| 222233_s_at | 12.3 | 6.80E-06 | 1.171342736 | NM_001033855| | NA |  |  |  |
| 203941_at | 12.3 | 6.80E-06 | 1.081759895 | NM_018250| | FLJ10871,hypothetical protein FLJ10871 | | | |
| 212399_s_at | 12.29 | 6.86E-06 | 1.074433992 | NM_014667| | VGLL4,vestigial like 4 | |  |  |
| 233446_at | 12.28 | 6.89E-06 | 1.335960879 | NM_004852| | ONECUT2,one cut domain, family member 2 | | | |
| 200711_s_at | 12.28 | 6.87E-06 | 1.042245566 | NM_006930| | SKP1A,S-phase kinase-associated protein 1A isoform a | | | |
| 39248_at | 12.27 | 6.91E-06 | 1.244031164 | NM_004925| | AQP3,aquaporin 3 | |  |  |
| 200979_at | 12.27 | 6.91E-06 | 1.107251011 | NM_000284| | PDHA1,pyruvate dehydrogenase (lipoamide) alpha 1 | | | |
| 202075_s_at | 12.27 | 6.92E-06 | 1.11445169 | NM_006227| | PLTP,phospholipid transfer protein isoform a | | | |
| 212254_s_at | 12.27 | 6.91E-06 | 1.067695887 | NM_001723| | DST,dystonin isoform 1e precursor | | |  |
| 242838_at | 12.27 | 6.91E-06 | 1.281705619 | NM_024871| | FLJ12748,hypothetical protein FLJ12748 | | | |
| 221844_x_at | 12.26 | 6.94E-06 | 1.047949582 | NA |  |  |  |  |
| AFFX-HUMRGE/M10098_5_at | 12.26 | 6.95E-06 | 1.043749368 | NA |  |  |  |  |
| 228711_at | 12.26 | 6.95E-06 | 1.327573603 | NM_001007094| | ZNF37A,zinc finger protein 37a | | |  |
| 209001_s_at | 12.26 | 6.97E-06 | 1.070304202 | NM_015391| | ANAPC13,anaphase promoting complex subunit 13 | | | |
| 204361_s_at | 12.25 | 6.99E-06 | 1.346622784 | NM_003930| | SCAP2,src family associated phosphoprotein 2 | | | |
| 214247_s_at | 12.24 | 7.02E-06 | 1.086899043 | NM_001018057| | NA |  |  |  |
| 213145_at | 12.24 | 7.05E-06 | 1.098858596 | NM_152441| | FBXL14,F-box and leucine-rich repeat protein 14 | | | |
| 204508_s_at | 12.23 | 7.06E-06 | 1.263694985 | NM_001218| | CA12,carbonic anhydrase XII isoform 1 precursor | | | |
| 221813_at | 12.22 | 7.11E-06 | 1.051014354 | NM_018994| | NA |  |  |  |
| 237386_at | 12.21 | 7.15E-06 | 1.158438651 | NA |  |  |  |  |
| 210762_s_at | 12.21 | 7.15E-06 | 1.3453436 | NM_006094| | DLC1,deleted in liver cancer 1 isoform 2 | | | |
| 238949_at | 12.21 | 7.18E-06 | 1.182872832 | NM_144726| | FLJ31951,hypothetical protein FLJ31951 | | | |
| 226947_at | 12.21 | 7.17E-06 | 1.340554737 | NA |  |  |  |  |
| 223340_at | 12.2 | 7.19E-06 | 1.133114009 | NM_015915| | SPG3A,atlastin | |  |  |
| 203303_at | 12.2 | 7.22E-06 | 1.09052434 | NM_006520| | TCTE1L,t-complex-associated-testis-expressed 1-like | | | |
| 227277_at | 12.19 | 7.25E-06 | 1.250700255 | NA |  |  |  |  |
| 227904_at | 12.18 | 7.31E-06 | 1.155170545 | NM_022461| | AZI2,5-azacytidine induced 2 isoform a | | |  |
| 222753_s_at | 12.17 | 7.32E-06 | 1.135423111 | NM_021928| | SPCS3,signal peptidase complex subunit 3 homolog | | | |
| 235801_at | 12.17 | 7.36E-06 | 1.12952358 | NA |  |  |  |  |
| 230746_s_at | 12.17 | 7.36E-06 | 1.342878226 | NM_003155| | STC1,stanniocalcin 1 | |  |  |
| 235562_at | 12.16 | 7.38E-06 | 1.402286894 | NM_001025266| | NA |  |  |  |
| 1558368_s_at | 12.16 | 7.36E-06 | 1.105085667 | NM_198545| | LOC374946,hypothetical gene supported by AK075558; | | | |
| 238915_at | 12.16 | 7.38E-06 | 1.193075855 | NA |  |  |  |  |
| 214706_at | 12.15 | 7.41E-06 | 1.138622562 | NM_003454| | ZNF200,zinc finger protein 200 | | |  |
| 213397_x_at | 12.15 | 7.44E-06 | 1.203123752 | NM_002937| | RNASE4,ribonuclease, RNase A family, 4 precursor | | | |
| 202541_at | 12.15 | 7.42E-06 | 1.06061517 | NM_004757| | SCYE1,small inducible cytokine subfamily E, member 1 | | | |
| 230372_at | 12.14 | 7.46E-06 | 1.449173991 | NM_005328| | HAS2,hyaluronan synthase 2 | | |  |
| 208927_at | 12.14 | 7.46E-06 | 1.08883411 | NM_001007226| | SPOP,speckle-type POZ protein | | |  |
| 204813_at | 12.14 | 7.46E-06 | 1.13943733 | NM_002753| | MAPK10,mitogen-activated protein kinase 10 isoform 1 | | | |
| 208881_x_at | 12.13 | 7.54E-06 | 1.124735576 | NM_004508| | IDI1,isopentenyl-diphosphate delta isomerase | | | |
| 50374_at | 12.13 | 7.52E-06 | 1.067800915 | NM_001039842| | NA |  |  |  |
| 209286_at | 12.12 | 7.55E-06 | 1.248708316 | NM_006449| | CDC42EP3,Cdc42 effector protein 3 | | |  |
| 64488_at | 12.12 | 7.54E-06 | 1.070253503 | NA |  |  |  |  |
| 202051_s_at | 12.12 | 7.55E-06 | 1.051595253 | NM_005095| | ZNF262,zinc finger protein 262 | | |  |
| 225445_at | 12.12 | 7.54E-06 | 1.043690691 | NM_173569| | NA |  |  |  |
| 218357_s_at | 12.12 | 7.54E-06 | 1.075173744 | NM_012459| | TIMM8B,translocase of inner mitochondrial membrane 8 | | | |
| 1558292_s_at | 12.1 | 7.62E-06 | 1.173148245 | NM_178517| | PIGW,phosphatidylinositol glycan, class W | | | |
| 228805_at | 12.1 | 7.62E-06 | 1.084650243 | NM_198567| | FLJ44216,FLJ44216 protein | | |  |
| 236714_at | 12.1 | 7.62E-06 | 1.837135174 | NA |  |  |  |  |
| 235764_at | 12.09 | 7.66E-06 | 1.135939248 | NA |  |  |  |  |
| 211828_s_at | 12.09 | 7.68E-06 | 1.654954378 | NM_015028| | TNIK,TRAF2 and NCK interacting kinase | | |  |
| 244362_at | 12.09 | 7.66E-06 | 1.124238285 | NA |  |  |  |  |
| 200632_s_at | 12.09 | 7.69E-06 | 1.246206748 | NM_006096| | NDRG1,N-myc downstream regulated gene 1 | | | |
| 236297_at | 12.08 | 7.70E-06 | 1.155387232 | NA |  |  |  |  |
| 208858_s_at | 12.08 | 7.71E-06 | 1.067709317 | NM_015292| | MBC2,KIAA0747 protein | |  |  |
| 222982_x_at | 12.08 | 7.71E-06 | 1.034480131 | NM_018976| | SLC38A2,solute carrier family 38, member 2 | | | |
| 233078_at | 12.07 | 7.74E-06 | 1.50203659 | NM_006595| | API5,apoptosis inhibitor 5 | |  |  |
| 228764_s_at | 12.07 | 7.74E-06 | 1.105286857 | NM_014169| | C14orf123,Snf7 homologue associated with Alix 2 | | | |
| 215691_x_at | 12.07 | 7.74E-06 | 1.066278529 | NM_016126| | C1orf41,chromosome 1 open reading frame 41 | | | |
| 229464_at | 12.07 | 7.76E-06 | 1.073267977 | NM_016132| | MYEF2,myelin gene expression factor 2 | | |  |
| 220949_s_at | 12.06 | 7.80E-06 | 1.105373887 | NM_024033| | MGC5242,hypothetical protein MGC5242 | | | |
| 221646_s_at | 12.06 | 7.79E-06 | 1.107393627 | NM_024786| | ZDHHC11,zinc finger, DHHC domain containing 11 | | | |
| 230964_at | 12.06 | 7.77E-06 | 1.087578694 | NM_207361| | FREM2,FRAS1 related extracellular matrix protein 2 | | | |
| 212325_at | 12.06 | 7.80E-06 | 1.164025233 | NM_001112717| | NA |  |  |  |
| 218350_s_at | 12.05 | 7.85E-06 | 1.063020373 | NM_015895| | GMNN,geminin | |  |  |
| 238166_s_at | 12.05 | 7.82E-06 | 1.369940674 | NM_001113434| | NA |  |  |  |
| 217047_s_at | 12.04 | 7.87E-06 | 1.122806056 | NM_001015045| | NA |  |  |  |
| 202586_at | 12.03 | 7.95E-06 | 1.129384375 | NM_021128| | POLR2L,DNA directed RNA polymerase II polypeptide L | | | |
| 213911_s_at | 12.03 | 7.93E-06 | 1.028857226 | NM_002106| | H2AFZ,H2A histone family, member Z | | |  |
| 224839_s_at | 12.03 | 7.93E-06 | 1.093634 | NM_133443| | GPT2,alanine aminotransferase 2 | | |  |
| 218891_at | 12.02 | 8.00E-06 | 1.100529931 | NM_024541| | C10orf76,chromosome 10 open reading frame 76 | | | |
| 215165_x_at | 12.02 | 7.99E-06 | 1.038910942 | NM_000373| | UMPS,uridine monophosphate synthase | | | |
| 217769_s_at | 12.02 | 7.97E-06 | 1.028569912 | NM_015932| | C13orf12,chromosome 13 open reading frame 12 | | | |
| 202644_s_at | 12.02 | 7.97E-06 | 1.14194747 | NM_006290| | TNFAIP3,tumor necrosis factor, alpha-induced protein 3 | | | |
| 222958_s_at | 12.02 | 7.97E-06 | 1.061033382 | NM_001114120| | NA |  |  |  |
| 221582_at | 11.97 | 8.24E-06 | 1.1359677 | NM_033445| | HIST3H2A,histone H2a | |  |  |
| 239292_at | 11.97 | 8.24E-06 | 1.160108969 | NA |  |  |  |  |
| 221667_s_at | 11.97 | 8.24E-06 | 1.192516648 | NM_014365| | HSPB8,heat shock 27kDa protein 8 | | |  |
| 206702_at | 11.96 | 8.31E-06 | 1.289176251 | NM_000459| | TEK,TEK tyrosine kinase, endothelial | | |  |
| 227566_at | 11.96 | 8.29E-06 | 1.212037851 | NM_001048209| | NA |  |  |  |
| 218003_s_at | 11.96 | 8.31E-06 | 1.038864985 | NM_002013| | FKBP3,FK506-binding protein 3 | | |  |
| 202732_at | 11.95 | 8.38E-06 | 1.08170638 | NM_007066| | PKIG,cAMP-dependent protein kinase inhibitor gamma | | | |
| 209890_at | 11.95 | 8.38E-06 | 1.098582194 | NM_005723| | TM4SF9,transmembrane 4 superfamily member 9 | | | |
| 201664_at | 11.94 | 8.39E-06 | 1.062307429 | NM_001002800| | SMC4L1,SMC4 structural maintenance of chromosomes | | | |
| 209585_s_at | 11.94 | 8.39E-06 | 1.050022888 | NM_004897| | MINPP1,multiple inositol polyphosphate histidine | | | |
| 217845_x_at | 11.94 | 8.40E-06 | 1.039602347 | NM_001099668| | NA |  |  |  |
| 225800_at | 11.94 | 8.38E-06 | 1.115678271 | NM_175061| | JAZF1,juxtaposed with another zinc finger gene 1 | | | |
| 204396_s_at | 11.94 | 8.38E-06 | 1.248481334 | NM_005308| | GRK5,G protein-coupled receptor kinase 5 | | | |
| 227935_s_at | 11.94 | 8.38E-06 | 1.088433449 | NM_032373| | PCGF5,polycomb group ring finger 5 | | |  |
| 40562_at | 11.94 | 8.39E-06 | 1.063836333 | NM_002067| | GNA11,guanine nucleotide binding protein (G protein), | | | |
| 225793_at | 11.94 | 8.39E-06 | 1.137784284 | NM_153713| | LIX1L,Lix1 homolog (mouse) like | | |  |
| 226625_at | 11.93 | 8.43E-06 | 1.109807867 | NM_003243| | TGFBR3,transforming growth factor, beta receptor III | | | |
| 206440_at | 11.92 | 8.50E-06 | 1.140273768 | NM_004664| | LIN7A,lin-7 homolog A | |  |  |
| 225893_at | 11.92 | 8.51E-06 | 1.047319426 | NM_172071| | NA |  |  |  |
| 1558164_s_at | 11.92 | 8.50E-06 | 1.096061746 | NM_002618| | PEX13,peroxisome biogenesis factor 13 | | | |
| 201425_at | 11.92 | 8.51E-06 | 1.05084386 | NM_000690| | ALDH2,mitochondrial aldehyde dehydrogenase 2 | | | |
| 221606_s_at | 11.92 | 8.50E-06 | 1.146685707 | NM_030763| | NSBP1,nucleosomal binding protein 1 | | |  |
| 223839_s_at | 11.92 | 8.50E-06 | 1.152287745 | NA |  |  |  |  |
| 244835_at | 11.91 | 8.51E-06 | 1.26752041 | NM_173501| | LOC146174,hypothetical protein LOC146174 | | | |
| 232569_at | 11.9 | 8.62E-06 | 1.276394566 | NA |  |  |  |  |
| 227929_at | 11.9 | 8.58E-06 | 1.202537392 | NA |  |  |  |  |
| 227110_at | 11.9 | 8.57E-06 | 1.076851093 | NM_001077442| | NA |  |  |  |
| 223618_at | 11.88 | 8.70E-06 | 1.273142026 | NM_020066| | FMN2,formin 2 | |  |  |
| 219990_at | 11.88 | 8.70E-06 | 1.164577123 | NM_024680| | FLJ23311,FLJ23311 protein | | |  |
| 202478_at | 11.88 | 8.70E-06 | 1.169063716 | NM_021643| | TRIB2,tribbles homolog 2 | |  |  |
| 226082_s_at | 11.88 | 8.69E-06 | 1.050496271 | NM_020706| | SFRS15,splicing factor, arginine/serine-rich 15 | | | |
| 226190_at | 11.88 | 8.70E-06 | 1.112582234 | NA |  |  |  |  |
| 238015_at | 11.88 | 8.70E-06 | 1.095153902 | NM_001008393| | LOC201725,hypothetical protein LOC201725 | | | |
| 211958_at | 11.88 | 8.70E-06 | 1.193033725 | NM_000599| | IGFBP5,insulin-like growth factor binding protein 5 | | | |
| 218516_s_at | 11.88 | 8.69E-06 | 1.212946601 | NM_017813| | IMPA3,myo-inositol monophosphatase A3 | | | |
| 227959_at | 11.87 | 8.76E-06 | 1.06249298 | NA |  |  |  |  |
| 1556283_s_at | 11.87 | 8.73E-06 | 1.290258885 | NM_015633| | FGFR1OP2,FGFR1 oncogene partner 2 | | |  |
| 1566303_s_at | 11.87 | 8.76E-06 | 1.089777012 | NM_021959| | PPP1R11,protein phosphatase 1, regulatory (inhibitor) | | | |
| 244111_at | 11.87 | 8.74E-06 | 1.486149309 | NM_152349| | MGC45562,hypothetical protein MGC45562 | | | |
| 229614_at | 11.86 | 8.76E-06 | 1.109112964 | NM_207333| | NA |  |  |  |
| 202969_at | 11.86 | 8.76E-06 | 1.072266266 | NM_003583| | DYRK2,dual-specificity tyrosine-(Y)-phosphorylation | | | |
| 225368_at | 11.86 | 8.77E-06 | 1.031046095 | NM_001113239| | NA |  |  |  |
| 243309_at | 11.86 | 8.76E-06 | 1.335621289 | NA |  |  |  |  |
| 219017_at | 11.85 | 8.85E-06 | 1.163764977 | NM_001039481| | NA |  |  |  |
| 218578_at | 11.85 | 8.83E-06 | 1.100521078 | NM_024529| | HRPT2,parafibromin | |  |  |
| 203008_x_at | 11.85 | 8.84E-06 | 1.051877052 | NM_005783| | TXNDC9,ATP binding protein associated with cell | | | |
| 201626_at | 11.84 | 8.87E-06 | 1.192302067 | NM_005542| | INSIG1,insulin induced gene 1 isoform 1 | | | |
| 215051_x_at | 11.84 | 8.89E-06 | 1.138771414 | NM_001623| | AIF1,allograft inflammatory factor 1 isoform 3 | | | |
| 213838_at | 11.84 | 8.90E-06 | 1.10768603 | NM_016167| | NOL7,nucleolar protein 7, 27kDa | | |  |
| 230445_at | 11.83 | 8.96E-06 | 1.304740356 | NM_001080466| | NA |  |  |  |
| 202511_s_at | 11.83 | 8.96E-06 | 1.042556461 | NM_004849| | APG5L,APG5 autophagy 5-like | | |  |
| 212973_at | 11.83 | 8.94E-06 | 1.05753252 | NM_144563| | RPIA,ribose 5-phosphate isomerase A (ribose | | | |
| 207023_x_at | 11.82 | 9.03E-06 | 1.130512772 | NM_000421| | KRT10,keratin 10 | |  |  |
| 221710_x_at | 11.82 | 8.98E-06 | 1.104711514 | NM_018166| | FLJ10647,hypothetical protein FLJ10647 | | | |
| 229606_at | 11.81 | 9.04E-06 | 1.189094045 | NA |  |  |  |  |
| 209563_x_at | 11.8 | 9.09E-06 | 1.02728355 | NM_001743| | CALM2,calmodulin 2 | |  |  |
| 1562921_at | 11.8 | 9.09E-06 | 1.274314192 | NA |  |  |  |  |
| 225144_at | 11.79 | 9.15E-06 | 1.118918416 | NM_001204| | BMPR2,bone morphogenetic protein receptor, type II | | | |
| 224702_at | 11.79 | 9.19E-06 | 1.049903713 | NM_174909| | MGC23909,hypothetical protein MGC23909 | | | |
| 232254_at | 11.79 | 9.16E-06 | 1.135397139 | NA |  |  |  |  |
| 204780_s_at | 11.78 | 9.20E-06 | 1.333519044 | NM_000043| | FAS,tumor necrosis factor receptor superfamily, | | | |
| 238467_at | 11.78 | 9.24E-06 | 1.261810874 | NA |  |  |  |  |
| 224774_s_at | 11.78 | 9.20E-06 | 1.165653741 | NM_020443| | NAV1,neuron navigator 1 | |  |  |
| 217905_at | 11.78 | 9.21E-06 | 1.100809242 | NM_024834| | C10orf119,chromosome 10 open reading frame 119 | | | |
| 237719_x_at | 11.78 | 9.21E-06 | 1.258784238 | NM_001029875| | NA |  |  |  |
| 232760_at | 11.77 | 9.25E-06 | 1.104472515 | NM_031271| | TEX15,testis expressed sequence 15 | | |  |
| 224962_at | 11.77 | 9.27E-06 | 1.049909046 | NM_152833| | NA |  |  |  |
| 236004_at | 11.77 | 9.26E-06 | 1.092587783 | NA |  |  |  |  |
| 227847_at | 11.77 | 9.25E-06 | 1.169280762 | NM_014805| | EPM2AIP1,EPM2A interacting protein 1 | | |  |
| 203139_at | 11.77 | 9.25E-06 | 1.028194901 | NM_004938| | DAPK1,death-associated protein kinase 1 | | | |
| 236278_at | 11.76 | 9.33E-06 | 1.507430608 | NM_003532| | HIST1H3E,H3 histone family, member D | | | |
| 232136_s_at | 11.76 | 9.35E-06 | 1.148120274 | NM_033427| | CTTNBP2,cortactin binding protein 2 | | |  |
| 201586_s_at | 11.75 | 9.37E-06 | 1.073985944 | NM_005066| | SFPQ,splicing factor proline/glutamine rich | | | |
| 225496_s_at | 11.75 | 9.37E-06 | 1.448936247 | NM_032379| | SYTL2,synaptotagmin-like 2 isoform b | | |  |
| 202492_at | 11.75 | 9.38E-06 | 1.076998507 | NM_001077198| | NA |  |  |  |
| 218170_at | 11.75 | 9.37E-06 | 1.104366059 | NM_016048| | ISOC1,isochorismatase domain containing 1 | | | |
| 225174_at | 11.74 | 9.46E-06 | 1.045192526 | NM_018981| | DNAJC10,DnaJ (Hsp40) homolog, subfamily C, member 10 | | | |
| 221729_at | 11.74 | 9.41E-06 | 1.243416867 | NM_000393| | COL5A2,alpha 2 type V collagen preproprotein | | | |
| 202076_at | 11.74 | 9.45E-06 | 1.030436668 | NM_001166| | BIRC2,baculoviral IAP repeat-containing protein 2 | | | |
| 209497_s_at | 11.73 | 9.51E-06 | 1.108798371 | NM_031492| | RBM30,RNA binding motif protein 30 | | |  |
| 225639_at | 11.73 | 9.46E-06 | 1.310702904 | NM_003930| | SCAP2,src family associated phosphoprotein 2 | | | |
| 229888_at | 11.72 | 9.55E-06 | 1.221976596 | NM_175874| | MGC47869,hypothetical protein MGC47869 | | | |
| 206026_s_at | 11.72 | 9.53E-06 | 1.365292225 | NM_007115| | TNFAIP6,tumor necrosis factor, alpha-induced protein 6 | | | |
| 210174_at | 11.71 | 9.60E-06 | 1.449951168 | NM_003822| | NR5A2,nuclear receptor subfamily 5, group A, member 2 | | | |
| 212956_at | 11.71 | 9.61E-06 | 1.153455014 | NM_015130| | KIAA0882,KIAA0882 protein | | |  |
| 228073_at | 11.71 | 9.60E-06 | 1.121895186 | NM_152667| | HDHD4,haloacid dehalogenase-like hydrolase domain | | | |
| 222028_at | 11.69 | 9.77E-06 | 1.103005628 | NM_003425| | ZNF45,zinc finger protein 45 | | |  |
| 202311_s_at | 11.68 | 9.82E-06 | 1.142191398 | NM_000088| | COL1A1,alpha 1 type I collagen preproprotein | | | |
| 212415_at | 11.67 | 9.85E-06 | 1.120754276 | NM_015129| | SEPT6,septin 6 isoform B | |  |  |
| 228574_at | 11.67 | 9.84E-06 | 1.09354426 | NM_152588| | DKFZp762A217,hypothetical protein DKFZp762A217 | | | |
| 205733_at | 11.67 | 9.88E-06 | 1.063588785 | NM_000057| | BLM,Bloom syndrome protein | | |  |
| 212083_at | 11.67 | 9.87E-06 | 1.142757886 | NM_144582| | TEX261,testis expressed gene 261 | | |  |
| 225575_at | 11.66 | 9.89E-06 | 1.124599405 | NM_002310| | LIFR, |  |  |  |
| 219670_at | 11.66 | 9.90E-06 | 1.213313419 | NM_024603| | FLJ11588,hypothetical protein FLJ11588 | | | |
| 221471_at | 11.65 | 9.97E-06 | 1.058961188 | NM_006811| | TDE1,tumor differentially expressed protein 1 | | | |
| 233675_s_at | 11.64 | 1.01E-05 | 1.279985716 | NA |  |  |  |  |
| 203359_s_at | 11.64 | 1.01E-05 | 1.041057528 | NM_012333| | MYCBP,c-myc binding protein | | |  |
| 1558152_at | 11.63 | 1.01E-05 | 1.152564138 | NA |  |  |  |  |
| 218987_at | 11.63 | 1.01E-05 | 1.074712926 | NM_018179| | ATF7IP,activating transcription factor 7 interacting | | | |
| 227022_at | 11.63 | 1.01E-05 | 1.107416277 | NM_138335| | GNPDA2,glucosamine-6-phosphate deaminase 2 | | | |
| 224996_at | 11.62 | 1.02E-05 | 1.077500894 | NA |  |  |  |  |
| 224446_at | 11.62 | 1.02E-05 | 1.051389999 | NM_032338| | MGC14817,hypothetical protein MGC14817 | | | |
| 224909_s_at | 11.62 | 1.01E-05 | 1.159509154 | NM_020820| | PREX1,PREX1 protein | |  |  |
| 222549_at | 11.61 | 1.02E-05 | 1.163154802 | NM_021101| | CLDN1,claudin 1 | |  |  |
| 220486_x_at | 11.61 | 1.02E-05 | 1.063631482 | NM_032227| | FLJ22679,hypothetical protein FLJ22679 | | | |
| 1556060_a_at | 11.61 | 1.02E-05 | 1.094593787 | NA |  |  |  |  |
| 238067_at | 11.61 | 1.02E-05 | 1.30369006 | NM_017752| | FLJ20298,FLJ20298 protein isoform a | | |  |
| 218333_at | 11.6 | 1.03E-05 | 1.082736265 | NM_016041| | DERL2,Der1-like domain family, member 2 | | | |
| 238439_at | 11.6 | 1.03E-05 | 1.498423602 | NM_144590| | ANKRD22,ankyrin repeat domain 22 | | |  |
| 220281_at | 11.6 | 1.03E-05 | 1.711477606 | NM_000338| | SLC12A1,sodium potassium chloride cotransporter 2 | | | |
| 222406_s_at | 11.6 | 1.03E-05 | 1.091141747 | NM_017761| | PNRC2,proline-rich nuclear receptor coactivator 2 | | | |
| 218100_s_at | 11.6 | 1.03E-05 | 1.121062833 | NM_018010| | ESRRBL1,estrogen-related receptor beta like 1 | | | |
| 204944_at | 11.58 | 1.04E-05 | 1.046419848 | NM_002841| | PTPRG,protein tyrosine phosphatase, receptor type, G | | | |
| 209194_at | 11.58 | 1.04E-05 | 1.055974184 | NM_004344| | CETN2,caltractin | |  |  |
| 230240_at | 11.58 | 1.04E-05 | 1.164913138 | NA |  |  |  |  |
| 214664_at | 11.57 | 1.05E-05 | 1.248364694 | NM_001079524| | NA |  |  |  |
| 204080_at | 11.57 | 1.05E-05 | 1.086953326 | NM_025077| | TOE1,target of EGR1, member 1 (nuclear) | | | |
| 227385_at | 11.57 | 1.05E-05 | 1.077433187 | NM_203453| | LOC403313,hypothetical LOC403313 | | |  |
| 218681_s_at | 11.57 | 1.05E-05 | 1.094980085 | NM_022044| | SDF2L1,stromal cell-derived factor 2-like 1 precursor | | | |
| 207009_at | 11.56 | 1.05E-05 | 1.139066158 | NM_003924| | PHOX2B,paired-like homeobox 2b | | |  |
| 209550_at | 11.56 | 1.05E-05 | 1.079498747 | NM_002487| | NDN,necdin |  |  |  |
| 243372_at | 11.55 | 1.06E-05 | 1.167551702 | NM_002156| | HSPD1,chaperonin | |  |  |
| 218875_s_at | 11.54 | 1.07E-05 | 1.065870618 | NM_012177| | FBXO5,F-box only protein 5 | | |  |
| 228060_at | 11.53 | 1.08E-05 | 1.074984689 | NM_001029858| | NA |  |  |  |
| 200665_s_at | 11.53 | 1.08E-05 | 1.039590366 | NM_003118| | SPARC,secreted protein, acidic, cysteine-rich | | | |
| 1552309_a_at | 11.53 | 1.08E-05 | 1.193362893 | NM_144573| | NEXN,nexilin (F actin binding protein) | | |  |
| 209045_at | 11.53 | 1.08E-05 | 1.055052774 | NM_020383| | XPNPEP1,X-prolyl aminopeptidase (aminopeptidase P) 1, | | | |
| 202626_s_at | 11.53 | 1.08E-05 | 1.088331526 | NM_001111097| | NA |  |  |  |
| 214695_at | 11.52 | 1.08E-05 | 1.17889916 | NM_014847| | UBAP2L,ubiquitin associated protein 2-like | | | |
| 235581_at | 11.52 | 1.08E-05 | 1.28407018 | NA |  |  |  |  |
| AFFX-M27830_5_at | 11.52 | 1.08E-05 | 1.060271506 | NA |  |  |  |  |
| 209514_s_at | 11.52 | 1.08E-05 | 1.158753725 | NM_004580| | RAB27A,Ras-related protein Rab-27A | | |  |
| 226800_at | 11.52 | 1.08E-05 | 1.184928417 | NM_032437| | KIAA1799,KIAA1799 protein | | |  |
| 564_at | 11.51 | 1.08E-05 | 1.052678713 | NM_002067| | GNA11,guanine nucleotide binding protein (G protein), | | | |
| 200875_s_at | 11.5 | 1.09E-05 | 1.043614842 | NM_006392| | NOL5A,nucleolar protein 5A | | |  |
| 202972_s_at | 11.5 | 1.10E-05 | 1.137691394 | NM_001015045| | NA |  |  |  |
| 219446_at | 11.5 | 1.10E-05 | 1.060840414 | NM_018157| | hSyn,brain synembryn | |  |  |
| 1556301_at | 11.5 | 1.09E-05 | 1.121177496 | NA |  |  |  |  |
| 220345_at | 11.5 | 1.09E-05 | 1.235232761 | NM_024993| | LRRTM4,leucine rich repeat transmembrane neuronal 4 | | | |
| 225387_at | 11.49 | 1.10E-05 | 1.103482729 | NM_005723| | TM4SF9,transmembrane 4 superfamily member 9 | | | |
| 200906_s_at | 11.49 | 1.10E-05 | 1.082376199 | NM_016081| | KIAA0992,palladin | |  |  |
| 213008_at | 11.49 | 1.10E-05 | 1.029501711 | NM_001113378| | NA |  |  |  |
| 210910_s_at | 11.49 | 1.10E-05 | 1.195926022 | NM_012230| | POMZP3,POMZP3 fusion protein isoform 1 | | | |
| 204066_s_at | 11.49 | 1.10E-05 | 1.048425459 | NM_001037131| | NA |  |  |  |
| 218534_s_at | 11.49 | 1.10E-05 | 1.071690329 | NM_018046| | AGGF1,angiogenic factor VG5Q | | |  |
| 208941_s_at | 11.49 | 1.10E-05 | 1.035452279 | NM_012247| | SEPHS1,selenophosphate synthetase | | |  |
| 208322_s_at | 11.48 | 1.10E-05 | 1.132502747 | NM_003033| | ST3GAL1,sialyltransferase 4A | | |  |
| 230248_x_at | 11.48 | 1.10E-05 | 1.305939965 | NA |  |  |  |  |
| 212126_at | 11.47 | 1.11E-05 | 1.044629322 | NA |  |  |  |  |
| 208732_at | 11.47 | 1.11E-05 | 1.069639771 | NM_002865| | RAB2,RAB2, member RAS oncogene family | | | |
| 216606_x_at | 11.46 | 1.12E-05 | 1.052203968 | NA |  |  |  |  |
| 225855_at | 11.46 | 1.12E-05 | 1.110619805 | NM_020909| | EPB41L5,erythrocyte membrane protein band 4.1 like 5 | | | |
| 217404_s_at | 11.46 | 1.12E-05 | 1.449624185 | NM_001844| | COL2A1,alpha 1 type II collagen isoform 1 | | | |
| 204042_at | 11.46 | 1.12E-05 | 1.045848884 | NM_006646| | WASF3,WAS protein family, member 3 | | |  |
| 208555_x_at | 11.46 | 1.12E-05 | 1.110699129 | NM_001322| | CST2,cystatin SA precursor | | |  |
| 224959_at | 11.46 | 1.12E-05 | 1.093639732 | NM_000112| | SLC26A2,solute carrier family 26 member 2 | | | |
| 204043_at | 11.46 | 1.12E-05 | 1.089767441 | NM_000355| | TCN2,transcobalamin II precursor | | |  |
| 224717_s_at | 11.46 | 1.12E-05 | 1.079385073 | NM_024104| | MGC2747,hypothetical protein MGC2747 | | | |
| 209711_at | 11.45 | 1.13E-05 | 1.180199408 | NM_015139| | SLC35D1,solute carrier family 35 (UDP-glucuronic | | | |
| 210560_at | 11.45 | 1.13E-05 | 1.322918236 | NM_001485| | GBX2,gastrulation brain homeo box 2 | | |  |
| 224341_x_at | 11.45 | 1.13E-05 | 1.311203905 | NM_138554| | TLR4,toll-like receptor 4 isoform A | | |  |
| 213934_s_at | 11.45 | 1.13E-05 | 1.078280137 | NM_145911| | ZNF23,zinc finger protein 23 | | |  |
| 232051_at | 11.44 | 1.13E-05 | 1.142383203 | NM_033212| | MGC10992,hypothetical protein LOC92922 | | | |
| 215146_s_at | 11.44 | 1.13E-05 | 1.115763571 | NA |  |  |  |  |
| 201016_at | 11.44 | 1.13E-05 | 1.078973539 | NM_001412| | EIF1AX,X-linked eukaryotic translation initiation | | | |
| 200043_at | 11.44 | 1.13E-05 | 1.025094716 | NM_004450| | ERH,enhancer of rudimentary homolog | | |  |
| 214862_x_at | 11.44 | 1.13E-05 | 1.282096008 | NA |  |  |  |  |
| 213677_s_at | 11.43 | 1.14E-05 | 1.032616153 | NM_000534| | PMS1,postmeiotic segregation 1 | | |  |
| 239138_at | 11.43 | 1.14E-05 | 1.371272789 | NA |  |  |  |  |
| 212442_s_at | 11.43 | 1.14E-05 | 1.044816915 | NM_203463| | LASS6,longevity assurance homolog 6 | | |  |
| 217738_at | 11.42 | 1.15E-05 | 1.048716995 | NM_005746| | PBEF1,pre-B-cell colony enhancing factor 1 isoform a | | | |
| 226439_s_at | 11.42 | 1.15E-05 | 1.093139786 | NM_015678| | NBEA,neurobeachin | |  |  |
| 218540_at | 11.42 | 1.15E-05 | 1.064506304 | NM_001126339| | NA |  |  |  |
| 1553167_a_at | 11.42 | 1.15E-05 | 1.214143782 | NM_016955| | SLA/LP,soluble liver antigen/liver pancreas antigen | | | |
| 235424_at | 11.41 | 1.15E-05 | 1.249412669 | NA |  |  |  |  |
| 230795_at | 11.41 | 1.15E-05 | 1.406348091 | NA |  |  |  |  |
| 202196_s_at | 11.41 | 1.15E-05 | 1.100682641 | NM_001018057| | NA |  |  |  |
| 1552310_at | 11.41 | 1.15E-05 | 1.078570972 | NM_144597| | MGC29937,hypothetical protein MGC29937 | | | |
| 1569788_at | 11.4 | 1.16E-05 | 1.205889453 | NM_003034| | ST8SIA1,ST8 alpha-N-acetyl-neuraminide | | | |
| 218482_at | 11.4 | 1.16E-05 | 1.054893739 | NM_020189| | e(y)2,e(y)2 protein | |  |  |
| 237706_at | 11.4 | 1.16E-05 | 1.151288342 | NM_178509| | STXBP4,syntaxin binding protein 4 | | |  |
| 227921_at | 11.4 | 1.16E-05 | 1.212603856 | NA |  |  |  |  |
| 200832_s_at | 11.4 | 1.16E-05 | 1.072881116 | NM_005063| | SCD,stearoyl-CoA desaturase | | |  |
| 209901_x_at | 11.39 | 1.17E-05 | 1.141601474 | NM_001623| | AIF1,allograft inflammatory factor 1 isoform 3 | | | |
| 215535_s_at | 11.39 | 1.16E-05 | 1.125174514 | NM_006411| | AGPAT1,1-acylglycerol-3-phosphate O-acyltransferase 1 | | | |
| 212901_s_at | 11.39 | 1.17E-05 | 1.04117503 | NM_015235| | CSTF2T,cleavage stimulation factor, 3' pre-RNA, subunit | | | |
| 203440_at | 11.39 | 1.17E-05 | 1.108785481 | NM_001792| | CDH2,cadherin 2, type 1 preproprotein | | |  |
| 218570_at | 11.39 | 1.16E-05 | 1.08854781 | NM_016506| | KBTBD4,kelch repeat and BTB (POZ) domain containing 4 | | | |
| 218139_s_at | 11.38 | 1.17E-05 | 1.073610277 | NM_018229| | C14orf108,chromosome 14 open reading frame 108 | | | |
| 1566509_s_at | 11.37 | 1.18E-05 | 1.14240992 | NM_012347| | FBXO9,F-box only protein 9 isoform 1 | | |  |
| 235690_at | 11.37 | 1.18E-05 | 1.265187401 | NM_032530| | NA |  |  |  |
| 212855_at | 11.36 | 1.19E-05 | 1.112119449 | NM_001040402| | NA |  |  |  |
| 239710_at | 11.36 | 1.19E-05 | 1.074289555 | NM_018086| | FIGN,fidgetin | |  |  |
| 221778_at | 11.36 | 1.19E-05 | 1.041032219 | NM_030647| | NA |  |  |  |
| 209683_at | 11.35 | 1.20E-05 | 1.438064385 | NM_030797| | FAM49A,family with sequence similarity 49, member A | | | |
| 203291_at | 11.35 | 1.19E-05 | 1.064595673 | NM_001008225| | CNOT4,CCR4-NOT transcription complex, subunit 4 | | | |
| 32099_at | 11.34 | 1.20E-05 | 1.093311524 | NM_014649| | SAFB2,scaffold attachment factor B2 | | |  |
| 242539_at | 11.34 | 1.20E-05 | 1.320593182 | NA |  |  |  |  |
| 204604_at | 11.33 | 1.21E-05 | 1.089918607 | NM_012395| | PFTK1,PFTAIRE protein kinase 1 | | |  |
| 212205_at | 11.33 | 1.21E-05 | 1.068154636 | NM_012412| | H2AFV,H2A histone family, member V isoform 1 | | | |
| 201248_s_at | 11.33 | 1.21E-05 | 1.056295078 | NM_004599| | SREBF2,sterol regulatory element-binding transcription | | | |
| 220651_s_at | 11.33 | 1.21E-05 | 1.081760751 | NM_018518| | MCM10,minichromosome maintenance protein 10 isoform 2 | | | |
| 226269_at | 11.32 | 1.22E-05 | 1.135116113 | NM_001040875| | NA |  |  |  |
| 218193_s_at | 11.32 | 1.22E-05 | 1.091615066 | NM_016072| | GOLT1B,golgi transport 1 homolog B | | |  |
| 224597_at | 11.32 | 1.22E-05 | 1.058978749 | NA |  |  |  |  |
| 202294_at | 11.32 | 1.22E-05 | 1.181343246 | NM_005862| | STAG1,stromal antigen 1 | |  |  |
| 202735_at | 11.32 | 1.22E-05 | 1.085030783 | NM_006579| | EBP,emopamil binding protein (sterol isomerase) | | | |
| 205702_at | 11.32 | 1.22E-05 | 1.141550478 | NM_006608| | PHTF1,putative homeodomain transcription factor 1 | | | |
| 227856_at | 11.31 | 1.23E-05 | 1.426009709 | NM_152400| | FLJ39370,hypothetical protein FLJ39370 | | | |
| 36552_at | 11.31 | 1.22E-05 | 1.073363941 | NM_015531| | DKFZP586P0123,DKFZP586P0123 protein | | | |
| 226762_at | 11.3 | 1.23E-05 | 1.062847051 | NM_033224| | PURB,purine-rich element binding protein B | | | |
| 225200_at | 11.3 | 1.23E-05 | 1.078078139 | NM_001047434| | NA |  |  |  |
| 203422_at | 11.3 | 1.23E-05 | 1.047302053 | NM_002691| | POLD1,polymerase (DNA directed), delta 1, catalytic | | | |
| 201915_at | 11.3 | 1.23E-05 | 1.08990354 | NM_007214| | SEC63,SEC63-like protein | |  |  |
| 233461_x_at | 11.3 | 1.23E-05 | 1.060064492 | NM_001032372| | NA |  |  |  |
| 226942_at | 11.3 | 1.23E-05 | 1.113806895 | NM_016018| | PHF20L1,PHD finger protein 20-like 1 isoform 1 | | | |
| 229359_at | 11.3 | 1.23E-05 | 1.141751325 | NA |  |  |  |  |
| 200679_x_at | 11.3 | 1.23E-05 | 1.027580588 | NM_002128| | HMGB1,high-mobility group box 1 | | |  |
| 232437_at | 11.29 | 1.24E-05 | 1.202664182 | NM_017871| | FLJ20542,hypothetical protein FLJ20542 | | | |
| 204615_x_at | 11.29 | 1.24E-05 | 1.116740254 | NM_004508| | IDI1,isopentenyl-diphosphate delta isomerase | | | |
| 213109_at | 11.28 | 1.24E-05 | 1.767902853 | NM_015028| | TNIK,TRAF2 and NCK interacting kinase | | |  |
| 1558512_at | 11.28 | 1.24E-05 | 1.330867904 | NA |  |  |  |  |
| 229875_at | 11.27 | 1.25E-05 | 1.30529084 | NM_174976| | ZDHHC22,zinc finger, DHHC domain containing 22 | | | |
| 205578_at | 11.27 | 1.25E-05 | 1.289632897 | NM_004560| | ROR2,receptor tyrosine kinase-like orphan receptor 2 | | | |
| 203029_s_at | 11.27 | 1.25E-05 | 1.240101967 | NM_002847| | PTPRN2,protein tyrosine phosphatase, receptor type, N | | | |
| 202422_s_at | 11.26 | 1.26E-05 | 1.217456638 | NM_004458| | ACSL4,acyl-CoA synthetase long-chain family member 4 | | | |
| 46323_at | 11.26 | 1.26E-05 | 1.106941118 | NM_138793| | CANT1,calcium activated nucleotidase 1 | | |  |
| 226985_at | 11.26 | 1.27E-05 | 1.115605443 | NM_152536| | FGD5,FYVE, RhoGEF and PH domain containing 5 | | | |
| 213712_at | 11.25 | 1.27E-05 | 1.177743681 | NM_017770| | ELOVL2,elongation of very long chain fatty acids | | | |
| 218660_at | 11.25 | 1.27E-05 | 1.103112377 | NM_003494| | DYSF,dysferlin | |  |  |
| 224817_at | 11.25 | 1.27E-05 | 1.062791794 | NM_014631| | SH3MD1,SH3 multiple domains 1 | | |  |
| 209604_s_at | 11.24 | 1.28E-05 | 1.351053085 | NM_001002295| | GATA3,GATA binding protein 3 isoform 1 | | | |
| 208765_s_at | 11.24 | 1.28E-05 | 1.046006269 | NM_001102397| | NA |  |  |  |
| 201628_s_at | 11.24 | 1.28E-05 | 1.04853183 | NM_006570| | RRAGA,Ras-related GTP binding A | | |  |
| 227394_at | 11.24 | 1.28E-05 | 1.382452778 | NM_000615| | NCAM1,neural cell adhesion molecule 1 | | |  |
| 229029_at | 11.24 | 1.28E-05 | 1.197869969 | NA |  |  |  |  |
| 209537_at | 11.24 | 1.28E-05 | 1.044483908 | NM_001033025| | NA |  |  |  |
| 214878_at | 11.23 | 1.29E-05 | 1.35059495 | NM_001007094| | ZNF37A,zinc finger protein 37a | | |  |
| 209056_s_at | 11.23 | 1.29E-05 | 1.033882494 | NM_001253| | CDC5L,CDC5-like | |  |  |
| 235285_at | 11.23 | 1.28E-05 | 1.194406145 | NA |  |  |  |  |
| 226799_at | 11.23 | 1.29E-05 | 1.07734562 | NM_018351| | FGD6,FYVE, RhoGEF and PH domain containing 6 | | | |
| 213197_at | 11.23 | 1.28E-05 | 1.287123523 | NM_004319| | ASTN,astrotactin isoform 1 | | |  |
| 207992_s_at | 11.23 | 1.28E-05 | 1.129044142 | NM_000480| | AMPD3,adenosine monophosphate deaminase (isoform E) | | | |
| 216962_at | 11.22 | 1.29E-05 | 1.09729854 | NM_001033002| | NA |  |  |  |
| 202341_s_at | 11.21 | 1.30E-05 | 1.127778391 | NM_015271| | TRIM2,tripartite motif-containing 2 | | |  |
| 242618_at | 11.21 | 1.30E-05 | 1.117625305 | NM_003449| | TRIM26,tripartite motif-containing 26 | | |  |
| 229068_at | 11.21 | 1.30E-05 | 1.077322579 | NM_012073| | CCT5,chaperonin containing TCP1, subunit 5 (epsilon) | | | |
| 207011_s_at | 11.2 | 1.31E-05 | 1.042000792 | NM_002821| | PTK7,PTK7 protein tyrosine kinase 7 isoform a | | | |
| 217763_s_at | 11.2 | 1.31E-05 | 1.0927025 | NM_006868| | RAB31,RAB31, member RAS oncogene family | | | |
| 213679_at | 11.2 | 1.31E-05 | 1.218170656 | NM_152275| | FLJ13946,hypothetical protein FLJ13946 | | | |
| 235155_at | 11.2 | 1.31E-05 | 1.373272551 | NM_020139| | DHRS6,dehydrogenase/reductase (SDR family) member 6 | | | |
| 201829_at | 11.2 | 1.31E-05 | 1.051849889 | NM_001047160| | NA |  |  |  |
| 228164_at | 11.2 | 1.31E-05 | 1.070322706 | NM_007347| | AP4E1,adaptor-related protein complex 4, epsilon 1 | | | |
| 203434_s_at | 11.2 | 1.31E-05 | 1.163004916 | NM_000902| | MME,membrane metallo-endopeptidase | | | |
| 219450_at | 11.2 | 1.31E-05 | 1.249522658 | NM_001104629| | NA |  |  |  |
| 209082_s_at | 11.19 | 1.32E-05 | 1.071229239 | NM_030582| | COL18A1,alpha 1 type XVIII collagen isoform 1 precursor | | | |
| 226919_at | 11.19 | 1.32E-05 | 1.122203347 | NM_020466| | DJ122O8.2,hypothetical protein dJ122O8.2 | | | |
| 228776_at | 11.19 | 1.31E-05 | 1.080725512 | NM_001080383| | NA |  |  |  |
| 202856_s_at | 11.18 | 1.32E-05 | 1.388026862 | NM_001042422| | NA |  |  |  |
| 202598_at | 11.17 | 1.33E-05 | 1.084361044 | NM_001024210| | NA |  |  |  |
| 223218_s_at | 11.17 | 1.33E-05 | 1.220343412 | NM_001005474| | NFKBIZ,nuclear factor of kappa light polypeptide gene | | | |
| 200785_s_at | 11.17 | 1.33E-05 | 1.089770444 | NM_002332| | LRP1,low density lipoprotein-related protein 1 | | | |
| 230098_at | 11.16 | 1.34E-05 | 1.190074491 | NM_016018| | PHF20L1,PHD finger protein 20-like 1 isoform 1 | | | |
| 204588_s_at | 11.16 | 1.34E-05 | 1.120634438 | NM_001126105| | NA |  |  |  |
| 201239_s_at | 11.16 | 1.34E-05 | 1.066739637 | NM_014752| | SPCS2,signal peptidase complex subunit 2 homolog | | | |
| 1556826_s_at | 11.16 | 1.34E-05 | 1.127912644 | NM_198545| | LOC374946,hypothetical gene supported by AK075558; | | | |
| 231123_at | 11.16 | 1.34E-05 | 1.37508371 | NM_001017397| | NA |  |  |  |
| 215071_s_at | 11.15 | 1.35E-05 | 1.28986162 | NM_003512| | HIST1H2AC,H2A histone family, member L | | | |
| 226106_at | 11.15 | 1.35E-05 | 1.053144771 | NM_016422| | RNF141,ring finger protein 141 | | |  |
| 201577_at | 11.15 | 1.35E-05 | 1.077613582 | NM_000269| | NME1,nucleoside-diphosphate kinase 1 isoform b | | | |
| 212063_at | 11.15 | 1.35E-05 | 1.322205565 | NM_000610| | CD44,CD44 antigen isoform 1 precursor | | | |
| 203043_at | 11.15 | 1.34E-05 | 1.032973271 | NM_004729| | ZBED1,Ac-like transposable element | | |  |
| 219030_at | 11.14 | 1.36E-05 | 1.085667978 | NM_016058| | CGI-121,CGI-121 protein | |  |  |
| 235174_s_at | 11.14 | 1.36E-05 | 1.335069609 | NA |  |  |  |  |
| 232679_at | 11.14 | 1.35E-05 | 1.239197326 | NA |  |  |  |  |
| 204143_s_at | 11.13 | 1.37E-05 | 1.091819857 | NM_001126123| | NA |  |  |  |
| 225773_at | 11.13 | 1.36E-05 | 1.059982191 | NM_133368| | KIAA1972,KIAA1972 protein | | |  |
| 229178_at | 11.12 | 1.37E-05 | 1.301817134 | NA |  |  |  |  |
| 234986_at | 11.11 | 1.38E-05 | 1.09615015 | NA |  |  |  |  |
| 206045_s_at | 11.11 | 1.38E-05 | 1.480176154 | NM_003787| | NOL4,nucleolar protein 4 | |  |  |
| 229492_at | 11.11 | 1.39E-05 | 1.191925844 | NM_138959| | VANGL1,vang-like 1 | |  |  |
| 226298_at | 11.1 | 1.39E-05 | 1.154711077 | NM_173079| | RUNDC1,RUN domain containing 1 | | |  |
| 226777_at | 11.1 | 1.39E-05 | 1.396250074 | NA |  |  |  |  |
| 232034_at | 11.1 | 1.39E-05 | 1.324012093 | NA |  |  |  |  |
| 221797_at | 11.09 | 1.40E-05 | 1.066398762 | NM_001039842| | NA |  |  |  |
| 227306_at | 11.09 | 1.40E-05 | 1.100291829 | NA |  |  |  |  |
| 202228_s_at | 11.09 | 1.40E-05 | 1.035686589 | NM_012428| | SDFR1,stromal cell derived factor receptor 1 isoform | | | |
| 212149_at | 11.09 | 1.40E-05 | 1.218574942 | NM_015137| | KIAA0143,KIAA0143 protein | | |  |
| 213940_s_at | 11.08 | 1.41E-05 | 1.046178983 | NM_015033| | FNBP1,formin-binding protein 17 | | |  |
| 201249_at | 11.08 | 1.41E-05 | 1.236227761 | NM_006516| | SLC2A1,solute carrier family 2 (facilitated glucose | | | |
| 205061_s_at | 11.07 | 1.42E-05 | 1.035727342 | NM_001034194| | NA |  |  |  |
| 212013_at | 11.07 | 1.42E-05 | 1.047693537 | NM_012293| | NA |  |  |  |
| 213295_at | 11.07 | 1.42E-05 | 1.124285044 | NM_001042355| | NA |  |  |  |
| 216326_s_at | 11.06 | 1.43E-05 | 1.059682771 | NM_003883| | HDAC3,histone deacetylase 3 | | |  |
| 228667_at | 11.06 | 1.43E-05 | 1.181666415 | NM_020133| | AGPAT4,1-acylglycerol-3-phosphate O-acyltransferase 4 | | | |
| 228763_at | 11.06 | 1.43E-05 | 1.115683166 | NM_014169| | C14orf123,Snf7 homologue associated with Alix 2 | | | |
| 222030_at | 11.06 | 1.42E-05 | 1.077897176 | NM_006427| | SIVA,CD27-binding (Siva) protein isoform 1 | | | |
| 212603_at | 11.05 | 1.43E-05 | 1.061407989 | NM_005830| | MRPS31,mitochondrial ribosomal protein S31 | | | |
| 229532_at | 11.05 | 1.43E-05 | 1.125070403 | NM_033210| | ZNF502,zinc finger protein 502 | | |  |
| 223136_at | 11.05 | 1.44E-05 | 1.139253543 | NM_016108| | AIG1,androgen-induced 1 | |  |  |
| 222772_at | 11.04 | 1.45E-05 | 1.068604779 | NM_016132| | MYEF2,myelin gene expression factor 2 | | |  |
| 235852_at | 11.04 | 1.45E-05 | 1.395350224 | NM_033104| | STN2,stonin 2 | |  |  |
| 241803_s_at | 11.04 | 1.45E-05 | 1.274802982 | NA |  |  |  |  |
| 232579_at | 11.04 | 1.44E-05 | 1.117964613 | NA |  |  |  |  |
| 212947_at | 11.04 | 1.45E-05 | 1.067984904 | NM_015266| | SLC9A8,Na+/H+ exchanger isoform 8 | | |  |
| 226590_at | 11.03 | 1.45E-05 | 1.108604386 | NM_133374| | NA |  |  |  |
| 203424_s_at | 11.03 | 1.45E-05 | 1.154254237 | NM_000599| | IGFBP5,insulin-like growth factor binding protein 5 | | | |
| 225852_at | 11.03 | 1.45E-05 | 1.031160221 | NM_032217| | ANKRD17,ankyrin repeat domain protein 17 isoform a | | | |
| 229084_at | 11.03 | 1.45E-05 | 1.211414086 | NM_175607| | CNTN4,contactin 4 isoform a precursor | | |  |
| 241871_at | 11.02 | 1.46E-05 | 1.189773669 | NM_001744| | CAMK4,calcium/calmodulin-dependent protein kinase IV | | | |
| 202060_at | 11.02 | 1.46E-05 | 1.037153932 | NM_014633| | SH2BP1,SH2 domain binding protein 1 | | |  |
| 207606_s_at | 11.02 | 1.46E-05 | 1.087762278 | NM_018287| | ARHGAP12,Rho GTPase activating protein 12 | | | |
| 227435_at | 11.02 | 1.46E-05 | 1.09925315 | NM_001009899| | KIAA2018,hypothetical LOC205717 | | |  |
| 204897_at | 11.02 | 1.46E-05 | 1.338923825 | NM_000958| | PTGER4,prostaglandin E receptor 4, subtype EP4 | | | |
| 1554557_at | 11.02 | 1.46E-05 | 1.177814752 | NM_014616| | NA |  |  |  |
| 209160_at | 11.01 | 1.46E-05 | 1.369250585 | NM_003739| | AKR1C3,aldo-keto reductase family 1, member C3 | | | |
| 231832_at | 11 | 1.48E-05 | 1.263287028 | NM_003774| | GALNT4,polypeptide N-acetylgalactosaminyltransferase 4 | | | |
| 225701_at | 11 | 1.48E-05 | 1.054815218 | NM_030767| | AKNA,AT-hook transcription factor | | |  |
| 204900_x_at | 11 | 1.47E-05 | 1.147592868 | NM_003864| | SAP30,sin3 associated polypeptide p30 | | |  |
| 201393_s_at | 10.99 | 1.49E-05 | 1.047070901 | NM_000876| | IGF2R,insulin-like growth factor 2 receptor | | | |
| 217979_at | 10.99 | 1.48E-05 | 1.04370585 | NM_014399| | TM4SF13,tetraspan NET-6 | |  |  |
| 208405_s_at | 10.99 | 1.48E-05 | 1.035511536 | NM_006016| | CD164,CD164 antigen, sialomucin | | |  |
| 228796_at | 10.98 | 1.49E-05 | 1.335304909 | NM_130808| | CPNE4,copine IV | |  |  |
| 215093_at | 10.98 | 1.49E-05 | 1.07973101 | NM_015922| | NSDHL,NAD(P) dependent steroid dehydrogenase-like | | | |
| 227523_s_at | 10.98 | 1.50E-05 | 1.131192061 | NM_016018| | PHF20L1,PHD finger protein 20-like 1 isoform 1 | | | |
| 244738_at | 10.98 | 1.49E-05 | 1.116860191 | NM_153252| | BRWD3,bromo domain-containing protein disrupted in | | | |
| 202450_s_at | 10.97 | 1.51E-05 | 1.230332171 | NM_000396| | CTSK,cathepsin K preproprotein | | |  |
| 219974_x_at | 10.97 | 1.51E-05 | 1.09090402 | NM_001002030| | NA |  |  |  |
| 214046_at | 10.97 | 1.51E-05 | 1.197841054 | NM_006581| | FUT9,fucosyltransferase 9 (alpha (1,3) | | |  |
| 204260_at | 10.96 | 1.52E-05 | 1.183177989 | NM_001819| | CHGB,chromogranin B precursor | | |  |
| 202468_s_at | 10.96 | 1.51E-05 | 1.037210619 | NM_003798| | CTNNAL1,catenin, alpha-like 1 | | |  |
| 229072_at | 10.96 | 1.52E-05 | 1.238267254 | NA |  |  |  |  |
| 200854_at | 10.96 | 1.51E-05 | 1.026798099 | NM_006311| | NCOR1,nuclear receptor co-repressor 1 | | |  |
| 200957_s_at | 10.96 | 1.52E-05 | 1.032603529 | NM_003146| | SSRP1,structure specific recognition protein 1 | | | |
| 241698_at | 10.95 | 1.52E-05 | 1.398958749 | NM_144629| | C2orf11,chromosome 2 open reading frame 11 | | | |
| 218392_x_at | 10.93 | 1.55E-05 | 1.089903046 | NM_022754| | SFXN1,sideroflexin 1 | |  |  |
| 217122_s_at | 10.93 | 1.55E-05 | 1.058043765 | NM_001110781| | NA |  |  |  |
| 229134_at | 10.93 | 1.54E-05 | 1.2854604 | NM_138959| | VANGL1,vang-like 1 | |  |  |
| 204291_at | 10.93 | 1.55E-05 | 1.066377085 | NM_014803| | ZNF518,zinc finger protein 518 | | |  |
| 228851_s_at | 10.91 | 1.56E-05 | 1.082412382 | NM_004436| | ENSA,endosulfine alpha isoform 3 | | |  |
| 217766_s_at | 10.91 | 1.57E-05 | 1.037916256 | NM_014313| | SMP1,small membrane protein 1 | | |  |
| 229778_at | 10.91 | 1.56E-05 | 1.482890591 | NM_030572| | MGC10946,hypothetical protein MGC10946 | | | |
| 228377_at | 10.9 | 1.58E-05 | 1.569884194 | NM_020805| | KLHL14,kelch-like 14 | |  |  |
| 227905_s_at | 10.9 | 1.57E-05 | 1.216003458 | NM_022461| | AZI2,5-azacytidine induced 2 isoform a | | |  |
| 210381_s_at | 10.89 | 1.59E-05 | 1.131403795 | NM_176875| | CCKBR,cholecystokinin B receptor | | |  |
| 222646_s_at | 10.89 | 1.59E-05 | 1.081426092 | NM_014584| | ERO1L,ERO1-like | |  |  |
| 221847_at | 10.89 | 1.58E-05 | 1.0795407 | NA |  |  |  |  |
| 214439_x_at | 10.88 | 1.60E-05 | 1.072090353 | NM_004305| | BIN1,bridging integrator 1 isoform 8 | | |  |
| 235456_at | 10.88 | 1.60E-05 | 1.258768853 | NA |  |  |  |  |
| 218794_s_at | 10.88 | 1.59E-05 | 1.070521129 | NM_017853| | TXNL4B,thioredoxin-like 4B | | |  |
| 228093_at | 10.88 | 1.60E-05 | 1.095459818 | NM_001007248| | ZNF599,zinc finger protein 599 isoform a | | | |
| 217885_at | 10.88 | 1.59E-05 | 1.036464496 | NM_018085| | IPO9,importin 9 | |  |  |
| 237215_s_at | 10.88 | 1.60E-05 | 1.260970411 | NM_003234| | TFRC,transferrin receptor | |  |  |
| 209806_at | 10.87 | 1.61E-05 | 1.178395499 | NM_080593| | HIST1H2BK,H2B histone family, member T | | | |
| 229615_at | 10.87 | 1.61E-05 | 1.088258923 | NA |  |  |  |  |
| 202973_x_at | 10.87 | 1.60E-05 | 1.124457319 | NM_001015045| | NA |  |  |  |
| 206288_at | 10.87 | 1.61E-05 | 1.080918256 | NM_005023| | PGGT1B,protein geranylgeranyltransferase type I, beta | | | |
| 206108_s_at | 10.86 | 1.62E-05 | 1.158708714 | NM_006275| | SFRS6,arginine/serine-rich splicing factor 6 | | | |
| 244546_at | 10.86 | 1.62E-05 | 1.343087791 | NM_018947| | CYCS,cytochrome c | |  |  |
| 36936_at | 10.86 | 1.62E-05 | 1.05845875 | NM_003313| | TSTA3,tissue specific transplantation antigen P35B | | | |
| 229018_at | 10.85 | 1.63E-05 | 1.096323906 | NM_032230| | FLJ22789,hypothetical protein FLJ22789 | | | |
| 228289_at | 10.85 | 1.63E-05 | 1.143916406 | NA |  |  |  |  |
| 210320_s_at | 10.84 | 1.64E-05 | 1.074591103 | NM_007010| | DDX52,ATP-dependent RNA helicase ROK1 isoform a | | | |
| 212570_at | 10.84 | 1.63E-05 | 1.287622864 | NM_015036| | NA |  |  |  |
| 210589_s_at | 10.84 | 1.64E-05 | 1.111045004 | NM_000157| | GBA,glucocerebrosidase precursor | | |  |
| 219230_at | 10.84 | 1.64E-05 | 1.347852772 | NM_001099640| | NA |  |  |  |
| 218976_at | 10.83 | 1.65E-05 | 1.2974113 | NM_021800| | DNAJC12,J domain containing protein 1 isoform a | | | |
| 241926_s_at | 10.82 | 1.66E-05 | 1.504489023 | NM_004449| | ERG,v-ets erythroblastosis virus E26 oncogene like | | | |
| 212698_s_at | 10.82 | 1.66E-05 | 1.045410429 | NM_144710| | SEPT10,septin 10 isoform 1 | | |  |
| 202342_s_at | 10.82 | 1.66E-05 | 1.12438207 | NM_015271| | TRIM2,tripartite motif-containing 2 | | |  |
| 213344_s_at | 10.82 | 1.66E-05 | 1.095199311 | NM_002105| | H2AFX,H2A histone family, member X | | |  |
| 222740_at | 10.82 | 1.66E-05 | 1.086650257 | NM_014109| | ATAD2,two AAA domain containing protein | | | |
| 1552794_a_at | 10.82 | 1.66E-05 | 1.365069235 | NM_173631| | ZNF547,zinc finger protein 547 | | |  |
| 219672_at | 10.82 | 1.66E-05 | 1.272309761 | NM_016633| | ERAF,erythroid associated factor | | |  |
| 208708_x_at | 10.82 | 1.66E-05 | 1.130919135 | NM_001969| | EIF5,eukaryotic translation initiation factor 5 | | | |
| 203960_s_at | 10.82 | 1.66E-05 | 1.096830488 | NM_016126| | C1orf41,chromosome 1 open reading frame 41 | | | |
| 229928_at | 10.81 | 1.67E-05 | 1.350986557 | NA |  |  |  |  |
| 202855_s_at | 10.81 | 1.67E-05 | 1.344327353 | NM_001042422| | NA |  |  |  |
| 205769_at | 10.81 | 1.67E-05 | 1.149714705 | NM_003645| | SLC27A2,solute carrier family 27 (fatty acid | | | |
| 202836_s_at | 10.8 | 1.68E-05 | 1.034462972 | NM_006701| | TXNL4A,thioredoxin-like 4A | | |  |
| 201170_s_at | 10.8 | 1.68E-05 | 1.444646662 | NM_003670| | BHLHB2,differentiated embryo chondrocyte expressed gene | | | |
| 221483_s_at | 10.8 | 1.68E-05 | 1.034121162 | NM_006628| | ARPP-19,cyclic AMP phosphoprotein, 19 kD | | | |
| 228370_at | 10.8 | 1.68E-05 | 1.036878901 | NM_003097| | SNRPN,small nuclear ribonucleoprotein polypeptide N | | | |
| 214214_s_at | 10.8 | 1.68E-05 | 1.039386199 | NM_001212| | C1QBP,complement component 1, q subcomponent binding | | | |
| 229693_at | 10.8 | 1.68E-05 | 1.102656508 | NM_001004313| | LOC388335,similar to RIKEN cDNA A730055C05 gene | | | |
| 220117_at | 10.8 | 1.68E-05 | 1.19743879 | NM_024697| | ZNF659,zinc finger protein 659 | | |  |
| 225036_at | 10.79 | 1.69E-05 | 1.045611356 | NM_001001790| | C9orf105,chromosome 9 open reading frame 105 | | | |
| 225936_at | 10.79 | 1.69E-05 | 1.071668325 | NM_153232| | CRI2,CREBBP/EP300 inhibitor 2 | | |  |
| 230280_at | 10.78 | 1.70E-05 | 1.319982905 | NM_015163| | TRIM9,tripartite motif protein 9 isoform 1 | | | |
| 235219_at | 10.78 | 1.70E-05 | 1.241899884 | NM_138464| | NA |  |  |  |
| 242705_x_at | 10.78 | 1.70E-05 | 1.075596532 | NA |  |  |  |  |
| 211009_s_at | 10.78 | 1.70E-05 | 1.0759813 | NM_001112663| | NA |  |  |  |
| 225766_s_at | 10.78 | 1.70E-05 | 1.040057308 | NM_002270| | TNPO1,transportin 1 | |  |  |
| 225433_at | 10.78 | 1.70E-05 | 1.041539825 | NM_015859| | GTF2A1,TFIIA alpha, p55 isoform 1 | | |  |
| 203545_at | 10.77 | 1.71E-05 | 1.03603068 | NM_001007027| | ALG8,alpha-1,3-glucosyltransferase ALG8 isoform b | | | |
| 202353_s_at | 10.77 | 1.71E-05 | 1.08494031 | NM_002816| | PSMD12,proteasome 26S non-ATPase subunit 12 isoform 1 | | | |
| 239144_at | 10.76 | 1.72E-05 | 1.44628741 | NM_080742| | B3GAT2,beta-1,3-glucuronyltransferase 2 | | | |
| 231705_at | 10.76 | 1.72E-05 | 1.073631249 | NM_005836| | HRSP12,heat-responsive protein 12 | | |  |
| 225297_at | 10.75 | 1.74E-05 | 1.065235076 | NM_138443| | CCDC5,coiled-coil domain containing 5 (spindle | | | |
| 225091_at | 10.75 | 1.74E-05 | 1.069478481 | NM_033089| | ZCCHC3,zinc finger, CCHC domain containing 3 | | | |
| 218929_at | 10.75 | 1.74E-05 | 1.070660182 | NM_017632| | CARF,collaborates/cooperates with ARF (alternate | | | |
| 244008_at | 10.75 | 1.74E-05 | 1.410883651 | NA |  |  |  |  |
| 219384_s_at | 10.74 | 1.75E-05 | 1.089016297 | NM_012091| | ADAT1,adenosine deaminase, tRNA-specific 1 | | | |
| 236532_at | 10.74 | 1.75E-05 | 1.286371715 | NM_207645| | LOC399947,similar to expressed sequence AI593442 | | | |
| 208986_at | 10.74 | 1.75E-05 | 1.044173092 | NM_003205| | TCF12,transcription factor 12 isoform b | | |  |
| 229088_at | 10.73 | 1.76E-05 | 1.095758484 | NM_006208| | ENPP1,ectonucleotide pyrophosphatase/phosphodiesterase | | | |
| 201286_at | 10.73 | 1.76E-05 | 1.086857637 | NM_001006946| | SDC1,syndecan 1 precursor | | |  |
| 212640_at | 10.73 | 1.76E-05 | 1.110696104 | NM_198402| | PTPLB,protein tyrosine phosphatase-like (proline | | | |
| 236242_at | 10.72 | 1.78E-05 | 1.350449578 | NA |  |  |  |  |
| 204521_at | 10.72 | 1.78E-05 | 1.064394845 | NM_013300| | HSU79274,protein predicted by clone 23733 | | | |
| 220060_s_at | 10.72 | 1.78E-05 | 1.127684327 | NM_017915| | FLJ20641,hypothetical protein FLJ20641 | | | |
| 243242_at | 10.72 | 1.78E-05 | 1.193129299 | NA |  |  |  |  |
| 210638_s_at | 10.71 | 1.79E-05 | 1.139170911 | NM_012347| | FBXO9,F-box only protein 9 isoform 1 | | |  |
| 224687_at | 10.7 | 1.80E-05 | 1.039039844 | NM_019004| | NA |  |  |  |
| 221796_at | 10.7 | 1.80E-05 | 1.526451012 | NM_001007097| | NTRK2,neurotrophic tyrosine kinase, receptor, type 2 | | | |
| 201438_at | 10.69 | 1.81E-05 | 1.439024514 | NM_004369| | COL6A3,alpha 3 type VI collagen isoform 1 precursor | | | |
| 214508_x_at | 10.68 | 1.83E-05 | 1.061331607 | NM_001881| | CREM,cAMP responsive element modulator isoform b | | | |
| 227048_at | 10.68 | 1.83E-05 | 1.045947915 | NM_005559| | LAMA1,laminin, alpha 1 precursor | | |  |
| 223225_s_at | 10.66 | 1.85E-05 | 1.05817032 | NM_001013437| | NA |  |  |  |
| 223290_at | 10.66 | 1.85E-05 | 1.091607666 | NM_020315| | PDXP,pyridoxal (pyridoxine, vitamin B6) phosphatase | | | |
| 205475_at | 10.66 | 1.86E-05 | 1.323576969 | NM_007281| | SCRG1,scrapie responsive protein 1 | | |  |
| 203798_s_at | 10.66 | 1.85E-05 | 1.312323138 | NM_003385| | VSNL1,visinin-like 1 | |  |  |
| 243023_at | 10.65 | 1.86E-05 | 1.298708218 | NA |  |  |  |  |
| 217911_s_at | 10.65 | 1.87E-05 | 1.038259162 | NM_004281| | BAG3,BCL2-associated athanogene 3 | | |  |
| 232899_at | 10.65 | 1.86E-05 | 1.227439771 | NM_203302| | MGC70863,similar to RPL23AP7 protein | | |  |
| 205055_at | 10.65 | 1.87E-05 | 1.062087671 | NM_002208| | ITGAE,integrin, alpha E (antigen CD103, human mucosal | | | |
| 222101_s_at | 10.65 | 1.86E-05 | 1.126434572 | NM_003737| | DCHS1,dachsous 1 precursor | | |  |
| 207543_s_at | 10.65 | 1.87E-05 | 1.118770368 | NM_000917| | P4HA1,procollagen-proline, 2-oxoglutarate | | | |
| 204114_at | 10.64 | 1.88E-05 | 1.200325465 | NM_007361| | NID2,nidogen 2 | |  |  |
| 213129_s_at | 10.64 | 1.87E-05 | 1.02124939 | NM_004483| | GCSH,glycine cleavage system protein H (aminomethyl | | | |
| 238825_at | 10.64 | 1.88E-05 | 1.213019757 | NM_052957| | ACRC,ACRC protein | |  |  |
| 203521_s_at | 10.64 | 1.88E-05 | 1.066462051 | NM_014345| | ZNF318,zinc finger protein 318 | | |  |
| 228188_at | 10.64 | 1.88E-05 | 1.258774461 | NM_005253| | FOSL2,FOS-like antigen 2 | |  |  |
| 236449_at | 10.63 | 1.88E-05 | 1.213282062 | NM_000100| | CSTB,cystatin B | |  |  |
| 231863_at | 10.63 | 1.89E-05 | 1.264399724 | NM_019071| | ING3,inhibitor of growth family, member 3 isoform 1 | | | |
| 209654_at | 10.63 | 1.88E-05 | 1.023481802 | NM_015325| | NA |  |  |  |
| 212922_s_at | 10.63 | 1.88E-05 | 1.157005182 | NM_020197| | SMYD2,SET and MYND domain containing 2 | | | |
| 228201_at | 10.63 | 1.88E-05 | 1.138064442 | NM_144996| | ARL2L1,ADP-ribosylation factor-like 2-like 1 isoform 2 | | | |
| 220018_at | 10.62 | 1.90E-05 | 1.091919912 | NM_024814| | CBLL1,Cas-Br-M (murine) ecotropic retroviral | | | |
| 218161_s_at | 10.62 | 1.90E-05 | 1.096249938 | NM_017882| | CLN6,CLN6 protein | |  |  |
| 205528_s_at | 10.62 | 1.90E-05 | 1.072432893 | NM_004349| | RUNX1T1,acute myelogenous leukemia 1 translocation 1 | | | |
| 223063_at | 10.62 | 1.89E-05 | 1.052627615 | NM_032800| | FLJ14525,hypothetical protein FLJ14525 | | | |
| 203485_at | 10.62 | 1.90E-05 | 1.306565669 | NM_021136| | RTN1,reticulon 1 isoform A | | |  |
| 223170_at | 10.61 | 1.90E-05 | 1.068451073 | NM_001033504| | NA |  |  |  |
| 225962_at | 10.61 | 1.91E-05 | 1.141150725 | NM_032268| | ZNRF1,zinc and ring finger protein 1 | | |  |
| 201362_at | 10.61 | 1.91E-05 | 1.040060209 | NM_006469| | IVNS1ABP,influenza virus NS1A binding protein isoform a | | | |
| 226499_at | 10.61 | 1.90E-05 | 1.042864563 | NM_001004354| | MGC61598,similar to ankyrin-repeat protein Nrarp | | | |
| 221826_at | 10.61 | 1.91E-05 | 1.074855513 | NM_144567| | LOC90806,similar to RIKEN cDNA 2610307I21 | | | |
| 231411_at | 10.61 | 1.90E-05 | 1.376178393 | NM_005780| | LHFP,lipoma HMGIC fusion partner | | |  |
| 243492_at | 10.61 | 1.91E-05 | 1.180973694 | NM_053055| | CTMP,carboxyl-terminal modulator protein isoform a | | | |
| 218203_at | 10.59 | 1.94E-05 | 1.064810814 | NM_013338| | ALG5,dolichyl phosphate glucosyltransferase | | | |
| 231876_at | 10.59 | 1.93E-05 | 1.090556872 | NM_030961| | TRIM56,tripartite motif-containing 56 | | |  |
| 219338_s_at | 10.59 | 1.94E-05 | 1.082580789 | NM_017691| | FLJ20156,hypothetical protein FLJ20156 | | | |
| 204278_s_at | 10.59 | 1.94E-05 | 1.082200068 | NM_004215| | EBAG9,estrogen receptor binding site associated | | | |
| 219274_at | 10.58 | 1.95E-05 | 1.119261512 | NM_012338| | TM4SF12,transmembrane 4 superfamily member 12 | | | |
| 230724_s_at | 10.58 | 1.95E-05 | 1.091208751 | NM_001082969| | NA |  |  |  |
| 209507_at | 10.58 | 1.95E-05 | 1.0452467 | NM_002947| | RPA3,replication protein A3, 14kDa | | |  |
| 225788_at | 10.58 | 1.95E-05 | 1.058587065 | NM_033112| | C6orf153,hypothetical protein LOC88745 | | | |
| 211725_s_at | 10.58 | 1.94E-05 | 1.081451172 | NM_001196| | BID,BH3 interacting domain death agonist isoform 2 | | | |
| 242635_s_at | 10.58 | 1.94E-05 | 1.301922732 | NM_001122838| | NA |  |  |  |
| 218073_s_at | 10.58 | 1.94E-05 | 1.06719372 | NM_018087| | FLJ10407,hypothetical protein FLJ10407 | | | |
| 221946_at | 10.57 | 1.95E-05 | 1.13306104 | NM_001048265| | NA |  |  |  |
| 204462_s_at | 10.57 | 1.95E-05 | 1.065738786 | NM_006517| | SLC16A2,solute carrier family 16, member 2 | | | |
| 241801_at | 10.56 | 1.97E-05 | 1.131698791 | NM_024989| | PGAP1,GPI deacylase | |  |  |
| 213508_at | 10.56 | 1.97E-05 | 1.044064361 | NM_138288| | C14orf147,chromosome 14 open reading frame 147 | | | |
| 240236_at | 10.56 | 1.97E-05 | 1.420688429 | NM_014980| | NA |  |  |  |
| 201938_at | 10.55 | 1.99E-05 | 1.045144657 | NM_004642| | CDK2AP1,CDK2-associated protein 1 | | |  |
| 1553810_a_at | 10.55 | 1.99E-05 | 1.128905334 | NM_020890| | KIAA1524,KIAA1524 | |  |  |
| 224840_at | 10.55 | 1.99E-05 | 1.064458087 | NM_004117| | FKBP5,FK506 binding protein 5 | | |  |
| 205637_s_at | 10.55 | 1.98E-05 | 1.055437687 | NM_003027| | SH3GL3,SH3-domain GRB2-like 3 | | |  |
| 239252_at | 10.54 | 2.01E-05 | 1.285974556 | NA |  |  |  |  |
| 203054_s_at | 10.54 | 2.01E-05 | 1.102121389 | NM_022171| | TCTA,T-cell leukemia translocation altered gene | | | |
| 210258_at | 10.54 | 2.01E-05 | 1.609475525 | NM_002927| | RGS13,regulator of G-protein signalling 13 | | | |
| 227066_at | 10.54 | 2.01E-05 | 1.276239805 | NM_145279| | MOBKL2C,MOB1, Mps One Binder kinase activator-like 2C | | | |
| 219821_s_at | 10.54 | 2.00E-05 | 1.089164592 | NM_018988| | GFOD1,glucose-fructose oxidoreductase domain | | | |
| 208296_x_at | 10.54 | 2.01E-05 | 1.068204473 | NM_001077654| | NA |  |  |  |
| 214672_at | 10.53 | 2.02E-05 | 1.104705642 | NM_015072| | KIAA0998,KIAA0998 | |  |  |
| 1555725_a_at | 10.53 | 2.02E-05 | 1.255023997 | NM_003617| | RGS5,regulator of G-protein signalling 5 | | | |
| 209761_s_at | 10.52 | 2.03E-05 | 1.148105392 | NM_004509| | SP110,SP110 nuclear body protein isoform a | | | |
| 225798_at | 10.52 | 2.02E-05 | 1.114728105 | NM_175061| | JAZF1,juxtaposed with another zinc finger gene 1 | | | |
| 240000_at | 10.52 | 2.02E-05 | 1.244197166 | NA |  |  |  |  |
| 218259_at | 10.52 | 2.02E-05 | 1.058448959 | NM_014048| | MKL2,megakaryoblastic leukemia 2 protein | | | |
| 206866_at | 10.52 | 2.03E-05 | 1.154158648 | NM_001794| | CDH4,cadherin 4, type 1 preproprotein | | |  |
| 203031_s_at | 10.51 | 2.04E-05 | 1.075958595 | NM_000375| | UROS,uroporphyrinogen III synthase | | |  |
| 219659_at | 10.51 | 2.03E-05 | 1.194057127 | NM_016529| | ATP8A2,ATPase, aminophospholipid transporter-like, | | | |
| 229992_at | 10.51 | 2.04E-05 | 1.255213242 | NA |  |  |  |  |
| 205352_at | 10.51 | 2.04E-05 | 1.082418703 | NM_001122752| | NA |  |  |  |
| 226387_at | 10.49 | 2.08E-05 | 1.080412252 | NM_198467| | RSBN1L,round spermatid basic protein 1-like | | | |
| 223266_at | 10.48 | 2.09E-05 | 1.04172177 | NM_018571| | ALS2CR2,amyotrophic lateral sclerosis 2 (juvenile) | | | |
| 211509_s_at | 10.48 | 2.09E-05 | 1.03010943 | NM_007008| | RTN4,reticulon 4 isoform C | | |  |
| 228812_at | 10.46 | 2.12E-05 | 1.120312262 | NA |  |  |  |  |
| 1554080_at | 10.46 | 2.13E-05 | 1.095672819 | NM_005444| | RQCD1,RCD1 required for cell differentiation1 homolog | | | |
| 229705_at | 10.46 | 2.13E-05 | 1.346082788 | NA |  |  |  |  |
| 214164_x_at | 10.44 | 2.15E-05 | 1.149151746 | NM_001218| | CA12,carbonic anhydrase XII isoform 1 precursor | | | |
| 205296_at | 10.44 | 2.15E-05 | 1.105163723 | NM_002895| | RBL1,retinoblastoma-like protein 1 isoform a | | | |
| 203710_at | 10.44 | 2.16E-05 | 1.331871184 | NM_001099952| | NA |  |  |  |
| 219726_at | 10.44 | 2.15E-05 | 1.145892762 | NM_018977| | NLGN3,neuroligin 3 | |  |  |
| 206994_at | 10.44 | 2.16E-05 | 1.164583459 | NM_001899| | CST4,cystatin S precursor | |  |  |
| 207522_s_at | 10.44 | 2.16E-05 | 1.124538757 | NM_005173| | ATP2A3,sarco/endoplasmic reticulum Ca2+ -ATPase isoform | | | |
| 201790_s_at | 10.43 | 2.17E-05 | 1.140066137 | NM_001360| | DHCR7,7-dehydrocholesterol reductase | | |  |
| 226576_at | 10.43 | 2.17E-05 | 1.303560683 | NM_015071| | ARHGAP26,GTPase regulator associated with the focal | | | |
| 212833_at | 10.43 | 2.16E-05 | 1.03315922 | NM_138773| | LOC91137,hypothetical protein BC017169 | | | |
| 200945_s_at | 10.43 | 2.17E-05 | 1.049110629 | NM_001077206| | NA |  |  |  |
| 217841_s_at | 10.42 | 2.18E-05 | 1.073140558 | NM_016147| | PME-1,protein phosphatase methylesterase-1 | | | |
| 202960_s_at | 10.42 | 2.18E-05 | 1.054071258 | NM_000255| | MUT,methylmalonyl Coenzyme A mutase precursor | | | |
| 238447_at | 10.42 | 2.18E-05 | 1.233297223 | NM_001003792| | RBMS3,RNA binding motif, single stranded interacting | | | |
| 226771_at | 10.42 | 2.19E-05 | 1.040859562 | NM_001005855| | ATP8B2,ATPase, Class I, type 8B, member 2 isoform b | | | |
| 219374_s_at | 10.42 | 2.18E-05 | 1.048913389 | NM_001077690| | NA |  |  |  |
| 226448_at | 10.41 | 2.20E-05 | 1.367274719 | NM_198552| | MGC15887,hypothetical gene supported by BC009447 | | | |
| 202668_at | 10.41 | 2.20E-05 | 1.052877128 | NM_004093| | EFNB2,ephrin B2 | |  |  |
| 235942_at | 10.4 | 2.21E-05 | 1.348729922 | NA |  |  |  |  |
| 204761_at | 10.4 | 2.21E-05 | 1.053182382 | NM_001080491| | NA |  |  |  |
| 235195_at | 10.4 | 2.21E-05 | 1.164243309 | NM_012164| | FBXW2,F-box and WD-40 domain protein 2 | | | |
| 221497_x_at | 10.4 | 2.21E-05 | 1.257906122 | NM_022051| | EGLN1,egl nine homolog 1 | | |  |
| 210766_s_at | 10.4 | 2.22E-05 | 1.025170141 | NM_001316| | CSE1L,CSE1 chromosome segregation 1-like protein | | | |
| 219820_at | 10.4 | 2.22E-05 | 1.1781104 | NM_014037| | SLC6A16,solute carrier family 6, member 16 | | | |
| 209450_at | 10.39 | 2.22E-05 | 1.082723178 | NM_017807| | OSGEP,O-sialoglycoprotein endopeptidase | | | |
| 225491_at | 10.38 | 2.24E-05 | 1.262463515 | NM_004171| | SLC1A2,solute carrier family 1, member 2 | | | |
| 224715_at | 10.38 | 2.24E-05 | 1.082361057 | NM_052844| | WDR34,WD repeat domain 34 | | |  |
| 205327_s_at | 10.38 | 2.24E-05 | 1.071206623 | NM_001616| | ACVR2,activin A type II receptor precursor | | | |
| 222335_at | 10.38 | 2.24E-05 | 1.147347458 | NA |  |  |  |  |
| 222769_at | 10.38 | 2.24E-05 | 1.084038967 | NM_022764| | FLJ12998,hypothetical protein FLJ12998 | | | |
| 244641_at | 10.38 | 2.24E-05 | 1.212961181 | NM_138446| | C7orf30,chromosome 7 open reading frame 30 | | | |
| 209871_s_at | 10.37 | 2.25E-05 | 1.138742107 | NM_005503| | APBA2,amyloid beta A4 precursor protein-binding, | | | |
| 221877_at | 10.37 | 2.25E-05 | 1.050516716 | NA |  |  |  |  |
| 230319_at | 10.36 | 2.27E-05 | 1.537861114 | NA |  |  |  |  |
| 224400_s_at | 10.36 | 2.27E-05 | 1.098865737 | NM_031422| | CHST9,GalNAc-4-sulfotransferase 2 | | |  |
| 225050_at | 10.36 | 2.27E-05 | 1.025430003 | NM_032434| | ZNF512,zinc finger protein 512 | | |  |
| 202425_x_at | 10.36 | 2.28E-05 | 1.12432168 | NM_000944| | PPP3CA,protein phosphatase 3 (formerly 2B), catalytic | | | |
| 217590_s_at | 10.35 | 2.28E-05 | 1.305288743 | NM_007332| | TRPA1,ankyrin-like protein 1 | | |  |
| 223543_at | 10.35 | 2.28E-05 | 1.139357604 | NM_032512| | PDZK4,PDZ domain containing 4 | | |  |
| 227798_at | 10.35 | 2.28E-05 | 1.074772806 | NM_001003688| | SMAD1,Sma- and Mad-related protein 1 | | | |
| 217940_s_at | 10.34 | 2.31E-05 | 1.054694185 | NM_018210| | FLJ10769,hypothetical protein FLJ10769 | | | |
| 222489_s_at | 10.34 | 2.29E-05 | 1.037233587 | NM_020135| | WRNIP1,Werner helicase interacting protein isoform 1 | | | |
| 203164_at | 10.33 | 2.31E-05 | 1.104991164 | NM_004733| | SLC33A1,acetyl-coenzyme A transporter | | | |
| 225702_at | 10.33 | 2.31E-05 | 1.053846764 | NM_032847| | FLJ14825,hypothetical protein FLJ14825 | | | |
| 200847_s_at | 10.33 | 2.31E-05 | 1.041906928 | NM_016127| | MGC8721,hypothetical protein MGC8721 | | | |
| 225685_at | 10.33 | 2.31E-05 | 1.21710339 | NA |  |  |  |  |
| 204014_at | 10.32 | 2.32E-05 | 1.163333751 | NM_001394| | DUSP4,dual specificity phosphatase 4 isoform 1 | | | |
| 227699_at | 10.32 | 2.33E-05 | 1.124204412 | NM_144581| | C14orf149,chromosome 14 open reading frame 149 | | | |
| 203153_at | 10.32 | 2.32E-05 | 1.143248538 | NM_001548| | IFIT1,interferon-induced protein with | | |  |
| 234472_at | 10.31 | 2.35E-05 | 1.07567913 | NM_052917| | GALNT13,UDP-N-acetyl-alpha-D-galactosamine:polypeptide | | | |
| 225308_s_at | 10.31 | 2.35E-05 | 1.206752359 | NM_033394| | TANC,TPR domain, ankyrin-repeat and | | |  |
| 223666_at | 10.31 | 2.34E-05 | 1.075642698 | NM_014426| | SNX5,sorting nexin 5 | |  |  |
| 204975_at | 10.3 | 2.36E-05 | 1.111191154 | NM_001424| | EMP2,epithelial membrane protein 2 | | |  |
| 206756_at | 10.3 | 2.35E-05 | 1.082847188 | NM_019886| | CHST7,carbohydrate (N-acetylglucosamine 6-O) | | | |
| 218663_at | 10.3 | 2.35E-05 | 1.113755588 | NM_022346| | HCAP-G,chromosome condensation protein G | | | |
| 208657_s_at | 10.3 | 2.35E-05 | 1.076655466 | NM_001113491| | NA |  |  |  |
| 220350_at | 10.3 | 2.36E-05 | 1.186710268 | NM_004234| | ZNF235,zinc finger protein 93 homolog | | |  |
| 223980_s_at | 10.3 | 2.35E-05 | 1.11796897 | NM_004509| | SP110,SP110 nuclear body protein isoform a | | | |
| 219650_at | 10.3 | 2.35E-05 | 1.049203031 | NM_017669| | FLJ20105,FLJ20105 protein isoform a | | |  |
| 210201_x_at | 10.29 | 2.37E-05 | 1.068753398 | NM_004305| | BIN1,bridging integrator 1 isoform 8 | | |  |
| 229509_at | 10.29 | 2.37E-05 | 1.093629974 | NM_152778| | MGC33302,hypothetical protein MGC33302 | | | |
| 229371_at | 10.29 | 2.37E-05 | 1.128012394 | NA |  |  |  |  |
| 1569190_at | 10.28 | 2.39E-05 | 1.162975963 | NM_144643| | FLJ30655,hypothetical protein FLJ30655 | | | |
| 218386_x_at | 10.28 | 2.39E-05 | 1.047434185 | NM_001001992| | USP16,ubiquitin specific protease 16 isoform b | | | |
| 202038_at | 10.28 | 2.39E-05 | 1.03454077 | NM_004788| | UBE4A,ubiquitination factor E4A | | |  |
| 235102_x_at | 10.28 | 2.40E-05 | 1.373101506 | NA |  |  |  |  |
| 227476_at | 10.28 | 2.39E-05 | 1.171059276 | NA |  |  |  |  |
| 52005_at | 10.27 | 2.42E-05 | 1.051814261 | NM_021241| | NA |  |  |  |
| 202078_at | 10.27 | 2.42E-05 | 1.037489442 | NM_003653| | COPS3,COP9 constitutive photomorphogenic homolog | | | |
| 216834_at | 10.26 | 2.43E-05 | 1.312130928 | NM_002922| | RGS1,regulator of G-protein signalling 1 | | | |
| 207084_at | 10.26 | 2.43E-05 | 1.544446619 | NM_005604| | POU3F2,POU domain, class 3, transcription factor 2 | | | |
| 232238_at | 10.26 | 2.43E-05 | 1.150106023 | NM_018136| | ASPM,asp (abnormal spindle)-like, microcephaly | | | |
| 1558163_at | 10.26 | 2.43E-05 | 1.223112222 | NM_002618| | PEX13,peroxisome biogenesis factor 13 | | | |
| 225770_at | 10.26 | 2.43E-05 | 1.066714907 | NM_133368| | KIAA1972,KIAA1972 protein | | |  |
| 220327_at | 10.26 | 2.43E-05 | 1.545052771 | NM_016206| | VGL-3,colon carcinoma related protein | | |  |
| 230339_at | 10.26 | 2.43E-05 | 1.20517626 | NM_144978| | FLJ32745,hypothetical protein FLJ32745 | | | |
| 213624_at | 10.25 | 2.44E-05 | 1.284486435 | NM_006714| | SMPDL3A,acid sphingomyelinase-like phosphodiesterase 3A | | | |
| 234979_at | 10.25 | 2.45E-05 | 1.107072614 | NM_181708| | LOC144233,hypothetical protein LOC144233 | | | |
| 209628_at | 10.25 | 2.45E-05 | 1.053661473 | NM_018698| | NXT2,nuclear transport factor 2-like export factor 2 | | | |
| 217809_at | 10.25 | 2.45E-05 | 1.041792329 | NM_014038| | BZW2,basic leucine zipper and W2 domains 2 | | | |
| 226365_at | 10.25 | 2.45E-05 | 1.141832044 | NA |  |  |  |  |
| 213626_at | 10.25 | 2.43E-05 | 1.06233848 | NM_032783| | CBR4,carbonic reductase 4 | | |  |
| 223693_s_at | 10.24 | 2.46E-05 | 1.074044384 | NM_018059| | FLJ10324,hypothetical protein FLJ10324 | | | |
| 220397_at | 10.24 | 2.45E-05 | 1.255532756 | NM_017440| | MDM1,Mdm4, transformed 3T3 cell double minute 1, p53 | | | |
| 209602_s_at | 10.23 | 2.47E-05 | 1.512865883 | NM_001002295| | GATA3,GATA binding protein 3 isoform 1 | | | |
| 213989_x_at | 10.23 | 2.47E-05 | 1.121305257 | NM_001007259| | C21orf18,chromosome 21 open reading frame 18 isoform c | | | |
| 226911_at | 10.23 | 2.47E-05 | 1.180224228 | NM_152403| | FLJ39155,hypothetical protein FLJ39155 isoform 1 | | | |
| 229774_at | 10.23 | 2.48E-05 | 1.278995802 | NM_025212| | CXXC4,CXXC finger 4 | |  |  |
| 235874_at | 10.23 | 2.47E-05 | 1.181668727 | NM_153362| | PRSS35,protease, serine, 35 | | |  |
| 218770_s_at | 10.22 | 2.50E-05 | 1.158038983 | NM_018056| | TMEM39B,transmembrane protein 39B | | |  |
| 222732_at | 10.22 | 2.49E-05 | 1.022051327 | NM_021253| | TRIM39,tripartite motif-containing 39 isoform 1 | | | |
| 217717_s_at | 10.21 | 2.51E-05 | 1.056076566 | NM_003404| | YWHAB,tyrosine 3-monooxygenase/tryptophan | | | |
| 202314_at | 10.21 | 2.52E-05 | 1.141742072 | NM_000786| | CYP51A1,cytochrome P450, family 51 | | |  |
| 205932_s_at | 10.21 | 2.50E-05 | 1.497568654 | NM_002448| | MSX1,msh homeo box homolog 1 | | |  |
| 205316_at | 10.21 | 2.52E-05 | 1.286084847 | NA |  |  |  |  |
| 1557077_a_at | 10.2 | 2.52E-05 | 1.178749499 | NA |  |  |  |  |
| 218072_at | 10.2 | 2.52E-05 | 1.080248697 | NM_001101653| | NA |  |  |  |
| 222797_at | 10.2 | 2.52E-05 | 1.121197986 | NM_020134| | DPYSL5,dihydropyrimidinase-like 5 | | |  |
| 204314_s_at | 10.19 | 2.55E-05 | 1.07717361 | NM_004379| | CREB1,cAMP responsive element binding protein 1 | | | |
| 243998_at | 10.19 | 2.54E-05 | 1.292367228 | NM_152349| | MGC45562,hypothetical protein MGC45562 | | | |
| 208290_s_at | 10.19 | 2.55E-05 | 1.150017541 | NM_001969| | EIF5,eukaryotic translation initiation factor 5 | | | |
| 222482_at | 10.19 | 2.54E-05 | 1.05572381 | NM_001009955| | SSBP3,single stranded DNA binding protein 3 isoform c | | | |
| 205501_at | 10.19 | 2.55E-05 | 1.154602651 | NM_006661| | PDE10A,phosphodiesterase 10A | | |  |
| 222582_at | 10.18 | 2.56E-05 | 1.10404685 | NM_001040633| | NA |  |  |  |
| 230416_at | 10.18 | 2.56E-05 | 1.161403089 | NA |  |  |  |  |
| 206845_s_at | 10.18 | 2.56E-05 | 1.060919593 | NM_014771| | RNF40,ring finger protein 40 isoform 1 | | |  |
| 227932_at | 10.18 | 2.57E-05 | 1.101054979 | NM_006321| | ARIH2,ariadne homolog 2 | |  |  |
| 223299_at | 10.18 | 2.57E-05 | 1.091213918 | NM_033280| | SEC11L3,SEC11-like 3 | |  |  |
| 201985_at | 10.18 | 2.56E-05 | 1.047298011 | NM_014846| | KIAA0196,KIAA0196 gene product | | |  |
| 227900_at | 10.17 | 2.58E-05 | 1.264870718 | NM_170662| | CBLB,Cas-Br-M (murine) ecotropic retroviral | | | |
| 207742_s_at | 10.17 | 2.57E-05 | 1.14988495 | NM_001489| | NR6A1,nuclear receptor subfamily 6, group A, member 1 | | | |
| 228097_at | 10.17 | 2.58E-05 | 1.47958956 | NM_013262| | MYLIP,myosin regulatory light chain interacting | | | |
| 214430_at | 10.17 | 2.59E-05 | 1.051668364 | NM_000169| | GLA,galactosidase, alpha | |  |  |
| 244623_at | 10.16 | 2.60E-05 | 1.395695808 | NM_019842| | KCNQ5,potassium voltage-gated channel, KQT-like | | | |
| 224861_at | 10.16 | 2.59E-05 | 1.077995088 | NM_002072| | GNAQ,guanine nucleotide binding protein (G protein), | | | |
| 227037_at | 10.16 | 2.60E-05 | 1.130483938 | NM_178836| | LOC201164,similar to CG12314 gene product | | | |
| 228256_s_at | 10.16 | 2.59E-05 | 1.109029981 | NM_022140| | EPB41L4A,erythrocyte protein band 4.1-like 4 | | | |
| 227516_at | 10.16 | 2.59E-05 | 1.061911421 | NM_001005409| | SF3A1,splicing factor 3a, subunit 1, 120kDa isoform 2 | | | |
| 213034_at | 10.15 | 2.62E-05 | 1.03393882 | NM_025164| | KIAA0999,KIAA0999 protein | | |  |
| 219196_at | 10.15 | 2.63E-05 | 1.214103595 | NM_013243| | SCG3,secretogranin III | |  |  |
| 218024_at | 10.15 | 2.61E-05 | 1.094548073 | NM_016098| | BRP44L,brain protein 44-like | | |  |
| 204370_at | 10.15 | 2.62E-05 | 1.039895213 | NM_006831| | HEAB,ATP/GTP-binding protein | | |  |
| 220637_at | 10.15 | 2.61E-05 | 1.149968343 | NM_001122779| | NA |  |  |  |
| 225720_at | 10.14 | 2.64E-05 | 1.158345307 | NM_133477| | SYNPO2,synaptopodin 2 | |  |  |
| 236691_at | 10.14 | 2.64E-05 | 1.24400778 | NA |  |  |  |  |
| 244350_at | 10.14 | 2.63E-05 | 1.334405866 | NM_012334| | MYO10,myosin X | |  |  |
| 232212_at | 10.14 | 2.64E-05 | 1.101789743 | NM_032639| | PLEKHA8,pleckstrin homology domain containing, family A | | | |
| 211965_at | 10.14 | 2.63E-05 | 1.158313783 | NM_004926| | ZFP36L1,butyrate response factor 1 | | |  |
| 222947_at | 10.13 | 2.66E-05 | 1.354982723 | NA |  |  |  |  |
| 225662_at | 10.13 | 2.66E-05 | 1.146605486 | NM_016653| | ZAK,sterile-alpha motif and leucine zipper | | | |
| 218223_s_at | 10.13 | 2.65E-05 | 1.07582882 | NM_016274| | CKIP-1,CK2 interacting protein 1; HQ0024c protein | | | |
| 235092_at | 10.13 | 2.66E-05 | 1.183588777 | NA |  |  |  |  |
| 200014_s_at | 10.12 | 2.67E-05 | 1.024276419 | NM_001077442| | NA |  |  |  |
| 202630_at | 10.12 | 2.66E-05 | 1.112052412 | NM_006380| | APPBP2,amyloid beta precursor protein-binding protein | | | |
| 202733_at | 10.12 | 2.67E-05 | 1.111813278 | NM_001017973| | NA |  |  |  |
| 221196_x_at | 10.12 | 2.68E-05 | 1.102262082 | NM_001018055| | NA |  |  |  |
| 208180_s_at | 10.12 | 2.67E-05 | 1.186971633 | NM_001034077| | NA |  |  |  |
| 208863_s_at | 10.11 | 2.69E-05 | 1.059030084 | NM_001078166| | NA |  |  |  |
| 39650_s_at | 10.11 | 2.70E-05 | 1.122753042 | NM_014801| | PCNXL2,pecanex-like 2 | |  |  |
| 218314_s_at | 10.11 | 2.69E-05 | 1.068911459 | NM_001082969| | NA |  |  |  |
| 218358_at | 10.11 | 2.68E-05 | 1.058102086 | NM_024324| | MGC11256,hypothetical protein MGC11256 | | | |
| 203186_s_at | 10.1 | 2.71E-05 | 1.114749765 | NM_002961| | S100A4,S100 calcium-binding protein A4 | | | |
| 214787_at | 10.1 | 2.72E-05 | 1.23263771 | NM_005848| | MYCPBP,c-myc promoter binding protein | | | |
| 219041_s_at | 10.1 | 2.71E-05 | 1.029829735 | NM_001099695| | NA |  |  |  |
| 241706_at | 10.09 | 2.73E-05 | 1.127991395 | NM_153634| | CPNE8,copine VIII | |  |  |
| 226889_at | 10.09 | 2.72E-05 | 1.046914201 | NM_001006657| | WDR35,WD repeat domain 35 isoform 1 | | | |
| 204299_at | 10.09 | 2.73E-05 | 1.100791344 | NM_006625| | FUSIP1,FUS interacting protein (serine-arginine rich) 1 | | | |
| 200621_at | 10.09 | 2.73E-05 | 1.247998947 | NM_004078| | CSRP1,cysteine and glycine-rich protein 1 | | | |
| 203489_at | 10.09 | 2.74E-05 | 1.066702923 | NM_006427| | SIVA,CD27-binding (Siva) protein isoform 1 | | | |
| 205133_s_at | 10.08 | 2.75E-05 | 1.03709785 | NM_002157| | HSPE1,heat shock 10kDa protein 1 (chaperonin 10) | | | |
| 218676_s_at | 10.07 | 2.78E-05 | 1.069145251 | NM_001102402| | NA |  |  |  |
| 238129_s_at | 10.07 | 2.77E-05 | 1.152932929 | NA |  |  |  |  |
| 230927_at | 10.07 | 2.76E-05 | 1.251536094 | NA |  |  |  |  |
| 202428_x_at | 10.07 | 2.78E-05 | 1.048439224 | NM_001079862| | NA |  |  |  |
| 214632_at | 10.06 | 2.80E-05 | 1.218429186 | NM_003872| | NRP2,neuropilin 2 isoform 2 precursor | | |  |
| 225156_at | 10.06 | 2.80E-05 | 1.045813714 | NM_032377| | MGC4549,hypothetical protein MGC4549 | | | |
| 209556_at | 10.06 | 2.80E-05 | 1.157569472 | NM_001014839| | NA |  |  |  |
| 1555461_at | 10.06 | 2.80E-05 | 1.166659111 | NA |  |  |  |  |
| 208735_s_at | 10.05 | 2.81E-05 | 1.044262273 | NM_005730| | CTDSP2,nuclear LIM interactor-interacting factor 2 | | | |
| 222024_s_at | 10.05 | 2.81E-05 | 1.091171004 | NM_006738| | AKAP13,A-kinase anchor protein 13 isoform 1 | | | |
| 212604_at | 10.05 | 2.81E-05 | 1.063257515 | NM_005830| | MRPS31,mitochondrial ribosomal protein S31 | | | |
| 200718_s_at | 10.05 | 2.81E-05 | 1.013106959 | NM_006930| | SKP1A,S-phase kinase-associated protein 1A isoform a | | | |
| 214938_x_at | 10.05 | 2.82E-05 | 1.018025428 | NM_002128| | HMGB1,high-mobility group box 1 | | |  |
| 223610_at | 10.04 | 2.83E-05 | 1.08991634 | NM_001031702| | NA |  |  |  |
| 204731_at | 10.04 | 2.83E-05 | 1.213061493 | NM_003243| | TGFBR3,transforming growth factor, beta receptor III | | | |
| 218717_s_at | 10.04 | 2.83E-05 | 1.119663689 | NM_018192| | LEPREL1,leprecan-like 1 | |  |  |
| 224811_at | 10.03 | 2.86E-05 | 1.068957235 | NA |  |  |  |  |
| 224906_at | 10.02 | 2.88E-05 | 1.067922626 | NM_001025356| | NA |  |  |  |
| 228674_s_at | 10.02 | 2.86E-05 | 1.127862691 | NM_019063| | EML4,echinoderm microtubule associated protein like | | | |
| 226183_at | 10.02 | 2.86E-05 | 1.074035102 | NA |  |  |  |  |
| 213963_s_at | 10.02 | 2.88E-05 | 1.138453053 | NM_003864| | SAP30,sin3 associated polypeptide p30 | | |  |
| 231270_at | 10 | 2.91E-05 | 1.208038172 | NM_198584| | CA13,carbonic anhydrase XIII | | |  |
| 226032_at | 10 | 2.91E-05 | 1.065796825 | NM_032982| | CASP2,caspase 2 isoform 1 preproprotein | | | |
| 230165_at | 10 | 2.91E-05 | 1.07111926 | NM_152524| | SGOL2,shugoshin-like 2 | |  |  |
| 203643_at | 9.99 | 2.94E-05 | 1.049868512 | NM_006494| | ERF,Ets2 repressor factor | |  |  |
| 226034_at | 9.99 | 2.93E-05 | 1.112017589 | NA |  |  |  |  |
| 225564_at | 9.98 | 2.96E-05 | 1.106010779 | NM_153023| | SPATA13,spermatogenesis associated 13 | | | |
| 225037_at | 9.98 | 2.95E-05 | 1.05774649 | NM_015945| | SLC35C2,ovarian cancer overexpressed 1 isoform a | | | |
| 635_s_at | 9.98 | 2.95E-05 | 1.133438147 | NM_006244| | PPP2R5B,beta isoform of regulatory subunit B56, protein | | | |
| 209389_x_at | 9.98 | 2.95E-05 | 1.057771595 | NM_001079862| | NA |  |  |  |
| 238333_s_at | 9.98 | 2.95E-05 | 1.219896626 | NM_138384| | GTP,GTP_binding protein | |  |  |
| 218719_s_at | 9.97 | 2.98E-05 | 1.124741628 | NM_001126129| | NA |  |  |  |
| 201914_s_at | 9.97 | 2.98E-05 | 1.054835777 | NM_007214| | SEC63,SEC63-like protein | |  |  |
| 203675_at | 9.97 | 2.98E-05 | 1.11966898 | NM_005013| | NUCB2,nucleobindin 2 | |  |  |
| 212364_at | 9.97 | 2.98E-05 | 1.126480965 | NM_012223| | MYO1B,myosin IB | |  |  |
| 226981_at | 9.97 | 2.97E-05 | 1.103876425 | NM_005933| | MLL,myeloid/lymphoid or mixed-lineage leukemia | | | |
| 1552521_a_at | 9.97 | 2.98E-05 | 1.155353278 | NM_153015| | FLJ30668,hypothetical protein FLJ30668 | | | |
| 233124_s_at | 9.96 | 2.99E-05 | 1.070517549 | NM_001002030| | NA |  |  |  |
| 202146_at | 9.96 | 3.00E-05 | 1.111419157 | NM_001007245| | IFRD1,interferon-related developmental regulator 1 | | | |
| 213179_at | 9.96 | 2.99E-05 | 1.043895404 | NM_005444| | RQCD1,RCD1 required for cell differentiation1 homolog | | | |
| 1555443_at | 9.96 | 3.00E-05 | 1.127571591 | NM_198515| | NA |  |  |  |
| 238574_at | 9.96 | 2.99E-05 | 1.108077421 | NM_033412| | MCART1,mitochondrial carrier triple repeat 1 | | | |
| 227263_at | 9.96 | 3.00E-05 | 1.159547166 | NM_001013842| | NA |  |  |  |
| 243887_at | 9.95 | 3.00E-05 | 1.109950967 | NA |  |  |  |  |
| 225282_at | 9.95 | 3.00E-05 | 1.119489235 | NM_022733| | LOC64744,hypothetical protein AL133206 | | | |
| 223046_at | 9.95 | 3.02E-05 | 1.249165896 | NM_022051| | EGLN1,egl nine homolog 1 | | |  |
| 205110_s_at | 9.95 | 3.01E-05 | 1.041294655 | NM_004114| | FGF13,fibroblast growth factor 13 isoform 1A | | | |
| 223367_at | 9.95 | 3.02E-05 | 1.084111303 | NM_032317| | WBSCR18,Williams Beuren syndrome chromosome region 18 | | | |
| 205771_s_at | 9.95 | 3.01E-05 | 1.100853615 | NM_004842| | AKAP7,A-kinase anchor protein 7 isoform alpha | | | |
| 215358_x_at | 9.95 | 3.00E-05 | 1.232308907 | NA |  |  |  |  |
| 204283_at | 9.95 | 3.02E-05 | 1.044645778 | NM_006567| | FARS2,phenylalanine-tRNA synthetase 2 | | | |
| 236517_at | 9.94 | 3.03E-05 | 1.202938628 | NM_032446| | MEGF10,MEGF10 protein | |  |  |
| 202173_s_at | 9.94 | 3.03E-05 | 1.076434184 | NM_007146| | ZNF161,zinc finger protein 161 | | |  |
| 236622_at | 9.94 | 3.02E-05 | 1.282527284 | NM_145167| | PIGM,PIG-M mannosyltransferase | | |  |
| 209966_x_at | 9.94 | 3.03E-05 | 1.124549193 | NM_001438| | ESRRG,estrogen-related receptor gamma isoform 1 | | | |
| 206698_at | 9.93 | 3.05E-05 | 1.3111215 | NM_021083| | XK,McLeod syndrome-associated, Kell blood group | | | |
| 215843_s_at | 9.93 | 3.07E-05 | 1.179249939 | NM_012465| | TLL2,tolloid-like 2 | |  |  |
| 225307_at | 9.93 | 3.04E-05 | 1.060163419 | NM_145806| | ZNF511,zinc finger protein 511 | | |  |
| 218562_s_at | 9.92 | 3.08E-05 | 1.097418775 | NM_018202| | FLJ10747,hypothetical protein FLJ10747 | | | |
| 204933_s_at | 9.92 | 3.08E-05 | 1.230559591 | NM_002546| | TNFRSF11B,osteoprotegerin precursor | | |  |
| 200007_at | 9.92 | 3.08E-05 | 1.033010551 | NM_003134| | SRP14,signal recognition particle 14kDa (homologous | | | |
| 222020_s_at | 9.92 | 3.07E-05 | 1.291556877 | NM_001048209| | NA |  |  |  |
| 233070_at | 9.92 | 3.07E-05 | 1.104016263 | NM_001024855| | NA |  |  |  |
| 210046_s_at | 9.92 | 3.08E-05 | 1.071017831 | NM_002168| | IDH2,isocitrate dehydrogenase 2 (NADP+), | | | |
| 239897_at | 9.92 | 3.07E-05 | 1.175697226 | NM_001077440| | NA |  |  |  |
| 225168_at | 9.92 | 3.08E-05 | 1.192674857 | NM_018027| | FRMD4A,FERM domain containing 4A | | |  |
| 1555500_s_at | 9.91 | 3.09E-05 | 1.1229865 | NM_020062| | SLC2A4RG,SLC2A4 regulator | | |  |
| 207984_s_at | 9.9 | 3.11E-05 | 1.101203208 | NM_005374| | MPP2,palmitoylated membrane protein 2 | | | |
| 219439_at | 9.9 | 3.13E-05 | 1.070577364 | NM_020156| | C1GALT1,core 1 synthase, | | |  |
| 213379_at | 9.9 | 3.11E-05 | 1.059214471 | NM_015697| | CL640,hypothetical protein CL640 | | |  |
| 225210_s_at | 9.9 | 3.13E-05 | 1.032358834 | NM_031452| | MGC2560,hypothetical LOC83640 | | |  |
| 225114_at | 9.9 | 3.12E-05 | 1.093852108 | NM_003659| | AGPS,alkylglycerone phosphate synthase precursor | | | |
| 241364_at | 9.89 | 3.14E-05 | 1.121212638 | NM_018202| | FLJ10747,hypothetical protein FLJ10747 | | | |
| 205591_at | 9.89 | 3.14E-05 | 1.099841244 | NM_006334| | OLFM1,olfactomedin related ER localized protein | | | |
| 228694_at | 9.89 | 3.14E-05 | 1.052698469 | NA |  |  |  |  |
| 207002_s_at | 9.89 | 3.14E-05 | 1.427099757 | NM_001080951| | NA |  |  |  |
| 200961_at | 9.89 | 3.14E-05 | 1.03858979 | NM_012248| | SEPHS2,selenophosphate synthetase 2 | | |  |
| 228478_at | 9.89 | 3.13E-05 | 1.132869094 | NA |  |  |  |  |
| 215022_x_at | 9.88 | 3.17E-05 | 1.08194464 | NM_006955| | ZNF11B,zinc finger protein 11b (KOX 2) | | |  |
| 229640_x_at | 9.88 | 3.15E-05 | 1.036699098 | NA |  |  |  |  |
| 228378_at | 9.87 | 3.20E-05 | 1.140860805 | NM_001009894| | DKFZp434N2030,hypothetical protein DKFZp434N2030 | | | |
| 1552754_a_at | 9.87 | 3.18E-05 | 1.176201501 | NM_153184| | IGSF4D,immunoglobulin superfamily, member 4D | | | |
| 212622_at | 9.87 | 3.18E-05 | 1.057324597 | NM_015012| | NA |  |  |  |
| 227628_at | 9.86 | 3.21E-05 | 1.065139883 | NM_001008397| | LOC493869,similar to 2310016C16Rik protein | | | |
| 214803_at | 9.86 | 3.20E-05 | 1.186339768 | NA |  |  |  |  |
| 228369_at | 9.86 | 3.21E-05 | 1.139305499 | NM_006586| | TNRC5,trinucleotide repeat containing 5 | | | |
| 228996_at | 9.86 | 3.22E-05 | 1.15445166 | NM_172071| | NA |  |  |  |
| 205151_s_at | 9.85 | 3.22E-05 | 1.18205503 | NM_014817| | NA |  |  |  |
| 1552518_s_at | 9.85 | 3.22E-05 | 1.136253847 | NM_022045| | MTBP,Mdm2, transformed 3T3 cell double minute 2, p53 | | | |
| 211070_x_at | 9.85 | 3.23E-05 | 1.057104283 | NM_001079862| | NA |  |  |  |
| 213189_at | 9.84 | 3.26E-05 | 1.320119021 | NM_001042533| | NA |  |  |  |
| 210139_s_at | 9.84 | 3.27E-05 | 1.215604106 | NM_000304| | PMP22,peripheral myelin protein 22 | | |  |
| 220238_s_at | 9.84 | 3.25E-05 | 1.06892252 | NM_001031710| | NA |  |  |  |
| 210933_s_at | 9.84 | 3.25E-05 | 1.100672736 | NM_003088| | FSCN1,fascin 1 | |  |  |
| 226592_at | 9.83 | 3.27E-05 | 1.122555244 | NM_133374| | NA |  |  |  |
| 220999_s_at | 9.83 | 3.28E-05 | 1.247668229 | NM_001037332| | NA |  |  |  |
| 215952_s_at | 9.83 | 3.27E-05 | 1.025906957 | NM_004152| | OAZ1,ornithine decarboxylase antizyme 1 | | | |
| 213561_at | 9.82 | 3.29E-05 | 1.12927645 | NM_014034| | ASF1A,ASF1 anti-silencing function 1 homolog A | | | |
| 200831_s_at | 9.82 | 3.31E-05 | 1.105006319 | NM_005063| | SCD,stearoyl-CoA desaturase | | |  |
| 213093_at | 9.81 | 3.34E-05 | 1.121138236 | NM_002737| | PRKCA,protein kinase C, alpha | | |  |
| 208963_x_at | 9.81 | 3.33E-05 | 1.08203961 | NM_013402| | FADS1,fatty acid desaturase 1 | | |  |
| 209179_s_at | 9.81 | 3.32E-05 | 1.046815415 | NM_024298| | LENG4,leukocyte receptor cluster (LRC) member 4 | | | |
| 200941_at | 9.81 | 3.34E-05 | 1.066278661 | NM_001537| | HSBP1,heat shock factor binding protein 1 | | | |
| 239671_at | 9.81 | 3.34E-05 | 1.119449296 | NA |  |  |  |  |
| 1554588_a_at | 9.8 | 3.34E-05 | 1.141309271 | NM_152517| | FLJ30990,hypothetical protein FLJ30990 | | | |
| 243502_at | 9.79 | 3.38E-05 | 1.125721735 | NM_001080383| | NA |  |  |  |
| 221127_s_at | 9.79 | 3.38E-05 | 1.221552879 | NA |  |  |  |  |
| 219770_at | 9.79 | 3.37E-05 | 1.046084524 | NM_001006636| | GTDC1,glycosyltransferase-like domain containing 1 | | | |
| 213742_at | 9.79 | 3.37E-05 | 1.423161021 | NM_004768| | SFRS11,splicing factor p54 | | |  |
| 215947_s_at | 9.79 | 3.37E-05 | 1.044767322 | NM_032822| | FLJ14668,hypothetical protein FLJ14668 | | | |
| 222376_at | 9.78 | 3.41E-05 | 1.259645241 | NA |  |  |  |  |
| 207856_s_at | 9.78 | 3.41E-05 | 1.037741215 | NM_017751| | FLJ20297,hypothetical protein FLJ20297 | | | |
| 229173_at | 9.77 | 3.43E-05 | 1.152160319 | NM_030650| | KIAA1715,Lunapark | |  |  |
| 203685_at | 9.77 | 3.42E-05 | 1.267812246 | NM_000633| | BCL2,B-cell lymphoma protein 2 alpha isoform | | | |
| 217761_at | 9.77 | 3.44E-05 | 1.207693058 | NM_018269| | MTCBP-1,membrane-type 1 matrix metalloproteinase | | | |
| 225682_s_at | 9.77 | 3.44E-05 | 1.05289312 | NM_001018050| | NA |  |  |  |
| 226113_at | 9.77 | 3.42E-05 | 1.309472494 | NM_001077195| | NA |  |  |  |
| 232282_at | 9.76 | 3.44E-05 | 1.061888006 | NM_001002838| | WNK3,WNK lysine deficient protein kinase 3 isoform 2 | | | |
| 205631_at | 9.75 | 3.48E-05 | 1.076094475 | NM_014749| | KIAA0586,KIAA0586 | |  |  |
| 219551_at | 9.75 | 3.49E-05 | 1.092871047 | NM_018456| | EAF2,ELL associated factor 2 | | |  |
| 210616_s_at | 9.75 | 3.47E-05 | 1.035991659 | NM_001077206| | NA |  |  |  |
| 201503_at | 9.75 | 3.48E-05 | 1.049375546 | NM_005754| | G3BP,Ras-GTPase-activating protein SH3-domain-binding | | | |
| 235230_at | 9.75 | 3.49E-05 | 1.165367488 | NA |  |  |  |  |
| 205351_at | 9.74 | 3.49E-05 | 1.085253921 | NM_000821| | GGCX,gamma-glutamyl carboxylase | | |  |
| 201795_at | 9.74 | 3.50E-05 | 1.038480694 | NM_002296| | LBR,lamin B receptor | |  |  |
| 208818_s_at | 9.74 | 3.51E-05 | 1.135432217 | NM_000754| | COMT,catechol-O-methyltransferase isoform MB-COMT | | | |
| 235132_at | 9.74 | 3.50E-05 | 1.169016428 | NA |  |  |  |  |
| 231946_at | 9.74 | 3.50E-05 | 1.141814638 | NA |  |  |  |  |
| 220311_at | 9.74 | 3.50E-05 | 1.094623908 | NM_013240| | C21orf127,N6-DNA-methyltransferase isoform 1 | | | |
| 1552736_a_at | 9.74 | 3.50E-05 | 1.168051899 | NM_138966| | NETO1,neuropilin- and tolloid-like protein 1 isoform 3 | | | |
| 213329_at | 9.73 | 3.52E-05 | 1.106517587 | NM_001042758| | NA |  |  |  |
| 229362_at | 9.73 | 3.53E-05 | 1.093242072 | NM_144709| | FLJ32312,hypothetical protein FLJ32312 | | | |
| 242209_at | 9.73 | 3.52E-05 | 1.142337235 | NM_182608| | ANKRD33,ankyrin repeat domain 33 | | |  |
| 221737_at | 9.73 | 3.51E-05 | 1.136084503 | NM_007353| | GNA12,guanine nucleotide binding protein (G protein) | | | |
| 202647_s_at | 9.73 | 3.52E-05 | 1.052389048 | NM_002524| | NRAS,neuroblastoma RAS viral (v-ras) oncogene | | | |
| 228620_at | 9.73 | 3.52E-05 | 1.136817266 | NA |  |  |  |  |
| 208747_s_at | 9.73 | 3.52E-05 | 1.094795062 | NM_001734| | C1S,complement component 1, s subcomponent | | | |
| 203603_s_at | 9.72 | 3.56E-05 | 1.285527924 | NM_014795| | ZFHX1B,zinc finger homeobox 1b | | |  |
| 211098_x_at | 9.72 | 3.55E-05 | 1.075557368 | NM_019026| | LOC54499,putative membrane protein | | |  |
| 214006_s_at | 9.72 | 3.54E-05 | 1.066589016 | NM_000821| | GGCX,gamma-glutamyl carboxylase | | |  |
| 227230_s_at | 9.71 | 3.56E-05 | 1.142748946 | NM_020722| | NA |  |  |  |
| 210135_s_at | 9.71 | 3.57E-05 | 1.44656511 | NM_003030| | SHOX2,short stature homeobox 2 isoform b | | | |
| 211597_s_at | 9.71 | 3.56E-05 | 1.317468638 | NM_032495| | HOP,homeodomain-only protein | | |  |
| 227318_at | 9.71 | 3.57E-05 | 1.075463067 | NA |  |  |  |  |
| 235205_at | 9.7 | 3.61E-05 | 1.191073561 | NA |  |  |  |  |
| 220956_s_at | 9.7 | 3.61E-05 | 1.036621313 | NM_053046| | EGLN2,EGL nine (C.elegans) homolog 2 isoform 1 | | | |
| 235154_at | 9.7 | 3.60E-05 | 1.098926435 | NM_031923| | NA |  |  |  |
| 225026_at | 9.7 | 3.60E-05 | 1.090767037 | NM_032221| | CHD6,chromodomain helicase DNA binding protein 6 | | | |
| 209281_s_at | 9.69 | 3.62E-05 | 1.068993035 | NM_001001323| | ATP2B1,plasma membrane calcium ATPase 1 isoform 1a | | | |
| 238835_at | 9.69 | 3.62E-05 | 1.309514875 | NA |  |  |  |  |
| 212740_at | 9.69 | 3.62E-05 | 1.035613688 | NM_014602| | PIK3R4,phosphoinositide-3-kinase, regulatory subunit 4, | | | |
| 222402_at | 9.69 | 3.63E-05 | 1.124005214 | NM_015932| | C13orf12,chromosome 13 open reading frame 12 | | | |
| 214390_s_at | 9.68 | 3.64E-05 | 1.102760038 | NM_005504| | BCAT1,branched chain aminotransferase 1, cytosolic | | | |
| 230968_at | 9.68 | 3.64E-05 | 1.206916754 | NA |  |  |  |  |
| 244552_at | 9.68 | 3.64E-05 | 1.066094699 | NA |  |  |  |  |
| 200788_s_at | 9.68 | 3.64E-05 | 1.062039275 | NM_003768| | PEA15,phosphoprotein enriched in astrocytes 15 | | | |
| 212359_s_at | 9.68 | 3.66E-05 | 1.076266386 | NM_015037| | KIAA0913,KIAA0913 | |  |  |
| 228558_at | 9.67 | 3.68E-05 | 1.14023517 | NM_173608| | C14orf80,chromosome 14 open reading frame 80 | | | |
| 1557966_x_at | 9.67 | 3.68E-05 | 1.038448346 | NM_182501| | MGC61716,hypothetical protein MGC61716 | | | |
| 213497_at | 9.67 | 3.68E-05 | 1.104197815 | NM_145804| | ABTB2,ankyrin repeat and BTB (POZ) domain containing | | | |
| 229453_at | 9.67 | 3.68E-05 | 1.117794952 | NM_005313| | GRP58,glucose regulated protein, 58kDa | | | |
| 218566_s_at | 9.67 | 3.67E-05 | 1.06046745 | NM_012124| | CHORDC1,cysteine and histidine-rich domain | | | |
| 221008_s_at | 9.66 | 3.71E-05 | 1.398823711 | NM_031279| | AGXT2L1,alanine-glyoxylate aminotransferase 2-like 1 | | | |
| 221727_at | 9.66 | 3.70E-05 | 1.078178124 | NM_006713| | PC4,activated RNA polymerase II transcription | | | |
| 212333_at | 9.66 | 3.70E-05 | 1.028991229 | NM_015475| | DKFZP564F0522,DKFZP564F0522 protein | | | |
| 1568951_at | 9.66 | 3.70E-05 | 1.454198154 | NM_001002843| | SUHW4,suppressor of hairy wing homolog 4 isoform 2 | | | |
| 201595_s_at | 9.66 | 3.71E-05 | 1.045512453 | NM_018471| | LEREPO4,erythropoietin 4 immediate early response | | | |
| 222067_x_at | 9.66 | 3.69E-05 | 1.111575681 | NM_021063| | HIST1H2BD,H2B histone family, member B | | | |
| 202819_s_at | 9.65 | 3.72E-05 | 1.070497686 | NM_003198| | TCEB3,elongin A | |  |  |
| 225927_at | 9.65 | 3.71E-05 | 1.080288661 | NM_005921| | NA |  |  |  |
| 221987_s_at | 9.65 | 3.71E-05 | 1.066877642 | NM_018128| | FLJ10534,hypothetical protein FLJ10534 | | | |
| 238047_at | 9.64 | 3.74E-05 | 1.422364712 | NM_144967| | FLJ30058,hypothetical protein FLJ30058 | | | |
| 235692_at | 9.64 | 3.75E-05 | 1.180490265 | NM_001024666| | NA |  |  |  |
| 218577_at | 9.64 | 3.75E-05 | 1.033440207 | NM_017768| | FLJ20331,hypothetical protein FLJ20331 | | | |
| 47550_at | 9.63 | 3.77E-05 | 1.097298264 | NM_021020| | LZTS1,leucine zipper, putative tumor suppressor 1 | | | |
| 201348_at | 9.63 | 3.79E-05 | 1.104335469 | NM_002084| | GPX3,plasma glutathione peroxidase 3 precursor | | | |
| 218150_at | 9.63 | 3.78E-05 | 1.040047779 | NM_001037174| | NA |  |  |  |
| 214043_at | 9.63 | 3.79E-05 | 1.0414187 | NM_001040712| | NA |  |  |  |
| 205115_s_at | 9.63 | 3.77E-05 | 1.101825242 | NM_016196| | RBM19,RNA binding motif protein 19 | | |  |
| 220134_x_at | 9.62 | 3.82E-05 | 1.115641518 | NM_018166| | FLJ10647,hypothetical protein FLJ10647 | | | |
| 218981_at | 9.62 | 3.82E-05 | 1.111166805 | NM_020186| | ACN9,ACN9 homolog | |  |  |
| 221541_at | 9.61 | 3.84E-05 | 1.093614385 | NM_031476| | CRISPLD2,cysteine-rich secretory protein LCCL domain | | | |
| 226242_at | 9.61 | 3.84E-05 | 1.056190136 | NM_152379| | DKFZp547B1713,hypothetical protein DKFZp547B1713 | | | |
| 202375_at | 9.6 | 3.89E-05 | 1.126868749 | NM_014822| | SEC24D,Sec24-related protein D | | |  |
| 210024_s_at | 9.6 | 3.89E-05 | 1.05318039 | NM_006357| | UBE2E3,ubiquitin-conjugating enzyme E2E 3 | | | |
| 227838_at | 9.6 | 3.87E-05 | 1.255055142 | NA |  |  |  |  |
| 227618_at | 9.6 | 3.89E-05 | 1.111267857 | NA |  |  |  |  |
| 202854_at | 9.6 | 3.87E-05 | 1.023248979 | NM_000194| | HPRT1,hypoxanthine phosphoribosyltransferase 1 | | | |
| 223497_at | 9.59 | 3.91E-05 | 1.068461351 | NM_001105531| | NA |  |  |  |
| 202427_s_at | 9.59 | 3.89E-05 | 1.069668524 | NM_015415| | DKFZP564B167,DKFZP564B167 protein | | |  |
| 40687_at | 9.59 | 3.91E-05 | 1.152420728 | NM_002060| | GJA4,connexin 37 | |  |  |
| 208668_x_at | 9.59 | 3.91E-05 | 1.02194551 | NM_005517| | HMGN2,high-mobility group nucleosomal binding domain | | | |
| 201730_s_at | 9.59 | 3.91E-05 | 1.039917213 | NM_003292| | TPR,translocated promoter region (to activated MET | | | |
| 210757_x_at | 9.59 | 3.89E-05 | 1.241358386 | NM_001343| | DAB2,disabled homolog 2 | |  |  |
| 218782_s_at | 9.59 | 3.91E-05 | 1.0661519 | NM_014109| | ATAD2,two AAA domain containing protein | | | |
| 225485_at | 9.59 | 3.91E-05 | 1.132976693 | NM_018718| | TSGA14,testis specific, 14 | |  |  |
| 205825_at | 9.59 | 3.91E-05 | 1.249406517 | NM_000439| | PCSK1,proprotein convertase subtilisin/kexin type 1 | | | |
| 225006_x_at | 9.59 | 3.89E-05 | 1.114358365 | NM_198976| | TH1L,TH1-like protein | |  |  |
| 210691_s_at | 9.59 | 3.91E-05 | 1.050369693 | NM_001007214| | CACYBP,calcyclin binding protein isoform 2 | | | |
| 227330_x_at | 9.59 | 3.91E-05 | 1.246110471 | NM_001033515| | NA |  |  |  |
| 220432_s_at | 9.58 | 3.92E-05 | 1.479825986 | NM_016593| | CYP39A1,cytochrome P450, family 39, subfamily A, | | | |
| 217869_at | 9.58 | 3.92E-05 | 1.032561555 | NM_016142| | HSD17B12,steroid dehydrogenase homolog | | | |
| 229460_at | 9.58 | 3.92E-05 | 1.070714095 | NA |  |  |  |  |
| 202520_s_at | 9.58 | 3.92E-05 | 1.038471278 | NM_000249| | MLH1,MutL protein homolog 1 | | |  |
| 212353_at | 9.57 | 3.96E-05 | 1.45829823 | NM_015170| | SULF1,sulfatase 1 | |  |  |
| 236219_at | 9.57 | 3.95E-05 | 1.312024888 | NA |  |  |  |  |
| 201275_at | 9.57 | 3.97E-05 | 1.079881516 | NM_002004| | FDPS,farnesyl diphosphate synthase | | |  |
| 222843_at | 9.56 | 3.98E-05 | 1.243387256 | NM_001042762| | NA |  |  |  |
| 218276_s_at | 9.56 | 3.99E-05 | 1.046832254 | NM_021818| | SAV1,WW45 protein | |  |  |
| 213133_s_at | 9.56 | 3.99E-05 | 1.019942754 | NM_004483| | GCSH,glycine cleavage system protein H (aminomethyl | | | |
| 1569334_at | 9.56 | 3.98E-05 | 1.308718693 | NM_022369| | STRA6,stimulated by retinoic acid gene 6 homolog | | | |
| 229281_at | 9.56 | 3.97E-05 | 1.338492551 | NM_022123| | NPAS3,neuronal PAS domain protein 3 | | |  |
| 221002_s_at | 9.55 | 4.01E-05 | 1.054225498 | NM_030927| | TM4SF14,transmembrane 4 superfamily member 14 | | | |
| 225219_at | 9.55 | 4.02E-05 | 1.058716466 | NM_001001419| | SMAD5,SMAD, mothers against DPP homolog 5 | | | |
| 212649_at | 9.55 | 4.01E-05 | 1.105011362 | NM_019030| | DHX29,DEAH (Asp-Glu-Ala-His) box polypeptide 29 | | | |
| 222962_s_at | 9.55 | 4.02E-05 | 1.051830198 | NM_018518| | MCM10,minichromosome maintenance protein 10 isoform 2 | | | |
| 221732_at | 9.54 | 4.03E-05 | 1.082355207 | NM_138793| | CANT1,calcium activated nucleotidase 1 | | |  |
| 201662_s_at | 9.54 | 4.03E-05 | 1.126454811 | NM_004457| | ACSL3,acyl-CoA synthetase long-chain family member 3 | | | |
| 209276_s_at | 9.54 | 4.05E-05 | 1.112240917 | NM_001118890| | NA |  |  |  |
| 201454_s_at | 9.54 | 4.05E-05 | 1.043646755 | NM_006310| | NPEPPS,aminopeptidase puromycin sensitive | | | |
| 226580_at | 9.54 | 4.05E-05 | 1.072870866 | NM_032352| | BRMS1L,breast cancer metastasis-suppressor 1-like | | | |
| 221814_at | 9.54 | 4.05E-05 | 1.140336942 | NM_032777| | GPR124,G protein-coupled receptor 124 | | | |
| 220330_s_at | 9.54 | 4.05E-05 | 1.635072864 | NM_022136| | SAMSN1,SAM domain, SH3 domain and nuclear localisation | | | |
| 202532_s_at | 9.54 | 4.05E-05 | 1.232869379 | NM_000791| | DHFR,dihydrofolate reductase | | |  |
| 242324_x_at | 9.53 | 4.07E-05 | 1.191728404 | NM_133459| | CCBE1,collagen and calcium binding EGF domains 1 | | | |
| 217943_s_at | 9.53 | 4.08E-05 | 1.083771315 | NM_018067| | FLJ10350,hypothetical protein FLJ10350 | | | |
| 244194_at | 9.53 | 4.06E-05 | 1.16522461 | NM_004194| | ADAM22,a disintegrin and metalloproteinase domain 22 | | | |
| 227663_at | 9.53 | 4.07E-05 | 1.177360259 | NA |  |  |  |  |
| 226029_at | 9.53 | 4.05E-05 | 1.065667443 | NM_020335| | NA |  |  |  |
| 214789_x_at | 9.52 | 4.11E-05 | 1.042957281 | NM_032102| | SRP46,Splicing factor, arginine/serine-rich, 46kD | | | |
| 222568_at | 9.51 | 4.14E-05 | 1.084580236 | NM_001025777| | NA |  |  |  |
| 235409_at | 9.51 | 4.12E-05 | 1.056774198 | NM_001080541| | NA |  |  |  |
| 221437_s_at | 9.5 | 4.17E-05 | 1.044930013 | NM_031280| | MRPS15,mitochondrial ribosomal protein S15 | | | |
| 231361_at | 9.5 | 4.16E-05 | 1.47614045 | NM_014932| | NLGN1,neuroligin 1 | |  |  |
| 217640_x_at | 9.5 | 4.15E-05 | 1.063877173 | NM_001039535| | NA |  |  |  |
| 201022_s_at | 9.5 | 4.15E-05 | 1.036726296 | NM_001011546| | DSTN,destrin isoform b | |  |  |
| 204850_s_at | 9.5 | 4.16E-05 | 1.296936609 | NM_000555| | DCX,doublecortin isoform a | | |  |
| 1569607_s_at | 9.49 | 4.19E-05 | 1.317775634 | NM_001012419| | LOC441425,OTTHUMP00000015360 | | |  |
| 205935_at | 9.48 | 4.22E-05 | 1.374346402 | NM_001451| | FOXF1,forkhead box F1 | |  |  |
| 242630_at | 9.48 | 4.23E-05 | 1.19151727 | NA |  |  |  |  |
| 214582_at | 9.48 | 4.22E-05 | 1.228105979 | NM_000922| | PDE3B,phosphodiesterase 3B, cGMP-inhibited | | | |
| 218465_at | 9.48 | 4.22E-05 | 1.077544858 | NM_018126| | TMEM33,transmembrane protein 33 | | |  |
| 206541_at | 9.47 | 4.25E-05 | 1.118462597 | NM_000892| | KLKB1,plasma kallikrein B1 precursor | | |  |
| 210849_s_at | 9.47 | 4.26E-05 | 1.072054012 | NM_014396| | VPS41,vacuolar protein sorting 41 (yeast homolog) | | | |
| 237061_at | 9.47 | 4.27E-05 | 1.198246389 | NM_032584| | ZNF347,zinc finger protein 347 | | |  |
| 225086_at | 9.47 | 4.26E-05 | 1.026146777 | NM_001042429| | NA |  |  |  |
| 244631_at | 9.46 | 4.30E-05 | 1.224361154 | NM_001013655| | NA |  |  |  |
| 209610_s_at | 9.46 | 4.28E-05 | 1.210428522 | NM_003038| | SLC1A4,solute carrier family 1, member 4 | | | |
| 212864_at | 9.46 | 4.28E-05 | 1.055755427 | NM_003818| | CDS2,phosphatidate cytidylyltransferase 2 | | | |
| 223984_s_at | 9.45 | 4.31E-05 | 1.173277914 | NM_001008564| | NUPL1,nucleoporin like 1 isoform b | | |  |
| 235803_at | 9.45 | 4.34E-05 | 1.224910112 | NA |  |  |  |  |
| 242538_at | 9.45 | 4.34E-05 | 1.258469718 | NM_007111| | TFDP1,transcription factor Dp-1 | | |  |
| 225567_at | 9.44 | 4.37E-05 | 1.127949695 | NA |  |  |  |  |
| 224732_at | 9.44 | 4.37E-05 | 1.049753623 | NM_001039690| | NA |  |  |  |
| 228185_at | 9.43 | 4.39E-05 | 1.214450324 | NM_145011| | ZNF25,zinc finger protein 25 | | |  |
| 223814_at | 9.43 | 4.39E-05 | 1.269705049 | NM_182916| | TRNT1,CGI-47 protein | |  |  |
| 230859_at | 9.43 | 4.39E-05 | 1.186294049 | NA |  |  |  |  |
| 203797_at | 9.43 | 4.39E-05 | 1.255285463 | NM_003385| | VSNL1,visinin-like 1 | |  |  |
| 206051_at | 9.42 | 4.43E-05 | 1.280858833 | NM_021952| | ELAVL4,ELAV (embryonic lethal, abnormal vision, | | | |
| 235109_at | 9.42 | 4.43E-05 | 1.111330273 | NA |  |  |  |  |
| 229511_at | 9.41 | 4.48E-05 | 1.35801244 | NM_003079| | SMARCE1,SWI/SNF-related matrix-associated | | | |
| 204860_s_at | 9.41 | 4.46E-05 | 1.157841568 | NM_004536| | BIRC1,baculoviral IAP repeat-containing 1 | | | |
| 206001_at | 9.4 | 4.52E-05 | 1.408885901 | NM_000905| | NPY,neuropeptide Y | |  |  |
| 211991_s_at | 9.4 | 4.51E-05 | 1.184820627 | NM_033554| | HLA-DPA1,major histocompatibility complex, class II, DP | | | |
| 205034_at | 9.4 | 4.51E-05 | 1.139390303 | NM_057749| | CCNE2,cyclin E2 isoform 1 | |  |  |
| 208851_s_at | 9.4 | 4.50E-05 | 1.130492788 | NM_006288| | THY1,Thy-1 cell surface antigen | | |  |
| 203283_s_at | 9.4 | 4.49E-05 | 1.078648357 | NM_012262| | HS2ST1,heparan sulfate 2-O-sulfotransferase 1 | | | |
| 203580_s_at | 9.4 | 4.50E-05 | 1.043033943 | NM_001076785| | NA |  |  |  |
| 210792_x_at | 9.4 | 4.51E-05 | 1.059015188 | NM_006427| | SIVA,CD27-binding (Siva) protein isoform 1 | | | |
| 225501_at | 9.39 | 4.53E-05 | 1.056668844 | NM_001015877| | NA |  |  |  |
| 219124_at | 9.39 | 4.55E-05 | 1.04172406 | NM_001102401| | NA |  |  |  |
| 241912_at | 9.39 | 4.53E-05 | 1.109998747 | NA |  |  |  |  |
| 217746_s_at | 9.39 | 4.55E-05 | 1.014111056 | NM_013374| | PDCD6IP,programmed cell death 6 interacting protein | | | |
| 208002_s_at | 9.39 | 4.54E-05 | 1.073048485 | NM_007274| | BACH,brain acyl-CoA hydrolase isoform hBACHa | | | |
| 214737_x_at | 9.39 | 4.53E-05 | 1.019283572 | NM_001077442| | NA |  |  |  |
| 212163_at | 9.39 | 4.52E-05 | 1.04784899 | NM_020738| | KIDINS220,kinase D-interacting substance of 220 kDa | | | |
| 208838_at | 9.39 | 4.52E-05 | 1.033934415 | NM_018448| | TIP120A,TIP120 protein | |  |  |
| 206777_s_at | 9.38 | 4.56E-05 | 1.272877396 | NM_000496| | CRYBB2,crystallin, beta B2 | | |  |
| 209849_s_at | 9.38 | 4.57E-05 | 1.108718118 | NM_002876| | RAD51C,RAD51 homolog C isoform 2 | | |  |
| 240806_at | 9.38 | 4.57E-05 | 1.126341075 | NM_002948| | RPL15,ribosomal protein L15 | | |  |
| 208190_s_at | 9.38 | 4.58E-05 | 1.10322359 | NM_015925| | LISCH7,LISCH protein isoform 1 | | |  |
| 202614_at | 9.38 | 4.58E-05 | 1.027775205 | NM_006345| | SLC30A9,solute carrier family 30 (zinc transporter), | | | |
| 205530_at | 9.37 | 4.59E-05 | 1.135735688 | NM_004453| | ETFDH,electron-transferring-flavoprotein | | | |
| 222037_at | 9.37 | 4.60E-05 | 1.05458233 | NM_005914| | MCM4,minichromosome maintenance protein 4 | | | |
| 219504_s_at | 9.37 | 4.60E-05 | 1.116847867 | NM_024813| | FLJ13150,hypothetical protein FLJ13150 | | | |
| 223319_at | 9.36 | 4.64E-05 | 1.086858911 | NM_001024218| | NA |  |  |  |
| 224586_x_at | 9.36 | 4.64E-05 | 1.020885248 | NM_006713| | PC4,activated RNA polymerase II transcription | | | |
| 219324_at | 9.35 | 4.67E-05 | 1.08229551 | NM_001039141| | NA |  |  |  |
| 234295_at | 9.35 | 4.68E-05 | 1.107251224 | NM_016216| | DBR1,debranching enzyme homolog 1 | | |  |
| 225261_x_at | 9.35 | 4.68E-05 | 1.112743358 | NM_198976| | TH1L,TH1-like protein | |  |  |
| 213160_at | 9.35 | 4.67E-05 | 1.1882238 | NM_004946| | DOCK2,dedicator of cytokinesis 2 | | |  |
| 228774_at | 9.35 | 4.66E-05 | 1.115809576 | NM_001098802| | NA |  |  |  |
| 235129_at | 9.34 | 4.72E-05 | 1.181331456 | NM_006741| | PPP1R1A,protein phosphatase 1, regulatory (inhibitor) | | | |
| 203647_s_at | 9.34 | 4.72E-05 | 1.046262389 | NM_004109| | FDX1,ferredoxin 1 precursor | | |  |
| 212110_at | 9.34 | 4.69E-05 | 1.07961131 | NM_015359| | SLC39A14,solute carrier family 39 (zinc transporter), | | | |
| 227337_at | 9.34 | 4.69E-05 | 1.316012684 | NM_181726| | ANKRD37,ankyrin repeat domain 37 | | |  |
| 212441_at | 9.33 | 4.75E-05 | 1.054558629 | NM_001100590| | NA |  |  |  |
| 202384_s_at | 9.33 | 4.74E-05 | 1.062022103 | NM_000356| | TCOF1,Treacher Collins-Franceschetti syndrome 1 | | | |
| 227345_at | 9.33 | 4.75E-05 | 1.24317084 | NM_003840| | TNFRSF10D,tumor necrosis factor receptor superfamily, | | | |
| 218692_at | 9.33 | 4.76E-05 | 1.173138799 | NM_001099743| | NA |  |  |  |
| 213682_at | 9.33 | 4.74E-05 | 1.036784276 | NM_007172| | NUP50,nucleoporin 50kDa isoform b | | |  |
| 229523_at | 9.32 | 4.79E-05 | 1.101554862 | NM_001080209| | NA |  |  |  |
| 202651_at | 9.32 | 4.78E-05 | 1.099007268 | NM_014873| | LPGAT1,lysophosphatidylglycerol acyltransferase 1 | | | |
| 211072_x_at | 9.32 | 4.78E-05 | 1.015673662 | NM_006082| | K-ALPHA-1,tubulin, alpha, ubiquitous | | |  |
| 220643_s_at | 9.31 | 4.84E-05 | 1.123063527 | NM_001033030| | NA |  |  |  |
| 228390_at | 9.31 | 4.83E-05 | 1.295694541 | NA |  |  |  |  |
| 229073_at | 9.31 | 4.83E-05 | 1.257453739 | NA |  |  |  |  |
| 229267_at | 9.31 | 4.83E-05 | 1.052487029 | NA |  |  |  |  |
| 226709_at | 9.31 | 4.81E-05 | 1.247926304 | NM_002942| | NA |  |  |  |
| 202047_s_at | 9.31 | 4.81E-05 | 1.092488602 | NM_014292| | CBX6,chromobox homolog 6 | | |  |
| 212150_at | 9.31 | 4.81E-05 | 1.235657363 | NM_015137| | KIAA0143,KIAA0143 protein | | |  |
| 202338_at | 9.31 | 4.83E-05 | 1.029540221 | NM_003258| | TK1,thymidine kinase 1, soluble | | |  |
| 55662_at | 9.31 | 4.83E-05 | 1.133385887 | NM_024541| | C10orf76,chromosome 10 open reading frame 76 | | | |
| 235545_at | 9.3 | 4.87E-05 | 1.080135854 | NM_001114120| | NA |  |  |  |
| 224576_at | 9.3 | 4.86E-05 | 1.039882913 | NM_001031711| | NA |  |  |  |
| 242607_at | 9.3 | 4.86E-05 | 1.20119824 | NA |  |  |  |  |
| 201338_x_at | 9.3 | 4.86E-05 | 1.037544442 | NM_002097| | GTF3A,general transcription factor IIIA | | |  |
| 230031_at | 9.3 | 4.87E-05 | 1.119080946 | NM_005347| | HSPA5,heat shock 70kDa protein 5 (glucose-regulated | | | |
| 225377_at | 9.29 | 4.89E-05 | 1.086421515 | NM_017995| | NA |  |  |  |
| 227995_at | 9.29 | 4.90E-05 | 1.173991437 | NA |  |  |  |  |
| 235915_at | 9.29 | 4.88E-05 | 1.200398747 | NA |  |  |  |  |
| 201972_at | 9.29 | 4.88E-05 | 1.027870683 | NM_001690| | ATP6V1A,ATPase, H+ transporting, lysosomal 70kD, V1 | | | |
| 209511_at | 9.29 | 4.90E-05 | 1.053068686 | NM_021974| | POLR2F,DNA directed RNA polymerase II polypeptide F | | | |
| 231532_at | 9.29 | 4.88E-05 | 1.124123017 | NM_000615| | NCAM1,neural cell adhesion molecule 1 | | |  |
| 228190_at | 9.28 | 4.93E-05 | 1.049700078 | NM_014633| | SH2BP1,SH2 domain binding protein 1 | | |  |
| 205350_at | 9.28 | 4.94E-05 | 1.072705851 | NM_004378| | CRABP1,cellular retinoic acid binding protein 1 | | | |
| 226276_at | 9.28 | 4.94E-05 | 1.027026476 | NM_174909| | MGC23909,hypothetical protein MGC23909 | | | |
| 238755_at | 9.28 | 4.92E-05 | 1.293119901 | NA |  |  |  |  |
| 230556_at | 9.28 | 4.93E-05 | 1.117610114 | NM_144981| | FLJ25059,hypothetical protein FLJ25059 | | | |
| 219267_at | 9.27 | 4.96E-05 | 1.250296038 | NM_016433| | GLTP,glycolipid transfer protein | | |  |
| 201261_x_at | 9.27 | 4.97E-05 | 1.183531214 | NM_001711| | BGN,biglycan preproprotein | | |  |
| 212731_at | 9.27 | 4.96E-05 | 1.041852831 | NM_198401| | LOC157567,hypothetical protein LOC157567 | | | |
| 235949_at | 9.27 | 4.96E-05 | 1.13415827 | NA |  |  |  |  |
| 212282_at | 9.27 | 4.97E-05 | 1.065428455 | NM_014573| | MAC30,hypothetical protein MAC30 | | |  |
| 229004_at | 9.27 | 4.96E-05 | 1.223096021 | NA |  |  |  |  |
| 242055_at | 9.27 | 4.96E-05 | 1.195323415 | NA |  |  |  |  |
| 212281_s_at | 9.27 | 4.96E-05 | 1.071917168 | NM_014573| | MAC30,hypothetical protein MAC30 | | |  |
| 212523_s_at | 9.26 | 4.98E-05 | 1.157147582 | NM_001080394| | NA |  |  |  |
| 213438_at | 9.26 | 4.98E-05 | 1.333713611 | NM_015090| | NFASC,neurofascin isoform 2 | | |  |
| 238078_at | 9.26 | 4.99E-05 | 1.106666188 | NA |  |  |  |  |
| 218793_s_at | 9.25 | 5.06E-05 | 1.41408495 | NM_001037535| | NA |  |  |  |
| 221983_at | 9.25 | 5.05E-05 | 1.068451186 | NM_024293| | C2orf17,chromosome 2 open reading frame 17 | | | |
| 212681_at | 9.25 | 5.05E-05 | 1.139827199 | NM_012307| | EPB41L3,erythrocyte membrane protein band 4.1-like 3 | | | |
| 239733_at | 9.25 | 5.06E-05 | 1.130342212 | NM_032372| | MGC16186,hypothetical protein MGC16186 | | | |
| 201054_at | 9.25 | 5.06E-05 | 1.021604364 | NM_006805| | HNRPA0,heterogeneous nuclear ribonucleoprotein A0 | | | |
| 228710_at | 9.24 | 5.10E-05 | 1.144750286 | NA |  |  |  |  |
| 229664_at | 9.24 | 5.09E-05 | 1.049948553 | NM_002750| | MAPK8,mitogen-activated protein kinase 8 isoform 2 | | | |
| 205625_s_at | 9.24 | 5.06E-05 | 1.14611085 | NM_004929| | CALB1,calbindin 1 | |  |  |
| 220235_s_at | 9.23 | 5.13E-05 | 1.071300876 | NM_001006945| | RIF1,receptor-interacting factor 1 isoform 2 | | | |
| 223039_at | 9.23 | 5.14E-05 | 1.043206832 | NM_031444| | C22orf13,chromosome 22 open reading frame 13 | | | |
| 228736_at | 9.23 | 5.14E-05 | 1.257399383 | NM_133636| | HEL308,DNA helicase HEL308 | | |  |
| 227025_at | 9.23 | 5.14E-05 | 1.080762388 | NM_016488| | PPHLN1,periphilin 1 isoform 1 | | |  |
| 216457_s_at | 9.23 | 5.14E-05 | 1.03599102 | NM_001005409| | SF3A1,splicing factor 3a, subunit 1, 120kDa isoform 2 | | | |
| 212461_at | 9.23 | 5.14E-05 | 1.054234418 | NM_015878| | OAZIN,ornithine decarboxylase antizyme inhibitor | | | |
| 205709_s_at | 9.22 | 5.17E-05 | 1.099379527 | NM_001263| | CDS1,phosphatidate cytidylyltransferase 1 | | | |
| 225365_at | 9.22 | 5.17E-05 | 1.083662648 | NM_153251| | FLJ25952,DHHC-containing protein 20 | | |  |
| 202093_s_at | 9.22 | 5.16E-05 | 1.074747716 | NM_019088| | PD2,hypothetical protein F23149_1 | | |  |
| 223181_at | 9.22 | 5.14E-05 | 1.060091409 | NM_014177| | C18orf55,chromosome 18 open reading frame 55 | | | |
| 225521_at | 9.22 | 5.14E-05 | 1.294082658 | NM_016238| | ANAPC7,anaphase-promoting complex subunit 7 | | | |
| 201853_s_at | 9.22 | 5.17E-05 | 1.051074994 | NM_004358| | CDC25B,cell division cycle 25B isoform 1 | | | |
| 239834_at | 9.21 | 5.19E-05 | 1.161722839 | NA |  |  |  |  |
| 1554655_a_at | 9.21 | 5.20E-05 | 1.179032698 | NM_203400| | LOC388394,similar to candidate mediator of the | | | |
| 203760_s_at | 9.21 | 5.18E-05 | 1.133231296 | NM_001045556| | NA |  |  |  |
| 221548_s_at | 9.21 | 5.18E-05 | 1.067369105 | NM_030768| | ILKAP,integrin-linked kinase-associated protein | | | |
| 228096_at | 9.21 | 5.18E-05 | 1.262894318 | NM_001032363| | NA |  |  |  |
| 227844_at | 9.2 | 5.22E-05 | 1.217017404 | NM_175736| | FMNL3,formin-like 3 isoform 1 | | |  |
| 228832_at | 9.2 | 5.22E-05 | 1.166943057 | NA |  |  |  |  |
| 224484_s_at | 9.2 | 5.22E-05 | 1.18815681 | NM_032352| | BRMS1L,breast cancer metastasis-suppressor 1-like | | | |
| 218060_s_at | 9.2 | 5.22E-05 | 1.100684465 | NM_024598| | FLJ13154,hypothetical protein FLJ13154 | | | |
| 217764_s_at | 9.2 | 5.24E-05 | 1.058273801 | NM_006868| | RAB31,RAB31, member RAS oncogene family | | | |
| 214920_at | 9.2 | 5.21E-05 | 1.365510964 | NM_015204| | NA |  |  |  |
| 210002_at | 9.19 | 5.27E-05 | 1.488876521 | NM_005257| | GATA6,GATA binding protein 6 | | |  |
| 242871_at | 9.19 | 5.25E-05 | 1.206280285 | NM_001104554| | NA |  |  |  |
| 1566603_s_at | 9.18 | 5.32E-05 | 1.148805961 | NM_173659| | RPUSD3,RNA pseudouridylate synthase domain containing | | | |
| 212739_s_at | 9.18 | 5.32E-05 | 1.035008797 | NM_005009| | NME4,nucleoside-diphosphate kinase 4 | | |  |
| 221481_x_at | 9.18 | 5.32E-05 | 1.013850958 | NM_001003810| | HNRPD,heterogeneous nuclear ribonucleoprotein D | | | |
| 228008_at | 9.18 | 5.32E-05 | 1.032459266 | NA |  |  |  |  |
| 213390_at | 9.17 | 5.35E-05 | 1.070745846 | NM_015168| | NA |  |  |  |
| 225548_at | 9.17 | 5.36E-05 | 1.0409318 | NM_020859| | ShrmL,Shroom-related protein | | |  |
| 224862_at | 9.17 | 5.32E-05 | 1.05712549 | NM_002072| | GNAQ,guanine nucleotide binding protein (G protein), | | | |
| 208670_s_at | 9.17 | 5.33E-05 | 1.046086985 | NM_014335| | CRI1,CREBBP/EP300 inhibitor 1 | | |  |
| 205344_at | 9.17 | 5.36E-05 | 1.158055951 | NM_006574| | CSPG5,chondroitin sulfate proteoglycan 5 (neuroglycan | | | |
| 205434_s_at | 9.17 | 5.35E-05 | 1.084094418 | NM_014911| | AAK1,AP2 associated kinase 1 | | |  |
| 204274_at | 9.17 | 5.33E-05 | 1.076834398 | NM_004215| | EBAG9,estrogen receptor binding site associated | | | |
| 218588_s_at | 9.17 | 5.33E-05 | 1.08226803 | NM_018691| | C5orf3,chromosome 5 open reading frame 3 | | | |
| 212687_at | 9.16 | 5.39E-05 | 1.061412965 | NM_004987| | LIMS1,LIM and senescent cell antigen-like domains 1 | | | |
| 203444_s_at | 9.15 | 5.40E-05 | 1.205998825 | NM_004739| | MTA2,metastasis-associated protein 2 | | |  |
| 224709_s_at | 9.15 | 5.43E-05 | 1.038318621 | NM_001038702| | NA |  |  |  |
| 229300_at | 9.15 | 5.42E-05 | 1.207334176 | NA |  |  |  |  |
| 32209_at | 9.15 | 5.41E-05 | 1.057975859 | NM_001098784| | NA |  |  |  |
| 224847_at | 9.15 | 5.43E-05 | 1.159796891 | NM_001259| | CDK6,cyclin-dependent kinase 6 | | |  |
| 1570032_at | 9.14 | 5.48E-05 | 1.346928802 | NM_004644| | AP3B2,adaptor-related protein complex 3, beta 2 | | | |
| 225133_at | 9.14 | 5.49E-05 | 1.103715073 | NM_016531| | KLF3,Kruppel-like factor 3 (basic) | | |  |
| 217733_s_at | 9.14 | 5.48E-05 | 1.028997183 | NM_021103| | TMSB10,thymosin, beta 10 | | |  |
| 221952_x_at | 9.14 | 5.49E-05 | 1.027224681 | NM_020810| | KIAA1393,tRNA-(N1G37) methyltransferase | | | |
| 201310_s_at | 9.14 | 5.48E-05 | 1.050185115 | NM_004772| | C5orf13,neuronal protein 3.1 | | |  |
| 213423_x_at | 9.14 | 5.46E-05 | 1.03739598 | NM_006765| | TUSC3,tumor suppressor candidate 3 isoform a | | | |
| 204415_at | 9.13 | 5.50E-05 | 1.137766112 | NM_002038| | G1P3,interferon induced 6-16 protein isoform a | | | |
| 218280_x_at | 9.13 | 5.50E-05 | 1.157765909 | NM_001040874| | NA |  |  |  |
| 209525_at | 9.13 | 5.50E-05 | 1.071288721 | NM_016073| | HDGFRP3,hepatoma-derived growth factor, related protein | | | |
| 216247_at | 9.13 | 5.51E-05 | 1.25177687 | NM_001023| | RPS20,ribosomal protein S20 | | |  |
| 219593_at | 9.13 | 5.50E-05 | 1.115092062 | NM_016582| | SLC15A3,solute carrier family 15, member 3 | | | |
| 201202_at | 9.12 | 5.53E-05 | 1.038126136 | NM_002592| | PCNA,proliferating cell nuclear antigen | | |  |
| 219932_at | 9.12 | 5.57E-05 | 1.137410543 | NM_001017372| | NA |  |  |  |
| 209318_x_at | 9.12 | 5.55E-05 | 1.503906022 | NM_001080951| | NA |  |  |  |
| 213766_x_at | 9.12 | 5.54E-05 | 1.053057689 | NM_002067| | GNA11,guanine nucleotide binding protein (G protein), | | | |
| 213606_s_at | 9.12 | 5.55E-05 | 1.139406893 | NM_004309| | ARHGDIA,Rho GDP dissociation inhibitor (GDI) alpha | | | |
| 222118_at | 9.12 | 5.54E-05 | 1.217501883 | NM_001100624| | NA |  |  |  |
| 53968_at | 9.12 | 5.54E-05 | 1.044149262 | NM_030628| | KIAA1698,KIAA1698 protein | | |  |
| 224002_s_at | 9.11 | 5.59E-05 | 1.208716213 | NM_181342| | FKBP7,FK506-binding protein 7 isoform 2 precursor | | | |
| 236591_at | 9.11 | 5.58E-05 | 1.333412503 | NA |  |  |  |  |
| 208628_s_at | 9.11 | 5.57E-05 | 1.014507859 | NM_004559| | NSEP1,nuclease sensitive element binding protein 1 | | | |
| 208788_at | 9.11 | 5.61E-05 | 1.02728367 | NM_021814| | ELOVL5,homolog of yeast long chain polyunsaturated | | | |
| 238605_at | 9.11 | 5.58E-05 | 1.527861169 | NM_003787| | NOL4,nucleolar protein 4 | |  |  |
| 221830_at | 9.1 | 5.64E-05 | 1.073460528 | NM_021033| | RAP2A,RAP2A, member of RAS oncogene family | | | |
| 212199_at | 9.1 | 5.61E-05 | 1.033230972 | NM_152301| | PP784,PP784 | |  |  |
| 214807_at | 9.09 | 5.68E-05 | 1.149310179 | NA |  |  |  |  |
| 221730_at | 9.09 | 5.70E-05 | 1.215898648 | NM_000393| | COL5A2,alpha 2 type V collagen preproprotein | | | |
| 219210_s_at | 9.09 | 5.68E-05 | 1.152210038 | NM_016530| | RAB8B,RAB8B, member RAS oncogene family | | | |
| 201449_at | 9.09 | 5.70E-05 | 1.111578164 | NM_022037| | TIA1,TIA1 protein isoform 1 | | |  |
| 229014_at | 9.09 | 5.66E-05 | 1.1646724 | NA |  |  |  |  |
| 208857_s_at | 9.09 | 5.68E-05 | 1.03183062 | NM_005389| | PCMT1,protein-L-isoaspartate (D-aspartate) | | | |
| 219146_at | 9.09 | 5.68E-05 | 1.034410417 | NM_024683| | FLJ22729,hypothetical protein FLJ22729 | | | |
| 239002_at | 9.08 | 5.75E-05 | 1.131478893 | NM_018136| | ASPM,asp (abnormal spindle)-like, microcephaly | | | |
| 219495_s_at | 9.08 | 5.70E-05 | 1.128475325 | NM_013256| | ZNF180,zinc finger protein 180 (HHZ168) | | | |
| 206364_at | 9.08 | 5.74E-05 | 1.060508519 | NM_014875| | NA |  |  |  |
| 238775_at | 9.08 | 5.74E-05 | 1.129768689 | NA |  |  |  |  |
| 224859_at | 9.07 | 5.78E-05 | 1.056218723 | NM_001024736| | NA |  |  |  |
| 202413_s_at | 9.07 | 5.75E-05 | 1.02817361 | NM_001017415| | NA |  |  |  |
| 201627_s_at | 9.07 | 5.76E-05 | 1.185556023 | NM_005542| | INSIG1,insulin induced gene 1 isoform 1 | | | |
| 233132_at | 9.07 | 5.78E-05 | 1.09286205 | NA |  |  |  |  |
| 200599_s_at | 9.06 | 5.84E-05 | 1.028633987 | NM_003299| | TRA1,tumor rejection antigen (gp96) 1 | | |  |
| 203500_at | 9.06 | 5.84E-05 | 1.051047882 | NM_000159| | GCDH,glutaryl-Coenzyme A dehydrogenase isoform a | | | |
| 203966_s_at | 9.06 | 5.81E-05 | 1.042526796 | NM_021003| | PPM1A,protein phosphatase 1A isoform 1 | | | |
| 228743_at | 9.06 | 5.82E-05 | 1.167785445 | NM_032731| | TXNL5,thioredoxin-like 5 | |  |  |
| 236356_at | 9.05 | 5.87E-05 | 1.063985379 | NM_005006| | NDUFS1,NADH dehydrogenase (ubiquinone) Fe-S protein 1, | | | |
| 227852_at | 9.05 | 5.85E-05 | 1.076653705 | NM_203288| | RP9,retinitis pigmentosa 9 protein | | |  |
| 225561_at | 9.05 | 5.86E-05 | 1.061670757 | NM_016275| | SELT,selenoprotein T | |  |  |
| 221935_s_at | 9.05 | 5.85E-05 | 1.337073344 | NM_173654| | AER61,AER61 glycosyltransferase | | |  |
| 219403_s_at | 9.04 | 5.89E-05 | 1.161994938 | NM_001098540| | NA |  |  |  |
| 238496_at | 9.04 | 5.93E-05 | 1.09941089 | NA |  |  |  |  |
| 210428_s_at | 9.04 | 5.89E-05 | 1.044498424 | NM_004712| | HGS,hepatocyte growth factor-regulated tyrosine | | | |
| 216450_x_at | 9.04 | 5.89E-05 | 1.022943316 | NM_003299| | TRA1,tumor rejection antigen (gp96) 1 | | |  |
| 227012_at | 9.04 | 5.90E-05 | 1.063739624 | NM_018843| | MCFP,mitochondrial carrier family protein | | | |
| 209534_x_at | 9.04 | 5.93E-05 | 1.056453696 | NM_006738| | AKAP13,A-kinase anchor protein 13 isoform 1 | | | |
| 205375_at | 9.03 | 5.98E-05 | 1.100953595 | NM_005586| | MDFI,MyoD family inhibitor | | |  |
| 223267_at | 9.03 | 5.98E-05 | 1.0410182 | NM_017819| | RG9MTD1,RNA (guanine-9-) methyltransferase domain | | | |
| 205626_s_at | 9.03 | 5.98E-05 | 1.11842848 | NM_004929| | CALB1,calbindin 1 | |  |  |
| 231119_at | 9.02 | 5.99E-05 | 1.150497315 | NM_002915| | RFC3,replication factor C 3 isoform 1 | | |  |
| 224944_at | 9.02 | 6.00E-05 | 1.028742616 | NM_001032283| | NA |  |  |  |
| 201673_s_at | 9.02 | 6.00E-05 | 1.077296664 | NM_002103| | GYS1,glycogen synthase 1 (muscle) | | |  |
| 209853_s_at | 9.02 | 6.02E-05 | 1.057052965 | NM_005789| | PSME3,proteasome activator subunit 3 isoform 1 | | | |
| 220615_s_at | 9.02 | 6.00E-05 | 1.047785747 | NM_018099| | MLSTD1,male sterility domain containing 1 | | | |
| 213785_at | 9.02 | 6.00E-05 | 1.074667974 | NM_018085| | IPO9,importin 9 | |  |  |
| 235123_at | 9.02 | 6.01E-05 | 1.130697409 | NA |  |  |  |  |
| 226831_at | 9.01 | 6.07E-05 | 1.115040264 | NM_138773| | LOC91137,hypothetical protein BC017169 | | | |
| 230243_at | 9.01 | 6.03E-05 | 1.110117525 | NM_152292| | RG9MTD2,RNA (guanine-9-) methyltransferase domain | | | |
| 202718_at | 9.01 | 6.03E-05 | 1.151804088 | NM_000597| | IGFBP2,insulin-like growth factor binding protein 2, | | | |
| 222448_s_at | 9.01 | 6.03E-05 | 1.057694837 | NM_016308| | UMP-CMPK,UMP-CMP kinase | | |  |
| 209982_s_at | 9 | 6.12E-05 | 1.189452202 | NM_015080| | NRXN2,neurexin 2 isoform alpha-1 precursor | | | |
| 230179_at | 9 | 6.09E-05 | 1.130310875 | NA |  |  |  |  |
| 200040_at | 9 | 6.07E-05 | 1.013110967 | NM_006559| | KHDRBS1,KH domain containing, RNA binding, signal | | | |
| 1568618_a_at | 9 | 6.09E-05 | 1.040933423 | NM_020474| | GALNT1,polypeptide N-acetylgalactosaminyltransferase 1 | | | |
| 211948_x_at | 9 | 6.09E-05 | 1.054064797 | NM_015172| | BAT2D1,HBxAg transactivated protein 2 | | |  |
| 221207_s_at | 9 | 6.09E-05 | 1.135547704 | NM_015678| | NBEA,neurobeachin | |  |  |
| 202828_s_at | 9 | 6.10E-05 | 1.088821547 | NM_004995| | MMP14,matrix metalloproteinase 14 preproprotein | | | |
| 202318_s_at | 8.99 | 6.17E-05 | 1.046427201 | NM_001100409| | NA |  |  |  |
| 235286_at | 8.99 | 6.16E-05 | 1.189270489 | NA |  |  |  |  |
| 242157_at | 8.99 | 6.17E-05 | 1.250705818 | NM_025134| | CHD9,chromodomain helicase DNA binding protein 9 | | | |
| 228039_at | 8.99 | 6.18E-05 | 1.088627905 | NM_014829| | DDX46,DEAD (Asp-Glu-Ala-Asp) box polypeptide 46 | | | |
| 213095_x_at | 8.98 | 6.22E-05 | 1.097520158 | NM_001623| | AIF1,allograft inflammatory factor 1 isoform 3 | | | |
| 223608_at | 8.98 | 6.22E-05 | 1.107770338 | NM_032328| | MGC12458,hypothetical protein MGC12458 | | | |
| 227915_at | 8.98 | 6.20E-05 | 1.131836218 | NM_016150| | ASB2,ankyrin repeat and SOCS box-containing protein | | | |
| 221591_s_at | 8.97 | 6.23E-05 | 1.051073457 | NM_019013| | FLJ10156,hypothetical protein FLJ10156 | | | |
| 224058_s_at | 8.97 | 6.26E-05 | 1.331233821 | NA |  |  |  |  |
| 207695_s_at | 8.97 | 6.24E-05 | 1.154490585 | NM_001555| | IGSF1,immunoglobulin superfamily, member 1 isoform 1 | | | |
| 209811_at | 8.97 | 6.24E-05 | 1.10803887 | NM_032982| | CASP2,caspase 2 isoform 1 preproprotein | | | |
| 235607_at | 8.97 | 6.23E-05 | 1.164344669 | NA |  |  |  |  |
| 218659_at | 8.96 | 6.28E-05 | 1.038855273 | NM_018263| | ASXL2,additional sex combs like 2 | | |  |
| 200951_s_at | 8.96 | 6.28E-05 | 1.083153112 | NM_001759| | CCND2,cyclin D2 | |  |  |
| 202800_at | 8.96 | 6.28E-05 | 1.32992613 | NM_004172| | SLC1A3,solute carrier family 1 (glial high affinity | | | |
| 231913_s_at | 8.96 | 6.29E-05 | 1.128733393 | NM_001018055| | NA |  |  |  |
| 210882_s_at | 8.95 | 6.37E-05 | 1.088322232 | NM_001039705| | NA |  |  |  |
| 223721_s_at | 8.95 | 6.33E-05 | 1.223550424 | NM_021800| | DNAJC12,J domain containing protein 1 isoform a | | | |
| 229034_at | 8.95 | 6.35E-05 | 1.142704168 | NM_018013| | NA |  |  |  |
| 208900_s_at | 8.95 | 6.33E-05 | 1.060700166 | NM_003286| | TOP1,DNA topoisomerase I | | |  |
| 201582_at | 8.95 | 6.35E-05 | 1.069263189 | NM_006363| | SEC23B,Sec23 (S. cerevisiae) homolog B | | | |
| 201620_at | 8.95 | 6.35E-05 | 1.053903322 | NM_003791| | MBTPS1,membrane-bound transcription factor site-1 | | | |
| 207037_at | 8.94 | 6.41E-05 | 1.222397308 | NM_003839| | TNFRSF11A,tumor necrosis factor receptor superfamily, | | | |
| 209218_at | 8.94 | 6.42E-05 | 1.111596417 | NM_003129| | SQLE,squalene monooxygenase | | |  |
| 224744_at | 8.94 | 6.39E-05 | 1.136377869 | NM_017813| | IMPA3,myo-inositol monophosphatase A3 | | | |
| 204671_s_at | 8.94 | 6.41E-05 | 1.172329812 | NM_014942| | ANKRD6,ankyrin repeat domain 6 | | |  |
| 209686_at | 8.94 | 6.38E-05 | 1.2422302 | NM_006272| | S100B,S100 calcium-binding protein, beta | | | |
| 236179_at | 8.94 | 6.40E-05 | 1.597819575 | NM_001797| | CDH11,cadherin 11, type 2 isoform 1 preproprotein | | | |
| 230261_at | 8.93 | 6.44E-05 | 1.24009854 | NM_005668| | ST8SIA4,ST8 alpha-N-acetyl-neuraminide | | | |
| 207425_s_at | 8.93 | 6.44E-05 | 1.177778042 | NM_001113491| | NA |  |  |  |
| 205560_at | 8.93 | 6.44E-05 | 1.130521159 | NM_006200| | PCSK5,proprotein convertase subtilisin/kexin type 5 | | | |
| 201426_s_at | 8.93 | 6.43E-05 | 1.048981307 | NM_003380| | VIM,vimentin | |  |  |
| 223461_at | 8.93 | 6.44E-05 | 1.046762954 | NM_016495| | TBC1D7,TBC1 domain family, member 7 | | | |
| 203605_at | 8.93 | 6.44E-05 | 1.048515906 | NM_003136| | SRP54,signal recognition particle 54kDa | | | |
| 212946_at | 8.92 | 6.49E-05 | 1.049554964 | NM_001009814| | KIAA0564,KIAA0564 protein isoform b | | |  |
| 1560433_at | 8.92 | 6.46E-05 | 1.135541909 | NA |  |  |  |  |
| 202927_at | 8.92 | 6.47E-05 | 1.049020261 | NM_006221| | PIN1,protein (peptidyl-prolyl cis/trans isomerase) | | | |
| 230427_s_at | 8.92 | 6.49E-05 | 1.058116474 | NM_001015048| | NA |  |  |  |
| 200987_x_at | 8.92 | 6.48E-05 | 1.050681271 | NM_005789| | PSME3,proteasome activator subunit 3 isoform 1 | | | |
| 225688_s_at | 8.92 | 6.48E-05 | 1.307404356 | NM_145753| | PHLDB2,pleckstrin homology-like domain, family B, | | | |
| 203133_at | 8.92 | 6.50E-05 | 1.029431588 | NM_006808| | SEC61B,Sec61 beta subunit | | |  |
| 225303_at | 8.92 | 6.47E-05 | 1.195337569 | NM_018240| | KIRREL,kin of IRRE like | |  |  |
| 236704_at | 8.91 | 6.55E-05 | 1.213382732 | NA |  |  |  |  |
| 218070_s_at | 8.91 | 6.54E-05 | 1.093739736 | NM_013335| | GMPPA,GDP-mannose pyrophosphorylase A | | | |
| 219424_at | 8.91 | 6.54E-05 | 1.176155905 | NM_005755| | EBI3,Epstein-Barr virus induced gene 3 precursor | | | |
| 221514_at | 8.91 | 6.53E-05 | 1.100688753 | NM_006649| | UTP14A,UTP14, U3 small nucleolar ribonucleoprotein, | | | |
| 238350_at | 8.91 | 6.51E-05 | 1.156418631 | NM_173569| | NA |  |  |  |
| 227682_at | 8.91 | 6.51E-05 | 1.129862606 | NA |  |  |  |  |
| 235494_at | 8.9 | 6.60E-05 | 1.213849283 | NA |  |  |  |  |
| 227804_at | 8.9 | 6.59E-05 | 1.076044362 | NM_138463| | LOC116238,hypothetical protein BC014072 | | | |
| 232007_at | 8.9 | 6.58E-05 | 1.051793002 | NM_018361| | AGPAT5,1-acylglycerol-3-phosphate O-acyltransferase 5 | | | |
| 227100_at | 8.9 | 6.59E-05 | 1.088722235 | NM_194318| | B3GTL,beta 3-glycosyltransferase-like | | |  |
| 210811_s_at | 8.89 | 6.61E-05 | 1.055843643 | NM_019070| | DDX49,DEAD (Asp-Glu-Ala-Asp) box polypeptide 49 | | | |
| 228361_at | 8.89 | 6.62E-05 | 1.136171794 | NM_004091| | E2F2,E2F transcription factor 2 | | |  |
| 202549_at | 8.89 | 6.63E-05 | 1.227913132 | NM_004738| | VAPB,VAMP-associated protein B/C | | |  |
| 218662_s_at | 8.89 | 6.63E-05 | 1.105500849 | NM_022346| | HCAP-G,chromosome condensation protein G | | | |
| 238637_at | 8.89 | 6.64E-05 | 1.10696939 | NA |  |  |  |  |
| 214055_x_at | 8.89 | 6.62E-05 | 1.053503838 | NM_015172| | BAT2D1,HBxAg transactivated protein 2 | | |  |
| 208064_s_at | 8.89 | 6.62E-05 | 1.380619215 | NM_015879| | ST8SIA3,ST8 alpha-N-acetyl-neuraminide | | | |
| 214014_at | 8.89 | 6.63E-05 | 1.133072281 | NM_006779| | CDC42EP2,Cdc42 effector protein 2 | | |  |
| 201588_at | 8.88 | 6.69E-05 | 1.018636524 | NM_004786| | TXNL1,thioredoxin-like 1 | |  |  |
| 210835_s_at | 8.88 | 6.66E-05 | 1.014187687 | NM_001083914| | NA |  |  |  |
| 228329_at | 8.88 | 6.70E-05 | 1.099252346 | NM_021080| | DAB1,disabled homolog 1 | |  |  |
| 203162_s_at | 8.88 | 6.66E-05 | 1.063544481 | NM_005886| | KATNB1,katanin p80 subunit B 1 | | |  |
| 204859_s_at | 8.88 | 6.66E-05 | 1.102097671 | NM_001160| | APAF1,apoptotic protease activating factor isoform b | | | |
| 227869_at | 8.88 | 6.69E-05 | 1.135231021 | NM_138362| | CXorf44,chromosome X open reading frame 44 | | | |
| 222872_x_at | 8.87 | 6.71E-05 | 1.143991102 | NM_001031716| | NA |  |  |  |
| 226575_at | 8.87 | 6.73E-05 | 1.028108237 | NM_021224| | ZNF462,zinc finger protein 462 | | |  |
| 204929_s_at | 8.87 | 6.74E-05 | 1.101509824 | NM_006634| | VAMP5,vesicle-associated membrane protein 5 | | | |
| 244802_at | 8.87 | 6.74E-05 | 1.286307948 | NM_005271| | GLUD1,glutamate dehydrogenase 1 | | |  |
| 227247_at | 8.87 | 6.75E-05 | 1.03763152 | NA |  |  |  |  |
| 212836_at | 8.87 | 6.74E-05 | 1.033426813 | NM_006591| | POLD3,polymerase (DNA directed), delta 3 | | | |
| 224397_s_at | 8.87 | 6.74E-05 | 1.171289696 | NM_175861| | ARG99,ARG99 protein | |  |  |
| 223422_s_at | 8.87 | 6.74E-05 | 1.193060918 | NM_001025616| | NA |  |  |  |
| 219363_s_at | 8.86 | 6.80E-05 | 1.048526293 | NM_015942| | CGI-12,CGI-12 protein | |  |  |
| 236480_at | 8.86 | 6.79E-05 | 1.318145634 | NA |  |  |  |  |
| 225750_at | 8.85 | 6.84E-05 | 1.047601908 | NA |  |  |  |  |
| 207008_at | 8.85 | 6.82E-05 | 1.457054703 | NM_001557| | IL8RB,interleukin 8 receptor beta | | |  |
| 225202_at | 8.85 | 6.83E-05 | 1.160930318 | NM_014899| | RHOBTB3,rho-related BTB domain containing 3 | | | |
| 41220_at | 8.85 | 6.84E-05 | 1.031849955 | NM_001113491| | NA |  |  |  |
| 228281_at | 8.84 | 6.88E-05 | 1.057735504 | NM_145018| | FLJ25416,hypothetical protein FLJ25416 | | | |
| 201043_s_at | 8.84 | 6.87E-05 | 1.046176094 | NM_006305| | ANP32A,acidic (leucine-rich) nuclear phosphoprotein 32 | | | |
| 229952_at | 8.84 | 6.90E-05 | 1.265517679 | NA |  |  |  |  |
| 225617_at | 8.84 | 6.91E-05 | 1.124361685 | NM_002540| | ODF2,outer dense fiber of sperm tails 2 isoform 1 | | | |
| 227280_s_at | 8.84 | 6.90E-05 | 1.09688356 | NM_152523| | FLJ40432,hypothetical protein FLJ40432 | | | |
| 213385_at | 8.84 | 6.90E-05 | 1.291675955 | NM_001039936| | NA |  |  |  |
| 201072_s_at | 8.84 | 6.87E-05 | 1.030599928 | NM_003074| | SMARCC1,SWI/SNF-related matrix-associated | | | |
| 209989_at | 8.84 | 6.87E-05 | 1.120264877 | NM_003415| | ZNF268,zinc finger protein 268 | | |  |
| 218163_at | 8.84 | 6.87E-05 | 1.046991628 | NM_014060| | MCTS1,malignant T cell amplified sequence 1 | | | |
| 210749_x_at | 8.84 | 6.89E-05 | 1.026411888 | NM_001954| | DDR1,discoidin receptor tyrosine kinase isoform b | | | |
| 232242_at | 8.83 | 6.96E-05 | 1.320035823 | NA |  |  |  |  |
| 210517_s_at | 8.83 | 6.95E-05 | 1.019506981 | NM_005100| | AKAP12,A-kinase anchor protein 12 isoform 1 | | | |
| 225616_at | 8.83 | 6.95E-05 | 1.088212579 | NM_207344| | LOC283377,hypothetical protein LOC283377 | | | |
| 45633_at | 8.83 | 6.94E-05 | 1.134065589 | NM_001126129| | NA |  |  |  |
| 208012_x_at | 8.83 | 6.97E-05 | 1.138708988 | NM_004509| | SP110,SP110 nuclear body protein isoform a | | | |
| 204240_s_at | 8.83 | 6.95E-05 | 1.049182418 | NM_001042550| | NA |  |  |  |
| 230057_at | 8.82 | 6.97E-05 | 1.214096634 | NA |  |  |  |  |
| 228835_at | 8.82 | 6.99E-05 | 1.081349065 | NA |  |  |  |  |
| 200623_s_at | 8.82 | 6.97E-05 | 1.038089543 | NM_001743| | CALM2,calmodulin 2 | |  |  |
| 1568611_at | 8.82 | 7.01E-05 | 1.204817119 | NA |  |  |  |  |
| 229236_s_at | 8.82 | 6.99E-05 | 1.07686569 | NM_213649| | SFXN4,sideroflexin 4 isoform 1 | | |  |
| 236236_at | 8.82 | 6.97E-05 | 1.039241839 | NA |  |  |  |  |
| 203895_at | 8.82 | 6.97E-05 | 1.040553798 | NM_000933| | PLCB4,phospholipase C beta 4 isoform a | | | |
| 204849_at | 8.82 | 6.97E-05 | 1.036473495 | NM_006602| | TCFL5,transcription factor-like 5 protein | | |  |
| 206006_s_at | 8.82 | 6.97E-05 | 1.22096037 | NM_014895| | C6orf84,KIAA1009 protein | |  |  |
| 201041_s_at | 8.82 | 7.01E-05 | 1.093065919 | NM_004417| | DUSP1,dual specificity phosphatase 1 | | |  |
| 214510_at | 8.81 | 7.06E-05 | 1.137425778 | NM_005293| | GPR20,G protein-coupled receptor 20 | | |  |
| 227384_s_at | 8.81 | 7.04E-05 | 1.093938822 | NA |  |  |  |  |
| 211378_x_at | 8.81 | 7.04E-05 | 1.008806905 | NM_021130| | PPIA,peptidylprolyl isomerase A isoform 1 | | | |
| 1553179_at | 8.81 | 7.04E-05 | 1.092014747 | NM_133638| | ADAMTS19,a disintegrin-like and metalloprotease | | | |
| 208779_x_at | 8.8 | 7.10E-05 | 1.028983593 | NM_001954| | DDR1,discoidin receptor tyrosine kinase isoform b | | | |
| 229426_at | 8.8 | 7.08E-05 | 1.075668533 | NM_004255| | COX5A,cytochrome c oxidase subunit Va precursor | | | |
| 236800_at | 8.79 | 7.15E-05 | 1.251930687 | NM_145314| | C10orf49,chromosome 10 open reading frame 49 | | | |
| 229410_at | 8.79 | 7.17E-05 | 1.094621445 | NM_024881| | SLC35E1,solute carrier family 35, member E1 | | | |
| 244758_at | 8.79 | 7.18E-05 | 1.138928544 | NM_052923| | ZNF452,zinc finger protein 452 | | |  |
| 219694_at | 8.79 | 7.15E-05 | 1.148610213 | NM_019018| | FLJ11127,hypothetical protein FLJ11127 | | | |
| 229647_at | 8.79 | 7.19E-05 | 1.086292555 | NA |  |  |  |  |
| 221447_s_at | 8.79 | 7.14E-05 | 1.092115941 | NM_031302| | GLT8D2,glycosyltransferase 8 domain containing 2 | | | |
| 243688_at | 8.78 | 7.23E-05 | 1.192658869 | NA |  |  |  |  |
| 209274_s_at | 8.78 | 7.21E-05 | 1.086238324 | NM_030940| | HBLD2,HESB like domain containing 2 | | |  |
| 226320_at | 8.78 | 7.23E-05 | 1.053901985 | NM_005782| | THOC4,THO complex 4 | |  |  |
| 235603_at | 8.78 | 7.22E-05 | 1.146280298 | NM_004501| | HNRPU,heterogeneous nuclear ribonucleoprotein U | | | |
| 227140_at | 8.78 | 7.24E-05 | 1.302010317 | NA |  |  |  |  |
| 206095_s_at | 8.77 | 7.27E-05 | 1.052788675 | NM_006625| | FUSIP1,FUS interacting protein (serine-arginine rich) 1 | | | |
| 238455_at | 8.77 | 7.29E-05 | 1.152222371 | NA |  |  |  |  |
| 213364_s_at | 8.77 | 7.26E-05 | 1.075157872 | NM_003099| | SNX1,sorting nexin 1 isoform a | | |  |
| 230154_at | 8.77 | 7.30E-05 | 1.215185927 | NA |  |  |  |  |
| 219211_at | 8.77 | 7.25E-05 | 1.229471763 | NM_017414| | USP18,ubiquitin specific protease 18 | | |  |
| 209576_at | 8.77 | 7.25E-05 | 1.070610654 | NM_002069| | GNAI1,guanine nucleotide binding protein (G protein), | | | |
| 208970_s_at | 8.77 | 7.25E-05 | 1.033902887 | NM_000374| | UROD,uroporphyrinogen decarboxylase | | |  |
| 230752_at | 8.77 | 7.25E-05 | 1.168118165 | NA |  |  |  |  |
| 202306_at | 8.77 | 7.30E-05 | 1.018112787 | NM_002696| | POLR2G,DNA directed RNA polymerase II polypeptide G | | | |
| 220355_s_at | 8.77 | 7.30E-05 | 1.051930159 | NM_018165| | PB1,polybromo 1 | |  |  |
| 219060_at | 8.77 | 7.30E-05 | 1.034869081 | NM_018024| | FLJ10204,hypothetical protein FLJ10204 | | | |
| 218703_at | 8.76 | 7.36E-05 | 1.064948374 | NM_012430| | SEC22L2,SEC22 vesicle trafficking protein-like 2 | | | |
| 229054_at | 8.76 | 7.36E-05 | 1.163535911 | NM_207442| | FLJ39779,FLJ39779 protein | | |  |
| 230149_at | 8.76 | 7.35E-05 | 1.341869145 | NA |  |  |  |  |
| 1552502_s_at | 8.76 | 7.35E-05 | 1.182759481 | NM_017821| | RHBDL2,rhomboid-related protein 2 | | |  |
| 201448_at | 8.76 | 7.33E-05 | 1.066883037 | NM_022037| | TIA1,TIA1 protein isoform 1 | | |  |
| 209866_s_at | 8.76 | 7.33E-05 | 1.081780688 | NM_015236| | LPHN3,latrophilin 3 precursor | | |  |
| 238462_at | 8.75 | 7.41E-05 | 1.384439459 | NM_032873| | STS-1,Cbl-interacting protein Sts-1 | | |  |
| 223201_s_at | 8.75 | 7.39E-05 | 1.118016627 | NM_032227| | FLJ22679,hypothetical protein FLJ22679 | | | |
| 205140_at | 8.75 | 7.39E-05 | 1.160328129 | NM_003838| | FPGT,fucose-1-phosphate guanyltransferase | | | |
| 239196_at | 8.75 | 7.38E-05 | 1.100141927 | NM_144590| | ANKRD22,ankyrin repeat domain 22 | | |  |
| 201251_at | 8.75 | 7.42E-05 | 1.076197744 | NM_002654| | PKM2,pyruvate kinase 3 isoform 1 | | |  |
| 210414_at | 8.74 | 7.46E-05 | 1.124522537 | NM_013280| | FLRT1,fibronectin leucine rich transmembrane protein | | | |
| 225834_at | 8.74 | 7.46E-05 | 1.022329985 | NM_001100910| | NA |  |  |  |
| 217604_at | 8.73 | 7.50E-05 | 1.136987242 | NA |  |  |  |  |
| 217299_s_at | 8.73 | 7.51E-05 | 1.134545257 | NM_001024688| | NA |  |  |  |
| 215160_x_at | 8.73 | 7.54E-05 | 1.114880854 | NA |  |  |  |  |
| 225922_at | 8.73 | 7.49E-05 | 1.040097176 | NM_020840| | NA |  |  |  |
| 222673_x_at | 8.73 | 7.53E-05 | 1.12285275 | NM_018202| | FLJ10747,hypothetical protein FLJ10747 | | | |
| 237105_at | 8.73 | 7.54E-05 | 1.200376354 | NA |  |  |  |  |
| 222455_s_at | 8.72 | 7.57E-05 | 1.157710227 | NM_018222| | PARVA,parvin, alpha | |  |  |
| 1553954_at | 8.72 | 7.57E-05 | 1.11879597 | NM_144988| | MGC19780,hypothetical protein MGC19780 | | | |
| 202692_s_at | 8.72 | 7.55E-05 | 1.081457403 | NM_001076683| | NA |  |  |  |
| 232825_s_at | 8.72 | 7.55E-05 | 1.137423066 | NM_032160| | C18orf4,chromosome 18 open reading frame 4 | | | |
| 1553593_a_at | 8.72 | 7.56E-05 | 1.206155578 | NM_005421| | TAL2,T-cell acute lymphocytic leukemia 2 | | | |
| 219972_s_at | 8.72 | 7.56E-05 | 1.118133538 | NM_022495| | C14orf135,chromosome 14 open reading frame 135 | | | |
| 224982_at | 8.71 | 7.60E-05 | 1.114251974 | NM_001098632| | NA |  |  |  |
| 209891_at | 8.71 | 7.63E-05 | 1.074909021 | NM_020675| | Spc25,kinetochore protein Spc25 | | |  |
| 230326_s_at | 8.71 | 7.61E-05 | 1.043663121 | NM_016401| | HSPC138,hypothetical protein HSPC138 | | |  |
| 219747_at | 8.71 | 7.61E-05 | 1.240598878 | NM_024574| | FLJ23191,hypothetical protein FLJ23191 | | | |
| 1557326_at | 8.71 | 7.62E-05 | 1.337658323 | NA |  |  |  |  |
| 203817_at | 8.7 | 7.67E-05 | 1.129106984 | NM_000857| | GUCY1B3,guanylate cyclase 1, soluble, beta 3 | | | |
| 225895_at | 8.7 | 7.68E-05 | 1.513680312 | NM_133477| | SYNPO2,synaptopodin 2 | |  |  |
| 203432_at | 8.7 | 7.67E-05 | 1.065136677 | NM_001032283| | NA |  |  |  |
| 212040_at | 8.7 | 7.68E-05 | 1.044140763 | NM_006464| | TGOLN2,trans-golgi network protein 2 | | |  |
| 219002_at | 8.7 | 7.70E-05 | 1.023310896 | NM_024622| | FLJ21901,hypothetical protein FLJ21901 | | | |
| 212070_at | 8.7 | 7.69E-05 | 1.208997832 | NM_005682| | GPR56,G protein-coupled receptor 56 isoform a | | | |
| 227579_at | 8.69 | 7.73E-05 | 1.132536528 | NA |  |  |  |  |
| 222757_s_at | 8.69 | 7.76E-05 | 1.240503077 | NM_016653| | ZAK,sterile-alpha motif and leucine zipper | | | |
| 226510_at | 8.69 | 7.77E-05 | 1.066096759 | NM_015473| | NA |  |  |  |
| 202565_s_at | 8.69 | 7.74E-05 | 1.08844244 | NM_003174| | SVIL,supervillin isoform 1 | |  |  |
| 218569_s_at | 8.69 | 7.76E-05 | 1.06482516 | NM_016506| | KBTBD4,kelch repeat and BTB (POZ) domain containing 4 | | | |
| 215527_at | 8.69 | 7.77E-05 | 1.148900246 | NM_152688| | KHDRBS2,KH domain-containing, RNA-binding, signal | | | |
| 222036_s_at | 8.69 | 7.76E-05 | 1.033018817 | NM_005914| | MCM4,minichromosome maintenance protein 4 | | | |
| 202918_s_at | 8.69 | 7.75E-05 | 1.033516314 | NM_001100819| | NA |  |  |  |
| 206033_s_at | 8.69 | 7.74E-05 | 1.257028173 | NM_001941| | DSC3,desmocollin 3 isoform Dsc3a preproprotein | | | |
| 233085_s_at | 8.69 | 7.75E-05 | 1.267391565 | NM_001031716| | NA |  |  |  |
| 218617_at | 8.69 | 7.76E-05 | 1.036808566 | NM_017646| | TRIT1,tRNA isopentenyltransferase 1 | | |  |
| 231037_at | 8.68 | 7.80E-05 | 1.497529586 | NA |  |  |  |  |
| 219083_at | 8.68 | 7.79E-05 | 1.031444674 | NM_018130| | SHQ1,SHQ1 homolog | |  |  |
| 223538_at | 8.68 | 7.83E-05 | 1.058095223 | NM_021967| | SERF1A,small EDRK-rich factor 1A, telomeric | | | |
| 44654_at | 8.68 | 7.83E-05 | 1.047143413 | NM_138387| | G6PC3,glucose-6-phosphatase catalytic subunit 3 | | | |
| 230025_at | 8.68 | 7.80E-05 | 1.099918908 | NM_152219| | GJC1,gap junction protein, chi 1, 31.9kDa (connexin | | | |
| 205405_at | 8.67 | 7.90E-05 | 1.060303713 | NM_003966| | SEMA5A,semaphorin 5A | |  |  |
| 219882_at | 8.67 | 7.84E-05 | 1.090652042 | NM_024686| | FLJ23033,hypothetical protein FLJ23033 | | | |
| 218109_s_at | 8.67 | 7.89E-05 | 1.069971086 | NM_022736| | MFSD1,major facilitator superfamily domain containing | | | |
| 220679_s_at | 8.67 | 7.88E-05 | 1.144577637 | NM_004361| | CDH7,cadherin 7, type 2 preproprotein | | |  |
| 232451_at | 8.66 | 7.95E-05 | 1.309156816 | NA |  |  |  |  |
| 207886_s_at | 8.66 | 7.90E-05 | 1.204059733 | NM_001742| | CALCR,calcitonin receptor | |  |  |
| 202815_s_at | 8.66 | 7.95E-05 | 1.093953496 | NM_006460| | HIS1,HMBA-inducible | |  |  |
| 225155_at | 8.66 | 7.93E-05 | 1.020070821 | NA |  |  |  |  |
| 207943_x_at | 8.66 | 7.95E-05 | 1.370280441 | NM_001080951| | NA |  |  |  |
| 243982_at | 8.65 | 7.97E-05 | 1.241708897 | NM_017658| | BTBD5,BTB (POZ) domain containing 5 | | |  |
| 217106_x_at | 8.65 | 8.02E-05 | 1.03640999 | NM_014473| | HSA9761,putative dimethyladenosine transferase | | | |
| 203279_at | 8.65 | 8.00E-05 | 1.133845019 | NM_014674| | EDEM1,ER degradation enhancer, mannosidase alpha-like | | | |
| 213165_at | 8.65 | 7.97E-05 | 1.076711659 | NM_014810| | CAP350,centrosome-associated protein 350 | | | |
| 203053_at | 8.65 | 8.02E-05 | 1.025704035 | NM_005872| | BCAS2,breast carcinoma amplified sequence 2 | | | |
| 242346_x_at | 8.65 | 7.98E-05 | 1.131575797 | NA |  |  |  |  |
| 222599_s_at | 8.65 | 7.97E-05 | 1.08655982 | NM_001111018| | NA |  |  |  |
| 205484_at | 8.65 | 8.00E-05 | 1.115547963 | NM_014450| | SIT,SHP2-interacting transmembrane adaptor protein | | | |
| 203106_s_at | 8.64 | 8.08E-05 | 1.171964751 | NM_014396| | VPS41,vacuolar protein sorting 41 (yeast homolog) | | | |
| 214030_at | 8.64 | 8.08E-05 | 1.11312487 | NM_153605| | NA |  |  |  |
| 201687_s_at | 8.64 | 8.03E-05 | 1.032823321 | NM_006595| | API5,apoptosis inhibitor 5 | |  |  |
| 229436_x_at | 8.64 | 8.05E-05 | 1.107383856 | NM_001018055| | NA |  |  |  |
| 244177_at | 8.64 | 8.04E-05 | 1.066413936 | NA |  |  |  |  |
| 224737_x_at | 8.64 | 8.05E-05 | 1.094264882 | NM_018237| | CCAR1,cell-cycle and apoptosis regulatory protein 1 | | | |
| 225047_at | 8.64 | 8.08E-05 | 1.144760655 | NM_001008564| | NUPL1,nucleoporin like 1 isoform b | | |  |
| 202664_at | 8.63 | 8.10E-05 | 1.131683299 | NM_001077269| | NA |  |  |  |
| 230479_at | 8.63 | 8.12E-05 | 1.323826337 | NA |  |  |  |  |
| 200919_at | 8.63 | 8.10E-05 | 1.108717009 | NM_004427| | PHC2,polyhomeotic 2-like isoform b | | |  |
| 229878_at | 8.63 | 8.14E-05 | 1.102904572 | NM_033395| | NA |  |  |  |
| 1553587_a_at | 8.62 | 8.21E-05 | 1.070551588 | NM_019896| | POLE4,DNA polymerase epsilon subunit 4 | | | |
| 210907_s_at | 8.62 | 8.16E-05 | 1.020533687 | NM_007217| | PDCD10,programmed cell death 10 | | |  |
| 224587_at | 8.62 | 8.15E-05 | 1.074218984 | NM_006713| | PC4,activated RNA polymerase II transcription | | | |
| 202726_at | 8.62 | 8.15E-05 | 1.059906404 | NM_000234| | LIG1,DNA ligase I | |  |  |
| 227599_at | 8.61 | 8.28E-05 | 1.058720187 | NM_178496| | LOC151963,similar to BcDNA:GH11415 gene product | | | |
| 231045_x_at | 8.61 | 8.28E-05 | 1.026958375 | NM_170746| | C11orf31,selenoprotein H | |  |  |
| 232095_at | 8.61 | 8.26E-05 | 1.089139528 | NA |  |  |  |  |
| 236594_at | 8.61 | 8.23E-05 | 1.043777782 | NM_004140| | LLGL1,lethal giant larvae homolog 1 | | |  |
| 228168_at | 8.6 | 8.31E-05 | 1.044960111 | NM_001002258| | ATP5G3,ATP synthase, H+ transporting, mitochondrial F0 | | | |
| 209751_s_at | 8.6 | 8.34E-05 | 1.084536385 | NM_001011658| | TRAPPC2,trafficking protein particle complex 2 | | | |
| 223513_at | 8.6 | 8.29E-05 | 1.045067738 | NM_018451| | CENPJ,centromere protein J | | |  |
| 203138_at | 8.6 | 8.33E-05 | 1.040175827 | NM_001033085| | NA |  |  |  |
| 219648_at | 8.6 | 8.31E-05 | 1.0431264 | NM_018000| | DSU,likely ortholog of mouse dilute suppressor | | | |
| 225666_at | 8.6 | 8.30E-05 | 1.07963658 | NM_001079669| | NA |  |  |  |
| 226490_at | 8.59 | 8.36E-05 | 1.091785941 | NA |  |  |  |  |
| 237054_at | 8.59 | 8.38E-05 | 1.149879932 | NM_021572| | ENPP5,ectonucleotide pyrophosphatase/phosphodiesterase | | | |
| 204720_s_at | 8.59 | 8.41E-05 | 1.084380932 | NM_014787| | DNAJC6,DnaJ (Hsp40) homolog, subfamily C, member 6 | | | |
| 211402_x_at | 8.58 | 8.45E-05 | 1.126817591 | NM_001489| | NR6A1,nuclear receptor subfamily 6, group A, member 1 | | | |
| 206175_x_at | 8.58 | 8.47E-05 | 1.11635054 | NM_013360| | ZNF222,zinc finger protein 222 | | |  |
| 201272_at | 8.58 | 8.47E-05 | 1.038769154 | NM_001628| | AKR1B1,aldo-keto reductase family 1, member B1 | | | |
| 232739_at | 8.58 | 8.47E-05 | 1.246927276 | NM_003121| | SPIB,Spi-B transcription factor (Spi-1/PU.1 related) | | | |
| 235845_at | 8.58 | 8.49E-05 | 1.333450758 | NM_001003845| | SP5,Sp5 transcription factor | | |  |
| 228063_s_at | 8.58 | 8.48E-05 | 1.164117198 | NM_153757| | NAP1L5,nucleosome assembly protein 1-like 5 | | | |
| 204400_at | 8.58 | 8.45E-05 | 1.056909027 | NM_005864| | EFS,embryonal Fyn-associated substrate isoform 1 | | | |
| 204225_at | 8.58 | 8.49E-05 | 1.086920778 | NM_006037| | HDAC4,histone deacetylase 4 | | |  |
| 224918_x_at | 8.57 | 8.50E-05 | 1.03622428 | NM_020300| | MGST1,microsomal glutathione S-transferase 1 | | | |
| 232385_x_at | 8.57 | 8.52E-05 | 1.133902543 | NA |  |  |  |  |
| 211071_s_at | 8.57 | 8.51E-05 | 1.103050283 | NM_006818| | AF1Q,AF1Q protein | |  |  |
| 204488_at | 8.57 | 8.52E-05 | 1.046602963 | NM_014908| | TMEM15,transmembrane protein 15 | | |  |
| 224593_at | 8.57 | 8.51E-05 | 1.06553601 | NM_152437| | ZFOC1,zinc finger protein ZFOC1 | | |  |
| 1553276_at | 8.57 | 8.52E-05 | 1.124033006 | NM_152476| | ZNF560,zinc finger protein 560 | | |  |
| 226761_at | 8.57 | 8.50E-05 | 1.059758422 | NM_022465| | ZNFN1A4,zinc finger protein, subfamily 1A, 4 | | | |
| 210392_x_at | 8.57 | 8.54E-05 | 1.137112467 | NM_001489| | NR6A1,nuclear receptor subfamily 6, group A, member 1 | | | |
| 232080_at | 8.57 | 8.53E-05 | 1.079466847 | NM_020760| | HECW2,HECT, C2 and WW domain containing E3 ubiquitin | | | |
| 223166_x_at | 8.56 | 8.62E-05 | 1.067149081 | NM_017995| | NA |  |  |  |
| 212546_s_at | 8.56 | 8.59E-05 | 1.049892785 | NM_015030| | NA |  |  |  |
| 227133_at | 8.56 | 8.58E-05 | 1.060455483 | NM_207318| | CXorf39,chromosome X open reading frame 39 | | | |
| 242100_at | 8.56 | 8.57E-05 | 1.189608938 | NM_175856| | CSS3,chondroitin sulfate synthase 3 | | |  |
| 1558722_at | 8.55 | 8.68E-05 | 1.293671116 | NA |  |  |  |  |
| 217779_s_at | 8.55 | 8.66E-05 | 1.060028839 | NM_017761| | PNRC2,proline-rich nuclear receptor coactivator 2 | | | |
| 205818_at | 8.55 | 8.67E-05 | 1.107066923 | NM_014618| | DBC1,deleted in bladder cancer 1 | | |  |
| 201589_at | 8.55 | 8.68E-05 | 1.035420867 | NM_006306| | SMC1L1,SMC1 structural maintenance of chromosomes | | | |
| 235938_at | 8.54 | 8.71E-05 | 1.156375318 | NA |  |  |  |  |
| 230591_at | 8.54 | 8.72E-05 | 1.167110515 | NA |  |  |  |  |
| 214848_at | 8.54 | 8.75E-05 | 1.106931995 | NA |  |  |  |  |
| 219112_at | 8.54 | 8.76E-05 | 1.03881763 | NM_016340| | RAPGEF6,PDZ domain-containing guanine nucleotide | | | |
| 222010_at | 8.54 | 8.71E-05 | 1.102503625 | NM_001008897| | TCP1,T-complex protein 1 isoform b | | |  |
| 235509_at | 8.54 | 8.74E-05 | 1.074229937 | NM_152416| | MGC40214,hypothetical protein MGC40214 | | | |
| 204711_at | 8.53 | 8.82E-05 | 1.06436423 | NM_014804| | KIAA0753,KIAA0753 gene product | | |  |
| 1557348_at | 8.53 | 8.83E-05 | 1.175128464 | NA |  |  |  |  |
| 228829_at | 8.53 | 8.80E-05 | 1.162042393 | NM_006856| | ATF7,activating transcription factor 7 | | |  |
| 222640_at | 8.53 | 8.78E-05 | 1.028340632 | NM_022552| | DNMT3A,DNA cytosine methyltransferase 3 alpha isoform | | | |
| 236665_at | 8.52 | 8.84E-05 | 1.093921473 | NM_206886| | NY-SAR-41,sarcoma antigen NY-SAR-41 | | | |
| 210448_s_at | 8.52 | 8.90E-05 | 1.098651233 | NM_002561| | P2RX5,purinergic receptor P2X5 isoform A | | | |
| 1556328_at | 8.52 | 8.88E-05 | 1.272069758 | NA |  |  |  |  |
| 227780_s_at | 8.52 | 8.84E-05 | 1.224682 | NM_001077693| | NA |  |  |  |
| 222500_at | 8.52 | 8.84E-05 | 1.043637482 | NM_016059| | PPIL1,peptidylprolyl isomerase-like 1 | | |  |
| 208669_s_at | 8.52 | 8.89E-05 | 1.053345007 | NM_014335| | CRI1,CREBBP/EP300 inhibitor 1 | | |  |
| 200968_s_at | 8.52 | 8.84E-05 | 1.054243873 | NM_000942| | PPIB,peptidylprolyl isomerase B precursor | | | |
| 226185_at | 8.52 | 8.88E-05 | 1.056919915 | NA |  |  |  |  |
| 212012_at | 8.52 | 8.84E-05 | 1.031418086 | NM_012293| | NA |  |  |  |
| 228499_at | 8.51 | 8.93E-05 | 1.301946481 | NM_004567| | PFKFB4,6-phosphofructo-2-kinase/fructose-2, | | | |
| 203225_s_at | 8.51 | 8.94E-05 | 1.113205776 | NM_018339| | RFK,riboflavin kinase | |  |  |
| 213150_at | 8.51 | 8.93E-05 | 1.282120584 | NM_018951| | HOXA10,homeobox protein A10 isoform a | | | |
| 204236_at | 8.51 | 8.93E-05 | 1.27577126 | NM_002017| | FLI1,Friend leukemia virus integration 1 | | | |
| 229795_at | 8.51 | 8.95E-05 | 1.258228275 | NA |  |  |  |  |
| 203970_s_at | 8.51 | 8.93E-05 | 1.088522214 | NM_003630| | PEX3,peroxisomal biogenesis factor 3 | | |  |
| 209109_s_at | 8.51 | 8.94E-05 | 1.047847057 | NM_003270| | TM4SF6,transmembrane 4 superfamily member 6 | | | |
| 225935_at | 8.51 | 8.95E-05 | 1.109515818 | NA |  |  |  |  |
| 1560116_a_at | 8.5 | 8.99E-05 | 1.059201702 | NM_152905| | NEDD1,neural precursor cell expressed, developmentally | | | |
| 244519_at | 8.5 | 8.99E-05 | 1.14125191 | NM_015338| | ASXL1,additional sex combs like 1 | | |  |
| 230184_at | 8.5 | 9.01E-05 | 1.321026505 | NA |  |  |  |  |
| 226487_at | 8.5 | 9.02E-05 | 1.105554438 | NM_032829| | FLJ14721,hypothetical protein FLJ14721 | | | |
| 203394_s_at | 8.5 | 9.02E-05 | 1.109699194 | NM_005524| | HES1,hairy and enhancer of split 1 | | |  |
| 235203_at | 8.49 | 9.07E-05 | 1.099855412 | NA |  |  |  |  |
| 211671_s_at | 8.49 | 9.13E-05 | 1.130277928 | NM_000176| | NR3C1,nuclear receptor subfamily 3, group C, member 1 | | | |
| 241986_at | 8.48 | 9.20E-05 | 1.47570249 | NM_133468| | BMPER,BMP-binding endothelial regulator precursor | | | |
| 202746_at | 8.48 | 9.15E-05 | 1.124562236 | NM_004867| | ITM2A,integral membrane protein 2A | | |  |
| 213321_at | 8.48 | 9.17E-05 | 1.08057524 | NM_000056| | BCKDHB,branched chain keto acid dehydrogenase E1, beta | | | |
| 217624_at | 8.48 | 9.19E-05 | 1.216196367 | NM_014891| | PDAP1,PDGFA associated protein 1 | | |  |
| 239036_at | 8.48 | 9.20E-05 | 1.107580562 | NA |  |  |  |  |
| 228809_at | 8.48 | 9.20E-05 | 1.061831816 | NM_178124| | CXorf40,chromosome X open reading frame 40 | | | |
| 205364_at | 8.48 | 9.18E-05 | 1.207777213 | NM_003500| | ACOX2,acyl-Coenzyme A oxidase 2, branched chain | | | |
| 225712_at | 8.48 | 9.14E-05 | 1.026001503 | NM_015465| | GEMIN5,gemin 5 | |  |  |
| 202462_s_at | 8.48 | 9.21E-05 | 1.024532104 | NM_014829| | DDX46,DEAD (Asp-Glu-Ala-Asp) box polypeptide 46 | | | |
| 235465_at | 8.47 | 9.29E-05 | 1.419656271 | NM_152704| | FLJ25477,hypothetical protein FLJ25477 isoform 1 | | | |
| 227768_at | 8.47 | 9.29E-05 | 1.073590572 | NM_017757| | ZNF407,zinc finger protein 407 | | |  |
| 225356_at | 8.47 | 9.23E-05 | 1.04194079 | NA |  |  |  |  |
| 211058_x_at | 8.46 | 9.35E-05 | 1.013591007 | NM_006082| | K-ALPHA-1,tubulin, alpha, ubiquitous | | |  |
| 206382_s_at | 8.46 | 9.33E-05 | 1.07461083 | NM_001709| | BDNF,brain-derived neurotrophic factor isoform a | | | |
| 230634_x_at | 8.46 | 9.33E-05 | 1.11362101 | NM_138422| | LOC113179,hypothetical protein BC011824 | | | |
| 228266_s_at | 8.46 | 9.37E-05 | 1.029614068 | NM_016073| | HDGFRP3,hepatoma-derived growth factor, related protein | | | |
| 201532_at | 8.45 | 9.44E-05 | 1.01707693 | NM_002788| | PSMA3,proteasome alpha 3 subunit isoform 1 | | | |
| 222162_s_at | 8.45 | 9.42E-05 | 1.176034189 | NM_006988| | ADAMTS1,a disintegrin and metalloprotease with | | | |
| 201508_at | 8.45 | 9.39E-05 | 1.110877457 | NM_001552| | IGFBP4,insulin-like growth factor binding protein 4 | | | |
| 201731_s_at | 8.45 | 9.42E-05 | 1.029910009 | NM_003292| | TPR,translocated promoter region (to activated MET | | | |
| 205854_at | 8.45 | 9.42E-05 | 1.075651757 | NM_003324| | TULP3,tubby like protein 3 | | |  |
| 217828_at | 8.45 | 9.44E-05 | 1.050038756 | NM_001013843| | NA |  |  |  |
| 218049_s_at | 8.45 | 9.44E-05 | 1.047885166 | NM_014078| | MRPL13,mitochondrial ribosomal protein L13 | | | |
| 209778_at | 8.44 | 9.51E-05 | 1.104957751 | NM_004239| | TRIP11,thyroid hormone receptor interactor 11 | | | |
| 214871_x_at | 8.44 | 9.48E-05 | 1.138748404 | NA |  |  |  |  |
| 212032_s_at | 8.44 | 9.49E-05 | 1.046964746 | NM_017432| | PTOV1,prostate tumor overexpressed gene 1 | | | |
| 203637_s_at | 8.44 | 9.47E-05 | 1.062551035 | NM_000381| | MID1,midline 1 isoform alpha | | |  |
| 1555821_a_at | 8.44 | 9.50E-05 | 1.120527397 | NM_001098632| | NA |  |  |  |
| 203017_s_at | 8.43 | 9.54E-05 | 1.078663157 | NM_014021| | SSX2IP,synovial sarcoma, X breakpoint 2 interacting | | | |
| 212987_at | 8.43 | 9.56E-05 | 1.100885345 | NM_012347| | FBXO9,F-box only protein 9 isoform 1 | | |  |
| 1553169_at | 8.43 | 9.54E-05 | 1.152206236 | NM_152611| | C20orf75,chromosome 20 open reading frame 75 | | | |
| 213822_s_at | 8.43 | 9.57E-05 | 1.079913121 | NM_130466| | UBE3B,ubiquitin protein ligase E3B isoform a | | | |
| 223539_s_at | 8.43 | 9.56E-05 | 1.049879827 | NM_021967| | SERF1A,small EDRK-rich factor 1A, telomeric | | | |
| 201019_s_at | 8.43 | 9.58E-05 | 1.034548273 | NM_001412| | EIF1AX,X-linked eukaryotic translation initiation | | | |
| 202631_s_at | 8.43 | 9.54E-05 | 1.0784551 | NM_006380| | APPBP2,amyloid beta precursor protein-binding protein | | | |
| 201719_s_at | 8.42 | 9.68E-05 | 1.057882366 | NM_001431| | EPB41L2,erythrocyte membrane protein band 4.1-like 2 | | | |
| 228563_at | 8.42 | 9.67E-05 | 1.110790138 | NM_001080383| | NA |  |  |  |
| 235420_at | 8.42 | 9.67E-05 | 1.141713879 | NM_023002| | HAPLN4,brain link protein 2 | | |  |
| 200878_at | 8.42 | 9.67E-05 | 1.234176385 | NM_001430| | EPAS1,endothelial PAS domain protein 1 | | | |
| 223758_s_at | 8.42 | 9.68E-05 | 1.048698666 | NM_001515| | GTF2H2,general transcription factor IIH, polypeptide 2, | | | |
| 206465_at | 8.42 | 9.63E-05 | 1.285927418 | NM_015162| | BG1,lipidosin | |  |  |
| 212953_x_at | 8.42 | 9.67E-05 | 1.061102005 | NM_004343| | CALR,calreticulin precursor | | |  |
| 219247_s_at | 8.42 | 9.67E-05 | 1.177132779 | NM_024630| | ZDHHC14,NEW1 domain containing protein | | | |
| 230418_s_at | 8.42 | 9.66E-05 | 1.125582449 | NM_020692| | NA |  |  |  |
| 205353_s_at | 8.41 | 9.70E-05 | 1.032464082 | NM_002567| | PBP,prostatic binding protein | | |  |
| 203156_at | 8.41 | 9.72E-05 | 1.066725409 | NM_016248| | AKAP11,A-kinase anchor protein 11 isoform 1 | | | |
| 224755_at | 8.41 | 9.72E-05 | 1.089597372 | NA |  |  |  |  |
| 209704_at | 8.41 | 9.72E-05 | 1.023067267 | NM_007358| | M96,putative DNA binding protein | | |  |
| 212104_s_at | 8.41 | 9.71E-05 | 1.038694414 | NM_001031695| | NA |  |  |  |
| 221521_s_at | 8.41 | 9.73E-05 | 1.046466319 | NM_016095| | Pfs2,DNA replication complex GINS protein PSF2 | | | |
| 208368_s_at | 8.41 | 9.72E-05 | 1.141483777 | NM_000059| | BRCA2,breast cancer 2, early onset | | |  |
| 204416_x_at | 8.4 | 9.84E-05 | 1.093586134 | NM_001645| | APOC1,apolipoprotein C-I precursor | | |  |
| 205257_s_at | 8.4 | 9.79E-05 | 1.104843815 | NM_001635| | AMPH,amphiphysin isoform 1 | | |  |
| 206533_at | 8.4 | 9.77E-05 | 1.138877933 | NM_000745| | CHRNA5,cholinergic receptor, nicotinic, alpha | | | |
| 205880_at | 8.4 | 9.79E-05 | 1.237055101 | NM_002742| | PRKD1,protein kinase D1 | |  |  |
| 214095_at | 8.4 | 9.80E-05 | 1.070444207 | NM_005412| | SHMT2,serine hydroxymethyltransferase 2 | | | |
| 1568605_at | 8.4 | 9.79E-05 | 1.15884795 | NM_001077527| | NA |  |  |  |
| 232636_at | 8.4 | 9.82E-05 | 1.162938064 | NM_173078| | SLITRK4,slit and trk like 4 protein | | |  |
| 219646_at | 8.4 | 9.79E-05 | 1.091406439 | NM_017702| | FLJ20186,differentially expressed in FDCP 8 isoform 2 | | | |
| 209708_at | 8.4 | 9.77E-05 | 1.12363993 | NM_015529| | MOXD1,monooxygenase, DBH-like 1 | | |  |
| 231736_x_at | 8.39 | 9.85E-05 | 1.0348892 | NM_020300| | MGST1,microsomal glutathione S-transferase 1 | | | |
| 225921_at | 8.39 | 9.85E-05 | 1.063158031 | NM_016350| | NIN,ninein isoform 4 | |  |  |
| 225865_x_at | 8.39 | 9.87E-05 | 1.104623778 | NM_198976| | TH1L,TH1-like protein | |  |  |
| 231861_at | 8.39 | 9.87E-05 | 1.153838563 | NM_014045| | LRP10,low density lipoprotein receptor-related protein | | | |
| AFFX-HUMRGE/M10098_M_at | 8.39 | 9.85E-05 | 1.040822841 | NA |  |  |  |  |
| 214744_s_at | 8.39 | 9.87E-05 | 1.101880006 | NM_000978| | RPL23,ribosomal protein L23 | | |  |
| 208476_s_at | 8.38 | 9.94E-05 | 1.178850888 | NM_018027| | FRMD4A,FERM domain containing 4A | | |  |
| 228126_x_at | 8.38 | 9.98E-05 | 1.08644772 | NM_206833| | CTXN1,cortexin | |  |  |
| 215283_at | 8.38 | 9.94E-05 | 1.142070841 | NA |  |  |  |  |
| 217956_s_at | 8.38 | 9.96E-05 | 1.020025856 | NM_021204| | MASA,E-1 enzyme | |  |  |
| 228941_at | 8.38 | 9.94E-05 | 1.148393371 | NM_001013620| | NA |  |  |  |
| 212094_at | 8.38 | 9.94E-05 | 1.128554755 | NM_001040152| | NA |  |  |  |
| 200084_at | 8.38 | 9.98E-05 | 1.034514306 | NM_014267| | SMAP,small acidic protein | |  |  |
| 243805_at | 8.38 | 9.94E-05 | 1.186710146 | NM_133459| | CCBE1,collagen and calcium binding EGF domains 1 | | | |
|  |  |  |  |  |  |  |  |  |
