# Supplementary material for: Transcriptional Signature and Memory Retention of Human-Induced Pluripotent Stem Cells
Source: PLoS One. 2009 Sep 18;4(9):e7076. doi: 10.1371/journal.pone.0007076 (PMC2741600; doi:10.1371/journal.pone.0007076)
Supplement: Table S2 — ES-enriched probes in IPSC versus ES. Probesets enriched in group-wise comparisons: Column headings are probeset identifiers, T-statistic, P-value, Fold-Change (log2), Refseq identifier and Description of the gene. (NA indicates no Refseq annotation). (2.66 MB DOC) [file pone.0007076.s006.doc]

| Probeset | T-statistic | P-value | Fold-Change (log2) | Refseq | Description |
| --- | --- | --- | --- | --- | --- |
| 209448_at | 100.1 | 1.33E-11 | 2.120858772 | NM_001098520| | NA |
| 243161_x_at | 59.78 | 3.05E-10 | 3.336176165 | NM_174900| | ZFP42,zinc finger protein 42 |
| 1554777_at | 54.86 | 4.10E-10 | 3.45135307 | NM_174900| | ZFP42,zinc finger protein 42 |
| 226482_s_at | 54.47 | 4.10E-10 | 1.991133406 | NM_001113205| | NA |
| 214806_at | 48.92 | 8.41E-10 | 1.282041754 | NM_001003398| | BICD1,bicaudal D homolog 1 isoform 2 |
| 203607_at | 48.13 | 9.42E-10 | 1.338575723 | NM_014937| | INPP5F,inositol polyphosphate-5-phosphatase F isoform |
| 225314_at | 45.66 | 1.39E-09 | 1.847451799 | NM_001014446| | NA |
| 1554776_at | 45.48 | 1.39E-09 | 3.09970864 | NM_174900| | ZFP42,zinc finger protein 42 |
| 206309_at | 43.57 | 1.72E-09 | 1.996565695 | NM_001011705| | LECT1,leukocyte cell derived chemotaxin 1 isoform 2 |
| 216405_at | 43.56 | 1.72E-09 | 3.236689094 | NA |  |
| 207180_s_at | 42.94 | 1.92E-09 | 1.562861784 | NM_001098520| | NA |
| 201099_at | 41.72 | 2.29E-09 | 1.099072809 | NM_001039590| | NA |
| 241981_at | 40.75 | 2.73E-09 | 2.300235514 | NM_017565| | FAM20A,family with sequence similarity 20, member A |
| 208690_s_at | 39.51 | 3.33E-09 | 1.103558339 | NM_020992| | PDLIM1,PDZ and LIM domain 1 (elfin) |
| 201100_s_at | 39.24 | 3.33E-09 | 1.085182923 | NM_001039590| | NA |
| 229223_at | 38.47 | 3.75E-09 | 1.879377415 | NA |  |
| 206268_at | 37.33 | 4.50E-09 | 1.745591509 | NM_020997| | LEFTY1,left-right determination, factor B |
| 220013_at | 35.4 | 6.57E-09 | 1.620723914 | NM_024794| | ABHD9,abhydrolase domain containing 9 |
| 224301_x_at | 34.78 | 7.59E-09 | 1.374649195 | NM_177925| | H2AFJ,H2A histone family, member J isoform 2 |
| 204984_at | 34.72 | 7.59E-09 | 1.124544537 | NM_001448| | GPC4,glypican 4 |
| 207627_s_at | 34.41 | 7.94E-09 | 1.373497031 | NM_005653| | TFCP2,transcription factor CP2 |
| 223169_s_at | 33.9 | 8.84E-09 | 1.245941426 | NM_021205| | RHOU,ras homolog gene family, member U |
| 219509_at | 33.61 | 9.25E-09 | 1.292402757 | NM_021245| | MYOZ1,myozenin 1 |
| 224344_at | 33.37 | 9.62E-09 | 2.274442792 | NM_004373| | COX6A1,cytochrome c oxidase subunit VIa polypeptide 1 |
| 219395_at | 33.33 | 9.62E-09 | 1.679754474 | NM_024939| | FLJ21918,hypothetical protein FLJ21918 |
| 226804_at | 32.81 | 1.04E-08 | 1.879573271 | NM_017565| | FAM20A,family with sequence similarity 20, member A |
| 214203_s_at | 32.51 | 1.06E-08 | 1.391479125 | NM_016335| | PRODH,proline dehydrogenase (oxidase) 1 |
| 231310_at | 31.88 | 1.21E-08 | 1.051637363 | NA |  |
| 209869_at | 31.87 | 1.21E-08 | 1.17734923 | NM_000681| | ADRA2A,alpha-2A-adrenergic receptor |
| 225245_x_at | 31.6 | 1.29E-08 | 1.385437346 | NM_177925| | H2AFJ,H2A histone family, member J isoform 2 |
| 205135_s_at | 31.55 | 1.30E-08 | 1.118550544 | NM_012345| | NUFIP1,nuclear fragile X mental retardation protein |
| 212226_s_at | 31.48 | 1.32E-08 | 1.101770143 | NM_003713| | PPAP2B,phosphatidic acid phosphatase type 2B |
| 209297_at | 31.19 | 1.36E-08 | 1.093415854 | NM_001001132| | ITSN1,intersectin 1 isoform ITSN-s |
| 210852_s_at | 31.13 | 1.37E-08 | 1.265545206 | NM_005763| | AASS,aminoadipate-semialdehyde synthase |
| 223551_at | 30.99 | 1.38E-08 | 2.03034864 | NM_032471| | PKIB,cAMP-dependent protein kinase inhibitor beta |
| 237192_at | 30.68 | 1.46E-08 | 1.287111027 | NA |  |
| 221245_s_at | 30.49 | 1.52E-08 | 1.162114299 | NM_003468| | FZD5,frizzled 5 |
| 223580_at | 30.48 | 1.52E-08 | 1.188698554 | NM_032641| | GRCC9,SPRY domain-containing SOCS box protein SSB-2 |
| 221502_at | 30.45 | 1.52E-08 | 1.070103207 | NM_002267| | KPNA3,karyopherin alpha 3 |
| 210042_s_at | 30.38 | 1.52E-08 | 2.052680742 | NM_001336| | CTSZ,cathepsin Z preproprotein |
| 209193_at | 29.8 | 1.69E-08 | 1.159694573 | NM_002648| | PIM1,pim-1 oncogene |
| 231628_s_at | 29.16 | 1.99E-08 | 3.010194014 | NA |  |
| 201462_at | 28.74 | 2.19E-08 | 1.088659266 | NM_014766| | SCRN1,secernin 1 |
| 227837_at | 28.64 | 2.24E-08 | 1.083020733 | NA |  |
| 65517_at | 28.61 | 2.25E-08 | 1.138981222 | NM_005498| | AP1M2,adaptor-related protein complex 1, mu 2 subunit |
| 206417_at | 28.41 | 2.35E-08 | 1.593861806 | NM_000087| | CNGA1,cyclic nucleotide gated channel alpha 1 |
| 205286_at | 28.32 | 2.39E-08 | 1.344725687 | NM_003222| | TFAP2C,transcription factor AP-2 gamma |
| 218261_at | 28.23 | 2.44E-08 | 1.13819651 | NM_005498| | AP1M2,adaptor-related protein complex 1, mu 2 subunit |
| 238697_at | 28.06 | 2.54E-08 | 1.355977793 | NA |  |
| 219359_at | 28.02 | 2.57E-08 | 1.174090654 | NM_025092| | FLJ22635,hypothetical protein FLJ22635 |
| 206012_at | 27.97 | 2.59E-08 | 1.514663712 | NM_003240| | LEFTY2,endometrial bleeding associated factor |
| 204379_s_at | 27.94 | 2.61E-08 | 1.227616317 | NM_000142| | FGFR3,fibroblast growth factor receptor 3 isoform 1 |
| 1556824_at | 27.86 | 2.66E-08 | 2.017541321 | NA |  |
| 31846_at | 27.77 | 2.67E-08 | 1.089938895 | NM_014578| | RHOD,ras homolog D |
| 206424_at | 27.76 | 2.67E-08 | 1.24159322 | NM_000783| | CYP26A1,cytochrome P450, family 26, subfamily A, |
| 218282_at | 27.28 | 3.00E-08 | 1.204351119 | NM_018217| | C20orf31,chromosome 20 open reading frame 31 |
| 223125_s_at | 27.28 | 3.00E-08 | 1.08175283 | NM_030806| | C1orf21,chromosome 1 open reading frame 21 |
| 204121_at | 27.2 | 3.07E-08 | 1.396314229 | NM_006705| | GADD45G,growth arrest and DNA-damage-inducible, gamma |
| 211204_at | 27.17 | 3.09E-08 | 1.943412459 | NM_002395| | ME1,cytosolic malic enzyme 1 |
| 205134_s_at | 27 | 3.19E-08 | 1.172823978 | NM_012345| | NUFIP1,nuclear fragile X mental retardation protein |
| 208613_s_at | 26.72 | 3.42E-08 | 1.126419584 | NM_001457| | FLNB,filamin B, beta (actin binding protein 278) |
| 200701_at | 26.6 | 3.53E-08 | 1.077083568 | NM_006432| | NPC2,Niemann-Pick disease, type C2 precursor |
| 224767_at | 26.44 | 3.67E-08 | 1.263653249 | NM_000997| | RPL37,ribosomal protein L37 |
| 204020_at | 26.33 | 3.74E-08 | 1.172758928 | NM_005859| | PURA,purine-rich element binding protein A |
| 212203_x_at | 26.18 | 3.93E-08 | 1.101336597 | NM_021034| | IFITM3,interferon-induced transmembrane protein 3 |
| 218243_at | 26.15 | 3.95E-08 | 1.097549923 | NM_001040451| | NA |
| 242945_at | 26.1 | 3.98E-08 | 1.717793322 | NM_017565| | FAM20A,family with sequence similarity 20, member A |
| 231594_at | 26.05 | 4.03E-08 | 2.022900938 | NA |  |
| 226754_at | 25.97 | 4.14E-08 | 1.298042689 | NM_138367| | NA |
| 201061_s_at | 25.95 | 4.14E-08 | 1.192836513 | NM_004099| | STOM,stomatin isoform a |
| 1558143_a_at | 25.7 | 4.44E-08 | 1.211693835 | NM_006538| | BCL2L11,BCL2-like 11 isoform 6 |
| 218412_s_at | 25.67 | 4.45E-08 | 1.071690622 | NM_005685| | GTF2IRD1,GTF2I repeat domain containing 1 isoform 2 |
| 237193_s_at | 25.58 | 4.48E-08 | 1.233357681 | NA |  |
| 201601_x_at | 25.55 | 4.50E-08 | 1.128919559 | NM_003641| | IFITM1,interferon induced transmembrane protein 1 |
| 1552792_at | 25.32 | 4.81E-08 | 1.347796937 | NM_080867| | SOCS4,suppressor of cytokine signaling 4 |
| 218957_s_at | 25.28 | 4.84E-08 | 1.133455884 | NM_025155| | FLJ11848,hypothetical protein FLJ11848 |
| 204728_s_at | 25.21 | 4.94E-08 | 1.117674584 | NM_001008396| | WDHD1,WD repeat and HMG-box DNA binding protein 1 |
| 231120_x_at | 25.01 | 5.20E-08 | 2.496147473 | NM_032471| | PKIB,cAMP-dependent protein kinase inhibitor beta |
| 222759_at | 24.71 | 5.69E-08 | 1.119242623 | NM_016028| | SUV420H1,suppressor of variegation 4-20 homolog 1 isoform |
| 218341_at | 24.63 | 5.82E-08 | 1.172647189 | NM_001077447| | NA |
| 209338_at | 24.45 | 6.20E-08 | 1.231665465 | NM_005653| | TFCP2,transcription factor CP2 |
| 202178_at | 24.33 | 6.43E-08 | 1.097636579 | NM_001033581| | NA |
| 209122_at | 24.27 | 6.52E-08 | 1.08941378 | NM_001122| | ADFP,adipose differentiation-related protein |
| 209009_at | 24.26 | 6.54E-08 | 1.097045801 | NM_001984| | ESD,esterase D/formylglutathione hydrolase |
| 201976_s_at | 24.07 | 6.88E-08 | 1.051361365 | NM_012334| | MYO10,myosin X |
| 243_g_at | 24.03 | 6.91E-08 | 1.031750018 | NM_002375| | MAP4,microtubule-associated protein 4 isoform 1 |
| 226934_at | 23.74 | 7.51E-08 | 1.10342913 | NM_007007| | CPSF6,cleavage and polyadenylation specific factor 6, |
| 243221_at | 23.74 | 7.51E-08 | 1.984273843 | NA |  |
| 235339_at | 23.51 | 8.03E-08 | 1.256513346 | NM_031915| | SETDB2,CLLL8 protein |
| 237291_at | 23.48 | 8.06E-08 | 1.980118053 | NA |  |
| 241535_at | 23.25 | 8.61E-08 | 1.188233811 | NA |  |
| 1554043_a_at | 23.1 | 9.13E-08 | 1.123425137 | NA |  |
| 231517_at | 22.94 | 9.63E-08 | 1.301163246 | NA |  |
| 214022_s_at | 22.89 | 9.79E-08 | 1.107722818 | NM_003641| | IFITM1,interferon induced transmembrane protein 1 |
| 242814_at | 22.89 | 9.79E-08 | 1.571408435 | NM_004155| | SERPINB9,serine (or cysteine) proteinase inhibitor, clade |
| 228713_s_at | 22.74 | 1.02E-07 | 1.120808898 | NM_016246| | DHRS10,dehydrogenase/reductase (SDR family) member 10 |
| 218536_at | 22.73 | 1.02E-07 | 1.22040914 | NM_020662| | MRS2L,MRS2-like, magnesium homeostasis factor |
| 221519_at | 22.72 | 1.02E-07 | 1.087453222 | NM_022039| | SHFM3,split hand/foot malformation (ectrodactyly) type |
| 236525_at | 22.65 | 1.05E-07 | 1.218699885 | NM_174899| | FBXO36,F-box protein 36 |
| 213709_at | 22.47 | 1.11E-07 | 1.181243867 | NM_030639| | BHLHB9,basic helix-loop-helix domain containing, class |
| 213208_at | 22.43 | 1.11E-07 | 1.043349432 | NM_015349| | KIAA0240,KIAA0240 |
| 227978_s_at | 22.43 | 1.11E-07 | 1.649428447 | NM_175907| | ZADH2,zinc binding alcohol dehydrogenase, domain |
| 215028_at | 22.41 | 1.12E-07 | 1.230390255 | NM_020796| | SEMA6A,semaphorin 6A1 |
| 221588_x_at | 22.37 | 1.13E-07 | 1.08250374 | NM_005589| | ALDH6A1,aldehyde dehydrogenase 6A1 precursor |
| 225536_at | 22.29 | 1.17E-07 | 1.348095569 | NM_033504| | BCLP,beta-casein-like protein |
| 223592_s_at | 22.27 | 1.17E-07 | 1.165523144 | NM_032322| | RNF135,ring finger protein 135 isoform 1 |
| 209147_s_at | 22.21 | 1.20E-07 | 1.189477802 | NM_003711| | PPAP2A,phosphatidic acid phosphatase type 2A isoform 1 |
| 204319_s_at | 22.18 | 1.20E-07 | 1.139293195 | NM_001005339| | RGS10,regulator of G-protein signaling 10 isoform a |
| 209210_s_at | 22.17 | 1.20E-07 | 1.046274403 | NM_006832| | PLEKHC1,pleckstrin homology domain containing, family C |
| 230097_at | 22.14 | 1.21E-07 | 1.396877469 | NM_000819| | GART,phosphoribosylglycinamide formyltransferase, |
| 227129_x_at | 22.02 | 1.26E-07 | 1.152162455 | NA |  |
| 224998_at | 21.97 | 1.28E-07 | 1.100111476 | NM_178818| | CKLFSF4,chemokine-like factor superfamily 4 isoform 1 |
| 228415_at | 21.81 | 1.35E-07 | 1.070941141 | NM_003916| | AP1S2,adaptor-related protein complex 1 sigma 2 |
| 216565_x_at | 21.7 | 1.40E-07 | 1.144095154 | NA |  |
| 209477_at | 21.62 | 1.43E-07 | 1.115752721 | NM_000117| | EMD,emerin |
| 207836_s_at | 21.6 | 1.44E-07 | 1.099391452 | NM_001008710| | RBPMS,RNA-binding protein with multiple splicing |
| 210946_at | 21.59 | 1.44E-07 | 1.1445322 | NM_003711| | PPAP2A,phosphatidic acid phosphatase type 2A isoform 1 |
| 39705_at | 21.55 | 1.46E-07 | 1.125806993 | NM_015260| | SIN3B,SIN3 homolog B, transcription regulator |
| 226132_s_at | 21.35 | 1.56E-07 | 1.240949758 | NM_001031740| | NA |
| 203638_s_at | 21.28 | 1.59E-07 | 1.091629509 | NM_000141| | FGFR2,fibroblast growth factor receptor 2 isoform 1 |
| 229095_s_at | 21.23 | 1.61E-07 | 1.690945672 | NA |  |
| 203614_at | 21.22 | 1.61E-07 | 1.08972166 | NM_021645| | UTP14C,UTP14, U3 small nucleolar ribonucleoprotein, |
| 219259_at | 21.16 | 1.64E-07 | 1.15940045 | NM_022367| | SEMA4A,semaphorin B |
| 204290_s_at | 21.13 | 1.65E-07 | 1.101925077 | NM_005589| | ALDH6A1,aldehyde dehydrogenase 6A1 precursor |
| 228968_at | 21.09 | 1.67E-07 | 1.212918938 | NM_152695| | ZNF449,zinc finger protein 449 |
| 210431_at | 21.07 | 1.67E-07 | 1.381287258 | NM_031313| | ALPPL2,placental-like alkaline phosphatase |
| 217938_s_at | 21.07 | 1.67E-07 | 1.066620752 | NM_020122| | KCMF1,potassium channel modulatory factor 1 |
| 242054_s_at | 21.04 | 1.69E-07 | 1.904416507 | NA |  |
| 222523_at | 20.84 | 1.80E-07 | 1.090550865 | NM_021627| | SENP2,SUMO1/sentrin/SMT3 specific protease 2 |
| 208822_s_at | 20.82 | 1.82E-07 | 1.055830643 | NM_004632| | DAP3,death-associated protein 3 |
| 212873_at | 20.79 | 1.84E-07 | 1.064012009 | NM_012292| | HA-1,minor histocompatibility antigen HA-1 |
| 212068_s_at | 20.63 | 1.96E-07 | 1.072852981 | NM_013318| | NA |
| 203407_at | 20.54 | 2.02E-07 | 1.091188498 | NM_002705| | PPL,periplakin |
| 211062_s_at | 20.54 | 2.02E-07 | 1.151552562 | NM_001014447| | NA |
| 217676_at | 20.52 | 2.02E-07 | 2.024864949 | NA |  |
| 227909_at | 20.5 | 2.02E-07 | 1.137661131 | NM_001031705| | NA |
| 213171_s_at | 20.41 | 2.08E-07 | 1.207442864 | NM_006690| | MMP24,matrix metalloproteinase 24 (membrane-inserted) |
| 213546_at | 20.34 | 2.13E-07 | 1.210478591 | NA |  |
| 213279_at | 20.2 | 2.26E-07 | 1.210765574 | NM_138452| | DHRS1,dehydrogenase/reductase (SDR family) member 1 |
| 213301_x_at | 20 | 2.43E-07 | 1.05196755 | NM_003852| | TIF1,transcriptional intermediary factor 1 alpha |
| 203633_at | 19.98 | 2.45E-07 | 1.349462861 | NM_001031847| | NA |
| 207305_s_at | 19.97 | 2.45E-07 | 1.055670854 | NM_014939| | KIAA1012,KIAA1012 |
| 210150_s_at | 19.88 | 2.54E-07 | 1.085011889 | NM_005560| | LAMA5,laminin alpha 5 |
| 41660_at | 19.85 | 2.56E-07 | 1.065912798 | NM_014246| | CELSR1,cadherin EGF LAG seven-pass G-type receptor 1 |
| 224215_s_at | 19.84 | 2.57E-07 | 1.168946501 | NM_005618| | DLL1,delta-like 1 |
| 236337_at | 19.83 | 2.57E-07 | 1.29652045 | NM_001040274| | NA |
| 202597_at | 19.77 | 2.63E-07 | 1.152594998 | NM_006147| | IRF6,interferon regulatory factor 6 |
| 203389_at | 19.73 | 2.67E-07 | 1.057021743 | NM_002254| | KIF3C,kinesin family member 3C |
| 223179_at | 19.72 | 2.67E-07 | 1.103048189 | NM_031477| | YPEL3,yippee-like 3 |
| 202234_s_at | 19.71 | 2.67E-07 | 1.105166953 | NM_003051| | SLC16A1,solute carrier family 16, member 1 |
| 212838_at | 19.71 | 2.68E-07 | 1.130365887 | NM_015221| | DNMBP,dynamin binding protein |
| 45749_at | 19.67 | 2.71E-07 | 1.139657315 | NM_024519| | FLJ13725,hypothetical protein FLJ13725 |
| 1564154_at | 19.66 | 2.73E-07 | 1.332170506 | NA |  |
| 1559280_a_at | 19.63 | 2.75E-07 | 1.122835471 | NA |  |
| 219118_at | 19.6 | 2.77E-07 | 1.123110502 | NM_016594| | FKBP11,FK506 binding protein precursor |
| 1554248_at | 19.55 | 2.82E-07 | 1.21015124 | NM_001014972| | NA |
| 206322_at | 19.5 | 2.88E-07 | 1.395678 | NM_003490| | SYN3,synapsin III isoform IIIa |
| 229433_at | 19.48 | 2.90E-07 | 1.174022693 | NM_022118| | C13orf10,cutaneous T-cell lymphoma tumor antigen se70-2 |
| 224763_at | 19.27 | 3.10E-07 | 1.166660498 | NM_000997| | RPL37,ribosomal protein L37 |
| 220341_s_at | 19.19 | 3.20E-07 | 1.104810042 | NM_001017987| | NA |
| 210774_s_at | 19.18 | 3.20E-07 | 1.057871342 | NM_005437| | NCOA4,nuclear receptor coactivator 4 |
| 229971_at | 19.1 | 3.27E-07 | 1.472572087 | NM_153837| | GPR114,G-protein coupled receptor 114 |
| 223251_s_at | 19.06 | 3.32E-07 | 1.114928488 | NM_017664| | ANKRD10,ankyrin repeat domain 10 |
| 203714_s_at | 19.03 | 3.33E-07 | 1.047364335 | NM_001079515| | NA |
| 202930_s_at | 18.98 | 3.39E-07 | 1.072584053 | NM_003850| | SUCLA2,succinate-CoA ligase, ADP-forming, beta subunit |
| 204391_x_at | 18.93 | 3.46E-07 | 1.055371292 | NM_003852| | TIF1,transcriptional intermediary factor 1 alpha |
| 211800_s_at | 18.92 | 3.47E-07 | 1.069080168 | NM_003363| | USP4,ubiquitin specific protease, proto-oncogene |
| 201060_x_at | 18.9 | 3.48E-07 | 1.171446341 | NM_004099| | STOM,stomatin isoform a |
| 242751_at | 18.9 | 3.48E-07 | 1.192331261 | NA |  |
| 209873_s_at | 18.78 | 3.68E-07 | 1.218392519 | NM_007183| | PKP3,plakophilin 3 |
| 224995_at | 18.76 | 3.72E-07 | 1.143521383 | NM_020148| | SPIRE1,spire homolog 1 |
| 1570107_at | 18.73 | 3.75E-07 | 1.84217593 | NA |  |
| 226240_at | 18.66 | 3.86E-07 | 1.108452899 | NM_152293| | NA |
| 209590_at | 18.63 | 3.91E-07 | 1.30088734 | NM_001719| | BMP7,bone morphogenetic protein 7 precursor |
| 225478_at | 18.63 | 3.91E-07 | 1.067625713 | NM_004225| | MFHAS1,malignant fibrous histiocytoma amplified |
| 203639_s_at | 18.56 | 4.04E-07 | 1.296909177 | NM_000141| | FGFR2,fibroblast growth factor receptor 2 isoform 1 |
| 225009_at | 18.54 | 4.05E-07 | 1.151568228 | NM_178818| | CKLFSF4,chemokine-like factor superfamily 4 isoform 1 |
| 220822_at | 18.48 | 4.18E-07 | 1.731338238 | NM_017973| | NA |
| 207170_s_at | 18.47 | 4.19E-07 | 1.18056613 | NM_001024668| | NA |
| 217732_s_at | 18.4 | 4.32E-07 | 1.090777871 | NM_021999| | ITM2B,integral membrane protein 2B |
| 36475_at | 18.39 | 4.32E-07 | 1.102036357 | NM_014291| | GCAT,glycine C-acetyltransferase precursor |
| 219303_at | 18.32 | 4.39E-07 | 1.19160503 | NM_024546| | C13orf7,chromosome 13 open reading frame 7 |
| 203324_s_at | 18.27 | 4.49E-07 | 1.225579634 | NM_001233| | CAV2,caveolin 2 isoform a and b |
| 226249_at | 18.07 | 4.89E-07 | 1.087099763 | NM_001012994| | NA |
| 202789_at | 17.96 | 5.10E-07 | 1.074888187 | NM_002660| | PLCG1,phospholipase C gamma 1 isoform a |
| 1559827_at | 17.96 | 5.10E-07 | 1.218410333 | NA |  |
| 204092_s_at | 17.96 | 5.10E-07 | 1.034178258 | NM_003600| | STK6,serine/threonine protein kinase 6 |
| 225840_at | 17.94 | 5.11E-07 | 1.197347935 | NM_003216| | TEF,thyrotrophic embryonic factor |
| 1555713_at | 17.94 | 5.11E-07 | 1.529125262 | NM_001077399| | NA |
| 222595_s_at | 17.87 | 5.26E-07 | 1.210607532 | NM_022105| | DATF1,death associated transcription factor 1 isoform |
| 210281_s_at | 17.85 | 5.28E-07 | 1.260197006 | NM_003453| | ZNF198,zinc finger protein 198 |
| 210372_s_at | 17.81 | 5.36E-07 | 1.247194206 | NM_001003395| | TPD52L1,tumor protein D52-like 1 isoform 2 |
| 232180_at | 17.81 | 5.36E-07 | 1.246676808 | NM_001001521| | UGP2,UDP-glucose pyrophosphorylase 2 isoform b |
| 212232_at | 17.8 | 5.37E-07 | 1.085857951 | NM_015308| | FNBP4,formin binding protein 4 |
| 210822_at | 17.75 | 5.48E-07 | 1.159139681 | NA |  |
| 208228_s_at | 17.74 | 5.50E-07 | 1.189820901 | NM_000141| | FGFR2,fibroblast growth factor receptor 2 isoform 1 |
| 225915_at | 17.6 | 5.85E-07 | 1.394824135 | NM_001079670| | NA |
| 225301_s_at | 17.59 | 5.87E-07 | 1.150825562 | NM_001080467| | NA |
| 225569_at | 17.54 | 5.98E-07 | 1.045934847 | NM_012154| | EIF2C2,eukaryotic translation initiation factor 2C, 2 |
| 238688_at | 17.52 | 6.04E-07 | 1.241604462 | NM_000366| | TPM1,tropomyosin 1 (alpha) |
| 209185_s_at | 17.5 | 6.11E-07 | 1.075481562 | NM_003749| | IRS2,insulin receptor substrate 2 |
| 210416_s_at | 17.49 | 6.11E-07 | 1.088421101 | NM_001005735| | CHEK2,protein kinase CHK2 isoform c |
| 205164_at | 17.42 | 6.31E-07 | 1.100987938 | NM_014291| | GCAT,glycine C-acetyltransferase precursor |
| 201285_at | 17.41 | 6.34E-07 | 1.084766489 | NM_013446| | MKRN1,makorin, ring finger protein, 1 |
| 243332_at | 17.38 | 6.41E-07 | 1.319691512 | NA |  |
| 218029_at | 17.38 | 6.41E-07 | 1.18006806 | NM_024519| | FLJ13725,hypothetical protein FLJ13725 |
| 227072_at | 17.32 | 6.62E-07 | 1.056697262 | NM_173630| | RTTN,rotatin |
| 225977_at | 17.31 | 6.62E-07 | 1.119882236 | NM_019035| | PCDH18,protocadherin 18 precursor |
| 1560738_at | 17.3 | 6.65E-07 | 1.811823562 | NA |  |
| 226627_at | 17.3 | 6.65E-07 | 1.106835877 | NM_001098811| | NA |
| 204982_at | 17.27 | 6.68E-07 | 1.227467751 | NM_014776| | GIT2,G protein-coupled receptor kinase-interactor 2 |
| 226568_at | 17.23 | 6.78E-07 | 1.154104246 | NM_001010883| | LOC284611,hypothetical protein LOC284611 |
| 225132_at | 17.22 | 6.80E-07 | 1.103847474 | NM_012158| | FBXL3,F-box and leucine-rich repeat protein 3 |
| 231713_s_at | 17.22 | 6.80E-07 | 1.047064846 | NM_018255| | STATIP1,elongator protein 2 |
| 226140_s_at | 17.19 | 6.93E-07 | 1.101461415 | NA |  |
| 209238_at | 17.17 | 6.97E-07 | 1.086371774 | NM_004177| | STX3A,syntaxin 3A |
| 219801_at | 17.16 | 6.99E-07 | 1.177659679 | NM_030580| | ZNF34,zinc finger protein 34 (KOX 32) |
| 226316_at | 17.15 | 6.99E-07 | 1.156780732 | NA |  |
| 212463_at | 17.1 | 7.14E-07 | 1.100303009 | NM_000611| | CD59,CD59 antigen p18-20 |
| 236125_at | 17.09 | 7.15E-07 | 1.1959414 | NA |  |
| 222258_s_at | 17.05 | 7.26E-07 | 1.064948919 | NM_014521| | SH3BP4,SH3-domain binding protein 4 |
| 204981_at | 17.03 | 7.32E-07 | 1.20783874 | NM_002555| | SLC22A18,tumor suppressing subtransferable candidate 5 |
| 221045_s_at | 17.01 | 7.41E-07 | 1.136946058 | NM_016831| | PER3,period 3 |
| 209018_s_at | 17 | 7.42E-07 | 1.160526515 | NM_032409| | PINK1,PTEN induced putative kinase 1 |
| 200022_at | 16.97 | 7.49E-07 | 1.018253172 | NM_000979| | RPL18,ribosomal protein L18 |
| 227934_at | 16.97 | 7.49E-07 | 1.105968663 | NM_002269| | KPNA5,karyopherin alpha 5 (importin alpha 6) |
| 1553987_at | 16.95 | 7.57E-07 | 1.084792005 | NA |  |
| 206018_at | 16.95 | 7.55E-07 | 1.41643625 | NM_005249| | FOXG1B,forkhead box G1B |
| 218552_at | 16.93 | 7.62E-07 | 1.290608424 | NM_018281| | ECHDC2,enoyl Coenzyme A hydratase domain containing 2 |
| 212869_x_at | 16.9 | 7.69E-07 | 1.022415117 | NM_003295| | TPT1,tumor protein, translationally-controlled 1 |
| 214790_at | 16.86 | 7.79E-07 | 1.190523741 | NM_001100409| | NA |
| 208536_s_at | 16.85 | 7.84E-07 | 1.221601063 | NM_006538| | BCL2L11,BCL2-like 11 isoform 6 |
| 220419_s_at | 16.79 | 8.07E-07 | 1.095582945 | NM_013396| | USP25,ubiquitin specific protease 25 |
| 225822_at | 16.75 | 8.23E-07 | 1.063563216 | NM_144626| | MGC17299,hypothetical protein MGC17299 |
| 209306_s_at | 16.74 | 8.23E-07 | 1.091791764 | NM_015055| | SWAP70,SWAP-70 protein |
| 209411_s_at | 16.73 | 8.28E-07 | 1.067381136 | NM_014001| | GGA3,ADP-ribosylation factor binding protein 3 |
| 201998_at | 16.69 | 8.41E-07 | 1.075431726 | NM_003032| | ST6GAL1,sialyltransferase 1 isoform a |
| 1564155_x_at | 16.64 | 8.60E-07 | 1.247306967 | NA |  |
| 200815_s_at | 16.62 | 8.67E-07 | 1.060613785 | NM_000430| | PAFAH1B1,platelet-activating factor acetylhydrolase, |
| 216520_s_at | 16.62 | 8.68E-07 | 1.041494063 | NM_003295| | TPT1,tumor protein, translationally-controlled 1 |
| 223591_at | 16.6 | 8.73E-07 | 1.202351558 | NM_032322| | RNF135,ring finger protein 135 isoform 1 |
| 213280_at | 16.57 | 8.83E-07 | 1.094208749 | NM_001100398| | NA |
| 203634_s_at | 16.56 | 8.85E-07 | 1.543146034 | NM_001031847| | NA |
| 209313_at | 16.5 | 9.12E-07 | 1.039386697 | NM_007266| | XAB1,XPA binding protein 1 |
| 209679_s_at | 16.48 | 9.15E-07 | 1.130741877 | NM_001031628| | NA |
| 235089_at | 16.48 | 9.13E-07 | 1.156368954 | NM_032875| | FBXL20,F-box and leucine-rich repeat protein 20 |
| 221981_s_at | 16.46 | 9.23E-07 | 1.130721635 | NM_030581| | FLJ12270,FLJ12270 protein |
| 203277_at | 16.46 | 9.21E-07 | 1.084277593 | NM_004401| | DFFA,DNA fragmentation factor, 45kDa, alpha |
| 225068_at | 16.45 | 9.25E-07 | 1.051355944 | NM_021633| | KLHL12,kelch-like 12 |
| 217114_at | 16.44 | 9.25E-07 | 1.162945423 | NA |  |
| 225007_at | 16.43 | 9.30E-07 | 1.058590156 | NM_005754| | G3BP,Ras-GTPase-activating protein SH3-domain-binding |
| 226842_at | 16.42 | 9.32E-07 | 1.0927844 | NA |  |
| 219809_at | 16.41 | 9.36E-07 | 1.073078768 | NM_017706| | FLJ20195,hypothetical protein FLJ20195 |
| 221499_s_at | 16.37 | 9.53E-07 | 1.125656125 | NM_001001433| | STX16,syntaxin 16 isoform a |
| 213412_at | 16.36 | 9.56E-07 | 1.179274114 | NM_014428| | TJP3,tight junction protein 3 (zona occludens 3) |
| 1568593_a_at | 16.34 | 9.66E-07 | 1.77880802 | NA |  |
| 241363_at | 16.25 | 1.01E-06 | 1.22384562 | NM_017820| | FLJ20433,hypothetical protein FLJ20433 |
| 241510_at | 16.21 | 1.03E-06 | 1.514729109 | NA |  |
| 203046_s_at | 16.19 | 1.03E-06 | 1.052232591 | NM_003920| | TIMELESS,timeless homolog |
| 215672_s_at | 16.18 | 1.04E-06 | 1.1555207 | NM_015328| | KIAA0828,KIAA0828 protein |
| 217609_at | 16.17 | 1.05E-06 | 1.189117924 | NM_006992| | B7,leucine-rich B7 protein isoform 2 |
| 235911_at | 16.13 | 1.06E-06 | 1.064825756 | NA |  |
| 218056_at | 16.12 | 1.06E-06 | 1.045059731 | NM_016561| | BFAR,apoptosis regulator |
| 212402_at | 16.11 | 1.06E-06 | 1.095716778 | NM_015070| | KIAA0853,KIAA0853 |
| 205355_at | 16.09 | 1.08E-06 | 1.361034424 | NM_001609| | ACADSB,acyl-Coenzyme A dehydrogenase, short/branched |
| 213630_at | 16.07 | 1.09E-06 | 1.142949319 | NA |  |
| 225964_at | 16.06 | 1.09E-06 | 1.104452716 | NM_001040653| | NA |
| 225974_at | 16.06 | 1.09E-06 | 1.255546109 | NM_001008495| | DKFZp762C1112,hypothetical protein DKFZp762C1112 |
| 206782_s_at | 16.02 | 1.11E-06 | 1.196743497 | NM_005528| | DNAJC4,DnaJ (Hsp40) homolog, subfamily C, member 4 |
| 226086_at | 15.99 | 1.13E-06 | 1.057837031 | NM_020826| | SYT13,synaptotagmin XIII |
| 231973_s_at | 15.96 | 1.14E-06 | 1.061692089 | NM_022662| | ANAPC1,anaphase promoting complex subunit 1 |
| 209782_s_at | 15.95 | 1.14E-06 | 1.155577587 | NM_001352| | DBP,D site of albumin promoter (albumin D-box) |
| 221883_at | 15.95 | 1.14E-06 | 1.158246866 | NM_004571| | PKNOX1,PBX/knotted 1 homeobox 1 isoform 1 |
| 205593_s_at | 15.92 | 1.16E-06 | 1.104508049 | NM_001001567| | PDE9A,phosphodiesterase 9A isoform b |
| 223017_at | 15.91 | 1.16E-06 | 1.045200632 | NM_015913| | TLP19,endoplasmic reticulum thioredoxin superfamily |
| 226744_at | 15.91 | 1.16E-06 | 1.073111044 | NM_024086| | MGC3329,hypothetical protein MGC3329 |
| 212959_s_at | 15.9 | 1.17E-06 | 1.151405359 | NM_024312| | MGC4170,MGC4170 protein |
| 231964_at | 15.89 | 1.17E-06 | 1.093178204 | NA |  |
| 208249_s_at | 15.88 | 1.18E-06 | 1.121361312 | NM_014305| | TGDS,TDP-glucose 4,6-dehydratase |
| 204285_s_at | 15.81 | 1.22E-06 | 1.113098083 | NM_021127| | PMAIP1,phorbol-12-myristate-13-acetate-induced protein |
| 201696_at | 15.81 | 1.22E-06 | 1.068371661 | NM_005626| | SFRS4,splicing factor, arginine/serine-rich 4 |
| 204021_s_at | 15.77 | 1.23E-06 | 1.166366504 | NM_005859| | PURA,purine-rich element binding protein A |
| 231079_at | 15.74 | 1.25E-06 | 1.160600559 | NA |  |
| 205136_s_at | 15.73 | 1.26E-06 | 1.206855297 | NM_012345| | NUFIP1,nuclear fragile X mental retardation protein |
| 219411_at | 15.73 | 1.25E-06 | 1.296848489 | NM_024712| | ELMO3,engulfment and cell motility 3 |
| 211937_at | 15.72 | 1.26E-06 | 1.046203931 | NM_001417| | EIF4B,eukaryotic translation initiation factor 4B |
| 215111_s_at | 15.72 | 1.26E-06 | 1.112811186 | NM_006022| | TGFB1I4,transforming growth factor beta 1 induced |
| 1559065_a_at | 15.71 | 1.26E-06 | 1.499715177 | NM_198492| | CLEC4G,C-type lectin superfamily 4, member G |
| 204085_s_at | 15.69 | 1.27E-06 | 1.233951862 | NM_006493| | CLN5,ceroid-lipofuscinosis, neuronal 5 |
| 203773_x_at | 15.65 | 1.29E-06 | 1.073437292 | NM_000712| | BLVRA,biliverdin reductase A |
| 214092_x_at | 15.64 | 1.30E-06 | 1.051572405 | NM_001017392| | NA |
| 229131_at | 15.63 | 1.30E-06 | 1.069882586 | NA |  |
| 238931_at | 15.56 | 1.34E-06 | 1.13345216 | NM_024086| | MGC3329,hypothetical protein MGC3329 |
| 220085_at | 15.56 | 1.34E-06 | 1.058931017 | NM_018063| | HELLS,helicase, lymphoid-specific |
| 211259_s_at | 15.54 | 1.36E-06 | 1.16865574 | NM_001719| | BMP7,bone morphogenetic protein 7 precursor |
| 212832_s_at | 15.47 | 1.40E-06 | 1.054675094 | NM_001008938| | ch-TOG,colonic and hepatic tumor over-expressed protein |
| 222099_s_at | 15.46 | 1.40E-06 | 1.062453087 | NM_001114093| | NA |
| 211729_x_at | 15.46 | 1.41E-06 | 1.069495534 | NM_000712| | BLVRA,biliverdin reductase A |
| 239319_at | 15.45 | 1.41E-06 | 1.655415495 | NA |  |
| 218956_s_at | 15.42 | 1.43E-06 | 1.081043578 | NM_015545| | PTCD1,pentatricopeptide repeat domain 1 |
| 225265_at | 15.41 | 1.44E-06 | 1.050769628 | NM_002897| | RBMS1,RNA binding motif, single stranded interacting |
| 218795_at | 15.36 | 1.47E-06 | 1.083814018 | NM_016361| | ACP6,acid phosphatase 6, lysophosphatidic |
| 223013_at | 15.35 | 1.48E-06 | 1.037642946 | NM_024665| | TBL1XR1,nuclear receptor co-repressor/HDAC3 complex |
| 214493_s_at | 15.31 | 1.51E-06 | 1.31299043 | NM_176877| | INADL,InaD-like protein isoform 2 |
| 237465_at | 15.29 | 1.52E-06 | 1.333606551 | NM_019050| | USP53,ubiquitin specific protease 53 |
| 213065_at | 15.26 | 1.54E-06 | 1.077540801 | NM_144982| | MGC23401,hypothetical protein MGC23401 |
| 206634_at | 15.24 | 1.56E-06 | 1.328871328 | NM_005413| | SIX3,sine oculis homeobox homolog 3 |
| 208810_at | 15.24 | 1.56E-06 | 1.117447799 | NM_005494| | DNAJB6,DnaJ (Hsp40) homolog, subfamily B, member 6 |
| 227641_at | 15.23 | 1.57E-06 | 1.140521973 | NM_153350| | FBXL16,F-box and leucine-rich repeat protein 16 |
| 208718_at | 15.23 | 1.57E-06 | 1.035651713 | NM_001098504| | NA |
| 223467_at | 15.22 | 1.58E-06 | 1.164234042 | NM_016084| | RASD1,RAS, dexamethasone-induced 1 |
| 208819_at | 15.19 | 1.60E-06 | 1.083178182 | NM_005370| | RAB8A,mel transforming oncogene |
| 200992_at | 15.19 | 1.60E-06 | 1.058712422 | NM_006391| | IPO7,importin 7 |
| 209463_s_at | 15.15 | 1.63E-06 | 1.103833806 | NM_005644| | TAF12,TAF12 RNA polymerase II, TATA box binding |
| 209019_s_at | 15.13 | 1.64E-06 | 1.148270923 | NM_032409| | PINK1,PTEN induced putative kinase 1 |
| 200883_at | 15.13 | 1.64E-06 | 1.08499442 | NM_003366| | UQCRC2,ubiquinol-cytochrome c reductase core protein |
| 223360_at | 15.13 | 1.64E-06 | 1.171516518 | NM_032261| | C21orf56,chromosome 21 open reading frame 56 |
| 210282_at | 15.11 | 1.66E-06 | 1.326080703 | NM_003453| | ZNF198,zinc finger protein 198 |
| 222700_at | 15.11 | 1.66E-06 | 1.05672963 | NM_022374| | ARL6IP2,ADP-ribosylation factor-like 6 interacting |
| 223450_s_at | 15.1 | 1.66E-06 | 1.179707243 | NM_031431| | COG3,component of golgi transport complex 3 |
| 224217_s_at | 15.1 | 1.66E-06 | 1.053255568 | NM_007051| | FAF1,FAS-associated factor 1 isoform a |
| 242052_at | 15.1 | 1.66E-06 | 1.229837464 | NA |  |
| 212557_at | 15.1 | 1.66E-06 | 1.067424361 | NM_001031623| | NA |
| 206783_at | 15.09 | 1.67E-06 | 1.244569812 | NM_002007| | FGF4,fibroblast growth factor 4 precursor |
| 238549_at | 15.07 | 1.69E-06 | 1.18227248 | NM_001032999| | NA |
| 227827_at | 15.03 | 1.72E-06 | 1.21990625 | NA |  |
| 203894_at | 15.02 | 1.74E-06 | 1.166572484 | NM_016437| | TUBG2,tubulin, gamma 2 |
| 206801_at | 14.97 | 1.77E-06 | 1.417472471 | NM_002521| | NPPB,natriuretic peptide precursor B |
| 232341_x_at | 14.96 | 1.78E-06 | 1.151015576 | NM_014282| | HABP4,hyaluronan binding protein 4 |
| 219214_s_at | 14.95 | 1.79E-06 | 1.061067492 | NM_014595| | NT5C,5',3'-nucleotidase, cytosolic |
| 204053_x_at | 14.94 | 1.79E-06 | 1.072150525 | NM_000314| | PTEN,phosphatase and tensin homolog |
| 212284_x_at | 14.94 | 1.80E-06 | 1.022206433 | NM_003295| | TPT1,tumor protein, translationally-controlled 1 |
| 226429_at | 14.93 | 1.80E-06 | 1.196368923 | NM_018559| | KIAA1704,KIAA1704 |
| 225312_at | 14.89 | 1.83E-06 | 1.090956747 | NM_203495| | COMMD6,COMM domain containing 6 isoform b |
| 203215_s_at | 14.87 | 1.84E-06 | 1.07677673 | NM_004999| | MYO6,myosin VI |
| 231149_s_at | 14.86 | 1.85E-06 | 1.283627586 | NM_017886| | NA |
| 218972_at | 14.84 | 1.87E-06 | 1.102063239 | NM_018259| | TTC17,tetratricopeptide repeat domain 17 |
| 223053_x_at | 14.83 | 1.88E-06 | 1.053170553 | NM_014188| | HSPC182,HSPC182 protein |
| 202301_s_at | 14.77 | 1.94E-06 | 1.055054726 | NM_023012| | FLJ11021,similar to splicing factor, arginine/serine-rich |
| 213505_s_at | 14.77 | 1.94E-06 | 1.064056882 | NM_001017392| | NA |
| 200597_at | 14.75 | 1.95E-06 | 1.059601247 | NM_003750| | EIF3S10,eukaryotic translation initiation factor 3, |
| 203095_at | 14.74 | 1.96E-06 | 1.079878441 | NM_001005369| | MTIF2,mitochondrial translational initiation factor 2 |
| 1554472_a_at | 14.74 | 1.96E-06 | 1.193680654 | NM_016018| | PHF20L1,PHD finger protein 20-like 1 isoform 1 |
| 240269_at | 14.73 | 1.97E-06 | 1.452131761 | NA |  |
| 209581_at | 14.71 | 1.98E-06 | 1.237910192 | NM_007069| | HRASLS3,HRAS-like suppressor 3 |
| 207076_s_at | 14.69 | 2.01E-06 | 1.082794404 | NM_000050| | ASS,argininosuccinate synthetase |
| 231698_at | 14.67 | 2.03E-06 | 1.10527552 | NA |  |
| 208623_s_at | 14.66 | 2.05E-06 | 1.107772152 | NM_001111077| | NA |
| 232596_at | 14.65 | 2.06E-06 | 1.187589414 | NM_001042517| | NA |
| 1554020_at | 14.63 | 2.07E-06 | 1.291350473 | NM_001003398| | BICD1,bicaudal D homolog 1 isoform 2 |
| 230979_at | 14.62 | 2.07E-06 | 1.144318643 | NA |  |
| 1558440_at | 14.6 | 2.10E-06 | 1.503533644 | NA |  |
| 202845_s_at | 14.58 | 2.11E-06 | 1.052539329 | NM_006788| | RALBP1,ralA binding protein 1 |
| 209814_at | 14.54 | 2.15E-06 | 1.069561809 | NM_014487| | ZNF330,zinc finger protein 330 |
| 1556911_at | 14.53 | 2.17E-06 | 1.234654744 | NA |  |
| 219429_at | 14.52 | 2.17E-06 | 1.548293245 | NM_024306| | FA2H,fatty acid 2-hydroxylase |
| 225914_s_at | 14.49 | 2.22E-06 | 1.387784915 | NM_001079670| | NA |
| 237911_at | 14.49 | 2.21E-06 | 1.184770179 | NA |  |
| 202197_at | 14.49 | 2.22E-06 | 1.088991184 | NM_021090| | MTMR3,myotubularin-related protein 3 isoform c |
| 1556035_s_at | 14.48 | 2.22E-06 | 1.143369185 | NM_001032293| | NA |
| 223038_s_at | 14.46 | 2.24E-06 | 1.049773551 | NM_021238| | C12orf14,chromosome 12 open reading frame 14 |
| 214132_at | 14.45 | 2.26E-06 | 1.258032785 | NM_001001973| | ATP5C1,ATP synthase, H+ transporting, mitochondrial F1 |
| 203771_s_at | 14.44 | 2.26E-06 | 1.154767412 | NM_000712| | BLVRA,biliverdin reductase A |
| 218211_s_at | 14.44 | 2.26E-06 | 1.145958672 | NM_001042467| | NA |
| 205645_at | 14.43 | 2.27E-06 | 1.181036983 | NM_001080975| | NA |
| 226912_at | 14.41 | 2.29E-06 | 1.124821285 | NM_173570| | ZDHHC23,zinc finger, DHHC domain containing 23 |
| 244242_at | 14.4 | 2.31E-06 | 1.271582454 | NA |  |
| 225401_at | 14.39 | 2.32E-06 | 1.053863551 | NM_144580| | MGC31963,kidney predominant protein NCU-G1 |
| 38703_at | 14.38 | 2.33E-06 | 1.032030692 | NM_012100| | DNPEP,aspartyl aminopeptidase |
| 231407_s_at | 14.38 | 2.33E-06 | 1.078263786 | NM_003923| | FOXH1,forkhead box H1 |
| 1560739_a_at | 14.38 | 2.33E-06 | 1.670371008 | NA |  |
| 222361_at | 14.38 | 2.32E-06 | 1.204264437 | NA |  |
| 208945_s_at | 14.37 | 2.34E-06 | 1.070338937 | NM_003766| | BECN1,beclin 1 |
| 241436_at | 14.37 | 2.34E-06 | 1.530071558 | NM_001039| | SCNN1G,sodium channel, nonvoltage-gated 1, gamma |
| 203656_at | 14.36 | 2.34E-06 | 1.049894836 | NM_014845| | KIAA0274,Sac domain-containing inositol phosphatase 3 |
| 207929_at | 14.36 | 2.34E-06 | 1.529125012 | NM_005314| | GRPR,gastrin-releasing peptide receptor |
| 213026_at | 14.35 | 2.35E-06 | 1.049864939 | NM_004707| | APG12L,APG12 autophagy 12-like |
| 200610_s_at | 14.32 | 2.39E-06 | 1.026695193 | NM_005381| | NCL,nucleolin |
| 224609_at | 14.32 | 2.39E-06 | 1.101488553 | NM_020428| | CTL2,CTL2 gene |
| 35148_at | 14.32 | 2.39E-06 | 1.184150124 | NM_014428| | TJP3,tight junction protein 3 (zona occludens 3) |
| 225074_at | 14.31 | 2.40E-06 | 1.072494327 | NM_032846| | RAB2B,RAB2B protein |
| 203786_s_at | 14.3 | 2.41E-06 | 1.136177569 | NM_001003395| | TPD52L1,tumor protein D52-like 1 isoform 2 |
| 225416_at | 14.29 | 2.43E-06 | 1.051407506 | NM_016120| | RNF12,ring finger protein 12 |
| 213969_x_at | 14.27 | 2.45E-06 | 1.020036748 | NM_000992| | RPL29,ribosomal protein L29 |
| 57082_at | 14.26 | 2.45E-06 | 1.101686313 | NM_015627| | ARH,LDL receptor adaptor protein |
| 203173_s_at | 14.26 | 2.45E-06 | 1.064163225 | NM_020314| | MGC16824,esophageal cancer associated protein |
| 200823_x_at | 14.22 | 2.50E-06 | 1.030781354 | NM_000992| | RPL29,ribosomal protein L29 |
| 204294_at | 14.22 | 2.51E-06 | 1.41181165 | NM_000481| | AMT,aminomethyltransferase (glycine cleavage system |
| 221848_at | 14.21 | 2.51E-06 | 1.099151504 | NM_001083113| | NA |
| 203905_at | 14.19 | 2.54E-06 | 1.051622425 | NM_002582| | PARN,poly(A)-specific ribonuclease (deadenylation |
| 212230_at | 14.19 | 2.54E-06 | 1.074384879 | NM_003713| | PPAP2B,phosphatidic acid phosphatase type 2B |
| 200074_s_at | 14.17 | 2.56E-06 | 1.01901646 | NM_001034996| | NA |
| 240284_x_at | 14.17 | 2.57E-06 | 1.167423718 | NM_006331| | C2F,C2f protein |
| 238983_at | 14.15 | 2.59E-06 | 1.184489542 | NM_024677| | FLJ14001,hypothetical protein FLJ14001 |
| 232293_at | 14.12 | 2.63E-06 | 1.163185299 | NM_153686| | MLR1,transcription factor MLR1 |
| 223034_s_at | 14.11 | 2.65E-06 | 1.028009496 | NM_001098616| | NA |
| 227425_at | 14.08 | 2.69E-06 | 1.305938244 | NM_001080975| | NA |
| 201924_at | 14.08 | 2.69E-06 | 1.06642592 | NM_005935| | MLLT2,myeloid/lymphoid or mixed-lineage leukemia |
| 229064_s_at | 14.07 | 2.71E-06 | 1.144083264 | NM_013441| | DSCR1L2,Down syndrome critical region gene 1-like 2 |
| 210153_s_at | 14.07 | 2.71E-06 | 1.074758491 | NM_002396| | ME2,malic enzyme 2, NAD(+)-dependent, mitochondrial |
| 226839_at | 14.05 | 2.73E-06 | 1.064195084 | NM_176880| | TRA16,TR4 orphan receptor associated protein TRA16 |
| 213760_s_at | 14.01 | 2.79E-06 | 1.151446591 | NM_014487| | ZNF330,zinc finger protein 330 |
| 219357_at | 14.01 | 2.78E-06 | 1.05635925 | NM_004286| | GTPBP1,GTP binding protein 1 |
| 209222_s_at | 13.98 | 2.83E-06 | 1.11579831 | NM_014835| | OSBPL2,oxysterol-binding protein-like protein 2 isoform |
| 200868_s_at | 13.98 | 2.83E-06 | 1.086090574 | NM_018683| | ZNF313,zinc finger protein 313 |
| 241530_at | 13.96 | 2.84E-06 | 1.837692739 | NA |  |
| 225756_at | 13.96 | 2.84E-06 | 1.085518518 | NM_001894| | CSNK1E,casein kinase 1 epsilon |
| 221011_s_at | 13.96 | 2.84E-06 | 1.08418314 | NM_030915| | LBH,hypothetical protein DKFZp566J091 |
| 232017_at | 13.94 | 2.86E-06 | 1.25310136 | NM_004817| | TJP2,tight junction protein 2 (zona occludens 2) |
| 229673_at | 13.93 | 2.88E-06 | 1.10022372 | NA |  |
| 41577_at | 13.92 | 2.89E-06 | 1.169453555 | NM_015568| | PPP1R16B,protein phosphatase 1 regulatory inhibitor |
| 227220_at | 13.92 | 2.89E-06 | 1.05811881 | NM_152995| | HOZFP,ovarian zinc finger protein |
| 226994_at | 13.92 | 2.89E-06 | 1.063185325 | NM_005880| | DNAJA2,DnaJ subfamily A member 2 |
| 203955_at | 13.9 | 2.91E-06 | 1.056353705 | NM_014811| | KIAA0649,KIAA0649 gene product |
| 236574_at | 13.9 | 2.91E-06 | 1.282476682 | NA |  |
| 224321_at | 13.9 | 2.91E-06 | 1.130428225 | NM_016192| | TMEFF2,transmembrane protein with EGF-like and two |
| 225886_at | 13.9 | 2.91E-06 | 1.113489974 | NA |  |
| 230835_at | 13.89 | 2.93E-06 | 1.193387165 | NM_207392| | UNQ467,KIPV467 |
| 223376_s_at | 13.88 | 2.94E-06 | 1.069853247 | NM_015379| | BRI3,brain protein I3 |
| 209298_s_at | 13.87 | 2.95E-06 | 1.103468018 | NM_001001132| | ITSN1,intersectin 1 isoform ITSN-s |
| 227347_x_at | 13.86 | 2.98E-06 | 1.201102143 | NM_021170| | HES4,hairy and enhancer of split 4 |
| 239947_at | 13.85 | 2.99E-06 | 1.192839296 | NA |  |
| 219817_at | 13.84 | 2.99E-06 | 1.076218944 | NA |  |
| 242272_at | 13.84 | 2.99E-06 | 1.242426777 | NM_152458| | FLJ32130,hypothetical protein FLJ32130 |
| 46665_at | 13.81 | 3.03E-06 | 1.049399698 | NM_017789| | SEMA4C,semaphorin 4C |
| 226135_at | 13.81 | 3.03E-06 | 1.068855989 | NM_017754| | C6orf107,ICBP90 binding protein 1 |
| 227742_at | 13.8 | 3.06E-06 | 1.134860705 | NM_053277| | CLIC6,chloride intracellular channel 6 |
| 225975_at | 13.78 | 3.09E-06 | 1.057268113 | NM_019035| | PCDH18,protocadherin 18 precursor |
| 210053_at | 13.77 | 3.10E-06 | 1.050129659 | NM_006951| | TAF5,TBP-associated factor 5 |
| 228824_s_at | 13.77 | 3.09E-06 | 1.087831938 | NM_012212| | LTB4DH,NADP-dependent leukotriene B4 |
| 224482_s_at | 13.76 | 3.11E-06 | 1.138688608 | NM_032932| | RAB11FIP4,RAB11 family interacting protein 4 (class II) |
| 219914_at | 13.74 | 3.15E-06 | 1.223779259 | NM_004826| | ECEL1,endothelin converting enzyme-like 1 |
| 203827_at | 13.72 | 3.17E-06 | 1.134117532 | NM_017983| | WIPI49,hypothetical protein FLJ10055 |
| 206731_at | 13.72 | 3.18E-06 | 1.242005557 | NM_014927| | CNKSR2,connector enhancer of kinase suppressor of Ras |
| 222607_s_at | 13.72 | 3.17E-06 | 1.12766939 | NM_014953| | KIAA1008,KIAA1008 |
| 51192_at | 13.71 | 3.20E-06 | 1.105077765 | NM_017857| | SSH3,slingshot homolog 3 |
| 214060_at | 13.7 | 3.22E-06 | 1.243984027 | NM_003143| | SSBP1,single-stranded DNA binding protein 1 |
| 226216_at | 13.68 | 3.25E-06 | 1.110218324 | NM_000208| | INSR,insulin receptor |
| 224746_at | 13.67 | 3.26E-06 | 1.049659478 | NM_020888| | NA |
| 200940_s_at | 13.67 | 3.25E-06 | 1.036766563 | NM_001042681| | NA |
| 204343_at | 13.66 | 3.27E-06 | 1.168080958 | NM_001089| | ABCA3,ATP-binding cassette, sub-family A member 3 |
| 241453_at | 13.66 | 3.27E-06 | 1.18891814 | NM_005607| | PTK2,PTK2 protein tyrosine kinase 2 isoform b |
| 201800_s_at | 13.63 | 3.33E-06 | 1.041909009 | NM_002556| | OSBP,oxysterol binding protein |
| 226795_at | 13.63 | 3.32E-06 | 1.160577921 | NA |  |
| 202893_at | 13.6 | 3.39E-06 | 1.063476331 | NM_006377| | UNC13B,UNC13 (C. elegans)-like |
| 233528_s_at | 13.58 | 3.42E-06 | 1.217759508 | NM_001037666| | NA |
| 204809_at | 13.58 | 3.42E-06 | 1.09793337 | NM_006660| | CLPX,ClpX caseinolytic protease X homolog |
| 228916_at | 13.57 | 3.43E-06 | 1.056491006 | NM_152434| | CWF19L2,CWF19-like 2, cell cycle control |
| 219849_at | 13.57 | 3.43E-06 | 1.189593929 | NM_024833| | FLJ23506,hypothetical protein FLJ23506 |
| 225949_at | 13.56 | 3.44E-06 | 1.139743521 | NM_178564| | NRBP2,nuclear receptor binding protein 2 |
| 218080_x_at | 13.53 | 3.50E-06 | 1.057766008 | NM_007051| | FAF1,FAS-associated factor 1 isoform a |
| 222804_x_at | 13.51 | 3.54E-06 | 1.096751596 | NM_024345| | MGC10765,hypothetical protein MGC10765 |
| 216525_x_at | 13.5 | 3.57E-06 | 1.047779853 | NM_001003686| | PMS2L3,postmeiotic segregation increased 2-like 3 |
| 227822_at | 13.5 | 3.57E-06 | 1.158648165 | NM_183238| | ZNF605,zinc finger protein 605 |
| 209591_s_at | 13.5 | 3.57E-06 | 1.492642813 | NM_001719| | BMP7,bone morphogenetic protein 7 precursor |
| 235024_at | 13.49 | 3.58E-06 | 1.100222565 | NM_024900| | PHF17,Jade1 protein short isoform |
| 202838_at | 13.48 | 3.60E-06 | 1.077591056 | NM_000147| | FUCA1,fucosidase, alpha-L- 1, tissue |
| 225972_at | 13.48 | 3.59E-06 | 1.351409076 | NM_001008495| | DKFZp762C1112,hypothetical protein DKFZp762C1112 |
| 227374_at | 13.47 | 3.62E-06 | 1.070402878 | NM_001083614| | NA |
| 209088_s_at | 13.47 | 3.62E-06 | 1.054712003 | NM_001079514| | NA |
| 239142_at | 13.44 | 3.67E-06 | 1.164172316 | NM_173362| | LOC317671,LOC317671 |
| 202697_at | 13.44 | 3.66E-06 | 1.073137633 | NM_007006| | CPSF5,cleavage and polyadenylation specific factor 5 |
| 227826_s_at | 13.43 | 3.68E-06 | 1.198813214 | NA |  |
| 242196_at | 13.41 | 3.74E-06 | 1.138481426 | NA |  |
| 200765_x_at | 13.4 | 3.74E-06 | 1.032719498 | NM_001903| | CTNNA1,catenin, alpha 1 |
| 36553_at | 13.4 | 3.74E-06 | 1.06518361 | NM_004192| | ASMTL,acetylserotonin O-methyltransferase-like |
| 220721_at | 13.39 | 3.77E-06 | 1.137548091 | NM_014650| | ZNF432,zinc finger protein 432 |
| 216500_at | 13.38 | 3.80E-06 | 1.454881458 | NA |  |
| 212600_s_at | 13.37 | 3.82E-06 | 1.030546122 | NM_003366| | UQCRC2,ubiquinol-cytochrome c reductase core protein |
| 205691_at | 13.37 | 3.82E-06 | 1.101232838 | NM_004209| | SYNGR3,synaptogyrin 3 |
| 219961_s_at | 13.36 | 3.82E-06 | 1.062716175 | NM_018474| | C20orf19,uncharacterized hypothalamus protein HT013 |
| 201989_s_at | 13.36 | 3.83E-06 | 1.053761319 | NM_001310| | CREBL2,cAMP responsive element binding protein-like 2 |
| 237885_at | 13.35 | 3.86E-06 | 1.351447815 | NA |  |
| 222125_s_at | 13.35 | 3.85E-06 | 1.104169564 | NM_177938| | PH-4,hypoxia-inducible factor prolyl 4-hydroxylase |
| 210154_at | 13.35 | 3.85E-06 | 1.125621378 | NM_002396| | ME2,malic enzyme 2, NAD(+)-dependent, mitochondrial |
| 202201_at | 13.31 | 3.93E-06 | 1.112096371 | NM_000713| | BLVRB,biliverdin reductase B (flavin reductase |
| 218031_s_at | 13.31 | 3.93E-06 | 1.099409681 | NM_001085471| | NA |
| 230764_at | 13.31 | 3.93E-06 | 1.236678481 | NA |  |
| 211401_s_at | 13.3 | 3.95E-06 | 1.291516544 | NM_000141| | FGFR2,fibroblast growth factor receptor 2 isoform 1 |
| 224761_at | 13.3 | 3.95E-06 | 1.030645018 | NM_006572| | GNA13,guanine nucleotide binding protein (G protein), |
| 230563_at | 13.3 | 3.95E-06 | 1.07826313 | NM_145313| | RASGEF1A,RasGEF domain family, member 1A |
| 227260_at | 13.27 | 4.01E-06 | 1.318253173 | NM_017664| | ANKRD10,ankyrin repeat domain 10 |
| 214212_x_at | 13.26 | 4.02E-06 | 1.077713175 | NM_006832| | PLEKHC1,pleckstrin homology domain containing, family C |
| 225169_at | 13.26 | 4.03E-06 | 1.051541643 | NM_033547| | MGC16733,MGC16733 protein |
| 227213_at | 13.26 | 4.03E-06 | 1.136125161 | NM_182503| | DEADC1,deaminase domain containing 1 |
| 224566_at | 13.25 | 4.05E-06 | 1.379597774 | NA |  |
| 218918_at | 13.24 | 4.07E-06 | 1.193531576 | NM_020379| | MAN1C1,mannosidase, alpha, class 1C, member 1 |
| 225204_at | 13.23 | 4.10E-06 | 1.110282506 | NM_139283| | TA-PP2C,T-cell activation protein phosphatase 2C |
| 230287_at | 13.22 | 4.11E-06 | 1.107946343 | NM_001039948| | NA |
| 217886_at | 13.21 | 4.14E-06 | 1.054164947 | NM_001981| | EPS15,epidermal growth factor receptor pathway |
| 202085_at | 13.18 | 4.19E-06 | 1.050951007 | NM_004817| | TJP2,tight junction protein 2 (zona occludens 2) |
| 226770_at | 13.18 | 4.19E-06 | 1.120351003 | NM_152900| | MAGI-3,membrane-associated guanylate kinase-related 3 |
| 1570534_a_at | 13.18 | 4.19E-06 | 1.485019545 | NM_001007169| | ZNF483,zinc finger protein 483 isoform b |
| 222666_s_at | 13.16 | 4.24E-06 | 1.071798261 | NM_005772| | RCL1,RNA cyclase homolog |
| 235060_at | 13.16 | 4.24E-06 | 1.105483176 | NA |  |
| 218352_at | 13.15 | 4.27E-06 | 1.135929853 | NM_018191| | RCBTB1,regulator of chromosome condensation (RCC1) and |
| 210827_s_at | 13.15 | 4.26E-06 | 1.160357961 | NM_001114309| | NA |
| 218973_at | 13.13 | 4.30E-06 | 1.053881797 | NM_001040610| | NA |
| 229344_x_at | 13.13 | 4.30E-06 | 1.104616354 | NM_020734| | KIAA1238,KIAA1238 protein |
| 209592_s_at | 13.13 | 4.30E-06 | 1.128060481 | NM_005828| | HAN11,WD-repeat protein |
| 203815_at | 13.13 | 4.30E-06 | 1.088082279 | NM_000853| | GSTT1,glutathione S-transferase theta 1 |
| 230532_at | 13.12 | 4.33E-06 | 1.128810824 | NM_144970| | CXorf38,chromosome X open reading frame 38 |
| 231466_at | 13.12 | 4.31E-06 | 1.311939021 | NM_032599| | NYD-SP18,testes development-related NYD-SP18 |
| 57539_at | 13.1 | 4.37E-06 | 1.077594183 | NM_001083113| | NA |
| 227992_s_at | 13.1 | 4.37E-06 | 1.17330824 | NA |  |
| 225456_at | 13.08 | 4.41E-06 | 1.05028511 | NM_004774| | PPARBP,peroxisome proliferator-activated receptor |
| 229292_at | 13.07 | 4.44E-06 | 1.07966925 | NM_020909| | EPB41L5,erythrocyte membrane protein band 4.1 like 5 |
| 205873_at | 13.05 | 4.48E-06 | 1.175816696 | NM_004278| | PIGL,phosphatidylinositol glycan, class L |
| 222764_at | 13.04 | 4.51E-06 | 1.079055275 | NM_001083926| | NA |
| 213407_at | 13.03 | 4.56E-06 | 1.089551659 | NM_015020| | KIAA0931,KIAA0931 protein |
| 221762_s_at | 13.03 | 4.54E-06 | 1.130308478 | NM_022104| | C20orf67,phosphorylated CTD interacting factor 1 |
| 202512_s_at | 13.03 | 4.56E-06 | 1.109658054 | NM_004849| | APG5L,APG5 autophagy 5-like |
| 208361_s_at | 13.02 | 4.58E-06 | 1.084335656 | NM_001722| | POLR3D,RNA polymerase III 53 kDa subunit RPC4 |
| 219039_at | 13.01 | 4.60E-06 | 1.061832286 | NM_017789| | SEMA4C,semaphorin 4C |
| 37796_at | 13.01 | 4.60E-06 | 1.066961997 | NM_002319| | LRCH4,leucine-rich repeats and calponin homology (CH) |
| 209786_at | 13.01 | 4.60E-06 | 1.07335679 | NM_006353| | HMGN4,high mobility group nucleosomal binding domain |
| 202680_at | 12.99 | 4.65E-06 | 1.070285178 | NM_002095| | GTF2E2,general transcription factor IIE, polypeptide 2, |
| 200770_s_at | 12.97 | 4.70E-06 | 1.090205081 | NM_002293| | LAMC1,laminin, gamma 1 precursor |
| 212693_at | 12.97 | 4.70E-06 | 1.047380004 | NM_014611| | MDN1,MDN1, midasin homolog |
| 222896_at | 12.96 | 4.71E-06 | 1.144231385 | NM_024074| | TMEM38A,transmembrane protein 38A |
| 209240_at | 12.95 | 4.74E-06 | 1.079966673 | NM_181672| | OGT,O-linked GlcNAc transferase isoform 1 |
| 212177_at | 12.95 | 4.73E-06 | 1.096366718 | NM_015491| | NA |
| 205942_s_at | 12.95 | 4.73E-06 | 1.264380082 | NM_005622| | SAH,SA hypertension-associated homolog isoform 1 |
| 217843_s_at | 12.94 | 4.76E-06 | 1.086799093 | NM_014166| | MED4,mediator of RNA polymerase II transcription, |
| 207783_x_at | 12.92 | 4.81E-06 | 1.021732897 | NM_031407| | NA |
| 210552_s_at | 12.92 | 4.80E-06 | 1.18410324 | NM_014636| | RALGPS1,Ral GEF with PH domain and SH3 binding motif 1 |
| 221526_x_at | 12.92 | 4.81E-06 | 1.048767174 | NM_019619| | PARD3,partitioning-defective protein 3 homolog |
| 204727_at | 12.89 | 4.89E-06 | 1.132678973 | NM_001008396| | WDHD1,WD repeat and HMG-box DNA binding protein 1 |
| 238440_at | 12.89 | 4.90E-06 | 1.139115963 | NM_206808| | CLYBL,citrate lyase beta like |
| 209013_x_at | 12.89 | 4.89E-06 | 1.185158994 | NM_007118| | TRIO,triple functional domain (PTPRF interacting) |
| 212756_s_at | 12.88 | 4.92E-06 | 1.143190525 | NM_015255| | UBR2,ubiquitin protein ligase E3 component n-recognin |
| 226033_at | 12.87 | 4.95E-06 | 1.057838631 | NM_020718| | USP31,ubiquitin specific protease 31 |
| 209180_at | 12.87 | 4.95E-06 | 1.063030142 | NM_004582| | RABGGTB,Rab geranylgeranyltransferase, beta subunit |
| 37278_at | 12.87 | 4.95E-06 | 1.053707917 | NM_000116| | TAZ,tafazzin isoform 1 |
| 226718_at | 12.87 | 4.95E-06 | 1.180313876 | NM_020703| | AMIGO,AMIGO protein |
| 212564_at | 12.87 | 4.96E-06 | 1.060783566 | NM_015353| | KCTD2,potassium channel tetramerisation domain |
| 1553138_a_at | 12.86 | 4.96E-06 | 1.210966811 | NM_152363| | FLJ39369,hypothetical protein FLJ39369 |
| 211089_s_at | 12.85 | 5.01E-06 | 1.213366555 | NM_002498| | NEK3,NIMA-related kinase 3 |
| 209355_s_at | 12.84 | 5.03E-06 | 1.13486078 | NM_003713| | PPAP2B,phosphatidic acid phosphatase type 2B |
| 226798_at | 12.83 | 5.04E-06 | 1.084568704 | NM_015367| | BCL2L13,BCL2-like 13 (apoptosis facilitator) |
| 1563369_at | 12.83 | 5.06E-06 | 1.153211795 | NM_207436| | FLJ42957,FLJ42957 protein |
| 203040_s_at | 12.82 | 5.10E-06 | 1.045435171 | NM_000190| | HMBS,hydroxymethylbilane synthase |
| 217741_s_at | 12.79 | 5.17E-06 | 1.105501738 | NM_001102420| | NA |
| 241968_at | 12.79 | 5.17E-06 | 1.08658724 | NA |  |
| 201937_s_at | 12.79 | 5.18E-06 | 1.073410206 | NM_012100| | DNPEP,aspartyl aminopeptidase |
| 229691_at | 12.79 | 5.18E-06 | 1.16110287 | NA |  |
| 235071_at | 12.79 | 5.17E-06 | 1.042356718 | NM_138458| | LOC116143,hypothetical protein BC014022 |
| 229097_at | 12.78 | 5.21E-06 | 1.085325515 | NM_001042517| | NA |
| 201510_at | 12.77 | 5.23E-06 | 1.201940738 | NM_001114309| | NA |
| 218546_at | 12.76 | 5.26E-06 | 1.085803137 | NM_024709| | FLJ14146,hypothetical protein FLJ14146 |
| 1570266_x_at | 12.76 | 5.26E-06 | 1.076979411 | NA |  |
| 242228_at | 12.76 | 5.26E-06 | 1.280107797 | NA |  |
| 209927_s_at | 12.74 | 5.31E-06 | 1.097559506 | NM_015607| | DKFZP547E1010,DKFZP547E1010 protein |
| 214779_s_at | 12.74 | 5.31E-06 | 1.084137925 | NM_015705| | RUTBC3,RUN and TBC1 domain containing 3 |
| 213464_at | 12.74 | 5.31E-06 | 1.192518999 | NM_012435| | NA |
| 1569906_s_at | 12.74 | 5.31E-06 | 1.117769585 | NM_016436| | PHF20,PHD finger protein 20 |
| 208811_s_at | 12.73 | 5.33E-06 | 1.144087048 | NM_005494| | DNAJB6,DnaJ (Hsp40) homolog, subfamily B, member 6 |
| 225183_at | 12.73 | 5.34E-06 | 1.026976651 | NM_014117| | PRO0149,PRO0149 protein |
| 212997_s_at | 12.73 | 5.33E-06 | 1.057688769 | NM_001112707| | NA |
| 216228_s_at | 12.73 | 5.34E-06 | 1.090551183 | NM_001008396| | WDHD1,WD repeat and HMG-box DNA binding protein 1 |
| 203912_s_at | 12.72 | 5.37E-06 | 1.069979146 | NM_001009932| | DNASE1L1,deoxyribonuclease I-like 1 precursor |
| 201431_s_at | 12.7 | 5.42E-06 | 1.067964355 | NM_001387| | DPYSL3,dihydropyrimidinase-like 3 |
| 212340_at | 12.68 | 5.49E-06 | 1.090463181 | NM_173834| | MGC21416,hypothetical protein MGC21416 |
| 200962_at | 12.67 | 5.51E-06 | 1.079711235 | NM_000993| | RPL31,ribosomal protein L31 |
| 214829_at | 12.65 | 5.60E-06 | 1.104561554 | NM_005763| | AASS,aminoadipate-semialdehyde synthase |
| 221726_at | 12.65 | 5.59E-06 | 1.058304656 | NM_000983| | RPL22,ribosomal protein L22 proprotein |
| 206200_s_at | 12.64 | 5.62E-06 | 1.064136543 | NM_001157| | ANXA11,annexin A11 |
| 203397_s_at | 12.63 | 5.64E-06 | 1.451986999 | NM_004482| | GALNT3,polypeptide N-acetylgalactosaminyltransferase 3 |
| 204398_s_at | 12.62 | 5.69E-06 | 1.081846853 | NM_012155| | EML2,echinoderm microtubule associated protein like |
| 220638_s_at | 12.62 | 5.67E-06 | 1.155157607 | NM_012116| | CBLC,Cas-Br-M (murine) ecotropic retroviral |
| 235148_at | 12.61 | 5.72E-06 | 1.104631245 | NM_173853| | KRTCAP3,keratinocyte associated protein 3 |
| 203072_at | 12.61 | 5.72E-06 | 1.140235655 | NM_004998| | MYO1E,myosin IE |
| 204934_s_at | 12.6 | 5.74E-06 | 1.189065744 | NM_002151| | HPN,hepsin (transmembrane protease, serine 1) |
| 227248_at | 12.59 | 5.77E-06 | 1.073927646 | NM_024927| | FLJ21019,hypothetical protein FLJ21019 |
| 212729_at | 12.58 | 5.81E-06 | 1.122250915 | NM_020730| | NA |
| 200936_at | 12.58 | 5.81E-06 | 1.01449003 | NM_000973| | RPL8,ribosomal protein L8 |
| 202559_x_at | 12.57 | 5.85E-06 | 1.05598034 | NM_015607| | DKFZP547E1010,DKFZP547E1010 protein |
| 226419_s_at | 12.57 | 5.84E-06 | 1.131117911 | NA |  |
| 215286_s_at | 12.57 | 5.86E-06 | 1.195684592 | NM_020432| | PHTF2,putative homeodomain transcription factor 2 |
| 35776_at | 12.55 | 5.91E-06 | 1.05388863 | NM_001001132| | ITSN1,intersectin 1 isoform ITSN-s |
| 230055_at | 12.54 | 5.95E-06 | 1.178672431 | NM_030568| | C6orf148,chromosome 6 open reading frame 148 |
| 226782_at | 12.54 | 5.94E-06 | 1.193108096 | NM_001010875| | SLC25A30,solute carrier family 25, member 30 |
| 212584_at | 12.54 | 5.94E-06 | 1.055685997 | NM_014691| | AQR,aquarius |
| 202211_at | 12.53 | 5.97E-06 | 1.069003919 | NM_014570| | ARFGAP3,ADP-ribosylation factor GTPase activating |
| 232094_at | 12.51 | 6.05E-06 | 1.256361404 | NM_024713| | C15orf29,chromosome 15 open reading frame 29 |
| 210686_x_at | 12.51 | 6.05E-06 | 1.06501733 | NM_152707| | SLC25A16,solute carrier family 25, member 16 |
| 221503_s_at | 12.5 | 6.10E-06 | 1.051979219 | NM_002267| | KPNA3,karyopherin alpha 3 |
| 235315_at | 12.5 | 6.10E-06 | 1.260808335 | NM_006022| | TGFB1I4,transforming growth factor beta 1 induced |
| 1553225_s_at | 12.47 | 6.20E-06 | 1.553104442 | NM_007131| | ZNF75,zinc finger protein 75 |
| 226403_at | 12.46 | 6.24E-06 | 1.266595295 | NM_144686| | TMC4,transmembrane channel-like 4 |
| 210513_s_at | 12.46 | 6.24E-06 | 1.101372926 | NM_001025366| | NA |
| 236019_at | 12.45 | 6.26E-06 | 1.142951521 | NM_001025300| | NA |
| 209907_s_at | 12.45 | 6.26E-06 | 1.110572652 | NM_006277| | ITSN2,intersectin 2 isoform 1 |
| 210235_s_at | 12.44 | 6.32E-06 | 1.17646561 | NM_003626| | PPFIA1,PTPRF interacting protein alpha 1 isoform b |
| 229644_at | 12.43 | 6.32E-06 | 1.133061542 | NM_002726| | PREP,prolyl endopeptidase |
| 239169_at | 12.42 | 6.39E-06 | 1.1700929 | NM_001034836| | NA |
| 201578_at | 12.41 | 6.41E-06 | 1.035279672 | NM_001018111| | NA |
| 1557521_a_at | 12.41 | 6.42E-06 | 1.111323 | NA |  |
| 219340_s_at | 12.41 | 6.41E-06 | 1.088246324 | NM_018941| | CLN8,CLN8 protein |
| 37028_at | 12.4 | 6.43E-06 | 1.05825063 | NM_014330| | PPP1R15A,protein phosphatase 1, regulatory subunit 15A |
| 231871_at | 12.4 | 6.43E-06 | 1.174198128 | NM_180989| | ITR,intimal thickness-related receptor |
| 237461_at | 12.38 | 6.54E-06 | 1.315457871 | NM_139176| | NALP7,NACHT, leucine rich repeat and PYD containing 7 |
| 232913_at | 12.38 | 6.54E-06 | 1.061214784 | NM_213601| | TMED8,transmembrane emp24 domain containing 8 |
| 208748_s_at | 12.36 | 6.58E-06 | 1.137508926 | NM_005803| | FLOT1,flotillin 1 |
| 204741_at | 12.36 | 6.58E-06 | 1.130379379 | NM_001003398| | BICD1,bicaudal D homolog 1 isoform 2 |
| 204455_at | 12.35 | 6.64E-06 | 1.46665286 | NM_001723| | DST,dystonin isoform 1e precursor |
| 212750_at | 12.34 | 6.67E-06 | 1.131951601 | NM_015568| | PPP1R16B,protein phosphatase 1 regulatory inhibitor |
| 238751_at | 12.34 | 6.68E-06 | 1.202957942 | NA |  |
| 223234_at | 12.33 | 6.70E-06 | 1.08019593 | NM_006341| | MAD2L2,MAD2 homolog |
| 217492_s_at | 12.33 | 6.70E-06 | 1.077571571 | NM_000314| | PTEN,phosphatase and tensin homolog |
| 231050_at | 12.32 | 6.75E-06 | 1.433486247 | NM_054108| | HRLP5,H-rev107-like protein 5 |
| 239752_at | 12.31 | 6.78E-06 | 1.052202488 | NA |  |
| 202658_at | 12.31 | 6.77E-06 | 1.092853289 | NM_003846| | PEX11B,peroxisomal biogenesis factor 11B |
| 224983_at | 12.31 | 6.76E-06 | 1.049156195 | NM_005506| | SCARB2,scavenger receptor class B, member 2 |
| 64883_at | 12.3 | 6.79E-06 | 1.0555071 | NM_152581| | MOSPD2,motile sperm domain containing 2 |
| 213988_s_at | 12.29 | 6.85E-06 | 1.113242195 | NM_002970| | SAT,spermidine/spermine N1-acetyltransferase |
| 209394_at | 12.29 | 6.86E-06 | 1.086509568 | NM_004192| | ASMTL,acetylserotonin O-methyltransferase-like |
| 208692_at | 12.29 | 6.83E-06 | 1.021593995 | NM_001005| | RPS3,ribosomal protein S3 |
| 225138_at | 12.28 | 6.88E-06 | 1.053256774 | NM_017580| | ZRANB1,TRABID protein |
| 213116_at | 12.26 | 6.94E-06 | 1.094532319 | NM_002498| | NEK3,NIMA-related kinase 3 |
| 202790_at | 12.26 | 6.94E-06 | 1.068230045 | NM_001307| | CLDN7,claudin 7 |
| 34858_at | 12.26 | 6.94E-06 | 1.044680097 | NM_015353| | KCTD2,potassium channel tetramerisation domain |
| 1569069_s_at | 12.25 | 6.98E-06 | 1.166279258 | NM_030794| | TDRD3,tudor domain containing 3 |
| 225107_at | 12.22 | 7.11E-06 | 1.134682636 | NM_002137| | HNRPA2B1,heterogeneous nuclear ribonucleoprotein A2/B1 |
| 204985_s_at | 12.22 | 7.14E-06 | 1.094200477 | NM_024108| | MGC2650,hypothetical protein MGC2650 |
| 226507_at | 12.22 | 7.13E-06 | 1.029640553 | NM_002576| | PAK1,p21-activated kinase 1 |
| 203196_at | 12.2 | 7.22E-06 | 1.063252237 | NM_001105515| | NA |
| 227778_at | 12.19 | 7.27E-06 | 1.147434298 | NM_001099280| | NA |
| 226485_at | 12.19 | 7.25E-06 | 1.056600221 | NA |  |
| 205719_s_at | 12.19 | 7.24E-06 | 1.197264654 | NM_000277| | PAH,phenylalanine hydroxylase |
| 214494_s_at | 12.18 | 7.31E-06 | 1.048343118 | NM_003119| | SPG7,paraplegin isoform 1 |
| 214813_at | 12.18 | 7.28E-06 | 1.345407322 | NM_007131| | ZNF75,zinc finger protein 75 |
| 219188_s_at | 12.16 | 7.40E-06 | 1.152122863 | NM_014067| | LRP16,LRP16 protein |
| 208749_x_at | 12.15 | 7.44E-06 | 1.049448328 | NM_005803| | FLOT1,flotillin 1 |
| 227864_s_at | 12.13 | 7.53E-06 | 1.067490837 | NM_138401| | LOC93343,hypothetical protein BC011840 |
| 200844_s_at | 12.13 | 7.54E-06 | 1.035045511 | NM_004905| | PRDX6,peroxiredoxin 6 |
| 225862_at | 12.12 | 7.54E-06 | 1.065404033 | NM_173471| | SLC25A26,solute carrier family 25, member 26 isoform b |
| 226538_at | 12.11 | 7.60E-06 | 1.05267303 | NM_002372| | MAN2A1,mannosidase, alpha, class 2A, member 1 |
| 78047_s_at | 12.11 | 7.58E-06 | 1.057636203 | NA |  |
| 208089_s_at | 12.11 | 7.61E-06 | 1.145673257 | NM_030794| | TDRD3,tudor domain containing 3 |
| 223084_s_at | 12.1 | 7.62E-06 | 1.061209446 | NM_012142| | CCNDBP1,cyclin D-type binding-protein 1 |
| 225390_s_at | 12.1 | 7.66E-06 | 1.045654669 | NM_015995| | KLF13,Kruppel-like factor 13 |
| 200959_at | 12.08 | 7.71E-06 | 1.012775774 | NM_004960| | FUS,fusion (involved in t(12;16) in malignant |
| 203584_at | 12.08 | 7.72E-06 | 1.035040296 | NM_014673| | KIAA0103,KIAA0103 |
| 225742_at | 12.08 | 7.70E-06 | 1.231510362 | NA |  |
| 231837_at | 12.08 | 7.71E-06 | 1.114725448 | NM_020886| | USP28,ubiquitin specific protease 28 |
| 202235_at | 12.07 | 7.74E-06 | 1.099707027 | NM_003051| | SLC16A1,solute carrier family 16, member 1 |
| 209417_s_at | 12.07 | 7.74E-06 | 1.127276328 | NM_005533| | IFI35,interferon-induced protein 35 |
| 1557169_x_at | 12.03 | 7.94E-06 | 1.177427852 | NA |  |
| 1553823_a_at | 12.03 | 7.95E-06 | 1.460928587 | NM_153708| | RTP1,receptor transporting protein 1 |
| 200802_at | 12.02 | 8.00E-06 | 1.058120188 | NM_006513| | SARS,seryl-tRNA synthetase |
| 217984_at | 12.02 | 8.00E-06 | 1.147691696 | NM_003730| | RNASET2,ribonuclease 6 precursor |
| 212100_s_at | 12.02 | 8.00E-06 | 1.051357856 | NM_032311| | POLDIP3,DNA polymerase delta interacting protein 3 |
| 213198_at | 12.01 | 8.04E-06 | 1.06207505 | NM_004302| | ACVR1B,activin A type IB receptor isoform a precursor |
| 228875_at | 12 | 8.10E-06 | 1.126726596 | NM_001085480| | NA |
| 217983_s_at | 12 | 8.10E-06 | 1.168194299 | NM_003730| | RNASET2,ribonuclease 6 precursor |
| 220714_at | 11.99 | 8.15E-06 | 1.171943128 | NM_024504| | PRDM14,PR domain containing 14 |
| 221906_at | 11.98 | 8.20E-06 | 1.126956021 | NA |  |
| 235623_at | 11.98 | 8.21E-06 | 1.092284977 | NM_018255| | STATIP1,elongator protein 2 |
| 225899_x_at | 11.97 | 8.26E-06 | 1.148884561 | NA |  |
| 1560741_at | 11.96 | 8.28E-06 | 1.109736153 | NM_003097| | SNRPN,small nuclear ribonucleoprotein polypeptide N |
| 226334_s_at | 11.94 | 8.39E-06 | 1.15322881 | NM_152392| | AHSA2,AHA1, activator of heat shock 90kDa protein |
| 204518_s_at | 11.94 | 8.40E-06 | 1.17436462 | NM_000943| | PPIC,peptidylprolyl isomerase C |
| 243631_at | 11.94 | 8.40E-06 | 1.19734439 | NA |  |
| 1569157_s_at | 11.91 | 8.51E-06 | 1.253743603 | NM_001077624| | NA |
| 201900_s_at | 11.9 | 8.56E-06 | 1.037404949 | NM_006066| | AKR1A1,aldo-keto reductase family 1, member A1 |
| 225746_at | 11.88 | 8.68E-06 | 1.154063204 | NM_032932| | RAB11FIP4,RAB11 family interacting protein 4 (class II) |
| 1569629_x_at | 11.88 | 8.69E-06 | 1.117331372 | NA |  |
| 218454_at | 11.86 | 8.76E-06 | 1.112298406 | NM_024829| | FLJ22662,hypothetical protein FLJ22662 |
| 243264_s_at | 11.85 | 8.86E-06 | 1.240313302 | NM_001033578| | NA |
| 210601_at | 11.84 | 8.87E-06 | 1.167204343 | NM_004932| | CDH6,cadherin 6, type 2 preproprotein |
| 200648_s_at | 11.83 | 8.96E-06 | 1.156524828 | NM_001033044| | NA |
| 210715_s_at | 11.81 | 9.09E-06 | 1.050716283 | NM_021102| | SPINT2,serine protease inhibitor, Kunitz type, 2 |
| 204760_s_at | 11.81 | 9.08E-06 | 1.205059505 | NM_003250| | THRA,thyroid hormone receptor, alpha isoform 2 |
| 220027_s_at | 11.81 | 9.05E-06 | 1.173574831 | NM_017805| | RASIP1,Ras-interacting protein 1 |
| 224676_at | 11.8 | 9.09E-06 | 1.068795548 | NM_182547| | TMED4,transmembrane emp24 protein transport domain |
| 1555882_at | 11.8 | 9.10E-06 | 1.160675392 | NM_001010862| | SPIN3,spindlin family, member 3 |
| 1558097_at | 11.79 | 9.14E-06 | 1.058128387 | NM_173566| | MGC50372,hypothetical protein MGC50372 |
| 220233_at | 11.78 | 9.20E-06 | 1.084425202 | NM_024907| | FBXO17,F-box protein FBG4 isoform 2 |
| 228787_s_at | 11.76 | 9.29E-06 | 1.072003422 | NM_001010974| | BCAS4,breast carcinoma amplified sequence 4 isoform c |
| 218505_at | 11.75 | 9.37E-06 | 1.042271669 | NM_030581| | FLJ12270,FLJ12270 protein |
| 226656_at | 11.75 | 9.37E-06 | 1.078296839 | NM_006371| | CRTAP,cartilage associated protein precursor |
| 230348_at | 11.73 | 9.51E-06 | 1.325649426 | NM_014572| | LATS2,LATS, large tumor suppressor, homolog 2 |
| 226354_at | 11.73 | 9.49E-06 | 1.080807596 | NM_032857| | LACTB,lactamase, beta isoform a |
| 224705_s_at | 11.72 | 9.54E-06 | 1.089791091 | NM_014494| | TNRC6A,trinucleotide repeat containing 6A |
| 228885_at | 11.72 | 9.54E-06 | 1.601277668 | NM_153267| | MAMDC2,MAM domain containing 2 |
| 219113_x_at | 11.71 | 9.60E-06 | 1.115863398 | NM_016246| | DHRS10,dehydrogenase/reductase (SDR family) member 10 |
| 213982_s_at | 11.7 | 9.71E-06 | 1.13476195 | NM_001035230| | NA |
| 225901_at | 11.69 | 9.77E-06 | 1.091008379 | NM_175732| | NA |
| 219831_at | 11.69 | 9.77E-06 | 1.066763571 | NM_001113575| | NA |
| 210540_s_at | 11.68 | 9.78E-06 | 1.095549864 | NM_003778| | B4GALT4,UDP-Gal:betaGlcNAc beta 1,4- |
| 221742_at | 11.68 | 9.80E-06 | 1.021556161 | NM_001025596| | NA |
| 229803_s_at | 11.68 | 9.83E-06 | 1.030891429 | NA |  |
| 224678_at | 11.67 | 9.88E-06 | 1.079770822 | NM_020336| | KIAA1219,KIAA1219 protein |
| 1559510_at | 11.67 | 9.84E-06 | 1.239392213 | NM_030639| | BHLHB9,basic helix-loop-helix domain containing, class |
| 208776_at | 11.65 | 9.99E-06 | 1.092660953 | NM_002815| | PSMD11,proteasome 26S non-ATPase subunit 11 |
| 203590_at | 11.64 | 1.01E-05 | 1.046392086 | NM_006141| | DNCLI2,dynein, cytoplasmic, light intermediate |
| 202323_s_at | 11.64 | 1.01E-05 | 1.085454187 | NM_022735| | ACBD3,golgi complex associated protein 1 |
| 1566152_a_at | 11.63 | 1.01E-05 | 1.195904892 | NM_006831| | HEAB,ATP/GTP-binding protein |
| 218538_s_at | 11.63 | 1.01E-05 | 1.173988334 | NM_020662| | MRS2L,MRS2-like, magnesium homeostasis factor |
| 202100_at | 11.63 | 1.01E-05 | 1.17011861 | NM_002881| | RALB,v-ral simian leukemia viral oncogene homolog B |
| 1566207_at | 11.62 | 1.02E-05 | 1.10626766 | NM_006756| | TCEA1,transcription elongation factor A 1 isoform 1 |
| 1558778_s_at | 11.61 | 1.02E-05 | 1.42138996 | NM_014048| | MKL2,megakaryoblastic leukemia 2 protein |
| 200042_at | 11.6 | 1.03E-05 | 1.032863062 | NM_014306| | HSPC117,hypothetical protein HSPC117 |
| 224447_s_at | 11.59 | 1.03E-05 | 1.090345824 | NM_032339| | C17orf37,chromosome 17 open reading frame 37 |
| 226110_at | 11.59 | 1.03E-05 | 1.109753087 | NM_001099666| | NA |
| 217202_s_at | 11.58 | 1.04E-05 | 1.180806625 | NM_001033044| | NA |
| 205287_s_at | 11.58 | 1.04E-05 | 1.4706997 | NM_003222| | TFAP2C,transcription factor AP-2 gamma |
| 218706_s_at | 11.55 | 1.06E-05 | 1.192105827 | NM_023927| | NS3TP2,HCV NS3-transactivated protein 2 |
| 223770_x_at | 11.54 | 1.07E-05 | 1.124889344 | NM_001031727| | NA |
| 229468_at | 11.54 | 1.07E-05 | 1.154141368 | NM_001258| | CDK3,cyclin-dependent kinase 3 |
| 200904_at | 11.54 | 1.07E-05 | 1.079118006 | NM_005516| | HLA-E,major histocompatibility complex, class I, E |
| 219627_at | 11.53 | 1.08E-05 | 1.128396182 | NM_024910| | FLJ12700,hypothetical protein FLJ12700 |
| 210502_s_at | 11.53 | 1.08E-05 | 1.079868345 | NM_006112| | PPIE,peptidylprolyl isomerase E isoform 1 |
| 213920_at | 11.53 | 1.08E-05 | 1.055919063 | NM_015267| | CUTL2,cut-like 2 |
| 217805_at | 11.53 | 1.08E-05 | 1.043117704 | NM_004516| | ILF3,interleukin enhancer binding factor 3 isoform b |
| 205594_at | 11.52 | 1.08E-05 | 1.093636375 | NM_014897| | ZNF652,zinc finger protein 652 |
| 218451_at | 11.52 | 1.08E-05 | 1.124313575 | NM_022842| | CDCP1,CUB domain-containing protein 1 isoform 1 |
| 215470_at | 11.52 | 1.08E-05 | 1.171211624 | NM_001098729| | NA |
| 229849_at | 11.5 | 1.09E-05 | 1.117844694 | NA |  |
| 239951_at | 11.49 | 1.10E-05 | 1.782685725 | NA |  |
| 201122_x_at | 11.47 | 1.11E-05 | 1.08265425 | NM_001970| | EIF5A,eukaryotic translation initiation factor 5A |
| 233380_s_at | 11.47 | 1.11E-05 | 1.100593036 | NM_001040451| | NA |
| 235184_at | 11.47 | 1.12E-05 | 1.317713938 | NM_001114176| | NA |
| 209166_s_at | 11.44 | 1.13E-05 | 1.050344996 | NM_000528| | MAN2B1,mannosidase, alpha, class 2B, member 1 |
| 212069_s_at | 11.42 | 1.15E-05 | 1.085905054 | NM_013318| | NA |
| 1555278_a_at | 11.39 | 1.17E-05 | 1.096393591 | NM_001008938| | ch-TOG,colonic and hepatic tumor over-expressed protein |
| 228077_at | 11.39 | 1.16E-05 | 1.083587148 | NM_001031727| | NA |
| 1553778_at | 11.38 | 1.18E-05 | 1.203190405 | NM_152559| | WBSCR27,Williams-Beuren syndrome chromosome region 27 |
| 203974_at | 11.37 | 1.18E-05 | 1.042196492 | NM_012080| | HDHD1A,haloacid dehalogenase-like hydrolase domain |
| 221934_s_at | 11.37 | 1.18E-05 | 1.047089995 | NM_001009996| | DALRD3,DALR anticodon binding domain containing 3 |
| 203065_s_at | 11.37 | 1.18E-05 | 1.120349931 | NM_001753| | CAV1,caveolin 1 |
| 204517_at | 11.37 | 1.18E-05 | 1.111093106 | NM_000943| | PPIC,peptidylprolyl isomerase C |
| 219241_x_at | 11.36 | 1.19E-05 | 1.107620172 | NM_017857| | SSH3,slingshot homolog 3 |
| 218343_s_at | 11.35 | 1.19E-05 | 1.083064339 | NM_012086| | GTF3C3,general transcription factor IIIC, polypeptide |
| 201949_x_at | 11.34 | 1.20E-05 | 1.027112238 | NM_004930| | CAPZB,F-actin capping protein beta subunit |
| 203624_at | 11.34 | 1.20E-05 | 1.042605861 | NM_005088| | DXYS155E,DNA segment on chromosome X and Y (unique) 155 |
| 210826_x_at | 11.34 | 1.20E-05 | 1.086832392 | NM_002873| | RAD17,RAD17 homolog isoform 1 |
| 212059_s_at | 11.32 | 1.22E-05 | 1.093131018 | NM_015638| | TRPC4AP,TRPC4-associated protein isoform a |
| 229396_at | 11.32 | 1.22E-05 | 1.178650594 | NM_004561| | OVOL1,OVO-like 1 binding protein |
| 228120_at | 11.31 | 1.22E-05 | 1.022671212 | NA |  |
| 210480_s_at | 11.31 | 1.22E-05 | 1.206111295 | NM_004999| | MYO6,myosin VI |
| 241687_at | 11.29 | 1.24E-05 | 1.139882901 | NA |  |
| 243745_at | 11.29 | 1.24E-05 | 1.14964961 | NA |  |
| 204798_at | 11.29 | 1.24E-05 | 1.115075336 | NM_005375| | MYB,v-myb myeloblastosis viral oncogene homolog |
| 228716_at | 11.29 | 1.24E-05 | 1.412126737 | NM_000461| | THRB,thyroid hormone receptor, beta |
| 241575_at | 11.28 | 1.25E-05 | 1.515185207 | NA |  |
| 212036_s_at | 11.27 | 1.26E-05 | 1.057576718 | NM_002687| | PNN,pinin, desmosome associated protein |
| 201871_s_at | 11.25 | 1.27E-05 | 1.044356803 | NM_015853| | LOC51035,unknown protein LOC51035 |
| 224064_s_at | 11.25 | 1.27E-05 | 1.130860941 | NM_024887| | DHDDS,dehydrodolichyl diphosphate synthase isoform a |
| 227999_at | 11.25 | 1.27E-05 | 1.082773695 | NM_001098637| | NA |
| 212130_x_at | 11.24 | 1.28E-05 | 1.026905782 | NM_005801| | SUI1,putative translation initiation factor |
| 211057_at | 11.24 | 1.28E-05 | 1.086102032 | NM_001083592| | NA |
| 201637_s_at | 11.23 | 1.28E-05 | 1.029880535 | NM_001013438| | NA |
| 211943_x_at | 11.22 | 1.29E-05 | 1.027425485 | NM_003295| | TPT1,tumor protein, translationally-controlled 1 |
| 205778_at | 11.22 | 1.29E-05 | 1.275376585 | NM_005046| | KLK7,stratum corneum chymotryptic enzyme |
| 206397_x_at | 11.22 | 1.30E-05 | 1.106421548 | NM_001492| | GDF1,growth differentiation factor 1 |
| 37117_at | 11.2 | 1.31E-05 | 1.113815564 | NM_001017526| | NA |
| 214955_at | 11.19 | 1.32E-05 | 1.066825496 | NM_153609| | TMPRSS6,transmembrane protease, serine 6 |
| 225956_at | 11.19 | 1.32E-05 | 1.189344872 | NM_153607| | LOC153222,adult retina protein |
| 225902_at | 11.18 | 1.32E-05 | 1.067244174 | NA |  |
| 231457_at | 11.17 | 1.34E-05 | 1.135604843 | NA |  |
| 212748_at | 11.17 | 1.33E-05 | 1.043721449 | NM_020831| | MKL1,megakaryoblastic leukemia 1 protein |
| 209772_s_at | 11.17 | 1.33E-05 | 1.046541241 | NM_013230| | CD24,CD24 antigen |
| 228719_at | 11.17 | 1.33E-05 | 1.270404934 | NM_001042697| | NA |
| 225362_at | 11.16 | 1.34E-05 | 1.087071341 | NM_145284| | LOC159090,similar to hypothetical protein MGC17347 |
| 238714_at | 11.16 | 1.34E-05 | 1.16840999 | NA |  |
| 210821_x_at | 11.15 | 1.35E-05 | 1.084030679 | NM_001042426| | NA |
| 222494_at | 11.15 | 1.34E-05 | 1.04579047 | NM_001085471| | NA |
| 221094_s_at | 11.14 | 1.36E-05 | 1.092577667 | NM_018091| | ELP3,elongation protein 3 homolog |
| 213659_at | 11.14 | 1.36E-05 | 1.248383074 | NM_007131| | ZNF75,zinc finger protein 75 |
| 213653_at | 11.13 | 1.36E-05 | 1.165219952 | NM_019852| | METTL3,methyltransferase like 3 |
| 242354_at | 11.13 | 1.36E-05 | 1.095619382 | NA |  |
| 229078_s_at | 11.12 | 1.37E-05 | 1.176328145 | NM_018559| | KIAA1704,KIAA1704 |
| 236305_at | 11.12 | 1.37E-05 | 1.132551484 | NM_173362| | LOC317671,LOC317671 |
| 224494_x_at | 11.12 | 1.37E-05 | 1.136067128 | NM_016246| | DHRS10,dehydrogenase/reductase (SDR family) member 10 |
| 232057_at | 11.12 | 1.37E-05 | 1.060597367 | NM_032178| | FLJ13291,hypothetical protein FLJ13291 |
| 223536_at | 11.12 | 1.37E-05 | 1.119566325 | NM_032289| | PSD2,pleckstrin and Sec7 domain containing 2 |
| 1555148_a_at | 11.11 | 1.38E-05 | 1.679735373 | NM_138804| | LOC130951,hypothetical protein BC014602 |
| 228723_at | 11.1 | 1.39E-05 | 1.138013187 | NA |  |
| 208117_s_at | 11.09 | 1.40E-05 | 1.055509004 | NM_031206| | LAS1L,LAS1-like |
| 219019_at | 11.08 | 1.41E-05 | 1.074519038 | NM_018494| | LRDD,leucine rich repeat and death domain containing |
| 218383_at | 11.07 | 1.42E-05 | 1.047623406 | NM_017815| | C14orf94,chromosome 14 open reading frame 94 |
| 232116_at | 11.06 | 1.43E-05 | 1.232820982 | NM_021180| | TFCP2L4,sister-of-mammalian grainyhead protein isoform |
| 216551_x_at | 11.06 | 1.43E-05 | 1.10142944 | NM_002660| | PLCG1,phospholipase C gamma 1 isoform a |
| 224684_at | 11.06 | 1.43E-05 | 1.051130139 | NM_013346| | SNX12,sorting nexin 12 |
| 242917_at | 11.06 | 1.43E-05 | 1.134868054 | NM_145313| | RASGEF1A,RasGEF domain family, member 1A |
| 1560153_at | 11.06 | 1.43E-05 | 1.23307468 | NM_025074| | FRAS1,Fraser syndrome 1 isoform 1 |
| 222343_at | 11.06 | 1.43E-05 | 1.164722152 | NM_006538| | BCL2L11,BCL2-like 11 isoform 6 |
| 223912_s_at | 11.05 | 1.44E-05 | 1.092217543 | NM_018941| | CLN8,CLN8 protein |
| 1553538_s_at | 11.05 | 1.44E-05 | 1.027825343 | NA |  |
| 224497_x_at | 11.05 | 1.44E-05 | 1.153434694 | NM_016246| | DHRS10,dehydrogenase/reductase (SDR family) member 10 |
| 217827_s_at | 11.05 | 1.43E-05 | 1.043578286 | NM_016630| | SPG21,acid cluster protein 33 |
| 201765_s_at | 11.04 | 1.44E-05 | 1.052545684 | NM_000520| | HEXA,hexosaminidase A preproprotein |
| 207056_s_at | 11.04 | 1.45E-05 | 1.184007658 | NM_001039960| | NA |
| 229899_s_at | 11.03 | 1.45E-05 | 1.147981059 | NA |  |
| 224568_x_at | 11.03 | 1.45E-05 | 1.229597613 | NA |  |
| 209600_s_at | 11.02 | 1.46E-05 | 1.053915863 | NM_004035| | ACOX1,acyl-Coenzyme A oxidase isoform a |
| 226792_s_at | 11.02 | 1.46E-05 | 1.109008693 | NM_145754| | KIFC2,kinesin family member C2 |
| 1555310_a_at | 11.02 | 1.46E-05 | 1.158551031 | NM_020168| | PAK6,p21-activated kinase 6 |
| 211748_x_at | 11.02 | 1.46E-05 | 1.097584835 | NM_000954| | PTGDS,prostaglandin D2 synthase 21kDa |
| 64432_at | 11.02 | 1.46E-05 | 1.073688368 | NA |  |
| 223311_s_at | 11.01 | 1.46E-05 | 1.100855511 | NM_020744| | MTA3,metastasis associated 1 family, member 3 |
| 203678_at | 11.01 | 1.46E-05 | 1.059350709 | NM_014967| | KIAA1018,KIAA1018 protein |
| 213890_x_at | 11 | 1.47E-05 | 1.010236179 | NM_001020| | RPS16,ribosomal protein S16 |
| 207368_at | 10.99 | 1.48E-05 | 1.173254227 | NM_000864| | HTR1D,5-hydroxytryptamine (serotonin) receptor 1D |
| 225355_at | 10.98 | 1.49E-05 | 1.056683398 | NA |  |
| 46256_at | 10.98 | 1.49E-05 | 1.064266052 | NM_080861| | SSB3,SPRY domain-containing SOCS box protein SSB-3 |
| 206695_x_at | 10.98 | 1.49E-05 | 1.115277425 | NM_003423| | ZNF43,zinc finger protein 43 (HTF6) |
| 203962_s_at | 10.98 | 1.50E-05 | 1.15488534 | NM_006393| | NEBL,nebulette sarcomeric isoform |
| 210094_s_at | 10.97 | 1.51E-05 | 1.032586708 | NM_019619| | PARD3,partitioning-defective protein 3 homolog |
| 201157_s_at | 10.96 | 1.52E-05 | 1.053823705 | NM_021079| | NMT1,N-myristoyltransferase 1 |
| 231195_at | 10.96 | 1.52E-05 | 1.050608661 | NM_198508| | FLJ44186,FLJ44186 protein |
| 221790_s_at | 10.95 | 1.52E-05 | 1.08476295 | NM_015627| | ARH,LDL receptor adaptor protein |
| 237856_at | 10.95 | 1.52E-05 | 1.344578545 | NM_001100426| | NA |
| 209595_at | 10.95 | 1.52E-05 | 1.093283245 | NM_004128| | GTF2F2,general transcription factor IIF, polypeptide 2 |
| 216620_s_at | 10.94 | 1.54E-05 | 1.04897917 | NM_014629| | ARHGEF10,Rho guanine nucleotide exchange factor 10 |
| 207206_s_at | 10.94 | 1.54E-05 | 1.180330878 | NM_000697| | ALOX12,arachidonate 12-lipoxygenase |
| 226488_at | 10.93 | 1.54E-05 | 1.114607329 | NM_001017919| | NA |
| 207129_at | 10.93 | 1.54E-05 | 1.210105785 | NM_007220| | CA5B,carbonic anhydrase VB, mitochondrial precursor |
| 235747_at | 10.92 | 1.56E-05 | 1.264901475 | NM_152707| | SLC25A16,solute carrier family 25, member 16 |
| 219020_at | 10.9 | 1.58E-05 | 1.103784043 | NM_022460| | FLJ14249,HS1-binding protein 3 isoform 1 |
| 201123_s_at | 10.9 | 1.58E-05 | 1.090996005 | NM_001970| | EIF5A,eukaryotic translation initiation factor 5A |
| 203715_at | 10.89 | 1.59E-05 | 1.048611677 | NM_001079515| | NA |
| 234660_s_at | 10.89 | 1.59E-05 | 1.121861327 | NM_014953| | KIAA1008,KIAA1008 |
| 219228_at | 10.89 | 1.59E-05 | 1.147696083 | NM_001079906| | NA |
| 205953_at | 10.89 | 1.59E-05 | 1.153690224 | NM_014813| | LRIG2,leucine-rich repeats and immunoglobulin-like |
| 224431_s_at | 10.89 | 1.58E-05 | 1.07244873 | NM_032701| | SUV420H2,suppressor of variegation 4-20 homolog 2 |
| 200715_x_at | 10.88 | 1.59E-05 | 1.023888453 | NM_012423| | RPL13A,ribosomal protein L13a |
| 226663_at | 10.87 | 1.60E-05 | 1.143872117 | NM_017664| | ANKRD10,ankyrin repeat domain 10 |
| 217749_at | 10.87 | 1.60E-05 | 1.057109451 | NM_016128| | COPG,coatomer protein complex, subunit gamma 1 |
| 224739_at | 10.87 | 1.60E-05 | 1.078843496 | NM_001001852| | PIM3,pim-3 oncogene |
| 227861_at | 10.86 | 1.61E-05 | 1.051555284 | NM_153354| | MGC33214,hypothetical protein MGC33214 |
| 244227_at | 10.86 | 1.61E-05 | 1.143796126 | NM_205848| | SYT6,synaptotagmin VI |
| 201592_at | 10.85 | 1.63E-05 | 1.029910259 | NM_003756| | EIF3S3,eukaryotic translation initiation factor 3, |
| 209220_at | 10.84 | 1.64E-05 | 1.072716414 | NM_004484| | GPC3,glypican 3 |
| 209002_s_at | 10.84 | 1.64E-05 | 1.062888058 | NM_020898| | KIAA1536,coiled-coil transcriptional coactivator |
| 223702_x_at | 10.83 | 1.65E-05 | 1.110400936 | NM_006657| | FTCD,formiminotransferase cyclodeaminase |
| 235012_at | 10.82 | 1.66E-05 | 1.259577749 | NM_015116| | LRCH1,leucine-rich repeats and calponin homology (CH) |
| 227371_at | 10.81 | 1.67E-05 | 1.106261405 | NM_018842| | BAIAP2L1,BAI1-associated protein 2-like 1 |
| 213501_at | 10.8 | 1.68E-05 | 1.090431931 | NM_004035| | ACOX1,acyl-Coenzyme A oxidase isoform a |
| 231385_at | 10.8 | 1.68E-05 | 1.503276496 | NM_199286| | DPPA3,stella |
| 45572_s_at | 10.79 | 1.70E-05 | 1.042181432 | NM_001001560| | GGA1,golgi associated, gamma adaptin ear containing, |
| 223282_at | 10.79 | 1.69E-05 | 1.445476646 | NM_005786| | SDCCAG33,serologically defined colon cancer antigen 33 |
| 229241_at | 10.78 | 1.70E-05 | 1.650112518 | NM_153486| | LDHD,D-lactate dehydrogenase isoform 1 precursor |
| 241601_at | 10.75 | 1.74E-05 | 1.137812878 | NA |  |
| 227429_at | 10.75 | 1.74E-05 | 1.135829954 | NM_173584| | MGC45840,hypothetical protein MGC45840 |
| 206460_at | 10.74 | 1.75E-05 | 1.16195752 | NM_001042478| | NA |
| 223601_at | 10.74 | 1.75E-05 | 1.137366932 | NM_058164| | OLFM2,olfactomedin 2 |
| 227123_at | 10.73 | 1.76E-05 | 1.071018284 | NM_002867| | RAB3B,RAB3B, member RAS oncogene family |
| 200905_x_at | 10.72 | 1.77E-05 | 1.06681337 | NM_005516| | HLA-E,major histocompatibility complex, class I, E |
| 208947_s_at | 10.72 | 1.78E-05 | 1.088971875 | NM_002911| | RENT1,regulator of nonsense transcripts 1 |
| 218878_s_at | 10.72 | 1.78E-05 | 1.07307472 | NM_012238| | SIRT1,sirtuin 1 |
| 221500_s_at | 10.71 | 1.79E-05 | 1.085208928 | NM_001001433| | STX16,syntaxin 16 isoform a |
| 209090_s_at | 10.71 | 1.79E-05 | 1.041793085 | NM_016009| | SH3GLB1,SH3-containing protein SH3GLB1 |
| 223442_at | 10.7 | 1.79E-05 | 1.040821207 | NM_032316| | NICN1,nicolin 1 |
| 213413_at | 10.69 | 1.82E-05 | 1.082174676 | NM_006873| | SBLF,stoned B-like factor |
| 223348_x_at | 10.68 | 1.82E-05 | 1.098256313 | NM_032853| | MUM1,melanoma ubiquitous mutated protein |
| 224565_at | 10.67 | 1.85E-05 | 1.30418726 | NA |  |
| 226155_at | 10.67 | 1.85E-05 | 1.07917752 | NM_020940| | KIAA1600,KIAA1600 |
| 203822_s_at | 10.67 | 1.84E-05 | 1.055031623 | NM_006874| | ELF2,E74-like factor 2 (ets domain transcription |
| 230508_at | 10.66 | 1.85E-05 | 1.097926852 | NM_001018057| | NA |
| 203658_at | 10.66 | 1.86E-05 | 1.101164951 | NM_000387| | SLC25A20,carnitine/acylcarnitine translocase |
| 225203_at | 10.66 | 1.86E-05 | 1.148169889 | NM_032902| | PPP1R16A,protein phosphatase 1, regulatory (inhibitor) |
| 200023_s_at | 10.66 | 1.85E-05 | 1.022845517 | NM_003754| | EIF3S5,eukaryotic translation initiation factor 3, |
| 221735_at | 10.65 | 1.87E-05 | 1.053477737 | NM_020839| | WDR48,WD repeat domain 48 |
| 212728_at | 10.65 | 1.86E-05 | 1.094587355 | NM_020730| | NA |
| 1553551_s_at | 10.64 | 1.88E-05 | 1.051496562 | NA |  |
| 31637_s_at | 10.63 | 1.89E-05 | 1.101633265 | NM_003250| | THRA,thyroid hormone receptor, alpha isoform 2 |
| 226970_at | 10.63 | 1.89E-05 | 1.080841851 | NM_203301| | FBXO33,F-box protein 33 |
| 221486_at | 10.62 | 1.90E-05 | 1.032353611 | NM_004436| | ENSA,endosulfine alpha isoform 3 |
| 213288_at | 10.62 | 1.90E-05 | 1.094625917 | NM_138799| | OACT2,O-acyltransferase (membrane bound) domain |
| 226986_at | 10.61 | 1.91E-05 | 1.055728193 | NM_001033518| | NA |
| 208061_at | 10.61 | 1.91E-05 | 1.305492638 | NA |  |
| 228640_at | 10.61 | 1.91E-05 | 1.244025918 | NM_002589| | PCDH7,protocadherin 7 isoform a precursor |
| 219061_s_at | 10.6 | 1.91E-05 | 1.044982256 | NM_006014| | DXS9879E,ESO3 protein |
| 225296_at | 10.6 | 1.92E-05 | 1.056125758 | NM_020933| | ZNF317,zinc finger protein 317 |
| 1554113_a_at | 10.58 | 1.94E-05 | 1.263370252 | NM_001039960| | NA |
| 213792_s_at | 10.58 | 1.95E-05 | 1.087904062 | NM_000208| | INSR,insulin receptor |
| 232280_at | 10.57 | 1.95E-05 | 1.277561983 | NM_001039355| | NA |
| 1556069_s_at | 10.57 | 1.96E-05 | 1.112704838 | NM_022462| | HIF3A,hypoxia-inducible factor-3 alpha isoform b |
| 227017_at | 10.54 | 2.00E-05 | 1.044222144 | NM_207332| | LOC157697,hypothetical protein LOC157697 |
| 226883_at | 10.54 | 2.01E-05 | 1.041065213 | NA |  |
| 205980_s_at | 10.54 | 2.01E-05 | 1.132249475 | NM_001017526| | NA |
| 229518_at | 10.53 | 2.02E-05 | 1.071058382 | NM_052943| | FAM46B,family with sequence similarity 46, member B |
| 237275_at | 10.52 | 2.02E-05 | 1.248531131 | NA |  |
| 208717_at | 10.52 | 2.02E-05 | 1.055748281 | NM_005015| | OXA1L,oxidase (cytochrome c) assembly 1-like |
| 204556_s_at | 10.52 | 2.03E-05 | 1.051310622 | NM_014934| | DZIP1,DAZ interacting protein 1 |
| 228396_at | 10.48 | 2.10E-05 | 1.10354613 | NM_001098512| | NA |
| 213482_at | 10.47 | 2.12E-05 | 1.215422119 | NM_004947| | DOCK3,dedicator of cytokinesis 3 |
| 202848_s_at | 10.47 | 2.11E-05 | 1.086573457 | NM_001004105| | GRK6,G protein-coupled receptor kinase 6 isoform C |
| 215471_s_at | 10.47 | 2.12E-05 | 1.096576347 | NM_003980| | MAP7,microtubule-associated protein 7 |
| 226939_at | 10.46 | 2.12E-05 | 1.283745836 | NM_182485| | CPEB2,cytoplasmic polyadenylation element binding |
| 217965_s_at | 10.46 | 2.12E-05 | 1.054919638 | NM_013260| | HCNGP,transcriptional regulator protein |
| 202804_at | 10.45 | 2.15E-05 | 1.029475858 | NM_004996| | ABCC1,ATP-binding cassette, sub-family C, member 1 |
| 1557616_at | 10.45 | 2.15E-05 | 1.128162344 | NM_032752| | ZNF496,zinc finger protein 496 |
| 1554780_a_at | 10.45 | 2.13E-05 | 1.160725173 | NM_020432| | PHTF2,putative homeodomain transcription factor 2 |
| 226450_at | 10.44 | 2.15E-05 | 1.105019867 | NM_000208| | INSR,insulin receptor |
| 209455_at | 10.44 | 2.15E-05 | 1.050809448 | NM_012300| | FBXW11,F-box and WD-40 domain protein 1B isoform C |
| 212374_at | 10.44 | 2.16E-05 | 1.091233161 | NM_015322| | FEM1B,fem-1 homolog b |
| 225130_at | 10.42 | 2.18E-05 | 1.162720754 | NM_017580| | ZRANB1,TRABID protein |
| 236321_at | 10.41 | 2.20E-05 | 1.148714005 | NA |  |
| 210592_s_at | 10.41 | 2.19E-05 | 1.070027918 | NM_002970| | SAT,spermidine/spermine N1-acetyltransferase |
| 229497_at | 10.41 | 2.19E-05 | 1.148822218 | NM_182703| | LOC348094,hypothetical protein LOC348094 |
| 203766_s_at | 10.4 | 2.21E-05 | 1.110889547 | NM_012134| | LMOD1,leiomodin 1 (smooth muscle) |
| 209622_at | 10.4 | 2.21E-05 | 1.06728817 | NM_001008910| | STK16,serine/threonine kinase 16 |
| 244292_at | 10.4 | 2.21E-05 | 1.213126191 | NA |  |
| 225889_at | 10.4 | 2.21E-05 | 1.073805871 | NM_001114176| | NA |
| 218184_at | 10.4 | 2.21E-05 | 1.060610271 | NM_001007466| | TULP4,tubby like protein 4 isoform 2 |
| 216308_x_at | 10.39 | 2.22E-05 | 1.044280951 | NM_012203| | GRHPR,glyoxylate reductase/hydroxypyruvate reductase |
| 231836_at | 10.39 | 2.22E-05 | 1.110280887 | NM_181786| | HKR1,GLI-Kruppel family member HKR1 |
| 222386_s_at | 10.38 | 2.24E-05 | 1.050781167 | NM_016057| | COPZ1,coatomer protein complex, subunit zeta 1 |
| 222418_s_at | 10.38 | 2.23E-05 | 1.145258359 | NM_024334| | TMEM43,transmembrane protein 43 |
| 203695_s_at | 10.38 | 2.24E-05 | 1.08295468 | NM_004403| | DFNA5,deafness, autosomal dominant 5 protein |
| 236331_at | 10.38 | 2.23E-05 | 1.252338508 | NM_003948| | CDKL2,cyclin-dependent kinase-like 2 |
| 218393_s_at | 10.38 | 2.24E-05 | 1.08727159 | NM_018225| | SMU1,smu-1 suppressor of mec-8 and unc-52 homolog |
| 208359_s_at | 10.38 | 2.23E-05 | 1.221133029 | NM_004981| | KCNJ4,potassium inwardly-rectifying channel J4 |
| 216111_x_at | 10.37 | 2.25E-05 | 1.055004057 | NM_001003686| | PMS2L3,postmeiotic segregation increased 2-like 3 |
| 1553286_at | 10.37 | 2.25E-05 | 1.075086054 | NM_152791| | ZNF555,zinc finger protein 555 |
| 233503_at | 10.36 | 2.27E-05 | 1.198446456 | NM_017826| | FLJ20449,hypothetical protein FLJ20449 |
| 226977_at | 10.36 | 2.27E-05 | 1.203630912 | NM_001007189| | LOC492311,similar to bovine IgA regulatory protein |
| 228590_at | 10.35 | 2.28E-05 | 1.053747738 | NM_017952| | FLJ20758,FLJ20758 protein |
| 225957_at | 10.35 | 2.28E-05 | 1.192513326 | NM_153607| | LOC153222,adult retina protein |
| 212627_s_at | 10.35 | 2.28E-05 | 1.040587582 | NM_015004| | EXOSC7,exosome component 7 |
| 235061_at | 10.35 | 2.29E-05 | 1.14580042 | NM_152542| | PPM1K,protein phosphatase 1K (PP2C domain containing) |
| 226297_at | 10.34 | 2.30E-05 | 1.057137671 | NA |  |
| 210567_s_at | 10.34 | 2.29E-05 | 1.129576501 | NM_005983| | SKP2,S-phase kinase-associated protein 2 isoform 1 |
| 223049_at | 10.34 | 2.29E-05 | 1.024426073 | NM_002086| | GRB2,growth factor receptor-bound protein 2 isoform |
| 229813_x_at | 10.34 | 2.31E-05 | 1.02039278 | NM_018959| | DAZAP1,DAZ associated protein 1 isoform b |
| 215167_at | 10.33 | 2.31E-05 | 1.1737023 | NM_004229| | CRSP2,cofactor required for Sp1 transcriptional |
| 235528_at | 10.33 | 2.31E-05 | 1.110138354 | NM_002098| | GUCA1B,guanylate cyclase activator 1B (retina) |
| 228953_at | 10.33 | 2.31E-05 | 1.093706074 | NM_001080435| | NA |
| 220588_at | 10.33 | 2.31E-05 | 1.082569962 | NM_001010974| | BCAS4,breast carcinoma amplified sequence 4 isoform c |
| 1563983_at | 10.32 | 2.32E-05 | 1.112585331 | NA |  |
| 225507_at | 10.32 | 2.33E-05 | 1.061018709 | NM_015491| | NA |
| 213753_x_at | 10.31 | 2.35E-05 | 1.073221465 | NM_001970| | EIF5A,eukaryotic translation initiation factor 5A |
| 206822_s_at | 10.31 | 2.35E-05 | 1.230208056 | NM_015478| | L3MBTL,l(3)mbt-like isoform I |
| 208836_at | 10.31 | 2.34E-05 | 1.022919526 | NM_001679| | ATP1B3,Na+/K+ -ATPase beta 3 subunit |
| 219461_at | 10.3 | 2.35E-05 | 1.175119748 | NM_020168| | PAK6,p21-activated kinase 6 |
| 232881_at | 10.29 | 2.37E-05 | 1.552235384 | NA |  |
| 223437_at | 10.29 | 2.37E-05 | 1.155205046 | NM_001001928| | PPARA,peroxisome proliferative activated receptor, |
| 226212_s_at | 10.29 | 2.38E-05 | 1.213922334 | NM_000208| | INSR,insulin receptor |
| 232092_at | 10.28 | 2.39E-05 | 1.110214867 | NM_033412| | MCART1,mitochondrial carrier triple repeat 1 |
| 219117_s_at | 10.27 | 2.40E-05 | 1.068530551 | NM_016594| | FKBP11,FK506 binding protein precursor |
| 223329_x_at | 10.26 | 2.43E-05 | 1.06301905 | NM_006704| | SUGT1,suppressor of G2 allele of SKP1 |
| 208178_x_at | 10.26 | 2.43E-05 | 1.165764038 | NM_007118| | TRIO,triple functional domain (PTPRF interacting) |
| 241829_at | 10.25 | 2.44E-05 | 1.231707401 | NM_145019| | FLJ30707,hypothetical protein FLJ30707 |
| 1552678_a_at | 10.25 | 2.45E-05 | 1.156295166 | NM_020886| | USP28,ubiquitin specific protease 28 |
| 218855_at | 10.24 | 2.45E-05 | 1.110569002 | NM_016372| | GPR175,G protein-coupled receptor 175 |
| 204019_s_at | 10.24 | 2.45E-05 | 1.056886018 | NM_015677| | SH3YL1,SH3 domain containing, Ysc84-like 1 |
| 211955_at | 10.23 | 2.48E-05 | 1.042931536 | NM_002271| | RANBP5,RAN binding protein 5 |
| 232165_at | 10.23 | 2.48E-05 | 1.128305263 | NM_031308| | EPPK1,epiplakin 1 |
| 205181_at | 10.22 | 2.49E-05 | 1.087078495 | NM_006299| | ZNF193,zinc finger protein 193 |
| 228983_at | 10.22 | 2.49E-05 | 1.135269825 | NA |  |
| 212227_x_at | 10.22 | 2.49E-05 | 1.027067102 | NM_005801| | SUI1,putative translation initiation factor |
| 1559194_a_at | 10.21 | 2.51E-05 | 1.2163323 | NA |  |
| 233753_at | 10.2 | 2.54E-05 | 1.20216844 | NM_020706| | SFRS15,splicing factor, arginine/serine-rich 15 |
| 223114_at | 10.2 | 2.52E-05 | 1.065400211 | NM_032314| | MGC4767,hypothetical protein MGC4767 |
| 228622_s_at | 10.19 | 2.54E-05 | 1.119048746 | NM_005528| | DNAJC4,DnaJ (Hsp40) homolog, subfamily C, member 4 |
| 200058_s_at | 10.17 | 2.58E-05 | 1.032157901 | NM_014014| | ASCC3L1,activating signal cointegrator 1 complex subunit |
| 228730_s_at | 10.17 | 2.59E-05 | 1.205877444 | NM_138355| | SCRN2,secernin 2 |
| 204879_at | 10.16 | 2.59E-05 | 1.073393211 | NM_001006624| | T1A-2,lung type-I cell membrane-associated |
| 206042_x_at | 10.16 | 2.59E-05 | 1.055752682 | NM_003097| | SNRPN,small nuclear ribonucleoprotein polypeptide N |
| 208865_at | 10.16 | 2.60E-05 | 1.027701628 | NM_001025105| | NA |
| 203014_x_at | 10.16 | 2.60E-05 | 1.068651408 | NM_015705| | RUTBC3,RUN and TBC1 domain containing 3 |
| 217728_at | 10.14 | 2.64E-05 | 1.694704756 | NM_014624| | S100A6,S100 calcium-binding protein A6 |
| 1554637_a_at | 10.13 | 2.66E-05 | 1.24305042 | NM_001032999| | NA |
| 214078_at | 10.12 | 2.68E-05 | 1.136524378 | NA |  |
| 203919_at | 10.12 | 2.68E-05 | 1.072715764 | NM_003195| | TCEA2,transcription elongation factor A protein 2 |
| 1559861_at | 10.12 | 2.68E-05 | 1.132732737 | NA |  |
| 210169_at | 10.11 | 2.69E-05 | 1.163116468 | NM_014692| | NA |
| 219926_at | 10.1 | 2.70E-05 | 1.156028887 | NM_022361| | POPDC3,popeye protein 3 |
| 225131_at | 10.1 | 2.72E-05 | 1.07849842 | NM_017580| | ZRANB1,TRABID protein |
| 243880_at | 10.09 | 2.74E-05 | 1.42098217 | NM_001012511| | GOSR2,golgi SNAP receptor complex member 2 isoform C |
| 227492_at | 10.06 | 2.79E-05 | 1.032260258 | NA |  |
| 244393_x_at | 10.06 | 2.79E-05 | 1.334470373 | NA |  |
| 204554_at | 10.06 | 2.79E-05 | 1.089118887 | NM_006242| | PPP1R3D,protein phosphatase 1, regulatory subunit 3D |
| 224606_at | 10.05 | 2.81E-05 | 1.065425154 | NM_001300| | KLF6,Kruppel-like factor 6 |
| 226734_at | 10.05 | 2.81E-05 | 1.04140665 | NM_004846| | EIF4E2,eukaryotic translation initiation factor 4E |
| 215548_s_at | 10.05 | 2.81E-05 | 1.031498106 | NM_016106| | SCFD1,vesicle transport-related protein isoform a |
| 202825_at | 10.04 | 2.83E-05 | 1.07281449 | NM_001151| | SLC25A4,solute carrier family 25 (mitochondrial carrier; |
| 217673_x_at | 10.04 | 2.83E-05 | 1.076238367 | NM_000516| | GNAS,guanine nucleotide binding protein, alpha |
| 201714_at | 10.03 | 2.86E-05 | 1.033261908 | NM_001070| | TUBG1,tubulin, gamma 1 |
| 202313_at | 10.03 | 2.85E-05 | 1.026284097 | NM_002717| | PPP2R2A,alpha isoform of regulatory subunit B55, protein |
| 224452_s_at | 10.03 | 2.86E-05 | 1.051376359 | NM_001037163| | NA |
| 201020_at | 10.03 | 2.86E-05 | 1.056344995 | NM_003405| | YWHAH,tyrosine 3/tryptophan 5 -monooxygenase |
| 203554_x_at | 10.02 | 2.88E-05 | 1.040818104 | NM_004219| | PTTG1,pituitary tumor-transforming protein 1 |
| 209916_at | 10.02 | 2.87E-05 | 1.047459817 | NM_018706| | DHTKD1,dehydrogenase E1 and transketolase domain |
| 240206_at | 10.02 | 2.86E-05 | 1.2661967 | NM_152295| | TARS,threonyl-tRNA synthetase |
| 232389_at | 10.01 | 2.89E-05 | 1.204288562 | NM_001080529| | NA |
| 227961_at | 10.01 | 2.89E-05 | 1.086951885 | NM_001908| | CTSB,cathepsin B preproprotein |
| 1555847_a_at | 10.01 | 2.90E-05 | 1.090650328 | NA |  |
| 211938_at | 10 | 2.91E-05 | 1.046941141 | NM_001417| | EIF4B,eukaryotic translation initiation factor 4B |
| 230407_at | 10 | 2.92E-05 | 1.03615873 | NA |  |
| 214327_x_at | 10 | 2.91E-05 | 1.023954239 | NM_003295| | TPT1,tumor protein, translationally-controlled 1 |
| 206600_s_at | 10 | 2.91E-05 | 1.110857689 | NM_004695| | SLC16A5,solute carrier family 16, member 5 |
| 1564359_a_at | 10 | 2.91E-05 | 1.533514716 | NA |  |
| 34406_at | 10 | 2.91E-05 | 1.040974571 | NM_001100913| | NA |
| 223802_s_at | 10 | 2.91E-05 | 1.063199154 | NM_006910| | RBBP6,retinoblastoma-binding protein 6 isoform 1 |
| 215306_at | 9.99 | 2.93E-05 | 1.201493137 | NA |  |
| 206896_s_at | 9.99 | 2.94E-05 | 1.168665891 | NM_052847| | GNG7,guanine nucleotide binding protein (G protein), |
| 1552503_at | 9.99 | 2.93E-05 | 1.172297856 | NM_025074| | FRAS1,Fraser syndrome 1 isoform 1 |
| 203713_s_at | 9.99 | 2.94E-05 | 1.075601287 | NM_001015002| | NA |
| 243770_at | 9.98 | 2.95E-05 | 1.377400249 | NM_001007169| | ZNF483,zinc finger protein 483 isoform b |
| 217810_x_at | 9.98 | 2.95E-05 | 1.055711813 | NM_020117| | LARS,leucyl-tRNA synthetase |
| 213804_at | 9.98 | 2.95E-05 | 1.066529955 | NM_005540| | INPP5B,inositol polyphosphate-5-phosphatase, 75kDa |
| 244317_at | 9.98 | 2.95E-05 | 1.259657592 | NM_152748| | FLJ31340,hypothetical protein FLJ31340 |
| 218476_at | 9.98 | 2.95E-05 | 1.085451536 | NM_001077365| | NA |
| 239246_at | 9.97 | 2.97E-05 | 1.132736438 | NM_001001715| | FARP1,FERM, RhoGEF, and pleckstrin domain protein 1 |
| 230818_at | 9.96 | 2.99E-05 | 1.308462374 | NA |  |
| 222021_x_at | 9.96 | 2.99E-05 | 1.038017522 | NA |  |
| 225398_at | 9.96 | 2.99E-05 | 1.047242337 | NM_032795| | RPUSD4,RNA pseudouridylate synthase domain containing |
| 228778_at | 9.96 | 2.99E-05 | 1.091014208 | NA |  |
| 227996_at | 9.95 | 3.01E-05 | 1.207747835 | NM_001001715| | FARP1,FERM, RhoGEF, and pleckstrin domain protein 1 |
| 214535_s_at | 9.94 | 3.02E-05 | 1.19418561 | NM_014244| | ADAMTS2,a disintegrin and metalloprotease with |
| 232251_at | 9.94 | 3.03E-05 | 1.547489544 | NA |  |
| 238662_at | 9.92 | 3.08E-05 | 1.07756948 | NM_080650| | MGC14798,hypothetical protein MGC14798 |
| 218278_at | 9.92 | 3.07E-05 | 1.035230633 | NM_018093| | FLJ10439,hypothetical protein FLJ10439 |
| 207064_s_at | 9.91 | 3.11E-05 | 1.142691353 | NM_001158| | AOC2,amine oxidase, copper containing 2 isoform a |
| 239223_s_at | 9.91 | 3.10E-05 | 1.21640662 | NM_032875| | FBXL20,F-box and leucine-rich repeat protein 20 |
| 210377_at | 9.91 | 3.11E-05 | 1.21823543 | NM_005622| | SAH,SA hypertension-associated homolog isoform 1 |
| 214252_s_at | 9.9 | 3.13E-05 | 1.115058061 | NM_006493| | CLN5,ceroid-lipofuscinosis, neuronal 5 |
| 223940_x_at | 9.9 | 3.12E-05 | 1.203388415 | NA |  |
| 224366_s_at | 9.89 | 3.13E-05 | 1.045086066 | NM_031922| | REPS1,RALBP1 associated Eps domain containing 1 |
| 222529_at | 9.89 | 3.14E-05 | 1.062424148 | NM_016612| | MSCP,mitochondrial solute carrier protein |
| 213050_at | 9.88 | 3.15E-05 | 1.111253045 | NM_015198| | COBL,cordon-bleu homolog |
| 210306_at | 9.88 | 3.17E-05 | 1.295853221 | NM_015478| | L3MBTL,l(3)mbt-like isoform I |
| 235271_s_at | 9.88 | 3.16E-05 | 1.122979699 | NM_032347| | ZNF397,zinc finger protein 397 |
| 209425_at | 9.88 | 3.17E-05 | 1.100207493 | NM_014324| | AMACR,alpha-methylacyl-CoA racemase isoform 1 |
| 232183_at | 9.87 | 3.18E-05 | 1.134476514 | NM_032861| | SERAC1,serine active site containing 1 |
| 223060_at | 9.87 | 3.20E-05 | 1.031670432 | NM_017924| | C14orf119,chromosome 14 open reading frame 119 |
| 223954_x_at | 9.86 | 3.22E-05 | 1.054387571 | NM_031231| | APBA2BP,amyloid beta (A4) precursor protein-binding, |
| 36554_at | 9.86 | 3.21E-05 | 1.1092201 | NM_004192| | ASMTL,acetylserotonin O-methyltransferase-like |
| 218609_s_at | 9.86 | 3.21E-05 | 1.061571884 | NM_001161| | NUDT2,nudix-type motif 2 |
| 236214_at | 9.85 | 3.23E-05 | 1.175572258 | NM_032599| | NYD-SP18,testes development-related NYD-SP18 |
| 225279_s_at | 9.84 | 3.26E-05 | 1.355087506 | NM_001025072| | NA |
| 239334_at | 9.84 | 3.27E-05 | 1.20446244 | NM_020728| | CHR2SYT,chr2 synaptotagmin |
| 225326_at | 9.84 | 3.27E-05 | 1.032068754 | NM_018989| | NA |
| 224765_at | 9.84 | 3.25E-05 | 1.058406003 | NM_001012241| | NA |
| 212131_at | 9.84 | 3.26E-05 | 1.036596421 | NM_001114093| | NA |
| 214988_s_at | 9.83 | 3.28E-05 | 1.01842698 | NM_032195| | SON,SON DNA-binding protein isoform B |
| 1569077_x_at | 9.83 | 3.29E-05 | 1.124958399 | NM_001102657| | NA |
| 225578_at | 9.83 | 3.28E-05 | 1.069516985 | NM_001071775| | NA |
| 221003_s_at | 9.83 | 3.29E-05 | 1.189244536 | NM_001079670| | NA |
| 227074_at | 9.83 | 3.27E-05 | 1.213185629 | NA |  |
| 1552496_a_at | 9.82 | 3.29E-05 | 1.238170211 | NM_015198| | COBL,cordon-bleu homolog |
| 38157_at | 9.81 | 3.33E-05 | 1.067274161 | NM_005510| | DOM3Z,DOM-3 homolog Z |
| 1554464_a_at | 9.81 | 3.33E-05 | 1.123840434 | NM_006371| | CRTAP,cartilage associated protein precursor |
| 229669_at | 9.8 | 3.36E-05 | 1.580305567 | NA |  |
| 203853_s_at | 9.8 | 3.35E-05 | 1.054564548 | NM_012296| | GAB2,GRB2-associated binding protein 2 isoform b |
| 1553952_at | 9.8 | 3.35E-05 | 1.076065601 | NM_001039617| | NA |
| 1554424_at | 9.8 | 3.36E-05 | 1.201209616 | NM_030917| | FIP1L1,FIP1-like 1 |
| 213540_at | 9.8 | 3.35E-05 | 1.060213411 | NM_014234| | HSD17B8,estradiol 17 beta-dehydrogenase 8 |
| 227908_at | 9.79 | 3.38E-05 | 1.065431614 | NM_020705| | NA |
| 1554309_at | 9.79 | 3.38E-05 | 1.490815813 | NM_003760| | EIF4G3,eukaryotic translation initiation factor 4 |
| 228831_s_at | 9.79 | 3.37E-05 | 1.054602038 | NM_052847| | GNG7,guanine nucleotide binding protein (G protein), |
| 206862_at | 9.78 | 3.41E-05 | 1.117630741 | NM_203282| | ZNF539,zinc finger protein 539 |
| 235338_s_at | 9.78 | 3.41E-05 | 1.187117725 | NM_031915| | SETDB2,CLLL8 protein |
| 203026_at | 9.78 | 3.40E-05 | 1.05086472 | NM_014872| | ZBTB5,zinc finger and BTB domain containing 5 |
| 202032_s_at | 9.78 | 3.41E-05 | 1.098170826 | NM_006122| | MAN2A2,mannosidase, alpha, class 2A, member 2 |
| 201225_s_at | 9.77 | 3.42E-05 | 1.015377275 | NM_005839| | SRRM1,serine/arginine repetitive matrix 1 |
| 221611_s_at | 9.77 | 3.43E-05 | 1.154336382 | NM_016483| | PHF7,PHD finger protein 7 isoform 1 |
| 1552685_a_at | 9.76 | 3.46E-05 | 1.186921562 | NM_014552| | TFCP2L2,leader-binding protein 32 isoform 1 |
| 226738_at | 9.76 | 3.44E-05 | 1.040256619 | NM_152348| | FLJ33817,hypothetical protein FLJ33817 |
| 1555343_at | 9.76 | 3.45E-05 | 1.181245483 | NM_032446| | MEGF10,MEGF10 protein |
| 202361_at | 9.76 | 3.45E-05 | 1.031328408 | NM_004922| | SEC24C,SEC24-related protein C |
| 228855_at | 9.75 | 3.47E-05 | 1.120031846 | NM_001105663| | NA |
| 213118_at | 9.75 | 3.49E-05 | 1.063596432 | NM_001006947| | KIAA0701,KIAA0701 protein isoform b |
| 233764_s_at | 9.74 | 3.50E-05 | 1.126046297 | NM_018078| | FLJ10378,FLJ10378 protein isoform 1 |
| 204216_s_at | 9.74 | 3.51E-05 | 1.05134699 | NM_024824| | FLJ11806,nuclear protein UKp68 isoform 1 |
| 238951_at | 9.74 | 3.50E-05 | 1.154190721 | NA |  |
| 231035_s_at | 9.74 | 3.49E-05 | 1.095848977 | NA |  |
| 221115_s_at | 9.74 | 3.50E-05 | 1.122922709 | NM_018655| | LENEP,lens epithelial protein |
| 213554_s_at | 9.74 | 3.50E-05 | 1.053125886 | NM_017548| | H41,hypothetical protein H41 |
| 241611_s_at | 9.73 | 3.51E-05 | 1.148618579 | NM_001079673| | NA |
| 1554006_a_at | 9.73 | 3.52E-05 | 1.148105785 | NM_001015002| | NA |
| 1562497_at | 9.73 | 3.52E-05 | 1.336620153 | NA |  |
| 200604_s_at | 9.72 | 3.54E-05 | 1.035725029 | NM_002734| | PRKAR1A,cAMP-dependent protein kinase, regulatory |
| 209184_s_at | 9.71 | 3.57E-05 | 1.051855413 | NM_003749| | IRS2,insulin receptor substrate 2 |
| 230916_at | 9.71 | 3.58E-05 | 1.197655184 | NM_018055| | NODAL,nodal-related protein |
| 202772_at | 9.71 | 3.57E-05 | 1.059646684 | NM_000191| | HMGCL,3-hydroxymethyl-3-methylglutaryl-Coenzyme A |
| 204930_s_at | 9.7 | 3.61E-05 | 1.103500648 | NM_001205| | BNIP1,BCL2/adenovirus E1B 19kD interacting protein 1 |
| 1560652_at | 9.7 | 3.60E-05 | 1.084903545 | NA |  |
| 228562_at | 9.7 | 3.61E-05 | 1.090272693 | NA |  |
| 208407_s_at | 9.7 | 3.60E-05 | 1.05676388 | NM_001085458| | NA |
| 238032_at | 9.69 | 3.62E-05 | 1.136050058 | NA |  |
| 222490_at | 9.69 | 3.63E-05 | 1.071179138 | NM_018119| | POLR3E,polymerase (RNA) III (DNA directed) polypeptide |
| 228145_s_at | 9.69 | 3.62E-05 | 1.047225886 | NM_020781| | ZNF398,zinc finger 398 isoform b |
| 1556007_s_at | 9.69 | 3.62E-05 | 1.124377896 | NA |  |
| 210078_s_at | 9.68 | 3.64E-05 | 1.134705257 | NM_003471| | KCNAB1,potassium voltage-gated channel, shaker-related |
| 226620_x_at | 9.68 | 3.64E-05 | 1.02789703 | NM_018959| | DAZAP1,DAZ associated protein 1 isoform b |
| 208202_s_at | 9.67 | 3.67E-05 | 1.122153329 | NM_015288| | PHF15,PHD finger protein 15 |
| 201832_s_at | 9.67 | 3.68E-05 | 1.032392066 | NM_003715| | VDP,vesicle docking protein p115 |
| 200900_s_at | 9.67 | 3.68E-05 | 1.060842494 | NM_002355| | M6PR,cation-dependent mannose-6-phosphate receptor |
| 217466_x_at | 9.67 | 3.69E-05 | 1.026820702 | NM_002952| | RPS2,ribosomal protein S2 |
| 227653_at | 9.66 | 3.71E-05 | 1.042768339 | NM_020810| | KIAA1393,tRNA-(N1G37) methyltransferase |
| 206122_at | 9.66 | 3.70E-05 | 1.214039809 | NM_006942| | SOX15,SRY-box 15 |
| 1558678_s_at | 9.66 | 3.70E-05 | 1.127116251 | NA |  |
| 242323_at | 9.66 | 3.69E-05 | 1.08187921 | NA |  |
| 212936_at | 9.65 | 3.71E-05 | 1.099979882 | NM_032042| | DKFZP564D172,hypothetical protein DKFZp564D172 |
| 1729_at | 9.65 | 3.74E-05 | 1.084610651 | NM_003789| | TRADD,TNFRSF1A-associated via death domain isoform 1 |
| 1558693_s_at | 9.64 | 3.74E-05 | 1.059321207 | NM_144580| | MGC31963,kidney predominant protein NCU-G1 |
| 224647_at | 9.64 | 3.75E-05 | 1.03779549 | NM_145012| | C10orf9,cyclin fold protein 1 |
| 211208_s_at | 9.63 | 3.79E-05 | 1.022027997 | NM_001126054| | NA |
| 219154_at | 9.63 | 3.80E-05 | 1.074944686 | NM_019034| | RHOF,ras homolog gene family, member F |
| 215096_s_at | 9.62 | 3.80E-05 | 1.072960837 | NM_001984| | ESD,esterase D/formylglutathione hydrolase |
| 203542_s_at | 9.62 | 3.81E-05 | 1.286690164 | NM_001206| | KLF9,Kruppel-like factor 9 |
| 205105_at | 9.62 | 3.80E-05 | 1.044003577 | NM_002372| | MAN2A1,mannosidase, alpha, class 2A, member 1 |
| 226369_at | 9.62 | 3.81E-05 | 1.088955129 | NA |  |
| 221309_at | 9.61 | 3.84E-05 | 1.214411622 | NM_032905| | RBM17,RNA binding motif protein 17 |
| 211530_x_at | 9.6 | 3.87E-05 | 1.044518949 | NM_002127| | HLA-G,major histocompatibility complex, class I, G |
| 200764_s_at | 9.6 | 3.89E-05 | 1.055964177 | NM_001903| | CTNNA1,catenin, alpha 1 |
| 223606_x_at | 9.59 | 3.91E-05 | 1.12714499 | NM_018559| | KIAA1704,KIAA1704 |
| 216320_x_at | 9.58 | 3.92E-05 | 1.143824955 | NM_020998| | MST1,macrophage stimulating 1 (hepatocyte growth |
| 202996_at | 9.58 | 3.93E-05 | 1.088835797 | NM_021173| | POLD4,polymerase (DNA-directed), delta 4 |
| 212455_at | 9.58 | 3.93E-05 | 1.027552366 | NM_001031732| | NA |
| 208918_s_at | 9.58 | 3.92E-05 | 1.057981946 | NM_023018| | FLJ13052,NAD kinase |
| 244822_at | 9.56 | 3.99E-05 | 1.451303514 | NM_000819| | GART,phosphoribosylglycinamide formyltransferase, |
| 217307_at | 9.55 | 4.00E-05 | 1.120392675 | NM_001098| | ACO2,aconitase 2 precursor |
| 208734_x_at | 9.55 | 4.00E-05 | 1.028998251 | NM_002865| | RAB2,RAB2, member RAS oncogene family |
| 229141_at | 9.55 | 4.00E-05 | 1.14844716 | NM_001006622| | WDR33,WD repeat domain 33 isoform 2 |
| 215789_s_at | 9.55 | 4.02E-05 | 1.104643551 | NM_001042478| | NA |
| 200615_s_at | 9.54 | 4.03E-05 | 1.061883254 | NM_001030006| | NA |
| 222723_at | 9.54 | 4.03E-05 | 1.09444098 | NA |  |
| 223485_at | 9.54 | 4.03E-05 | 1.129219189 | NM_032304| | HAGHL,hydroxyacylglutathione hydrolase-like isoform 2 |
| 202481_at | 9.54 | 4.04E-05 | 1.111173137 | NM_004753| | DHRS3,dehydrogenase/reductase (SDR family) member 3 |
| 208862_s_at | 9.53 | 4.08E-05 | 1.031217083 | NM_001085458| | NA |
| 201347_x_at | 9.52 | 4.11E-05 | 1.044359232 | NM_012203| | GRHPR,glyoxylate reductase/hydroxypyruvate reductase |
| 218871_x_at | 9.52 | 4.09E-05 | 1.106525193 | NM_018590| | GALNACT-2,chondroitin beta1,4 |
| 215001_s_at | 9.52 | 4.11E-05 | 1.043612211 | NM_001033044| | NA |
| 210844_x_at | 9.52 | 4.11E-05 | 1.030250509 | NM_001903| | CTNNA1,catenin, alpha 1 |
| 242463_x_at | 9.51 | 4.13E-05 | 1.117244556 | NM_198457| | ZNF600,zinc finger protein 600 |
| 219305_x_at | 9.51 | 4.14E-05 | 1.117194714 | NM_012168| | FBXO2,F-box only protein 2 |
| 212208_at | 9.5 | 4.15E-05 | 1.068410057 | NM_015335| | THRAP2,thyroid hormone receptor associated protein 2 |
| 225591_at | 9.5 | 4.16E-05 | 1.090933398 | NM_012173| | FBXO25,F-box only protein 25 isoform 3 |
| 219639_x_at | 9.5 | 4.17E-05 | 1.054795452 | NM_020214| | PARP6,poly (ADP-ribose) polymerase family, member 6 |
| 212849_at | 9.5 | 4.17E-05 | 1.080077396 | NM_003502| | AXIN1,axin 1 isoform a |
| 202889_x_at | 9.49 | 4.19E-05 | 1.109871273 | NM_003980| | MAP7,microtubule-associated protein 7 |
| 218099_at | 9.49 | 4.20E-05 | 1.062004929 | NM_018469| | HT008,uncharacterized hypothalamus protein HT008 |
| 238449_at | 9.49 | 4.19E-05 | 1.105758786 | NA |  |
| 209669_s_at | 9.49 | 4.19E-05 | 1.021589707 | NM_001018067| | NA |
| 202545_at | 9.48 | 4.22E-05 | 1.106550712 | NM_006254| | PRKCD,protein kinase C, delta |
| 217242_at | 9.48 | 4.24E-05 | 1.263508879 | NM_001085384| | NA |
| 1555226_s_at | 9.48 | 4.23E-05 | 1.080232575 | NM_001098616| | NA |
| 219329_s_at | 9.48 | 4.22E-05 | 1.027999833 | NM_016085| | C2orf28,apoptosis related protein 3 isoform a |
| 218143_s_at | 9.48 | 4.22E-05 | 1.045572234 | NM_005697| | SCAMP2,secretory carrier membrane protein 2 |
| 212468_at | 9.48 | 4.23E-05 | 1.074755734 | NM_003971| | SPAG9,sperm associated antigen 9 isoform 1 |
| 225743_at | 9.46 | 4.28E-05 | 1.115415826 | NM_173659| | RPUSD3,RNA pseudouridylate synthase domain containing |
| 204622_x_at | 9.46 | 4.28E-05 | 1.321383115 | NM_006186| | NR4A2,nuclear receptor subfamily 4, group A, member 2 |
| 1554322_a_at | 9.46 | 4.29E-05 | 1.195359076 | NM_006037| | HDAC4,histone deacetylase 4 |
| 33814_at | 9.45 | 4.32E-05 | 1.041563753 | NM_001014831| | NA |
| 213010_at | 9.45 | 4.32E-05 | 1.452961418 | NM_145040| | PRKCDBP,protein kinase C, delta binding protein |
| 218325_s_at | 9.45 | 4.33E-05 | 1.115470676 | NM_022105| | DATF1,death associated transcription factor 1 isoform |
| 1557385_at | 9.44 | 4.37E-05 | 1.038860288 | NM_032180| | NA |
| 212543_at | 9.44 | 4.37E-05 | 1.159863429 | NM_001624| | NA |
| 213632_at | 9.44 | 4.37E-05 | 1.076499869 | NM_001361| | DHODH,dihydroorotate dehydrogenase precursor |
| 231142_at | 9.44 | 4.36E-05 | 1.115342374 | NA |  |
| 221664_s_at | 9.44 | 4.35E-05 | 1.080959201 | NM_016946| | F11R,F11 receptor isoform a precursor |
| 217925_s_at | 9.42 | 4.43E-05 | 1.041243237 | NM_022758| | C6orf106,chromosome 6 open reading frame 106 isoform b |
| 207831_x_at | 9.41 | 4.46E-05 | 1.058391886 | NM_001930| | DHPS,deoxyhypusine synthase isoform a |
| 219765_at | 9.41 | 4.49E-05 | 1.103283509 | NM_024620| | ZNF329,zinc finger protein 329 |
| 226202_at | 9.4 | 4.51E-05 | 1.101473718 | NM_020781| | ZNF398,zinc finger 398 isoform b |
| 205641_s_at | 9.4 | 4.51E-05 | 1.06729635 | NM_003789| | TRADD,TNFRSF1A-associated via death domain isoform 1 |
| 211951_at | 9.4 | 4.50E-05 | 1.027245187 | NM_004741| | NOLC1,nucleolar and coiled-body phosphoprotein 1 |
| 204070_at | 9.39 | 4.55E-05 | 1.241617809 | NM_004585| | RARRES3,retinoic acid receptor responder (tazarotene |
| 1557167_at | 9.39 | 4.53E-05 | 1.230153133 | NA |  |
| 207829_s_at | 9.38 | 4.56E-05 | 1.102381254 | NM_001205| | BNIP1,BCL2/adenovirus E1B 19kD interacting protein 1 |
| 223373_s_at | 9.38 | 4.58E-05 | 1.066998584 | NM_030821| | PLA2G12A,phospholipase A2, group XIIA |
| 202096_s_at | 9.37 | 4.61E-05 | 1.071758341 | NM_000714| | BZRP,peripheral benzodiazapine receptor isoform PBR |
| 235301_at | 9.37 | 4.59E-05 | 1.101772942 | NM_152748| | FLJ31340,hypothetical protein FLJ31340 |
| 1554677_s_at | 9.37 | 4.61E-05 | 1.127404312 | NM_178818| | CKLFSF4,chemokine-like factor superfamily 4 isoform 1 |
| 209787_s_at | 9.37 | 4.59E-05 | 1.0479495 | NM_006353| | HMGN4,high mobility group nucleosomal binding domain |
| 226613_at | 9.36 | 4.66E-05 | 1.149972311 | NM_001037666| | NA |
| 201892_s_at | 9.36 | 4.65E-05 | 1.036410432 | NM_000884| | IMPDH2,IMP (inosine monophosphate) dehydrogenase 2 |
| 211956_s_at | 9.36 | 4.65E-05 | 1.010555165 | NM_005801| | SUI1,putative translation initiation factor |
| 238477_at | 9.35 | 4.68E-05 | 1.055028533 | NA |  |
| 203831_at | 9.35 | 4.66E-05 | 1.091224909 | NM_014925| | KIAA1002,KIAA1002 protein |
| 214755_at | 9.35 | 4.66E-05 | 1.263389166 | NM_207309| | UAP1L1,UDP-N-acteylglucosamine pyrophosphorylase 1-like |
| 204286_s_at | 9.35 | 4.68E-05 | 1.094636002 | NM_021127| | PMAIP1,phorbol-12-myristate-13-acetate-induced protein |
| 1555775_a_at | 9.35 | 4.68E-05 | 1.14034439 | NM_175619| | ZAR1,zygote arrest 1 |
| 34846_at | 9.34 | 4.71E-05 | 1.055382132 | NM_001220| | CAMK2B,calcium/calmodulin-dependent protein kinase IIB |
| 202575_at | 9.34 | 4.72E-05 | 1.121517216 | NM_001878| | CRABP2,cellular retinoic acid binding protein 2 |
| 208678_at | 9.33 | 4.75E-05 | 1.040855694 | NM_001039366| | NA |
| 212714_at | 9.33 | 4.76E-05 | 1.043179616 | NM_052879| | LOC113251,c-Mpl binding protein isoform a |
| 210028_s_at | 9.33 | 4.75E-05 | 1.023385272 | NM_012381| | ORC3L,origin recognition complex, subunit 3 isoform 2 |
| 231829_at | 9.32 | 4.79E-05 | 1.109305386 | NM_020746| | KIAA1271,KIAA1271 protein |
| 212231_at | 9.32 | 4.80E-05 | 1.041529395 | NM_015002| | FBXO21,F-box only protein 21 isoform 2 |
| 227569_at | 9.31 | 4.84E-05 | 1.038284111 | NM_153371| | LNX2,PDZ domain containing ring finger 1 |
| 212770_at | 9.31 | 4.82E-05 | 1.063522895 | NM_001105192| | NA |
| 235484_at | 9.3 | 4.87E-05 | 1.240208242 | NM_001099666| | NA |
| 223110_at | 9.29 | 4.91E-05 | 1.048654646 | NM_015496| | DKFZP434I116,DKFZP434I116 protein isoform 1 |
| 209212_s_at | 9.29 | 4.89E-05 | 1.240637408 | NM_001730| | KLF5,Kruppel-like factor 5 |
| 214323_s_at | 9.29 | 4.90E-05 | 1.038174458 | NM_023011| | UPF3A,UPF3 regulator of nonsense transcripts homolog A |
| 202116_at | 9.29 | 4.88E-05 | 1.047323529 | NM_006268| | DPF2,D4, zinc and double PHD fingers family 2 |
| 209794_at | 9.29 | 4.88E-05 | 1.072949291 | NM_001033117| | NA |
| 208937_s_at | 9.28 | 4.93E-05 | 1.210486427 | NM_002165| | ID1,inhibitor of DNA binding 1 isoform a |
| 211474_s_at | 9.28 | 4.93E-05 | 1.053460872 | NM_004568| | SERPINB6,serine (or cysteine) proteinase inhibitor, clade |
| 219298_at | 9.28 | 4.92E-05 | 1.065695808 | NM_024693| | ECHDC3,enoyl Coenzyme A hydratase domain containing 3 |
| 1557961_s_at | 9.27 | 4.96E-05 | 1.109788796 | NA |  |
| 236314_at | 9.26 | 5.00E-05 | 1.173996981 | NA |  |
| 218431_at | 9.26 | 4.98E-05 | 1.045766322 | NM_022067| | C14orf133,chromosome 14 open reading frame 133 |
| 222852_at | 9.25 | 5.04E-05 | 1.123282842 | NM_024942| | C10orf88,chromosome 10 open reading frame 88 |
| 208855_s_at | 9.24 | 5.06E-05 | 1.028176771 | NM_001032296| | NA |
| 201690_s_at | 9.24 | 5.06E-05 | 1.023230903 | NM_001025252| | NA |
| 209858_x_at | 9.23 | 5.13E-05 | 1.075682797 | NM_023075| | MPPE1,metallophosphoesterase 1 isoform a precursor |
| 236832_at | 9.23 | 5.14E-05 | 1.264767554 | NA |  |
| 50277_at | 9.22 | 5.14E-05 | 1.036924116 | NM_001001560| | GGA1,golgi associated, gamma adaptin ear containing, |
| 227709_at | 9.22 | 5.15E-05 | 1.196062862 | NA |  |
| 203380_x_at | 9.22 | 5.17E-05 | 1.036865851 | NM_001039465| | NA |
| 209522_s_at | 9.22 | 5.16E-05 | 1.067399351 | NM_000755| | CRAT,carnitine acetyltransferase isoform 1 precursor |
| 226750_at | 9.21 | 5.20E-05 | 1.102243673 | NM_018078| | FLJ10378,FLJ10378 protein isoform 1 |
| 210142_x_at | 9.21 | 5.20E-05 | 1.042050843 | NM_005803| | FLOT1,flotillin 1 |
| 65635_at | 9.21 | 5.20E-05 | 1.06586865 | NM_001042573| | NA |
| 225272_at | 9.21 | 5.19E-05 | 1.042233809 | NM_133491| | SAT2,polyamine N-acetyltransferase |
| 209433_s_at | 9.21 | 5.18E-05 | 1.020823794 | NM_002703| | PPAT,phosphoribosyl pyrophosphate amidotransferase |
| 226345_at | 9.21 | 5.20E-05 | 1.035213045 | NA |  |
| 216241_s_at | 9.2 | 5.21E-05 | 1.089342729 | NM_006756| | TCEA1,transcription elongation factor A 1 isoform 1 |
| 228295_at | 9.2 | 5.23E-05 | 1.233549328 | NM_030581| | FLJ12270,FLJ12270 protein |
| 209352_s_at | 9.2 | 5.22E-05 | 1.090899968 | NM_015260| | SIN3B,SIN3 homolog B, transcription regulator |
| 205546_s_at | 9.2 | 5.24E-05 | 1.089248355 | NM_003331| | TYK2,tyrosine kinase 2 |
| 213338_at | 9.2 | 5.24E-05 | 1.120170947 | NM_015444| | RIS1,Ras-induced senescence 1 |
| 229790_at | 9.2 | 5.21E-05 | 1.057557204 | NM_005652| | TERF2,telomeric repeat binding factor 2 |
| 1555238_at | 9.2 | 5.21E-05 | 1.084473005 | NM_178449| | TIP39,tuberoinfundibular 39 residue protein precursor |
| 1560271_at | 9.19 | 5.28E-05 | 1.134438444 | NA |  |
| 220751_s_at | 9.19 | 5.25E-05 | 1.118528568 | NM_016348| | C5orf4,chromosome 5 open reading frame 4 |
| 229672_at | 9.19 | 5.27E-05 | 1.207105339 | NM_018244| | C20orf44,basic FGF-repressed Zic binding protein isoform |
| 227093_at | 9.19 | 5.29E-05 | 1.033787789 | NM_025090| | USP36,ubiquitin specific protease 36 |
| 201959_s_at | 9.19 | 5.27E-05 | 1.066324509 | NM_015057| | MYCBP2,MYC binding protein 2 |
| 219562_at | 9.18 | 5.31E-05 | 1.11042439 | NM_014353| | RAB26,RAB26, member RAS oncogene family |
| 217562_at | 9.18 | 5.32E-05 | 1.438879147 | NM_199051| | DBCCR1L,DBCCR1-like |
| 200993_at | 9.18 | 5.32E-05 | 1.033603362 | NM_006391| | IPO7,importin 7 |
| 208037_s_at | 9.18 | 5.29E-05 | 1.128618459 | NM_130760| | MADCAM1,mucosal vascular addressin cell adhesion |
| 1553155_x_at | 9.18 | 5.32E-05 | 1.239880633 | NM_152565| | ATP6V0D2,ATPase, H+ transporting, lysosomal 38kDa, V0 |
| 225986_x_at | 9.18 | 5.32E-05 | 1.083726573 | NM_017437| | CPSF2,cleavage and polyadenylation specific factor 2 |
| 206764_x_at | 9.18 | 5.29E-05 | 1.073161549 | NM_023075| | MPPE1,metallophosphoesterase 1 isoform a precursor |
| 203386_at | 9.17 | 5.32E-05 | 1.075659252 | NM_014832| | TBC1D4,TBC1 domain family, member 4 |
| 226956_at | 9.17 | 5.34E-05 | 1.095434514 | NM_021090| | MTMR3,myotubularin-related protein 3 isoform c |
| 203642_s_at | 9.17 | 5.33E-05 | 1.144306956 | NM_014900| | COBLL1,COBL-like 1 |
| 1556937_at | 9.17 | 5.35E-05 | 1.09114332 | NA |  |
| 1564360_a_at | 9.16 | 5.39E-05 | 1.498483229 | NA |  |
| 209984_at | 9.16 | 5.40E-05 | 1.053659241 | NM_015061| | JMJD2C,jumonji domain containing 2C |
| 200053_at | 9.16 | 5.37E-05 | 1.041921278 | NM_004890| | SPAG7,sperm associated antigen 7 |
| 201911_s_at | 9.15 | 5.44E-05 | 1.05163951 | NM_001001715| | FARP1,FERM, RhoGEF, and pleckstrin domain protein 1 |
| 221768_at | 9.13 | 5.49E-05 | 1.124330616 | NM_005066| | SFPQ,splicing factor proline/glutamine rich |
| 222212_s_at | 9.13 | 5.50E-05 | 1.024464762 | NM_022075| | LASS2,LAG1 longevity assurance homolog 2 isoform 1 |
| 204992_s_at | 9.13 | 5.50E-05 | 1.02825899 | NM_002628| | PFN2,profilin 2 isoform b |
| 225069_at | 9.13 | 5.49E-05 | 1.05318746 | NM_005017| | PCYT1A,phosphate cytidylyltransferase 1, choline, alpha |
| 217945_at | 9.13 | 5.49E-05 | 1.03416895 | NM_001011885| | BTBD1,BTB (POZ) domain containing 1 isoform 2 |
| 212599_at | 9.12 | 5.54E-05 | 1.076680209 | NM_001127231| | NA |
| 202579_x_at | 9.12 | 5.54E-05 | 1.069931679 | NM_006353| | HMGN4,high mobility group nucleosomal binding domain |
| 206656_s_at | 9.12 | 5.57E-05 | 1.023919948 | NM_020531| | C20orf3,chromosome 20 open reading frame 3 |
| 204446_s_at | 9.12 | 5.55E-05 | 1.27867471 | NM_000698| | ALOX5,arachidonate 5-lipoxygenase |
| 216222_s_at | 9.11 | 5.59E-05 | 1.064098422 | NM_012334| | MYO10,myosin X |
| 230951_at | 9.11 | 5.59E-05 | 1.191293147 | NA |  |
| 235069_at | 9.11 | 5.58E-05 | 1.113798712 | NM_001042552| | NA |
| 217216_x_at | 9.11 | 5.57E-05 | 1.076737276 | NM_001040108| | NA |
| 206959_s_at | 9.1 | 5.64E-05 | 1.093594385 | NM_023011| | UPF3A,UPF3 regulator of nonsense transcripts homolog A |
| 1557987_at | 9.1 | 5.63E-05 | 1.083677485 | NA |  |
| 241973_x_at | 9.1 | 5.64E-05 | 1.154349121 | NM_013379| | DPP7,dipeptidyl peptidase 7 preproprotein |
| 204483_at | 9.09 | 5.68E-05 | 1.04189469 | NM_001976| | ENO3,enolase 3 |
| 1553570_x_at | 9.09 | 5.68E-05 | 1.009393451 | NM_022340| | ZFYVE20,FYVE-finger-containing Rab5 effector protein |
| 233642_s_at | 9.08 | 5.72E-05 | 1.051746661 | NM_019024| | KIAA1414,KIAA1414 protein |
| 231292_at | 9.08 | 5.74E-05 | 1.508619722 | NM_001008394| | LOC493861,similar to hypothetical protein 1700027M21Rik |
| 221802_s_at | 9.07 | 5.75E-05 | 1.043536667 | NM_001127211| | NA |
| 222884_at | 9.07 | 5.78E-05 | 1.056099293 | NM_012279| | ZNF346,zinc finger protein 346 |
| 210198_s_at | 9.07 | 5.78E-05 | 1.171712306 | NM_000533| | PLP1,proteolipid protein 1 isoform 1 |
| 203216_s_at | 9.07 | 5.78E-05 | 1.062179294 | NM_004999| | MYO6,myosin VI |
| 224893_at | 9.07 | 5.75E-05 | 1.06163574 | NM_015459| | DKFZP564J0863,DKFZP564J0863 protein |
| 226938_at | 9.06 | 5.84E-05 | 1.112108149 | NM_015604| | WDR21,WD repeat domain 21 isoform 1 |
| 231550_at | 9.06 | 5.82E-05 | 1.116756961 | NA |  |
| 214448_x_at | 9.05 | 5.87E-05 | 1.058912925 | NM_001001716| | NFKBIB,nuclear factor of kappa light polypeptide gene |
| 207188_at | 9.05 | 5.86E-05 | 1.134286291 | NM_001258| | CDK3,cyclin-dependent kinase 3 |
| 202513_s_at | 9.04 | 5.89E-05 | 1.039483453 | NM_006245| | PPP2R5D,delta isoform of regulatory subunit B56, protein |
| 1553743_at | 9.04 | 5.92E-05 | 1.180808652 | NM_145280| | LOC151194,hepatocellular carcinoma-associated antigen |
| 217731_s_at | 9.03 | 5.97E-05 | 1.066794258 | NM_021999| | ITM2B,integral membrane protein 2B |
| 214936_at | 9.03 | 5.98E-05 | 1.276849382 | NM_015116| | LRCH1,leucine-rich repeats and calponin homology (CH) |
| 218767_at | 9.03 | 5.98E-05 | 1.032574055 | NM_020385| | XPMC2H,XPMC2 prevents mitotic catastrophe 2 homolog |
| 216248_s_at | 9.03 | 5.98E-05 | 1.405176994 | NM_006186| | NR4A2,nuclear receptor subfamily 4, group A, member 2 |
| 200860_s_at | 9.02 | 6.02E-05 | 1.030163002 | NM_016284| | CNOT1,CCR4-NOT transcription complex, subunit 1 |
| 206136_at | 9.02 | 6.01E-05 | 1.132891514 | NM_003468| | FZD5,frizzled 5 |
| 227200_at | 9.02 | 5.99E-05 | 1.034322759 | NA |  |
| 212420_at | 9.02 | 6.01E-05 | 1.113849581 | NM_172373| | ELF1,E74-like factor 1 (ets domain transcription |
| 222821_s_at | 9.01 | 6.05E-05 | 1.038297473 | NM_001007269| | GEMIN7,gemin 7 |
| 223628_at | 9.01 | 6.03E-05 | 1.083419054 | NA |  |
| 222235_s_at | 9.01 | 6.07E-05 | 1.11869447 | NM_018590| | GALNACT-2,chondroitin beta1,4 |
| 228291_s_at | 9 | 6.09E-05 | 1.055865055 | NM_018474| | C20orf19,uncharacterized hypothalamus protein HT013 |
| 221882_s_at | 8.99 | 6.14E-05 | 1.081254345 | NM_021259| | TMEM8,transmembrane protein 8 (five membrane-spanning |
| 1555811_at | 8.98 | 6.21E-05 | 1.063526689 | NM_001175| | ARHGDIB,Rho GDP dissociation inhibitor (GDI) beta |
| 201258_at | 8.98 | 6.22E-05 | 1.020292282 | NM_001020| | RPS16,ribosomal protein S16 |
| 202529_at | 8.98 | 6.21E-05 | 1.03689017 | NM_002766| | PRPSAP1,phosphoribosyl pyrophosphate |
| 223167_s_at | 8.98 | 6.21E-05 | 1.085597663 | NM_013396| | USP25,ubiquitin specific protease 25 |
| 204233_s_at | 8.97 | 6.23E-05 | 1.094114217 | NM_001277| | CHKA,choline kinase alpha isoform a |
| 1556051_a_at | 8.97 | 6.24E-05 | 1.121655344 | NM_001003398| | BICD1,bicaudal D homolog 1 isoform 2 |
| 208093_s_at | 8.97 | 6.23E-05 | 1.03709806 | NM_001025579| | NA |
| 229325_at | 8.97 | 6.25E-05 | 1.04902242 | NM_015534| | ZZZ3,zinc finger, ZZ domain containing 3 |
| 224833_at | 8.96 | 6.32E-05 | 1.087171683 | NM_005238| | ETS1,v-ets erythroblastosis virus E26 oncogene |
| 204977_at | 8.96 | 6.32E-05 | 1.043857895 | NM_004398| | DDX10,DEAD (Asp-Glu-Ala-Asp) box polypeptide 10 |
| 203256_at | 8.95 | 6.36E-05 | 1.053760067 | NM_001793| | CDH3,cadherin 3, type 1 preproprotein |
| 229317_at | 8.95 | 6.35E-05 | 1.098590557 | NM_002269| | KPNA5,karyopherin alpha 5 (importin alpha 6) |
| 40472_at | 8.94 | 6.41E-05 | 1.073001083 | NM_153613| | LOC254531,PLSC domain containing protein |
| 238476_at | 8.94 | 6.42E-05 | 1.232383225 | NM_153607| | LOC153222,adult retina protein |
| 37012_at | 8.94 | 6.38E-05 | 1.031020162 | NM_004930| | CAPZB,F-actin capping protein beta subunit |
| 229438_at | 8.94 | 6.38E-05 | 1.157780505 | NA |  |
| 232077_s_at | 8.94 | 6.40E-05 | 1.124689141 | NM_031477| | YPEL3,yippee-like 3 |
| 208911_s_at | 8.94 | 6.42E-05 | 1.071939916 | NM_000925| | PDHB,pyruvate dehydrogenase (lipoamide) beta |
| 206958_s_at | 8.93 | 6.44E-05 | 1.060051402 | NM_023011| | UPF3A,UPF3 regulator of nonsense transcripts homolog A |
| 239754_at | 8.93 | 6.43E-05 | 1.161971415 | NM_152350| | MGC40157,hypothetical protein MGC40157 |
| 202272_s_at | 8.93 | 6.44E-05 | 1.031658205 | NM_015176| | FBXO28,F-box protein 28 |
| 201781_s_at | 8.93 | 6.44E-05 | 1.075207044 | NM_003977| | AIP,aryl hydrocarbon receptor interacting protein |
| 225269_s_at | 8.93 | 6.45E-05 | 1.024478609 | NA |  |
| 225516_at | 8.93 | 6.44E-05 | 1.072161653 | NM_001008539| | SLC7A2,solute carrier family 7, member 2 isoform 1 |
| 220184_at | 8.93 | 6.44E-05 | 1.056436716 | NM_024865| | NANOG,Nanog homeobox |
| 213715_s_at | 8.92 | 6.49E-05 | 1.239783117 | NM_198471| | FLJ46061,FLJ46061 protein |
| 203140_at | 8.92 | 6.49E-05 | 1.099527538 | NM_001706| | BCL6,B-cell lymphoma 6 protein |
| 207222_at | 8.92 | 6.47E-05 | 1.183634788 | NM_003561| | PLA2G10,phospholipase A2, group X |
| 1554112_a_at | 8.92 | 6.50E-05 | 1.241047011 | NM_014683| | ULK2,unc-51-like kinase 2 |
| 228627_at | 8.91 | 6.54E-05 | 1.134454495 | NM_001112707| | NA |
| 1552897_a_at | 8.91 | 6.54E-05 | 1.193977567 | NM_133329| | KCNG3,potassium voltage-gated channel, subfamily G, |
| 205100_at | 8.91 | 6.52E-05 | 1.063089724 | NM_005110| | GFPT2,glutamine-fructose-6-phosphate transaminase 2 |
| 230856_at | 8.91 | 6.55E-05 | 1.202769541 | NA |  |
| 231789_at | 8.9 | 6.57E-05 | 1.595621391 | NM_018935| | PCDHB15,protocadherin beta 15 precursor |
| 229845_at | 8.9 | 6.56E-05 | 1.105545062 | NM_001006617| | MAPKAP1,mitogen-activated protein kinase associated |
| 231022_at | 8.9 | 6.61E-05 | 1.053702406 | NA |  |
| 232708_at | 8.89 | 6.63E-05 | 1.11392016 | NM_000155| | GALT,galactose-1-phosphate uridylyltransferase |
| 224280_s_at | 8.89 | 6.62E-05 | 1.05969528 | NM_001099625| | NA |
| 32259_at | 8.88 | 6.68E-05 | 1.085005799 | NM_001991| | EZH1,enhancer of zeste homolog 1 |
| 237741_at | 8.88 | 6.69E-05 | 1.198401839 | NM_001104647| | NA |
| 1555078_at | 8.88 | 6.66E-05 | 1.095699799 | NA |  |
| 1553096_s_at | 8.88 | 6.69E-05 | 1.070070018 | NM_006538| | BCL2L11,BCL2-like 11 isoform 6 |
| 225636_at | 8.87 | 6.72E-05 | 1.05314148 | NM_005419| | STAT2,signal transducer and activator of transcription |
| 219597_s_at | 8.87 | 6.71E-05 | 1.19271909 | NM_017434| | DUOX1,dual oxidase 1 precursor |
| 239324_at | 8.87 | 6.74E-05 | 1.113761285 | NA |  |
| 218857_s_at | 8.87 | 6.73E-05 | 1.051231702 | NM_001083926| | NA |
| 225579_at | 8.87 | 6.74E-05 | 1.060317019 | NM_152391| | C2orf22,hypothetical protein MGC33602 |
| 218088_s_at | 8.87 | 6.71E-05 | 1.050429658 | NM_022157| | RRAGC,Ras-related GTP binding C |
| 215501_s_at | 8.87 | 6.74E-05 | 1.191927242 | NM_007207| | DUSP10,dual specificity phosphatase 10 isoform a |
| 226489_at | 8.86 | 6.79E-05 | 1.125868905 | NM_020698| | TMCC3,transmembrane and coiled-coil domains 3 |
| 227219_x_at | 8.86 | 6.80E-05 | 1.073152215 | NM_032514| | MAP1LC3A,microtubule-associated protein 1 light chain 3 |
| 218991_at | 8.86 | 6.80E-05 | 1.070209582 | NM_022070| | ABC1,amplified in breast cancer 1 |
| 221820_s_at | 8.86 | 6.80E-05 | 1.047718184 | NM_032188| | MYST1,MYST histone acetyltransferase 1 |
| 203650_at | 8.86 | 6.80E-05 | 1.083189704 | NM_006404| | PROCR,endothelial protein C receptor precursor |
| 203445_s_at | 8.85 | 6.85E-05 | 1.043405329 | NM_005730| | CTDSP2,nuclear LIM interactor-interacting factor 2 |
| 1560099_at | 8.84 | 6.87E-05 | 1.615493819 | NA |  |
| 220197_at | 8.84 | 6.89E-05 | 1.2949068 | NM_020632| | ATP6V0A4,ATPase, H+ transporting, lysosomal V0 subunit a |
| 234734_s_at | 8.84 | 6.87E-05 | 1.026042787 | NM_014494| | TNRC6A,trinucleotide repeat containing 6A |
| 219664_s_at | 8.83 | 6.95E-05 | 1.045078245 | NM_020664| | DECR2,2,4-dienoyl CoA reductase 2, peroxisomal |
| 212683_at | 8.83 | 6.92E-05 | 1.056105234 | NM_014655| | KIAA0446,KIAA0446 gene product |
| 224481_s_at | 8.83 | 6.93E-05 | 1.034854882 | NM_015382| | HECTD1,HECT domain containing 1 |
| 202929_s_at | 8.82 | 6.98E-05 | 1.048290045 | NM_001084392| | NA |
| 226662_at | 8.82 | 6.99E-05 | 1.057122991 | NM_017919| | STX17,syntaxin 17 |
| 243439_at | 8.82 | 6.97E-05 | 1.070854672 | NM_133460| | ZNF418,zinc finger protein 418 |
| 213681_at | 8.82 | 6.97E-05 | 1.075063484 | NM_032687| | CYHR1,cysteine and histidine rich 1 |
| 217150_s_at | 8.82 | 7.01E-05 | 1.088978159 | NM_000268| | NF2,neurofibromin 2 isoform 1 |
| 202337_at | 8.81 | 7.03E-05 | 1.059851631 | NM_007221| | PMF1,polyamine-modulated factor 1 |
| 206567_s_at | 8.8 | 7.08E-05 | 1.032160643 | NM_016436| | PHF20,PHD finger protein 20 |
| 212026_s_at | 8.8 | 7.14E-05 | 1.100238759 | NM_001013839| | NA |
| 224616_at | 8.8 | 7.08E-05 | 1.048026076 | NM_006141| | DNCLI2,dynein, cytoplasmic, light intermediate |
| 204229_at | 8.79 | 7.14E-05 | 1.137333401 | NM_020309| | SLC17A7,solute carrier family 17, member 7 |
| 221992_at | 8.79 | 7.15E-05 | 1.166551086 | NM_001018059| | NA |
| 212760_at | 8.79 | 7.16E-05 | 1.053818591 | NM_015255| | UBR2,ubiquitin protein ligase E3 component n-recognin |
| 214102_at | 8.79 | 7.15E-05 | 1.092613049 | NM_015230| | CENTD1,centaurin delta 1 isoform a |
| 222427_s_at | 8.79 | 7.19E-05 | 1.040974005 | NM_020117| | LARS,leucyl-tRNA synthetase |
| 212112_s_at | 8.78 | 7.22E-05 | 1.092544711 | NM_177424| | STX12,syntaxin 12 |
| 217736_s_at | 8.77 | 7.30E-05 | 1.029774629 | NM_014413| | EIF2AK1,heme-regulated initiation factor 2-alpha kinase |
| 211065_x_at | 8.77 | 7.30E-05 | 1.038646181 | NM_001002021| | PFKL,liver phosphofructokinase isoform a |
| 219922_s_at | 8.77 | 7.29E-05 | 1.075089934 | NM_021070| | LTBP3,latent transforming growth factor beta binding |
| 240221_at | 8.76 | 7.34E-05 | 1.135691648 | NM_001025105| | NA |
| 229694_at | 8.76 | 7.36E-05 | 1.216799975 | NM_018117| | WDR11,WD repeat domain 11 |
| 1560622_at | 8.76 | 7.34E-05 | 1.10432872 | NA |  |
| 226505_x_at | 8.75 | 7.40E-05 | 1.045728054 | NM_032582| | USP32,ubiquitin specific protease 32 |
| 213607_x_at | 8.75 | 7.40E-05 | 1.095543371 | NM_023018| | FLJ13052,NAD kinase |
| 214194_at | 8.75 | 7.41E-05 | 1.11267377 | NM_014953| | KIAA1008,KIAA1008 |
| 1554555_a_at | 8.75 | 7.37E-05 | 1.143518343 | NM_024860| | FLJ21148,hypothetical protein FLJ21148 |
| 213375_s_at | 8.74 | 7.45E-05 | 1.295481972 | NM_001079691| | NA |
| 204445_s_at | 8.74 | 7.45E-05 | 1.156881241 | NM_000698| | ALOX5,arachidonate 5-lipoxygenase |
| 226866_at | 8.73 | 7.54E-05 | 1.029988077 | NM_052911| | ESCO1,establishment of cohesion 1 homolog 1 |
| 216232_s_at | 8.73 | 7.52E-05 | 1.060926681 | NM_006836| | GCN1L1,GCN1 general control of amino-acid synthesis |
| 205017_s_at | 8.73 | 7.51E-05 | 1.334012626 | NM_144778| | MBNL2,muscleblind-like 2 isoform 1 |
| 1558249_s_at | 8.72 | 7.58E-05 | 1.205732816 | NM_001001433| | STX16,syntaxin 16 isoform a |
| 227040_at | 8.72 | 7.59E-05 | 1.047425982 | NM_001012754| | NA |
| 202194_at | 8.72 | 7.55E-05 | 1.035193819 | NM_016040| | TMED5,transmembrane emp24 protein transport domain |
| 212350_at | 8.72 | 7.57E-05 | 1.044875055 | NM_015173| | TBC1D1,TBC1 (tre-2/USP6, BUB2, cdc16) domain family, |
| 213842_x_at | 8.72 | 7.60E-05 | 1.038711138 | NM_001039487| | NA |
| 208094_s_at | 8.71 | 7.63E-05 | 1.062688117 | NM_030818| | MGC10471,hypothetical protein MGC10471 |
| 222842_at | 8.71 | 7.60E-05 | 1.178753992 | NM_017629| | EIF2C4,eukaryotic translation initiation factor 2C, 4 |
| 205238_at | 8.71 | 7.61E-05 | 1.214393846 | NM_024917| | CXorf34,chromosome X open reading frame 34 |
| 59631_at | 8.71 | 7.61E-05 | 1.12383732 | NA |  |
| 200622_x_at | 8.71 | 7.60E-05 | 1.040271265 | NM_001743| | CALM2,calmodulin 2 |
| 230430_at | 8.71 | 7.60E-05 | 1.146737069 | NM_001246| | ENTPD2,ectonucleoside triphosphate diphosphohydrolase 2 |
| 229980_s_at | 8.71 | 7.65E-05 | 1.023214196 | NM_014426| | SNX5,sorting nexin 5 |
| 214846_s_at | 8.7 | 7.69E-05 | 1.309195685 | NM_020778| | ALPK3,alpha-kinase 3 |
| 202021_x_at | 8.7 | 7.66E-05 | 1.024944949 | NM_005801| | SUI1,putative translation initiation factor |
| 38964_r_at | 8.7 | 7.68E-05 | 1.063923162 | NM_000377| | WAS,Wiskott-Aldrich syndrome protein |
| 227613_at | 8.69 | 7.74E-05 | 1.122240842 | NM_001079906| | NA |
| 206053_at | 8.68 | 7.82E-05 | 1.098349723 | NM_014930| | ZNF510,zinc finger protein 510 |
| 202802_at | 8.68 | 7.80E-05 | 1.061569924 | NM_001930| | DHPS,deoxyhypusine synthase isoform a |
| 235729_at | 8.68 | 7.82E-05 | 1.130736263 | NM_032788| | ZNF514,zinc finger protein 514 |
| 204578_at | 8.68 | 7.82E-05 | 1.052930646 | NM_001024463| | NA |
| 212164_at | 8.68 | 7.78E-05 | 1.128695281 | NM_138391| | C1orf37,chromosome 1 open reading frame 37 |
| 228566_at | 8.67 | 7.88E-05 | 1.067799435 | NM_018170| | P15RS,hypothetical protein FLJ10656 |
| 209558_s_at | 8.67 | 7.90E-05 | 1.090491276 | NM_003959| | HIP1R,huntingtin interacting protein-1-related |
| 226529_at | 8.67 | 7.90E-05 | 1.081181618 | NM_018374| | FLJ11273,hypothetical protein FLJ11273 |
| 203319_s_at | 8.67 | 7.90E-05 | 1.08570826 | NM_021964| | ZNF148,zinc finger protein 148 (pHZ-52) |
| 204679_at | 8.67 | 7.89E-05 | 1.064851262 | NM_002245| | KCNK1,potassium channel, subfamily K, member 1 |
| 229876_at | 8.67 | 7.89E-05 | 1.136693168 | NM_001122670| | NA |
| 205408_at | 8.67 | 7.85E-05 | 1.174332264 | NM_001009569| | MLLT10,myeloid/lymphoid or mixed-lineage leukemia |
| 224655_at | 8.66 | 7.95E-05 | 1.045659881 | NM_016282| | AK3L1,adenylate kinase 3 alpha like |
| 213727_x_at | 8.66 | 7.95E-05 | 1.06903286 | NM_023075| | MPPE1,metallophosphoesterase 1 isoform a precursor |
| 242073_at | 8.66 | 7.96E-05 | 1.126433711 | NA |  |
| 203503_s_at | 8.65 | 8.00E-05 | 1.063376438 | NM_004565| | PEX14,peroxisomal biogenesis factor 14 |
| 232291_at | 8.65 | 8.01E-05 | 1.209450483 | NA |  |
| 225576_at | 8.65 | 7.96E-05 | 1.03550467 | NM_138785| | C6orf72,chromosome 6 open reading frame 72 |
| 222921_s_at | 8.65 | 7.96E-05 | 1.124826772 | NM_012259| | HEY2,hairy/enhancer-of-split related with YRPW motif |
| 211320_s_at | 8.65 | 7.98E-05 | 1.08204237 | NM_005704| | PTPRU,protein tyrosine phosphatase, receptor type, U |
| 217789_at | 8.64 | 8.07E-05 | 1.05286924 | NM_021249| | SNX6,sorting nexin 6 isoform a |
| 203387_s_at | 8.64 | 8.08E-05 | 1.099994449 | NM_014832| | TBC1D4,TBC1 domain family, member 4 |
| 243673_at | 8.63 | 8.12E-05 | 1.288257228 | NA |  |
| 212539_at | 8.63 | 8.12E-05 | 1.031236478 | NM_004284| | CHD1L,chromodomain helicase DNA binding protein |
| 210544_s_at | 8.63 | 8.14E-05 | 1.143716338 | NM_000382| | ALDH3A2,aldehyde dehydrogenase 3A2 |
| 230313_at | 8.63 | 8.12E-05 | 1.106066102 | NA |  |
| 37226_at | 8.63 | 8.10E-05 | 1.105316282 | NM_001205| | BNIP1,BCL2/adenovirus E1B 19kD interacting protein 1 |
| 202564_x_at | 8.63 | 8.13E-05 | 1.031332988 | NM_001667| | ARL2,ADP-ribosylation factor-like 2 |
| 1556274_at | 8.63 | 8.12E-05 | 1.092541891 | NA |  |
| 203249_at | 8.62 | 8.18E-05 | 1.08930597 | NM_001991| | EZH1,enhancer of zeste homolog 1 |
| 212533_at | 8.62 | 8.21E-05 | 1.027335195 | NM_003390| | WEE1,wee1 tyrosine kinase |
| 90265_at | 8.61 | 8.26E-05 | 1.074087652 | NM_006869| | CENTA1,centaurin, alpha 1 |
| 235103_at | 8.61 | 8.22E-05 | 1.152022595 | NM_002372| | MAN2A1,mannosidase, alpha, class 2A, member 1 |
| 212933_x_at | 8.61 | 8.28E-05 | 1.011240355 | NM_000977| | RPL13,ribosomal protein L13 |
| 232207_at | 8.61 | 8.26E-05 | 1.22882113 | NA |  |
| 205480_s_at | 8.61 | 8.28E-05 | 1.030117731 | NM_001001521| | UGP2,UDP-glucose pyrophosphorylase 2 isoform b |
| 225846_at | 8.61 | 8.27E-05 | 1.038169148 | NM_001034915| | NA |
| 208946_s_at | 8.59 | 8.41E-05 | 1.046410113 | NM_003766| | BECN1,beclin 1 |
| 1569905_at | 8.59 | 8.36E-05 | 1.089311552 | NM_198533| | SCDR10,short-chain dehydrogenase/reductase 10 isoform |
| 228994_at | 8.59 | 8.41E-05 | 1.065420167 | NM_152499| | MGC45441,hypothetical protein MGC45441 |
| 1554445_at | 8.58 | 8.47E-05 | 1.190096027 | NM_003429| | ZNF85,zinc finger protein 85 (HPF4, HTF1) |
| 212263_at | 8.58 | 8.47E-05 | 1.019682906 | NM_006775| | QKI,quaking homolog, KH domain RNA binding isoform |
| 237145_at | 8.57 | 8.50E-05 | 1.199497646 | NM_001013703| | NA |
| 229417_at | 8.57 | 8.54E-05 | 1.087092639 | NA |  |
| 205022_s_at | 8.57 | 8.52E-05 | 1.052076494 | NM_001085471| | NA |
| 221590_s_at | 8.57 | 8.52E-05 | 1.107764717 | NA |  |
| 201039_s_at | 8.56 | 8.58E-05 | 1.07031351 | NM_005053| | RAD23A,UV excision repair protein RAD23 homolog A |
| 218488_at | 8.56 | 8.62E-05 | 1.056297817 | NM_020365| | EIF2B3,eukaryotic translation initiation factor 2B, |
| 219431_at | 8.56 | 8.57E-05 | 1.132784565 | NM_024605| | ARHGAP10,Rho GTPase activating protein 10 |
| 220631_at | 8.56 | 8.63E-05 | 1.09158584 | NM_022353| | OSGEPL1,O-sialoglycoprotein endopeptidase-like 1 |
| 239303_at | 8.55 | 8.69E-05 | 1.157098382 | NA |  |
| 235749_at | 8.55 | 8.69E-05 | 1.278241479 | NM_020121| | UGCGL2,UDP-glucose:glycoprotein glucosyltransferase 2 |
| 213996_at | 8.55 | 8.70E-05 | 1.064029629 | NM_013313| | YPEL1,yippee-like 1 |
| 235099_at | 8.55 | 8.69E-05 | 1.053095211 | NM_178868| | CKLFSF8,chemokine-like factor superfamily 8 |
| 228250_at | 8.54 | 8.75E-05 | 1.179551961 | NM_001008738| | KIAA1961,KIAA1961 protein isoform 2 |
| 238149_at | 8.54 | 8.74E-05 | 1.193566097 | NA |  |
| 214831_at | 8.54 | 8.75E-05 | 1.21542568 | NM_025205| | MED28,mediator of RNA polymerase II transcription, |
| 224573_at | 8.54 | 8.71E-05 | 1.033029514 | NM_001004333| | MGC71993,similar to DNA segment, Chr 11, Brigham & Womens |
| 213468_at | 8.54 | 8.74E-05 | 1.088846571 | NM_000400| | ERCC2,excision repair cross-complementing rodent |
| 1555561_a_at | 8.54 | 8.71E-05 | 1.303766132 | NM_020121| | UGCGL2,UDP-glucose:glycoprotein glucosyltransferase 2 |
| 240681_at | 8.54 | 8.74E-05 | 1.089231853 | NA |  |
| 209898_x_at | 8.53 | 8.78E-05 | 1.058577694 | NM_006277| | ITSN2,intersectin 2 isoform 1 |
| 201554_x_at | 8.53 | 8.83E-05 | 1.067918533 | NM_004130| | GYG,glycogenin |
| 225275_at | 8.53 | 8.82E-05 | 1.048009824 | NM_005711| | EDIL3,EGF-like repeats and discoidin I-like |
| 227708_at | 8.53 | 8.83E-05 | 1.048500203 | NM_001402| | EEF1A1,eukaryotic translation elongation factor 1 alpha |
| 206723_s_at | 8.53 | 8.78E-05 | 1.037584234 | NM_004720| | EDG4,endothelial differentiation, lysophosphatidic |
| 200005_at | 8.53 | 8.81E-05 | 1.034554758 | NM_003753| | EIF3S7,eukaryotic translation initiation factor 3 |
| 212139_at | 8.53 | 8.83E-05 | 1.035683899 | NM_006836| | GCN1L1,GCN1 general control of amino-acid synthesis |
| 218603_at | 8.52 | 8.88E-05 | 1.095482815 | NM_016217| | HECA,headcase homolog |
| 203467_at | 8.52 | 8.88E-05 | 1.063864827 | NM_002676| | PMM1,phosphomannomutase 1 |
| 227031_at | 8.51 | 8.97E-05 | 1.07032177 | NA |  |
| 218946_at | 8.51 | 8.93E-05 | 1.045645972 | NM_001002755| | HIRIP5,HIRA interacting protein 5 isoform 2 |
| 203155_at | 8.5 | 8.98E-05 | 1.052892408 | NM_012432| | SETDB1,SET domain, bifurcated 1 |
| 212401_s_at | 8.5 | 9.01E-05 | 1.02720418 | NM_024011| | CDC2L2,cell division cycle 2-like 2 isoform 1 |
| 229294_at | 8.49 | 9.13E-05 | 1.028562439 | NM_020655| | JPH3,junctophilin 3 |
| 224843_at | 8.49 | 9.11E-05 | 1.143557279 | NM_020846| | NA |
| 226267_at | 8.49 | 9.06E-05 | 1.137537747 | NM_130469| | JDP2,Jun dimerization protein |
| 204522_at | 8.49 | 9.11E-05 | 1.101960093 | NM_005510| | DOM3Z,DOM-3 homolog Z |
| 200758_s_at | 8.48 | 9.16E-05 | 1.030954183 | NM_003204| | NFE2L1,nuclear factor (erythroid-derived 2)-like 1 |
| 221173_at | 8.48 | 9.14E-05 | 1.105997604 | NM_005709| | USH1C,harmonin |
| 204842_x_at | 8.47 | 9.29E-05 | 1.027493633 | NM_004157| | PRKAR2A,cAMP-dependent protein kinase, regulatory |
| 201950_x_at | 8.47 | 9.23E-05 | 1.061988154 | NM_004930| | CAPZB,F-actin capping protein beta subunit |
| 229321_s_at | 8.47 | 9.25E-05 | 1.102145343 | NA |  |
| 207625_s_at | 8.47 | 9.29E-05 | 1.078076712 | NM_001032999| | NA |
| 222649_at | 8.47 | 9.21E-05 | 1.036571821 | NM_022459| | XPO4,exportin 4 |
| 224614_at | 8.47 | 9.29E-05 | 1.050466548 | NM_006141| | DNCLI2,dynein, cytoplasmic, light intermediate |
| 201827_at | 8.46 | 9.33E-05 | 1.041319203 | NM_001098426| | NA |
| 200912_s_at | 8.46 | 9.35E-05 | 1.030152802 | NM_001967| | EIF4A2,eukaryotic translation initiation factor 4A, |
| 235037_at | 8.46 | 9.35E-05 | 1.045604344 | NM_080652| | TMEM41A,transmembrane protein 41A |
| 227372_s_at | 8.45 | 9.40E-05 | 1.077431197 | NM_018842| | BAIAP2L1,BAI1-associated protein 2-like 1 |
| 211228_s_at | 8.44 | 9.51E-05 | 1.093001064 | NM_002873| | RAD17,RAD17 homolog isoform 1 |
| 219341_at | 8.44 | 9.52E-05 | 1.072055996 | NM_018941| | CLN8,CLN8 protein |
| 215982_s_at | 8.43 | 9.61E-05 | 1.111068402 | NM_005510| | DOM3Z,DOM-3 homolog Z |
| 217968_at | 8.43 | 9.56E-05 | 1.045496885 | NM_003310| | TSSC1,tumor suppressing subtransferable candidate 1 |
| 221904_at | 8.43 | 9.55E-05 | 1.05368087 | NM_144635| | MGC21688,hypothetical protein MGC21688 |
| 230394_at | 8.42 | 9.63E-05 | 1.15150566 | NM_144659| | TCP10L,T-complex 10A-2 |
| 231940_at | 8.42 | 9.63E-05 | 1.058224378 | NM_020951| | ZNF529,zinc finger protein 529 |
| 213902_at | 8.42 | 9.63E-05 | 1.040522075 | NM_004315| | ASAH1,N-acylsphingosine amidohydrolase (acid |
| 211600_at | 8.42 | 9.67E-05 | 1.062218902 | NM_002848| | PTPRO,receptor-type protein tyrosine phosphatase O |
| 202180_s_at | 8.42 | 9.63E-05 | 1.178684514 | NM_005115| | MVP,major vault protein |
| 212176_at | 8.41 | 9.75E-05 | 1.069667158 | NM_015491| | NA |
| 212009_s_at | 8.41 | 9.72E-05 | 1.142298187 | NM_006819| | STIP1,stress-induced-phosphoprotein 1 |
| 200710_at | 8.41 | 9.72E-05 | 1.075928223 | NM_000018| | ACADVL,acyl-Coenzyme A dehydrogenase, very long chain |
| 242087_x_at | 8.4 | 9.77E-05 | 1.121371794 | NA |  |
| 219036_at | 8.4 | 9.79E-05 | 1.033431272 | NM_024491| | Cep70,centrosomal protein 70 kDa |
| 1569871_at | 8.4 | 9.77E-05 | 1.085418598 | NA |  |
| 1553174_at | 8.39 | 9.85E-05 | 1.075835071 | NM_020433| | JPH2,junctophilin 2 isoform 1 |
| 233982_x_at | 8.39 | 9.85E-05 | 1.059341176 | NM_016086| | DUSP24,map kinase phosphatase-like protein MK-STYX |
| 213812_s_at | 8.39 | 9.85E-05 | 1.083136454 | NM_006549| | CAMKK2,calcium/calmodulin-dependent protein kinase |
| 216018_at | 8.39 | 9.84E-05 | 1.240429309 | NM_006913| | RNF5,ring finger protein 5 |
| 209265_s_at | 8.39 | 9.89E-05 | 1.042212861 | NM_019852| | METTL3,methyltransferase like 3 |
| 220147_s_at | 8.39 | 9.88E-05 | 1.029473067 | NM_021238| | C12orf14,chromosome 12 open reading frame 14 |
| 211240_x_at | 8.38 | 9.96E-05 | 1.030106625 | NM_001085458| | NA |
| 201383_s_at | 8.38 | 9.96E-05 | 1.122425504 | NM_005899| | NBR1,neighbor of BRCA1 gene 1 |
| 1553961_s_at | 8.38 | 9.94E-05 | 1.075459643 | NM_001042632| | NA |
