# Supplementary material for: Transcriptional Signature and Memory Retention of Human-Induced Pluripotent Stem Cells
Source: PLoS One. 2009 Sep 18;4(9):e7076. doi: 10.1371/journal.pone.0007076 (PMC2741600; doi:10.1371/journal.pone.0007076)
Supplement: Table S3 — IPSC-enriched probes in IPSC versus NSC. Probesets enriched in group-wise comparisons: Column headings are probeset identifiers, T-statistic, P-value, Fold-Change (log2), Refseq identifier and Description of the gene. (NA indicates no Refseq annotation). (8.67 MB DOC) [file pone.0007076.s007.doc]

| Probeset | T-statistic | P-value | Fold-Change (log2) | Refseq | Description |
| --- | --- | --- | --- | --- | --- |
| 227705_at | 198.81 | 5.70E-10 | 3.504480387 | NM_152278| | TCEAL7,transcription elongation factor A (SII)-like 7 |
| 230493_at | 188.45 | 5.70E-10 | 3.861798103 | NM_001007538| | LOC387914,WGAR9166 |
| 228894_at | 167.21 | 8.21E-10 | 2.144018145 | NM_001489| | NR6A1,nuclear receptor subfamily 6, group A, member 1 |
| 209822_s_at | 163.53 | 8.21E-10 | 1.844294356 | NM_001018056| | NA |
| 209757_s_at | 161.99 | 8.21E-10 | 1.582492655 | NM_005378| | MYCN,v-myc myelocytomatosis viral related oncogene, |
| 226507_at | 155.63 | 8.21E-10 | 1.512544731 | NM_002576| | PAK1,p21-activated kinase 1 |
| 227566_at | 154.84 | 8.21E-10 | 2.98153756 | NM_001048209| | NA |
| 206247_at | 146.3 | 9.92E-10 | 2.781857112 | NM_000247| | MICA,MHC class I chain-related gene A protein |
| 200701_at | 142.61 | 1.00E-09 | 1.335068761 | NM_006432| | NPC2,Niemann-Pick disease, type C2 precursor |
| 212589_at | 140.82 | 1.01E-09 | 1.308915621 | NM_001102669| | NA |
| 239552_at | 133.57 | 1.23E-09 | 2.152935792 | NA |  |
| 209488_s_at | 133.53 | 1.23E-09 | 1.690706504 | NM_001008710| | RBPMS,RNA-binding protein with multiple splicing |
| 230988_at | 131.14 | 1.25E-09 | 1.470580222 | NA |  |
| 219368_at | 129.19 | 1.25E-09 | 2.892984912 | NM_021963| | NAP1L2,nucleosome assembly protein 1-like 2 |
| 210643_at | 129.02 | 1.25E-09 | 2.843550293 | NM_003701| | TNFSF11,tumor necrosis factor ligand superfamily, member |
| 228565_at | 123.52 | 1.35E-09 | 1.500542272 | NM_032435| | KIAA1804,mixed lineage kinase 4 |
| 226497_s_at | 120.75 | 1.41E-09 | 2.602127132 | NA |  |
| 204679_at | 120.29 | 1.41E-09 | 1.848510005 | NM_002245| | KCNK1,potassium channel, subfamily K, member 1 |
| 226847_at | 119.13 | 1.41E-09 | 3.297678373 | NM_006350| | FST,follistatin isoform FST317 precursor |
| 228415_at | 115.06 | 1.64E-09 | 1.310046902 | NM_003916| | AP1S2,adaptor-related protein complex 1 sigma 2 |
| 202037_s_at | 111.18 | 1.96E-09 | 1.428011226 | NM_003012| | SFRP1,secreted frizzled-related protein 1 |
| 204784_s_at | 110.62 | 1.98E-09 | 1.253667395 | NM_022443| | MLF1,myeloid leukemia factor 1 |
| 214974_x_at | 106.87 | 2.26E-09 | 3.45073145 | NM_002994| | CXCL5,chemokine (C-X-C motif) ligand 5 precursor |
| 219121_s_at | 105.39 | 2.28E-09 | 2.567359664 | NM_001034915| | NA |
| 203233_at | 105.21 | 2.28E-09 | 1.581126043 | NM_000418| | IL4R,interleukin 4 receptor alpha chain isoform a |
| 203417_at | 104.62 | 2.28E-09 | 1.75931918 | NM_002403| | MFAP2,microfibrillar-associated protein 2 precursor |
| 217967_s_at | 104.47 | 2.28E-09 | 2.685996455 | NM_052966| | C1orf24,niban protein |
| 1555623_at | 104.46 | 2.28E-09 | 3.913712531 | NA |  |
| 227760_at | 102.11 | 2.49E-09 | 1.711358137 | NM_001007563| | IGFBPL1,insulin-like growth factor binding protein-like |
| 220786_s_at | 101.4 | 2.56E-09 | 3.208915927 | NM_018018| | SLC38A4,solute carrier family 38, member 4 |
| 230560_at | 100.83 | 2.56E-09 | 2.816071801 | NM_014178| | STXBP6,amisyn |
| 229292_at | 100.73 | 2.56E-09 | 2.297758752 | NM_020909| | EPB41L5,erythrocyte membrane protein band 4.1 like 5 |
| 221245_s_at | 100.62 | 2.56E-09 | 1.394725375 | NM_003468| | FZD5,frizzled 5 |
| 203917_at | 99.52 | 2.71E-09 | 1.224408029 | NM_001338| | CXADR,coxsackie virus and adenovirus receptor |
| 224910_at | 98.81 | 2.80E-09 | 1.25429489 | NM_001042476| | NA |
| 213258_at | 98.2 | 2.87E-09 | 2.268696654 | NM_001032281| | NA |
| 205479_s_at | 97.69 | 2.91E-09 | 1.557065344 | NM_002658| | PLAU,urokinase plasminogen activator preproprotein |
| 205480_s_at | 97.52 | 2.91E-09 | 1.296842924 | NM_001001521| | UGP2,UDP-glucose pyrophosphorylase 2 isoform b |
| 237336_at | 97.15 | 2.94E-09 | 1.544847131 | NM_001617| | ADD2,adducin 2 isoform a |
| 209771_x_at | 96.02 | 3.02E-09 | 1.173425521 | NM_013230| | CD24,CD24 antigen |
| 211654_x_at | 95.97 | 3.02E-09 | 1.651915048 | NM_002123| | HLA-DQB1,major histocompatibility complex, class II, DQ |
| 205541_s_at | 95.9 | 3.02E-09 | 1.179882387 | NM_018094| | GSPT2,peptide chain release factor 3 |
| 202912_at | 95.87 | 3.02E-09 | 1.33252994 | NM_001124| | ADM,adrenomedullin |
| 227177_at | 94.8 | 3.17E-09 | 2.503014468 | NM_003389| | CORO2A,coronin, actin binding protein, 2A |
| 1569886_a_at | 94.14 | 3.19E-09 | 2.314774218 | NM_001080407| | NA |
| 204595_s_at | 91.47 | 3.55E-09 | 1.901421567 | NM_003155| | STC1,stanniocalcin 1 |
| 204079_at | 91.44 | 3.55E-09 | 1.326011042 | NM_001008566| | TPST2,tyrosylprotein sulfotransferase 2 |
| 232985_s_at | 90.88 | 3.61E-09 | 3.126797417 | NM_018189| | DPPA4,developmental pluripotency associated 4 |
| 219073_s_at | 90.6 | 3.61E-09 | 1.668474594 | NM_017784| | OSBPL10,oxysterol-binding protein-like protein 10 |
| 203889_at | 90.53 | 3.61E-09 | 2.799555804 | NM_003020| | SGNE1,secretory granule, neuroendocrine protein 1 (7B2 |
| 236894_at | 89.95 | 3.63E-09 | 3.52554353 | NM_019079| | FLJ10884,hypothetical protein FLJ10884 |
| 227371_at | 89.79 | 3.63E-09 | 2.012130566 | NM_018842| | BAIAP2L1,BAI1-associated protein 2-like 1 |
| 203638_s_at | 89.52 | 3.63E-09 | 1.194874013 | NM_000141| | FGFR2,fibroblast growth factor receptor 2 isoform 1 |
| 211126_s_at | 89.31 | 3.63E-09 | 1.153801257 | NM_001321| | CSRP2,cysteine and glycine-rich protein 2 |
| 208940_at | 89.24 | 3.63E-09 | 1.278775226 | NM_012247| | SEPHS1,selenophosphate synthetase |
| 218883_s_at | 89.24 | 3.63E-09 | 1.102664648 | NM_024629| | MLF1IP,MLF1 interacting protein |
| 226587_at | 87.81 | 3.83E-09 | 1.337185802 | NA |  |
| 1554593_s_at | 87.2 | 3.95E-09 | 2.495709527 | NM_005071| | SLC1A6,solute carrier family 1 (high affinity |
| 229256_at | 86.75 | 4.04E-09 | 1.922054264 | NM_173582| | PGM2L1,phosphoglucomutase 2-like 1 |
| 203397_s_at | 86.56 | 4.04E-09 | 2.515250961 | NM_004482| | GALNT3,polypeptide N-acetylgalactosaminyltransferase 3 |
| 231192_at | 86.53 | 4.04E-09 | 1.67883931 | NA |  |
| 219489_s_at | 86.06 | 4.15E-09 | 1.202106 | NM_022463| | NXN,nucleoredoxin |
| 217853_at | 85.74 | 4.15E-09 | 1.605528017 | NM_022748| | TENS1,tensin-like SH2 domain containing 1 |
| 203413_at | 85.71 | 4.15E-09 | 1.499328918 | NM_006159| | NELL2,nel-like 2 |
| 227372_s_at | 85.01 | 4.36E-09 | 2.291469649 | NM_018842| | BAIAP2L1,BAI1-associated protein 2-like 1 |
| 227830_at | 84.87 | 4.37E-09 | 2.407415016 | NM_000814| | GABRB3,gamma-aminobutyric acid (GABA) A receptor, beta |
| 204235_s_at | 84.14 | 4.55E-09 | 1.296776226 | NM_016315| | GULP1,GULP, engulfment adaptor PTB domain containing |
| 205514_at | 83.55 | 4.72E-09 | 2.431510161 | NM_018355| | ZNF415,zinc finger protein 415 |
| 227911_at | 83.48 | 4.72E-09 | 2.388154961 | NM_001010000| | ARHGAP28,Rho GTPase activating protein 28 isoform a |
| 210074_at | 83.01 | 4.81E-09 | 1.360627855 | NM_001333| | CTSL2,cathepsin L2 preproprotein |
| 203903_s_at | 82.92 | 4.81E-09 | 2.679487455 | NM_014799| | HEPH,hephaestin isoform b |
| 221868_at | 82.12 | 5.02E-09 | 1.800740099 | NM_020459| | NA |
| 225958_at | 81.61 | 5.08E-09 | 1.377018508 | NM_004426| | PHC1,polyhomeotic 1-like |
| 221011_s_at | 81.61 | 5.08E-09 | 1.451652599 | NM_030915| | LBH,hypothetical protein DKFZp566J091 |
| 203297_s_at | 81.57 | 5.08E-09 | 1.307748199 | NM_004973| | JARID2,jumonji, AT rich interactive domain 2 protein |
| 206116_s_at | 81.15 | 5.10E-09 | 1.389076164 | NM_000366| | TPM1,tropomyosin 1 (alpha) |
| 230195_at | 81.12 | 5.10E-09 | 2.955353838 | NA |  |
| 218735_s_at | 80.61 | 5.25E-09 | 1.599193264 | NM_014480| | ZNF544,zinc finger protein 544 |
| 209536_s_at | 79.42 | 5.65E-09 | 1.218005712 | NM_139265| | EHD4,EH-domain containing 4 |
| 204589_at | 79.34 | 5.65E-09 | 1.295083618 | NM_014840| | ARK5,AMPK-related protein kinase 5 |
| 202497_x_at | 78.59 | 5.84E-09 | 1.9420628 | NM_006931| | SLC2A3,solute carrier family 2 (facilitated glucose |
| 228992_at | 77.63 | 6.19E-09 | 1.165606979 | NM_025205| | MED28,mediator of RNA polymerase II transcription, |
| 229390_at | 77.35 | 6.19E-09 | 2.092609472 | NM_001010919| | NA |
| 240301_at | 77.3 | 6.19E-09 | 1.994615756 | NM_138815| | DPPA2,developmental pluripotency associated 2 |
| 224458_at | 77.27 | 6.19E-09 | 1.896062924 | NM_032342| | C9orf125,chromosome 9 open reading frame 125 |
| 205066_s_at | 77.27 | 6.19E-09 | 1.583824228 | NM_006208| | ENPP1,ectonucleotide pyrophosphatase/phosphodiesterase |
| 210002_at | 77.07 | 6.19E-09 | 2.641685433 | NM_005257| | GATA6,GATA binding protein 6 |
| 210517_s_at | 77.06 | 6.19E-09 | 1.235411946 | NM_005100| | AKAP12,A-kinase anchor protein 12 isoform 1 |
| 201100_s_at | 76.52 | 6.34E-09 | 1.14822669 | NM_001039590| | NA |
| 216836_s_at | 76.42 | 6.34E-09 | 1.308138735 | NM_001005862| | ERBB2,erbB-2 isoform b |
| 239975_at | 76.06 | 6.40E-09 | 2.774224076 | NA |  |
| 228906_at | 76.03 | 6.40E-09 | 1.298233611 | NM_030625| | CXXC6,CXXC finger 6 |
| 203298_s_at | 75.48 | 6.52E-09 | 1.29323192 | NM_004973| | JARID2,jumonji, AT rich interactive domain 2 protein |
| 225846_at | 75.46 | 6.52E-09 | 3.032423912 | NM_001034915| | NA |
| 219505_at | 75.34 | 6.52E-09 | 1.477873224 | NM_017424| | CECR1,cat eye syndrome critical region protein 1 |
| 202722_s_at | 74.94 | 6.65E-09 | 1.304588523 | NM_002056| | GFPT1,glucosamine-fructose-6-phosphate |
| 226846_at | 74.31 | 6.85E-09 | 1.591374747 | NM_001100876| | NA |
| 215145_s_at | 74.24 | 6.85E-09 | 2.332587484 | NM_014141| | CNTNAP2,cell recognition molecule Caspr2 precursor |
| 201596_x_at | 74.23 | 6.85E-09 | 1.94952775 | NM_000224| | KRT18,keratin 18 |
| 228275_at | 74.04 | 6.93E-09 | 1.264216724 | NA |  |
| 204048_s_at | 73.77 | 7.03E-09 | 1.248994914 | NM_001100164| | NA |
| 205814_at | 73.3 | 7.31E-09 | 1.8726251 | NM_000840| | GRM3,glutamate receptor, metabotropic 3 precursor |
| 232069_at | 73.06 | 7.37E-09 | 1.379048364 | NM_015656| | NA |
| 228598_at | 73.04 | 7.37E-09 | 2.348049905 | NM_001004360| | DPP10,dipeptidylpeptidase 10 isoform 2 |
| 202036_s_at | 72.39 | 7.72E-09 | 1.526275784 | NM_003012| | SFRP1,secreted frizzled-related protein 1 |
| 205347_s_at | 72.26 | 7.76E-09 | 1.196266305 | NM_021992| | TMSL8,thymosin-like 8 |
| 238606_at | 71.71 | 8.06E-09 | 1.972184219 | NM_023931| | MGC2474,hypothetical protein MGC2474 |
| 203748_x_at | 71.43 | 8.15E-09 | 1.301441496 | NM_002897| | RBMS1,RNA binding motif, single stranded interacting |
| 210605_s_at | 71.19 | 8.29E-09 | 1.65699177 | NM_001114614| | NA |
| 223435_s_at | 70.91 | 8.46E-09 | 3.268979856 | NM_014005| | PCDHA9,protocadherin alpha 9 isoform 2 precursor |
| 223514_at | 70.88 | 8.46E-09 | 1.325028643 | NM_032415| | CARD11,caspase recruitment domain family, member 11 |
| 205547_s_at | 70.69 | 8.53E-09 | 2.118175551 | NM_001001522| | TAGLN,transgelin |
| 209487_at | 70.58 | 8.58E-09 | 1.494068278 | NM_001008710| | RBPMS,RNA-binding protein with multiple splicing |
| 208690_s_at | 70.5 | 8.59E-09 | 1.318096929 | NM_020992| | PDLIM1,PDZ and LIM domain 1 (elfin) |
| 235515_at | 70.23 | 8.75E-09 | 1.818464377 | NM_001039876| | NA |
| 205930_at | 70.06 | 8.84E-09 | 1.306470157 | NM_005513| | GTF2E1,general transcription factor IIE, polypeptide 1 |
| 212151_at | 70.03 | 8.84E-09 | 1.248711684 | NM_002585| | PBX1,pre-B-cell leukemia transcription factor 1 |
| 224097_s_at | 69.23 | 9.29E-09 | 1.743469406 | NM_016946| | F11R,F11 receptor isoform a precursor |
| 217938_s_at | 68.43 | 9.75E-09 | 1.123013418 | NM_020122| | KCMF1,potassium channel modulatory factor 1 |
| 221805_at | 68.36 | 9.75E-09 | 1.802865628 | NM_006158| | NEFL,neurofilament, light polypeptide 68kDa |
| 232164_s_at | 68.32 | 9.75E-09 | 2.264172195 | NM_031308| | EPPK1,epiplakin 1 |
| 208025_s_at | 68.3 | 9.75E-09 | 2.416194573 | NM_003483| | HMGA2,high mobility group AT-hook 2 |
| 235874_at | 68.28 | 9.75E-09 | 2.045419699 | NM_153362| | PRSS35,protease, serine, 35 |
| 223217_s_at | 68.21 | 9.77E-09 | 1.4297556 | NM_001005474| | NFKBIZ,nuclear factor of kappa light polypeptide gene |
| 208296_x_at | 67.9 | 1.00E-08 | 1.542945176 | NM_001077654| | NA |
| 209071_s_at | 67.79 | 1.01E-08 | 2.161793425 | NM_003617| | RGS5,regulator of G-protein signalling 5 |
| 223599_at | 67.53 | 1.03E-08 | 2.944465632 | NM_001003818| | TRIM6,tripartite motif-containing 6 isoform 1 |
| 202876_s_at | 67.53 | 1.03E-08 | 1.219801213 | NM_002586| | PBX2,pre-B-cell leukemia transcription factor 2 |
| 209868_s_at | 67.4 | 1.04E-08 | 1.309649475 | NM_002897| | RBMS1,RNA binding motif, single stranded interacting |
| 206756_at | 67.35 | 1.04E-08 | 1.417453591 | NM_019886| | CHST7,carbohydrate (N-acetylglucosamine 6-O) |
| 201289_at | 66.97 | 1.07E-08 | 1.220503706 | NM_001554| | CYR61,cysteine-rich, angiogenic inducer, 61 |
| 219901_at | 66.89 | 1.07E-08 | 1.638740736 | NM_018351| | FGD6,FYVE, RhoGEF and PH domain containing 6 |
| 214823_at | 66.89 | 1.07E-08 | 2.031572984 | NA |  |
| 218951_s_at | 66.75 | 1.08E-08 | 1.241691962 | NM_018390| | PLCXD1,phosphatidylinositol-specific phospholipase C, X |
| 227628_at | 66.62 | 1.08E-08 | 1.193591751 | NM_001008397| | LOC493869,similar to 2310016C16Rik protein |
| 202546_at | 66.57 | 1.08E-08 | 2.866754089 | NM_003761| | VAMP8,vesicle-associated membrane protein 8 |
| 228933_at | 66.02 | 1.11E-08 | 1.49574086 | NM_198270| | NHS,Nance-Horan syndrome protein |
| 229484_at | 65.78 | 1.14E-08 | 1.364908248 | NM_005167| | PPP2CZ,protein phosphatase 2a, catalytic subunit, zeta |
| 206224_at | 65.74 | 1.14E-08 | 1.941824609 | NM_001898| | CST1,cystatin SN precursor |
| 203474_at | 65.19 | 1.18E-08 | 1.401178674 | NM_006633| | IQGAP2,IQ motif containing GTPase activating protein 2 |
| 211781_x_at | 64.99 | 1.19E-08 | 2.312280343 | NA |  |
| 225275_at | 64.8 | 1.21E-08 | 3.430563539 | NM_005711| | EDIL3,EGF-like repeats and discoidin I-like |
| 223245_at | 64.77 | 1.21E-08 | 1.22472474 | NM_018387| | STRBP,spermatid perinuclear RNA-binding protein |
| 205352_at | 64.6 | 1.22E-08 | 1.921610866 | NM_001122752| | NA |
| 205698_s_at | 64.38 | 1.24E-08 | 1.361501145 | NM_002758| | MAP2K6,mitogen-activated protein kinase kinase 6 |
| 234973_at | 64.35 | 1.24E-08 | 1.894947192 | NM_033518| | SLC38A5,amino acid transport system N2 |
| 226926_at | 64.31 | 1.24E-08 | 2.814182235 | NM_001035516| | NA |
| 224252_s_at | 63.93 | 1.28E-08 | 1.473309938 | NM_014164| | FXYD5,FXYD domain-containing ion transport regulator |
| 229349_at | 63.68 | 1.31E-08 | 3.642009158 | NM_001004317| | FLJ16517,FLJ16517 protein |
| 226473_at | 63.66 | 1.31E-08 | 1.157129479 | NM_005189| | NA |
| 202746_at | 63.59 | 1.31E-08 | 3.275398727 | NM_004867| | ITM2A,integral membrane protein 2A |
| 208398_s_at | 63.56 | 1.31E-08 | 1.178864558 | NM_004865| | TBPL1,TBP-like 1 |
| 242127_at | 63.54 | 1.31E-08 | 2.330963759 | NA |  |
| 211057_at | 63.14 | 1.34E-08 | 1.463705685 | NM_001083592| | NA |
| 209369_at | 63.06 | 1.35E-08 | 2.989374208 | NM_005139| | ANXA3,annexin A3 |
| 204684_at | 63.02 | 1.35E-08 | 2.605503519 | NM_002522| | NPTX1,neuronal pentraxin I precursor |
| 218276_s_at | 62.89 | 1.37E-08 | 1.244907195 | NM_021818| | SAV1,WW45 protein |
| 216379_x_at | 62.78 | 1.37E-08 | 1.184592481 | NM_013230| | CD24,CD24 antigen |
| 209632_at | 62.62 | 1.38E-08 | 1.209754482 | NM_002718| | PPP2R3A,alpha isoform of regulatory subunit B'', protein |
| 219740_at | 62.58 | 1.38E-08 | 1.480590149 | NM_024749| | FLJ12505,hypothetical protein FLJ12505 |
| 203778_at | 62.21 | 1.42E-08 | 1.150553424 | NM_005908| | MANBA,mannosidase, beta A, lysosomal |
| 224817_at | 62.06 | 1.44E-08 | 1.293056884 | NM_014631| | SH3MD1,SH3 multiple domains 1 |
| 206023_at | 61.98 | 1.44E-08 | 1.842182611 | NM_006681| | NMU,neuromedin U |
| 244552_at | 61.8 | 1.46E-08 | 1.38647831 | NA |  |
| 210001_s_at | 61.7 | 1.46E-08 | 2.094119684 | NM_003745| | SOCS1,suppressor of cytokine signaling 1 |
| 230785_at | 61.7 | 1.46E-08 | 1.269451781 | NA |  |
| 206373_at | 61.69 | 1.46E-08 | 1.250584217 | NM_003412| | ZIC1,zinc finger protein of the cerebellum 1 |
| 218384_at | 61.55 | 1.47E-08 | 1.223109088 | NM_001042476| | NA |
| 205399_at | 61.54 | 1.47E-08 | 1.460057105 | NM_004734| | DCAMKL1,doublecortin and CaM kinase-like 1 |
| 1555963_x_at | 61.46 | 1.47E-08 | 2.433375154 | NM_145236| | B3GNT7,UDP-GlcNAc:betaGal |
| 203313_s_at | 61.17 | 1.50E-08 | 1.241723252 | NM_003244| | TGIF,TG-interacting factor isoform c |
| 228821_at | 60.92 | 1.54E-08 | 2.772243085 | NM_032528| | ST6GAL2,beta-galactoside alpha-2,6-sialyltransferase II |
| 206572_x_at | 60.87 | 1.54E-08 | 1.184266364 | NM_003429| | ZNF85,zinc finger protein 85 (HPF4, HTF1) |
| 242919_at | 60.78 | 1.55E-08 | 1.42236416 | NM_021047| | ZNF253,DNA-binding protein |
| 218964_at | 60.65 | 1.56E-08 | 1.868917153 | NM_006465| | ARID3B,AT rich interactive domain 3B (BRIGHT- like) |
| 238035_at | 60.61 | 1.56E-08 | 1.113503254 | NM_001017371| | NA |
| 219987_at | 60.36 | 1.60E-08 | 1.972626585 | NA |  |
| 201846_s_at | 60.14 | 1.63E-08 | 1.21845269 | NM_012234| | RYBP,RING1 and YY1 binding protein |
| 219681_s_at | 59.98 | 1.64E-08 | 1.492741011 | NM_001002233| | RAB11FIP1,Rab coupling protein isoform 2 |
| 204675_at | 59.61 | 1.69E-08 | 1.508094056 | NM_001047| | SRD5A1,steroid-5-alpha-reductase 1 |
| 229872_s_at | 59.38 | 1.72E-08 | 1.504304507 | NA |  |
| 205805_s_at | 59.36 | 1.72E-08 | 1.870654319 | NM_001083592| | NA |
| 218729_at | 59.31 | 1.72E-08 | 2.887448013 | NM_020169| | LXN,latexin |
| 208939_at | 59.25 | 1.73E-08 | 1.21618347 | NM_012247| | SEPHS1,selenophosphate synthetase |
| 209496_at | 59.04 | 1.74E-08 | 2.252064045 | NM_002889| | RARRES2,retinoic acid receptor responder (tazarotene |
| 201278_at | 58.93 | 1.76E-08 | 1.677325501 | NM_001343| | DAB2,disabled homolog 2 |
| 202071_at | 58.75 | 1.78E-08 | 1.354644138 | NM_002999| | SDC4,syndecan 4 precursor |
| 226899_at | 58.61 | 1.80E-08 | 1.240674563 | NM_170744| | UNC5B,unc-5 homolog B |
| 223734_at | 58.53 | 1.81E-08 | 1.36411777 | NM_032623| | OSAP,ovary-specific acidic protein |
| 242979_at | 58.46 | 1.81E-08 | 1.493651465 | NM_005544| | IRS1,insulin receptor substrate 1 |
| 200618_at | 58.26 | 1.85E-08 | 1.179852612 | NM_006148| | LASP1,LIM and SH3 protein 1 |
| 209081_s_at | 58.22 | 1.85E-08 | 1.324960016 | NM_030582| | COL18A1,alpha 1 type XVIII collagen isoform 1 precursor |
| 223529_at | 58.09 | 1.87E-08 | 1.820386551 | NM_020783| | SYT4,synaptotagmin IV |
| 225817_at | 58.04 | 1.87E-08 | 2.150943214 | NM_032866| | CGNL1,cingulin-like 1 |
| 214591_at | 57.84 | 1.91E-08 | 2.215677408 | NM_019117| | KLHL4,kelch-like 4 isoform 1 |
| 1561101_at | 57.52 | 1.97E-08 | 2.246379397 | NA |  |
| 201998_at | 57.41 | 1.99E-08 | 1.121417023 | NM_003032| | ST6GAL1,sialyltransferase 1 isoform a |
| 236519_at | 57.24 | 2.02E-08 | 2.163281997 | NM_001010940| | LOC138255,OTTHUMP00000021439 |
| 205020_s_at | 57.23 | 2.02E-08 | 1.267329363 | NM_001037164| | NA |
| 203881_s_at | 57.19 | 2.02E-08 | 1.94962821 | NM_000109| | DMD,dystrophin Dp427c isoform |
| 229313_at | 57.08 | 2.04E-08 | 1.717159109 | NM_213599| | TMEM16E,transmembrane protein 16E |
| 225295_at | 56.76 | 2.08E-08 | 1.1416192 | NM_001127257| | NA |
| 205110_s_at | 56.75 | 2.08E-08 | 2.749285567 | NM_004114| | FGF13,fibroblast growth factor 13 isoform 1A |
| 37117_at | 56.74 | 2.08E-08 | 1.761774586 | NM_001017526| | NA |
| 205924_at | 56.67 | 2.08E-08 | 1.37206282 | NM_002867| | RAB3B,RAB3B, member RAS oncogene family |
| 216442_x_at | 56.59 | 2.10E-08 | 1.832768439 | NM_002026| | FN1,fibronectin 1 isoform 3 preproprotein |
| 214397_at | 56.56 | 2.10E-08 | 1.840078706 | NM_003927| | MBD2,methyl-CpG binding domain protein 2 isoform 1 |
| 224768_at | 56.49 | 2.11E-08 | 1.12466875 | NM_017969| | FLJ10006,hypothetical protein FLJ10006 |
| 1554256_a_at | 56.42 | 2.12E-08 | 1.449791524 | NM_014801| | PCNXL2,pecanex-like 2 |
| 226863_at | 55.91 | 2.23E-08 | 2.467680644 | NM_001077710| | NA |
| 230788_at | 55.88 | 2.23E-08 | 1.321841819 | NM_001491| | GCNT2,glucosaminyl (N-acetyl) transferase 2 isoform B |
| 229498_at | 55.86 | 2.23E-08 | 1.628686574 | NA |  |
| 224617_at | 55.8 | 2.23E-08 | 1.1622458 | NM_005156| | ROD1,ROD1 regulator of differentiation 1 |
| 1552897_a_at | 55.57 | 2.28E-08 | 2.477290074 | NM_133329| | KCNG3,potassium voltage-gated channel, subfamily G, |
| 231061_at | 55.43 | 2.29E-08 | 1.768429663 | NA |  |
| 223038_s_at | 55.15 | 2.33E-08 | 1.178222969 | NM_021238| | C12orf14,chromosome 12 open reading frame 14 |
| 209297_at | 55.08 | 2.33E-08 | 1.119892038 | NM_001001132| | ITSN1,intersectin 1 isoform ITSN-s |
| 207030_s_at | 55.06 | 2.33E-08 | 1.128246705 | NM_001321| | CSRP2,cysteine and glycine-rich protein 2 |
| 244227_at | 54.84 | 2.38E-08 | 1.571136482 | NM_205848| | SYT6,synaptotagmin VI |
| 219302_s_at | 54.8 | 2.38E-08 | 2.589892375 | NM_014141| | CNTNAP2,cell recognition molecule Caspr2 precursor |
| 202499_s_at | 54.72 | 2.40E-08 | 2.190010044 | NM_006931| | SLC2A3,solute carrier family 2 (facilitated glucose |
| 229963_at | 54.63 | 2.42E-08 | 2.521211722 | NM_001012978| | NA |
| 244565_at | 54.47 | 2.45E-08 | 1.685614368 | NM_005519| | NA |
| 204983_s_at | 54.41 | 2.45E-08 | 1.438405744 | NM_001448| | GPC4,glypican 4 |
| 206291_at | 54.35 | 2.45E-08 | 3.355597192 | NM_006183| | NTS,neurotensin/neuromedin N preproprotein |
| 204790_at | 54.35 | 2.45E-08 | 1.966727702 | NM_005904| | SMAD7,MAD, mothers against decapentaplegic homolog 7 |
| 1553875_s_at | 54.29 | 2.46E-08 | 1.416207668 | NM_032805| | ZNF206,zinc finger protein 206 |
| 206481_s_at | 54.26 | 2.46E-08 | 2.609330878 | NM_001290| | LDB2,LIM domain binding 2 |
| 219147_s_at | 54.23 | 2.47E-08 | 1.376527031 | NM_017881| | C9orf95,nicotinamide riboside kinase 1 |
| 201744_s_at | 54.21 | 2.47E-08 | 2.747772145 | NM_002345| | LUM,lumican |
| 205268_s_at | 54.14 | 2.47E-08 | 1.423274469 | NM_001617| | ADD2,adducin 2 isoform a |
| 202626_s_at | 54.13 | 2.47E-08 | 1.525878648 | NM_001111097| | NA |
| 231690_at | 53.99 | 2.50E-08 | 2.619319188 | NA |  |
| 213308_at | 53.88 | 2.53E-08 | 1.775875745 | NM_012309| | SHANK2,SH3 and multiple ankyrin repeat domains 2 |
| 205138_s_at | 53.84 | 2.53E-08 | 1.408546877 | NM_005715| | UST,uronyl-2-sulfotransferase |
| 203632_s_at | 53.82 | 2.53E-08 | 1.085632746 | NM_016235| | GPRC5B,G protein-coupled receptor, family C, group 5, |
| 205637_s_at | 53.76 | 2.53E-08 | 1.942268632 | NM_003027| | SH3GL3,SH3-domain GRB2-like 3 |
| 1552487_a_at | 53.73 | 2.54E-08 | 2.153076063 | NM_001717| | BNC1,basonuclin 1 |
| 1552736_a_at | 53.68 | 2.54E-08 | 1.867586341 | NM_138966| | NETO1,neuropilin- and tolloid-like protein 1 isoform 3 |
| 218717_s_at | 53.61 | 2.55E-08 | 2.131664914 | NM_018192| | LEPREL1,leprecan-like 1 |
| 211458_s_at | 53.51 | 2.57E-08 | 1.46805004 | NM_031412| | GABARAPL1,GABA(A) receptor-associated protein like 1 |
| 1553179_at | 53.5 | 2.57E-08 | 2.102107295 | NM_133638| | ADAMTS19,a disintegrin-like and metalloprotease |
| 226658_at | 53.43 | 2.59E-08 | 1.336633601 | NM_001006624| | T1A-2,lung type-I cell membrane-associated |
| 203697_at | 53.38 | 2.60E-08 | 2.227495466 | NM_001463| | FRZB,frizzled-related protein |
| 1570515_a_at | 53.29 | 2.62E-08 | 2.600463427 | NM_015687| | FILIP1,filamin A interacting protein 1 |
| 222496_s_at | 53.23 | 2.63E-08 | 2.513712507 | NM_001098634| | NA |
| 218491_s_at | 53.22 | 2.63E-08 | 1.069631192 | NM_001037304| | NA |
| 207705_s_at | 53.17 | 2.64E-08 | 1.248374484 | NM_025176| | KIAA0980,KIAA0980 protein |
| 226267_at | 53.12 | 2.64E-08 | 1.606618584 | NM_130469| | JDP2,Jun dimerization protein |
| 241535_at | 53.02 | 2.66E-08 | 2.913898839 | NA |  |
| 213249_at | 53.01 | 2.66E-08 | 1.160766652 | NM_012304| | FBXL7,F-box and leucine-rich repeat protein 7 |
| 226439_s_at | 52.95 | 2.66E-08 | 1.892639412 | NM_015678| | NBEA,neurobeachin |
| 219287_at | 52.93 | 2.67E-08 | 1.946306637 | NM_014505| | KCNMB4,calcium-activated potassium channel beta 4 |
| 207037_at | 52.77 | 2.70E-08 | 1.799735342 | NM_003839| | TNFRSF11A,tumor necrosis factor receptor superfamily, |
| 208131_s_at | 52.76 | 2.70E-08 | 2.067301042 | NM_000961| | PTGIS,prostaglandin I2 (prostacyclin) synthase |
| 201650_at | 52.71 | 2.70E-08 | 3.453537708 | NM_002276| | KRT19,keratin 19 |
| 212727_at | 52.71 | 2.70E-08 | 1.1666772 | NM_020730| | NA |
| 210986_s_at | 52.59 | 2.74E-08 | 1.435414729 | NM_000366| | TPM1,tropomyosin 1 (alpha) |
| 207266_x_at | 52.57 | 2.74E-08 | 1.288725659 | NM_002897| | RBMS1,RNA binding motif, single stranded interacting |
| 223178_s_at | 52.53 | 2.75E-08 | 1.514256625 | NM_152729| | NT5C2L1,5'-nucleotidase, cytosolic II-like 1 protein |
| 201641_at | 52.41 | 2.76E-08 | 1.959363258 | NM_004335| | BST2,bone marrow stromal cell antigen 2 |
| 226007_at | 52.4 | 2.76E-08 | 1.136456298 | NM_194279| | HBLD1,HESB like domain containing 1 |
| 211202_s_at | 52.39 | 2.76E-08 | 1.165958603 | NM_006618| | JARID1B,Jumonji, AT rich interactive domain 1B |
| 211297_s_at | 52.39 | 2.76E-08 | 1.166848056 | NM_001799| | CDK7,cyclin-dependent kinase 7 |
| 202341_s_at | 52.37 | 2.76E-08 | 1.144143098 | NM_015271| | TRIM2,tripartite motif-containing 2 |
| 208851_s_at | 52.36 | 2.76E-08 | 1.204342304 | NM_006288| | THY1,Thy-1 cell surface antigen |
| 201309_x_at | 52.29 | 2.78E-08 | 1.15047243 | NM_004772| | C5orf13,neuronal protein 3.1 |
| 209421_at | 52.25 | 2.79E-08 | 1.165724761 | NM_000251| | MSH2,mutS homolog 2 |
| 228329_at | 52.12 | 2.83E-08 | 2.202198913 | NM_021080| | DAB1,disabled homolog 1 |
| 209829_at | 51.76 | 2.92E-08 | 1.623078056 | NM_014722| | NA |
| 208119_s_at | 51.74 | 2.93E-08 | 1.346301253 | NM_031218| | ZNF505,zinc finger protein 505 isoform a |
| 213172_at | 51.64 | 2.96E-08 | 2.775209666 | NM_015351| | NA |
| 227492_at | 51.55 | 2.96E-08 | 1.43631275 | NA |  |
| 220994_s_at | 51.55 | 2.96E-08 | 2.381937207 | NM_014178| | STXBP6,amisyn |
| 201667_at | 51.48 | 2.98E-08 | 1.210969708 | NM_000165| | GJA1,connexin 43 |
| 242346_x_at | 51.35 | 3.02E-08 | 2.571195485 | NA |  |
| 203974_at | 51.26 | 3.04E-08 | 1.136497212 | NM_012080| | HDHD1A,haloacid dehalogenase-like hydrolase domain |
| 213137_s_at | 51.23 | 3.05E-08 | 1.147281811 | NM_002828| | PTPN2,protein tyrosine phosphatase, non-receptor type |
| 221207_s_at | 51.09 | 3.09E-08 | 2.050624038 | NM_015678| | NBEA,neurobeachin |
| 205405_at | 51.07 | 3.09E-08 | 1.287670808 | NM_003966| | SEMA5A,semaphorin 5A |
| 202446_s_at | 51.01 | 3.11E-08 | 1.210891918 | NM_021105| | PLSCR1,phospholipid scramblase 1 |
| 209687_at | 51 | 3.11E-08 | 3.063183599 | NM_000609| | CXCL12,chemokine (C-X-C motif) ligand 12 (stromal |
| 208286_x_at | 50.98 | 3.11E-08 | 2.098175875 | NM_002701| | POU5F1,POU domain, class 5, transcription factor 1 |
| 213924_at | 50.94 | 3.12E-08 | 1.610229696 | NM_023075| | MPPE1,metallophosphoesterase 1 isoform a precursor |
| 227702_at | 50.88 | 3.13E-08 | 2.073441296 | NM_178033| | CYP4X1,cytochrome P450, family 4, subfamily X, |
| 206117_at | 50.87 | 3.13E-08 | 1.525371897 | NM_000366| | TPM1,tropomyosin 1 (alpha) |
| 223541_at | 50.83 | 3.14E-08 | 1.517463423 | NM_005329| | HAS3,hyaluronan synthase 3 isoform a |
| 209772_s_at | 50.72 | 3.17E-08 | 1.440664306 | NM_013230| | CD24,CD24 antigen |
| 203020_at | 50.59 | 3.22E-08 | 1.498938925 | NM_001035230| | NA |
| 202697_at | 50.51 | 3.24E-08 | 1.159216568 | NM_007006| | CPSF5,cleavage and polyadenylation specific factor 5 |
| 206653_at | 50.5 | 3.24E-08 | 1.361234241 | NM_006467| | POLR3G,polymerase (RNA) III (DNA directed) polypeptide |
| 224367_at | 50.35 | 3.29E-08 | 1.4649514 | NM_032621| | BEX2,brain expressed X-linked 2 |
| 206891_at | 50.2 | 3.35E-08 | 1.690335245 | NM_001104| | ACTN3,skeletal muscle specific actinin, alpha 3 |
| 213293_s_at | 50.15 | 3.36E-08 | 1.31596786 | NM_006074| | TRIM22,tripartite motif-containing 22 |
| 202973_x_at | 50.14 | 3.36E-08 | 1.071514125 | NM_001015045| | NA |
| 201215_at | 50.12 | 3.37E-08 | 1.25904895 | NM_005032| | PLS3,plastin 3 |
| 212966_at | 50.02 | 3.38E-08 | 1.346389054 | NM_015094| | HIC2,hypermethylated in cancer 2 |
| 221489_s_at | 50.01 | 3.38E-08 | 1.24677357 | NM_030964| | SPRY4,sprouty homolog 4 |
| 218341_at | 49.98 | 3.39E-08 | 1.300042266 | NM_001077447| | NA |
| 223130_s_at | 49.96 | 3.39E-08 | 1.571199611 | NM_013262| | MYLIP,myosin regulatory light chain interacting |
| 201250_s_at | 49.95 | 3.39E-08 | 1.401080933 | NM_006516| | SLC2A1,solute carrier family 2 (facilitated glucose |
| 200606_at | 49.75 | 3.46E-08 | 2.730320161 | NM_001008844| | DSP,desmoplakin isoform II |
| 225660_at | 49.7 | 3.46E-08 | 1.135077854 | NM_020796| | SEMA6A,semaphorin 6A1 |
| 227399_at | 49.68 | 3.46E-08 | 2.349997441 | NM_016206| | VGL-3,colon carcinoma related protein |
| 201431_s_at | 49.63 | 3.46E-08 | 1.310043887 | NM_001387| | DPYSL3,dihydropyrimidinase-like 3 |
| 200931_s_at | 49.59 | 3.47E-08 | 1.234787623 | NM_003373| | VCL,vinculin isoform VCL |
| 214240_at | 49.29 | 3.57E-08 | 1.24856366 | NM_015973| | GAL,galanin preproprotein |
| 201413_at | 49.19 | 3.60E-08 | 1.170157264 | NM_000414| | HSD17B4,hydroxysteroid (17-beta) dehydrogenase 4 |
| 206343_s_at | 49.13 | 3.61E-08 | 2.055052415 | NM_004495| | NRG1,neuregulin 1 isoform HRG-gamma |
| 208190_s_at | 49.09 | 3.62E-08 | 2.046625746 | NM_015925| | LISCH7,LISCH protein isoform 1 |
| 228802_at | 49.06 | 3.63E-08 | 1.354083699 | NM_194272| | NA |
| 214264_s_at | 48.97 | 3.66E-08 | 1.261392388 | NM_145231| | C14orf143,chromosome 14 open reading frame 143 |
| 208782_at | 48.94 | 3.66E-08 | 1.251635535 | NM_007085| | FSTL1,follistatin-like 1 precursor |
| 1554539_a_at | 48.89 | 3.68E-08 | 1.318063037 | NM_019034| | RHOF,ras homolog gene family, member F |
| 32836_at | 48.86 | 3.69E-08 | 1.081529429 | NM_006411| | AGPAT1,1-acylglycerol-3-phosphate O-acyltransferase 1 |
| 204565_at | 48.82 | 3.69E-08 | 1.163880468 | NM_018473| | THEM2,thioesterase superfamily member 2 |
| 216236_s_at | 48.81 | 3.69E-08 | 1.863383243 | NM_006931| | SLC2A3,solute carrier family 2 (facilitated glucose |
| 202350_s_at | 48.74 | 3.71E-08 | 1.676279531 | NM_002380| | MATN2,matrilin 2 precursor |
| 220780_at | 48.69 | 3.73E-08 | 1.193489781 | NM_015715| | PLA2G3,phospholipase A2, group III precursor |
| 212372_at | 48.66 | 3.73E-08 | 1.090173541 | NM_005964| | MYH10,myosin, heavy polypeptide 10, non-muscle |
| 203698_s_at | 48.65 | 3.73E-08 | 2.254038453 | NM_001463| | FRZB,frizzled-related protein |
| 227623_at | 48.58 | 3.76E-08 | 2.420454366 | NA |  |
| 231325_at | 48.54 | 3.77E-08 | 2.618087779 | NM_080872| | UNC5D,netrin receptor Unc5h4 |
| 219814_at | 48.44 | 3.79E-08 | 1.811720702 | NM_018388| | MBNL3,muscleblind-like 3 isoform G |
| 242414_at | 48.43 | 3.79E-08 | 2.013252944 | NM_014298| | QPRT,quinolinate phosphoribosyltransferase |
| 219010_at | 48.41 | 3.79E-08 | 1.224158166 | NM_018265| | FLJ10901,hypothetical protein FLJ10901 |
| 228499_at | 48.39 | 3.79E-08 | 1.367058633 | NM_004567| | PFKFB4,6-phosphofructo-2-kinase/fructose-2, |
| 203448_s_at | 48.39 | 3.79E-08 | 1.347133929 | NM_003218| | TERF1,telomeric repeat binding factor 1 isoform 2 |
| 203028_s_at | 48.33 | 3.81E-08 | 1.818044845 | NM_000101| | CYBA,cytochrome b, alpha polypeptide |
| 212148_at | 48.29 | 3.83E-08 | 1.387177025 | NM_002585| | PBX1,pre-B-cell leukemia transcription factor 1 |
| 231195_at | 48.23 | 3.84E-08 | 1.583637782 | NM_198508| | FLJ44186,FLJ44186 protein |
| 226538_at | 48.22 | 3.84E-08 | 1.141056849 | NM_002372| | MAN2A1,mannosidase, alpha, class 2A, member 1 |
| 223000_s_at | 48.13 | 3.87E-08 | 2.183799775 | NM_016946| | F11R,F11 receptor isoform a precursor |
| 200961_at | 48.06 | 3.91E-08 | 1.083158345 | NM_012248| | SEPHS2,selenophosphate synthetase 2 |
| 219863_at | 48.04 | 3.91E-08 | 2.45658611 | NM_016323| | HERC5,hect domain and RLD 5 |
| 200863_s_at | 48.01 | 3.92E-08 | 1.110395238 | NM_004663| | RAB11A,Ras-related protein Rab-11A |
| 207345_at | 47.97 | 3.93E-08 | 2.177992857 | NM_006350| | FST,follistatin isoform FST317 precursor |
| 201578_at | 47.79 | 4.03E-08 | 1.414719158 | NM_001018111| | NA |
| 225688_s_at | 47.73 | 4.04E-08 | 1.491509434 | NM_145753| | PHLDB2,pleckstrin homology-like domain, family B, |
| 201348_at | 47.69 | 4.06E-08 | 1.382280957 | NM_002084| | GPX3,plasma glutathione peroxidase 3 precursor |
| 217966_s_at | 47.68 | 4.06E-08 | 2.124302741 | NM_052966| | C1orf24,niban protein |
| 211161_s_at | 47.62 | 4.07E-08 | 2.335336515 | NM_000090| | COL3A1,alpha 1 type III collagen |
| 209284_s_at | 47.61 | 4.07E-08 | 1.210630258 | NM_001112736| | NA |
| 203984_s_at | 47.61 | 4.07E-08 | 1.234311263 | NM_001229| | CASP9,caspase 9 isoform alpha preproprotein |
| 228547_at | 47.61 | 4.07E-08 | 2.175929426 | NM_004801| | NRXN1,neurexin 1 isoform alpha precursor |
| 222008_at | 47.54 | 4.10E-08 | 1.245096013 | NM_001851| | COL9A1,alpha 1 type IX collagen isoform 1 precursor |
| 238846_at | 47.5 | 4.12E-08 | 2.569094697 | NM_003839| | TNFRSF11A,tumor necrosis factor receptor superfamily, |
| 1553186_x_at | 47.49 | 4.12E-08 | 1.345930632 | NM_152573| | RASEF,RAS and EF hand domain containing |
| 1553180_at | 47.47 | 4.12E-08 | 2.716872716 | NM_133638| | ADAMTS19,a disintegrin-like and metalloprotease |
| 205531_s_at | 47.43 | 4.12E-08 | 1.619297905 | NM_013267| | GLS2,glutaminase GA isoform a |
| 208456_s_at | 47.4 | 4.12E-08 | 1.387670101 | NM_001102669| | NA |
| 221667_s_at | 47.4 | 4.12E-08 | 1.685886005 | NM_014365| | HSPB8,heat shock 27kDa protein 8 |
| 209049_s_at | 47.22 | 4.19E-08 | 1.158040841 | NM_012408| | PRKCBP1,protein kinase C binding protein 1 isoform b |
| 222619_at | 47.21 | 4.19E-08 | 1.167754881 | NM_012482| | ZNF281,zinc finger protein 281 |
| 209875_s_at | 47.19 | 4.19E-08 | 1.363088809 | NM_000582| | SPP1,secreted phosphoprotein 1 (osteopontin, bone |
| 221916_at | 47.16 | 4.21E-08 | 1.689857338 | NM_006158| | NEFL,neurofilament, light polypeptide 68kDa |
| 206683_at | 47.09 | 4.23E-08 | 1.586072567 | NM_003447| | ZNF165,zinc finger protein 165 |
| 233002_at | 46.98 | 4.28E-08 | 2.25353787 | NM_020958| | KIAA1622,HEAT-like repeat-containing protein isoform 2 |
| 226069_at | 46.92 | 4.30E-08 | 2.901743292 | NM_153026| | PRICKLE1,prickle-like 1 |
| 213518_at | 46.92 | 4.30E-08 | 1.127528758 | NM_002740| | PRKCI,protein kinase C, iota |
| 205206_at | 46.92 | 4.30E-08 | 1.838489342 | NM_000216| | KAL1,Kallmann syndrome 1 protein |
| 218507_at | 46.87 | 4.32E-08 | 1.169280515 | NM_001098786| | NA |
| 222162_s_at | 46.86 | 4.32E-08 | 2.37434945 | NM_006988| | ADAMTS1,a disintegrin and metalloprotease with |
| 206857_s_at | 46.85 | 4.32E-08 | 1.309104813 | NM_004116| | FKBP1B,FK506-binding protein 1B isoform a |
| 205047_s_at | 46.84 | 4.32E-08 | 1.196707038 | NM_001673| | ASNS,asparagine synthetase |
| 231407_s_at | 46.84 | 4.32E-08 | 1.759495785 | NM_003923| | FOXH1,forkhead box H1 |
| 217678_at | 46.77 | 4.34E-08 | 1.496561919 | NM_014331| | SLC7A11,solute carrier family 7, (cationic amino acid |
| 229875_at | 46.7 | 4.38E-08 | 1.428281888 | NM_174976| | ZDHHC22,zinc finger, DHHC domain containing 22 |
| 219622_at | 46.67 | 4.38E-08 | 1.526142507 | NM_017817| | RAB20,RAB20, member RAS oncogene family |
| 226215_s_at | 46.67 | 4.38E-08 | 1.106232092 | NM_001005366| | FBXL10,F-box and leucine-rich repeat protein 10 isoform |
| 215076_s_at | 46.64 | 4.38E-08 | 2.499705054 | NM_000090| | COL3A1,alpha 1 type III collagen |
| 218667_at | 46.64 | 4.38E-08 | 1.164848229 | NM_001032396| | NA |
| 228754_at | 46.6 | 4.40E-08 | 1.127918707 | NM_003043| | SLC6A6,solute carrier family 6 (neurotransmitter |
| 200758_s_at | 46.49 | 4.44E-08 | 1.145130464 | NM_003204| | NFE2L1,nuclear factor (erythroid-derived 2)-like 1 |
| 204284_at | 46.36 | 4.48E-08 | 1.625316388 | NM_005398| | PPP1R3C,protein phosphatase 1, regulatory (inhibitor) |
| 203814_s_at | 46.32 | 4.48E-08 | 1.198139586 | NM_000904| | NQO2,NAD(P)H dehydrogenase, quinone 2 |
| 212788_x_at | 46.31 | 4.48E-08 | 1.042839387 | NM_000146| | FTL,ferritin, light polypeptide |
| 218404_at | 46.29 | 4.48E-08 | 1.173453327 | NM_013322| | SNX10,sorting nexin 10 |
| 41577_at | 46.29 | 4.48E-08 | 1.400534459 | NM_015568| | PPP1R16B,protein phosphatase 1 regulatory inhibitor |
| 211276_at | 46.22 | 4.51E-08 | 2.225595561 | NM_080390| | TCEAL2,transcription elongation factor A (SII)-like 2 |
| 209817_at | 46.19 | 4.52E-08 | 1.138409056 | NM_021132| | PPP3CB,protein phosphatase 3 (formerly 2B), catalytic |
| 229513_at | 46.17 | 4.52E-08 | 1.246586373 | NM_018387| | STRBP,spermatid perinuclear RNA-binding protein |
| 236124_at | 46.16 | 4.52E-08 | 1.352682338 | NA |  |
| 224048_at | 46.12 | 4.54E-08 | 3.150767518 | NM_001042403| | NA |
| 1553105_s_at | 46.06 | 4.56E-08 | 2.816224261 | NM_001943| | DSG2,desmoglein 2 preproprotein |
| 202822_at | 46.06 | 4.56E-08 | 1.203094028 | NM_005578| | LPP,LIM domain containing preferred translocation |
| 227494_at | 46.01 | 4.58E-08 | 1.903232653 | NM_001489| | NR6A1,nuclear receptor subfamily 6, group A, member 1 |
| 224354_at | 46.01 | 4.58E-08 | 3.451996119 | NA |  |
| 223714_at | 45.97 | 4.60E-08 | 1.259521592 | NM_005773| | ZNF256,zinc finger protein 256 |
| 212013_at | 45.92 | 4.61E-08 | 1.146318185 | NM_012293| | NA |
| 204620_s_at | 45.91 | 4.61E-08 | 1.251824444 | NM_001126336| | NA |
| 228441_s_at | 45.87 | 4.63E-08 | 2.398295731 | NA |  |
| 219033_at | 45.86 | 4.63E-08 | 2.217458519 | NM_024615| | PARP8,poly (ADP-ribose) polymerase family, member 8 |
| 227690_at | 45.64 | 4.75E-08 | 2.555465648 | NM_000814| | GABRB3,gamma-aminobutyric acid (GABA) A receptor, beta |
| 1553185_at | 45.59 | 4.77E-08 | 1.318416877 | NM_152573| | RASEF,RAS and EF hand domain containing |
| 225265_at | 45.57 | 4.79E-08 | 1.332623474 | NM_002897| | RBMS1,RNA binding motif, single stranded interacting |
| 204984_at | 45.52 | 4.79E-08 | 1.378735554 | NM_001448| | GPC4,glypican 4 |
| 209755_at | 45.52 | 4.79E-08 | 1.751309971 | NM_015039| | NMNAT2,nicotinamide mononucleotide adenylyltransferase |
| 208898_at | 45.44 | 4.84E-08 | 1.074696839 | NM_015994| | ATP6V1D,ATPase, H+ transporting, lysosomal 34kD, V1 |
| 205742_at | 45.44 | 4.84E-08 | 2.211256647 | NM_000363| | TNNI3,troponin I, cardiac |
| 204359_at | 45.39 | 4.86E-08 | 2.068808025 | NM_013231| | FLRT2,fibronectin leucine rich transmembrane protein |
| 209705_at | 45.38 | 4.86E-08 | 1.27371356 | NM_007358| | M96,putative DNA binding protein |
| 212689_s_at | 45.37 | 4.86E-08 | 1.065650615 | NM_018433| | JMJD1A,jumonji domain containing 1A |
| 225820_at | 45.3 | 4.90E-08 | 1.234372807 | NM_024900| | PHF17,Jade1 protein short isoform |
| 227931_at | 45.29 | 4.90E-08 | 1.270448892 | NM_017759| | FLJ20309,hypothetical protein FLJ20309 |
| 209785_s_at | 45.18 | 4.98E-08 | 2.37949786 | NM_003706| | PLA2G4C,phospholipase A2, group IVC |
| 201611_s_at | 45.05 | 5.07E-08 | 1.144156325 | NM_012405| | ICMT,isoprenylcysteine carboxyl methyltransferase |
| 202670_at | 45.04 | 5.07E-08 | 1.119024865 | NM_002755| | MAP2K1,mitogen-activated protein kinase kinase 1 |
| 1552754_a_at | 45.01 | 5.09E-08 | 1.834160577 | NM_153184| | IGSF4D,immunoglobulin superfamily, member 4D |
| 202241_at | 44.94 | 5.13E-08 | 1.226557169 | NM_025195| | TRIB1,G-protein-coupled receptor induced protein |
| 205968_at | 44.88 | 5.15E-08 | 2.788445733 | NM_002252| | KCNS3,potassium voltage-gated channel |
| 219685_at | 44.84 | 5.17E-08 | 1.378576783 | NM_021637| | TMEM35,transmembrane protein 35 |
| 217722_s_at | 44.79 | 5.18E-08 | 1.073869427 | NM_001033088| | NA |
| 35666_at | 44.79 | 5.18E-08 | 1.17490883 | NM_004186| | SEMA3F,semaphorin 3F |
| 203408_s_at | 44.76 | 5.18E-08 | 1.266205778 | NM_002971| | SATB1,special AT-rich sequence binding protein 1 |
| 229294_at | 44.75 | 5.18E-08 | 2.785660747 | NM_020655| | JPH3,junctophilin 3 |
| 210026_s_at | 44.71 | 5.21E-08 | 1.34185251 | NM_014550| | CARD10,caspase recruitment domain protein 10 |
| 205625_s_at | 44.69 | 5.23E-08 | 2.612827213 | NM_004929| | CALB1,calbindin 1 |
| 209765_at | 44.62 | 5.27E-08 | 1.108149221 | NM_023038| | ADAM19,a disintegrin and metalloproteinase domain 19 |
| 210987_x_at | 44.61 | 5.27E-08 | 1.455309894 | NM_000366| | TPM1,tropomyosin 1 (alpha) |
| 225566_at | 44.55 | 5.31E-08 | 1.305634373 | NM_003872| | NRP2,neuropilin 2 isoform 2 precursor |
| 210540_s_at | 44.54 | 5.31E-08 | 1.282141741 | NM_003778| | B4GALT4,UDP-Gal:betaGlcNAc beta 1,4- |
| 208742_s_at | 44.53 | 5.31E-08 | 1.071556914 | NM_005870| | SAP18,sin3 associated polypeptide p18 |
| 228320_x_at | 44.51 | 5.33E-08 | 1.431418467 | NM_207311| | LOC92558,hypothetical protein LOC92558 |
| 204464_s_at | 44.49 | 5.34E-08 | 1.24588758 | NM_001957| | EDNRA,endothelin receptor type A |
| 212464_s_at | 44.4 | 5.40E-08 | 1.885320711 | NM_002026| | FN1,fibronectin 1 isoform 3 preproprotein |
| 231945_at | 44.39 | 5.40E-08 | 1.714560196 | NM_015687| | FILIP1,filamin A interacting protein 1 |
| 228120_at | 44.34 | 5.44E-08 | 1.108684256 | NA |  |
| 239858_at | 44.32 | 5.44E-08 | 1.704833229 | NA |  |
| 204326_x_at | 43.97 | 5.69E-08 | 1.188321569 | NM_005952| | MT1X,metallothionein 1X |
| 227365_at | 43.93 | 5.72E-08 | 1.303414962 | NM_033064| | ATCAY,caytaxin |
| 201839_s_at | 43.92 | 5.72E-08 | 3.053383261 | NM_002354| | TACSTD1,tumor-associated calcium signal transducer 1 |
| 244362_at | 43.81 | 5.80E-08 | 1.428596522 | NA |  |
| 226277_at | 43.81 | 5.80E-08 | 1.181052186 | NM_005713| | COL4A3BP,alpha 3 type IV collagen binding protein isoform |
| 226530_at | 43.79 | 5.81E-08 | 1.410408183 | NM_001003940| | BMF,Bcl2 modifying factor isoform bmf-1 |
| 226799_at | 43.7 | 5.87E-08 | 1.686858916 | NM_018351| | FGD6,FYVE, RhoGEF and PH domain containing 6 |
| 232136_s_at | 43.66 | 5.90E-08 | 1.814329508 | NM_033427| | CTTNBP2,cortactin binding protein 2 |
| 212203_x_at | 43.65 | 5.90E-08 | 1.100422728 | NM_021034| | IFITM3,interferon-induced transmembrane protein 3 |
| 202672_s_at | 43.6 | 5.92E-08 | 1.569221585 | NM_001030287| | NA |
| 205053_at | 43.59 | 5.92E-08 | 1.121557282 | NM_000946| | PRIM1,DNA primase small subunit, 49kDa |
| 202875_s_at | 43.57 | 5.93E-08 | 1.317275378 | NM_002586| | PBX2,pre-B-cell leukemia transcription factor 2 |
| 218454_at | 43.55 | 5.94E-08 | 1.865590522 | NM_024829| | FLJ22662,hypothetical protein FLJ22662 |
| 201032_at | 43.48 | 5.99E-08 | 1.115867756 | NM_006698| | BLCAP,bladder cancer associated protein |
| 203404_at | 43.45 | 6.01E-08 | 1.440858363 | NM_014782| | ARMCX2,ALEX2 protein |
| 219806_s_at | 43.43 | 6.01E-08 | 1.335549152 | NM_020179| | FN5,FN5 protein |
| 221249_s_at | 43.36 | 6.05E-08 | 1.360564391 | NM_030802| | LOC81558,C/EBP-induced protein |
| 243610_at | 43.33 | 6.07E-08 | 3.149595764 | NM_001010940| | LOC138255,OTTHUMP00000021439 |
| 209111_at | 43.31 | 6.07E-08 | 1.101868501 | NM_006913| | RNF5,ring finger protein 5 |
| 225269_s_at | 43.3 | 6.07E-08 | 1.253835467 | NA |  |
| 202035_s_at | 43.27 | 6.09E-08 | 1.424033192 | NM_003012| | SFRP1,secreted frizzled-related protein 1 |
| 222640_at | 43.27 | 6.09E-08 | 1.189696663 | NM_022552| | DNMT3A,DNA cytosine methyltransferase 3 alpha isoform |
| 206385_s_at | 43.25 | 6.10E-08 | 1.398015294 | NM_001149| | ANK3,ankyrin 3 isoform 2 |
| 230563_at | 43.23 | 6.10E-08 | 2.832853184 | NM_145313| | RASGEF1A,RasGEF domain family, member 1A |
| 201904_s_at | 43.2 | 6.12E-08 | 1.162161473 | NM_001008392| | CTDSPL,small CTD phosphatase 3 isoform 1 |
| 227247_at | 43.19 | 6.12E-08 | 1.164437573 | NA |  |
| 231731_at | 43.15 | 6.13E-08 | 2.676728013 | NM_021728| | OTX2,orthodenticle 2 isoform a |
| 204288_s_at | 43.1 | 6.18E-08 | 1.233800854 | NM_003603| | ARGBP2,Arg/Abl-interacting protein 2 isoform 1 |
| 205107_s_at | 43.06 | 6.22E-08 | 1.334370927 | NM_005227| | EFNA4,ephrin A4 isoform a |
| 206710_s_at | 43.05 | 6.22E-08 | 2.241364675 | NM_012307| | EPB41L3,erythrocyte membrane protein band 4.1-like 3 |
| 209210_s_at | 43.02 | 6.24E-08 | 1.057793188 | NM_006832| | PLEKHC1,pleckstrin homology domain containing, family C |
| 227013_at | 43 | 6.25E-08 | 1.260983139 | NM_014572| | LATS2,LATS, large tumor suppressor, homolog 2 |
| 227202_at | 42.96 | 6.28E-08 | 2.065814967 | NM_001843| | CNTN1,contactin 1 isoform 1 precursor |
| 235763_at | 42.96 | 6.28E-08 | 1.764208349 | NM_152697| | MGC34032,hypothetical protein MGC34032 |
| 202856_s_at | 42.9 | 6.32E-08 | 2.158500576 | NM_001042422| | NA |
| 1552947_x_at | 42.89 | 6.32E-08 | 1.299373285 | NM_153608| | MGC17986,hypothetical protein MGC17986 |
| 205523_at | 42.87 | 6.32E-08 | 3.051495377 | NM_001884| | HAPLN1,cartilage linking protein 1 |
| 205051_s_at | 42.85 | 6.32E-08 | 2.479318087 | NM_000222| | KIT,v-kit Hardy-Zuckerman 4 feline sarcoma viral |
| 213107_at | 42.84 | 6.32E-08 | 1.293407317 | NM_015028| | TNIK,TRAF2 and NCK interacting kinase |
| 228587_at | 42.82 | 6.33E-08 | 1.303902328 | NM_001039999| | NA |
| 202747_s_at | 42.77 | 6.37E-08 | 2.160832053 | NM_004867| | ITM2A,integral membrane protein 2A |
| 214844_s_at | 42.74 | 6.39E-08 | 1.218228673 | NM_018431| | DOK5,DOK5 protein isoform a |
| 201310_s_at | 42.72 | 6.40E-08 | 1.121863422 | NM_004772| | C5orf13,neuronal protein 3.1 |
| 220474_at | 42.7 | 6.42E-08 | 2.012202014 | NM_030631| | SLC25A21,solute carrier family 25 (mitochondrial |
| 213283_s_at | 42.67 | 6.43E-08 | 1.181685796 | NM_005407| | SALL2,sal-like 2 |
| 219937_at | 42.58 | 6.51E-08 | 1.566591047 | NM_013381| | TRHDE,thyrotropin-releasing hormone degrading |
| 205463_s_at | 42.55 | 6.53E-08 | 1.18074011 | NM_002607| | NA |
| 203436_at | 42.54 | 6.53E-08 | 1.093509901 | NM_001104546| | NA |
| 220520_s_at | 42.53 | 6.53E-08 | 1.808539325 | NM_017681| | FLJ20130,hypothetical protein FLJ20130 |
| 223361_at | 42.53 | 6.53E-08 | 1.274613246 | NM_021243| | NA |
| 213869_x_at | 42.53 | 6.53E-08 | 1.217787878 | NM_006288| | THY1,Thy-1 cell surface antigen |
| 222494_at | 42.5 | 6.54E-08 | 1.135670154 | NM_001085471| | NA |
| 227314_at | 42.42 | 6.60E-08 | 1.602011561 | NM_002203| | ITGA2,integrin alpha 2 precursor |
| 223174_at | 42.41 | 6.60E-08 | 1.078274124 | NM_032320| | GMRP-1,K+ channel tetramerization protein |
| 229553_at | 42.4 | 6.61E-08 | 1.750467308 | NM_173582| | PGM2L1,phosphoglucomutase 2-like 1 |
| 202087_s_at | 42.38 | 6.62E-08 | 1.099795103 | NM_001912| | CTSL,cathepsin L preproprotein |
| 213237_at | 42.37 | 6.62E-08 | 1.103901266 | NM_001012991| | NA |
| 203966_s_at | 42.29 | 6.69E-08 | 1.128631629 | NM_021003| | PPM1A,protein phosphatase 1A isoform 1 |
| 201193_at | 42.29 | 6.69E-08 | 1.093516842 | NM_005896| | IDH1,isocitrate dehydrogenase 1 (NADP+), soluble |
| 202022_at | 42.28 | 6.69E-08 | 1.27488676 | NM_005165| | ALDOC,aldolase C, fructose-bisphosphate |
| 200752_s_at | 42.23 | 6.72E-08 | 1.203598043 | NM_005186| | CAPN1,calpain 1, large subunit |
| 218517_at | 42.21 | 6.74E-08 | 1.233928012 | NM_024900| | PHF17,Jade1 protein short isoform |
| 219945_at | 42.16 | 6.78E-08 | 1.427906561 | NM_013264| | DDX25,DEAD (Asp-Glu-Ala-Asp) box polypeptide 25 |
| 221731_x_at | 42.15 | 6.78E-08 | 1.248313169 | NM_001126336| | NA |
| 223121_s_at | 42.14 | 6.78E-08 | 2.65932421 | NM_003013| | SFRP2,secreted frizzled-related protein 2 precursor |
| 1554541_a_at | 42.14 | 6.78E-08 | 1.403236548 | NM_014696| | KIAA0514,KIAA0514 |
| 202998_s_at | 42.12 | 6.79E-08 | 1.822143641 | NM_002318| | LOXL2,lysyl oxidase-like 2 |
| 1558693_s_at | 42.1 | 6.80E-08 | 1.459775003 | NM_144580| | MGC31963,kidney predominant protein NCU-G1 |
| 230425_at | 41.98 | 6.91E-08 | 1.426497619 | NM_004441| | EPHB1,ephrin receptor EphB1 precursor |
| 207992_s_at | 41.97 | 6.91E-08 | 1.681150969 | NM_000480| | AMPD3,adenosine monophosphate deaminase (isoform E) |
| 219976_at | 41.91 | 6.93E-08 | 2.30324455 | NM_015888| | HOOK1,hook homolog 1 |
| 202347_s_at | 41.76 | 7.06E-08 | 1.079328215 | NM_001111112| | NA |
| 214853_s_at | 41.74 | 7.07E-08 | 1.084252181 | NM_003029| | SHC1,SHC (Src homology 2 domain containing) |
| 217744_s_at | 41.73 | 7.07E-08 | 2.521385556 | NM_022121| | PERP,PERP, TP53 apoptosis effector |
| 209848_s_at | 41.6 | 7.20E-08 | 2.116990592 | NM_006928| | SILV,silver homolog |
| 218338_at | 41.6 | 7.20E-08 | 1.264895099 | NM_004426| | PHC1,polyhomeotic 1-like |
| 236126_at | 41.49 | 7.29E-08 | 1.295698054 | NM_001106| | ACVR2B,activin A type IIB receptor precursor |
| 219352_at | 41.39 | 7.37E-08 | 1.490411393 | NM_017912| | HERC6,hect domain and RLD 6 |
| 227070_at | 41.39 | 7.37E-08 | 2.712421979 | NM_031302| | GLT8D2,glycosyltransferase 8 domain containing 2 |
| 204597_x_at | 41.37 | 7.39E-08 | 2.236691338 | NM_003155| | STC1,stanniocalcin 1 |
| 214071_at | 41.36 | 7.39E-08 | 2.059205797 | NM_023075| | MPPE1,metallophosphoesterase 1 isoform a precursor |
| 65438_at | 41.36 | 7.39E-08 | 1.382446223 | NM_020947| | KIAA1609,KIAA1609 protein |
| 222821_s_at | 41.3 | 7.44E-08 | 1.079337493 | NM_001007269| | GEMIN7,gemin 7 |
| 226771_at | 41.24 | 7.51E-08 | 1.193419029 | NM_001005855| | ATP8B2,ATPase, Class I, type 8B, member 2 isoform b |
| 205980_s_at | 41.23 | 7.51E-08 | 1.778905391 | NM_001017526| | NA |
| 216917_s_at | 41.21 | 7.53E-08 | 2.002482557 | NM_003176| | SYCP1,synaptonemal complex protein 1 |
| 228819_at | 41.14 | 7.60E-08 | 1.409106263 | NM_001031730| | NA |
| 216295_s_at | 41.14 | 7.60E-08 | 1.045203458 | NM_001076677| | NA |
| 204976_s_at | 41.13 | 7.60E-08 | 1.113036501 | NM_001025580| | NA |
| 219932_at | 41.07 | 7.66E-08 | 2.426845283 | NM_001017372| | NA |
| 243000_at | 40.99 | 7.75E-08 | 1.246881048 | NM_001259| | CDK6,cyclin-dependent kinase 6 |
| 225258_at | 40.97 | 7.77E-08 | 1.31759213 | NM_001024215| | NA |
| 225114_at | 40.93 | 7.80E-08 | 1.217729275 | NM_003659| | AGPS,alkylglycerone phosphate synthase precursor |
| 223218_s_at | 40.88 | 7.84E-08 | 1.56295989 | NM_001005474| | NFKBIZ,nuclear factor of kappa light polypeptide gene |
| 237193_s_at | 40.78 | 7.93E-08 | 2.420923365 | NA |  |
| 223177_at | 40.74 | 7.96E-08 | 1.38878285 | NM_152729| | NT5C2L1,5'-nucleotidase, cytosolic II-like 1 protein |
| 235334_at | 40.58 | 8.13E-08 | 1.815421994 | NM_152996| | ST6GALNAC3,ST6 |
| 203892_at | 40.49 | 8.20E-08 | 1.556005543 | NM_006103| | WFDC2,WAP four-disulfide core domain 2 isoform 1 |
| 213629_x_at | 40.48 | 8.22E-08 | 1.384756873 | NM_005949| | MT1F,metallothionein 1F |
| 1555370_a_at | 40.47 | 8.22E-08 | 1.284506409 | NM_015215| | CAMTA1,calmodulin-binding transcription activator 1 |
| 218614_at | 40.45 | 8.23E-08 | 1.275628444 | NM_018169| | FLJ10652,hypothetical protein FLJ10652 |
| 222001_x_at | 40.45 | 8.23E-08 | 1.447920546 | NA |  |
| 209108_at | 40.37 | 8.31E-08 | 1.108787355 | NM_003270| | TM4SF6,transmembrane 4 superfamily member 6 |
| 227297_at | 40.3 | 8.39E-08 | 1.92288505 | NM_002207| | ITGA9,integrin, alpha 9 precursor |
| 201121_s_at | 40.09 | 8.65E-08 | 1.137319415 | NM_006667| | PGRMC1,progesterone receptor membrane component 1 |
| 203066_at | 40.08 | 8.65E-08 | 1.270269173 | NM_015892| | GALNAC4S-6ST,B cell RAG associated protein |
| 212055_at | 40.04 | 8.69E-08 | 1.059878308 | NM_015476| | C18orf10,chromosome 18 open reading frame 10 |
| 205709_s_at | 40.03 | 8.69E-08 | 2.161497668 | NM_001263| | CDS1,phosphatidate cytidylyltransferase 1 |
| 219397_at | 40.02 | 8.69E-08 | 1.177310395 | NM_025147| | FLJ13448,hypothetical protein FLJ13448 |
| 213131_at | 40.01 | 8.70E-08 | 2.561962496 | NM_006334| | OLFM1,olfactomedin related ER localized protein |
| 218651_s_at | 39.99 | 8.71E-08 | 1.132145688 | NM_018357| | FLJ11196,acheron isoform 1 |
| 212964_at | 39.98 | 8.71E-08 | 1.416063801 | NM_015094| | HIC2,hypermethylated in cancer 2 |
| 1564083_at | 39.96 | 8.72E-08 | 2.079942992 | NA |  |
| 206074_s_at | 39.89 | 8.81E-08 | 1.229817226 | NM_002131| | HMGA1,high mobility group AT-hook 1 isoform b |
| 203798_s_at | 39.79 | 8.95E-08 | 2.840872567 | NM_003385| | VSNL1,visinin-like 1 |
| 222698_s_at | 39.78 | 8.95E-08 | 1.160652176 | NM_018439| | IMPACT,hypothetical protein IMPACT |
| 205008_s_at | 39.78 | 8.95E-08 | 1.225101586 | NM_006383| | CIB2,DNA-dependent protein kinase catalytic |
| 209581_at | 39.72 | 9.04E-08 | 1.306972073 | NM_007069| | HRASLS3,HRAS-like suppressor 3 |
| 231079_at | 39.7 | 9.06E-08 | 2.059433789 | NA |  |
| 233110_s_at | 39.69 | 9.06E-08 | 1.176391882 | NM_001040668| | NA |
| 222482_at | 39.67 | 9.09E-08 | 1.199918543 | NM_001009955| | SSBP3,single stranded DNA binding protein 3 isoform c |
| 210381_s_at | 39.64 | 9.13E-08 | 1.650429315 | NM_176875| | CCKBR,cholecystokinin B receptor |
| 227732_at | 39.6 | 9.16E-08 | 1.537731058 | NM_020725| | NA |
| 232647_at | 39.59 | 9.17E-08 | 1.260737829 | NM_152465| | PROCA1,proline-rich cyclin A1-interacting protein |
| 35617_at | 39.5 | 9.31E-08 | 1.229045527 | NM_002749| | MAPK7,mitogen-activated protein kinase 7 isoform 1 |
| 219412_at | 39.49 | 9.32E-08 | 1.548445638 | NM_022337| | RAB38,RAB38 |
| 208626_s_at | 39.45 | 9.35E-08 | 1.195682385 | NM_006373| | VAT1,vesicle amine transport protein 1 |
| 203946_s_at | 39.44 | 9.35E-08 | 1.711736376 | NM_001172| | ARG2,arginase, type II precursor |
| 229492_at | 39.4 | 9.39E-08 | 1.347823347 | NM_138959| | VANGL1,vang-like 1 |
| 209792_s_at | 39.4 | 9.39E-08 | 1.410502187 | NM_001077500| | NA |
| 231725_at | 39.37 | 9.40E-08 | 1.971621475 | NM_018936| | PCDHB2,protocadherin beta 2 precursor |
| 228260_at | 39.36 | 9.40E-08 | 2.567040103 | NM_004432| | ELAVL2,ELAV (embryonic lethal, abnormal vision, |
| 201130_s_at | 39.36 | 9.40E-08 | 1.581981791 | NM_004360| | CDH1,cadherin 1, type 1 preproprotein |
| 201069_at | 39.34 | 9.42E-08 | 1.492317047 | NM_004530| | MMP2,matrix metalloproteinase 2 preproprotein |
| 222431_at | 39.33 | 9.43E-08 | 1.104951432 | NM_006717| | SPIN,spindlin |
| 217901_at | 39.28 | 9.47E-08 | 3.076686702 | NM_001943| | DSG2,desmoglein 2 preproprotein |
| 233142_at | 39.23 | 9.52E-08 | 1.260730156 | NA |  |
| 203097_s_at | 39.23 | 9.52E-08 | 1.124105573 | NM_014247| | NA |
| 227803_at | 39.19 | 9.55E-08 | 1.770325317 | NM_021572| | ENPP5,ectonucleotide pyrophosphatase/phosphodiesterase |
| 227677_at | 39.19 | 9.55E-08 | 1.667496807 | NM_000215| | JAK3,Janus kinase 3 |
| 201842_s_at | 39.12 | 9.65E-08 | 2.201547436 | NM_001039348| | NA |
| 201832_s_at | 39.01 | 9.81E-08 | 1.15148022 | NM_003715| | VDP,vesicle docking protein p115 |
| 214532_x_at | 39 | 9.81E-08 | 1.945195246 | NA |  |
| 222732_at | 39 | 9.81E-08 | 1.163020361 | NM_021253| | TRIM39,tripartite motif-containing 39 isoform 1 |
| 229288_at | 39 | 9.81E-08 | 1.77007114 | NA |  |
| 203961_at | 38.99 | 9.81E-08 | 1.755136853 | NM_006393| | NEBL,nebulette sarcomeric isoform |
| 204867_at | 38.94 | 9.88E-08 | 1.557962694 | NM_005258| | GCHFR,GTP cyclohydrolase I feedback regulatory |
| 227014_at | 38.89 | 9.95E-08 | 1.352068912 | NM_020437| | LOC57168,similar to aspartate beta hydroxylase (ASPH) |
| 203706_s_at | 38.89 | 9.95E-08 | 1.270315493 | NM_003507| | FZD7,frizzled 7 |
| 219179_at | 38.83 | 1.00E-07 | 1.511432844 | NM_001079520| | NA |
| 225029_at | 38.79 | 1.01E-07 | 1.1536116 | NA |  |
| 227827_at | 38.75 | 1.01E-07 | 2.16505288 | NA |  |
| 234623_x_at | 38.73 | 1.01E-07 | 3.289377733 | NA |  |
| 210220_at | 38.71 | 1.02E-07 | 1.148945334 | NM_001466| | FZD2,frizzled 2 |
| 201659_s_at | 38.7 | 1.02E-07 | 1.078498544 | NM_001177| | ARL1,ADP-ribosylation factor-like 1 |
| 203705_s_at | 38.67 | 1.02E-07 | 1.262487101 | NM_003507| | FZD7,frizzled 7 |
| 213671_s_at | 38.62 | 1.02E-07 | 1.08598652 | NM_004990| | MARS,methionine-tRNA synthetase |
| 209008_x_at | 38.6 | 1.03E-07 | 1.801736858 | NM_002273| | KRT8,keratin 8 |
| 201926_s_at | 38.59 | 1.03E-07 | 1.800172239 | NM_000574| | DAF,decay accelerating factor for complement (CD55, |
| 203381_s_at | 38.54 | 1.03E-07 | 1.942489264 | NM_000041| | APOE,apolipoprotein E precursor |
| 202686_s_at | 38.51 | 1.04E-07 | 1.196524156 | NM_001699| | AXL,AXL receptor tyrosine kinase isoform 2 |
| 203345_s_at | 38.49 | 1.04E-07 | 1.206006915 | NM_007358| | M96,putative DNA binding protein |
| 227533_at | 38.43 | 1.05E-07 | 2.177811493 | NA |  |
| 226801_s_at | 38.35 | 1.06E-07 | 1.082239644 | NM_022831| | FLJ12806,hypothetical protein FLJ12806 |
| 224650_at | 38.32 | 1.07E-07 | 3.699731187 | NM_052886| | MAL2,mal, T-cell differentiation protein 2 |
| 202346_at | 38.3 | 1.07E-07 | 1.102355144 | NM_001111112| | NA |
| 205542_at | 38.3 | 1.07E-07 | 2.131499157 | NM_012449| | STEAP,six transmembrane epithelial antigen of the |
| 236756_at | 38.28 | 1.07E-07 | 1.985441665 | NA |  |
| 206002_at | 38.28 | 1.07E-07 | 2.515262012 | NM_001079858| | NA |
| 205093_at | 38.26 | 1.07E-07 | 1.202549624 | NM_014935| | PLEKHA6,phosphoinositol 3-phosphate-binding protein-3 |
| 219650_at | 38.2 | 1.09E-07 | 1.224790605 | NM_017669| | FLJ20105,FLJ20105 protein isoform a |
| 242128_at | 38.12 | 1.10E-07 | 4.196603564 | NM_021728| | OTX2,orthodenticle 2 isoform a |
| 223466_x_at | 38.12 | 1.10E-07 | 1.139000238 | NM_005713| | COL4A3BP,alpha 3 type IV collagen binding protein isoform |
| 212728_at | 38.12 | 1.10E-07 | 1.210540425 | NM_020730| | NA |
| 208796_s_at | 38.09 | 1.10E-07 | 1.112188552 | NM_004060| | CCNG1,cyclin G1 |
| 207121_s_at | 38.08 | 1.10E-07 | 1.091544116 | NM_002748| | MAPK6,mitogen-activated protein kinase 6 |
| 206382_s_at | 38.07 | 1.10E-07 | 2.026437727 | NM_001709| | BDNF,brain-derived neurotrophic factor isoform a |
| 221843_s_at | 37.97 | 1.12E-07 | 1.248610465 | NM_020947| | KIAA1609,KIAA1609 protein |
| 205738_s_at | 37.97 | 1.12E-07 | 1.636435233 | NM_004102| | FABP3,fatty acid binding protein 3 |
| 228370_at | 37.96 | 1.12E-07 | 1.37736619 | NM_003097| | SNRPN,small nuclear ribonucleoprotein polypeptide N |
| 218361_at | 37.95 | 1.12E-07 | 1.187032662 | NM_018178| | GOLPH3L,GPP34-related protein |
| 230356_at | 37.94 | 1.12E-07 | 3.263328327 | NA |  |
| 201487_at | 37.93 | 1.12E-07 | 1.176681577 | NM_001114173| | NA |
| 225030_at | 37.81 | 1.14E-07 | 1.052307304 | NM_138369| | FAM44B,family with sequence similarity 44, member B |
| 205100_at | 37.79 | 1.14E-07 | 1.790510608 | NM_005110| | GFPT2,glutamine-fructose-6-phosphate transaminase 2 |
| 228489_at | 37.76 | 1.15E-07 | 2.203688091 | NM_138786| | LOC116441,hypothetical protein BC014339 |
| 227262_at | 37.75 | 1.15E-07 | 1.231995903 | NM_178232| | HAPLN3,hyaluronan and proteoglycan link protein 3 |
| 204044_at | 37.74 | 1.15E-07 | 2.025177044 | NM_014298| | QPRT,quinolinate phosphoribosyltransferase |
| 227599_at | 37.74 | 1.15E-07 | 1.309361224 | NM_178496| | LOC151963,similar to BcDNA:GH11415 gene product |
| 225579_at | 37.74 | 1.15E-07 | 1.151985867 | NM_152391| | C2orf22,hypothetical protein MGC33602 |
| 201313_at | 37.69 | 1.16E-07 | 1.227280207 | NM_001975| | ENO2,enolase 2 |
| 223193_x_at | 37.69 | 1.16E-07 | 1.120220089 | NM_014367| | E2IG5,growth and transformation-dependent protein |
| 205977_s_at | 37.67 | 1.16E-07 | 2.306688491 | NM_005232| | EPHA1,ephrin receptor EphA1 |
| 227794_at | 37.62 | 1.17E-07 | 1.892267304 | NM_080661| | MGC15937,hypothetical protein MGC15937 similar to |
| 226549_at | 37.58 | 1.17E-07 | 1.319043519 | NM_001024401| | NA |
| 201362_at | 37.57 | 1.17E-07 | 1.096155497 | NM_006469| | IVNS1ABP,influenza virus NS1A binding protein isoform a |
| 219660_s_at | 37.56 | 1.17E-07 | 1.503006529 | NM_016529| | ATP8A2,ATPase, aminophospholipid transporter-like, |
| 208096_s_at | 37.55 | 1.17E-07 | 1.385395322 | NM_030820| | COL21A1,alpha 1 type XXI collagen precursor |
| 209726_at | 37.53 | 1.18E-07 | 1.56782302 | NM_001217| | CA11,carbonic anhydrase XI precursor |
| 207305_s_at | 37.53 | 1.18E-07 | 1.085593165 | NM_014939| | KIAA1012,KIAA1012 |
| 1568609_s_at | 37.52 | 1.18E-07 | 2.022173583 | NA |  |
| 203139_at | 37.49 | 1.18E-07 | 1.144827425 | NM_004938| | DAPK1,death-associated protein kinase 1 |
| 226103_at | 37.49 | 1.18E-07 | 1.961649913 | NM_144573| | NEXN,nexilin (F actin binding protein) |
| 242890_at | 37.47 | 1.18E-07 | 1.180737405 | NA |  |
| 207186_s_at | 37.44 | 1.19E-07 | 1.081086775 | NM_004459| | FALZ,fetal Alzheimer antigen isoform 2 |
| 205440_s_at | 37.41 | 1.19E-07 | 2.545527953 | NM_000909| | NPY1R,neuropeptide Y receptor Y1 |
| 214369_s_at | 37.4 | 1.19E-07 | 2.017722143 | NM_001098670| | NA |
| 1255_g_at | 37.38 | 1.19E-07 | 2.772772795 | NM_000409| | GUCA1A,guanylate cyclase activator 1A (retina) |
| 228776_at | 37.38 | 1.19E-07 | 1.101672087 | NM_001080383| | NA |
| 202733_at | 37.38 | 1.19E-07 | 1.283894873 | NM_001017973| | NA |
| 224467_s_at | 37.35 | 1.20E-07 | 1.086606097 | NM_032346| | MGC13096,hypothetical protein MGC13096 |
| 209398_at | 37.32 | 1.20E-07 | 1.24762085 | NM_005319| | HIST1H1C,H1 histone family, member 2 |
| 1559280_a_at | 37.24 | 1.22E-07 | 3.354905257 | NA |  |
| 239292_at | 37.24 | 1.22E-07 | 1.911247608 | NA |  |
| 219439_at | 37.17 | 1.23E-07 | 1.414848462 | NM_020156| | C1GALT1,core 1 synthase, |
| 1555724_s_at | 37.11 | 1.23E-07 | 1.719046776 | NM_001001522| | TAGLN,transgelin |
| 226456_at | 37.09 | 1.24E-07 | 1.25553726 | NM_152308| | MGC24665,hypothetical protein MGC24665 |
| 209296_at | 37.06 | 1.24E-07 | 1.235284495 | NM_001033556| | NA |
| 224156_x_at | 37.03 | 1.25E-07 | 1.177519351 | NM_018725| | IL17RB,interleukin 17B receptor isoform 1 precursor |
| 203295_s_at | 37.01 | 1.25E-07 | 1.317262734 | NM_000702| | ATP1A2,Na+/K+ -ATPase alpha 2 subunit proprotein |
| 213400_s_at | 36.97 | 1.26E-07 | 1.110938674 | NM_005647| | TBL1X,transducin beta-like 1X |
| 232282_at | 36.96 | 1.26E-07 | 1.284855518 | NM_001002838| | WNK3,WNK lysine deficient protein kinase 3 isoform 2 |
| 202052_s_at | 36.96 | 1.26E-07 | 1.081154696 | NM_015577| | RAI14,retinoic acid induced 14 |
| 226778_at | 36.91 | 1.27E-07 | 1.267314424 | NM_175075| | INM01,hypothetical protein INM01 |
| 222361_at | 36.9 | 1.27E-07 | 1.524482951 | NA |  |
| 221810_at | 36.88 | 1.27E-07 | 1.254549637 | NM_198686| | RAB15,Ras-related protein Rab-15 |
| 227284_at | 36.83 | 1.28E-07 | 1.128696637 | NM_001010851| | LOC90321,hypothetical protein LOC90321 |
| 236480_at | 36.78 | 1.29E-07 | 1.270228477 | NA |  |
| 214058_at | 36.78 | 1.29E-07 | 1.35947547 | NM_001033081| | NA |
| 230623_x_at | 36.73 | 1.30E-07 | 1.213275943 | NM_020886| | USP28,ubiquitin specific protease 28 |
| 231991_at | 36.71 | 1.30E-07 | 1.920199961 | NM_080625| | C20orf160,chromosome 20 open reading frame 160 |
| 211990_at | 36.7 | 1.30E-07 | 1.547211696 | NM_033554| | HLA-DPA1,major histocompatibility complex, class II, DP |
| 222877_at | 36.68 | 1.30E-07 | 1.533830073 | NA |  |
| 201601_x_at | 36.67 | 1.30E-07 | 2.048439457 | NM_003641| | IFITM1,interferon induced transmembrane protein 1 |
| 219134_at | 36.62 | 1.31E-07 | 2.015900062 | NM_022159| | NA |
| 239503_at | 36.62 | 1.31E-07 | 1.473422446 | NA |  |
| 219143_s_at | 36.6 | 1.31E-07 | 1.268385724 | NM_017793| | RPP25,ribonuclease P 25kDa subunit |
| 230497_at | 36.58 | 1.32E-07 | 1.791022064 | NM_021938| | BRUNOL5,bruno-like 5, RNA binding protein |
| 214440_at | 36.55 | 1.32E-07 | 1.367869831 | NM_000662| | NAT1,N-acetyltransferase 1 |
| 201852_x_at | 36.55 | 1.32E-07 | 2.290163442 | NM_000090| | COL3A1,alpha 1 type III collagen |
| 1570266_x_at | 36.54 | 1.33E-07 | 1.742673072 | NA |  |
| 212943_at | 36.51 | 1.33E-07 | 1.088881474 | NM_014802| | KIAA0528,KIAA0528 gene product |
| 206555_s_at | 36.47 | 1.34E-07 | 1.093800996 | NM_017736| | THUMPD1,THUMP domain containing 1 |
| 202540_s_at | 36.46 | 1.34E-07 | 1.054669265 | NM_000859| | HMGCR,3-hydroxy-3-methylglutaryl-Coenzyme A reductase |
| 202081_at | 36.45 | 1.34E-07 | 1.096002245 | NM_004907| | IER2,immediate early response 2 |
| 234994_at | 36.45 | 1.34E-07 | 3.178456528 | NM_052913| | KIAA1913,KIAA1913 |
| 235144_at | 36.39 | 1.35E-07 | 1.752435989 | NA |  |
| 208650_s_at | 36.36 | 1.36E-07 | 1.39643512 | NM_013230| | CD24,CD24 antigen |
| 212012_at | 36.36 | 1.36E-07 | 1.13438484 | NM_012293| | NA |
| 205429_s_at | 36.33 | 1.37E-07 | 1.206962126 | NM_016447| | MPP6,membrane protein, palmitoylated 6 |
| 1553411_s_at | 36.29 | 1.37E-07 | 1.256352615 | NM_171999| | SALL3,sal-like 3 |
| 203517_at | 36.29 | 1.37E-07 | 1.1603004 | NM_001006635| | MTX2,metaxin 2 isoform b |
| 215101_s_at | 36.29 | 1.37E-07 | 2.856848253 | NM_002994| | CXCL5,chemokine (C-X-C motif) ligand 5 precursor |
| 206932_at | 36.28 | 1.37E-07 | 1.867668184 | NM_003956| | CH25H,cholesterol 25-hydroxylase |
| 203438_at | 36.24 | 1.37E-07 | 1.962084629 | NM_003714| | STC2,stanniocalcin 2 |
| 232202_at | 36.23 | 1.37E-07 | 1.74086965 | NA |  |
| 222701_s_at | 36.22 | 1.38E-07 | 1.491909579 | NM_001011667| | CHCHD7,coiled-coil-helix-coiled-coil-helix domain |
| 224933_s_at | 36.16 | 1.39E-07 | 1.138369232 | NM_004241| | JMJD1C,jumonji domain containing 1C |
| 218617_at | 36.15 | 1.39E-07 | 1.054120427 | NM_017646| | TRIT1,tRNA isopentenyltransferase 1 |
| 201939_at | 36.11 | 1.40E-07 | 1.360017286 | NM_006622| | PLK2,polo-like kinase 2 |
| 225816_at | 36.11 | 1.40E-07 | 1.233494466 | NM_024900| | PHF17,Jade1 protein short isoform |
| 227254_at | 36.09 | 1.41E-07 | 1.230835098 | NM_002697| | POU2F1,POU domain, class 2, transcription factor 1 |
| 229796_at | 36.07 | 1.41E-07 | 1.228316251 | NM_017420| | SIX4,sine oculis homeobox homolog 4 |
| 238417_at | 36.04 | 1.41E-07 | 1.53058186 | NM_173582| | PGM2L1,phosphoglucomutase 2-like 1 |
| 228291_s_at | 36.03 | 1.41E-07 | 1.256075052 | NM_018474| | C20orf19,uncharacterized hypothalamus protein HT013 |
| 210260_s_at | 36.02 | 1.42E-07 | 1.51056115 | NM_001077654| | NA |
| 219573_at | 35.98 | 1.43E-07 | 1.231586346 | NM_017640| | LRRC16,leucine rich repeat containing 16 |
| 236297_at | 35.97 | 1.43E-07 | 1.83759328 | NA |  |
| 223503_at | 35.91 | 1.44E-07 | 2.220774299 | NM_030923| | DKFZP566N034,hypothetical protein DKFZp566N034 |
| 215342_s_at | 35.88 | 1.45E-07 | 1.797832587 | NM_001035230| | NA |
| 213301_x_at | 35.88 | 1.45E-07 | 1.151549179 | NM_003852| | TIF1,transcriptional intermediary factor 1 alpha |
| 226185_at | 35.86 | 1.45E-07 | 2.213557809 | NA |  |
| 208855_s_at | 35.83 | 1.45E-07 | 1.045522502 | NM_001032296| | NA |
| 220239_at | 35.83 | 1.45E-07 | 1.176876129 | NM_001031710| | NA |
| 220147_s_at | 35.8 | 1.46E-07 | 1.240806876 | NM_021238| | C12orf14,chromosome 12 open reading frame 14 |
| 209493_at | 35.79 | 1.46E-07 | 1.96424687 | NM_178140| | PDZK3,PDZ domain containing 3 isoform a |
| 200007_at | 35.78 | 1.46E-07 | 1.063024386 | NM_003134| | SRP14,signal recognition particle 14kDa (homologous |
| 212314_at | 35.78 | 1.46E-07 | 1.236963153 | NM_015187| | KIAA0746,KIAA0746 protein |
| 205751_at | 35.77 | 1.46E-07 | 1.207714417 | NM_003026| | SH3GL2,SH3-domain GRB2-like 2 |
| 226161_at | 35.75 | 1.46E-07 | 1.120219294 | NM_017964| | SLC30A6,solute carrier family 30 (zinc transporter), |
| 202723_s_at | 35.75 | 1.46E-07 | 1.195230539 | NM_002015| | FOXO1A,forkhead box O1A |
| 202498_s_at | 35.74 | 1.47E-07 | 2.097852401 | NM_006931| | SLC2A3,solute carrier family 2 (facilitated glucose |
| 220536_at | 35.73 | 1.47E-07 | 1.795034251 | NM_018228| | C14orf115,chromosome 14 open reading frame 115 |
| 201430_s_at | 35.72 | 1.47E-07 | 1.57843479 | NM_001387| | DPYSL3,dihydropyrimidinase-like 3 |
| 227349_at | 35.72 | 1.47E-07 | 1.147849864 | NM_018063| | HELLS,helicase, lymphoid-specific |
| 201365_at | 35.7 | 1.47E-07 | 1.202283656 | NM_002537| | OAZ2,ornithine decarboxylase antizyme 2 |
| 212371_at | 35.68 | 1.47E-07 | 1.089441813 | NM_016076| | PNAS-4,CGI-146 protein |
| 1553132_a_at | 35.68 | 1.47E-07 | 2.191027128 | NM_152332| | MTAC2D1,membrane targeting (tandem) C2 domain containing |
| 223681_s_at | 35.66 | 1.47E-07 | 1.950386331 | NM_176877| | INADL,InaD-like protein isoform 2 |
| 212912_at | 35.62 | 1.48E-07 | 1.206318219 | NM_001006932| | RPS6KA2,ribosomal protein S6 kinase, 90kDa, polypeptide |
| 222033_s_at | 35.61 | 1.49E-07 | 2.234715497 | NA |  |
| 212859_x_at | 35.57 | 1.50E-07 | 1.408818109 | NM_175617| | MT1E,metallothionein 1E |
| 238956_at | 35.55 | 1.50E-07 | 1.304426656 | NA |  |
| 219955_at | 35.51 | 1.51E-07 | 3.505281986 | NM_019079| | FLJ10884,hypothetical protein FLJ10884 |
| 226817_at | 35.51 | 1.51E-07 | 2.824004471 | NM_004949| | DSC2,desmocollin 2 isoform Dsc2b preproprotein |
| 220161_s_at | 35.5 | 1.51E-07 | 1.463799932 | NM_018424| | EPB41L4B,erythrocyte membrane protein band 4.1 like 4B |
| 221690_s_at | 35.5 | 1.51E-07 | 1.435169657 | NM_017852| | NALP2,NACHT, leucine rich repeat and PYD containing 2 |
| 202536_at | 35.5 | 1.51E-07 | 1.133509129 | NM_014043| | DKFZP564O123,DKFZP564O123 protein |
| 203286_at | 35.47 | 1.51E-07 | 1.116481606 | NM_014901| | RNF44,ring finger protein 44 |
| 204005_s_at | 35.46 | 1.51E-07 | 1.168005079 | NM_002583| | PAWR,PRKC, apoptosis, WT1, regulator |
| 205052_at | 35.43 | 1.52E-07 | 1.140469256 | NM_001698| | AUH,AU RNA-binding protein/enoyl-Coenzyme A |
| 213590_at | 35.43 | 1.52E-07 | 1.737374008 | NM_004695| | SLC16A5,solute carrier family 16, member 5 |
| 205126_at | 35.42 | 1.52E-07 | 1.232124777 | NM_006296| | VRK2,vaccinia related kinase 2 |
| 1552712_a_at | 35.38 | 1.53E-07 | 1.469027152 | NM_015039| | NMNAT2,nicotinamide mononucleotide adenylyltransferase |
| 207076_s_at | 35.3 | 1.55E-07 | 1.191535606 | NM_000050| | ASS,argininosuccinate synthetase |
| 200640_at | 35.3 | 1.55E-07 | 1.149892295 | NM_003406| | YWHAZ,tyrosine 3/tryptophan 5 -monooxygenase |
| 203605_at | 35.27 | 1.56E-07 | 1.112111372 | NM_003136| | SRP54,signal recognition particle 54kDa |
| 204416_x_at | 35.26 | 1.56E-07 | 1.79633801 | NM_001645| | APOC1,apolipoprotein C-I precursor |
| 206552_s_at | 35.25 | 1.56E-07 | 2.904721002 | NM_003182| | TAC1,tachykinin 1 isoform beta precursor |
| 204278_s_at | 35.21 | 1.57E-07 | 1.090379142 | NM_004215| | EBAG9,estrogen receptor binding site associated |
| 201548_s_at | 35.16 | 1.58E-07 | 1.227623432 | NM_006618| | JARID1B,Jumonji, AT rich interactive domain 1B |
| 213698_at | 35.11 | 1.59E-07 | 1.17728626 | NM_007167| | ZNF258,zinc finger protein 258 |
| 211546_x_at | 35.1 | 1.59E-07 | 1.271059585 | NM_000345| | SNCA,alpha-synuclein isoform NACP140 |
| 206204_at | 35.09 | 1.60E-07 | 1.271651767 | NM_004490| | GRB14,growth factor receptor-bound protein 14 |
| 228959_at | 35.07 | 1.60E-07 | 1.08521905 | NA |  |
| 210029_at | 35.03 | 1.61E-07 | 1.993246451 | NM_002164| | INDO,indoleamine-pyrrole 2,3 dioxygenase |
| 227645_at | 35.02 | 1.61E-07 | 1.477073147 | NM_014308| | PIK3R5,phosphoinositide-3-kinase, regulatory subunit 5, |
| 203382_s_at | 35.01 | 1.61E-07 | 1.913400183 | NM_000041| | APOE,apolipoprotein E precursor |
| 201512_s_at | 34.98 | 1.62E-07 | 1.077223172 | NM_014820| | TOMM70A,translocase of outer mitochondrial membrane 70 |
| 229518_at | 34.91 | 1.64E-07 | 2.164422684 | NM_052943| | FAM46B,family with sequence similarity 46, member B |
| 205532_s_at | 34.9 | 1.64E-07 | 1.902974327 | NM_004932| | CDH6,cadherin 6, type 2 preproprotein |
| 201590_x_at | 34.9 | 1.64E-07 | 1.103109497 | NM_001002857| | ANXA2,annexin A2 isoform 2 |
| 204596_s_at | 34.88 | 1.65E-07 | 1.531608608 | NM_003155| | STC1,stanniocalcin 1 |
| 202465_at | 34.84 | 1.66E-07 | 1.56509133 | NM_002593| | PCOLCE,procollagen C-endopeptidase enhancer |
| 227276_at | 34.83 | 1.66E-07 | 1.654912955 | NM_032812| | PLXDC2,plexin domain containing 2 precursor |
| 201844_s_at | 34.82 | 1.66E-07 | 1.192038877 | NM_012234| | RYBP,RING1 and YY1 binding protein |
| 204447_at | 34.81 | 1.66E-07 | 1.292292973 | NM_014731| | ProSAPiP1,ProSAPiP1 protein |
| 215543_s_at | 34.79 | 1.66E-07 | 1.263258932 | NM_004737| | LARGE,like-glycosyltransferase |
| 202388_at | 34.77 | 1.67E-07 | 1.258939898 | NM_002923| | RGS2,regulator of G-protein signalling 2, 24kDa |
| 1554592_a_at | 34.74 | 1.68E-07 | 2.271796292 | NM_005071| | SLC1A6,solute carrier family 1 (high affinity |
| 231914_at | 34.73 | 1.68E-07 | 1.318179365 | NM_177533| | NUDT14,nudix -type motif 14 |
| 226809_at | 34.72 | 1.68E-07 | 1.732027079 | NA |  |
| 208941_s_at | 34.7 | 1.69E-07 | 1.163662851 | NM_012247| | SEPHS1,selenophosphate synthetase |
| 233064_at | 34.62 | 1.71E-07 | 1.604904773 | NA |  |
| 228153_at | 34.61 | 1.71E-07 | 1.352368424 | NM_182757| | IBRDC2,IBR domain containing 2 |
| 218450_at | 34.61 | 1.71E-07 | 1.146640741 | NM_015987| | HEBP1,heme binding protein 1 |
| 213447_at | 34.6 | 1.71E-07 | 1.351697617 | NA |  |
| 226455_at | 34.59 | 1.71E-07 | 1.258221828 | NM_130898| | CREB3L4,cAMP responsive element binding protein 3-like |
| 201318_s_at | 34.58 | 1.71E-07 | 1.054892618 | NM_006471| | MRCL3,myosin regulatory light chain MRCL3 |
| 214442_s_at | 34.56 | 1.72E-07 | 1.121450784 | NM_004671| | PIAS2,protein inhibitor of activated STAT X isoform |
| 203945_at | 34.56 | 1.72E-07 | 1.286904359 | NM_001172| | ARG2,arginase, type II precursor |
| 222750_s_at | 34.51 | 1.73E-07 | 1.113550263 | NM_024592| | SRD5A2L,steroid 5 alpha-reductase 2-like |
| 235301_at | 34.5 | 1.74E-07 | 1.866524776 | NM_152748| | FLJ31340,hypothetical protein FLJ31340 |
| 219466_s_at | 34.48 | 1.74E-07 | 1.876057914 | NM_001643| | APOA2,apolipoprotein A-II precursor |
| 225731_at | 34.48 | 1.74E-07 | 1.128596746 | NM_020337| | NA |
| 229461_x_at | 34.44 | 1.75E-07 | 2.492509227 | NM_173808| | NEGR1,neuronal growth regulator 1 |
| 203895_at | 34.42 | 1.75E-07 | 1.337392763 | NM_000933| | PLCB4,phospholipase C beta 4 isoform a |
| 1556499_s_at | 34.39 | 1.76E-07 | 2.805591231 | NM_000088| | COL1A1,alpha 1 type I collagen preproprotein |
| 203000_at | 34.35 | 1.76E-07 | 1.929098832 | NM_007029| | STMN2,superiorcervical ganglia, neural specific 10 |
| 203574_at | 34.34 | 1.77E-07 | 1.302108446 | NM_005384| | NFIL3,nuclear factor, interleukin 3 regulated |
| 220677_s_at | 34.33 | 1.77E-07 | 1.525120203 | NM_007037| | ADAMTS8,a disintegrin and metalloprotease with |
| 203325_s_at | 34.31 | 1.77E-07 | 1.914247642 | NM_000093| | COL5A1,alpha 1 type V collagen preproprotein |
| 222870_s_at | 34.29 | 1.78E-07 | 1.421003082 | NM_006577| | B3GNT1,beta-1,3-N-acetylglucosaminyltransferase bGnT-1 |
| 202455_at | 34.28 | 1.78E-07 | 1.179828512 | NM_001015053| | NA |
| 218854_at | 34.28 | 1.78E-07 | 1.582016008 | NM_001080976| | NA |
| 232060_at | 34.25 | 1.79E-07 | 1.910194036 | NM_001083592| | NA |
| 209442_x_at | 34.23 | 1.79E-07 | 1.407309825 | NM_001149| | ANK3,ankyrin 3 isoform 2 |
| 216565_x_at | 34.2 | 1.80E-07 | 1.189201488 | NA |  |
| 65517_at | 34.2 | 1.80E-07 | 1.569316145 | NM_005498| | AP1M2,adaptor-related protein complex 1, mu 2 subunit |
| 239302_s_at | 34.18 | 1.80E-07 | 1.514243974 | NA |  |
| 219949_at | 34.17 | 1.80E-07 | 2.086037563 | NM_024512| | LRRC2,leucine rich repeat containing 2 |
| 211767_at | 34.14 | 1.81E-07 | 1.107710525 | NM_032336| | SLD5,SLD5 |
| 233297_s_at | 34.14 | 1.81E-07 | 1.581449832 | NA |  |
| 221260_s_at | 34.14 | 1.81E-07 | 1.132716616 | NM_030809| | C12orf22,TGF-beta induced apotosis protein 12 |
| 229523_at | 34.08 | 1.82E-07 | 2.030303254 | NM_001080209| | NA |
| 223246_s_at | 34.07 | 1.82E-07 | 1.239282489 | NM_018387| | STRBP,spermatid perinuclear RNA-binding protein |
| 205505_at | 34.06 | 1.83E-07 | 1.886035203 | NM_001097633| | NA |
| 238066_at | 34.06 | 1.83E-07 | 1.521450561 | NM_052960| | RBP7,retinol binding protein 7, cellular |
| 212686_at | 34.05 | 1.83E-07 | 2.438725307 | NM_020700| | NA |
| 202074_s_at | 34.02 | 1.83E-07 | 1.361003876 | NM_001008211| | OPTN,optineurin |
| 208737_at | 33.98 | 1.84E-07 | 1.078405441 | NM_004888| | ATP6V1G1,ATPase, H+ transporting, lysosomal, V1 subunit G |
| 218286_s_at | 33.98 | 1.84E-07 | 1.082996028 | NM_014245| | RNF7,ring finger protein 7 isoform 1 |
| 222401_s_at | 33.98 | 1.84E-07 | 1.072040887 | NM_014313| | SMP1,small membrane protein 1 |
| 230903_s_at | 33.93 | 1.86E-07 | 1.243817291 | NM_175075| | INM01,hypothetical protein INM01 |
| 218434_s_at | 33.91 | 1.87E-07 | 1.067246865 | NM_023928| | AACS,acetoacetyl-CoA synthetase |
| 224702_at | 33.83 | 1.89E-07 | 1.201674338 | NM_174909| | MGC23909,hypothetical protein MGC23909 |
| 226148_at | 33.79 | 1.90E-07 | 1.095878908 | NM_014155| | HSPC063,HSPC063 protein |
| 209659_s_at | 33.79 | 1.90E-07 | 1.049773971 | NM_001078645| | NA |
| 206261_at | 33.77 | 1.91E-07 | 1.261771804 | NM_001099282| | NA |
| 204686_at | 33.74 | 1.91E-07 | 1.333382548 | NM_005544| | IRS1,insulin receptor substrate 1 |
| 209048_s_at | 33.73 | 1.92E-07 | 1.170603082 | NM_012408| | PRKCBP1,protein kinase C binding protein 1 isoform b |
| 214889_at | 33.73 | 1.92E-07 | 1.250599014 | NM_001006655| | DKFZP564J102,DKFZP564J102 protein |
| 213920_at | 33.72 | 1.92E-07 | 2.013556783 | NM_015267| | CUTL2,cut-like 2 |
| 1554485_s_at | 33.69 | 1.92E-07 | 1.875772543 | NM_183240| | TMEM37,transmembrane protein 37 |
| 214463_x_at | 33.69 | 1.92E-07 | 1.197101292 | NM_021968| | HIST1H4J,H4 histone family, member E |
| 223402_at | 33.67 | 1.93E-07 | 1.149939284 | NM_017823| | DUSP23,dual specificity phosphatase 23 |
| 223305_at | 33.66 | 1.93E-07 | 1.194703704 | NM_016499| | MGC13379,HSPC244 |
| 212884_x_at | 33.65 | 1.93E-07 | 1.639314758 | NM_000041| | APOE,apolipoprotein E precursor |
| 226866_at | 33.62 | 1.94E-07 | 1.13674115 | NM_052911| | ESCO1,establishment of cohesion 1 homolog 1 |
| 217574_at | 33.61 | 1.94E-07 | 1.619467362 | NM_001796| | CDH8,cadherin 8, type 2 preproprotein |
| 227955_s_at | 33.59 | 1.95E-07 | 1.478465369 | NA |  |
| 237810_at | 33.58 | 1.95E-07 | 2.750867686 | NM_021195| | CLDN6,claudin 6 |
| 226421_at | 33.56 | 1.96E-07 | 1.131714812 | NM_001025580| | NA |
| 225775_at | 33.54 | 1.96E-07 | 1.168526611 | NM_178562| | MGC50844,hypothetical protein MGC50844 |
| 209631_s_at | 33.51 | 1.97E-07 | 2.471184888 | NM_005302| | GPR37,G protein-coupled receptor 37 |
| 207836_s_at | 33.5 | 1.97E-07 | 1.63514684 | NM_001008710| | RBPMS,RNA-binding protein with multiple splicing |
| 202517_at | 33.5 | 1.97E-07 | 1.158854628 | NM_001014809| | NA |
| 220272_at | 33.5 | 1.97E-07 | 1.809711812 | NM_017637| | BNC2,basonuclin 2 |
| 1559470_at | 33.5 | 1.97E-07 | 2.259057341 | NA |  |
| 218333_at | 33.48 | 1.98E-07 | 1.093286382 | NM_016041| | DERL2,Der1-like domain family, member 2 |
| 240479_at | 33.48 | 1.97E-07 | 1.645630898 | NM_153612| | HS3ST5,heparan sulfate (glucosamine) |
| 240382_at | 33.47 | 1.98E-07 | 1.857397531 | NA |  |
| 231514_at | 33.46 | 1.98E-07 | 1.517650955 | NM_032884| | MGC15882,hypothetical protein MGC15882 |
| 209064_x_at | 33.45 | 1.98E-07 | 1.127009506 | NM_006451| | PAIP1,poly(A) binding protein interacting protein 1 |
| 203058_s_at | 33.42 | 1.98E-07 | 1.736689554 | NM_001015880| | NA |
| 228145_s_at | 33.42 | 1.98E-07 | 1.115540722 | NM_020781| | ZNF398,zinc finger 398 isoform b |
| 230269_at | 33.41 | 1.99E-07 | 1.223703647 | NA |  |
| 224967_at | 33.41 | 1.98E-07 | 1.097896994 | NM_003358| | UGCG,ceramide glucosyltransferase |
| 238974_at | 33.41 | 1.99E-07 | 1.106389304 | NM_153689| | FLJ38973,hypothetical protein FLJ38973 |
| 209278_s_at | 33.4 | 1.99E-07 | 1.83615091 | NM_006528| | TFPI2,tissue factor pathway inhibitor 2 |
| 213075_at | 33.37 | 1.99E-07 | 1.933670199 | NM_182487| | OLFML2A,olfactomedin-like 2A |
| 227933_at | 33.37 | 1.99E-07 | 1.228840219 | NM_032808| | LRRN6A,leucine-rich repeat neuronal 6A |
| 228785_at | 33.37 | 1.99E-07 | 1.214196162 | NM_012482| | ZNF281,zinc finger protein 281 |
| 226435_at | 33.36 | 1.99E-07 | 2.225592557 | NM_173462| | PAPLN,papilin |
| 209147_s_at | 33.31 | 2.01E-07 | 1.355248822 | NM_003711| | PPAP2A,phosphatidic acid phosphatase type 2A isoform 1 |
| 201158_at | 33.3 | 2.01E-07 | 1.075202429 | NM_021079| | NMT1,N-myristoyltransferase 1 |
| 219271_at | 33.29 | 2.01E-07 | 1.433227957 | NM_024572| | GALNT14,UDP-N-acetyl-alpha-D-galactosamine:polypeptide |
| 203430_at | 33.26 | 2.02E-07 | 1.105244585 | NM_014320| | HEBP2,heme binding protein 2 |
| 201351_s_at | 33.25 | 2.02E-07 | 1.048157558 | NM_014263| | YME1L1,YME1-like 1 isoform 3 |
| 231188_at | 33.25 | 2.02E-07 | 1.212663326 | NM_001007072| | ZSCAN2,zinc finger protein 29 isoform 3 |
| 203528_at | 33.24 | 2.03E-07 | 1.274713715 | NM_006378| | SEMA4D,semaphorin 4D |
| 209704_at | 33.22 | 2.03E-07 | 1.320025095 | NM_007358| | M96,putative DNA binding protein |
| 228041_at | 33.21 | 2.03E-07 | 1.044975882 | NM_181806| | NRPS998,2-aminoadipic 6-semialdehyde dehydrogenase |
| 212881_at | 33.2 | 2.04E-07 | 1.144817781 | NM_015897| | PIAS4,protein inhibitor of activated STAT, 4 |
| 218945_at | 33.13 | 2.06E-07 | 1.094027432 | NM_024109| | MGC2654,hypothetical protein MGC2654 |
| AFFX-M27830_5_at | 33.11 | 2.06E-07 | 1.058635174 | NA |  |
| 235048_at | 33.07 | 2.08E-07 | 1.279231863 | NM_015566| | NA |
| 238360_s_at | 33.03 | 2.09E-07 | 1.982497818 | NA |  |
| 233337_s_at | 33.03 | 2.09E-07 | 1.604110069 | NM_001114099| | NA |
| 202311_s_at | 33.01 | 2.10E-07 | 2.17176951 | NM_000088| | COL1A1,alpha 1 type I collagen preproprotein |
| 235148_at | 33 | 2.10E-07 | 1.707964966 | NM_173853| | KRTCAP3,keratinocyte associated protein 3 |
| 201427_s_at | 32.98 | 2.10E-07 | 2.14027241 | NM_001085486| | NA |
| 213676_at | 32.96 | 2.11E-07 | 1.646769628 | NA |  |
| 218983_at | 32.96 | 2.11E-07 | 1.96021835 | NM_016546| | C1RL,complement component 1, r subcomponent-like |
| 1560019_at | 32.96 | 2.11E-07 | 2.253835784 | NA |  |
| 222982_x_at | 32.94 | 2.11E-07 | 1.086423686 | NM_018976| | SLC38A2,solute carrier family 38, member 2 |
| 204285_s_at | 32.94 | 2.11E-07 | 1.204778408 | NM_021127| | PMAIP1,phorbol-12-myristate-13-acetate-induced protein |
| 208770_s_at | 32.93 | 2.11E-07 | 1.142860477 | NM_004096| | EIF4EBP2,eukaryotic translation initiation factor 4E |
| 205113_at | 32.88 | 2.13E-07 | 1.890892973 | NM_001105541| | NA |
| 203453_at | 32.85 | 2.14E-07 | 2.445443555 | NM_001038| | SCNN1A,sodium channel, nonvoltage-gated 1 alpha |
| 238751_at | 32.82 | 2.15E-07 | 1.885662947 | NA |  |
| 223658_at | 32.81 | 2.15E-07 | 1.770728764 | NM_004823| | KCNK6,potassium channel, subfamily K, member 6 |
| 229521_at | 32.8 | 2.15E-07 | 1.317798121 | NM_175884| | FLJ36031,hypothetical protein FLJ36031 |
| 204865_at | 32.77 | 2.16E-07 | 2.026915133 | NM_005181| | CA3,carbonic anhydrase III |
| 1555630_a_at | 32.77 | 2.16E-07 | 1.189359785 | NM_031934| | RAB34,RAB39 |
| 219155_at | 32.76 | 2.16E-07 | 1.167638283 | NM_012417| | PITPNC1,phosphatidylinositol transfer protein, |
| 211203_s_at | 32.69 | 2.19E-07 | 1.305129567 | NM_001843| | CNTN1,contactin 1 isoform 1 precursor |
| 207069_s_at | 32.67 | 2.19E-07 | 1.50126255 | NM_005585| | SMAD6,MAD, mothers against decapentaplegic homolog 6 |
| 225434_at | 32.65 | 2.20E-07 | 1.168053437 | NM_133328| | DEDD2,death effector domain-containing DNA binding |
| 227224_at | 32.63 | 2.21E-07 | 2.332340716 | NM_018037| | RALGPS2,Ral GEF with PH domain and SH3 binding motif 2 |
| 204400_at | 32.63 | 2.21E-07 | 1.635133005 | NM_005864| | EFS,embryonal Fyn-associated substrate isoform 1 |
| 226548_at | 32.61 | 2.21E-07 | 1.300898984 | NM_001024401| | NA |
| 202883_s_at | 32.61 | 2.21E-07 | 1.20390201 | NM_002716| | PPP2R1B,beta isoform of regulatory subunit A, protein |
| 244758_at | 32.6 | 2.21E-07 | 1.66514359 | NM_052923| | ZNF452,zinc finger protein 452 |
| 227377_at | 32.59 | 2.22E-07 | 1.908004923 | NM_006546| | IMP-1,IGF-II mRNA-binding protein 1 |
| 213307_at | 32.58 | 2.22E-07 | 1.574796238 | NM_012309| | SHANK2,SH3 and multiple ankyrin repeat domains 2 |
| 1554016_a_at | 32.56 | 2.22E-07 | 1.103010031 | NM_024598| | FLJ13154,hypothetical protein FLJ13154 |
| 226982_at | 32.55 | 2.23E-07 | 1.294435692 | NM_012081| | ELL2,elongation factor, RNA polymerase II, 2 |
| 212759_s_at | 32.54 | 2.23E-07 | 1.198911021 | NM_030756| | TCF7L2,transcription factor 7-like 2 (T-cell specific, |
| 224699_s_at | 32.53 | 2.23E-07 | 1.109462491 | NM_020728| | CHR2SYT,chr2 synaptotagmin |
| 229073_at | 32.51 | 2.24E-07 | 1.473641925 | NA |  |
| 203126_at | 32.51 | 2.24E-07 | 1.180588005 | NM_014214| | IMPA2,inositol(myo)-1(or 4)-monophosphatase 2 |
| 204715_at | 32.5 | 2.24E-07 | 1.164262058 | NM_015368| | PANX1,pannexin 1 |
| 202710_at | 32.48 | 2.24E-07 | 1.126466861 | NM_005868| | BET1,BET1 homolog |
| 202890_at | 32.44 | 2.26E-07 | 2.553637889 | NM_003980| | MAP7,microtubule-associated protein 7 |
| 1562484_at | 32.44 | 2.26E-07 | 2.468388455 | NM_001033659| | NA |
| 209620_s_at | 32.4 | 2.28E-07 | 1.097615785 | NM_004299| | ABCB7,ATP-binding cassette, sub-family B, member 7 |
| 202911_at | 32.4 | 2.28E-07 | 1.095441952 | NM_000179| | MSH6,mutS homolog 6 |
| 200977_s_at | 32.35 | 2.29E-07 | 1.120077262 | NM_001079864| | NA |
| 206506_s_at | 32.35 | 2.29E-07 | 1.194095 | NM_003599| | SUPT3H,suppressor of Ty 3 homolog |
| 213503_x_at | 32.29 | 2.31E-07 | 1.104063736 | NM_001002857| | ANXA2,annexin A2 isoform 2 |
| 201636_at | 32.26 | 2.32E-07 | 1.09144699 | NM_001013438| | NA |
| 204269_at | 32.26 | 2.32E-07 | 1.381025056 | NM_006875| | PIM2,pim-2 oncogene |
| 200639_s_at | 32.24 | 2.33E-07 | 1.140275196 | NM_003406| | YWHAZ,tyrosine 3/tryptophan 5 -monooxygenase |
| 209277_at | 32.22 | 2.33E-07 | 1.579578932 | NM_006528| | TFPI2,tissue factor pathway inhibitor 2 |
| 202886_s_at | 32.2 | 2.34E-07 | 1.112232418 | NM_002716| | PPP2R1B,beta isoform of regulatory subunit A, protein |
| 215913_s_at | 32.18 | 2.34E-07 | 1.43425283 | NM_016315| | GULP1,GULP, engulfment adaptor PTB domain containing |
| 209337_at | 32.18 | 2.34E-07 | 1.111844501 | NM_021144| | PSIP1,PC4 and SFRS1 interacting protein 1 |
| 205825_at | 32.17 | 2.35E-07 | 1.613373917 | NM_000439| | PCSK1,proprotein convertase subtilisin/kexin type 1 |
| 230986_at | 32.13 | 2.37E-07 | 1.855360199 | NM_007250| | KLF8,Kruppel-like factor 8 |
| 219874_at | 32.11 | 2.37E-07 | 1.437534138 | NM_024628| | SLC12A8,solute carrier family 12, member 8 |
| 227080_at | 32.11 | 2.37E-07 | 1.364838826 | NM_001080470| | NA |
| 222610_s_at | 32.1 | 2.37E-07 | 1.150846964 | NM_001017406| | NA |
| 227496_at | 32.1 | 2.37E-07 | 1.969446958 | NM_001489| | NR6A1,nuclear receptor subfamily 6, group A, member 1 |
| 1560587_s_at | 32.08 | 2.38E-07 | 1.056227127 | NM_012094| | PRDX5,peroxiredoxin 5 precursor, isoform a |
| 218399_s_at | 32.08 | 2.38E-07 | 1.102623035 | NM_017955| | CDCA4,cell division cycle associated 4 |
| AFFX-M27830_M_at | 32.05 | 2.39E-07 | 1.12542433 | NA |  |
| 204567_s_at | 32.04 | 2.40E-07 | 1.649786131 | NM_004915| | ABCG1,ATP-binding cassette sub-family G member 1 |
| 203449_s_at | 32.01 | 2.41E-07 | 1.358739227 | NM_003218| | TERF1,telomeric repeat binding factor 1 isoform 2 |
| 242328_at | 31.99 | 2.41E-07 | 1.602674796 | NM_138453| | RAB3C,RAB3C, member RAS oncogene family |
| 202391_at | 31.98 | 2.42E-07 | 2.890392812 | NM_006317| | BASP1,brain abundant, membrane attached signal protein |
| 219446_at | 31.97 | 2.42E-07 | 1.114161277 | NM_018157| | hSyn,brain synembryn |
| 222847_s_at | 31.96 | 2.42E-07 | 1.381457758 | NM_022073| | EGLN3,egl nine homolog 3 |
| 239155_at | 31.91 | 2.44E-07 | 1.583078189 | NA |  |
| 244008_at | 31.88 | 2.45E-07 | 1.780885397 | NA |  |
| 236456_at | 31.87 | 2.45E-07 | 1.640249888 | NM_001039970| | NA |
| 202947_s_at | 31.86 | 2.46E-07 | 1.521491873 | NM_002101| | GYPC,glycophorin C isoform 1 |
| 206363_at | 31.85 | 2.46E-07 | 1.578469358 | NM_001031804| | NA |
| 205802_at | 31.82 | 2.48E-07 | 1.279484711 | NM_003304| | TRPC1,transient receptor potential cation channel, |
| 235285_at | 31.81 | 2.48E-07 | 1.573507118 | NA |  |
| 226265_at | 31.8 | 2.48E-07 | 1.033820371 | NM_001076786| | NA |
| 206062_at | 31.78 | 2.49E-07 | 2.05894581 | NM_000409| | GUCA1A,guanylate cyclase activator 1A (retina) |
| 205803_s_at | 31.76 | 2.50E-07 | 1.267145385 | NM_003304| | TRPC1,transient receptor potential cation channel, |
| 224596_at | 31.76 | 2.50E-07 | 1.224772558 | NM_080546| | CDW92,CDW92 antigen |
| 201631_s_at | 31.75 | 2.50E-07 | 1.249269501 | NM_003897| | IER3,immediate early response 3 isoform short |
| 227916_x_at | 31.74 | 2.50E-07 | 1.06684021 | NM_001002269| | EXOSC3,exosome component 3 isoform 2 |
| 227846_at | 31.68 | 2.53E-07 | 1.638142725 | NM_007223| | GPR,putative G protein coupled receptor |
| 220116_at | 31.68 | 2.53E-07 | 1.955007015 | NM_021614| | KCNN2,small conductance calcium-activated potassium |
| 210479_s_at | 31.66 | 2.54E-07 | 1.50132092 | NM_002943| | RORA,RAR-related orphan receptor A isoform c |
| 213992_at | 31.66 | 2.54E-07 | 1.2965858 | NM_001847| | COL4A6,type IV alpha 6 collagen isoform A precursor |
| 208862_s_at | 31.66 | 2.54E-07 | 1.1132167 | NM_001085458| | NA |
| 241749_at | 31.65 | 2.54E-07 | 1.551161305 | NM_001018116| | NA |
| 204425_at | 31.65 | 2.54E-07 | 1.304877331 | NM_001666| | ARHGAP4,Rho GTPase activating protein 4 |
| 229429_x_at | 31.64 | 2.54E-07 | 1.376465628 | NA |  |
| 236465_at | 31.63 | 2.54E-07 | 1.343439468 | NM_173662| | RNF175,ring finger protein 175 |
| 36711_at | 31.62 | 2.55E-07 | 1.19625999 | NM_012323| | MAFF,transcription factor MAFF |
| 221543_s_at | 31.62 | 2.55E-07 | 1.107644976 | NM_001003790| | SPFH2,SPFH domain family, member 2 isoform 2 |
| 225601_at | 31.57 | 2.57E-07 | 1.12022655 | NM_005342| | HMGB3,high-mobility group box 3 |
| 240616_at | 31.55 | 2.58E-07 | 1.205773502 | NA |  |
| 236236_at | 31.55 | 2.58E-07 | 1.220325862 | NA |  |
| 239205_s_at | 31.54 | 2.58E-07 | 1.939584043 | NM_000573| | CR1,complement component (3b/4b) receptor 1 isoform |
| 221051_s_at | 31.53 | 2.59E-07 | 2.185000977 | NM_170678| | ITGB1BP3,integrin beta 1 binding protein 3 |
| 219651_at | 31.51 | 2.60E-07 | 2.70244733 | NM_018189| | DPPA4,developmental pluripotency associated 4 |
| 219222_at | 31.49 | 2.61E-07 | 1.210272094 | NM_022128| | RBKS,ribokinase |
| 224832_at | 31.48 | 2.61E-07 | 1.167495535 | NM_030640| | DUSP16,dual specificity phosphatase 16 |
| 210715_s_at | 31.47 | 2.61E-07 | 2.67664795 | NM_021102| | SPINT2,serine protease inhibitor, Kunitz type, 2 |
| 203160_s_at | 31.44 | 2.62E-07 | 1.108170477 | NM_003958| | RNF8,ring finger protein 8 isoform 1 |
| 219697_at | 31.42 | 2.63E-07 | 1.475719193 | NM_006043| | HS3ST2,heparan sulfate D-glucosaminyl |
| 217768_at | 31.41 | 2.63E-07 | 1.034967137 | NM_016039| | C14orf166,chromosome 14 open reading frame 166 |
| 213322_at | 31.4 | 2.64E-07 | 1.059520242 | NM_145063| | C6orf130,chromosome 6 open reading frame 130 |
| 223194_s_at | 31.37 | 2.65E-07 | 1.215698047 | NM_015482| | NA |
| 218546_at | 31.3 | 2.68E-07 | 1.756138364 | NM_024709| | FLJ14146,hypothetical protein FLJ14146 |
| 218401_s_at | 31.26 | 2.70E-07 | 1.150032967 | NM_012482| | ZNF281,zinc finger protein 281 |
| 230205_at | 31.24 | 2.71E-07 | 1.142522817 | NM_152289| | ZNF561,zinc finger protein 561 |
| 220085_at | 31.21 | 2.72E-07 | 1.117014696 | NM_018063| | HELLS,helicase, lymphoid-specific |
| 213553_x_at | 31.18 | 2.74E-07 | 1.219667999 | NM_001645| | APOC1,apolipoprotein C-I precursor |
| 218946_at | 31.18 | 2.74E-07 | 1.07476777 | NM_001002755| | HIRIP5,HIRA interacting protein 5 isoform 2 |
| 202437_s_at | 31.12 | 2.78E-07 | 2.419796609 | NM_000104| | CYP1B1,cytochrome P450, family 1, subfamily B, |
| 218091_at | 31.1 | 2.79E-07 | 1.139370604 | NM_004504| | HRB,HIV-1 Rev binding protein |
| 238547_at | 31.09 | 2.79E-07 | 1.18913905 | NM_144608| | FLJ32384,hypothetical protein MGC39389 |
| 229519_at | 31.08 | 2.79E-07 | 1.133150443 | NM_001013438| | NA |
| 219370_at | 31.08 | 2.79E-07 | 1.741539979 | NM_019845| | RPRM,reprimo, TP53 dependant G2 arrest mediator |
| 218809_at | 31.04 | 2.81E-07 | 1.086869576 | NM_024960| | PANK2,pantothenate kinase 2 isoform 3 |
| 200886_s_at | 31.02 | 2.82E-07 | 1.046321014 | NM_002629| | PGAM1,phosphoglycerate mutase 1 (brain) |
| 201099_at | 31 | 2.83E-07 | 1.170503739 | NM_001039590| | NA |
| 223885_at | 30.99 | 2.83E-07 | 1.911900421 | NM_001017440| | NA |
| 212024_x_at | 30.93 | 2.86E-07 | 1.124406995 | NM_002018| | FLII,flightless I homolog |
| 210753_s_at | 30.92 | 2.86E-07 | 1.238009929 | NM_004441| | EPHB1,ephrin receptor EphB1 precursor |
| 203205_at | 30.91 | 2.87E-07 | 1.137036365 | NM_014663| | JMJD2A,jumonji domain containing 2A |
| 203498_at | 30.82 | 2.92E-07 | 1.543611092 | NM_005822| | DSCR1L1,Down syndrome critical region gene 1-like 1 |
| 237911_at | 30.8 | 2.93E-07 | 3.044946291 | NA |  |
| 201286_at | 30.78 | 2.93E-07 | 1.101632297 | NM_001006946| | SDC1,syndecan 1 precursor |
| 227167_s_at | 30.78 | 2.93E-07 | 1.200267013 | NA |  |
| 205818_at | 30.77 | 2.93E-07 | 2.564411485 | NM_014618| | DBC1,deleted in bladder cancer 1 |
| 209921_at | 30.76 | 2.94E-07 | 1.509319523 | NM_014331| | SLC7A11,solute carrier family 7, (cationic amino acid |
| 219961_s_at | 30.76 | 2.94E-07 | 1.248563816 | NM_018474| | C20orf19,uncharacterized hypothalamus protein HT013 |
| 201058_s_at | 30.75 | 2.94E-07 | 1.82258126 | NM_006097| | MYL9,myosin regulatory light polypeptide 9 isoform a |
| 201739_at | 30.75 | 2.94E-07 | 1.356859673 | NM_005627| | SGK,serum/glucocorticoid regulated kinase |
| 214597_at | 30.75 | 2.94E-07 | 1.66950612 | NM_001050| | SSTR2,somatostatin receptor 2 |
| 200799_at | 30.72 | 2.96E-07 | 1.238798817 | NM_005345| | HSPA1A,heat shock 70kDa protein 1A |
| 219125_s_at | 30.67 | 2.98E-07 | 1.221825945 | NM_001122837| | NA |
| 242053_at | 30.64 | 3.00E-07 | 1.789222862 | NA |  |
| 202315_s_at | 30.63 | 3.00E-07 | 1.155596363 | NM_004327| | BCR,breakpoint cluster region isoform 1 |
| 202603_at | 30.6 | 3.02E-07 | 1.065193939 | NA |  |
| 234970_at | 30.6 | 3.02E-07 | 1.795083821 | NM_152332| | MTAC2D1,membrane targeting (tandem) C2 domain containing |
| 226282_at | 30.59 | 3.02E-07 | 1.121688062 | NA |  |
| 202295_s_at | 30.59 | 3.02E-07 | 1.236041522 | NM_004390| | CTSH,cathepsin H isoform a preproprotein |
| 218226_s_at | 30.58 | 3.02E-07 | 1.043521998 | NM_004547| | NDUFB4,NADH dehydrogenase (ubiquinone) 1 beta |
| 203714_s_at | 30.58 | 3.02E-07 | 1.077503484 | NM_001079515| | NA |
| 231776_at | 30.57 | 3.02E-07 | 1.916186836 | NM_005442| | EOMES,eomesodermin |
| 202511_s_at | 30.57 | 3.02E-07 | 1.126908312 | NM_004849| | APG5L,APG5 autophagy 5-like |
| 219132_at | 30.56 | 3.02E-07 | 1.360677278 | NM_021255| | PELI2,pellino 2 |
| 226834_at | 30.53 | 3.04E-07 | 1.974697777 | NA |  |
| 223122_s_at | 30.48 | 3.07E-07 | 2.735201258 | NM_003013| | SFRP2,secreted frizzled-related protein 2 precursor |
| 224250_s_at | 30.41 | 3.11E-07 | 1.076155669 | NM_024077| | SECISBP2,SECIS binding protein 2 |
| 239835_at | 30.41 | 3.11E-07 | 1.432040037 | NM_032505| | TA-KRP,T-cell activation kelch repeat protein |
| 208708_x_at | 30.41 | 3.11E-07 | 1.088311811 | NM_001969| | EIF5,eukaryotic translation initiation factor 5 |
| 221804_s_at | 30.37 | 3.13E-07 | 1.09242587 | NM_018472| | FAM45B,family with sequence similarity 45, member B |
| 203263_s_at | 30.37 | 3.13E-07 | 1.199334645 | NM_015185| | ARHGEF9,Cdc42 guanine exchange factor 9 |
| 209570_s_at | 30.37 | 3.13E-07 | 1.723613431 | NM_001040101| | NA |
| 218559_s_at | 30.31 | 3.17E-07 | 2.011961297 | NM_005461| | MAFB,transcription factor MAFB |
| 201506_at | 30.3 | 3.18E-07 | 1.732434574 | NM_000358| | TGFBI,transforming growth factor, beta-induced, 68kDa |
| 207559_s_at | 30.29 | 3.18E-07 | 1.117525533 | NM_005096| | ZNF261,zinc finger protein 261 |
| 211921_x_at | 30.25 | 3.20E-07 | 1.034718927 | NM_001099285| | NA |
| 223276_at | 30.25 | 3.20E-07 | 1.16345131 | NM_032947| | NID67,putative small membrane protein NID67 |
| 210265_x_at | 30.23 | 3.21E-07 | 2.361465771 | NA |  |
| 228827_at | 30.2 | 3.23E-07 | 1.140834692 | NA |  |
| 203879_at | 30.2 | 3.23E-07 | 1.610333196 | NM_005026| | PIK3CD,phosphoinositide-3-kinase, catalytic, delta |
| 230698_at | 30.18 | 3.24E-07 | 2.110775345 | NM_001017440| | NA |
| 224710_at | 30.18 | 3.24E-07 | 1.18415588 | NM_031934| | RAB34,RAB39 |
| 231530_s_at | 30.17 | 3.24E-07 | 1.161494602 | NM_022761| | C11orf1,hypothetical protein FLJ23499 |
| 207034_s_at | 30.12 | 3.27E-07 | 1.269379664 | NM_005270| | GLI2,GLI-Kruppel family member GLI2 isoform delta |
| 214825_at | 30.12 | 3.27E-07 | 1.894848314 | NM_001080396| | NA |
| 217982_s_at | 30.09 | 3.29E-07 | 1.053454831 | NM_006791| | MORF4L1,MORF-related gene 15 isoform 1 |
| 242517_at | 30.07 | 3.30E-07 | 2.632773396 | NM_032551| | GPR54,G protein-coupled receptor 54 |
| 201204_s_at | 30.06 | 3.30E-07 | 1.221921385 | NM_001042576| | NA |
| 227006_at | 30.06 | 3.30E-07 | 1.480930718 | NM_033256| | PPP1R14A,protein phosphatase 1, regulatory (inhibitor) |
| 230669_at | 30.05 | 3.31E-07 | 1.186652673 | NM_006506| | RASA2,RAS p21 protein activator 2 |
| 235830_at | 30.04 | 3.31E-07 | 1.531937128 | NA |  |
| 204260_at | 30.02 | 3.32E-07 | 1.419681615 | NM_001819| | CHGB,chromogranin B precursor |
| 1554689_a_at | 30.02 | 3.32E-07 | 1.279713779 | NM_020742| | NLGN4X,X-linked neuroligin 4 |
| 202272_s_at | 29.97 | 3.35E-07 | 1.139995066 | NM_015176| | FBXO28,F-box protein 28 |
| 226625_at | 29.97 | 3.35E-07 | 1.268519805 | NM_003243| | TGFBR3,transforming growth factor, beta receptor III |
| 229399_at | 29.95 | 3.36E-07 | 1.51605039 | NM_018017| | C10orf118,CTCL tumor antigen L14-2 |
| 206588_at | 29.93 | 3.37E-07 | 1.71114729 | NM_001351| | DAZL,deleted in azoospermia-like |
| 35776_at | 29.93 | 3.37E-07 | 1.164136152 | NM_001001132| | ITSN1,intersectin 1 isoform ITSN-s |
| 1558368_s_at | 29.91 | 3.38E-07 | 1.331200225 | NM_198545| | LOC374946,hypothetical gene supported by AK075558; |
| 213256_at | 29.87 | 3.41E-07 | 1.472579678 | NM_178450| | MARCH3,membrane-associated ring finger (C3HC4) 3 |
| 227684_at | 29.85 | 3.42E-07 | 1.210354064 | NM_004230| | EDG5,endothelial differentiation, sphingolipid |
| 207127_s_at | 29.83 | 3.43E-07 | 1.052068228 | NM_012207| | HNRPH3,heterogeneous nuclear ribonucleoprotein H3 |
| 212489_at | 29.83 | 3.43E-07 | 1.69765795 | NM_000093| | COL5A1,alpha 1 type V collagen preproprotein |
| 222071_s_at | 29.83 | 3.43E-07 | 2.213166597 | NM_180991| | SLCO4C1,solute carrier organic anion transporter family, |
| 1554863_s_at | 29.83 | 3.43E-07 | 1.245429125 | NM_018431| | DOK5,DOK5 protein isoform a |
| 234432_at | 29.83 | 3.43E-07 | 2.905547276 | NA |  |
| 200704_at | 29.82 | 3.43E-07 | 1.175023927 | NM_004862| | LITAF,LPS-induced TNF-alpha factor |
| 210758_at | 29.82 | 3.43E-07 | 1.14699583 | NM_021144| | PSIP1,PC4 and SFRS1 interacting protein 1 |
| 228707_at | 29.82 | 3.43E-07 | 1.931350197 | NM_194284| | CLDN23,claudin 23 |
| 203738_at | 29.81 | 3.43E-07 | 1.087044683 | NM_018356| | FLJ11193,hypothetical protein FLJ11193 |
| 220668_s_at | 29.8 | 3.44E-07 | 1.698596505 | NM_006892| | DNMT3B,DNA cytosine-5 methyltransferase 3 beta isoform |
| 225651_at | 29.78 | 3.45E-07 | 1.346669501 | NM_152653| | UBE2E2,ubiquitin-conjugating enzyme E2E 2 (UBC4/5 |
| 204064_at | 29.75 | 3.46E-07 | 1.088001221 | NM_005131| | THOC1,nuclear matrix protein p84 |
| 208614_s_at | 29.73 | 3.47E-07 | 1.105115932 | NM_001457| | FLNB,filamin B, beta (actin binding protein 278) |
| 237192_at | 29.72 | 3.49E-07 | 1.909155418 | NA |  |
| 205309_at | 29.7 | 3.49E-07 | 2.000120423 | NM_001009568| | SMPDL3B,acid sphingomyelinase-like phosphodiesterase 3B |
| 227980_at | 29.69 | 3.50E-07 | 1.084494344 | NM_024639| | ZNF322A,zinc finger protein 322A |
| 205153_s_at | 29.68 | 3.50E-07 | 1.270398689 | NM_001250| | CD40,CD40 antigen isoform 1 precursor |
| 213348_at | 29.68 | 3.50E-07 | 1.312849066 | NM_000076| | CDKN1C,cyclin-dependent kinase inhibitor 1C |
| 1553955_at | 29.67 | 3.50E-07 | 1.110170395 | NM_152994| | LOC129285,smooth muscle myosin heavy chain 11 isoform |
| 210495_x_at | 29.63 | 3.52E-07 | 1.891952373 | NM_002026| | FN1,fibronectin 1 isoform 3 preproprotein |
| 1552946_at | 29.6 | 3.55E-07 | 1.326960232 | NM_153608| | MGC17986,hypothetical protein MGC17986 |
| 201403_s_at | 29.58 | 3.56E-07 | 1.095784932 | NM_004528| | MGST3,microsomal glutathione S-transferase 3 |
| 225354_s_at | 29.57 | 3.57E-07 | 1.160511743 | NM_031469| | SH3BGRL2,SH3 domain binding glutamic acid-rich protein |
| 215193_x_at | 29.57 | 3.57E-07 | 1.821232653 | NM_001023561| | NA |
| 202621_at | 29.55 | 3.58E-07 | 1.099052001 | NM_001571| | IRF3,interferon regulatory factor 3 |
| 228266_s_at | 29.55 | 3.58E-07 | 1.099742757 | NM_016073| | HDGFRP3,hepatoma-derived growth factor, related protein |
| 227662_at | 29.54 | 3.58E-07 | 2.13887997 | NM_133477| | SYNPO2,synaptopodin 2 |
| 227337_at | 29.54 | 3.59E-07 | 1.210609939 | NM_181726| | ANKRD37,ankyrin repeat domain 37 |
| 218764_at | 29.52 | 3.59E-07 | 1.537199494 | NM_006255| | PRKCH,protein kinase C, eta |
| 226749_at | 29.52 | 3.59E-07 | 1.07101691 | NM_182640| | MRPS9,mitochondrial ribosomal protein S9 |
| 200604_s_at | 29.49 | 3.61E-07 | 1.114844006 | NM_002734| | PRKAR1A,cAMP-dependent protein kinase, regulatory |
| 203896_s_at | 29.48 | 3.62E-07 | 1.277153071 | NM_000933| | PLCB4,phospholipase C beta 4 isoform a |
| 225051_at | 29.47 | 3.62E-07 | 1.134505776 | NM_004437| | EPB41,erythrocyte membrane protein band 4.1 |
| 201438_at | 29.42 | 3.65E-07 | 2.42312406 | NM_004369| | COL6A3,alpha 3 type VI collagen isoform 1 precursor |
| 223386_at | 29.42 | 3.65E-07 | 1.125448575 | NM_024556| | FLJ21103,hypothetical protein FLJ21103 |
| 204944_at | 29.4 | 3.67E-07 | 1.200321418 | NM_002841| | PTPRG,protein tyrosine phosphatase, receptor type, G |
| 203179_at | 29.37 | 3.69E-07 | 1.339831984 | NM_000155| | GALT,galactose-1-phosphate uridylyltransferase |
| 223610_at | 29.35 | 3.70E-07 | 1.184758999 | NM_001031702| | NA |
| 1555841_at | 29.34 | 3.70E-07 | 1.123689345 | NM_003692| | TMEFF1,transmembrane protein with EGF-like and two |
| 201005_at | 29.34 | 3.70E-07 | 1.106886708 | NM_001769| | CD9,CD9 antigen |
| 200759_x_at | 29.32 | 3.72E-07 | 1.145963299 | NM_003204| | NFE2L1,nuclear factor (erythroid-derived 2)-like 1 |
| 224595_at | 29.3 | 3.74E-07 | 1.142921252 | NM_080546| | CDW92,CDW92 antigen |
| 1559471_s_at | 29.28 | 3.75E-07 | 1.736563781 | NA |  |
| 242344_at | 29.28 | 3.75E-07 | 1.972931228 | NM_000813| | GABRB2,gamma-aminobutyric acid (GABA) A receptor, beta |
| 222154_s_at | 29.25 | 3.77E-07 | 1.069192995 | NM_001100422| | NA |
| 201098_at | 29.25 | 3.76E-07 | 1.052292007 | NM_004766| | COPB2,coatomer protein complex, subunit beta 2 (beta |
| 201137_s_at | 29.21 | 3.80E-07 | 1.445201005 | NM_002121| | HLA-DPB1,major histocompatibility complex, class II, DP |
| 1552400_a_at | 29.2 | 3.80E-07 | 1.53338691 | NM_152335| | C15orf27,chromosome 15 open reading frame 27 |
| 208165_s_at | 29.2 | 3.80E-07 | 2.103260319 | NM_005865| | PRSS16,protease, serine, 16 |
| 223423_at | 29.2 | 3.80E-07 | 2.844697649 | NM_014373| | GPR160,G protein-coupled receptor 160 |
| 209723_at | 29.19 | 3.81E-07 | 2.908634477 | NM_004155| | SERPINB9,serine (or cysteine) proteinase inhibitor, clade |
| 225769_at | 29.19 | 3.81E-07 | 1.077957766 | NM_020751| | COG6,component of oligomeric golgi complex 6 |
| 220231_at | 29.16 | 3.82E-07 | 2.034867618 | NM_006658| | C7orf16,G-substrate |
| 219625_s_at | 29.15 | 3.83E-07 | 1.135172669 | NM_005713| | COL4A3BP,alpha 3 type IV collagen binding protein isoform |
| 202847_at | 29.15 | 3.83E-07 | 1.724148957 | NM_001018073| | NA |
| 1553423_a_at | 29.13 | 3.84E-07 | 1.730081371 | NM_144682| | FLJ31952,hypothetical protein FLJ31952 |
| 226051_at | 29.13 | 3.84E-07 | 1.267302816 | NM_080430| | SELM,selenoprotein M precursor |
| 213135_at | 29.13 | 3.84E-07 | 1.205577653 | NM_003253| | TIAM1,T-cell lymphoma invasion and metastasis 1 |
| 224345_x_at | 29.12 | 3.84E-07 | 1.117454617 | NM_014367| | E2IG5,growth and transformation-dependent protein |
| 219196_at | 29.11 | 3.84E-07 | 2.599490013 | NM_013243| | SCG3,secretogranin III |
| 226374_at | 29.11 | 3.85E-07 | 1.241754319 | NA |  |
| 229649_at | 29.1 | 3.85E-07 | 1.866767723 | NM_001105250| | NA |
| 240189_at | 29.1 | 3.85E-07 | 1.70262684 | NA |  |
| 218472_s_at | 29.07 | 3.87E-07 | 1.13987148 | NM_015946| | PELO,CGI-17 protein |
| 228176_at | 29.07 | 3.87E-07 | 1.286185936 | NM_005226| | EDG3,endothelial differentiation, sphingolipid |
| 212865_s_at | 29.07 | 3.87E-07 | 2.205023282 | NM_021110| | COL14A1,collagen, type XIV, alpha 1 |
| 227133_at | 29.07 | 3.87E-07 | 1.145783053 | NM_207318| | CXorf39,chromosome X open reading frame 39 |
| 229817_at | 29.05 | 3.88E-07 | 1.18512445 | NM_020747| | ZNF608,zinc finger protein 608 |
| 228337_at | 29.03 | 3.89E-07 | 1.167367957 | NM_052927| | NA |
| 222908_at | 29.03 | 3.89E-07 | 1.704334622 | NM_022068| | FAM38B,family with sequence similarity 38, member B |
| 226276_at | 29.01 | 3.90E-07 | 1.092156505 | NM_174909| | MGC23909,hypothetical protein MGC23909 |
| 223232_s_at | 28.99 | 3.91E-07 | 1.39180729 | NM_020770| | CGN,cingulin |
| 204712_at | 28.99 | 3.91E-07 | 2.787355916 | NM_007191| | WIF1,Wnt inhibitory factor-1 precursor |
| 218035_s_at | 28.98 | 3.92E-07 | 2.532549246 | NM_001098634| | NA |
| 220184_at | 28.97 | 3.92E-07 | 3.244533211 | NM_024865| | NANOG,Nanog homeobox |
| 204224_s_at | 28.95 | 3.94E-07 | 1.324273006 | NM_000161| | GCH1,GTP cyclohydrolase 1 (dopa-responsive dystonia) |
| 201673_s_at | 28.94 | 3.94E-07 | 1.103473493 | NM_002103| | GYS1,glycogen synthase 1 (muscle) |
| 204584_at | 28.94 | 3.95E-07 | 1.635787042 | NM_000425| | L1CAM,L1 cell adhesion molecule isoform 1 precursor |
| 225639_at | 28.91 | 3.96E-07 | 2.041455631 | NM_003930| | SCAP2,src family associated phosphoprotein 2 |
| 212571_at | 28.91 | 3.97E-07 | 1.087019022 | NM_020920| | CHD8,chromodomain helicase DNA binding protein 8 |
| 225831_at | 28.9 | 3.97E-07 | 1.141824265 | NM_033631| | LUZP1,leucine zipper protein 1 |
| 206091_at | 28.89 | 3.97E-07 | 1.782566445 | NM_002381| | MATN3,matrilin 3 precursor |
| 232914_s_at | 28.89 | 3.98E-07 | 1.654881551 | NM_032379| | SYTL2,synaptotagmin-like 2 isoform b |
| 235343_at | 28.83 | 4.01E-07 | 1.580993865 | NM_024749| | FLJ12505,hypothetical protein FLJ12505 |
| 201057_s_at | 28.83 | 4.01E-07 | 1.143845057 | NM_004487| | GOLGB1,golgi autoantigen, golgin subfamily b, |
| 37012_at | 28.82 | 4.02E-07 | 1.078894904 | NM_004930| | CAPZB,F-actin capping protein beta subunit |
| 204281_at | 28.82 | 4.02E-07 | 1.072112864 | NM_003213| | TEAD4,TEA domain family member 4 isoform 1 |
| 218162_at | 28.81 | 4.03E-07 | 2.332903939 | NM_020190| | OLFML3,olfactomedin-like 3 |
| 200738_s_at | 28.81 | 4.03E-07 | 1.058026385 | NM_000291| | PGK1,phosphoglycerate kinase 1 |
| 231271_x_at | 28.79 | 4.03E-07 | 1.217906883 | NM_020677| | HSCARG,HSCARG protein |
| 214022_s_at | 28.78 | 4.04E-07 | 2.225741833 | NM_003641| | IFITM1,interferon induced transmembrane protein 1 |
| 203379_at | 28.78 | 4.04E-07 | 1.225415616 | NM_001006665| | RPS6KA1,ribosomal protein S6 kinase, 90kDa, polypeptide |
| 235152_at | 28.77 | 4.05E-07 | 1.651733127 | NA |  |
| 205263_at | 28.75 | 4.06E-07 | 1.101094317 | NM_003921| | BCL10,B-cell CLL/lymphoma 10 |
| 219123_at | 28.75 | 4.06E-07 | 1.193236505 | NM_014519| | ZNF232,zinc finger protein 232 |
| 229202_at | 28.74 | 4.06E-07 | 1.29767148 | NA |  |
| 236377_at | 28.74 | 4.06E-07 | 1.550090175 | NM_133448| | KIAA1944,KIAA1944 protein |
| 226325_at | 28.73 | 4.07E-07 | 1.442982598 | NM_152328| | ADSSL1,adenylosuccinate synthase-like 1 isoform 2 |
| 223233_s_at | 28.71 | 4.08E-07 | 1.313417586 | NM_020770| | CGN,cingulin |
| 212747_at | 28.71 | 4.08E-07 | 1.132828368 | NM_015245| | ANKS1,ankyrin repeat and sterile alpha motif domain |
| 222088_s_at | 28.71 | 4.08E-07 | 1.997862941 | NM_006931| | SLC2A3,solute carrier family 2 (facilitated glucose |
| 229751_s_at | 28.67 | 4.10E-07 | 1.219719996 | NM_001098614| | NA |
| 205807_s_at | 28.66 | 4.11E-07 | 1.249392071 | NM_001126337| | NA |
| 204891_s_at | 28.65 | 4.12E-07 | 2.403363711 | NM_001042771| | NA |
| 235085_at | 28.65 | 4.12E-07 | 1.205933962 | NM_001080826| | NA |
| 202619_s_at | 28.64 | 4.13E-07 | 1.142018342 | NM_000935| | PLOD2,procollagen-lysine, 2-oxoglutarate 5-dioxygenase |
| 235944_at | 28.62 | 4.15E-07 | 2.358249216 | NM_031935| | FIBL-6,hemicentin |
| 223557_s_at | 28.59 | 4.17E-07 | 1.611215636 | NM_016192| | TMEFF2,transmembrane protein with EGF-like and two |
| 208636_at | 28.54 | 4.21E-07 | 1.064344391 | NM_001102| | ACTN1,actinin, alpha 1 |
| 203373_at | 28.54 | 4.21E-07 | 1.095251004 | NM_003877| | SOCS2,suppressor of cytokine signaling-2 |
| 227329_at | 28.53 | 4.21E-07 | 1.751613048 | NM_025224| | BTBD4,BTB (POZ) domain containing 4 |
| 201831_s_at | 28.53 | 4.21E-07 | 1.164004292 | NM_003715| | VDP,vesicle docking protein p115 |
| 211267_at | 28.53 | 4.21E-07 | 1.481610359 | NM_003865| | HESX1,homeo box (expressed in ES cells) 1 |
| 219454_at | 28.53 | 4.21E-07 | 1.820038389 | NM_015507| | EGFL6,epidermal growth factor-like protein 6 |
| 209377_s_at | 28.51 | 4.23E-07 | 1.034403808 | NM_004242| | HMGN3,high mobility group nucleosomal binding domain 3 |
| 205981_s_at | 28.5 | 4.23E-07 | 1.153787535 | NM_001564| | ING2,inhibitor of growth family, member 1-like |
| 228098_s_at | 28.48 | 4.25E-07 | 1.465791476 | NM_013262| | MYLIP,myosin regulatory light chain interacting |
| 239482_x_at | 28.43 | 4.30E-07 | 1.213352084 | NM_021269| | ZNF15L1,zinc finger protein 15-like 1 (KOX 8) |
| 207695_s_at | 28.41 | 4.31E-07 | 2.104078249 | NM_001555| | IGSF1,immunoglobulin superfamily, member 1 isoform 1 |
| 202766_s_at | 28.4 | 4.32E-07 | 1.380858645 | NM_000138| | FBN1,fibrillin 1 |
| 201602_s_at | 28.39 | 4.32E-07 | 1.215336384 | NM_002480| | PPP1R12A,protein phosphatase 1, regulatory (inhibitor) |
| 210905_x_at | 28.38 | 4.33E-07 | 2.133771107 | NA |  |
| 242348_at | 28.37 | 4.34E-07 | 1.845949755 | NM_001005527| | FAM19A4,family with sequence similarity 19 (chemokine |
| 213659_at | 28.37 | 4.34E-07 | 1.152996233 | NM_007131| | ZNF75,zinc finger protein 75 |
| 202538_s_at | 28.35 | 4.35E-07 | 1.146961732 | NM_014043| | DKFZP564O123,DKFZP564O123 protein |
| 212495_at | 28.35 | 4.35E-07 | 1.116126389 | NM_015015| | JMJD2B,jumonji domain containing 2B |
| 204905_s_at | 28.34 | 4.36E-07 | 1.067766641 | NM_004280| | EEF1E1,eukaryotic translation elongation factor 1 |
| 206109_at | 28.33 | 4.36E-07 | 1.553218449 | NM_000148| | FUT1,fucosyltransferase 1 |
| 202575_at | 28.33 | 4.36E-07 | 1.466777079 | NM_001878| | CRABP2,cellular retinoic acid binding protein 2 |
| 1561775_at | 28.31 | 4.38E-07 | 2.431947595 | NA |  |
| 203934_at | 28.3 | 4.40E-07 | 1.963162615 | NM_002253| | KDR,kinase insert domain receptor (a type III |
| 238510_at | 28.29 | 4.41E-07 | 1.191259994 | NM_001004300| | LOC124411,hypothetical protein LOC124411 |
| 212104_s_at | 28.28 | 4.41E-07 | 1.113429535 | NM_001031695| | NA |
| 209101_at | 28.25 | 4.44E-07 | 1.336265626 | NM_001901| | CTGF,connective tissue growth factor |
| 239781_at | 28.24 | 4.45E-07 | 2.148027605 | NA |  |
| 226553_at | 28.22 | 4.47E-07 | 1.215745313 | NM_005656| | TMPRSS2,transmembrane protease, serine 2 |
| 220992_s_at | 28.22 | 4.46E-07 | 1.27315118 | NM_030934| | C1orf25,N2,N2-dimethylguanosine tRNA |
| 213039_at | 28.19 | 4.49E-07 | 1.054935311 | NM_015318| | ARHGEF18,Rho-specific guanine nucleotide exchange factor |
| 57540_at | 28.19 | 4.49E-07 | 1.326817542 | NM_022128| | RBKS,ribokinase |
| 201752_s_at | 28.17 | 4.51E-07 | 1.104189354 | NM_001121| | NA |
| 227236_at | 28.17 | 4.50E-07 | 2.766989217 | NM_005725| | TSPAN2,tetraspan 2 |
| 243439_at | 28.16 | 4.51E-07 | 1.193137633 | NM_133460| | ZNF418,zinc finger protein 418 |
| 217769_s_at | 28.16 | 4.51E-07 | 1.036930597 | NM_015932| | C13orf12,chromosome 13 open reading frame 12 |
| 213664_at | 28.16 | 4.51E-07 | 2.435863071 | NM_004170| | SLC1A1,solute carrier family 1, member 1 |
| 218922_s_at | 28.15 | 4.51E-07 | 1.13654421 | NM_024552| | LASS4,LAG1 longevity assurance homolog 4 |
| 202712_s_at | 28.13 | 4.53E-07 | 1.277179147 | NM_001015001| | NA |
| 228336_at | 28.12 | 4.54E-07 | 1.168218495 | NM_052927| | NA |
| 204804_at | 28.12 | 4.54E-07 | 1.177987301 | NM_003141| | TRIM21,52kD Ro/SSA autoantigen |
| 226462_at | 28.09 | 4.57E-07 | 1.851011674 | NM_014178| | STXBP6,amisyn |
| 230869_at | 28.09 | 4.57E-07 | 2.881829196 | NM_001080396| | NA |
| 211719_x_at | 28.07 | 4.59E-07 | 1.932097198 | NM_002026| | FN1,fibronectin 1 isoform 3 preproprotein |
| 230087_at | 28.06 | 4.59E-07 | 1.158883213 | NM_178013| | PRIMA1,proline rich membrane anchor 1 |
| 214179_s_at | 28.04 | 4.61E-07 | 1.168511003 | NM_003204| | NFE2L1,nuclear factor (erythroid-derived 2)-like 1 |
| 221933_at | 28.02 | 4.62E-07 | 1.280152599 | NM_020742| | NLGN4X,X-linked neuroligin 4 |
| 211796_s_at | 28.02 | 4.62E-07 | 2.008527478 | NA |  |
| 212445_s_at | 27.99 | 4.65E-07 | 1.183061281 | NM_015277| | NEDD4L,ubiquitin-protein ligase NEDD4-like |
| 202889_x_at | 27.98 | 4.66E-07 | 1.768867124 | NM_003980| | MAP7,microtubule-associated protein 7 |
| 228492_at | 27.96 | 4.67E-07 | 1.304128421 | NM_004654| | USP9Y,ubiquitin specific protease 9, Y-linked |
| 207153_s_at | 27.96 | 4.67E-07 | 1.198054407 | NM_053274| | GLMN,glomulin isoform FAP68 |
| 202425_x_at | 27.96 | 4.67E-07 | 1.100775198 | NM_000944| | PPP3CA,protein phosphatase 3 (formerly 2B), catalytic |
| 224918_x_at | 27.93 | 4.69E-07 | 1.074306912 | NM_020300| | MGST1,microsomal glutathione S-transferase 1 |
| 221803_s_at | 27.92 | 4.70E-07 | 1.126772437 | NM_030759| | NRBF2,nuclear receptor binding factor 2 |
| 209658_at | 27.92 | 4.70E-07 | 1.074912124 | NM_001078645| | NA |
| 222785_x_at | 27.91 | 4.71E-07 | 1.188671502 | NM_022761| | C11orf1,hypothetical protein FLJ23499 |
| 206336_at | 27.9 | 4.71E-07 | 3.181744684 | NM_002993| | CXCL6,chemokine (C-X-C motif) ligand 6 (granulocyte |
| 229088_at | 27.89 | 4.72E-07 | 1.628741222 | NM_006208| | ENPP1,ectonucleotide pyrophosphatase/phosphodiesterase |
| 202982_s_at | 27.89 | 4.72E-07 | 1.143443008 | NM_001037161| | NA |
| 207197_at | 27.88 | 4.73E-07 | 2.5277609 | NM_003413| | ZIC3,zinc finger protein of the cerebellum 3 |
| 32137_at | 27.87 | 4.74E-07 | 1.2179436 | NM_002226| | JAG2,jagged 2 isoform a precursor |
| 225309_at | 27.87 | 4.73E-07 | 1.079438099 | NM_032758| | PHF5A,PHD-finger 5A |
| 1563022_at | 27.82 | 4.79E-07 | 1.792058443 | NM_001101357| | NA |
| 208869_s_at | 27.8 | 4.81E-07 | 1.601771184 | NM_031412| | GABARAPL1,GABA(A) receptor-associated protein like 1 |
| 212492_s_at | 27.75 | 4.86E-07 | 1.119380937 | NM_015015| | JMJD2B,jumonji domain containing 2B |
| 235044_at | 27.72 | 4.88E-07 | 2.883401377 | NM_052954| | CYYR1,cysteine and tyrosine-rich 1 protein precursor |
| 203013_at | 27.72 | 4.88E-07 | 1.094424779 | NM_007265| | HSGT1,suppressor of S. cerevisiae gcr2 |
| 214636_at | 27.72 | 4.88E-07 | 1.943496002 | NM_000728| | CALCB,calcitonin-related polypeptide, beta |
| 203434_s_at | 27.72 | 4.88E-07 | 2.215340375 | NM_000902| | MME,membrane metallo-endopeptidase |
| 226270_at | 27.71 | 4.88E-07 | 1.158980753 | NM_018303| | SEC5L1,Sec5 protein |
| 1570153_at | 27.69 | 4.90E-07 | 1.358890788 | NM_017826| | FLJ20449,hypothetical protein FLJ20449 |
| 228010_at | 27.69 | 4.91E-07 | 1.73675828 | NM_020416| | PPP2R2C,gamma isoform of regulatory subunit B55, protein |
| 213278_at | 27.67 | 4.93E-07 | 1.130908407 | NM_015458| | MTMR9,myotubularin-related protein 9 |
| 212872_s_at | 27.65 | 4.95E-07 | 1.086648345 | NM_004275| | USP49,TRF-proximal protein |
| 219410_at | 27.65 | 4.94E-07 | 3.111795614 | NM_018004| | TMEM45A,transmembrane protein 45A |
| 221556_at | 27.63 | 4.97E-07 | 1.18816431 | NM_001077181| | NA |
| 218248_at | 27.63 | 4.96E-07 | 1.349060543 | NM_022074| | FLJ22794,FLJ22794 protein |
| 210835_s_at | 27.62 | 4.98E-07 | 1.081382288 | NM_001083914| | NA |
| 205022_s_at | 27.6 | 4.99E-07 | 1.122032321 | NM_001085471| | NA |
| 217864_s_at | 27.59 | 5.00E-07 | 1.097044345 | NM_016166| | PIAS1,protein inhibitor of activated STAT, 1 |
| 229782_at | 27.58 | 5.01E-07 | 1.242730601 | NA |  |
| 211343_s_at | 27.57 | 5.01E-07 | 2.100239082 | NM_005203| | COL13A1,alpha 1 type XIII collagen isoform 1 |
| 225664_at | 27.56 | 5.02E-07 | 1.466890892 | NM_004370| | COL12A1,alpha 1 type XII collagen long isoform |
| 227180_at | 27.56 | 5.02E-07 | 2.397494555 | NM_001104558| | NA |
| 203628_at | 27.55 | 5.02E-07 | 1.133877871 | NM_000875| | IGF1R,insulin-like growth factor 1 receptor precursor |
| 214036_at | 27.55 | 5.02E-07 | 1.388534798 | NA |  |
| 200986_at | 27.49 | 5.09E-07 | 1.824709831 | NM_000062| | SERPING1,complement component 1 inhibitor precursor |
| 226602_s_at | 27.48 | 5.09E-07 | 1.101887684 | NM_004327| | BCR,breakpoint cluster region isoform 1 |
| 231953_at | 27.47 | 5.10E-07 | 1.36814645 | NM_004459| | FALZ,fetal Alzheimer antigen isoform 2 |
| 218412_s_at | 27.47 | 5.10E-07 | 1.090708098 | NM_005685| | GTF2IRD1,GTF2I repeat domain containing 1 isoform 2 |
| 226443_at | 27.46 | 5.11E-07 | 1.126188266 | NM_138333| | C9orf42,chromosome 9 open reading frame 42 |
| 228374_at | 27.44 | 5.12E-07 | 1.151803692 | NM_014472| | C10orf28,growth inhibition and differentiation related |
| 208858_s_at | 27.44 | 5.12E-07 | 1.151733716 | NM_015292| | MBC2,KIAA0747 protein |
| 241612_at | 27.44 | 5.12E-07 | 2.308803611 | NM_012183| | FOXD3,forkhead box D3 |
| 219968_at | 27.38 | 5.19E-07 | 1.187998067 | NM_016089| | ZNF589,zinc finger protein 589 |
| 1553858_at | 27.35 | 5.21E-07 | 1.299399888 | NM_024784| | ZBTB3,zinc finger and BTB domain containing 3 |
| 203041_s_at | 27.29 | 5.28E-07 | 1.106571099 | NM_001122606| | NA |
| 239770_at | 27.27 | 5.30E-07 | 1.48548474 | NM_031913| | CHR3SYT,chr3 synaptotagmin |
| 213652_at | 27.27 | 5.30E-07 | 2.080437411 | NM_006200| | PCSK5,proprotein convertase subtilisin/kexin type 5 |
| 218330_s_at | 27.25 | 5.31E-07 | 1.089655541 | NM_001111018| | NA |
| 201559_s_at | 27.25 | 5.31E-07 | 1.245622683 | NM_013943| | CLIC4,chloride intracellular channel 4 |
| 231597_x_at | 27.25 | 5.31E-07 | 1.633002792 | NA |  |
| 226390_at | 27.24 | 5.32E-07 | 1.156664392 | NM_139164| | STARD4,START domain containing 4, sterol regulated |
| 235696_at | 27.23 | 5.33E-07 | 1.470327856 | NA |  |
| 200815_s_at | 27.18 | 5.37E-07 | 1.085021156 | NM_000430| | PAFAH1B1,platelet-activating factor acetylhydrolase, |
| 1569191_at | 27.17 | 5.38E-07 | 2.554555716 | NM_001039884| | NA |
| 235773_at | 27.17 | 5.38E-07 | 1.223838019 | NM_001010879| | ZIK1,zinc finger protein interacting with K protein |
| 228859_at | 27.17 | 5.38E-07 | 1.133074197 | NM_001099776| | NA |
| 204043_at | 27.14 | 5.41E-07 | 1.404548858 | NM_000355| | TCN2,transcobalamin II precursor |
| 244523_at | 27.13 | 5.42E-07 | 1.138368927 | NM_012329| | MMD,monocyte to macrophage |
| 218780_at | 27.13 | 5.42E-07 | 1.184217078 | NM_001100176| | NA |
| 209892_at | 27.1 | 5.45E-07 | 1.587520746 | NM_002033| | FUT4,fucosyltransferase 4 |
| 210391_at | 27.09 | 5.46E-07 | 1.449578449 | NM_001489| | NR6A1,nuclear receptor subfamily 6, group A, member 1 |
| 200778_s_at | 27.09 | 5.46E-07 | 1.102500135 | NM_001008491| | SEPT2,septin 2 |
| 203352_at | 27.08 | 5.46E-07 | 1.192300893 | NM_002552| | ORC4L,origin recognition complex subunit 4 |
| 218928_s_at | 27.08 | 5.46E-07 | 1.508556068 | NM_018964| | SLC37A1,solute carrier family 37 member 1 |
| 208474_at | 27.06 | 5.48E-07 | 1.407610705 | NM_021195| | CLDN6,claudin 6 |
| 239670_at | 27.06 | 5.48E-07 | 1.157509395 | NA |  |
| 218950_at | 27.05 | 5.49E-07 | 1.066252356 | NM_022481| | CENTD3,centaurin, delta 3 |
| 207644_at | 27.04 | 5.50E-07 | 1.329298105 | NM_003923| | FOXH1,forkhead box H1 |
| 219403_s_at | 27.02 | 5.52E-07 | 1.664897686 | NM_001098540| | NA |
| 209220_at | 27.01 | 5.53E-07 | 1.87961541 | NM_004484| | GPC3,glypican 3 |
| 203256_at | 27.01 | 5.53E-07 | 1.813900453 | NM_001793| | CDH3,cadherin 3, type 1 preproprotein |
| 206461_x_at | 27 | 5.54E-07 | 1.137467597 | NM_005951| | MT1H,metallothionein 1H |
| 236279_at | 27 | 5.54E-07 | 1.971853489 | NA |  |
| 204114_at | 26.99 | 5.55E-07 | 2.546422303 | NM_007361| | NID2,nidogen 2 |
| 201848_s_at | 26.99 | 5.55E-07 | 1.111507147 | NM_004052| | BNIP3,BCL2/adenovirus E1B 19kD-interacting protein 3 |
| 226145_s_at | 26.98 | 5.55E-07 | 3.273462342 | NM_025074| | FRAS1,Fraser syndrome 1 isoform 1 |
| 201906_s_at | 26.98 | 5.55E-07 | 1.152509145 | NM_001008392| | CTDSPL,small CTD phosphatase 3 isoform 1 |
| 239697_x_at | 26.97 | 5.56E-07 | 1.728469343 | NM_198463| | FLJ42117,FLJ42117 protein |
| 218104_at | 26.97 | 5.56E-07 | 1.07518501 | NM_017746| | TEX10,testis expressed sequence 10 |
| 218457_s_at | 26.96 | 5.57E-07 | 1.170587809 | NM_022552| | DNMT3A,DNA cytosine methyltransferase 3 alpha isoform |
| 45714_at | 26.95 | 5.58E-07 | 1.22758593 | NM_001002017| | HCFC1R1,host cell factor C1 regulator 1 (XPO1 dependant) |
| 212461_at | 26.95 | 5.58E-07 | 1.145684783 | NM_015878| | OAZIN,ornithine decarboxylase antizyme inhibitor |
| 40687_at | 26.94 | 5.60E-07 | 1.201632767 | NM_002060| | GJA4,connexin 37 |
| 205350_at | 26.91 | 5.63E-07 | 2.53811122 | NM_004378| | CRABP1,cellular retinoic acid binding protein 1 |
| 229618_at | 26.91 | 5.63E-07 | 1.330120832 | NM_022133| | SNX16,sorting nexin 16 isoform a |
| 227909_at | 26.91 | 5.62E-07 | 1.50554986 | NM_001031705| | NA |
| 214396_s_at | 26.88 | 5.66E-07 | 1.839371262 | NM_003927| | MBD2,methyl-CpG binding domain protein 2 isoform 1 |
| 1560652_at | 26.88 | 5.65E-07 | 2.327078933 | NA |  |
| 212590_at | 26.85 | 5.70E-07 | 1.364483552 | NM_001102669| | NA |
| 204217_s_at | 26.82 | 5.73E-07 | 1.269147966 | NM_005619| | RTN2,reticulon 2 isoform A |
| 223404_s_at | 26.78 | 5.78E-07 | 1.297827401 | NM_030934| | C1orf25,N2,N2-dimethylguanosine tRNA |
| 212692_s_at | 26.76 | 5.80E-07 | 1.137234757 | NM_006726| | LRBA,LPS-responsive vesicle trafficking, beach and |
| 227997_at | 26.76 | 5.80E-07 | 1.145937421 | NM_001080973| | NA |
| 203786_s_at | 26.74 | 5.82E-07 | 1.351438639 | NM_001003395| | TPD52L1,tumor protein D52-like 1 isoform 2 |
| 222392_x_at | 26.72 | 5.85E-07 | 2.690186348 | NM_022121| | PERP,PERP, TP53 apoptosis effector |
| 203964_at | 26.71 | 5.86E-07 | 1.342815925 | NM_004688| | NMI,N-myc and STAT interactor |
| 211423_s_at | 26.71 | 5.85E-07 | 1.097677082 | NM_001024956| | NA |
| 223711_s_at | 26.67 | 5.91E-07 | 1.101739748 | NM_001037304| | NA |
| 223358_s_at | 26.67 | 5.91E-07 | 1.204936927 | NA |  |
| 213506_at | 26.64 | 5.94E-07 | 2.170577013 | NM_005242| | F2RL1,coagulation factor II (thrombin) receptor-like 1 |
| 209082_s_at | 26.64 | 5.93E-07 | 1.446532891 | NM_030582| | COL18A1,alpha 1 type XVIII collagen isoform 1 precursor |
| 206299_at | 26.62 | 5.96E-07 | 1.459441958 | NM_015686| | TMEM28,transmembrane protein 28 |
| 1553972_a_at | 26.61 | 5.96E-07 | 1.088990102 | NM_000071| | CBS,cystathionine-beta-synthase |
| 214632_at | 26.6 | 5.97E-07 | 1.337481651 | NM_003872| | NRP2,neuropilin 2 isoform 2 precursor |
| 209152_s_at | 26.57 | 6.01E-07 | 1.116723994 | NM_003200| | TCF3,transcription factor 3 |
| 205139_s_at | 26.57 | 6.01E-07 | 1.274715511 | NM_005715| | UST,uronyl-2-sulfotransferase |
| 219983_at | 26.57 | 6.01E-07 | 1.235429722 | NM_020386| | HRASLS,HRAS-like suppressor |
| 204853_at | 26.57 | 6.01E-07 | 1.147801669 | NM_006190| | ORC2L,origin recognition complex, subunit 2 |
| 212317_at | 26.56 | 6.02E-07 | 1.142999366 | NM_012470| | TNPO3,transportin 3 |
| 209586_s_at | 26.55 | 6.03E-07 | 1.096160774 | NM_021222| | PRUNE,prune homolog |
| 219335_at | 26.55 | 6.03E-07 | 1.150109057 | NM_022838| | ARMCX5,armadillo repeat containing, X-linked 5 |
| 206654_s_at | 26.54 | 6.03E-07 | 1.41898493 | NM_006467| | POLR3G,polymerase (RNA) III (DNA directed) polypeptide |
| 206296_x_at | 26.54 | 6.03E-07 | 1.502731842 | NM_001042600| | NA |
| 210136_at | 26.54 | 6.03E-07 | 1.956566491 | NM_001025081| | NA |
| 218574_s_at | 26.52 | 6.05E-07 | 1.481886972 | NM_014583| | LMCD1,LIM and cysteine-rich domains 1 |
| 227526_at | 26.52 | 6.06E-07 | 1.481579944 | NM_016952| | CDON,surface glycoprotein, Ig superfamily member |
| 241772_at | 26.51 | 6.07E-07 | 2.485942334 | NA |  |
| 204751_x_at | 26.51 | 6.07E-07 | 2.389654152 | NM_004949| | DSC2,desmocollin 2 isoform Dsc2b preproprotein |
| 228378_at | 26.48 | 6.09E-07 | 1.085696305 | NM_001009894| | DKFZp434N2030,hypothetical protein DKFZp434N2030 |
| 203439_s_at | 26.48 | 6.09E-07 | 1.859089657 | NM_003714| | STC2,stanniocalcin 2 |
| 244052_at | 26.48 | 6.09E-07 | 1.16698734 | NM_032783| | CBR4,carbonic reductase 4 |
| 228245_s_at | 26.48 | 6.09E-07 | 1.323189895 | NM_001080502| | NA |
| 205123_s_at | 26.46 | 6.11E-07 | 1.332512459 | NM_003692| | TMEFF1,transmembrane protein with EGF-like and two |
| 202303_x_at | 26.44 | 6.14E-07 | 1.091236234 | NM_003601| | SMARCA5,SWI/SNF-related matrix-associated |
| 202625_at | 26.44 | 6.14E-07 | 1.673286695 | NM_001111097| | NA |
| 222351_at | 26.43 | 6.15E-07 | 1.312521643 | NM_002716| | PPP2R1B,beta isoform of regulatory subunit A, protein |
| 203476_at | 26.43 | 6.15E-07 | 1.205879981 | NM_006670| | TPBG,5T4 oncofetal trophoblast glycoprotein |
| 235129_at | 26.42 | 6.16E-07 | 1.678196678 | NM_006741| | PPP1R1A,protein phosphatase 1, regulatory (inhibitor) |
| 202946_s_at | 26.41 | 6.18E-07 | 1.052573018 | NM_014962| | BTBD3,BTB/POZ domain containing protein 3 isoform a |
| 37950_at | 26.4 | 6.18E-07 | 1.09602094 | NM_002726| | PREP,prolyl endopeptidase |
| 225422_at | 26.4 | 6.18E-07 | 1.075675772 | NM_139286| | CDC26,CDC26 subunit of anaphase promoting complex |
| 204750_s_at | 26.38 | 6.22E-07 | 2.460374062 | NM_004949| | DSC2,desmocollin 2 isoform Dsc2b preproprotein |
| 207014_at | 26.37 | 6.23E-07 | 1.454867367 | NM_000807| | GABRA2,gamma-aminobutyric acid A receptor, alpha 2 |
| 220446_s_at | 26.34 | 6.26E-07 | 1.820210577 | NM_005769| | CHST4,carbohydrate (N-acetylglucosamine 6-O) |
| 226354_at | 26.34 | 6.27E-07 | 1.19026264 | NM_032857| | LACTB,lactamase, beta isoform a |
| 211926_s_at | 26.34 | 6.26E-07 | 1.073100156 | NM_002473| | MYH9,myosin, heavy polypeptide 9, non-muscle |
| 230960_at | 26.34 | 6.27E-07 | 1.475089801 | NM_004884| | PUNC,putative neuronal cell adhesion molecule |
| 215127_s_at | 26.32 | 6.28E-07 | 1.254436823 | NM_002897| | RBMS1,RNA binding motif, single stranded interacting |
| 202720_at | 26.3 | 6.30E-07 | 1.375264142 | NM_015641| | TES,testin isoform 1 |
| 214430_at | 26.28 | 6.33E-07 | 1.107266012 | NM_000169| | GLA,galactosidase, alpha |
| 222609_s_at | 26.27 | 6.33E-07 | 1.103475509 | NM_016046| | EXOSC1,exosomal core protein CSL4 |
| 207957_s_at | 26.25 | 6.36E-07 | 2.834132774 | NM_002738| | PRKCB1,protein kinase C, beta isoform 2 |
| 212353_at | 26.24 | 6.38E-07 | 1.982887959 | NM_015170| | SULF1,sulfatase 1 |
| 239206_at | 26.23 | 6.38E-07 | 1.776118574 | NM_175710| | NA |
| 229305_at | 26.23 | 6.38E-07 | 1.169450734 | NM_024629| | MLF1IP,MLF1 interacting protein |
| 1555846_a_at | 26.21 | 6.41E-07 | 1.175429917 | NA |  |
| 204568_at | 26.21 | 6.42E-07 | 1.168555646 | NM_014924| | KIAA0831,KIAA0831 |
| 204730_at | 26.19 | 6.44E-07 | 1.287196792 | NM_014747| | RIMS3,regulating synaptic membrane exocytosis 3 |
| 236800_at | 26.18 | 6.45E-07 | 1.54498244 | NM_145314| | C10orf49,chromosome 10 open reading frame 49 |
| 225519_at | 26.18 | 6.45E-07 | 1.12463648 | NM_174907| | PPP4R2,protein phosphatase 4, regulatory subunit 2 |
| 238005_s_at | 26.16 | 6.48E-07 | 1.115866951 | NM_015477| | SIN3A,transcriptional co-repressor Sin3A |
| 218910_at | 26.16 | 6.48E-07 | 1.232822391 | NM_018075| | FLJ10375,hypothetical protein FLJ10375 |
| 223068_at | 26.16 | 6.48E-07 | 1.112117806 | NM_019063| | EML4,echinoderm microtubule associated protein like |
| 239492_at | 26.14 | 6.49E-07 | 1.678650234 | NM_174977| | SEC14L4,SEC14p-like protein TAP3 |
| 200887_s_at | 26.14 | 6.49E-07 | 1.085741111 | NM_007315| | STAT1,signal transducer and activator of transcription |
| 204466_s_at | 26.11 | 6.54E-07 | 2.478337385 | NM_000345| | SNCA,alpha-synuclein isoform NACP140 |
| 221437_s_at | 26.09 | 6.56E-07 | 1.079448844 | NM_031280| | MRPS15,mitochondrial ribosomal protein S15 |
| 242477_at | 26.07 | 6.59E-07 | 1.970843338 | NM_152574| | C9orf52,hypothetical protein FLJ33868 |
| 208065_at | 26.06 | 6.59E-07 | 1.910225119 | NM_015879| | ST8SIA3,ST8 alpha-N-acetyl-neuraminide |
| 1556012_at | 26.05 | 6.62E-07 | 1.775611684 | NM_152375| | FLJ38753,hypothetical protein FLJ38753 |
| 1555950_a_at | 26.04 | 6.63E-07 | 1.622915251 | NM_000574| | DAF,decay accelerating factor for complement (CD55, |
| 209864_at | 26.02 | 6.65E-07 | 1.097339512 | NM_012083| | FRAT2,GSK-3 binding protein FRAT2 |
| 228051_at | 26.01 | 6.68E-07 | 2.519847 | NM_020340| | KIAA1244,KIAA1244 |
| 34206_at | 26.01 | 6.68E-07 | 1.07907313 | NM_001040118| | NA |
| 201973_s_at | 26 | 6.69E-07 | 1.06015442 | NM_015622| | C7orf28A,chromosome 7 open reading frame 28A |
| 212367_at | 25.99 | 6.70E-07 | 1.071209418 | NM_015322| | FEM1B,fem-1 homolog b |
| 221778_at | 25.99 | 6.71E-07 | 1.288723266 | NM_030647| | NA |
| 202857_at | 25.98 | 6.72E-07 | 1.031453725 | NM_014255| | TMEM4,transmembrane protein 4 |
| 1555495_a_at | 25.98 | 6.72E-07 | 1.078718058 | NM_005869| | SDCCAG10,serologically defined colon cancer antigen 10 |
| 1560562_a_at | 25.97 | 6.72E-07 | 1.352414495 | NM_182609| | MGC48625,hypothetical protein MGC48625 |
| 208580_x_at | 25.96 | 6.75E-07 | 1.183740207 | NM_001034077| | NA |
| 225093_at | 25.94 | 6.78E-07 | 1.283249908 | NM_007124| | UTRN,utrophin |
| 206789_s_at | 25.93 | 6.78E-07 | 1.286515146 | NM_002697| | POU2F1,POU domain, class 2, transcription factor 1 |
| 228503_at | 25.93 | 6.79E-07 | 1.57211597 | NA |  |
| 209678_s_at | 25.93 | 6.79E-07 | 1.15244037 | NM_002740| | PRKCI,protein kinase C, iota |
| 213523_at | 25.93 | 6.79E-07 | 1.153585509 | NM_001238| | CCNE1,cyclin E1 isoform 1 |
| 239761_at | 25.91 | 6.81E-07 | 1.800543987 | NM_001097633| | NA |
| 49452_at | 25.91 | 6.82E-07 | 1.306871961 | NM_001093| | ACACB,acetyl-Coenzyme A carboxylase beta |
| 225445_at | 25.89 | 6.85E-07 | 1.084421193 | NM_173569| | NA |
| 225720_at | 25.89 | 6.84E-07 | 1.296178815 | NM_133477| | SYNPO2,synaptopodin 2 |
| 239752_at | 25.89 | 6.85E-07 | 1.219074232 | NA |  |
| 223592_s_at | 25.87 | 6.87E-07 | 1.261732122 | NM_032322| | RNF135,ring finger protein 135 isoform 1 |
| 218917_s_at | 25.85 | 6.90E-07 | 1.074717866 | NM_006015| | ARID1A,AT rich interactive domain 1A (SWI- like) |
| 239108_at | 25.84 | 6.92E-07 | 1.420363135 | NM_018099| | MLSTD1,male sterility domain containing 1 |
| 222065_s_at | 25.84 | 6.92E-07 | 1.113969868 | NM_002018| | FLII,flightless I homolog |
| 242871_at | 25.82 | 6.95E-07 | 2.06380853 | NM_001104554| | NA |
| 208581_x_at | 25.8 | 6.97E-07 | 1.125258837 | NM_005952| | MT1X,metallothionein 1X |
| 225809_at | 25.79 | 6.99E-07 | 1.734061277 | NM_015393| | DKFZP564O0823,DKFZP564O0823 protein |
| 231808_at | 25.72 | 7.10E-07 | 1.141055283 | NM_033061| | KRTAP4-7,keratin associated protein 4-7 |
| 204237_at | 25.72 | 7.10E-07 | 1.338478977 | NM_016315| | GULP1,GULP, engulfment adaptor PTB domain containing |
| 227163_at | 25.7 | 7.12E-07 | 1.526404276 | NM_183239| | GSTO2,glutathione S-transferase omega 2 |
| 223349_s_at | 25.7 | 7.13E-07 | 1.31791598 | NM_032515| | BOK,BCL2-related ovarian killer |
| 221378_at | 25.68 | 7.15E-07 | 1.721706621 | NM_005454| | CER1,cerberus 1 |
| 219670_at | 25.67 | 7.16E-07 | 1.12150289 | NM_024603| | FLJ11588,hypothetical protein FLJ11588 |
| 223250_at | 25.66 | 7.18E-07 | 1.215216598 | NM_001031710| | NA |
| 1553764_a_at | 25.66 | 7.18E-07 | 1.405206056 | NM_032876| | JUB,jub, ajuba homolog isoform 1 |
| 204619_s_at | 25.66 | 7.18E-07 | 1.406820542 | NM_001126336| | NA |
| 205559_s_at | 25.65 | 7.18E-07 | 2.234441708 | NM_006200| | PCSK5,proprotein convertase subtilisin/kexin type 5 |
| 227461_at | 25.65 | 7.19E-07 | 1.263735381 | NM_033104| | STN2,stonin 2 |
| 222635_s_at | 25.63 | 7.22E-07 | 1.178043607 | NM_025205| | MED28,mediator of RNA polymerase II transcription, |
| 238653_at | 25.61 | 7.24E-07 | 1.15879483 | NM_014813| | LRIG2,leucine-rich repeats and immunoglobulin-like |
| 205588_s_at | 25.61 | 7.24E-07 | 1.191299513 | NM_007045| | FGFR1OP,FGFR1 oncogene partner isoform a |
| 227348_at | 25.6 | 7.26E-07 | 1.16097939 | NM_152268| | DKFZp727A071,similar to tRNA synthetase class II |
| 212542_s_at | 25.55 | 7.34E-07 | 1.063385343 | NM_017934| | PHIP,pleckstrin homology domain interacting protein |
| 222675_s_at | 25.55 | 7.34E-07 | 1.351331016 | NM_018842| | BAIAP2L1,BAI1-associated protein 2-like 1 |
| 227271_at | 25.55 | 7.34E-07 | 1.253363404 | NM_004112| | FGF11,fibroblast growth factor 11 |
| 223373_s_at | 25.54 | 7.36E-07 | 1.115661086 | NM_030821| | PLA2G12A,phospholipase A2, group XIIA |
| 213061_s_at | 25.53 | 7.37E-07 | 1.054958976 | NM_173474| | NTAN1,N-terminal Asn amidase |
| 205733_at | 25.52 | 7.39E-07 | 1.090889892 | NM_000057| | BLM,Bloom syndrome protein |
| 216985_s_at | 25.51 | 7.40E-07 | 1.853461159 | NM_004177| | STX3A,syntaxin 3A |
| 217165_x_at | 25.5 | 7.41E-07 | 1.20324238 | NM_005949| | MT1F,metallothionein 1F |
| 1568678_s_at | 25.5 | 7.41E-07 | 1.173205083 | NM_007045| | FGFR1OP,FGFR1 oncogene partner isoform a |
| 229700_at | 25.47 | 7.46E-07 | 1.188363229 | NA |  |
| 229485_x_at | 25.46 | 7.48E-07 | 1.809335075 | NM_001080505| | NA |
| 226498_at | 25.46 | 7.47E-07 | 3.318673256 | NA |  |
| 212681_at | 25.45 | 7.49E-07 | 2.327085344 | NM_012307| | EPB41L3,erythrocyte membrane protein band 4.1-like 3 |
| 201850_at | 25.41 | 7.55E-07 | 1.379277283 | NM_001747| | CAPG,capping protein (actin filament), gelsolin-like |
| 205538_at | 25.4 | 7.57E-07 | 2.052830416 | NM_003389| | CORO2A,coronin, actin binding protein, 2A |
| 205578_at | 25.4 | 7.57E-07 | 1.710748708 | NM_004560| | ROR2,receptor tyrosine kinase-like orphan receptor 2 |
| 235141_at | 25.39 | 7.60E-07 | 1.54078045 | NM_001038603| | NA |
| 202635_s_at | 25.37 | 7.63E-07 | 1.065092849 | NM_005034| | POLR2K,DNA directed RNA polymerase II polypeptide K |
| 210547_x_at | 25.35 | 7.67E-07 | 1.201420884 | NM_004968| | ICA1,islet cell autoantigen 1 isoform 2 |
| 223380_s_at | 25.34 | 7.67E-07 | 1.154159047 | NM_014572| | LATS2,LATS, large tumor suppressor, homolog 2 |
| 212812_at | 25.3 | 7.74E-07 | 1.68295293 | NA |  |
| 1555793_a_at | 25.29 | 7.76E-07 | 1.569398064 | NM_133466| | ZNF545,zinc finger protein 545 |
| 202950_at | 25.28 | 7.78E-07 | 1.051792231 | NM_001889| | CRYZ,crystallin, zeta |
| 201924_at | 25.27 | 7.79E-07 | 1.176754805 | NM_005935| | MLLT2,myeloid/lymphoid or mixed-lineage leukemia |
| 225447_at | 25.25 | 7.83E-07 | 1.189885843 | NM_000408| | GPD2,glycerol-3-phosphate dehydrogenase 2 |
| 208613_s_at | 25.25 | 7.83E-07 | 1.173580345 | NM_001457| | FLNB,filamin B, beta (actin binding protein 278) |
| 233559_s_at | 25.24 | 7.84E-07 | 1.187475885 | NM_020830| | WDFY1,WD repeat and FYVE domain containing 1 |
| 213927_at | 25.24 | 7.84E-07 | 2.103673072 | NM_033141| | MAP3K9,mitogen-activated protein kinase kinase kinase |
| 201034_at | 25.23 | 7.85E-07 | 1.102245171 | NM_001121| | NA |
| 218781_at | 25.22 | 7.86E-07 | 1.133619714 | NM_024624| | SMC6L1,SMC6 protein |
| 219578_s_at | 25.21 | 7.88E-07 | 1.354129623 | NM_001079533| | NA |
| 205415_s_at | 25.2 | 7.90E-07 | 1.21556996 | NM_004993| | ATXN3,ataxin 3 isoform 1 |
| 224329_s_at | 25.2 | 7.90E-07 | 1.694703068 | NM_032488| | CNFN,cornifelin |
| 223457_at | 25.18 | 7.94E-07 | 1.081523098 | NM_012133| | COPG2,coatomer protein complex, subunit gamma 2 |
| 212344_at | 25.18 | 7.93E-07 | 1.389347021 | NM_015170| | SULF1,sulfatase 1 |
| 241353_s_at | 25.16 | 7.97E-07 | 1.285815181 | NA |  |
| 229432_at | 25.14 | 7.99E-07 | 1.487886245 | NM_153006| | NAGS,N-acetylglutamate synthase |
| 226865_at | 25.14 | 8.00E-07 | 1.291850939 | NA |  |
| 223383_at | 25.13 | 8.02E-07 | 1.083641762 | NM_032268| | ZNRF1,zinc and ring finger protein 1 |
| 206286_s_at | 25.13 | 8.01E-07 | 3.023751365 | NM_003212| | TDGF1,teratocarcinoma-derived growth factor 1 |
| 229139_at | 25.13 | 8.01E-07 | 1.180863311 | NM_020647| | JPH1,junctophilin 1 |
| 226747_at | 25.11 | 8.03E-07 | 1.156168388 | NM_020784| | KIAA1344,KIAA1344 |
| 222646_s_at | 25.11 | 8.03E-07 | 1.164045166 | NM_014584| | ERO1L,ERO1-like |
| 203433_at | 25.11 | 8.03E-07 | 1.18605761 | NM_001100879| | NA |
| 221019_s_at | 25.1 | 8.05E-07 | 2.202937456 | NM_130386| | COLEC12,collectin sub-family member 12 isoform I |
| 227804_at | 25.09 | 8.07E-07 | 1.152069238 | NM_138463| | LOC116238,hypothetical protein BC014072 |
| 232037_at | 25.09 | 8.07E-07 | 1.312033031 | NM_004884| | PUNC,putative neuronal cell adhesion molecule |
| 230839_at | 25.03 | 8.18E-07 | 1.217818055 | NM_019854| | HRMT1L4,protein arginine N-methyltransferase 4 |
| 203296_s_at | 25.03 | 8.18E-07 | 1.289061356 | NM_000702| | ATP1A2,Na+/K+ -ATPase alpha 2 subunit proprotein |
| 219786_at | 25 | 8.24E-07 | 1.748574681 | NM_001039656| | NA |
| 202535_at | 25 | 8.23E-07 | 1.107512036 | NM_003824| | FADD,Fas-associated via death domain |
| 230746_s_at | 25 | 8.24E-07 | 2.695022962 | NM_003155| | STC1,stanniocalcin 1 |
| 220942_x_at | 24.99 | 8.25E-07 | 1.102143248 | NM_014367| | E2IG5,growth and transformation-dependent protein |
| 204462_s_at | 24.99 | 8.25E-07 | 1.125123662 | NM_006517| | SLC16A2,solute carrier family 16, member 2 |
| 226313_at | 24.96 | 8.29E-07 | 1.380144109 | NM_145306| | C10orf35,chromosome 10 open reading frame 35 |
| 203704_s_at | 24.96 | 8.30E-07 | 1.146600832 | NM_001003698| | RREB1,ras responsive element binding protein 1 isoform |
| 219959_at | 24.95 | 8.30E-07 | 2.000927268 | NM_017947| | MOCOS,molybdenum cofactor sulfurase |
| 213558_at | 24.95 | 8.31E-07 | 1.699896322 | NM_014510| | NA |
| 226567_at | 24.95 | 8.30E-07 | 1.066979955 | NM_001037334| | NA |
| 239231_at | 24.94 | 8.32E-07 | 1.350100429 | NA |  |
| 221898_at | 24.93 | 8.33E-07 | 1.456316882 | NM_001006624| | T1A-2,lung type-I cell membrane-associated |
| 243252_at | 24.92 | 8.36E-07 | 1.551636471 | NA |  |
| 229360_at | 24.91 | 8.38E-07 | 1.244204755 | NM_080764| | SUHW2,suppressor of hairy wing homolog 2 |
| 224413_s_at | 24.91 | 8.37E-07 | 1.094590659 | NM_001024380| | NA |
| 210427_x_at | 24.9 | 8.38E-07 | 1.096535627 | NM_001002857| | ANXA2,annexin A2 isoform 2 |
| 224871_at | 24.9 | 8.38E-07 | 1.212550372 | NM_182752| | LOC127262,hypothetical protein LOC127262 |
| 205861_at | 24.84 | 8.50E-07 | 1.667506405 | NM_003121| | SPIB,Spi-B transcription factor (Spi-1/PU.1 related) |
| 207727_s_at | 24.84 | 8.51E-07 | 1.082683666 | NM_001048171| | NA |
| 200860_s_at | 24.83 | 8.52E-07 | 1.040033598 | NM_016284| | CNOT1,CCR4-NOT transcription complex, subunit 1 |
| 217234_s_at | 24.83 | 8.52E-07 | 1.172852372 | NM_001111077| | NA |
| 206355_at | 24.81 | 8.56E-07 | 1.839807907 | NM_002071| | GNAL,guanine nucleotide binding protein (G protein), |
| 201243_s_at | 24.81 | 8.56E-07 | 1.041657928 | NM_001001787| | ATP1B1,Na+/K+ -ATPase beta 1 subunit isoform b |
| 236163_at | 24.8 | 8.58E-07 | 1.609196066 | NM_153234| | LIX1,limb expression 1 |
| 224800_at | 24.8 | 8.57E-07 | 1.14514009 | NM_020830| | WDFY1,WD repeat and FYVE domain containing 1 |
| 202620_s_at | 24.78 | 8.61E-07 | 1.169978294 | NM_000935| | PLOD2,procollagen-lysine, 2-oxoglutarate 5-dioxygenase |
| 215594_at | 24.77 | 8.61E-07 | 1.765047811 | NA |  |
| 59697_at | 24.77 | 8.61E-07 | 1.245348719 | NM_198686| | RAB15,Ras-related protein Rab-15 |
| 225380_at | 24.77 | 8.61E-07 | 1.568941181 | NM_138370| | NA |
| 239148_at | 24.76 | 8.63E-07 | 2.32660021 | NM_001017967| | NA |
| 203090_at | 24.72 | 8.71E-07 | 1.057876863 | NM_006923| | SDF2,stromal cell-derived factor 2 precursor |
| 222234_s_at | 24.72 | 8.71E-07 | 1.132923569 | NM_001042610| | NA |
| 204749_at | 24.72 | 8.71E-07 | 1.510969341 | NM_004538| | NAP1L3,nucleosome assembly protein 1-like 3 |
| 209109_s_at | 24.72 | 8.71E-07 | 1.093398381 | NM_003270| | TM4SF6,transmembrane 4 superfamily member 6 |
| 41037_at | 24.71 | 8.72E-07 | 1.107935995 | NM_003213| | TEAD4,TEA domain family member 4 isoform 1 |
| 232636_at | 24.71 | 8.72E-07 | 2.358972626 | NM_173078| | SLITRK4,slit and trk like 4 protein |
| 207071_s_at | 24.71 | 8.72E-07 | 1.133180542 | NM_002197| | ACO1,aconitase 1 |
| 229178_at | 24.71 | 8.72E-07 | 1.490792873 | NA |  |
| 224920_x_at | 24.69 | 8.75E-07 | 1.202308776 | NM_001020818| | NA |
| 225548_at | 24.69 | 8.76E-07 | 1.109982836 | NM_020859| | ShrmL,Shroom-related protein |
| 224439_x_at | 24.67 | 8.78E-07 | 1.083792138 | NM_014245| | RNF7,ring finger protein 7 isoform 1 |
| 227920_at | 24.67 | 8.78E-07 | 1.157161118 | NM_001080450| | NA |
| 230047_at | 24.66 | 8.80E-07 | 1.315878015 | NA |  |
| 209267_s_at | 24.65 | 8.80E-07 | 1.467555451 | NM_022154| | SLC39A8,solute carrier family 39 (zinc transporter), |
| 225792_at | 24.64 | 8.83E-07 | 2.712167896 | NM_015888| | HOOK1,hook homolog 1 |
| 212864_at | 24.63 | 8.84E-07 | 1.254417985 | NM_003818| | CDS2,phosphatidate cytidylyltransferase 2 |
| 228850_s_at | 24.61 | 8.87E-07 | 1.935966621 | NA |  |
| 218987_at | 24.61 | 8.88E-07 | 1.152026421 | NM_018179| | ATF7IP,activating transcription factor 7 interacting |
| 240152_at | 24.6 | 8.88E-07 | 1.415199102 | NA |  |
| 232172_at | 24.6 | 8.89E-07 | 1.213838026 | NA |  |
| 203127_s_at | 24.59 | 8.90E-07 | 1.207850445 | NM_004863| | SPTLC2,serine palmitoyltransferase, long chain base |
| 225806_at | 24.57 | 8.94E-07 | 1.346679552 | NM_032876| | JUB,jub, ajuba homolog isoform 1 |
| 210754_s_at | 24.57 | 8.95E-07 | 1.425506094 | NM_001111097| | NA |
| 223531_x_at | 24.56 | 8.96E-07 | 1.052296551 | NM_001097612| | NA |
| 202117_at | 24.56 | 8.97E-07 | 1.029919833 | NM_004308| | ARHGAP1,Rho GTPase activating protein 1 |
| 223239_at | 24.55 | 8.98E-07 | 1.172459059 | NM_016472| | C14orf129,chromosome 14 open reading frame 129 |
| 228665_at | 24.54 | 8.99E-07 | 2.473684812 | NM_052954| | CYYR1,cysteine and tyrosine-rich 1 protein precursor |
| 221486_at | 24.52 | 9.05E-07 | 1.050959563 | NM_004436| | ENSA,endosulfine alpha isoform 3 |
| 46665_at | 24.5 | 9.07E-07 | 1.100291793 | NM_017789| | SEMA4C,semaphorin 4C |
| 205483_s_at | 24.5 | 9.08E-07 | 1.324426589 | NM_005101| | G1P2,interferon, alpha-inducible protein (clone |
| 203346_s_at | 24.5 | 9.09E-07 | 1.207839186 | NM_007358| | M96,putative DNA binding protein |
| 202918_s_at | 24.49 | 9.11E-07 | 1.137697637 | NM_001100819| | NA |
| 206662_at | 24.48 | 9.13E-07 | 1.38306705 | NM_001118890| | NA |
| 218205_s_at | 24.48 | 9.12E-07 | 1.087769947 | NM_017572| | MKNK2,MAP kinase-interacting serine/threonine kinase |
| 202752_x_at | 24.46 | 9.15E-07 | 1.662936529 | NM_012244| | SLC7A8,solute carrier family 7 (cationic amino acid |
| 214285_at | 24.45 | 9.18E-07 | 2.039429876 | NM_004102| | FABP3,fatty acid binding protein 3 |
| 212888_at | 24.44 | 9.19E-07 | 1.17931663 | NM_030621| | DICER1,dicer1 |
| 231867_at | 24.44 | 9.19E-07 | 1.481365699 | NM_001080428| | NA |
| 214913_at | 24.44 | 9.20E-07 | 1.289802785 | NM_014243| | ADAMTS3,a disintegrin-like and metalloprotease |
| 32502_at | 24.41 | 9.25E-07 | 1.083414553 | NM_030792| | PP1665,hypothetical protein PP1665 |
| 231856_at | 24.37 | 9.35E-07 | 2.110855374 | NM_020340| | KIAA1244,KIAA1244 |
| 210372_s_at | 24.36 | 9.38E-07 | 1.503661441 | NM_001003395| | TPD52L1,tumor protein D52-like 1 isoform 2 |
| 219045_at | 24.35 | 9.39E-07 | 1.446950477 | NM_019034| | RHOF,ras homolog gene family, member F |
| 212110_at | 24.34 | 9.42E-07 | 1.155993314 | NM_015359| | SLC39A14,solute carrier family 39 (zinc transporter), |
| 227647_at | 24.33 | 9.42E-07 | 1.700094262 | NM_005472| | KCNE3,potassium voltage-gated channel, Isk-related |
| 208343_s_at | 24.32 | 9.44E-07 | 1.857172213 | NM_003822| | NR5A2,nuclear receptor subfamily 5, group A, member 2 |
| 211700_s_at | 24.32 | 9.45E-07 | 1.157140164 | NM_001039705| | NA |
| 224899_s_at | 24.31 | 9.45E-07 | 1.086454248 | NM_032121| | DKFZp564K142,implantation-associated protein |
| 237203_at | 24.31 | 9.46E-07 | 1.885249029 | NA |  |
| 212770_at | 24.31 | 9.46E-07 | 1.115303633 | NM_001105192| | NA |
| 203975_s_at | 24.28 | 9.53E-07 | 1.080240273 | NM_005483| | CHAF1A,chromatin assembly factor 1, subunit A (p150) |
| 213541_s_at | 24.26 | 9.57E-07 | 1.565598938 | NM_004449| | ERG,v-ets erythroblastosis virus E26 oncogene like |
| 206729_at | 24.26 | 9.57E-07 | 1.677775432 | NM_001243| | TNFRSF8,tumor necrosis factor receptor superfamily, |
| 218720_x_at | 24.26 | 9.57E-07 | 1.498442873 | NM_001114099| | NA |
| 235241_at | 24.23 | 9.63E-07 | 1.290132526 | NM_173514| | FLJ90709,hypothetical protein FLJ90709 |
| 219602_s_at | 24.21 | 9.65E-07 | 2.229607081 | NM_022068| | FAM38B,family with sequence similarity 38, member B |
| 228277_at | 24.21 | 9.65E-07 | 1.100650442 | NM_001099784| | NA |
| 243672_at | 24.21 | 9.66E-07 | 1.172302508 | NM_171999| | SALL3,sal-like 3 |
| 221864_at | 24.18 | 9.72E-07 | 1.14422448 | NM_152288| | MGC13024,hypothetical protein MGC13024 |
| 207714_s_at | 24.18 | 9.72E-07 | 1.15367454 | NM_001235| | SERPINH1,serine (or cysteine) proteinase inhibitor, clade |
| 218671_s_at | 24.18 | 9.73E-07 | 1.101952324 | NM_016311| | ATPIF1,ATPase inhibitory factor 1 isoform 1 precursor |
| 201418_s_at | 24.17 | 9.74E-07 | 1.082719662 | NM_003107| | SOX4,SRY (sex determining region Y)-box 4 |
| 221957_at | 24.15 | 9.80E-07 | 1.282085876 | NM_005391| | PDK3,pyruvate dehydrogenase kinase, isoenzyme 3 |
| 205603_s_at | 24.14 | 9.81E-07 | 1.213343682 | NM_006729| | DIAPH2,diaphanous 2 isoform 156 |
| 221903_s_at | 24.14 | 9.81E-07 | 1.409425613 | NM_001042355| | NA |
| 221065_s_at | 24.14 | 9.81E-07 | 1.23971484 | NM_022467| | CHST8,carbohydrate (N-acetylgalactosamine 4-0) |
| 220952_s_at | 24.13 | 9.82E-07 | 1.387530871 | NM_019012| | PLEKHA5,pleckstrin homology domain containing, family A |
| 204368_at | 24.13 | 9.82E-07 | 1.463926741 | NM_005630| | SLCO2A1,solute carrier organic anion transporter family, |
| 244370_at | 24.12 | 9.86E-07 | 1.626727686 | NM_001008537| | KIAA2022,KIAA2022 protein |
| 201880_at | 24.1 | 9.90E-07 | 1.076185683 | NM_005744| | ARIH1,ariadne homolog, ubiquitin-conjugating enzyme E2 |
| 235258_at | 24.09 | 9.91E-07 | 1.157019463 | NM_152624| | DCP2,DCP2 decapping enzyme |
| 229264_at | 24.09 | 9.91E-07 | 1.540395811 | NA |  |
| 225080_at | 24.08 | 9.94E-07 | 1.134858354 | NM_001080779| | NA |
| 229205_at | 24.08 | 9.94E-07 | 1.656839819 | NA |  |
| 209566_at | 24.06 | 9.98E-07 | 1.165180769 | NM_016133| | INSIG2,insulin induced protein 2 |
| 214079_at | 24.03 | 1.00E-06 | 2.140331305 | NM_005794| | DHRS2,dehydrogenase/reductase (SDR family) member 2 |
| 242592_at | 24.02 | 1.01E-06 | 1.80748237 | NM_001099652| | NA |
| 220108_at | 24.01 | 1.01E-06 | 2.225130606 | NM_004297| | GNA14,guanine nucleotide binding protein (G protein), |
| 201560_at | 24.01 | 1.01E-06 | 1.148999922 | NM_013943| | CLIC4,chloride intracellular channel 4 |
| 236030_at | 23.99 | 1.01E-06 | 1.170426066 | NM_173587| | RCOR2,REST corepressor 2 |
| 201605_x_at | 23.98 | 1.01E-06 | 1.29392408 | NM_004368| | CNN2,calponin 2 isoform a |
| 232254_at | 23.97 | 1.02E-06 | 1.33991581 | NA |  |
| 1564413_at | 23.96 | 1.02E-06 | 1.700852496 | NA |  |
| 223628_at | 23.96 | 1.02E-06 | 1.425597637 | NA |  |
| 208959_s_at | 23.96 | 1.02E-06 | 1.086807482 | NM_015051| | TXNDC4,thioredoxin domain containing 4 (endoplasmic |
| 212769_at | 23.95 | 1.02E-06 | 1.278764827 | NM_001105192| | NA |
| 218441_s_at | 23.94 | 1.02E-06 | 1.113110374 | NM_015540| | RPAP1,RNA polymerase II associated protein 1 |
| 220615_s_at | 23.93 | 1.02E-06 | 1.463086577 | NM_018099| | MLSTD1,male sterility domain containing 1 |
| 1553575_at | 23.91 | 1.03E-06 | 1.082541634 | NA |  |
| 205373_at | 23.91 | 1.03E-06 | 1.718608516 | NM_004389| | CTNNA2,catenin, alpha 2 |
| 209015_s_at | 23.9 | 1.03E-06 | 1.280774167 | NM_005494| | DNAJB6,DnaJ (Hsp40) homolog, subfamily B, member 6 |
| 225320_at | 23.9 | 1.03E-06 | 1.144892959 | NM_138357| | C10orf42,chromosome 10 open reading frame 42 |
| 207522_s_at | 23.89 | 1.03E-06 | 1.557347065 | NM_005173| | ATP2A3,sarco/endoplasmic reticulum Ca2+ -ATPase isoform |
| 228658_at | 23.88 | 1.03E-06 | 1.17372067 | NA |  |
| 225478_at | 23.87 | 1.04E-06 | 1.089445975 | NM_004225| | MFHAS1,malignant fibrous histiocytoma amplified |
| 205416_s_at | 23.85 | 1.04E-06 | 1.185049646 | NM_004993| | ATXN3,ataxin 3 isoform 1 |
| 213801_x_at | 23.85 | 1.04E-06 | 1.013446358 | NM_001005472| | LOC388524,similar to Laminin receptor 1 |
| 219185_at | 23.85 | 1.04E-06 | 1.124286762 | NM_012241| | SIRT5,sirtuin 5 isoform 1 |
| 214933_at | 23.85 | 1.04E-06 | 1.730741313 | NM_000068| | CACNA1A,calcium channel, alpha 1A subunit isoform 1 |
| 219301_s_at | 23.84 | 1.04E-06 | 1.904040101 | NM_014141| | CNTNAP2,cell recognition molecule Caspr2 precursor |
| 227068_at | 23.83 | 1.05E-06 | 1.090630541 | NM_000291| | PGK1,phosphoglycerate kinase 1 |
| 201131_s_at | 23.82 | 1.05E-06 | 3.707862481 | NM_004360| | CDH1,cadherin 1, type 1 preproprotein |
| 202562_s_at | 23.82 | 1.05E-06 | 1.089345989 | NM_007176| | C14orf1,chromosome 14 open reading frame 1 |
| 212626_x_at | 23.82 | 1.05E-06 | 1.049640756 | NM_001077442| | NA |
| 212295_s_at | 23.81 | 1.05E-06 | 1.047654916 | NM_003045| | SLC7A1,solute carrier family 7 (cationic amino acid |
| 206696_at | 23.81 | 1.05E-06 | 1.536946338 | NM_000273| | GPR143,G protein-coupled receptor 143 |
| 213139_at | 23.8 | 1.05E-06 | 2.124636005 | NM_003068| | SNAI2,snail 2 |
| 225646_at | 23.78 | 1.06E-06 | 1.285518462 | NM_001114173| | NA |
| 207984_s_at | 23.76 | 1.06E-06 | 1.199676984 | NM_005374| | MPP2,palmitoylated membrane protein 2 |
| 219000_s_at | 23.75 | 1.07E-06 | 1.133517719 | NM_024094| | DCC1,hypothetical protein MGC5528 |
| 210511_s_at | 23.73 | 1.07E-06 | 1.74145997 | NM_002192| | INHBA,inhibin beta A subunit precursor |
| 208290_s_at | 23.72 | 1.07E-06 | 1.096756318 | NM_001969| | EIF5,eukaryotic translation initiation factor 5 |
| 224465_s_at | 23.72 | 1.07E-06 | 1.142772302 | NM_032345| | PYM,PYM protein |
| 40359_at | 23.71 | 1.07E-06 | 1.075036557 | NM_003475| | C11orf13,HRAS1-related cluster-1 |
| 218499_at | 23.68 | 1.08E-06 | 1.302115129 | NM_001042452| | NA |
| 224561_s_at | 23.65 | 1.09E-06 | 1.082797022 | NM_006791| | MORF4L1,MORF-related gene 15 isoform 1 |
| 219736_at | 23.65 | 1.09E-06 | 1.333759316 | NM_001017397| | NA |
| 205857_at | 23.65 | 1.09E-06 | 1.698830164 | NM_003054| | SLC18A2,solute carrier family 18 (vesicular monoamine), |
| 217794_at | 23.62 | 1.10E-06 | 1.086660143 | NM_001005354| | DKFZp564J157,DKFZp564J157 protein isoform 2 |
| 221729_at | 23.62 | 1.10E-06 | 1.95560792 | NM_000393| | COL5A2,alpha 2 type V collagen preproprotein |
| 209242_at | 23.61 | 1.10E-06 | 1.126466007 | NM_006210| | PEG3,paternally expressed 3 |
| 219326_s_at | 23.61 | 1.10E-06 | 1.465249433 | NM_006577| | B3GNT1,beta-1,3-N-acetylglucosaminyltransferase bGnT-1 |
| 203407_at | 23.59 | 1.11E-06 | 1.56669744 | NM_002705| | PPL,periplakin |
| 207513_s_at | 23.58 | 1.11E-06 | 1.093889144 | NM_003452| | ZNF189,zinc finger protein 189 |
| 214761_at | 23.58 | 1.11E-06 | 1.215659217 | NM_015069| | ZNF423,zinc finger protein 423 |
| 244406_at | 23.58 | 1.11E-06 | 1.17280661 | NM_021143| | ZNF20,zinc finger protein 20 (KOX 13) |
| 201253_s_at | 23.58 | 1.11E-06 | 1.064432251 | NM_006319| | CDIPT,CDP-diacylglycerol--inositol |
| 205011_at | 23.57 | 1.11E-06 | 1.160387288 | NM_014622| | LOH11CR2A,BCSC-1 isoform 1 |
| 229377_at | 23.57 | 1.11E-06 | 1.851526504 | NM_024719| | GRTP1,growth hormone regulated TBC protein 1 |
| 228952_at | 23.54 | 1.12E-06 | 1.410938368 | NM_006208| | ENPP1,ectonucleotide pyrophosphatase/phosphodiesterase |
| 202826_at | 23.53 | 1.12E-06 | 1.775615306 | NM_001032367| | NA |
| 204866_at | 23.53 | 1.12E-06 | 1.239140736 | NM_001077445| | NA |
| 209891_at | 23.53 | 1.12E-06 | 1.08721275 | NM_020675| | Spc25,kinetochore protein Spc25 |
| 1553581_s_at | 23.51 | 1.12E-06 | 1.119688921 | NM_173829| | FLJ36754,hypothetical protein FLJ36754 |
| 222946_s_at | 23.5 | 1.13E-06 | 1.143875706 | NM_024037| | MGC2603,hypothetical protein MGC2603 |
| 226723_at | 23.5 | 1.13E-06 | 1.17963366 | NM_199342| | LOC374969,hypothetical protein LOC374969 |
| 225750_at | 23.48 | 1.13E-06 | 1.124725318 | NA |  |
| 203650_at | 23.47 | 1.14E-06 | 2.01127853 | NM_006404| | PROCR,endothelial protein C receptor precursor |
| 213921_at | 23.46 | 1.14E-06 | 1.266934593 | NM_001048| | SST,somatostatin |
| 203797_at | 23.45 | 1.14E-06 | 2.653849997 | NM_003385| | VSNL1,visinin-like 1 |
| 227568_at | 23.45 | 1.14E-06 | 1.315352134 | NM_173497| | HECTD2,HECT domain containing 2 isoform b |
| 225418_at | 23.44 | 1.14E-06 | 1.225354023 | NM_001042724| | NA |
| 204184_s_at | 23.43 | 1.14E-06 | 1.445752232 | NM_005160| | ADRBK2,beta adrenergic receptor kinase 2 |
| 223522_at | 23.43 | 1.15E-06 | 1.175887744 | NA |  |
| 225227_at | 23.42 | 1.15E-06 | 1.38425265 | NA |  |
| 209348_s_at | 23.42 | 1.15E-06 | 1.523615484 | NM_001031804| | NA |
| 201549_x_at | 23.41 | 1.15E-06 | 1.179270947 | NM_006618| | JARID1B,Jumonji, AT rich interactive domain 1B |
| 225648_at | 23.4 | 1.15E-06 | 1.137326627 | NM_080836| | STK35,serine/threonine kinase 35 |
| 203414_at | 23.4 | 1.15E-06 | 1.13983919 | NM_012329| | MMD,monocyte to macrophage |
| 202956_at | 23.4 | 1.15E-06 | 1.156844074 | NM_006421| | ARFGEF1,brefeldin A-inhibited guanine |
| 224445_s_at | 23.39 | 1.15E-06 | 1.104270316 | NM_024071| | ZFYVE21,zinc finger, FYVE domain containing 21 |
| 228799_at | 23.35 | 1.17E-06 | 1.18409073 | NA |  |
| 217821_s_at | 23.34 | 1.17E-06 | 1.066728168 | NM_016312| | WBP11,WW domain binding protein 11 |
| 224397_s_at | 23.33 | 1.17E-06 | 1.239988482 | NM_175861| | ARG99,ARG99 protein |
| 1552575_a_at | 23.32 | 1.17E-06 | 1.581222039 | NA |  |
| 201811_x_at | 23.32 | 1.17E-06 | 1.094634347 | NM_001018009| | NA |
| 220334_at | 23.32 | 1.17E-06 | 1.157299675 | NM_012419| | RGS17,regulator of G-protein signalling 17 |
| 208771_s_at | 23.32 | 1.17E-06 | 1.085344414 | NM_000895| | LTA4H,leukotriene A4 hydrolase |
| 206743_s_at | 23.3 | 1.18E-06 | 1.628313401 | NM_001671| | ASGR1,asialoglycoprotein receptor 1 |
| 208998_at | 23.3 | 1.18E-06 | 1.226728964 | NM_003355| | UCP2,uncoupling protein 2 |
| 226720_at | 23.29 | 1.18E-06 | 1.283940624 | NM_052927| | NA |
| 229724_at | 23.29 | 1.18E-06 | 2.355577296 | NM_000814| | GABRB3,gamma-aminobutyric acid (GABA) A receptor, beta |
| 220367_s_at | 23.28 | 1.18E-06 | 1.072127911 | NM_024545| | SAP130,mSin3A-associated protein 130 |
| 224819_at | 23.26 | 1.19E-06 | 1.259755572 | NM_001006684| | TCEAL8,transcription elongation factor A (SII)-like 8 |
| 235333_at | 23.25 | 1.19E-06 | 1.238658044 | NM_004775| | B4GALT6,UDP-Gal:betaGlcNAc beta 1,4- |
| 214239_x_at | 23.25 | 1.19E-06 | 1.163036179 | NM_007144| | PCGF2,ring finger protein 110 |
| 225068_at | 23.24 | 1.20E-06 | 1.053564928 | NM_021633| | KLHL12,kelch-like 12 |
| 231896_s_at | 23.23 | 1.20E-06 | 1.034352169 | NM_003677| | DENR,density-regulated protein |
| 218133_s_at | 23.23 | 1.20E-06 | 1.040723268 | NM_021824| | NIF3L1,NIF3 NGG1 interacting factor 3-like 1 |
| 204694_at | 23.22 | 1.20E-06 | 1.677404516 | NM_001134| | AFP,alpha-fetoprotein precursor |
| 204483_at | 23.22 | 1.20E-06 | 1.23327977 | NM_001976| | ENO3,enolase 3 |
| 228189_at | 23.22 | 1.20E-06 | 1.122217344 | NM_004874| | BAG4,BCL2-associated athanogene 4 |
| 208022_s_at | 23.22 | 1.20E-06 | 1.194891639 | NM_001077181| | NA |
| 228912_at | 23.22 | 1.20E-06 | 1.556091748 | NM_007127| | VIL1,villin 1 |
| 212345_s_at | 23.2 | 1.20E-06 | 1.140879108 | NM_194071| | CREB3L2,cAMP responsive element binding protein 3-like |
| 201280_s_at | 23.18 | 1.21E-06 | 1.621542794 | NM_001343| | DAB2,disabled homolog 2 |
| 206984_s_at | 23.17 | 1.21E-06 | 1.915581075 | NM_002930| | RIT2,Ras-like without CAAX 2 |
| 223264_at | 23.15 | 1.22E-06 | 1.109949162 | NM_022566| | MESDC1,mesoderm development candidate 1 |
| 203304_at | 23.14 | 1.22E-06 | 1.275125361 | NM_012342| | BAMBI,BMP and activin membrane-bound inhibitor |
| 202085_at | 23.14 | 1.22E-06 | 1.119720277 | NM_004817| | TJP2,tight junction protein 2 (zona occludens 2) |
| 218353_at | 23.13 | 1.22E-06 | 1.856917747 | NM_003617| | RGS5,regulator of G-protein signalling 5 |
| 205281_s_at | 23.11 | 1.23E-06 | 1.085251866 | NM_002641| | PIGA,phosphatidylinositol |
| 212632_at | 23.11 | 1.23E-06 | 1.119970169 | NM_003569| | STX7,syntaxin 7 |
| 205664_at | 23.11 | 1.23E-06 | 1.137981214 | NM_012311| | KIN,HsKin17 protein |
| 201332_s_at | 23.1 | 1.23E-06 | 1.268063594 | NM_003153| | STAT6,signal transducer and activator of transcription |
| 214843_s_at | 23.1 | 1.23E-06 | 1.100837872 | NM_015017| | USP33,ubiquitin specific protease 33 isoform 1 |
| 209931_s_at | 23.09 | 1.23E-06 | 1.316328401 | NM_004116| | FKBP1B,FK506-binding protein 1B isoform a |
| 217990_at | 23.04 | 1.25E-06 | 1.084092657 | NM_001002000| | GMPR2,guanosine monophosphate reductase 2 isoform 2 |
| 212731_at | 23.04 | 1.25E-06 | 1.17911884 | NM_198401| | LOC157567,hypothetical protein LOC157567 |
| 244246_at | 23.04 | 1.25E-06 | 1.400927229 | NM_138731| | MIPOL1,mirror-image polydactyly 1 |
| 220157_x_at | 23.03 | 1.25E-06 | 1.187481103 | NM_015899| | PLEKHA9,pleckstrin homology domain containing, family A |
| 200960_x_at | 23.02 | 1.25E-06 | 1.037534996 | NM_001076677| | NA |
| 225195_at | 23 | 1.26E-06 | 1.305730277 | NM_001047434| | NA |
| 236896_at | 23 | 1.26E-06 | 1.845950551 | NM_003412| | ZIC1,zinc finger protein of the cerebellum 1 |
| 1569023_a_at | 23 | 1.26E-06 | 2.371619234 | NA |  |
| 221832_s_at | 23 | 1.26E-06 | 1.151600849 | NM_033631| | LUZP1,leucine zipper protein 1 |
| 223556_at | 22.99 | 1.26E-06 | 1.209880114 | NM_018063| | HELLS,helicase, lymphoid-specific |
| 210809_s_at | 22.99 | 1.26E-06 | 1.972661606 | NM_006475| | POSTN,periostin, osteoblast specific factor |
| 229573_at | 22.95 | 1.27E-06 | 1.189176126 | NM_001039590| | NA |
| 203752_s_at | 22.94 | 1.28E-06 | 1.082210843 | NM_005354| | JUND,jun D proto-oncogene |
| 228537_at | 22.94 | 1.28E-06 | 1.182503583 | NM_005270| | GLI2,GLI-Kruppel family member GLI2 isoform delta |
| 1554887_at | 22.94 | 1.28E-06 | 1.968818244 | NA |  |
| 203351_s_at | 22.93 | 1.28E-06 | 1.093961843 | NM_002552| | ORC4L,origin recognition complex subunit 4 |
| 1554628_at | 22.93 | 1.28E-06 | 1.164375965 | NM_173480| | LOC126295,hypothetical protein LOC126295 |
| 208724_s_at | 22.93 | 1.28E-06 | 1.033715933 | NM_004161| | RAB1A,RAB1A, member RAS oncogene family |
| 234341_x_at | 22.89 | 1.29E-06 | 1.086246761 | NA |  |
| 202442_at | 22.89 | 1.29E-06 | 1.045396029 | NM_001284| | AP3S1,adaptor-related protein complex 3, sigma 1 |
| 1555971_s_at | 22.89 | 1.29E-06 | 1.150382033 | NM_015176| | FBXO28,F-box protein 28 |
| 218537_at | 22.89 | 1.29E-06 | 1.227933253 | NM_001002017| | HCFC1R1,host cell factor C1 regulator 1 (XPO1 dependant) |
| 203851_at | 22.87 | 1.30E-06 | 1.578340124 | NM_002178| | IGFBP6,insulin-like growth factor binding protein 6 |
| 206440_at | 22.85 | 1.30E-06 | 1.21582945 | NM_004664| | LIN7A,lin-7 homolog A |
| 220419_s_at | 22.85 | 1.30E-06 | 1.146708903 | NM_013396| | USP25,ubiquitin specific protease 25 |
| 207966_s_at | 22.84 | 1.30E-06 | 1.04151803 | NM_012201| | GLG1,golgi apparatus protein 1 |
| 201849_at | 22.83 | 1.31E-06 | 1.090313321 | NM_004052| | BNIP3,BCL2/adenovirus E1B 19kD-interacting protein 3 |
| 231736_x_at | 22.83 | 1.31E-06 | 1.068391813 | NM_020300| | MGST1,microsomal glutathione S-transferase 1 |
| 227692_at | 22.82 | 1.31E-06 | 1.222109842 | NM_002069| | GNAI1,guanine nucleotide binding protein (G protein), |
| 203510_at | 22.82 | 1.31E-06 | 1.763460995 | NM_000245| | MET,met proto-oncogene precursor |
| 204370_at | 22.81 | 1.31E-06 | 1.080292459 | NM_006831| | HEAB,ATP/GTP-binding protein |
| 232111_at | 22.8 | 1.32E-06 | 2.152967532 | NA |  |
| 203666_at | 22.77 | 1.33E-06 | 1.495988716 | NM_000609| | CXCL12,chemokine (C-X-C motif) ligand 12 (stromal |
| 220040_x_at | 22.77 | 1.32E-06 | 1.124371705 | NM_018684| | KIAA1166,KIAA1166 |
| 218186_at | 22.77 | 1.33E-06 | 2.183677239 | NM_020387| | RAB25,RAB25 |
| 227417_at | 22.76 | 1.33E-06 | 1.253526559 | NM_017898| | FLJ20605,hypothetical protein FLJ20605 |
| 209102_s_at | 22.75 | 1.33E-06 | 1.077916639 | NM_012257| | HBP1,HMG-box transcription factor 1 |
| 228377_at | 22.74 | 1.33E-06 | 2.557461423 | NM_020805| | KLHL14,kelch-like 14 |
| 202372_at | 22.73 | 1.34E-06 | 1.049676299 | NM_012414| | RAB3-GAP150,rab3 GTPase-activating protein, non-catalytic |
| 226312_at | 22.73 | 1.34E-06 | 1.114098008 | NM_152756| | AVO3,rapamycin-insensitive companion of mTOR |
| 209533_s_at | 22.72 | 1.34E-06 | 1.089140865 | NM_001031689| | NA |
| 231118_at | 22.72 | 1.34E-06 | 1.580434768 | NM_144698| | ANKRD35,ankyrin repeat domain 35 |
| 218878_s_at | 22.71 | 1.34E-06 | 1.295015401 | NM_012238| | SIRT1,sirtuin 1 |
| 227131_at | 22.71 | 1.34E-06 | 1.133034321 | NM_002401| | MAP3K3,mitogen-activated protein kinase kinase kinase 3 |
| 230061_at | 22.7 | 1.34E-06 | 2.141421704 | NM_138786| | LOC116441,hypothetical protein BC014339 |
| 223206_s_at | 22.7 | 1.34E-06 | 1.184237717 | NM_020677| | HSCARG,HSCARG protein |
| 227917_at | 22.7 | 1.34E-06 | 1.209600035 | NA |  |
| 201897_s_at | 22.69 | 1.35E-06 | 1.050258893 | NM_001826| | CKS1B,CDC28 protein kinase 1B |
| 202338_at | 22.69 | 1.35E-06 | 1.107289813 | NM_003258| | TK1,thymidine kinase 1, soluble |
| 201898_s_at | 22.67 | 1.35E-06 | 1.082242974 | NM_003336| | UBE2A,ubiquitin-conjugating enzyme E2A isoform 1 |
| 1557143_at | 22.67 | 1.35E-06 | 1.481190129 | NM_052896| | CSMD2,CUB and Sushi multiple domains 2 |
| 1559827_at | 22.67 | 1.35E-06 | 1.604848902 | NA |  |
| 230100_x_at | 22.67 | 1.35E-06 | 1.758276678 | NM_002576| | PAK1,p21-activated kinase 1 |
| 228909_at | 22.65 | 1.36E-06 | 1.169580222 | NA |  |
| 223311_s_at | 22.65 | 1.36E-06 | 1.383636245 | NM_020744| | MTA3,metastasis associated 1 family, member 3 |
| 201502_s_at | 22.64 | 1.36E-06 | 1.195950175 | NM_020529| | NFKBIA,nuclear factor of kappa light polypeptide gene |
| 203588_s_at | 22.64 | 1.36E-06 | 1.150101334 | NM_006286| | TFDP2,transcription factor Dp-2 (E2F dimerization |
| 235810_at | 22.63 | 1.36E-06 | 1.207258685 | NM_001007088| | ZNF21,zinc finger protein 21 isoform 2 |
| 203447_at | 22.63 | 1.36E-06 | 1.057528505 | NM_005047| | PSMD5,proteasome 26S non-ATPase subunit 5 |
| 221646_s_at | 22.63 | 1.36E-06 | 1.140502134 | NM_024786| | ZDHHC11,zinc finger, DHHC domain containing 11 |
| 205105_at | 22.63 | 1.36E-06 | 1.12613906 | NM_002372| | MAN2A1,mannosidase, alpha, class 2A, member 1 |
| 219499_at | 22.62 | 1.37E-06 | 1.113985473 | NM_018144| | SEC61A2,Sec61 alpha form 2 |
| 201981_at | 22.6 | 1.37E-06 | 1.26823976 | NM_002581| | PAPPA,pregnancy-associated plasma protein A |
| 218464_s_at | 22.59 | 1.37E-06 | 1.0752255 | NM_001077498| | NA |
| 212158_at | 22.59 | 1.38E-06 | 1.272837999 | NM_002998| | SDC2,syndecan 2 precursor |
| 222395_s_at | 22.58 | 1.38E-06 | 1.080302565 | NM_023079| | FLJ13855,hypothetical protein FLJ13855 |
| 217665_at | 22.57 | 1.38E-06 | 1.432924002 | NA |  |
| 200039_s_at | 22.54 | 1.39E-06 | 1.0414462 | NM_002794| | PSMB2,proteasome beta 2 subunit |
| 236448_at | 22.52 | 1.40E-06 | 1.755251314 | NM_133369| | UNC5A,netrin receptor Unc5h1 |
| 214697_s_at | 22.52 | 1.40E-06 | 1.239707717 | NM_005156| | ROD1,ROD1 regulator of differentiation 1 |
| 210788_s_at | 22.51 | 1.40E-06 | 1.080048665 | NM_016029| | DHRS7,dehydrogenase/reductase (SDR family) member 7 |
| 208146_s_at | 22.5 | 1.41E-06 | 1.108266636 | NM_019029| | CPVL,serine carboxypeptidase vitellogenic-like |
| 208785_s_at | 22.48 | 1.41E-06 | 1.098862191 | NM_022818| | MAP1LC3B,microtubule-associated proteins 1A/1B light |
| 244071_at | 22.46 | 1.42E-06 | 1.138380426 | NA |  |
| 208815_x_at | 22.46 | 1.42E-06 | 1.097567017 | NM_002154| | HSPA4,heat shock 70kDa protein 4 isoform a |
| 226925_at | 22.46 | 1.42E-06 | 1.110276101 | NM_001037172| | NA |
| 204602_at | 22.45 | 1.42E-06 | 2.112072124 | NM_012242| | DKK1,dickkopf homolog 1 |
| 218694_at | 22.45 | 1.42E-06 | 1.290278825 | NM_016608| | ARMCX1,armadillo repeat containing, X-linked 1 |
| 226597_at | 22.44 | 1.43E-06 | 1.24788343 | NM_138393| | C19orf32,polyposis locus protein 1-like 1 |
| 232165_at | 22.44 | 1.42E-06 | 2.044501727 | NM_031308| | EPPK1,epiplakin 1 |
| 213067_at | 22.42 | 1.44E-06 | 1.132468284 | NM_005964| | MYH10,myosin, heavy polypeptide 10, non-muscle |
| 209089_at | 22.41 | 1.44E-06 | 1.030226648 | NM_004162| | RAB5A,RAB5A, member RAS oncogene family |
| 1558212_at | 22.41 | 1.44E-06 | 1.573768536 | NA |  |
| 209366_x_at | 22.4 | 1.44E-06 | 1.078432483 | NM_001914| | CYB5,cytochrome b-5 isoform 2 |
| 218041_x_at | 22.38 | 1.45E-06 | 1.1076164 | NM_018976| | SLC38A2,solute carrier family 38, member 2 |
| 215440_s_at | 22.38 | 1.45E-06 | 1.255817946 | NM_001080425| | NA |
| 1554340_a_at | 22.36 | 1.46E-06 | 1.545625904 | NM_198545| | LOC374946,hypothetical gene supported by AK075558; |
| 238194_at | 22.36 | 1.46E-06 | 1.787332462 | NA |  |
| 235045_at | 22.36 | 1.46E-06 | 1.165807907 | NM_016090| | RBM7,RNA binding motif protein 7 |
| 204164_at | 22.33 | 1.47E-06 | 1.166038604 | NM_006747| | SIPA1,signal-induced proliferation-associated protein |
| 228861_at | 22.33 | 1.47E-06 | 1.127031816 | NA |  |
| 229687_s_at | 22.32 | 1.47E-06 | 1.219842977 | NA |  |
| 217883_at | 22.31 | 1.47E-06 | 1.046052552 | NM_015702| | C2orf25,chromosome 2 open reading frame 25 |
| 226555_at | 22.31 | 1.47E-06 | 1.212629087 | NM_017759| | FLJ20309,hypothetical protein FLJ20309 |
| 225175_s_at | 22.3 | 1.48E-06 | 1.124973763 | NM_020428| | CTL2,CTL2 gene |
| 228497_at | 22.3 | 1.48E-06 | 1.643059907 | NM_018420| | SLC22A15,solute carrier family 22 (organic cation |
| 228463_at | 22.29 | 1.48E-06 | 1.729685247 | NM_004497| | FOXA3,forkhead box A3 |
| 219506_at | 22.29 | 1.48E-06 | 1.209786367 | NM_024579| | FLJ23221,hypothetical protein FLJ23221 |
| 205961_s_at | 22.27 | 1.49E-06 | 1.117082773 | NM_021144| | PSIP1,PC4 and SFRS1 interacting protein 1 |
| 207703_at | 22.27 | 1.49E-06 | 1.244866139 | NM_014893| | NLGN4Y,neuroligin 4, Y-linked |
| 223786_at | 22.25 | 1.50E-06 | 1.388605821 | NM_021615| | CHST6,carbohydrate (N-acetylglucosamine 6-O) |
| 39650_s_at | 22.25 | 1.49E-06 | 1.318929045 | NM_014801| | PCNXL2,pecanex-like 2 |
| 219080_s_at | 22.24 | 1.50E-06 | 1.222762815 | NM_019857| | CTPS2,cytidine triphosphate synthase II |
| 214804_at | 22.24 | 1.50E-06 | 1.178765808 | NM_006733| | FSHPRH1,follicle-stimulating hormone primary response |
| 238175_at | 22.23 | 1.50E-06 | 1.747535565 | NA |  |
| 206214_at | 22.23 | 1.50E-06 | 1.896804766 | NM_005084| | PLA2G7,phospholipase A2, group VII |
| 204678_s_at | 22.22 | 1.51E-06 | 1.725916003 | NM_002245| | KCNK1,potassium channel, subfamily K, member 1 |
| 209141_at | 22.22 | 1.51E-06 | 1.075360716 | NM_003342| | UBE2G1,ubiquitin-conjugating enzyme E2G 1 isoform 1 |
| 203585_at | 22.22 | 1.51E-06 | 1.299615978 | NM_007150| | ZNF185,zinc finger protein 185 (LIM domain) |
| 225177_at | 22.21 | 1.51E-06 | 1.345753491 | NM_001002233| | RAB11FIP1,Rab coupling protein isoform 2 |
| 206142_at | 22.2 | 1.51E-06 | 1.47718058 | NM_003436| | ZNF135,zinc finger protein 135 (clone pHZ-17) |
| 219520_s_at | 22.19 | 1.51E-06 | 1.073054601 | NM_015691| | KIAA1280,KIAA1280 protein |
| 216867_s_at | 22.19 | 1.51E-06 | 1.191371168 | NM_002607| | NA |
| 239250_at | 22.19 | 1.51E-06 | 1.367718396 | NA |  |
| 202567_at | 22.18 | 1.52E-06 | 1.026920373 | NM_004175| | SNRPD3,small nuclear ribonucleoprotein polypeptide D3 |
| 212430_at | 22.17 | 1.52E-06 | 1.123780822 | NM_017495| | RNPC1,RNA-binding region containing protein 1 isoform |
| 238765_at | 22.16 | 1.52E-06 | 1.164047507 | NM_004888| | ATP6V1G1,ATPase, H+ transporting, lysosomal, V1 subunit G |
| 226657_at | 22.15 | 1.53E-06 | 1.198014941 | NM_152914| | MGC33894,transcript expressed during hematopoiesis 2 |
| 1553799_at | 22.15 | 1.53E-06 | 1.63900084 | NM_152647| | FLJ32800,hypothetical protein FLJ32800 |
| 205528_s_at | 22.14 | 1.53E-06 | 1.1736327 | NM_004349| | RUNX1T1,acute myelogenous leukemia 1 translocation 1 |
| 219773_at | 22.11 | 1.54E-06 | 1.551646724 | NM_016931| | NOX4,NADPH oxidase 4 |
| 235687_at | 22.11 | 1.54E-06 | 1.486564257 | NM_001076675| | NA |
| 227063_at | 22.1 | 1.55E-06 | 1.105303502 | NM_152766| | MGC40107,hypothetical protein MGC40107 |
| 209276_s_at | 22.1 | 1.55E-06 | 1.472049532 | NM_001118890| | NA |
| 236037_at | 22.08 | 1.56E-06 | 1.597067388 | NM_020340| | KIAA1244,KIAA1244 |
| 218865_at | 22.08 | 1.55E-06 | 1.120881345 | NM_022746| | FLJ22390,hypothetical protein FLJ22390 |
| 238025_at | 22.07 | 1.56E-06 | 1.512117409 | NM_152649| | MLKL,mixed lineage kinase domain-like |
| 210416_s_at | 22.06 | 1.56E-06 | 1.098074665 | NM_001005735| | CHEK2,protein kinase CHK2 isoform c |
| 222020_s_at | 22.05 | 1.56E-06 | 1.888471366 | NM_001048209| | NA |
| 224675_at | 22.05 | 1.56E-06 | 1.041431287 | NM_015154| | NA |
| 229427_at | 22.05 | 1.56E-06 | 1.533883536 | NM_003966| | SEMA5A,semaphorin 5A |
| 204411_at | 22.04 | 1.57E-06 | 1.522397123 | NM_017596| | NA |
| 205068_s_at | 22.03 | 1.57E-06 | 1.667640305 | NM_015071| | ARHGAP26,GTPase regulator associated with the focal |
| 208848_at | 22.02 | 1.57E-06 | 1.105749753 | NM_000671| | ADH5,class III alcohol dehydrogenase 5 chi subunit |
| 210880_s_at | 22.01 | 1.58E-06 | 1.35491793 | NM_005864| | EFS,embryonal Fyn-associated substrate isoform 1 |
| 201787_at | 22 | 1.58E-06 | 1.093982005 | NM_001996| | FBLN1,fibulin 1 isoform C precursor |
| 210674_s_at | 22 | 1.58E-06 | 2.588408209 | NM_014005| | PCDHA9,protocadherin alpha 9 isoform 2 precursor |
| 206042_x_at | 21.98 | 1.59E-06 | 1.178145743 | NM_003097| | SNRPN,small nuclear ribonucleoprotein polypeptide N |
| 228750_at | 21.98 | 1.59E-06 | 1.739257116 | NA |  |
| 212350_at | 21.98 | 1.59E-06 | 1.082740919 | NM_015173| | TBC1D1,TBC1 (tre-2/USP6, BUB2, cdc16) domain family, |
| 204695_at | 21.98 | 1.59E-06 | 1.106865612 | NM_001789| | CDC25A,cell division cycle 25A isoform a |
| 230708_at | 21.98 | 1.59E-06 | 1.862006016 | NM_153026| | PRICKLE1,prickle-like 1 |
| 212082_s_at | 21.97 | 1.59E-06 | 1.074764382 | NM_002475| | MLC1SA,myosin alkali light chain 1 slow a |
| 205308_at | 21.96 | 1.60E-06 | 1.173035139 | NM_016010| | CGI-62,CGI-62 protein |
| 230895_at | 21.96 | 1.59E-06 | 2.330047631 | NA |  |
| 220038_at | 21.96 | 1.59E-06 | 1.645035725 | NM_001033578| | NA |
| 212154_at | 21.95 | 1.60E-06 | 1.284702024 | NM_002998| | SDC2,syndecan 2 precursor |
| 212114_at | 21.95 | 1.60E-06 | 1.06897155 | NA |  |
| 205368_at | 21.95 | 1.60E-06 | 1.385105664 | NM_001031690| | NA |
| 225480_at | 21.93 | 1.61E-06 | 1.044981861 | NM_198446| | FLJ45459,FLJ45459 protein |
| 209238_at | 21.93 | 1.60E-06 | 2.250264933 | NM_004177| | STX3A,syntaxin 3A |
| 236281_x_at | 21.93 | 1.60E-06 | 1.909318569 | NM_000872| | HTR7,5-hydroxytryptamine receptor 7 isoform a |
| 226000_at | 21.91 | 1.61E-06 | 1.139242668 | NM_018704| | DKFZp547A023,hypothetical protein DKFZp547A023 |
| 223294_at | 21.91 | 1.61E-06 | 1.079429747 | NM_016500| | CXorf26,chromosome X open reading frame 26 |
| 210038_at | 21.9 | 1.62E-06 | 1.529130228 | NM_006257| | PRKCQ,protein kinase C, theta |
| 231202_at | 21.89 | 1.62E-06 | 1.791062779 | NM_001034173| | NA |
| 209900_s_at | 21.88 | 1.62E-06 | 1.104882282 | NM_003051| | SLC16A1,solute carrier family 16, member 1 |
| 204604_at | 21.88 | 1.62E-06 | 1.131710677 | NM_012395| | PFTK1,PFTAIRE protein kinase 1 |
| 223526_at | 21.88 | 1.62E-06 | 1.050277848 | NM_031446| | C18orf21,chromosome 18 open reading frame 21 |
| 204342_at | 21.87 | 1.62E-06 | 1.220915487 | NM_013386| | SLC25A24,solute carrier family 25 member 24 isoform 1 |
| 211571_s_at | 21.87 | 1.63E-06 | 1.258406586 | NM_001126336| | NA |
| 211538_s_at | 21.85 | 1.63E-06 | 1.377535093 | NM_021979| | HSPA2,heat shock 70kDa protein 2 |
| 224646_x_at | 21.84 | 1.64E-06 | 1.488099948 | NA |  |
| 201482_at | 21.81 | 1.65E-06 | 1.172099945 | NM_001004128| | QSCN6,quiescin Q6 isoform b |
| 225359_at | 21.81 | 1.65E-06 | 1.057042995 | NM_145261| | TIM14,homolog of yeast TIM14 isoform a |
| 200772_x_at | 21.8 | 1.65E-06 | 1.028689781 | NM_001099285| | NA |
| 205626_s_at | 21.8 | 1.65E-06 | 2.664415863 | NM_004929| | CALB1,calbindin 1 |
| 217893_s_at | 21.79 | 1.66E-06 | 1.127447531 | NM_024595| | FLJ12666,hypothetical protein FLJ12666 |
| 204391_x_at | 21.79 | 1.66E-06 | 1.15084883 | NM_003852| | TIF1,transcriptional intermediary factor 1 alpha |
| 202436_s_at | 21.78 | 1.66E-06 | 1.801162464 | NM_000104| | CYP1B1,cytochrome P450, family 1, subfamily B, |
| 221963_x_at | 21.78 | 1.66E-06 | 1.176072923 | NA |  |
| 206472_s_at | 21.77 | 1.66E-06 | 1.258728092 | NM_001105192| | NA |
| 204485_s_at | 21.77 | 1.66E-06 | 1.169644376 | NM_005486| | TOM1L1,target of myb1-like 1 |
| 204837_at | 21.77 | 1.66E-06 | 1.082285529 | NM_015458| | MTMR9,myotubularin-related protein 9 |
| 212132_at | 21.75 | 1.67E-06 | 1.04210227 | NM_001114093| | NA |
| 212647_at | 21.74 | 1.67E-06 | 1.215822006 | NM_006270| | RRAS,related RAS viral (r-ras) oncogene homolog |
| 243357_at | 21.74 | 1.67E-06 | 1.633288725 | NM_173808| | NEGR1,neuronal growth regulator 1 |
| 208847_s_at | 21.71 | 1.69E-06 | 1.073238864 | NM_000671| | ADH5,class III alcohol dehydrogenase 5 chi subunit |
| 231385_at | 21.69 | 1.70E-06 | 1.768121359 | NM_199286| | DPPA3,stella |
| 225155_at | 21.69 | 1.70E-06 | 1.03807538 | NA |  |
| 47550_at | 21.68 | 1.70E-06 | 1.795329724 | NM_021020| | LZTS1,leucine zipper, putative tumor suppressor 1 |
| 226503_at | 21.67 | 1.70E-06 | 1.111437416 | NM_018151| | RIF1,RAP1 interacting factor 1 |
| 217789_at | 21.67 | 1.70E-06 | 1.085180813 | NM_021249| | SNX6,sorting nexin 6 isoform a |
| 238453_at | 21.67 | 1.70E-06 | 1.046544972 | NM_152429| | C10orf13,hypothetical protein MGC39320 |
| 227033_at | 21.67 | 1.70E-06 | 1.080013528 | NM_005313| | GRP58,glucose regulated protein, 58kDa |
| 233847_x_at | 21.67 | 1.70E-06 | 2.914367415 | NA |  |
| 215227_x_at | 21.65 | 1.71E-06 | 1.047306831 | NM_001040649| | NA |
| 213106_at | 21.65 | 1.71E-06 | 1.766680405 | NM_001105529| | NA |
| 235387_at | 21.65 | 1.71E-06 | 1.098346605 | NM_001031720| | NA |
| 226415_at | 21.64 | 1.71E-06 | 3.195411702 | NM_020927| | KIAA1576,KIAA1576 protein |
| 213661_at | 21.64 | 1.71E-06 | 1.355861762 | NM_001001991| | DKFZP586H2123,regeneration associated muscle protease isoform |
| 222158_s_at | 21.63 | 1.72E-06 | 1.091625051 | NM_016076| | PNAS-4,CGI-146 protein |
| 238467_at | 21.62 | 1.72E-06 | 1.161386606 | NA |  |
| 213285_at | 21.62 | 1.72E-06 | 2.043908447 | NM_001017970| | NA |
| 226054_at | 21.6 | 1.73E-06 | 1.079992318 | NM_014299| | BRD4,bromodomain-containing protein 4 isoform short |
| 219464_at | 21.6 | 1.73E-06 | 1.259806764 | NM_012113| | CA14,carbonic anhydrase XIV precursor |
| 221497_x_at | 21.6 | 1.73E-06 | 1.047963343 | NM_022051| | EGLN1,egl nine homolog 1 |
| 203616_at | 21.59 | 1.73E-06 | 1.115491559 | NM_002690| | POLB,polymerase (DNA directed), beta |
| 224857_s_at | 21.59 | 1.73E-06 | 1.066798886 | NM_015972| | POLR1D,RNA polymerase I 16 kDa subunit |
| 230896_at | 21.59 | 1.73E-06 | 2.43159678 | NM_207406| | FLJ43965,FLJ43965 protein |
| 218319_at | 21.58 | 1.74E-06 | 1.154630808 | NM_020651| | PELI1,pellino protein |
| 210683_at | 21.57 | 1.74E-06 | 1.441457551 | NM_004558| | NRTN,neurturin precursor |
| 218321_x_at | 21.55 | 1.75E-06 | 1.086346982 | NM_016086| | DUSP24,map kinase phosphatase-like protein MK-STYX |
| 200627_at | 21.55 | 1.75E-06 | 1.036010105 | NM_006601| | TEBP,unactive progesterone receptor, 23 kD |
| 229177_at | 21.53 | 1.76E-06 | 1.69461619 | NM_001098514| | NA |
| 211947_s_at | 21.52 | 1.76E-06 | 1.118384693 | NM_015172| | BAT2D1,HBxAg transactivated protein 2 |
| 228184_at | 21.51 | 1.77E-06 | 1.386653232 | NM_032890| | DISP1,dispatched A |
| 224846_at | 21.5 | 1.77E-06 | 1.139327487 | NM_138392| | SHKBP1,SH3KBP1 binding protein 1 |
| 219545_at | 21.49 | 1.78E-06 | 1.254532368 | NM_023930| | KCTD14,potassium channel tetramerisation domain |
| 203665_at | 21.49 | 1.77E-06 | 1.395721591 | NM_002133| | HMOX1,heme oxygenase (decyclizing) 1 |
| 219004_s_at | 21.48 | 1.78E-06 | 1.062806765 | NM_018944| | C21orf45,chromosome 21 open reading frame 45 |
| 201311_s_at | 21.48 | 1.78E-06 | 1.204492989 | NM_003022| | SH3BGRL,SH3 domain binding glutamic acid-rich protein |
| 201315_x_at | 21.47 | 1.79E-06 | 1.20282259 | NM_006435| | IFITM2,interferon induced transmembrane protein 2 |
| 227921_at | 21.47 | 1.79E-06 | 1.105482314 | NA |  |
| 202003_s_at | 21.44 | 1.80E-06 | 1.093653233 | NM_006111| | ACAA2,acetyl-coenzyme A acyltransferase 2 |
| 222883_at | 21.44 | 1.80E-06 | 1.09312065 | NM_023077| | FLJ12439,hypothetical protein FLJ12439 |
| 201054_at | 21.44 | 1.80E-06 | 1.02912937 | NM_006805| | HNRPA0,heterogeneous nuclear ribonucleoprotein A0 |
| 225007_at | 21.43 | 1.80E-06 | 1.090107809 | NM_005754| | G3BP,Ras-GTPase-activating protein SH3-domain-binding |
| 221666_s_at | 21.43 | 1.80E-06 | 2.079080091 | NM_013258| | PYCARD,PYD and CARD domain containing isoform a |
| 225401_at | 21.42 | 1.80E-06 | 1.439490086 | NM_144580| | MGC31963,kidney predominant protein NCU-G1 |
| 202235_at | 21.41 | 1.81E-06 | 1.126200826 | NM_003051| | SLC16A1,solute carrier family 16, member 1 |
| 222906_at | 21.41 | 1.81E-06 | 1.156814966 | NM_014053| | FLVCR,feline leukemia virus subgroup C cellular |
| 210110_x_at | 21.41 | 1.81E-06 | 1.03240158 | NM_012207| | HNRPH3,heterogeneous nuclear ribonucleoprotein H3 |
| 222024_s_at | 21.4 | 1.81E-06 | 1.11842895 | NM_006738| | AKAP13,A-kinase anchor protein 13 isoform 1 |
| 206702_at | 21.4 | 1.81E-06 | 1.789751707 | NM_000459| | TEK,TEK tyrosine kinase, endothelial |
| 214061_at | 21.4 | 1.81E-06 | 1.090448982 | NM_145647| | MGC21654,unknown MGC21654 product |
| 218905_at | 21.39 | 1.82E-06 | 1.055299809 | NM_017864| | FLJ20530,hypothetical protein FLJ20530 |
| 218848_at | 21.37 | 1.83E-06 | 1.128027555 | NM_024339| | MGC2655,hypothetical protein MGC2655 |
| 225728_at | 21.36 | 1.83E-06 | 1.230081589 | NM_003603| | ARGBP2,Arg/Abl-interacting protein 2 isoform 1 |
| 200978_at | 21.35 | 1.83E-06 | 1.065545841 | NM_005917| | MDH1,cytosolic malate dehydrogenase |
| 226591_at | 21.34 | 1.85E-06 | 1.382940501 | NA |  |
| 225231_at | 21.32 | 1.85E-06 | 1.142145415 | NM_005188| | CBL,Cas-Br-M (murine) ecotropic retroviral |
| 225439_at | 21.32 | 1.85E-06 | 1.069651593 | NM_032869| | NUDCD1,NudC domain containing 1 |
| 211944_at | 21.31 | 1.86E-06 | 1.072726644 | NM_015172| | BAT2D1,HBxAg transactivated protein 2 |
| 207843_x_at | 21.31 | 1.86E-06 | 1.079464899 | NM_001914| | CYB5,cytochrome b-5 isoform 2 |
| 210095_s_at | 21.3 | 1.86E-06 | 1.202689508 | NM_000598| | IGFBP3,insulin-like growth factor binding protein 3 |
| 213136_at | 21.3 | 1.86E-06 | 1.133674645 | NM_002828| | PTPN2,protein tyrosine phosphatase, non-receptor type |
| 206740_x_at | 21.29 | 1.87E-06 | 1.651357004 | NM_003176| | SYCP1,synaptonemal complex protein 1 |
| 202201_at | 21.29 | 1.87E-06 | 1.282405552 | NM_000713| | BLVRB,biliverdin reductase B (flavin reductase |
| 203594_at | 21.26 | 1.88E-06 | 1.143457986 | NM_003729| | RTCD1,RNA terminal phosphate cyclase domain 1 |
| 218313_s_at | 21.25 | 1.89E-06 | 1.08148623 | NM_017423| | GALNT7,polypeptide N-acetylgalactosaminyltransferase 7 |
| 227123_at | 21.24 | 1.89E-06 | 1.384308508 | NM_002867| | RAB3B,RAB3B, member RAS oncogene family |
| 222479_s_at | 21.24 | 1.89E-06 | 1.084065877 | NM_016141| | DNCLI1,dynein light chain-A |
| 209685_s_at | 21.23 | 1.90E-06 | 2.249072487 | NM_002738| | PRKCB1,protein kinase C, beta isoform 2 |
| 221935_s_at | 21.22 | 1.90E-06 | 1.438869454 | NM_173654| | AER61,AER61 glycosyltransferase |
| 227423_at | 21.2 | 1.91E-06 | 1.081090399 | NM_144598| | LRRC28,leucine rich repeat containing 28 |
| 201649_at | 21.2 | 1.91E-06 | 1.105130741 | NM_004223| | UBE2L6,ubiquitin-conjugating enzyme E2L 6 isoform 1 |
| 219635_at | 21.2 | 1.91E-06 | 1.187694078 | NM_025027| | ZNF606,zinc finger protein 606 |
| 202542_s_at | 21.19 | 1.92E-06 | 1.036565194 | NM_004757| | SCYE1,small inducible cytokine subfamily E, member 1 |
| 233982_x_at | 21.18 | 1.92E-06 | 1.084331703 | NM_016086| | DUSP24,map kinase phosphatase-like protein MK-STYX |
| 208747_s_at | 21.17 | 1.92E-06 | 1.450688202 | NM_001734| | C1S,complement component 1, s subcomponent |
| 223710_at | 21.17 | 1.92E-06 | 2.052358177 | NM_006072| | CCL26,chemokine (C-C motif) ligand 26 precursor |
| 200862_at | 21.17 | 1.92E-06 | 1.051813746 | NM_014762| | DHCR24,24-dehydrocholesterol reductase precursor |
| 212998_x_at | 21.16 | 1.93E-06 | 1.801589549 | NM_001023561| | NA |
| 227220_at | 21.16 | 1.93E-06 | 1.171665911 | NM_152995| | HOZFP,ovarian zinc finger protein |
| 38290_at | 21.15 | 1.93E-06 | 1.293657367 | NM_006480| | RGS14,regulator of G-protein signalling 14 |
| 212053_at | 21.15 | 1.93E-06 | 1.110248099 | NM_015027| | KIAA0251,KIAA0251 protein |
| 53720_at | 21.14 | 1.93E-06 | 1.268978096 | NM_018381| | FLJ11286,hypothetical protein FLJ11286 |
| 205768_s_at | 21.14 | 1.94E-06 | 1.518716137 | NM_003645| | SLC27A2,solute carrier family 27 (fatty acid |
| 207183_at | 21.13 | 1.94E-06 | 1.112184375 | NM_006143| | GPR19,G protein-coupled receptor 19 |
| 230009_at | 21.13 | 1.94E-06 | 1.134881863 | NM_024556| | FLJ21103,hypothetical protein FLJ21103 |
| 239202_at | 21.13 | 1.94E-06 | 1.500698154 | NA |  |
| 217546_at | 21.12 | 1.94E-06 | 1.457304225 | NM_176870| | MT1K,metallothionein 1K |
| 1562848_at | 21.12 | 1.94E-06 | 1.137446623 | NA |  |
| 210665_at | 21.12 | 1.94E-06 | 2.073068722 | NM_001032281| | NA |
| 224990_at | 21.12 | 1.94E-06 | 1.256452616 | NM_174921| | LOC201895,hypothetical protein LOC201895 |
| 210766_s_at | 21.12 | 1.94E-06 | 1.053742072 | NM_001316| | CSE1L,CSE1 chromosome segregation 1-like protein |
| 1552477_a_at | 21.1 | 1.95E-06 | 1.45475296 | NM_006147| | IRF6,interferon regulatory factor 6 |
| 203428_s_at | 21.1 | 1.96E-06 | 1.045913568 | NM_014034| | ASF1A,ASF1 anti-silencing function 1 homolog A |
| 224813_at | 21.09 | 1.96E-06 | 1.057822946 | NM_003941| | WASL,Wiskott-Aldrich syndrome gene-like protein |
| 204049_s_at | 21.08 | 1.96E-06 | 1.188566025 | NM_001100164| | NA |
| 236578_at | 21.06 | 1.98E-06 | 1.32449352 | NA |  |
| 201074_at | 21.06 | 1.98E-06 | 1.047585208 | NM_003074| | SMARCC1,SWI/SNF-related matrix-associated |
| 212666_at | 21.06 | 1.97E-06 | 1.106789437 | NM_020429| | SMURF1,Smad ubiquitination regulatory factor 1 isoform |
| 204083_s_at | 21.04 | 1.99E-06 | 1.079030361 | NM_003289| | TPM2,tropomyosin 2 (beta) isoform 1 |
| 210186_s_at | 21.04 | 1.98E-06 | 1.102653728 | NM_000801| | FKBP1A,FK506-binding protein 1A |
| 1555962_at | 21.02 | 2.00E-06 | 2.214102495 | NM_145236| | B3GNT7,UDP-GlcNAc:betaGal |
| 214698_at | 21.02 | 2.00E-06 | 1.185049536 | NM_005156| | ROD1,ROD1 regulator of differentiation 1 |
| 203992_s_at | 21.02 | 2.00E-06 | 1.339534189 | NM_021140| | UTX,ubiquitously transcribed tetratricopeptide |
| 223559_s_at | 21.02 | 2.00E-06 | 1.05136633 | NM_021218| | C9orf80,chromosome 9 open reading frame 80 |
| 212745_s_at | 21.02 | 2.00E-06 | 1.133085458 | NM_033028| | BBS4,Bardet-Biedl syndrome 4 |
| 209781_s_at | 21 | 2.00E-06 | 1.192894711 | NM_006558| | KHDRBS3,KH domain containing, RNA binding, signal |
| 203868_s_at | 21 | 2.01E-06 | 1.559576666 | NM_001078| | VCAM1,vascular cell adhesion molecule 1 isoform a |
| 201843_s_at | 21 | 2.00E-06 | 2.049976633 | NM_001039348| | NA |
| 229351_at | 20.98 | 2.01E-06 | 1.152115039 | NM_018561| | USP49,ubiquitin specific protease 49 |
| 214633_at | 20.97 | 2.02E-06 | 1.083124127 | NM_005634| | SOX3,SRY (sex determining region Y)-box 3 |
| 210174_at | 20.95 | 2.03E-06 | 1.778079908 | NM_003822| | NR5A2,nuclear receptor subfamily 5, group A, member 2 |
| 238477_at | 20.94 | 2.03E-06 | 1.109619395 | NA |  |
| 213325_at | 20.94 | 2.04E-06 | 1.39694623 | NM_015480| | PVRL3,nectin 3 |
| 235126_at | 20.93 | 2.04E-06 | 1.227562319 | NA |  |
| 221969_at | 20.92 | 2.05E-06 | 1.521284827 | NA |  |
| 242800_at | 20.91 | 2.05E-06 | 1.257138852 | NM_198270| | NHS,Nance-Horan syndrome protein |
| 218536_at | 20.91 | 2.05E-06 | 1.201558843 | NM_020662| | MRS2L,MRS2-like, magnesium homeostasis factor |
| 202352_s_at | 20.91 | 2.05E-06 | 1.092369878 | NM_002816| | PSMD12,proteasome 26S non-ATPase subunit 12 isoform 1 |
| 228205_at | 20.9 | 2.05E-06 | 1.248303136 | NM_001064| | TKT,transketolase |
| 223318_s_at | 20.89 | 2.06E-06 | 1.113628225 | NM_032306| | SPATA11,spermatogenesis associated 11 |
| 217766_s_at | 20.89 | 2.06E-06 | 1.113067055 | NM_014313| | SMP1,small membrane protein 1 |
| 219526_at | 20.88 | 2.07E-06 | 1.151961884 | NM_024644| | C14orf169,chromosome 14 open reading frame 169 |
| 235781_at | 20.88 | 2.06E-06 | 1.524551106 | NM_000718| | CACNA1B,calcium channel, voltage-dependent, L type, |
| 218047_at | 20.88 | 2.06E-06 | 1.087209468 | NM_024586| | OSBPL9,oxysterol-binding protein-like protein 9 isoform |
| 215783_s_at | 20.88 | 2.06E-06 | 1.841338061 | NM_000478| | ALPL,tissue non-specific alkaline phosphatase |
| 218111_s_at | 20.87 | 2.07E-06 | 1.177740504 | NM_018686| | CMAS,cytidine 5'-monophosphate N-acetylneuraminic |
| 210721_s_at | 20.87 | 2.07E-06 | 1.553181817 | NM_020341| | PAK7,p21-activated kinase 7 |
| 225739_at | 20.87 | 2.07E-06 | 1.383488993 | NM_032932| | RAB11FIP4,RAB11 family interacting protein 4 (class II) |
| 223642_at | 20.86 | 2.07E-06 | 3.538116585 | NM_007129| | ZIC2,zinc finger protein of the cerebellum 2 |
| 207981_s_at | 20.86 | 2.07E-06 | 1.705585402 | NM_001438| | ESRRG,estrogen-related receptor gamma isoform 1 |
| 217991_x_at | 20.85 | 2.08E-06 | 1.249853922 | NM_001009955| | SSBP3,single stranded DNA binding protein 3 isoform c |
| 223331_s_at | 20.85 | 2.08E-06 | 1.042703088 | NM_007204| | DDX20,DEAD (Asp-Glu-Ala-Asp) box polypeptide 20 |
| 218530_at | 20.85 | 2.08E-06 | 1.158587544 | NM_013241| | FHOD1,formin homology 2 domain containing 1 |
| 217952_x_at | 20.84 | 2.08E-06 | 1.06908418 | NM_015153| | PHF3,PHD finger protein 3 |
| 223125_s_at | 20.83 | 2.09E-06 | 1.049115018 | NM_030806| | C1orf21,chromosome 1 open reading frame 21 |
| 224129_s_at | 20.83 | 2.09E-06 | 1.068120315 | NM_032574| | LOC84661,dpy-30-like protein |
| 204465_s_at | 20.81 | 2.10E-06 | 1.160579887 | NM_032727| | INA,internexin neuronal intermediate filament |
| 218757_s_at | 20.81 | 2.10E-06 | 1.084074745 | NM_023010| | UPF3B,UPF3 regulator of nonsense transcripts homolog B |
| 201015_s_at | 20.8 | 2.10E-06 | 1.557820679 | NM_002230| | JUP,junction plakoglobin |
| 228624_at | 20.8 | 2.11E-06 | 1.738979982 | NM_018342| | FLJ11155,hypothetical protein FLJ11155 |
| 216905_s_at | 20.78 | 2.12E-06 | 1.703598754 | NM_021978| | ST14,matriptase |
| 229298_at | 20.77 | 2.12E-06 | 1.132442112 | NM_032138| | KBTBD7,kelch repeat and BTB (POZ) domain containing 7 |
| 201134_x_at | 20.76 | 2.13E-06 | 1.047231844 | NM_001867| | COX7C,cytochrome c oxidase subunit VIIc precursor |
| 201109_s_at | 20.76 | 2.13E-06 | 1.796662179 | NM_003246| | THBS1,thrombospondin 1 precursor |
| 218482_at | 20.75 | 2.13E-06 | 1.052877533 | NM_020189| | e(y)2,e(y)2 protein |
| 228008_at | 20.75 | 2.13E-06 | 2.047998064 | NA |  |
| 220588_at | 20.75 | 2.13E-06 | 1.288546439 | NM_001010974| | BCAS4,breast carcinoma amplified sequence 4 isoform c |
| 1558173_a_at | 20.75 | 2.13E-06 | 1.177087506 | NM_033631| | LUZP1,leucine zipper protein 1 |
| 221016_s_at | 20.75 | 2.13E-06 | 1.327214522 | NM_031283| | TCF7L1,HMG-box transcription factor TCF-3 |
| 203529_at | 20.74 | 2.14E-06 | 1.062323574 | NM_001123355| | NA |
| 211948_x_at | 20.74 | 2.14E-06 | 1.063740362 | NM_015172| | BAT2D1,HBxAg transactivated protein 2 |
| 215195_at | 20.73 | 2.14E-06 | 1.201741793 | NM_002737| | PRKCA,protein kinase C, alpha |
| 210907_s_at | 20.73 | 2.14E-06 | 1.06948589 | NM_007217| | PDCD10,programmed cell death 10 |
| 203872_at | 20.72 | 2.15E-06 | 1.982232533 | NM_001100| | ACTA1,alpha 1 actin precursor |
| 202637_s_at | 20.72 | 2.15E-06 | 1.18448814 | NM_000201| | ICAM1,intercellular adhesion molecule 1 precursor |
| 202760_s_at | 20.71 | 2.15E-06 | 1.126449941 | NM_007203| | PALM2-AKAP2,PALM2-AKAP2 protein isoform 1 |
| 219992_at | 20.71 | 2.15E-06 | 1.542587603 | NM_001006667| | TAC3,tachykinin 3 |
| 201206_s_at | 20.7 | 2.15E-06 | 1.192128035 | NM_001042576| | NA |
| 229978_at | 20.68 | 2.17E-06 | 1.54068796 | NA |  |
| 214129_at | 20.68 | 2.17E-06 | 1.293551241 | NM_001002810| | PDE4DIP,phosphodiesterase 4D interacting protein isoform |
| 205171_at | 20.68 | 2.17E-06 | 1.146172661 | NM_002830| | PTPN4,protein tyrosine phosphatase, non-receptor type |
| 209897_s_at | 20.67 | 2.17E-06 | 1.473232691 | NM_004787| | SLIT2,slit homolog 2 |
| 208228_s_at | 20.66 | 2.18E-06 | 1.16568224 | NM_000141| | FGFR2,fibroblast growth factor receptor 2 isoform 1 |
| 218217_at | 20.65 | 2.18E-06 | 1.179950757 | NM_021626| | SCPEP1,serine carboxypeptidase 1 precursor protein |
| 1568604_a_at | 20.65 | 2.18E-06 | 1.722091951 | NM_003716| | CADPS,Ca2+-dependent secretion activator isoform 1 |
| 206442_at | 20.65 | 2.18E-06 | 2.052834047 | NM_003007| | SEMG1,semenogelin I isoform a preproprotein |
| 204243_at | 20.64 | 2.19E-06 | 1.102689048 | NM_012421| | RLF,rearranged L-myc fusion sequence |
| 58780_s_at | 20.64 | 2.19E-06 | 1.083683466 | NM_018071| | FLJ10357,hypothetical protein FLJ10357 |
| 207988_s_at | 20.64 | 2.19E-06 | 1.050892792 | NM_005731| | ARPC2,actin related protein 2/3 complex subunit 2 |
| 203960_s_at | 20.64 | 2.19E-06 | 1.107134711 | NM_016126| | C1orf41,chromosome 1 open reading frame 41 |
| 235720_at | 20.63 | 2.19E-06 | 1.338958222 | NM_206922| | CRIP3,cysteine-rich protein 3 |
| 224509_s_at | 20.62 | 2.20E-06 | 1.115515232 | NM_032730| | RTN4IP1,reticulon 4 interacting protein 1 |
| 224325_at | 20.62 | 2.20E-06 | 2.036389451 | NM_031866| | FZD8,frizzled 8 |
| 203001_s_at | 20.62 | 2.20E-06 | 2.347928133 | NM_007029| | STMN2,superiorcervical ganglia, neural specific 10 |
| 239007_at | 20.62 | 2.20E-06 | 1.303148836 | NM_178523| | ZNF616,zinc finger protein 616 |
| 212653_s_at | 20.61 | 2.20E-06 | 1.115270408 | NM_015252| | EHBP1,EH domain binding protein 1 |
| 207173_x_at | 20.61 | 2.20E-06 | 1.362608636 | NM_001797| | CDH11,cadherin 11, type 2 isoform 1 preproprotein |
| 208886_at | 20.6 | 2.21E-06 | 1.414246127 | NM_005318| | H1F0,H1 histone family, member 0 |
| 212816_s_at | 20.6 | 2.21E-06 | 1.077621368 | NM_000071| | CBS,cystathionine-beta-synthase |
| 214920_at | 20.6 | 2.21E-06 | 1.545469904 | NM_015204| | NA |
| 205229_s_at | 20.6 | 2.21E-06 | 1.169640659 | NM_004086| | COCH,coagulation factor C homolog, cochlin precursor |
| 231035_s_at | 20.6 | 2.21E-06 | 1.230564922 | NA |  |
| 217783_s_at | 20.59 | 2.21E-06 | 1.115050254 | NM_016061| | YPEL5,yippee-like 5 |
| 227749_at | 20.58 | 2.22E-06 | 1.33279159 | NA |  |
| 202147_s_at | 20.58 | 2.22E-06 | 1.196343316 | NM_001007245| | IFRD1,interferon-related developmental regulator 1 |
| 209262_s_at | 20.57 | 2.22E-06 | 1.130983011 | NM_005234| | NR2F6,nuclear receptor subfamily 2, group F, member 6 |
| 203593_at | 20.56 | 2.23E-06 | 1.303642978 | NM_012120| | CD2AP,CD2-associated protein |
| 223737_x_at | 20.56 | 2.23E-06 | 2.246649356 | NM_031422| | CHST9,GalNAc-4-sulfotransferase 2 |
| 244261_at | 20.56 | 2.23E-06 | 1.637581277 | NM_170743| | IL28RA,interleukin 28 receptor, alpha isoform 1 |
| 233841_s_at | 20.55 | 2.24E-06 | 1.132131253 | NM_022491| | SDS3,hypothetical protein FLJ00052 |
| 217223_s_at | 20.55 | 2.24E-06 | 1.172687803 | NM_004327| | BCR,breakpoint cluster region isoform 1 |
| 209392_at | 20.55 | 2.23E-06 | 1.43699129 | NM_001040092| | NA |
| 213556_at | 20.54 | 2.24E-06 | 1.661594902 | NA |  |
| 200797_s_at | 20.53 | 2.24E-06 | 1.043116857 | NM_021960| | MCL1,myeloid cell leukemia sequence 1 isoform 1 |
| 226231_at | 20.53 | 2.25E-06 | 1.220546522 | NA |  |
| 229641_at | 20.52 | 2.25E-06 | 1.61997006 | NA |  |
| 203341_at | 20.51 | 2.25E-06 | 1.052398333 | NM_005760| | CEBPZ,CCAAT/enhancer binding protein zeta |
| 217057_s_at | 20.51 | 2.25E-06 | 1.641457324 | NM_000516| | GNAS,guanine nucleotide binding protein, alpha |
| 202429_s_at | 20.49 | 2.27E-06 | 1.085402027 | NM_000944| | PPP3CA,protein phosphatase 3 (formerly 2B), catalytic |
| 215566_x_at | 20.46 | 2.29E-06 | 1.079401735 | NM_007260| | LYPLA2,lysophospholipase II |
| 219551_at | 20.45 | 2.29E-06 | 1.218650575 | NM_018456| | EAF2,ELL associated factor 2 |
| 204450_x_at | 20.45 | 2.30E-06 | 1.407858988 | NM_000039| | APOA1,apolipoprotein A-I precursor |
| 203389_at | 20.45 | 2.30E-06 | 1.135752777 | NM_002254| | KIF3C,kinesin family member 3C |
| 201543_s_at | 20.44 | 2.30E-06 | 1.075260802 | NM_020150| | SARA1,SAR1a gene homolog 1 |
| 225010_at | 20.43 | 2.31E-06 | 1.061003094 | NM_005436| | CCDC6,coiled-coil domain containing 6 |
| 217975_at | 20.43 | 2.31E-06 | 1.099922342 | NM_001006612| | WBP5,WW domain binding protein 5 |
| 203491_s_at | 20.42 | 2.31E-06 | 1.127477867 | NM_014679| | PIG8,translokin |
| 201071_x_at | 20.42 | 2.31E-06 | 1.042737175 | NM_001005526| | SF3B1,splicing factor 3b, subunit 1 isoform 2 |
| 201364_s_at | 20.42 | 2.31E-06 | 1.190343839 | NM_002537| | OAZ2,ornithine decarboxylase antizyme 2 |
| 202808_at | 20.42 | 2.31E-06 | 1.114236714 | NM_001083913| | NA |
| 227052_at | 20.4 | 2.32E-06 | 1.10679716 | NA |  |
| 202550_s_at | 20.4 | 2.32E-06 | 1.066645901 | NM_004738| | VAPB,VAMP-associated protein B/C |
| 210639_s_at | 20.39 | 2.33E-06 | 1.194833148 | NM_004849| | APG5L,APG5 autophagy 5-like |
| 1553979_at | 20.37 | 2.34E-06 | 1.072523517 | NA |  |
| 239237_at | 20.36 | 2.34E-06 | 1.509604094 | NA |  |
| 228422_at | 20.36 | 2.35E-06 | 1.413545007 | NM_198560| | LOC375323,lipoma HMGIC fusion partner-like protein 4 |
| 219534_x_at | 20.35 | 2.35E-06 | 1.378991779 | NM_000076| | CDKN1C,cyclin-dependent kinase inhibitor 1C |
| 202724_s_at | 20.35 | 2.35E-06 | 1.218235097 | NM_002015| | FOXO1A,forkhead box O1A |
| 222690_s_at | 20.35 | 2.35E-06 | 1.160329945 | NM_018266| | TMEM39A,transmembrane protein 39A |
| 230710_at | 20.34 | 2.36E-06 | 1.251392551 | NA |  |
| 209282_at | 20.34 | 2.35E-06 | 1.142634369 | NM_001079880| | NA |
| 223794_at | 20.33 | 2.37E-06 | 1.347970422 | NM_018076| | ARMC4,armadillo repeat containing 4 |
| 244353_s_at | 20.33 | 2.36E-06 | 1.330005646 | NM_145176| | SLC2A12,solute carrier family 2 (facilitated glucose |
| 219292_at | 20.33 | 2.36E-06 | 1.119496833 | NM_018105| | THAP1,THAP domain containing, apoptosis associated |
| 232615_at | 20.32 | 2.37E-06 | 1.749467944 | NA |  |
| 224895_at | 20.32 | 2.37E-06 | 1.12048222 | NM_006106| | YAP1,Yes-associated protein 1, 65 kD |
| 202644_s_at | 20.3 | 2.38E-06 | 1.348998939 | NM_006290| | TNFAIP3,tumor necrosis factor, alpha-induced protein 3 |
| 222356_at | 20.3 | 2.38E-06 | 1.629474291 | NA |  |
| 228937_at | 20.29 | 2.38E-06 | 1.567605228 | NM_153218| | FLJ38725,hypothetical protein FLJ38725 |
| 202719_s_at | 20.29 | 2.39E-06 | 1.34031265 | NM_015641| | TES,testin isoform 1 |
| 231018_at | 20.29 | 2.38E-06 | 1.573507127 | NA |  |
| 203342_at | 20.28 | 2.39E-06 | 1.080737274 | NM_005834| | TIMM17B,translocase of inner mitochondrial membrane 17 |
| 220924_s_at | 20.27 | 2.40E-06 | 1.108342223 | NM_018976| | SLC38A2,solute carrier family 38, member 2 |
| 212488_at | 20.26 | 2.40E-06 | 1.756558608 | NM_000093| | COL5A1,alpha 1 type V collagen preproprotein |
| 226150_at | 20.26 | 2.40E-06 | 1.142757306 | NM_001102559| | NA |
| 235723_at | 20.26 | 2.41E-06 | 2.037060043 | NM_017637| | BNC2,basonuclin 2 |
| 214829_at | 20.23 | 2.42E-06 | 1.21726044 | NM_005763| | AASS,aminoadipate-semialdehyde synthase |
| 235857_at | 20.22 | 2.43E-06 | 1.189095014 | NM_001002914| | KCTD11,potassium channel tetramerisation domain |
| 201603_at | 20.22 | 2.43E-06 | 1.170436324 | NM_002480| | PPP1R12A,protein phosphatase 1, regulatory (inhibitor) |
| 240181_at | 20.21 | 2.43E-06 | 1.388363973 | NA |  |
| 202011_at | 20.2 | 2.44E-06 | 1.097424791 | NM_003257| | TJP1,tight junction protein 1 isoform a |
| 205037_at | 20.19 | 2.45E-06 | 1.198843734 | NM_006860| | RABL4,RAB, member of RAS oncogene family-like 4 |
| 240261_at | 20.19 | 2.45E-06 | 1.223705123 | NM_005486| | TOM1L1,target of myb1-like 1 |
| 201637_s_at | 20.19 | 2.45E-06 | 1.06480004 | NM_001013438| | NA |
| 216035_x_at | 20.17 | 2.46E-06 | 1.112782611 | NM_030756| | TCF7L2,transcription factor 7-like 2 (T-cell specific, |
| 203713_s_at | 20.17 | 2.46E-06 | 1.522124303 | NM_001015002| | NA |
| AFFX-HUMRGE/M10098_3_at | 20.16 | 2.47E-06 | 1.042496082 | NA |  |
| 207571_x_at | 20.16 | 2.47E-06 | 1.434394671 | NM_001039477| | NA |
| 1566766_a_at | 20.15 | 2.47E-06 | 1.357050272 | NM_182762| | 7A5,putative binding protein 7a5 |
| 214047_s_at | 20.15 | 2.47E-06 | 1.074322975 | NM_003925| | MBD4,methyl-CpG binding domain protein 4 |
| 226199_at | 20.15 | 2.47E-06 | 1.268181897 | NM_145052| | MGC23937,hypothetical protein MGC23937 similar to CG4798 |
| 1552938_at | 20.14 | 2.47E-06 | 1.910099585 | NM_033132| | ZIC5,zinc finger protein of the cerebellum 5 |
| 230789_at | 20.14 | 2.48E-06 | 1.29428586 | NM_080764| | SUHW2,suppressor of hairy wing homolog 2 |
| 229930_at | 20.13 | 2.48E-06 | 1.070233835 | NA |  |
| 223524_s_at | 20.13 | 2.48E-06 | 1.625260606 | NM_023943| | MGC3040,hypothetical protein MGC3040 |
| 212318_at | 20.13 | 2.48E-06 | 1.08082398 | NM_012470| | TNPO3,transportin 3 |
| 204897_at | 20.12 | 2.49E-06 | 1.67813086 | NM_000958| | PTGER4,prostaglandin E receptor 4, subtype EP4 |
| 32069_at | 20.11 | 2.50E-06 | 1.141946431 | NM_153029| | N4BP1,Nedd4 binding protein 1 |
| 1556047_s_at | 20.11 | 2.50E-06 | 1.325614546 | NM_020932| | MAGEE1,melanoma antigen family E, 1 |
| 226869_at | 20.1 | 2.50E-06 | 1.827226011 | NM_001409| | EGFL3,EGF-like-domain, multiple 3 |
| 218302_at | 20.09 | 2.51E-06 | 1.074386628 | NM_172341| | PSENEN,presenilin enhancer 2 |
| 227290_at | 20.08 | 2.52E-06 | 1.325838838 | NA |  |
| 235466_s_at | 20.08 | 2.51E-06 | 1.155968854 | NM_032890| | DISP1,dispatched A |
| 220887_at | 20.08 | 2.51E-06 | 1.210037101 | NA |  |
| 239911_at | 20.07 | 2.52E-06 | 1.426720888 | NM_004852| | ONECUT2,one cut domain, family member 2 |
| 228974_at | 20.05 | 2.54E-06 | 1.374534841 | NA |  |
| 41858_at | 20.05 | 2.54E-06 | 1.090771904 | NM_014489| | FRAG1,FGF receptor activating protein 1 |
| 212311_at | 20.04 | 2.54E-06 | 1.310608595 | NM_015187| | KIAA0746,KIAA0746 protein |
| 239710_at | 20.04 | 2.54E-06 | 1.232058243 | NM_018086| | FIGN,fidgetin |
| 202543_s_at | 20.04 | 2.54E-06 | 1.162672942 | NM_004124| | GMFB,glia maturation factor, beta |
| 225582_at | 20.04 | 2.54E-06 | 1.270645684 | NM_033397| | KIAA1754,KIAA1754 |
| 204199_at | 20.03 | 2.55E-06 | 1.458625299 | NM_014636| | RALGPS1,Ral GEF with PH domain and SH3 binding motif 1 |
| 225005_at | 20.02 | 2.55E-06 | 1.070740009 | NM_153812| | PHF13,PHD finger protein 13 |
| 206675_s_at | 20 | 2.57E-06 | 1.934770571 | NM_005414| | SKIL,SKI-like |
| 241703_at | 20 | 2.57E-06 | 1.947205503 | NM_138290| | RPIB9,Rap2-binding protein 9 |
| 225647_s_at | 20 | 2.57E-06 | 1.201323401 | NM_001114173| | NA |
| 1554452_a_at | 19.99 | 2.58E-06 | 1.177724167 | NM_001098786| | NA |
| 203060_s_at | 19.99 | 2.58E-06 | 1.572760903 | NM_001015880| | NA |
| 243681_at | 19.97 | 2.59E-06 | 1.66258341 | NM_012309| | SHANK2,SH3 and multiple ankyrin repeat domains 2 |
| 204702_s_at | 19.97 | 2.59E-06 | 1.499463697 | NM_004289| | NFE2L3,nuclear factor (erythroid-derived 2)-like 3 |
| 209911_x_at | 19.94 | 2.61E-06 | 1.307879843 | NM_021063| | HIST1H2BD,H2B histone family, member B |
| 235518_at | 19.94 | 2.61E-06 | 1.787255664 | NM_001112800| | NA |
| 212599_at | 19.93 | 2.62E-06 | 1.065243737 | NM_001127231| | NA |
| 203637_s_at | 19.93 | 2.62E-06 | 1.101658703 | NM_000381| | MID1,midline 1 isoform alpha |
| 202214_s_at | 19.93 | 2.61E-06 | 1.082716189 | NM_001079872| | NA |
| 236798_at | 19.92 | 2.62E-06 | 1.29234966 | NA |  |
| 222939_s_at | 19.92 | 2.62E-06 | 1.182430263 | NM_018593| | SLC16A10,solute carrier family 16, member 10 |
| 219208_at | 19.92 | 2.62E-06 | 1.213949785 | NM_012167| | FBXO11,F-box only protein 11 isoform 3 |
| 226216_at | 19.91 | 2.63E-06 | 1.114950485 | NM_000208| | INSR,insulin receptor |
| 211015_s_at | 19.91 | 2.63E-06 | 1.136371254 | NM_002154| | HSPA4,heat shock 70kDa protein 4 isoform a |
| 227386_s_at | 19.9 | 2.64E-06 | 1.452593267 | NM_001003682| | DKFZp434C184,cDNA DKFZp434C184 gene |
| 207949_s_at | 19.89 | 2.65E-06 | 1.201232097 | NM_004968| | ICA1,islet cell autoantigen 1 isoform 2 |
| 213894_at | 19.87 | 2.66E-06 | 1.706536353 | NM_015204| | NA |
| 1556826_s_at | 19.87 | 2.66E-06 | 1.342951468 | NM_198545| | LOC374946,hypothetical gene supported by AK075558; |
| 227618_at | 19.86 | 2.67E-06 | 1.281574741 | NA |  |
| 1559957_a_at | 19.85 | 2.67E-06 | 1.210266397 | NA |  |
| 202027_at | 19.84 | 2.69E-06 | 1.085246573 | NM_012264| | C22orf5,chromosome 22 open reading frame 5 |
| 207194_s_at | 19.83 | 2.69E-06 | 1.34948085 | NM_001039132| | NA |
| 229016_s_at | 19.83 | 2.69E-06 | 1.318190116 | NM_033502| | TRERF1,transcriptional regulating factor 1 isoform 1 |
| 238205_at | 19.83 | 2.69E-06 | 1.786203309 | NM_178470| | WDR40B,WD repeat domain 40B |
| 209576_at | 19.82 | 2.70E-06 | 1.357549093 | NM_002069| | GNAI1,guanine nucleotide binding protein (G protein), |
| 210609_s_at | 19.82 | 2.69E-06 | 1.070847316 | NM_004881| | TP53I3,tumor protein p53 inducible protein 3 |
| 238047_at | 19.81 | 2.71E-06 | 1.928519886 | NM_144967| | FLJ30058,hypothetical protein FLJ30058 |
| 222903_s_at | 19.81 | 2.70E-06 | 1.341088519 | NM_001079533| | NA |
| 212455_at | 19.81 | 2.70E-06 | 1.059884512 | NM_001031732| | NA |
| 226682_at | 19.81 | 2.70E-06 | 1.415354063 | NA |  |
| 203918_at | 19.79 | 2.72E-06 | 1.451794147 | NM_002587| | PCDH1,protocadherin 1 isoform 1 precursor |
| 213793_s_at | 19.79 | 2.72E-06 | 1.08200601 | NM_004272| | HOMER1,homer 1 |
| 204879_at | 19.78 | 2.73E-06 | 1.371514611 | NM_001006624| | T1A-2,lung type-I cell membrane-associated |
| 219227_at | 19.77 | 2.73E-06 | 1.281979118 | NM_024565| | FLJ14166,hypothetical protein FLJ14166 |
| 202385_s_at | 19.76 | 2.74E-06 | 1.116723201 | NM_000356| | TCOF1,Treacher Collins-Franceschetti syndrome 1 |
| 204579_at | 19.75 | 2.75E-06 | 1.53608523 | NM_002011| | FGFR4,fibroblast growth factor receptor 4 isoform 1 |
| 221606_s_at | 19.75 | 2.75E-06 | 1.65957402 | NM_030763| | NSBP1,nucleosomal binding protein 1 |
| 238865_at | 19.75 | 2.74E-06 | 1.486413848 | NM_001114734| | NA |
| 204526_s_at | 19.75 | 2.75E-06 | 1.109892425 | NM_001102426| | NA |
| 206490_at | 19.74 | 2.75E-06 | 1.70472899 | NM_001003809| | DLGAP1,discs large homolog-associated protein 1 isoform |
| 206495_s_at | 19.74 | 2.76E-06 | 1.078533188 | NM_015517| | MIZF,MBD2 (methyl-CpG-binding protein)-interacting |
| 206541_at | 19.73 | 2.76E-06 | 1.578345829 | NM_000892| | KLKB1,plasma kallikrein B1 precursor |
| 224608_s_at | 19.73 | 2.76E-06 | 1.029174999 | NM_032353| | MGC10540,hypothetical protein MGC10540 |
| 219036_at | 19.71 | 2.78E-06 | 1.143922083 | NM_024491| | Cep70,centrosomal protein 70 kDa |
| 237282_s_at | 19.71 | 2.78E-06 | 1.546759934 | NM_001008534| | AKAP14,A kinase (PRKA) anchor protein 14 isoform b |
| 223506_at | 19.7 | 2.79E-06 | 1.075412144 | NM_032494| | ZC3HDC8,zinc finger CCCH type domain containing 8 |
| 236501_at | 19.7 | 2.78E-06 | 2.09087798 | NM_020436| | SALL4,sal-like 4 |
| 217739_s_at | 19.7 | 2.79E-06 | 1.149270027 | NM_005746| | PBEF1,pre-B-cell colony enhancing factor 1 isoform a |
| 210457_x_at | 19.7 | 2.78E-06 | 1.278588684 | NM_002131| | HMGA1,high mobility group AT-hook 1 isoform b |
| 230359_at | 19.7 | 2.78E-06 | 1.328321006 | NM_152643| | KNDC1,kinase non-catalytic C-lobe domain (KIND) |
| 209526_s_at | 19.69 | 2.79E-06 | 1.092193992 | NM_016073| | HDGFRP3,hepatoma-derived growth factor, related protein |
| 205060_at | 19.69 | 2.79E-06 | 1.081217651 | NM_003631| | PARG,poly (ADP-ribose) glycohydrolase |
| 213496_at | 19.68 | 2.80E-06 | 1.519383032 | NM_014839| | LPPR4,plasticity related gene 1 |
| 224721_at | 19.66 | 2.81E-06 | 1.059552295 | NM_032168| | FLJ12519,hypothetical protein FLJ12519 |
| 236824_at | 19.66 | 2.82E-06 | 1.418719921 | NM_052907| | KIAA1906,KIAA1906 protein |
| 202421_at | 19.65 | 2.82E-06 | 1.10484055 | NM_001007237| | IGSF3,immunoglobulin superfamily, member 3 isoform 2 |
| 231470_at | 19.64 | 2.83E-06 | 2.01302687 | NA |  |
| 205209_at | 19.64 | 2.83E-06 | 1.077657491 | NM_004302| | ACVR1B,activin A type IB receptor isoform a precursor |
| 1557348_at | 19.63 | 2.83E-06 | 1.573764113 | NA |  |
| 208816_x_at | 19.63 | 2.83E-06 | 1.111804231 | NA |  |
| 219316_s_at | 19.63 | 2.83E-06 | 1.335514353 | NM_017791| | C14orf58,chromosome 14 open reading frame 58 |
| 205560_at | 19.62 | 2.84E-06 | 1.861303083 | NM_006200| | PCSK5,proprotein convertase subtilisin/kexin type 5 |
| 205529_s_at | 19.62 | 2.85E-06 | 1.218833662 | NM_004349| | RUNX1T1,acute myelogenous leukemia 1 translocation 1 |
| 203011_at | 19.61 | 2.85E-06 | 1.076144206 | NM_005536| | IMPA1,inositol(myo)-1(or 4)-monophosphatase 1 |
| 223497_at | 19.59 | 2.86E-06 | 1.090487321 | NM_001105531| | NA |
| 214130_s_at | 19.59 | 2.86E-06 | 1.340049084 | NM_001002810| | PDE4DIP,phosphodiesterase 4D interacting protein isoform |
| 228340_at | 19.59 | 2.86E-06 | 1.256689319 | NM_001105192| | NA |
| 201219_at | 19.57 | 2.88E-06 | 1.099761957 | NM_001083914| | NA |
| 204168_at | 19.56 | 2.89E-06 | 1.157492206 | NM_002413| | MGST2,microsomal glutathione S-transferase 2 |
| 204697_s_at | 19.56 | 2.89E-06 | 1.628209762 | NM_001275| | CHGA,chromogranin A |
| 202951_at | 19.55 | 2.89E-06 | 1.09251917 | NM_007271| | STK38,serine/threonine kinase 38 |
| 207893_at | 19.55 | 2.90E-06 | 1.831288039 | NM_003140| | SRY,sex determining region Y |
| 226638_at | 19.54 | 2.90E-06 | 1.213436973 | NA |  |
| 205235_s_at | 19.53 | 2.91E-06 | 1.083966192 | NM_016195| | MPHOSPH1,M-phase phosphoprotein 1 |
| 214157_at | 19.52 | 2.93E-06 | 1.187930135 | NM_000516| | GNAS,guanine nucleotide binding protein, alpha |
| 223582_at | 19.51 | 2.93E-06 | 1.100892268 | NM_032119| | MASS1,very large G-protein coupled receptor 1 |
| 228577_x_at | 19.51 | 2.93E-06 | 1.363129931 | NM_001007022| | KIAA1229,KIAA1229 protein isoform b |
| 1557170_at | 19.51 | 2.93E-06 | 1.10389473 | NM_178170| | NEK8,NIMA-related kinase 8 |
| 211421_s_at | 19.49 | 2.94E-06 | 2.088417934 | NM_020630| | RET,ret proto-oncogene isoform c |
| 227493_s_at | 19.48 | 2.96E-06 | 1.090956765 | NM_020696| | KIAA1143,KIAA1143 protein |
| 238983_at | 19.48 | 2.95E-06 | 2.129100001 | NM_024677| | FLJ14001,hypothetical protein FLJ14001 |
| 210149_s_at | 19.48 | 2.96E-06 | 1.019838598 | NM_001003785| | ATP5H,ATP synthase, H+ transporting, mitochondrial F0 |
| 209642_at | 19.48 | 2.95E-06 | 1.119418505 | NM_004336| | BUB1,BUB1 budding uninhibited by benzimidazoles 1 |
| 222606_at | 19.47 | 2.96E-06 | 1.077685434 | NM_017975| | FLJ10036,Zwilch |
| 236471_at | 19.47 | 2.96E-06 | 1.512249152 | NM_004289| | NFE2L3,nuclear factor (erythroid-derived 2)-like 3 |
| 201615_x_at | 19.47 | 2.96E-06 | 1.112605007 | NM_004342| | CALD1,caldesmon 1 isoform 2 |
| 237289_at | 19.47 | 2.96E-06 | 1.136116932 | NM_004379| | CREB1,cAMP responsive element binding protein 1 |
| 222641_s_at | 19.47 | 2.96E-06 | 1.106842022 | NM_001077498| | NA |
| 204588_s_at | 19.46 | 2.97E-06 | 1.492391192 | NM_001126105| | NA |
| 219984_s_at | 19.45 | 2.98E-06 | 1.355364358 | NM_020386| | HRASLS,HRAS-like suppressor |
| 204257_at | 19.45 | 2.97E-06 | 1.112798447 | NM_021727| | FADS3,fatty acid desaturase 3 |
| 230889_at | 19.45 | 2.97E-06 | 1.200016736 | NA |  |
| 225235_at | 19.44 | 2.98E-06 | 1.106591807 | NM_001006616| | TM4SF17,transmembrane 4 superfamily member 17 isoform c |
| 228987_at | 19.44 | 2.98E-06 | 1.121276956 | NA |  |
| 200713_s_at | 19.43 | 2.99E-06 | 1.023977274 | NM_012325| | MAPRE1,microtubule-associated protein, RP/EB family, |
| 220391_at | 19.43 | 2.99E-06 | 1.419369061 | NM_024784| | ZBTB3,zinc finger and BTB domain containing 3 |
| 56197_at | 19.42 | 3.00E-06 | 1.08948343 | NM_020360| | PLSCR3,phospholipid scramblase 3 |
| 203909_at | 19.42 | 3.00E-06 | 1.058181361 | NM_001042537| | NA |
| 212658_at | 19.41 | 3.00E-06 | 1.175634956 | NM_005779| | LHFPL2,lipoma HMGIC fusion partner-like 2 |
| 202735_at | 19.41 | 3.01E-06 | 1.097146016 | NM_006579| | EBP,emopamil binding protein (sterol isomerase) |
| 212660_at | 19.41 | 3.01E-06 | 1.22169802 | NM_015288| | PHF15,PHD finger protein 15 |
| 227627_at | 19.4 | 3.01E-06 | 1.46866607 | NM_001033578| | NA |
| 224336_s_at | 19.4 | 3.01E-06 | 1.396168484 | NM_030640| | DUSP16,dual specificity phosphatase 16 |
| 213229_at | 19.39 | 3.02E-06 | 1.16276874 | NM_030621| | DICER1,dicer1 |
| 225174_at | 19.39 | 3.02E-06 | 1.080521727 | NM_018981| | DNAJC10,DnaJ (Hsp40) homolog, subfamily C, member 10 |
| 207080_s_at | 19.38 | 3.03E-06 | 1.601205239 | NM_004160| | PYY,peptide YY |
| 212288_at | 19.37 | 3.04E-06 | 1.033403824 | NM_015033| | FNBP1,formin-binding protein 17 |
| 219888_at | 19.37 | 3.04E-06 | 1.673491871 | NM_003116| | SPAG4,sperm associated antigen 4 |
| 222819_at | 19.35 | 3.06E-06 | 1.09528009 | NM_019857| | CTPS2,cytidine triphosphate synthase II |
| 216215_s_at | 19.34 | 3.07E-06 | 1.082646842 | NM_001031695| | NA |
| 209579_s_at | 19.33 | 3.07E-06 | 1.050631134 | NM_003925| | MBD4,methyl-CpG binding domain protein 4 |
| 233252_s_at | 19.33 | 3.08E-06 | 1.21458842 | NM_018387| | STRBP,spermatid perinuclear RNA-binding protein |
| 226803_at | 19.32 | 3.08E-06 | 1.834642435 | NM_152284| | Shax3,Snf7 homologue associated with Alix 3 |
| 202076_at | 19.32 | 3.08E-06 | 1.030209237 | NM_001166| | BIRC2,baculoviral IAP repeat-containing protein 2 |
| 232549_at | 19.32 | 3.08E-06 | 1.684099232 | NM_144770| | RBM11,RNA binding motif protein 11 |
| 213647_at | 19.31 | 3.09E-06 | 1.144045418 | NM_001080449| | NA |
| 215017_s_at | 19.31 | 3.09E-06 | 1.076171105 | NM_001024948| | NA |
| 222976_s_at | 19.31 | 3.09E-06 | 1.076380388 | NM_001043351| | NA |
| 201522_x_at | 19.3 | 3.10E-06 | 1.154974857 | NM_003097| | SNRPN,small nuclear ribonucleoprotein polypeptide N |
| 203350_at | 19.29 | 3.11E-06 | 1.102983304 | NM_001030007| | NA |
| 213182_x_at | 19.27 | 3.12E-06 | 1.370988419 | NM_000076| | CDKN1C,cyclin-dependent kinase inhibitor 1C |
| 205478_at | 19.27 | 3.13E-06 | 2.060206034 | NM_006741| | PPP1R1A,protein phosphatase 1, regulatory (inhibitor) |
| 205213_at | 19.27 | 3.13E-06 | 1.446489329 | NM_014716| | CENTB1,centaurin beta1 |
| 201829_at | 19.27 | 3.13E-06 | 1.187913697 | NM_001047160| | NA |
| 244738_at | 19.27 | 3.12E-06 | 1.265132397 | NM_153252| | BRWD3,bromo domain-containing protein disrupted in |
| 219654_at | 19.26 | 3.14E-06 | 1.060107978 | NM_014241| | PTPLA,protein tyrosine phosphatase-like, member a |
| 218943_s_at | 19.25 | 3.15E-06 | 1.606885487 | NM_014314| | DDX58,DEAD/H (Asp-Glu-Ala-Asp/His) box polypeptide |
| 200706_s_at | 19.24 | 3.16E-06 | 1.173702037 | NM_004862| | LITAF,LPS-induced TNF-alpha factor |
| 226213_at | 19.24 | 3.16E-06 | 1.139826883 | NM_001005915| | ERBB3,erbB-3 isoform s precursor |
| 213010_at | 19.24 | 3.16E-06 | 1.664005249 | NM_145040| | PRKCDBP,protein kinase C, delta binding protein |
| 211656_x_at | 19.24 | 3.15E-06 | 1.21911574 | NM_002123| | HLA-DQB1,major histocompatibility complex, class II, DQ |
| 224863_at | 19.23 | 3.16E-06 | 1.079181035 | NM_002072| | GNAQ,guanine nucleotide binding protein (G protein), |
| 202268_s_at | 19.23 | 3.16E-06 | 1.041438231 | NM_001018159| | NA |
| 221760_at | 19.23 | 3.16E-06 | 1.896497449 | NM_005907| | MAN1A1,mannosidase, alpha, class 1A, member 1 |
| 220408_x_at | 19.23 | 3.16E-06 | 1.068495483 | NM_001014286| | NA |
| 1556911_at | 19.22 | 3.17E-06 | 1.301825211 | NA |  |
| 227230_s_at | 19.21 | 3.18E-06 | 1.067177755 | NM_020722| | NA |
| 202558_s_at | 19.21 | 3.18E-06 | 1.159592294 | NM_006948| | STCH,stress 70 protein chaperone, |
| 209070_s_at | 19.21 | 3.18E-06 | 1.845333658 | NM_003617| | RGS5,regulator of G-protein signalling 5 |
| 205041_s_at | 19.2 | 3.19E-06 | 1.767717623 | NM_000607| | ORM1,orosomucoid 1 precursor |
| 227506_at | 19.2 | 3.19E-06 | 1.133264395 | NM_194298| | SLC16A9,solute carrier family 16 (monocarboxylic acid |
| 201604_s_at | 19.2 | 3.19E-06 | 1.179390856 | NM_002480| | PPP1R12A,protein phosphatase 1, regulatory (inhibitor) |
| 201883_s_at | 19.19 | 3.20E-06 | 1.142474029 | NM_001497| | B4GALT1,UDP-Gal:betaGlcNAc beta 1,4- |
| 223686_at | 19.18 | 3.20E-06 | 1.332365147 | NM_001042482| | NA |
| 229355_at | 19.18 | 3.21E-06 | 1.146689423 | NA |  |
| 228440_at | 19.18 | 3.20E-06 | 2.060349271 | NA |  |
| 230588_s_at | 19.16 | 3.22E-06 | 1.093101386 | NA |  |
| 218061_at | 19.16 | 3.22E-06 | 1.069312565 | NM_014623| | MEA,male-enhanced antigen |
| 204791_at | 19.15 | 3.23E-06 | 1.135097014 | NM_001032287| | NA |
| 208850_s_at | 19.15 | 3.23E-06 | 1.145121432 | NM_006288| | THY1,Thy-1 cell surface antigen |
| 244050_at | 19.15 | 3.23E-06 | 1.694579256 | NM_001010915| | LOC401494,similar to RIKEN 4933428I03 |
| 219593_at | 19.15 | 3.23E-06 | 1.157572723 | NM_016582| | SLC15A3,solute carrier family 15, member 3 |
| 209647_s_at | 19.14 | 3.24E-06 | 1.112227303 | NM_014011| | SOCS5,suppressor of cytokine signaling 5 |
| 226485_at | 19.14 | 3.24E-06 | 1.14286558 | NA |  |
| 218642_s_at | 19.14 | 3.24E-06 | 1.338243752 | NM_001011667| | CHCHD7,coiled-coil-helix-coiled-coil-helix domain |
| 238478_at | 19.13 | 3.25E-06 | 1.66045518 | NM_017637| | BNC2,basonuclin 2 |
| 38269_at | 19.11 | 3.27E-06 | 1.094903119 | NM_001079880| | NA |
| 203764_at | 19.11 | 3.27E-06 | 1.084161507 | NM_014750| | DLG7,discs large homolog 7 |
| 223255_at | 19.1 | 3.28E-06 | 1.098933074 | NM_017769| | KIAA1333,KIAA1333 |
| 210587_at | 19.09 | 3.28E-06 | 2.583593122 | NM_031479| | INHBE,activin beta E |
| 213457_at | 19.09 | 3.28E-06 | 1.094860423 | NM_004225| | MFHAS1,malignant fibrous histiocytoma amplified |
| 223208_at | 19.08 | 3.30E-06 | 1.110628803 | NM_031954| | KCTD10,potassium channel tetramerisation domain |
| 228298_at | 19.07 | 3.30E-06 | 1.440823619 | NM_138371| | MGC16044,hypothetical protein MGC16044 |
| 210664_s_at | 19.06 | 3.32E-06 | 2.49853357 | NM_001032281| | NA |
| 239247_at | 19.06 | 3.32E-06 | 1.177016203 | NA |  |
| 213668_s_at | 19.06 | 3.32E-06 | 1.123486959 | NM_003107| | SOX4,SRY (sex determining region Y)-box 4 |
| 1555037_a_at | 19.05 | 3.32E-06 | 1.100918872 | NM_005896| | IDH1,isocitrate dehydrogenase 1 (NADP+), soluble |
| 217738_at | 19.05 | 3.32E-06 | 1.151720595 | NM_005746| | PBEF1,pre-B-cell colony enhancing factor 1 isoform a |
| 219298_at | 19.05 | 3.32E-06 | 1.248686846 | NM_024693| | ECHDC3,enoyl Coenzyme A hydratase domain containing 3 |
| 266_s_at | 19.04 | 3.34E-06 | 1.24942268 | NM_013230| | CD24,CD24 antigen |
| 223427_s_at | 19.04 | 3.34E-06 | 1.58397291 | NM_018424| | EPB41L4B,erythrocyte membrane protein band 4.1 like 4B |
| 201349_at | 19.04 | 3.33E-06 | 1.073648417 | NM_004252| | SLC9A3R1,solute carrier family 9 (sodium/hydrogen |
| 205327_s_at | 19.03 | 3.34E-06 | 1.091784361 | NM_001616| | ACVR2,activin A type II receptor precursor |
| 219764_at | 19.02 | 3.35E-06 | 2.142967744 | NM_007197| | FZD10,frizzled 10 |
| 215084_s_at | 19.01 | 3.36E-06 | 1.166459109 | NM_052940| | MGC8974,hypothetical protein MGC8974 |
| 229661_at | 19.01 | 3.36E-06 | 2.06835469 | NM_020436| | SALL4,sal-like 4 |
| 225091_at | 19.01 | 3.37E-06 | 1.126466632 | NM_033089| | ZCCHC3,zinc finger, CCHC domain containing 3 |
| 235413_at | 19.01 | 3.37E-06 | 1.137246534 | NM_000821| | GGCX,gamma-glutamyl carboxylase |
| 203702_s_at | 19 | 3.37E-06 | 1.116559238 | NM_014640| | TTLL4,tubulin tyrosine ligase-like family, member 4 |
| 222603_at | 19 | 3.37E-06 | 1.202360635 | NM_024896| | KIAA1815,KIAA1815 |
| 221880_s_at | 19 | 3.37E-06 | 1.326328918 | NM_207446| | LOC400451,hypothetical gene supported by AK075564; |
| 218098_at | 18.99 | 3.38E-06 | 1.061947015 | NM_006420| | ARFGEF2,ADP-ribosylation factor guanine |
| 213235_at | 18.99 | 3.38E-06 | 1.084900715 | NM_001012991| | NA |
| 200629_at | 18.99 | 3.38E-06 | 1.078421435 | NM_004184| | WARS,tryptophanyl-tRNA synthetase isoform a |
| 217750_s_at | 18.99 | 3.38E-06 | 1.091903287 | NM_023079| | FLJ13855,hypothetical protein FLJ13855 |
| 223382_s_at | 18.98 | 3.39E-06 | 1.071165872 | NM_032268| | ZNRF1,zinc and ring finger protein 1 |
| 211936_at | 18.98 | 3.39E-06 | 1.047098111 | NM_005347| | HSPA5,heat shock 70kDa protein 5 (glucose-regulated |
| 226468_at | 18.98 | 3.39E-06 | 1.05966043 | NA |  |
| 232629_at | 18.98 | 3.39E-06 | 1.391915468 | NM_001126128| | NA |
| 219875_s_at | 18.97 | 3.40E-06 | 1.148118435 | NM_016076| | PNAS-4,CGI-146 protein |
| 225917_at | 18.96 | 3.40E-06 | 1.082648322 | NA |  |
| 226048_at | 18.96 | 3.40E-06 | 1.172976001 | NM_002750| | MAPK8,mitogen-activated protein kinase 8 isoform 2 |
| 219405_at | 18.95 | 3.42E-06 | 1.117607144 | NM_018073| | TRIM68,ring finger protein 137 |
| 208623_s_at | 18.95 | 3.41E-06 | 1.080394224 | NM_001111077| | NA |
| 236656_s_at | 18.94 | 3.42E-06 | 1.312638248 | NA |  |
| 235244_at | 18.94 | 3.42E-06 | 1.076848904 | NM_001017928| | NA |
| 209424_s_at | 18.93 | 3.43E-06 | 1.222451508 | NM_014324| | AMACR,alpha-methylacyl-CoA racemase isoform 1 |
| 1559266_s_at | 18.93 | 3.43E-06 | 1.156425394 | NM_207371| | FLJ45187,FLJ45187 protein |
| 205524_s_at | 18.93 | 3.43E-06 | 1.841419422 | NM_001884| | HAPLN1,cartilage linking protein 1 |
| 214091_s_at | 18.91 | 3.45E-06 | 1.377992453 | NM_002084| | GPX3,plasma glutathione peroxidase 3 precursor |
| 207223_s_at | 18.91 | 3.45E-06 | 1.332901636 | NM_005156| | ROD1,ROD1 regulator of differentiation 1 |
| 234725_s_at | 18.91 | 3.45E-06 | 1.101177751 | NM_020210| | SEMA4B,semaphorin 4B precursor |
| 211724_x_at | 18.9 | 3.46E-06 | 1.072406607 | NM_019005| | FLJ20323,hypothetical protein FLJ20323 |
| 225784_s_at | 18.9 | 3.46E-06 | 1.162285318 | NM_018684| | KIAA1166,KIAA1166 |
| 201955_at | 18.89 | 3.47E-06 | 1.073323597 | NM_001013399| | NA |
| 223475_at | 18.89 | 3.47E-06 | 1.12286141 | NM_031461| | CRISPLD1,cocoacrisp |
| 201972_at | 18.89 | 3.47E-06 | 1.09609421 | NM_001690| | ATP6V1A,ATPase, H+ transporting, lysosomal 70kD, V1 |
| 201616_s_at | 18.88 | 3.48E-06 | 1.092658286 | NM_004342| | CALD1,caldesmon 1 isoform 2 |
| 232856_at | 18.88 | 3.48E-06 | 1.588131338 | NM_001005210| | FLJ45686,FLJ45686 protein |
| 202130_at | 18.88 | 3.48E-06 | 1.037986342 | NM_003831| | RIOK3,sudD suppressor of bimD6 homolog isoform 1 |
| 203322_at | 18.87 | 3.49E-06 | 1.061203826 | NM_014913| | KIAA0863,KIAA0863 protein |
| 226271_at | 18.87 | 3.49E-06 | 1.142090785 | NM_001040875| | NA |
| 231257_at | 18.85 | 3.51E-06 | 1.680176242 | NM_174937| | TCERG1L,transcription elongation regulator 1-like |
| 202310_s_at | 18.84 | 3.52E-06 | 2.120243701 | NM_000088| | COL1A1,alpha 1 type I collagen preproprotein |
| 201110_s_at | 18.83 | 3.54E-06 | 2.502528891 | NM_003246| | THBS1,thrombospondin 1 precursor |
| 235358_at | 18.83 | 3.53E-06 | 1.220444 | NA |  |
| 209982_s_at | 18.81 | 3.56E-06 | 1.585818006 | NM_015080| | NRXN2,neurexin 2 isoform alpha-1 precursor |
| 1552580_at | 18.8 | 3.57E-06 | 1.696062632 | NM_173553| | FLJ25801,hypothetical protein FLJ25801 |
| 221774_x_at | 18.8 | 3.56E-06 | 1.066494541 | NM_001014286| | NA |
| 235355_at | 18.8 | 3.57E-06 | 1.73720447 | NA |  |
| 214909_s_at | 18.79 | 3.57E-06 | 1.131549585 | NM_013974| | DDAH2,dimethylarginine dimethylaminohydrolase 2 |
| 210776_x_at | 18.79 | 3.57E-06 | 1.050644975 | NM_003200| | TCF3,transcription factor 3 |
| 227547_at | 18.79 | 3.57E-06 | 1.10440195 | NA |  |
| 222411_s_at | 18.78 | 3.59E-06 | 1.039500338 | NM_007107| | SSR3,signal sequence receptor gamma subunit |
| 1554609_at | 18.77 | 3.59E-06 | 1.365111173 | NM_006591| | POLD3,polymerase (DNA directed), delta 3 |
| 218261_at | 18.77 | 3.59E-06 | 1.435687997 | NM_005498| | AP1M2,adaptor-related protein complex 1, mu 2 subunit |
| 219425_at | 18.77 | 3.59E-06 | 1.044208503 | NM_014351| | SULT4A1,sulfotransferase family 4A, member 1 isoform a |
| 206818_s_at | 18.77 | 3.59E-06 | 1.168390969 | NM_017649| | CNNM2,cyclin M2 isoform 1 |
| 225698_at | 18.76 | 3.60E-06 | 1.050341067 | NA |  |
| 223060_at | 18.74 | 3.63E-06 | 1.07679832 | NM_017924| | C14orf119,chromosome 14 open reading frame 119 |
| 209885_at | 18.74 | 3.63E-06 | 1.196670847 | NM_014578| | RHOD,ras homolog D |
| 203098_at | 18.74 | 3.63E-06 | 1.075576948 | NM_004824| | CDYL,chromodomain protein, Y chromosome-like isoform |
| 205953_at | 18.73 | 3.64E-06 | 1.203548362 | NM_014813| | LRIG2,leucine-rich repeats and immunoglobulin-like |
| 223543_at | 18.72 | 3.65E-06 | 1.340175911 | NM_032512| | PDZK4,PDZ domain containing 4 |
| 228191_at | 18.71 | 3.65E-06 | 1.165104129 | NA |  |
| 220345_at | 18.69 | 3.68E-06 | 1.983532578 | NM_024993| | LRRTM4,leucine rich repeat transmembrane neuronal 4 |
| 203936_s_at | 18.68 | 3.69E-06 | 1.270289138 | NM_004994| | MMP9,matrix metalloproteinase 9 preproprotein |
| 201881_s_at | 18.67 | 3.70E-06 | 1.072707717 | NM_005744| | ARIH1,ariadne homolog, ubiquitin-conjugating enzyme E2 |
| 208427_s_at | 18.66 | 3.71E-06 | 1.867618258 | NM_004432| | ELAVL2,ELAV (embryonic lethal, abnormal vision, |
| 238819_at | 18.66 | 3.71E-06 | 1.308407285 | NM_032584| | ZNF347,zinc finger protein 347 |
| 1556308_at | 18.66 | 3.72E-06 | 1.17096933 | NM_207351| | FLJ33674,hypothetical protein FLJ33674 |
| 228100_at | 18.66 | 3.72E-06 | 1.378288341 | NM_181643| | LOC128344,hypothetical protein LOC128344 |
| 207717_s_at | 18.65 | 3.72E-06 | 2.507455999 | NM_001005242| | PKP2,plakophilin 2 isoform 2a |
| 227878_s_at | 18.64 | 3.74E-06 | 1.139107057 | NM_032306| | SPATA11,spermatogenesis associated 11 |
| 200782_at | 18.64 | 3.74E-06 | 1.082699408 | NM_001154| | ANXA5,annexin 5 |
| 225944_at | 18.62 | 3.76E-06 | 1.092822271 | NM_020726| | NLN,neurolysin |
| 232306_at | 18.61 | 3.76E-06 | 1.309431388 | NM_021810| | CDH26,cadherin-like 26 isoform b |
| 209163_at | 18.6 | 3.78E-06 | 1.070998838 | NM_001017916| | NA |
| 1568611_at | 18.59 | 3.79E-06 | 1.225082143 | NA |  |
| 209146_at | 18.59 | 3.79E-06 | 1.033285168 | NM_001017369| | NA |
| 204613_at | 18.59 | 3.78E-06 | 1.232322045 | NM_002661| | PLCG2,phospholipase C, gamma 2 |
| 202832_at | 18.58 | 3.80E-06 | 1.152763558 | NM_014635| | GCC2,GRIP coiled-coil protein GCC185 isoform b |
| 211974_x_at | 18.58 | 3.80E-06 | 1.050075151 | NM_005349| | RBPSUH,recombining binding protein suppressor of |
| 219382_at | 18.58 | 3.80E-06 | 1.200930482 | NM_013368| | SERTAD3,RPA-binding trans-activator |
| 209580_s_at | 18.57 | 3.81E-06 | 1.088884866 | NM_003925| | MBD4,methyl-CpG binding domain protein 4 |
| 228194_s_at | 18.56 | 3.82E-06 | 1.577928885 | NM_001013031| | NA |
| 201216_at | 18.56 | 3.82E-06 | 1.095136633 | NM_001034025| | NA |
| 230900_at | 18.56 | 3.82E-06 | 1.516317619 | NM_152775| | KM-HN-1,KM-HN-1 protein |
| 201930_at | 18.55 | 3.83E-06 | 1.061026984 | NM_005915| | MCM6,minichromosome maintenance protein 6 |
| 91826_at | 18.55 | 3.83E-06 | 1.427573276 | NM_017729| | EPS8L1,epidermal growth factor receptor pathway |
| 224940_s_at | 18.54 | 3.84E-06 | 1.725052576 | NM_002581| | PAPPA,pregnancy-associated plasma protein A |
| 219463_at | 18.5 | 3.89E-06 | 1.2576895 | NM_012261| | C20orf103,chromosome 20 open reading frame 103 precursor |
| 221008_s_at | 18.5 | 3.89E-06 | 1.849519544 | NM_031279| | AGXT2L1,alanine-glyoxylate aminotransferase 2-like 1 |
| 227027_at | 18.5 | 3.89E-06 | 1.350862263 | NA |  |
| 209529_at | 18.5 | 3.89E-06 | 1.380691119 | NM_003712| | PPAP2C,phosphatidic acid phosphatase type 2C isoform 1 |
| 206460_at | 18.5 | 3.89E-06 | 1.721935156 | NM_001042478| | NA |
| 221215_s_at | 18.48 | 3.90E-06 | 1.420950903 | NM_020639| | RIPK4,ankyrin repeat domain 3 |
| 222713_s_at | 18.48 | 3.90E-06 | 1.167249072 | NM_022725| | FANCF,Fanconi anemia, complementation group F |
| 211456_x_at | 18.48 | 3.91E-06 | 1.116535627 | NA |  |
| 232667_at | 18.48 | 3.90E-06 | 1.262578576 | NA |  |
| 209560_s_at | 18.45 | 3.95E-06 | 2.039636827 | NM_003836| | DLK1,delta-like homolog |
| 222909_s_at | 18.43 | 3.97E-06 | 1.17057236 | NM_004874| | BAG4,BCL2-associated athanogene 4 |
| 226397_s_at | 18.43 | 3.97E-06 | 1.809577771 | NA |  |
| 236180_at | 18.42 | 3.98E-06 | 1.510002845 | NA |  |
| 227388_at | 18.42 | 3.98E-06 | 1.307388319 | NM_001004125| | TUSC1,tumor suppressor candidate 1 |
| 201970_s_at | 18.42 | 3.98E-06 | 1.062278262 | NM_002482| | NASP,nuclear autoantigenic sperm protein isoform 2 |
| 227172_at | 18.41 | 3.99E-06 | 1.134796812 | NM_138341| | LOC89894,hypothetical protein BC000282 |
| 242565_x_at | 18.41 | 3.99E-06 | 1.138355772 | NM_001006114| | C21orf57,chromosome 21 open reading frame 57 isoform 2 |
| 227862_at | 18.41 | 3.99E-06 | 1.186129809 | NM_001013642| | NA |
| 206356_s_at | 18.4 | 4.00E-06 | 1.670591206 | NM_002071| | GNAL,guanine nucleotide binding protein (G protein), |
| 220372_at | 18.4 | 4.00E-06 | 1.452485706 | NM_001040192| | NA |
| 224395_s_at | 18.4 | 4.00E-06 | 1.088078988 | NM_014245| | RNF7,ring finger protein 7 isoform 1 |
| 205372_at | 18.4 | 4.00E-06 | 2.485727187 | NM_001114634| | NA |
| 201952_at | 18.39 | 4.01E-06 | 1.081232232 | NM_001627| | ALCAM,activated leukocyte cell adhesion molecule |
| 230204_at | 18.39 | 4.01E-06 | 2.909007166 | NA |  |
| 222807_at | 18.37 | 4.03E-06 | 1.083963936 | NM_020193| | C11orf30,EMSY protein |
| 220956_s_at | 18.37 | 4.04E-06 | 1.090798265 | NM_053046| | EGLN2,EGL nine (C.elegans) homolog 2 isoform 1 |
| 205942_s_at | 18.37 | 4.03E-06 | 1.449352248 | NM_005622| | SAH,SA hypertension-associated homolog isoform 1 |
| 225522_at | 18.36 | 4.05E-06 | 1.122832221 | NM_014911| | AAK1,AP2 associated kinase 1 |
| 211149_at | 18.36 | 4.05E-06 | 1.118073737 | NM_007125| | UTY,tetratricopeptide repeat protein isoform 3 |
| 201949_x_at | 18.35 | 4.06E-06 | 1.059953414 | NM_004930| | CAPZB,F-actin capping protein beta subunit |
| 226886_at | 18.35 | 4.06E-06 | 1.321534565 | NA |  |
| 1555725_a_at | 18.33 | 4.09E-06 | 1.621634914 | NM_003617| | RGS5,regulator of G-protein signalling 5 |
| 214150_x_at | 18.33 | 4.08E-06 | 1.077575506 | NM_003945| | ATP6V0E,ATPase, H+ transporting, lysosomal, V0 subunit |
| 227111_at | 18.32 | 4.10E-06 | 1.216107994 | NM_001099270| | NA |
| 217047_s_at | 18.32 | 4.10E-06 | 1.062298957 | NM_001015045| | NA |
| 209486_at | 18.31 | 4.10E-06 | 1.032515507 | NM_020368| | SAS10,disrupter of silencing 10 |
| 225154_at | 18.31 | 4.10E-06 | 1.078496336 | NM_032796| | SYAP1,SYAP1 protein |
| 215603_x_at | 18.3 | 4.12E-06 | 1.158780063 | NM_001032364| | NA |
| 201263_at | 18.3 | 4.12E-06 | 1.122120649 | NM_152295| | TARS,threonyl-tRNA synthetase |
| 214339_s_at | 18.3 | 4.12E-06 | 1.523690319 | NM_001042600| | NA |
| 218006_s_at | 18.3 | 4.12E-06 | 1.062062206 | NM_006963| | ZNF22,zinc finger protein 22 (KOX 15) |
| 222895_s_at | 18.3 | 4.12E-06 | 1.840203432 | NM_022898| | BCL11B,B-cell CLL/lymphoma 11B isoform 2 |
| 226955_at | 18.3 | 4.12E-06 | 1.368357094 | NM_152406| | FLJ36748,hypothetical protein FLJ36748 |
| 225274_at | 18.29 | 4.12E-06 | 1.147536293 | NM_016297| | PCYOX1,prenylcysteine oxidase 1 |
| 205190_at | 18.29 | 4.12E-06 | 1.263192115 | NM_002670| | PLS1,plastin 1 |
| 236915_at | 18.29 | 4.12E-06 | 1.51364795 | NM_001114357| | NA |
| 223689_at | 18.28 | 4.14E-06 | 1.787263686 | NM_006546| | IMP-1,IGF-II mRNA-binding protein 1 |
| 213567_at | 18.28 | 4.14E-06 | 1.12894029 | NA |  |
| 203035_s_at | 18.28 | 4.14E-06 | 1.085575868 | NM_006099| | PIAS3,protein inhibitor of activated STAT, 3 |
| 236826_at | 18.27 | 4.15E-06 | 1.271584321 | NM_152574| | C9orf52,hypothetical protein FLJ33868 |
| 218683_at | 18.27 | 4.15E-06 | 1.218399116 | NM_021190| | PTBP2,polypyrimidine tract binding protein 2 |
| 225925_s_at | 18.27 | 4.15E-06 | 1.099003095 | NM_001032730| | NA |
| 227190_at | 18.26 | 4.17E-06 | 1.568017928 | NM_183240| | TMEM37,transmembrane protein 37 |
| 211417_x_at | 18.26 | 4.17E-06 | 1.14163393 | NM_001032364| | NA |
| 211240_x_at | 18.25 | 4.17E-06 | 1.116966478 | NM_001085458| | NA |
| 221522_at | 18.25 | 4.17E-06 | 1.074843058 | NM_032139| | ANKRD27,ankyrin repeat domain 27 (VPS9 domain) |
| 201588_at | 18.24 | 4.19E-06 | 1.036430909 | NM_004786| | TXNL1,thioredoxin-like 1 |
| 222203_s_at | 18.23 | 4.20E-06 | 1.112101724 | NM_001002006| | NT5C1B,5' nucleotidase, cytosolic IB isoform 1 |
| 214119_s_at | 18.22 | 4.22E-06 | 1.10810408 | NM_000801| | FKBP1A,FK506-binding protein 1A |
| 204423_at | 18.22 | 4.22E-06 | 1.170739111 | NM_013255| | MKLN1,muskelin 1, intracellular mediator containing |
| 206854_s_at | 18.22 | 4.22E-06 | 1.064142798 | NM_003188| | MAP3K7,mitogen-activated protein kinase kinase kinase 7 |
| 209154_at | 18.22 | 4.22E-06 | 1.080933049 | NM_014604| | TAX1BP3,Tax1 (human T-cell leukemia virus type I) |
| 217687_at | 18.21 | 4.22E-06 | 1.319760864 | NM_020546| | ADCY2,adenylate cyclase 2 |
| 225111_s_at | 18.21 | 4.22E-06 | 1.096246828 | NM_022080| | NAPB,N-ethylmaleimide-sensitive factor attachment |
| 225517_at | 18.21 | 4.22E-06 | 1.141609659 | NM_014106| | FLJ20582,hypothetical protein FLJ20582 |
| 219494_at | 18.2 | 4.24E-06 | 1.089367288 | NM_012415| | RAD54B,RAD54 homolog B isoform 1 |
| 223807_at | 18.2 | 4.24E-06 | 1.663183996 | NM_001555| | IGSF1,immunoglobulin superfamily, member 1 isoform 1 |
| 224602_at | 18.2 | 4.23E-06 | 1.087621998 | NM_001001701| | LOC401152,HCV F-transactivated protein 1 |
| 228223_at | 18.19 | 4.25E-06 | 1.171092955 | NM_080752| | ZSWIM3,zinc finger, SWIM domain containing 3 |
| 221479_s_at | 18.19 | 4.25E-06 | 1.100722467 | NM_004331| | BNIP3L,BCL2/adenovirus E1B 19kD-interacting protein |
| 212080_at | 18.17 | 4.29E-06 | 1.046145312 | NM_005933| | MLL,myeloid/lymphoid or mixed-lineage leukemia |
| 230166_at | 18.17 | 4.28E-06 | 1.122468751 | NM_133465| | KIAA1958,KIAA1958 |
| 209524_at | 18.16 | 4.29E-06 | 1.084808142 | NM_016073| | HDGFRP3,hepatoma-derived growth factor, related protein |
| 219470_x_at | 18.15 | 4.31E-06 | 1.094593967 | NM_019084| | CCNJ,cyclin J |
| 218775_s_at | 18.15 | 4.30E-06 | 1.182404462 | NM_024949| | BOMB,BH3-only member B protein |
| 212354_at | 18.13 | 4.34E-06 | 1.526590772 | NM_015170| | SULF1,sulfatase 1 |
| 223705_s_at | 18.13 | 4.33E-06 | 1.077168601 | NM_001127235| | NA |
| 228563_at | 18.13 | 4.34E-06 | 1.08352369 | NM_001080383| | NA |
| 218689_at | 18.12 | 4.34E-06 | 1.220757002 | NM_022725| | FANCF,Fanconi anemia, complementation group F |
| 215535_s_at | 18.11 | 4.36E-06 | 1.104465969 | NM_006411| | AGPAT1,1-acylglycerol-3-phosphate O-acyltransferase 1 |
| 209893_s_at | 18.1 | 4.37E-06 | 1.474609838 | NM_002033| | FUT4,fucosyltransferase 4 |
| 229168_at | 18.1 | 4.37E-06 | 1.378140676 | NM_173465| | COL23A1,collagen, type XXIII, alpha 1 |
| 202677_at | 18.1 | 4.38E-06 | 1.102663887 | NM_002890| | RASA1,RAS p21 protein activator 1 isoform 1 |
| 225822_at | 18.09 | 4.38E-06 | 1.667305195 | NM_144626| | MGC17299,hypothetical protein MGC17299 |
| 239468_at | 18.08 | 4.40E-06 | 1.370457631 | NM_173576| | C10orf48,chromosome 10 open reading frame 48 |
| 206374_at | 18.08 | 4.40E-06 | 1.264534006 | NM_004420| | DUSP8,dual specificity phosphatase 8 |
| 226344_at | 18.07 | 4.41E-06 | 1.305474407 | NM_001011657| | ZMAT1,zinc finger, matrin type 1 isoform 1 |
| 225297_at | 18.06 | 4.43E-06 | 1.101492423 | NM_138443| | CCDC5,coiled-coil domain containing 5 (spindle |
| 216054_x_at | 18.06 | 4.43E-06 | 1.25590978 | NM_001002841| | MYL4,atrial/embryonic alkali myosin light chain |
| 210964_s_at | 18.06 | 4.43E-06 | 1.133263379 | NM_001079855| | NA |
| 222810_s_at | 18.06 | 4.42E-06 | 1.246132046 | NM_004841| | RASAL2,RAS protein activator like 2 isoform 1 |
| 229080_at | 18.05 | 4.44E-06 | 1.565898537 | NM_133457| | EMID2,putative emu2 |
| 218368_s_at | 18.03 | 4.46E-06 | 1.108175461 | NM_016639| | TNFRSF12A,type I transmembrane protein Fn14 |
| 214589_at | 18.02 | 4.48E-06 | 1.364624702 | NM_004113| | FGF12,fibroblast growth factor 12 isoform 2 |
| 58916_at | 18.01 | 4.49E-06 | 1.327791369 | NM_023930| | KCTD14,potassium channel tetramerisation domain |
| 242629_at | 18.01 | 4.49E-06 | 1.726835131 | NA |  |
| 201544_x_at | 18.01 | 4.49E-06 | 1.030400114 | NM_004643| | PABPN1,poly(A) binding protein, nuclear 1 |
| 211776_s_at | 18 | 4.50E-06 | 1.804691534 | NM_012307| | EPB41L3,erythrocyte membrane protein band 4.1-like 3 |
| 208813_at | 18 | 4.51E-06 | 1.07994614 | NM_002079| | GOT1,aspartate aminotransferase 1 |
| 64486_at | 18 | 4.51E-06 | 1.086629201 | NM_001018070| | NA |
| 201845_s_at | 18 | 4.51E-06 | 1.213021809 | NM_012234| | RYBP,RING1 and YY1 binding protein |
| 226878_at | 18 | 4.50E-06 | 1.933108771 | NM_002119| | HLA-DOA,major histocompatibility complex, class II, DO |
| 227385_at | 18 | 4.50E-06 | 1.129542881 | NM_203453| | LOC403313,hypothetical LOC403313 |
| 211954_s_at | 17.99 | 4.52E-06 | 1.034306979 | NM_002271| | RANBP5,RAN binding protein 5 |
| 231233_at | 17.99 | 4.52E-06 | 1.459901947 | NA |  |
| 208728_s_at | 17.99 | 4.51E-06 | 1.064021917 | NM_001039802| | NA |
| 211385_x_at | 17.99 | 4.52E-06 | 1.082783265 | NM_001054| | SULT1A2,sulfotransferase family, cytosolic, 1A, |
| 221046_s_at | 17.98 | 4.52E-06 | 1.064770637 | NM_014170| | HSPC135,HSPC135 protein isoform 1 |
| 210946_at | 17.98 | 4.53E-06 | 1.390835371 | NM_003711| | PPAP2A,phosphatidic acid phosphatase type 2A isoform 1 |
| 221763_at | 17.97 | 4.54E-06 | 1.160089719 | NM_004241| | JMJD1C,jumonji domain containing 1C |
| 218117_at | 17.96 | 4.55E-06 | 1.035872688 | NM_014248| | RBX1,ring-box 1 |
| 215726_s_at | 17.94 | 4.58E-06 | 1.096191448 | NM_001914| | CYB5,cytochrome b-5 isoform 2 |
| 244170_at | 17.94 | 4.58E-06 | 1.660146147 | NA |  |
| 200921_s_at | 17.94 | 4.59E-06 | 1.102672657 | NM_001731| | BTG1,B-cell translocation protein 1 |
| 229693_at | 17.94 | 4.59E-06 | 1.59262061 | NM_001004313| | LOC388335,similar to RIKEN cDNA A730055C05 gene |
| 205704_s_at | 17.93 | 4.59E-06 | 1.140652495 | NM_012463| | ATP6V0A2,ATPase, H+ transporting, lysosomal V0 subunit a |
| 204361_s_at | 17.92 | 4.60E-06 | 1.786270479 | NM_003930| | SCAP2,src family associated phosphoprotein 2 |
| 202894_at | 17.92 | 4.60E-06 | 1.129783592 | NM_004444| | EPHB4,ephrin receptor EphB4 precursor |
| 232523_at | 17.92 | 4.60E-06 | 1.130259747 | NM_032446| | MEGF10,MEGF10 protein |
| 202481_at | 17.92 | 4.60E-06 | 1.682293105 | NM_004753| | DHRS3,dehydrogenase/reductase (SDR family) member 3 |
| 33304_at | 17.92 | 4.60E-06 | 1.249267165 | NM_002201| | ISG20,interferon stimulated gene 20kDa |
| 202363_at | 17.92 | 4.60E-06 | 1.464503117 | NM_004598| | SPOCK,sparc/osteonectin, cwcv and kazal-like domains |
| 235649_at | 17.92 | 4.60E-06 | 1.767134452 | NM_007037| | ADAMTS8,a disintegrin and metalloprotease with |
| 209457_at | 17.91 | 4.62E-06 | 1.260614871 | NM_004419| | DUSP5,dual specificity phosphatase 5 |
| 205489_at | 17.91 | 4.62E-06 | 1.11037844 | NM_001014444| | NA |
| 226119_at | 17.9 | 4.62E-06 | 1.229624839 | NM_052937| | LOC115294,similar to hypothetical protein FLJ10883 |
| 235420_at | 17.9 | 4.62E-06 | 1.355584969 | NM_023002| | HAPLN4,brain link protein 2 |
| 209682_at | 17.9 | 4.63E-06 | 1.1777975 | NM_170662| | CBLB,Cas-Br-M (murine) ecotropic retroviral |
| 237061_at | 17.9 | 4.62E-06 | 1.403208775 | NM_032584| | ZNF347,zinc finger protein 347 |
| 201425_at | 17.89 | 4.64E-06 | 1.190734253 | NM_000690| | ALDH2,mitochondrial aldehyde dehydrogenase 2 |
| 206026_s_at | 17.89 | 4.64E-06 | 1.627609591 | NM_007115| | TNFAIP6,tumor necrosis factor, alpha-induced protein 6 |
| 227274_at | 17.89 | 4.64E-06 | 1.092606256 | NM_018373| | SYNJ2BP,synaptojanin 2 binding protein |
| 204117_at | 17.89 | 4.64E-06 | 1.07721986 | NM_002726| | PREP,prolyl endopeptidase |
| 1555579_s_at | 17.88 | 4.65E-06 | 1.585091476 | NM_001105244| | NA |
| 209958_s_at | 17.87 | 4.67E-06 | 1.210183229 | NM_001033604| | NA |
| 204047_s_at | 17.87 | 4.67E-06 | 1.279091751 | NM_001100164| | NA |
| 238752_at | 17.86 | 4.68E-06 | 1.255119573 | NA |  |
| 210785_s_at | 17.85 | 4.69E-06 | 1.423721735 | NM_001039477| | NA |
| 205577_at | 17.85 | 4.69E-06 | 1.381281996 | NM_005609| | PYGM,glycogen phosphorylase |
| 218556_at | 17.85 | 4.69E-06 | 1.066116142 | NM_014182| | ORMDL2,ORMDL2 |
| 201867_s_at | 17.84 | 4.71E-06 | 1.149252348 | NM_005647| | TBL1X,transducin beta-like 1X |
| 1560741_at | 17.84 | 4.71E-06 | 1.197507356 | NM_003097| | SNRPN,small nuclear ribonucleoprotein polypeptide N |
| 1556469_s_at | 17.83 | 4.73E-06 | 1.87364162 | NA |  |
| 223441_at | 17.83 | 4.73E-06 | 1.182007327 | NM_012434| | SLC17A5,solute carrier family 17 (anion/sugar |
| 224911_s_at | 17.83 | 4.72E-06 | 1.105307736 | NM_080927| | DCBLD2,discoidin, CUB and LCCL domain containing 2 |
| 229391_s_at | 17.83 | 4.72E-06 | 1.580788062 | NM_001010919| | NA |
| 202891_at | 17.82 | 4.73E-06 | 1.086656689 | NM_005600| | NIT1,nitrilase 1 |
| 226541_at | 17.82 | 4.73E-06 | 1.106867708 | NM_032145| | FBXO30,F-box only protein 30 |
| 212622_at | 17.82 | 4.74E-06 | 1.033255237 | NM_015012| | NA |
| 202925_s_at | 17.8 | 4.76E-06 | 1.156525651 | NM_002657| | PLAGL2,pleiomorphic adenoma gene-like 2 |
| 219360_s_at | 17.8 | 4.77E-06 | 1.422333009 | NM_017636| | TRPM4,transient receptor potential cation channel, |
| 1552390_a_at | 17.8 | 4.76E-06 | 1.600731133 | NM_173549| | FLJ39553,hypothetical protein FLJ39553 |
| 208926_at | 17.79 | 4.79E-06 | 1.127647723 | NM_000434| | NEU1,neuraminidase precursor |
| 217605_at | 17.79 | 4.78E-06 | 1.214868021 | NA |  |
| 204298_s_at | 17.79 | 4.78E-06 | 1.577571111 | NM_002317| | LOX,lysyl oxidase preproprotein |
| 226915_s_at | 17.78 | 4.79E-06 | 1.077766994 | NM_030978| | ARPC5L,actin related protein 2/3 complex, subunit |
| 1553957_at | 17.78 | 4.80E-06 | 1.163319931 | NM_144976| | ZNF564,zinc finger protein 564 |
| 238778_at | 17.78 | 4.79E-06 | 2.497975459 | NM_173496| | MPP7,palmitoylated membrane protein 7 |
| 206085_s_at | 17.77 | 4.80E-06 | 1.193215243 | NM_001902| | CTH,cystathionase isoform 1 |
| 204937_s_at | 17.76 | 4.82E-06 | 1.148078595 | NM_016324| | ZNF274,zinc finger protein 274 isoform b |
| 228647_at | 17.76 | 4.81E-06 | 1.405320448 | NA |  |
| 210757_x_at | 17.75 | 4.83E-06 | 1.403394753 | NM_001343| | DAB2,disabled homolog 2 |
| 203266_s_at | 17.73 | 4.86E-06 | 1.072745214 | NM_003010| | MAP2K4,mitogen-activated protein kinase kinase 4 |
| 230441_at | 17.73 | 4.87E-06 | 1.3783903 | NM_052909| | KIAA1909,KIAA1909 protein |
| 218127_at | 17.72 | 4.88E-06 | 1.073685759 | NM_006166| | NFYB,nuclear transcription factor Y, beta |
| 207219_at | 17.72 | 4.87E-06 | 1.214194233 | NM_023070| | ZNF643,zinc finger protein 643 |
| 204178_s_at | 17.72 | 4.89E-06 | 1.039819404 | NM_006328| | RBM14,RNA binding motif protein 14 |
| 202274_at | 17.72 | 4.88E-06 | 1.673564023 | NM_001615| | ACTG2,actin, gamma 2 propeptide |
| 213397_x_at | 17.71 | 4.89E-06 | 1.435920456 | NM_002937| | RNASE4,ribonuclease, RNase A family, 4 precursor |
| 214474_at | 17.71 | 4.90E-06 | 1.185044333 | NM_005399| | PRKAB2,AMP-activated protein kinase beta 2 |
| 205016_at | 17.71 | 4.89E-06 | 1.533243912 | NM_001099691| | NA |
| 244497_at | 17.71 | 4.89E-06 | 1.23211615 | NA |  |
| 226985_at | 17.71 | 4.89E-06 | 1.80360024 | NM_152536| | FGD5,FYVE, RhoGEF and PH domain containing 5 |
| 202919_at | 17.7 | 4.90E-06 | 1.08502739 | NM_001100819| | NA |
| 204668_at | 17.7 | 4.90E-06 | 1.21492381 | NM_007219| | RNF24,ring finger protein 24 |
| 204880_at | 17.7 | 4.90E-06 | 1.168538146 | NM_002412| | MGMT,O-6-methylguanine-DNA methyltransferase |
| 205632_s_at | 17.69 | 4.92E-06 | 2.208196721 | NM_003558| | PIP5K1B,phosphatidylinositol-4-phosphate 5-kinase, type |
| 228970_at | 17.69 | 4.92E-06 | 1.12674401 | NM_178547| | ARCH,archease |
| 206233_at | 17.69 | 4.92E-06 | 1.279257858 | NM_004775| | B4GALT6,UDP-Gal:betaGlcNAc beta 1,4- |
| 220289_s_at | 17.69 | 4.92E-06 | 1.81505149 | NM_001039775| | NA |
| 235645_at | 17.69 | 4.91E-06 | 1.199727087 | NM_052911| | ESCO1,establishment of cohesion 1 homolog 1 |
| 209608_s_at | 17.69 | 4.92E-06 | 1.041619005 | NM_005891| | ACAT2,acetyl-Coenzyme A acetyltransferase 2 |
| 230619_at | 17.68 | 4.93E-06 | 1.121401899 | NM_001668| | ARNT,aryl hydrocarbon receptor nuclear translocator |
| 217828_at | 17.67 | 4.95E-06 | 1.099007817 | NM_001013843| | NA |
| 226129_at | 17.67 | 4.95E-06 | 1.977340203 | NM_198488| | FLJ46072,FLJ46072 protein |
| 220028_at | 17.67 | 4.94E-06 | 1.165325449 | NM_001106| | ACVR2B,activin A type IIB receptor precursor |
| 218228_s_at | 17.67 | 4.95E-06 | 1.091443267 | NM_025235| | TNKS2,tankyrase, TRF1-interacting ankyrin-related |
| 229630_s_at | 17.66 | 4.97E-06 | 1.068739585 | NM_004906| | WTAP,Wilms' tumour 1-associating protein isoform 1 |
| 200976_s_at | 17.65 | 4.97E-06 | 1.067265023 | NM_001079864| | NA |
| 202610_s_at | 17.65 | 4.97E-06 | 1.107525902 | NM_004229| | CRSP2,cofactor required for Sp1 transcriptional |
| 205789_at | 17.65 | 4.98E-06 | 2.003720859 | NM_001766| | CD1D,CD1D antigen, d polypeptide |
| 224792_at | 17.64 | 5.00E-06 | 1.206573662 | NM_033396| | TNKS1BP1,tankyrase 1-binding protein of 182 kDa |
| 203875_at | 17.63 | 5.00E-06 | 1.107557217 | NM_003069| | SMARCA1,SWI/SNF-related matrix-associated |
| 217972_at | 17.61 | 5.04E-06 | 1.044062038 | NM_017812| | CHCHD3,coiled-coil-helix-coiled-coil-helix domain |
| 204951_at | 17.61 | 5.04E-06 | 1.552699652 | NM_004310| | RHOH,ras homolog gene family, member H |
| 206055_s_at | 17.61 | 5.03E-06 | 1.047023078 | NM_003090| | SNRPA1,small nuclear ribonucleoprotein polypeptide A' |
| 209033_s_at | 17.59 | 5.07E-06 | 1.05836923 | NM_001396| | DYRK1A,dual-specificity tyrosine-(Y)-phosphorylation |
| 213169_at | 17.58 | 5.09E-06 | 1.494876126 | NM_003966| | SEMA5A,semaphorin 5A |
| 211464_x_at | 17.58 | 5.09E-06 | 1.196920779 | NM_001226| | CASP6,caspase 6 isoform alpha preproprotein |
| 230597_at | 17.57 | 5.10E-06 | 2.517417901 | NM_001048164| | NA |
| 242515_x_at | 17.57 | 5.11E-06 | 1.086065847 | NM_020642| | C11orf17,chromosome 11 open reading frame 17 |
| 203274_at | 17.56 | 5.11E-06 | 1.057087465 | NM_001007523| | F8A2,coagulation factor VIII-associated (intronic |
| 201368_at | 17.56 | 5.11E-06 | 1.079244666 | NM_006887| | ZFP36L2,butyrate response factor 2 |
| 1569469_a_at | 17.56 | 5.11E-06 | 1.438216291 | NM_001001933| | LHX8,LIM homeobox 8 |
| 201810_s_at | 17.56 | 5.11E-06 | 1.075231081 | NM_001018009| | NA |
| 218274_s_at | 17.56 | 5.11E-06 | 1.122117451 | NM_001042410| | NA |
| 220275_at | 17.56 | 5.11E-06 | 2.360886278 | NM_022034| | CUZD1,CUB and zona pellucida-like domains 1 |
| 228561_at | 17.56 | 5.11E-06 | 1.171683907 | NM_017913| | CDC37L1,cell division cycle 37 homolog (S. |
| 209545_s_at | 17.55 | 5.12E-06 | 1.06573637 | NM_003821| | RIPK2,receptor-interacting serine-threonine kinase 2 |
| 212744_at | 17.55 | 5.13E-06 | 1.141407183 | NM_033028| | BBS4,Bardet-Biedl syndrome 4 |
| 201434_at | 17.54 | 5.14E-06 | 1.081474299 | NM_003314| | TTC1,tetratricopeptide repeat domain 1 |
| 208840_s_at | 17.53 | 5.16E-06 | 1.103317187 | NM_012297| | G3BP2,Ras-GTPase activating protein SH3 domain-binding |
| 205774_at | 17.53 | 5.16E-06 | 1.196763575 | NM_000505| | F12,coagulation factor XII precursor |
| 213217_at | 17.53 | 5.15E-06 | 1.669964565 | NM_020546| | ADCY2,adenylate cyclase 2 |
| 215071_s_at | 17.52 | 5.17E-06 | 1.450516482 | NM_003512| | HIST1H2AC,H2A histone family, member L |
| 220065_at | 17.5 | 5.20E-06 | 1.795758045 | NM_022144| | TNMD,tenomodulin |
| 226914_at | 17.49 | 5.22E-06 | 1.035290636 | NM_030978| | ARPC5L,actin related protein 2/3 complex, subunit |
| 224562_at | 17.49 | 5.21E-06 | 1.061318177 | NM_006990| | WASF2,WAS protein family, member 2 |
| 202638_s_at | 17.49 | 5.22E-06 | 1.507031029 | NM_000201| | ICAM1,intercellular adhesion molecule 1 precursor |
| 214109_at | 17.48 | 5.23E-06 | 1.212575225 | NM_006726| | LRBA,LPS-responsive vesicle trafficking, beach and |
| 204825_at | 17.47 | 5.26E-06 | 1.03451032 | NM_014791| | MELK,maternal embryonic leucine zipper kinase |
| 234974_at | 17.47 | 5.26E-06 | 1.149238944 | NM_138801| | GALM,galactose mutarotase (aldose 1-epimerase) |
| 225807_at | 17.46 | 5.28E-06 | 1.476499075 | NM_032876| | JUB,jub, ajuba homolog isoform 1 |
| 210692_s_at | 17.45 | 5.30E-06 | 1.344182858 | NM_014096| | SLC43A3,solute carrier family 43, member 3 |
| 203935_at | 17.45 | 5.29E-06 | 1.476309007 | NM_001105| | ACVR1,activin A type I receptor precursor |
| 219296_at | 17.44 | 5.31E-06 | 1.106902052 | NM_001001483| | ZDHHC13,zinc finger, DHHC domain containing 13 isoform |
| 235177_at | 17.44 | 5.31E-06 | 1.111825934 | NM_145280| | LOC151194,hepatocellular carcinoma-associated antigen |
| 208680_at | 17.44 | 5.30E-06 | 1.029108682 | NM_002574| | PRDX1,peroxiredoxin 1 |
| 212737_at | 17.44 | 5.31E-06 | 1.189400297 | NM_000405| | GM2A,GM2 ganglioside activator precursor |
| 216606_x_at | 17.43 | 5.31E-06 | 1.085704256 | NA |  |
| 210426_x_at | 17.43 | 5.32E-06 | 1.668929212 | NM_002943| | RORA,RAR-related orphan receptor A isoform c |
| 214983_at | 17.43 | 5.31E-06 | 1.170962628 | NA |  |
| 226689_at | 17.41 | 5.35E-06 | 1.068943937 | NM_001008388| | LOC493856,similar to mouse 1500009M05Rik protein |
| 209962_at | 17.41 | 5.36E-06 | 1.327509432 | NM_000121| | EPOR,erythropoietin receptor precursor |
| 205850_s_at | 17.41 | 5.36E-06 | 2.409419737 | NM_000814| | GABRB3,gamma-aminobutyric acid (GABA) A receptor, beta |
| 206882_at | 17.4 | 5.37E-06 | 1.214780734 | NM_005071| | SLC1A6,solute carrier family 1 (high affinity |
| 227140_at | 17.4 | 5.36E-06 | 2.166810116 | NA |  |
| 200899_s_at | 17.39 | 5.38E-06 | 1.090927702 | NM_012215| | MGEA5,meningioma expressed antigen 5 (hyaluronidase) |
| 212496_s_at | 17.39 | 5.38E-06 | 1.106641369 | NM_015015| | JMJD2B,jumonji domain containing 2B |
| 232353_s_at | 17.38 | 5.40E-06 | 1.090531491 | NM_016086| | DUSP24,map kinase phosphatase-like protein MK-STYX |
| 234103_at | 17.37 | 5.41E-06 | 1.216200469 | NM_198503| | SLICK,sodium- and chloride-activated ATP-sensitive |
| 205771_s_at | 17.37 | 5.42E-06 | 1.152920846 | NM_004842| | AKAP7,A-kinase anchor protein 7 isoform alpha |
| 239648_at | 17.37 | 5.41E-06 | 1.229166908 | NM_173475| | MGC48972,hypothetical protein MGC48972 |
| 220011_at | 17.36 | 5.44E-06 | 1.118897198 | NM_024037| | MGC2603,hypothetical protein MGC2603 |
| 220387_s_at | 17.36 | 5.44E-06 | 1.319559412 | NM_001031693| | NA |
| 231382_at | 17.34 | 5.47E-06 | 1.56327199 | NM_003862| | FGF18,fibroblast growth factor 18 precursor |
| 204453_at | 17.34 | 5.49E-06 | 1.12067683 | NM_003428| | ZNF84,zinc finger protein 84 (HPF2) |
| 201706_s_at | 17.33 | 5.50E-06 | 1.100606129 | NM_002857| | PEX19,peroxisomal biogenesis factor 19 |
| 202068_s_at | 17.33 | 5.49E-06 | 1.077479168 | NM_000527| | LDLR,low density lipoprotein receptor precursor |
| 201573_s_at | 17.33 | 5.50E-06 | 1.063254009 | NM_004730| | ETF1,eukaryotic translation termination factor 1 |
| 228961_at | 17.32 | 5.51E-06 | 1.097474043 | NM_152622| | FLJ35954,hypothetical protein FLJ35954 |
| 229854_at | 17.32 | 5.51E-06 | 1.333243598 | NM_001098623| | NA |
| 208648_at | 17.31 | 5.53E-06 | 1.056827516 | NM_007126| | VCP,valosin-containing protein |
| 202591_s_at | 17.31 | 5.53E-06 | 1.029724567 | NM_003143| | SSBP1,single-stranded DNA binding protein 1 |
| 222981_s_at | 17.29 | 5.57E-06 | 1.094211033 | NM_016131| | RAB10,ras-related GTP-binding protein RAB10 |
| 218816_at | 17.29 | 5.56E-06 | 1.216496876 | NM_018214| | LRRC1,leucine rich repeat containing 1 |
| 1562701_at | 17.29 | 5.57E-06 | 1.455144631 | NA |  |
| 201181_at | 17.29 | 5.57E-06 | 1.055029613 | NM_006496| | GNAI3,guanine nucleotide binding protein (G protein), |
| 219343_at | 17.27 | 5.60E-06 | 1.235824428 | NM_017913| | CDC37L1,cell division cycle 37 homolog (S. |
| 202814_s_at | 17.27 | 5.61E-06 | 1.119126719 | NM_006460| | HIS1,HMBA-inducible |
| 209823_x_at | 17.26 | 5.63E-06 | 1.253992033 | NM_002123| | HLA-DQB1,major histocompatibility complex, class II, DQ |
| 209904_at | 17.25 | 5.64E-06 | 1.54798432 | NM_003280| | TNNC1,troponin C, slow |
| 238600_at | 17.25 | 5.63E-06 | 1.491554633 | NM_001099433| | NA |
| 35820_at | 17.25 | 5.64E-06 | 1.252146414 | NM_000405| | GM2A,GM2 ganglioside activator precursor |
| 204811_s_at | 17.25 | 5.63E-06 | 1.467911637 | NM_001005505| | CACNA2D2,calcium channel, voltage-dependent, alpha |
| 1556242_a_at | 17.25 | 5.63E-06 | 1.219716499 | NA |  |
| 213187_x_at | 17.24 | 5.65E-06 | 1.047120503 | NM_000146| | FTL,ferritin, light polypeptide |
| 204890_s_at | 17.23 | 5.67E-06 | 2.038447858 | NM_001042771| | NA |
| 202855_s_at | 17.23 | 5.66E-06 | 1.742020015 | NM_001042422| | NA |
| 225082_at | 17.23 | 5.67E-06 | 1.041953709 | NM_016207| | CPSF3,cleavage and polyadenylation specific factor 3, |
| 228899_at | 17.22 | 5.69E-06 | 1.078203747 | NA |  |
| 200949_x_at | 17.22 | 5.69E-06 | 1.021626672 | NM_001023| | RPS20,ribosomal protein S20 |
| 208621_s_at | 17.22 | 5.69E-06 | 1.16359444 | NM_001111077| | NA |
| 236451_at | 17.22 | 5.69E-06 | 1.662632785 | NA |  |
| 231946_at | 17.2 | 5.72E-06 | 1.554260858 | NA |  |
| 223151_at | 17.2 | 5.71E-06 | 1.074042819 | NM_032299| | MGC2714,hypothetical protein MGC2714 |
| 218139_s_at | 17.2 | 5.71E-06 | 1.100046223 | NM_018229| | C14orf108,chromosome 14 open reading frame 108 |
| 201000_at | 17.2 | 5.71E-06 | 1.068234748 | NM_001605| | AARS,alanyl-tRNA synthetase |
| 218394_at | 17.19 | 5.74E-06 | 1.219339702 | NM_024589| | FLJ22386,leucine zipper domain protein |
| 219474_at | 17.17 | 5.77E-06 | 1.292867758 | NM_024616| | FLJ23186,hypothetical protein FLJ23186 |
| 212977_at | 17.16 | 5.81E-06 | 1.061514309 | NM_020311| | CMKOR1,chemokine orphan receptor 1 |
| 219822_at | 17.15 | 5.83E-06 | 1.066986549 | NM_004294| | MTRF1,mitochondrial translational release factor 1 |
| 235390_at | 17.15 | 5.83E-06 | 1.13208695 | NM_173829| | FLJ36754,hypothetical protein FLJ36754 |
| 210056_at | 17.14 | 5.85E-06 | 1.290465009 | NM_014470| | RND1,GTP-binding protein RHO6 |
| 226440_at | 17.14 | 5.84E-06 | 1.098385015 | NM_020185| | DUSP22,dual specificity phosphatase 22 |
| 213982_s_at | 17.14 | 5.84E-06 | 1.758339675 | NM_001035230| | NA |
| 218470_at | 17.13 | 5.85E-06 | 1.071787723 | NM_001040436| | NA |
| 31845_at | 17.13 | 5.86E-06 | 1.122310585 | NM_001127197| | NA |
| 202342_s_at | 17.13 | 5.85E-06 | 1.156100372 | NM_015271| | TRIM2,tripartite motif-containing 2 |
| 203301_s_at | 17.12 | 5.87E-06 | 1.078646986 | NM_021145| | DMTF1,cyclin D binding myb-like transcription factor |
| 207268_x_at | 17.12 | 5.87E-06 | 1.062715765 | NM_005759| | ABI2,abl interactor 2 |
| 239422_at | 17.11 | 5.89E-06 | 1.125447161 | NM_152742| | GPC2,glypican 2 |
| 205253_at | 17.1 | 5.90E-06 | 1.667838539 | NM_002585| | PBX1,pre-B-cell leukemia transcription factor 1 |
| 205591_at | 17.1 | 5.89E-06 | 1.979630641 | NM_006334| | OLFM1,olfactomedin related ER localized protein |
| 227174_at | 17.09 | 5.93E-06 | 2.226499334 | NM_182758| | FLJ38736,hypothetical protein FLJ38736 |
| 203860_at | 17.09 | 5.92E-06 | 1.131463636 | NM_000282| | PCCA,propionyl-Coenzyme A carboxylase, alpha |
| 205882_x_at | 17.08 | 5.94E-06 | 1.12110302 | NM_001121| | NA |
| 223389_s_at | 17.06 | 6.00E-06 | 1.09492346 | NM_016535| | ZNF581,zinc finger protein 581 |
| 205702_at | 17.06 | 5.98E-06 | 1.092327106 | NM_006608| | PHTF1,putative homeodomain transcription factor 1 |
| 225871_at | 17.06 | 5.98E-06 | 1.511053971 | NM_001040665| | NA |
| 224169_at | 17.05 | 6.00E-06 | 1.779174003 | NM_004885| | GPR74,G protein-coupled receptor 74 |
| 212650_at | 17.05 | 6.01E-06 | 1.122909836 | NM_015252| | EHBP1,EH domain binding protein 1 |
| 203680_at | 17.04 | 6.02E-06 | 1.123706132 | NM_002736| | PRKAR2B,cAMP-dependent protein kinase, regulatory |
| 226918_at | 17.04 | 6.02E-06 | 1.833293957 | NM_032452| | JPH4,junctophilin 4 |
| 220274_at | 17.03 | 6.05E-06 | 1.343801471 | NM_024726| | IQCA,IQ motif containing with AAA domain |
| 202006_at | 17.02 | 6.07E-06 | 1.089050485 | NM_002835| | PTPN12,protein tyrosine phosphatase, non-receptor type |
| 203186_s_at | 17.01 | 6.07E-06 | 1.285656606 | NM_002961| | S100A4,S100 calcium-binding protein A4 |
| 206494_s_at | 17.01 | 6.09E-06 | 1.239055055 | NM_000419| | ITGA2B,integrin alpha 2b precursor |
| 201802_at | 17.01 | 6.08E-06 | 1.141566626 | NM_001078174| | NA |
| 230793_at | 17 | 6.10E-06 | 1.3366513 | NM_017640| | LRRC16,leucine rich repeat containing 16 |
| 230075_at | 17 | 6.11E-06 | 1.161169554 | NM_171998| | RAB39B,RAB39B, member RAS oncogene family |
| 232679_at | 16.99 | 6.13E-06 | 1.625709133 | NA |  |
| 225444_at | 16.99 | 6.13E-06 | 1.102356446 | NM_173569| | NA |
| 226329_s_at | 16.99 | 6.12E-06 | 1.085714546 | NM_138798| | LOC129531,hypothetical protein BC018453 |
| 203775_at | 16.99 | 6.13E-06 | 1.112450389 | NM_014251| | SLC25A13,solute carrier family 25, member 13 (citrin) |
| 218241_at | 16.98 | 6.15E-06 | 1.040694299 | NM_005113| | GOLGA5,Golgi autoantigen, golgin subfamily a, 5 |
| 37512_at | 16.98 | 6.14E-06 | 1.343715045 | NM_003725| | RODH,3-hydroxysteroid epimerase |
| 214890_s_at | 16.98 | 6.14E-06 | 1.125178709 | NM_001006655| | DKFZP564J102,DKFZP564J102 protein |
| 214106_s_at | 16.97 | 6.17E-06 | 1.097275416 | NM_001500| | GMDS,GDP-mannose 4,6-dehydratase |
| 227781_x_at | 16.97 | 6.16E-06 | 1.160038162 | NM_031478| | DKFZP434I2117,hypothetical protein DKFZp434I2117 |
| 203636_at | 16.96 | 6.17E-06 | 1.101884026 | NM_000381| | MID1,midline 1 isoform alpha |
| 202435_s_at | 16.96 | 6.17E-06 | 1.495203653 | NM_000104| | CYP1B1,cytochrome P450, family 1, subfamily B, |
| 214519_s_at | 16.96 | 6.17E-06 | 2.235642715 | NM_005059| | RLN2,relaxin 2 isoform 2 |
| 203999_at | 16.96 | 6.17E-06 | 1.138813865 | NM_005639| | SYT1,synaptotagmin I |
| 226513_at | 16.95 | 6.20E-06 | 1.085567728 | NA |  |
| 202384_s_at | 16.95 | 6.19E-06 | 1.132883066 | NM_000356| | TCOF1,Treacher Collins-Franceschetti syndrome 1 |
| 1552370_at | 16.95 | 6.20E-06 | 1.208546836 | NM_001099783| | NA |
| 243356_at | 16.94 | 6.21E-06 | 1.495926118 | NA |  |
| 214695_at | 16.94 | 6.22E-06 | 1.205387375 | NM_014847| | UBAP2L,ubiquitin associated protein 2-like |
| 227606_s_at | 16.94 | 6.22E-06 | 1.389644348 | NM_020799| | AMSH-LP,associated molecule with the SH3 domain of STAM |
| 1564031_a_at | 16.94 | 6.22E-06 | 1.187150307 | NM_173828| | C5orf16,chromosome 5 open reading frame 16 |
| 218909_at | 16.94 | 6.22E-06 | 1.06930323 | NM_012424| | RPS6KC1,ribosomal protein S6 kinase, 52kDa, polypeptide |
| 218615_s_at | 16.94 | 6.22E-06 | 1.193262741 | NM_018266| | TMEM39A,transmembrane protein 39A |
| 232180_at | 16.93 | 6.24E-06 | 1.426196169 | NM_001001521| | UGP2,UDP-glucose pyrophosphorylase 2 isoform b |
| 206268_at | 16.93 | 6.23E-06 | 1.743380205 | NM_020997| | LEFTY1,left-right determination, factor B |
| 222459_at | 16.93 | 6.23E-06 | 1.175673958 | NM_024595| | FLJ12666,hypothetical protein FLJ12666 |
| 231013_at | 16.92 | 6.25E-06 | 1.518327575 | NA |  |
| 223901_at | 16.91 | 6.26E-06 | 1.327007779 | NM_032298| | SYT3,synaptotagmin 3 |
| 203819_s_at | 16.91 | 6.26E-06 | 1.051515407 | NM_006547| | IMP-3,IGF-II mRNA-binding protein 3 |
| 221801_x_at | 16.91 | 6.27E-06 | 1.934855016 | NM_006158| | NEFL,neurofilament, light polypeptide 68kDa |
| 213270_at | 16.89 | 6.31E-06 | 1.191177212 | NM_005374| | MPP2,palmitoylated membrane protein 2 |
| 223249_at | 16.88 | 6.32E-06 | 1.164573531 | NM_012129| | CLDN12,claudin 12 |
| 222271_at | 16.88 | 6.32E-06 | 1.501474608 | NA |  |
| 202102_s_at | 16.88 | 6.32E-06 | 1.08592003 | NM_014299| | BRD4,bromodomain-containing protein 4 isoform short |
| 218129_s_at | 16.88 | 6.32E-06 | 1.187362471 | NM_006166| | NFYB,nuclear transcription factor Y, beta |
| 213113_s_at | 16.88 | 6.32E-06 | 1.417093223 | NM_014096| | SLC43A3,solute carrier family 43, member 3 |
| 212842_x_at | 16.88 | 6.32E-06 | 1.080498018 | NM_001123363| | NA |
| 236517_at | 16.85 | 6.39E-06 | 1.409341776 | NM_032446| | MEGF10,MEGF10 protein |
| 212436_at | 16.85 | 6.38E-06 | 1.143690486 | NM_015906| | TRIM33,tripartite motif-containing 33 protein |
| 239457_at | 16.85 | 6.39E-06 | 1.560263611 | NM_138813| | ATP8B3,ATPase, Class I, type 8B, member 3 |
| 232151_at | 16.85 | 6.38E-06 | 1.689617346 | NM_182762| | 7A5,putative binding protein 7a5 |
| 208093_s_at | 16.85 | 6.39E-06 | 1.050308624 | NM_001025579| | NA |
| 241574_s_at | 16.83 | 6.42E-06 | 1.756685522 | NM_006546| | IMP-1,IGF-II mRNA-binding protein 1 |
| 1557014_a_at | 16.83 | 6.42E-06 | 1.741234082 | NA |  |
| 226140_s_at | 16.83 | 6.42E-06 | 1.149428643 | NA |  |
| 225959_s_at | 16.83 | 6.42E-06 | 1.050431176 | NM_032268| | ZNRF1,zinc and ring finger protein 1 |
| 216615_s_at | 16.82 | 6.45E-06 | 1.658271935 | NM_000869| | HTR3A,5-hydroxytryptamine (serotonin) receptor 3A |
| 204421_s_at | 16.82 | 6.45E-06 | 1.381512456 | NM_002006| | FGF2,fibroblast growth factor 2 |
| 206376_at | 16.82 | 6.44E-06 | 1.254752641 | NM_018057| | SLC6A15,solute carrier family 6, member 15 isoform 2 |
| 205474_at | 16.81 | 6.48E-06 | 1.089044084 | NM_015986| | CRLF3,cytokine receptor-like factor 3 |
| 221515_s_at | 16.8 | 6.49E-06 | 1.087279445 | NM_001032391| | NA |
| 224503_s_at | 16.8 | 6.49E-06 | 1.189392383 | NM_017742| | ZCCHC2,zinc finger, CCHC domain containing 2 |
| 226015_at | 16.8 | 6.49E-06 | 1.046990322 | NM_006956| | ZNF12,zinc finger protein 12 (KOX 3) |
| 203954_x_at | 16.78 | 6.53E-06 | 1.290890283 | NM_001306| | CLDN3,claudin 3 |
| 205290_s_at | 16.78 | 6.52E-06 | 1.112143167 | NM_001200| | BMP2,bone morphogenetic protein 2 precursor |
| 214212_x_at | 16.77 | 6.56E-06 | 1.089481139 | NM_006832| | PLEKHC1,pleckstrin homology domain containing, family C |
| 211153_s_at | 16.76 | 6.59E-06 | 1.360123845 | NM_003701| | TNFSF11,tumor necrosis factor ligand superfamily, member |
| 200634_at | 16.76 | 6.59E-06 | 1.044501811 | NM_005022| | PFN1,profilin 1 |
| 227445_at | 16.76 | 6.58E-06 | 1.079564881 | NM_138447| | LOC115509,hypothetical protein BC014000 |
| 231039_at | 16.76 | 6.58E-06 | 1.27695076 | NA |  |
| 223267_at | 16.75 | 6.60E-06 | 1.034667274 | NM_017819| | RG9MTD1,RNA (guanine-9-) methyltransferase domain |
| 202763_at | 16.75 | 6.60E-06 | 1.10540548 | NM_004346| | CASP3,caspase 3 preproprotein |
| 218180_s_at | 16.74 | 6.62E-06 | 1.548072003 | NM_022772| | EPS8L2,epidermal growth factor receptor pathway |
| 221622_s_at | 16.74 | 6.63E-06 | 1.052305305 | NM_018480| | HT007,uncharacterized hypothalamus protein HT007 |
| 1555355_a_at | 16.73 | 6.63E-06 | 1.526678452 | NM_005238| | ETS1,v-ets erythroblastosis virus E26 oncogene |
| 221637_s_at | 16.73 | 6.63E-06 | 1.06089183 | NM_024099| | MGC2477,hypothetical protein MGC2477 |
| 225766_s_at | 16.73 | 6.63E-06 | 1.096168256 | NM_002270| | TNPO1,transportin 1 |
| 217834_s_at | 16.72 | 6.66E-06 | 1.095557274 | NM_006372| | SYNCRIP,synaptotagmin binding, cytoplasmic RNA |
| 226065_at | 16.72 | 6.66E-06 | 2.192933384 | NM_153026| | PRICKLE1,prickle-like 1 |
| 203492_x_at | 16.72 | 6.65E-06 | 1.099418442 | NM_014679| | PIG8,translokin |
| 204354_at | 16.72 | 6.65E-06 | 1.084487593 | NM_001042594| | NA |
| 215075_s_at | 16.72 | 6.65E-06 | 1.053094732 | NM_002086| | GRB2,growth factor receptor-bound protein 2 isoform |
| 226922_at | 16.72 | 6.66E-06 | 1.106685739 | NM_006267| | RANBP2,RAN binding protein 2 |
| 223784_at | 16.71 | 6.67E-06 | 1.519786086 | NM_020665| | TMEM27,transmembrane protein 27 |
| 1569039_s_at | 16.71 | 6.68E-06 | 1.526629496 | NM_182609| | MGC48625,hypothetical protein MGC48625 |
| 215022_x_at | 16.71 | 6.67E-06 | 1.255147567 | NM_006955| | ZNF11B,zinc finger protein 11b (KOX 2) |
| 217925_s_at | 16.71 | 6.67E-06 | 1.136700263 | NM_022758| | C6orf106,chromosome 6 open reading frame 106 isoform b |
| 232265_at | 16.71 | 6.67E-06 | 1.816773017 | NM_020725| | NA |
| 228713_s_at | 16.7 | 6.70E-06 | 1.224621194 | NM_016246| | DHRS10,dehydrogenase/reductase (SDR family) member 10 |
| 218931_at | 16.69 | 6.72E-06 | 1.341099015 | NM_022449| | RAB17,RAB17, member RAS oncogene family |
| 209507_at | 16.69 | 6.70E-06 | 1.069202982 | NM_002947| | RPA3,replication protein A3, 14kDa |
| 222636_at | 16.69 | 6.70E-06 | 1.22531985 | NM_025205| | MED28,mediator of RNA polymerase II transcription, |
| 226575_at | 16.67 | 6.75E-06 | 1.062463121 | NM_021224| | ZNF462,zinc finger protein 462 |
| 217798_at | 16.67 | 6.75E-06 | 1.056654455 | NM_014515| | CNOT2,CCR4-NOT transcription complex, subunit 2 |
| 226986_at | 16.67 | 6.75E-06 | 1.141094084 | NM_001033518| | NA |
| 40284_at | 16.64 | 6.81E-06 | 1.493246696 | NM_021784| | FOXA2,forkhead box A2 |
| 203087_s_at | 16.63 | 6.86E-06 | 1.093651099 | NM_001098511| | NA |
| 225232_at | 16.63 | 6.85E-06 | 1.131467212 | NM_001040446| | NA |
| 226052_at | 16.62 | 6.88E-06 | 1.090440322 | NM_014299| | BRD4,bromodomain-containing protein 4 isoform short |
| 212157_at | 16.61 | 6.89E-06 | 1.344824542 | NM_002998| | SDC2,syndecan 2 precursor |
| 203288_at | 16.61 | 6.90E-06 | 1.061782871 | NM_014686| | KIAA0355,KIAA0355 |
| 220047_at | 16.61 | 6.89E-06 | 1.309955503 | NM_012240| | SIRT4,sirtuin 4 |
| 205466_s_at | 16.61 | 6.90E-06 | 1.962725637 | NM_005114| | HS3ST1,heparan sulfate D-glucosaminyl |
| 231876_at | 16.61 | 6.91E-06 | 1.062219069 | NM_030961| | TRIM56,tripartite motif-containing 56 |
| 224681_at | 16.6 | 6.91E-06 | 1.073226267 | NM_007353| | GNA12,guanine nucleotide binding protein (G protein) |
| 200011_s_at | 16.58 | 6.96E-06 | 1.044550765 | NM_001659| | ARF3,ADP-ribosylation factor 3 |
| 225494_at | 16.57 | 6.99E-06 | 1.049454875 | NA |  |
| 209436_at | 16.57 | 7.00E-06 | 1.257636537 | NM_006108| | SPON1,spondin 1, extracellular matrix protein |
| 242463_x_at | 16.56 | 7.01E-06 | 1.407369819 | NM_198457| | ZNF600,zinc finger protein 600 |
| 228855_at | 16.56 | 7.01E-06 | 1.232267075 | NM_001105663| | NA |
| 220144_s_at | 16.55 | 7.03E-06 | 1.376239248 | NM_022096| | ANKRD5,ankyrin repeat domain protein 5 |
| 202647_s_at | 16.54 | 7.05E-06 | 1.048531235 | NM_002524| | NRAS,neuroblastoma RAS viral (v-ras) oncogene |
| 219376_at | 16.53 | 7.09E-06 | 1.180931406 | NM_199005| | ZNF322B,zinc finger protein 322B |
| 218429_s_at | 16.53 | 7.08E-06 | 1.136300728 | NM_018381| | FLJ11286,hypothetical protein FLJ11286 |
| 208783_s_at | 16.53 | 7.08E-06 | 1.10085942 | NM_002389| | MCP,membrane cofactor protein isoform 1 precursor |
| 231310_at | 16.51 | 7.12E-06 | 3.037323814 | NA |  |
| 209583_s_at | 16.51 | 7.13E-06 | 1.160854582 | NM_001004196| | CD200,CD200 antigen isoform b |
| 207447_s_at | 16.51 | 7.12E-06 | 2.120629119 | NM_013244| | HGNT-IV-H,UDP-N-acetylglucosamine:a-1,3-D-mannoside |
| 217988_at | 16.51 | 7.13E-06 | 1.055576189 | NM_021178| | CCNB1IP1,cyclin B1 interacting protein 1 isoform a |
| 206712_at | 16.51 | 7.14E-06 | 1.45975155 | NM_024719| | GRTP1,growth hormone regulated TBC protein 1 |
| 217916_s_at | 16.5 | 7.15E-06 | 1.078113997 | NM_016623| | FAM49B,family with sequence similarity 49, member B |
| 229155_at | 16.5 | 7.16E-06 | 1.432144298 | NA |  |
| 206960_at | 16.5 | 7.16E-06 | 1.594012413 | NM_005296| | GPR23,G protein-coupled receptor 23 |
| 224841_x_at | 16.49 | 7.18E-06 | 1.033248009 | NA |  |
| 229559_at | 16.48 | 7.21E-06 | 1.504289287 | NM_001080401| | NA |
| 212481_s_at | 16.48 | 7.22E-06 | 1.125174534 | NM_003290| | TPM4,tropomyosin 4 |
| 219714_s_at | 16.48 | 7.21E-06 | 1.278134064 | NM_018398| | CACNA2D3,calcium channel, voltage-dependent, alpha |
| 223138_s_at | 16.45 | 7.27E-06 | 1.055131407 | NM_001114397| | NA |
| 201331_s_at | 16.45 | 7.28E-06 | 1.53358127 | NM_003153| | STAT6,signal transducer and activator of transcription |
| 220138_at | 16.43 | 7.33E-06 | 1.798787067 | NM_004821| | HAND1,basic helix-loop-helix transcription factor |
| 224605_at | 16.43 | 7.34E-06 | 1.058669741 | NM_001001701| | LOC401152,HCV F-transactivated protein 1 |
| 206698_at | 16.42 | 7.37E-06 | 1.946568255 | NM_021083| | XK,McLeod syndrome-associated, Kell blood group |
| 212876_at | 16.42 | 7.35E-06 | 1.295145997 | NM_003778| | B4GALT4,UDP-Gal:betaGlcNAc beta 1,4- |
| 223248_at | 16.42 | 7.35E-06 | 1.121234914 | NM_031463| | HSDL1,hydroxysteroid dehydrogenase like 1 |
| 224845_s_at | 16.41 | 7.38E-06 | 1.143552879 | NM_020846| | NA |
| 207307_at | 16.41 | 7.38E-06 | 1.519302762 | NM_000868| | HTR2C,5-hydroxytryptamine (serotonin) receptor 2C |
| 228330_at | 16.41 | 7.39E-06 | 1.12581276 | NM_145062| | C6orf113,chromosome 6 open reading frame 113 |
| 216563_at | 16.41 | 7.38E-06 | 1.114655648 | NM_001083625| | NA |
| 225152_at | 16.4 | 7.39E-06 | 1.039060591 | NM_033414| | ZNF622,zinc finger protein 622 |
| 37547_at | 16.4 | 7.41E-06 | 1.323437371 | NM_001033604| | NA |
| 224679_at | 16.39 | 7.43E-06 | 1.070483631 | NM_015154| | NA |
| 201854_s_at | 16.39 | 7.44E-06 | 1.089847849 | NM_015251| | KIAA0431,KIAA0431 protein |
| 201475_x_at | 16.39 | 7.43E-06 | 1.089010736 | NM_004990| | MARS,methionine-tRNA synthetase |
| 223665_at | 16.38 | 7.45E-06 | 1.710720581 | NM_032487| | ARPM1,actin related protein M1 |
| 225164_s_at | 16.38 | 7.45E-06 | 1.10238574 | NM_001013703| | NA |
| 219924_s_at | 16.38 | 7.45E-06 | 1.133641851 | NM_007167| | ZNF258,zinc finger protein 258 |
| 210028_s_at | 16.37 | 7.48E-06 | 1.070718023 | NM_012381| | ORC3L,origin recognition complex, subunit 3 isoform 2 |
| 213725_x_at | 16.37 | 7.49E-06 | 2.635054704 | NM_022166| | XYLT1,xylosyltransferase I |
| 229304_s_at | 16.37 | 7.48E-06 | 1.146812771 | NM_024629| | MLF1IP,MLF1 interacting protein |
| 234665_x_at | 16.36 | 7.50E-06 | 1.194736987 | NM_001031693| | NA |
| 202981_x_at | 16.36 | 7.51E-06 | 1.053277475 | NM_001006610| | SIAH1,seven in absentia homolog 1 isoform b |
| 203971_at | 16.35 | 7.52E-06 | 1.137642911 | NM_001859| | SLC31A1,solute carrier family 31 (copper transporters), |
| 219232_s_at | 16.34 | 7.56E-06 | 1.672615738 | NM_022073| | EGLN3,egl nine homolog 3 |
| 226434_at | 16.34 | 7.55E-06 | 1.060012865 | NM_145030| | MGC22793,hypothetical protein MGC22793 |
| 221540_x_at | 16.34 | 7.55E-06 | 1.093632437 | NM_001042490| | NA |
| 222599_s_at | 16.33 | 7.60E-06 | 1.128926706 | NM_001111018| | NA |
| 203435_s_at | 16.32 | 7.61E-06 | 1.4759555 | NM_000902| | MME,membrane metallo-endopeptidase |
| 211535_s_at | 16.31 | 7.65E-06 | 1.048876311 | NM_015850| | FGFR1,fibroblast growth factor receptor 1 isoform 2 |
| 202721_s_at | 16.31 | 7.64E-06 | 1.230151045 | NM_002056| | GFPT1,glucosamine-fructose-6-phosphate |
| 222839_s_at | 16.3 | 7.65E-06 | 1.169471015 | NM_022894| | PAPOLG,poly(A) polymerase gamma |
| 204512_at | 16.3 | 7.65E-06 | 1.216108212 | NM_002114| | HIVEP1,human immunodeficiency virus type I enhancer |
| 203656_at | 16.29 | 7.68E-06 | 1.11101006 | NM_014845| | KIAA0274,Sac domain-containing inositol phosphatase 3 |
| 212945_s_at | 16.28 | 7.71E-06 | 1.080526356 | NM_001080541| | NA |
| 206033_s_at | 16.27 | 7.75E-06 | 1.438228042 | NM_001941| | DSC3,desmocollin 3 isoform Dsc3a preproprotein |
| 1555845_at | 16.26 | 7.78E-06 | 1.19449259 | NA |  |
| 203485_at | 16.25 | 7.80E-06 | 1.315956004 | NM_021136| | RTN1,reticulon 1 isoform A |
| 1569108_a_at | 16.25 | 7.81E-06 | 1.254079496 | NM_016089| | ZNF589,zinc finger protein 589 |
| 227320_at | 16.24 | 7.84E-06 | 1.259490838 | NM_181709| | LOC144347,hypothetical protein LOC144347 |
| 208158_s_at | 16.24 | 7.82E-06 | 1.039418812 | NM_018030| | OSBPL1A,oxysterol-binding protein-like 1A isoform A |
| 209357_at | 16.23 | 7.87E-06 | 1.204281263 | NM_006079| | CITED2,Cbp/p300-interacting transactivator, with |
| 217963_s_at | 16.23 | 7.86E-06 | 1.045731185 | NM_014380| | NGFRAP1,nerve growth factor receptor (TNFRSF16) |
| 204023_at | 16.23 | 7.84E-06 | 1.056891144 | NM_002916| | RFC4,replication factor C 4 |
| 201287_s_at | 16.21 | 7.91E-06 | 1.15611425 | NM_001006946| | SDC1,syndecan 1 precursor |
| 210069_at | 16.21 | 7.92E-06 | 1.493354786 | NM_004377| | CPT1B,carnitine palmitoyltransferase 1B isoform a |
| 230519_at | 16.21 | 7.92E-06 | 1.409923217 | NM_145019| | FLJ30707,hypothetical protein FLJ30707 |
| 219793_at | 16.21 | 7.90E-06 | 1.544673523 | NM_022133| | SNX16,sorting nexin 16 isoform a |
| 227541_at | 16.21 | 7.92E-06 | 1.074229257 | NM_144574| | WDR20,WD repeat domain 20 isoform 2 |
| 219823_at | 16.21 | 7.91E-06 | 3.454668109 | NM_024674| | LIN28,lin-28 homolog |
| 227166_at | 16.2 | 7.93E-06 | 1.082306129 | NM_152686| | MGC29463,hypothetical protein MGC29463 |
| 228407_at | 16.2 | 7.95E-06 | 1.292589921 | NM_152753| | SCUBE3,signal peptide, CUB domain, EGF-like 3 |
| 223254_s_at | 16.19 | 7.97E-06 | 1.10263311 | NM_017769| | KIAA1333,KIAA1333 |
| 226029_at | 16.19 | 7.96E-06 | 1.08831216 | NM_020335| | NA |
| 206137_at | 16.19 | 7.98E-06 | 1.125206518 | NM_001100117| | NA |
| 219255_x_at | 16.19 | 7.96E-06 | 1.174537594 | NM_018725| | IL17RB,interleukin 17B receptor isoform 1 precursor |
| 224482_s_at | 16.18 | 7.99E-06 | 1.238496356 | NM_032932| | RAB11FIP4,RAB11 family interacting protein 4 (class II) |
| 200944_s_at | 16.18 | 7.98E-06 | 1.038160462 | NM_004965| | HMGN1,high-mobility group nucleosome binding domain 1 |
| 229013_at | 16.18 | 8.00E-06 | 1.264475444 | NA |  |
| 212465_at | 16.18 | 7.99E-06 | 1.088718674 | NM_032233| | C14orf154,chromosome 14 open reading frame 154 isoform a |
| 226107_at | 16.18 | 8.00E-06 | 1.169518173 | NA |  |
| 236714_at | 16.18 | 8.00E-06 | 2.058801581 | NA |  |
| 230085_at | 16.17 | 8.01E-06 | 1.248641852 | NA |  |
| 218231_at | 16.16 | 8.04E-06 | 1.073316491 | NM_017567| | NAGK,N-Acetylglucosamine kinase |
| 218285_s_at | 16.16 | 8.04E-06 | 1.053208821 | NM_020139| | DHRS6,dehydrogenase/reductase (SDR family) member 6 |
| 219167_at | 16.16 | 8.04E-06 | 1.496887093 | NM_016563| | RASL12,RAS-like, family 12 protein |
| 205719_s_at | 16.16 | 8.05E-06 | 1.642354537 | NM_000277| | PAH,phenylalanine hydroxylase |
| 230423_at | 16.15 | 8.06E-06 | 2.037191589 | NA |  |
| 219993_at | 16.15 | 8.06E-06 | 1.747490734 | NM_022454| | SOX17,SRY-box 17 |
| 242923_at | 16.15 | 8.07E-06 | 1.145252732 | NM_178549| | MGC42493,hypothetical protein MGC42493 |
| 213272_s_at | 16.15 | 8.06E-06 | 1.064770172 | NM_020422| | LOC57146,promethin |
| 204362_at | 16.13 | 8.12E-06 | 1.901749155 | NM_003930| | SCAP2,src family associated phosphoprotein 2 |
| 211404_s_at | 16.12 | 8.14E-06 | 1.05658241 | NM_001642| | APLP2,amyloid beta (A4) precursor-like protein 2 |
| 204759_at | 16.12 | 8.16E-06 | 1.232993695 | NM_001268| | CHC1L,RCC1-like G exchanging factor RLG |
| 222662_at | 16.12 | 8.16E-06 | 1.278658041 | NM_024607| | PPP1R3B,protein phosphatase 1, regulatory (inhibitor) |
| 204479_at | 16.1 | 8.22E-06 | 1.153909302 | NM_012383| | OSTF1,osteoclast stimulating factor 1 |
| 204948_s_at | 16.1 | 8.22E-06 | 1.620796597 | NM_006350| | FST,follistatin isoform FST317 precursor |
| 231296_at | 16.09 | 8.25E-06 | 1.151141326 | NA |  |
| 224451_x_at | 16.08 | 8.25E-06 | 1.412226944 | NM_001080156| | NA |
| 206456_at | 16.08 | 8.27E-06 | 1.678927632 | NM_000810| | GABRA5,gamma-aminobutyric acid (GABA) A receptor, alpha |
| 235052_at | 16.08 | 8.27E-06 | 1.209165006 | NM_175872| | FLJ38451,FLJ38451 protein |
| 222991_s_at | 16.08 | 8.25E-06 | 1.108892052 | NM_013438| | UBQLN1,ubiquilin 1 isoform 1 |
| 219704_at | 16.07 | 8.28E-06 | 1.262580546 | NM_015982| | YBX2,germ cell specific Y-box binding protein |
| 203830_at | 16.07 | 8.30E-06 | 1.138839587 | NM_022344| | NJMU-R1,protein kinase Njmu-R1 |
| 219765_at | 16.07 | 8.29E-06 | 1.179761948 | NM_024620| | ZNF329,zinc finger protein 329 |
| 235840_at | 16.06 | 8.31E-06 | 1.137401474 | NM_144597| | MGC29937,hypothetical protein MGC29937 |
| 227468_at | 16.05 | 8.35E-06 | 1.277939213 | NM_152359| | CPT1C,carnitine palmitoyltransferase 1C |
| 238147_at | 16.05 | 8.34E-06 | 1.200003819 | NM_025058| | TRIM46,tripartite motif-containing 46 |
| 212805_at | 16.05 | 8.34E-06 | 1.421383882 | NM_015225| | NA |
| 242828_at | 16.05 | 8.34E-06 | 1.134019247 | NM_018086| | FIGN,fidgetin |
| 202761_s_at | 16.04 | 8.36E-06 | 1.108077147 | NM_015180| | SYNE2,synaptic nuclei expressed gene 2 isoform a |
| 1555241_at | 16.02 | 8.44E-06 | 1.157416034 | NM_001099670| | NA |
| 231963_at | 16.02 | 8.44E-06 | 1.500010912 | NA |  |
| 227088_at | 16.01 | 8.46E-06 | 2.461230963 | NM_001083| | PDE5A,phosphodiesterase 5A isoform 1 |
| 235165_at | 16.01 | 8.47E-06 | 1.504472555 | NM_032521| | NA |
| 218092_s_at | 16 | 8.48E-06 | 1.157770727 | NM_004504| | HRB,HIV-1 Rev binding protein |
| 202073_at | 16 | 8.49E-06 | 1.497157612 | NM_001008211| | OPTN,optineurin |
| 214352_s_at | 15.99 | 8.53E-06 | 1.128081399 | NM_004985| | KRAS,c-K-ras2 protein isoform b |
| 234734_s_at | 15.99 | 8.52E-06 | 1.040411921 | NM_014494| | TNRC6A,trinucleotide repeat containing 6A |
| 213186_at | 15.98 | 8.53E-06 | 1.13928522 | NM_014648| | DZIP3,zinc finger DAZ interacting protein 3 |
| 228771_at | 15.97 | 8.59E-06 | 1.777242962 | NM_005160| | ADRBK2,beta adrenergic receptor kinase 2 |
| 221900_at | 15.97 | 8.58E-06 | 1.284045234 | NM_005202| | COL8A2,collagen, type VIII, alpha 2 |
| 214055_x_at | 15.97 | 8.57E-06 | 1.068582925 | NM_015172| | BAT2D1,HBxAg transactivated protein 2 |
| 227209_at | 15.97 | 8.57E-06 | 2.114075945 | NM_001843| | CNTN1,contactin 1 isoform 1 precursor |
| 224985_at | 15.96 | 8.62E-06 | 1.047541106 | NM_002524| | NRAS,neuroblastoma RAS viral (v-ras) oncogene |
| 218448_at | 15.96 | 8.60E-06 | 1.091855025 | NM_017896| | C20orf11,chromosome 20 open reading frame 11 |
| 227694_at | 15.96 | 8.62E-06 | 1.174008617 | NM_178122| | LOC90529,hypothetical protein LOC90529 |
| 224991_at | 15.95 | 8.64E-06 | 1.149833633 | NM_030629| | CMIP,c-Maf-inducing protein Tc-mip isoform |
| 203166_at | 15.94 | 8.65E-06 | 1.065074042 | NM_006324| | CFDP1,craniofacial development protein 1 |
| 203488_at | 15.93 | 8.68E-06 | 1.097214334 | NM_001008701| | LPHN1,latrophilin 1 isoform 1 precursor |
| 224361_s_at | 15.93 | 8.68E-06 | 1.143805195 | NM_018725| | IL17RB,interleukin 17B receptor isoform 1 precursor |
| 218535_s_at | 15.93 | 8.69E-06 | 1.074517021 | NM_018343| | RIOK2,RIO kinase 2 |
| 219310_at | 15.92 | 8.73E-06 | 1.422482165 | NM_024893| | C20orf39,chromosome 20 open reading frame 39 |
| 227530_at | 15.92 | 8.72E-06 | 1.183910039 | NM_005100| | AKAP12,A-kinase anchor protein 12 isoform 1 |
| 208679_s_at | 15.91 | 8.75E-06 | 1.032700768 | NM_005731| | ARPC2,actin related protein 2/3 complex subunit 2 |
| 239355_at | 15.9 | 8.78E-06 | 1.070022213 | NA |  |
| 229534_at | 15.9 | 8.78E-06 | 1.604849865 | NM_152331| | PTE2B,peroxisomal acyl-CoA thioesterase 2B |
| 1555137_a_at | 15.89 | 8.84E-06 | 1.714449296 | NM_018351| | FGD6,FYVE, RhoGEF and PH domain containing 6 |
| 209933_s_at | 15.88 | 8.86E-06 | 1.55558571 | NM_007261| | CD300A,leukocyte membrane antigen |
| 201385_at | 15.88 | 8.84E-06 | 1.033360301 | NM_001358| | DHX15,DEAH (Asp-Glu-Ala-His) box polypeptide 15 |
| 201798_s_at | 15.88 | 8.86E-06 | 1.876778075 | NM_013451| | FER1L3,myoferlin isoform a |
| 227422_at | 15.88 | 8.87E-06 | 1.071793061 | NA |  |
| 200615_s_at | 15.87 | 8.88E-06 | 1.088368963 | NM_001030006| | NA |
| 56256_at | 15.87 | 8.89E-06 | 1.066746195 | NM_001040455| | NA |
| 233305_at | 15.86 | 8.91E-06 | 1.392068217 | NM_022351| | EFCBP1,EF hand calcium binding protein 1 |
| 224407_s_at | 15.86 | 8.91E-06 | 1.41619591 | NM_001042452| | NA |
| 223140_s_at | 15.85 | 8.95E-06 | 1.080240527 | NM_001114397| | NA |
| 240681_at | 15.85 | 8.95E-06 | 3.056018511 | NA |  |
| 201369_s_at | 15.85 | 8.94E-06 | 1.180270589 | NM_006887| | ZFP36L2,butyrate response factor 2 |
| 210882_s_at | 15.84 | 8.96E-06 | 1.304803172 | NM_001039705| | NA |
| 1555443_at | 15.84 | 8.96E-06 | 1.564140213 | NM_198515| | NA |
| 219300_s_at | 15.83 | 9.00E-06 | 2.255691844 | NM_014141| | CNTNAP2,cell recognition molecule Caspr2 precursor |
| 215718_s_at | 15.83 | 9.01E-06 | 1.105435488 | NM_015153| | PHF3,PHD finger protein 3 |
| 213201_s_at | 15.83 | 9.00E-06 | 2.087460011 | NM_001126132| | NA |
| 201345_s_at | 15.82 | 9.05E-06 | 1.055756004 | NM_003339| | UBE2D2,ubiquitin-conjugating enzyme E2D 2 isoform 1 |
| 213954_at | 15.82 | 9.02E-06 | 1.361079464 | NM_015566| | NA |
| 215691_x_at | 15.82 | 9.02E-06 | 1.085884531 | NM_016126| | C1orf41,chromosome 1 open reading frame 41 |
| 203765_at | 15.81 | 9.08E-06 | 1.587366916 | NM_012198| | GCA,grancalcin, EF-hand calcium binding protein |
| 229234_at | 15.8 | 9.09E-06 | 1.329658332 | NM_001010888| | CXorf32,chromosome X open reading frame 32 |
| 227856_at | 15.8 | 9.11E-06 | 1.575662277 | NM_152400| | FLJ39370,hypothetical protein FLJ39370 |
| 213849_s_at | 15.8 | 9.10E-06 | 1.936424154 | NM_004576| | PPP2R2B,beta isoform of regulatory subunit B55, protein |
| 209652_s_at | 15.8 | 9.09E-06 | 1.329171472 | NM_002632| | PGF,placental growth factor, vascular endothelial |
| 220022_at | 15.79 | 9.13E-06 | 1.420378428 | NM_018102| | ZNF334,zinc finger protein 334 isoform a |
| 213592_at | 15.79 | 9.14E-06 | 2.077747187 | NM_005161| | AGTRL1,angiotensin II receptor-like 1 |
| 228256_s_at | 15.79 | 9.12E-06 | 1.817268321 | NM_022140| | EPB41L4A,erythrocyte protein band 4.1-like 4 |
| 204379_s_at | 15.78 | 9.18E-06 | 1.15597555 | NM_000142| | FGFR3,fibroblast growth factor receptor 3 isoform 1 |
| 218676_s_at | 15.78 | 9.16E-06 | 1.410082403 | NM_001102402| | NA |
| 210088_x_at | 15.77 | 9.20E-06 | 1.184229613 | NM_001002841| | MYL4,atrial/embryonic alkali myosin light chain |
| 225222_at | 15.77 | 9.18E-06 | 1.054564962 | NM_033055| | HIAT1,hippocampus abundant transcript 1 |
| 203091_at | 15.76 | 9.22E-06 | 1.054900255 | NM_003902| | FUBP1,far upstream element-binding protein |
| 226963_at | 15.74 | 9.32E-06 | 1.057433188 | NM_152265| | MGC23908,similar to transcription factor BTF3 |
| 227651_at | 15.74 | 9.31E-06 | 1.140925008 | NM_052876| | BTBD14B,transcriptional repressor NAC1 |
| 236454_at | 15.73 | 9.32E-06 | 1.131941663 | NM_194439| | LOC285498,hypothetical protein LOC285498 |
| 228082_at | 15.73 | 9.33E-06 | 1.680479082 | NM_024769| | ASAM,adipocyte-specific adhesion molecule |
| 243154_at | 15.73 | 9.34E-06 | 1.601890911 | NA |  |
| 219662_at | 15.72 | 9.38E-06 | 1.137069029 | NM_024093| | MGC5509,hypothetical protein MGC5509 |
| 204590_x_at | 15.72 | 9.36E-06 | 1.154098876 | NM_022916| | VPS33A,vacuolar protein sorting 33A |
| 224664_at | 15.72 | 9.38E-06 | 1.03724903 | NM_173473| | C10orf104,chromosome 10 open reading frame 104 |
| 221041_s_at | 15.72 | 9.35E-06 | 1.135544851 | NM_012434| | SLC17A5,solute carrier family 17 (anion/sugar |
| 57588_at | 15.72 | 9.36E-06 | 1.274416785 | NM_020689| | SLC24A3,solute carrier family 24 |
| 218379_at | 15.71 | 9.39E-06 | 1.149945857 | NM_016090| | RBM7,RNA binding motif protein 7 |
| 230201_at | 15.71 | 9.39E-06 | 1.149582399 | NA |  |
| 222820_at | 15.71 | 9.40E-06 | 1.121903245 | NM_018996| | TNRC6C,trinucleotide repeat containing 6C |
| 219288_at | 15.71 | 9.41E-06 | 1.062757962 | NM_020685| | C3orf14,chromosome 3 open reading frame 14 |
| 222209_s_at | 15.7 | 9.44E-06 | 1.095645124 | NM_022918| | FLJ22104,hypothetical protein FLJ22104 |
| 203874_s_at | 15.7 | 9.44E-06 | 1.160391643 | NM_003069| | SMARCA1,SWI/SNF-related matrix-associated |
| 235915_at | 15.69 | 9.46E-06 | 1.324266656 | NA |  |
| 213051_at | 15.69 | 9.46E-06 | 1.031140849 | NM_020119| | ZC3HAV1,zinc finger antiviral protein isoform 1 |
| 227004_at | 15.69 | 9.46E-06 | 2.214232481 | NA |  |
| 220760_x_at | 15.69 | 9.47E-06 | 1.112303991 | NM_024733| | FLJ14345,hypothetical protein FLJ14345 |
| 1557369_a_at | 15.69 | 9.45E-06 | 1.83253567 | NA |  |
| 1556061_at | 15.69 | 9.47E-06 | 1.177072245 | NM_001104546| | NA |
| 203745_at | 15.68 | 9.47E-06 | 1.059307587 | NM_001122608| | NA |
| 203396_at | 15.68 | 9.47E-06 | 1.043266576 | NM_001102667| | NA |
| 211668_s_at | 15.67 | 9.52E-06 | 1.456604616 | NM_002658| | PLAU,urokinase plasminogen activator preproprotein |
| 210751_s_at | 15.67 | 9.53E-06 | 1.411240511 | NM_004683| | RGN,regucalcin |
| 229065_at | 15.66 | 9.55E-06 | 1.328411252 | NM_173508| | SLC35F3,solute carrier family 35, member F3 |
| 202923_s_at | 15.66 | 9.54E-06 | 1.127487177 | NM_001498| | GCLC,glutamate-cysteine ligase, catalytic subunit |
| 1555716_a_at | 15.65 | 9.58E-06 | 1.233889777 | NM_001338| | CXADR,coxsackie virus and adenovirus receptor |
| 212113_at | 15.64 | 9.62E-06 | 1.092477418 | NA |  |
| 218258_at | 15.64 | 9.60E-06 | 1.049313233 | NM_015972| | POLR1D,RNA polymerase I 16 kDa subunit |
| 221826_at | 15.64 | 9.61E-06 | 1.129560381 | NM_144567| | LOC90806,similar to RIKEN cDNA 2610307I21 |
| 227168_at | 15.64 | 9.62E-06 | 1.101869904 | NA |  |
| 231609_at | 15.63 | 9.65E-06 | 1.607735613 | NM_144661| | C10orf82,chromosome 10 open reading frame 82 |
| 37652_at | 15.63 | 9.63E-06 | 1.072685573 | NM_012295| | CABIN1,calcineurin binding protein 1 |
| 203006_at | 15.62 | 9.68E-06 | 1.089855977 | NM_005539| | INPP5A,inositol polyphosphate-5-phosphatase A |
| 218152_at | 15.61 | 9.72E-06 | 1.05291993 | NM_018200| | HMG20A,high-mobility group 20A |
| 232416_at | 15.61 | 9.72E-06 | 1.51396083 | NM_021938| | BRUNOL5,bruno-like 5, RNA binding protein |
| 204274_at | 15.61 | 9.72E-06 | 1.086518365 | NM_004215| | EBAG9,estrogen receptor binding site associated |
| 218551_at | 15.61 | 9.72E-06 | 1.083434739 | NM_021933| | FLJ12438,IGFBP-2-Binding Protein, IIp45 |
| AFFX-HUMRGE/M10098_5_at | 15.61 | 9.72E-06 | 1.033923287 | NA |  |
| 240069_at | 15.61 | 9.72E-06 | 1.155980867 | NA |  |
| 209966_x_at | 15.61 | 9.71E-06 | 1.456972819 | NM_001438| | ESRRG,estrogen-related receptor gamma isoform 1 |
| 205485_at | 15.61 | 9.71E-06 | 1.339784309 | NM_000540| | RYR1,ryanodine receptor 1 (skeletal) |
| 223591_at | 15.6 | 9.75E-06 | 1.281817967 | NM_032322| | RNF135,ring finger protein 135 isoform 1 |
| 221584_s_at | 15.6 | 9.73E-06 | 1.431773124 | NM_001014797| | NA |
| 212817_at | 15.59 | 9.77E-06 | 1.131017004 | NM_012266| | DNAJB5,DnaJ (Hsp40) homolog, subfamily B, member 5 |
| 218845_at | 15.59 | 9.77E-06 | 1.060446356 | NM_020185| | DUSP22,dual specificity phosphatase 22 |
| 231807_at | 15.59 | 9.77E-06 | 1.274408416 | NM_001098500| | NA |
| 213234_at | 15.59 | 9.77E-06 | 1.164118132 | NM_020853| | NA |
| 201172_x_at | 15.58 | 9.80E-06 | 1.05379206 | NM_003945| | ATP6V0E,ATPase, H+ transporting, lysosomal, V0 subunit |
| 244334_at | 15.58 | 9.81E-06 | 1.166434385 | NM_152402| | TRAM1L1,translocation associated membrane protein 1-like |
| 218350_s_at | 15.58 | 9.81E-06 | 1.042324434 | NM_015895| | GMNN,geminin |
| 226875_at | 15.58 | 9.80E-06 | 1.169982808 | NM_144658| | DOCK11,dedicator of cytokinesis 11 |
| 209363_s_at | 15.58 | 9.80E-06 | 1.034416523 | NM_004264| | SURB7,SRB7 suppressor of RNA polymerase B homolog |
| 222549_at | 15.57 | 9.83E-06 | 1.83185609 | NM_021101| | CLDN1,claudin 1 |
| 232087_at | 15.57 | 9.85E-06 | 1.21876898 | NM_198279| | CXorf23,chromosome X open reading frame 23 |
| 231859_at | 15.56 | 9.89E-06 | 1.061259726 | NA |  |
| 221730_at | 15.56 | 9.89E-06 | 2.171821152 | NM_000393| | COL5A2,alpha 2 type V collagen preproprotein |
| 225900_at | 15.56 | 9.88E-06 | 1.173139025 | NM_015189| | NA |
| 201220_x_at | 15.54 | 9.95E-06 | 1.071835251 | NM_001083914| | NA |
| 210102_at | 15.54 | 9.96E-06 | 1.082125849 | NM_014622| | LOH11CR2A,BCSC-1 isoform 1 |
| 224704_at | 15.54 | 9.94E-06 | 1.051398407 | NM_014494| | TNRC6A,trinucleotide repeat containing 6A |
| 205407_at | 15.53 | 1.00E-05 | 1.268654818 | NM_021111| | RECK,RECK protein precursor |
| 204502_at | 15.53 | 1.00E-05 | 1.143855652 | NM_015474| | SAMHD1,SAM domain- and HD domain-containing protein 1 |
| 36553_at | 15.53 | 1.00E-05 | 1.078010923 | NM_004192| | ASMTL,acetylserotonin O-methyltransferase-like |
| 235529_x_at | 15.53 | 9.99E-06 | 1.138794359 | NA |  |
| 220840_s_at | 15.53 | 9.98E-06 | 1.113533975 | NM_018186| | FLJ10706,hypothetical protein FLJ10706 |
| 224049_at | 15.52 | 1.00E-05 | 1.293616343 | NM_031460| | KCNK17,potassium channel, subfamily K, member 17 |
| 210389_x_at | 15.52 | 1.00E-05 | 1.07203715 | NM_016261| | TUBD1,delta-tubulin |
| 222408_s_at | 15.52 | 1.00E-05 | 1.08589197 | NM_016061| | YPEL5,yippee-like 5 |
| 205174_s_at | 15.52 | 1.00E-05 | 1.31721688 | NM_012413| | QPCT,glutaminyl-peptide cyclotransferase precursor |
| 222589_at | 15.51 | 1.00E-05 | 1.214113704 | NM_016231| | NLK,nemo like kinase |
| 218804_at | 15.51 | 1.01E-05 | 1.798464577 | NM_018043| | TMEM16A,transmembrane protein 16A |
| 211971_s_at | 15.5 | 1.01E-05 | 1.023488296 | NM_133259| | LRPPRC,leucine-rich PPR motif-containing protein |
| 227569_at | 15.5 | 1.01E-05 | 1.197469823 | NM_153371| | LNX2,PDZ domain containing ring finger 1 |
| 228954_at | 15.5 | 1.01E-05 | 1.163016025 | NM_152449| | FLJ33008,hypothetical protein FLJ33008 |
| 235507_at | 15.5 | 1.01E-05 | 1.150453298 | NM_052937| | LOC115294,similar to hypothetical protein FLJ10883 |
| 209925_at | 15.49 | 1.01E-05 | 1.918290205 | NM_002538| | OCLN,occludin |
| 205244_s_at | 15.49 | 1.01E-05 | 1.363273564 | NM_001011554| | SLC13A3,solute carrier family 13 member 3 isoform b |
| 219162_s_at | 15.49 | 1.01E-05 | 1.048899688 | NM_016050| | MRPL11,mitochondrial ribosomal protein L11 isoform a |
| 209619_at | 15.48 | 1.02E-05 | 1.47228409 | NM_001025158| | NA |
| 228782_at | 15.48 | 1.02E-05 | 1.613856042 | NM_054023| | SCGB3A2,secretoglobin, family 3A, member 2 |
| 209368_at | 15.47 | 1.02E-05 | 1.652339775 | NM_001979| | EPHX2,epoxide hydrolase 2, cytoplasmic |
| 232028_at | 15.47 | 1.02E-05 | 1.089251138 | NM_178549| | MGC42493,hypothetical protein MGC42493 |
| 223523_at | 15.47 | 1.02E-05 | 1.668092849 | NM_023943| | MGC3040,hypothetical protein MGC3040 |
| 227687_at | 15.47 | 1.02E-05 | 1.109151401 | NM_145014| | FLJ32915,hypothetical protein FLJ32915 |
| 227801_at | 15.46 | 1.02E-05 | 1.146183863 | NM_173084| | TRIM59,tripartite motif-containing 59 |
| 218304_s_at | 15.46 | 1.02E-05 | 1.140336519 | NM_022776| | OSBPL11,oxysterol-binding protein-like protein 11 |
| 222101_s_at | 15.46 | 1.02E-05 | 1.32975353 | NM_003737| | DCHS1,dachsous 1 precursor |
| 219304_s_at | 15.46 | 1.02E-05 | 1.704939972 | NM_025208| | PDGFD,platelet derived growth factor D isoform 1 |
| 202994_s_at | 15.46 | 1.02E-05 | 1.079687171 | NM_001996| | FBLN1,fibulin 1 isoform C precursor |
| 210395_x_at | 15.45 | 1.03E-05 | 1.287260426 | NM_001002841| | MYL4,atrial/embryonic alkali myosin light chain |
| 205132_at | 15.45 | 1.03E-05 | 1.664404395 | NM_005159| | ACTC,cardiac muscle alpha actin proprotein |
| 1555851_s_at | 15.45 | 1.03E-05 | 1.057360664 | NM_003009| | SEPW1,selenoprotein W, 1 |
| 208536_s_at | 15.44 | 1.03E-05 | 1.229133946 | NM_006538| | BCL2L11,BCL2-like 11 isoform 6 |
| 209633_at | 15.44 | 1.03E-05 | 1.187295831 | NM_002718| | PPP2R3A,alpha isoform of regulatory subunit B'', protein |
| 213101_s_at | 15.44 | 1.03E-05 | 1.031235628 | NM_005721| | ACTR3,ARP3 actin-related protein 3 homolog |
| 213198_at | 15.43 | 1.03E-05 | 1.063928871 | NM_004302| | ACVR1B,activin A type IB receptor isoform a precursor |
| 229039_at | 15.43 | 1.03E-05 | 1.266417898 | NM_003178| | SYN2,synapsin II isoform IIb |
| 202828_s_at | 15.42 | 1.04E-05 | 1.128790953 | NM_004995| | MMP14,matrix metalloproteinase 14 preproprotein |
| 229664_at | 15.41 | 1.04E-05 | 1.153037291 | NM_002750| | MAPK8,mitogen-activated protein kinase 8 isoform 2 |
| 210775_x_at | 15.41 | 1.04E-05 | 1.180727937 | NM_001229| | CASP9,caspase 9 isoform alpha preproprotein |
| 212999_x_at | 15.41 | 1.04E-05 | 1.374980017 | NM_001023561| | NA |
| 219165_at | 15.41 | 1.04E-05 | 1.075005483 | NM_021630| | PDLIM2,PDZ and LIM domain 2 isoform 2 |
| 227296_at | 15.4 | 1.05E-05 | 1.187826814 | NM_138431| | LOC113655,hypothetical protein BC011982 |
| 226666_at | 15.39 | 1.05E-05 | 1.061381796 | NM_014992| | DAAM1,dishevelled-associated activator of |
| 1553991_s_at | 15.39 | 1.05E-05 | 1.221744018 | NM_019086| | FLJ20674,hypothetical protein FLJ20674 |
| 236901_at | 15.39 | 1.05E-05 | 1.435136498 | NA |  |
| 204280_at | 15.38 | 1.05E-05 | 1.178419065 | NM_006480| | RGS14,regulator of G-protein signalling 14 |
| 222323_at | 15.37 | 1.05E-05 | 1.645845138 | NA |  |
| 229888_at | 15.37 | 1.06E-05 | 1.125972252 | NM_175874| | MGC47869,hypothetical protein MGC47869 |
| 242957_at | 15.37 | 1.06E-05 | 1.225549438 | NM_152718| | FLJ32009,hypothetical protein FLJ32009 |
| 221261_x_at | 15.37 | 1.06E-05 | 1.07437867 | NM_001098800| | NA |
| 204068_at | 15.36 | 1.06E-05 | 1.118170713 | NM_006281| | STK3,serine/threonine kinase 3 (STE20 homolog, |
| 218031_s_at | 15.36 | 1.06E-05 | 1.088163744 | NM_001085471| | NA |
| 223568_s_at | 15.36 | 1.06E-05 | 1.078302019 | NM_001102559| | NA |
| 203967_at | 15.36 | 1.06E-05 | 1.090984587 | NM_001254| | CDC6,CDC6 homolog |
| 201329_s_at | 15.36 | 1.06E-05 | 1.186386789 | NM_005239| | ETS2,v-ets erythroblastosis virus E26 oncogene |
| 224811_at | 15.35 | 1.06E-05 | 1.209703857 | NA |  |
| 222531_s_at | 15.35 | 1.06E-05 | 1.103626271 | NM_018229| | C14orf108,chromosome 14 open reading frame 108 |
| 224984_at | 15.35 | 1.06E-05 | 1.093377186 | NM_001113178| | NA |
| 207753_at | 15.35 | 1.06E-05 | 1.093345185 | NM_020657| | ZNF304,zinc finger protein 304 |
| 205122_at | 15.35 | 1.06E-05 | 1.299475932 | NM_003692| | TMEFF1,transmembrane protein with EGF-like and two |
| 204740_at | 15.35 | 1.06E-05 | 1.221261651 | NM_006314| | CNKSR1,connector enhancer of kinase suppressor of Ras |
| 224725_at | 15.34 | 1.07E-05 | 1.093016634 | NM_020774| | MIB1,mindbomb homolog 1 |
| 1560853_x_at | 15.34 | 1.07E-05 | 1.807324061 | NM_001039884| | NA |
| 202351_at | 15.33 | 1.07E-05 | 1.10995738 | NM_002210| | ITGAV,integrin alpha-V precursor |
| 209130_at | 15.33 | 1.07E-05 | 1.161133095 | NM_003825| | SNAP23,synaptosomal-associated protein 23 isoform |
| 1560537_at | 15.33 | 1.07E-05 | 1.393273941 | NA |  |
| 217478_s_at | 15.33 | 1.07E-05 | 1.678706427 | NM_006120| | HLA-DMA,major histocompatibility complex, class II, DM |
| 236118_at | 15.32 | 1.07E-05 | 1.298210635 | NA |  |
| 202193_at | 15.32 | 1.07E-05 | 1.202636469 | NM_001031801| | NA |
| 219212_at | 15.32 | 1.07E-05 | 1.050502953 | NM_016299| | HSPA14,heat shock protein hsp70-related protein |
| 225890_at | 15.32 | 1.07E-05 | 1.087222344 | NM_052865| | C20orf72,chromosome 20 open reading frame 72 |
| 218935_at | 15.31 | 1.08E-05 | 1.146963546 | NM_014600| | EHD3,EH-domain containing 3 |
| 204731_at | 15.3 | 1.08E-05 | 1.25653052 | NM_003243| | TGFBR3,transforming growth factor, beta receptor III |
| 219229_at | 15.3 | 1.08E-05 | 1.244377991 | NM_013272| | SLCO3A1,solute carrier organic anion transporter family, |
| 214116_at | 15.29 | 1.08E-05 | 1.296465159 | NM_000060| | BTD,biotinidase precursor |
| 207749_s_at | 15.29 | 1.09E-05 | 1.215748981 | NM_002718| | PPP2R3A,alpha isoform of regulatory subunit B'', protein |
| 225117_at | 15.28 | 1.09E-05 | 1.066797477 | NM_015443| | LOC284058,hypothetical protein LOC284058 |
| 233080_s_at | 15.28 | 1.09E-05 | 1.049745593 | NM_017892| | NA |
| 201564_s_at | 15.28 | 1.09E-05 | 1.060077526 | NM_003088| | FSCN1,fascin 1 |
| 209751_s_at | 15.28 | 1.09E-05 | 1.07296345 | NM_001011658| | TRAPPC2,trafficking protein particle complex 2 |
| 201532_at | 15.27 | 1.09E-05 | 1.028416339 | NM_002788| | PSMA3,proteasome alpha 3 subunit isoform 1 |
| 227529_s_at | 15.26 | 1.10E-05 | 1.237965663 | NM_005100| | AKAP12,A-kinase anchor protein 12 isoform 1 |
| 222573_s_at | 15.26 | 1.10E-05 | 1.118575378 | NM_021818| | SAV1,WW45 protein |
| 225234_at | 15.26 | 1.10E-05 | 1.097998116 | NM_005188| | CBL,Cas-Br-M (murine) ecotropic retroviral |
| 201417_at | 15.26 | 1.10E-05 | 1.059498674 | NM_003107| | SOX4,SRY (sex determining region Y)-box 4 |
| 217707_x_at | 15.26 | 1.10E-05 | 1.889628572 | NM_003070| | SMARCA2,SWI/SNF-related matrix-associated |
| 221613_s_at | 15.25 | 1.10E-05 | 1.110072652 | NM_019006| | ZA20D3,zinc finger, A20 domain containing 3 |
| 223490_s_at | 15.25 | 1.10E-05 | 1.073553854 | NM_001002269| | EXOSC3,exosome component 3 isoform 2 |
| 242283_at | 15.25 | 1.10E-05 | 1.241841878 | NM_144989| | NA |
| 202399_s_at | 15.24 | 1.10E-05 | 1.054360119 | NM_005829| | AP3S2,adaptor-related protein complex 3, sigma 2 |
| 203002_at | 15.22 | 1.11E-05 | 1.338349728 | NM_016201| | AMOTL2,angiomotin like 2 |
| 1557248_at | 15.22 | 1.11E-05 | 1.218827952 | NM_032828| | ZNF587,zinc finger protein 587 |
| 208037_s_at | 15.22 | 1.11E-05 | 1.091201601 | NM_130760| | MADCAM1,mucosal vascular addressin cell adhesion |
| 227139_s_at | 15.22 | 1.11E-05 | 1.152521689 | NM_032383| | HPS3,Hermansky-Pudlak syndrome 3 protein |
| 225919_s_at | 15.21 | 1.12E-05 | 1.132944848 | NM_018325| | C9orf72,hypothetical protein MGC23980 isoform a |
| 244455_at | 15.19 | 1.12E-05 | 1.285917675 | NM_198503| | SLICK,sodium- and chloride-activated ATP-sensitive |
| 225183_at | 15.19 | 1.12E-05 | 1.102965171 | NM_014117| | PRO0149,PRO0149 protein |
| 213859_x_at | 15.19 | 1.12E-05 | 1.097859315 | NM_003601| | SMARCA5,SWI/SNF-related matrix-associated |
| 229829_at | 15.18 | 1.13E-05 | 1.117519702 | NA |  |
| 211945_s_at | 15.18 | 1.13E-05 | 1.035276291 | NM_002211| | ITGB1,integrin beta 1 isoform 1A precursor |
| 226521_s_at | 15.17 | 1.13E-05 | 1.131417554 | NM_139076| | FLJ13614,hypothetical protein FLJ13614 |
| 222492_at | 15.17 | 1.13E-05 | 1.125922829 | NM_003681| | PDXK,pyridoxal kinase |
| 215446_s_at | 15.17 | 1.13E-05 | 1.520012381 | NM_002317| | LOX,lysyl oxidase preproprotein |
| 206751_s_at | 15.17 | 1.13E-05 | 1.23325613 | NM_004845| | PCYT1B,CTP:phosphocholine cytidylyltransferase b |
| 203988_s_at | 15.16 | 1.14E-05 | 1.063978507 | NM_004480| | FUT8,fucosyltransferase 8 isoform b |
| 224774_s_at | 15.16 | 1.13E-05 | 1.195291586 | NM_020443| | NAV1,neuron navigator 1 |
| 1558404_at | 15.16 | 1.14E-05 | 1.812587362 | NA |  |
| 213467_at | 15.16 | 1.14E-05 | 1.165608187 | NM_005440| | RND2,GTP-binding protein Rho7 |
| 210275_s_at | 15.13 | 1.15E-05 | 1.037265867 | NM_001102420| | NA |
| 212823_s_at | 15.13 | 1.15E-05 | 1.179252361 | NM_015549| | PLEKHG3,pleckstrin homology domain containing, family G, |
| 213295_at | 15.13 | 1.15E-05 | 1.244079481 | NM_001042355| | NA |
| 218977_s_at | 15.13 | 1.15E-05 | 1.120431651 | NM_017846| | SECP43,tRNA selenocysteine associated protein |
| 225597_at | 15.13 | 1.15E-05 | 1.150559816 | NM_001080431| | NA |
| 220297_at | 15.13 | 1.15E-05 | 1.227970216 | NM_001002860| | BTBD7,BTB (POZ) domain containing 7 isoform 1 |
| 207011_s_at | 15.12 | 1.15E-05 | 1.076235607 | NM_002821| | PTK7,PTK7 protein tyrosine kinase 7 isoform a |
| 234085_at | 15.12 | 1.15E-05 | 1.615470554 | NA |  |
| 209615_s_at | 15.12 | 1.15E-05 | 2.008290823 | NM_002576| | PAK1,p21-activated kinase 1 |
| 215537_x_at | 15.12 | 1.15E-05 | 1.1214909 | NM_013974| | DDAH2,dimethylarginine dimethylaminohydrolase 2 |
| 213268_at | 15.12 | 1.15E-05 | 1.616810484 | NM_015215| | CAMTA1,calmodulin-binding transcription activator 1 |
| 225538_at | 15.11 | 1.16E-05 | 1.104550803 | NM_032280| | ZCCHC9,zinc finger, CCHC domain containing 9 |
| 1556619_at | 15.11 | 1.16E-05 | 1.314569097 | NA |  |
| 231698_at | 15.11 | 1.16E-05 | 2.071717758 | NA |  |
| 227109_at | 15.11 | 1.16E-05 | 1.243479649 | NM_024514| | CYP2R1,cytochrome P450, family 2, subfamily R, |
| 213938_at | 15.1 | 1.16E-05 | 1.283434579 | NM_015576| | CAST,cytomatrix protein p110 |
| 204681_s_at | 15.09 | 1.17E-05 | 1.266082766 | NM_012294| | RAPGEF5,Rap guanine nucleotide exchange factor (GEF) 5 |
| 227112_at | 15.09 | 1.17E-05 | 1.076881638 | NM_001017395| | NA |
| 223235_s_at | 15.07 | 1.17E-05 | 2.191666271 | NM_022138| | SMOC2,secreted modular calcium-binding protein 2 |
| 219420_s_at | 15.07 | 1.17E-05 | 1.074906595 | NM_023077| | FLJ12439,hypothetical protein FLJ12439 |
| 227995_at | 15.07 | 1.18E-05 | 1.309133538 | NA |  |
| 221786_at | 15.07 | 1.18E-05 | 1.05059946 | NM_001029863| | NA |
| 219894_at | 15.07 | 1.18E-05 | 1.47832561 | NM_019066| | MAGEL2,MAGE-like protein 2 |
| 218973_at | 15.07 | 1.18E-05 | 1.075705739 | NM_001040610| | NA |
| 221847_at | 15.06 | 1.18E-05 | 1.056206854 | NA |  |
| 224364_s_at | 15.06 | 1.18E-05 | 1.035509186 | NM_032472| | PPIL3,peptidylprolyl isomerase-like protein 3 isoform |
| 235181_at | 15.06 | 1.18E-05 | 1.195977796 | NM_001039693| | NA |
| 225864_at | 15.05 | 1.18E-05 | 1.129076105 | NM_174911| | NSE2,breast cancer membrane protein 101 |
| 212873_at | 15.05 | 1.18E-05 | 1.464261005 | NM_012292| | HA-1,minor histocompatibility antigen HA-1 |
| 33814_at | 15.05 | 1.18E-05 | 1.061080022 | NM_001014831| | NA |
| 220436_at | 15.04 | 1.19E-05 | 1.139922914 | NA |  |
| 225976_at | 15.03 | 1.19E-05 | 1.03567923 | NM_152265| | MGC23908,similar to transcription factor BTF3 |
| 206348_s_at | 15.03 | 1.19E-05 | 1.333657005 | NM_005391| | PDK3,pyruvate dehydrogenase kinase, isoenzyme 3 |
| 205007_s_at | 15.03 | 1.19E-05 | 1.192247855 | NM_006383| | CIB2,DNA-dependent protein kinase catalytic |
| 235904_at | 15.03 | 1.19E-05 | 1.986320363 | NM_152404| | FLJ34658,hypothetical protein FLJ34658 |
| 229582_at | 15.03 | 1.19E-05 | 1.060349711 | NM_001098817| | NA |
| 211702_s_at | 15.03 | 1.19E-05 | 1.146757332 | NM_032582| | USP32,ubiquitin specific protease 32 |
| 1568603_at | 15.03 | 1.19E-05 | 1.734628956 | NM_003716| | CADPS,Ca2+-dependent secretion activator isoform 1 |
| 244811_at | 15.03 | 1.19E-05 | 1.162730666 | NA |  |
| 241436_at | 15.03 | 1.19E-05 | 1.406195988 | NM_001039| | SCNN1G,sodium channel, nonvoltage-gated 1, gamma |
| 228994_at | 15.02 | 1.19E-05 | 1.148583761 | NM_152499| | MGC45441,hypothetical protein MGC45441 |
| 231260_at | 15.02 | 1.19E-05 | 1.324160693 | NA |  |
| 206044_s_at | 15.02 | 1.20E-05 | 1.383727245 | NM_004333| | BRAF,v-raf murine sarcoma viral oncogene homolog B1 |
| 229278_at | 15.02 | 1.20E-05 | 1.383274845 | NA |  |
| 204417_at | 15.01 | 1.20E-05 | 1.150972495 | NM_000153| | GALC,galactosylceramidase precursor |
| 224212_s_at | 15.01 | 1.20E-05 | 1.841262865 | NM_014005| | PCDHA9,protocadherin alpha 9 isoform 2 precursor |
| 218318_s_at | 15 | 1.20E-05 | 1.179266886 | NM_016231| | NLK,nemo like kinase |
| 228345_at | 15 | 1.20E-05 | 1.257693919 | NM_001039840| | NA |
| 243174_at | 15 | 1.20E-05 | 1.458496176 | NA |  |
| 244264_at | 15 | 1.21E-05 | 1.590410339 | NM_198508| | FLJ44186,FLJ44186 protein |
| 228944_at | 14.99 | 1.21E-05 | 1.209859785 | NA |  |
| 219270_at | 14.99 | 1.21E-05 | 1.48302194 | NM_024111| | MGC4504,hypothetical protein MGC4504 |
| 226353_at | 14.99 | 1.21E-05 | 1.06283762 | NM_032802| | SPPL2A,putative intramembrane cleaving protease |
| 218209_s_at | 14.97 | 1.22E-05 | 1.102915461 | NM_018170| | P15RS,hypothetical protein FLJ10656 |
| 39248_at | 14.97 | 1.22E-05 | 1.362867883 | NM_004925| | AQP3,aquaporin 3 |
| 204745_x_at | 14.97 | 1.22E-05 | 1.150795262 | NM_005950| | MT1G,metallothionein 1G |
| 223062_s_at | 14.96 | 1.22E-05 | 1.077549755 | NM_021154| | PSAT1,phosphoserine aminotransferase isoform 2 |
| 213280_at | 14.96 | 1.22E-05 | 1.769281155 | NM_001100398| | NA |
| 209967_s_at | 14.96 | 1.22E-05 | 1.164802193 | NM_001881| | CREM,cAMP responsive element modulator isoform b |
| 203728_at | 14.96 | 1.22E-05 | 1.082268026 | NM_001188| | BAK1,BCL2-antagonist/killer 1 |
| 209790_s_at | 14.95 | 1.23E-05 | 1.214420279 | NM_001226| | CASP6,caspase 6 isoform alpha preproprotein |
| 216548_x_at | 14.95 | 1.23E-05 | 1.042030987 | NA |  |
| 220137_at | 14.94 | 1.23E-05 | 1.200415944 | NM_019086| | FLJ20674,hypothetical protein FLJ20674 |
| 229300_at | 14.94 | 1.23E-05 | 1.298033313 | NA |  |
| 225378_at | 14.93 | 1.24E-05 | 1.156875101 | NM_152415| | FLJ32642,hypothetical protein FLJ32642 |
| 209426_s_at | 14.93 | 1.23E-05 | 1.166160135 | NM_014324| | AMACR,alpha-methylacyl-CoA racemase isoform 1 |
| 210315_at | 14.93 | 1.23E-05 | 1.304039965 | NM_003178| | SYN2,synapsin II isoform IIb |
| 215646_s_at | 14.93 | 1.23E-05 | 1.295791205 | NM_001126336| | NA |
| 207819_s_at | 14.93 | 1.23E-05 | 1.199031374 | NM_000443| | ABCB4,ATP-binding cassette, subfamily B, member 4 |
| 212699_at | 14.92 | 1.24E-05 | 1.158391895 | NM_138967| | SCAMP5,secretory carrier membrane protein 5 |
| 202729_s_at | 14.92 | 1.24E-05 | 1.113126115 | NM_000627| | LTBP1,latent transforming growth factor beta binding |
| 218060_s_at | 14.92 | 1.24E-05 | 1.097282134 | NM_024598| | FLJ13154,hypothetical protein FLJ13154 |
| 225463_x_at | 14.92 | 1.24E-05 | 1.05582372 | NM_001097612| | NA |
| 200920_s_at | 14.92 | 1.24E-05 | 1.058300354 | NM_001731| | BTG1,B-cell translocation protein 1 |
| 209999_x_at | 14.92 | 1.24E-05 | 1.372646048 | NM_003745| | SOCS1,suppressor of cytokine signaling 1 |
| 211257_x_at | 14.91 | 1.24E-05 | 1.04285551 | NM_001014972| | NA |
| 223560_s_at | 14.91 | 1.24E-05 | 1.110994327 | NM_001083946| | NA |
| 209582_s_at | 14.91 | 1.24E-05 | 1.136086936 | NM_001004196| | CD200,CD200 antigen isoform b |
| 215321_at | 14.91 | 1.24E-05 | 1.590001275 | NM_138290| | RPIB9,Rap2-binding protein 9 |
| 208841_s_at | 14.9 | 1.25E-05 | 1.063574653 | NM_012297| | G3BP2,Ras-GTPase activating protein SH3 domain-binding |
| 213097_s_at | 14.9 | 1.25E-05 | 1.034939294 | NM_014377| | NA |
| 218828_at | 14.9 | 1.25E-05 | 1.128749999 | NM_020360| | PLSCR3,phospholipid scramblase 3 |
| 228398_at | 14.9 | 1.25E-05 | 1.111385534 | NM_145204| | SENP8,SUMO/sentrin specific protease family member 8 |
| 201120_s_at | 14.89 | 1.25E-05 | 1.179186235 | NM_006667| | PGRMC1,progesterone receptor membrane component 1 |
| 206723_s_at | 14.88 | 1.26E-05 | 1.451927528 | NM_004720| | EDG4,endothelial differentiation, lysophosphatidic |
| 224279_s_at | 14.88 | 1.26E-05 | 1.207300989 | NM_012189| | CABYR,calcium-binding tyrosine |
| 201165_s_at | 14.87 | 1.26E-05 | 1.032908248 | NM_001020658| | NA |
| 225351_at | 14.86 | 1.27E-05 | 1.149785997 | NM_018472| | FAM45B,family with sequence similarity 45, member B |
| 242462_at | 14.86 | 1.26E-05 | 1.202475076 | NA |  |
| 204470_at | 14.86 | 1.27E-05 | 1.409602425 | NM_001511| | CXCL1,chemokine (C-X-C motif) ligand 1 |
| 225627_s_at | 14.86 | 1.26E-05 | 1.116663833 | NM_020925| | KIAA1573,KIAA1573 protein |
| 200616_s_at | 14.85 | 1.27E-05 | 1.053730476 | NM_014730| | KIAA0152,KIAA0152 gene product |
| 205570_at | 14.85 | 1.27E-05 | 1.181110588 | NM_005028| | PIP5K2A,phosphatidylinositol-4-phosphate 5-kinase type |
| 1555864_s_at | 14.84 | 1.27E-05 | 1.038287877 | NM_000284| | PDHA1,pyruvate dehydrogenase (lipoamide) alpha 1 |
| 204776_at | 14.84 | 1.27E-05 | 1.114939175 | NM_003248| | THBS4,thrombospondin 4 precursor |
| 230951_at | 14.84 | 1.27E-05 | 1.872891111 | NA |  |
| 212584_at | 14.84 | 1.27E-05 | 1.053911624 | NM_014691| | AQR,aquarius |
| 217455_s_at | 14.84 | 1.27E-05 | 1.259970881 | NM_001050| | SSTR2,somatostatin receptor 2 |
| 223625_at | 14.83 | 1.28E-05 | 1.205024114 | NM_032581| | DRCTNNB1A,down-regulated by Ctnnb1, a |
| 210445_at | 14.83 | 1.28E-05 | 2.026877273 | NM_001040442| | NA |
| 202260_s_at | 14.83 | 1.28E-05 | 1.087918689 | NM_001032221| | NA |
| 208726_s_at | 14.83 | 1.28E-05 | 1.031006695 | NM_003908| | EIF2S2,eukaryotic translation initiation factor 2 beta |
| 223845_at | 14.82 | 1.29E-05 | 1.160557837 | NA |  |
| 206230_at | 14.82 | 1.29E-05 | 1.653951659 | NM_005568| | LHX1,LIM homeobox protein 1 |
| 226099_at | 14.82 | 1.28E-05 | 1.737697407 | NM_012081| | ELL2,elongation factor, RNA polymerase II, 2 |
| 226279_at | 14.81 | 1.29E-05 | 1.171970206 | NM_007173| | PRSS23,protease, serine, 23 precursor |
| 215228_at | 14.81 | 1.29E-05 | 1.813829121 | NM_001111061| | NA |
| 201194_at | 14.81 | 1.29E-05 | 1.063757187 | NM_003009| | SEPW1,selenoprotein W, 1 |
| 230835_at | 14.81 | 1.29E-05 | 1.216633007 | NM_207392| | UNQ467,KIPV467 |
| 224452_s_at | 14.81 | 1.29E-05 | 1.084008099 | NM_001037163| | NA |
| 205670_at | 14.81 | 1.29E-05 | 1.37037156 | NM_004861| | GAL3ST1,galactose-3-O-sulfotransferase 1 |
| 227429_at | 14.81 | 1.29E-05 | 1.274551362 | NM_173584| | MGC45840,hypothetical protein MGC45840 |
| 1552277_a_at | 14.8 | 1.29E-05 | 1.077340008 | NM_003692| | TMEFF1,transmembrane protein with EGF-like and two |
| 227383_at | 14.8 | 1.29E-05 | 1.612552257 | NA |  |
| 213940_s_at | 14.8 | 1.29E-05 | 1.082980583 | NM_015033| | FNBP1,formin-binding protein 17 |
| 203983_at | 14.8 | 1.29E-05 | 1.076665423 | NM_005999| | TSNAX,translin-associated factor X |
| 222595_s_at | 14.79 | 1.30E-05 | 1.101482616 | NM_022105| | DATF1,death associated transcription factor 1 isoform |
| 219061_s_at | 14.79 | 1.29E-05 | 1.078486413 | NM_006014| | DXS9879E,ESO3 protein |
| 227959_at | 14.78 | 1.30E-05 | 1.069588256 | NA |  |
| 217388_s_at | 14.78 | 1.30E-05 | 1.818792352 | NM_001032998| | NA |
| 229160_at | 14.77 | 1.31E-05 | 1.631665474 | NM_152423| | MUM1L1,melanoma associated antigen (mutated) 1-like 1 |
| 230943_at | 14.77 | 1.30E-05 | 1.873083202 | NM_022454| | SOX17,SRY-box 17 |
| 209715_at | 14.77 | 1.30E-05 | 1.051443924 | NM_012117| | CBX5,chromobox homolog 5 (HP1 alpha homolog, |
| 219330_at | 14.77 | 1.31E-05 | 1.432508334 | NM_138959| | VANGL1,vang-like 1 |
| 228810_at | 14.77 | 1.31E-05 | 1.167866721 | NM_152523| | FLJ40432,hypothetical protein FLJ40432 |
| 236468_at | 14.76 | 1.31E-05 | 1.27263858 | NA |  |
| 219527_at | 14.76 | 1.31E-05 | 1.245830865 | NM_017898| | FLJ20605,hypothetical protein FLJ20605 |
| 210187_at | 14.76 | 1.31E-05 | 1.1599617 | NM_000801| | FKBP1A,FK506-binding protein 1A |
| 203827_at | 14.75 | 1.32E-05 | 1.163892695 | NM_017983| | WIPI49,hypothetical protein FLJ10055 |
| 205689_at | 14.74 | 1.32E-05 | 1.270397611 | NM_014801| | PCNXL2,pecanex-like 2 |
| 226272_at | 14.74 | 1.32E-05 | 1.236966753 | NM_013441| | DSCR1L2,Down syndrome critical region gene 1-like 2 |
| 241834_at | 14.73 | 1.33E-05 | 1.341723911 | NA |  |
| 229276_at | 14.73 | 1.33E-05 | 1.247407117 | NM_020789| | IGSF9,immunoglobulin superfamily, member 9 |
| 202846_s_at | 14.72 | 1.33E-05 | 1.060669353 | NM_002642| | PIGC,phosphatidylinositol glycan, class C |
| 207610_s_at | 14.72 | 1.33E-05 | 1.374585021 | NM_013447| | EMR2,egf-like module containing, mucin-like, hormone |
| 201055_s_at | 14.7 | 1.34E-05 | 1.083831554 | NM_006805| | HNRPA0,heterogeneous nuclear ribonucleoprotein A0 |
| 227280_s_at | 14.7 | 1.34E-05 | 1.156643357 | NM_152523| | FLJ40432,hypothetical protein FLJ40432 |
| 221974_at | 14.7 | 1.34E-05 | 1.339037474 | NM_003097| | SNRPN,small nuclear ribonucleoprotein polypeptide N |
| 201328_at | 14.7 | 1.34E-05 | 1.313689032 | NM_005239| | ETS2,v-ets erythroblastosis virus E26 oncogene |
| 217746_s_at | 14.7 | 1.34E-05 | 1.032345491 | NM_013374| | PDCD6IP,programmed cell death 6 interacting protein |
| 208906_at | 14.7 | 1.34E-05 | 1.085732621 | NM_001079559| | NA |
| 227352_at | 14.69 | 1.35E-05 | 1.179126734 | NM_175871| | FLJ35119,hypothetical protein FLJ35119 |
| 208622_s_at | 14.69 | 1.35E-05 | 1.132659314 | NM_001111077| | NA |
| 228570_at | 14.69 | 1.34E-05 | 1.456904128 | NM_001017523| | NA |
| 215695_s_at | 14.69 | 1.35E-05 | 1.159004168 | NM_001079855| | NA |
| 213792_s_at | 14.69 | 1.35E-05 | 1.169001816 | NM_000208| | INSR,insulin receptor |
| 217590_s_at | 14.68 | 1.35E-05 | 1.472828757 | NM_007332| | TRPA1,ankyrin-like protein 1 |
| 210112_at | 14.68 | 1.35E-05 | 1.115634615 | NM_000195| | HPS1,Hermansky-Pudlak syndrome 1 protein isoform a |
| 212264_s_at | 14.68 | 1.35E-05 | 1.103152543 | NM_015045| | KIAA0261,KIAA0261 |
| 221735_at | 14.68 | 1.35E-05 | 1.061515177 | NM_020839| | WDR48,WD repeat domain 48 |
| 216945_x_at | 14.68 | 1.35E-05 | 1.065985424 | NM_015148| | PASK,PAS domain containing serine/threonine kinase |
| 209552_at | 14.67 | 1.35E-05 | 1.15446106 | NM_003466| | PAX8,paired box gene 8 isoform PAX8A |
| 244694_at | 14.67 | 1.36E-05 | 1.783521066 | NM_001101372| | NA |
| 208943_s_at | 14.67 | 1.35E-05 | 1.064158781 | NM_003262| | TLOC1,translocation protein 1 |
| 219616_at | 14.66 | 1.36E-05 | 1.097447644 | NM_024560| | FLJ21963,FLJ21963 protein |
| 220987_s_at | 14.66 | 1.36E-05 | 1.383837249 | NM_020642| | C11orf17,chromosome 11 open reading frame 17 |
| 212761_at | 14.66 | 1.36E-05 | 1.092117851 | NM_030756| | TCF7L2,transcription factor 7-like 2 (T-cell specific, |
| 239330_at | 14.66 | 1.36E-05 | 1.642547825 | NA |  |
| 210123_s_at | 14.65 | 1.36E-05 | 1.28482138 | NM_000746| | CHRNA7,cholinergic receptor, nicotinic, alpha |
| 212483_at | 14.65 | 1.36E-05 | 1.056311944 | NM_015384| | NIPBL,delangin isoform B |
| 204467_s_at | 14.65 | 1.36E-05 | 1.574124 | NM_000345| | SNCA,alpha-synuclein isoform NACP140 |
| 37802_r_at | 14.65 | 1.36E-05 | 1.112528218 | NM_001040450| | NA |
| 221802_s_at | 14.64 | 1.37E-05 | 1.15180413 | NM_001127211| | NA |
| 205083_at | 14.63 | 1.37E-05 | 1.554966112 | NM_001159| | AOX1,aldehyde oxidase 1 |
| 201218_at | 14.62 | 1.38E-05 | 1.086040401 | NM_001083914| | NA |
| 1552626_a_at | 14.62 | 1.38E-05 | 1.685756576 | NM_030923| | DKFZP566N034,hypothetical protein DKFZp566N034 |
| 225490_at | 14.62 | 1.38E-05 | 1.058993462 | NM_152641| | ARID2,AT rich interactive domain 2 (ARID, RFX-like) |
| 201712_s_at | 14.62 | 1.38E-05 | 1.153802028 | NM_006267| | RANBP2,RAN binding protein 2 |
| 224625_x_at | 14.62 | 1.38E-05 | 1.026371627 | NM_001018108| | NA |
| 219327_s_at | 14.61 | 1.39E-05 | 1.496651923 | NM_018653| | GPRC5C,G protein-coupled receptor family C, group 5, |
| 231381_at | 14.61 | 1.39E-05 | 3.559887948 | NA |  |
| 212296_at | 14.61 | 1.38E-05 | 1.02331678 | NM_005805| | PSMD14,26S proteasome-associated pad1 homolog |
| 228141_at | 14.61 | 1.38E-05 | 1.499785719 | NM_001008397| | LOC493869,similar to 2310016C16Rik protein |
| 228186_s_at | 14.6 | 1.39E-05 | 2.011099039 | NM_032784| | THSD2,thrombospondin, type I, domain containing 2 |
| 43427_at | 14.59 | 1.39E-05 | 1.298119783 | NM_001093| | ACACB,acetyl-Coenzyme A carboxylase beta |
| 228596_at | 14.59 | 1.40E-05 | 1.959465234 | NA |  |
| 235947_at | 14.59 | 1.39E-05 | 1.62096397 | NA |  |
| 203857_s_at | 14.59 | 1.40E-05 | 1.077218657 | NM_006810| | PDIR,for protein disulfide isomerase-related |
| 232780_s_at | 14.59 | 1.40E-05 | 1.123470178 | NM_015911| | LOC51058,hypothetical protein LOC51058 |
| 235149_at | 14.58 | 1.40E-05 | 1.672810995 | NM_173582| | PGM2L1,phosphoglucomutase 2-like 1 |
| 211999_at | 14.57 | 1.40E-05 | 1.046526834 | NM_002107| | H3F3A,H3 histone, family 3A |
| 220199_s_at | 14.57 | 1.41E-05 | 1.126414333 | NM_022831| | FLJ12806,hypothetical protein FLJ12806 |
| 236782_at | 14.57 | 1.41E-05 | 1.933046393 | NM_001017373| | NA |
| 216092_s_at | 14.57 | 1.40E-05 | 1.657531899 | NM_012244| | SLC7A8,solute carrier family 7 (cationic amino acid |
| 204327_s_at | 14.57 | 1.41E-05 | 1.107721703 | NM_003455| | ZNF202,zinc finger protein 202 |
| 219928_s_at | 14.57 | 1.41E-05 | 1.149039581 | NM_012189| | CABYR,calcium-binding tyrosine |
| 220750_s_at | 14.56 | 1.41E-05 | 1.161468945 | NM_022356| | LEPRE1,leucine proline-enriched proteoglycan (leprecan) |
| 223199_at | 14.56 | 1.41E-05 | 1.124316866 | NM_017572| | MKNK2,MAP kinase-interacting serine/threonine kinase |
| 224648_at | 14.56 | 1.41E-05 | 1.04858846 | NM_001127235| | NA |
| 202205_at | 14.56 | 1.41E-05 | 1.243331618 | NM_003370| | VASP,vasodilator-stimulated phosphoprotein isoform 1 |
| 226994_at | 14.56 | 1.41E-05 | 1.106333913 | NM_005880| | DNAJA2,DnaJ subfamily A member 2 |
| 226305_at | 14.55 | 1.42E-05 | 1.188867663 | NM_023946| | LYNX1,Ly-6 neurotoxin-like protein 1 isoform a |
| 220230_s_at | 14.55 | 1.42E-05 | 1.795799137 | NM_016229| | CYB5R2,cytochrome b5 reductase b5R.2 isoform 1 |
| 241379_at | 14.55 | 1.42E-05 | 1.779116229 | NM_173545| | C2orf13,chromosome 2 open reading frame 13 |
| 234192_s_at | 14.55 | 1.41E-05 | 1.119039913 | NM_025211| | GKAP1,G kinase anchoring protein 1 |
| 206046_at | 14.55 | 1.42E-05 | 1.083977361 | NM_003812| | ADAM23,a disintegrin and metalloproteinase domain 23 |
| 202375_at | 14.54 | 1.42E-05 | 1.517274885 | NM_014822| | SEC24D,Sec24-related protein D |
| 1553830_s_at | 14.54 | 1.42E-05 | 1.115260535 | NM_005361| | MAGEA2,melanoma antigen family A, 2 |
| 219572_at | 14.54 | 1.42E-05 | 1.175692522 | NM_001009571| | CADPS2,Ca2+-dependent activator protein for secretion 2 |
| 238576_at | 14.53 | 1.43E-05 | 1.416192646 | NA |  |
| 218486_at | 14.53 | 1.42E-05 | 1.107313817 | NM_003597| | KLF11,Kruppel-like factor 11 |
| 222354_at | 14.53 | 1.43E-05 | 1.702576143 | NM_016946| | F11R,F11 receptor isoform a precursor |
| 45288_at | 14.52 | 1.43E-05 | 1.098464897 | NM_020676| | ABHD6,abhydrolase domain containing 6 |
| 218084_x_at | 14.52 | 1.43E-05 | 1.47165057 | NM_014164| | FXYD5,FXYD domain-containing ion transport regulator |
| 1555751_a_at | 14.52 | 1.43E-05 | 1.061081017 | NM_001007269| | GEMIN7,gemin 7 |
| 203204_s_at | 14.52 | 1.43E-05 | 1.123577206 | NM_014663| | JMJD2A,jumonji domain containing 2A |
| 225824_at | 14.51 | 1.44E-05 | 1.059610972 | NM_001099402| | NA |
| 230312_at | 14.5 | 1.44E-05 | 1.106624756 | NA |  |
| 34408_at | 14.5 | 1.44E-05 | 1.323028134 | NM_005619| | RTN2,reticulon 2 isoform A |
| 204446_s_at | 14.5 | 1.44E-05 | 1.728316552 | NM_000698| | ALOX5,arachidonate 5-lipoxygenase |
| 212860_at | 14.48 | 1.45E-05 | 1.074048171 | NM_032283| | ZDHHC18,zinc finger, DHHC domain containing 18 |
| 1557385_at | 14.48 | 1.45E-05 | 1.053102672 | NM_032180| | NA |
| 212242_at | 14.48 | 1.45E-05 | 1.311769019 | NM_006000| | TUBA1,tubulin, alpha 1 |
| 213533_at | 14.47 | 1.46E-05 | 1.395580119 | NM_001040101| | NA |
| 222696_at | 14.47 | 1.46E-05 | 1.466230969 | NM_004655| | AXIN2,axin 2 |
| 228408_s_at | 14.47 | 1.46E-05 | 1.045716604 | NM_018115| | SDAD1,SDA1 domain containing 1 |
| 204162_at | 14.47 | 1.46E-05 | 1.054948722 | NM_006101| | KNTC2,kinetochore associated 2 |
| 221555_x_at | 14.46 | 1.46E-05 | 1.147278691 | NM_001077181| | NA |
| 238521_at | 14.46 | 1.47E-05 | 1.674834032 | NA |  |
| 210053_at | 14.45 | 1.47E-05 | 1.041527038 | NM_006951| | TAF5,TBP-associated factor 5 |
| 211828_s_at | 14.45 | 1.47E-05 | 1.200319513 | NM_015028| | TNIK,TRAF2 and NCK interacting kinase |
| 212978_at | 14.45 | 1.47E-05 | 1.081057545 | NM_015350| | TA-LRRP,T-cell activation leucine repeat-rich protein |
| 205769_at | 14.45 | 1.47E-05 | 1.449334741 | NM_003645| | SLC27A2,solute carrier family 27 (fatty acid |
| 224492_s_at | 14.44 | 1.48E-05 | 1.091502686 | NM_145295| | ZNF627,zinc finger protein 627 |
| 51158_at | 14.44 | 1.48E-05 | 1.542903144 | NM_207446| | LOC400451,hypothetical gene supported by AK075564; |
| 227826_s_at | 14.43 | 1.48E-05 | 2.115804026 | NA |  |
| 229566_at | 14.43 | 1.48E-05 | 1.185033227 | NA |  |
| 220188_at | 14.43 | 1.49E-05 | 1.231290097 | NM_020655| | JPH3,junctophilin 3 |
| 232080_at | 14.43 | 1.48E-05 | 1.307657816 | NM_020760| | HECW2,HECT, C2 and WW domain containing E3 ubiquitin |
| 204783_at | 14.43 | 1.49E-05 | 1.175047939 | NM_022443| | MLF1,myeloid leukemia factor 1 |
| 202908_at | 14.43 | 1.49E-05 | 1.073676584 | NM_006005| | WFS1,wolframin |
| 216996_s_at | 14.42 | 1.49E-05 | 1.04715043 | NM_014929| | KIAA0971,KIAA0971 |
| 228057_at | 14.42 | 1.49E-05 | 1.63756478 | NM_145244| | DDIT4L,DNA-damage-inducible transcript 4-like |
| 225084_at | 14.42 | 1.49E-05 | 1.082952782 | NM_006544| | SEC10L1,brain secretory protein SEC10P |
| 52940_at | 14.41 | 1.49E-05 | 1.161864469 | NM_021805| | SIGIRR,single Ig IL-1R-related molecule |
| 229085_at | 14.41 | 1.50E-05 | 1.276302889 | NM_052953| | LRRC3B,leucine rich repeat containing 3B |
| 209735_at | 14.41 | 1.49E-05 | 1.48165827 | NM_004827| | ABCG2,ATP-binding cassette, sub-family G, member 2 |
| 222900_at | 14.41 | 1.49E-05 | 1.265583808 | NA |  |
| 225969_at | 14.41 | 1.50E-05 | 1.131751596 | NM_001039876| | NA |
| 203677_s_at | 14.4 | 1.50E-05 | 1.057139564 | NM_004178| | TARBP2,TAR RNA binding protein 2 isoform c |
| 205414_s_at | 14.4 | 1.50E-05 | 1.637024033 | NM_014859| | KIAA0672,KIAA0672 gene product |
| 201841_s_at | 14.4 | 1.50E-05 | 1.06453431 | NM_001540| | HSPB1,heat shock 27kDa protein 1 |
| 223424_s_at | 14.39 | 1.50E-05 | 1.089640271 | NM_145914| | ZNF38,zinc finger protein 38 |
| 209984_at | 14.39 | 1.51E-05 | 1.080255958 | NM_015061| | JMJD2C,jumonji domain containing 2C |
| 213349_at | 14.38 | 1.51E-05 | 1.131295563 | NM_001017395| | NA |
| 228430_at | 14.38 | 1.51E-05 | 1.1172679 | NA |  |
| 203120_at | 14.37 | 1.52E-05 | 1.039397515 | NM_001031685| | NA |
| 227141_at | 14.37 | 1.52E-05 | 1.056960869 | NM_138467| | LOC127253,hypothetical protein BC009514 |
| 203981_s_at | 14.36 | 1.52E-05 | 1.057789477 | NM_005050| | ABCD4,ATP-binding cassette, sub-family D, member 4 |
| 214550_s_at | 14.35 | 1.53E-05 | 1.077498608 | NM_012470| | TNPO3,transportin 3 |
| 228373_at | 14.34 | 1.53E-05 | 1.069093582 | NM_014117| | PRO0149,PRO0149 protein |
| 211712_s_at | 14.33 | 1.54E-05 | 1.356510935 | NM_003568| | ANXA9,annexin A9 |
| 217902_s_at | 14.33 | 1.54E-05 | 1.069726141 | NM_004667| | HERC2,hect domain and RLD 2 |
| 225673_at | 14.32 | 1.55E-05 | 1.089594804 | NM_001020818| | NA |
| 206631_at | 14.32 | 1.55E-05 | 1.332945602 | NM_000956| | PTGER2,prostaglandin E receptor 2 (subtype EP2), 53kDa |
| 235121_at | 14.31 | 1.55E-05 | 1.292618187 | NA |  |
| 202537_s_at | 14.31 | 1.55E-05 | 1.15152235 | NM_014043| | DKFZP564O123,DKFZP564O123 protein |
| 226707_at | 14.31 | 1.56E-05 | 1.146389 | NM_145201| | PP3856,similar to CG3714 gene product |
| 225407_at | 14.3 | 1.56E-05 | 1.569841652 | NM_001025081| | NA |
| 202053_s_at | 14.3 | 1.56E-05 | 1.089989945 | NM_000382| | ALDH3A2,aldehyde dehydrogenase 3A2 |
| 227453_at | 14.3 | 1.56E-05 | 1.089338235 | NM_001080421| | NA |
| 201312_s_at | 14.3 | 1.56E-05 | 1.197503427 | NM_003022| | SH3BGRL,SH3 domain binding glutamic acid-rich protein |
| 232235_at | 14.29 | 1.57E-05 | 1.20581776 | NM_032160| | C18orf4,chromosome 18 open reading frame 4 |
| 227551_at | 14.29 | 1.57E-05 | 1.086630822 | NM_001025780| | NA |
| 227697_at | 14.28 | 1.58E-05 | 1.897981921 | NM_003955| | SOCS3,suppressor of cytokine signaling 3 |
| 208549_x_at | 14.28 | 1.58E-05 | 1.057513158 | NA |  |
| 238663_x_at | 14.28 | 1.58E-05 | 1.818363126 | NM_000829| | GRIA4,glutamate receptor, ionotrophic |
| 55705_at | 14.28 | 1.57E-05 | 1.041730694 | NM_138774| | C19orf22,chromosome 19 open reading frame 22 |
| 223530_at | 14.28 | 1.57E-05 | 1.074733273 | NM_001083963| | NA |
| 203635_at | 14.28 | 1.58E-05 | 1.060635118 | NM_006052| | DSCR3,Down syndrome critical region protein 3 |
| 218600_at | 14.28 | 1.57E-05 | 1.097028422 | NM_030576| | MGC10986,hypothetical protein MGC10986 |
| 223160_s_at | 14.28 | 1.57E-05 | 1.088664251 | NM_032560| | KIAA2010,KIAA2010 isoform 1 |
| 223616_at | 14.27 | 1.58E-05 | 1.186614376 | NM_023074| | FLJ12644,hypothetical protein FLJ12644 |
| 236115_at | 14.27 | 1.58E-05 | 1.518084221 | NA |  |
| 225273_at | 14.26 | 1.59E-05 | 1.082521439 | NM_015691| | KIAA1280,KIAA1280 protein |
| 204130_at | 14.25 | 1.59E-05 | 1.784999567 | NM_000196| | HSD11B2,hydroxysteroid (11-beta) dehydrogenase 2 |
| 210657_s_at | 14.25 | 1.59E-05 | 1.401814603 | NM_004574| | SEPT4,septin 4 isoform 1 |
| 224400_s_at | 14.24 | 1.60E-05 | 2.542228346 | NM_031422| | CHST9,GalNAc-4-sulfotransferase 2 |
| 236385_at | 14.24 | 1.60E-05 | 1.324104968 | NA |  |
| 227607_at | 14.24 | 1.60E-05 | 1.43017655 | NM_020799| | AMSH-LP,associated molecule with the SH3 domain of STAM |
| 209679_s_at | 14.24 | 1.60E-05 | 1.200090003 | NM_001031628| | NA |
| 225614_at | 14.24 | 1.60E-05 | 1.048023607 | NM_138421| | LOC113174,hypothetical protein BC012010 |
| 228346_at | 14.23 | 1.60E-05 | 1.19374243 | NA |  |
| 218490_s_at | 14.23 | 1.60E-05 | 1.061177495 | NM_001012320| | ZNF302,zinc finger protein 302 |
| 235552_at | 14.23 | 1.60E-05 | 1.099140634 | NM_020961| | KIAA1627,KIAA1627 protein |
| 206424_at | 14.23 | 1.61E-05 | 2.180936519 | NM_000783| | CYP26A1,cytochrome P450, family 26, subfamily A, |
| 201317_s_at | 14.23 | 1.61E-05 | 1.041846692 | NM_002787| | PSMA2,proteasome alpha 2 subunit |
| 223234_at | 14.22 | 1.61E-05 | 1.11820378 | NM_006341| | MAD2L2,MAD2 homolog |
| 221677_s_at | 14.22 | 1.61E-05 | 1.088601033 | NM_017613| | DONSON,downstream neighbor of SON isoform a |
| 203135_at | 14.22 | 1.61E-05 | 1.043938085 | NM_003194| | TBP,TATA box binding protein |
| 213627_at | 14.22 | 1.61E-05 | 1.12420083 | NM_014599| | MAGED2,melanoma antigen family D, 2 |
| 239403_at | 14.22 | 1.61E-05 | 1.10544897 | NM_033626| | JM11,JM11 protein |
| 229084_at | 14.22 | 1.61E-05 | 1.485159361 | NM_175607| | CNTN4,contactin 4 isoform a precursor |
| 37986_at | 14.21 | 1.61E-05 | 1.196445514 | NM_000121| | EPOR,erythropoietin receptor precursor |
| 202683_s_at | 14.21 | 1.61E-05 | 1.064168071 | NM_003799| | RNMT,RNA (guanine-7-) methyltransferase |
| 228608_at | 14.21 | 1.61E-05 | 1.172643443 | NM_052867| | VGCNL1,voltage gated channel like 1 |
| 218433_at | 14.2 | 1.62E-05 | 1.127714679 | NM_024594| | PANK3,pantothenate kinase 3 |
| 203038_at | 14.2 | 1.62E-05 | 1.04410436 | NM_002844| | PTPRK,protein tyrosine phosphatase, receptor type, K |
| 222062_at | 14.2 | 1.62E-05 | 1.159531483 | NM_004843| | IL27RA,class I cytokine receptor |
| 227294_at | 14.2 | 1.62E-05 | 1.109840239 | NM_138447| | LOC115509,hypothetical protein BC014000 |
| 212624_s_at | 14.2 | 1.62E-05 | 1.046765656 | NM_001025201| | NA |
| 202842_s_at | 14.2 | 1.62E-05 | 1.063762457 | NM_012328| | DNAJB9,DnaJ (Hsp40) homolog, subfamily B, member 9 |
| 226560_at | 14.19 | 1.63E-05 | 1.309138804 | NA |  |
| 160020_at | 14.19 | 1.63E-05 | 1.110267068 | NM_004995| | MMP14,matrix metalloproteinase 14 preproprotein |
| 206513_at | 14.18 | 1.63E-05 | 1.543657662 | NM_004833| | AIM2,absent in melanoma 2 |
| 220673_s_at | 14.18 | 1.64E-05 | 1.458466648 | NM_020958| | KIAA1622,HEAT-like repeat-containing protein isoform 2 |
| 41512_at | 14.17 | 1.64E-05 | 1.07158708 | NA |  |
| 205881_at | 14.17 | 1.64E-05 | 1.091706866 | NM_003426| | ZNF74,zinc finger protein 74 (Cos52) |
| 209035_at | 14.17 | 1.64E-05 | 1.16663137 | NM_001012333| | MDK,midkine |
| 221035_s_at | 14.17 | 1.64E-05 | 1.793517785 | NM_031272| | TEX14,testis expressed sequence 14 isoform b |
| 201925_s_at | 14.16 | 1.65E-05 | 1.737538286 | NM_000574| | DAF,decay accelerating factor for complement (CD55, |
| 224518_s_at | 14.16 | 1.65E-05 | 1.081358808 | NM_032497| | ZNF559,zinc finger protein 559 |
| 1553103_at | 14.16 | 1.65E-05 | 1.103121461 | NM_002504| | NFX1,nuclear transcription factor, X-box binding 1 |
| 200737_at | 14.15 | 1.65E-05 | 1.0802417 | NM_000291| | PGK1,phosphoglycerate kinase 1 |
| 37254_at | 14.15 | 1.65E-05 | 1.048472811 | NM_001083330| | NA |
| 233536_at | 14.14 | 1.66E-05 | 1.549083637 | NM_030632| | NA |
| 208734_x_at | 14.14 | 1.66E-05 | 1.029650633 | NM_002865| | RAB2,RAB2, member RAS oncogene family |
| 202887_s_at | 14.13 | 1.67E-05 | 1.069870185 | NM_019058| | DDIT4,RTP801 |
| 64488_at | 14.12 | 1.67E-05 | 1.031766415 | NA |  |
| 200798_x_at | 14.12 | 1.67E-05 | 1.110668062 | NM_021960| | MCL1,myeloid cell leukemia sequence 1 isoform 1 |
| 224563_at | 14.12 | 1.67E-05 | 1.079849781 | NM_006990| | WASF2,WAS protein family, member 2 |
| 1557816_a_at | 14.11 | 1.68E-05 | 1.237621126 | NA |  |
| 226187_at | 14.11 | 1.68E-05 | 1.464359302 | NA |  |
| 220060_s_at | 14.11 | 1.68E-05 | 1.142990219 | NM_017915| | FLJ20641,hypothetical protein FLJ20641 |
| 237322_at | 14.11 | 1.68E-05 | 1.165653177 | NA |  |
| 202228_s_at | 14.11 | 1.68E-05 | 1.095870798 | NM_012428| | SDFR1,stromal cell derived factor receptor 1 isoform |
| 202431_s_at | 14.11 | 1.68E-05 | 2.015848276 | NM_002467| | MYC,v-myc myelocytomatosis viral oncogene homolog |
| 229569_at | 14.1 | 1.69E-05 | 1.422525512 | NA |  |
| 210058_at | 14.1 | 1.68E-05 | 1.378889177 | NM_002754| | MAPK13,mitogen-activated protein kinase 13 |
| 204050_s_at | 14.1 | 1.69E-05 | 1.0461506 | NM_001076677| | NA |
| 1557621_at | 14.09 | 1.70E-05 | 1.323497883 | NA |  |
| 222580_at | 14.09 | 1.69E-05 | 1.110360942 | NM_016620| | ZNF644,zinc finger protein 644 isoform 2 |
| 211137_s_at | 14.09 | 1.70E-05 | 1.132225196 | NM_001001485| | ATP2C1,calcium-transporting ATPase 2C1 isoform 1c |
| 232382_s_at | 14.08 | 1.70E-05 | 1.263856062 | NM_052937| | LOC115294,similar to hypothetical protein FLJ10883 |
| 222986_s_at | 14.08 | 1.70E-05 | 1.060928538 | NM_016479| | SCOTIN,scotin |
| 226345_at | 14.08 | 1.70E-05 | 1.044435243 | NA |  |
| 225330_at | 14.07 | 1.71E-05 | 1.082957954 | NM_000875| | IGF1R,insulin-like growth factor 1 receptor precursor |
| 215758_x_at | 14.07 | 1.71E-05 | 1.242675627 | NM_031218| | ZNF505,zinc finger protein 505 isoform a |
| 1567107_s_at | 14.07 | 1.71E-05 | 1.160899021 | NM_003290| | TPM4,tropomyosin 4 |
| 213916_at | 14.05 | 1.72E-05 | 1.160004432 | NM_021143| | ZNF20,zinc finger protein 20 (KOX 13) |
| 208091_s_at | 14.05 | 1.72E-05 | 1.079719453 | NM_030796| | DKFZP564K0822,hypothetical protein DKFZp564K0822 |
| 235199_at | 14.04 | 1.72E-05 | 1.584154039 | NM_017831| | RNF125,ring finger protein 125 |
| 213761_at | 14.04 | 1.72E-05 | 1.054931418 | NM_017440| | MDM1,Mdm4, transformed 3T3 cell double minute 1, p53 |
| 244463_at | 14.04 | 1.72E-05 | 1.249913349 | NM_003812| | ADAM23,a disintegrin and metalloproteinase domain 23 |
| 44696_at | 14.04 | 1.72E-05 | 1.082434821 | NM_018201| | TBC1D13,TBC1 domain family, member 13 |
| 201599_at | 14.03 | 1.73E-05 | 1.08703862 | NM_000274| | OAT,ornithine aminotransferase precursor |
| 202454_s_at | 14.03 | 1.73E-05 | 1.115993293 | NM_001005915| | ERBB3,erbB-3 isoform s precursor |
| 200055_at | 14.03 | 1.74E-05 | 1.023349365 | NM_006284| | TAF10,TBP-related factor 10 |
| 223595_at | 14.03 | 1.73E-05 | 1.192551576 | NM_032021| | AD031,AD031 protein |
| 237585_at | 14.03 | 1.73E-05 | 1.347444208 | NM_001114357| | NA |
| 227263_at | 14.02 | 1.74E-05 | 1.2929721 | NM_001013842| | NA |
| 238546_at | 14.01 | 1.75E-05 | 1.296822406 | NM_001112800| | NA |
| 213044_at | 14.01 | 1.74E-05 | 1.081984333 | NM_005406| | ROCK1,Rho-associated, coiled-coil containing protein |
| 214414_x_at | 14 | 1.75E-05 | 1.343694511 | NM_000517| | HBA2,alpha 2 globin |
| 222857_s_at | 14 | 1.75E-05 | 1.713765196 | NM_014505| | KCNMB4,calcium-activated potassium channel beta 4 |
| 221269_s_at | 14 | 1.75E-05 | 1.103420917 | NM_031286| | SH3BGRL3,SH3 domain binding glutamic acid-rich protein |
| 219030_at | 14 | 1.76E-05 | 1.051673854 | NM_016058| | CGI-121,CGI-121 protein |
| 225943_at | 13.99 | 1.76E-05 | 1.078861734 | NM_020726| | NLN,neurolysin |
| 208712_at | 13.98 | 1.76E-05 | 1.044687037 | NM_053056| | CCND1,cyclin D1 |
| 223338_s_at | 13.97 | 1.78E-05 | 1.067553108 | NM_016311| | ATPIF1,ATPase inhibitory factor 1 isoform 1 precursor |
| 219465_at | 13.96 | 1.78E-05 | 1.680135867 | NM_001643| | APOA2,apolipoprotein A-II precursor |
| 208758_at | 13.96 | 1.78E-05 | 1.054778964 | NM_004044| | ATIC,5-aminoimidazole-4-carboxamide ribonucleotide |
| 227334_at | 13.96 | 1.79E-05 | 1.062166625 | NM_152586| | USP54,ubiquitin specific protease 54 |
| 221217_s_at | 13.96 | 1.79E-05 | 1.426690581 | NM_018723| | A2BP1,ataxin 2-binding protein 1 isoform 4 |
| 240117_at | 13.95 | 1.79E-05 | 1.589035274 | NM_032447| | FBN3,fibrillin 3 precursor |
| 205484_at | 13.95 | 1.79E-05 | 1.296641411 | NM_014450| | SIT,SHP2-interacting transmembrane adaptor protein |
| 228338_at | 13.95 | 1.79E-05 | 1.594094598 | NA |  |
| 226974_at | 13.95 | 1.79E-05 | 1.139101834 | NA |  |
| 221425_s_at | 13.95 | 1.79E-05 | 1.065059872 | NM_030940| | HBLD2,HESB like domain containing 2 |
| 227449_at | 13.95 | 1.79E-05 | 1.270355525 | NM_004438| | EPHA4,ephrin receptor EphA4 |
| 203985_at | 13.95 | 1.79E-05 | 1.040767184 | NM_012256| | ZNF212,zinc finger protein 212 |
| 203374_s_at | 13.94 | 1.80E-05 | 1.084549629 | NM_003291| | TPP2,tripeptidyl peptidase II |
| 209569_x_at | 13.93 | 1.81E-05 | 1.903484056 | NM_001040101| | NA |
| 63009_at | 13.93 | 1.81E-05 | 1.045954278 | NM_018130| | SHQ1,SHQ1 homolog |
| 225765_at | 13.92 | 1.81E-05 | 1.121016454 | NM_002270| | TNPO1,transportin 1 |
| 229545_at | 13.92 | 1.81E-05 | 1.206164022 | NM_017671| | C20orf42,chromosome 20 open reading frame 42 |
| 1552678_a_at | 13.92 | 1.81E-05 | 1.212126781 | NM_020886| | USP28,ubiquitin specific protease 28 |
| 200614_at | 13.92 | 1.81E-05 | 1.019787133 | NM_004859| | CLTC,clathrin heavy chain 1 |
| 221831_at | 13.91 | 1.82E-05 | 1.141066933 | NM_033631| | LUZP1,leucine zipper protein 1 |
| 223337_at | 13.91 | 1.82E-05 | 1.100279132 | NM_005869| | SDCCAG10,serologically defined colon cancer antigen 10 |
| 204435_at | 13.91 | 1.82E-05 | 1.157445127 | NM_001008564| | NUPL1,nucleoporin like 1 isoform b |
| 226873_at | 13.9 | 1.83E-05 | 1.177341232 | NA |  |
| 201508_at | 13.89 | 1.84E-05 | 1.37247615 | NM_001552| | IGFBP4,insulin-like growth factor binding protein 4 |
| 205944_s_at | 13.89 | 1.84E-05 | 1.115579256 | NM_007098| | CLTCL1,clathrin, heavy polypeptide-like 1 isoform b |
| 237281_at | 13.88 | 1.85E-05 | 1.308617458 | NM_001008534| | AKAP14,A kinase (PRKA) anchor protein 14 isoform b |
| 228922_at | 13.88 | 1.84E-05 | 1.287931251 | NM_138356| | LOC90525,hypothetical protein BC007586 |
| 206777_s_at | 13.88 | 1.85E-05 | 1.089798019 | NM_000496| | CRYBB2,crystallin, beta B2 |
| 202795_x_at | 13.88 | 1.85E-05 | 1.088053562 | NM_001039141| | NA |
| 33322_i_at | 13.88 | 1.85E-05 | 1.117161632 | NM_006142| | SFN,stratifin |
| 235845_at | 13.87 | 1.85E-05 | 1.574197232 | NM_001003845| | SP5,Sp5 transcription factor |
| 204329_s_at | 13.87 | 1.85E-05 | 1.13600982 | NM_003455| | ZNF202,zinc finger protein 202 |
| 224741_x_at | 13.86 | 1.86E-05 | 1.026789051 | NA |  |
| 210621_s_at | 13.86 | 1.86E-05 | 1.138704447 | NM_002890| | RASA1,RAS p21 protein activator 1 isoform 1 |
| 223729_at | 13.86 | 1.86E-05 | 1.285891074 | NM_031413| | NA |
| 218128_at | 13.85 | 1.86E-05 | 1.130036445 | NM_006166| | NFYB,nuclear transcription factor Y, beta |
| 225667_s_at | 13.84 | 1.87E-05 | 1.68005199 | NM_145175| | NSE1,NSE1 |
| 218622_at | 13.84 | 1.87E-05 | 1.038780494 | NM_024057| | NUP37,nucleoporin 37kDa |
| 229172_at | 13.84 | 1.87E-05 | 1.539662129 | NM_052970| | HSPA12B,heat shock 70kD protein 12B |
| 218538_s_at | 13.83 | 1.88E-05 | 1.193305043 | NM_020662| | MRS2L,MRS2-like, magnesium homeostasis factor |
| 218885_s_at | 13.83 | 1.88E-05 | 1.336730921 | NM_024642| | GALNT12,UDP-N-acetyl-alpha-D-galactosamine:polypeptide |
| 204000_at | 13.83 | 1.88E-05 | 1.074817798 | NM_006578| | GNB5,guanine nucleotide-binding protein, beta-5 |
| 228252_at | 13.83 | 1.88E-05 | 1.082282792 | NM_025049| | C15orf20,DNA helicase homolog PIF1 |
| 213095_x_at | 13.82 | 1.89E-05 | 1.247035496 | NM_001623| | AIF1,allograft inflammatory factor 1 isoform 3 |
| 213610_s_at | 13.82 | 1.88E-05 | 1.061818944 | NM_144711| | MGC2610,hypothetical protein MGC2610 |
| 221881_s_at | 13.82 | 1.88E-05 | 1.372668466 | NM_013943| | CLIC4,chloride intracellular channel 4 |
| 1553874_a_at | 13.82 | 1.88E-05 | 1.838903551 | NM_032805| | ZNF206,zinc finger protein 206 |
| 213608_s_at | 13.82 | 1.89E-05 | 1.057846268 | NM_001013694| | NA |
| 218283_at | 13.82 | 1.88E-05 | 1.079361717 | NM_016305| | SS18L2,synovial sarcoma translocation gene on |
| 209179_s_at | 13.81 | 1.89E-05 | 1.0556377 | NM_024298| | LENG4,leukocyte receptor cluster (LRC) member 4 |
| 236513_at | 13.8 | 1.90E-05 | 1.350671261 | NA |  |
| 220448_at | 13.8 | 1.90E-05 | 1.523322536 | NM_022055| | KCNK12,potassium channel, subfamily K, member 12 |
| 214969_at | 13.8 | 1.90E-05 | 1.193076206 | NM_033141| | MAP3K9,mitogen-activated protein kinase kinase kinase |
| 224574_at | 13.79 | 1.91E-05 | 1.051414878 | NM_001004333| | MGC71993,similar to DNA segment, Chr 11, Brigham & Womens |
| 202252_at | 13.78 | 1.92E-05 | 1.058036228 | NM_002870| | RAB13,RAB13, member RAS oncogene family |
| 238455_at | 13.78 | 1.91E-05 | 1.679427296 | NA |  |
| 201772_at | 13.78 | 1.91E-05 | 1.154765409 | NM_015878| | OAZIN,ornithine decarboxylase antizyme inhibitor |
| 203791_at | 13.78 | 1.92E-05 | 1.148641004 | NM_005509| | DMXL1,Dmx-like 1 |
| 218451_at | 13.77 | 1.92E-05 | 1.28499809 | NM_022842| | CDCP1,CUB domain-containing protein 1 isoform 1 |
| 204422_s_at | 13.77 | 1.93E-05 | 1.318591008 | NM_002006| | FGF2,fibroblast growth factor 2 |
| 235957_at | 13.77 | 1.92E-05 | 1.686561095 | NA |  |
| 209175_at | 13.77 | 1.92E-05 | 1.104250705 | NM_007190| | SEC23IP,Sec23-interacting protein p125 |
| 204500_s_at | 13.76 | 1.93E-05 | 1.200087709 | NM_015239| | AGTPBP1,ATP/GTP binding protein 1 |
| 1553252_a_at | 13.76 | 1.93E-05 | 1.177363524 | NM_153252| | BRWD3,bromo domain-containing protein disrupted in |
| 211950_at | 13.76 | 1.93E-05 | 1.074083967 | NM_020765| | RBAF600,retinoblastoma-associated factor 600 |
| 220911_s_at | 13.76 | 1.93E-05 | 1.357783752 | NM_025081| | NA |
| 1552524_at | 13.75 | 1.94E-05 | 1.207362984 | NM_001079536| | NA |
| 209160_at | 13.75 | 1.94E-05 | 1.388004928 | NM_003739| | AKR1C3,aldo-keto reductase family 1, member C3 |
| 229997_at | 13.75 | 1.94E-05 | 1.200903806 | NM_138959| | VANGL1,vang-like 1 |
| 225165_at | 13.75 | 1.94E-05 | 1.440261747 | NM_032192| | PPP1R1B,protein phosphatase 1, regulatory (inhibitor) |
| 211965_at | 13.75 | 1.94E-05 | 1.319857121 | NM_004926| | ZFP36L1,butyrate response factor 1 |
| 201316_at | 13.75 | 1.94E-05 | 1.079031316 | NM_002787| | PSMA2,proteasome alpha 2 subunit |
| 222730_s_at | 13.75 | 1.94E-05 | 1.090024328 | NM_016353| | ZDHHC2,rec |
| 225278_at | 13.75 | 1.94E-05 | 1.114673714 | NM_005399| | PRKAB2,AMP-activated protein kinase beta 2 |
| 235226_at | 13.74 | 1.95E-05 | 1.161832418 | NM_015076| | CDC2L6,cyclin-dependent kinase (CDC2-like) 11 |
| 211097_s_at | 13.74 | 1.95E-05 | 1.287654384 | NM_002586| | PBX2,pre-B-cell leukemia transcription factor 2 |
| 231929_at | 13.73 | 1.95E-05 | 1.244954987 | NM_001079526| | NA |
| 1565162_s_at | 13.73 | 1.95E-05 | 1.087843821 | NM_020300| | MGST1,microsomal glutathione S-transferase 1 |
| 226512_at | 13.72 | 1.96E-05 | 1.155848397 | NM_003453| | ZNF198,zinc finger protein 198 |
| 221664_s_at | 13.72 | 1.96E-05 | 2.079411618 | NM_016946| | F11R,F11 receptor isoform a precursor |
| 1556301_at | 13.72 | 1.96E-05 | 1.164296589 | NA |  |
| 212557_at | 13.72 | 1.96E-05 | 1.043799203 | NM_001031623| | NA |
| 215672_s_at | 13.72 | 1.96E-05 | 1.124375127 | NM_015328| | KIAA0828,KIAA0828 protein |
| 222936_s_at | 13.72 | 1.96E-05 | 1.198428749 | NM_016076| | PNAS-4,CGI-146 protein |
| 202075_s_at | 13.72 | 1.96E-05 | 1.059005753 | NM_006227| | PLTP,phospholipid transfer protein isoform a |
| 219084_at | 13.71 | 1.97E-05 | 1.177727241 | NM_022455| | NSD1,nuclear receptor binding SET domain protein 1 |
| 218002_s_at | 13.71 | 1.97E-05 | 1.505880568 | NM_004887| | CXCL14,small inducible cytokine B14 precursor |
| 201914_s_at | 13.71 | 1.97E-05 | 1.040183004 | NM_007214| | SEC63,SEC63-like protein |
| 202922_at | 13.7 | 1.98E-05 | 1.090280583 | NM_001498| | GCLC,glutamate-cysteine ligase, catalytic subunit |
| 210278_s_at | 13.7 | 1.97E-05 | 1.16489693 | NM_007077| | AP4S1,adaptor-related protein complex 4, sigma 1 |
| 205691_at | 13.7 | 1.97E-05 | 1.134201678 | NM_004209| | SYNGR3,synaptogyrin 3 |
| 220319_s_at | 13.69 | 1.98E-05 | 1.35067206 | NM_013262| | MYLIP,myosin regulatory light chain interacting |
| 227306_at | 13.69 | 1.99E-05 | 1.359305852 | NA |  |
| 217553_at | 13.69 | 1.98E-05 | 1.678396326 | NA |  |
| 200632_s_at | 13.68 | 1.99E-05 | 1.341475338 | NM_006096| | NDRG1,N-myc downstream regulated gene 1 |
| 217127_at | 13.68 | 1.99E-05 | 1.219378801 | NM_001902| | CTH,cystathionase isoform 1 |
| 227701_at | 13.68 | 1.99E-05 | 1.40404602 | NM_018017| | C10orf118,CTCL tumor antigen L14-2 |
| 219424_at | 13.68 | 1.99E-05 | 1.426639055 | NM_005755| | EBI3,Epstein-Barr virus induced gene 3 precursor |
| 227444_at | 13.68 | 1.99E-05 | 1.18403308 | NM_152583| | ARMCX4,armadillo repeat containing, X-linked 4 |
| 200681_at | 13.67 | 1.99E-05 | 1.045603361 | NM_006708| | GLO1,glyoxalase I |
| 205441_at | 13.67 | 2.00E-05 | 1.160498461 | NM_024578| | FLJ22709,hypothetical protein FLJ22709 |
| 202013_s_at | 13.66 | 2.01E-05 | 1.066719114 | NM_000401| | EXT2,exostosin 2 |
| 205249_at | 13.66 | 2.01E-05 | 1.211520428 | NM_000399| | EGR2,early growth response 2 protein |
| 203522_at | 13.65 | 2.01E-05 | 1.097973907 | NM_005125| | CCS,copper chaperone for superoxide dismutase |
| 228151_at | 13.65 | 2.02E-05 | 1.106877584 | NA |  |
| 1555281_x_at | 13.65 | 2.01E-05 | 1.115140166 | NM_014154| | ARMC8,armadillo repeat containing 8 |
| 225135_at | 13.65 | 2.01E-05 | 1.076681617 | NM_015477| | SIN3A,transcriptional co-repressor Sin3A |
| 227796_at | 13.64 | 2.02E-05 | 1.077957312 | NM_152283| | ZFP62,zinc finger protein 62 homolog |
| 204328_at | 13.63 | 2.03E-05 | 1.376765943 | NM_001127198| | NA |
| 210601_at | 13.63 | 2.03E-05 | 1.318163383 | NM_004932| | CDH6,cadherin 6, type 2 preproprotein |
| 208651_x_at | 13.62 | 2.04E-05 | 1.274809288 | NM_013230| | CD24,CD24 antigen |
| 209992_at | 13.62 | 2.04E-05 | 1.309765624 | NM_001018053| | NA |
| 205001_s_at | 13.62 | 2.04E-05 | 1.106570895 | NM_001122665| | NA |
| 229280_s_at | 13.62 | 2.04E-05 | 1.30417402 | NA |  |
| 225730_s_at | 13.62 | 2.04E-05 | 1.044670832 | NM_001114092| | NA |
| 224180_x_at | 13.62 | 2.04E-05 | 1.217485985 | NM_001042692| | NA |
| 204459_at | 13.61 | 2.05E-05 | 1.053279299 | NM_001325| | CSTF2,cleavage stimulation factor subunit 2 |
| 218980_at | 13.6 | 2.06E-05 | 1.070621409 | NM_025135| | FHOD3,formin homology 2 domain containing 3 |
| 219438_at | 13.6 | 2.06E-05 | 1.14020961 | NM_024522| | FLJ12650,hypothetical protein FLJ12650 |
| 1555274_a_at | 13.59 | 2.07E-05 | 1.130948611 | NM_033505| | SELI,selenoprotein I |
| 228004_at | 13.58 | 2.08E-05 | 1.321280409 | NA |  |
| 214737_x_at | 13.58 | 2.07E-05 | 1.049189867 | NM_001077442| | NA |
| 230067_at | 13.58 | 2.07E-05 | 1.577088391 | NM_145019| | FLJ30707,hypothetical protein FLJ30707 |
| 231798_at | 13.58 | 2.07E-05 | 1.85082946 | NM_005450| | NOG,noggin precursor |
| 220030_at | 13.57 | 2.08E-05 | 1.487563268 | NM_018423| | STYK1,serine/threonine/tyrosine kinase 1 |
| 231919_at | 13.57 | 2.08E-05 | 1.125947074 | NM_001918| | DBT,dihydrolipoamide branched chain transacylase |
| 242481_at | 13.56 | 2.09E-05 | 1.637468066 | NA |  |
| 228360_at | 13.56 | 2.09E-05 | 1.418216977 | NM_177964| | LOC130576,hypothetical protein LOC130576 |
| 212920_at | 13.56 | 2.09E-05 | 1.180326532 | NA |  |
| 213384_x_at | 13.55 | 2.10E-05 | 1.068323633 | NM_000932| | PLCB3,phospholipase C, beta 3 |
| 1560250_s_at | 13.55 | 2.10E-05 | 1.341834878 | NA |  |
| 205141_at | 13.55 | 2.10E-05 | 1.209372857 | NM_001097577| | NA |
| 202728_s_at | 13.54 | 2.11E-05 | 1.103538339 | NM_000627| | LTBP1,latent transforming growth factor beta binding |
| 241968_at | 13.54 | 2.11E-05 | 1.116542827 | NA |  |
| 1556069_s_at | 13.54 | 2.11E-05 | 1.558666736 | NM_022462| | HIF3A,hypoxia-inducible factor-3 alpha isoform b |
| 235791_x_at | 13.54 | 2.11E-05 | 1.074531741 | NM_001270| | CHD1,chromodomain helicase DNA binding protein 1 |
| 1552388_at | 13.54 | 2.11E-05 | 1.220695266 | NA |  |
| 209709_s_at | 13.53 | 2.11E-05 | 1.144760584 | NM_012484| | HMMR,hyaluronan-mediated motility receptor isoform a |
| 209480_at | 13.53 | 2.12E-05 | 2.169461312 | NM_002123| | HLA-DQB1,major histocompatibility complex, class II, DQ |
| 204510_at | 13.53 | 2.12E-05 | 1.039689903 | NM_003503| | CDC7,CDC7 cell division cycle 7 |
| 205401_at | 13.53 | 2.12E-05 | 1.190551772 | NM_003659| | AGPS,alkylglycerone phosphate synthase precursor |
| 217608_at | 13.52 | 2.12E-05 | 1.069014202 | NM_173829| | FLJ36754,hypothetical protein FLJ36754 |
| 205162_at | 13.51 | 2.14E-05 | 1.052600211 | NM_000082| | ERCC8,excision repair cross-complementing rodent |
| 237719_x_at | 13.51 | 2.13E-05 | 1.415008872 | NM_001029875| | NA |
| 226338_at | 13.51 | 2.13E-05 | 1.18259921 | NM_018710| | DKFZp762O076,hypothetical protein DKFZp762O076 |
| 1558097_at | 13.51 | 2.14E-05 | 1.068424591 | NM_173566| | MGC50372,hypothetical protein MGC50372 |
| 226274_at | 13.51 | 2.13E-05 | 1.104645135 | NM_000084| | CLCN5,chloride channel 5 |
| 217718_s_at | 13.51 | 2.13E-05 | 1.029680302 | NM_003404| | YWHAB,tyrosine 3-monooxygenase/tryptophan |
| 202108_at | 13.5 | 2.14E-05 | 1.093003532 | NM_000285| | PEPD,Xaa-Pro dipeptidase |
| 213572_s_at | 13.5 | 2.14E-05 | 1.333455164 | NM_030666| | SERPINB1,serine (or cysteine) proteinase inhibitor, clade |
| 218195_at | 13.5 | 2.14E-05 | 1.20601189 | NM_024573| | C6orf211,chromosome 6 open reading frame 211 |
| 204670_x_at | 13.5 | 2.14E-05 | 1.347763174 | NM_001023561| | NA |
| 222835_at | 13.5 | 2.14E-05 | 1.459165707 | NM_024817| | FLJ13710,hypothetical protein FLJ13710 |
| 239147_at | 13.49 | 2.15E-05 | 1.131988927 | NM_198150| | DKFZp313G1735,hypothetical protein DKFZp313G1735 |
| 213865_at | 13.49 | 2.15E-05 | 1.147938388 | NM_080927| | DCBLD2,discoidin, CUB and LCCL domain containing 2 |
| 244533_at | 13.49 | 2.15E-05 | 1.179407366 | NA |  |
| 207501_s_at | 13.48 | 2.15E-05 | 1.394492477 | NM_004113| | FGF12,fibroblast growth factor 12 isoform 2 |
| 235017_s_at | 13.48 | 2.16E-05 | 1.575576212 | NA |  |
| 201518_at | 13.47 | 2.17E-05 | 1.068608849 | NM_006807| | CBX1,chromobox homolog 1 (HP1 beta homolog Drosophila |
| 219025_at | 13.47 | 2.17E-05 | 1.133327039 | NM_020404| | CD248,tumor endothelial marker 1 precursor |
| 214224_s_at | 13.47 | 2.17E-05 | 1.089732888 | NM_006223| | PIN4,protein (peptidyl-prolyl cis/trans isomerase) |
| 204208_at | 13.47 | 2.17E-05 | 1.10038178 | NM_003800| | RNGTT,RNA guanylyltransferase and 5'-phosphatase |
| 229982_at | 13.47 | 2.17E-05 | 1.121257265 | NM_001076786| | NA |
| 209930_s_at | 13.46 | 2.18E-05 | 1.285217829 | NM_006163| | NFE2,nuclear factor (erythroid-derived 2), 45kDa |
| 225800_at | 13.46 | 2.17E-05 | 1.315400623 | NM_175061| | JAZF1,juxtaposed with another zinc finger gene 1 |
| 225365_at | 13.46 | 2.18E-05 | 1.153231861 | NM_153251| | FLJ25952,DHHC-containing protein 20 |
| 203688_at | 13.46 | 2.18E-05 | 1.118979795 | NM_000297| | PKD2,polycystin 2 |
| 221047_s_at | 13.44 | 2.20E-05 | 1.119401722 | NM_018650| | MARK1,MAP/microtubule affinity-regulating kinase 1 |
| 202234_s_at | 13.44 | 2.19E-05 | 1.108965957 | NM_003051| | SLC16A1,solute carrier family 16, member 1 |
| 1555742_at | 13.44 | 2.19E-05 | 1.333877832 | NA |  |
| 215051_x_at | 13.44 | 2.19E-05 | 1.328166541 | NM_001623| | AIF1,allograft inflammatory factor 1 isoform 3 |
| 204832_s_at | 13.43 | 2.20E-05 | 1.128303644 | NM_004329| | BMPR1A,bone morphogenetic protein receptor, type IA |
| 229302_at | 13.43 | 2.20E-05 | 1.233725406 | NM_152390| | MGC33926,hypothetical protein MGC33926 |
| 243805_at | 13.43 | 2.20E-05 | 1.396788243 | NM_133459| | CCBE1,collagen and calcium binding EGF domains 1 |
| 201078_at | 13.43 | 2.20E-05 | 1.077411152 | NM_004800| | TM9SF2,transmembrane 9 superfamily member 2 |
| 218522_s_at | 13.43 | 2.21E-05 | 1.109643461 | NM_018174| | BPY2IP1,BPY2 interacting protein 1 |
| 225210_s_at | 13.43 | 2.20E-05 | 1.133938287 | NM_031452| | MGC2560,hypothetical LOC83640 |
| 227015_at | 13.42 | 2.21E-05 | 1.293593036 | NM_020437| | LOC57168,similar to aspartate beta hydroxylase (ASPH) |
| 220089_at | 13.42 | 2.21E-05 | 1.0839969 | NM_024884| | C14orf160,chromosome 14 open reading frame 160 |
| 229012_at | 13.42 | 2.21E-05 | 1.253427298 | NM_032596| | C9orf24,testes development-related NYD-SP22 isoform 1 |
| 223426_s_at | 13.42 | 2.21E-05 | 1.1344916 | NM_018424| | EPB41L4B,erythrocyte membrane protein band 4.1 like 4B |
| 238860_at | 13.41 | 2.21E-05 | 1.113939273 | NM_145063| | C6orf130,chromosome 6 open reading frame 130 |
| 223211_at | 13.41 | 2.22E-05 | 1.112802988 | NM_012260| | HPCL2,2-hydroxyphytanoyl-CoA lyase |
| 226870_at | 13.41 | 2.22E-05 | 1.232126514 | NM_144589| | COMTD1,catechol-O-methyltransferase domain containing |
| 206114_at | 13.41 | 2.22E-05 | 1.170370404 | NM_004438| | EPHA4,ephrin receptor EphA4 |
| 219342_at | 13.4 | 2.23E-05 | 1.166827031 | NM_022900| | CAS1,O-acetyltransferase |
| 229018_at | 13.4 | 2.23E-05 | 1.12514132 | NM_032230| | FLJ22789,hypothetical protein FLJ22789 |
| 202449_s_at | 13.4 | 2.23E-05 | 1.043065034 | NM_002957| | RXRA,retinoid X receptor, alpha |
| 202468_s_at | 13.39 | 2.24E-05 | 1.047026753 | NM_003798| | CTNNAL1,catenin, alpha-like 1 |
| 221841_s_at | 13.39 | 2.24E-05 | 1.337121925 | NM_004235| | KLF4,Kruppel-like factor 4 (gut) |
| 216550_x_at | 13.38 | 2.24E-05 | 1.114716845 | NM_001083625| | NA |
| 228249_at | 13.38 | 2.25E-05 | 1.118957045 | NM_138787| | LOC119710,hypothetical protein BC009561 |
| 225694_at | 13.37 | 2.26E-05 | 1.043525969 | NM_015083| | NA |
| 225217_s_at | 13.37 | 2.25E-05 | 1.117816451 | NM_015695| | BRPF3,bromodomain and PHD finger containing, 3 |
| 232687_at | 13.37 | 2.26E-05 | 1.539398714 | NA |  |
| 235253_at | 13.36 | 2.26E-05 | 1.096041828 | NM_002853| | RAD1,RAD1 homolog isoform 1 |
| 221928_at | 13.36 | 2.26E-05 | 1.1346439 | NM_001093| | ACACB,acetyl-Coenzyme A carboxylase beta |
| 225954_s_at | 13.36 | 2.27E-05 | 1.086504405 | NM_177401| | MIDN,midnolin |
| 202450_s_at | 13.35 | 2.28E-05 | 1.409639082 | NM_000396| | CTSK,cathepsin K preproprotein |
| 216304_x_at | 13.35 | 2.28E-05 | 1.04086658 | NM_014263| | YME1L1,YME1-like 1 isoform 3 |
| 224894_at | 13.35 | 2.28E-05 | 1.13119436 | NM_006106| | YAP1,Yes-associated protein 1, 65 kD |
| 239344_at | 13.34 | 2.29E-05 | 1.198817241 | NA |  |
| 210551_s_at | 13.34 | 2.29E-05 | 1.305607734 | NM_004043| | ASMT,acetylserotonin O-methyltransferase |
| 215167_at | 13.34 | 2.29E-05 | 1.123440587 | NM_004229| | CRSP2,cofactor required for Sp1 transcriptional |
| 209945_s_at | 13.34 | 2.29E-05 | 1.08067757 | NM_002093| | GSK3B,glycogen synthase kinase 3 beta |
| 224665_at | 13.33 | 2.30E-05 | 1.048095921 | NM_173473| | C10orf104,chromosome 10 open reading frame 104 |
| 229830_at | 13.33 | 2.30E-05 | 1.210350509 | NA |  |
| 209016_s_at | 13.33 | 2.30E-05 | 1.774520618 | NM_005556| | KRT7,keratin 7 |
| 219079_at | 13.33 | 2.30E-05 | 1.086317324 | NM_016230| | NCB5OR,NADPH cytochrome B5 oxidoreductase |
| 204009_s_at | 13.33 | 2.30E-05 | 1.084219046 | NM_004985| | KRAS,c-K-ras2 protein isoform b |
| 218604_at | 13.32 | 2.31E-05 | 1.090924415 | NM_014319| | LEMD3,LEM domain containing 3 |
| 220195_at | 13.32 | 2.31E-05 | 1.155767061 | NM_018328| | MBD5,methyl-CpG binding domain protein 5 |
| 224671_at | 13.32 | 2.30E-05 | 1.051031214 | NM_145255| | MRPL10,mitochondrial ribosomal protein L10 isoform a |
| 202596_at | 13.32 | 2.30E-05 | 1.080208665 | NM_004436| | ENSA,endosulfine alpha isoform 3 |
| 201900_s_at | 13.32 | 2.31E-05 | 1.05105452 | NM_006066| | AKR1A1,aldo-keto reductase family 1, member A1 |
| 228611_s_at | 13.31 | 2.32E-05 | 1.152149006 | NA |  |
| 221648_s_at | 13.31 | 2.31E-05 | 1.530907553 | NA |  |
| 201555_at | 13.31 | 2.31E-05 | 1.062641343 | NM_002388| | MCM3,minichromosome maintenance protein 3 |
| 209076_s_at | 13.31 | 2.31E-05 | 1.033016807 | NM_019613| | WDR45L,WDR45-like |
| 209174_s_at | 13.31 | 2.31E-05 | 1.040233489 | NM_017730| | FLJ20259,FLJ20259 protein |
| 212603_at | 13.31 | 2.31E-05 | 1.033033951 | NM_005830| | MRPS31,mitochondrial ribosomal protein S31 |
| 227408_s_at | 13.31 | 2.31E-05 | 1.169937933 | NM_031953| | SNX25,sorting nexin 25 |
| 209472_at | 13.31 | 2.31E-05 | 1.106964267 | NM_001008661| | KAT3,kynurenine aminotransferase III isoform 1 |
| 219114_at | 13.3 | 2.32E-05 | 1.129200809 | NM_016210| | C3orf18,chromosome 3 open reading frame 18 |
| 204601_at | 13.3 | 2.32E-05 | 1.130909042 | NM_153029| | N4BP1,Nedd4 binding protein 1 |
| 213900_at | 13.3 | 2.32E-05 | 1.406221248 | NM_004816| | C9orf61,chromosome 9 open reading frame 61 |
| 208836_at | 13.3 | 2.32E-05 | 1.027588879 | NM_001679| | ATP1B3,Na+/K+ -ATPase beta 3 subunit |
| 241416_at | 13.3 | 2.32E-05 | 1.267223397 | NA |  |
| 208306_x_at | 13.3 | 2.32E-05 | 1.286937948 | NM_002124| | HLA-DRB1,major histocompatibility complex, class II, DR |
| 210101_x_at | 13.3 | 2.32E-05 | 1.052039619 | NM_016009| | SH3GLB1,SH3-containing protein SH3GLB1 |
| 204454_at | 13.29 | 2.33E-05 | 1.132397853 | NM_012317| | LDOC1,leucine zipper, down-regulated in cancer 1 |
| 229263_at | 13.29 | 2.33E-05 | 1.198861711 | NM_001080973| | NA |
| 229933_at | 13.29 | 2.33E-05 | 1.271889637 | NM_152485| | FLJ25078,hypothetical protein FLJ25078 |
| 202965_s_at | 13.27 | 2.35E-05 | 1.760846711 | NM_014289| | CAPN6,calpain 6 |
| 229373_at | 13.27 | 2.36E-05 | 1.36614945 | NA |  |
| 213484_at | 13.27 | 2.36E-05 | 1.504226085 | NA |  |
| 222244_s_at | 13.27 | 2.35E-05 | 1.058873647 | NA |  |
| 213682_at | 13.26 | 2.37E-05 | 1.035470505 | NM_007172| | NUP50,nucleoporin 50kDa isoform b |
| 235269_at | 13.26 | 2.37E-05 | 1.527794271 | NM_138435| | LOC113828,hypothetical protein BC011204 |
| 209205_s_at | 13.26 | 2.37E-05 | 1.034656169 | NM_006769| | LMO4,LIM domain only 4 |
| 212513_s_at | 13.25 | 2.37E-05 | 1.046375861 | NM_015017| | USP33,ubiquitin specific protease 33 isoform 1 |
| 42361_g_at | 13.24 | 2.39E-05 | 1.110818846 | NM_001105563| | NA |
| 218456_at | 13.24 | 2.38E-05 | 1.088954001 | NM_001002259| | C1QDC1,C1q domain containing 1 isoform 1 |
| 238551_at | 13.24 | 2.39E-05 | 1.106238354 | NM_173540| | FUT11,fucosyltransferase 11 (alpha (1,3) |
| 210554_s_at | 13.24 | 2.38E-05 | 1.072727142 | NM_001083914| | NA |
| 222067_x_at | 13.23 | 2.39E-05 | 1.12582178 | NM_021063| | HIST1H2BD,H2B histone family, member B |
| 221025_x_at | 13.23 | 2.39E-05 | 1.167306854 | NM_001098614| | NA |
| 208857_s_at | 13.22 | 2.40E-05 | 1.077121014 | NM_005389| | PCMT1,protein-L-isoaspartate (D-aspartate) |
| 218847_at | 13.22 | 2.40E-05 | 1.092801937 | NM_001007225| | IMP-2,IGF-II mRNA-binding protein 2 isoform b |
| 219966_x_at | 13.22 | 2.41E-05 | 1.067818401 | NM_017869| | BANP,BTG3 associated nuclear protein isoform a |
| 224707_at | 13.22 | 2.40E-05 | 1.055909985 | NM_032412| | ORF1-FL49,putative nuclear protein ORF1-FL49 |
| 244650_at | 13.22 | 2.41E-05 | 1.239485698 | NA |  |
| 228856_at | 13.22 | 2.41E-05 | 1.641602934 | NM_023931| | MGC2474,hypothetical protein MGC2474 |
| 207267_s_at | 13.21 | 2.42E-05 | 1.360134221 | NM_018962| | DSCR6,Down syndrome critical region protein 6 |
| 204173_at | 13.21 | 2.42E-05 | 1.06517082 | NM_002475| | MLC1SA,myosin alkali light chain 1 slow a |
| 227947_at | 13.21 | 2.41E-05 | 1.259226845 | NM_001100164| | NA |
| 229944_at | 13.21 | 2.42E-05 | 1.895338641 | NM_000912| | OPRK1,opioid receptor, kappa 1 |
| 220091_at | 13.21 | 2.42E-05 | 1.13490389 | NM_017585| | SLC2A6,solute carrier family 2 (facilitated glucose |
| 225239_at | 13.21 | 2.42E-05 | 1.236597502 | NA |  |
| 223461_at | 13.21 | 2.41E-05 | 1.047322806 | NM_016495| | TBC1D7,TBC1 domain family, member 7 |
| 228868_x_at | 13.19 | 2.43E-05 | 1.08368291 | NM_030928| | CDT1,DNA replication factor |
| 212931_at | 13.19 | 2.43E-05 | 1.097370974 | NM_005650| | TCF20,transcription factor 20 isoform 1 |
| 213802_at | 13.18 | 2.45E-05 | 1.298862931 | NA |  |
| 222955_s_at | 13.18 | 2.44E-05 | 1.106552303 | NM_018472| | FAM45B,family with sequence similarity 45, member B |
| 201026_at | 13.18 | 2.44E-05 | 1.037082159 | NM_015904| | EIF5B,translation initiation factor IF2 |
| 222590_s_at | 13.18 | 2.45E-05 | 1.158807025 | NM_016231| | NLK,nemo like kinase |
| 232263_at | 13.17 | 2.45E-05 | 1.444132274 | NM_018057| | SLC6A15,solute carrier family 6, member 15 isoform 2 |
| 223433_at | 13.17 | 2.45E-05 | 1.047937932 | NM_020192| | C7orf36,chromosome 7 open reading frame 36 |
| 221475_s_at | 13.16 | 2.47E-05 | 1.014348478 | NM_002948| | RPL15,ribosomal protein L15 |
| 241808_at | 13.16 | 2.47E-05 | 1.350727993 | NA |  |
| 210839_s_at | 13.15 | 2.48E-05 | 1.387242496 | NM_001040092| | NA |
| 227727_at | 13.15 | 2.48E-05 | 1.667648814 | NM_001098515| | NA |
| 216693_x_at | 13.15 | 2.48E-05 | 1.094493286 | NM_016073| | HDGFRP3,hepatoma-derived growth factor, related protein |
| 230466_s_at | 13.14 | 2.48E-05 | 1.27393454 | NA |  |
| 64432_at | 13.13 | 2.49E-05 | 1.048502086 | NA |  |
| 200971_s_at | 13.13 | 2.50E-05 | 1.047576086 | NM_014445| | SERP1,stress-associated endoplasmic reticulum protein |
| 235648_at | 13.13 | 2.50E-05 | 1.091712603 | NM_152603| | ZNF567,zinc finger protein 567 |
| 224578_at | 13.13 | 2.50E-05 | 1.043753786 | NM_018715| | TD-60,RCC1-like |
| 225755_at | 13.13 | 2.50E-05 | 1.075821405 | NM_173546| | MGC35097,hypothetical protein MGC35097 |
| 229874_x_at | 13.12 | 2.50E-05 | 1.241309904 | NA |  |
| 217028_at | 13.12 | 2.50E-05 | 1.126435914 | NM_001008540| | CXCR4,chemokine (C-X-C motif) receptor 4 isoform a |
| 235969_at | 13.12 | 2.51E-05 | 1.225946838 | NA |  |
| 205700_at | 13.12 | 2.51E-05 | 1.247715154 | NM_003725| | RODH,3-hydroxysteroid epimerase |
| 229778_at | 13.11 | 2.52E-05 | 1.575362267 | NM_030572| | MGC10946,hypothetical protein MGC10946 |
| 232124_at | 13.11 | 2.52E-05 | 1.414934773 | NA |  |
| 218479_s_at | 13.11 | 2.52E-05 | 1.103272168 | NM_022459| | XPO4,exportin 4 |
| 213729_at | 13.1 | 2.53E-05 | 1.052968376 | NM_017892| | NA |
| AFFX-HUMISGF3A/M97935_3_at | 13.1 | 2.52E-05 | 1.096699997 | NM_007315| | STAT1,signal transducer and activator of transcription |
| 1564383_s_at | 13.1 | 2.52E-05 | 1.605061466 | NA |  |
| 227413_at | 13.1 | 2.53E-05 | 1.073324081 | NM_145049| | MGC10067,hypothetical protein MGC10067 |
| 219517_at | 13.1 | 2.53E-05 | 1.17205653 | NM_025165| | ELL3,elongation factor RNA polymerase II-like 3 |
| 201833_at | 13.09 | 2.54E-05 | 1.066487347 | NM_001527| | HDAC2,histone deacetylase 2 |
| 35148_at | 13.09 | 2.54E-05 | 1.474016201 | NM_014428| | TJP3,tight junction protein 3 (zona occludens 3) |
| 204350_s_at | 13.09 | 2.54E-05 | 1.059268013 | NM_001100816| | NA |
| 1553169_at | 13.08 | 2.55E-05 | 1.246482555 | NM_152611| | C20orf75,chromosome 20 open reading frame 75 |
| 235348_at | 13.08 | 2.55E-05 | 1.164326355 | NM_032859| | C13orf6,chromosome 13 open reading frame 6 |
| 237054_at | 13.08 | 2.55E-05 | 1.713507038 | NM_021572| | ENPP5,ectonucleotide pyrophosphatase/phosphodiesterase |
| 213438_at | 13.07 | 2.56E-05 | 1.266260351 | NM_015090| | NFASC,neurofascin isoform 2 |
| 244111_at | 13.07 | 2.57E-05 | 1.534073532 | NM_152349| | MGC45562,hypothetical protein MGC45562 |
| 239647_at | 13.07 | 2.56E-05 | 1.411200387 | NM_152889| | CHST13,carbohydrate (chondroitin 4) sulfotransferase |
| 233543_s_at | 13.06 | 2.57E-05 | 1.136899239 | NM_139076| | FLJ13614,hypothetical protein FLJ13614 |
| 201847_at | 13.06 | 2.58E-05 | 1.030091017 | NM_000235| | LIPA,lipase A precursor |
| 221156_x_at | 13.06 | 2.57E-05 | 1.244907631 | NM_004748| | CCPG1,cell cycle progression 1 |
| 210582_s_at | 13.05 | 2.58E-05 | 1.137415715 | NM_001031801| | NA |
| 233205_at | 13.05 | 2.59E-05 | 1.303818956 | NA |  |
| 228109_at | 13.04 | 2.60E-05 | 1.295618943 | NM_006909| | RASGRF2,Ras protein-specific guanine |
| 214111_at | 13.04 | 2.60E-05 | 1.511001201 | NM_001012393| | OPCML,opioid binding protein/cell adhesion |
| 228812_at | 13.04 | 2.59E-05 | 1.153675366 | NA |  |
| 226472_at | 13.04 | 2.59E-05 | 1.107205739 | NM_139126| | PPIL4,peptidylprolyl isomerase-like 4 |
| 210740_s_at | 13.04 | 2.59E-05 | 1.092274919 | NM_014216| | ITPK1,inositol 1,3,4-triphosphate 5/6 kinase |
| 219426_at | 13.04 | 2.60E-05 | 1.074085564 | NM_024852| | EIF2C3,eukaryotic translation initiation factor 2C, 3 |
| 235176_at | 13.03 | 2.60E-05 | 1.390429155 | NM_133466| | ZNF545,zinc finger protein 545 |
| 226759_at | 13.03 | 2.60E-05 | 1.18419265 | NM_022465| | ZNFN1A4,zinc finger protein, subfamily 1A, 4 |
| 235609_at | 13.03 | 2.61E-05 | 1.071971444 | NA |  |
| 59999_at | 13.03 | 2.60E-05 | 1.096266862 | NM_017902| | HIF1AN,hypoxia-inducible factor 1, alpha subunit |
| 202146_at | 13.02 | 2.61E-05 | 1.168041386 | NM_001007245| | IFRD1,interferon-related developmental regulator 1 |
| 226789_at | 13.02 | 2.61E-05 | 2.273132477 | NA |  |
| 224624_at | 13.02 | 2.61E-05 | 1.070468853 | NM_001127244| | NA |
| 214244_s_at | 13.02 | 2.62E-05 | 1.123607136 | NM_003945| | ATP6V0E,ATPase, H+ transporting, lysosomal, V0 subunit |
| 224521_s_at | 13.02 | 2.62E-05 | 1.052202663 | NM_032358| | MGC13183,hypothetical protein MGC13183 |
| 220327_at | 13.01 | 2.63E-05 | 1.762104808 | NM_016206| | VGL-3,colon carcinoma related protein |
| 204309_at | 13.01 | 2.63E-05 | 1.303195793 | NM_000781| | CYP11A1,cytochrome P450, subfamily XIA precursor |
| 214714_at | 13.01 | 2.62E-05 | 1.093156394 | NM_032164| | ZNF394,zinc finger protein 99 |
| 213358_at | 13.01 | 2.62E-05 | 1.120008805 | NM_015210| | KIAA0802,KIAA0802 |
| 219319_at | 13 | 2.64E-05 | 1.246424942 | NM_022462| | HIF3A,hypoxia-inducible factor-3 alpha isoform b |
| 219649_at | 13 | 2.63E-05 | 1.074192298 | NM_013339| | ALG6,dolichyl-P-Glc:Man9GlcNAc2-PP- |
| 226330_s_at | 13 | 2.63E-05 | 1.066106467 | NM_001014286| | NA |
| 203153_at | 13 | 2.64E-05 | 1.509163597 | NM_001548| | IFIT1,interferon-induced protein with |
| 33323_r_at | 12.98 | 2.66E-05 | 1.396722426 | NM_006142| | SFN,stratifin |
| 222466_s_at | 12.98 | 2.66E-05 | 1.062140015 | NM_014050| | MRPL42,mitochondrial ribosomal protein L42 isoform a |
| 229327_s_at | 12.98 | 2.66E-05 | 1.586617241 | NA |  |
| 205761_s_at | 12.98 | 2.66E-05 | 1.069099656 | NM_181581| | PP35,protein similar to E.coli yhdg and R. capsulatus |
| 1568617_a_at | 12.98 | 2.67E-05 | 1.673867136 | NM_001080429| | NA |
| 202525_at | 12.98 | 2.66E-05 | 1.810987062 | NM_002773| | PRSS8,prostasin preproprotein |
| 219988_s_at | 12.98 | 2.66E-05 | 1.053479067 | NM_018150| | FLJ10597,hypothetical protein FLJ10597 |
| 227653_at | 12.97 | 2.67E-05 | 1.043047327 | NM_020810| | KIAA1393,tRNA-(N1G37) methyltransferase |
| 238750_at | 12.97 | 2.67E-05 | 1.238002022 | NA |  |
| 205899_at | 12.97 | 2.67E-05 | 1.321775661 | NM_001111045| | NA |
| 202054_s_at | 12.97 | 2.67E-05 | 1.098118166 | NM_000382| | ALDH3A2,aldehyde dehydrogenase 3A2 |
| 226576_at | 12.96 | 2.69E-05 | 1.640752138 | NM_015071| | ARHGAP26,GTPase regulator associated with the focal |
| 217778_at | 12.95 | 2.69E-05 | 1.145825565 | NM_014437| | SLC39A1,solute carrier family 39 (zinc transporter), |
| 242560_at | 12.95 | 2.70E-05 | 1.060340863 | NM_001018115| | NA |
| 203998_s_at | 12.95 | 2.70E-05 | 1.212131005 | NM_005639| | SYT1,synaptotagmin I |
| 229289_at | 12.95 | 2.70E-05 | 1.324412247 | NM_138411| | NA |
| 230006_s_at | 12.95 | 2.70E-05 | 1.387847285 | NM_148893| | NA |
| 1558794_at | 12.94 | 2.70E-05 | 1.268510526 | NA |  |
| 238740_at | 12.94 | 2.71E-05 | 1.458351661 | NM_025267| | MGC2744,hypothetical protein MGC2744 |
| 219615_s_at | 12.93 | 2.72E-05 | 1.765058479 | NM_003740| | KCNK5,potassium channel, subfamily K, member 5 |
| 208761_s_at | 12.93 | 2.72E-05 | 1.063665307 | NM_001005781| | SUMO1,small ubiquitin-like modifier 1 isoform a |
| 212762_s_at | 12.93 | 2.71E-05 | 1.153021023 | NM_030756| | TCF7L2,transcription factor 7-like 2 (T-cell specific, |
| 203905_at | 12.92 | 2.73E-05 | 1.030102116 | NM_002582| | PARN,poly(A)-specific ribonuclease (deadenylation |
| 236045_x_at | 12.92 | 2.74E-05 | 1.447382863 | NA |  |
| 204076_at | 12.92 | 2.74E-05 | 1.108395652 | NM_004901| | ENTPD4,ectonucleoside triphosphate diphosphohydrolase |
| 208755_x_at | 12.91 | 2.74E-05 | 1.017011205 | NM_002107| | H3F3A,H3 histone, family 3A |
| 218967_s_at | 12.91 | 2.74E-05 | 1.154007437 | NM_001001484| | PTER,phosphotriesterase related |
| 206924_at | 12.9 | 2.75E-05 | 1.354229609 | NM_000641| | IL11,interleukin 11 precursor |
| 207008_at | 12.9 | 2.75E-05 | 1.49872755 | NM_001557| | IL8RB,interleukin 8 receptor beta |
| 238654_at | 12.9 | 2.75E-05 | 1.412811544 | NA |  |
| 213926_s_at | 12.9 | 2.76E-05 | 1.15401891 | NM_004504| | HRB,HIV-1 Rev binding protein |
| 214866_at | 12.89 | 2.76E-05 | 1.178342052 | NM_001005376| | PLAUR,plasminogen activator, urokinase receptor |
| 213073_at | 12.89 | 2.76E-05 | 1.05779316 | NM_015346| | ZFYVE26,zinc finger, FYVE domain containing 26 |
| 238537_at | 12.89 | 2.76E-05 | 1.074159522 | NA |  |
| 232720_at | 12.89 | 2.76E-05 | 1.737673155 | NM_152570| | FLJ31810,hypothetical protein FLJ31810 |
| 228567_at | 12.88 | 2.78E-05 | 1.064927557 | NA |  |
| 212591_at | 12.88 | 2.78E-05 | 1.038155546 | NM_015014| | KIAA0117,KIAA0117 protein |
| 200726_at | 12.87 | 2.79E-05 | 1.034023002 | NM_002710| | PPP1CC,protein phosphatase 1, catalytic subunit, gamma |
| 212886_at | 12.86 | 2.80E-05 | 1.78668388 | NM_015621| | DKFZP434C171,DKFZP434C171 protein |
| 238122_at | 12.86 | 2.80E-05 | 1.141657599 | NM_203390| | LOC389677,similar to RIKEN cDNA 3000004N20 |
| 1553171_x_at | 12.86 | 2.80E-05 | 1.331577354 | NM_152611| | C20orf75,chromosome 20 open reading frame 75 |
| 233911_s_at | 12.86 | 2.80E-05 | 1.419514568 | NM_020700| | NA |
| 233413_at | 12.86 | 2.80E-05 | 1.652425603 | NA |  |
| 203494_s_at | 12.86 | 2.80E-05 | 1.101098294 | NM_014679| | PIG8,translokin |
| 206359_at | 12.86 | 2.80E-05 | 1.352213897 | NM_003955| | SOCS3,suppressor of cytokine signaling 3 |
| 209243_s_at | 12.86 | 2.80E-05 | 1.105102947 | NM_006210| | PEG3,paternally expressed 3 |
| 231775_at | 12.85 | 2.81E-05 | 1.64258978 | NM_003844| | TNFRSF10A,tumor necrosis factor receptor superfamily, |
| 213907_at | 12.85 | 2.81E-05 | 1.100133302 | NM_004280| | EEF1E1,eukaryotic translation elongation factor 1 |
| 219870_at | 12.85 | 2.81E-05 | 1.092990042 | NM_024997| | ATF7IP2,activating transcription factor 7 interacting |
| 215354_s_at | 12.85 | 2.81E-05 | 1.112401404 | NM_014389| | PELP1,proline-, glutamic acid-, leucine-rich protein |
| 226917_s_at | 12.84 | 2.82E-05 | 1.054396413 | NM_013367| | ANAPC4,anaphase-promoting complex subunit 4 |
| 204286_s_at | 12.84 | 2.82E-05 | 1.274988045 | NM_021127| | PMAIP1,phorbol-12-myristate-13-acetate-induced protein |
| 230136_at | 12.84 | 2.83E-05 | 1.169740333 | NA |  |
| 207558_s_at | 12.84 | 2.82E-05 | 1.924934903 | NM_000325| | PITX2,paired-like homeodomain transcription factor 2 |
| 229225_at | 12.84 | 2.82E-05 | 1.313324616 | NM_003872| | NRP2,neuropilin 2 isoform 2 precursor |
| 240293_at | 12.83 | 2.83E-05 | 1.307531949 | NM_001033658| | NA |
| 200951_s_at | 12.83 | 2.83E-05 | 1.127269274 | NM_001759| | CCND2,cyclin D2 |
| 212809_at | 12.83 | 2.83E-05 | 1.079918921 | NM_032815| | NFATC2IP,nuclear factor of activated T-cells, |
| 204618_s_at | 12.83 | 2.83E-05 | 1.07170357 | NM_002041| | GABPB2,GA binding protein transcription factor, beta |
| 205858_at | 12.83 | 2.84E-05 | 1.255666107 | NM_002507| | NGFR,nerve growth factor receptor precursor |
| 241879_at | 12.82 | 2.84E-05 | 1.219422817 | NA |  |
| 203920_at | 12.82 | 2.85E-05 | 1.11369707 | NM_005693| | NR1H3,nuclear receptor subfamily 1, group H, member 3 |
| 204901_at | 12.82 | 2.85E-05 | 1.099751673 | NM_003939| | BTRC,beta-transducin repeat containing protein |
| 200851_s_at | 12.82 | 2.85E-05 | 1.039270305 | NM_014761| | KIAA0174,KIAA0174 gene product |
| 228018_at | 12.81 | 2.86E-05 | 1.180889279 | NM_152864| | C20orf58,chromosome 20 open reading frame 58 |
| 224753_at | 12.81 | 2.86E-05 | 1.017227885 | NM_080668| | CDCA5,cell division cycle associated 5 |
| 206987_x_at | 12.8 | 2.88E-05 | 1.468208973 | NM_003862| | FGF18,fibroblast growth factor 18 precursor |
| 1554125_a_at | 12.79 | 2.88E-05 | 1.714763272 | NM_014893| | NLGN4Y,neuroligin 4, Y-linked |
| 231897_at | 12.79 | 2.89E-05 | 1.063054326 | NM_012212| | LTB4DH,NADP-dependent leukotriene B4 |
| 219837_s_at | 12.79 | 2.89E-05 | 1.354603862 | NM_018659| | CYTL1,cytokine-like 1 |
| 242141_at | 12.78 | 2.90E-05 | 1.134288931 | NM_001527| | HDAC2,histone deacetylase 2 |
| 205747_at | 12.78 | 2.90E-05 | 1.348761298 | NM_004352| | CBLN1,cerebellin |
| 215722_s_at | 12.78 | 2.90E-05 | 1.049929272 | NM_003090| | SNRPA1,small nuclear ribonucleoprotein polypeptide A' |
| 201276_at | 12.78 | 2.89E-05 | 1.072720033 | NM_002868| | RAB5B,RAB5B, member RAS oncogene family |
| 223146_at | 12.78 | 2.90E-05 | 1.140951688 | NM_001006622| | WDR33,WD repeat domain 33 isoform 2 |
| 227432_s_at | 12.77 | 2.92E-05 | 1.191119407 | NA |  |
| 201344_at | 12.77 | 2.92E-05 | 1.088173819 | NM_003339| | UBE2D2,ubiquitin-conjugating enzyme E2D 2 isoform 1 |
| 230464_at | 12.76 | 2.92E-05 | 1.297420507 | NM_030760| | EDG8,endothelial differentiation, sphingolipid |
| 218325_s_at | 12.76 | 2.92E-05 | 1.142889223 | NM_022105| | DATF1,death associated transcription factor 1 isoform |
| 204415_at | 12.75 | 2.94E-05 | 1.259800604 | NM_002038| | G1P3,interferon induced 6-16 protein isoform a |
| 207785_s_at | 12.75 | 2.93E-05 | 1.0873291 | NM_005349| | RBPSUH,recombining binding protein suppressor of |
| 228190_at | 12.73 | 2.97E-05 | 1.123342247 | NM_014633| | SH2BP1,SH2 domain binding protein 1 |
| 206619_at | 12.73 | 2.97E-05 | 1.453213124 | NM_014420| | DKK4,dickkopf homolog 4 |
| 218853_s_at | 12.73 | 2.97E-05 | 1.101009699 | NM_019556| | MOSPD1,motile sperm domain containing 1 |
| 1559072_a_at | 12.73 | 2.97E-05 | 1.104254799 | NM_052906| | KIAA1904,KIAA1904 protein |
| 225798_at | 12.73 | 2.96E-05 | 1.173144555 | NM_175061| | JAZF1,juxtaposed with another zinc finger gene 1 |
| 228027_at | 12.73 | 2.97E-05 | 1.097581335 | NM_001004051| | GPRASP2,G protein-coupled receptor associated sorting |
| 212911_at | 12.72 | 2.97E-05 | 1.11409286 | NM_015291| | KIAA0962,KIAA0962 protein |
| 226901_at | 12.72 | 2.97E-05 | 1.052610275 | NM_181655| | LOC284018,hypothetical protein LOC284018 isoform a |
| 205894_at | 12.72 | 2.97E-05 | 1.30549308 | NM_000047| | ARSE,arylsulfatase E precursor |
| 218660_at | 12.71 | 3.00E-05 | 1.215176396 | NM_003494| | DYSF,dysferlin |
| 230920_at | 12.7 | 3.01E-05 | 1.441850927 | NA |  |
| 209304_x_at | 12.7 | 3.00E-05 | 1.07937173 | NM_015675| | GADD45B,growth arrest and DNA-damage-inducible, beta |
| 221447_s_at | 12.7 | 3.01E-05 | 1.713006514 | NM_031302| | GLT8D2,glycosyltransferase 8 domain containing 2 |
| 209602_s_at | 12.69 | 3.02E-05 | 1.683104033 | NM_001002295| | GATA3,GATA binding protein 3 isoform 1 |
| 217949_s_at | 12.69 | 3.03E-05 | 1.03052504 | NM_024006| | VKORC1,vitamin K epoxide reductase complex, subunit 1 |
| 223231_at | 12.69 | 3.02E-05 | 1.051108758 | NM_032026| | TATDN1,TatD DNase domain containing 1 |
| 213183_s_at | 12.68 | 3.03E-05 | 1.409247567 | NM_000076| | CDKN1C,cyclin-dependent kinase inhibitor 1C |
| 222586_s_at | 12.68 | 3.03E-05 | 1.100677984 | NM_022776| | OSBPL11,oxysterol-binding protein-like protein 11 |
| 232847_at | 12.68 | 3.04E-05 | 1.224965879 | NM_171999| | SALL3,sal-like 3 |
| 219349_s_at | 12.68 | 3.03E-05 | 1.14225573 | NM_018303| | SEC5L1,Sec5 protein |
| 226222_at | 12.68 | 3.04E-05 | 1.119672242 | NM_020829| | NA |
| 224793_s_at | 12.67 | 3.04E-05 | 1.092383151 | NM_004612| | TGFBR1,transforming growth factor, beta receptor I |
| 244640_at | 12.67 | 3.04E-05 | 1.360473344 | NA |  |
| 219311_at | 12.67 | 3.05E-05 | 1.070737648 | NM_024899| | C18orf9,chromosome 18 open reading frame 9 |
| 212529_at | 12.67 | 3.04E-05 | 1.051609556 | NM_152344| | FLJ30656,hypothetical protein FLJ30656 |
| 201569_s_at | 12.67 | 3.04E-05 | 1.027600988 | NM_015380| | CGI-51,CGI-51 protein |
| 202223_at | 12.67 | 3.04E-05 | 1.030590633 | NM_152713| | ITM1,integral membrane protein 1 |
| 220115_s_at | 12.66 | 3.06E-05 | 1.565356337 | NM_006727| | CDH10,cadherin 10, type 2 preproprotein |
| 234924_s_at | 12.66 | 3.06E-05 | 1.108817078 | NM_020832| | KIAA1441,KIAA1441 protein |
| 210242_x_at | 12.65 | 3.07E-05 | 1.123751703 | NM_001100879| | NA |
| 202667_s_at | 12.65 | 3.07E-05 | 1.042078276 | NM_001077516| | NA |
| 219081_at | 12.65 | 3.07E-05 | 1.070451176 | NM_003732| | EIF4EBP3,eukaryotic translation initiation factor 4E |
| 205627_at | 12.64 | 3.09E-05 | 1.735833979 | NM_001785| | CDA,cytidine deaminase |
| 217765_at | 12.64 | 3.08E-05 | 1.071711562 | NM_013392| | NRBP,nuclear receptor binding protein |
| 220638_s_at | 12.64 | 3.09E-05 | 1.566140166 | NM_012116| | CBLC,Cas-Br-M (murine) ecotropic retroviral |
| 212520_s_at | 12.64 | 3.08E-05 | 1.044559727 | NM_003072| | SMARCA4,SWI/SNF-related matrix-associated |
| 207480_s_at | 12.63 | 3.09E-05 | 1.38690437 | NM_002399| | MEIS2,homeobox protein Meis2 isoform f |
| 236835_at | 12.63 | 3.10E-05 | 1.265044724 | NA |  |
| 238350_at | 12.63 | 3.09E-05 | 1.198586334 | NM_173569| | NA |
| 207850_at | 12.63 | 3.10E-05 | 1.79709914 | NM_002090| | CXCL3,chemokine (C-X-C motif) ligand 3 |
| 227058_at | 12.63 | 3.09E-05 | 1.401913221 | NM_032849| | FLJ14834,hypothetical protein FLJ14834 |
| 212914_at | 12.63 | 3.10E-05 | 1.324153336 | NM_175709| | CBX7,chromobox homolog 7 |
| 222798_at | 12.62 | 3.11E-05 | 1.178211622 | NM_001001484| | PTER,phosphotriesterase related |
| 223084_s_at | 12.62 | 3.10E-05 | 1.07375548 | NM_012142| | CCNDBP1,cyclin D-type binding-protein 1 |
| 1568780_at | 12.62 | 3.11E-05 | 1.568256593 | NA |  |
| 210287_s_at | 12.62 | 3.10E-05 | 1.726867803 | NM_002019| | FLT1,fms-related tyrosine kinase 1 (vascular |
| 239132_at | 12.61 | 3.12E-05 | 1.421498553 | NM_000620| | NOS1,nitric oxide synthase 1 (neuronal) |
| 220742_s_at | 12.61 | 3.12E-05 | 1.056372582 | NM_018297| | NGLY1,N-glycanase 1 |
| 227985_at | 12.61 | 3.13E-05 | 1.82574235 | NA |  |
| 226269_at | 12.61 | 3.11E-05 | 1.209938215 | NM_001040875| | NA |
| 212749_s_at | 12.61 | 3.11E-05 | 1.063585261 | NM_001008925| | RCHY1,ring finger and CHY zinc finger domain |
| 220160_s_at | 12.61 | 3.12E-05 | 1.169741941 | NM_007059| | KPTN,kaptin (actin binding protein) |
| 202777_at | 12.61 | 3.12E-05 | 1.028145125 | NM_007373| | SHOC2,soc-2 suppressor of clear homolog |
| 205795_at | 12.6 | 3.14E-05 | 1.425957094 | NM_001105250| | NA |
| 213122_at | 12.6 | 3.13E-05 | 1.28216881 | NM_033512| | TSPYL5,TSPY-like 5 |
| 231022_at | 12.59 | 3.15E-05 | 1.514972005 | NA |  |
| 242070_at | 12.59 | 3.14E-05 | 1.545677267 | NA |  |
| 213271_s_at | 12.59 | 3.15E-05 | 1.081859655 | NM_015018| | KIAA1117,KIAA1117 protein |
| 226773_at | 12.59 | 3.15E-05 | 1.144081326 | NA |  |
| 203133_at | 12.59 | 3.15E-05 | 1.022041493 | NM_006808| | SEC61B,Sec61 beta subunit |
| 221542_s_at | 12.58 | 3.16E-05 | 1.093107795 | NM_001003790| | SPFH2,SPFH domain family, member 2 isoform 2 |
| 228787_s_at | 12.58 | 3.16E-05 | 1.227311048 | NM_001010974| | BCAS4,breast carcinoma amplified sequence 4 isoform c |
| 219361_s_at | 12.58 | 3.15E-05 | 1.046631672 | NM_022767| | FLJ12484,hypothetical protein FLJ12484 |
| 230230_at | 12.57 | 3.18E-05 | 1.19147648 | NA |  |
| 206314_at | 12.56 | 3.18E-05 | 1.408025712 | NM_018651| | ZNF167,zinc finger protein ZFP isoform 1 |
| 202838_at | 12.56 | 3.18E-05 | 1.177174619 | NM_000147| | FUCA1,fucosidase, alpha-L- 1, tissue |
| 204823_at | 12.56 | 3.19E-05 | 1.356668867 | NM_014903| | NAV3,neuron navigator 3 |
| 206542_s_at | 12.55 | 3.21E-05 | 2.032270972 | NM_003070| | SMARCA2,SWI/SNF-related matrix-associated |
| 224779_s_at | 12.55 | 3.21E-05 | 1.04207891 | NM_001014812| | NA |
| 226291_at | 12.55 | 3.21E-05 | 1.082431979 | NM_020919| | ALS2,alsin |
| 228976_at | 12.55 | 3.20E-05 | 1.10818526 | NM_015259| | ICOSLG,inducible T-cell co-stimulator ligand |
| 220382_s_at | 12.55 | 3.21E-05 | 1.446343298 | NM_001010000| | ARHGAP28,Rho GTPase activating protein 28 isoform a |
| 218342_s_at | 12.54 | 3.21E-05 | 1.13156861 | NM_024896| | KIAA1815,KIAA1815 |
| 220215_at | 12.54 | 3.22E-05 | 1.114217759 | NM_024804| | FLJ12606,hypothetical protein FLJ12606 |
| 222637_at | 12.54 | 3.22E-05 | 1.081972801 | NM_016144| | COMMD10,COMM domain containing 10 |
| 222103_at | 12.54 | 3.21E-05 | 1.172578209 | NM_005171| | ATF1,activating transcription factor 1 |
| 208647_at | 12.53 | 3.24E-05 | 1.019570425 | NM_004462| | FDFT1,farnesyl-diphosphate farnesyltransferase 1 |
| 226659_at | 12.53 | 3.23E-05 | 1.397984325 | NM_022047| | DEF6,differentially expressed in FDCP 6 homolog |
| 212168_at | 12.52 | 3.25E-05 | 1.044608841 | NM_006047| | RBM12,RNA binding motif protein 12 |
| 206673_at | 12.52 | 3.24E-05 | 1.406790586 | NM_007223| | GPR,putative G protein coupled receptor |
| 211707_s_at | 12.51 | 3.26E-05 | 1.089900927 | NM_001023570| | NA |
| 1557135_at | 12.51 | 3.27E-05 | 1.363555785 | NA |  |
| 213843_x_at | 12.51 | 3.27E-05 | 1.053656556 | NM_005629| | SLC6A8,solute carrier family 6 (neurotransmitter |
| 236214_at | 12.5 | 3.27E-05 | 1.375330125 | NM_032599| | NYD-SP18,testes development-related NYD-SP18 |
| 217877_s_at | 12.5 | 3.27E-05 | 1.028813686 | NM_021639| | SP192,hypothetical protein SP192 |
| 203718_at | 12.5 | 3.28E-05 | 1.095011026 | NM_006702| | NTE,neuropathy target esterase |
| 241550_at | 12.5 | 3.27E-05 | 1.449847911 | NM_001025290| | NA |
| 223064_at | 12.49 | 3.29E-05 | 1.083341231 | NM_016494| | LOC51255,hypothetical protein LOC51255 |
| 218986_s_at | 12.49 | 3.30E-05 | 1.190907643 | NM_017631| | FLJ20035,hypothetical protein FLJ20035 |
| 240184_at | 12.49 | 3.29E-05 | 1.204041312 | NA |  |
| 223700_at | 12.48 | 3.31E-05 | 1.055142029 | NM_032117| | GAJ,GAJ protein |
| 220255_at | 12.48 | 3.31E-05 | 1.086337016 | NM_021922| | FANCE,Fanconi anemia, complementation group E |
| 226730_s_at | 12.48 | 3.31E-05 | 1.095707968 | NM_020935| | USP37,ubiquitin specific protease 37 |
| 223960_s_at | 12.48 | 3.30E-05 | 1.069261591 | NM_013399| | C16orf5,chromosome 16 open reading frame 5 |
| 203968_s_at | 12.48 | 3.30E-05 | 1.068209662 | NM_001254| | CDC6,CDC6 homolog |
| 208899_x_at | 12.48 | 3.31E-05 | 1.067730038 | NM_015994| | ATP6V1D,ATPase, H+ transporting, lysosomal 34kD, V1 |
| 221636_s_at | 12.48 | 3.31E-05 | 1.260012117 | NM_017898| | FLJ20605,hypothetical protein FLJ20605 |
| 237451_x_at | 12.47 | 3.32E-05 | 1.172313465 | NA |  |
| 230326_s_at | 12.47 | 3.32E-05 | 1.045068593 | NM_016401| | HSPC138,hypothetical protein HSPC138 |
| 213018_at | 12.46 | 3.33E-05 | 1.066225562 | NM_021167| | ODAG,ocular development-associated gene |
| 229054_at | 12.46 | 3.34E-05 | 1.403215422 | NM_207442| | FLJ39779,FLJ39779 protein |
| 203653_s_at | 12.46 | 3.33E-05 | 1.070910904 | NM_004645| | COIL,coilin |
| 241888_at | 12.45 | 3.36E-05 | 1.423879383 | NA |  |
| 204612_at | 12.45 | 3.35E-05 | 1.161235015 | NM_006823| | PKIA,cAMP-dependent protein kinase inhibitor alpha |
| 225142_at | 12.45 | 3.35E-05 | 1.609500913 | NM_030647| | NA |
| 219979_s_at | 12.45 | 3.36E-05 | 1.081003534 | NM_016401| | HSPC138,hypothetical protein HSPC138 |
| 222243_s_at | 12.44 | 3.37E-05 | 1.114964092 | NM_016272| | TOB2,transducer of ERBB2, 2 |
| 223275_at | 12.44 | 3.37E-05 | 1.10856796 | NM_018137| | HRMT1L6,HMT1 hnRNP methyltransferase-like 6 |
| 205865_at | 12.44 | 3.36E-05 | 1.731769073 | NM_005224| | ARID3A,AT rich interactive domain 3A (BRIGHT- like) |
| 242093_at | 12.43 | 3.38E-05 | 1.526900488 | NM_138780| | SYTL5,synaptotagmin-like 5 |
| 1320_at | 12.43 | 3.39E-05 | 1.074364588 | NM_007039| | PTPN21,protein tyrosine phosphatase, non-receptor type |
| 221218_s_at | 12.43 | 3.37E-05 | 1.479479248 | NM_001042482| | NA |
| 219676_at | 12.43 | 3.38E-05 | 1.120107943 | NM_025231| | ZNF435,zinc finger protein 435 |
| 210222_s_at | 12.42 | 3.40E-05 | 1.274008472 | NM_021136| | RTN1,reticulon 1 isoform A |
| 209874_x_at | 12.42 | 3.39E-05 | 1.170403019 | NM_017649| | CNNM2,cyclin M2 isoform 1 |
| 217887_s_at | 12.42 | 3.40E-05 | 1.104410094 | NM_001981| | EPS15,epidermal growth factor receptor pathway |
| 224974_at | 12.42 | 3.39E-05 | 1.194905795 | NM_022491| | SDS3,hypothetical protein FLJ00052 |
| 242136_x_at | 12.42 | 3.40E-05 | 1.178328519 | NA |  |
| 223796_at | 12.42 | 3.39E-05 | 1.561232903 | NM_033655| | CNTNAP3,cell recognition molecule CASPR3 |
| 224150_s_at | 12.41 | 3.40E-05 | 1.171787486 | NM_024491| | Cep70,centrosomal protein 70 kDa |
| 215812_s_at | 12.41 | 3.41E-05 | 1.041844426 | NM_005629| | SLC6A8,solute carrier family 6 (neurotransmitter |
| 223026_s_at | 12.41 | 3.41E-05 | 1.0271988 | NM_016226| | VPS29,vacuolar protein sorting 29 isoform 1 |
| 216202_s_at | 12.41 | 3.41E-05 | 1.185867635 | NM_004863| | SPTLC2,serine palmitoyltransferase, long chain base |
| 235476_at | 12.41 | 3.41E-05 | 1.193340633 | NM_173084| | TRIM59,tripartite motif-containing 59 |
| 206593_s_at | 12.41 | 3.40E-05 | 1.105855105 | NM_133640| | SURF5,surfeit 5 isoform b |
| 40093_at | 12.41 | 3.41E-05 | 1.103228529 | NM_001013257| | NA |
| 206557_at | 12.4 | 3.42E-05 | 1.677493285 | NA |  |
| 229927_at | 12.4 | 3.43E-05 | 1.146332103 | NM_001001552| | LEMD1,LEM domain containing 1 |
| 213225_at | 12.4 | 3.42E-05 | 1.291136692 | NM_001033556| | NA |
| 206032_at | 12.4 | 3.42E-05 | 1.430115222 | NM_001941| | DSC3,desmocollin 3 isoform Dsc3a preproprotein |
| 230185_at | 12.4 | 3.42E-05 | 1.213327973 | NM_024672| | THAP9,THAP domain containing 9 |
| 203530_s_at | 12.39 | 3.45E-05 | 1.060568385 | NM_004604| | STX4A,syntaxin 4A (placental) |
| 228335_at | 12.39 | 3.44E-05 | 1.682933741 | NM_005602| | CLDN11,claudin 11 |
| 224618_at | 12.39 | 3.44E-05 | 1.229244072 | NM_005156| | ROD1,ROD1 regulator of differentiation 1 |
| 221794_at | 12.38 | 3.45E-05 | 1.068241924 | NM_020812| | DOCK6,dedicator of cytokinesis 6 |
| 225834_at | 12.38 | 3.45E-05 | 1.074314447 | NM_001100910| | NA |
| 216834_at | 12.38 | 3.45E-05 | 1.353165082 | NM_002922| | RGS1,regulator of G-protein signalling 1 |
| 214508_x_at | 12.38 | 3.45E-05 | 1.143273267 | NM_001881| | CREM,cAMP responsive element modulator isoform b |
| 218469_at | 12.38 | 3.45E-05 | 1.374262927 | NM_013372| | GREM1,cysteine knot superfamily 1, BMP antagonist 1 |
| 1555679_a_at | 12.36 | 3.48E-05 | 1.076882939 | NM_032730| | RTN4IP1,reticulon 4 interacting protein 1 |
| 225046_at | 12.36 | 3.49E-05 | 1.085897452 | NA |  |
| 231837_at | 12.35 | 3.49E-05 | 1.169850637 | NM_020886| | USP28,ubiquitin specific protease 28 |
| 212838_at | 12.35 | 3.49E-05 | 1.059043336 | NM_015221| | DNMBP,dynamin binding protein |
| 200623_s_at | 12.34 | 3.51E-05 | 1.059990992 | NM_001743| | CALM2,calmodulin 2 |
| 218005_at | 12.34 | 3.51E-05 | 1.060742842 | NM_006963| | ZNF22,zinc finger protein 22 (KOX 15) |
| 218263_s_at | 12.34 | 3.51E-05 | 1.043691748 | NA |  |
| 219248_at | 12.34 | 3.52E-05 | 1.095738728 | NM_025264| | THUMPD2,THUMP domain containing 2 |
| 205193_at | 12.34 | 3.51E-05 | 1.101307899 | NM_012323| | MAFF,transcription factor MAFF |
| 230644_at | 12.33 | 3.53E-05 | 1.424998201 | NM_152447| | LRFN5,leucine rich repeat and fibronectin type III |
| 235955_at | 12.33 | 3.53E-05 | 1.803795055 | NM_001038603| | NA |
| 206981_at | 12.32 | 3.55E-05 | 1.126159389 | NM_000334| | SCN4A,sodium channel, voltage-gated, type IV, alpha |
| 212906_at | 12.31 | 3.56E-05 | 1.14673713 | NM_020716| | NA |
| 205165_at | 12.3 | 3.58E-05 | 1.069670709 | NM_001040454| | NA |
| 224609_at | 12.29 | 3.61E-05 | 1.074998026 | NM_020428| | CTL2,CTL2 gene |
| 238183_at | 12.28 | 3.61E-05 | 1.41812531 | NA |  |
| 201354_s_at | 12.28 | 3.62E-05 | 1.095441468 | NM_013449| | BAZ2A,bromodomain adjacent to zinc finger domain, 2A |
| 212441_at | 12.27 | 3.64E-05 | 1.055916468 | NM_001100590| | NA |
| 220281_at | 12.27 | 3.63E-05 | 1.809813845 | NM_000338| | SLC12A1,sodium potassium chloride cotransporter 2 |
| 213578_at | 12.25 | 3.68E-05 | 1.117159627 | NM_004329| | BMPR1A,bone morphogenetic protein receptor, type IA |
| 229752_at | 12.25 | 3.67E-05 | 1.117632248 | NM_022465| | ZNFN1A4,zinc finger protein, subfamily 1A, 4 |
| 214857_at | 12.24 | 3.68E-05 | 1.137519273 | NA |  |
| 243489_at | 12.24 | 3.69E-05 | 1.352895485 | NA |  |
| 241371_at | 12.24 | 3.69E-05 | 1.375131035 | NA |  |
| 222405_at | 12.24 | 3.69E-05 | 1.021530919 | NM_016395| | HSPC121,butyrate-induced transcript 1 |
| 210981_s_at | 12.23 | 3.70E-05 | 1.102905271 | NM_001004105| | GRK6,G protein-coupled receptor kinase 6 isoform C |
| 1558942_at | 12.23 | 3.70E-05 | 1.082110272 | NM_001040185| | NA |
| 209273_s_at | 12.22 | 3.72E-05 | 1.044074409 | NM_030940| | HBLD2,HESB like domain containing 2 |
| 225074_at | 12.21 | 3.74E-05 | 1.046711831 | NM_032846| | RAB2B,RAB2B protein |
| 210619_s_at | 12.21 | 3.75E-05 | 1.318810599 | NM_007312| | HYAL1,hyaluronoglucosaminidase 1 isoform 1 |
| 1552389_at | 12.21 | 3.75E-05 | 1.42370113 | NM_173549| | FLJ39553,hypothetical protein FLJ39553 |
| 236113_at | 12.21 | 3.74E-05 | 1.249417055 | NA |  |
| 208420_x_at | 12.21 | 3.74E-05 | 1.087006272 | NM_003170| | SUPT6H,suppressor of Ty 6 homolog |
| 236266_at | 12.2 | 3.76E-05 | 1.461160747 | NA |  |
| 235088_at | 12.2 | 3.75E-05 | 1.227632877 | NM_001008393| | LOC201725,hypothetical protein LOC201725 |
| 212479_s_at | 12.2 | 3.76E-05 | 1.067132132 | NM_022780| | FLJ13910,hypothetical protein FLJ13910 |
| 224709_s_at | 12.2 | 3.76E-05 | 1.040040958 | NM_001038702| | NA |
| 226085_at | 12.2 | 3.76E-05 | 1.109344858 | NA |  |
| 212959_s_at | 12.2 | 3.76E-05 | 1.186001729 | NM_024312| | MGC4170,MGC4170 protein |
| 228437_at | 12.2 | 3.75E-05 | 1.21436813 | NM_014184| | HSPC163,HSPC163 protein |
| 242387_at | 12.2 | 3.76E-05 | 1.437390791 | NM_175075| | INM01,hypothetical protein INM01 |
| 226202_at | 12.19 | 3.77E-05 | 1.07978501 | NM_020781| | ZNF398,zinc finger 398 isoform b |
| 230876_at | 12.18 | 3.79E-05 | 1.363676128 | NM_001101338| | NA |
| 219014_at | 12.18 | 3.78E-05 | 1.460592206 | NM_016619| | PLAC8,placenta-specific 8 |
| 223478_at | 12.18 | 3.79E-05 | 1.050259894 | NM_012459| | TIMM8B,translocase of inner mitochondrial membrane 8 |
| 208711_s_at | 12.18 | 3.79E-05 | 1.050782834 | NM_053056| | CCND1,cyclin D1 |
| 214819_at | 12.18 | 3.78E-05 | 1.242543808 | NM_001111125| | NA |
| 223989_s_at | 12.17 | 3.81E-05 | 1.035538579 | NM_015523| | DKFZP566E144,small fragment nuclease |
| 227273_at | 12.17 | 3.81E-05 | 1.042153498 | NA |  |
| 214426_x_at | 12.17 | 3.80E-05 | 1.047900172 | NM_005483| | CHAF1A,chromatin assembly factor 1, subunit A (p150) |
| 209040_s_at | 12.16 | 3.82E-05 | 1.087273089 | NM_004159| | PSMB8,proteasome beta 8 subunit isoform E1 proprotein |
| 203715_at | 12.16 | 3.83E-05 | 1.110813601 | NM_001079515| | NA |
| 226562_at | 12.15 | 3.85E-05 | 1.064102198 | NM_152455| | FLJ35867,hypothetical protein FLJ35867 |
| 201951_at | 12.15 | 3.84E-05 | 1.109949292 | NM_001627| | ALCAM,activated leukocyte cell adhesion molecule |
| 225991_at | 12.15 | 3.83E-05 | 1.081220312 | NM_080652| | TMEM41A,transmembrane protein 41A |
| 210951_x_at | 12.15 | 3.85E-05 | 1.122501551 | NM_004580| | RAB27A,Ras-related protein Rab-27A |
| 214014_at | 12.15 | 3.85E-05 | 1.180459345 | NM_006779| | CDC42EP2,Cdc42 effector protein 2 |
| 219418_at | 12.14 | 3.86E-05 | 1.061748871 | NM_024782| | FLJ12610,similar to mouse 1700029B21Rik protein |
| 223458_at | 12.14 | 3.87E-05 | 1.332624168 | NM_001114099| | NA |
| 37425_g_at | 12.14 | 3.87E-05 | 1.086974042 | NM_001105563| | NA |
| 239177_at | 12.14 | 3.86E-05 | 1.158172411 | NA |  |
| 222674_at | 12.14 | 3.87E-05 | 1.059727392 | NM_016390| | C9orf114,chromosome 9 open reading frame 114 |
| 230319_at | 12.13 | 3.87E-05 | 1.60690417 | NA |  |
| 224366_s_at | 12.13 | 3.88E-05 | 1.051366869 | NM_031922| | REPS1,RALBP1 associated Eps domain containing 1 |
| 225455_at | 12.12 | 3.90E-05 | 1.101023582 | NM_053053| | STAF42,SPT3-associated factor 42 |
| 223495_at | 12.12 | 3.90E-05 | 1.13767005 | NM_032040| | CCDC8,coiled-coil domain containing 8 |
| 1569796_s_at | 12.12 | 3.90E-05 | 1.461117856 | NM_207303| | ATRNL1,attractin-like 1 |
| 213413_at | 12.11 | 3.91E-05 | 1.163244019 | NM_006873| | SBLF,stoned B-like factor |
| 203556_at | 12.11 | 3.92E-05 | 1.099680768 | NM_014943| | ZHX2,zinc fingers and homeoboxes 2 |
| 204352_at | 12.11 | 3.91E-05 | 1.131796415 | NM_001033910| | NA |
| 227522_at | 12.11 | 3.92E-05 | 1.045955049 | NM_138809| | LOC134147,similar to mouse 2310016A09Rik protein |
| 207318_s_at | 12.1 | 3.93E-05 | 1.059164492 | NM_003718| | CDC2L5,cell division cycle 2-like 5 isoform 1 |
| 223753_s_at | 12.1 | 3.94E-05 | 1.894562547 | NM_001079530| | NA |
| 207700_s_at | 12.1 | 3.94E-05 | 1.081659966 | NM_006534| | NCOA3,nuclear receptor coactivator 3 isoform b |
| 1563658_a_at | 12.1 | 3.93E-05 | 1.176395658 | NM_175733| | SYT9,synaptotagmin IX |
| 205340_at | 12.1 | 3.94E-05 | 1.06497241 | NM_014797| | ZBTB24,zinc finger and BTB domain containing 24 |
| 233675_s_at | 12.09 | 3.95E-05 | 1.496925845 | NA |  |
| 201656_at | 12.09 | 3.95E-05 | 1.09882604 | NM_000210| | ITGA6,integrin alpha chain, alpha 6 |
| 208739_x_at | 12.09 | 3.95E-05 | 1.029757253 | NM_001005849| | SUMO2,small ubiquitin-like modifier 2 isoform b |
| 222738_at | 12.09 | 3.95E-05 | 1.294180563 | NM_024949| | BOMB,BH3-only member B protein |
| 202409_at | 12.08 | 3.97E-05 | 1.647841294 | NM_000612| | IGF2,insulin-like growth factor 2 (somatomedin A) |
| 212448_at | 12.08 | 3.97E-05 | 1.025232155 | NM_015277| | NEDD4L,ubiquitin-protein ligase NEDD4-like |
| 222458_s_at | 12.08 | 3.98E-05 | 1.131990329 | NM_024595| | FLJ12666,hypothetical protein FLJ12666 |
| 208995_s_at | 12.08 | 3.96E-05 | 1.097574411 | NM_004792| | PPIG,peptidyl-prolyl isomerase G (cyclophilin G) |
| 223327_x_at | 12.08 | 3.96E-05 | 1.068587948 | NA |  |
| 1559322_at | 12.07 | 3.98E-05 | 1.321423981 | NA |  |
| 202302_s_at | 12.07 | 3.99E-05 | 1.028967705 | NM_023012| | FLJ11021,similar to splicing factor, arginine/serine-rich |
| 205215_at | 12.07 | 3.98E-05 | 1.115439402 | NM_007212| | RNF2,ring finger protein 2 |
| 221572_s_at | 12.07 | 3.98E-05 | 1.100567969 | NM_001040454| | NA |
| 222052_at | 12.06 | 4.00E-05 | 1.14661366 | NM_198476| | FLJ41131,FLJ41131 protein |
| 220053_at | 12.06 | 4.01E-05 | 1.620660184 | NM_020634| | GDF3,growth differentiation factor 3 precursor |
| 205659_at | 12.06 | 4.01E-05 | 1.209683181 | NM_014707| | HDAC9,histone deacetylase 9 isoform 3 |
| 226038_at | 12.06 | 4.00E-05 | 1.061622197 | NM_152271| | FLJ23749,hypothetical protein FLJ23749 |
| 222621_at | 12.05 | 4.01E-05 | 1.074476529 | NM_022365| | DNAJC1,DnaJ (Hsp40) homolog, subfamily C, member 1 |
| 241776_at | 12.05 | 4.01E-05 | 1.510788854 | NA |  |
| 1555229_a_at | 12.05 | 4.01E-05 | 1.166288028 | NM_001734| | C1S,complement component 1, s subcomponent |
| 228487_s_at | 12.05 | 4.02E-05 | 1.207101714 | NA |  |
| 200631_s_at | 12.05 | 4.01E-05 | 1.022405656 | NM_001122821| | NA |
| 209225_x_at | 12.04 | 4.04E-05 | 1.039285529 | NM_002270| | TNPO1,transportin 1 |
| 204067_at | 12.04 | 4.04E-05 | 1.086537543 | NM_000456| | SUOX, |
| 209654_at | 12.04 | 4.05E-05 | 1.046238854 | NM_015325| | NA |
| 201436_at | 12.04 | 4.05E-05 | 1.117037459 | NM_001968| | EIF4E,eukaryotic translation initiation factor 4E |
| 211962_s_at | 12.04 | 4.04E-05 | 1.126618532 | NM_004926| | ZFP36L1,butyrate response factor 1 |
| 217822_at | 12.04 | 4.04E-05 | 1.04110515 | NM_016312| | WBP11,WW domain binding protein 11 |
| 206213_at | 12.04 | 4.05E-05 | 1.129030271 | NM_003394| | WNT10B,wingless-type MMTV integration site family, |
| 40273_at | 12.03 | 4.05E-05 | 1.070272225 | NM_020126| | SPHK2,sphingosine kinase type 2 isoform |
| 222994_at | 12.03 | 4.06E-05 | 1.065095108 | NM_012094| | PRDX5,peroxiredoxin 5 precursor, isoform a |
| 222016_s_at | 12.03 | 4.05E-05 | 1.147096934 | NM_030899| | ZNF323,zinc finger protein 323 |
| 239765_at | 12.03 | 4.06E-05 | 1.243259553 | NA |  |
| 208913_at | 12.03 | 4.06E-05 | 1.052193511 | NM_015044| | GGA2,ADP-ribosylation factor binding protein 2 |
| 241926_s_at | 12.03 | 4.05E-05 | 1.529699288 | NM_004449| | ERG,v-ets erythroblastosis virus E26 oncogene like |
| 203990_s_at | 12.03 | 4.05E-05 | 1.414721721 | NM_021140| | UTX,ubiquitously transcribed tetratricopeptide |
| 219176_at | 12.02 | 4.08E-05 | 1.022650472 | NM_024520| | FLJ22555,hypothetical protein FLJ22555 |
| 229974_at | 12.02 | 4.07E-05 | 1.631546257 | NM_147127| | EVC2,limbin |
| 226599_at | 12.02 | 4.08E-05 | 1.543528591 | NM_033393| | KIAA1727,KIAA1727 protein |
| 229644_at | 12.01 | 4.09E-05 | 1.109097609 | NM_002726| | PREP,prolyl endopeptidase |
| 209535_s_at | 12.01 | 4.10E-05 | 1.246141555 | NA |  |
| 205498_at | 12.01 | 4.08E-05 | 1.21495361 | NM_000163| | GHR,growth hormone receptor precursor |
| 202149_at | 12.01 | 4.10E-05 | 1.143537902 | NM_006403| | NEDD9,neural precursor cell expressed, developmentally |
| 209308_s_at | 12.01 | 4.09E-05 | 1.123356795 | NM_004330| | BNIP2,BCL2/adenovirus E1B 19kD interacting protein 2 |
| 230008_at | 12 | 4.11E-05 | 1.438357959 | NM_015204| | NA |
| 201916_s_at | 12 | 4.11E-05 | 1.098524954 | NM_007214| | SEC63,SEC63-like protein |
| 204366_s_at | 12 | 4.11E-05 | 1.042370338 | NM_001035521| | NA |
| 223539_s_at | 12 | 4.11E-05 | 1.08236241 | NM_021967| | SERF1A,small EDRK-rich factor 1A, telomeric |
| 235037_at | 12 | 4.12E-05 | 1.050017495 | NM_080652| | TMEM41A,transmembrane protein 41A |
| 200801_x_at | 11.99 | 4.12E-05 | 1.013449268 | NM_001101| | ACTB,beta actin |
| 206949_s_at | 11.99 | 4.13E-05 | 1.085581044 | NM_001105203| | NA |
| 203406_at | 11.99 | 4.12E-05 | 1.049186154 | NM_005926| | MFAP1,microfibrillar-associated protein 1 |
| 214820_at | 11.99 | 4.13E-05 | 1.109533986 | NM_001007246| | WDR9,WD repeat domain 9 isoform C |
| 231864_at | 11.99 | 4.13E-05 | 1.151009114 | NM_006954| | NA |
| 238975_at | 11.98 | 4.14E-05 | 1.104019225 | NM_052845| | MMAB,cob(I)alamin adenosyltransferase |
| 204108_at | 11.98 | 4.14E-05 | 1.068386147 | NM_002505| | NFYA,nuclear transcription factor Y, alpha isoform 1 |
| 231973_s_at | 11.98 | 4.15E-05 | 1.053183359 | NM_022662| | ANAPC1,anaphase promoting complex subunit 1 |
| 225845_at | 11.98 | 4.14E-05 | 1.096656972 | NM_014155| | HSPC063,HSPC063 protein |
| 206747_at | 11.98 | 4.14E-05 | 1.20073229 | NM_014696| | KIAA0514,KIAA0514 |
| 204881_s_at | 11.98 | 4.14E-05 | 1.087748589 | NM_003358| | UGCG,ceramide glucosyltransferase |
| 225606_at | 11.97 | 4.16E-05 | 1.060029234 | NM_006538| | BCL2L11,BCL2-like 11 isoform 6 |
| 229901_at | 11.97 | 4.16E-05 | 1.400487161 | NM_153034| | ZNF488,zinc finger protein 488 |
| 230865_at | 11.97 | 4.16E-05 | 1.939567819 | NM_153234| | LIX1,limb expression 1 |
| 208064_s_at | 11.97 | 4.16E-05 | 1.494491048 | NM_015879| | ST8SIA3,ST8 alpha-N-acetyl-neuraminide |
| 227475_at | 11.97 | 4.16E-05 | 1.640074575 | NM_033260| | FOXQ1,forkhead box Q1 |
| 209069_s_at | 11.97 | 4.17E-05 | 1.043366502 | NM_002107| | H3F3A,H3 histone, family 3A |
| 238169_at | 11.97 | 4.16E-05 | 1.400975103 | NA |  |
| 229105_at | 11.97 | 4.16E-05 | 1.158023656 | NM_001508| | GPR39,G protein-coupled receptor 39 |
| 223993_s_at | 11.97 | 4.17E-05 | 1.045422747 | NM_014184| | HSPC163,HSPC163 protein |
| 238964_at | 11.97 | 4.16E-05 | 1.185969683 | NM_018086| | FIGN,fidgetin |
| 1552727_s_at | 11.97 | 4.17E-05 | 1.569530057 | NM_139057| | ADAMTS17,a disintegrin-like and metalloprotease |
| 201950_x_at | 11.96 | 4.18E-05 | 1.066408821 | NM_004930| | CAPZB,F-actin capping protein beta subunit |
| 213370_s_at | 11.96 | 4.18E-05 | 1.102484504 | NM_001005158| | SFMBT1,Scm-like with four mbt domains 1 |
| 204979_s_at | 11.96 | 4.18E-05 | 1.243371746 | NM_001001713| | SH3BGR,SH3-binding domain and glutamic acid-rich |
| 223589_at | 11.96 | 4.17E-05 | 1.076342847 | NM_017879| | ZNF416,zinc finger protein 416 |
| 213025_at | 11.96 | 4.18E-05 | 1.105197544 | NM_017736| | THUMPD1,THUMP domain containing 1 |
| 235049_at | 11.96 | 4.19E-05 | 1.081517785 | NM_021116| | ADCY1,brain adenylate cyclase 1 |
| 213150_at | 11.96 | 4.17E-05 | 1.425371091 | NM_018951| | HOXA10,homeobox protein A10 isoform a |
| 215491_at | 11.96 | 4.17E-05 | 1.247194719 | NM_001033081| | NA |
| 219580_s_at | 11.95 | 4.20E-05 | 1.329803918 | NM_001105248| | NA |
| 214749_s_at | 11.95 | 4.19E-05 | 1.068362923 | NM_001009584| | ARMCX6,armadillo repeat containing, X-linked 6 |
| 218900_at | 11.94 | 4.22E-05 | 1.082417352 | NM_020184| | CNNM4,cyclin M4 |
| 221954_at | 11.94 | 4.23E-05 | 1.032848936 | NM_016470| | C20orf111,chromosome 20 open reading frame 111 |
| 1563327_a_at | 11.94 | 4.22E-05 | 1.302152383 | NA |  |
| 216037_x_at | 11.94 | 4.22E-05 | 1.11885251 | NM_030756| | TCF7L2,transcription factor 7-like 2 (T-cell specific, |
| 227829_at | 11.94 | 4.22E-05 | 1.424981236 | NM_152312| | GYLTL1B,glycosyltransferase-like 1B |
| 201786_s_at | 11.93 | 4.23E-05 | 1.047406004 | NM_001025107| | NA |
| 220111_s_at | 11.93 | 4.24E-05 | 1.344251265 | NM_020373| | TMEM16B,transmembrane protein 16B |
| 1555270_a_at | 11.92 | 4.27E-05 | 1.068845127 | NM_006005| | WFS1,wolframin |
| 220432_s_at | 11.92 | 4.27E-05 | 1.640459623 | NM_016593| | CYP39A1,cytochrome P450, family 39, subfamily A, |
| 226763_at | 11.92 | 4.27E-05 | 1.024498965 | NM_178123| | SESTD1,SEC14 and spectrin domains 1 |
| 202005_at | 11.92 | 4.27E-05 | 1.244422441 | NM_021978| | ST14,matriptase |
| 226027_at | 11.92 | 4.26E-05 | 1.066213246 | NM_001040011| | NA |
| 226833_at | 11.91 | 4.28E-05 | 1.047126225 | NM_144607| | FLJ32499,hypothetical protein FLJ32499 |
| 244407_at | 11.91 | 4.29E-05 | 1.4453217 | NM_016593| | CYP39A1,cytochrome P450, family 39, subfamily A, |
| 238992_at | 11.91 | 4.29E-05 | 1.099671846 | NM_007195| | POLI,polymerase (DNA directed) iota |
| 223542_at | 11.91 | 4.29E-05 | 1.073977869 | NM_032290| | ANKRD32,ankyrin repeat domain 32 |
| 210713_at | 11.9 | 4.31E-05 | 1.125739358 | NM_001001132| | ITSN1,intersectin 1 isoform ITSN-s |
| 218264_at | 11.9 | 4.31E-05 | 1.037641495 | NM_016567| | BCCIP,BRCA2 and CDKN1A-interacting protein isoform |
| 243225_at | 11.9 | 4.31E-05 | 1.182172894 | NA |  |
| 237053_at | 11.9 | 4.31E-05 | 1.279595334 | NM_001105579| | NA |
| 212368_at | 11.9 | 4.31E-05 | 1.063586257 | NM_015021| | NA |
| 1554043_a_at | 11.89 | 4.33E-05 | 1.165197287 | NA |  |
| 1569453_a_at | 11.89 | 4.32E-05 | 1.25510343 | NA |  |
| 203755_at | 11.89 | 4.33E-05 | 1.036894302 | NM_001211| | BUB1B,BUB1 budding uninhibited by benzimidazoles 1 |
| 217954_s_at | 11.88 | 4.34E-05 | 1.070819475 | NM_015153| | PHF3,PHD finger protein 3 |
| 201773_at | 11.88 | 4.34E-05 | 1.038309368 | NM_015339| | ADNP,activity-dependent neuroprotector |
| 226884_at | 11.88 | 4.35E-05 | 1.128674383 | NM_020873| | LRRN1,leucine rich repeat neuronal 1 |
| 1560119_at | 11.88 | 4.34E-05 | 1.155642446 | NA |  |
| 236369_at | 11.88 | 4.35E-05 | 1.124036178 | NA |  |
| 220487_at | 11.87 | 4.37E-05 | 1.566190055 | NM_018968| | SNTG2,syntrophin, gamma 2 |
| 203307_at | 11.87 | 4.35E-05 | 1.071297354 | NM_005275| | GNL1,guanine nucleotide binding protein-like 1 |
| 235423_at | 11.87 | 4.36E-05 | 1.120454697 | NA |  |
| 230083_at | 11.87 | 4.36E-05 | 1.472501283 | NM_019050| | USP53,ubiquitin specific protease 53 |
| 224416_s_at | 11.86 | 4.38E-05 | 1.173066783 | NM_025205| | MED28,mediator of RNA polymerase II transcription, |
| 201112_s_at | 11.86 | 4.38E-05 | 1.033723725 | NM_001316| | CSE1L,CSE1 chromosome segregation 1-like protein |
| 224752_at | 11.86 | 4.37E-05 | 1.029183416 | NM_012450| | SLC13A4,solute carrier family 13 (sodium/sulfate |
| 1555231_a_at | 11.86 | 4.39E-05 | 1.252912724 | NA |  |
| 201769_at | 11.85 | 4.40E-05 | 1.075818546 | NM_014666| | ENTH,enthoprotin |
| 235964_x_at | 11.85 | 4.40E-05 | 1.146811522 | NA |  |
| 223362_s_at | 11.85 | 4.40E-05 | 1.048598999 | NM_019106| | SEPT3,septin 3 isoform B |
| 208393_s_at | 11.84 | 4.42E-05 | 1.109597705 | NM_005732| | RAD50,RAD50 homolog isoform 1 |
| 227384_s_at | 11.84 | 4.42E-05 | 1.415100725 | NA |  |
| 206149_at | 11.84 | 4.42E-05 | 1.291862177 | NM_022097| | LOC63928,hepatocellular carcinoma antigen gene 520 |
| 228622_s_at | 11.83 | 4.44E-05 | 1.106616716 | NM_005528| | DNAJC4,DnaJ (Hsp40) homolog, subfamily C, member 4 |
| 227815_at | 11.83 | 4.44E-05 | 1.049430982 | NA |  |
| 208490_x_at | 11.83 | 4.44E-05 | 1.080517631 | NM_003518| | HIST1H2BG,H2B histone family, member A |
| 233487_s_at | 11.83 | 4.44E-05 | 1.095164347 | NM_001127244| | NA |
| 205791_x_at | 11.82 | 4.46E-05 | 1.087064322 | NM_006300| | ZNF230,zinc finger protein 230 |
| 1554747_a_at | 11.82 | 4.46E-05 | 1.126250033 | NM_001008491| | SEPT2,septin 2 |
| 213943_at | 11.81 | 4.49E-05 | 1.557133502 | NM_000474| | TWIST1,twist |
| 212136_at | 11.81 | 4.49E-05 | 1.100771118 | NM_001001396| | ATP2B4,plasma membrane calcium ATPase 4 isoform 4b |
| 209578_s_at | 11.81 | 4.50E-05 | 1.074352402 | NM_015227| | POFUT2,protein O-fucosyltransferase 2 isoform A |
| 239733_at | 11.81 | 4.50E-05 | 1.45649067 | NM_032372| | MGC16186,hypothetical protein MGC16186 |
| 208812_x_at | 11.8 | 4.52E-05 | 1.055810456 | NM_002117| | HLA-C,major histocompatibility complex, class I, C |
| 221010_s_at | 11.8 | 4.51E-05 | 1.109394498 | NM_012241| | SIRT5,sirtuin 5 isoform 1 |
| 209306_s_at | 11.8 | 4.52E-05 | 1.095374305 | NM_015055| | SWAP70,SWAP-70 protein |
| 213828_x_at | 11.8 | 4.50E-05 | 1.016011027 | NM_002107| | H3F3A,H3 histone, family 3A |
| 203343_at | 11.8 | 4.51E-05 | 1.064415371 | NM_003359| | UGDH,UDP-glucose dehydrogenase |
| 225386_s_at | 11.79 | 4.54E-05 | 1.064665942 | NM_138394| | HNRPLL,heterogeneous nuclear ribonucleoprotein L-like |
| 205506_at | 11.79 | 4.54E-05 | 1.632541533 | NM_007127| | VIL1,villin 1 |
| 241367_at | 11.78 | 4.55E-05 | 1.137894738 | NM_207459| | FLJ35767,FLJ35767 protein |
| 201963_at | 11.77 | 4.58E-05 | 1.682858363 | NM_001995| | ACSL1,acyl-CoA synthetase long-chain family member 1 |
| 204927_at | 11.77 | 4.58E-05 | 1.22456559 | NM_003475| | C11orf13,HRAS1-related cluster-1 |
| 226612_at | 11.77 | 4.57E-05 | 1.252695664 | NA |  |
| 213245_at | 11.77 | 4.58E-05 | 1.037758555 | NM_021116| | ADCY1,brain adenylate cyclase 1 |
| 205297_s_at | 11.76 | 4.60E-05 | 1.171464975 | NM_000626| | CD79B,CD79B antigen isoform 1 precursor |
| 223804_s_at | 11.76 | 4.60E-05 | 1.034628171 | NM_001114092| | NA |
| 200929_at | 11.76 | 4.60E-05 | 1.060578069 | NM_006827| | TMP21,transmembrane trafficking protein |
| 202266_at | 11.76 | 4.59E-05 | 1.066427673 | NM_016614| | TTRAP,TRAF and TNF receptor-associated protein |
| 203044_at | 11.76 | 4.60E-05 | 1.085557251 | NM_014918| | CHSY1,carbohydrate (chondroitin) synthase 1 |
| 212564_at | 11.76 | 4.59E-05 | 1.084886236 | NM_015353| | KCTD2,potassium channel tetramerisation domain |
| 239036_at | 11.76 | 4.60E-05 | 1.211257685 | NA |  |
| 227601_at | 11.76 | 4.59E-05 | 1.082687361 | NM_020961| | KIAA1627,KIAA1627 protein |
| 241704_x_at | 11.75 | 4.61E-05 | 1.261958993 | NM_207333| | NA |
| 218375_at | 11.75 | 4.63E-05 | 1.05472107 | NM_024047| | NUDT9,nudix -type motif 9 isoform a |
| 211689_s_at | 11.74 | 4.64E-05 | 1.222274616 | NM_005656| | TMPRSS2,transmembrane protease, serine 2 |
| 218059_at | 11.74 | 4.65E-05 | 1.021211169 | NM_001042510| | NA |
| 235146_at | 11.74 | 4.65E-05 | 1.123897142 | NA |  |
| 1554481_a_at | 11.74 | 4.64E-05 | 1.202445087 | NM_004437| | EPB41,erythrocyte membrane protein band 4.1 |
| 225525_at | 11.74 | 4.63E-05 | 1.047259809 | NA |  |
| 225577_at | 11.73 | 4.67E-05 | 1.109649365 | NA |  |
| 225464_at | 11.73 | 4.66E-05 | 1.054223339 | NM_001042481| | NA |
| 203234_at | 11.73 | 4.66E-05 | 1.228554832 | NM_003364| | UPP1,uridine phosphorylase 1 |
| 202934_at | 11.73 | 4.66E-05 | 1.028843603 | NM_000189| | HK2,hexokinase 2 |
| 228449_at | 11.72 | 4.68E-05 | 1.227461653 | NA |  |
| 1554795_a_at | 11.72 | 4.68E-05 | 1.386943304 | NM_001024215| | NA |
| 228400_at | 11.72 | 4.68E-05 | 1.091786086 | NM_020859| | ShrmL,Shroom-related protein |
| 1555272_at | 11.71 | 4.70E-05 | 1.118131058 | NM_001099697| | NA |
| 229500_at | 11.71 | 4.71E-05 | 1.103786874 | NM_006345| | SLC30A9,solute carrier family 30 (zinc transporter), |
| 205583_s_at | 11.71 | 4.70E-05 | 1.07121796 | NA |  |
| 226857_at | 11.71 | 4.71E-05 | 1.215726678 | NM_153213| | ARHGEF19,Rho guanine nucleotide exchange factor (GEF) 19 |
| 226084_at | 11.71 | 4.70E-05 | 1.056963348 | NM_005909| | MAP1B,microtubule-associated protein 1B isoform 1 |
| 204922_at | 11.7 | 4.73E-05 | 1.147884684 | NM_024650| | FLJ22531,hypothetical protein FLJ22531 |
| 1555491_a_at | 11.7 | 4.73E-05 | 1.165882561 | NM_018381| | FLJ11286,hypothetical protein FLJ11286 |
| 200791_s_at | 11.7 | 4.73E-05 | 1.047826097 | NM_003870| | IQGAP1,IQ motif containing GTPase activating protein 1 |
| 225062_at | 11.69 | 4.75E-05 | 1.114012544 | NA |  |
| 205335_s_at | 11.69 | 4.75E-05 | 1.064088126 | NM_003135| | SRP19,signal recognition particle 19kDa |
| 238716_at | 11.69 | 4.76E-05 | 1.253507256 | NA |  |
| 228773_at | 11.69 | 4.75E-05 | 1.296159672 | NA |  |
| 201343_at | 11.68 | 4.77E-05 | 1.07167916 | NM_003339| | UBE2D2,ubiquitin-conjugating enzyme E2D 2 isoform 1 |
| 1553138_a_at | 11.68 | 4.78E-05 | 1.38880013 | NM_152363| | FLJ39369,hypothetical protein FLJ39369 |
| 225697_at | 11.67 | 4.80E-05 | 1.038521043 | NM_015083| | NA |
| 238632_at | 11.67 | 4.79E-05 | 1.786591438 | NA |  |
| 227430_at | 11.67 | 4.80E-05 | 1.096250256 | NM_032786| | FLJ14451,hypothetical protein FLJ14451 |
| 204291_at | 11.67 | 4.79E-05 | 1.288406523 | NM_014803| | ZNF518,zinc finger protein 518 |
| 217950_at | 11.67 | 4.81E-05 | 1.064072877 | NM_015953| | NOSIP,eNOS interacting protein |
| 216060_s_at | 11.67 | 4.81E-05 | 1.117239196 | NM_014992| | DAAM1,dishevelled-associated activator of |
| 223650_s_at | 11.67 | 4.80E-05 | 1.085668618 | NM_030759| | NRBF2,nuclear receptor binding factor 2 |
| 218397_at | 11.66 | 4.82E-05 | 1.086945882 | NM_001114636| | NA |
[truncated: 60,026 more chars]
