# Supplementary material for: Transcriptional Signature and Memory Retention of Human-Induced Pluripotent Stem Cells
Source: PLoS One. 2009 Sep 18;4(9):e7076. doi: 10.1371/journal.pone.0007076 (PMC2741600; doi:10.1371/journal.pone.0007076)
Supplement: Table S4 — NSC-enriched probes in IPSC versus NSC. Probesets enriched in group-wise comparisons: Column headings are probeset identifiers, T-statistic, P-value, Fold-Change (log2), Refseq identifier and Description of the gene. (NA indicates no Refseq annotation). (9.01 MB DOC) [file pone.0007076.s008.doc]

| Probeset | T-statistic | P-value | Fold-Change (log2) | Refseq | Description |
| --- | --- | --- | --- | --- | --- |
| 236761_at | 218.34 | 5.70E-10 | 2.603756675 | NM_199000| | LHFPL3,lipoma HMGIC fusion partner-like 3 |
| 227221_at | 152.89 | 8.21E-10 | 1.276675774 | NA |  |
| 209539_at | 144.6 | 9.92E-10 | 1.414634246 | NM_004840| | ARHGEF6,Rac/Cdc42 guanine nucleotide exchange factor 6 |
| 207327_at | 132.95 | 1.23E-09 | 3.194046339 | NM_004100| | EYA4,eyes absent 4 isoform a |
| 232095_at | 126.88 | 1.25E-09 | 1.221625081 | NA |  |
| 213029_at | 126.81 | 1.25E-09 | 1.817539054 | NM_005596| | NFIB,nuclear factor I/B |
| 209859_at | 126.64 | 1.25E-09 | 1.77067214 | NM_015163| | TRIM9,tripartite motif protein 9 isoform 1 |
| 244797_at | 124.54 | 1.35E-09 | 1.623898665 | NA |  |
| 238877_at | 123.07 | 1.35E-09 | 3.757797911 | NM_004100| | EYA4,eyes absent 4 isoform a |
| 218966_at | 122.31 | 1.35E-09 | 1.246704462 | NM_018728| | MYO5C,myosin VC |
| 209289_at | 119.15 | 1.41E-09 | 1.713101494 | NM_005596| | NFIB,nuclear factor I/B |
| 227805_at | 118.63 | 1.41E-09 | 1.31045891 | NA |  |
| 219415_at | 115.81 | 1.61E-09 | 1.405439275 | NM_001005367| | TTYH1,tweety 1 isoform 2 |
| 202800_at | 114.48 | 1.65E-09 | 1.384318792 | NM_004172| | SLC1A3,solute carrier family 1 (glial high affinity |
| 203540_at | 109.33 | 2.09E-09 | 3.115490761 | NM_002055| | GFAP,glial fibrillary acidic protein |
| 218613_at | 108.76 | 2.11E-09 | 1.25580379 | NM_015310| | PSD3,ADP-ribosylation factor guanine nucleotide |
| 218309_at | 107.35 | 2.25E-09 | 1.334593861 | NM_018584| | CaMKIINalpha,calcium/calmodulin-dependent protein kinase II |
| 235977_at | 106.19 | 2.28E-09 | 2.173423533 | NM_198461| | FLJ45273,FLJ45273 protein |
| 220316_at | 104.35 | 2.28E-09 | 2.133536233 | NM_022123| | NPAS3,neuronal PAS domain protein 3 |
| 205103_at | 103.14 | 2.42E-09 | 2.110501069 | NM_006365| | CROC4,transcriptional activator of the c-fos promoter |
| 202484_s_at | 102.23 | 2.49E-09 | 2.589895765 | NM_003927| | MBD2,methyl-CpG binding domain protein 2 isoform 1 |
| 225182_at | 95.22 | 3.12E-09 | 1.411414849 | NM_006134| | C21orf4,HCV p7-transregulated protein 3 |
| 223741_s_at | 94.55 | 3.18E-09 | 1.307074906 | NM_032646| | TTYH2,tweety 2 isoform 1 |
| 213714_at | 94.16 | 3.19E-09 | 1.506875802 | NM_000724| | CACNB2,calcium channel, voltage-dependent, beta 2 |
| 234985_at | 93.32 | 3.33E-09 | 1.223242289 | NM_174902| | LOC143458,hypothetical protein LOC143458 |
| 235829_at | 93.15 | 3.33E-09 | 1.588067206 | NA |  |
| 239230_at | 92.7 | 3.39E-09 | 1.209925969 | NM_001010926| | HES5,hairy and enhancer of split 5 |
| 235308_at | 92.56 | 3.39E-09 | 1.861893242 | NM_015642| | ZBTB20,zinc finger and BTB domain containing 20 |
| 232195_at | 91.68 | 3.55E-09 | 2.605384306 | NM_020752| | GPR158,G protein-coupled receptor 158 |
| 212251_at | 90.87 | 3.61E-09 | 1.103146713 | NM_178812| | LYRIC,LYRIC/3D3 |
| 209469_at | 90.04 | 3.63E-09 | 1.635297942 | NM_005277| | GPM6A,glycoprotein M6A isoform 1 |
| 206701_x_at | 89.82 | 3.63E-09 | 1.258735178 | NM_000115| | EDNRB,endothelin receptor type B isoform 1 |
| 227666_at | 89 | 3.64E-09 | 2.256610578 | NM_001040260| | NA |
| 238058_at | 88.93 | 3.64E-09 | 3.025070427 | NA |  |
| 205112_at | 88.2 | 3.81E-09 | 2.183491009 | NM_016341| | PLCE1,pancreas-enriched phospholipase C |
| 230184_at | 87.86 | 3.83E-09 | 1.542452641 | NA |  |
| 217870_s_at | 87.71 | 3.83E-09 | 1.190270382 | NM_016308| | UMP-CMPK,UMP-CMP kinase |
| 200621_at | 85.78 | 4.15E-09 | 1.264460291 | NM_004078| | CSRP1,cysteine and glycine-rich protein 1 |
| 201152_s_at | 84.63 | 4.41E-09 | 1.405809697 | NM_021038| | MBNL1,muscleblind-like 1 isoform a |
| 202096_s_at | 83.1 | 4.81E-09 | 1.422637893 | NM_000714| | BZRP,peripheral benzodiazapine receptor isoform PBR |
| 214805_at | 82.27 | 5.02E-09 | 1.18550659 | NM_001416| | EIF4A1,eukaryotic translation initiation factor 4A, |
| 203963_at | 82.1 | 5.02E-09 | 1.523091203 | NM_001218| | CA12,carbonic anhydrase XII isoform 1 precursor |
| 210138_at | 81.59 | 5.08E-09 | 2.182290725 | NM_003702| | RGS20,regulator of G-protein signalling 20 |
| 222871_at | 81.3 | 5.10E-09 | 1.785295177 | NM_018203| | FLJ10748,hypothetical protein FLJ10748 |
| 212387_at | 81.26 | 5.10E-09 | 1.166306553 | NM_001083962| | NA |
| 212558_at | 81.02 | 5.11E-09 | 1.202306522 | NM_005841| | SPRY1,sprouty homolog 1, antagonist of FGF signaling |
| 209598_at | 80.4 | 5.27E-09 | 1.303661156 | NM_007257| | NA |
| 226377_at | 80.39 | 5.27E-09 | 2.2416681 | NA |  |
| 212510_at | 79.32 | 5.65E-09 | 1.561109917 | NM_015141| | GPD1L,glycerol-3-phosphate dehydrogenase 1-like |
| 209988_s_at | 79.04 | 5.74E-09 | 1.779604799 | NM_004316| | ASCL1,achaete-scute complex homolog-like 1 |
| 238850_at | 78.96 | 5.74E-09 | 2.643058601 | NA |  |
| 205646_s_at | 78.67 | 5.84E-09 | 1.728112088 | NM_000280| | PAX6,paired box gene 6 isoform a |
| 218718_at | 77.82 | 6.19E-09 | 1.424412117 | NM_016205| | PDGFC,platelet-derived growth factor C precursor |
| 202070_s_at | 77.51 | 6.19E-09 | 1.229208378 | NM_005530| | IDH3A,isocitrate dehydrogenase 3 (NAD+) alpha |
| 205412_at | 77.5 | 6.19E-09 | 1.144291028 | NM_000019| | ACAT1,acetyl-Coenzyme A acetyltransferase 1 precursor |
| 201136_at | 77.1 | 6.19E-09 | 1.376477747 | NM_002668| | PLP2,proteolipid protein 2 (colonic |
| 219051_x_at | 76.75 | 6.29E-09 | 1.504963145 | NM_024042| | METRN,meteorin, glial cell differentiation regulator |
| 219148_at | 76.73 | 6.29E-09 | 1.362750576 | NM_018492| | PBK,T-LAK cell-originated protein kinase |
| 219511_s_at | 76.46 | 6.34E-09 | 1.433144672 | NM_005460| | SNCAIP,synuclein alpha interacting protein |
| 212830_at | 76.2 | 6.40E-09 | 1.775023944 | NM_001080497| | NA |
| 230272_at | 76.05 | 6.40E-09 | 3.33694501 | NA |  |
| 209301_at | 75.67 | 6.52E-09 | 1.214884682 | NM_000067| | CA2,carbonic anhydrase II |
| 213395_at | 75.57 | 6.52E-09 | 2.606744965 | NM_015166| | MLC1,megalencephalic leukoencephalopathy with |
| 222217_s_at | 75.55 | 6.52E-09 | 1.31986465 | NM_024330| | SLC27A3,solute carrier family 27 member 3 |
| 205143_at | 75.39 | 6.52E-09 | 1.906432176 | NM_004386| | CSPG3,chondroitin sulfate proteoglycan 3 (neurocan) |
| 203232_s_at | 75.13 | 6.61E-09 | 1.544086115 | NM_000332| | ATXN1,ataxin 1 |
| 211467_s_at | 75.06 | 6.61E-09 | 2.209471005 | NM_005596| | NFIB,nuclear factor I/B |
| 223594_at | 74.8 | 6.69E-09 | 1.595072555 | NM_032256| | DKFZp434K2435,hypothetical protein DKFZp434K2435 |
| 225381_at | 74.74 | 6.69E-09 | 2.570836571 | NA |  |
| 204508_s_at | 74.52 | 6.79E-09 | 1.532715539 | NM_001218| | CA12,carbonic anhydrase XII isoform 1 precursor |
| 200916_at | 73.93 | 6.97E-09 | 1.175525625 | NM_003564| | TAGLN2,transgelin 2 |
| 201464_x_at | 73.23 | 7.32E-09 | 1.209262996 | NM_002228| | JUN,v-jun avian sarcoma virus 17 oncogene homolog |
| 208630_at | 72.89 | 7.43E-09 | 1.038679474 | NM_000182| | HADHA,hydroxyacyl dehydrogenase, subunit A |
| 210869_s_at | 72.43 | 7.72E-09 | 1.266808929 | NM_006500| | MCAM,melanoma cell adhesion molecule |
| 226806_s_at | 72.02 | 7.91E-09 | 2.418574463 | NA |  |
| 209470_s_at | 71.87 | 7.97E-09 | 1.741259937 | NM_005277| | GPM6A,glycoprotein M6A isoform 1 |
| 203661_s_at | 71.47 | 8.15E-09 | 1.472636578 | NM_003275| | TMOD1,tropomodulin 1 |
| 215867_x_at | 71.47 | 8.15E-09 | 1.489271138 | NM_001218| | CA12,carbonic anhydrase XII isoform 1 precursor |
| 212895_s_at | 70.71 | 8.53E-09 | 1.212788867 | NM_001092| | ABR,active breakpoint cluster region-related |
| 202132_at | 70.46 | 8.59E-09 | 1.344696084 | NM_015472| | WWTR1,WW domain containing transcription regulator 1 |
| 228461_at | 69.91 | 8.86E-09 | 1.752607322 | NM_001099289| | NA |
| 244403_at | 69.9 | 8.86E-09 | 2.476882809 | NM_201253| | CRB1,crumbs homolog 1 isoform II precursor |
| 202935_s_at | 69.83 | 8.87E-09 | 1.44212941 | NM_000346| | SOX9,transcription factor SOX9 |
| 202910_s_at | 69.79 | 8.87E-09 | 1.87911187 | NM_001025160| | NA |
| 212070_at | 69.4 | 9.18E-09 | 1.77389178 | NM_005682| | GPR56,G protein-coupled receptor 56 isoform a |
| 227082_at | 69.04 | 9.42E-09 | 2.624418589 | NA |  |
| 203662_s_at | 68.97 | 9.45E-09 | 1.583123353 | NM_003275| | TMOD1,tropomodulin 1 |
| 203708_at | 68.59 | 9.75E-09 | 1.269318192 | NM_001037339| | NA |
| 227400_at | 68.5 | 9.75E-09 | 2.018493418 | NM_002501| | NFIX,nuclear factor I/X (CCAAT-binding transcription |
| 203231_s_at | 68.4 | 9.75E-09 | 1.674501375 | NM_000332| | ATXN1,ataxin 1 |
| 209121_x_at | 67.34 | 1.04E-08 | 1.443324114 | NM_021005| | NR2F2,nuclear receptor subfamily 2, group F, member 2 |
| 209029_at | 66.98 | 1.07E-08 | 1.092374765 | NM_016319| | COPS7A,COP9 complex subunit 7a |
| 200663_at | 66.67 | 1.08E-08 | 1.057698026 | NM_001040034| | NA |
| 209644_x_at | 66.49 | 1.09E-08 | 1.470753075 | NM_000077| | CDKN2A,cyclin-dependent kinase inhibitor 2A isoform 1 |
| 200794_x_at | 66.42 | 1.09E-08 | 1.260452141 | NM_014764| | DAZAP2,DAZ associated protein 2 |
| 217143_s_at | 66.29 | 1.10E-08 | 2.345642969 | NA |  |
| 209686_at | 66.28 | 1.10E-08 | 2.175695289 | NM_006272| | S100B,S100 calcium-binding protein, beta |
| 227452_at | 66.17 | 1.11E-08 | 1.944519632 | NA |  |
| 212063_at | 66.08 | 1.11E-08 | 1.580224506 | NM_000610| | CD44,CD44 antigen isoform 1 precursor |
| 213158_at | 65.61 | 1.14E-08 | 2.644787611 | NA |  |
| 226134_s_at | 65.59 | 1.14E-08 | 1.265277118 | NA |  |
| 230864_at | 65.58 | 1.14E-08 | 2.31766582 | NM_153361| | MGC42105,hypothetical protein MGC42105 |
| 214164_x_at | 65.55 | 1.14E-08 | 1.529994889 | NM_001218| | CA12,carbonic anhydrase XII isoform 1 precursor |
| 204457_s_at | 65.49 | 1.14E-08 | 1.447574463 | NM_002048| | GAS1,growth arrest-specific 1 |
| 207017_at | 65.02 | 1.19E-08 | 1.691634842 | NM_004163| | RAB27B,RAB27B, member RAS oncogene family |
| 213500_at | 64.76 | 1.21E-08 | 1.346326029 | NA |  |
| 227049_at | 64.35 | 1.24E-08 | 1.694580772 | NM_175907| | ZADH2,zinc binding alcohol dehydrogenase, domain |
| 227124_at | 64.15 | 1.26E-08 | 1.238691647 | NA |  |
| 201462_at | 64.09 | 1.26E-08 | 1.147749655 | NM_014766| | SCRN1,secernin 1 |
| 231810_at | 63.36 | 1.33E-08 | 1.201613383 | NM_080626| | BRI3BP,BRI3-binding protein |
| 209834_at | 63.32 | 1.33E-08 | 1.31951673 | NM_004273| | CHST3,carbohydrate (chondroitin 6) sulfotransferase 3 |
| 1559966_a_at | 63.31 | 1.33E-08 | 2.288579655 | NA |  |
| 242086_at | 63.18 | 1.34E-08 | 1.318794673 | NA |  |
| 209789_at | 62.86 | 1.37E-08 | 1.563844197 | NM_006091| | CORO2B,coronin, actin binding protein, 2B |
| 201143_s_at | 62.8 | 1.37E-08 | 1.290998577 | NM_004094| | EIF2S1,eukaryotic translation initiation factor 2, |
| 213032_at | 62.7 | 1.38E-08 | 1.961441373 | NM_005596| | NFIB,nuclear factor I/B |
| 228375_at | 62.61 | 1.38E-08 | 1.64051888 | NM_001015887| | NA |
| 201338_x_at | 62.33 | 1.41E-08 | 1.10290498 | NM_002097| | GTF3A,general transcription factor IIIA |
| 224690_at | 62.26 | 1.42E-08 | 1.219150608 | NM_080821| | C20orf108,chromosome 20 open reading frame 108 |
| 207084_at | 62.13 | 1.43E-08 | 1.485986272 | NM_005604| | POU3F2,POU domain, class 3, transcription factor 2 |
| 232269_x_at | 61.87 | 1.46E-08 | 1.47576832 | NM_024042| | METRN,meteorin, glial cell differentiation regulator |
| 209197_at | 61.84 | 1.46E-08 | 1.182512421 | NM_152280| | SYT11,synaptotagmin 12 |
| 222774_s_at | 61.7 | 1.46E-08 | 1.213359419 | NM_018092| | NETO2,neuropilin- and tolloid-like protein 2 |
| 213939_s_at | 61.57 | 1.47E-08 | 1.349192316 | NM_001037442| | NA |
| 202284_s_at | 61.36 | 1.49E-08 | 1.394314616 | NM_000389| | CDKN1A,cyclin-dependent kinase inhibitor 1A |
| 32094_at | 61.28 | 1.49E-08 | 1.285075612 | NM_004273| | CHST3,carbohydrate (chondroitin 6) sulfotransferase 3 |
| 228708_at | 61.17 | 1.50E-08 | 2.571155759 | NM_004163| | RAB27B,RAB27B, member RAS oncogene family |
| 210393_at | 60.95 | 1.54E-08 | 1.73108227 | NM_003667| | LGR5,leucine-rich repeat-containing G protein-coupled |
| 218055_s_at | 60.66 | 1.56E-08 | 1.11556582 | NM_018268| | WDR41,WD repeat domain 41 |
| 228060_at | 60.6 | 1.56E-08 | 1.236775026 | NM_001029858| | NA |
| 225334_at | 60.3 | 1.61E-08 | 1.251781489 | NM_144591| | C10orf32,hypothetical protein MGC27171 |
| 226250_at | 60.13 | 1.63E-08 | 1.937135277 | NA |  |
| 209184_s_at | 60.09 | 1.63E-08 | 1.148012468 | NM_003749| | IRS2,insulin receptor substrate 2 |
| 213288_at | 60.08 | 1.63E-08 | 1.473253503 | NM_138799| | OACT2,O-acyltransferase (membrane bound) domain |
| 209883_at | 60.01 | 1.64E-08 | 1.51104248 | NM_015101| | GLT25D2,glycosyltransferase 25 domain containing 2 |
| 206071_s_at | 59.88 | 1.65E-08 | 2.171092572 | NM_005233| | EPHA3,ephrin receptor EphA3 isoform a precursor |
| 205304_s_at | 59.88 | 1.65E-08 | 1.487091081 | NM_004982| | KCNJ8,potassium inwardly-rectifying channel J8 |
| 225127_at | 59.5 | 1.71E-08 | 1.190187519 | NM_020823| | NA |
| 230258_at | 59.39 | 1.72E-08 | 2.174740975 | NM_001042413| | NA |
| 226740_x_at | 59.38 | 1.72E-08 | 1.143549225 | NM_001037501| | NA |
| 200774_at | 59.19 | 1.73E-08 | 1.172578134 | NM_014612| | C9orf10,C9orf10 protein |
| 215290_at | 59.15 | 1.73E-08 | 2.261597598 | NA |  |
| 225533_at | 59.15 | 1.73E-08 | 1.232291828 | NM_001009936| | PHF19,PHD finger protein 19 isoform b |
| 203185_at | 59.05 | 1.74E-08 | 1.398251518 | NM_014737| | RASSF2,Ras association domain family 2 isoform 1 |
| 200884_at | 58.87 | 1.77E-08 | 1.162285152 | NM_001823| | CKB,brain creatine kinase |
| 223092_at | 58.86 | 1.77E-08 | 1.431830641 | NM_054027| | ANKH,ankylosis, progressive homolog |
| 208798_x_at | 58.68 | 1.79E-08 | 1.16838517 | NM_181077| | GOLGIN-67,golgin-67 isoform c |
| 218203_at | 58.59 | 1.80E-08 | 1.07056675 | NM_013338| | ALG5,dolichyl phosphate glucosyltransferase |
| 224976_at | 58.46 | 1.81E-08 | 2.246117821 | NM_005595| | NFIA,nuclear factor I/A |
| 222301_at | 58.2 | 1.85E-08 | 2.130037192 | NM_006365| | CROC4,transcriptional activator of the c-fos promoter |
| 1568629_s_at | 58.12 | 1.87E-08 | 1.125854033 | NM_005027| | PIK3R2,phosphoinositide-3-kinase, regulatory subunit 2 |
| 203324_s_at | 57.98 | 1.88E-08 | 1.541798639 | NM_001233| | CAV2,caveolin 2 isoform a and b |
| 226913_s_at | 57.79 | 1.92E-08 | 1.4842708 | NM_014587| | SOX8,SRY (sex determining region Y)-box 8 |
| 53991_at | 57.39 | 1.99E-08 | 1.358491402 | NM_015689| | KIAA1277,KIAA1277 protein |
| 226018_at | 57.23 | 2.02E-08 | 1.541684563 | NM_152793| | Ells1,hypothetical protein Ells1 |
| 223673_at | 57.08 | 2.04E-08 | 1.569746854 | NM_002920| | RFX4,regulatory factor X4 isoform b |
| 204685_s_at | 56.9 | 2.07E-08 | 1.515097441 | NM_001001331| | ATP2B2,plasma membrane calcium ATPase 2 isoform a |
| 228837_at | 56.88 | 2.07E-08 | 1.285841042 | NM_001083962| | NA |
| 226895_at | 56.81 | 2.08E-08 | 1.684997165 | NM_005597| | NFIC,nuclear factor I/C isoform 1 |
| 213830_at | 56.81 | 2.08E-08 | 2.000491612 | NA |  |
| 228315_at | 56.73 | 2.08E-08 | 1.314842283 | NA |  |
| 222146_s_at | 56.7 | 2.08E-08 | 1.158980402 | NM_001083962| | NA |
| 222403_at | 56.41 | 2.12E-08 | 1.290178375 | NM_014342| | MTCH2,mitochondrial carrier homolog 2 |
| 202554_s_at | 55.98 | 2.23E-08 | 1.403049672 | NM_000849| | GSTM3,glutathione S-transferase M3 |
| 235457_at | 55.9 | 2.23E-08 | 1.308291601 | NM_032427| | MAML2,mastermind-like 2 |
| 205794_s_at | 55.82 | 2.23E-08 | 1.368975096 | NM_002515| | NOVA1,neuro-oncological ventral antigen 1 isoform 1 |
| 211474_s_at | 55.82 | 2.23E-08 | 1.157441303 | NM_004568| | SERPINB6,serine (or cysteine) proteinase inhibitor, clade |
| 218888_s_at | 55.8 | 2.23E-08 | 1.229801259 | NM_018092| | NETO2,neuropilin- and tolloid-like protein 2 |
| 225214_at | 55.8 | 2.23E-08 | 1.25015997 | NM_002799| | PSMB7,proteasome beta 7 subunit proprotein |
| 219107_at | 55.63 | 2.27E-08 | 1.596341968 | NM_021948| | BCAN,brevican isoform 1 |
| 200814_at | 55.57 | 2.28E-08 | 1.079248927 | NM_006263| | PSME1,proteasome activator subunit 1 isoform 1 |
| 219249_s_at | 55.54 | 2.28E-08 | 1.102620462 | NM_021939| | FKBP10,FK506 binding protein 10, 65 kDa |
| 1557852_at | 55.47 | 2.29E-08 | 1.586722768 | NA |  |
| 228581_at | 55.43 | 2.29E-08 | 1.858873584 | NM_002241| | KCNJ10,potassium inwardly-rectifying channel J10 |
| 222803_at | 55.38 | 2.30E-08 | 1.249602833 | NM_020200| | PRTFDC1,phosphoribosyl transferase domain containing 1 |
| 205030_at | 55.37 | 2.30E-08 | 1.326797566 | NM_001446| | FABP7,fatty acid binding protein 7, brain |
| 225144_at | 55.34 | 2.30E-08 | 1.278728213 | NM_001204| | BMPR2,bone morphogenetic protein receptor, type II |
| 212543_at | 55.31 | 2.30E-08 | 1.298944314 | NM_001624| | NA |
| 224593_at | 55.21 | 2.33E-08 | 1.133772229 | NM_152437| | ZFOC1,zinc finger protein ZFOC1 |
| 242455_at | 55.12 | 2.33E-08 | 1.477043996 | NM_005604| | POU3F2,POU domain, class 3, transcription factor 2 |
| 209167_at | 55.08 | 2.33E-08 | 1.213193524 | NM_001001994| | GPM6B,glycoprotein M6B isoform 4 |
| 205996_s_at | 55.06 | 2.33E-08 | 1.519960176 | NM_001625| | AK2,adenylate kinase 2 isoform a |
| 226016_at | 55.05 | 2.33E-08 | 1.39820329 | NM_001025079| | NA |
| 209466_x_at | 54.86 | 2.38E-08 | 1.196027166 | NM_002825| | PTN,pleiotrophin |
| 229127_at | 54.85 | 2.38E-08 | 1.361427199 | NM_021219| | JAM2,junctional adhesion molecule 2 precursor |
| 226810_at | 54.82 | 2.38E-08 | 1.512181916 | NM_024576| | OGFRL1,opioid growth factor receptor-like 1 |
| 212357_at | 54.62 | 2.42E-08 | 1.407126822 | NM_015159| | NA |
| 208818_s_at | 54.54 | 2.44E-08 | 1.164561688 | NM_000754| | COMT,catechol-O-methyltransferase isoform MB-COMT |
| 241998_at | 54.51 | 2.44E-08 | 1.494319609 | NM_001099334| | NA |
| 229281_at | 54.42 | 2.45E-08 | 2.371165985 | NM_022123| | NPAS3,neuronal PAS domain protein 3 |
| 236834_at | 54.42 | 2.45E-08 | 1.482473362 | NM_152540| | SCFD2,sec1 family domain containing 2 |
| 229266_at | 54.32 | 2.46E-08 | 1.700661024 | NA |  |
| 206070_s_at | 54.19 | 2.47E-08 | 2.427539877 | NM_005233| | EPHA3,ephrin receptor EphA3 isoform a precursor |
| 208920_at | 54.17 | 2.47E-08 | 1.458104866 | NM_003130| | SRI,sorcin isoform a |
| 222797_at | 54.15 | 2.47E-08 | 1.2968709 | NM_020134| | DPYSL5,dihydropyrimidinase-like 5 |
| 223184_s_at | 53.97 | 2.50E-08 | 1.739986489 | NM_001037553| | NA |
| 225725_at | 53.83 | 2.53E-08 | 1.412231286 | NA |  |
| 205164_at | 53.79 | 2.53E-08 | 1.222247154 | NM_014291| | GCAT,glycine C-acetyltransferase precursor |
| 231430_at | 53.76 | 2.53E-08 | 1.829676985 | NM_175885| | MGC33846,hypothetical protein MGC33846 |
| 226780_s_at | 53.71 | 2.54E-08 | 1.146501126 | NM_197964| | HSPC268,hypothetical protein HSPC268 |
| 215014_at | 53.65 | 2.55E-08 | 2.912593643 | NM_004980| | KCND3,potassium voltage-gated channel, Shal-related |
| 218611_at | 53.52 | 2.57E-08 | 1.189589821 | NM_016545| | IER5,immediate early response 5 |
| 209987_s_at | 53.45 | 2.58E-08 | 1.914745609 | NM_004316| | ASCL1,achaete-scute complex homolog-like 1 |
| 217762_s_at | 53.35 | 2.60E-08 | 1.208808526 | NM_006868| | RAB31,RAB31, member RAS oncogene family |
| 201983_s_at | 53.14 | 2.64E-08 | 1.676215073 | NM_005228| | EGFR,epidermal growth factor receptor isoform a |
| 205450_at | 53.06 | 2.66E-08 | 1.241103338 | NM_001122670| | NA |
| 205475_at | 53.03 | 2.66E-08 | 2.251993611 | NM_007281| | SCRG1,scrapie responsive protein 1 |
| 226656_at | 53.01 | 2.66E-08 | 1.35288178 | NM_006371| | CRTAP,cartilage associated protein precursor |
| 31874_at | 52.96 | 2.66E-08 | 1.273680637 | NM_006478| | GAS2L1,growth arrest-specific 2 like 1 isoform a |
| 232125_at | 52.81 | 2.70E-08 | 2.862239638 | NA |  |
| 225325_at | 52.74 | 2.70E-08 | 1.392599883 | NM_017694| | FLJ20160,FLJ20160 protein |
| 211964_at | 52.73 | 2.70E-08 | 1.107195427 | NM_001846| | COL4A2,alpha 2 type IV collagen preproprotein |
| 213768_s_at | 52.41 | 2.76E-08 | 1.816929767 | NM_004316| | ASCL1,achaete-scute complex homolog-like 1 |
| 225842_at | 52.39 | 2.76E-08 | 1.169590883 | NM_007350| | PHLDA1,pleckstrin homology-like domain, family A, |
| 208949_s_at | 52.36 | 2.76E-08 | 1.276180105 | NM_002306| | LGALS3,galectin-3 |
| 204556_s_at | 52.11 | 2.83E-08 | 1.221030954 | NM_014934| | DZIP1,DAZ interacting protein 1 |
| 223282_at | 52.1 | 2.83E-08 | 2.59884505 | NM_005786| | SDCCAG33,serologically defined colon cancer antigen 33 |
| 206765_at | 52.06 | 2.84E-08 | 1.822070666 | NM_000891| | KCNJ2,potassium inwardly-rectifying channel J2 |
| 228953_at | 52.02 | 2.85E-08 | 1.392813746 | NM_001080435| | NA |
| 238906_s_at | 51.9 | 2.89E-08 | 2.400860029 | NM_020663| | RHOJ,TC10-like Rho GTPase |
| 1552621_at | 51.86 | 2.90E-08 | 1.283382403 | NM_032959| | POLR2J2,DNA directed RNA polymerase II polypeptide |
| 225996_at | 51.78 | 2.92E-08 | 2.767202451 | NM_198461| | FLJ45273,FLJ45273 protein |
| 224716_at | 51.7 | 2.94E-08 | 1.151610177 | NM_178148| | SLC35B2,solute carrier family 35, member B2 |
| 218024_at | 51.61 | 2.96E-08 | 1.247658256 | NM_016098| | BRP44L,brain protein 44-like |
| 226883_at | 51.59 | 2.96E-08 | 1.13019796 | NA |  |
| 212758_s_at | 51.57 | 2.96E-08 | 1.574197822 | NM_030751| | TCF8,transcription factor 8 (represses interleukin 2 |
| 228218_at | 51.49 | 2.98E-08 | 1.388046804 | NA |  |
| 203336_s_at | 51.38 | 3.01E-08 | 1.168227841 | NM_004763| | ITGB1BP1,integrin cytoplasmic domain-associated protein 1 |
| 227055_at | 51.28 | 3.04E-08 | 1.430965265 | NM_152637| | MGC17301,hypothetical protein MGC17301 |
| 202693_s_at | 51.22 | 3.05E-08 | 1.325646982 | NM_004760| | STK17A,serine/threonine kinase 17a |
| 212828_at | 51.11 | 3.09E-08 | 1.221530127 | NM_003898| | SYNJ2,synaptojanin 2 |
| 223013_at | 50.93 | 3.12E-08 | 1.080362198 | NM_024665| | TBL1XR1,nuclear receptor co-repressor/HDAC3 complex |
| 225240_s_at | 50.86 | 3.13E-08 | 1.183265669 | NM_138962| | MSI2,musashi 2 isoform a |
| 226368_at | 50.82 | 3.14E-08 | 1.621215342 | NM_018413| | CHST11,carbohydrate (chondroitin 4) sulfotransferase |
| 216331_at | 50.72 | 3.17E-08 | 1.298268884 | NM_002206| | ITGA7,integrin alpha 7 precursor |
| 227178_at | 50.51 | 3.24E-08 | 1.332889363 | NM_001025076| | NA |
| 227625_s_at | 50.51 | 3.24E-08 | 1.089680137 | NM_005861| | STUB1,STIP1 homology and U-box containing protein 1 |
| 201764_at | 50.38 | 3.29E-08 | 1.264889497 | NM_024056| | MGC5576,hypothetical protein MGC5576 |
| 225617_at | 50.3 | 3.31E-08 | 1.293455981 | NM_002540| | ODF2,outer dense fiber of sperm tails 2 isoform 1 |
| 206140_at | 50.09 | 3.38E-08 | 2.138334602 | NM_004789| | LHX2,LIM homeobox protein 2 |
| 215411_s_at | 50.05 | 3.38E-08 | 1.228179837 | NM_147200| | C6orf4,chromosome 6 open reading frame 4 isoform 1 |
| 227121_at | 50.04 | 3.38E-08 | 2.350691338 | NA |  |
| 242985_x_at | 50.01 | 3.38E-08 | 1.843349182 | NM_001113561| | NA |
| 209185_s_at | 49.95 | 3.39E-08 | 1.140488116 | NM_003749| | IRS2,insulin receptor substrate 2 |
| 227954_at | 49.86 | 3.42E-08 | 1.390888474 | NM_001034841| | NA |
| 222446_s_at | 49.85 | 3.42E-08 | 1.289089065 | NM_012105| | BACE2,beta-site APP-cleaving enzyme 2 isoform A |
| 225198_at | 49.74 | 3.46E-08 | 1.107660654 | NM_003574| | VAPA,vesicle-associated membrane protein-associated |
| 242837_at | 49.72 | 3.46E-08 | 1.342649139 | NM_005626| | SFRS4,splicing factor, arginine/serine-rich 4 |
| 215259_s_at | 49.7 | 3.46E-08 | 1.596581037 | NM_145296| | IGSF4C,immunoglobulin superfamily, member 4C |
| 231319_x_at | 49.69 | 3.46E-08 | 1.293556161 | NM_022342| | KIF9,kinesin family member 9 |
| 223071_at | 49.66 | 3.46E-08 | 1.087677128 | NM_016097| | IER3IP1,immediate early response 3 interacting protein |
| 224995_at | 49.65 | 3.46E-08 | 1.255309686 | NM_020148| | SPIRE1,spire homolog 1 |
| 218548_x_at | 49.59 | 3.47E-08 | 1.208431206 | NM_015926| | TEX264,testis expressed sequence 264 |
| 201185_at | 49.59 | 3.47E-08 | 1.383507262 | NM_002775| | PRSS11,protease, serine, 11 |
| 218834_s_at | 49.57 | 3.47E-08 | 1.235299303 | NM_017870| | HSPA5BP1,GBP protein isoform a |
| 209290_s_at | 49.54 | 3.47E-08 | 1.754069934 | NM_005596| | NFIB,nuclear factor I/B |
| 225685_at | 49.54 | 3.47E-08 | 1.232519368 | NA |  |
| 229380_at | 49.38 | 3.54E-08 | 1.591332241 | NA |  |
| 226923_at | 49.37 | 3.54E-08 | 1.261358543 | NM_152540| | SCFD2,sec1 family domain containing 2 |
| 211976_at | 49.34 | 3.55E-08 | 1.126853181 | NA |  |
| 223035_s_at | 49.28 | 3.57E-08 | 1.12552379 | NM_005687| | FARSLB,phenylalanine-tRNA synthetase-like, beta |
| 213157_s_at | 49.24 | 3.58E-08 | 1.201645511 | NM_015253| | KIAA0523,KIAA0523 protein |
| 206935_at | 49.15 | 3.61E-08 | 2.226621671 | NM_002590| | PCDH8,protocadherin 8 isoform 1 precursor |
| 227617_at | 49.13 | 3.61E-08 | 1.316629743 | NM_001010866| | RP13-15M17.2,novel protein |
| 217837_s_at | 49.12 | 3.61E-08 | 1.194276372 | NM_001005753| | VPS24,vacuolar protein sorting 24 isoform 2 |
| 212174_at | 49.01 | 3.65E-08 | 1.532556308 | NM_001625| | AK2,adenylate kinase 2 isoform a |
| 206022_at | 48.97 | 3.66E-08 | 2.231493993 | NM_000266| | NDP,Norrie disease protein |
| 224215_s_at | 48.94 | 3.66E-08 | 1.423002484 | NM_005618| | DLL1,delta-like 1 |
| 205472_s_at | 48.86 | 3.69E-08 | 1.922475444 | NM_004392| | DACH1,dachshund homolog 1 isoform c |
| 212022_s_at | 48.82 | 3.69E-08 | 1.170084134 | NM_002417| | MKI67,antigen identified by monoclonal antibody Ki-67 |
| 202936_s_at | 48.77 | 3.71E-08 | 1.40334924 | NM_000346| | SOX9,transcription factor SOX9 |
| 212991_at | 48.75 | 3.71E-08 | 1.748685996 | NM_012347| | FBXO9,F-box only protein 9 isoform 1 |
| 235795_at | 48.65 | 3.73E-08 | 2.124345583 | NM_000280| | PAX6,paired box gene 6 isoform a |
| 206397_x_at | 48.64 | 3.73E-08 | 1.360241149 | NM_001492| | GDF1,growth differentiation factor 1 |
| 205481_at | 48.62 | 3.74E-08 | 1.425467974 | NM_000674| | ADORA1,adenosine A1 receptor |
| 221216_s_at | 48.53 | 3.77E-08 | 1.269863399 | NM_001031694| | NA |
| 217763_s_at | 48.49 | 3.78E-08 | 1.1901657 | NM_006868| | RAB31,RAB31, member RAS oncogene family |
| 209094_at | 48.48 | 3.78E-08 | 1.126122997 | NM_012137| | DDAH1,dimethylarginine dimethylaminohydrolase 1 |
| 1569303_s_at | 48.46 | 3.79E-08 | 1.306932489 | NM_003702| | RGS20,regulator of G-protein signalling 20 |
| 222761_at | 48.42 | 3.79E-08 | 1.300814608 | NM_017693| | BIVM,basic, immunoglobulin-like variable motif |
| 242138_at | 48.27 | 3.83E-08 | 2.40454666 | NM_001038493| | NA |
| 213601_at | 48.25 | 3.83E-08 | 1.941427398 | NM_003061| | SLIT1,slit homolog 1 |
| 205638_at | 48.25 | 3.83E-08 | 2.165038541 | NM_001704| | BAI3,brain-specific angiogenesis inhibitor 3 |
| 212294_at | 48.18 | 3.86E-08 | 1.256477397 | NM_018841| | GNG12,G-protein gamma-12 subunit |
| 1552256_a_at | 48.04 | 3.91E-08 | 1.190448367 | NM_001082959| | NA |
| 204105_s_at | 47.84 | 4.00E-08 | 1.532199751 | NM_001037132| | NA |
| 226547_at | 47.76 | 4.04E-08 | 1.153392652 | NM_001099412| | NA |
| 236261_at | 47.73 | 4.04E-08 | 1.492040293 | NM_032523| | OSBPL6,oxysterol-binding protein-like protein 6 isoform |
| 1559477_s_at | 47.63 | 4.07E-08 | 2.332875655 | NM_002398| | MEIS1,Meis1 homolog |
| 205383_s_at | 47.55 | 4.10E-08 | 1.935003064 | NM_015642| | ZBTB20,zinc finger and BTB domain containing 20 |
| 208912_s_at | 47.47 | 4.12E-08 | 1.129388707 | NM_033133| | CNP,2',3'-cyclic nucleotide 3' phosphodiesterase |
| 226760_at | 47.45 | 4.12E-08 | 1.139774308 | NA |  |
| 218633_x_at | 47.4 | 4.12E-08 | 1.175619119 | NM_018394| | ABHD10,abhydrolase domain containing 10 |
| 225288_at | 47.39 | 4.12E-08 | 1.325369097 | NM_032888| | COL27A1,collagen, type XXVII, alpha 1 |
| 204456_s_at | 47.36 | 4.14E-08 | 1.681223812 | NM_002048| | GAS1,growth arrest-specific 1 |
| 212385_at | 47.3 | 4.16E-08 | 1.215442408 | NM_001083962| | NA |
| 239952_at | 47.28 | 4.17E-08 | 1.913146241 | NM_030751| | TCF8,transcription factor 8 (represses interleukin 2 |
| 225132_at | 47.25 | 4.18E-08 | 1.317121958 | NM_012158| | FBXL3,F-box and leucine-rich repeat protein 3 |
| 213411_at | 47.2 | 4.19E-08 | 1.424449499 | NM_004194| | ADAM22,a disintegrin and metalloproteinase domain 22 |
| 229715_at | 47.12 | 4.22E-08 | 2.017139657 | NA |  |
| 224826_at | 47.12 | 4.22E-08 | 1.286989454 | NM_019593| | KIAA1434,hypothetical protein KIAA1434 |
| 238041_at | 47.04 | 4.26E-08 | 2.011292017 | NA |  |
| 224715_at | 47.02 | 4.26E-08 | 1.168061583 | NM_052844| | WDR34,WD repeat domain 34 |
| 203908_at | 46.95 | 4.29E-08 | 1.872523042 | NM_001098484| | NA |
| 235289_at | 46.81 | 4.33E-08 | 1.678711389 | NM_020390| | EIF5A2,eIF-5A2 protein |
| 230728_at | 46.78 | 4.34E-08 | 1.149280615 | NA |  |
| 44790_s_at | 46.68 | 4.38E-08 | 1.799978289 | NM_025113| | C13orf18,chromosome 13 open reading frame 18 |
| 201162_at | 46.67 | 4.38E-08 | 1.324544205 | NM_001553| | IGFBP7,insulin-like growth factor binding protein 7 |
| 205880_at | 46.56 | 4.42E-08 | 1.407453855 | NM_002742| | PRKD1,protein kinase D1 |
| 217882_at | 46.52 | 4.43E-08 | 1.049584529 | NM_018447| | LOC55831,30 kDa protein |
| 37408_at | 46.52 | 4.43E-08 | 1.255350648 | NM_006039| | MRC2,mannose receptor, C type 2 |
| 228780_at | 46.47 | 4.44E-08 | 1.677801775 | NA |  |
| 212509_s_at | 46.47 | 4.44E-08 | 1.197448186 | NM_001008528| | TMAP1,transmembrane anchor protein 1 isoform 1 |
| 203354_s_at | 46.47 | 4.44E-08 | 1.245224902 | NM_015310| | PSD3,ADP-ribosylation factor guanine nucleotide |
| 226779_at | 46.42 | 4.47E-08 | 1.156144439 | NA |  |
| 224928_at | 46.35 | 4.48E-08 | 1.405292512 | NM_030648| | SET7,SET domain-containing protein 7 |
| 1556194_a_at | 46.34 | 4.48E-08 | 1.84940062 | NA |  |
| 230412_at | 46.33 | 4.48E-08 | 2.788232479 | NM_022123| | NPAS3,neuronal PAS domain protein 3 |
| 223468_s_at | 46.32 | 4.48E-08 | 1.203403415 | NM_020211| | RGMA,RGM domain family, member A |
| 202202_s_at | 46.32 | 4.48E-08 | 2.352541166 | NM_001105206| | NA |
| 229512_at | 46.31 | 4.48E-08 | 1.376886716 | NM_017848| | CXorf17,chromosome X open reading frame 17 |
| 209590_at | 46.29 | 4.48E-08 | 1.436319543 | NM_001719| | BMP7,bone morphogenetic protein 7 precursor |
| 204271_s_at | 46.24 | 4.51E-08 | 1.19205899 | NM_000115| | EDNRB,endothelin receptor type B isoform 1 |
| 228170_at | 46.21 | 4.51E-08 | 1.520980746 | NM_138983| | OLIG1,oligodendrocyte transcription factor 1 |
| 210345_s_at | 46.16 | 4.52E-08 | 1.425948899 | NM_001372| | DNAH9,dynein, axonemal, heavy polypeptide 9 isoform 2 |
| 201029_s_at | 46.1 | 4.55E-08 | 1.211409838 | NM_001122898| | NA |
| 1554251_at | 46.07 | 4.56E-08 | 1.344728328 | NM_016287| | HP1-BP74,HP1-BP74 |
| 224994_at | 46.01 | 4.58E-08 | 1.454496979 | NM_001221| | CAMK2D,calcium/calmodulin-dependent protein kinase II |
| 213832_at | 45.93 | 4.61E-08 | 2.41135327 | NM_004980| | KCND3,potassium voltage-gated channel, Shal-related |
| 201432_at | 45.87 | 4.63E-08 | 1.14453398 | NM_001752| | CAT,catalase |
| 229259_at | 45.83 | 4.65E-08 | 2.095955722 | NM_002055| | GFAP,glial fibrillary acidic protein |
| 204035_at | 45.82 | 4.65E-08 | 2.20824933 | NM_003469| | SCG2,secretogranin II precursor |
| 208804_s_at | 45.78 | 4.67E-08 | 1.11156132 | NM_006275| | SFRS6,arginine/serine-rich splicing factor 6 |
| 223182_s_at | 45.73 | 4.70E-08 | 1.841398543 | NM_001037553| | NA |
| 203065_s_at | 45.72 | 4.70E-08 | 1.389899428 | NM_001753| | CAV1,caveolin 1 |
| 211737_x_at | 45.67 | 4.73E-08 | 1.163834563 | NM_002825| | PTN,pleiotrophin |
| 202920_at | 45.55 | 4.79E-08 | 1.316526452 | NM_001148| | ANK2,ankyrin 2 isoform 1 |
| 218776_s_at | 45.52 | 4.79E-08 | 1.48370151 | NM_024956| | FLJ23375,hypothetical protein FLJ23375 |
| 205363_at | 45.42 | 4.85E-08 | 2.108930302 | NM_003986| | BBOX1,gamma-butyrobetaine hydroxylase |
| 240122_at | 45.39 | 4.86E-08 | 2.344594761 | NM_017594| | DIRAS2,Di-Ras2 |
| 214175_x_at | 45.29 | 4.90E-08 | 1.363644103 | NM_003687| | PDLIM4,PDZ and LIM domain 4 |
| 201142_at | 45.16 | 4.99E-08 | 1.231710795 | NM_004094| | EIF2S1,eukaryotic translation initiation factor 2, |
| 208662_s_at | 44.96 | 5.11E-08 | 1.219307533 | NM_001001894| | TTC3,tetratricopeptide repeat domain 3 |
| 204085_s_at | 44.88 | 5.15E-08 | 1.64814298 | NM_006493| | CLN5,ceroid-lipofuscinosis, neuronal 5 |
| 209794_at | 44.88 | 5.15E-08 | 1.549367235 | NM_001033117| | NA |
| 200788_s_at | 44.86 | 5.16E-08 | 1.146211219 | NM_003768| | PEA15,phosphoprotein enriched in astrocytes 15 |
| 229656_s_at | 44.82 | 5.18E-08 | 1.962722294 | NA |  |
| 221495_s_at | 44.81 | 5.18E-08 | 1.14968094 | NM_014972| | KIAA1049,KIAA1049 protein |
| 224738_x_at | 44.81 | 5.18E-08 | 1.082167897 | NM_198486| | RPL7L1,ribosomal protein L7-like 1 |
| 215091_s_at | 44.77 | 5.18E-08 | 1.106437393 | NM_002097| | GTF3A,general transcription factor IIIA |
| 218692_at | 44.58 | 5.29E-08 | 1.884500088 | NM_001099743| | NA |
| 208661_s_at | 44.36 | 5.43E-08 | 1.210621975 | NM_001001894| | TTC3,tetratricopeptide repeat domain 3 |
| 217904_s_at | 44.29 | 5.46E-08 | 1.422297006 | NM_012104| | BACE1,beta-site APP-cleaving enzyme 1 isoform A |
| 213574_s_at | 44.26 | 5.47E-08 | 1.123031273 | NA |  |
| 212504_at | 44.26 | 5.47E-08 | 1.163766535 | NM_014974| | KIAA0934,KIAA0934 |
| 213289_at | 44.22 | 5.50E-08 | 1.226207266 | NM_198450| | CXorf33,chromosome X open reading frame 33 |
| 212361_s_at | 44.21 | 5.50E-08 | 1.165078077 | NM_001681| | ATP2A2,ATPase, Ca++ transporting, cardiac muscle, slow |
| 204343_at | 44.13 | 5.56E-08 | 1.379672567 | NM_001089| | ABCA3,ATP-binding cassette, sub-family A member 3 |
| 205677_s_at | 44.06 | 5.62E-08 | 1.187849347 | NA |  |
| 238753_at | 43.93 | 5.72E-08 | 1.146099921 | NM_014286| | FREQ,frequenin homolog |
| 219628_at | 43.88 | 5.75E-08 | 1.396526923 | NM_022470| | WIG1,p53 target zinc finger protein isoform 1 |
| 224520_s_at | 43.78 | 5.81E-08 | 1.927520173 | NM_032735| | MGC13168,hypothetical protein MGC13168 |
| 223214_s_at | 43.76 | 5.83E-08 | 1.682316757 | NM_001017926| | NA |
| 220265_at | 43.7 | 5.87E-08 | 1.5242451 | NM_020960| | GPR107,G protein-coupled receptor 107 |
| 236120_at | 43.64 | 5.90E-08 | 1.648068511 | NA |  |
| 228574_at | 43.6 | 5.92E-08 | 1.332083377 | NM_152588| | DKFZp762A217,hypothetical protein DKFZp762A217 |
| 209199_s_at | 43.55 | 5.94E-08 | 1.742805086 | NM_002397| | MEF2C,MADS box transcription enhancer factor 2, |
| 208663_s_at | 43.53 | 5.95E-08 | 1.218443066 | NM_001001894| | TTC3,tetratricopeptide repeat domain 3 |
| 226623_at | 43.53 | 5.95E-08 | 1.91707657 | NM_032439| | PHYHIPL,phytanoyl-CoA hydroxylase interacting |
| 221571_at | 43.42 | 6.02E-08 | 1.305596245 | NM_003300| | TRAF3,TNF receptor-associated factor 3 isoform 1 |
| 209990_s_at | 43.39 | 6.04E-08 | 1.748836421 | NM_005458| | GPR51,G protein-coupled receptor 51 |
| 232113_at | 43.38 | 6.05E-08 | 2.2253576 | NA |  |
| 201941_at | 43.35 | 6.06E-08 | 1.126374293 | NM_001304| | CPD,carboxypeptidase D precursor |
| 201729_s_at | 43.31 | 6.07E-08 | 1.122628791 | NM_014680| | KIAA0100,antigen MLAA-22 |
| 204916_at | 43.3 | 6.07E-08 | 1.476762397 | NM_005855| | RAMP1,receptor (calcitonin) activity modifying protein |
| 222784_at | 43.23 | 6.10E-08 | 1.453519514 | NM_001034852| | NA |
| 204069_at | 43.2 | 6.12E-08 | 2.005019352 | NM_002398| | MEIS1,Meis1 homolog |
| 212486_s_at | 43.18 | 6.12E-08 | 1.200823968 | NM_002037| | FYN,protein-tyrosine kinase fyn isoform a |
| 224801_at | 43.17 | 6.13E-08 | 1.439509975 | NM_019080| | NDFIP2,Nedd4 family interacting protein 2 |
| 219038_at | 42.89 | 6.32E-08 | 1.609726511 | NM_001085354| | NA |
| 224755_at | 42.88 | 6.32E-08 | 1.171851641 | NA |  |
| 201819_at | 42.87 | 6.32E-08 | 1.22558968 | NM_001082959| | NA |
| 204319_s_at | 42.85 | 6.32E-08 | 1.166158856 | NM_001005339| | RGS10,regulator of G-protein signaling 10 isoform a |
| 206050_s_at | 42.85 | 6.32E-08 | 1.181358985 | NM_002939| | RNH,ribonuclease/angiogenin inhibitor |
| 226751_at | 42.8 | 6.35E-08 | 1.30801674 | NM_001111101| | NA |
| 211066_x_at | 42.77 | 6.37E-08 | 1.205293794 | NM_002588| | PCDHGC3,protocadherin gamma subfamily C, 3 isoform 1 |
| 214608_s_at | 42.68 | 6.43E-08 | 1.428931363 | NM_000503| | EYA1,eyes absent 1 isoform b |
| 209291_at | 42.63 | 6.46E-08 | 1.432677682 | NM_001546| | ID4,inhibitor of DNA binding 4, dominant negative |
| 232421_at | 42.54 | 6.53E-08 | 1.797011423 | NM_001082959| | NA |
| 226132_s_at | 42.47 | 6.57E-08 | 1.399029549 | NM_001031740| | NA |
| 210045_at | 42.45 | 6.59E-08 | 1.233140359 | NM_002168| | IDH2,isocitrate dehydrogenase 2 (NADP+), |
| 227467_at | 42.43 | 6.60E-08 | 1.422906451 | NM_172037| | RDH10,retinol dehydrogenase 10 |
| 235990_at | 42.39 | 6.62E-08 | 1.489884622 | NA |  |
| 213587_s_at | 42.29 | 6.69E-08 | 1.380800732 | NM_001100592| | NA |
| 225578_at | 42.23 | 6.72E-08 | 1.242511797 | NM_001071775| | NA |
| 233364_s_at | 42.23 | 6.72E-08 | 2.249347431 | NA |  |
| 228915_at | 42.17 | 6.77E-08 | 1.728578231 | NM_004392| | DACH1,dachshund homolog 1 isoform c |
| 226113_at | 42.1 | 6.80E-08 | 1.689974666 | NM_001077195| | NA |
| 227610_at | 42.09 | 6.80E-08 | 1.461687295 | NA |  |
| 209506_s_at | 42.06 | 6.83E-08 | 1.368236535 | NM_005654| | NR2F1,nuclear receptor subfamily 2, group F, member 1 |
| 225000_at | 42.04 | 6.84E-08 | 1.088822997 | NM_004157| | PRKAR2A,cAMP-dependent protein kinase, regulatory |
| 202125_s_at | 41.95 | 6.92E-08 | 1.163785699 | NM_015049| | ALS2CR3,amyotrophic lateral sclerosis 2 (juvenile) |
| 204600_at | 41.94 | 6.93E-08 | 1.344756108 | NM_004443| | EPHB3,ephrin receptor EphB3 precursor |
| 211060_x_at | 41.93 | 6.93E-08 | 1.133143111 | NM_003801| | GPAA1,anchor attachment protein 1 |
| 40562_at | 41.91 | 6.93E-08 | 1.147685983 | NM_002067| | GNA11,guanine nucleotide binding protein (G protein), |
| 202136_at | 41.91 | 6.93E-08 | 1.164863015 | NM_006624| | ZMYND11,zinc finger, MYND domain containing 11 isoform |
| 217848_s_at | 41.89 | 6.93E-08 | 1.063304904 | NM_021129| | PP,inorganic pyrophosphatase |
| 229975_at | 41.89 | 6.93E-08 | 1.941756084 | NM_001203| | BMPR1B,bone morphogenetic protein receptor, type IB |
| 202048_s_at | 41.89 | 6.93E-08 | 1.154766968 | NM_014292| | CBX6,chromobox homolog 6 |
| 201896_s_at | 41.81 | 7.01E-08 | 1.111931337 | NM_001005290| | DDA3,p53-regulated DDA3 isoform b |
| 229176_at | 41.8 | 7.02E-08 | 1.455345474 | NM_054027| | ANKH,ankylosis, progressive homolog |
| 212419_at | 41.67 | 7.15E-08 | 1.247366489 | NM_153367| | C10orf56,chromosome 10 open reading frame 56 |
| 225562_at | 41.66 | 7.15E-08 | 1.179078953 | NM_007368| | RASA3,RAS p21 protein activator 3 |
| 202133_at | 41.59 | 7.20E-08 | 1.286832776 | NM_015472| | WWTR1,WW domain containing transcription regulator 1 |
| 209286_at | 41.57 | 7.22E-08 | 1.281097398 | NM_006449| | CDC42EP3,Cdc42 effector protein 3 |
| 224818_at | 41.56 | 7.23E-08 | 1.146652296 | NM_002959| | SORT1,sortilin 1 preproprotein |
| 213435_at | 41.54 | 7.25E-08 | 1.250315011 | NM_015265| | SATB2,SATB family member 2 |
| 224970_at | 41.51 | 7.28E-08 | 1.882697607 | NM_005595| | NFIA,nuclear factor I/A |
| 210749_x_at | 41.47 | 7.31E-08 | 1.124998841 | NM_001954| | DDR1,discoidin receptor tyrosine kinase isoform b |
| 213628_at | 41.45 | 7.32E-08 | 1.166471712 | NM_001048210| | NA |
| 222780_s_at | 41.39 | 7.37E-08 | 1.732356998 | NM_001024372| | NA |
| 228278_at | 41.35 | 7.40E-08 | 1.669478761 | NM_002501| | NFIX,nuclear factor I/X (CCAAT-binding transcription |
| 226781_at | 41.3 | 7.44E-08 | 1.18644853 | NM_197964| | HSPC268,hypothetical protein HSPC268 |
| 230291_s_at | 41.13 | 7.60E-08 | 2.28717971 | NM_005596| | NFIB,nuclear factor I/B |
| 203037_s_at | 41.12 | 7.60E-08 | 1.2241915 | NM_014751| | MTSS1,metastasis suppressor 1 |
| 208923_at | 40.98 | 7.77E-08 | 1.138092589 | NM_001033028| | NA |
| 204364_s_at | 40.96 | 7.77E-08 | 1.662894597 | NM_022912| | C2orf23,receptor expression enhancing protein 1 |
| 227326_at | 40.95 | 7.78E-08 | 1.254276609 | NM_001008528| | TMAP1,transmembrane anchor protein 1 isoform 1 |
| 229590_at | 40.92 | 7.80E-08 | 1.102852757 | NM_000977| | RPL13,ribosomal protein L13 |
| 214334_x_at | 40.9 | 7.82E-08 | 1.221289166 | NM_014764| | DAZAP2,DAZ associated protein 2 |
| 228049_x_at | 40.88 | 7.83E-08 | 1.183016394 | NA |  |
| 235743_at | 40.84 | 7.87E-08 | 2.135665907 | NA |  |
| 212411_at | 40.82 | 7.89E-08 | 1.142023063 | NM_033416| | IMP4,IMP4, U3 small nucleolar ribonucleoprotein, |
| 220559_at | 40.81 | 7.89E-08 | 2.773905273 | NM_001426| | EN1,engrailed homolog 1 |
| 229744_at | 40.75 | 7.96E-08 | 1.431394522 | NM_006751| | SSFA2,sperm specific antigen 2 |
| 228831_s_at | 40.74 | 7.96E-08 | 1.349456643 | NM_052847| | GNG7,guanine nucleotide binding protein (G protein), |
| 223093_at | 40.66 | 8.07E-08 | 1.647374275 | NM_054027| | ANKH,ankylosis, progressive homolog |
| 224814_at | 40.64 | 8.09E-08 | 1.598030827 | NM_013379| | DPP7,dipeptidyl peptidase 7 preproprotein |
| 228480_at | 40.63 | 8.09E-08 | 1.795063012 | NM_003574| | VAPA,vesicle-associated membrane protein-associated |
| 227197_at | 40.62 | 8.09E-08 | 1.1505058 | NM_015595| | SGEF,DKFZP434D146 protein |
| 204011_at | 40.6 | 8.11E-08 | 1.251444799 | NM_005842| | SPRY2,sprouty 2 |
| 205012_s_at | 40.58 | 8.13E-08 | 1.152224242 | NM_001040427| | NA |
| 221766_s_at | 40.55 | 8.15E-08 | 1.397747564 | NM_017633| | FAM46A,family with sequence similarity 46, member A |
| 239221_at | 40.55 | 8.15E-08 | 1.56346809 | NM_001083909| | NA |
| 212993_at | 40.5 | 8.20E-08 | 1.224535527 | NM_144653| | BTBD14A,BTB (POZ) domain containing 14A |
| 37892_at | 40.49 | 8.20E-08 | 1.170417886 | NM_001854| | COL11A1,alpha 1 type XI collagen isoform A |
| 224999_at | 40.43 | 8.25E-08 | 2.057910117 | NA |  |
| 226874_at | 40.41 | 8.26E-08 | 1.160948825 | NM_020803| | KLHL8,kelch-like 8 |
| 225793_at | 40.41 | 8.26E-08 | 1.278292591 | NM_153713| | LIX1L,Lix1 homolog (mouse) like |
| 239069_s_at | 40.38 | 8.30E-08 | 1.264372094 | NA |  |
| 203318_s_at | 40.34 | 8.34E-08 | 1.160826259 | NM_021964| | ZNF148,zinc finger protein 148 (pHZ-52) |
| 219468_s_at | 40.24 | 8.46E-08 | 1.298704704 | NM_017949| | CUEDC1,CUE domain-containing 1 |
| 200767_s_at | 40.11 | 8.64E-08 | 1.266163317 | NM_014612| | C9orf10,C9orf10 protein |
| 201028_s_at | 40.11 | 8.64E-08 | 1.282523319 | NM_001122898| | NA |
| 231128_at | 40.1 | 8.64E-08 | 1.926247208 | NM_175885| | MGC33846,hypothetical protein MGC33846 |
| 204646_at | 40.09 | 8.65E-08 | 2.78439315 | NM_000110| | DPYD,dihydropyrimidine dehydrogenase |
| 242825_at | 40.05 | 8.69E-08 | 1.280981278 | NM_001010861| | NA |
| 225897_at | 40.03 | 8.69E-08 | 1.151757916 | NM_002356| | MARCKS,myristoylated alanine-rich protein kinase C |
| 227605_at | 39.99 | 8.71E-08 | 1.143484048 | NM_004757| | SCYE1,small inducible cytokine subfamily E, member 1 |
| 202478_at | 39.99 | 8.71E-08 | 1.200909718 | NM_021643| | TRIB2,tribbles homolog 2 |
| 202157_s_at | 39.99 | 8.71E-08 | 1.118814987 | NM_001025076| | NA |
| 229435_at | 39.97 | 8.72E-08 | 2.488411492 | NM_001042413| | NA |
| 222719_s_at | 39.91 | 8.79E-08 | 1.897178433 | NM_016205| | PDGFC,platelet-derived growth factor C precursor |
| 213156_at | 39.86 | 8.85E-08 | 2.44232966 | NA |  |
| 215111_s_at | 39.63 | 9.13E-08 | 1.158776741 | NM_006022| | TGFB1I4,transforming growth factor beta 1 induced |
| 1438_at | 39.6 | 9.16E-08 | 1.363993764 | NM_004443| | EPHB3,ephrin receptor EphB3 precursor |
| 216191_s_at | 39.48 | 9.32E-08 | 3.008116565 | NA |  |
| 202695_s_at | 39.45 | 9.35E-08 | 1.644344169 | NM_004760| | STK17A,serine/threonine kinase 17a |
| 219737_s_at | 39.45 | 9.35E-08 | 2.010859705 | NM_020403| | PCDH9,protocadherin 9 isoform 2 precursor |
| 201876_at | 39.4 | 9.39E-08 | 1.163118799 | NM_000305| | PON2,paraoxonase 2 |
| 233587_s_at | 39.4 | 9.39E-08 | 1.217936227 | NM_020808| | SIPA1L2,signal-induced proliferation-associated 1 like |
| 238009_at | 39.38 | 9.40E-08 | 1.554006583 | NA |  |
| 219911_s_at | 39.37 | 9.40E-08 | 1.225941451 | NM_016354| | SLCO4A1,solute carrier organic anion transporter family |
| 1555326_a_at | 39.36 | 9.40E-08 | 1.931638623 | NM_001005845| | ADAM9,a disintegrin and metalloproteinase domain 9 |
| 210735_s_at | 39.32 | 9.44E-08 | 1.61362328 | NM_001218| | CA12,carbonic anhydrase XII isoform 1 precursor |
| 222437_s_at | 39.28 | 9.47E-08 | 1.230109476 | NM_001005753| | VPS24,vacuolar protein sorting 24 isoform 2 |
| 213119_at | 39.28 | 9.47E-08 | 1.080531404 | NM_078483| | SLC36A1,solute carrier family 36 member 1 |
| 225002_s_at | 39.25 | 9.50E-08 | 1.109052689 | NM_001042468| | NA |
| 203799_at | 39.22 | 9.54E-08 | 1.719648329 | NM_014880| | CD302,CD302 antigen |
| 242488_at | 39.19 | 9.55E-08 | 1.735469856 | NA |  |
| 205273_s_at | 39.19 | 9.55E-08 | 1.176464695 | NM_014889| | PITRM1,metalloprotease 1 |
| 223319_at | 39.13 | 9.64E-08 | 1.146714127 | NM_001024218| | NA |
| 228302_x_at | 39 | 9.81E-08 | 1.480319296 | NM_018584| | CaMKIINalpha,calcium/calmodulin-dependent protein kinase II |
| 204863_s_at | 38.99 | 9.81E-08 | 1.399068972 | NM_002184| | IL6ST,interleukin 6 signal transducer isoform 1 |
| 226713_at | 38.97 | 9.85E-08 | 1.566199098 | NM_174908| | C3orf6,Ymer protein short isoform |
| 217867_x_at | 38.84 | 1.00E-07 | 1.373310264 | NM_012105| | BACE2,beta-site APP-cleaving enzyme 2 isoform A |
| 225921_at | 38.82 | 1.00E-07 | 1.338192763 | NM_016350| | NIN,ninein isoform 4 |
| 204519_s_at | 38.82 | 1.00E-07 | 1.263343177 | NM_015993| | TM4SF11,plasmolipin |
| 203217_s_at | 38.8 | 1.01E-07 | 1.402721934 | NM_001042437| | NA |
| 212764_at | 38.79 | 1.01E-07 | 1.554641816 | NM_030751| | TCF8,transcription factor 8 (represses interleukin 2 |
| 218899_s_at | 38.77 | 1.01E-07 | 1.630630453 | NM_001024372| | NA |
| 222834_s_at | 38.76 | 1.01E-07 | 1.312320429 | NM_018841| | GNG12,G-protein gamma-12 subunit |
| 218086_at | 38.73 | 1.01E-07 | 1.287285832 | NM_015392| | NPDC1,neural proliferation, differentiation and |
| 201053_s_at | 38.7 | 1.02E-07 | 1.192058821 | NM_006814| | PSMF1,proteasome inhibitor subunit 1 isoform 1 |
| 237833_s_at | 38.63 | 1.02E-07 | 1.59778592 | NM_005460| | SNCAIP,synuclein alpha interacting protein |
| 212774_at | 38.62 | 1.02E-07 | 1.194083027 | NM_006352| | ZNF238,zinc finger protein 238 isoform 2 |
| 204030_s_at | 38.62 | 1.02E-07 | 1.216230133 | NM_014575| | SCHIP1,schwannomin interacting protein 1 |
| 1553984_s_at | 38.61 | 1.03E-07 | 1.094460094 | NM_012145| | DTYMK,deoxythymidylate kinase (thymidylate kinase) |
| 213224_s_at | 38.59 | 1.03E-07 | 1.149158205 | NA |  |
| 218243_at | 38.55 | 1.03E-07 | 1.085992235 | NM_001040451| | NA |
| 216231_s_at | 38.47 | 1.05E-07 | 1.09744714 | NM_004048| | B2M,beta-2-microglobulin precursor |
| 228555_at | 38.44 | 1.05E-07 | 1.63394986 | NM_001221| | CAMK2D,calcium/calmodulin-dependent protein kinase II |
| 223024_at | 38.38 | 1.06E-07 | 1.096777945 | NM_032493| | AP1M1,adaptor-related protein complex 1, mu 1 subunit |
| 225170_at | 38.37 | 1.06E-07 | 1.105159366 | NM_017588| | WDR5,WD repeat domain 5 protein |
| 208221_s_at | 38.21 | 1.08E-07 | 1.779618657 | NM_003061| | SLIT1,slit homolog 1 |
| 228429_x_at | 38.14 | 1.10E-07 | 1.304577268 | NM_022342| | KIF9,kinesin family member 9 |
| 226621_at | 38.11 | 1.10E-07 | 1.34617543 | NA |  |
| 225006_x_at | 38.09 | 1.10E-07 | 1.133182652 | NM_198976| | TH1L,TH1-like protein |
| 213618_at | 38.01 | 1.11E-07 | 2.13631819 | NM_015230| | CENTD1,centaurin delta 1 isoform a |
| 1007_s_at | 37.98 | 1.12E-07 | 1.10381004 | NM_001954| | DDR1,discoidin receptor tyrosine kinase isoform b |
| 217897_at | 37.91 | 1.13E-07 | 1.148164975 | NM_022003| | FXYD6,FXYD domain-containing ion transport regulator |
| 221569_at | 37.9 | 1.13E-07 | 1.174299742 | NM_017651| | AHI1,jouberin |
| 222651_s_at | 37.9 | 1.13E-07 | 1.40145948 | NM_014112| | TRPS1,zinc finger transcription factor TRPS1 |
| 219076_s_at | 37.89 | 1.13E-07 | 1.311619047 | NM_018663| | PXMP2,peroxisomal membrane protein 2, 22kDa |
| 1555945_s_at | 37.88 | 1.13E-07 | 1.209776813 | NM_014612| | C9orf10,C9orf10 protein |
| 201174_s_at | 37.86 | 1.13E-07 | 1.169229299 | NM_018975| | TERF2IP,TRF2-interacting telomeric RAP1 protein |
| 218132_s_at | 37.86 | 1.13E-07 | 1.140317433 | NM_001077446| | NA |
| 213712_at | 37.75 | 1.15E-07 | 1.968627855 | NM_017770| | ELOVL2,elongation of very long chain fatty acids |
| 201778_s_at | 37.69 | 1.16E-07 | 1.064749895 | NM_014774| | KIAA0494,KIAA0494 gene product |
| 205970_at | 37.64 | 1.16E-07 | 1.272752992 | NM_005954| | MT3,metallothionein 3 |
| 228395_at | 37.62 | 1.17E-07 | 1.333896962 | NM_001010983| | GLT8D1,glycosyltransferase 8 domain containing 1 |
| 203349_s_at | 37.57 | 1.17E-07 | 1.246680782 | NM_004454| | ETV5,ets variant gene 5 (ets-related molecule) |
| 200761_s_at | 37.55 | 1.17E-07 | 1.090416537 | NM_006407| | ARL6IP5,ADP-ribosylation-like factor 6 interacting |
| 208903_at | 37.55 | 1.17E-07 | 1.247328164 | NM_001031| | RPS28,ribosomal protein S28 |
| 205672_at | 37.52 | 1.18E-07 | 1.302336784 | NM_000380| | XPA,xeroderma pigmentosum, complementation group A |
| 238736_at | 37.49 | 1.18E-07 | 1.958627792 | NM_002912| | REV3L,REV3-like, catalytic subunit of DNA polymerase |
| 204021_s_at | 37.46 | 1.18E-07 | 1.279220174 | NM_005859| | PURA,purine-rich element binding protein A |
| 226193_x_at | 37.43 | 1.19E-07 | 1.064212572 | NM_001024916| | NA |
| 226748_at | 37.4 | 1.19E-07 | 1.295203655 | NM_153374| | MGC35274,hypothetical protein MGC35274 |
| 204341_at | 37.39 | 1.19E-07 | 1.310308541 | NM_006470| | TRIM16,tripartite motif-containing 16 |
| 218589_at | 37.39 | 1.19E-07 | 1.594870186 | NM_005767| | P2RY5,G-protein coupled purinergic receptor P2Y5 |
| 223419_at | 37.32 | 1.20E-07 | 1.215773898 | NM_032301| | FBXW9,F-box and WD-40 domain protein 9 |
| 227978_s_at | 37.31 | 1.20E-07 | 1.942629736 | NM_175907| | ZADH2,zinc binding alcohol dehydrogenase, domain |
| 212169_at | 37.31 | 1.20E-07 | 1.281459323 | NM_007270| | FKBP9,FK506 binding protein 9 |
| 241741_at | 37.28 | 1.21E-07 | 1.340988515 | NM_019095| | C20orf155,chromosome 20 open reading frame 155 |
| 217771_at | 37.22 | 1.22E-07 | 1.198384822 | NM_016548| | GOLPH2,golgi phosphoprotein 2 |
| 221885_at | 37.21 | 1.22E-07 | 1.363601448 | NM_015689| | KIAA1277,KIAA1277 protein |
| 213838_at | 37.18 | 1.22E-07 | 1.240442608 | NM_016167| | NOL7,nucleolar protein 7, 27kDa |
| 200795_at | 37.15 | 1.23E-07 | 1.629907414 | NM_004684| | SPARCL1,SPARC-like 1 |
| 221840_at | 37.14 | 1.23E-07 | 1.49678059 | NM_006504| | PTPRE,protein tyrosine phosphatase, receptor type, E |
| 228679_at | 37.14 | 1.23E-07 | 2.306458797 | NA |  |
| 235051_at | 37.14 | 1.23E-07 | 1.250818383 | NM_174908| | C3orf6,Ymer protein short isoform |
| 217529_at | 37.12 | 1.23E-07 | 1.368616254 | NA |  |
| 201115_at | 37.03 | 1.25E-07 | 1.089819848 | NM_001127218| | NA |
| 217077_s_at | 36.93 | 1.27E-07 | 1.63375402 | NM_005458| | GPR51,G protein-coupled receptor 51 |
| 202042_at | 36.91 | 1.27E-07 | 1.08996431 | NM_002109| | HARS,histidyl-tRNA synthetase |
| 218599_at | 36.88 | 1.27E-07 | 1.197255468 | NM_001048205| | NA |
| 226021_at | 36.87 | 1.27E-07 | 1.317745628 | NM_172037| | RDH10,retinol dehydrogenase 10 |
| 212277_at | 36.86 | 1.28E-07 | 1.164149672 | NM_004687| | MTMR4,myotubularin related protein 4 |
| 225018_at | 36.84 | 1.28E-07 | 1.298765642 | NM_020148| | SPIRE1,spire homolog 1 |
| 202171_at | 36.83 | 1.28E-07 | 1.06252305 | NM_007146| | ZNF161,zinc finger protein 161 |
| 203729_at | 36.81 | 1.29E-07 | 1.182461943 | NM_001425| | EMP3,epithelial membrane protein 3 |
| 225242_s_at | 36.79 | 1.29E-07 | 1.327831767 | NM_199511| | URB,steroid-sensitive protein 1 |
| 226726_at | 36.77 | 1.29E-07 | 1.18341045 | NM_138799| | OACT2,O-acyltransferase (membrane bound) domain |
| 210508_s_at | 36.76 | 1.29E-07 | 1.23177201 | NM_004518| | KCNQ2,potassium voltage-gated channel KQT-like protein |
| 1569872_a_at | 36.75 | 1.29E-07 | 1.387769171 | NA |  |
| 205344_at | 36.72 | 1.30E-07 | 1.353295036 | NM_006574| | CSPG5,chondroitin sulfate proteoglycan 5 (neuroglycan |
| 224463_s_at | 36.72 | 1.30E-07 | 1.314399072 | NM_032930| | MGC13040,hypothetical protein MGC13040 |
| 209871_s_at | 36.7 | 1.30E-07 | 1.208166358 | NM_005503| | APBA2,amyloid beta A4 precursor protein-binding, |
| 1555233_at | 36.69 | 1.30E-07 | 2.12976055 | NM_020663| | RHOJ,TC10-like Rho GTPase |
| 205029_s_at | 36.66 | 1.30E-07 | 1.417131704 | NM_001446| | FABP7,fatty acid binding protein 7, brain |
| 209460_at | 36.63 | 1.31E-07 | 1.225437322 | NM_000663| | ABAT,4-aminobutyrate aminotransferase precursor |
| 225932_s_at | 36.61 | 1.31E-07 | 1.056817439 | NM_002137| | HNRPA2B1,heterogeneous nuclear ribonucleoprotein A2/B1 |
| 219154_at | 36.52 | 1.33E-07 | 1.241574001 | NM_019034| | RHOF,ras homolog gene family, member F |
| 227558_at | 36.48 | 1.34E-07 | 1.317835668 | NM_003655| | CBX4,chromobox homolog 4 |
| 1554807_a_at | 36.45 | 1.34E-07 | 1.406636925 | NM_020148| | SPIRE1,spire homolog 1 |
| 235192_at | 36.37 | 1.36E-07 | 1.230021065 | NM_033550| | TP53RK,p53-related protein kinase |
| 238711_s_at | 36.3 | 1.37E-07 | 1.26282305 | NM_021964| | ZNF148,zinc finger protein 148 (pHZ-52) |
| 219973_at | 36.28 | 1.37E-07 | 1.605810782 | NM_024590| | ARSJ,arylsulfatase J |
| 204142_at | 36.26 | 1.37E-07 | 1.164551377 | NM_001126123| | NA |
| 225368_at | 36.25 | 1.37E-07 | 1.078617402 | NM_001113239| | NA |
| 220940_at | 36.24 | 1.37E-07 | 1.396341401 | NM_025190| | NA |
| 213880_at | 36.24 | 1.37E-07 | 1.659405973 | NM_003667| | LGR5,leucine-rich repeat-containing G protein-coupled |
| 226938_at | 36.24 | 1.37E-07 | 1.206634525 | NM_015604| | WDR21,WD repeat domain 21 isoform 1 |
| 228628_at | 36.24 | 1.37E-07 | 1.167642377 | NA |  |
| 1556341_s_at | 36.17 | 1.39E-07 | 1.24459478 | NM_002969| | MAPK12,mitogen-activated protein kinase 12 |
| 228259_s_at | 36.09 | 1.41E-07 | 1.318582403 | NM_022140| | EPB41L4A,erythrocyte protein band 4.1-like 4 |
| 226508_at | 36.07 | 1.41E-07 | 1.07976734 | NM_024947| | PHC3,polyhomeotic like 3 |
| 204724_s_at | 36.06 | 1.41E-07 | 1.130101042 | NM_001853| | COL9A3,alpha 3 type IX collagen |
| 202471_s_at | 35.94 | 1.43E-07 | 1.107314971 | NM_004135| | IDH3G,isocitrate dehydrogenase 3 (NAD+) gamma isoform |
| 235676_at | 35.93 | 1.44E-07 | 1.472824203 | NA |  |
| 202479_s_at | 35.9 | 1.44E-07 | 1.276675219 | NM_021643| | TRIB2,tribbles homolog 2 |
| 217806_s_at | 35.85 | 1.45E-07 | 1.060900049 | NM_015584| | POLDIP2,DNA polymerase delta interacting protein 2 |
| 1559965_at | 35.83 | 1.45E-07 | 3.067021831 | NA |  |
| 219779_at | 35.83 | 1.45E-07 | 1.366057272 | NM_024721| | ZFHX4,zinc finger homeodomain 4 |
| 213194_at | 35.83 | 1.45E-07 | 1.126660136 | NM_002941| | ROBO1,roundabout 1 isoform a |
| 208890_s_at | 35.79 | 1.46E-07 | 1.140226711 | NM_012401| | NA |
| 212456_at | 35.78 | 1.46E-07 | 1.134449973 | NM_015229| | KIAA0664,KIAA0664 protein |
| 222517_at | 35.75 | 1.46E-07 | 1.248943371 | NM_012095| | AP3M1,adaptor-related protein complex 3, mu 1 subunit |
| 238472_at | 35.72 | 1.47E-07 | 1.850635866 | NM_012347| | FBXO9,F-box only protein 9 isoform 1 |
| 226372_at | 35.72 | 1.47E-07 | 1.531855233 | NM_018413| | CHST11,carbohydrate (chondroitin 4) sulfotransferase |
| 227084_at | 35.7 | 1.47E-07 | 1.146142312 | NM_001390| | DTNA,dystrobrevin alpha isoform 1 |
| 227998_at | 35.7 | 1.47E-07 | 1.319948679 | NM_080388| | S100A16,S100 calcium binding protein A16 |
| 218187_s_at | 35.7 | 1.47E-07 | 1.146571472 | NM_023080| | FLJ20989,hypothetical protein FLJ20989 |
| 225729_at | 35.69 | 1.47E-07 | 1.200055148 | NM_152734| | C6orf89,hypothetical protein FLJ25357 |
| 1568763_s_at | 35.58 | 1.49E-07 | 1.074201064 | NM_013232| | PDCD6,programmed cell death 6 |
| 1556035_s_at | 35.57 | 1.50E-07 | 1.256524858 | NM_001032293| | NA |
| 1557433_at | 35.56 | 1.50E-07 | 1.505378284 | NA |  |
| 206670_s_at | 35.46 | 1.51E-07 | 1.459066622 | NM_000817| | GAD1,glutamate decarboxylase 1 isoform GAD67 |
| 223283_s_at | 35.46 | 1.51E-07 | 2.396770741 | NM_005786| | SDCCAG33,serologically defined colon cancer antigen 33 |
| 231898_x_at | 35.46 | 1.51E-07 | 1.887516886 | NA |  |
| 236207_at | 35.45 | 1.51E-07 | 1.807285125 | NM_006751| | SSFA2,sperm specific antigen 2 |
| 212909_at | 35.42 | 1.52E-07 | 1.5359726 | NM_001077427| | NA |
| 212111_at | 35.38 | 1.53E-07 | 1.180493369 | NM_177424| | STX12,syntaxin 12 |
| 218508_at | 35.34 | 1.54E-07 | 1.08906976 | NM_018403| | DCP1A,decapping enzyme |
| 213259_s_at | 35.32 | 1.55E-07 | 1.236521665 | NM_015077| | SARM1,sterile alpha and TIR motif containing 1 |
| 204099_at | 35.28 | 1.55E-07 | 1.388453298 | NM_001003801| | SMARCD3,SWI/SNF related, matrix associated, actin |
| 225157_at | 35.27 | 1.56E-07 | 1.126309463 | NA |  |
| 217764_s_at | 35.26 | 1.56E-07 | 1.206264159 | NM_006868| | RAB31,RAB31, member RAS oncogene family |
| 222423_at | 35.25 | 1.56E-07 | 1.169112589 | NM_030571| | NDFIP1,Nedd4 family interacting protein 1 |
| 218648_at | 35.21 | 1.57E-07 | 1.206302627 | NM_001042574| | NA |
| 1556051_a_at | 35.17 | 1.58E-07 | 1.251434768 | NM_001003398| | BICD1,bicaudal D homolog 1 isoform 2 |
| 213103_at | 35.17 | 1.58E-07 | 1.545989645 | NM_052851| | STARD13,START domain containing 13 isoform gamma |
| 235489_at | 35.13 | 1.59E-07 | 2.391794622 | NM_020663| | RHOJ,TC10-like Rho GTPase |
| 228605_at | 35.05 | 1.60E-07 | 1.092375185 | NA |  |
| 215667_x_at | 35.01 | 1.61E-07 | 1.12496689 | NA |  |
| 218199_s_at | 34.97 | 1.63E-07 | 1.095173319 | NM_022917| | NOL6,nucleolar RNA-associated protein alpha isoform |
| 222503_s_at | 34.87 | 1.65E-07 | 1.13453366 | NM_018268| | WDR41,WD repeat domain 41 |
| 235775_at | 34.85 | 1.66E-07 | 1.403891545 | NM_152588| | DKFZp762A217,hypothetical protein DKFZp762A217 |
| 214460_at | 34.84 | 1.66E-07 | 1.219809112 | NM_002338| | LSAMP,limbic system-associated membrane protein |
| 1553108_at | 34.84 | 1.66E-07 | 1.4754499 | NM_152409| | FLJ37562,hypothetical protein FLJ37562 |
| 218793_s_at | 34.84 | 1.66E-07 | 1.466708219 | NM_001037535| | NA |
| 223041_at | 34.82 | 1.66E-07 | 1.241223039 | NM_031462| | CD99L2,CD99 antigen-like 2 |
| 224666_at | 34.81 | 1.66E-07 | 1.090598951 | NM_145080| | NSMCE1,non-SMC element 1 homolog |
| 213508_at | 34.79 | 1.66E-07 | 1.096122887 | NM_138288| | C14orf147,chromosome 14 open reading frame 147 |
| 242307_at | 34.76 | 1.67E-07 | 1.169761508 | NM_001013258| | NA |
| 219619_at | 34.71 | 1.68E-07 | 2.423738334 | NM_017594| | DIRAS2,Di-Ras2 |
| 224882_at | 34.69 | 1.69E-07 | 1.64079055 | NM_032501| | ACAS2L,acetyl-CoA synthetase 2-like |
| 225516_at | 34.67 | 1.69E-07 | 1.164755667 | NM_001008539| | SLC7A2,solute carrier family 7, member 2 isoform 1 |
| 211275_s_at | 34.66 | 1.70E-07 | 1.100386237 | NM_004130| | GYG,glycogenin |
| 212730_at | 34.61 | 1.71E-07 | 1.220269932 | NM_015286| | DMN,desmuslin isoform B |
| 212097_at | 34.6 | 1.71E-07 | 1.421456737 | NM_001753| | CAV1,caveolin 1 |
| 201149_s_at | 34.51 | 1.73E-07 | 1.351725069 | NM_000362| | TIMP3,tissue inhibitor of metalloproteinase 3 |
| 212636_at | 34.49 | 1.74E-07 | 1.361724049 | NM_006775| | QKI,quaking homolog, KH domain RNA binding isoform |
| 212613_at | 34.48 | 1.74E-07 | 1.255923773 | NM_007047| | BTN3A2,butyrophilin, subfamily 3, member A2 |
| 203897_at | 34.46 | 1.74E-07 | 1.111469313 | NM_020424| | LOC57149,hypothetical protein A-211C6.1 |
| 225383_at | 34.46 | 1.74E-07 | 1.160085495 | NM_001080485| | NA |
| 204084_s_at | 34.44 | 1.75E-07 | 1.342827799 | NM_006493| | CLN5,ceroid-lipofuscinosis, neuronal 5 |
| 224983_at | 34.44 | 1.75E-07 | 1.104299698 | NM_005506| | SCARB2,scavenger receptor class B, member 2 |
| 225316_at | 34.44 | 1.75E-07 | 1.162504442 | NM_032793| | FLJ14490,hypothetical protein FLJ14490 |
| 1555867_at | 34.43 | 1.75E-07 | 1.279420904 | NM_001098721| | NA |
| 212021_s_at | 34.38 | 1.76E-07 | 1.196311332 | NM_002417| | MKI67,antigen identified by monoclonal antibody Ki-67 |
| 207039_at | 34.37 | 1.76E-07 | 1.750329992 | NM_000077| | CDKN2A,cyclin-dependent kinase inhibitor 2A isoform 1 |
| 205111_s_at | 34.36 | 1.76E-07 | 2.068617587 | NM_016341| | PLCE1,pancreas-enriched phospholipase C |
| 225163_at | 34.36 | 1.76E-07 | 1.182726676 | NM_018027| | FRMD4A,FERM domain containing 4A |
| 203758_at | 34.33 | 1.77E-07 | 1.360383107 | NM_001334| | CTSO,cathepsin O preproprotein |
| 238532_at | 34.32 | 1.77E-07 | 2.211571649 | NM_012074| | DPF3,cer-d4 (mouse) homolog |
| 202092_s_at | 34.27 | 1.78E-07 | 1.291096806 | NM_012106| | ARL2BP,binder of Arl Two |
| 209009_at | 34.27 | 1.78E-07 | 1.141028718 | NM_001984| | ESD,esterase D/formylglutathione hydrolase |
| 243579_at | 34.25 | 1.79E-07 | 1.250672015 | NM_138962| | MSI2,musashi 2 isoform a |
| 243664_at | 34.24 | 1.79E-07 | 1.336728556 | NM_004786| | TXNL1,thioredoxin-like 1 |
| 224783_at | 34.23 | 1.79E-07 | 1.154128004 | NM_182565| | MGC29814,hypothetical protein MGC29814 |
| 217485_x_at | 34.22 | 1.79E-07 | 1.119635222 | NA |  |
| 223040_at | 34.2 | 1.80E-07 | 1.080663855 | NM_016100| | NAT5,N-acetyltransferase 5 isoform a |
| 205251_at | 34.2 | 1.80E-07 | 1.285309373 | NM_022817| | PER2,period 2 isoform 1 |
| 205471_s_at | 34.19 | 1.80E-07 | 2.023023364 | NM_004392| | DACH1,dachshund homolog 1 isoform c |
| 209617_s_at | 34.18 | 1.80E-07 | 1.36812399 | NM_001332| | CTNND2,catenin (cadherin-associated protein), delta 2 |
| 226889_at | 34.17 | 1.80E-07 | 1.17033838 | NM_001006657| | WDR35,WD repeat domain 35 isoform 1 |
| 229404_at | 34.15 | 1.81E-07 | 1.177509669 | NM_057179| | TWIST2,twist homolog 2 |
| 209589_s_at | 34.11 | 1.81E-07 | 1.192594255 | NM_004442| | EPHB2,ephrin receptor EphB2 isoform 2 precursor |
| 201067_at | 34.1 | 1.82E-07 | 1.165117899 | NM_002803| | PSMC2,proteasome 26S ATPase subunit 2 |
| 211564_s_at | 34.08 | 1.82E-07 | 1.536123917 | NM_003687| | PDLIM4,PDZ and LIM domain 4 |
| 209293_x_at | 34.07 | 1.83E-07 | 1.540136904 | NM_001546| | ID4,inhibitor of DNA binding 4, dominant negative |
| 229447_x_at | 34.06 | 1.83E-07 | 1.139012827 | NM_001037501| | NA |
| 49329_at | 34.05 | 1.83E-07 | 1.156231902 | NM_032775| | KELCHL,kelch-like |
| 239999_at | 34.01 | 1.84E-07 | 1.71149797 | NM_001005732| | C21orf34,chromosome 21 open reading frame 34 isoform a |
| 234977_at | 33.98 | 1.84E-07 | 1.78774075 | NM_175907| | ZADH2,zinc binding alcohol dehydrogenase, domain |
| 212605_s_at | 33.95 | 1.85E-07 | 1.124042006 | NA |  |
| 200699_at | 33.92 | 1.86E-07 | 1.138014663 | NM_001100603| | NA |
| 242263_at | 33.89 | 1.87E-07 | 1.764173537 | NM_016040| | TMED5,transmembrane emp24 protein transport domain |
| 208817_at | 33.88 | 1.87E-07 | 1.198341193 | NM_000754| | COMT,catechol-O-methyltransferase isoform MB-COMT |
| 208664_s_at | 33.84 | 1.89E-07 | 1.302048325 | NM_001001894| | TTC3,tetratricopeptide repeat domain 3 |
| 224993_at | 33.84 | 1.89E-07 | 1.110030785 | NM_005934| | MLLT1,myeloid/lymphoid or mixed-lineage leukemia |
| 219481_at | 33.8 | 1.90E-07 | 1.184837584 | NM_001122835| | NA |
| 218862_at | 33.79 | 1.90E-07 | 1.171074036 | NM_024701| | ASB13,ankyrin repeat and SOCS box-containing protein |
| 205109_s_at | 33.79 | 1.90E-07 | 1.280407537 | NM_015320| | ARHGEF4,Rho guanine nucleotide exchange factor 4 isoform |
| 212386_at | 33.74 | 1.91E-07 | 1.112483319 | NM_001083962| | NA |
| 212195_at | 33.74 | 1.91E-07 | 1.235360855 | NM_002184| | IL6ST,interleukin 6 signal transducer isoform 1 |
| 213825_at | 33.71 | 1.92E-07 | 1.2903855 | NM_005806| | OLIG2,oligodendrocyte lineage transcription factor 2 |
| 204967_at | 33.71 | 1.92E-07 | 1.166375571 | NM_001649| | APXL,apical protein of Xenopus-like |
| 206083_at | 33.71 | 1.92E-07 | 1.436867179 | NM_001702| | BAI1,brain-specific angiogenesis inhibitor 1 |
| 230304_at | 33.7 | 1.92E-07 | 1.270010322 | NA |  |
| 214501_s_at | 33.69 | 1.92E-07 | 1.135331057 | NM_001040158| | NA |
| 209104_s_at | 33.69 | 1.92E-07 | 1.060341772 | NM_001034833| | NA |
| 236154_at | 33.68 | 1.92E-07 | 2.271145398 | NM_006775| | QKI,quaking homolog, KH domain RNA binding isoform |
| 226652_at | 33.65 | 1.93E-07 | 1.25224733 | NM_006537| | USP3,ubiquitin specific protease 3 |
| 212360_at | 33.59 | 1.95E-07 | 1.164853869 | NM_004037| | AMPD2,adenosine monophosphate deaminase 2 (isoform L) |
| 204521_at | 33.57 | 1.95E-07 | 1.08985228 | NM_013300| | HSU79274,protein predicted by clone 23733 |
| 212646_at | 33.57 | 1.95E-07 | 1.286426054 | NM_015150| | RAFTLIN,raft-linking protein |
| 213155_at | 33.52 | 1.97E-07 | 1.164881981 | NM_015253| | KIAA0523,KIAA0523 protein |
| 202191_s_at | 33.5 | 1.97E-07 | 1.293655228 | NM_003644| | GAS7,growth arrest-specific 7 isoform a |
| 200022_at | 33.47 | 1.98E-07 | 1.021612173 | NM_000979| | RPL18,ribosomal protein L18 |
| 229126_at | 33.47 | 1.98E-07 | 1.301555061 | NM_018279| | TMEM19,transmembrane protein 19 |
| 1554239_s_at | 33.45 | 1.98E-07 | 1.567656529 | NM_175907| | ZADH2,zinc binding alcohol dehydrogenase, domain |
| 200923_at | 33.44 | 1.98E-07 | 1.277919944 | NM_005567| | LGALS3BP,galectin 3 binding protein |
| 214722_at | 33.43 | 1.98E-07 | 1.19500932 | NM_203458| | NOTCH2NL,Notch homolog 2 N-terminal like protein |
| 209435_s_at | 33.43 | 1.98E-07 | 1.106109044 | NM_004723| | ARHGEF2,rho/rac guanine nucleotide exchange factor 2 |
| 218270_at | 33.42 | 1.98E-07 | 1.213224625 | NM_024540| | MRPL24,mitochondrial ribosomal protein L24 |
| 209317_at | 33.4 | 1.99E-07 | 1.109642665 | NM_004875| | POLR1C,RNA polymerase I subunit isoform 2 |
| 226114_at | 33.4 | 1.99E-07 | 1.683122488 | NM_001077195| | NA |
| 213134_x_at | 33.39 | 1.99E-07 | 1.130377009 | NM_006806| | BTG3,B-cell translocation gene 3 |
| 235164_at | 33.38 | 1.99E-07 | 1.70588107 | NM_145011| | ZNF25,zinc finger protein 25 |
| 209292_at | 33.38 | 1.99E-07 | 1.69106143 | NM_001546| | ID4,inhibitor of DNA binding 4, dominant negative |
| 202305_s_at | 33.36 | 1.99E-07 | 1.108777221 | NM_001042548| | NA |
| 223623_at | 33.34 | 2.00E-07 | 1.167079084 | NM_032411| | ECRG4,esophageal cancer related gene 4 protein |
| 213132_s_at | 33.32 | 2.01E-07 | 1.226233349 | NM_014507| | MT,mitochondrial malonyltransferase isoform b |
| 238462_at | 33.3 | 2.01E-07 | 1.812587617 | NM_032873| | STS-1,Cbl-interacting protein Sts-1 |
| 241701_at | 33.29 | 2.01E-07 | 1.447959468 | NM_020824| | ARHGAP21,Rho GTPase activating protein 21 |
| 204647_at | 33.27 | 2.02E-07 | 1.178436248 | NM_004838| | HOMER3,Homer, neuronal immediate early gene, 3 |
| 239144_at | 33.25 | 2.02E-07 | 1.365116849 | NM_080742| | B3GAT2,beta-1,3-glucuronyltransferase 2 |
| 223165_s_at | 33.22 | 2.03E-07 | 1.069853786 | NM_001005909| | IHPK2,inositol hexaphosphate kinase 2 isoform a |
| 231716_at | 33.19 | 2.04E-07 | 1.099557245 | NM_001100588| | NA |
| 201751_at | 33.18 | 2.04E-07 | 1.126940435 | NM_014876| | KIAA0063,KIAA0063 gene product |
| 233562_at | 33.18 | 2.04E-07 | 1.97524103 | NA |  |
| 202662_s_at | 33.17 | 2.04E-07 | 1.336848536 | NM_002223| | ITPR2,inositol 1,4,5-triphosphate receptor, type 2 |
| 225129_at | 33.16 | 2.05E-07 | 1.236805842 | NM_152727| | CPNE2,copine II |
| 203192_at | 33.15 | 2.05E-07 | 1.081045767 | NM_005689| | ABCB6,ATP-binding cassette, sub-family B, member 6 |
| 212586_at | 33.12 | 2.06E-07 | 1.189290089 | NM_001042440| | NA |
| 204541_at | 33.07 | 2.08E-07 | 1.429180167 | NM_012429| | SEC14L2,SEC14-like 2 |
| 226919_at | 33.04 | 2.09E-07 | 1.214859407 | NM_020466| | DJ122O8.2,hypothetical protein dJ122O8.2 |
| 239562_at | 33.03 | 2.09E-07 | 1.253790698 | NM_001004346| | MTHFD2L,methylenetetrahydrofolate dehydrogenase (NADP+ |
| 228635_at | 33.03 | 2.09E-07 | 1.306245239 | NM_020815| | PCDH10,protocadherin 10 isoform 2 precursor |
| 206453_s_at | 33.03 | 2.09E-07 | 1.258286692 | NM_016250| | NDRG2,N-myc downstream-regulated gene 2 isoform b |
| 232921_at | 32.99 | 2.10E-07 | 1.288141731 | NM_020910| | NA |
| 224641_at | 32.98 | 2.10E-07 | 1.197299322 | NM_001011537| | FYTTD1,forty-two-three domain containing 1 isoform 2 |
| 208902_s_at | 32.97 | 2.11E-07 | 1.344080101 | NM_001031| | RPS28,ribosomal protein S28 |
| 235174_s_at | 32.96 | 2.11E-07 | 1.513022483 | NA |  |
| 210830_s_at | 32.94 | 2.11E-07 | 1.187011747 | NM_000305| | PON2,paraoxonase 2 |
| 238898_at | 32.93 | 2.11E-07 | 1.761238278 | NA |  |
| 224184_s_at | 32.91 | 2.12E-07 | 1.397630789 | NM_033254| | BOC,brother of CDO |
| 204736_s_at | 32.89 | 2.13E-07 | 1.475027831 | NM_001897| | CSPG4,melanoma-associated chondroitin sulfate |
| 202985_s_at | 32.88 | 2.13E-07 | 1.127375863 | NM_001015048| | NA |
| 212083_at | 32.86 | 2.14E-07 | 1.220390639 | NM_144582| | TEX261,testis expressed gene 261 |
| 204273_at | 32.86 | 2.14E-07 | 1.76793915 | NM_000115| | EDNRB,endothelin receptor type B isoform 1 |
| 201734_at | 32.85 | 2.14E-07 | 1.104983911 | NM_001829| | CLCN3,chloride channel 3 isoform b |
| 211984_at | 32.85 | 2.14E-07 | 1.106397771 | NM_001743| | CALM2,calmodulin 2 |
| 207147_at | 32.85 | 2.14E-07 | 1.826951816 | NM_004405| | DLX2,distal-less homeo box 2 |
| 212913_at | 32.84 | 2.14E-07 | 1.393662614 | NM_001039651| | NA |
| 1556601_a_at | 32.82 | 2.15E-07 | 1.239970437 | NM_153023| | SPATA13,spermatogenesis associated 13 |
| 222021_x_at | 32.8 | 2.15E-07 | 1.112680413 | NA |  |
| 228479_at | 32.79 | 2.15E-07 | 1.40593622 | NA |  |
| 218332_at | 32.77 | 2.16E-07 | 1.052891312 | NM_018476| | BEX1,brain expressed, X-linked 1 |
| 225718_at | 32.74 | 2.17E-07 | 1.178831957 | NM_030650| | KIAA1715,Lunapark |
| 224621_at | 32.74 | 2.17E-07 | 1.072677007 | NM_002745| | MAPK1,mitogen-activated protein kinase 1 |
| 244826_at | 32.73 | 2.17E-07 | 1.300366341 | NA |  |
| 203441_s_at | 32.71 | 2.18E-07 | 1.238597056 | NM_001792| | CDH2,cadherin 2, type 1 preproprotein |
| 228310_at | 32.68 | 2.19E-07 | 1.160745975 | NM_001008493| | ENAH,enabled homolog isoform a |
| 203845_at | 32.66 | 2.20E-07 | 2.214841286 | NM_003884| | PCAF,p300/CBP-associated factor |
| 224847_at | 32.65 | 2.20E-07 | 1.19088919 | NM_001259| | CDK6,cyclin-dependent kinase 6 |
| 212522_at | 32.6 | 2.21E-07 | 1.256675164 | NM_002605| | PDE8A,phosphodiesterase 8A isoform 1 |
| 227035_x_at | 32.58 | 2.22E-07 | 1.127853862 | NA |  |
| 204401_at | 32.58 | 2.22E-07 | 1.94490865 | NM_002250| | KCNN4,intermediate conductance calcium-activated |
| 202730_s_at | 32.57 | 2.22E-07 | 1.272578224 | NM_014456| | PDCD4,programmed cell death 4 isoform 1 |
| 238034_at | 32.55 | 2.23E-07 | 1.155597925 | NM_001024649| | NA |
| 209030_s_at | 32.54 | 2.23E-07 | 1.202240849 | NM_001098517| | NA |
| 214202_at | 32.53 | 2.23E-07 | 1.235418967 | NA |  |
| 210875_s_at | 32.52 | 2.24E-07 | 2.277174842 | NM_030751| | TCF8,transcription factor 8 (represses interleukin 2 |
| 212240_s_at | 32.5 | 2.24E-07 | 1.258084422 | NM_181504| | PIK3R1,phosphoinositide-3-kinase, regulatory subunit, |
| 225553_at | 32.49 | 2.24E-07 | 1.198496645 | NA |  |
| 214541_s_at | 32.47 | 2.25E-07 | 1.733684038 | NM_006775| | QKI,quaking homolog, KH domain RNA binding isoform |
| 207776_s_at | 32.46 | 2.25E-07 | 1.451809076 | NM_000724| | CACNB2,calcium channel, voltage-dependent, beta 2 |
| 217825_s_at | 32.42 | 2.27E-07 | 1.3225291 | NM_016021| | UBE2J1,ubiquitin-conjugating enzyme E2, J1 |
| 205379_at | 32.4 | 2.28E-07 | 1.219417443 | NM_001236| | CBR3,carbonyl reductase 3 |
| 222409_at | 32.39 | 2.28E-07 | 1.111264626 | NM_014325| | CORO1C,coronin, actin binding protein, 1C |
| 213891_s_at | 32.35 | 2.29E-07 | 1.120180914 | NM_001083962| | NA |
| 227498_at | 32.34 | 2.30E-07 | 1.922429837 | NA |  |
| 222231_s_at | 32.34 | 2.29E-07 | 1.106571994 | NM_018509| | PRO1855,hypothetical protein PRO1855 |
| 223847_s_at | 32.33 | 2.30E-07 | 1.102540388 | NM_001031711| | NA |
| 211763_s_at | 32.33 | 2.30E-07 | 1.171292394 | NM_003337| | UBE2B,ubiquitin-conjugating enzyme E2B |
| 201103_x_at | 32.33 | 2.30E-07 | 1.141431427 | NM_001037501| | NA |
| 209409_at | 32.31 | 2.30E-07 | 1.116378832 | NM_001001549| | GRB10,growth factor receptor-bound protein 10 isoform |
| 208978_at | 32.3 | 2.31E-07 | 1.562723708 | NM_001312| | CRIP2,cysteine-rich protein 2 |
| 212005_at | 32.29 | 2.31E-07 | 1.120202622 | NM_001114600| | NA |
| 221860_at | 32.29 | 2.31E-07 | 1.191168066 | NM_001005335| | HNRPL,heterogeneous nuclear ribonucleoprotein L |
| 244354_at | 32.23 | 2.33E-07 | 1.462695454 | NA |  |
| 1554472_a_at | 32.23 | 2.33E-07 | 1.366425435 | NM_016018| | PHF20L1,PHD finger protein 20-like 1 isoform 1 |
| 214247_s_at | 32.21 | 2.34E-07 | 1.12209604 | NM_001018057| | NA |
| 235315_at | 32.21 | 2.34E-07 | 1.417448425 | NM_006022| | TGFB1I4,transforming growth factor beta 1 induced |
| 222435_s_at | 32.2 | 2.34E-07 | 1.27295365 | NM_016021| | UBE2J1,ubiquitin-conjugating enzyme E2, J1 |
| 222125_s_at | 32.19 | 2.34E-07 | 1.21982976 | NM_177938| | PH-4,hypoxia-inducible factor prolyl 4-hydroxylase |
| 1559402_a_at | 32.18 | 2.34E-07 | 1.645076739 | NM_006365| | CROC4,transcriptional activator of the c-fos promoter |
| 203723_at | 32.12 | 2.37E-07 | 1.436833584 | NM_002221| | ITPKB,1D-myo-inositol-trisphosphate 3-kinase B |
| 238008_at | 32.09 | 2.38E-07 | 1.437010729 | NM_175922| | MGC35308,hypothetical protein MGC35308 |
| 213189_at | 32.05 | 2.39E-07 | 1.203771536 | NM_001042533| | NA |
| 209504_s_at | 32.04 | 2.40E-07 | 1.286728384 | NM_021200| | PLEKHB1,pleckstrin homology domain containing, family B |
| 227646_at | 32.04 | 2.40E-07 | 1.626708092 | NM_024007| | EBF,early B-cell factor |
| 203225_s_at | 32.04 | 2.40E-07 | 1.115539208 | NM_018339| | RFK,riboflavin kinase |
| 227067_x_at | 32.02 | 2.40E-07 | 1.235892431 | NM_203458| | NOTCH2NL,Notch homolog 2 N-terminal like protein |
| 228716_at | 31.98 | 2.42E-07 | 1.460669048 | NM_000461| | THRB,thyroid hormone receptor, beta |
| 209000_s_at | 31.97 | 2.42E-07 | 1.241217657 | NM_001098811| | NA |
| 200947_s_at | 31.96 | 2.42E-07 | 1.08880559 | NM_005271| | GLUD1,glutamate dehydrogenase 1 |
| 212017_at | 31.96 | 2.42E-07 | 1.094002031 | NM_001009993| | LOC130074,hypothetical protein LOC130074 |
| 214194_at | 31.95 | 2.43E-07 | 1.33935297 | NM_014953| | KIAA1008,KIAA1008 |
| 212708_at | 31.95 | 2.43E-07 | 1.089702273 | NM_001012241| | NA |
| 215489_x_at | 31.94 | 2.43E-07 | 1.255874796 | NM_004838| | HOMER3,Homer, neuronal immediate early gene, 3 |
| 212121_at | 31.94 | 2.43E-07 | 1.095089531 | NM_015631| | C10orf61,chromosome 10 open reading frame 61 |
| 214053_at | 31.93 | 2.43E-07 | 1.43380907 | NM_001042599| | NA |
| 225143_at | 31.91 | 2.44E-07 | 1.155807452 | NM_213649| | SFXN4,sideroflexin 4 isoform 1 |
| 230403_at | 31.89 | 2.45E-07 | 1.260648801 | NA |  |
| 204493_at | 31.89 | 2.45E-07 | 1.112063113 | NM_001196| | BID,BH3 interacting domain death agonist isoform 2 |
| 226594_at | 31.84 | 2.46E-07 | 1.20279456 | NA |  |
| 226933_s_at | 31.78 | 2.49E-07 | 1.685267781 | NM_001546| | ID4,inhibitor of DNA binding 4, dominant negative |
| 230212_at | 31.77 | 2.50E-07 | 1.290363808 | NA |  |
| 1554600_s_at | 31.71 | 2.52E-07 | 1.34583854 | NM_005572| | LMNA,lamin A/C isoform 2 |
| 213188_s_at | 31.69 | 2.53E-07 | 1.146286777 | NM_001042533| | NA |
| 203188_at | 31.69 | 2.53E-07 | 1.130108209 | NM_006876| | B3GNT6,beta-1,3-N-acetylglucosaminyltransferase bGnT-6 |
| 229347_at | 31.68 | 2.53E-07 | 1.177492089 | NA |  |
| 229572_at | 31.67 | 2.53E-07 | 1.265495562 | NA |  |
| 227699_at | 31.62 | 2.55E-07 | 1.42857695 | NM_144581| | C14orf149,chromosome 14 open reading frame 149 |
| 209663_s_at | 31.6 | 2.55E-07 | 1.391394215 | NM_002206| | ITGA7,integrin alpha 7 precursor |
| 203725_at | 31.6 | 2.56E-07 | 1.156082422 | NM_001924| | GADD45A,growth arrest and DNA-damage-inducible, alpha |
| 209170_s_at | 31.58 | 2.57E-07 | 1.196122272 | NM_001001994| | GPM6B,glycoprotein M6B isoform 4 |
| 220299_at | 31.54 | 2.58E-07 | 1.317144193 | NM_019073| | SPATA6,spermatogenesis associated 6 |
| 214773_x_at | 31.5 | 2.60E-07 | 1.098594236 | NM_001031800| | NA |
| 238012_at | 31.49 | 2.60E-07 | 1.342611934 | NM_013379| | DPP7,dipeptidyl peptidase 7 preproprotein |
| 227514_at | 31.44 | 2.62E-07 | 1.435221666 | NM_001034841| | NA |
| 225237_s_at | 31.42 | 2.63E-07 | 1.2486117 | NM_138962| | MSI2,musashi 2 isoform a |
| 226946_at | 31.42 | 2.63E-07 | 1.220617865 | NM_001085411| | NA |
| 225293_at | 31.42 | 2.63E-07 | 1.250754194 | NM_032888| | COL27A1,collagen, type XXVII, alpha 1 |
| 221567_at | 31.42 | 2.63E-07 | 1.199913752 | NM_003946| | NOL3,nucleolar protein 3 |
| 224767_at | 31.39 | 2.64E-07 | 1.188358338 | NM_000997| | RPL37,ribosomal protein L37 |
| 227894_at | 31.37 | 2.65E-07 | 1.204583343 | NM_145294| | LOC197336,similar to RIKEN cDNA 3230401M21 [Mus musculus] |
| 219213_at | 31.33 | 2.67E-07 | 1.145704675 | NM_021219| | JAM2,junctional adhesion molecule 2 precursor |
| 226252_at | 31.33 | 2.67E-07 | 2.205370616 | NA |  |
| 1557384_at | 31.32 | 2.68E-07 | 1.270087716 | NM_003432| | NA |
| 217826_s_at | 31.32 | 2.67E-07 | 1.266474939 | NM_016021| | UBE2J1,ubiquitin-conjugating enzyme E2, J1 |
| 213405_at | 31.31 | 2.68E-07 | 1.197202044 | NM_020673| | RAB22A,RAS-related protein RAB-22A |
| 209985_s_at | 31.31 | 2.68E-07 | 1.865876455 | NM_004316| | ASCL1,achaete-scute complex homolog-like 1 |
| 205741_s_at | 31.3 | 2.68E-07 | 1.271516999 | NM_001390| | DTNA,dystrobrevin alpha isoform 1 |
| 205240_at | 31.3 | 2.68E-07 | 1.263847002 | NM_013296| | GPSM2,G-protein signalling modulator 2 (AGS3-like, C. |
| 224636_at | 31.23 | 2.72E-07 | 1.113244496 | NM_053023| | ZFP91,zinc finger protein 91 isoform 1 |
| 212546_s_at | 31.22 | 2.72E-07 | 1.145794645 | NM_015030| | NA |
| 224937_at | 31.13 | 2.77E-07 | 1.115935675 | NM_020440| | PTGFRN,prostaglandin F2 receptor negative regulator |
| 230577_at | 31.08 | 2.79E-07 | 2.543198412 | NA |  |
| 212237_at | 31.08 | 2.79E-07 | 1.209444976 | NM_015338| | ASXL1,additional sex combs like 1 |
| 227138_at | 31.05 | 2.81E-07 | 1.382642611 | NM_006371| | CRTAP,cartilage associated protein precursor |
| 203580_s_at | 31.04 | 2.81E-07 | 1.117160574 | NM_001076785| | NA |
| 205548_s_at | 31.02 | 2.82E-07 | 1.130604427 | NM_006806| | BTG3,B-cell translocation gene 3 |
| 212813_at | 30.99 | 2.83E-07 | 1.092049665 | NM_032801| | JAM3,junctional adhesion molecule 3 precursor |
| 218408_at | 30.98 | 2.84E-07 | 1.133372626 | NM_012456| | TIMM10,translocase of inner mitochondrial membrane 10 |
| 201153_s_at | 30.97 | 2.85E-07 | 1.375762238 | NM_021038| | MBNL1,muscleblind-like 1 isoform a |
| 224472_x_at | 30.96 | 2.85E-07 | 1.092671736 | NM_016176| | Cab45,calcium binding protein Cab45 precursor |
| 201535_at | 30.96 | 2.85E-07 | 1.149281368 | NM_007106| | UBL3,ubiquitin-like 3 |
| 202386_s_at | 30.94 | 2.86E-07 | 1.141500939 | NM_014647| | LKAP,limkain b1 isoform 1 |
| 221989_at | 30.93 | 2.86E-07 | 1.163296672 | NM_006013| | RPL10,ribosomal protein L10 |
| 228299_at | 30.92 | 2.87E-07 | 1.135140158 | NM_173562| | C6orf69,hypothetical protein MGC14254 |
| 217933_s_at | 30.92 | 2.86E-07 | 1.101641636 | NM_015907| | LAP3,leucine aminopeptidase |
| 225526_at | 30.9 | 2.87E-07 | 1.105610508 | NM_013255| | MKLN1,muskelin 1, intracellular mediator containing |
| 232015_at | 30.9 | 2.87E-07 | 1.325152509 | NA |  |
| 200787_s_at | 30.9 | 2.87E-07 | 1.217705619 | NM_003768| | PEA15,phosphoprotein enriched in astrocytes 15 |
| 203323_at | 30.87 | 2.89E-07 | 1.880811303 | NM_001233| | CAV2,caveolin 2 isoform a and b |
| 214252_s_at | 30.86 | 2.90E-07 | 1.463018533 | NM_006493| | CLN5,ceroid-lipofuscinosis, neuronal 5 |
| 225799_at | 30.85 | 2.90E-07 | 1.20584161 | NA |  |
| 219663_s_at | 30.85 | 2.90E-07 | 1.360230357 | NM_025268| | MGC4659,hole protein |
| 225590_at | 30.81 | 2.92E-07 | 1.14937621 | NM_020870| | SH3MD2,SH3 multiple domains 2 |
| 218009_s_at | 30.79 | 2.93E-07 | 1.141829357 | NM_003981| | PRC1,protein regulator of cytokinesis 1 isoform 1 |
| 202585_s_at | 30.77 | 2.93E-07 | 1.170326028 | NM_002504| | NFX1,nuclear transcription factor, X-box binding 1 |
| 220298_s_at | 30.76 | 2.94E-07 | 1.392561263 | NM_019073| | SPATA6,spermatogenesis associated 6 |
| 227143_s_at | 30.75 | 2.94E-07 | 1.187720621 | NM_001196| | BID,BH3 interacting domain death agonist isoform 2 |
| 227125_at | 30.72 | 2.96E-07 | 1.157182068 | NA |  |
| 213451_x_at | 30.7 | 2.97E-07 | 1.157125959 | NM_019105| | TNXB,tenascin XB isoform 1 |
| 227407_at | 30.7 | 2.97E-07 | 1.169733928 | NM_153365| | FLJ90013,hypothetical protein FLJ90013 |
| 226712_at | 30.69 | 2.97E-07 | 1.15237858 | NM_003144| | SSR1,signal sequence receptor, alpha |
| 1552474_a_at | 30.67 | 2.98E-07 | 1.209718709 | NM_000156| | GAMT,guanidinoacetate N-methyltransferase isoform a |
| 226365_at | 30.66 | 2.99E-07 | 1.135185164 | NA |  |
| 212020_s_at | 30.64 | 3.00E-07 | 1.19943202 | NM_002417| | MKI67,antigen identified by monoclonal antibody Ki-67 |
| 225546_at | 30.63 | 3.00E-07 | 1.275854225 | NM_013302| | EEF2K,elongation factor-2 kinase |
| 220029_at | 30.6 | 3.02E-07 | 1.942937035 | NM_017770| | ELOVL2,elongation of very long chain fatty acids |
| 225050_at | 30.59 | 3.02E-07 | 1.058391786 | NM_032434| | ZNF512,zinc finger protein 512 |
| 202364_at | 30.57 | 3.02E-07 | 1.099780169 | NM_001008541| | MXI1,MAX interactor 1 isoform c |
| 239082_at | 30.57 | 3.02E-07 | 1.147846183 | NA |  |
| 204836_at | 30.57 | 3.02E-07 | 1.052871043 | NM_000170| | GLDC,glycine dehydrogenase (decarboxylating; glycine |
| 210410_s_at | 30.54 | 3.04E-07 | 1.378987551 | NM_001039651| | NA |
| 204642_at | 30.52 | 3.05E-07 | 1.344989324 | NM_001400| | EDG1,endothelial differentiation, sphingolipid |
| 227212_s_at | 30.52 | 3.05E-07 | 1.17691783 | NM_001009936| | PHF19,PHD finger protein 19 isoform b |
| 200911_s_at | 30.51 | 3.05E-07 | 1.252587492 | NM_001122824| | NA |
| 218997_at | 30.5 | 3.06E-07 | 1.105239981 | NM_022490| | PAF53,RNA polymerase I associated factor 53 |
| 222752_s_at | 30.5 | 3.06E-07 | 1.102182645 | NM_018252| | FLJ10874,hypothetical protein FLJ10874 |
| 200732_s_at | 30.48 | 3.07E-07 | 1.078155194 | NM_003463| | PTP4A1,protein tyrosine phosphatase type IVA, member 1 |
| 211000_s_at | 30.45 | 3.09E-07 | 1.349369946 | NM_002184| | IL6ST,interleukin 6 signal transducer isoform 1 |
| 226808_at | 30.42 | 3.11E-07 | 1.270156728 | NM_001099220| | NA |
| 233167_at | 30.41 | 3.11E-07 | 1.236098353 | NM_031454| | SELO,selenoprotein O |
| 233350_s_at | 30.38 | 3.12E-07 | 1.26946633 | NM_015926| | TEX264,testis expressed sequence 264 |
| 204795_at | 30.3 | 3.18E-07 | 1.200224841 | NM_001077497| | NA |
| 209200_at | 30.3 | 3.18E-07 | 1.725363157 | NM_002397| | MEF2C,MADS box transcription enhancer factor 2, |
| 36612_at | 30.29 | 3.18E-07 | 1.346768541 | NM_015159| | NA |
| 1569854_at | 30.28 | 3.18E-07 | 2.100341384 | NA |  |
| 202283_at | 30.28 | 3.18E-07 | 1.11796962 | NM_002615| | SERPINF1,serine (or cysteine) proteinase inhibitor, clade |
| 243591_at | 30.28 | 3.18E-07 | 1.571435066 | NA |  |
| 201577_at | 30.26 | 3.19E-07 | 1.069229118 | NM_000269| | NME1,nucleoside-diphosphate kinase 1 isoform b |
| 226490_at | 30.25 | 3.20E-07 | 1.206509204 | NA |  |
| 231964_at | 30.17 | 3.24E-07 | 1.267626506 | NA |  |
| 211110_s_at | 30.16 | 3.25E-07 | 1.209759356 | NM_000044| | AR,androgen receptor isoform 1 |
| 211814_s_at | 30.15 | 3.26E-07 | 1.679198303 | NM_057749| | CCNE2,cyclin E2 isoform 1 |
| 226373_at | 30.13 | 3.26E-07 | 1.112250235 | NM_144579| | SFXN5,sideroflexin 5 |
| 218109_s_at | 30.11 | 3.28E-07 | 1.148698992 | NM_022736| | MFSD1,major facilitator superfamily domain containing |
| 225019_at | 30.1 | 3.28E-07 | 1.283700946 | NM_001221| | CAMK2D,calcium/calmodulin-dependent protein kinase II |
| 223707_at | 30.09 | 3.29E-07 | 1.428692102 | NM_000990| | RPL27A,ribosomal protein L27a |
| 203048_s_at | 30.08 | 3.29E-07 | 1.043716497 | NM_014639| | KIAA0372,KIAA0372 |
| 228752_at | 30.07 | 3.30E-07 | 1.436608027 | NA |  |
| 227249_at | 30.07 | 3.29E-07 | 1.169097002 | NM_017668| | NDE1,nuclear distribution gene E homolog 1 |
| 212936_at | 30.06 | 3.30E-07 | 1.291822383 | NM_032042| | DKFZP564D172,hypothetical protein DKFZp564D172 |
| 213338_at | 30 | 3.34E-07 | 1.070704009 | NM_015444| | RIS1,Ras-induced senescence 1 |
| 219331_s_at | 30 | 3.33E-07 | 1.867418445 | NM_018203| | FLJ10748,hypothetical protein FLJ10748 |
| 201910_at | 29.99 | 3.34E-07 | 1.062512993 | NM_001001715| | FARP1,FERM, RhoGEF, and pleckstrin domain protein 1 |
| 226395_at | 29.98 | 3.34E-07 | 1.190825444 | NM_032410| | HOOK3,golgi-associated microtubule-binding protein |
| 45572_s_at | 29.98 | 3.34E-07 | 1.069001852 | NM_001001560| | GGA1,golgi associated, gamma adaptin ear containing, |
| 225363_at | 29.94 | 3.36E-07 | 1.103061459 | NM_000314| | PTEN,phosphatase and tensin homolog |
| 241710_at | 29.94 | 3.36E-07 | 1.444840863 | NM_001101330| | NA |
| 208073_x_at | 29.94 | 3.36E-07 | 1.202555789 | NM_001001894| | TTC3,tetratricopeptide repeat domain 3 |
| 219182_at | 29.92 | 3.38E-07 | 1.312660315 | NM_001077416| | NA |
| 203029_s_at | 29.89 | 3.40E-07 | 1.20077953 | NM_002847| | PTPRN2,protein tyrosine phosphatase, receptor type, N |
| 220547_s_at | 29.88 | 3.40E-07 | 1.108964659 | NM_019054| | FAM35A,family with sequence similarity 35, member A |
| 218099_at | 29.83 | 3.43E-07 | 1.191016508 | NM_018469| | HT008,uncharacterized hypothalamus protein HT008 |
| 230958_s_at | 29.83 | 3.43E-07 | 2.04342776 | NA |  |
| 212362_at | 29.8 | 3.44E-07 | 1.278369305 | NM_001681| | ATP2A2,ATPase, Ca++ transporting, cardiac muscle, slow |
| 207060_at | 29.79 | 3.44E-07 | 1.272178077 | NM_001427| | EN2,engrailed homolog 2 |
| 226072_at | 29.78 | 3.45E-07 | 1.220129321 | NM_145059| | FUK,fucokinase |
| 1559633_a_at | 29.77 | 3.46E-07 | 1.634304504 | NM_000740| | CHRM3,cholinergic receptor, muscarinic 3 |
| 226419_s_at | 29.75 | 3.46E-07 | 1.254961001 | NA |  |
| 209288_s_at | 29.75 | 3.46E-07 | 1.115061168 | NM_006449| | CDC42EP3,Cdc42 effector protein 3 |
| 224909_s_at | 29.74 | 3.47E-07 | 1.265331505 | NM_020820| | PREX1,PREX1 protein |
| 202370_s_at | 29.74 | 3.47E-07 | 1.227121906 | NM_001755| | CBFB,core-binding factor, beta subunit isoform 2 |
| 202836_s_at | 29.73 | 3.47E-07 | 1.044586434 | NM_006701| | TXNL4A,thioredoxin-like 4A |
| 218208_at | 29.71 | 3.49E-07 | 1.167649335 | NM_025078| | PQLC1,PQ loop repeat containing 1 |
| 228843_at | 29.71 | 3.49E-07 | 1.189061956 | NA |  |
| 228131_at | 29.7 | 3.49E-07 | 1.37880475 | NM_001983| | ERCC1,excision repair cross-complementing 1 isofrom 2 |
| 228495_at | 29.69 | 3.50E-07 | 1.202391731 | NM_174931| | FLJ38348,hypothetical protein FLJ38348 |
| 204808_s_at | 29.67 | 3.50E-07 | 1.15086423 | NM_014254| | TMEM5,transmembrane protein 5 |
| 212023_s_at | 29.67 | 3.50E-07 | 1.211818885 | NM_002417| | MKI67,antigen identified by monoclonal antibody Ki-67 |
| 232377_at | 29.66 | 3.51E-07 | 2.463940814 | NM_152745| | NXPH1,neurexophilin 1 |
| 204743_at | 29.66 | 3.51E-07 | 1.258755617 | NM_001008272| | TAGLN3,transgelin 3 |
| 227543_at | 29.65 | 3.51E-07 | 1.285094475 | NM_032193| | AYP1,AYP1 protein |
| 227524_at | 29.64 | 3.52E-07 | 1.202598643 | NA |  |
| 239229_at | 29.63 | 3.52E-07 | 1.87156547 | NA |  |
| 225612_s_at | 29.58 | 3.56E-07 | 1.191821154 | NM_032047| | B3GNT5,beta-1,3-N-acetylglucosaminyltransferase bGnT-5 |
| 207233_s_at | 29.54 | 3.59E-07 | 1.4049495 | NM_000248| | MITF,microphthalmia-associated transcription factor |
| 219569_s_at | 29.53 | 3.59E-07 | 1.311001681 | NM_001097599| | NA |
| 212970_at | 29.5 | 3.61E-07 | 1.161747949 | NA |  |
| 226336_at | 29.49 | 3.61E-07 | 1.072477849 | NM_021130| | PPIA,peptidylprolyl isomerase A isoform 1 |
| 217819_at | 29.49 | 3.61E-07 | 1.069928536 | NM_001002296| | GOLGA7,golgi autoantigen, golgin subfamily a, 7 |
| 38157_at | 29.48 | 3.62E-07 | 1.094819348 | NM_005510| | DOM3Z,DOM-3 homolog Z |
| 225435_at | 29.47 | 3.62E-07 | 1.140241232 | NM_003144| | SSR1,signal sequence receptor, alpha |
| 210006_at | 29.46 | 3.62E-07 | 1.285878427 | NM_015407| | DKFZP564O243,DKFZP564O243 protein |
| 218918_at | 29.46 | 3.62E-07 | 1.275965069 | NM_020379| | MAN1C1,mannosidase, alpha, class 1C, member 1 |
| 235798_at | 29.44 | 3.64E-07 | 1.427563929 | NM_001100829| | NA |
| 225355_at | 29.41 | 3.66E-07 | 1.208618162 | NA |  |
| 219133_at | 29.41 | 3.66E-07 | 1.154269102 | NM_017897| | FLJ20604,hypothetical protein FLJ20604 |
| 224901_at | 29.39 | 3.67E-07 | 1.623203685 | NM_001037582| | NA |
| 217874_at | 29.36 | 3.70E-07 | 1.133373762 | NM_003849| | SUCLG1,succinate-CoA ligase, GDP-forming, alpha |
| 204225_at | 29.36 | 3.70E-07 | 1.101486864 | NM_006037| | HDAC4,histone deacetylase 4 |
| 235106_at | 29.35 | 3.70E-07 | 1.561135906 | NM_032427| | MAML2,mastermind-like 2 |
| 227061_at | 29.35 | 3.70E-07 | 1.671462504 | NA |  |
| 217824_at | 29.32 | 3.72E-07 | 1.351179444 | NM_016021| | UBE2J1,ubiquitin-conjugating enzyme E2, J1 |
| 219135_s_at | 29.3 | 3.74E-07 | 1.32413533 | NM_022773| | FLJ12681,hypothetical protein FLJ12681 |
| 225136_at | 29.28 | 3.75E-07 | 1.280759256 | NM_021623| | NA |
| 235405_at | 29.27 | 3.75E-07 | 1.30054415 | NM_001512| | GSTA4,glutathione S-transferase A4 |
| 217890_s_at | 29.25 | 3.77E-07 | 1.165957746 | NM_018222| | PARVA,parvin, alpha |
| 228720_at | 29.23 | 3.78E-07 | 1.482825922 | NM_020777| | SORCS2,VPS10 domain receptor protein SORCS 2 |
| 227051_at | 29.18 | 3.81E-07 | 1.870010225 | NA |  |
| 224592_x_at | 29.18 | 3.81E-07 | 1.170671936 | NM_016287| | HP1-BP74,HP1-BP74 |
| 211520_s_at | 29.16 | 3.82E-07 | 1.951569834 | NM_000827| | GRIA1,glutamate receptor, ionotropic, AMPA 1 |
| 201581_at | 29.16 | 3.82E-07 | 1.206419337 | NM_021156| | DJ971N18.2,hypothetical protein DJ971N18.2 |
| 210105_s_at | 29.14 | 3.84E-07 | 1.061748657 | NM_002037| | FYN,protein-tyrosine kinase fyn isoform a |
| 229228_at | 29.14 | 3.84E-07 | 1.691820342 | NM_001011666| | CREB5,cAMP responsive element binding protein 5 |
| 210046_s_at | 29.12 | 3.84E-07 | 1.133317093 | NM_002168| | IDH2,isocitrate dehydrogenase 2 (NADP+), |
| 204159_at | 29.12 | 3.84E-07 | 2.43334393 | NM_001262| | CDKN2C,cyclin-dependent kinase inhibitor 2C |
| 224973_at | 29.1 | 3.85E-07 | 1.595661575 | NM_017633| | FAM46A,family with sequence similarity 46, member A |
| 210978_s_at | 29.1 | 3.85E-07 | 1.21438622 | NM_003564| | TAGLN2,transgelin 2 |
| 57163_at | 29.07 | 3.87E-07 | 1.135291343 | NM_022821| | ELOVL1,elongation of very long chain fatty acids |
| 209013_x_at | 29.05 | 3.88E-07 | 1.292099706 | NM_007118| | TRIO,triple functional domain (PTPRF interacting) |
| 209079_x_at | 29.05 | 3.88E-07 | 1.222634289 | NM_002588| | PCDHGC3,protocadherin gamma subfamily C, 3 isoform 1 |
| 210089_s_at | 29.04 | 3.89E-07 | 1.918477143 | NM_001105206| | NA |
| 36129_at | 29.04 | 3.88E-07 | 1.146986224 | NM_001098509| | NA |
| 202986_at | 29.03 | 3.89E-07 | 1.393849754 | NM_014862| | ARNT2,aryl-hydrocarbon receptor nuclear translocator |
| 226774_at | 29.02 | 3.90E-07 | 1.24934455 | NM_032448| | KIAA1838,KIAA1838 |
| 209618_at | 29.01 | 3.90E-07 | 1.396930091 | NM_001332| | CTNND2,catenin (cadherin-associated protein), delta 2 |
| 228162_at | 29 | 3.91E-07 | 1.305729144 | NM_001984| | ESD,esterase D/formylglutathione hydrolase |
| 227135_at | 28.99 | 3.91E-07 | 1.382278635 | NM_001042402| | NA |
| 201266_at | 28.99 | 3.91E-07 | 1.061860762 | NM_001093771| | NA |
| 223228_at | 28.98 | 3.92E-07 | 1.10772978 | NM_032287| | LDOC1L,leucine zipper, down-regulated in cancer 1-like |
| 201666_at | 28.95 | 3.94E-07 | 1.135816448 | NM_003254| | TIMP1,tissue inhibitor of metalloproteinase 1 |
| 217792_at | 28.93 | 3.95E-07 | 1.073877523 | NM_014426| | SNX5,sorting nexin 5 |
| 213298_at | 28.93 | 3.95E-07 | 1.543279165 | NM_005597| | NFIC,nuclear factor I/C isoform 1 |
| 226066_at | 28.92 | 3.95E-07 | 1.403033495 | NM_000248| | MITF,microphthalmia-associated transcription factor |
| 205449_at | 28.89 | 3.97E-07 | 1.096483417 | NM_013299| | SHD1,Sac3 homology domain 1 |
| 209046_s_at | 28.88 | 3.98E-07 | 1.089414087 | NM_007285| | GABARAPL2,GABA(A) receptor-associated protein-like 2 |
| 1553976_a_at | 28.87 | 3.99E-07 | 1.117470863 | NM_015448| | DPCD,DPCD protein |
| 230023_at | 28.87 | 3.99E-07 | 1.207181035 | NM_199044| | NSUN4,NOL1/NOP2/Sun domain family 4 protein |
| 1568983_a_at | 28.86 | 3.99E-07 | 1.212700994 | NA |  |
| 228540_at | 28.84 | 4.01E-07 | 1.436983028 | NM_006775| | QKI,quaking homolog, KH domain RNA binding isoform |
| 1568598_at | 28.84 | 4.01E-07 | 1.566158998 | NM_030929| | KAZALD1,Kazal-type serine protease inhibitor domain 1 |
| 1553111_a_at | 28.84 | 4.01E-07 | 1.270482236 | NM_152903| | KBTBD6,kelch repeat and BTB (POZ) domain-containing 6 |
| 212607_at | 28.82 | 4.02E-07 | 1.081621036 | NM_005465| | AKT3,v-akt murine thymoma viral oncogene homolog 3 |
| 211219_s_at | 28.8 | 4.03E-07 | 1.863004389 | NM_004789| | LHX2,LIM homeobox protein 2 |
| 1558164_s_at | 28.8 | 4.03E-07 | 1.103382827 | NM_002618| | PEX13,peroxisome biogenesis factor 13 |
| 200919_at | 28.8 | 4.03E-07 | 1.190423716 | NM_004427| | PHC2,polyhomeotic 2-like isoform b |
| 226902_at | 28.79 | 4.03E-07 | 1.147818369 | NA |  |
| 223158_s_at | 28.79 | 4.03E-07 | 1.224837501 | NM_014397| | NEK6,putative serine-threonine protein kinase |
| 218837_s_at | 28.78 | 4.04E-07 | 1.214966122 | NM_015983| | UBE2D4,ubiquitin-conjugating enzyme E2D 4 (putative) |
| 223299_at | 28.77 | 4.04E-07 | 1.086619285 | NM_033280| | SEC11L3,SEC11-like 3 |
| 203355_s_at | 28.75 | 4.06E-07 | 1.214633175 | NM_015310| | PSD3,ADP-ribosylation factor guanine nucleotide |
| 204249_s_at | 28.73 | 4.07E-07 | 1.445556797 | NM_005574| | LMO2,LIM domain only 2 |
| 224694_at | 28.73 | 4.07E-07 | 1.172758865 | NM_018153| | ANTXR1,tumor endothelial marker 8 isoform 3 precursor |
| 201959_s_at | 28.72 | 4.08E-07 | 1.21784771 | NM_015057| | MYCBP2,MYC binding protein 2 |
| 208683_at | 28.71 | 4.08E-07 | 1.162480086 | NM_001748| | CAPN2,calpain 2, large subunit |
| 223034_s_at | 28.71 | 4.08E-07 | 1.071579329 | NM_001098616| | NA |
| 222006_at | 28.7 | 4.09E-07 | 1.103059174 | NM_012318| | LETM1,leucine zipper-EF-hand containing transmembrane |
| 208779_x_at | 28.69 | 4.09E-07 | 1.117458028 | NM_001954| | DDR1,discoidin receptor tyrosine kinase isoform b |
| 228067_at | 28.69 | 4.09E-07 | 1.518738264 | NM_207362| | MGC42367,similar to 2010300C02Rik protein |
| 220576_at | 28.65 | 4.12E-07 | 1.437241542 | NM_024989| | PGAP1,GPI deacylase |
| 225682_s_at | 28.64 | 4.13E-07 | 1.125720809 | NM_001018050| | NA |
| 1555847_a_at | 28.6 | 4.16E-07 | 1.204632359 | NA |  |
| 204363_at | 28.59 | 4.17E-07 | 1.309745456 | NM_001993| | F3,coagulation factor III precursor |
| 206580_s_at | 28.57 | 4.19E-07 | 1.217879193 | NM_016938| | EFEMP2,EGF-containing fibulin-like extracellular matrix |
| 210645_s_at | 28.56 | 4.20E-07 | 1.200381311 | NM_001001894| | TTC3,tetratricopeptide repeat domain 3 |
| 224851_at | 28.55 | 4.21E-07 | 1.208482025 | NM_001259| | CDK6,cyclin-dependent kinase 6 |
| 243768_at | 28.55 | 4.21E-07 | 1.167931327 | NA |  |
| 209090_s_at | 28.55 | 4.21E-07 | 1.135882143 | NM_016009| | SH3GLB1,SH3-containing protein SH3GLB1 |
| 220155_s_at | 28.52 | 4.22E-07 | 1.096418752 | NM_001009877| | BRD9,bromodomain containing 9 isoform 2 |
| 217922_at | 28.5 | 4.23E-07 | 1.166397076 | NA |  |
| 203132_at | 28.48 | 4.25E-07 | 1.282111764 | NM_000321| | RB1,retinoblastoma 1 |
| 209793_at | 28.47 | 4.26E-07 | 2.192022252 | NM_000827| | GRIA1,glutamate receptor, ionotropic, AMPA 1 |
| 226009_at | 28.46 | 4.26E-07 | 1.106139207 | NM_015448| | DPCD,DPCD protein |
| 205278_at | 28.41 | 4.31E-07 | 1.496563922 | NM_000817| | GAD1,glutamate decarboxylase 1 isoform GAD67 |
| 226446_at | 28.4 | 4.32E-07 | 1.353272131 | NM_018645| | HES6,hairy and enhancer of split 6 |
| 218678_at | 28.39 | 4.32E-07 | 1.094103534 | NM_006617| | NES,nestin |
| 218691_s_at | 28.37 | 4.34E-07 | 2.008011647 | NM_003687| | PDLIM4,PDZ and LIM domain 4 |
| 205152_at | 28.37 | 4.34E-07 | 2.399652904 | NM_003042| | SLC6A1,solute carrier family 6 (neurotransmitter |
| 235471_at | 28.36 | 4.35E-07 | 1.312494493 | NM_001031746| | NA |
| 202651_at | 28.35 | 4.36E-07 | 1.140441231 | NM_014873| | LPGAT1,lysophosphatidylglycerol acyltransferase 1 |
| 238963_at | 28.34 | 4.36E-07 | 1.302007352 | NA |  |
| 208919_s_at | 28.27 | 4.42E-07 | 1.093096013 | NM_023018| | FLJ13052,NAD kinase |
| 823_at | 28.24 | 4.45E-07 | 1.305193755 | NM_002996| | CX3CL1,chemokine (C-X3-C motif) ligand 1 |
| 213392_at | 28.22 | 4.46E-07 | 1.188029316 | NM_153208| | MGC35048,hypothetical protein MGC35048 |
| 202207_at | 28.21 | 4.47E-07 | 1.14663316 | NM_005737| | ARL7,ADP-ribosylation factor-like 7 |
| 219600_s_at | 28.18 | 4.50E-07 | 1.111141385 | NM_006134| | C21orf4,HCV p7-transregulated protein 3 |
| 239780_at | 28.17 | 4.50E-07 | 1.541136885 | NA |  |
| 228977_at | 28.16 | 4.51E-07 | 1.263123311 | NA |  |
| 221858_at | 28.13 | 4.53E-07 | 1.293087778 | NM_015188| | NA |
| 1553959_a_at | 28.07 | 4.59E-07 | 1.112763181 | NM_080605| | B3GALT6,UDP-Gal:betaGal beta 1,3-galactosyltransferase |
| 213353_at | 28.06 | 4.59E-07 | 1.575296754 | NM_018672| | ABCA5,ATP-binding cassette, sub-family A , member 5 |
| 221069_s_at | 28.06 | 4.59E-07 | 1.076170126 | NM_016360| | LOC51204,clone HQ0477 PRO0477p |
| 224796_at | 28.06 | 4.59E-07 | 1.110228518 | NM_018482| | DDEF1,development and differentiation enhancing factor |
| 235428_at | 28.05 | 4.60E-07 | 1.559471956 | NA |  |
| 201554_x_at | 28.03 | 4.62E-07 | 1.147866603 | NM_004130| | GYG,glycogenin |
| 231899_at | 28.03 | 4.62E-07 | 1.240175742 | NM_033390| | NA |
| 201150_s_at | 28.03 | 4.62E-07 | 1.451802484 | NM_000362| | TIMP3,tissue inhibitor of metalloproteinase 3 |
| 204610_s_at | 28.02 | 4.62E-07 | 1.17987217 | NM_006848| | DIPA,hepatitis delta antigen-interacting protein A |
| 1557996_at | 28.02 | 4.62E-07 | 1.189320883 | NA |  |
| 215000_s_at | 28 | 4.64E-07 | 1.116148077 | NM_001042548| | NA |
| 209087_x_at | 27.99 | 4.65E-07 | 1.252702344 | NM_006500| | MCAM,melanoma cell adhesion molecule |
| 226691_at | 27.98 | 4.65E-07 | 1.440978734 | NM_001013722| | NA |
| 243619_at | 27.97 | 4.66E-07 | 1.362239399 | NM_015633| | FGFR1OP2,FGFR1 oncogene partner 2 |
| 212563_at | 27.97 | 4.66E-07 | 1.125418776 | NM_015201| | BOP1,block of proliferation 1 |
| 224642_at | 27.96 | 4.67E-07 | 1.259022311 | NM_001011537| | FYTTD1,forty-two-three domain containing 1 isoform 2 |
| 227448_at | 27.94 | 4.68E-07 | 1.434933343 | NM_018011| | FLJ10154,hypothetical protein FLJ10154 |
| 205439_at | 27.94 | 4.68E-07 | 1.18797191 | NM_000854| | GSTT2,glutathione S-transferase theta 2 |
| 222478_at | 27.93 | 4.69E-07 | 1.324783219 | NM_016075| | C13orf9,CGI-145 protein |
| 45749_at | 27.9 | 4.71E-07 | 1.195279009 | NM_024519| | FLJ13725,hypothetical protein FLJ13725 |
| 205088_at | 27.9 | 4.71E-07 | 1.621972709 | NM_005491| | CXorf6,chromosome X open reading frame 6 |
| 224848_at | 27.9 | 4.72E-07 | 1.213930122 | NM_001259| | CDK6,cyclin-dependent kinase 6 |
| 243514_at | 27.87 | 4.74E-07 | 1.205569746 | NA |  |
| 212843_at | 27.83 | 4.77E-07 | 1.230624827 | NM_000615| | NCAM1,neural cell adhesion molecule 1 |
| 212234_at | 27.8 | 4.81E-07 | 1.206062985 | NM_015338| | ASXL1,additional sex combs like 1 |
| 201466_s_at | 27.79 | 4.83E-07 | 1.350149966 | NM_002228| | JUN,v-jun avian sarcoma virus 17 oncogene homolog |
| 214473_x_at | 27.77 | 4.84E-07 | 1.131772041 | NM_001003686| | PMS2L3,postmeiotic segregation increased 2-like 3 |
| 232884_s_at | 27.75 | 4.86E-07 | 1.321029284 | NA |  |
| 228897_at | 27.75 | 4.86E-07 | 1.348747763 | NM_001002862| | DERL3,derlin-3 protein isoform b |
| 223203_at | 27.75 | 4.86E-07 | 1.139699831 | NM_001099684| | NA |
| 205279_s_at | 27.75 | 4.86E-07 | 1.900311889 | NM_000824| | GLRB,glycine receptor, beta |
| 229608_at | 27.75 | 4.86E-07 | 1.239041806 | NM_019099| | LOC55924,hypothetical protein LOC55924 isoform 1 |
| 217911_s_at | 27.73 | 4.88E-07 | 1.156553851 | NM_004281| | BAG3,BCL2-associated athanogene 3 |
| 225575_at | 27.72 | 4.88E-07 | 1.243912432 | NM_002310| | LIFR, |
| 227394_at | 27.69 | 4.91E-07 | 1.22061785 | NM_000615| | NCAM1,neural cell adhesion molecule 1 |
| 1559993_at | 27.66 | 4.94E-07 | 1.445142114 | NM_030971| | SFXN3,sideroflexin 3 |
| 201645_at | 27.65 | 4.95E-07 | 1.478824323 | NM_002160| | TNC,tenascin C (hexabrachion) |
| 226013_at | 27.61 | 4.98E-07 | 1.25386429 | NM_001042646| | NA |
| 242939_at | 27.6 | 4.99E-07 | 1.382878203 | NM_007111| | TFDP1,transcription factor Dp-1 |
| 207515_s_at | 27.6 | 4.99E-07 | 1.134322009 | NM_004875| | POLR1C,RNA polymerase I subunit isoform 2 |
| 218616_at | 27.6 | 4.99E-07 | 1.108206098 | NM_020395| | LOC57117,hypothetical nuclear factor SBBI22 |
| 203527_s_at | 27.6 | 4.99E-07 | 1.380521252 | NM_000038| | APC,adenomatosis polyposis coli |
| 220443_s_at | 27.59 | 5.00E-07 | 1.647189711 | NM_012476| | VAX2,ventral anterior homeobox 2 |
| 229876_at | 27.59 | 5.00E-07 | 1.575272573 | NM_001122670| | NA |
| 214717_at | 27.57 | 5.01E-07 | 1.237936217 | NA |  |
| 218140_x_at | 27.56 | 5.02E-07 | 1.170491793 | NM_021203| | SRPRB,signal recognition particle receptor, beta |
| 227119_at | 27.55 | 5.02E-07 | 1.358680592 | NM_144571| | CNOT6L,CCR4-NOT transcription complex, subunit 6-like |
| 219639_x_at | 27.55 | 5.02E-07 | 1.140058587 | NM_020214| | PARP6,poly (ADP-ribose) polymerase family, member 6 |
| 203089_s_at | 27.54 | 5.03E-07 | 1.14140105 | NM_013247| | PRSS25,protease, serine, 25 isoform 1 preproprotein |
| 229674_at | 27.5 | 5.08E-07 | 1.226329679 | NM_019605| | SERTAD4,SERTA domain containing 4 |
| 204476_s_at | 27.5 | 5.08E-07 | 1.219521754 | NM_000920| | PC,pyruvate carboxylase precursor |
| 226197_at | 27.5 | 5.08E-07 | 1.164867122 | NA |  |
| 222617_s_at | 27.48 | 5.09E-07 | 1.088183561 | NM_022063| | C10orf84,chromosome 10 open reading frame 84 |
| 225540_at | 27.47 | 5.10E-07 | 1.278337754 | NM_001039538| | NA |
| 213257_at | 27.47 | 5.10E-07 | 1.454572172 | NM_015077| | SARM1,sterile alpha and TIR motif containing 1 |
| 218328_at | 27.47 | 5.10E-07 | 1.074960729 | NM_016035| | COQ4,CGI-92 protein |
| 218488_at | 27.45 | 5.12E-07 | 1.095921517 | NM_020365| | EIF2B3,eukaryotic translation initiation factor 2B, |
| 1557137_at | 27.45 | 5.12E-07 | 1.205499217 | NM_198276| | TMEM17,transmembrane protein 17 |
| 209763_at | 27.44 | 5.12E-07 | 1.258028176 | NM_145234| | CHRDL1,chordin-like 1 |
| 201190_s_at | 27.44 | 5.12E-07 | 1.119101016 | NM_006224| | PITPNA,phosphatidylinositol transfer protein, alpha |
| 203759_at | 27.41 | 5.15E-07 | 1.246982147 | NM_006278| | ST3GAL4,ST3 beta-galactoside alpha-2,3-sialyltransferase |
| 207336_at | 27.41 | 5.15E-07 | 1.618375832 | NM_006940| | SOX5,SRY (sex determining region Y)-box 5 isoform a |
| 227347_x_at | 27.39 | 5.17E-07 | 1.305688121 | NM_021170| | HES4,hairy and enhancer of split 4 |
| 242033_at | 27.38 | 5.18E-07 | 2.372017027 | NM_001113561| | NA |
| 222669_s_at | 27.38 | 5.18E-07 | 1.096128859 | NM_016038| | SBDS,Shwachman-Bodian-Diamond syndrome protein |
| 230655_at | 27.38 | 5.18E-07 | 1.189653361 | NA |  |
| 205280_at | 27.35 | 5.22E-07 | 2.15648234 | NM_000824| | GLRB,glycine receptor, beta |
| 202196_s_at | 27.35 | 5.21E-07 | 1.170169153 | NM_001018057| | NA |
| 224952_at | 27.34 | 5.23E-07 | 1.351371997 | NM_025185| | NA |
| 203264_s_at | 27.33 | 5.24E-07 | 1.163801114 | NM_015185| | ARHGEF9,Cdc42 guanine exchange factor 9 |
| 223805_at | 27.31 | 5.26E-07 | 1.241541198 | NM_032523| | OSBPL6,oxysterol-binding protein-like protein 6 isoform |
| 203275_at | 27.3 | 5.27E-07 | 1.128748759 | NM_002199| | IRF2,interferon regulatory factor 2 |
| 213598_at | 27.3 | 5.27E-07 | 1.11721018 | NM_014473| | HSA9761,putative dimethyladenosine transferase |
| 228159_at | 27.28 | 5.29E-07 | 1.089188408 | NA |  |
| 229086_at | 27.27 | 5.30E-07 | 1.362139688 | NM_001008896| | LOC148898,hypothetical protein BC007899 isoform 2 |
| 233168_s_at | 27.27 | 5.30E-07 | 1.165326345 | NM_031454| | SELO,selenoprotein O |
| 212458_at | 27.27 | 5.30E-07 | 1.164513411 | NM_181784| | SPRED2,sprouty-related protein with EVH-1 domain 2 |
| 225096_at | 27.25 | 5.31E-07 | 1.087822459 | NM_018405| | HSA272196,hypothetical protein, clone 2746033 |
| 202182_at | 27.24 | 5.32E-07 | 1.129181616 | NM_021078| | GCN5L2,GCN5 general control of amino-acid synthesis |
| 204962_s_at | 27.24 | 5.32E-07 | 1.112294544 | NM_001042426| | NA |
| 218532_s_at | 27.24 | 5.32E-07 | 1.399876056 | NM_001034850| | NA |
| 1555594_a_at | 27.24 | 5.32E-07 | 1.288036743 | NM_021038| | MBNL1,muscleblind-like 1 isoform a |
| 223230_at | 27.24 | 5.32E-07 | 1.123468855 | NM_032864| | FLJ14936,hypothetical protein FLJ14936 |
| 229269_x_at | 27.23 | 5.32E-07 | 1.227941305 | NM_001009998| | SSBP4,single stranded DNA binding protein 4 isoform b |
| 203063_at | 27.22 | 5.33E-07 | 1.166204046 | NM_014634| | PPM1F,protein phosphatase 1F |
| 226728_at | 27.22 | 5.33E-07 | 1.229867863 | NM_198580| | SLC27A1,solute carrier family 27 (fatty acid |
| 209012_at | 27.21 | 5.34E-07 | 1.146544688 | NM_007118| | TRIO,triple functional domain (PTPRF interacting) |
| 231720_s_at | 27.21 | 5.34E-07 | 1.20268264 | NM_032801| | JAM3,junctional adhesion molecule 3 precursor |
| 209867_s_at | 27.19 | 5.36E-07 | 1.118316019 | NM_015236| | LPHN3,latrophilin 3 precursor |
| 227667_at | 27.19 | 5.37E-07 | 1.285012236 | NM_017949| | CUEDC1,CUE domain-containing 1 |
| 237449_at | 27.17 | 5.38E-07 | 1.279122441 | NM_182700| | SP8,Sp8 transcription factor isoform 1 |
| 220227_at | 27.17 | 5.38E-07 | 1.140432925 | NM_001794| | CDH4,cadherin 4, type 1 preproprotein |
| 229354_at | 27.16 | 5.39E-07 | 1.745718509 | NM_013232| | PDCD6,programmed cell death 6 |
| 221909_at | 27.15 | 5.40E-07 | 1.095374902 | NM_001109903| | NA |
| 225233_at | 27.14 | 5.41E-07 | 1.441025823 | NM_138962| | MSI2,musashi 2 isoform a |
| 204688_at | 27.13 | 5.41E-07 | 1.077624672 | NM_001099400| | NA |
| 36475_at | 27.13 | 5.42E-07 | 1.203029479 | NM_014291| | GCAT,glycine C-acetyltransferase precursor |
| 206307_s_at | 27.12 | 5.42E-07 | 1.128261352 | NM_004472| | FOXD1,forkhead box D1 |
| 229666_s_at | 27.11 | 5.44E-07 | 1.111019557 | NM_001033505| | NA |
| 204504_s_at | 27.1 | 5.44E-07 | 1.094293078 | NM_003609| | HIRIP3,HIRA interacting protein 3 |
| 204033_at | 27.09 | 5.46E-07 | 1.110699956 | NM_004237| | TRIP13,thyroid hormone receptor interactor 13 |
| 203410_at | 27.09 | 5.46E-07 | 1.117920414 | NM_006803| | AP3M2,adaptor-related protein complex 3, mu 2 subunit |
| 221796_at | 27.08 | 5.46E-07 | 1.494486652 | NM_001007097| | NTRK2,neurotrophic tyrosine kinase, receptor, type 2 |
| 225491_at | 27.03 | 5.52E-07 | 1.574656259 | NM_004171| | SLC1A2,solute carrier family 1, member 2 |
| 209587_at | 27.03 | 5.52E-07 | 1.315743647 | NM_002653| | PITX1,paired-like homeodomain transcription factor 1 |
| 209123_at | 27.02 | 5.52E-07 | 1.097932187 | NM_000320| | QDPR,quinoid dihydropteridine reductase |
| 209341_s_at | 27.02 | 5.52E-07 | 1.153510972 | NM_001556| | IKBKB,inhibitor of kappa light polypeptide gene |
| 221214_s_at | 26.99 | 5.55E-07 | 1.160708241 | NM_015537| | NELF,nasal embryonic LHRH factor |
| 227665_at | 26.98 | 5.55E-07 | 1.211091681 | NA |  |
| 218557_at | 26.96 | 5.58E-07 | 1.085957519 | NM_020202| | NIT2,nitrilase family, member 2 |
| 219236_at | 26.95 | 5.58E-07 | 1.360753773 | NM_024897| | PAQR6,progestin and adipoQ receptor family member VI |
| 209505_at | 26.95 | 5.58E-07 | 1.495326305 | NM_005654| | NR2F1,nuclear receptor subfamily 2, group F, member 1 |
| 239425_at | 26.93 | 5.60E-07 | 1.428988619 | NA |  |
| 226445_s_at | 26.93 | 5.60E-07 | 1.07214503 | NM_033549| | TRIM41,tripartite motif-containing 41 isform 1 |
| 234005_x_at | 26.91 | 5.63E-07 | 1.095411113 | NM_015690| | STK36,serine/threonine kinase 36 (fused homolog, |
| 227737_at | 26.9 | 5.63E-07 | 1.210657864 | NM_021203| | SRPRB,signal recognition particle receptor, beta |
| 217945_at | 26.9 | 5.64E-07 | 1.081290544 | NM_001011885| | BTBD1,BTB (POZ) domain containing 1 isoform 2 |
| 236350_at | 26.9 | 5.63E-07 | 1.417568274 | NA |  |
| 225441_x_at | 26.89 | 5.64E-07 | 1.130438121 | NM_032356| | MGC14151,hypothetical protein MGC14151 |
| 226840_at | 26.87 | 5.67E-07 | 1.393001448 | NM_001040158| | NA |
| 228738_at | 26.83 | 5.72E-07 | 1.204520842 | NM_152783| | MGC25181,hypothetical protein MGC25181 |
| 213489_at | 26.81 | 5.75E-07 | 1.100852667 | NM_014268| | MAPRE2,microtubule-associated protein, RP/EB family, |
| 226242_at | 26.81 | 5.75E-07 | 1.083513475 | NM_152379| | DKFZp547B1713,hypothetical protein DKFZp547B1713 |
| 210150_s_at | 26.8 | 5.75E-07 | 1.135098783 | NM_005560| | LAMA5,laminin alpha 5 |
| 205931_s_at | 26.79 | 5.76E-07 | 1.761195744 | NM_001011666| | CREB5,cAMP responsive element binding protein 5 |
| 213058_at | 26.79 | 5.77E-07 | 1.14627969 | NA |  |
| 202584_at | 26.77 | 5.78E-07 | 1.31551314 | NM_002504| | NFX1,nuclear transcription factor, X-box binding 1 |
| 219279_at | 26.76 | 5.80E-07 | 2.281692643 | NM_014689| | DOCK10,dedicator of cytokinesis 10 |
| 217910_x_at | 26.76 | 5.80E-07 | 1.071610693 | NM_170607| | MLX,transcription factor-like protein 4 isoform |
| 204126_s_at | 26.75 | 5.80E-07 | 1.149932273 | NM_003504| | CDC45L,CDC45-like |
| 203411_s_at | 26.74 | 5.81E-07 | 1.261371906 | NM_005572| | LMNA,lamin A/C isoform 2 |
| 225886_at | 26.72 | 5.84E-07 | 1.196469056 | NA |  |
| 209281_s_at | 26.7 | 5.87E-07 | 1.154388291 | NM_001001323| | ATP2B1,plasma membrane calcium ATPase 1 isoform 1a |
| 1560425_s_at | 26.7 | 5.87E-07 | 1.354492386 | NA |  |
| 236766_at | 26.69 | 5.88E-07 | 1.584170444 | NA |  |
| 204032_at | 26.67 | 5.91E-07 | 1.124738968 | NM_003567| | BCAR3,breast cancer antiestrogen resistance 3 |
| 218048_at | 26.66 | 5.91E-07 | 1.367997955 | NM_012071| | COMMD3,COMM domain containing 3 |
| 1558044_s_at | 26.66 | 5.91E-07 | 1.140037709 | NM_058219| | EXOSC6,homolog of yeast mRNA transport regulator 3 |
| 202194_at | 26.64 | 5.93E-07 | 1.111860319 | NM_016040| | TMED5,transmembrane emp24 protein transport domain |
| 204320_at | 26.62 | 5.96E-07 | 1.178536293 | NM_001854| | COL11A1,alpha 1 type XI collagen isoform A |
| 218714_at | 26.62 | 5.96E-07 | 1.194181881 | NM_024031| | MGC3121,hypothetical protein MGC3121 |
| 237885_at | 26.62 | 5.96E-07 | 1.762601035 | NA |  |
| 221654_s_at | 26.62 | 5.96E-07 | 1.114960834 | NM_006537| | USP3,ubiquitin specific protease 3 |
| 226941_at | 26.61 | 5.96E-07 | 1.171577391 | NA |  |
| 224693_at | 26.61 | 5.97E-07 | 1.284785507 | NM_080821| | C20orf108,chromosome 20 open reading frame 108 |
| 235747_at | 26.59 | 5.99E-07 | 1.530450709 | NM_152707| | SLC25A16,solute carrier family 25, member 16 |
| 212274_at | 26.59 | 5.99E-07 | 1.2921128 | NM_145693| | LPIN1,lipin 1 |
| 221739_at | 26.58 | 6.00E-07 | 1.117826218 | NM_019107| | C19orf10,chromosome 19 open reading frame 10 |
| 204966_at | 26.57 | 6.01E-07 | 1.257922578 | NM_001703| | BAI2,brain-specific angiogenesis inhibitor 2 |
| 217895_at | 26.57 | 6.01E-07 | 1.08972584 | NM_017952| | FLJ20758,FLJ20758 protein |
| 205241_at | 26.56 | 6.01E-07 | 1.110220611 | NM_005138| | SCO2,cytochrome oxidase deficient homolog 2 |
| 242826_at | 26.55 | 6.03E-07 | 1.202967315 | NA |  |
| 235060_at | 26.54 | 6.03E-07 | 1.194724206 | NA |  |
| 208877_at | 26.52 | 6.05E-07 | 1.13576476 | NM_002577| | PAK2,p21-activated kinase 2 |
| 244260_at | 26.52 | 6.05E-07 | 1.379997731 | NA |  |
| 224764_at | 26.5 | 6.08E-07 | 1.253239871 | NM_020824| | ARHGAP21,Rho GTPase activating protein 21 |
| 205354_at | 26.5 | 6.07E-07 | 1.238074047 | NM_000156| | GAMT,guanidinoacetate N-methyltransferase isoform a |
| 1553954_at | 26.5 | 6.07E-07 | 1.244065029 | NM_144988| | MGC19780,hypothetical protein MGC19780 |
| 200957_s_at | 26.49 | 6.08E-07 | 1.030736627 | NM_003146| | SSRP1,structure specific recognition protein 1 |
| 204653_at | 26.47 | 6.10E-07 | 1.552328466 | NM_001032280| | NA |
| 201360_at | 26.41 | 6.18E-07 | 1.2831997 | NM_000099| | CST3,cystatin C precursor |
| 203440_at | 26.4 | 6.18E-07 | 1.099700196 | NM_001792| | CDH2,cadherin 2, type 1 preproprotein |
| 205005_s_at | 26.39 | 6.20E-07 | 1.145427577 | NM_004808| | NMT2,glycylpeptide N-tetradecanoyltransferase 2 |
| 212595_s_at | 26.38 | 6.22E-07 | 1.372396612 | NM_014764| | DAZAP2,DAZ associated protein 2 |
| 225723_at | 26.35 | 6.25E-07 | 1.191361569 | NM_138493| | NA |
| 230257_s_at | 26.34 | 6.26E-07 | 1.118031063 | NM_052965| | C1orf19,chromosome 1 open reading frame 19 |
| 231989_s_at | 26.34 | 6.26E-07 | 1.252624276 | NA |  |
| 228967_at | 26.33 | 6.27E-07 | 1.113754808 | NM_005801| | SUI1,putative translation initiation factor |
| 209158_s_at | 26.32 | 6.29E-07 | 1.118259556 | NM_004228| | PSCD2,pleckstrin homology, Sec7 and coiled/coil |
| 239118_at | 26.32 | 6.28E-07 | 2.332831269 | NM_004974| | KCNA2,potassium voltage-gated channel, shaker-related |
| 216242_x_at | 26.32 | 6.28E-07 | 1.109902651 | NM_001097615| | NA |
| 35626_at | 26.31 | 6.29E-07 | 1.128301718 | NM_000199| | SGSH,N-sulfoglucosamine sulfohydrolase (sulfamidase) |
| 218175_at | 26.3 | 6.30E-07 | 1.431141186 | NM_025140| | FLJ22471,limkain beta 2 |
| 212498_at | 26.3 | 6.30E-07 | 1.049864118 | NA |  |
| 37590_g_at | 26.3 | 6.30E-07 | 1.337055301 | NA |  |
| 229111_at | 26.29 | 6.31E-07 | 1.332113482 | NA |  |
| 203633_at | 26.29 | 6.31E-07 | 1.349232822 | NM_001031847| | NA |
| 226324_s_at | 26.29 | 6.31E-07 | 1.217776061 | NM_015662| | SLB,selective LIM binding factor, rat homolog |
| 239432_at | 26.28 | 6.32E-07 | 1.526518485 | NA |  |
| 37860_at | 26.26 | 6.34E-07 | 1.136187598 | NM_015655| | ZNF337,zinc finger protein 337 |
| 227517_s_at | 26.24 | 6.38E-07 | 1.075565227 | NA |  |
| 201148_s_at | 26.24 | 6.37E-07 | 1.23588643 | NM_000362| | TIMP3,tissue inhibitor of metalloproteinase 3 |
| 213052_at | 26.23 | 6.38E-07 | 1.110559042 | NM_004157| | PRKAR2A,cAMP-dependent protein kinase, regulatory |
| 235111_at | 26.23 | 6.38E-07 | 1.72186161 | NA |  |
| 232226_at | 26.19 | 6.44E-07 | 1.504848379 | NM_020929| | NGL-1,netrin-G1 ligand |
| 229382_at | 26.17 | 6.47E-07 | 1.294954787 | NM_019099| | LOC55924,hypothetical protein LOC55924 isoform 1 |
| 201426_s_at | 26.17 | 6.47E-07 | 1.090971787 | NM_003380| | VIM,vimentin |
| 200816_s_at | 26.17 | 6.46E-07 | 1.047538465 | NM_000430| | PAFAH1B1,platelet-activating factor acetylhydrolase, |
| 203424_s_at | 26.15 | 6.49E-07 | 1.233195511 | NM_000599| | IGFBP5,insulin-like growth factor binding protein 5 |
| 224835_at | 26.14 | 6.50E-07 | 1.251971912 | NM_019593| | KIAA1434,hypothetical protein KIAA1434 |
| 244214_at | 26.14 | 6.50E-07 | 1.16949516 | NM_001098510| | NA |
| 228077_at | 26.13 | 6.50E-07 | 1.137612344 | NM_001031727| | NA |
| 212552_at | 26.11 | 6.54E-07 | 1.201647583 | NM_002149| | HPCAL1,hippocalcin-like 1 |
| 204246_s_at | 26.11 | 6.54E-07 | 1.160203842 | NM_007234| | DCTN3,dynactin 3 isoform 1 |
| 229803_s_at | 26.1 | 6.54E-07 | 1.12996788 | NA |  |
| 1568597_at | 26.1 | 6.55E-07 | 1.20884913 | NA |  |
| 225665_at | 26.09 | 6.56E-07 | 1.191858134 | NM_016653| | ZAK,sterile-alpha motif and leucine zipper |
| 206833_s_at | 26.07 | 6.59E-07 | 1.28935393 | NM_138448| | ACYP2,muscle-type acylphosphatase 2 |
| 31807_at | 26.06 | 6.61E-07 | 1.086129088 | NM_019070| | DDX49,DEAD (Asp-Glu-Ala-Asp) box polypeptide 49 |
| 31799_at | 25.99 | 6.70E-07 | 1.388615698 | NA |  |
| 218584_at | 25.98 | 6.71E-07 | 1.286379458 | NM_001082537| | NA |
| 39966_at | 25.94 | 6.78E-07 | 1.282113734 | NM_006574| | CSPG5,chondroitin sulfate proteoglycan 5 (neuroglycan |
| 222574_s_at | 25.89 | 6.84E-07 | 1.13529458 | NM_024612| | DHX40,DEAH (Asp-Glu-Ala-His) box polypeptide 40 |
| 210239_at | 25.88 | 6.86E-07 | 1.550057108 | NM_005853| | IRX5,iroquois homeobox protein 5 |
| 229487_at | 25.84 | 6.92E-07 | 1.612994258 | NM_024007| | EBF,early B-cell factor |
| 208370_s_at | 25.83 | 6.93E-07 | 1.265036233 | NM_004414| | DSCR1,calcipressin 1 isoform a |
| 224606_at | 25.81 | 6.97E-07 | 1.097391052 | NM_001300| | KLF6,Kruppel-like factor 6 |
| 238549_at | 25.81 | 6.96E-07 | 1.160206582 | NM_001032999| | NA |
| 234140_s_at | 25.81 | 6.97E-07 | 1.231461005 | NM_020860| | STIM2,stromal interaction molecule 2 |
| 214671_s_at | 25.78 | 7.01E-07 | 1.319854537 | NM_001092| | ABR,active breakpoint cluster region-related |
| 227620_at | 25.77 | 7.03E-07 | 1.30591356 | NM_080546| | CDW92,CDW92 antigen |
| 210448_s_at | 25.76 | 7.05E-07 | 1.22636951 | NM_002561| | P2RX5,purinergic receptor P2X5 isoform A |
| 237314_at | 25.74 | 7.07E-07 | 1.98420297 | NM_145010| | C10orf63,enkurin |
| 204143_s_at | 25.74 | 7.07E-07 | 1.200483083 | NM_001126123| | NA |
| 229335_at | 25.72 | 7.10E-07 | 1.374722597 | NM_145296| | IGSF4C,immunoglobulin superfamily, member 4C |
| 209086_x_at | 25.72 | 7.10E-07 | 1.242987058 | NM_006500| | MCAM,melanoma cell adhesion molecule |
| 238853_at | 25.72 | 7.10E-07 | 1.363887478 | NM_001024647| | NA |
| 223306_at | 25.71 | 7.11E-07 | 1.081885287 | NM_032565| | EBPL,emopamil binding related protein, delta8-delta7 |
| 202084_s_at | 25.69 | 7.14E-07 | 1.130652366 | NM_001039573| | NA |
| 201668_x_at | 25.69 | 7.15E-07 | 1.181585077 | NM_002356| | MARCKS,myristoylated alanine-rich protein kinase C |
| 201825_s_at | 25.68 | 7.15E-07 | 1.144540189 | NM_016002| | CGI-49,CGI-49 protein |
| 225469_at | 25.68 | 7.15E-07 | 1.198054232 | NM_001001660| | LOC144363,hypothetical protein LOC144363 |
| 200760_s_at | 25.67 | 7.17E-07 | 1.127709071 | NM_006407| | ARL6IP5,ADP-ribosylation-like factor 6 interacting |
| 240105_at | 25.66 | 7.18E-07 | 1.249731314 | NA |  |
| 225311_at | 25.65 | 7.19E-07 | 1.103040859 | NM_002225| | IVD,isovaleryl Coenzyme A dehydrogenase |
| 207163_s_at | 25.64 | 7.20E-07 | 1.199473346 | NM_001014431| | NA |
| 1559419_at | 25.63 | 7.21E-07 | 1.818423093 | NM_000724| | CACNB2,calcium channel, voltage-dependent, beta 2 |
| 226043_at | 25.63 | 7.22E-07 | 1.186536812 | NM_015597| | GPSM1,G-protein signalling modulator 1 (AGS3-like, C. |
| 229454_at | 25.62 | 7.23E-07 | 1.387916041 | NM_001077440| | NA |
| 207169_x_at | 25.61 | 7.24E-07 | 1.11638515 | NM_001954| | DDR1,discoidin receptor tyrosine kinase isoform b |
| 221264_s_at | 25.59 | 7.27E-07 | 1.175711757 | NM_007375| | TARDBP,TAR DNA binding protein |
| 212775_at | 25.57 | 7.32E-07 | 1.095591825 | NM_015311| | NA |
| 226844_at | 25.57 | 7.31E-07 | 1.111944856 | NM_024761| | MOBKL2B,MOB1, Mps One Binder kinase activator-like 2B |
| 201465_s_at | 25.56 | 7.33E-07 | 1.25716139 | NM_002228| | JUN,v-jun avian sarcoma virus 17 oncogene homolog |
| 219471_at | 25.54 | 7.36E-07 | 1.549750343 | NM_025113| | C13orf18,chromosome 13 open reading frame 18 |
| 236664_at | 25.52 | 7.39E-07 | 1.44878858 | NM_001626| | AKT2,v-akt murine thymoma viral oncogene homolog 2 |
| 201953_at | 25.51 | 7.39E-07 | 1.117044604 | NM_006384| | CIB1,calcium and integrin binding 1 (calmyrin) |
| 201826_s_at | 25.5 | 7.41E-07 | 1.219084397 | NM_016002| | CGI-49,CGI-49 protein |
| 220688_s_at | 25.5 | 7.41E-07 | 1.114158554 | NM_016183| | C1orf33,ribosomal protein P0-like protein |
| 225261_x_at | 25.49 | 7.43E-07 | 1.133111351 | NM_198976| | TH1L,TH1-like protein |
| 202203_s_at | 25.48 | 7.45E-07 | 1.32741561 | NM_001144| | AMFR,autocrine motility factor receptor isoform a |
| 201007_at | 25.46 | 7.48E-07 | 1.067745237 | NM_000183| | HADHB,hydroxyacyl dehydrogenase, subunit B |
| 201236_s_at | 25.44 | 7.51E-07 | 1.300980295 | NM_006763| | BTG2,B-cell translocation gene 2 |
| 225276_at | 25.44 | 7.51E-07 | 1.122603753 | NA |  |
| 235635_at | 25.43 | 7.52E-07 | 1.346511956 | NM_001030055| | NA |
| 219477_s_at | 25.4 | 7.57E-07 | 1.502676037 | NM_018676| | THSD1,thrombospondin type I domain-containing 1 |
| 224975_at | 25.37 | 7.63E-07 | 1.981381383 | NM_005595| | NFIA,nuclear factor I/A |
| 209866_s_at | 25.36 | 7.64E-07 | 1.181525325 | NM_015236| | LPHN3,latrophilin 3 precursor |
| 203557_s_at | 25.36 | 7.64E-07 | 1.123241901 | NM_000281| | PCBD1,pterin-4 alpha-carbinolamine dehydratase isoform |
| 225716_at | 25.35 | 7.65E-07 | 1.164185535 | NA |  |
| 224663_s_at | 25.35 | 7.66E-07 | 1.138937276 | NM_021914| | CFL2,cofilin 2 |
| 212206_s_at | 25.34 | 7.67E-07 | 1.166479536 | NM_012412| | H2AFV,H2A histone family, member V isoform 1 |
| 211800_s_at | 25.34 | 7.67E-07 | 1.098643819 | NM_003363| | USP4,ubiquitin specific protease, proto-oncogene |
| 208979_at | 25.33 | 7.70E-07 | 1.157957266 | NM_014071| | NCOA6,nuclear receptor coactivator 6 |
| 203045_at | 25.28 | 7.78E-07 | 1.181571144 | NM_004148| | NINJ1,ninjurin 1 |
| 242389_at | 25.27 | 7.79E-07 | 1.199482194 | NA |  |
| 211075_s_at | 25.26 | 7.81E-07 | 1.106915428 | NM_001025079| | NA |
| 202743_at | 25.25 | 7.83E-07 | 1.15914497 | NM_001114172| | NA |
| 219188_s_at | 25.25 | 7.83E-07 | 1.250237107 | NM_014067| | LRP16,LRP16 protein |
| 218039_at | 25.23 | 7.86E-07 | 1.051923843 | NM_016359| | NUSAP1,nucleolar and spindle associated protein 1 |
| 225695_at | 25.23 | 7.86E-07 | 1.116990205 | NM_017877| | C2orf18,chromosome 2 open reading frame 18 |
| 203885_at | 25.22 | 7.87E-07 | 1.067130811 | NM_014999| | RAB21,RAB21, member RAS oncogene family |
| 213897_s_at | 25.2 | 7.90E-07 | 1.075006242 | NM_021134| | MRPL23,mitochondrial ribosomal protein L23 |
| 201660_at | 25.2 | 7.90E-07 | 1.09013785 | NM_004457| | ACSL3,acyl-CoA synthetase long-chain family member 3 |
| 204933_s_at | 25.18 | 7.94E-07 | 1.23818838 | NM_002546| | TNFRSF11B,osteoprotegerin precursor |
| 225745_at | 25.18 | 7.93E-07 | 1.060177011 | NM_002336| | LRP6,low density lipoprotein receptor-related protein |
| 204522_at | 25.17 | 7.94E-07 | 1.129037474 | NM_005510| | DOM3Z,DOM-3 homolog Z |
| 202032_s_at | 25.15 | 7.98E-07 | 1.268866136 | NM_006122| | MAN2A2,mannosidase, alpha, class 2A, member 2 |
| 1564746_at | 25.15 | 7.98E-07 | 1.603457197 | NM_178833| | LOC133308,hypothetical protein BC009732 |
| 202088_at | 25.14 | 7.99E-07 | 1.094190911 | NM_001099406| | NA |
| 1555764_s_at | 25.12 | 8.03E-07 | 1.10306282 | NM_012456| | TIMM10,translocase of inner mitochondrial membrane 10 |
| 230482_at | 25.12 | 8.03E-07 | 1.206940445 | NM_030965| | ST6GALNAC5,sialyltransferase 7E |
| 235350_at | 25.12 | 8.03E-07 | 1.689846445 | NM_001104629| | NA |
| 215429_s_at | 25.12 | 8.03E-07 | 1.220108465 | NM_182498| | MGC51082,hypothetical protein MGC51082 |
| 223472_at | 25.11 | 8.03E-07 | 1.405190608 | NM_001042424| | NA |
| 204993_at | 25.11 | 8.03E-07 | 1.190707736 | NM_002073| | GNAZ,guanine nucleotide binding protein, alpha z |
| 241355_at | 25.08 | 8.08E-07 | 1.483420187 | NM_005144| | HR,hairless protein isoform a |
| 224869_s_at | 25.08 | 8.08E-07 | 1.094701874 | NM_022497| | MRPS25,mitochondrial ribosomal protein S25 |
| 208819_at | 25.06 | 8.12E-07 | 1.105755114 | NM_005370| | RAB8A,mel transforming oncogene |
| 221623_at | 25.06 | 8.13E-07 | 1.504859687 | NM_021948| | BCAN,brevican isoform 1 |
| 209902_at | 25.05 | 8.13E-07 | 1.086715976 | NM_001184| | ATR,ataxia telangiectasia and Rad3 related protein |
| 1558034_s_at | 25.03 | 8.17E-07 | 1.823498545 | NM_000096| | CP,ceruloplasmin (ferroxidase) |
| 202694_at | 25.01 | 8.21E-07 | 1.918157422 | NM_004760| | STK17A,serine/threonine kinase 17a |
| 204639_at | 24.99 | 8.25E-07 | 1.103331999 | NM_000022| | ADA,adenosine deaminase |
| 218699_at | 24.99 | 8.24E-07 | 1.093502004 | NM_003929| | RAB7L1,RAB7, member RAS oncogene family-like 1 |
| 204317_at | 24.99 | 8.24E-07 | 1.20867809 | NM_016426| | GTSE1,G-2 and S-phase expressed 1 |
| 37384_at | 24.97 | 8.27E-07 | 1.164131981 | NM_014634| | PPM1F,protein phosphatase 1F |
| 223253_at | 24.97 | 8.28E-07 | 1.083952361 | NM_017549| | EPDR1,upregulated in colorectal cancer gene 1 protein |
| 1559822_s_at | 24.96 | 8.29E-07 | 1.254120875 | NA |  |
| 226412_at | 24.96 | 8.30E-07 | 1.200926098 | NM_015491| | NA |
| 210638_s_at | 24.95 | 8.30E-07 | 1.096785236 | NM_012347| | FBXO9,F-box only protein 9 isoform 1 |
| 242579_at | 24.94 | 8.33E-07 | 1.85279164 | NM_001203| | BMPR1B,bone morphogenetic protein receptor, type IB |
| 213606_s_at | 24.94 | 8.31E-07 | 1.240111757 | NM_004309| | ARHGDIA,Rho GDP dissociation inhibitor (GDI) alpha |
| 244623_at | 24.89 | 8.41E-07 | 1.785970577 | NM_019842| | KCNQ5,potassium voltage-gated channel, KQT-like |
| 218007_s_at | 24.88 | 8.44E-07 | 1.078406695 | NM_015920| | RPS27L,ribosomal protein S27-like protein |
| 212831_at | 24.88 | 8.43E-07 | 1.813539069 | NM_001080497| | NA |
| 225371_at | 24.87 | 8.45E-07 | 1.127523339 | NM_001003722| | GLE1L,GLE1-like, RNA export mediator isoform 1 |
| 201125_s_at | 24.87 | 8.44E-07 | 1.068839734 | NM_002213| | ITGB5,integrin, beta 5 |
| 203542_s_at | 24.86 | 8.46E-07 | 1.821640731 | NM_001206| | KLF9,Kruppel-like factor 9 |
| 213803_at | 24.85 | 8.48E-07 | 1.145168349 | NA |  |
| 206408_at | 24.82 | 8.54E-07 | 1.784718522 | NM_015564| | LRRTM2,leucine rich repeat transmembrane neuronal 2 |
| 231793_s_at | 24.82 | 8.54E-07 | 1.539115337 | NM_001221| | CAMK2D,calcium/calmodulin-dependent protein kinase II |
| 225120_at | 24.81 | 8.56E-07 | 1.09047165 | NM_033224| | PURB,purine-rich element binding protein B |
| 224784_at | 24.81 | 8.56E-07 | 1.580148347 | NM_005937| | MLLT6,myeloid/lymphoid or mixed-lineage leukemia |
| 205590_at | 24.8 | 8.56E-07 | 1.353631462 | NM_005739| | RASGRP1,RAS guanyl releasing protein 1 |
| 219634_at | 24.79 | 8.58E-07 | 1.194382561 | NM_018413| | CHST11,carbohydrate (chondroitin 4) sulfotransferase |
| 225655_at | 24.78 | 8.61E-07 | 1.136057722 | NM_001048201| | NA |
| 225626_at | 24.78 | 8.61E-07 | 1.723600427 | NM_018440| | PAG,phosphoprotein associated with |
| 203973_s_at | 24.78 | 8.60E-07 | 1.14756858 | NM_005195| | CEBPD,CCAAT/enhancer binding protein delta |
| 1559283_a_at | 24.77 | 8.61E-07 | 1.643196725 | NM_001103176| | NA |
| 209522_s_at | 24.76 | 8.64E-07 | 1.187820917 | NM_000755| | CRAT,carnitine acetyltransferase isoform 1 precursor |
| 227719_at | 24.74 | 8.67E-07 | 1.268766511 | NM_001127217| | NA |
| 212112_s_at | 24.72 | 8.71E-07 | 1.226105729 | NM_177424| | STX12,syntaxin 12 |
| 203469_s_at | 24.72 | 8.71E-07 | 1.142590319 | NM_001098533| | NA |
| 225492_at | 24.71 | 8.72E-07 | 1.078190761 | NM_018126| | TMEM33,transmembrane protein 33 |
| 224917_at | 24.71 | 8.72E-07 | 1.206564383 | NA |  |
| 209031_at | 24.71 | 8.72E-07 | 1.1890553 | NM_001098517| | NA |
| 202636_at | 24.68 | 8.78E-07 | 1.176050621 | NM_005667| | RNF103,ring finger protein 103 |
| 207401_at | 24.67 | 8.78E-07 | 1.312253067 | NM_002763| | PROX1,prospero-related homeobox 1 |
| 206811_at | 24.67 | 8.78E-07 | 1.555947595 | NM_001115| | ADCY8,adenylate cyclase 8 |
| 227229_at | 24.67 | 8.78E-07 | 1.058281468 | NA |  |
| 212973_at | 24.67 | 8.78E-07 | 1.089595738 | NM_144563| | RPIA,ribose 5-phosphate isomerase A (ribose |
| 225823_at | 24.67 | 8.78E-07 | 1.178180149 | NM_205767| | QIL1,QIL1 protein |
| 227100_at | 24.67 | 8.78E-07 | 1.189620391 | NM_194318| | B3GTL,beta 3-glycosyltransferase-like |
| 211070_x_at | 24.66 | 8.80E-07 | 1.067940165 | NM_001079862| | NA |
| 217860_at | 24.66 | 8.80E-07 | 1.089665953 | NM_004544| | NDUFA10,NADH dehydrogenase (ubiquinone) 1 alpha |
| 203526_s_at | 24.65 | 8.80E-07 | 1.38063535 | NM_000038| | APC,adenomatosis polyposis coli |
| 218812_s_at | 24.65 | 8.81E-07 | 1.280252232 | NM_001126340| | NA |
| 202095_s_at | 24.64 | 8.82E-07 | 1.057649954 | NM_001012270| | BIRC5,baculoviral IAP repeat-containing protein 5 |
| 218447_at | 24.64 | 8.82E-07 | 1.053824525 | NM_020188| | DC13,DC13 protein |
| 203543_s_at | 24.63 | 8.84E-07 | 2.245917307 | NM_001206| | KLF9,Kruppel-like factor 9 |
| 210993_s_at | 24.63 | 8.83E-07 | 1.116578234 | NM_001003688| | SMAD1,Sma- and Mad-related protein 1 |
| 211569_s_at | 24.63 | 8.84E-07 | 1.09942267 | NM_005327| | HADHSC,L-3-hydroxyacyl-Coenzyme A dehydrogenase, short |
| 211930_at | 24.62 | 8.85E-07 | 1.156794967 | NM_194247| | HNRPA3,heterogeneous nuclear ribonucleoprotein A3 |
| 1554102_a_at | 24.61 | 8.87E-07 | 1.29798827 | NM_001079669| | NA |
| 225865_x_at | 24.61 | 8.87E-07 | 1.132310978 | NM_198976| | TH1L,TH1-like protein |
| 232032_x_at | 24.6 | 8.89E-07 | 1.10415106 | NM_016176| | Cab45,calcium binding protein Cab45 precursor |
| 213281_at | 24.58 | 8.91E-07 | 1.3422901 | NM_002228| | JUN,v-jun avian sarcoma virus 17 oncogene homolog |
| 226180_at | 24.56 | 8.97E-07 | 1.065225558 | NM_139281| | WDR36,WD repeat domain 36 |
| 232793_at | 24.55 | 8.98E-07 | 1.609954719 | NA |  |
| 207168_s_at | 24.54 | 8.99E-07 | 1.106287742 | NM_001040158| | NA |
| 205303_at | 24.54 | 8.99E-07 | 1.383091923 | NM_004982| | KCNJ8,potassium inwardly-rectifying channel J8 |
| 230280_at | 24.51 | 9.06E-07 | 1.343308198 | NM_015163| | TRIM9,tripartite motif protein 9 isoform 1 |
| 205046_at | 24.51 | 9.07E-07 | 1.040793735 | NM_001813| | CENPE,centromere protein E |
| 233049_x_at | 24.5 | 9.08E-07 | 1.102480035 | NM_005861| | STUB1,STIP1 homology and U-box containing protein 1 |
| 237675_at | 24.48 | 9.12E-07 | 1.348934146 | NA |  |
| 200046_at | 24.47 | 9.15E-07 | 1.039741314 | NM_001344| | DAD1,defender against cell death 1 |
| 222633_at | 24.47 | 9.13E-07 | 1.074270579 | NM_024665| | TBL1XR1,nuclear receptor co-repressor/HDAC3 complex |
| 216120_s_at | 24.46 | 9.15E-07 | 1.540037677 | NM_001001331| | ATP2B2,plasma membrane calcium ATPase 2 isoform a |
| 203319_s_at | 24.44 | 9.19E-07 | 1.253400824 | NM_021964| | ZNF148,zinc finger protein 148 (pHZ-52) |
| 216044_x_at | 24.43 | 9.23E-07 | 1.104351026 | NM_001006605| | LOC388650,hypothetical LOC388650 |
| 238735_at | 24.42 | 9.25E-07 | 2.090304565 | NA |  |
| 50277_at | 24.41 | 9.25E-07 | 1.065542741 | NM_001001560| | GGA1,golgi associated, gamma adaptin ear containing, |
| 209840_s_at | 24.39 | 9.30E-07 | 1.235280151 | NM_001099658| | NA |
| 230653_at | 24.39 | 9.30E-07 | 1.352107811 | NA |  |
| 200661_at | 24.38 | 9.32E-07 | 1.127728888 | NM_000308| | PPGB,protective protein for beta-galactosidase |
| 229800_at | 24.36 | 9.38E-07 | 1.122815565 | NM_004734| | DCAMKL1,doublecortin and CaM kinase-like 1 |
| 208873_s_at | 24.34 | 9.42E-07 | 1.111207508 | NM_005669| | C5orf18,deleted in polyposis 1 |
| 228739_at | 24.34 | 9.41E-07 | 1.431446787 | NM_001037160| | NA |
| 205717_x_at | 24.34 | 9.42E-07 | 1.305690409 | NM_002588| | PCDHGC3,protocadherin gamma subfamily C, 3 isoform 1 |
| 225259_at | 24.32 | 9.45E-07 | 1.10440258 | NM_016577| | RAB6B,RAB6B, member RAS oncogene family |
| 218071_s_at | 24.32 | 9.45E-07 | 1.164649027 | NM_014160| | MKRN2,makorin, ring finger protein, 2 |
| 227158_at | 24.32 | 9.45E-07 | 1.087042133 | NM_080664| | C14orf126,hypothetical protein MGC9912 |
| 212528_at | 24.3 | 9.49E-07 | 1.162267116 | NA |  |
| 218032_at | 24.29 | 9.50E-07 | 1.076732538 | NM_003498| | SNN,Stannin |
| 225777_at | 24.28 | 9.53E-07 | 1.198241917 | NM_178448| | C9orf140,chromosome 9 open reading frame 140 |
| 224512_s_at | 24.28 | 9.53E-07 | 1.130687771 | NM_032356| | MGC14151,hypothetical protein MGC14151 |
| 220050_at | 24.27 | 9.55E-07 | 1.371614538 | NM_018956| | C9orf9,chromosome 9 open reading frame 9 |
| 231866_at | 24.27 | 9.55E-07 | 1.248440002 | NM_005575| | LNPEP,leucyl/cystinyl aminopeptidase |
| 225246_at | 24.25 | 9.59E-07 | 1.277541048 | NM_020860| | STIM2,stromal interaction molecule 2 |
| 203608_at | 24.25 | 9.57E-07 | 1.4479463 | NM_001080| | ALDH5A1,aldehyde dehydrogenase 5A1 precursor, isoform 2 |
| 241198_s_at | 24.25 | 9.57E-07 | 1.34380221 | NM_032930| | MGC13040,hypothetical protein MGC13040 |
| 230528_s_at | 24.25 | 9.57E-07 | 1.131872275 | NA |  |
| 224743_at | 24.24 | 9.60E-07 | 1.109233112 | NM_017813| | IMPA3,myo-inositol monophosphatase A3 |
| 1559603_at | 24.23 | 9.63E-07 | 1.679503928 | NA |  |
| 226831_at | 24.23 | 9.63E-07 | 1.265031384 | NM_138773| | LOC91137,hypothetical protein BC017169 |
| 227840_at | 24.22 | 9.63E-07 | 1.115111137 | NM_001017927| | NA |
| 1552622_s_at | 24.22 | 9.63E-07 | 1.362054489 | NM_032959| | POLR2J2,DNA directed RNA polymerase II polypeptide |
| 243707_at | 24.22 | 9.63E-07 | 1.285991232 | NA |  |
| 205675_at | 24.21 | 9.65E-07 | 1.702443452 | NM_000253| | MTP,microsomal triglyceride transfer protein large |
| 228381_at | 24.19 | 9.69E-07 | 1.177286031 | NM_024997| | ATF7IP2,activating transcription factor 7 interacting |
| 204908_s_at | 24.16 | 9.78E-07 | 1.26500404 | NM_005178| | BCL3,B-cell CLL/lymphoma 3 |
| 215001_s_at | 24.14 | 9.81E-07 | 1.06157421 | NM_001033044| | NA |
| 225669_at | 24.13 | 9.83E-07 | 1.386833969 | NM_000629| | IFNAR1,interferon-alpha receptor 1 precursor |
| 208795_s_at | 24.11 | 9.87E-07 | 1.063301084 | NM_005916| | MCM7,minichromosome maintenance protein 7 isoform 1 |
| 241840_at | 24.11 | 9.89E-07 | 1.231023191 | NA |  |
| 205283_at | 24.1 | 9.90E-07 | 1.214296143 | NM_001079802| | NA |
| 225159_s_at | 24.1 | 9.89E-07 | 1.141271719 | NA |  |
| 219825_at | 24.09 | 9.91E-07 | 1.413838578 | NM_019885| | CYP26B1,cytochrome P450, family 26, subfamily b, |
| 222030_at | 24.09 | 9.92E-07 | 1.125162244 | NM_006427| | SIVA,CD27-binding (Siva) protein isoform 1 |
| 209729_at | 24.07 | 9.95E-07 | 1.223792754 | NM_006478| | GAS2L1,growth arrest-specific 2 like 1 isoform a |
| 201104_x_at | 24.06 | 9.98E-07 | 1.213919931 | NM_001037501| | NA |
| 209050_s_at | 24.06 | 9.98E-07 | 1.193799492 | NM_001042368| | NA |
| 238438_at | 24.06 | 9.98E-07 | 1.419959603 | NM_144571| | CNOT6L,CCR4-NOT transcription complex, subunit 6-like |
| 231042_s_at | 24.05 | 9.99E-07 | 1.669346793 | NA |  |
| 230002_at | 24.04 | 1.00E-06 | 1.351273678 | NM_001048210| | NA |
| 204027_s_at | 24.04 | 1.00E-06 | 1.090551141 | NM_005371| | METTL1,methyltransferase-like protein 1 isoform a |
| 202945_at | 24.04 | 1.00E-06 | 1.045470788 | NM_001018078| | NA |
| 209120_at | 24.03 | 1.00E-06 | 1.550545794 | NM_021005| | NR2F2,nuclear receptor subfamily 2, group F, member 2 |
| 202170_s_at | 24.03 | 1.00E-06 | 1.081736008 | NM_015423| | AASDHPPT,aminoadipate-semialdehyde |
| 238022_at | 24.02 | 1.01E-06 | 1.179879868 | NA |  |
| 221853_s_at | 24.02 | 1.01E-06 | 1.068864321 | NM_001004060| | NOMO2,nodal modulator 2 isoform 1 |
| 227771_at | 24.01 | 1.01E-06 | 1.318031022 | NM_002310| | LIFR, |
| 231967_at | 24.01 | 1.01E-06 | 1.613430669 | NM_016018| | PHF20L1,PHD finger protein 20-like 1 isoform 1 |
| 564_at | 24 | 1.01E-06 | 1.154823882 | NM_002067| | GNA11,guanine nucleotide binding protein (G protein), |
| 229549_at | 24 | 1.01E-06 | 1.203392613 | NA |  |
| 214297_at | 23.98 | 1.01E-06 | 1.657899936 | NM_001897| | CSPG4,melanoma-associated chondroitin sulfate |
| 219709_x_at | 23.97 | 1.02E-06 | 1.122157112 | NM_023933| | MGC2494,hypothetical protein MGC2494 |
| 244690_at | 23.95 | 1.02E-06 | 1.385703286 | NA |  |
| 1564907_s_at | 23.95 | 1.02E-06 | 1.130219264 | NM_018834| | MATR3,matrin 3 |
| 201346_at | 23.95 | 1.02E-06 | 1.079879781 | NM_024551| | ADIPOR2,adiponectin receptor 2 |
| 202830_s_at | 23.95 | 1.02E-06 | 1.086428106 | NM_001467| | SLC37A4,solute carrier family 37 (glycerol-6-phosphate |
| 219450_at | 23.95 | 1.02E-06 | 1.505087583 | NM_001104629| | NA |
| 226848_at | 23.94 | 1.02E-06 | 1.283907819 | NA |  |
| 213750_at | 23.94 | 1.02E-06 | 1.145207544 | NM_015659| | RSL1D1,ribosomal L1 domain containing 1 |
| 218502_s_at | 23.93 | 1.02E-06 | 1.489323642 | NM_014112| | TRPS1,zinc finger transcription factor TRPS1 |
| 218146_at | 23.93 | 1.02E-06 | 1.075891305 | NM_001010983| | GLT8D1,glycosyltransferase 8 domain containing 1 |
| 213459_at | 23.91 | 1.03E-06 | 1.233974736 | NM_000998| | RPL37A,ribosomal protein L37a |
| 212753_at | 23.91 | 1.03E-06 | 1.118740899 | NM_006315| | PCGF3,ring finger protein 3 |
| 212987_at | 23.85 | 1.04E-06 | 1.108206865 | NM_012347| | FBXO9,F-box only protein 9 isoform 1 |
| 228930_at | 23.84 | 1.04E-06 | 1.35842208 | NA |  |
| 219746_at | 23.83 | 1.05E-06 | 1.39519987 | NM_012074| | DPF3,cer-d4 (mouse) homolog |
| 202461_at | 23.83 | 1.05E-06 | 1.056641079 | NM_014239| | EIF2B2,eukaryotic translation initiation factor 2B, |
| 236208_at | 23.83 | 1.05E-06 | 1.562598464 | NM_004531| | MOCS2,molybdopterin synthase large subunit MOCS2B |
| 202443_x_at | 23.82 | 1.05E-06 | 1.10280896 | NM_024408| | NOTCH2,notch 2 preproprotein |
| 237482_s_at | 23.81 | 1.05E-06 | 1.304859048 | NM_025247| | ACAD10,acyl-Coenzyme A dehydrogenase family, member 10 |
| 222995_s_at | 23.8 | 1.05E-06 | 1.160269486 | NM_001040456| | NA |
| 204640_s_at | 23.8 | 1.05E-06 | 1.129678168 | NM_001007226| | SPOP,speckle-type POZ protein |
| 218124_at | 23.79 | 1.05E-06 | 1.146290986 | NM_017750| | RetSat,all-trans-13,14-dihydroretinol saturase |
| 59437_at | 23.77 | 1.06E-06 | 1.110834253 | NM_001048265| | NA |
| 202762_at | 23.76 | 1.06E-06 | 1.171724153 | NM_004850| | ROCK2,Rho-associated, coiled-coil containing protein |
| 212608_s_at | 23.75 | 1.06E-06 | 1.122843545 | NA |  |
| 217997_at | 23.74 | 1.07E-06 | 1.071950487 | NM_007350| | PHLDA1,pleckstrin homology-like domain, family A, |
| 230026_at | 23.71 | 1.07E-06 | 1.233881903 | NM_032112| | MRPL43,mitochondrial ribosomal protein L43 isoform a |
| 218839_at | 23.68 | 1.08E-06 | 1.310678307 | NM_001040708| | NA |
| 216870_x_at | 23.68 | 1.08E-06 | 1.357576741 | NA |  |
| 225536_at | 23.68 | 1.08E-06 | 1.407996766 | NM_033504| | BCLP,beta-casein-like protein |
| 208178_x_at | 23.67 | 1.09E-06 | 1.25064267 | NM_007118| | TRIO,triple functional domain (PTPRF interacting) |
| 209497_s_at | 23.67 | 1.09E-06 | 1.11796764 | NM_031492| | RBM30,RNA binding motif protein 30 |
| 217526_at | 23.65 | 1.09E-06 | 1.102862045 | NM_032815| | NFATC2IP,nuclear factor of activated T-cells, |
| 217934_x_at | 23.65 | 1.09E-06 | 1.106598279 | NM_005861| | STUB1,STIP1 homology and U-box containing protein 1 |
| 232231_at | 23.64 | 1.09E-06 | 1.939380202 | NM_001015051| | NA |
| 243010_at | 23.64 | 1.09E-06 | 1.22605059 | NM_138962| | MSI2,musashi 2 isoform a |
| 206920_s_at | 23.64 | 1.09E-06 | 1.240661917 | NM_001003722| | GLE1L,GLE1-like, RNA export mediator isoform 1 |
| 1556329_a_at | 23.61 | 1.10E-06 | 1.648371245 | NA |  |
| 52975_at | 23.59 | 1.11E-06 | 1.185418973 | NM_001011703| | C9orf28,chromosome 9 open reading frame 28 isoform 2 |
| 208668_x_at | 23.58 | 1.11E-06 | 1.035187746 | NM_005517| | HMGN2,high-mobility group nucleosomal binding domain |
| 227962_at | 23.57 | 1.11E-06 | 1.116915969 | NM_004035| | ACOX1,acyl-Coenzyme A oxidase isoform a |
| 222875_at | 23.57 | 1.11E-06 | 1.041966658 | NM_020162| | DHX33,DEAH (Asp-Glu-Ala-His) box polypeptide 33 |
| 217823_s_at | 23.57 | 1.11E-06 | 1.23120213 | NM_016021| | UBE2J1,ubiquitin-conjugating enzyme E2, J1 |
| 203031_s_at | 23.56 | 1.11E-06 | 1.149673923 | NM_000375| | UROS,uroporphyrinogen III synthase |
| 202975_s_at | 23.56 | 1.11E-06 | 1.198147611 | NM_014899| | RHOBTB3,rho-related BTB domain containing 3 |
| 227977_at | 23.56 | 1.11E-06 | 1.656911128 | NM_175907| | ZADH2,zinc binding alcohol dehydrogenase, domain |
| 213713_s_at | 23.53 | 1.12E-06 | 1.214539473 | NM_138342| | LOC89944,hypothetical protein BC008326 |
| 213701_at | 23.53 | 1.12E-06 | 1.171342804 | NM_001009894| | DKFZp434N2030,hypothetical protein DKFZp434N2030 |
| 216205_s_at | 23.53 | 1.12E-06 | 1.13913043 | NM_014874| | MFN2,mitofusin 2 |
| 214543_x_at | 23.52 | 1.12E-06 | 1.145000308 | NM_006775| | QKI,quaking homolog, KH domain RNA binding isoform |
| 224977_at | 23.52 | 1.12E-06 | 1.261991739 | NM_152734| | C6orf89,hypothetical protein FLJ25357 |
| 238459_x_at | 23.51 | 1.12E-06 | 1.585437876 | NM_019073| | SPATA6,spermatogenesis associated 6 |
| 218653_at | 23.51 | 1.12E-06 | 1.124174984 | NM_014252| | SLC25A15,solute carrier family 25 (mitochondrial carrier; |
| 1553705_a_at | 23.45 | 1.14E-06 | 1.385187441 | NM_000740| | CHRM3,cholinergic receptor, muscarinic 3 |
| 201391_at | 23.45 | 1.14E-06 | 1.115570172 | NM_016292| | TRAP1,TNF receptor-associated protein 1 |
| 208972_s_at | 23.45 | 1.14E-06 | 1.121648463 | NM_001002027| | ATP5G1,ATP synthase, H+ transporting, mitochondrial F0 |
| 209032_s_at | 23.43 | 1.14E-06 | 1.248388452 | NM_001098517| | NA |
| 205543_at | 23.43 | 1.14E-06 | 1.345161592 | NM_014278| | HSPA4L,heat shock 70kDa protein 4-like |
| 214004_s_at | 23.43 | 1.14E-06 | 1.084690447 | NM_014667| | VGLL4,vestigial like 4 |
| 211471_s_at | 23.42 | 1.15E-06 | 1.461016494 | NM_004914| | RAB36,RAB36, member RAS oncogene family |
| 211165_x_at | 23.42 | 1.15E-06 | 1.222318721 | NM_004442| | EPHB2,ephrin receptor EphB2 isoform 2 precursor |
| 220939_s_at | 23.42 | 1.15E-06 | 1.08139711 | NM_017743| | DPP8,dipeptidyl peptidase 8 isoform 2 |
| 244788_at | 23.41 | 1.15E-06 | 1.458835157 | NA |  |
| 202566_s_at | 23.39 | 1.15E-06 | 1.275944714 | NM_003174| | SVIL,supervillin isoform 1 |
| 228185_at | 23.39 | 1.15E-06 | 1.526400414 | NM_145011| | ZNF25,zinc finger protein 25 |
| 212222_at | 23.38 | 1.16E-06 | 1.115776449 | NM_014614| | PSME4,proteasome (prosome, macropain) activator |
| 221879_at | 23.35 | 1.17E-06 | 1.075960188 | NM_001031733| | NA |
| 204020_at | 23.35 | 1.17E-06 | 1.290577026 | NM_005859| | PURA,purine-rich element binding protein A |
| 224591_at | 23.34 | 1.17E-06 | 1.138232418 | NM_016287| | HP1-BP74,HP1-BP74 |
| 227395_at | 23.33 | 1.17E-06 | 1.141361368 | NA |  |
| 230591_at | 23.32 | 1.17E-06 | 1.233954466 | NA |  |
| 218097_s_at | 23.32 | 1.17E-06 | 1.124356187 | NM_024040| | CUEDC2,CUE domain containing 2 |
| 218028_at | 23.31 | 1.17E-06 | 1.152617417 | NM_022821| | ELOVL1,elongation of very long chain fatty acids |
| 222668_at | 23.31 | 1.18E-06 | 1.104156327 | NM_024076| | KCTD15,potassium channel tetramerisation domain |
| 223066_at | 23.31 | 1.17E-06 | 1.08883186 | NM_012437| | SNAPAP,SNAP-associated protein |
| 201795_at | 23.27 | 1.19E-06 | 1.19192349 | NM_002296| | LBR,lamin B receptor |
| 223302_s_at | 23.24 | 1.20E-06 | 1.174637116 | NM_001009958| | ZNF655,zinc finger protein 655 isoform c |
| 226861_at | 23.23 | 1.20E-06 | 1.162753064 | NM_024095| | ASB8,ankyrin repeat and SOCS box-containing 8 |
| 212869_x_at | 23.23 | 1.20E-06 | 1.018554675 | NM_003295| | TPT1,tumor protein, translationally-controlled 1 |
| 231979_at | 23.22 | 1.20E-06 | 1.278613828 | NA |  |
| 222824_at | 23.21 | 1.20E-06 | 1.18829282 | NA |  |
| 202565_s_at | 23.19 | 1.21E-06 | 1.183986273 | NM_003174| | SVIL,supervillin isoform 1 |
| 216933_x_at | 23.19 | 1.21E-06 | 1.439953579 | NM_000038| | APC,adenomatosis polyposis coli |
| 1554690_a_at | 23.18 | 1.21E-06 | 1.21478275 | NM_001122824| | NA |
| 209991_x_at | 23.18 | 1.21E-06 | 1.610783631 | NM_005458| | GPR51,G protein-coupled receptor 51 |
| 205265_s_at | 23.17 | 1.21E-06 | 1.169341016 | NM_005876| | APEG1,aortic preferentially expressed gene 1 |
| 228391_at | 23.16 | 1.22E-06 | 2.00576523 | NM_207352| | CYP4V2,cytochrome P450, family 4, subfamily v, |
| 236442_at | 23.16 | 1.22E-06 | 1.939114974 | NM_012074| | DPF3,cer-d4 (mouse) homolog |
| 200810_s_at | 23.15 | 1.22E-06 | 1.072926539 | NM_001280| | CIRBP,cold inducible RNA binding protein |
| 212019_at | 23.15 | 1.22E-06 | 1.173481832 | NM_015659| | RSL1D1,ribosomal L1 domain containing 1 |
| 225118_at | 23.14 | 1.22E-06 | 1.286245891 | NM_020382| | SET8,SET domain-containing protein 8 |
| 205737_at | 23.13 | 1.22E-06 | 1.364887135 | NM_004518| | KCNQ2,potassium voltage-gated channel KQT-like protein |
| 241774_at | 23.13 | 1.22E-06 | 1.685373156 | NA |  |
| 210434_x_at | 23.13 | 1.22E-06 | 1.079281025 | NM_006694| | JTB,jumping translocation breakpoint |
| 217998_at | 23.12 | 1.22E-06 | 1.213102365 | NM_007350| | PHLDA1,pleckstrin homology-like domain, family A, |
| 238948_at | 23.12 | 1.23E-06 | 1.130609948 | NM_001014842| | NA |
| 211852_s_at | 23.11 | 1.23E-06 | 1.176736462 | NM_139321| | ATRN,attractin isoform 1 |
| 209234_at | 23.1 | 1.23E-06 | 1.108018934 | NM_015074| | KIF1B,kinesin family member 1B isoform b |
| 223407_at | 23.1 | 1.23E-06 | 1.219825825 | NM_032140| | C16orf48,chromosome 16 open reading frame 48 |
| 205963_s_at | 23.09 | 1.23E-06 | 1.100799663 | NM_005147| | DNAJA3,DnaJ (Hsp40) homolog, subfamily A, member 3 |
| 203039_s_at | 23.09 | 1.23E-06 | 1.029780517 | NM_005006| | NDUFS1,NADH dehydrogenase (ubiquinone) Fe-S protein 1, |
| 203292_s_at | 23.08 | 1.24E-06 | 1.088124745 | NM_021729| | VPS11,vacuolar protein sorting 11 (yeast homolog) |
| 212863_x_at | 23.07 | 1.24E-06 | 1.052918987 | NM_001012614| | NA |
| 224806_at | 23.06 | 1.24E-06 | 1.110533547 | NM_005082| | TRIM25,tripartite motif-containing 25 |
| 219525_at | 23.05 | 1.25E-06 | 1.317797066 | NM_018242| | FLJ10847,hypothetical protein FLJ10847 |
| 228545_at | 23.05 | 1.25E-06 | 1.228760374 | NM_021964| | ZNF148,zinc finger protein 148 (pHZ-52) |
| 200839_s_at | 23.05 | 1.25E-06 | 1.124058336 | NM_001908| | CTSB,cathepsin B preproprotein |
| 200896_x_at | 23.02 | 1.25E-06 | 1.020116682 | NM_001126050| | NA |
| 202185_at | 23 | 1.26E-06 | 1.175286651 | NM_001084| | PLOD3,procollagen-lysine, 2-oxoglutarate 5-dioxygenase |
| 227259_at | 23 | 1.26E-06 | 1.434330978 | NM_001025079| | NA |
| 227146_at | 22.99 | 1.26E-06 | 1.116458123 | NM_181701| | QSCN6L1,quiescin Q6-like 1 |
| 219549_s_at | 22.99 | 1.26E-06 | 1.060916254 | NM_006054| | RTN3,reticulon 3 isoform a |
| 210927_x_at | 22.98 | 1.26E-06 | 1.082862922 | NM_006694| | JTB,jumping translocation breakpoint |
| 229535_at | 22.97 | 1.27E-06 | 1.440536105 | NM_175732| | NA |
| 221951_at | 22.97 | 1.27E-06 | 1.229407143 | NM_001042463| | NA |
| 204193_at | 22.96 | 1.27E-06 | 1.150035722 | NM_005198| | CHKB,choline/ethanolamine kinase isoform a |
| 223571_at | 22.96 | 1.27E-06 | 1.176836288 | NM_031910| | C1QTNF6,C1q and tumor necrosis factor related protein 6 |
| 202897_at | 22.96 | 1.27E-06 | 1.203155795 | NM_001040022| | NA |
| 212468_at | 22.95 | 1.27E-06 | 1.182118987 | NM_003971| | SPAG9,sperm associated antigen 9 isoform 1 |
| 215171_s_at | 22.94 | 1.28E-06 | 1.049494875 | NM_006335| | TIMM17A,translocase of inner mitochondrial membrane 17 |
| 230079_at | 22.92 | 1.28E-06 | 1.301713827 | NM_017744| | ST7L,suppression of tumorigenicity 7-like isoform 1 |
| 235260_s_at | 22.92 | 1.28E-06 | 1.197437746 | NM_145048| | MGC29898,hypothetical protein MGC29898 |
| 203595_s_at | 22.91 | 1.29E-06 | 1.253220929 | NM_012420| | IFIT5,interferon-induced protein with |
| 225795_at | 22.9 | 1.29E-06 | 1.087301698 | NM_033318| | LOC91689,hypothetical protein supported by AL449243 |
| 212326_at | 22.9 | 1.29E-06 | 1.150700686 | NM_015378| | VPS13D,vacuolar protein sorting 13D isoform 1 |
| 212534_at | 22.89 | 1.29E-06 | 1.095559232 | NA |  |
| 210015_s_at | 22.88 | 1.29E-06 | 1.299746181 | NM_001039538| | NA |
| 206718_at | 22.88 | 1.29E-06 | 1.41458899 | NM_002315| | LMO1,LIM domain only 1 |
| 201259_s_at | 22.88 | 1.29E-06 | 1.113447547 | NM_006754| | SYPL,synaptophysin-like protein isoform a |
| 202661_at | 22.88 | 1.29E-06 | 1.623919738 | NM_002223| | ITPR2,inositol 1,4,5-triphosphate receptor, type 2 |
| 202089_s_at | 22.87 | 1.30E-06 | 1.137255174 | NM_001099406| | NA |
| 233257_at | 22.86 | 1.30E-06 | 1.921842948 | NA |  |
| 218841_at | 22.85 | 1.30E-06 | 1.205675964 | NM_024095| | ASB8,ankyrin repeat and SOCS box-containing 8 |
| 201857_at | 22.84 | 1.31E-06 | 1.038329021 | NM_016107| | ZFR,zinc finger RNA binding protein |
| 213164_at | 22.84 | 1.31E-06 | 1.107852093 | NM_006933| | SLC5A3,solute carrier family 5 (inositol transporters), |
| 209053_s_at | 22.83 | 1.31E-06 | 1.049303371 | NM_001042424| | NA |
| 227932_at | 22.82 | 1.31E-06 | 1.146446058 | NM_006321| | ARIH2,ariadne homolog 2 |
| 212625_at | 22.82 | 1.31E-06 | 1.15320982 | NM_003765| | STX10,syntaxin 10 |
| 214279_s_at | 22.81 | 1.31E-06 | 1.445320979 | NM_016250| | NDRG2,N-myc downstream-regulated gene 2 isoform b |
| 211958_at | 22.8 | 1.32E-06 | 1.178718837 | NM_000599| | IGFBP5,insulin-like growth factor binding protein 5 |
| 223159_s_at | 22.8 | 1.32E-06 | 1.357843144 | NM_014397| | NEK6,putative serine-threonine protein kinase |
| 209413_at | 22.78 | 1.32E-06 | 1.088824222 | NM_001005417| | B4GALT2,UDP-Gal:betaGlcNAc beta 1,4- |
| 203724_s_at | 22.78 | 1.32E-06 | 1.475674023 | NM_001037442| | NA |
| 202834_at | 22.77 | 1.33E-06 | 1.53113756 | NM_000029| | AGT,angiotensinogen precursor |
| 203907_s_at | 22.77 | 1.32E-06 | 1.07535948 | NM_014869| | IQSEC1,IQ motif and Sec7 domain 1 |
| 210251_s_at | 22.77 | 1.32E-06 | 1.064765635 | NM_001037442| | NA |
| 239483_at | 22.76 | 1.33E-06 | 1.18469997 | NA |  |
| 227903_x_at | 22.76 | 1.33E-06 | 1.160582727 | NM_033513| | C19orf20,chromosome 19 open reading frame 20 |
| 207081_s_at | 22.75 | 1.33E-06 | 1.111917061 | NM_002650| | PIK4CA,phosphatidylinositol 4-kinase, catalytic, alpha |
| 204276_at | 22.74 | 1.33E-06 | 1.351979037 | NM_004614| | TK2,thymidine kinase 2, mitochondrial |
| 230755_at | 22.74 | 1.33E-06 | 1.172412467 | NM_138328| | RHBDL4,rhomboid, veinlet-like 4 |
| 225990_at | 22.73 | 1.34E-06 | 1.315140396 | NM_033254| | BOC,brother of CDO |
| 219785_s_at | 22.73 | 1.34E-06 | 1.103781596 | NM_024735| | FBXO31,F-box protein 31 |
| 223176_at | 22.73 | 1.34E-06 | 1.168655999 | NM_173562| | C6orf69,hypothetical protein MGC14254 |
| 241700_at | 22.72 | 1.34E-06 | 1.506926362 | NM_024721| | ZFHX4,zinc finger homeodomain 4 |
| 229014_at | 22.72 | 1.34E-06 | 1.177837424 | NA |  |
| 219269_at | 22.72 | 1.34E-06 | 1.138212541 | NM_024567| | FLJ21616,hypothetical protein FLJ21616 |
| 213509_x_at | 22.71 | 1.34E-06 | 1.100366427 | NM_003869| | CES2,carboxylesterase 2 isoform 1 |
| 209171_at | 22.7 | 1.34E-06 | 1.092990877 | NM_033453| | ITPA,inosine triphosphatase isoform a |
| 223056_s_at | 22.68 | 1.35E-06 | 1.071217212 | NM_020750| | XPO5,exportin 5 |
| 211679_x_at | 22.67 | 1.35E-06 | 1.494254948 | NM_005458| | GPR51,G protein-coupled receptor 51 |
| 227640_s_at | 22.67 | 1.35E-06 | 1.125283058 | NM_203288| | RP9,retinitis pigmentosa 9 protein |
| 213448_at | 22.65 | 1.36E-06 | 1.171318963 | NA |  |
| 201178_at | 22.64 | 1.36E-06 | 1.028798978 | NM_001033024| | NA |
| 225056_at | 22.64 | 1.36E-06 | 1.184472683 | NM_020808| | SIPA1L2,signal-induced proliferation-associated 1 like |
| 203773_x_at | 22.63 | 1.36E-06 | 1.094088494 | NM_000712| | BLVRA,biliverdin reductase A |
| 223370_at | 22.63 | 1.36E-06 | 1.091471948 | NM_019091| | PLEKHA3,pleckstrin homology domain containing, family A |
| 230872_s_at | 22.62 | 1.37E-06 | 1.217253011 | NM_001025930| | NA |
| 223061_at | 22.62 | 1.37E-06 | 1.118220675 | NM_023947| | MGC3234,hypothetical protein MGC3234 |
| 225384_at | 22.6 | 1.37E-06 | 1.114024386 | NM_033407| | DOCK7,dedicator of cytokinesis 7 |
| 221706_s_at | 22.6 | 1.37E-06 | 1.135452345 | NM_018467| | MDS032,uncharacterized hematopoietic stem/progenitor |
| 239654_at | 22.59 | 1.37E-06 | 1.351616172 | NM_025134| | CHD9,chromodomain helicase DNA binding protein 9 |
| 212098_at | 22.55 | 1.39E-06 | 1.09808464 | NA |  |
| 230596_at | 22.54 | 1.39E-06 | 1.343392636 | NA |  |
| 225948_at | 22.54 | 1.40E-06 | 1.04879364 | NM_032374| | C14orf153,chromosome 14 open reading frame 153 |
| 222398_s_at | 22.54 | 1.40E-06 | 1.073028885 | NM_004247| | U5-116KD,U5 snRNP-specific protein, 116 kD |
| 204066_s_at | 22.53 | 1.40E-06 | 1.065902151 | NM_001037131| | NA |
| 212434_at | 22.53 | 1.40E-06 | 1.065594052 | NM_025196| | GRPEL1,GrpE-like 1, mitochondrial |
| 1552439_s_at | 22.52 | 1.40E-06 | 1.715452221 | NM_032445| | MEGF11,MEGF11 protein |
| 228028_at | 22.52 | 1.40E-06 | 1.218474244 | NA |  |
| 203314_at | 22.52 | 1.40E-06 | 1.127082889 | NM_012227| | GTPBP6,pseudoautosomal GTP-binding protein-like |
| 200973_s_at | 22.52 | 1.40E-06 | 1.190144824 | NM_005724| | TM4SF8,transmembrane 4 superfamily member 8 isoform 1 |
| 230071_at | 22.5 | 1.41E-06 | 1.821656126 | NM_018243| | SEPT11,septin 11 |
| 241505_at | 22.49 | 1.41E-06 | 1.39866204 | NA |  |
| 201335_s_at | 22.49 | 1.41E-06 | 1.302804098 | NM_015313| | ARHGEF12,Rho guanine nucleotide exchange factor (GEF) 12 |
| 240339_at | 22.48 | 1.41E-06 | 1.246022335 | NA |  |
| 222747_s_at | 22.48 | 1.42E-06 | 1.4124187 | NM_001037535| | NA |
| 221675_s_at | 22.47 | 1.42E-06 | 1.087927547 | NM_020244| | CHPT1,choline phosphotransferase 1 |
| 227624_at | 22.46 | 1.42E-06 | 1.339191212 | NM_001127208| | NA |
| 224280_s_at | 22.46 | 1.42E-06 | 1.130912159 | NM_001099625| | NA |
| 227354_at | 22.41 | 1.44E-06 | 1.988897273 | NM_018440| | PAG,phosphoprotein associated with |
| 231406_at | 22.39 | 1.44E-06 | 1.325330024 | NA |  |
| 220999_s_at | 22.37 | 1.45E-06 | 1.197988147 | NM_001037332| | NA |
| 238587_at | 22.37 | 1.45E-06 | 1.521124748 | NM_032873| | STS-1,Cbl-interacting protein Sts-1 |
| 228486_at | 22.36 | 1.46E-06 | 1.48983028 | NM_080546| | CDW92,CDW92 antigen |
| 214500_at | 22.35 | 1.46E-06 | 1.632476952 | NM_001040158| | NA |
| 38703_at | 22.35 | 1.46E-06 | 1.057369614 | NM_012100| | DNPEP,aspartyl aminopeptidase |
| 216959_x_at | 22.35 | 1.46E-06 | 1.36331748 | NM_001037132| | NA |
| 238003_at | 22.35 | 1.46E-06 | 1.429316595 | NM_152722| | FLJ25530,hypothetical protein FLJ25530 |
| 230782_at | 22.32 | 1.47E-06 | 1.452700461 | NM_003104| | SORD,sorbitol dehydrogenase |
| 219137_s_at | 22.32 | 1.47E-06 | 1.068532426 | NM_020194| | C2orf33,chromosome 2 open reading frame 33 |
| 226425_at | 22.31 | 1.47E-06 | 1.990749362 | NM_024692| | RSNL2,restin-like 2 |
| 218065_s_at | 22.31 | 1.47E-06 | 1.142278947 | NM_020644| | C11orf15,chromosome 11 open reading frame 15 |
| 233647_s_at | 22.3 | 1.48E-06 | 1.253218594 | NM_030911| | CDADC1,cytidine and dCMP deaminase domain containing 1 |
| 1552790_a_at | 22.29 | 1.48E-06 | 1.193737812 | NM_003262| | TLOC1,translocation protein 1 |
| 201341_at | 22.28 | 1.48E-06 | 1.157225952 | NM_003633| | ENC1,ectodermal-neural cortex (with BTB-like domain) |
| 212875_s_at | 22.27 | 1.49E-06 | 1.082708221 | NM_015500| | NA |
| 204577_s_at | 22.27 | 1.49E-06 | 1.492508525 | NM_015041| | CLUAP1,clusterin associated protein 1 |
| 226156_at | 22.24 | 1.50E-06 | 1.284845656 | NM_001626| | AKT2,v-akt murine thymoma viral oncogene homolog 2 |
| 209605_at | 22.24 | 1.50E-06 | 1.072206227 | NM_003312| | TST,thiosulfate sulfurtransferase |
| 218494_s_at | 22.23 | 1.50E-06 | 1.104198905 | NM_020062| | SLC2A4RG,SLC2A4 regulator |
| 227812_at | 22.22 | 1.50E-06 | 1.224589445 | NM_018647| | TNFRSF19,tumor necrosis factor receptor superfamily, |
| 202921_s_at | 22.21 | 1.51E-06 | 1.396265141 | NM_001148| | ANK2,ankyrin 2 isoform 1 |
| 212276_at | 22.2 | 1.51E-06 | 1.239782832 | NM_145693| | LPIN1,lipin 1 |
| 231397_at | 22.2 | 1.51E-06 | 1.46838028 | NM_001010861| | NA |
| 225908_at | 22.19 | 1.51E-06 | 1.198706207 | NM_001039613| | NA |
| 203795_s_at | 22.18 | 1.52E-06 | 1.137548425 | NM_001024808| | NA |
| 1555609_a_at | 22.17 | 1.52E-06 | 1.711471254 | NM_022470| | WIG1,p53 target zinc finger protein isoform 1 |
| 213094_at | 22.17 | 1.52E-06 | 1.262477607 | NM_001032394| | NA |
| 223455_at | 22.17 | 1.52E-06 | 1.213724827 | NM_032300| | MGC10854,hypothetical protein MGC10854 |
| 225851_at | 22.16 | 1.53E-06 | 1.072199311 | NM_002028| | FNTB,farnesyltransferase, CAAX box, beta |
| 215836_s_at | 22.16 | 1.52E-06 | 1.224233759 | NM_002588| | PCDHGC3,protocadherin gamma subfamily C, 3 isoform 1 |
| 215258_at | 22.15 | 1.53E-06 | 1.33766034 | NM_145296| | IGSF4C,immunoglobulin superfamily, member 4C |
| 225704_at | 22.15 | 1.53E-06 | 1.30404296 | NA |  |
| 242133_s_at | 22.15 | 1.53E-06 | 1.239980312 | NA |  |
| 227374_at | 22.13 | 1.54E-06 | 1.050657811 | NM_001083614| | NA |
| 236291_at | 22.12 | 1.54E-06 | 1.262505569 | NM_002905| | RDH5,retinol dehydrogenase 5 (11-cis and 9-cis) |
| 222462_s_at | 22.11 | 1.54E-06 | 1.16077244 | NM_012104| | BACE1,beta-site APP-cleaving enzyme 1 isoform A |
| 1552287_s_at | 22.1 | 1.55E-06 | 1.213165252 | NA |  |
| 230098_at | 22.1 | 1.55E-06 | 1.149994951 | NM_016018| | PHF20L1,PHD finger protein 20-like 1 isoform 1 |
| 235112_at | 22.09 | 1.55E-06 | 1.081688544 | NA |  |
| 228359_at | 22.09 | 1.55E-06 | 1.225388862 | NM_032873| | STS-1,Cbl-interacting protein Sts-1 |
| 228062_at | 22.09 | 1.55E-06 | 1.112657735 | NM_153757| | NAP1L5,nucleosome assembly protein 1-like 5 |
| 227401_at | 22.08 | 1.56E-06 | 1.493732899 | NM_138284| | IL17D,interleukin 17D precursor |
| 213239_at | 22.08 | 1.56E-06 | 1.205102307 | NM_006346| | C13orf24,chromosome 13 open reading frame 24 |
| 242473_at | 22.07 | 1.56E-06 | 1.241680961 | NM_004295| | TRAF4,TNF receptor-associated factor 4 isoform 1 |
| 226742_at | 22.07 | 1.56E-06 | 1.191584993 | NA |  |
| 202206_at | 22.06 | 1.56E-06 | 1.160982021 | NM_005737| | ARL7,ADP-ribosylation factor-like 7 |
| 217985_s_at | 22.06 | 1.56E-06 | 1.175742839 | NM_013448| | BAZ1A,bromodomain adjacent to zinc finger domain, 1A |
| 220744_s_at | 22.06 | 1.56E-06 | 1.103538252 | NM_018262| | WDR10,WD repeat domain 10 isoform 3 |
| 229434_at | 22.06 | 1.56E-06 | 1.240214612 | NA |  |
| 244509_at | 22.06 | 1.56E-06 | 1.429044322 | NM_001033045| | NA |
| 224733_at | 22.05 | 1.56E-06 | 1.081417813 | NM_001048251| | NA |
| 212734_x_at | 22.05 | 1.56E-06 | 1.010171808 | NM_000977| | RPL13,ribosomal protein L13 |
| 211729_x_at | 22.04 | 1.57E-06 | 1.083325414 | NM_000712| | BLVRA,biliverdin reductase A |
| 212719_at | 22.04 | 1.56E-06 | 1.094217522 | NM_194449| | PLEKHE1,suprachiasmatic nucleus circadian oscillatory |
| 241434_at | 22.04 | 1.57E-06 | 1.264664937 | NA |  |
| 208921_s_at | 22.03 | 1.57E-06 | 1.100630199 | NM_003130| | SRI,sorcin isoform a |
| 218277_s_at | 22.03 | 1.57E-06 | 1.166776784 | NM_024612| | DHX40,DEAH (Asp-Glu-Ala-His) box polypeptide 40 |
| 218898_at | 22.01 | 1.58E-06 | 1.098882676 | NM_024792| | CT120,membrane protein expressed in epithelial-like |
| 213429_at | 22.01 | 1.58E-06 | 1.984980622 | NM_001080512| | NA |
| 233842_x_at | 22 | 1.58E-06 | 1.074748551 | NM_016407| | C20orf43,chromosome 20 open reading frame 43 |
| 212630_at | 22 | 1.58E-06 | 1.100314576 | NM_007277| | SEC6L1,Sec6 protein |
| 204279_at | 21.99 | 1.58E-06 | 1.175233688 | NM_002800| | PSMB9,proteasome beta 9 subunit isoform 1 proprotein |
| 204306_s_at | 21.98 | 1.59E-06 | 1.217010471 | NM_001039490| | NA |
| 212733_at | 21.97 | 1.59E-06 | 1.169770778 | NM_014687| | NA |
| 201306_s_at | 21.97 | 1.59E-06 | 1.070355476 | NM_006401| | ANP32B,acidic (leucine-rich) nuclear phosphoprotein 32 |
| 229949_at | 21.97 | 1.59E-06 | 1.158472216 | NA |  |
| 236958_at | 21.96 | 1.59E-06 | 1.160384465 | NA |  |
| 230418_s_at | 21.96 | 1.59E-06 | 1.173545685 | NM_020692| | NA |
| 200015_s_at | 21.94 | 1.60E-06 | 1.048046559 | NM_001008491| | SEPT2,septin 2 |
| 203458_at | 21.93 | 1.60E-06 | 1.070933395 | NM_003124| | SPR,sepiapterin reductase |
| 213581_at | 21.93 | 1.61E-06 | 1.066031641 | NM_002598| | PDCD2,programmed cell death 2 isoform 1 |
| 212230_at | 21.93 | 1.60E-06 | 1.093326483 | NM_003713| | PPAP2B,phosphatidic acid phosphatase type 2B |
| 229860_x_at | 21.92 | 1.61E-06 | 1.141705917 | NA |  |
| 210756_s_at | 21.92 | 1.61E-06 | 1.232032347 | NM_024408| | NOTCH2,notch 2 preproprotein |
| 208072_s_at | 21.92 | 1.61E-06 | 1.113757233 | NM_003648| | DGKD,diacylglycerol kinase, delta 130kDa isoform 1 |
| 203921_at | 21.91 | 1.61E-06 | 1.143785322 | NM_004267| | CHST2,carbohydrate (N-acetylglucosamine-6-O) |
| 221910_at | 21.91 | 1.61E-06 | 1.394357164 | NM_004956| | ETV1,ets variant gene 1 |
| 225191_at | 21.9 | 1.61E-06 | 1.169907404 | NM_001280| | CIRBP,cold inducible RNA binding protein |
| 235494_at | 21.9 | 1.61E-06 | 1.718868787 | NA |  |
| 204065_at | 21.9 | 1.62E-06 | 1.068098543 | NM_004854| | CHST10,HNK-1 sulfotransferase |
| 221503_s_at | 21.89 | 1.62E-06 | 1.094191455 | NM_002267| | KPNA3,karyopherin alpha 3 |
| 203683_s_at | 21.88 | 1.62E-06 | 1.135170128 | NM_003377| | VEGFB,vascular endothelial growth factor B |
| 203823_at | 21.88 | 1.62E-06 | 1.131541681 | NM_017790| | RGS3,regulator of G-protein signalling 3 isoform 3 |
| 225484_at | 21.85 | 1.63E-06 | 1.153327063 | NM_018718| | TSGA14,testis specific, 14 |
| 201207_at | 21.85 | 1.63E-06 | 1.108807096 | NM_021137| | TNFAIP1,tumor necrosis factor, alpha-induced protein 1 |
| 225674_at | 21.85 | 1.63E-06 | 1.067178219 | NM_001008405| | BCAP29,B-cell receptor-associated protein BAP29 isoform |
| 223593_at | 21.83 | 1.64E-06 | 1.175840656 | NM_016228| | AADAT,alpha-aminoadipate aminotransferase |
| 227250_at | 21.83 | 1.64E-06 | 1.433683311 | NM_001039570| | NA |
| 91920_at | 21.82 | 1.64E-06 | 1.245394674 | NM_021948| | BCAN,brevican isoform 1 |
| 232990_at | 21.82 | 1.64E-06 | 1.394926702 | NM_138362| | CXorf44,chromosome X open reading frame 44 |
| 209177_at | 21.81 | 1.65E-06 | 1.111598612 | NM_199069| | DKFZP564J0123,nuclear protein E3-3 isoform a |
| 204576_s_at | 21.81 | 1.65E-06 | 1.120504152 | NM_015041| | CLUAP1,clusterin associated protein 1 |
| 226839_at | 21.81 | 1.65E-06 | 1.20366791 | NM_176880| | TRA16,TR4 orphan receptor associated protein TRA16 |
| 225440_at | 21.81 | 1.65E-06 | 1.300034917 | NM_001037553| | NA |
| 217122_s_at | 21.8 | 1.65E-06 | 1.042435647 | NM_001110781| | NA |
| 225782_at | 21.79 | 1.66E-06 | 1.307226721 | NM_001031679| | NA |
| 225098_at | 21.79 | 1.66E-06 | 1.077367826 | NM_005759| | ABI2,abl interactor 2 |
| 225389_at | 21.79 | 1.66E-06 | 1.11620513 | NM_033271| | BTBD6,BTB domain protein BDPL |
| 228349_at | 21.77 | 1.66E-06 | 1.153959102 | NA |  |
| 202159_at | 21.77 | 1.67E-06 | 1.0752072 | NM_004461| | FARSLA,phenylalanine-tRNA synthetase-like protein |
| 226745_at | 21.76 | 1.67E-06 | 1.607745417 | NM_207352| | CYP4V2,cytochrome P450, family 4, subfamily v, |
| 221727_at | 21.76 | 1.67E-06 | 1.164604911 | NM_006713| | PC4,activated RNA polymerase II transcription |
| 238880_at | 21.75 | 1.67E-06 | 1.27406428 | NM_002097| | GTF3A,general transcription factor IIIA |
| 233255_s_at | 21.73 | 1.68E-06 | 1.344749141 | NM_017693| | BIVM,basic, immunoglobulin-like variable motif |
| 225619_at | 21.73 | 1.68E-06 | 1.30384791 | NM_001040153| | NA |
| 210270_at | 21.72 | 1.68E-06 | 1.344122612 | NM_004296| | RGS6,regulator of G-protein signalling 6 |
| 241363_at | 21.72 | 1.68E-06 | 1.191954993 | NM_017820| | FLJ20433,hypothetical protein FLJ20433 |
| 239657_x_at | 21.71 | 1.69E-06 | 1.147837813 | NA |  |
| 212899_at | 21.71 | 1.69E-06 | 1.127228548 | NM_015076| | CDC2L6,cyclin-dependent kinase (CDC2-like) 11 |
| 218974_at | 21.71 | 1.69E-06 | 1.174887553 | NM_018013| | NA |
| 200078_s_at | 21.7 | 1.69E-06 | 1.074029412 | NM_001039457| | NA |
| 202868_s_at | 21.68 | 1.70E-06 | 1.073506345 | NM_006627| | POP4,POP4 (processing of precursor , S. cerevisiae) |
| 225980_at | 21.68 | 1.70E-06 | 1.128472016 | NM_001043318| | NA |
| 230300_at | 21.68 | 1.70E-06 | 1.401833953 | NA |  |
| 220807_at | 21.68 | 1.70E-06 | 1.255964414 | NM_005331| | HBQ1,theta 1 globin |
| 212001_at | 21.68 | 1.70E-06 | 1.12343051 | NM_001017392| | NA |
| 214102_at | 21.67 | 1.70E-06 | 1.22569991 | NM_015230| | CENTD1,centaurin delta 1 isoform a |
| 201870_at | 21.67 | 1.70E-06 | 1.114876136 | NM_006809| | TOMM34,translocase of outer mitochondrial membrane 34 |
| 208094_s_at | 21.66 | 1.71E-06 | 1.143163048 | NM_030818| | MGC10471,hypothetical protein MGC10471 |
| 205436_s_at | 21.66 | 1.71E-06 | 1.050637389 | NM_002105| | H2AFX,H2A histone family, member X |
| 232204_at | 21.66 | 1.71E-06 | 1.813548637 | NM_024007| | EBF,early B-cell factor |
| 207144_s_at | 21.65 | 1.71E-06 | 1.439450781 | NM_004143| | CITED1,Cbp/p300-interacting transactivator, with |
| 226907_at | 21.65 | 1.71E-06 | 1.543180806 | NM_030949| | PPP1R14C,protein phosphatase 1, regulatory (inhibitor) |
| 1554783_s_at | 21.65 | 1.71E-06 | 1.218805293 | NM_004723| | ARHGEF2,rho/rac guanine nucleotide exchange factor 2 |
| 229430_at | 21.65 | 1.71E-06 | 1.676464312 | NM_152765| | MGC33510,hypothetical protein MGC33510 |
| 231403_at | 21.65 | 1.71E-06 | 1.684526933 | NM_007118| | TRIO,triple functional domain (PTPRF interacting) |
| 241464_s_at | 21.65 | 1.71E-06 | 1.608772193 | NA |  |
| 204311_at | 21.64 | 1.71E-06 | 1.268506759 | NM_001678| | ATP1B2,Na+/K+ -ATPase beta 2 subunit |
| 231166_at | 21.64 | 1.71E-06 | 1.849963504 | NM_001033045| | NA |
| 201066_at | 21.64 | 1.71E-06 | 1.089380248 | NM_001916| | CYC1,cytochrome c-1 |
| 212609_s_at | 21.62 | 1.72E-06 | 1.209384022 | NM_005465| | AKT3,v-akt murine thymoma viral oncogene homolog 3 |
| 205034_at | 21.61 | 1.73E-06 | 1.347730831 | NM_057749| | CCNE2,cyclin E2 isoform 1 |
| 204824_at | 21.6 | 1.73E-06 | 1.15731625 | NM_004435| | ENDOG,endonuclease G precursor |
| 212397_at | 21.6 | 1.73E-06 | 1.040806515 | NM_002906| | RDX,radixin |
| 235316_at | 21.59 | 1.73E-06 | 1.270109962 | NM_178557| | FLJ37478,hypothetical protein FLJ37478 |
| 204247_s_at | 21.57 | 1.74E-06 | 1.067673769 | NM_004935| | CDK5,cyclin-dependent kinase 5 |
| 203261_at | 21.57 | 1.74E-06 | 1.078416962 | NM_006571| | DCTN6,dynactin 6 |
| 243071_at | 21.56 | 1.75E-06 | 1.428761181 | NA |  |
| 1553974_at | 21.56 | 1.74E-06 | 1.135430774 | NM_173793| | LOC128977,hypothetical protein LOC128977 |
| 226660_at | 21.55 | 1.75E-06 | 1.102020908 | NM_003161| | RPS6KB1,ribosomal protein S6 kinase, 70kDa, polypeptide |
| 223025_s_at | 21.54 | 1.75E-06 | 1.16053319 | NM_032493| | AP1M1,adaptor-related protein complex 1, mu 1 subunit |
| 225530_at | 21.54 | 1.76E-06 | 1.256633172 | NM_130807| | MOBKL2A,MOB-LAK |
| 204238_s_at | 21.53 | 1.76E-06 | 1.154103746 | NM_006443| | C6orf108,putative c-Myc-responsive isoform 1 |
| 228880_at | 21.53 | 1.76E-06 | 1.247326313 | NA |  |
| 1553538_s_at | 21.53 | 1.76E-06 | 1.04635087 | NA |  |
| 227590_at | 21.52 | 1.76E-06 | 1.065914912 | NM_207327| | LOC150383,similar to RIKEN cDNA 2210021J22 isoform 1 |
| 225458_at | 21.52 | 1.76E-06 | 1.218423215 | NA |  |
| 236024_at | 21.51 | 1.77E-06 | 1.613501818 | NM_005277| | GPM6A,glycoprotein M6A isoform 1 |
| 221815_at | 21.5 | 1.77E-06 | 1.132747971 | NM_007011| | ABHD2,alpha/beta hydrolase domain containing protein |
| 237008_at | 21.48 | 1.78E-06 | 1.569845728 | NA |  |
| 220178_at | 21.47 | 1.79E-06 | 1.097637846 | NM_001042680| | NA |
| 226135_at | 21.46 | 1.79E-06 | 1.042323204 | NM_017754| | C6orf107,ICBP90 binding protein 1 |
| 209250_at | 21.46 | 1.79E-06 | 1.0695853 | NM_003676| | DEGS1,degenerative spermatocyte homolog 1, lipid |
| 224865_at | 21.46 | 1.79E-06 | 1.133419048 | NM_032228| | MLSTD2,male sterility domain containing 2 |
| 211488_s_at | 21.46 | 1.79E-06 | 1.516807255 | NM_002214| | ITGB8,integrin, beta 8 |
| 203303_at | 21.45 | 1.79E-06 | 1.145757161 | NM_006520| | TCTE1L,t-complex-associated-testis-expressed 1-like |
| 207672_at | 21.45 | 1.79E-06 | 1.942887952 | NA |  |
| 1560792_at | 21.44 | 1.80E-06 | 1.813851814 | NA |  |
| 218529_at | 21.44 | 1.80E-06 | 1.144635697 | NM_016579| | CD320,8D6 antigen |
| 228661_s_at | 21.43 | 1.80E-06 | 1.17257298 | NA |  |
| 213645_at | 21.43 | 1.80E-06 | 1.194484261 | NM_001126123| | NA |
| 201716_at | 21.43 | 1.80E-06 | 1.102409481 | NM_003099| | SNX1,sorting nexin 1 isoform a |
| 238469_at | 21.41 | 1.81E-06 | 2.003680372 | NM_024576| | OGFRL1,opioid growth factor receptor-like 1 |
| 209846_s_at | 21.4 | 1.81E-06 | 1.273706714 | NM_007047| | BTN3A2,butyrophilin, subfamily 3, member A2 |
| 218358_at | 21.4 | 1.81E-06 | 1.080959047 | NM_024324| | MGC11256,hypothetical protein MGC11256 |
| 244411_at | 21.39 | 1.82E-06 | 1.285823651 | NA |  |
| 244627_at | 21.37 | 1.83E-06 | 1.193526795 | NM_015533| | DKFZP586B1621,DKFZP586B1621 protein |
| 201992_s_at | 21.36 | 1.83E-06 | 1.139598594 | NM_004521| | KIF5B,kinesin family member 5B |
| 202156_s_at | 21.36 | 1.83E-06 | 1.124684522 | NM_001025076| | NA |
| 206197_at | 21.36 | 1.83E-06 | 1.20363358 | NM_003551| | NME5,non-metastatic cells 5, protein expressed in |
| 209356_x_at | 21.36 | 1.83E-06 | 1.258559303 | NM_016938| | EFEMP2,EGF-containing fibulin-like extracellular matrix |
| 227476_at | 21.33 | 1.85E-06 | 1.345048788 | NA |  |
| 242323_at | 21.32 | 1.85E-06 | 1.157800352 | NA |  |
| 202616_s_at | 21.3 | 1.86E-06 | 1.182381331 | NM_001110792| | NA |
| 202047_s_at | 21.3 | 1.86E-06 | 1.163882292 | NM_014292| | CBX6,chromobox homolog 6 |
| 221059_s_at | 21.29 | 1.87E-06 | 1.036809172 | NM_021149| | COTL1,coactosin-like 1 |
| 209447_at | 21.29 | 1.87E-06 | 1.372109344 | NM_015293| | SYNE1,nesprin 1 isoform beta |
| 202799_at | 21.28 | 1.87E-06 | 1.062563802 | NM_006012| | CLPP,endopeptidase Clp precursor |
| 225656_at | 21.28 | 1.87E-06 | 1.209261017 | NM_018100| | EFHC1,EF-hand domain (C-terminal) containing 1 |
| 225390_s_at | 21.27 | 1.88E-06 | 1.083487584 | NM_015995| | KLF13,Kruppel-like factor 13 |
| 224711_at | 21.27 | 1.88E-06 | 1.059343313 | NM_003403| | YY1,YY1 transcription factor |
| 208070_s_at | 21.27 | 1.88E-06 | 1.385408992 | NM_002912| | REV3L,REV3-like, catalytic subunit of DNA polymerase |
| 214046_at | 21.27 | 1.88E-06 | 1.21276136 | NM_006581| | FUT9,fucosyltransferase 9 (alpha (1,3) |
| 214930_at | 21.27 | 1.88E-06 | 1.108536909 | NM_015567| | SLITRK5,SLIT and NTRK-like family, member 5 |
| 224160_s_at | 21.26 | 1.88E-06 | 1.11870103 | NM_014049| | ACAD9,acyl-Coenzyme A dehydrogenase family, member 9 |
| 212186_at | 21.24 | 1.89E-06 | 1.062633393 | NM_198834| | ACACA,acetyl-Coenzyme A carboxylase alpha isoform 1 |
| 221792_at | 21.24 | 1.89E-06 | 1.27884486 | NM_016577| | RAB6B,RAB6B, member RAS oncogene family |
| 212068_s_at | 21.23 | 1.89E-06 | 1.073615854 | NM_013318| | NA |
| 206154_at | 21.21 | 1.90E-06 | 1.401303483 | NM_000326| | RLBP1,retinaldehyde binding protein 1 |
| 204365_s_at | 21.21 | 1.91E-06 | 1.426531731 | NM_022912| | C2orf23,receptor expression enhancing protein 1 |
| 225483_at | 21.21 | 1.90E-06 | 1.166317174 | NM_052875| | MGC10485,hypothetical protein MGC10485 |
| 201923_at | 21.21 | 1.91E-06 | 1.047986303 | NM_006406| | PRDX4,thioredoxin peroxidase |
| 218473_s_at | 21.21 | 1.90E-06 | 1.11797964 | NM_024656| | GLT25D1,glycosyltransferase 25 domain containing 1 |
| 201155_s_at | 21.2 | 1.91E-06 | 1.075707309 | NM_014874| | MFN2,mitofusin 2 |
| 1558369_at | 21.2 | 1.91E-06 | 1.115648038 | NM_022782| | MPHOSPH9,M-phase phosphoprotein 9 |
| 204820_s_at | 21.19 | 1.91E-06 | 1.236939144 | NM_006994| | BTN3A3,butyrophilin, subfamily 3, member A3 isoform a |
| 226255_at | 21.17 | 1.92E-06 | 1.111072868 | NM_006777| | ZBTB33,kaiso |
| 1558189_a_at | 21.16 | 1.93E-06 | 1.54061403 | NA |  |
| 230724_s_at | 21.15 | 1.93E-06 | 1.144431974 | NM_001082969| | NA |
| 1554724_at | 21.14 | 1.93E-06 | 2.230393912 | NM_014229| | SLC6A11,solute carrier family 6 (neurotransmitter |
| 218855_at | 21.14 | 1.93E-06 | 1.181783837 | NM_016372| | GPR175,G protein-coupled receptor 175 |
| 201489_at | 21.13 | 1.94E-06 | 1.099787099 | NM_005729| | PPIF,peptidylprolyl isomerase F precursor |
| 206342_x_at | 21.13 | 1.94E-06 | 1.199188357 | NM_000202| | IDS,iduronate-2-sulfatase isoform a precursor |
| 224925_at | 21.12 | 1.94E-06 | 1.216461497 | NM_020820| | PREX1,PREX1 protein |
| 227325_at | 21.11 | 1.95E-06 | 1.111349956 | NA |  |
| 210821_x_at | 21.1 | 1.95E-06 | 1.148259336 | NM_001042426| | NA |
| 228990_at | 21.09 | 1.96E-06 | 1.134442675 | NA |  |
| 223099_s_at | 21.08 | 1.96E-06 | 1.126185091 | NM_031490| | LONP,peroxisomal lon protease |
| 228515_at | 21.07 | 1.97E-06 | 1.152282817 | NA |  |
| 230776_at | 21.07 | 1.97E-06 | 1.380865868 | NM_052916| | RNF157,ring finger protein 157 |
| 217721_at | 21.06 | 1.98E-06 | 1.244431012 | NA |  |
| 225421_at | 21.05 | 1.98E-06 | 1.288829363 | NM_001010853| | ACY1L2,aminoacylase 1-like 2 |
| 210415_s_at | 21.04 | 1.99E-06 | 1.349247209 | NM_002540| | ODF2,outer dense fiber of sperm tails 2 isoform 1 |
| 1559517_a_at | 21.04 | 1.99E-06 | 1.556727459 | NM_020148| | SPIRE1,spire homolog 1 |
| 227403_at | 21.03 | 1.99E-06 | 1.330074073 | NM_017861| | PIGX,GPI-mannosyltransferase subunit |
| 229058_at | 21.02 | 2.00E-06 | 1.146501288 | NM_001009941| | ANKRD16,ankyrin repeat domain 16 isoform a |
| 201095_at | 21.02 | 2.00E-06 | 1.084918985 | NM_004394| | DAP,death-associated protein |
| 214073_at | 21 | 2.00E-06 | 1.202837387 | NM_005231| | CTTN,cortactin isoform a |
| 226436_at | 21 | 2.01E-06 | 1.116617646 | NM_032023| | RASSF4,Ras association domain family 4 isoform a |
| 202204_s_at | 20.99 | 2.01E-06 | 1.215356394 | NM_001144| | AMFR,autocrine motility factor receptor isoform a |
| 218624_s_at | 20.99 | 2.01E-06 | 1.153597781 | NA |  |
| 236146_at | 20.98 | 2.01E-06 | 1.238926618 | NM_006372| | SYNCRIP,synaptotagmin binding, cytoplasmic RNA |
| 225591_at | 20.97 | 2.02E-06 | 1.098568056 | NM_012173| | FBXO25,F-box only protein 25 isoform 3 |
| 203212_s_at | 20.97 | 2.02E-06 | 1.102960728 | NM_016156| | MTMR2,myotubularin-related protein 2 isoform 1 |
| 231848_x_at | 20.96 | 2.03E-06 | 1.237638128 | NM_001032293| | NA |
| 225661_at | 20.96 | 2.03E-06 | 1.467306209 | NM_000629| | IFNAR1,interferon-alpha receptor 1 precursor |
| 222778_s_at | 20.95 | 2.03E-06 | 1.1794403 | NM_001042424| | NA |
| 233303_at | 20.94 | 2.04E-06 | 1.482499449 | NA |  |
| 201433_s_at | 20.94 | 2.04E-06 | 1.073094576 | NM_014754| | PTDSS1,phosphatidylserine synthase 1 |
| 51774_s_at | 20.94 | 2.04E-06 | 1.112383681 | NA |  |
| 208967_s_at | 20.94 | 2.04E-06 | 1.056244465 | NM_001625| | AK2,adenylate kinase 2 isoform a |
| 37966_at | 20.94 | 2.04E-06 | 1.250307015 | NM_001003828| | PARVB,parvin, beta isoform a |
| 1556606_at | 20.93 | 2.04E-06 | 1.756910011 | NM_001111018| | NA |
| 202993_at | 20.93 | 2.04E-06 | 1.090728362 | NM_006844| | ILVBL,ilvB (bacterial acetolactate synthase)-like |
| 218135_at | 20.92 | 2.05E-06 | 1.053441892 | NM_016570| | PTX1,CDA14 |
| 202395_at | 20.92 | 2.05E-06 | 1.077281185 | NM_006178| | NSF,N-ethylmaleimide-sensitive factor |
| 235587_at | 20.92 | 2.04E-06 | 1.129359246 | NA |  |
| 217896_s_at | 20.92 | 2.05E-06 | 1.139595592 | NM_024946| | NIP30,NEFA-interacting nuclear protein NIP30 |
| 226610_at | 20.91 | 2.05E-06 | 1.034714157 | NM_181716| | PRR6,proline rich 6 |
| 230761_at | 20.9 | 2.06E-06 | 1.163960146 | NA |  |
| 208249_s_at | 20.88 | 2.07E-06 | 1.108579264 | NM_014305| | TGDS,TDP-glucose 4,6-dehydratase |
| 219581_at | 20.87 | 2.07E-06 | 1.132165975 | NM_025265| | SEN2L,hypothetical protein MGC2776 |
| 209459_s_at | 20.86 | 2.07E-06 | 1.238932882 | NM_000663| | ABAT,4-aminobutyrate aminotransferase precursor |
| 226428_at | 20.84 | 2.08E-06 | 1.098641425 | NM_013433| | TNPO2,transportin 2 (importin 3, karyopherin beta 2b) |
| 217855_x_at | 20.84 | 2.09E-06 | 1.096281642 | NM_016176| | Cab45,calcium binding protein Cab45 precursor |
| 221892_at | 20.83 | 2.09E-06 | 1.226512907 | NM_004285| | H6PD,hexose-6-phosphate dehydrogenase precursor |
| 227272_at | 20.83 | 2.09E-06 | 1.589644145 | NM_207380| | FLJ43339,FLJ43339 protein |
| 228148_at | 20.82 | 2.10E-06 | 1.203850359 | NM_173548| | ZNF584,zinc finger protein 584 |
| 1553743_at | 20.82 | 2.09E-06 | 1.396818698 | NM_145280| | LOC151194,hepatocellular carcinoma-associated antigen |
| 204764_at | 20.82 | 2.10E-06 | 1.227782951 | NM_002028| | FNTB,farnesyltransferase, CAAX box, beta |
| 224790_at | 20.81 | 2.10E-06 | 1.145611755 | NM_018482| | DDEF1,development and differentiation enhancing factor |
| 216520_s_at | 20.81 | 2.10E-06 | 1.031083761 | NM_003295| | TPT1,tumor protein, translationally-controlled 1 |
| 228419_at | 20.81 | 2.10E-06 | 1.356464603 | NM_024042| | METRN,meteorin, glial cell differentiation regulator |
| 225633_at | 20.81 | 2.10E-06 | 1.083715703 | NM_207325| | LOC147991,hypothetical protein LOC147991 |
| 223307_at | 20.81 | 2.10E-06 | 1.069897086 | NM_031299| | CDCA3,trigger of mitotic entry 1 |
| 219918_s_at | 20.78 | 2.12E-06 | 1.039719924 | NM_018136| | ASPM,asp (abnormal spindle)-like, microcephaly |
| 228606_at | 20.78 | 2.12E-06 | 1.112816083 | NM_138461| | LOC116211,hypothetical protein BC013113 |
| 225804_at | 20.78 | 2.12E-06 | 1.147338895 | NM_144611| | MGC32124,hypothetical protein MGC32124 |
| 203668_at | 20.76 | 2.13E-06 | 1.18739991 | NM_006715| | MAN2C1,mannosidase, alpha, class 2C, member 1 |
| 238164_at | 20.75 | 2.13E-06 | 1.334854477 | NM_001080491| | NA |
| 202589_at | 20.74 | 2.14E-06 | 1.071488809 | NM_001071| | TYMS,thymidylate synthetase |
| 219816_s_at | 20.73 | 2.14E-06 | 1.058985838 | NM_001077351| | NA |
| 227359_at | 20.72 | 2.15E-06 | 1.282357282 | NM_145047| | NOR1,oxidored-nitro domain-containing protein isoform |
| 227293_at | 20.72 | 2.15E-06 | 1.094248605 | NA |  |
| 1565681_s_at | 20.71 | 2.15E-06 | 1.269293372 | NM_014974| | KIAA0934,KIAA0934 |
| 206066_s_at | 20.71 | 2.15E-06 | 1.156129363 | NM_002876| | RAD51C,RAD51 homolog C isoform 2 |
| 217833_at | 20.71 | 2.15E-06 | 1.065011947 | NM_006372| | SYNCRIP,synaptotagmin binding, cytoplasmic RNA |
| 203805_s_at | 20.7 | 2.16E-06 | 1.141614743 | NM_000135| | FANCA,Fanconi anemia, complementation group A |
| 203285_s_at | 20.7 | 2.15E-06 | 1.114729531 | NM_012262| | HS2ST1,heparan sulfate 2-O-sulfotransferase 1 |
| 201853_s_at | 20.7 | 2.16E-06 | 1.131513682 | NM_004358| | CDC25B,cell division cycle 25B isoform 1 |
| 227193_at | 20.7 | 2.15E-06 | 1.41505427 | NA |  |
| 223398_at | 20.7 | 2.16E-06 | 1.167472551 | NM_032310| | C9orf89,chromosome 9 open reading frame 89 |
| 200811_at | 20.69 | 2.16E-06 | 1.127384646 | NM_001280| | CIRBP,cold inducible RNA binding protein |
| 225670_at | 20.69 | 2.16E-06 | 1.108264161 | NM_199133| | LOC134145,hypothetical protein LOC134145 |
| 213708_s_at | 20.68 | 2.17E-06 | 1.070439324 | NM_170607| | MLX,transcription factor-like protein 4 isoform |
| 223012_at | 20.68 | 2.17E-06 | 1.128994151 | NM_025241| | UBXD1,UBX domain containing 1 |
| 226192_at | 20.67 | 2.17E-06 | 1.20943035 | NA |  |
| 211152_s_at | 20.67 | 2.17E-06 | 1.136298081 | NM_013247| | PRSS25,protease, serine, 25 isoform 1 preproprotein |
| 1559942_at | 20.67 | 2.17E-06 | 1.18539094 | NM_199072| | HIC,I-mfa domain-containing protein isoform p40 |
| 229653_at | 20.67 | 2.17E-06 | 1.252794017 | NA |  |
| 203150_at | 20.66 | 2.18E-06 | 1.117148501 | NM_005833| | RAB9P40,Rab9 effector p40 |
| 225875_s_at | 20.66 | 2.18E-06 | 1.201329958 | NM_020448| | DJ462O23.2,hypothetical protein dJ462O23.2 |
| 200700_s_at | 20.64 | 2.19E-06 | 1.038756448 | NM_001100603| | NA |
| 226429_at | 20.64 | 2.18E-06 | 1.299188106 | NM_018559| | KIAA1704,KIAA1704 |
| 219200_at | 20.61 | 2.20E-06 | 1.067103672 | NM_024091| | MGC5297,hypothetical protein MGC5297 |
| 225460_at | 20.61 | 2.20E-06 | 1.058881306 | NM_004206| | SEC22L3,vesicle trafficking protein isoform b |
| 207360_s_at | 20.61 | 2.20E-06 | 1.178268348 | NM_002531| | NTSR1,neurotensin receptor 1 |
| 225415_at | 20.6 | 2.21E-06 | 1.165243215 | NM_138287| | DTX3L,deltex 3-like |
| 220607_x_at | 20.58 | 2.22E-06 | 1.153866874 | NM_198976| | TH1L,TH1-like protein |
| 225742_at | 20.57 | 2.23E-06 | 1.348227434 | NA |  |
| 203196_at | 20.57 | 2.23E-06 | 1.172918304 | NM_001105515| | NA |
| 226334_s_at | 20.57 | 2.23E-06 | 1.133222084 | NM_152392| | AHSA2,AHA1, activator of heat shock 90kDa protein |
| 221039_s_at | 20.56 | 2.23E-06 | 1.180510364 | NM_018482| | DDEF1,development and differentiation enhancing factor |
| 228108_at | 20.56 | 2.23E-06 | 1.161726925 | NA |  |
| 204482_at | 20.56 | 2.23E-06 | 1.450279801 | NM_003277| | CLDN5,claudin 5 |
| 241470_x_at | 20.56 | 2.23E-06 | 1.73340545 | NA |  |
| 212319_at | 20.54 | 2.24E-06 | 1.196578513 | NM_001098509| | NA |
| 233648_at | 20.54 | 2.24E-06 | 1.318370279 | NA |  |
| 232295_at | 20.53 | 2.25E-06 | 1.423360428 | NM_024996| | GFM1,G elongation factor, mitochondrial 1 |
| 226484_at | 20.52 | 2.25E-06 | 1.376507441 | NM_145166| | ZNF651,zinc finger protein 651 |
| 213031_s_at | 20.51 | 2.26E-06 | 1.10067089 | NM_032856| | FLJ14888,hypothetical protein FLJ14888 |
| 218386_x_at | 20.5 | 2.27E-06 | 1.084458074 | NM_001001992| | USP16,ubiquitin specific protease 16 isoform b |
| 207828_s_at | 20.5 | 2.26E-06 | 1.073088102 | NM_016343| | CENPF,centromere protein F (350/400kD) |
| 239741_at | 20.49 | 2.27E-06 | 1.850309416 | NA |  |
| 235949_at | 20.48 | 2.28E-06 | 1.120825221 | NA |  |
| 211725_s_at | 20.48 | 2.27E-06 | 1.086523178 | NM_001196| | BID,BH3 interacting domain death agonist isoform 2 |
| 218074_at | 20.47 | 2.28E-06 | 1.07312679 | NM_016062| | CGI-128,CGI-128 protein |
| 206052_s_at | 20.47 | 2.29E-06 | 1.084247855 | NM_006527| | SLBP,histone stem-loop binding protein |
| 226237_at | 20.46 | 2.29E-06 | 1.60411257 | NA |  |
| 230210_at | 20.45 | 2.30E-06 | 1.302634209 | NM_025154| | UNC84A,unc-84 homolog A |
| 224688_at | 20.44 | 2.30E-06 | 1.124797948 | NM_017994| | FLJ10099,hypothetical protein FLJ10099 |
| 239297_at | 20.44 | 2.30E-06 | 1.242519253 | NM_001099677| | NA |
| 201816_s_at | 20.43 | 2.31E-06 | 1.129811684 | NM_001483| | GBAS,nipsnap homolog 2 |
| 219097_x_at | 20.42 | 2.31E-06 | 1.047478832 | NM_024104| | MGC2747,hypothetical protein MGC2747 |
| 1569243_at | 20.42 | 2.31E-06 | 1.309197488 | NA |  |
| 204591_at | 20.42 | 2.31E-06 | 2.660024426 | NM_006614| | CHL1,cell adhesion molecule with homology to L1CAM |
| 222453_at | 20.41 | 2.32E-06 | 1.306247891 | NM_024843| | CYBRD1,cytochrome b reductase 1 |
| 200972_at | 20.41 | 2.32E-06 | 1.114895083 | NM_005724| | TM4SF8,transmembrane 4 superfamily member 8 isoform 1 |
| 228273_at | 20.4 | 2.32E-06 | 1.12722664 | NA |  |
| 201294_s_at | 20.4 | 2.32E-06 | 1.128438478 | NM_015626| | WSB1,WD SOCS-box protein 1 isoform 1 |
| 218526_s_at | 20.4 | 2.33E-06 | 1.046836839 | NM_016492| | RANGNRF,RAN guanine nucleotide release factor |
| 206929_s_at | 20.37 | 2.34E-06 | 1.480847746 | NM_005597| | NFIC,nuclear factor I/C isoform 1 |
| 207959_s_at | 20.37 | 2.34E-06 | 1.675235929 | NM_001372| | DNAH9,dynein, axonemal, heavy polypeptide 9 isoform 2 |
| 208828_at | 20.37 | 2.34E-06 | 1.129697773 | NM_017443| | POLE3,DNA polymerase epsilon subunit 3 |
| 227153_at | 20.37 | 2.34E-06 | 1.131414966 | NM_032549| | IMMP2L,IMP2 inner mitochondrial membrane protease-like |
| 221886_at | 20.37 | 2.34E-06 | 1.582573227 | NM_015689| | KIAA1277,KIAA1277 protein |
| 40149_at | 20.37 | 2.34E-06 | 1.096750657 | NM_015503| | SH2B,SH2-B homolog |
| 213296_at | 20.37 | 2.34E-06 | 1.120627012 | NM_007033| | RER1,RER1 retention in endoplasmic reticulum 1 |
| 226805_at | 20.36 | 2.35E-06 | 1.317417659 | NM_001080472| | NA |
| 1555870_at | 20.36 | 2.35E-06 | 1.427906524 | NM_207396| | FLJ46380,FLJ46380 protein |
| 201580_s_at | 20.35 | 2.35E-06 | 1.197094444 | NM_021156| | DJ971N18.2,hypothetical protein DJ971N18.2 |
| 238575_at | 20.35 | 2.35E-06 | 1.237502906 | NM_032523| | OSBPL6,oxysterol-binding protein-like protein 6 isoform |
| 224517_at | 20.35 | 2.35E-06 | 1.184923672 | NA |  |
| 206235_at | 20.34 | 2.35E-06 | 1.295757404 | NM_001098268| | NA |
| 202506_at | 20.34 | 2.35E-06 | 1.457047764 | NM_006751| | SSFA2,sperm specific antigen 2 |
| 223519_at | 20.33 | 2.36E-06 | 1.280754596 | NM_016653| | ZAK,sterile-alpha motif and leucine zipper |
| 202082_s_at | 20.32 | 2.37E-06 | 1.098684407 | NM_001039573| | NA |
| 242476_at | 20.31 | 2.38E-06 | 1.979764576 | NA |  |
| 217909_s_at | 20.31 | 2.37E-06 | 1.120854238 | NM_170607| | MLX,transcription factor-like protein 4 isoform |
| 222532_at | 20.3 | 2.38E-06 | 1.129802587 | NM_021203| | SRPRB,signal recognition particle receptor, beta |
| 220773_s_at | 20.3 | 2.38E-06 | 1.278930054 | NM_001024218| | NA |
| 218355_at | 20.3 | 2.38E-06 | 1.046294124 | NM_012310| | KIF4A,kinesin family member 4 |
| 227792_at | 20.3 | 2.38E-06 | 1.265541626 | NM_001034841| | NA |
| 244801_at | 20.3 | 2.38E-06 | 1.357041145 | NM_002799| | PSMB7,proteasome beta 7 subunit proprotein |
| 225430_at | 20.29 | 2.38E-06 | 1.210782424 | NM_176818| | 15E1.2,hypothetical protein 15E1.2 |
| 219944_at | 20.28 | 2.39E-06 | 1.502112545 | NM_024692| | RSNL2,restin-like 2 |
| 226459_at | 20.28 | 2.39E-06 | 1.219673483 | NM_152309| | PIK3AP1,phosphoinositide-3-kinase adaptor protein 1 |
| 1554078_s_at | 20.28 | 2.39E-06 | 1.086861893 | NM_005147| | DNAJA3,DnaJ (Hsp40) homolog, subfamily A, member 3 |
| 212238_at | 20.27 | 2.40E-06 | 1.190234451 | NM_015338| | ASXL1,additional sex combs like 1 |
| 223945_x_at | 20.26 | 2.40E-06 | 1.153584046 | NA |  |
| 242725_at | 20.26 | 2.40E-06 | 1.295086134 | NA |  |
| 219056_at | 20.26 | 2.40E-06 | 1.337987304 | NM_024570| | FLJ11712,hypothetical protein FLJ11712 |
| 213046_at | 20.25 | 2.41E-06 | 1.181537944 | NM_004643| | PABPN1,poly(A) binding protein, nuclear 1 |
| 209339_at | 20.24 | 2.41E-06 | 1.128983036 | NM_005067| | SIAH2,seven in absentia homolog 2 |
| 210157_at | 20.24 | 2.42E-06 | 1.129693736 | NM_003796| | C19orf2,RPB5-mediating protein isoform a |
| 204202_at | 20.24 | 2.42E-06 | 1.3002885 | NM_001100390| | NA |
| 201516_at | 20.23 | 2.42E-06 | 1.105129971 | NM_003132| | SRM,spermidine synthase |
| 202345_s_at | 20.23 | 2.42E-06 | 1.065238943 | NM_001444| | FABP5,fatty acid binding protein 5 |
| 220935_s_at | 20.23 | 2.42E-06 | 1.11852671 | NM_001011649| | CDK5RAP2,CDK5 regulatory subunit associated protein 2 |
| 229157_at | 20.23 | 2.42E-06 | 1.300164724 | NA |  |
| 229981_at | 20.22 | 2.43E-06 | 1.269534512 | NM_014426| | SNX5,sorting nexin 5 |
| 227784_s_at | 20.22 | 2.43E-06 | 1.094656667 | NM_018714| | COG1,low density lipoprotein B |
| 207809_s_at | 20.22 | 2.43E-06 | 1.082358012 | NM_001183| | ATP6AP1,ATPase, H+ transporting, lysosomal accessory |
| 226003_at | 20.2 | 2.44E-06 | 1.096883363 | NM_017641| | KIF21A,kinesin family member 21A |
| 64371_at | 20.2 | 2.44E-06 | 1.160025801 | NM_001017392| | NA |
| 226690_at | 20.2 | 2.44E-06 | 1.127300972 | NM_001118| | ADCYAP1R1,type I adenylate cyclase activating polypeptide |
| 205356_at | 20.2 | 2.44E-06 | 1.083212046 | NM_003940| | USP13,ubiquitin specific protease 13 (isopeptidase |
| 230667_at | 20.2 | 2.44E-06 | 1.25196089 | NA |  |
| 222916_s_at | 20.19 | 2.45E-06 | 1.264556492 | NM_005336| | HDLBP,high density lipoprotein binding protein |
| 225397_at | 20.19 | 2.44E-06 | 1.121934568 | NM_001080791| | NA |
| 242590_at | 20.19 | 2.45E-06 | 1.162288009 | NA |  |
| 230477_at | 20.19 | 2.45E-06 | 1.222003509 | NA |  |
| 217727_x_at | 20.18 | 2.45E-06 | 1.044209909 | NM_018206| | VPS35,vacuolar protein sorting 35 |
| 225625_at | 20.17 | 2.46E-06 | 1.116731402 | NM_001001655| | MGC90512,similar to hypothetical protein 9530023G02 |
| 221965_at | 20.16 | 2.46E-06 | 1.166692051 | NM_022782| | MPHOSPH9,M-phase phosphoprotein 9 |
| 243481_at | 20.16 | 2.46E-06 | 2.012919713 | NM_020663| | RHOJ,TC10-like Rho GTPase |
| 220525_s_at | 20.16 | 2.46E-06 | 1.08529688 | NM_181575| | AUP1,ancient ubiquitous protein 1 isoform 2 |
| 240153_at | 20.16 | 2.46E-06 | 1.377442557 | NA |  |
| 223220_s_at | 20.15 | 2.47E-06 | 1.202760324 | NM_031458| | PARP9,B aggressive lymphoma gene |
| 212995_x_at | 20.13 | 2.48E-06 | 1.058916769 | NM_001085365| | NA |
| 210383_at | 20.13 | 2.48E-06 | 1.835821769 | NM_006920| | SCN1A,sodium channel, voltage-gated, type I, alpha |
| 207629_s_at | 20.13 | 2.48E-06 | 1.179887488 | NM_004723| | ARHGEF2,rho/rac guanine nucleotide exchange factor 2 |
| 64900_at | 20.13 | 2.48E-06 | 1.321166705 | NM_001077416| | NA |
| 201960_s_at | 20.13 | 2.48E-06 | 1.157572585 | NM_015057| | MYCBP2,MYC binding protein 2 |
| 212946_at | 20.12 | 2.49E-06 | 1.080971522 | NM_001009814| | KIAA0564,KIAA0564 protein isoform b |
| 218481_at | 20.11 | 2.49E-06 | 1.063387509 | NM_020158| | EXOSC5,exosome component Rrp46 |
| 227536_at | 20.1 | 2.50E-06 | 1.05203299 | NM_015070| | KIAA0853,KIAA0853 |
| 202265_at | 20.1 | 2.50E-06 | 1.250105346 | NM_005180| | PCGF4,polycomb group ring finger 4 |
| 235036_at | 20.08 | 2.51E-06 | 1.595521175 | NM_153713| | LIX1L,Lix1 homolog (mouse) like |
| 221506_s_at | 20.08 | 2.52E-06 | 1.089423878 | NM_013433| | TNPO2,transportin 2 (importin 3, karyopherin beta 2b) |
| 233825_s_at | 20.08 | 2.52E-06 | 1.299898016 | NM_031462| | CD99L2,CD99 antigen-like 2 |
| 200048_s_at | 20.08 | 2.52E-06 | 1.065516797 | NM_006694| | JTB,jumping translocation breakpoint |
| 222476_at | 20.07 | 2.52E-06 | 1.081077407 | NM_015455| | CNOT6,CCR4-NOT transcription complex, subunit 6 |
| 228333_at | 20.07 | 2.52E-06 | 1.349863502 | NA |  |
| 226363_at | 20.07 | 2.52E-06 | 1.147164239 | NM_001023587| | NA |
| 224218_s_at | 20.05 | 2.53E-06 | 1.635004505 | NM_014112| | TRPS1,zinc finger transcription factor TRPS1 |
| 229053_at | 20.04 | 2.54E-06 | 1.140389115 | NM_016524| | LOC51760,B/K protein |
| 216483_s_at | 20.04 | 2.54E-06 | 1.121377751 | NM_019107| | C19orf10,chromosome 19 open reading frame 10 |
| 218168_s_at | 20.04 | 2.54E-06 | 1.115477135 | NM_020247| | CABC1,chaperone, ABC1 activity of bc1 complex like |
| 211755_s_at | 20.02 | 2.55E-06 | 1.024443229 | NM_001688| | ATP5F1,ATP synthase, H+ transporting, mitochondrial F0 |
| 235147_at | 20.01 | 2.56E-06 | 1.447389927 | NA |  |
| 228955_at | 20 | 2.57E-06 | 1.186119083 | NA |  |
| 202813_at | 19.99 | 2.58E-06 | 1.081380731 | NM_005646| | TARBP1,TAR RNA binding protein 1 |
| 222681_at | 19.98 | 2.58E-06 | 1.071524457 | NM_020231| | MDS010,x 010 protein |
| 226487_at | 19.98 | 2.58E-06 | 1.278859027 | NM_032829| | FLJ14721,hypothetical protein FLJ14721 |
| 1553106_at | 19.98 | 2.58E-06 | 1.273351106 | NM_152409| | FLJ37562,hypothetical protein FLJ37562 |
| 227983_at | 19.97 | 2.59E-06 | 1.11811676 | NM_145058| | MGC7036,hypothetical protein MGC7036 |
| 214434_at | 19.96 | 2.60E-06 | 1.54292366 | NM_025015| | NA |
| 212088_at | 19.96 | 2.59E-06 | 1.097761047 | NM_015160| | PMPCA,mitochondrial matrix processing protease, alpha |
| 214558_at | 19.96 | 2.59E-06 | 1.833925239 | NM_005288| | GPR12,G protein-coupled receptor 12 |
| 229000_at | 19.96 | 2.60E-06 | 1.09666574 | NM_021217| | ZNF77,zinc finger protein 77 |
| 212577_at | 19.96 | 2.60E-06 | 1.128818269 | NM_015295| | NA |
| 236322_at | 19.95 | 2.61E-06 | 1.533669835 | NA |  |
| 229645_at | 19.95 | 2.60E-06 | 1.531735032 | NM_001044369| | NA |
| 222557_at | 19.95 | 2.61E-06 | 1.098512994 | NM_015894| | STMN3,SCG10-like-protein |
| 235985_at | 19.94 | 2.61E-06 | 1.45507287 | NA |  |
| 213969_x_at | 19.93 | 2.61E-06 | 1.020970784 | NM_000992| | RPL29,ribosomal protein L29 |
| 223227_at | 19.93 | 2.62E-06 | 1.191807422 | NM_031885| | BBS2,Bardet-Biedl syndrome 2 protein |
| 231423_s_at | 19.92 | 2.62E-06 | 1.139168407 | NM_001009941| | ANKRD16,ankyrin repeat domain 16 isoform a |
| 204119_s_at | 19.92 | 2.62E-06 | 1.139511505 | NM_001123| | ADK,adenosine kinase isoform a |
| 212327_at | 19.9 | 2.64E-06 | 1.113118793 | NM_001112717| | NA |
| 220739_s_at | 19.9 | 2.64E-06 | 1.12582821 | NM_017623| | CNNM3,cyclin M3 isoform 1 |
| 203152_at | 19.89 | 2.65E-06 | 1.127031344 | NM_003776| | MRPL40,mitochondrial ribosomal protein L40 |
| 218915_at | 19.88 | 2.65E-06 | 1.084972338 | NM_000268| | NF2,neurofibromin 2 isoform 1 |
| 204139_x_at | 19.88 | 2.65E-06 | 1.108084305 | NM_003422| | ZNF42,zinc finger protein 42 isoform 1 |
| 212802_s_at | 19.87 | 2.66E-06 | 1.092053163 | NM_015635| | DKFZP434C212,DKFZP434C212 protein |
| 1555460_a_at | 19.87 | 2.66E-06 | 1.224494098 | NM_001099406| | NA |
| 201908_at | 19.87 | 2.66E-06 | 1.105950858 | NM_004423| | DVL3,dishevelled 3 |
| 224558_s_at | 19.86 | 2.67E-06 | 1.189077509 | NA |  |
| 208707_at | 19.86 | 2.67E-06 | 1.302129424 | NM_001969| | EIF5,eukaryotic translation initiation factor 5 |
| 208764_s_at | 19.85 | 2.68E-06 | 1.031303119 | NM_001002031| | ATP5G2,ATP synthase, H+ transporting, mitochondrial F0 |
| 223852_s_at | 19.84 | 2.69E-06 | 1.18480435 | NM_032017| | MGC4796,SINK-homologous serine/threonine kinase |
| 203202_at | 19.83 | 2.69E-06 | 1.078943757 | NM_007043| | HRB2,HIV-1 rev binding protein 2 |
| 222193_at | 19.83 | 2.69E-06 | 1.071392789 | NM_021925| | FLJ21820,hypothetical protein FLJ21820 |
| 1558088_a_at | 19.82 | 2.70E-06 | 1.19069516 | NM_003345| | UBE2I,ubiquitin-conjugating enzyme E2I |
| 223485_at | 19.82 | 2.70E-06 | 1.207470263 | NM_032304| | HAGHL,hydroxyacylglutathione hydrolase-like isoform 2 |
| 39817_s_at | 19.81 | 2.71E-06 | 1.123743383 | NM_006443| | C6orf108,putative c-Myc-responsive isoform 1 |
| 212049_at | 19.81 | 2.70E-06 | 1.076930125 | NM_133264| | WIRE,WIRE protein |
| 231877_at | 19.79 | 2.72E-06 | 1.226309716 | NM_152292| | RG9MTD2,RNA (guanine-9-) methyltransferase domain |
| 222222_s_at | 19.79 | 2.72E-06 | 1.235055953 | NM_004838| | HOMER3,Homer, neuronal immediate early gene, 3 |
| 1555595_at | 19.78 | 2.72E-06 | 1.23247956 | NM_024583| | SCRN3,secernin 3 |
| 229459_at | 19.78 | 2.73E-06 | 1.156292241 | NM_001082967| | NA |
| 1553569_at | 19.77 | 2.73E-06 | 1.023891139 | NM_022340| | ZFYVE20,FYVE-finger-containing Rab5 effector protein |
| 206692_at | 19.76 | 2.74E-06 | 1.460545951 | NM_002241| | KCNJ10,potassium inwardly-rectifying channel J10 |
| 219118_at | 19.76 | 2.74E-06 | 1.059645163 | NM_016594| | FKBP11,FK506 binding protein precursor |
| 227816_at | 19.76 | 2.74E-06 | 1.394133165 | NM_004822| | NTN1,netrin 1 |
| 221911_at | 19.76 | 2.74E-06 | 1.11818111 | NM_004956| | ETV1,ets variant gene 1 |
| 225615_at | 19.75 | 2.75E-06 | 1.197470735 | NA |  |
| 233518_at | 19.74 | 2.76E-06 | 1.418734429 | NA |  |
| 209077_at | 19.73 | 2.76E-06 | 1.066828656 | NM_012473| | TXN2,thioredoxin 2 precursor |
| 225810_at | 19.73 | 2.76E-06 | 1.128299215 | NM_017762| | FLJ20313,hypothetical protein FLJ20313 |
| 223500_at | 19.72 | 2.77E-06 | 1.587018841 | NM_006651| | CPLX1,complexin 1 |
| 226935_s_at | 19.71 | 2.77E-06 | 1.080054575 | NM_030782| | CRR9,cisplatin resistance related protein CRR9p |
| 226627_at | 19.71 | 2.78E-06 | 1.744732702 | NM_001098811| | NA |
| 240221_at | 19.71 | 2.78E-06 | 1.176362922 | NM_001025105| | NA |
| 208359_s_at | 19.71 | 2.77E-06 | 1.319560735 | NM_004981| | KCNJ4,potassium inwardly-rectifying channel J4 |
| 215436_at | 19.7 | 2.79E-06 | 1.506960697 | NM_032303| | HSDL2,hydroxysteroid dehydrogenase like 2 |
| 1554774_at | 19.69 | 2.79E-06 | 1.41430206 | NM_001042533| | NA |
| 236640_at | 19.69 | 2.79E-06 | 1.585122112 | NA |  |
| 238919_at | 19.68 | 2.80E-06 | 2.757527398 | NA |  |
| 222653_at | 19.67 | 2.80E-06 | 1.117339353 | NM_018129| | PNPO,pyridoxine 5'-phosphate oxidase |
| 1558750_a_at | 19.67 | 2.80E-06 | 1.233121881 | NA |  |
| 226968_at | 19.67 | 2.80E-06 | 1.075490716 | NM_015074| | KIF1B,kinesin family member 1B isoform b |
| 222266_at | 19.66 | 2.81E-06 | 1.229420889 | NM_003796| | C19orf2,RPB5-mediating protein isoform a |
| 239632_at | 19.66 | 2.81E-06 | 1.476261024 | NA |  |
| 1557165_s_at | 19.66 | 2.81E-06 | 1.161354477 | NM_025010| | KLHL18,kelch-like 18 |
| 218826_at | 19.65 | 2.82E-06 | 1.079052352 | NM_017515| | SLC35F2,solute carrier family 35, member F2 |
| 223213_s_at | 19.65 | 2.82E-06 | 1.558681663 | NM_001017926| | NA |
| 202678_at | 19.64 | 2.83E-06 | 1.017909686 | NM_004492| | GTF2A2,general transcription factor IIA, 2 (12kD |
| 205710_at | 19.64 | 2.83E-06 | 1.32384537 | NM_004525| | LRP2,low density lipoprotein-related protein 2 |
| 216048_s_at | 19.62 | 2.85E-06 | 1.310176525 | NM_014899| | RHOBTB3,rho-related BTB domain containing 3 |
| 226034_at | 19.6 | 2.86E-06 | 1.152722395 | NA |  |
| 233852_at | 19.6 | 2.86E-06 | 1.318568428 | NM_006502| | POLH,polymerase (DNA directed), eta |
| 212248_at | 19.6 | 2.86E-06 | 1.152523822 | NM_178812| | LYRIC,LYRIC/3D3 |
| 238756_at | 19.6 | 2.86E-06 | 1.197697602 | NM_174942| | GAS2L3,growth arrest-specific 2 like 3 |
| 209812_x_at | 19.6 | 2.86E-06 | 1.142755811 | NM_032982| | CASP2,caspase 2 isoform 1 preproprotein |
| 201163_s_at | 19.6 | 2.86E-06 | 1.506172611 | NM_001553| | IGFBP7,insulin-like growth factor binding protein 7 |
| 202381_at | 19.59 | 2.86E-06 | 1.317411352 | NM_001005845| | ADAM9,a disintegrin and metalloproteinase domain 9 |
| 206469_x_at | 19.59 | 2.86E-06 | 1.159514982 | NM_012067| | AKR7A3,aldo-keto reductase family 7, member A3 |
| 212109_at | 19.59 | 2.87E-06 | 1.052429573 | NM_144570| | C16orf34,chromosome 16 open reading frame 34 |
| 214526_x_at | 19.59 | 2.86E-06 | 1.132673601 | NA |  |
| 213657_s_at | 19.58 | 2.87E-06 | 1.288472474 | NA |  |
| 213649_at | 19.57 | 2.88E-06 | 1.101890694 | NM_001031684| | NA |
| 222445_at | 19.57 | 2.88E-06 | 1.082821183 | NM_018375| | SLC39A9,solute carrier family 39 (zinc transporter), |
| 228548_at | 19.56 | 2.88E-06 | 1.310443536 | NA |  |
| 224955_at | 19.56 | 2.88E-06 | 1.050878011 | NM_021961| | TEAD1,TEA domain family member 1 |
| 214756_x_at | 19.55 | 2.90E-06 | 1.136394519 | NA |  |
| 1554101_a_at | 19.54 | 2.91E-06 | 1.284550102 | NM_001079669| | NA |
| 226764_at | 19.53 | 2.91E-06 | 1.064266147 | NM_178835| | LOC152485,hypothetical protein LOC152485 |
| 221908_at | 19.53 | 2.91E-06 | 1.171245648 | NM_001109903| | NA |
| 212377_s_at | 19.53 | 2.91E-06 | 1.105667323 | NM_024408| | NOTCH2,notch 2 preproprotein |
| 238613_at | 19.51 | 2.93E-06 | 1.361045316 | NM_016653| | ZAK,sterile-alpha motif and leucine zipper |
| 212923_s_at | 19.51 | 2.93E-06 | 1.319740929 | NM_183373| | C6orf145,chromosome 6 open reading frame 145 |
| 222664_at | 19.51 | 2.93E-06 | 1.133036179 | NM_024076| | KCTD15,potassium channel tetramerisation domain |
| 217367_s_at | 19.48 | 2.96E-06 | 1.293245909 | NM_015035| | ZHX3,zinc fingers and homeoboxes 3 protein |
| 242065_x_at | 19.48 | 2.96E-06 | 1.128844819 | NM_004508| | IDI1,isopentenyl-diphosphate delta isomerase |
| 240312_at | 19.48 | 2.96E-06 | 1.298382686 | NA |  |
| 212038_s_at | 19.48 | 2.96E-06 | 1.09495135 | NM_003374| | VDAC1,voltage-dependent anion channel 1 |
| 225652_at | 19.48 | 2.95E-06 | 1.149187636 | NA |  |
| 218723_s_at | 19.48 | 2.96E-06 | 1.536091077 | NM_014059| | RGC32,response gene to complement 32 |
| 223378_at | 19.47 | 2.96E-06 | 1.085067561 | NM_032575| | GLIS2,GLIS family zinc finger 2 |
| 1558487_a_at | 19.47 | 2.96E-06 | 1.127069781 | NM_182547| | TMED4,transmembrane emp24 protein transport domain |
| 212726_at | 19.47 | 2.96E-06 | 1.243691356 | NM_005392| | PHF2,PHD finger protein 2 isoform a |
| 215164_at | 19.46 | 2.97E-06 | 1.319887653 | NA |  |
| 214964_at | 19.45 | 2.98E-06 | 1.36544469 | NA |  |
| 205875_s_at | 19.45 | 2.97E-06 | 1.39566631 | NM_016381| | TREX1,three prime repair exonuclease 1 isoform a |
| 202115_s_at | 19.45 | 2.98E-06 | 1.065708174 | NM_015658| | DKFZP564C186,DKFZP564C186 protein |
| 209168_at | 19.45 | 2.98E-06 | 1.131009326 | NM_001001994| | GPM6B,glycoprotein M6B isoform 4 |
| 1557049_at | 19.45 | 2.97E-06 | 1.354353594 | NA |  |
| 228949_at | 19.43 | 2.99E-06 | 1.318533071 | NM_001002292| | FLJ23091,putative NFkB activating protein 373 isoform 2 |
| 222526_at | 19.43 | 2.99E-06 | 1.057363377 | NM_017660| | p66alpha,p66 alpha |
| 222138_s_at | 19.42 | 3.00E-06 | 1.198753228 | NM_017883| | WDR13,WD repeat domain 13 protein |
| 241938_at | 19.4 | 3.02E-06 | 1.477118264 | NM_006775| | QKI,quaking homolog, KH domain RNA binding isoform |
| 218252_at | 19.4 | 3.01E-06 | 1.169364355 | NM_001098525| | NA |
| 230030_at | 19.4 | 3.01E-06 | 1.269455128 | NM_001077188| | NA |
| 225504_at | 19.4 | 3.02E-06 | 1.121375625 | NA |  |
| 206669_at | 19.4 | 3.01E-06 | 1.308394908 | NM_000817| | GAD1,glutamate decarboxylase 1 isoform GAD67 |
| 215169_at | 19.39 | 3.02E-06 | 1.170870403 | NM_001110781| | NA |
| 211651_s_at | 19.36 | 3.05E-06 | 1.104920838 | NM_002291| | LAMB1,laminin, beta 1 precursor |
| 238431_at | 19.36 | 3.05E-06 | 1.192627822 | NA |  |
| 1553677_a_at | 19.36 | 3.05E-06 | 1.106187652 | NM_001031800| | NA |
| 219999_at | 19.35 | 3.05E-06 | 1.361683601 | NM_006122| | MAN2A2,mannosidase, alpha, class 2A, member 2 |
| 201076_at | 19.35 | 3.06E-06 | 1.060779067 | NM_001003796| | NHP2L1,NHP2 non-histone chromosome protein 2-like 1 |
| 219564_at | 19.35 | 3.05E-06 | 1.677342164 | NM_018658| | KCNJ16,potassium inwardly-rectifying channel J16 |
| 235709_at | 19.35 | 3.05E-06 | 1.280490321 | NM_174942| | GAS2L3,growth arrest-specific 2 like 3 |
| 224478_s_at | 19.35 | 3.06E-06 | 1.138471231 | NM_032350| | MGC11257,hypothetical protein MGC11257 |
| 221335_x_at | 19.35 | 3.06E-06 | 1.178533002 | NM_019108| | FLJ12886,hypothetical protein FLJ12886 |
| 228516_at | 19.33 | 3.08E-06 | 1.114349794 | NM_138477| | CDAN1,codanin 1 |
| 225479_at | 19.32 | 3.09E-06 | 1.188928898 | NM_001099678| | NA |
| 201361_at | 19.32 | 3.09E-06 | 1.08037821 | NM_024092| | MGC5508,hypothetical protein MGC5508 |
| 205413_at | 19.29 | 3.11E-06 | 1.134307536 | NM_001584| | C11orf8,chromosome 11 open reading frame 8 |
| 227022_at | 19.28 | 3.12E-06 | 1.203183744 | NM_138335| | GNPDA2,glucosamine-6-phosphate deaminase 2 |
| 213369_at | 19.28 | 3.12E-06 | 1.195814251 | NM_033100| | PCDH21,protocadherin 21 precursor |
| 204254_s_at | 19.25 | 3.14E-06 | 1.380634751 | NM_000376| | VDR,vitamin D (1,25- dihydroxyvitamin D3) receptor |
| 202771_at | 19.25 | 3.14E-06 | 1.156370847 | NA |  |
| 220495_s_at | 19.24 | 3.15E-06 | 1.075516182 | NM_024715| | C5orf14,disulfide isomerase |
| 206238_s_at | 19.24 | 3.16E-06 | 1.321064079 | NM_005748| | YAF2,YY1 associated factor 2 isoform a |
| 1555091_at | 19.22 | 3.17E-06 | 1.510333537 | NM_014634| | PPM1F,protein phosphatase 1F |
| 230142_s_at | 19.22 | 3.17E-06 | 1.253714091 | NM_001280| | CIRBP,cold inducible RNA binding protein |
| 209042_s_at | 19.22 | 3.18E-06 | 1.08315047 | NM_003343| | UBE2G2,ubiquitin-conjugating enzyme E2G 2 isoform 1 |
| 231106_at | 19.2 | 3.19E-06 | 1.209055454 | NA |  |
| 244659_at | 19.2 | 3.19E-06 | 1.308991133 | NA |  |
| 204914_s_at | 19.19 | 3.19E-06 | 1.084853794 | NM_003108| | SOX11,SRY-box 11 |
| 235131_at | 19.19 | 3.20E-06 | 1.492927715 | NM_020663| | RHOJ,TC10-like Rho GTPase |
| 242989_at | 19.18 | 3.20E-06 | 1.514755733 | NA |  |
| 237315_at | 19.18 | 3.20E-06 | 1.409349159 | NA |  |
| 213033_s_at | 19.17 | 3.21E-06 | 2.008620243 | NM_005596| | NFIB,nuclear factor I/B |
| 242051_at | 19.17 | 3.22E-06 | 1.71770889 | NA |  |
| 1555680_a_at | 19.17 | 3.21E-06 | 1.212766177 | NM_175839| | SMOX,polyamine oxidase isoform 1 |
| 227641_at | 19.17 | 3.22E-06 | 1.154352072 | NM_153350| | FBXL16,F-box and leucine-rich repeat protein 16 |
| 222742_s_at | 19.16 | 3.22E-06 | 1.057487141 | NM_022777| | RABL5,RAB, member RAS oncogene family-like 5 |
| 231056_at | 19.16 | 3.22E-06 | 1.118843164 | NA |  |
| 1563014_at | 19.15 | 3.23E-06 | 1.268462019 | NM_001018| | RPS15,ribosomal protein S15 |
| 1557953_at | 19.13 | 3.25E-06 | 1.194427483 | NM_003439| | ZKSCAN1,zinc finger protein 36 |
| 223368_s_at | 19.13 | 3.25E-06 | 1.048660792 | NM_014064| | AD-003,AD-003 protein |
| 215073_s_at | 19.12 | 3.26E-06 | 1.872676025 | NM_021005| | NR2F2,nuclear receptor subfamily 2, group F, member 2 |
| 218510_x_at | 19.12 | 3.26E-06 | 1.328750816 | NM_001034850| | NA |
| 229774_at | 19.12 | 3.26E-06 | 1.354893277 | NM_025212| | CXXC4,CXXC finger 4 |
| 222830_at | 19.12 | 3.26E-06 | 1.296957817 | NM_014552| | TFCP2L2,leader-binding protein 32 isoform 1 |
| 243718_at | 19.11 | 3.27E-06 | 1.577965027 | NA |  |
| 37232_at | 19.1 | 3.27E-06 | 1.099721076 | NM_014749| | KIAA0586,KIAA0586 |
| 202508_s_at | 19.1 | 3.28E-06 | 1.589887611 | NM_003081| | SNAP25,synaptosomal-associated protein 25 isoform |
| 235433_at | 19.09 | 3.29E-06 | 1.197633252 | NM_198450| | CXorf33,chromosome X open reading frame 33 |
| 203997_at | 19.09 | 3.28E-06 | 1.323520165 | NM_002829| | PTPN3,protein tyrosine phosphatase, non-receptor type |
| 213005_s_at | 19.09 | 3.28E-06 | 1.132050619 | NM_015158| | ANKRD15,ankyrin repeat domain protein 15 |
| 229132_at | 19.08 | 3.30E-06 | 1.322735483 | NM_001042533| | NA |
| 235736_at | 19.07 | 3.31E-06 | 1.555191847 | NA |  |
| 203771_s_at | 19.07 | 3.30E-06 | 1.154832769 | NM_000712| | BLVRA,biliverdin reductase A |
| 226254_s_at | 19.05 | 3.32E-06 | 1.090049018 | NM_020827| | NA |
| 229022_at | 19.05 | 3.32E-06 | 1.137613212 | NM_003410| | ZFX,zinc finger protein, X-linked |
| 201620_at | 19.04 | 3.33E-06 | 1.06446163 | NM_003791| | MBTPS1,membrane-bound transcription factor site-1 |
| 218440_at | 19.03 | 3.34E-06 | 1.103817483 | NM_020166| | MCCC1,methylcrotonoyl-Coenzyme A carboxylase 1 |
| 232420_x_at | 19.03 | 3.34E-06 | 1.087636923 | NA |  |
| 225238_at | 19.01 | 3.36E-06 | 1.300014897 | NM_138962| | MSI2,musashi 2 isoform a |
| 212561_at | 19 | 3.37E-06 | 1.075347758 | NM_015213| | RAB6IP1,RAB6 interacting protein 1 |
| 211715_s_at | 19 | 3.37E-06 | 1.132521844 | NM_004051| | BDH,3-hydroxybutyrate dehydrogenase precursor |
| 227497_at | 19 | 3.37E-06 | 1.591690358 | NA |  |
| 212334_at | 18.99 | 3.38E-06 | 1.09485315 | NM_002076| | GNS,glucosamine (N-acetyl)-6-sulfatase precursor |
| 242492_at | 18.99 | 3.38E-06 | 1.247224119 | NA |  |
| 233445_at | 18.99 | 3.38E-06 | 1.11328624 | NA |  |
| 203911_at | 18.98 | 3.38E-06 | 1.130958198 | NM_002885| | RAP1GA1,RAP1, GTPase activating protein 1 |
| 212226_s_at | 18.98 | 3.39E-06 | 1.110786255 | NM_003713| | PPAP2B,phosphatidic acid phosphatase type 2B |
| 229994_at | 18.97 | 3.40E-06 | 1.498788765 | NA |  |
| 221834_at | 18.97 | 3.39E-06 | 1.5217504 | NM_031490| | LONP,peroxisomal lon protease |
| 227604_at | 18.97 | 3.40E-06 | 1.182211816 | NA |  |
| 235468_at | 18.97 | 3.40E-06 | 1.162259753 | NM_001082575| | NA |
| 225545_at | 18.96 | 3.41E-06 | 1.080157809 | NM_013302| | EEF2K,elongation factor-2 kinase |
| 230494_at | 18.96 | 3.40E-06 | 1.359885863 | NM_005415| | SLC20A1,solute carrier family 20 (phosphate |
| 231954_at | 18.96 | 3.40E-06 | 1.16170654 | NA |  |
| 230119_at | 18.94 | 3.42E-06 | 1.315653702 | NA |  |
| 225100_at | 18.94 | 3.42E-06 | 1.053384225 | NM_001105573| | NA |
| 225967_s_at | 18.94 | 3.42E-06 | 1.068866046 | NM_001086521| | NA |
| 217538_at | 18.94 | 3.42E-06 | 1.359085657 | NM_001098509| | NA |
| 242251_at | 18.93 | 3.43E-06 | 1.392224271 | NM_145207| | SPATA5,spermatogenesis associated factor SPAF |
| 224576_at | 18.93 | 3.43E-06 | 1.058485558 | NM_001031711| | NA |
| 221676_s_at | 18.92 | 3.44E-06 | 1.073315122 | NM_014325| | CORO1C,coronin, actin binding protein, 1C |
| 1556082_a_at | 18.91 | 3.45E-06 | 1.641769994 | NA |  |
| 201797_s_at | 18.91 | 3.45E-06 | 1.083554947 | NM_006295| | VARS2,valyl-tRNA synthetase 2 |
| 236961_at | 18.9 | 3.46E-06 | 1.250984039 | NA |  |
| 219544_at | 18.89 | 3.47E-06 | 1.124473481 | NM_024808| | FLJ22624,FLJ22624 protein |
| 217124_at | 18.89 | 3.47E-06 | 1.372179857 | NM_001100390| | NA |
| 227415_at | 18.89 | 3.47E-06 | 1.144764542 | NA |  |
| 1556646_at | 18.88 | 3.48E-06 | 1.068829844 | NA |  |
| 206016_at | 18.87 | 3.49E-06 | 1.105994965 | NM_014008| | CXorf37,chromosome X open reading frame 37 |
| 222697_s_at | 18.87 | 3.49E-06 | 1.147390029 | NM_018394| | ABHD10,abhydrolase domain containing 10 |
| 241867_at | 18.87 | 3.49E-06 | 1.378299191 | NA |  |
| 1558724_at | 18.86 | 3.50E-06 | 1.856275693 | NA |  |
| 211302_s_at | 18.86 | 3.50E-06 | 1.578387727 | NM_001037339| | NA |
| 226736_at | 18.84 | 3.52E-06 | 1.204128871 | NM_145165| | CHURC1,churchill domain containing 1 |
| 218750_at | 18.84 | 3.52E-06 | 1.098567954 | NM_024116| | MGC5306,hypothetical protein MGC5306 |
| 225137_at | 18.84 | 3.52E-06 | 1.152964807 | NA |  |
| 212399_s_at | 18.83 | 3.53E-06 | 1.086459282 | NM_014667| | VGLL4,vestigial like 4 |
| 212427_at | 18.82 | 3.55E-06 | 1.397174912 | NM_001080398| | NA |
| 226501_at | 18.82 | 3.55E-06 | 1.134197466 | NM_022098| | LOC63929,hypothetical protein LOC63929 |
| 236882_at | 18.79 | 3.57E-06 | 1.375744685 | NA |  |
| 1552767_a_at | 18.79 | 3.57E-06 | 1.239290842 | NM_001077188| | NA |
| 225308_s_at | 18.78 | 3.59E-06 | 1.160748257 | NM_033394| | TANC,TPR domain, ankyrin-repeat and |
| 203284_s_at | 18.78 | 3.59E-06 | 1.128833367 | NM_012262| | HS2ST1,heparan sulfate 2-O-sulfotransferase 1 |
| 209870_s_at | 18.78 | 3.58E-06 | 1.105327871 | NM_005503| | APBA2,amyloid beta A4 precursor protein-binding, |
| 1555240_s_at | 18.78 | 3.58E-06 | 1.386775055 | NM_018841| | GNG12,G-protein gamma-12 subunit |
| 213785_at | 18.78 | 3.59E-06 | 1.179611664 | NM_018085| | IPO9,importin 9 |
| 232710_at | 18.77 | 3.59E-06 | 1.150301314 | NA |  |
| 213436_at | 18.77 | 3.59E-06 | 1.831496167 | NM_016083| | CNR1,central cannabinoid receptor isoform a |
| 203466_at | 18.77 | 3.59E-06 | 1.083895381 | NM_002437| | MPV17,MpV17 transgene, murine homolog, |
| 227188_at | 18.77 | 3.59E-06 | 1.280451555 | NM_058187| | C21orf63,chromosome 21 open reading frame 63 |
| 212792_at | 18.76 | 3.60E-06 | 1.091346588 | NM_015283| | NA |
| 213244_at | 18.76 | 3.60E-06 | 1.12560226 | NM_079834| | SCAMP4,secretory carrier membrane protein 4 |
| 235421_at | 18.75 | 3.61E-06 | 1.291235464 | NM_005204| | MAP3K8,mitogen-activated protein kinase kinase kinase |
| 219355_at | 18.74 | 3.63E-06 | 1.19397004 | NM_018015| | FLJ10178,hypothetical protein FLJ10178 |
| 230102_at | 18.73 | 3.64E-06 | 1.2014116 | NM_004454| | ETV5,ets variant gene 5 (ets-related molecule) |
| 221231_s_at | 18.73 | 3.64E-06 | 1.206189932 | NM_017970| | C14orf102,chromosome 14 open reading frame 102 isoform 1 |
| 225115_at | 18.72 | 3.65E-06 | 1.145559609 | NM_001113239| | NA |
| 224900_at | 18.72 | 3.65E-06 | 1.062704088 | NM_016376| | ANKFY1,ankyrin repeat and FYVE domain containing 1 |
| 1560029_a_at | 18.71 | 3.65E-06 | 1.415405041 | NM_001082969| | NA |
| 204992_s_at | 18.71 | 3.65E-06 | 1.053957663 | NM_002628| | PFN2,profilin 2 isoform b |
| 219843_at | 18.7 | 3.67E-06 | 1.271524824 | NM_005897| | IPP,intracisternal A particle-promoted polypeptide |
| 225528_at | 18.69 | 3.68E-06 | 1.043419572 | NM_006390| | IPO8,importin 8 |
| 204807_at | 18.69 | 3.69E-06 | 1.316729621 | NM_014254| | TMEM5,transmembrane protein 5 |
| 224391_s_at | 18.68 | 3.70E-06 | 1.474957807 | NM_170601| | CSE-C,cytosolic sialic acid 9-O-acetylesterase |
| 225291_at | 18.68 | 3.69E-06 | 1.046583186 | NM_033109| | PNPT1,polyribonucleotide nucleotidyltransferase 1 |
| 209591_s_at | 18.66 | 3.72E-06 | 1.676225457 | NM_001719| | BMP7,bone morphogenetic protein 7 precursor |
| 212676_at | 18.66 | 3.71E-06 | 1.089549435 | NM_000267| | NF1,neurofibromin |
| 219831_at | 18.66 | 3.71E-06 | 1.105478921 | NM_001113575| | NA |
| 243432_at | 18.66 | 3.71E-06 | 1.289885933 | NA |  |
| 229742_at | 18.66 | 3.71E-06 | 1.246314887 | NA |  |
| 221899_at | 18.65 | 3.72E-06 | 1.178049342 | NM_014887| | PFAAP5,phosphonoformate immuno-associated protein 5 |
| 235309_at | 18.65 | 3.72E-06 | 1.158741215 | NM_001019| | RPS15A,ribosomal protein S15a |
| 227926_s_at | 18.64 | 3.74E-06 | 1.135024176 | NM_001037501| | NA |
| 211596_s_at | 18.63 | 3.75E-06 | 1.049018981 | NM_015541| | LRIG1,leucine-rich repeats and immunoglobulin-like |
| 1554317_s_at | 18.63 | 3.75E-06 | 1.156092093 | NM_152307| | C14orf172,chromosome 14 open reading frame 172 |
| 221406_s_at | 18.63 | 3.74E-06 | 1.37283557 | NM_001039651| | NA |
| 211804_s_at | 18.63 | 3.74E-06 | 1.306604314 | NM_001798| | CDK2,cyclin-dependent kinase 2 isoform 1 |
| 201133_s_at | 18.62 | 3.75E-06 | 1.125585715 | NM_014819| | PJA2,praja 2, RING-H2 motif containing |
| 202197_at | 18.62 | 3.75E-06 | 1.091995797 | NM_021090| | MTMR3,myotubularin-related protein 3 isoform c |
| 238824_at | 18.61 | 3.77E-06 | 1.131414415 | NA |  |
| 231952_at | 18.61 | 3.76E-06 | 1.18912585 | NA |  |
| 212629_s_at | 18.6 | 3.77E-06 | 1.062649776 | NM_006256| | PKN2,protein kinase N2 |
| 209708_at | 18.6 | 3.78E-06 | 1.140875925 | NM_015529| | MOXD1,monooxygenase, DBH-like 1 |
| 223226_x_at | 18.59 | 3.79E-06 | 1.187735526 | NM_001009998| | SSBP4,single stranded DNA binding protein 4 isoform b |
| 227251_at | 18.59 | 3.78E-06 | 1.180360582 | NM_003861| | WDR22,Breakpoint cluster region protein, uterine |
| 224763_at | 18.58 | 3.80E-06 | 1.194106834 | NM_000997| | RPL37,ribosomal protein L37 |
| 1558002_at | 18.58 | 3.80E-06 | 1.202437928 | NM_007178| | STRAP,serine/threonine kinase receptor associated |
| 213262_at | 18.57 | 3.81E-06 | 1.130590484 | NM_014363| | SACS,sacsin |
| 206465_at | 18.57 | 3.81E-06 | 1.357389805 | NM_015162| | BG1,lipidosin |
| 228093_at | 18.57 | 3.81E-06 | 1.177968197 | NM_001007248| | ZNF599,zinc finger protein 599 isoform a |
| 211985_s_at | 18.56 | 3.81E-06 | 1.128962389 | NM_001743| | CALM2,calmodulin 2 |
| 235067_at | 18.56 | 3.82E-06 | 1.143879692 | NM_013255| | MKLN1,muskelin 1, intracellular mediator containing |
| 218153_at | 18.55 | 3.83E-06 | 1.087069154 | NM_024537| | FLJ12118,hypothetical protein FLJ12118 |
| 39835_at | 18.55 | 3.83E-06 | 1.056800747 | NM_002972| | SBF1,SET binding factor 1 isoform a |
| 212549_at | 18.55 | 3.83E-06 | 1.052621537 | NM_012448| | STAT5B,signal transducer and activator of transcription |
| 64438_at | 18.55 | 3.83E-06 | 1.102862718 | NM_024648| | FLJ22222,hypothetical protein FLJ22222 |
| 222163_s_at | 18.55 | 3.83E-06 | 1.071250431 | NM_024063| | SPATA5L1,spermatogenesis associated 5-like 1 |
| 218827_s_at | 18.54 | 3.84E-06 | 1.06556549 | NM_032142| | Cep192,centrosomal protein 192 kDa isoform 1 |
| 49485_at | 18.54 | 3.84E-06 | 1.082263464 | NM_012406| | PRDM4,PR domain containing 4 |
| 224560_at | 18.54 | 3.84E-06 | 1.083673442 | NM_003255| | TIMP2,tissue inhibitor of metalloproteinase 2 |
| 212423_at | 18.53 | 3.85E-06 | 1.232705386 | NM_153367| | C10orf56,chromosome 10 open reading frame 56 |
| 239053_at | 18.53 | 3.85E-06 | 1.102591614 | NM_004804| | WDR39,WD repeat domain 39 |
| 214670_at | 18.52 | 3.86E-06 | 1.185285548 | NM_003439| | ZKSCAN1,zinc finger protein 36 |
| 211259_s_at | 18.52 | 3.87E-06 | 1.24923649 | NM_001719| | BMP7,bone morphogenetic protein 7 precursor |
| 203235_at | 18.5 | 3.89E-06 | 1.104694506 | NM_003249| | THOP1,thimet oligopeptidase 1 |
| 229428_at | 18.5 | 3.89E-06 | 1.039205782 | NA |  |
| 1556473_at | 18.5 | 3.89E-06 | 1.466029758 | NA |  |
| 222783_s_at | 18.49 | 3.90E-06 | 1.460050218 | NM_001034852| | NA |
| 213069_at | 18.49 | 3.90E-06 | 1.117482875 | NM_020733| | NA |
| 204029_at | 18.48 | 3.91E-06 | 1.149635881 | NM_001408| | CELSR2,cadherin EGF LAG seven-pass G-type receptor 2 |
| 225250_at | 18.48 | 3.91E-06 | 1.497149117 | NM_020860| | STIM2,stromal interaction molecule 2 |
| 206106_at | 18.48 | 3.91E-06 | 1.331198831 | NM_002969| | MAPK12,mitogen-activated protein kinase 12 |
| 234975_at | 18.47 | 3.92E-06 | 1.544145621 | NA |  |
| 212593_s_at | 18.47 | 3.92E-06 | 1.17035726 | NM_014456| | PDCD4,programmed cell death 4 isoform 1 |
| 209247_s_at | 18.46 | 3.93E-06 | 1.128221612 | NM_005692| | ABCF2,ATP-binding cassette, sub-family F, member 2 |
| 203068_at | 18.45 | 3.94E-06 | 1.109032261 | NM_014851| | KLHL21,kelch-like 21 |
| 226316_at | 18.45 | 3.95E-06 | 1.156496626 | NA |  |
| 235954_at | 18.45 | 3.94E-06 | 1.214902771 | NA |  |
| 218496_at | 18.44 | 3.95E-06 | 1.101074748 | NM_002936| | RNASEH1,ribonuclease H1 |
| 230570_at | 18.44 | 3.96E-06 | 1.128508417 | NA |  |
| 229272_at | 18.44 | 3.96E-06 | 1.350152808 | NM_015308| | FNBP4,formin binding protein 4 |
| 201063_at | 18.43 | 3.97E-06 | 1.033565039 | NM_002901| | RCN1,reticulocalbin 1 precursor |
| 202414_at | 18.43 | 3.97E-06 | 1.066035078 | NM_000123| | ERCC5,XPG-complementing protein |
| 208433_s_at | 18.42 | 3.98E-06 | 1.061493967 | NM_001018054| | NA |
| 231316_at | 18.42 | 3.98E-06 | 1.168196519 | NA |  |
| 225473_at | 18.42 | 3.98E-06 | 1.206361923 | NM_199181| | FLJ44670,FLJ44670 protein |
| 213304_at | 18.42 | 3.98E-06 | 1.111047004 | NM_015091| | KIAA0423,KIAA0423 |
| 221127_s_at | 18.41 | 3.99E-06 | 1.206226425 | NA |  |
| 226433_at | 18.41 | 3.99E-06 | 1.147639183 | NM_052916| | RNF157,ring finger protein 157 |
| 217737_x_at | 18.4 | 4.00E-06 | 1.06606564 | NM_016407| | C20orf43,chromosome 20 open reading frame 43 |
| 226063_at | 18.39 | 4.01E-06 | 1.096980798 | NM_003371| | VAV2,vav 2 oncogene |
| 217511_at | 18.38 | 4.02E-06 | 1.235672976 | NM_030929| | KAZALD1,Kazal-type serine protease inhibitor domain 1 |
| 218225_at | 18.38 | 4.03E-06 | 1.099625914 | NM_016581| | SITPEC,evolutionarily conserved signaling intermediate |
| 242705_x_at | 18.37 | 4.04E-06 | 1.101080813 | NA |  |
| 1559139_at | 18.37 | 4.03E-06 | 1.308411636 | NM_015658| | DKFZP564C186,DKFZP564C186 protein |
| 226343_at | 18.36 | 4.05E-06 | 1.147842667 | NA |  |
| 203115_at | 18.36 | 4.05E-06 | 1.11111131 | NM_000140| | FECH,ferrochelatase isoform b precursor |
| 238905_at | 18.36 | 4.05E-06 | 1.672275049 | NM_020663| | RHOJ,TC10-like Rho GTPase |
| 229029_at | 18.36 | 4.05E-06 | 1.242284223 | NA |  |
| 214093_s_at | 18.35 | 4.05E-06 | 1.117910096 | NM_003902| | FUBP1,far upstream element-binding protein |
| 224674_at | 18.35 | 4.06E-06 | 1.068383373 | NM_025250| | TTYH3,tweety 3 |
| 212832_s_at | 18.35 | 4.06E-06 | 1.076833308 | NM_001008938| | ch-TOG,colonic and hepatic tumor over-expressed protein |
| 221704_s_at | 18.35 | 4.06E-06 | 1.104225392 | NM_024667| | FLJ12750,hypothetical protein FLJ12750 |
| 222671_s_at | 18.34 | 4.06E-06 | 1.118586073 | NM_023007| | FLJ12517,hypothetical protein FLJ12517 |
| 214092_x_at | 18.34 | 4.07E-06 | 1.062941108 | NM_001017392| | NA |
| 205546_s_at | 18.34 | 4.06E-06 | 1.095771903 | NM_003331| | TYK2,tyrosine kinase 2 |
| 225471_s_at | 18.33 | 4.08E-06 | 1.12947477 | NM_001626| | AKT2,v-akt murine thymoma viral oncogene homolog 2 |
| 219383_at | 18.33 | 4.08E-06 | 1.332243508 | NM_024841| | FLJ14213,hypothetical protein FLJ14213 |
| 225107_at | 18.32 | 4.10E-06 | 1.094337793 | NM_002137| | HNRPA2B1,heterogeneous nuclear ribonucleoprotein A2/B1 |
| 222570_at | 18.31 | 4.10E-06 | 1.14557092 | NM_014286| | FREQ,frequenin homolog |
| 230058_at | 18.31 | 4.10E-06 | 1.188253726 | NM_001039707| | NA |
| 225327_at | 18.27 | 4.15E-06 | 1.194270596 | NM_019600| | FLJ10980,hypothetical protein FLJ10980 |
| 203427_at | 18.27 | 4.15E-06 | 1.078910081 | NM_014034| | ASF1A,ASF1 anti-silencing function 1 homolog A |
| 201415_at | 18.27 | 4.15E-06 | 1.104157981 | NM_000178| | GSS,glutathione synthetase |
| 222131_x_at | 18.27 | 4.15E-06 | 1.041689832 | NM_138769| | RHOT2,ras homolog gene family, member T2 |
| 213434_at | 18.26 | 4.16E-06 | 1.086963836 | NM_001980| | EPIM,epimorphin isoform 1 |
| 232590_at | 18.25 | 4.17E-06 | 1.49718443 | NA |  |
| 203596_s_at | 18.25 | 4.17E-06 | 1.290478695 | NM_012420| | IFIT5,interferon-induced protein with |
| 1563983_at | 18.25 | 4.17E-06 | 1.041635054 | NA |  |
| 223104_at | 18.25 | 4.17E-06 | 1.083955109 | NM_032492| | JAGN1,jagunal homolog 1 |
| 210651_s_at | 18.24 | 4.18E-06 | 1.178008733 | NM_004442| | EPHB2,ephrin receptor EphB2 isoform 2 precursor |
| 218952_at | 18.24 | 4.19E-06 | 1.324280692 | NM_013271| | PCSK1N,proprotein convertase subtilisin/kexin type 1 |
| 202423_at | 18.24 | 4.19E-06 | 1.136040094 | NM_001099412| | NA |
| 219152_at | 18.24 | 4.18E-06 | 1.233131466 | NM_015720| | PODXL2,endoglycan |
| 224966_s_at | 18.23 | 4.19E-06 | 1.201272345 | NM_020175| | LOC56931,hypothetical protein from EUROIMAGE 1967720 |
| 200006_at | 18.22 | 4.21E-06 | 1.037556637 | NM_001123377| | NA |
| 238885_at | 18.21 | 4.22E-06 | 1.270942748 | NM_020910| | NA |
| 242186_x_at | 18.21 | 4.22E-06 | 1.879330684 | NM_015236| | LPHN3,latrophilin 3 precursor |
| 230998_at | 18.17 | 4.28E-06 | 1.281545632 | NA |  |
| 206448_at | 18.16 | 4.30E-06 | 1.45115964 | NM_014951| | NA |
| 235980_at | 18.15 | 4.31E-06 | 1.310513695 | NM_006218| | PIK3CA,phosphoinositide-3-kinase, catalytic, alpha |
| 210073_at | 18.14 | 4.33E-06 | 1.211833999 | NM_003034| | ST8SIA1,ST8 alpha-N-acetyl-neuraminide |
| 223183_at | 18.14 | 4.33E-06 | 1.748113518 | NM_001037553| | NA |
| 226184_at | 18.13 | 4.34E-06 | 1.089389417 | NM_052905| | FMNL2,formin-like 2 isoform B |
| 209268_at | 18.12 | 4.35E-06 | 1.084245259 | NM_007259| | VPS45A,vacuolar protein sorting 45A |
| 209068_at | 18.12 | 4.35E-06 | 1.095475373 | NM_031372| | HNRPDL,heterogeneous nuclear ribonucleoprotein D-like |
| 203456_at | 18.12 | 4.34E-06 | 1.199029905 | NM_007213| | PRAF2,JM4 protein |
| 225733_at | 18.12 | 4.34E-06 | 1.137592795 | NM_080605| | B3GALT6,UDP-Gal:betaGal beta 1,3-galactosyltransferase |
| 222516_at | 18.11 | 4.36E-06 | 1.035086567 | NM_012095| | AP3M1,adaptor-related protein complex 3, mu 1 subunit |
| 224662_at | 18.11 | 4.37E-06 | 1.116075677 | NM_004521| | KIF5B,kinesin family member 5B |
| 212850_s_at | 18.1 | 4.37E-06 | 1.18696371 | NM_002334| | NA |
| 203242_s_at | 18.1 | 4.37E-06 | 1.169674356 | NM_001011513| | PDLIM5,PDZ and LIM domain 5 isoform b |
| 221973_at | 18.1 | 4.38E-06 | 1.47017747 | NA |  |
| 221002_s_at | 18.1 | 4.37E-06 | 1.04790796 | NM_030927| | TM4SF14,transmembrane 4 superfamily member 14 |
| 212172_at | 18.09 | 4.39E-06 | 1.295707137 | NM_001625| | AK2,adenylate kinase 2 isoform a |
| 219522_at | 18.09 | 4.38E-06 | 1.126758404 | NM_014344| | FJX1,four jointed box 1 |
| 226696_at | 18.08 | 4.39E-06 | 1.071973432 | NM_006606| | RBBP9,retinoblastoma binding protein 9 |
| 203384_s_at | 18.08 | 4.40E-06 | 1.324154456 | NM_002077| | GOLGA1,golgin 97 |
| 213724_s_at | 18.07 | 4.41E-06 | 1.133613098 | NM_002611| | PDK2,pyruvate dehydrogenase kinase, isoenzyme 2 |
| 208030_s_at | 18.06 | 4.43E-06 | 1.082881361 | NM_001119| | ADD1,adducin 1 (alpha) isoform a |
| 212272_at | 18.05 | 4.44E-06 | 1.685033244 | NM_145693| | LPIN1,lipin 1 |
| 218437_s_at | 18.05 | 4.44E-06 | 1.24325033 | NM_020347| | LZTFL1,leucine zipper transcription factor-like 1 |
| 236836_at | 18.05 | 4.44E-06 | 1.210147221 | NA |  |
| 224513_s_at | 18.04 | 4.45E-06 | 1.047456212 | NM_020131| | UBQLN4,ataxin-1 ubiquitin-like interacting protein |
| 217720_at | 18.04 | 4.46E-06 | 1.069710001 | NM_016139| | CHCHD2,coiled-coil-helix-coiled-coil-helix domain |
| 227010_at | 18.03 | 4.46E-06 | 1.116108129 | NA |  |
| 212854_x_at | 18.03 | 4.46E-06 | 1.105488706 | NM_001039703| | NA |
| 235278_at | 18.03 | 4.47E-06 | 1.309575299 | NM_001033087| | NA |
| 233289_at | 18.02 | 4.48E-06 | 1.608128722 | NA |  |
| 229989_at | 18.02 | 4.48E-06 | 1.285666711 | NM_138378| | NA |
| 224893_at | 18.01 | 4.50E-06 | 1.116878981 | NM_015459| | DKFZP564J0863,DKFZP564J0863 protein |
| 208985_s_at | 18 | 4.51E-06 | 1.056065098 | NM_003758| | EIF3S1,eukaryotic translation initiation factor 3, |
| 227081_at | 18 | 4.50E-06 | 1.240504993 | NM_003462| | DNALI1,axonemal dynein light chain |
| 227580_s_at | 18 | 4.50E-06 | 1.169948372 | NM_015395| | DKFZP434B0335,DKFZP434B0335 protein |
| 202976_s_at | 17.99 | 4.52E-06 | 1.202674373 | NM_014899| | RHOBTB3,rho-related BTB domain containing 3 |
| 239014_at | 17.99 | 4.52E-06 | 1.435284783 | NM_018237| | CCAR1,cell-cycle and apoptosis regulatory protein 1 |
| 209355_s_at | 17.98 | 4.52E-06 | 1.158782343 | NM_003713| | PPAP2B,phosphatidic acid phosphatase type 2B |
| 205496_at | 17.98 | 4.52E-06 | 1.43161552 | NM_014702| | KIAA0408,KIAA0408 protein |
| 204175_at | 17.98 | 4.52E-06 | 1.062015561 | NM_015871| | ZNF593,zinc finger protein LOC51042 |
| 228904_at | 17.98 | 4.52E-06 | 1.769306733 | NM_002146| | HOXB3,homeo box B3 |
| 201046_s_at | 17.98 | 4.53E-06 | 1.116130931 | NM_005053| | RAD23A,UV excision repair protein RAD23 homolog A |
| 217832_at | 17.98 | 4.53E-06 | 1.078821735 | NM_006372| | SYNCRIP,synaptotagmin binding, cytoplasmic RNA |
| 213540_at | 17.98 | 4.52E-06 | 1.111971556 | NM_014234| | HSD17B8,estradiol 17 beta-dehydrogenase 8 |
| 205652_s_at | 17.97 | 4.54E-06 | 1.147333271 | NM_001008572| | TTLL1,tubulin tyrosine ligase-like family, member 1 |
| 201105_at | 17.97 | 4.54E-06 | 1.236885928 | NM_002305| | LGALS1,beta-galactosidase binding lectin precursor |
| 223042_s_at | 17.97 | 4.54E-06 | 1.03420815 | NM_023934| | FUNDC2,FUN14 domain containing 2 |
| 232053_x_at | 17.96 | 4.55E-06 | 1.28557889 | NM_001040456| | NA |
| 218664_at | 17.96 | 4.56E-06 | 1.111587085 | NM_001024732| | NA |
| 217973_at | 17.95 | 4.56E-06 | 1.148093804 | NM_016286| | DCXR,dicarbonyl/L-xylulose reductase |
| 212402_at | 17.94 | 4.59E-06 | 1.071901017 | NM_015070| | KIAA0853,KIAA0853 |
| 207160_at | 17.94 | 4.59E-06 | 1.276742058 | NM_000882| | IL12A,interleukin 12A precursor |
| 1557543_at | 17.94 | 4.58E-06 | 1.586116412 | NA |  |
| 230811_at | 17.93 | 4.59E-06 | 1.142228007 | NM_153025| | FLJ31606,hypothetical protein FLJ31606 |
| 209944_at | 17.93 | 4.59E-06 | 1.109058959 | NM_021188| | ZNF410,clones 23667 and 23775 zinc finger protein |
| 214723_x_at | 17.93 | 4.59E-06 | 1.306792995 | NM_025190| | NA |
| 201334_s_at | 17.93 | 4.59E-06 | 1.181195695 | NM_015313| | ARHGEF12,Rho guanine nucleotide exchange factor (GEF) 12 |
| 224525_s_at | 17.93 | 4.59E-06 | 1.521570821 | NM_001011708| | PTD004,GTP-binding protein PTD004 isoform 2 |
| 207761_s_at | 17.93 | 4.59E-06 | 1.057244501 | NM_014033| | DKFZP586A0522,DKFZP586A0522 protein |
| 235688_s_at | 17.92 | 4.60E-06 | 1.206477264 | NM_004295| | TRAF4,TNF receptor-associated factor 4 isoform 1 |
| 218337_at | 17.92 | 4.60E-06 | 1.114972647 | NM_022749| | RAI16,retinoic acid induced 16 |
| 225282_at | 17.92 | 4.60E-06 | 1.238098796 | NM_022733| | LOC64744,hypothetical protein AL133206 |
| 227499_at | 17.92 | 4.60E-06 | 1.159518979 | NA |  |
| 225521_at | 17.91 | 4.62E-06 | 1.296471759 | NM_016238| | ANAPC7,anaphase-promoting complex subunit 7 |
| 204318_s_at | 17.9 | 4.63E-06 | 1.065094148 | NM_016426| | GTSE1,G-2 and S-phase expressed 1 |
| 221922_at | 17.9 | 4.63E-06 | 1.251097681 | NM_013296| | GPSM2,G-protein signalling modulator 2 (AGS3-like, C. |
| 222805_at | 17.9 | 4.63E-06 | 1.219981214 | NM_024641| | MANEA,mannosidase, endo-alpha |
| 201765_s_at | 17.89 | 4.64E-06 | 1.110405322 | NM_000520| | HEXA,hexosaminidase A preproprotein |
| 226579_at | 17.88 | 4.65E-06 | 1.257165185 | NA |  |
| 226877_at | 17.88 | 4.65E-06 | 1.265639797 | NA |  |
| 214007_s_at | 17.87 | 4.66E-06 | 1.292526528 | NM_002822| | PTK9,twinfilin isoform 1 |
| 203518_at | 17.87 | 4.66E-06 | 1.656497804 | NM_000081| | LYST,lysosomal trafficking regulator isoform 1 |
| 238070_at | 17.87 | 4.67E-06 | 1.376364831 | NM_004284| | CHD1L,chromodomain helicase DNA binding protein |
| 41160_at | 17.87 | 4.67E-06 | 1.075399601 | NM_003926| | MBD3,methyl-CpG binding domain protein 3 |
| 224820_at | 17.87 | 4.67E-06 | 1.164266136 | NM_198076| | FAM36A,family with sequence similarity 36, member A |
| 221838_at | 17.86 | 4.69E-06 | 1.239758685 | NM_032775| | KELCHL,kelch-like |
| 203270_at | 17.86 | 4.67E-06 | 1.115264961 | NM_012145| | DTYMK,deoxythymidylate kinase (thymidylate kinase) |
| 242208_at | 17.86 | 4.68E-06 | 1.432986693 | NA |  |
| 216804_s_at | 17.85 | 4.69E-06 | 1.130134295 | NM_001011513| | PDLIM5,PDZ and LIM domain 5 isoform b |
| 214869_x_at | 17.85 | 4.69E-06 | 1.097227433 | NM_015635| | DKFZP434C212,DKFZP434C212 protein |
| 213346_at | 17.84 | 4.70E-06 | 1.085352239 | NM_138779| | LOC93081,hypothetical protein BC015148 |
| 217226_s_at | 17.82 | 4.74E-06 | 1.718160416 | NM_030971| | SFXN3,sideroflexin 3 |
| 223207_x_at | 17.82 | 4.73E-06 | 1.072328274 | NM_014172| | PHPT1,phosphohistidine phosphatase 1 |
| 212351_at | 17.82 | 4.73E-06 | 1.096468553 | NM_003907| | EIF2B5,eukaryotic translation initiation factor 2B, |
| 228743_at | 17.81 | 4.75E-06 | 1.244268442 | NM_032731| | TXNL5,thioredoxin-like 5 |
| 202731_at | 17.8 | 4.76E-06 | 1.283566006 | NM_014456| | PDCD4,programmed cell death 4 isoform 1 |
| 217944_at | 17.8 | 4.76E-06 | 1.109515448 | NM_017739| | FLJ20277,O-linked mannose |
| 212382_at | 17.79 | 4.78E-06 | 1.144167547 | NM_001083962| | NA |
| 1555471_a_at | 17.79 | 4.78E-06 | 1.520360759 | NM_020066| | FMN2,formin 2 |
| 210127_at | 17.79 | 4.78E-06 | 1.11271759 | NM_016577| | RAB6B,RAB6B, member RAS oncogene family |
| 233540_s_at | 17.78 | 4.80E-06 | 1.118057849 | NM_001011649| | CDK5RAP2,CDK5 regulatory subunit associated protein 2 |
| 201700_at | 17.78 | 4.80E-06 | 1.12305902 | NM_001760| | CCND3,cyclin D3 |
| 240344_x_at | 17.78 | 4.80E-06 | 1.104780174 | NM_181705| | LOC90624,hypothetical protein LOC90624 |
| 224331_s_at | 17.77 | 4.80E-06 | 1.07967767 | NM_032479| | MRPL36,mitochondrial ribosomal protein L36 |
| 1559946_s_at | 17.77 | 4.80E-06 | 1.038632753 | NM_006666| | RUVBL2,RuvB-like 2 |
| 224896_s_at | 17.77 | 4.80E-06 | 1.06282424 | NM_153712| | TTL,tubulin tyrosine ligase |
| 228790_at | 17.77 | 4.80E-06 | 1.225350591 | NM_147189| | MGC39325,hypothetical protein MGC39325 |
| 226416_at | 17.77 | 4.80E-06 | 1.131075527 | NM_153332| | 3'HEXO,3' exoribonuclease |
| 213931_at | 17.77 | 4.80E-06 | 1.172002906 | NM_002166| | ID2,inhibitor of DNA binding 2 |
| 243868_at | 17.77 | 4.80E-06 | 1.202672102 | NA |  |
| 1566509_s_at | 17.75 | 4.84E-06 | 1.10917671 | NM_012347| | FBXO9,F-box only protein 9 isoform 1 |
| 218601_at | 17.74 | 4.85E-06 | 1.089528833 | NM_001077663| | NA |
| 1557123_a_at | 17.74 | 4.86E-06 | 1.204498815 | NA |  |
| 208502_s_at | 17.73 | 4.87E-06 | 1.611667068 | NM_002653| | PITX1,paired-like homeodomain transcription factor 1 |
| 238861_at | 17.73 | 4.87E-06 | 1.354850552 | NA |  |
| 230821_at | 17.72 | 4.88E-06 | 1.313654961 | NM_021964| | ZNF148,zinc finger protein 148 (pHZ-52) |
| 201646_at | 17.72 | 4.87E-06 | 1.220345098 | NM_005506| | SCARB2,scavenger receptor class B, member 2 |
| 242052_at | 17.71 | 4.90E-06 | 1.329962834 | NA |  |
| 218380_at | 17.71 | 4.90E-06 | 1.096746708 | NM_001033053| | NA |
| 226077_at | 17.71 | 4.89E-06 | 1.041814989 | NM_144726| | FLJ31951,hypothetical protein FLJ31951 |
| 227549_x_at | 17.7 | 4.91E-06 | 1.097070559 | NM_207340| | LOC254359,hypothetical protein LOC254359 |
| 215942_s_at | 17.7 | 4.90E-06 | 1.084180883 | NM_016426| | GTSE1,G-2 and S-phase expressed 1 |
| 207048_at | 17.69 | 4.91E-06 | 1.342077797 | NM_014229| | SLC6A11,solute carrier family 6 (neurotransmitter |
| 200946_x_at | 17.68 | 4.92E-06 | 1.138701121 | NM_005271| | GLUD1,glutamate dehydrogenase 1 |
| 205078_at | 17.68 | 4.92E-06 | 1.057249528 | NM_002643| | PIGF,phosphatidylinositol glycan, class F isoform 1 |
| 1555009_a_at | 17.68 | 4.94E-06 | 1.380299725 | NM_003898| | SYNJ2,synaptojanin 2 |
| 226934_at | 17.66 | 4.97E-06 | 1.118714893 | NM_007007| | CPSF6,cleavage and polyadenylation specific factor 6, |
| 1557081_at | 17.65 | 4.98E-06 | 1.155635071 | NM_021239| | RBM25,RNA binding motif protein 25 |
| 210612_s_at | 17.65 | 4.98E-06 | 1.24569769 | NM_003898| | SYNJ2,synaptojanin 2 |
| 236866_at | 17.65 | 4.98E-06 | 1.155412723 | NM_000512| | GALNS,N-acetylgalactosamine-6-sulfatase precursor |
| 226189_at | 17.65 | 4.98E-06 | 1.4292119 | NM_002214| | ITGB8,integrin, beta 8 |
| 235890_at | 17.64 | 4.99E-06 | 1.249466062 | NA |  |
| 229153_at | 17.64 | 4.99E-06 | 1.155412531 | NM_032178| | FLJ13291,hypothetical protein FLJ13291 |
| 230286_at | 17.64 | 5.00E-06 | 1.646764678 | NA |  |
| 236976_at | 17.63 | 5.01E-06 | 1.264502987 | NM_000135| | FANCA,Fanconi anemia, complementation group A |
| 225722_at | 17.63 | 5.01E-06 | 1.099040053 | NA |  |
| 213008_at | 17.63 | 5.00E-06 | 1.08330935 | NM_001113378| | NA |
| 204424_s_at | 17.63 | 5.01E-06 | 1.17100666 | NM_001001395| | LMO3,LIM domain only 3 |
| 210424_s_at | 17.63 | 5.01E-06 | 1.19419922 | NM_001023567| | NA |
| 202118_s_at | 17.61 | 5.04E-06 | 1.168514806 | NM_003909| | CPNE3,copine III |
| 1560371_at | 17.61 | 5.05E-06 | 1.392906415 | NA |  |
| 228542_at | 17.6 | 5.05E-06 | 1.057116456 | NM_020662| | MRS2L,MRS2-like, magnesium homeostasis factor |
| 212922_s_at | 17.6 | 5.06E-06 | 1.134745192 | NM_020197| | SMYD2,SET and MYND domain containing 2 |
| 208999_at | 17.6 | 5.06E-06 | 1.12762115 | NM_001098811| | NA |
| 219384_s_at | 17.59 | 5.07E-06 | 1.068485637 | NM_012091| | ADAT1,adenosine deaminase, tRNA-specific 1 |
| 212710_at | 17.59 | 5.07E-06 | 1.068267991 | NM_015447| | CAMSAP1,calmodulin regulated spectrin-associated protein |
| 37226_at | 17.59 | 5.07E-06 | 1.098748598 | NM_001205| | BNIP1,BCL2/adenovirus E1B 19kD interacting protein 1 |
| 226031_at | 17.58 | 5.09E-06 | 1.063338459 | NM_017667| | NA |
| 1561691_at | 17.58 | 5.09E-06 | 1.734488587 | NA |  |
| 223614_at | 17.58 | 5.09E-06 | 1.159910741 | NA |  |
| 209575_at | 17.58 | 5.09E-06 | 1.387842922 | NM_000628| | IL10RB,interleukin 10 receptor, beta precursor |
| 204381_at | 17.58 | 5.09E-06 | 1.139981853 | NM_002333| | LRP3,low density lipoprotein receptor-related protein |
| 201860_s_at | 17.57 | 5.09E-06 | 1.208655508 | NM_000930| | PLAT,plasminogen activator, tissue type isoform 1 |
| 201805_at | 17.57 | 5.11E-06 | 1.082570141 | NM_002733| | PRKAG1,AMP-activated protein kinase, noncatalytic |
| 224860_at | 17.57 | 5.10E-06 | 1.197749178 | NM_033428| | NA |
| 214744_s_at | 17.57 | 5.11E-06 | 1.166415561 | NM_000978| | RPL23,ribosomal protein L23 |
| 229531_at | 17.56 | 5.12E-06 | 1.349252761 | NA |  |
| 226842_at | 17.56 | 5.12E-06 | 1.162850083 | NA |  |
| 1555912_at | 17.56 | 5.12E-06 | 1.243088276 | NA |  |
| 219254_at | 17.56 | 5.11E-06 | 1.253319645 | NM_024648| | FLJ22222,hypothetical protein FLJ22222 |
| 221845_s_at | 17.55 | 5.12E-06 | 1.224304165 | NM_030813| | SKD3,suppressor of potassium transport defect 3 |
| 219041_s_at | 17.55 | 5.12E-06 | 1.078100665 | NM_001099695| | NA |
| 239266_at | 17.54 | 5.15E-06 | 1.161268295 | NA |  |
| 223606_x_at | 17.52 | 5.17E-06 | 1.18490034 | NM_018559| | KIAA1704,KIAA1704 |
| 212388_at | 17.52 | 5.17E-06 | 1.076869176 | NM_015306| | NA |
| 205130_at | 17.52 | 5.18E-06 | 1.347161965 | NM_014226| | RAGE,MAPK/MAK/MRK overlapping kinase |
| 212954_at | 17.52 | 5.17E-06 | 1.212688831 | NM_003845| | DYRK4,dual-specificity tyrosine-(Y)-phosphorylation |
| 203719_at | 17.52 | 5.18E-06 | 1.088333279 | NM_001983| | ERCC1,excision repair cross-complementing 1 isofrom 2 |
| 208425_s_at | 17.52 | 5.17E-06 | 1.445977104 | NM_025185| | NA |
| 202850_at | 17.51 | 5.19E-06 | 1.137799655 | NM_001122674| | NA |
| 211052_s_at | 17.51 | 5.18E-06 | 1.061944683 | NM_005993| | TBCD,beta-tubulin cofactor D |
| 201911_s_at | 17.51 | 5.20E-06 | 1.090139487 | NM_001001715| | FARP1,FERM, RhoGEF, and pleckstrin domain protein 1 |
| 244391_at | 17.51 | 5.18E-06 | 1.115527416 | NM_025265| | SEN2L,hypothetical protein MGC2776 |
| 225139_at | 17.5 | 5.20E-06 | 1.098196974 | NA |  |
| 1555948_s_at | 17.5 | 5.20E-06 | 1.241423002 | NM_014612| | C9orf10,C9orf10 protein |
| 204842_x_at | 17.5 | 5.21E-06 | 1.075822783 | NM_004157| | PRKAR2A,cAMP-dependent protein kinase, regulatory |
| 217164_at | 17.5 | 5.20E-06 | 1.128619241 | NA |  |
| 231714_s_at | 17.48 | 5.23E-06 | 1.135614309 | NM_006594| | AP4B1,adaptor-related protein complex 4, beta 1 |
| 234583_at | 17.48 | 5.24E-06 | 1.536341784 | NM_006614| | CHL1,cell adhesion molecule with homology to L1CAM |
| 219324_at | 17.47 | 5.25E-06 | 1.116946553 | NM_001039141| | NA |
| 209513_s_at | 17.47 | 5.26E-06 | 1.127018141 | NM_032303| | HSDL2,hydroxysteroid dehydrogenase like 2 |
| 36499_at | 17.46 | 5.27E-06 | 1.122622065 | NM_001408| | CELSR2,cadherin EGF LAG seven-pass G-type receptor 2 |
| 224908_s_at | 17.45 | 5.29E-06 | 1.11205967 | NM_153712| | TTL,tubulin tyrosine ligase |
| 244015_at | 17.44 | 5.31E-06 | 1.206667895 | NA |  |
| 201093_x_at | 17.44 | 5.31E-06 | 1.125673247 | NM_004168| | SDHA,succinate dehydrogenase complex, subunit A, |
| 1559960_x_at | 17.44 | 5.30E-06 | 1.196539938 | NA |  |
| 225221_at | 17.44 | 5.31E-06 | 1.148654404 | NA |  |
| 213493_at | 17.43 | 5.32E-06 | 1.79661902 | NM_001080437| | NA |
| 232683_s_at | 17.43 | 5.31E-06 | 1.163856973 | NM_020214| | PARP6,poly (ADP-ribose) polymerase family, member 6 |
| 235803_at | 17.43 | 5.32E-06 | 1.631174581 | NA |  |
| 228841_at | 17.43 | 5.31E-06 | 1.108612436 | NM_181705| | LOC90624,hypothetical protein LOC90624 |
| 228760_at | 17.43 | 5.31E-06 | 1.069639626 | NM_032102| | SRP46,Splicing factor, arginine/serine-rich, 46kD |
| 225241_at | 17.42 | 5.34E-06 | 1.265020499 | NM_199511| | URB,steroid-sensitive protein 1 |
| 239841_at | 17.41 | 5.35E-06 | 1.131582903 | NA |  |
| 220669_at | 17.41 | 5.36E-06 | 1.341691597 | NM_001102653| | NA |
| 214596_at | 17.41 | 5.35E-06 | 1.310397211 | NM_000740| | CHRM3,cholinergic receptor, muscarinic 3 |
| 223095_at | 17.41 | 5.35E-06 | 1.165827236 | NA |  |
| 209637_s_at | 17.4 | 5.37E-06 | 1.46121211 | NM_002926| | RGS12,regulator of G-protein signalling 12 isoform 2 |
| 204248_at | 17.4 | 5.37E-06 | 1.146764406 | NM_002067| | GNA11,guanine nucleotide binding protein (G protein), |
| 221850_x_at | 17.39 | 5.38E-06 | 1.083596182 | NM_001077665| | NA |
| 210243_s_at | 17.38 | 5.40E-06 | 1.069172767 | NM_003779| | B4GALT3,UDP-Gal:betaGlcNAc beta 1,4- |
| 207618_s_at | 17.38 | 5.40E-06 | 1.142522965 | NM_001079866| | NA |
| 225055_at | 17.37 | 5.41E-06 | 1.234064061 | NA |  |
| 229885_at | 17.37 | 5.41E-06 | 1.112354388 | NA |  |
| 218188_s_at | 17.37 | 5.41E-06 | 1.033136029 | NM_012458| | TIMM13,translocase of inner mitochondrial membrane 13 |
| 242260_at | 17.35 | 5.47E-06 | 1.183793573 | NM_018834| | MATR3,matrin 3 |
| 214274_s_at | 17.34 | 5.48E-06 | 1.115157135 | NM_001607| | ACAA1,acetyl-Coenzyme A acyltransferase 1 |
| 241722_x_at | 17.33 | 5.50E-06 | 1.207292635 | NA |  |
| 227085_at | 17.33 | 5.50E-06 | 1.065044678 | NM_012412| | H2AFV,H2A histone family, member V isoform 1 |
| 237094_at | 17.32 | 5.51E-06 | 1.14423365 | NM_001082967| | NA |
| 228584_at | 17.32 | 5.51E-06 | 1.160558326 | NM_000232| | SGCB,sarcoglycan, beta (43kDa dystrophin-associated |
| 213653_at | 17.31 | 5.52E-06 | 1.197841298 | NM_019852| | METTL3,methyltransferase like 3 |
| 1552310_at | 17.3 | 5.54E-06 | 1.096365064 | NM_144597| | MGC29937,hypothetical protein MGC29937 |
| 1556110_at | 17.29 | 5.57E-06 | 1.170312105 | NA |  |
| 202650_s_at | 17.29 | 5.57E-06 | 1.091356665 | NM_014738| | KIAA0195,KIAA0195 gene product |
| 226488_at | 17.29 | 5.57E-06 | 1.116332819 | NM_001017919| | NA |
| 219354_at | 17.29 | 5.56E-06 | 1.165242955 | NM_018316| | FLJ11078,hypothetical protein FLJ11078 |
| 213359_at | 17.28 | 5.58E-06 | 1.156320892 | NM_001003810| | HNRPD,heterogeneous nuclear ribonucleoprotein D |
| 209711_at | 17.27 | 5.61E-06 | 1.170599034 | NM_015139| | SLC35D1,solute carrier family 35 (UDP-glucuronic |
| 1555154_a_at | 17.27 | 5.60E-06 | 1.203076522 | NM_006775| | QKI,quaking homolog, KH domain RNA binding isoform |
| 232064_at | 17.27 | 5.60E-06 | 1.344368756 | NA |  |
| 204227_s_at | 17.26 | 5.63E-06 | 1.17705928 | NM_004614| | TK2,thymidine kinase 2, mitochondrial |
| 1556346_at | 17.26 | 5.62E-06 | 1.263936832 | NA |  |
| 224615_x_at | 17.25 | 5.63E-06 | 1.068942576 | NM_030789| | HM13,minor histocompatibility antigen 13 isoform 1 |
| 202792_s_at | 17.25 | 5.63E-06 | 1.31973723 | NM_014678| | KIAA0685,KIAA0685 gene product |
| 225514_at | 17.25 | 5.63E-06 | 1.102962914 | NM_174913| | C14orf21,chromosome 14 open reading frame 21 |
| 219537_x_at | 17.24 | 5.65E-06 | 1.203539427 | NM_016941| | DLL3,delta-like 3 protein isoform 1 precursor |
| 219373_at | 17.24 | 5.65E-06 | 1.113760091 | NM_018973| | DPM3,dolichyl-phosphate mannosyltransferase |
| 242611_at | 17.23 | 5.67E-06 | 1.221173942 | NA |  |
| 202358_s_at | 17.23 | 5.66E-06 | 1.090972187 | NM_014758| | NA |
| 222941_at | 17.23 | 5.67E-06 | 1.142383826 | NA |  |
| 233341_s_at | 17.23 | 5.67E-06 | 1.096566997 | NM_019014| | POLR1B,RNA polymerase I polypeptide B |
| 211752_s_at | 17.23 | 5.68E-06 | 1.057860396 | NM_024407| | NDUFS7,NADH-ubiquinone oxidoreductase Fe-S protein 7 |
| 230088_at | 17.22 | 5.69E-06 | 1.488411507 | NA |  |
| 221307_at | 17.22 | 5.69E-06 | 1.811720907 | NM_001034837| | NA |
| 1569905_at | 17.21 | 5.71E-06 | 1.138574944 | NM_198533| | SCDR10,short-chain dehydrogenase/reductase 10 isoform |
| 211749_s_at | 17.21 | 5.70E-06 | 1.111648459 | NM_004781| | VAMP3,vesicle-associated membrane protein 3 |
| 239515_at | 17.21 | 5.69E-06 | 1.412621182 | NA |  |
| 215287_at | 17.2 | 5.71E-06 | 1.526814364 | NA |  |
| 219521_at | 17.2 | 5.72E-06 | 1.069034549 | NM_018644| | B3GAT1,beta-1,3-glucuronyltransferase 1 |
| 221507_at | 17.2 | 5.71E-06 | 1.105253571 | NM_013433| | TNPO2,transportin 2 (importin 3, karyopherin beta 2b) |
| 225594_at | 17.2 | 5.71E-06 | 1.107956056 | NM_001039618| | NA |
| 223020_at | 17.19 | 5.74E-06 | 1.095929182 | NM_030782| | CRR9,cisplatin resistance related protein CRR9p |
| 230904_at | 17.19 | 5.74E-06 | 1.429311685 | NM_031919| | CCDC10,cystatin and DUF19 domain-containing protein 1 |
| 233496_s_at | 17.18 | 5.75E-06 | 1.150059496 | NM_021914| | CFL2,cofilin 2 |
| 232910_at | 17.18 | 5.75E-06 | 1.240646602 | NA |  |
| 38340_at | 17.18 | 5.76E-06 | 1.149815582 | NM_003959| | HIP1R,huntingtin interacting protein-1-related |
| 200768_s_at | 17.17 | 5.77E-06 | 1.077364936 | NM_005911| | MAT2A,methionine adenosyltransferase II, alpha |
| 65884_at | 17.16 | 5.79E-06 | 1.108792454 | NM_016219| | MAN1B1,alpha 1,2-mannosidase |
| 209385_s_at | 17.15 | 5.83E-06 | 1.089536721 | NM_007198| | PROSC,proline synthetase co-transcribed homolog |
| 200790_at | 17.14 | 5.83E-06 | 1.104679384 | NM_002539| | ODC1,ornithine decarboxylase 1 |
| 1554547_at | 17.14 | 5.83E-06 | 1.589320953 | NM_001001971| | FAM13C1,family with sequence similarity 13, member C1 |
| 235079_at | 17.13 | 5.85E-06 | 1.413851843 | NA |  |
| 211685_s_at | 17.13 | 5.85E-06 | 1.206242418 | NM_001040624| | NA |
| 224417_at | 17.13 | 5.86E-06 | 1.426392045 | NA |  |
| 235672_at | 17.13 | 5.85E-06 | 1.203836176 | NM_033063| | MAP6,microtubule-associated protein 6 isoform 1 |
| 1558795_at | 17.13 | 5.86E-06 | 1.748793242 | NA |  |
| 226762_at | 17.12 | 5.87E-06 | 1.105841558 | NM_033224| | PURB,purine-rich element binding protein B |
| 214268_s_at | 17.12 | 5.87E-06 | 1.142936191 | NM_004687| | MTMR4,myotubularin related protein 4 |
| 226418_at | 17.12 | 5.87E-06 | 1.23943556 | NM_016570| | PTX1,CDA14 |
| 221883_at | 17.12 | 5.87E-06 | 1.146380815 | NM_004571| | PKNOX1,PBX/knotted 1 homeobox 1 isoform 1 |
| 226070_at | 17.12 | 5.86E-06 | 1.1191686 | NM_183241| | LOC286257,hypothetical protein LOC286257 |
| 220476_s_at | 17.11 | 5.89E-06 | 1.296174331 | NM_019099| | LOC55924,hypothetical protein LOC55924 isoform 1 |
| 201571_s_at | 17.11 | 5.89E-06 | 1.091861252 | NM_001012732| | NA |
| 231214_at | 17.11 | 5.89E-06 | 1.470975018 | NA |  |
| 227961_at | 17.11 | 5.88E-06 | 1.175348991 | NM_001908| | CTSB,cathepsin B preproprotein |
| 229448_at | 17.11 | 5.89E-06 | 1.263800673 | NM_021267| | LASS1,longevity assurance gene 1 isoform 1 |
| 223296_at | 17.1 | 5.90E-06 | 1.071698338 | NM_032315| | MGC4399,mitochondrial carrier protein MGC4399 |
| 232890_at | 17.08 | 5.94E-06 | 1.306999549 | NA |  |
| 218474_s_at | 17.08 | 5.96E-06 | 1.059457856 | NM_018992| | KCTD5,potassium channel tetramerisation domain |
| 227579_at | 17.07 | 5.98E-06 | 1.173235456 | NA |  |
| 220460_at | 17.07 | 5.97E-06 | 1.834899931 | NM_017435| | SLCO1C1,solute carrier organic anion transporter family, |
| 234049_at | 17.06 | 5.99E-06 | 1.244333911 | NA |  |
| 240258_at | 17.05 | 6.02E-06 | 1.363357654 | NM_001428| | ENO1,enolase 1 |
| 201491_at | 17.05 | 6.01E-06 | 1.050826498 | NM_012111| | AHSA1,AHA1, activator of heat shock 90kDa protein |
| 219001_s_at | 17.05 | 6.00E-06 | 1.125010218 | NM_024345| | MGC10765,hypothetical protein MGC10765 |
| 1554080_at | 17.04 | 6.03E-06 | 1.141833767 | NM_005444| | RQCD1,RCD1 required for cell differentiation1 homolog |
| 231883_at | 17.04 | 6.02E-06 | 1.219916637 | NM_012174| | FBXW8,F-box and WD-40 domain protein 8 isoform 2 |
| 232478_at | 17.04 | 6.02E-06 | 1.38775395 | NA |  |
| 202010_s_at | 17.03 | 6.05E-06 | 1.119512529 | NM_021188| | ZNF410,clones 23667 and 23775 zinc finger protein |
| 213656_s_at | 17.03 | 6.06E-06 | 1.06577797 | NM_005552| | KNS2,kinesin 2 60/70kDa |
| 201039_s_at | 17.02 | 6.06E-06 | 1.152972937 | NM_005053| | RAD23A,UV excision repair protein RAD23 homolog A |
| 36545_s_at | 17.02 | 6.07E-06 | 1.181380742 | NM_001007467| | SFI1,spindle assembly associated Sfi1 homolog isoform |
| 226795_at | 17.02 | 6.07E-06 | 1.219906922 | NA |  |
| 219377_at | 17.02 | 6.06E-06 | 1.140285458 | NM_022751| | C18orf11,chromosome 18 open reading frame 11 |
| 211499_s_at | 17.01 | 6.08E-06 | 1.321606952 | NM_002751| | MAPK11,mitogen-activated protein kinase 11 |
| 223286_at | 17.01 | 6.08E-06 | 1.031872097 | NM_015362| | DERP6,S-phase 2 protein |
| 53071_s_at | 17 | 6.10E-06 | 1.223999173 | NM_024648| | FLJ22222,hypothetical protein FLJ22222 |
| 228314_at | 17 | 6.11E-06 | 1.176094841 | NA |  |
| 222477_s_at | 16.99 | 6.13E-06 | 1.055352035 | NM_016551| | TM7SF3,transmembrane 7 superfamily member 3 |
| 208660_at | 16.98 | 6.14E-06 | 1.063479854 | NM_004077| | CS,citrate synthase precursor, isoform a |
| 233380_s_at | 16.97 | 6.17E-06 | 1.102545425 | NM_001040451| | NA |
| 225204_at | 16.97 | 6.16E-06 | 1.141491922 | NM_139283| | TA-PP2C,T-cell activation protein phosphatase 2C |
| 218476_at | 16.96 | 6.19E-06 | 1.099097028 | NM_001077365| | NA |
| 221942_s_at | 16.96 | 6.19E-06 | 1.541326994 | NM_000856| | GUCY1A3,guanylate cyclase 1, soluble, alpha 3 |
| 206748_s_at | 16.96 | 6.18E-06 | 1.332745095 | NM_003971| | SPAG9,sperm associated antigen 9 isoform 1 |
| 200941_at | 16.96 | 6.19E-06 | 1.046959403 | NM_001537| | HSBP1,heat shock factor binding protein 1 |
| 201408_at | 16.95 | 6.19E-06 | 1.175947582 | NM_002709| | PPP1CB,protein phosphatase 1, catalytic subunit, beta |
| 238617_at | 16.95 | 6.20E-06 | 1.385911447 | NA |  |
| 1553551_s_at | 16.94 | 6.21E-06 | 1.069549549 | NA |  |
| 201940_at | 16.94 | 6.21E-06 | 1.11293165 | NM_001304| | CPD,carboxypeptidase D precursor |
| 205055_at | 16.94 | 6.22E-06 | 1.065142749 | NM_002208| | ITGAE,integrin, alpha E (antigen CD103, human mucosal |
| 225571_at | 16.94 | 6.22E-06 | 1.344258831 | NM_002310| | LIFR, |
| 243134_at | 16.94 | 6.21E-06 | 1.609939538 | NA |  |
| 227424_x_at | 16.93 | 6.23E-06 | 1.226295116 | NA |  |
| 204187_at | 16.93 | 6.24E-06 | 1.414147707 | NM_006877| | GMPR,guanosine monophosphate reductase |
| 238554_at | 16.93 | 6.24E-06 | 1.135685325 | NM_030579| | CYB5-M,cytochrome b5 outer mitochondrial membrane |
| 217735_s_at | 16.93 | 6.23E-06 | 1.113733671 | NM_014413| | EIF2AK1,heme-regulated initiation factor 2-alpha kinase |
| 225382_at | 16.93 | 6.24E-06 | 1.254316514 | NM_001080485| | NA |
| 236219_at | 16.92 | 6.24E-06 | 1.283082059 | NA |  |
| 230791_at | 16.92 | 6.24E-06 | 2.173260656 | NA |  |
| 207551_s_at | 16.92 | 6.25E-06 | 1.110223616 | NM_006800| | MSL3L1,male-specific lethal 3-like 1 isoform c |
| 203570_at | 16.91 | 6.27E-06 | 1.187112796 | NM_005576| | LOXL1,lysyl oxidase-like 1 |
| 1552822_at | 16.91 | 6.27E-06 | 1.599988195 | NM_019022| | TXNDC10,thioredoxin domain containing 10 |
| 212256_at | 16.9 | 6.29E-06 | 1.156733638 | NM_017540| | GALNT10,GalNAc transferase 10 isoform b |
| 218229_s_at | 16.9 | 6.28E-06 | 1.101468404 | NM_017542| | POGK,pogo transposable element with KRAB domain |
| 228890_at | 16.89 | 6.31E-06 | 1.256087679 | NM_032827| | ATOH8,atonal homolog 8 |
| 239978_at | 16.89 | 6.31E-06 | 1.454725315 | NA |  |
| 213365_at | 16.89 | 6.31E-06 | 1.114012639 | NM_080663| | MGC16943,hypothetical protein MGC16943 |
| 212432_at | 16.88 | 6.33E-06 | 1.065130106 | NM_025196| | GRPEL1,GrpE-like 1, mitochondrial |
| 213241_at | 16.88 | 6.32E-06 | 1.27090153 | NM_005761| | PLXNC1,plexin C1 |
| 206501_x_at | 16.88 | 6.32E-06 | 1.200721199 | NM_004956| | ETV1,ets variant gene 1 |
| 218592_s_at | 16.88 | 6.32E-06 | 1.037628363 | NM_017829| | CECR5,cat eye syndrome chromosome region, candidate 5 |
| 211067_s_at | 16.88 | 6.32E-06 | 1.296182058 | NM_003644| | GAS7,growth arrest-specific 7 isoform a |
| 209236_at | 16.87 | 6.35E-06 | 1.127975015 | NM_005116| | SLC23A2,solute carrier family 23 (nucleobase |
| 220741_s_at | 16.87 | 6.35E-06 | 1.038667091 | NM_001034191| | NA |
| 218396_at | 16.87 | 6.33E-06 | 1.162177681 | NM_001018088| | NA |
| 201704_at | 16.87 | 6.35E-06 | 1.128711505 | NM_001114089| | NA |
| 219683_at | 16.87 | 6.34E-06 | 1.150974915 | NM_017412| | FZD3,frizzled 3 |
| 220346_at | 16.86 | 6.36E-06 | 1.359203555 | NM_001004346| | MTHFD2L,methylenetetrahydrofolate dehydrogenase (NADP+ |
| 211299_s_at | 16.85 | 6.39E-06 | 1.200467138 | NM_004475| | FLOT2,flotillin 2 |
| 204376_at | 16.85 | 6.39E-06 | 1.433247389 | NM_014703| | VprBP,Vpr-binding protein |
| 218198_at | 16.84 | 6.40E-06 | 1.097379045 | NM_018180| | DHX32,DEAD/H (Asp-Glu-Ala-Asp/His) box polypeptide 32 |
| 241425_at | 16.84 | 6.40E-06 | 1.244149826 | NM_001008564| | NUPL1,nucleoporin like 1 isoform b |
| 222666_s_at | 16.84 | 6.42E-06 | 1.121515514 | NM_005772| | RCL1,RNA cyclase homolog |
| 231806_s_at | 16.83 | 6.42E-06 | 1.09999497 | NM_015690| | STK36,serine/threonine kinase 36 (fused homolog, |
| 1554355_a_at | 16.83 | 6.43E-06 | 1.840060829 | NM_170601| | CSE-C,cytosolic sialic acid 9-O-acetylesterase |
| 219281_at | 16.83 | 6.42E-06 | 1.221597428 | NM_012331| | MSRA,methionine sulfoxide reductase A |
| 219646_at | 16.83 | 6.42E-06 | 1.103105421 | NM_017702| | FLJ20186,differentially expressed in FDCP 8 isoform 2 |
| 239238_at | 16.81 | 6.47E-06 | 1.277742157 | NA |  |
| 204843_s_at | 16.81 | 6.46E-06 | 1.189219659 | NM_004157| | PRKAR2A,cAMP-dependent protein kinase, regulatory |
| 238021_s_at | 16.8 | 6.49E-06 | 1.154014327 | NA |  |
| 228737_at | 16.8 | 6.49E-06 | 1.176020682 | NM_001098796| | NA |
| 201568_at | 16.79 | 6.52E-06 | 1.067010471 | NM_014402| | QP-C,low molecular mass ubiquinone-binding protein |
| 225193_at | 16.79 | 6.52E-06 | 1.104059244 | NA |  |
| 227908_at | 16.79 | 6.52E-06 | 1.119132283 | NM_020705| | NA |
| 220522_at | 16.79 | 6.50E-06 | 1.49537782 | NM_201253| | CRB1,crumbs homolog 1 isoform II precursor |
| 218515_at | 16.79 | 6.51E-06 | 1.069590462 | NM_013329| | C21orf66,GC-rich sequence DNA-binding factor candidate |
| 203687_at | 16.77 | 6.55E-06 | 1.183305895 | NM_002996| | CX3CL1,chemokine (C-X3-C motif) ligand 1 |
| 231650_s_at | 16.77 | 6.55E-06 | 1.440285793 | NM_021115| | SEZ6L,seizure related 6 homolog (mouse)-like |
| 202789_at | 16.76 | 6.59E-06 | 1.087604461 | NM_002660| | PLCG1,phospholipase C gamma 1 isoform a |
| 230884_s_at | 16.76 | 6.59E-06 | 1.149959968 | NM_003119| | SPG7,paraplegin isoform 1 |
| 226727_at | 16.75 | 6.60E-06 | 1.081505319 | NA |  |
| 202630_at | 16.75 | 6.60E-06 | 1.199667166 | NM_006380| | APPBP2,amyloid beta precursor protein-binding protein |
| 1555952_at | 16.75 | 6.60E-06 | 1.169722698 | NM_194255| | SLC19A1,solute carrier family 19 member 1 isoform a |
| 236229_at | 16.75 | 6.60E-06 | 1.563229233 | NA |  |
| 208609_s_at | 16.75 | 6.60E-06 | 1.190451225 | NM_019105| | TNXB,tenascin XB isoform 1 |
| 210560_at | 16.75 | 6.60E-06 | 1.297548743 | NM_001485| | GBX2,gastrulation brain homeo box 2 |
| 226125_at | 16.75 | 6.60E-06 | 1.283485983 | NA |  |
| 227584_at | 16.75 | 6.60E-06 | 1.073352518 | NM_020443| | NAV1,neuron navigator 1 |
| 231149_s_at | 16.74 | 6.62E-06 | 1.310392025 | NM_017886| | NA |
| 205972_at | 16.73 | 6.64E-06 | 1.254998713 | NM_006841| | SLC38A3,solute carrier family 38, member 3 |
| 216033_s_at | 16.73 | 6.64E-06 | 1.137105013 | NM_002037| | FYN,protein-tyrosine kinase fyn isoform a |
| 204131_s_at | 16.72 | 6.65E-06 | 1.064695503 | NM_001455| | FOXO3A,forkhead box O3A |
| 231779_at | 16.72 | 6.65E-06 | 1.341162521 | NM_001570| | IRAK2,interleukin-1 receptor-associated kinase 2 |
| 224631_at | 16.72 | 6.65E-06 | 1.132249564 | NM_053023| | ZFP91,zinc finger protein 91 isoform 1 |
| 229733_s_at | 16.72 | 6.65E-06 | 1.214251062 | NA |  |
| 209371_s_at | 16.72 | 6.65E-06 | 1.413098383 | NM_001122681| | NA |
| 221436_s_at | 16.72 | 6.65E-06 | 1.08763375 | NM_031299| | CDCA3,trigger of mitotic entry 1 |
| 229034_at | 16.71 | 6.67E-06 | 1.220249122 | NM_018013| | NA |
| 209265_s_at | 16.71 | 6.66E-06 | 1.064931558 | NM_019852| | METTL3,methyltransferase like 3 |
| 214765_s_at | 16.71 | 6.67E-06 | 1.277204205 | NM_001042402| | NA |
| 1569607_s_at | 16.71 | 6.67E-06 | 1.214027666 | NM_001012419| | LOC441425,OTTHUMP00000015360 |
| 230777_s_at | 16.7 | 6.69E-06 | 1.094920254 | NM_001040424| | NA |
| 222551_s_at | 16.7 | 6.70E-06 | 1.113065364 | NM_023080| | FLJ20989,hypothetical protein FLJ20989 |
| 226447_at | 16.7 | 6.68E-06 | 1.114203615 | NM_018489| | ASH1L,ash1 (absent, small, or homeotic)-like |
| 214110_s_at | 16.69 | 6.70E-06 | 1.25808498 | NA |  |
| 217986_s_at | 16.69 | 6.72E-06 | 1.217582696 | NM_013448| | BAZ1A,bromodomain adjacent to zinc finger domain, 1A |
| 244519_at | 16.68 | 6.74E-06 | 1.210460573 | NM_015338| | ASXL1,additional sex combs like 1 |
| 223743_s_at | 16.68 | 6.73E-06 | 1.145610127 | NM_015956| | MRPL4,mitochondrial ribosomal protein L4 isoform a |
| 210707_x_at | 16.68 | 6.73E-06 | 1.096998834 | NA |  |
| 215360_at | 16.68 | 6.72E-06 | 1.147490798 | NA |  |
| 213893_x_at | 16.67 | 6.74E-06 | 1.101934954 | NM_174930| | PMS2L5,postmeiotic segregation increased 2-like 5 |
| 231171_at | 16.66 | 6.79E-06 | 1.250675127 | NA |  |
| 205381_at | 16.66 | 6.78E-06 | 1.070518194 | NM_001031692| | NA |
| 239280_at | 16.66 | 6.78E-06 | 1.348610261 | NA |  |
| 213658_at | 16.65 | 6.80E-06 | 1.39824619 | NA |  |
| 232541_at | 16.65 | 6.79E-06 | 1.897093746 | NA |  |
| 203257_s_at | 16.65 | 6.79E-06 | 1.113266366 | NM_001003676| | MGC4707,MGC4707 protein isoform 1 |
| 1557370_s_at | 16.65 | 6.80E-06 | 1.270479484 | NM_015057| | MYCBP2,MYC binding protein 2 |
| 227101_at | 16.65 | 6.80E-06 | 1.472603491 | NM_176814| | LOC168850,hypothetical protein LOC168850 |
| 205151_s_at | 16.64 | 6.83E-06 | 1.130616674 | NM_014817| | NA |
| 227021_at | 16.63 | 6.84E-06 | 1.33808444 | NM_153042| | NA |
| 213812_s_at | 16.62 | 6.88E-06 | 1.069169442 | NM_006549| | CAMKK2,calcium/calmodulin-dependent protein kinase |
| 225939_at | 16.62 | 6.88E-06 | 1.212311295 | NM_173359| | NA |
| 217791_s_at | 16.61 | 6.90E-06 | 1.111521615 | NM_001017423| | NA |
| 208927_at | 16.61 | 6.89E-06 | 1.110878103 | NM_001007226| | SPOP,speckle-type POZ protein |
| 201774_s_at | 16.61 | 6.90E-06 | 1.091941635 | NM_014865| | CNAP1,chromosome condensation-related SMC-associated |
| 212570_at | 16.61 | 6.90E-06 | 1.203286951 | NM_015036| | NA |
| 205316_at | 16.61 | 6.90E-06 | 1.248901275 | NA |  |
| 205184_at | 16.6 | 6.92E-06 | 1.08469606 | NM_001098721| | NA |
| 228530_at | 16.6 | 6.91E-06 | 1.317748787 | NM_001071775| | NA |
| 225202_at | 16.59 | 6.95E-06 | 1.154902557 | NM_014899| | RHOBTB3,rho-related BTB domain containing 3 |
| 224791_at | 16.59 | 6.93E-06 | 1.065870014 | NM_018482| | DDEF1,development and differentiation enhancing factor |
| 224428_s_at | 16.58 | 6.96E-06 | 1.036342983 | NM_031942| | CDCA7,cell division cycle associated protein 7 isoform |
| 230150_at | 16.58 | 6.96E-06 | 1.444335862 | NM_001008405| | BCAP29,B-cell receptor-associated protein BAP29 isoform |
| 235201_at | 16.58 | 6.96E-06 | 1.493629094 | NM_014491| | FOXP2,forkhead box P2 isoform I |
| 241950_at | 16.57 | 7.00E-06 | 1.312961102 | NA |  |
| 209001_s_at | 16.57 | 6.99E-06 | 1.060721099 | NM_015391| | ANAPC13,anaphase promoting complex subunit 13 |
| 214000_s_at | 16.56 | 7.02E-06 | 1.170472092 | NM_001005339| | RGS10,regulator of G-protein signaling 10 isoform a |
| 212330_at | 16.56 | 7.01E-06 | 1.080388585 | NM_007111| | TFDP1,transcription factor Dp-1 |
| 228180_at | 16.56 | 7.01E-06 | 1.36951899 | NA |  |
| 222914_s_at | 16.56 | 7.01E-06 | 1.447393769 | NM_025268| | MGC4659,hole protein |
| 226179_at | 16.55 | 7.04E-06 | 1.110339608 | NM_016612| | MSCP,mitochondrial solute carrier protein |
| 1554640_at | 16.55 | 7.03E-06 | 1.377913087 | NM_001037293| | NA |
| 201191_at | 16.55 | 7.03E-06 | 1.141630883 | NM_006224| | PITPNA,phosphatidylinositol transfer protein, alpha |
| 230948_at | 16.55 | 7.04E-06 | 1.31309713 | NA |  |
| 203445_s_at | 16.55 | 7.04E-06 | 1.03619534 | NM_005730| | CTDSP2,nuclear LIM interactor-interacting factor 2 |
| 220500_s_at | 16.54 | 7.05E-06 | 1.23012116 | NM_001003789| | RABL2B,RAB, member of RAS oncogene family-like 2B |
| 213334_x_at | 16.53 | 7.08E-06 | 1.054479114 | NM_017518| | NA |
| 209389_x_at | 16.53 | 7.09E-06 | 1.072282444 | NM_001079862| | NA |
| 224759_s_at | 16.53 | 7.08E-06 | 1.066267165 | NM_152261| | MGC17943,hypothetical protein MGC17943 |
| 243417_at | 16.52 | 7.10E-06 | 1.864615945 | NA |  |
| 219122_s_at | 16.51 | 7.14E-06 | 1.110064231 | NM_017872| | ICF45,interphase cyctoplasmic foci protein 45 |
| 201012_at | 16.5 | 7.16E-06 | 1.231815785 | NM_000700| | ANXA1,annexin I |
| 236267_at | 16.49 | 7.17E-06 | 1.214262198 | NM_012279| | ZNF346,zinc finger protein 346 |
| 221946_at | 16.49 | 7.19E-06 | 1.117905445 | NM_001048265| | NA |
| 1564339_a_at | 16.48 | 7.21E-06 | 1.303268198 | NM_000740| | CHRM3,cholinergic receptor, muscarinic 3 |
| 226280_at | 16.48 | 7.21E-06 | 1.140733234 | NA |  |
| 224655_at | 16.47 | 7.23E-06 | 1.062391667 | NM_016282| | AK3L1,adenylate kinase 3 alpha like |
| 214693_x_at | 16.47 | 7.24E-06 | 1.183658177 | NM_001037501| | NA |
| 1559901_s_at | 16.46 | 7.26E-06 | 1.72702943 | NM_001005732| | C21orf34,chromosome 21 open reading frame 34 isoform a |
| 213726_x_at | 16.46 | 7.25E-06 | 1.051791983 | NM_006088| | TUBB2,tubulin, beta, 2 |
| 202181_at | 16.46 | 7.25E-06 | 1.155742321 | NM_014734| | KIAA0247,KIAA0247 |
| 1555953_at | 16.46 | 7.26E-06 | 1.242125503 | NM_030582| | COL18A1,alpha 1 type XVIII collagen isoform 1 precursor |
| 225840_at | 16.46 | 7.26E-06 | 1.163112945 | NM_003216| | TEF,thyrotrophic embryonic factor |
| 1559496_at | 16.46 | 7.26E-06 | 1.49497589 | NM_001034191| | NA |
| 228361_at | 16.45 | 7.28E-06 | 1.139343081 | NM_004091| | E2F2,E2F transcription factor 2 |
| 230465_at | 16.45 | 7.27E-06 | 1.652609626 | NM_012262| | HS2ST1,heparan sulfate 2-O-sulfotransferase 1 |
| 1554085_at | 16.45 | 7.28E-06 | 1.151083732 | NM_175066| | DDX51,DEAD (Asp-Glu-Ala-Asp) box polypeptide 51 |
| 202119_s_at | 16.45 | 7.29E-06 | 1.057729844 | NM_003909| | CPNE3,copine III |
| 225915_at | 16.43 | 7.34E-06 | 1.266980922 | NM_001079670| | NA |
| 217770_at | 16.43 | 7.35E-06 | 1.059699071 | NM_015937| | PIGT,phosphatidylinositol glycan, class T |
| 239848_at | 16.42 | 7.35E-06 | 1.833882835 | NA |  |
| 222364_at | 16.41 | 7.38E-06 | 1.344519468 | NM_080546| | CDW92,CDW92 antigen |
| 213130_at | 16.41 | 7.38E-06 | 1.09704372 | NM_001006656| | ZNF473,zinc finger protein 473 |
| 211922_s_at | 16.41 | 7.38E-06 | 1.193654823 | NM_001752| | CAT,catalase |
| 225560_at | 16.41 | 7.38E-06 | 1.155044421 | NM_013382| | POMT2,putative protein O-mannosyltransferase |
| 243931_at | 16.4 | 7.42E-06 | 1.3136435 | NA |  |
| 214126_at | 16.4 | 7.39E-06 | 1.156657479 | NA |  |
| 209149_s_at | 16.4 | 7.39E-06 | 1.144773456 | NM_001014842| | NA |
| 1554089_s_at | 16.39 | 7.42E-06 | 1.088459478 | NM_016038| | SBDS,Shwachman-Bodian-Diamond syndrome protein |
| 231117_at | 16.38 | 7.45E-06 | 1.479887991 | NM_138344| | C14orf152,chromosome 14 open reading frame 152 |
| 215434_x_at | 16.38 | 7.46E-06 | 1.16528581 | NM_001037501| | NA |
| 239512_at | 16.38 | 7.47E-06 | 1.483928449 | NM_005626| | SFRS4,splicing factor, arginine/serine-rich 4 |
| 222463_s_at | 16.36 | 7.50E-06 | 1.15153849 | NM_012104| | BACE1,beta-site APP-cleaving enzyme 1 isoform A |
| 1555920_at | 16.36 | 7.50E-06 | 1.270227181 | NM_007276| | CBX3,chromobox homolog 3 |
| 205433_at | 16.36 | 7.51E-06 | 1.38486736 | NM_000055| | BCHE,butyrylcholinesterase precursor |
| 228656_at | 16.35 | 7.53E-06 | 1.42416426 | NM_002763| | PROX1,prospero-related homeobox 1 |
| 1553218_a_at | 16.35 | 7.54E-06 | 1.123299958 | NM_032434| | ZNF512,zinc finger protein 512 |
| 243740_at | 16.35 | 7.52E-06 | 1.1829439 | NA |  |
| 226821_at | 16.34 | 7.55E-06 | 1.119484696 | NA |  |
| 221837_at | 16.33 | 7.58E-06 | 1.284573963 | NM_032775| | KELCHL,kelch-like |
| 212656_at | 16.33 | 7.59E-06 | 1.062989613 | NM_005726| | TSFM,Ts translation elongation factor, mitochondrial |
| 202069_s_at | 16.33 | 7.60E-06 | 1.118478557 | NM_005530| | IDH3A,isocitrate dehydrogenase 3 (NAD+) alpha |
| 207627_s_at | 16.32 | 7.62E-06 | 1.122167111 | NM_005653| | TFCP2,transcription factor CP2 |
| 224680_at | 16.32 | 7.61E-06 | 1.032192711 | NM_182547| | TMED4,transmembrane emp24 protein transport domain |
| 209558_s_at | 16.31 | 7.65E-06 | 1.258348889 | NM_003959| | HIP1R,huntingtin interacting protein-1-related |
| 226166_x_at | 16.31 | 7.64E-06 | 1.097580435 | NM_015690| | STK36,serine/threonine kinase 36 (fused homolog, |
| 207000_s_at | 16.3 | 7.67E-06 | 1.188437604 | NM_005605| | PPP3CC,protein phosphatase 3 (formerly 2B), catalytic |
| 226663_at | 16.3 | 7.67E-06 | 1.154222459 | NM_017664| | ANKRD10,ankyrin repeat domain 10 |
| 217800_s_at | 16.3 | 7.67E-06 | 1.132936923 | NM_030571| | NDFIP1,Nedd4 family interacting protein 1 |
| 239024_at | 16.29 | 7.70E-06 | 1.26973101 | NM_021964| | ZNF148,zinc finger protein 148 (pHZ-52) |
| 203467_at | 16.29 | 7.68E-06 | 1.074679584 | NM_002676| | PMM1,phosphomannomutase 1 |
| 205521_at | 16.29 | 7.68E-06 | 1.16809781 | NM_005107| | ENDOGL1,endonuclease G-like 1 |
| 201490_s_at | 16.28 | 7.72E-06 | 1.152739033 | NM_005729| | PPIF,peptidylprolyl isomerase F precursor |
| 235376_at | 16.28 | 7.71E-06 | 1.349893667 | NA |  |
| 225843_at | 16.27 | 7.73E-06 | 1.076647335 | NM_001077268| | NA |
| 1552378_s_at | 16.27 | 7.73E-06 | 1.455035347 | NM_172037| | RDH10,retinol dehydrogenase 10 |
| 236511_at | 16.27 | 7.74E-06 | 1.528850803 | NA |  |
| 226777_at | 16.26 | 7.79E-06 | 1.302176948 | NA |  |
| 221713_s_at | 16.25 | 7.79E-06 | 1.16364997 | NM_024871| | FLJ12748,hypothetical protein FLJ12748 |
| 207621_s_at | 16.25 | 7.80E-06 | 1.070578507 | NM_007169| | PEMT,phosphatidylethanolamine N-methyltransferase |
| 1558388_a_at | 16.24 | 7.83E-06 | 1.672312164 | NA |  |
| 234926_s_at | 16.23 | 7.84E-06 | 1.074471187 | NM_016407| | C20orf43,chromosome 20 open reading frame 43 |
| 217844_at | 16.22 | 7.88E-06 | 1.108919524 | NM_021198| | CTDSP1,CTD (carboxy-terminal domain, RNA polymerase II, |
| 203367_at | 16.22 | 7.90E-06 | 1.089386488 | NM_007026| | DUSP14,dual specificity phosphatase 14 |
| 210425_x_at | 16.21 | 7.92E-06 | 1.214791291 | NM_001023567| | NA |
| 1561660_at | 16.21 | 7.92E-06 | 1.44988783 | NM_001012755| | NA |
| 200974_at | 16.2 | 7.93E-06 | 1.076898506 | NM_001613| | ACTA2,alpha 2 actin |
| 227148_at | 16.2 | 7.94E-06 | 1.352831676 | NM_172069| | PLEKHH2,pleckstrin homology domain containing, family H |
| 224855_at | 16.19 | 7.98E-06 | 1.07663355 | NM_013328| | PYCR2,pyrroline-5-carboxylate reductase family, member |
| 202967_at | 16.19 | 7.96E-06 | 1.077361743 | NM_001512| | GSTA4,glutathione S-transferase A4 |
| 215707_s_at | 16.18 | 7.98E-06 | 1.261616313 | NM_000311| | PRNP,prion protein preproprotein |
| 219019_at | 16.18 | 8.00E-06 | 1.113619249 | NM_018494| | LRDD,leucine rich repeat and death domain containing |
| 215785_s_at | 16.18 | 8.00E-06 | 1.05587616 | NM_001037332| | NA |
| 213604_at | 16.17 | 8.01E-06 | 1.072126859 | NA |  |
| 207805_s_at | 16.17 | 8.00E-06 | 1.053074001 | NM_002813| | PSMD9,proteasome 26S non-ATPase subunit 9 |
| 236298_at | 16.16 | 8.04E-06 | 1.136455227 | NM_014317| | TPRT,trans-prenyltransferase |
| 204489_s_at | 16.16 | 8.04E-06 | 1.236901622 | NM_000610| | CD44,CD44 antigen isoform 1 precursor |
| 235606_at | 16.16 | 8.04E-06 | 1.390423992 | NA |  |
| 205227_at | 16.15 | 8.06E-06 | 1.24618018 | NM_002182| | IL1RAP,interleukin 1 receptor accessory protein isoform |
| 231767_at | 16.15 | 8.06E-06 | 1.256840402 | NM_024015| | HOXB4,homeo box B4 |
| 1555226_s_at | 16.15 | 8.06E-06 | 1.10585796 | NM_001098616| | NA |
| 1558678_s_at | 16.14 | 8.10E-06 | 1.132327052 | NA |  |
| 206502_s_at | 16.14 | 8.10E-06 | 1.618648271 | NM_002196| | INSM1,insulinoma-associated 1 |
| 232473_at | 16.14 | 8.11E-06 | 1.500831592 | NM_003675| | PRPF18,PRP18 pre-mRNA processing factor 18 homolog |
| 212416_at | 16.14 | 8.10E-06 | 1.058454489 | NM_004866| | SCAMP1,secretory carrier membrane protein 1 isoform 1 |
| 224584_at | 16.13 | 8.12E-06 | 1.032577346 | NM_001009923| | C20orf30,chromosome 20 open reading frame 30 isoform 1 |
| 216551_x_at | 16.13 | 8.14E-06 | 1.102775545 | NM_002660| | PLCG1,phospholipase C gamma 1 isoform a |
| 235381_at | 16.12 | 8.14E-06 | 1.124812716 | NA |  |
| 235543_at | 16.12 | 8.14E-06 | 1.234599733 | NA |  |
| 225236_at | 16.12 | 8.14E-06 | 1.110094209 | NM_033117| | RBM18,RNA binding motif protein 18 |
| 38241_at | 16.11 | 8.17E-06 | 1.54386204 | NM_006994| | BTN3A3,butyrophilin, subfamily 3, member A3 isoform a |
| 226120_at | 16.11 | 8.17E-06 | 1.109428805 | NM_144596| | TTC8,tetratricopeptide repeat domain 8 isoform A |
| 201494_at | 16.11 | 8.18E-06 | 1.049225131 | NM_005040| | PRCP,prolylcarboxypeptidase isoform 1 preproprotein |
| 225361_x_at | 16.1 | 8.20E-06 | 1.13512764 | NM_145284| | LOC159090,similar to hypothetical protein MGC17347 |
| 229319_at | 16.1 | 8.20E-06 | 1.167144105 | NA |  |
| 227189_at | 16.1 | 8.20E-06 | 1.43012405 | NM_020939| | CPNE5,copine V |
| 202701_at | 16.09 | 8.24E-06 | 1.203746707 | NM_001199| | BMP1,bone morphogenetic protein 1 isoform 1, |
| 208640_at | 16.09 | 8.23E-06 | 1.027843682 | NM_006908| | RAC1,ras-related C3 botulinum toxin substrate 1 |
| 1568795_at | 16.09 | 8.23E-06 | 1.61708945 | NA |  |
| 203660_s_at | 16.08 | 8.26E-06 | 1.063401697 | NM_006031| | PCNT2,pericentrin B |
| 229537_at | 16.08 | 8.25E-06 | 1.341470777 | NA |  |
| 244058_at | 16.08 | 8.27E-06 | 1.170442007 | NM_001031746| | NA |
| 1554260_a_at | 16.07 | 8.28E-06 | 1.190120806 | NM_015030| | NA |
| 201618_x_at | 16.07 | 8.28E-06 | 1.124742092 | NM_003801| | GPAA1,anchor attachment protein 1 |
| 221620_s_at | 16.07 | 8.28E-06 | 1.053647297 | NM_024122| | MGC4825,hypothetical protein MGC4825 |
| 235427_at | 16.07 | 8.30E-06 | 1.388106903 | NA |  |
| 204930_s_at | 16.06 | 8.32E-06 | 1.11588297 | NM_001205| | BNIP1,BCL2/adenovirus E1B 19kD interacting protein 1 |
| 1556340_at | 16.06 | 8.32E-06 | 1.107306876 | NM_002969| | MAPK12,mitogen-activated protein kinase 12 |
| 201481_s_at | 16.05 | 8.35E-06 | 1.077775377 | NM_002862| | PYGB,brain glycogen phosphorylase |
| 218362_s_at | 16.05 | 8.34E-06 | 1.753023896 | NM_014953| | KIAA1008,KIAA1008 |
| 205453_at | 16.05 | 8.35E-06 | 1.379104883 | NM_002145| | HOXB2,homeo box B2 |
| 212933_x_at | 16.04 | 8.36E-06 | 1.019433394 | NM_000977| | RPL13,ribosomal protein L13 |
| 201966_at | 16.04 | 8.36E-06 | 1.038926451 | NM_004550| | NDUFS2,NADH dehydrogenase (ubiquinone) Fe-S protein 2, |
| 222258_s_at | 16.04 | 8.38E-06 | 1.056055666 | NM_014521| | SH3BP4,SH3-domain binding protein 4 |
| 224057_s_at | 16.03 | 8.39E-06 | 1.059832551 | NM_015963| | THAP4,THAP domain containing 4 |
| 228950_s_at | 16.03 | 8.40E-06 | 1.351507634 | NM_001002292| | FLJ23091,putative NFkB activating protein 373 isoform 2 |
| 238929_at | 16.03 | 8.40E-06 | 1.170675693 | NM_032102| | SRP46,Splicing factor, arginine/serine-rich, 46kD |
| 238802_at | 16.02 | 8.43E-06 | 1.155043138 | NM_001040273| | NA |
| 218874_s_at | 16.02 | 8.42E-06 | 1.287497737 | NM_001031722| | NA |
| 229610_at | 16.01 | 8.45E-06 | 1.091443379 | NM_152515| | FLJ40629,hypothetical protein FLJ40629 |
| 203117_s_at | 16.01 | 8.47E-06 | 1.166676783 | NM_014871| | USP52,ubiquitin specific protease 52 |
| 202959_at | 16.01 | 8.47E-06 | 1.043839921 | NM_000255| | MUT,methylmalonyl Coenzyme A mutase precursor |
| 223641_at | 16.01 | 8.45E-06 | 1.22032713 | NA |  |
| 209625_at | 16 | 8.49E-06 | 1.225512417 | NM_004569| | PIGH,phosphatidylinositol glycan, class H |
| 1563111_a_at | 15.99 | 8.52E-06 | 1.192309479 | NM_017861| | PIGX,GPI-mannosyltransferase subunit |
| 224534_at | 15.99 | 8.52E-06 | 1.624960753 | NM_001039570| | NA |
| 217772_s_at | 15.99 | 8.52E-06 | 1.037973998 | NM_014342| | MTCH2,mitochondrial carrier homolog 2 |
| 212191_x_at | 15.99 | 8.53E-06 | 1.013658101 | NM_000977| | RPL13,ribosomal protein L13 |
| 227750_at | 15.99 | 8.53E-06 | 1.108740116 | NM_001024660| | NA |
| 240606_at | 15.99 | 8.53E-06 | 1.197203886 | NA |  |
| 203357_s_at | 15.99 | 8.52E-06 | 1.185670075 | NM_014296| | CAPN7,calpain 7 |
| 226606_s_at | 15.98 | 8.53E-06 | 1.41038123 | NM_015666| | GTPBP5,GTP binding protein 5 |
| 203247_s_at | 15.98 | 8.53E-06 | 1.080246923 | NM_006965| | ZNF24,zinc finger protein 24 (KOX 17) |
| 212250_at | 15.97 | 8.58E-06 | 1.096608543 | NM_178812| | LYRIC,LYRIC/3D3 |
| 226790_at | 15.96 | 8.61E-06 | 1.117912326 | NM_194270| | MOPT,protein containing single MORN motif in testis |
| 215012_at | 15.96 | 8.61E-06 | 1.192604636 | NM_001031623| | NA |
| 1555848_at | 15.95 | 8.64E-06 | 1.308238312 | NA |  |
| 236916_at | 15.95 | 8.63E-06 | 1.222096098 | NA |  |
| 1555906_s_at | 15.95 | 8.65E-06 | 1.121215813 | NM_001029839| | NA |
| 236004_at | 15.95 | 8.63E-06 | 1.139056687 | NA |  |
| 202867_s_at | 15.94 | 8.65E-06 | 1.129997134 | NM_001002762| | DNAJB12,DnaJ (Hsp40) homolog, subfamily B, member 12 |
| 1554410_a_at | 15.94 | 8.68E-06 | 1.13751715 | NM_030798| | WBSCR16,RCC1-like G exchanging factor-like isoform 1 |
| 223348_x_at | 15.94 | 8.66E-06 | 1.159541252 | NM_032853| | MUM1,melanoma ubiquitous mutated protein |
| 227567_at | 15.93 | 8.68E-06 | 1.161506411 | NM_001033569| | NA |
| 214531_s_at | 15.93 | 8.68E-06 | 1.1015368 | NM_003099| | SNX1,sorting nexin 1 isoform a |
| 1558796_a_at | 15.93 | 8.68E-06 | 1.538029337 | NA |  |
| 45687_at | 15.93 | 8.70E-06 | 1.140892329 | NM_024031| | MGC3121,hypothetical protein MGC3121 |
| 1554159_a_at | 15.93 | 8.70E-06 | 1.220529849 | NM_006624| | ZMYND11,zinc finger, MYND domain containing 11 isoform |
| 220183_s_at | 15.92 | 8.74E-06 | 1.151243619 | NM_007083| | NUDT6,nudix-type motif 6 isoform a |
| 233224_at | 15.92 | 8.73E-06 | 1.422282188 | NA |  |
| 244680_at | 15.92 | 8.71E-06 | 1.448745634 | NM_000824| | GLRB,glycine receptor, beta |
| 227776_at | 15.9 | 8.81E-06 | 1.099030423 | NM_018367| | PHCA,phytoceramidase, alkaline |
| 224335_s_at | 15.9 | 8.78E-06 | 1.222656913 | NM_012104| | BACE1,beta-site APP-cleaving enzyme 1 isoform A |
| 235023_at | 15.9 | 8.78E-06 | 1.495039806 | NM_001018088| | NA |
| 203720_s_at | 15.89 | 8.84E-06 | 1.106459957 | NM_001983| | ERCC1,excision repair cross-complementing 1 isofrom 2 |
| 242239_at | 15.88 | 8.86E-06 | 1.477166585 | NA |  |
| 236270_at | 15.86 | 8.93E-06 | 1.123728199 | NM_004554| | NFATC4,cytoplasmic nuclear factor of activated T-cells |
| 223320_s_at | 15.86 | 8.91E-06 | 1.233224305 | NM_012089| | ABCB10,ATP-binding cassette, sub-family B, member 10 |
| 208066_s_at | 15.86 | 8.91E-06 | 1.046142754 | NM_001514| | GTF2B,general transcription factor IIB |
| 228830_s_at | 15.86 | 8.91E-06 | 1.12265895 | NM_006856| | ATF7,activating transcription factor 7 |
| 213449_at | 15.86 | 8.92E-06 | 1.204666961 | NM_015029| | POP1,processing of precursor 1, ribonuclease P/MRP |
| 229287_at | 15.86 | 8.91E-06 | 1.203926945 | NM_014982| | PCNX,pecanex homolog |
| 229986_at | 15.86 | 8.91E-06 | 1.39030161 | NA |  |
| 203116_s_at | 15.85 | 8.95E-06 | 1.067932778 | NM_000140| | FECH,ferrochelatase isoform b precursor |
| 221566_s_at | 15.85 | 8.95E-06 | 1.280521183 | NM_003946| | NOL3,nucleolar protein 3 |
| 201661_s_at | 15.85 | 8.94E-06 | 1.072390277 | NM_004457| | ACSL3,acyl-CoA synthetase long-chain family member 3 |
| 236821_at | 15.85 | 8.94E-06 | 1.216804463 | NA |  |
| 215123_at | 15.85 | 8.94E-06 | 1.236148755 | NA |  |
| 211630_s_at | 15.85 | 8.94E-06 | 1.122577482 | NM_000178| | GSS,glutathione synthetase |
| 232632_at | 15.84 | 8.96E-06 | 1.628381351 | NA |  |
| 244803_at | 15.84 | 8.97E-06 | 1.284274208 | NA |  |
| 230999_at | 15.83 | 8.99E-06 | 1.101405541 | NA |  |
| 204955_at | 15.83 | 9.00E-06 | 1.129644837 | NM_006307| | SRPX,sushi-repeat-containing protein, X-linked |
| 207396_s_at | 15.83 | 9.01E-06 | 1.064834967 | NM_005787| | ALG3,alpha-1,3-mannosyltransferase ALG3 isoform a |
| 220354_at | 15.82 | 9.02E-06 | 1.267422104 | NA |  |
| 208878_s_at | 15.81 | 9.05E-06 | 1.127468909 | NM_002577| | PAK2,p21-activated kinase 2 |
| 237400_at | 15.81 | 9.06E-06 | 1.436859346 | NM_001003803| | ATP5S,ATP synthase, H+ transporting, mitochondrial F0 |
| 235259_at | 15.8 | 9.09E-06 | 1.364797942 | NM_145048| | MGC29898,hypothetical protein MGC29898 |
| 229163_at | 15.8 | 9.09E-06 | 1.401369216 | NM_018584| | CaMKIINalpha,calcium/calmodulin-dependent protein kinase II |
| 241091_at | 15.79 | 9.12E-06 | 1.200512857 | NA |  |
| 206752_s_at | 15.79 | 9.14E-06 | 1.115043812 | NM_004402| | DFFB,DNA fragmentation factor, 40 kD, beta |
| 201864_at | 15.79 | 9.12E-06 | 1.106077492 | NM_001493| | GDI1,GDP dissociation inhibitor 1 |
| 225391_at | 15.78 | 9.18E-06 | 1.039531423 | NA |  |
| 222233_s_at | 15.78 | 9.18E-06 | 1.078755265 | NM_001033855| | NA |
| 223494_at | 15.77 | 9.21E-06 | 1.188964512 | NM_012215| | MGEA5,meningioma expressed antigen 5 (hyaluronidase) |
| 216997_x_at | 15.77 | 9.18E-06 | 1.413141695 | NM_007005| | TLE4,transducin-like enhancer protein 4 |
| 235652_at | 15.77 | 9.21E-06 | 1.729135851 | NA |  |
| 220605_s_at | 15.76 | 9.24E-06 | 1.15860114 | NM_012237| | SIRT2,sirtuin 2 isoform 1 |
| 213416_at | 15.76 | 9.23E-06 | 1.457345752 | NM_000885| | ITGA4,integrin alpha 4 precursor |
| 205079_s_at | 15.76 | 9.23E-06 | 1.072606192 | NM_003829| | MPDZ,multiple PDZ domain protein |
| 211998_at | 15.75 | 9.26E-06 | 1.074359006 | NM_002107| | H3F3A,H3 histone, family 3A |
| 203943_at | 15.75 | 9.25E-06 | 1.101170555 | NM_004798| | KIF3B,kinesin family member 3B |
| 223288_at | 15.74 | 9.29E-06 | 1.086362499 | NM_032557| | USP38,ubiquitin specific protease 38 |
| 1556328_at | 15.74 | 9.31E-06 | 1.754401571 | NA |  |
| 239585_at | 15.73 | 9.32E-06 | 1.449178424 | NM_003884| | PCAF,p300/CBP-associated factor |
| 241702_at | 15.72 | 9.35E-06 | 1.319872369 | NA |  |
| 224068_x_at | 15.72 | 9.38E-06 | 1.066073607 | NM_018047| | RBM22,RNA binding motif protein 22 |
| 218253_s_at | 15.71 | 9.39E-06 | 1.065337394 | NM_006893| | LGTN,ligatin |
| 206414_s_at | 15.71 | 9.40E-06 | 1.080836382 | NM_003887| | DDEF2,development- and differentiation-enhancing |
| 229395_at | 15.71 | 9.40E-06 | 1.121855218 | NM_004604| | STX4A,syntaxin 4A (placental) |
| 223499_at | 15.7 | 9.44E-06 | 1.224081188 | NM_015645| | C1QTNF5,C1q and tumor necrosis factor related protein 5 |
| 1555882_at | 15.7 | 9.44E-06 | 1.181932079 | NM_001010862| | SPIN3,spindlin family, member 3 |
| 240066_at | 15.7 | 9.44E-06 | 1.749414275 | NA |  |
| 203016_s_at | 15.69 | 9.45E-06 | 1.06927989 | NM_014021| | SSX2IP,synovial sarcoma, X breakpoint 2 interacting |
| 225876_at | 15.69 | 9.47E-06 | 1.152625734 | NM_020448| | DJ462O23.2,hypothetical protein dJ462O23.2 |
| 239300_at | 15.69 | 9.46E-06 | 1.273193897 | NA |  |
| 227964_at | 15.68 | 9.48E-06 | 1.085976424 | NM_031904| | FKSG44,FKSG44 protein |
| 1557987_at | 15.68 | 9.47E-06 | 1.155221128 | NA |  |
| 213222_at | 15.68 | 9.49E-06 | 1.235578079 | NM_015192| | PLCB1,phosphoinositide-specific phospholipase C beta 1 |
| 223094_s_at | 15.68 | 9.48E-06 | 1.340736789 | NM_054027| | ANKH,ankylosis, progressive homolog |
| 225949_at | 15.68 | 9.47E-06 | 1.215963015 | NM_178564| | NRBP2,nuclear receptor binding protein 2 |
| 202784_s_at | 15.68 | 9.48E-06 | 1.311125478 | NM_012343| | NNT,nicotinamide nucleotide transhydrogenase |
| 229593_at | 15.67 | 9.52E-06 | 1.348033095 | NA |  |
| 200771_at | 15.67 | 9.50E-06 | 1.056850433 | NM_002293| | LAMC1,laminin, gamma 1 precursor |
| 201540_at | 15.67 | 9.51E-06 | 1.056884418 | NM_001449| | FHL1,four and a half LIM domains 1 |
| 209408_at | 15.67 | 9.52E-06 | 1.03997672 | NM_006845| | KIF2C,kinesin family member 2C |
| 238844_s_at | 15.67 | 9.53E-06 | 1.192111405 | NM_000272| | NPHP1,nephrocystin isoform 1 |
| 213685_at | 15.66 | 9.55E-06 | 1.0969415 | NA |  |
| 225901_at | 15.66 | 9.55E-06 | 1.057844228 | NM_175732| | NA |
| 225797_at | 15.66 | 9.54E-06 | 1.057424145 | NM_172251| | MRPL54,mitochondrial ribosomal protein L54 |
| 202942_at | 15.66 | 9.55E-06 | 1.077076561 | NM_001014763| | NA |
| 203524_s_at | 15.65 | 9.58E-06 | 1.076571622 | NM_001013436| | NA |
| 241905_at | 15.65 | 9.58E-06 | 1.176835667 | NM_002645| | PIK3C2A,phosphoinositide-3-kinase, class 2, alpha |
| 212968_at | 15.65 | 9.58E-06 | 1.060961607 | NM_002917| | NA |
| 226897_s_at | 15.65 | 9.59E-06 | 1.056639127 | NM_014153| | ZC3HDC7,zinc finger CCCH type domain containing 7 |
| 214910_s_at | 15.64 | 9.60E-06 | 1.181257244 | NM_019101| | APOM,apolipoprotein M |
| 230388_s_at | 15.64 | 9.60E-06 | 1.140628532 | NA |  |
| 218463_s_at | 15.64 | 9.61E-06 | 1.05132902 | NM_025128| | MUS81,MUS81 endonuclease homolog |
| 228702_at | 15.64 | 9.60E-06 | 1.572946355 | NA |  |
| 202304_at | 15.63 | 9.65E-06 | 1.095549389 | NM_001079673| | NA |
| 241408_at | 15.62 | 9.69E-06 | 1.088906275 | NM_175918| | FLJ34443,hypothetical protein FLJ34443 |
| 225103_at | 15.62 | 9.68E-06 | 1.127230519 | NM_032478| | MRPL38,mitochondrial ribosomal protein L38 |
| 203151_at | 15.61 | 9.70E-06 | 1.218109136 | NM_002373| | MAP1A,microtubule-associated protein 1A |
| 214331_at | 15.61 | 9.72E-06 | 1.329739914 | NM_005726| | TSFM,Ts translation elongation factor, mitochondrial |
| 218407_x_at | 15.61 | 9.72E-06 | 1.085261856 | NM_013349| | SCIRP10,SCIRP10-related protein |
| 216028_at | 15.6 | 9.75E-06 | 1.17595108 | NA |  |
| 232004_at | 15.6 | 9.75E-06 | 1.206619949 | NM_001102397| | NA |
| 204649_at | 15.6 | 9.72E-06 | 1.08866564 | NM_001100620| | NA |
| 207839_s_at | 15.6 | 9.74E-06 | 1.221438666 | NM_001042589| | NA |
| 226394_at | 15.59 | 9.77E-06 | 1.134305232 | NM_017824| | MARCH5,ring finger protein 153 |
| 223892_s_at | 15.59 | 9.78E-06 | 1.031186473 | NM_016056| | CGI-119,CGI-119 protein |
| 212194_s_at | 15.58 | 9.81E-06 | 1.140067131 | NM_014742| | TM9SF4,transmembrane 9 superfamily protein member 4 |
| 227345_at | 15.57 | 9.85E-06 | 1.126508106 | NM_003840| | TNFRSF10D,tumor necrosis factor receptor superfamily, |
| 221532_s_at | 15.56 | 9.88E-06 | 1.044578934 | NM_025234| | REC14,recombination protein REC14 |
| 223605_at | 15.56 | 9.88E-06 | 1.140089775 | NM_031481| | SLC25A18,solute carrier |
| 218656_s_at | 15.56 | 9.89E-06 | 1.170569887 | NM_005780| | LHFP,lipoma HMGIC fusion partner |
| 212667_at | 15.54 | 9.96E-06 | 1.150108698 | NM_003118| | SPARC,secreted protein, acidic, cysteine-rich |
| 212594_at | 15.53 | 9.99E-06 | 1.206720124 | NM_014456| | PDCD4,programmed cell death 4 isoform 1 |
| 217943_s_at | 15.53 | 9.99E-06 | 1.069937289 | NM_018067| | FLJ10350,hypothetical protein FLJ10350 |
| 44783_s_at | 15.53 | 1.00E-05 | 1.266232669 | NM_001040708| | NA |
| 209511_at | 15.52 | 1.00E-05 | 1.057616105 | NM_021974| | POLR2F,DNA directed RNA polymerase II polypeptide F |
| 1555216_a_at | 15.52 | 1.00E-05 | 2.058991933 | NA |  |
| 225251_at | 15.52 | 1.00E-05 | 1.114144205 | NM_001031677| | NA |
| 232480_at | 15.52 | 1.00E-05 | 1.508480771 | NA |  |
| 1558111_at | 15.52 | 1.00E-05 | 1.594366085 | NM_021038| | MBNL1,muscleblind-like 1 isoform a |
| 1562012_at | 15.51 | 1.01E-05 | 1.502689714 | NA |  |
| 203994_s_at | 15.51 | 1.01E-05 | 1.140493245 | NM_004928| | C21orf2,chromosome 21 open reading frame 2 |
| 203327_at | 15.5 | 1.01E-05 | 1.074814593 | NM_004969| | IDE,insulysin |
| 1556476_at | 15.5 | 1.01E-05 | 1.513928468 | NA |  |
| 215090_x_at | 15.5 | 1.01E-05 | 1.129592396 | NA |  |
| 204331_s_at | 15.49 | 1.01E-05 | 1.060213163 | NM_021107| | MRPS12,mitochondrial ribosomal protein S12 precursor |
| 209361_s_at | 15.49 | 1.01E-05 | 1.204392524 | NM_020418| | PCBP4,poly(rC) binding protein 4 isoform a |
| 202447_at | 15.49 | 1.01E-05 | 1.098598427 | NM_001359| | DECR1,2,4-dienoyl CoA reductase 1 precursor |
| 203333_at | 15.48 | 1.02E-05 | 1.155658936 | NM_014970| | KIFAP3,kinesin-associated protein 3 |
| 202128_at | 15.48 | 1.02E-05 | 1.073315245 | NM_001039479| | NA |
| 224766_at | 15.48 | 1.02E-05 | 1.45409197 | NM_000997| | RPL37,ribosomal protein L37 |
| 32209_at | 15.48 | 1.02E-05 | 1.115990266 | NM_001098784| | NA |
| 225094_at | 15.47 | 1.02E-05 | 1.202668728 | NM_020382| | SET8,SET domain-containing protein 8 |
| 202134_s_at | 15.47 | 1.02E-05 | 1.150418715 | NM_015472| | WWTR1,WW domain containing transcription regulator 1 |
| 206330_s_at | 15.47 | 1.02E-05 | 1.170476083 | NM_016848| | SHC3,src homology 2 domain containing transforming |
| 201468_s_at | 15.46 | 1.02E-05 | 1.118148738 | NM_000903| | NQO1,NAD(P)H menadione oxidoreductase 1, |
| 208977_x_at | 15.46 | 1.02E-05 | 1.05009382 | NM_006088| | TUBB2,tubulin, beta, 2 |
| 239272_at | 15.46 | 1.02E-05 | 1.738568799 | NM_001032278| | NA |
| 1773_at | 15.46 | 1.02E-05 | 1.179880598 | NM_002028| | FNTB,farnesyltransferase, CAAX box, beta |
| 210896_s_at | 15.45 | 1.03E-05 | 1.139979747 | NM_004318| | ASPH,aspartate beta-hydroxylase isoform a |
| 218587_s_at | 15.45 | 1.03E-05 | 1.077968856 | NM_020231| | MDS010,x 010 protein |
| 205651_x_at | 15.45 | 1.03E-05 | 1.65796384 | NM_001100397| | NA |
| 208918_s_at | 15.44 | 1.03E-05 | 1.101314726 | NM_023018| | FLJ13052,NAD kinase |
| 211337_s_at | 15.44 | 1.03E-05 | 1.134016848 | NM_014444| | 76P,gamma tubulin ring complex protein (76p gene) |
| 215634_at | 15.43 | 1.03E-05 | 1.877041095 | NA |  |
| 200918_s_at | 15.43 | 1.03E-05 | 1.127938178 | NM_003139| | SRPR,signal recognition particle receptor ('docking |
| 227059_at | 15.43 | 1.03E-05 | 1.182822423 | NM_005708| | GPC6,glypican 6 precursor |
| 228443_s_at | 15.42 | 1.04E-05 | 1.226889569 | NA |  |
| 242723_at | 15.42 | 1.04E-05 | 1.217554918 | NA |  |
| 213632_at | 15.41 | 1.04E-05 | 1.145780597 | NM_001361| | DHODH,dihydroorotate dehydrogenase precursor |
| 221311_x_at | 15.41 | 1.04E-05 | 1.12208628 | NM_020466| | DJ122O8.2,hypothetical protein dJ122O8.2 |
| 220661_s_at | 15.41 | 1.04E-05 | 1.115808286 | NM_017865| | FLJ20531,hypothetical protein FLJ20531 |
| 229824_at | 15.4 | 1.05E-05 | 1.207822373 | NA |  |
| 1554010_at | 15.4 | 1.05E-05 | 1.148177406 | NM_001543| | NDST1,N-deacetylase/N-sulfotransferase (heparan |
| 204545_at | 15.4 | 1.04E-05 | 1.087847977 | NM_000287| | PEX6,peroxisomal biogenesis factor 6 |
| 209360_s_at | 15.39 | 1.05E-05 | 1.705078104 | NM_001001890| | RUNX1,runt-related transcription factor 1 isoform b |
| 217736_s_at | 15.39 | 1.05E-05 | 1.042116816 | NM_014413| | EIF2AK1,heme-regulated initiation factor 2-alpha kinase |
| 235302_at | 15.38 | 1.05E-05 | 1.075419902 | NA |  |
| 1562699_at | 15.38 | 1.05E-05 | 1.318225356 | NA |  |
| 200883_at | 15.38 | 1.05E-05 | 1.070145288 | NM_003366| | UQCRC2,ubiquinol-cytochrome c reductase core protein |
| 209321_s_at | 15.37 | 1.06E-05 | 1.149492803 | NM_004036| | ADCY3,adenylate cyclase 3 |
| 1563808_at | 15.37 | 1.05E-05 | 1.183304895 | NM_001112732| | NA |
| 227649_s_at | 15.37 | 1.06E-05 | 1.312970421 | NM_001042758| | NA |
| 225926_at | 15.37 | 1.05E-05 | 1.157262281 | NM_006370| | VTI1B,vesicle transport through interaction with |
| 213742_at | 15.37 | 1.06E-05 | 1.173011878 | NM_004768| | SFRS11,splicing factor p54 |
| 239289_x_at | 15.36 | 1.06E-05 | 1.087229212 | NM_014967| | KIAA1018,KIAA1018 protein |
| 218939_at | 15.36 | 1.06E-05 | 1.12249423 | NM_012318| | LETM1,leucine zipper-EF-hand containing transmembrane |
| 228693_at | 15.35 | 1.06E-05 | 1.440875008 | NM_174908| | C3orf6,Ymer protein short isoform |
| 214724_at | 15.35 | 1.06E-05 | 1.057536636 | NM_001037954| | NA |
| 228379_at | 15.35 | 1.06E-05 | 1.206891929 | NM_005796| | NUTF2,nuclear transport factor 2 |
| 223261_at | 15.35 | 1.06E-05 | 1.156546319 | NM_016218| | POLK,polymerase (DNA directed) kappa |
| 235935_at | 15.34 | 1.06E-05 | 1.239124539 | NM_001012974| | NA |
| 1569788_at | 15.34 | 1.06E-05 | 1.558098622 | NM_003034| | ST8SIA1,ST8 alpha-N-acetyl-neuraminide |
| 202522_at | 15.33 | 1.07E-05 | 1.053757951 | NM_012399| | PITPNB,phosphatidylinositol transfer protein, beta |
| 201782_s_at | 15.33 | 1.07E-05 | 1.109149487 | NM_003977| | AIP,aryl hydrocarbon receptor interacting protein |
| 203916_at | 15.32 | 1.07E-05 | 1.077268818 | NM_003635| | NDST2,N-deacetylase/N-sulfotransferase (heparan |
| 239033_at | 15.32 | 1.07E-05 | 1.070209769 | NA |  |
| 225012_at | 15.32 | 1.07E-05 | 1.171817188 | NM_005336| | HDLBP,high density lipoprotein binding protein |
| 238207_at | 15.32 | 1.07E-05 | 1.227145257 | NM_001124767| | NA |
| 225772_s_at | 15.31 | 1.07E-05 | 1.079327978 | NM_032901| | MGC14288,hypothetical protein MGC14288 |
| 233304_at | 15.3 | 1.08E-05 | 1.783331166 | NM_005596| | NFIB,nuclear factor I/B |
| 224950_at | 15.3 | 1.08E-05 | 1.179066462 | NM_020440| | PTGFRN,prostaglandin F2 receptor negative regulator |
| 212217_at | 15.3 | 1.08E-05 | 1.094891918 | NM_001042385| | NA |
| 207346_at | 15.3 | 1.08E-05 | 1.052157187 | NM_001980| | EPIM,epimorphin isoform 1 |
| 218029_at | 15.29 | 1.09E-05 | 1.268114094 | NM_024519| | FLJ13725,hypothetical protein FLJ13725 |
| 217550_at | 15.29 | 1.09E-05 | 1.161238097 | NM_007348| | ATF6,activating transcription factor 6 |
| 201295_s_at | 15.29 | 1.08E-05 | 1.290800979 | NM_015626| | WSB1,WD SOCS-box protein 1 isoform 1 |
| 201584_s_at | 15.28 | 1.09E-05 | 1.048250956 | NM_005804| | DDX39,DEAD (Asp-Glu-Ala-Asp) box polypeptide 39 |
| 203353_s_at | 15.28 | 1.09E-05 | 1.062455515 | NM_002384| | MBD1,methyl-CpG binding domain protein 1 isoform 4 |
| 222310_at | 15.28 | 1.09E-05 | 1.263003517 | NM_020706| | SFRS15,splicing factor, arginine/serine-rich 15 |
| 208563_x_at | 15.28 | 1.09E-05 | 1.334257337 | NM_006236| | POU3F3,POU domain, class 3, transcription factor 3 |
| 214736_s_at | 15.28 | 1.09E-05 | 1.115932312 | NM_001119| | ADD1,adducin 1 (alpha) isoform a |
| 211792_s_at | 15.27 | 1.09E-05 | 1.152024758 | NM_001262| | CDKN2C,cyclin-dependent kinase inhibitor 2C |
| 204160_s_at | 15.27 | 1.09E-05 | 1.17425305 | NM_014936| | ENPP4,ectonucleotide pyrophosphatase/phosphodiesterase |
| 218233_s_at | 15.27 | 1.09E-05 | 1.022801684 | NM_013397| | C6orf49,over-expressed breast tumor protein |
| 223843_at | 15.26 | 1.10E-05 | 1.545014053 | NM_016240| | SCARA3,scavenger receptor class A, member 3 isoform 1 |
| 235296_at | 15.26 | 1.10E-05 | 1.231726482 | NM_020390| | EIF5A2,eIF-5A2 protein |
| 1568815_a_at | 15.26 | 1.10E-05 | 1.213697543 | NM_024045| | DDX50,nucleolar protein GU2 |
| 202254_at | 15.25 | 1.10E-05 | 1.182589382 | NM_015556| | SIPA1L1,signal-induced proliferation-associated 1 like |
| 211466_at | 15.25 | 1.10E-05 | 1.499170535 | NM_005596| | NFIB,nuclear factor I/B |
| 227696_at | 15.25 | 1.10E-05 | 1.104616111 | NM_058219| | EXOSC6,homolog of yeast mRNA transport regulator 3 |
| 238714_at | 15.25 | 1.10E-05 | 1.095609112 | NA |  |
| 232800_at | 15.24 | 1.10E-05 | 1.531537061 | NA |  |
| 1568594_s_at | 15.24 | 1.10E-05 | 1.167385973 | NM_032765| | TRIM52,tripartite motif-containing 52 |
| 206788_s_at | 15.24 | 1.10E-05 | 1.194480846 | NM_001755| | CBFB,core-binding factor, beta subunit isoform 2 |
| 228369_at | 15.24 | 1.10E-05 | 1.314020365 | NM_006586| | TNRC5,trinucleotide repeat containing 5 |
| 218244_at | 15.23 | 1.11E-05 | 1.098019397 | NM_017948| | NOL8,nucleolar protein 8 |
| 204041_at | 15.23 | 1.11E-05 | 1.228314752 | NM_000898| | MAOB,amine oxidase (flavin-containing) |
| 211959_at | 15.23 | 1.11E-05 | 1.125578223 | NM_000599| | IGFBP5,insulin-like growth factor binding protein 5 |
| 200823_x_at | 15.23 | 1.11E-05 | 1.028415157 | NM_000992| | RPL29,ribosomal protein L29 |
| 215128_at | 15.23 | 1.11E-05 | 1.107176573 | NA |  |
| 218901_at | 15.22 | 1.11E-05 | 1.316705089 | NM_020353| | PLSCR4,phospholipid scramblase 4 |
| 205320_at | 15.22 | 1.11E-05 | 1.269109468 | NM_005883| | APC2,adenomatosis polyposis coli 2 |
| 235673_at | 15.22 | 1.11E-05 | 1.231241033 | NA |  |
| 216375_s_at | 15.22 | 1.11E-05 | 1.331580145 | NM_004454| | ETV5,ets variant gene 5 (ets-related molecule) |
| 212856_at | 15.22 | 1.11E-05 | 1.177440261 | NM_015124| | NA |
| 226692_at | 15.22 | 1.11E-05 | 1.14779578 | NM_001018108| | NA |
| 230741_at | 15.22 | 1.11E-05 | 1.400825256 | NA |  |
| 200098_s_at | 15.22 | 1.11E-05 | 1.058512456 | NM_016237| | ANAPC5,anaphase-promoting complex subunit 5 |
| 223712_at | 15.21 | 1.11E-05 | 1.17606891 | NM_032151| | PCBD2,dimerization cofactor of hepatocyte nuclear |
| 240128_at | 15.21 | 1.12E-05 | 1.292626271 | NA |  |
| 241631_at | 15.2 | 1.12E-05 | 1.246890479 | NA |  |
| 235628_x_at | 15.2 | 1.12E-05 | 1.22700292 | NA |  |
| 217869_at | 15.2 | 1.12E-05 | 1.014503794 | NM_016142| | HSD17B12,steroid dehydrogenase homolog |
| 201175_at | 15.2 | 1.12E-05 | 1.048043601 | NM_015959| | TMX2,thioredoxin-related transmembrane protein 2 |
| 202791_s_at | 15.19 | 1.12E-05 | 1.068195962 | NM_014678| | KIAA0685,KIAA0685 gene product |
| 202705_at | 15.19 | 1.12E-05 | 1.035467976 | NM_004701| | CCNB2,cyclin B2 |
| 222270_at | 15.19 | 1.12E-05 | 1.292681323 | NM_001122964| | NA |
| 204514_at | 15.19 | 1.12E-05 | 1.128364473 | NM_001039589| | NA |
| 226458_at | 15.18 | 1.13E-05 | 1.135051541 | NA |  |
| 210139_s_at | 15.16 | 1.14E-05 | 1.271158548 | NM_000304| | PMP22,peripheral myelin protein 22 |
| 238161_at | 15.16 | 1.13E-05 | 1.222519782 | NA |  |
| 222777_s_at | 15.15 | 1.14E-05 | 1.254641408 | NM_001042424| | NA |
| 205885_s_at | 15.14 | 1.15E-05 | 1.511484017 | NM_000885| | ITGA4,integrin alpha 4 precursor |
| 230571_at | 15.14 | 1.14E-05 | 1.305459076 | NA |  |
| 216858_x_at | 15.14 | 1.14E-05 | 1.066104618 | NA |  |
| 219205_at | 15.14 | 1.14E-05 | 1.161296156 | NM_021947| | SRR,serine racemase |
| 209230_s_at | 15.13 | 1.15E-05 | 1.281907412 | NM_001042483| | NA |
| 201151_s_at | 15.13 | 1.15E-05 | 1.652918448 | NM_021038| | MBNL1,muscleblind-like 1 isoform a |
| 1559132_at | 15.13 | 1.15E-05 | 1.151551687 | NM_001042463| | NA |
| 224691_at | 15.12 | 1.15E-05 | 1.10077508 | NA |  |
| 212715_s_at | 15.12 | 1.15E-05 | 1.168327562 | NM_001122731| | NA |
| 222880_at | 15.1 | 1.16E-05 | 1.419188898 | NM_005465| | AKT3,v-akt murine thymoma viral oncogene homolog 3 |
| 202632_at | 15.1 | 1.16E-05 | 1.08275619 | NM_001383| | DPH2L1,diptheria toxin resistance protein required for |
| 212776_s_at | 15.1 | 1.16E-05 | 1.095943746 | NM_015311| | NA |
| 230652_at | 15.09 | 1.17E-05 | 1.341016798 | NM_001654| | ARAF,v-raf murine sarcoma 3611 viral oncogene |
| 230863_at | 15.09 | 1.17E-05 | 1.290374619 | NM_004525| | LRP2,low density lipoprotein-related protein 2 |
| 230831_at | 15.08 | 1.17E-05 | 1.111150476 | NA |  |
| 224804_s_at | 15.08 | 1.17E-05 | 1.074600862 | NM_020447| | C15orf17,chromosome 15 open reading frame 17 |
| 213088_s_at | 15.08 | 1.17E-05 | 1.071297877 | NM_015190| | DNAJC9,DnaJ homolog, subfamily C, member 9 |
| 203162_s_at | 15.08 | 1.17E-05 | 1.062137756 | NM_005886| | KATNB1,katanin p80 subunit B 1 |
| 1555234_a_at | 15.07 | 1.17E-05 | 1.309229994 | NM_020663| | RHOJ,TC10-like Rho GTPase |
| 200005_at | 15.07 | 1.18E-05 | 1.03887788 | NM_003753| | EIF3S7,eukaryotic translation initiation factor 3 |
| 223312_at | 15.06 | 1.18E-05 | 1.123912164 | NM_032319| | C2orf7,chromosome 2 open reading frame 7 |
| 229078_s_at | 15.06 | 1.18E-05 | 1.276109981 | NM_018559| | KIAA1704,KIAA1704 |
| 241792_x_at | 15.06 | 1.18E-05 | 1.128362045 | NA |  |
| 206499_s_at | 15.06 | 1.18E-05 | 1.071573219 | NM_001048194| | NA |
| 226838_at | 15.06 | 1.18E-05 | 1.103297596 | NM_001008237| | LOC130502,similar to CG14894-PA |
| 228558_at | 15.05 | 1.18E-05 | 1.092854879 | NM_173608| | C14orf80,chromosome 14 open reading frame 80 |
| 1556283_s_at | 15.05 | 1.18E-05 | 1.163934694 | NM_015633| | FGFR1OP2,FGFR1 oncogene partner 2 |
| 1552476_s_at | 15.05 | 1.18E-05 | 1.208925052 | NM_133373| | PLCD3,phospholipase C delta 3 |
| 233442_at | 15.04 | 1.19E-05 | 1.446307092 | NA |  |
| 208714_at | 15.04 | 1.19E-05 | 1.094529599 | NM_007103| | NDUFV1,NADH dehydrogenase (ubiquinone) flavoprotein 1, |
| 207107_at | 15.04 | 1.18E-05 | 2.28685577 | NM_000329| | RPE65,retinal pigment epithelium-specific protein |
| 232149_s_at | 15.04 | 1.19E-05 | 1.204067805 | NM_003580| | NSMAF,neutral sphingomyelinase (N-SMase) activation |
| 1557286_at | 15.04 | 1.19E-05 | 1.480932294 | NA |  |
| 50314_i_at | 15.03 | 1.19E-05 | 1.071099496 | NM_001039140| | NA |
| 231847_at | 15.01 | 1.20E-05 | 1.177021913 | NM_138797| | LOC129138,hypothetical protein BC014641 |
| 230039_at | 15.01 | 1.20E-05 | 1.169847319 | NA |  |
| 216232_s_at | 15.01 | 1.20E-05 | 1.075715216 | NM_006836| | GCN1L1,GCN1 general control of amino-acid synthesis |
| 212439_at | 15.01 | 1.20E-05 | 1.088588424 | NM_001006115| | IHPK1,inositol hexaphosphate kinase 1 isoform 2 |
| 215114_at | 15 | 1.21E-05 | 1.195741332 | NM_015670| | SENP3,SUMO1/sentrin/SMT3 specific protease 3 |
| 202294_at | 14.99 | 1.21E-05 | 1.096719445 | NM_005862| | STAG1,stromal antigen 1 |
| 231034_s_at | 14.99 | 1.21E-05 | 1.204586745 | NA |  |
| 221428_s_at | 14.99 | 1.21E-05 | 1.204814357 | NM_024665| | TBL1XR1,nuclear receptor co-repressor/HDAC3 complex |
| 225255_at | 14.98 | 1.21E-05 | 1.22266832 | NM_016622| | MRPL35,mitochondrial ribosomal protein L35 isoform a |
| 236168_at | 14.98 | 1.21E-05 | 1.304756027 | NA |  |
| 218017_s_at | 14.98 | 1.21E-05 | 1.224160314 | NM_152419| | NA |
| 218284_at | 14.98 | 1.21E-05 | 1.143869332 | NM_005902| | SMAD3,MAD, mothers against decapentaplegic homolog 3 |
| 232244_at | 14.98 | 1.21E-05 | 1.314050487 | NM_020702| | KIAA1161,KIAA1161 |
| 240111_at | 14.97 | 1.22E-05 | 1.354027595 | NM_014899| | RHOBTB3,rho-related BTB domain containing 3 |
| 228900_at | 14.96 | 1.22E-05 | 1.138242039 | NM_001033553| | NA |
| 218953_s_at | 14.96 | 1.22E-05 | 1.114143483 | NM_024028| | MGC3265,hypothetical protein MGC3265 |
| 225623_at | 14.95 | 1.23E-05 | 1.072097679 | NM_033426| | KIAA1737,KIAA1737 protein |
| 212263_at | 14.95 | 1.22E-05 | 1.079360849 | NM_006775| | QKI,quaking homolog, KH domain RNA binding isoform |
| 1554789_a_at | 14.95 | 1.23E-05 | 1.576857396 | NM_001029851| | NA |
| 201018_at | 14.95 | 1.22E-05 | 1.046697508 | NM_001412| | EIF1AX,X-linked eukaryotic translation initiation |
| 202551_s_at | 14.95 | 1.23E-05 | 1.099956442 | NM_016441| | CRIM1,cysteine-rich motor neuron 1 |
| 215412_x_at | 14.95 | 1.23E-05 | 1.144409051 | NA |  |
| 201186_at | 14.94 | 1.23E-05 | 1.116155775 | NM_002337| | LRPAP1,low density lipoprotein receptor-related protein |
| 221952_x_at | 14.94 | 1.23E-05 | 1.053900782 | NM_020810| | KIAA1393,tRNA-(N1G37) methyltransferase |
| 218590_at | 14.94 | 1.23E-05 | 1.080799987 | NM_021830| | PEO1,twinkle |
| 235031_at | 14.94 | 1.23E-05 | 1.603439234 | NA |  |
| 215690_x_at | 14.93 | 1.23E-05 | 1.118876906 | NM_003801| | GPAA1,anchor attachment protein 1 |
| 235010_at | 14.93 | 1.24E-05 | 1.192510293 | NA |  |
| 232589_at | 14.93 | 1.23E-05 | 1.171477219 | NA |  |
| 238670_at | 14.93 | 1.23E-05 | 1.362545785 | NA |  |
| 213311_s_at | 14.93 | 1.23E-05 | 1.087674239 | NM_014972| | KIAA1049,KIAA1049 protein |
| 212711_at | 14.92 | 1.24E-05 | 1.05385358 | NM_015447| | CAMSAP1,calmodulin regulated spectrin-associated protein |
| 229884_s_at | 14.92 | 1.24E-05 | 1.189820976 | NM_015950| | MRPL2,mitochondrial ribosomal protein L2 |
| 1556060_a_at | 14.92 | 1.24E-05 | 1.108523922 | NA |  |
| 51176_at | 14.92 | 1.24E-05 | 1.052988135 | NM_004269| | CRSP8,cofactor required for Sp1 transcriptional |
| 242673_at | 14.92 | 1.24E-05 | 1.345734768 | NA |  |
| 204206_at | 14.92 | 1.24E-05 | 1.085824627 | NM_020310| | MNT,MAX binding protein |
| 201865_x_at | 14.9 | 1.25E-05 | 1.076920892 | NM_000176| | NR3C1,nuclear receptor subfamily 3, group C, member 1 |
| 239219_at | 14.9 | 1.25E-05 | 1.151374116 | NM_004217| | AURKB,aurora kinase B |
| 229321_s_at | 14.9 | 1.25E-05 | 1.143355449 | NA |  |
| 224765_at | 14.9 | 1.25E-05 | 1.077162145 | NM_001012241| | NA |
| 215185_at | 14.9 | 1.25E-05 | 1.341251431 | NA |  |
| 204868_at | 14.9 | 1.25E-05 | 1.034917174 | NM_001545| | ICT1,immature colon carcinoma transcript 1 |
| 223532_at | 14.9 | 1.25E-05 | 1.153235026 | NM_016466| | MGC41816,hypothetical protein MGC41816 |
| 238935_at | 14.9 | 1.25E-05 | 1.330292258 | NM_015920| | RPS27L,ribosomal protein S27-like protein |
| 213650_at | 14.89 | 1.25E-05 | 1.210523131 | NM_001023567| | NA |
| 221878_at | 14.89 | 1.25E-05 | 1.095737682 | NM_001013649| | NA |
| 236250_at | 14.89 | 1.25E-05 | 1.338275184 | NA |  |
| 200962_at | 14.88 | 1.26E-05 | 1.115598275 | NM_000993| | RPL31,ribosomal protein L31 |
| 214743_at | 14.88 | 1.25E-05 | 1.075223185 | NM_001913| | CUTL1,CCAAT displacement protein isoform b |
| 201986_at | 14.88 | 1.26E-05 | 1.074437996 | NM_005121| | THRAP1,thyroid hormone receptor associated protein 1 |
| 200028_s_at | 14.88 | 1.26E-05 | 1.035956285 | NM_020151| | STARD7,START domain containing 7 |
| 227970_at | 14.87 | 1.26E-05 | 1.178012645 | NM_024980| | GPR157,G protein-coupled receptor 157 |
| 216705_s_at | 14.87 | 1.26E-05 | 1.116957034 | NM_000022| | ADA,adenosine deaminase |
| 227219_x_at | 14.86 | 1.27E-05 | 1.077071836 | NM_032514| | MAP1LC3A,microtubule-associated protein 1 light chain 3 |
| 213763_at | 14.86 | 1.27E-05 | 1.157601651 | NM_001113239| | NA |
| 231055_at | 14.86 | 1.26E-05 | 1.467677131 | NA |  |
| 220543_at | 14.85 | 1.27E-05 | 1.485785409 | NM_019596| | C21orf62,chromosome 21 open reading frame 62 |
| 1552689_at | 14.85 | 1.27E-05 | 1.097013582 | NM_020764| | CASKIN1,CASK interacting protein 1 |
| 228084_at | 14.85 | 1.27E-05 | 1.132772373 | NA |  |
| 236594_at | 14.85 | 1.27E-05 | 1.038914382 | NM_004140| | LLGL1,lethal giant larvae homolog 1 |
| 225310_at | 14.85 | 1.27E-05 | 1.069445292 | NM_002139| | RBMX,RNA binding motif protein, X-linked |
| 208715_at | 14.84 | 1.28E-05 | 1.049395066 | NM_019026| | LOC54499,putative membrane protein |
| 244114_x_at | 14.84 | 1.27E-05 | 1.367370483 | NA |  |
| 212556_at | 14.84 | 1.27E-05 | 1.095812577 | NM_015356| | SCRIB,scribble isoform b |
| 216525_x_at | 14.84 | 1.27E-05 | 1.123726071 | NM_001003686| | PMS2L3,postmeiotic segregation increased 2-like 3 |
| 203817_at | 14.83 | 1.28E-05 | 1.182560181 | NM_000857| | GUCY1B3,guanylate cyclase 1, soluble, beta 3 |
| 210175_at | 14.83 | 1.28E-05 | 1.350053678 | NM_003203| | C2orf3,chromosome 2 open reading frame 3 |
| 201324_at | 14.82 | 1.28E-05 | 1.482437836 | NM_001423| | EMP1,epithelial membrane protein 1 |
| 217912_at | 14.81 | 1.29E-05 | 1.068135404 | NM_022156| | PP3111,PP3111 protein |
| 225576_at | 14.81 | 1.29E-05 | 1.088181349 | NM_138785| | C6orf72,chromosome 6 open reading frame 72 |
| 214788_x_at | 14.81 | 1.29E-05 | 1.274699348 | NM_015086| | NA |
| 225336_at | 14.81 | 1.29E-05 | 1.075243626 | NM_004719| | SFRS2IP,splicing factor, arginine/serine-rich 2, |
| 212765_at | 14.81 | 1.29E-05 | 1.085356706 | NM_203459| | KIAA1078,KIAA1078 protein |
| 214107_x_at | 14.8 | 1.29E-05 | 1.136236861 | NA |  |
| 235513_at | 14.79 | 1.29E-05 | 1.120335607 | NA |  |
| 222396_at | 14.79 | 1.29E-05 | 1.078865819 | NM_001002032| | HN1,hematological and neurological expressed 1 |
| 227984_at | 14.79 | 1.30E-05 | 1.51457384 | NA |  |
| 222844_s_at | 14.79 | 1.29E-05 | 1.154235439 | NM_021947| | SRR,serine racemase |
| 203836_s_at | 14.79 | 1.29E-05 | 1.157095922 | NM_005923| | MAP3K5,mitogen-activated protein kinase kinase kinase |
| 212847_at | 14.79 | 1.30E-05 | 1.164508644 | NM_003902| | FUBP1,far upstream element-binding protein |
| 209444_at | 14.79 | 1.29E-05 | 1.073442965 | NM_001100426| | NA |
| 217061_s_at | 14.79 | 1.30E-05 | 1.191550867 | NM_004956| | ETV1,ets variant gene 1 |
| 241699_at | 14.78 | 1.30E-05 | 1.451363033 | NA |  |
| 201146_at | 14.77 | 1.30E-05 | 1.072464999 | NM_006164| | NFE2L2,nuclear factor (erythroid-derived 2)-like 2 |
| 230404_at | 14.77 | 1.30E-05 | 1.39703929 | NA |  |
| 227720_at | 14.77 | 1.30E-05 | 1.100731475 | NM_152345| | FLJ25555,hypothetical protein FLJ25555 |
| 202245_at | 14.77 | 1.31E-05 | 1.047880361 | NM_001001438| | LSS,lanosterol synthase |
| 225878_at | 14.76 | 1.31E-05 | 1.073136437 | NM_015074| | KIF1B,kinesin family member 1B isoform b |
| 214259_s_at | 14.76 | 1.31E-05 | 1.078409319 | NM_003689| | AKR7A2,aldo-keto reductase family 7, member A2 |
| 239084_at | 14.76 | 1.31E-05 | 1.292116152 | NA |  |
| 222165_x_at | 14.76 | 1.31E-05 | 1.085240694 | NM_024112| | C9orf16,chromosome 9 open reading frame 16 |
| 230651_at | 14.76 | 1.31E-05 | 1.333189375 | NA |  |
| 227907_at | 14.76 | 1.31E-05 | 1.245651595 | NA |  |
| 1553570_x_at | 14.76 | 1.31E-05 | 1.024479431 | NM_022340| | ZFYVE20,FYVE-finger-containing Rab5 effector protein |
| 218105_s_at | 14.75 | 1.32E-05 | 1.096185535 | NM_015956| | MRPL4,mitochondrial ribosomal protein L4 isoform a |
| 201632_at | 14.75 | 1.32E-05 | 1.049550901 | NM_001414| | EIF2B1,eukaryotic translation initiation factor 2B, |
| 1553268_at | 14.75 | 1.32E-05 | 1.222431423 | NM_138328| | RHBDL4,rhomboid, veinlet-like 4 |
| 239273_s_at | 14.74 | 1.32E-05 | 1.595343626 | NM_001032278| | NA |
| 201340_s_at | 14.74 | 1.32E-05 | 1.126675406 | NM_003633| | ENC1,ectodermal-neural cortex (with BTB-like domain) |
| 205729_at | 14.73 | 1.32E-05 | 1.452845567 | NM_003999| | OSMR,oncostatin M receptor |
| 223940_x_at | 14.73 | 1.32E-05 | 1.216774683 | NA |  |
| 228630_at | 14.73 | 1.33E-05 | 1.196559055 | NM_003428| | ZNF84,zinc finger protein 84 (HPF2) |
| 52169_at | 14.72 | 1.33E-05 | 1.071530191 | NM_001003786| | LYK5,protein kinase LYK5 isoform 2 |
| 244881_at | 14.72 | 1.33E-05 | 1.086129256 | NM_033029| | LMLN,leishmanolysin-like (metallopeptidase M8 |
| 217604_at | 14.71 | 1.34E-05 | 1.186374648 | NA |  |
| 236903_at | 14.71 | 1.34E-05 | 1.17859304 | NA |  |
| 224301_x_at | 14.71 | 1.34E-05 | 1.101282217 | NM_177925| | H2AFJ,H2A histone family, member J isoform 2 |
| 210137_s_at | 14.7 | 1.34E-05 | 1.074182978 | NM_001012732| | NA |
| 223080_at | 14.7 | 1.34E-05 | 1.301025661 | NM_014905| | GLS,glutaminase C |
| 216005_at | 14.7 | 1.34E-05 | 1.578753833 | NM_002160| | TNC,tenascin C (hexabrachion) |
| 211340_s_at | 14.69 | 1.35E-05 | 1.208741961 | NM_006500| | MCAM,melanoma cell adhesion molecule |
| 46142_at | 14.69 | 1.34E-05 | 1.391175585 | NM_022773| | FLJ12681,hypothetical protein FLJ12681 |
| 204420_at | 14.68 | 1.35E-05 | 1.318176145 | NM_005438| | FOSL1,FOS-like antigen 1 |
| 212228_s_at | 14.68 | 1.35E-05 | 1.135169031 | NM_020312| | DKFZP434K046,hypothetical protein DKFZp434K046 |
| 209054_s_at | 14.68 | 1.35E-05 | 1.05124332 | NM_001042424| | NA |
| 241343_at | 14.67 | 1.36E-05 | 1.253256141 | NM_002936| | RNASEH1,ribonuclease H1 |
| 209198_s_at | 14.67 | 1.36E-05 | 1.186253185 | NM_152280| | SYT11,synaptotagmin 12 |
| 204014_at | 14.67 | 1.35E-05 | 1.090934636 | NM_001394| | DUSP4,dual specificity phosphatase 4 isoform 1 |
| 212205_at | 14.66 | 1.36E-05 | 1.09379438 | NM_012412| | H2AFV,H2A histone family, member V isoform 1 |
| 218329_at | 14.66 | 1.36E-05 | 1.069447953 | NM_012406| | PRDM4,PR domain containing 4 |
| 218554_s_at | 14.66 | 1.36E-05 | 1.264328318 | NM_018489| | ASH1L,ash1 (absent, small, or homeotic)-like |
| 217968_at | 14.66 | 1.36E-05 | 1.06847283 | NM_003310| | TSSC1,tumor suppressing subtransferable candidate 1 |
| 235213_at | 14.66 | 1.36E-05 | 1.647726119 | NM_002221| | ITPKB,1D-myo-inositol-trisphosphate 3-kinase B |
| 226554_at | 14.66 | 1.36E-05 | 1.071977591 | NM_015898| | ZBTB7,zinc finger and BTB domain-containing 7 |
| 211558_s_at | 14.65 | 1.36E-05 | 1.085272194 | NM_001930| | DHPS,deoxyhypusine synthase isoform a |
| 1569312_at | 14.65 | 1.37E-05 | 1.296925357 | NA |  |
| 217527_s_at | 14.65 | 1.36E-05 | 1.080083658 | NM_032815| | NFATC2IP,nuclear factor of activated T-cells, |
| 225197_at | 14.65 | 1.36E-05 | 1.063268406 | NA |  |
| 204377_s_at | 14.64 | 1.37E-05 | 1.202031878 | NM_014703| | VprBP,Vpr-binding protein |
| 219072_at | 14.63 | 1.38E-05 | 1.113483994 | NM_004765| | BCL7C,B-cell CLL/lymphoma 7C |
| 220200_s_at | 14.63 | 1.37E-05 | 1.288617303 | NM_020382| | SET8,SET domain-containing protein 8 |
| 231931_at | 14.63 | 1.37E-05 | 1.172492348 | NM_001040424| | NA |
| 204594_s_at | 14.63 | 1.37E-05 | 1.081720527 | NM_019008| | FLJ20232,hypothetical protein FLJ20232 |
| 212328_at | 14.63 | 1.37E-05 | 1.093534634 | NM_001112717| | NA |
| 208958_at | 14.62 | 1.38E-05 | 1.385941465 | NM_015051| | TXNDC4,thioredoxin domain containing 4 (endoplasmic |
| 234321_x_at | 14.62 | 1.38E-05 | 1.195362136 | NA |  |
| 235989_at | 14.62 | 1.38E-05 | 1.280218416 | NA |  |
| 203259_s_at | 14.62 | 1.38E-05 | 1.027627886 | NM_016063| | C6orf74,chromosome 6 open reading frame 74 |
| 227581_at | 14.62 | 1.38E-05 | 1.11494657 | NM_015395| | DKFZP434B0335,DKFZP434B0335 protein |
| 211621_at | 14.61 | 1.39E-05 | 1.237528997 | NM_000044| | AR,androgen receptor isoform 1 |
| 1554670_at | 14.61 | 1.38E-05 | 1.30333356 | NM_001001560| | GGA1,golgi associated, gamma adaptin ear containing, |
| 202783_at | 14.61 | 1.39E-05 | 1.1987488 | NM_012343| | NNT,nicotinamide nucleotide transhydrogenase |
| 1555905_a_at | 14.6 | 1.39E-05 | 1.253586861 | NM_001029839| | NA |
| 227966_s_at | 14.6 | 1.39E-05 | 1.053357463 | NM_138770| | LOC90557,hypothetical protein BC016861 |
| 205493_s_at | 14.6 | 1.39E-05 | 1.070161325 | NM_006426| | DPYSL4,dihydropyrimidinase-like 4 |
| 242550_at | 14.59 | 1.39E-05 | 1.116607269 | NM_001037283| | NA |
| 208966_x_at | 14.58 | 1.40E-05 | 1.329484438 | NM_005531| | IFI16,interferon, gamma-inducible protein 16 |
| 1555288_s_at | 14.58 | 1.40E-05 | 1.074985387 | NM_001080542| | NA |
| 216958_s_at | 14.58 | 1.40E-05 | 1.127658869 | NM_002225| | IVD,isovaleryl Coenzyme A dehydrogenase |
| 227656_at | 14.58 | 1.40E-05 | 1.111728378 | NM_018341| | NA |
| 212678_at | 14.57 | 1.40E-05 | 1.154330933 | NM_000267| | NF1,neurofibromin |
| 220753_s_at | 14.57 | 1.41E-05 | 1.372557471 | NM_015974| | CRYL1,lambda-crystallin |
| 229943_at | 14.57 | 1.40E-05 | 1.108195715 | NM_001007278| | RFP2,ret finger protein 2 isoform 2 |
| 219416_at | 14.57 | 1.41E-05 | 1.205846102 | NM_016240| | SCARA3,scavenger receptor class A, member 3 isoform 1 |
| 201528_at | 14.56 | 1.41E-05 | 1.022296412 | NM_002945| | RPA1,replication protein A1, 70kDa |
| 1555278_a_at | 14.56 | 1.41E-05 | 1.121054432 | NM_001008938| | ch-TOG,colonic and hepatic tumor over-expressed protein |
| 224626_at | 14.56 | 1.41E-05 | 1.047484758 | NM_080670| | SLC35A4,solute carrier family 35, member A4 |
| 228849_at | 14.55 | 1.41E-05 | 1.111589613 | NM_001007156| | NTRK3,neurotrophic tyrosine kinase, receptor, type 3 |
| 1554574_a_at | 14.54 | 1.42E-05 | 1.084602865 | NM_000398| | DIA1,cytochrome b5 reductase membrane-bound isoform |
| 212232_at | 14.54 | 1.42E-05 | 1.103416667 | NM_015308| | FNBP4,formin binding protein 4 |
| 223429_x_at | 14.54 | 1.42E-05 | 1.080079883 | NM_020701| | KIAA1160,KIAA1160 protein |
| 212714_at | 14.54 | 1.42E-05 | 1.085006987 | NM_052879| | LOC113251,c-Mpl binding protein isoform a |
| 227712_at | 14.54 | 1.42E-05 | 1.15044692 | NM_020466| | DJ122O8.2,hypothetical protein dJ122O8.2 |
| 1554696_s_at | 14.54 | 1.42E-05 | 1.119362459 | NM_001071| | TYMS,thymidylate synthetase |
| 217780_at | 14.53 | 1.42E-05 | 1.053254067 | NM_016145| | PTD008,PTD008 protein |
| 1554740_a_at | 14.53 | 1.42E-05 | 1.272530771 | NM_005897| | IPP,intracisternal A particle-promoted polypeptide |
| 222538_s_at | 14.52 | 1.43E-05 | 1.114144969 | NM_012096| | APPL,adaptor protein containing pH domain, PTB domain |
| 228844_at | 14.5 | 1.44E-05 | 1.443041337 | NM_177550| | SLC13A5,solute carrier family 13 (sodium-dependent |
| 1556761_at | 14.5 | 1.44E-05 | 1.365317303 | NA |  |
| 218126_at | 14.5 | 1.44E-05 | 1.098165286 | NM_018145| | FLJ10579,hypothetical protein FLJ10579 |
| 203669_s_at | 14.49 | 1.45E-05 | 1.112937419 | NM_012079| | DGAT1,diacylglycerol O-acyltransferase 1 |
| 238190_at | 14.49 | 1.45E-05 | 1.177471364 | NM_003321| | TUFM,Tu translation elongation factor, mitochondrial |
| 230274_s_at | 14.49 | 1.45E-05 | 1.221876555 | NM_002532| | NUP88,nucleoporin 88kDa |
| 212321_at | 14.49 | 1.45E-05 | 1.056896518 | NM_003901| | SGPL1,sphingosine-1-phosphate lyase 1 |
| 214006_s_at | 14.49 | 1.45E-05 | 1.072092617 | NM_000821| | GGCX,gamma-glutamyl carboxylase |
| 63825_at | 14.48 | 1.46E-05 | 1.137139677 | NM_007011| | ABHD2,alpha/beta hydrolase domain containing protein |
| 212508_at | 14.47 | 1.46E-05 | 1.045334104 | NM_022151| | MOAP1,modulator of apoptosis 1 |
| 218227_at | 14.47 | 1.46E-05 | 1.053207393 | NM_012225| | NUBP2,nucleotide binding protein 2 (MinD homolog, E. |
| 203776_at | 14.46 | 1.46E-05 | 1.084469171 | NM_015698| | GPKOW,G patch domain and KOW motifs |
| 221267_s_at | 14.46 | 1.47E-05 | 1.092523605 | NM_031213| | C19orf27,chromosome 19 open reading frame 27 |
| 201409_s_at | 14.46 | 1.47E-05 | 1.102459469 | NM_002709| | PPP1CB,protein phosphatase 1, catalytic subunit, beta |
| 200648_s_at | 14.44 | 1.48E-05 | 1.106607264 | NM_001033044| | NA |
| 223110_at | 14.44 | 1.48E-05 | 1.04484123 | NM_015496| | DKFZP434I116,DKFZP434I116 protein isoform 1 |
| 215517_at | 14.44 | 1.48E-05 | 1.433613616 | NM_015617| | PYGO1,pygopus homolog 1 |
| 1558101_at | 14.44 | 1.48E-05 | 1.820462167 | NA |  |
| 221516_s_at | 14.44 | 1.48E-05 | 1.059140475 | NM_019008| | FLJ20232,hypothetical protein FLJ20232 |
| 222757_s_at | 14.43 | 1.49E-05 | 1.138076249 | NM_016653| | ZAK,sterile-alpha motif and leucine zipper |
| 222613_at | 14.41 | 1.50E-05 | 1.123704659 | NM_020374| | C12orf4,chromosome 12 open reading frame 4 |
| 222441_x_at | 14.41 | 1.49E-05 | 1.053270832 | NM_016045| | C20orf45,CGI-107 protein |
| 211595_s_at | 14.4 | 1.50E-05 | 1.057002337 | NM_022839| | MRPS11,mitochondrial ribosomal protein S11 isoform a |
| 217964_at | 14.4 | 1.50E-05 | 1.028312888 | NM_017775| | TTC19,tetratricopeptide repeat domain 19 |
| 217959_s_at | 14.4 | 1.50E-05 | 1.027164349 | NM_016146| | TRAPPC4,trafficking protein particle complex 4 |
| 241937_s_at | 14.4 | 1.50E-05 | 1.068613562 | NM_018669| | WDR4,WD repeat domain 4 protein |
| 212140_at | 14.38 | 1.52E-05 | 1.034496049 | NM_001100399| | NA |
| 218628_at | 14.38 | 1.51E-05 | 1.069266458 | NM_016053| | CGI-116,CGI-116 protein |
| 231348_s_at | 14.38 | 1.51E-05 | 1.234516627 | NM_001001395| | LMO3,LIM domain only 3 |
| 234971_x_at | 14.38 | 1.51E-05 | 1.11741733 | NM_133373| | PLCD3,phospholipase C delta 3 |
| 219543_at | 14.38 | 1.51E-05 | 1.311771409 | NM_001033083| | NA |
| 209324_s_at | 14.37 | 1.52E-05 | 1.114888408 | NM_002928| | RGS16,regulator of G-protein signalling 16 |
| 1556021_at | 14.37 | 1.52E-05 | 1.575989383 | NA |  |
| 241396_at | 14.37 | 1.52E-05 | 1.108417462 | NM_015277| | NEDD4L,ubiquitin-protein ligase NEDD4-like |
| 230712_at | 14.37 | 1.52E-05 | 1.166361441 | NM_001037501| | NA |
| 1565833_at | 14.36 | 1.52E-05 | 1.278380264 | NA |  |
| 219596_at | 14.36 | 1.53E-05 | 1.085387853 | NM_020147| | THAP10,THAP domain containing 10 |
| 224447_s_at | 14.36 | 1.53E-05 | 1.098983442 | NM_032339| | C17orf37,chromosome 17 open reading frame 37 |
| 216913_s_at | 14.36 | 1.52E-05 | 1.100989269 | NM_015179| | KIAA0690,KIAA0690 |
| 241989_at | 14.35 | 1.53E-05 | 1.236357723 | NM_001004056| | GRK4,G protein-coupled receptor kinase 4 isoform |
| 235793_at | 14.35 | 1.53E-05 | 1.153190876 | NA |  |
| 212239_at | 14.35 | 1.53E-05 | 1.220199575 | NM_181504| | PIK3R1,phosphoinositide-3-kinase, regulatory subunit, |
| 210811_s_at | 14.35 | 1.53E-05 | 1.108003275 | NM_019070| | DDX49,DEAD (Asp-Glu-Ala-Asp) box polypeptide 49 |
| 230729_at | 14.35 | 1.53E-05 | 1.341723302 | NA |  |
| 223284_at | 14.34 | 1.54E-05 | 1.070825109 | NM_020378| | KLP1,K562 cell-derived leucine-zipper-like protein 1 |
| 215143_at | 14.34 | 1.54E-05 | 1.419530244 | NA |  |
| 217597_x_at | 14.34 | 1.54E-05 | 1.123521967 | NM_006822| | RAB40B,RAB40B, member RAS oncogene family |
| 209610_s_at | 14.33 | 1.54E-05 | 1.209343273 | NM_003038| | SLC1A4,solute carrier family 1, member 4 |
| 205364_at | 14.33 | 1.54E-05 | 1.132811598 | NM_003500| | ACOX2,acyl-Coenzyme A oxidase 2, branched chain |
| 225414_at | 14.33 | 1.54E-05 | 1.057477266 | NM_173647| | RNF149,ring finger protein 149 |
| 219392_x_at | 14.33 | 1.54E-05 | 1.071291286 | NM_018304| | FLJ11029,hypothetical protein FLJ11029 |
| 218742_at | 14.33 | 1.54E-05 | 1.069942168 | NM_022493| | NARFL,nuclear prelamin A recognition factor-like |
| 219538_at | 14.32 | 1.55E-05 | 1.207771821 | NM_019069| | WDR5B,WD repeat domain 5B |
| 239801_at | 14.32 | 1.55E-05 | 1.152851994 | NA |  |
| 217053_x_at | 14.3 | 1.56E-05 | 1.195244318 | NM_004956| | ETV1,ets variant gene 1 |
| 224523_s_at | 14.3 | 1.56E-05 | 1.054261716 | NM_032359| | MGC4308,hypothetical protein MGC4308 |
| 1569110_x_at | 14.29 | 1.56E-05 | 1.061025599 | NA |  |
| 201113_at | 14.28 | 1.57E-05 | 1.039595996 | NM_003321| | TUFM,Tu translation elongation factor, mitochondrial |
| 228032_s_at | 14.28 | 1.57E-05 | 1.232505046 | NA |  |
| 240637_at | 14.28 | 1.58E-05 | 1.214792846 | NM_018268| | WDR41,WD repeat domain 41 |
| 202871_at | 14.27 | 1.58E-05 | 1.034508758 | NM_004295| | TRAF4,TNF receptor-associated factor 4 isoform 1 |
| 202106_at | 14.27 | 1.58E-05 | 1.080266725 | NM_005895| | GOLGA3,Golgi autoantigen, golgin subfamily a, 3 |
| 201305_x_at | 14.26 | 1.58E-05 | 1.052563656 | NM_006401| | ANP32B,acidic (leucine-rich) nuclear phosphoprotein 32 |
| 212674_s_at | 14.26 | 1.58E-05 | 1.065009829 | NM_014966| | DHX30,DEAH (Asp-Glu-Ala-His) box polypeptide 30 |
| 219469_at | 14.26 | 1.59E-05 | 1.128672368 | NM_001080463| | NA |
| 228645_at | 14.26 | 1.58E-05 | 1.349540939 | NA |  |
| 230508_at | 14.26 | 1.58E-05 | 1.176347437 | NM_001018057| | NA |
| 202408_s_at | 14.26 | 1.58E-05 | 1.077968016 | NM_015629| | PRPF31,pre-mRNA processing factor 31 homolog |
| 203452_at | 14.25 | 1.59E-05 | 1.164897106 | NM_012200| | B3GAT3,beta-1,3-glucuronyltransferase 3 |
| 242172_at | 14.25 | 1.59E-05 | 1.984687299 | NM_002398| | MEIS1,Meis1 homolog |
| 226556_at | 14.25 | 1.59E-05 | 1.085656659 | NA |  |
| 200820_at | 14.24 | 1.60E-05 | 1.038071198 | NM_002812| | PSMD8,proteasome 26S non-ATPase subunit 8 |
| 236649_at | 14.23 | 1.61E-05 | 1.223436382 | NM_020234| | MDS009,x 009 protein |
| 213379_at | 14.23 | 1.60E-05 | 1.039813822 | NM_015697| | CL640,hypothetical protein CL640 |
| 224496_s_at | 14.22 | 1.61E-05 | 1.095703557 | NM_032354| | MGC10744,hypothetical protein MGC10744 isoform 1 |
| 202055_at | 14.22 | 1.61E-05 | 1.086402197 | NM_002264| | KPNA1,karyopherin alpha 1 |
| 226644_at | 14.22 | 1.61E-05 | 1.125754318 | NM_080875| | ZZANK1,zinc finger, ZZ type with ankyrin repeat domain |
| 201977_s_at | 14.21 | 1.61E-05 | 1.165544786 | NM_014773| | KIAA0141,KIAA0141 |
| 213861_s_at | 14.21 | 1.62E-05 | 1.167215628 | NM_015433| | DKFZP586D0919,hepatocellularcarcinoma-associated antigen |
| 201477_s_at | 14.21 | 1.62E-05 | 1.043982664 | NM_001033| | RRM1,ribonucleoside-diphosphate reductase M1 chain |
| 209407_s_at | 14.21 | 1.61E-05 | 1.165874292 | NM_021008| | DEAF1,suppressin |
| 204703_at | 14.21 | 1.62E-05 | 1.067021501 | NM_006531| | TTC10,Tg737 protein isoform 2 |
| 225119_at | 14.21 | 1.62E-05 | 1.066473339 | NM_176812| | C20orf178,Snf7 homologue associated with Alix 1 |
| 211973_at | 14.21 | 1.61E-05 | 1.174881967 | NA |  |
| 200058_s_at | 14.2 | 1.62E-05 | 1.043100601 | NM_014014| | ASCC3L1,activating signal cointegrator 1 complex subunit |
| 224963_at | 14.2 | 1.62E-05 | 1.051596367 | NM_000112| | SLC26A2,solute carrier family 26 member 2 |
| 37965_at | 14.2 | 1.62E-05 | 1.193368554 | NM_001003828| | PARVB,parvin, beta isoform a |
| 208445_s_at | 14.2 | 1.62E-05 | 1.029734149 | NM_032408| | BAZ1B,bromodomain adjacent to zinc finger domain, 1B |
| 1554167_a_at | 14.2 | 1.62E-05 | 1.089192604 | NM_001002296| | GOLGA7,golgi autoantigen, golgin subfamily a, 7 |
| 226410_at | 14.2 | 1.62E-05 | 1.118365451 | NM_001012759| | NA |
| 230401_at | 14.2 | 1.62E-05 | 1.25863846 | NA |  |
| 203525_s_at | 14.19 | 1.63E-05 | 1.191490638 | NM_000038| | APC,adenomatosis polyposis coli |
| 200665_s_at | 14.18 | 1.63E-05 | 1.081065487 | NM_003118| | SPARC,secreted protein, acidic, cysteine-rich |
| 225574_at | 14.18 | 1.64E-05 | 1.050186732 | NM_152682| | MGC10198,hypothetical protein MGC10198 |
| 222416_at | 14.18 | 1.64E-05 | 1.060760557 | NM_001017423| | NA |
| 218786_at | 14.18 | 1.63E-05 | 1.117625348 | NM_001031701| | NA |
| 214255_at | 14.16 | 1.65E-05 | 1.190242795 | NM_024490| | ATP10A,ATPase, Class V, type 10A |
| 226017_at | 14.15 | 1.65E-05 | 1.055010407 | NM_138410| | CKLFSF7,chemokine-like factor superfamily 7 isoform a |
| 200871_s_at | 14.15 | 1.66E-05 | 1.036840166 | NM_001042465| | NA |
| 1553909_x_at | 14.15 | 1.65E-05 | 1.136153913 | NM_018121| | C10orf6,chromosome 10 open reading frame 6 |
| 225377_at | 14.15 | 1.66E-05 | 1.030891422 | NM_017995| | NA |
| 203796_s_at | 14.15 | 1.65E-05 | 1.152923 | NM_001024808| | NA |
| 214753_at | 14.14 | 1.66E-05 | 1.213785261 | NM_014887| | PFAAP5,phosphonoformate immuno-associated protein 5 |
| 212786_at | 14.14 | 1.66E-05 | 1.135339642 | NM_015226| | KIAA0350,KIAA0350 protein |
| 239283_at | 14.14 | 1.66E-05 | 1.369721953 | NM_016040| | TMED5,transmembrane emp24 protein transport domain |
| 201077_s_at | 14.14 | 1.66E-05 | 1.036038702 | NM_001003796| | NHP2L1,NHP2 non-histone chromosome protein 2-like 1 |
| 204566_at | 14.14 | 1.66E-05 | 1.058951234 | NM_003620| | PPM1D,protein phosphatase 1D |
| 207711_at | 14.14 | 1.66E-05 | 1.393066363 | NM_199181| | FLJ44670,FLJ44670 protein |
| 228045_at | 14.14 | 1.66E-05 | 1.167177017 | NA |  |
| 224303_x_at | 14.13 | 1.67E-05 | 1.121313196 | NM_016350| | NIN,ninein isoform 4 |
| 1558906_a_at | 14.13 | 1.66E-05 | 1.109652803 | NA |  |
| 230306_at | 14.13 | 1.67E-05 | 1.179821847 | NM_052875| | MGC10485,hypothetical protein MGC10485 |
| 208986_at | 14.12 | 1.67E-05 | 1.181572436 | NM_003205| | TCF12,transcription factor 12 isoform b |
| 202172_at | 14.12 | 1.67E-05 | 1.099938999 | NM_007146| | ZNF161,zinc finger protein 161 |
| 226448_at | 14.11 | 1.68E-05 | 1.203096293 | NM_198552| | MGC15887,hypothetical gene supported by BC009447 |
| 229815_at | 14.11 | 1.68E-05 | 1.180731996 | NA |  |
| 203206_at | 14.11 | 1.68E-05 | 1.134232449 | NM_014661| | FAM53B,family with sequence similarity 53, member B |
| 224568_x_at | 14.11 | 1.68E-05 | 1.244320701 | NA |  |
| 33307_at | 14.1 | 1.69E-05 | 1.096803126 | NM_015703| | CGI-96,CGI-96 protein |
| 214011_s_at | 14.09 | 1.69E-05 | 1.068002269 | NM_016391| | HSPC111,hypothetical protein HSPC111 |
| 202937_x_at | 14.08 | 1.70E-05 | 1.09243125 | NM_015703| | CGI-96,CGI-96 protein |
| 230449_x_at | 14.08 | 1.70E-05 | 1.184506288 | NA |  |
| 1557804_at | 14.08 | 1.70E-05 | 1.324180123 | NA |  |
| 202239_at | 14.08 | 1.70E-05 | 1.042592811 | NM_006437| | PARP4,poly (ADP-ribose) polymerase family, member 4 |
| 230097_at | 14.07 | 1.70E-05 | 1.334504028 | NM_000819| | GART,phosphoribosylglycinamide formyltransferase, |
| 220914_at | 14.07 | 1.71E-05 | 1.190220474 | NA |  |
| 217786_at | 14.06 | 1.71E-05 | 1.060383144 | NM_001039619| | NA |
| 204839_at | 14.06 | 1.71E-05 | 1.045316768 | NM_015918| | POP5,processing of precursor 5, ribonuclease P/MRP |
| 1556551_s_at | 14.06 | 1.71E-05 | 1.205605148 | NM_001099406| | NA |
| 221270_s_at | 14.06 | 1.71E-05 | 1.125371728 | NM_031209| | QTRT1,queuine tRNA-ribosyltransferase 1 (tRNA-guanine |
| 209680_s_at | 14.05 | 1.72E-05 | 1.020069287 | NM_002263| | NA |
| 207494_s_at | 14.05 | 1.72E-05 | 1.10868975 | NM_003427| | ZNF76,zinc finger protein 76 (expressed in testis) |
| 229632_s_at | 14.05 | 1.72E-05 | 1.068307062 | NM_018142| | FLJ10569,hypothetical protein FLJ10569 |
| 218143_s_at | 14.05 | 1.72E-05 | 1.090942383 | NM_005697| | SCAMP2,secretory carrier membrane protein 2 |
| 217809_at | 14.05 | 1.72E-05 | 1.027030594 | NM_014038| | BZW2,basic leucine zipper and W2 domains 2 |
| 214043_at | 14.05 | 1.72E-05 | 1.077228627 | NM_001040712| | NA |
| 200698_at | 14.04 | 1.73E-05 | 1.104580401 | NM_001100603| | NA |
| 212000_at | 14.04 | 1.72E-05 | 1.141656228 | NM_001017392| | NA |
| 227564_at | 14.04 | 1.72E-05 | 1.179608461 | NM_152419| | NA |
| 230168_at | 14.04 | 1.72E-05 | 1.241494443 | NA |  |
| 40569_at | 14.04 | 1.72E-05 | 1.101051412 | NM_003422| | ZNF42,zinc finger protein 42 isoform 1 |
| 233564_s_at | 14.04 | 1.72E-05 | 1.260345254 | NM_030911| | CDADC1,cytidine and dCMP deaminase domain containing 1 |
| 242888_at | 14.03 | 1.73E-05 | 1.549998386 | NA |  |
| 239718_at | 14.02 | 1.74E-05 | 1.247372674 | NA |  |
| 230031_at | 14.02 | 1.74E-05 | 1.121376943 | NM_005347| | HSPA5,heat shock 70kDa protein 5 (glucose-regulated |
| 202183_s_at | 14.02 | 1.74E-05 | 1.039660329 | NM_007317| | KIF22,kinesin family member 22 |
| 228664_at | 14.02 | 1.74E-05 | 1.102365248 | NA |  |
| 1557239_at | 14.01 | 1.74E-05 | 1.194375542 | NM_020235| | BBX,HMG-BOX transcription factor BBX |
| 223842_s_at | 14.01 | 1.74E-05 | 1.4015865 | NM_016240| | SCARA3,scavenger receptor class A, member 3 isoform 1 |
| 225498_at | 14.01 | 1.75E-05 | 1.066807783 | NM_176812| | C20orf178,Snf7 homologue associated with Alix 1 |
| 225209_s_at | 14.01 | 1.74E-05 | 1.037324541 | NM_058167| | UBE2J2,ubiquitin conjugating enzyme E2, J2 isoform 2 |
| 225826_at | 14 | 1.76E-05 | 1.199367784 | NM_052845| | MMAB,cob(I)alamin adenosyltransferase |
| 239581_at | 14 | 1.75E-05 | 1.245831889 | NA |  |
| 225588_s_at | 14 | 1.75E-05 | 1.096805634 | NM_001127266| | NA |
| 221844_x_at | 14 | 1.76E-05 | 1.067809358 | NA |  |
| 244196_at | 13.99 | 1.76E-05 | 1.199951861 | NA |  |
| 201539_s_at | 13.99 | 1.76E-05 | 1.182460664 | NM_001449| | FHL1,four and a half LIM domains 1 |
| 212956_at | 13.99 | 1.76E-05 | 1.167121517 | NM_015130| | KIAA0882,KIAA0882 protein |
| 225300_at | 13.99 | 1.76E-05 | 1.047801906 | NM_033286| | C15orf23,chromosome 15 open reading frame 23 |
| 52741_at | 13.98 | 1.76E-05 | 1.066061471 | NM_152307| | C14orf172,chromosome 14 open reading frame 172 |
| 218920_at | 13.98 | 1.77E-05 | 1.034271689 | NM_019057| | FLJ10404,hypothetical protein FLJ10404 |
| 203450_at | 13.98 | 1.77E-05 | 1.092794789 | NM_001002880| | PGEA1,PKD2 interactor, golgi and endoplasmic reticulum |
| 215574_at | 13.97 | 1.78E-05 | 1.229585039 | NA |  |
| 216843_x_at | 13.97 | 1.78E-05 | 1.142205251 | NA |  |
| 225898_at | 13.96 | 1.79E-05 | 1.045252638 | NM_032118| | WDR54,WD repeat domain 54 |
| 229966_at | 13.95 | 1.79E-05 | 1.197615008 | NM_005243| | EWSR1,Ewing sarcoma breakpoint region 1 isoform EWS |
| 218801_at | 13.95 | 1.79E-05 | 1.327098697 | NM_020121| | UGCGL2,UDP-glucose:glycoprotein glucosyltransferase 2 |
| 215731_s_at | 13.95 | 1.79E-05 | 1.159968412 | NM_022782| | MPHOSPH9,M-phase phosphoprotein 9 |
| 226186_at | 13.94 | 1.80E-05 | 1.419681728 | NA |  |
| 224616_at | 13.94 | 1.80E-05 | 1.059979789 | NM_006141| | DNCLI2,dynein, cytoplasmic, light intermediate |
| 209465_x_at | 13.94 | 1.79E-05 | 1.166026631 | NM_002825| | PTN,pleiotrophin |
| 227722_at | 13.93 | 1.80E-05 | 1.203947645 | NM_001025| | RPS23,ribosomal protein S23 |
| 204846_at | 13.93 | 1.80E-05 | 1.342758092 | NM_000096| | CP,ceruloplasmin (ferroxidase) |
| 225079_at | 13.92 | 1.81E-05 | 1.071665197 | NM_001424| | EMP2,epithelial membrane protein 2 |
| 1552685_a_at | 13.92 | 1.81E-05 | 1.428085693 | NM_014552| | TFCP2L2,leader-binding protein 32 isoform 1 |
| 203156_at | 13.91 | 1.82E-05 | 1.066375114 | NM_016248| | AKAP11,A-kinase anchor protein 11 isoform 1 |
| 1554906_a_at | 13.91 | 1.82E-05 | 1.456593717 | NM_005792| | MPHOSPH6,M-phase phosphoprotein 6 |
| 225099_at | 13.91 | 1.82E-05 | 1.044146602 | NM_001105573| | NA |
| 229870_at | 13.91 | 1.82E-05 | 1.141741595 | NA |  |
| 226143_at | 13.91 | 1.82E-05 | 1.227717675 | NM_030665| | RAI1,retinoic acid induced 1 |
| 221206_at | 13.91 | 1.82E-05 | 1.100460383 | NM_000535| | PMS2,PMS2 |
| 211065_x_at | 13.9 | 1.82E-05 | 1.038453202 | NM_001002021| | PFKL,liver phosphofructokinase isoform a |
| 204977_at | 13.89 | 1.83E-05 | 1.024198576 | NM_004398| | DDX10,DEAD (Asp-Glu-Ala-Asp) box polypeptide 10 |
| 232528_at | 13.89 | 1.83E-05 | 1.438090366 | NA |  |
| 236337_at | 13.89 | 1.83E-05 | 1.261954511 | NM_001040274| | NA |
| 244585_at | 13.89 | 1.83E-05 | 1.139453618 | NM_014714| | KIAA0590,KIAA0590 gene product |
| 221828_s_at | 13.88 | 1.84E-05 | 1.15123433 | NM_001011703| | C9orf28,chromosome 9 open reading frame 28 isoform 2 |
| 244766_at | 13.87 | 1.85E-05 | 1.161255145 | NM_015092| | SMG1,PI-3-kinase-related kinase SMG-1 isoform 1 |
| 235551_at | 13.87 | 1.85E-05 | 1.318135413 | NM_018669| | WDR4,WD repeat domain 4 protein |
| 226479_at | 13.87 | 1.85E-05 | 1.142886175 | NM_152903| | KBTBD6,kelch repeat and BTB (POZ) domain-containing 6 |
| 215096_s_at | 13.87 | 1.85E-05 | 1.090930893 | NM_001984| | ESD,esterase D/formylglutathione hydrolase |
| 231411_at | 13.87 | 1.85E-05 | 1.219306207 | NM_005780| | LHFP,lipoma HMGIC fusion partner |
| 219742_at | 13.87 | 1.85E-05 | 1.121012899 | NM_030567| | PRR7,proline rich 7 (synaptic) |
| 221427_s_at | 13.87 | 1.85E-05 | 1.112975553 | NM_001039577| | NA |
| 232899_at | 13.86 | 1.86E-05 | 1.145305318 | NM_203302| | MGC70863,similar to RPL23AP7 protein |
| 226684_at | 13.86 | 1.86E-05 | 1.068857213 | NM_018036| | C14orf103,chromosome 14 open reading frame 103 |
| 228765_at | 13.86 | 1.86E-05 | 1.064423278 | NM_173537| | GTF2IRD2,GTF2I repeat domain containing 2 |
| 209143_s_at | 13.86 | 1.86E-05 | 1.022869787 | NM_001293| | CLNS1A,chloride channel, nucleotide-sensitive, 1A |
| 234135_x_at | 13.86 | 1.86E-05 | 1.129348898 | NA |  |
| 239252_at | 13.86 | 1.86E-05 | 1.225287723 | NA |  |
| 227562_at | 13.86 | 1.86E-05 | 1.136015765 | NA |  |
| 227746_at | 13.85 | 1.86E-05 | 1.330114308 | NM_001419| | ELAVL1,ELAV-like 1 |
| 200965_s_at | 13.85 | 1.86E-05 | 1.06600634 | NM_001003407| | ABLIM1,actin-binding LIM protein 1 isoform b |
| 230605_at | 13.85 | 1.86E-05 | 1.213100746 | NA |  |
| 213573_at | 13.84 | 1.87E-05 | 1.105949296 | NA |  |
| 203657_s_at | 13.84 | 1.87E-05 | 1.061857466 | NM_003793| | CTSF,cathepsin F |
| 225085_at | 13.84 | 1.87E-05 | 1.150535094 | NM_018218| | USP40,ubiquitin specific protease 40 |
| 1552766_at | 13.83 | 1.88E-05 | 1.753958472 | NM_001077188| | NA |
| 1557950_at | 13.83 | 1.88E-05 | 1.047773267 | NM_004713| | SDCCAG1,serologically defined colon cancer antigen 1 |
| 1557360_at | 13.82 | 1.89E-05 | 1.139262021 | NM_133259| | LRPPRC,leucine-rich PPR motif-containing protein |
| 209692_at | 13.82 | 1.88E-05 | 1.260139933 | NM_005244| | EYA2,eyes absent 2 isoform a |
| 227585_at | 13.81 | 1.89E-05 | 1.065341615 | NA |  |
| 1552931_a_at | 13.81 | 1.90E-05 | 1.488672532 | NM_002605| | PDE8A,phosphodiesterase 8A isoform 1 |
| 203931_s_at | 13.81 | 1.89E-05 | 1.046981386 | NM_002949| | MRPL12,mitochondrial ribosomal protein L12 |
| 234299_s_at | 13.8 | 1.90E-05 | 1.438523797 | NM_016350| | NIN,ninein isoform 4 |
| 228239_at | 13.8 | 1.90E-05 | 1.265620875 | NM_058182| | C21orf51,chromosome 21 open reading frame 51 |
| 227203_at | 13.8 | 1.90E-05 | 1.26380095 | NM_022824| | FBXL17,F-box and leucine-rich repeat protein 17 |
| 1557270_at | 13.8 | 1.90E-05 | 1.263329292 | NA |  |
| 227968_at | 13.79 | 1.90E-05 | 1.12502657 | NM_182612| | FLJ34283,hypothetical protein FLJ34283 |
| 227890_at | 13.79 | 1.91E-05 | 1.16383532 | NM_001005209| | MGC99813,similar to RIKEN cDNA A230078I05 gene |
| 203536_s_at | 13.79 | 1.91E-05 | 1.057346361 | NM_004804| | WDR39,WD repeat domain 39 |
| 210268_at | 13.79 | 1.91E-05 | 1.266388011 | NM_002504| | NFX1,nuclear transcription factor, X-box binding 1 |
| 218278_at | 13.78 | 1.91E-05 | 1.036695197 | NM_018093| | FLJ10439,hypothetical protein FLJ10439 |
| 226091_s_at | 13.78 | 1.91E-05 | 1.02276006 | NM_033296| | PGR1,protein associated with MRG, 14 kDa |
| 229092_at | 13.78 | 1.91E-05 | 1.581941685 | NM_021005| | NR2F2,nuclear receptor subfamily 2, group F, member 2 |
| 211891_s_at | 13.78 | 1.92E-05 | 1.379630359 | NM_015320| | ARHGEF4,Rho guanine nucleotide exchange factor 4 isoform |
| 208829_at | 13.77 | 1.93E-05 | 1.059254089 | NM_003190| | TAPBP,tapasin isoform 1 precursor |
| 204915_s_at | 13.77 | 1.92E-05 | 1.031959622 | NM_003108| | SOX11,SRY-box 11 |
| 223482_at | 13.77 | 1.93E-05 | 1.091992449 | NM_031925| | TMPIT,transmembrane protein induced by tumor necrosis |
| 203245_s_at | 13.76 | 1.93E-05 | 1.10531222 | NA |  |
| 239533_at | 13.76 | 1.93E-05 | 1.915605109 | NM_001033045| | NA |
| 203511_s_at | 13.76 | 1.93E-05 | 1.058520973 | NM_014408| | TRAPPC3,BET3 homolog |
| 1552651_a_at | 13.76 | 1.93E-05 | 1.0911238 | NM_001017368| | NA |
| 235240_at | 13.76 | 1.93E-05 | 1.06653895 | NM_004993| | ATXN3,ataxin 3 isoform 1 |
| 209325_s_at | 13.75 | 1.94E-05 | 1.144678241 | NM_002928| | RGS16,regulator of G-protein signalling 16 |
| 204241_at | 13.75 | 1.94E-05 | 1.166029614 | NM_001101667| | NA |
| 233575_s_at | 13.74 | 1.95E-05 | 1.509428068 | NM_007005| | TLE4,transducin-like enhancer protein 4 |
| 202169_s_at | 13.74 | 1.95E-05 | 1.099841055 | NM_015423| | AASDHPPT,aminoadipate-semialdehyde |
| 236591_at | 13.74 | 1.95E-05 | 1.470926768 | NA |  |
| 211981_at | 13.73 | 1.95E-05 | 1.134697384 | NM_001845| | COL4A1,alpha 1 type IV collagen preproprotein |
| 204161_s_at | 13.73 | 1.96E-05 | 1.14924131 | NM_014936| | ENPP4,ectonucleotide pyrophosphatase/phosphodiesterase |
| 204251_s_at | 13.73 | 1.95E-05 | 1.1229227 | NM_014956| | Cep164,KIAA1052 protein |
| 242068_at | 13.73 | 1.95E-05 | 1.298905512 | NA |  |
| 220173_at | 13.73 | 1.95E-05 | 1.435255289 | NM_025057| | C14orf45,chromosome 14 open reading frame 45 |
| 225321_s_at | 13.73 | 1.95E-05 | 1.109127209 | NM_013440| | PILRB,paired immunoglobulin-like type 2 receptor beta |
| 203380_x_at | 13.73 | 1.96E-05 | 1.052280325 | NM_001039465| | NA |
| 221897_at | 13.73 | 1.95E-05 | 1.103015123 | NM_032765| | TRIM52,tripartite motif-containing 52 |
| 209078_s_at | 13.72 | 1.96E-05 | 1.072933639 | NM_012473| | TXN2,thioredoxin 2 precursor |
| 212284_x_at | 13.72 | 1.96E-05 | 1.017763559 | NM_003295| | TPT1,tumor protein, translationally-controlled 1 |
| 235760_at | 13.72 | 1.96E-05 | 1.357805384 | NM_022455| | NSD1,nuclear receptor binding SET domain protein 1 |
| 48580_at | 13.71 | 1.96E-05 | 1.035480123 | NM_001101654| | NA |
| 235926_at | 13.71 | 1.97E-05 | 1.258153766 | NA |  |
| 243704_at | 13.71 | 1.97E-05 | 1.191562728 | NA |  |
| 203148_s_at | 13.7 | 1.97E-05 | 1.075105375 | NM_014788| | TRIM14,tripartite motif protein TRIM14 isoform alpha |
| 223109_at | 13.7 | 1.97E-05 | 1.075787337 | NM_015679| | TRUB2,TruB pseudouridine (psi) synthase homolog 2 |
| 208050_s_at | 13.7 | 1.97E-05 | 1.138639073 | NM_032982| | CASP2,caspase 2 isoform 1 preproprotein |
| 235347_at | 13.7 | 1.97E-05 | 1.108300435 | NM_032773| | LRCH3,leucine-rich repeats and calponin homology (CH) |
| 1553947_at | 13.7 | 1.97E-05 | 1.150466938 | NM_058219| | EXOSC6,homolog of yeast mRNA transport regulator 3 |
| 212601_at | 13.7 | 1.98E-05 | 1.116086776 | NM_015113| | ZZEF1,zinc finger, ZZ-type with EF hand domain 1 |
| 208721_s_at | 13.7 | 1.97E-05 | 1.139025119 | NM_016237| | ANAPC5,anaphase-promoting complex subunit 5 |
| 226112_at | 13.69 | 1.99E-05 | 1.094092808 | NM_000232| | SGCB,sarcoglycan, beta (43kDa dystrophin-associated |
| 233360_at | 13.69 | 1.98E-05 | 1.103150298 | NM_003345| | UBE2I,ubiquitin-conjugating enzyme E2I |
| 238146_at | 13.69 | 1.98E-05 | 1.369468454 | NA |  |
| 238597_at | 13.69 | 1.99E-05 | 1.200002002 | NM_030816| | DKFZP566D1346,hypothetical protein DKFZp566D1346 |
| 220865_s_at | 13.68 | 1.99E-05 | 1.052739102 | NM_014317| | TPRT,trans-prenyltransferase |
| 221972_s_at | 13.68 | 1.99E-05 | 1.126933695 | NM_016176| | Cab45,calcium binding protein Cab45 precursor |
| 227563_at | 13.68 | 1.99E-05 | 1.193878969 | NM_175923| | MGC42630,hypothetical protein MGC42630 |
| 1558426_x_at | 13.67 | 2.00E-05 | 1.177404417 | NM_001126340| | NA |
| 223742_at | 13.67 | 2.00E-05 | 1.211593869 | NM_015956| | MRPL4,mitochondrial ribosomal protein L4 isoform a |
| 202179_at | 13.67 | 2.00E-05 | 1.092944543 | NM_000386| | BLMH,bleomycin hydrolase |
| 232256_s_at | 13.67 | 2.00E-05 | 1.523922444 | NA |  |
| 242648_at | 13.67 | 2.00E-05 | 1.115136055 | NM_020803| | KLHL8,kelch-like 8 |
| 227751_at | 13.67 | 2.00E-05 | 1.303954765 | NM_004708| | PDCD5,programmed cell death 5 |
| 235925_at | 13.66 | 2.01E-05 | 1.440193512 | NM_003205| | TCF12,transcription factor 12 isoform b |
| 220076_at | 13.66 | 2.01E-05 | 1.948876656 | NM_054027| | ANKH,ankylosis, progressive homolog |
| 1566482_at | 13.66 | 2.00E-05 | 1.275833902 | NA |  |
| 222999_s_at | 13.64 | 2.02E-05 | 1.084480822 | NM_001039577| | NA |
| 238932_at | 13.64 | 2.03E-05 | 1.216026344 | NA |  |
| 227935_s_at | 13.64 | 2.03E-05 | 1.161504212 | NM_032373| | PCGF5,polycomb group ring finger 5 |
| 228769_at | 13.63 | 2.03E-05 | 1.14980141 | NM_181846| | HKR2,GLI-Kruppel family member HKR2 |
| 207018_s_at | 13.63 | 2.03E-05 | 2.366770807 | NM_004163| | RAB27B,RAB27B, member RAS oncogene family |
| 227406_at | 13.63 | 2.03E-05 | 1.073789126 | NM_002041| | GABPB2,GA binding protein transcription factor, beta |
| 238461_at | 13.63 | 2.03E-05 | 1.274226187 | NM_173359| | NA |
| 222275_at | 13.63 | 2.03E-05 | 1.042348474 | NA |  |
| 223774_at | 13.63 | 2.03E-05 | 1.086127279 | NA |  |
| 222415_at | 13.62 | 2.04E-05 | 1.045765032 | NM_170606| | MLL3,myeloid/lymphoid or mixed-lineage leukemia 3 |
| 1566513_a_at | 13.62 | 2.04E-05 | 1.430356465 | NM_001098721| | NA |
| 202246_s_at | 13.62 | 2.04E-05 | 1.070331167 | NM_000075| | CDK4,cyclin-dependent kinase 4 |
| 226611_s_at | 13.61 | 2.05E-05 | 1.048819624 | NM_181716| | PRR6,proline rich 6 |
| 224948_at | 13.61 | 2.05E-05 | 1.053479248 | NM_032014| | MRPS24,mitochondrial ribosomal protein S24 |
| 236038_at | 13.61 | 2.05E-05 | 1.221522236 | NA |  |
| 213609_s_at | 13.6 | 2.06E-05 | 1.479880956 | NM_021115| | SEZ6L,seizure related 6 homolog (mouse)-like |
| 208968_s_at | 13.6 | 2.06E-05 | 1.026997435 | NM_020313| | CIAPIN1,cytokine induced apoptosis inhibitor 1 |
| 202139_at | 13.59 | 2.07E-05 | 1.12119598 | NM_003689| | AKR7A2,aldo-keto reductase family 7, member A2 |
| 227992_s_at | 13.59 | 2.07E-05 | 1.262924611 | NA |  |
| 203926_x_at | 13.59 | 2.07E-05 | 1.073306034 | NM_001001975| | ATP5D,ATP synthase, H+ transporting, mitochondrial F1 |
| 218483_s_at | 13.59 | 2.07E-05 | 1.073648256 | NM_020153| | FLJ21827,hypothetical protein FLJ21827 |
| 203209_at | 13.58 | 2.07E-05 | 1.068349079 | NM_007370| | RFC5,replication factor C 5 isoform 1 |
| 200769_s_at | 13.58 | 2.08E-05 | 1.195792376 | NM_005911| | MAT2A,methionine adenosyltransferase II, alpha |
| 225712_at | 13.58 | 2.07E-05 | 1.037265638 | NM_015465| | GEMIN5,gemin 5 |
| 244414_at | 13.58 | 2.07E-05 | 1.522171774 | NA |  |
| 220441_at | 13.58 | 2.07E-05 | 1.472687462 | NM_024902| | FLJ13236,hypothetical protein FLJ13236 |
| 209192_x_at | 13.57 | 2.08E-05 | 1.108743193 | NM_006388| | HTATIP,HIV-1 Tat interactive protein, 60kDa isoform 2 |
| 243683_at | 13.57 | 2.09E-05 | 1.198726796 | NM_012286| | MORF4L2,MORF-related gene X |
| 212002_at | 13.56 | 2.10E-05 | 1.130846222 | NM_001114600| | NA |
| 235878_at | 13.56 | 2.09E-05 | 1.106068917 | NM_005680| | TAF1B,TBP-associated factor 1B |
| 202660_at | 13.56 | 2.09E-05 | 1.264624865 | NM_002223| | ITPR2,inositol 1,4,5-triphosphate receptor, type 2 |
| 204530_s_at | 13.55 | 2.10E-05 | 1.240960036 | NM_014729| | TOX,thymus high mobility group box protein TOX |
| 210592_s_at | 13.55 | 2.10E-05 | 1.050939378 | NM_002970| | SAT,spermidine/spermine N1-acetyltransferase |
| 206993_at | 13.55 | 2.10E-05 | 1.152382886 | NM_001003803| | ATP5S,ATP synthase, H+ transporting, mitochondrial F0 |
| 232395_x_at | 13.55 | 2.10E-05 | 1.581792179 | NA |  |
| 203855_at | 13.54 | 2.11E-05 | 1.156695401 | NM_014969| | WDR47,WD repeat domain 47 |
| 227213_at | 13.54 | 2.11E-05 | 1.13908061 | NM_182503| | DEADC1,deaminase domain containing 1 |
| 236609_at | 13.54 | 2.11E-05 | 1.284796213 | NA |  |
| 225097_at | 13.54 | 2.11E-05 | 1.07819695 | NM_001113239| | NA |
| 222667_s_at | 13.53 | 2.11E-05 | 1.114513082 | NM_018489| | ASH1L,ash1 (absent, small, or homeotic)-like |
| 47083_at | 13.53 | 2.12E-05 | 1.039943361 | NM_024067| | C7orf26,chromosome 7 open reading frame 26 |
| 211966_at | 13.53 | 2.11E-05 | 1.196773983 | NM_001846| | COL4A2,alpha 2 type IV collagen preproprotein |
| 225443_at | 13.53 | 2.11E-05 | 1.094265366 | NM_018403| | DCP1A,decapping enzyme |
| 220633_s_at | 13.53 | 2.11E-05 | 1.166150143 | NM_016287| | HP1-BP74,HP1-BP74 |
| 221767_x_at | 13.53 | 2.12E-05 | 1.050395532 | NM_005336| | HDLBP,high density lipoprotein binding protein |
| 216583_x_at | 13.52 | 2.13E-05 | 1.093399245 | NM_001034833| | NA |
| 41660_at | 13.52 | 2.12E-05 | 1.060168213 | NM_014246| | CELSR1,cadherin EGF LAG seven-pass G-type receptor 1 |
| 206866_at | 13.52 | 2.12E-05 | 1.098393866 | NM_001794| | CDH4,cadherin 4, type 1 preproprotein |
| 201664_at | 13.52 | 2.12E-05 | 1.043610143 | NM_001002800| | SMC4L1,SMC4 structural maintenance of chromosomes |
| 204034_at | 13.52 | 2.13E-05 | 1.060973325 | NM_014297| | ETHE1,ETHE1 protein |
| 212985_at | 13.52 | 2.13E-05 | 1.091122856 | NA |  |
| 228023_x_at | 13.52 | 2.12E-05 | 1.090797477 | NM_020978| | AMY2B,amylase, alpha 2B; pancreatic precursor |
| 201587_s_at | 13.52 | 2.13E-05 | 1.077703634 | NM_001025242| | NA |
| 218234_at | 13.51 | 2.13E-05 | 1.117038856 | NM_016162| | ING4,inhibitor of growth family, member 4 isoform 1 |
| 203883_s_at | 13.51 | 2.13E-05 | 1.054646035 | NM_014904| | RAB11FIP2,RAB11 family interacting protein 2 (class I) |
| 233019_at | 13.51 | 2.13E-05 | 1.288194813 | NM_013354| | CNOT7,CCR4-NOT transcription complex, subunit 7 |
| 228573_at | 13.51 | 2.13E-05 | 1.232631383 | NA |  |
| 235224_s_at | 13.5 | 2.14E-05 | 1.53601302 | NA |  |
| 221158_at | 13.5 | 2.14E-05 | 1.073733585 | NM_013329| | C21orf66,GC-rich sequence DNA-binding factor candidate |
| 239246_at | 13.5 | 2.14E-05 | 1.225723835 | NM_001001715| | FARP1,FERM, RhoGEF, and pleckstrin domain protein 1 |
| 243278_at | 13.49 | 2.15E-05 | 1.57683363 | NM_014491| | FOXP2,forkhead box P2 isoform I |
| 223516_s_at | 13.49 | 2.15E-05 | 1.022661078 | NM_013397| | C6orf49,over-expressed breast tumor protein |
| 212573_at | 13.49 | 2.15E-05 | 1.230447958 | NM_015036| | NA |
| 212808_at | 13.49 | 2.15E-05 | 1.197068038 | NM_032815| | NFATC2IP,nuclear factor of activated T-cells, |
| 218609_s_at | 13.48 | 2.16E-05 | 1.063561585 | NM_001161| | NUDT2,nudix-type motif 2 |
| 203839_s_at | 13.47 | 2.17E-05 | 1.089257247 | NM_001010938| | TNK2,tyrosine kinase, non-receptor, 2 isoform 2 |
| 225176_at | 13.47 | 2.17E-05 | 1.220323702 | NA |  |
| 238672_at | 13.47 | 2.17E-05 | 1.224562575 | NA |  |
| 232330_at | 13.47 | 2.17E-05 | 1.851308885 | NM_018224| | FLJ10803,hypothetical protein FLJ10803 |
| 211865_s_at | 13.47 | 2.17E-05 | 1.134005004 | NM_016263| | FZR1,Fzr1 protein |
| 203753_at | 13.47 | 2.17E-05 | 1.0972797 | NM_001083962| | NA |
| 201771_at | 13.46 | 2.18E-05 | 1.10109246 | NM_005698| | SCAMP3,secretory carrier membrane protein 3 isoform 1 |
| 225877_at | 13.46 | 2.17E-05 | 1.070330477 | NM_001040273| | NA |
| 203658_at | 13.45 | 2.19E-05 | 1.190141473 | NM_000387| | SLC25A20,carnitine/acylcarnitine translocase |
| 201999_s_at | 13.45 | 2.19E-05 | 1.03432287 | NM_006519| | TCTEL1,t-complex-associated-testis-expressed 1-like 1 |
| 228512_at | 13.45 | 2.19E-05 | 1.065153056 | NM_017952| | FLJ20758,FLJ20758 protein |
| 224739_at | 13.45 | 2.18E-05 | 1.032067522 | NM_001001852| | PIM3,pim-3 oncogene |
| 224988_at | 13.45 | 2.18E-05 | 1.184149905 | NM_152734| | C6orf89,hypothetical protein FLJ25357 |
| 236264_at | 13.44 | 2.19E-05 | 1.663847018 | NM_015236| | LPHN3,latrophilin 3 precursor |
| 208965_s_at | 13.44 | 2.20E-05 | 1.385808605 | NM_005531| | IFI16,interferon, gamma-inducible protein 16 |
| 217624_at | 13.44 | 2.19E-05 | 1.23775281 | NM_014891| | PDAP1,PDGFA associated protein 1 |
| 223456_s_at | 13.44 | 2.19E-05 | 1.336993849 | NM_032300| | MGC10854,hypothetical protein MGC10854 |
| 208803_s_at | 13.43 | 2.20E-05 | 1.030292217 | NM_006947| | SRP72,signal recognition particle 72kDa |
| 228655_at | 13.43 | 2.20E-05 | 1.197653827 | NA |  |
| 217755_at | 13.43 | 2.20E-05 | 1.05579091 | NM_001002032| | HN1,hematological and neurological expressed 1 |
| 227150_at | 13.43 | 2.20E-05 | 1.095792373 | NM_005955| | MTF1,metal-regulatory transcription factor 1 |
| 222740_at | 13.43 | 2.21E-05 | 1.108905404 | NM_014109| | ATAD2,two AAA domain containing protein |
| 208212_s_at | 13.42 | 2.21E-05 | 1.453817618 | NM_004304| | ALK,anaplastic lymphoma kinase Ki-1 |
| 242108_at | 13.42 | 2.21E-05 | 1.160447783 | NA |  |
| 214040_s_at | 13.42 | 2.21E-05 | 1.1548387 | NM_000177| | GSN,gelsolin isoform a |
| 222809_x_at | 13.42 | 2.21E-05 | 1.070880645 | NA |  |
| 218336_at | 13.42 | 2.21E-05 | 1.070384608 | NM_012394| | PFDN2,prefoldin 2 |
| 219587_at | 13.42 | 2.21E-05 | 1.074551235 | NM_017868| | TTC12,tetratricopeptide repeat domain 12 |
| 222026_at | 13.42 | 2.21E-05 | 1.102695435 | NM_006743| | RBM3,RNA binding motif (RNP1, RRM) protein 3 |
| 217907_at | 13.42 | 2.21E-05 | 1.059824035 | NM_014161| | MRPL18,mitochondrial ribosomal protein L18 |
| 218565_at | 13.41 | 2.21E-05 | 1.13248401 | NM_016390| | C9orf114,chromosome 9 open reading frame 114 |
| 224573_at | 13.41 | 2.22E-05 | 1.023452075 | NM_001004333| | MGC71993,similar to DNA segment, Chr 11, Brigham & Womens |
| 201536_at | 13.41 | 2.22E-05 | 1.053042602 | NM_004090| | DUSP3,dual specificity phosphatase 3 |
| 216602_s_at | 13.4 | 2.23E-05 | 1.108877822 | NM_004461| | FARSLA,phenylalanine-tRNA synthetase-like protein |
| 222644_s_at | 13.4 | 2.23E-05 | 1.275278013 | NM_024656| | GLT25D1,glycosyltransferase 25 domain containing 1 |
| 223871_x_at | 13.4 | 2.23E-05 | 1.203153987 | NM_032329| | ING5,inhibitor of growth family, member 5 |
| 1555573_at | 13.39 | 2.24E-05 | 1.304562765 | NM_173572| | C10orf93,chromosome 10 open reading frame 93 |
| 209468_at | 13.39 | 2.24E-05 | 1.114148281 | NM_002335| | LRP5,low density lipoprotein receptor-related protein |
| 217927_at | 13.39 | 2.24E-05 | 1.038610173 | NM_014041| | SPCS1,signal peptidase complex subunit 1 homolog |
| 1559739_at | 13.38 | 2.25E-05 | 1.475725748 | NM_020244| | CHPT1,choline phosphotransferase 1 |
| 225108_at | 13.38 | 2.25E-05 | 1.112088019 | NA |  |
| 231069_at | 13.37 | 2.25E-05 | 1.191881834 | NA |  |
| 223618_at | 13.36 | 2.27E-05 | 1.360226044 | NM_020066| | FMN2,formin 2 |
| 221779_at | 13.36 | 2.27E-05 | 1.068485191 | NM_033386| | MICAL-L1,molecule interacting with Rab13 |
| 222781_s_at | 13.36 | 2.26E-05 | 1.040735947 | NM_017998| | C9orf40,chromosome 9 open reading frame 40 |
| 218902_at | 13.36 | 2.27E-05 | 1.06487806 | NM_017617| | NOTCH1,notch1 preproprotein |
| 217729_s_at | 13.35 | 2.28E-05 | 1.055714571 | NM_001130| | AES,amino-terminal enhancer of split isoform b |
| 203340_s_at | 13.34 | 2.29E-05 | 1.104092704 | NM_003705| | SLC25A12,solute carrier family 25 (mitochondrial carrier, |
| 237208_at | 13.34 | 2.29E-05 | 1.380021515 | NM_025234| | REC14,recombination protein REC14 |
| 215747_s_at | 13.33 | 2.30E-05 | 1.128358755 | NM_001048194| | NA |
| 212282_at | 13.33 | 2.30E-05 | 1.024927252 | NM_014573| | MAC30,hypothetical protein MAC30 |
| 235683_at | 13.33 | 2.30E-05 | 1.143386212 | NM_144665| | SESN3,sestrin 3 |
| 228450_at | 13.32 | 2.31E-05 | 1.127545082 | NM_175058| | PLEKHA7,pleckstrin homology domain containing, family A |
| 226783_at | 13.32 | 2.31E-05 | 1.15884691 | NM_153373| | MGC15875,hypothetical protein MGC15875 |
| 219733_s_at | 13.32 | 2.31E-05 | 1.110151121 | NM_012254| | SLC27A5,solute carrier family 27 (fatty acid |
| 217992_s_at | 13.31 | 2.31E-05 | 1.064829557 | NM_024329| | EFHD2,EF hand domain family, member D2 |
| 214754_at | 13.31 | 2.31E-05 | 1.137622588 | NM_144993| | NA |
| 1566257_at | 13.3 | 2.32E-05 | 1.370054428 | NA |  |
| 201308_s_at | 13.29 | 2.33E-05 | 1.19417504 | NM_018243| | SEPT11,septin 11 |
| 212095_s_at | 13.29 | 2.33E-05 | 1.216282505 | NM_001001924| | MTUS1,mitochondrial tumor suppressor 1 isoform 1 |
| 217106_x_at | 13.29 | 2.33E-05 | 1.044213659 | NM_014473| | HSA9761,putative dimethyladenosine transferase |
| 210589_s_at | 13.29 | 2.33E-05 | 1.085582351 | NM_000157| | GBA,glucocerebrosidase precursor |
| 207628_s_at | 13.29 | 2.33E-05 | 1.081784624 | NM_017528| | WBSCR22,Williams Beuren syndrome chromosome region 22 |
| 228931_at | 13.28 | 2.34E-05 | 1.140074135 | NM_016035| | COQ4,CGI-92 protein |
| 229366_at | 13.28 | 2.34E-05 | 1.181651439 | NA |  |
| 220954_s_at | 13.27 | 2.36E-05 | 1.155848361 | NM_013440| | PILRB,paired immunoglobulin-like type 2 receptor beta |
| 208832_at | 13.27 | 2.35E-05 | 1.071724005 | NM_013236| | ATXN10,ataxin 10 |
| 201740_at | 13.27 | 2.36E-05 | 1.042277341 | NM_004551| | NDUFS3,NADH dehydrogenase (ubiquinone) Fe-S protein 3, |
| 230144_at | 13.26 | 2.36E-05 | 1.872059769 | NM_000828| | GRIA3,glutamate receptor 3 isoform flop precursor |
| 222889_at | 13.26 | 2.36E-05 | 1.120657828 | NM_022836| | DCLRE1B,DNA cross-link repair 1B (PSO2 homolog, S. |
| 201337_s_at | 13.26 | 2.37E-05 | 1.136523307 | NM_004781| | VAMP3,vesicle-associated membrane protein 3 |
| 228847_at | 13.25 | 2.38E-05 | 1.247991765 | NM_007277| | SEC6L1,Sec6 protein |
| 221000_s_at | 13.25 | 2.37E-05 | 1.252814533 | NM_030929| | KAZALD1,Kazal-type serine protease inhibitor domain 1 |
| 217645_at | 13.25 | 2.37E-05 | 1.089717632 | NM_016468| | C14orf112,chromosome 14 open reading frame 112 |
| 243030_at | 13.24 | 2.39E-05 | 1.328767401 | NA |  |
| 236196_at | 13.24 | 2.39E-05 | 1.222184264 | NA |  |
| 208776_at | 13.24 | 2.39E-05 | 1.138411323 | NM_002815| | PSMD11,proteasome 26S non-ATPase subunit 11 |
| 231513_at | 13.24 | 2.39E-05 | 1.657097742 | NA |  |
| 221502_at | 13.24 | 2.39E-05 | 1.088286967 | NM_002267| | KPNA3,karyopherin alpha 3 |
| 1554423_a_at | 13.24 | 2.39E-05 | 1.083615841 | NM_001033024| | NA |
| 238452_at | 13.23 | 2.39E-05 | 1.454605908 | NM_001002901| | FCRL2,hypothetical protein FLJ31052 isoform a |
| 239692_at | 13.23 | 2.39E-05 | 1.245637594 | NA |  |
| 230416_at | 13.23 | 2.39E-05 | 1.150002593 | NA |  |
| 203230_at | 13.23 | 2.39E-05 | 1.093076231 | NM_004421| | DVL1,dishevelled 1 isoform a |
| 213703_at | 13.22 | 2.40E-05 | 1.480738534 | NA |  |
| 218266_s_at | 13.22 | 2.40E-05 | 1.082582873 | NM_014286| | FREQ,frequenin homolog |
| 215794_x_at | 13.22 | 2.40E-05 | 1.194706989 | NM_012084| | GLUD2,glutamate dehydrogenase 2 |
| 214789_x_at | 13.21 | 2.42E-05 | 1.050940087 | NM_032102| | SRP46,Splicing factor, arginine/serine-rich, 46kD |
| 217759_at | 13.21 | 2.42E-05 | 1.03389386 | NM_017583| | TRIM44,DIPB protein |
| 220702_at | 13.2 | 2.43E-05 | 1.472420873 | NM_018616| | NA |
| 235167_at | 13.2 | 2.43E-05 | 1.355816161 | NA |  |
| 209712_at | 13.2 | 2.42E-05 | 1.131559161 | NM_015139| | SLC35D1,solute carrier family 35 (UDP-glucuronic |
| 204662_at | 13.2 | 2.42E-05 | 1.089325417 | NM_014711| | CP110,CP110 protein |
| 212164_at | 13.19 | 2.44E-05 | 1.186929533 | NM_138391| | C1orf37,chromosome 1 open reading frame 37 |
| 203392_s_at | 13.19 | 2.43E-05 | 1.053002772 | NM_001012614| | NA |
| 227996_at | 13.18 | 2.45E-05 | 1.327739025 | NM_001001715| | FARP1,FERM, RhoGEF, and pleckstrin domain protein 1 |
| 203030_s_at | 13.18 | 2.45E-05 | 1.257342587 | NM_002847| | PTPRN2,protein tyrosine phosphatase, receptor type, N |
| 205823_at | 13.18 | 2.45E-05 | 1.115899493 | NM_002926| | RGS12,regulator of G-protein signalling 12 isoform 2 |
| 225812_at | 13.17 | 2.45E-05 | 1.060723602 | NM_001033564| | NA |
| 229331_at | 13.17 | 2.45E-05 | 1.613490074 | NM_145263| | LOC132671,LOC132671 |
| 213512_at | 13.17 | 2.46E-05 | 1.164084727 | NM_174891| | C14orf79,chromosome 14 open reading frame 79 |
| 217140_s_at | 13.17 | 2.46E-05 | 1.121894288 | NM_003374| | VDAC1,voltage-dependent anion channel 1 |
| 202896_s_at | 13.17 | 2.46E-05 | 1.182817561 | NM_001040022| | NA |
| 225835_at | 13.17 | 2.45E-05 | 1.158801076 | NM_001046| | SLC12A2,solute carrier family 12 |
| 1554127_s_at | 13.17 | 2.45E-05 | 1.253289053 | NM_001031679| | NA |
| 228857_at | 13.16 | 2.47E-05 | 1.202142092 | NM_005275| | GNL1,guanine nucleotide binding protein-like 1 |
| 221512_at | 13.15 | 2.48E-05 | 1.086989364 | NM_032125| | DKFZP564D0478,hypothetical protein DKFZp564D0478 |
| 209006_s_at | 13.15 | 2.48E-05 | 1.205497924 | NM_020317| | NPD014,NPD014 protein isoform 2 |
| 200710_at | 13.15 | 2.48E-05 | 1.072917293 | NM_000018| | ACADVL,acyl-Coenzyme A dehydrogenase, very long chain |
| 230329_s_at | 13.15 | 2.47E-05 | 1.141668461 | NM_007083| | NUDT6,nudix-type motif 6 isoform a |
| 219098_at | 13.15 | 2.48E-05 | 1.081609858 | NM_001105538| | NA |
| 209150_s_at | 13.15 | 2.48E-05 | 1.062300659 | NM_001014842| | NA |
| 219325_s_at | 13.15 | 2.48E-05 | 1.404953681 | NM_018696| | ELAC1,elaC homolog 1 |
| 226545_at | 13.15 | 2.48E-05 | 1.116042965 | NM_133493| | CD109,CD109 |
| 214703_s_at | 13.14 | 2.49E-05 | 1.170206266 | NM_015274| | MAN2B2,mannosidase, alpha, class 2B, member 2 |
| 207375_s_at | 13.14 | 2.48E-05 | 1.23064696 | NM_002189| | IL15RA,interleukin 15 receptor, alpha isoform 1 |
| 222618_at | 13.14 | 2.48E-05 | 1.058728002 | NM_018225| | SMU1,smu-1 suppressor of mec-8 and unc-52 homolog |
| 207616_s_at | 13.13 | 2.50E-05 | 1.096724839 | NM_004180| | TANK,TRAF interacting protein TANK isoform a |
| 222843_at | 13.13 | 2.50E-05 | 1.048629946 | NM_001042762| | NA |
| 1554878_a_at | 13.13 | 2.50E-05 | 1.332643671 | NM_001122674| | NA |
| 219262_at | 13.13 | 2.49E-05 | 1.223037235 | NM_024670| | SUV39H2,suppressor of variegation 3-9 homolog 2 |
| 217854_s_at | 13.13 | 2.49E-05 | 1.0358055 | NM_002695| | POLR2E,DNA directed RNA polymerase II polypeptide E |
| 235068_at | 13.12 | 2.51E-05 | 1.163229771 | NM_178566| | ZDHHC21,zinc finger, DHHC domain containing 21 |
| 212996_s_at | 13.12 | 2.51E-05 | 1.114185627 | NA |  |
| 227056_at | 13.12 | 2.51E-05 | 1.134053423 | NM_014773| | KIAA0141,KIAA0141 |
| 213013_at | 13.12 | 2.51E-05 | 1.107284962 | NM_005456| | MAPK8IP1,mitogen-activated protein kinase 8 interacting |
| 1552364_s_at | 13.12 | 2.51E-05 | 1.110917434 | NM_138962| | MSI2,musashi 2 isoform a |
| 229530_at | 13.11 | 2.52E-05 | 1.722019171 | NA |  |
| 240451_at | 13.11 | 2.51E-05 | 1.195023594 | NA |  |
| 222507_s_at | 13.11 | 2.52E-05 | 1.174917953 | NM_020644| | C11orf15,chromosome 11 open reading frame 15 |
| 214351_x_at | 13.11 | 2.52E-05 | 1.016073242 | NM_000977| | RPL13,ribosomal protein L13 |
| 227255_at | 13.11 | 2.51E-05 | 1.314783466 | NM_152835| | PDIK1L,PDLIM1 interacting kinase 1 like |
| 200838_at | 13.11 | 2.51E-05 | 1.123042916 | NM_001908| | CTSB,cathepsin B preproprotein |
| 203168_at | 13.1 | 2.53E-05 | 1.142830967 | NM_004381| | CREBL1,cAMP responsive element binding protein-like 1 |
| 1552925_at | 13.1 | 2.52E-05 | 1.443141317 | NM_020815| | PCDH10,protocadherin 10 isoform 2 precursor |
| 217942_at | 13.09 | 2.54E-05 | 1.034767416 | NM_021821| | MRPS35,mitochondrial ribosomal protein S35 |
| 212144_at | 13.09 | 2.54E-05 | 1.192519969 | NM_015374| | UNC84B,unc-84 homolog B |
| 65770_at | 13.08 | 2.55E-05 | 1.023309634 | NM_138769| | RHOT2,ras homolog gene family, member T2 |
| 208453_s_at | 13.08 | 2.55E-05 | 1.099085247 | NM_020383| | XPNPEP1,X-prolyl aminopeptidase (aminopeptidase P) 1, |
| 231930_at | 13.08 | 2.55E-05 | 1.574333074 | NM_018712| | ELMOD1,ELMO domain containing 1 |
| 242518_at | 13.07 | 2.56E-05 | 1.170799248 | NA |  |
| 235626_at | 13.07 | 2.56E-05 | 1.08678558 | NM_020397| | CAMK1D,calcium/calmodulin-dependent protein kinase ID |
| 205134_s_at | 13.07 | 2.56E-05 | 1.089133104 | NM_012345| | NUFIP1,nuclear fragile X mental retardation protein |
| 213612_x_at | 13.07 | 2.56E-05 | 1.129415497 | NM_001037501| | NA |
| 219706_at | 13.06 | 2.57E-05 | 1.197441675 | NM_018347| | C20orf29,chromosome 20 open reading frame 29 |
| 241044_x_at | 13.06 | 2.58E-05 | 1.21906515 | NA |  |
| 205077_s_at | 13.06 | 2.57E-05 | 1.043513168 | NM_002643| | PIGF,phosphatidylinositol glycan, class F isoform 1 |
| 201783_s_at | 13.05 | 2.58E-05 | 1.149264567 | NM_021975| | RELA,v-rel reticuloendotheliosis viral oncogene |
| 1555476_at | 13.05 | 2.59E-05 | 1.306314546 | NM_004136| | IREB2,iron-responsive element binding protein 2 |
| 211136_s_at | 13.05 | 2.59E-05 | 1.11961514 | NM_001294| | CLPTM1,cleft lip and palate associated transmembrane |
| 226229_s_at | 13.05 | 2.58E-05 | 1.160134304 | NM_014188| | HSPC182,HSPC182 protein |
| 213362_at | 13.05 | 2.58E-05 | 1.109214195 | NM_001040712| | NA |
| 226782_at | 13.04 | 2.60E-05 | 1.275422515 | NM_001010875| | SLC25A30,solute carrier family 25, member 30 |
| 232276_at | 13.04 | 2.60E-05 | 1.385276995 | NM_153456| | HS6ST3,heparan sulfate 6-O-sulfotransferase 3 |
| 1563629_a_at | 13.04 | 2.59E-05 | 1.3746127 | NA |  |
| 226665_at | 13.03 | 2.60E-05 | 1.258919855 | NM_152392| | AHSA2,AHA1, activator of heat shock 90kDa protein |
| 224730_at | 13.03 | 2.60E-05 | 1.082604943 | NM_005828| | HAN11,WD-repeat protein |
| 228020_at | 13.03 | 2.61E-05 | 1.043391905 | NM_017952| | FLJ20758,FLJ20758 protein |
| 227557_at | 13.03 | 2.61E-05 | 1.190356941 | NM_153334| | SCARF2,scavenger receptor class F, member 2 isoform 1 |
| 231715_s_at | 13.03 | 2.61E-05 | 1.103872533 | NM_013328| | PYCR2,pyrroline-5-carboxylate reductase family, member |
| 201421_s_at | 13.02 | 2.61E-05 | 1.075456296 | NM_024102| | MEP50,methylosome protein 50 |
| 1556602_at | 13.02 | 2.61E-05 | 1.244597281 | NA |  |
| 214258_x_at | 13.02 | 2.62E-05 | 1.116059665 | NM_006388| | HTATIP,HIV-1 Tat interactive protein, 60kDa isoform 2 |
| 203801_at | 13.01 | 2.63E-05 | 1.206616828 | NM_022100| | MRPS14,mitochondrial ribosomal protein S14 |
| 201476_s_at | 13.01 | 2.63E-05 | 1.16962759 | NM_001033| | RRM1,ribonucleoside-diphosphate reductase M1 chain |
| 229244_at | 13 | 2.64E-05 | 1.234420743 | NA |  |
| 237262_at | 13 | 2.64E-05 | 1.198614571 | NA |  |
| 213584_s_at | 13 | 2.63E-05 | 1.217912938 | NM_001039618| | NA |
| 238810_at | 13 | 2.63E-05 | 1.427027712 | NM_002919| | RFX3,regulatory factor X3 isoform a |
| 226230_at | 13 | 2.64E-05 | 1.092710199 | NM_001122964| | NA |
| 235754_at | 13 | 2.63E-05 | 1.280137489 | NM_000410| | HFE,hemochromatosis protein isoform 1 precursor |
| 231904_at | 13 | 2.64E-05 | 1.203403515 | NM_001025203| | NA |
| 228943_at | 12.99 | 2.65E-05 | 1.097376875 | NM_033063| | MAP6,microtubule-associated protein 6 isoform 1 |
| 219175_s_at | 12.98 | 2.66E-05 | 1.096257851 | NM_001008485| | SLC41A3,solute carrier family 41, member 3 isoform 1 |
| 230748_at | 12.97 | 2.67E-05 | 1.611926244 | NM_004694| | SLC16A6,solute carrier family 16, member 6 |
| 200927_s_at | 12.97 | 2.67E-05 | 1.074432544 | NM_016322| | RAB14,GTPase Rab14 |
| 242201_at | 12.97 | 2.67E-05 | 1.182469455 | NM_174930| | PMS2L5,postmeiotic segregation increased 2-like 5 |
| 202329_at | 12.97 | 2.67E-05 | 1.070863901 | NM_001127190| | NA |
| 219978_s_at | 12.96 | 2.68E-05 | 1.049873553 | NM_016359| | NUSAP1,nucleolar and spindle associated protein 1 |
| 201642_at | 12.96 | 2.69E-05 | 1.072943513 | NM_005534| | IFNGR2,interferon-gamma receptor beta chain precursor |
| 223773_s_at | 12.96 | 2.68E-05 | 1.057082882 | NA |  |
| 235058_at | 12.96 | 2.69E-05 | 1.140887905 | NA |  |
| 212249_at | 12.96 | 2.68E-05 | 1.508993387 | NM_181504| | PIK3R1,phosphoinositide-3-kinase, regulatory subunit, |
| 59625_at | 12.96 | 2.68E-05 | 1.196683759 | NM_003946| | NOL3,nucleolar protein 3 |
| 226655_at | 12.95 | 2.70E-05 | 1.27765849 | NM_017919| | STX17,syntaxin 17 |
| 235501_at | 12.95 | 2.69E-05 | 1.142797179 | NA |  |
| 213988_s_at | 12.95 | 2.70E-05 | 1.047924826 | NM_002970| | SAT,spermidine/spermine N1-acetyltransferase |
| 226807_at | 12.94 | 2.71E-05 | 1.113016039 | NM_153688| | ZFP1,zinc finger protein 1 homolog |
| 212640_at | 12.94 | 2.71E-05 | 1.033378377 | NM_198402| | PTPLB,protein tyrosine phosphatase-like (proline |
| 205406_s_at | 12.94 | 2.71E-05 | 1.106268551 | NM_017425| | SPA17,sperm autoantigenic protein 17 |
| 235783_at | 12.94 | 2.70E-05 | 1.134113759 | NM_016183| | C1orf33,ribosomal protein P0-like protein |
| 203554_x_at | 12.93 | 2.72E-05 | 1.045804836 | NM_004219| | PTTG1,pituitary tumor-transforming protein 1 |
| 212506_at | 12.93 | 2.72E-05 | 1.04327915 | NM_001008660| | PICALM,phosphatidylinositol-binding clathrin assembly |
| 224372_at | 12.93 | 2.72E-05 | 1.018783234 | NM_001017977| | NA |
| 217289_s_at | 12.93 | 2.72E-05 | 1.181546515 | NM_001467| | SLC37A4,solute carrier family 37 (glycerol-6-phosphate |
| 229375_at | 12.91 | 2.74E-05 | 1.101527588 | NA |  |
| 225340_s_at | 12.91 | 2.75E-05 | 1.043594596 | NM_005898| | M11S1,membrane component, chromosome 11, surface |
| 223119_s_at | 12.91 | 2.74E-05 | 1.129797816 | NM_017944| | USP47,ubiquitin specific protease 47 |
| 1553510_s_at | 12.91 | 2.74E-05 | 1.082164733 | NM_005444| | RQCD1,RCD1 required for cell differentiation1 homolog |
| 208837_at | 12.91 | 2.74E-05 | 1.087905761 | NM_007364| | TMED3,transmembrane emp24 domain containing 3 |
| 201792_at | 12.91 | 2.74E-05 | 1.087937729 | NM_001129| | AEBP1,adipocyte enhancer binding protein 1 precursor |
| 209093_s_at | 12.9 | 2.76E-05 | 1.128919495 | NM_000157| | GBA,glucocerebrosidase precursor |
| 221726_at | 12.9 | 2.75E-05 | 1.061822473 | NM_000983| | RPL22,ribosomal protein L22 proprotein |
| 209036_s_at | 12.9 | 2.76E-05 | 1.031940179 | NM_005918| | MDH2,mitochondrial malate dehydrogenase precursor |
| 229757_at | 12.9 | 2.76E-05 | 1.056034398 | NA |  |
| 202681_at | 12.9 | 2.76E-05 | 1.071513385 | NM_003363| | USP4,ubiquitin specific protease, proto-oncogene |
| 221290_s_at | 12.9 | 2.76E-05 | 1.126333586 | NM_032853| | MUM1,melanoma ubiquitous mutated protein |
| 235391_at | 12.9 | 2.76E-05 | 1.11142872 | NM_145269| | LOC137392,similar to CG6405 gene product |
| 35179_at | 12.9 | 2.76E-05 | 1.137863561 | NM_012200| | B3GAT3,beta-1,3-glucuronyltransferase 3 |
| 227491_at | 12.89 | 2.76E-05 | 1.119658782 | NM_024090| | ELOVL6,ELOVL family member 6, elongation of long chain |
| 219034_at | 12.89 | 2.77E-05 | 1.060447788 | NM_017851| | PARP16,poly (ADP-ribose) polymerase family, member 16 |
| 228722_at | 12.89 | 2.76E-05 | 1.190709485 | NM_001535| | HRMT1L1,HMT1 hnRNP methyltransferase-like 1 |
| 1553588_at | 12.88 | 2.78E-05 | 1.018455521 | NM_001024666| | NA |
| 202598_at | 12.88 | 2.78E-05 | 1.071608371 | NM_001024210| | NA |
| 239611_at | 12.88 | 2.78E-05 | 1.606038747 | NA |  |
| 1565706_at | 12.88 | 2.78E-05 | 1.196423435 | NA |  |
| 216305_s_at | 12.87 | 2.78E-05 | 1.190116238 | NM_003203| | C2orf3,chromosome 2 open reading frame 3 |
| 227037_at | 12.87 | 2.79E-05 | 1.249377667 | NM_178836| | LOC201164,similar to CG12314 gene product |
| 208676_s_at | 12.87 | 2.79E-05 | 1.057195929 | NM_006191| | PA2G4,proliferation-associated 2G4, 38kDa |
| 204133_at | 12.87 | 2.79E-05 | 1.12821647 | NM_004704| | RNU3IP2,RNA, U3 small nucleolar interacting protein 2 |
| 228817_at | 12.87 | 2.79E-05 | 1.080792935 | NM_001077690| | NA |
| 220233_at | 12.86 | 2.80E-05 | 1.107021717 | NM_024907| | FBXO17,F-box protein FBG4 isoform 2 |
| 1557353_at | 12.86 | 2.80E-05 | 1.430772338 | NA |  |
| 221235_s_at | 12.86 | 2.80E-05 | 1.11226917 | NA |  |
| 202968_s_at | 12.86 | 2.80E-05 | 1.028052535 | NM_003583| | DYRK2,dual-specificity tyrosine-(Y)-phosphorylation |
| 203337_x_at | 12.86 | 2.80E-05 | 1.057682341 | NM_004763| | ITGB1BP1,integrin cytoplasmic domain-associated protein 1 |
| 215714_s_at | 12.85 | 2.81E-05 | 1.067005639 | NM_003072| | SMARCA4,SWI/SNF-related matrix-associated |
| 1557507_at | 12.85 | 2.82E-05 | 1.294559848 | NA |  |
| 218210_at | 12.85 | 2.81E-05 | 1.060116522 | NM_024619| | FN3KRP,fructosamine-3-kinase-related protein |
| 238045_at | 12.84 | 2.82E-05 | 1.126528905 | NM_194291| | LOC157378,hypothetical protein BC017881 |
| 209124_at | 12.84 | 2.83E-05 | 1.039401952 | NM_002468| | MYD88,myeloid differentiation primary response gene |
| 243016_at | 12.84 | 2.82E-05 | 1.662240981 | NA |  |
| 211611_s_at | 12.84 | 2.83E-05 | 1.224207789 | NM_004381| | CREBL1,cAMP responsive element binding protein-like 1 |
| 208797_s_at | 12.84 | 2.82E-05 | 1.142303729 | NM_181077| | GOLGIN-67,golgin-67 isoform c |
| 208865_at | 12.84 | 2.82E-05 | 1.057370854 | NM_001025105| | NA |
| 216397_s_at | 12.83 | 2.84E-05 | 1.180747347 | NM_015201| | BOP1,block of proliferation 1 |
| 221413_at | 12.83 | 2.83E-05 | 1.312149922 | NM_004732| | KCNAB3,potassium voltage-gated channel, shaker-related |
| 213944_x_at | 12.83 | 2.83E-05 | 1.12061216 | NM_002067| | GNA11,guanine nucleotide binding protein (G protein), |
| 217501_at | 12.83 | 2.83E-05 | 1.084290035 | NM_004804| | WDR39,WD repeat domain 39 |
| 203611_at | 12.82 | 2.85E-05 | 1.093019266 | NM_005652| | TERF2,telomeric repeat binding factor 2 |
| 212099_at | 12.82 | 2.85E-05 | 1.081361463 | NM_004040| | RHOB,ras homolog gene family, member B |
| 231863_at | 12.82 | 2.85E-05 | 1.352910813 | NM_019071| | ING3,inhibitor of growth family, member 3 isoform 1 |
| 225794_s_at | 12.81 | 2.86E-05 | 1.078268091 | NM_033318| | LOC91689,hypothetical protein supported by AL449243 |
| 213488_at | 12.81 | 2.86E-05 | 1.491152108 | NM_001080437| | NA |
| 209694_at | 12.81 | 2.85E-05 | 1.020836161 | NM_000317| | PTS,6-pyruvoyltetrahydropterin synthase |
| 224282_s_at | 12.81 | 2.85E-05 | 1.199080077 | NM_001037553| | NA |
| 238099_at | 12.81 | 2.86E-05 | 1.156872165 | NA |  |
| 212347_x_at | 12.8 | 2.88E-05 | 1.119293692 | NM_006454| | MXD4,MAD4 |
| 204547_at | 12.8 | 2.87E-05 | 1.201160619 | NM_006822| | RAB40B,RAB40B, member RAS oncogene family |
| 204529_s_at | 12.8 | 2.87E-05 | 1.129284828 | NM_014729| | TOX,thymus high mobility group box protein TOX |
| 239329_at | 12.8 | 2.87E-05 | 1.109755758 | NA |  |
| 221432_s_at | 12.79 | 2.89E-05 | 1.112408354 | NM_031212| | SLC25A28,solute carrier family 25, member 28 |
| 1569001_at | 12.79 | 2.89E-05 | 1.142362341 | NM_001199| | BMP1,bone morphogenetic protein 1 isoform 1, |
| 225604_s_at | 12.79 | 2.89E-05 | 1.151414179 | NM_022343| | C9orf19,chromosome 9 open reading frame 19 |
| 213043_s_at | 12.78 | 2.90E-05 | 1.064028716 | NM_001079518| | NA |
| 225446_at | 12.78 | 2.90E-05 | 1.099188644 | NM_001007246| | WDR9,WD repeat domain 9 isoform C |
| 1556039_s_at | 12.78 | 2.89E-05 | 1.116622215 | NM_018969| | GPR173,G-protein coupled receptor 173 |
| 1558483_at | 12.78 | 2.90E-05 | 1.141645947 | NM_030626| | LRRC27,leucine rich repeat containing 27 |
| 1569472_s_at | 12.77 | 2.91E-05 | 1.525477154 | NM_001001894| | TTC3,tetratricopeptide repeat domain 3 |
| 218550_s_at | 12.77 | 2.91E-05 | 1.079422269 | NM_018205| | LRRC20,leucine rich repeat containing 20 isoform 3 |
| 214960_at | 12.76 | 2.92E-05 | 1.184886012 | NM_006595| | API5,apoptosis inhibitor 5 |
| 218242_s_at | 12.76 | 2.92E-05 | 1.081909439 | NM_016028| | SUV420H1,suppressor of variegation 4-20 homolog 1 isoform |
| 205760_s_at | 12.76 | 2.93E-05 | 1.185353233 | NM_002542| | OGG1,8-oxoguanine DNA glycosylase isoform 1a |
| 222705_s_at | 12.76 | 2.92E-05 | 1.159154487 | NM_014252| | SLC25A15,solute carrier family 25 (mitochondrial carrier; |
| 206144_at | 12.76 | 2.93E-05 | 1.229844596 | NM_001033057| | NA |
| 202104_s_at | 12.75 | 2.94E-05 | 1.049902084 | NM_003119| | SPG7,paraplegin isoform 1 |
| 230270_at | 12.75 | 2.93E-05 | 1.206581989 | NM_018061| | FLJ10330,sarcoma antigen NY-SAR-27 |
| 225089_at | 12.75 | 2.94E-05 | 1.115403529 | NM_018218| | USP40,ubiquitin specific protease 40 |
| 66053_at | 12.75 | 2.93E-05 | 1.153815661 | NM_001079559| | NA |
| 220757_s_at | 12.75 | 2.93E-05 | 1.154850432 | NM_025241| | UBXD1,UBX domain containing 1 |
| 213041_s_at | 12.74 | 2.95E-05 | 1.065374768 | NM_001001975| | ATP5D,ATP synthase, H+ transporting, mitochondrial F1 |
| 212073_at | 12.74 | 2.95E-05 | 1.057324482 | NM_001895| | CSNK2A1,casein kinase II alpha 1 subunit isoform a |
| 235266_at | 12.74 | 2.96E-05 | 1.121787513 | NM_014109| | ATAD2,two AAA domain containing protein |
| 1558467_a_at | 12.74 | 2.96E-05 | 1.271687108 | NA |  |
| 233528_s_at | 12.72 | 2.98E-05 | 1.159754818 | NM_001037666| | NA |
| 213152_s_at | 12.72 | 2.98E-05 | 1.091588941 | NM_032102| | SRP46,Splicing factor, arginine/serine-rich, 46kD |
| 200832_s_at | 12.72 | 2.98E-05 | 1.036591013 | NM_005063| | SCD,stearoyl-CoA desaturase |
| 226238_at | 12.71 | 2.99E-05 | 1.073156359 | NM_032601| | MCEE,methylmalonyl-CoA epimerase |
| 219868_s_at | 12.71 | 3.00E-05 | 1.257649621 | NM_016376| | ANKFY1,ankyrin repeat and FYVE domain containing 1 |
| 232865_at | 12.71 | 2.99E-05 | 1.159366214 | NM_014423| | AF5Q31,ALL1 fused gene from 5q31 |
| 227356_at | 12.7 | 3.00E-05 | 1.090387828 | NA |  |
| 209239_at | 12.7 | 3.01E-05 | 1.206843901 | NM_003998| | NFKB1,nuclear factor kappa-B, subunit 1 |
| 244165_at | 12.7 | 3.01E-05 | 1.26955667 | NM_017782| | NA |
| 214924_s_at | 12.7 | 3.00E-05 | 1.108038826 | NM_001042646| | NA |
| 235534_at | 12.69 | 3.02E-05 | 1.226933133 | NA |  |
| 218427_at | 12.68 | 3.04E-05 | 1.092155707 | NM_001039707| | NA |
| 1555408_at | 12.68 | 3.03E-05 | 1.327936244 | NM_181704| | BAGE4,B melanoma antigen family, member 4 |
| 227318_at | 12.68 | 3.04E-05 | 1.104862868 | NA |  |
| 202212_at | 12.68 | 3.04E-05 | 1.090021784 | NM_014303| | PES1,pescadillo homolog 1, containing BRCT domain |
| 225292_at | 12.67 | 3.04E-05 | 1.24077645 | NM_032888| | COL27A1,collagen, type XXVII, alpha 1 |
| 212139_at | 12.67 | 3.04E-05 | 1.045625533 | NM_006836| | GCN1L1,GCN1 general control of amino-acid synthesis |
| 201932_at | 12.67 | 3.04E-05 | 1.101283985 | NM_006369| | MUF1,MUF1 protein |
| 201161_s_at | 12.66 | 3.06E-05 | 1.081952465 | NM_003651| | CSDA,cold shock domain protein A |
| 239174_at | 12.66 | 3.06E-05 | 1.225697004 | NA |  |
| 228142_at | 12.66 | 3.06E-05 | 1.066910506 | NM_001003684| | HSPC051,ubiquinol-cytochrome c reductase complex 7.2kDa |
| 1558568_a_at | 12.66 | 3.06E-05 | 1.268675369 | NA |  |
| 235729_at | 12.65 | 3.08E-05 | 1.081834089 | NM_032788| | ZNF514,zinc finger protein 514 |
| 210655_s_at | 12.65 | 3.07E-05 | 1.16578017 | NM_001455| | FOXO3A,forkhead box O3A |
| 218785_s_at | 12.65 | 3.07E-05 | 1.153593672 | NM_022777| | RABL5,RAB, member RAS oncogene family-like 5 |
| 1559049_a_at | 12.65 | 3.07E-05 | 1.225332992 | NA |  |
| 200854_at | 12.65 | 3.07E-05 | 1.028077813 | NM_006311| | NCOR1,nuclear receptor co-repressor 1 |
| 213890_x_at | 12.65 | 3.07E-05 | 1.011581795 | NM_001020| | RPS16,ribosomal protein S16 |
| 208907_s_at | 12.65 | 3.07E-05 | 1.082629538 | NM_014046| | MRPS18B,mitochondrial ribosomal protein S18B |
| 229384_at | 12.65 | 3.07E-05 | 1.10558374 | NA |  |
| 214664_at | 12.64 | 3.09E-05 | 1.166607847 | NM_001079524| | NA |
| 225940_at | 12.64 | 3.08E-05 | 1.110072013 | NM_173359| | NA |
| 218736_s_at | 12.64 | 3.09E-05 | 1.661072611 | NM_017734| | PALMD,palmdelphin |
| 234351_x_at | 12.64 | 3.09E-05 | 1.168927686 | NM_014112| | TRPS1,zinc finger transcription factor TRPS1 |
| 244457_at | 12.64 | 3.09E-05 | 1.290108166 | NA |  |
| 212858_at | 12.64 | 3.09E-05 | 1.092812831 | NM_152341| | PAQR4,progestin and adipoQ receptor family member IV |
| 225523_at | 12.64 | 3.08E-05 | 1.062297773 | NM_053050| | MRPL53,mitochondrial ribosomal protein L53 |
| 231845_at | 12.63 | 3.09E-05 | 1.039964215 | NM_020745| | AARSL,alanyl-tRNA synthetase like |
| 242304_at | 12.63 | 3.10E-05 | 1.078392669 | NM_032345| | PYM,PYM protein |
| 238659_at | 12.63 | 3.10E-05 | 1.29912674 | NA |  |
| 242134_at | 12.63 | 3.10E-05 | 1.319964381 | NA |  |
| 241816_at | 12.63 | 3.09E-05 | 1.168892975 | NM_018353| | C14orf106,chromosome 14 open reading frame 106 |
| 214263_x_at | 12.63 | 3.10E-05 | 1.011507982 | NM_032940| | POLR2C,DNA directed RNA polymerase II polypeptide C |
| 236627_at | 12.63 | 3.10E-05 | 1.674148423 | NA |  |
| 202656_s_at | 12.62 | 3.11E-05 | 1.066844868 | NM_014755| | SERTAD2,SERTA domain containing 2 |
| 1556186_s_at | 12.62 | 3.11E-05 | 1.116303844 | NM_015047| | KIAA0090,KIAA0090 protein |
| 1560100_at | 12.62 | 3.11E-05 | 1.315159208 | NM_001038493| | NA |
| 220720_x_at | 12.62 | 3.10E-05 | 1.061115065 | NM_025029| | FLJ14346,hypothetical protein FLJ14346 |
| 228698_at | 12.62 | 3.11E-05 | 1.314205128 | NM_031439| | SOX7,SRY-box 7 |
| 209329_x_at | 12.61 | 3.11E-05 | 1.047800607 | NM_138820| | MGC2198,hypothetical protein MGC2198 |
| 235206_at | 12.61 | 3.11E-05 | 1.195936942 | NM_016558| | SCAND1,SCAN domain containing protein 1 |
| 201214_s_at | 12.61 | 3.11E-05 | 1.04267597 | NM_002712| | PPP1R7,protein phosphatase 1, regulatory subunit 7 |
| 225569_at | 12.61 | 3.12E-05 | 1.025540777 | NM_012154| | EIF2C2,eukaryotic translation initiation factor 2C, 2 |
| 213758_at | 12.61 | 3.11E-05 | 1.151266089 | NM_001861| | COX4I1,cytochrome c oxidase subunit IV isoform 1 |
| 1564911_at | 12.6 | 3.13E-05 | 1.314925493 | NA |  |
| 226500_at | 12.6 | 3.14E-05 | 1.169066367 | NM_145166| | ZNF651,zinc finger protein 651 |
| 223369_at | 12.59 | 3.15E-05 | 1.0525902 | NM_014064| | AD-003,AD-003 protein |
| 226477_at | 12.59 | 3.15E-05 | 1.063456175 | NM_014703| | VprBP,Vpr-binding protein |
| 201714_at | 12.59 | 3.15E-05 | 1.024430472 | NM_001070| | TUBG1,tubulin, gamma 1 |
| 230408_at | 12.59 | 3.15E-05 | 1.080305539 | NA |  |
| 229236_s_at | 12.59 | 3.15E-05 | 1.1430664 | NM_213649| | SFXN4,sideroflexin 4 isoform 1 |
| 210622_x_at | 12.59 | 3.15E-05 | 1.153656633 | NM_001098533| | NA |
| 212062_at | 12.59 | 3.15E-05 | 1.14676002 | NM_006045| | NA |
| 201598_s_at | 12.59 | 3.14E-05 | 1.113760878 | NM_001567| | INPPL1,inositol polyphosphate phosphatase-like 1 |
| 202451_at | 12.58 | 3.16E-05 | 1.110138553 | NM_005316| | GTF2H1,general transcription factor IIH, polypeptide 1 |
| 239236_at | 12.58 | 3.16E-05 | 1.258597601 | NA |  |
| 213050_at | 12.58 | 3.16E-05 | 1.07010235 | NM_015198| | COBL,cordon-bleu homolog |
| 213727_x_at | 12.58 | 3.15E-05 | 1.089072918 | NM_023075| | MPPE1,metallophosphoesterase 1 isoform a precursor |
| 200647_x_at | 12.58 | 3.16E-05 | 1.029373633 | NM_001037808| | NA |
| 202780_at | 12.58 | 3.16E-05 | 1.089137427 | NM_000436| | OXCT1,3-oxoacid CoA transferase 1 precursor |
| 237169_at | 12.58 | 3.17E-05 | 1.930485951 | NA |  |
| 1555900_at | 12.57 | 3.17E-05 | 1.058722128 | NA |  |
| 202682_s_at | 12.57 | 3.17E-05 | 1.07482816 | NM_003363| | USP4,ubiquitin specific protease, proto-oncogene |
| 32625_at | 12.57 | 3.18E-05 | 1.171571873 | NM_000906| | NPR1,natriuretic peptide receptor A/guanylate cyclase |
| 238890_at | 12.57 | 3.18E-05 | 1.248753834 | NM_003720| | DSCR2,Down syndrome critical region protein 2 isoform |
| 225116_at | 12.56 | 3.19E-05 | 1.075900815 | NM_001113239| | NA |
| 235595_at | 12.56 | 3.19E-05 | 1.151344386 | NM_004723| | ARHGEF2,rho/rac guanine nucleotide exchange factor 2 |
| 210153_s_at | 12.56 | 3.19E-05 | 1.060371562 | NM_002396| | ME2,malic enzyme 2, NAD(+)-dependent, mitochondrial |
| 213333_at | 12.56 | 3.19E-05 | 1.048660212 | NM_005918| | MDH2,mitochondrial malate dehydrogenase precursor |
| 214747_at | 12.56 | 3.19E-05 | 1.29440466 | NM_014838| | ZBED4,zinc finger, BED domain containing 4 |
| 236408_at | 12.55 | 3.21E-05 | 1.24397445 | NA |  |
| 212551_at | 12.55 | 3.21E-05 | 1.089979488 | NM_006366| | CAP2,adenylyl cyclase-associated protein 2 |
| 207826_s_at | 12.54 | 3.21E-05 | 1.074133579 | NM_002167| | ID3,inhibitor of DNA binding 3 |
| 218597_s_at | 12.54 | 3.22E-05 | 1.044244711 | NM_018464| | C10orf70,chromosome 10 open reading frame 70 |
| 230885_at | 12.54 | 3.21E-05 | 1.187075546 | NM_003119| | SPG7,paraplegin isoform 1 |
| 201124_at | 12.54 | 3.21E-05 | 1.23294768 | NM_002213| | ITGB5,integrin, beta 5 |
| 232412_at | 12.54 | 3.22E-05 | 1.372454481 | NA |  |
| 230720_at | 12.54 | 3.21E-05 | 1.07254555 | NM_152737| | RNF182,ring finger protein 182 |
| 208961_s_at | 12.53 | 3.23E-05 | 1.096535467 | NM_001300| | KLF6,Kruppel-like factor 6 |
| 225927_at | 12.53 | 3.24E-05 | 1.079660595 | NM_005921| | NA |
| 237834_at | 12.53 | 3.24E-05 | 1.362315341 | NM_005460| | SNCAIP,synuclein alpha interacting protein |
| 208908_s_at | 12.52 | 3.25E-05 | 1.068223132 | NM_001042440| | NA |
| 204315_s_at | 12.52 | 3.24E-05 | 1.074821111 | NM_016426| | GTSE1,G-2 and S-phase expressed 1 |
| 202629_at | 12.51 | 3.27E-05 | 1.094373165 | NM_006380| | APPBP2,amyloid beta precursor protein-binding protein |
| 224889_at | 12.51 | 3.27E-05 | 1.058911872 | NM_001455| | FOXO3A,forkhead box O3A |
| 225352_at | 12.51 | 3.27E-05 | 1.079875289 | NM_003262| | TLOC1,translocation protein 1 |
| 1553971_a_at | 12.51 | 3.26E-05 | 1.18696843 | NM_178831| | GATS,opposite strand transcription unit to STAG3 |
| 213980_s_at | 12.51 | 3.27E-05 | 1.042715356 | NM_001012614| | NA |
| 202041_s_at | 12.5 | 3.28E-05 | 1.047568786 | NM_004214| | FIBP,FGF intracellular binding protein isoform b |
| 218821_at | 12.5 | 3.27E-05 | 1.065341256 | NM_024663| | NPEPL1,aminopeptidase-like 1 |
| 219763_at | 12.5 | 3.27E-05 | 1.053321486 | NM_020946| | KIAA1608,KIAA1608 protein isoform 1 |
| 235488_at | 12.5 | 3.28E-05 | 1.062413054 | NM_033315| | RASL10B,RAS-like, family 10, member B |
| 203224_at | 12.5 | 3.28E-05 | 1.158780782 | NM_018339| | RFK,riboflavin kinase |
| 201350_at | 12.49 | 3.30E-05 | 1.062607809 | NM_004475| | FLOT2,flotillin 2 |
| 211058_x_at | 12.48 | 3.30E-05 | 1.016734112 | NM_006082| | K-ALPHA-1,tubulin, alpha, ubiquitous |
| 227634_at | 12.48 | 3.31E-05 | 1.169076209 | NM_173575| | STK32C,serine/threonine kinase 32C |
| 222077_s_at | 12.47 | 3.32E-05 | 1.082312787 | NM_001126103| | NA |
| 1556656_at | 12.47 | 3.32E-05 | 1.308254703 | NA |  |
| 202940_at | 12.47 | 3.32E-05 | 1.196208352 | NM_018979| | WNK1,WNK lysine deficient protein kinase 1 |
| 235172_at | 12.47 | 3.32E-05 | 1.204204597 | NA |  |
| 218025_s_at | 12.46 | 3.34E-05 | 1.028343352 | NM_006117| | PECI,peroxisomal D3,D2-enoyl-CoA isomerase isoform 1 |
| 216192_at | 12.46 | 3.33E-05 | 1.640426452 | NM_001446| | FABP7,fatty acid binding protein 7, brain |
| 226956_at | 12.46 | 3.34E-05 | 1.182072306 | NM_021090| | MTMR3,myotubularin-related protein 3 isoform c |
| 204821_at | 12.45 | 3.35E-05 | 1.611344576 | NM_006994| | BTN3A3,butyrophilin, subfamily 3, member A3 isoform a |
| 228173_at | 12.45 | 3.34E-05 | 1.173130493 | NM_000516| | GNAS,guanine nucleotide binding protein, alpha |
| 213857_s_at | 12.45 | 3.36E-05 | 1.079592672 | NM_001025079| | NA |
| 222436_s_at | 12.45 | 3.36E-05 | 1.067246734 | NM_001005753| | VPS24,vacuolar protein sorting 24 isoform 2 |
| 237626_at | 12.44 | 3.37E-05 | 1.103780719 | NA |  |
| 239271_at | 12.44 | 3.36E-05 | 1.172574106 | NM_001003652| | SMAD2,Sma- and Mad-related protein 2 |
| 219957_at | 12.44 | 3.37E-05 | 1.185251269 | NM_001042417| | NA |
| 235015_at | 12.44 | 3.37E-05 | 1.195231498 | NA |  |
| 213383_at | 12.44 | 3.36E-05 | 1.147271931 | NA |  |
| 1559214_at | 12.44 | 3.36E-05 | 1.423790156 | NA |  |
| 220917_s_at | 12.43 | 3.38E-05 | 1.10252512 | NM_025132| | WDR19,WD repeat domain 19 |
| 217913_at | 12.43 | 3.39E-05 | 1.049731063 | NM_013245| | VPS4A,vacuolar protein sorting factor 4A |
| 212014_x_at | 12.43 | 3.39E-05 | 1.155186291 | NM_000610| | CD44,CD44 antigen isoform 1 precursor |
| 209568_s_at | 12.43 | 3.39E-05 | 1.098561407 | NM_015149| | RGL1,ral guanine nucleotide dissociation |
| 235193_at | 12.42 | 3.39E-05 | 1.738313526 | NA |  |
| 209275_s_at | 12.42 | 3.40E-05 | 1.091287929 | NM_000086| | CLN3,CLN3 protein |
| 202658_at | 12.42 | 3.40E-05 | 1.11529066 | NM_003846| | PEX11B,peroxisomal biogenesis factor 11B |
| 201943_s_at | 12.42 | 3.39E-05 | 1.117374266 | NM_001304| | CPD,carboxypeptidase D precursor |
| 1556818_at | 12.41 | 3.40E-05 | 1.180423528 | NA |  |
| 225112_at | 12.41 | 3.41E-05 | 1.074390094 | NM_005759| | ABI2,abl interactor 2 |
| 214661_s_at | 12.41 | 3.42E-05 | 1.087505089 | NM_003703| | C4orf9,gene near HD on 4p16.3 with homology to |
| 222625_s_at | 12.4 | 3.42E-05 | 1.269088877 | NM_017668| | NDE1,nuclear distribution gene E homolog 1 |
| 206093_x_at | 12.4 | 3.43E-05 | 1.136791535 | NM_019105| | TNXB,tenascin XB isoform 1 |
| 231769_at | 12.4 | 3.42E-05 | 1.450768769 | NM_018438| | FBXO6,F-box only protein 6 |
| 242857_at | 12.4 | 3.42E-05 | 1.308744355 | NA |  |
| 205284_at | 12.39 | 3.44E-05 | 1.096538043 | NM_014777| | KIAA0133,KIAA0133 |
| 39549_at | 12.39 | 3.44E-05 | 1.236860964 | NM_002518| | NPAS2,neuronal PAS domain protein 2 |
| 210752_s_at | 12.39 | 3.43E-05 | 1.126846122 | NM_170607| | MLX,transcription factor-like protein 4 isoform |
| 239476_at | 12.39 | 3.44E-05 | 1.237434819 | NA |  |
| 225884_s_at | 12.39 | 3.43E-05 | 1.051295519 | NM_022482| | ZNF336,zinc finger protein 336 |
| 226872_at | 12.39 | 3.44E-05 | 1.215218885 | NM_000635| | RFX2,regulatory factor X2 isoform a |
| 217202_s_at | 12.38 | 3.45E-05 | 1.111292197 | NM_001033044| | NA |
| 242182_x_at | 12.38 | 3.45E-05 | 1.17449386 | NA |  |
| 209716_at | 12.38 | 3.45E-05 | 1.129800285 | NM_000757| | CSF1,colony stimulating factor 1 isoform a precursor |
| 1556103_at | 12.38 | 3.45E-05 | 1.108198435 | NA |  |
| 223917_s_at | 12.38 | 3.45E-05 | 1.084026762 | NM_144564| | SLC39A3,solute carrier family 39 (zinc transporter), |
| 212852_s_at | 12.38 | 3.45E-05 | 1.051398477 | NM_001042369| | NA |
| 223223_at | 12.38 | 3.45E-05 | 1.093545738 | NM_022786| | ARV1,ARV1 homolog |
| 203509_at | 12.38 | 3.45E-05 | 1.04614691 | NM_003105| | SORL1,sortilin-related receptor containing LDLR class |
| 1558673_s_at | 12.38 | 3.45E-05 | 1.199721975 | NM_021217| | ZNF77,zinc finger protein 77 |
| 209588_at | 12.38 | 3.45E-05 | 1.131873612 | NM_004442| | EPHB2,ephrin receptor EphB2 isoform 2 precursor |
| 232759_at | 12.38 | 3.45E-05 | 1.181477916 | NA |  |
| 202093_s_at | 12.37 | 3.47E-05 | 1.062332613 | NM_019088| | PD2,hypothetical protein F23149_1 |
| 222628_s_at | 12.37 | 3.47E-05 | 1.044896235 | NM_001037872| | NA |
| 225219_at | 12.37 | 3.46E-05 | 1.095636254 | NM_001001419| | SMAD5,SMAD, mothers against DPP homolog 5 |
| 202428_x_at | 12.37 | 3.46E-05 | 1.060357488 | NM_001079862| | NA |
| 229007_at | 12.37 | 3.47E-05 | 1.648301825 | NA |  |
| 208853_s_at | 12.37 | 3.47E-05 | 1.10153094 | NM_001024649| | NA |
| 202439_s_at | 12.36 | 3.48E-05 | 1.100148653 | NM_000202| | IDS,iduronate-2-sulfatase isoform a precursor |
| 209597_s_at | 12.36 | 3.48E-05 | 1.23704143 | NM_007257| | NA |
| 201818_at | 12.36 | 3.49E-05 | 1.034989488 | NM_024830| | FLJ12443,hypothetical protein FLJ12443 |
| 229194_at | 12.36 | 3.48E-05 | 1.238935009 | NM_032373| | PCGF5,polycomb group ring finger 5 |
| 213790_at | 12.36 | 3.49E-05 | 1.320664606 | NA |  |
| 1557133_at | 12.36 | 3.49E-05 | 1.227869581 | NM_001126492| | NA |
| 210417_s_at | 12.35 | 3.51E-05 | 1.1590928 | NM_002651| | PIK4CB,phosphatidylinositol 4-kinase, catalytic, beta |
| 222725_s_at | 12.35 | 3.50E-05 | 1.425549213 | NM_017734| | PALMD,palmdelphin |
| 209370_s_at | 12.35 | 3.50E-05 | 1.224554725 | NM_001122681| | NA |
| 202757_at | 12.35 | 3.51E-05 | 1.014955994 | NM_015456| | COBRA1,cofactor of BRCA1 |
| 208405_s_at | 12.35 | 3.49E-05 | 1.056271381 | NM_006016| | CD164,CD164 antigen, sialomucin |
| 217838_s_at | 12.35 | 3.50E-05 | 1.072149872 | NM_016337| | EVL,Enah/Vasp-like |
| 214494_s_at | 12.34 | 3.52E-05 | 1.030893191 | NM_003119| | SPG7,paraplegin isoform 1 |
| 213689_x_at | 12.34 | 3.51E-05 | 1.081237533 | NM_001006605| | LOC388650,hypothetical LOC388650 |
| 219204_s_at | 12.34 | 3.51E-05 | 1.095551563 | NM_021947| | SRR,serine racemase |
| 212600_s_at | 12.34 | 3.52E-05 | 1.032003669 | NM_003366| | UQCRC2,ubiquinol-cytochrome c reductase core protein |
| 221548_s_at | 12.34 | 3.52E-05 | 1.047368618 | NM_030768| | ILKAP,integrin-linked kinase-associated protein |
| 203057_s_at | 12.34 | 3.52E-05 | 1.079992681 | NM_001007257| | PRDM2,retinoblastoma protein-binding zinc finger |
| 212501_at | 12.33 | 3.53E-05 | 1.049158375 | NM_005194| | CEBPB,CCAAT/enhancer binding protein beta |
| 229843_at | 12.33 | 3.53E-05 | 1.253501422 | NM_016033| | CGI-90,CGI-90 protein |
| 242191_at | 12.32 | 3.55E-05 | 1.59381995 | NM_001039703| | NA |
| 204106_at | 12.32 | 3.55E-05 | 1.057230567 | NM_006285| | TESK1,testis-specific protein kinase 1 |
| 229425_at | 12.32 | 3.55E-05 | 1.097847542 | NA |  |
| 235677_at | 12.31 | 3.56E-05 | 1.117947044 | NM_021947| | SRR,serine racemase |
| 235211_at | 12.31 | 3.56E-05 | 1.214937978 | NA |  |
| 237270_at | 12.31 | 3.56E-05 | 1.325305265 | NA |  |
| 227740_at | 12.3 | 3.58E-05 | 1.093608501 | NM_175866| | UHMK1,kinase interacting stathmin |
| 203395_s_at | 12.3 | 3.58E-05 | 1.04132421 | NM_005524| | HES1,hairy and enhancer of split 1 |
| 232293_at | 12.29 | 3.60E-05 | 1.115258707 | NM_153686| | MLR1,transcription factor MLR1 |
| 218760_at | 12.29 | 3.60E-05 | 1.14914253 | NM_182476| | COQ6,CGI-10 protein isoform a |
| 206874_s_at | 12.29 | 3.60E-05 | 1.091955806 | NA |  |
| 201894_s_at | 12.29 | 3.61E-05 | 1.042406526 | NM_003144| | SSR1,signal sequence receptor, alpha |
| 241392_at | 12.28 | 3.61E-05 | 1.284885121 | NM_018266| | TMEM39A,transmembrane protein 39A |
| 208917_x_at | 12.28 | 3.62E-05 | 1.11559487 | NM_023018| | FLJ13052,NAD kinase |
| 201320_at | 12.28 | 3.61E-05 | 1.138031504 | NM_003075| | SMARCC2,SWI/SNF-related matrix-associated |
| 228932_at | 12.28 | 3.61E-05 | 1.223683242 | NA |  |
| 225662_at | 12.28 | 3.61E-05 | 1.201796707 | NM_016653| | ZAK,sterile-alpha motif and leucine zipper |
| 227797_x_at | 12.28 | 3.62E-05 | 1.062537487 | NM_020466| | DJ122O8.2,hypothetical protein dJ122O8.2 |
| 238401_at | 12.28 | 3.62E-05 | 1.091584106 | NM_173627| | FLJ35220,hypothetical protein FLJ35220 |
| 228305_at | 12.28 | 3.62E-05 | 1.103466597 | NM_001042474| | NA |
| 1555831_s_at | 12.27 | 3.63E-05 | 1.163888146 | NM_006369| | MUF1,MUF1 protein |
| 239049_at | 12.27 | 3.63E-05 | 1.094077136 | NA |  |
| 232086_at | 12.26 | 3.66E-05 | 1.286316519 | NA |  |
| 226891_at | 12.26 | 3.66E-05 | 1.033825862 | NM_152531| | C3orf21,chromosome 3 open reading frame 21 |
| 219015_s_at | 12.26 | 3.64E-05 | 1.159272833 | NM_018466| | GLT28D1,glycosyltransferase 28 domain containing 1 |
| 226882_x_at | 12.26 | 3.64E-05 | 1.202465268 | NM_018669| | WDR4,WD repeat domain 4 protein |
| 215248_at | 12.25 | 3.66E-05 | 1.285547827 | NM_001001549| | GRB10,growth factor receptor-bound protein 10 isoform |
| 226101_at | 12.25 | 3.67E-05 | 1.242390035 | NM_005400| | PRKCE,protein kinase C, epsilon |
| 218708_at | 12.25 | 3.67E-05 | 1.080543508 | NM_013248| | NXT1,NTF2-like export factor 1 |
| 224891_at | 12.25 | 3.67E-05 | 1.0372031 | NM_001455| | FOXO3A,forkhead box O3A |
| 1556821_x_at | 12.24 | 3.68E-05 | 2.018654074 | NA |  |
| 223260_s_at | 12.24 | 3.69E-05 | 1.204521001 | NM_016218| | POLK,polymerase (DNA directed) kappa |
| 200675_at | 12.24 | 3.69E-05 | 1.028517878 | NM_004356| | CD81,CD81 antigen |
| 202984_s_at | 12.24 | 3.69E-05 | 1.118858836 | NM_001015048| | NA |
| 243367_at | 12.23 | 3.71E-05 | 1.308924971 | NA |  |
| 227331_at | 12.23 | 3.71E-05 | 1.036106377 | NM_001004304| | LOC283337,hypothetical protein LOC283337 |
| 208089_s_at | 12.23 | 3.70E-05 | 1.125746634 | NM_030794| | TDRD3,tudor domain containing 3 |
| 227986_at | 12.23 | 3.70E-05 | 1.138273293 | NM_024325| | ZNF343,zinc finger protein 343 |
| 212009_s_at | 12.23 | 3.70E-05 | 1.146581257 | NM_006819| | STIP1,stress-induced-phosphoprotein 1 |
| 201410_at | 12.22 | 3.73E-05 | 1.038170613 | NM_001100623| | NA |
| 212192_at | 12.22 | 3.73E-05 | 1.167618501 | NM_138444| | KCTD12,potassium channel tetramerisation domain |
| 220202_s_at | 12.22 | 3.72E-05 | 1.12233104 | NM_001100588| | NA |
| 227170_at | 12.22 | 3.73E-05 | 1.112213438 | NA |  |
| 213398_s_at | 12.21 | 3.74E-05 | 1.031112277 | NM_020195| | C14orf124,HCDI protein |
| 1555894_s_at | 12.21 | 3.74E-05 | 1.316469108 | NM_138383| | LOC92154,hypothetical protein BC002770 |
| 227756_at | 12.21 | 3.75E-05 | 1.395211118 | NM_152450| | MGC26690,hypothetical protein MGC26690 |
| 1553685_s_at | 12.2 | 3.75E-05 | 1.325843493 | NM_138473| | SP1,Sp1 transcription factor |
| 1552680_a_at | 12.19 | 3.77E-05 | 1.10120487 | NM_144508| | AF15Q14,AF15q14 protein isoform 2 |
| 222760_at | 12.19 | 3.78E-05 | 1.264747797 | NM_025069| | FLJ14299,hypothetical protein FLJ14299 |
| 227766_at | 12.19 | 3.77E-05 | 1.17139091 | NM_001098268| | NA |
| 218774_at | 12.19 | 3.77E-05 | 1.079729804 | NM_014026| | DCPS,mRNA decapping enzyme |
| 205160_at | 12.18 | 3.80E-05 | 1.170353695 | NM_003847| | PEX11A,peroxisomal biogenesis factor 11A |
| 223674_s_at | 12.18 | 3.78E-05 | 1.377876854 | NM_001038707| | NA |
| 243366_s_at | 12.18 | 3.78E-05 | 1.537100296 | NA |  |
| 203260_at | 12.17 | 3.80E-05 | 1.131135659 | NM_016063| | C6orf74,chromosome 6 open reading frame 74 |
| 231300_at | 12.17 | 3.81E-05 | 1.33867701 | NM_001014979| | NA |
| 221253_s_at | 12.17 | 3.80E-05 | 1.020982733 | NM_022085| | TXNDC5,thioredoxin domain containing 5 isoform 2 |
| 209777_s_at | 12.17 | 3.80E-05 | 1.117145278 | NM_194255| | SLC19A1,solute carrier family 19 member 1 isoform a |
| 217445_s_at | 12.17 | 3.80E-05 | 1.202907545 | NM_000819| | GART,phosphoribosylglycinamide formyltransferase, |
| 234710_s_at | 12.17 | 3.81E-05 | 1.227536817 | NM_020214| | PARP6,poly (ADP-ribose) polymerase family, member 6 |
| 213279_at | 12.17 | 3.80E-05 | 1.114454682 | NM_138452| | DHRS1,dehydrogenase/reductase (SDR family) member 1 |
| 239392_s_at | 12.16 | 3.83E-05 | 1.174429348 | NM_017542| | POGK,pogo transposable element with KRAB domain |
| 1558802_at | 12.16 | 3.82E-05 | 1.369937314 | NA |  |
| 223243_s_at | 12.16 | 3.82E-05 | 1.120864484 | NM_025191| | C1orf22,chromosome 1 open reading frame 22 |
| 201647_s_at | 12.16 | 3.83E-05 | 1.235362631 | NM_005506| | SCARB2,scavenger receptor class B, member 2 |
| 230069_at | 12.16 | 3.82E-05 | 1.128633555 | NM_022754| | SFXN1,sideroflexin 1 |
| 1557915_s_at | 12.15 | 3.85E-05 | 1.019242527 | NM_004832| | GSTO1,glutathione-S-transferase omega 1 |
| 201979_s_at | 12.15 | 3.84E-05 | 1.106115333 | NM_006247| | PPP5C,protein phosphatase 5, catalytic subunit |
| 221889_at | 12.15 | 3.85E-05 | 1.122756577 | NM_178863| | KCTD13,potassium channel tetramerisation domain |
| 222113_s_at | 12.15 | 3.85E-05 | 1.098718628 | NM_021235| | EPS15L1,epidermal growth factor receptor pathway |
| 235461_at | 12.14 | 3.86E-05 | 1.24158463 | NM_001127208| | NA |
| 219419_at | 12.13 | 3.89E-05 | 1.090256079 | NM_024805| | C18orf22,chromosome 18 open reading frame 22 |
| 240467_at | 12.13 | 3.89E-05 | 1.356563421 | NA |  |
| 204860_s_at | 12.12 | 3.89E-05 | 1.350701013 | NM_004536| | BIRC1,baculoviral IAP repeat-containing 1 |
| 206320_s_at | 12.12 | 3.89E-05 | 1.132881922 | NM_001127217| | NA |
| 201835_s_at | 12.12 | 3.90E-05 | 1.108224108 | NM_006253| | PRKAB1,AMP-activated protein kinase beta 1 |
| 215364_s_at | 12.12 | 3.90E-05 | 1.144675559 | NM_015284| | KIAA0467,KIAA0467 protein |
| 211914_x_at | 12.11 | 3.92E-05 | 1.103865789 | NM_000267| | NF1,neurofibromin |
| 218467_at | 12.11 | 3.91E-05 | 1.024361542 | NM_020232| | TNFSF5IP1,tumor necrosis factor superfamily, member |
| 218034_at | 12.1 | 3.93E-05 | 1.059890486 | NM_016068| | TTC11,tetratricopeptide repeat domain 11 |
| 233803_s_at | 12.1 | 3.93E-05 | 1.140115334 | NM_001105538| | NA |
| 227982_at | 12.1 | 3.93E-05 | 1.139965372 | NM_016955| | SLA/LP,soluble liver antigen/liver pancreas antigen |
| 210983_s_at | 12.1 | 3.93E-05 | 1.084138092 | NM_005916| | MCM7,minichromosome maintenance protein 7 isoform 1 |
| 223706_at | 12.1 | 3.93E-05 | 1.239353019 | NM_032561| | C22orf23,EVG1 protein |
| 204127_at | 12.1 | 3.94E-05 | 1.037255271 | NM_002915| | RFC3,replication factor C 3 isoform 1 |
| 241798_at | 12.1 | 3.94E-05 | 1.195255779 | NA |  |
| 216247_at | 12.1 | 3.93E-05 | 1.242360911 | NM_001023| | RPS20,ribosomal protein S20 |
| 229192_s_at | 12.1 | 3.94E-05 | 1.137878998 | NM_005993| | TBCD,beta-tubulin cofactor D |
| 215236_s_at | 12.1 | 3.93E-05 | 1.222680672 | NM_001008660| | PICALM,phosphatidylinositol-binding clathrin assembly |
| 214211_at | 12.09 | 3.96E-05 | 1.069371226 | NM_002032| | FTH1,ferritin, heavy polypeptide 1 |
| 225356_at | 12.09 | 3.95E-05 | 1.10085897 | NA |  |
| 235693_at | 12.09 | 3.94E-05 | 1.206946365 | NA |  |
| 227925_at | 12.09 | 3.96E-05 | 1.270963638 | NA |  |
| 225302_at | 12.09 | 3.96E-05 | 1.138325148 | NM_019022| | TXNDC10,thioredoxin domain containing 10 |
| 215716_s_at | 12.09 | 3.95E-05 | 1.11577651 | NM_001001323| | ATP2B1,plasma membrane calcium ATPase 1 isoform 1a |
| 200746_s_at | 12.08 | 3.96E-05 | 1.036012522 | NM_002074| | GNB1,guanine nucleotide-binding protein, beta-1 |
| 201991_s_at | 12.08 | 3.96E-05 | 1.074574629 | NM_004521| | KIF5B,kinesin family member 5B |
| 232758_s_at | 12.08 | 3.97E-05 | 1.214795416 | NA |  |
| 1562460_at | 12.08 | 3.97E-05 | 1.164115111 | NM_018235| | CNDP2,CNDP dipeptidase 2 (metallopeptidase M20 |
| 220264_s_at | 12.08 | 3.97E-05 | 1.149411985 | NM_020960| | GPR107,G protein-coupled receptor 107 |
| 226208_at | 12.08 | 3.97E-05 | 1.05802249 | NA |  |
| 204497_at | 12.08 | 3.96E-05 | 1.110229804 | NM_001116| | ADCY9,adenylate cyclase 9 |
| 201556_s_at | 12.07 | 3.98E-05 | 1.246461938 | NM_014232| | VAMP2,vesicle-associated membrane protein 2 |
| 212503_s_at | 12.07 | 3.98E-05 | 1.11749324 | NM_014974| | KIAA0934,KIAA0934 |
| 221768_at | 12.06 | 4.00E-05 | 1.119726735 | NM_005066| | SFPQ,splicing factor proline/glutamine rich |
| 211943_x_at | 12.06 | 4.00E-05 | 1.021142648 | NM_003295| | TPT1,tumor protein, translationally-controlled 1 |
| 236358_at | 12.06 | 4.01E-05 | 1.214931992 | NA |  |
| 228043_at | 12.06 | 4.01E-05 | 1.288686518 | NM_032175| | FLJ12787,Src-associated protein SAW |
| 230213_at | 12.05 | 4.01E-05 | 1.149858411 | NM_024038| | MGC2803,hypothetical protein MGC2803 |
| 215307_at | 12.05 | 4.01E-05 | 1.140708354 | NM_020951| | ZNF529,zinc finger protein 529 |
| 201230_s_at | 12.05 | 4.01E-05 | 1.044739463 | NM_006321| | ARIH2,ariadne homolog 2 |
| 1552610_a_at | 12.05 | 4.03E-05 | 1.167428021 | NM_002227| | JAK1,janus kinase 1 |
| 241407_at | 12.05 | 4.01E-05 | 1.191279414 | NA |  |
| 210198_s_at | 12.05 | 4.02E-05 | 1.083027567 | NM_000533| | PLP1,proteolipid protein 1 isoform 1 |
| 228619_x_at | 12.05 | 4.01E-05 | 1.091330278 | NM_001031800| | NA |
| 213273_at | 12.04 | 4.04E-05 | 1.129299934 | NM_001098816| | NA |
| 223036_at | 12.04 | 4.04E-05 | 1.559961714 | NM_005687| | FARSLB,phenylalanine-tRNA synthetase-like, beta |
| 228040_at | 12.04 | 4.04E-05 | 1.097003089 | NA |  |
| 206103_at | 12.03 | 4.06E-05 | 1.065031738 | NM_005052| | RAC3,ras-related C3 botulinum toxin substrate 3 (rho |
| 208822_s_at | 12.03 | 4.06E-05 | 1.042658962 | NM_004632| | DAP3,death-associated protein 3 |
| 218802_at | 12.02 | 4.07E-05 | 1.077223597 | NM_017918| | FLJ20647,hypothetical protein FLJ20647 |
| 208867_s_at | 12.02 | 4.07E-05 | 1.063350829 | NM_001025105| | NA |
| 1553348_a_at | 12.02 | 4.08E-05 | 1.210902722 | NM_002504| | NFX1,nuclear transcription factor, X-box binding 1 |
| 209826_at | 12.02 | 4.07E-05 | 1.120640074 | NM_005155| | PPT2,palmitoyl-protein thioesterase 2 isoform a |
| 213471_at | 12.02 | 4.08E-05 | 1.103760718 | NM_015102| | NPHP4,nephroretinin |
| 226965_at | 12.02 | 4.07E-05 | 1.098871853 | NM_152678| | FLJ34969,hypothetical protein FLJ34969 |
| 241017_at | 12.02 | 4.07E-05 | 1.210129509 | NM_001102426| | NA |
| 224284_x_at | 12.01 | 4.10E-05 | 1.091278858 | NA |  |
| 225341_at | 12.01 | 4.09E-05 | 1.115422908 | NM_001033050| | NA |
| 212680_x_at | 12.01 | 4.10E-05 | 1.035985992 | NM_138689| | NA |
| 204195_s_at | 12.01 | 4.09E-05 | 1.054690469 | NM_004571| | PKNOX1,PBX/knotted 1 homeobox 1 isoform 1 |
| 221822_at | 12 | 4.11E-05 | 1.062745234 | NM_138414| | LOC112869,hypothetical protein BC011981 |
| 228006_at | 12 | 4.11E-05 | 1.169126826 | NA |  |
| 216113_at | 12 | 4.12E-05 | 1.098687498 | NM_005759| | ABI2,abl interactor 2 |
| 226234_at | 12 | 4.11E-05 | 1.113589287 | NA |  |
| 205010_at | 11.99 | 4.13E-05 | 1.088018255 | NM_019067| | GNL3L,guanine nucleotide binding protein-like 3 |
| 214538_x_at | 11.99 | 4.13E-05 | 1.287596551 | NM_004296| | RGS6,regulator of G-protein signalling 6 |
| 200917_s_at | 11.99 | 4.13E-05 | 1.056234711 | NM_003139| | SRPR,signal recognition particle receptor ('docking |
| 222869_s_at | 11.99 | 4.13E-05 | 1.09720034 | NM_018696| | ELAC1,elaC homolog 1 |
| 226977_at | 11.99 | 4.13E-05 | 1.168878657 | NM_001007189| | LOC492311,similar to bovine IgA regulatory protein |
| 222040_at | 11.99 | 4.13E-05 | 1.135594296 | NM_002136| | HNRPA1,heterogeneous nuclear ribonucleoprotein A1 |
| 240078_at | 11.99 | 4.12E-05 | 1.215348764 | NM_004592| | SFRS8,splicing factor, arginine/serine-rich 8 isoform |
| 1555486_a_at | 11.98 | 4.14E-05 | 1.193876569 | NM_024841| | FLJ14213,hypothetical protein FLJ14213 |
| 243943_x_at | 11.98 | 4.15E-05 | 1.07516011 | NA |  |
| 201956_s_at | 11.98 | 4.15E-05 | 1.018656589 | NM_014236| | GNPAT,glyceronephosphate O-acyltransferase |
| 234312_s_at | 11.98 | 4.14E-05 | 1.053290304 | NM_001076552| | NA |
| 209693_at | 11.98 | 4.15E-05 | 1.05958382 | NM_014010| | ASTN2,astrotactin 2 isoform a |
| 212412_at | 11.97 | 4.17E-05 | 1.099600071 | NM_001011513| | PDLIM5,PDZ and LIM domain 5 isoform b |
| 212346_s_at | 11.97 | 4.16E-05 | 1.085988887 | NM_006454| | MXD4,MAD4 |
| 228822_s_at | 11.97 | 4.17E-05 | 1.082229117 | NM_001001992| | USP16,ubiquitin specific protease 16 isoform b |
| 1556696_s_at | 11.97 | 4.16E-05 | 1.426470772 | NA |  |
| 212188_at | 11.97 | 4.17E-05 | 1.21805126 | NM_138444| | KCTD12,potassium channel tetramerisation domain |
| 221272_s_at | 11.96 | 4.18E-05 | 1.194952354 | NM_030806| | C1orf21,chromosome 1 open reading frame 21 |
| 229413_s_at | 11.96 | 4.18E-05 | 1.23625439 | NA |  |
| 221139_s_at | 11.96 | 4.18E-05 | 1.248273356 | NM_015989| | CSAD,cysteine sulfinic acid decarboxylase-related |
| 1553107_s_at | 11.96 | 4.18E-05 | 1.143778096 | NM_152409| | FLJ37562,hypothetical protein FLJ37562 |
| 207469_s_at | 11.95 | 4.20E-05 | 1.051998898 | NM_001018109| | NA |
| 205182_s_at | 11.95 | 4.21E-05 | 1.117340876 | NM_014347| | ZNF324,zinc finger protein 324 |
| 204015_s_at | 11.95 | 4.20E-05 | 1.091881138 | NM_001394| | DUSP4,dual specificity phosphatase 4 isoform 1 |
| 235919_at | 11.95 | 4.20E-05 | 1.07775343 | NA |  |
| 1559227_s_at | 11.95 | 4.19E-05 | 1.111263484 | NM_000551| | VHL,von Hippel-Lindau tumor suppressor isoform 1 |
| 213045_at | 11.95 | 4.19E-05 | 1.120150685 | NM_015016| | NA |
| 229544_at | 11.95 | 4.20E-05 | 1.112106968 | NA |  |
| 230656_s_at | 11.94 | 4.22E-05 | 1.059740283 | NM_032830| | CIRH1A,cirhin |
| 236310_at | 11.94 | 4.22E-05 | 1.419257484 | NA |  |
| 202192_s_at | 11.94 | 4.21E-05 | 1.236067729 | NM_003644| | GAS7,growth arrest-specific 7 isoform a |
| 226032_at | 11.94 | 4.22E-05 | 1.037699303 | NM_032982| | CASP2,caspase 2 isoform 1 preproprotein |
| 228399_at | 11.94 | 4.22E-05 | 1.204381848 | NM_145260| | OSR1,odd-skipped related 1 |
| 208629_s_at | 11.94 | 4.22E-05 | 1.024777636 | NM_000182| | HADHA,hydroxyacyl dehydrogenase, subunit A |
| 202866_at | 11.94 | 4.22E-05 | 1.155731341 | NM_001002762| | DNAJB12,DnaJ (Hsp40) homolog, subfamily B, member 12 |
| 1558787_a_at | 11.93 | 4.23E-05 | 1.281303728 | NA |  |
| 209195_s_at | 11.92 | 4.26E-05 | 1.102975623 | NM_015270| | ADCY6,adenylate cyclase 6 isoform a |
| 239810_at | 11.91 | 4.27E-05 | 1.261883345 | NM_014909| | KIAA1036,KIAA1036 |
| 226693_at | 11.91 | 4.28E-05 | 1.1738342 | NA |  |
| 213850_s_at | 11.91 | 4.27E-05 | 1.074719221 | NM_004719| | SFRS2IP,splicing factor, arginine/serine-rich 2, |
| 209169_at | 11.9 | 4.30E-05 | 1.10614452 | NM_001001994| | GPM6B,glycoprotein M6B isoform 4 |
| 213209_at | 11.9 | 4.30E-05 | 1.215966779 | NM_006473| | TAF6L,TAF6-like RNA polymerase II |
| 219890_at | 11.89 | 4.32E-05 | 1.10131912 | NM_013252| | CLEC5A,C-type lectin, superfamily member 5 |
| 232309_at | 11.89 | 4.33E-05 | 1.199577463 | NA |  |
| 214786_at | 11.89 | 4.31E-05 | 1.414215457 | NM_005921| | NA |
| 232659_at | 11.88 | 4.33E-05 | 1.220817501 | NA |  |
| 217940_s_at | 11.88 | 4.34E-05 | 1.060874441 | NM_018210| | FLJ10769,hypothetical protein FLJ10769 |
| 228615_at | 11.88 | 4.33E-05 | 1.240806852 | NA |  |
| 202675_at | 11.88 | 4.33E-05 | 1.054327938 | NM_003000| | SDHB,succinate dehydrogenase complex, subunit B, iron |
| 239151_at | 11.87 | 4.36E-05 | 1.274608493 | NA |  |
| 1558714_at | 11.87 | 4.36E-05 | 1.433955077 | NA |  |
| 242785_at | 11.86 | 4.38E-05 | 1.292950516 | NA |  |
| 214180_at | 11.86 | 4.38E-05 | 1.230805306 | NM_020379| | MAN1C1,mannosidase, alpha, class 1C, member 1 |
| 242337_at | 11.86 | 4.38E-05 | 1.208077464 | NA |  |
| 225312_at | 11.86 | 4.38E-05 | 1.048051342 | NM_203495| | COMMD6,COMM domain containing 6 isoform b |
| 1553666_at | 11.85 | 4.41E-05 | 1.912221041 | NM_030771| | NA |
| 219802_at | 11.85 | 4.40E-05 | 1.155079825 | NM_024854| | FLJ22028,hypothetical protein FLJ22028 |
| 210461_s_at | 11.85 | 4.41E-05 | 1.074164994 | NM_001003407| | ABLIM1,actin-binding LIM protein 1 isoform b |
| 212054_x_at | 11.85 | 4.40E-05 | 1.063763063 | NM_015043| | KIAA0676,KIAA0676 protein isoform b |
| 238333_s_at | 11.85 | 4.41E-05 | 1.151728257 | NM_138384| | GTP,GTP_binding protein |
| 240575_at | 11.84 | 4.43E-05 | 1.279213373 | NA |  |
| 215519_x_at | 11.84 | 4.43E-05 | 1.102609779 | NM_015705| | RUTBC3,RUN and TBC1 domain containing 3 |
| 209067_s_at | 11.84 | 4.41E-05 | 1.026632764 | NM_031372| | HNRPDL,heterogeneous nuclear ribonucleoprotein D-like |
| 224872_at | 11.84 | 4.42E-05 | 1.116680102 | NM_173602| | KIAA1463,KIAA1463 protein |
| 204877_s_at | 11.84 | 4.42E-05 | 1.166575893 | NM_004783| | TAOK2,TAO kinase 2 |
| 236202_at | 11.84 | 4.41E-05 | 1.414905451 | NA |  |
| 239793_at | 11.83 | 4.46E-05 | 1.587300607 | NA |  |
| 232951_at | 11.83 | 4.44E-05 | 1.100018274 | NA |  |
| 214210_at | 11.83 | 4.44E-05 | 1.077516049 | NM_006358| | SLC25A17,solute carrier family 25 (mitochondrial carrier; |
| 218679_s_at | 11.83 | 4.44E-05 | 1.095816967 | NM_016208| | VPS28,vacuolar protein sorting 28 isoform 1 |
| 221745_at | 11.82 | 4.46E-05 | 1.080249383 | NM_005828| | HAN11,WD-repeat protein |
| 203785_s_at | 11.82 | 4.46E-05 | 1.04962774 | NM_018380| | DDX28,DEAD (Asp-Glu-Ala-Asp) box polypeptide 28 |
| 241100_at | 11.81 | 4.50E-05 | 1.23516362 | NA |  |
| 204490_s_at | 11.81 | 4.50E-05 | 1.14334972 | NM_000610| | CD44,CD44 antigen isoform 1 precursor |
| 200840_at | 11.81 | 4.50E-05 | 1.012159997 | NM_005548| | KARS,lysyl-tRNA synthetase |
| 209256_s_at | 11.81 | 4.48E-05 | 1.079737336 | NM_014997| | KIAA0265,KIAA0265 protein |
| 235123_at | 11.81 | 4.49E-05 | 1.100976482 | NA |  |
| 215099_s_at | 11.81 | 4.49E-05 | 1.34085984 | NM_021976| | RXRB,retinoid X receptor, beta |
| 222032_s_at | 11.8 | 4.52E-05 | 1.06927406 | NM_003470| | USP7,ubiquitin specific protease 7 (herpes |
| 238762_at | 11.79 | 4.53E-05 | 1.193243734 | NM_001004346| | MTHFD2L,methylenetetrahydrofolate dehydrogenase (NADP+ |
| 212252_at | 11.79 | 4.53E-05 | 1.072389615 | NM_006549| | CAMKK2,calcium/calmodulin-dependent protein kinase |
| 214512_s_at | 11.79 | 4.54E-05 | 1.028161921 | NM_006713| | PC4,activated RNA polymerase II transcription |
| 236153_at | 11.79 | 4.53E-05 | 1.343307041 | NA |  |
| 225758_s_at | 11.79 | 4.53E-05 | 1.162811636 | NM_020461| | TUBGCP6,tubulin, gamma complex associated protein 6 |
| 236000_s_at | 11.79 | 4.53E-05 | 1.298812681 | NA |  |
| 204242_s_at | 11.78 | 4.55E-05 | 1.182047475 | NM_001101667| | NA |
| 226854_at | 11.78 | 4.54E-05 | 1.092506223 | NA |  |
| 1554635_a_at | 11.78 | 4.55E-05 | 1.411395245 | NM_022123| | NPAS3,neuronal PAS domain protein 3 |
| 1558365_at | 11.78 | 4.56E-05 | 1.416779004 | NA |  |
| 201937_s_at | 11.78 | 4.54E-05 | 1.067118247 | NM_012100| | DNPEP,aspartyl aminopeptidase |
| 212173_at | 11.77 | 4.57E-05 | 1.188033372 | NM_001625| | AK2,adenylate kinase 2 isoform a |
| 222425_s_at | 11.77 | 4.58E-05 | 1.11509438 | NM_015584| | POLDIP2,DNA polymerase delta interacting protein 2 |
| 1560082_at | 11.77 | 4.58E-05 | 1.333895799 | NA |  |
| 1557350_at | 11.77 | 4.58E-05 | 1.213210957 | NM_005754| | G3BP,Ras-GTPase-activating protein SH3-domain-binding |
| 203695_s_at | 11.76 | 4.59E-05 | 1.126347991 | NM_004403| | DFNA5,deafness, autosomal dominant 5 protein |
| 214361_s_at | 11.76 | 4.59E-05 | 1.217565736 | NM_002926| | RGS12,regulator of G-protein signalling 12 isoform 2 |
| 202271_at | 11.76 | 4.59E-05 | 1.038524732 | NM_015176| | FBXO28,F-box protein 28 |
| 228274_at | 11.76 | 4.60E-05 | 1.351104773 | NM_138432| | SDSL,serine dehydratase-like |
| 209745_at | 11.76 | 4.60E-05 | 1.081611728 | NM_016138| | COQ7,COQ7 protein |
| 209858_x_at | 11.76 | 4.60E-05 | 1.066484173 | NM_023075| | MPPE1,metallophosphoesterase 1 isoform a precursor |
| 218560_s_at | 11.76 | 4.60E-05 | 1.124602764 | NM_023007| | FLJ12517,hypothetical protein FLJ12517 |
| 212694_s_at | 11.75 | 4.62E-05 | 1.057258695 | NM_000532| | PCCB,propionyl Coenzyme A carboxylase, beta |
| 1557315_a_at | 11.75 | 4.61E-05 | 1.178660735 | NA |  |
| 239042_at | 11.75 | 4.63E-05 | 1.107067079 | NM_018128| | FLJ10534,hypothetical protein FLJ10534 |
| 201779_s_at | 11.75 | 4.61E-05 | 1.066400314 | NM_007282| | RNF13,ring finger protein 13 isoform 1 |
| 231852_at | 11.75 | 4.63E-05 | 1.232738252 | NM_153332| | 3'HEXO,3' exoribonuclease |
| 209695_at | 11.75 | 4.63E-05 | 1.07032648 | NM_007079| | PTP4A3,protein tyrosine phosphatase type IVA, member 3 |
| 224601_at | 11.74 | 4.65E-05 | 1.081185436 | NA |  |
| 202758_s_at | 11.74 | 4.63E-05 | 1.062478241 | NM_003721| | RFXANK,regulatory factor X-associated |
| 216799_at | 11.74 | 4.65E-05 | 1.39119221 | NA |  |
| 228214_at | 11.74 | 4.64E-05 | 1.323851469 | NA |  |
| 236531_at | 11.74 | 4.63E-05 | 1.364624127 | NA |  |
| 1561759_at | 11.74 | 4.63E-05 | 1.192953393 | NA |  |
| 205682_x_at | 11.73 | 4.66E-05 | 1.183836622 | NM_019101| | APOM,apolipoprotein M |
| 226198_at | 11.73 | 4.67E-05 | 1.117743131 | NM_001033551| | NA |
[truncated: 66,208 more chars]
