# Supplementary material for: Transcriptional Signature and Memory Retention of Human-Induced Pluripotent Stem Cells
Source: PLoS One. 2009 Sep 18;4(9):e7076. doi: 10.1371/journal.pone.0007076 (PMC2741600; doi:10.1371/journal.pone.0007076)
Supplement: Table S5 — ES-enriched probes in ES versus NSC. Probesets enriched in group-wise comparisons: Column headings are probeset identifiers, T-statistic, P-value, Fold-Change (log2), Refseq identifier and Description of the gene. (NA indicates no Refseq annotation). (6.06 MB DOC) [file pone.0007076.s009.doc]

| Probeset | T-statistic | P-value | Fold-Change (log2) | Refseq | Description |
| --- | --- | --- | --- | --- | --- |
| 226507_at | 164.64 | 4.40E-09 | 1.557377393 | NM_002576| | PAK1,p21-activated kinase 1 |
| 209757_s_at | 137.47 | 6.37E-09 | 1.523691083 | NM_005378| | MYCN,v-myc myelocytomatosis viral related oncogene, |
| 205514_at | 113.67 | 6.37E-09 | 2.330761105 | NM_018355| | ZNF415,zinc finger protein 415 |
| 201100_s_at | 113.38 | 6.37E-09 | 1.246035995 | NM_001039590| | NA |
| 216836_s_at | 110.66 | 6.37E-09 | 1.339873909 | NM_001005862| | ERBB2,erbB-2 isoform b |
| 219073_s_at | 110.56 | 6.37E-09 | 1.658905081 | NM_017784| | OSBPL10,oxysterol-binding protein-like protein 10 |
| 200701_at | 108.19 | 6.37E-09 | 1.437980625 | NM_006432| | NPC2,Niemann-Pick disease, type C2 precursor |
| 203917_at | 106.06 | 6.37E-09 | 1.204535134 | NM_001338| | CXADR,coxsackie virus and adenovirus receptor |
| 227705_at | 103.64 | 6.63E-09 | 3.095547642 | NM_152278| | TCEAL7,transcription elongation factor A (SII)-like 7 |
| 208690_s_at | 103.58 | 6.63E-09 | 1.454596857 | NM_020992| | PDLIM1,PDZ and LIM domain 1 (elfin) |
| 229292_at | 102.28 | 6.84E-09 | 2.480819468 | NM_020909| | EPB41L5,erythrocyte membrane protein band 4.1 like 5 |
| 219368_at | 99.7 | 7.75E-09 | 2.654529966 | NM_021963| | NAP1L2,nucleosome assembly protein 1-like 2 |
| 205480_s_at | 94.15 | 8.72E-09 | 1.33590089 | NM_001001521| | UGP2,UDP-glucose pyrophosphorylase 2 isoform b |
| 226497_s_at | 92.83 | 8.72E-09 | 2.536891972 | NA |  |
| 232985_s_at | 92.52 | 8.72E-09 | 3.24749578 | NM_018189| | DPPA4,developmental pluripotency associated 4 |
| 206247_at | 92.18 | 8.72E-09 | 2.84311194 | NM_000247| | MICA,MHC class I chain-related gene A protein |
| 226587_at | 90.98 | 8.72E-09 | 1.327464012 | NA |  |
| 201832_s_at | 89.51 | 8.99E-09 | 1.188779043 | NM_003715| | VDP,vesicle docking protein p115 |
| 208862_s_at | 89.15 | 8.99E-09 | 1.147968078 | NM_001085458| | NA |
| 219121_s_at | 89.07 | 8.99E-09 | 2.674643527 | NM_001034915| | NA |
| 203417_at | 88.64 | 9.05E-09 | 1.711302456 | NM_002403| | MFAP2,microfibrillar-associated protein 2 precursor |
| 208456_s_at | 84.8 | 1.16E-08 | 1.452172733 | NM_001102669| | NA |
| 228415_at | 84.62 | 1.16E-08 | 1.402983124 | NM_003916| | AP1S2,adaptor-related protein complex 1 sigma 2 |
| 228565_at | 84.12 | 1.16E-08 | 1.499077172 | NM_032435| | KIAA1804,mixed lineage kinase 4 |
| 209297_at | 83.71 | 1.16E-08 | 1.22450771 | NM_001001132| | ITSN1,intersectin 1 isoform ITSN-s |
| 209771_x_at | 83.22 | 1.16E-08 | 1.173920428 | NM_013230| | CD24,CD24 antigen |
| 209488_s_at | 81.76 | 1.25E-08 | 1.712872892 | NM_001008710| | RBPMS,RNA-binding protein with multiple splicing |
| 221245_s_at | 81.32 | 1.28E-08 | 1.620830302 | NM_003468| | FZD5,frizzled 5 |
| 225314_at | 77.55 | 1.60E-08 | 1.821194635 | NM_001014446| | NA |
| 217853_at | 76.99 | 1.60E-08 | 1.644839684 | NM_022748| | TENS1,tensin-like SH2 domain containing 1 |
| 219681_s_at | 75.07 | 1.75E-08 | 1.485670044 | NM_001002233| | RAB11FIP1,Rab coupling protein isoform 2 |
| 218735_s_at | 74.96 | 1.75E-08 | 1.56417055 | NM_014480| | ZNF544,zinc finger protein 544 |
| 221810_at | 74.9 | 1.75E-08 | 1.291561467 | NM_198686| | RAB15,Ras-related protein Rab-15 |
| 225846_at | 74.33 | 1.81E-08 | 3.14816895 | NM_001034915| | NA |
| 229256_at | 74.23 | 1.81E-08 | 1.823901787 | NM_173582| | PGM2L1,phosphoglucomutase 2-like 1 |
| 228894_at | 73.68 | 1.81E-08 | 1.88581215 | NM_001489| | NR6A1,nuclear receptor subfamily 6, group A, member 1 |
| 225958_at | 73.26 | 1.81E-08 | 1.373559192 | NM_004426| | PHC1,polyhomeotic 1-like |
| 220419_s_at | 72.87 | 1.81E-08 | 1.256314716 | NM_013396| | USP25,ubiquitin specific protease 25 |
| 225275_at | 71.3 | 2.05E-08 | 3.595264289 | NM_005711| | EDIL3,EGF-like repeats and discoidin I-like |
| 220085_at | 71.11 | 2.06E-08 | 1.182841507 | NM_018063| | HELLS,helicase, lymphoid-specific |
| 219489_s_at | 69.8 | 2.30E-08 | 1.230643208 | NM_022463| | NXN,nucleoredoxin |
| 1564359_a_at | 68.2 | 2.51E-08 | 2.502195196 | NA |  |
| 239552_at | 67.71 | 2.53E-08 | 1.925043625 | NA |  |
| 223435_s_at | 66.8 | 2.68E-08 | 3.183243093 | NM_014005| | PCDHA9,protocadherin alpha 9 isoform 2 precursor |
| 209487_at | 66.73 | 2.68E-08 | 1.526986582 | NM_001008710| | RBPMS,RNA-binding protein with multiple splicing |
| 227760_at | 66.19 | 2.74E-08 | 1.557052139 | NM_001007563| | IGFBPL1,insulin-like growth factor binding protein-like |
| 230560_at | 65.88 | 2.78E-08 | 2.835738478 | NM_014178| | STXBP6,amisyn |
| 218341_at | 65.63 | 2.81E-08 | 1.524490909 | NM_001077447| | NA |
| 207186_s_at | 65.58 | 2.81E-08 | 1.099574727 | NM_004459| | FALZ,fetal Alzheimer antigen isoform 2 |
| 228120_at | 65.15 | 2.89E-08 | 1.133819472 | NA |  |
| 203638_s_at | 65.12 | 2.89E-08 | 1.304359732 | NM_000141| | FGFR2,fibroblast growth factor receptor 2 isoform 1 |
| 210074_at | 64.89 | 2.94E-08 | 1.39947003 | NM_001333| | CTSL2,cathepsin L2 preproprotein |
| 227830_at | 64.51 | 2.97E-08 | 2.354279892 | NM_000814| | GABRB3,gamma-aminobutyric acid (GABA) A receptor, beta |
| 227372_s_at | 64.33 | 2.97E-08 | 2.468900887 | NM_018842| | BAIAP2L1,BAI1-associated protein 2-like 1 |
| 210517_s_at | 64.22 | 2.98E-08 | 1.211773897 | NM_005100| | AKAP12,A-kinase anchor protein 12 isoform 1 |
| 219901_at | 64.12 | 2.98E-08 | 1.671650728 | NM_018351| | FGD6,FYVE, RhoGEF and PH domain containing 6 |
| 223038_s_at | 63.92 | 3.01E-08 | 1.23686731 | NM_021238| | C12orf14,chromosome 12 open reading frame 14 |
| 204984_at | 63.8 | 3.01E-08 | 1.550449535 | NM_001448| | GPC4,glypican 4 |
| 221868_at | 63.79 | 3.01E-08 | 1.8815981 | NM_020459| | NA |
| 204679_at | 63.66 | 3.03E-08 | 1.968388211 | NM_002245| | KCNK1,potassium channel, subfamily K, member 1 |
| 237336_at | 63.54 | 3.03E-08 | 1.542118531 | NM_001617| | ADD2,adducin 2 isoform a |
| 241535_at | 63.43 | 3.03E-08 | 3.462393121 | NA |  |
| 229349_at | 63.35 | 3.03E-08 | 3.64777806 | NM_001004317| | FLJ16517,FLJ16517 protein |
| 216379_x_at | 63.17 | 3.05E-08 | 1.185193998 | NM_013230| | CD24,CD24 antigen |
| 218454_at | 63.05 | 3.05E-08 | 2.075093364 | NM_024829| | FLJ22662,hypothetical protein FLJ22662 |
| 227365_at | 62.06 | 3.29E-08 | 1.362092281 | NM_033064| | ATCAY,caytaxin |
| 201596_x_at | 61.99 | 3.29E-08 | 1.888397038 | NM_000224| | KRT18,keratin 18 |
| 217967_s_at | 61.06 | 3.46E-08 | 2.259600765 | NM_052966| | C1orf24,niban protein |
| 221011_s_at | 60.49 | 3.67E-08 | 1.573857272 | NM_030915| | LBH,hypothetical protein DKFZp566J091 |
| 212589_at | 60.13 | 3.70E-08 | 1.321961875 | NM_001102669| | NA |
| 203413_at | 59.85 | 3.75E-08 | 1.454603445 | NM_006159| | NELL2,nel-like 2 |
| 227371_at | 58.54 | 4.12E-08 | 2.225942387 | NM_018842| | BAIAP2L1,BAI1-associated protein 2-like 1 |
| 219010_at | 58.17 | 4.21E-08 | 1.184504324 | NM_018265| | FLJ10901,hypothetical protein FLJ10901 |
| 225269_s_at | 57.46 | 4.36E-08 | 1.284527615 | NA |  |
| 205924_at | 57.27 | 4.38E-08 | 1.396593533 | NM_002867| | RAB3B,RAB3B, member RAS oncogene family |
| 225817_at | 57.02 | 4.44E-08 | 2.186342616 | NM_032866| | CGNL1,cingulin-like 1 |
| 206268_at | 56.8 | 4.50E-08 | 3.043229683 | NM_020997| | LEFTY1,left-right determination, factor B |
| 211781_x_at | 56.26 | 4.59E-08 | 2.308450217 | NA |  |
| 212203_x_at | 56.21 | 4.59E-08 | 1.211935822 | NM_021034| | IFITM3,interferon-induced transmembrane protein 3 |
| 217938_s_at | 56.06 | 4.62E-08 | 1.197829415 | NM_020122| | KCMF1,potassium channel modulatory factor 1 |
| 211057_at | 55.99 | 4.62E-08 | 1.589733719 | NM_001083592| | NA |
| 1553105_s_at | 55.77 | 4.71E-08 | 3.028383304 | NM_001943| | DSG2,desmoglein 2 preproprotein |
| 209705_at | 55.7 | 4.71E-08 | 1.311153929 | NM_007358| | M96,putative DNA binding protein |
| 226482_s_at | 55.29 | 4.88E-08 | 2.596998705 | NM_001113205| | NA |
| 209210_s_at | 54.87 | 5.11E-08 | 1.106741937 | NM_006832| | PLEKHC1,pleckstrin homology domain containing, family C |
| 231061_at | 54.85 | 5.11E-08 | 1.758874689 | NA |  |
| 207305_s_at | 54.55 | 5.13E-08 | 1.146029064 | NM_014939| | KIAA1012,KIAA1012 |
| 208119_s_at | 54.53 | 5.13E-08 | 1.354892962 | NM_031218| | ZNF505,zinc finger protein 505 isoform a |
| 218412_s_at | 54.44 | 5.13E-08 | 1.168901639 | NM_005685| | GTF2IRD1,GTF2I repeat domain containing 1 isoform 2 |
| 209772_s_at | 54.41 | 5.13E-08 | 1.507714611 | NM_013230| | CD24,CD24 antigen |
| 224097_s_at | 53.88 | 5.34E-08 | 1.812547867 | NM_016946| | F11R,F11 receptor isoform a precursor |
| 237193_s_at | 53.87 | 5.34E-08 | 2.985864429 | NA |  |
| 227492_at | 53.79 | 5.36E-08 | 1.48264857 | NA |  |
| 214397_at | 53.46 | 5.49E-08 | 1.855356919 | NM_003927| | MBD2,methyl-CpG binding domain protein 2 isoform 1 |
| 208286_x_at | 53.4 | 5.49E-08 | 2.126441243 | NM_002701| | POU5F1,POU domain, class 5, transcription factor 1 |
| 204675_at | 53.39 | 5.49E-08 | 1.503409106 | NM_001047| | SRD5A1,steroid-5-alpha-reductase 1 |
| 205399_at | 53.17 | 5.62E-08 | 1.480136372 | NM_004734| | DCAMKL1,doublecortin and CaM kinase-like 1 |
| 203697_at | 53.08 | 5.66E-08 | 2.089839904 | NM_001463| | FRZB,frizzled-related protein |
| 230788_at | 53.02 | 5.66E-08 | 1.341014042 | NM_001491| | GCNT2,glucosaminyl (N-acetyl) transferase 2 isoform B |
| 230493_at | 52.73 | 5.73E-08 | 3.559440256 | NM_001007538| | LOC387914,WGAR9166 |
| 207705_s_at | 52.57 | 5.79E-08 | 1.229573632 | NM_025176| | KIAA0980,KIAA0980 protein |
| 201099_at | 52.23 | 5.95E-08 | 1.286468833 | NM_001039590| | NA |
| 230988_at | 51.84 | 6.13E-08 | 1.484444329 | NA |  |
| 216565_x_at | 51.82 | 6.13E-08 | 1.360559659 | NA |  |
| 235773_at | 51.55 | 6.32E-08 | 1.263873896 | NM_001010879| | ZIK1,zinc finger protein interacting with K protein |
| 208131_s_at | 51.27 | 6.49E-08 | 2.053805775 | NM_000961| | PTGIS,prostaglandin I2 (prostacyclin) synthase |
| 201578_at | 50.8 | 6.80E-08 | 1.464629986 | NM_001018111| | NA |
| 226847_at | 50.78 | 6.80E-08 | 3.21039829 | NM_006350| | FST,follistatin isoform FST317 precursor |
| 200752_s_at | 50.72 | 6.81E-08 | 1.256248202 | NM_005186| | CAPN1,calpain 1, large subunit |
| 1558693_s_at | 50.67 | 6.81E-08 | 1.546370619 | NM_144580| | MGC31963,kidney predominant protein NCU-G1 |
| 218964_at | 50.64 | 6.82E-08 | 1.836543356 | NM_006465| | ARID3B,AT rich interactive domain 3B (BRIGHT- like) |
| 203903_s_at | 50.57 | 6.82E-08 | 2.758501781 | NM_014799| | HEPH,hephaestin isoform b |
| 205110_s_at | 50.48 | 6.82E-08 | 2.640257064 | NM_004114| | FGF13,fibroblast growth factor 13 isoform 1A |
| 206309_at | 50.43 | 6.82E-08 | 2.258967251 | NM_001011705| | LECT1,leukocyte cell derived chemotaxin 1 isoform 2 |
| 202546_at | 50.4 | 6.82E-08 | 2.695164872 | NM_003761| | VAMP8,vesicle-associated membrane protein 8 |
| 208025_s_at | 50.39 | 6.82E-08 | 2.288100577 | NM_003483| | HMGA2,high mobility group AT-hook 2 |
| 210643_at | 50.29 | 6.85E-08 | 2.300715302 | NM_003701| | TNFSF11,tumor necrosis factor ligand superfamily, member |
| 200758_s_at | 50.25 | 6.86E-08 | 1.180577042 | NM_003204| | NFE2L1,nuclear factor (erythroid-derived 2)-like 1 |
| 222494_at | 50.17 | 6.89E-08 | 1.187673025 | NM_001085471| | NA |
| 202272_s_at | 50.1 | 6.89E-08 | 1.176085264 | NM_015176| | FBXO28,F-box protein 28 |
| 205930_at | 49.99 | 6.98E-08 | 1.28246863 | NM_005513| | GTF2E1,general transcription factor IIE, polypeptide 1 |
| 226804_at | 49.94 | 6.98E-08 | 1.808227335 | NM_017565| | FAM20A,family with sequence similarity 20, member A |
| 201559_s_at | 49.91 | 6.98E-08 | 1.258076591 | NM_013943| | CLIC4,chloride intracellular channel 4 |
| 234973_at | 49.87 | 6.99E-08 | 1.550126 | NM_033518| | SLC38A5,amino acid transport system N2 |
| 231407_s_at | 49.63 | 7.12E-08 | 1.897200587 | NM_003923| | FOXH1,forkhead box H1 |
| 203889_at | 49.41 | 7.27E-08 | 2.852478256 | NM_003020| | SGNE1,secretory granule, neuroendocrine protein 1 (7B2 |
| 203786_s_at | 49.33 | 7.27E-08 | 1.535474267 | NM_001003395| | TPD52L1,tumor protein D52-like 1 isoform 2 |
| 225265_at | 49.22 | 7.36E-08 | 1.400280273 | NM_002897| | RBMS1,RNA binding motif, single stranded interacting |
| 1554256_a_at | 48.92 | 7.58E-08 | 1.53167896 | NM_014801| | PCNXL2,pecanex-like 2 |
| 223599_at | 48.8 | 7.65E-08 | 2.817891094 | NM_001003818| | TRIM6,tripartite motif-containing 6 isoform 1 |
| 232164_s_at | 48.66 | 7.75E-08 | 2.54577132 | NM_031308| | EPPK1,epiplakin 1 |
| 203448_s_at | 48.49 | 7.86E-08 | 1.348139522 | NM_003218| | TERF1,telomeric repeat binding factor 1 isoform 2 |
| 205805_s_at | 48.43 | 7.87E-08 | 1.812041518 | NM_001083592| | NA |
| 231195_at | 48.16 | 8.04E-08 | 1.663783571 | NM_198508| | FLJ44186,FLJ44186 protein |
| 35776_at | 47.98 | 8.20E-08 | 1.226869855 | NM_001001132| | ITSN1,intersectin 1 isoform ITSN-s |
| 204285_s_at | 47.93 | 8.23E-08 | 1.341036537 | NM_021127| | PMAIP1,phorbol-12-myristate-13-acetate-induced protein |
| 215145_s_at | 47.86 | 8.24E-08 | 2.444790252 | NM_014141| | CNTNAP2,cell recognition molecule Caspr2 precursor |
| 201641_at | 47.38 | 8.61E-08 | 1.920314115 | NM_004335| | BST2,bone marrow stromal cell antigen 2 |
| 223000_s_at | 47.31 | 8.64E-08 | 2.269936121 | NM_016946| | F11R,F11 receptor isoform a precursor |
| 201846_s_at | 47.31 | 8.64E-08 | 1.22358191 | NM_012234| | RYBP,RING1 and YY1 binding protein |
| 227980_at | 47.26 | 8.68E-08 | 1.10327795 | NM_024639| | ZNF322A,zinc finger protein 322A |
| 219454_at | 47.21 | 8.71E-08 | 1.880079663 | NM_015507| | EGFL6,epidermal growth factor-like protein 6 |
| 202499_s_at | 47.02 | 8.85E-08 | 2.115883199 | NM_006931| | SLC2A3,solute carrier family 2 (facilitated glucose |
| 229294_at | 46.82 | 9.03E-08 | 2.865226014 | NM_020655| | JPH3,junctophilin 3 |
| 224458_at | 46.7 | 9.16E-08 | 1.831306892 | NM_032342| | C9orf125,chromosome 9 open reading frame 125 |
| 236894_at | 46.55 | 9.31E-08 | 3.387168652 | NM_019079| | FLJ10884,hypothetical protein FLJ10884 |
| 230205_at | 46.43 | 9.46E-08 | 1.179504168 | NM_152289| | ZNF561,zinc finger protein 561 |
| 1555630_a_at | 46.22 | 9.70E-08 | 1.158751179 | NM_031934| | RAB34,RAB39 |
| 203028_s_at | 46.14 | 9.74E-08 | 1.804712807 | NM_000101| | CYBA,cytochrome b, alpha polypeptide |
| 209496_at | 46.1 | 9.76E-08 | 2.227176495 | NM_002889| | RARRES2,retinoic acid receptor responder (tazarotene |
| 212964_at | 46.04 | 9.78E-08 | 1.419821979 | NM_015094| | HIC2,hypermethylated in cancer 2 |
| 231079_at | 46.03 | 9.78E-08 | 2.390180006 | NA |  |
| 230563_at | 45.9 | 9.87E-08 | 3.05456114 | NM_145313| | RASGEF1A,RasGEF domain family, member 1A |
| 203748_x_at | 45.84 | 9.92E-08 | 1.299808805 | NM_002897| | RBMS1,RNA binding motif, single stranded interacting |
| 222361_at | 45.71 | 1.00E-07 | 1.835880604 | NA |  |
| 209868_s_at | 45.71 | 1.00E-07 | 1.2987854 | NM_002897| | RBMS1,RNA binding motif, single stranded interacting |
| 204379_s_at | 45.7 | 1.00E-07 | 1.419094447 | NM_000142| | FGFR3,fibroblast growth factor receptor 3 isoform 1 |
| 1561101_at | 45.57 | 1.01E-07 | 2.245897567 | NA |  |
| 214974_x_at | 45.46 | 1.01E-07 | 2.164533315 | NM_002994| | CXCL5,chemokine (C-X-C motif) ligand 5 precursor |
| 37117_at | 45.38 | 1.02E-07 | 1.962291954 | NM_001017526| | NA |
| 1554776_at | 45.38 | 1.02E-07 | 3.043288986 | NM_174900| | ZFP42,zinc finger protein 42 |
| 203974_at | 45.26 | 1.03E-07 | 1.184453407 | NM_012080| | HDHD1A,haloacid dehalogenase-like hydrolase domain |
| 208782_at | 45.2 | 1.04E-07 | 1.277969076 | NM_007085| | FSTL1,follistatin-like 1 precursor |
| 237192_at | 45.15 | 1.04E-07 | 2.457294991 | NA |  |
| 227177_at | 45.09 | 1.04E-07 | 2.424394382 | NM_003389| | CORO2A,coronin, actin binding protein, 2A |
| 219287_at | 44.93 | 1.07E-07 | 1.877983158 | NM_014505| | KCNMB4,calcium-activated potassium channel beta 4 |
| 226926_at | 44.73 | 1.09E-07 | 2.865906096 | NM_001035516| | NA |
| 243161_x_at | 44.61 | 1.11E-07 | 3.345764861 | NM_174900| | ZFP42,zinc finger protein 42 |
| 205637_s_at | 44.55 | 1.12E-07 | 1.840249458 | NM_003027| | SH3GL3,SH3-domain GRB2-like 3 |
| 222496_s_at | 44.33 | 1.14E-07 | 2.407289122 | NM_001098634| | NA |
| 208536_s_at | 44.28 | 1.15E-07 | 1.501511335 | NM_006538| | BCL2L11,BCL2-like 11 isoform 6 |
| 205942_s_at | 44.13 | 1.17E-07 | 1.832532114 | NM_005622| | SAH,SA hypertension-associated homolog isoform 1 |
| 201431_s_at | 44.08 | 1.17E-07 | 1.399080175 | NM_001387| | DPYSL3,dihydropyrimidinase-like 3 |
| 213301_x_at | 43.91 | 1.19E-07 | 1.211392369 | NM_003852| | TIF1,transcriptional intermediary factor 1 alpha |
| 204784_s_at | 43.87 | 1.19E-07 | 1.252636362 | NM_022443| | MLF1,myeloid leukemia factor 1 |
| 207121_s_at | 43.83 | 1.19E-07 | 1.087891576 | NM_002748| | MAPK6,mitogen-activated protein kinase 6 |
| 201601_x_at | 43.79 | 1.19E-07 | 2.312523368 | NM_003641| | IFITM1,interferon induced transmembrane protein 1 |
| 231725_at | 43.77 | 1.19E-07 | 1.970977454 | NM_018936| | PCDHB2,protocadherin beta 2 precursor |
| 242414_at | 43.7 | 1.20E-07 | 2.068802352 | NM_014298| | QPRT,quinolinate phosphoribosyltransferase |
| 228441_s_at | 43.68 | 1.20E-07 | 2.450142476 | NA |  |
| 65517_at | 43.48 | 1.23E-07 | 1.787421621 | NM_005498| | AP1M2,adaptor-related protein complex 1, mu 2 subunit |
| 204790_at | 43.38 | 1.25E-07 | 2.066464966 | NM_005904| | SMAD7,MAD, mothers against decapentaplegic homolog 7 |
| 235141_at | 43.36 | 1.25E-07 | 1.701805227 | NM_001038603| | NA |
| 214240_at | 43.29 | 1.26E-07 | 1.263767083 | NM_015973| | GAL,galanin preproprotein |
| 203066_at | 43.27 | 1.26E-07 | 1.295169105 | NM_015892| | GALNAC4S-6ST,B cell RAG associated protein |
| 214264_s_at | 43.21 | 1.27E-07 | 1.255588806 | NM_145231| | C14orf143,chromosome 14 open reading frame 143 |
| 213308_at | 42.98 | 1.30E-07 | 1.646485682 | NM_012309| | SHANK2,SH3 and multiple ankyrin repeat domains 2 |
| 224048_at | 42.95 | 1.30E-07 | 3.245028964 | NM_001042403| | NA |
| 233110_s_at | 42.94 | 1.30E-07 | 1.14931992 | NM_001040668| | NA |
| 204983_s_at | 42.65 | 1.33E-07 | 1.557044115 | NM_001448| | GPC4,glypican 4 |
| 209147_s_at | 42.54 | 1.35E-07 | 1.61203839 | NM_003711| | PPAP2A,phosphatidic acid phosphatase type 2A isoform 1 |
| 202446_s_at | 42.53 | 1.35E-07 | 1.204300334 | NM_021105| | PLSCR1,phospholipid scramblase 1 |
| 201839_s_at | 42.43 | 1.36E-07 | 2.997830584 | NM_002354| | TACSTD1,tumor-associated calcium signal transducer 1 |
| 203404_at | 42.32 | 1.37E-07 | 1.444417652 | NM_014782| | ARMCX2,ALEX2 protein |
| 224768_at | 42.24 | 1.39E-07 | 1.14353212 | NM_017969| | FLJ10006,hypothetical protein FLJ10006 |
| 208296_x_at | 42.21 | 1.39E-07 | 1.444428679 | NM_001077654| | NA |
| 204447_at | 42.2 | 1.39E-07 | 1.361638055 | NM_014731| | ProSAPiP1,ProSAPiP1 protein |
| 205153_s_at | 42.2 | 1.39E-07 | 1.347580037 | NM_001250| | CD40,CD40 antigen isoform 1 precursor |
| 223824_at | 42.15 | 1.39E-07 | 1.702879615 | NM_001031709| | NA |
| 218729_at | 42.09 | 1.40E-07 | 2.751251567 | NM_020169| | LXN,latexin |
| 210540_s_at | 42.06 | 1.40E-07 | 1.404650209 | NM_003778| | B4GALT4,UDP-Gal:betaGlcNAc beta 1,4- |
| 203698_s_at | 42.05 | 1.40E-07 | 2.155999385 | NM_001463| | FRZB,frizzled-related protein |
| 203607_at | 41.95 | 1.41E-07 | 1.315251202 | NM_014937| | INPP5F,inositol polyphosphate-5-phosphatase F isoform |
| 1559280_a_at | 41.84 | 1.43E-07 | 3.767006623 | NA |  |
| 208613_s_at | 41.83 | 1.43E-07 | 1.321943884 | NM_001457| | FLNB,filamin B, beta (actin binding protein 278) |
| 243610_at | 41.83 | 1.43E-07 | 3.311542326 | NM_001010940| | LOC138255,OTTHUMP00000021439 |
| 201998_at | 41.76 | 1.44E-07 | 1.206007445 | NM_003032| | ST6GAL1,sialyltransferase 1 isoform a |
| 218361_at | 41.75 | 1.44E-07 | 1.209053689 | NM_018178| | GOLPH3L,GPP34-related protein |
| 228587_at | 41.74 | 1.44E-07 | 1.343314661 | NM_001039999| | NA |
| 220721_at | 41.7 | 1.45E-07 | 1.297071903 | NM_014650| | ZNF432,zinc finger protein 432 |
| 205742_at | 41.66 | 1.45E-07 | 2.394796293 | NM_000363| | TNNI3,troponin I, cardiac |
| 207076_s_at | 41.63 | 1.45E-07 | 1.290188086 | NM_000050| | ASS,argininosuccinate synthetase |
| 229288_at | 41.54 | 1.47E-07 | 1.768439487 | NA |  |
| 232069_at | 41.28 | 1.51E-07 | 1.325870439 | NM_015656| | NA |
| 227533_at | 41.18 | 1.52E-07 | 2.301521309 | NA |  |
| 203381_s_at | 41.18 | 1.52E-07 | 2.030762899 | NM_000041| | APOE,apolipoprotein E precursor |
| 214532_x_at | 41.15 | 1.52E-07 | 1.978053866 | NA |  |
| 207836_s_at | 40.97 | 1.56E-07 | 1.797666458 | NM_001008710| | RBPMS,RNA-binding protein with multiple splicing |
| 237911_at | 40.9 | 1.57E-07 | 3.607561562 | NA |  |
| 1558643_s_at | 40.89 | 1.57E-07 | 1.443619719 | NM_005711| | EDIL3,EGF-like repeats and discoidin I-like |
| 220147_s_at | 40.88 | 1.57E-07 | 1.27737726 | NM_021238| | C12orf14,chromosome 12 open reading frame 14 |
| 227827_at | 40.77 | 1.59E-07 | 2.64116154 | NA |  |
| 33814_at | 40.73 | 1.59E-07 | 1.105182491 | NM_001014831| | NA |
| 225478_at | 40.72 | 1.59E-07 | 1.163120535 | NM_004225| | MFHAS1,malignant fibrous histiocytoma amplified |
| 202071_at | 40.64 | 1.60E-07 | 1.384449982 | NM_002999| | SDC4,syndecan 4 precursor |
| 1570266_x_at | 40.61 | 1.60E-07 | 1.876823018 | NA |  |
| 225007_at | 40.58 | 1.60E-07 | 1.153977395 | NM_005754| | G3BP,Ras-GTPase-activating protein SH3-domain-binding |
| 222640_at | 40.54 | 1.60E-07 | 1.156909127 | NM_022552| | DNMT3A,DNA cytosine methyltransferase 3 alpha isoform |
| 213924_at | 40.22 | 1.68E-07 | 1.67477816 | NM_023075| | MPPE1,metallophosphoesterase 1 isoform a precursor |
| 238606_at | 40.13 | 1.70E-07 | 1.867714443 | NM_023931| | MGC2474,hypothetical protein MGC2474 |
| 222759_at | 39.96 | 1.74E-07 | 1.161150584 | NM_016028| | SUV420H1,suppressor of variegation 4-20 homolog 1 isoform |
| 226538_at | 39.91 | 1.75E-07 | 1.201159771 | NM_002372| | MAN2A1,mannosidase, alpha, class 2A, member 1 |
| 223592_s_at | 39.91 | 1.75E-07 | 1.47057799 | NM_032322| | RNF135,ring finger protein 135 isoform 1 |
| 216442_x_at | 39.91 | 1.75E-07 | 1.554012934 | NM_002026| | FN1,fibronectin 1 isoform 3 preproprotein |
| 210665_at | 39.85 | 1.76E-07 | 2.16803102 | NM_001032281| | NA |
| 203714_s_at | 39.82 | 1.76E-07 | 1.12853872 | NM_001079515| | NA |
| 211297_s_at | 39.81 | 1.76E-07 | 1.176999492 | NM_001799| | CDK7,cyclin-dependent kinase 7 |
| 1555963_x_at | 39.76 | 1.77E-07 | 2.461371289 | NM_145236| | B3GNT7,UDP-GlcNAc:betaGal |
| 219976_at | 39.71 | 1.78E-07 | 2.340567356 | NM_015888| | HOOK1,hook homolog 1 |
| 204294_at | 39.62 | 1.80E-07 | 1.607911319 | NM_000481| | AMT,aminomethyltransferase (glycine cleavage system |
| 231325_at | 39.59 | 1.80E-07 | 2.408842314 | NM_080872| | UNC5D,netrin receptor Unc5h4 |
| 205968_at | 39.55 | 1.80E-07 | 2.814197878 | NM_002252| | KCNS3,potassium voltage-gated channel |
| 201650_at | 39.54 | 1.80E-07 | 3.222981696 | NM_002276| | KRT19,keratin 19 |
| 207266_x_at | 39.51 | 1.80E-07 | 1.287154573 | NM_002897| | RBMS1,RNA binding motif, single stranded interacting |
| 223125_s_at | 39.45 | 1.81E-07 | 1.13488314 | NM_030806| | C1orf21,chromosome 1 open reading frame 21 |
| 214823_at | 39.4 | 1.82E-07 | 2.205642735 | NA |  |
| 228968_at | 39.31 | 1.83E-07 | 1.301339568 | NM_152695| | ZNF449,zinc finger protein 449 |
| 205352_at | 39.28 | 1.83E-07 | 1.775293481 | NM_001122752| | NA |
| 213258_at | 39.25 | 1.84E-07 | 1.715312623 | NM_001032281| | NA |
| 208939_at | 39.05 | 1.90E-07 | 1.135189674 | NM_012247| | SEPHS1,selenophosphate synthetase |
| 236126_at | 38.92 | 1.94E-07 | 1.292843341 | NM_001106| | ACVR2B,activin A type IIB receptor precursor |
| 203298_s_at | 38.9 | 1.94E-07 | 1.338001943 | NM_004973| | JARID2,jumonji, AT rich interactive domain 2 protein |
| 1569886_a_at | 38.84 | 1.95E-07 | 2.248385342 | NM_001080407| | NA |
| 206857_s_at | 38.83 | 1.95E-07 | 1.338971334 | NM_004116| | FKBP1B,FK506-binding protein 1B isoform a |
| 220994_s_at | 38.73 | 1.97E-07 | 2.392107119 | NM_014178| | STXBP6,amisyn |
| 212966_at | 38.71 | 1.97E-07 | 1.36393912 | NM_015094| | HIC2,hypermethylated in cancer 2 |
| 204044_at | 38.62 | 1.99E-07 | 2.102058047 | NM_014298| | QPRT,quinolinate phosphoribosyltransferase |
| 209848_s_at | 38.61 | 1.99E-07 | 2.097606945 | NM_006928| | SILV,silver homolog |
| 207180_s_at | 38.6 | 1.99E-07 | 1.618769868 | NM_001098520| | NA |
| 227909_at | 38.59 | 1.99E-07 | 1.712805557 | NM_001031705| | NA |
| 225977_at | 38.58 | 1.99E-07 | 1.117470582 | NM_019035| | PCDH18,protocadherin 18 precursor |
| 200797_s_at | 38.52 | 2.00E-07 | 1.059282806 | NM_021960| | MCL1,myeloid cell leukemia sequence 1 isoform 1 |
| 205051_s_at | 38.52 | 2.00E-07 | 2.553109467 | NM_000222| | KIT,v-kit Hardy-Zuckerman 4 feline sarcoma viral |
| 236519_at | 38.47 | 2.01E-07 | 2.254139692 | NM_001010940| | LOC138255,OTTHUMP00000021439 |
| 208761_s_at | 38.23 | 2.08E-07 | 1.058140792 | NM_001005781| | SUMO1,small ubiquitin-like modifier 1 isoform a |
| 226456_at | 38.09 | 2.12E-07 | 1.28295695 | NM_152308| | MGC24665,hypothetical protein MGC24665 |
| 203233_at | 38.05 | 2.13E-07 | 1.432928841 | NM_000418| | IL4R,interleukin 4 receptor alpha chain isoform a |
| 204464_s_at | 38.01 | 2.13E-07 | 1.300216738 | NM_001957| | EDNRA,endothelin receptor type A |
| 244227_at | 37.93 | 2.17E-07 | 1.797059821 | NM_205848| | SYT6,synaptotagmin VI |
| 226555_at | 37.72 | 2.23E-07 | 1.273939583 | NM_017759| | FLJ20309,hypothetical protein FLJ20309 |
| 205100_at | 37.66 | 2.25E-07 | 1.903473429 | NM_005110| | GFPT2,glutamine-fructose-6-phosphate transaminase 2 |
| 201637_s_at | 37.61 | 2.26E-07 | 1.096616835 | NM_001013438| | NA |
| 228291_s_at | 37.6 | 2.26E-07 | 1.326245754 | NM_018474| | C20orf19,uncharacterized hypothalamus protein HT013 |
| 41577_at | 37.56 | 2.27E-07 | 1.637860002 | NM_015568| | PPP1R16B,protein phosphatase 1 regulatory inhibitor |
| 213131_at | 37.54 | 2.27E-07 | 2.636727411 | NM_006334| | OLFM1,olfactomedin related ER localized protein |
| 204686_at | 37.51 | 2.28E-07 | 1.380691129 | NM_005544| | IRS1,insulin receptor substrate 1 |
| 203961_at | 37.49 | 2.29E-07 | 1.89038357 | NM_006393| | NEBL,nebulette sarcomeric isoform |
| 226754_at | 37.45 | 2.30E-07 | 1.296179929 | NM_138367| | NA |
| 217901_at | 37.44 | 2.30E-07 | 3.019226968 | NM_001943| | DSG2,desmoglein 2 preproprotein |
| 219863_at | 37.36 | 2.32E-07 | 2.464066287 | NM_016323| | HERC5,hect domain and RLD 5 |
| 235148_at | 37.25 | 2.36E-07 | 1.886671467 | NM_173853| | KRTCAP3,keratinocyte associated protein 3 |
| 208770_s_at | 37.23 | 2.36E-07 | 1.147537754 | NM_004096| | EIF4EBP2,eukaryotic translation initiation factor 4E |
| 221249_s_at | 37.21 | 2.36E-07 | 1.340867966 | NM_030802| | LOC81558,C/EBP-induced protein |
| 1569023_a_at | 37.19 | 2.37E-07 | 2.635586439 | NA |  |
| 229518_at | 37.09 | 2.40E-07 | 2.318223057 | NM_052943| | FAM46B,family with sequence similarity 46, member B |
| 226866_at | 36.98 | 2.45E-07 | 1.170829831 | NM_052911| | ESCO1,establishment of cohesion 1 homolog 1 |
| 236377_at | 36.8 | 2.48E-07 | 1.589672824 | NM_133448| | KIAA1944,KIAA1944 protein |
| 223246_s_at | 36.71 | 2.50E-07 | 1.232916298 | NM_018387| | STRBP,spermatid perinuclear RNA-binding protein |
| 221843_s_at | 36.7 | 2.50E-07 | 1.274088844 | NM_020947| | KIAA1609,KIAA1609 protein |
| 223177_at | 36.67 | 2.51E-07 | 1.417247903 | NM_152729| | NT5C2L1,5'-nucleotidase, cytosolic II-like 1 protein |
| 213447_at | 36.64 | 2.52E-07 | 1.342417646 | NA |  |
| 202697_at | 36.58 | 2.54E-07 | 1.243998925 | NM_007006| | CPSF5,cleavage and polyadenylation specific factor 5 |
| 218614_at | 36.56 | 2.54E-07 | 1.295860533 | NM_018169| | FLJ10652,hypothetical protein FLJ10652 |
| 208650_s_at | 36.5 | 2.55E-07 | 1.412197767 | NM_013230| | CD24,CD24 antigen |
| 202498_s_at | 36.5 | 2.55E-07 | 2.093826886 | NM_006931| | SLC2A3,solute carrier family 2 (facilitated glucose |
| 225320_at | 36.48 | 2.55E-07 | 1.180457886 | NM_138357| | C10orf42,chromosome 10 open reading frame 42 |
| 218338_at | 36.46 | 2.55E-07 | 1.316418538 | NM_004426| | PHC1,polyhomeotic 1-like |
| 213920_at | 36.45 | 2.55E-07 | 2.126152992 | NM_015267| | CUTL2,cut-like 2 |
| 1553179_at | 36.45 | 2.55E-07 | 1.924980684 | NM_133638| | ADAMTS19,a disintegrin-like and metalloprotease |
| 226439_s_at | 36.43 | 2.55E-07 | 1.73137913 | NM_015678| | NBEA,neurobeachin |
| 226799_at | 36.38 | 2.57E-07 | 1.565754651 | NM_018351| | FGD6,FYVE, RhoGEF and PH domain containing 6 |
| 226658_at | 36.34 | 2.58E-07 | 1.438863862 | NM_001006624| | T1A-2,lung type-I cell membrane-associated |
| 223178_s_at | 36.29 | 2.60E-07 | 1.507496021 | NM_152729| | NT5C2L1,5'-nucleotidase, cytosolic II-like 1 protein |
| 202746_at | 36.24 | 2.62E-07 | 2.912598895 | NM_004867| | ITM2A,integral membrane protein 2A |
| 233064_at | 36.24 | 2.62E-07 | 1.643003643 | NA |  |
| 1552947_x_at | 36.23 | 2.62E-07 | 1.29937414 | NM_153608| | MGC17986,hypothetical protein MGC17986 |
| 224617_at | 36.18 | 2.62E-07 | 1.146933951 | NM_005156| | ROD1,ROD1 regulator of differentiation 1 |
| 1554593_s_at | 36.1 | 2.65E-07 | 2.58725296 | NM_005071| | SLC1A6,solute carrier family 1 (high affinity |
| 202951_at | 36.09 | 2.65E-07 | 1.072153127 | NM_007271| | STK38,serine/threonine kinase 38 |
| 213709_at | 36.07 | 2.66E-07 | 1.250815153 | NM_030639| | BHLHB9,basic helix-loop-helix domain containing, class |
| 209008_x_at | 36.03 | 2.67E-07 | 1.709055399 | NM_002273| | KRT8,keratin 8 |
| 227349_at | 35.96 | 2.70E-07 | 1.166297506 | NM_018063| | HELLS,helicase, lymphoid-specific |
| 202876_s_at | 35.95 | 2.70E-07 | 1.224271345 | NM_002586| | PBX2,pre-B-cell leukemia transcription factor 2 |
| 227911_at | 35.93 | 2.70E-07 | 2.010787068 | NM_001010000| | ARHGAP28,Rho GTPase activating protein 28 isoform a |
| 202436_s_at | 35.77 | 2.77E-07 | 1.687368223 | NM_000104| | CYP1B1,cytochrome P450, family 1, subfamily B, |
| 202911_at | 35.74 | 2.77E-07 | 1.10723471 | NM_000179| | MSH6,mutS homolog 6 |
| 231690_at | 35.66 | 2.79E-07 | 2.335481963 | NA |  |
| 226846_at | 35.65 | 2.79E-07 | 1.648044722 | NM_001100876| | NA |
| 205691_at | 35.65 | 2.79E-07 | 1.249020134 | NM_004209| | SYNGR3,synaptogyrin 3 |
| 219949_at | 35.52 | 2.85E-07 | 1.986265531 | NM_024512| | LRRC2,leucine rich repeat containing 2 |
| 202875_s_at | 35.51 | 2.85E-07 | 1.310089705 | NM_002586| | PBX2,pre-B-cell leukemia transcription factor 2 |
| 205980_s_at | 35.5 | 2.86E-07 | 2.014164694 | NM_001017526| | NA |
| 201005_at | 35.45 | 2.87E-07 | 1.104329141 | NM_001769| | CD9,CD9 antigen |
| 65438_at | 35.36 | 2.90E-07 | 1.383767186 | NM_020947| | KIAA1609,KIAA1609 protein |
| 203382_s_at | 35.32 | 2.92E-07 | 2.007924545 | NM_000041| | APOE,apolipoprotein E precursor |
| 203408_s_at | 35.2 | 2.98E-07 | 1.291229662 | NM_002971| | SATB1,special AT-rich sequence binding protein 1 |
| 213518_at | 35.11 | 3.02E-07 | 1.127463401 | NM_002740| | PRKCI,protein kinase C, iota |
| 212884_x_at | 35.07 | 3.04E-07 | 1.70226513 | NM_000041| | APOE,apolipoprotein E precursor |
| 206710_s_at | 35.06 | 3.04E-07 | 1.964871385 | NM_012307| | EPB41L3,erythrocyte membrane protein band 4.1-like 3 |
| 229872_s_at | 34.78 | 3.18E-07 | 1.525342711 | NA |  |
| 218546_at | 34.77 | 3.18E-07 | 1.906820545 | NM_024709| | FLJ14146,hypothetical protein FLJ14146 |
| 203892_at | 34.75 | 3.19E-07 | 1.469504762 | NM_006103| | WFDC2,WAP four-disulfide core domain 2 isoform 1 |
| 219961_s_at | 34.73 | 3.19E-07 | 1.326868963 | NM_018474| | C20orf19,uncharacterized hypothalamus protein HT013 |
| 228819_at | 34.73 | 3.19E-07 | 1.359972218 | NM_001031730| | NA |
| 216405_at | 34.64 | 3.24E-07 | 3.113026946 | NA |  |
| 202497_x_at | 34.58 | 3.27E-07 | 1.94995754 | NM_006931| | SLC2A3,solute carrier family 2 (facilitated glucose |
| 214022_s_at | 34.58 | 3.27E-07 | 2.465505015 | NM_003641| | IFITM1,interferon induced transmembrane protein 1 |
| 222946_s_at | 34.54 | 3.27E-07 | 1.183463959 | NM_024037| | MGC2603,hypothetical protein MGC2603 |
| 201413_at | 34.53 | 3.28E-07 | 1.180076681 | NM_000414| | HSD17B4,hydroxysteroid (17-beta) dehydrogenase 4 |
| 203345_s_at | 34.52 | 3.28E-07 | 1.200035033 | NM_007358| | M96,putative DNA binding protein |
| 210715_s_at | 34.45 | 3.31E-07 | 2.812397585 | NM_021102| | SPINT2,serine protease inhibitor, Kunitz type, 2 |
| 213237_at | 34.43 | 3.31E-07 | 1.11389951 | NM_001012991| | NA |
| 1553875_s_at | 34.28 | 3.41E-07 | 1.467375363 | NM_032805| | ZNF206,zinc finger protein 206 |
| 200640_at | 34.25 | 3.41E-07 | 1.149707198 | NM_003406| | YWHAZ,tyrosine 3/tryptophan 5 -monooxygenase |
| 203215_s_at | 34.23 | 3.42E-07 | 1.116555069 | NM_004999| | MYO6,myosin VI |
| 219395_at | 34.07 | 3.51E-07 | 2.031657399 | NM_024939| | FLJ21918,hypothetical protein FLJ21918 |
| 223121_s_at | 34.05 | 3.53E-07 | 2.360516214 | NM_003013| | SFRP2,secreted frizzled-related protein 2 precursor |
| 201430_s_at | 34.01 | 3.54E-07 | 1.580176076 | NM_001387| | DPYSL3,dihydropyrimidinase-like 3 |
| 225068_at | 33.97 | 3.55E-07 | 1.10767175 | NM_021633| | KLHL12,kelch-like 12 |
| 1552897_a_at | 33.97 | 3.55E-07 | 2.957828776 | NM_133329| | KCNG3,potassium voltage-gated channel, subfamily G, |
| 202295_s_at | 33.96 | 3.55E-07 | 1.276582166 | NM_004390| | CTSH,cathepsin H isoform a preproprotein |
| 1559827_at | 33.85 | 3.61E-07 | 1.955364485 | NA |  |
| 207069_s_at | 33.82 | 3.62E-07 | 1.500944108 | NM_005585| | SMAD6,MAD, mothers against decapentaplegic homolog 6 |
| 202403_s_at | 33.68 | 3.70E-07 | 2.191400038 | NM_000089| | COL1A2,alpha 2 type I collagen |
| 200815_s_at | 33.55 | 3.74E-07 | 1.150788396 | NM_000430| | PAFAH1B1,platelet-activating factor acetylhydrolase, |
| 212464_s_at | 33.55 | 3.74E-07 | 1.597858294 | NM_002026| | FN1,fibronectin 1 isoform 3 preproprotein |
| 228329_at | 33.52 | 3.75E-07 | 2.003360666 | NM_021080| | DAB1,disabled homolog 1 |
| 235301_at | 33.46 | 3.78E-07 | 2.056486493 | NM_152748| | FLJ31340,hypothetical protein FLJ31340 |
| 219955_at | 33.44 | 3.78E-07 | 3.464224235 | NM_019079| | FLJ10884,hypothetical protein FLJ10884 |
| 238477_at | 33.44 | 3.78E-07 | 1.170680122 | NA |  |
| 204079_at | 33.42 | 3.79E-07 | 1.413016784 | NM_001008566| | TPST2,tyrosylprotein sulfotransferase 2 |
| 226473_at | 33.42 | 3.79E-07 | 1.132049284 | NM_005189| | NA |
| 219932_at | 33.32 | 3.84E-07 | 2.133658157 | NM_001017372| | NA |
| 212686_at | 33.31 | 3.84E-07 | 2.435062805 | NM_020700| | NA |
| 222065_s_at | 33.3 | 3.84E-07 | 1.138236149 | NM_002018| | FLII,flightless I homolog |
| 222619_at | 33.22 | 3.89E-07 | 1.149684782 | NM_012482| | ZNF281,zinc finger protein 281 |
| 218401_s_at | 33.21 | 3.89E-07 | 1.141322137 | NM_012482| | ZNF281,zinc finger protein 281 |
| 204595_s_at | 33.19 | 3.89E-07 | 1.554052688 | NM_003155| | STC1,stanniocalcin 1 |
| 204596_s_at | 33.19 | 3.89E-07 | 1.593261698 | NM_003155| | STC1,stanniocalcin 1 |
| 226771_at | 33.18 | 3.89E-07 | 1.146570655 | NM_001005855| | ATP8B2,ATPase, Class I, type 8B, member 2 isoform b |
| 222674_at | 33.18 | 3.89E-07 | 1.083154537 | NM_016390| | C9orf114,chromosome 9 open reading frame 114 |
| 241981_at | 33.12 | 3.93E-07 | 2.393061707 | NM_017565| | FAM20A,family with sequence similarity 20, member A |
| 226185_at | 33.08 | 3.96E-07 | 2.094347715 | NA |  |
| 1554777_at | 33.06 | 3.96E-07 | 3.346894679 | NM_174900| | ZFP42,zinc finger protein 42 |
| 202391_at | 33.05 | 3.97E-07 | 3.071790704 | NM_006317| | BASP1,brain abundant, membrane attached signal protein |
| 231713_s_at | 33.04 | 3.97E-07 | 1.081843318 | NM_018255| | STATIP1,elongator protein 2 |
| 210372_s_at | 33.03 | 3.97E-07 | 1.875357836 | NM_001003395| | TPD52L1,tumor protein D52-like 1 isoform 2 |
| 205022_s_at | 33.02 | 3.97E-07 | 1.180463831 | NM_001085471| | NA |
| 203313_s_at | 33.02 | 3.97E-07 | 1.252118797 | NM_003244| | TGIF,TG-interacting factor isoform c |
| 224301_x_at | 32.99 | 3.97E-07 | 1.248226089 | NM_177925| | H2AFJ,H2A histone family, member J isoform 2 |
| 234994_at | 32.97 | 3.99E-07 | 3.08640619 | NM_052913| | KIAA1913,KIAA1913 |
| 221804_s_at | 32.96 | 3.99E-07 | 1.104268431 | NM_018472| | FAM45B,family with sequence similarity 45, member B |
| 227566_at | 32.93 | 4.00E-07 | 2.459937665 | NM_001048209| | NA |
| 202889_x_at | 32.9 | 4.01E-07 | 1.963214806 | NM_003980| | MAP7,microtubule-associated protein 7 |
| 212881_at | 32.9 | 4.01E-07 | 1.142104802 | NM_015897| | PIAS4,protein inhibitor of activated STAT, 4 |
| 226216_at | 32.81 | 4.06E-07 | 1.237838459 | NM_000208| | INSR,insulin receptor |
| 208190_s_at | 32.77 | 4.08E-07 | 1.855132327 | NM_015925| | LISCH7,LISCH protein isoform 1 |
| 218536_at | 32.75 | 4.09E-07 | 1.466393394 | NM_020662| | MRS2L,MRS2-like, magnesium homeostasis factor |
| 231973_s_at | 32.71 | 4.10E-07 | 1.118156441 | NM_022662| | ANAPC1,anaphase promoting complex subunit 1 |
| 226069_at | 32.65 | 4.13E-07 | 2.981999016 | NM_153026| | PRICKLE1,prickle-like 1 |
| 206382_s_at | 32.64 | 4.13E-07 | 1.885741024 | NM_001709| | BDNF,brain-derived neurotrophic factor isoform a |
| 202085_at | 32.63 | 4.13E-07 | 1.176771152 | NM_004817| | TJP2,tight junction protein 2 (zona occludens 2) |
| 35666_at | 32.45 | 4.27E-07 | 1.145544348 | NM_004186| | SEMA3F,semaphorin 3F |
| 201313_at | 32.44 | 4.27E-07 | 1.254490711 | NM_001975| | ENO2,enolase 2 |
| 213590_at | 32.42 | 4.29E-07 | 1.813249932 | NM_004695| | SLC16A5,solute carrier family 16, member 5 |
| 200759_x_at | 32.37 | 4.32E-07 | 1.168810504 | NM_003204| | NFE2L1,nuclear factor (erythroid-derived 2)-like 1 |
| 226267_at | 32.33 | 4.34E-07 | 1.827589284 | NM_130469| | JDP2,Jun dimerization protein |
| 203528_at | 32.31 | 4.35E-07 | 1.306314884 | NM_006378| | SEMA4D,semaphorin 4D |
| 200618_at | 32.26 | 4.39E-07 | 1.192249148 | NM_006148| | LASP1,LIM and SH3 protein 1 |
| 235339_at | 32.19 | 4.43E-07 | 1.225046269 | NM_031915| | SETDB2,CLLL8 protein |
| 233337_s_at | 32.14 | 4.47E-07 | 1.674269716 | NM_001114099| | NA |
| 208626_s_at | 32.09 | 4.52E-07 | 1.251046261 | NM_006373| | VAT1,vesicle amine transport protein 1 |
| 244552_at | 32.07 | 4.53E-07 | 1.30052078 | NA |  |
| 203453_at | 32.06 | 4.54E-07 | 2.530964659 | NM_001038| | SCNN1A,sodium channel, nonvoltage-gated 1 alpha |
| 202719_s_at | 32.05 | 4.54E-07 | 1.24091793 | NM_015641| | TES,testin isoform 1 |
| 208228_s_at | 32.04 | 4.54E-07 | 1.386953092 | NM_000141| | FGFR2,fibroblast growth factor receptor 2 isoform 1 |
| 226374_at | 32.04 | 4.54E-07 | 1.223670615 | NA |  |
| 59697_at | 32.03 | 4.55E-07 | 1.279422581 | NM_198686| | RAB15,Ras-related protein Rab-15 |
| 226801_s_at | 31.97 | 4.58E-07 | 1.069197994 | NM_022831| | FLJ12806,hypothetical protein FLJ12806 |
| 224817_at | 31.97 | 4.58E-07 | 1.216660582 | NM_014631| | SH3MD1,SH3 multiple domains 1 |
| 220668_s_at | 31.95 | 4.59E-07 | 1.741409332 | NM_006892| | DNMT3B,DNA cytosine-5 methyltransferase 3 beta isoform |
| 223130_s_at | 31.91 | 4.61E-07 | 1.535795075 | NM_013262| | MYLIP,myosin regulatory light chain interacting |
| 209586_s_at | 31.85 | 4.64E-07 | 1.10114133 | NM_021222| | PRUNE,prune homolog |
| 228370_at | 31.85 | 4.64E-07 | 1.328377103 | NM_003097| | SNRPN,small nuclear ribonucleoprotein polypeptide N |
| 238751_at | 31.79 | 4.68E-07 | 2.268373218 | NA |  |
| 212557_at | 31.77 | 4.69E-07 | 1.114176698 | NM_001031623| | NA |
| 210265_x_at | 31.77 | 4.69E-07 | 2.405925659 | NA |  |
| 203778_at | 31.77 | 4.69E-07 | 1.193327053 | NM_005908| | MANBA,mannosidase, beta A, lysosomal |
| 210416_s_at | 31.74 | 4.71E-07 | 1.195167636 | NM_001005735| | CHEK2,protein kinase CHK2 isoform c |
| 228754_at | 31.72 | 4.72E-07 | 1.125625777 | NM_003043| | SLC6A6,solute carrier family 6 (neurotransmitter |
| 217966_s_at | 31.71 | 4.73E-07 | 1.792523478 | NM_052966| | C1orf24,niban protein |
| 203286_at | 31.71 | 4.73E-07 | 1.141978347 | NM_014901| | RNF44,ring finger protein 44 |
| 220161_s_at | 31.7 | 4.73E-07 | 1.457316081 | NM_018424| | EPB41L4B,erythrocyte membrane protein band 4.1 like 4B |
| 241510_at | 31.68 | 4.73E-07 | 1.872313441 | NA |  |
| 227224_at | 31.68 | 4.73E-07 | 2.294701072 | NM_018037| | RALGPS2,Ral GEF with PH domain and SH3 binding motif 2 |
| 242053_at | 31.63 | 4.76E-07 | 1.91030813 | NA |  |
| 222732_at | 31.63 | 4.76E-07 | 1.137927548 | NM_021253| | TRIM39,tripartite motif-containing 39 isoform 1 |
| 205709_s_at | 31.62 | 4.76E-07 | 1.966106895 | NM_001263| | CDS1,phosphatidate cytidylyltransferase 1 |
| 206023_at | 31.61 | 4.76E-07 | 1.806590972 | NM_006681| | NMU,neuromedin U |
| 239752_at | 31.6 | 4.76E-07 | 1.28271294 | NA |  |
| 205625_s_at | 31.59 | 4.76E-07 | 2.279733424 | NM_004929| | CALB1,calbindin 1 |
| 242945_at | 31.58 | 4.76E-07 | 1.656981211 | NM_017565| | FAM20A,family with sequence similarity 20, member A |
| 226277_at | 31.58 | 4.76E-07 | 1.180563767 | NM_005713| | COL4A3BP,alpha 3 type IV collagen binding protein isoform |
| 231188_at | 31.58 | 4.76E-07 | 1.173422696 | NM_001007072| | ZSCAN2,zinc finger protein 29 isoform 3 |
| 209015_s_at | 31.57 | 4.76E-07 | 1.336522813 | NM_005494| | DNAJB6,DnaJ (Hsp40) homolog, subfamily B, member 6 |
| 230785_at | 31.54 | 4.76E-07 | 1.325720976 | NA |  |
| 225579_at | 31.54 | 4.76E-07 | 1.221470221 | NM_152391| | C2orf22,hypothetical protein MGC33602 |
| 203297_s_at | 31.48 | 4.81E-07 | 1.326182466 | NM_004973| | JARID2,jumonji, AT rich interactive domain 2 protein |
| 214179_s_at | 31.46 | 4.82E-07 | 1.183743176 | NM_003204| | NFE2L1,nuclear factor (erythroid-derived 2)-like 1 |
| 223245_at | 31.31 | 4.93E-07 | 1.205406659 | NM_018387| | STRBP,spermatid perinuclear RNA-binding protein |
| 224609_at | 31.29 | 4.94E-07 | 1.18409802 | NM_020428| | CTL2,CTL2 gene |
| 225245_x_at | 31.25 | 4.97E-07 | 1.24857373 | NM_177925| | H2AFJ,H2A histone family, member J isoform 2 |
| 206385_s_at | 31.25 | 4.97E-07 | 1.42674408 | NM_001149| | ANK3,ankyrin 3 isoform 2 |
| 231192_at | 31.24 | 4.97E-07 | 1.583421246 | NA |  |
| 224650_at | 31.22 | 4.98E-07 | 3.648148212 | NM_052886| | MAL2,mal, T-cell differentiation protein 2 |
| 226403_at | 31.21 | 4.99E-07 | 1.358115592 | NM_144686| | TMC4,transmembrane channel-like 4 |
| 224344_at | 31.16 | 5.03E-07 | 2.323454853 | NM_004373| | COX6A1,cytochrome c oxidase subunit VIa polypeptide 1 |
| 234734_s_at | 31.16 | 5.03E-07 | 1.067507147 | NM_014494| | TNRC6A,trinucleotide repeat containing 6A |
| 209049_s_at | 31.16 | 5.03E-07 | 1.154172212 | NM_012408| | PRKCBP1,protein kinase C binding protein 1 isoform b |
| 1553180_at | 31.15 | 5.03E-07 | 2.322722732 | NM_133638| | ADAMTS19,a disintegrin-like and metalloprotease |
| 46665_at | 31.14 | 5.03E-07 | 1.154645876 | NM_017789| | SEMA4C,semaphorin 4C |
| 227846_at | 31.14 | 5.03E-07 | 1.662945235 | NM_007223| | GPR,putative G protein coupled receptor |
| 229399_at | 31.13 | 5.03E-07 | 1.550612638 | NM_018017| | C10orf118,CTCL tumor antigen L14-2 |
| 218384_at | 31.12 | 5.04E-07 | 1.239636551 | NM_001042476| | NA |
| 1562484_at | 31.03 | 5.10E-07 | 2.590011 | NM_001033659| | NA |
| 235085_at | 30.96 | 5.18E-07 | 1.242211937 | NM_001080826| | NA |
| 209220_at | 30.94 | 5.19E-07 | 2.016294302 | NM_004484| | GPC3,glypican 3 |
| 227329_at | 30.86 | 5.26E-07 | 1.853129051 | NM_025224| | BTBD4,BTB (POZ) domain containing 4 |
| 226007_at | 30.83 | 5.29E-07 | 1.147590368 | NM_194279| | HBLD1,HESB like domain containing 1 |
| 209448_at | 30.81 | 5.30E-07 | 2.033894485 | NM_001098520| | NA |
| 220184_at | 30.79 | 5.30E-07 | 3.42764401 | NM_024865| | NANOG,Nanog homeobox |
| 205977_s_at | 30.76 | 5.33E-07 | 2.154837805 | NM_005232| | EPHA1,ephrin receptor EphA1 |
| 202350_s_at | 30.74 | 5.34E-07 | 1.651450054 | NM_002380| | MATN2,matrilin 2 precursor |
| 242128_at | 30.74 | 5.34E-07 | 4.423508341 | NM_021728| | OTX2,orthodenticle 2 isoform a |
| 228145_s_at | 30.73 | 5.34E-07 | 1.168223121 | NM_020781| | ZNF398,zinc finger 398 isoform b |
| 225301_s_at | 30.72 | 5.35E-07 | 1.160878872 | NM_001080467| | NA |
| 220272_at | 30.69 | 5.38E-07 | 1.82447363 | NM_017637| | BNC2,basonuclin 2 |
| 203438_at | 30.69 | 5.38E-07 | 1.911191368 | NM_003714| | STC2,stanniocalcin 2 |
| 235334_at | 30.68 | 5.38E-07 | 1.650862819 | NM_152996| | ST6GALNAC3,ST6 |
| 206756_at | 30.65 | 5.40E-07 | 1.309006115 | NM_019886| | CHST7,carbohydrate (N-acetylglucosamine 6-O) |
| 205802_at | 30.62 | 5.43E-07 | 1.32089726 | NM_003304| | TRPC1,transient receptor potential cation channel, |
| 228260_at | 30.6 | 5.45E-07 | 2.277522201 | NM_004432| | ELAVL2,ELAV (embryonic lethal, abnormal vision, |
| 204269_at | 30.6 | 5.45E-07 | 1.453472595 | NM_006875| | PIM2,pim-2 oncogene |
| 218286_s_at | 30.53 | 5.51E-07 | 1.077457246 | NM_014245| | RNF7,ring finger protein 7 isoform 1 |
| 232180_at | 30.51 | 5.52E-07 | 1.778005688 | NM_001001521| | UGP2,UDP-glucose pyrophosphorylase 2 isoform b |
| 1554689_a_at | 30.51 | 5.52E-07 | 1.207695431 | NM_020742| | NLGN4X,X-linked neuroligin 4 |
| 229313_at | 30.5 | 5.52E-07 | 1.898605306 | NM_213599| | TMEM16E,transmembrane protein 16E |
| 218276_s_at | 30.5 | 5.52E-07 | 1.18921364 | NM_021818| | SAV1,WW45 protein |
| 214071_at | 30.49 | 5.53E-07 | 2.095305078 | NM_023075| | MPPE1,metallophosphoesterase 1 isoform a precursor |
| 215672_s_at | 30.46 | 5.55E-07 | 1.299238734 | NM_015328| | KIAA0828,KIAA0828 protein |
| 208836_at | 30.39 | 5.60E-07 | 1.051140729 | NM_001679| | ATP1B3,Na+/K+ -ATPase beta 3 subunit |
| 204867_at | 30.38 | 5.60E-07 | 1.547151196 | NM_005258| | GCHFR,GTP cyclohydrolase I feedback regulatory |
| 203389_at | 30.38 | 5.60E-07 | 1.20051538 | NM_002254| | KIF3C,kinesin family member 3C |
| 220536_at | 30.34 | 5.63E-07 | 1.753088981 | NM_018228| | C14orf115,chromosome 14 open reading frame 115 |
| 232060_at | 30.32 | 5.65E-07 | 1.891151221 | NM_001083592| | NA |
| 1560587_s_at | 30.3 | 5.66E-07 | 1.065310427 | NM_012094| | PRDX5,peroxiredoxin 5 precursor, isoform a |
| 221051_s_at | 30.28 | 5.67E-07 | 2.365195863 | NM_170678| | ITGB1BP3,integrin beta 1 binding protein 3 |
| 229553_at | 30.28 | 5.67E-07 | 1.718805003 | NM_173582| | PGM2L1,phosphoglucomutase 2-like 1 |
| 222701_s_at | 30.28 | 5.67E-07 | 1.434247399 | NM_001011667| | CHCHD7,coiled-coil-helix-coiled-coil-helix domain |
| 210029_at | 30.26 | 5.69E-07 | 2.224187161 | NM_002164| | INDO,indoleamine-pyrrole 2,3 dioxygenase |
| 227837_at | 30.25 | 5.69E-07 | 1.102295701 | NA |  |
| 201487_at | 30.25 | 5.69E-07 | 1.150903748 | NM_001114173| | NA |
| 201831_s_at | 30.25 | 5.69E-07 | 1.21258135 | NM_003715| | VDP,vesicle docking protein p115 |
| 204391_x_at | 30.24 | 5.69E-07 | 1.214572817 | NM_003852| | TIF1,transcriptional intermediary factor 1 alpha |
| 203407_at | 30.21 | 5.71E-07 | 1.709562227 | NM_002705| | PPL,periplakin |
| 201924_at | 30.21 | 5.71E-07 | 1.254921825 | NM_005935| | MLLT2,myeloid/lymphoid or mixed-lineage leukemia |
| 228906_at | 30.18 | 5.75E-07 | 1.333766822 | NM_030625| | CXXC6,CXXC finger 6 |
| 209442_x_at | 30.16 | 5.75E-07 | 1.416567685 | NM_001149| | ANK3,ankyrin 3 isoform 2 |
| 219132_at | 30.16 | 5.75E-07 | 1.369525717 | NM_021255| | PELI2,pellino 2 |
| 226817_at | 30.16 | 5.75E-07 | 2.706305682 | NM_004949| | DSC2,desmocollin 2 isoform Dsc2b preproprotein |
| 202538_s_at | 30.13 | 5.77E-07 | 1.135007492 | NM_014043| | DKFZP564O123,DKFZP564O123 protein |
| 211276_at | 30.11 | 5.78E-07 | 2.064272978 | NM_080390| | TCEAL2,transcription elongation factor A (SII)-like 2 |
| 218951_s_at | 30.08 | 5.81E-07 | 1.157200419 | NM_018390| | PLCXD1,phosphatidylinositol-specific phospholipase C, X |
| 209081_s_at | 30.08 | 5.81E-07 | 1.274976661 | NM_030582| | COL18A1,alpha 1 type XVIII collagen isoform 1 precursor |
| 202575_at | 30.07 | 5.82E-07 | 1.645015746 | NM_001878| | CRABP2,cellular retinoic acid binding protein 2 |
| 206891_at | 30.05 | 5.83E-07 | 1.897349101 | NM_001104| | ACTN3,skeletal muscle specific actinin, alpha 3 |
| 236756_at | 30.01 | 5.88E-07 | 1.81169356 | NA |  |
| 222698_s_at | 29.97 | 5.90E-07 | 1.128443558 | NM_018439| | IMPACT,hypothetical protein IMPACT |
| 200799_at | 29.97 | 5.90E-07 | 1.231960921 | NM_005345| | HSPA1A,heat shock 70kDa protein 1A |
| 57540_at | 29.96 | 5.91E-07 | 1.363066137 | NM_022128| | RBKS,ribokinase |
| 227494_at | 29.94 | 5.92E-07 | 1.664078292 | NM_001489| | NR6A1,nuclear receptor subfamily 6, group A, member 1 |
| 214212_x_at | 29.94 | 5.91E-07 | 1.174148177 | NM_006832| | PLEKHC1,pleckstrin homology domain containing, family C |
| 215485_s_at | 29.91 | 5.95E-07 | 1.131938577 | NM_000201| | ICAM1,intercellular adhesion molecule 1 precursor |
| 219359_at | 29.89 | 5.97E-07 | 1.178850676 | NM_025092| | FLJ22635,hypothetical protein FLJ22635 |
| 202747_s_at | 29.89 | 5.97E-07 | 1.981032715 | NM_004867| | ITM2A,integral membrane protein 2A |
| 236456_at | 29.86 | 5.99E-07 | 1.711063294 | NM_001039970| | NA |
| 218854_at | 29.79 | 6.07E-07 | 1.502454316 | NM_001080976| | NA |
| 203139_at | 29.78 | 6.07E-07 | 1.113434256 | NM_004938| | DAPK1,death-associated protein kinase 1 |
| 231257_at | 29.77 | 6.07E-07 | 1.690180773 | NM_174937| | TCERG1L,transcription elongation regulator 1-like |
| 203205_at | 29.76 | 6.08E-07 | 1.159952001 | NM_014663| | JMJD2A,jumonji domain containing 2A |
| 219370_at | 29.76 | 6.08E-07 | 1.708312945 | NM_019845| | RPRM,reprimo, TP53 dependant G2 arrest mediator |
| 223466_x_at | 29.75 | 6.09E-07 | 1.135282328 | NM_005713| | COL4A3BP,alpha 3 type IV collagen binding protein isoform |
| 219397_at | 29.74 | 6.09E-07 | 1.225974247 | NM_025147| | FLJ13448,hypothetical protein FLJ13448 |
| 202626_s_at | 29.71 | 6.13E-07 | 1.402034777 | NM_001111097| | NA |
| 239781_at | 29.71 | 6.13E-07 | 2.398689277 | NA |  |
| 210905_x_at | 29.7 | 6.14E-07 | 2.174058633 | NA |  |
| 225651_at | 29.66 | 6.17E-07 | 1.346715074 | NM_152653| | UBE2E2,ubiquitin-conjugating enzyme E2E 2 (UBC4/5 |
| 226215_s_at | 29.63 | 6.21E-07 | 1.093443194 | NM_001005366| | FBXL10,F-box and leucine-rich repeat protein 10 isoform |
| 223232_s_at | 29.62 | 6.22E-07 | 1.471191184 | NM_020770| | CGN,cingulin |
| 227254_at | 29.59 | 6.24E-07 | 1.232068312 | NM_002697| | POU2F1,POU domain, class 2, transcription factor 1 |
| 219740_at | 29.55 | 6.28E-07 | 1.41334705 | NM_024749| | FLJ12505,hypothetical protein FLJ12505 |
| 212590_at | 29.5 | 6.34E-07 | 1.40107268 | NM_001102669| | NA |
| 211089_s_at | 29.47 | 6.38E-07 | 1.240359591 | NM_002498| | NEK3,NIMA-related kinase 3 |
| 224465_s_at | 29.47 | 6.38E-07 | 1.157954852 | NM_032345| | PYM,PYM protein |
| 219651_at | 29.46 | 6.39E-07 | 2.773354148 | NM_018189| | DPPA4,developmental pluripotency associated 4 |
| 224933_s_at | 29.42 | 6.41E-07 | 1.143427848 | NM_004241| | JMJD1C,jumonji domain containing 1C |
| 227677_at | 29.42 | 6.41E-07 | 1.714827145 | NM_000215| | JAK3,Janus kinase 3 |
| 209704_at | 29.41 | 6.42E-07 | 1.290262272 | NM_007358| | M96,putative DNA binding protein |
| 202890_at | 29.37 | 6.47E-07 | 2.535682411 | NM_003980| | MAP7,microtubule-associated protein 7 |
| 207992_s_at | 29.37 | 6.47E-07 | 1.489003757 | NM_000480| | AMPD3,adenosine monophosphate deaminase (isoform E) |
| 214825_at | 29.34 | 6.49E-07 | 1.874373168 | NM_001080396| | NA |
| 225051_at | 29.34 | 6.49E-07 | 1.161144155 | NM_004437| | EPB41,erythrocyte membrane protein band 4.1 |
| 227955_s_at | 29.24 | 6.62E-07 | 1.444620732 | NA |  |
| 203397_s_at | 29.24 | 6.62E-07 | 3.652111695 | NM_004482| | GALNT3,polypeptide N-acetylgalactosaminyltransferase 3 |
| 222482_at | 29.23 | 6.62E-07 | 1.136583765 | NM_001009955| | SSBP3,single stranded DNA binding protein 3 isoform c |
| 1554539_a_at | 29.23 | 6.62E-07 | 1.415609902 | NM_019034| | RHOF,ras homolog gene family, member F |
| 218717_s_at | 29.23 | 6.62E-07 | 1.903843927 | NM_018192| | LEPREL1,leprecan-like 1 |
| 202465_at | 29.18 | 6.67E-07 | 1.515658816 | NM_002593| | PCOLCE,procollagen C-endopeptidase enhancer |
| 205008_s_at | 29.14 | 6.70E-07 | 1.232795752 | NM_006383| | CIB2,DNA-dependent protein kinase catalytic |
| 206042_x_at | 29.14 | 6.70E-07 | 1.243830528 | NM_003097| | SNRPN,small nuclear ribonucleoprotein polypeptide N |
| 219298_at | 29.14 | 6.71E-07 | 1.330720337 | NM_024693| | ECHDC3,enoyl Coenzyme A hydratase domain containing 3 |
| 203449_s_at | 29.13 | 6.71E-07 | 1.368991065 | NM_003218| | TERF1,telomeric repeat binding factor 1 isoform 2 |
| 219412_at | 29.12 | 6.72E-07 | 1.440305581 | NM_022337| | RAB38,RAB38 |
| 205803_s_at | 29.1 | 6.74E-07 | 1.302996846 | NM_003304| | TRPC1,transient receptor potential cation channel, |
| 214239_x_at | 29.09 | 6.75E-07 | 1.195739627 | NM_007144| | PCGF2,ring finger protein 110 |
| 231953_at | 29.06 | 6.79E-07 | 1.391840061 | NM_004459| | FALZ,fetal Alzheimer antigen isoform 2 |
| 1560652_at | 29.03 | 6.81E-07 | 2.524656183 | NA |  |
| 203256_at | 29.03 | 6.81E-07 | 1.911415863 | NM_001793| | CDH3,cadherin 3, type 1 preproprotein |
| 216035_x_at | 29.01 | 6.84E-07 | 1.136747184 | NM_030756| | TCF7L2,transcription factor 7-like 2 (T-cell specific, |
| 227276_at | 28.98 | 6.87E-07 | 1.544436116 | NM_032812| | PLXDC2,plexin domain containing 2 precursor |
| 212445_s_at | 28.94 | 6.90E-07 | 1.235350789 | NM_015277| | NEDD4L,ubiquitin-protein ligase NEDD4-like |
| 203814_s_at | 28.93 | 6.92E-07 | 1.179037895 | NM_000904| | NQO2,NAD(P)H dehydrogenase, quinone 2 |
| 227314_at | 28.88 | 6.98E-07 | 1.515853583 | NM_002203| | ITGA2,integrin alpha 2 precursor |
| 220038_at | 28.87 | 7.00E-07 | 1.588417564 | NM_001033578| | NA |
| 219625_s_at | 28.85 | 7.02E-07 | 1.127242857 | NM_005713| | COL4A3BP,alpha 3 type IV collagen binding protein isoform |
| 209048_s_at | 28.85 | 7.02E-07 | 1.158186717 | NM_012408| | PRKCBP1,protein kinase C binding protein 1 isoform b |
| 231731_at | 28.85 | 7.02E-07 | 2.958024815 | NM_021728| | OTX2,orthodenticle 2 isoform a |
| 242054_s_at | 28.83 | 7.02E-07 | 2.064801528 | NA |  |
| 208941_s_at | 28.83 | 7.02E-07 | 1.123820842 | NM_012247| | SEPHS1,selenophosphate synthetase |
| 200604_s_at | 28.83 | 7.02E-07 | 1.15467184 | NM_002734| | PRKAR1A,cAMP-dependent protein kinase, regulatory |
| 216960_s_at | 28.82 | 7.02E-07 | 1.101640858 | NM_001083330| | NA |
| 223658_at | 28.82 | 7.02E-07 | 1.793970087 | NM_004823| | KCNK6,potassium channel, subfamily K, member 6 |
| 220013_at | 28.78 | 7.08E-07 | 2.055097581 | NM_024794| | ABHD9,abhydrolase domain containing 9 |
| 202201_at | 28.77 | 7.09E-07 | 1.426158561 | NM_000713| | BLVRB,biliverdin reductase B (flavin reductase |
| 209424_s_at | 28.74 | 7.13E-07 | 1.264267162 | NM_014324| | AMACR,alpha-methylacyl-CoA racemase isoform 1 |
| 227006_at | 28.74 | 7.13E-07 | 1.574670485 | NM_033256| | PPP1R14A,protein phosphatase 1, regulatory (inhibitor) |
| 201926_s_at | 28.7 | 7.17E-07 | 2.033249995 | NM_000574| | DAF,decay accelerating factor for complement (CD55, |
| 203827_at | 28.7 | 7.18E-07 | 1.319991111 | NM_017983| | WIPI49,hypothetical protein FLJ10055 |
| 227623_at | 28.63 | 7.26E-07 | 2.362895002 | NA |  |
| 220588_at | 28.6 | 7.28E-07 | 1.39494167 | NM_001010974| | BCAS4,breast carcinoma amplified sequence 4 isoform c |
| 209369_at | 28.59 | 7.30E-07 | 3.161357611 | NM_005139| | ANXA3,annexin A3 |
| 203160_s_at | 28.55 | 7.36E-07 | 1.143910176 | NM_003958| | RNF8,ring finger protein 8 isoform 1 |
| 223373_s_at | 28.54 | 7.36E-07 | 1.190408799 | NM_030821| | PLA2G12A,phospholipase A2, group XIIA |
| 222610_s_at | 28.54 | 7.36E-07 | 1.197671492 | NM_001017406| | NA |
| 218031_s_at | 28.53 | 7.37E-07 | 1.196337754 | NM_001085471| | NA |
| 228821_at | 28.47 | 7.45E-07 | 2.618054129 | NM_032528| | ST6GAL2,beta-galactoside alpha-2,6-sialyltransferase II |
| 207627_s_at | 28.47 | 7.46E-07 | 1.223968353 | NM_005653| | TFCP2,transcription factor CP2 |
| 203498_at | 28.4 | 7.56E-07 | 1.492479194 | NM_005822| | DSCR1L1,Down syndrome critical region gene 1-like 1 |
| 208940_at | 28.38 | 7.59E-07 | 1.119526322 | NM_012247| | SEPHS1,selenophosphate synthetase |
| 201636_at | 28.28 | 7.74E-07 | 1.102833565 | NM_001013438| | NA |
| 230497_at | 28.27 | 7.76E-07 | 1.732116876 | NM_021938| | BRUNOL5,bruno-like 5, RNA binding protein |
| 210479_s_at | 28.23 | 7.81E-07 | 1.583745754 | NM_002943| | RORA,RAR-related orphan receptor A isoform c |
| 229155_at | 28.21 | 7.85E-07 | 1.419489498 | NA |  |
| 227803_at | 28.14 | 7.95E-07 | 1.67449602 | NM_021572| | ENPP5,ectonucleotide pyrophosphatase/phosphodiesterase |
| 226354_at | 28.13 | 7.97E-07 | 1.286444902 | NM_032857| | LACTB,lactamase, beta isoform a |
| 205105_at | 28.12 | 7.97E-07 | 1.175693207 | NM_002372| | MAN2A1,mannosidase, alpha, class 2A, member 1 |
| 242346_x_at | 28.11 | 7.99E-07 | 2.272225591 | NA |  |
| 208398_s_at | 28.1 | 8.00E-07 | 1.105416815 | NM_004865| | TBPL1,TBP-like 1 |
| 1553132_a_at | 28.1 | 8.00E-07 | 2.211195609 | NM_152332| | MTAC2D1,membrane targeting (tandem) C2 domain containing |
| 1556824_at | 28.09 | 8.00E-07 | 2.111099432 | NA |  |
| 227377_at | 28.09 | 8.00E-07 | 1.845144917 | NM_006546| | IMP-1,IGF-II mRNA-binding protein 1 |
| 201667_at | 28.08 | 8.00E-07 | 1.205764817 | NM_000165| | GJA1,connexin 43 |
| 230623_x_at | 28.08 | 8.00E-07 | 1.286077437 | NM_020886| | USP28,ubiquitin specific protease 28 |
| 229796_at | 28.07 | 8.01E-07 | 1.226421473 | NM_017420| | SIX4,sine oculis homeobox homolog 4 |
| 208796_s_at | 28.04 | 8.06E-07 | 1.132658821 | NM_004060| | CCNG1,cyclin G1 |
| 225295_at | 28.03 | 8.06E-07 | 1.138689257 | NM_001127257| | NA |
| 1555370_a_at | 28.02 | 8.07E-07 | 1.281224836 | NM_015215| | CAMTA1,calmodulin-binding transcription activator 1 |
| 227180_at | 27.98 | 8.12E-07 | 2.606500168 | NM_001104558| | NA |
| 200606_at | 27.95 | 8.16E-07 | 2.83525002 | NM_001008844| | DSP,desmoplakin isoform II |
| 207197_at | 27.94 | 8.16E-07 | 2.562556912 | NM_003413| | ZIC3,zinc finger protein of the cerebellum 3 |
| 208742_s_at | 27.88 | 8.24E-07 | 1.096939377 | NM_005870| | SAP18,sin3 associated polypeptide p18 |
| 241612_at | 27.87 | 8.25E-07 | 2.444529954 | NM_012183| | FOXD3,forkhead box D3 |
| 212838_at | 27.84 | 8.28E-07 | 1.19710646 | NM_015221| | DNMBP,dynamin binding protein |
| 226140_s_at | 27.84 | 8.28E-07 | 1.2660513 | NA |  |
| 208623_s_at | 27.83 | 8.28E-07 | 1.196830635 | NM_001111077| | NA |
| 202712_s_at | 27.83 | 8.28E-07 | 1.303934413 | NM_001015001| | NA |
| 219650_at | 27.8 | 8.32E-07 | 1.167353285 | NM_017669| | FLJ20105,FLJ20105 protein isoform a |
| 213135_at | 27.8 | 8.32E-07 | 1.192003077 | NM_003253| | TIAM1,T-cell lymphoma invasion and metastasis 1 |
| 209581_at | 27.79 | 8.32E-07 | 1.61791405 | NM_007069| | HRASLS3,HRAS-like suppressor 3 |
| 238035_at | 27.78 | 8.35E-07 | 1.144730098 | NM_001017371| | NA |
| 218973_at | 27.76 | 8.37E-07 | 1.133666698 | NM_001040610| | NA |
| 239292_at | 27.74 | 8.39E-07 | 1.647472486 | NA |  |
| 231233_at | 27.74 | 8.39E-07 | 1.674451378 | NA |  |
| 230047_at | 27.68 | 8.49E-07 | 1.406138024 | NA |  |
| 212728_at | 27.66 | 8.52E-07 | 1.325042242 | NM_020730| | NA |
| 221486_at | 27.64 | 8.56E-07 | 1.084961899 | NM_004436| | ENSA,endosulfine alpha isoform 3 |
| 226325_at | 27.59 | 8.64E-07 | 1.466494741 | NM_152328| | ADSSL1,adenylosuccinate synthase-like 1 isoform 2 |
| 214036_at | 27.58 | 8.65E-07 | 1.32106563 | NA |  |
| 228933_at | 27.53 | 8.73E-07 | 1.426109068 | NM_198270| | NHS,Nance-Horan syndrome protein |
| 227220_at | 27.53 | 8.73E-07 | 1.23976174 | NM_152995| | HOZFP,ovarian zinc finger protein |
| 203895_at | 27.52 | 8.74E-07 | 1.285270176 | NM_000933| | PLCB4,phospholipase C beta 4 isoform a |
| 225074_at | 27.51 | 8.75E-07 | 1.122592501 | NM_032846| | RAB2B,RAB2B protein |
| 228713_s_at | 27.51 | 8.75E-07 | 1.372566332 | NM_016246| | DHRS10,dehydrogenase/reductase (SDR family) member 10 |
| 223591_at | 27.47 | 8.83E-07 | 1.54119583 | NM_032322| | RNF135,ring finger protein 135 isoform 1 |
| 225354_s_at | 27.42 | 8.92E-07 | 1.190919354 | NM_031469| | SH3BGRL2,SH3 domain binding glutamic acid-rich protein |
| 222033_s_at | 27.39 | 8.95E-07 | 1.988376598 | NA |  |
| 227236_at | 27.33 | 9.07E-07 | 2.809875866 | NM_005725| | TSPAN2,tetraspan 2 |
| 202437_s_at | 27.31 | 9.09E-07 | 2.089304044 | NM_000104| | CYP1B1,cytochrome P450, family 1, subfamily B, |
| 210986_s_at | 27.31 | 9.09E-07 | 1.469585436 | NM_000366| | TPM1,tropomyosin 1 (alpha) |
| 242328_at | 27.29 | 9.12E-07 | 1.478241583 | NM_138453| | RAB3C,RAB3C, member RAS oncogene family |
| 210852_s_at | 27.28 | 9.12E-07 | 1.462534438 | NM_005763| | AASS,aminoadipate-semialdehyde synthase |
| 208165_s_at | 27.27 | 9.14E-07 | 2.050827869 | NM_005865| | PRSS16,protease, serine, 16 |
| 236279_at | 27.24 | 9.20E-07 | 2.03756203 | NA |  |
| 36553_at | 27.22 | 9.23E-07 | 1.148279567 | NM_004192| | ASMTL,acetylserotonin O-methyltransferase-like |
| 219271_at | 27.21 | 9.24E-07 | 1.374793 | NM_024572| | GALNT14,UDP-N-acetyl-alpha-D-galactosamine:polypeptide |
| 230588_s_at | 27.16 | 9.34E-07 | 1.135213785 | NA |  |
| 226485_at | 27.15 | 9.36E-07 | 1.207552024 | NA |  |
| 227247_at | 27.14 | 9.37E-07 | 1.12220721 | NA |  |
| 227690_at | 27.13 | 9.38E-07 | 2.366488152 | NM_000814| | GABRB3,gamma-aminobutyric acid (GABA) A receptor, beta |
| 222595_s_at | 27.12 | 9.38E-07 | 1.333463151 | NM_022105| | DATF1,death associated transcription factor 1 isoform |
| 219045_at | 27.11 | 9.41E-07 | 1.486524681 | NM_019034| | RHOF,ras homolog gene family, member F |
| 223714_at | 27.09 | 9.41E-07 | 1.292063168 | NM_005773| | ZNF256,zinc finger protein 256 |
| 232165_at | 27.09 | 9.41E-07 | 2.306822059 | NM_031308| | EPPK1,epiplakin 1 |
| 238956_at | 27.04 | 9.51E-07 | 1.294231124 | NA |  |
| 223233_s_at | 27.03 | 9.51E-07 | 1.374841778 | NM_020770| | CGN,cingulin |
| 206291_at | 27.02 | 9.52E-07 | 2.182025165 | NM_006183| | NTS,neurotensin/neuromedin N preproprotein |
| 222343_at | 26.99 | 9.57E-07 | 1.314192635 | NM_006538| | BCL2L11,BCL2-like 11 isoform 6 |
| 218261_at | 26.98 | 9.57E-07 | 1.634095067 | NM_005498| | AP1M2,adaptor-related protein complex 1, mu 2 subunit |
| 218162_at | 26.92 | 9.69E-07 | 2.317316065 | NM_020190| | OLFML3,olfactomedin-like 3 |
| 205953_at | 26.92 | 9.68E-07 | 1.388521979 | NM_014813| | LRIG2,leucine-rich repeats and immunoglobulin-like |
| 210136_at | 26.91 | 9.70E-07 | 2.05668341 | NM_001025081| | NA |
| 204981_at | 26.9 | 9.71E-07 | 1.304376684 | NM_002555| | SLC22A18,tumor suppressing subtransferable candidate 5 |
| 236297_at | 26.89 | 9.71E-07 | 1.590456627 | NA |  |
| 207034_s_at | 26.89 | 9.71E-07 | 1.267616621 | NM_005270| | GLI2,GLI-Kruppel family member GLI2 isoform delta |
| 211343_s_at | 26.86 | 9.77E-07 | 1.99847829 | NM_005203| | COL13A1,alpha 1 type XIII collagen isoform 1 |
| 223628_at | 26.85 | 9.77E-07 | 1.544519644 | NA |  |
| 231035_s_at | 26.85 | 9.77E-07 | 1.34851331 | NA |  |
| 205206_at | 26.83 | 9.79E-07 | 1.688587144 | NM_000216| | KAL1,Kallmann syndrome 1 protein |
| 219945_at | 26.77 | 9.91E-07 | 1.441976463 | NM_013264| | DDX25,DEAD (Asp-Glu-Ala-Asp) box polypeptide 25 |
| 230669_at | 26.77 | 9.91E-07 | 1.194238279 | NM_006506| | RASA2,RAS p21 protein activator 2 |
| 40359_at | 26.71 | 1.00E-06 | 1.115340494 | NM_003475| | C11orf13,HRAS1-related cluster-1 |
| 228992_at | 26.7 | 1.00E-06 | 1.17718113 | NM_025205| | MED28,mediator of RNA polymerase II transcription, |
| 212224_at | 26.68 | 1.01E-06 | 1.18690884 | NM_000689| | ALDH1A1,aldehyde dehydrogenase 1A1 |
| 214790_at | 26.67 | 1.01E-06 | 1.224242424 | NM_001100409| | NA |
| 1552736_a_at | 26.66 | 1.01E-06 | 1.598889863 | NM_138966| | NETO1,neuropilin- and tolloid-like protein 1 isoform 3 |
| 202883_s_at | 26.64 | 1.02E-06 | 1.214923509 | NM_002716| | PPP2R1B,beta isoform of regulatory subunit A, protein |
| 213075_at | 26.63 | 1.02E-06 | 1.772575097 | NM_182487| | OLFML2A,olfactomedin-like 2A |
| 213172_at | 26.62 | 1.02E-06 | 2.252037536 | NM_015351| | NA |
| 1566766_a_at | 26.61 | 1.02E-06 | 1.464415018 | NM_182762| | 7A5,putative binding protein 7a5 |
| 214396_s_at | 26.6 | 1.02E-06 | 1.833553585 | NM_003927| | MBD2,methyl-CpG binding domain protein 2 isoform 1 |
| 201134_x_at | 26.59 | 1.02E-06 | 1.051285046 | NM_001867| | COX7C,cytochrome c oxidase subunit VIIc precursor |
| 224367_at | 26.59 | 1.02E-06 | 1.335192754 | NM_032621| | BEX2,brain expressed X-linked 2 |
| 217991_x_at | 26.58 | 1.02E-06 | 1.228356644 | NM_001009955| | SSBP3,single stranded DNA binding protein 3 isoform c |
| 222088_s_at | 26.57 | 1.02E-06 | 1.979333743 | NM_006931| | SLC2A3,solute carrier family 2 (facilitated glucose |
| 207127_s_at | 26.55 | 1.03E-06 | 1.054435182 | NM_012207| | HNRPH3,heterogeneous nuclear ribonucleoprotein H3 |
| 203656_at | 26.53 | 1.03E-06 | 1.166443725 | NM_014845| | KIAA0274,Sac domain-containing inositol phosphatase 3 |
| 214203_s_at | 26.52 | 1.03E-06 | 1.60382865 | NM_016335| | PRODH,proline dehydrogenase (oxidase) 1 |
| 218399_s_at | 26.51 | 1.04E-06 | 1.088265792 | NM_017955| | CDCA4,cell division cycle associated 4 |
| 214829_at | 26.5 | 1.04E-06 | 1.344539083 | NM_005763| | AASS,aminoadipate-semialdehyde synthase |
| 217574_at | 26.49 | 1.04E-06 | 1.927240726 | NM_001796| | CDH8,cadherin 8, type 2 preproprotein |
| 227702_at | 26.49 | 1.04E-06 | 1.682975146 | NM_178033| | CYP4X1,cytochrome P450, family 4, subfamily X, |
| 238417_at | 26.48 | 1.04E-06 | 1.486041267 | NM_173582| | PGM2L1,phosphoglucomutase 2-like 1 |
| 202722_s_at | 26.46 | 1.04E-06 | 1.341548167 | NM_002056| | GFPT1,glucosamine-fructose-6-phosphate |
| 220446_s_at | 26.45 | 1.04E-06 | 1.834942997 | NM_005769| | CHST4,carbohydrate (N-acetylglucosamine 6-O) |
| 229669_at | 26.45 | 1.04E-06 | 2.614854943 | NA |  |
| 229618_at | 26.42 | 1.05E-06 | 1.356416975 | NM_022133| | SNX16,sorting nexin 16 isoform a |
| 229223_at | 26.39 | 1.06E-06 | 2.043665368 | NA |  |
| 223311_s_at | 26.37 | 1.06E-06 | 1.523183585 | NM_020744| | MTA3,metastasis associated 1 family, member 3 |
| 202672_s_at | 26.36 | 1.06E-06 | 1.577027587 | NM_001030287| | NA |
| 212812_at | 26.33 | 1.07E-06 | 1.743810489 | NA |  |
| 1552754_a_at | 26.29 | 1.08E-06 | 1.559393161 | NM_153184| | IGSF4D,immunoglobulin superfamily, member 4D |
| 225820_at | 26.25 | 1.09E-06 | 1.148112007 | NM_024900| | PHF17,Jade1 protein short isoform |
| 229523_at | 26.24 | 1.09E-06 | 1.843124954 | NM_001080209| | NA |
| 203946_s_at | 26.24 | 1.09E-06 | 1.63912109 | NM_001172| | ARG2,arginase, type II precursor |
| 212340_at | 26.21 | 1.09E-06 | 1.155002751 | NM_173834| | MGC21416,hypothetical protein MGC21416 |
| 226161_at | 26.21 | 1.09E-06 | 1.093636259 | NM_017964| | SLC30A6,solute carrier family 30 (zinc transporter), |
| 221803_s_at | 26.19 | 1.10E-06 | 1.134766906 | NM_030759| | NRBF2,nuclear receptor binding factor 2 |
| 207644_at | 26.16 | 1.10E-06 | 1.414790264 | NM_003923| | FOXH1,forkhead box H1 |
| 230356_at | 26.15 | 1.10E-06 | 3.138094602 | NA |  |
| 206460_at | 26.13 | 1.11E-06 | 2.000815504 | NM_001042478| | NA |
| 37012_at | 26.13 | 1.11E-06 | 1.112362399 | NM_004930| | CAPZB,F-actin capping protein beta subunit |
| 208621_s_at | 26.12 | 1.11E-06 | 1.255862071 | NM_001111077| | NA |
| 223208_at | 26.11 | 1.11E-06 | 1.099949413 | NM_031954| | KCTD10,potassium channel tetramerisation domain |
| 238523_at | 26.1 | 1.11E-06 | 1.08156878 | NM_024731| | C16orf44,chromosome 16 open reading frame 44 |
| 218909_at | 26.06 | 1.12E-06 | 1.107277403 | NM_012424| | RPS6KC1,ribosomal protein S6 kinase, 52kDa, polypeptide |
| 222713_s_at | 26.06 | 1.12E-06 | 1.187664307 | NM_022725| | FANCF,Fanconi anemia, complementation group F |
| 238983_at | 26.04 | 1.13E-06 | 2.521896686 | NM_024677| | FLJ14001,hypothetical protein FLJ14001 |
| 216215_s_at | 26.04 | 1.13E-06 | 1.080257668 | NM_001031695| | NA |
| 205814_at | 26.02 | 1.13E-06 | 1.327264184 | NM_000840| | GRM3,glutamate receptor, metabotropic 3 precursor |
| 205047_s_at | 25.98 | 1.14E-06 | 1.174804573 | NM_001673| | ASNS,asparagine synthetase |
| 206653_at | 25.98 | 1.14E-06 | 1.287948827 | NM_006467| | POLR3G,polymerase (RNA) III (DNA directed) polypeptide |
| 225831_at | 25.96 | 1.14E-06 | 1.160452549 | NM_033631| | LUZP1,leucine zipper protein 1 |
| 218035_s_at | 25.95 | 1.15E-06 | 2.39048813 | NM_001098634| | NA |
| 205373_at | 25.92 | 1.15E-06 | 1.858643534 | NM_004389| | CTNNA2,catenin, alpha 2 |
| 219033_at | 25.92 | 1.15E-06 | 1.825832377 | NM_024615| | PARP8,poly (ADP-ribose) polymerase family, member 8 |
| 237810_at | 25.89 | 1.16E-06 | 2.800050878 | NM_021195| | CLDN6,claudin 6 |
| 222001_x_at | 25.89 | 1.16E-06 | 1.396731938 | NA |  |
| 222821_s_at | 25.88 | 1.16E-06 | 1.120673391 | NM_001007269| | GEMIN7,gemin 7 |
| 204224_s_at | 25.87 | 1.16E-06 | 1.34647123 | NM_000161| | GCH1,GTP cyclohydrolase 1 (dopa-responsive dystonia) |
| 206481_s_at | 25.79 | 1.18E-06 | 2.403653672 | NM_001290| | LDB2,LIM domain binding 2 |
| 217744_s_at | 25.77 | 1.19E-06 | 2.51133845 | NM_022121| | PERP,PERP, TP53 apoptosis effector |
| 212053_at | 25.76 | 1.19E-06 | 1.126800159 | NM_015027| | KIAA0251,KIAA0251 protein |
| 243000_at | 25.76 | 1.19E-06 | 1.329777543 | NM_001259| | CDK6,cyclin-dependent kinase 6 |
| 210431_at | 25.76 | 1.19E-06 | 1.548041023 | NM_031313| | ALPPL2,placental-like alkaline phosphatase |
| 202847_at | 25.75 | 1.19E-06 | 1.807513158 | NM_001018073| | NA |
| 228051_at | 25.74 | 1.19E-06 | 2.509199121 | NM_020340| | KIAA1244,KIAA1244 |
| 218983_at | 25.73 | 1.19E-06 | 1.898401909 | NM_016546| | C1RL,complement component 1, r subcomponent-like |
| 204483_at | 25.72 | 1.20E-06 | 1.284947643 | NM_001976| | ENO3,enolase 3 |
| 231120_x_at | 25.71 | 1.20E-06 | 2.66744791 | NM_032471| | PKIB,cAMP-dependent protein kinase inhibitor beta |
| 230869_at | 25.7 | 1.20E-06 | 2.803557554 | NM_001080396| | NA |
| 212693_at | 25.7 | 1.20E-06 | 1.081563736 | NM_014611| | MDN1,MDN1, midasin homolog |
| 231628_s_at | 25.68 | 1.20E-06 | 2.829600476 | NA |  |
| 221588_x_at | 25.66 | 1.21E-06 | 1.157446166 | NM_005589| | ALDH6A1,aldehyde dehydrogenase 6A1 precursor |
| 209723_at | 25.62 | 1.22E-06 | 3.505998998 | NM_004155| | SERPINB9,serine (or cysteine) proteinase inhibitor, clade |
| 226462_at | 25.6 | 1.22E-06 | 1.874066495 | NM_014178| | STXBP6,amisyn |
| 1556499_s_at | 25.6 | 1.22E-06 | 2.636008622 | NM_000088| | COL1A1,alpha 1 type I collagen preproprotein |
| 233559_s_at | 25.58 | 1.23E-06 | 1.203327565 | NM_020830| | WDFY1,WD repeat and FYVE domain containing 1 |
| 1554485_s_at | 25.58 | 1.23E-06 | 1.822508562 | NM_183240| | TMEM37,transmembrane protein 37 |
| 204678_s_at | 25.57 | 1.23E-06 | 1.860467168 | NM_002245| | KCNK1,potassium channel, subfamily K, member 1 |
| 214369_s_at | 25.56 | 1.23E-06 | 1.898156175 | NM_001098670| | NA |
| 218878_s_at | 25.55 | 1.23E-06 | 1.389648289 | NM_012238| | SIRT1,sirtuin 1 |
| 227167_s_at | 25.54 | 1.24E-06 | 1.200978731 | NA |  |
| 229829_at | 25.52 | 1.24E-06 | 1.113242578 | NA |  |
| 244362_at | 25.5 | 1.25E-06 | 1.270723957 | NA |  |
| 203439_s_at | 25.47 | 1.25E-06 | 2.017026325 | NM_003714| | STC2,stanniocalcin 2 |
| 209296_at | 25.47 | 1.25E-06 | 1.247348891 | NM_001033556| | NA |
| 203896_s_at | 25.47 | 1.25E-06 | 1.227097694 | NM_000933| | PLCB4,phospholipase C beta 4 isoform a |
| 58916_at | 25.46 | 1.25E-06 | 1.358049243 | NM_023930| | KCTD14,potassium channel tetramerisation domain |
| 203650_at | 25.46 | 1.25E-06 | 2.178596195 | NM_006404| | PROCR,endothelial protein C receptor precursor |
| 210946_at | 25.46 | 1.25E-06 | 1.591855867 | NM_003711| | PPAP2A,phosphatidic acid phosphatase type 2A isoform 1 |
| 204879_at | 25.46 | 1.25E-06 | 1.472174473 | NM_001006624| | T1A-2,lung type-I cell membrane-associated |
| 243439_at | 25.42 | 1.26E-06 | 1.277677009 | NM_133460| | ZNF418,zinc finger protein 418 |
| 203706_s_at | 25.41 | 1.26E-06 | 1.271627626 | NM_003507| | FZD7,frizzled 7 |
| 1255_g_at | 25.41 | 1.26E-06 | 2.816048812 | NM_000409| | GUCA1A,guanylate cyclase activator 1A (retina) |
| 206286_s_at | 25.39 | 1.27E-06 | 3.06021029 | NM_003212| | TDGF1,teratocarcinoma-derived growth factor 1 |
| 202558_s_at | 25.37 | 1.27E-06 | 1.21378495 | NM_006948| | STCH,stress 70 protein chaperone, |
| 227599_at | 25.34 | 1.28E-06 | 1.236739641 | NM_178496| | LOC151963,similar to BcDNA:GH11415 gene product |
| 218809_at | 25.34 | 1.28E-06 | 1.090140788 | NM_024960| | PANK2,pantothenate kinase 2 isoform 3 |
| 206818_s_at | 25.34 | 1.28E-06 | 1.225411047 | NM_017649| | CNNM2,cyclin M2 isoform 1 |
| 225648_at | 25.32 | 1.28E-06 | 1.168499648 | NM_080836| | STK35,serine/threonine kinase 35 |
| 204400_at | 25.3 | 1.29E-06 | 1.547089638 | NM_005864| | EFS,embryonal Fyn-associated substrate isoform 1 |
| 209536_s_at | 25.27 | 1.30E-06 | 1.250037278 | NM_139265| | EHD4,EH-domain containing 4 |
| 224452_s_at | 25.27 | 1.29E-06 | 1.139700489 | NM_001037163| | NA |
| 217988_at | 25.26 | 1.30E-06 | 1.096003317 | NM_021178| | CCNB1IP1,cyclin B1 interacting protein 1 isoform a |
| 212371_at | 25.23 | 1.30E-06 | 1.119477418 | NM_016076| | PNAS-4,CGI-146 protein |
| 201880_at | 25.22 | 1.30E-06 | 1.099437086 | NM_005744| | ARIH1,ariadne homolog, ubiquitin-conjugating enzyme E2 |
| 240269_at | 25.22 | 1.30E-06 | 1.738083447 | NA |  |
| 214058_at | 25.19 | 1.31E-06 | 1.373012548 | NM_001033081| | NA |
| 218720_x_at | 25.17 | 1.31E-06 | 1.570687006 | NM_001114099| | NA |
| 205350_at | 25.12 | 1.33E-06 | 2.366083132 | NM_004378| | CRABP1,cellular retinoic acid binding protein 1 |
| 202241_at | 25.11 | 1.33E-06 | 1.208619204 | NM_025195| | TRIB1,G-protein-coupled receptor induced protein |
| 212747_at | 25.11 | 1.33E-06 | 1.109663268 | NM_015245| | ANKS1,ankyrin repeat and sterile alpha motif domain |
| 208855_s_at | 25.1 | 1.33E-06 | 1.07498195 | NM_001032296| | NA |
| 207957_s_at | 25.1 | 1.33E-06 | 3.010376303 | NM_002738| | PRKCB1,protein kinase C, beta isoform 2 |
| 209238_at | 25.07 | 1.33E-06 | 2.444624307 | NM_004177| | STX3A,syntaxin 3A |
| 219222_at | 25.07 | 1.33E-06 | 1.254369795 | NM_022128| | RBKS,ribokinase |
| 222936_s_at | 25.07 | 1.33E-06 | 1.240654816 | NM_016076| | PNAS-4,CGI-146 protein |
| 239148_at | 25.05 | 1.34E-06 | 2.379797314 | NM_001017967| | NA |
| 228189_at | 25.05 | 1.34E-06 | 1.15098425 | NM_004874| | BAG4,BCL2-associated athanogene 4 |
| 201060_x_at | 25.04 | 1.34E-06 | 1.213649274 | NM_004099| | STOM,stomatin isoform a |
| 224871_at | 25.03 | 1.34E-06 | 1.243117258 | NM_182752| | LOC127262,hypothetical protein LOC127262 |
| 202347_s_at | 25.02 | 1.34E-06 | 1.086424846 | NM_001111112| | NA |
| 223467_at | 25.01 | 1.35E-06 | 1.268243079 | NM_016084| | RASD1,RAS, dexamethasone-induced 1 |
| 229644_at | 24.99 | 1.35E-06 | 1.256675847 | NM_002726| | PREP,prolyl endopeptidase |
| 218928_s_at | 24.98 | 1.35E-06 | 1.414042025 | NM_018964| | SLC37A1,solute carrier family 37 member 1 |
| 206296_x_at | 24.98 | 1.35E-06 | 1.677005763 | NM_001042600| | NA |
| 203474_at | 24.98 | 1.35E-06 | 1.226486952 | NM_006633| | IQGAP2,IQ motif containing GTPase activating protein 2 |
| 220387_s_at | 24.97 | 1.36E-06 | 1.385073492 | NM_001031693| | NA |
| 235044_at | 24.96 | 1.36E-06 | 2.662522019 | NM_052954| | CYYR1,cysteine and tyrosine-rich 1 protein precursor |
| 209631_s_at | 24.96 | 1.36E-06 | 2.265033592 | NM_005302| | GPR37,G protein-coupled receptor 37 |
| 219545_at | 24.95 | 1.36E-06 | 1.295410739 | NM_023930| | KCTD14,potassium channel tetramerisation domain |
| 202234_s_at | 24.93 | 1.36E-06 | 1.225592528 | NM_003051| | SLC16A1,solute carrier family 16, member 1 |
| 208310_s_at | 24.93 | 1.36E-06 | 1.09997113 | NM_015622| | C7orf28A,chromosome 7 open reading frame 28A |
| 201131_s_at | 24.92 | 1.36E-06 | 3.871498958 | NM_004360| | CDH1,cadherin 1, type 1 preproprotein |
| 225566_at | 24.91 | 1.37E-06 | 1.131505656 | NM_003872| | NRP2,neuropilin 2 isoform 2 precursor |
| 212770_at | 24.91 | 1.37E-06 | 1.186150948 | NM_001105192| | NA |
| 212455_at | 24.89 | 1.37E-06 | 1.089086838 | NM_001031732| | NA |
| 227123_at | 24.88 | 1.38E-06 | 1.482619723 | NM_002867| | RAB3B,RAB3B, member RAS oncogene family |
| 209687_at | 24.87 | 1.38E-06 | 2.950913068 | NM_000609| | CXCL12,chemokine (C-X-C motif) ligand 12 (stromal |
| 206654_s_at | 24.87 | 1.38E-06 | 1.349355364 | NM_006467| | POLR3G,polymerase (RNA) III (DNA directed) polypeptide |
| 225401_at | 24.84 | 1.39E-06 | 1.517026135 | NM_144580| | MGC31963,kidney predominant protein NCU-G1 |
| 219987_at | 24.82 | 1.39E-06 | 1.67189481 | NA |  |
| 205126_at | 24.82 | 1.39E-06 | 1.223112651 | NM_006296| | VRK2,vaccinia related kinase 2 |
| 225227_at | 24.81 | 1.39E-06 | 1.42672562 | NA |  |
| 228598_at | 24.8 | 1.40E-06 | 2.744183032 | NM_001004360| | DPP10,dipeptidylpeptidase 10 isoform 2 |
| 206299_at | 24.8 | 1.40E-06 | 1.466978834 | NM_015686| | TMEM28,transmembrane protein 28 |
| 236448_at | 24.79 | 1.40E-06 | 1.78246054 | NM_133369| | UNC5A,netrin receptor Unc5h1 |
| 203822_s_at | 24.78 | 1.40E-06 | 1.120170276 | NM_006874| | ELF2,E74-like factor 2 (ets domain transcription |
| 210835_s_at | 24.78 | 1.40E-06 | 1.066254602 | NM_001083914| | NA |
| 243_g_at | 24.74 | 1.41E-06 | 1.053271964 | NM_002375| | MAP4,microtubule-associated protein 4 isoform 1 |
| 218651_s_at | 24.72 | 1.42E-06 | 1.186736779 | NM_018357| | FLJ11196,acheron isoform 1 |
| 202404_s_at | 24.69 | 1.42E-06 | 2.478583789 | NM_000089| | COL1A2,alpha 2 type I collagen |
| 228994_at | 24.69 | 1.42E-06 | 1.223724303 | NM_152499| | MGC45441,hypothetical protein MGC45441 |
| 201844_s_at | 24.68 | 1.42E-06 | 1.193564859 | NM_012234| | RYBP,RING1 and YY1 binding protein |
| 223396_at | 24.65 | 1.44E-06 | 1.07596931 | NM_032936| | C7orf35,chromosome 7 open reading frame 35 |
| 214440_at | 24.64 | 1.44E-06 | 1.307945452 | NM_000662| | NAT1,N-acetyltransferase 1 |
| 219000_s_at | 24.64 | 1.44E-06 | 1.098652339 | NM_024094| | DCC1,hypothetical protein MGC5528 |
| 228665_at | 24.62 | 1.45E-06 | 2.357256081 | NM_052954| | CYYR1,cysteine and tyrosine-rich 1 protein precursor |
| 201900_s_at | 24.6 | 1.45E-06 | 1.090369161 | NM_006066| | AKR1A1,aldo-keto reductase family 1, member A1 |
| 228802_at | 24.58 | 1.45E-06 | 1.336670099 | NM_194272| | NA |
| 219302_s_at | 24.56 | 1.46E-06 | 2.690381107 | NM_014141| | CNTNAP2,cell recognition molecule Caspr2 precursor |
| 215227_x_at | 24.54 | 1.47E-06 | 1.038783392 | NM_001040649| | NA |
| 210605_s_at | 24.53 | 1.47E-06 | 1.575323993 | NM_001114614| | NA |
| 205538_at | 24.53 | 1.47E-06 | 2.125365782 | NM_003389| | CORO2A,coronin, actin binding protein, 2A |
| 231517_at | 24.49 | 1.48E-06 | 1.253405776 | NA |  |
| 218667_at | 24.48 | 1.48E-06 | 1.145176435 | NM_001032396| | NA |
| 207727_s_at | 24.46 | 1.49E-06 | 1.092561049 | NM_001048171| | NA |
| 226498_at | 24.46 | 1.49E-06 | 3.249699837 | NA |  |
| 221207_s_at | 24.45 | 1.49E-06 | 1.805845787 | NM_015678| | NBEA,neurobeachin |
| 234970_at | 24.4 | 1.50E-06 | 1.832686553 | NM_152332| | MTAC2D1,membrane targeting (tandem) C2 domain containing |
| 223423_at | 24.4 | 1.50E-06 | 2.868354642 | NM_014373| | GPR160,G protein-coupled receptor 160 |
| 226778_at | 24.38 | 1.51E-06 | 1.324232746 | NM_175075| | INM01,hypothetical protein INM01 |
| 205483_s_at | 24.36 | 1.52E-06 | 1.391419915 | NM_005101| | G1P2,interferon, alpha-inducible protein (clone |
| 223060_at | 24.35 | 1.52E-06 | 1.110900988 | NM_017924| | C14orf119,chromosome 14 open reading frame 119 |
| 223122_s_at | 24.34 | 1.52E-06 | 2.399548684 | NM_003013| | SFRP2,secreted frizzled-related protein 2 precursor |
| 212457_at | 24.34 | 1.52E-06 | 1.069385877 | NM_006521| | TFE3,transcription factor binding to IGHM enhancer 3 |
| 230835_at | 24.32 | 1.53E-06 | 1.451914216 | NM_207392| | UNQ467,KIPV467 |
| 201032_at | 24.31 | 1.53E-06 | 1.091477112 | NM_006698| | BLCAP,bladder cancer associated protein |
| 229088_at | 24.31 | 1.53E-06 | 1.486405304 | NM_006208| | ENPP1,ectonucleotide pyrophosphatase/phosphodiesterase |
| 228340_at | 24.3 | 1.53E-06 | 1.288993356 | NM_001105192| | NA |
| 221735_at | 24.27 | 1.54E-06 | 1.118282606 | NM_020839| | WDR48,WD repeat domain 48 |
| 1553987_at | 24.25 | 1.55E-06 | 1.107847075 | NA |  |
| 201061_s_at | 24.23 | 1.55E-06 | 1.169151513 | NM_004099| | STOM,stomatin isoform a |
| 228010_at | 24.23 | 1.55E-06 | 1.915656625 | NM_020416| | PPP2R2C,gamma isoform of regulatory subunit B55, protein |
| 1556911_at | 24.22 | 1.55E-06 | 1.607304672 | NA |  |
| 220116_at | 24.22 | 1.55E-06 | 1.944955562 | NM_021614| | KCNN2,small conductance calcium-activated potassium |
| 202311_s_at | 24.21 | 1.56E-06 | 1.901405942 | NM_000088| | COL1A1,alpha 1 type I collagen preproprotein |
| 212151_at | 24.2 | 1.56E-06 | 1.267528562 | NM_002585| | PBX1,pre-B-cell leukemia transcription factor 1 |
| 205107_s_at | 24.18 | 1.56E-06 | 1.321411148 | NM_005227| | EFNA4,ephrin A4 isoform a |
| 219806_s_at | 24.18 | 1.56E-06 | 1.320110316 | NM_020179| | FN5,FN5 protein |
| 224595_at | 24.17 | 1.57E-06 | 1.190465019 | NM_080546| | CDW92,CDW92 antigen |
| 226313_at | 24.16 | 1.57E-06 | 1.392503922 | NM_145306| | C10orf35,chromosome 10 open reading frame 35 |
| 206074_s_at | 24.15 | 1.57E-06 | 1.194973109 | NM_002131| | HMGA1,high mobility group AT-hook 1 isoform b |
| 227933_at | 24.14 | 1.57E-06 | 1.212492134 | NM_032808| | LRRN6A,leucine-rich repeat neuronal 6A |
| 224596_at | 24.1 | 1.58E-06 | 1.264829031 | NM_080546| | CDW92,CDW92 antigen |
| 205857_at | 24.1 | 1.58E-06 | 1.74345742 | NM_003054| | SLC18A2,solute carrier family 18 (vesicular monoamine), |
| 212013_at | 24.09 | 1.59E-06 | 1.094135016 | NM_012293| | NA |
| 203992_s_at | 24.08 | 1.59E-06 | 1.350963777 | NM_021140| | UTX,ubiquitously transcribed tetratricopeptide |
| 212024_x_at | 24.07 | 1.60E-06 | 1.127682239 | NM_002018| | FLII,flightless I homolog |
| 209931_s_at | 24.06 | 1.60E-06 | 1.389407933 | NM_004116| | FKBP1B,FK506-binding protein 1B isoform a |
| 1553955_at | 24.05 | 1.60E-06 | 1.132767269 | NM_152994| | LOC129285,smooth muscle myosin heavy chain 11 isoform |
| 203351_s_at | 24.02 | 1.61E-06 | 1.087078603 | NM_002552| | ORC4L,origin recognition complex subunit 4 |
| 208022_s_at | 24.01 | 1.61E-06 | 1.203676092 | NM_001077181| | NA |
| 1560562_a_at | 23.99 | 1.62E-06 | 1.390696677 | NM_182609| | MGC48625,hypothetical protein MGC48625 |
| 206552_s_at | 23.96 | 1.63E-06 | 2.294565943 | NM_003182| | TAC1,tachykinin 1 isoform beta precursor |
| 202826_at | 23.95 | 1.64E-06 | 1.848637135 | NM_001032367| | NA |
| 225258_at | 23.94 | 1.64E-06 | 1.276218426 | NM_001024215| | NA |
| 236124_at | 23.92 | 1.64E-06 | 1.270926667 | NA |  |
| 210053_at | 23.89 | 1.65E-06 | 1.093738433 | NM_006951| | TAF5,TBP-associated factor 5 |
| 235126_at | 23.84 | 1.67E-06 | 1.269961068 | NA |  |
| 219765_at | 23.82 | 1.68E-06 | 1.301611902 | NM_024620| | ZNF329,zinc finger protein 329 |
| 218957_s_at | 23.76 | 1.71E-06 | 1.103497644 | NM_025155| | FLJ11848,hypothetical protein FLJ11848 |
| 240479_at | 23.74 | 1.72E-06 | 1.540765718 | NM_153612| | HS3ST5,heparan sulfate (glucosamine) |
| 1552678_a_at | 23.73 | 1.72E-06 | 1.401576338 | NM_020886| | USP28,ubiquitin specific protease 28 |
| 202235_at | 23.73 | 1.72E-06 | 1.238490962 | NM_003051| | SLC16A1,solute carrier family 16, member 1 |
| 204416_x_at | 23.7 | 1.73E-06 | 1.642612276 | NM_001645| | APOC1,apolipoprotein C-I precursor |
| 228275_at | 23.69 | 1.73E-06 | 1.207096787 | NA |  |
| 226720_at | 23.66 | 1.75E-06 | 1.333163167 | NM_052927| | NA |
| 219588_s_at | 23.66 | 1.75E-06 | 1.049689893 | NM_017760| | MTB,more than blood homolog |
| 231837_at | 23.65 | 1.75E-06 | 1.304062275 | NM_020886| | USP28,ubiquitin specific protease 28 |
| 1554592_a_at | 23.65 | 1.75E-06 | 2.422793051 | NM_005071| | SLC1A6,solute carrier family 1 (high affinity |
| 210260_s_at | 23.63 | 1.75E-06 | 1.40472174 | NM_001077654| | NA |
| 1552946_at | 23.63 | 1.76E-06 | 1.300943911 | NM_153608| | MGC17986,hypothetical protein MGC17986 |
| 222162_s_at | 23.6 | 1.77E-06 | 2.018945938 | NM_006988| | ADAMTS1,a disintegrin and metalloprotease with |
| 1560741_at | 23.6 | 1.77E-06 | 1.328917207 | NM_003097| | SNRPN,small nuclear ribonucleoprotein polypeptide N |
| 231856_at | 23.6 | 1.77E-06 | 2.042462852 | NM_020340| | KIAA1244,KIAA1244 |
| 205559_s_at | 23.59 | 1.77E-06 | 2.107701441 | NM_006200| | PCSK5,proprotein convertase subtilisin/kexin type 5 |
| 205405_at | 23.58 | 1.77E-06 | 1.214435818 | NM_003966| | SEMA5A,semaphorin 5A |
| 205286_at | 23.57 | 1.78E-06 | 1.230676893 | NM_003222| | TFAP2C,transcription factor AP-2 gamma |
| 232136_s_at | 23.55 | 1.78E-06 | 1.580260839 | NM_033427| | CTTNBP2,cortactin binding protein 2 |
| 231470_at | 23.55 | 1.78E-06 | 2.44077391 | NA |  |
| 218398_at | 23.53 | 1.79E-06 | 1.054968349 | NM_016640| | MRPS30,mitochondrial ribosomal protein S30 |
| 213283_s_at | 23.53 | 1.79E-06 | 1.213270812 | NM_005407| | SALL2,sal-like 2 |
| 211546_x_at | 23.52 | 1.79E-06 | 1.234990719 | NM_000345| | SNCA,alpha-synuclein isoform NACP140 |
| 1555950_a_at | 23.51 | 1.80E-06 | 1.802168778 | NM_000574| | DAF,decay accelerating factor for complement (CD55, |
| 223234_at | 23.48 | 1.81E-06 | 1.207879172 | NM_006341| | MAD2L2,MAD2 homolog |
| 219697_at | 23.48 | 1.81E-06 | 1.333644644 | NM_006043| | HS3ST2,heparan sulfate D-glucosaminyl |
| 225235_at | 23.44 | 1.82E-06 | 1.130536451 | NM_001006616| | TM4SF17,transmembrane 4 superfamily member 17 isoform c |
| 212842_x_at | 23.43 | 1.83E-06 | 1.066338064 | NM_001123363| | NA |
| 230903_s_at | 23.41 | 1.84E-06 | 1.286034054 | NM_175075| | INM01,hypothetical protein INM01 |
| 235874_at | 23.41 | 1.84E-06 | 1.730958644 | NM_153362| | PRSS35,protease, serine, 35 |
| 225792_at | 23.4 | 1.84E-06 | 2.659158345 | NM_015888| | HOOK1,hook homolog 1 |
| 206109_at | 23.39 | 1.85E-06 | 1.515779423 | NM_000148| | FUT1,fucosyltransferase 1 |
| 203879_at | 23.38 | 1.85E-06 | 1.575863005 | NM_005026| | PIK3CD,phosphoinositide-3-kinase, catalytic, delta |
| 222431_at | 23.35 | 1.86E-06 | 1.120178657 | NM_006717| | SPIN,spindlin |
| 224710_at | 23.35 | 1.86E-06 | 1.146525795 | NM_031934| | RAB34,RAB39 |
| 225177_at | 23.35 | 1.86E-06 | 1.384356939 | NM_001002233| | RAB11FIP1,Rab coupling protein isoform 2 |
| 202550_s_at | 23.35 | 1.86E-06 | 1.077618505 | NM_004738| | VAPB,VAMP-associated protein B/C |
| 205818_at | 23.3 | 1.88E-06 | 2.316401504 | NM_014618| | DBC1,deleted in bladder cancer 1 |
| 238547_at | 23.28 | 1.89E-06 | 1.206245314 | NM_144608| | FLJ32384,hypothetical protein MGC39389 |
| 207345_at | 23.27 | 1.89E-06 | 2.063510023 | NM_006350| | FST,follistatin isoform FST317 precursor |
| 201403_s_at | 23.26 | 1.90E-06 | 1.091234261 | NM_004528| | MGST3,microsomal glutathione S-transferase 3 |
| 227526_at | 23.25 | 1.90E-06 | 1.458954145 | NM_016952| | CDON,surface glycoprotein, Ig superfamily member |
| 227070_at | 23.23 | 1.91E-06 | 2.238934331 | NM_031302| | GLT8D2,glycosyltransferase 8 domain containing 2 |
| 203705_s_at | 23.22 | 1.91E-06 | 1.283596136 | NM_003507| | FZD7,frizzled 7 |
| 219439_at | 23.22 | 1.91E-06 | 1.32157517 | NM_020156| | C1GALT1,core 1 synthase, |
| 209493_at | 23.21 | 1.92E-06 | 2.001468953 | NM_178140| | PDZK3,PDZ domain containing 3 isoform a |
| 205719_s_at | 23.21 | 1.91E-06 | 1.966333036 | NM_000277| | PAH,phenylalanine hydroxylase |
| 205309_at | 23.2 | 1.92E-06 | 2.116171527 | NM_001009568| | SMPDL3B,acid sphingomyelinase-like phosphodiesterase 3B |
| 201137_s_at | 23.19 | 1.92E-06 | 1.387137775 | NM_002121| | HLA-DPB1,major histocompatibility complex, class II, DP |
| 213285_at | 23.18 | 1.93E-06 | 2.308742215 | NM_001017970| | NA |
| 226443_at | 23.16 | 1.93E-06 | 1.143115459 | NM_138333| | C9orf42,chromosome 9 open reading frame 42 |
| 203713_s_at | 23.14 | 1.94E-06 | 1.63719886 | NM_001015002| | NA |
| 210042_s_at | 23.12 | 1.95E-06 | 2.28654182 | NM_001336| | CTSZ,cathepsin Z preproprotein |
| 225647_s_at | 23.09 | 1.96E-06 | 1.229905667 | NM_001114173| | NA |
| 218517_at | 23.07 | 1.97E-06 | 1.157206599 | NM_024900| | PHF17,Jade1 protein short isoform |
| 201976_s_at | 23.03 | 1.98E-06 | 1.097222618 | NM_012334| | MYO10,myosin X |
| 201071_x_at | 23.02 | 1.99E-06 | 1.050616437 | NM_001005526| | SF3B1,splicing factor 3b, subunit 1 isoform 2 |
| 209344_at | 23.01 | 1.99E-06 | 1.123180493 | NM_003290| | TPM4,tropomyosin 4 |
| 203020_at | 23.01 | 1.99E-06 | 1.493159924 | NM_001035230| | NA |
| 223017_at | 23 | 1.99E-06 | 1.062139889 | NM_015913| | TLP19,endoplasmic reticulum thioredoxin superfamily |
| 209545_s_at | 22.98 | 2.01E-06 | 1.083437417 | NM_003821| | RIPK2,receptor-interacting serine-threonine kinase 2 |
| 1555793_a_at | 22.96 | 2.02E-06 | 1.599364058 | NM_133466| | ZNF545,zinc finger protein 545 |
| 218404_at | 22.93 | 2.03E-06 | 1.078116718 | NM_013322| | SNX10,sorting nexin 10 |
| 219036_at | 22.92 | 2.03E-06 | 1.182164853 | NM_024491| | Cep70,centrosomal protein 70 kDa |
| 206675_s_at | 22.91 | 2.03E-06 | 1.98435463 | NM_005414| | SKIL,SKI-like |
| 226107_at | 22.9 | 2.04E-06 | 1.213545122 | NA |  |
| 205123_s_at | 22.89 | 2.04E-06 | 1.376064995 | NM_003692| | TMEFF1,transmembrane protein with EGF-like and two |
| 209529_at | 22.88 | 2.05E-06 | 1.469454074 | NM_003712| | PPAP2C,phosphatidic acid phosphatase type 2C isoform 1 |
| 226986_at | 22.87 | 2.05E-06 | 1.204685195 | NM_001033518| | NA |
| 1552938_at | 22.87 | 2.05E-06 | 2.060338471 | NM_033132| | ZIC5,zinc finger protein of the cerebellum 5 |
| 215440_s_at | 22.86 | 2.06E-06 | 1.243249104 | NM_001080425| | NA |
| 213198_at | 22.83 | 2.07E-06 | 1.129972309 | NM_004302| | ACVR1B,activin A type IB receptor isoform a precursor |
| 226000_at | 22.8 | 2.08E-06 | 1.144065593 | NM_018704| | DKFZp547A023,hypothetical protein DKFZp547A023 |
| 205531_s_at | 22.79 | 2.09E-06 | 1.843964395 | NM_013267| | GLS2,glutaminase GA isoform a |
| 220011_at | 22.79 | 2.09E-06 | 1.165971214 | NM_024037| | MGC2603,hypothetical protein MGC2603 |
| 225380_at | 22.78 | 2.09E-06 | 1.5680535 | NM_138370| | NA |
| 204730_at | 22.78 | 2.09E-06 | 1.258830238 | NM_014747| | RIMS3,regulating synaptic membrane exocytosis 3 |
| 206116_s_at | 22.77 | 2.09E-06 | 1.527020935 | NM_000366| | TPM1,tropomyosin 1 (alpha) |
| 210674_s_at | 22.75 | 2.10E-06 | 2.640610654 | NM_014005| | PCDHA9,protocadherin alpha 9 isoform 2 precursor |
| 224252_s_at | 22.74 | 2.11E-06 | 1.520865221 | NM_014164| | FXYD5,FXYD domain-containing ion transport regulator |
| 203588_s_at | 22.73 | 2.11E-06 | 1.212029195 | NM_006286| | TFDP2,transcription factor Dp-2 (E2F dimerization |
| 210774_s_at | 22.72 | 2.11E-06 | 1.049119099 | NM_005437| | NCOA4,nuclear receptor coactivator 4 |
| 217234_s_at | 22.72 | 2.11E-06 | 1.250763737 | NM_001111077| | NA |
| 228547_at | 22.71 | 2.11E-06 | 2.431299428 | NM_004801| | NRXN1,neurexin 1 isoform alpha precursor |
| 207206_s_at | 22.7 | 2.11E-06 | 1.280738733 | NM_000697| | ALOX12,arachidonate 12-lipoxygenase |
| 231897_at | 22.69 | 2.12E-06 | 1.082214503 | NM_012212| | LTB4DH,NADP-dependent leukotriene B4 |
| 209620_s_at | 22.68 | 2.12E-06 | 1.137084789 | NM_004299| | ABCB7,ATP-binding cassette, sub-family B, member 7 |
| 216236_s_at | 22.68 | 2.12E-06 | 1.864206489 | NM_006931| | SLC2A3,solute carrier family 2 (facilitated glucose |
| 218451_at | 22.65 | 2.14E-06 | 1.444740797 | NM_022842| | CDCP1,CUB domain-containing protein 1 isoform 1 |
| 201611_s_at | 22.63 | 2.15E-06 | 1.110604884 | NM_012405| | ICMT,isoprenylcysteine carboxyl methyltransferase |
| 201253_s_at | 22.63 | 2.15E-06 | 1.05995119 | NM_006319| | CDIPT,CDP-diacylglycerol--inositol |
| 221898_at | 22.62 | 2.15E-06 | 1.497990113 | NM_001006624| | T1A-2,lung type-I cell membrane-associated |
| 228785_at | 22.61 | 2.16E-06 | 1.214227368 | NM_012482| | ZNF281,zinc finger protein 281 |
| 203346_s_at | 22.59 | 2.17E-06 | 1.206520432 | NM_007358| | M96,putative DNA binding protein |
| 225434_at | 22.59 | 2.17E-06 | 1.169468895 | NM_133328| | DEDD2,death effector domain-containing DNA binding |
| 219526_at | 22.58 | 2.17E-06 | 1.153564963 | NM_024644| | C14orf169,chromosome 14 open reading frame 169 |
| 241968_at | 22.58 | 2.17E-06 | 1.213221189 | NA |  |
| 1552712_a_at | 22.57 | 2.17E-06 | 1.459594847 | NM_015039| | NMNAT2,nicotinamide mononucleotide adenylyltransferase |
| 218946_at | 22.57 | 2.17E-06 | 1.123826589 | NM_001002755| | HIRIP5,HIRA interacting protein 5 isoform 2 |
| 218464_s_at | 22.54 | 2.19E-06 | 1.088013341 | NM_001077498| | NA |
| 226657_at | 22.53 | 2.19E-06 | 1.308793726 | NM_152914| | MGC33894,transcript expressed during hematopoiesis 2 |
| 204199_at | 22.52 | 2.19E-06 | 1.580542048 | NM_014636| | RALGPS1,Ral GEF with PH domain and SH3 binding motif 1 |
| 207080_s_at | 22.52 | 2.19E-06 | 1.598138237 | NM_004160| | PYY,peptide YY |
| 204423_at | 22.51 | 2.20E-06 | 1.240699973 | NM_013255| | MKLN1,muskelin 1, intracellular mediator containing |
| 235045_at | 22.51 | 2.20E-06 | 1.209334045 | NM_016090| | RBM7,RNA binding motif protein 7 |
| 232151_at | 22.51 | 2.20E-06 | 1.877080013 | NM_182762| | 7A5,putative binding protein 7a5 |
| 226744_at | 22.5 | 2.20E-06 | 1.102577831 | NM_024086| | MGC3329,hypothetical protein MGC3329 |
| 237291_at | 22.5 | 2.20E-06 | 1.892282017 | NA |  |
| 202455_at | 22.49 | 2.21E-06 | 1.25198728 | NM_001015053| | NA |
| 208926_at | 22.49 | 2.21E-06 | 1.1515186 | NM_000434| | NEU1,neuraminidase precursor |
| 229377_at | 22.49 | 2.21E-06 | 1.881317111 | NM_024719| | GRTP1,growth hormone regulated TBC protein 1 |
| 230195_at | 22.48 | 2.21E-06 | 3.518797419 | NA |  |
| 228974_at | 22.46 | 2.22E-06 | 1.4555832 | NA |  |
| 239202_at | 22.45 | 2.23E-06 | 1.54857675 | NA |  |
| 209679_s_at | 22.44 | 2.23E-06 | 1.356992023 | NM_001031628| | NA |
| 228098_s_at | 22.42 | 2.24E-06 | 1.430475889 | NM_013262| | MYLIP,myosin regulatory light chain interacting |
| 224561_s_at | 22.4 | 2.25E-06 | 1.066522787 | NM_006791| | MORF4L1,MORF-related gene 15 isoform 1 |
| 218186_at | 22.39 | 2.26E-06 | 2.286901535 | NM_020387| | RAB25,RAB25 |
| 227749_at | 22.39 | 2.26E-06 | 1.297874783 | NA |  |
| 1567107_s_at | 22.39 | 2.26E-06 | 1.174169972 | NM_003290| | TPM4,tropomyosin 4 |
| 235687_at | 22.33 | 2.29E-06 | 1.583165798 | NM_001076675| | NA |
| 228463_at | 22.33 | 2.29E-06 | 1.739570392 | NM_004497| | FOXA3,forkhead box A3 |
| 222392_x_at | 22.31 | 2.30E-06 | 2.66125847 | NM_022121| | PERP,PERP, TP53 apoptosis effector |
| 223885_at | 22.3 | 2.31E-06 | 1.769528879 | NM_001017440| | NA |
| 235696_at | 22.27 | 2.32E-06 | 1.419864082 | NA |  |
| 209792_s_at | 22.25 | 2.33E-06 | 1.533666669 | NM_001077500| | NA |
| 218450_at | 22.22 | 2.35E-06 | 1.129739959 | NM_015987| | HEBP1,heme binding protein 1 |
| 217893_s_at | 22.21 | 2.35E-06 | 1.16711003 | NM_024595| | FLJ12666,hypothetical protein FLJ12666 |
| 205440_s_at | 22.2 | 2.36E-06 | 2.645611895 | NM_000909| | NPY1R,neuropeptide Y receptor Y1 |
| 229344_x_at | 22.19 | 2.36E-06 | 1.139067868 | NM_020734| | KIAA1238,KIAA1238 protein |
| 219959_at | 22.18 | 2.37E-06 | 1.901674593 | NM_017947| | MOCOS,molybdenum cofactor sulfurase |
| 238697_at | 22.17 | 2.38E-06 | 1.953885231 | NA |  |
| 200931_s_at | 22.16 | 2.38E-06 | 1.256455215 | NM_003373| | VCL,vinculin isoform VCL |
| 225731_at | 22.16 | 2.38E-06 | 1.150097952 | NM_020337| | NA |
| 242477_at | 22.16 | 2.38E-06 | 1.912139098 | NM_152574| | C9orf52,hypothetical protein FLJ33868 |
| 226548_at | 22.15 | 2.38E-06 | 1.292397802 | NM_001024401| | NA |
| 219352_at | 22.12 | 2.40E-06 | 1.667681146 | NM_017912| | HERC6,hect domain and RLD 6 |
| 206683_at | 22.11 | 2.40E-06 | 1.493552028 | NM_003447| | ZNF165,zinc finger protein 165 |
| 211458_s_at | 22.11 | 2.40E-06 | 1.567863896 | NM_031412| | GABARAPL1,GABA(A) receptor-associated protein like 1 |
| 206355_at | 22.1 | 2.40E-06 | 2.015735514 | NM_002071| | GNAL,guanine nucleotide binding protein (G protein), |
| 1554887_at | 22.1 | 2.41E-06 | 2.037438375 | NA |  |
| 222976_s_at | 22.1 | 2.40E-06 | 1.085531847 | NM_001043351| | NA |
| 204466_s_at | 22.1 | 2.41E-06 | 2.270243224 | NM_000345| | SNCA,alpha-synuclein isoform NACP140 |
| 32137_at | 22.09 | 2.41E-06 | 1.296327906 | NM_002226| | JAG2,jagged 2 isoform a precursor |
| 219635_at | 22.08 | 2.41E-06 | 1.184302209 | NM_025027| | ZNF606,zinc finger protein 606 |
| 227934_at | 22.08 | 2.41E-06 | 1.127575313 | NM_002269| | KPNA5,karyopherin alpha 5 (importin alpha 6) |
| 224800_at | 22.08 | 2.41E-06 | 1.158484087 | NM_020830| | WDFY1,WD repeat and FYVE domain containing 1 |
| 1554043_a_at | 22.07 | 2.41E-06 | 1.309011922 | NA |  |
| 227692_at | 22.07 | 2.41E-06 | 1.244593772 | NM_002069| | GNAI1,guanine nucleotide binding protein (G protein), |
| 209166_s_at | 22.07 | 2.41E-06 | 1.081621195 | NM_000528| | MAN2B1,mannosidase, alpha, class 2B, member 1 |
| 215127_s_at | 22.05 | 2.42E-06 | 1.276875789 | NM_002897| | RBMS1,RNA binding motif, single stranded interacting |
| 229817_at | 22.04 | 2.43E-06 | 1.162995811 | NM_020747| | ZNF608,zinc finger protein 608 |
| 227063_at | 22.03 | 2.43E-06 | 1.128132956 | NM_152766| | MGC40107,hypothetical protein MGC40107 |
| 210987_x_at | 22.03 | 2.43E-06 | 1.515977559 | NM_000366| | TPM1,tropomyosin 1 (alpha) |
| 215342_s_at | 22.02 | 2.44E-06 | 1.873546677 | NM_001035230| | NA |
| 201158_at | 22.02 | 2.44E-06 | 1.068106632 | NM_021079| | NMT1,N-myristoyltransferase 1 |
| 226749_at | 22.01 | 2.45E-06 | 1.067487859 | NM_182640| | MRPS9,mitochondrial ribosomal protein S9 |
| 201949_x_at | 22 | 2.45E-06 | 1.088691124 | NM_004930| | CAPZB,F-actin capping protein beta subunit |
| 222158_s_at | 22 | 2.45E-06 | 1.133279201 | NM_016076| | PNAS-4,CGI-146 protein |
| 224671_at | 21.99 | 2.45E-06 | 1.084852439 | NM_145255| | MRPL10,mitochondrial ribosomal protein L10 isoform a |
| 212997_s_at | 21.99 | 2.45E-06 | 1.086289889 | NM_001112707| | NA |
| 224503_s_at | 21.98 | 2.46E-06 | 1.174239369 | NM_017742| | ZCCHC2,zinc finger, CCHC domain containing 2 |
| 241436_at | 21.98 | 2.46E-06 | 2.151580485 | NM_001039| | SCNN1G,sodium channel, nonvoltage-gated 1, gamma |
| 222675_s_at | 21.96 | 2.47E-06 | 1.350740642 | NM_018842| | BAIAP2L1,BAI1-associated protein 2-like 1 |
| 236236_at | 21.96 | 2.47E-06 | 1.174246279 | NA |  |
| 213792_s_at | 21.94 | 2.48E-06 | 1.271761824 | NM_000208| | INSR,insulin receptor |
| 226834_at | 21.94 | 2.49E-06 | 1.750645144 | NA |  |
| 201560_at | 21.93 | 2.49E-06 | 1.156981463 | NM_013943| | CLIC4,chloride intracellular channel 4 |
| 226145_s_at | 21.92 | 2.49E-06 | 3.154619149 | NM_025074| | FRAS1,Fraser syndrome 1 isoform 1 |
| 226549_at | 21.9 | 2.50E-06 | 1.289445683 | NM_001024401| | NA |
| 201057_s_at | 21.9 | 2.51E-06 | 1.130849536 | NM_004487| | GOLGB1,golgi autoantigen, golgin subfamily b, |
| 1559510_at | 21.88 | 2.52E-06 | 1.4967721 | NM_030639| | BHLHB9,basic helix-loop-helix domain containing, class |
| 243221_at | 21.88 | 2.52E-06 | 2.110261824 | NA |  |
| 216295_s_at | 21.88 | 2.52E-06 | 1.044694232 | NM_001076677| | NA |
| 1555971_s_at | 21.85 | 2.54E-06 | 1.18148582 | NM_015176| | FBXO28,F-box protein 28 |
| 204891_s_at | 21.84 | 2.54E-06 | 2.145156532 | NM_001042771| | NA |
| 225816_at | 21.84 | 2.54E-06 | 1.15929477 | NM_024900| | PHF17,Jade1 protein short isoform |
| 210601_at | 21.83 | 2.55E-06 | 1.538566025 | NM_004932| | CDH6,cadherin 6, type 2 preproprotein |
| 56197_at | 21.82 | 2.55E-06 | 1.118985821 | NM_020360| | PLSCR3,phospholipid scramblase 3 |
| 206506_s_at | 21.81 | 2.56E-06 | 1.251745093 | NM_003599| | SUPT3H,suppressor of Ty 3 homolog |
| 227432_s_at | 21.8 | 2.56E-06 | 1.281496708 | NA |  |
| 230100_x_at | 21.79 | 2.56E-06 | 1.756162313 | NM_002576| | PAK1,p21-activated kinase 1 |
| 226051_at | 21.77 | 2.58E-06 | 1.31031697 | NM_080430| | SELM,selenoprotein M precursor |
| 209658_at | 21.75 | 2.59E-06 | 1.060119346 | NM_001078645| | NA |
| 203517_at | 21.75 | 2.59E-06 | 1.139062757 | NM_001006635| | MTX2,metaxin 2 isoform b |
| 224129_s_at | 21.75 | 2.59E-06 | 1.078728212 | NM_032574| | LOC84661,dpy-30-like protein |
| 244261_at | 21.74 | 2.60E-06 | 1.694543657 | NM_170743| | IL28RA,interleukin 28 receptor, alpha isoform 1 |
| 206018_at | 21.72 | 2.61E-06 | 1.793852038 | NM_005249| | FOXG1B,forkhead box G1B |
| 224482_s_at | 21.71 | 2.61E-06 | 1.410261692 | NM_032932| | RAB11FIP4,RAB11 family interacting protein 4 (class II) |
| 226455_at | 21.7 | 2.62E-06 | 1.213907603 | NM_130898| | CREB3L4,cAMP responsive element binding protein 3-like |
| 226567_at | 21.7 | 2.62E-06 | 1.064137421 | NM_001037334| | NA |
| 213506_at | 21.7 | 2.62E-06 | 2.156520861 | NM_005242| | F2RL1,coagulation factor II (thrombin) receptor-like 1 |
| 206117_at | 21.66 | 2.64E-06 | 1.744133973 | NM_000366| | TPM1,tropomyosin 1 (alpha) |
| 1558173_a_at | 21.64 | 2.65E-06 | 1.172531201 | NM_033631| | LUZP1,leucine zipper protein 1 |
| 209477_at | 21.64 | 2.65E-06 | 1.130725424 | NM_000117| | EMD,emerin |
| 1554541_a_at | 21.63 | 2.65E-06 | 1.399958098 | NM_014696| | KIAA0514,KIAA0514 |
| 1558368_s_at | 21.62 | 2.67E-06 | 1.204612697 | NM_198545| | LOC374946,hypothetical gene supported by AK075558; |
| 232282_at | 21.61 | 2.67E-06 | 1.209972719 | NM_001002838| | WNK3,WNK lysine deficient protein kinase 3 isoform 2 |
| 211204_at | 21.61 | 2.67E-06 | 1.923414714 | NM_002395| | ME1,cytosolic malic enzyme 1 |
| 224721_at | 21.59 | 2.68E-06 | 1.050156659 | NM_032168| | FLJ12519,hypothetical protein FLJ12519 |
| 209033_s_at | 21.59 | 2.68E-06 | 1.071158837 | NM_001396| | DYRK1A,dual-specificity tyrosine-(Y)-phosphorylation |
| 45714_at | 21.58 | 2.69E-06 | 1.272611587 | NM_001002017| | HCFC1R1,host cell factor C1 regulator 1 (XPO1 dependant) |
| 223686_at | 21.57 | 2.69E-06 | 1.349703944 | NM_001042482| | NA |
| 1557385_at | 21.53 | 2.73E-06 | 1.094026546 | NM_032180| | NA |
| 239975_at | 21.52 | 2.73E-06 | 2.576320203 | NA |  |
| 209739_s_at | 21.5 | 2.74E-06 | 2.12008135 | NM_004650| | PNPLA4,GS2 gene |
| 223068_at | 21.5 | 2.74E-06 | 1.094198625 | NM_019063| | EML4,echinoderm microtubule associated protein like |
| 202087_s_at | 21.47 | 2.76E-06 | 1.153382768 | NM_001912| | CTSL,cathepsin L preproprotein |
| 209685_s_at | 21.47 | 2.76E-06 | 2.298398738 | NM_002738| | PRKCB1,protein kinase C, beta isoform 2 |
| 210776_x_at | 21.47 | 2.76E-06 | 1.060740374 | NM_003200| | TCF3,transcription factor 3 |
| 228503_at | 21.46 | 2.76E-06 | 1.582374843 | NA |  |
| 204750_s_at | 21.45 | 2.77E-06 | 2.162477992 | NM_004949| | DSC2,desmocollin 2 isoform Dsc2b preproprotein |
| 232382_s_at | 21.43 | 2.78E-06 | 1.278175723 | NM_052937| | LOC115294,similar to hypothetical protein FLJ10883 |
| 205570_at | 21.43 | 2.78E-06 | 1.183698476 | NM_005028| | PIP5K2A,phosphatidylinositol-4-phosphate 5-kinase type |
| 55705_at | 21.4 | 2.80E-06 | 1.073277431 | NM_138774| | C19orf22,chromosome 19 open reading frame 22 |
| 219382_at | 21.39 | 2.80E-06 | 1.306927107 | NM_013368| | SERTAD3,RPA-binding trans-activator |
| 229429_x_at | 21.39 | 2.80E-06 | 1.307821356 | NA |  |
| 213982_s_at | 21.37 | 2.82E-06 | 1.995296958 | NM_001035230| | NA |
| 203179_at | 21.36 | 2.82E-06 | 1.462389674 | NM_000155| | GALT,galactose-1-phosphate uridylyltransferase |
| 233841_s_at | 21.32 | 2.85E-06 | 1.171956878 | NM_022491| | SDS3,hypothetical protein FLJ00052 |
| 202637_s_at | 21.32 | 2.86E-06 | 1.255348968 | NM_000201| | ICAM1,intercellular adhesion molecule 1 precursor |
| 225029_at | 21.32 | 2.86E-06 | 1.140521513 | NA |  |
| 204684_at | 21.31 | 2.86E-06 | 1.974593077 | NM_002522| | NPTX1,neuronal pentraxin I precursor |
| 222896_at | 21.29 | 2.88E-06 | 1.265423592 | NM_024074| | TMEM38A,transmembrane protein 38A |
| 201285_at | 21.28 | 2.88E-06 | 1.124049736 | NM_013446| | MKRN1,makorin, ring finger protein, 1 |
| 236501_at | 21.27 | 2.88E-06 | 2.173065055 | NM_020436| | SALL4,sal-like 4 |
| 200860_s_at | 21.26 | 2.88E-06 | 1.071404134 | NM_016284| | CNOT1,CCR4-NOT transcription complex, subunit 1 |
| 204184_s_at | 21.24 | 2.89E-06 | 1.419564793 | NM_005160| | ADRBK2,beta adrenergic receptor kinase 2 |
| 200986_at | 21.22 | 2.90E-06 | 1.69901164 | NM_000062| | SERPING1,complement component 1 inhibitor precursor |
| 1569287_at | 21.21 | 2.91E-06 | 1.201627467 | NA |  |
| 242517_at | 21.21 | 2.91E-06 | 2.126012753 | NM_032551| | GPR54,G protein-coupled receptor 54 |
| 231122_x_at | 21.21 | 2.91E-06 | 1.261329961 | NM_001039617| | NA |
| 201263_at | 21.2 | 2.92E-06 | 1.140935539 | NM_152295| | TARS,threonyl-tRNA synthetase |
| 1564083_at | 21.2 | 2.92E-06 | 2.418501176 | NA |  |
| 230698_at | 21.18 | 2.92E-06 | 1.971205187 | NM_001017440| | NA |
| 233297_s_at | 21.18 | 2.92E-06 | 1.746885348 | NA |  |
| 226568_at | 21.18 | 2.92E-06 | 1.253754169 | NM_001010883| | LOC284611,hypothetical protein LOC284611 |
| 218499_at | 21.17 | 2.93E-06 | 1.280359406 | NM_001042452| | NA |
| 236030_at | 21.16 | 2.94E-06 | 1.169875284 | NM_173587| | RCOR2,REST corepressor 2 |
| 217883_at | 21.15 | 2.95E-06 | 1.045062311 | NM_015702| | C2orf25,chromosome 2 open reading frame 25 |
| 212959_s_at | 21.15 | 2.95E-06 | 1.365568747 | NM_024312| | MGC4170,MGC4170 protein |
| 207066_at | 21.14 | 2.95E-06 | 1.231140048 | NM_002152| | HRC,histidine-rich calcium-binding protein |
| 221666_s_at | 21.14 | 2.95E-06 | 2.080526423 | NM_013258| | PYCARD,PYD and CARD domain containing isoform a |
| 239947_at | 21.14 | 2.95E-06 | 1.175702806 | NA |  |
| 238066_at | 21.13 | 2.96E-06 | 1.619449545 | NM_052960| | RBP7,retinol binding protein 7, cellular |
| 208886_at | 21.12 | 2.97E-06 | 1.426149748 | NM_005318| | H1F0,H1 histone family, member 0 |
| 203988_s_at | 21.11 | 2.97E-06 | 1.101441484 | NM_004480| | FUT8,fucosyltransferase 8 isoform b |
| 223642_at | 21.1 | 2.98E-06 | 3.609199386 | NM_007129| | ZIC2,zinc finger protein of the cerebellum 2 |
| 227386_s_at | 21.09 | 2.98E-06 | 1.524493297 | NM_001003682| | DKFZp434C184,cDNA DKFZp434C184 gene |
| 243745_at | 21.07 | 3.00E-06 | 1.310808308 | NA |  |
| 231772_x_at | 21.05 | 3.02E-06 | 1.114206469 | NM_022909| | CENPH,centromere protein H |
| 218848_at | 21.04 | 3.02E-06 | 1.126045376 | NM_024339| | MGC2655,hypothetical protein MGC2655 |
| 241897_at | 21.04 | 3.03E-06 | 1.382827424 | NA |  |
| 214339_s_at | 21.03 | 3.03E-06 | 1.693781155 | NM_001042600| | NA |
| 236901_at | 21.03 | 3.03E-06 | 1.599192301 | NA |  |
| 201603_at | 21.01 | 3.04E-06 | 1.199279068 | NM_002480| | PPP1R12A,protein phosphatase 1, regulatory (inhibitor) |
| 218559_s_at | 20.97 | 3.07E-06 | 2.015916903 | NM_005461| | MAFB,transcription factor MAFB |
| 64486_at | 20.97 | 3.07E-06 | 1.122980474 | NM_001018070| | NA |
| 227732_at | 20.97 | 3.07E-06 | 1.288829195 | NM_020725| | NA |
| 201069_at | 20.95 | 3.08E-06 | 1.205396072 | NM_004530| | MMP2,matrix metalloproteinase 2 preproprotein |
| 201522_x_at | 20.93 | 3.10E-06 | 1.189584979 | NM_003097| | SNRPN,small nuclear ribonucleoprotein polypeptide N |
| 200868_s_at | 20.92 | 3.11E-06 | 1.139556216 | NM_018683| | ZNF313,zinc finger protein 313 |
| 225822_at | 20.92 | 3.11E-06 | 1.773284475 | NM_144626| | MGC17299,hypothetical protein MGC17299 |
| 223376_s_at | 20.91 | 3.12E-06 | 1.115306464 | NM_015379| | BRI3,brain protein I3 |
| 200704_at | 20.9 | 3.12E-06 | 1.230577882 | NM_004862| | LITAF,LPS-induced TNF-alpha factor |
| 210495_x_at | 20.89 | 3.13E-06 | 1.606301552 | NM_002026| | FN1,fibronectin 1 isoform 3 preproprotein |
| 211240_x_at | 20.87 | 3.14E-06 | 1.150594569 | NM_001085458| | NA |
| 218671_s_at | 20.87 | 3.14E-06 | 1.098810866 | NM_016311| | ATPIF1,ATPase inhibitory factor 1 isoform 1 precursor |
| 219410_at | 20.86 | 3.15E-06 | 2.624006737 | NM_018004| | TMEM45A,transmembrane protein 45A |
| 228855_at | 20.86 | 3.15E-06 | 1.380178366 | NM_001105663| | NA |
| 229724_at | 20.84 | 3.16E-06 | 2.25238482 | NM_000814| | GABRB3,gamma-aminobutyric acid (GABA) A receptor, beta |
| 225030_at | 20.83 | 3.17E-06 | 1.05319109 | NM_138369| | FAM44B,family with sequence similarity 44, member B |
| 203905_at | 20.81 | 3.18E-06 | 1.083278485 | NM_002582| | PARN,poly(A)-specific ribonuclease (deadenylation |
| 206424_at | 20.8 | 3.18E-06 | 2.707835995 | NM_000783| | CYP26A1,cytochrome P450, family 26, subfamily A, |
| 229064_s_at | 20.78 | 3.20E-06 | 1.274928043 | NM_013441| | DSCR1L2,Down syndrome critical region gene 1-like 2 |
| 231896_s_at | 20.78 | 3.20E-06 | 1.039715962 | NM_003677| | DENR,density-regulated protein |
| 211203_s_at | 20.77 | 3.21E-06 | 1.432951672 | NM_001843| | CNTN1,contactin 1 isoform 1 precursor |
| 239697_x_at | 20.74 | 3.23E-06 | 1.69697478 | NM_198463| | FLJ42117,FLJ42117 protein |
| 202442_at | 20.73 | 3.25E-06 | 1.040514075 | NM_001284| | AP3S1,adaptor-related protein complex 3, sigma 1 |
| 204944_at | 20.73 | 3.24E-06 | 1.147074399 | NM_002841| | PTPRG,protein tyrosine phosphatase, receptor type, G |
| 202178_at | 20.7 | 3.27E-06 | 1.186785968 | NM_001033581| | NA |
| 225775_at | 20.7 | 3.27E-06 | 1.166228091 | NM_178562| | MGC50844,hypothetical protein MGC50844 |
| 224678_at | 20.69 | 3.28E-06 | 1.120488198 | NM_020336| | KIAA1219,KIAA1219 protein |
| 228778_at | 20.68 | 3.28E-06 | 1.134812496 | NA |  |
| 244050_at | 20.67 | 3.29E-06 | 1.73484697 | NM_001010915| | LOC401494,similar to RIKEN 4933428I03 |
| 203127_s_at | 20.66 | 3.30E-06 | 1.220381384 | NM_004863| | SPTLC2,serine palmitoyltransferase, long chain base |
| 220952_s_at | 20.65 | 3.30E-06 | 1.381887928 | NM_019012| | PLEKHA5,pleckstrin homology domain containing, family A |
| 203414_at | 20.64 | 3.31E-06 | 1.128165608 | NM_012329| | MMD,monocyte to macrophage |
| 206789_s_at | 20.63 | 3.32E-06 | 1.256445632 | NM_002697| | POU2F1,POU domain, class 2, transcription factor 1 |
| 223514_at | 20.63 | 3.32E-06 | 1.253091733 | NM_032415| | CARD11,caspase recruitment domain family, member 11 |
| 236826_at | 20.62 | 3.33E-06 | 1.364173788 | NM_152574| | C9orf52,hypothetical protein FLJ33868 |
| 222603_at | 20.61 | 3.33E-06 | 1.217665621 | NM_024896| | KIAA1815,KIAA1815 |
| 212769_at | 20.61 | 3.33E-06 | 1.323191563 | NM_001105192| | NA |
| 224369_s_at | 20.61 | 3.33E-06 | 1.078476154 | NM_030793| | FBXO38,F-box protein 38 isoform a |
| 225646_at | 20.6 | 3.34E-06 | 1.314527032 | NM_001114173| | NA |
| 222895_s_at | 20.6 | 3.34E-06 | 2.032441462 | NM_022898| | BCL11B,B-cell CLL/lymphoma 11B isoform 2 |
| 205542_at | 20.59 | 3.34E-06 | 1.731468696 | NM_012449| | STEAP,six transmembrane epithelial antigen of the |
| 226249_at | 20.59 | 3.34E-06 | 1.142632201 | NM_001012994| | NA |
| 223361_at | 20.59 | 3.34E-06 | 1.22289599 | NM_021243| | NA |
| 200778_s_at | 20.58 | 3.35E-06 | 1.11611592 | NM_001008491| | SEPT2,septin 2 |
| 223631_s_at | 20.57 | 3.35E-06 | 2.30221685 | NM_033520| | C19orf33,chromosome 19 open reading frame 33 |
| 230896_at | 20.57 | 3.35E-06 | 2.465104534 | NM_207406| | FLJ43965,FLJ43965 protein |
| 1555962_at | 20.56 | 3.36E-06 | 2.267887117 | NM_145236| | B3GNT7,UDP-GlcNAc:betaGal |
| 1554340_a_at | 20.56 | 3.36E-06 | 1.489289648 | NM_198545| | LOC374946,hypothetical gene supported by AK075558; |
| 204740_at | 20.56 | 3.36E-06 | 1.255552371 | NM_006314| | CNKSR1,connector enhancer of kinase suppressor of Ras |
| 207571_x_at | 20.56 | 3.36E-06 | 1.467565783 | NM_001039477| | NA |
| 206062_at | 20.56 | 3.36E-06 | 2.122025353 | NM_000409| | GUCA1A,guanylate cyclase activator 1A (retina) |
| 232306_at | 20.56 | 3.36E-06 | 1.435578522 | NM_021810| | CDH26,cadherin-like 26 isoform b |
| 204751_x_at | 20.55 | 3.36E-06 | 2.392834069 | NM_004949| | DSC2,desmocollin 2 isoform Dsc2b preproprotein |
| 212314_at | 20.55 | 3.36E-06 | 1.226178993 | NM_015187| | KIAA0746,KIAA0746 protein |
| 228320_x_at | 20.52 | 3.40E-06 | 1.350406335 | NM_207311| | LOC92558,hypothetical protein LOC92558 |
| 202481_at | 20.52 | 3.39E-06 | 1.869318907 | NM_004753| | DHRS3,dehydrogenase/reductase (SDR family) member 3 |
| 211528_x_at | 20.52 | 3.40E-06 | 1.103549481 | NM_002127| | HLA-G,major histocompatibility complex, class I, G |
| 223358_s_at | 20.51 | 3.40E-06 | 1.168399867 | NA |  |
| 232636_at | 20.5 | 3.41E-06 | 2.028459382 | NM_173078| | SLITRK4,slit and trk like 4 protein |
| 202763_at | 20.49 | 3.42E-06 | 1.114944898 | NM_004346| | CASP3,caspase 3 preproprotein |
| 240189_at | 20.48 | 3.43E-06 | 1.704927846 | NA |  |
| 201842_s_at | 20.48 | 3.42E-06 | 1.735721058 | NM_001039348| | NA |
| 221778_at | 20.47 | 3.43E-06 | 1.237928321 | NM_030647| | NA |
| 210381_s_at | 20.44 | 3.46E-06 | 1.458744723 | NM_176875| | CCKBR,cholecystokinin B receptor |
| 226597_at | 20.44 | 3.46E-06 | 1.305213733 | NM_138393| | C19orf32,polyposis locus protein 1-like 1 |
| 222903_s_at | 20.43 | 3.47E-06 | 1.353197644 | NM_001079533| | NA |
| 232111_at | 20.42 | 3.47E-06 | 2.161233523 | NA |  |
| 232116_at | 20.42 | 3.47E-06 | 1.39941607 | NM_021180| | TFCP2L4,sister-of-mammalian grainyhead protein isoform |
| 220108_at | 20.39 | 3.50E-06 | 2.041923554 | NM_004297| | GNA14,guanine nucleotide binding protein (G protein), |
| 242565_x_at | 20.38 | 3.51E-06 | 1.199185991 | NM_001006114| | C21orf57,chromosome 21 open reading frame 57 isoform 2 |
| 206695_x_at | 20.38 | 3.51E-06 | 1.209474753 | NM_003423| | ZNF43,zinc finger protein 43 (HTF6) |
| 212148_at | 20.36 | 3.52E-06 | 1.393827423 | NM_002585| | PBX1,pre-B-cell leukemia transcription factor 1 |
| 233142_at | 20.34 | 3.54E-06 | 1.222516051 | NA |  |
| 226994_at | 20.34 | 3.55E-06 | 1.176237981 | NM_005880| | DNAJA2,DnaJ subfamily A member 2 |
| 1553138_a_at | 20.33 | 3.55E-06 | 1.681790865 | NM_152363| | FLJ39369,hypothetical protein FLJ39369 |
| 223427_s_at | 20.31 | 3.57E-06 | 1.654588049 | NM_018424| | EPB41L4B,erythrocyte membrane protein band 4.1 like 4B |
| 205328_at | 20.31 | 3.58E-06 | 2.190604771 | NM_006984| | CLDN10,claudin 10 isoform b |
| 209306_s_at | 20.31 | 3.58E-06 | 1.195920644 | NM_015055| | SWAP70,SWAP-70 protein |
| 212584_at | 20.31 | 3.58E-06 | 1.112599744 | NM_014691| | AQR,aquarius |
| 205547_s_at | 20.3 | 3.58E-06 | 2.062416029 | NM_001001522| | TAGLN,transgelin |
| 227425_at | 20.28 | 3.59E-06 | 1.894586747 | NM_001080975| | NA |
| 214843_s_at | 20.28 | 3.59E-06 | 1.121217443 | NM_015017| | USP33,ubiquitin specific protease 33 isoform 1 |
| 242463_x_at | 20.26 | 3.61E-06 | 1.572376269 | NM_198457| | ZNF600,zinc finger protein 600 |
| 218781_at | 20.26 | 3.61E-06 | 1.147905172 | NM_024624| | SMC6L1,SMC6 protein |
| 229396_at | 20.25 | 3.62E-06 | 1.34696069 | NM_004561| | OVOL1,OVO-like 1 binding protein |
| 202893_at | 20.23 | 3.63E-06 | 1.102067231 | NM_006377| | UNC13B,UNC13 (C. elegans)-like |
| 223305_at | 20.21 | 3.65E-06 | 1.195729585 | NM_016499| | MGC13379,HSPC244 |
| 202894_at | 20.21 | 3.66E-06 | 1.119494062 | NM_004444| | EPHB4,ephrin receptor EphB4 precursor |
| 231018_at | 20.21 | 3.65E-06 | 1.585095952 | NA |  |
| 218694_at | 20.21 | 3.65E-06 | 1.279826815 | NM_016608| | ARMCX1,armadillo repeat containing, X-linked 1 |
| 217794_at | 20.19 | 3.67E-06 | 1.112672411 | NM_001005354| | DKFZp564J157,DKFZp564J157 protein isoform 2 |
| 203934_at | 20.19 | 3.67E-06 | 1.906703947 | NM_002253| | KDR,kinase insert domain receptor (a type III |
| 224336_s_at | 20.17 | 3.69E-06 | 1.462710841 | NM_030640| | DUSP16,dual specificity phosphatase 16 |
| 209282_at | 20.14 | 3.72E-06 | 1.160682544 | NM_001079880| | NA |
| 227496_at | 20.14 | 3.71E-06 | 1.670127826 | NM_001489| | NR6A1,nuclear receptor subfamily 6, group A, member 1 |
| 219316_s_at | 20.14 | 3.71E-06 | 1.347855248 | NM_017791| | C14orf58,chromosome 14 open reading frame 58 |
| 225183_at | 20.13 | 3.72E-06 | 1.132719478 | NM_014117| | PRO0149,PRO0149 protein |
| 220297_at | 20.13 | 3.72E-06 | 1.289501795 | NM_001002860| | BTBD7,BTB (POZ) domain containing 7 isoform 1 |
| 242629_at | 20.12 | 3.73E-06 | 1.714841216 | NA |  |
| 209822_s_at | 20.1 | 3.75E-06 | 1.863109232 | NM_001018056| | NA |
| 217925_s_at | 20.08 | 3.77E-06 | 1.183581461 | NM_022758| | C6orf106,chromosome 6 open reading frame 106 isoform b |
| 210028_s_at | 20.08 | 3.77E-06 | 1.095757055 | NM_012381| | ORC3L,origin recognition complex, subunit 3 isoform 2 |
| 201950_x_at | 20.08 | 3.77E-06 | 1.132513535 | NM_004930| | CAPZB,F-actin capping protein beta subunit |
| 243174_at | 20.06 | 3.79E-06 | 1.370094831 | NA |  |
| 217750_s_at | 20.06 | 3.79E-06 | 1.108642675 | NM_023079| | FLJ13855,hypothetical protein FLJ13855 |
| 226809_at | 20.06 | 3.79E-06 | 1.462600747 | NA |  |
| 209193_at | 20.05 | 3.80E-06 | 1.468928624 | NM_002648| | PIM1,pim-1 oncogene |
| 221016_s_at | 20.05 | 3.80E-06 | 1.341177665 | NM_031283| | TCF7L1,HMG-box transcription factor TCF-3 |
| 202822_at | 20.05 | 3.80E-06 | 1.145998236 | NM_005578| | LPP,LIM domain containing preferred translocation |
| 216985_s_at | 20.04 | 3.80E-06 | 2.062334606 | NM_004177| | STX3A,syntaxin 3A |
| 225639_at | 20.04 | 3.80E-06 | 1.557527358 | NM_003930| | SCAP2,src family associated phosphoprotein 2 |
| 219143_s_at | 20.02 | 3.82E-06 | 1.222483551 | NM_017793| | RPP25,ribonuclease P 25kDa subunit |
| 227290_at | 20.01 | 3.83E-06 | 1.426343232 | NA |  |
| 206204_at | 19.96 | 3.88E-06 | 1.29216963 | NM_004490| | GRB14,growth factor receptor-bound protein 14 |
| 218574_s_at | 19.95 | 3.89E-06 | 1.417844333 | NM_014583| | LMCD1,LIM and cysteine-rich domains 1 |
| 212350_at | 19.95 | 3.89E-06 | 1.131328977 | NM_015173| | TBC1D1,TBC1 (tre-2/USP6, BUB2, cdc16) domain family, |
| 208680_at | 19.94 | 3.91E-06 | 1.034471416 | NM_002574| | PRDX1,peroxiredoxin 1 |
| 209726_at | 19.94 | 3.91E-06 | 1.472469461 | NM_001217| | CA11,carbonic anhydrase XI precursor |
| 205268_s_at | 19.93 | 3.91E-06 | 1.426569369 | NM_001617| | ADD2,adducin 2 isoform a |
| 242919_at | 19.92 | 3.92E-06 | 1.349559992 | NM_021047| | ZNF253,DNA-binding protein |
| 213400_s_at | 19.92 | 3.92E-06 | 1.125953043 | NM_005647| | TBL1X,transducin beta-like 1X |
| 223681_s_at | 19.91 | 3.93E-06 | 1.798741639 | NM_176877| | INADL,InaD-like protein isoform 2 |
| 235358_at | 19.88 | 3.97E-06 | 1.348508622 | NA |  |
| 225944_at | 19.88 | 3.97E-06 | 1.093707604 | NM_020726| | NLN,neurolysin |
| 227348_at | 19.86 | 3.98E-06 | 1.166119082 | NM_152268| | DKFZp727A071,similar to tRNA synthetase class II |
| 223794_at | 19.85 | 4.00E-06 | 1.24382382 | NM_018076| | ARMC4,armadillo repeat containing 4 |
| 201315_x_at | 19.84 | 4.00E-06 | 1.312095651 | NM_006435| | IFITM2,interferon induced transmembrane protein 2 |
| 235947_at | 19.84 | 4.00E-06 | 1.721537229 | NA |  |
| 218047_at | 19.84 | 4.00E-06 | 1.113597257 | NM_024586| | OSBPL9,oxysterol-binding protein-like protein 9 isoform |
| 229095_s_at | 19.84 | 4.00E-06 | 1.760796655 | NA |  |
| 223556_at | 19.84 | 4.00E-06 | 1.181883328 | NM_018063| | HELLS,helicase, lymphoid-specific |
| 212461_at | 19.84 | 4.00E-06 | 1.086745759 | NM_015878| | OAZIN,ornithine decarboxylase antizyme inhibitor |
| 235830_at | 19.83 | 4.00E-06 | 1.537164399 | NA |  |
| 1569906_s_at | 19.82 | 4.01E-06 | 1.171172745 | NM_016436| | PHF20,PHD finger protein 20 |
| 224702_at | 19.81 | 4.03E-06 | 1.144556708 | NM_174909| | MGC23909,hypothetical protein MGC23909 |
| 229484_at | 19.8 | 4.04E-06 | 1.413309821 | NM_005167| | PPP2CZ,protein phosphatase 2a, catalytic subunit, zeta |
| 213992_at | 19.8 | 4.04E-06 | 1.269915385 | NM_001847| | COL4A6,type IV alpha 6 collagen isoform A precursor |
| 226415_at | 19.79 | 4.04E-06 | 3.044532447 | NM_020927| | KIAA1576,KIAA1576 protein |
| 225974_at | 19.79 | 4.04E-06 | 1.318409904 | NM_001008495| | DKFZp762C1112,hypothetical protein DKFZp762C1112 |
| 212012_at | 19.78 | 4.06E-06 | 1.099830278 | NM_012293| | NA |
| 213546_at | 19.77 | 4.07E-06 | 1.207104443 | NA |  |
| 204579_at | 19.75 | 4.08E-06 | 1.541009878 | NM_002011| | FGFR4,fibroblast growth factor receptor 4 isoform 1 |
| 213556_at | 19.75 | 4.08E-06 | 1.673887001 | NA |  |
| 243332_at | 19.75 | 4.08E-06 | 1.453898964 | NA |  |
| 219704_at | 19.75 | 4.08E-06 | 1.333939392 | NM_015982| | YBX2,germ cell specific Y-box binding protein |
| 203097_s_at | 19.75 | 4.08E-06 | 1.103778986 | NM_014247| | NA |
| 212681_at | 19.74 | 4.09E-06 | 2.0416124 | NM_012307| | EPB41L3,erythrocyte membrane protein band 4.1-like 3 |
| 205577_at | 19.72 | 4.11E-06 | 1.494782097 | NM_005609| | PYGM,glycogen phosphorylase |
| 220615_s_at | 19.72 | 4.11E-06 | 1.39636045 | NM_018099| | MLSTD1,male sterility domain containing 1 |
| 209421_at | 19.7 | 4.13E-06 | 1.116177742 | NM_000251| | MSH2,mutS homolog 2 |
| 226682_at | 19.7 | 4.13E-06 | 1.437867977 | NA |  |
| 212564_at | 19.7 | 4.13E-06 | 1.150829489 | NM_015353| | KCTD2,potassium channel tetramerisation domain |
| 203881_s_at | 19.66 | 4.17E-06 | 1.856001963 | NM_000109| | DMD,dystrophin Dp427c isoform |
| 205532_s_at | 19.66 | 4.17E-06 | 1.810008339 | NM_004932| | CDH6,cadherin 6, type 2 preproprotein |
| 212660_at | 19.66 | 4.17E-06 | 1.190657248 | NM_015288| | PHF15,PHD finger protein 15 |
| 207749_s_at | 19.64 | 4.19E-06 | 1.277083332 | NM_002718| | PPP2R3A,alpha isoform of regulatory subunit B'', protein |
| 53720_at | 19.64 | 4.19E-06 | 1.26500086 | NM_018381| | FLJ11286,hypothetical protein FLJ11286 |
| 212082_s_at | 19.63 | 4.20E-06 | 1.06402675 | NM_002475| | MLC1SA,myosin alkali light chain 1 slow a |
| 203718_at | 19.6 | 4.24E-06 | 1.134484219 | NM_006702| | NTE,neuropathy target esterase |
| 213413_at | 19.59 | 4.24E-06 | 1.258833219 | NM_006873| | SBLF,stoned B-like factor |
| 203325_s_at | 19.57 | 4.27E-06 | 1.43014062 | NM_000093| | COL5A1,alpha 1 type V collagen preproprotein |
| 211719_x_at | 19.55 | 4.29E-06 | 1.630681036 | NM_002026| | FN1,fibronectin 1 isoform 3 preproprotein |
| 212481_s_at | 19.54 | 4.30E-06 | 1.156073502 | NM_003290| | TPM4,tropomyosin 4 |
| 204164_at | 19.52 | 4.32E-06 | 1.211247607 | NM_006747| | SIPA1,signal-induced proliferation-associated protein |
| 212943_at | 19.5 | 4.34E-06 | 1.064897878 | NM_014802| | KIAA0528,KIAA0528 gene product |
| 218865_at | 19.5 | 4.34E-06 | 1.110917039 | NM_022746| | FLJ22390,hypothetical protein FLJ22390 |
| 209984_at | 19.49 | 4.35E-06 | 1.138221673 | NM_015061| | JMJD2C,jumonji domain containing 2C |
| 223206_s_at | 19.49 | 4.35E-06 | 1.179447515 | NM_020677| | HSCARG,HSCARG protein |
| 226312_at | 19.49 | 4.35E-06 | 1.152643471 | NM_152756| | AVO3,rapamycin-insensitive companion of mTOR |
| 225739_at | 19.48 | 4.35E-06 | 1.428320498 | NM_032932| | RAB11FIP4,RAB11 family interacting protein 4 (class II) |
| 38269_at | 19.47 | 4.37E-06 | 1.128422169 | NM_001079880| | NA |
| 212647_at | 19.47 | 4.37E-06 | 1.224083566 | NM_006270| | RRAS,related RAS viral (r-ras) oncogene homolog |
| 240301_at | 19.45 | 4.39E-06 | 2.197108977 | NM_138815| | DPPA2,developmental pluripotency associated 2 |
| 216945_x_at | 19.45 | 4.39E-06 | 1.088739629 | NM_015148| | PASK,PAS domain containing serine/threonine kinase |
| 223582_at | 19.44 | 4.40E-06 | 1.107052333 | NM_032119| | MASS1,very large G-protein coupled receptor 1 |
| 224812_at | 19.44 | 4.40E-06 | 1.091705712 | NM_152740| | HIBADH,3-hydroxyisobutyrate dehydrogenase |
| 1558212_at | 19.43 | 4.41E-06 | 1.787738831 | NA |  |
| 1558097_at | 19.42 | 4.42E-06 | 1.130530388 | NM_173566| | MGC50372,hypothetical protein MGC50372 |
| 218910_at | 19.42 | 4.41E-06 | 1.258816586 | NM_018075| | FLJ10375,hypothetical protein FLJ10375 |
| 220022_at | 19.42 | 4.42E-06 | 1.493915656 | NM_018102| | ZNF334,zinc finger protein 334 isoform a |
| 204092_s_at | 19.41 | 4.42E-06 | 1.04325822 | NM_003600| | STK6,serine/threonine protein kinase 6 |
| 223580_at | 19.4 | 4.43E-06 | 1.464374797 | NM_032641| | GRCC9,SPRY domain-containing SOCS box protein SSB-2 |
| 232202_at | 19.38 | 4.46E-06 | 1.690924001 | NA |  |
| 202512_s_at | 19.37 | 4.48E-06 | 1.210678387 | NM_004849| | APG5L,APG5 autophagy 5-like |
| 204425_at | 19.37 | 4.48E-06 | 1.280013409 | NM_001666| | ARHGAP4,Rho GTPase activating protein 4 |
| 204712_at | 19.35 | 4.49E-06 | 2.24293524 | NM_007191| | WIF1,Wnt inhibitory factor-1 precursor |
| 204446_s_at | 19.35 | 4.50E-06 | 2.209954665 | NM_000698| | ALOX5,arachidonate 5-lipoxygenase |
| 228008_at | 19.35 | 4.49E-06 | 1.98361149 | NA |  |
| 227645_at | 19.34 | 4.51E-06 | 1.383183394 | NM_014308| | PIK3R5,phosphoinositide-3-kinase, regulatory subunit 5, |
| 209082_s_at | 19.33 | 4.52E-06 | 1.350348589 | NM_030582| | COL18A1,alpha 1 type XVIII collagen isoform 1 precursor |
| 204043_at | 19.33 | 4.52E-06 | 1.28885192 | NM_000355| | TCN2,transcobalamin II precursor |
| 209570_s_at | 19.33 | 4.52E-06 | 1.686602996 | NM_001040101| | NA |
| 235184_at | 19.33 | 4.52E-06 | 1.402412793 | NM_001114176| | NA |
| 1553528_a_at | 19.32 | 4.52E-06 | 1.095197857 | NM_006951| | TAF5,TBP-associated factor 5 |
| 218552_at | 19.32 | 4.53E-06 | 1.501413943 | NM_018281| | ECHDC2,enoyl Coenzyme A hydratase domain containing 2 |
| 91826_at | 19.31 | 4.53E-06 | 1.453725494 | NM_017729| | EPS8L1,epidermal growth factor receptor pathway |
| 204568_at | 19.29 | 4.56E-06 | 1.213991337 | NM_014924| | KIAA0831,KIAA0831 |
| 215758_x_at | 19.27 | 4.58E-06 | 1.300435492 | NM_031218| | ZNF505,zinc finger protein 505 isoform a |
| 231385_at | 19.26 | 4.59E-06 | 2.657975281 | NM_199286| | DPPA3,stella |
| 201348_at | 19.26 | 4.59E-06 | 1.251685739 | NM_002084| | GPX3,plasma glutathione peroxidase 3 precursor |
| 229432_at | 19.25 | 4.61E-06 | 1.400050926 | NM_153006| | NAGS,N-acetylglutamate synthase |
| 237465_at | 19.25 | 4.61E-06 | 1.594453393 | NM_019050| | USP53,ubiquitin specific protease 53 |
| 223424_s_at | 19.24 | 4.62E-06 | 1.132525277 | NM_145914| | ZNF38,zinc finger protein 38 |
| 217678_at | 19.23 | 4.63E-06 | 1.438133915 | NM_014331| | SLC7A11,solute carrier family 7, (cationic amino acid |
| 208785_s_at | 19.23 | 4.63E-06 | 1.110763418 | NM_022818| | MAP1LC3B,microtubule-associated proteins 1A/1B light |
| 208737_at | 19.22 | 4.64E-06 | 1.070112584 | NM_004888| | ATP6V1G1,ATPase, H+ transporting, lysosomal, V1 subunit G |
| 203126_at | 19.22 | 4.64E-06 | 1.186653817 | NM_014214| | IMPA2,inositol(myo)-1(or 4)-monophosphatase 2 |
| 219061_s_at | 19.2 | 4.67E-06 | 1.126999165 | NM_006014| | DXS9879E,ESO3 protein |
| 227429_at | 19.2 | 4.67E-06 | 1.447673615 | NM_173584| | MGC45840,hypothetical protein MGC45840 |
| 212372_at | 19.19 | 4.67E-06 | 1.057335835 | NM_005964| | MYH10,myosin, heavy polypeptide 10, non-muscle |
| 223522_at | 19.19 | 4.68E-06 | 1.175839544 | NA |  |
| 203966_s_at | 19.18 | 4.69E-06 | 1.082592441 | NM_021003| | PPM1A,protein phosphatase 1A isoform 1 |
| 201417_at | 19.17 | 4.69E-06 | 1.082833911 | NM_003107| | SOX4,SRY (sex determining region Y)-box 4 |
| 222479_s_at | 19.17 | 4.69E-06 | 1.079002107 | NM_016141| | DNCLI1,dynein light chain-A |
| 213927_at | 19.14 | 4.74E-06 | 1.844527662 | NM_033141| | MAP3K9,mitogen-activated protein kinase kinase kinase |
| 214442_s_at | 19.13 | 4.75E-06 | 1.1536855 | NM_004671| | PIAS2,protein inhibitor of activated STAT X isoform |
| 226899_at | 19.13 | 4.75E-06 | 1.168749574 | NM_170744| | UNC5B,unc-5 homolog B |
| 211015_s_at | 19.12 | 4.76E-06 | 1.12755962 | NM_002154| | HSPA4,heat shock 70kDa protein 4 isoform a |
| 213171_s_at | 19.11 | 4.77E-06 | 1.405880103 | NM_006690| | MMP24,matrix metalloproteinase 24 (membrane-inserted) |
| 202808_at | 19.09 | 4.80E-06 | 1.099071109 | NM_001083913| | NA |
| 225636_at | 19.08 | 4.81E-06 | 1.141442014 | NM_005419| | STAT2,signal transducer and activator of transcription |
| 225195_at | 19.07 | 4.83E-06 | 1.288746431 | NM_001047434| | NA |
| 201216_at | 19.04 | 4.87E-06 | 1.120820282 | NM_001034025| | NA |
| 210088_x_at | 19.03 | 4.88E-06 | 1.196884423 | NM_001002841| | MYL4,atrial/embryonic alkali myosin light chain |
| 213039_at | 19.02 | 4.88E-06 | 1.07387286 | NM_015318| | ARHGEF18,Rho-specific guanine nucleotide exchange factor |
| 208734_x_at | 19.02 | 4.89E-06 | 1.0595087 | NM_002865| | RAB2,RAB2, member RAS oncogene family |
| 218537_at | 19.02 | 4.88E-06 | 1.290572698 | NM_001002017| | HCFC1R1,host cell factor C1 regulator 1 (XPO1 dependant) |
| 227014_at | 19 | 4.92E-06 | 1.314020431 | NM_020437| | LOC57168,similar to aspartate beta hydroxylase (ASPH) |
| 206924_at | 19 | 4.92E-06 | 1.476352003 | NM_000641| | IL11,interleukin 11 precursor |
| 218258_at | 19 | 4.91E-06 | 1.071566127 | NM_015972| | POLR1D,RNA polymerase I 16 kDa subunit |
| 237275_at | 18.98 | 4.94E-06 | 1.97335187 | NA |  |
| 217741_s_at | 18.98 | 4.94E-06 | 1.142812933 | NM_001102420| | NA |
| 205807_s_at | 18.96 | 4.96E-06 | 1.232947465 | NM_001126337| | NA |
| 35148_at | 18.96 | 4.96E-06 | 1.745456468 | NM_014428| | TJP3,tight junction protein 3 (zona occludens 3) |
| 203204_s_at | 18.96 | 4.96E-06 | 1.153377226 | NM_014663| | JMJD2A,jumonji domain containing 2A |
| 218282_at | 18.96 | 4.96E-06 | 1.223631423 | NM_018217| | C20orf31,chromosome 20 open reading frame 31 |
| 1556047_s_at | 18.96 | 4.96E-06 | 1.301079437 | NM_020932| | MAGEE1,melanoma antigen family E, 1 |
| 203984_s_at | 18.95 | 4.97E-06 | 1.181392384 | NM_001229| | CASP9,caspase 9 isoform alpha preproprotein |
| 214969_at | 18.95 | 4.97E-06 | 1.192310827 | NM_033141| | MAP3K9,mitogen-activated protein kinase kinase kinase |
| 219301_s_at | 18.94 | 4.98E-06 | 1.857563745 | NM_014141| | CNTNAP2,cell recognition molecule Caspr2 precursor |
| 208407_s_at | 18.94 | 4.97E-06 | 1.109510298 | NM_001085458| | NA |
| 228537_at | 18.93 | 4.99E-06 | 1.220746242 | NM_005270| | GLI2,GLI-Kruppel family member GLI2 isoform delta |
| 202313_at | 18.93 | 4.99E-06 | 1.051778338 | NM_002717| | PPP2R2A,alpha isoform of regulatory subunit B55, protein |
| 203628_at | 18.91 | 5.01E-06 | 1.104838839 | NM_000875| | IGF1R,insulin-like growth factor 1 receptor precursor |
| 203639_s_at | 18.91 | 5.01E-06 | 1.468598412 | NM_000141| | FGFR2,fibroblast growth factor receptor 2 isoform 1 |
| 1553778_at | 18.91 | 5.01E-06 | 1.242689068 | NM_152559| | WBSCR27,Williams-Beuren syndrome chromosome region 27 |
| 202214_s_at | 18.91 | 5.01E-06 | 1.088426364 | NM_001079872| | NA |
| 228647_at | 18.9 | 5.03E-06 | 1.492082992 | NA |  |
| 206472_s_at | 18.9 | 5.03E-06 | 1.286250507 | NM_001105192| | NA |
| 207717_s_at | 18.87 | 5.07E-06 | 2.585178822 | NM_001005242| | PKP2,plakophilin 2 isoform 2a |
| 204597_x_at | 18.87 | 5.08E-06 | 1.781745603 | NM_003155| | STC1,stanniocalcin 1 |
| 203715_at | 18.86 | 5.08E-06 | 1.164812113 | NM_001079515| | NA |
| 205704_s_at | 18.85 | 5.11E-06 | 1.116669759 | NM_012463| | ATP6V0A2,ATPase, H+ transporting, lysosomal V0 subunit a |
| 205668_at | 18.84 | 5.11E-06 | 1.893868104 | NM_002349| | LY75,lymphocyte antigen 75 |
| 201604_s_at | 18.84 | 5.12E-06 | 1.224174796 | NM_002480| | PPP1R12A,protein phosphatase 1, regulatory (inhibitor) |
| 1554628_at | 18.83 | 5.13E-06 | 1.180340441 | NM_173480| | LOC126295,hypothetical protein LOC126295 |
| 242890_at | 18.83 | 5.14E-06 | 1.141243676 | NA |  |
| 212699_at | 18.82 | 5.15E-06 | 1.139933048 | NM_138967| | SCAMP5,secretory carrier membrane protein 5 |
| 229661_at | 18.81 | 5.16E-06 | 2.069498609 | NM_020436| | SALL4,sal-like 4 |
| 200863_s_at | 18.81 | 5.16E-06 | 1.105313582 | NM_004663| | RAB11A,Ras-related protein Rab-11A |
| 208869_s_at | 18.79 | 5.19E-06 | 1.722648259 | NM_031412| | GABARAPL1,GABA(A) receptor-associated protein like 1 |
| 229205_at | 18.79 | 5.19E-06 | 1.642722144 | NA |  |
| 209071_s_at | 18.78 | 5.21E-06 | 1.630773154 | NM_003617| | RGS5,regulator of G-protein signalling 5 |
| 227931_at | 18.76 | 5.23E-06 | 1.239839095 | NM_017759| | FLJ20309,hypothetical protein FLJ20309 |
| 206002_at | 18.76 | 5.23E-06 | 2.503982148 | NM_001079858| | NA |
| 230986_at | 18.76 | 5.23E-06 | 1.901101564 | NM_007250| | KLF8,Kruppel-like factor 8 |
| 212737_at | 18.75 | 5.23E-06 | 1.194977292 | NM_000405| | GM2A,GM2 ganglioside activator precursor |
| 209209_s_at | 18.75 | 5.24E-06 | 1.197730401 | NM_006832| | PLEKHC1,pleckstrin homology domain containing, family C |
| 206743_s_at | 18.75 | 5.24E-06 | 1.587941443 | NM_001671| | ASGR1,asialoglycoprotein receptor 1 |
| 227080_at | 18.75 | 5.24E-06 | 1.33280345 | NM_001080470| | NA |
| 210426_x_at | 18.74 | 5.25E-06 | 1.754781992 | NM_002943| | RORA,RAR-related orphan receptor A isoform c |
| 211417_x_at | 18.74 | 5.25E-06 | 1.169102333 | NM_001032364| | NA |
| 215783_s_at | 18.74 | 5.24E-06 | 1.766151948 | NM_000478| | ALPL,tissue non-specific alkaline phosphatase |
| 205068_s_at | 18.74 | 5.24E-06 | 1.636228078 | NM_015071| | ARHGAP26,GTPase regulator associated with the focal |
| 239231_at | 18.73 | 5.27E-06 | 1.304971859 | NA |  |
| 1552643_at | 18.73 | 5.27E-06 | 1.438650946 | NM_001076675| | NA |
| 227971_at | 18.73 | 5.26E-06 | 1.620303601 | NM_198465| | NRK,Nik related kinase |
| AFFX-M27830_M_at | 18.71 | 5.30E-06 | 1.093152115 | NA |  |
| 227826_s_at | 18.7 | 5.31E-06 | 2.536453824 | NA |  |
| 238147_at | 18.7 | 5.31E-06 | 1.219875422 | NM_025058| | TRIM46,tripartite motif-containing 46 |
| 207966_s_at | 18.7 | 5.31E-06 | 1.036846598 | NM_012201| | GLG1,golgi apparatus protein 1 |
| 213664_at | 18.7 | 5.31E-06 | 2.40261733 | NM_004170| | SLC1A1,solute carrier family 1, member 1 |
| 219714_s_at | 18.69 | 5.32E-06 | 1.307729309 | NM_018398| | CACNA2D3,calcium channel, voltage-dependent, alpha |
| 229630_s_at | 18.68 | 5.34E-06 | 1.070088181 | NM_004906| | WTAP,Wilms' tumour 1-associating protein isoform 1 |
| 200912_s_at | 18.68 | 5.33E-06 | 1.081750066 | NM_001967| | EIF4A2,eukaryotic translation initiation factor 4A, |
| 226553_at | 18.67 | 5.36E-06 | 1.310097034 | NM_005656| | TMPRSS2,transmembrane protease, serine 2 |
| 227653_at | 18.66 | 5.37E-06 | 1.087656728 | NM_020810| | KIAA1393,tRNA-(N1G37) methyltransferase |
| 204237_at | 18.66 | 5.36E-06 | 1.342735816 | NM_016315| | GULP1,GULP, engulfment adaptor PTB domain containing |
| 223084_s_at | 18.66 | 5.36E-06 | 1.139479458 | NM_012142| | CCNDBP1,cyclin D-type binding-protein 1 |
| 203098_at | 18.62 | 5.43E-06 | 1.079872751 | NM_004824| | CDYL,chromodomain protein, Y chromosome-like isoform |
| 223786_at | 18.61 | 5.45E-06 | 1.44804513 | NM_021615| | CHST6,carbohydrate (N-acetylglucosamine 6-O) |
| 202670_at | 18.61 | 5.45E-06 | 1.089608305 | NM_002755| | MAP2K1,mitogen-activated protein kinase kinase 1 |
| 202011_at | 18.6 | 5.47E-06 | 1.09312157 | NM_003257| | TJP1,tight junction protein 1 isoform a |
| 1555137_a_at | 18.6 | 5.46E-06 | 1.892865808 | NM_018351| | FGD6,FYVE, RhoGEF and PH domain containing 6 |
| 226863_at | 18.58 | 5.50E-06 | 2.114666926 | NM_001077710| | NA |
| 229427_at | 18.57 | 5.51E-06 | 1.5544195 | NM_003966| | SEMA5A,semaphorin 5A |
| 233252_s_at | 18.57 | 5.51E-06 | 1.194383835 | NM_018387| | STRBP,spermatid perinuclear RNA-binding protein |
| 205751_at | 18.57 | 5.53E-06 | 1.213817676 | NM_003026| | SH3GL2,SH3-domain GRB2-like 2 |
| 1569979_at | 18.56 | 5.54E-06 | 1.061369282 | NM_181786| | HKR1,GLI-Kruppel family member HKR1 |
| 222099_s_at | 18.56 | 5.53E-06 | 1.074740626 | NM_001114093| | NA |
| 238974_at | 18.55 | 5.55E-06 | 1.080939445 | NM_153689| | FLJ38973,hypothetical protein FLJ38973 |
| 223251_s_at | 18.55 | 5.55E-06 | 1.103554481 | NM_017664| | ANKRD10,ankyrin repeat domain 10 |
| 208943_s_at | 18.54 | 5.56E-06 | 1.064242623 | NM_003262| | TLOC1,translocation protein 1 |
| 233208_x_at | 18.54 | 5.56E-06 | 1.08010215 | NM_017437| | CPSF2,cleavage and polyadenylation specific factor 2 |
| 206142_at | 18.54 | 5.56E-06 | 1.549267076 | NM_003436| | ZNF135,zinc finger protein 135 (clone pHZ-17) |
| 217789_at | 18.53 | 5.56E-06 | 1.142553498 | NM_021249| | SNX6,sorting nexin 6 isoform a |
| 210547_x_at | 18.53 | 5.57E-06 | 1.244101739 | NM_004968| | ICA1,islet cell autoantigen 1 isoform 2 |
| 221499_s_at | 18.53 | 5.56E-06 | 1.078695949 | NM_001001433| | STX16,syntaxin 16 isoform a |
| 202352_s_at | 18.53 | 5.56E-06 | 1.082725554 | NM_002816| | PSMD12,proteasome 26S non-ATPase subunit 12 isoform 1 |
| 211530_x_at | 18.52 | 5.59E-06 | 1.074413598 | NM_002127| | HLA-G,major histocompatibility complex, class I, G |
| 35617_at | 18.52 | 5.59E-06 | 1.17990676 | NM_002749| | MAPK7,mitogen-activated protein kinase 7 isoform 1 |
| 205083_at | 18.49 | 5.63E-06 | 1.656839797 | NM_001159| | AOX1,aldehyde oxidase 1 |
| 204290_s_at | 18.45 | 5.70E-06 | 1.175836832 | NM_005589| | ALDH6A1,aldehyde dehydrogenase 6A1 precursor |
| 218091_at | 18.45 | 5.71E-06 | 1.155615483 | NM_004504| | HRB,HIV-1 Rev binding protein |
| 244406_at | 18.44 | 5.72E-06 | 1.194165292 | NM_021143| | ZNF20,zinc finger protein 20 (KOX 13) |
| 266_s_at | 18.44 | 5.72E-06 | 1.248692078 | NM_013230| | CD24,CD24 antigen |
| 1555238_at | 18.43 | 5.74E-06 | 1.123373433 | NM_178449| | TIP39,tuberoinfundibular 39 residue protein precursor |
| 1555751_a_at | 18.43 | 5.74E-06 | 1.110385652 | NM_001007269| | GEMIN7,gemin 7 |
| 204613_at | 18.43 | 5.74E-06 | 1.205544026 | NM_002661| | PLCG2,phospholipase C, gamma 2 |
| 203616_at | 18.4 | 5.78E-06 | 1.109428195 | NM_002690| | POLB,polymerase (DNA directed), beta |
| 201850_at | 18.39 | 5.80E-06 | 1.594223445 | NM_001747| | CAPG,capping protein (actin filament), gelsolin-like |
| 235037_at | 18.39 | 5.80E-06 | 1.097902854 | NM_080652| | TMEM41A,transmembrane protein 41A |
| 207173_x_at | 18.38 | 5.81E-06 | 1.298423566 | NM_001797| | CDH11,cadherin 11, type 2 isoform 1 preproprotein |
| 244353_s_at | 18.34 | 5.88E-06 | 1.277858246 | NM_145176| | SLC2A12,solute carrier family 2 (facilitated glucose |
| 236465_at | 18.34 | 5.88E-06 | 1.188157629 | NM_173662| | RNF175,ring finger protein 175 |
| 239169_at | 18.33 | 5.90E-06 | 1.188001933 | NM_001034836| | NA |
| 225853_at | 18.32 | 5.92E-06 | 1.083656881 | NM_198066| | GNPNAT1,glucosamine-phosphate N-acetyltransferase 1 |
| 203635_at | 18.32 | 5.91E-06 | 1.072378623 | NM_006052| | DSCR3,Down syndrome critical region protein 3 |
| 203091_at | 18.31 | 5.93E-06 | 1.069758372 | NM_003902| | FUBP1,far upstream element-binding protein |
| 221731_x_at | 18.3 | 5.93E-06 | 1.184099516 | NM_001126336| | NA |
| 1570515_a_at | 18.3 | 5.93E-06 | 2.826011371 | NM_015687| | FILIP1,filamin A interacting protein 1 |
| 228771_at | 18.29 | 5.95E-06 | 1.927748075 | NM_005160| | ADRBK2,beta adrenergic receptor kinase 2 |
| 218180_s_at | 18.28 | 5.97E-06 | 1.618122846 | NM_022772| | EPS8L2,epidermal growth factor receptor pathway |
| 217609_at | 18.28 | 5.98E-06 | 1.381490488 | NM_006992| | B7,leucine-rich B7 protein isoform 2 |
| 207559_s_at | 18.27 | 6.00E-06 | 1.123793476 | NM_005096| | ZNF261,zinc finger protein 261 |
| 201204_s_at | 18.27 | 6.00E-06 | 1.136985609 | NM_001042576| | NA |
| 227569_at | 18.27 | 5.99E-06 | 1.24331389 | NM_153371| | LNX2,PDZ domain containing ring finger 1 |
| 200615_s_at | 18.26 | 6.02E-06 | 1.155720777 | NM_001030006| | NA |
| 213661_at | 18.25 | 6.03E-06 | 1.553023549 | NM_001001991| | DKFZP586H2123,regeneration associated muscle protease isoform |
| 223524_s_at | 18.24 | 6.05E-06 | 1.590691773 | NM_023943| | MGC3040,hypothetical protein MGC3040 |
| 222209_s_at | 18.23 | 6.06E-06 | 1.090548444 | NM_022918| | FLJ22104,hypothetical protein FLJ22104 |
| 220822_at | 18.23 | 6.06E-06 | 1.683385107 | NM_017973| | NA |
| 231698_at | 18.23 | 6.06E-06 | 2.289818922 | NA |  |
| 228266_s_at | 18.23 | 6.06E-06 | 1.068111626 | NM_016073| | HDGFRP3,hepatoma-derived growth factor, related protein |
| 203954_x_at | 18.2 | 6.12E-06 | 1.339387404 | NM_001306| | CLDN3,claudin 3 |
| 201845_s_at | 18.19 | 6.14E-06 | 1.211574854 | NM_012234| | RYBP,RING1 and YY1 binding protein |
| 238440_at | 18.18 | 6.16E-06 | 1.165260265 | NM_206808| | CLYBL,citrate lyase beta like |
| 227027_at | 18.17 | 6.16E-06 | 1.365228835 | NA |  |
| 228707_at | 18.16 | 6.19E-06 | 1.848868664 | NM_194284| | CLDN23,claudin 23 |
| 223318_s_at | 18.15 | 6.20E-06 | 1.150539424 | NM_032306| | SPATA11,spermatogenesis associated 11 |
| 218775_s_at | 18.15 | 6.20E-06 | 1.187067837 | NM_024949| | BOMB,BH3-only member B protein |
| 213280_at | 18.15 | 6.20E-06 | 1.935962919 | NM_001100398| | NA |
| 212873_at | 18.15 | 6.20E-06 | 1.557991294 | NM_012292| | HA-1,minor histocompatibility antigen HA-1 |
| 226886_at | 18.15 | 6.20E-06 | 1.329784184 | NA |  |
| 233982_x_at | 18.14 | 6.22E-06 | 1.148677222 | NM_016086| | DUSP24,map kinase phosphatase-like protein MK-STYX |
| 217478_s_at | 18.13 | 6.24E-06 | 1.803828265 | NM_006120| | HLA-DMA,major histocompatibility complex, class II, DM |
| 208474_at | 18.13 | 6.24E-06 | 1.437935907 | NM_021195| | CLDN6,claudin 6 |
| 210822_at | 18.12 | 6.26E-06 | 1.182256542 | NA |  |
| 224445_s_at | 18.12 | 6.26E-06 | 1.123799191 | NM_024071| | ZFYVE21,zinc finger, FYVE domain containing 21 |
| 223217_s_at | 18.1 | 6.30E-06 | 1.225202516 | NM_001005474| | NFKBIZ,nuclear factor of kappa light polypeptide gene |
| 204114_at | 18.09 | 6.31E-06 | 2.121443206 | NM_007361| | NID2,nidogen 2 |
| 218538_s_at | 18.09 | 6.31E-06 | 1.400926199 | NM_020662| | MRS2L,MRS2-like, magnesium homeostasis factor |
| 209992_at | 18.07 | 6.35E-06 | 1.45429789 | NM_001018053| | NA |
| 209921_at | 18.04 | 6.40E-06 | 1.464683312 | NM_014331| | SLC7A11,solute carrier family 7, (cationic amino acid |
| 208093_s_at | 18.01 | 6.46E-06 | 1.089273037 | NM_001025579| | NA |
| 208712_at | 18 | 6.49E-06 | 1.073961103 | NM_053056| | CCND1,cyclin D1 |
| 222870_s_at | 17.99 | 6.50E-06 | 1.561721873 | NM_006577| | B3GNT1,beta-1,3-N-acetylglucosaminyltransferase bGnT-1 |
| 219988_s_at | 17.99 | 6.49E-06 | 1.078278814 | NM_018150| | FLJ10597,hypothetical protein FLJ10597 |
| 1553858_at | 17.99 | 6.50E-06 | 1.247763675 | NM_024784| | ZBTB3,zinc finger and BTB domain containing 3 |
| 214493_s_at | 17.99 | 6.50E-06 | 1.736434828 | NM_176877| | INADL,InaD-like protein isoform 2 |
| 225175_s_at | 17.98 | 6.52E-06 | 1.194383358 | NM_020428| | CTL2,CTL2 gene |
| 206712_at | 17.98 | 6.52E-06 | 1.536877222 | NM_024719| | GRTP1,growth hormone regulated TBC protein 1 |
| 201034_at | 17.98 | 6.51E-06 | 1.075378481 | NM_001121| | NA |
| 201622_at | 17.97 | 6.52E-06 | 1.043558851 | NM_014390| | SND1,staphylococcal nuclease domain containing 1 |
| 224329_s_at | 17.97 | 6.52E-06 | 1.466178454 | NM_032488| | CNFN,cornifelin |
| 214636_at | 17.97 | 6.53E-06 | 1.638614441 | NM_000728| | CALCB,calcitonin-related polypeptide, beta |
| 208771_s_at | 17.96 | 6.53E-06 | 1.107165221 | NM_000895| | LTA4H,leukotriene A4 hydrolase |
| 64432_at | 17.94 | 6.57E-06 | 1.125764493 | NA |  |
| 226329_s_at | 17.93 | 6.59E-06 | 1.096708478 | NM_138798| | LOC129531,hypothetical protein BC018453 |
| 227878_s_at | 17.93 | 6.58E-06 | 1.17145534 | NM_032306| | SPATA11,spermatogenesis associated 11 |
| 206729_at | 17.93 | 6.59E-06 | 1.492188364 | NM_001243| | TNFRSF8,tumor necrosis factor receptor superfamily, |
| 202274_at | 17.91 | 6.62E-06 | 1.751419165 | NM_001615| | ACTG2,actin, gamma 2 propeptide |
| 221522_at | 17.91 | 6.63E-06 | 1.096704517 | NM_032139| | ANKRD27,ankyrin repeat domain 27 (VPS9 domain) |
| 213652_at | 17.91 | 6.62E-06 | 1.894380795 | NM_006200| | PCSK5,proprotein convertase subtilisin/kexin type 5 |
| 1553991_s_at | 17.9 | 6.65E-06 | 1.281320499 | NM_019086| | FLJ20674,hypothetical protein FLJ20674 |
| 226345_at | 17.9 | 6.63E-06 | 1.081212988 | NA |  |
| 210785_s_at | 17.89 | 6.65E-06 | 1.448148568 | NM_001039477| | NA |
| 206343_s_at | 17.89 | 6.65E-06 | 1.993880731 | NM_004495| | NRG1,neuregulin 1 isoform HRG-gamma |
| 231594_at | 17.88 | 6.67E-06 | 2.086821426 | NA |  |
| 237461_at | 17.87 | 6.68E-06 | 1.508974434 | NM_139176| | NALP7,NACHT, leucine rich repeat and PYD containing 7 |
| 204411_at | 17.86 | 6.71E-06 | 1.484442112 | NM_017596| | NA |
| 235024_at | 17.86 | 6.72E-06 | 1.252965635 | NM_024900| | PHF17,Jade1 protein short isoform |
| 228561_at | 17.85 | 6.74E-06 | 1.142885458 | NM_017913| | CDC37L1,cell division cycle 37 homolog (S. |
| 204811_s_at | 17.85 | 6.74E-06 | 1.663502094 | NM_001005505| | CACNA2D2,calcium channel, voltage-dependent, alpha |
| 221802_s_at | 17.83 | 6.77E-06 | 1.201949843 | NM_001127211| | NA |
| 1554555_a_at | 17.83 | 6.76E-06 | 1.156965747 | NM_024860| | FLJ21148,hypothetical protein FLJ21148 |
| 230951_at | 17.81 | 6.80E-06 | 2.231162345 | NA |  |
| 232615_at | 17.8 | 6.84E-06 | 1.486434359 | NA |  |
| 238846_at | 17.8 | 6.85E-06 | 1.65405322 | NM_003839| | TNFRSF11A,tumor necrosis factor receptor superfamily, |
| 221519_at | 17.78 | 6.88E-06 | 1.139588774 | NM_022039| | SHFM3,split hand/foot malformation (ectrodactyly) type |
| 226202_at | 17.78 | 6.89E-06 | 1.18935481 | NM_020781| | ZNF398,zinc finger 398 isoform b |
| 231310_at | 17.78 | 6.87E-06 | 3.194163206 | NA |  |
| 223993_s_at | 17.77 | 6.89E-06 | 1.071627662 | NM_014184| | HSPC163,HSPC163 protein |
| 223239_at | 17.77 | 6.91E-06 | 1.146108686 | NM_016472| | C14orf129,chromosome 14 open reading frame 129 |
| 236163_at | 17.77 | 6.89E-06 | 1.493136918 | NM_153234| | LIX1,limb expression 1 |
| 227139_s_at | 17.76 | 6.91E-06 | 1.171499431 | NM_032383| | HPS3,Hermansky-Pudlak syndrome 3 protein |
| 203593_at | 17.76 | 6.91E-06 | 1.284494046 | NM_012120| | CD2AP,CD2-associated protein |
| 1555495_a_at | 17.74 | 6.96E-06 | 1.088383596 | NM_005869| | SDCCAG10,serologically defined colon cancer antigen 10 |
| 207929_at | 17.74 | 6.95E-06 | 1.642626582 | NM_005314| | GRPR,gastrin-releasing peptide receptor |
| 202435_s_at | 17.73 | 6.98E-06 | 1.388450554 | NM_000104| | CYP1B1,cytochrome P450, family 1, subfamily B, |
| 224895_at | 17.72 | 7.00E-06 | 1.164403664 | NM_006106| | YAP1,Yes-associated protein 1, 65 kD |
| 210281_s_at | 17.71 | 7.03E-06 | 1.187793749 | NM_003453| | ZNF198,zinc finger protein 198 |
| 213849_s_at | 17.71 | 7.04E-06 | 2.087833058 | NM_004576| | PPP2R2B,beta isoform of regulatory subunit B55, protein |
| 204620_s_at | 17.7 | 7.04E-06 | 1.183078655 | NM_001126336| | NA |
| 217990_at | 17.68 | 7.10E-06 | 1.121199238 | NM_001002000| | GMPR2,guanosine monophosphate reductase 2 isoform 2 |
| 225232_at | 17.68 | 7.09E-06 | 1.142396614 | NM_001040446| | NA |
| 64942_at | 17.67 | 7.10E-06 | 1.083125551 | NM_207370| | GPR153,G protein-coupled receptor 153 |
| 212871_at | 17.67 | 7.11E-06 | 1.064936207 | NM_003668| | MAPKAPK5,mitogen-activated protein kinase-activated |
| 203945_at | 17.67 | 7.11E-06 | 1.250318836 | NM_001172| | ARG2,arginase, type II precursor |
| 213676_at | 17.67 | 7.11E-06 | 1.502526343 | NA |  |
| 230519_at | 17.66 | 7.13E-06 | 1.46121588 | NM_145019| | FLJ30707,hypothetical protein FLJ30707 |
| 203434_s_at | 17.66 | 7.13E-06 | 1.904841798 | NM_000902| | MME,membrane metallo-endopeptidase |
| 219343_at | 17.65 | 7.14E-06 | 1.209006197 | NM_017913| | CDC37L1,cell division cycle 37 homolog (S. |
| 208622_s_at | 17.65 | 7.15E-06 | 1.239065241 | NM_001111077| | NA |
| 240681_at | 17.65 | 7.14E-06 | 3.328712705 | NA |  |
| 212745_s_at | 17.64 | 7.16E-06 | 1.111887252 | NM_033028| | BBS4,Bardet-Biedl syndrome 4 |
| 204727_at | 17.64 | 7.16E-06 | 1.35538309 | NM_001008396| | WDHD1,WD repeat and HMG-box DNA binding protein 1 |
| 206085_s_at | 17.64 | 7.16E-06 | 1.314764506 | NM_001902| | CTH,cystathionase isoform 1 |
| 205020_s_at | 17.62 | 7.20E-06 | 1.302338234 | NM_001037164| | NA |
| 219573_at | 17.62 | 7.20E-06 | 1.22287046 | NM_017640| | LRRC16,leucine rich repeat containing 16 |
| 203918_at | 17.62 | 7.21E-06 | 1.386002538 | NM_002587| | PCDH1,protocadherin 1 isoform 1 precursor |
| 233002_at | 17.61 | 7.21E-06 | 1.927395786 | NM_020958| | KIAA1622,HEAT-like repeat-containing protein isoform 2 |
| 206662_at | 17.61 | 7.21E-06 | 1.266385243 | NM_001118890| | NA |
| 213534_s_at | 17.59 | 7.27E-06 | 1.081594522 | NM_015148| | PASK,PAS domain containing serine/threonine kinase |
| 213725_x_at | 17.58 | 7.28E-06 | 2.835867897 | NM_022166| | XYLT1,xylosyltransferase I |
| 232057_at | 17.58 | 7.28E-06 | 1.110160547 | NM_032178| | FLJ13291,hypothetical protein FLJ13291 |
| 209823_x_at | 17.58 | 7.27E-06 | 1.217251596 | NM_002123| | HLA-DQB1,major histocompatibility complex, class II, DQ |
| 212865_s_at | 17.58 | 7.28E-06 | 1.817483901 | NM_021110| | COL14A1,collagen, type XIV, alpha 1 |
| 205626_s_at | 17.57 | 7.31E-06 | 2.382285422 | NM_004929| | CALB1,calbindin 1 |
| 229974_at | 17.57 | 7.31E-06 | 1.839818264 | NM_147127| | EVC2,limbin |
| 209426_s_at | 17.55 | 7.36E-06 | 1.216947889 | NM_014324| | AMACR,alpha-methylacyl-CoA racemase isoform 1 |
| 201506_at | 17.55 | 7.34E-06 | 1.405002727 | NM_000358| | TGFBI,transforming growth factor, beta-induced, 68kDa |
| 225005_at | 17.54 | 7.38E-06 | 1.098854356 | NM_153812| | PHF13,PHD finger protein 13 |
| 226625_at | 17.54 | 7.38E-06 | 1.143008482 | NM_003243| | TGFBR3,transforming growth factor, beta receptor III |
| 204866_at | 17.53 | 7.39E-06 | 1.193016172 | NM_001077445| | NA |
| 204354_at | 17.53 | 7.39E-06 | 1.113334154 | NM_001042594| | NA |
| 226119_at | 17.52 | 7.42E-06 | 1.26024687 | NM_052937| | LOC115294,similar to hypothetical protein FLJ10883 |
| 1556069_s_at | 17.51 | 7.44E-06 | 1.734336019 | NM_022462| | HIF3A,hypoxia-inducible factor-3 alpha isoform b |
| 228787_s_at | 17.5 | 7.46E-06 | 1.315681644 | NM_001010974| | BCAS4,breast carcinoma amplified sequence 4 isoform c |
| 223386_at | 17.49 | 7.48E-06 | 1.127156007 | NM_024556| | FLJ21103,hypothetical protein FLJ21103 |
| 202686_s_at | 17.48 | 7.49E-06 | 1.253656269 | NM_001699| | AXL,AXL receptor tyrosine kinase isoform 2 |
| 223541_at | 17.48 | 7.49E-06 | 1.258022069 | NM_005329| | HAS3,hyaluronan synthase 3 isoform a |
| 206696_at | 17.48 | 7.49E-06 | 1.819996274 | NM_000273| | GPR143,G protein-coupled receptor 143 |
| 221774_x_at | 17.48 | 7.50E-06 | 1.071459494 | NM_001014286| | NA |
| 212367_at | 17.46 | 7.55E-06 | 1.105855283 | NM_015322| | FEM1B,fem-1 homolog b |
| 228810_at | 17.46 | 7.54E-06 | 1.180881797 | NM_152523| | FLJ40432,hypothetical protein FLJ40432 |
| 209781_s_at | 17.45 | 7.56E-06 | 1.163653963 | NM_006558| | KHDRBS3,KH domain containing, RNA binding, signal |
| 221648_s_at | 17.44 | 7.58E-06 | 1.612267489 | NA |  |
| 203704_s_at | 17.44 | 7.57E-06 | 1.136100272 | NM_001003698| | RREB1,ras responsive element binding protein 1 isoform |
| 221526_x_at | 17.43 | 7.60E-06 | 1.09408953 | NM_019619| | PARD3,partitioning-defective protein 3 homolog |
| 210275_s_at | 17.42 | 7.62E-06 | 1.046441567 | NM_001102420| | NA |
| 220638_s_at | 17.42 | 7.62E-06 | 1.809138726 | NM_012116| | CBLC,Cas-Br-M (murine) ecotropic retroviral |
| 35820_at | 17.42 | 7.62E-06 | 1.248042531 | NM_000405| | GM2A,GM2 ganglioside activator precursor |
| 220040_x_at | 17.42 | 7.62E-06 | 1.176372134 | NM_018684| | KIAA1166,KIAA1166 |
| 205415_s_at | 17.4 | 7.66E-06 | 1.201398468 | NM_004993| | ATXN3,ataxin 3 isoform 1 |
| 204370_at | 17.4 | 7.66E-06 | 1.03884742 | NM_006831| | HEAB,ATP/GTP-binding protein |
| 226052_at | 17.4 | 7.66E-06 | 1.107747809 | NM_014299| | BRD4,bromodomain-containing protein 4 isoform short |
| 204791_at | 17.4 | 7.66E-06 | 1.147092869 | NM_001032287| | NA |
| 205253_at | 17.39 | 7.68E-06 | 1.756762622 | NM_002585| | PBX1,pre-B-cell leukemia transcription factor 1 |
| 1557521_a_at | 17.39 | 7.67E-06 | 1.26044045 | NA |  |
| 201752_s_at | 17.39 | 7.67E-06 | 1.087287568 | NM_001121| | NA |
| 226591_at | 17.39 | 7.67E-06 | 1.338115882 | NA |  |
| 207695_s_at | 17.39 | 7.67E-06 | 1.822516594 | NM_001555| | IGSF1,immunoglobulin superfamily, member 1 isoform 1 |
| 228440_at | 17.39 | 7.68E-06 | 2.044455587 | NA |  |
| 202832_at | 17.38 | 7.69E-06 | 1.177639867 | NM_014635| | GCC2,GRIP coiled-coil protein GCC185 isoform b |
| 201020_at | 17.38 | 7.70E-06 | 1.149372959 | NM_003405| | YWHAH,tyrosine 3/tryptophan 5 -monooxygenase |
| 202838_at | 17.37 | 7.71E-06 | 1.268512841 | NM_000147| | FUCA1,fucosidase, alpha-L- 1, tissue |
| 206214_at | 17.37 | 7.71E-06 | 1.796854712 | NM_005084| | PLA2G7,phospholipase A2, group VII |
| 239155_at | 17.37 | 7.71E-06 | 1.451547391 | NA |  |
| 225991_at | 17.36 | 7.71E-06 | 1.125399358 | NM_080652| | TMEM41A,transmembrane protein 41A |
| 225131_at | 17.36 | 7.71E-06 | 1.098000048 | NM_017580| | ZRANB1,TRABID protein |
| 244071_at | 17.35 | 7.75E-06 | 1.184532326 | NA |  |
| 225694_at | 17.34 | 7.76E-06 | 1.052339028 | NM_015083| | NA |
| 205416_s_at | 17.34 | 7.76E-06 | 1.233593964 | NM_004993| | ATXN3,ataxin 3 isoform 1 |
| 219534_x_at | 17.34 | 7.76E-06 | 1.354722364 | NM_000076| | CDKN1C,cyclin-dependent kinase inhibitor 1C |
| 241834_at | 17.34 | 7.76E-06 | 1.428859537 | NA |  |
| 219685_at | 17.34 | 7.76E-06 | 1.258029017 | NM_021637| | TMEM35,transmembrane protein 35 |
| 1568609_s_at | 17.33 | 7.77E-06 | 1.807837573 | NA |  |
| 229016_s_at | 17.31 | 7.83E-06 | 1.474436759 | NM_033502| | TRERF1,transcriptional regulating factor 1 isoform 1 |
| 219340_s_at | 17.31 | 7.83E-06 | 1.130189585 | NM_018941| | CLN8,CLN8 protein |
| 220239_at | 17.31 | 7.84E-06 | 1.13575712 | NM_001031710| | NA |
| 209678_s_at | 17.3 | 7.85E-06 | 1.137684308 | NM_002740| | PRKCI,protein kinase C, iota |
| 204217_s_at | 17.3 | 7.85E-06 | 1.245987583 | NM_005619| | RTN2,reticulon 2 isoform A |
| 212864_at | 17.3 | 7.85E-06 | 1.188171004 | NM_003818| | CDS2,phosphatidate cytidylyltransferase 2 |
| 218379_at | 17.29 | 7.87E-06 | 1.187484772 | NM_016090| | RBM7,RNA binding motif protein 7 |
| 218905_at | 17.29 | 7.87E-06 | 1.046326659 | NM_017864| | FLJ20530,hypothetical protein FLJ20530 |
| 223051_at | 17.28 | 7.90E-06 | 1.077916212 | NM_014188| | HSPC182,HSPC182 protein |
| 226270_at | 17.27 | 7.91E-06 | 1.16371472 | NM_018303| | SEC5L1,Sec5 protein |
| 209141_at | 17.27 | 7.93E-06 | 1.136708338 | NM_003342| | UBE2G1,ubiquitin-conjugating enzyme E2G 1 isoform 1 |
| 222271_at | 17.27 | 7.91E-06 | 1.580515559 | NA |  |
| 215167_at | 17.26 | 7.94E-06 | 1.318584801 | NM_004229| | CRSP2,cofactor required for Sp1 transcriptional |
| 202511_s_at | 17.26 | 7.94E-06 | 1.080908665 | NM_004849| | APG5L,APG5 autophagy 5-like |
| 224684_at | 17.26 | 7.93E-06 | 1.10340454 | NM_013346| | SNX12,sorting nexin 12 |
| 223160_s_at | 17.26 | 7.94E-06 | 1.104962257 | NM_032560| | KIAA2010,KIAA2010 isoform 1 |
| 236656_s_at | 17.25 | 7.96E-06 | 1.320399982 | NA |  |
| 225750_at | 17.24 | 7.99E-06 | 1.073619005 | NA |  |
| 221555_x_at | 17.24 | 7.99E-06 | 1.162458006 | NM_001077181| | NA |
| 37950_at | 17.22 | 8.03E-06 | 1.07323554 | NM_002726| | PREP,prolyl endopeptidase |
| 222955_s_at | 17.22 | 8.03E-06 | 1.149568745 | NM_018472| | FAM45B,family with sequence similarity 45, member B |
| 238931_at | 17.21 | 8.05E-06 | 1.141250892 | NM_024086| | MGC3329,hypothetical protein MGC3329 |
| 227627_at | 17.21 | 8.05E-06 | 1.453381973 | NM_001033578| | NA |
| 1564360_a_at | 17.2 | 8.09E-06 | 2.132620969 | NA |  |
| 210657_s_at | 17.2 | 8.08E-06 | 1.437773 | NM_004574| | SEPT4,septin 4 isoform 1 |
| 201250_s_at | 17.18 | 8.13E-06 | 1.131613662 | NM_006516| | SLC2A1,solute carrier family 2 (facilitated glucose |
| 205372_at | 17.17 | 8.17E-06 | 2.41260597 | NM_001114634| | NA |
| 212436_at | 17.17 | 8.16E-06 | 1.125262737 | NM_015906| | TRIM33,tripartite motif-containing 33 protein |
| 217676_at | 17.17 | 8.17E-06 | 1.986776231 | NA |  |
| 218129_s_at | 17.14 | 8.24E-06 | 1.174851052 | NM_006166| | NFYB,nuclear transcription factor Y, beta |
| 241772_at | 17.14 | 8.24E-06 | 2.029176688 | NA |  |
| 1560250_s_at | 17.14 | 8.23E-06 | 1.476554005 | NA |  |
| 223551_at | 17.14 | 8.24E-06 | 2.87369987 | NM_032471| | PKIB,cAMP-dependent protein kinase inhibitor beta |
| 201015_s_at | 17.13 | 8.27E-06 | 1.49575723 | NM_002230| | JUP,junction plakoglobin |
| 243356_at | 17.11 | 8.33E-06 | 1.517068292 | NA |  |
| 236214_at | 17.1 | 8.36E-06 | 1.61679994 | NM_032599| | NYD-SP18,testes development-related NYD-SP18 |
| 37254_at | 17.09 | 8.39E-06 | 1.088938741 | NM_001083330| | NA |
| 230916_at | 17.08 | 8.41E-06 | 1.587271206 | NM_018055| | NODAL,nodal-related protein |
| 212876_at | 17.08 | 8.40E-06 | 1.392492893 | NM_003778| | B4GALT4,UDP-Gal:betaGlcNAc beta 1,4- |
| 226384_at | 17.07 | 8.43E-06 | 1.107849897 | NM_001102559| | NA |
| 200851_s_at | 17.07 | 8.43E-06 | 1.073221593 | NM_014761| | KIAA0174,KIAA0174 gene product |
| 238510_at | 17.07 | 8.43E-06 | 1.156410772 | NM_001004300| | LOC124411,hypothetical protein LOC124411 |
| 221933_at | 17.07 | 8.43E-06 | 1.18741702 | NM_020742| | NLGN4X,X-linked neuroligin 4 |
| 236037_at | 17.06 | 8.44E-06 | 1.543155882 | NM_020340| | KIAA1244,KIAA1244 |
| 201989_s_at | 17.06 | 8.43E-06 | 1.10101671 | NM_001310| | CREBL2,cAMP responsive element binding protein-like 2 |
| 234665_x_at | 17.06 | 8.43E-06 | 1.238597368 | NM_001031693| | NA |
| 226103_at | 17.06 | 8.44E-06 | 1.512385158 | NM_144573| | NEXN,nexilin (F actin binding protein) |
| 201605_x_at | 17.05 | 8.47E-06 | 1.294527735 | NM_004368| | CNN2,calponin 2 isoform a |
| 202981_x_at | 17.05 | 8.47E-06 | 1.056530441 | NM_001006610| | SIAH1,seven in absentia homolog 1 isoform b |
| 209755_at | 17.05 | 8.46E-06 | 1.780078817 | NM_015039| | NMNAT2,nicotinamide mononucleotide adenylyltransferase |
| 223389_s_at | 17.04 | 8.49E-06 | 1.16216234 | NM_016535| | ZNF581,zinc finger protein 581 |
| 212727_at | 17.04 | 8.49E-06 | 1.225049186 | NM_020730| | NA |
| 203574_at | 17.04 | 8.49E-06 | 1.23509217 | NM_005384| | NFIL3,nuclear factor, interleukin 3 regulated |
| 200706_s_at | 17.02 | 8.54E-06 | 1.220103606 | NM_004862| | LITAF,LPS-induced TNF-alpha factor |
| 213348_at | 17.02 | 8.54E-06 | 1.278914244 | NM_000076| | CDKN1C,cyclin-dependent kinase inhibitor 1C |
| 205505_at | 17.01 | 8.54E-06 | 2.220219048 | NM_001097633| | NA |
| 202610_s_at | 17.01 | 8.54E-06 | 1.144046681 | NM_004229| | CRSP2,cofactor required for Sp1 transcriptional |
| 1557169_x_at | 17.01 | 8.54E-06 | 1.327437108 | NA |  |
| 221261_x_at | 17 | 8.59E-06 | 1.118571715 | NM_001098800| | NA |
| 209874_x_at | 17 | 8.59E-06 | 1.235278652 | NM_017649| | CNNM2,cyclin M2 isoform 1 |
| 220137_at | 17 | 8.57E-06 | 1.283393395 | NM_019086| | FLJ20674,hypothetical protein FLJ20674 |
| 38290_at | 16.99 | 8.59E-06 | 1.29875527 | NM_006480| | RGS14,regulator of G-protein signalling 14 |
| 201215_at | 16.99 | 8.60E-06 | 1.300211242 | NM_005032| | PLS3,plastin 3 |
| 204584_at | 16.99 | 8.59E-06 | 1.38423545 | NM_000425| | L1CAM,L1 cell adhesion molecule isoform 1 precursor |
| 213182_x_at | 16.99 | 8.60E-06 | 1.345897493 | NM_000076| | CDKN1C,cyclin-dependent kinase inhibitor 1C |
| 235052_at | 16.99 | 8.59E-06 | 1.214575665 | NM_175872| | FLJ38451,FLJ38451 protein |
| 228396_at | 16.98 | 8.62E-06 | 1.358789739 | NM_001098512| | NA |
| 210664_s_at | 16.96 | 8.68E-06 | 2.352188808 | NM_001032281| | NA |
| 222641_s_at | 16.96 | 8.70E-06 | 1.111636218 | NM_001077498| | NA |
| 202346_at | 16.95 | 8.72E-06 | 1.104486464 | NM_001111112| | NA |
| 205229_s_at | 16.95 | 8.71E-06 | 1.289122111 | NM_004086| | COCH,coagulation factor C homolog, cochlin precursor |
| 210026_s_at | 16.94 | 8.74E-06 | 1.251808654 | NM_014550| | CARD10,caspase recruitment domain protein 10 |
| 203072_at | 16.94 | 8.72E-06 | 1.267448655 | NM_004998| | MYO1E,myosin IE |
| 202998_s_at | 16.93 | 8.77E-06 | 1.6013443 | NM_002318| | LOXL2,lysyl oxidase-like 2 |
| 222700_at | 16.93 | 8.75E-06 | 1.163575327 | NM_022374| | ARL6IP2,ADP-ribosylation factor-like 6 interacting |
| 1560537_at | 16.93 | 8.77E-06 | 1.28924598 | NA |  |
| 218429_s_at | 16.89 | 8.86E-06 | 1.146157957 | NM_018381| | FLJ11286,hypothetical protein FLJ11286 |
| 214890_s_at | 16.88 | 8.88E-06 | 1.167793108 | NM_001006655| | DKFZP564J102,DKFZP564J102 protein |
| 202006_at | 16.88 | 8.88E-06 | 1.089359142 | NM_002835| | PTPN12,protein tyrosine phosphatase, non-receptor type |
| 224565_at | 16.87 | 8.92E-06 | 1.651629028 | NA |  |
| 213659_at | 16.87 | 8.92E-06 | 1.439380983 | NM_007131| | ZNF75,zinc finger protein 75 |
| 205738_s_at | 16.87 | 8.92E-06 | 1.636513744 | NM_004102| | FABP3,fatty acid binding protein 3 |
| 212692_s_at | 16.86 | 8.95E-06 | 1.143488884 | NM_006726| | LRBA,LPS-responsive vesicle trafficking, beach and |
| 205007_s_at | 16.86 | 8.95E-06 | 1.252383269 | NM_006383| | CIB2,DNA-dependent protein kinase catalytic |
| 236125_at | 16.85 | 8.96E-06 | 1.34721095 | NA |  |
| 212761_at | 16.85 | 8.96E-06 | 1.117112957 | NM_030756| | TCF7L2,transcription factor 7-like 2 (T-cell specific, |
| 224819_at | 16.85 | 8.97E-06 | 1.170719595 | NM_001006684| | TCEAL8,transcription elongation factor A (SII)-like 8 |
| 235955_at | 16.84 | 8.99E-06 | 2.01202921 | NM_001038603| | NA |
| 231945_at | 16.84 | 8.99E-06 | 1.862122087 | NM_015687| | FILIP1,filamin A interacting protein 1 |
| 1552390_a_at | 16.83 | 9.03E-06 | 1.616251168 | NM_173549| | FLJ39553,hypothetical protein FLJ39553 |
| 202804_at | 16.81 | 9.08E-06 | 1.096426868 | NM_004996| | ABCC1,ATP-binding cassette, sub-family C, member 1 |
| 226212_s_at | 16.81 | 9.10E-06 | 1.316156373 | NM_000208| | INSR,insulin receptor |
| 222635_s_at | 16.81 | 9.08E-06 | 1.203384522 | NM_025205| | MED28,mediator of RNA polymerase II transcription, |
| 226434_at | 16.79 | 9.14E-06 | 1.093772681 | NM_145030| | MGC22793,hypothetical protein MGC22793 |
| 226065_at | 16.79 | 9.14E-06 | 2.262812734 | NM_153026| | PRICKLE1,prickle-like 1 |
| 219155_at | 16.79 | 9.15E-06 | 1.198104213 | NM_012417| | PITPNC1,phosphatidylinositol transfer protein, |
| 238778_at | 16.78 | 9.17E-06 | 2.451866319 | NM_173496| | MPP7,palmitoylated membrane protein 7 |
| 203955_at | 16.77 | 9.21E-06 | 1.103712159 | NM_014811| | KIAA0649,KIAA0649 gene product |
| 209338_at | 16.77 | 9.20E-06 | 1.169773309 | NM_005653| | TFCP2,transcription factor CP2 |
| 221667_s_at | 16.77 | 9.20E-06 | 1.413721149 | NM_014365| | HSPB8,heat shock 27kDa protein 8 |
| 204235_s_at | 16.77 | 9.20E-06 | 1.31541909 | NM_016315| | GULP1,GULP, engulfment adaptor PTB domain containing |
| 205478_at | 16.75 | 9.26E-06 | 1.96952971 | NM_006741| | PPP1R1A,protein phosphatase 1, regulatory (inhibitor) |
| 221556_at | 16.75 | 9.26E-06 | 1.13720051 | NM_001077181| | NA |
| 213010_at | 16.75 | 9.26E-06 | 2.417735426 | NM_145040| | PRKCDBP,protein kinase C, delta binding protein |
| 221832_s_at | 16.75 | 9.27E-06 | 1.166581242 | NM_033631| | LUZP1,leucine zipper protein 1 |
| 212599_at | 16.74 | 9.29E-06 | 1.14692685 | NM_001127231| | NA |
| 225230_at | 16.74 | 9.29E-06 | 1.104450865 | NM_178454| | MGC54289,hypothetical protein MGC54289 |
| 215548_s_at | 16.74 | 9.28E-06 | 1.109445931 | NM_016106| | SCFD1,vesicle transport-related protein isoform a |
| 228336_at | 16.72 | 9.34E-06 | 1.206635011 | NM_052927| | NA |
| 204759_at | 16.72 | 9.34E-06 | 1.266374218 | NM_001268| | CHC1L,RCC1-like G exchanging factor RLG |
| 219125_s_at | 16.72 | 9.34E-06 | 1.208873815 | NM_001122837| | NA |
| 205698_s_at | 16.72 | 9.33E-06 | 1.290537122 | NM_002758| | MAP2K6,mitogen-activated protein kinase kinase 6 |
| 228422_at | 16.71 | 9.36E-06 | 1.605437557 | NM_198560| | LOC375323,lipoma HMGIC fusion partner-like protein 4 |
| 244264_at | 16.71 | 9.36E-06 | 1.657225056 | NM_198508| | FLJ44186,FLJ44186 protein |
| 231271_x_at | 16.69 | 9.41E-06 | 1.162261228 | NM_020677| | HSCARG,HSCARG protein |
| 223650_s_at | 16.69 | 9.41E-06 | 1.110673952 | NM_030759| | NRBF2,nuclear receptor binding factor 2 |
| 203594_at | 16.69 | 9.41E-06 | 1.126067064 | NM_003729| | RTCD1,RNA terminal phosphate cyclase domain 1 |
| 203026_at | 16.69 | 9.41E-06 | 1.072140863 | NM_014872| | ZBTB5,zinc finger and BTB domain containing 5 |
| 202891_at | 16.67 | 9.47E-06 | 1.115048442 | NM_005600| | NIT1,nitrilase 1 |
| 201925_s_at | 16.66 | 9.51E-06 | 2.005032999 | NM_000574| | DAF,decay accelerating factor for complement (CD55, |
| 210754_s_at | 16.66 | 9.52E-06 | 1.324604371 | NM_001111097| | NA |
| 227297_at | 16.65 | 9.53E-06 | 1.672678323 | NM_002207| | ITGA9,integrin, alpha 9 precursor |
| 227111_at | 16.65 | 9.54E-06 | 1.237470636 | NM_001099270| | NA |
| 226015_at | 16.64 | 9.56E-06 | 1.053141873 | NM_006956| | ZNF12,zinc finger protein 12 (KOX 3) |
| 204702_s_at | 16.63 | 9.60E-06 | 1.49266983 | NM_004289| | NFE2L3,nuclear factor (erythroid-derived 2)-like 3 |
| 201635_s_at | 16.62 | 9.63E-06 | 1.125444165 | NM_001013438| | NA |
| 235241_at | 16.62 | 9.62E-06 | 1.206494865 | NM_173514| | FLJ90709,hypothetical protein FLJ90709 |
| 1552477_a_at | 16.61 | 9.64E-06 | 1.711667554 | NM_006147| | IRF6,interferon regulatory factor 6 |
| 202677_at | 16.61 | 9.65E-06 | 1.090877657 | NM_002890| | RASA1,RAS p21 protein activator 1 isoform 1 |
| 244317_at | 16.6 | 9.69E-06 | 1.418628644 | NM_152748| | FLJ31340,hypothetical protein FLJ31340 |
| 202638_s_at | 16.6 | 9.69E-06 | 1.545159273 | NM_000201| | ICAM1,intercellular adhesion molecule 1 precursor |
| 213217_at | 16.6 | 9.69E-06 | 1.653866047 | NM_020546| | ADCY2,adenylate cyclase 2 |
| 218945_at | 16.59 | 9.69E-06 | 1.129289244 | NM_024109| | MGC2654,hypothetical protein MGC2654 |
| 201365_at | 16.59 | 9.69E-06 | 1.174917172 | NM_002537| | OAZ2,ornithine decarboxylase antizyme 2 |
| 217605_at | 16.58 | 9.73E-06 | 1.215766404 | NA |  |
| 204565_at | 16.57 | 9.77E-06 | 1.22577471 | NM_018473| | THEM2,thioesterase superfamily member 2 |
| 215076_s_at | 16.57 | 9.78E-06 | 1.872488306 | NM_000090| | COL3A1,alpha 1 type III collagen |
| 202536_at | 16.57 | 9.77E-06 | 1.119664162 | NM_014043| | DKFZP564O123,DKFZP564O123 protein |
| 239142_at | 16.56 | 9.79E-06 | 1.347154272 | NM_173362| | LOC317671,LOC317671 |
| 221584_s_at | 16.55 | 9.84E-06 | 1.4721876 | NM_001014797| | NA |
| 203301_s_at | 16.54 | 9.87E-06 | 1.080581891 | NM_021145| | DMTF1,cyclin D binding myb-like transcription factor |
| 223382_s_at | 16.54 | 9.87E-06 | 1.122493637 | NM_032268| | ZNRF1,zinc and ring finger protein 1 |
| 235343_at | 16.54 | 9.87E-06 | 1.473510537 | NM_024749| | FLJ12505,hypothetical protein FLJ12505 |
| 219084_at | 16.53 | 9.89E-06 | 1.221817421 | NM_022455| | NSD1,nuclear receptor binding SET domain protein 1 |
| 206012_at | 16.53 | 9.88E-06 | 2.106011493 | NM_003240| | LEFTY2,endometrial bleeding associated factor |
| 211776_s_at | 16.51 | 9.96E-06 | 1.734143307 | NM_012307| | EPB41L3,erythrocyte membrane protein band 4.1-like 3 |
| 224439_x_at | 16.5 | 9.98E-06 | 1.07883715 | NM_014245| | RNF7,ring finger protein 7 isoform 1 |
| 226033_at | 16.5 | 9.99E-06 | 1.169325154 | NM_020718| | USP31,ubiquitin specific protease 31 |
| 219196_at | 16.5 | 9.97E-06 | 2.141077601 | NM_013243| | SCG3,secretogranin III |
| 204934_s_at | 16.5 | 9.97E-06 | 1.411909525 | NM_002151| | HPN,hepsin (transmembrane protease, serine 1) |
| 218056_at | 16.5 | 9.99E-06 | 1.065991597 | NM_016561| | BFAR,apoptosis regulator |
| 227694_at | 16.49 | 9.99E-06 | 1.229293782 | NM_178122| | LOC90529,hypothetical protein LOC90529 |
| 206723_s_at | 16.49 | 1.00E-05 | 1.506497112 | NM_004720| | EDG4,endothelial differentiation, lysophosphatidic |
| 228782_at | 16.48 | 1.00E-05 | 1.711512227 | NM_054023| | SCGB3A2,secretoglobin, family 3A, member 2 |
| 235618_at | 16.48 | 1.00E-05 | 1.150205624 | NM_014910| | ZNF507,zinc finger protein 507 |
| 227862_at | 16.48 | 1.00E-05 | 1.171357161 | NM_001013642| | NA |
| 212025_s_at | 16.47 | 1.01E-05 | 1.14765165 | NM_002018| | FLII,flightless I homolog |
| 220028_at | 16.47 | 1.00E-05 | 1.175730298 | NM_001106| | ACVR2B,activin A type IIB receptor precursor |
| 231039_at | 16.47 | 1.00E-05 | 1.250901278 | NA |  |
| 220289_s_at | 16.47 | 1.00E-05 | 1.765177453 | NM_001039775| | NA |
| 204047_s_at | 16.46 | 1.01E-05 | 1.304806424 | NM_001100164| | NA |
| 208202_s_at | 16.46 | 1.01E-05 | 1.271880205 | NM_015288| | PHF15,PHD finger protein 15 |
| 209526_s_at | 16.46 | 1.01E-05 | 1.058128337 | NM_016073| | HDGFRP3,hepatoma-derived growth factor, related protein |
| 231991_at | 16.45 | 1.01E-05 | 1.856132042 | NM_080625| | C20orf160,chromosome 20 open reading frame 160 |
| 202752_x_at | 16.45 | 1.01E-05 | 1.449837722 | NM_012244| | SLC7A8,solute carrier family 7 (cationic amino acid |
| 57082_at | 16.45 | 1.01E-05 | 1.227628185 | NM_015627| | ARH,LDL receptor adaptor protein |
| 34206_at | 16.45 | 1.01E-05 | 1.087502116 | NM_001040118| | NA |
| 222071_s_at | 16.45 | 1.01E-05 | 1.950982638 | NM_180991| | SLCO4C1,solute carrier organic anion transporter family, |
| 219823_at | 16.45 | 1.01E-05 | 3.49884687 | NM_024674| | LIN28,lin-28 homolog |
| 230166_at | 16.45 | 1.01E-05 | 1.163798065 | NM_133465| | KIAA1958,KIAA1958 |
| 202074_s_at | 16.43 | 1.02E-05 | 1.575972702 | NM_001008211| | OPTN,optineurin |
| 201898_s_at | 16.42 | 1.02E-05 | 1.080969402 | NM_003336| | UBE2A,ubiquitin-conjugating enzyme E2A isoform 1 |
| 52940_at | 16.42 | 1.02E-05 | 1.237089183 | NM_021805| | SIGIRR,single Ig IL-1R-related molecule |
| 235723_at | 16.41 | 1.02E-05 | 2.099650264 | NM_017637| | BNC2,basonuclin 2 |
| 208739_x_at | 16.4 | 1.03E-05 | 1.044781637 | NM_001005849| | SUMO2,small ubiquitin-like modifier 2 isoform b |
| 229971_at | 16.39 | 1.03E-05 | 1.500764221 | NM_153837| | GPR114,G-protein coupled receptor 114 |
| 225080_at | 16.39 | 1.03E-05 | 1.166593033 | NM_001080779| | NA |
| 209615_s_at | 16.38 | 1.03E-05 | 2.138907608 | NM_002576| | PAK1,p21-activated kinase 1 |
| 218457_s_at | 16.38 | 1.03E-05 | 1.136692728 | NM_022552| | DNMT3A,DNA cytosine methyltransferase 3 alpha isoform |
| 235355_at | 16.37 | 1.04E-05 | 1.655478855 | NA |  |
| 220992_s_at | 16.37 | 1.04E-05 | 1.22670774 | NM_030934| | C1orf25,N2,N2-dimethylguanosine tRNA |
| 209560_s_at | 16.36 | 1.04E-05 | 2.542643703 | NM_003836| | DLK1,delta-like homolog |
| 220642_x_at | 16.35 | 1.04E-05 | 1.062037511 | NM_001097612| | NA |
| 225274_at | 16.34 | 1.05E-05 | 1.136706269 | NM_016297| | PCYOX1,prenylcysteine oxidase 1 |
| 209647_s_at | 16.34 | 1.05E-05 | 1.163432012 | NM_014011| | SOCS5,suppressor of cytokine signaling 5 |
| 1555846_a_at | 16.34 | 1.05E-05 | 1.185080674 | NA |  |
| 201309_x_at | 16.33 | 1.05E-05 | 1.066074751 | NM_004772| | C5orf13,neuronal protein 3.1 |
| 217127_at | 16.31 | 1.06E-05 | 1.293682755 | NM_001902| | CTH,cystathionase isoform 1 |
| 1553286_at | 16.31 | 1.06E-05 | 1.197602861 | NM_152791| | ZNF555,zinc finger protein 555 |
| 223737_x_at | 16.31 | 1.06E-05 | 2.011405227 | NM_031422| | CHST9,GalNAc-4-sulfotransferase 2 |
| 222606_at | 16.31 | 1.06E-05 | 1.069068494 | NM_017975| | FLJ10036,Zwilch |
| 227684_at | 16.3 | 1.06E-05 | 1.17679308 | NM_004230| | EDG5,endothelial differentiation, sphingolipid |
| 218133_s_at | 16.3 | 1.06E-05 | 1.046520043 | NM_021824| | NIF3L1,NIF3 NGG1 interacting factor 3-like 1 |
| 231609_at | 16.29 | 1.06E-05 | 1.644669177 | NM_144661| | C10orf82,chromosome 10 open reading frame 82 |
| 216511_s_at | 16.29 | 1.06E-05 | 1.139520767 | NM_030756| | TCF7L2,transcription factor 7-like 2 (T-cell specific, |
| 203935_at | 16.29 | 1.06E-05 | 1.454797126 | NM_001105| | ACVR1,activin A type I receptor precursor |
| 213693_s_at | 16.29 | 1.06E-05 | 1.221212249 | NM_001018016| | NA |
| 235269_at | 16.29 | 1.06E-05 | 1.593104397 | NM_138435| | LOC113828,hypothetical protein BC011204 |
| 224467_s_at | 16.28 | 1.06E-05 | 1.12397775 | NM_032346| | MGC13096,hypothetical protein MGC13096 |
| 212805_at | 16.27 | 1.07E-05 | 1.389914765 | NM_015225| | NA |
| 217739_s_at | 16.27 | 1.07E-05 | 1.133969018 | NM_005746| | PBEF1,pre-B-cell colony enhancing factor 1 isoform a |
| 200639_s_at | 16.26 | 1.07E-05 | 1.111806706 | NM_003406| | YWHAZ,tyrosine 3/tryptophan 5 -monooxygenase |
| 229139_at | 16.26 | 1.07E-05 | 1.171545292 | NM_020647| | JPH1,junctophilin 1 |
| 218757_s_at | 16.25 | 1.08E-05 | 1.126412666 | NM_023010| | UPF3B,UPF3 regulator of nonsense transcripts homolog B |
| 224366_s_at | 16.24 | 1.08E-05 | 1.098768865 | NM_031922| | REPS1,RALBP1 associated Eps domain containing 1 |
| 212749_s_at | 16.23 | 1.08E-05 | 1.08645234 | NM_001008925| | RCHY1,ring finger and CHY zinc finger domain |
| 202956_at | 16.23 | 1.08E-05 | 1.167688697 | NM_006421| | ARFGEF1,brefeldin A-inhibited guanine |
| 203350_at | 16.22 | 1.09E-05 | 1.087631214 | NM_001030007| | NA |
| 209152_s_at | 16.22 | 1.09E-05 | 1.117787414 | NM_003200| | TCF3,transcription factor 3 |
| 205308_at | 16.22 | 1.09E-05 | 1.175880273 | NM_016010| | CGI-62,CGI-62 protein |
| 212374_at | 16.21 | 1.09E-05 | 1.213352758 | NM_015322| | FEM1B,fem-1 homolog b |
| 204465_s_at | 16.21 | 1.09E-05 | 1.148886005 | NM_032727| | INA,internexin neuronal intermediate filament |
| 207513_s_at | 16.2 | 1.09E-05 | 1.061965906 | NM_003452| | ZNF189,zinc finger protein 189 |
| 213235_at | 16.2 | 1.09E-05 | 1.09279361 | NM_001012991| | NA |
| 228577_x_at | 16.19 | 1.10E-05 | 1.377386859 | NM_001007022| | KIAA1229,KIAA1229 protein isoform b |
| 215789_s_at | 16.19 | 1.10E-05 | 1.40067364 | NM_001042478| | NA |
| 213503_x_at | 16.18 | 1.10E-05 | 1.130918236 | NM_001002857| | ANXA2,annexin A2 isoform 2 |
| 205213_at | 16.18 | 1.10E-05 | 1.453275635 | NM_014716| | CENTB1,centaurin beta1 |
| 1568604_a_at | 16.18 | 1.10E-05 | 1.60147601 | NM_003716| | CADPS,Ca2+-dependent secretion activator isoform 1 |
| 217149_x_at | 16.17 | 1.10E-05 | 1.159124334 | NM_003985| | TNK1,tyrosine kinase, non-receptor, 1 |
| 201318_s_at | 16.17 | 1.10E-05 | 1.058586249 | NM_006471| | MRCL3,myosin regulatory light chain MRCL3 |
| 204121_at | 16.16 | 1.11E-05 | 1.2356714 | NM_006705| | GADD45G,growth arrest and DNA-damage-inducible, gamma |
| 203304_at | 16.16 | 1.11E-05 | 1.170616711 | NM_012342| | BAMBI,BMP and activin membrane-bound inhibitor |
| 34858_at | 16.16 | 1.11E-05 | 1.118118391 | NM_015353| | KCTD2,potassium channel tetramerisation domain |
| 209873_s_at | 16.15 | 1.11E-05 | 1.247872892 | NM_007183| | PKP3,plakophilin 3 |
| 235645_at | 16.14 | 1.11E-05 | 1.204483117 | NM_052911| | ESCO1,establishment of cohesion 1 homolog 1 |
| 206747_at | 16.13 | 1.12E-05 | 1.25355913 | NM_014696| | KIAA0514,KIAA0514 |
| 239503_at | 16.13 | 1.12E-05 | 1.479989138 | NA |  |
| 241416_at | 16.13 | 1.12E-05 | 1.331221938 | NA |  |
| 203096_s_at | 16.13 | 1.12E-05 | 1.186884209 | NM_014247| | NA |
| 200764_s_at | 16.12 | 1.12E-05 | 1.089331558 | NM_001903| | CTNNA1,catenin, alpha 1 |
| 202053_s_at | 16.12 | 1.12E-05 | 1.119037014 | NM_000382| | ALDH3A2,aldehyde dehydrogenase 3A2 |
| 209262_s_at | 16.12 | 1.12E-05 | 1.114582527 | NM_005234| | NR2F6,nuclear receptor subfamily 2, group F, member 6 |
| 201329_s_at | 16.12 | 1.12E-05 | 1.190996832 | NM_005239| | ETS2,v-ets erythroblastosis virus E26 oncogene |
| 235810_at | 16.11 | 1.12E-05 | 1.191324562 | NM_001007088| | ZNF21,zinc finger protein 21 isoform 2 |
| 226869_at | 16.1 | 1.13E-05 | 1.735400837 | NM_001409| | EGFL3,EGF-like-domain, multiple 3 |
| 204286_s_at | 16.09 | 1.13E-05 | 1.395647816 | NM_021127| | PMAIP1,phorbol-12-myristate-13-acetate-induced protein |
| 218321_x_at | 16.09 | 1.13E-05 | 1.141460039 | NM_016086| | DUSP24,map kinase phosphatase-like protein MK-STYX |
| 201758_at | 16.08 | 1.13E-05 | 1.046138415 | NM_006292| | TSG101,tumor susceptibility gene 101 |
| 1552524_at | 16.08 | 1.13E-05 | 1.231827946 | NM_001079536| | NA |
| 209122_at | 16.08 | 1.13E-05 | 1.096968832 | NM_001122| | ADFP,adipose differentiation-related protein |
| 227628_at | 16.07 | 1.14E-05 | 1.120596243 | NM_001008397| | LOC493869,similar to 2310016C16Rik protein |
| 212859_x_at | 16.07 | 1.13E-05 | 1.449436916 | NM_175617| | MT1E,metallothionein 1E |
| 235129_at | 16.07 | 1.14E-05 | 1.42059764 | NM_006741| | PPP1R1A,protein phosphatase 1, regulatory (inhibitor) |
| 215177_s_at | 16.07 | 1.14E-05 | 1.175822181 | NM_000210| | ITGA6,integrin alpha chain, alpha 6 |
| 220089_at | 16.06 | 1.14E-05 | 1.13727681 | NM_024884| | C14orf160,chromosome 14 open reading frame 160 |
| 221515_s_at | 16.05 | 1.14E-05 | 1.140177405 | NM_001032391| | NA |
| 200976_s_at | 16.04 | 1.15E-05 | 1.084723534 | NM_001079864| | NA |
| 220030_at | 16.04 | 1.15E-05 | 1.422548517 | NM_018423| | STYK1,serine/threonine/tyrosine kinase 1 |
| 224967_at | 16.04 | 1.15E-05 | 1.102177666 | NM_003358| | UGCG,ceramide glucosyltransferase |
| 205774_at | 16.04 | 1.15E-05 | 1.18664777 | NM_000505| | F12,coagulation factor XII precursor |
| 228248_at | 16.04 | 1.15E-05 | 1.175770991 | NM_152756| | AVO3,rapamycin-insensitive companion of mTOR |
| 222458_s_at | 16.03 | 1.15E-05 | 1.213809845 | NM_024595| | FLJ12666,hypothetical protein FLJ12666 |
| 1553979_at | 16.02 | 1.15E-05 | 1.066012681 | NA |  |
| 200887_s_at | 16.01 | 1.16E-05 | 1.074047566 | NM_007315| | STAT1,signal transducer and activator of transcription |
| 1556242_a_at | 16.01 | 1.16E-05 | 1.277842314 | NA |  |
| 207981_s_at | 16 | 1.16E-05 | 1.497070106 | NM_001438| | ESRRG,estrogen-related receptor gamma isoform 1 |
| 235216_at | 15.99 | 1.17E-05 | 1.255008769 | NM_052911| | ESCO1,establishment of cohesion 1 homolog 1 |
| 230708_at | 15.99 | 1.16E-05 | 1.979919874 | NM_153026| | PRICKLE1,prickle-like 1 |
| 222900_at | 15.99 | 1.16E-05 | 1.316251451 | NA |  |
| 214933_at | 15.98 | 1.17E-05 | 1.611107173 | NM_000068| | CACNA1A,calcium channel, alpha 1A subunit isoform 1 |
| 244565_at | 15.97 | 1.17E-05 | 1.481702647 | NM_005519| | NA |
| 223710_at | 15.97 | 1.17E-05 | 1.877507014 | NM_006072| | CCL26,chemokine (C-C motif) ligand 26 precursor |
| 217768_at | 15.97 | 1.18E-05 | 1.026020106 | NM_016039| | C14orf166,chromosome 14 open reading frame 166 |
| 237203_at | 15.96 | 1.18E-05 | 1.609270203 | NA |  |
| 232353_s_at | 15.96 | 1.18E-05 | 1.13042587 | NM_016086| | DUSP24,map kinase phosphatase-like protein MK-STYX |
| 210511_s_at | 15.95 | 1.18E-05 | 1.473147101 | NM_002192| | INHBA,inhibin beta A subunit precursor |
| 232146_at | 15.95 | 1.18E-05 | 1.155323513 | NM_002494| | NDUFC1,NADH dehydrogenase (ubiquinone) 1, subcomplex |
| 222657_s_at | 15.93 | 1.19E-05 | 1.069797208 | NM_001001481| | FLJ11011,hypothetical protein FLJ11011 isoform 1 |
| 242979_at | 15.93 | 1.19E-05 | 1.44388781 | NM_005544| | IRS1,insulin receptor substrate 1 |
| 224325_at | 15.93 | 1.19E-05 | 1.94856778 | NM_031866| | FZD8,frizzled 8 |
| 223169_s_at | 15.93 | 1.19E-05 | 1.627378052 | NM_021205| | RHOU,ras homolog gene family, member U |
| 219300_s_at | 15.93 | 1.19E-05 | 2.313899037 | NM_014141| | CNTNAP2,cell recognition molecule Caspr2 precursor |
| 211702_s_at | 15.93 | 1.19E-05 | 1.24163083 | NM_032582| | USP32,ubiquitin specific protease 32 |
| 215603_x_at | 15.92 | 1.19E-05 | 1.182144373 | NM_001032364| | NA |
| 230597_at | 15.91 | 1.19E-05 | 2.445955458 | NM_001048164| | NA |
| 204976_s_at | 15.91 | 1.19E-05 | 1.049691336 | NM_001025580| | NA |
| 206782_s_at | 15.91 | 1.19E-05 | 1.270606966 | NM_005528| | DNAJC4,DnaJ (Hsp40) homolog, subfamily C, member 4 |
| 205165_at | 15.9 | 1.20E-05 | 1.105614954 | NM_001040454| | NA |
| 230406_at | 15.89 | 1.20E-05 | 1.372291099 | NA |  |
| 47550_at | 15.89 | 1.20E-05 | 1.636136485 | NM_021020| | LZTS1,leucine zipper, putative tumor suppressor 1 |
| 202625_at | 15.89 | 1.20E-05 | 1.508559596 | NM_001111097| | NA |
| 208682_s_at | 15.89 | 1.20E-05 | 1.128294027 | NM_014599| | MAGED2,melanoma antigen family D, 2 |
| 210115_at | 15.89 | 1.20E-05 | 1.141417675 | NM_052969| | RPL39L,ribosomal protein L39-like protein |
| 206634_at | 15.88 | 1.21E-05 | 1.427884237 | NM_005413| | SIX3,sine oculis homeobox homolog 3 |
| 228622_s_at | 15.87 | 1.21E-05 | 1.238358048 | NM_005528| | DNAJC4,DnaJ (Hsp40) homolog, subfamily C, member 4 |
| 218325_s_at | 15.87 | 1.21E-05 | 1.274859414 | NM_022105| | DATF1,death associated transcription factor 1 isoform |
| 226129_at | 15.86 | 1.21E-05 | 1.899241691 | NM_198488| | FLJ46072,FLJ46072 protein |
| 230534_at | 15.86 | 1.21E-05 | 1.252196723 | NA |  |
| 201256_at | 15.86 | 1.21E-05 | 1.055035976 | NM_004718| | COX7A2L,cytochrome c oxidase subunit VIIa polypeptide 2 |
| 1559065_a_at | 15.85 | 1.22E-05 | 1.457430707 | NM_198492| | CLEC4G,C-type lectin superfamily 4, member G |
| 211937_at | 15.85 | 1.22E-05 | 1.08845979 | NM_001417| | EIF4B,eukaryotic translation initiation factor 4B |
| 219466_s_at | 15.84 | 1.22E-05 | 1.792787934 | NM_001643| | APOA2,apolipoprotein A-II precursor |
| 225919_s_at | 15.83 | 1.22E-05 | 1.125973702 | NM_018325| | C9orf72,hypothetical protein MGC23980 isoform a |
| 206490_at | 15.83 | 1.23E-05 | 1.833023413 | NM_001003809| | DLGAP1,discs large homolog-associated protein 1 isoform |
| 209222_s_at | 15.82 | 1.23E-05 | 1.109561159 | NM_014835| | OSBPL2,oxysterol-binding protein-like protein 2 isoform |
| 206356_s_at | 15.81 | 1.23E-05 | 1.831709527 | NM_002071| | GNAL,guanine nucleotide binding protein (G protein), |
| 207949_s_at | 15.81 | 1.23E-05 | 1.235221237 | NM_004968| | ICA1,islet cell autoantigen 1 isoform 2 |
| 203492_x_at | 15.81 | 1.23E-05 | 1.106686808 | NM_014679| | PIG8,translokin |
| 204502_at | 15.81 | 1.23E-05 | 1.277365088 | NM_015474| | SAMHD1,SAM domain- and HD domain-containing protein 1 |
| 226878_at | 15.79 | 1.24E-05 | 2.005138835 | NM_002119| | HLA-DOA,major histocompatibility complex, class II, DO |
| 206417_at | 15.79 | 1.24E-05 | 1.805625664 | NM_000087| | CNGA1,cyclic nucleotide gated channel alpha 1 |
| 229513_at | 15.79 | 1.24E-05 | 1.184492381 | NM_018387| | STRBP,spermatid perinuclear RNA-binding protein |
| 209892_at | 15.78 | 1.25E-05 | 1.50821767 | NM_002033| | FUT4,fucosyltransferase 4 |
| 218205_s_at | 15.78 | 1.25E-05 | 1.084601965 | NM_017572| | MKNK2,MAP kinase-interacting serine/threonine kinase |
| 222401_s_at | 15.77 | 1.25E-05 | 1.07696561 | NM_014313| | SMP1,small membrane protein 1 |
| 225135_at | 15.77 | 1.25E-05 | 1.149521302 | NM_015477| | SIN3A,transcriptional co-repressor Sin3A |
| 1564154_at | 15.76 | 1.25E-05 | 1.867503097 | NA |  |
| 1564662_at | 15.75 | 1.26E-05 | 1.223956195 | NA |  |
| 226982_at | 15.75 | 1.26E-05 | 1.427109773 | NM_012081| | ELL2,elongation factor, RNA polymerase II, 2 |
| 202323_s_at | 15.73 | 1.27E-05 | 1.147366296 | NM_022735| | ACBD3,golgi complex associated protein 1 |
| 219114_at | 15.72 | 1.27E-05 | 1.173870572 | NM_016210| | C3orf18,chromosome 3 open reading frame 18 |
| 226963_at | 15.72 | 1.27E-05 | 1.075045516 | NM_152265| | MGC23908,similar to transcription factor BTF3 |
| 204922_at | 15.71 | 1.28E-05 | 1.275991518 | NM_024650| | FLJ22531,hypothetical protein FLJ22531 |
| 234725_s_at | 15.71 | 1.27E-05 | 1.102598817 | NM_020210| | SEMA4B,semaphorin 4B precursor |
| 212571_at | 15.71 | 1.28E-05 | 1.08172216 | NM_020920| | CHD8,chromodomain helicase DNA binding protein 8 |
| 219793_at | 15.68 | 1.29E-05 | 1.532410828 | NM_022133| | SNX16,sorting nexin 16 isoform a |
| 225278_at | 15.68 | 1.29E-05 | 1.156583901 | NM_005399| | PRKAB2,AMP-activated protein kinase beta 2 |
| 224990_at | 15.67 | 1.29E-05 | 1.359570126 | NM_174921| | LOC201895,hypothetical protein LOC201895 |
| 1558143_a_at | 15.66 | 1.30E-05 | 1.301193449 | NM_006538| | BCL2L11,BCL2-like 11 isoform 6 |
| 210069_at | 15.66 | 1.30E-05 | 1.504502808 | NM_004377| | CPT1B,carnitine palmitoyltransferase 1B isoform a |
| 218441_s_at | 15.66 | 1.30E-05 | 1.094053376 | NM_015540| | RPAP1,RNA polymerase II associated protein 1 |
| 218486_at | 15.66 | 1.30E-05 | 1.165286685 | NM_003597| | KLF11,Kruppel-like factor 11 |
| 220195_at | 15.65 | 1.30E-05 | 1.162295632 | NM_018328| | MBD5,methyl-CpG binding domain protein 5 |
| 225900_at | 15.65 | 1.30E-05 | 1.21826682 | NM_015189| | NA |
| 37802_r_at | 15.65 | 1.30E-05 | 1.137292487 | NM_001040450| | NA |
| 228824_s_at | 15.64 | 1.31E-05 | 1.11678431 | NM_012212| | LTB4DH,NADP-dependent leukotriene B4 |
| 202117_at | 15.64 | 1.31E-05 | 1.038672627 | NM_004308| | ARHGAP1,Rho GTPase activating protein 1 |
| 37005_at | 15.63 | 1.31E-05 | 1.149908627 | NM_005380| | NBL1,neuroblastoma, suppression of tumorigenicity 1 |
| 207183_at | 15.63 | 1.31E-05 | 1.158793345 | NM_006143| | GPR19,G protein-coupled receptor 19 |
| 235763_at | 15.63 | 1.31E-05 | 1.55098555 | NM_152697| | MGC34032,hypothetical protein MGC34032 |
| 55065_at | 15.63 | 1.31E-05 | 1.135250697 | NM_031417| | MARK4,MAP/microtubule affinity-regulating kinase 4 |
| 1560739_a_at | 15.62 | 1.31E-05 | 1.605508077 | NA |  |
| 222523_at | 15.61 | 1.32E-05 | 1.159363823 | NM_021627| | SENP2,SUMO1/sentrin/SMT3 specific protease 2 |
| 221664_s_at | 15.61 | 1.32E-05 | 2.24775912 | NM_016946| | F11R,F11 receptor isoform a precursor |
| 227647_at | 15.58 | 1.33E-05 | 1.464298449 | NM_005472| | KCNE3,potassium voltage-gated channel, Isk-related |
| 232780_s_at | 15.58 | 1.33E-05 | 1.124039184 | NM_015911| | LOC51058,hypothetical protein LOC51058 |
| 221065_s_at | 15.58 | 1.33E-05 | 1.301294038 | NM_022467| | CHST8,carbohydrate (N-acetylgalactosamine 4-0) |
| 232647_at | 15.58 | 1.33E-05 | 1.268932582 | NM_152465| | PROCA1,proline-rich cyclin A1-interacting protein |
| 227172_at | 15.57 | 1.34E-05 | 1.16964164 | NM_138341| | LOC89894,hypothetical protein BC000282 |
| 213698_at | 15.56 | 1.34E-05 | 1.118398755 | NM_007167| | ZNF258,zinc finger protein 258 |
| 229264_at | 15.56 | 1.34E-05 | 1.406558774 | NA |  |
| 205414_s_at | 15.56 | 1.34E-05 | 1.689025098 | NM_014859| | KIAA0672,KIAA0672 gene product |
| 227004_at | 15.56 | 1.34E-05 | 2.216750287 | NA |  |
| 1560738_at | 15.55 | 1.34E-05 | 1.687780278 | NA |  |
| 219113_x_at | 15.55 | 1.35E-05 | 1.32502515 | NM_016246| | DHRS10,dehydrogenase/reductase (SDR family) member 10 |
| 228952_at | 15.55 | 1.34E-05 | 1.424159215 | NM_006208| | ENPP1,ectonucleotide pyrophosphatase/phosphodiesterase |
| 242602_x_at | 15.55 | 1.34E-05 | 1.120893016 | NM_203282| | ZNF539,zinc finger protein 539 |
| 224910_at | 15.54 | 1.35E-05 | 1.28808638 | NM_001042476| | NA |
| 221790_s_at | 15.54 | 1.35E-05 | 1.248568861 | NM_015627| | ARH,LDL receptor adaptor protein |
| 1555679_a_at | 15.54 | 1.35E-05 | 1.122175065 | NM_032730| | RTN4IP1,reticulon 4 interacting protein 1 |
| 219826_at | 15.54 | 1.35E-05 | 1.124719497 | NM_001098491| | NA |
| 244758_at | 15.53 | 1.35E-05 | 1.462026393 | NM_052923| | ZNF452,zinc finger protein 452 |
| 222459_at | 15.53 | 1.36E-05 | 1.207669884 | NM_024595| | FLJ12666,hypothetical protein FLJ12666 |
| 232687_at | 15.52 | 1.36E-05 | 1.471223188 | NA |  |
| 1555724_s_at | 15.51 | 1.36E-05 | 1.698653606 | NM_001001522| | TAGLN,transgelin |
| 218088_s_at | 15.51 | 1.36E-05 | 1.105830053 | NM_022157| | RRAGC,Ras-related GTP binding C |
| 223235_s_at | 15.5 | 1.37E-05 | 2.288698444 | NM_022138| | SMOC2,secreted modular calcium-binding protein 2 |
| 236113_at | 15.5 | 1.37E-05 | 1.37344091 | NA |  |
| 219929_s_at | 15.48 | 1.38E-05 | 1.163066938 | NM_024071| | ZFYVE21,zinc finger, FYVE domain containing 21 |
| 203830_at | 15.47 | 1.38E-05 | 1.127127996 | NM_022344| | NJMU-R1,protein kinase Njmu-R1 |
| 205778_at | 15.46 | 1.39E-05 | 1.450581898 | NM_005046| | KLK7,stratum corneum chymotryptic enzyme |
| 37986_at | 15.46 | 1.39E-05 | 1.270103063 | NM_000121| | EPOR,erythropoietin receptor precursor |
| 213279_at | 15.45 | 1.39E-05 | 1.086419747 | NM_138452| | DHRS1,dehydrogenase/reductase (SDR family) member 1 |
| 239761_at | 15.45 | 1.39E-05 | 2.037703754 | NM_001097633| | NA |
| 223383_at | 15.45 | 1.39E-05 | 1.114312032 | NM_032268| | ZNRF1,zinc and ring finger protein 1 |
| 220624_s_at | 15.44 | 1.40E-05 | 1.119775621 | NM_001422| | ELF5,E74-like factor 5 ESE-2b |
| 218906_x_at | 15.44 | 1.39E-05 | 1.068923001 | NM_022822| | KLC2,likely ortholog of kinesin light chain 2 |
| 239770_at | 15.44 | 1.39E-05 | 1.472862458 | NM_031913| | CHR3SYT,chr3 synaptotagmin |
| 226512_at | 15.43 | 1.40E-05 | 1.226749514 | NM_003453| | ZNF198,zinc finger protein 198 |
| 205011_at | 15.43 | 1.40E-05 | 1.110944687 | NM_014622| | LOH11CR2A,BCSC-1 isoform 1 |
| 217984_at | 15.43 | 1.40E-05 | 1.217753233 | NM_003730| | RNASET2,ribonuclease 6 precursor |
| 212744_at | 15.43 | 1.40E-05 | 1.123114707 | NM_033028| | BBS4,Bardet-Biedl syndrome 4 |
| 213106_at | 15.42 | 1.40E-05 | 1.705669794 | NM_001105529| | NA |
| 223665_at | 15.42 | 1.40E-05 | 1.595321619 | NM_032487| | ARPM1,actin related protein M1 |
| 230746_s_at | 15.42 | 1.40E-05 | 2.006900485 | NM_003155| | STC1,stanniocalcin 1 |
| 237306_at | 15.4 | 1.41E-05 | 1.088908345 | NM_001037232| | NA |
| 233543_s_at | 15.39 | 1.42E-05 | 1.118701886 | NM_139076| | FLJ13614,hypothetical protein FLJ13614 |
| 225784_s_at | 15.39 | 1.42E-05 | 1.21757354 | NM_018684| | KIAA1166,KIAA1166 |
| 229649_at | 15.39 | 1.42E-05 | 1.722289711 | NM_001105250| | NA |
| 205593_s_at | 15.39 | 1.42E-05 | 1.306390347 | NM_001001567| | PDE9A,phosphodiesterase 9A isoform b |
| 222839_s_at | 15.38 | 1.42E-05 | 1.158162389 | NM_022894| | PAPOLG,poly(A) polymerase gamma |
| 244052_at | 15.38 | 1.43E-05 | 1.110749251 | NM_032783| | CBR4,carbonic reductase 4 |
| 227618_at | 15.37 | 1.43E-05 | 1.153254576 | NA |  |
| 224846_at | 15.37 | 1.43E-05 | 1.164953324 | NM_138392| | SHKBP1,SH3KBP1 binding protein 1 |
| 226435_at | 15.35 | 1.44E-05 | 2.103215837 | NM_173462| | PAPLN,papilin |
| 1555742_at | 15.35 | 1.44E-05 | 1.425517146 | NA |  |
| 205578_at | 15.35 | 1.44E-05 | 1.32653929 | NM_004560| | ROR2,receptor tyrosine kinase-like orphan receptor 2 |
| 202721_s_at | 15.34 | 1.44E-05 | 1.335577762 | NM_002056| | GFPT1,glucosamine-fructose-6-phosphate |
| 216222_s_at | 15.34 | 1.44E-05 | 1.114171756 | NM_012334| | MYO10,myosin X |
| 221881_s_at | 15.34 | 1.44E-05 | 1.419117167 | NM_013943| | CLIC4,chloride intracellular channel 4 |
| 202338_at | 15.34 | 1.44E-05 | 1.075518752 | NM_003258| | TK1,thymidine kinase 1, soluble |
| 203013_at | 15.34 | 1.44E-05 | 1.100205009 | NM_007265| | HSGT1,suppressor of S. cerevisiae gcr2 |
| 219801_at | 15.33 | 1.45E-05 | 1.240613802 | NM_030580| | ZNF34,zinc finger protein 34 (KOX 32) |
| 202385_s_at | 15.33 | 1.44E-05 | 1.09594229 | NM_000356| | TCOF1,Treacher Collins-Franceschetti syndrome 1 |
| 225234_at | 15.33 | 1.44E-05 | 1.114245049 | NM_005188| | CBL,Cas-Br-M (murine) ecotropic retroviral |
| 243672_at | 15.33 | 1.45E-05 | 1.287744758 | NM_171999| | SALL3,sal-like 3 |
| 228245_s_at | 15.32 | 1.45E-05 | 1.269788458 | NM_001080502| | NA |
| 216228_s_at | 15.32 | 1.45E-05 | 1.252935957 | NM_001008396| | WDHD1,WD repeat and HMG-box DNA binding protein 1 |
| 223404_s_at | 15.3 | 1.46E-05 | 1.231325836 | NM_030934| | C1orf25,N2,N2-dimethylguanosine tRNA |
| 228742_at | 15.3 | 1.46E-05 | 1.472410682 | NA |  |
| 242923_at | 15.3 | 1.46E-05 | 1.125773531 | NM_178549| | MGC42493,hypothetical protein MGC42493 |
| 208614_s_at | 15.3 | 1.46E-05 | 1.182812027 | NM_001457| | FLNB,filamin B, beta (actin binding protein 278) |
| 207268_x_at | 15.3 | 1.46E-05 | 1.071131598 | NM_005759| | ABI2,abl interactor 2 |
| 1554980_a_at | 15.29 | 1.47E-05 | 1.234149971 | NM_001030287| | NA |
| 203860_at | 15.29 | 1.47E-05 | 1.187751137 | NM_000282| | PCCA,propionyl-Coenzyme A carboxylase, alpha |
| 208813_at | 15.29 | 1.47E-05 | 1.135584154 | NM_002079| | GOT1,aspartate aminotransferase 1 |
| 234085_at | 15.28 | 1.47E-05 | 1.694294822 | NA |  |
| 221985_at | 15.28 | 1.47E-05 | 1.240471734 | NM_017644| | DRE1,DRE1 protein |
| 228430_at | 15.28 | 1.47E-05 | 1.090170005 | NA |  |
| 212317_at | 15.27 | 1.48E-05 | 1.12941928 | NM_012470| | TNPO3,transportin 3 |
| 209962_at | 15.26 | 1.48E-05 | 1.392626814 | NM_000121| | EPOR,erythropoietin receptor precursor |
| 212311_at | 15.26 | 1.48E-05 | 1.358389914 | NM_015187| | KIAA0746,KIAA0746 protein |
| 1557369_a_at | 15.25 | 1.49E-05 | 1.926319093 | NA |  |
| 212264_s_at | 15.25 | 1.49E-05 | 1.137769217 | NM_015045| | KIAA0261,KIAA0261 |
| 219992_at | 15.25 | 1.49E-05 | 1.4791735 | NM_001006667| | TAC3,tachykinin 3 |
| 218638_s_at | 15.24 | 1.49E-05 | 1.202536342 | NM_012445| | SPON2,spondin 2, extracellular matrix protein |
| 241829_at | 15.24 | 1.49E-05 | 1.992469503 | NM_145019| | FLJ30707,hypothetical protein FLJ30707 |
| 203522_at | 15.23 | 1.49E-05 | 1.16074732 | NM_005125| | CCS,copper chaperone for superoxide dismutase |
| 210058_at | 15.23 | 1.49E-05 | 1.351428853 | NM_002754| | MAPK13,mitogen-activated protein kinase 13 |
| 209698_at | 15.22 | 1.50E-05 | 1.123516564 | NM_001105563| | NA |
| 206231_at | 15.22 | 1.50E-05 | 1.134317298 | NM_002248| | KCNN1,potassium intermediate/small conductance |
| 203912_s_at | 15.21 | 1.50E-05 | 1.244839556 | NM_001009932| | DNASE1L1,deoxyribonuclease I-like 1 precursor |
| 203266_s_at | 15.21 | 1.51E-05 | 1.056185069 | NM_003010| | MAP2K4,mitogen-activated protein kinase kinase 4 |
| 204985_s_at | 15.21 | 1.51E-05 | 1.1461797 | NM_024108| | MGC2650,hypothetical protein MGC2650 |
| 210287_s_at | 15.21 | 1.51E-05 | 1.926754154 | NM_002019| | FLT1,fms-related tyrosine kinase 1 (vascular |
| 213047_x_at | 15.21 | 1.50E-05 | 1.043821317 | NM_001122821| | NA |
| 235103_at | 15.2 | 1.51E-05 | 1.265997378 | NM_002372| | MAN2A1,mannosidase, alpha, class 2A, member 1 |
| 228624_at | 15.2 | 1.51E-05 | 1.695732225 | NM_018342| | FLJ11155,hypothetical protein FLJ11155 |
| 201798_s_at | 15.19 | 1.52E-05 | 1.810919503 | NM_013451| | FER1L3,myoferlin isoform a |
| 213249_at | 15.19 | 1.51E-05 | 1.154959616 | NM_012304| | FBXL7,F-box and leucine-rich repeat protein 7 |
| 1566152_a_at | 15.19 | 1.52E-05 | 1.359944846 | NM_006831| | HEAB,ATP/GTP-binding protein |
| 209102_s_at | 15.18 | 1.52E-05 | 1.117669721 | NM_012257| | HBP1,HMG-box transcription factor 1 |
| 205981_s_at | 15.18 | 1.52E-05 | 1.142185362 | NM_001564| | ING2,inhibitor of growth family, member 1-like |
| 229849_at | 15.18 | 1.52E-05 | 1.638831556 | NA |  |
| 223426_s_at | 15.17 | 1.53E-05 | 1.18380352 | NM_018424| | EPB41L4B,erythrocyte membrane protein band 4.1 like 4B |
| 214820_at | 15.16 | 1.53E-05 | 1.143987567 | NM_001007246| | WDR9,WD repeat domain 9 isoform C |
| 224704_at | 15.16 | 1.53E-05 | 1.086302389 | NM_014494| | TNRC6A,trinucleotide repeat containing 6A |
| 204309_at | 15.16 | 1.53E-05 | 1.289791992 | NM_000781| | CYP11A1,cytochrome P450, subfamily XIA precursor |
| 226276_at | 15.16 | 1.53E-05 | 1.063416114 | NM_174909| | MGC23909,hypothetical protein MGC23909 |
| 223503_at | 15.16 | 1.53E-05 | 1.546812417 | NM_030923| | DKFZP566N034,hypothetical protein DKFZp566N034 |
| 227131_at | 15.16 | 1.53E-05 | 1.136398386 | NM_002401| | MAP3K3,mitogen-activated protein kinase kinase kinase 3 |
| 210639_s_at | 15.15 | 1.54E-05 | 1.164851427 | NM_004849| | APG5L,APG5 autophagy 5-like |
| 232549_at | 15.14 | 1.54E-05 | 1.498211885 | NM_144770| | RBM11,RNA binding motif protein 11 |
| 208091_s_at | 15.14 | 1.54E-05 | 1.089595906 | NM_030796| | DKFZP564K0822,hypothetical protein DKFZp564K0822 |
| 231381_at | 15.14 | 1.54E-05 | 3.677151183 | NA |  |
| 242515_x_at | 15.14 | 1.54E-05 | 1.066541768 | NM_020642| | C11orf17,chromosome 11 open reading frame 17 |
| 201176_s_at | 15.13 | 1.55E-05 | 1.093383558 | NM_001655| | ARCN1,archain |
| 203985_at | 15.12 | 1.55E-05 | 1.067378224 | NM_012256| | ZNF212,zinc finger protein 212 |
| 239302_s_at | 15.11 | 1.56E-05 | 1.370560321 | NA |  |
| 227163_at | 15.11 | 1.56E-05 | 1.491592133 | NM_183239| | GSTO2,glutathione S-transferase omega 2 |
| 206981_at | 15.1 | 1.56E-05 | 1.146252928 | NM_000334| | SCN4A,sodium channel, voltage-gated, type IV, alpha |
| 216202_s_at | 15.09 | 1.57E-05 | 1.243530179 | NM_004863| | SPTLC2,serine palmitoyltransferase, long chain base |
| 216054_x_at | 15.09 | 1.57E-05 | 1.243943371 | NM_001002841| | MYL4,atrial/embryonic alkali myosin light chain |
| 205850_s_at | 15.09 | 1.57E-05 | 2.23359862 | NM_000814| | GABRB3,gamma-aminobutyric acid (GABA) A receptor, beta |
| 220382_s_at | 15.09 | 1.57E-05 | 1.518891473 | NM_001010000| | ARHGAP28,Rho GTPase activating protein 28 isoform a |
| 211450_s_at | 15.08 | 1.57E-05 | 1.156056614 | NM_000179| | MSH6,mutS homolog 6 |
| 214761_at | 15.08 | 1.57E-05 | 1.16290947 | NM_015069| | ZNF423,zinc finger protein 423 |
| 202525_at | 15.08 | 1.57E-05 | 1.961796309 | NM_002773| | PRSS8,prostasin preproprotein |
| 205489_at | 15.08 | 1.57E-05 | 1.150879654 | NM_001014444| | NA |
| 210907_s_at | 15.07 | 1.58E-05 | 1.047967258 | NM_007217| | PDCD10,programmed cell death 10 |
| 1558692_at | 15.07 | 1.58E-05 | 1.935740534 | NM_144580| | MGC31963,kidney predominant protein NCU-G1 |
| 1552486_s_at | 15.07 | 1.58E-05 | 1.322293114 | NM_032857| | LACTB,lactamase, beta isoform a |
| 205441_at | 15.07 | 1.58E-05 | 1.219753278 | NM_024578| | FLJ22709,hypothetical protein FLJ22709 |
| 203798_s_at | 15.07 | 1.58E-05 | 2.164766044 | NM_003385| | VSNL1,visinin-like 1 |
| 243880_at | 15.06 | 1.58E-05 | 1.762482686 | NM_001012511| | GOSR2,golgi SNAP receptor complex member 2 isoform C |
| 217982_s_at | 15.06 | 1.58E-05 | 1.042257367 | NM_006791| | MORF4L1,MORF-related gene 15 isoform 1 |
| 204832_s_at | 15.06 | 1.58E-05 | 1.137196668 | NM_004329| | BMPR1A,bone morphogenetic protein receptor, type IA |
| 213572_s_at | 15.05 | 1.59E-05 | 1.424888125 | NM_030666| | SERPINB1,serine (or cysteine) proteinase inhibitor, clade |
| 233911_s_at | 15.05 | 1.59E-05 | 1.344775449 | NM_020700| | NA |
| 230793_at | 15.03 | 1.60E-05 | 1.434051078 | NM_017640| | LRRC16,leucine rich repeat containing 16 |
| 216037_x_at | 15.03 | 1.60E-05 | 1.14425887 | NM_030756| | TCF7L2,transcription factor 7-like 2 (T-cell specific, |
| 202113_s_at | 15.03 | 1.60E-05 | 1.105889926 | NM_003100| | SNX2,sorting nexin 2 |
| 226659_at | 15.03 | 1.60E-05 | 1.522040877 | NM_022047| | DEF6,differentially expressed in FDCP 6 homolog |
| 228859_at | 15.03 | 1.60E-05 | 1.183308148 | NM_001099776| | NA |
| 1557167_at | 15.03 | 1.60E-05 | 1.357052709 | NA |  |
| 1557014_a_at | 15.02 | 1.60E-05 | 1.632658946 | NA |  |
| 204257_at | 15.02 | 1.61E-05 | 1.152410473 | NM_021727| | FADS3,fatty acid desaturase 3 |
| 213859_x_at | 15.02 | 1.60E-05 | 1.074031389 | NM_003601| | SMARCA5,SWI/SNF-related matrix-associated |
| 218967_s_at | 15.01 | 1.61E-05 | 1.159312828 | NM_001001484| | PTER,phosphotriesterase related |
| 213926_s_at | 15.01 | 1.61E-05 | 1.181528586 | NM_004504| | HRB,HIV-1 Rev binding protein |
| 208037_s_at | 15.01 | 1.61E-05 | 1.231550269 | NM_130760| | MADCAM1,mucosal vascular addressin cell adhesion |
| 203058_s_at | 15.01 | 1.61E-05 | 1.53277653 | NM_001015880| | NA |
| 202193_at | 15.01 | 1.61E-05 | 1.176480079 | NM_001031801| | NA |
| 226450_at | 15 | 1.61E-05 | 1.286087667 | NM_000208| | INSR,insulin receptor |
| 209394_at | 15 | 1.62E-05 | 1.143626256 | NM_004192| | ASMTL,acetylserotonin O-methyltransferase-like |
| 220381_at | 15 | 1.61E-05 | 1.323741278 | NM_001010000| | ARHGAP28,Rho GTPase activating protein 28 isoform a |
| 211689_s_at | 14.99 | 1.62E-05 | 1.437419717 | NM_005656| | TMPRSS2,transmembrane protease, serine 2 |
| 208724_s_at | 14.99 | 1.62E-05 | 1.043300321 | NM_004161| | RAB1A,RAB1A, member RAS oncogene family |
| 201602_s_at | 14.99 | 1.62E-05 | 1.167051409 | NM_002480| | PPP1R12A,protein phosphatase 1, regulatory (inhibitor) |
| 218949_s_at | 14.98 | 1.62E-05 | 1.125560477 | NM_018292| | QRSL1,glutaminyl-tRNA synthase |
| 225601_at | 14.97 | 1.63E-05 | 1.144480504 | NM_005342| | HMGB3,high-mobility group box 3 |
| 222847_s_at | 14.97 | 1.63E-05 | 1.383579666 | NM_022073| | EGLN3,egl nine homolog 3 |
| 209357_at | 14.97 | 1.63E-05 | 1.211584748 | NM_006079| | CITED2,Cbp/p300-interacting transactivator, with |
| 218622_at | 14.97 | 1.63E-05 | 1.048268628 | NM_024057| | NUP37,nucleoporin 37kDa |
| 208758_at | 14.95 | 1.64E-05 | 1.071513741 | NM_004044| | ATIC,5-aminoimidazole-4-carboxamide ribonucleotide |
| 226620_x_at | 14.95 | 1.64E-05 | 1.048355768 | NM_018959| | DAZAP1,DAZ associated protein 1 isoform b |
| 1559957_a_at | 14.95 | 1.64E-05 | 1.205604327 | NA |  |
| 227468_at | 14.94 | 1.64E-05 | 1.273538593 | NM_152359| | CPT1C,carnitine palmitoyltransferase 1C |
| 215925_s_at | 14.94 | 1.64E-05 | 1.588701072 | NM_001782| | CD72,CD72 antigen |
| 203632_s_at | 14.93 | 1.65E-05 | 1.095501261 | NM_016235| | GPRC5B,G protein-coupled receptor, family C, group 5, |
| 209632_at | 14.91 | 1.66E-05 | 1.1647243 | NM_002718| | PPP2R3A,alpha isoform of regulatory subunit B'', protein |
| 201696_at | 14.91 | 1.67E-05 | 1.044074091 | NM_005626| | SFRS4,splicing factor, arginine/serine-rich 4 |
| 227417_at | 14.9 | 1.67E-05 | 1.253789785 | NM_017898| | FLJ20605,hypothetical protein FLJ20605 |
| 218489_s_at | 14.9 | 1.67E-05 | 1.151059639 | NM_000031| | ALAD,delta-aminolevulinic acid dehydratase isoform b |
| 204023_at | 14.88 | 1.69E-05 | 1.056748477 | NM_002916| | RFC4,replication factor C 4 |
| 228596_at | 14.87 | 1.69E-05 | 1.975289228 | NA |  |
| 1560853_x_at | 14.87 | 1.69E-05 | 1.985277194 | NM_001039884| | NA |
| 208811_s_at | 14.86 | 1.70E-05 | 1.11746815 | NM_005494| | DNAJB6,DnaJ (Hsp40) homolog, subfamily B, member 6 |
| 201276_at | 14.86 | 1.70E-05 | 1.099954492 | NM_002868| | RAB5B,RAB5B, member RAS oncogene family |
| 1557961_s_at | 14.86 | 1.70E-05 | 1.204062243 | NA |  |
| 219418_at | 14.85 | 1.70E-05 | 1.076961586 | NM_024782| | FLJ12610,similar to mouse 1700029B21Rik protein |
| 203680_at | 14.85 | 1.70E-05 | 1.127074514 | NM_002736| | PRKAR2B,cAMP-dependent protein kinase, regulatory |
| 222395_s_at | 14.84 | 1.71E-05 | 1.105390091 | NM_023079| | FLJ13855,hypothetical protein FLJ13855 |
| 203677_s_at | 14.83 | 1.71E-05 | 1.072928959 | NM_004178| | TARBP2,TAR RNA binding protein 2 isoform c |
| 223729_at | 14.83 | 1.72E-05 | 1.319586965 | NM_031413| | NA |
| 226530_at | 14.83 | 1.71E-05 | 1.46552701 | NM_001003940| | BMF,Bcl2 modifying factor isoform bmf-1 |
| 202603_at | 14.82 | 1.72E-05 | 1.057254627 | NA |  |
| 217665_at | 14.81 | 1.73E-05 | 1.392530871 | NA |  |
| 223327_x_at | 14.81 | 1.73E-05 | 1.113022339 | NA |  |
| 235165_at | 14.8 | 1.73E-05 | 1.48124883 | NM_032521| | NA |
| 202720_at | 14.8 | 1.73E-05 | 1.218609872 | NM_015641| | TES,testin isoform 1 |
| 218885_s_at | 14.8 | 1.73E-05 | 1.362998523 | NM_024642| | GALNT12,UDP-N-acetyl-alpha-D-galactosamine:polypeptide |
| 230423_at | 14.8 | 1.73E-05 | 1.947478728 | NA |  |
| 204728_s_at | 14.8 | 1.73E-05 | 1.285238379 | NM_001008396| | WDHD1,WD repeat and HMG-box DNA binding protein 1 |
| 1553103_at | 14.8 | 1.73E-05 | 1.14118149 | NM_002504| | NFX1,nuclear transcription factor, X-box binding 1 |
| 231050_at | 14.79 | 1.74E-05 | 1.748224134 | NM_054108| | HRLP5,H-rev107-like protein 5 |
| 227017_at | 14.79 | 1.74E-05 | 1.061990556 | NM_207332| | LOC157697,hypothetical protein LOC157697 |
| 204853_at | 14.79 | 1.74E-05 | 1.136981984 | NM_006190| | ORC2L,origin recognition complex, subunit 2 |
| 203585_at | 14.79 | 1.74E-05 | 1.363059028 | NM_007150| | ZNF185,zinc finger protein 185 (LIM domain) |
| 238662_at | 14.78 | 1.75E-05 | 1.156769519 | NM_080650| | MGC14798,hypothetical protein MGC14798 |
| 219420_s_at | 14.77 | 1.75E-05 | 1.095007475 | NM_023077| | FLJ12439,hypothetical protein FLJ12439 |
| 230067_at | 14.77 | 1.75E-05 | 1.655262332 | NM_145019| | FLJ30707,hypothetical protein FLJ30707 |
| 223589_at | 14.75 | 1.76E-05 | 1.077496 | NM_017879| | ZNF416,zinc finger protein 416 |
| 227088_at | 14.75 | 1.77E-05 | 2.41124444 | NM_001083| | PDE5A,phosphodiesterase 5A isoform 1 |
| 202918_s_at | 14.75 | 1.76E-05 | 1.100802786 | NM_001100819| | NA |
| 202003_s_at | 14.74 | 1.77E-05 | 1.103882424 | NM_006111| | ACAA2,acetyl-coenzyme A acyltransferase 2 |
| 235199_at | 14.73 | 1.78E-05 | 1.619341653 | NM_017831| | RNF125,ring finger protein 125 |
| 211950_at | 14.73 | 1.78E-05 | 1.087811634 | NM_020765| | RBAF600,retinoblastoma-associated factor 600 |
| 222981_s_at | 14.72 | 1.79E-05 | 1.101880053 | NM_016131| | RAB10,ras-related GTP-binding protein RAB10 |
| 209278_s_at | 14.72 | 1.79E-05 | 1.500206148 | NM_006528| | TFPI2,tissue factor pathway inhibitor 2 |
| 225164_s_at | 14.72 | 1.79E-05 | 1.134133933 | NM_001013703| | NA |
| 208490_x_at | 14.72 | 1.79E-05 | 1.103360565 | NM_003518| | HIST1H2BG,H2B histone family, member A |
| 201331_s_at | 14.71 | 1.79E-05 | 1.486820505 | NM_003153| | STAT6,signal transducer and activator of transcription |
| 227262_at | 14.71 | 1.79E-05 | 1.161642606 | NM_178232| | HAPLN3,hyaluronan and proteoglycan link protein 3 |
| 225925_s_at | 14.71 | 1.79E-05 | 1.111865932 | NM_001032730| | NA |
| 202337_at | 14.71 | 1.79E-05 | 1.132775798 | NM_007221| | PMF1,polyamine-modulated factor 1 |
| 206702_at | 14.7 | 1.79E-05 | 1.388290938 | NM_000459| | TEK,TEK tyrosine kinase, endothelial |
| 230441_at | 14.7 | 1.79E-05 | 1.322442189 | NM_052909| | KIAA1909,KIAA1909 protein |
| 225899_x_at | 14.7 | 1.80E-05 | 1.300749045 | NA |  |
| 230972_at | 14.7 | 1.79E-05 | 1.077110847 | NM_152326| | ANKRD9,ankyrin repeat domain 9 |
| 223167_s_at | 14.69 | 1.80E-05 | 1.300977119 | NM_013396| | USP25,ubiquitin specific protease 25 |
| 229497_at | 14.69 | 1.80E-05 | 1.234152905 | NM_182703| | LOC348094,hypothetical protein LOC348094 |
| 225519_at | 14.68 | 1.81E-05 | 1.081231609 | NM_174907| | PPP4R2,protein phosphatase 4, regulatory subunit 2 |
| 218530_at | 14.68 | 1.81E-05 | 1.135381151 | NM_013241| | FHOD1,formin homology 2 domain containing 1 |
| 201569_s_at | 14.68 | 1.81E-05 | 1.042397027 | NM_015380| | CGI-51,CGI-51 protein |
| 210826_x_at | 14.68 | 1.81E-05 | 1.125485455 | NM_002873| | RAD17,RAD17 homolog isoform 1 |
| 238819_at | 14.68 | 1.81E-05 | 1.244338074 | NM_032584| | ZNF347,zinc finger protein 347 |
| 227551_at | 14.67 | 1.82E-05 | 1.120210797 | NM_001025780| | NA |
| 231514_at | 14.67 | 1.81E-05 | 1.308789627 | NM_032884| | MGC15882,hypothetical protein MGC15882 |
| 218127_at | 14.67 | 1.81E-05 | 1.087660502 | NM_006166| | NFYB,nuclear transcription factor Y, beta |
| 212731_at | 14.66 | 1.82E-05 | 1.131751822 | NM_198401| | LOC157567,hypothetical protein LOC157567 |
| 226747_at | 14.66 | 1.82E-05 | 1.137592073 | NM_020784| | KIAA1344,KIAA1344 |
| 219968_at | 14.66 | 1.82E-05 | 1.201018526 | NM_016089| | ZNF589,zinc finger protein 589 |
| 203430_at | 14.65 | 1.82E-05 | 1.100970541 | NM_014320| | HEBP2,heme binding protein 2 |
| 201368_at | 14.65 | 1.82E-05 | 1.141976975 | NM_006887| | ZFP36L2,butyrate response factor 2 |
| 201881_s_at | 14.65 | 1.83E-05 | 1.073131818 | NM_005744| | ARIH1,ariadne homolog, ubiquitin-conjugating enzyme E2 |
| 239007_at | 14.65 | 1.82E-05 | 1.23292665 | NM_178523| | ZNF616,zinc finger protein 616 |
| 219403_s_at | 14.64 | 1.83E-05 | 1.432792546 | NM_001098540| | NA |
| 209019_s_at | 14.63 | 1.84E-05 | 1.162861398 | NM_032409| | PINK1,PTEN induced putative kinase 1 |
| 219660_s_at | 14.63 | 1.84E-05 | 1.495041778 | NM_016529| | ATP8A2,ATPase, aminophospholipid transporter-like, |
| 206149_at | 14.63 | 1.84E-05 | 1.461922522 | NM_022097| | LOC63928,hepatocellular carcinoma antigen gene 520 |
| 225746_at | 14.63 | 1.84E-05 | 1.436937384 | NM_032932| | RAB11FIP4,RAB11 family interacting protein 4 (class II) |
| 203962_s_at | 14.63 | 1.84E-05 | 1.996090472 | NM_006393| | NEBL,nebulette sarcomeric isoform |
| 214285_at | 14.62 | 1.84E-05 | 1.91100868 | NM_004102| | FABP3,fatty acid binding protein 3 |
| 219319_at | 14.62 | 1.85E-05 | 1.333050237 | NM_022462| | HIF3A,hypoxia-inducible factor-3 alpha isoform b |
| 208815_x_at | 14.62 | 1.85E-05 | 1.098947801 | NM_002154| | HSPA4,heat shock 70kDa protein 4 isoform a |
| 207843_x_at | 14.62 | 1.85E-05 | 1.065737582 | NM_001914| | CYB5,cytochrome b-5 isoform 2 |
| 219464_at | 14.61 | 1.85E-05 | 1.250696713 | NM_012113| | CA14,carbonic anhydrase XIV precursor |
| 227226_at | 14.61 | 1.85E-05 | 1.231215758 | NM_138409| | C6orf117,chromosome 6 open reading frame 117 |
| 227742_at | 14.61 | 1.85E-05 | 1.184121833 | NM_053277| | CLIC6,chloride intracellular channel 6 |
| 225447_at | 14.6 | 1.86E-05 | 1.119579543 | NM_000408| | GPD2,glycerol-3-phosphate dehydrogenase 2 |
| 223402_at | 14.6 | 1.85E-05 | 1.107382686 | NM_017823| | DUSP23,dual specificity phosphatase 23 |
| 201098_at | 14.6 | 1.86E-05 | 1.058628677 | NM_004766| | COPB2,coatomer protein complex, subunit beta 2 (beta |
| 227530_at | 14.6 | 1.86E-05 | 1.174753775 | NM_005100| | AKAP12,A-kinase anchor protein 12 isoform 1 |
| 221309_at | 14.59 | 1.86E-05 | 1.217758716 | NM_032905| | RBM17,RNA binding motif protein 17 |
| 225407_at | 14.58 | 1.87E-05 | 1.637369918 | NM_001025081| | NA |
| 231022_at | 14.58 | 1.87E-05 | 1.596329647 | NA |  |
| 209313_at | 14.57 | 1.88E-05 | 1.049043286 | NM_007266| | XAB1,XPA binding protein 1 |
| 228750_at | 14.57 | 1.88E-05 | 1.53669781 | NA |  |
| 216905_s_at | 14.57 | 1.88E-05 | 1.600155325 | NM_021978| | ST14,matriptase |
| 222212_s_at | 14.56 | 1.88E-05 | 1.058394491 | NM_022075| | LASS2,LAG1 longevity assurance homolog 2 isoform 1 |
| 227166_at | 14.56 | 1.88E-05 | 1.126169402 | NM_152686| | MGC29463,hypothetical protein MGC29463 |
| 203173_s_at | 14.56 | 1.88E-05 | 1.05975365 | NM_020314| | MGC16824,esophageal cancer associated protein |
| 218472_s_at | 14.55 | 1.89E-05 | 1.115229759 | NM_015946| | PELO,CGI-17 protein |
| 228226_s_at | 14.55 | 1.89E-05 | 1.190242635 | NM_173680| | MGC33584,hypothetical protein MGC33584 |
| 224395_s_at | 14.54 | 1.90E-05 | 1.092634469 | NM_014245| | RNF7,ring finger protein 7 isoform 1 |
| 216091_s_at | 14.54 | 1.89E-05 | 1.189479804 | NM_003939| | BTRC,beta-transducin repeat containing protein |
| 209875_s_at | 14.54 | 1.90E-05 | 1.331972596 | NM_000582| | SPP1,secreted phosphoprotein 1 (osteopontin, bone |
| 229168_at | 14.54 | 1.90E-05 | 1.509960284 | NM_173465| | COL23A1,collagen, type XXIII, alpha 1 |
| 220367_s_at | 14.53 | 1.90E-05 | 1.070951074 | NM_024545| | SAP130,mSin3A-associated protein 130 |
| 231296_at | 14.53 | 1.90E-05 | 1.150333999 | NA |  |
| 1563327_a_at | 14.53 | 1.90E-05 | 1.393612183 | NA |  |
| 41512_at | 14.52 | 1.91E-05 | 1.075334069 | NA |  |
| 229559_at | 14.51 | 1.92E-05 | 1.587648487 | NM_001080401| | NA |
| 212632_at | 14.51 | 1.92E-05 | 1.176907113 | NM_003569| | STX7,syntaxin 7 |
| 222690_s_at | 14.51 | 1.92E-05 | 1.205991183 | NM_018266| | TMEM39A,transmembrane protein 39A |
| 205768_s_at | 14.51 | 1.92E-05 | 1.339506962 | NM_003645| | SLC27A2,solute carrier family 27 (fatty acid |
| 209174_s_at | 14.49 | 1.94E-05 | 1.055847227 | NM_017730| | FLJ20259,FLJ20259 protein |
| 209463_s_at | 14.49 | 1.94E-05 | 1.117584948 | NM_005644| | TAF12,TAF12 RNA polymerase II, TATA box binding |
| 202286_s_at | 14.49 | 1.94E-05 | 1.543523736 | NM_002353| | TACSTD2,tumor-associated calcium signal transducer 2 |
| 209298_s_at | 14.49 | 1.94E-05 | 1.314410806 | NM_001001132| | ITSN1,intersectin 1 isoform ITSN-s |
| 226048_at | 14.48 | 1.94E-05 | 1.166747453 | NM_002750| | MAPK8,mitogen-activated protein kinase 8 isoform 2 |
| 232092_at | 14.47 | 1.95E-05 | 1.145302423 | NM_033412| | MCART1,mitochondrial carrier triple repeat 1 |
| 40020_at | 14.47 | 1.95E-05 | 1.109885082 | NM_001040454| | NA |
| 212658_at | 14.47 | 1.95E-05 | 1.150516887 | NM_005779| | LHFPL2,lipoma HMGIC fusion partner-like 2 |
| 206322_at | 14.47 | 1.95E-05 | 1.717563604 | NM_003490| | SYN3,synapsin III isoform IIIa |
| 227529_s_at | 14.46 | 1.96E-05 | 1.224596744 | NM_005100| | AKAP12,A-kinase anchor protein 12 isoform 1 |
| 225231_at | 14.46 | 1.95E-05 | 1.120438749 | NM_005188| | CBL,Cas-Br-M (murine) ecotropic retroviral |
| 210880_s_at | 14.45 | 1.96E-05 | 1.275342959 | NM_005864| | EFS,embryonal Fyn-associated substrate isoform 1 |
| 222982_x_at | 14.45 | 1.96E-05 | 1.05021223 | NM_018976| | SLC38A2,solute carrier family 38, member 2 |
| 205591_at | 14.44 | 1.97E-05 | 1.79992399 | NM_006334| | OLFM1,olfactomedin related ER localized protein |
| 206044_s_at | 14.44 | 1.97E-05 | 1.405566948 | NM_004333| | BRAF,v-raf murine sarcoma viral oncogene homolog B1 |
| 206495_s_at | 14.43 | 1.98E-05 | 1.088345254 | NM_015517| | MIZF,MBD2 (methyl-CpG-binding protein)-interacting |
| 203872_at | 14.43 | 1.98E-05 | 2.258289538 | NM_001100| | ACTA1,alpha 1 actin precursor |
| 241477_at | 14.43 | 1.98E-05 | 1.084411936 | NM_024608| | NEIL1,nei endonuclease VIII-like 1 |
| 213201_s_at | 14.43 | 1.98E-05 | 2.131702189 | NM_001126132| | NA |
| 203352_at | 14.42 | 1.99E-05 | 1.115521964 | NM_002552| | ORC4L,origin recognition complex subunit 4 |
| 201800_s_at | 14.42 | 1.98E-05 | 1.063946117 | NM_002556| | OSBP,oxysterol binding protein |
| 206456_at | 14.42 | 1.99E-05 | 1.687503608 | NM_000810| | GABRA5,gamma-aminobutyric acid (GABA) A receptor, alpha |
| 235071_at | 14.42 | 1.98E-05 | 1.060299231 | NM_138458| | LOC116143,hypothetical protein BC014022 |
| 232077_s_at | 14.41 | 1.99E-05 | 1.198123874 | NM_031477| | YPEL3,yippee-like 3 |
| 218221_at | 14.41 | 2.00E-05 | 1.089474207 | NM_001668| | ARNT,aryl hydrocarbon receptor nuclear translocator |
| 209307_at | 14.4 | 2.00E-05 | 1.119200264 | NM_015055| | SWAP70,SWAP-70 protein |
| 214116_at | 14.4 | 2.00E-05 | 1.359687493 | NM_000060| | BTD,biotinidase precursor |
| 213137_s_at | 14.4 | 2.00E-05 | 1.133973765 | NM_002828| | PTPN2,protein tyrosine phosphatase, non-receptor type |
| 217813_s_at | 14.39 | 2.01E-05 | 1.14767092 | NM_006717| | SPIN,spindlin |
| 206600_s_at | 14.39 | 2.01E-05 | 1.906400655 | NM_004695| | SLC16A5,solute carrier family 16, member 5 |
| 211999_at | 14.38 | 2.02E-05 | 1.04524808 | NM_002107| | H3F3A,H3 histone, family 3A |
| 224832_at | 14.37 | 2.02E-05 | 1.259894099 | NM_030640| | DUSP16,dual specificity phosphatase 16 |
| 201311_s_at | 14.37 | 2.02E-05 | 1.190265676 | NM_003022| | SH3BGRL,SH3 domain binding glutamic acid-rich protein |
| 221613_s_at | 14.37 | 2.02E-05 | 1.112561689 | NM_019006| | ZA20D3,zinc finger, A20 domain containing 3 |
| 32069_at | 14.37 | 2.02E-05 | 1.146563906 | NM_153029| | N4BP1,Nedd4 binding protein 1 |
| 202054_s_at | 14.36 | 2.03E-05 | 1.129118774 | NM_000382| | ALDH3A2,aldehyde dehydrogenase 3A2 |
| 202180_s_at | 14.36 | 2.03E-05 | 1.190681104 | NM_005115| | MVP,major vault protein |
| 206588_at | 14.35 | 2.03E-05 | 1.594694088 | NM_001351| | DAZL,deleted in azoospermia-like |
| 1553952_at | 14.35 | 2.04E-05 | 1.07574963 | NM_001039617| | NA |
| 1562988_at | 14.35 | 2.04E-05 | 1.417379162 | NM_001112734| | NA |
| 210587_at | 14.35 | 2.03E-05 | 2.261536786 | NM_031479| | INHBE,activin beta E |
| 212904_at | 14.33 | 2.06E-05 | 1.065938114 | NM_020710| | KIAA1185,KIAA1185 protein |
| 236385_at | 14.33 | 2.05E-05 | 1.323514383 | NA |  |
| 203095_at | 14.33 | 2.05E-05 | 1.082846575 | NM_001005369| | MTIF2,mitochondrial translational initiation factor 2 |
| 204479_at | 14.33 | 2.05E-05 | 1.140804896 | NM_012383| | OSTF1,osteoclast stimulating factor 1 |
| 1553972_a_at | 14.32 | 2.06E-05 | 1.107926 | NM_000071| | CBS,cystathionine-beta-synthase |
| 206801_at | 14.32 | 2.06E-05 | 1.690980278 | NM_002521| | NPPB,natriuretic peptide precursor B |
| 215543_s_at | 14.32 | 2.06E-05 | 1.251730614 | NM_004737| | LARGE,like-glycosyltransferase |
| 236454_at | 14.31 | 2.06E-05 | 1.104178499 | NM_194439| | LOC285498,hypothetical protein LOC285498 |
| 1564155_x_at | 14.31 | 2.07E-05 | 1.602456946 | NA |  |
| 219764_at | 14.29 | 2.09E-05 | 1.339628538 | NM_007197| | FZD10,frizzled 10 |
| 221019_s_at | 14.29 | 2.09E-05 | 1.705326839 | NM_130386| | COLEC12,collectin sub-family member 12 isoform I |
| 1569039_s_at | 14.29 | 2.08E-05 | 1.566137369 | NM_182609| | MGC48625,hypothetical protein MGC48625 |
| 238205_at | 14.28 | 2.09E-05 | 1.560544423 | NM_178470| | WDR40B,WD repeat domain 40B |
| 214844_s_at | 14.28 | 2.09E-05 | 1.079203453 | NM_018431| | DOK5,DOK5 protein isoform a |
| 203120_at | 14.28 | 2.09E-05 | 1.038415538 | NM_001031685| | NA |
| 220127_s_at | 14.27 | 2.10E-05 | 1.107933067 | NM_017703| | FBXL12,F-box and leucine-rich repeat protein 12 |
| 217150_s_at | 14.27 | 2.10E-05 | 1.386067332 | NM_000268| | NF2,neurofibromin 2 isoform 1 |
| 203857_s_at | 14.27 | 2.10E-05 | 1.105245547 | NM_006810| | PDIR,for protein disulfide isomerase-related |
| 205670_at | 14.27 | 2.10E-05 | 1.352656188 | NM_004861| | GAL3ST1,galactose-3-O-sulfotransferase 1 |
| 222696_at | 14.26 | 2.10E-05 | 1.492682953 | NM_004655| | AXIN2,axin 2 |
| 227209_at | 14.26 | 2.10E-05 | 2.242130068 | NM_001843| | CNTN1,contactin 1 isoform 1 precursor |
| 240181_at | 14.26 | 2.10E-05 | 1.297153749 | NA |  |
| 209276_s_at | 14.26 | 2.10E-05 | 1.323498811 | NM_001118890| | NA |
| 213136_at | 14.26 | 2.10E-05 | 1.133725811 | NM_002828| | PTPN2,protein tyrosine phosphatase, non-receptor type |
| 222636_at | 14.26 | 2.10E-05 | 1.267608964 | NM_025205| | MED28,mediator of RNA polymerase II transcription, |
| 226099_at | 14.25 | 2.11E-05 | 1.762937749 | NM_012081| | ELL2,elongation factor, RNA polymerase II, 2 |
| 225756_at | 14.25 | 2.11E-05 | 1.084317942 | NM_001894| | CSNK1E,casein kinase 1 epsilon |
| 228885_at | 14.25 | 2.11E-05 | 2.249959448 | NM_153267| | MAMDC2,MAM domain containing 2 |
| 208747_s_at | 14.25 | 2.11E-05 | 1.325077407 | NM_001734| | C1S,complement component 1, s subcomponent |
| 211535_s_at | 14.24 | 2.12E-05 | 1.085720665 | NM_015850| | FGFR1,fibroblast growth factor receptor 1 isoform 2 |
| 232265_at | 14.24 | 2.12E-05 | 1.717474346 | NM_020725| | NA |
| 244246_at | 14.24 | 2.12E-05 | 1.339562706 | NM_138731| | MIPOL1,mirror-image polydactyly 1 |
| 228944_at | 14.23 | 2.13E-05 | 1.232727537 | NA |  |
| 1552400_a_at | 14.23 | 2.13E-05 | 1.253621912 | NM_152335| | C15orf27,chromosome 15 open reading frame 27 |
| 214889_at | 14.22 | 2.13E-05 | 1.300640144 | NM_001006655| | DKFZP564J102,DKFZP564J102 protein |
| 201973_s_at | 14.21 | 2.14E-05 | 1.056322525 | NM_015622| | C7orf28A,chromosome 7 open reading frame 28A |
| 219470_x_at | 14.21 | 2.14E-05 | 1.070846239 | NM_019084| | CCNJ,cyclin J |
| 205132_at | 14.21 | 2.14E-05 | 1.697616484 | NM_005159| | ACTC,cardiac muscle alpha actin proprotein |
| 204049_s_at | 14.2 | 2.15E-05 | 1.212898702 | NM_001100164| | NA |
| 209422_at | 14.2 | 2.15E-05 | 1.102977004 | NM_016436| | PHF20,PHD finger protein 20 |
| 228407_at | 14.2 | 2.15E-05 | 1.269179646 | NM_152753| | SCUBE3,signal peptide, CUB domain, EGF-like 3 |
| 217886_at | 14.2 | 2.15E-05 | 1.127297148 | NM_001981| | EPS15,epidermal growth factor receptor pathway |
| 225660_at | 14.19 | 2.16E-05 | 1.208636552 | NM_020796| | SEMA6A,semaphorin 6A1 |
| 244242_at | 14.19 | 2.16E-05 | 1.535357304 | NA |  |
| 233503_at | 14.19 | 2.16E-05 | 1.216008469 | NM_017826| | FLJ20449,hypothetical protein FLJ20449 |
| 227284_at | 14.19 | 2.16E-05 | 1.115279124 | NM_001010851| | LOC90321,hypothetical protein LOC90321 |
| 238478_at | 14.19 | 2.15E-05 | 1.525590806 | NM_017637| | BNC2,basonuclin 2 |
| 203981_s_at | 14.18 | 2.17E-05 | 1.055528985 | NM_005050| | ABCD4,ATP-binding cassette, sub-family D, member 4 |
| 210683_at | 14.17 | 2.18E-05 | 1.424968488 | NM_004558| | NRTN,neurturin precursor |
| 209831_x_at | 14.17 | 2.18E-05 | 1.08885522 | NM_001375| | DNASE2,deoxyribonuclease II, lysosomal precursor |
| 203851_at | 14.17 | 2.18E-05 | 1.348894468 | NM_002178| | IGFBP6,insulin-like growth factor binding protein 6 |
| 230889_at | 14.16 | 2.18E-05 | 1.376675629 | NA |  |
| 218217_at | 14.15 | 2.19E-05 | 1.128764439 | NM_021626| | SCPEP1,serine carboxypeptidase 1 precursor protein |
| 202301_s_at | 14.14 | 2.20E-05 | 1.082981835 | NM_023012| | FLJ11021,similar to splicing factor, arginine/serine-rich |
| 221880_s_at | 14.14 | 2.20E-05 | 1.251038224 | NM_207446| | LOC400451,hypothetical gene supported by AK075564; |
| 202543_s_at | 14.12 | 2.22E-05 | 1.124877803 | NM_004124| | GMFB,glia maturation factor, beta |
| 205122_at | 14.11 | 2.23E-05 | 1.318007795 | NM_003692| | TMEFF1,transmembrane protein with EGF-like and two |
| 203379_at | 14.11 | 2.23E-05 | 1.218965838 | NM_001006665| | RPS6KA1,ribosomal protein S6 kinase, 90kDa, polypeptide |
| 213061_s_at | 14.11 | 2.23E-05 | 1.076824516 | NM_173474| | NTAN1,N-terminal Asn amidase |
| 203277_at | 14.11 | 2.23E-05 | 1.106919621 | NM_004401| | DFFA,DNA fragmentation factor, 45kDa, alpha |
| 205479_s_at | 14.11 | 2.23E-05 | 1.610378522 | NM_002658| | PLAU,urokinase plasminogen activator preproprotein |
| 208652_at | 14.1 | 2.24E-05 | 1.030297837 | NM_002715| | PPP2CA,protein phosphatase 2, catalytic subunit, alpha |
| 227794_at | 14.1 | 2.24E-05 | 1.72714577 | NM_080661| | MGC15937,hypothetical protein MGC15937 similar to |
| 218092_s_at | 14.1 | 2.24E-05 | 1.155896525 | NM_004504| | HRB,HIV-1 Rev binding protein |
| 239636_at | 14.09 | 2.25E-05 | 1.533459007 | NM_001112732| | NA |
| 226857_at | 14.09 | 2.25E-05 | 1.353314448 | NM_153213| | ARHGEF19,Rho guanine nucleotide exchange factor (GEF) 19 |
| 223114_at | 14.09 | 2.25E-05 | 1.087930612 | NM_032314| | MGC4767,hypothetical protein MGC4767 |
| 1569108_a_at | 14.08 | 2.25E-05 | 1.348727815 | NM_016089| | ZNF589,zinc finger protein 589 |
| 225111_s_at | 14.08 | 2.25E-05 | 1.118074318 | NM_022080| | NAPB,N-ethylmaleimide-sensitive factor attachment |
| 218342_s_at | 14.08 | 2.25E-05 | 1.181995742 | NM_024896| | KIAA1815,KIAA1815 |
| 215084_s_at | 14.07 | 2.26E-05 | 1.141929344 | NM_052940| | MGC8974,hypothetical protein MGC8974 |
| 203853_s_at | 14.07 | 2.26E-05 | 1.184956238 | NM_012296| | GAB2,GRB2-associated binding protein 2 isoform b |
| 203140_at | 14.07 | 2.26E-05 | 1.134698484 | NM_001706| | BCL6,B-cell lymphoma 6 protein |
| 242070_at | 14.07 | 2.26E-05 | 1.676835963 | NA |  |
| 204927_at | 14.07 | 2.26E-05 | 1.276235697 | NM_003475| | C11orf13,HRAS1-related cluster-1 |
| 209337_at | 14.06 | 2.27E-05 | 1.099157482 | NM_021144| | PSIP1,PC4 and SFRS1 interacting protein 1 |
| 201328_at | 14.05 | 2.28E-05 | 1.328826139 | NM_005239| | ETS2,v-ets erythroblastosis virus E26 oncogene |
| 217877_s_at | 14.05 | 2.28E-05 | 1.056756356 | NM_021639| | SP192,hypothetical protein SP192 |
| 235285_at | 14.05 | 2.28E-05 | 1.317397039 | NA |  |
| 202947_s_at | 14.05 | 2.28E-05 | 1.232875862 | NM_002101| | GYPC,glycophorin C isoform 1 |
| 232263_at | 14.04 | 2.29E-05 | 1.584569981 | NM_018057| | SLC6A15,solute carrier family 6, member 15 isoform 2 |
| 228337_at | 14.04 | 2.29E-05 | 1.228194347 | NM_052927| | NA |
| 228151_at | 14.03 | 2.30E-05 | 1.086584605 | NA |  |
| 201369_s_at | 14.03 | 2.30E-05 | 1.26850772 | NM_006887| | ZFP36L2,butyrate response factor 2 |
| 237896_at | 14.03 | 2.30E-05 | 1.459943117 | NM_018055| | NODAL,nodal-related protein |
| 51192_at | 14.03 | 2.30E-05 | 1.256184839 | NM_017857| | SSH3,slingshot homolog 3 |
| 218551_at | 14 | 2.33E-05 | 1.136229501 | NM_021933| | FLJ12438,IGFBP-2-Binding Protein, IIp45 |
| 244497_at | 13.99 | 2.33E-05 | 1.302233002 | NA |  |
| 227190_at | 13.99 | 2.34E-05 | 1.479082905 | NM_183240| | TMEM37,transmembrane protein 37 |
| 223457_at | 13.99 | 2.33E-05 | 1.093322341 | NM_012133| | COPG2,coatomer protein complex, subunit gamma 2 |
| 201897_s_at | 13.98 | 2.34E-05 | 1.045394502 | NM_001826| | CKS1B,CDC28 protein kinase 1B |
| 211997_x_at | 13.98 | 2.34E-05 | 1.046297893 | NM_002107| | H3F3A,H3 histone, family 3A |
| 207522_s_at | 13.98 | 2.34E-05 | 1.384876292 | NM_005173| | ATP2A3,sarco/endoplasmic reticulum Ca2+ -ATPase isoform |
| 220275_at | 13.97 | 2.35E-05 | 2.289415719 | NM_022034| | CUZD1,CUB and zona pellucida-like domains 1 |
| 209925_at | 13.96 | 2.36E-05 | 1.869967579 | NM_002538| | OCLN,occludin |
| 221378_at | 13.96 | 2.36E-05 | 2.100253222 | NM_005454| | CER1,cerberus 1 |
| 228027_at | 13.96 | 2.36E-05 | 1.131062044 | NM_001004051| | GPRASP2,G protein-coupled receptor associated sorting |
| 230839_at | 13.96 | 2.36E-05 | 1.141282504 | NM_019854| | HRMT1L4,protein arginine N-methyltransferase 4 |
| 224212_s_at | 13.95 | 2.36E-05 | 1.784407648 | NM_014005| | PCDHA9,protocadherin alpha 9 isoform 2 precursor |
| 225326_at | 13.95 | 2.37E-05 | 1.089684091 | NM_018989| | NA |
| 1560099_at | 13.94 | 2.38E-05 | 2.063790054 | NA |  |
| 228338_at | 13.94 | 2.38E-05 | 1.595783781 | NA |  |
| 49452_at | 13.94 | 2.38E-05 | 1.354430121 | NM_001093| | ACACB,acetyl-Coenzyme A carboxylase beta |
| 210389_x_at | 13.94 | 2.38E-05 | 1.094780173 | NM_016261| | TUBD1,delta-tubulin |
| 203234_at | 13.94 | 2.37E-05 | 1.286294065 | NM_003364| | UPP1,uridine phosphorylase 1 |
| 222986_s_at | 13.94 | 2.37E-05 | 1.0747795 | NM_016479| | SCOTIN,scotin |
| 228875_at | 13.94 | 2.38E-05 | 2.039330602 | NM_001085480| | NA |
| 203041_s_at | 13.93 | 2.38E-05 | 1.107722928 | NM_001122606| | NA |
| 228961_at | 13.92 | 2.40E-05 | 1.092150939 | NM_152622| | FLJ35954,hypothetical protein FLJ35954 |
| 208009_s_at | 13.91 | 2.41E-05 | 1.133537471 | NM_014448| | ARHGEF16,Rho guanine exchange factor 16 |
| 202923_s_at | 13.91 | 2.40E-05 | 1.112527484 | NM_001498| | GCLC,glutamate-cysteine ligase, catalytic subunit |
| 204167_at | 13.91 | 2.41E-05 | 1.213470549 | NM_000060| | BTD,biotinidase precursor |
| 213116_at | 13.9 | 2.42E-05 | 1.126859661 | NM_002498| | NEK3,NIMA-related kinase 3 |
| 219335_at | 13.89 | 2.42E-05 | 1.10885322 | NM_022838| | ARMCX5,armadillo repeat containing, X-linked 5 |
| 218228_s_at | 13.89 | 2.43E-05 | 1.076730271 | NM_025235| | TNKS2,tankyrase, TRF1-interacting ankyrin-related |
| 210395_x_at | 13.88 | 2.44E-05 | 1.262636124 | NM_001002841| | MYL4,atrial/embryonic alkali myosin light chain |
| 219429_at | 13.88 | 2.44E-05 | 1.718819217 | NM_024306| | FA2H,fatty acid 2-hydroxylase |
| 223279_s_at | 13.87 | 2.44E-05 | 1.197425339 | NM_001008224| | UACA,uveal autoantigen with coiled-coil domains and |
| 204053_x_at | 13.87 | 2.45E-05 | 1.076391752 | NM_000314| | PTEN,phosphatase and tensin homolog |
| 238765_at | 13.87 | 2.44E-05 | 1.15685315 | NM_004888| | ATP6V1G1,ATPase, H+ transporting, lysosomal, V1 subunit G |
| 1555355_a_at | 13.86 | 2.45E-05 | 1.474634406 | NM_005238| | ETS1,v-ets erythroblastosis virus E26 oncogene |
| 213464_at | 13.86 | 2.45E-05 | 1.255762274 | NM_012435| | NA |
| 243445_at | 13.85 | 2.46E-05 | 1.374091244 | NM_017637| | BNC2,basonuclin 2 |
| 206783_at | 13.84 | 2.47E-05 | 1.568606186 | NM_002007| | FGF4,fibroblast growth factor 4 precursor |
| 223179_at | 13.84 | 2.48E-05 | 1.233278409 | NM_031477| | YPEL3,yippee-like 3 |
| 227729_at | 13.84 | 2.48E-05 | 1.126566053 | NA |  |
| 236266_at | 13.83 | 2.49E-05 | 1.501471833 | NA |  |
| 204776_at | 13.83 | 2.48E-05 | 1.153972645 | NM_003248| | THBS4,thrombospondin 4 precursor |
| 1556469_s_at | 13.82 | 2.49E-05 | 1.686543768 | NA |  |
| 219894_at | 13.82 | 2.49E-05 | 1.429930654 | NM_019066| | MAGEL2,MAGE-like protein 2 |
| 212729_at | 13.81 | 2.50E-05 | 1.325297566 | NM_020730| | NA |
| 214305_s_at | 13.81 | 2.50E-05 | 1.083025219 | NM_001005526| | SF3B1,splicing factor 3b, subunit 1 isoform 2 |
| 239422_at | 13.81 | 2.50E-05 | 1.128145704 | NM_152742| | GPC2,glypican 2 |
| 202766_s_at | 13.8 | 2.52E-05 | 1.167741205 | NM_000138| | FBN1,fibrillin 1 |
| 200765_x_at | 13.79 | 2.52E-05 | 1.07371491 | NM_001903| | CTNNA1,catenin, alpha 1 |
| 203765_at | 13.79 | 2.52E-05 | 1.532216024 | NM_012198| | GCA,grancalcin, EF-hand calcium binding protein |
| 200751_s_at | 13.79 | 2.52E-05 | 1.043187598 | NM_001077442| | NA |
| 218226_s_at | 13.78 | 2.53E-05 | 1.0342145 | NM_004547| | NDUFB4,NADH dehydrogenase (ubiquinone) 1 beta |
| 208812_x_at | 13.78 | 2.53E-05 | 1.078687175 | NM_002117| | HLA-C,major histocompatibility complex, class I, C |
| 207170_s_at | 13.77 | 2.54E-05 | 1.103245203 | NM_001024668| | NA |
| 202073_at | 13.77 | 2.54E-05 | 1.709398262 | NM_001008211| | OPTN,optineurin |
| 241879_at | 13.76 | 2.55E-05 | 1.27408982 | NA |  |
| 239132_at | 13.75 | 2.56E-05 | 1.496127649 | NM_000620| | NOS1,nitric oxide synthase 1 (neuronal) |
| 244024_at | 13.75 | 2.56E-05 | 1.137186711 | NM_001007088| | ZNF21,zinc finger protein 21 isoform 2 |
| 222764_at | 13.74 | 2.57E-05 | 1.079193399 | NM_001083926| | NA |
| 220319_s_at | 13.74 | 2.57E-05 | 1.374552274 | NM_013262| | MYLIP,myosin regulatory light chain interacting |
| 220408_x_at | 13.74 | 2.57E-05 | 1.077343698 | NM_001014286| | NA |
| 225959_s_at | 13.74 | 2.57E-05 | 1.061075724 | NM_032268| | ZNRF1,zinc and ring finger protein 1 |
| 210427_x_at | 13.73 | 2.58E-05 | 1.127189142 | NM_001002857| | ANXA2,annexin A2 isoform 2 |
| 211956_s_at | 13.73 | 2.58E-05 | 1.023354229 | NM_005801| | SUI1,putative translation initiation factor |
| 204421_s_at | 13.73 | 2.57E-05 | 1.556105925 | NM_002006| | FGF2,fibroblast growth factor 2 |
| 228082_at | 13.73 | 2.57E-05 | 1.635633523 | NM_024769| | ASAM,adipocyte-specific adhesion molecule |
| 233080_s_at | 13.73 | 2.58E-05 | 1.036149364 | NM_017892| | NA |
| 213412_at | 13.72 | 2.59E-05 | 1.475769702 | NM_014428| | TJP3,tight junction protein 3 (zona occludens 3) |
| 208651_x_at | 13.72 | 2.59E-05 | 1.280728747 | NM_013230| | CD24,CD24 antigen |
| 221217_s_at | 13.72 | 2.59E-05 | 1.359927836 | NM_018723| | A2BP1,ataxin 2-binding protein 1 isoform 4 |
| 211062_s_at | 13.71 | 2.60E-05 | 1.387687018 | NM_001014447| | NA |
| 219123_at | 13.71 | 2.59E-05 | 1.137703648 | NM_014519| | ZNF232,zinc finger protein 232 |
| 224761_at | 13.7 | 2.61E-05 | 1.09311878 | NM_006572| | GNA13,guanine nucleotide binding protein (G protein), |
| 203436_at | 13.7 | 2.61E-05 | 1.057038971 | NM_001104546| | NA |
| 222812_s_at | 13.7 | 2.61E-05 | 1.367191117 | NM_019034| | RHOF,ras homolog gene family, member F |
| 225871_at | 13.7 | 2.60E-05 | 1.428802219 | NM_001040665| | NA |
| 220372_at | 13.69 | 2.62E-05 | 1.390451865 | NM_001040192| | NA |
| 227616_at | 13.69 | 2.61E-05 | 1.254740677 | NM_182557| | BCL9L,B-cell CLL/lymphoma 9-like |
| 1554795_a_at | 13.69 | 2.61E-05 | 1.420317595 | NM_001024215| | NA |
| 217864_s_at | 13.68 | 2.63E-05 | 1.091679673 | NM_016166| | PIAS1,protein inhibitor of activated STAT, 1 |
| 213457_at | 13.68 | 2.63E-05 | 1.153406427 | NM_004225| | MFHAS1,malignant fibrous histiocytoma amplified |
| 201379_s_at | 13.68 | 2.62E-05 | 1.044268616 | NM_003288| | TPD52L2,tumor protein D52-like 2 isoform e |
| 221763_at | 13.67 | 2.64E-05 | 1.149119133 | NM_004241| | JMJD1C,jumonji domain containing 1C |
| 226541_at | 13.67 | 2.64E-05 | 1.089823016 | NM_032145| | FBXO30,F-box only protein 30 |
| 222580_at | 13.67 | 2.64E-05 | 1.080809597 | NM_016620| | ZNF644,zinc finger protein 644 isoform 2 |
| 230359_at | 13.67 | 2.64E-05 | 1.405608644 | NM_152643| | KNDC1,kinase non-catalytic C-lobe domain (KIND) |
| 204260_at | 13.66 | 2.64E-05 | 1.199888459 | NM_001819| | CHGB,chromogranin B precursor |
| 212488_at | 13.66 | 2.65E-05 | 1.511695223 | NM_000093| | COL5A1,alpha 1 type V collagen preproprotein |
| 200011_s_at | 13.66 | 2.65E-05 | 1.060726026 | NM_001659| | ARF3,ADP-ribosylation factor 3 |
| 215028_at | 13.65 | 2.65E-05 | 1.136450138 | NM_020796| | SEMA6A,semaphorin 6A1 |
| 206200_s_at | 13.65 | 2.65E-05 | 1.158683352 | NM_001157| | ANXA11,annexin A11 |
| 238688_at | 13.65 | 2.66E-05 | 1.332070062 | NM_000366| | TPM1,tropomyosin 1 (alpha) |
| 224775_at | 13.65 | 2.65E-05 | 1.188972822 | NM_017969| | FLJ10006,hypothetical protein FLJ10006 |
| 64883_at | 13.65 | 2.65E-05 | 1.126285754 | NM_152581| | MOSPD2,motile sperm domain containing 2 |
| 230900_at | 13.65 | 2.65E-05 | 1.699516662 | NM_152775| | KM-HN-1,KM-HN-1 protein |
| 230083_at | 13.64 | 2.66E-05 | 1.549490543 | NM_019050| | USP53,ubiquitin specific protease 53 |
| 205789_at | 13.64 | 2.67E-05 | 1.847019519 | NM_001766| | CD1D,CD1D antigen, d polypeptide |
| 218972_at | 13.63 | 2.67E-05 | 1.146509585 | NM_018259| | TTC17,tetratricopeptide repeat domain 17 |
| 212653_s_at | 13.62 | 2.68E-05 | 1.081351931 | NM_015252| | EHBP1,EH domain binding protein 1 |
| 235048_at | 13.62 | 2.68E-05 | 1.269502376 | NM_015566| | NA |
| 203744_at | 13.62 | 2.68E-05 | 1.063312662 | NM_005342| | HMGB3,high-mobility group box 3 |
| 1566764_at | 13.61 | 2.69E-05 | 1.446834061 | NM_182762| | 7A5,putative binding protein 7a5 |
| 1555845_at | 13.61 | 2.69E-05 | 1.187918775 | NA |  |
| 209907_s_at | 13.6 | 2.71E-05 | 1.084130002 | NM_006277| | ITSN2,intersectin 2 isoform 1 |
| 203791_at | 13.6 | 2.71E-05 | 1.176781215 | NM_005509| | DMXL1,Dmx-like 1 |
| 212114_at | 13.6 | 2.70E-05 | 1.072008742 | NA |  |
| 34408_at | 13.58 | 2.72E-05 | 1.32430875 | NM_005619| | RTN2,reticulon 2 isoform A |
| 229693_at | 13.58 | 2.73E-05 | 1.444348805 | NM_001004313| | LOC388335,similar to RIKEN cDNA A730055C05 gene |
| 222991_s_at | 13.58 | 2.72E-05 | 1.096226597 | NM_013438| | UBQLN1,ubiquilin 1 isoform 1 |
| 206026_s_at | 13.58 | 2.72E-05 | 1.192132762 | NM_007115| | TNFAIP6,tumor necrosis factor, alpha-induced protein 6 |
| 205129_at | 13.58 | 2.73E-05 | 1.058338497 | NM_006993| | NPM3,nucleophosmin/nucleoplasmin 3 |
| 209735_at | 13.57 | 2.73E-05 | 1.482213613 | NM_004827| | ABCG2,ATP-binding cassette, sub-family G, member 2 |
| 213558_at | 13.56 | 2.75E-05 | 1.465474194 | NM_014510| | NA |
| 203815_at | 13.56 | 2.75E-05 | 1.130786721 | NM_000853| | GSTT1,glutathione S-transferase theta 1 |
| 218847_at | 13.55 | 2.76E-05 | 1.117348202 | NM_001007225| | IMP-2,IGF-II mRNA-binding protein 2 isoform b |
| 218195_at | 13.55 | 2.76E-05 | 1.219376668 | NM_024573| | C6orf211,chromosome 6 open reading frame 211 |
| 224064_s_at | 13.54 | 2.77E-05 | 1.125102345 | NM_024887| | DHDDS,dehydrodolichyl diphosphate synthase isoform a |
| 218152_at | 13.54 | 2.77E-05 | 1.03539406 | NM_018200| | HMG20A,high-mobility group 20A |
| 209642_at | 13.54 | 2.77E-05 | 1.12487559 | NM_004336| | BUB1,BUB1 budding uninhibited by benzimidazoles 1 |
| 229080_at | 13.54 | 2.77E-05 | 1.526676866 | NM_133457| | EMID2,putative emu2 |
| 222466_s_at | 13.52 | 2.79E-05 | 1.056827537 | NM_014050| | MRPL42,mitochondrial ribosomal protein L42 isoform a |
| 227333_at | 13.52 | 2.79E-05 | 1.126353859 | NA |  |
| 235099_at | 13.52 | 2.79E-05 | 1.192663966 | NM_178868| | CKLFSF8,chemokine-like factor superfamily 8 |
| 209576_at | 13.51 | 2.80E-05 | 1.268013809 | NM_002069| | GNAI1,guanine nucleotide binding protein (G protein), |
| 228912_at | 13.51 | 2.80E-05 | 1.382022102 | NM_007127| | VIL1,villin 1 |
| 209380_s_at | 13.51 | 2.80E-05 | 1.122656719 | NM_001023587| | NA |
| 202205_at | 13.51 | 2.80E-05 | 1.213824458 | NM_003370| | VASP,vasodilator-stimulated phosphoprotein isoform 1 |
| 1553411_s_at | 13.5 | 2.82E-05 | 1.29093882 | NM_171999| | SALL3,sal-like 3 |
| 200899_s_at | 13.5 | 2.81E-05 | 1.123719953 | NM_012215| | MGEA5,meningioma expressed antigen 5 (hyaluronidase) |
| 212627_s_at | 13.5 | 2.81E-05 | 1.051141192 | NM_015004| | EXOSC7,exosome component 7 |
| 205190_at | 13.49 | 2.82E-05 | 1.213216236 | NM_002670| | PLS1,plastin 1 |
| 209682_at | 13.49 | 2.82E-05 | 1.186234554 | NM_170662| | CBLB,Cas-Br-M (murine) ecotropic retroviral |
| 205368_at | 13.49 | 2.82E-05 | 1.318150212 | NM_001031690| | NA |
| 222857_s_at | 13.48 | 2.84E-05 | 1.717780575 | NM_014505| | KCNMB4,calcium-activated potassium channel beta 4 |
| 212750_at | 13.47 | 2.84E-05 | 1.676725435 | NM_015568| | PPP1R16B,protein phosphatase 1 regulatory inhibitor |
| 203752_s_at | 13.47 | 2.85E-05 | 1.080328669 | NM_005354| | JUND,jun D proto-oncogene |
| 220150_s_at | 13.47 | 2.84E-05 | 1.161603477 | NM_001100411| | NA |
| 209035_at | 13.46 | 2.85E-05 | 1.191252809 | NM_001012333| | MDK,midkine |
| 210457_x_at | 13.46 | 2.85E-05 | 1.215533142 | NM_002131| | HMGA1,high mobility group AT-hook 1 isoform b |
| 214813_at | 13.46 | 2.85E-05 | 1.423728803 | NM_007131| | ZNF75,zinc finger protein 75 |
| 225649_s_at | 13.46 | 2.85E-05 | 1.128884241 | NM_080836| | STK35,serine/threonine kinase 35 |
| 202322_s_at | 13.46 | 2.85E-05 | 1.085088772 | NM_001037277| | NA |
| 224833_at | 13.45 | 2.86E-05 | 1.657579865 | NM_005238| | ETS1,v-ets erythroblastosis virus E26 oncogene |
| 218686_s_at | 13.45 | 2.86E-05 | 1.170565013 | NM_022450| | RHBDF1,rhomboid family 1 |
| 205560_at | 13.44 | 2.87E-05 | 1.646411541 | NM_006200| | PCSK5,proprotein convertase subtilisin/kexin type 5 |
| 206858_s_at | 13.44 | 2.88E-05 | 2.282512996 | NM_004503| | HOXC6,homeo box C6 isoform 1 |
| 205340_at | 13.44 | 2.87E-05 | 1.093600351 | NM_014797| | ZBTB24,zinc finger and BTB domain containing 24 |
| 219025_at | 13.44 | 2.87E-05 | 1.108195037 | NM_020404| | CD248,tumor endothelial marker 1 precursor |
| 213793_s_at | 13.44 | 2.87E-05 | 1.06254137 | NM_004272| | HOMER1,homer 1 |
| 218084_x_at | 13.43 | 2.89E-05 | 1.512746456 | NM_014164| | FXYD5,FXYD domain-containing ion transport regulator |
| 1554020_at | 13.43 | 2.89E-05 | 1.283320327 | NM_001003398| | BICD1,bicaudal D homolog 1 isoform 2 |
| 229289_at | 13.43 | 2.89E-05 | 1.507413193 | NM_138411| | NA |
| 228223_at | 13.43 | 2.89E-05 | 1.174656307 | NM_080752| | ZSWIM3,zinc finger, SWIM domain containing 3 |
| 205632_s_at | 13.42 | 2.89E-05 | 1.938308222 | NM_003558| | PIP5K1B,phosphatidylinositol-4-phosphate 5-kinase, type |
| 244178_at | 13.42 | 2.89E-05 | 1.692686607 | NM_001099339| | NA |
| 1554747_a_at | 13.41 | 2.91E-05 | 1.157837761 | NM_001008491| | SEPT2,septin 2 |
| 203060_s_at | 13.41 | 2.91E-05 | 1.453104119 | NM_001015880| | NA |
| 208816_x_at | 13.41 | 2.91E-05 | 1.165192068 | NA |  |
| 227296_at | 13.4 | 2.91E-05 | 1.235092894 | NM_138431| | LOC113655,hypothetical protein BC011982 |
| 227040_at | 13.4 | 2.91E-05 | 1.265908246 | NM_001012754| | NA |
| 44822_s_at | 13.4 | 2.91E-05 | 1.157536239 | NM_017550| | KIAA1193,KIAA1193 |
| 228228_at | 13.39 | 2.92E-05 | 1.137669702 | NM_145056| | MGC15476,thymus expressed gene 3-like |
| 219167_at | 13.39 | 2.92E-05 | 1.413373345 | NM_016563| | RASL12,RAS-like, family 12 protein |
| 211421_s_at | 13.39 | 2.93E-05 | 2.026840635 | NM_020630| | RET,ret proto-oncogene isoform c |
| 203975_s_at | 13.37 | 2.95E-05 | 1.062554587 | NM_005483| | CHAF1A,chromatin assembly factor 1, subunit A (p150) |
| 223689_at | 13.37 | 2.95E-05 | 1.624217566 | NM_006546| | IMP-1,IGF-II mRNA-binding protein 1 |
| 221974_at | 13.37 | 2.95E-05 | 1.312832323 | NM_003097| | SNRPN,small nuclear ribonucleoprotein polypeptide N |
| 228497_at | 13.36 | 2.96E-05 | 1.549532908 | NM_018420| | SLC22A15,solute carrier family 22 (organic cation |
| 219572_at | 13.36 | 2.96E-05 | 1.183411933 | NM_001009571| | CADPS2,Ca2+-dependent activator protein for secretion 2 |
| 232172_at | 13.36 | 2.97E-05 | 1.256187704 | NA |  |
| 202310_s_at | 13.35 | 2.98E-05 | 2.005618215 | NM_000088| | COL1A1,alpha 1 type I collagen preproprotein |
| 204328_at | 13.35 | 2.97E-05 | 1.378771943 | NM_001127198| | NA |
| 201416_at | 13.34 | 2.98E-05 | 1.091784277 | NM_003107| | SOX4,SRY (sex determining region Y)-box 4 |
| 218178_s_at | 13.34 | 2.98E-05 | 1.087839356 | NM_020412| | CHMP1.5,CHMP1.5 protein |
| 242348_at | 13.34 | 2.99E-05 | 1.455339782 | NM_001005527| | FAM19A4,family with sequence similarity 19 (chemokine |
| 1554966_a_at | 13.34 | 2.99E-05 | 1.363886119 | NM_001042459| | NA |
| 217591_at | 13.34 | 2.99E-05 | 1.416001673 | NA |  |
| 238654_at | 13.34 | 2.99E-05 | 1.451436484 | NA |  |
| 242283_at | 13.34 | 2.98E-05 | 1.218986513 | NM_144989| | NA |
| 201709_s_at | 13.33 | 3.00E-05 | 1.071529055 | NM_003634| | NIPSNAP1,nipsnap homolog 1 |
| 1564706_s_at | 13.33 | 3.00E-05 | 1.687684671 | NM_013267| | GLS2,glutaminase GA isoform a |
| 230466_s_at | 13.33 | 3.00E-05 | 1.267470331 | NA |  |
| 229234_at | 13.32 | 3.01E-05 | 1.415729162 | NM_001010888| | CXorf32,chromosome X open reading frame 32 |
| 201206_s_at | 13.32 | 3.00E-05 | 1.138998353 | NM_001042576| | NA |
| 218921_at | 13.32 | 3.01E-05 | 1.270007527 | NM_021805| | SIGIRR,single Ig IL-1R-related molecule |
| 209569_x_at | 13.32 | 3.01E-05 | 1.903254065 | NM_001040101| | NA |
| 215566_x_at | 13.31 | 3.02E-05 | 1.030686562 | NM_007260| | LYPLA2,lysophospholipase II |
| 209927_s_at | 13.31 | 3.02E-05 | 1.083299462 | NM_015607| | DKFZP547E1010,DKFZP547E1010 protein |
| 241530_at | 13.31 | 3.02E-05 | 1.957157978 | NA |  |
| 218780_at | 13.31 | 3.02E-05 | 1.201201776 | NM_001100176| | NA |
| 217765_at | 13.3 | 3.03E-05 | 1.060325598 | NM_013392| | NRBP,nuclear receptor binding protein |
| 203764_at | 13.3 | 3.03E-05 | 1.062732626 | NM_014750| | DLG7,discs large homolog 7 |
| 227013_at | 13.3 | 3.04E-05 | 1.291376588 | NM_014572| | LATS2,LATS, large tumor suppressor, homolog 2 |
| 239350_at | 13.29 | 3.04E-05 | 1.793319537 | NM_001017967| | NA |
| 204353_s_at | 13.29 | 3.04E-05 | 1.154018901 | NM_001042594| | NA |
| 210813_s_at | 13.28 | 3.06E-05 | 1.122452675 | NM_003401| | XRCC4,X-ray repair cross complementing protein 4 |
| 208718_at | 13.28 | 3.06E-05 | 1.032561059 | NM_001098504| | NA |
| 205135_s_at | 13.28 | 3.05E-05 | 1.074722107 | NM_012345| | NUFIP1,nuclear fragile X mental retardation protein |
| 235149_at | 13.27 | 3.07E-05 | 1.639074683 | NM_173582| | PGM2L1,phosphoglucomutase 2-like 1 |
| 222583_s_at | 13.27 | 3.07E-05 | 1.146636689 | NM_007172| | NUP50,nucleoporin 50kDa isoform b |
| 217216_x_at | 13.27 | 3.07E-05 | 1.102685212 | NM_001040108| | NA |
| 203624_at | 13.26 | 3.08E-05 | 1.063700626 | NM_005088| | DXYS155E,DNA segment on chromosome X and Y (unique) 155 |
| 1553957_at | 13.25 | 3.10E-05 | 1.17060987 | NM_144976| | ZNF564,zinc finger protein 564 |
| 210215_at | 13.25 | 3.09E-05 | 1.11394909 | NM_003227| | TFR2,transferrin receptor 2 |
| 37278_at | 13.25 | 3.10E-05 | 1.040837853 | NM_000116| | TAZ,tafazzin isoform 1 |
| 200622_x_at | 13.25 | 3.09E-05 | 1.075965044 | NM_001743| | CALM2,calmodulin 2 |
| 227701_at | 13.24 | 3.10E-05 | 1.403349093 | NM_018017| | C10orf118,CTCL tumor antigen L14-2 |
| 214400_at | 13.23 | 3.12E-05 | 1.298217127 | NM_000215| | JAK3,Janus kinase 3 |
| 211748_x_at | 13.23 | 3.12E-05 | 1.124373668 | NM_000954| | PTGDS,prostaglandin D2 synthase 21kDa |
| 202842_s_at | 13.23 | 3.12E-05 | 1.071528385 | NM_012328| | DNAJB9,DnaJ (Hsp40) homolog, subfamily B, member 9 |
| 221916_at | 13.22 | 3.13E-05 | 1.539399169 | NM_006158| | NEFL,neurofilament, light polypeptide 68kDa |
| 240616_at | 13.22 | 3.13E-05 | 1.190307671 | NA |  |
| 219349_s_at | 13.22 | 3.13E-05 | 1.195870994 | NM_018303| | SEC5L1,Sec5 protein |
| 225864_at | 13.22 | 3.13E-05 | 1.203341577 | NM_174911| | NSE2,breast cancer membrane protein 101 |
| 235144_at | 13.21 | 3.14E-05 | 1.478414805 | NA |  |
| 202303_x_at | 13.2 | 3.16E-05 | 1.078589435 | NM_003601| | SMARCA5,SWI/SNF-related matrix-associated |
| 212788_x_at | 13.2 | 3.15E-05 | 1.045042901 | NM_000146| | FTL,ferritin, light polypeptide |
| 202996_at | 13.2 | 3.16E-05 | 1.420018314 | NM_021173| | POLD4,polymerase (DNA-directed), delta 4 |
| 218111_s_at | 13.2 | 3.15E-05 | 1.128609459 | NM_018686| | CMAS,cytidine 5'-monophosphate N-acetylneuraminic |
| 225992_at | 13.18 | 3.19E-05 | 1.092693705 | NM_001009569| | MLLT10,myeloid/lymphoid or mixed-lineage leukemia |
| 219926_at | 13.18 | 3.18E-05 | 1.398307733 | NM_022361| | POPDC3,popeye protein 3 |
| 224629_at | 13.18 | 3.19E-05 | 1.038187228 | NM_005570| | LMAN1,lectin, mannose-binding, 1 precursor |
| 200726_at | 13.17 | 3.20E-05 | 1.039817495 | NM_002710| | PPP1CC,protein phosphatase 1, catalytic subunit, gamma |
| 219081_at | 13.17 | 3.20E-05 | 1.139004603 | NM_003732| | EIF4EBP3,eukaryotic translation initiation factor 4E |
| 220230_s_at | 13.16 | 3.22E-05 | 1.839911801 | NM_016229| | CYB5R2,cytochrome b5 reductase b5R.2 isoform 1 |
| 214474_at | 13.16 | 3.21E-05 | 1.210014497 | NM_005399| | PRKAB2,AMP-activated protein kinase beta 2 |
| 226723_at | 13.16 | 3.21E-05 | 1.181095534 | NM_199342| | LOC374969,hypothetical protein LOC374969 |
| 242871_at | 13.16 | 3.21E-05 | 1.710886397 | NM_001104554| | NA |
| 224705_s_at | 13.15 | 3.22E-05 | 1.121726434 | NM_014494| | TNRC6A,trinucleotide repeat containing 6A |
| 207153_s_at | 13.15 | 3.22E-05 | 1.125567326 | NM_053274| | GLMN,glomulin isoform FAP68 |
| 206555_s_at | 13.15 | 3.23E-05 | 1.084641756 | NM_017736| | THUMPD1,THUMP domain containing 1 |
| 205138_s_at | 13.15 | 3.22E-05 | 1.296028572 | NM_005715| | UST,uronyl-2-sulfotransferase |
| 206290_s_at | 13.15 | 3.22E-05 | 1.390289673 | NM_002924| | RGS7,regulator of G-protein signalling 7 |
| 204695_at | 13.15 | 3.22E-05 | 1.099326935 | NM_001789| | CDC25A,cell division cycle 25A isoform a |
| 216493_s_at | 13.14 | 3.24E-05 | 1.186563983 | NM_006547| | IMP-3,IGF-II mRNA-binding protein 3 |
| 211538_s_at | 13.14 | 3.23E-05 | 1.484491871 | NM_021979| | HSPA2,heat shock 70kDa protein 2 |
| 209411_s_at | 13.14 | 3.24E-05 | 1.099075341 | NM_014001| | GGA3,ADP-ribosylation factor binding protein 3 |
| 226397_s_at | 13.13 | 3.25E-05 | 1.725650145 | NA |  |
| 242064_at | 13.13 | 3.25E-05 | 1.192204305 | NM_019064| | SDK2,sidekick 2 |
| 202147_s_at | 13.13 | 3.24E-05 | 1.12011815 | NM_001007245| | IFRD1,interferon-related developmental regulator 1 |
| 200936_at | 13.12 | 3.26E-05 | 1.021024461 | NM_000973| | RPL8,ribosomal protein L8 |
| 210552_s_at | 13.12 | 3.27E-05 | 1.388620403 | NM_014636| | RALGPS1,Ral GEF with PH domain and SH3 binding motif 1 |
| 219527_at | 13.1 | 3.29E-05 | 1.191954885 | NM_017898| | FLJ20605,hypothetical protein FLJ20605 |
| 239835_at | 13.1 | 3.29E-05 | 1.297363169 | NM_032505| | TA-KRP,T-cell activation kelch repeat protein |
| 201000_at | 13.1 | 3.28E-05 | 1.111797765 | NM_001605| | AARS,alanyl-tRNA synthetase |
| 36554_at | 13.09 | 3.30E-05 | 1.134875281 | NM_004192| | ASMTL,acetylserotonin O-methyltransferase-like |
| 235634_at | 13.08 | 3.31E-05 | 1.110179719 | NM_001015508| | NA |
| 223254_s_at | 13.07 | 3.32E-05 | 1.074241336 | NM_017769| | KIAA1333,KIAA1333 |
| 222394_at | 13.07 | 3.32E-05 | 1.069138155 | NM_013374| | PDCD6IP,programmed cell death 6 interacting protein |
| 225335_at | 13.07 | 3.32E-05 | 1.055441606 | NM_032752| | ZNF496,zinc finger protein 496 |
| 211700_s_at | 13.07 | 3.33E-05 | 1.124351347 | NM_001039705| | NA |
| 225239_at | 13.06 | 3.33E-05 | 1.655776591 | NA |  |
| 1563022_at | 13.05 | 3.35E-05 | 1.593490605 | NM_001101357| | NA |
| 226918_at | 13.05 | 3.35E-05 | 1.649293684 | NM_032452| | JPH4,junctophilin 4 |
| 242127_at | 13.04 | 3.37E-05 | 1.816352272 | NA |  |
| 212268_at | 13.04 | 3.37E-05 | 1.321902031 | NM_030666| | SERPINB1,serine (or cysteine) proteinase inhibitor, clade |
| 231819_at | 13.04 | 3.37E-05 | 1.09388503 | NA |  |
| 201708_s_at | 13.03 | 3.39E-05 | 1.073035942 | NM_003634| | NIPSNAP1,nipsnap homolog 1 |
| 1568780_at | 13.03 | 3.38E-05 | 1.624847108 | NA |  |
| 209180_at | 13.03 | 3.38E-05 | 1.043702038 | NM_004582| | RABGGTB,Rab geranylgeranyltransferase, beta subunit |
| 217972_at | 13.02 | 3.40E-05 | 1.036762575 | NM_017812| | CHCHD3,coiled-coil-helix-coiled-coil-helix domain |
| 220484_at | 13.02 | 3.40E-05 | 1.31293028 | NM_018298| | MCOLN3,mucolipin 3 |
| 201397_at | 13.02 | 3.40E-05 | 1.09178142 | NM_006623| | PHGDH,phosphoglycerate dehydrogenase |
| 202363_at | 13.02 | 3.40E-05 | 1.418413012 | NM_004598| | SPOCK,sparc/osteonectin, cwcv and kazal-like domains |
| 202027_at | 13.01 | 3.41E-05 | 1.094995573 | NM_012264| | C22orf5,chromosome 22 open reading frame 5 |
| 1553874_a_at | 13.01 | 3.41E-05 | 1.904372668 | NM_032805| | ZNF206,zinc finger protein 206 |
| 206631_at | 13.01 | 3.41E-05 | 1.193639328 | NM_000956| | PTGER2,prostaglandin E receptor 2 (subtype EP2), 53kDa |
| 220714_at | 13.01 | 3.41E-05 | 1.808331926 | NM_024504| | PRDM14,PR domain containing 14 |
| 236574_at | 13.01 | 3.41E-05 | 1.279156776 | NA |  |
| 201364_s_at | 13.01 | 3.41E-05 | 1.168040529 | NM_002537| | OAZ2,ornithine decarboxylase antizyme 2 |
| 217887_s_at | 13.01 | 3.41E-05 | 1.126573795 | NM_001981| | EPS15,epidermal growth factor receptor pathway |
| 1553186_x_at | 13.01 | 3.41E-05 | 1.304620562 | NM_152573| | RASEF,RAS and EF hand domain containing |
| 225548_at | 13 | 3.42E-05 | 1.066335792 | NM_020859| | ShrmL,Shroom-related protein |
| 210582_s_at | 13 | 3.42E-05 | 1.145403468 | NM_001031801| | NA |
| 222590_s_at | 13 | 3.42E-05 | 1.170060844 | NM_016231| | NLK,nemo like kinase |
| 212168_at | 13 | 3.41E-05 | 1.040424351 | NM_006047| | RBM12,RNA binding motif protein 12 |
| 209885_at | 12.99 | 3.43E-05 | 1.332750812 | NM_014578| | RHOD,ras homolog D |
| 219817_at | 12.99 | 3.43E-05 | 1.141130647 | NA |  |
| 213225_at | 12.98 | 3.45E-05 | 1.347357846 | NM_001033556| | NA |
| 203376_at | 12.98 | 3.46E-05 | 1.052335056 | NM_015891| | CDC40,pre-mRNA splicing factor 17 |
| 204588_s_at | 12.98 | 3.45E-05 | 1.331737756 | NM_001126105| | NA |
| 228856_at | 12.98 | 3.45E-05 | 1.613668605 | NM_023931| | MGC2474,hypothetical protein MGC2474 |
| 217738_at | 12.98 | 3.46E-05 | 1.098218681 | NM_005746| | PBEF1,pre-B-cell colony enhancing factor 1 isoform a |
| 205037_at | 12.98 | 3.46E-05 | 1.160291592 | NM_006860| | RABL4,RAB, member of RAS oncogene family-like 4 |
| 201548_s_at | 12.97 | 3.47E-05 | 1.196782074 | NM_006618| | JARID1B,Jumonji, AT rich interactive domain 1B |
| 228705_at | 12.96 | 3.48E-05 | 1.43822423 | NM_144691| | CAPN12,calpain 12 |
| 201829_at | 12.96 | 3.48E-05 | 1.129356678 | NM_001047160| | NA |
| 211571_s_at | 12.96 | 3.48E-05 | 1.210288627 | NM_001126336| | NA |
| 202621_at | 12.95 | 3.49E-05 | 1.102840765 | NM_001571| | IRF3,interferon regulatory factor 3 |
| 212591_at | 12.95 | 3.50E-05 | 1.050274975 | NM_015014| | KIAA0117,KIAA0117 protein |
| 215239_x_at | 12.95 | 3.50E-05 | 1.208804401 | NM_021148| | ZNF273,zinc finger protein 273 |
| 1556176_at | 12.95 | 3.50E-05 | 1.087787463 | NM_138572| | TBN,taube nuss |
| 203819_s_at | 12.95 | 3.50E-05 | 1.093742072 | NM_006547| | IMP-3,IGF-II mRNA-binding protein 3 |
| 217983_s_at | 12.95 | 3.50E-05 | 1.231363043 | NM_003730| | RNASET2,ribonuclease 6 precursor |
| 1557616_at | 12.94 | 3.50E-05 | 1.174637426 | NM_032752| | ZNF496,zinc finger protein 496 |
| 1554006_a_at | 12.94 | 3.51E-05 | 1.247578118 | NM_001015002| | NA |
| 234974_at | 12.93 | 3.52E-05 | 1.125924366 | NM_138801| | GALM,galactose mutarotase (aldose 1-epimerase) |
| 201482_at | 12.93 | 3.53E-05 | 1.119092467 | NM_001004128| | QSCN6,quiescin Q6 isoform b |
| 206673_at | 12.93 | 3.52E-05 | 1.562327798 | NM_007223| | GPR,putative G protein coupled receptor |
| 213229_at | 12.93 | 3.52E-05 | 1.120462527 | NM_030621| | DICER1,dicer1 |
| 234103_at | 12.92 | 3.53E-05 | 1.302272736 | NM_198503| | SLICK,sodium- and chloride-activated ATP-sensitive |
| 225378_at | 12.92 | 3.54E-05 | 1.138231696 | NM_152415| | FLJ32642,hypothetical protein FLJ32642 |
| 227399_at | 12.92 | 3.53E-05 | 1.932107112 | NM_016206| | VGL-3,colon carcinoma related protein |
| 215913_s_at | 12.92 | 3.54E-05 | 1.471455932 | NM_016315| | GULP1,GULP, engulfment adaptor PTB domain containing |
| 201418_s_at | 12.92 | 3.53E-05 | 1.098578317 | NM_003107| | SOX4,SRY (sex determining region Y)-box 4 |
| 200904_at | 12.92 | 3.54E-05 | 1.168705211 | NM_005516| | HLA-E,major histocompatibility complex, class I, E |
| 1569191_at | 12.91 | 3.54E-05 | 2.027128287 | NM_001039884| | NA |
| 232389_at | 12.91 | 3.55E-05 | 1.4257964 | NM_001080529| | NA |
| 209578_s_at | 12.91 | 3.55E-05 | 1.096749073 | NM_015227| | POFUT2,protein O-fucosyltransferase 2 isoform A |
| 1568593_a_at | 12.91 | 3.56E-05 | 1.687685482 | NA |  |
| 228959_at | 12.9 | 3.57E-05 | 1.134189999 | NA |  |
| 224451_x_at | 12.9 | 3.56E-05 | 1.444733463 | NM_001080156| | NA |
| 222730_s_at | 12.9 | 3.57E-05 | 1.083463514 | NM_016353| | ZDHHC2,rec |
| 220079_s_at | 12.9 | 3.56E-05 | 1.064591414 | NM_001032730| | NA |
| 236045_x_at | 12.89 | 3.58E-05 | 1.426991783 | NA |  |
| 1568887_at | 12.89 | 3.58E-05 | 1.120859837 | NA |  |
| 212104_s_at | 12.89 | 3.58E-05 | 1.071951018 | NM_001031695| | NA |
| 228109_at | 12.89 | 3.58E-05 | 1.284219242 | NM_006909| | RASGRF2,Ras protein-specific guanine |
| 205134_s_at | 12.89 | 3.58E-05 | 1.076841732 | NM_012345| | NUFIP1,nuclear fragile X mental retardation protein |
| 203584_at | 12.88 | 3.59E-05 | 1.067639341 | NM_014673| | KIAA0103,KIAA0103 |
| 205081_at | 12.88 | 3.60E-05 | 1.372228566 | NM_001311| | CRIP1,cysteine-rich protein 1 (intestinal) |
| 209982_s_at | 12.88 | 3.60E-05 | 1.333233907 | NM_015080| | NRXN2,neurexin 2 isoform alpha-1 precursor |
| 227444_at | 12.88 | 3.60E-05 | 1.168382294 | NM_152583| | ARMCX4,armadillo repeat containing, X-linked 4 |
| 228194_s_at | 12.88 | 3.59E-05 | 1.529055164 | NM_001013031| | NA |
| 235527_at | 12.87 | 3.60E-05 | 2.326783975 | NM_001003809| | DLGAP1,discs large homolog-associated protein 1 isoform |
| 226985_at | 12.87 | 3.61E-05 | 1.616700825 | NM_152536| | FGD5,FYVE, RhoGEF and PH domain containing 5 |
| 202302_s_at | 12.87 | 3.60E-05 | 1.050188951 | NM_023012| | FLJ11021,similar to splicing factor, arginine/serine-rich |
| 224647_at | 12.86 | 3.63E-05 | 1.071621356 | NM_145012| | C10orf9,cyclin fold protein 1 |
| 215017_s_at | 12.86 | 3.62E-05 | 1.080875151 | NM_001024948| | NA |
| 200039_s_at | 12.85 | 3.63E-05 | 1.036312581 | NM_002794| | PSMB2,proteasome beta 2 subunit |
| 240261_at | 12.85 | 3.63E-05 | 1.169419201 | NM_005486| | TOM1L1,target of myb1-like 1 |
| 209619_at | 12.85 | 3.65E-05 | 1.356290237 | NM_001025158| | NA |
| 58367_s_at | 12.84 | 3.65E-05 | 1.087859818 | NM_001098491| | NA |
| 201590_x_at | 12.84 | 3.65E-05 | 1.129540531 | NM_001002857| | ANXA2,annexin A2 isoform 2 |
| 222433_at | 12.83 | 3.67E-05 | 1.06827317 | NM_001008493| | ENAH,enabled homolog isoform a |
| 203038_at | 12.83 | 3.68E-05 | 1.078710334 | NM_002844| | PTPRK,protein tyrosine phosphatase, receptor type, K |
| 214909_s_at | 12.82 | 3.68E-05 | 1.121708049 | NM_013974| | DDAH2,dimethylarginine dimethylaminohydrolase 2 |
| 225517_at | 12.82 | 3.69E-05 | 1.12923659 | NM_014106| | FLJ20582,hypothetical protein FLJ20582 |
| 236331_at | 12.81 | 3.70E-05 | 1.697004586 | NM_003948| | CDKL2,cyclin-dependent kinase-like 2 |
| 231118_at | 12.81 | 3.70E-05 | 1.479845368 | NM_144698| | ANKRD35,ankyrin repeat domain 35 |
| 228437_at | 12.81 | 3.70E-05 | 1.232203696 | NM_014184| | HSPC163,HSPC163 protein |
| 209869_at | 12.81 | 3.70E-05 | 2.037614744 | NM_000681| | ADRA2A,alpha-2A-adrenergic receptor |
| 1554007_at | 12.81 | 3.70E-05 | 1.087323255 | NA |  |
| 223568_s_at | 12.81 | 3.70E-05 | 1.0993186 | NM_001102559| | NA |
| 238600_at | 12.81 | 3.69E-05 | 1.35405125 | NM_001099433| | NA |
| 204948_s_at | 12.81 | 3.69E-05 | 1.598748544 | NM_006350| | FST,follistatin isoform FST317 precursor |
| 235423_at | 12.8 | 3.71E-05 | 1.135252519 | NA |  |
| 210621_s_at | 12.8 | 3.72E-05 | 1.115802543 | NM_002890| | RASA1,RAS p21 protein activator 1 isoform 1 |
| 210844_x_at | 12.8 | 3.71E-05 | 1.05752337 | NM_001903| | CTNNA1,catenin, alpha 1 |
| 1553823_a_at | 12.8 | 3.71E-05 | 1.535760278 | NM_153708| | RTP1,receptor transporting protein 1 |
| 225486_at | 12.8 | 3.72E-05 | 1.089334929 | NM_152641| | ARID2,AT rich interactive domain 2 (ARID, RFX-like) |
| 224492_s_at | 12.79 | 3.72E-05 | 1.086630008 | NM_145295| | ZNF627,zinc finger protein 627 |
| 208726_s_at | 12.79 | 3.72E-05 | 1.039227391 | NM_003908| | EIF2S2,eukaryotic translation initiation factor 2 beta |
| 219185_at | 12.79 | 3.74E-05 | 1.091538302 | NM_012241| | SIRT5,sirtuin 5 isoform 1 |
| 227129_x_at | 12.78 | 3.74E-05 | 1.217019695 | NA |  |
| 204783_at | 12.78 | 3.75E-05 | 1.145320534 | NM_022443| | MLF1,myeloid leukemia factor 1 |
| 242045_at | 12.78 | 3.74E-05 | 1.32048455 | NA |  |
| 219360_s_at | 12.77 | 3.76E-05 | 1.464916324 | NM_017636| | TRPM4,transient receptor potential cation channel, |
| 203953_s_at | 12.77 | 3.76E-05 | 1.795641209 | NM_001306| | CLDN3,claudin 3 |
| 225679_at | 12.77 | 3.76E-05 | 1.06333022 | NM_001011713| | C14orf35,chromosome 14 open reading frame 35 |
| 235957_at | 12.77 | 3.76E-05 | 1.642151666 | NA |  |
| 226734_at | 12.76 | 3.77E-05 | 1.091695563 | NM_004846| | EIF4E2,eukaryotic translation initiation factor 4E |
| 219786_at | 12.76 | 3.78E-05 | 2.00333001 | NM_001039656| | NA |
| 203315_at | 12.76 | 3.78E-05 | 1.088455703 | NM_001004720| | NCK2,NCK adaptor protein 2 isoform A |
| 213118_at | 12.75 | 3.79E-05 | 1.105839386 | NM_001006947| | KIAA0701,KIAA0701 protein isoform b |
| 202081_at | 12.75 | 3.80E-05 | 1.096371681 | NM_004907| | IER2,immediate early response 2 |
| 212756_s_at | 12.74 | 3.81E-05 | 1.191885401 | NM_015255| | UBR2,ubiquitin protein ligase E3 component n-recognin |
| 223616_at | 12.74 | 3.81E-05 | 1.347363827 | NM_023074| | FLJ12644,hypothetical protein FLJ12644 |
| 236798_at | 12.73 | 3.82E-05 | 1.213211833 | NA |  |
| 222773_s_at | 12.73 | 3.82E-05 | 1.475709056 | NM_024642| | GALNT12,UDP-N-acetyl-alpha-D-galactosamine:polypeptide |
| 210001_s_at | 12.73 | 3.83E-05 | 2.006900496 | NM_003745| | SOCS1,suppressor of cytokine signaling 1 |
| 1554153_a_at | 12.73 | 3.83E-05 | 1.065456655 | NM_001101802| | NA |
| 223140_s_at | 12.72 | 3.83E-05 | 1.071985548 | NM_001114397| | NA |
| 224486_s_at | 12.72 | 3.83E-05 | 1.221930779 | NM_032499| | HH114,hypothetical protein HH114 |
| 238576_at | 12.72 | 3.83E-05 | 1.310583886 | NA |  |
| 201351_s_at | 12.72 | 3.84E-05 | 1.02902431 | NM_014263| | YME1L1,YME1-like 1 isoform 3 |
| 221882_s_at | 12.72 | 3.84E-05 | 1.173757328 | NM_021259| | TMEM8,transmembrane protein 8 (five membrane-spanning |
| 1554242_a_at | 12.71 | 3.86E-05 | 1.357174172 | NM_004086| | COCH,coagulation factor C homolog, cochlin precursor |
| 201854_s_at | 12.71 | 3.85E-05 | 1.075026077 | NM_015251| | KIAA0431,KIAA0431 protein |
| 224725_at | 12.7 | 3.87E-05 | 1.116540895 | NM_020774| | MIB1,mindbomb homolog 1 |
| 235177_at | 12.7 | 3.86E-05 | 1.082033762 | NM_145280| | LOC151194,hepatocellular carcinoma-associated antigen |
| 231466_at | 12.7 | 3.87E-05 | 1.895785996 | NM_032599| | NYD-SP18,testes development-related NYD-SP18 |
| 229790_at | 12.7 | 3.87E-05 | 1.097586439 | NM_005652| | TERF2,telomeric repeat binding factor 2 |
| 206562_s_at | 12.69 | 3.89E-05 | 1.057854159 | NM_001025105| | NA |
| 223529_at | 12.69 | 3.88E-05 | 1.215756674 | NM_020783| | SYT4,synaptotagmin IV |
| 207785_s_at | 12.69 | 3.88E-05 | 1.131990952 | NM_005349| | RBPSUH,recombining binding protein suppressor of |
| 221475_s_at | 12.67 | 3.91E-05 | 1.01604136 | NM_002948| | RPL15,ribosomal protein L15 |
| 212154_at | 12.67 | 3.93E-05 | 1.238819102 | NM_002998| | SDC2,syndecan 2 precursor |
| 218942_at | 12.67 | 3.92E-05 | 1.099995503 | NM_024779| | PIP5K2C,phosphatidylinositol-4-phosphate 5-kinase, type |
| 212463_at | 12.67 | 3.92E-05 | 1.252996156 | NM_000611| | CD59,CD59 antigen p18-20 |
| 218241_at | 12.66 | 3.94E-05 | 1.077658426 | NM_005113| | GOLGA5,Golgi autoantigen, golgin subfamily a, 5 |
| 217767_at | 12.66 | 3.93E-05 | 1.30095677 | NM_000064| | C3,complement component 3 precursor |
| 200977_s_at | 12.66 | 3.93E-05 | 1.114191993 | NM_001079864| | NA |
| 235017_s_at | 12.66 | 3.94E-05 | 1.586212181 | NA |  |
| 1553252_a_at | 12.65 | 3.95E-05 | 1.175176218 | NM_153252| | BRWD3,bromo domain-containing protein disrupted in |
| 233234_at | 12.65 | 3.96E-05 | 1.137268792 | NM_020768| | KCTD16,potassium channel tetramerisation domain |
| 210235_s_at | 12.65 | 3.96E-05 | 1.157128358 | NM_003626| | PPFIA1,PTPRF interacting protein alpha 1 isoform b |
| 228252_at | 12.64 | 3.97E-05 | 1.086800545 | NM_025049| | C15orf20,DNA helicase homolog PIF1 |
| 227200_at | 12.64 | 3.97E-05 | 1.135010589 | NA |  |
| 204681_s_at | 12.63 | 3.98E-05 | 1.234455721 | NM_012294| | RAPGEF5,Rap guanine nucleotide exchange factor (GEF) 5 |
| 213835_x_at | 12.63 | 3.98E-05 | 1.110061131 | NM_032620| | GTPBP3,GTP binding protein 3 (mitochondrial) isoform V |
| 206541_at | 12.62 | 4.00E-05 | 1.41117444 | NM_000892| | KLKB1,plasma kallikrein B1 precursor |
| 225272_at | 12.62 | 4.00E-05 | 1.060351482 | NM_133491| | SAT2,polyamine N-acetyltransferase |
| 244650_at | 12.62 | 4.00E-05 | 1.198776508 | NA |  |
| 223784_at | 12.62 | 4.00E-05 | 1.466925908 | NM_020665| | TMEM27,transmembrane protein 27 |
| 221606_s_at | 12.62 | 4.00E-05 | 1.447278893 | NM_030763| | NSBP1,nucleosomal binding protein 1 |
| 227985_at | 12.61 | 4.01E-05 | 1.842905192 | NA |  |
| 225014_at | 12.61 | 4.02E-05 | 1.081360629 | NA |  |
| 201070_x_at | 12.61 | 4.02E-05 | 1.092833453 | NM_001005526| | SF3B1,splicing factor 3b, subunit 1 isoform 2 |
| 1553185_at | 12.61 | 4.01E-05 | 1.276979604 | NM_152573| | RASEF,RAS and EF hand domain containing |
| 204890_s_at | 12.61 | 4.01E-05 | 1.816656467 | NM_001042771| | NA |
| 213203_at | 12.61 | 4.01E-05 | 1.080221599 | NM_006049| | SNAPC5,small nuclear RNA activating complex, |
| 212673_at | 12.6 | 4.02E-05 | 1.050937065 | NM_015143| | METAP1,methionyl aminopeptidase 1 |
| 228755_at | 12.6 | 4.03E-05 | 1.076136597 | NM_022574| | PERQ1,PERQ amino acid rich, with GYF domain 1 |
| 1555279_at | 12.6 | 4.03E-05 | 1.139926986 | NM_014154| | ARMC8,armadillo repeat containing 8 |
| 223174_at | 12.6 | 4.04E-05 | 1.096533532 | NM_032320| | GMRP-1,K+ channel tetramerization protein |
| 209524_at | 12.59 | 4.05E-05 | 1.053904342 | NM_016073| | HDGFRP3,hepatoma-derived growth factor, related protein |
| 241339_at | 12.59 | 4.06E-05 | 1.160505102 | NA |  |
| 215195_at | 12.59 | 4.05E-05 | 1.173455445 | NM_002737| | PRKCA,protein kinase C, alpha |
| 48659_at | 12.59 | 4.05E-05 | 1.136486015 | NM_021933| | FLJ12438,IGFBP-2-Binding Protein, IIp45 |
| 202315_s_at | 12.58 | 4.06E-05 | 1.159614154 | NM_004327| | BCR,breakpoint cluster region isoform 1 |
| 203046_s_at | 12.58 | 4.07E-05 | 1.058082997 | NM_003920| | TIMELESS,timeless homolog |
| 211320_s_at | 12.58 | 4.07E-05 | 1.261327896 | NM_005704| | PTPRU,protein tyrosine phosphatase, receptor type, U |
| 213938_at | 12.58 | 4.06E-05 | 1.267237377 | NM_015576| | CAST,cytomatrix protein p110 |
| 217223_s_at | 12.57 | 4.09E-05 | 1.169764245 | NM_004327| | BCR,breakpoint cluster region isoform 1 |
| 204937_s_at | 12.57 | 4.09E-05 | 1.110341711 | NM_016324| | ZNF274,zinc finger protein 274 isoform b |
| 237289_at | 12.57 | 4.08E-05 | 1.114740286 | NM_004379| | CREB1,cAMP responsive element binding protein 1 |
| 207071_s_at | 12.57 | 4.08E-05 | 1.082468494 | NM_002197| | ACO1,aconitase 1 |
| 203288_at | 12.57 | 4.08E-05 | 1.069860925 | NM_014686| | KIAA0355,KIAA0355 |
| 218370_s_at | 12.56 | 4.09E-05 | 1.1519592 | NM_001017406| | NA |
| 223008_s_at | 12.56 | 4.10E-05 | 1.047967671 | NM_001099734| | NA |
| 205474_at | 12.56 | 4.10E-05 | 1.101945268 | NM_015986| | CRLF3,cytokine receptor-like factor 3 |
| 215780_s_at | 12.56 | 4.10E-05 | 1.071158935 | NM_001122821| | NA |
| 217562_at | 12.55 | 4.11E-05 | 1.537603346 | NM_199051| | DBCCR1L,DBCCR1-like |
| 202066_at | 12.55 | 4.11E-05 | 1.128199312 | NM_003626| | PPFIA1,PTPRF interacting protein alpha 1 isoform b |
| 206182_at | 12.55 | 4.12E-05 | 1.186728383 | NM_003435| | ZNF134,zinc finger protein 134 |
| 209533_s_at | 12.55 | 4.11E-05 | 1.074792695 | NM_001031689| | NA |
| 1558027_s_at | 12.54 | 4.13E-05 | 1.194095659 | NM_005399| | PRKAB2,AMP-activated protein kinase beta 2 |
| 208393_s_at | 12.53 | 4.15E-05 | 1.166760665 | NM_005732| | RAD50,RAD50 homolog isoform 1 |
| 213916_at | 12.53 | 4.16E-05 | 1.168037441 | NM_021143| | ZNF20,zinc finger protein 20 (KOX 13) |
| 213186_at | 12.53 | 4.15E-05 | 1.152124791 | NM_014648| | DZIP3,zinc finger DAZ interacting protein 3 |
| 207837_at | 12.53 | 4.16E-05 | 1.273283402 | NM_001008710| | RBPMS,RNA-binding protein with multiple splicing |
| 229687_s_at | 12.53 | 4.16E-05 | 1.167251441 | NA |  |
| 239208_s_at | 12.52 | 4.18E-05 | 1.232596145 | NM_001006114| | C21orf57,chromosome 21 open reading frame 57 isoform 2 |
| 226025_at | 12.51 | 4.20E-05 | 1.073481822 | NM_015199| | ANKRD28,ankyrin repeat domain 28 |
| 1555851_s_at | 12.51 | 4.18E-05 | 1.048511244 | NM_003009| | SEPW1,selenoprotein W, 1 |
| 220391_at | 12.51 | 4.20E-05 | 1.325742022 | NM_024784| | ZBTB3,zinc finger and BTB domain containing 3 |
| 226421_at | 12.5 | 4.20E-05 | 1.065136961 | NM_001025580| | NA |
| 224940_s_at | 12.5 | 4.21E-05 | 1.571472907 | NM_002581| | PAPPA,pregnancy-associated plasma protein A |
| 1564413_at | 12.49 | 4.23E-05 | 1.6386124 | NA |  |
| 223049_at | 12.49 | 4.22E-05 | 1.047810649 | NM_002086| | GRB2,growth factor receptor-bound protein 2 isoform |
| 221805_at | 12.49 | 4.23E-05 | 1.519067269 | NM_006158| | NEFL,neurofilament, light polypeptide 68kDa |
| 215594_at | 12.49 | 4.23E-05 | 1.530476397 | NA |  |
| 202267_at | 12.49 | 4.23E-05 | 1.37048381 | NM_005562| | LAMC2,laminin, gamma 2 isoform a precursor |
| 207037_at | 12.48 | 4.24E-05 | 1.472299825 | NM_003839| | TNFRSF11A,tumor necrosis factor receptor superfamily, |
| 227109_at | 12.48 | 4.24E-05 | 1.211033074 | NM_024514| | CYP2R1,cytochrome P450, family 2, subfamily R, |
| 201345_s_at | 12.48 | 4.25E-05 | 1.073557972 | NM_003339| | UBE2D2,ubiquitin-conjugating enzyme E2D 2 isoform 1 |
| 223349_s_at | 12.47 | 4.26E-05 | 1.191068453 | NM_032515| | BOK,BCL2-related ovarian killer |
| 214057_at | 12.47 | 4.26E-05 | 1.080396657 | NM_021960| | MCL1,myeloid cell leukemia sequence 1 isoform 1 |
| 220144_s_at | 12.46 | 4.27E-05 | 1.276411799 | NM_022096| | ANKRD5,ankyrin repeat domain protein 5 |
| 225957_at | 12.46 | 4.27E-05 | 1.293845704 | NM_153607| | LOC153222,adult retina protein |
| 218394_at | 12.46 | 4.27E-05 | 1.163114016 | NM_024589| | FLJ22386,leucine zipper domain protein |
| 227662_at | 12.46 | 4.27E-05 | 1.737396338 | NM_133477| | SYNPO2,synaptopodin 2 |
| 212626_x_at | 12.46 | 4.27E-05 | 1.031630501 | NM_001077442| | NA |
| 230819_at | 12.45 | 4.29E-05 | 1.216338778 | NA |  |
| 228256_s_at | 12.45 | 4.30E-05 | 1.638610635 | NM_022140| | EPB41L4A,erythrocyte protein band 4.1-like 4 |
| 203510_at | 12.45 | 4.29E-05 | 1.431032831 | NM_000245| | MET,met proto-oncogene precursor |
| 215157_x_at | 12.44 | 4.31E-05 | 1.01010138 | NM_002568| | PABPC1,poly(A) binding protein, cytoplasmic 1 |
| 1555281_x_at | 12.44 | 4.31E-05 | 1.128422588 | NM_014154| | ARMC8,armadillo repeat containing 8 |
| 221900_at | 12.44 | 4.32E-05 | 1.317334625 | NM_005202| | COL8A2,collagen, type VIII, alpha 2 |
| 216468_s_at | 12.44 | 4.31E-05 | 1.318003539 | NM_001077349| | NA |
| 223329_x_at | 12.44 | 4.32E-05 | 1.08291743 | NM_006704| | SUGT1,suppressor of G2 allele of SKP1 |
| 225862_at | 12.44 | 4.31E-05 | 1.105352204 | NM_173471| | SLC25A26,solute carrier family 25, member 26 isoform b |
| 226086_at | 12.43 | 4.33E-05 | 2.073452733 | NM_020826| | SYT13,synaptotagmin XIII |
| 222514_at | 12.43 | 4.34E-05 | 1.126483976 | NM_022157| | RRAGC,Ras-related GTP binding C |
| 205882_x_at | 12.43 | 4.33E-05 | 1.101067073 | NM_001121| | NA |
| 226105_at | 12.43 | 4.34E-05 | 1.2499145 | NA |  |
| 214117_s_at | 12.41 | 4.37E-05 | 1.335919512 | NM_000060| | BTD,biotinidase precursor |
| 216319_at | 12.41 | 4.37E-05 | 1.914617597 | NA |  |
| 201963_at | 12.4 | 4.38E-05 | 1.738389988 | NM_001995| | ACSL1,acyl-CoA synthetase long-chain family member 1 |
| 223337_at | 12.4 | 4.40E-05 | 1.114927407 | NM_005869| | SDCCAG10,serologically defined colon cancer antigen 10 |
| 208061_at | 12.39 | 4.40E-05 | 1.318066669 | NA |  |
| 225351_at | 12.38 | 4.43E-05 | 1.145114312 | NM_018472| | FAM45B,family with sequence similarity 45, member B |
| 202919_at | 12.38 | 4.43E-05 | 1.097238626 | NM_001100819| | NA |
| 220073_s_at | 12.38 | 4.43E-05 | 1.312215524 | NM_018173| | FLJ10665,hypothetical protein FLJ10665 |
| 229241_at | 12.37 | 4.45E-05 | 1.762768432 | NM_153486| | LDHD,D-lactate dehydrogenase isoform 1 precursor |
| 208679_s_at | 12.37 | 4.44E-05 | 1.021675494 | NM_005731| | ARPC2,actin related protein 2/3 complex subunit 2 |
| 222909_s_at | 12.37 | 4.45E-05 | 1.14752773 | NM_004874| | BAG4,BCL2-associated athanogene 4 |
| 218676_s_at | 12.36 | 4.48E-05 | 1.31888759 | NM_001102402| | NA |
| 240117_at | 12.36 | 4.47E-05 | 1.576744312 | NM_032447| | FBN3,fibrillin 3 precursor |
| 227094_at | 12.35 | 4.49E-05 | 1.093361459 | NM_018706| | DHTKD1,dehydrogenase E1 and transketolase domain |
| 209489_at | 12.35 | 4.49E-05 | 1.069688436 | NM_001025596| | NA |
| 200009_at | 12.35 | 4.49E-05 | 1.042123532 | NM_001115156| | NA |
| 225114_at | 12.35 | 4.48E-05 | 1.113248551 | NM_003659| | AGPS,alkylglycerone phosphate synthase precursor |
| 1552389_at | 12.34 | 4.51E-05 | 1.461328084 | NM_173549| | FLJ39553,hypothetical protein FLJ39553 |
| 221677_s_at | 12.34 | 4.51E-05 | 1.078278447 | NM_017613| | DONSON,downstream neighbor of SON isoform a |
| 205961_s_at | 12.33 | 4.53E-05 | 1.121490279 | NM_021144| | PSIP1,PC4 and SFRS1 interacting protein 1 |
| 1563321_s_at | 12.33 | 4.54E-05 | 1.165160899 | NM_001009569| | MLLT10,myeloid/lymphoid or mixed-lineage leukemia |
| 222810_s_at | 12.33 | 4.53E-05 | 1.234695373 | NM_004841| | RASAL2,RAS protein activator like 2 isoform 1 |
| 225890_at | 12.32 | 4.55E-05 | 1.073159379 | NM_052865| | C20orf72,chromosome 20 open reading frame 72 |
| 220474_at | 12.32 | 4.55E-05 | 1.873239443 | NM_030631| | SLC25A21,solute carrier family 25 (mitochondrial |
| 230075_at | 12.32 | 4.56E-05 | 1.167492498 | NM_171998| | RAB39B,RAB39B, member RAS oncogene family |
| 229612_at | 12.32 | 4.55E-05 | 1.192928303 | NA |  |
| 232847_at | 12.31 | 4.57E-05 | 1.303255394 | NM_171999| | SALL3,sal-like 3 |
| 224625_x_at | 12.31 | 4.58E-05 | 1.033880369 | NM_001018108| | NA |
| 219326_s_at | 12.3 | 4.59E-05 | 1.712440797 | NM_006577| | B3GNT1,beta-1,3-N-acetylglucosaminyltransferase bGnT-1 |
| 201220_x_at | 12.3 | 4.58E-05 | 1.058022173 | NM_001083914| | NA |
| 229734_at | 12.3 | 4.59E-05 | 1.145395793 | NA |  |
| 202597_at | 12.3 | 4.59E-05 | 1.485654803 | NM_006147| | IRF6,interferon regulatory factor 6 |
| 240293_at | 12.29 | 4.60E-05 | 1.341427408 | NM_001033658| | NA |
| 213625_at | 12.28 | 4.63E-05 | 1.123267708 | NM_019110| | ZNF307,zinc finger protein 307 |
| 37796_at | 12.28 | 4.62E-05 | 1.100998592 | NM_002319| | LRCH4,leucine-rich repeats and calponin homology (CH) |
| 203797_at | 12.28 | 4.63E-05 | 2.114140629 | NM_003385| | VSNL1,visinin-like 1 |
| 204618_s_at | 12.28 | 4.63E-05 | 1.049379084 | NM_002041| | GABPB2,GA binding protein transcription factor, beta |
| 235152_at | 12.27 | 4.66E-05 | 1.547979802 | NA |  |
| 229830_at | 12.27 | 4.66E-05 | 1.17569488 | NA |  |
| 213268_at | 12.26 | 4.68E-05 | 1.637321005 | NM_015215| | CAMTA1,calmodulin-binding transcription activator 1 |
| 228868_x_at | 12.26 | 4.68E-05 | 1.081988239 | NM_030928| | CDT1,DNA replication factor |
| 210123_s_at | 12.25 | 4.69E-05 | 1.286354141 | NM_000746| | CHRNA7,cholinergic receptor, nicotinic, alpha |
| 232206_at | 12.25 | 4.70E-05 | 1.154878444 | NM_017886| | NA |
| 229797_at | 12.25 | 4.70E-05 | 1.498091621 | NM_018298| | MCOLN3,mucolipin 3 |
| 223625_at | 12.25 | 4.68E-05 | 1.193150522 | NM_032581| | DRCTNNB1A,down-regulated by Ctnnb1, a |
| 229933_at | 12.25 | 4.69E-05 | 1.232789547 | NM_152485| | FLJ25078,hypothetical protein FLJ25078 |
| 203433_at | 12.24 | 4.72E-05 | 1.209812902 | NM_001100879| | NA |
| 208636_at | 12.24 | 4.71E-05 | 1.047968585 | NM_001102| | ACTN1,actinin, alpha 1 |
| 213234_at | 12.24 | 4.70E-05 | 1.132307833 | NM_020853| | NA |
| 218803_at | 12.24 | 4.70E-05 | 1.061101593 | NM_018223| | CHFR,checkpoint with forkhead and ring finger |
| 1568617_a_at | 12.23 | 4.73E-05 | 1.658961612 | NM_001080429| | NA |
| 228041_at | 12.23 | 4.74E-05 | 1.067568561 | NM_181806| | NRPS998,2-aminoadipic 6-semialdehyde dehydrogenase |
| 220448_at | 12.22 | 4.75E-05 | 1.923269262 | NM_022055| | KCNK12,potassium channel, subfamily K, member 12 |
| 220426_at | 12.22 | 4.76E-05 | 1.276368128 | NM_024059| | MGC5356,hypothetical protein MGC5356 |
| 235226_at | 12.21 | 4.77E-05 | 1.226975533 | NM_015076| | CDC2L6,cyclin-dependent kinase (CDC2-like) 11 |
| 213553_x_at | 12.21 | 4.77E-05 | 1.140848609 | NM_001645| | APOC1,apolipoprotein C-I precursor |
| 218283_at | 12.21 | 4.77E-05 | 1.077418607 | NM_016305| | SS18L2,synovial sarcoma translocation gene on |
| 236305_at | 12.21 | 4.77E-05 | 1.564379971 | NM_173362| | LOC317671,LOC317671 |
| 242387_at | 12.21 | 4.78E-05 | 1.520779826 | NM_175075| | INM01,hypothetical protein INM01 |
| 203263_s_at | 12.2 | 4.80E-05 | 1.275377501 | NM_015185| | ARHGEF9,Cdc42 guanine exchange factor 9 |
| 220987_s_at | 12.19 | 4.82E-05 | 1.525229578 | NM_020642| | C11orf17,chromosome 11 open reading frame 17 |
| 209945_s_at | 12.19 | 4.82E-05 | 1.116856194 | NM_002093| | GSK3B,glycogen synthase kinase 3 beta |
| 223103_at | 12.18 | 4.84E-05 | 1.277526263 | NM_006645| | STARD10,START domain containing 10 |
| 229355_at | 12.18 | 4.84E-05 | 1.132323517 | NA |  |
| 213533_at | 12.18 | 4.84E-05 | 1.404430369 | NM_001040101| | NA |
| 1557203_at | 12.18 | 4.84E-05 | 1.072310689 | NM_001012977| | NA |
| 201786_s_at | 12.17 | 4.86E-05 | 1.060734552 | NM_001025107| | NA |
| 227521_at | 12.17 | 4.85E-05 | 1.072228195 | NM_203301| | FBXO33,F-box protein 33 |
| 1552575_a_at | 12.16 | 4.89E-05 | 1.316809655 | NA |  |
| 214656_x_at | 12.16 | 4.88E-05 | 1.087225777 | NM_001080779| | NA |
| 204814_at | 12.16 | 4.88E-05 | 1.182835777 | NM_003716| | CADPS,Ca2+-dependent secretion activator isoform 1 |
| 200055_at | 12.15 | 4.91E-05 | 1.046408572 | NM_006284| | TAF10,TBP-related factor 10 |
| 236172_at | 12.15 | 4.91E-05 | 1.216764794 | NM_181657| | LTB4R,leukotriene B4 receptor |
| 231822_at | 12.15 | 4.91E-05 | 1.221008955 | NM_018704| | DKFZp547A023,hypothetical protein DKFZp547A023 |
| 209016_s_at | 12.15 | 4.91E-05 | 1.734036015 | NM_005556| | KRT7,keratin 7 |
| 203342_at | 12.14 | 4.93E-05 | 1.087492396 | NM_005834| | TIMM17B,translocase of inner mitochondrial membrane 17 |
| 223385_at | 12.14 | 4.92E-05 | 2.047741869 | NM_030622| | CYP2S1,cytochrome P450, family 2, subfamily S, |
| 226750_at | 12.14 | 4.93E-05 | 1.149047777 | NM_018078| | FLJ10378,FLJ10378 protein isoform 1 |
| 210101_x_at | 12.14 | 4.93E-05 | 1.061125264 | NM_016009| | SH3GLB1,SH3-containing protein SH3GLB1 |
| 237040_at | 12.13 | 4.95E-05 | 1.179213392 | NM_152434| | CWF19L2,CWF19-like 2, cell cycle control |
| 222819_at | 12.13 | 4.94E-05 | 1.207874589 | NM_019857| | CTPS2,cytidine triphosphate synthase II |
| 238149_at | 12.12 | 4.97E-05 | 1.560297442 | NA |  |
| 219305_x_at | 12.12 | 4.98E-05 | 1.952578397 | NM_012168| | FBXO2,F-box only protein 2 |
| 223053_x_at | 12.12 | 4.97E-05 | 1.083659894 | NM_014188| | HSPC182,HSPC182 protein |
| 213358_at | 12.12 | 4.96E-05 | 1.137049408 | NM_015210| | KIAA0802,KIAA0802 |
| 207368_at | 12.11 | 5.00E-05 | 1.263776842 | NM_000864| | HTR1D,5-hydroxytryptamine (serotonin) receptor 1D |
| 226330_s_at | 12.11 | 5.00E-05 | 1.065940197 | NM_001014286| | NA |
| 212059_s_at | 12.1 | 5.01E-05 | 1.074093471 | NM_015638| | TRPC4AP,TRPC4-associated protein isoform a |
| 207700_s_at | 12.1 | 5.03E-05 | 1.16083672 | NM_006534| | NCOA3,nuclear receptor coactivator 3 isoform b |
| 228345_at | 12.1 | 5.01E-05 | 1.271843936 | NM_001039840| | NA |
| 224400_s_at | 12.1 | 5.01E-05 | 2.313502242 | NM_031422| | CHST9,GalNAc-4-sulfotransferase 2 |
| 202680_at | 12.1 | 5.01E-05 | 1.079716108 | NM_002095| | GTF2E2,general transcription factor IIE, polypeptide 2, |
| 224416_s_at | 12.09 | 5.03E-05 | 1.179875945 | NM_025205| | MED28,mediator of RNA polymerase II transcription, |
| 219193_at | 12.09 | 5.04E-05 | 1.049477337 | NM_018034| | FLJ10233,hypothetical protein FLJ10233 |
| 201193_at | 12.09 | 5.04E-05 | 1.023293867 | NM_005896| | IDH1,isocitrate dehydrogenase 1 (NADP+), soluble |
| 214853_s_at | 12.09 | 5.04E-05 | 1.051248721 | NM_003029| | SHC1,SHC (Src homology 2 domain containing) |
| 207318_s_at | 12.08 | 5.05E-05 | 1.05807444 | NM_003718| | CDC2L5,cell division cycle 2-like 5 isoform 1 |
| 1554445_at | 12.08 | 5.06E-05 | 1.309130073 | NM_003429| | ZNF85,zinc finger protein 85 (HPF4, HTF1) |
| 201425_at | 12.08 | 5.05E-05 | 1.133121959 | NM_000690| | ALDH2,mitochondrial aldehyde dehydrogenase 2 |
| 209696_at | 12.08 | 5.06E-05 | 1.198235437 | NM_000507| | FBP1,fructose-1,6-bisphosphatase 1 |
| 220677_s_at | 12.07 | 5.09E-05 | 1.353503616 | NM_007037| | ADAMTS8,a disintegrin and metalloprotease with |
| 222723_at | 12.07 | 5.08E-05 | 1.339637304 | NA |  |
| 201179_s_at | 12.06 | 5.11E-05 | 1.089630106 | NM_006496| | GNAI3,guanine nucleotide binding protein (G protein), |
| 214047_s_at | 12.06 | 5.11E-05 | 1.079314901 | NM_003925| | MBD4,methyl-CpG binding domain protein 4 |
| 225463_x_at | 12.06 | 5.11E-05 | 1.05509207 | NM_001097612| | NA |
| 206949_s_at | 12.06 | 5.12E-05 | 1.118110439 | NM_001105203| | NA |
| 203322_at | 12.05 | 5.14E-05 | 1.054421236 | NM_014913| | KIAA0863,KIAA0863 protein |
| 201867_s_at | 12.05 | 5.13E-05 | 1.163370092 | NM_005647| | TBL1X,transducin beta-like 1X |
| 218642_s_at | 12.05 | 5.14E-05 | 1.257183367 | NM_001011667| | CHCHD7,coiled-coil-helix-coiled-coil-helix domain |
| 204048_s_at | 12.04 | 5.16E-05 | 1.290574591 | NM_001100164| | NA |
| 201110_s_at | 12.04 | 5.16E-05 | 2.724244402 | NM_003246| | THBS1,thrombospondin 1 precursor |
| 202733_at | 12.03 | 5.18E-05 | 1.154775625 | NM_001017973| | NA |
| 209154_at | 12.02 | 5.20E-05 | 1.057751196 | NM_014604| | TAX1BP3,Tax1 (human T-cell leukemia virus type I) |
| 235704_at | 12.02 | 5.21E-05 | 1.492210556 | NM_014764| | DAZAP2,DAZ associated protein 2 |
| 228976_at | 12.02 | 5.21E-05 | 1.200208908 | NM_015259| | ICOSLG,inducible T-cell co-stimulator ligand |
| 204809_at | 12.01 | 5.24E-05 | 1.125151141 | NM_006660| | CLPX,ClpX caseinolytic protease X homolog |
| 221729_at | 12.01 | 5.22E-05 | 1.572769336 | NM_000393| | COL5A2,alpha 2 type V collagen preproprotein |
| 203087_s_at | 12 | 5.24E-05 | 1.07136039 | NM_001098511| | NA |
| 1554093_a_at | 12 | 5.25E-05 | 1.076721818 | NM_006049| | SNAPC5,small nuclear RNA activating complex, |
| 224746_at | 11.99 | 5.28E-05 | 1.549448973 | NM_020888| | NA |
| 209472_at | 11.99 | 5.27E-05 | 1.109603077 | NM_001008661| | KAT3,kynurenine aminotransferase III isoform 1 |
| 203494_s_at | 11.98 | 5.29E-05 | 1.104694018 | NM_014679| | PIG8,translokin |
| 201761_at | 11.98 | 5.30E-05 | 1.074726079 | NM_001040409| | NA |
| 1570107_at | 11.98 | 5.29E-05 | 1.73459964 | NA |  |
| 203166_at | 11.98 | 5.30E-05 | 1.077221507 | NM_006324| | CFDP1,craniofacial development protein 1 |
| 1555399_a_at | 11.98 | 5.29E-05 | 1.191126837 | NM_030640| | DUSP16,dual specificity phosphatase 16 |
| 201518_at | 11.98 | 5.29E-05 | 1.086520552 | NM_006807| | CBX1,chromobox homolog 1 (HP1 beta homolog Drosophila |
| 213715_s_at | 11.97 | 5.32E-05 | 1.390392204 | NM_198471| | FLJ46061,FLJ46061 protein |
| 206055_s_at | 11.97 | 5.33E-05 | 1.03730491 | NM_003090| | SNRPA1,small nuclear ribonucleoprotein polypeptide A' |
| 239319_at | 11.97 | 5.32E-05 | 1.57698473 | NA |  |
| 201362_at | 11.96 | 5.36E-05 | 1.053934654 | NM_006469| | IVNS1ABP,influenza virus NS1A binding protein isoform a |
| 230619_at | 11.96 | 5.34E-05 | 1.171575458 | NM_001668| | ARNT,aryl hydrocarbon receptor nuclear translocator |
| 225747_at | 11.95 | 5.36E-05 | 1.105091559 | NM_001099337| | NA |
| 205141_at | 11.95 | 5.38E-05 | 1.209595274 | NM_001097577| | NA |
| 217827_s_at | 11.95 | 5.38E-05 | 1.041050549 | NM_016630| | SPG21,acid cluster protein 33 |
| 226503_at | 11.94 | 5.40E-05 | 1.122631628 | NM_018151| | RIF1,RAP1 interacting factor 1 |
| 227046_at | 11.94 | 5.40E-05 | 1.075629337 | NM_139177| | SLC39A11,solute carrier family 39 (metal ion |
| 202542_s_at | 11.93 | 5.42E-05 | 1.062707033 | NM_004757| | SCYE1,small inducible cytokine subfamily E, member 1 |
| 227872_at | 11.93 | 5.42E-05 | 1.062704513 | NM_007055| | POLR3A,polymerase (RNA) III (DNA directed) polypeptide |
| 218564_at | 11.93 | 5.42E-05 | 1.069561787 | NM_018124| | NA |
| 209130_at | 11.93 | 5.41E-05 | 1.141215772 | NM_003825| | SNAP23,synaptosomal-associated protein 23 isoform |
| 241754_at | 11.92 | 5.45E-05 | 1.49792786 | NM_173690| | C9orf126,chromosome 9 open reading frame 126 |
| 218211_s_at | 11.92 | 5.44E-05 | 1.235423084 | NM_001042467| | NA |
| 43427_at | 11.91 | 5.46E-05 | 1.350891042 | NM_001093| | ACACB,acetyl-Coenzyme A carboxylase beta |
| 235904_at | 11.91 | 5.45E-05 | 1.790051683 | NM_152404| | FLJ34658,hypothetical protein FLJ34658 |
| 232087_at | 11.91 | 5.45E-05 | 1.182469269 | NM_198279| | CXorf23,chromosome X open reading frame 23 |
| 208911_s_at | 11.9 | 5.49E-05 | 1.099160032 | NM_000925| | PDHB,pyruvate dehydrogenase (lipoamide) beta |
| 226155_at | 11.89 | 5.51E-05 | 1.112086546 | NM_020940| | KIAA1600,KIAA1600 |
| 202537_s_at | 11.89 | 5.51E-05 | 1.130909289 | NM_014043| | DKFZP564O123,DKFZP564O123 protein |
| 204453_at | 11.89 | 5.50E-05 | 1.09841864 | NM_003428| | ZNF84,zinc finger protein 84 (HPF2) |
| 203278_s_at | 11.88 | 5.52E-05 | 1.04338006 | NM_001101802| | NA |
| 213067_at | 11.88 | 5.53E-05 | 1.083218232 | NM_005964| | MYH10,myosin, heavy polypeptide 10, non-muscle |
| 226870_at | 11.88 | 5.54E-05 | 1.239890679 | NM_144589| | COMTD1,catechol-O-methyltransferase domain containing |
| 212465_at | 11.87 | 5.55E-05 | 1.098113925 | NM_032233| | C14orf154,chromosome 14 open reading frame 154 isoform a |
| 1555579_s_at | 11.86 | 5.58E-05 | 1.590767577 | NM_001105244| | NA |
| 218535_s_at | 11.86 | 5.58E-05 | 1.079114467 | NM_018343| | RIOK2,RIO kinase 2 |
| 211401_s_at | 11.85 | 5.62E-05 | 1.371404178 | NM_000141| | FGFR2,fibroblast growth factor receptor 2 isoform 1 |
| 226116_at | 11.85 | 5.61E-05 | 1.083143741 | NA |  |
| 213307_at | 11.85 | 5.62E-05 | 1.2769017 | NM_012309| | SHANK2,SH3 and multiple ankyrin repeat domains 2 |
| 217805_at | 11.85 | 5.61E-05 | 1.039869338 | NM_004516| | ILF3,interleukin enhancer binding factor 3 isoform b |
| 212489_at | 11.84 | 5.64E-05 | 1.302248528 | NM_000093| | COL5A1,alpha 1 type V collagen preproprotein |
| 205865_at | 11.84 | 5.64E-05 | 1.698075325 | NM_005224| | ARID3A,AT rich interactive domain 3A (BRIGHT- like) |
| 225769_at | 11.84 | 5.65E-05 | 1.088884597 | NM_020751| | COG6,component of oligomeric golgi complex 6 |
| 228799_at | 11.83 | 5.67E-05 | 1.145113047 | NA |  |
| 223705_s_at | 11.83 | 5.66E-05 | 1.074854365 | NM_001127235| | NA |
| 1556236_at | 11.83 | 5.65E-05 | 1.327345183 | NA |  |
| 209257_s_at | 11.83 | 5.66E-05 | 1.058704954 | NM_005445| | CSPG6,chondroitin sulfate proteoglycan 6 (bamacan) |
| 243764_at | 11.83 | 5.67E-05 | 1.426968213 | NM_182607| | VSIG1,V-set and immunoglobulin domain containing 1 |
| 1553603_s_at | 11.83 | 5.66E-05 | 1.231617493 | NM_022374| | ARL6IP2,ADP-ribosylation factor-like 6 interacting |
| 236578_at | 11.83 | 5.66E-05 | 1.215089428 | NA |  |
| 1553288_a_at | 11.83 | 5.66E-05 | 1.328982735 | NM_173564| | FLJ37538,hypothetical protein FLJ37538 |
| 244738_at | 11.82 | 5.70E-05 | 1.132758073 | NM_153252| | BRWD3,bromo domain-containing protein disrupted in |
| 205486_at | 11.81 | 5.71E-05 | 1.338184503 | NM_007170| | TESK2,testis-specific protein kinase 2 |
| 208275_x_at | 11.81 | 5.72E-05 | 1.498670167 | NM_003577| | UTF1,undifferentiated embryonic cell transcription |
| 205485_at | 11.81 | 5.70E-05 | 1.28325371 | NM_000540| | RYR1,ryanodine receptor 1 (skeletal) |
| 1558440_at | 11.8 | 5.73E-05 | 1.480935903 | NA |  |
| 201972_at | 11.79 | 5.75E-05 | 1.066373649 | NM_001690| | ATP6V1A,ATPase, H+ transporting, lysosomal 70kD, V1 |
| 235759_at | 11.78 | 5.79E-05 | 1.224234488 | NA |  |
| 210169_at | 11.78 | 5.80E-05 | 1.516164095 | NM_014692| | NA |
| 235110_at | 11.78 | 5.80E-05 | 1.199129477 | NM_007069| | HRASLS3,HRAS-like suppressor 3 |
| 232183_at | 11.77 | 5.83E-05 | 1.330563418 | NM_032861| | SERAC1,serine active site containing 1 |
| 218274_s_at | 11.77 | 5.81E-05 | 1.095144046 | NM_001042410| | NA |
| 235072_s_at | 11.77 | 5.82E-05 | 1.080211059 | NA |  |
| 212757_s_at | 11.77 | 5.83E-05 | 1.042685861 | NM_001222| | CAMK2G,calcium/calmodulin-dependent protein kinase II |
| 228360_at | 11.77 | 5.81E-05 | 1.32096822 | NM_177964| | LOC130576,hypothetical protein LOC130576 |
| 205992_s_at | 11.77 | 5.81E-05 | 1.336206274 | NM_000585| | IL15,interleukin 15 isoform 1 precursor |
| 222573_s_at | 11.76 | 5.84E-05 | 1.130793171 | NM_021818| | SAV1,WW45 protein |
| 241575_at | 11.75 | 5.87E-05 | 1.54489859 | NA |  |
| 219622_at | 11.75 | 5.88E-05 | 1.41766344 | NM_017817| | RAB20,RAB20, member RAS oncogene family |
| 209904_at | 11.75 | 5.87E-05 | 1.510737778 | NM_003280| | TNNC1,troponin C, slow |
| 209814_at | 11.75 | 5.86E-05 | 1.085574996 | NM_014487| | ZNF330,zinc finger protein 330 |
| 203100_s_at | 11.75 | 5.87E-05 | 1.076337279 | NM_004824| | CDYL,chromodomain protein, Y chromosome-like isoform |
| 210094_s_at | 11.74 | 5.91E-05 | 1.091601472 | NM_019619| | PARD3,partitioning-defective protein 3 homolog |
| 209790_s_at | 11.74 | 5.90E-05 | 1.153826382 | NM_001226| | CASP6,caspase 6 isoform alpha preproprotein |
| 206136_at | 11.74 | 5.90E-05 | 1.44685538 | NM_003468| | FZD5,frizzled 5 |
| 210075_at | 11.74 | 5.91E-05 | 1.117310578 | NM_001005415| | MARCH2,membrane-associated ring finger (C3HC4) 2 |
| 226562_at | 11.74 | 5.90E-05 | 1.061819034 | NM_152455| | FLJ35867,hypothetical protein FLJ35867 |
| 232612_s_at | 11.74 | 5.90E-05 | 1.105826304 | NM_017974| | APG16L,APG16 autophagy 16-like isoform 2 |
| 211228_s_at | 11.73 | 5.92E-05 | 1.135818557 | NM_002873| | RAD17,RAD17 homolog isoform 1 |
| 229039_at | 11.73 | 5.94E-05 | 1.258880261 | NM_003178| | SYN2,synapsin II isoform IIb |
| 216548_x_at | 11.73 | 5.93E-05 | 1.054490469 | NA |  |
| 218248_at | 11.73 | 5.92E-05 | 1.198115366 | NM_022074| | FLJ22794,FLJ22794 protein |
| 223807_at | 11.72 | 5.96E-05 | 1.501076815 | NM_001555| | IGSF1,immunoglobulin superfamily, member 1 isoform 1 |
| 205355_at | 11.71 | 6.00E-05 | 1.392415625 | NM_001609| | ACADSB,acyl-Coenzyme A dehydrogenase, short/branched |
| 210231_x_at | 11.71 | 5.97E-05 | 1.03891808 | NM_001122821| | NA |
| 229545_at | 11.71 | 6.00E-05 | 1.169914295 | NM_017671| | C20orf42,chromosome 20 open reading frame 42 |
| 204398_s_at | 11.71 | 5.99E-05 | 1.232284474 | NM_012155| | EML2,echinoderm microtubule associated protein like |
| 211662_s_at | 11.71 | 5.98E-05 | 1.023400171 | NM_003375| | VDAC2,voltage-dependent anion channel 2 |
| 229813_x_at | 11.71 | 5.99E-05 | 1.046756895 | NM_018959| | DAZAP1,DAZ associated protein 1 isoform b |
| 218645_at | 11.71 | 5.99E-05 | 1.098076368 | NM_021994| | ZNF277,zinc finger protein (C2H2 type) 277 |
| 235245_at | 11.71 | 5.99E-05 | 1.522588923 | NM_153229| | FLJ33318,hypothetical protein FLJ33318 |
| 217766_s_at | 11.7 | 6.02E-05 | 1.072405455 | NM_014313| | SMP1,small membrane protein 1 |
| 215419_at | 11.7 | 6.02E-05 | 1.193547908 | NM_015174| | NA |
| 209263_x_at | 11.7 | 6.00E-05 | 1.113522944 | NM_001025234| | NA |
| 1555443_at | 11.7 | 6.00E-05 | 1.387175968 | NM_198515| | NA |
| 220157_x_at | 11.7 | 6.02E-05 | 1.124408506 | NM_015899| | PLEKHA9,pleckstrin homology domain containing, family A |
| 228482_at | 11.7 | 6.00E-05 | 1.707850503 | NM_145301| | LOC201158,similar to CGI-148 protein |
| 213467_at | 11.69 | 6.05E-05 | 1.204220834 | NM_005440| | RND2,GTP-binding protein Rho7 |
| 237145_at | 11.69 | 6.03E-05 | 1.493383957 | NM_001013703| | NA |
| 224049_at | 11.69 | 6.05E-05 | 1.276183803 | NM_031460| | KCNK17,potassium channel, subfamily K, member 17 |
| 233634_at | 11.69 | 6.04E-05 | 1.595374187 | NM_001017967| | NA |
| 239710_at | 11.69 | 6.05E-05 | 1.146858626 | NM_018086| | FIGN,fidgetin |
| 205066_s_at | 11.69 | 6.03E-05 | 1.543718075 | NM_006208| | ENPP1,ectonucleotide pyrophosphatase/phosphodiesterase |
| 202324_s_at | 11.68 | 6.06E-05 | 1.114828881 | NM_022735| | ACBD3,golgi complex associated protein 1 |
| 235338_s_at | 11.68 | 6.06E-05 | 1.20393269 | NM_031915| | SETDB2,CLLL8 protein |
| 207223_s_at | 11.68 | 6.08E-05 | 1.221100146 | NM_005156| | ROD1,ROD1 regulator of differentiation 1 |
| 1552617_a_at | 11.67 | 6.09E-05 | 1.094742122 | NM_001001740| | RFWD2,ring finger and WD repeat domain 2 isoform d24 |
| 211954_s_at | 11.67 | 6.08E-05 | 1.035065075 | NM_002271| | RANBP5,RAN binding protein 5 |
| 219465_at | 11.66 | 6.11E-05 | 1.63443387 | NM_001643| | APOA2,apolipoprotein A-II precursor |
| 218615_s_at | 11.66 | 6.12E-05 | 1.209898823 | NM_018266| | TMEM39A,transmembrane protein 39A |
| 227651_at | 11.66 | 6.12E-05 | 1.127891805 | NM_052876| | BTBD14B,transcriptional repressor NAC1 |
| 214382_at | 11.66 | 6.11E-05 | 1.192690752 | NM_018974| | UNC93A,unc-93 homolog A |
| 223450_s_at | 11.66 | 6.13E-05 | 1.063966736 | NM_031431| | COG3,component of golgi transport complex 3 |
| 218383_at | 11.66 | 6.13E-05 | 1.089355408 | NM_017815| | C14orf94,chromosome 14 open reading frame 94 |
| 230633_at | 11.66 | 6.12E-05 | 1.211020224 | NM_178518| | FLJ36878,hypothetical protein FLJ36878 |
| 232713_at | 11.65 | 6.16E-05 | 1.195518254 | NA |  |
| 207980_s_at | 11.65 | 6.14E-05 | 1.222147708 | NM_006079| | CITED2,Cbp/p300-interacting transactivator, with |
| 209531_at | 11.65 | 6.14E-05 | 1.082702079 | NM_001513| | GSTZ1,glutathione transferase zeta 1 isoform 3 |
| 235648_at | 11.65 | 6.15E-05 | 1.089086371 | NM_152603| | ZNF567,zinc finger protein 567 |
| 209864_at | 11.65 | 6.14E-05 | 1.264670444 | NM_012083| | FRAT2,GSK-3 binding protein FRAT2 |
| 227917_at | 11.65 | 6.14E-05 | 1.235768462 | NA |  |
| 227829_at | 11.65 | 6.16E-05 | 1.471198494 | NM_152312| | GYLTL1B,glycosyltransferase-like 1B |
| 214597_at | 11.65 | 6.15E-05 | 1.491325607 | NM_001050| | SSTR2,somatostatin receptor 2 |
| 210315_at | 11.64 | 6.17E-05 | 1.264375932 | NM_003178| | SYN2,synapsin II isoform IIb |
| 219327_s_at | 11.64 | 6.16E-05 | 1.404609065 | NM_018653| | GPRC5C,G protein-coupled receptor family C, group 5, |
| 218012_at | 11.64 | 6.16E-05 | 1.08971158 | NM_022117| | TSPYL2,TSPY-like 2 |
| 230185_at | 11.64 | 6.17E-05 | 1.239468778 | NM_024672| | THAP9,THAP domain containing 9 |
| 213954_at | 11.64 | 6.18E-05 | 1.334346757 | NM_015566| | NA |
| 213627_at | 11.63 | 6.21E-05 | 1.10695281 | NM_014599| | MAGED2,melanoma antigen family D, 2 |
| 208810_at | 11.63 | 6.19E-05 | 1.119554929 | NM_005494| | DNAJB6,DnaJ (Hsp40) homolog, subfamily B, member 6 |
| 34210_at | 11.63 | 6.20E-05 | 1.72937995 | NM_001803| | CD52,CD52 antigen (CAMPATH-1 antigen) |
| 212131_at | 11.63 | 6.19E-05 | 1.048402751 | NM_001114093| | NA |
| 225093_at | 11.62 | 6.23E-05 | 1.254854656 | NM_007124| | UTRN,utrophin |
| 207732_s_at | 11.62 | 6.23E-05 | 1.296817236 | NM_020730| | NA |
| 241574_s_at | 11.61 | 6.26E-05 | 1.576012773 | NM_006546| | IMP-1,IGF-II mRNA-binding protein 1 |
| 1558116_x_at | 11.61 | 6.26E-05 | 1.228435974 | NM_021111| | RECK,RECK protein precursor |
| 219270_at | 11.61 | 6.26E-05 | 1.52742574 | NM_024111| | MGC4504,hypothetical protein MGC4504 |
| 201121_s_at | 11.61 | 6.24E-05 | 1.06058658 | NM_006667| | PGRMC1,progesterone receptor membrane component 1 |
| 221962_s_at | 11.61 | 6.27E-05 | 1.145939081 | NM_003344| | UBE2H,ubiquitin-conjugating enzyme E2H isoform 1 |
| 209212_s_at | 11.6 | 6.29E-05 | 1.251501364 | NM_001730| | KLF5,Kruppel-like factor 5 |
| 205388_at | 11.6 | 6.30E-05 | 1.493373595 | NM_003279| | TNNC2,fast skeletal muscle troponin C |
| 226187_at | 11.6 | 6.28E-05 | 1.372522705 | NA |  |
| 228658_at | 11.6 | 6.30E-05 | 1.30539027 | NA |  |
| 223886_s_at | 11.59 | 6.31E-05 | 1.037342762 | NM_030963| | RNF146,ring finger protein 146 |
| 211778_s_at | 11.59 | 6.32E-05 | 1.632521096 | NM_021220| | ZNF339,zinc finger protein 339 |
| 211971_s_at | 11.58 | 6.34E-05 | 1.041136019 | NM_133259| | LRPPRC,leucine-rich PPR motif-containing protein |
| 202848_s_at | 11.58 | 6.34E-05 | 1.113840151 | NM_001004105| | GRK6,G protein-coupled receptor kinase 6 isoform C |
| 203216_s_at | 11.58 | 6.34E-05 | 1.147057153 | NM_004999| | MYO6,myosin VI |
| 223734_at | 11.58 | 6.33E-05 | 1.396496274 | NM_032623| | OSAP,ovary-specific acidic protein |
| 201970_s_at | 11.58 | 6.34E-05 | 1.057943592 | NM_002482| | NASP,nuclear autoantigenic sperm protein isoform 2 |
| 235333_at | 11.58 | 6.33E-05 | 1.235147751 | NM_004775| | B4GALT6,UDP-Gal:betaGlcNAc beta 1,4- |
| 222471_s_at | 11.58 | 6.35E-05 | 1.112420984 | NM_020122| | KCMF1,potassium channel modulatory factor 1 |
| 228206_at | 11.57 | 6.36E-05 | 1.526364262 | NM_006040| | HS3ST4,heparan sulfate D-glucosaminyl |
| 221500_s_at | 11.57 | 6.37E-05 | 1.079642451 | NM_001001433| | STX16,syntaxin 16 isoform a |
| 203968_s_at | 11.56 | 6.39E-05 | 1.045862846 | NM_001254| | CDC6,CDC6 homolog |
| 229910_at | 11.56 | 6.40E-05 | 1.271727456 | NM_001010846| | LOC126669,hypothetical protein LOC126669 |
| 214253_s_at | 11.56 | 6.40E-05 | 1.130276517 | NM_021907| | DTNB,dystrobrevin, beta isoform 1 |
| 236525_at | 11.55 | 6.44E-05 | 1.178694828 | NM_174899| | FBXO36,F-box protein 36 |
| 227058_at | 11.55 | 6.42E-05 | 1.478589912 | NM_032849| | FLJ14834,hypothetical protein FLJ14834 |
| 236832_at | 11.55 | 6.42E-05 | 1.33913792 | NA |  |
| 201688_s_at | 11.55 | 6.42E-05 | 1.077993649 | NM_001025252| | NA |
| 226521_s_at | 11.54 | 6.46E-05 | 1.109647915 | NM_139076| | FLJ13614,hypothetical protein FLJ13614 |
| 224845_s_at | 11.54 | 6.46E-05 | 1.129786337 | NM_020846| | NA |
| 1554677_s_at | 11.54 | 6.45E-05 | 1.170904766 | NM_178818| | CKLFSF4,chemokine-like factor superfamily 4 isoform 1 |
| 1554646_at | 11.54 | 6.47E-05 | 1.255645951 | NM_018030| | OSBPL1A,oxysterol-binding protein-like 1A isoform A |
| 216623_x_at | 11.53 | 6.47E-05 | 1.082707674 | NM_001080430| | NA |
| 223313_s_at | 11.53 | 6.47E-05 | 1.121857091 | NM_001098800| | NA |
| 200905_x_at | 11.52 | 6.51E-05 | 1.108126838 | NM_005516| | HLA-E,major histocompatibility complex, class I, E |
| 202793_at | 11.52 | 6.51E-05 | 1.101910112 | NM_005768| | C3F,gene rich cluster, C3f gene |
| 225439_at | 11.52 | 6.52E-05 | 1.059693281 | NM_032869| | NUDCD1,NudC domain containing 1 |
| 210319_x_at | 11.52 | 6.52E-05 | 1.410975291 | NM_002449| | MSX2,msh homeo box homolog 2 |
| 238476_at | 11.51 | 6.56E-05 | 1.525783154 | NM_153607| | LOC153222,adult retina protein |
| 1565558_at | 11.51 | 6.55E-05 | 1.139350629 | NA |  |
| 212420_at | 11.51 | 6.55E-05 | 1.113576453 | NM_172373| | ELF1,E74-like factor 1 (ets domain transcription |
| 201852_x_at | 11.51 | 6.56E-05 | 1.307697957 | NM_000090| | COL3A1,alpha 1 type III collagen |
| 235380_at | 11.51 | 6.54E-05 | 1.124128267 | NA |  |
| 200960_x_at | 11.51 | 6.54E-05 | 1.037280769 | NM_001076677| | NA |
| 204694_at | 11.51 | 6.56E-05 | 1.328882247 | NM_001134| | AFP,alpha-fetoprotein precursor |
| 241353_s_at | 11.51 | 6.56E-05 | 1.241084409 | NA |  |
| 229854_at | 11.51 | 6.56E-05 | 1.292034116 | NM_001098623| | NA |
| 218779_x_at | 11.5 | 6.58E-05 | 1.379448745 | NM_017729| | EPS8L1,epidermal growth factor receptor pathway |
| 218041_x_at | 11.5 | 6.57E-05 | 1.069489766 | NM_018976| | SLC38A2,solute carrier family 38, member 2 |
| 202431_s_at | 11.5 | 6.58E-05 | 2.088792763 | NM_002467| | MYC,v-myc myelocytomatosis viral oncogene homolog |
| 224509_s_at | 11.5 | 6.59E-05 | 1.111528475 | NM_032730| | RTN4IP1,reticulon 4 interacting protein 1 |
| 209069_s_at | 11.5 | 6.59E-05 | 1.044961601 | NM_002107| | H3F3A,H3 histone, family 3A |
| 239344_at | 11.49 | 6.63E-05 | 1.292869628 | NA |  |
| 225525_at | 11.49 | 6.63E-05 | 1.072983471 | NA |  |
| 204838_s_at | 11.48 | 6.66E-05 | 1.147320466 | NM_001040108| | NA |
| 205689_at | 11.48 | 6.63E-05 | 1.146373792 | NM_014801| | PCNXL2,pecanex-like 2 |
| 226789_at | 11.48 | 6.63E-05 | 2.132153006 | NA |  |
| 1570534_a_at | 11.48 | 6.64E-05 | 1.355977429 | NM_001007169| | ZNF483,zinc finger protein 483 isoform b |
| 223249_at | 11.47 | 6.66E-05 | 1.130761216 | NM_012129| | CLDN12,claudin 12 |
| 213523_at | 11.47 | 6.67E-05 | 1.146288545 | NM_001238| | CCNE1,cyclin E1 isoform 1 |
| 241985_at | 11.47 | 6.67E-05 | 1.175765012 | NM_152405| | JMY,junction-mediating and regulatory protein |
| 234341_x_at | 11.46 | 6.72E-05 | 1.139930632 | NA |  |
| 203006_at | 11.46 | 6.72E-05 | 1.068448561 | NM_005539| | INPP5A,inositol polyphosphate-5-phosphatase A |
| 229582_at | 11.46 | 6.69E-05 | 1.064717363 | NM_001098817| | NA |
| 228719_at | 11.46 | 6.71E-05 | 1.327291031 | NM_001042697| | NA |
| 225914_s_at | 11.45 | 6.74E-05 | 1.125027845 | NM_001079670| | NA |
| 218343_s_at | 11.45 | 6.75E-05 | 1.078822604 | NM_012086| | GTF3C3,general transcription factor IIIC, polypeptide |
| 207735_at | 11.45 | 6.75E-05 | 1.481556653 | NM_017831| | RNF125,ring finger protein 125 |
| 1558695_at | 11.44 | 6.76E-05 | 1.122317982 | NA |  |
| 214246_x_at | 11.44 | 6.78E-05 | 1.078696825 | NM_001024937| | NA |
| 210149_s_at | 11.43 | 6.82E-05 | 1.044682098 | NM_001003785| | ATP5H,ATP synthase, H+ transporting, mitochondrial F0 |
| 221841_s_at | 11.43 | 6.82E-05 | 1.8492338 | NM_004235| | KLF4,Kruppel-like factor 4 (gut) |
| 1557176_a_at | 11.43 | 6.80E-05 | 1.107646487 | NM_001001872| | C14orf37,chromosome 14 open reading frame 37 |
| 200042_at | 11.43 | 6.81E-05 | 1.04564009 | NM_014306| | HSPC117,hypothetical protein HSPC117 |
| 225309_at | 11.43 | 6.79E-05 | 1.064485777 | NM_032758| | PHF5A,PHD-finger 5A |
| 201385_at | 11.42 | 6.84E-05 | 1.030976388 | NM_001358| | DHX15,DEAH (Asp-Glu-Ala-His) box polypeptide 15 |
| 206310_at | 11.42 | 6.82E-05 | 1.597125064 | NM_021114| | SPINK2,serine protease inhibitor, Kazal type 2 |
| 209662_at | 11.42 | 6.85E-05 | 1.074606287 | NM_004365| | CETN3,centrin 3 |
| 222852_at | 11.42 | 6.82E-05 | 1.150582713 | NM_024942| | C10orf88,chromosome 10 open reading frame 88 |
| 228373_at | 11.42 | 6.84E-05 | 1.088391693 | NM_014117| | PRO0149,PRO0149 protein |
| 200800_s_at | 11.41 | 6.88E-05 | 1.081637796 | NM_005345| | HSPA1A,heat shock 70kDa protein 1A |
| 208711_s_at | 11.41 | 6.87E-05 | 1.05914897 | NM_053056| | CCND1,cyclin D1 |
| 1554266_at | 11.4 | 6.91E-05 | 1.343768804 | NA |  |
| 205645_at | 11.4 | 6.91E-05 | 1.281325169 | NM_001080975| | NA |
| 219376_at | 11.4 | 6.91E-05 | 1.179507217 | NM_199005| | ZNF322B,zinc finger protein 322B |
| 209366_x_at | 11.4 | 6.89E-05 | 1.067473804 | NM_001914| | CYB5,cytochrome b-5 isoform 2 |
| 225824_at | 11.4 | 6.90E-05 | 1.059337867 | NM_001099402| | NA |
| 204359_at | 11.39 | 6.94E-05 | 1.77635848 | NM_013231| | FLRT2,fibronectin leucine rich transmembrane protein |
| 229438_at | 11.39 | 6.94E-05 | 1.199633546 | NA |  |
| 223531_x_at | 11.39 | 6.92E-05 | 1.054931493 | NM_001097612| | NA |
| 206032_at | 11.39 | 6.93E-05 | 1.216888622 | NM_001941| | DSC3,desmocollin 3 isoform Dsc3a preproprotein |
| 222203_s_at | 11.38 | 6.96E-05 | 1.082063939 | NM_001002006| | NT5C1B,5' nucleotidase, cytosolic IB isoform 1 |
| 1552792_at | 11.38 | 6.98E-05 | 1.340020981 | NM_080867| | SOCS4,suppressor of cytokine signaling 4 |
| 218858_at | 11.38 | 6.96E-05 | 1.79317893 | NM_022783| | DEPDC6,DEP domain containing 6 |
| 208549_x_at | 11.38 | 6.95E-05 | 1.061981174 | NA |  |
| 218873_at | 11.38 | 6.96E-05 | 1.038213047 | NM_001037533| | NA |
| 200053_at | 11.38 | 6.97E-05 | 1.05156676 | NM_004890| | SPAG7,sperm associated antigen 7 |
| 223255_at | 11.37 | 7.00E-05 | 1.065948498 | NM_017769| | KIAA1333,KIAA1333 |
| 225972_at | 11.37 | 6.99E-05 | 1.279996663 | NM_001008495| | DKFZp762C1112,hypothetical protein DKFZp762C1112 |
| 212914_at | 11.37 | 7.00E-05 | 1.308367672 | NM_175709| | CBX7,chromobox homolog 7 |
| 238504_at | 11.37 | 7.00E-05 | 1.151198479 | NM_145267| | C6orf57,chromosome 6 open reading frame 57 |
| 218640_s_at | 11.36 | 7.04E-05 | 1.172120166 | NM_024613| | PLEKHF2,phafin 2 |
| 202116_at | 11.36 | 7.03E-05 | 1.06631495 | NM_006268| | DPF2,D4, zinc and double PHD fingers family 2 |
| 242957_at | 11.36 | 7.02E-05 | 1.21324157 | NM_152718| | FLJ32009,hypothetical protein FLJ32009 |
| 220215_at | 11.35 | 7.06E-05 | 1.107711561 | NM_024804| | FLJ12606,hypothetical protein FLJ12606 |
| 37512_at | 11.35 | 7.06E-05 | 1.234978828 | NM_003725| | RODH,3-hydroxysteroid epimerase |
| 209073_s_at | 11.35 | 7.06E-05 | 1.160369273 | NM_001005743| | NUMB,numb homolog isoform 1 |
| 201427_s_at | 11.35 | 7.05E-05 | 2.091433108 | NM_001085486| | NA |
| 225490_at | 11.35 | 7.06E-05 | 1.081046275 | NM_152641| | ARID2,AT rich interactive domain 2 (ARID, RFX-like) |
| 223211_at | 11.34 | 7.09E-05 | 1.133795697 | NM_012260| | HPCL2,2-hydroxyphytanoyl-CoA lyase |
| 226423_at | 11.34 | 7.09E-05 | 1.121987423 | NM_133367| | C6orf33,chromosome 6 open reading frame 33 |
| 209681_at | 11.33 | 7.13E-05 | 1.14582519 | NM_006996| | SLC19A2,solute carrier family 19, member 2 |
| 218845_at | 11.33 | 7.14E-05 | 1.086193491 | NM_020185| | DUSP22,dual specificity phosphatase 22 |
| 230287_at | 11.33 | 7.14E-05 | 1.555136413 | NM_001039948| | NA |
| 213836_s_at | 11.33 | 7.14E-05 | 1.214969041 | NM_017983| | WIPI49,hypothetical protein FLJ10055 |
| 222835_at | 11.33 | 7.13E-05 | 1.469628463 | NM_024817| | FLJ13710,hypothetical protein FLJ13710 |
| 222609_s_at | 11.33 | 7.14E-05 | 1.089314916 | NM_016046| | EXOSC1,exosomal core protein CSL4 |
| 212132_at | 11.33 | 7.12E-05 | 1.05607417 | NM_001114093| | NA |
| 210652_s_at | 11.33 | 7.14E-05 | 1.697277052 | NM_001080494| | NA |
| 1559059_s_at | 11.32 | 7.18E-05 | 1.136547009 | NM_030972| | ZNF611,zinc finger protein 611 |
| 218631_at | 11.32 | 7.18E-05 | 1.12421809 | NM_021732| | AVPI1,vasopressin-induced protein, 32kDa |
| 205541_s_at | 11.31 | 7.20E-05 | 1.131120193 | NM_018094| | GSPT2,peptide chain release factor 3 |
| 207405_s_at | 11.31 | 7.21E-05 | 1.116869276 | NM_002873| | RAD17,RAD17 homolog isoform 1 |
| 206217_at | 11.31 | 7.21E-05 | 1.383902988 | NM_001005609| | EDA,ectodysplasin A isoform EDA-A2 |
| 213187_x_at | 11.31 | 7.20E-05 | 1.054991138 | NM_000146| | FTL,ferritin, light polypeptide |
| 205780_at | 11.31 | 7.20E-05 | 1.829380897 | NM_001197| | BIK,BCL2-interacting killer |
| 212945_s_at | 11.3 | 7.22E-05 | 1.058036826 | NM_001080541| | NA |
| 227319_at | 11.3 | 7.24E-05 | 1.102679741 | NA |  |
| 201930_at | 11.3 | 7.22E-05 | 1.048649329 | NM_005915| | MCM6,minichromosome maintenance protein 6 |
| 219736_at | 11.3 | 7.24E-05 | 1.223764851 | NM_001017397| | NA |
| 202016_at | 11.3 | 7.22E-05 | 1.058570283 | NM_002402| | MEST,mesoderm specific transcript isoform a |
| 226912_at | 11.29 | 7.29E-05 | 1.132353345 | NM_173570| | ZDHHC23,zinc finger, DHHC domain containing 23 |
| 1555801_s_at | 11.27 | 7.33E-05 | 1.831183059 | NM_001113397| | NA |
| 232902_s_at | 11.27 | 7.33E-05 | 1.080189849 | NM_020320| | RARSL,arginyl-tRNA synthetase-like |
| 204928_s_at | 11.27 | 7.35E-05 | 1.110026447 | NM_019848| | SLC10A3,solute carrier family 10, member 3 |
| 216266_s_at | 11.27 | 7.34E-05 | 1.169481914 | NM_006421| | ARFGEF1,brefeldin A-inhibited guanine |
| 226612_at | 11.27 | 7.36E-05 | 1.419584611 | NA |  |
| 201310_s_at | 11.27 | 7.33E-05 | 1.068253022 | NM_004772| | C5orf13,neuronal protein 3.1 |
| 235533_at | 11.26 | 7.37E-05 | 1.220582758 | NM_001031617| | NA |
| 240152_at | 11.26 | 7.38E-05 | 1.321308492 | NA |  |
| 206348_s_at | 11.26 | 7.38E-05 | 1.429256141 | NM_005391| | PDK3,pyruvate dehydrogenase kinase, isoenzyme 3 |
| 213256_at | 11.26 | 7.39E-05 | 1.479732722 | NM_178450| | MARCH3,membrane-associated ring finger (C3HC4) 3 |
| 210514_x_at | 11.26 | 7.38E-05 | 1.098153353 | NM_002127| | HLA-G,major histocompatibility complex, class I, G |
| 206429_at | 11.26 | 7.38E-05 | 1.590190702 | NM_005242| | F2RL1,coagulation factor II (thrombin) receptor-like 1 |
| 207714_s_at | 11.26 | 7.39E-05 | 1.09817794 | NM_001235| | SERPINH1,serine (or cysteine) proteinase inhibitor, clade |
| 215101_s_at | 11.26 | 7.38E-05 | 1.664189579 | NM_002994| | CXCL5,chemokine (C-X-C motif) ligand 5 precursor |
| 204123_at | 11.25 | 7.40E-05 | 1.082020913 | NM_002311| | LIG3,ligase III, DNA, ATP-dependent isoform beta |
| 200980_s_at | 11.25 | 7.42E-05 | 1.036067889 | NM_000284| | PDHA1,pyruvate dehydrogenase (lipoamide) alpha 1 |
| 204212_at | 11.25 | 7.40E-05 | 1.123199222 | NM_005469| | PTE1,peroxisomal acyl-CoA thioesterase isoform a |
| 206900_x_at | 11.25 | 7.40E-05 | 1.323344448 | NM_021047| | ZNF253,DNA-binding protein |
| 219773_at | 11.24 | 7.46E-05 | 1.346096961 | NM_016931| | NOX4,NADPH oxidase 4 |
| 235657_at | 11.24 | 7.46E-05 | 1.546190586 | NA |  |
| 235061_at | 11.24 | 7.43E-05 | 1.221783269 | NM_152542| | PPM1K,protein phosphatase 1K (PP2C domain containing) |
| 221957_at | 11.24 | 7.43E-05 | 1.374525465 | NM_005391| | PDK3,pyruvate dehydrogenase kinase, isoenzyme 3 |
| 221260_s_at | 11.24 | 7.45E-05 | 1.089755732 | NM_030809| | C12orf22,TGF-beta induced apotosis protein 12 |
| 201738_at | 11.24 | 7.45E-05 | 1.108809034 | NM_005875| | GC20,translation factor sui1 homolog |
| 212829_at | 11.23 | 7.48E-05 | 1.076912315 | NM_005028| | PIP5K2A,phosphatidylinositol-4-phosphate 5-kinase type |
| 1556826_s_at | 11.23 | 7.49E-05 | 1.19065202 | NM_198545| | LOC374946,hypothetical gene supported by AK075558; |
| 231975_s_at | 11.23 | 7.48E-05 | 1.109601422 | NM_152622| | FLJ35954,hypothetical protein FLJ35954 |
| 230201_at | 11.22 | 7.50E-05 | 1.131427233 | NA |  |
| 218986_s_at | 11.22 | 7.50E-05 | 1.188223086 | NM_017631| | FLJ20035,hypothetical protein FLJ20035 |
| 205925_s_at | 11.22 | 7.52E-05 | 1.394440019 | NM_002867| | RAB3B,RAB3B, member RAS oncogene family |
| 204619_s_at | 11.22 | 7.49E-05 | 1.275978108 | NM_001126336| | NA |
| 243295_at | 11.21 | 7.56E-05 | 1.084777331 | NM_018989| | NA |
| 214992_s_at | 11.21 | 7.53E-05 | 1.116691773 | NM_001375| | DNASE2,deoxyribonuclease II, lysosomal precursor |
| 215346_at | 11.21 | 7.56E-05 | 1.533402996 | NM_001250| | CD40,CD40 antigen isoform 1 precursor |
| 229278_at | 11.21 | 7.55E-05 | 1.329167042 | NA |  |
| 209433_s_at | 11.2 | 7.57E-05 | 1.046932914 | NM_002703| | PPAT,phosphoribosyl pyrophosphate amidotransferase |
| 202187_s_at | 11.2 | 7.57E-05 | 1.053508741 | NM_006243| | PPP2R5A,alpha isoform of regulatory subunit B56, protein |
| 200060_s_at | 11.2 | 7.59E-05 | 1.02937402 | NM_006711| | RNPS1,RNA-binding protein S1, serine-rich domain |
| 227601_at | 11.2 | 7.58E-05 | 1.084247083 | NM_020961| | KIAA1627,KIAA1627 protein |
| 235552_at | 11.2 | 7.59E-05 | 1.06642972 | NM_020961| | KIAA1627,KIAA1627 protein |
| 206239_s_at | 11.2 | 7.60E-05 | 1.510423285 | NM_003122| | SPINK1,serine protease inhibitor, Kazal type 1 |
| 227018_at | 11.19 | 7.63E-05 | 1.109571782 | NM_017743| | DPP8,dipeptidyl peptidase 8 isoform 2 |
| 227052_at | 11.19 | 7.62E-05 | 1.197466089 | NA |  |
| 220716_at | 11.19 | 7.60E-05 | 1.166033997 | NA |  |
| 224618_at | 11.18 | 7.67E-05 | 1.223278154 | NM_005156| | ROD1,ROD1 regulator of differentiation 1 |
| 234924_s_at | 11.18 | 7.66E-05 | 1.146084455 | NM_020832| | KIAA1441,KIAA1441 protein |
| 234192_s_at | 11.18 | 7.64E-05 | 1.149324825 | NM_025211| | GKAP1,G kinase anchoring protein 1 |
| 35436_at | 11.17 | 7.68E-05 | 1.092450039 | NM_004486| | GOLGA2,Golgi autoantigen, golgin subfamily a, 2 |
| 223360_at | 11.17 | 7.70E-05 | 1.715196699 | NM_032261| | C21orf56,chromosome 21 open reading frame 56 |
| 38918_at | 11.16 | 7.74E-05 | 1.298564273 | NM_005686| | SOX13,SRY-box 13 |
| 1562701_at | 11.16 | 7.73E-05 | 1.314622516 | NA |  |
| 216996_s_at | 11.16 | 7.73E-05 | 1.038579783 | NM_014929| | KIAA0971,KIAA0971 |
| 213349_at | 11.16 | 7.75E-05 | 1.12221268 | NM_001017395| | NA |
| 235176_at | 11.15 | 7.76E-05 | 1.355963255 | NM_133466| | ZNF545,zinc finger protein 545 |
| 220956_s_at | 11.15 | 7.77E-05 | 1.052263012 | NM_053046| | EGLN2,EGL nine (C.elegans) homolog 2 isoform 1 |
| 208427_s_at | 11.15 | 7.76E-05 | 1.542456201 | NM_004432| | ELAVL2,ELAV (embryonic lethal, abnormal vision, |
| 236657_at | 11.15 | 7.76E-05 | 1.340814659 | NA |  |
| 204327_s_at | 11.14 | 7.81E-05 | 1.086194878 | NM_003455| | ZNF202,zinc finger protein 202 |
| 220174_at | 11.14 | 7.82E-05 | 1.252160923 | NM_025061| | FLJ23420,hypothetical protein FLJ23420 |
| 212158_at | 11.14 | 7.83E-05 | 1.204014593 | NM_002998| | SDC2,syndecan 2 precursor |
| 1556166_x_at | 11.14 | 7.80E-05 | 1.834423919 | NA |  |
| 211921_x_at | 11.13 | 7.86E-05 | 1.037340719 | NM_001099285| | NA |
| 218762_at | 11.13 | 7.87E-05 | 1.109972008 | NM_022752| | ZNF574,zinc finger protein 574 |
| 223250_at | 11.12 | 7.88E-05 | 1.096658491 | NM_001031710| | NA |
| 1553764_a_at | 11.12 | 7.90E-05 | 1.454295078 | NM_032876| | JUB,jub, ajuba homolog isoform 1 |
| 226272_at | 11.12 | 7.89E-05 | 1.306921358 | NM_013441| | DSCR1L2,Down syndrome critical region gene 1-like 2 |
| 1554553_s_at | 11.11 | 7.93E-05 | 1.092204374 | NM_001039671| | NA |
| 217862_at | 11.11 | 7.93E-05 | 1.04943638 | NM_016166| | PIAS1,protein inhibitor of activated STAT, 1 |
| 1554441_a_at | 11.11 | 7.93E-05 | 1.152799567 | NM_015045| | KIAA0261,KIAA0261 |
| 1558404_at | 11.11 | 7.93E-05 | 1.704999455 | NA |  |
| 218764_at | 11.1 | 7.95E-05 | 1.434164685 | NM_006255| | PRKCH,protein kinase C, eta |
| 209919_x_at | 11.1 | 7.96E-05 | 1.263128162 | NM_001032364| | NA |
| 212759_s_at | 11.1 | 7.96E-05 | 1.157421881 | NM_030756| | TCF7L2,transcription factor 7-like 2 (T-cell specific, |
| 202037_s_at | 11.09 | 8.02E-05 | 1.320601157 | NM_003012| | SFRP1,secreted frizzled-related protein 1 |
| 216241_s_at | 11.09 | 8.03E-05 | 1.10257686 | NM_006756| | TCEA1,transcription elongation factor A 1 isoform 1 |
| 218710_at | 11.08 | 8.04E-05 | 1.085076419 | NM_017735| | FLJ20272,hypothetical protein FLJ20272 |
| 204068_at | 11.08 | 8.03E-05 | 1.133396026 | NM_006281| | STK3,serine/threonine kinase 3 (STE20 homolog, |
| 220953_s_at | 11.08 | 8.03E-05 | 1.227593564 | NM_001040446| | NA |
| 220603_s_at | 11.08 | 8.04E-05 | 1.180443464 | NM_018349| | MCTP2,multiple C2-domains with two transmembrane |
| 212242_at | 11.08 | 8.06E-05 | 1.261129515 | NM_006000| | TUBA1,tubulin, alpha 1 |
| 206336_at | 11.08 | 8.04E-05 | 2.14776412 | NM_002993| | CXCL6,chemokine (C-X-C motif) ligand 6 (granulocyte |
| 233177_s_at | 11.08 | 8.03E-05 | 1.133806256 | NM_001077399| | NA |
| 212794_s_at | 11.08 | 8.04E-05 | 1.039418361 | NM_015275| | KIAA1033,KIAA1033 protein |
| 203001_s_at | 11.07 | 8.07E-05 | 2.310634925 | NM_007029| | STMN2,superiorcervical ganglia, neural specific 10 |
| 243924_at | 11.07 | 8.07E-05 | 1.160125195 | NA |  |
| 200013_at | 11.06 | 8.12E-05 | 1.011458634 | NM_000986| | RPL24,ribosomal protein L24 |
| 223458_at | 11.06 | 8.14E-05 | 1.336637073 | NM_001114099| | NA |
| 205429_s_at | 11.05 | 8.16E-05 | 1.101138503 | NM_016447| | MPP6,membrane protein, palmitoylated 6 |
| 221423_s_at | 11.05 | 8.17E-05 | 1.083672975 | NM_001024947| | NA |
| 203605_at | 11.05 | 8.17E-05 | 1.060652839 | NM_003136| | SRP54,signal recognition particle 54kDa |
| 208937_s_at | 11.04 | 8.22E-05 | 1.259043394 | NM_002165| | ID1,inhibitor of DNA binding 1 isoform a |
| 218683_at | 11.04 | 8.20E-05 | 1.189979321 | NM_021190| | PTBP2,polypyrimidine tract binding protein 2 |
| 221025_x_at | 11.04 | 8.20E-05 | 1.151900658 | NM_001098614| | NA |
| 234987_at | 11.04 | 8.20E-05 | 1.293157526 | NA |  |
| 1559361_at | 11.03 | 8.24E-05 | 1.33949869 | NM_182762| | 7A5,putative binding protein 7a5 |
| 230464_at | 11.03 | 8.26E-05 | 1.362032997 | NM_030760| | EDG8,endothelial differentiation, sphingolipid |
| 225082_at | 11.03 | 8.26E-05 | 1.028363082 | NM_016207| | CPSF3,cleavage and polyadenylation specific factor 3, |
| 222994_at | 11.02 | 8.29E-05 | 1.06796015 | NM_012094| | PRDX5,peroxiredoxin 5 precursor, isoform a |
| 201649_at | 11.02 | 8.29E-05 | 1.110486005 | NM_004223| | UBE2L6,ubiquitin-conjugating enzyme E2L 6 isoform 1 |
| 1556029_s_at | 11.02 | 8.29E-05 | 1.415960311 | NM_015039| | NMNAT2,nicotinamide mononucleotide adenylyltransferase |
| 225956_at | 11.02 | 8.29E-05 | 1.381340681 | NM_153607| | LOC153222,adult retina protein |
| 218900_at | 11.02 | 8.29E-05 | 1.150890163 | NM_020184| | CNNM4,cyclin M4 |
| 203491_s_at | 11.02 | 8.29E-05 | 1.121189391 | NM_014679| | PIG8,translokin |
| 227850_x_at | 11.02 | 8.27E-05 | 2.059811936 | NM_145057| | CDC42EP5,CDC42 effector protein 5 |
| 222549_at | 11.01 | 8.31E-05 | 1.574903089 | NM_021101| | CLDN1,claudin 1 |
| 229730_at | 11.01 | 8.34E-05 | 1.768075979 | NM_001114974| | NA |
| 224741_x_at | 11 | 8.38E-05 | 1.073150076 | NA |  |
| 227015_at | 11 | 8.38E-05 | 1.244528996 | NM_020437| | LOC57168,similar to aspartate beta hydroxylase (ASPH) |
| 201222_s_at | 11 | 8.38E-05 | 1.034418243 | NM_002874| | RAD23B,UV excision repair protein RAD23 homolog B |
| 222234_s_at | 11 | 8.37E-05 | 1.140364613 | NM_001042610| | NA |
| 244370_at | 10.99 | 8.39E-05 | 1.53623103 | NM_001008537| | KIAA2022,KIAA2022 protein |
| 237322_at | 10.99 | 8.40E-05 | 1.30819315 | NA |  |
| 228970_at | 10.99 | 8.43E-05 | 1.114373134 | NM_178547| | ARCH,archease |
| 225583_at | 10.99 | 8.43E-05 | 1.09342254 | NM_025076| | UXS1,UDP-glucuronate decarboxylase 1 |
| 243347_at | 10.98 | 8.47E-05 | 1.924955764 | NA |  |
| 204038_s_at | 10.98 | 8.44E-05 | 1.133489956 | NM_001401| | EDG2,endothelial differentiation, lysophosphatidic |
| 221047_s_at | 10.97 | 8.47E-05 | 1.11362748 | NM_018650| | MARK1,MAP/microtubule affinity-regulating kinase 1 |
| 210981_s_at | 10.96 | 8.54E-05 | 1.127251936 | NM_001004105| | GRK6,G protein-coupled receptor kinase 6 isoform C |
| 203666_at | 10.96 | 8.52E-05 | 1.495974751 | NM_000609| | CXCL12,chemokine (C-X-C motif) ligand 12 (stromal |
| 211974_x_at | 10.96 | 8.53E-05 | 1.082763708 | NM_005349| | RBPSUH,recombining binding protein suppressor of |
| 219822_at | 10.96 | 8.53E-05 | 1.113031697 | NM_004294| | MTRF1,mitochondrial translational release factor 1 |
| 228916_at | 10.96 | 8.54E-05 | 1.225494905 | NM_152434| | CWF19L2,CWF19-like 2, cell cycle control |
| 218765_at | 10.96 | 8.52E-05 | 1.090985515 | NM_001040455| | NA |
| 204749_at | 10.96 | 8.55E-05 | 1.447617153 | NM_004538| | NAP1L3,nucleosome assembly protein 1-like 3 |
| 223559_s_at | 10.96 | 8.54E-05 | 1.077516835 | NM_021218| | C9orf80,chromosome 9 open reading frame 80 |
| 201706_s_at | 10.96 | 8.52E-05 | 1.085019746 | NM_002857| | PEX19,peroxisomal biogenesis factor 19 |
| 219814_at | 10.95 | 8.56E-05 | 1.270660252 | NM_018388| | MBNL3,muscleblind-like 3 isoform G |
| 225664_at | 10.95 | 8.57E-05 | 1.314554296 | NM_004370| | COL12A1,alpha 1 type XII collagen long isoform |
| 1557918_s_at | 10.95 | 8.59E-05 | 1.155531996 | NM_003051| | SLC16A1,solute carrier family 16, member 1 |
| 217546_at | 10.95 | 8.57E-05 | 1.800786723 | NM_176870| | MT1K,metallothionein 1K |
| 222512_at | 10.94 | 8.60E-05 | 1.063533035 | NM_016118| | NYREN18,NEDD8 ultimate buster-1 |
| 219232_s_at | 10.94 | 8.62E-05 | 1.580209926 | NM_022073| | EGLN3,egl nine homolog 3 |
| 202886_s_at | 10.94 | 8.62E-05 | 1.09378822 | NM_002716| | PPP2R1B,beta isoform of regulatory subunit A, protein |
| 225806_at | 10.93 | 8.65E-05 | 1.373312005 | NM_032876| | JUB,jub, ajuba homolog isoform 1 |
| 208612_at | 10.93 | 8.68E-05 | 1.016877233 | NM_005313| | GRP58,glucose regulated protein, 58kDa |
| 240419_at | 10.93 | 8.68E-05 | 1.382229251 | NM_018057| | SLC6A15,solute carrier family 6, member 15 isoform 2 |
| 224427_s_at | 10.93 | 8.66E-05 | 1.205468484 | NM_022894| | PAPOLG,poly(A) polymerase gamma |
| 201510_at | 10.93 | 8.65E-05 | 1.496314102 | NM_001114309| | NA |
| 201058_s_at | 10.93 | 8.66E-05 | 1.7760817 | NM_006097| | MYL9,myosin regulatory light polypeptide 9 isoform a |
| 227133_at | 10.92 | 8.68E-05 | 1.080463132 | NM_207318| | CXorf39,chromosome X open reading frame 39 |
| 201165_s_at | 10.92 | 8.69E-05 | 1.042407706 | NM_001020658| | NA |
| 208678_at | 10.92 | 8.72E-05 | 1.046644196 | NM_001039366| | NA |
| 238513_at | 10.91 | 8.73E-05 | 1.696946076 | NM_024081| | PRRG4,proline rich Gla (G-carboxyglutamic acid) 4 |
| 229177_at | 10.91 | 8.73E-05 | 1.468619198 | NM_001098514| | NA |
| 225577_at | 10.91 | 8.74E-05 | 1.104673079 | NA |  |
| 228141_at | 10.91 | 8.75E-05 | 1.513508652 | NM_001008397| | LOC493869,similar to 2310016C16Rik protein |
| 203167_at | 10.9 | 8.81E-05 | 1.181653206 | NM_003255| | TIMP2,tissue inhibitor of metalloproteinase 2 |
| 209454_s_at | 10.9 | 8.78E-05 | 1.39112278 | NM_003214| | TEAD3,TEA domain family member 3 |
| 207030_s_at | 10.89 | 8.82E-05 | 1.050103866 | NM_001321| | CSRP2,cysteine and glycine-rich protein 2 |
| 212886_at | 10.89 | 8.84E-05 | 1.681422831 | NM_015621| | DKFZP434C171,DKFZP434C171 protein |
| 226732_at | 10.89 | 8.84E-05 | 1.061573198 | NM_001008408| | LOC155435,hypothetical protein LOC155435 |
| 236835_at | 10.89 | 8.84E-05 | 1.377897258 | NA |  |
| 238482_at | 10.89 | 8.84E-05 | 1.371371129 | NM_003709| | KLF7,Kruppel-like factor 7 (ubiquitous) |
| 215537_x_at | 10.89 | 8.84E-05 | 1.112628101 | NM_013974| | DDAH2,dimethylarginine dimethylaminohydrolase 2 |
| 235089_at | 10.89 | 8.84E-05 | 1.158996492 | NM_032875| | FBXL20,F-box and leucine-rich repeat protein 20 |
| 208079_s_at | 10.88 | 8.88E-05 | 1.043549354 | NM_003600| | STK6,serine/threonine protein kinase 6 |
| 232322_x_at | 10.88 | 8.89E-05 | 1.258411407 | NM_006645| | STARD10,START domain containing 10 |
| 203488_at | 10.88 | 8.89E-05 | 1.106300398 | NM_001008701| | LPHN1,latrophilin 1 isoform 1 precursor |
| 208847_s_at | 10.88 | 8.86E-05 | 1.059238263 | NM_000671| | ADH5,class III alcohol dehydrogenase 5 chi subunit |
| 1555864_s_at | 10.87 | 8.93E-05 | 1.049514677 | NM_000284| | PDHA1,pyruvate dehydrogenase (lipoamide) alpha 1 |
| 220760_x_at | 10.87 | 8.93E-05 | 1.148954579 | NM_024733| | FLJ14345,hypothetical protein FLJ14345 |
| 202517_at | 10.86 | 8.96E-05 | 1.115411407 | NM_001014809| | NA |
| 227920_at | 10.86 | 8.95E-05 | 1.112103429 | NM_001080450| | NA |
| 227405_s_at | 10.86 | 8.98E-05 | 1.743826608 | NM_031866| | FZD8,frizzled 8 |
| 215022_x_at | 10.85 | 9.00E-05 | 1.160084833 | NM_006955| | ZNF11B,zinc finger protein 11b (KOX 2) |
| 201739_at | 10.85 | 9.04E-05 | 1.344430285 | NM_005627| | SGK,serum/glucocorticoid regulated kinase |
| 228036_s_at | 10.85 | 9.00E-05 | 1.151959044 | NM_012168| | FBXO2,F-box only protein 2 |
| 244694_at | 10.85 | 9.04E-05 | 1.622158426 | NM_001101372| | NA |
| 225887_at | 10.84 | 9.05E-05 | 1.050879249 | NM_025138| | C13orf23,hypothetical protein FLJ12661 |
| 226291_at | 10.84 | 9.06E-05 | 1.076560319 | NM_020919| | ALS2,alsin |
| 226588_at | 10.84 | 9.04E-05 | 1.12760218 | NM_020943| | KIAA1604,KIAA1604 protein |
| 242462_at | 10.83 | 9.11E-05 | 1.258327347 | NA |  |
| 242800_at | 10.83 | 9.09E-05 | 1.184677708 | NM_198270| | NHS,Nance-Horan syndrome protein |
| 219928_s_at | 10.83 | 9.09E-05 | 1.166255646 | NM_012189| | CABYR,calcium-binding tyrosine |
| 214219_x_at | 10.82 | 9.17E-05 | 1.717593493 | NM_001042600| | NA |
| 203435_s_at | 10.82 | 9.17E-05 | 1.33959515 | NM_000902| | MME,membrane metallo-endopeptidase |
| 201906_s_at | 10.82 | 9.14E-05 | 1.151631142 | NM_001008392| | CTDSPL,small CTD phosphatase 3 isoform 1 |
| 239648_at | 10.82 | 9.16E-05 | 1.156882427 | NM_173475| | MGC48972,hypothetical protein MGC48972 |
| 206864_s_at | 10.82 | 9.14E-05 | 1.205285687 | NM_003806| | HRK,harakiri |
| 235088_at | 10.81 | 9.21E-05 | 1.215913218 | NM_001008393| | LOC201725,hypothetical protein LOC201725 |
| 1565909_at | 10.8 | 9.26E-05 | 1.114063449 | NA |  |
| 222312_s_at | 10.8 | 9.26E-05 | 1.241569006 | NA |  |
| 1557223_at | 10.8 | 9.25E-05 | 1.235270383 | NA |  |
| 223331_s_at | 10.79 | 9.28E-05 | 1.041754571 | NM_007204| | DDX20,DEAD (Asp-Glu-Ala-Asp) box polypeptide 20 |
| 228211_at | 10.78 | 9.35E-05 | 1.120155572 | NM_001010895| | LOC375748,RAD26L hypothetical protein |
| 230467_at | 10.78 | 9.33E-05 | 1.177449352 | NM_178545| | LOC339456,hypothetical protein LOC339456 |
| 242522_at | 10.77 | 9.38E-05 | 1.579041126 | NA |  |
| 226875_at | 10.77 | 9.41E-05 | 1.201634433 | NM_144658| | DOCK11,dedicator of cytokinesis 11 |
| 218824_at | 10.76 | 9.44E-05 | 1.055021233 | NM_001103149| | NA |
| 223611_s_at | 10.76 | 9.43E-05 | 1.721593731 | NM_001126328| | NA |
| 220199_s_at | 10.76 | 9.47E-05 | 1.094636524 | NM_022831| | FLJ12806,hypothetical protein FLJ12806 |
| 224494_x_at | 10.76 | 9.45E-05 | 1.515672055 | NM_016246| | DHRS10,dehydrogenase/reductase (SDR family) member 10 |
| 200791_s_at | 10.76 | 9.43E-05 | 1.094719031 | NM_003870| | IQGAP1,IQ motif containing GTPase activating protein 1 |
| 219557_s_at | 10.75 | 9.51E-05 | 1.362846748 | NM_020645| | NRIP3,nuclear receptor interacting protein 3 |
| 227422_at | 10.75 | 9.48E-05 | 1.173257201 | NA |  |
| 40093_at | 10.75 | 9.48E-05 | 1.166553875 | NM_001013257| | NA |
| 219296_at | 10.74 | 9.54E-05 | 1.066270732 | NM_001001483| | ZDHHC13,zinc finger, DHHC domain containing 13 isoform |
| 202955_s_at | 10.74 | 9.56E-05 | 1.167892447 | NM_006421| | ARFGEF1,brefeldin A-inhibited guanine |
| 225475_at | 10.74 | 9.55E-05 | 1.1184105 | NM_001077700| | NA |
| 215471_s_at | 10.74 | 9.55E-05 | 1.846780402 | NM_003980| | MAP7,microtubule-associated protein 7 |
| 212920_at | 10.74 | 9.54E-05 | 1.202115692 | NA |  |
| 1557044_at | 10.73 | 9.57E-05 | 1.337004668 | NA |  |
| 210588_x_at | 10.73 | 9.58E-05 | 1.041240043 | NM_012207| | HNRPH3,heterogeneous nuclear ribonucleoprotein H3 |
| 224813_at | 10.73 | 9.59E-05 | 1.091716709 | NM_003941| | WASL,Wiskott-Aldrich syndrome gene-like protein |
| 227168_at | 10.73 | 9.58E-05 | 1.260062477 | NA |  |
| 222411_s_at | 10.73 | 9.60E-05 | 1.05056425 | NM_007107| | SSR3,signal sequence receptor gamma subunit |
| 213208_at | 10.72 | 9.65E-05 | 1.097686616 | NM_015349| | KIAA0240,KIAA0240 |
| 234976_x_at | 10.72 | 9.65E-05 | 1.202570349 | NM_021196| | SLC4A5,sodium bicarbonate transporter 4 isoform a |
| 233354_at | 10.72 | 9.64E-05 | 1.107944016 | NA |  |
| 204500_s_at | 10.72 | 9.64E-05 | 1.179328105 | NM_015239| | AGTPBP1,ATP/GTP binding protein 1 |
| 229842_at | 10.72 | 9.63E-05 | 1.581480231 | NM_001114309| | NA |
| 207890_s_at | 10.72 | 9.64E-05 | 1.204240009 | NM_022468| | MMP25,matrix metalloproteinase 25 |
| 212072_s_at | 10.71 | 9.71E-05 | 1.09143879 | NM_001895| | CSNK2A1,casein kinase II alpha 1 subunit isoform a |
| 209443_at | 10.71 | 9.70E-05 | 1.087531181 | NM_000624| | SERPINA5,serine (or cysteine) proteinase inhibitor, clade |
| 204182_s_at | 10.71 | 9.69E-05 | 1.05883742 | NM_014007| | ZNF297B,zinc finger protein 297B |
| 205401_at | 10.71 | 9.70E-05 | 1.182530079 | NM_003659| | AGPS,alkylglycerone phosphate synthase precursor |
| 203951_at | 10.71 | 9.68E-05 | 1.653607244 | NM_001299| | CNN1,calponin 1, basic, smooth muscle |
| 209284_s_at | 10.7 | 9.75E-05 | 1.085584643 | NM_001112736| | NA |
| 217491_x_at | 10.7 | 9.71E-05 | 1.048633969 | NM_001867| | COX7C,cytochrome c oxidase subunit VIIc precursor |
| 219241_x_at | 10.69 | 9.78E-05 | 1.22339334 | NM_017857| | SSH3,slingshot homolog 3 |
| 210237_at | 10.69 | 9.80E-05 | 1.238981255 | NM_003976| | ARTN,neurotrophic factor artemin isoform 1, |
| 231955_s_at | 10.69 | 9.77E-05 | 1.128899461 | NM_152740| | HIBADH,3-hydroxyisobutyrate dehydrogenase |
| 218318_s_at | 10.69 | 9.78E-05 | 1.148699713 | NM_016231| | NLK,nemo like kinase |
| 223495_at | 10.69 | 9.78E-05 | 1.142185141 | NM_032040| | CCDC8,coiled-coil domain containing 8 |
| 200961_at | 10.68 | 9.85E-05 | 1.042912568 | NM_012248| | SEPHS2,selenophosphate synthetase 2 |
| 1569796_s_at | 10.67 | 9.85E-05 | 1.43204536 | NM_207303| | ATRNL1,attractin-like 1 |
| 219342_at | 10.67 | 9.86E-05 | 1.131331225 | NM_022900| | CAS1,O-acetyltransferase |
| 202929_s_at | 10.67 | 9.86E-05 | 1.055950716 | NM_001084392| | NA |
| 231940_at | 10.66 | 9.94E-05 | 1.097978901 | NM_020951| | ZNF529,zinc finger protein 529 |
| 202022_at | 10.66 | 9.94E-05 | 1.219558462 | NM_005165| | ALDOC,aldolase C, fructose-bisphosphate |
| 236118_at | 10.66 | 9.92E-05 | 1.133735194 | NA |  |
| 220256_s_at | 10.66 | 9.96E-05 | 1.151961197 | NM_022120| | OXCT2,3-oxoacid CoA transferase 2 |
| 217492_s_at | 10.66 | 9.91E-05 | 1.071647157 | NM_000314| | PTEN,phosphatase and tensin homolog |
| 210156_s_at | 10.65 | 9.98E-05 | 1.050077355 | NM_005389| | PCMT1,protein-L-isoaspartate (D-aspartate) |
| 1552727_s_at | 10.65 | 9.99E-05 | 1.314063095 | NM_139057| | ADAMTS17,a disintegrin-like and metalloprotease |
