# Supplementary material for: Transcriptional Signature and Memory Retention of Human-Induced Pluripotent Stem Cells
Source: PLoS One. 2009 Sep 18;4(9):e7076. doi: 10.1371/journal.pone.0007076 (PMC2741600; doi:10.1371/journal.pone.0007076)
Supplement: Table S6 — NSC-enriched probes in ES versus NSC. Probesets enriched in group-wise comparisons: Column headings are probeset identifiers, T-statistic, P-value, Fold-Change (log2), Refseq identifier and Description of the gene. (NA indicates no Refseq annotation). (7.45 MB DOC) [file pone.0007076.s010.doc]

| Probeset | T-statistic | P-value | Fold-Change (log2) | Refseq | Description |
| --- | --- | --- | --- | --- | --- |
| 229281_at | 132.96 | 6.37E-09 | 3.173788009 | NM_022123| | NPAS3,neuronal PAS domain protein 3 |
| 230272_at | 125.01 | 6.37E-09 | 3.102486455 | NA |  |
| 205103_at | 118.85 | 6.37E-09 | 1.924059886 | NM_006365| | CROC4,transcriptional activator of the c-fos promoter |
| 228904_at | 113.83 | 6.37E-09 | 2.491749287 | NM_002146| | HOXB3,homeo box B3 |
| 225182_at | 108.56 | 6.37E-09 | 1.373037168 | NM_006134| | C21orf4,HCV p7-transregulated protein 3 |
| 238877_at | 106.64 | 6.37E-09 | 3.732985196 | NM_004100| | EYA4,eyes absent 4 isoform a |
| 222146_s_at | 106.21 | 6.37E-09 | 1.234084916 | NM_001083962| | NA |
| 203540_at | 98.27 | 8.14E-09 | 2.849776625 | NM_002055| | GFAP,glial fibrillary acidic protein |
| 209988_s_at | 95.81 | 8.72E-09 | 3.138717835 | NM_004316| | ASCL1,achaete-scute complex homolog-like 1 |
| 202132_at | 95.72 | 8.72E-09 | 1.346735426 | NM_015472| | WWTR1,WW domain containing transcription regulator 1 |
| 242455_at | 94.4 | 8.72E-09 | 2.911366141 | NM_005604| | POU3F2,POU domain, class 3, transcription factor 2 |
| 211467_s_at | 94.33 | 8.72E-09 | 2.123986468 | NM_005596| | NFIB,nuclear factor I/B |
| 206070_s_at | 91.89 | 8.72E-09 | 4.385676111 | NM_005233| | EPHA3,ephrin receptor EphA3 isoform a precursor |
| 227666_at | 91.78 | 8.72E-09 | 2.158966629 | NM_001040260| | NA |
| 37408_at | 91.19 | 8.72E-09 | 1.24175819 | NM_006039| | MRC2,mannose receptor, C type 2 |
| 207327_at | 89.33 | 8.99E-09 | 3.076504068 | NM_004100| | EYA4,eyes absent 4 isoform a |
| 201860_s_at | 84.17 | 1.16E-08 | 2.306463038 | NM_000930| | PLAT,plasminogen activator, tissue type isoform 1 |
| 226114_at | 83.88 | 1.16E-08 | 1.938110337 | NM_001077195| | NA |
| 238058_at | 83.37 | 1.16E-08 | 2.845249751 | NA |  |
| 232195_at | 82.17 | 1.24E-08 | 2.744453187 | NM_020752| | GPR158,G protein-coupled receptor 158 |
| 227082_at | 79.17 | 1.51E-08 | 3.010381151 | NA |  |
| 208920_at | 78.65 | 1.55E-08 | 1.694855577 | NM_003130| | SRI,sorcin isoform a |
| 219148_at | 78.35 | 1.56E-08 | 1.402205477 | NM_018492| | PBK,T-LAK cell-originated protein kinase |
| 205030_at | 77.92 | 1.59E-08 | 1.992871895 | NM_001446| | FABP7,fatty acid binding protein 7, brain |
| 226368_at | 77.13 | 1.60E-08 | 1.493789197 | NM_018413| | CHST11,carbohydrate (chondroitin 4) sulfotransferase |
| 205638_at | 77.06 | 1.60E-08 | 2.477907949 | NM_001704| | BAI3,brain-specific angiogenesis inhibitor 3 |
| 204069_at | 76.7 | 1.62E-08 | 2.025835665 | NM_002398| | MEIS1,Meis1 homolog |
| 225575_at | 76.26 | 1.65E-08 | 1.398903181 | NM_002310| | LIFR, |
| 229608_at | 75.97 | 1.67E-08 | 1.330010233 | NM_019099| | LOC55924,hypothetical protein LOC55924 isoform 1 |
| 209987_s_at | 73.96 | 1.81E-08 | 2.718493241 | NM_004316| | ASCL1,achaete-scute complex homolog-like 1 |
| 203963_at | 73.87 | 1.81E-08 | 2.047647758 | NM_001218| | CA12,carbonic anhydrase XII isoform 1 precursor |
| 202936_s_at | 73.44 | 1.81E-08 | 1.665796413 | NM_000346| | SOX9,transcription factor SOX9 |
| 241998_at | 73 | 1.81E-08 | 2.896099899 | NM_001099334| | NA |
| 212169_at | 72.96 | 1.81E-08 | 1.417362806 | NM_007270| | FKBP9,FK506 binding protein 9 |
| 202484_s_at | 72.92 | 1.81E-08 | 3.082567208 | NM_003927| | MBD2,methyl-CpG binding domain protein 2 isoform 1 |
| 202950_at | 72.17 | 1.91E-08 | 2.734602024 | NM_001889| | CRYZ,crystallin, zeta |
| 228574_at | 69.74 | 2.30E-08 | 1.45669213 | NM_152588| | DKFZp762A217,hypothetical protein DKFZp762A217 |
| 244403_at | 69.62 | 2.30E-08 | 2.72522622 | NM_201253| | CRB1,crumbs homolog 1 isoform II precursor |
| 223184_s_at | 69.46 | 2.30E-08 | 1.703445774 | NM_001037553| | NA |
| 226377_at | 68.62 | 2.48E-08 | 2.083643927 | NA |  |
| 228780_at | 68.3 | 2.51E-08 | 3.077814185 | NA |  |
| 215867_x_at | 68.05 | 2.51E-08 | 1.707921027 | NM_001218| | CA12,carbonic anhydrase XII isoform 1 precursor |
| 209466_x_at | 68 | 2.51E-08 | 1.510326611 | NM_002825| | PTN,pleiotrophin |
| 209686_at | 67.92 | 2.51E-08 | 2.702714394 | NM_006272| | S100B,S100 calcium-binding protein, beta |
| 228708_at | 66.9 | 2.68E-08 | 2.428626327 | NM_004163| | RAB27B,RAB27B, member RAS oncogene family |
| 225381_at | 66.83 | 2.68E-08 | 2.940778674 | NA |  |
| 238850_at | 66.22 | 2.74E-08 | 2.466087103 | NA |  |
| 218888_s_at | 66.2 | 2.74E-08 | 1.457714064 | NM_018092| | NETO2,neuropilin- and tolloid-like protein 2 |
| 205111_s_at | 65.93 | 2.78E-08 | 2.319165254 | NM_016341| | PLCE1,pancreas-enriched phospholipase C |
| 229975_at | 64.7 | 2.95E-08 | 2.058313839 | NM_001203| | BMPR1B,bone morphogenetic protein receptor, type IB |
| 226558_at | 64.66 | 2.95E-08 | 2.269478637 | NA |  |
| 1559477_s_at | 64.34 | 2.97E-08 | 2.484134584 | NM_002398| | MEIS1,Meis1 homolog |
| 225368_at | 63.34 | 3.03E-08 | 1.112104259 | NM_001113239| | NA |
| 230212_at | 63.12 | 3.05E-08 | 1.404006305 | NA |  |
| 229127_at | 62.92 | 3.06E-08 | 1.845752747 | NM_021219| | JAM2,junctional adhesion molecule 2 precursor |
| 231128_at | 62.53 | 3.18E-08 | 1.841776871 | NM_175885| | MGC33846,hypothetical protein MGC33846 |
| 220316_at | 62.16 | 3.28E-08 | 2.253768321 | NM_022123| | NPAS3,neuronal PAS domain protein 3 |
| 204556_s_at | 61.89 | 3.30E-08 | 1.161436903 | NM_014934| | DZIP1,DAZ interacting protein 1 |
| 235977_at | 61.77 | 3.32E-08 | 2.082405649 | NM_198461| | FLJ45273,FLJ45273 protein |
| 230412_at | 61.25 | 3.46E-08 | 3.373677274 | NM_022123| | NPAS3,neuronal PAS domain protein 3 |
| 211737_x_at | 61.2 | 3.46E-08 | 1.4706341 | NM_002825| | PTN,pleiotrophin |
| 209199_s_at | 61.17 | 3.46E-08 | 1.935278545 | NM_002397| | MEF2C,MADS box transcription enhancer factor 2, |
| 224463_s_at | 61.14 | 3.46E-08 | 1.62344354 | NM_032930| | MGC13040,hypothetical protein MGC13040 |
| 224520_s_at | 60.28 | 3.70E-08 | 1.878902321 | NM_032735| | MGC13168,hypothetical protein MGC13168 |
| 228837_at | 60.25 | 3.70E-08 | 1.556577848 | NM_001083962| | NA |
| 207168_s_at | 60.09 | 3.70E-08 | 1.330615792 | NM_001040158| | NA |
| 206765_at | 60.08 | 3.70E-08 | 1.807089395 | NM_000891| | KCNJ2,potassium inwardly-rectifying channel J2 |
| 209859_at | 60.04 | 3.70E-08 | 2.625937036 | NM_015163| | TRIM9,tripartite motif protein 9 isoform 1 |
| 222803_at | 59.73 | 3.78E-08 | 1.522394344 | NM_020200| | PRTFDC1,phosphoribosyl transferase domain containing 1 |
| 218966_at | 59.63 | 3.79E-08 | 1.413967946 | NM_018728| | MYO5C,myosin VC |
| 228635_at | 59.49 | 3.83E-08 | 1.899384361 | NM_020815| | PCDH10,protocadherin 10 isoform 2 precursor |
| 201029_s_at | 59.29 | 3.87E-08 | 1.254658346 | NM_001122898| | NA |
| 213395_at | 59.28 | 3.87E-08 | 2.6549474 | NM_015166| | MLC1,megalencephalic leukoencephalopathy with |
| 217143_s_at | 58.97 | 3.98E-08 | 2.323271538 | NA |  |
| 225129_at | 58.93 | 3.98E-08 | 1.318587856 | NM_152727| | CPNE2,copine II |
| 222557_at | 58.85 | 3.99E-08 | 1.274516301 | NM_015894| | STMN3,SCG10-like-protein |
| 237449_at | 58.43 | 4.14E-08 | 1.699840617 | NM_182700| | SP8,Sp8 transcription factor isoform 1 |
| 201142_at | 58.24 | 4.21E-08 | 1.235844 | NM_004094| | EIF2S1,eukaryotic translation initiation factor 2, |
| 218692_at | 58 | 4.24E-08 | 2.21078017 | NM_001099743| | NA |
| 218309_at | 57.94 | 4.24E-08 | 1.546030804 | NM_018584| | CaMKIINalpha,calcium/calmodulin-dependent protein kinase II |
| 205029_s_at | 57.91 | 4.24E-08 | 2.415248057 | NM_001446| | FABP7,fatty acid binding protein 7, brain |
| 242263_at | 57.84 | 4.24E-08 | 2.015990675 | NM_016040| | TMED5,transmembrane emp24 protein transport domain |
| 202910_s_at | 57.84 | 4.24E-08 | 1.905564465 | NM_001025160| | NA |
| 222231_s_at | 57.79 | 4.24E-08 | 1.100271065 | NM_018509| | PRO1855,hypothetical protein PRO1855 |
| 242138_at | 57.69 | 4.27E-08 | 2.855039329 | NM_001038493| | NA |
| 201143_s_at | 57.38 | 4.38E-08 | 1.260007753 | NM_004094| | EIF2S1,eukaryotic translation initiation factor 2, |
| 213601_at | 57.26 | 4.38E-08 | 1.747053848 | NM_003061| | SLIT1,slit homolog 1 |
| 202935_s_at | 57.23 | 4.38E-08 | 1.771901913 | NM_000346| | SOX9,transcription factor SOX9 |
| 201028_s_at | 57.14 | 4.40E-08 | 1.320960974 | NM_001122898| | NA |
| 219415_at | 56.92 | 4.47E-08 | 1.441798219 | NM_001005367| | TTYH1,tweety 1 isoform 2 |
| 213158_at | 56.63 | 4.51E-08 | 2.688747977 | NA |  |
| 217870_s_at | 56.59 | 4.51E-08 | 1.194811474 | NM_016308| | UMP-CMPK,UMP-CMP kinase |
| 227617_at | 56.56 | 4.51E-08 | 1.465301624 | NM_001010866| | RP13-15M17.2,novel protein |
| 226810_at | 56.54 | 4.51E-08 | 1.795260374 | NM_024576| | OGFRL1,opioid growth factor receptor-like 1 |
| 228060_at | 56.52 | 4.51E-08 | 1.329514216 | NM_001029858| | NA |
| 225533_at | 56.51 | 4.51E-08 | 1.219484908 | NM_001009936| | PHF19,PHD finger protein 19 isoform b |
| 219511_s_at | 56.26 | 4.59E-08 | 1.487625064 | NM_005460| | SNCAIP,synuclein alpha interacting protein |
| 221654_s_at | 56.06 | 4.62E-08 | 1.454053398 | NM_006537| | USP3,ubiquitin specific protease 3 |
| 200795_at | 56.01 | 4.62E-08 | 2.935344638 | NM_004684| | SPARCL1,SPARC-like 1 |
| 1557852_at | 55.69 | 4.71E-08 | 1.637277693 | NA |  |
| 201152_s_at | 55.65 | 4.71E-08 | 1.490885261 | NM_021038| | MBNL1,muscleblind-like 1 isoform a |
| 211984_at | 55.43 | 4.82E-08 | 1.191349216 | NM_001743| | CALM2,calmodulin 2 |
| 203661_s_at | 54.72 | 5.13E-08 | 1.539033316 | NM_003275| | TMOD1,tropomodulin 1 |
| 213768_s_at | 54.63 | 5.13E-08 | 1.930966547 | NM_004316| | ASCL1,achaete-scute complex homolog-like 1 |
| 210138_at | 54.55 | 5.13E-08 | 2.095628772 | NM_003702| | RGS20,regulator of G-protein signalling 20 |
| 216331_at | 54.52 | 5.13E-08 | 1.283518873 | NM_002206| | ITGA7,integrin alpha 7 precursor |
| 227141_at | 54.49 | 5.13E-08 | 2.521871392 | NM_138467| | LOC127253,hypothetical protein BC009514 |
| 219763_at | 54.48 | 5.13E-08 | 1.188701343 | NM_020946| | KIAA1608,KIAA1608 protein isoform 1 |
| 217762_s_at | 54.37 | 5.13E-08 | 1.308929495 | NM_006868| | RAB31,RAB31, member RAS oncogene family |
| 225163_at | 54.24 | 5.19E-08 | 1.446695414 | NM_018027| | FRMD4A,FERM domain containing 4A |
| 206307_s_at | 54.1 | 5.25E-08 | 1.634777258 | NM_004472| | FOXD1,forkhead box D1 |
| 215290_at | 53.96 | 5.33E-08 | 2.241689855 | NA |  |
| 223182_s_at | 53.72 | 5.38E-08 | 1.851735099 | NM_001037553| | NA |
| 202096_s_at | 53.69 | 5.38E-08 | 1.327386817 | NM_000714| | BZRP,peripheral benzodiazapine receptor isoform PBR |
| 205475_at | 53.43 | 5.49E-08 | 2.980686878 | NM_007281| | SCRG1,scrapie responsive protein 1 |
| 206022_at | 53.04 | 5.66E-08 | 2.773433047 | NM_000266| | NDP,Norrie disease protein |
| 210830_s_at | 52.94 | 5.70E-08 | 1.230095886 | NM_000305| | PON2,paraoxonase 2 |
| 225325_at | 52.88 | 5.70E-08 | 1.383066741 | NM_017694| | FLJ20160,FLJ20160 protein |
| 236761_at | 52.86 | 5.70E-08 | 2.801582489 | NM_199000| | LHFPL3,lipoma HMGIC fusion partner-like 3 |
| 205383_s_at | 52.74 | 5.73E-08 | 1.85906382 | NM_015642| | ZBTB20,zinc finger and BTB domain containing 20 |
| 213832_at | 52.68 | 5.73E-08 | 2.496988311 | NM_004980| | KCND3,potassium voltage-gated channel, Shal-related |
| 227719_at | 52.68 | 5.73E-08 | 1.984140503 | NM_001127217| | NA |
| 214608_s_at | 52.51 | 5.80E-08 | 1.882123789 | NM_000503| | EYA1,eyes absent 1 isoform b |
| 209789_at | 52.48 | 5.80E-08 | 2.13163589 | NM_006091| | CORO2B,coronin, actin binding protein, 2B |
| 229266_at | 52.45 | 5.80E-08 | 1.85289483 | NA |  |
| 212070_at | 52.17 | 5.97E-08 | 2.144631316 | NM_005682| | GPR56,G protein-coupled receptor 56 isoform a |
| 209598_at | 51.97 | 6.11E-08 | 1.487002122 | NM_007257| | NA |
| 215014_at | 51.86 | 6.13E-08 | 2.917247156 | NM_004980| | KCND3,potassium voltage-gated channel, Shal-related |
| 226372_at | 51.81 | 6.13E-08 | 1.520290096 | NM_018413| | CHST11,carbohydrate (chondroitin 4) sulfotransferase |
| 212360_at | 51.52 | 6.33E-08 | 1.194148312 | NM_004037| | AMPD2,adenosine monophosphate deaminase 2 (isoform L) |
| 231810_at | 51.42 | 6.39E-08 | 1.401381885 | NM_080626| | BRI3BP,BRI3-binding protein |
| 210749_x_at | 51.01 | 6.69E-08 | 1.154712184 | NM_001954| | DDR1,discoidin receptor tyrosine kinase isoform b |
| 214717_at | 50.9 | 6.77E-08 | 1.731470789 | NA |  |
| 213032_at | 50.8 | 6.80E-08 | 2.388578126 | NM_005596| | NFIB,nuclear factor I/B |
| 210393_at | 50.68 | 6.81E-08 | 1.820879122 | NM_003667| | LGR5,leucine-rich repeat-containing G protein-coupled |
| 209470_s_at | 50.53 | 6.82E-08 | 2.743458601 | NM_005277| | GPM6A,glycoprotein M6A isoform 1 |
| 208221_s_at | 50.47 | 6.82E-08 | 1.668664872 | NM_003061| | SLIT1,slit homolog 1 |
| 203185_at | 50.38 | 6.82E-08 | 1.619329698 | NM_014737| | RASSF2,Ras association domain family 2 isoform 1 |
| 228381_at | 50.38 | 6.82E-08 | 1.519632001 | NM_024997| | ATF7IP2,activating transcription factor 7 interacting |
| 201535_at | 50.33 | 6.83E-08 | 1.229626743 | NM_007106| | UBL3,ubiquitin-like 3 |
| 232921_at | 50.16 | 6.89E-08 | 1.396510519 | NM_020910| | NA |
| 213435_at | 50.11 | 6.89E-08 | 1.53971805 | NM_015265| | SATB2,SATB family member 2 |
| 231430_at | 49.9 | 6.98E-08 | 1.873721353 | NM_175885| | MGC33846,hypothetical protein MGC33846 |
| 204493_at | 49.69 | 7.12E-08 | 1.287680915 | NM_001196| | BID,BH3 interacting domain death agonist isoform 2 |
| 214164_x_at | 49.64 | 7.12E-08 | 1.758196298 | NM_001218| | CA12,carbonic anhydrase XII isoform 1 precursor |
| 223203_at | 49.64 | 7.12E-08 | 1.112628468 | NM_001099684| | NA |
| 204743_at | 49.54 | 7.17E-08 | 1.544139007 | NM_001008272| | TAGLN3,transgelin 3 |
| 223283_s_at | 49.53 | 7.17E-08 | 2.336631642 | NM_005786| | SDCCAG33,serologically defined colon cancer antigen 33 |
| 1568598_at | 49.38 | 7.27E-08 | 1.530795648 | NM_030929| | KAZALD1,Kazal-type serine protease inhibitor domain 1 |
| 212830_at | 49.35 | 7.27E-08 | 2.001827514 | NM_001080497| | NA |
| 221059_s_at | 49.09 | 7.47E-08 | 1.112725536 | NM_021149| | COTL1,coactosin-like 1 |
| 222301_at | 48.94 | 7.58E-08 | 1.972415906 | NM_006365| | CROC4,transcriptional activator of the c-fos promoter |
| 227326_at | 48.9 | 7.58E-08 | 1.278384585 | NM_001008528| | TMAP1,transmembrane anchor protein 1 isoform 1 |
| 204030_s_at | 48.88 | 7.58E-08 | 1.255206045 | NM_014575| | SCHIP1,schwannomin interacting protein 1 |
| 224925_at | 48.72 | 7.71E-08 | 1.430453837 | NM_020820| | PREX1,PREX1 protein |
| 223214_s_at | 48.62 | 7.77E-08 | 1.808217948 | NM_001017926| | NA |
| 212546_s_at | 48.49 | 7.86E-08 | 1.202961531 | NM_015030| | NA |
| 1560425_s_at | 48.43 | 7.87E-08 | 1.952753586 | NA |  |
| 1438_at | 48.41 | 7.87E-08 | 1.471297901 | NM_004443| | EPHB3,ephrin receptor EphB3 precursor |
| 212486_s_at | 48.36 | 7.89E-08 | 1.439735504 | NM_002037| | FYN,protein-tyrosine kinase fyn isoform a |
| 1552256_a_at | 48.23 | 8.02E-08 | 1.287598107 | NM_001082959| | NA |
| 219051_x_at | 48.21 | 8.02E-08 | 1.450259995 | NM_024042| | METRN,meteorin, glial cell differentiation regulator |
| 229259_at | 48.14 | 8.04E-08 | 1.926062964 | NM_002055| | GFAP,glial fibrillary acidic protein |
| 239952_at | 47.89 | 8.24E-08 | 1.931055635 | NM_030751| | TCF8,transcription factor 8 (represses interleukin 2 |
| 239432_at | 47.87 | 8.24E-08 | 1.521540919 | NA |  |
| 202202_s_at | 47.82 | 8.26E-08 | 2.615520236 | NM_001105206| | NA |
| 201941_at | 47.79 | 8.26E-08 | 1.114514045 | NM_001304| | CPD,carboxypeptidase D precursor |
| 230291_s_at | 47.66 | 8.39E-08 | 2.501210758 | NM_005596| | NFIB,nuclear factor I/B |
| 32094_at | 47.56 | 8.49E-08 | 1.302752493 | NM_004273| | CHST3,carbohydrate (chondroitin 6) sulfotransferase 3 |
| 226250_at | 47.52 | 8.51E-08 | 1.94974334 | NA |  |
| 230864_at | 47.48 | 8.53E-08 | 2.276790713 | NM_153361| | MGC42105,hypothetical protein MGC42105 |
| 215649_s_at | 47.39 | 8.61E-08 | 1.277533669 | NM_000431| | MVK,mevalonate kinase |
| 212206_s_at | 47.19 | 8.71E-08 | 1.211914789 | NM_012412| | H2AFV,H2A histone family, member V isoform 1 |
| 1555867_at | 47.13 | 8.77E-08 | 1.673250921 | NM_001098721| | NA |
| 225197_at | 47.03 | 8.85E-08 | 1.179302237 | NA |  |
| 233562_at | 46.98 | 8.87E-08 | 1.862140033 | NA |  |
| 227498_at | 46.89 | 8.97E-08 | 1.99912391 | NA |  |
| 210415_s_at | 46.55 | 9.31E-08 | 1.433405092 | NM_002540| | ODF2,outer dense fiber of sperm tails 2 isoform 1 |
| 220227_at | 46.26 | 9.68E-08 | 1.342619153 | NM_001794| | CDH4,cadherin 4, type 1 preproprotein |
| 218024_at | 46.14 | 9.74E-08 | 1.36562194 | NM_016098| | BRP44L,brain protein 44-like |
| 228218_at | 46.13 | 9.74E-08 | 1.848541409 | NA |  |
| 222774_s_at | 46.07 | 9.77E-08 | 1.332386503 | NM_018092| | NETO2,neuropilin- and tolloid-like protein 2 |
| 235798_at | 45.92 | 9.87E-08 | 1.911575066 | NM_001100829| | NA |
| 224963_at | 45.91 | 9.87E-08 | 1.140970957 | NM_000112| | SLC26A2,solute carrier family 26 member 2 |
| 204508_s_at | 45.87 | 9.89E-08 | 1.936884941 | NM_001218| | CA12,carbonic anhydrase XII isoform 1 precursor |
| 205143_at | 45.73 | 1.00E-07 | 2.08340799 | NM_004386| | CSPG3,chondroitin sulfate proteoglycan 3 (neurocan) |
| 215091_s_at | 45.65 | 1.01E-07 | 1.144572341 | NM_002097| | GTF3A,general transcription factor IIIA |
| 227452_at | 45.62 | 1.01E-07 | 2.119951818 | NA |  |
| 232113_at | 45.61 | 1.01E-07 | 2.259180523 | NA |  |
| 204646_at | 45.55 | 1.01E-07 | 3.281389996 | NM_000110| | DPYD,dihydropyrimidine dehydrogenase |
| 40562_at | 45.5 | 1.01E-07 | 1.220950047 | NM_002067| | GNA11,guanine nucleotide binding protein (G protein), |
| 205363_at | 45.48 | 1.01E-07 | 2.000372033 | NM_003986| | BBOX1,gamma-butyrobetaine hydroxylase |
| 226907_at | 45.45 | 1.01E-07 | 1.99446025 | NM_030949| | PPP1R14C,protein phosphatase 1, regulatory (inhibitor) |
| 232125_at | 45.39 | 1.02E-07 | 2.927490749 | NA |  |
| 238041_at | 45.34 | 1.02E-07 | 2.081924484 | NA |  |
| 205278_at | 45.17 | 1.04E-07 | 1.642811819 | NM_000817| | GAD1,glutamate decarboxylase 1 isoform GAD67 |
| 227049_at | 44.88 | 1.07E-07 | 1.863147512 | NM_175907| | ZADH2,zinc binding alcohol dehydrogenase, domain |
| 213712_at | 44.55 | 1.12E-07 | 2.318539016 | NM_017770| | ELOVL2,elongation of very long chain fatty acids |
| 212387_at | 44.5 | 1.12E-07 | 1.206358598 | NM_001083962| | NA |
| 203708_at | 44.39 | 1.14E-07 | 1.780252486 | NM_001037339| | NA |
| 227121_at | 44.35 | 1.14E-07 | 2.375867203 | NA |  |
| 207060_at | 44.32 | 1.14E-07 | 2.155722844 | NM_001427| | EN2,engrailed homolog 2 |
| 201983_s_at | 44.26 | 1.15E-07 | 1.634091922 | NM_005228| | EGFR,epidermal growth factor receptor isoform a |
| 226919_at | 44.18 | 1.16E-07 | 1.363319292 | NM_020466| | DJ122O8.2,hypothetical protein dJ122O8.2 |
| 209840_s_at | 43.94 | 1.19E-07 | 2.21277103 | NM_001099658| | NA |
| 204767_s_at | 43.9 | 1.19E-07 | 1.042683029 | NM_004111| | FEN1,flap structure-specific endonuclease 1 |
| 225793_at | 43.89 | 1.19E-07 | 1.45442122 | NM_153713| | LIX1L,Lix1 homolog (mouse) like |
| 225921_at | 43.86 | 1.19E-07 | 1.422710383 | NM_016350| | NIN,ninein isoform 4 |
| 212195_at | 43.83 | 1.19E-07 | 1.527437655 | NM_002184| | IL6ST,interleukin 6 signal transducer isoform 1 |
| 213058_at | 43.79 | 1.19E-07 | 1.343393693 | NA |  |
| 209469_at | 43.68 | 1.20E-07 | 2.399421635 | NM_005277| | GPM6A,glycoprotein M6A isoform 1 |
| 242837_at | 43.61 | 1.21E-07 | 1.361602173 | NM_005626| | SFRS4,splicing factor, arginine/serine-rich 4 |
| 224909_s_at | 43.59 | 1.21E-07 | 1.467163463 | NM_020820| | PREX1,PREX1 protein |
| 213939_s_at | 43.39 | 1.25E-07 | 1.28533121 | NM_001037442| | NA |
| 235743_at | 43.31 | 1.26E-07 | 2.060787069 | NA |  |
| 203115_at | 43.16 | 1.27E-07 | 1.204119279 | NM_000140| | FECH,ferrochelatase isoform b precursor |
| 235147_at | 43.12 | 1.28E-07 | 2.355197647 | NA |  |
| 205112_at | 43.04 | 1.29E-07 | 2.283215685 | NM_016341| | PLCE1,pancreas-enriched phospholipase C |
| 213029_at | 42.92 | 1.30E-07 | 1.85836221 | NM_005596| | NFIB,nuclear factor I/B |
| 227143_s_at | 42.91 | 1.30E-07 | 1.308623715 | NM_001196| | BID,BH3 interacting domain death agonist isoform 2 |
| 232269_x_at | 42.89 | 1.30E-07 | 1.41417683 | NM_024042| | METRN,meteorin, glial cell differentiation regulator |
| 223419_at | 42.89 | 1.30E-07 | 1.218265668 | NM_032301| | FBXW9,F-box and WD-40 domain protein 9 |
| 212509_s_at | 42.85 | 1.30E-07 | 1.167632963 | NM_001008528| | TMAP1,transmembrane anchor protein 1 isoform 1 |
| 228902_at | 42.82 | 1.31E-07 | 1.239873648 | NM_005085| | NUP214,nucleoporin 214kDa |
| 224215_s_at | 42.69 | 1.33E-07 | 1.217337562 | NM_005618| | DLL1,delta-like 1 |
| 225144_at | 42.67 | 1.33E-07 | 1.430792547 | NM_001204| | BMPR2,bone morphogenetic protein receptor, type II |
| 238008_at | 42.65 | 1.33E-07 | 1.466327838 | NM_175922| | MGC35308,hypothetical protein MGC35308 |
| 223253_at | 42.58 | 1.34E-07 | 1.186630648 | NM_017549| | EPDR1,upregulated in colorectal cancer gene 1 protein |
| 207826_s_at | 42.56 | 1.35E-07 | 1.276104008 | NM_002167| | ID3,inhibitor of DNA binding 3 |
| 223455_at | 42.5 | 1.35E-07 | 1.462347851 | NM_032300| | MGC10854,hypothetical protein MGC10854 |
| 204685_s_at | 42.45 | 1.36E-07 | 1.584719821 | NM_001001331| | ATP2B2,plasma membrane calcium ATPase 2 isoform a |
| 205794_s_at | 42.37 | 1.37E-07 | 1.78516084 | NM_002515| | NOVA1,neuro-oncological ventral antigen 1 isoform 1 |
| 219133_at | 42.33 | 1.37E-07 | 1.416645888 | NM_017897| | FLJ20604,hypothetical protein FLJ20604 |
| 229656_s_at | 42.2 | 1.39E-07 | 2.11492692 | NA |  |
| 224694_at | 42.16 | 1.39E-07 | 1.273727054 | NM_018153| | ANTXR1,tumor endothelial marker 8 isoform 3 precursor |
| 229347_at | 42.14 | 1.39E-07 | 1.97248773 | NA |  |
| 228953_at | 42.14 | 1.39E-07 | 1.273480854 | NM_001080435| | NA |
| 238906_s_at | 42.01 | 1.40E-07 | 2.44409124 | NM_020663| | RHOJ,TC10-like Rho GTPase |
| 204364_s_at | 41.76 | 1.44E-07 | 1.622126042 | NM_022912| | C2orf23,receptor expression enhancing protein 1 |
| 213288_at | 41.75 | 1.44E-07 | 1.345896786 | NM_138799| | OACT2,O-acyltransferase (membrane bound) domain |
| 204142_at | 41.68 | 1.45E-07 | 1.253593697 | NM_001126123| | NA |
| 225670_at | 41.65 | 1.45E-07 | 1.280183539 | NM_199133| | LOC134145,hypothetical protein LOC134145 |
| 228555_at | 41.52 | 1.47E-07 | 1.781830682 | NM_001221| | CAMK2D,calcium/calmodulin-dependent protein kinase II |
| 211964_at | 41.47 | 1.48E-07 | 1.210357997 | NM_001846| | COL4A2,alpha 2 type IV collagen preproprotein |
| 204457_s_at | 41.44 | 1.48E-07 | 1.447798375 | NM_002048| | GAS1,growth arrest-specific 1 |
| 203595_s_at | 41.42 | 1.49E-07 | 1.561900795 | NM_012420| | IFIT5,interferon-induced protein with |
| 212357_at | 41.33 | 1.50E-07 | 1.400042523 | NM_015159| | NA |
| 204341_at | 41.23 | 1.52E-07 | 1.324256956 | NM_006470| | TRIM16,tripartite motif-containing 16 |
| 226656_at | 41.22 | 1.52E-07 | 1.254646895 | NM_006371| | CRTAP,cartilage associated protein precursor |
| 207017_at | 41.2 | 1.52E-07 | 1.677646192 | NM_004163| | RAB27B,RAB27B, member RAS oncogene family |
| 223035_s_at | 41.19 | 1.52E-07 | 1.127796357 | NM_005687| | FARSLB,phenylalanine-tRNA synthetase-like, beta |
| 205436_s_at | 41.17 | 1.52E-07 | 1.140968588 | NM_002105| | H2AFX,H2A histone family, member X |
| 223228_at | 41.15 | 1.52E-07 | 1.177437647 | NM_032287| | LDOC1L,leucine zipper, down-regulated in cancer 1-like |
| 213156_at | 41.13 | 1.52E-07 | 2.665583978 | NA |  |
| 1559966_a_at | 41.04 | 1.54E-07 | 2.236477295 | NA |  |
| 244797_at | 40.84 | 1.58E-07 | 1.668455336 | NA |  |
| 207147_at | 40.79 | 1.58E-07 | 2.332973839 | NM_004405| | DLX2,distal-less homeo box 2 |
| 225050_at | 40.79 | 1.58E-07 | 1.085306693 | NM_032434| | ZNF512,zinc finger protein 512 |
| 202662_s_at | 40.64 | 1.60E-07 | 1.80250589 | NM_002223| | ITPR2,inositol 1,4,5-triphosphate receptor, type 2 |
| 204424_s_at | 40.62 | 1.60E-07 | 1.801289608 | NM_001001395| | LMO3,LIM domain only 3 |
| 233364_s_at | 40.62 | 1.60E-07 | 2.271041265 | NA |  |
| 218187_s_at | 40.58 | 1.60E-07 | 1.128194969 | NM_023080| | FLJ20989,hypothetical protein FLJ20989 |
| 214501_s_at | 40.56 | 1.60E-07 | 1.321373171 | NM_001040158| | NA |
| 212294_at | 40.56 | 1.60E-07 | 1.251454057 | NM_018841| | GNG12,G-protein gamma-12 subunit |
| 227977_at | 40.56 | 1.60E-07 | 1.660627388 | NM_175907| | ZADH2,zinc binding alcohol dehydrogenase, domain |
| 201645_at | 40.52 | 1.61E-07 | 1.899065241 | NM_002160| | TNC,tenascin C (hexabrachion) |
| 222270_at | 40.49 | 1.61E-07 | 1.79648574 | NM_001122964| | NA |
| 208683_at | 40.41 | 1.63E-07 | 1.193244858 | NM_001748| | CAPN2,calpain 2, large subunit |
| 235990_at | 40.39 | 1.64E-07 | 1.522132055 | NA |  |
| 200862_at | 40.24 | 1.68E-07 | 1.147184367 | NM_014762| | DHCR24,24-dehydrocholesterol reductase precursor |
| 224976_at | 40.15 | 1.70E-07 | 2.418275815 | NM_005595| | NFIA,nuclear factor I/A |
| 225716_at | 40.05 | 1.72E-07 | 1.338580272 | NA |  |
| 210735_s_at | 39.82 | 1.76E-07 | 1.905039026 | NM_001218| | CA12,carbonic anhydrase XII isoform 1 precursor |
| 209170_s_at | 39.8 | 1.76E-07 | 1.367434371 | NM_001001994| | GPM6B,glycoprotein M6B isoform 4 |
| 222446_s_at | 39.79 | 1.76E-07 | 1.354166305 | NM_012105| | BACE2,beta-site APP-cleaving enzyme 2 isoform A |
| 223673_at | 39.73 | 1.77E-07 | 3.222197731 | NM_002920| | RFX4,regulatory factor X4 isoform b |
| 204348_s_at | 39.61 | 1.80E-07 | 1.192668263 | NM_001002921| | AK3L2,adenylate kinase 3-like 2 |
| 209506_s_at | 39.57 | 1.80E-07 | 1.549235936 | NM_005654| | NR2F1,nuclear receptor subfamily 2, group F, member 1 |
| 212063_at | 39.54 | 1.80E-07 | 2.089381636 | NM_000610| | CD44,CD44 antigen isoform 1 precursor |
| 229126_at | 39.54 | 1.80E-07 | 1.329205503 | NM_018279| | TMEM19,transmembrane protein 19 |
| 216191_s_at | 39.51 | 1.80E-07 | 2.937674334 | NA |  |
| 225473_at | 39.5 | 1.80E-07 | 1.321967094 | NM_199181| | FLJ44670,FLJ44670 protein |
| 230264_s_at | 39.49 | 1.80E-07 | 1.189011465 | NM_003916| | AP1S2,adaptor-related protein complex 1 sigma 2 |
| 237675_at | 39.44 | 1.81E-07 | 1.74739127 | NA |  |
| 205996_s_at | 39.43 | 1.81E-07 | 1.556040796 | NM_001625| | AK2,adenylate kinase 2 isoform a |
| 235919_at | 39.34 | 1.83E-07 | 1.321480688 | NA |  |
| 213838_at | 39.32 | 1.83E-07 | 1.374020948 | NM_016167| | NOL7,nucleolar protein 7, 27kDa |
| 229512_at | 39.3 | 1.83E-07 | 1.421595706 | NM_017848| | CXorf17,chromosome X open reading frame 17 |
| 209841_s_at | 39.29 | 1.83E-07 | 1.806830321 | NM_001099658| | NA |
| 217848_s_at | 39.29 | 1.83E-07 | 1.079864492 | NM_021129| | PP,inorganic pyrophosphatase |
| 225342_at | 39.09 | 1.89E-07 | 1.245897454 | NM_001002921| | AK3L2,adenylate kinase 3-like 2 |
| 206197_at | 38.99 | 1.92E-07 | 1.454897166 | NM_003551| | NME5,non-metastatic cells 5, protein expressed in |
| 226652_at | 38.87 | 1.95E-07 | 1.659054802 | NM_006537| | USP3,ubiquitin specific protease 3 |
| 204011_at | 38.86 | 1.95E-07 | 1.274047064 | NM_005842| | SPRY2,sprouty 2 |
| 223282_at | 38.84 | 1.95E-07 | 1.797915627 | NM_005786| | SDCCAG33,serologically defined colon cancer antigen 33 |
| 217763_s_at | 38.74 | 1.97E-07 | 1.300497036 | NM_006868| | RAB31,RAB31, member RAS oncogene family |
| 209794_at | 38.72 | 1.97E-07 | 1.444026523 | NM_001033117| | NA |
| 227124_at | 38.71 | 1.97E-07 | 1.253721268 | NA |  |
| 210105_s_at | 38.7 | 1.97E-07 | 1.115241746 | NM_002037| | FYN,protein-tyrosine kinase fyn isoform a |
| 227699_at | 38.65 | 1.98E-07 | 1.606012511 | NM_144581| | C14orf149,chromosome 14 open reading frame 149 |
| 236207_at | 38.65 | 1.98E-07 | 1.918403463 | NM_006751| | SSFA2,sperm specific antigen 2 |
| 244623_at | 38.64 | 1.98E-07 | 2.492671648 | NM_019842| | KCNQ5,potassium voltage-gated channel, KQT-like |
| 242985_x_at | 38.55 | 1.99E-07 | 1.860373183 | NM_001113561| | NA |
| 1554640_at | 38.54 | 1.99E-07 | 1.559957371 | NM_001037293| | NA |
| 201338_x_at | 38.41 | 2.03E-07 | 1.144312932 | NM_002097| | GTF3A,general transcription factor IIIA |
| 211763_s_at | 38.35 | 2.05E-07 | 1.209970263 | NM_003337| | UBE2B,ubiquitin-conjugating enzyme E2B |
| 223071_at | 38.33 | 2.05E-07 | 1.10939799 | NM_016097| | IER3IP1,immediate early response 3 interacting protein |
| 205493_s_at | 38.3 | 2.06E-07 | 1.217867725 | NM_006426| | DPYSL4,dihydropyrimidinase-like 4 |
| 228302_x_at | 38.16 | 2.10E-07 | 1.655272039 | NM_018584| | CaMKIINalpha,calcium/calmodulin-dependent protein kinase II |
| 206071_s_at | 38.15 | 2.10E-07 | 2.081984165 | NM_005233| | EPHA3,ephrin receptor EphA3 isoform a precursor |
| 204099_at | 38.11 | 2.12E-07 | 1.40743434 | NM_001003801| | SMARCD3,SWI/SNF related, matrix associated, actin |
| 225990_at | 38.05 | 2.13E-07 | 1.448811409 | NM_033254| | BOC,brother of CDO |
| 218714_at | 38.03 | 2.13E-07 | 1.263684639 | NM_024031| | MGC3121,hypothetical protein MGC3121 |
| 228628_at | 37.92 | 2.17E-07 | 1.29984601 | NA |  |
| 235381_at | 37.89 | 2.17E-07 | 1.412921396 | NA |  |
| 235164_at | 37.89 | 2.17E-07 | 1.920949029 | NM_145011| | ZNF25,zinc finger protein 25 |
| 224851_at | 37.77 | 2.21E-07 | 1.441199521 | NM_001259| | CDK6,cyclin-dependent kinase 6 |
| 202589_at | 37.68 | 2.24E-07 | 1.16065386 | NM_001071| | TYMS,thymidylate synthetase |
| 227178_at | 37.6 | 2.26E-07 | 1.288729245 | NM_001025076| | NA |
| 223024_at | 37.56 | 2.27E-07 | 1.219764017 | NM_032493| | AP1M1,adaptor-related protein complex 1, mu 1 subunit |
| 244075_at | 37.47 | 2.29E-07 | 1.719708308 | NA |  |
| 228679_at | 37.4 | 2.31E-07 | 2.430646875 | NA |  |
| 228581_at | 37.36 | 2.32E-07 | 2.151147862 | NM_002241| | KCNJ10,potassium inwardly-rectifying channel J10 |
| 209505_at | 37.34 | 2.33E-07 | 1.89486245 | NM_005654| | NR2F1,nuclear receptor subfamily 2, group F, member 1 |
| 219918_s_at | 37.23 | 2.36E-07 | 1.134938797 | NM_018136| | ASPM,asp (abnormal spindle)-like, microcephaly |
| 222778_s_at | 37.23 | 2.36E-07 | 1.196919062 | NM_001042424| | NA |
| 238009_at | 37.17 | 2.37E-07 | 1.747994225 | NA |  |
| 228955_at | 37.09 | 2.40E-07 | 1.687049128 | NA |  |
| 1568629_s_at | 37 | 2.44E-07 | 1.171020531 | NM_005027| | PIK3R2,phosphoinositide-3-kinase, regulatory subunit 2 |
| 200774_at | 36.91 | 2.47E-07 | 1.15104922 | NM_014612| | C9orf10,C9orf10 protein |
| 200794_x_at | 36.91 | 2.47E-07 | 1.242198857 | NM_014764| | DAZAP2,DAZ associated protein 2 |
| 224716_at | 36.89 | 2.47E-07 | 1.208201088 | NM_178148| | SLC35B2,solute carrier family 35, member B2 |
| 234985_at | 36.89 | 2.47E-07 | 1.245793783 | NM_174902| | LOC143458,hypothetical protein LOC143458 |
| 203424_s_at | 36.88 | 2.47E-07 | 1.423421143 | NM_000599| | IGFBP5,insulin-like growth factor binding protein 5 |
| 236004_at | 36.87 | 2.47E-07 | 1.244519421 | NA |  |
| 235829_at | 36.86 | 2.47E-07 | 1.634718285 | NA |  |
| 205675_at | 36.85 | 2.47E-07 | 2.197485962 | NM_000253| | MTP,microsomal triglyceride transfer protein large |
| 239229_at | 36.81 | 2.48E-07 | 1.821571878 | NA |  |
| 225897_at | 36.81 | 2.48E-07 | 1.214005914 | NM_002356| | MARCKS,myristoylated alanine-rich protein kinase C |
| 1556329_a_at | 36.79 | 2.48E-07 | 2.779192614 | NA |  |
| 218048_at | 36.78 | 2.48E-07 | 1.434082198 | NM_012071| | COMMD3,COMM domain containing 3 |
| 205344_at | 36.77 | 2.49E-07 | 1.567191369 | NM_006574| | CSPG5,chondroitin sulfate proteoglycan 5 (neuroglycan |
| 225492_at | 36.72 | 2.50E-07 | 1.055764588 | NM_018126| | TMEM33,transmembrane protein 33 |
| 213094_at | 36.71 | 2.50E-07 | 1.705670886 | NM_001032394| | NA |
| 223092_at | 36.62 | 2.53E-07 | 1.34291649 | NM_054027| | ANKH,ankylosis, progressive homolog |
| 204035_at | 36.58 | 2.54E-07 | 2.436857496 | NM_003469| | SCG2,secretogranin II precursor |
| 226113_at | 36.58 | 2.54E-07 | 2.212975342 | NM_001077195| | NA |
| 223707_at | 36.52 | 2.55E-07 | 1.666096779 | NM_000990| | RPL27A,ribosomal protein L27a |
| 238472_at | 36.5 | 2.55E-07 | 1.844056408 | NM_012347| | FBXO9,F-box only protein 9 isoform 1 |
| 210045_at | 36.49 | 2.55E-07 | 1.462094856 | NM_002168| | IDH2,isocitrate dehydrogenase 2 (NADP+), |
| 235350_at | 36.49 | 2.55E-07 | 1.884605813 | NM_001104629| | NA |
| 217764_s_at | 36.46 | 2.55E-07 | 1.276557756 | NM_006868| | RAB31,RAB31, member RAS oncogene family |
| 1557433_at | 36.44 | 2.55E-07 | 1.725905351 | NA |  |
| 209763_at | 36.42 | 2.55E-07 | 1.539516294 | NM_145234| | CHRDL1,chordin-like 1 |
| 212774_at | 36.34 | 2.58E-07 | 1.217088592 | NM_006352| | ZNF238,zinc finger protein 238 isoform 2 |
| 211959_at | 36.33 | 2.58E-07 | 1.436495102 | NM_000599| | IGFBP5,insulin-like growth factor binding protein 5 |
| 1559965_at | 36.22 | 2.62E-07 | 2.981498443 | NA |  |
| 225617_at | 36.2 | 2.62E-07 | 1.454312347 | NM_002540| | ODF2,outer dense fiber of sperm tails 2 isoform 1 |
| 218589_at | 36.19 | 2.62E-07 | 1.771981551 | NM_005767| | P2RY5,G-protein coupled purinergic receptor P2Y5 |
| 225127_at | 36.19 | 2.62E-07 | 1.167367489 | NM_020823| | NA |
| 240122_at | 36.12 | 2.65E-07 | 2.413524283 | NM_017594| | DIRAS2,Di-Ras2 |
| 224937_at | 36.12 | 2.65E-07 | 1.202608999 | NM_020440| | PTGFRN,prostaglandin F2 receptor negative regulator |
| 227524_at | 36.04 | 2.67E-07 | 1.318458315 | NA |  |
| 213825_at | 35.95 | 2.70E-07 | 2.109034641 | NM_005806| | OLIG2,oligodendrocyte lineage transcription factor 2 |
| 224818_at | 35.94 | 2.70E-07 | 1.12701152 | NM_002959| | SORT1,sortilin 1 preproprotein |
| 219107_at | 35.91 | 2.71E-07 | 1.561876577 | NM_021948| | BCAN,brevican isoform 1 |
| 222797_at | 35.82 | 2.75E-07 | 1.454049041 | NM_020134| | DPYSL5,dihydropyrimidinase-like 5 |
| 228049_x_at | 35.81 | 2.75E-07 | 1.262052033 | NA |  |
| 206157_at | 35.76 | 2.77E-07 | 2.412866959 | NM_002852| | PTX3,pentaxin-related gene, rapidly induced by IL-1 |
| 220559_at | 35.75 | 2.77E-07 | 2.42605156 | NM_001426| | EN1,engrailed homolog 1 |
| 203758_at | 35.75 | 2.77E-07 | 1.417634921 | NM_001334| | CTSO,cathepsin O preproprotein |
| 200788_s_at | 35.75 | 2.77E-07 | 1.217321332 | NM_003768| | PEA15,phosphoprotein enriched in astrocytes 15 |
| 200923_at | 35.67 | 2.79E-07 | 1.34999766 | NM_005567| | LGALS3BP,galectin 3 binding protein |
| 53991_at | 35.66 | 2.79E-07 | 1.366663917 | NM_015689| | KIAA1277,KIAA1277 protein |
| 209866_s_at | 35.66 | 2.79E-07 | 1.27815128 | NM_015236| | LPHN3,latrophilin 3 precursor |
| 213257_at | 35.65 | 2.79E-07 | 1.613815181 | NM_015077| | SARM1,sterile alpha and TIR motif containing 1 |
| 213830_at | 35.62 | 2.81E-07 | 1.852561063 | NA |  |
| 209301_at | 35.54 | 2.84E-07 | 1.255200568 | NM_000067| | CA2,carbonic anhydrase II |
| 228170_at | 35.48 | 2.86E-07 | 2.507684663 | NM_138983| | OLIG1,oligodendrocyte transcription factor 1 |
| 201266_at | 35.45 | 2.87E-07 | 1.090868127 | NM_001093771| | NA |
| 31874_at | 35.43 | 2.88E-07 | 1.286103354 | NM_006478| | GAS2L1,growth arrest-specific 2 like 1 isoform a |
| 207169_x_at | 35.4 | 2.89E-07 | 1.159233665 | NM_001954| | DDR1,discoidin receptor tyrosine kinase isoform b |
| 1007_s_at | 35.4 | 2.89E-07 | 1.125648317 | NM_001954| | DDR1,discoidin receptor tyrosine kinase isoform b |
| 202136_at | 35.21 | 2.98E-07 | 1.167843385 | NM_006624| | ZMYND11,zinc finger, MYND domain containing 11 isoform |
| 202133_at | 35.19 | 2.98E-07 | 1.303165885 | NM_015472| | WWTR1,WW domain containing transcription regulator 1 |
| 209460_at | 35.19 | 2.98E-07 | 1.280553617 | NM_000663| | ABAT,4-aminobutyrate aminotransferase precursor |
| 221216_s_at | 35.1 | 3.03E-07 | 1.25329069 | NM_001031694| | NA |
| 212646_at | 34.99 | 3.08E-07 | 1.239238172 | NM_015150| | RAFTLIN,raft-linking protein |
| 212895_s_at | 34.99 | 3.08E-07 | 1.168767093 | NM_001092| | ABR,active breakpoint cluster region-related |
| 226806_s_at | 34.85 | 3.16E-07 | 2.550662456 | NA |  |
| 200916_at | 34.82 | 3.17E-07 | 1.188155562 | NM_003564| | TAGLN2,transgelin 2 |
| 226623_at | 34.81 | 3.17E-07 | 2.01564504 | NM_032439| | PHYHIPL,phytanoyl-CoA hydroxylase interacting |
| 221885_at | 34.8 | 3.18E-07 | 1.385102786 | NM_015689| | KIAA1277,KIAA1277 protein |
| 204863_s_at | 34.78 | 3.18E-07 | 1.886664246 | NM_002184| | IL6ST,interleukin 6 signal transducer isoform 1 |
| 214175_x_at | 34.75 | 3.19E-07 | 1.361095965 | NM_003687| | PDLIM4,PDZ and LIM domain 4 |
| 229487_at | 34.73 | 3.19E-07 | 1.749465939 | NM_024007| | EBF,early B-cell factor |
| 226621_at | 34.71 | 3.20E-07 | 1.778137247 | NA |  |
| 224621_at | 34.63 | 3.24E-07 | 1.075085486 | NM_002745| | MAPK1,mitogen-activated protein kinase 1 |
| 206397_x_at | 34.56 | 3.27E-07 | 1.229405873 | NM_001492| | GDF1,growth differentiation factor 1 |
| 208779_x_at | 34.54 | 3.27E-07 | 1.149845976 | NM_001954| | DDR1,discoidin receptor tyrosine kinase isoform b |
| 231397_at | 34.49 | 3.30E-07 | 2.308927703 | NM_001010861| | NA |
| 210875_s_at | 34.47 | 3.30E-07 | 2.305521537 | NM_030751| | TCF8,transcription factor 8 (represses interleukin 2 |
| 204143_s_at | 34.45 | 3.31E-07 | 1.310711268 | NM_001126123| | NA |
| 57163_at | 34.44 | 3.31E-07 | 1.146225924 | NM_022821| | ELOVL1,elongation of very long chain fatty acids |
| 213714_at | 34.31 | 3.39E-07 | 1.635397564 | NM_000724| | CACNB2,calcium channel, voltage-dependent, beta 2 |
| 238736_at | 34.28 | 3.41E-07 | 1.888940416 | NM_002912| | REV3L,REV3-like, catalytic subunit of DNA polymerase |
| 202364_at | 34.25 | 3.41E-07 | 1.270576722 | NM_001008541| | MXI1,MAX interactor 1 isoform c |
| 213618_at | 34.25 | 3.41E-07 | 2.303146019 | NM_015230| | CENTD1,centaurin delta 1 isoform a |
| 225484_at | 34.12 | 3.49E-07 | 1.310115321 | NM_018718| | TSGA14,testis specific, 14 |
| 242086_at | 34.1 | 3.50E-07 | 1.455982797 | NA |  |
| 217529_at | 34.09 | 3.51E-07 | 1.369493775 | NA |  |
| 218899_s_at | 34.02 | 3.54E-07 | 2.174953977 | NM_001024372| | NA |
| 203625_x_at | 34.01 | 3.54E-07 | 1.076544588 | NM_005983| | SKP2,S-phase kinase-associated protein 2 isoform 1 |
| 239562_at | 34.01 | 3.54E-07 | 1.654123358 | NM_001004346| | MTHFD2L,methylenetetrahydrofolate dehydrogenase (NADP+ |
| 203318_s_at | 33.98 | 3.55E-07 | 1.120396018 | NM_021964| | ZNF148,zinc finger protein 148 (pHZ-52) |
| 203580_s_at | 33.95 | 3.55E-07 | 1.165236398 | NM_001076785| | NA |
| 209281_s_at | 33.86 | 3.61E-07 | 1.234033042 | NM_001001323| | ATP2B1,plasma membrane calcium ATPase 1 isoform 1a |
| 222834_s_at | 33.86 | 3.61E-07 | 1.361379579 | NM_018841| | GNG12,G-protein gamma-12 subunit |
| 238670_at | 33.85 | 3.61E-07 | 1.310566055 | NA |  |
| 219619_at | 33.82 | 3.62E-07 | 2.481692703 | NM_017594| | DIRAS2,Di-Ras2 |
| 224715_at | 33.81 | 3.62E-07 | 1.26426437 | NM_052844| | WDR34,WD repeat domain 34 |
| 226889_at | 33.74 | 3.67E-07 | 1.22524387 | NM_001006657| | WDR35,WD repeat domain 35 isoform 1 |
| 1555233_at | 33.73 | 3.67E-07 | 2.18747236 | NM_020663| | RHOJ,TC10-like Rho GTPase |
| 209680_s_at | 33.72 | 3.68E-07 | 1.047416937 | NM_002263| | NA |
| 225978_at | 33.72 | 3.68E-07 | 1.263991476 | NM_020734| | KIAA1238,KIAA1238 protein |
| 224560_at | 33.67 | 3.71E-07 | 1.184855051 | NM_003255| | TIMP2,tissue inhibitor of metalloproteinase 2 |
| 234977_at | 33.66 | 3.71E-07 | 1.721006805 | NM_175907| | ZADH2,zinc binding alcohol dehydrogenase, domain |
| 224733_at | 33.65 | 3.71E-07 | 1.161319146 | NM_001048251| | NA |
| 204916_at | 33.64 | 3.71E-07 | 1.505633987 | NM_005855| | RAMP1,receptor (calcitonin) activity modifying protein |
| 231166_at | 33.63 | 3.71E-07 | 2.220244332 | NM_001033045| | NA |
| 225655_at | 33.63 | 3.71E-07 | 1.339616103 | NM_001048201| | NA |
| 226713_at | 33.6 | 3.73E-07 | 1.646043002 | NM_174908| | C3orf6,Ymer protein short isoform |
| 212361_s_at | 33.59 | 3.73E-07 | 1.158706276 | NM_001681| | ATP2A2,ATPase, Ca++ transporting, cardiac muscle, slow |
| 226072_at | 33.58 | 3.73E-07 | 1.197396264 | NM_145059| | FUK,fucokinase |
| 227998_at | 33.57 | 3.73E-07 | 1.318506961 | NM_080388| | S100A16,S100 calcium binding protein A16 |
| 226255_at | 33.57 | 3.73E-07 | 1.136920377 | NM_006777| | ZBTB33,kaiso |
| 238704_at | 33.57 | 3.73E-07 | 1.233416342 | NA |  |
| 209663_s_at | 33.54 | 3.74E-07 | 1.343917566 | NM_002206| | ITGA7,integrin alpha 7 precursor |
| 209834_at | 33.52 | 3.75E-07 | 1.35136324 | NM_004273| | CHST3,carbohydrate (chondroitin 6) sulfotransferase 3 |
| 229697_at | 33.45 | 3.78E-07 | 1.218150456 | NM_003609| | HIRIP3,HIRA interacting protein 3 |
| 228333_at | 33.45 | 3.78E-07 | 1.599668464 | NA |  |
| 224848_at | 33.4 | 3.80E-07 | 1.392677407 | NM_001259| | CDK6,cyclin-dependent kinase 6 |
| 219355_at | 33.38 | 3.81E-07 | 1.728016936 | NM_018015| | FLJ10178,hypothetical protein FLJ10178 |
| 226808_at | 33.36 | 3.82E-07 | 1.293947181 | NM_001099220| | NA |
| 201178_at | 33.35 | 3.82E-07 | 1.04190794 | NM_001033024| | NA |
| 219450_at | 33.3 | 3.84E-07 | 1.880641038 | NM_001104629| | NA |
| 211072_x_at | 33.3 | 3.84E-07 | 1.031039324 | NM_006082| | K-ALPHA-1,tubulin, alpha, ubiquitous |
| 236834_at | 33.28 | 3.85E-07 | 1.539300562 | NM_152540| | SCFD2,sec1 family domain containing 2 |
| 235109_at | 33.27 | 3.86E-07 | 1.2350148 | NA |  |
| 225967_s_at | 33.24 | 3.88E-07 | 1.149012977 | NM_001086521| | NA |
| 203192_at | 33.19 | 3.89E-07 | 1.211207514 | NM_005689| | ABCB6,ATP-binding cassette, sub-family B, member 6 |
| 231040_at | 33.12 | 3.93E-07 | 1.853847139 | NA |  |
| 235489_at | 33.08 | 3.96E-07 | 2.724402911 | NM_020663| | RHOJ,TC10-like Rho GTPase |
| 223093_at | 33.05 | 3.97E-07 | 1.613667487 | NM_054027| | ANKH,ankylosis, progressive homolog |
| 218332_at | 33.01 | 3.97E-07 | 1.142752283 | NM_018476| | BEX1,brain expressed, X-linked 1 |
| 212991_at | 33 | 3.97E-07 | 1.828298105 | NM_012347| | FBXO9,F-box only protein 9 isoform 1 |
| 214247_s_at | 32.99 | 3.97E-07 | 1.219605111 | NM_001018057| | NA |
| 204273_at | 32.99 | 3.97E-07 | 1.869991088 | NM_000115| | EDNRB,endothelin receptor type B isoform 1 |
| 204246_s_at | 32.95 | 3.99E-07 | 1.242140736 | NM_007234| | DCTN3,dynactin 3 isoform 1 |
| 230724_s_at | 32.94 | 3.99E-07 | 1.248814185 | NM_001082969| | NA |
| 218529_at | 32.9 | 4.01E-07 | 1.195579018 | NM_016579| | CD320,8D6 antigen |
| 211302_s_at | 32.9 | 4.01E-07 | 1.810139577 | NM_001037339| | NA |
| 203662_s_at | 32.89 | 4.01E-07 | 1.627461072 | NM_003275| | TMOD1,tropomodulin 1 |
| 203845_at | 32.88 | 4.01E-07 | 2.132156228 | NM_003884| | PCAF,p300/CBP-associated factor |
| 226895_at | 32.87 | 4.01E-07 | 1.814198716 | NM_005597| | NFIC,nuclear factor I/C isoform 1 |
| 201185_at | 32.86 | 4.02E-07 | 1.407237178 | NM_002775| | PRSS11,protease, serine, 11 |
| 203384_s_at | 32.83 | 4.04E-07 | 1.41559808 | NM_002077| | GOLGA1,golgin 97 |
| 212020_s_at | 32.76 | 4.09E-07 | 1.269976545 | NM_002417| | MKI67,antigen identified by monoclonal antibody Ki-67 |
| 229535_at | 32.76 | 4.08E-07 | 1.379325575 | NM_175732| | NA |
| 238762_at | 32.74 | 4.09E-07 | 1.86217122 | NM_001004346| | MTHFD2L,methylenetetrahydrofolate dehydrogenase (NADP+ |
| 204456_s_at | 32.73 | 4.09E-07 | 1.478227481 | NM_002048| | GAS1,growth arrest-specific 1 |
| 201826_s_at | 32.71 | 4.10E-07 | 1.230300074 | NM_016002| | CGI-49,CGI-49 protein |
| 224928_at | 32.7 | 4.10E-07 | 1.465177754 | NM_030648| | SET7,SET domain-containing protein 7 |
| 227100_at | 32.68 | 4.12E-07 | 1.295166171 | NM_194318| | B3GTL,beta 3-glycosyltransferase-like |
| 203729_at | 32.66 | 4.12E-07 | 1.384501467 | NM_001425| | EMP3,epithelial membrane protein 3 |
| 202196_s_at | 32.66 | 4.12E-07 | 1.287984874 | NM_001018057| | NA |
| 203231_s_at | 32.64 | 4.13E-07 | 1.67431992 | NM_000332| | ATXN1,ataxin 1 |
| 201105_at | 32.6 | 4.16E-07 | 1.306495229 | NM_002305| | LGALS1,beta-galactosidase binding lectin precursor |
| 220865_s_at | 32.51 | 4.22E-07 | 1.164162463 | NM_014317| | TPRT,trans-prenyltransferase |
| 230577_at | 32.51 | 4.22E-07 | 2.573231212 | NA |  |
| 228062_at | 32.51 | 4.22E-07 | 1.290826093 | NM_153757| | NAP1L5,nucleosome assembly protein 1-like 5 |
| 224964_s_at | 32.47 | 4.26E-07 | 1.504364228 | NM_053064| | GNG2,guanine nucleotide binding protein (G protein), |
| 224583_at | 32.39 | 4.31E-07 | 1.135163157 | NM_021149| | COTL1,coactosin-like 1 |
| 205182_s_at | 32.38 | 4.32E-07 | 1.163216856 | NM_014347| | ZNF324,zinc finger protein 324 |
| 226740_x_at | 32.34 | 4.34E-07 | 1.182634845 | NM_001037501| | NA |
| 218639_s_at | 32.34 | 4.34E-07 | 1.206547387 | NM_001040653| | NA |
| 214004_s_at | 32.3 | 4.36E-07 | 1.175766461 | NM_014667| | VGLL4,vestigial like 4 |
| 226691_at | 32.27 | 4.38E-07 | 1.423287653 | NM_001013722| | NA |
| 203908_at | 32.23 | 4.41E-07 | 2.04388668 | NM_001098484| | NA |
| 203336_s_at | 32.21 | 4.42E-07 | 1.213160328 | NM_004763| | ITGB1BP1,integrin cytoplasmic domain-associated protein 1 |
| 238462_at | 32.11 | 4.50E-07 | 2.509417821 | NM_032873| | STS-1,Cbl-interacting protein Sts-1 |
| 236261_at | 32.01 | 4.56E-07 | 1.606363839 | NM_032523| | OSBPL6,oxysterol-binding protein-like protein 6 isoform |
| 218723_s_at | 32 | 4.57E-07 | 1.825970725 | NM_014059| | RGC32,response gene to complement 32 |
| 232095_at | 31.99 | 4.57E-07 | 1.330520164 | NA |  |
| 228831_s_at | 31.95 | 4.59E-07 | 1.279588503 | NM_052847| | GNG7,guanine nucleotide binding protein (G protein), |
| 218648_at | 31.95 | 4.59E-07 | 1.15949866 | NM_001042574| | NA |
| 225132_at | 31.94 | 4.59E-07 | 1.193210103 | NM_012158| | FBXL3,F-box and leucine-rich repeat protein 3 |
| 225540_at | 31.92 | 4.60E-07 | 1.87830253 | NM_001039538| | NA |
| 225996_at | 31.9 | 4.62E-07 | 2.874044808 | NM_198461| | FLJ45273,FLJ45273 protein |
| 217911_s_at | 31.89 | 4.62E-07 | 1.200802632 | NM_004281| | BAG3,BCL2-associated athanogene 3 |
| 222503_s_at | 31.89 | 4.62E-07 | 1.123258425 | NM_018268| | WDR41,WD repeat domain 41 |
| 224999_at | 31.87 | 4.63E-07 | 2.124673133 | NA |  |
| 200787_s_at | 31.84 | 4.65E-07 | 1.279894721 | NM_003768| | PEA15,phosphoprotein enriched in astrocytes 15 |
| 218242_s_at | 31.83 | 4.65E-07 | 1.188914537 | NM_016028| | SUV420H1,suppressor of variegation 4-20 homolog 1 isoform |
| 209465_x_at | 31.79 | 4.68E-07 | 1.486373121 | NM_002825| | PTN,pleiotrophin |
| 225704_at | 31.76 | 4.69E-07 | 1.407490292 | NA |  |
| 203065_s_at | 31.7 | 4.73E-07 | 1.240594023 | NM_001753| | CAV1,caveolin 1 |
| 202743_at | 31.68 | 4.73E-07 | 1.269021786 | NM_001114172| | NA |
| 212956_at | 31.67 | 4.73E-07 | 1.346222166 | NM_015130| | KIAA0882,KIAA0882 protein |
| 228278_at | 31.63 | 4.76E-07 | 1.615054377 | NM_002501| | NFIX,nuclear factor I/X (CCAAT-binding transcription |
| 227932_at | 31.63 | 4.76E-07 | 1.26230014 | NM_006321| | ARIH2,ariadne homolog 2 |
| 39966_at | 31.6 | 4.76E-07 | 1.448567765 | NM_006574| | CSPG5,chondroitin sulfate proteoglycan 5 (neuroglycan |
| 207039_at | 31.6 | 4.76E-07 | 1.62590324 | NM_000077| | CDKN2A,cyclin-dependent kinase inhibitor 2A isoform 1 |
| 235174_s_at | 31.59 | 4.76E-07 | 2.019990335 | NA |  |
| 200884_at | 31.57 | 4.76E-07 | 1.156918437 | NM_001823| | CKB,brain creatine kinase |
| 201067_at | 31.57 | 4.76E-07 | 1.206831609 | NM_002803| | PSMC2,proteasome 26S ATPase subunit 2 |
| 208979_at | 31.55 | 4.76E-07 | 1.21093827 | NM_014071| | NCOA6,nuclear receptor coactivator 6 |
| 203232_s_at | 31.54 | 4.76E-07 | 1.515019359 | NM_000332| | ATXN1,ataxin 1 |
| 213411_at | 31.52 | 4.78E-07 | 1.602755863 | NM_004194| | ADAM22,a disintegrin and metalloproteinase domain 22 |
| 201101_s_at | 31.49 | 4.80E-07 | 1.120200238 | NM_001077440| | NA |
| 213880_at | 31.47 | 4.82E-07 | 3.029082032 | NM_003667| | LGR5,leucine-rich repeat-containing G protein-coupled |
| 1554260_a_at | 31.46 | 4.82E-07 | 1.26234595 | NM_015030| | NA |
| 208962_s_at | 31.46 | 4.82E-07 | 1.142324427 | NM_013402| | FADS1,fatty acid desaturase 1 |
| 213508_at | 31.45 | 4.82E-07 | 1.144422842 | NM_138288| | C14orf147,chromosome 14 open reading frame 147 |
| 204320_at | 31.42 | 4.85E-07 | 1.691193613 | NM_001854| | COL11A1,alpha 1 type XI collagen isoform A |
| 225491_at | 31.4 | 4.87E-07 | 1.987946076 | NM_004171| | SLC1A2,solute carrier family 1, member 2 |
| 205152_at | 31.39 | 4.87E-07 | 2.626986229 | NM_003042| | SLC6A1,solute carrier family 6 (neurotransmitter |
| 202565_s_at | 31.38 | 4.88E-07 | 1.288700908 | NM_003174| | SVIL,supervillin isoform 1 |
| 209167_at | 31.36 | 4.89E-07 | 1.405090142 | NM_001001994| | GPM6B,glycoprotein M6B isoform 4 |
| 205741_s_at | 31.35 | 4.90E-07 | 1.282768247 | NM_001390| | DTNA,dystrobrevin alpha isoform 1 |
| 204451_at | 31.34 | 4.90E-07 | 1.399738021 | NM_003505| | FZD1,frizzled 1 |
| 242385_at | 31.3 | 4.94E-07 | 1.694502501 | NM_006914| | RORB,RAR-related orphan receptor B |
| 229774_at | 31.28 | 4.94E-07 | 1.732902814 | NM_025212| | CXXC4,CXXC finger 4 |
| 235111_at | 31.25 | 4.97E-07 | 1.864961073 | NA |  |
| 238532_at | 31.24 | 4.97E-07 | 2.216971482 | NM_012074| | DPF3,cer-d4 (mouse) homolog |
| 209289_at | 31.23 | 4.97E-07 | 2.08456251 | NM_005596| | NFIB,nuclear factor I/B |
| 212186_at | 31.15 | 5.03E-07 | 1.125700482 | NM_198834| | ACACA,acetyl-Coenzyme A carboxylase alpha isoform 1 |
| 226281_at | 31.13 | 5.03E-07 | 1.653261332 | NM_139072| | DNER,delta-notch-like EGF repeat-containing |
| 223623_at | 31.1 | 5.05E-07 | 1.534948268 | NM_032411| | ECRG4,esophageal cancer related gene 4 protein |
| 209290_s_at | 31.09 | 5.05E-07 | 1.92444308 | NM_005596| | NFIB,nuclear factor I/B |
| 225682_s_at | 31.09 | 5.06E-07 | 1.185263695 | NM_001018050| | NA |
| 203354_s_at | 31.08 | 5.07E-07 | 1.261851974 | NM_015310| | PSD3,ADP-ribosylation factor guanine nucleotide |
| 232015_at | 31.07 | 5.07E-07 | 1.364150755 | NA |  |
| 225553_at | 31.06 | 5.07E-07 | 1.149702966 | NA |  |
| 209236_at | 30.9 | 5.23E-07 | 1.187368069 | NM_005116| | SLC23A2,solute carrier family 23 (nucleobase |
| 226790_at | 30.9 | 5.23E-07 | 1.335005206 | NM_194270| | MOPT,protein containing single MORN motif in testis |
| 228738_at | 30.9 | 5.23E-07 | 1.201185108 | NM_152783| | MGC25181,hypothetical protein MGC25181 |
| 228259_s_at | 30.87 | 5.26E-07 | 1.400162352 | NM_022140| | EPB41L4A,erythrocyte protein band 4.1-like 4 |
| 222217_s_at | 30.86 | 5.26E-07 | 1.341892422 | NM_024330| | SLC27A3,solute carrier family 27 member 3 |
| 201426_s_at | 30.84 | 5.27E-07 | 1.144409011 | NM_003380| | VIM,vimentin |
| 225851_at | 30.82 | 5.29E-07 | 1.214711496 | NM_002028| | FNTB,farnesyltransferase, CAAX box, beta |
| 1557953_at | 30.82 | 5.29E-07 | 1.235575896 | NM_003439| | ZKSCAN1,zinc finger protein 36 |
| 212083_at | 30.8 | 5.30E-07 | 1.394611026 | NM_144582| | TEX261,testis expressed gene 261 |
| 241464_s_at | 30.75 | 5.34E-07 | 1.48888835 | NA |  |
| 239425_at | 30.74 | 5.34E-07 | 1.52508164 | NA |  |
| 222476_at | 30.68 | 5.38E-07 | 1.103403102 | NM_015455| | CNOT6,CCR4-NOT transcription complex, subunit 6 |
| 222995_s_at | 30.68 | 5.38E-07 | 1.19845614 | NM_001040456| | NA |
| 201053_s_at | 30.6 | 5.45E-07 | 1.219054839 | NM_006814| | PSMF1,proteasome inhibitor subunit 1 isoform 1 |
| 235457_at | 30.59 | 5.46E-07 | 1.475833115 | NM_032427| | MAML2,mastermind-like 2 |
| 207828_s_at | 30.57 | 5.48E-07 | 1.113243623 | NM_016343| | CENPF,centromere protein F (350/400kD) |
| 201163_s_at | 30.55 | 5.50E-07 | 2.51669708 | NM_001553| | IGFBP7,insulin-like growth factor binding protein 7 |
| 227022_at | 30.54 | 5.50E-07 | 1.332425263 | NM_138335| | GNPDA2,glucosamine-6-phosphate deaminase 2 |
| 225729_at | 30.52 | 5.52E-07 | 1.272856066 | NM_152734| | C6orf89,hypothetical protein FLJ25357 |
| 226034_at | 30.51 | 5.52E-07 | 1.281847579 | NA |  |
| 211564_s_at | 30.48 | 5.53E-07 | 1.529766648 | NM_003687| | PDLIM4,PDZ and LIM domain 4 |
| 232231_at | 30.45 | 5.55E-07 | 2.174922899 | NM_001015051| | NA |
| 241741_at | 30.45 | 5.55E-07 | 1.524305212 | NM_019095| | C20orf155,chromosome 20 open reading frame 155 |
| 202048_s_at | 30.44 | 5.56E-07 | 1.18484741 | NM_014292| | CBX6,chromobox homolog 6 |
| 227061_at | 30.41 | 5.58E-07 | 1.951601066 | NA |  |
| 204159_at | 30.41 | 5.58E-07 | 2.499419973 | NM_001262| | CDKN2C,cyclin-dependent kinase inhibitor 2C |
| 1559402_a_at | 30.38 | 5.60E-07 | 1.629774043 | NM_006365| | CROC4,transcriptional activator of the c-fos promoter |
| 214334_x_at | 30.36 | 5.62E-07 | 1.190111716 | NM_014764| | DAZAP2,DAZ associated protein 2 |
| 227665_at | 30.36 | 5.62E-07 | 1.221114228 | NA |  |
| 213392_at | 30.35 | 5.62E-07 | 1.236717717 | NM_153208| | MGC35048,hypothetical protein MGC35048 |
| 235775_at | 30.31 | 5.66E-07 | 1.516997604 | NM_152588| | DKFZp762A217,hypothetical protein DKFZp762A217 |
| 204105_s_at | 30.28 | 5.67E-07 | 1.655283479 | NM_001037132| | NA |
| 229435_at | 30.28 | 5.67E-07 | 2.439921664 | NM_001042413| | NA |
| 226779_at | 30.24 | 5.69E-07 | 1.162331998 | NA |  |
| 224184_s_at | 30.23 | 5.70E-07 | 1.43590762 | NM_033254| | BOC,brother of CDO |
| 226923_at | 30.17 | 5.75E-07 | 1.300953625 | NM_152540| | SCFD2,sec1 family domain containing 2 |
| 202370_s_at | 30.17 | 5.75E-07 | 1.22161954 | NM_001755| | CBFB,core-binding factor, beta subunit isoform 2 |
| 213606_s_at | 30.16 | 5.75E-07 | 1.412991884 | NM_004309| | ARHGDIA,Rho GDP dissociation inhibitor (GDI) alpha |
| 210073_at | 30.14 | 5.76E-07 | 2.016787528 | NM_003034| | ST8SIA1,ST8 alpha-N-acetyl-neuraminide |
| 213132_s_at | 30.14 | 5.76E-07 | 1.238676706 | NM_014507| | MT,mitochondrial malonyltransferase isoform b |
| 232990_at | 30.07 | 5.82E-07 | 1.55117169 | NM_138362| | CXorf44,chromosome X open reading frame 44 |
| 208923_at | 30.03 | 5.86E-07 | 1.119270571 | NM_001033028| | NA |
| 200761_s_at | 30.03 | 5.86E-07 | 1.111898452 | NM_006407| | ARL6IP5,ADP-ribosylation-like factor 6 interacting |
| 205381_at | 30 | 5.88E-07 | 1.583737574 | NM_001031692| | NA |
| 212173_at | 29.99 | 5.89E-07 | 1.333975123 | NM_001625| | AK2,adenylate kinase 2 isoform a |
| 236350_at | 29.97 | 5.90E-07 | 1.529091841 | NA |  |
| 226252_at | 29.97 | 5.90E-07 | 2.292501851 | NA |  |
| 225460_at | 29.96 | 5.91E-07 | 1.064486447 | NM_004206| | SEC22L3,vesicle trafficking protein isoform b |
| 232377_at | 29.95 | 5.91E-07 | 2.562362655 | NM_152745| | NXPH1,neurexophilin 1 |
| 220029_at | 29.88 | 5.98E-07 | 1.898613582 | NM_017770| | ELOVL2,elongation of very long chain fatty acids |
| 210089_s_at | 29.86 | 5.99E-07 | 2.013913127 | NM_001105206| | NA |
| 222030_at | 29.86 | 5.99E-07 | 1.212809206 | NM_006427| | SIVA,CD27-binding (Siva) protein isoform 1 |
| 210015_s_at | 29.81 | 6.06E-07 | 1.76904896 | NM_001039538| | NA |
| 238898_at | 29.79 | 6.07E-07 | 1.902061856 | NA |  |
| 232590_at | 29.78 | 6.07E-07 | 1.425816218 | NA |  |
| 233255_s_at | 29.77 | 6.07E-07 | 1.452557334 | NM_017693| | BIVM,basic, immunoglobulin-like variable motif |
| 217904_s_at | 29.76 | 6.08E-07 | 1.555218116 | NM_012104| | BACE1,beta-site APP-cleaving enzyme 1 isoform A |
| 213628_at | 29.69 | 6.15E-07 | 1.191516566 | NM_001048210| | NA |
| 209625_at | 29.67 | 6.17E-07 | 1.539762766 | NM_004569| | PIGH,phosphatidylinositol glycan, class H |
| 210239_at | 29.61 | 6.22E-07 | 1.387045252 | NM_005853| | IRX5,iroquois homeobox protein 5 |
| 209200_at | 29.61 | 6.22E-07 | 1.900673432 | NM_002397| | MEF2C,MADS box transcription enhancer factor 2, |
| 235068_at | 29.59 | 6.24E-07 | 1.651610814 | NM_178566| | ZDHHC21,zinc finger, DHHC domain containing 21 |
| 37590_g_at | 29.59 | 6.24E-07 | 1.345389625 | NA |  |
| 219182_at | 29.58 | 6.24E-07 | 1.371587354 | NM_001077416| | NA |
| 222780_s_at | 29.57 | 6.25E-07 | 2.043792049 | NM_001024372| | NA |
| 222914_s_at | 29.52 | 6.32E-07 | 1.516292392 | NM_025268| | MGC4659,hole protein |
| 202478_at | 29.46 | 6.39E-07 | 1.403939978 | NM_021643| | TRIB2,tribbles homolog 2 |
| 218109_s_at | 29.45 | 6.39E-07 | 1.229074707 | NM_022736| | MFSD1,major facilitator superfamily domain containing |
| 208817_at | 29.44 | 6.40E-07 | 1.270435923 | NM_000754| | COMT,catechol-O-methyltransferase isoform MB-COMT |
| 222651_s_at | 29.44 | 6.40E-07 | 1.548115979 | NM_014112| | TRPS1,zinc finger transcription factor TRPS1 |
| 224592_x_at | 29.41 | 6.42E-07 | 1.198515772 | NM_016287| | HP1-BP74,HP1-BP74 |
| 227605_at | 29.35 | 6.49E-07 | 1.145713941 | NM_004757| | SCYE1,small inducible cytokine subfamily E, member 1 |
| 209090_s_at | 29.33 | 6.50E-07 | 1.090314534 | NM_016009| | SH3GLB1,SH3-containing protein SH3GLB1 |
| 209871_s_at | 29.32 | 6.52E-07 | 1.375789905 | NM_005503| | APBA2,amyloid beta A4 precursor protein-binding, |
| 203527_s_at | 29.26 | 6.61E-07 | 1.573122258 | NM_000038| | APC,adenomatosis polyposis coli |
| 221796_at | 29.25 | 6.61E-07 | 2.281260662 | NM_001007097| | NTRK2,neurotrophic tyrosine kinase, receptor, type 2 |
| 214541_s_at | 29.23 | 6.62E-07 | 1.662013192 | NM_006775| | QKI,quaking homolog, KH domain RNA binding isoform |
| 218691_s_at | 29.21 | 6.64E-07 | 2.046905272 | NM_003687| | PDLIM4,PDZ and LIM domain 4 |
| 202986_at | 29.2 | 6.64E-07 | 1.345981263 | NM_014862| | ARNT2,aryl-hydrocarbon receptor nuclear translocator |
| 230023_at | 29.2 | 6.64E-07 | 1.218972097 | NM_199044| | NSUN4,NOL1/NOP2/Sun domain family 4 protein |
| 1556060_a_at | 29.19 | 6.66E-07 | 1.213383399 | NA |  |
| 219331_s_at | 29.17 | 6.67E-07 | 1.933560612 | NM_018203| | FLJ10748,hypothetical protein FLJ10748 |
| 210046_s_at | 29.12 | 6.72E-07 | 1.213802815 | NM_002168| | IDH2,isocitrate dehydrogenase 2 (NADP+), |
| 202245_at | 29.08 | 6.76E-07 | 1.14517087 | NM_001001438| | LSS,lanosterol synthase |
| 242033_at | 29.06 | 6.79E-07 | 2.352436368 | NM_001113561| | NA |
| 229885_at | 29.04 | 6.81E-07 | 1.496449319 | NA |  |
| 218009_s_at | 29.03 | 6.81E-07 | 1.136769082 | NM_003981| | PRC1,protein regulator of cytokinesis 1 isoform 1 |
| 206670_s_at | 28.99 | 6.87E-07 | 1.587466143 | NM_000817| | GAD1,glutamate decarboxylase 1 isoform GAD67 |
| 205265_s_at | 28.98 | 6.87E-07 | 1.244371209 | NM_005876| | APEG1,aortic preferentially expressed gene 1 |
| 218584_at | 28.97 | 6.88E-07 | 1.350371919 | NM_001082537| | NA |
| 209793_at | 28.97 | 6.88E-07 | 2.491435454 | NM_000827| | GRIA1,glutamate receptor, ionotropic, AMPA 1 |
| 218208_at | 28.96 | 6.89E-07 | 1.179048929 | NM_025078| | PQLC1,PQ loop repeat containing 1 |
| 213069_at | 28.96 | 6.89E-07 | 1.233687815 | NM_020733| | NA |
| 204085_s_at | 28.94 | 6.91E-07 | 1.335662298 | NM_006493| | CLN5,ceroid-lipofuscinosis, neuronal 5 |
| 235494_at | 28.91 | 6.94E-07 | 2.086447644 | NA |  |
| 36566_at | 28.83 | 7.02E-07 | 1.13240892 | NM_001031681| | NA |
| 217819_at | 28.83 | 7.02E-07 | 1.06559235 | NM_001002296| | GOLGA7,golgi autoantigen, golgin subfamily a, 7 |
| 239999_at | 28.82 | 7.02E-07 | 1.692011881 | NM_001005732| | C21orf34,chromosome 21 open reading frame 34 isoform a |
| 222617_s_at | 28.75 | 7.12E-07 | 1.124178542 | NM_022063| | C10orf84,chromosome 10 open reading frame 84 |
| 202554_s_at | 28.71 | 7.17E-07 | 1.491451933 | NM_000849| | GSTM3,glutathione S-transferase M3 |
| 225143_at | 28.69 | 7.19E-07 | 1.237722898 | NM_213649| | SFXN4,sideroflexin 4 isoform 1 |
| 203063_at | 28.68 | 7.19E-07 | 1.197494186 | NM_014634| | PPM1F,protein phosphatase 1F |
| 219600_s_at | 28.68 | 7.19E-07 | 1.120598339 | NM_006134| | C21orf4,HCV p7-transregulated protein 3 |
| 212758_s_at | 28.67 | 7.20E-07 | 1.859741198 | NM_030751| | TCF8,transcription factor 8 (represses interleukin 2 |
| 209644_x_at | 28.67 | 7.21E-07 | 1.40574487 | NM_000077| | CDKN2A,cyclin-dependent kinase inhibitor 2A isoform 1 |
| 204249_s_at | 28.65 | 7.23E-07 | 1.594160912 | NM_005574| | LMO2,LIM domain only 2 |
| 222669_s_at | 28.64 | 7.25E-07 | 1.131265661 | NM_016038| | SBDS,Shwachman-Bodian-Diamond syndrome protein |
| 235308_at | 28.62 | 7.27E-07 | 1.951490969 | NM_015642| | ZBTB20,zinc finger and BTB domain containing 20 |
| 206083_at | 28.6 | 7.28E-07 | 1.388499133 | NM_001702| | BAI1,brain-specific angiogenesis inhibitor 1 |
| 208668_x_at | 28.54 | 7.36E-07 | 1.057905469 | NM_005517| | HMGN2,high-mobility group nucleosomal binding domain |
| 209985_s_at | 28.53 | 7.37E-07 | 1.92147219 | NM_004316| | ASCL1,achaete-scute complex homolog-like 1 |
| 219279_at | 28.52 | 7.39E-07 | 2.554910286 | NM_014689| | DOCK10,dedicator of cytokinesis 10 |
| 212419_at | 28.51 | 7.40E-07 | 1.341884045 | NM_153367| | C10orf56,chromosome 10 open reading frame 56 |
| 205433_at | 28.46 | 7.47E-07 | 3.125947467 | NM_000055| | BCHE,butyrylcholinesterase precursor |
| 204482_at | 28.46 | 7.47E-07 | 1.441378044 | NM_003277| | CLDN5,claudin 5 |
| 224593_at | 28.42 | 7.53E-07 | 1.208075138 | NM_152437| | ZFOC1,zinc finger protein ZFOC1 |
| 224690_at | 28.37 | 7.59E-07 | 1.281829242 | NM_080821| | C20orf108,chromosome 20 open reading frame 108 |
| 228545_at | 28.37 | 7.59E-07 | 1.183662821 | NM_021964| | ZNF148,zinc finger protein 148 (pHZ-52) |
| 228720_at | 28.32 | 7.68E-07 | 1.493667959 | NM_020777| | SORCS2,VPS10 domain receptor protein SORCS 2 |
| 244881_at | 28.31 | 7.69E-07 | 1.259748894 | NM_033029| | LMLN,leishmanolysin-like (metallopeptidase M8 |
| 229430_at | 28.3 | 7.72E-07 | 1.681427494 | NM_152765| | MGC33510,hypothetical protein MGC33510 |
| 204600_at | 28.24 | 7.80E-07 | 1.526331466 | NM_004443| | EPHB3,ephrin receptor EphB3 precursor |
| 226726_at | 28.2 | 7.86E-07 | 1.207422975 | NM_138799| | OACT2,O-acyltransferase (membrane bound) domain |
| 230728_at | 28.19 | 7.87E-07 | 1.213023727 | NA |  |
| 228185_at | 28.18 | 7.89E-07 | 1.853737478 | NM_145011| | ZNF25,zinc finger protein 25 |
| 204476_s_at | 28.15 | 7.94E-07 | 1.234559815 | NM_000920| | PC,pyruvate carboxylase precursor |
| 227476_at | 28.13 | 7.97E-07 | 1.57513186 | NA |  |
| 1555471_a_at | 28.09 | 8.00E-07 | 1.896754155 | NM_020066| | FMN2,formin 2 |
| 230280_at | 28.08 | 8.00E-07 | 1.773143857 | NM_015163| | TRIM9,tripartite motif protein 9 isoform 1 |
| 241701_at | 28.08 | 8.00E-07 | 1.530974001 | NM_020824| | ARHGAP21,Rho GTPase activating protein 21 |
| 225391_at | 28.03 | 8.07E-07 | 1.110868677 | NA |  |
| 221623_at | 28.03 | 8.07E-07 | 1.580952854 | NM_021948| | BCAN,brevican isoform 1 |
| 205354_at | 28.02 | 8.07E-07 | 1.277440612 | NM_000156| | GAMT,guanidinoacetate N-methyltransferase isoform a |
| 202920_at | 28.01 | 8.08E-07 | 1.433587698 | NM_001148| | ANK2,ankyrin 2 isoform 1 |
| 223472_at | 28 | 8.10E-07 | 1.400784936 | NM_001042424| | NA |
| 228131_at | 27.98 | 8.13E-07 | 1.477678666 | NM_001983| | ERCC1,excision repair cross-complementing 1 isofrom 2 |
| 221727_at | 27.97 | 8.13E-07 | 1.255651539 | NM_006713| | PC4,activated RNA polymerase II transcription |
| 223075_s_at | 27.95 | 8.16E-07 | 1.050429366 | NM_031426| | C9orf58,chromosome 9 open reading frame 58 isoform 1 |
| 200790_at | 27.94 | 8.16E-07 | 1.180227337 | NM_002539| | ODC1,ornithine decarboxylase 1 |
| 220298_s_at | 27.94 | 8.16E-07 | 1.411871207 | NM_019073| | SPATA6,spermatogenesis associated 6 |
| 242825_at | 27.92 | 8.18E-07 | 1.434862643 | NM_001010861| | NA |
| 204347_at | 27.92 | 8.18E-07 | 1.188080394 | NM_001002921| | AK3L2,adenylate kinase 3-like 2 |
| 213007_at | 27.92 | 8.18E-07 | 1.124974729 | NM_001113378| | NA |
| 224970_at | 27.92 | 8.18E-07 | 1.957379162 | NM_005595| | NFIA,nuclear factor I/A |
| 226242_at | 27.92 | 8.18E-07 | 1.144396245 | NM_152379| | DKFZp547B1713,hypothetical protein DKFZp547B1713 |
| 564_at | 27.91 | 8.18E-07 | 1.215658518 | NM_002067| | GNA11,guanine nucleotide binding protein (G protein), |
| 204521_at | 27.91 | 8.18E-07 | 1.160033149 | NM_013300| | HSU79274,protein predicted by clone 23733 |
| 227407_at | 27.91 | 8.18E-07 | 1.134048887 | NM_153365| | FLJ90013,hypothetical protein FLJ90013 |
| 210139_s_at | 27.86 | 8.26E-07 | 1.54522555 | NM_000304| | PMP22,peripheral myelin protein 22 |
| 37892_at | 27.86 | 8.26E-07 | 1.659719006 | NM_001854| | COL11A1,alpha 1 type XI collagen isoform A |
| 209286_at | 27.85 | 8.26E-07 | 1.599716974 | NM_006449| | CDC42EP3,Cdc42 effector protein 3 |
| 229228_at | 27.83 | 8.28E-07 | 1.908572688 | NM_001011666| | CREB5,cAMP responsive element binding protein 5 |
| 201876_at | 27.8 | 8.32E-07 | 1.232346187 | NM_000305| | PON2,paraoxonase 2 |
| 201581_at | 27.8 | 8.32E-07 | 1.299782401 | NM_021156| | DJ971N18.2,hypothetical protein DJ971N18.2 |
| 1558487_a_at | 27.77 | 8.36E-07 | 1.409800445 | NM_182547| | TMED4,transmembrane emp24 protein transport domain |
| 212190_at | 27.76 | 8.37E-07 | 1.084463786 | NM_006216| | SERPINE2,plasminogen activator inhibitor type 1, member |
| 218678_at | 27.72 | 8.44E-07 | 1.121453779 | NM_006617| | NES,nestin |
| 202661_at | 27.7 | 8.47E-07 | 1.944710124 | NM_002223| | ITPR2,inositol 1,4,5-triphosphate receptor, type 2 |
| 230720_at | 27.69 | 8.47E-07 | 1.246812777 | NM_152737| | RNF182,ring finger protein 182 |
| 213224_s_at | 27.64 | 8.56E-07 | 1.204307222 | NA |  |
| 239069_s_at | 27.64 | 8.56E-07 | 1.635590551 | NA |  |
| 227610_at | 27.64 | 8.56E-07 | 1.608191681 | NA |  |
| 244519_at | 27.63 | 8.56E-07 | 1.381440441 | NM_015338| | ASXL1,additional sex combs like 1 |
| 203217_s_at | 27.62 | 8.57E-07 | 1.3733897 | NM_001042437| | NA |
| 201007_at | 27.62 | 8.57E-07 | 1.076831117 | NM_000183| | HADHB,hydroxyacyl dehydrogenase, subunit B |
| 206701_x_at | 27.58 | 8.64E-07 | 1.276490089 | NM_000115| | EDNRB,endothelin receptor type B isoform 1 |
| 221952_x_at | 27.57 | 8.65E-07 | 1.082592894 | NM_020810| | KIAA1393,tRNA-(N1G37) methyltransferase |
| 227197_at | 27.54 | 8.73E-07 | 1.186659317 | NM_015595| | SGEF,DKFZP434D146 protein |
| 218613_at | 27.5 | 8.76E-07 | 1.325196874 | NM_015310| | PSD3,ADP-ribosylation factor guanine nucleotide |
| 225145_at | 27.48 | 8.80E-07 | 1.261614075 | NM_020967| | NCOA5,nuclear receptor coactivator 5 |
| 206414_s_at | 27.45 | 8.86E-07 | 1.187325364 | NM_003887| | DDEF2,development- and differentiation-enhancing |
| 208985_s_at | 27.44 | 8.87E-07 | 1.097899768 | NM_003758| | EIF3S1,eukaryotic translation initiation factor 3, |
| 204401_at | 27.41 | 8.93E-07 | 1.878790339 | NM_002250| | KCNN4,intermediate conductance calcium-activated |
| 241699_at | 27.41 | 8.93E-07 | 1.795326187 | NA |  |
| 223319_at | 27.4 | 8.94E-07 | 1.246316467 | NM_001024218| | NA |
| 212005_at | 27.39 | 8.95E-07 | 1.148824833 | NM_001114600| | NA |
| 225961_at | 27.38 | 8.97E-07 | 1.220720375 | NM_020782| | KLHDC5,kelch domain containing 5 |
| 1554251_at | 27.37 | 8.99E-07 | 1.330865303 | NM_016287| | HP1-BP74,HP1-BP74 |
| 219038_at | 27.32 | 9.08E-07 | 1.540232623 | NM_001085354| | NA |
| 224755_at | 27.31 | 9.09E-07 | 1.276846468 | NA |  |
| 210869_s_at | 27.3 | 9.10E-07 | 1.265159995 | NM_006500| | MCAM,melanoma cell adhesion molecule |
| 203799_at | 27.29 | 9.11E-07 | 1.797990951 | NM_014880| | CD302,CD302 antigen |
| 1553613_s_at | 27.24 | 9.20E-07 | 1.76642878 | NM_001453| | FOXC1,forkhead box C1 |
| 1554239_s_at | 27.24 | 9.20E-07 | 1.616692132 | NM_175907| | ZADH2,zinc binding alcohol dehydrogenase, domain |
| 209883_at | 27.22 | 9.23E-07 | 1.780195201 | NM_015101| | GLT25D2,glycosyltransferase 25 domain containing 2 |
| 239082_at | 27.21 | 9.24E-07 | 1.225099455 | NA |  |
| 217867_x_at | 27.16 | 9.34E-07 | 1.362461464 | NM_012105| | BACE2,beta-site APP-cleaving enzyme 2 isoform A |
| 228375_at | 27.15 | 9.35E-07 | 1.787857414 | NM_001015887| | NA |
| 205672_at | 27.13 | 9.38E-07 | 1.38354203 | NM_000380| | XPA,xeroderma pigmentosum, complementation group A |
| 205240_at | 27.13 | 9.38E-07 | 1.278704808 | NM_013296| | GPSM2,G-protein signalling modulator 2 (AGS3-like, C. |
| 209589_s_at | 27.11 | 9.41E-07 | 1.222525284 | NM_004442| | EPHB2,ephrin receptor EphB2 isoform 2 precursor |
| 213346_at | 27.1 | 9.41E-07 | 1.187660111 | NM_138779| | LOC93081,hypothetical protein BC015148 |
| 214531_s_at | 27.09 | 9.41E-07 | 1.172156844 | NM_003099| | SNX1,sorting nexin 1 isoform a |
| 201361_at | 27.09 | 9.41E-07 | 1.208072262 | NM_024092| | MGC5508,hypothetical protein MGC5508 |
| 230184_at | 27.08 | 9.43E-07 | 2.037620821 | NA |  |
| 203300_x_at | 27.08 | 9.43E-07 | 1.207874322 | NM_003916| | AP1S2,adaptor-related protein complex 1 sigma 2 |
| 218377_s_at | 27.03 | 9.51E-07 | 1.210369336 | NM_016940| | C21orf6,chromosome 21 open reading frame 6 |
| 31807_at | 27.03 | 9.51E-07 | 1.107113233 | NM_019070| | DDX49,DEAD (Asp-Glu-Ala-Asp) box polypeptide 49 |
| 204202_at | 27.03 | 9.51E-07 | 1.301817035 | NM_001100390| | NA |
| 229715_at | 27.02 | 9.52E-07 | 2.068271747 | NA |  |
| 213103_at | 27.02 | 9.52E-07 | 1.621564454 | NM_052851| | STARD13,START domain containing 13 isoform gamma |
| 204917_s_at | 27.02 | 9.52E-07 | 1.332253761 | NM_004529| | MLLT3,myeloid/lymphoid or mixed-lineage leukemia |
| 212386_at | 27.01 | 9.52E-07 | 1.141015267 | NM_001083962| | NA |
| 227805_at | 27.01 | 9.53E-07 | 1.379028336 | NA |  |
| 227448_at | 26.99 | 9.57E-07 | 1.487659489 | NM_018011| | FLJ10154,hypothetical protein FLJ10154 |
| 201153_s_at | 26.98 | 9.57E-07 | 1.535493884 | NM_021038| | MBNL1,muscleblind-like 1 isoform a |
| 221495_s_at | 26.95 | 9.64E-07 | 1.209005993 | NM_014972| | KIAA1049,KIAA1049 protein |
| 208716_s_at | 26.93 | 9.68E-07 | 1.099499829 | NM_019026| | LOC54499,putative membrane protein |
| 1552439_s_at | 26.92 | 9.68E-07 | 1.975566897 | NM_032445| | MEGF11,MEGF11 protein |
| 220940_at | 26.9 | 9.71E-07 | 1.489761276 | NM_025190| | NA |
| 213713_s_at | 26.9 | 9.71E-07 | 1.209059578 | NM_138342| | LOC89944,hypothetical protein BC008326 |
| 218656_s_at | 26.89 | 9.71E-07 | 1.910331835 | NM_005780| | LHFP,lipoma HMGIC fusion partner |
| 226897_s_at | 26.89 | 9.71E-07 | 1.085020815 | NM_014153| | ZC3HDC7,zinc finger CCCH type domain containing 7 |
| 219581_at | 26.87 | 9.73E-07 | 1.152634367 | NM_025265| | SEN2L,hypothetical protein MGC2776 |
| 200760_s_at | 26.85 | 9.77E-07 | 1.126471464 | NM_006407| | ARL6IP5,ADP-ribosylation-like factor 6 interacting |
| 201464_x_at | 26.84 | 9.79E-07 | 1.192271771 | NM_002228| | JUN,v-jun avian sarcoma virus 17 oncogene homolog |
| 223302_s_at | 26.84 | 9.79E-07 | 1.284937971 | NM_001009958| | ZNF655,zinc finger protein 655 isoform c |
| 239118_at | 26.83 | 9.79E-07 | 2.491190935 | NM_004974| | KCNA2,potassium voltage-gated channel, shaker-related |
| 212328_at | 26.83 | 9.79E-07 | 1.303556742 | NM_001112717| | NA |
| 228515_at | 26.81 | 9.84E-07 | 1.232356836 | NA |  |
| 218508_at | 26.79 | 9.87E-07 | 1.094587471 | NM_018403| | DCP1A,decapping enzyme |
| 229029_at | 26.75 | 9.94E-07 | 1.488094963 | NA |  |
| 230304_at | 26.74 | 9.98E-07 | 1.395041954 | NA |  |
| 241505_at | 26.72 | 1.00E-06 | 1.345062306 | NA |  |
| 209867_s_at | 26.71 | 1.00E-06 | 1.185347338 | NM_015236| | LPHN3,latrophilin 3 precursor |
| 214252_s_at | 26.69 | 1.01E-06 | 1.312055922 | NM_006493| | CLN5,ceroid-lipofuscinosis, neuronal 5 |
| 213194_at | 26.69 | 1.01E-06 | 1.127375675 | NM_002941| | ROBO1,roundabout 1 isoform a |
| 229824_at | 26.67 | 1.01E-06 | 2.240146818 | NA |  |
| 203303_at | 26.66 | 1.01E-06 | 1.249476073 | NM_006520| | TCTE1L,t-complex-associated-testis-expressed 1-like |
| 1569854_at | 26.63 | 1.02E-06 | 2.079463412 | NA |  |
| 218625_at | 26.62 | 1.02E-06 | 1.834472193 | NM_016588| | NRN1,neuritin precursor |
| 226844_at | 26.61 | 1.02E-06 | 1.127010832 | NM_024761| | MOBKL2B,MOB1, Mps One Binder kinase activator-like 2B |
| 222871_at | 26.6 | 1.02E-06 | 1.835504684 | NM_018203| | FLJ10748,hypothetical protein FLJ10748 |
| 219973_at | 26.6 | 1.02E-06 | 1.860351751 | NM_024590| | ARSJ,arylsulfatase J |
| 228299_at | 26.59 | 1.02E-06 | 1.191473283 | NM_173562| | C6orf69,hypothetical protein MGC14254 |
| 205453_at | 26.58 | 1.02E-06 | 1.623318179 | NM_002145| | HOXB2,homeo box B2 |
| 212248_at | 26.56 | 1.02E-06 | 1.335137145 | NM_178812| | LYRIC,LYRIC/3D3 |
| 225732_at | 26.55 | 1.03E-06 | 1.144385127 | NM_020782| | KLHDC5,kelch domain containing 5 |
| 214722_at | 26.52 | 1.03E-06 | 1.196163238 | NM_203458| | NOTCH2NL,Notch homolog 2 N-terminal like protein |
| 202042_at | 26.5 | 1.04E-06 | 1.097043295 | NM_002109| | HARS,histidyl-tRNA synthetase |
| 212251_at | 26.49 | 1.04E-06 | 1.127185645 | NM_178812| | LYRIC,LYRIC/3D3 |
| 1559283_a_at | 26.48 | 1.04E-06 | 1.981162448 | NM_001103176| | NA |
| 229132_at | 26.48 | 1.04E-06 | 1.468129256 | NM_001042533| | NA |
| 201162_at | 26.48 | 1.04E-06 | 1.255701802 | NM_001553| | IGFBP7,insulin-like growth factor binding protein 7 |
| 240467_at | 26.43 | 1.05E-06 | 1.718991956 | NA |  |
| 212399_s_at | 26.4 | 1.05E-06 | 1.167328783 | NM_014667| | VGLL4,vestigial like 4 |
| 37384_at | 26.36 | 1.06E-06 | 1.165503285 | NM_014634| | PPM1F,protein phosphatase 1F |
| 212327_at | 26.35 | 1.07E-06 | 1.349073576 | NM_001112717| | NA |
| 205280_at | 26.33 | 1.07E-06 | 2.301728332 | NM_000824| | GLRB,glycine receptor, beta |
| 1552790_a_at | 26.33 | 1.07E-06 | 1.302631374 | NM_003262| | TLOC1,translocation protein 1 |
| 204066_s_at | 26.31 | 1.07E-06 | 1.117518952 | NM_001037131| | NA |
| 205304_s_at | 26.31 | 1.07E-06 | 1.784493154 | NM_004982| | KCNJ8,potassium inwardly-rectifying channel J8 |
| 213416_at | 26.3 | 1.07E-06 | 1.813414217 | NM_000885| | ITGA4,integrin alpha 4 precursor |
| 218599_at | 26.29 | 1.08E-06 | 1.216516443 | NM_001048205| | NA |
| 233167_at | 26.27 | 1.08E-06 | 1.229989487 | NM_031454| | SELO,selenoprotein O |
| 221840_at | 26.24 | 1.09E-06 | 1.677327732 | NM_006504| | PTPRE,protein tyrosine phosphatase, receptor type, E |
| 201795_at | 26.2 | 1.10E-06 | 1.237789533 | NM_002296| | LBR,lamin B receptor |
| 214930_at | 26.2 | 1.10E-06 | 1.542960264 | NM_015567| | SLITRK5,SLIT and NTRK-like family, member 5 |
| 212636_at | 26.19 | 1.10E-06 | 1.4435441 | NM_006775| | QKI,quaking homolog, KH domain RNA binding isoform |
| 202088_at | 26.16 | 1.10E-06 | 1.123413377 | NM_001099406| | NA |
| 212973_at | 26.15 | 1.10E-06 | 1.152282927 | NM_144563| | RPIA,ribose 5-phosphate isomerase A (ribose |
| 203031_s_at | 26.15 | 1.10E-06 | 1.23700154 | NM_000375| | UROS,uroporphyrinogen III synthase |
| 1553984_s_at | 26.14 | 1.10E-06 | 1.139207114 | NM_012145| | DTYMK,deoxythymidylate kinase (thymidylate kinase) |
| 211569_s_at | 26.14 | 1.11E-06 | 1.143227321 | NM_005327| | HADHSC,L-3-hydroxyacyl-Coenzyme A dehydrogenase, short |
| 200816_s_at | 26.14 | 1.10E-06 | 1.126800539 | NM_000430| | PAFAH1B1,platelet-activating factor acetylhydrolase, |
| 228310_at | 26.08 | 1.12E-06 | 1.258204046 | NM_001008493| | ENAH,enabled homolog isoform a |
| 229447_x_at | 26.08 | 1.12E-06 | 1.19248403 | NM_001037501| | NA |
| 231579_s_at | 26.07 | 1.12E-06 | 1.176888037 | NM_003255| | TIMP2,tissue inhibitor of metalloproteinase 2 |
| 1553954_at | 26.04 | 1.13E-06 | 1.391854942 | NM_144988| | MGC19780,hypothetical protein MGC19780 |
| 201433_s_at | 26.02 | 1.13E-06 | 1.115087721 | NM_014754| | PTDSS1,phosphatidylserine synthase 1 |
| 222526_at | 26.01 | 1.13E-06 | 1.153148812 | NM_017660| | p66alpha,p66 alpha |
| 239221_at | 25.99 | 1.14E-06 | 1.508412747 | NM_001083909| | NA |
| 228630_at | 25.97 | 1.14E-06 | 1.337302924 | NM_003428| | ZNF84,zinc finger protein 84 (HPF2) |
| 218132_s_at | 25.93 | 1.15E-06 | 1.125195394 | NM_001077446| | NA |
| 211755_s_at | 25.93 | 1.15E-06 | 1.03829666 | NM_001688| | ATP5F1,ATP synthase, H+ transporting, mitochondrial F0 |
| 202464_s_at | 25.93 | 1.15E-06 | 1.320141947 | NM_004566| | PFKFB3,6-phosphofructo-2-kinase/fructose-2, |
| 205880_at | 25.92 | 1.15E-06 | 1.741097971 | NM_002742| | PRKD1,protein kinase D1 |
| 236154_at | 25.92 | 1.15E-06 | 2.205798772 | NM_006775| | QKI,quaking homolog, KH domain RNA binding isoform |
| 204271_s_at | 25.91 | 1.15E-06 | 1.20855988 | NM_000115| | EDNRB,endothelin receptor type B isoform 1 |
| 205097_at | 25.9 | 1.16E-06 | 1.190822553 | NM_000112| | SLC26A2,solute carrier family 26 member 2 |
| 1559942_at | 25.89 | 1.16E-06 | 1.809716529 | NM_199072| | HIC,I-mfa domain-containing protein isoform p40 |
| 227035_x_at | 25.82 | 1.17E-06 | 1.154180039 | NA |  |
| 212049_at | 25.82 | 1.17E-06 | 1.110786849 | NM_133264| | WIRE,WIRE protein |
| 1558164_s_at | 25.81 | 1.18E-06 | 1.209375707 | NM_002618| | PEX13,peroxisome biogenesis factor 13 |
| 224993_at | 25.8 | 1.18E-06 | 1.143833447 | NM_005934| | MLLT1,myeloid/lymphoid or mixed-lineage leukemia |
| 219468_s_at | 25.78 | 1.18E-06 | 1.432605115 | NM_017949| | CUEDC1,CUE domain-containing 1 |
| 218140_x_at | 25.77 | 1.19E-06 | 1.15105889 | NM_021203| | SRPRB,signal recognition particle receptor, beta |
| 226556_at | 25.71 | 1.20E-06 | 1.230176071 | NA |  |
| 219249_s_at | 25.7 | 1.20E-06 | 1.161708867 | NM_021939| | FKBP10,FK506 binding protein 10, 65 kDa |
| 209870_s_at | 25.69 | 1.20E-06 | 1.189856886 | NM_005503| | APBA2,amyloid beta A4 precursor protein-binding, |
| 224882_at | 25.69 | 1.20E-06 | 1.58461836 | NM_032501| | ACAS2L,acetyl-CoA synthetase 2-like |
| 219250_s_at | 25.68 | 1.20E-06 | 1.178322075 | NM_013281| | FLRT3,fibronectin leucine rich transmembrane protein 3 |
| 208912_s_at | 25.68 | 1.20E-06 | 1.110707126 | NM_033133| | CNP,2',3'-cyclic nucleotide 3' phosphodiesterase |
| 219779_at | 25.64 | 1.21E-06 | 1.412437554 | NM_024721| | ZFHX4,zinc finger homeodomain 4 |
| 202414_at | 25.64 | 1.21E-06 | 1.122722465 | NM_000123| | ERCC5,XPG-complementing protein |
| 201207_at | 25.63 | 1.21E-06 | 1.129595012 | NM_021137| | TNFAIP1,tumor necrosis factor, alpha-induced protein 1 |
| 230630_at | 25.62 | 1.22E-06 | 1.301840349 | NA |  |
| 208978_at | 25.57 | 1.23E-06 | 1.594070497 | NM_001312| | CRIP2,cysteine-rich protein 2 |
| 1558034_s_at | 25.55 | 1.23E-06 | 2.013185562 | NM_000096| | CP,ceruloplasmin (ferroxidase) |
| 218829_s_at | 25.55 | 1.23E-06 | 1.060752362 | NM_017780| | CHD7,chromodomain helicase DNA binding protein 7 |
| 227624_at | 25.54 | 1.23E-06 | 1.503429154 | NM_001127208| | NA |
| 241938_at | 25.52 | 1.24E-06 | 1.520553987 | NM_006775| | QKI,quaking homolog, KH domain RNA binding isoform |
| 230591_at | 25.47 | 1.25E-06 | 1.440161232 | NA |  |
| 202985_s_at | 25.46 | 1.25E-06 | 1.100218286 | NM_001015048| | NA |
| 215429_s_at | 25.46 | 1.25E-06 | 1.299024668 | NM_182498| | MGC51082,hypothetical protein MGC51082 |
| 227543_at | 25.45 | 1.25E-06 | 1.395509303 | NM_032193| | AYP1,AYP1 protein |
| 205717_x_at | 25.45 | 1.25E-06 | 1.312064686 | NM_002588| | PCDHGC3,protocadherin gamma subfamily C, 3 isoform 1 |
| 225282_at | 25.43 | 1.26E-06 | 1.386038274 | NM_022733| | LOC64744,hypothetical protein AL133206 |
| 226902_at | 25.41 | 1.26E-06 | 1.128603322 | NA |  |
| 227212_s_at | 25.4 | 1.26E-06 | 1.248913128 | NM_001009936| | PHF19,PHD finger protein 19 isoform b |
| 225626_at | 25.39 | 1.27E-06 | 2.080024299 | NM_018440| | PAG,phosphoprotein associated with |
| 228573_at | 25.39 | 1.27E-06 | 1.589431671 | NA |  |
| 241198_s_at | 25.38 | 1.27E-06 | 1.454873606 | NM_032930| | MGC13040,hypothetical protein MGC13040 |
| 226781_at | 25.38 | 1.27E-06 | 1.258586969 | NM_197964| | HSPC268,hypothetical protein HSPC268 |
| 205251_at | 25.36 | 1.27E-06 | 1.235581629 | NM_022817| | PER2,period 2 isoform 1 |
| 232421_at | 25.36 | 1.27E-06 | 1.621490412 | NM_001082959| | NA |
| 221886_at | 25.35 | 1.27E-06 | 1.912378928 | NM_015689| | KIAA1277,KIAA1277 protein |
| 220265_at | 25.33 | 1.28E-06 | 1.602312776 | NM_020960| | GPR107,G protein-coupled receptor 107 |
| 228897_at | 25.33 | 1.28E-06 | 1.496882125 | NM_001002862| | DERL3,derlin-3 protein isoform b |
| 225316_at | 25.32 | 1.28E-06 | 1.428605126 | NM_032793| | FLJ14490,hypothetical protein FLJ14490 |
| 224784_at | 25.28 | 1.29E-06 | 1.609692195 | NM_005937| | MLLT6,myeloid/lymphoid or mixed-lineage leukemia |
| 200919_at | 25.28 | 1.29E-06 | 1.319843022 | NM_004427| | PHC2,polyhomeotic 2-like isoform b |
| 201462_at | 25.26 | 1.30E-06 | 1.05427813 | NM_014766| | SCRN1,secernin 1 |
| 1553108_at | 25.24 | 1.30E-06 | 1.490248285 | NM_152409| | FLJ37562,hypothetical protein FLJ37562 |
| 213008_at | 25.24 | 1.30E-06 | 1.11526883 | NM_001113378| | NA |
| 226016_at | 25.23 | 1.30E-06 | 1.368232231 | NM_001025079| | NA |
| 212983_at | 25.23 | 1.30E-06 | 1.192174246 | NM_005343| | HRAS,v-Ha-ras Harvey rat sarcoma viral oncogene |
| 225723_at | 25.23 | 1.30E-06 | 1.179277527 | NM_138493| | NA |
| 226542_at | 25.21 | 1.31E-06 | 1.189164547 | NA |  |
| 203608_at | 25.21 | 1.31E-06 | 1.510697263 | NM_001080| | ALDH5A1,aldehyde dehydrogenase 5A1 precursor, isoform 2 |
| 211675_s_at | 25.2 | 1.31E-06 | 1.627438896 | NM_199072| | HIC,I-mfa domain-containing protein isoform p40 |
| 59437_at | 25.2 | 1.31E-06 | 1.253679801 | NM_001048265| | NA |
| 237833_s_at | 25.2 | 1.31E-06 | 1.583963036 | NM_005460| | SNCAIP,synuclein alpha interacting protein |
| 204363_at | 25.2 | 1.31E-06 | 1.329152361 | NM_001993| | F3,coagulation factor III precursor |
| 238613_at | 25.19 | 1.31E-06 | 1.503028749 | NM_016653| | ZAK,sterile-alpha motif and leucine zipper |
| 216870_x_at | 25.19 | 1.31E-06 | 1.473894243 | NA |  |
| 205164_at | 25.19 | 1.31E-06 | 1.110136734 | NM_014291| | GCAT,glycine C-acetyltransferase precursor |
| 209123_at | 25.18 | 1.31E-06 | 1.137556206 | NM_000320| | QDPR,quinoid dihydropteridine reductase |
| 226487_at | 25.15 | 1.32E-06 | 1.413848273 | NM_032829| | FLJ14721,hypothetical protein FLJ14721 |
| 219098_at | 25.15 | 1.32E-06 | 1.099030462 | NM_001105538| | NA |
| 213587_s_at | 25.15 | 1.32E-06 | 1.344022376 | NM_001100592| | NA |
| 224693_at | 25.13 | 1.32E-06 | 1.314625693 | NM_080821| | C20orf108,chromosome 20 open reading frame 108 |
| 223594_at | 25.13 | 1.32E-06 | 1.78552709 | NM_032256| | DKFZp434K2435,hypothetical protein DKFZp434K2435 |
| 202694_at | 25.11 | 1.33E-06 | 1.956396966 | NM_004760| | STK17A,serine/threonine kinase 17a |
| 206243_at | 25.1 | 1.33E-06 | 1.321085253 | NM_003256| | TIMP4,tissue inhibitor of metalloproteinase 4 |
| 212510_at | 25.09 | 1.33E-06 | 1.535985509 | NM_015141| | GPD1L,glycerol-3-phosphate dehydrogenase 1-like |
| 224218_s_at | 25.08 | 1.33E-06 | 1.446657539 | NM_014112| | TRPS1,zinc finger transcription factor TRPS1 |
| 239780_at | 25.08 | 1.33E-06 | 1.503563164 | NA |  |
| 1553106_at | 25.08 | 1.33E-06 | 1.448184332 | NM_152409| | FLJ37562,hypothetical protein FLJ37562 |
| 230958_s_at | 25.07 | 1.33E-06 | 1.979972317 | NA |  |
| 219154_at | 25.04 | 1.34E-06 | 1.155011989 | NM_019034| | RHOF,ras homolog gene family, member F |
| 210448_s_at | 25.03 | 1.34E-06 | 1.347352375 | NM_002561| | P2RX5,purinergic receptor P2X5 isoform A |
| 230570_at | 25.03 | 1.34E-06 | 1.29460067 | NA |  |
| 222761_at | 25.01 | 1.35E-06 | 1.410829396 | NM_017693| | BIVM,basic, immunoglobulin-like variable motif |
| 209990_s_at | 25 | 1.35E-06 | 1.558329432 | NM_005458| | GPR51,G protein-coupled receptor 51 |
| 202836_s_at | 24.99 | 1.35E-06 | 1.080585986 | NM_006701| | TXNL4A,thioredoxin-like 4A |
| 202506_at | 24.98 | 1.35E-06 | 1.757831655 | NM_006751| | SSFA2,sperm specific antigen 2 |
| 217077_s_at | 24.97 | 1.35E-06 | 1.608759562 | NM_005458| | GPR51,G protein-coupled receptor 51 |
| 220299_at | 24.96 | 1.36E-06 | 1.380830091 | NM_019073| | SPATA6,spermatogenesis associated 6 |
| 221910_at | 24.96 | 1.36E-06 | 1.513346889 | NM_004956| | ETV1,ets variant gene 1 |
| 233955_x_at | 24.95 | 1.36E-06 | 1.06543225 | NM_016463| | CXXC5,CXXC finger 5 |
| 228480_at | 24.95 | 1.36E-06 | 1.816475369 | NM_003574| | VAPA,vesicle-associated membrane protein-associated |
| 1557996_at | 24.94 | 1.36E-06 | 1.272098947 | NA |  |
| 220773_s_at | 24.92 | 1.37E-06 | 1.262015811 | NM_001024218| | NA |
| 222517_at | 24.9 | 1.37E-06 | 1.409870325 | NM_012095| | AP3M1,adaptor-related protein complex 3, mu 1 subunit |
| 204027_s_at | 24.87 | 1.38E-06 | 1.133461806 | NM_005371| | METTL1,methyltransferase-like protein 1 isoform a |
| 221942_s_at | 24.84 | 1.39E-06 | 1.57712029 | NM_000856| | GUCY1A3,guanylate cyclase 1, soluble, alpha 3 |
| 213365_at | 24.82 | 1.39E-06 | 1.191948465 | NM_080663| | MGC16943,hypothetical protein MGC16943 |
| 209610_s_at | 24.81 | 1.39E-06 | 1.46382359 | NM_003038| | SLC1A4,solute carrier family 1, member 4 |
| 221911_at | 24.81 | 1.39E-06 | 1.216303754 | NM_004956| | ETV1,ets variant gene 1 |
| 226490_at | 24.79 | 1.40E-06 | 1.317249786 | NA |  |
| 204933_s_at | 24.78 | 1.40E-06 | 1.523664586 | NM_002546| | TNFRSF11B,osteoprotegerin precursor |
| 226831_at | 24.78 | 1.40E-06 | 1.410560929 | NM_138773| | LOC91137,hypothetical protein BC017169 |
| 211784_s_at | 24.77 | 1.40E-06 | 1.058500181 | NM_001078166| | NA |
| 217721_at | 24.76 | 1.40E-06 | 1.397559662 | NA |  |
| 223213_s_at | 24.74 | 1.41E-06 | 1.879085292 | NM_001017926| | NA |
| 211985_s_at | 24.74 | 1.41E-06 | 1.193214626 | NM_001743| | CALM2,calmodulin 2 |
| 207401_at | 24.73 | 1.41E-06 | 1.44517715 | NM_002763| | PROX1,prospero-related homeobox 1 |
| 228752_at | 24.73 | 1.41E-06 | 1.639087649 | NA |  |
| 202386_s_at | 24.73 | 1.41E-06 | 1.138738468 | NM_014647| | LKAP,limkain b1 isoform 1 |
| 205737_at | 24.7 | 1.42E-06 | 1.392042968 | NM_004518| | KCNQ2,potassium voltage-gated channel KQT-like protein |
| 204703_at | 24.69 | 1.42E-06 | 1.156776611 | NM_006531| | TTC10,Tg737 protein isoform 2 |
| 213321_at | 24.69 | 1.42E-06 | 1.182089511 | NM_000056| | BCKDHB,branched chain keto acid dehydrogenase E1, beta |
| 241950_at | 24.68 | 1.43E-06 | 1.469486054 | NA |  |
| 212513_s_at | 24.66 | 1.43E-06 | 1.092502205 | NM_015017| | USP33,ubiquitin specific protease 33 isoform 1 |
| 224663_s_at | 24.65 | 1.43E-06 | 1.277285165 | NM_021914| | CFL2,cofilin 2 |
| 203285_s_at | 24.63 | 1.44E-06 | 1.127229305 | NM_012262| | HS2ST1,heparan sulfate 2-O-sulfotransferase 1 |
| 209386_at | 24.61 | 1.45E-06 | 1.514952451 | NM_014220| | TM4SF1,transmembrane 4 superfamily member 1 |
| 218793_s_at | 24.61 | 1.45E-06 | 2.074050018 | NM_001037535| | NA |
| 201584_s_at | 24.59 | 1.45E-06 | 1.058672218 | NM_005804| | DDX39,DEAD (Asp-Glu-Ala-Asp) box polypeptide 39 |
| 214046_at | 24.58 | 1.46E-06 | 1.452695346 | NM_006581| | FUT9,fucosyltransferase 9 (alpha (1,3) |
| 203037_s_at | 24.57 | 1.46E-06 | 1.369827224 | NM_014751| | MTSS1,metastasis suppressor 1 |
| 212558_at | 24.57 | 1.46E-06 | 1.282806729 | NM_005841| | SPRY1,sprouty homolog 1, antagonist of FGF signaling |
| 226077_at | 24.55 | 1.46E-06 | 1.167151059 | NM_144726| | FLJ31951,hypothetical protein FLJ31951 |
| 224814_at | 24.54 | 1.47E-06 | 1.475708137 | NM_013379| | DPP7,dipeptidyl peptidase 7 preproprotein |
| 201896_s_at | 24.54 | 1.47E-06 | 1.152560449 | NM_001005290| | DDA3,p53-regulated DDA3 isoform b |
| 211814_s_at | 24.53 | 1.47E-06 | 1.832028277 | NM_057749| | CCNE2,cyclin E2 isoform 1 |
| 202630_at | 24.52 | 1.47E-06 | 1.334092766 | NM_006380| | APPBP2,amyloid beta precursor protein-binding protein |
| 236208_at | 24.51 | 1.48E-06 | 1.751378922 | NM_004531| | MOCS2,molybdopterin synthase large subunit MOCS2B |
| 201819_at | 24.49 | 1.48E-06 | 1.339904943 | NM_001082959| | NA |
| 205525_at | 24.49 | 1.48E-06 | 1.19455196 | NM_004342| | CALD1,caldesmon 1 isoform 2 |
| 202976_s_at | 24.48 | 1.48E-06 | 1.388450425 | NM_014899| | RHOBTB3,rho-related BTB domain containing 3 |
| 212613_at | 24.47 | 1.48E-06 | 1.25627584 | NM_007047| | BTN3A2,butyrophilin, subfamily 3, member A2 |
| 206330_s_at | 24.47 | 1.48E-06 | 1.436924872 | NM_016848| | SHC3,src homology 2 domain containing transforming |
| 229572_at | 24.47 | 1.48E-06 | 1.400330097 | NA |  |
| 221532_s_at | 24.46 | 1.49E-06 | 1.072760122 | NM_025234| | REC14,recombination protein REC14 |
| 222654_at | 24.45 | 1.49E-06 | 1.407003708 | NM_017813| | IMPA3,myo-inositol monophosphatase A3 |
| 238469_at | 24.45 | 1.49E-06 | 2.030702848 | NM_024576| | OGFRL1,opioid growth factor receptor-like 1 |
| 230300_at | 24.44 | 1.49E-06 | 1.95558447 | NA |  |
| 204248_at | 24.44 | 1.49E-06 | 1.187587277 | NM_002067| | GNA11,guanine nucleotide binding protein (G protein), |
| 225214_at | 24.42 | 1.50E-06 | 1.297738805 | NM_002799| | PSMB7,proteasome beta 7 subunit proprotein |
| 238431_at | 24.42 | 1.50E-06 | 1.286385274 | NA |  |
| 213645_at | 24.39 | 1.51E-06 | 1.273827264 | NM_001126123| | NA |
| 225514_at | 24.37 | 1.52E-06 | 1.13602238 | NM_174913| | C14orf21,chromosome 14 open reading frame 21 |
| 202503_s_at | 24.36 | 1.52E-06 | 1.08352024 | NM_001029989| | NA |
| 227667_at | 24.36 | 1.52E-06 | 1.341946767 | NM_017949| | CUEDC1,CUE domain-containing 1 |
| 225018_at | 24.34 | 1.52E-06 | 1.210912576 | NM_020148| | SPIRE1,spire homolog 1 |
| 212174_at | 24.34 | 1.52E-06 | 1.769503186 | NM_001625| | AK2,adenylate kinase 2 isoform a |
| 218611_at | 24.34 | 1.52E-06 | 1.270318774 | NM_016545| | IER5,immediate early response 5 |
| 226840_at | 24.34 | 1.52E-06 | 1.525561083 | NM_001040158| | NA |
| 212240_s_at | 24.33 | 1.52E-06 | 1.373907991 | NM_181504| | PIK3R1,phosphoinositide-3-kinase, regulatory subunit, |
| 207084_at | 24.33 | 1.52E-06 | 2.295026474 | NM_005604| | POU3F2,POU domain, class 3, transcription factor 2 |
| 238735_at | 24.31 | 1.53E-06 | 2.189788366 | NA |  |
| 236358_at | 24.31 | 1.53E-06 | 1.499527865 | NA |  |
| 231954_at | 24.31 | 1.53E-06 | 1.254578897 | NA |  |
| 1554690_a_at | 24.29 | 1.53E-06 | 1.280766119 | NM_001122824| | NA |
| 207551_s_at | 24.28 | 1.54E-06 | 1.184643528 | NM_006800| | MSL3L1,male-specific lethal 3-like 1 isoform c |
| 1554807_a_at | 24.27 | 1.54E-06 | 1.288472281 | NM_020148| | SPIRE1,spire homolog 1 |
| 1554080_at | 24.26 | 1.54E-06 | 1.251076223 | NM_005444| | RQCD1,RCD1 required for cell differentiation1 homolog |
| 209497_s_at | 24.25 | 1.55E-06 | 1.239600697 | NM_031492| | RBM30,RNA binding motif protein 30 |
| 223230_at | 24.25 | 1.55E-06 | 1.165180835 | NM_032864| | FLJ14936,hypothetical protein FLJ14936 |
| 238929_at | 24.25 | 1.55E-06 | 1.221124977 | NM_032102| | SRP46,Splicing factor, arginine/serine-rich, 46kD |
| 225483_at | 24.22 | 1.55E-06 | 1.180221039 | NM_052875| | MGC10485,hypothetical protein MGC10485 |
| 224820_at | 24.22 | 1.55E-06 | 1.248189793 | NM_198076| | FAM36A,family with sequence similarity 36, member A |
| 239144_at | 24.21 | 1.55E-06 | 1.974351312 | NM_080742| | B3GAT2,beta-1,3-glucuronyltransferase 2 |
| 1554774_at | 24.2 | 1.56E-06 | 1.471256513 | NM_001042533| | NA |
| 1553111_a_at | 24.2 | 1.56E-06 | 1.320478587 | NM_152903| | KBTBD6,kelch repeat and BTB (POZ) domain-containing 6 |
| 205046_at | 24.19 | 1.56E-06 | 1.145189155 | NM_001813| | CENPE,centromere protein E |
| 205885_s_at | 24.18 | 1.56E-06 | 1.522764565 | NM_000885| | ITGA4,integrin alpha 4 precursor |
| 219825_at | 24.16 | 1.57E-06 | 1.537903341 | NM_019885| | CYP26B1,cytochrome P450, family 26, subfamily b, |
| 204836_at | 24.16 | 1.57E-06 | 1.06234634 | NM_000170| | GLDC,glycine dehydrogenase (decarboxylating; glycine |
| 214512_s_at | 24.15 | 1.57E-06 | 1.063002661 | NM_006713| | PC4,activated RNA polymerase II transcription |
| 230098_at | 24.15 | 1.57E-06 | 1.368579657 | NM_016018| | PHF20L1,PHD finger protein 20-like 1 isoform 1 |
| 219798_s_at | 24.14 | 1.57E-06 | 1.0932169 | NM_019606| | FLJ20257,hypothetical protein FLJ20257 |
| 217792_at | 24.13 | 1.58E-06 | 1.06460131 | NM_014426| | SNX5,sorting nexin 5 |
| 227354_at | 24.12 | 1.58E-06 | 2.169952675 | NM_018440| | PAG,phosphoprotein associated with |
| 230026_at | 24.11 | 1.58E-06 | 1.367033128 | NM_032112| | MRPL43,mitochondrial ribosomal protein L43 isoform a |
| 235635_at | 24.11 | 1.58E-06 | 1.347478001 | NM_001030055| | NA |
| 213657_s_at | 24.11 | 1.58E-06 | 1.354624017 | NA |  |
| 219151_s_at | 24.1 | 1.58E-06 | 1.291766332 | NM_001003789| | RABL2B,RAB, member of RAS oncogene family-like 2B |
| 236330_at | 24.09 | 1.59E-06 | 1.45752513 | NA |  |
| 226742_at | 24.06 | 1.60E-06 | 1.241179633 | NA |  |
| 202897_at | 24.06 | 1.60E-06 | 1.203534199 | NM_001040022| | NA |
| 236120_at | 24.05 | 1.60E-06 | 1.858800581 | NA |  |
| 242770_at | 24.03 | 1.61E-06 | 1.946506235 | NA |  |
| 238617_at | 24.02 | 1.61E-06 | 1.700759452 | NA |  |
| 213001_at | 24.02 | 1.61E-06 | 1.224942549 | NM_012098| | ANGPTL2,angiopoietin-like 2 precursor |
| 225685_at | 24.01 | 1.61E-06 | 1.500103501 | NA |  |
| 226360_at | 23.96 | 1.63E-06 | 1.242341097 | NM_032173| | NA |
| 211958_at | 23.94 | 1.64E-06 | 1.406251325 | NM_000599| | IGFBP5,insulin-like growth factor binding protein 5 |
| 208476_s_at | 23.94 | 1.64E-06 | 1.35015342 | NM_018027| | FRMD4A,FERM domain containing 4A |
| 231319_x_at | 23.93 | 1.64E-06 | 1.372475875 | NM_022342| | KIF9,kinesin family member 9 |
| 203029_s_at | 23.93 | 1.64E-06 | 1.489089057 | NM_002847| | PTPRN2,protein tyrosine phosphatase, receptor type, N |
| 231866_at | 23.93 | 1.64E-06 | 1.24980655 | NM_005575| | LNPEP,leucyl/cystinyl aminopeptidase |
| 201174_s_at | 23.92 | 1.64E-06 | 1.166862545 | NM_018975| | TERF2IP,TRF2-interacting telomeric RAP1 protein |
| 205933_at | 23.92 | 1.64E-06 | 1.343324426 | NM_015559| | SETBP1,SET binding protein 1 |
| 230782_at | 23.92 | 1.64E-06 | 1.514117911 | NM_003104| | SORD,sorbitol dehydrogenase |
| 219564_at | 23.92 | 1.64E-06 | 2.075363899 | NM_018658| | KCNJ16,potassium inwardly-rectifying channel J16 |
| 218175_at | 23.9 | 1.65E-06 | 1.664695054 | NM_025140| | FLJ22471,limkain beta 2 |
| 221858_at | 23.89 | 1.65E-06 | 1.373855001 | NM_015188| | NA |
| 226775_at | 23.89 | 1.65E-06 | 1.249810951 | NM_020189| | e(y)2,e(y)2 protein |
| 237314_at | 23.85 | 1.67E-06 | 1.903118584 | NM_145010| | C10orf63,enkurin |
| 203440_at | 23.85 | 1.67E-06 | 1.21933161 | NM_001792| | CDH2,cadherin 2, type 1 preproprotein |
| 222532_at | 23.84 | 1.67E-06 | 1.145747052 | NM_021203| | SRPRB,signal recognition particle receptor, beta |
| 1558044_s_at | 23.84 | 1.67E-06 | 1.152462318 | NM_058219| | EXOSC6,homolog of yeast mRNA transport regulator 3 |
| 222875_at | 23.83 | 1.68E-06 | 1.060656981 | NM_020162| | DHX33,DEAH (Asp-Glu-Ala-His) box polypeptide 33 |
| 1561660_at | 23.8 | 1.69E-06 | 1.614366057 | NM_001012755| | NA |
| 204577_s_at | 23.79 | 1.69E-06 | 1.681978456 | NM_015041| | CLUAP1,clusterin associated protein 1 |
| 242826_at | 23.79 | 1.69E-06 | 1.22263394 | NA |  |
| 218386_x_at | 23.74 | 1.71E-06 | 1.135898459 | NM_001001992| | USP16,ubiquitin specific protease 16 isoform b |
| 216120_s_at | 23.73 | 1.72E-06 | 1.664616155 | NM_001001331| | ATP2B2,plasma membrane calcium ATPase 2 isoform a |
| 204918_s_at | 23.73 | 1.72E-06 | 1.659715481 | NM_004529| | MLLT3,myeloid/lymphoid or mixed-lineage leukemia |
| 1555945_s_at | 23.72 | 1.72E-06 | 1.207049306 | NM_014612| | C9orf10,C9orf10 protein |
| 226395_at | 23.68 | 1.74E-06 | 1.221752816 | NM_032410| | HOOK3,golgi-associated microtubule-binding protein |
| 202479_s_at | 23.66 | 1.75E-06 | 1.507592546 | NM_021643| | TRIB2,tribbles homolog 2 |
| 1555240_s_at | 23.65 | 1.75E-06 | 1.382142848 | NM_018841| | GNG12,G-protein gamma-12 subunit |
| 226748_at | 23.65 | 1.75E-06 | 1.354336968 | NM_153374| | MGC35274,hypothetical protein MGC35274 |
| 224636_at | 23.63 | 1.76E-06 | 1.114911188 | NM_053023| | ZFP91,zinc finger protein 91 isoform 1 |
| 228108_at | 23.63 | 1.75E-06 | 1.249999815 | NA |  |
| 214960_at | 23.61 | 1.76E-06 | 1.29641615 | NM_006595| | API5,apoptosis inhibitor 5 |
| 203897_at | 23.6 | 1.77E-06 | 1.139815653 | NM_020424| | LOC57149,hypothetical protein A-211C6.1 |
| 201306_s_at | 23.59 | 1.77E-06 | 1.101800689 | NM_006401| | ANP32B,acidic (leucine-rich) nuclear phosphoprotein 32 |
| 213429_at | 23.58 | 1.77E-06 | 2.189536781 | NM_001080512| | NA |
| 212843_at | 23.56 | 1.78E-06 | 1.874985874 | NM_000615| | NCAM1,neural cell adhesion molecule 1 |
| 200988_s_at | 23.56 | 1.78E-06 | 1.294249081 | NM_005789| | PSME3,proteasome activator subunit 3 isoform 1 |
| 204084_s_at | 23.55 | 1.78E-06 | 1.235333439 | NM_006493| | CLN5,ceroid-lipofuscinosis, neuronal 5 |
| 224847_at | 23.55 | 1.78E-06 | 1.381189581 | NM_001259| | CDK6,cyclin-dependent kinase 6 |
| 204254_s_at | 23.54 | 1.78E-06 | 1.461512512 | NM_000376| | VDR,vitamin D (1,25- dihydroxyvitamin D3) receptor |
| 215073_s_at | 23.49 | 1.80E-06 | 2.316566707 | NM_021005| | NR2F2,nuclear receptor subfamily 2, group F, member 2 |
| 211466_at | 23.49 | 1.80E-06 | 1.53766473 | NM_005596| | NFIB,nuclear factor I/B |
| 200621_at | 23.49 | 1.80E-06 | 1.578045112 | NM_004078| | CSRP1,cysteine and glycine-rich protein 1 |
| 204820_s_at | 23.47 | 1.81E-06 | 1.224235709 | NM_006994| | BTN3A3,butyrophilin, subfamily 3, member A3 isoform a |
| 212915_at | 23.46 | 1.82E-06 | 1.344387647 | NM_015009| | PDZRN3,PDZ domain containing RING finger 3 |
| 206811_at | 23.44 | 1.82E-06 | 1.833859107 | NM_001115| | ADCY8,adenylate cyclase 8 |
| 1552474_a_at | 23.44 | 1.82E-06 | 1.218973039 | NM_000156| | GAMT,guanidinoacetate N-methyltransferase isoform a |
| 200854_at | 23.44 | 1.82E-06 | 1.055628344 | NM_006311| | NCOR1,nuclear receptor co-repressor 1 |
| 37232_at | 23.42 | 1.83E-06 | 1.256011473 | NM_014749| | KIAA0586,KIAA0586 |
| 203043_at | 23.38 | 1.85E-06 | 1.039469286 | NM_004729| | ZBED1,Ac-like transposable element |
| 203260_at | 23.35 | 1.86E-06 | 1.313429604 | NM_016063| | C6orf74,chromosome 6 open reading frame 74 |
| 221311_x_at | 23.34 | 1.86E-06 | 1.264943589 | NM_020466| | DJ122O8.2,hypothetical protein dJ122O8.2 |
| 212321_at | 23.34 | 1.86E-06 | 1.057715071 | NM_003901| | SGPL1,sphingosine-1-phosphate lyase 1 |
| 238333_s_at | 23.33 | 1.87E-06 | 1.404989415 | NM_138384| | GTP,GTP_binding protein |
| 205034_at | 23.3 | 1.88E-06 | 1.53559144 | NM_057749| | CCNE2,cyclin E2 isoform 1 |
| 218203_at | 23.3 | 1.88E-06 | 1.139951052 | NM_013338| | ALG5,dolichyl phosphate glucosyltransferase |
| 236591_at | 23.3 | 1.88E-06 | 1.961352144 | NA |  |
| 230071_at | 23.29 | 1.89E-06 | 1.924132696 | NM_018243| | SEPT11,septin 11 |
| 200957_s_at | 23.29 | 1.89E-06 | 1.064342278 | NM_003146| | SSRP1,structure specific recognition protein 1 |
| 211520_s_at | 23.28 | 1.89E-06 | 1.984359149 | NM_000827| | GRIA1,glutamate receptor, ionotropic, AMPA 1 |
| 223614_at | 23.27 | 1.89E-06 | 1.296672148 | NA |  |
| 203548_s_at | 23.24 | 1.91E-06 | 1.65600279 | NM_000237| | LPL,lipoprotein lipase precursor |
| 235289_at | 23.23 | 1.91E-06 | 1.738589706 | NM_020390| | EIF5A2,eIF-5A2 protein |
| 230418_s_at | 23.23 | 1.91E-06 | 1.320922425 | NM_020692| | NA |
| 204795_at | 23.21 | 1.92E-06 | 1.269139471 | NM_001077497| | NA |
| 214670_at | 23.21 | 1.91E-06 | 1.263280584 | NM_003439| | ZKSCAN1,zinc finger protein 36 |
| 206154_at | 23.21 | 1.91E-06 | 1.34503986 | NM_000326| | RLBP1,retinaldehyde binding protein 1 |
| 224641_at | 23.21 | 1.91E-06 | 1.236465437 | NM_001011537| | FYTTD1,forty-two-three domain containing 1 isoform 2 |
| 211058_x_at | 23.2 | 1.92E-06 | 1.030552553 | NM_006082| | K-ALPHA-1,tubulin, alpha, ubiquitous |
| 212775_at | 23.19 | 1.92E-06 | 1.157200574 | NM_015311| | NA |
| 224901_at | 23.17 | 1.93E-06 | 2.100238722 | NM_001037582| | NA |
| 202975_s_at | 23.17 | 1.93E-06 | 1.390905506 | NM_014899| | RHOBTB3,rho-related BTB domain containing 3 |
| 209171_at | 23.17 | 1.93E-06 | 1.132898065 | NM_033453| | ITPA,inosine triphosphatase isoform a |
| 218862_at | 23.14 | 1.94E-06 | 1.217440492 | NM_024701| | ASB13,ankyrin repeat and SOCS box-containing protein |
| 227198_at | 23.13 | 1.95E-06 | 2.196200287 | NM_001025108| | NA |
| 225293_at | 23.13 | 1.95E-06 | 1.291822937 | NM_032888| | COL27A1,collagen, type XXVII, alpha 1 |
| 212458_at | 23.13 | 1.95E-06 | 1.21419526 | NM_181784| | SPRED2,sprouty-related protein with EVH-1 domain 2 |
| 227056_at | 23.12 | 1.95E-06 | 1.201693469 | NM_014773| | KIAA0141,KIAA0141 |
| 227400_at | 23.11 | 1.95E-06 | 1.703200153 | NM_002501| | NFIX,nuclear factor I/X (CCAAT-binding transcription |
| 225562_at | 23.1 | 1.96E-06 | 1.190788895 | NM_007368| | RASA3,RAS p21 protein activator 3 |
| 217922_at | 23.09 | 1.96E-06 | 1.175896787 | NA |  |
| 236024_at | 23.09 | 1.96E-06 | 1.713333325 | NM_005277| | GPM6A,glycoprotein M6A isoform 1 |
| 228067_at | 23.08 | 1.97E-06 | 1.540709946 | NM_207362| | MGC42367,similar to 2010300C02Rik protein |
| 204591_at | 23.08 | 1.97E-06 | 3.264525177 | NM_006614| | CHL1,cell adhesion molecule with homology to L1CAM |
| 212822_at | 23.08 | 1.97E-06 | 1.2560554 | NM_020733| | NA |
| 211725_s_at | 23.08 | 1.97E-06 | 1.175021765 | NM_001196| | BID,BH3 interacting domain death agonist isoform 2 |
| 207776_s_at | 23.08 | 1.97E-06 | 1.415139884 | NM_000724| | CACNB2,calcium channel, voltage-dependent, beta 2 |
| 214744_s_at | 23.07 | 1.97E-06 | 1.285249986 | NM_000978| | RPL23,ribosomal protein L23 |
| 202660_at | 23.06 | 1.97E-06 | 1.747755335 | NM_002223| | ITPR2,inositol 1,4,5-triphosphate receptor, type 2 |
| 242705_x_at | 23.05 | 1.98E-06 | 1.184318704 | NA |  |
| 229754_at | 23.05 | 1.98E-06 | 1.182810346 | NA |  |
| 203723_at | 23.04 | 1.98E-06 | 1.448830744 | NM_002221| | ITPKB,1D-myo-inositol-trisphosphate 3-kinase B |
| 201115_at | 23.04 | 1.98E-06 | 1.110990458 | NM_001127218| | NA |
| 214743_at | 23.03 | 1.98E-06 | 1.072581005 | NM_001913| | CUTL1,CCAAT displacement protein isoform b |
| 226762_at | 23.02 | 1.99E-06 | 1.175340439 | NM_033224| | PURB,purine-rich element binding protein B |
| 202800_at | 23.01 | 1.99E-06 | 1.841041733 | NM_004172| | SLC1A3,solute carrier family 1 (glial high affinity |
| 209030_s_at | 23 | 2.00E-06 | 1.281900944 | NM_001098517| | NA |
| 1552621_at | 22.96 | 2.02E-06 | 1.341071273 | NM_032959| | POLR2J2,DNA directed RNA polymerase II polypeptide |
| 217874_at | 22.96 | 2.02E-06 | 1.127240865 | NM_003849| | SUCLG1,succinate-CoA ligase, GDP-forming, alpha |
| 225383_at | 22.96 | 2.01E-06 | 1.164674112 | NM_001080485| | NA |
| 1560792_at | 22.95 | 2.02E-06 | 1.846992613 | NA |  |
| 231130_at | 22.95 | 2.02E-06 | 1.917456416 | NA |  |
| 221965_at | 22.94 | 2.03E-06 | 1.184697595 | NM_022782| | MPHOSPH9,M-phase phosphoprotein 9 |
| 201853_s_at | 22.93 | 2.03E-06 | 1.189305737 | NM_004358| | CDC25B,cell division cycle 25B isoform 1 |
| 204610_s_at | 22.93 | 2.03E-06 | 1.197869938 | NM_006848| | DIPA,hepatitis delta antigen-interacting protein A |
| 230653_at | 22.92 | 2.03E-06 | 1.61266437 | NA |  |
| 201890_at | 22.92 | 2.03E-06 | 1.134378027 | NM_001034| | RRM2,ribonucleotide reductase M2 polypeptide |
| 238711_s_at | 22.92 | 2.03E-06 | 1.245448129 | NM_021964| | ZNF148,zinc finger protein 148 (pHZ-52) |
| 227019_at | 22.9 | 2.04E-06 | 1.079855072 | NM_001085375| | NA |
| 212614_at | 22.9 | 2.04E-06 | 1.668524777 | NM_032199| | NA |
| 1552310_at | 22.9 | 2.04E-06 | 1.182507533 | NM_144597| | MGC29937,hypothetical protein MGC29937 |
| 202069_s_at | 22.9 | 2.04E-06 | 1.09832915 | NM_005530| | IDH3A,isocitrate dehydrogenase 3 (NAD+) alpha |
| 226459_at | 22.88 | 2.04E-06 | 1.391084994 | NM_152309| | PIK3AP1,phosphoinositide-3-kinase adaptor protein 1 |
| 226780_s_at | 22.86 | 2.06E-06 | 1.206357142 | NM_197964| | HSPC268,hypothetical protein HSPC268 |
| 210410_s_at | 22.85 | 2.06E-06 | 1.303806513 | NM_001039651| | NA |
| 202834_at | 22.83 | 2.07E-06 | 1.530149342 | NM_000029| | AGT,angiotensinogen precursor |
| 219469_at | 22.82 | 2.07E-06 | 1.273996178 | NM_001080463| | NA |
| 202047_s_at | 22.82 | 2.07E-06 | 1.271528139 | NM_014292| | CBX6,chromobox homolog 6 |
| 218314_s_at | 22.81 | 2.08E-06 | 1.120865483 | NM_001082969| | NA |
| 206408_at | 22.79 | 2.09E-06 | 1.787999594 | NM_015564| | LRRTM2,leucine rich repeat transmembrane neuronal 2 |
| 202598_at | 22.78 | 2.09E-06 | 1.162010372 | NM_001024210| | NA |
| 208818_s_at | 22.78 | 2.09E-06 | 1.322280859 | NM_000754| | COMT,catechol-O-methyltransferase isoform MB-COMT |
| 218358_at | 22.78 | 2.09E-06 | 1.143765023 | NM_024324| | MGC11256,hypothetical protein MGC11256 |
| 228841_at | 22.76 | 2.10E-06 | 1.189888699 | NM_181705| | LOC90624,hypothetical protein LOC90624 |
| 208903_at | 22.76 | 2.10E-06 | 1.286848502 | NM_001031| | RPS28,ribosomal protein S28 |
| 200633_at | 22.76 | 2.10E-06 | 1.02699156 | NM_002954| | RPS27A,ubiquitin and ribosomal protein S27a precursor |
| 212850_s_at | 22.74 | 2.11E-06 | 1.230729129 | NM_002334| | NA |
| 1559822_s_at | 22.74 | 2.11E-06 | 1.352355234 | NA |  |
| 226891_at | 22.73 | 2.11E-06 | 1.128788912 | NM_152531| | C3orf21,chromosome 3 open reading frame 21 |
| 226969_at | 22.73 | 2.11E-06 | 1.223586332 | NM_000254| | MTR,5-methyltetrahydrofolate-homocysteine |
| 202830_s_at | 22.73 | 2.11E-06 | 1.088057392 | NM_001467| | SLC37A4,solute carrier family 37 (glycerol-6-phosphate |
| 236882_at | 22.73 | 2.11E-06 | 1.525797714 | NA |  |
| 204908_s_at | 22.72 | 2.11E-06 | 1.344150496 | NM_005178| | BCL3,B-cell CLL/lymphoma 3 |
| 242630_at | 22.72 | 2.11E-06 | 1.331185951 | NA |  |
| 203886_s_at | 22.72 | 2.11E-06 | 1.216613409 | NM_001004019| | FBLN2,fibulin 2 isoform a precursor |
| 212274_at | 22.72 | 2.11E-06 | 1.206537047 | NM_145693| | LPIN1,lipin 1 |
| 202762_at | 22.72 | 2.11E-06 | 1.196900034 | NM_004850| | ROCK2,Rho-associated, coiled-coil containing protein |
| 241700_at | 22.71 | 2.11E-06 | 1.533174214 | NM_024721| | ZFHX4,zinc finger homeodomain 4 |
| 224994_at | 22.7 | 2.11E-06 | 1.531251813 | NM_001221| | CAMK2D,calcium/calmodulin-dependent protein kinase II |
| 202566_s_at | 22.7 | 2.11E-06 | 1.29326525 | NM_003174| | SVIL,supervillin isoform 1 |
| 209079_x_at | 22.69 | 2.12E-06 | 1.189147575 | NM_002588| | PCDHGC3,protocadherin gamma subfamily C, 3 isoform 1 |
| 1560916_a_at | 22.68 | 2.12E-06 | 1.221986669 | NM_015283| | NA |
| 220576_at | 22.66 | 2.13E-06 | 1.481123245 | NM_024989| | PGAP1,GPI deacylase |
| 229176_at | 22.66 | 2.13E-06 | 1.395612057 | NM_054027| | ANKH,ankylosis, progressive homolog |
| 238810_at | 22.66 | 2.14E-06 | 1.53819437 | NM_002919| | RFX3,regulatory factor X3 isoform a |
| 47530_at | 22.65 | 2.14E-06 | 1.155622608 | NM_016481| | C9orf156,chromosome 9 open reading frame 156 |
| 208804_s_at | 22.65 | 2.14E-06 | 1.144531466 | NM_006275| | SFRS6,arginine/serine-rich splicing factor 6 |
| 212970_at | 22.65 | 2.14E-06 | 1.214848218 | NA |  |
| 204642_at | 22.61 | 2.16E-06 | 1.494663777 | NM_001400| | EDG1,endothelial differentiation, sphingolipid |
| 238022_at | 22.58 | 2.17E-06 | 1.185957098 | NA |  |
| 226745_at | 22.58 | 2.17E-06 | 1.72220342 | NM_207352| | CYP4V2,cytochrome P450, family 4, subfamily v, |
| 201465_s_at | 22.58 | 2.17E-06 | 1.174917699 | NM_002228| | JUN,v-jun avian sarcoma virus 17 oncogene homolog |
| 1554906_a_at | 22.56 | 2.18E-06 | 1.851064079 | NM_005792| | MPHOSPH6,M-phase phosphoprotein 6 |
| 211070_x_at | 22.55 | 2.19E-06 | 1.128924123 | NM_001079862| | NA |
| 209729_at | 22.55 | 2.19E-06 | 1.233612186 | NM_006478| | GAS2L1,growth arrest-specific 2 like 1 isoform a |
| 212097_at | 22.54 | 2.19E-06 | 1.284224672 | NM_001753| | CAV1,caveolin 1 |
| 236664_at | 22.53 | 2.19E-06 | 1.406967564 | NM_001626| | AKT2,v-akt murine thymoma viral oncogene homolog 2 |
| 210383_at | 22.52 | 2.19E-06 | 2.121830407 | NM_006920| | SCN1A,sodium channel, voltage-gated, type I, alpha |
| 210993_s_at | 22.52 | 2.20E-06 | 1.167664091 | NM_001003688| | SMAD1,Sma- and Mad-related protein 1 |
| 236291_at | 22.52 | 2.19E-06 | 1.26725577 | NM_002905| | RDH5,retinol dehydrogenase 5 (11-cis and 9-cis) |
| 209087_x_at | 22.52 | 2.19E-06 | 1.308649376 | NM_006500| | MCAM,melanoma cell adhesion molecule |
| 229371_at | 22.51 | 2.20E-06 | 1.245178878 | NA |  |
| 208927_at | 22.48 | 2.21E-06 | 1.209561971 | NM_001007226| | SPOP,speckle-type POZ protein |
| 209032_s_at | 22.48 | 2.21E-06 | 1.216995936 | NM_001098517| | NA |
| 1555953_at | 22.47 | 2.21E-06 | 1.349909877 | NM_030582| | COL18A1,alpha 1 type XVIII collagen isoform 1 precursor |
| 213891_s_at | 22.46 | 2.22E-06 | 1.154893895 | NM_001083962| | NA |
| 222784_at | 22.46 | 2.22E-06 | 1.429153137 | NM_001034852| | NA |
| 222867_s_at | 22.44 | 2.23E-06 | 1.250600363 | NM_016060| | MED31,mediator of RNA polymerase II transcription, |
| 214297_at | 22.43 | 2.23E-06 | 1.758358843 | NM_001897| | CSPG4,melanoma-associated chondroitin sulfate |
| 215836_s_at | 22.42 | 2.24E-06 | 1.200838024 | NM_002588| | PCDHGC3,protocadherin gamma subfamily C, 3 isoform 1 |
| 214558_at | 22.37 | 2.27E-06 | 1.772144263 | NM_005288| | GPR12,G protein-coupled receptor 12 |
| 210508_s_at | 22.37 | 2.27E-06 | 1.272316969 | NM_004518| | KCNQ2,potassium voltage-gated channel KQT-like protein |
| 205088_at | 22.37 | 2.27E-06 | 1.6074722 | NM_005491| | CXorf6,chromosome X open reading frame 6 |
| 228028_at | 22.36 | 2.27E-06 | 1.243917215 | NA |  |
| 206718_at | 22.36 | 2.27E-06 | 1.365218551 | NM_002315| | LMO1,LIM domain only 1 |
| 219944_at | 22.35 | 2.28E-06 | 1.451390502 | NM_024692| | RSNL2,restin-like 2 |
| 234140_s_at | 22.34 | 2.28E-06 | 1.302239566 | NM_020860| | STIM2,stromal interaction molecule 2 |
| 212831_at | 22.34 | 2.29E-06 | 1.892840326 | NM_001080497| | NA |
| 222740_at | 22.31 | 2.30E-06 | 1.204992343 | NM_014109| | ATAD2,two AAA domain containing protein |
| 225202_at | 22.3 | 2.31E-06 | 1.340761392 | NM_014899| | RHOBTB3,rho-related BTB domain containing 3 |
| 218473_s_at | 22.3 | 2.31E-06 | 1.127299396 | NM_024656| | GLT25D1,glycosyltransferase 25 domain containing 1 |
| 213188_s_at | 22.28 | 2.32E-06 | 1.430995056 | NM_001042533| | NA |
| 202584_at | 22.28 | 2.32E-06 | 1.286104405 | NM_002504| | NFX1,nuclear transcription factor, X-box binding 1 |
| 226861_at | 22.27 | 2.32E-06 | 1.280554605 | NM_024095| | ASB8,ankyrin repeat and SOCS box-containing 8 |
| 221571_at | 22.27 | 2.32E-06 | 1.300383435 | NM_003300| | TRAF3,TNF receptor-associated factor 3 isoform 1 |
| 209046_s_at | 22.27 | 2.32E-06 | 1.126100355 | NM_007285| | GABARAPL2,GABA(A) receptor-associated protein-like 2 |
| 212946_at | 22.26 | 2.32E-06 | 1.134539027 | NM_001009814| | KIAA0564,KIAA0564 protein isoform b |
| 206692_at | 22.26 | 2.32E-06 | 1.506527004 | NM_002241| | KCNJ10,potassium inwardly-rectifying channel J10 |
| 201103_x_at | 22.24 | 2.34E-06 | 1.181237581 | NM_001037501| | NA |
| 202171_at | 22.23 | 2.34E-06 | 1.093049105 | NM_007146| | ZNF161,zinc finger protein 161 |
| 226021_at | 22.23 | 2.34E-06 | 1.442272632 | NM_172037| | RDH10,retinol dehydrogenase 10 |
| 219521_at | 22.21 | 2.35E-06 | 1.172628175 | NM_018644| | B3GAT1,beta-1,3-glucuronyltransferase 1 |
| 218841_at | 22.19 | 2.36E-06 | 1.328352112 | NM_024095| | ASB8,ankyrin repeat and SOCS box-containing 8 |
| 238753_at | 22.17 | 2.38E-06 | 1.175713079 | NM_014286| | FREQ,frequenin homolog |
| 205055_at | 22.16 | 2.38E-06 | 1.131274981 | NM_002208| | ITGAE,integrin, alpha E (antigen CD103, human mucosal |
| 228093_at | 22.16 | 2.38E-06 | 1.290416827 | NM_001007248| | ZNF599,zinc finger protein 599 isoform a |
| 200767_s_at | 22.15 | 2.38E-06 | 1.273253122 | NM_014612| | C9orf10,C9orf10 protein |
| 230210_at | 22.15 | 2.38E-06 | 1.507133567 | NM_025154| | UNC84A,unc-84 homolog A |
| 230031_at | 22.14 | 2.38E-06 | 1.254911569 | NM_005347| | HSPA5,heat shock 70kDa protein 5 (glucose-regulated |
| 231403_at | 22.14 | 2.38E-06 | 1.801102603 | NM_007118| | TRIO,triple functional domain (PTPRF interacting) |
| 64900_at | 22.13 | 2.40E-06 | 1.447624698 | NM_001077416| | NA |
| 209708_at | 22.13 | 2.40E-06 | 1.281933745 | NM_015529| | MOXD1,monooxygenase, DBH-like 1 |
| 228146_at | 22.12 | 2.40E-06 | 1.542544475 | NM_001113434| | NA |
| 205931_s_at | 22.12 | 2.40E-06 | 1.747697115 | NM_001011666| | CREB5,cAMP responsive element binding protein 5 |
| 1556606_at | 22.11 | 2.40E-06 | 1.876541844 | NM_001111018| | NA |
| 218502_s_at | 22.11 | 2.40E-06 | 1.522201928 | NM_014112| | TRPS1,zinc finger transcription factor TRPS1 |
| 228461_at | 22.1 | 2.40E-06 | 1.804167747 | NM_001099289| | NA |
| 209712_at | 22.09 | 2.41E-06 | 1.268412519 | NM_015139| | SLC35D1,solute carrier family 35 (UDP-glucuronic |
| 209587_at | 22.09 | 2.41E-06 | 1.309944492 | NM_002653| | PITX1,paired-like homeodomain transcription factor 1 |
| 241425_at | 22.07 | 2.41E-06 | 1.399547843 | NM_001008564| | NUPL1,nucleoporin like 1 isoform b |
| 212330_at | 22.07 | 2.41E-06 | 1.191961854 | NM_007111| | TFDP1,transcription factor Dp-1 |
| 238554_at | 22.07 | 2.41E-06 | 1.431071745 | NM_030579| | CYB5-M,cytochrome b5 outer mitochondrial membrane |
| 202508_s_at | 22.07 | 2.41E-06 | 1.867438413 | NM_003081| | SNAP25,synaptosomal-associated protein 25 isoform |
| 235676_at | 22.06 | 2.42E-06 | 1.709888258 | NA |  |
| 204736_s_at | 22.06 | 2.42E-06 | 1.583433487 | NM_001897| | CSPG4,melanoma-associated chondroitin sulfate |
| 218055_s_at | 22.01 | 2.45E-06 | 1.117458029 | NM_018268| | WDR41,WD repeat domain 41 |
| 1557487_at | 22.01 | 2.45E-06 | 1.91176976 | NA |  |
| 226018_at | 22 | 2.45E-06 | 1.467944529 | NM_152793| | Ells1,hypothetical protein Ells1 |
| 224591_at | 21.99 | 2.46E-06 | 1.164485896 | NM_016287| | HP1-BP74,HP1-BP74 |
| 225019_at | 21.98 | 2.46E-06 | 1.321584444 | NM_001221| | CAMK2D,calcium/calmodulin-dependent protein kinase II |
| 223805_at | 21.96 | 2.47E-06 | 1.24483028 | NM_032523| | OSBPL6,oxysterol-binding protein-like protein 6 isoform |
| 209104_s_at | 21.96 | 2.47E-06 | 1.06649547 | NM_001034833| | NA |
| 205283_at | 21.95 | 2.47E-06 | 1.260010057 | NM_001079802| | NA |
| 203355_s_at | 21.92 | 2.49E-06 | 1.259765921 | NM_015310| | PSD3,ADP-ribosylation factor guanine nucleotide |
| 227055_at | 21.92 | 2.49E-06 | 1.610454539 | NM_152637| | MGC17301,hypothetical protein MGC17301 |
| 219553_at | 21.91 | 2.50E-06 | 1.121805349 | NM_013330| | NME7,nucleoside-diphosphate kinase 7 isoform a |
| 201716_at | 21.9 | 2.50E-06 | 1.140239992 | NM_003099| | SNX1,sorting nexin 1 isoform a |
| 219383_at | 21.88 | 2.52E-06 | 1.659017615 | NM_024841| | FLJ14213,hypothetical protein FLJ14213 |
| 228395_at | 21.87 | 2.52E-06 | 1.407228144 | NM_001010983| | GLT8D1,glycosyltransferase 8 domain containing 1 |
| 205521_at | 21.87 | 2.52E-06 | 1.22742435 | NM_005107| | ENDOGL1,endonuclease G-like 1 |
| 200881_s_at | 21.85 | 2.54E-06 | 1.069950347 | NM_001539| | DNAJA1,DnaJ (Hsp40) homolog, subfamily A, member 1 |
| 242579_at | 21.84 | 2.54E-06 | 1.943502694 | NM_001203| | BMPR1B,bone morphogenetic protein receptor, type IB |
| 227646_at | 21.81 | 2.56E-06 | 1.819674436 | NM_024007| | EBF,early B-cell factor |
| 41160_at | 21.81 | 2.56E-06 | 1.118817674 | NM_003926| | MBD3,methyl-CpG binding domain protein 3 |
| 218834_s_at | 21.8 | 2.56E-06 | 1.269257279 | NM_017870| | HSPA5BP1,GBP protein isoform a |
| 239283_at | 21.8 | 2.56E-06 | 1.390731545 | NM_016040| | TMED5,transmembrane emp24 protein transport domain |
| 44783_s_at | 21.8 | 2.56E-06 | 1.537571151 | NM_001040708| | NA |
| 219262_at | 21.8 | 2.56E-06 | 1.470362366 | NM_024670| | SUV39H2,suppressor of variegation 3-9 homolog 2 |
| 204131_s_at | 21.79 | 2.56E-06 | 1.085522011 | NM_001455| | FOXO3A,forkhead box O3A |
| 225006_x_at | 21.79 | 2.57E-06 | 1.262771567 | NM_198976| | TH1L,TH1-like protein |
| 225098_at | 21.78 | 2.57E-06 | 1.087159436 | NM_005759| | ABI2,abl interactor 2 |
| 227584_at | 21.76 | 2.59E-06 | 1.103513846 | NM_020443| | NAV1,neuron navigator 1 |
| 219525_at | 21.75 | 2.59E-06 | 1.326354238 | NM_018242| | FLJ10847,hypothetical protein FLJ10847 |
| 235201_at | 21.75 | 2.59E-06 | 1.838901365 | NM_014491| | FOXP2,forkhead box P2 isoform I |
| 227903_x_at | 21.75 | 2.59E-06 | 1.190018972 | NM_033513| | C19orf20,chromosome 19 open reading frame 20 |
| 211713_x_at | 21.73 | 2.60E-06 | 1.279979538 | NM_001029989| | NA |
| 203719_at | 21.73 | 2.61E-06 | 1.073423381 | NM_001983| | ERCC1,excision repair cross-complementing 1 isofrom 2 |
| 204187_at | 21.72 | 2.61E-06 | 1.587930417 | NM_006877| | GMPR,guanosine monophosphate reductase |
| 224160_s_at | 21.72 | 2.61E-06 | 1.136911657 | NM_014049| | ACAD9,acyl-Coenzyme A dehydrogenase family, member 9 |
| 213658_at | 21.7 | 2.62E-06 | 1.447094777 | NA |  |
| 203759_at | 21.7 | 2.62E-06 | 1.276859605 | NM_006278| | ST3GAL4,ST3 beta-galactoside alpha-2,3-sialyltransferase |
| 52975_at | 21.7 | 2.62E-06 | 1.200304145 | NM_001011703| | C9orf28,chromosome 9 open reading frame 28 isoform 2 |
| 230258_at | 21.68 | 2.63E-06 | 2.013643049 | NM_001042413| | NA |
| 222734_at | 21.68 | 2.63E-06 | 1.164917803 | NM_015836| | WARS2,mitochondrial tryptophanyl tRNA synthetase 2 |
| 204966_at | 21.67 | 2.64E-06 | 1.456337973 | NM_001703| | BAI2,brain-specific angiogenesis inhibitor 2 |
| 227737_at | 21.66 | 2.64E-06 | 1.158700234 | NM_021203| | SRPRB,signal recognition particle receptor, beta |
| 225384_at | 21.66 | 2.64E-06 | 1.161767465 | NM_033407| | DOCK7,dedicator of cytokinesis 7 |
| 225261_x_at | 21.66 | 2.64E-06 | 1.26086213 | NM_198976| | TH1L,TH1-like protein |
| 229744_at | 21.65 | 2.65E-06 | 1.854255106 | NM_006751| | SSFA2,sperm specific antigen 2 |
| 205189_s_at | 21.65 | 2.65E-06 | 1.216506504 | NM_000136| | FANCC,Fanconi anemia, complementation group C |
| 202693_s_at | 21.65 | 2.65E-06 | 1.389352659 | NM_004760| | STK17A,serine/threonine kinase 17a |
| 1564521_x_at | 21.64 | 2.65E-06 | 1.061411889 | NM_001039619| | NA |
| 230079_at | 21.62 | 2.67E-06 | 1.238522967 | NM_017744| | ST7L,suppression of tumorigenicity 7-like isoform 1 |
| 203724_s_at | 21.62 | 2.66E-06 | 1.395448817 | NM_001037442| | NA |
| 222437_s_at | 21.59 | 2.68E-06 | 1.302486877 | NM_001005753| | VPS24,vacuolar protein sorting 24 isoform 2 |
| 209094_at | 21.59 | 2.69E-06 | 1.1069583 | NM_012137| | DDAH1,dimethylarginine dimethylaminohydrolase 1 |
| 231406_at | 21.58 | 2.69E-06 | 1.325115501 | NA |  |
| 232204_at | 21.58 | 2.69E-06 | 1.757194934 | NM_024007| | EBF,early B-cell factor |
| 208765_s_at | 21.57 | 2.70E-06 | 1.06430418 | NM_001102397| | NA |
| 218249_at | 21.55 | 2.71E-06 | 1.150992209 | NM_022494| | ZDHHC6,zinc finger, DHHC domain containing 6 |
| 201360_at | 21.54 | 2.72E-06 | 1.314160366 | NM_000099| | CST3,cystatin C precursor |
| 202616_s_at | 21.54 | 2.72E-06 | 1.251382392 | NM_001110792| | NA |
| 227712_at | 21.54 | 2.72E-06 | 1.272030907 | NM_020466| | DJ122O8.2,hypothetical protein dJ122O8.2 |
| 213296_at | 21.54 | 2.72E-06 | 1.114565117 | NM_007033| | RER1,RER1 retention in endoplasmic reticulum 1 |
| 235192_at | 21.51 | 2.74E-06 | 1.259818504 | NM_033550| | TP53RK,p53-related protein kinase |
| 222163_s_at | 21.5 | 2.74E-06 | 1.075675456 | NM_024063| | SPATA5L1,spermatogenesis associated 5-like 1 |
| 224472_x_at | 21.5 | 2.74E-06 | 1.096949737 | NM_016176| | Cab45,calcium binding protein Cab45 precursor |
| 205150_s_at | 21.49 | 2.75E-06 | 1.31477139 | NM_014817| | NA |
| 225440_at | 21.49 | 2.75E-06 | 1.335401481 | NM_001037553| | NA |
| 215143_at | 21.48 | 2.75E-06 | 1.912067016 | NA |  |
| 212985_at | 21.48 | 2.75E-06 | 1.176727411 | NA |  |
| 210560_at | 21.48 | 2.75E-06 | 1.716550894 | NM_001485| | GBX2,gastrulation brain homeo box 2 |
| 213785_at | 21.48 | 2.75E-06 | 1.267690878 | NM_018085| | IPO9,importin 9 |
| 219737_s_at | 21.48 | 2.75E-06 | 2.007032197 | NM_020403| | PCDH9,protocadherin 9 isoform 2 precursor |
| 225718_at | 21.47 | 2.76E-06 | 1.275359207 | NM_030650| | KIAA1715,Lunapark |
| 203785_s_at | 21.47 | 2.76E-06 | 1.098486434 | NM_018380| | DDX28,DEAD (Asp-Glu-Ala-Asp) box polypeptide 28 |
| 241840_at | 21.47 | 2.76E-06 | 1.282958527 | NA |  |
| 218974_at | 21.47 | 2.76E-06 | 1.297728856 | NM_018013| | NA |
| 243864_at | 21.45 | 2.77E-06 | 1.365572727 | NM_199511| | URB,steroid-sensitive protein 1 |
| 221815_at | 21.45 | 2.77E-06 | 1.242322838 | NM_007011| | ABHD2,alpha/beta hydrolase domain containing protein |
| 231042_s_at | 21.45 | 2.77E-06 | 1.701577873 | NA |  |
| 225246_at | 21.44 | 2.77E-06 | 1.296654005 | NM_020860| | STIM2,stromal interaction molecule 2 |
| 235803_at | 21.44 | 2.77E-06 | 1.99804224 | NA |  |
| 200699_at | 21.42 | 2.79E-06 | 1.130462066 | NM_001100603| | NA |
| 217226_s_at | 21.42 | 2.79E-06 | 1.791341185 | NM_030971| | SFXN3,sideroflexin 3 |
| 203683_s_at | 21.41 | 2.79E-06 | 1.139665477 | NM_003377| | VEGFB,vascular endothelial growth factor B |
| 229748_x_at | 21.41 | 2.79E-06 | 1.290150689 | NM_001033515| | NA |
| 204640_s_at | 21.41 | 2.79E-06 | 1.17298994 | NM_001007226| | SPOP,speckle-type POZ protein |
| 235461_at | 21.4 | 2.80E-06 | 1.342448162 | NM_001127208| | NA |
| 237034_at | 21.38 | 2.81E-06 | 1.704580837 | NA |  |
| 219477_s_at | 21.38 | 2.81E-06 | 1.38394493 | NM_018676| | THSD1,thrombospondin type I domain-containing 1 |
| 201563_at | 21.37 | 2.82E-06 | 1.068929431 | NM_003104| | SORD,sorbitol dehydrogenase |
| 213262_at | 21.34 | 2.85E-06 | 1.202159056 | NM_014363| | SACS,sacsin |
| 224826_at | 21.33 | 2.85E-06 | 1.388452851 | NM_019593| | KIAA1434,hypothetical protein KIAA1434 |
| 208873_s_at | 21.32 | 2.85E-06 | 1.17461354 | NM_005669| | C5orf18,deleted in polyposis 1 |
| 228930_at | 21.32 | 2.85E-06 | 1.421654727 | NA |  |
| 201577_at | 21.31 | 2.86E-06 | 1.15221582 | NM_000269| | NME1,nucleoside-diphosphate kinase 1 isoform b |
| 217882_at | 21.31 | 2.86E-06 | 1.076307545 | NM_018447| | LOC55831,30 kDa protein |
| 202651_at | 21.31 | 2.86E-06 | 1.253353202 | NM_014873| | LPGAT1,lysophosphatidylglycerol acyltransferase 1 |
| 209121_x_at | 21.29 | 2.87E-06 | 1.47261246 | NM_021005| | NR2F2,nuclear receptor subfamily 2, group F, member 2 |
| 211471_s_at | 21.29 | 2.87E-06 | 1.490622337 | NM_004914| | RAB36,RAB36, member RAS oncogene family |
| 227394_at | 21.29 | 2.87E-06 | 1.687446537 | NM_000615| | NCAM1,neural cell adhesion molecule 1 |
| 238164_at | 21.28 | 2.88E-06 | 1.507380894 | NM_001080491| | NA |
| 228391_at | 21.28 | 2.88E-06 | 2.042432528 | NM_207352| | CYP4V2,cytochrome P450, family 4, subfamily v, |
| 224512_s_at | 21.28 | 2.88E-06 | 1.197208157 | NM_032356| | MGC14151,hypothetical protein MGC14151 |
| 1556328_at | 21.28 | 2.88E-06 | 2.231721181 | NA |  |
| 226365_at | 21.28 | 2.88E-06 | 1.296190797 | NA |  |
| 229537_at | 21.27 | 2.88E-06 | 1.462718224 | NA |  |
| 204807_at | 21.27 | 2.88E-06 | 1.87491953 | NM_014254| | TMEM5,transmembrane protein 5 |
| 226189_at | 21.27 | 2.88E-06 | 1.547206878 | NM_002214| | ITGB8,integrin, beta 8 |
| 230596_at | 21.26 | 2.88E-06 | 1.409216479 | NA |  |
| 213724_s_at | 21.26 | 2.89E-06 | 1.17518597 | NM_002611| | PDK2,pyruvate dehydrogenase kinase, isoenzyme 2 |
| 213454_at | 21.25 | 2.89E-06 | 1.134538299 | NM_198544| | NA |
| 202631_s_at | 21.25 | 2.89E-06 | 1.134769581 | NM_006380| | APPBP2,amyloid beta precursor protein-binding protein |
| 202921_s_at | 21.25 | 2.89E-06 | 1.406007871 | NM_001148| | ANK2,ankyrin 2 isoform 1 |
| 226425_at | 21.24 | 2.89E-06 | 1.965456014 | NM_024692| | RSNL2,restin-like 2 |
| 211980_at | 21.24 | 2.89E-06 | 1.128656193 | NM_001845| | COL4A1,alpha 1 type IV collagen preproprotein |
| 223307_at | 21.24 | 2.89E-06 | 1.09740519 | NM_031299| | CDCA3,trigger of mitotic entry 1 |
| 215716_s_at | 21.24 | 2.89E-06 | 1.264649741 | NM_001001323| | ATP2B1,plasma membrane calcium ATPase 1 isoform 1a |
| 1554317_s_at | 21.23 | 2.90E-06 | 1.172739399 | NM_152307| | C14orf172,chromosome 14 open reading frame 172 |
| 227249_at | 21.23 | 2.90E-06 | 1.15843005 | NM_017668| | NDE1,nuclear distribution gene E homolog 1 |
| 231411_at | 21.23 | 2.90E-06 | 1.677982857 | NM_005780| | LHFP,lipoma HMGIC fusion partner |
| 224975_at | 21.23 | 2.90E-06 | 2.230862168 | NM_005595| | NFIA,nuclear factor I/A |
| 222409_at | 21.22 | 2.90E-06 | 1.094903204 | NM_014325| | CORO1C,coronin, actin binding protein, 1C |
| 236766_at | 21.22 | 2.90E-06 | 1.536912523 | NA |  |
| 211708_s_at | 21.21 | 2.91E-06 | 1.263980754 | NM_005063| | SCD,stearoyl-CoA desaturase |
| 225669_at | 21.2 | 2.92E-06 | 1.472436192 | NM_000629| | IFNAR1,interferon-alpha receptor 1 precursor |
| 214194_at | 21.19 | 2.92E-06 | 1.203724763 | NM_014953| | KIAA1008,KIAA1008 |
| 209341_s_at | 21.19 | 2.92E-06 | 1.175604029 | NM_001556| | IKBKB,inhibitor of kappa light polypeptide gene |
| 208933_s_at | 21.19 | 2.92E-06 | 1.411157189 | NM_006499| | LGALS8,galectin 8 isoform a |
| 203526_s_at | 21.18 | 2.92E-06 | 1.390619094 | NM_000038| | APC,adenomatosis polyposis coli |
| 240312_at | 21.18 | 2.92E-06 | 1.463445346 | NA |  |
| 227164_at | 21.18 | 2.92E-06 | 1.210990017 | NM_001078166| | NA |
| 203324_s_at | 21.15 | 2.95E-06 | 1.258015878 | NM_001233| | CAV2,caveolin 2 isoform a and b |
| 206580_s_at | 21.14 | 2.95E-06 | 1.229167897 | NM_016938| | EFEMP2,EGF-containing fibulin-like extracellular matrix |
| 223056_s_at | 21.13 | 2.95E-06 | 1.08564685 | NM_020750| | XPO5,exportin 5 |
| 209031_at | 21.13 | 2.96E-06 | 1.313623784 | NM_001098517| | NA |
| 225241_at | 21.11 | 2.97E-06 | 1.498259676 | NM_199511| | URB,steroid-sensitive protein 1 |
| 237400_at | 21.09 | 2.98E-06 | 1.498345266 | NM_001003803| | ATP5S,ATP synthase, H+ transporting, mitochondrial F0 |
| 202669_s_at | 21.08 | 2.99E-06 | 1.134408217 | NM_004093| | EFNB2,ephrin B2 |
| 226777_at | 21.08 | 3.00E-06 | 1.818164661 | NA |  |
| 219041_s_at | 21.08 | 2.99E-06 | 1.110260122 | NM_001099695| | NA |
| 202289_s_at | 21.08 | 2.99E-06 | 1.079130676 | NM_006997| | TACC2,transforming, acidic coiled-coil containing |
| 224738_x_at | 21.04 | 3.02E-06 | 1.076986031 | NM_198486| | RPL7L1,ribosomal protein L7-like 1 |
| 202089_s_at | 21.03 | 3.04E-06 | 1.131138572 | NM_001099406| | NA |
| 213311_s_at | 21.03 | 3.04E-06 | 1.167540402 | NM_014972| | KIAA1049,KIAA1049 protein |
| 214633_at | 21.02 | 3.04E-06 | 1.198997422 | NM_005634| | SOX3,SRY (sex determining region Y)-box 3 |
| 221127_s_at | 21.02 | 3.04E-06 | 1.473469362 | NA |  |
| 213298_at | 21.02 | 3.04E-06 | 1.620697681 | NM_005597| | NFIC,nuclear factor I/C isoform 1 |
| 230401_at | 21.02 | 3.04E-06 | 1.217008578 | NA |  |
| 217122_s_at | 21.01 | 3.04E-06 | 1.102942536 | NM_001110781| | NA |
| 222752_s_at | 21 | 3.05E-06 | 1.095427902 | NM_018252| | FLJ10874,hypothetical protein FLJ10874 |
| 36612_at | 21 | 3.05E-06 | 1.345044217 | NM_015159| | NA |
| 209385_s_at | 21 | 3.05E-06 | 1.13218103 | NM_007198| | PROSC,proline synthetase co-transcribed homolog |
| 228361_at | 20.99 | 3.05E-06 | 1.294489473 | NM_004091| | E2F2,E2F transcription factor 2 |
| 225546_at | 20.99 | 3.05E-06 | 1.256834793 | NM_013302| | EEF2K,elongation factor-2 kinase |
| 238756_at | 20.99 | 3.05E-06 | 1.27011443 | NM_174942| | GAS2L3,growth arrest-specific 2 like 3 |
| 209459_s_at | 20.97 | 3.07E-06 | 1.309826079 | NM_000663| | ABAT,4-aminobutyrate aminotransferase precursor |
| 222574_s_at | 20.97 | 3.07E-06 | 1.183407114 | NM_024612| | DHX40,DEAH (Asp-Glu-Ala-His) box polypeptide 40 |
| 237472_at | 20.96 | 3.08E-06 | 1.496558886 | NM_005986| | SOX1,SRY (sex determining region Y)-box 1 |
| 212468_at | 20.96 | 3.08E-06 | 1.099895492 | NM_003971| | SPAG9,sperm associated antigen 9 isoform 1 |
| 201791_s_at | 20.95 | 3.09E-06 | 1.139779986 | NM_001360| | DHCR7,7-dehydrocholesterol reductase |
| 223299_at | 20.95 | 3.09E-06 | 1.185734088 | NM_033280| | SEC11L3,SEC11-like 3 |
| 230741_at | 20.94 | 3.09E-06 | 1.483789174 | NA |  |
| 202308_at | 20.93 | 3.10E-06 | 1.343573325 | NM_001005291| | SREBF1,sterol regulatory element binding transcription |
| 202170_s_at | 20.92 | 3.11E-06 | 1.076665538 | NM_015423| | AASDHPPT,aminoadipate-semialdehyde |
| 216933_x_at | 20.91 | 3.12E-06 | 1.511996489 | NM_000038| | APC,adenomatosis polyposis coli |
| 219152_at | 20.91 | 3.12E-06 | 1.323107205 | NM_015720| | PODXL2,endoglycan |
| 211621_at | 20.9 | 3.12E-06 | 1.321041822 | NM_000044| | AR,androgen receptor isoform 1 |
| 222747_s_at | 20.9 | 3.12E-06 | 1.633229523 | NM_001037535| | NA |
| 212411_at | 20.89 | 3.13E-06 | 1.117287833 | NM_033416| | IMP4,IMP4, U3 small nucleolar ribonucleoprotein, |
| 209001_s_at | 20.89 | 3.13E-06 | 1.135294249 | NM_015391| | ANAPC13,anaphase promoting complex subunit 13 |
| 229803_s_at | 20.87 | 3.14E-06 | 1.096107551 | NA |  |
| 230150_at | 20.87 | 3.14E-06 | 1.502717496 | NM_001008405| | BCAP29,B-cell receptor-associated protein BAP29 isoform |
| 204033_at | 20.86 | 3.15E-06 | 1.095367018 | NM_004237| | TRIP13,thyroid hormone receptor interactor 13 |
| 202191_s_at | 20.86 | 3.15E-06 | 1.288573565 | NM_003644| | GAS7,growth arrest-specific 7 isoform a |
| 207336_at | 20.86 | 3.15E-06 | 1.725203524 | NM_006940| | SOX5,SRY (sex determining region Y)-box 5 isoform a |
| 201466_s_at | 20.85 | 3.16E-06 | 1.365795428 | NM_002228| | JUN,v-jun avian sarcoma virus 17 oncogene homolog |
| 1552622_s_at | 20.85 | 3.16E-06 | 1.401718435 | NM_032959| | POLR2J2,DNA directed RNA polymerase II polypeptide |
| 212423_at | 20.84 | 3.17E-06 | 1.273926461 | NM_153367| | C10orf56,chromosome 10 open reading frame 56 |
| 218065_s_at | 20.84 | 3.16E-06 | 1.134866212 | NM_020644| | C11orf15,chromosome 11 open reading frame 15 |
| 214878_at | 20.83 | 3.17E-06 | 1.608914378 | NM_001007094| | ZNF37A,zinc finger protein 37a |
| 209384_at | 20.83 | 3.17E-06 | 1.156480813 | NM_007198| | PROSC,proline synthetase co-transcribed homolog |
| 231967_at | 20.83 | 3.17E-06 | 1.398967649 | NM_016018| | PHF20L1,PHD finger protein 20-like 1 isoform 1 |
| 244788_at | 20.83 | 3.17E-06 | 1.55883971 | NA |  |
| 231714_s_at | 20.82 | 3.18E-06 | 1.203029654 | NM_006594| | AP4B1,adaptor-related protein complex 4, beta 1 |
| 1557799_at | 20.82 | 3.17E-06 | 1.216785942 | NM_170746| | C11orf31,selenoprotein H |
| 219596_at | 20.82 | 3.17E-06 | 1.175478985 | NM_020147| | THAP10,THAP domain containing 10 |
| 1556601_a_at | 20.82 | 3.17E-06 | 1.223690557 | NM_153023| | SPATA13,spermatogenesis associated 13 |
| 233257_at | 20.81 | 3.18E-06 | 1.84017182 | NA |  |
| 1554696_s_at | 20.81 | 3.18E-06 | 1.193232313 | NM_001071| | TYMS,thymidylate synthetase |
| 201923_at | 20.8 | 3.18E-06 | 1.071406704 | NM_006406| | PRDX4,thioredoxin peroxidase |
| 212108_at | 20.79 | 3.19E-06 | 1.043300962 | NM_014613| | ETEA,protein expressed in T-cells and eosinophils in |
| 1559419_at | 20.78 | 3.20E-06 | 1.904838787 | NM_000724| | CACNB2,calcium channel, voltage-dependent, beta 2 |
| 223215_s_at | 20.78 | 3.21E-06 | 1.090286382 | NM_001098625| | NA |
| 226883_at | 20.78 | 3.20E-06 | 1.085616872 | NA |  |
| 209711_at | 20.77 | 3.21E-06 | 1.381540288 | NM_015139| | SLC35D1,solute carrier family 35 (UDP-glucuronic |
| 211042_x_at | 20.77 | 3.21E-06 | 1.082241653 | NM_006500| | MCAM,melanoma cell adhesion molecule |
| 200911_s_at | 20.77 | 3.21E-06 | 1.211197853 | NM_001122824| | NA |
| 200972_at | 20.76 | 3.21E-06 | 1.119819101 | NM_005724| | TM4SF8,transmembrane 4 superfamily member 8 isoform 1 |
| 226610_at | 20.75 | 3.22E-06 | 1.108096277 | NM_181716| | PRR6,proline rich 6 |
| 227051_at | 20.74 | 3.24E-06 | 1.901129814 | NA |  |
| 229382_at | 20.72 | 3.25E-06 | 1.297707992 | NM_019099| | LOC55924,hypothetical protein LOC55924 isoform 1 |
| 213353_at | 20.71 | 3.26E-06 | 1.474890052 | NM_018672| | ABCA5,ATP-binding cassette, sub-family A , member 5 |
| 207144_s_at | 20.7 | 3.27E-06 | 1.591081934 | NM_004143| | CITED1,Cbp/p300-interacting transactivator, with |
| 212534_at | 20.7 | 3.27E-06 | 1.092099836 | NA |  |
| 241470_x_at | 20.7 | 3.27E-06 | 1.786021842 | NA |  |
| 225665_at | 20.68 | 3.28E-06 | 1.331441256 | NM_016653| | ZAK,sterile-alpha motif and leucine zipper |
| 235106_at | 20.68 | 3.28E-06 | 1.539764948 | NM_032427| | MAML2,mastermind-like 2 |
| 240128_at | 20.68 | 3.28E-06 | 1.32246187 | NA |  |
| 212023_s_at | 20.67 | 3.29E-06 | 1.289135559 | NM_002417| | MKI67,antigen identified by monoclonal antibody Ki-67 |
| 203235_at | 20.65 | 3.30E-06 | 1.11821123 | NM_003249| | THOP1,thimet oligopeptidase 1 |
| 209902_at | 20.65 | 3.30E-06 | 1.12305769 | NM_001184| | ATR,ataxia telangiectasia and Rad3 related protein |
| 225458_at | 20.65 | 3.30E-06 | 1.181995806 | NA |  |
| 823_at | 20.64 | 3.32E-06 | 1.442192798 | NM_002996| | CX3CL1,chemokine (C-X3-C motif) ligand 1 |
| 202125_s_at | 20.63 | 3.32E-06 | 1.223613568 | NM_015049| | ALS2CR3,amyotrophic lateral sclerosis 2 (juvenile) |
| 225355_at | 20.63 | 3.32E-06 | 1.143784566 | NA |  |
| 232793_at | 20.62 | 3.33E-06 | 1.612966605 | NA |  |
| 218839_at | 20.62 | 3.32E-06 | 1.522936909 | NM_001040708| | NA |
| 1568597_at | 20.62 | 3.32E-06 | 1.206602736 | NA |  |
| 230403_at | 20.62 | 3.32E-06 | 1.441539699 | NA |  |
| 200953_s_at | 20.62 | 3.33E-06 | 1.044949457 | NM_001759| | CCND2,cyclin D2 |
| 206052_s_at | 20.62 | 3.33E-06 | 1.074908177 | NM_006527| | SLBP,histone stem-loop binding protein |
| 211852_s_at | 20.61 | 3.33E-06 | 1.221901916 | NM_139321| | ATRN,attractin isoform 1 |
| 227579_at | 20.6 | 3.33E-06 | 1.328732009 | NA |  |
| 221908_at | 20.59 | 3.34E-06 | 1.15760919 | NM_001109903| | NA |
| 200897_s_at | 20.58 | 3.35E-06 | 1.081773976 | NM_016081| | KIAA0992,palladin |
| 213170_at | 20.58 | 3.35E-06 | 1.116018377 | NM_015696| | GPX7,glutathione peroxidase 7 |
| 238034_at | 20.58 | 3.35E-06 | 1.179154709 | NM_001024649| | NA |
| 225935_at | 20.58 | 3.35E-06 | 1.193942491 | NA |  |
| 220939_s_at | 20.56 | 3.36E-06 | 1.078497854 | NM_017743| | DPP8,dipeptidyl peptidase 8 isoform 2 |
| 243071_at | 20.55 | 3.37E-06 | 1.393911944 | NA |  |
| 203103_s_at | 20.52 | 3.39E-06 | 1.087329141 | NM_014502| | PRP19,PRP19/PSO4 homolog |
| 203151_at | 20.51 | 3.40E-06 | 1.308173066 | NM_002373| | MAP1A,microtubule-associated protein 1A |
| 235954_at | 20.51 | 3.40E-06 | 1.211677246 | NA |  |
| 201666_at | 20.5 | 3.41E-06 | 1.155512987 | NM_003254| | TIMP1,tissue inhibitor of metalloproteinase 1 |
| 210811_s_at | 20.5 | 3.41E-06 | 1.169878214 | NM_019070| | DDX49,DEAD (Asp-Glu-Ala-Asp) box polypeptide 49 |
| 223470_at | 20.49 | 3.41E-06 | 1.117935757 | NM_145167| | PIGM,PIG-M mannosyltransferase |
| 229969_at | 20.49 | 3.42E-06 | 1.259661474 | NA |  |
| 212121_at | 20.49 | 3.41E-06 | 1.081881462 | NM_015631| | C10orf61,chromosome 10 open reading frame 61 |
| 238536_at | 20.48 | 3.43E-06 | 1.225092703 | NA |  |
| 207233_s_at | 20.47 | 3.43E-06 | 1.449253304 | NM_000248| | MITF,microphthalmia-associated transcription factor |
| 213155_at | 20.47 | 3.43E-06 | 1.218642303 | NM_015253| | KIAA0523,KIAA0523 protein |
| 200644_at | 20.46 | 3.44E-06 | 1.061447557 | NM_023009| | MARCKSL1,MARCKS-like 1 |
| 231898_x_at | 20.46 | 3.44E-06 | 2.522573263 | NA |  |
| 229236_s_at | 20.46 | 3.44E-06 | 1.230928987 | NM_213649| | SFXN4,sideroflexin 4 isoform 1 |
| 230465_at | 20.45 | 3.45E-06 | 2.00054195 | NM_012262| | HS2ST1,heparan sulfate 2-O-sulfotransferase 1 |
| 223041_at | 20.43 | 3.47E-06 | 1.338278853 | NM_031462| | CD99L2,CD99 antigen-like 2 |
| 243481_at | 20.43 | 3.46E-06 | 2.050338847 | NM_020663| | RHOJ,TC10-like Rho GTPase |
| 219325_s_at | 20.42 | 3.47E-06 | 1.647192484 | NM_018696| | ELAC1,elaC homolog 1 |
| 217824_at | 20.41 | 3.48E-06 | 1.509961723 | NM_016021| | UBE2J1,ubiquitin-conjugating enzyme E2, J1 |
| 203333_at | 20.41 | 3.48E-06 | 1.179466043 | NM_014970| | KIFAP3,kinesin-associated protein 3 |
| 208370_s_at | 20.4 | 3.49E-06 | 1.387223875 | NM_004414| | DSCR1,calcipressin 1 isoform a |
| 226132_s_at | 20.4 | 3.49E-06 | 1.127386133 | NM_001031740| | NA |
| 201956_s_at | 20.4 | 3.49E-06 | 1.077863535 | NM_014236| | GNPAT,glyceronephosphate O-acyltransferase |
| 239632_at | 20.39 | 3.50E-06 | 1.526548506 | NA |  |
| 219384_s_at | 20.38 | 3.51E-06 | 1.163598272 | NM_012091| | ADAT1,adenosine deaminase, tRNA-specific 1 |
| 223593_at | 20.38 | 3.51E-06 | 1.223957606 | NM_016228| | AADAT,alpha-aminoadipate aminotransferase |
| 238885_at | 20.38 | 3.51E-06 | 1.346124547 | NM_020910| | NA |
| 205316_at | 20.37 | 3.51E-06 | 1.606193006 | NA |  |
| 226177_at | 20.37 | 3.51E-06 | 1.261511248 | NM_016433| | GLTP,glycolipid transfer protein |
| 223339_at | 20.33 | 3.55E-06 | 1.359715587 | NM_016311| | ATPIF1,ATPase inhibitory factor 1 isoform 1 precursor |
| 205481_at | 20.33 | 3.56E-06 | 1.577970177 | NM_000674| | ADORA1,adenosine A1 receptor |
| 200665_s_at | 20.31 | 3.57E-06 | 1.123865264 | NM_003118| | SPARC,secreted protein, acidic, cysteine-rich |
| 227840_at | 20.3 | 3.58E-06 | 1.176229225 | NM_001017927| | NA |
| 203148_s_at | 20.3 | 3.58E-06 | 1.10178579 | NM_014788| | TRIM14,tripartite motif protein TRIM14 isoform alpha |
| 201104_x_at | 20.29 | 3.59E-06 | 1.238626782 | NM_001037501| | NA |
| 209435_s_at | 20.29 | 3.59E-06 | 1.134288675 | NM_004723| | ARHGEF2,rho/rac guanine nucleotide exchange factor 2 |
| 219663_s_at | 20.29 | 3.59E-06 | 1.481438208 | NM_025268| | MGC4659,hole protein |
| 226947_at | 20.29 | 3.59E-06 | 1.477028035 | NA |  |
| 225578_at | 20.28 | 3.59E-06 | 1.161750411 | NM_001071775| | NA |
| 212764_at | 20.28 | 3.59E-06 | 2.456933213 | NM_030751| | TCF8,transcription factor 8 (represses interleukin 2 |
| 203410_at | 20.28 | 3.59E-06 | 1.076028791 | NM_006803| | AP3M2,adaptor-related protein complex 3, mu 2 subunit |
| 39549_at | 20.27 | 3.60E-06 | 1.361490607 | NM_002518| | NPAS2,neuronal PAS domain protein 2 |
| 208863_s_at | 20.27 | 3.60E-06 | 1.06098095 | NM_001078166| | NA |
| 226446_at | 20.26 | 3.61E-06 | 1.401317471 | NM_018645| | HES6,hairy and enhancer of split 6 |
| 226766_at | 20.26 | 3.61E-06 | 1.357647986 | NM_002942| | NA |
| 225826_at | 20.25 | 3.62E-06 | 1.405367993 | NM_052845| | MMAB,cob(I)alamin adenosyltransferase |
| 225421_at | 20.25 | 3.61E-06 | 1.313505636 | NM_001010853| | ACY1L2,aminoacylase 1-like 2 |
| 229157_at | 20.25 | 3.62E-06 | 1.426298867 | NA |  |
| 244260_at | 20.25 | 3.62E-06 | 1.37758402 | NA |  |
| 222432_s_at | 20.24 | 3.62E-06 | 1.122279312 | NM_020198| | GK001,GK001 protein |
| 223154_at | 20.23 | 3.64E-06 | 1.137209735 | NM_020236| | MRPL1,mitochondrial ribosomal protein L1 |
| 225471_s_at | 20.22 | 3.64E-06 | 1.153324986 | NM_001626| | AKT2,v-akt murine thymoma viral oncogene homolog 2 |
| 204781_s_at | 20.21 | 3.66E-06 | 1.461231906 | NM_000043| | FAS,tumor necrosis factor receptor superfamily, |
| 224801_at | 20.2 | 3.66E-06 | 1.340528654 | NM_019080| | NDFIP2,Nedd4 family interacting protein 2 |
| 201334_s_at | 20.2 | 3.66E-06 | 1.210306183 | NM_015313| | ARHGEF12,Rho guanine nucleotide exchange factor (GEF) 12 |
| 206554_x_at | 20.2 | 3.66E-06 | 1.301909062 | NM_006515| | SETMAR,SET domain and mariner transposase fusion gene |
| 1569472_s_at | 20.19 | 3.67E-06 | 1.984829814 | NM_001001894| | TTC3,tetratricopeptide repeat domain 3 |
| 1558111_at | 20.19 | 3.67E-06 | 1.660581538 | NM_021038| | MBNL1,muscleblind-like 1 isoform a |
| 231847_at | 20.18 | 3.68E-06 | 1.235716802 | NM_138797| | LOC129138,hypothetical protein BC014641 |
| 227816_at | 20.18 | 3.68E-06 | 1.450125649 | NM_004822| | NTN1,netrin 1 |
| 217940_s_at | 20.18 | 3.68E-06 | 1.118898104 | NM_018210| | FLJ10769,hypothetical protein FLJ10769 |
| 231964_at | 20.17 | 3.69E-06 | 1.159579015 | NA |  |
| 223300_s_at | 20.16 | 3.70E-06 | 1.129359586 | NM_024725| | FLJ23518,hypothetical protein FLJ23518 |
| 228743_at | 20.16 | 3.69E-06 | 1.453038577 | NM_032731| | TXNL5,thioredoxin-like 5 |
| 202010_s_at | 20.16 | 3.69E-06 | 1.115604577 | NM_021188| | ZNF410,clones 23667 and 23775 zinc finger protein |
| 208660_at | 20.14 | 3.71E-06 | 1.06581094 | NM_004077| | CS,citrate synthase precursor, isoform a |
| 1560371_at | 20.14 | 3.71E-06 | 1.49093376 | NA |  |
| 226448_at | 20.13 | 3.72E-06 | 1.644963146 | NM_198552| | MGC15887,hypothetical gene supported by BC009447 |
| 207959_s_at | 20.12 | 3.73E-06 | 1.764341123 | NM_001372| | DNAH9,dynein, axonemal, heavy polypeptide 9 isoform 2 |
| 232295_at | 20.12 | 3.73E-06 | 1.493559211 | NM_024996| | GFM1,G elongation factor, mitochondrial 1 |
| 214664_at | 20.11 | 3.74E-06 | 1.456352048 | NM_001079524| | NA |
| 229014_at | 20.11 | 3.74E-06 | 1.371794739 | NA |  |
| 201175_at | 20.11 | 3.74E-06 | 1.067893705 | NM_015959| | TMX2,thioredoxin-related transmembrane protein 2 |
| 229876_at | 20.09 | 3.76E-06 | 1.385837988 | NM_001122670| | NA |
| 226166_x_at | 20.09 | 3.76E-06 | 1.154728801 | NM_015690| | STK36,serine/threonine kinase 36 (fused homolog, |
| 211966_at | 20.07 | 3.78E-06 | 1.275967883 | NM_001846| | COL4A2,alpha 2 type IV collagen preproprotein |
| 227847_at | 20.07 | 3.78E-06 | 1.278940315 | NM_014805| | EPM2AIP1,EPM2A interacting protein 1 |
| 212382_at | 20.06 | 3.79E-06 | 1.212041978 | NM_001083962| | NA |
| 212385_at | 20.06 | 3.79E-06 | 1.279617783 | NM_001083962| | NA |
| 244509_at | 20.05 | 3.80E-06 | 1.38823703 | NM_001033045| | NA |
| 223034_s_at | 20.05 | 3.80E-06 | 1.042382715 | NM_001098616| | NA |
| 235793_at | 20.01 | 3.83E-06 | 1.232434765 | NA |  |
| 228839_s_at | 20.01 | 3.83E-06 | 1.139387813 | NA |  |
| 242447_at | 20.01 | 3.84E-06 | 1.449794063 | NM_001025266| | NA |
| 222006_at | 20 | 3.84E-06 | 1.144744009 | NM_012318| | LETM1,leucine zipper-EF-hand containing transmembrane |
| 242476_at | 20 | 3.84E-06 | 2.045631661 | NA |  |
| 225242_s_at | 20 | 3.85E-06 | 1.519435144 | NM_199511| | URB,steroid-sensitive protein 1 |
| 224977_at | 20 | 3.85E-06 | 1.31181577 | NM_152734| | C6orf89,hypothetical protein FLJ25357 |
| 213164_at | 19.99 | 3.85E-06 | 1.13971953 | NM_006933| | SLC5A3,solute carrier family 5 (inositol transporters), |
| 203162_s_at | 19.99 | 3.85E-06 | 1.129630748 | NM_005886| | KATNB1,katanin p80 subunit B 1 |
| 219933_at | 19.98 | 3.87E-06 | 1.28543107 | NM_016066| | GLRX2,glutaredoxin 2 isoform 1 |
| 225371_at | 19.97 | 3.87E-06 | 1.140411417 | NM_001003722| | GLE1L,GLE1-like, RNA export mediator isoform 1 |
| 1554724_at | 19.96 | 3.89E-06 | 2.261129518 | NM_014229| | SLC6A11,solute carrier family 6 (neurotransmitter |
| 204343_at | 19.96 | 3.89E-06 | 1.181144644 | NM_001089| | ABCA3,ATP-binding cassette, sub-family A member 3 |
| 203270_at | 19.96 | 3.88E-06 | 1.157059213 | NM_012145| | DTYMK,deoxythymidylate kinase (thymidylate kinase) |
| 209389_x_at | 19.95 | 3.89E-06 | 1.134229911 | NM_001079862| | NA |
| 202551_s_at | 19.95 | 3.89E-06 | 1.158452311 | NM_016441| | CRIM1,cysteine-rich motor neuron 1 |
| 1558345_a_at | 19.92 | 3.93E-06 | 1.526370836 | NA |  |
| 228615_at | 19.92 | 3.92E-06 | 1.194062217 | NA |  |
| 204251_s_at | 19.91 | 3.93E-06 | 1.175103867 | NM_014956| | Cep164,KIAA1052 protein |
| 206373_at | 19.91 | 3.93E-06 | 1.260636008 | NM_003412| | ZIC1,zinc finger protein of the cerebellum 1 |
| 201339_s_at | 19.91 | 3.93E-06 | 1.060780959 | NM_001007098| | SCP2,sterol carrier protein X isoform 2 |
| 204311_at | 19.89 | 3.95E-06 | 1.254155734 | NM_001678| | ATP1B2,Na+/K+ -ATPase beta 2 subunit |
| 223159_s_at | 19.89 | 3.95E-06 | 1.595958657 | NM_014397| | NEK6,putative serine-threonine protein kinase |
| 1558388_a_at | 19.89 | 3.95E-06 | 1.847619246 | NA |  |
| 229549_at | 19.88 | 3.96E-06 | 1.177383626 | NA |  |
| 218017_s_at | 19.87 | 3.98E-06 | 1.226846009 | NM_152419| | NA |
| 225865_x_at | 19.87 | 3.98E-06 | 1.250777631 | NM_198976| | TH1L,TH1-like protein |
| 39729_at | 19.87 | 3.98E-06 | 1.051622907 | NM_005809| | PRDX2,peroxiredoxin 2 isoform a |
| 204225_at | 19.86 | 3.99E-06 | 1.197228959 | NM_006037| | HDAC4,histone deacetylase 4 |
| 202599_s_at | 19.85 | 3.99E-06 | 1.339817099 | NM_003489| | NRIP1,receptor interacting protein 140 |
| 1566509_s_at | 19.85 | 3.99E-06 | 1.267134477 | NM_012347| | FBXO9,F-box only protein 9 isoform 1 |
| 217727_x_at | 19.85 | 4.00E-06 | 1.053531226 | NM_018206| | VPS35,vacuolar protein sorting 35 |
| 219683_at | 19.84 | 4.00E-06 | 1.236375039 | NM_017412| | FZD3,frizzled 3 |
| 225530_at | 19.83 | 4.00E-06 | 1.296648221 | NM_130807| | MOBKL2A,MOB-LAK |
| 224576_at | 19.82 | 4.01E-06 | 1.100701045 | NM_001031711| | NA |
| 221002_s_at | 19.81 | 4.03E-06 | 1.104731291 | NM_030927| | TM4SF14,transmembrane 4 superfamily member 14 |
| 235471_at | 19.8 | 4.04E-06 | 1.413787368 | NM_001031746| | NA |
| 209012_at | 19.8 | 4.04E-06 | 1.142280876 | NM_007118| | TRIO,triple functional domain (PTPRF interacting) |
| 212730_at | 19.79 | 4.05E-06 | 1.394160762 | NM_015286| | DMN,desmuslin isoform B |
| 1569607_s_at | 19.79 | 4.05E-06 | 1.599816077 | NM_001012419| | LOC441425,OTTHUMP00000015360 |
| 225571_at | 19.78 | 4.05E-06 | 1.523203598 | NM_002310| | LIFR, |
| 212205_at | 19.77 | 4.07E-06 | 1.168341538 | NM_012412| | H2AFV,H2A histone family, member V isoform 1 |
| 235031_at | 19.77 | 4.07E-06 | 1.888541397 | NA |  |
| 205590_at | 19.76 | 4.08E-06 | 1.50901939 | NM_005739| | RASGRP1,RAS guanyl releasing protein 1 |
| 226676_at | 19.76 | 4.08E-06 | 1.108948391 | NM_015461| | ZNF521,zinc finger protein 521 |
| 244690_at | 19.75 | 4.08E-06 | 1.40419644 | NA |  |
| 223099_s_at | 19.75 | 4.08E-06 | 1.139126748 | NM_031490| | LONP,peroxisomal lon protease |
| 222742_s_at | 19.75 | 4.08E-06 | 1.181303682 | NM_022777| | RABL5,RAB, member RAS oncogene family-like 5 |
| 209184_s_at | 19.75 | 4.08E-06 | 1.091416609 | NM_003749| | IRS2,insulin receptor substrate 2 |
| 228369_at | 19.75 | 4.08E-06 | 1.497070628 | NM_006586| | TNRC5,trinucleotide repeat containing 5 |
| 229034_at | 19.74 | 4.08E-06 | 1.394383758 | NM_018013| | NA |
| 227359_at | 19.74 | 4.09E-06 | 1.4619106 | NM_145047| | NOR1,oxidored-nitro domain-containing protein isoform |
| 201664_at | 19.73 | 4.10E-06 | 1.108634808 | NM_001002800| | SMC4L1,SMC4 structural maintenance of chromosomes |
| 1569349_at | 19.72 | 4.11E-06 | 1.883706378 | NM_020193| | C11orf30,EMSY protein |
| 201146_at | 19.72 | 4.11E-06 | 1.093918712 | NM_006164| | NFE2L2,nuclear factor (erythroid-derived 2)-like 2 |
| 240451_at | 19.72 | 4.11E-06 | 1.331099047 | NA |  |
| 219092_s_at | 19.71 | 4.12E-06 | 1.046647776 | NM_022755| | C9orf12,chromosome 9 open reading frame 12 |
| 229354_at | 19.71 | 4.12E-06 | 1.587709852 | NM_013232| | PDCD6,programmed cell death 6 |
| 201150_s_at | 19.71 | 4.13E-06 | 1.88802377 | NM_000362| | TIMP3,tissue inhibitor of metalloproteinase 3 |
| 201012_at | 19.7 | 4.13E-06 | 1.315319641 | NM_000700| | ANXA1,annexin I |
| 236219_at | 19.69 | 4.14E-06 | 1.683435595 | NA |  |
| 225927_at | 19.69 | 4.14E-06 | 1.166345098 | NM_005921| | NA |
| 220443_s_at | 19.68 | 4.15E-06 | 1.563812248 | NM_012476| | VAX2,ventral anterior homeobox 2 |
| 215259_s_at | 19.68 | 4.15E-06 | 1.564313212 | NM_145296| | IGSF4C,immunoglobulin superfamily, member 4C |
| 201959_s_at | 19.68 | 4.15E-06 | 1.142098583 | NM_015057| | MYCBP2,MYC binding protein 2 |
| 1553167_a_at | 19.67 | 4.15E-06 | 1.314638518 | NM_016955| | SLA/LP,soluble liver antigen/liver pancreas antigen |
| 212936_at | 19.67 | 4.15E-06 | 1.174405463 | NM_032042| | DKFZP564D172,hypothetical protein DKFZp564D172 |
| 231863_at | 19.66 | 4.17E-06 | 1.710620058 | NM_019071| | ING3,inhibitor of growth family, member 3 isoform 1 |
| 212629_s_at | 19.66 | 4.17E-06 | 1.075398029 | NM_006256| | PKN2,protein kinase N2 |
| 203817_at | 19.64 | 4.19E-06 | 1.335236959 | NM_000857| | GUCY1B3,guanylate cyclase 1, soluble, beta 3 |
| 202695_s_at | 19.64 | 4.20E-06 | 1.72253295 | NM_004760| | STK17A,serine/threonine kinase 17a |
| 224517_at | 19.64 | 4.19E-06 | 1.170866016 | NA |  |
| 221844_x_at | 19.64 | 4.19E-06 | 1.11901037 | NA |  |
| 231793_s_at | 19.63 | 4.20E-06 | 1.721369659 | NM_001221| | CAMK2D,calcium/calmodulin-dependent protein kinase II |
| 208707_at | 19.63 | 4.20E-06 | 1.369277272 | NM_001969| | EIF5,eukaryotic translation initiation factor 5 |
| 201324_at | 19.63 | 4.20E-06 | 2.145737583 | NM_001423| | EMP1,epithelial membrane protein 1 |
| 1557545_s_at | 19.62 | 4.21E-06 | 1.647565566 | NM_152470| | C18orf23,chromosome 18 open reading frame 23 |
| 212256_at | 19.61 | 4.22E-06 | 1.189048978 | NM_017540| | GALNT10,GalNAc transferase 10 isoform b |
| 219076_s_at | 19.61 | 4.22E-06 | 1.379402553 | NM_018663| | PXMP2,peroxisomal membrane protein 2, 22kDa |
| 223839_s_at | 19.61 | 4.22E-06 | 1.161922349 | NA |  |
| 203795_s_at | 19.6 | 4.23E-06 | 1.195737228 | NM_001024808| | NA |
| 212022_s_at | 19.59 | 4.24E-06 | 1.164058475 | NM_002417| | MKI67,antigen identified by monoclonal antibody Ki-67 |
| 238919_at | 19.59 | 4.24E-06 | 2.752813392 | NA |  |
| 1555326_a_at | 19.59 | 4.25E-06 | 1.587458544 | NM_001005845| | ADAM9,a disintegrin and metalloproteinase domain 9 |
| 226419_s_at | 19.59 | 4.25E-06 | 1.109487339 | NA |  |
| 234005_x_at | 19.58 | 4.26E-06 | 1.154953114 | NM_015690| | STK36,serine/threonine kinase 36 (fused homolog, |
| 213500_at | 19.58 | 4.25E-06 | 1.3667993 | NA |  |
| 229710_at | 19.58 | 4.25E-06 | 1.186258206 | NA |  |
| 222869_s_at | 19.57 | 4.26E-06 | 1.422301748 | NM_018696| | ELAC1,elaC homolog 1 |
| 1568795_at | 19.57 | 4.27E-06 | 1.860281554 | NA |  |
| 221834_at | 19.56 | 4.27E-06 | 1.588951678 | NM_031490| | LONP,peroxisomal lon protease |
| 208502_s_at | 19.56 | 4.27E-06 | 1.787311015 | NM_002653| | PITX1,paired-like homeodomain transcription factor 1 |
| 209617_s_at | 19.56 | 4.27E-06 | 1.308487968 | NM_001332| | CTNND2,catenin (cadherin-associated protein), delta 2 |
| 208661_s_at | 19.56 | 4.28E-06 | 1.300158365 | NM_001001894| | TTC3,tetratricopeptide repeat domain 3 |
| 227250_at | 19.56 | 4.28E-06 | 1.346824473 | NM_001039570| | NA |
| 204519_s_at | 19.56 | 4.28E-06 | 1.354119514 | NM_015993| | TM4SF11,plasmolipin |
| 243579_at | 19.55 | 4.29E-06 | 1.272116736 | NM_138962| | MSI2,musashi 2 isoform a |
| 204824_at | 19.53 | 4.30E-06 | 1.169272916 | NM_004435| | ENDOG,endonuclease G precursor |
| 227746_at | 19.52 | 4.32E-06 | 1.433290954 | NM_001419| | ELAVL1,ELAV-like 1 |
| 214043_at | 19.52 | 4.32E-06 | 1.121846036 | NM_001040712| | NA |
| 238853_at | 19.52 | 4.32E-06 | 1.533763422 | NM_001024647| | NA |
| 212922_s_at | 19.51 | 4.32E-06 | 1.312906068 | NM_020197| | SMYD2,SET and MYND domain containing 2 |
| 204112_s_at | 19.51 | 4.32E-06 | 1.626312142 | NM_001024074| | NA |
| 219416_at | 19.51 | 4.32E-06 | 1.219336314 | NM_016240| | SCARA3,scavenger receptor class A, member 3 isoform 1 |
| 212552_at | 19.5 | 4.34E-06 | 1.240184378 | NM_002149| | HPCAL1,hippocalcin-like 1 |
| 238453_at | 19.5 | 4.34E-06 | 1.24632767 | NM_152429| | C10orf13,hypothetical protein MGC39320 |
| 204484_at | 19.5 | 4.34E-06 | 1.113897483 | NM_002646| | PIK3C2B,phosphoinositide-3-kinase, class 2, beta |
| 202032_s_at | 19.5 | 4.34E-06 | 1.155436026 | NM_006122| | MAN2A2,mannosidase, alpha, class 2A, member 2 |
| 242300_at | 19.5 | 4.34E-06 | 1.391778926 | NA |  |
| 239913_at | 19.48 | 4.35E-06 | 1.606063162 | NM_152679| | SLC10A4,solute carrier family 10 (sodium/bile acid |
| 223066_at | 19.48 | 4.35E-06 | 1.1370585 | NM_012437| | SNAPAP,SNAP-associated protein |
| 203542_s_at | 19.47 | 4.37E-06 | 1.415757097 | NM_001206| | KLF9,Kruppel-like factor 9 |
| 223641_at | 19.47 | 4.37E-06 | 1.305924897 | NA |  |
| 242989_at | 19.46 | 4.38E-06 | 1.761824438 | NA |  |
| 231894_at | 19.45 | 4.39E-06 | 1.277271245 | NA |  |
| 242981_at | 19.45 | 4.39E-06 | 1.550034959 | NA |  |
| 228314_at | 19.45 | 4.39E-06 | 1.272418989 | NA |  |
| 212355_at | 19.44 | 4.40E-06 | 1.164603279 | NM_015299| | KIAA0323,KIAA0323 |
| 223945_x_at | 19.43 | 4.41E-06 | 1.216286462 | NA |  |
| 228349_at | 19.43 | 4.41E-06 | 1.232345724 | NA |  |
| 227119_at | 19.43 | 4.41E-06 | 1.265613519 | NM_144571| | CNOT6L,CCR4-NOT transcription complex, subunit 6-like |
| 208405_s_at | 19.42 | 4.41E-06 | 1.0937812 | NM_006016| | CD164,CD164 antigen, sialomucin |
| 208073_x_at | 19.42 | 4.42E-06 | 1.291013025 | NM_001001894| | TTC3,tetratricopeptide repeat domain 3 |
| 220741_s_at | 19.41 | 4.42E-06 | 1.059233205 | NM_001034191| | NA |
| 202632_at | 19.41 | 4.42E-06 | 1.129855263 | NM_001383| | DPH2L1,diptheria toxin resistance protein required for |
| 1569303_s_at | 19.4 | 4.44E-06 | 1.240010325 | NM_003702| | RGS20,regulator of G-protein signalling 20 |
| 225327_at | 19.4 | 4.44E-06 | 1.25317232 | NM_019600| | FLJ10980,hypothetical protein FLJ10980 |
| 214789_x_at | 19.4 | 4.44E-06 | 1.096085616 | NM_032102| | SRP46,Splicing factor, arginine/serine-rich, 46kD |
| 208949_s_at | 19.4 | 4.44E-06 | 1.174323381 | NM_002306| | LGALS3,galectin-3 |
| 219639_x_at | 19.4 | 4.44E-06 | 1.080833809 | NM_020214| | PARP6,poly (ADP-ribose) polymerase family, member 6 |
| 202754_at | 19.4 | 4.44E-06 | 1.068924235 | NM_015361| | R3HDM,R3H domain (binds single-stranded nucleic acids) |
| 32625_at | 19.39 | 4.44E-06 | 1.397503148 | NM_000906| | NPR1,natriuretic peptide receptor A/guanylate cyclase |
| 1569788_at | 19.38 | 4.46E-06 | 1.878894694 | NM_003034| | ST8SIA1,ST8 alpha-N-acetyl-neuraminide |
| 219855_at | 19.38 | 4.46E-06 | 1.044179171 | NM_018159| | NUDT11,nudix (nucleoside diphosphate linked moiety |
| 227478_at | 19.37 | 4.47E-06 | 1.373097162 | NM_015559| | SETBP1,SET binding protein 1 |
| 223468_s_at | 19.36 | 4.49E-06 | 1.132328231 | NM_020211| | RGMA,RGM domain family, member A |
| 213259_s_at | 19.35 | 4.49E-06 | 1.412699117 | NM_015077| | SARM1,sterile alpha and TIR motif containing 1 |
| 202381_at | 19.34 | 4.50E-06 | 1.323457767 | NM_001005845| | ADAM9,a disintegrin and metalloproteinase domain 9 |
| 225619_at | 19.33 | 4.52E-06 | 1.263293715 | NM_001040153| | NA |
| 243931_at | 19.33 | 4.52E-06 | 1.504797152 | NA |  |
| 229448_at | 19.33 | 4.52E-06 | 1.18088937 | NM_021267| | LASS1,longevity assurance gene 1 isoform 1 |
| 225521_at | 19.32 | 4.53E-06 | 1.677741619 | NM_016238| | ANAPC7,anaphase-promoting complex subunit 7 |
| 214964_at | 19.31 | 4.54E-06 | 1.317390978 | NA |  |
| 203048_s_at | 19.31 | 4.54E-06 | 1.080905337 | NM_014639| | KIAA0372,KIAA0372 |
| 204012_s_at | 19.31 | 4.53E-06 | 1.11623162 | NM_014793| | LCMT2,leucine carboxyl methyltransferase 2 |
| 224730_at | 19.3 | 4.55E-06 | 1.11358522 | NM_005828| | HAN11,WD-repeat protein |
| 221750_at | 19.3 | 4.54E-06 | 1.196708555 | NM_001098272| | NA |
| 1552822_at | 19.3 | 4.55E-06 | 1.713583246 | NM_019022| | TXNDC10,thioredoxin domain containing 10 |
| 200046_at | 19.3 | 4.54E-06 | 1.048051729 | NM_001344| | DAD1,defender against cell death 1 |
| 212362_at | 19.29 | 4.56E-06 | 1.218949054 | NM_001681| | ATP2A2,ATPase, Ca++ transporting, cardiac muscle, slow |
| 228429_x_at | 19.29 | 4.56E-06 | 1.376266689 | NM_022342| | KIF9,kinesin family member 9 |
| 227259_at | 19.29 | 4.55E-06 | 1.578428971 | NM_001025079| | NA |
| 1556194_a_at | 19.29 | 4.56E-06 | 2.018327419 | NA |  |
| 241387_at | 19.28 | 4.56E-06 | 1.208074895 | NA |  |
| 212570_at | 19.28 | 4.56E-06 | 1.549379789 | NM_015036| | NA |
| 235497_at | 19.26 | 4.59E-06 | 1.496692342 | NA |  |
| 210768_x_at | 19.26 | 4.59E-06 | 1.103423188 | NM_019026| | LOC54499,putative membrane protein |
| 203156_at | 19.25 | 4.60E-06 | 1.13752943 | NM_016248| | AKAP11,A-kinase anchor protein 11 isoform 1 |
| 217604_at | 19.24 | 4.62E-06 | 1.348892839 | NA |  |
| 218952_at | 19.24 | 4.62E-06 | 1.359530455 | NM_013271| | PCSK1N,proprotein convertase subtilisin/kexin type 1 |
| 202294_at | 19.22 | 4.64E-06 | 1.295602108 | NM_005862| | STAG1,stromal antigen 1 |
| 205279_s_at | 19.22 | 4.64E-06 | 1.910748285 | NM_000824| | GLRB,glycine receptor, beta |
| 231899_at | 19.22 | 4.64E-06 | 1.325012976 | NM_033390| | NA |
| 226156_at | 19.21 | 4.65E-06 | 1.271517427 | NM_001626| | AKT2,v-akt murine thymoma viral oncogene homolog 2 |
| 212019_at | 19.2 | 4.66E-06 | 1.300887697 | NM_015659| | RSL1D1,ribosomal L1 domain containing 1 |
| 223158_s_at | 19.2 | 4.66E-06 | 1.279582131 | NM_014397| | NEK6,putative serine-threonine protein kinase |
| 222423_at | 19.19 | 4.67E-06 | 1.210469851 | NM_030571| | NDFIP1,Nedd4 family interacting protein 1 |
| 1564520_s_at | 19.19 | 4.68E-06 | 1.072140408 | NM_001039619| | NA |
| 227467_at | 19.19 | 4.67E-06 | 1.524379415 | NM_172037| | RDH10,retinol dehydrogenase 10 |
| 201587_s_at | 19.18 | 4.69E-06 | 1.238438937 | NM_001025242| | NA |
| 223095_at | 19.18 | 4.69E-06 | 1.218818051 | NA |  |
| 224516_s_at | 19.17 | 4.70E-06 | 1.078810276 | NM_016463| | CXXC5,CXXC finger 5 |
| 228790_at | 19.17 | 4.69E-06 | 1.377985647 | NM_147189| | MGC39325,hypothetical protein MGC39325 |
| 225661_at | 19.16 | 4.72E-06 | 1.511091414 | NM_000629| | IFNAR1,interferon-alpha receptor 1 precursor |
| 212736_at | 19.15 | 4.72E-06 | 1.184926283 | NM_033201| | C16orf45,chromosome 16 open reading frame 45 |
| 212021_s_at | 19.15 | 4.72E-06 | 1.245129318 | NM_002417| | MKI67,antigen identified by monoclonal antibody Ki-67 |
| 201857_at | 19.15 | 4.72E-06 | 1.064769236 | NM_016107| | ZFR,zinc finger RNA binding protein |
| 233289_at | 19.15 | 4.72E-06 | 1.711720154 | NA |  |
| 227138_at | 19.14 | 4.74E-06 | 1.385013499 | NM_006371| | CRTAP,cartilage associated protein precursor |
| 209268_at | 19.13 | 4.74E-06 | 1.132097033 | NM_007259| | VPS45A,vacuolar protein sorting 45A |
| 91920_at | 19.12 | 4.76E-06 | 1.212800357 | NM_021948| | BCAN,brevican isoform 1 |
| 224764_at | 19.12 | 4.76E-06 | 1.261685438 | NM_020824| | ARHGAP21,Rho GTPase activating protein 21 |
| 1556283_s_at | 19.12 | 4.75E-06 | 1.501777081 | NM_015633| | FGFR1OP2,FGFR1 oncogene partner 2 |
| 207672_at | 19.12 | 4.76E-06 | 1.98863273 | NA |  |
| 210006_at | 19.11 | 4.77E-06 | 1.321670261 | NM_015407| | DKFZP564O243,DKFZP564O243 protein |
| 207048_at | 19.11 | 4.76E-06 | 1.251904671 | NM_014229| | SLC6A11,solute carrier family 6 (neurotransmitter |
| 1552506_at | 19.11 | 4.77E-06 | 1.256090825 | NM_173689| | CRB2,crumbs homolog 2 |
| 204014_at | 19.1 | 4.78E-06 | 1.269121082 | NM_001394| | DUSP4,dual specificity phosphatase 4 isoform 1 |
| 240344_x_at | 19.1 | 4.79E-06 | 1.201926785 | NM_181705| | LOC90624,hypothetical protein LOC90624 |
| 234583_at | 19.09 | 4.80E-06 | 1.552302205 | NM_006614| | CHL1,cell adhesion molecule with homology to L1CAM |
| 206866_at | 19.09 | 4.80E-06 | 1.267720779 | NM_001794| | CDH4,cadherin 4, type 1 preproprotein |
| 233132_at | 19.08 | 4.80E-06 | 1.198473294 | NA |  |
| 205173_x_at | 19.07 | 4.82E-06 | 1.257907722 | NM_001779| | CD58,CD58 antigen, (lymphocyte function-associated |
| 218298_s_at | 19.07 | 4.82E-06 | 1.13223448 | NM_001102366| | NA |
| 224743_at | 19.06 | 4.83E-06 | 1.158762769 | NM_017813| | IMPA3,myo-inositol monophosphatase A3 |
| 241774_at | 19.06 | 4.83E-06 | 1.573754748 | NA |  |
| 218794_s_at | 19.05 | 4.85E-06 | 1.198688683 | NM_017853| | TXNL4B,thioredoxin-like 4B |
| 238587_at | 19.05 | 4.85E-06 | 1.63142893 | NM_032873| | STS-1,Cbl-interacting protein Sts-1 |
| 225120_at | 19.04 | 4.86E-06 | 1.148520334 | NM_033224| | PURB,purine-rich element binding protein B |
| 227193_at | 19.03 | 4.88E-06 | 1.463248138 | NA |  |
| 210638_s_at | 19.03 | 4.88E-06 | 1.249425837 | NM_012347| | FBXO9,F-box only protein 9 isoform 1 |
| 226942_at | 19.02 | 4.88E-06 | 1.197707789 | NM_016018| | PHF20L1,PHD finger protein 20-like 1 isoform 1 |
| 223220_s_at | 19.02 | 4.89E-06 | 1.328021395 | NM_031458| | PARP9,B aggressive lymphoma gene |
| 205005_s_at | 19.02 | 4.89E-06 | 1.138660509 | NM_004808| | NMT2,glycylpeptide N-tetradecanoyltransferase 2 |
| 220441_at | 19.02 | 4.89E-06 | 1.440640127 | NM_024902| | FLJ13236,hypothetical protein FLJ13236 |
| 238003_at | 19.01 | 4.89E-06 | 1.400272252 | NM_152722| | FLJ25530,hypothetical protein FLJ25530 |
| 202159_at | 19 | 4.92E-06 | 1.101997305 | NM_004461| | FARSLA,phenylalanine-tRNA synthetase-like protein |
| 230002_at | 18.99 | 4.93E-06 | 1.452026491 | NM_001048210| | NA |
| 215000_s_at | 18.99 | 4.92E-06 | 1.10601738 | NM_001042548| | NA |
| 211110_s_at | 18.98 | 4.94E-06 | 1.277156404 | NM_000044| | AR,androgen receptor isoform 1 |
| 1555091_at | 18.98 | 4.94E-06 | 1.541363106 | NM_014634| | PPM1F,protein phosphatase 1F |
| 212625_at | 18.98 | 4.94E-06 | 1.155690936 | NM_003765| | STX10,syntaxin 10 |
| 212667_at | 18.97 | 4.94E-06 | 1.209215107 | NM_003118| | SPARC,secreted protein, acidic, cysteine-rich |
| 231806_s_at | 18.97 | 4.94E-06 | 1.167876498 | NM_015690| | STK36,serine/threonine kinase 36 (fused homolog, |
| 226336_at | 18.97 | 4.94E-06 | 1.066021982 | NM_021130| | PPIA,peptidylprolyl isomerase A isoform 1 |
| 218124_at | 18.97 | 4.94E-06 | 1.225788911 | NM_017750| | RetSat,all-trans-13,14-dihydroretinol saturase |
| 225139_at | 18.96 | 4.95E-06 | 1.139474813 | NA |  |
| 216044_x_at | 18.96 | 4.96E-06 | 1.150000201 | NM_001006605| | LOC388650,hypothetical LOC388650 |
| 201628_s_at | 18.96 | 4.96E-06 | 1.072407412 | NM_006570| | RRAGA,Ras-related GTP binding A |
| 223183_at | 18.96 | 4.96E-06 | 1.71646807 | NM_001037553| | NA |
| 1552378_s_at | 18.94 | 4.97E-06 | 1.442180386 | NM_172037| | RDH10,retinol dehydrogenase 10 |
| 209725_at | 18.94 | 4.97E-06 | 1.094740201 | NM_014503| | DRIM,down-regulated in metastasis |
| 239741_at | 18.94 | 4.98E-06 | 1.799449577 | NA |  |
| 236640_at | 18.94 | 4.98E-06 | 1.549956903 | NA |  |
| 1562699_at | 18.93 | 4.99E-06 | 1.548320234 | NA |  |
| 1557137_at | 18.93 | 4.98E-06 | 1.239591053 | NM_198276| | TMEM17,transmembrane protein 17 |
| 225288_at | 18.92 | 5.00E-06 | 1.337457913 | NM_032888| | COL27A1,collagen, type XXVII, alpha 1 |
| 225187_at | 18.91 | 5.01E-06 | 1.068639408 | NM_021174| | KIAA1967,p30 DBC protein |
| 1555948_s_at | 18.91 | 5.01E-06 | 1.22599161 | NM_014612| | C9orf10,C9orf10 protein |
| 236594_at | 18.91 | 5.01E-06 | 1.084395749 | NM_004140| | LLGL1,lethal giant larvae homolog 1 |
| 227514_at | 18.88 | 5.06E-06 | 1.488762158 | NM_001034841| | NA |
| 203941_at | 18.88 | 5.06E-06 | 1.149293342 | NM_018250| | FLJ10871,hypothetical protein FLJ10871 |
| 211000_s_at | 18.88 | 5.06E-06 | 1.816848028 | NM_002184| | IL6ST,interleukin 6 signal transducer isoform 1 |
| 225308_s_at | 18.87 | 5.08E-06 | 1.400735697 | NM_033394| | TANC,TPR domain, ankyrin-repeat and |
| 226805_at | 18.86 | 5.09E-06 | 1.312849177 | NM_001080472| | NA |
| 202540_s_at | 18.85 | 5.11E-06 | 1.086326121 | NM_000859| | HMGCR,3-hydroxy-3-methylglutaryl-Coenzyme A reductase |
| 201620_at | 18.84 | 5.12E-06 | 1.121839648 | NM_003791| | MBTPS1,membrane-bound transcription factor site-1 |
| 242939_at | 18.81 | 5.16E-06 | 1.633933254 | NM_007111| | TFDP1,transcription factor Dp-1 |
| 202070_s_at | 18.81 | 5.17E-06 | 1.164893666 | NM_005530| | IDH3A,isocitrate dehydrogenase 3 (NAD+) alpha |
| 235688_s_at | 18.8 | 5.18E-06 | 1.28725187 | NM_004295| | TRAF4,TNF receptor-associated factor 4 isoform 1 |
| 212573_at | 18.79 | 5.19E-06 | 1.341875147 | NM_015036| | NA |
| 242208_at | 18.79 | 5.20E-06 | 1.760697341 | NA |  |
| 217825_s_at | 18.79 | 5.19E-06 | 1.357900941 | NM_016021| | UBE2J1,ubiquitin-conjugating enzyme E2, J1 |
| 226484_at | 18.78 | 5.20E-06 | 1.421315963 | NM_145166| | ZNF651,zinc finger protein 651 |
| 224865_at | 18.78 | 5.20E-06 | 1.249155649 | NM_032228| | MLSTD2,male sterility domain containing 2 |
| 234111_at | 18.77 | 5.21E-06 | 1.259786167 | NA |  |
| 210589_s_at | 18.77 | 5.22E-06 | 1.206130847 | NM_000157| | GBA,glucocerebrosidase precursor |
| 215287_at | 18.76 | 5.23E-06 | 1.866335067 | NA |  |
| 212987_at | 18.76 | 5.23E-06 | 1.220008697 | NM_012347| | FBXO9,F-box only protein 9 isoform 1 |
| 235543_at | 18.76 | 5.22E-06 | 1.351452583 | NA |  |
| 220607_x_at | 18.75 | 5.24E-06 | 1.251411395 | NM_198976| | TH1L,TH1-like protein |
| 233168_s_at | 18.75 | 5.23E-06 | 1.142321488 | NM_031454| | SELO,selenoprotein O |
| 44790_s_at | 18.73 | 5.27E-06 | 1.722634936 | NM_025113| | C13orf18,chromosome 13 open reading frame 18 |
| 235935_at | 18.71 | 5.29E-06 | 1.191874554 | NM_001012974| | NA |
| 213189_at | 18.71 | 5.29E-06 | 1.589121702 | NM_001042533| | NA |
| 226501_at | 18.71 | 5.29E-06 | 1.151247363 | NM_022098| | LOC63929,hypothetical protein LOC63929 |
| 219324_at | 18.7 | 5.31E-06 | 1.208866239 | NM_001039141| | NA |
| 208963_x_at | 18.7 | 5.31E-06 | 1.135355177 | NM_013402| | FADS1,fatty acid desaturase 1 |
| 226066_at | 18.67 | 5.34E-06 | 1.577204983 | NM_000248| | MITF,microphthalmia-associated transcription factor |
| 200875_s_at | 18.67 | 5.35E-06 | 1.063130315 | NM_006392| | NOL5A,nucleolar protein 5A |
| 200880_at | 18.67 | 5.34E-06 | 1.117259363 | NM_001539| | DNAJA1,DnaJ (Hsp40) homolog, subfamily A, member 1 |
| 209444_at | 18.63 | 5.43E-06 | 1.092255368 | NM_001100426| | NA |
| 200048_s_at | 18.63 | 5.43E-06 | 1.060849569 | NM_006694| | JTB,jumping translocation breakpoint |
| 226946_at | 18.61 | 5.46E-06 | 1.285325795 | NM_001085411| | NA |
| 218138_at | 18.61 | 5.45E-06 | 1.106163806 | NM_018848| | MKKS,McKusick-Kaufman syndrome protein |
| 231650_s_at | 18.6 | 5.47E-06 | 1.583429201 | NM_021115| | SEZ6L,seizure related 6 homolog (mouse)-like |
| 202541_at | 18.59 | 5.49E-06 | 1.148416469 | NM_004757| | SCYE1,small inducible cytokine subfamily E, member 1 |
| 209292_at | 18.59 | 5.48E-06 | 1.583259951 | NM_001546| | ID4,inhibitor of DNA binding 4, dominant negative |
| 228548_at | 18.56 | 5.54E-06 | 1.350727728 | NA |  |
| 218633_x_at | 18.56 | 5.53E-06 | 1.194562275 | NM_018394| | ABHD10,abhydrolase domain containing 10 |
| 203323_at | 18.55 | 5.55E-06 | 1.592916198 | NM_001233| | CAV2,caveolin 2 isoform a and b |
| 31799_at | 18.55 | 5.54E-06 | 1.425345269 | NA |  |
| 212866_at | 18.54 | 5.56E-06 | 1.20087133 | NA |  |
| 229000_at | 18.54 | 5.56E-06 | 1.123313651 | NM_021217| | ZNF77,zinc finger protein 77 |
| 215983_s_at | 18.54 | 5.56E-06 | 1.252666931 | NM_005671| | D8S2298E,reproduction 8 |
| 226143_at | 18.53 | 5.57E-06 | 1.29380799 | NM_030665| | RAI1,retinoic acid induced 1 |
| 223519_at | 18.53 | 5.56E-06 | 1.544209392 | NM_016653| | ZAK,sterile-alpha motif and leucine zipper |
| 205151_s_at | 18.52 | 5.59E-06 | 1.336451126 | NM_014817| | NA |
| 212456_at | 18.51 | 5.59E-06 | 1.170317996 | NM_015229| | KIAA0664,KIAA0664 protein |
| 221530_s_at | 18.51 | 5.60E-06 | 1.452421673 | NM_030762| | BHLHB3,basic helix-loop-helix domain containing, class |
| 204279_at | 18.51 | 5.60E-06 | 1.172858828 | NM_002800| | PSMB9,proteasome beta 9 subunit isoform 1 proprotein |
| 204962_s_at | 18.48 | 5.65E-06 | 1.078505708 | NM_001042426| | NA |
| 218785_s_at | 18.48 | 5.66E-06 | 1.256259955 | NM_022777| | RABL5,RAB, member RAS oncogene family-like 5 |
| 1564746_at | 18.48 | 5.66E-06 | 1.44427156 | NM_178833| | LOC133308,hypothetical protein BC009732 |
| 223104_at | 18.48 | 5.65E-06 | 1.119663582 | NM_032492| | JAGN1,jagunal homolog 1 |
| 232226_at | 18.48 | 5.65E-06 | 1.795719227 | NM_020929| | NGL-1,netrin-G1 ligand |
| 243278_at | 18.46 | 5.69E-06 | 2.034643829 | NM_014491| | FOXP2,forkhead box P2 isoform I |
| 222462_s_at | 18.46 | 5.69E-06 | 1.149409538 | NM_012104| | BACE1,beta-site APP-cleaving enzyme 1 isoform A |
| 238273_at | 18.46 | 5.69E-06 | 1.263157858 | NM_012450| | SLC13A4,solute carrier family 13 (sodium/sulfate |
| 220688_s_at | 18.46 | 5.69E-06 | 1.118108447 | NM_016183| | C1orf33,ribosomal protein P0-like protein |
| 227095_at | 18.44 | 5.72E-06 | 1.360286353 | NA |  |
| 239252_at | 18.44 | 5.72E-06 | 1.575688835 | NA |  |
| 228693_at | 18.43 | 5.74E-06 | 1.616606437 | NM_174908| | C3orf6,Ymer protein short isoform |
| 232473_at | 18.43 | 5.74E-06 | 1.721312849 | NM_003675| | PRPF18,PRP18 pre-mRNA processing factor 18 homolog |
| 229335_at | 18.42 | 5.74E-06 | 1.428230553 | NM_145296| | IGSF4C,immunoglobulin superfamily, member 4C |
| 203753_at | 18.42 | 5.75E-06 | 1.149536514 | NM_001083962| | NA |
| 228516_at | 18.41 | 5.77E-06 | 1.10744423 | NM_138477| | CDAN1,codanin 1 |
| 221946_at | 18.41 | 5.76E-06 | 1.266655106 | NM_001048265| | NA |
| 1558189_a_at | 18.4 | 5.78E-06 | 1.487532876 | NA |  |
| 235709_at | 18.4 | 5.78E-06 | 1.334038509 | NM_174942| | GAS2L3,growth arrest-specific 2 like 3 |
| 201149_s_at | 18.4 | 5.78E-06 | 1.412294842 | NM_000362| | TIMP3,tissue inhibitor of metalloproteinase 3 |
| 217624_at | 18.4 | 5.78E-06 | 1.505350471 | NM_014891| | PDAP1,PDGFA associated protein 1 |
| 225167_at | 18.39 | 5.80E-06 | 1.312559191 | NM_018027| | FRMD4A,FERM domain containing 4A |
| 205530_at | 18.38 | 5.81E-06 | 1.221029992 | NM_004453| | ETFDH,electron-transferring-flavoprotein |
| 238146_at | 18.36 | 5.85E-06 | 1.520544244 | NA |  |
| 220999_s_at | 18.36 | 5.85E-06 | 1.49469175 | NM_001037332| | NA |
| 210645_s_at | 18.36 | 5.85E-06 | 1.304709343 | NM_001001894| | TTC3,tetratricopeptide repeat domain 3 |
| 226968_at | 18.36 | 5.85E-06 | 1.078840692 | NM_015074| | KIF1B,kinesin family member 1B isoform b |
| 205510_s_at | 18.35 | 5.86E-06 | 1.240927655 | NA |  |
| 235989_at | 18.35 | 5.87E-06 | 1.303333436 | NA |  |
| 228645_at | 18.35 | 5.87E-06 | 1.374559386 | NA |  |
| 204376_at | 18.34 | 5.88E-06 | 1.390028606 | NM_014703| | VprBP,Vpr-binding protein |
| 221821_s_at | 18.33 | 5.89E-06 | 1.031381643 | NM_017822| | FLJ20436,hypothetical LOC54934 |
| 226031_at | 18.33 | 5.90E-06 | 1.102387342 | NM_017667| | NA |
| 213493_at | 18.33 | 5.90E-06 | 1.768954661 | NM_001080437| | NA |
| 229860_x_at | 18.33 | 5.90E-06 | 1.19651856 | NA |  |
| 204317_at | 18.32 | 5.92E-06 | 1.243799937 | NM_016426| | GTSE1,G-2 and S-phase expressed 1 |
| 213379_at | 18.32 | 5.92E-06 | 1.101385848 | NM_015697| | CL640,hypothetical protein CL640 |
| 209852_x_at | 18.31 | 5.92E-06 | 1.059617421 | NM_005789| | PSME3,proteasome activator subunit 3 isoform 1 |
| 32099_at | 18.31 | 5.92E-06 | 1.116917065 | NM_014649| | SAFB2,scaffold attachment factor B2 |
| 209086_x_at | 18.31 | 5.92E-06 | 1.237670207 | NM_006500| | MCAM,melanoma cell adhesion molecule |
| 207761_s_at | 18.31 | 5.92E-06 | 1.400512553 | NM_014033| | DKFZP586A0522,DKFZP586A0522 protein |
| 222551_s_at | 18.31 | 5.93E-06 | 1.154264546 | NM_023080| | FLJ20989,hypothetical protein FLJ20989 |
| 227558_at | 18.3 | 5.95E-06 | 1.228380864 | NM_003655| | CBX4,chromobox homolog 4 |
| 244659_at | 18.3 | 5.93E-06 | 1.344857298 | NA |  |
| 202084_s_at | 18.28 | 5.98E-06 | 1.108737605 | NM_001039573| | NA |
| 1554089_s_at | 18.28 | 5.97E-06 | 1.109797703 | NM_016038| | SBDS,Shwachman-Bodian-Diamond syndrome protein |
| 223618_at | 18.24 | 6.06E-06 | 1.731760942 | NM_020066| | FMN2,formin 2 |
| 226579_at | 18.24 | 6.05E-06 | 1.323055343 | NA |  |
| 214074_s_at | 18.24 | 6.06E-06 | 1.17016162 | NM_005231| | CTTN,cortactin isoform a |
| 219537_x_at | 18.23 | 6.06E-06 | 1.199835561 | NM_016941| | DLL3,delta-like 3 protein isoform 1 precursor |
| 230791_at | 18.23 | 6.06E-06 | 2.305358213 | NA |  |
| 221567_at | 18.23 | 6.06E-06 | 1.239668059 | NM_003946| | NOL3,nucleolar protein 3 |
| 214006_s_at | 18.23 | 6.06E-06 | 1.14348221 | NM_000821| | GGCX,gamma-glutamyl carboxylase |
| 201648_at | 18.21 | 6.10E-06 | 1.114664678 | NM_002227| | JAK1,janus kinase 1 |
| 228273_at | 18.2 | 6.12E-06 | 1.12479444 | NA |  |
| 211066_x_at | 18.2 | 6.12E-06 | 1.210103385 | NM_002588| | PCDHGC3,protocadherin gamma subfamily C, 3 isoform 1 |
| 218548_x_at | 18.2 | 6.13E-06 | 1.238425724 | NM_015926| | TEX264,testis expressed sequence 264 |
| 1559603_at | 18.18 | 6.16E-06 | 1.621338038 | NA |  |
| 232899_at | 18.18 | 6.16E-06 | 1.405793297 | NM_203302| | MGC70863,similar to RPL23AP7 protein |
| 214661_s_at | 18.18 | 6.16E-06 | 1.127313119 | NM_003703| | C4orf9,gene near HD on 4p16.3 with homology to |
| 212522_at | 18.16 | 6.19E-06 | 1.3838595 | NM_002605| | PDE8A,phosphodiesterase 8A isoform 1 |
| 218380_at | 18.16 | 6.20E-06 | 1.158425459 | NM_001033053| | NA |
| 209511_at | 18.15 | 6.20E-06 | 1.113742401 | NM_021974| | POLR2F,DNA directed RNA polymerase II polypeptide F |
| 217933_s_at | 18.14 | 6.22E-06 | 1.120365155 | NM_015907| | LAP3,leucine aminopeptidase |
| 222233_s_at | 18.13 | 6.24E-06 | 1.263592144 | NM_001033855| | NA |
| 201700_at | 18.13 | 6.25E-06 | 1.12594076 | NM_001760| | CCND3,cyclin D3 |
| 203131_at | 18.12 | 6.26E-06 | 1.427364769 | NM_006206| | PDGFRA,platelet-derived growth factor receptor alpha |
| 1552287_s_at | 18.11 | 6.28E-06 | 1.240708444 | NA |  |
| 213030_s_at | 18.11 | 6.28E-06 | 1.299338797 | NM_025179| | PLXNA2,plexin A2 |
| 222760_at | 18.1 | 6.30E-06 | 1.379028479 | NM_025069| | FLJ14299,hypothetical protein FLJ14299 |
| 227335_at | 18.1 | 6.30E-06 | 1.463441654 | NM_022105| | DATF1,death associated transcription factor 1 isoform |
| 223012_at | 18.1 | 6.30E-06 | 1.163367511 | NM_025241| | UBXD1,UBX domain containing 1 |
| 52005_at | 18.09 | 6.31E-06 | 1.079188511 | NM_021241| | NA |
| 227696_at | 18.09 | 6.31E-06 | 1.141670944 | NM_058219| | EXOSC6,homolog of yeast mRNA transport regulator 3 |
| 219281_at | 18.09 | 6.31E-06 | 1.222888536 | NM_012331| | MSRA,methionine sulfoxide reductase A |
| 227210_at | 18.09 | 6.31E-06 | 1.55975929 | NM_001029880| | NA |
| 229454_at | 18.08 | 6.34E-06 | 1.33782043 | NM_001077440| | NA |
| 32209_at | 18.08 | 6.33E-06 | 1.18069076 | NM_001098784| | NA |
| 1555952_at | 18.07 | 6.34E-06 | 1.225326103 | NM_194255| | SLC19A1,solute carrier family 19 member 1 isoform a |
| 225662_at | 18.07 | 6.35E-06 | 1.377986697 | NM_016653| | ZAK,sterile-alpha motif and leucine zipper |
| 227415_at | 18.07 | 6.35E-06 | 1.170627291 | NA |  |
| 209890_at | 18.05 | 6.40E-06 | 1.139233336 | NM_005723| | TM4SF9,transmembrane 4 superfamily member 9 |
| 226120_at | 18.05 | 6.38E-06 | 1.144750066 | NM_144596| | TTC8,tetratricopeptide repeat domain 8 isoform A |
| 221214_s_at | 18.04 | 6.41E-06 | 1.201476022 | NM_015537| | NELF,nasal embryonic LHRH factor |
| 201477_s_at | 18.04 | 6.40E-06 | 1.079037194 | NM_001033| | RRM1,ribonucleoside-diphosphate reductase M1 chain |
| 215489_x_at | 18.04 | 6.41E-06 | 1.188917543 | NM_004838| | HOMER3,Homer, neuronal immediate early gene, 3 |
| 1554841_at | 18.04 | 6.41E-06 | 1.227617757 | NM_001004346| | MTHFD2L,methylenetetrahydrofolate dehydrogenase (NADP+ |
| 200006_at | 18.04 | 6.41E-06 | 1.049466959 | NM_001123377| | NA |
| 227557_at | 18.04 | 6.41E-06 | 1.219796573 | NM_153334| | SCARF2,scavenger receptor class F, member 2 isoform 1 |
| 211474_s_at | 18.04 | 6.40E-06 | 1.098703648 | NM_004568| | SERPINB6,serine (or cysteine) proteinase inhibitor, clade |
| 218708_at | 18.04 | 6.40E-06 | 1.147885576 | NM_013248| | NXT1,NTF2-like export factor 1 |
| 240339_at | 18.03 | 6.41E-06 | 1.281773898 | NA |  |
| 227935_s_at | 18.03 | 6.42E-06 | 1.264220036 | NM_032373| | PCGF5,polycomb group ring finger 5 |
| 209745_at | 18.02 | 6.43E-06 | 1.118609859 | NM_016138| | COQ7,COQ7 protein |
| 208563_x_at | 18.01 | 6.47E-06 | 1.414631924 | NM_006236| | POU3F3,POU domain, class 3, transcription factor 3 |
| 205303_at | 18.01 | 6.47E-06 | 1.585414602 | NM_004982| | KCNJ8,potassium inwardly-rectifying channel J8 |
| 223852_s_at | 17.99 | 6.49E-06 | 1.348078142 | NM_032017| | MGC4796,SINK-homologous serine/threonine kinase |
| 213145_at | 17.99 | 6.50E-06 | 1.225162062 | NM_152441| | FBXL14,F-box and leucine-rich repeat protein 14 |
| 202741_at | 17.98 | 6.51E-06 | 1.064435707 | NM_002731| | PRKACB,cAMP-dependent protein kinase catalytic subunit |
| 209694_at | 17.98 | 6.52E-06 | 1.090606493 | NM_000317| | PTS,6-pyruvoyltetrahydropterin synthase |
| 1553978_at | 17.98 | 6.52E-06 | 1.1656648 | NM_005919| | MEF2B,MADS box transcription enhancer factor 2, |
| 208964_s_at | 17.97 | 6.52E-06 | 1.139330378 | NM_013402| | FADS1,fatty acid desaturase 1 |
| 202094_at | 17.97 | 6.53E-06 | 1.209794751 | NM_001012270| | BIRC5,baculoviral IAP repeat-containing protein 5 |
| 1554789_a_at | 17.97 | 6.52E-06 | 1.653723808 | NM_001029851| | NA |
| 214053_at | 17.97 | 6.52E-06 | 1.819289385 | NM_001042599| | NA |
| 203089_s_at | 17.97 | 6.52E-06 | 1.104269526 | NM_013247| | PRSS25,protease, serine, 25 isoform 1 preproprotein |
| 204115_at | 17.96 | 6.55E-06 | 1.368589415 | NM_004126| | GNG11,guanine nucleotide binding protein (G protein), |
| 218244_at | 17.95 | 6.57E-06 | 1.138481016 | NM_017948| | NOL8,nucleolar protein 8 |
| 203225_s_at | 17.95 | 6.56E-06 | 1.24182469 | NM_018339| | RFK,riboflavin kinase |
| 220500_s_at | 17.95 | 6.56E-06 | 1.350301788 | NM_001003789| | RABL2B,RAB, member of RAS oncogene family-like 2B |
| 221264_s_at | 17.95 | 6.56E-06 | 1.18887751 | NM_007375| | TARDBP,TAR DNA binding protein |
| 208756_at | 17.95 | 6.56E-06 | 1.049427853 | NM_003757| | EIF3S2,eukaryotic translation initiation factor 3, |
| 204813_at | 17.94 | 6.57E-06 | 1.177396894 | NM_002753| | MAPK10,mitogen-activated protein kinase 10 isoform 1 |
| 201778_s_at | 17.94 | 6.57E-06 | 1.046057086 | NM_014774| | KIAA0494,KIAA0494 gene product |
| 206465_at | 17.94 | 6.57E-06 | 1.745504767 | NM_015162| | BG1,lipidosin |
| 241721_at | 17.94 | 6.57E-06 | 1.209128349 | NA |  |
| 226611_s_at | 17.93 | 6.59E-06 | 1.111081661 | NM_181716| | PRR6,proline rich 6 |
| 243718_at | 17.93 | 6.59E-06 | 1.681816846 | NA |  |
| 213362_at | 17.93 | 6.59E-06 | 1.176198502 | NM_001040712| | NA |
| 235036_at | 17.92 | 6.60E-06 | 1.568966827 | NM_153713| | LIX1L,Lix1 homolog (mouse) like |
| 224642_at | 17.92 | 6.60E-06 | 1.313449077 | NM_001011537| | FYTTD1,forty-two-three domain containing 1 isoform 2 |
| 235428_at | 17.92 | 6.60E-06 | 1.398887155 | NA |  |
| 226347_at | 17.91 | 6.62E-06 | 1.169741913 | NA |  |
| 217106_x_at | 17.91 | 6.62E-06 | 1.082233468 | NM_014473| | HSA9761,putative dimethyladenosine transferase |
| 208967_s_at | 17.9 | 6.64E-06 | 1.079424643 | NM_001625| | AK2,adenylate kinase 2 isoform a |
| 1557129_a_at | 17.9 | 6.65E-06 | 1.198309298 | NM_198947| | CANP,cancer-associated nucleoprotein |
| 223020_at | 17.89 | 6.65E-06 | 1.116595738 | NM_030782| | CRR9,cisplatin resistance related protein CRR9p |
| 232478_at | 17.89 | 6.65E-06 | 1.309151806 | NA |  |
| 221675_s_at | 17.88 | 6.67E-06 | 1.103399894 | NM_020244| | CHPT1,choline phosphotransferase 1 |
| 227497_at | 17.88 | 6.67E-06 | 1.614620321 | NA |  |
| 227272_at | 17.88 | 6.68E-06 | 1.548751277 | NM_207380| | FLJ43339,FLJ43339 protein |
| 228486_at | 17.87 | 6.69E-06 | 1.534128826 | NM_080546| | CDW92,CDW92 antigen |
| 242051_at | 17.87 | 6.69E-06 | 1.948490964 | NA |  |
| 228558_at | 17.86 | 6.70E-06 | 1.246111568 | NM_173608| | C14orf80,chromosome 14 open reading frame 80 |
| 230652_at | 17.86 | 6.72E-06 | 1.379974932 | NM_001654| | ARAF,v-raf murine sarcoma 3611 viral oncogene |
| 229163_at | 17.85 | 6.74E-06 | 1.623943229 | NM_018584| | CaMKIINalpha,calcium/calmodulin-dependent protein kinase II |
| 209716_at | 17.84 | 6.75E-06 | 1.153447693 | NM_000757| | CSF1,colony stimulating factor 1 isoform a precursor |
| 213689_x_at | 17.84 | 6.75E-06 | 1.118341267 | NM_001006605| | LOC388650,hypothetical LOC388650 |
| 225356_at | 17.84 | 6.75E-06 | 1.147029865 | NA |  |
| 209846_s_at | 17.84 | 6.75E-06 | 1.244629378 | NM_007047| | BTN3A2,butyrophilin, subfamily 3, member A2 |
| 204377_s_at | 17.83 | 6.76E-06 | 1.188338525 | NM_014703| | VprBP,Vpr-binding protein |
| 1554547_at | 17.82 | 6.78E-06 | 2.118280355 | NM_001001971| | FAM13C1,family with sequence similarity 13, member C1 |
| 209168_at | 17.81 | 6.81E-06 | 1.175713974 | NM_001001994| | GPM6B,glycoprotein M6B isoform 4 |
| 223013_at | 17.79 | 6.86E-06 | 1.04116951 | NM_024665| | TBL1XR1,nuclear receptor co-repressor/HDAC3 complex |
| 219746_at | 17.77 | 6.89E-06 | 1.306492305 | NM_012074| | DPF3,cer-d4 (mouse) homolog |
| 221566_s_at | 17.77 | 6.89E-06 | 1.288361542 | NM_003946| | NOL3,nucleolar protein 3 |
| 222554_s_at | 17.76 | 6.93E-06 | 1.107671613 | NM_022917| | NOL6,nucleolar RNA-associated protein alpha isoform |
| 206669_at | 17.76 | 6.92E-06 | 1.393502797 | NM_000817| | GAD1,glutamate decarboxylase 1 isoform GAD67 |
| 203257_s_at | 17.75 | 6.95E-06 | 1.092607067 | NM_001003676| | MGC4707,MGC4707 protein isoform 1 |
| 202119_s_at | 17.75 | 6.95E-06 | 1.077299267 | NM_003909| | CPNE3,copine III |
| 209339_at | 17.74 | 6.96E-06 | 1.155298903 | NM_005067| | SIAH2,seven in absentia homolog 2 |
| 210793_s_at | 17.74 | 6.96E-06 | 1.409511788 | NM_005387| | NUP98,nucleoporin 98kD isoform 3 |
| 1552330_at | 17.74 | 6.96E-06 | 1.298935364 | NM_145039| | MGC16385,hypothetical protein MGC16385 |
| 206993_at | 17.73 | 6.99E-06 | 1.275035187 | NM_001003803| | ATP5S,ATP synthase, H+ transporting, mitochondrial F0 |
| 227101_at | 17.72 | 7.00E-06 | 1.442508073 | NM_176814| | LOC168850,hypothetical protein LOC168850 |
| 212854_x_at | 17.71 | 7.03E-06 | 1.13836628 | NM_001039703| | NA |
| 223398_at | 17.71 | 7.03E-06 | 1.176765155 | NM_032310| | C9orf89,chromosome 9 open reading frame 89 |
| 242473_at | 17.7 | 7.06E-06 | 1.384130426 | NM_004295| | TRAF4,TNF receptor-associated factor 4 isoform 1 |
| 203319_s_at | 17.7 | 7.06E-06 | 1.154454534 | NM_021964| | ZNF148,zinc finger protein 148 (pHZ-52) |
| 226190_at | 17.69 | 7.07E-06 | 1.173998766 | NA |  |
| 219634_at | 17.69 | 7.08E-06 | 1.193979615 | NM_018413| | CHST11,carbohydrate (chondroitin 4) sulfotransferase |
| 227081_at | 17.68 | 7.10E-06 | 1.19485925 | NM_003462| | DNALI1,axonemal dynein light chain |
| 218891_at | 17.67 | 7.11E-06 | 1.138801465 | NM_024541| | C10orf76,chromosome 10 open reading frame 76 |
| 210927_x_at | 17.67 | 7.11E-06 | 1.066648742 | NM_006694| | JTB,jumping translocation breakpoint |
| 228791_at | 17.67 | 7.11E-06 | 1.285437394 | NA |  |
| 242389_at | 17.67 | 7.11E-06 | 1.333954244 | NA |  |
| 210612_s_at | 17.66 | 7.13E-06 | 1.454365578 | NM_003898| | SYNJ2,synaptojanin 2 |
| 212277_at | 17.64 | 7.16E-06 | 1.12635834 | NM_004687| | MTMR4,myotubularin related protein 4 |
| 205412_at | 17.64 | 7.16E-06 | 1.09732861 | NM_000019| | ACAT1,acetyl-Coenzyme A acetyltransferase 1 precursor |
| 241434_at | 17.64 | 7.16E-06 | 1.258073576 | NA |  |
| 223176_at | 17.64 | 7.17E-06 | 1.262475082 | NM_173562| | C6orf69,hypothetical protein MGC14254 |
| 219569_s_at | 17.63 | 7.19E-06 | 1.246393197 | NM_001097599| | NA |
| 227037_at | 17.63 | 7.17E-06 | 1.412401386 | NM_178836| | LOC201164,similar to CG12314 gene product |
| 1558796_a_at | 17.63 | 7.18E-06 | 1.578414247 | NA |  |
| 239581_at | 17.63 | 7.18E-06 | 1.207495636 | NA |  |
| 230069_at | 17.63 | 7.18E-06 | 1.163779117 | NM_022754| | SFXN1,sideroflexin 1 |
| 226192_at | 17.6 | 7.25E-06 | 1.22952845 | NA |  |
| 219709_x_at | 17.6 | 7.25E-06 | 1.152367759 | NM_023933| | MGC2494,hypothetical protein MGC2494 |
| 201764_at | 17.6 | 7.25E-06 | 1.396811538 | NM_024056| | MGC5576,hypothetical protein MGC5576 |
| 236264_at | 17.59 | 7.26E-06 | 2.004884967 | NM_015236| | LPHN3,latrophilin 3 precursor |
| 226032_at | 17.59 | 7.27E-06 | 1.105976623 | NM_032982| | CASP2,caspase 2 isoform 1 preproprotein |
| 224772_at | 17.59 | 7.27E-06 | 1.081603918 | NM_020443| | NAV1,neuron navigator 1 |
| 210434_x_at | 17.59 | 7.26E-06 | 1.066701503 | NM_006694| | JTB,jumping translocation breakpoint |
| 225712_at | 17.57 | 7.31E-06 | 1.064236104 | NM_015465| | GEMIN5,gemin 5 |
| 206531_at | 17.57 | 7.31E-06 | 1.117772336 | NM_004647| | DPF1,Neuro-d4 (rat) homolog |
| 204337_at | 17.56 | 7.33E-06 | 1.78397525 | NM_001102445| | NA |
| 203509_at | 17.56 | 7.33E-06 | 1.077627791 | NM_003105| | SORL1,sortilin-related receptor containing LDLR class |
| 203687_at | 17.56 | 7.33E-06 | 1.235295055 | NM_002996| | CX3CL1,chemokine (C-X3-C motif) ligand 1 |
| 207616_s_at | 17.55 | 7.34E-06 | 1.103697949 | NM_004180| | TANK,TRAF interacting protein TANK isoform a |
| 243591_at | 17.55 | 7.34E-06 | 1.341090565 | NA |  |
| 225794_s_at | 17.53 | 7.39E-06 | 1.095763889 | NM_033318| | LOC91689,hypothetical protein supported by AL449243 |
| 212913_at | 17.53 | 7.39E-06 | 1.31580592 | NM_001039651| | NA |
| 213033_s_at | 17.52 | 7.42E-06 | 1.927909575 | NM_005596| | NFIB,nuclear factor I/B |
| 239273_s_at | 17.52 | 7.41E-06 | 1.64729973 | NM_001032278| | NA |
| 201412_at | 17.52 | 7.42E-06 | 1.173646183 | NM_014045| | LRP10,low density lipoprotein receptor-related protein |
| 233575_s_at | 17.51 | 7.42E-06 | 1.494397886 | NM_007005| | TLE4,transducin-like enhancer protein 4 |
| 209325_s_at | 17.51 | 7.42E-06 | 1.288192129 | NM_002928| | RGS16,regulator of G-protein signalling 16 |
| 227771_at | 17.5 | 7.45E-06 | 1.426613255 | NM_002310| | LIFR, |
| 205543_at | 17.5 | 7.47E-06 | 1.383382242 | NM_014278| | HSPA4L,heat shock 70kDa protein 4-like |
| 225652_at | 17.5 | 7.45E-06 | 1.133407631 | NA |  |
| 203596_s_at | 17.49 | 7.47E-06 | 1.443275094 | NM_012420| | IFIT5,interferon-induced protein with |
| 202600_s_at | 17.49 | 7.48E-06 | 1.542945488 | NM_003489| | NRIP1,receptor interacting protein 140 |
| 202172_at | 17.49 | 7.47E-06 | 1.160556583 | NM_007146| | ZNF161,zinc finger protein 161 |
| 230494_at | 17.48 | 7.50E-06 | 1.415544164 | NM_005415| | SLC20A1,solute carrier family 20 (phosphate |
| 211976_at | 17.48 | 7.51E-06 | 1.149101136 | NA |  |
| 202428_x_at | 17.47 | 7.53E-06 | 1.111720382 | NM_001079862| | NA |
| 243432_at | 17.46 | 7.54E-06 | 1.25397573 | NA |  |
| 227499_at | 17.46 | 7.54E-06 | 1.286943649 | NA |  |
| 201084_s_at | 17.45 | 7.57E-06 | 1.111275683 | NM_001077440| | NA |
| 233648_at | 17.45 | 7.57E-06 | 1.322843908 | NA |  |
| 210978_s_at | 17.45 | 7.56E-06 | 1.186864774 | NM_003564| | TAGLN2,transgelin 2 |
| 201489_at | 17.45 | 7.57E-06 | 1.142740388 | NM_005729| | PPIF,peptidylprolyl isomerase F precursor |
| 219646_at | 17.45 | 7.57E-06 | 1.20393636 | NM_017702| | FLJ20186,differentially expressed in FDCP 8 isoform 2 |
| 211075_s_at | 17.44 | 7.57E-06 | 1.207910604 | NM_001025079| | NA |
| 240111_at | 17.43 | 7.60E-06 | 1.368951692 | NM_014899| | RHOBTB3,rho-related BTB domain containing 3 |
| 227926_s_at | 17.43 | 7.60E-06 | 1.310817475 | NM_001037501| | NA |
| 204127_at | 17.43 | 7.60E-06 | 1.054209901 | NM_002915| | RFC3,replication factor C 3 isoform 1 |
| 206238_s_at | 17.43 | 7.60E-06 | 1.652770505 | NM_005748| | YAF2,YY1 associated factor 2 isoform a |
| 1557353_at | 17.42 | 7.62E-06 | 1.471506838 | NA |  |
| 223273_at | 17.42 | 7.62E-06 | 1.264991685 | NM_032490| | C14orf142,chromosome 14 open reading frame 142 |
| 210157_at | 17.41 | 7.63E-06 | 1.090442632 | NM_003796| | C19orf2,RPB5-mediating protein isoform a |
| 205875_s_at | 17.4 | 7.66E-06 | 1.367584501 | NM_016381| | TREX1,three prime repair exonuclease 1 isoform a |
| 227585_at | 17.4 | 7.67E-06 | 1.105399257 | NA |  |
| 235593_at | 17.4 | 7.66E-06 | 1.34814162 | NM_014795| | ZFHX1B,zinc finger homeobox 1b |
| 221507_at | 17.4 | 7.67E-06 | 1.079966803 | NM_013433| | TNPO2,transportin 2 (importin 3, karyopherin beta 2b) |
| 217986_s_at | 17.4 | 7.67E-06 | 1.401906887 | NM_013448| | BAZ1A,bromodomain adjacent to zinc finger domain, 1A |
| 218980_at | 17.4 | 7.67E-06 | 1.116952847 | NM_025135| | FHOD3,formin homology 2 domain containing 3 |
| 224469_s_at | 17.39 | 7.67E-06 | 1.172802115 | NM_001031714| | NA |
| 208958_at | 17.39 | 7.68E-06 | 1.581761363 | NM_015051| | TXNDC4,thioredoxin domain containing 4 (endoplasmic |
| 221972_s_at | 17.39 | 7.67E-06 | 1.142754386 | NM_016176| | Cab45,calcium binding protein Cab45 precursor |
| 212296_at | 17.39 | 7.67E-06 | 1.054374468 | NM_005805| | PSMD14,26S proteasome-associated pad1 homolog |
| 236029_at | 17.38 | 7.69E-06 | 1.390518865 | NM_001008781| | NA |
| 212733_at | 17.38 | 7.69E-06 | 1.168844408 | NM_014687| | NA |
| 226990_at | 17.38 | 7.69E-06 | 1.061689634 | NM_005898| | M11S1,membrane component, chromosome 11, surface |
| 215310_at | 17.38 | 7.69E-06 | 1.532202737 | NM_000038| | APC,adenomatosis polyposis coli |
| 204992_s_at | 17.37 | 7.71E-06 | 1.024992413 | NM_002628| | PFN2,profilin 2 isoform b |
| 227954_at | 17.37 | 7.71E-06 | 1.379273322 | NM_001034841| | NA |
| 240121_x_at | 17.36 | 7.73E-06 | 1.284811519 | NA |  |
| 239272_at | 17.36 | 7.73E-06 | 1.90400229 | NM_001032278| | NA |
| 236442_at | 17.36 | 7.72E-06 | 1.859364521 | NM_012074| | DPF3,cer-d4 (mouse) homolog |
| 225340_s_at | 17.36 | 7.71E-06 | 1.047891704 | NM_005898| | M11S1,membrane component, chromosome 11, surface |
| 202093_s_at | 17.34 | 7.76E-06 | 1.14173955 | NM_019088| | PD2,hypothetical protein F23149_1 |
| 1562062_at | 17.34 | 7.76E-06 | 1.10876287 | NM_001037501| | NA |
| 238890_at | 17.34 | 7.76E-06 | 1.470310035 | NM_003720| | DSCR2,Down syndrome critical region protein 2 isoform |
| 216247_at | 17.34 | 7.76E-06 | 1.555158653 | NM_001023| | RPS20,ribosomal protein S20 |
| 221773_at | 17.34 | 7.76E-06 | 1.281609442 | NM_005230| | ELK3,ELK3 protein |
| 201346_at | 17.33 | 7.78E-06 | 1.103335683 | NM_024551| | ADIPOR2,adiponectin receptor 2 |
| 236322_at | 17.32 | 7.81E-06 | 1.66910296 | NA |  |
| 229674_at | 17.31 | 7.83E-06 | 1.247957487 | NM_019605| | SERTAD4,SERTA domain containing 4 |
| 230787_at | 17.31 | 7.84E-06 | 1.249712834 | NA |  |
| 228280_at | 17.3 | 7.86E-06 | 1.472491385 | NM_080660| | MGC14289,hypothetical protein MGC14289 |
| 229193_at | 17.3 | 7.85E-06 | 1.547310722 | NA |  |
| 215942_s_at | 17.3 | 7.86E-06 | 1.175483714 | NM_016426| | GTSE1,G-2 and S-phase expressed 1 |
| 211597_s_at | 17.3 | 7.85E-06 | 1.785928798 | NM_032495| | HOP,homeodomain-only protein |
| 224968_at | 17.29 | 7.87E-06 | 1.165942065 | NM_080667| | MGC15407,hypothetical protein MGC15407 |
| 235131_at | 17.29 | 7.87E-06 | 1.384612554 | NM_020663| | RHOJ,TC10-like Rho GTPase |
| 213763_at | 17.28 | 7.90E-06 | 1.168498195 | NM_001113239| | NA |
| 238722_x_at | 17.28 | 7.90E-06 | 1.297120473 | NM_001122838| | NA |
| 208663_s_at | 17.28 | 7.91E-06 | 1.288351675 | NM_001001894| | TTC3,tetratricopeptide repeat domain 3 |
| 221922_at | 17.27 | 7.91E-06 | 1.301283171 | NM_013296| | GPSM2,G-protein signalling modulator 2 (AGS3-like, C. |
| 36552_at | 17.26 | 7.94E-06 | 1.083194515 | NM_015531| | DKFZP586P0123,DKFZP586P0123 protein |
| 216048_s_at | 17.26 | 7.94E-06 | 1.545409782 | NM_014899| | RHOBTB3,rho-related BTB domain containing 3 |
| 217943_s_at | 17.26 | 7.94E-06 | 1.159567342 | NM_018067| | FLJ10350,hypothetical protein FLJ10350 |
| 225097_at | 17.26 | 7.94E-06 | 1.140725747 | NM_001113239| | NA |
| 228698_at | 17.26 | 7.94E-06 | 1.535573906 | NM_031439| | SOX7,SRY-box 7 |
| 206039_at | 17.26 | 7.94E-06 | 1.261800667 | NM_004794| | RAB33A,Ras-related protein Rab-33A |
| 208828_at | 17.24 | 7.97E-06 | 1.13981464 | NM_017443| | POLE3,DNA polymerase epsilon subunit 3 |
| 204649_at | 17.24 | 7.98E-06 | 1.07135336 | NM_001100620| | NA |
| 222463_s_at | 17.23 | 8.00E-06 | 1.160845133 | NM_012104| | BACE1,beta-site APP-cleaving enzyme 1 isoform A |
| 232064_at | 17.23 | 8.00E-06 | 1.478692004 | NA |  |
| 230413_s_at | 17.23 | 8.01E-06 | 1.235884714 | NA |  |
| 201409_s_at | 17.22 | 8.03E-06 | 1.136764344 | NM_002709| | PPP1CB,protein phosphatase 1, catalytic subunit, beta |
| 225441_x_at | 17.21 | 8.06E-06 | 1.193592847 | NM_032356| | MGC14151,hypothetical protein MGC14151 |
| 205379_at | 17.19 | 8.10E-06 | 1.288348959 | NM_001236| | CBR3,carbonyl reductase 3 |
| 224280_s_at | 17.19 | 8.11E-06 | 1.067205055 | NM_001099625| | NA |
| 203685_at | 17.19 | 8.11E-06 | 1.537266973 | NM_000633| | BCL2,B-cell lymphoma protein 2 alpha isoform |
| 202440_s_at | 17.18 | 8.14E-06 | 1.188074836 | NM_005418| | ST5,suppression of tumorigenicity 5 isoform 1 |
| 209185_s_at | 17.18 | 8.13E-06 | 1.060444137 | NM_003749| | IRS2,insulin receptor substrate 2 |
| 228148_at | 17.18 | 8.13E-06 | 1.290080173 | NM_173548| | ZNF584,zinc finger protein 584 |
| 230427_s_at | 17.18 | 8.12E-06 | 1.156863992 | NM_001015048| | NA |
| 217404_s_at | 17.17 | 8.17E-06 | 1.638281533 | NM_001844| | COL2A1,alpha 1 type II collagen isoform 1 |
| 231877_at | 17.16 | 8.19E-06 | 1.327997177 | NM_152292| | RG9MTD2,RNA (guanine-9-) methyltransferase domain |
| 225255_at | 17.16 | 8.17E-06 | 1.224176201 | NM_016622| | MRPL35,mitochondrial ribosomal protein L35 isoform a |
| 218587_s_at | 17.16 | 8.18E-06 | 1.146432068 | NM_020231| | MDS010,x 010 protein |
| 222047_s_at | 17.15 | 8.23E-06 | 1.036108273 | NM_015908| | ARS2,arsenate resistance protein ARS2 isoform a |
| 235949_at | 17.14 | 8.25E-06 | 1.271193194 | NA |  |
| 228932_at | 17.13 | 8.28E-06 | 1.317758953 | NA |  |
| 225558_at | 17.12 | 8.29E-06 | 1.178351836 | NM_014776| | GIT2,G protein-coupled receptor kinase-interactor 2 |
| 1558152_at | 17.11 | 8.32E-06 | 1.222403086 | NA |  |
| 226776_at | 17.11 | 8.33E-06 | 1.332804222 | NM_020189| | e(y)2,e(y)2 protein |
| 213302_at | 17.1 | 8.36E-06 | 1.064301343 | NM_012393| | PFAS,phosphoribosylformylglycinamidine synthase |
| 201580_s_at | 17.09 | 8.37E-06 | 1.211961406 | NM_021156| | DJ971N18.2,hypothetical protein DJ971N18.2 |
| 203284_s_at | 17.09 | 8.38E-06 | 1.140744408 | NM_012262| | HS2ST1,heparan sulfate 2-O-sulfotransferase 1 |
| 1558724_at | 17.09 | 8.37E-06 | 1.765915164 | NA |  |
| 209522_s_at | 17.09 | 8.37E-06 | 1.112817725 | NM_000755| | CRAT,carnitine acetyltransferase isoform 1 precursor |
| 217124_at | 17.08 | 8.41E-06 | 1.381807964 | NM_001100390| | NA |
| 225219_at | 17.08 | 8.39E-06 | 1.159968143 | NM_001001419| | SMAD5,SMAD, mothers against DPP homolog 5 |
| 225002_s_at | 17.08 | 8.40E-06 | 1.086271117 | NM_001042468| | NA |
| 227188_at | 17.08 | 8.41E-06 | 1.291988322 | NM_058187| | C21orf63,chromosome 21 open reading frame 63 |
| 230286_at | 17.07 | 8.43E-06 | 1.681272465 | NA |  |
| 218516_s_at | 17.07 | 8.42E-06 | 1.31521837 | NM_017813| | IMPA3,myo-inositol monophosphatase A3 |
| 219952_s_at | 17.05 | 8.47E-06 | 1.128039384 | NM_020533| | MCOLN1,mucolipin 1 |
| 211744_s_at | 17.05 | 8.47E-06 | 1.295098973 | NM_001779| | CD58,CD58 antigen, (lymphocyte function-associated |
| 228365_at | 17.05 | 8.46E-06 | 1.514016703 | NM_153634| | CPNE8,copine VIII |
| 201953_at | 17.04 | 8.50E-06 | 1.152860201 | NM_006384| | CIB1,calcium and integrin binding 1 (calmyrin) |
| 202265_at | 17.04 | 8.48E-06 | 1.257313616 | NM_005180| | PCGF4,polycomb group ring finger 4 |
| 202528_at | 17.03 | 8.52E-06 | 1.098478066 | NM_000403| | GALE,UDP-galactose-4-epimerase |
| 225615_at | 17.03 | 8.50E-06 | 1.185688046 | NA |  |
| 201847_at | 17.03 | 8.50E-06 | 1.080781759 | NM_000235| | LIPA,lipase A precursor |
| 212434_at | 17.03 | 8.52E-06 | 1.119319177 | NM_025196| | GRPEL1,GrpE-like 1, mitochondrial |
| 225556_at | 17.02 | 8.54E-06 | 1.078724343 | NM_001017980| | NA |
| 1553976_a_at | 17.02 | 8.54E-06 | 1.187995522 | NM_015448| | DPCD,DPCD protein |
| 244411_at | 17.01 | 8.54E-06 | 1.252723739 | NA |  |
| 225276_at | 17.01 | 8.54E-06 | 1.15615805 | NA |  |
| 217826_s_at | 17 | 8.59E-06 | 1.420552198 | NM_016021| | UBE2J1,ubiquitin-conjugating enzyme E2, J1 |
| 223500_at | 17 | 8.59E-06 | 1.633505698 | NM_006651| | CPLX1,complexin 1 |
| 243294_at | 17 | 8.59E-06 | 1.175919525 | NM_001005851| | LOC163131,hypothetical BC331191_1 |
| 222435_s_at | 17 | 8.57E-06 | 1.349702429 | NM_016021| | UBE2J1,ubiquitin-conjugating enzyme E2, J1 |
| 213598_at | 16.98 | 8.64E-06 | 1.079971326 | NM_014473| | HSA9761,putative dimethyladenosine transferase |
| 235405_at | 16.96 | 8.70E-06 | 1.259419557 | NM_001512| | GSTA4,glutathione S-transferase A4 |
| 207360_s_at | 16.96 | 8.70E-06 | 1.234697224 | NM_002531| | NTSR1,neurotensin receptor 1 |
| 221710_x_at | 16.96 | 8.70E-06 | 1.189311244 | NM_018166| | FLJ10647,hypothetical protein FLJ10647 |
| 212172_at | 16.95 | 8.71E-06 | 1.269346685 | NM_001625| | AK2,adenylate kinase 2 isoform a |
| 223741_s_at | 16.95 | 8.71E-06 | 1.359124948 | NM_032646| | TTYH2,tweety 2 isoform 1 |
| 212721_at | 16.95 | 8.71E-06 | 1.095718749 | NM_001077199| | NA |
| 202297_s_at | 16.95 | 8.71E-06 | 1.088397178 | NM_007033| | RER1,RER1 retention in endoplasmic reticulum 1 |
| 213031_s_at | 16.95 | 8.71E-06 | 1.128709848 | NM_032856| | FLJ14888,hypothetical protein FLJ14888 |
| 229111_at | 16.94 | 8.74E-06 | 1.318594857 | NA |  |
| 233825_s_at | 16.94 | 8.72E-06 | 1.365975674 | NM_031462| | CD99L2,CD99 antigen-like 2 |
| 235168_at | 16.94 | 8.72E-06 | 1.445403131 | NM_145167| | PIGM,PIG-M mannosyltransferase |
| 236821_at | 16.94 | 8.72E-06 | 1.299093449 | NA |  |
| 212608_s_at | 16.93 | 8.75E-06 | 1.163285838 | NA |  |
| 212767_at | 16.92 | 8.79E-06 | 1.117364597 | NM_138384| | GTP,GTP_binding protein |
| 228274_at | 16.92 | 8.77E-06 | 1.561887938 | NM_138432| | SDSL,serine dehydratase-like |
| 219522_at | 16.91 | 8.81E-06 | 1.218099996 | NM_014344| | FJX1,four jointed box 1 |
| 226373_at | 16.91 | 8.81E-06 | 1.20521135 | NM_144579| | SFXN5,sideroflexin 5 |
| 219377_at | 16.91 | 8.80E-06 | 1.125734693 | NM_022751| | C18orf11,chromosome 18 open reading frame 11 |
| 226043_at | 16.9 | 8.84E-06 | 1.201375597 | NM_015597| | GPSM1,G-protein signalling modulator 1 (AGS3-like, C. |
| 227983_at | 16.9 | 8.85E-06 | 1.153240872 | NM_145058| | MGC7036,hypothetical protein MGC7036 |
| 230729_at | 16.9 | 8.85E-06 | 1.563813593 | NA |  |
| 228661_s_at | 16.89 | 8.87E-06 | 1.223386236 | NA |  |
| 1555967_at | 16.89 | 8.87E-06 | 1.545868317 | NA |  |
| 212605_s_at | 16.89 | 8.87E-06 | 1.187375948 | NA |  |
| 205227_at | 16.89 | 8.87E-06 | 1.288500755 | NM_002182| | IL1RAP,interleukin 1 receptor accessory protein isoform |
| 225118_at | 16.89 | 8.87E-06 | 1.193489742 | NM_020382| | SET8,SET domain-containing protein 8 |
| 221139_s_at | 16.89 | 8.87E-06 | 1.369522359 | NM_015989| | CSAD,cysteine sulfinic acid decarboxylase-related |
| 238905_at | 16.89 | 8.87E-06 | 1.703191175 | NM_020663| | RHOJ,TC10-like Rho GTPase |
| 212909_at | 16.88 | 8.89E-06 | 1.513303467 | NM_001077427| | NA |
| 224688_at | 16.87 | 8.91E-06 | 1.135319085 | NM_017994| | FLJ10099,hypothetical protein FLJ10099 |
| 1554085_at | 16.87 | 8.92E-06 | 1.190474493 | NM_175066| | DDX51,DEAD (Asp-Glu-Ala-Asp) box polypeptide 51 |
| 226627_at | 16.86 | 8.95E-06 | 1.576324672 | NM_001098811| | NA |
| 228402_at | 16.86 | 8.95E-06 | 1.183523448 | NM_032367| | ZBED3,zinc finger, BED domain containing 3 |
| 213451_x_at | 16.86 | 8.93E-06 | 1.136581625 | NM_019105| | TNXB,tenascin XB isoform 1 |
| 202408_s_at | 16.86 | 8.95E-06 | 1.101958742 | NM_015629| | PRPF31,pre-mRNA processing factor 31 homolog |
| 1554355_a_at | 16.85 | 8.97E-06 | 1.804948962 | NM_170601| | CSE-C,cytosolic sialic acid 9-O-acetylesterase |
| 226324_s_at | 16.85 | 8.95E-06 | 1.239872333 | NM_015662| | SLB,selective LIM binding factor, rat homolog |
| 1554878_a_at | 16.82 | 9.04E-06 | 1.652974049 | NM_001122674| | NA |
| 209068_at | 16.81 | 9.10E-06 | 1.066998967 | NM_031372| | HNRPDL,heterogeneous nuclear ribonucleoprotein D-like |
| 238190_at | 16.81 | 9.09E-06 | 1.249271699 | NM_003321| | TUFM,Tu translation elongation factor, mitochondrial |
| 213052_at | 16.8 | 9.13E-06 | 1.079904684 | NM_004157| | PRKAR2A,cAMP-dependent protein kinase, regulatory |
| 212813_at | 16.8 | 9.11E-06 | 1.144948596 | NM_032801| | JAM3,junctional adhesion molecule 3 precursor |
| 202413_s_at | 16.8 | 9.11E-06 | 1.040788256 | NM_001017415| | NA |
| 227318_at | 16.79 | 9.15E-06 | 1.188239209 | NA |  |
| 217895_at | 16.78 | 9.18E-06 | 1.070034776 | NM_017952| | FLJ20758,FLJ20758 protein |
| 40569_at | 16.78 | 9.17E-06 | 1.119835883 | NM_003422| | ZNF42,zinc finger protein 42 isoform 1 |
| 217927_at | 16.78 | 9.17E-06 | 1.043956027 | NM_014041| | SPCS1,signal peptidase complex subunit 1 homolog |
| 230763_at | 16.77 | 9.20E-06 | 1.717367542 | NM_138796| | LOC128153,hypothetical protein BC014608 |
| 201908_at | 16.76 | 9.22E-06 | 1.112490564 | NM_004423| | DVL3,dishevelled 3 |
| 224717_s_at | 16.76 | 9.23E-06 | 1.152551139 | NM_024104| | MGC2747,hypothetical protein MGC2747 |
| 219785_s_at | 16.74 | 9.28E-06 | 1.175500064 | NM_024735| | FBXO31,F-box protein 31 |
| 201421_s_at | 16.74 | 9.29E-06 | 1.117370805 | NM_024102| | MEP50,methylosome protein 50 |
| 212928_at | 16.74 | 9.29E-06 | 1.146074764 | NM_021648| | TSPYL4,KIAA0721 protein |
| 220632_s_at | 16.74 | 9.28E-06 | 1.08500368 | NM_013382| | POMT2,putative protein O-mannosyltransferase |
| 208986_at | 16.74 | 9.29E-06 | 1.233766144 | NM_003205| | TCF12,transcription factor 12 isoform b |
| 218592_s_at | 16.73 | 9.30E-06 | 1.073960837 | NM_017829| | CECR5,cat eye syndrome chromosome region, candidate 5 |
| 205972_at | 16.73 | 9.30E-06 | 1.230773446 | NM_006841| | SLC38A3,solute carrier family 38, member 3 |
| 225612_s_at | 16.72 | 9.34E-06 | 1.229748334 | NM_032047| | B3GNT5,beta-1,3-N-acetylglucosaminyltransferase bGnT-5 |
| 202420_s_at | 16.72 | 9.34E-06 | 1.066193556 | NM_001357| | DHX9,DEAH (Asp-Glu-Ala-His) box polypeptide 9 isoform |
| 204929_s_at | 16.72 | 9.34E-06 | 1.200630297 | NM_006634| | VAMP5,vesicle-associated membrane protein 5 |
| 232244_at | 16.72 | 9.34E-06 | 1.371257071 | NM_020702| | KIAA1161,KIAA1161 |
| 231957_s_at | 16.72 | 9.34E-06 | 1.170528498 | NM_139159| | DPP9,dipeptidylpeptidase 9 |
| 211679_x_at | 16.71 | 9.37E-06 | 1.379187156 | NM_005458| | GPR51,G protein-coupled receptor 51 |
| 201825_s_at | 16.7 | 9.38E-06 | 1.120683463 | NM_016002| | CGI-49,CGI-49 protein |
| 202701_at | 16.7 | 9.39E-06 | 1.189439321 | NM_001199| | BMP1,bone morphogenetic protein 1 isoform 1, |
| 214545_s_at | 16.7 | 9.39E-06 | 1.150870664 | NM_007198| | PROSC,proline synthetase co-transcribed homolog |
| 208921_s_at | 16.69 | 9.41E-06 | 1.10007887 | NM_003130| | SRI,sorcin isoform a |
| 232890_at | 16.69 | 9.42E-06 | 1.206278957 | NA |  |
| 228820_at | 16.69 | 9.43E-06 | 1.111056671 | NM_022098| | LOC63929,hypothetical protein LOC63929 |
| 233540_s_at | 16.68 | 9.45E-06 | 1.134220048 | NM_001011649| | CDK5RAP2,CDK5 regulatory subunit associated protein 2 |
| 214460_at | 16.67 | 9.47E-06 | 1.444237722 | NM_002338| | LSAMP,limbic system-associated membrane protein |
| 232884_s_at | 16.67 | 9.47E-06 | 1.303956383 | NA |  |
| 228095_at | 16.67 | 9.47E-06 | 1.137255797 | NM_001007157| | PHF14,PHD finger protein 14 isoform 1 |
| 224626_at | 16.67 | 9.47E-06 | 1.093480089 | NM_080670| | SLC35A4,solute carrier family 35, member A4 |
| 209230_s_at | 16.66 | 9.51E-06 | 1.245140898 | NM_001042483| | NA |
| 219072_at | 16.66 | 9.50E-06 | 1.153948405 | NM_004765| | BCL7C,B-cell CLL/lymphoma 7C |
| 232541_at | 16.66 | 9.52E-06 | 1.868988698 | NA |  |
| 230463_at | 16.65 | 9.54E-06 | 1.414487338 | NA |  |
| 221897_at | 16.65 | 9.53E-06 | 1.217281437 | NM_032765| | TRIM52,tripartite motif-containing 52 |
| 202813_at | 16.65 | 9.53E-06 | 1.121045088 | NM_005646| | TARBP1,TAR RNA binding protein 1 |
| 210251_s_at | 16.65 | 9.54E-06 | 1.04716128 | NM_001037442| | NA |
| 226874_at | 16.64 | 9.56E-06 | 1.276311606 | NM_020803| | KLHL8,kelch-like 8 |
| 224983_at | 16.64 | 9.58E-06 | 1.05255986 | NM_005506| | SCARB2,scavenger receptor class B, member 2 |
| 220807_at | 16.63 | 9.59E-06 | 1.207138131 | NM_005331| | HBQ1,theta 1 globin |
| 222613_at | 16.63 | 9.60E-06 | 1.202620982 | NM_020374| | C12orf4,chromosome 12 open reading frame 4 |
| 235980_at | 16.63 | 9.60E-06 | 1.190714449 | NM_006218| | PIK3CA,phosphoinositide-3-kinase, catalytic, alpha |
| 217809_at | 16.62 | 9.62E-06 | 1.069952594 | NM_014038| | BZW2,basic leucine zipper and W2 domains 2 |
| 243023_at | 16.61 | 9.64E-06 | 1.516237378 | NA |  |
| 244680_at | 16.61 | 9.66E-06 | 1.471417299 | NM_000824| | GLRB,glycine receptor, beta |
| 218653_at | 16.61 | 9.64E-06 | 1.125831157 | NM_014252| | SLC25A15,solute carrier family 25 (mitochondrial carrier; |
| 227277_at | 16.6 | 9.67E-06 | 1.542655877 | NA |  |
| 225250_at | 16.6 | 9.69E-06 | 1.599327868 | NM_020860| | STIM2,stromal interaction molecule 2 |
| 220964_s_at | 16.59 | 9.69E-06 | 1.056387852 | NM_030981| | RAB1B,RAB1B, member RAS oncogene family |
| 212828_at | 16.59 | 9.72E-06 | 1.3903542 | NM_003898| | SYNJ2,synaptojanin 2 |
| 213227_at | 16.58 | 9.76E-06 | 1.082749182 | NM_006320| | PGRMC2,progesterone membrane binding protein |
| 223181_at | 16.58 | 9.72E-06 | 1.093457603 | NM_014177| | C18orf55,chromosome 18 open reading frame 55 |
| 217890_s_at | 16.57 | 9.77E-06 | 1.314615966 | NM_018222| | PARVA,parvin, alpha |
| 213701_at | 16.57 | 9.77E-06 | 1.206265509 | NM_001009894| | DKFZp434N2030,hypothetical protein DKFZp434N2030 |
| 221973_at | 16.57 | 9.78E-06 | 1.458357267 | NA |  |
| 203150_at | 16.57 | 9.77E-06 | 1.11118591 | NM_005833| | RAB9P40,Rab9 effector p40 |
| 200967_at | 16.57 | 9.76E-06 | 1.070000688 | NM_000942| | PPIB,peptidylprolyl isomerase B precursor |
| 204276_at | 16.56 | 9.80E-06 | 1.332245434 | NM_004614| | TK2,thymidine kinase 2, mitochondrial |
| 235213_at | 16.56 | 9.80E-06 | 1.642172347 | NM_002221| | ITPKB,1D-myo-inositol-trisphosphate 3-kinase B |
| 206929_s_at | 16.55 | 9.85E-06 | 1.499017567 | NM_005597| | NFIC,nuclear factor I/C isoform 1 |
| 225733_at | 16.54 | 9.87E-06 | 1.250643796 | NM_080605| | B3GALT6,UDP-Gal:betaGal beta 1,3-galactosyltransferase |
| 51176_at | 16.54 | 9.87E-06 | 1.07858254 | NM_004269| | CRSP8,cofactor required for Sp1 transcriptional |
| 37860_at | 16.54 | 9.87E-06 | 1.093841953 | NM_015655| | ZNF337,zinc finger protein 337 |
| 213509_x_at | 16.53 | 9.89E-06 | 1.142359288 | NM_003869| | CES2,carboxylesterase 2 isoform 1 |
| 217823_s_at | 16.53 | 9.88E-06 | 1.373103551 | NM_016021| | UBE2J1,ubiquitin-conjugating enzyme E2, J1 |
| 212792_at | 16.53 | 9.88E-06 | 1.161526428 | NM_015283| | NA |
| 222129_at | 16.53 | 9.89E-06 | 1.587738591 | NM_024293| | C2orf17,chromosome 2 open reading frame 17 |
| 236016_at | 16.52 | 9.92E-06 | 1.327747491 | NA |  |
| 227562_at | 16.52 | 9.90E-06 | 1.277261373 | NA |  |
| 224068_x_at | 16.51 | 9.96E-06 | 1.098574775 | NM_018047| | RBM22,RNA binding motif protein 22 |
| 203138_at | 16.51 | 9.95E-06 | 1.026681723 | NM_001033085| | NA |
| 230416_at | 16.51 | 9.95E-06 | 1.335616563 | NA |  |
| 215434_x_at | 16.51 | 9.95E-06 | 1.213337944 | NM_001037501| | NA |
| 202185_at | 16.5 | 9.97E-06 | 1.152470716 | NM_001084| | PLOD3,procollagen-lysine, 2-oxoglutarate 5-dioxygenase |
| 209811_at | 16.5 | 9.98E-06 | 1.190878088 | NM_032982| | CASP2,caspase 2 isoform 1 preproprotein |
| 1569652_at | 16.49 | 1.00E-05 | 1.369095213 | NM_004529| | MLLT3,myeloid/lymphoid or mixed-lineage leukemia |
| 210345_s_at | 16.49 | 1.00E-05 | 1.416585626 | NM_001372| | DNAH9,dynein, axonemal, heavy polypeptide 9 isoform 2 |
| 225430_at | 16.49 | 1.00E-05 | 1.134612182 | NM_176818| | 15E1.2,hypothetical protein 15E1.2 |
| 215411_s_at | 16.48 | 1.00E-05 | 1.142712469 | NM_147200| | C6orf4,chromosome 6 open reading frame 4 isoform 1 |
| 225199_at | 16.48 | 1.00E-05 | 1.079592159 | NA |  |
| 238963_at | 16.48 | 1.00E-05 | 1.365267399 | NA |  |
| 236404_at | 16.48 | 1.00E-05 | 1.300882298 | NA |  |
| 203525_s_at | 16.48 | 1.00E-05 | 1.251220282 | NM_000038| | APC,adenomatosis polyposis coli |
| 1568594_s_at | 16.47 | 1.00E-05 | 1.211083453 | NM_032765| | TRIM52,tripartite motif-containing 52 |
| 221107_at | 16.47 | 1.01E-05 | 1.526927804 | NM_017581| | CHRNA9,cholinergic receptor, nicotinic, alpha |
| 206833_s_at | 16.47 | 1.00E-05 | 1.241409919 | NM_138448| | ACYP2,muscle-type acylphosphatase 2 |
| 203885_at | 16.47 | 1.01E-05 | 1.083696942 | NM_014999| | RAB21,RAB21, member RAS oncogene family |
| 200832_s_at | 16.47 | 1.00E-05 | 1.112138922 | NM_005063| | SCD,stearoyl-CoA desaturase |
| 212017_at | 16.46 | 1.01E-05 | 1.104471328 | NM_001009993| | LOC130074,hypothetical protein LOC130074 |
| 224869_s_at | 16.46 | 1.01E-05 | 1.09642522 | NM_022497| | MRPS25,mitochondrial ribosomal protein S25 |
| 213790_at | 16.46 | 1.01E-05 | 1.582692033 | NA |  |
| 216602_s_at | 16.45 | 1.01E-05 | 1.10226315 | NM_004461| | FARSLA,phenylalanine-tRNA synthetase-like protein |
| 225560_at | 16.45 | 1.01E-05 | 1.180586182 | NM_013382| | POMT2,putative protein O-mannosyltransferase |
| 230339_at | 16.45 | 1.01E-05 | 1.281179591 | NM_144978| | FLJ32745,hypothetical protein FLJ32745 |
| 218550_s_at | 16.45 | 1.01E-05 | 1.131166128 | NM_018205| | LRRC20,leucine rich repeat containing 20 isoform 3 |
| 216959_x_at | 16.44 | 1.01E-05 | 1.370298124 | NM_001037132| | NA |
| 228495_at | 16.43 | 1.02E-05 | 1.280622056 | NM_174931| | FLJ38348,hypothetical protein FLJ38348 |
| 203560_at | 16.43 | 1.02E-05 | 1.24650825 | NM_003878| | GGH,gamma-glutamyl hydrolase precursor |
| 204764_at | 16.43 | 1.02E-05 | 1.271565911 | NM_002028| | FNTB,farnesyltransferase, CAAX box, beta |
| 234321_x_at | 16.42 | 1.02E-05 | 1.221217924 | NA |  |
| 203282_at | 16.42 | 1.02E-05 | 1.237582072 | NM_000158| | GBE1,glucan (1,4-alpha-), branching enzyme 1 |
| 218898_at | 16.42 | 1.02E-05 | 1.134719613 | NM_024792| | CT120,membrane protein expressed in epithelial-like |
| 228760_at | 16.42 | 1.02E-05 | 1.136438259 | NM_032102| | SRP46,Splicing factor, arginine/serine-rich, 46kD |
| 200973_s_at | 16.41 | 1.02E-05 | 1.128408932 | NM_005724| | TM4SF8,transmembrane 4 superfamily member 8 isoform 1 |
| 208795_s_at | 16.41 | 1.02E-05 | 1.060824223 | NM_005916| | MCM7,minichromosome maintenance protein 7 isoform 1 |
| 209637_s_at | 16.41 | 1.02E-05 | 1.454704634 | NM_002926| | RGS12,regulator of G-protein signalling 12 isoform 2 |
| 244641_at | 16.41 | 1.02E-05 | 1.266606893 | NM_138446| | C7orf30,chromosome 7 open reading frame 30 |
| 231214_at | 16.38 | 1.03E-05 | 1.463640149 | NA |  |
| 230057_at | 16.37 | 1.04E-05 | 1.509641539 | NA |  |
| 225804_at | 16.37 | 1.03E-05 | 1.202604931 | NM_144611| | MGC32124,hypothetical protein MGC32124 |
| 227401_at | 16.36 | 1.04E-05 | 1.717006733 | NM_138284| | IL17D,interleukin 17D precursor |
| 219097_x_at | 16.35 | 1.04E-05 | 1.067866733 | NM_024104| | MGC2747,hypothetical protein MGC2747 |
| 240592_at | 16.35 | 1.04E-05 | 1.176614177 | NM_153686| | MLR1,transcription factor MLR1 |
| 223627_at | 16.35 | 1.04E-05 | 1.088126316 | NM_032246| | RKHD3,ring finger and KH domain containing 3 |
| 218028_at | 16.34 | 1.05E-05 | 1.136507637 | NM_022821| | ELOVL1,elongation of very long chain fatty acids |
| 212395_s_at | 16.33 | 1.05E-05 | 1.098435784 | NM_015047| | KIAA0090,KIAA0090 protein |
| 238597_at | 16.33 | 1.05E-05 | 1.320355764 | NM_030816| | DKFZP566D1346,hypothetical protein DKFZp566D1346 |
| 45687_at | 16.33 | 1.05E-05 | 1.128374079 | NM_024031| | MGC3121,hypothetical protein MGC3121 |
| 212359_s_at | 16.33 | 1.05E-05 | 1.145868428 | NM_015037| | KIAA0913,KIAA0913 |
| 212498_at | 16.32 | 1.05E-05 | 1.064671248 | NA |  |
| 204080_at | 16.32 | 1.05E-05 | 1.114252161 | NM_025077| | TOE1,target of EGR1, member 1 (nuclear) |
| 218534_s_at | 16.31 | 1.06E-05 | 1.095996901 | NM_018046| | AGGF1,angiogenic factor VG5Q |
| 203030_s_at | 16.31 | 1.06E-05 | 1.424981726 | NM_002847| | PTPRN2,protein tyrosine phosphatase, receptor type, N |
| 218471_s_at | 16.29 | 1.06E-05 | 1.167970499 | NM_024649| | BBS1,Bardet-Biedl syndrome 1 |
| 220173_at | 16.29 | 1.06E-05 | 1.510339303 | NM_025057| | C14orf45,chromosome 14 open reading frame 45 |
| 209124_at | 16.29 | 1.06E-05 | 1.079949152 | NM_002468| | MYD88,myeloid differentiation primary response gene |
| 1552611_a_at | 16.28 | 1.07E-05 | 1.11228648 | NM_002227| | JAK1,janus kinase 1 |
| 236511_at | 16.28 | 1.07E-05 | 1.565864367 | NA |  |
| 213374_x_at | 16.27 | 1.07E-05 | 1.102358279 | NM_014362| | HIBCH,3-hydroxyisobutyryl-Coenzyme A hydrolase isoform |
| 222013_x_at | 16.27 | 1.07E-05 | 1.125634831 | NM_001083537| | NA |
| 216033_s_at | 16.27 | 1.07E-05 | 1.17263416 | NM_002037| | FYN,protein-tyrosine kinase fyn isoform a |
| 1556551_s_at | 16.27 | 1.07E-05 | 1.152308287 | NM_001099406| | NA |
| 222108_at | 16.27 | 1.07E-05 | 1.256619376 | NM_181847| | AMIGO2,amphoterin induced gene 2 |
| 219254_at | 16.27 | 1.07E-05 | 1.247598563 | NM_024648| | FLJ22222,hypothetical protein FLJ22222 |
| 202732_at | 16.26 | 1.07E-05 | 1.118493682 | NM_007066| | PKIG,cAMP-dependent protein kinase inhibitor gamma |
| 204639_at | 16.25 | 1.08E-05 | 1.082999947 | NM_000022| | ADA,adenosine deaminase |
| 215436_at | 16.25 | 1.08E-05 | 1.456540046 | NM_032303| | HSDL2,hydroxysteroid dehydrogenase like 2 |
| 232032_x_at | 16.25 | 1.08E-05 | 1.088286915 | NM_016176| | Cab45,calcium binding protein Cab45 precursor |
| 239848_at | 16.25 | 1.08E-05 | 1.960707266 | NA |  |
| 225633_at | 16.25 | 1.08E-05 | 1.117777033 | NM_207325| | LOC147991,hypothetical protein LOC147991 |
| 233304_at | 16.24 | 1.08E-05 | 1.843864756 | NM_005596| | NFIB,nuclear factor I/B |
| 212249_at | 16.24 | 1.08E-05 | 1.739169919 | NM_181504| | PIK3R1,phosphoinositide-3-kinase, regulatory subunit, |
| 200663_at | 16.24 | 1.08E-05 | 1.049336473 | NM_001040034| | NA |
| 226117_at | 16.23 | 1.08E-05 | 1.467581612 | NM_052864| | TIFA,TRAF-interacting protein with a |
| 225089_at | 16.22 | 1.09E-05 | 1.178998261 | NM_018218| | USP40,ubiquitin specific protease 40 |
| 236609_at | 16.22 | 1.09E-05 | 1.346141097 | NA |  |
| 239053_at | 16.21 | 1.09E-05 | 1.088703245 | NM_004804| | WDR39,WD repeat domain 39 |
| 209120_at | 16.21 | 1.09E-05 | 1.426301304 | NM_021005| | NR2F2,nuclear receptor subfamily 2, group F, member 2 |
| 218718_at | 16.21 | 1.09E-05 | 1.490694082 | NM_016205| | PDGFC,platelet-derived growth factor C precursor |
| 204541_at | 16.21 | 1.09E-05 | 1.514737905 | NM_012429| | SEC14L2,SEC14-like 2 |
| 227135_at | 16.2 | 1.09E-05 | 1.441015702 | NM_001042402| | NA |
| 219181_at | 16.2 | 1.09E-05 | 1.493045648 | NM_006033| | LIPG,endothelial lipase |
| 1561691_at | 16.2 | 1.09E-05 | 1.58866598 | NA |  |
| 204489_s_at | 16.2 | 1.09E-05 | 1.272904156 | NM_000610| | CD44,CD44 antigen isoform 1 precursor |
| 218500_at | 16.19 | 1.09E-05 | 1.460050652 | NM_016647| | LOC51337,mesenchymal stem cell protein DSCD75 |
| 207839_s_at | 16.19 | 1.10E-05 | 1.279193847 | NM_001042589| | NA |
| 235433_at | 16.18 | 1.10E-05 | 1.205075122 | NM_198450| | CXorf33,chromosome X open reading frame 33 |
| 229007_at | 16.18 | 1.10E-05 | 2.157659639 | NA |  |
| 242186_x_at | 16.18 | 1.10E-05 | 2.156747952 | NM_015236| | LPHN3,latrophilin 3 precursor |
| 213129_s_at | 16.17 | 1.10E-05 | 1.038059662 | NM_004483| | GCSH,glycine cleavage system protein H (aminomethyl |
| 209239_at | 16.17 | 1.10E-05 | 1.273038191 | NM_003998| | NFKB1,nuclear factor kappa-B, subunit 1 |
| 216997_x_at | 16.17 | 1.10E-05 | 1.39823352 | NM_007005| | TLE4,transducin-like enhancer protein 4 |
| 201632_at | 16.17 | 1.10E-05 | 1.07056008 | NM_001414| | EIF2B1,eukaryotic translation initiation factor 2B, |
| 216942_s_at | 16.16 | 1.11E-05 | 1.3047854 | NM_001779| | CD58,CD58 antigen, (lymphocyte function-associated |
| 226479_at | 16.16 | 1.10E-05 | 1.181786552 | NM_152903| | KBTBD6,kelch repeat and BTB (POZ) domain-containing 6 |
| 1558795_at | 16.16 | 1.11E-05 | 1.774738979 | NA |  |
| 202092_s_at | 16.15 | 1.11E-05 | 1.287467922 | NM_012106| | ARL2BP,binder of Arl Two |
| 225805_at | 16.14 | 1.11E-05 | 1.059188701 | NM_004501| | HNRPU,heterogeneous nuclear ribonucleoprotein U |
| 242488_at | 16.13 | 1.12E-05 | 1.813912335 | NA |  |
| 227766_at | 16.13 | 1.12E-05 | 1.288594501 | NM_001098268| | NA |
| 225387_at | 16.13 | 1.12E-05 | 1.159093302 | NM_005723| | TM4SF9,transmembrane 4 superfamily member 9 |
| 229870_at | 16.13 | 1.12E-05 | 1.212446556 | NA |  |
| 213574_s_at | 16.12 | 1.12E-05 | 1.11655591 | NA |  |
| 228606_at | 16.12 | 1.12E-05 | 1.106024498 | NM_138461| | LOC116211,hypothetical protein BC013113 |
| 238459_x_at | 16.12 | 1.12E-05 | 1.671624124 | NM_019073| | SPATA6,spermatogenesis associated 6 |
| 227435_at | 16.11 | 1.12E-05 | 1.120980815 | NM_001009899| | KIAA2018,hypothetical LOC205717 |
| 1559121_s_at | 16.11 | 1.12E-05 | 1.438255105 | NM_006321| | ARIH2,ariadne homolog 2 |
| 212432_at | 16.1 | 1.12E-05 | 1.11290773 | NM_025196| | GRPEL1,GrpE-like 1, mitochondrial |
| 209944_at | 16.1 | 1.12E-05 | 1.144913872 | NM_021188| | ZNF410,clones 23667 and 23775 zinc finger protein |
| 1559922_at | 16.1 | 1.13E-05 | 1.215905488 | NA |  |
| 218984_at | 16.1 | 1.12E-05 | 1.068269165 | NM_019042| | FLJ20485,hypothetical protein FLJ20485 |
| 208744_x_at | 16.09 | 1.13E-05 | 1.095424874 | NM_006644| | HSPH1,heat shock 105kD |
| 225334_at | 16.09 | 1.13E-05 | 1.437634603 | NM_144591| | C10orf32,hypothetical protein MGC27171 |
| 201585_s_at | 16.09 | 1.13E-05 | 1.095962958 | NM_005066| | SFPQ,splicing factor proline/glutamine rich |
| 239810_at | 16.09 | 1.13E-05 | 1.261826072 | NM_014909| | KIAA1036,KIAA1036 |
| 218496_at | 16.09 | 1.13E-05 | 1.094965886 | NM_002936| | RNASEH1,ribonuclease H1 |
| 222445_at | 16.09 | 1.13E-05 | 1.096258162 | NM_018375| | SLC39A9,solute carrier family 39 (zinc transporter), |
| 203017_s_at | 16.08 | 1.13E-05 | 1.152881012 | NM_014021| | SSX2IP,synovial sarcoma, X breakpoint 2 interacting |
| 1556950_s_at | 16.08 | 1.13E-05 | 1.410836237 | NM_004568| | SERPINB6,serine (or cysteine) proteinase inhibitor, clade |
| 219122_s_at | 16.08 | 1.13E-05 | 1.159442401 | NM_017872| | ICF45,interphase cyctoplasmic foci protein 45 |
| 223284_at | 16.08 | 1.13E-05 | 1.114565828 | NM_020378| | KLP1,K562 cell-derived leucine-zipper-like protein 1 |
| 227640_s_at | 16.08 | 1.13E-05 | 1.198363285 | NM_203288| | RP9,retinitis pigmentosa 9 protein |
| 1557543_at | 16.07 | 1.14E-05 | 1.517759587 | NA |  |
| 1559901_s_at | 16.06 | 1.14E-05 | 1.791422213 | NM_001005732| | C21orf34,chromosome 21 open reading frame 34 isoform a |
| 208766_s_at | 16.06 | 1.14E-05 | 1.048165198 | NM_001102397| | NA |
| 209539_at | 16.06 | 1.14E-05 | 1.434971283 | NM_004840| | ARHGEF6,Rac/Cdc42 guanine nucleotide exchange factor 6 |
| 205651_x_at | 16.06 | 1.14E-05 | 1.847424555 | NM_001100397| | NA |
| 206845_s_at | 16.05 | 1.14E-05 | 1.14462916 | NM_014771| | RNF40,ring finger protein 40 isoform 1 |
| 230369_at | 16.04 | 1.15E-05 | 1.285475143 | NM_007369| | GPR161,G protein-coupled receptor 161 |
| 200941_at | 16.04 | 1.15E-05 | 1.116350471 | NM_001537| | HSBP1,heat shock factor binding protein 1 |
| 214331_at | 16.03 | 1.15E-05 | 1.294413027 | NM_005726| | TSFM,Ts translation elongation factor, mitochondrial |
| 220346_at | 16.03 | 1.15E-05 | 1.54648232 | NM_001004346| | MTHFD2L,methylenetetrahydrofolate dehydrogenase (NADP+ |
| 222021_x_at | 16.03 | 1.15E-05 | 1.071928354 | NA |  |
| 226186_at | 16.02 | 1.15E-05 | 1.617859982 | NA |  |
| 208935_s_at | 16.02 | 1.15E-05 | 1.269526638 | NM_006499| | LGALS8,galectin 8 isoform a |
| 218328_at | 16.02 | 1.15E-05 | 1.109805457 | NM_016035| | COQ4,CGI-92 protein |
| 206499_s_at | 16.02 | 1.15E-05 | 1.084831604 | NM_001048194| | NA |
| 209991_x_at | 16.01 | 1.16E-05 | 1.525261176 | NM_005458| | GPR51,G protein-coupled receptor 51 |
| 201241_at | 16 | 1.16E-05 | 1.036081357 | NM_004939| | DDX1,DEAD (Asp-Glu-Ala-Asp) box polypeptide 1 |
| 218616_at | 15.99 | 1.16E-05 | 1.150243686 | NM_020395| | LOC57117,hypothetical nuclear factor SBBI22 |
| 219706_at | 15.97 | 1.17E-05 | 1.257750041 | NM_018347| | C20orf29,chromosome 20 open reading frame 29 |
| 200711_s_at | 15.97 | 1.17E-05 | 1.067028666 | NM_006930| | SKP1A,S-phase kinase-associated protein 1A isoform a |
| 204283_at | 15.97 | 1.17E-05 | 1.077398224 | NM_006567| | FARS2,phenylalanine-tRNA synthetase 2 |
| 208910_s_at | 15.96 | 1.18E-05 | 1.04513574 | NM_001212| | C1QBP,complement component 1, q subcomponent binding |
| 231779_at | 15.95 | 1.18E-05 | 1.413680344 | NM_001570| | IRAK2,interleukin-1 receptor-associated kinase 2 |
| 220450_at | 15.95 | 1.18E-05 | 1.556084068 | NA |  |
| 210027_s_at | 15.95 | 1.18E-05 | 1.047633011 | NM_001641| | APEX1,APEX nuclease |
| 212377_s_at | 15.95 | 1.18E-05 | 1.130739079 | NM_024408| | NOTCH2,notch 2 preproprotein |
| 202560_s_at | 15.94 | 1.18E-05 | 1.068413659 | NM_015607| | DKFZP547E1010,DKFZP547E1010 protein |
| 224631_at | 15.94 | 1.18E-05 | 1.147491338 | NM_053023| | ZFP91,zinc finger protein 91 isoform 1 |
| 53071_s_at | 15.94 | 1.18E-05 | 1.246712752 | NM_024648| | FLJ22222,hypothetical protein FLJ22222 |
| 206108_s_at | 15.94 | 1.18E-05 | 1.17635643 | NM_006275| | SFRS6,arginine/serine-rich splicing factor 6 |
| 218071_s_at | 15.94 | 1.18E-05 | 1.15228271 | NM_014160| | MKRN2,makorin, ring finger protein, 2 |
| 226383_at | 15.94 | 1.18E-05 | 1.086037575 | NM_152316| | FLJ38968,hypothetical protein FLJ38968 |
| 235962_at | 15.93 | 1.19E-05 | 1.310376607 | NA |  |
| 231767_at | 15.93 | 1.19E-05 | 1.295020839 | NM_024015| | HOXB4,homeo box B4 |
| 208662_s_at | 15.93 | 1.19E-05 | 1.307443788 | NM_001001894| | TTC3,tetratricopeptide repeat domain 3 |
| 213152_s_at | 15.93 | 1.19E-05 | 1.134082637 | NM_032102| | SRP46,Splicing factor, arginine/serine-rich, 46kD |
| 241017_at | 15.93 | 1.19E-05 | 1.548361175 | NM_001102426| | NA |
| 204860_s_at | 15.92 | 1.19E-05 | 1.56389778 | NM_004536| | BIRC1,baculoviral IAP repeat-containing 1 |
| 229705_at | 15.92 | 1.19E-05 | 1.757451557 | NA |  |
| 243417_at | 15.92 | 1.19E-05 | 1.812874646 | NA |  |
| 208832_at | 15.92 | 1.19E-05 | 1.105320613 | NM_013236| | ATXN10,ataxin 10 |
| 228201_at | 15.92 | 1.19E-05 | 1.237812046 | NM_144996| | ARL2L1,ADP-ribosylation factor-like 2-like 1 isoform 2 |
| 204126_s_at | 15.92 | 1.19E-05 | 1.159430233 | NM_003504| | CDC45L,CDC45-like |
| 201307_at | 15.91 | 1.19E-05 | 1.103416397 | NM_018243| | SEPT11,septin 11 |
| 201887_at | 15.91 | 1.19E-05 | 1.112232316 | NM_001560| | IL13RA1,interleukin 13 receptor, alpha 1 precursor |
| 221850_x_at | 15.9 | 1.20E-05 | 1.066415231 | NM_001077665| | NA |
| 203247_s_at | 15.9 | 1.20E-05 | 1.116883869 | NM_006965| | ZNF24,zinc finger protein 24 (KOX 17) |
| 221335_x_at | 15.9 | 1.20E-05 | 1.166495633 | NM_019108| | FLJ12886,hypothetical protein FLJ12886 |
| 225485_at | 15.89 | 1.20E-05 | 1.227025538 | NM_018718| | TSGA14,testis specific, 14 |
| 243704_at | 15.89 | 1.20E-05 | 1.431267535 | NA |  |
| 208103_s_at | 15.88 | 1.21E-05 | 1.098082147 | NM_030920| | ANP32E,acidic (leucine-rich) nuclear phosphoprotein 32 |
| 202259_s_at | 15.88 | 1.21E-05 | 1.186667389 | NM_014887| | PFAAP5,phosphonoformate immuno-associated protein 5 |
| 230088_at | 15.88 | 1.20E-05 | 1.570893964 | NA |  |
| 203299_s_at | 15.87 | 1.21E-05 | 1.241595768 | NM_003916| | AP1S2,adaptor-related protein complex 1 sigma 2 |
| 230655_at | 15.87 | 1.21E-05 | 1.25093961 | NA |  |
| 222403_at | 15.86 | 1.21E-05 | 1.400286214 | NM_014342| | MTCH2,mitochondrial carrier homolog 2 |
| 1557036_at | 15.86 | 1.21E-05 | 1.35704128 | NM_001123329| | NA |
| 213758_at | 15.86 | 1.21E-05 | 1.211169856 | NM_001861| | COX4I1,cytochrome c oxidase subunit IV isoform 1 |
| 204808_s_at | 15.85 | 1.22E-05 | 1.172712316 | NM_014254| | TMEM5,transmembrane protein 5 |
| 1556063_s_at | 15.84 | 1.22E-05 | 1.296702462 | NM_001104546| | NA |
| 213238_at | 15.84 | 1.22E-05 | 1.334526621 | NM_020453| | ATP10D,ATPase, Class V, type 10D |
| 239515_at | 15.84 | 1.22E-05 | 1.473844521 | NA |  |
| 200907_s_at | 15.83 | 1.23E-05 | 1.092622362 | NM_016081| | KIAA0992,palladin |
| 221270_s_at | 15.83 | 1.23E-05 | 1.147252216 | NM_031209| | QTRT1,queuine tRNA-ribosyltransferase 1 (tRNA-guanine |
| 212276_at | 15.82 | 1.23E-05 | 1.208420797 | NM_145693| | LPIN1,lipin 1 |
| 209651_at | 15.81 | 1.23E-05 | 1.189116906 | NM_001042454| | NA |
| 1552344_s_at | 15.81 | 1.23E-05 | 1.1228466 | NM_013354| | CNOT7,CCR4-NOT transcription complex, subunit 7 |
| 212228_s_at | 15.81 | 1.23E-05 | 1.16425314 | NM_020312| | DKFZP434K046,hypothetical protein DKFZp434K046 |
| 202283_at | 15.81 | 1.23E-05 | 1.158145897 | NM_002615| | SERPINF1,serine (or cysteine) proteinase inhibitor, clade |
| 227146_at | 15.8 | 1.24E-05 | 1.075774552 | NM_181701| | QSCN6L1,quiescin Q6-like 1 |
| 203245_s_at | 15.8 | 1.24E-05 | 1.117806585 | NA |  |
| 244354_at | 15.8 | 1.24E-05 | 1.70841486 | NA |  |
| 225625_at | 15.79 | 1.24E-05 | 1.177301672 | NM_001001655| | MGC90512,similar to hypothetical protein 9530023G02 |
| 235925_at | 15.79 | 1.24E-05 | 1.442663065 | NM_003205| | TCF12,transcription factor 12 isoform b |
| 212593_s_at | 15.79 | 1.24E-05 | 1.144961988 | NM_014456| | PDCD4,programmed cell death 4 isoform 1 |
| 208638_at | 15.78 | 1.25E-05 | 1.040104812 | NM_005742| | TXNDC7,protein disulfide isomerase-related protein |
| 235123_at | 15.78 | 1.25E-05 | 1.244871256 | NA |  |
| 213239_at | 15.78 | 1.25E-05 | 1.180611554 | NM_006346| | C13orf24,chromosome 13 open reading frame 24 |
| 213289_at | 15.78 | 1.24E-05 | 1.152247372 | NM_198450| | CXorf33,chromosome X open reading frame 33 |
| 235079_at | 15.77 | 1.25E-05 | 1.561712623 | NA |  |
| 203447_at | 15.77 | 1.25E-05 | 1.074715914 | NM_005047| | PSMD5,proteasome 26S non-ATPase subunit 5 |
| 218277_s_at | 15.77 | 1.25E-05 | 1.194303631 | NM_024612| | DHX40,DEAH (Asp-Glu-Ala-His) box polypeptide 40 |
| 231930_at | 15.76 | 1.25E-05 | 1.774403979 | NM_018712| | ELMOD1,ELMO domain containing 1 |
| 213726_x_at | 15.75 | 1.26E-05 | 1.047332049 | NM_006088| | TUBB2,tubulin, beta, 2 |
| 209093_s_at | 15.74 | 1.26E-05 | 1.183701366 | NM_000157| | GBA,glucocerebrosidase precursor |
| 203640_at | 15.73 | 1.27E-05 | 1.58418114 | NM_144778| | MBNL2,muscleblind-like 2 isoform 1 |
| 1554655_a_at | 15.73 | 1.27E-05 | 1.402625949 | NM_203400| | LOC388394,similar to candidate mediator of the |
| 209042_s_at | 15.73 | 1.27E-05 | 1.090246455 | NM_003343| | UBE2G2,ubiquitin-conjugating enzyme E2G 2 isoform 1 |
| 216005_at | 15.73 | 1.27E-05 | 1.551969416 | NM_002160| | TNC,tenascin C (hexabrachion) |
| 222719_s_at | 15.72 | 1.27E-05 | 1.846754303 | NM_016205| | PDGFC,platelet-derived growth factor C precursor |
| 1558369_at | 15.72 | 1.27E-05 | 1.150519934 | NM_022782| | MPHOSPH9,M-phase phosphoprotein 9 |
| 209198_s_at | 15.71 | 1.28E-05 | 1.404845283 | NM_152280| | SYT11,synaptotagmin 12 |
| 239533_at | 15.71 | 1.27E-05 | 2.087043972 | NM_001033045| | NA |
| 224860_at | 15.71 | 1.28E-05 | 1.286634054 | NM_033428| | NA |
| 211611_s_at | 15.71 | 1.27E-05 | 1.257306848 | NM_004381| | CREBL1,cAMP responsive element binding protein-like 1 |
| 215185_at | 15.7 | 1.28E-05 | 1.311661666 | NA |  |
| 238551_at | 15.7 | 1.28E-05 | 1.184270095 | NM_173540| | FUT11,fucosyltransferase 11 (alpha (1,3) |
| 225823_at | 15.69 | 1.28E-05 | 1.174062823 | NM_205767| | QIL1,QIL1 protein |
| 212753_at | 15.69 | 1.28E-05 | 1.14817939 | NM_006315| | PCGF3,ring finger protein 3 |
| 204032_at | 15.69 | 1.28E-05 | 1.110992304 | NM_003567| | BCAR3,breast cancer antiestrogen resistance 3 |
| 1554472_a_at | 15.68 | 1.29E-05 | 1.144716077 | NM_016018| | PHF20L1,PHD finger protein 20-like 1 isoform 1 |
| 244414_at | 15.68 | 1.29E-05 | 1.654371708 | NA |  |
| 242648_at | 15.67 | 1.29E-05 | 1.215816616 | NM_020803| | KLHL8,kelch-like 8 |
| 203327_at | 15.66 | 1.30E-05 | 1.099871496 | NM_004969| | IDE,insulysin |
| 226060_at | 15.66 | 1.30E-05 | 1.088435592 | NM_052859| | RFT1,RFT1 homolog |
| 213056_at | 15.66 | 1.30E-05 | 1.476995726 | NM_015123| | NA |
| 226502_at | 15.64 | 1.31E-05 | 1.069452562 | NM_153702| | ELMOD2,ELMO domain containing 2 |
| 208070_s_at | 15.64 | 1.31E-05 | 1.396796484 | NM_002912| | REV3L,REV3-like, catalytic subunit of DNA polymerase |
| 233049_x_at | 15.64 | 1.31E-05 | 1.08351119 | NM_005861| | STUB1,STIP1 homology and U-box containing protein 1 |
| 229384_at | 15.64 | 1.31E-05 | 1.160188819 | NA |  |
| 236250_at | 15.64 | 1.31E-05 | 1.341320277 | NA |  |
| 219135_s_at | 15.63 | 1.31E-05 | 1.208130867 | NM_022773| | FLJ12681,hypothetical protein FLJ12681 |
| 217485_x_at | 15.63 | 1.31E-05 | 1.097633614 | NA |  |
| 223742_at | 15.63 | 1.31E-05 | 1.297234136 | NM_015956| | MRPL4,mitochondrial ribosomal protein L4 isoform a |
| 212234_at | 15.63 | 1.31E-05 | 1.280176494 | NM_015338| | ASXL1,additional sex combs like 1 |
| 1554010_at | 15.62 | 1.31E-05 | 1.237474775 | NM_001543| | NDST1,N-deacetylase/N-sulfotransferase (heparan |
| 231532_at | 15.62 | 1.31E-05 | 1.292149556 | NM_000615| | NCAM1,neural cell adhesion molecule 1 |
| 235652_at | 15.62 | 1.31E-05 | 1.741833424 | NA |  |
| 221582_at | 15.62 | 1.31E-05 | 1.172398517 | NM_033445| | HIST3H2A,histone H2a |
| 203543_s_at | 15.61 | 1.32E-05 | 1.710145771 | NM_001206| | KLF9,Kruppel-like factor 9 |
| 205070_at | 15.6 | 1.33E-05 | 1.127605325 | NM_019071| | ING3,inhibitor of growth family, member 3 isoform 1 |
| 1568763_s_at | 15.6 | 1.32E-05 | 1.07114125 | NM_013232| | PDCD6,programmed cell death 6 |
| 200777_s_at | 15.6 | 1.32E-05 | 1.044934247 | NM_014670| | BZW1,basic leucine zipper and W2 domains 1 |
| 232395_x_at | 15.6 | 1.32E-05 | 1.723590534 | NA |  |
| 201734_at | 15.59 | 1.33E-05 | 1.089070234 | NM_001829| | CLCN3,chloride channel 3 isoform b |
| 235060_at | 15.59 | 1.33E-05 | 1.080725815 | NA |  |
| 204975_at | 15.58 | 1.33E-05 | 1.172810876 | NM_001424| | EMP2,epithelial membrane protein 2 |
| 219372_at | 15.58 | 1.33E-05 | 1.308130559 | NM_014055| | CDV1,carnitine deficiency-associated, expressed in |
| 202443_x_at | 15.58 | 1.33E-05 | 1.118754483 | NM_024408| | NOTCH2,notch 2 preproprotein |
| 207808_s_at | 15.58 | 1.33E-05 | 1.473945995 | NM_000313| | PROS1,protein S (alpha) |
| 225666_at | 15.58 | 1.33E-05 | 1.243047233 | NM_001079669| | NA |
| 209288_s_at | 15.58 | 1.33E-05 | 1.241464528 | NM_006449| | CDC42EP3,Cdc42 effector protein 3 |
| 1553677_a_at | 15.58 | 1.33E-05 | 1.08258139 | NM_001031800| | NA |
| 225037_at | 15.57 | 1.34E-05 | 1.099353202 | NM_015945| | SLC35C2,ovarian cancer overexpressed 1 isoform a |
| 212315_s_at | 15.57 | 1.34E-05 | 1.128720252 | NM_024923| | NUP210,nucleoporin 210 |
| 235231_at | 15.57 | 1.33E-05 | 1.263526861 | NM_001013258| | NA |
| 222266_at | 15.56 | 1.34E-05 | 1.215712213 | NM_003796| | C19orf2,RPB5-mediating protein isoform a |
| 222824_at | 15.56 | 1.34E-05 | 1.216514284 | NA |  |
| 226229_s_at | 15.55 | 1.35E-05 | 1.299035399 | NM_014188| | HSPC182,HSPC182 protein |
| 206106_at | 15.54 | 1.35E-05 | 1.315890918 | NM_002969| | MAPK12,mitogen-activated protein kinase 12 |
| 212993_at | 15.53 | 1.35E-05 | 1.211389033 | NM_144653| | BTBD14A,BTB (POZ) domain containing 14A |
| 214499_s_at | 15.53 | 1.35E-05 | 1.133358239 | NM_001077440| | NA |
| 221959_at | 15.53 | 1.36E-05 | 1.197240748 | NM_147189| | MGC39325,hypothetical protein MGC39325 |
| 1562012_at | 15.52 | 1.36E-05 | 1.472342441 | NA |  |
| 224352_s_at | 15.52 | 1.36E-05 | 1.244791104 | NM_021914| | CFL2,cofilin 2 |
| 229395_at | 15.52 | 1.36E-05 | 1.196388877 | NM_004604| | STX4A,syntaxin 4A (placental) |
| 241355_at | 15.51 | 1.36E-05 | 1.441525173 | NM_005144| | HR,hairless protein isoform a |
| 224391_s_at | 15.5 | 1.37E-05 | 1.352493115 | NM_170601| | CSE-C,cytosolic sialic acid 9-O-acetylesterase |
| 213394_at | 15.5 | 1.37E-05 | 1.161230364 | NM_014994| | MAPKBP1,mitogen-activated protein kinase binding protein |
| 212250_at | 15.5 | 1.37E-05 | 1.131280609 | NM_178812| | LYRIC,LYRIC/3D3 |
| 221307_at | 15.5 | 1.37E-05 | 1.776205941 | NM_001034837| | NA |
| 203068_at | 15.49 | 1.37E-05 | 1.134020095 | NM_014851| | KLHL21,kelch-like 21 |
| 1552680_a_at | 15.49 | 1.37E-05 | 1.141114217 | NM_144508| | AF15Q14,AF15q14 protein isoform 2 |
| 234464_s_at | 15.49 | 1.37E-05 | 1.055683305 | NM_152463| | EME1,essential meiotic endonuclease 1 homolog 1 |
| 203261_at | 15.49 | 1.37E-05 | 1.087269156 | NM_006571| | DCTN6,dynactin 6 |
| 230306_at | 15.48 | 1.38E-05 | 1.145031663 | NM_052875| | MGC10485,hypothetical protein MGC10485 |
| 212397_at | 15.48 | 1.38E-05 | 1.052698603 | NM_002906| | RDX,radixin |
| 203283_s_at | 15.47 | 1.38E-05 | 1.206067985 | NM_012262| | HS2ST1,heparan sulfate 2-O-sulfotransferase 1 |
| 212528_at | 15.47 | 1.38E-05 | 1.139116124 | NA |  |
| 235008_at | 15.47 | 1.38E-05 | 1.155671618 | NA |  |
| 212442_s_at | 15.47 | 1.38E-05 | 1.0611448 | NM_203463| | LASS6,longevity assurance homolog 6 |
| 207196_s_at | 15.46 | 1.39E-05 | 1.103254604 | NM_006058| | TNIP1,Nef-associated factor 1 |
| 225469_at | 15.45 | 1.39E-05 | 1.27747045 | NM_001001660| | LOC144363,hypothetical protein LOC144363 |
| 218198_at | 15.45 | 1.39E-05 | 1.082752473 | NM_018180| | DHX32,DEAD/H (Asp-Glu-Ala-Asp/His) box polypeptide 32 |
| 205130_at | 15.45 | 1.39E-05 | 1.386414336 | NM_014226| | RAGE,MAPK/MAK/MRK overlapping kinase |
| 202636_at | 15.45 | 1.39E-05 | 1.154201333 | NM_005667| | RNF103,ring finger protein 103 |
| 217945_at | 15.45 | 1.39E-05 | 1.045564696 | NM_001011885| | BTBD1,BTB (POZ) domain containing 1 isoform 2 |
| 212680_x_at | 15.45 | 1.39E-05 | 1.059001708 | NM_138689| | NA |
| 202095_s_at | 15.44 | 1.40E-05 | 1.047275226 | NM_001012270| | BIRC5,baculoviral IAP repeat-containing protein 5 |
| 224995_at | 15.44 | 1.40E-05 | 1.097757947 | NM_020148| | SPIRE1,spire homolog 1 |
| 226872_at | 15.44 | 1.39E-05 | 1.350328091 | NM_000635| | RFX2,regulatory factor X2 isoform a |
| 1556054_at | 15.43 | 1.40E-05 | 1.408837938 | NA |  |
| 201408_at | 15.43 | 1.40E-05 | 1.226189755 | NM_002709| | PPP1CB,protein phosphatase 1, catalytic subunit, beta |
| 235051_at | 15.43 | 1.40E-05 | 1.327000282 | NM_174908| | C3orf6,Ymer protein short isoform |
| 229480_at | 15.43 | 1.40E-05 | 1.405046305 | NA |  |
| 227451_s_at | 15.43 | 1.40E-05 | 1.171798493 | NM_001031713| | NA |
| 213742_at | 15.42 | 1.40E-05 | 1.669384781 | NM_004768| | SFRS11,splicing factor p54 |
| 228160_at | 15.41 | 1.41E-05 | 1.274729698 | NA |  |
| 204318_s_at | 15.41 | 1.41E-05 | 1.11772301 | NM_016426| | GTSE1,G-2 and S-phase expressed 1 |
| 209000_s_at | 15.41 | 1.41E-05 | 1.285485838 | NM_001098811| | NA |
| 215517_at | 15.41 | 1.41E-05 | 1.467747886 | NM_015617| | PYGO1,pygopus homolog 1 |
| 203206_at | 15.41 | 1.41E-05 | 1.132135112 | NM_014661| | FAM53B,family with sequence similarity 53, member B |
| 214693_x_at | 15.4 | 1.41E-05 | 1.233438239 | NM_001037501| | NA |
| 218856_at | 15.4 | 1.41E-05 | 1.026446464 | NM_014452| | TNFRSF21,tumor necrosis factor receptor superfamily, |
| 201305_x_at | 15.39 | 1.42E-05 | 1.089985502 | NM_006401| | ANP32B,acidic (leucine-rich) nuclear phosphoprotein 32 |
| 203276_at | 15.38 | 1.42E-05 | 1.068557553 | NM_005573| | LMNB1,lamin B1 |
| 225055_at | 15.37 | 1.43E-05 | 1.228808528 | NA |  |
| 202758_s_at | 15.37 | 1.43E-05 | 1.123889573 | NM_003721| | RFXANK,regulatory factor X-associated |
| 212351_at | 15.37 | 1.43E-05 | 1.092629519 | NM_003907| | EIF2B5,eukaryotic translation initiation factor 2B, |
| 217832_at | 15.37 | 1.43E-05 | 1.055456238 | NM_006372| | SYNCRIP,synaptotagmin binding, cytoplasmic RNA |
| 208969_at | 15.37 | 1.43E-05 | 1.037722399 | NM_005002| | NDUFA9,NADH dehydrogenase (ubiquinone) 1 alpha |
| 219322_s_at | 15.36 | 1.43E-05 | 1.079350761 | NM_017818| | WDR8,WD repeat domain 8 protein |
| 206879_s_at | 15.35 | 1.44E-05 | 1.184966798 | NM_004883| | NRG2,neuregulin 2 isoform 1 |
| 227158_at | 15.35 | 1.44E-05 | 1.119128674 | NM_080664| | C14orf126,hypothetical protein MGC9912 |
| 243030_at | 15.34 | 1.44E-05 | 1.337595336 | NA |  |
| 208919_s_at | 15.34 | 1.44E-05 | 1.063723704 | NM_023018| | FLJ13052,NAD kinase |
| 231117_at | 15.34 | 1.44E-05 | 1.508384866 | NM_138344| | C14orf152,chromosome 14 open reading frame 152 |
| 224982_at | 15.34 | 1.44E-05 | 1.203396098 | NM_001098632| | NA |
| 206752_s_at | 15.33 | 1.44E-05 | 1.167353688 | NM_004402| | DFFB,DNA fragmentation factor, 40 kD, beta |
| 223290_at | 15.33 | 1.44E-05 | 1.142641775 | NM_020315| | PDXP,pyridoxal (pyridoxine, vitamin B6) phosphatase |
| 229666_s_at | 15.33 | 1.45E-05 | 1.159913344 | NM_001033505| | NA |
| 219213_at | 15.33 | 1.44E-05 | 1.249669683 | NM_021219| | JAM2,junctional adhesion molecule 2 precursor |
| 1555009_a_at | 15.33 | 1.44E-05 | 1.33936422 | NM_003898| | SYNJ2,synaptojanin 2 |
| 208893_s_at | 15.32 | 1.45E-05 | 1.677905608 | NM_001946| | DUSP6,dual specificity phosphatase 6 isoform a |
| 235785_at | 15.3 | 1.46E-05 | 1.222298258 | NA |  |
| 218542_at | 15.29 | 1.47E-05 | 1.122532285 | NM_001127182| | NA |
| 217855_x_at | 15.29 | 1.47E-05 | 1.09029605 | NM_016176| | Cab45,calcium binding protein Cab45 precursor |
| 203405_at | 15.29 | 1.46E-05 | 1.097316661 | NM_003720| | DSCR2,Down syndrome critical region protein 2 isoform |
| 228222_at | 15.27 | 1.48E-05 | 1.123738035 | NA |  |
| 220397_at | 15.27 | 1.48E-05 | 1.365572186 | NM_017440| | MDM1,Mdm4, transformed 3T3 cell double minute 1, p53 |
| 219137_s_at | 15.26 | 1.48E-05 | 1.094124222 | NM_020194| | C2orf33,chromosome 2 open reading frame 33 |
| 227330_x_at | 15.26 | 1.48E-05 | 1.28028177 | NM_001033515| | NA |
| 213329_at | 15.25 | 1.49E-05 | 1.195733886 | NM_001042758| | NA |
| 208972_s_at | 15.25 | 1.49E-05 | 1.111962833 | NM_001002027| | ATP5G1,ATP synthase, H+ transporting, mitochondrial F0 |
| 235155_at | 15.25 | 1.49E-05 | 1.474417568 | NM_020139| | DHRS6,dehydrogenase/reductase (SDR family) member 6 |
| 213947_s_at | 15.25 | 1.49E-05 | 1.089725544 | NM_024923| | NUP210,nucleoporin 210 |
| 221267_s_at | 15.24 | 1.49E-05 | 1.1292565 | NM_031213| | C19orf27,chromosome 19 open reading frame 27 |
| 202254_at | 15.24 | 1.49E-05 | 1.179552492 | NM_015556| | SIPA1L1,signal-induced proliferation-associated 1 like |
| 220476_s_at | 15.24 | 1.49E-05 | 1.310803966 | NM_019099| | LOC55924,hypothetical protein LOC55924 isoform 1 |
| 1553348_a_at | 15.24 | 1.49E-05 | 1.243474342 | NM_002504| | NFX1,nuclear transcription factor, X-box binding 1 |
| 238070_at | 15.24 | 1.49E-05 | 1.327668107 | NM_004284| | CHD1L,chromodomain helicase DNA binding protein |
| 217367_s_at | 15.24 | 1.49E-05 | 1.389952446 | NM_015035| | ZHX3,zinc fingers and homeoboxes 3 protein |
| 244391_at | 15.24 | 1.49E-05 | 1.190227943 | NM_025265| | SEN2L,hypothetical protein MGC2776 |
| 218072_at | 15.23 | 1.50E-05 | 1.134640854 | NM_001101653| | NA |
| 209777_s_at | 15.23 | 1.50E-05 | 1.178973638 | NM_194255| | SLC19A1,solute carrier family 19 member 1 isoform a |
| 213857_s_at | 15.23 | 1.50E-05 | 1.141071859 | NM_001025079| | NA |
| 226197_at | 15.22 | 1.50E-05 | 1.197214568 | NA |  |
| 229511_at | 15.21 | 1.51E-05 | 1.739298523 | NM_003079| | SMARCE1,SWI/SNF-related matrix-associated |
| 218168_s_at | 15.21 | 1.51E-05 | 1.079374977 | NM_020247| | CABC1,chaperone, ABC1 activity of bc1 complex like |
| 215169_at | 15.2 | 1.51E-05 | 1.212409381 | NM_001110781| | NA |
| 1555594_a_at | 15.2 | 1.51E-05 | 1.263159106 | NM_021038| | MBNL1,muscleblind-like 1 isoform a |
| 225094_at | 15.19 | 1.52E-05 | 1.14656313 | NM_020382| | SET8,SET domain-containing protein 8 |
| 224666_at | 15.19 | 1.51E-05 | 1.094785852 | NM_145080| | NSMCE1,non-SMC element 1 homolog |
| 218126_at | 15.19 | 1.52E-05 | 1.111163447 | NM_018145| | FLJ10579,hypothetical protein FLJ10579 |
| 218052_s_at | 15.18 | 1.52E-05 | 1.113579136 | NM_020410| | ATP13A1,ATPase type 13A1 |
| 220232_at | 15.18 | 1.52E-05 | 1.136427893 | NM_001037582| | NA |
| 35179_at | 15.18 | 1.52E-05 | 1.196536595 | NM_012200| | B3GAT3,beta-1,3-glucuronyltransferase 3 |
| 243367_at | 15.17 | 1.53E-05 | 1.604022191 | NA |  |
| 200965_s_at | 15.17 | 1.53E-05 | 1.14125612 | NM_001003407| | ABLIM1,actin-binding LIM protein 1 isoform b |
| 201240_s_at | 15.17 | 1.53E-05 | 1.06262197 | NM_014752| | SPCS2,signal peptidase complex subunit 2 homolog |
| 222489_s_at | 15.16 | 1.53E-05 | 1.076249272 | NM_020135| | WRNIP1,Werner helicase interacting protein isoform 1 |
| 223040_at | 15.16 | 1.53E-05 | 1.113721232 | NM_016100| | NAT5,N-acetyltransferase 5 isoform a |
| 228829_at | 15.16 | 1.53E-05 | 1.264285281 | NM_006856| | ATF7,activating transcription factor 7 |
| 223571_at | 15.16 | 1.53E-05 | 1.194345091 | NM_031910| | C1QTNF6,C1q and tumor necrosis factor related protein 6 |
| 220159_at | 15.15 | 1.54E-05 | 1.396596657 | NA |  |
| 231171_at | 15.14 | 1.54E-05 | 1.244656771 | NA |  |
| 201751_at | 15.14 | 1.54E-05 | 1.101005381 | NM_014876| | KIAA0063,KIAA0063 gene product |
| 214938_x_at | 15.14 | 1.54E-05 | 1.029715377 | NM_002128| | HMGB1,high-mobility group box 1 |
| 201148_s_at | 15.14 | 1.54E-05 | 1.249844901 | NM_000362| | TIMP3,tissue inhibitor of metalloproteinase 3 |
| 228805_at | 15.14 | 1.54E-05 | 1.131289264 | NM_198567| | FLJ44216,FLJ44216 protein |
| 221548_s_at | 15.13 | 1.55E-05 | 1.117928905 | NM_030768| | ILKAP,integrin-linked kinase-associated protein |
| 204587_at | 15.12 | 1.55E-05 | 1.132114928 | NM_003951| | SLC25A14,solute carrier family 25, member 14 isoform |
| 223063_at | 15.12 | 1.55E-05 | 1.119725375 | NM_032800| | FLJ14525,hypothetical protein FLJ14525 |
| 227203_at | 15.12 | 1.55E-05 | 1.516709102 | NM_022824| | FBXL17,F-box and leucine-rich repeat protein 17 |
| 206976_s_at | 15.12 | 1.55E-05 | 1.095288004 | NM_006644| | HSPH1,heat shock 105kD |
| 202664_at | 15.11 | 1.56E-05 | 1.345119516 | NM_001077269| | NA |
| 225361_x_at | 15.11 | 1.56E-05 | 1.239890017 | NM_145284| | LOC159090,similar to hypothetical protein MGC17347 |
| 209194_at | 15.11 | 1.56E-05 | 1.078788021 | NM_004344| | CETN2,caltractin |
| 202194_at | 15.11 | 1.56E-05 | 1.074060044 | NM_016040| | TMED5,transmembrane emp24 protein transport domain |
| 223340_at | 15.11 | 1.56E-05 | 1.156061134 | NM_015915| | SPG3A,atlastin |
| 227890_at | 15.11 | 1.55E-05 | 1.266866907 | NM_001005209| | MGC99813,similar to RIKEN cDNA A230078I05 gene |
| 231823_s_at | 15.1 | 1.56E-05 | 1.131249561 | NM_001017995| | NA |
| 241710_at | 15.1 | 1.56E-05 | 1.493210645 | NM_001101330| | NA |
| 221766_s_at | 15.09 | 1.57E-05 | 1.400419088 | NM_017633| | FAM46A,family with sequence similarity 46, member A |
| 222455_s_at | 15.09 | 1.57E-05 | 1.391511254 | NM_018222| | PARVA,parvin, alpha |
| 227625_s_at | 15.09 | 1.57E-05 | 1.072343927 | NM_005861| | STUB1,STIP1 homology and U-box containing protein 1 |
| 228739_at | 15.09 | 1.57E-05 | 1.264703883 | NM_001037160| | NA |
| 214452_at | 15.09 | 1.57E-05 | 1.143367465 | NM_005504| | BCAT1,branched chain aminotransferase 1, cytosolic |
| 223243_s_at | 15.08 | 1.57E-05 | 1.139876972 | NM_025191| | C1orf22,chromosome 1 open reading frame 22 |
| 239297_at | 15.07 | 1.58E-05 | 1.282816603 | NM_001099677| | NA |
| 203926_x_at | 15.06 | 1.58E-05 | 1.070305476 | NM_001001975| | ATP5D,ATP synthase, H+ transporting, mitochondrial F1 |
| 1557286_at | 15.06 | 1.58E-05 | 1.380214023 | NA |  |
| 226774_at | 15.05 | 1.59E-05 | 1.253716981 | NM_032448| | KIAA1838,KIAA1838 |
| 232569_at | 15.05 | 1.59E-05 | 1.37498417 | NA |  |
| 226319_s_at | 15.04 | 1.60E-05 | 1.082685606 | NM_005782| | THOC4,THO complex 4 |
| 204647_at | 15.03 | 1.60E-05 | 1.166018534 | NM_004838| | HOMER3,Homer, neuronal immediate early gene, 3 |
| 1557038_s_at | 15.03 | 1.60E-05 | 1.211649724 | NA |  |
| 230119_at | 15.03 | 1.60E-05 | 1.270470287 | NA |  |
| 226839_at | 15.03 | 1.60E-05 | 1.131059454 | NM_176880| | TRA16,TR4 orphan receptor associated protein TRA16 |
| 212038_s_at | 15.03 | 1.60E-05 | 1.045279408 | NM_003374| | VDAC1,voltage-dependent anion channel 1 |
| 218976_at | 15.02 | 1.60E-05 | 1.403489073 | NM_021800| | DNAJC12,J domain containing protein 1 isoform a |
| 203549_s_at | 15.02 | 1.60E-05 | 1.487879247 | NM_000237| | LPL,lipoprotein lipase precursor |
| 228714_at | 15.02 | 1.60E-05 | 1.22580472 | NA |  |
| 200853_at | 15.02 | 1.60E-05 | 1.033436531 | NM_002106| | H2AFZ,H2A histone family, member Z |
| 209100_at | 15.02 | 1.60E-05 | 1.093008038 | NM_006764| | IFRD2,interferon-related developmental regulator 2 |
| 202771_at | 15.02 | 1.60E-05 | 1.169159559 | NA |  |
| 213897_s_at | 15.01 | 1.61E-05 | 1.087890848 | NM_021134| | MRPL23,mitochondrial ribosomal protein L23 |
| 223298_s_at | 15.01 | 1.61E-05 | 1.094060949 | NM_001002009| | NA |
| 208881_x_at | 15 | 1.62E-05 | 1.149517081 | NM_004508| | IDI1,isopentenyl-diphosphate delta isomerase |
| 217737_x_at | 15 | 1.61E-05 | 1.061612123 | NM_016407| | C20orf43,chromosome 20 open reading frame 43 |
| 217800_s_at | 15 | 1.61E-05 | 1.219161805 | NM_030571| | NDFIP1,Nedd4 family interacting protein 1 |
| 202157_s_at | 15 | 1.61E-05 | 1.103137819 | NM_001025076| | NA |
| 235603_at | 14.99 | 1.62E-05 | 1.281205148 | NM_004501| | HNRPU,heterogeneous nuclear ribonucleoprotein U |
| 204065_at | 14.99 | 1.62E-05 | 1.096887955 | NM_004854| | CHST10,HNK-1 sulfotransferase |
| 213381_at | 14.98 | 1.62E-05 | 1.252225694 | NM_001031746| | NA |
| 227792_at | 14.98 | 1.62E-05 | 1.304579649 | NM_001034841| | NA |
| 202082_s_at | 14.97 | 1.63E-05 | 1.158196357 | NM_001039573| | NA |
| 223025_s_at | 14.97 | 1.63E-05 | 1.18824496 | NM_032493| | AP1M1,adaptor-related protein complex 1, mu 1 subunit |
| 49329_at | 14.97 | 1.63E-05 | 1.121763089 | NM_032775| | KELCHL,kelch-like |
| 202305_s_at | 14.97 | 1.63E-05 | 1.100605261 | NM_001042548| | NA |
| 1558568_a_at | 14.97 | 1.63E-05 | 1.357651137 | NA |  |
| 214773_x_at | 14.96 | 1.64E-05 | 1.11089833 | NM_001031800| | NA |
| 218407_x_at | 14.96 | 1.64E-05 | 1.094726248 | NM_013349| | SCIRP10,SCIRP10-related protein |
| 225302_at | 14.96 | 1.63E-05 | 1.222376642 | NM_019022| | TXNDC10,thioredoxin domain containing 10 |
| 208451_s_at | 14.95 | 1.64E-05 | 1.183857051 | NM_000592| | C4B,complement component 4B preproprotein |
| 207012_at | 14.95 | 1.64E-05 | 1.280190976 | NM_005941| | MMP16,matrix metalloproteinase 16 isoform 1 |
| 235470_at | 14.95 | 1.64E-05 | 1.095613352 | NA |  |
| 243236_at | 14.95 | 1.64E-05 | 1.358187964 | NA |  |
| 1555974_a_at | 14.95 | 1.64E-05 | 1.181974877 | NA |  |
| 200768_s_at | 14.94 | 1.65E-05 | 1.125419586 | NM_005911| | MAT2A,methionine adenosyltransferase II, alpha |
| 243531_at | 14.94 | 1.64E-05 | 1.500559423 | NM_153451| | ORAOV1,oral cancer overexpressed 1 |
| 229319_at | 14.93 | 1.65E-05 | 1.229748727 | NA |  |
| 225336_at | 14.92 | 1.66E-05 | 1.094707513 | NM_004719| | SFRS2IP,splicing factor, arginine/serine-rich 2, |
| 216231_s_at | 14.91 | 1.66E-05 | 1.135022172 | NM_004048| | B2M,beta-2-microglobulin precursor |
| 223212_at | 14.91 | 1.67E-05 | 1.066686502 | NM_032327| | ZDHHC16,Abl-philin 2 isoform 1 |
| 219471_at | 14.9 | 1.67E-05 | 1.504754988 | NM_025113| | C13orf18,chromosome 13 open reading frame 18 |
| 209291_at | 14.9 | 1.67E-05 | 1.387144915 | NM_001546| | ID4,inhibitor of DNA binding 4, dominant negative |
| 212002_at | 14.9 | 1.67E-05 | 1.140541041 | NM_001114600| | NA |
| 232297_at | 14.9 | 1.67E-05 | 1.488699018 | NA |  |
| 1555476_at | 14.89 | 1.68E-05 | 1.220248629 | NM_004136| | IREB2,iron-responsive element binding protein 2 |
| 227563_at | 14.89 | 1.68E-05 | 1.320177182 | NM_175923| | MGC42630,hypothetical protein MGC42630 |
| 221621_at | 14.89 | 1.68E-05 | 1.577412846 | NA |  |
| 1558215_s_at | 14.88 | 1.69E-05 | 1.136170565 | NM_001076683| | NA |
| 1555573_at | 14.88 | 1.68E-05 | 1.528814829 | NM_173572| | C10orf93,chromosome 10 open reading frame 93 |
| 225238_at | 14.88 | 1.68E-05 | 1.246806932 | NM_138962| | MSI2,musashi 2 isoform a |
| 239266_at | 14.86 | 1.70E-05 | 1.11729582 | NA |  |
| 207618_s_at | 14.86 | 1.70E-05 | 1.14908622 | NM_001079866| | NA |
| 211930_at | 14.86 | 1.70E-05 | 1.113819544 | NM_194247| | HNRPA3,heterogeneous nuclear ribonucleoprotein A3 |
| 201534_s_at | 14.85 | 1.70E-05 | 1.18575711 | NM_007106| | UBL3,ubiquitin-like 3 |
| 208891_at | 14.85 | 1.70E-05 | 1.5632489 | NM_001946| | DUSP6,dual specificity phosphatase 6 isoform a |
| 235112_at | 14.85 | 1.70E-05 | 1.154164415 | NA |  |
| 225016_at | 14.85 | 1.70E-05 | 1.353098852 | NM_153000| | APCDD1,adenomatosis polyposis coli down-regulated 1 |
| 1557384_at | 14.84 | 1.71E-05 | 1.258538583 | NM_003432| | NA |
| 234979_at | 14.84 | 1.71E-05 | 1.178211932 | NM_181708| | LOC144233,hypothetical protein LOC144233 |
| 222757_s_at | 14.84 | 1.71E-05 | 1.411787089 | NM_016653| | ZAK,sterile-alpha motif and leucine zipper |
| 233924_s_at | 14.84 | 1.71E-05 | 1.2297879 | NM_001013848| | NA |
| 212032_s_at | 14.84 | 1.71E-05 | 1.074364362 | NM_017432| | PTOV1,prostate tumor overexpressed gene 1 |
| 204932_at | 14.83 | 1.71E-05 | 1.711548711 | NM_002546| | TNFRSF11B,osteoprotegerin precursor |
| 226320_at | 14.83 | 1.71E-05 | 1.087870037 | NM_005782| | THOC4,THO complex 4 |
| 1553118_at | 14.83 | 1.71E-05 | 1.304213571 | NM_053055| | CTMP,carboxyl-terminal modulator protein isoform a |
| 207107_at | 14.83 | 1.71E-05 | 2.281105197 | NM_000329| | RPE65,retinal pigment epithelium-specific protein |
| 222478_at | 14.82 | 1.72E-05 | 1.210209961 | NM_016075| | C13orf9,CGI-145 protein |
| 243619_at | 14.82 | 1.72E-05 | 1.379636948 | NM_015633| | FGFR1OP2,FGFR1 oncogene partner 2 |
| 207163_s_at | 14.82 | 1.72E-05 | 1.187982916 | NM_001014431| | NA |
| 212899_at | 14.82 | 1.72E-05 | 1.131916454 | NM_015076| | CDC2L6,cyclin-dependent kinase (CDC2-like) 11 |
| 225358_at | 14.81 | 1.73E-05 | 1.099283096 | NM_145261| | TIM14,homolog of yeast TIM14 isoform a |
| 222668_at | 14.81 | 1.73E-05 | 1.128997451 | NM_024076| | KCTD15,potassium channel tetramerisation domain |
| 200680_x_at | 14.81 | 1.73E-05 | 1.021003081 | NM_002128| | HMGB1,high-mobility group box 1 |
| 212111_at | 14.81 | 1.73E-05 | 1.120354159 | NM_177424| | STX12,syntaxin 12 |
| 212548_s_at | 14.8 | 1.73E-05 | 1.145673288 | NM_015030| | NA |
| 218915_at | 14.8 | 1.73E-05 | 1.08402765 | NM_000268| | NF2,neurofibromin 2 isoform 1 |
| 215634_at | 14.79 | 1.74E-05 | 1.846987484 | NA |  |
| 222996_s_at | 14.79 | 1.74E-05 | 1.076381648 | NM_016463| | CXXC5,CXXC finger 5 |
| 227345_at | 14.78 | 1.74E-05 | 1.400442029 | NM_003840| | TNFRSF10D,tumor necrosis factor receptor superfamily, |
| 226217_at | 14.77 | 1.75E-05 | 1.122957296 | NM_133496| | SLC30A7,zinc transporter like 2 |
| 204396_s_at | 14.76 | 1.76E-05 | 1.302892532 | NM_005308| | GRK5,G protein-coupled receptor kinase 5 |
| 222277_at | 14.74 | 1.77E-05 | 1.158489676 | NM_001014442| | NA |
| 1558426_x_at | 14.74 | 1.77E-05 | 1.199186298 | NM_001126340| | NA |
| 218736_s_at | 14.74 | 1.77E-05 | 1.794122167 | NM_017734| | PALMD,palmdelphin |
| 212651_at | 14.74 | 1.77E-05 | 1.226388884 | NM_001032380| | NA |
| 217838_s_at | 14.74 | 1.77E-05 | 1.122879976 | NM_016337| | EVL,Enah/Vasp-like |
| 226348_at | 14.73 | 1.78E-05 | 1.130553966 | NA |  |
| 213708_s_at | 14.73 | 1.78E-05 | 1.064297759 | NM_170607| | MLX,transcription factor-like protein 4 isoform |
| 211162_x_at | 14.73 | 1.78E-05 | 1.243714622 | NM_005063| | SCD,stearoyl-CoA desaturase |
| 225000_at | 14.73 | 1.78E-05 | 1.073366568 | NM_004157| | PRKAR2A,cAMP-dependent protein kinase, regulatory |
| 213436_at | 14.72 | 1.79E-05 | 1.512818144 | NM_016083| | CNR1,central cannabinoid receptor isoform a |
| 213609_s_at | 14.72 | 1.78E-05 | 1.63510572 | NM_021115| | SEZ6L,seizure related 6 homolog (mouse)-like |
| 231873_at | 14.72 | 1.78E-05 | 1.434429257 | NM_001204| | BMPR2,bone morphogenetic protein receptor, type II |
| 224722_at | 14.72 | 1.78E-05 | 1.374196101 | NM_020774| | MIB1,mindbomb homolog 1 |
| 203132_at | 14.71 | 1.79E-05 | 1.23207641 | NM_000321| | RB1,retinoblastoma 1 |
| 208902_s_at | 14.71 | 1.79E-05 | 1.328954766 | NM_001031| | RPS28,ribosomal protein S28 |
| 208072_s_at | 14.71 | 1.79E-05 | 1.106171001 | NM_003648| | DGKD,diacylglycerol kinase, delta 130kDa isoform 1 |
| 225980_at | 14.7 | 1.79E-05 | 1.203374564 | NM_001043318| | NA |
| 221406_s_at | 14.69 | 1.80E-05 | 1.291970778 | NM_001039651| | NA |
| 226183_at | 14.69 | 1.80E-05 | 1.248667285 | NA |  |
| 217645_at | 14.69 | 1.80E-05 | 1.16044786 | NM_016468| | C14orf112,chromosome 14 open reading frame 112 |
| 204405_x_at | 14.68 | 1.81E-05 | 1.087867561 | NM_014473| | HSA9761,putative dimethyladenosine transferase |
| 219843_at | 14.68 | 1.81E-05 | 1.204990008 | NM_005897| | IPP,intracisternal A particle-promoted polypeptide |
| 236584_at | 14.68 | 1.81E-05 | 1.248865392 | NA |  |
| 221739_at | 14.68 | 1.81E-05 | 1.120122538 | NM_019107| | C19orf10,chromosome 19 open reading frame 10 |
| 226363_at | 14.68 | 1.81E-05 | 1.308868549 | NM_001023587| | NA |
| 240066_at | 14.68 | 1.81E-05 | 1.743103142 | NA |  |
| 205970_at | 14.67 | 1.81E-05 | 1.35777394 | NM_005954| | MT3,metallothionein 3 |
| 227741_at | 14.67 | 1.82E-05 | 1.148884421 | NM_198402| | PTPLB,protein tyrosine phosphatase-like (proline |
| 203212_s_at | 14.67 | 1.81E-05 | 1.089827068 | NM_016156| | MTMR2,myotubularin-related protein 2 isoform 1 |
| 204967_at | 14.67 | 1.81E-05 | 1.160100089 | NM_001649| | APXL,apical protein of Xenopus-like |
| 223456_s_at | 14.67 | 1.81E-05 | 1.42629774 | NM_032300| | MGC10854,hypothetical protein MGC10854 |
| 238452_at | 14.66 | 1.82E-05 | 1.538201219 | NM_001002901| | FCRL2,hypothetical protein FLJ31052 isoform a |
| 1555216_a_at | 14.66 | 1.82E-05 | 1.956690492 | NA |  |
| 209234_at | 14.66 | 1.82E-05 | 1.064086927 | NM_015074| | KIF1B,kinesin family member 1B isoform b |
| 211337_s_at | 14.66 | 1.82E-05 | 1.133610183 | NM_014444| | 76P,gamma tubulin ring complex protein (76p gene) |
| 228483_s_at | 14.66 | 1.82E-05 | 1.410645199 | NM_015975| | TAF9L,TBP-associated factor 9L |
| 227986_at | 14.65 | 1.82E-05 | 1.257658441 | NM_024325| | ZNF343,zinc finger protein 343 |
| 221830_at | 14.64 | 1.83E-05 | 1.128949981 | NM_021033| | RAP2A,RAP2A, member of RAS oncogene family |
| 203994_s_at | 14.64 | 1.83E-05 | 1.150348976 | NM_004928| | C21orf2,chromosome 21 open reading frame 2 |
| 205449_at | 14.64 | 1.83E-05 | 1.134505184 | NM_013299| | SHD1,Sac3 homology domain 1 |
| 239024_at | 14.63 | 1.84E-05 | 1.241335124 | NM_021964| | ZNF148,zinc finger protein 148 (pHZ-52) |
| 224950_at | 14.63 | 1.84E-05 | 1.241472803 | NM_020440| | PTGFRN,prostaglandin F2 receptor negative regulator |
| 205893_at | 14.63 | 1.84E-05 | 1.470704777 | NM_014932| | NLGN1,neuroligin 1 |
| 220495_s_at | 14.62 | 1.85E-05 | 1.087099192 | NM_024715| | C5orf14,disulfide isomerase |
| 204365_s_at | 14.62 | 1.84E-05 | 1.312836639 | NM_022912| | C2orf23,receptor expression enhancing protein 1 |
| 208892_s_at | 14.62 | 1.84E-05 | 1.671906174 | NM_001946| | DUSP6,dual specificity phosphatase 6 isoform a |
| 208999_at | 14.62 | 1.85E-05 | 1.093402526 | NM_001098811| | NA |
| 211596_s_at | 14.61 | 1.85E-05 | 1.050409196 | NM_015541| | LRIG1,leucine-rich repeats and immunoglobulin-like |
| 225585_at | 14.61 | 1.85E-05 | 1.098815474 | NM_021033| | RAP2A,RAP2A, member of RAS oncogene family |
| 211165_x_at | 14.6 | 1.85E-05 | 1.303016234 | NM_004442| | EPHB2,ephrin receptor EphB2 isoform 2 precursor |
| 1773_at | 14.6 | 1.85E-05 | 1.210384651 | NM_002028| | FNTB,farnesyltransferase, CAAX box, beta |
| 214268_s_at | 14.59 | 1.86E-05 | 1.10677446 | NM_004687| | MTMR4,myotubularin related protein 4 |
| 222138_s_at | 14.58 | 1.87E-05 | 1.16891819 | NM_017883| | WDR13,WD repeat domain 13 protein |
| 224417_at | 14.57 | 1.88E-05 | 1.461625134 | NA |  |
| 212640_at | 14.57 | 1.88E-05 | 1.147769338 | NM_198402| | PTPLB,protein tyrosine phosphatase-like (proline |
| 1553510_s_at | 14.57 | 1.88E-05 | 1.124652452 | NM_005444| | RQCD1,RCD1 required for cell differentiation1 homolog |
| 228046_at | 14.56 | 1.89E-05 | 1.116706972 | NM_178835| | LOC152485,hypothetical protein LOC152485 |
| 202784_s_at | 14.56 | 1.88E-05 | 1.273562475 | NM_012343| | NNT,nicotinamide nucleotide transhydrogenase |
| 215719_x_at | 14.56 | 1.88E-05 | 1.351196629 | NM_000043| | FAS,tumor necrosis factor receptor superfamily, |
| 225298_at | 14.56 | 1.88E-05 | 1.103621678 | NM_001077399| | NA |
| 204238_s_at | 14.55 | 1.89E-05 | 1.117258668 | NM_006443| | C6orf108,putative c-Myc-responsive isoform 1 |
| 202395_at | 14.55 | 1.89E-05 | 1.134856137 | NM_006178| | NSF,N-ethylmaleimide-sensitive factor |
| 219726_at | 14.55 | 1.89E-05 | 1.276031512 | NM_018977| | NLGN3,neuroligin 3 |
| 205496_at | 14.55 | 1.89E-05 | 1.438852866 | NM_014702| | KIAA0408,KIAA0408 protein |
| 237885_at | 14.54 | 1.90E-05 | 1.304231666 | NA |  |
| 231348_s_at | 14.54 | 1.90E-05 | 1.420768108 | NM_001001395| | LMO3,LIM domain only 3 |
| 218100_s_at | 14.54 | 1.90E-05 | 1.182611339 | NM_018010| | ESRRBL1,estrogen-related receptor beta like 1 |
| 229244_at | 14.53 | 1.91E-05 | 1.451168564 | NA |  |
| 225543_at | 14.52 | 1.91E-05 | 1.082923901 | NA |  |
| 227516_at | 14.51 | 1.92E-05 | 1.083969599 | NM_001005409| | SF3A1,splicing factor 3a, subunit 1, 120kDa isoform 2 |
| 238119_at | 14.5 | 1.93E-05 | 1.221468872 | NA |  |
| 203358_s_at | 14.5 | 1.93E-05 | 1.127691148 | NM_004456| | EZH2,enhancer of zeste 2 isoform a |
| 227580_s_at | 14.5 | 1.93E-05 | 1.186953223 | NM_015395| | DKFZP434B0335,DKFZP434B0335 protein |
| 239483_at | 14.48 | 1.94E-05 | 1.241538083 | NA |  |
| 33494_at | 14.47 | 1.95E-05 | 1.176676025 | NM_004453| | ETFDH,electron-transferring-flavoprotein |
| 213424_at | 14.47 | 1.95E-05 | 1.746517693 | NM_001100425| | NA |
| 207629_s_at | 14.47 | 1.95E-05 | 1.159704369 | NM_004723| | ARHGEF2,rho/rac guanine nucleotide exchange factor 2 |
| 228605_at | 14.47 | 1.95E-05 | 1.123071406 | NA |  |
| 218099_at | 14.47 | 1.95E-05 | 1.121479265 | NM_018469| | HT008,uncharacterized hypothalamus protein HT008 |
| 222582_at | 14.46 | 1.95E-05 | 1.136693369 | NM_001040633| | NA |
| 228063_s_at | 14.46 | 1.95E-05 | 1.283291503 | NM_153757| | NAP1L5,nucleosome assembly protein 1-like 5 |
| 224668_at | 14.45 | 1.96E-05 | 1.074048065 | NM_033542| | C20orf35,uncharacterized hypothalamus protein HSMNP1 |
| 218108_at | 14.45 | 1.96E-05 | 1.083065128 | NM_001100417| | NA |
| 209067_s_at | 14.45 | 1.96E-05 | 1.036661589 | NM_031372| | HNRPDL,heterogeneous nuclear ribonucleoprotein D-like |
| 36475_at | 14.43 | 1.98E-05 | 1.091642278 | NM_014291| | GCAT,glycine C-acetyltransferase precursor |
| 233496_s_at | 14.43 | 1.98E-05 | 1.300042907 | NM_021914| | CFL2,cofilin 2 |
| 1555225_at | 14.43 | 1.98E-05 | 1.248306074 | NM_001098616| | NA |
| 229359_at | 14.43 | 1.98E-05 | 1.248967984 | NA |  |
| 219374_s_at | 14.43 | 1.98E-05 | 1.121690119 | NM_001077690| | NA |
| 206364_at | 14.42 | 1.99E-05 | 1.123223129 | NM_014875| | NA |
| 226728_at | 14.42 | 1.99E-05 | 1.175547969 | NM_198580| | SLC27A1,solute carrier family 27 (fatty acid |
| 228822_s_at | 14.42 | 1.99E-05 | 1.132093335 | NM_001001992| | USP16,ubiquitin specific protease 16 isoform b |
| 239978_at | 14.4 | 2.00E-05 | 1.504865487 | NA |  |
| 224796_at | 14.4 | 2.00E-05 | 1.153718721 | NM_018482| | DDEF1,development and differentiation enhancing factor |
| 223087_at | 14.39 | 2.01E-05 | 1.127827077 | NM_001002030| | NA |
| 217869_at | 14.39 | 2.01E-05 | 1.047537615 | NM_016142| | HSD17B12,steroid dehydrogenase homolog |
| 215146_s_at | 14.39 | 2.01E-05 | 1.199531824 | NA |  |
| 232086_at | 14.38 | 2.01E-05 | 1.44885887 | NA |  |
| 203668_at | 14.38 | 2.01E-05 | 1.127842306 | NM_006715| | MAN2C1,mannosidase, alpha, class 2C, member 1 |
| 208676_s_at | 14.37 | 2.02E-05 | 1.094399098 | NM_006191| | PA2G4,proliferation-associated 2G4, 38kDa |
| 225674_at | 14.37 | 2.02E-05 | 1.12759956 | NM_001008405| | BCAP29,B-cell receptor-associated protein BAP29 isoform |
| 222816_s_at | 14.36 | 2.03E-05 | 1.077158083 | NM_017742| | ZCCHC2,zinc finger, CCHC domain containing 2 |
| 212040_at | 14.36 | 2.03E-05 | 1.056891175 | NM_006464| | TGOLN2,trans-golgi network protein 2 |
| 207711_at | 14.36 | 2.03E-05 | 1.3269229 | NM_199181| | FLJ44670,FLJ44670 protein |
| 202960_s_at | 14.36 | 2.03E-05 | 1.079904673 | NM_000255| | MUT,methylmalonyl Coenzyme A mutase precursor |
| 226201_at | 14.36 | 2.03E-05 | 1.070498809 | NM_032482| | DOT1L,DOT1-like, histone H3 methyltransferase |
| 202866_at | 14.35 | 2.04E-05 | 1.152871778 | NM_001002762| | DNAJB12,DnaJ (Hsp40) homolog, subfamily B, member 12 |
| 224952_at | 14.35 | 2.04E-05 | 1.288247665 | NM_025185| | NA |
| 227153_at | 14.34 | 2.04E-05 | 1.134438903 | NM_032549| | IMMP2L,IMP2 inner mitochondrial membrane protease-like |
| 213463_s_at | 14.34 | 2.04E-05 | 1.170164723 | NA |  |
| 224744_at | 14.34 | 2.04E-05 | 1.263118064 | NM_017813| | IMPA3,myo-inositol monophosphatase A3 |
| 218437_s_at | 14.33 | 2.05E-05 | 1.344470078 | NM_020347| | LZTFL1,leucine zipper transcription factor-like 1 |
| 217837_s_at | 14.33 | 2.05E-05 | 1.237790538 | NM_001005753| | VPS24,vacuolar protein sorting 24 isoform 2 |
| 225031_at | 14.33 | 2.06E-05 | 1.09273714 | NM_032221| | CHD6,chromodomain helicase DNA binding protein 6 |
| 230243_at | 14.32 | 2.06E-05 | 1.284029771 | NM_152292| | RG9MTD2,RNA (guanine-9-) methyltransferase domain |
| 209361_s_at | 14.32 | 2.06E-05 | 1.228710091 | NM_020418| | PCBP4,poly(rC) binding protein 4 isoform a |
| 212239_at | 14.32 | 2.06E-05 | 1.389102265 | NM_181504| | PIK3R1,phosphoinositide-3-kinase, regulatory subunit, |
| 224523_s_at | 14.32 | 2.06E-05 | 1.075775948 | NM_032359| | MGC4308,hypothetical protein MGC4308 |
| 201494_at | 14.32 | 2.06E-05 | 1.043422718 | NM_005040| | PRCP,prolylcarboxypeptidase isoform 1 preproprotein |
| 229211_at | 14.31 | 2.06E-05 | 1.257076838 | NM_001033575| | NA |
| 222916_s_at | 14.31 | 2.07E-05 | 1.200366041 | NM_005336| | HDLBP,high density lipoprotein binding protein |
| 201524_x_at | 14.3 | 2.08E-05 | 1.028091608 | NM_003348| | UBE2N,ubiquitin-conjugating enzyme E2N |
| 205706_s_at | 14.3 | 2.08E-05 | 1.110538193 | NM_014915| | ANKRD26,ankyrin repeat domain 26 |
| 233070_at | 14.3 | 2.07E-05 | 1.205786735 | NM_001024855| | NA |
| 212279_at | 14.29 | 2.09E-05 | 1.119545223 | NM_014573| | MAC30,hypothetical protein MAC30 |
| 242065_x_at | 14.29 | 2.09E-05 | 1.139409124 | NM_004508| | IDI1,isopentenyl-diphosphate delta isomerase |
| 233587_s_at | 14.29 | 2.09E-05 | 1.290485012 | NM_020808| | SIPA1L2,signal-induced proliferation-associated 1 like |
| 232330_at | 14.29 | 2.09E-05 | 1.854591623 | NM_018224| | FLJ10803,hypothetical protein FLJ10803 |
| 1552767_a_at | 14.29 | 2.08E-05 | 1.25052146 | NM_001077188| | NA |
| 208981_at | 14.28 | 2.09E-05 | 1.16958748 | NM_000442| | PECAM1,platelet/endothelial cell adhesion molecule |
| 239512_at | 14.28 | 2.09E-05 | 1.34680328 | NM_005626| | SFRS4,splicing factor, arginine/serine-rich 4 |
| 219999_at | 14.27 | 2.10E-05 | 1.272961996 | NM_006122| | MAN2A2,mannosidase, alpha, class 2A, member 2 |
| 217599_s_at | 14.27 | 2.10E-05 | 1.8156148 | NM_199072| | HIC,I-mfa domain-containing protein isoform p40 |
| 201938_at | 14.27 | 2.10E-05 | 1.093479182 | NM_004642| | CDK2AP1,CDK2-associated protein 1 |
| 213281_at | 14.27 | 2.10E-05 | 1.254846906 | NM_002228| | JUN,v-jun avian sarcoma virus 17 oncogene homolog |
| 201729_s_at | 14.26 | 2.10E-05 | 1.089640465 | NM_014680| | KIAA0100,antigen MLAA-22 |
| 219985_at | 14.26 | 2.10E-05 | 1.232591201 | NM_006042| | HS3ST3A1,heparan sulfate D-glucosaminyl |
| 207871_s_at | 14.26 | 2.11E-05 | 1.257408072 | NM_018412| | ST7,suppression of tumorigenicity 7 isoform a |
| 202522_at | 14.26 | 2.10E-05 | 1.029726196 | NM_012399| | PITPNB,phosphatidylinositol transfer protein, beta |
| 217896_s_at | 14.26 | 2.10E-05 | 1.13680514 | NM_024946| | NIP30,NEFA-interacting nuclear protein NIP30 |
| 200935_at | 14.25 | 2.11E-05 | 1.12707487 | NM_004343| | CALR,calreticulin precursor |
| 228977_at | 14.25 | 2.11E-05 | 1.333981803 | NA |  |
| 220460_at | 14.25 | 2.11E-05 | 1.782082615 | NM_017435| | SLCO1C1,solute carrier organic anion transporter family, |
| 222436_s_at | 14.25 | 2.11E-05 | 1.136632419 | NM_001005753| | VPS24,vacuolar protein sorting 24 isoform 2 |
| 232683_s_at | 14.24 | 2.11E-05 | 1.083250699 | NM_020214| | PARP6,poly (ADP-ribose) polymerase family, member 6 |
| 202780_at | 14.24 | 2.11E-05 | 1.141050342 | NM_000436| | OXCT1,3-oxoacid CoA transferase 1 precursor |
| 221517_s_at | 14.22 | 2.13E-05 | 1.064140954 | NM_004268| | CRSP6,cofactor required for Sp1 transcriptional |
| 213196_at | 14.22 | 2.14E-05 | 1.086232035 | NM_001080417| | NA |
| 225842_at | 14.22 | 2.13E-05 | 1.156390743 | NM_007350| | PHLDA1,pleckstrin homology-like domain, family A, |
| 228379_at | 14.22 | 2.13E-05 | 1.242898463 | NM_005796| | NUTF2,nuclear transport factor 2 |
| 218097_s_at | 14.22 | 2.14E-05 | 1.076179729 | NM_024040| | CUEDC2,CUE domain containing 2 |
| 209968_s_at | 14.21 | 2.14E-05 | 1.143044518 | NM_000615| | NCAM1,neural cell adhesion molecule 1 |
| 212726_at | 14.21 | 2.15E-05 | 1.196774906 | NM_005392| | PHF2,PHD finger protein 2 isoform a |
| 218032_at | 14.2 | 2.15E-05 | 1.120009698 | NM_003498| | SNN,Stannin |
| 227021_at | 14.2 | 2.15E-05 | 1.43715817 | NM_153042| | NA |
| 201190_s_at | 14.2 | 2.15E-05 | 1.089728157 | NM_006224| | PITPNA,phosphatidylinositol transfer protein, alpha |
| 218812_s_at | 14.19 | 2.15E-05 | 1.325058873 | NM_001126340| | NA |
| 229269_x_at | 14.19 | 2.16E-05 | 1.216043819 | NM_001009998| | SSBP4,single stranded DNA binding protein 4 isoform b |
| 212947_at | 14.19 | 2.16E-05 | 1.141843085 | NM_015266| | SLC9A8,Na+/H+ exchanger isoform 8 |
| 222121_at | 14.18 | 2.16E-05 | 1.262942304 | NM_015595| | SGEF,DKFZP434D146 protein |
| 215285_s_at | 14.17 | 2.17E-05 | 1.21094123 | NM_006608| | PHTF1,putative homeodomain transcription factor 1 |
| 218223_s_at | 14.17 | 2.17E-05 | 1.08391293 | NM_016274| | CKIP-1,CK2 interacting protein 1; HQ0024c protein |
| 202586_at | 14.16 | 2.19E-05 | 1.122465486 | NM_021128| | POLR2L,DNA directed RNA polymerase II polypeptide L |
| 242304_at | 14.15 | 2.19E-05 | 1.21300382 | NM_032345| | PYM,PYM protein |
| 224973_at | 14.15 | 2.19E-05 | 1.74393841 | NM_017633| | FAM46A,family with sequence similarity 46, member A |
| 236168_at | 14.15 | 2.19E-05 | 1.402634873 | NA |  |
| 217889_s_at | 14.15 | 2.19E-05 | 1.264908709 | NM_024843| | CYBRD1,cytochrome b reductase 1 |
| 239585_at | 14.15 | 2.19E-05 | 1.432143165 | NM_003884| | PCAF,p300/CBP-associated factor |
| 226800_at | 14.15 | 2.19E-05 | 1.23188194 | NM_032437| | KIAA1799,KIAA1799 protein |
| 203119_at | 14.14 | 2.21E-05 | 1.076343227 | NM_024098| | MGC2574,hypothetical protein MGC2574 |
| 224856_at | 14.13 | 2.21E-05 | 1.122447451 | NM_004117| | FKBP5,FK506 binding protein 5 |
| 203456_at | 14.13 | 2.21E-05 | 1.187890012 | NM_007213| | PRAF2,JM4 protein |
| 229943_at | 14.12 | 2.22E-05 | 1.237748032 | NM_001007278| | RFP2,ret finger protein 2 isoform 2 |
| 239392_s_at | 14.11 | 2.23E-05 | 1.214988203 | NM_017542| | POGK,pogo transposable element with KRAB domain |
| 202867_s_at | 14.1 | 2.24E-05 | 1.11117806 | NM_001002762| | DNAJB12,DnaJ (Hsp40) homolog, subfamily B, member 12 |
| 221748_s_at | 14.1 | 2.24E-05 | 1.409472702 | NM_022648| | TNS,tensin |
| 225157_at | 14.09 | 2.25E-05 | 1.118147809 | NA |  |
| 224957_at | 14.09 | 2.24E-05 | 1.06020192 | NM_001035005| | NA |
| 206320_s_at | 14.09 | 2.25E-05 | 1.23008673 | NM_001127217| | NA |
| 242172_at | 14.09 | 2.25E-05 | 1.938452042 | NM_002398| | MEIS1,Meis1 homolog |
| 203518_at | 14.08 | 2.25E-05 | 1.628718535 | NM_000081| | LYST,lysosomal trafficking regulator isoform 1 |
| 212954_at | 14.08 | 2.25E-05 | 1.176134883 | NM_003845| | DYRK4,dual-specificity tyrosine-(Y)-phosphorylation |
| 214526_x_at | 14.08 | 2.25E-05 | 1.108789386 | NA |  |
| 232480_at | 14.08 | 2.25E-05 | 1.447550398 | NA |  |
| 237315_at | 14.08 | 2.26E-05 | 1.34738672 | NA |  |
| 213646_x_at | 14.07 | 2.26E-05 | 1.039213793 | NM_006082| | K-ALPHA-1,tubulin, alpha, ubiquitous |
| 212776_s_at | 14.07 | 2.26E-05 | 1.175101555 | NM_015311| | NA |
| 203292_s_at | 14.06 | 2.27E-05 | 1.085674215 | NM_021729| | VPS11,vacuolar protein sorting 11 (yeast homolog) |
| 242486_at | 14.06 | 2.27E-05 | 1.469139846 | NA |  |
| 202218_s_at | 14.06 | 2.27E-05 | 1.218037467 | NM_004265| | FADS2,fatty acid desaturase 2 |
| 204514_at | 14.06 | 2.27E-05 | 1.136455214 | NM_001039589| | NA |
| 228844_at | 14.05 | 2.28E-05 | 1.470210615 | NM_177550| | SLC13A5,solute carrier family 13 (sodium-dependent |
| 205364_at | 14.05 | 2.28E-05 | 1.368184035 | NM_003500| | ACOX2,acyl-Coenzyme A oxidase 2, branched chain |
| 224476_s_at | 14.05 | 2.28E-05 | 1.262034891 | NM_018670| | MESP1,mesoderm posterior 1 |
| 213369_at | 14.05 | 2.27E-05 | 1.206095531 | NM_033100| | PCDH21,protocadherin 21 precursor |
| 1563014_at | 14.04 | 2.29E-05 | 1.228272056 | NM_001018| | RPS15,ribosomal protein S15 |
| 208030_s_at | 14.02 | 2.30E-05 | 1.083723707 | NM_001119| | ADD1,adducin 1 (alpha) isoform a |
| 226712_at | 14.02 | 2.31E-05 | 1.186143133 | NM_003144| | SSR1,signal sequence receptor, alpha |
| 1560029_a_at | 14.01 | 2.32E-05 | 1.414072343 | NM_001082969| | NA |
| 202896_s_at | 14.01 | 2.31E-05 | 1.16228731 | NM_001040022| | NA |
| 1556285_s_at | 14.01 | 2.31E-05 | 1.064788758 | NM_001034191| | NA |
| 235692_at | 14.01 | 2.31E-05 | 1.311650657 | NM_001024666| | NA |
| 1555847_a_at | 14.01 | 2.31E-05 | 1.104508318 | NA |  |
| 227189_at | 14.01 | 2.31E-05 | 1.422954044 | NM_020939| | CPNE5,copine V |
| 220010_at | 14.01 | 2.32E-05 | 1.308244725 | NM_012282| | KCNE1L,potassium voltage-gated channel, Isk-related |
| 236723_at | 14 | 2.32E-05 | 1.204233197 | NA |  |
| 222044_at | 13.99 | 2.33E-05 | 1.16740566 | NM_022104| | C20orf67,phosphorylated CTD interacting factor 1 |
| 223605_at | 13.99 | 2.33E-05 | 1.249901423 | NM_031481| | SLC25A18,solute carrier |
| 200947_s_at | 13.99 | 2.33E-05 | 1.056415617 | NM_005271| | GLUD1,glutamate dehydrogenase 1 |
| 218776_s_at | 13.99 | 2.33E-05 | 1.293254029 | NM_024956| | FLJ23375,hypothetical protein FLJ23375 |
| 242260_at | 13.99 | 2.33E-05 | 1.163252091 | NM_018834| | MATR3,matrin 3 |
| 235468_at | 13.99 | 2.33E-05 | 1.127567169 | NM_001082575| | NA |
| 36936_at | 13.99 | 2.33E-05 | 1.077477424 | NM_003313| | TSTA3,tissue specific transplantation antigen P35B |
| 222125_s_at | 13.98 | 2.34E-05 | 1.104748583 | NM_177938| | PH-4,hypoxia-inducible factor prolyl 4-hydroxylase |
| 202358_s_at | 13.98 | 2.34E-05 | 1.078736352 | NM_014758| | NA |
| 228620_at | 13.98 | 2.34E-05 | 1.300216191 | NA |  |
| 229531_at | 13.97 | 2.35E-05 | 1.429967057 | NA |  |
| 218270_at | 13.97 | 2.35E-05 | 1.163718848 | NM_024540| | MRPL24,mitochondrial ribosomal protein L24 |
| 229285_at | 13.97 | 2.35E-05 | 1.494442037 | NM_021133| | RNASEL,ribonuclease L |
| 235407_at | 13.97 | 2.35E-05 | 1.186302506 | NA |  |
| 239775_at | 13.96 | 2.36E-05 | 1.307186543 | NA |  |
| 220522_at | 13.96 | 2.36E-05 | 1.406396196 | NM_201253| | CRB1,crumbs homolog 1 isoform II precursor |
| 224729_s_at | 13.95 | 2.37E-05 | 1.070282907 | NM_001042546| | NA |
| 201792_at | 13.95 | 2.36E-05 | 1.101045715 | NM_001129| | AEBP1,adipocyte enhancer binding protein 1 precursor |
| 211499_s_at | 13.94 | 2.38E-05 | 1.281113744 | NM_002751| | MAPK11,mitogen-activated protein kinase 11 |
| 200698_at | 13.94 | 2.38E-05 | 1.107248728 | NM_001100603| | NA |
| 222681_at | 13.94 | 2.38E-05 | 1.185756152 | NM_020231| | MDS010,x 010 protein |
| 228915_at | 13.94 | 2.38E-05 | 1.469769307 | NM_004392| | DACH1,dachshund homolog 1 isoform c |
| 218329_at | 13.94 | 2.37E-05 | 1.073443472 | NM_012406| | PRDM4,PR domain containing 4 |
| 202020_s_at | 13.94 | 2.38E-05 | 1.065980082 | NM_006055| | LANCL1,lanthionine synthetase C-like protein 1 |
| 242673_at | 13.93 | 2.39E-05 | 1.309574735 | NA |  |
| 38241_at | 13.91 | 2.40E-05 | 1.51582982 | NM_006994| | BTN3A3,butyrophilin, subfamily 3, member A3 isoform a |
| 205631_at | 13.91 | 2.40E-05 | 1.174589728 | NM_014749| | KIAA0586,KIAA0586 |
| 205450_at | 13.91 | 2.41E-05 | 1.143641916 | NM_001122670| | NA |
| 214144_at | 13.9 | 2.42E-05 | 1.143944599 | NM_004805| | POLR2D,DNA directed RNA polymerase II polypeptide D |
| 228925_at | 13.9 | 2.41E-05 | 1.594193677 | NA |  |
| 212656_at | 13.9 | 2.41E-05 | 1.067994652 | NM_005726| | TSFM,Ts translation elongation factor, mitochondrial |
| 221039_s_at | 13.89 | 2.42E-05 | 1.203557744 | NM_018482| | DDEF1,development and differentiation enhancing factor |
| 213002_at | 13.88 | 2.43E-05 | 1.152887239 | NM_002356| | MARCKS,myristoylated alanine-rich protein kinase C |
| 35626_at | 13.88 | 2.44E-05 | 1.113037288 | NM_000199| | SGSH,N-sulfoglucosamine sulfohydrolase (sulfamidase) |
| 235498_at | 13.88 | 2.44E-05 | 1.76681983 | NM_001105659| | NA |
| 218783_at | 13.88 | 2.44E-05 | 1.174916508 | NM_015434| | DKFZP434B168,DKFZP434B168 protein |
| 213448_at | 13.88 | 2.43E-05 | 1.147151119 | NA |  |
| 218903_s_at | 13.88 | 2.43E-05 | 1.161016504 | NM_024068| | MGC2731,hypothetical protein MGC2731 |
| 239084_at | 13.87 | 2.44E-05 | 1.326398037 | NA |  |
| 212484_at | 13.87 | 2.44E-05 | 1.198968117 | NM_001098784| | NA |
| 227852_at | 13.87 | 2.44E-05 | 1.124659739 | NM_203288| | RP9,retinitis pigmentosa 9 protein |
| 202731_at | 13.86 | 2.45E-05 | 1.197979825 | NM_014456| | PDCD4,programmed cell death 4 isoform 1 |
| 228890_at | 13.86 | 2.45E-05 | 1.372695271 | NM_032827| | ATOH8,atonal homolog 8 |
| 209371_s_at | 13.86 | 2.45E-05 | 1.325094664 | NM_001122681| | NA |
| 225337_at | 13.85 | 2.46E-05 | 1.162344392 | NM_007011| | ABHD2,alpha/beta hydrolase domain containing protein |
| 203441_s_at | 13.84 | 2.47E-05 | 1.339080744 | NM_001792| | CDH2,cadherin 2, type 1 preproprotein |
| 228006_at | 13.84 | 2.47E-05 | 1.310935154 | NA |  |
| 209568_s_at | 13.84 | 2.47E-05 | 1.312261562 | NM_015149| | RGL1,ral guanine nucleotide dissociation |
| 215089_s_at | 13.84 | 2.47E-05 | 1.059377101 | NM_005676| | RBM10,RNA binding motif protein 10 isoform 1 |
| 203054_s_at | 13.83 | 2.48E-05 | 1.153557362 | NM_022171| | TCTA,T-cell leukemia translocation altered gene |
| 211922_s_at | 13.83 | 2.48E-05 | 1.59669528 | NM_001752| | CAT,catalase |
| 230479_at | 13.83 | 2.49E-05 | 1.701343547 | NA |  |
| 225479_at | 13.83 | 2.48E-05 | 1.221178424 | NM_001099678| | NA |
| 226935_s_at | 13.82 | 2.49E-05 | 1.108338133 | NM_030782| | CRR9,cisplatin resistance related protein CRR9p |
| 209029_at | 13.82 | 2.49E-05 | 1.100825477 | NM_016319| | COPS7A,COP9 complex subunit 7a |
| 218252_at | 13.82 | 2.49E-05 | 1.128175248 | NM_001098525| | NA |
| 204615_x_at | 13.82 | 2.49E-05 | 1.146939081 | NM_004508| | IDI1,isopentenyl-diphosphate delta isomerase |
| 202927_at | 13.82 | 2.49E-05 | 1.090842193 | NM_006221| | PIN1,protein (peptidyl-prolyl cis/trans isomerase) |
| 235067_at | 13.82 | 2.49E-05 | 1.13233203 | NM_013255| | MKLN1,muskelin 1, intracellular mediator containing |
| 202783_at | 13.82 | 2.49E-05 | 1.276961556 | NM_012343| | NNT,nicotinamide nucleotide transhydrogenase |
| 244184_at | 13.81 | 2.50E-05 | 1.267653035 | NA |  |
| 225415_at | 13.81 | 2.50E-05 | 1.18540042 | NM_138287| | DTX3L,deltex 3-like |
| 226394_at | 13.8 | 2.52E-05 | 1.150972546 | NM_017824| | MARCH5,ring finger protein 153 |
| 226121_at | 13.79 | 2.52E-05 | 1.143998619 | NM_144683| | MGC23280,hypothetical protein MGC23280 |
| 63825_at | 13.79 | 2.52E-05 | 1.178507727 | NM_007011| | ABHD2,alpha/beta hydrolase domain containing protein |
| 220311_at | 13.79 | 2.52E-05 | 1.148683418 | NM_013240| | C21orf127,N6-DNA-methyltransferase isoform 1 |
| 238824_at | 13.78 | 2.53E-05 | 1.19257015 | NA |  |
| 217806_s_at | 13.78 | 2.53E-05 | 1.066489597 | NM_015584| | POLDIP2,DNA polymerase delta interacting protein 2 |
| 241727_x_at | 13.78 | 2.53E-05 | 1.218386483 | NM_176815| | DHFRL1,dihydrofolate reductase-like 1 |
| 1559496_at | 13.78 | 2.53E-05 | 1.414455884 | NM_001034191| | NA |
| 230477_at | 13.78 | 2.53E-05 | 1.197955244 | NA |  |
| 222026_at | 13.78 | 2.53E-05 | 1.204623939 | NM_006743| | RBM3,RNA binding motif (RNP1, RRM) protein 3 |
| 1555916_at | 13.78 | 2.53E-05 | 1.099237549 | NM_173659| | RPUSD3,RNA pseudouridylate synthase domain containing |
| 209356_x_at | 13.78 | 2.53E-05 | 1.183826424 | NM_016938| | EFEMP2,EGF-containing fibulin-like extracellular matrix |
| 226642_s_at | 13.77 | 2.53E-05 | 1.046878631 | NM_145266| | NUDCD2,NudC domain containing 2 |
| 236268_at | 13.77 | 2.54E-05 | 1.215481399 | NM_004206| | SEC22L3,vesicle trafficking protein isoform b |
| 213405_at | 13.76 | 2.55E-05 | 1.232153036 | NM_020673| | RAB22A,RAS-related protein RAB-22A |
| 222222_s_at | 13.76 | 2.55E-05 | 1.180959577 | NM_004838| | HOMER3,Homer, neuronal immediate early gene, 3 |
| 241407_at | 13.76 | 2.55E-05 | 1.205928394 | NA |  |
| 201350_at | 13.76 | 2.55E-05 | 1.082123652 | NM_004475| | FLOT2,flotillin 2 |
| 226180_at | 13.75 | 2.56E-05 | 1.095860104 | NM_139281| | WDR36,WD repeat domain 36 |
| 212996_s_at | 13.75 | 2.56E-05 | 1.129357141 | NA |  |
| 221745_at | 13.75 | 2.55E-05 | 1.114652379 | NM_005828| | HAN11,WD-repeat protein |
| 1558365_at | 13.75 | 2.56E-05 | 1.638430231 | NA |  |
| 225389_at | 13.75 | 2.56E-05 | 1.220225062 | NM_033271| | BTBD6,BTB domain protein BDPL |
| 226003_at | 13.74 | 2.57E-05 | 1.196991057 | NM_017641| | KIF21A,kinesin family member 21A |
| 221506_s_at | 13.74 | 2.56E-05 | 1.074557201 | NM_013433| | TNPO2,transportin 2 (importin 3, karyopherin beta 2b) |
| 1556694_a_at | 13.74 | 2.57E-05 | 1.259451293 | NA |  |
| 209608_s_at | 13.74 | 2.57E-05 | 1.103329426 | NM_005891| | ACAT2,acetyl-Coenzyme A acetyltransferase 2 |
| 205320_at | 13.73 | 2.58E-05 | 1.292853265 | NM_005883| | APC2,adenomatosis polyposis coli 2 |
| 244425_at | 13.73 | 2.58E-05 | 1.318663577 | NA |  |
| 201516_at | 13.73 | 2.57E-05 | 1.123483475 | NM_003132| | SRM,spermidine synthase |
| 202730_s_at | 13.73 | 2.58E-05 | 1.196435754 | NM_014456| | PDCD4,programmed cell death 4 isoform 1 |
| 229415_at | 13.73 | 2.58E-05 | 1.361630551 | NM_018947| | CYCS,cytochrome c |
| 221683_s_at | 13.73 | 2.58E-05 | 1.267324225 | NM_025114| | Cep290,centrosome protein cep290 |
| 226128_at | 13.72 | 2.59E-05 | 1.086925745 | NA |  |
| 225799_at | 13.72 | 2.58E-05 | 1.271444472 | NA |  |
| 204724_s_at | 13.71 | 2.59E-05 | 1.107065229 | NM_001853| | COL9A3,alpha 3 type IX collagen |
| 203152_at | 13.7 | 2.61E-05 | 1.158380996 | NM_003776| | MRPL40,mitochondrial ribosomal protein L40 |
| 211382_s_at | 13.7 | 2.61E-05 | 1.074793073 | NM_006997| | TACC2,transforming, acidic coiled-coil containing |
| 203767_s_at | 13.7 | 2.61E-05 | 1.31961604 | NM_000351| | STS,steryl-sulfatase precursor |
| 221591_s_at | 13.69 | 2.61E-05 | 1.092245574 | NM_019013| | FLJ10156,hypothetical protein FLJ10156 |
| 243953_at | 13.69 | 2.61E-05 | 1.315733491 | NM_004265| | FADS2,fatty acid desaturase 2 |
| 227873_at | 13.69 | 2.61E-05 | 1.08799369 | NM_024715| | C5orf14,disulfide isomerase |
| 208735_s_at | 13.69 | 2.62E-05 | 1.049264679 | NM_005730| | CTDSP2,nuclear LIM interactor-interacting factor 2 |
| 226009_at | 13.69 | 2.61E-05 | 1.159800409 | NM_015448| | DPCD,DPCD protein |
| 238575_at | 13.68 | 2.63E-05 | 1.254689688 | NM_032523| | OSBPL6,oxysterol-binding protein-like protein 6 isoform |
| 227720_at | 13.68 | 2.63E-05 | 1.149355878 | NM_152345| | FLJ25555,hypothetical protein FLJ25555 |
| 230095_at | 13.67 | 2.64E-05 | 1.40598317 | NA |  |
| 217733_s_at | 13.66 | 2.64E-05 | 1.039653008 | NM_021103| | TMSB10,thymosin, beta 10 |
| 235581_at | 13.66 | 2.65E-05 | 1.409960267 | NA |  |
| 232053_x_at | 13.66 | 2.64E-05 | 1.198385839 | NM_001040456| | NA |
| 201999_s_at | 13.66 | 2.64E-05 | 1.044666711 | NM_006519| | TCTEL1,t-complex-associated-testis-expressed 1-like 1 |
| 200078_s_at | 13.66 | 2.65E-05 | 1.106370671 | NM_001039457| | NA |
| 226091_s_at | 13.66 | 2.64E-05 | 1.02649585 | NM_033296| | PGR1,protein associated with MRG, 14 kDa |
| 222507_s_at | 13.66 | 2.64E-05 | 1.15214876 | NM_020644| | C11orf15,chromosome 11 open reading frame 15 |
| 224840_at | 13.66 | 2.64E-05 | 1.104202697 | NM_004117| | FKBP5,FK506 binding protein 5 |
| 1557133_at | 13.66 | 2.64E-05 | 1.24341859 | NM_001126492| | NA |
| 213970_at | 13.65 | 2.65E-05 | 1.088014906 | NM_173825| | RABL3,RAB, member of RAS oncogene family-like 3 |
| 238880_at | 13.65 | 2.66E-05 | 1.174229601 | NM_002097| | GTF3A,general transcription factor IIIA |
| 1558964_at | 13.65 | 2.65E-05 | 1.314317 | NM_001008781| | NA |
| 220183_s_at | 13.64 | 2.66E-05 | 1.173427988 | NM_007083| | NUDT6,nudix-type motif 6 isoform a |
| 227255_at | 13.64 | 2.67E-05 | 1.359931041 | NM_152835| | PDIK1L,PDLIM1 interacting kinase 1 like |
| 219587_at | 13.64 | 2.66E-05 | 1.216754771 | NM_017868| | TTC12,tetratricopeptide repeat domain 12 |
| 226594_at | 13.64 | 2.66E-05 | 1.158201721 | NA |  |
| 212708_at | 13.64 | 2.66E-05 | 1.098814829 | NM_001012241| | NA |
| 235985_at | 13.63 | 2.67E-05 | 1.422190365 | NA |  |
| 242191_at | 13.63 | 2.67E-05 | 1.696294333 | NM_001039703| | NA |
| 227907_at | 13.63 | 2.67E-05 | 1.182899513 | NA |  |
| 242590_at | 13.63 | 2.68E-05 | 1.410551884 | NA |  |
| 225912_at | 13.63 | 2.67E-05 | 1.139778791 | NM_033285| | TP53INP1,tumor protein p53 inducible nuclear protein 1 |
| 237262_at | 13.62 | 2.68E-05 | 1.16566475 | NA |  |
| 203202_at | 13.62 | 2.68E-05 | 1.067084086 | NM_007043| | HRB2,HIV-1 rev binding protein 2 |
| 210651_s_at | 13.61 | 2.69E-05 | 1.18690852 | NM_004442| | EPHB2,ephrin receptor EphB2 isoform 2 precursor |
| 243707_at | 13.61 | 2.69E-05 | 1.188057423 | NA |  |
| 232668_at | 13.61 | 2.69E-05 | 1.33266266 | NA |  |
| 200837_at | 13.61 | 2.69E-05 | 1.063264031 | NM_005745| | BCAP31,B-cell receptor-associated protein 31 |
| 1557347_at | 13.6 | 2.71E-05 | 1.375687316 | NM_024596| | MCPH1,microcephalin |
| 35156_at | 13.6 | 2.71E-05 | 1.13791581 | NA |  |
| 228667_at | 13.6 | 2.71E-05 | 1.217332022 | NM_020133| | AGPAT4,1-acylglycerol-3-phosphate O-acyltransferase 4 |
| 219960_s_at | 13.6 | 2.71E-05 | 1.045960297 | NM_015984| | UCHL5,ubiquitin C-terminal hydrolase UCH37 |
| 201151_s_at | 13.59 | 2.72E-05 | 1.616924399 | NM_021038| | MBNL1,muscleblind-like 1 isoform a |
| 224713_at | 13.59 | 2.71E-05 | 1.080025231 | NM_032390| | MKI67IP,MKI67 (FHA domain) interacting nucleolar |
| 203349_s_at | 13.58 | 2.72E-05 | 1.352037932 | NM_004454| | ETV5,ets variant gene 5 (ets-related molecule) |
| 213944_x_at | 13.58 | 2.72E-05 | 1.161418261 | NM_002067| | GNA11,guanine nucleotide binding protein (G protein), |
| 210102_at | 13.58 | 2.72E-05 | 1.152587968 | NM_014622| | LOH11CR2A,BCSC-1 isoform 1 |
| 226517_at | 13.58 | 2.73E-05 | 1.082520435 | NM_005504| | BCAT1,branched chain aminotransferase 1, cytosolic |
| 236164_at | 13.58 | 2.73E-05 | 1.276805079 | NA |  |
| 232589_at | 13.57 | 2.74E-05 | 1.25651509 | NA |  |
| 238444_at | 13.57 | 2.73E-05 | 1.094667321 | NM_133374| | NA |
| 229004_at | 13.57 | 2.73E-05 | 1.299305081 | NA |  |
| 226684_at | 13.56 | 2.75E-05 | 1.06262396 | NM_018036| | C14orf103,chromosome 14 open reading frame 103 |
| 227750_at | 13.55 | 2.76E-05 | 1.150515897 | NM_001024660| | NA |
| 208825_x_at | 13.55 | 2.76E-05 | 1.010698823 | NM_000984| | RPL23A,ribosomal protein L23a |
| 212237_at | 13.55 | 2.76E-05 | 1.258449937 | NM_015338| | ASXL1,additional sex combs like 1 |
| 205260_s_at | 13.55 | 2.75E-05 | 1.110963409 | NM_001107| | ACYP1,erythrocyte acylphosphatase 1 isoform a |
| 215258_at | 13.54 | 2.77E-05 | 1.345786365 | NM_145296| | IGSF4C,immunoglobulin superfamily, member 4C |
| 226751_at | 13.54 | 2.77E-05 | 1.310676856 | NM_001111101| | NA |
| 36499_at | 13.54 | 2.77E-05 | 1.108100078 | NM_001408| | CELSR2,cadherin EGF LAG seven-pass G-type receptor 2 |
| 235302_at | 13.54 | 2.77E-05 | 1.191871657 | NA |  |
| 229800_at | 13.54 | 2.77E-05 | 1.162432991 | NM_004734| | DCAMKL1,doublecortin and CaM kinase-like 1 |
| 208664_s_at | 13.54 | 2.77E-05 | 1.325626647 | NM_001001894| | TTC3,tetratricopeptide repeat domain 3 |
| 204029_at | 13.53 | 2.79E-05 | 1.123597378 | NM_001408| | CELSR2,cadherin EGF LAG seven-pass G-type receptor 2 |
| 205967_at | 13.53 | 2.78E-05 | 1.022566615 | NM_001034077| | NA |
| 223407_at | 13.53 | 2.78E-05 | 1.30564026 | NM_032140| | C16orf48,chromosome 16 open reading frame 48 |
| 204078_at | 13.53 | 2.78E-05 | 1.144307762 | NM_006455| | SC65,synaptonemal complex protein SC65 |
| 228843_at | 13.52 | 2.79E-05 | 1.332609389 | NA |  |
| 213803_at | 13.52 | 2.80E-05 | 1.138363912 | NA |  |
| 225710_at | 13.52 | 2.80E-05 | 1.088785117 | NM_021629| | GNB4,guanine nucleotide-binding protein, beta-4 |
| 216483_s_at | 13.52 | 2.79E-05 | 1.108422848 | NM_019107| | C19orf10,chromosome 19 open reading frame 10 |
| 1554352_s_at | 13.52 | 2.80E-05 | 1.2863484 | NM_005848| | MYCPBP,c-myc promoter binding protein |
| 211067_s_at | 13.51 | 2.80E-05 | 1.181841803 | NM_003644| | GAS7,growth arrest-specific 7 isoform a |
| 226412_at | 13.51 | 2.80E-05 | 1.188740522 | NM_015491| | NA |
| 212300_at | 13.51 | 2.81E-05 | 1.090739097 | NM_175852| | DKFZp451J0118,taxilin |
| 237834_at | 13.51 | 2.80E-05 | 1.376630259 | NM_005460| | SNCAIP,synuclein alpha interacting protein |
| 226965_at | 13.5 | 2.81E-05 | 1.167983011 | NM_152678| | FLJ34969,hypothetical protein FLJ34969 |
| 202438_x_at | 13.5 | 2.81E-05 | 1.452393748 | NM_000202| | IDS,iduronate-2-sulfatase isoform a precursor |
| 212416_at | 13.5 | 2.82E-05 | 1.042684433 | NM_004866| | SCAMP1,secretory carrier membrane protein 1 isoform 1 |
| 232632_at | 13.49 | 2.82E-05 | 1.470493743 | NA |  |
| 219200_at | 13.49 | 2.82E-05 | 1.150299596 | NM_024091| | MGC5297,hypothetical protein MGC5297 |
| 210756_s_at | 13.49 | 2.82E-05 | 1.146393556 | NM_024408| | NOTCH2,notch 2 preproprotein |
| 202461_at | 13.48 | 2.83E-05 | 1.058524369 | NM_014239| | EIF2B2,eukaryotic translation initiation factor 2B, |
| 217905_at | 13.48 | 2.83E-05 | 1.099963394 | NM_024834| | C10orf119,chromosome 10 open reading frame 119 |
| 212080_at | 13.48 | 2.84E-05 | 1.067604312 | NM_005933| | MLL,myeloid/lymphoid or mixed-lineage leukemia |
| 1556476_at | 13.48 | 2.84E-05 | 1.478968324 | NA |  |
| 211152_s_at | 13.48 | 2.83E-05 | 1.105498642 | NM_013247| | PRSS25,protease, serine, 25 isoform 1 preproprotein |
| 224587_at | 13.48 | 2.83E-05 | 1.148077698 | NM_006713| | PC4,activated RNA polymerase II transcription |
| 225056_at | 13.48 | 2.84E-05 | 1.254485519 | NM_020808| | SIPA1L2,signal-induced proliferation-associated 1 like |
| 213573_at | 13.47 | 2.84E-05 | 1.135833094 | NA |  |
| 208669_s_at | 13.47 | 2.84E-05 | 1.066768946 | NM_014335| | CRI1,CREBBP/EP300 inhibitor 1 |
| 224791_at | 13.47 | 2.85E-05 | 1.141849761 | NM_018482| | DDEF1,development and differentiation enhancing factor |
| 226500_at | 13.47 | 2.85E-05 | 1.175918508 | NM_145166| | ZNF651,zinc finger protein 651 |
| 213488_at | 13.46 | 2.86E-05 | 1.481155587 | NM_001080437| | NA |
| 208977_x_at | 13.46 | 2.85E-05 | 1.039978639 | NM_006088| | TUBB2,tubulin, beta, 2 |
| 202124_s_at | 13.46 | 2.85E-05 | 1.217204245 | NM_015049| | ALS2CR3,amyotrophic lateral sclerosis 2 (juvenile) |
| 227656_at | 13.46 | 2.85E-05 | 1.1226826 | NM_018341| | NA |
| 236641_at | 13.46 | 2.85E-05 | 1.104758529 | NM_014875| | NA |
| 229201_at | 13.46 | 2.85E-05 | 1.22550836 | NA |  |
| 216724_at | 13.45 | 2.86E-05 | 1.236119983 | NM_001040260| | NA |
| 233350_s_at | 13.45 | 2.87E-05 | 1.271537319 | NM_015926| | TEX264,testis expressed sequence 264 |
| 218557_at | 13.45 | 2.86E-05 | 1.092259203 | NM_020202| | NIT2,nitrilase family, member 2 |
| 221951_at | 13.45 | 2.87E-05 | 1.179546863 | NM_001042463| | NA |
| 221103_s_at | 13.45 | 2.86E-05 | 1.354008349 | NM_018338| | WDR52,WD repeat domain 52 |
| 226123_at | 13.44 | 2.88E-05 | 1.08497571 | NM_017780| | CHD7,chromodomain helicase DNA binding protein 7 |
| 212949_at | 13.44 | 2.88E-05 | 1.064406718 | NM_015341| | BRRN1,barren |
| 236961_at | 13.43 | 2.88E-05 | 1.181391551 | NA |  |
| 227054_at | 13.43 | 2.88E-05 | 1.174601746 | NM_174928| | LOC221143,hypothetical protein LOC221143 |
| 1569519_at | 13.43 | 2.88E-05 | 1.288192901 | NM_001037501| | NA |
| 228200_at | 13.42 | 2.89E-05 | 1.176937624 | NA |  |
| 203452_at | 13.42 | 2.89E-05 | 1.182331544 | NM_012200| | B3GAT3,beta-1,3-glucuronyltransferase 3 |
| 233085_s_at | 13.42 | 2.90E-05 | 1.39293753 | NM_001031716| | NA |
| 204015_s_at | 13.42 | 2.89E-05 | 1.15206372 | NM_001394| | DUSP4,dual specificity phosphatase 4 isoform 1 |
| 204497_at | 13.42 | 2.90E-05 | 1.133168996 | NM_001116| | ADCY9,adenylate cyclase 9 |
| 205710_at | 13.42 | 2.89E-05 | 1.392211382 | NM_004525| | LRP2,low density lipoprotein-related protein 2 |
| 200075_s_at | 13.42 | 2.90E-05 | 1.09136216 | NM_000858| | GUK1,guanylate kinase 1 |
| 213051_at | 13.41 | 2.91E-05 | 1.090636939 | NM_020119| | ZC3HAV1,zinc finger antiviral protein isoform 1 |
| 224534_at | 13.41 | 2.90E-05 | 1.563185745 | NM_001039570| | NA |
| 223499_at | 13.41 | 2.90E-05 | 1.180902219 | NM_015645| | C1QTNF5,C1q and tumor necrosis factor related protein 5 |
| 239311_at | 13.41 | 2.90E-05 | 1.303703699 | NA |  |
| 231848_x_at | 13.4 | 2.91E-05 | 1.117437761 | NM_001032293| | NA |
| 228384_s_at | 13.4 | 2.92E-05 | 1.356145159 | NM_032709| | C10orf33,chromosome 10 open reading frame 33 |
| 219885_at | 13.4 | 2.92E-05 | 1.254560092 | NM_018042| | FLJ10260,hypothetical protein FLJ10260 |
| 210461_s_at | 13.4 | 2.92E-05 | 1.157004668 | NM_001003407| | ABLIM1,actin-binding LIM protein 1 isoform b |
| 213877_x_at | 13.39 | 2.93E-05 | 1.089415654 | NM_007108| | TCEB2,elongin B isoform a |
| 230329_s_at | 13.39 | 2.93E-05 | 1.190177971 | NM_007083| | NUDT6,nudix-type motif 6 isoform a |
| 228419_at | 13.39 | 2.92E-05 | 1.361995421 | NM_024042| | METRN,meteorin, glial cell differentiation regulator |
| 238672_at | 13.39 | 2.93E-05 | 1.221730735 | NA |  |
| 213134_x_at | 13.39 | 2.92E-05 | 1.138030078 | NM_006806| | BTG3,B-cell translocation gene 3 |
| 235347_at | 13.39 | 2.93E-05 | 1.318984073 | NM_032773| | LRCH3,leucine-rich repeats and calponin homology (CH) |
| 1553218_a_at | 13.39 | 2.93E-05 | 1.086812062 | NM_032434| | ZNF512,zinc finger protein 512 |
| 202850_at | 13.38 | 2.94E-05 | 1.175787094 | NM_001122674| | NA |
| 200049_at | 13.38 | 2.93E-05 | 1.074827722 | NM_007067| | MYST2,MYST histone acetyltransferase 2 |
| 243366_s_at | 13.38 | 2.94E-05 | 1.742244328 | NA |  |
| 203910_at | 13.37 | 2.95E-05 | 1.128381456 | NM_004815| | PARG1,PTPL1-associated RhoGAP 1 |
| 232276_at | 13.37 | 2.96E-05 | 1.419475328 | NM_153456| | HS6ST3,heparan sulfate 6-O-sulfotransferase 3 |
| 223429_x_at | 13.37 | 2.96E-05 | 1.090748504 | NM_020701| | KIAA1160,KIAA1160 protein |
| 203411_s_at | 13.37 | 2.95E-05 | 1.261983039 | NM_005572| | LMNA,lamin A/C isoform 2 |
| 202609_at | 13.36 | 2.96E-05 | 1.200358856 | NM_004447| | EPS8,epidermal growth factor receptor pathway |
| 213512_at | 13.36 | 2.96E-05 | 1.190439087 | NM_174891| | C14orf79,chromosome 14 open reading frame 79 |
| 239151_at | 13.36 | 2.97E-05 | 1.23732579 | NA |  |
| 1559214_at | 13.36 | 2.96E-05 | 1.522574805 | NA |  |
| 203921_at | 13.35 | 2.97E-05 | 1.207396396 | NM_004267| | CHST2,carbohydrate (N-acetylglucosamine-6-O) |
| 209773_s_at | 13.35 | 2.97E-05 | 1.089782372 | NM_001034| | RRM2,ribonucleotide reductase M2 polypeptide |
| 211685_s_at | 13.35 | 2.97E-05 | 1.335832552 | NM_001040624| | NA |
| 223555_at | 13.35 | 2.97E-05 | 1.067068745 | NM_001037533| | NA |
| 222826_at | 13.34 | 2.99E-05 | 1.066709318 | NM_012388| | PLDN,pallidin |
| 238574_at | 13.34 | 2.98E-05 | 1.175356112 | NM_033412| | MCART1,mitochondrial carrier triple repeat 1 |
| 221931_s_at | 13.33 | 2.99E-05 | 1.08825934 | NM_001013437| | NA |
| 1557123_a_at | 13.33 | 2.99E-05 | 1.132433898 | NA |  |
| 225797_at | 13.33 | 3.00E-05 | 1.085176777 | NM_172251| | MRPL54,mitochondrial ribosomal protein L54 |
| 209588_at | 13.33 | 2.99E-05 | 1.153387283 | NM_004442| | EPHB2,ephrin receptor EphB2 isoform 2 precursor |
| 222519_s_at | 13.33 | 3.00E-05 | 1.204872275 | NM_018010| | ESRRBL1,estrogen-related receptor beta like 1 |
| 208715_at | 13.32 | 3.01E-05 | 1.138971042 | NM_019026| | LOC54499,putative membrane protein |
| 217598_at | 13.32 | 3.00E-05 | 1.111068713 | NA |  |
| 208966_x_at | 13.3 | 3.04E-05 | 1.747836792 | NM_005531| | IFI16,interferon, gamma-inducible protein 16 |
| 224691_at | 13.3 | 3.04E-05 | 1.096405008 | NA |  |
| 235554_x_at | 13.3 | 3.04E-05 | 1.325948239 | NM_145048| | MGC29898,hypothetical protein MGC29898 |
| 203855_at | 13.29 | 3.04E-05 | 1.20739299 | NM_014969| | WDR47,WD repeat domain 47 |
| 203196_at | 13.29 | 3.05E-05 | 1.103142099 | NM_001105515| | NA |
| 212217_at | 13.29 | 3.04E-05 | 1.110326061 | NM_001042385| | NA |
| 202898_at | 13.29 | 3.05E-05 | 1.190085117 | NM_014654| | SDC3,syndecan 3 |
| 235587_at | 13.29 | 3.05E-05 | 1.181661099 | NA |  |
| 208212_s_at | 13.28 | 3.06E-05 | 1.409404089 | NM_004304| | ALK,anaplastic lymphoma kinase Ki-1 |
| 66053_at | 13.28 | 3.06E-05 | 1.126510925 | NM_001079559| | NA |
| 222122_s_at | 13.28 | 3.06E-05 | 1.070696348 | NM_001081550| | NA |
| 211340_s_at | 13.27 | 3.07E-05 | 1.246415146 | NM_006500| | MCAM,melanoma cell adhesion molecule |
| 223712_at | 13.27 | 3.07E-05 | 1.195041333 | NM_032151| | PCBD2,dimerization cofactor of hepatocyte nuclear |
| 1552789_at | 13.27 | 3.07E-05 | 1.365305072 | NM_003262| | TLOC1,translocation protein 1 |
| 202984_s_at | 13.27 | 3.07E-05 | 1.160349348 | NM_001015048| | NA |
| 228073_at | 13.27 | 3.07E-05 | 1.136833371 | NM_152667| | HDHD4,haloacid dehalogenase-like hydrolase domain |
| 225283_at | 13.26 | 3.08E-05 | 1.265798045 | NM_183376| | ARRDC4,arrestin domain containing 4 |
| 202182_at | 13.26 | 3.08E-05 | 1.08449704 | NM_021078| | GCN5L2,GCN5 general control of amino-acid synthesis |
| 208050_s_at | 13.26 | 3.09E-05 | 1.16335198 | NM_032982| | CASP2,caspase 2 isoform 1 preproprotein |
| 241706_at | 13.25 | 3.09E-05 | 1.237422415 | NM_153634| | CPNE8,copine VIII |
| 1558101_at | 13.25 | 3.09E-05 | 1.748890009 | NA |  |
| 230376_at | 13.25 | 3.09E-05 | 1.171748115 | NA |  |
| 221471_at | 13.25 | 3.09E-05 | 1.064887895 | NM_006811| | TDE1,tumor differentially expressed protein 1 |
| 234923_at | 13.25 | 3.09E-05 | 1.202807523 | NM_014990| | GARNL1,GTPase activating Rap/RanGAP domain-like 1 |
| 231716_at | 13.24 | 3.10E-05 | 1.082516525 | NM_001100588| | NA |
| 1558802_at | 13.24 | 3.11E-05 | 1.306606565 | NA |  |
| 218355_at | 13.24 | 3.10E-05 | 1.07203843 | NM_012310| | KIF4A,kinesin family member 4 |
| 1552925_at | 13.24 | 3.10E-05 | 1.526999276 | NM_020815| | PCDH10,protocadherin 10 isoform 2 precursor |
| 53968_at | 13.24 | 3.11E-05 | 1.066807194 | NM_030628| | KIAA1698,KIAA1698 protein |
| 243092_at | 13.23 | 3.12E-05 | 1.257114413 | NA |  |
| 203353_s_at | 13.22 | 3.13E-05 | 1.069618269 | NM_002384| | MBD1,methyl-CpG binding domain protein 1 isoform 4 |
| 214361_s_at | 13.22 | 3.13E-05 | 1.238894482 | NM_002926| | RGS12,regulator of G-protein signalling 12 isoform 2 |
| 225116_at | 13.22 | 3.14E-05 | 1.186273599 | NM_001113239| | NA |
| 223513_at | 13.22 | 3.13E-05 | 1.05609089 | NM_018451| | CENPJ,centromere protein J |
| 220155_s_at | 13.22 | 3.13E-05 | 1.078450136 | NM_001009877| | BRD9,bromodomain containing 9 isoform 2 |
| 226552_at | 13.22 | 3.14E-05 | 1.224437637 | NM_203434| | IER5L,immediate early response 5-like protein |
| 238844_s_at | 13.22 | 3.14E-05 | 1.322134212 | NM_000272| | NPHP1,nephrocystin isoform 1 |
| 242838_at | 13.22 | 3.14E-05 | 1.377443353 | NM_024871| | FLJ12748,hypothetical protein FLJ12748 |
| 228355_s_at | 13.21 | 3.14E-05 | 1.080533791 | NM_174889| | LOC91942,hypothetical protein LOC91942 |
| 223366_at | 13.21 | 3.14E-05 | 1.353213541 | NA |  |
| 218801_at | 13.21 | 3.14E-05 | 1.340569614 | NM_020121| | UGCGL2,UDP-glucose:glycoprotein glucosyltransferase 2 |
| 227479_at | 13.21 | 3.14E-05 | 1.096144563 | NM_020340| | KIAA1244,KIAA1244 |
| 223042_s_at | 13.21 | 3.14E-05 | 1.071035274 | NM_023934| | FUNDC2,FUN14 domain containing 2 |
| 227875_at | 13.2 | 3.15E-05 | 1.184269479 | NM_033495| | KLHL13,kelch-like 13 |
| 210896_s_at | 13.2 | 3.15E-05 | 1.22150378 | NM_004318| | ASPH,aspartate beta-hydroxylase isoform a |
| 225137_at | 13.2 | 3.15E-05 | 1.106938933 | NA |  |
| 1555460_a_at | 13.19 | 3.17E-05 | 1.15195348 | NM_001099406| | NA |
| 217957_at | 13.18 | 3.18E-05 | 1.032012895 | NM_013242| | GTL3,transcription factor IIB |
| 225749_at | 13.18 | 3.19E-05 | 1.105290125 | NM_001010878| | LOC283951,hypothetical protein LOC283951 |
| 229610_at | 13.17 | 3.20E-05 | 1.057301144 | NM_152515| | FLJ40629,hypothetical protein FLJ40629 |
| 207515_s_at | 13.17 | 3.19E-05 | 1.088473938 | NM_004875| | POLR1C,RNA polymerase I subunit isoform 2 |
| 221833_at | 13.17 | 3.20E-05 | 1.726841081 | NM_031490| | LONP,peroxisomal lon protease |
| 203314_at | 13.17 | 3.20E-05 | 1.10030552 | NM_012227| | GTPBP6,pseudoautosomal GTP-binding protein-like |
| 212907_at | 13.17 | 3.19E-05 | 1.104938114 | NM_021194| | SLC30A1,solute carrier family 30 (zinc transporter), |
| 240458_at | 13.16 | 3.22E-05 | 1.537229838 | NA |  |
| 204653_at | 13.16 | 3.21E-05 | 1.297964167 | NM_001032280| | NA |
| 212786_at | 13.15 | 3.22E-05 | 1.144496786 | NM_015226| | KIAA0350,KIAA0350 protein |
| 225725_at | 13.15 | 3.22E-05 | 1.4778136 | NA |  |
| 225377_at | 13.15 | 3.22E-05 | 1.11998262 | NM_017995| | NA |
| 202169_s_at | 13.15 | 3.22E-05 | 1.102294394 | NM_015423| | AASDHPPT,aminoadipate-semialdehyde |
| 204139_x_at | 13.15 | 3.22E-05 | 1.109419053 | NM_003422| | ZNF42,zinc finger protein 42 isoform 1 |
| 212282_at | 13.14 | 3.23E-05 | 1.091986659 | NM_014573| | MAC30,hypothetical protein MAC30 |
| 1554740_a_at | 13.14 | 3.23E-05 | 1.163578308 | NM_005897| | IPP,intracisternal A particle-promoted polypeptide |
| 229376_at | 13.14 | 3.23E-05 | 1.592392589 | NM_002763| | PROX1,prospero-related homeobox 1 |
| 221098_x_at | 13.14 | 3.24E-05 | 1.186390568 | NM_006649| | UTP14A,UTP14, U3 small nucleolar ribonucleoprotein, |
| 223185_s_at | 13.14 | 3.24E-05 | 1.504726467 | NM_030762| | BHLHB3,basic helix-loop-helix domain containing, class |
| 209009_at | 13.14 | 3.23E-05 | 1.040092142 | NM_001984| | ESD,esterase D/formylglutathione hydrolase |
| 211671_s_at | 13.13 | 3.25E-05 | 1.229932993 | NM_000176| | NR3C1,nuclear receptor subfamily 3, group C, member 1 |
| 235015_at | 13.13 | 3.25E-05 | 1.297189257 | NA |  |
| 200968_s_at | 13.13 | 3.25E-05 | 1.090079744 | NM_000942| | PPIB,peptidylprolyl isomerase B precursor |
| 204846_at | 13.13 | 3.24E-05 | 1.282426515 | NM_000096| | CP,ceruloplasmin (ferroxidase) |
| 229366_at | 13.12 | 3.26E-05 | 1.230428138 | NA |  |
| 230528_s_at | 13.12 | 3.26E-05 | 1.216272083 | NA |  |
| 209317_at | 13.11 | 3.28E-05 | 1.079701865 | NM_004875| | POLR1C,RNA polymerase I subunit isoform 2 |
| 228540_at | 13.11 | 3.27E-05 | 1.383634416 | NM_006775| | QKI,quaking homolog, KH domain RNA binding isoform |
| 236010_at | 13.11 | 3.27E-05 | 1.194000288 | NA |  |
| 235532_at | 13.11 | 3.27E-05 | 1.421973253 | NM_145167| | PIGM,PIG-M mannosyltransferase |
| 218086_at | 13.11 | 3.27E-05 | 1.206353415 | NM_015392| | NPDC1,neural proliferation, differentiation and |
| 202038_at | 13.11 | 3.27E-05 | 1.058999316 | NM_004788| | UBE4A,ubiquitination factor E4A |
| 201410_at | 13.1 | 3.29E-05 | 1.041668932 | NM_001100623| | NA |
| 212326_at | 13.1 | 3.29E-05 | 1.106580259 | NM_015378| | VPS13D,vacuolar protein sorting 13D isoform 1 |
| 229404_at | 13.1 | 3.29E-05 | 1.280162161 | NM_057179| | TWIST2,twist homolog 2 |
| 202345_s_at | 13.1 | 3.28E-05 | 1.066843555 | NM_001444| | FABP5,fatty acid binding protein 5 |
| 225875_s_at | 13.1 | 3.29E-05 | 1.133461629 | NM_020448| | DJ462O23.2,hypothetical protein dJ462O23.2 |
| 212594_at | 13.09 | 3.29E-05 | 1.223035274 | NM_014456| | PDCD4,programmed cell death 4 isoform 1 |
| 52169_at | 13.09 | 3.29E-05 | 1.060171703 | NM_001003786| | LYK5,protein kinase LYK5 isoform 2 |
| 229092_at | 13.09 | 3.29E-05 | 1.605410835 | NM_021005| | NR2F2,nuclear receptor subfamily 2, group F, member 2 |
| 228987_at | 13.09 | 3.30E-05 | 1.092269273 | NA |  |
| 220354_at | 13.09 | 3.29E-05 | 1.213755774 | NA |  |
| 244033_at | 13.09 | 3.30E-05 | 1.157448294 | NM_152446| | NA |
| 223044_at | 13.08 | 3.31E-05 | 1.520029912 | NM_014585| | SLC40A1,solute carrier family 40 (iron-regulated |
| 202368_s_at | 13.08 | 3.31E-05 | 1.156413574 | NM_012288| | TRAM2,translocation-associated membrane protein 2 |
| 1553107_s_at | 13.08 | 3.31E-05 | 1.173950054 | NM_152409| | FLJ37562,hypothetical protein FLJ37562 |
| 225390_s_at | 13.06 | 3.34E-05 | 1.036181079 | NM_015995| | KLF13,Kruppel-like factor 13 |
| 222398_s_at | 13.06 | 3.34E-05 | 1.069335821 | NM_004247| | U5-116KD,U5 snRNP-specific protein, 116 kD |
| 222830_at | 13.05 | 3.36E-05 | 1.188599257 | NM_014552| | TFCP2L2,leader-binding protein 32 isoform 1 |
| 222853_at | 13.04 | 3.37E-05 | 1.220721088 | NM_013281| | FLRT3,fibronectin leucine rich transmembrane protein 3 |
| 230143_at | 13.04 | 3.37E-05 | 1.419976587 | NM_152470| | C18orf23,chromosome 18 open reading frame 23 |
| 212994_at | 13.04 | 3.37E-05 | 1.069726793 | NM_001081550| | NA |
| 237008_at | 13.04 | 3.38E-05 | 1.497620655 | NA |  |
| 207018_s_at | 13.04 | 3.37E-05 | 2.18391781 | NM_004163| | RAB27B,RAB27B, member RAS oncogene family |
| 201391_at | 13.04 | 3.37E-05 | 1.097914773 | NM_016292| | TRAP1,TNF receptor-associated protein 1 |
| 227722_at | 13.04 | 3.37E-05 | 1.290374156 | NM_001025| | RPS23,ribosomal protein S23 |
| 215034_s_at | 13.04 | 3.37E-05 | 1.580137198 | NM_014220| | TM4SF1,transmembrane 4 superfamily member 1 |
| 219274_at | 13.03 | 3.39E-05 | 1.230157878 | NM_012338| | TM4SF12,transmembrane 4 superfamily member 12 |
| 218741_at | 13.03 | 3.39E-05 | 1.099479953 | NM_001002876| | C22orf18,proliferation associated nuclear element 1 |
| 212609_s_at | 13.02 | 3.40E-05 | 1.21524801 | NM_005465| | AKT3,v-akt murine thymoma viral oncogene homolog 3 |
| 209050_s_at | 13.02 | 3.40E-05 | 1.215404372 | NM_001042368| | NA |
| 203016_s_at | 13.02 | 3.40E-05 | 1.157129774 | NM_014021| | SSX2IP,synovial sarcoma, X breakpoint 2 interacting |
| 218588_s_at | 13.02 | 3.40E-05 | 1.17858306 | NM_018691| | C5orf3,chromosome 5 open reading frame 3 |
| 209591_s_at | 13.01 | 3.41E-05 | 1.122991678 | NM_001719| | BMP7,bone morphogenetic protein 7 precursor |
| 232659_at | 13.01 | 3.41E-05 | 1.258873636 | NA |  |
| 223312_at | 13.01 | 3.41E-05 | 1.116559325 | NM_032319| | C2orf7,chromosome 2 open reading frame 7 |
| 229743_at | 13.01 | 3.41E-05 | 1.186874751 | NM_182755| | LOC220929,hypothetical protein LOC220929 |
| 225594_at | 13.01 | 3.41E-05 | 1.089071549 | NM_001039618| | NA |
| 244546_at | 13.01 | 3.41E-05 | 1.438530103 | NM_018947| | CYCS,cytochrome c |
| 1558750_a_at | 13 | 3.42E-05 | 1.319330118 | NA |  |
| 204742_s_at | 13 | 3.42E-05 | 1.170183283 | NM_015032| | APRIN,androgen-induced prostate proliferative shutoff |
| 235588_at | 12.99 | 3.44E-05 | 1.235944064 | NM_001017420| | NA |
| 235882_at | 12.97 | 3.47E-05 | 1.71206325 | NA |  |
| 241713_s_at | 12.97 | 3.46E-05 | 1.320749206 | NM_001033559| | NA |
| 229884_s_at | 12.97 | 3.47E-05 | 1.164076267 | NM_015950| | MRPL2,mitochondrial ribosomal protein L2 |
| 204662_at | 12.96 | 3.48E-05 | 1.161505594 | NM_014711| | CP110,CP110 protein |
| 212934_at | 12.96 | 3.49E-05 | 1.071201523 | NM_001077619| | NA |
| 225036_at | 12.96 | 3.49E-05 | 1.053152749 | NM_001001790| | C9orf105,chromosome 9 open reading frame 105 |
| 230030_at | 12.95 | 3.49E-05 | 1.25932232 | NM_001077188| | NA |
| 219758_at | 12.95 | 3.49E-05 | 1.199641786 | NM_024926| | FLJ12571,hypothetical protein FLJ12571 |
| 222077_s_at | 12.94 | 3.50E-05 | 1.109977905 | NM_001126103| | NA |
| 217511_at | 12.94 | 3.50E-05 | 1.211284519 | NM_030929| | KAZALD1,Kazal-type serine protease inhibitor domain 1 |
| 211488_s_at | 12.94 | 3.50E-05 | 1.420115465 | NM_002214| | ITGB8,integrin, beta 8 |
| 239033_at | 12.93 | 3.53E-05 | 1.150573219 | NA |  |
| 222777_s_at | 12.93 | 3.53E-05 | 1.309855478 | NM_001042424| | NA |
| 229647_at | 12.93 | 3.52E-05 | 1.113239433 | NA |  |
| 232256_s_at | 12.93 | 3.52E-05 | 1.433494467 | NA |  |
| 213612_x_at | 12.92 | 3.54E-05 | 1.164405341 | NM_001037501| | NA |
| 1560028_at | 12.92 | 3.53E-05 | 1.196584883 | NM_001082969| | NA |
| 236038_at | 12.92 | 3.53E-05 | 1.187689688 | NA |  |
| 226580_at | 12.92 | 3.53E-05 | 1.095807348 | NM_032352| | BRMS1L,breast cancer metastasis-suppressor 1-like |
| 239654_at | 12.92 | 3.53E-05 | 1.389679908 | NM_025134| | CHD9,chromodomain helicase DNA binding protein 9 |
| 204026_s_at | 12.92 | 3.53E-05 | 1.045783876 | NM_001005413| | ZWINT,ZW10 interactor isoform b |
| 227491_at | 12.9 | 3.57E-05 | 1.377481365 | NM_024090| | ELOVL6,ELOVL family member 6, elongation of long chain |
| 242607_at | 12.9 | 3.57E-05 | 1.476326342 | NA |  |
| 203801_at | 12.9 | 3.56E-05 | 1.289176868 | NM_022100| | MRPS14,mitochondrial ribosomal protein S14 |
| 229530_at | 12.89 | 3.59E-05 | 1.764640491 | NA |  |
| 220200_s_at | 12.89 | 3.58E-05 | 1.158728196 | NM_020382| | SET8,SET domain-containing protein 8 |
| 209597_s_at | 12.88 | 3.59E-05 | 1.244402197 | NM_007257| | NA |
| 231925_at | 12.88 | 3.59E-05 | 1.280836936 | NA |  |
| 208965_s_at | 12.88 | 3.60E-05 | 1.779897555 | NM_005531| | IFI16,interferon, gamma-inducible protein 16 |
| 224577_at | 12.87 | 3.60E-05 | 1.120015387 | NM_001031711| | NA |
| 206448_at | 12.87 | 3.62E-05 | 1.436143468 | NM_014951| | NA |
| 210555_s_at | 12.87 | 3.60E-05 | 1.151715928 | NM_004555| | NFATC3,cytoplasmic nuclear factor of activated T-cells |
| 227604_at | 12.86 | 3.62E-05 | 1.195007196 | NA |  |
| 236278_at | 12.86 | 3.63E-05 | 1.257919376 | NM_003532| | HIST1H3E,H3 histone family, member D |
| 210268_at | 12.86 | 3.62E-05 | 1.252465285 | NM_002504| | NFX1,nuclear transcription factor, X-box binding 1 |
| 206110_at | 12.86 | 3.63E-05 | 1.328414036 | NM_003536| | HIST1H3H,H3 histone family, member K |
| 205012_s_at | 12.86 | 3.63E-05 | 1.096437656 | NM_001040427| | NA |
| 243740_at | 12.86 | 3.62E-05 | 1.383865227 | NA |  |
| 222671_s_at | 12.85 | 3.65E-05 | 1.195951314 | NM_023007| | FLJ12517,hypothetical protein FLJ12517 |
| 212595_s_at | 12.85 | 3.65E-05 | 1.235168926 | NM_014764| | DAZAP2,DAZ associated protein 2 |
| 217985_s_at | 12.85 | 3.64E-05 | 1.312290425 | NM_013448| | BAZ1A,bromodomain adjacent to zinc finger domain, 1A |
| 210270_at | 12.85 | 3.64E-05 | 1.293446561 | NM_004296| | RGS6,regulator of G-protein signalling 6 |
| 235754_at | 12.85 | 3.63E-05 | 1.332694385 | NM_000410| | HFE,hemochromatosis protein isoform 1 precursor |
| 243501_at | 12.84 | 3.66E-05 | 1.379418091 | NA |  |
| 205822_s_at | 12.84 | 3.65E-05 | 1.252762313 | NM_001098272| | NA |
| 209618_at | 12.84 | 3.65E-05 | 1.321369402 | NM_001332| | CTNND2,catenin (cadherin-associated protein), delta 2 |
| 209247_s_at | 12.83 | 3.67E-05 | 1.152818906 | NM_005692| | ABCF2,ATP-binding cassette, sub-family F, member 2 |
| 228656_at | 12.83 | 3.67E-05 | 1.521792923 | NM_002763| | PROX1,prospero-related homeobox 1 |
| 229271_x_at | 12.83 | 3.67E-05 | 2.349350525 | NM_001854| | COL11A1,alpha 1 type XI collagen isoform A |
| 1556473_at | 12.83 | 3.68E-05 | 1.483440482 | NA |  |
| 219112_at | 12.82 | 3.69E-05 | 1.051683304 | NM_016340| | RAPGEF6,PDZ domain-containing guanine nucleotide |
| 244457_at | 12.82 | 3.68E-05 | 1.31279461 | NA |  |
| 214671_s_at | 12.82 | 3.68E-05 | 1.240959154 | NM_001092| | ABR,active breakpoint cluster region-related |
| 228066_at | 12.82 | 3.68E-05 | 1.164815902 | NA |  |
| 235224_s_at | 12.81 | 3.71E-05 | 1.554911129 | NA |  |
| 218357_s_at | 12.81 | 3.71E-05 | 1.074122919 | NM_012459| | TIMM8B,translocase of inner mitochondrial membrane 8 |
| 226546_at | 12.81 | 3.70E-05 | 1.317838645 | NA |  |
| 200746_s_at | 12.8 | 3.71E-05 | 1.046361819 | NM_002074| | GNB1,guanine nucleotide-binding protein, beta-1 |
| 228830_s_at | 12.8 | 3.72E-05 | 1.336660768 | NM_006856| | ATF7,activating transcription factor 7 |
| 206935_at | 12.8 | 3.72E-05 | 1.542841839 | NM_002590| | PCDH8,protocadherin 8 isoform 1 precursor |
| 1563111_a_at | 12.79 | 3.73E-05 | 1.180525928 | NM_017861| | PIGX,GPI-mannosyltransferase subunit |
| 225779_at | 12.79 | 3.74E-05 | 1.158632945 | NM_005094| | SLC27A4,solute carrier family 27 (fatty acid |
| 217934_x_at | 12.79 | 3.72E-05 | 1.078171702 | NM_005861| | STUB1,STIP1 homology and U-box containing protein 1 |
| 229194_at | 12.79 | 3.72E-05 | 1.342740489 | NM_032373| | PCGF5,polycomb group ring finger 5 |
| 235760_at | 12.79 | 3.72E-05 | 1.413198766 | NM_022455| | NSD1,nuclear receptor binding SET domain protein 1 |
| 224002_s_at | 12.78 | 3.75E-05 | 1.272103976 | NM_181342| | FKBP7,FK506-binding protein 7 isoform 2 precursor |
| 222781_s_at | 12.77 | 3.76E-05 | 1.078934262 | NM_017998| | C9orf40,chromosome 9 open reading frame 40 |
| 221744_at | 12.77 | 3.76E-05 | 1.095939532 | NM_005828| | HAN11,WD-repeat protein |
| 1554102_a_at | 12.77 | 3.76E-05 | 1.219039161 | NM_001079669| | NA |
| 225292_at | 12.77 | 3.76E-05 | 1.241713028 | NM_032888| | COL27A1,collagen, type XXVII, alpha 1 |
| 224525_s_at | 12.77 | 3.76E-05 | 1.37097155 | NM_001011708| | PTD004,GTP-binding protein PTD004 isoform 2 |
| 212643_at | 12.77 | 3.76E-05 | 1.025822824 | NM_144578| | C14orf32,MAPK-interacting and spindle-stabilizing |
| 225523_at | 12.77 | 3.76E-05 | 1.082678829 | NM_053050| | MRPL53,mitochondrial ribosomal protein L53 |
| 238691_at | 12.76 | 3.78E-05 | 1.64651578 | NA |  |
| 201251_at | 12.76 | 3.78E-05 | 1.1055751 | NM_002654| | PKM2,pyruvate kinase 3 isoform 1 |
| 239897_at | 12.75 | 3.79E-05 | 1.253015837 | NM_001077440| | NA |
| 213364_s_at | 12.75 | 3.80E-05 | 1.221718338 | NM_003099| | SNX1,sorting nexin 1 isoform a |
| 203423_at | 12.75 | 3.79E-05 | 1.151297043 | NM_002899| | RBP1,retinol binding protein 1, cellular |
| 228724_at | 12.74 | 3.80E-05 | 1.477343806 | NA |  |
| 55662_at | 12.74 | 3.80E-05 | 1.164804273 | NM_024541| | C10orf76,chromosome 10 open reading frame 76 |
| 244114_x_at | 12.74 | 3.80E-05 | 1.290958554 | NA |  |
| 203188_at | 12.74 | 3.81E-05 | 1.136023085 | NM_006876| | B3GNT6,beta-1,3-N-acetylglucosaminyltransferase bGnT-6 |
| 234299_s_at | 12.73 | 3.83E-05 | 1.459083303 | NM_016350| | NIN,ninein isoform 4 |
| 206235_at | 12.73 | 3.83E-05 | 1.337873486 | NM_001098268| | NA |
| 235509_at | 12.73 | 3.82E-05 | 1.124256483 | NM_152416| | MGC40214,hypothetical protein MGC40214 |
| 203916_at | 12.72 | 3.83E-05 | 1.058876804 | NM_003635| | NDST2,N-deacetylase/N-sulfotransferase (heparan |
| 215707_s_at | 12.72 | 3.83E-05 | 1.252511101 | NM_000311| | PRNP,prion protein preproprotein |
| 228967_at | 12.72 | 3.83E-05 | 1.189496473 | NM_005801| | SUI1,putative translation initiation factor |
| 228162_at | 12.72 | 3.83E-05 | 1.186914488 | NM_001984| | ESD,esterase D/formylglutathione hydrolase |
| 212554_at | 12.72 | 3.84E-05 | 1.129373354 | NM_006366| | CAP2,adenylyl cyclase-associated protein 2 |
| 201790_s_at | 12.72 | 3.83E-05 | 1.164585141 | NM_001360| | DHCR7,7-dehydrocholesterol reductase |
| 205406_s_at | 12.72 | 3.84E-05 | 1.212124384 | NM_017425| | SPA17,sperm autoantigenic protein 17 |
| 222694_at | 12.71 | 3.86E-05 | 1.17940662 | NA |  |
| 238659_at | 12.71 | 3.86E-05 | 1.332619602 | NA |  |
| 221879_at | 12.71 | 3.85E-05 | 1.092021972 | NM_001031733| | NA |
| 1560082_at | 12.71 | 3.86E-05 | 1.324509272 | NA |  |
| 228214_at | 12.71 | 3.85E-05 | 1.322442717 | NA |  |
| 232528_at | 12.71 | 3.86E-05 | 1.469206561 | NA |  |
| 218287_s_at | 12.71 | 3.86E-05 | 1.107021097 | NM_012199| | EIF2C1,eukaryotic translation initiation factor 2C, 1 |
| 224900_at | 12.71 | 3.85E-05 | 1.060256229 | NM_016376| | ANKFY1,ankyrin repeat and FYVE domain containing 1 |
| 203394_s_at | 12.7 | 3.86E-05 | 1.137742131 | NM_005524| | HES1,hairy and enhancer of split 1 |
| 222889_at | 12.7 | 3.86E-05 | 1.115271099 | NM_022836| | DCLRE1B,DNA cross-link repair 1B (PSO2 homolog, S. |
| 204817_at | 12.7 | 3.87E-05 | 1.066451036 | NM_012291| | ESPL1,extra spindle poles like 1 |
| 210473_s_at | 12.69 | 3.88E-05 | 1.061094068 | NM_145290| | GPR125,G protein-coupled receptor 125 |
| 215248_at | 12.69 | 3.89E-05 | 1.289928846 | NM_001001549| | GRB10,growth factor receptor-bound protein 10 isoform |
| 201625_s_at | 12.68 | 3.90E-05 | 1.226008824 | NM_005542| | INSIG1,insulin induced gene 1 isoform 1 |
| 214805_at | 12.68 | 3.91E-05 | 1.202165716 | NM_001416| | EIF4A1,eukaryotic translation initiation factor 4A, |
| 204178_s_at | 12.68 | 3.90E-05 | 1.063445533 | NM_006328| | RBM14,RNA binding motif protein 14 |
| 1568815_a_at | 12.68 | 3.90E-05 | 1.403775152 | NM_024045| | DDX50,nucleolar protein GU2 |
| 79005_at | 12.67 | 3.92E-05 | 1.068270946 | NM_024881| | SLC35E1,solute carrier family 35, member E1 |
| 226802_s_at | 12.67 | 3.92E-05 | 1.132427455 | NA |  |
| 233341_s_at | 12.67 | 3.92E-05 | 1.086633723 | NM_019014| | POLR1B,RNA polymerase I polypeptide B |
| 229287_at | 12.67 | 3.93E-05 | 1.158180738 | NM_014982| | PCNX,pecanex homolog |
| 231034_s_at | 12.67 | 3.92E-05 | 1.23243273 | NA |  |
| 227565_at | 12.66 | 3.93E-05 | 1.377478669 | NA |  |
| 235309_at | 12.66 | 3.93E-05 | 1.13554322 | NM_001019| | RPS15A,ribosomal protein S15a |
| 59625_at | 12.66 | 3.94E-05 | 1.322899092 | NM_003946| | NOL3,nucleolar protein 3 |
| 1555848_at | 12.65 | 3.96E-05 | 1.222629063 | NA |  |
| 228304_at | 12.65 | 3.94E-05 | 1.239478401 | NA |  |
| 225346_at | 12.65 | 3.96E-05 | 1.179311012 | NM_001033050| | NA |
| 212178_s_at | 12.64 | 3.96E-05 | 1.054839975 | NM_001099415| | NA |
| 228702_at | 12.64 | 3.97E-05 | 1.389399408 | NA |  |
| 221235_s_at | 12.64 | 3.97E-05 | 1.140333555 | NA |  |
| 236247_at | 12.64 | 3.96E-05 | 1.161534769 | NM_199044| | NSUN4,NOL1/NOP2/Sun domain family 4 protein |
| 212858_at | 12.64 | 3.97E-05 | 1.129614655 | NM_152341| | PAQR4,progestin and adipoQ receptor family member IV |
| 1555912_at | 12.64 | 3.96E-05 | 1.423121052 | NA |  |
| 219544_at | 12.63 | 3.98E-05 | 1.106496599 | NM_024808| | FLJ22624,FLJ22624 protein |
| 209939_x_at | 12.63 | 3.99E-05 | 1.072567529 | NM_001127183| | NA |
| 230777_s_at | 12.63 | 3.98E-05 | 1.124171477 | NM_001040424| | NA |
| 212719_at | 12.63 | 3.98E-05 | 1.10918024 | NM_194449| | PLEKHE1,suprachiasmatic nucleus circadian oscillatory |
| 225012_at | 12.62 | 4.00E-05 | 1.135591106 | NM_005336| | HDLBP,high density lipoprotein binding protein |
| 46323_at | 12.62 | 4.01E-05 | 1.10941171 | NM_138793| | CANT1,calcium activated nucleotidase 1 |
| 243016_at | 12.62 | 4.00E-05 | 1.775065847 | NA |  |
| 233224_at | 12.62 | 4.00E-05 | 1.355974885 | NA |  |
| 212310_at | 12.61 | 4.01E-05 | 1.140077293 | NM_198551| | NA |
| 205042_at | 12.61 | 4.01E-05 | 1.227970473 | NM_005476| | GNE,UDP-N-acetylglucosamine-2-epimerase/N- |
| 207181_s_at | 12.61 | 4.01E-05 | 1.207487924 | NM_001227| | CASP7,caspase 7 isoform alpha precursor |
| 230904_at | 12.61 | 4.01E-05 | 1.414353368 | NM_031919| | CCDC10,cystatin and DUF19 domain-containing protein 1 |
| 201113_at | 12.6 | 4.02E-05 | 1.052311078 | NM_003321| | TUFM,Tu translation elongation factor, mitochondrial |
| 225341_at | 12.6 | 4.03E-05 | 1.212198382 | NM_001033050| | NA |
| 224756_s_at | 12.6 | 4.03E-05 | 1.100520615 | NM_021160| | BAT5,HLA-B associated transcript 5 |
| 220739_s_at | 12.6 | 4.03E-05 | 1.113850659 | NM_017623| | CNNM3,cyclin M3 isoform 1 |
| 236310_at | 12.59 | 4.05E-05 | 1.360405507 | NA |  |
| 226822_at | 12.59 | 4.05E-05 | 1.19722628 | NM_020225| | DKFZp762K222,hypothetical protein DKFZp762K222 |
| 200927_s_at | 12.59 | 4.04E-05 | 1.097180439 | NM_016322| | RAB14,GTPase Rab14 |
| 235205_at | 12.58 | 4.07E-05 | 1.256479777 | NA |  |
| 209275_s_at | 12.58 | 4.07E-05 | 1.108201025 | NM_000086| | CLN3,CLN3 protein |
| 231513_at | 12.58 | 4.06E-05 | 1.654591803 | NA |  |
| 212439_at | 12.57 | 4.09E-05 | 1.121430919 | NM_001006115| | IHPK1,inositol hexaphosphate kinase 1 isoform 2 |
| 229362_at | 12.57 | 4.08E-05 | 1.140863876 | NM_144709| | FLJ32312,hypothetical protein FLJ32312 |
| 218605_at | 12.56 | 4.11E-05 | 1.106963623 | NM_022366| | TFB2M,transcription factor B2, mitochondrial |
| 201626_at | 12.56 | 4.10E-05 | 1.229522534 | NM_005542| | INSIG1,insulin induced gene 1 isoform 1 |
| 221366_at | 12.56 | 4.11E-05 | 1.523675808 | NM_006168| | NKX6-1,NK6 transcription factor related, locus 1 |
| 203168_at | 12.56 | 4.10E-05 | 1.138566979 | NM_004381| | CREBL1,cAMP responsive element binding protein-like 1 |
| 212797_at | 12.56 | 4.10E-05 | 1.153668046 | NM_002959| | SORT1,sortilin 1 preproprotein |
| 218590_at | 12.56 | 4.09E-05 | 1.079680945 | NM_021830| | PEO1,twinkle |
| 201568_at | 12.54 | 4.14E-05 | 1.101630583 | NM_014402| | QP-C,low molecular mass ubiquinone-binding protein |
| 241989_at | 12.54 | 4.13E-05 | 1.286457505 | NM_001004056| | GRK4,G protein-coupled receptor kinase 4 isoform |
| 1555758_a_at | 12.54 | 4.13E-05 | 1.059710891 | NM_005192| | CDKN3,cyclin-dependent kinase inhibitor 3 |
| 228530_at | 12.54 | 4.14E-05 | 1.250411297 | NM_001071775| | NA |
| 206502_s_at | 12.54 | 4.13E-05 | 1.652555229 | NM_002196| | INSM1,insulinoma-associated 1 |
| 206443_at | 12.53 | 4.15E-05 | 1.248659209 | NM_006914| | RORB,RAR-related orphan receptor B |
| 203997_at | 12.53 | 4.16E-05 | 1.202040075 | NM_002829| | PTPN3,protein tyrosine phosphatase, non-receptor type |
| 218447_at | 12.52 | 4.17E-05 | 1.066059436 | NM_020188| | DC13,DC13 protein |
| 209054_s_at | 12.52 | 4.17E-05 | 1.104082634 | NM_001042424| | NA |
| 238785_at | 12.52 | 4.16E-05 | 1.219670544 | NA |  |
| 219001_s_at | 12.52 | 4.17E-05 | 1.118864677 | NM_024345| | MGC10765,hypothetical protein MGC10765 |
| 204821_at | 12.51 | 4.20E-05 | 1.518611426 | NM_006994| | BTN3A3,butyrophilin, subfamily 3, member A3 isoform a |
| 222748_s_at | 12.51 | 4.20E-05 | 1.344867492 | NM_017853| | TXNL4B,thioredoxin-like 4B |
| 214881_s_at | 12.51 | 4.20E-05 | 1.152392584 | NM_001076683| | NA |
| 213703_at | 12.5 | 4.22E-05 | 1.486934755 | NA |  |
| 204488_at | 12.5 | 4.21E-05 | 1.0975811 | NM_014908| | TMEM15,transmembrane protein 15 |
| 202181_at | 12.5 | 4.20E-05 | 1.277118627 | NM_014734| | KIAA0247,KIAA0247 |
| 229590_at | 12.5 | 4.21E-05 | 1.112740607 | NM_000977| | RPL13,ribosomal protein L13 |
| 212427_at | 12.5 | 4.20E-05 | 1.2692447 | NM_001080398| | NA |
| 226545_at | 12.5 | 4.20E-05 | 1.385286054 | NM_133493| | CD109,CD109 |
| 227211_at | 12.5 | 4.21E-05 | 1.218627209 | NM_001009936| | PHF19,PHD finger protein 19 isoform b |
| 229645_at | 12.49 | 4.23E-05 | 1.438274209 | NM_001044369| | NA |
| 206284_x_at | 12.49 | 4.22E-05 | 1.066724146 | NM_001834| | CLTB,clathrin, light polypeptide isoform a |
| 201490_s_at | 12.49 | 4.23E-05 | 1.144787875 | NM_005729| | PPIF,peptidylprolyl isomerase F precursor |
| 203816_at | 12.48 | 4.24E-05 | 1.111096263 | NM_080916| | DGUOK,deoxyguanosine kinase isoform a precursor |
| 242134_at | 12.48 | 4.25E-05 | 1.286757849 | NA |  |
| 1554038_at | 12.48 | 4.25E-05 | 1.137662093 | NM_018078| | FLJ10378,FLJ10378 protein isoform 1 |
| 222565_s_at | 12.48 | 4.24E-05 | 1.114895275 | NM_005813| | PRKD3,protein kinase D3 |
| 218791_s_at | 12.48 | 4.24E-05 | 1.206435772 | NM_024713| | C15orf29,chromosome 15 open reading frame 29 |
| 235626_at | 12.47 | 4.27E-05 | 1.092824505 | NM_020397| | CAMK1D,calcium/calmodulin-dependent protein kinase ID |
| 224660_at | 12.47 | 4.26E-05 | 1.086185981 | NM_001042616| | NA |
| 214274_s_at | 12.47 | 4.25E-05 | 1.09735568 | NM_001607| | ACAA1,acetyl-Coenzyme A acyltransferase 1 |
| 224013_s_at | 12.47 | 4.25E-05 | 1.538444451 | NM_031439| | SOX7,SRY-box 7 |
| 217881_s_at | 12.47 | 4.25E-05 | 1.089160204 | NM_001114091| | NA |
| 213088_s_at | 12.47 | 4.25E-05 | 1.094305459 | NM_015190| | DNAJC9,DnaJ homolog, subfamily C, member 9 |
| 240575_at | 12.46 | 4.27E-05 | 1.208531056 | NA |  |
| 1557053_s_at | 12.46 | 4.27E-05 | 1.072564612 | NM_003343| | UBE2G2,ubiquitin-conjugating enzyme E2G 2 isoform 1 |
| 230102_at | 12.46 | 4.28E-05 | 1.396863778 | NM_004454| | ETV5,ets variant gene 5 (ets-related molecule) |
| 229989_at | 12.46 | 4.27E-05 | 1.21690451 | NM_138378| | NA |
| 220076_at | 12.46 | 4.28E-05 | 1.833750643 | NM_054027| | ANKH,ankylosis, progressive homolog |
| 203275_at | 12.46 | 4.28E-05 | 1.116251027 | NM_002199| | IRF2,interferon regulatory factor 2 |
| 225221_at | 12.45 | 4.30E-05 | 1.161459833 | NA |  |
| 219549_s_at | 12.45 | 4.29E-05 | 1.066411916 | NM_006054| | RTN3,reticulon 3 isoform a |
| 209414_at | 12.45 | 4.29E-05 | 1.218501975 | NM_016263| | FZR1,Fzr1 protein |
| 201704_at | 12.44 | 4.32E-05 | 1.110185543 | NM_001114089| | NA |
| 219567_s_at | 12.44 | 4.31E-05 | 1.186419526 | NM_022774| | FLJ21144,hypothetical protein FLJ21144 |
| 208931_s_at | 12.44 | 4.31E-05 | 1.087086478 | NM_004516| | ILF3,interleukin enhancer binding factor 3 isoform b |
| 211061_s_at | 12.44 | 4.31E-05 | 1.099094179 | NM_002408| | MGAT2,alpha-1,6-mannosyl-glycoprotein |
| 229994_at | 12.43 | 4.33E-05 | 1.394363135 | NA |  |
| 212254_s_at | 12.43 | 4.33E-05 | 1.068859671 | NM_001723| | DST,dystonin isoform 1e precursor |
| 200745_s_at | 12.43 | 4.32E-05 | 1.067369928 | NM_002074| | GNB1,guanine nucleotide-binding protein, beta-1 |
| 243868_at | 12.43 | 4.33E-05 | 1.281563314 | NA |  |
| 238565_at | 12.42 | 4.35E-05 | 1.170111232 | NA |  |
| 227481_at | 12.42 | 4.34E-05 | 1.104946116 | NM_173515| | MAGI1,membrane associated guanylate kinase interacting |
| 225835_at | 12.42 | 4.34E-05 | 1.243877726 | NM_001046| | SLC12A2,solute carrier family 12 |
| 1570135_at | 12.42 | 4.35E-05 | 1.121693723 | NM_006300| | ZNF230,zinc finger protein 230 |
| 228736_at | 12.42 | 4.35E-05 | 1.344147584 | NM_133636| | HEL308,DNA helicase HEL308 |
| 238012_at | 12.42 | 4.34E-05 | 1.392258476 | NM_013379| | DPP7,dipeptidyl peptidase 7 preproprotein |
| 238021_s_at | 12.41 | 4.38E-05 | 1.144387778 | NA |  |
| 213757_at | 12.41 | 4.37E-05 | 1.136639833 | NA |  |
| 205247_at | 12.41 | 4.37E-05 | 1.185050287 | NM_004557| | NOTCH4,notch4 preproprotein |
| 221480_at | 12.4 | 4.38E-05 | 1.176382206 | NM_001003810| | HNRPD,heterogeneous nuclear ribonucleoprotein D |
| 213850_s_at | 12.4 | 4.40E-05 | 1.082257469 | NM_004719| | SFRS2IP,splicing factor, arginine/serine-rich 2, |
| 238829_at | 12.4 | 4.38E-05 | 1.299364212 | NA |  |
| 205729_at | 12.39 | 4.41E-05 | 1.511469134 | NM_003999| | OSMR,oncostatin M receptor |
| 239863_at | 12.39 | 4.41E-05 | 1.335521495 | NA |  |
| 221983_at | 12.39 | 4.40E-05 | 1.157918434 | NM_024293| | C2orf17,chromosome 2 open reading frame 17 |
| 204711_at | 12.38 | 4.43E-05 | 1.064997346 | NM_014804| | KIAA0753,KIAA0753 gene product |
| 238932_at | 12.38 | 4.44E-05 | 1.186728792 | NA |  |
| 244347_at | 12.38 | 4.43E-05 | 1.32549899 | NA |  |
| 221838_at | 12.38 | 4.43E-05 | 1.204042111 | NM_032775| | KELCHL,kelch-like |
| 230399_at | 12.38 | 4.44E-05 | 1.366860229 | NA |  |
| 224966_s_at | 12.37 | 4.44E-05 | 1.211095478 | NM_020175| | LOC56931,hypothetical protein from EUROIMAGE 1967720 |
| 217908_s_at | 12.37 | 4.45E-05 | 1.155309203 | NM_001017977| | NA |
| 230029_x_at | 12.37 | 4.45E-05 | 1.148844337 | NM_172070| | ZNF650,zinc finger protein 650 |
| 39817_s_at | 12.36 | 4.48E-05 | 1.093453044 | NM_006443| | C6orf108,putative c-Myc-responsive isoform 1 |
| 225259_at | 12.35 | 4.48E-05 | 1.120194763 | NM_016577| | RAB6B,RAB6B, member RAS oncogene family |
| 236958_at | 12.35 | 4.49E-05 | 1.160800821 | NA |  |
| 208803_s_at | 12.34 | 4.51E-05 | 1.041968119 | NM_006947| | SRP72,signal recognition particle 72kDa |
| 1554345_a_at | 12.34 | 4.50E-05 | 1.066609724 | NM_017676| | FLJ20125,hypothetical protein FLJ20125 |
| 218058_at | 12.34 | 4.52E-05 | 1.087234319 | NM_001101654| | NA |
| 236359_at | 12.34 | 4.50E-05 | 1.189706631 | NM_174934| | SCN4B,sodium channel, voltage-gated, type IV, beta |
| 200946_x_at | 12.33 | 4.54E-05 | 1.075057928 | NM_005271| | GLUD1,glutamate dehydrogenase 1 |
| 1559739_at | 12.33 | 4.52E-05 | 1.41389807 | NM_020244| | CHPT1,choline phosphotransferase 1 |
| 227925_at | 12.33 | 4.53E-05 | 1.286872848 | NA |  |
| 206501_x_at | 12.32 | 4.55E-05 | 1.163348585 | NM_004956| | ETV1,ets variant gene 1 |
| 218544_s_at | 12.32 | 4.55E-05 | 1.041093664 | NM_005772| | RCL1,RNA cyclase homolog |
| 203259_s_at | 12.32 | 4.56E-05 | 1.066589065 | NM_016063| | C6orf74,chromosome 6 open reading frame 74 |
| 226709_at | 12.32 | 4.55E-05 | 1.384010854 | NM_002942| | NA |
| 1563467_at | 12.31 | 4.56E-05 | 1.355857778 | NA |  |
| 236536_at | 12.31 | 4.57E-05 | 1.398429527 | NM_052917| | GALNT13,UDP-N-acetyl-alpha-D-galactosamine:polypeptide |
| 204744_s_at | 12.31 | 4.56E-05 | 1.013102693 | NM_002161| | IARS,isoleucine-tRNA synthetase |
| 219733_s_at | 12.31 | 4.58E-05 | 1.139259768 | NM_012254| | SLC27A5,solute carrier family 27 (fatty acid |
| 228813_at | 12.31 | 4.57E-05 | 1.289007324 | NM_006037| | HDAC4,histone deacetylase 4 |
| 231300_at | 12.3 | 4.59E-05 | 1.429317007 | NM_001014979| | NA |
| 212218_s_at | 12.3 | 4.58E-05 | 1.168185505 | NM_004104| | FASN,fatty acid synthase |
| 208890_s_at | 12.3 | 4.59E-05 | 1.132164762 | NM_012401| | NA |
| 213241_at | 12.29 | 4.60E-05 | 1.365511161 | NM_005761| | PLXNC1,plexin C1 |
| 205109_s_at | 12.29 | 4.60E-05 | 1.212590309 | NM_015320| | ARHGEF4,Rho guanine nucleotide exchange factor 4 isoform |
| 1566513_a_at | 12.29 | 4.60E-05 | 1.444125742 | NM_001098721| | NA |
| 235260_s_at | 12.29 | 4.62E-05 | 1.223602003 | NM_145048| | MGC29898,hypothetical protein MGC29898 |
| 209332_s_at | 12.28 | 4.64E-05 | 1.092004259 | NM_002382| | MAX,MAX protein isoform a |
| 222725_s_at | 12.28 | 4.64E-05 | 1.47100989 | NM_017734| | PALMD,palmdelphin |
| 227084_at | 12.28 | 4.63E-05 | 1.224847826 | NM_001390| | DTNA,dystrobrevin alpha isoform 1 |
| 220113_x_at | 12.28 | 4.63E-05 | 1.094409887 | NM_019014| | POLR1B,RNA polymerase I polypeptide B |
| 204955_at | 12.28 | 4.63E-05 | 1.111153858 | NM_006307| | SRPX,sushi-repeat-containing protein, X-linked |
| 213043_s_at | 12.27 | 4.65E-05 | 1.060872573 | NM_001079518| | NA |
| 1555486_a_at | 12.27 | 4.65E-05 | 1.237495464 | NM_024841| | FLJ14213,hypothetical protein FLJ14213 |
| 230240_at | 12.27 | 4.64E-05 | 1.165970424 | NA |  |
| 218742_at | 12.27 | 4.64E-05 | 1.077756103 | NM_022493| | NARFL,nuclear prelamin A recognition factor-like |
| 232527_at | 12.27 | 4.66E-05 | 1.138508146 | NA |  |
| 238712_at | 12.26 | 4.67E-05 | 1.309082556 | NA |  |
| 209360_s_at | 12.26 | 4.68E-05 | 1.666219676 | NM_001001890| | RUNX1,runt-related transcription factor 1 isoform b |
| 235193_at | 12.25 | 4.70E-05 | 1.689976314 | NA |  |
| 202423_at | 12.25 | 4.69E-05 | 1.105152255 | NM_001099412| | NA |
| 212930_at | 12.25 | 4.70E-05 | 1.229290106 | NM_001001323| | ATP2B1,plasma membrane calcium ATPase 1 isoform 1a |
| 224018_s_at | 12.24 | 4.70E-05 | 1.2768622 | NM_001037582| | NA |
| 214787_at | 12.24 | 4.71E-05 | 1.760833171 | NM_005848| | MYCPBP,c-myc promoter binding protein |
| 225108_at | 12.24 | 4.70E-05 | 1.238449017 | NA |  |
| 203163_at | 12.24 | 4.71E-05 | 1.240440893 | NM_005886| | KATNB1,katanin p80 subunit B 1 |
| 205548_s_at | 12.24 | 4.71E-05 | 1.137794959 | NM_006806| | BTG3,B-cell translocation gene 3 |
| 238068_at | 12.23 | 4.74E-05 | 1.189069437 | NM_006321| | ARIH2,ariadne homolog 2 |
| 227229_at | 12.23 | 4.72E-05 | 1.056434755 | NA |  |
| 223981_at | 12.23 | 4.74E-05 | 1.485584995 | NM_016350| | NIN,ninein isoform 4 |
| 238161_at | 12.23 | 4.74E-05 | 1.224387251 | NA |  |
| 37793_r_at | 12.23 | 4.73E-05 | 1.174352245 | NM_002878| | RAD51L3,RAD51-like 3 isoform 1 |
| 207160_at | 12.22 | 4.75E-05 | 1.270111803 | NM_000882| | IL12A,interleukin 12A precursor |
| 239657_x_at | 12.22 | 4.76E-05 | 1.08624883 | NA |  |
| 227523_s_at | 12.22 | 4.75E-05 | 1.164344774 | NM_016018| | PHF20L1,PHD finger protein 20-like 1 isoform 1 |
| 209208_at | 12.22 | 4.75E-05 | 1.080256453 | NM_004870| | MPDU1,mannose-P-dolichol utilization defect 1 |
| 224766_at | 12.22 | 4.75E-05 | 1.393276589 | NM_000997| | RPL37,ribosomal protein L37 |
| 204842_x_at | 12.22 | 4.75E-05 | 1.047035961 | NM_004157| | PRKAR2A,cAMP-dependent protein kinase, regulatory |
| 218874_s_at | 12.22 | 4.76E-05 | 1.317624694 | NM_001031722| | NA |
| 219742_at | 12.22 | 4.76E-05 | 1.134988141 | NM_030567| | PRR7,proline rich 7 (synaptic) |
| 225713_at | 12.22 | 4.75E-05 | 1.155856999 | NM_052902| | STK11IP,LKB1 interacting protein |
| 223166_x_at | 12.21 | 4.78E-05 | 1.11402296 | NM_017995| | NA |
| 202629_at | 12.21 | 4.77E-05 | 1.121885553 | NM_006380| | APPBP2,amyloid beta precursor protein-binding protein |
| 202041_s_at | 12.21 | 4.78E-05 | 1.061639503 | NM_004214| | FIBP,FGF intracellular binding protein isoform b |
| 233842_x_at | 12.21 | 4.77E-05 | 1.054848662 | NM_016407| | C20orf43,chromosome 20 open reading frame 43 |
| 220757_s_at | 12.21 | 4.78E-05 | 1.171245638 | NM_025241| | UBXD1,UBX domain containing 1 |
| 1553666_at | 12.2 | 4.79E-05 | 1.87512085 | NM_030771| | NA |
| 224789_at | 12.2 | 4.79E-05 | 1.120745302 | NM_015397| | WDR40A,WD repeat domain 40A |
| 220002_at | 12.2 | 4.78E-05 | 1.195516554 | NM_018012| | NA |
| 202173_s_at | 12.2 | 4.80E-05 | 1.13976385 | NM_007146| | ZNF161,zinc finger protein 161 |
| 226233_at | 12.2 | 4.80E-05 | 1.091281979 | NM_152490| | B3GALNT2,UDP-GalNAc:betaGlcNAc beta |
| 201341_at | 12.2 | 4.78E-05 | 1.310587129 | NM_003633| | ENC1,ectodermal-neural cortex (with BTB-like domain) |
| 203508_at | 12.19 | 4.81E-05 | 1.47921316 | NM_001066| | TNFRSF1B,tumor necrosis factor receptor 2 precursor |
| 202212_at | 12.19 | 4.81E-05 | 1.099939283 | NM_014303| | PES1,pescadillo homolog 1, containing BRCT domain |
| 201865_x_at | 12.17 | 4.85E-05 | 1.199191897 | NM_000176| | NR3C1,nuclear receptor subfamily 3, group C, member 1 |
| 223584_s_at | 12.17 | 4.85E-05 | 1.197988016 | NM_015483| | KBTBD2,kelch repeat and BTB (POZ) domain containing 2 |
| 201783_s_at | 12.17 | 4.85E-05 | 1.167210987 | NM_021975| | RELA,v-rel reticuloendotheliosis viral oncogene |
| 201213_at | 12.16 | 4.88E-05 | 1.144708926 | NM_002712| | PPP1R7,protein phosphatase 1, regulatory subunit 7 |
| 228847_at | 12.16 | 4.87E-05 | 1.236981196 | NM_007277| | SEC6L1,Sec6 protein |
| 209971_x_at | 12.16 | 4.87E-05 | 1.041521535 | NM_006303| | JTV1,JTV1 |
| 218624_s_at | 12.16 | 4.88E-05 | 1.163440037 | NA |  |
| 204240_s_at | 12.16 | 4.89E-05 | 1.075341345 | NM_001042550| | NA |
| 216135_at | 12.15 | 4.91E-05 | 1.351903945 | NM_153208| | MGC35048,hypothetical protein MGC35048 |
| 221809_at | 12.15 | 4.91E-05 | 1.087498543 | NM_020850| | RANBP10,RAN binding protein 10 |
| 209692_at | 12.15 | 4.91E-05 | 1.230365441 | NM_005244| | EYA2,eyes absent 2 isoform a |
| 225703_at | 12.15 | 4.91E-05 | 1.08735249 | NA |  |
| 218833_at | 12.14 | 4.94E-05 | 1.273811227 | NM_016653| | ZAK,sterile-alpha motif and leucine zipper |
| 223306_at | 12.14 | 4.92E-05 | 1.043768342 | NM_032565| | EBPL,emopamil binding related protein, delta8-delta7 |
| 222942_s_at | 12.14 | 4.92E-05 | 1.106665349 | NM_001010927| | TIAM2,T-cell lymphoma invasion and metastasis 2 |
| 203415_at | 12.14 | 4.93E-05 | 1.053326209 | NM_013232| | PDCD6,programmed cell death 6 |
| 1552278_a_at | 12.14 | 4.92E-05 | 1.215312814 | NM_080669| | MGC9564,hypothetical protein MGC9564 |
| 221713_s_at | 12.14 | 4.93E-05 | 1.233454811 | NM_024871| | FLJ12748,hypothetical protein FLJ12748 |
| 213624_at | 12.13 | 4.95E-05 | 1.386998199 | NM_006714| | SMPDL3A,acid sphingomyelinase-like phosphodiesterase 3A |
| 232605_s_at | 12.13 | 4.95E-05 | 1.28184361 | NA |  |
| 204119_s_at | 12.13 | 4.94E-05 | 1.109250234 | NM_001123| | ADK,adenosine kinase isoform a |
| 222400_s_at | 12.12 | 4.97E-05 | 1.127941695 | NM_018269| | MTCBP-1,membrane-type 1 matrix metalloproteinase |
| 212601_at | 12.12 | 4.98E-05 | 1.133574564 | NM_015113| | ZZEF1,zinc finger, ZZ-type with EF hand domain 1 |
| 236565_s_at | 12.12 | 4.97E-05 | 1.14259749 | NM_018357| | FLJ11196,acheron isoform 1 |
| 223261_at | 12.12 | 4.98E-05 | 1.12718758 | NM_016218| | POLK,polymerase (DNA directed) kappa |
| 228740_at | 12.11 | 4.99E-05 | 1.17891276 | NA |  |
| 201133_s_at | 12.11 | 4.99E-05 | 1.104757546 | NM_014819| | PJA2,praja 2, RING-H2 motif containing |
| 225079_at | 12.11 | 4.99E-05 | 1.166168989 | NM_001424| | EMP2,epithelial membrane protein 2 |
| 225112_at | 12.11 | 4.99E-05 | 1.086821913 | NM_005759| | ABI2,abl interactor 2 |
| 243664_at | 12.1 | 5.01E-05 | 1.274144629 | NM_004786| | TXNL1,thioredoxin-like 1 |
| 212188_at | 12.1 | 5.02E-05 | 1.358837499 | NM_138444| | KCTD12,potassium channel tetramerisation domain |
| 244801_at | 12.1 | 5.01E-05 | 1.321327234 | NM_002799| | PSMB7,proteasome beta 7 subunit proprotein |
| 239219_at | 12.09 | 5.04E-05 | 1.158977222 | NM_004217| | AURKB,aurora kinase B |
| 221909_at | 12.09 | 5.03E-05 | 1.081974947 | NM_001109903| | NA |
| 230144_at | 12.09 | 5.03E-05 | 1.75242019 | NM_000828| | GRIA3,glutamate receptor 3 isoform flop precursor |
| 1556082_a_at | 12.08 | 5.06E-05 | 1.606869386 | NA |  |
| 220295_x_at | 12.08 | 5.05E-05 | 1.071666039 | NM_001114120| | NA |
| 206103_at | 12.08 | 5.06E-05 | 1.097604262 | NM_005052| | RAC3,ras-related C3 botulinum toxin substrate 3 (rho |
| 220134_x_at | 12.08 | 5.06E-05 | 1.177612298 | NM_018166| | FLJ10647,hypothetical protein FLJ10647 |
| 234982_at | 12.08 | 5.06E-05 | 1.159529438 | NM_172070| | ZNF650,zinc finger protein 650 |
| 224748_at | 12.08 | 5.05E-05 | 1.07468666 | NM_005828| | HAN11,WD-repeat protein |
| 229459_at | 12.07 | 5.09E-05 | 1.121260073 | NM_001082967| | NA |
| 243309_at | 12.07 | 5.07E-05 | 1.232735543 | NA |  |
| 238861_at | 12.07 | 5.08E-05 | 1.381906546 | NA |  |
| 234032_at | 12.06 | 5.11E-05 | 1.337290715 | NA |  |
| 221704_s_at | 12.06 | 5.10E-05 | 1.081303496 | NM_024667| | FLJ12750,hypothetical protein FLJ12750 |
| 236242_at | 12.05 | 5.14E-05 | 1.462006242 | NA |  |
| 220007_at | 12.05 | 5.14E-05 | 1.141220291 | NM_024770| | FLJ13984,hypothetical protein FLJ13984 |
| 235850_at | 12.05 | 5.14E-05 | 1.444974202 | NM_019069| | WDR5B,WD repeat domain 5B |
| 227150_at | 12.05 | 5.13E-05 | 1.122444824 | NM_005955| | MTF1,metal-regulatory transcription factor 1 |
| 202282_at | 12.04 | 5.16E-05 | 1.061604123 | NM_001037811| | NA |
| 242273_at | 12.04 | 5.15E-05 | 1.232146937 | NA |  |
| 210175_at | 12.03 | 5.17E-05 | 1.225675487 | NM_003203| | C2orf3,chromosome 2 open reading frame 3 |
| 235211_at | 12.03 | 5.18E-05 | 1.27777821 | NA |  |
| 212124_at | 12.03 | 5.19E-05 | 1.059539115 | NM_020338| | RAI17,retinoic acid induced 17 |
| 205076_s_at | 12.03 | 5.19E-05 | 1.276592246 | NM_181873| | CRA,cisplatin resistance associated |
| 211595_s_at | 12.02 | 5.20E-05 | 1.099260956 | NM_022839| | MRPS11,mitochondrial ribosomal protein S11 isoform a |
| 218284_at | 12.02 | 5.21E-05 | 1.138064361 | NM_005902| | SMAD3,MAD, mothers against decapentaplegic homolog 3 |
| 221797_at | 12.02 | 5.21E-05 | 1.073669024 | NM_001039842| | NA |
| 211219_s_at | 12.02 | 5.19E-05 | 1.666759277 | NM_004789| | LHX2,LIM homeobox protein 2 |
| 225085_at | 12.02 | 5.20E-05 | 1.233769092 | NM_018218| | USP40,ubiquitin specific protease 40 |
| 214434_at | 12.01 | 5.23E-05 | 1.305736732 | NM_025015| | NA |
| 222429_at | 12.01 | 5.22E-05 | 1.108616397 | NM_001014436| | NA |
| 204247_s_at | 12.01 | 5.22E-05 | 1.110336475 | NM_004935| | CDK5,cyclin-dependent kinase 5 |
| 201627_s_at | 12.01 | 5.22E-05 | 1.230635085 | NM_005542| | INSIG1,insulin induced gene 1 isoform 1 |
| 218719_s_at | 12 | 5.25E-05 | 1.144704533 | NM_001126129| | NA |
| 218153_at | 12 | 5.26E-05 | 1.087049858 | NM_024537| | FLJ12118,hypothetical protein FLJ12118 |
| 213157_s_at | 12 | 5.24E-05 | 1.222371162 | NM_015253| | KIAA0523,KIAA0523 protein |
| 228039_at | 12 | 5.25E-05 | 1.152033048 | NM_014829| | DDX46,DEAD (Asp-Glu-Ala-Asp) box polypeptide 46 |
| 212050_at | 11.99 | 5.26E-05 | 1.090271512 | NM_133264| | WIRE,WIRE protein |
| 204288_s_at | 11.99 | 5.27E-05 | 1.125375357 | NM_003603| | ARGBP2,Arg/Abl-interacting protein 2 isoform 1 |
| 244766_at | 11.99 | 5.28E-05 | 1.210189585 | NM_015092| | SMG1,PI-3-kinase-related kinase SMG-1 isoform 1 |
| 215667_x_at | 11.99 | 5.26E-05 | 1.097002892 | NA |  |
| 229025_s_at | 11.99 | 5.26E-05 | 1.248248276 | NM_144981| | FLJ25059,hypothetical protein FLJ25059 |
| 226933_s_at | 11.99 | 5.28E-05 | 1.514004761 | NM_001546| | ID4,inhibitor of DNA binding 4, dominant negative |
| 210296_s_at | 11.99 | 5.27E-05 | 1.065067876 | NM_000318| | PXMP3,peroxisomal membrane protein 3 |
| 203911_at | 11.98 | 5.30E-05 | 1.103116484 | NM_002885| | RAP1GA1,RAP1, GTPase activating protein 1 |
| 241933_at | 11.98 | 5.29E-05 | 1.18777317 | NM_018292| | QRSL1,glutaminyl-tRNA synthase |
| 222697_s_at | 11.98 | 5.30E-05 | 1.175662499 | NM_018394| | ABHD10,abhydrolase domain containing 10 |
| 201670_s_at | 11.98 | 5.29E-05 | 1.168358361 | NM_002356| | MARCKS,myristoylated alanine-rich protein kinase C |
| 225531_at | 11.98 | 5.30E-05 | 1.232105042 | NM_001100619| | NA |
| 202903_at | 11.97 | 5.32E-05 | 1.154199116 | NM_012322| | LSM5,U6 snRNA-associated Sm-like protein 5 |
| 210762_s_at | 11.97 | 5.32E-05 | 1.334987789 | NM_006094| | DLC1,deleted in liver cancer 1 isoform 2 |
| 214005_at | 11.96 | 5.35E-05 | 1.157743853 | NM_000821| | GGCX,gamma-glutamyl carboxylase |
| 220117_at | 11.96 | 5.36E-05 | 1.382753752 | NM_024697| | ZNF659,zinc finger protein 659 |
| 218597_s_at | 11.95 | 5.37E-05 | 1.06086281 | NM_018464| | C10orf70,chromosome 10 open reading frame 70 |
| 202617_s_at | 11.95 | 5.36E-05 | 1.242999246 | NM_001110792| | NA |
| 227978_s_at | 11.94 | 5.40E-05 | 1.177759326 | NM_175907| | ZADH2,zinc binding alcohol dehydrogenase, domain |
| 232777_s_at | 11.94 | 5.40E-05 | 1.622728351 | NM_144980| | C6orf118,chromosome 6 open reading frame 118 |
| 239731_at | 11.94 | 5.40E-05 | 1.330267145 | NA |  |
| 209684_at | 11.94 | 5.38E-05 | 1.316658616 | NM_018993| | RIN2,RAB5 interacting protein 2 |
| 228323_at | 11.94 | 5.41E-05 | 1.066575005 | NM_144508| | AF15Q14,AF15q14 protein isoform 2 |
| 227896_at | 11.94 | 5.40E-05 | 1.119470674 | NM_016567| | BCCIP,BRCA2 and CDKN1A-interacting protein isoform |
| 217204_at | 11.93 | 5.41E-05 | 1.165538993 | NM_001114184| | NA |
| 212263_at | 11.93 | 5.43E-05 | 1.058525981 | NM_006775| | QKI,quaking homolog, KH domain RNA binding isoform |
| 227894_at | 11.93 | 5.42E-05 | 1.282754585 | NM_145294| | LOC197336,similar to RIKEN cDNA 3230401M21 [Mus musculus] |
| 201275_at | 11.93 | 5.41E-05 | 1.097488611 | NM_002004| | FDPS,farnesyl diphosphate synthase |
| 203989_x_at | 11.93 | 5.41E-05 | 1.25727063 | NM_001992| | F2R,coagulation factor II receptor precursor |
| 236229_at | 11.93 | 5.42E-05 | 1.495218095 | NA |  |
| 201022_s_at | 11.93 | 5.42E-05 | 1.045548757 | NM_001011546| | DSTN,destrin isoform b |
| 225905_s_at | 11.92 | 5.44E-05 | 1.117253035 | NM_006279| | ST3GAL3,sialyltransferase 6 isoform j |
| 233442_at | 11.92 | 5.43E-05 | 1.40612655 | NA |  |
| 211360_s_at | 11.92 | 5.44E-05 | 1.203126865 | NM_002223| | ITPR2,inositol 1,4,5-triphosphate receptor, type 2 |
| 202463_s_at | 11.92 | 5.44E-05 | 1.128859494 | NM_003926| | MBD3,methyl-CpG binding domain protein 3 |
| 225311_at | 11.92 | 5.44E-05 | 1.066335962 | NM_002225| | IVD,isovaleryl Coenzyme A dehydrogenase |
| 207693_at | 11.92 | 5.44E-05 | 1.230904303 | NM_000726| | CACNB4,calcium channel, voltage-dependent, beta 4 |
| 237169_at | 11.92 | 5.43E-05 | 1.87042955 | NA |  |
| 201063_at | 11.92 | 5.45E-05 | 1.092632712 | NM_002901| | RCN1,reticulocalbin 1 precursor |
| 244015_at | 11.91 | 5.45E-05 | 1.271382729 | NA |  |
| 219493_at | 11.91 | 5.45E-05 | 1.235219921 | NM_024745| | SHCBP1,SHC SH2-domain binding protein 1 |
| 212508_at | 11.91 | 5.46E-05 | 1.076417983 | NM_022151| | MOAP1,modulator of apoptosis 1 |
| 1555826_at | 11.91 | 5.46E-05 | 1.214342513 | NA |  |
| 225086_at | 11.91 | 5.47E-05 | 1.058451446 | NM_001042429| | NA |
| 200642_at | 11.9 | 5.49E-05 | 1.0526386 | NM_000454| | SOD1,superoxide dismutase 1, soluble |
| 238949_at | 11.9 | 5.48E-05 | 1.226493828 | NM_144726| | FLJ31951,hypothetical protein FLJ31951 |
| 222661_at | 11.9 | 5.48E-05 | 1.054381144 | NM_018046| | AGGF1,angiogenic factor VG5Q |
| 201414_s_at | 11.9 | 5.50E-05 | 1.05673954 | NM_005969| | NAP1L4,nucleosome assembly protein 1-like 4 |
| 220050_at | 11.9 | 5.48E-05 | 1.310881776 | NM_018956| | C9orf9,chromosome 9 open reading frame 9 |
| 1563541_at | 11.9 | 5.50E-05 | 1.144246725 | NA |  |
| 201186_at | 11.89 | 5.51E-05 | 1.132298231 | NM_002337| | LRPAP1,low density lipoprotein receptor-related protein |
| 242258_at | 11.89 | 5.50E-05 | 1.388178006 | NA |  |
| 209447_at | 11.89 | 5.51E-05 | 1.514808247 | NM_015293| | SYNE1,nesprin 1 isoform beta |
| 219938_s_at | 11.89 | 5.51E-05 | 1.528338204 | NM_024430| | PSTPIP2,proline-serine-threonine phosphatase interacting |
| 233746_x_at | 11.89 | 5.51E-05 | 1.08740956 | NM_016400| | HYPK,Huntingtin interacting protein K |
| 242293_at | 11.89 | 5.51E-05 | 1.144202302 | NM_019071| | ING3,inhibitor of growth family, member 3 isoform 1 |
| 222843_at | 11.89 | 5.51E-05 | 1.303853112 | NM_001042762| | NA |
| 221795_at | 11.89 | 5.51E-05 | 1.464234167 | NM_001007097| | NTRK2,neurotrophic tyrosine kinase, receptor, type 2 |
| 204081_at | 11.89 | 5.50E-05 | 1.23818656 | NM_001126181| | NA |
| 217942_at | 11.88 | 5.53E-05 | 1.025147896 | NM_021821| | MRPS35,mitochondrial ribosomal protein S35 |
| 242725_at | 11.88 | 5.53E-05 | 1.183495751 | NA |  |
| 201564_s_at | 11.88 | 5.53E-05 | 1.05936263 | NM_003088| | FSCN1,fascin 1 |
| 1557270_at | 11.88 | 5.54E-05 | 1.188908269 | NA |  |
| 216913_s_at | 11.88 | 5.55E-05 | 1.095787401 | NM_015179| | KIAA0690,KIAA0690 |
| 226760_at | 11.87 | 5.57E-05 | 1.162617809 | NA |  |
| 204648_at | 11.87 | 5.55E-05 | 1.145873803 | NM_000906| | NPR1,natriuretic peptide receptor A/guanylate cyclase |
| 219247_s_at | 11.87 | 5.56E-05 | 1.262078965 | NM_024630| | ZDHHC14,NEW1 domain containing protein |
| 205234_at | 11.87 | 5.55E-05 | 1.753417251 | NM_004696| | SLC16A4,solute carrier family 16, member 4 |
| 202447_at | 11.87 | 5.56E-05 | 1.091686431 | NM_001359| | DECR1,2,4-dienoyl CoA reductase 1 precursor |
| 218362_s_at | 11.86 | 5.59E-05 | 1.487749405 | NM_014953| | KIAA1008,KIAA1008 |
| 216192_at | 11.86 | 5.58E-05 | 1.586407277 | NM_001446| | FABP7,fatty acid binding protein 7, brain |
| 1557527_at | 11.85 | 5.61E-05 | 1.330443682 | NA |  |
| 229593_at | 11.85 | 5.62E-05 | 1.31871302 | NA |  |
| 224605_at | 11.85 | 5.60E-05 | 1.164132898 | NM_001001701| | LOC401152,HCV F-transactivated protein 1 |
| 203796_s_at | 11.85 | 5.61E-05 | 1.18829302 | NM_001024808| | NA |
| 229595_at | 11.85 | 5.60E-05 | 1.05251122 | NM_001098502| | NA |
| 213427_at | 11.84 | 5.64E-05 | 1.054467283 | NM_006638| | RPP40,ribonuclease P 40kDa subunit |
| 1556341_s_at | 11.84 | 5.64E-05 | 1.161048286 | NM_002969| | MAPK12,mitogen-activated protein kinase 12 |
| 222776_at | 11.84 | 5.63E-05 | 1.207790489 | NM_018048| | FLJ10292,mago-nashi homolog |
| 201662_s_at | 11.84 | 5.65E-05 | 1.182721615 | NM_004457| | ACSL3,acyl-CoA synthetase long-chain family member 3 |
| 212062_at | 11.84 | 5.64E-05 | 1.176196112 | NM_006045| | NA |
| 1558748_at | 11.84 | 5.65E-05 | 1.377243911 | NA |  |
| 202138_x_at | 11.83 | 5.66E-05 | 1.04389384 | NM_006303| | JTV1,JTV1 |
| 242307_at | 11.83 | 5.65E-05 | 1.141826711 | NM_001013258| | NA |
| 228239_at | 11.83 | 5.66E-05 | 1.296144064 | NM_058182| | C21orf51,chromosome 21 open reading frame 51 |
| 209407_s_at | 11.83 | 5.66E-05 | 1.13272909 | NM_021008| | DEAF1,suppressin |
| 229434_at | 11.83 | 5.66E-05 | 1.190666197 | NA |  |
| 208433_s_at | 11.82 | 5.70E-05 | 1.105864054 | NM_001018054| | NA |
| 221513_s_at | 11.82 | 5.69E-05 | 1.092997212 | NM_006649| | UTP14A,UTP14, U3 small nucleolar ribonucleoprotein, |
| 209853_s_at | 11.82 | 5.68E-05 | 1.083176536 | NM_005789| | PSME3,proteasome activator subunit 3 isoform 1 |
| 243887_at | 11.81 | 5.70E-05 | 1.133797955 | NA |  |
| 235876_at | 11.81 | 5.72E-05 | 1.408770822 | NA |  |
| 229491_at | 11.81 | 5.70E-05 | 1.29357868 | NM_178833| | LOC133308,hypothetical protein BC009732 |
| 213750_at | 11.81 | 5.72E-05 | 1.215901837 | NM_015659| | RSL1D1,ribosomal L1 domain containing 1 |
| 236524_at | 11.81 | 5.71E-05 | 1.394659536 | NA |  |
| 214214_s_at | 11.81 | 5.72E-05 | 1.043647977 | NM_001212| | C1QBP,complement component 1, q subcomponent binding |
| 230872_s_at | 11.81 | 5.72E-05 | 1.134317243 | NM_001025930| | NA |
| 200593_s_at | 11.81 | 5.72E-05 | 1.034682261 | NM_004501| | HNRPU,heterogeneous nuclear ribonucleoprotein U |
| 212144_at | 11.81 | 5.72E-05 | 1.175013595 | NM_015374| | UNC84B,unc-84 homolog B |
| 225896_at | 11.81 | 5.72E-05 | 1.158132444 | NA |  |
| 212565_at | 11.81 | 5.71E-05 | 1.134425685 | NM_015000| | STK38L,serine/threonine kinase 38 like |
| 200675_at | 11.8 | 5.74E-05 | 1.063095408 | NM_004356| | CD81,CD81 antigen |
| 218233_s_at | 11.8 | 5.74E-05 | 1.031598893 | NM_013397| | C6orf49,over-expressed breast tumor protein |
| 226807_at | 11.79 | 5.77E-05 | 1.093300581 | NM_153688| | ZFP1,zinc finger protein 1 homolog |
| 225348_at | 11.79 | 5.76E-05 | 1.103909057 | NM_006625| | FUSIP1,FUS interacting protein (serine-arginine rich) 1 |
| 231055_at | 11.79 | 5.76E-05 | 1.483103669 | NA |  |
| 230964_at | 11.79 | 5.77E-05 | 1.11840261 | NM_207361| | FREM2,FRAS1 related extracellular matrix protein 2 |
| 224304_x_at | 11.78 | 5.80E-05 | 1.098723695 | NM_016350| | NIN,ninein isoform 4 |
| 234926_s_at | 11.78 | 5.79E-05 | 1.054723291 | NM_016407| | C20orf43,chromosome 20 open reading frame 43 |
| 219833_s_at | 11.78 | 5.79E-05 | 1.128770151 | NM_018100| | EFHC1,EF-hand domain (C-terminal) containing 1 |
| 217771_at | 11.78 | 5.79E-05 | 1.198279151 | NM_016548| | GOLPH2,golgi phosphoprotein 2 |
| 222561_at | 11.78 | 5.80E-05 | 1.130580089 | NM_018697| | LANCL2,LanC lantibiotic synthetase component C-like 2 |
| 201736_s_at | 11.77 | 5.82E-05 | 1.067578989 | NM_005885| | MARCH6,membrane-associated ring finger (C3HC4) 6 |
| 1553569_at | 11.77 | 5.83E-05 | 1.014106918 | NM_022340| | ZFYVE20,FYVE-finger-containing Rab5 effector protein |
| 1557801_x_at | 11.77 | 5.82E-05 | 1.243787691 | NM_170746| | C11orf31,selenoprotein H |
| 243816_at | 11.77 | 5.82E-05 | 1.177757734 | NM_021916| | ZNF70,zinc finger protein 70 |
| 219175_s_at | 11.76 | 5.83E-05 | 1.084246787 | NM_001008485| | SLC41A3,solute carrier family 41, member 3 isoform 1 |
| 223226_x_at | 11.76 | 5.86E-05 | 1.171002256 | NM_001009998| | SSBP4,single stranded DNA binding protein 4 isoform b |
| 204241_at | 11.76 | 5.85E-05 | 1.195225886 | NM_001101667| | NA |
| 230667_at | 11.75 | 5.88E-05 | 1.233218694 | NA |  |
| 203102_s_at | 11.75 | 5.88E-05 | 1.10125599 | NM_002408| | MGAT2,alpha-1,6-mannosyl-glycoprotein |
| 229120_s_at | 11.74 | 5.91E-05 | 1.081661001 | NM_001038707| | NA |
| 226056_at | 11.73 | 5.93E-05 | 1.563024759 | NM_020754| | CDGAP,Cdc42 GTPase-activating protein |
| 1553743_at | 11.73 | 5.92E-05 | 1.182933997 | NM_145280| | LOC151194,hepatocellular carcinoma-associated antigen |
| 204041_at | 11.72 | 5.96E-05 | 1.220992099 | NM_000898| | MAOB,amine oxidase (flavin-containing) |
| 224822_at | 11.72 | 5.95E-05 | 1.278081483 | NM_006094| | DLC1,deleted in liver cancer 1 isoform 2 |
| 225388_at | 11.72 | 5.95E-05 | 1.11847253 | NM_005723| | TM4SF9,transmembrane 4 superfamily member 9 |
| 236435_at | 11.72 | 5.97E-05 | 1.118672589 | NA |  |
| 1555595_at | 11.71 | 5.97E-05 | 1.193775804 | NM_024583| | SCRN3,secernin 3 |
| 239611_at | 11.71 | 5.97E-05 | 1.560084167 | NA |  |
| 242538_at | 11.71 | 5.99E-05 | 1.515724348 | NM_007111| | TFDP1,transcription factor Dp-1 |
| 235219_at | 11.7 | 6.02E-05 | 1.484881182 | NM_138464| | NA |
| 238389_s_at | 11.7 | 6.00E-05 | 1.23536305 | NA |  |
| 244350_at | 11.7 | 6.02E-05 | 1.488403884 | NM_012334| | MYO10,myosin X |
| 222269_at | 11.69 | 6.05E-05 | 1.214797283 | NM_198450| | CXorf33,chromosome X open reading frame 33 |
| 37965_at | 11.69 | 6.05E-05 | 1.18518463 | NM_001003828| | PARVB,parvin, beta isoform a |
| 216252_x_at | 11.69 | 6.05E-05 | 1.350821063 | NM_000043| | FAS,tumor necrosis factor receptor superfamily, |
| 229653_at | 11.69 | 6.02E-05 | 1.275389068 | NA |  |
| 226326_at | 11.68 | 6.06E-05 | 1.99430828 | NM_032373| | PCGF5,polycomb group ring finger 5 |
| 231599_x_at | 11.68 | 6.06E-05 | 1.215099041 | NM_004647| | DPF1,Neuro-d4 (rat) homolog |
| 238603_at | 11.68 | 6.07E-05 | 1.32784343 | NA |  |
| 222527_s_at | 11.67 | 6.09E-05 | 1.086023921 | NM_018047| | RBM22,RNA binding motif protein 22 |
| 1553587_a_at | 11.66 | 6.13E-05 | 1.109542007 | NM_019896| | POLE4,DNA polymerase epsilon subunit 4 |
| 217780_at | 11.66 | 6.13E-05 | 1.06596396 | NM_016145| | PTD008,PTD008 protein |
| 209513_s_at | 11.66 | 6.13E-05 | 1.11504838 | NM_032303| | HSDL2,hydroxysteroid dehydrogenase like 2 |
| 222872_x_at | 11.66 | 6.13E-05 | 1.238100781 | NM_001031716| | NA |
| 223666_at | 11.66 | 6.12E-05 | 1.087360727 | NM_014426| | SNX5,sorting nexin 5 |
| 223367_at | 11.65 | 6.14E-05 | 1.137615034 | NM_032317| | WBSCR18,Williams Beuren syndrome chromosome region 18 |
| 38158_at | 11.65 | 6.16E-05 | 1.058436866 | NM_012291| | ESPL1,extra spindle poles like 1 |
| 235391_at | 11.65 | 6.14E-05 | 1.123569545 | NM_145269| | LOC137392,similar to CG6405 gene product |
| 242139_s_at | 11.64 | 6.16E-05 | 1.129337537 | NA |  |
| 225409_at | 11.64 | 6.17E-05 | 1.099867607 | NM_001008215| | MGC52110,hypothetical protein MGC52110 |
| 218199_s_at | 11.64 | 6.18E-05 | 1.100546952 | NM_022917| | NOL6,nucleolar RNA-associated protein alpha isoform |
| 201147_s_at | 11.64 | 6.17E-05 | 1.456555501 | NM_000362| | TIMP3,tissue inhibitor of metalloproteinase 3 |
| 201779_s_at | 11.64 | 6.18E-05 | 1.082480838 | NM_007282| | RNF13,ring finger protein 13 isoform 1 |
| 206020_at | 11.64 | 6.17E-05 | 1.310916817 | NM_004232| | SOCS6,suppressor of cytokine signaling 6 |
| 220651_s_at | 11.64 | 6.18E-05 | 1.092971824 | NM_018518| | MCM10,minichromosome maintenance protein 10 isoform 2 |
| 227581_at | 11.64 | 6.18E-05 | 1.123479474 | NM_015395| | DKFZP434B0335,DKFZP434B0335 protein |
| 212316_at | 11.63 | 6.21E-05 | 1.12498474 | NM_024923| | NUP210,nucleoporin 210 |
| 205090_s_at | 11.63 | 6.19E-05 | 1.150603961 | NM_016256| | NAGPA,N-acetylglucosamine-1-phosphodiester |
| 219204_s_at | 11.63 | 6.21E-05 | 1.179713658 | NM_021947| | SRR,serine racemase |
| 225695_at | 11.63 | 6.19E-05 | 1.162277859 | NM_017877| | C2orf18,chromosome 2 open reading frame 18 |
| 219408_at | 11.63 | 6.19E-05 | 1.131698424 | NM_019023| | PRMT7,protein arginine N-methyltransferase 7 |
| 218351_at | 11.63 | 6.20E-05 | 1.118426636 | NM_017845| | COMMD8,COMM domain containing 8 |
| 237482_s_at | 11.62 | 6.23E-05 | 1.201542862 | NM_025247| | ACAD10,acyl-Coenzyme A dehydrogenase family, member 10 |
| 228737_at | 11.62 | 6.23E-05 | 1.556505377 | NM_001098796| | NA |
| 237936_at | 11.62 | 6.21E-05 | 1.742876931 | NA |  |
| 212536_at | 11.61 | 6.26E-05 | 1.195662208 | NM_014616| | NA |
| 241091_at | 11.61 | 6.25E-05 | 1.386084734 | NA |  |
| 224100_s_at | 11.61 | 6.26E-05 | 1.307297265 | NM_020134| | DPYSL5,dihydropyrimidinase-like 5 |
| 220948_s_at | 11.61 | 6.26E-05 | 1.061181889 | NM_000701| | ATP1A1,Na+/K+ -ATPase alpha 1 subunit isoform a |
| 1557370_s_at | 11.6 | 6.28E-05 | 1.183493946 | NM_015057| | MYCBP2,MYC binding protein 2 |
| 221787_at | 11.6 | 6.28E-05 | 1.075559343 | NM_001029863| | NA |
| 209177_at | 11.6 | 6.28E-05 | 1.091528421 | NM_199069| | DKFZP564J0123,nuclear protein E3-3 isoform a |
| 212163_at | 11.6 | 6.27E-05 | 1.079664377 | NM_020738| | KIDINS220,kinase D-interacting substance of 220 kDa |
| 218235_s_at | 11.59 | 6.31E-05 | 1.044587116 | NM_016037| | CGI-94,CGI-94 protein |
| 228240_at | 11.59 | 6.30E-05 | 1.05740013 | NA |  |
| 226237_at | 11.59 | 6.32E-05 | 1.578521255 | NA |  |
| 214290_s_at | 11.59 | 6.31E-05 | 1.125326569 | NM_001040874| | NA |
| 226850_at | 11.59 | 6.31E-05 | 1.071226769 | NM_182760| | SUMF1,sulfatase modifying factor 1 |
| 213918_s_at | 11.59 | 6.31E-05 | 1.187844396 | NM_015384| | NIPBL,delangin isoform B |
| 242251_at | 11.58 | 6.35E-05 | 1.170417733 | NM_145207| | SPATA5,spermatogenesis associated factor SPAF |
| 208640_at | 11.58 | 6.35E-05 | 1.022212275 | NM_006908| | RAC1,ras-related C3 botulinum toxin substrate 1 |
| 209024_s_at | 11.58 | 6.33E-05 | 1.038370433 | NM_006372| | SYNCRIP,synaptotagmin binding, cytoplasmic RNA |
| 204303_s_at | 11.58 | 6.33E-05 | 1.170439954 | NM_014772| | KIAA0427,KIAA0427 |
| 242323_at | 11.57 | 6.37E-05 | 1.070175248 | NA |  |
| 212687_at | 11.57 | 6.37E-05 | 1.093760976 | NM_004987| | LIMS1,LIM and senescent cell antigen-like domains 1 |
| 209318_x_at | 11.57 | 6.36E-05 | 1.604596846 | NM_001080951| | NA |
| 219911_s_at | 11.56 | 6.41E-05 | 1.343955172 | NM_016354| | SLCO4A1,solute carrier organic anion transporter family |
| 230998_at | 11.56 | 6.41E-05 | 1.268923036 | NA |  |
| 239300_at | 11.56 | 6.39E-05 | 1.662649282 | NA |  |
| 212325_at | 11.56 | 6.39E-05 | 1.180826885 | NM_001112717| | NA |
| 219056_at | 11.56 | 6.41E-05 | 1.299769938 | NM_024570| | FLJ11712,hypothetical protein FLJ11712 |
| 206332_s_at | 11.56 | 6.39E-05 | 1.673438905 | NM_005531| | IFI16,interferon, gamma-inducible protein 16 |
| 218207_s_at | 11.55 | 6.42E-05 | 1.313202177 | NM_015894| | STMN3,SCG10-like-protein |
| 203570_at | 11.55 | 6.42E-05 | 1.225546842 | NM_005576| | LOXL1,lysyl oxidase-like 1 |
| 1558021_at | 11.55 | 6.44E-05 | 1.604695098 | NM_005833| | RAB9P40,Rab9 effector p40 |
| 202718_at | 11.55 | 6.42E-05 | 1.186810617 | NM_000597| | IGFBP2,insulin-like growth factor binding protein 2, |
| 209528_s_at | 11.55 | 6.42E-05 | 1.105725553 | NM_016111| | KIAA0683,KIAA0683 gene product |
| 231121_at | 11.55 | 6.44E-05 | 1.293782284 | NM_032383| | HPS3,Hermansky-Pudlak syndrome 3 protein |
| 235195_at | 11.55 | 6.42E-05 | 1.224690388 | NM_012164| | FBXW2,F-box and WD-40 domain protein 2 |
| 201598_s_at | 11.55 | 6.42E-05 | 1.130489652 | NM_001567| | INPPL1,inositol polyphosphate phosphatase-like 1 |
| 220633_s_at | 11.55 | 6.44E-05 | 1.183705471 | NM_016287| | HP1-BP74,HP1-BP74 |
| 203340_s_at | 11.54 | 6.45E-05 | 1.147449186 | NM_003705| | SLC25A12,solute carrier family 25 (mitochondrial carrier, |
| 235046_at | 11.54 | 6.47E-05 | 2.055843494 | NA |  |
| 210243_s_at | 11.54 | 6.44E-05 | 1.062265144 | NM_003779| | B4GALT3,UDP-Gal:betaGlcNAc beta 1,4- |
| 205322_s_at | 11.54 | 6.45E-05 | 1.085179654 | NM_005955| | MTF1,metal-regulatory transcription factor 1 |
| 230460_at | 11.54 | 6.46E-05 | 1.304497941 | NA |  |
| 226343_at | 11.53 | 6.48E-05 | 1.146001031 | NA |  |
| 222011_s_at | 11.53 | 6.48E-05 | 1.119176834 | NM_001008897| | TCP1,T-complex protein 1 isoform b |
| 228142_at | 11.53 | 6.50E-05 | 1.189723722 | NM_001003684| | HSPC051,ubiquinol-cytochrome c reductase complex 7.2kDa |
| 1559227_s_at | 11.53 | 6.49E-05 | 1.103395665 | NM_000551| | VHL,von Hippel-Lindau tumor suppressor isoform 1 |
| 228603_at | 11.52 | 6.52E-05 | 1.163341755 | NA |  |
| 218083_at | 11.51 | 6.55E-05 | 1.094566294 | NM_025072| | PTGES2,prostaglandin E synthase 2 isoform 1 |
| 224282_s_at | 11.51 | 6.56E-05 | 1.199138961 | NM_001037553| | NA |
| 203171_s_at | 11.51 | 6.56E-05 | 1.079585883 | NM_015324| | KIAA0409,KIAA0409 protein |
| 224959_at | 11.5 | 6.58E-05 | 1.101136474 | NM_000112| | SLC26A2,solute carrier family 26 member 2 |
| 210802_s_at | 11.49 | 6.63E-05 | 1.08442541 | NM_014473| | HSA9761,putative dimethyladenosine transferase |
| 215114_at | 11.49 | 6.61E-05 | 1.139800221 | NM_015670| | SENP3,SUMO1/sentrin/SMT3 specific protease 3 |
| 235545_at | 11.49 | 6.61E-05 | 1.130703766 | NM_001114120| | NA |
| 205471_s_at | 11.49 | 6.60E-05 | 1.897922312 | NM_004392| | DACH1,dachshund homolog 1 isoform c |
| 244433_at | 11.49 | 6.63E-05 | 1.560794189 | NA |  |
| 206144_at | 11.49 | 6.61E-05 | 1.301018254 | NM_001033057| | NA |
| 212192_at | 11.48 | 6.64E-05 | 1.415033633 | NM_138444| | KCTD12,potassium channel tetramerisation domain |
| 227406_at | 11.48 | 6.65E-05 | 1.104407148 | NM_002041| | GABPB2,GA binding protein transcription factor, beta |
| 227032_at | 11.48 | 6.65E-05 | 1.17652175 | NM_025179| | PLXNA2,plexin A2 |
| 227893_at | 11.48 | 6.64E-05 | 1.318335898 | NA |  |
| 1559993_at | 11.47 | 6.67E-05 | 1.712385016 | NM_030971| | SFXN3,sideroflexin 3 |
| 225656_at | 11.47 | 6.69E-05 | 1.269258336 | NM_018100| | EFHC1,EF-hand domain (C-terminal) containing 1 |
| 231912_s_at | 11.46 | 6.71E-05 | 1.177170126 | NM_015395| | DKFZP434B0335,DKFZP434B0335 protein |
| 205918_at | 11.46 | 6.72E-05 | 1.227126161 | NM_005070| | SLC4A3,solute carrier family 4, anion exchanger, member |
| 227462_at | 11.46 | 6.70E-05 | 1.466557636 | NA |  |
| 235266_at | 11.46 | 6.69E-05 | 1.219227771 | NM_014109| | ATAD2,two AAA domain containing protein |
| 235488_at | 11.46 | 6.70E-05 | 1.060768033 | NM_033315| | RASL10B,RAS-like, family 10, member B |
| 230448_at | 11.46 | 6.70E-05 | 1.242765992 | NM_001037984| | NA |
| 219858_s_at | 11.45 | 6.73E-05 | 1.485673062 | NM_017694| | FLJ20160,FLJ20160 protein |
| 230831_at | 11.45 | 6.74E-05 | 1.103256658 | NA |  |
| 217811_at | 11.45 | 6.73E-05 | 1.080703921 | NM_016275| | SELT,selenoprotein T |
| 204469_at | 11.45 | 6.74E-05 | 1.162159322 | NM_002851| | PTPRZ1,protein tyrosine phosphatase, receptor-type, Z |
| 201910_at | 11.45 | 6.72E-05 | 1.092949286 | NM_001001715| | FARP1,FERM, RhoGEF, and pleckstrin domain protein 1 |
| 207628_s_at | 11.45 | 6.73E-05 | 1.06757531 | NM_017528| | WBSCR22,Williams Beuren syndrome chromosome region 22 |
| 203790_s_at | 11.44 | 6.78E-05 | 1.066545006 | NM_005836| | HRSP12,heat-responsive protein 12 |
| 223036_at | 11.44 | 6.76E-05 | 1.53778336 | NM_005687| | FARSLB,phenylalanine-tRNA synthetase-like, beta |
| 220702_at | 11.44 | 6.76E-05 | 1.414335685 | NM_018616| | NA |
| 1568777_at | 11.44 | 6.77E-05 | 1.171476214 | NM_183387| | EML5,echinoderm microtubule associated protein like |
| 228857_at | 11.44 | 6.76E-05 | 1.249669504 | NM_005275| | GNL1,guanine nucleotide binding protein-like 1 |
| 206874_s_at | 11.44 | 6.76E-05 | 1.074621275 | NA |  |
| 202142_at | 11.44 | 6.77E-05 | 1.070367731 | NM_006710| | COPS8,COP9 signalosome subunit 8 isoform 1 |
| 202939_at | 11.43 | 6.79E-05 | 1.106203222 | NM_005857| | ZMPSTE24,zinc metalloproteinase STE24 homolog |
| 239329_at | 11.43 | 6.82E-05 | 1.10960364 | NA |  |
| 209169_at | 11.42 | 6.83E-05 | 1.145957995 | NM_001001994| | GPM6B,glycoprotein M6B isoform 4 |
| 208670_s_at | 11.42 | 6.83E-05 | 1.056268149 | NM_014335| | CRI1,CREBBP/EP300 inhibitor 1 |
| 244165_at | 11.41 | 6.89E-05 | 1.403577803 | NM_017782| | NA |
| 244291_x_at | 11.41 | 6.87E-05 | 1.284524034 | NA |  |
| 218680_x_at | 11.4 | 6.91E-05 | 1.082182927 | NM_016400| | HYPK,Huntingtin interacting protein K |
| 239678_at | 11.4 | 6.91E-05 | 1.259582708 | NA |  |
| 222193_at | 11.4 | 6.90E-05 | 1.114181731 | NM_021925| | FLJ21820,hypothetical protein FLJ21820 |
| 219375_at | 11.4 | 6.89E-05 | 1.082613608 | NM_001007794| | CEPT1,choline/ethanolaminephosphotransferase |
| 201136_at | 11.39 | 6.93E-05 | 1.325397751 | NM_002668| | PLP2,proteolipid protein 2 (colonic |
| 236294_at | 11.39 | 6.92E-05 | 1.203133871 | NM_031407| | NA |
| 222664_at | 11.39 | 6.92E-05 | 1.137433738 | NM_024076| | KCTD15,potassium channel tetramerisation domain |
| 1555870_at | 11.39 | 6.95E-05 | 1.243498225 | NM_207396| | FLJ46380,FLJ46380 protein |
| 227763_at | 11.39 | 6.95E-05 | 1.312436697 | NM_194317| | MGC52057,hypothetical protein MGC52057 |
| 201960_s_at | 11.39 | 6.94E-05 | 1.090230954 | NM_015057| | MYCBP2,MYC binding protein 2 |
| 226941_at | 11.38 | 6.96E-05 | 1.123471084 | NA |  |
| 209370_s_at | 11.38 | 6.95E-05 | 1.171423559 | NM_001122681| | NA |
| 217814_at | 11.38 | 6.97E-05 | 1.083069336 | NM_020198| | GK001,GK001 protein |
| 244532_x_at | 11.38 | 6.97E-05 | 1.495440472 | NA |  |
| 225844_at | 11.38 | 6.96E-05 | 1.095844412 | NM_019896| | POLE4,DNA polymerase epsilon subunit 4 |
| 224303_x_at | 11.37 | 6.99E-05 | 1.132567811 | NM_016350| | NIN,ninein isoform 4 |
| 228143_at | 11.37 | 7.01E-05 | 1.601029268 | NM_000096| | CP,ceruloplasmin (ferroxidase) |
| 221265_s_at | 11.37 | 7.00E-05 | 1.077189269 | NM_030800| | DKFZP564O1664,hypothetical protein DKFZp564O1664 |
| 217992_s_at | 11.37 | 7.01E-05 | 1.084099237 | NM_024329| | EFHD2,EF hand domain family, member D2 |
| 228177_at | 11.37 | 6.99E-05 | 1.123286423 | NM_001079846| | NA |
| 217801_at | 11.37 | 7.01E-05 | 1.068590381 | NM_001001977| | ATP5E,ATP synthase, H+ transporting, mitochondrial F1 |
| 236627_at | 11.37 | 6.99E-05 | 1.532321634 | NA |  |
| 220094_s_at | 11.36 | 7.02E-05 | 1.067964335 | NM_001031713| | NA |
| 201335_s_at | 11.36 | 7.04E-05 | 1.35367367 | NM_015313| | ARHGEF12,Rho guanine nucleotide exchange factor (GEF) 12 |
| 1554082_a_at | 11.36 | 7.04E-05 | 1.107703231 | NM_024654| | FLJ23323,hypothetical protein FLJ23323 |
| 220543_at | 11.35 | 7.05E-05 | 1.446434375 | NM_019596| | C21orf62,chromosome 21 open reading frame 62 |
| 242790_at | 11.35 | 7.05E-05 | 1.220273566 | NM_007241| | EAP30,EAP30 subunit of ELL complex |
| 239236_at | 11.35 | 7.05E-05 | 1.426636885 | NA |  |
| 234472_at | 11.35 | 7.05E-05 | 1.154717357 | NM_052917| | GALNT13,UDP-N-acetyl-alpha-D-galactosamine:polypeptide |
| 236353_at | 11.35 | 7.06E-05 | 1.170460571 | NA |  |
| 214202_at | 11.35 | 7.05E-05 | 1.35622009 | NA |  |
| 217775_s_at | 11.34 | 7.09E-05 | 1.058334172 | NM_016026| | RDH11,androgen-regulated short-chain |
| 234394_at | 11.34 | 7.10E-05 | 1.455974434 | NM_003431| | ZNF124,zinc finger protein 124 |
| 205330_at | 11.34 | 7.11E-05 | 1.193520014 | NM_002430| | MN1,meningioma 1 |
| 1559132_at | 11.34 | 7.11E-05 | 1.185857278 | NM_001042463| | NA |
| 1555004_a_at | 11.33 | 7.14E-05 | 1.280984004 | NM_002895| | RBL1,retinoblastoma-like protein 1 isoform a |
| 229333_at | 11.33 | 7.13E-05 | 1.151294563 | NA |  |
| 216799_at | 11.32 | 7.15E-05 | 1.379548735 | NA |  |
| 1561578_s_at | 11.32 | 7.18E-05 | 1.380661192 | NM_001012755| | NA |
| 220915_s_at | 11.31 | 7.18E-05 | 1.54436554 | NA |  |
| 232951_at | 11.31 | 7.20E-05 | 1.113704209 | NA |  |
| 235562_at | 11.31 | 7.19E-05 | 1.363608066 | NM_001025266| | NA |
| 220525_s_at | 11.31 | 7.21E-05 | 1.076256249 | NM_181575| | AUP1,ancient ubiquitous protein 1 isoform 2 |
| 230016_at | 11.31 | 7.21E-05 | 1.211448722 | NM_152416| | MGC40214,hypothetical protein MGC40214 |
| 202851_at | 11.31 | 7.18E-05 | 1.104597132 | NM_024666| | FLJ11506,hypothetical protein FLJ11506 |
| 226259_at | 11.3 | 7.21E-05 | 1.137728342 | NM_001013848| | NA |
| 226854_at | 11.3 | 7.22E-05 | 1.12739242 | NA |  |
| 235535_x_at | 11.3 | 7.22E-05 | 1.598811629 | NM_004477| | FRG1,FSHD region gene 1 |
| 1555705_a_at | 11.3 | 7.22E-05 | 1.165404001 | NM_001048251| | NA |
| 1558613_at | 11.3 | 7.24E-05 | 1.502704345 | NA |  |
| 214119_s_at | 11.28 | 7.32E-05 | 1.082315263 | NM_000801| | FKBP1A,FK506-binding protein 1A |
| 216375_s_at | 11.28 | 7.29E-05 | 1.379501872 | NM_004454| | ETV5,ets variant gene 5 (ets-related molecule) |
| 1555234_a_at | 11.28 | 7.32E-05 | 1.254866901 | NM_020663| | RHOJ,TC10-like Rho GTPase |
| 230343_at | 11.28 | 7.30E-05 | 1.456103756 | NA |  |
| 226458_at | 11.28 | 7.32E-05 | 1.172054837 | NA |  |
| 221514_at | 11.27 | 7.33E-05 | 1.124027899 | NM_006649| | UTP14A,UTP14, U3 small nucleolar ribonucleoprotein, |
| 206016_at | 11.26 | 7.36E-05 | 1.166074821 | NM_014008| | CXorf37,chromosome X open reading frame 37 |
| 212944_at | 11.26 | 7.39E-05 | 1.131181931 | NM_006933| | SLC5A3,solute carrier family 5 (inositol transporters), |
| 227663_at | 11.26 | 7.37E-05 | 1.270632956 | NA |  |
| 209083_at | 11.26 | 7.37E-05 | 1.126795882 | NM_007074| | CORO1A,coronin, actin binding protein, 1A |
| 204306_s_at | 11.26 | 7.37E-05 | 1.166945207 | NM_001039490| | NA |
| 225939_at | 11.25 | 7.42E-05 | 1.260253577 | NM_173359| | NA |
| 1558714_at | 11.25 | 7.42E-05 | 1.369800777 | NA |  |
| 223227_at | 11.25 | 7.40E-05 | 1.120403679 | NM_031885| | BBS2,Bardet-Biedl syndrome 2 protein |
| 226410_at | 11.25 | 7.40E-05 | 1.139618845 | NM_001012759| | NA |
| 1569243_at | 11.24 | 7.46E-05 | 1.339201844 | NA |  |
| 218998_at | 11.24 | 7.43E-05 | 1.09288457 | NM_017832| | FLJ20457,hypothetical protein FLJ20457 |
| 204780_s_at | 11.24 | 7.45E-05 | 1.31544852 | NM_000043| | FAS,tumor necrosis factor receptor superfamily, |
| 1556761_at | 11.24 | 7.46E-05 | 1.279772974 | NA |  |
| 223791_at | 11.24 | 7.46E-05 | 1.216334403 | NA |  |
| 202353_s_at | 11.24 | 7.43E-05 | 1.119656954 | NM_002816| | PSMD12,proteasome 26S non-ATPase subunit 12 isoform 1 |
| 225971_at | 11.23 | 7.49E-05 | 1.149278511 | NM_030637| | DDHD1,DDHD domain containing 1 |
| 217437_s_at | 11.23 | 7.47E-05 | 1.285720301 | NM_001122824| | NA |
| 208094_s_at | 11.23 | 7.47E-05 | 1.075727703 | NM_030818| | MGC10471,hypothetical protein MGC10471 |
| 213334_x_at | 11.23 | 7.48E-05 | 1.070830981 | NM_017518| | NA |
| 202756_s_at | 11.23 | 7.48E-05 | 1.149276429 | NM_002081| | GPC1,glypican 1 precursor |
| 203647_s_at | 11.23 | 7.47E-05 | 1.062971697 | NM_004109| | FDX1,ferredoxin 1 precursor |
| 202115_s_at | 11.23 | 7.47E-05 | 1.070629594 | NM_015658| | DKFZP564C186,DKFZP564C186 protein |
| 231883_at | 11.23 | 7.47E-05 | 1.251085602 | NM_012174| | FBXW8,F-box and WD-40 domain protein 8 isoform 2 |
| 213679_at | 11.23 | 7.47E-05 | 1.208248544 | NM_152275| | FLJ13946,hypothetical protein FLJ13946 |
| 202175_at | 11.23 | 7.49E-05 | 1.181744063 | NM_024536| | CHPF,chondroitin polymerizing factor |
| 1559946_s_at | 11.22 | 7.49E-05 | 1.049514656 | NM_006666| | RUVBL2,RuvB-like 2 |
| 210633_x_at | 11.22 | 7.49E-05 | 1.104129057 | NM_000421| | KRT10,keratin 10 |
| 235273_at | 11.22 | 7.50E-05 | 1.379772252 | NM_001033559| | NA |
| 201340_s_at | 11.22 | 7.52E-05 | 1.488184212 | NM_003633| | ENC1,ectodermal-neural cortex (with BTB-like domain) |
| 239022_at | 11.21 | 7.56E-05 | 1.150519056 | NA |  |
| 229346_at | 11.21 | 7.56E-05 | 1.136717186 | NM_006617| | NES,nestin |
| 235316_at | 11.21 | 7.55E-05 | 1.229308297 | NM_178557| | FLJ37478,hypothetical protein FLJ37478 |
| 211009_s_at | 11.21 | 7.53E-05 | 1.101396496 | NM_001112663| | NA |
| 233058_at | 11.21 | 7.53E-05 | 1.345014605 | NA |  |
| 230605_at | 11.2 | 7.58E-05 | 1.141465731 | NA |  |
| 1558686_at | 11.2 | 7.59E-05 | 2.024086818 | NA |  |
| 209577_at | 11.2 | 7.59E-05 | 1.111666579 | NM_002861| | PCYT2,phosphate cytidylyltransferase 2, ethanolamine |
| 242442_x_at | 11.2 | 7.57E-05 | 1.301908459 | NM_152292| | RG9MTD2,RNA (guanine-9-) methyltransferase domain |
| 218135_at | 11.19 | 7.62E-05 | 1.041861383 | NM_016570| | PTX1,CDA14 |
| 202595_s_at | 11.19 | 7.63E-05 | 1.05495938 | NM_015344| | LEPROTL1,leptin receptor overlapping transcript-like 1 |
| 236514_at | 11.19 | 7.60E-05 | 1.196168806 | NM_005469| | PTE1,peroxisomal acyl-CoA thioesterase isoform a |
| 218758_s_at | 11.19 | 7.62E-05 | 1.092246321 | NM_003683| | D21S2056E,nucleolar protein NOP52 |
| 208322_s_at | 11.18 | 7.65E-05 | 1.118147802 | NM_003033| | ST3GAL1,sialyltransferase 4A |
| 233124_s_at | 11.18 | 7.67E-05 | 1.106495235 | NM_001002030| | NA |
| 209635_at | 11.18 | 7.64E-05 | 1.062561029 | NM_001283| | AP1S1,adaptor-related protein complex 1, sigma 1 |
| 227009_at | 11.18 | 7.64E-05 | 1.214314838 | NA |  |
| 220202_s_at | 11.18 | 7.66E-05 | 1.107040701 | NM_001100588| | NA |
| 222206_s_at | 11.17 | 7.68E-05 | 1.103251826 | NM_020170| | NCLN,nicalin |
| 208628_s_at | 11.17 | 7.68E-05 | 1.023443921 | NM_004559| | NSEP1,nuclease sensitive element binding protein 1 |
| 228092_at | 11.17 | 7.69E-05 | 1.205618664 | NM_001881| | CREM,cAMP responsive element modulator isoform b |
| 212092_at | 11.17 | 7.68E-05 | 1.245987509 | NM_001040152| | NA |
| 219188_s_at | 11.16 | 7.74E-05 | 1.085159533 | NM_014067| | LRP16,LRP16 protein |
| 242405_at | 11.15 | 7.77E-05 | 1.303607326 | NA |  |
| 221896_s_at | 11.15 | 7.79E-05 | 1.051526338 | NM_001099668| | NA |
| 221521_s_at | 11.14 | 7.80E-05 | 1.057880608 | NM_016095| | Pfs2,DNA replication complex GINS protein PSF2 |
| 219390_at | 11.14 | 7.83E-05 | 1.133571681 | NM_017946| | FKBP14,FK506 binding protein 14, 22 kDa |
| 225315_at | 11.13 | 7.84E-05 | 1.071445089 | NM_181514| | MRPL21,mitochondrial ribosomal protein L21 isoform d |
| 232800_at | 11.13 | 7.86E-05 | 1.627282824 | NA |  |
| 231931_at | 11.13 | 7.84E-05 | 1.174321648 | NM_001040424| | NA |
| 209849_s_at | 11.13 | 7.86E-05 | 1.127384916 | NM_002876| | RAD51C,RAD51 homolog C isoform 2 |
| 217944_at | 11.13 | 7.84E-05 | 1.102494189 | NM_017739| | FLJ20277,O-linked mannose |
| 1555824_a_at | 11.13 | 7.87E-05 | 1.07202721 | NM_001100913| | NA |
| 1563076_x_at | 11.12 | 7.89E-05 | 1.110962834 | NA |  |
| 226106_at | 11.12 | 7.88E-05 | 1.091390155 | NM_016422| | RNF141,ring finger protein 141 |
| 235673_at | 11.12 | 7.89E-05 | 1.25605585 | NA |  |
| 219373_at | 11.12 | 7.89E-05 | 1.134408351 | NM_018973| | DPM3,dolichyl-phosphate mannosyltransferase |
| 227148_at | 11.12 | 7.91E-05 | 1.305514268 | NM_172069| | PLEKHH2,pleckstrin homology domain containing, family H |
| 226852_at | 11.12 | 7.90E-05 | 1.09696354 | NM_020744| | MTA3,metastasis associated 1 family, member 3 |
| 220748_s_at | 11.12 | 7.90E-05 | 1.12610115 | NM_016202| | ZNF580,zinc finger protein 580 |
| 226508_at | 11.11 | 7.94E-05 | 1.07248175 | NM_024947| | PHC3,polyhomeotic like 3 |
| 204964_s_at | 11.11 | 7.93E-05 | 1.245313257 | NM_005086| | SSPN,sarcospan |
| 221258_s_at | 11.11 | 7.94E-05 | 1.082090606 | NM_031217| | KIF18A,kinesin family member 18A |
| 202940_at | 11.11 | 7.93E-05 | 1.192903105 | NM_018979| | WNK1,WNK lysine deficient protein kinase 1 |
| 224988_at | 11.11 | 7.92E-05 | 1.167910363 | NM_152734| | C6orf89,hypothetical protein FLJ25357 |
| 222705_s_at | 11.1 | 7.98E-05 | 1.224281595 | NM_014252| | SLC25A15,solute carrier family 25 (mitochondrial carrier; |
| 222048_at | 11.1 | 7.97E-05 | 1.163349521 | NA |  |
| 212525_s_at | 11.1 | 7.96E-05 | 1.156912724 | NM_002105| | H2AFX,H2A histone family, member X |
| 226179_at | 11.09 | 8.03E-05 | 1.077634295 | NM_016612| | MSCP,mitochondrial solute carrier protein |
| 230999_at | 11.09 | 8.03E-05 | 1.140360291 | NA |  |
| 212923_s_at | 11.09 | 8.01E-05 | 1.211013527 | NM_183373| | C6orf145,chromosome 6 open reading frame 145 |
| 222387_s_at | 11.09 | 8.03E-05 | 1.063511008 | NM_018206| | VPS35,vacuolar protein sorting 35 |
| 203557_s_at | 11.09 | 8.00E-05 | 1.103103247 | NM_000281| | PCBD1,pterin-4 alpha-carbinolamine dehydratase isoform |
| 233530_at | 11.08 | 8.05E-05 | 1.23531347 | NA |  |
| 227518_at | 11.08 | 8.04E-05 | 1.082290086 | NM_024881| | SLC35E1,solute carrier family 35, member E1 |
| 209379_s_at | 11.08 | 8.03E-05 | 1.197938177 | NM_018999| | KIAA1128,KIAA1128 |
| 204087_s_at | 11.08 | 8.05E-05 | 1.052123166 | NM_021095| | SLC5A6,solute carrier family 5 (sodium-dependent |
| 228032_s_at | 11.08 | 8.04E-05 | 1.203136292 | NA |  |
| 38703_at | 11.08 | 8.04E-05 | 1.024552488 | NM_012100| | DNPEP,aspartyl aminopeptidase |
| 221792_at | 11.08 | 8.05E-05 | 1.189346994 | NM_016577| | RAB6B,RAB6B, member RAS oncogene family |
| 222481_at | 11.07 | 8.10E-05 | 1.118735956 | NM_012192| | FXC1,fracture callus 1 homolog |
| 214169_at | 11.07 | 8.09E-05 | 1.448368359 | NM_025154| | UNC84A,unc-84 homolog A |
| 223296_at | 11.07 | 8.08E-05 | 1.073853697 | NM_032315| | MGC4399,mitochondrial carrier protein MGC4399 |
| 212582_at | 11.07 | 8.08E-05 | 1.078543362 | NM_001003712| | OSBPL8,oxysterol-binding protein-like protein 8 isoform |
| 209135_at | 11.06 | 8.14E-05 | 1.192173148 | NM_004318| | ASPH,aspartate beta-hydroxylase isoform a |
| 226959_at | 11.06 | 8.14E-05 | 1.12560259 | NA |  |
| 217841_s_at | 11.06 | 8.15E-05 | 1.076366834 | NM_016147| | PME-1,protein phosphatase methylesterase-1 |
| 215364_s_at | 11.06 | 8.14E-05 | 1.201908878 | NM_015284| | KIAA0467,KIAA0467 protein |
| 225878_at | 11.05 | 8.17E-05 | 1.049668846 | NM_015074| | KIF1B,kinesin family member 1B isoform b |
| 214756_x_at | 11.05 | 8.17E-05 | 1.105842897 | NA |  |
| 227982_at | 11.05 | 8.17E-05 | 1.127437475 | NM_016955| | SLA/LP,soluble liver antigen/liver pancreas antigen |
| 238936_at | 11.05 | 8.17E-05 | 1.154298572 | NA |  |
| 217712_at | 11.04 | 8.20E-05 | 1.718141074 | NA |  |
| 213959_s_at | 11.04 | 8.20E-05 | 1.210979401 | NM_015272| | KIAA1005,KIAA1005 protein |
| 1560648_s_at | 11.04 | 8.20E-05 | 1.265287185 | NM_003309| | NA |
| 32541_at | 11.04 | 8.19E-05 | 1.253229953 | NM_005605| | PPP3CC,protein phosphatase 3 (formerly 2B), catalytic |
| 243176_at | 11.04 | 8.23E-05 | 1.224645371 | NA |  |
| 226381_at | 11.03 | 8.23E-05 | 1.175638422 | NA |  |
| 213766_x_at | 11.03 | 8.23E-05 | 1.18369147 | NM_002067| | GNA11,guanine nucleotide binding protein (G protein), |
| 225176_at | 11.03 | 8.26E-05 | 1.190812695 | NA |  |
| 220974_x_at | 11.03 | 8.23E-05 | 1.596285852 | NM_030971| | SFXN3,sideroflexin 3 |
| 229720_at | 11.03 | 8.25E-05 | 1.408673274 | NM_004323| | BAG1,BCL2-associated athanogene isoform 1L |
| 1554306_at | 11.03 | 8.24E-05 | 1.263364837 | NM_002221| | ITPKB,1D-myo-inositol-trisphosphate 3-kinase B |
| 226820_at | 11.03 | 8.24E-05 | 1.047768569 | NM_152493| | FLJ25476,FLJ25476 protein |
| 1555964_at | 11.02 | 8.28E-05 | 1.210393583 | NM_001039083| | NA |
| 1554600_s_at | 11.02 | 8.29E-05 | 1.345149925 | NM_005572| | LMNA,lamin A/C isoform 2 |
| 241865_at | 11.02 | 8.30E-05 | 1.161504462 | NA |  |
| 222618_at | 11.02 | 8.29E-05 | 1.053112839 | NM_018225| | SMU1,smu-1 suppressor of mec-8 and unc-52 homolog |
| 222632_s_at | 11.01 | 8.32E-05 | 1.141399856 | NM_020347| | LZTFL1,leucine zipper transcription factor-like 1 |
| 221069_s_at | 11.01 | 8.33E-05 | 1.079807068 | NM_016360| | LOC51204,clone HQ0477 PRO0477p |
| 214736_s_at | 11.01 | 8.32E-05 | 1.101720749 | NM_001119| | ADD1,adducin 1 (alpha) isoform a |
| 225628_s_at | 11 | 8.37E-05 | 1.265210086 | NM_005937| | MLLT6,myeloid/lymphoid or mixed-lineage leukemia |
| 206902_s_at | 11 | 8.38E-05 | 1.347326475 | NM_005107| | ENDOGL1,endonuclease G-like 1 |
| 224341_x_at | 11 | 8.38E-05 | 1.578149967 | NM_138554| | TLR4,toll-like receptor 4 isoform A |
| 226685_at | 11 | 8.38E-05 | 1.066815712 | NM_006750| | SNTB2,basic beta 2 syntrophin isoform a |
| 209118_s_at | 11 | 8.38E-05 | 1.102477108 | NM_006009| | TUBA3,tubulin, alpha 3 |
| 226913_s_at | 11 | 8.38E-05 | 1.292099968 | NM_014587| | SOX8,SRY (sex determining region Y)-box 8 |
| 214703_s_at | 10.99 | 8.39E-05 | 1.114463317 | NM_015274| | MAN2B2,mannosidase, alpha, class 2B, member 2 |
| 235215_at | 10.99 | 8.39E-05 | 1.312202388 | NM_005236| | ERCC4,excision repair cross-complementing rodent |
| 221483_s_at | 10.99 | 8.42E-05 | 1.040184727 | NM_006628| | ARPP-19,cyclic AMP phosphoprotein, 19 kD |
| 233401_at | 10.99 | 8.41E-05 | 1.784063789 | NA |  |
| 223061_at | 10.99 | 8.39E-05 | 1.073643271 | NM_023947| | MGC3234,hypothetical protein MGC3234 |
| 212238_at | 10.98 | 8.46E-05 | 1.158927402 | NM_015338| | ASXL1,additional sex combs like 1 |
| 223451_s_at | 10.98 | 8.43E-05 | 1.150644427 | NM_001040138| | NA |
| 204511_at | 10.98 | 8.44E-05 | 1.129976002 | NM_014808| | FARP2,FERM, RhoGEF and pleckstrin domain protein 2 |
| 218681_s_at | 10.98 | 8.44E-05 | 1.131233479 | NM_022044| | SDF2L1,stromal cell-derived factor 2-like 1 precursor |
| 237270_at | 10.98 | 8.44E-05 | 1.311811689 | NA |  |
| 204504_s_at | 10.97 | 8.48E-05 | 1.071903767 | NM_003609| | HIRIP3,HIRA interacting protein 3 |
| 224754_at | 10.97 | 8.48E-05 | 1.036091765 | NM_138473| | SP1,Sp1 transcription factor |
| 1566257_at | 10.97 | 8.49E-05 | 1.267461002 | NA |  |
| 225782_at | 10.97 | 8.50E-05 | 1.658942954 | NM_001031679| | NA |
| 204140_at | 10.97 | 8.49E-05 | 1.044064049 | NM_003596| | TPST1,tyrosylprotein sulfotransferase 1 |
| 204338_s_at | 10.96 | 8.54E-05 | 1.391237554 | NM_001102445| | NA |
| 219957_at | 10.96 | 8.55E-05 | 1.212869085 | NM_001042417| | NA |
| 223109_at | 10.96 | 8.52E-05 | 1.077878578 | NM_015679| | TRUB2,TruB pseudouridine (psi) synthase homolog 2 |
| 206788_s_at | 10.96 | 8.53E-05 | 1.182208544 | NM_001755| | CBFB,core-binding factor, beta subunit isoform 2 |
| 222783_s_at | 10.96 | 8.54E-05 | 1.522459948 | NM_001034852| | NA |
| 228043_at | 10.96 | 8.55E-05 | 1.165774249 | NM_032175| | FLJ12787,Src-associated protein SAW |
| 224876_at | 10.96 | 8.55E-05 | 1.187944662 | NM_152409| | FLJ37562,hypothetical protein FLJ37562 |
| 202532_s_at | 10.96 | 8.54E-05 | 1.290136419 | NM_000791| | DHFR,dihydrofolate reductase |
| 1556474_a_at | 10.95 | 8.59E-05 | 1.802700728 | NA |  |
| 230149_at | 10.95 | 8.56E-05 | 1.56217024 | NA |  |
| 213492_at | 10.95 | 8.59E-05 | 1.458293981 | NM_001844| | COL2A1,alpha 1 type II collagen isoform 1 |
| 226644_at | 10.95 | 8.59E-05 | 1.273443775 | NM_080875| | ZZANK1,zinc finger, ZZ type with ankyrin repeat domain |
| 217754_at | 10.94 | 8.63E-05 | 1.074351627 | NM_019082| | DDX56,DEAD (Asp-Glu-Ala-Asp) box polypeptide 56 |
| 1556051_a_at | 10.94 | 8.59E-05 | 1.115703477 | NM_001003398| | BICD1,bicaudal D homolog 1 isoform 2 |
| 211792_s_at | 10.94 | 8.60E-05 | 1.122678726 | NM_001262| | CDKN2C,cyclin-dependent kinase inhibitor 2C |
| 1559701_s_at | 10.93 | 8.67E-05 | 1.332757896 | NM_003635| | NDST2,N-deacetylase/N-sulfotransferase (heparan |
| 1566303_s_at | 10.93 | 8.67E-05 | 1.079881576 | NM_021959| | PPP1R11,protein phosphatase 1, regulatory (inhibitor) |
| 202192_s_at | 10.93 | 8.67E-05 | 1.214032175 | NM_003644| | GAS7,growth arrest-specific 7 isoform a |
| 221436_s_at | 10.93 | 8.66E-05 | 1.112674349 | NM_031299| | CDCA3,trigger of mitotic entry 1 |
| 238039_at | 10.92 | 8.68E-05 | 1.219041565 | NA |  |
| 201661_s_at | 10.92 | 8.72E-05 | 1.181136484 | NM_004457| | ACSL3,acyl-CoA synthetase long-chain family member 3 |
| 217856_at | 10.91 | 8.75E-05 | 1.216635325 | NM_005105| | RBM8A,RNA binding motif protein 8A |
| 201940_at | 10.91 | 8.73E-05 | 1.095439103 | NM_001304| | CPD,carboxypeptidase D precursor |
| 206441_s_at | 10.91 | 8.74E-05 | 1.063014317 | NM_017828| | COMMD4,COMM domain containing 4 |
| 217597_x_at | 10.91 | 8.76E-05 | 1.100228533 | NM_006822| | RAB40B,RAB40B, member RAS oncogene family |
| 213243_at | 10.91 | 8.76E-05 | 1.130569327 | NM_015243| | COH1,Cohen syndrome 1 protein isoform 3 |
| 239238_at | 10.9 | 8.79E-05 | 1.294550215 | NA |  |
| 201320_at | 10.9 | 8.78E-05 | 1.149282002 | NM_003075| | SMARCC2,SWI/SNF-related matrix-associated |
| 208430_s_at | 10.9 | 8.77E-05 | 1.33642933 | NM_001390| | DTNA,dystrobrevin alpha isoform 1 |
| 224232_s_at | 10.9 | 8.77E-05 | 1.062420593 | NM_013237| | PX19,px19-like protein |
| 219974_x_at | 10.89 | 8.85E-05 | 1.114425354 | NM_001002030| | NA |
| 211576_s_at | 10.89 | 8.84E-05 | 1.105244259 | NM_194255| | SLC19A1,solute carrier family 19 member 1 isoform a |
| 230948_at | 10.89 | 8.84E-05 | 1.330407849 | NA |  |
| 223266_at | 10.88 | 8.87E-05 | 1.053913914 | NM_018571| | ALS2CR2,amyotrophic lateral sclerosis 2 (juvenile) |
| 204191_at | 10.88 | 8.90E-05 | 1.206884212 | NM_000629| | IFNAR1,interferon-alpha receptor 1 precursor |
| 214786_at | 10.88 | 8.88E-05 | 1.486431873 | NM_005921| | NA |
| 222624_s_at | 10.88 | 8.89E-05 | 1.083253691 | NM_016331| | ZNF639,zinc finger protein 639 |
| 224872_at | 10.87 | 8.92E-05 | 1.144516159 | NM_173602| | KIAA1463,KIAA1463 protein |
| 226660_at | 10.87 | 8.95E-05 | 1.06210802 | NM_003161| | RPS6KB1,ribosomal protein S6 kinase, 70kDa, polypeptide |
| 217786_at | 10.86 | 8.96E-05 | 1.075287006 | NM_001039619| | NA |
| 212118_at | 10.86 | 8.98E-05 | 1.089827645 | NM_006510| | RFP,ret finger protein isoform alpha |
| 212388_at | 10.86 | 8.96E-05 | 1.076383882 | NM_015306| | NA |
| 215731_s_at | 10.86 | 8.95E-05 | 1.179131699 | NM_022782| | MPHOSPH9,M-phase phosphoprotein 9 |
| 201554_x_at | 10.86 | 8.97E-05 | 1.074863455 | NM_004130| | GYG,glycogenin |
| 205257_s_at | 10.85 | 9.00E-05 | 1.126609502 | NM_001635| | AMPH,amphiphysin isoform 1 |
| 238782_at | 10.85 | 9.01E-05 | 1.5451846 | NA |  |
| 222880_at | 10.85 | 9.00E-05 | 1.336463189 | NM_005465| | AKT3,v-akt murine thymoma viral oncogene homolog 3 |
| 216705_s_at | 10.85 | 9.01E-05 | 1.100035685 | NM_000022| | ADA,adenosine deaminase |
| 225443_at | 10.85 | 9.01E-05 | 1.08830902 | NM_018403| | DCP1A,decapping enzyme |
| 201864_at | 10.85 | 9.00E-05 | 1.091715553 | NM_001493| | GDI1,GDP dissociation inhibitor 1 |
| 234984_at | 10.84 | 9.09E-05 | 1.102851842 | NM_152905| | NEDD1,neural precursor cell expressed, developmentally |
| 235513_at | 10.84 | 9.07E-05 | 1.275848145 | NA |  |
| 223080_at | 10.84 | 9.06E-05 | 1.222833805 | NM_014905| | GLS,glutaminase C |
| 211769_x_at | 10.84 | 9.07E-05 | 1.046751017 | NM_006811| | TDE1,tumor differentially expressed protein 1 |
| 213449_at | 10.84 | 9.04E-05 | 1.1205344 | NM_015029| | POP1,processing of precursor 1, ribonuclease P/MRP |
| 203117_s_at | 10.83 | 9.09E-05 | 1.09711563 | NM_014871| | USP52,ubiquitin specific protease 52 |
| 215204_at | 10.83 | 9.10E-05 | 1.139322385 | NA |  |
| 222172_at | 10.83 | 9.13E-05 | 1.269076907 | NM_022123| | NPAS3,neuronal PAS domain protein 3 |
| 209563_x_at | 10.83 | 9.09E-05 | 1.029010404 | NM_001743| | CALM2,calmodulin 2 |
| 225510_at | 10.83 | 9.13E-05 | 1.185363968 | NM_178507| | NS5ATP13TP2,NS5ATP13TP2 protein |
| 235255_at | 10.82 | 9.16E-05 | 1.210019604 | NM_012463| | ATP6V0A2,ATPase, H+ transporting, lysosomal V0 subunit a |
| 213581_at | 10.82 | 9.16E-05 | 1.104637845 | NM_002598| | PDCD2,programmed cell death 2 isoform 1 |
| 213004_at | 10.82 | 9.14E-05 | 1.167495081 | NM_012098| | ANGPTL2,angiopoietin-like 2 precursor |
| 219703_at | 10.82 | 9.14E-05 | 1.22006031 | NM_018365| | MNS1,meiosis-specific nuclear structural protein 1 |
| 236476_at | 10.82 | 9.16E-05 | 1.537599447 | NA |  |
| 202585_s_at | 10.81 | 9.18E-05 | 1.205536922 | NM_002504| | NFX1,nuclear transcription factor, X-box binding 1 |
| 219447_s_at | 10.81 | 9.19E-05 | 1.110221156 | NM_015945| | SLC35C2,ovarian cancer overexpressed 1 isoform a |
| 233375_at | 10.81 | 9.20E-05 | 1.203486292 | NM_032328| | MGC12458,hypothetical protein MGC12458 |
| 215069_at | 10.81 | 9.19E-05 | 1.328398094 | NM_004808| | NMT2,glycylpeptide N-tetradecanoyltransferase 2 |
| 225168_at | 10.81 | 9.18E-05 | 1.20875476 | NM_018027| | FRMD4A,FERM domain containing 4A |
| 214383_x_at | 10.81 | 9.21E-05 | 1.044787028 | NM_057161| | KLHDC3,testis intracellular mediator protein |
| 229721_x_at | 10.81 | 9.19E-05 | 1.126385348 | NM_001002862| | DERL3,derlin-3 protein isoform b |
| 243303_at | 10.8 | 9.23E-05 | 1.50591368 | NA |  |
| 218827_s_at | 10.8 | 9.25E-05 | 1.099648053 | NM_032142| | Cep192,centrosomal protein 192 kDa isoform 1 |
| 241722_x_at | 10.8 | 9.24E-05 | 1.186836998 | NA |  |
| 222633_at | 10.8 | 9.23E-05 | 1.043008192 | NM_024665| | TBL1XR1,nuclear receptor co-repressor/HDAC3 complex |
| 210752_s_at | 10.79 | 9.28E-05 | 1.201525377 | NM_170607| | MLX,transcription factor-like protein 4 isoform |
| 210990_s_at | 10.79 | 9.28E-05 | 1.31555435 | NM_001105206| | NA |
| 202692_s_at | 10.78 | 9.36E-05 | 1.109707656 | NM_001076683| | NA |
| 220753_s_at | 10.78 | 9.36E-05 | 1.249330935 | NM_015974| | CRYL1,lambda-crystallin |
| 36545_s_at | 10.78 | 9.34E-05 | 1.136458591 | NM_001007467| | SFI1,spindle assembly associated Sfi1 homolog isoform |
| 201680_x_at | 10.78 | 9.36E-05 | 1.041159391 | NM_015908| | ARS2,arsenate resistance protein ARS2 isoform a |
| 219392_x_at | 10.78 | 9.36E-05 | 1.06497216 | NM_018304| | FLJ11029,hypothetical protein FLJ11029 |
| 1569181_x_at | 10.78 | 9.37E-05 | 1.294719161 | NA |  |
| 202959_at | 10.78 | 9.37E-05 | 1.100876731 | NM_000255| | MUT,methylmalonyl Coenzyme A mutase precursor |
| 231944_at | 10.78 | 9.37E-05 | 1.183220173 | NM_019891| | ERO1LB,endoplasmic reticulum oxidoreductin 1-Lbeta |
| 223094_s_at | 10.78 | 9.34E-05 | 1.239105053 | NM_054027| | ANKH,ankylosis, progressive homolog |
| 228692_at | 10.77 | 9.40E-05 | 1.268832902 | NA |  |
| 242440_at | 10.76 | 9.44E-05 | 1.324041134 | NA |  |
| 219816_s_at | 10.76 | 9.43E-05 | 1.124875731 | NM_001077351| | NA |
| 212639_x_at | 10.76 | 9.46E-05 | 1.034651767 | NM_006082| | K-ALPHA-1,tubulin, alpha, ubiquitous |
| 210910_s_at | 10.76 | 9.44E-05 | 1.241013293 | NM_012230| | POMZP3,POMZP3 fusion protein isoform 1 |
| 208012_x_at | 10.76 | 9.45E-05 | 1.154829631 | NM_004509| | SP110,SP110 nuclear body protein isoform a |
| 236436_at | 10.75 | 9.48E-05 | 1.329258338 | NM_001077241| | NA |
| 214788_x_at | 10.75 | 9.48E-05 | 1.192277676 | NM_015086| | NA |
| 200068_s_at | 10.75 | 9.49E-05 | 1.040565341 | NM_001024649| | NA |
| 211019_s_at | 10.75 | 9.51E-05 | 1.223124732 | NM_001001438| | LSS,lanosterol synthase |
| 244356_at | 10.74 | 9.54E-05 | 1.145643997 | NA |  |
| 233803_s_at | 10.74 | 9.54E-05 | 1.144391248 | NM_001105538| | NA |
| 207956_x_at | 10.74 | 9.56E-05 | 1.05448373 | NM_015032| | APRIN,androgen-induced prostate proliferative shutoff |
| 219762_s_at | 10.74 | 9.55E-05 | 1.025732697 | NM_015414| | RPL36,ribosomal protein L36 |
| 202942_at | 10.74 | 9.55E-05 | 1.092433444 | NM_001014763| | NA |
| 221123_x_at | 10.73 | 9.59E-05 | 1.117853233 | NM_018660| | ZNF395,zinc finger protein 395 |
| 203469_s_at | 10.73 | 9.62E-05 | 1.100687275 | NM_001098533| | NA |
| 219337_at | 10.73 | 9.57E-05 | 1.141959892 | NM_001114103| | NA |
| 1556821_x_at | 10.72 | 9.63E-05 | 1.943910331 | NA |  |
| 213499_at | 10.72 | 9.63E-05 | 1.119817864 | NM_004366| | CLCN2,chloride channel 2 |
| 201191_at | 10.72 | 9.62E-05 | 1.098224969 | NM_006224| | PITPNA,phosphatidylinositol transfer protein, alpha |
| 222516_at | 10.72 | 9.62E-05 | 1.073857111 | NM_012095| | AP3M1,adaptor-related protein complex 3, mu 1 subunit |
| 244517_x_at | 10.72 | 9.64E-05 | 1.498021883 | NA |  |
| 203720_s_at | 10.72 | 9.63E-05 | 1.076488172 | NM_001983| | ERCC1,excision repair cross-complementing 1 isofrom 2 |
| 204576_s_at | 10.72 | 9.63E-05 | 1.107111154 | NM_015041| | CLUAP1,clusterin associated protein 1 |
| 233490_at | 10.72 | 9.64E-05 | 1.249436577 | NM_016221| | DCTN4,dynactin 4 (p62) |
| 222616_s_at | 10.71 | 9.67E-05 | 1.090934499 | NM_001001992| | USP16,ubiquitin specific protease 16 isoform b |
| 224996_at | 10.71 | 9.70E-05 | 1.103768859 | NA |  |
| 226445_s_at | 10.71 | 9.70E-05 | 1.084806721 | NM_033549| | TRIM41,tripartite motif-containing 41 isform 1 |
| 204900_x_at | 10.71 | 9.71E-05 | 1.193984568 | NM_003864| | SAP30,sin3 associated polypeptide p30 |
| 205888_s_at | 10.7 | 9.75E-05 | 1.299858951 | NM_014790| | KIAA0555,KIAA0555 gene product |
| 212691_at | 10.7 | 9.74E-05 | 1.115207355 | NM_015354| | NA |
| 1554986_a_at | 10.7 | 9.76E-05 | 1.177060257 | NM_014758| | NA |
| 1566482_at | 10.7 | 9.72E-05 | 1.21458483 | NA |  |
| 228612_at | 10.69 | 9.78E-05 | 1.13898543 | NA |  |
| 239091_at | 10.69 | 9.78E-05 | 1.489466694 | NA |  |
| 226292_at | 10.69 | 9.77E-05 | 1.106391458 | NM_004055| | CAPN5,calpain 5 |
| 224783_at | 10.69 | 9.78E-05 | 1.111147256 | NM_182565| | MGC29814,hypothetical protein MGC29814 |
| 201018_at | 10.69 | 9.78E-05 | 1.078117959 | NM_001412| | EIF1AX,X-linked eukaryotic translation initiation |
| 205324_s_at | 10.69 | 9.77E-05 | 1.064369191 | NM_012280| | FTSJ1,FtsJ homolog 1 isoform a |
| 219267_at | 10.69 | 9.79E-05 | 1.298018638 | NM_016433| | GLTP,glycolipid transfer protein |
| 208778_s_at | 10.69 | 9.78E-05 | 1.030800022 | NM_001008897| | TCP1,T-complex protein 1 isoform b |
| 235482_at | 10.69 | 9.78E-05 | 1.160982931 | NA |  |
| 64438_at | 10.69 | 9.78E-05 | 1.094990321 | NM_024648| | FLJ22222,hypothetical protein FLJ22222 |
| 222629_at | 10.69 | 9.78E-05 | 1.345014428 | NM_001037872| | NA |
| 225357_s_at | 10.69 | 9.78E-05 | 1.068761686 | NM_017553| | INO80,yeast INO80-like protein |
| 229464_at | 10.69 | 9.78E-05 | 1.0609759 | NM_016132| | MYEF2,myelin gene expression factor 2 |
| 65884_at | 10.68 | 9.82E-05 | 1.088421559 | NM_016219| | MAN1B1,alpha 1,2-mannosidase |
| 202390_s_at | 10.68 | 9.83E-05 | 1.097976727 | NM_002111| | HD,huntingtin |
| 209667_at | 10.68 | 9.81E-05 | 1.187417026 | NM_003869| | CES2,carboxylesterase 2 isoform 1 |
| 204404_at | 10.67 | 9.85E-05 | 1.234596741 | NM_001046| | SLC12A2,solute carrier family 12 |
| 238115_at | 10.67 | 9.85E-05 | 1.348183143 | NA |  |
| 209256_s_at | 10.67 | 9.89E-05 | 1.052245474 | NM_014997| | KIAA0265,KIAA0265 protein |
| 203725_at | 10.67 | 9.86E-05 | 1.176542107 | NM_001924| | GADD45A,growth arrest and DNA-damage-inducible, alpha |
| 244660_at | 10.67 | 9.87E-05 | 1.328751807 | NM_001419| | ELAVL1,ELAV-like 1 |
| 214769_at | 10.66 | 9.96E-05 | 1.124475514 | NM_001830| | CLCN4,chloride channel 4 |
| 223433_at | 10.66 | 9.92E-05 | 1.102375578 | NM_020192| | C7orf36,chromosome 7 open reading frame 36 |
| 201745_at | 10.65 | 9.98E-05 | 1.158378708 | NM_002822| | PTK9,twinfilin isoform 1 |
| 225291_at | 10.65 | 9.99E-05 | 1.036803487 | NM_033109| | PNPT1,polyribonucleotide nucleotidyltransferase 1 |
