# Supplementary material for: Transcriptional Signature and Memory Retention of Human-Induced Pluripotent Stem Cells
Source: PLoS One. 2009 Sep 18;4(9):e7076. doi: 10.1371/journal.pone.0007076 (PMC2741600; doi:10.1371/journal.pone.0007076)
Supplement: Table S7 — IPSC, ES-enriched probes in IPSC, ES versus NSC. Probesets enriched in group-wise comparisons: Column headings are probeset identifiers, T-statistic, P-value, Fold-Change (log2), Refseq identifier and Description of the gene. (NA indicates no Refseq annotation). (10.18 MB DOC) [file pone.0007076.s011.doc]

| Probeset | T-statistic | P-value | Fold-Change (log2) | Refseq | Description |
| --- | --- | --- | --- | --- | --- |
| 206247_at | 115.62 | 8.45E-17 | 2.812484526 | NM_000247| | MICA,MHC class I chain-related gene A protein |
| 228565_at | 114.89 | 8.45E-17 | 1.499809722 | NM_032435| | KIAA1804,mixed lineage kinase 4 |
| 219073_s_at | 112.2 | 8.68E-17 | 1.663689838 | NM_017784| | OSBPL10,oxysterol-binding protein-like protein 10 |
| 209488_s_at | 105.32 | 1.16E-16 | 1.701789698 | NM_001008710| | RBPMS,RNA-binding protein with multiple splicing |
| 226497_s_at | 101.86 | 1.61E-16 | 2.569509552 | NA |  |
| 209771_x_at | 98.3 | 2.13E-16 | 1.173672974 | NM_013230| | CD24,CD24 antigen |
| 237336_at | 94.35 | 3.12E-16 | 1.543482831 | NM_001617| | ADD2,adducin 2 isoform a |
| 226587_at | 93.28 | 3.37E-16 | 1.332324907 | NA |  |
| 230988_at | 90.58 | 4.36E-16 | 1.477512276 | NA |  |
| 230560_at | 88.84 | 5.02E-16 | 2.82590514 | NM_014178| | STXBP6,amisyn |
| 212589_at | 84.83 | 7.89E-16 | 1.315438748 | NM_001102669| | NA |
| 203417_at | 82.11 | 1.10E-15 | 1.735310818 | NM_002403| | MFAP2,microfibrillar-associated protein 2 precursor |
| 204784_s_at | 80.87 | 1.20E-15 | 1.253151879 | NM_022443| | MLF1,myeloid leukemia factor 1 |
| 225958_at | 79.84 | 1.36E-15 | 1.37528885 | NM_004426| | PHC1,polyhomeotic 1-like |
| 226847_at | 77.03 | 1.82E-15 | 3.254038331 | NM_006350| | FST,follistatin isoform FST317 precursor |
| 219681_s_at | 76.91 | 1.82E-15 | 1.489205528 | NM_001002233| | RAB11FIP1,Rab coupling protein isoform 2 |
| 232985_s_at | 76.29 | 1.96E-15 | 3.187146599 | NM_018189| | DPPA4,developmental pluripotency associated 4 |
| 218735_s_at | 73.44 | 2.65E-15 | 1.581681907 | NM_014480| | ZNF544,zinc finger protein 544 |
| 219121_s_at | 73.42 | 2.65E-15 | 2.621001595 | NM_001034915| | NA |
| 226507_at | 73.37 | 2.65E-15 | 1.534961062 | NM_002576| | PAK1,p21-activated kinase 1 |
| 205514_at | 72.01 | 3.19E-15 | 2.381135633 | NM_018355| | ZNF415,zinc finger protein 415 |
| 227830_at | 71.96 | 3.19E-15 | 2.380847454 | NM_000814| | GABRB3,gamma-aminobutyric acid (GABA) A receptor, beta |
| 203889_at | 71.58 | 3.34E-15 | 2.82601703 | NM_003020| | SGNE1,secretory granule, neuroendocrine protein 1 (7B2 |
| 223435_s_at | 70.26 | 4.08E-15 | 3.226111475 | NM_014005| | PCDHA9,protocadherin alpha 9 isoform 2 precursor |
| 203748_x_at | 69.67 | 4.37E-15 | 1.30062515 | NM_002897| | RBMS1,RNA binding motif, single stranded interacting |
| 204675_at | 68.26 | 5.35E-15 | 1.505751581 | NM_001047| | SRD5A1,steroid-5-alpha-reductase 1 |
| 219901_at | 67.67 | 5.67E-15 | 1.655195732 | NM_018351| | FGD6,FYVE, RhoGEF and PH domain containing 6 |
| 217853_at | 67.08 | 6.07E-15 | 1.625183851 | NM_022748| | TENS1,tensin-like SH2 domain containing 1 |
| 209868_s_at | 65.36 | 7.98E-15 | 1.304217437 | NM_002897| | RBMS1,RNA binding motif, single stranded interacting |
| 225846_at | 64.96 | 8.27E-15 | 3.090296431 | NM_001034915| | NA |
| 216379_x_at | 64.95 | 8.27E-15 | 1.184893239 | NM_013230| | CD24,CD24 antigen |
| 205924_at | 64.35 | 8.75E-15 | 1.384328176 | NM_002867| | RAB3B,RAB3B, member RAS oncogene family |
| 211781_x_at | 64.27 | 8.75E-15 | 2.31036528 | NA |  |
| 203903_s_at | 64.24 | 8.75E-15 | 2.718994618 | NM_014799| | HEPH,hephaestin isoform b |
| 229349_at | 63.69 | 9.40E-15 | 3.644893609 | NM_001004317| | FLJ16517,FLJ16517 protein |
| 203917_at | 63.4 | 9.75E-15 | 1.214471582 | NM_001338| | CXADR,coxsackie virus and adenovirus receptor |
| 202876_s_at | 62.51 | 1.14E-14 | 1.222036279 | NM_002586| | PBX2,pre-B-cell leukemia transcription factor 2 |
| 227177_at | 62.34 | 1.16E-14 | 2.463704425 | NM_003389| | CORO2A,coronin, actin binding protein, 2A |
| 236894_at | 61.71 | 1.28E-14 | 3.456356091 | NM_019079| | FLJ10884,hypothetical protein FLJ10884 |
| 1569886_a_at | 61.47 | 1.28E-14 | 2.28157978 | NM_001080407| | NA |
| 202497_x_at | 59.8 | 1.78E-14 | 1.94601017 | NM_006931| | SLC2A3,solute carrier family 2 (facilitated glucose |
| 216836_s_at | 59.69 | 1.80E-14 | 1.324006322 | NM_001005862| | ERBB2,erbB-2 isoform b |
| 209487_at | 59.08 | 1.97E-14 | 1.51052743 | NM_001008710| | RBPMS,RNA-binding protein with multiple splicing |
| 205399_at | 58.56 | 2.18E-14 | 1.470096738 | NM_004734| | DCAMKL1,doublecortin and CaM kinase-like 1 |
| 209757_s_at | 58.52 | 2.18E-14 | 1.553091869 | NM_005378| | MYCN,v-myc myelocytomatosis viral related oncogene, |
| 201596_x_at | 58.34 | 2.25E-14 | 1.918962394 | NM_000224| | KRT18,keratin 18 |
| 225275_at | 57.94 | 2.43E-14 | 3.512913914 | NM_005711| | EDIL3,EGF-like repeats and discoidin I-like |
| 208119_s_at | 57.58 | 2.56E-14 | 1.350597107 | NM_031218| | ZNF505,zinc finger protein 505 isoform a |
| 231725_at | 57.53 | 2.56E-14 | 1.971299465 | NM_018936| | PCDHB2,protocadherin beta 2 precursor |
| 201846_s_at | 57.49 | 2.56E-14 | 1.2210173 | NM_012234| | RYBP,RING1 and YY1 binding protein |
| 207266_x_at | 57.21 | 2.64E-14 | 1.287940116 | NM_002897| | RBMS1,RNA binding motif, single stranded interacting |
| 225817_at | 57.19 | 2.64E-14 | 2.168642915 | NM_032866| | CGNL1,cingulin-like 1 |
| 214397_at | 57.13 | 2.65E-14 | 1.847717812 | NM_003927| | MBD2,methyl-CpG binding domain protein 2 isoform 1 |
| 214264_s_at | 56.51 | 2.97E-14 | 1.258490597 | NM_145231| | C14orf143,chromosome 14 open reading frame 143 |
| 226926_at | 56.28 | 3.11E-14 | 2.840044165 | NM_001035516| | NA |
| 231061_at | 55.71 | 3.37E-14 | 1.763652176 | NA |  |
| 224458_at | 55.33 | 3.62E-14 | 1.863684908 | NM_032342| | C9orf125,chromosome 9 open reading frame 125 |
| 218964_at | 55.23 | 3.66E-14 | 1.852730254 | NM_006465| | ARID3B,AT rich interactive domain 3B (BRIGHT- like) |
| 208131_s_at | 54.97 | 3.78E-14 | 2.060553409 | NM_000961| | PTGIS,prostaglandin I2 (prostacyclin) synthase |
| 209496_at | 54.61 | 4.06E-14 | 2.23962027 | NM_002889| | RARRES2,retinoic acid receptor responder (tazarotene |
| 1561101_at | 54.54 | 4.10E-14 | 2.246138482 | NA |  |
| 1554593_s_at | 54.25 | 4.26E-14 | 2.541481244 | NM_005071| | SLC1A6,solute carrier family 1 (high affinity |
| 1555963_x_at | 53.89 | 4.57E-14 | 2.447373222 | NM_145236| | B3GNT7,UDP-GlcNAc:betaGal |
| 230788_at | 53.46 | 4.92E-14 | 1.33142793 | NM_001491| | GCNT2,glucosaminyl (N-acetyl) transferase 2 isoform B |
| 203313_s_at | 53.27 | 4.99E-14 | 1.246921025 | NM_003244| | TGIF,TG-interacting factor isoform c |
| 221868_at | 53.19 | 5.05E-14 | 1.8411691 | NM_020459| | NA |
| 203413_at | 52.51 | 5.77E-14 | 1.476966181 | NM_006159| | NELL2,nel-like 2 |
| 223599_at | 52.43 | 5.79E-14 | 2.881178363 | NM_001003818| | TRIM6,tripartite motif-containing 6 isoform 1 |
| 215145_s_at | 52.14 | 6.04E-14 | 2.388688868 | NM_014141| | CNTNAP2,cell recognition molecule Caspr2 precursor |
| 208286_x_at | 52.07 | 6.11E-14 | 2.112308559 | NM_002701| | POU5F1,POU domain, class 5, transcription factor 1 |
| 210074_at | 52.02 | 6.13E-14 | 1.380048943 | NM_001333| | CTSL2,cathepsin L2 preproprotein |
| 230493_at | 51.7 | 6.53E-14 | 3.71061918 | NM_001007538| | LOC387914,WGAR9166 |
| 202446_s_at | 51.6 | 6.66E-14 | 1.207596126 | NM_021105| | PLSCR1,phospholipid scramblase 1 |
| 229872_s_at | 50.89 | 7.72E-14 | 1.514823609 | NA |  |
| 225295_at | 50.68 | 7.95E-14 | 1.140154228 | NM_001127257| | NA |
| 224097_s_at | 50.52 | 8.05E-14 | 1.778008636 | NM_016946| | F11R,F11 receptor isoform a precursor |
| 207705_s_at | 50.46 | 8.09E-14 | 1.238974058 | NM_025176| | KIAA0980,KIAA0980 protein |
| 229288_at | 50.46 | 8.09E-14 | 1.769255314 | NA |  |
| 205930_at | 50.13 | 8.77E-14 | 1.294469393 | NM_005513| | GTF2E1,general transcription factor IIE, polypeptide 1 |
| 205805_s_at | 50 | 8.93E-14 | 1.841347919 | NM_001083592| | NA |
| 205480_s_at | 49.99 | 8.93E-14 | 1.316371907 | NM_001001521| | UGP2,UDP-glucose pyrophosphorylase 2 isoform b |
| 203448_s_at | 49.8 | 9.28E-14 | 1.347636725 | NM_003218| | TERF1,telomeric repeat binding factor 1 isoform 2 |
| 201641_at | 49.41 | 1.02E-13 | 1.939838687 | NM_004335| | BST2,bone marrow stromal cell antigen 2 |
| 203297_s_at | 49.12 | 1.09E-13 | 1.316965333 | NM_004973| | JARID2,jumonji, AT rich interactive domain 2 protein |
| 210517_s_at | 48.97 | 1.12E-13 | 1.223592921 | NM_005100| | AKAP12,A-kinase anchor protein 12 isoform 1 |
| 229256_at | 48.79 | 1.17E-13 | 1.872978026 | NM_173582| | PGM2L1,phosphoglucomutase 2-like 1 |
| 212964_at | 48.62 | 1.21E-13 | 1.41794289 | NM_015094| | HIC2,hypermethylated in cancer 2 |
| 220994_s_at | 48.55 | 1.22E-13 | 2.387022163 | NM_014178| | STXBP6,amisyn |
| 213518_at | 48.43 | 1.25E-13 | 1.127496079 | NM_002740| | PRKCI,protein kinase C, iota |
| 228754_at | 48.39 | 1.26E-13 | 1.126772242 | NM_003043| | SLC6A6,solute carrier family 6 (neurotransmitter |
| 203028_s_at | 48.23 | 1.31E-13 | 1.811378826 | NM_000101| | CYBA,cytochrome b, alpha polypeptide |
| 212966_at | 48.19 | 1.31E-13 | 1.355164087 | NM_015094| | HIC2,hypermethylated in cancer 2 |
| 205110_s_at | 48.12 | 1.31E-13 | 2.694771315 | NM_004114| | FGF13,fibroblast growth factor 13 isoform 1A |
| 202071_at | 48.1 | 1.31E-13 | 1.36954706 | NM_002999| | SDC4,syndecan 4 precursor |
| 224048_at | 48.01 | 1.33E-13 | 3.197898241 | NM_001042403| | NA |
| 228441_s_at | 47.78 | 1.40E-13 | 2.424219104 | NA |  |
| 200618_at | 47.46 | 1.51E-13 | 1.18605088 | NM_006148| | LASP1,LIM and SH3 protein 1 |
| 208025_s_at | 47.43 | 1.52E-13 | 2.352147575 | NM_003483| | HMGA2,high mobility group AT-hook 2 |
| 226846_at | 47.41 | 1.52E-13 | 1.619709735 | NM_001100876| | NA |
| 223178_s_at | 47.29 | 1.56E-13 | 1.510876323 | NM_152729| | NT5C2L1,5'-nucleotidase, cytosolic II-like 1 protein |
| 236519_at | 46.9 | 1.70E-13 | 2.208710845 | NM_001010940| | LOC138255,OTTHUMP00000021439 |
| 202499_s_at | 46.63 | 1.82E-13 | 2.152946621 | NM_006931| | SLC2A3,solute carrier family 2 (facilitated glucose |
| 206023_at | 46.6 | 1.83E-13 | 1.824386792 | NM_006681| | NMU,neuromedin U |
| 226277_at | 46.52 | 1.85E-13 | 1.180807977 | NM_005713| | COL4A3BP,alpha 3 type IV collagen binding protein isoform |
| 229292_at | 46.41 | 1.90E-13 | 2.38928911 | NM_020909| | EPB41L5,erythrocyte membrane protein band 4.1 like 5 |
| 218729_at | 46.28 | 1.95E-13 | 2.81934979 | NM_020169| | LXN,latexin |
| 1552947_x_at | 46.19 | 1.97E-13 | 1.299373712 | NM_153608| | MGC17986,hypothetical protein MGC17986 |
| 202546_at | 46.09 | 2.02E-13 | 2.78095948 | NM_003761| | VAMP8,vesicle-associated membrane protein 8 |
| 209049_s_at | 45.85 | 2.13E-13 | 1.156106527 | NM_012408| | PRKCBP1,protein kinase C binding protein 1 isoform b |
| 219368_at | 45.84 | 2.13E-13 | 2.773757439 | NM_021963| | NAP1L2,nucleosome assembly protein 1-like 2 |
| 214240_at | 45.82 | 2.13E-13 | 1.256165371 | NM_015973| | GAL,galanin preproprotein |
| 223245_at | 45.64 | 2.20E-13 | 1.2150657 | NM_018387| | STRBP,spermatid perinuclear RNA-binding protein |
| 219287_at | 45.63 | 2.20E-13 | 1.912144897 | NM_014505| | KCNMB4,calcium-activated potassium channel beta 4 |
| 222496_s_at | 45.6 | 2.20E-13 | 2.460500814 | NM_001098634| | NA |
| 228992_at | 45.57 | 2.21E-13 | 1.171394055 | NM_025205| | MED28,mediator of RNA polymerase II transcription, |
| 211297_s_at | 45.26 | 2.41E-13 | 1.171923774 | NM_001799| | CDK7,cyclin-dependent kinase 7 |
| 242414_at | 45.16 | 2.46E-13 | 2.041027648 | NM_014298| | QPRT,quinolinate phosphoribosyltransferase |
| 219489_s_at | 45.03 | 2.53E-13 | 1.216374604 | NM_022463| | NXN,nucleoredoxin |
| 219863_at | 45.01 | 2.53E-13 | 2.460326199 | NM_016323| | HERC5,hect domain and RLD 5 |
| 205008_s_at | 44.8 | 2.67E-13 | 1.228948669 | NM_006383| | CIB2,DNA-dependent protein kinase catalytic |
| 204679_at | 44.59 | 2.79E-13 | 1.908449108 | NM_002245| | KCNK1,potassium channel, subfamily K, member 1 |
| 207121_s_at | 44.34 | 2.99E-13 | 1.089717846 | NM_002748| | MAPK6,mitogen-activated protein kinase 6 |
| 227372_s_at | 44.28 | 3.02E-13 | 2.380185268 | NM_018842| | BAIAP2L1,BAI1-associated protein 2-like 1 |
| 229294_at | 44.15 | 3.13E-13 | 2.82544338 | NM_020655| | JPH3,junctophilin 3 |
| 223000_s_at | 44.1 | 3.16E-13 | 2.226867948 | NM_016946| | F11R,F11 receptor isoform a precursor |
| 203404_at | 43.68 | 3.47E-13 | 1.442638007 | NM_014782| | ARMCX2,ALEX2 protein |
| 218384_at | 43.6 | 3.53E-13 | 1.23137282 | NM_001042476| | NA |
| 201413_at | 43.54 | 3.58E-13 | 1.175116972 | NM_000414| | HSD17B4,hydroxysteroid (17-beta) dehydrogenase 4 |
| 205968_at | 43.46 | 3.65E-13 | 2.801321806 | NM_002252| | KCNS3,potassium voltage-gated channel |
| 1553105_s_at | 43.26 | 3.83E-13 | 2.922303782 | NM_001943| | DSG2,desmoglein 2 preproprotein |
| 200640_at | 43.18 | 3.91E-13 | 1.149799747 | NM_003406| | YWHAZ,tyrosine 3/tryptophan 5 -monooxygenase |
| 201839_s_at | 42.95 | 4.17E-13 | 3.025606922 | NM_002354| | TACSTD1,tumor-associated calcium signal transducer 1 |
| 208782_at | 42.91 | 4.20E-13 | 1.264802305 | NM_007085| | FSTL1,follistatin-like 1 precursor |
| 238606_at | 42.78 | 4.32E-13 | 1.919949331 | NM_023931| | MGC2474,hypothetical protein MGC2474 |
| 204790_at | 42.62 | 4.49E-13 | 2.016596334 | NM_005904| | SMAD7,MAD, mothers against decapentaplegic homolog 7 |
| 213237_at | 42.58 | 4.53E-13 | 1.108900388 | NM_001012991| | NA |
| 236126_at | 42.5 | 4.56E-13 | 1.294270698 | NM_001106| | ACVR2B,activin A type IIB receptor precursor |
| 202350_s_at | 42.5 | 4.56E-13 | 1.663864792 | NM_002380| | MATN2,matrilin 2 precursor |
| 1555370_a_at | 42.37 | 4.71E-13 | 1.282865622 | NM_015215| | CAMTA1,calmodulin-binding transcription activator 1 |
| 226007_at | 42.35 | 4.71E-13 | 1.142023333 | NM_194279| | HBLD1,HESB like domain containing 1 |
| 203698_s_at | 42.34 | 4.71E-13 | 2.205018919 | NM_001463| | FRZB,frizzled-related protein |
| 227492_at | 42.11 | 5.00E-13 | 1.45948066 | NA |  |
| 218614_at | 42.04 | 5.07E-13 | 1.285744488 | NM_018169| | FLJ10652,hypothetical protein FLJ10652 |
| 212881_at | 41.9 | 5.27E-13 | 1.143461292 | NM_015897| | PIAS4,protein inhibitor of activated STAT, 4 |
| 202875_s_at | 41.79 | 5.41E-13 | 1.313682541 | NM_002586| | PBX2,pre-B-cell leukemia transcription factor 2 |
| 226069_at | 41.37 | 6.06E-13 | 2.941871154 | NM_153026| | PRICKLE1,prickle-like 1 |
| 201667_at | 41.17 | 6.40E-13 | 1.208367262 | NM_000165| | GJA1,connexin 43 |
| 219976_at | 41.11 | 6.43E-13 | 2.321905953 | NM_015888| | HOOK1,hook homolog 1 |
| 203697_at | 41.11 | 6.43E-13 | 2.158667685 | NM_001463| | FRZB,frizzled-related protein |
| 65438_at | 41.08 | 6.47E-13 | 1.383106705 | NM_020947| | KIAA1609,KIAA1609 protein |
| 209848_s_at | 41.02 | 6.55E-13 | 2.107298769 | NM_006928| | SILV,silver homolog |
| 209822_s_at | 40.92 | 6.75E-13 | 1.853701794 | NM_001018056| | NA |
| 223130_s_at | 40.87 | 6.80E-13 | 1.553497343 | NM_013262| | MYLIP,myosin regulatory light chain interacting |
| 228906_at | 40.73 | 7.07E-13 | 1.316000216 | NM_030625| | CXXC6,CXXC finger 6 |
| 205051_s_at | 40.59 | 7.37E-13 | 2.516213777 | NM_000222| | KIT,v-kit Hardy-Zuckerman 4 feline sarcoma viral |
| 212151_at | 40.51 | 7.51E-13 | 1.258120123 | NM_002585| | PBX1,pre-B-cell leukemia transcription factor 1 |
| 224617_at | 40.19 | 8.22E-13 | 1.154589875 | NM_005156| | ROD1,ROD1 regulator of differentiation 1 |
| 209369_at | 40.12 | 8.36E-13 | 3.07536591 | NM_005139| | ANXA3,annexin A3 |
| 213924_at | 40.12 | 8.36E-13 | 1.642503928 | NM_023075| | MPPE1,metallophosphoesterase 1 isoform a precursor |
| 201650_at | 40.01 | 8.64E-13 | 3.338259702 | NM_002276| | KRT19,keratin 19 |
| 201559_s_at | 39.83 | 9.03E-13 | 1.251849637 | NM_013943| | CLIC4,chloride intracellular channel 4 |
| 214532_x_at | 39.75 | 9.18E-13 | 1.961624556 | NA |  |
| 243610_at | 39.74 | 9.18E-13 | 3.230569045 | NM_001010940| | LOC138255,OTTHUMP00000021439 |
| 224933_s_at | 39.73 | 9.18E-13 | 1.14089854 | NM_004241| | JMJD1C,jumonji domain containing 1C |
| 1553875_s_at | 39.68 | 9.27E-13 | 1.441791515 | NM_032805| | ZNF206,zinc finger protein 206 |
| 213131_at | 39.67 | 9.27E-13 | 2.599344953 | NM_006334| | OLFM1,olfactomedin related ER localized protein |
| 227623_at | 39.51 | 9.67E-13 | 2.391674684 | NA |  |
| 228821_at | 39.48 | 9.74E-13 | 2.695148607 | NM_032528| | ST6GAL2,beta-galactoside alpha-2,6-sialyltransferase II |
| 221249_s_at | 39.47 | 9.76E-13 | 1.350716178 | NM_030802| | LOC81558,C/EBP-induced protein |
| 1554541_a_at | 39.17 | 1.06E-12 | 1.401597323 | NM_014696| | KIAA0514,KIAA0514 |
| 221810_at | 39.05 | 1.10E-12 | 1.273055552 | NM_198686| | RAB15,Ras-related protein Rab-15 |
| 225269_s_at | 39 | 1.11E-12 | 1.269181541 | NA |  |
| 218809_at | 38.92 | 1.13E-12 | 1.088505182 | NM_024960| | PANK2,pantothenate kinase 2 isoform 3 |
| 206857_s_at | 38.82 | 1.17E-12 | 1.324038074 | NM_004116| | FKBP1B,FK506-binding protein 1B isoform a |
| 203408_s_at | 38.75 | 1.18E-12 | 1.27871772 | NM_002971| | SATB1,special AT-rich sequence binding protein 1 |
| 216236_s_at | 38.74 | 1.18E-12 | 1.863794866 | NM_006931| | SLC2A3,solute carrier family 2 (facilitated glucose |
| 200606_at | 38.68 | 1.20E-12 | 2.78278509 | NM_001008844| | DSP,desmoplakin isoform II |
| 208456_s_at | 38.62 | 1.22E-12 | 1.419921417 | NM_001102669| | NA |
| 229553_at | 38.53 | 1.25E-12 | 1.734636156 | NM_173582| | PGM2L1,phosphoglucomutase 2-like 1 |
| 223177_at | 38.48 | 1.26E-12 | 1.403015376 | NM_152729| | NT5C2L1,5'-nucleotidase, cytosolic II-like 1 protein |
| 202498_s_at | 38.43 | 1.28E-12 | 2.095839643 | NM_006931| | SLC2A3,solute carrier family 2 (facilitated glucose |
| 219302_s_at | 38.39 | 1.29E-12 | 2.640136741 | NM_014141| | CNTNAP2,cell recognition molecule Caspr2 precursor |
| 201005_at | 38.34 | 1.30E-12 | 1.105607924 | NM_001769| | CD9,CD9 antigen |
| 201578_at | 38.32 | 1.31E-12 | 1.439674572 | NM_001018111| | NA |
| 204596_s_at | 38.27 | 1.32E-12 | 1.562435153 | NM_003155| | STC1,stanniocalcin 1 |
| 205637_s_at | 38.27 | 1.32E-12 | 1.891259045 | NM_003027| | SH3GL3,SH3-domain GRB2-like 3 |
| 202672_s_at | 38.21 | 1.33E-12 | 1.573124586 | NM_001030287| | NA |
| 223466_x_at | 38.2 | 1.34E-12 | 1.137141283 | NM_005713| | COL4A3BP,alpha 3 type IV collagen binding protein isoform |
| 217901_at | 38.15 | 1.35E-12 | 3.047956835 | NM_001943| | DSG2,desmoglein 2 preproprotein |
| 227349_at | 38.14 | 1.35E-12 | 1.157073685 | NM_018063| | HELLS,helicase, lymphoid-specific |
| 206385_s_at | 38.08 | 1.37E-12 | 1.412379687 | NM_001149| | ANK3,ankyrin 3 isoform 2 |
| 203066_at | 38.06 | 1.38E-12 | 1.282719139 | NM_015892| | GALNAC4S-6ST,B cell RAG associated protein |
| 219454_at | 38.05 | 1.38E-12 | 1.850059026 | NM_015507| | EGFL6,epidermal growth factor-like protein 6 |
| 207069_s_at | 38.02 | 1.39E-12 | 1.501103329 | NM_005585| | SMAD6,MAD, mothers against decapentaplegic homolog 6 |
| 210986_s_at | 37.98 | 1.41E-12 | 1.452500083 | NM_000366| | TPM1,tropomyosin 1 (alpha) |
| 209705_at | 37.91 | 1.43E-12 | 1.292433745 | NM_007358| | M96,putative DNA binding protein |
| 224768_at | 37.82 | 1.47E-12 | 1.134100435 | NM_017969| | FLJ10006,hypothetical protein FLJ10006 |
| 214071_at | 37.78 | 1.48E-12 | 2.077255437 | NM_023075| | MPPE1,metallophosphoesterase 1 isoform a precursor |
| 216295_s_at | 37.71 | 1.52E-12 | 1.044948845 | NM_001076677| | NA |
| 203345_s_at | 37.68 | 1.53E-12 | 1.203020974 | NM_007358| | M96,putative DNA binding protein |
| 223246_s_at | 37.39 | 1.66E-12 | 1.236099394 | NM_018387| | STRBP,spermatid perinuclear RNA-binding protein |
| 232069_at | 37.38 | 1.66E-12 | 1.352459401 | NM_015656| | NA |
| 203020_at | 37.35 | 1.67E-12 | 1.496049425 | NM_001035230| | NA |
| 228802_at | 37.29 | 1.71E-12 | 1.345376899 | NM_194272| | NA |
| 202722_s_at | 37.2 | 1.75E-12 | 1.323068345 | NM_002056| | GFPT1,glucosamine-fructose-6-phosphate |
| 219945_at | 37.19 | 1.75E-12 | 1.434941512 | NM_013264| | DDX25,DEAD (Asp-Glu-Ala-Asp) box polypeptide 25 |
| 203298_s_at | 37.18 | 1.76E-12 | 1.315616931 | NM_004973| | JARID2,jumonji, AT rich interactive domain 2 protein |
| 209687_at | 37.09 | 1.81E-12 | 3.007048334 | NM_000609| | CXCL12,chemokine (C-X-C motif) ligand 12 (stromal |
| 228587_at | 37.07 | 1.82E-12 | 1.323608495 | NM_001039999| | NA |
| 205547_s_at | 36.92 | 1.90E-12 | 2.09029579 | NM_001001522| | TAGLN,transgelin |
| 208650_s_at | 36.81 | 1.97E-12 | 1.404316444 | NM_013230| | CD24,CD24 antigen |
| 204867_at | 36.7 | 2.02E-12 | 1.552556945 | NM_005258| | GCHFR,GTP cyclohydrolase I feedback regulatory |
| 213447_at | 36.62 | 2.07E-12 | 1.347057631 | NA |  |
| 217744_s_at | 36.58 | 2.10E-12 | 2.516362003 | NM_022121| | PERP,PERP, TP53 apoptosis effector |
| 203381_s_at | 36.51 | 2.13E-12 | 1.986626082 | NM_000041| | APOE,apolipoprotein E precursor |
| 204044_at | 36.49 | 2.14E-12 | 2.063617546 | NM_014298| | QPRT,quinolinate phosphoribosyltransferase |
| 227705_at | 36.47 | 2.15E-12 | 3.300014015 | NM_152278| | TCEAL7,transcription elongation factor A (SII)-like 7 |
| 218361_at | 36.39 | 2.20E-12 | 1.198043176 | NM_018178| | GOLPH3L,GPP34-related protein |
| 201430_s_at | 36.27 | 2.29E-12 | 1.579305433 | NM_001387| | DPYSL3,dihydropyrimidinase-like 3 |
| 208770_s_at | 36.24 | 2.31E-12 | 1.145199115 | NM_004096| | EIF4EBP2,eukaryotic translation initiation factor 4E |
| 224650_at | 36.14 | 2.37E-12 | 3.673939699 | NM_052886| | MAL2,mal, T-cell differentiation protein 2 |
| 231195_at | 36.03 | 2.45E-12 | 1.623710676 | NM_198508| | FLJ44186,FLJ44186 protein |
| 230497_at | 36.02 | 2.45E-12 | 1.76156947 | NM_021938| | BRUNOL5,bruno-like 5, RNA binding protein |
| 209772_s_at | 35.99 | 2.48E-12 | 1.474189459 | NM_013230| | CD24,CD24 antigen |
| 218286_s_at | 35.95 | 2.51E-12 | 1.080226637 | NM_014245| | RNF7,ring finger protein 7 isoform 1 |
| 230563_at | 35.84 | 2.60E-12 | 2.943707162 | NM_145313| | RASGEF1A,RasGEF domain family, member 1A |
| 227254_at | 35.73 | 2.67E-12 | 1.231451705 | NM_002697| | POU2F1,POU domain, class 2, transcription factor 1 |
| 218401_s_at | 35.67 | 2.72E-12 | 1.145677552 | NM_012482| | ZNF281,zinc finger protein 281 |
| 225030_at | 35.61 | 2.76E-12 | 1.052749197 | NM_138369| | FAM44B,family with sequence similarity 44, member B |
| 205107_s_at | 35.59 | 2.77E-12 | 1.327891038 | NM_005227| | EFNA4,ephrin A4 isoform a |
| 227533_at | 35.59 | 2.77E-12 | 2.239666401 | NA |  |
| 1553132_a_at | 35.55 | 2.79E-12 | 2.201111368 | NM_152332| | MTAC2D1,membrane targeting (tandem) C2 domain containing |
| 219949_at | 35.52 | 2.82E-12 | 2.036151547 | NM_024512| | LRRC2,leucine rich repeat containing 2 |
| 229796_at | 35.47 | 2.87E-12 | 1.227368862 | NM_017420| | SIX4,sine oculis homeobox homolog 4 |
| 219806_s_at | 35.44 | 2.90E-12 | 1.327829734 | NM_020179| | FN5,FN5 protein |
| 221843_s_at | 35.27 | 3.05E-12 | 1.261349655 | NM_020947| | KIAA1609,KIAA1609 protein |
| 231192_at | 35.25 | 3.05E-12 | 1.631130278 | NA |  |
| 1552712_a_at | 35.18 | 3.12E-12 | 1.464311 | NM_015039| | NMNAT2,nicotinamide mononucleotide adenylyltransferase |
| 222619_at | 35.01 | 3.27E-12 | 1.158719831 | NM_012482| | ZNF281,zinc finger protein 281 |
| 214058_at | 35 | 3.28E-12 | 1.366244009 | NM_001033081| | NA |
| 202347_s_at | 34.97 | 3.32E-12 | 1.08287653 | NM_001111112| | NA |
| 214823_at | 34.91 | 3.37E-12 | 2.11860786 | NA |  |
| 205268_s_at | 34.9 | 3.37E-12 | 1.424921919 | NM_001617| | ADD2,adducin 2 isoform a |
| 202241_at | 34.88 | 3.39E-12 | 1.217588187 | NM_025195| | TRIB1,G-protein-coupled receptor induced protein |
| 224252_s_at | 34.83 | 3.45E-12 | 1.497087579 | NM_014164| | FXYD5,FXYD domain-containing ion transport regulator |
| 219955_at | 34.78 | 3.49E-12 | 3.48475311 | NM_019079| | FLJ10884,hypothetical protein FLJ10884 |
| 203706_s_at | 34.76 | 3.52E-12 | 1.27097156 | NM_003507| | FZD7,frizzled 7 |
| 210665_at | 34.73 | 3.54E-12 | 2.120549871 | NM_001032281| | NA |
| 233064_at | 34.55 | 3.77E-12 | 1.623954208 | NA |  |
| 234994_at | 34.5 | 3.82E-12 | 3.132431359 | NM_052913| | KIAA1913,KIAA1913 |
| 212148_at | 34.49 | 3.82E-12 | 1.390502224 | NM_002585| | PBX1,pre-B-cell leukemia transcription factor 1 |
| 1554256_a_at | 34.43 | 3.88E-12 | 1.490735242 | NM_014801| | PCNXL2,pecanex-like 2 |
| 227677_at | 34.39 | 3.93E-12 | 1.691161976 | NM_000215| | JAK3,Janus kinase 3 |
| 220161_s_at | 34.38 | 3.93E-12 | 1.460558007 | NM_018424| | EPB41L4B,erythrocyte membrane protein band 4.1 like 4B |
| 239975_at | 34.38 | 3.93E-12 | 2.67527214 | NA |  |
| 203438_at | 34.33 | 3.96E-12 | 1.936637999 | NM_003714| | STC2,stanniocalcin 2 |
| 202538_s_at | 34.24 | 4.07E-12 | 1.140984612 | NM_014043| | DKFZP564O123,DKFZP564O123 protein |
| 212686_at | 34.13 | 4.21E-12 | 2.436894056 | NM_020700| | NA |
| 242128_at | 34.12 | 4.22E-12 | 4.310055952 | NM_021728| | OTX2,orthodenticle 2 isoform a |
| 203814_s_at | 33.93 | 4.51E-12 | 1.188588741 | NM_000904| | NQO2,NAD(P)H dehydrogenase, quinone 2 |
| 209442_x_at | 33.87 | 4.59E-12 | 1.411938755 | NM_001149| | ANK3,ankyrin 3 isoform 2 |
| 205742_at | 33.86 | 4.60E-12 | 2.30302647 | NM_000363| | TNNI3,troponin I, cardiac |
| 209536_s_at | 33.78 | 4.70E-12 | 1.234021495 | NM_139265| | EHD4,EH-domain containing 4 |
| 201313_at | 33.74 | 4.75E-12 | 1.240885459 | NM_001975| | ENO2,enolase 2 |
| 210605_s_at | 33.7 | 4.82E-12 | 1.616157882 | NM_001114614| | NA |
| 230669_at | 33.69 | 4.83E-12 | 1.190445476 | NM_006506| | RASA2,RAS p21 protein activator 2 |
| 219740_at | 33.66 | 4.88E-12 | 1.446968599 | NM_024749| | FLJ12505,hypothetical protein FLJ12505 |
| 238956_at | 33.52 | 5.09E-12 | 1.29932889 | NA |  |
| 226456_at | 33.36 | 5.38E-12 | 1.269247105 | NM_152308| | MGC24665,hypothetical protein MGC24665 |
| 220536_at | 33.14 | 5.76E-12 | 1.774061616 | NM_018228| | C14orf115,chromosome 14 open reading frame 115 |
| 232060_at | 33.13 | 5.78E-12 | 1.900672628 | NM_001083592| | NA |
| 223714_at | 33.12 | 5.78E-12 | 1.27579238 | NM_005773| | ZNF256,zinc finger protein 256 |
| 1255_g_at | 33.1 | 5.82E-12 | 2.794410803 | NM_000409| | GUCA1A,guanylate cyclase activator 1A (retina) |
| 220272_at | 33.09 | 5.84E-12 | 1.817092721 | NM_017637| | BNC2,basonuclin 2 |
| 205047_s_at | 33.08 | 5.86E-12 | 1.185755805 | NM_001673| | ASNS,asparagine synthetase |
| 228819_at | 32.92 | 6.17E-12 | 1.38453924 | NM_001031730| | NA |
| 231325_at | 32.85 | 6.34E-12 | 2.513465047 | NM_080872| | UNC5D,netrin receptor Unc5h4 |
| 213590_at | 32.71 | 6.64E-12 | 1.77531197 | NM_004695| | SLC16A5,solute carrier family 16, member 5 |
| 205751_at | 32.59 | 6.86E-12 | 1.210766047 | NM_003026| | SH3GL2,SH3-domain GRB2-like 2 |
| 227365_at | 32.59 | 6.86E-12 | 1.332753621 | NM_033064| | ATCAY,caytaxin |
| 236377_at | 32.59 | 6.86E-12 | 1.5698815 | NM_133448| | KIAA1944,KIAA1944 protein |
| 206343_s_at | 32.58 | 6.86E-12 | 2.024466573 | NM_004495| | NRG1,neuregulin 1 isoform HRG-gamma |
| 219132_at | 32.47 | 7.13E-12 | 1.365101498 | NM_021255| | PELI2,pellino 2 |
| 226215_s_at | 32.45 | 7.18E-12 | 1.099837643 | NM_001005366| | FBXL10,F-box and leucine-rich repeat protein 10 isoform |
| 209296_at | 32.42 | 7.26E-12 | 1.241316693 | NM_001033556| | NA |
| 228933_at | 32.39 | 7.29E-12 | 1.460924964 | NM_198270| | NHS,Nance-Horan syndrome protein |
| 203382_s_at | 32.39 | 7.29E-12 | 1.960662364 | NM_000041| | APOE,apolipoprotein E precursor |
| 209493_at | 32.38 | 7.30E-12 | 1.982857912 | NM_178140| | PDZK3,PDZ domain containing 3 isoform a |
| 230356_at | 32.37 | 7.31E-12 | 3.200711465 | NA |  |
| 200931_s_at | 32.29 | 7.53E-12 | 1.245621419 | NM_003373| | VCL,vinculin isoform VCL |
| 200863_s_at | 32.29 | 7.53E-12 | 1.10785441 | NM_004663| | RAB11A,Ras-related protein Rab-11A |
| 209008_x_at | 32.28 | 7.54E-12 | 1.755396128 | NM_002273| | KRT8,keratin 8 |
| 203453_at | 32.2 | 7.73E-12 | 2.488204107 | NM_001038| | SCNN1A,sodium channel, nonvoltage-gated 1 alpha |
| 219573_at | 32.2 | 7.73E-12 | 1.227228403 | NM_017640| | LRRC16,leucine rich repeat containing 16 |
| 227371_at | 32.1 | 7.98E-12 | 2.119036477 | NM_018842| | BAIAP2L1,BAI1-associated protein 2-like 1 |
| 201403_s_at | 32.09 | 8.00E-12 | 1.093509596 | NM_004528| | MGST3,microsomal glutathione S-transferase 3 |
| 227224_at | 32.08 | 8.00E-12 | 2.313520894 | NM_018037| | RALGPS2,Ral GEF with PH domain and SH3 binding motif 2 |
| 205126_at | 32.08 | 8.00E-12 | 1.227618714 | NM_006296| | VRK2,vaccinia related kinase 2 |
| 207345_at | 32.07 | 8.01E-12 | 2.12075144 | NM_006350| | FST,follistatin isoform FST317 precursor |
| 212314_at | 32.01 | 8.17E-12 | 1.231571073 | NM_015187| | KIAA0746,KIAA0746 protein |
| 233110_s_at | 31.96 | 8.26E-12 | 1.162855901 | NM_001040668| | NA |
| 226817_at | 31.96 | 8.25E-12 | 2.765155077 | NM_004949| | DSC2,desmocollin 2 isoform Dsc2b preproprotein |
| 202346_at | 31.91 | 8.38E-12 | 1.103420804 | NM_001111112| | NA |
| 203705_s_at | 31.87 | 8.49E-12 | 1.273041618 | NM_003507| | FZD7,frizzled 7 |
| 200799_at | 31.85 | 8.53E-12 | 1.235379869 | NM_005345| | HSPA1A,heat shock 70kDa protein 1A |
| 57540_at | 31.83 | 8.57E-12 | 1.34494184 | NM_022128| | RBKS,ribokinase |
| 213135_at | 31.81 | 8.63E-12 | 1.198790365 | NM_003253| | TIAM1,T-cell lymphoma invasion and metastasis 1 |
| 223658_at | 31.77 | 8.73E-12 | 1.782349425 | NM_004823| | KCNK6,potassium channel, subfamily K, member 6 |
| 212024_x_at | 31.69 | 8.97E-12 | 1.126044617 | NM_002018| | FLII,flightless I homolog |
| 207034_s_at | 31.67 | 9.02E-12 | 1.268498143 | NM_005270| | GLI2,GLI-Kruppel family member GLI2 isoform delta |
| 201844_s_at | 31.66 | 9.03E-12 | 1.192801868 | NM_012234| | RYBP,RING1 and YY1 binding protein |
| 225434_at | 31.62 | 9.14E-12 | 1.168761166 | NM_133328| | DEDD2,death effector domain-containing DNA binding |
| 218983_at | 31.61 | 9.18E-12 | 1.92931013 | NM_016546| | C1RL,complement component 1, r subcomponent-like |
| 205440_s_at | 31.6 | 9.18E-12 | 2.595569924 | NM_000909| | NPY1R,neuropeptide Y receptor Y1 |
| 229399_at | 31.58 | 9.23E-12 | 1.533331514 | NM_018017| | C10orf118,CTCL tumor antigen L14-2 |
| 226549_at | 31.58 | 9.23E-12 | 1.304244601 | NM_001024401| | NA |
| 212884_x_at | 31.58 | 9.23E-12 | 1.670789944 | NM_000041| | APOE,apolipoprotein E precursor |
| 202890_at | 31.57 | 9.23E-12 | 2.54466015 | NM_003980| | MAP7,microtubule-associated protein 7 |
| 206481_s_at | 31.54 | 9.32E-12 | 2.506492275 | NM_001290| | LDB2,LIM domain binding 2 |
| 1554485_s_at | 31.54 | 9.32E-12 | 1.849140553 | NM_183240| | TMEM37,transmembrane protein 37 |
| 227846_at | 31.54 | 9.32E-12 | 1.65054398 | NM_007223| | GPR,putative G protein coupled receptor |
| 238547_at | 31.5 | 9.44E-12 | 1.197692182 | NM_144608| | FLJ32384,hypothetical protein MGC39389 |
| 204235_s_at | 31.5 | 9.44E-12 | 1.306097658 | NM_016315| | GULP1,GULP, engulfment adaptor PTB domain containing |
| 226473_at | 31.49 | 9.44E-12 | 1.144589382 | NM_005189| | NA |
| 218667_at | 31.49 | 9.44E-12 | 1.155012332 | NM_001032396| | NA |
| 202911_at | 31.45 | 9.55E-12 | 1.101338331 | NM_000179| | MSH6,mutS homolog 6 |
| 209048_s_at | 31.44 | 9.56E-12 | 1.1643949 | NM_012408| | PRKCBP1,protein kinase C binding protein 1 isoform b |
| 205352_at | 31.4 | 9.72E-12 | 1.848452174 | NM_001122752| | NA |
| 203449_s_at | 31.31 | 1.00E-11 | 1.363865146 | NM_003218| | TERF1,telomeric repeat binding factor 1 isoform 2 |
| 202883_s_at | 31.31 | 1.00E-11 | 1.209412759 | NM_002716| | PPP2R1B,beta isoform of regulatory subunit A, protein |
| 207127_s_at | 31.29 | 1.01E-11 | 1.053251705 | NM_012207| | HNRPH3,heterogeneous nuclear ribonucleoprotein H3 |
| 231407_s_at | 31.29 | 1.01E-11 | 1.828348186 | NM_003923| | FOXH1,forkhead box H1 |
| 203881_s_at | 31.27 | 1.01E-11 | 1.902815086 | NM_000109| | DMD,dystrophin Dp427c isoform |
| 235773_at | 31.21 | 1.03E-11 | 1.243855957 | NM_001010879| | ZIK1,zinc finger protein interacting with K protein |
| 237810_at | 31.21 | 1.04E-11 | 2.775459282 | NM_021195| | CLDN6,claudin 6 |
| 226749_at | 31.18 | 1.05E-11 | 1.069252385 | NM_182640| | MRPS9,mitochondrial ribosomal protein S9 |
| 202746_at | 31.13 | 1.07E-11 | 3.093998811 | NM_004867| | ITM2A,integral membrane protein 2A |
| 1562484_at | 31.04 | 1.10E-11 | 2.529199728 | NM_001033659| | NA |
| 213920_at | 31 | 1.12E-11 | 2.069854887 | NM_015267| | CUTL2,cut-like 2 |
| 221804_s_at | 30.97 | 1.13E-11 | 1.098347151 | NM_018472| | FAM45B,family with sequence similarity 45, member B |
| 210265_x_at | 30.97 | 1.13E-11 | 2.383695715 | NA |  |
| 226185_at | 30.96 | 1.13E-11 | 2.153952762 | NA |  |
| 201158_at | 30.94 | 1.14E-11 | 1.07165453 | NM_021079| | NMT1,N-myristoyltransferase 1 |
| 210715_s_at | 30.91 | 1.15E-11 | 2.744522768 | NM_021102| | SPINT2,serine protease inhibitor, Kunitz type, 2 |
| 205100_at | 30.87 | 1.16E-11 | 1.846992018 | NM_005110| | GFPT2,glutamine-fructose-6-phosphate transaminase 2 |
| 219651_at | 30.85 | 1.17E-11 | 2.737900739 | NM_018189| | DPPA4,developmental pluripotency associated 4 |
| 218399_s_at | 30.85 | 1.17E-11 | 1.095444413 | NM_017955| | CDCA4,cell division cycle associated 4 |
| 1558693_s_at | 30.83 | 1.18E-11 | 1.503072811 | NM_144580| | MGC31963,kidney predominant protein NCU-G1 |
| 220147_s_at | 30.78 | 1.20E-11 | 1.259092068 | NM_021238| | C12orf14,chromosome 12 open reading frame 14 |
| 211276_at | 30.78 | 1.20E-11 | 2.14493427 | NM_080390| | TCEAL2,transcription elongation factor A (SII)-like 2 |
| 219010_at | 30.78 | 1.20E-11 | 1.204331245 | NM_018265| | FLJ10901,hypothetical protein FLJ10901 |
| 211546_x_at | 30.71 | 1.22E-11 | 1.253025152 | NM_000345| | SNCA,alpha-synuclein isoform NACP140 |
| 227690_at | 30.71 | 1.22E-11 | 2.4609769 | NM_000814| | GABRB3,gamma-aminobutyric acid (GABA) A receptor, beta |
| 213992_at | 30.71 | 1.22E-11 | 1.283250593 | NM_001847| | COL4A6,type IV alpha 6 collagen isoform A precursor |
| 226567_at | 30.68 | 1.23E-11 | 1.065558688 | NM_001037334| | NA |
| 218671_s_at | 30.59 | 1.27E-11 | 1.100381595 | NM_016311| | ATPIF1,ATPase inhibitory factor 1 isoform 1 precursor |
| 204686_at | 30.55 | 1.29E-11 | 1.357036839 | NM_005544| | IRS1,insulin receptor substrate 1 |
| 227167_s_at | 30.55 | 1.29E-11 | 1.200622872 | NA |  |
| 209081_s_at | 30.54 | 1.29E-11 | 1.299968339 | NM_030582| | COL18A1,alpha 1 type XVIII collagen isoform 1 precursor |
| 209586_s_at | 30.53 | 1.29E-11 | 1.098651052 | NM_021222| | PRUNE,prune homolog |
| 227955_s_at | 30.51 | 1.30E-11 | 1.461543051 | NA |  |
| 225651_at | 30.47 | 1.32E-11 | 1.346692288 | NM_152653| | UBE2E2,ubiquitin-conjugating enzyme E2E 2 (UBC4/5 |
| 35666_at | 30.46 | 1.32E-11 | 1.160226589 | NM_004186| | SEMA3F,semaphorin 3F |
| 1555630_a_at | 30.43 | 1.33E-11 | 1.174055482 | NM_031934| | RAB34,RAB39 |
| 219370_at | 30.42 | 1.33E-11 | 1.724926462 | NM_019845| | RPRM,reprimo, TP53 dependant G2 arrest mediator |
| 229518_at | 30.4 | 1.33E-11 | 2.24132287 | NM_052943| | FAM46B,family with sequence similarity 46, member B |
| 238417_at | 30.4 | 1.33E-11 | 1.508311564 | NM_173582| | PGM2L1,phosphoglucomutase 2-like 1 |
| 202295_s_at | 30.38 | 1.34E-11 | 1.256311844 | NM_004390| | CTSH,cathepsin H isoform a preproprotein |
| 202465_at | 30.35 | 1.36E-11 | 1.540375073 | NM_002593| | PCOLCE,procollagen C-endopeptidase enhancer |
| 203946_s_at | 30.35 | 1.36E-11 | 1.675428733 | NM_001172| | ARG2,arginase, type II precursor |
| 226374_at | 30.33 | 1.37E-11 | 1.232712467 | NA |  |
| 205807_s_at | 30.3 | 1.38E-11 | 1.241169768 | NM_001126337| | NA |
| 201487_at | 30.28 | 1.39E-11 | 1.163792662 | NM_001114173| | NA |
| 225831_at | 30.27 | 1.39E-11 | 1.151138407 | NM_033631| | LUZP1,leucine zipper protein 1 |
| 222701_s_at | 30.24 | 1.41E-11 | 1.463078489 | NM_001011667| | CHCHD7,coiled-coil-helix-coiled-coil-helix domain |
| 217678_at | 30.23 | 1.41E-11 | 1.467347917 | NM_014331| | SLC7A11,solute carrier family 7, (cationic amino acid |
| 205803_s_at | 30.22 | 1.41E-11 | 1.285071115 | NM_003304| | TRPC1,transient receptor potential cation channel, |
| 232164_s_at | 30.21 | 1.41E-11 | 2.404971758 | NM_031308| | EPPK1,epiplakin 1 |
| 219625_s_at | 30.17 | 1.43E-11 | 1.131207763 | NM_005713| | COL4A3BP,alpha 3 type IV collagen binding protein isoform |
| 202391_at | 30.14 | 1.44E-11 | 2.981091758 | NM_006317| | BASP1,brain abundant, membrane attached signal protein |
| 228785_at | 30.14 | 1.45E-11 | 1.214211765 | NM_012482| | ZNF281,zinc finger protein 281 |
| 203528_at | 30.13 | 1.45E-11 | 1.290514299 | NM_006378| | SEMA4D,semaphorin 4D |
| 1553179_at | 30.06 | 1.49E-11 | 2.013543989 | NM_133638| | ADAMTS19,a disintegrin-like and metalloprotease |
| 214825_at | 30.05 | 1.49E-11 | 1.884610741 | NM_001080396| | NA |
| 222001_x_at | 30 | 1.52E-11 | 1.422326242 | NA |  |
| 220668_s_at | 29.98 | 1.53E-11 | 1.720002918 | NM_006892| | DNMT3B,DNA cytosine-5 methyltransferase 3 beta isoform |
| 201636_at | 29.94 | 1.55E-11 | 1.097140277 | NM_001013438| | NA |
| 226325_at | 29.93 | 1.56E-11 | 1.45473867 | NM_152328| | ADSSL1,adenylosuccinate synthase-like 1 isoform 2 |
| 229484_at | 29.93 | 1.56E-11 | 1.389109034 | NM_005167| | PPP2CZ,protein phosphatase 2a, catalytic subunit, zeta |
| 221803_s_at | 29.91 | 1.56E-11 | 1.130769671 | NM_030759| | NRBF2,nuclear receptor binding factor 2 |
| 233337_s_at | 29.91 | 1.56E-11 | 1.639189892 | NM_001114099| | NA |
| 236456_at | 29.89 | 1.57E-11 | 1.675656591 | NM_001039970| | NA |
| 201134_x_at | 29.78 | 1.65E-11 | 1.049258445 | NM_001867| | COX7C,cytochrome c oxidase subunit VIIc precursor |
| 203961_at | 29.77 | 1.65E-11 | 1.822760211 | NM_006393| | NEBL,nebulette sarcomeric isoform |
| 227348_at | 29.7 | 1.70E-11 | 1.163549236 | NM_152268| | DKFZp727A071,similar to tRNA synthetase class II |
| 227314_at | 29.69 | 1.70E-11 | 1.558932572 | NM_002203| | ITGA2,integrin alpha 2 precursor |
| 213249_at | 29.69 | 1.70E-11 | 1.157863134 | NM_012304| | FBXL7,F-box and leucine-rich repeat protein 7 |
| 208796_s_at | 29.62 | 1.74E-11 | 1.122423686 | NM_004060| | CCNG1,cyclin G1 |
| 231731_at | 29.61 | 1.74E-11 | 2.817376414 | NM_021728| | OTX2,orthodenticle 2 isoform a |
| 227377_at | 29.55 | 1.78E-11 | 1.87657492 | NM_006546| | IMP-1,IGF-II mRNA-binding protein 1 |
| 205977_s_at | 29.54 | 1.78E-11 | 2.230763148 | NM_005232| | EPHA1,ephrin receptor EphA1 |
| 227014_at | 29.53 | 1.78E-11 | 1.333044672 | NM_020437| | LOC57168,similar to aspartate beta hydroxylase (ASPH) |
| 220116_at | 29.5 | 1.81E-11 | 1.949981289 | NM_021614| | KCNN2,small conductance calcium-activated potassium |
| 213308_at | 29.47 | 1.83E-11 | 1.711180714 | NM_012309| | SHANK2,SH3 and multiple ankyrin repeat domains 2 |
| 242979_at | 29.44 | 1.85E-11 | 1.468769637 | NM_005544| | IRS1,insulin receptor substrate 1 |
| 218450_at | 29.43 | 1.86E-11 | 1.13819035 | NM_015987| | HEBP1,heme binding protein 1 |
| 227803_at | 29.38 | 1.89E-11 | 1.722410668 | NM_021572| | ENPP5,ectonucleotide pyrophosphatase/phosphodiesterase |
| 239552_at | 29.38 | 1.89E-11 | 2.038989708 | NA |  |
| 227933_at | 29.37 | 1.90E-11 | 1.220666177 | NM_032808| | LRRN6A,leucine-rich repeat neuronal 6A |
| 227931_at | 29.37 | 1.90E-11 | 1.255143994 | NM_017759| | FLJ20309,hypothetical protein FLJ20309 |
| 206002_at | 29.33 | 1.92E-11 | 2.50962208 | NM_001079858| | NA |
| 218338_at | 29.32 | 1.93E-11 | 1.290656818 | NM_004426| | PHC1,polyhomeotic 1-like |
| 235830_at | 29.3 | 1.95E-11 | 1.534550764 | NA |  |
| 208296_x_at | 29.29 | 1.95E-11 | 1.493686928 | NM_001077654| | NA |
| 225775_at | 29.28 | 1.96E-11 | 1.167377351 | NM_178562| | MGC50844,hypothetical protein MGC50844 |
| 230785_at | 29.15 | 2.05E-11 | 1.297586379 | NA |  |
| 209755_at | 29.13 | 2.07E-11 | 1.765694394 | NM_015039| | NMNAT2,nicotinamide mononucleotide adenylyltransferase |
| 231690_at | 29.12 | 2.07E-11 | 2.477400576 | NA |  |
| 225029_at | 29.11 | 2.08E-11 | 1.147066557 | NA |  |
| 225265_at | 29.11 | 2.08E-11 | 1.366451873 | NM_002897| | RBMS1,RNA binding motif, single stranded interacting |
| 207186_s_at | 29.1 | 2.08E-11 | 1.090330751 | NM_004459| | FALZ,fetal Alzheimer antigen isoform 2 |
| 224465_s_at | 29.06 | 2.12E-11 | 1.150363577 | NM_032345| | PYM,PYM protein |
| 204464_s_at | 29.05 | 2.12E-11 | 1.273052159 | NM_001957| | EDNRA,endothelin receptor type A |
| 203126_at | 29.02 | 2.15E-11 | 1.183620911 | NM_014214| | IMPA2,inositol(myo)-1(or 4)-monophosphatase 2 |
| 223305_at | 28.98 | 2.18E-11 | 1.195216644 | NM_016499| | MGC13379,HSPC244 |
| 215342_s_at | 28.97 | 2.19E-11 | 1.835689632 | NM_001035230| | NA |
| 226548_at | 28.96 | 2.19E-11 | 1.296648393 | NM_001024401| | NA |
| 232202_at | 28.95 | 2.20E-11 | 1.715896826 | NA |  |
| 210905_x_at | 28.94 | 2.21E-11 | 2.15391487 | NA |  |
| 209704_at | 28.85 | 2.28E-11 | 1.305143683 | NM_007358| | M96,putative DNA binding protein |
| 234970_at | 28.8 | 2.33E-11 | 1.813885187 | NM_152332| | MTAC2D1,membrane targeting (tandem) C2 domain containing |
| 1559280_a_at | 28.77 | 2.35E-11 | 3.56095594 | NA |  |
| 206204_at | 28.77 | 2.35E-11 | 1.281910699 | NM_004490| | GRB14,growth factor receptor-bound protein 14 |
| 206382_s_at | 28.75 | 2.37E-11 | 1.956089375 | NM_001709| | BDNF,brain-derived neurotrophic factor isoform a |
| 210987_x_at | 28.74 | 2.37E-11 | 1.485643727 | NM_000366| | TPM1,tropomyosin 1 (alpha) |
| 228329_at | 28.71 | 2.40E-11 | 2.102779789 | NM_021080| | DAB1,disabled homolog 1 |
| 203892_at | 28.68 | 2.42E-11 | 1.512755152 | NM_006103| | WFDC2,WAP four-disulfide core domain 2 isoform 1 |
| 228370_at | 28.67 | 2.43E-11 | 1.352871646 | NM_003097| | SNRPN,small nuclear ribonucleoprotein polypeptide N |
| 232647_at | 28.6 | 2.49E-11 | 1.264835205 | NM_152465| | PROCA1,proline-rich cyclin A1-interacting protein |
| 222088_s_at | 28.54 | 2.54E-11 | 1.988598342 | NM_006931| | SLC2A3,solute carrier family 2 (facilitated glucose |
| 211343_s_at | 28.5 | 2.58E-11 | 2.049358686 | NM_005203| | COL13A1,alpha 1 type XIII collagen isoform 1 |
| 228120_at | 28.48 | 2.60E-11 | 1.121251864 | NA |  |
| 226462_at | 28.47 | 2.60E-11 | 1.862539085 | NM_014178| | STXBP6,amisyn |
| 218162_at | 28.44 | 2.64E-11 | 2.325110002 | NM_020190| | OLFML3,olfactomedin-like 3 |
| 1556499_s_at | 28.44 | 2.63E-11 | 2.720799926 | NM_000088| | COL1A1,alpha 1 type I collagen preproprotein |
| 220184_at | 28.42 | 2.65E-11 | 3.336088611 | NM_024865| | NANOG,Nanog homeobox |
| 214369_s_at | 28.42 | 2.65E-11 | 1.957939159 | NM_001098670| | NA |
| 231953_at | 28.32 | 2.75E-11 | 1.379993256 | NM_004459| | FALZ,fetal Alzheimer antigen isoform 2 |
| 204224_s_at | 28.32 | 2.75E-11 | 1.335372118 | NM_000161| | GCH1,GTP cyclohydrolase 1 (dopa-responsive dystonia) |
| 1570266_x_at | 28.3 | 2.76E-11 | 1.809748045 | NA |  |
| 222640_at | 28.3 | 2.76E-11 | 1.173302895 | NM_022552| | DNMT3A,DNA cytosine methyltransferase 3 alpha isoform |
| 208165_s_at | 28.28 | 2.78E-11 | 2.077044094 | NM_005865| | PRSS16,protease, serine, 16 |
| 214179_s_at | 28.27 | 2.79E-11 | 1.17612709 | NM_003204| | NFE2L1,nuclear factor (erythroid-derived 2)-like 1 |
| 207197_at | 28.25 | 2.82E-11 | 2.545158906 | NM_003413| | ZIC3,zinc finger protein of the cerebellum 3 |
| 1554592_a_at | 28.24 | 2.82E-11 | 2.347294671 | NM_005071| | SLC1A6,solute carrier family 1 (high affinity |
| 216215_s_at | 28.17 | 2.90E-11 | 1.081452255 | NM_001031695| | NA |
| 203498_at | 28.13 | 2.95E-11 | 1.518045143 | NM_005822| | DSCR1L1,Down syndrome critical region gene 1-like 1 |
| 225258_at | 28.12 | 2.96E-11 | 1.296905278 | NM_001024215| | NA |
| 233559_s_at | 28.07 | 3.02E-11 | 1.195401725 | NM_020830| | WDFY1,WD repeat and FYVE domain containing 1 |
| 227236_at | 28.07 | 3.02E-11 | 2.788432542 | NM_005725| | TSPAN2,tetraspan 2 |
| 218848_at | 28.03 | 3.06E-11 | 1.127036466 | NM_024339| | MGC2655,hypothetical protein MGC2655 |
| 223423_at | 28.03 | 3.06E-11 | 2.856526146 | NM_014373| | GPR160,G protein-coupled receptor 160 |
| 205738_s_at | 28.02 | 3.07E-11 | 1.636474489 | NM_004102| | FABP3,fatty acid binding protein 3 |
| 217883_at | 27.99 | 3.11E-11 | 1.045557431 | NM_015702| | C2orf25,chromosome 2 open reading frame 25 |
| 203205_at | 27.99 | 3.11E-11 | 1.148494183 | NM_014663| | JMJD2A,jumonji domain containing 2A |
| 201057_s_at | 27.91 | 3.22E-11 | 1.137347296 | NM_004487| | GOLGB1,golgi autoantigen, golgin subfamily b, |
| 201832_s_at | 27.85 | 3.29E-11 | 1.170129632 | NM_003715| | VDP,vesicle docking protein p115 |
| 205020_s_at | 27.85 | 3.29E-11 | 1.284833799 | NM_001037164| | NA |
| 227980_at | 27.82 | 3.31E-11 | 1.093886147 | NM_024639| | ZNF322A,zinc finger protein 322A |
| 213137_s_at | 27.79 | 3.36E-11 | 1.140627788 | NM_002828| | PTPN2,protein tyrosine phosphatase, non-receptor type |
| 201318_s_at | 27.75 | 3.42E-11 | 1.056739434 | NM_006471| | MRCL3,myosin regulatory light chain MRCL3 |
| 222431_at | 27.72 | 3.46E-11 | 1.112565044 | NM_006717| | SPIN,spindlin |
| 213400_s_at | 27.69 | 3.50E-11 | 1.118445858 | NM_005647| | TBL1X,transducin beta-like 1X |
| 207727_s_at | 27.69 | 3.49E-11 | 1.087622358 | NM_001048171| | NA |
| 229817_at | 27.66 | 3.54E-11 | 1.174060131 | NM_020747| | ZNF608,zinc finger protein 608 |
| 223206_s_at | 27.63 | 3.56E-11 | 1.181842616 | NM_020677| | HSCARG,HSCARG protein |
| 219271_at | 27.61 | 3.58E-11 | 1.404010479 | NM_024572| | GALNT14,UDP-N-acetyl-alpha-D-galactosamine:polypeptide |
| 1552946_at | 27.61 | 3.59E-11 | 1.313952072 | NM_153608| | MGC17986,hypothetical protein MGC17986 |
| 1560587_s_at | 27.57 | 3.64E-11 | 1.060768777 | NM_012094| | PRDX5,peroxiredoxin 5 precursor, isoform a |
| 205802_at | 27.56 | 3.65E-11 | 1.300190985 | NM_003304| | TRPC1,transient receptor potential cation channel, |
| 202747_s_at | 27.54 | 3.67E-11 | 2.070932384 | NM_004867| | ITM2A,integral membrane protein 2A |
| 222713_s_at | 27.52 | 3.70E-11 | 1.17745669 | NM_022725| | FANCF,Fanconi anemia, complementation group F |
| 205123_s_at | 27.49 | 3.75E-11 | 1.354288727 | NM_003692| | TMEFF1,transmembrane protein with EGF-like and two |
| 200758_s_at | 27.48 | 3.76E-11 | 1.162853753 | NM_003204| | NFE2L1,nuclear factor (erythroid-derived 2)-like 1 |
| 223386_at | 27.43 | 3.84E-11 | 1.126302291 | NM_024556| | FLJ21103,hypothetical protein FLJ21103 |
| 205532_s_at | 27.42 | 3.85E-11 | 1.856491333 | NM_004932| | CDH6,cadherin 6, type 2 preproprotein |
| 205309_at | 27.42 | 3.85E-11 | 2.058145975 | NM_001009568| | SMPDL3B,acid sphingomyelinase-like phosphodiesterase 3B |
| 226801_s_at | 27.41 | 3.86E-11 | 1.075718819 | NM_022831| | FLJ12806,hypothetical protein FLJ12806 |
| 218454_at | 27.39 | 3.89E-11 | 1.970341943 | NM_024829| | FLJ22662,hypothetical protein FLJ22662 |
| 226439_s_at | 27.37 | 3.91E-11 | 1.812009271 | NM_015678| | NBEA,neurobeachin |
| 230869_at | 27.34 | 3.96E-11 | 2.842693375 | NM_001080396| | NA |
| 203879_at | 27.31 | 4.00E-11 | 1.593098101 | NM_005026| | PIK3CD,phosphoinositide-3-kinase, catalytic, delta |
| 218559_s_at | 27.24 | 4.11E-11 | 2.0139391 | NM_005461| | MAFB,transcription factor MAFB |
| 206299_at | 27.2 | 4.18E-11 | 1.463210396 | NM_015686| | TMEM28,transmembrane protein 28 |
| 206062_at | 27.12 | 4.31E-11 | 2.090485582 | NM_000409| | GUCA1A,guanylate cyclase activator 1A (retina) |
| 1570515_a_at | 27.12 | 4.31E-11 | 2.713237399 | NM_015687| | FILIP1,filamin A interacting protein 1 |
| 236279_at | 27.1 | 4.34E-11 | 2.004707759 | NA |  |
| 212571_at | 27.09 | 4.34E-11 | 1.084370591 | NM_020920| | CHD8,chromodomain helicase DNA binding protein 8 |
| 222732_at | 27.07 | 4.39E-11 | 1.150473954 | NM_021253| | TRIM39,tripartite motif-containing 39 isoform 1 |
| 242053_at | 27.06 | 4.39E-11 | 1.849765496 | NA |  |
| 202712_s_at | 27.05 | 4.41E-11 | 1.29055678 | NM_001015001| | NA |
| 214396_s_at | 27 | 4.50E-11 | 1.836462423 | NM_003927| | MBD2,methyl-CpG binding domain protein 2 isoform 1 |
| 204217_s_at | 26.99 | 4.51E-11 | 1.257567774 | NM_005619| | RTN2,reticulon 2 isoform A |
| 203517_at | 26.99 | 4.51E-11 | 1.149681578 | NM_001006635| | MTX2,metaxin 2 isoform b |
| 219660_s_at | 26.96 | 4.56E-11 | 1.499024153 | NM_016529| | ATP8A2,ATPase, aminophospholipid transporter-like, |
| 239503_at | 26.93 | 4.62E-11 | 1.476705792 | NA |  |
| 204751_x_at | 26.92 | 4.62E-11 | 2.391244111 | NM_004949| | DSC2,desmocollin 2 isoform Dsc2b preproprotein |
| 229155_at | 26.9 | 4.65E-11 | 1.425816898 | NA |  |
| 221051_s_at | 26.85 | 4.77E-11 | 2.27509842 | NM_170678| | ITGB1BP3,integrin beta 1 binding protein 3 |
| 210479_s_at | 26.82 | 4.83E-11 | 1.542533337 | NM_002943| | RORA,RAR-related orphan receptor A isoform c |
| 226799_at | 26.8 | 4.86E-11 | 1.626306784 | NM_018351| | FGD6,FYVE, RhoGEF and PH domain containing 6 |
| 204269_at | 26.78 | 4.90E-11 | 1.417248825 | NM_006875| | PIM2,pim-2 oncogene |
| 220446_s_at | 26.76 | 4.93E-11 | 1.827576787 | NM_005769| | CHST4,carbohydrate (N-acetylglucosamine 6-O) |
| 206074_s_at | 26.74 | 4.98E-11 | 1.212395167 | NM_002131| | HMGA1,high mobility group AT-hook 1 isoform b |
| 202436_s_at | 26.73 | 4.99E-11 | 1.744265344 | NM_000104| | CYP1B1,cytochrome P450, family 1, subfamily B, |
| 200752_s_at | 26.7 | 5.04E-11 | 1.229923123 | NM_005186| | CAPN1,calpain 1, large subunit |
| 227329_at | 26.67 | 5.10E-11 | 1.802371049 | NM_025224| | BTBD4,BTB (POZ) domain containing 4 |
| 240189_at | 26.67 | 5.09E-11 | 1.703777343 | NA |  |
| 203704_s_at | 26.63 | 5.16E-11 | 1.141350552 | NM_001003698| | RREB1,ras responsive element binding protein 1 isoform |
| 241612_at | 26.61 | 5.21E-11 | 2.376666783 | NM_012183| | FOXD3,forkhead box D3 |
| 213283_s_at | 26.61 | 5.20E-11 | 1.197478304 | NM_005407| | SALL2,sal-like 2 |
| 206109_at | 26.58 | 5.25E-11 | 1.534498936 | NM_000148| | FUT1,fucosyltransferase 1 |
| 209875_s_at | 26.58 | 5.25E-11 | 1.347530702 | NM_000582| | SPP1,secreted phosphoprotein 1 (osteopontin, bone |
| 209570_s_at | 26.58 | 5.25E-11 | 1.705108213 | NM_001040101| | NA |
| 200759_x_at | 26.56 | 5.29E-11 | 1.157386901 | NM_003204| | NFE2L1,nuclear factor (erythroid-derived 2)-like 1 |
| 206683_at | 26.55 | 5.30E-11 | 1.539812297 | NM_003447| | ZNF165,zinc finger protein 165 |
| 230986_at | 26.54 | 5.31E-11 | 1.878230881 | NM_007250| | KLF8,Kruppel-like factor 8 |
| 212590_at | 26.52 | 5.35E-11 | 1.382778116 | NM_001102669| | NA |
| 208190_s_at | 26.5 | 5.40E-11 | 1.950879036 | NM_015925| | LISCH7,LISCH protein isoform 1 |
| 220038_at | 26.45 | 5.51E-11 | 1.616726644 | NM_001033578| | NA |
| 222401_s_at | 26.45 | 5.50E-11 | 1.074503249 | NM_014313| | SMP1,small membrane protein 1 |
| 207080_s_at | 26.44 | 5.53E-11 | 1.599671738 | NM_004160| | PYY,peptide YY |
| 203097_s_at | 26.42 | 5.57E-11 | 1.113942279 | NM_014247| | NA |
| 219125_s_at | 26.39 | 5.65E-11 | 1.21534988 | NM_001122837| | NA |
| 201253_s_at | 26.39 | 5.64E-11 | 1.062191721 | NM_006319| | CDIPT,CDP-diacylglycerol--inositol |
| 229618_at | 26.37 | 5.69E-11 | 1.343268904 | NM_022133| | SNX16,sorting nexin 16 isoform a |
| 221011_s_at | 26.34 | 5.73E-11 | 1.512754935 | NM_030915| | LBH,hypothetical protein DKFZp566J091 |
| 205709_s_at | 26.33 | 5.76E-11 | 2.063802282 | NM_001263| | CDS1,phosphatidate cytidylyltransferase 1 |
| 236030_at | 26.33 | 5.75E-11 | 1.170150675 | NM_173587| | RCOR2,REST corepressor 2 |
| 204425_at | 26.29 | 5.84E-11 | 1.29244537 | NM_001666| | ARHGAP4,Rho GTPase activating protein 4 |
| 227760_at | 26.25 | 5.96E-11 | 1.634205138 | NM_001007563| | IGFBPL1,insulin-like growth factor binding protein-like |
| 218854_at | 26.24 | 5.97E-11 | 1.542235162 | NM_001080976| | NA |
| 213348_at | 26.21 | 6.04E-11 | 1.295881655 | NM_000076| | CDKN1C,cyclin-dependent kinase inhibitor 1C |
| 202847_at | 26.19 | 6.09E-11 | 1.765831058 | NM_001018073| | NA |
| 231991_at | 26.17 | 6.14E-11 | 1.888166001 | NM_080625| | C20orf160,chromosome 20 open reading frame 160 |
| 214440_at | 26.11 | 6.30E-11 | 1.337907641 | NM_000662| | NAT1,N-acetyltransferase 1 |
| 239770_at | 26.1 | 6.32E-11 | 1.479173599 | NM_031913| | CHR3SYT,chr3 synaptotagmin |
| 228051_at | 26.04 | 6.50E-11 | 2.51452306 | NM_020340| | KIAA1244,KIAA1244 |
| 211057_at | 26.04 | 6.50E-11 | 1.526719702 | NM_001083592| | NA |
| 203992_s_at | 26.03 | 6.51E-11 | 1.345248983 | NM_021140| | UTX,ubiquitously transcribed tetratricopeptide |
| 224910_at | 26.01 | 6.58E-11 | 1.271190635 | NM_001042476| | NA |
| 208737_at | 26.01 | 6.58E-11 | 1.074259013 | NM_004888| | ATP6V1G1,ATPase, H+ transporting, lysosomal, V1 subunit G |
| 226313_at | 25.97 | 6.69E-11 | 1.386324015 | NM_145306| | C10orf35,chromosome 10 open reading frame 35 |
| 222065_s_at | 25.97 | 6.68E-11 | 1.126103008 | NM_002018| | FLII,flightless I homolog |
| 1555724_s_at | 25.96 | 6.70E-11 | 1.708850191 | NM_001001522| | TAGLN,transgelin |
| 231257_at | 25.94 | 6.76E-11 | 1.685178508 | NM_174937| | TCERG1L,transcription elongation regulator 1-like |
| 205479_s_at | 25.93 | 6.80E-11 | 1.583721933 | NM_002658| | PLAU,urokinase plasminogen activator preproprotein |
| 208022_s_at | 25.92 | 6.81E-11 | 1.199283865 | NM_001077181| | NA |
| 231188_at | 25.92 | 6.82E-11 | 1.193043011 | NM_001007072| | ZSCAN2,zinc finger protein 29 isoform 3 |
| 225647_s_at | 25.86 | 6.96E-11 | 1.215614534 | NM_001114173| | NA |
| 230903_s_at | 25.81 | 7.11E-11 | 1.264925672 | NM_175075| | INM01,hypothetical protein INM01 |
| 207559_s_at | 25.78 | 7.21E-11 | 1.120659505 | NM_005096| | ZNF261,zinc finger protein 261 |
| 212859_x_at | 25.77 | 7.25E-11 | 1.429127513 | NM_175617| | MT1E,metallothionein 1E |
| 242919_at | 25.77 | 7.25E-11 | 1.385962076 | NM_021047| | ZNF253,DNA-binding protein |
| 222976_s_at | 25.76 | 7.27E-11 | 1.080956118 | NM_001043351| | NA |
| 222675_s_at | 25.74 | 7.33E-11 | 1.351035829 | NM_018842| | BAIAP2L1,BAI1-associated protein 2-like 1 |
| 210136_at | 25.74 | 7.32E-11 | 2.00662495 | NM_001025081| | NA |
| 219466_s_at | 25.73 | 7.36E-11 | 1.834422924 | NM_001643| | APOA2,apolipoprotein A-II precursor |
| 227526_at | 25.73 | 7.36E-11 | 1.470267045 | NM_016952| | CDON,surface glycoprotein, Ig superfamily member |
| 204447_at | 25.7 | 7.44E-11 | 1.326965514 | NM_014731| | ProSAPiP1,ProSAPiP1 protein |
| 203256_at | 25.66 | 7.57E-11 | 1.862658158 | NM_001793| | CDH3,cadherin 3, type 1 preproprotein |
| 204983_s_at | 25.64 | 7.60E-11 | 1.49772493 | NM_001448| | GPC4,glypican 4 |
| 224967_at | 25.64 | 7.61E-11 | 1.10003733 | NM_003358| | UGCG,ceramide glucosyltransferase |
| 59697_at | 25.63 | 7.63E-11 | 1.26238565 | NM_198686| | RAB15,Ras-related protein Rab-15 |
| 1560562_a_at | 25.62 | 7.66E-11 | 1.371555586 | NM_182609| | MGC48625,hypothetical protein MGC48625 |
| 218035_s_at | 25.61 | 7.70E-11 | 2.461518688 | NM_001098634| | NA |
| 228894_at | 25.59 | 7.77E-11 | 2.014915148 | NM_001489| | NR6A1,nuclear receptor subfamily 6, group A, member 1 |
| 229313_at | 25.58 | 7.78E-11 | 1.807882207 | NM_213599| | TMEM16E,transmembrane protein 16E |
| 209424_s_at | 25.57 | 7.83E-11 | 1.243359335 | NM_014324| | AMACR,alpha-methylacyl-CoA racemase isoform 1 |
| 227080_at | 25.55 | 7.88E-11 | 1.348821138 | NM_001080470| | NA |
| 37117_at | 25.52 | 8.00E-11 | 1.86203327 | NM_001017526| | NA |
| 203934_at | 25.49 | 8.11E-11 | 1.934933281 | NM_002253| | KDR,kinase insert domain receptor (a type III |
| 226270_at | 25.48 | 8.14E-11 | 1.161347736 | NM_018303| | SEC5L1,Sec5 protein |
| 228098_s_at | 25.47 | 8.18E-11 | 1.448133682 | NM_013262| | MYLIP,myosin regulatory light chain interacting |
| 225354_s_at | 25.46 | 8.20E-11 | 1.175715549 | NM_031469| | SH3BGRL2,SH3 domain binding glutamic acid-rich protein |
| 233142_at | 25.44 | 8.27E-11 | 1.241623104 | NA |  |
| 239697_x_at | 25.43 | 8.32E-11 | 1.712722062 | NM_198463| | FLJ42117,FLJ42117 protein |
| 205206_at | 25.43 | 8.32E-11 | 1.763538243 | NM_000216| | KAL1,Kallmann syndrome 1 protein |
| 203430_at | 25.39 | 8.45E-11 | 1.103107563 | NM_014320| | HEBP2,heme binding protein 2 |
| 235044_at | 25.37 | 8.52E-11 | 2.772961698 | NM_052954| | CYYR1,cysteine and tyrosine-rich 1 protein precursor |
| 204079_at | 25.36 | 8.52E-11 | 1.369513913 | NM_001008566| | TPST2,tyrosylprotein sulfotransferase 2 |
| 222392_x_at | 25.35 | 8.58E-11 | 2.675722409 | NM_022121| | PERP,PERP, TP53 apoptosis effector |
| 203632_s_at | 25.35 | 8.58E-11 | 1.090567003 | NM_016235| | GPRC5B,G protein-coupled receptor, family C, group 5, |
| 219045_at | 25.27 | 8.88E-11 | 1.466737579 | NM_019034| | RHOF,ras homolog gene family, member F |
| 219526_at | 25.27 | 8.88E-11 | 1.152763423 | NM_024644| | C14orf169,chromosome 14 open reading frame 169 |
| 223582_at | 25.27 | 8.88E-11 | 1.103972301 | NM_032119| | MASS1,very large G-protein coupled receptor 1 |
| 206286_s_at | 25.26 | 8.89E-11 | 3.041980828 | NM_003212| | TDGF1,teratocarcinoma-derived growth factor 1 |
| 228547_at | 25.26 | 8.90E-11 | 2.303614427 | NM_004801| | NRXN1,neurexin 1 isoform alpha precursor |
| 240301_at | 25.25 | 8.91E-11 | 2.095862366 | NM_138815| | DPPA2,developmental pluripotency associated 2 |
| 223121_s_at | 25.24 | 8.97E-11 | 2.509920212 | NM_003013| | SFRP2,secreted frizzled-related protein 2 precursor |
| 205538_at | 25.24 | 8.97E-11 | 2.089098099 | NM_003389| | CORO2A,coronin, actin binding protein, 2A |
| 239148_at | 25.23 | 8.97E-11 | 2.353198762 | NM_001017967| | NA |
| 1553955_at | 25.23 | 8.97E-11 | 1.121468832 | NM_152994| | LOC129285,smooth muscle myosin heavy chain 11 isoform |
| 211458_s_at | 25.22 | 9.01E-11 | 1.517956968 | NM_031412| | GABARAPL1,GABA(A) receptor-associated protein like 1 |
| 213506_at | 25.21 | 9.04E-11 | 2.163548937 | NM_005242| | F2RL1,coagulation factor II (thrombin) receptor-like 1 |
| 226443_at | 25.18 | 9.15E-11 | 1.134651862 | NM_138333| | C9orf42,chromosome 9 open reading frame 42 |
| 235085_at | 25.18 | 9.15E-11 | 1.224072949 | NM_001080826| | NA |
| 218091_at | 25.17 | 9.18E-11 | 1.147493043 | NM_004504| | HRB,HIV-1 Rev binding protein |
| 242346_x_at | 25.14 | 9.30E-11 | 2.421710538 | NA |  |
| 207957_s_at | 25.11 | 9.44E-11 | 2.922254538 | NM_002738| | PRKCB1,protein kinase C, beta isoform 2 |
| 203895_at | 25.09 | 9.53E-11 | 1.311331469 | NM_000933| | PLCB4,phospholipase C beta 4 isoform a |
| 209631_s_at | 25.08 | 9.56E-11 | 2.36810924 | NM_005302| | GPR37,G protein-coupled receptor 37 |
| 226778_at | 25.07 | 9.59E-11 | 1.295773585 | NM_175075| | INM01,hypothetical protein INM01 |
| 1555793_a_at | 25.04 | 9.71E-11 | 1.584381061 | NM_133466| | ZNF545,zinc finger protein 545 |
| 227180_at | 25.04 | 9.73E-11 | 2.501997361 | NM_001104558| | NA |
| 225731_at | 25.01 | 9.83E-11 | 1.139347349 | NM_020337| | NA |
| 228503_at | 25 | 9.88E-11 | 1.577245406 | NA |  |
| 218546_at | 24.96 | 1.01E-10 | 1.831479455 | NM_024709| | FLJ14146,hypothetical protein FLJ14146 |
| 226498_at | 24.95 | 1.01E-10 | 3.284186547 | NA |  |
| 212812_at | 24.94 | 1.01E-10 | 1.71338171 | NA |  |
| 203286_at | 24.92 | 1.02E-10 | 1.129229977 | NM_014901| | RNF44,ring finger protein 44 |
| 218910_at | 24.91 | 1.03E-10 | 1.245819489 | NM_018075| | FLJ10375,hypothetical protein FLJ10375 |
| 209220_at | 24.89 | 1.03E-10 | 1.947954856 | NM_004484| | GPC3,glypican 3 |
| 230698_at | 24.83 | 1.06E-10 | 2.040990266 | NM_001017440| | NA |
| 227749_at | 24.83 | 1.06E-10 | 1.315333186 | NA |  |
| 228415_at | 24.82 | 1.07E-10 | 1.356515013 | NM_003916| | AP1S2,adaptor-related protein complex 1 sigma 2 |
| 202536_at | 24.82 | 1.07E-10 | 1.126586645 | NM_014043| | DKFZP564O123,DKFZP564O123 protein |
| 206654_s_at | 24.8 | 1.07E-10 | 1.384170147 | NM_006467| | POLR3G,polymerase (RNA) III (DNA directed) polypeptide |
| 222847_s_at | 24.79 | 1.08E-10 | 1.382518712 | NM_022073| | EGLN3,egl nine homolog 3 |
| 226145_s_at | 24.77 | 1.09E-10 | 3.214040746 | NM_025074| | FRAS1,Fraser syndrome 1 isoform 1 |
| 204400_at | 24.74 | 1.10E-10 | 1.591111322 | NM_005864| | EFS,embryonal Fyn-associated substrate isoform 1 |
| 230205_at | 24.73 | 1.11E-10 | 1.161013493 | NM_152289| | ZNF561,zinc finger protein 561 |
| 225380_at | 24.73 | 1.10E-10 | 1.56849734 | NM_138370| | NA |
| 217991_x_at | 24.72 | 1.11E-10 | 1.239105283 | NM_001009955| | SSBP3,single stranded DNA binding protein 3 isoform c |
| 203945_at | 24.72 | 1.11E-10 | 1.268611598 | NM_001172| | ARG2,arginase, type II precursor |
| 213664_at | 24.72 | 1.11E-10 | 2.419240201 | NM_004170| | SLC1A1,solute carrier family 1, member 1 |
| 218205_s_at | 24.7 | 1.12E-10 | 1.086185956 | NM_017572| | MKNK2,MAP kinase-interacting serine/threonine kinase |
| 210001_s_at | 24.69 | 1.12E-10 | 2.05051009 | NM_003745| | SOCS1,suppressor of cytokine signaling 1 |
| 228665_at | 24.65 | 1.14E-10 | 2.415470446 | NM_052954| | CYYR1,cysteine and tyrosine-rich 1 protein precursor |
| 235301_at | 24.64 | 1.14E-10 | 1.961505635 | NM_152748| | FLJ31340,hypothetical protein FLJ31340 |
| 203351_s_at | 24.62 | 1.15E-10 | 1.090520223 | NM_002552| | ORC4L,origin recognition complex subunit 4 |
| 209921_at | 24.61 | 1.16E-10 | 1.487001418 | NM_014331| | SLC7A11,solute carrier family 7, (cationic amino acid |
| 229829_at | 24.6 | 1.17E-10 | 1.11538114 | NA |  |
| 203127_s_at | 24.6 | 1.17E-10 | 1.214115914 | NM_004863| | SPTLC2,serine palmitoyltransferase, long chain base |
| 236756_at | 24.58 | 1.17E-10 | 1.898567613 | NA |  |
| 236448_at | 24.57 | 1.18E-10 | 1.768855927 | NM_133369| | UNC5A,netrin receptor Unc5h1 |
| 201215_at | 24.57 | 1.18E-10 | 1.279630096 | NM_005032| | PLS3,plastin 3 |
| 200701_at | 24.57 | 1.18E-10 | 1.386524693 | NM_006432| | NPC2,Niemann-Pick disease, type C2 precursor |
| 1560652_at | 24.56 | 1.18E-10 | 2.425867558 | NA |  |
| 1555495_a_at | 24.53 | 1.20E-10 | 1.083550827 | NM_005869| | SDCCAG10,serologically defined colon cancer antigen 10 |
| 219545_at | 24.52 | 1.20E-10 | 1.274971554 | NM_023930| | KCTD14,potassium channel tetramerisation domain |
| 224596_at | 24.5 | 1.21E-10 | 1.244800794 | NM_080546| | CDW92,CDW92 antigen |
| 205857_at | 24.5 | 1.21E-10 | 1.721143792 | NM_003054| | SLC18A2,solute carrier family 18 (vesicular monoamine), |
| 235645_at | 24.48 | 1.22E-10 | 1.202105102 | NM_052911| | ESCO1,establishment of cohesion 1 homolog 1 |
| 205570_at | 24.46 | 1.23E-10 | 1.182404532 | NM_005028| | PIP5K2A,phosphatidylinositol-4-phosphate 5-kinase type |
| 212788_x_at | 24.46 | 1.23E-10 | 1.043941144 | NM_000146| | FTL,ferritin, light polypeptide |
| 215127_s_at | 24.42 | 1.25E-10 | 1.265656306 | NM_002897| | RBMS1,RNA binding motif, single stranded interacting |
| 203346_s_at | 24.42 | 1.25E-10 | 1.207179809 | NM_007358| | M96,putative DNA binding protein |
| 218717_s_at | 24.4 | 1.26E-10 | 2.01775442 | NM_018192| | LEPREL1,leprecan-like 1 |
| 203041_s_at | 24.38 | 1.27E-10 | 1.107147014 | NM_001122606| | NA |
| 1569023_a_at | 24.35 | 1.28E-10 | 2.503602836 | NA |  |
| 34206_at | 24.33 | 1.30E-10 | 1.083287623 | NM_001040118| | NA |
| 241535_at | 24.33 | 1.30E-10 | 3.18814598 | NA |  |
| 202272_s_at | 24.32 | 1.30E-10 | 1.158040165 | NM_015176| | FBXO28,F-box protein 28 |
| 242477_at | 24.32 | 1.30E-10 | 1.941491218 | NM_152574| | C9orf52,hypothetical protein FLJ33868 |
| 228707_at | 24.31 | 1.31E-10 | 1.89010943 | NM_194284| | CLDN23,claudin 23 |
| 228260_at | 24.27 | 1.33E-10 | 2.422281152 | NM_004432| | ELAVL2,ELAV (embryonic lethal, abnormal vision, |
| 214022_s_at | 24.24 | 1.35E-10 | 2.345623424 | NM_003641| | IFITM1,interferon induced transmembrane protein 1 |
| 238066_at | 24.22 | 1.36E-10 | 1.570450053 | NM_052960| | RBP7,retinol binding protein 7, cellular |
| 227276_at | 24.21 | 1.37E-10 | 1.599674535 | NM_032812| | PLXDC2,plexin domain containing 2 precursor |
| 235696_at | 24.18 | 1.39E-10 | 1.445095969 | NA |  |
| 227006_at | 24.15 | 1.41E-10 | 1.527800601 | NM_033256| | PPP1R14A,protein phosphatase 1, regulatory (inhibitor) |
| 224800_at | 24.14 | 1.41E-10 | 1.151812088 | NM_020830| | WDFY1,WD repeat and FYVE domain containing 1 |
| 223522_at | 24.12 | 1.43E-10 | 1.175863644 | NA |  |
| 228320_x_at | 24.11 | 1.43E-10 | 1.390912401 | NM_207311| | LOC92558,hypothetical protein LOC92558 |
| 221898_at | 24.11 | 1.43E-10 | 1.477153498 | NM_001006624| | T1A-2,lung type-I cell membrane-associated |
| 217864_s_at | 24.08 | 1.45E-10 | 1.094362009 | NM_016166| | PIAS1,protein inhibitor of activated STAT, 1 |
| 225792_at | 24.08 | 1.45E-10 | 2.68566312 | NM_015888| | HOOK1,hook homolog 1 |
| 227284_at | 24.06 | 1.47E-10 | 1.12198788 | NM_001010851| | LOC90321,hypothetical protein LOC90321 |
| 201601_x_at | 24.03 | 1.48E-10 | 2.180481412 | NM_003641| | IFITM1,interferon induced transmembrane protein 1 |
| 228275_at | 23.99 | 1.51E-10 | 1.235656755 | NA |  |
| 203778_at | 23.98 | 1.51E-10 | 1.171940239 | NM_005908| | MANBA,mannosidase, beta A, lysosomal |
| 215543_s_at | 23.98 | 1.51E-10 | 1.257494773 | NM_004737| | LARGE,like-glycosyltransferase |
| 201131_s_at | 23.97 | 1.51E-10 | 3.78968072 | NM_004360| | CDH1,cadherin 1, type 1 preproprotein |
| 219932_at | 23.97 | 1.51E-10 | 2.28025172 | NM_001017372| | NA |
| 201032_at | 23.97 | 1.51E-10 | 1.103672434 | NM_006698| | BLCAP,bladder cancer associated protein |
| 226530_at | 23.97 | 1.51E-10 | 1.437967596 | NM_001003940| | BMF,Bcl2 modifying factor isoform bmf-1 |
| 219222_at | 23.97 | 1.51E-10 | 1.232320945 | NM_022128| | RBKS,ribokinase |
| 205066_s_at | 23.97 | 1.51E-10 | 1.563771151 | NM_006208| | ENPP1,ectonucleotide pyrophosphatase/phosphodiesterase |
| 205625_s_at | 23.97 | 1.51E-10 | 2.446280319 | NM_004929| | CALB1,calbindin 1 |
| 231856_at | 23.97 | 1.51E-10 | 2.076659113 | NM_020340| | KIAA1244,KIAA1244 |
| 206653_at | 23.96 | 1.51E-10 | 1.324591534 | NM_006467| | POLR3G,polymerase (RNA) III (DNA directed) polypeptide |
| 226866_at | 23.96 | 1.51E-10 | 1.153785491 | NM_052911| | ESCO1,establishment of cohesion 1 homolog 1 |
| 229205_at | 23.96 | 1.51E-10 | 1.649780982 | NA |  |
| 201137_s_at | 23.96 | 1.51E-10 | 1.41616939 | NM_002121| | HLA-DPB1,major histocompatibility complex, class II, DP |
| 223232_s_at | 23.93 | 1.53E-10 | 1.431499237 | NM_020770| | CGN,cingulin |
| 224871_at | 23.93 | 1.53E-10 | 1.227833815 | NM_182752| | LOC127262,hypothetical protein LOC127262 |
| 223686_at | 23.91 | 1.54E-10 | 1.341034545 | NM_001042482| | NA |
| 219143_s_at | 23.91 | 1.54E-10 | 1.245434638 | NM_017793| | RPP25,ribonuclease P 25kDa subunit |
| 205350_at | 23.9 | 1.55E-10 | 2.452097176 | NM_004378| | CRABP1,cellular retinoic acid binding protein 1 |
| 204184_s_at | 23.89 | 1.55E-10 | 1.432658513 | NM_005160| | ADRBK2,beta adrenergic receptor kinase 2 |
| 222698_s_at | 23.88 | 1.55E-10 | 1.144547867 | NM_018439| | IMPACT,hypothetical protein IMPACT |
| 1553186_x_at | 23.86 | 1.57E-10 | 1.325275597 | NM_152573| | RASEF,RAS and EF hand domain containing |
| 204730_at | 23.85 | 1.57E-10 | 1.273013515 | NM_014747| | RIMS3,regulating synaptic membrane exocytosis 3 |
| 240479_at | 23.84 | 1.58E-10 | 1.593198308 | NM_153612| | HS3ST5,heparan sulfate (glucosamine) |
| 210029_at | 23.81 | 1.61E-10 | 2.108716806 | NM_002164| | INDO,indoleamine-pyrrole 2,3 dioxygenase |
| 208474_at | 23.78 | 1.63E-10 | 1.422773306 | NM_021195| | CLDN6,claudin 6 |
| 206675_s_at | 23.77 | 1.64E-10 | 1.959562601 | NM_005414| | SKIL,SKI-like |
| 204237_at | 23.74 | 1.66E-10 | 1.340607396 | NM_016315| | GULP1,GULP, engulfment adaptor PTB domain containing |
| 225051_at | 23.73 | 1.66E-10 | 1.147824965 | NM_004437| | EPB41,erythrocyte membrane protein band 4.1 |
| 244552_at | 23.71 | 1.68E-10 | 1.343499545 | NA |  |
| 223681_s_at | 23.64 | 1.74E-10 | 1.874563985 | NM_176877| | INADL,InaD-like protein isoform 2 |
| 1558173_a_at | 23.63 | 1.74E-10 | 1.174809353 | NM_033631| | LUZP1,leucine zipper protein 1 |
| 201560_at | 23.62 | 1.75E-10 | 1.152990692 | NM_013943| | CLIC4,chloride intracellular channel 4 |
| 225227_at | 23.58 | 1.78E-10 | 1.405489135 | NA |  |
| 209152_s_at | 23.57 | 1.78E-10 | 1.117255704 | NM_003200| | TCF3,transcription factor 3 |
| 201365_at | 23.57 | 1.78E-10 | 1.188600414 | NM_002537| | OAZ2,ornithine decarboxylase antizyme 2 |
| 226051_at | 23.56 | 1.79E-10 | 1.288809893 | NM_080430| | SELM,selenoprotein M precursor |
| 238035_at | 23.53 | 1.82E-10 | 1.129116676 | NM_001017371| | NA |
| 219397_at | 23.53 | 1.82E-10 | 1.201642321 | NM_025147| | FLJ13448,hypothetical protein FLJ13448 |
| 201431_s_at | 23.52 | 1.83E-10 | 1.354562031 | NM_001387| | DPYSL3,dihydropyrimidinase-like 3 |
| 223233_s_at | 23.52 | 1.83E-10 | 1.344129682 | NM_020770| | CGN,cingulin |
| 201898_s_at | 23.5 | 1.84E-10 | 1.081606188 | NM_003336| | UBE2A,ubiquitin-conjugating enzyme E2A isoform 1 |
| 220952_s_at | 23.5 | 1.84E-10 | 1.3847094 | NM_019012| | PLEKHA5,pleckstrin homology domain containing, family A |
| 214036_at | 23.45 | 1.89E-10 | 1.354800214 | NA |  |
| 226455_at | 23.44 | 1.89E-10 | 1.236064715 | NM_130898| | CREB3L4,cAMP responsive element binding protein 3-like |
| 229377_at | 23.42 | 1.91E-10 | 1.866421807 | NM_024719| | GRTP1,growth hormone regulated TBC protein 1 |
| 212692_s_at | 23.42 | 1.91E-10 | 1.14036182 | NM_006726| | LRBA,LPS-responsive vesicle trafficking, beach and |
| 210088_x_at | 23.37 | 1.95E-10 | 1.190557018 | NM_001002841| | MYL4,atrial/embryonic alkali myosin light chain |
| 45714_at | 23.36 | 1.96E-10 | 1.250098759 | NM_001002017| | HCFC1R1,host cell factor C1 regulator 1 (XPO1 dependant) |
| 227013_at | 23.36 | 1.96E-10 | 1.276179863 | NM_014572| | LATS2,LATS, large tumor suppressor, homolog 2 |
| 205698_s_at | 23.34 | 1.98E-10 | 1.326019133 | NM_002758| | MAP2K6,mitogen-activated protein kinase kinase 6 |
| 215913_s_at | 23.33 | 1.99E-10 | 1.452854381 | NM_016315| | GULP1,GULP, engulfment adaptor PTB domain containing |
| 205153_s_at | 23.3 | 2.02E-10 | 1.308989363 | NM_001250| | CD40,CD40 antigen isoform 1 precursor |
| 205559_s_at | 23.3 | 2.02E-10 | 2.171071575 | NM_006200| | PCSK5,proprotein convertase subtilisin/kexin type 5 |
| 226000_at | 23.29 | 2.02E-10 | 1.14165413 | NM_018704| | DKFZp547A023,hypothetical protein DKFZp547A023 |
| 226435_at | 23.29 | 2.02E-10 | 2.164404197 | NM_173462| | PAPLN,papilin |
| 209726_at | 23.25 | 2.05E-10 | 1.52014624 | NM_001217| | CA11,carbonic anhydrase XI precursor |
| 208862_s_at | 23.24 | 2.07E-10 | 1.130592389 | NM_001085458| | NA |
| 209658_at | 23.23 | 2.08E-10 | 1.067515735 | NM_001078645| | NA |
| 203013_at | 23.22 | 2.09E-10 | 1.097314894 | NM_007265| | HSGT1,suppressor of S. cerevisiae gcr2 |
| 223361_at | 23.21 | 2.09E-10 | 1.248754618 | NM_021243| | NA |
| 200778_s_at | 23.2 | 2.10E-10 | 1.109308027 | NM_001008491| | SEPT2,septin 2 |
| 207836_s_at | 23.19 | 2.11E-10 | 1.716406649 | NM_001008710| | RBPMS,RNA-binding protein with multiple splicing |
| 235334_at | 23.19 | 2.11E-10 | 1.733142407 | NM_152996| | ST6GALNAC3,ST6 |
| 203379_at | 23.19 | 2.11E-10 | 1.222190727 | NM_001006665| | RPS6KA1,ribosomal protein S6 kinase, 90kDa, polypeptide |
| 224503_s_at | 23.18 | 2.12E-10 | 1.181815876 | NM_017742| | ZCCHC2,zinc finger, CCHC domain containing 2 |
| 235148_at | 23.17 | 2.12E-10 | 1.797318217 | NM_173853| | KRTCAP3,keratinocyte associated protein 3 |
| 1553180_at | 23.17 | 2.12E-10 | 2.519797724 | NM_133638| | ADAMTS19,a disintegrin-like and metalloprotease |
| 222479_s_at | 23.17 | 2.12E-10 | 1.081533992 | NM_016141| | DNCLI1,dynein light chain-A |
| 228291_s_at | 23.15 | 2.14E-10 | 1.291160403 | NM_018474| | C20orf19,uncharacterized hypothalamus protein HT013 |
| 209015_s_at | 23.14 | 2.15E-10 | 1.30864849 | NM_005494| | DNAJB6,DnaJ (Hsp40) homolog, subfamily B, member 6 |
| 218276_s_at | 23.13 | 2.16E-10 | 1.217060418 | NM_021818| | SAV1,WW45 protein |
| 225646_at | 23.12 | 2.17E-10 | 1.300022747 | NM_001114173| | NA |
| 209282_at | 23.11 | 2.17E-10 | 1.151658457 | NM_001079880| | NA |
| 208761_s_at | 23.1 | 2.18E-10 | 1.060903049 | NM_001005781| | SUMO1,small ubiquitin-like modifier 1 isoform a |
| 1554887_at | 23.09 | 2.20E-10 | 2.00312831 | NA |  |
| 1554628_at | 23.07 | 2.21E-10 | 1.172358203 | NM_173480| | LOC126295,hypothetical protein LOC126295 |
| 229630_s_at | 23.04 | 2.25E-10 | 1.069413883 | NM_004906| | WTAP,Wilms' tumour 1-associating protein isoform 1 |
| 58916_at | 23.03 | 2.26E-10 | 1.342920306 | NM_023930| | KCTD14,potassium channel tetramerisation domain |
| 203414_at | 23.03 | 2.26E-10 | 1.134002399 | NM_012329| | MMD,monocyte to macrophage |
| 202442_at | 23.02 | 2.27E-10 | 1.042955052 | NM_001284| | AP3S1,adaptor-related protein complex 3, sigma 1 |
| 202826_at | 23.02 | 2.27E-10 | 1.812126221 | NM_001032367| | NA |
| 1553185_at | 23 | 2.28E-10 | 1.29769824 | NM_152573| | RASEF,RAS and EF hand domain containing |
| 229523_at | 22.99 | 2.29E-10 | 1.936714104 | NM_001080209| | NA |
| 213075_at | 22.97 | 2.31E-10 | 1.853122648 | NM_182487| | OLFML2A,olfactomedin-like 2A |
| 218781_at | 22.96 | 2.32E-10 | 1.140762443 | NM_024624| | SMC6L1,SMC6 protein |
| 219412_at | 22.95 | 2.33E-10 | 1.49437561 | NM_022337| | RAB38,RAB38 |
| 215440_s_at | 22.95 | 2.34E-10 | 1.249533525 | NM_001080425| | NA |
| 223208_at | 22.94 | 2.34E-10 | 1.105289108 | NM_031954| | KCTD10,potassium channel tetramerisation domain |
| 235048_at | 22.93 | 2.35E-10 | 1.27436712 | NM_015566| | NA |
| 202081_at | 22.93 | 2.35E-10 | 1.096186963 | NM_004907| | IER2,immediate early response 2 |
| 203492_x_at | 22.92 | 2.36E-10 | 1.103052625 | NM_014679| | PIG8,translokin |
| 219635_at | 22.91 | 2.37E-10 | 1.185998143 | NM_025027| | ZNF606,zinc finger protein 606 |
| 223885_at | 22.9 | 2.38E-10 | 1.84071465 | NM_001017440| | NA |
| 226658_at | 22.87 | 2.42E-10 | 1.387748732 | NM_001006624| | T1A-2,lung type-I cell membrane-associated |
| 201098_at | 22.86 | 2.43E-10 | 1.055460342 | NM_004766| | COPB2,coatomer protein complex, subunit beta 2 (beta |
| 205415_s_at | 22.85 | 2.44E-10 | 1.208484214 | NM_004993| | ATXN3,ataxin 3 isoform 1 |
| 227163_at | 22.83 | 2.46E-10 | 1.508998204 | NM_183239| | GSTO2,glutathione S-transferase omega 2 |
| 239781_at | 22.82 | 2.46E-10 | 2.273358441 | NA |  |
| 228463_at | 22.8 | 2.50E-10 | 1.734627819 | NM_004497| | FOXA3,forkhead box A3 |
| 201752_s_at | 22.78 | 2.52E-10 | 1.095738461 | NM_001121| | NA |
| 200887_s_at | 22.78 | 2.52E-10 | 1.079894338 | NM_007315| | STAT1,signal transducer and activator of transcription |
| 203439_s_at | 22.77 | 2.53E-10 | 1.938057991 | NM_003714| | STC2,stanniocalcin 2 |
| 230100_x_at | 22.77 | 2.52E-10 | 1.757219495 | NM_002576| | PAK1,p21-activated kinase 1 |
| 216035_x_at | 22.74 | 2.57E-10 | 1.124764898 | NM_030756| | TCF7L2,transcription factor 7-like 2 (T-cell specific, |
| 219968_at | 22.71 | 2.61E-10 | 1.194508296 | NM_016089| | ZNF589,zinc finger protein 589 |
| 202315_s_at | 22.7 | 2.61E-10 | 1.157605259 | NM_004327| | BCR,breakpoint cluster region isoform 1 |
| 218720_x_at | 22.68 | 2.64E-10 | 1.53456494 | NM_001114099| | NA |
| 227692_at | 22.68 | 2.64E-10 | 1.233351807 | NM_002069| | GNAI1,guanine nucleotide binding protein (G protein), |
| 212317_at | 22.68 | 2.64E-10 | 1.136209323 | NM_012470| | TNPO3,transportin 3 |
| 212747_at | 22.64 | 2.69E-10 | 1.121245818 | NM_015245| | ANKS1,ankyrin repeat and sterile alpha motif domain |
| 225320_at | 22.63 | 2.71E-10 | 1.162675422 | NM_138357| | C10orf42,chromosome 10 open reading frame 42 |
| 205818_at | 22.62 | 2.73E-10 | 2.440406494 | NM_014618| | DBC1,deleted in bladder cancer 1 |
| 1555846_a_at | 22.59 | 2.77E-10 | 1.180255296 | NA |  |
| 212769_at | 22.57 | 2.79E-10 | 1.300978195 | NM_001105192| | NA |
| 222903_s_at | 22.56 | 2.81E-10 | 1.347143081 | NM_001079533| | NA |
| 205981_s_at | 22.51 | 2.87E-10 | 1.147986448 | NM_001564| | ING2,inhibitor of growth family, member 1-like |
| 206743_s_at | 22.51 | 2.87E-10 | 1.608127422 | NM_001671| | ASGR1,asialoglycoprotein receptor 1 |
| 219959_at | 22.5 | 2.88E-10 | 1.951300931 | NM_017947| | MOCOS,molybdenum cofactor sulfurase |
| 223514_at | 22.5 | 2.88E-10 | 1.289060188 | NM_032415| | CARD11,caspase recruitment domain family, member 11 |
| 202603_at | 22.49 | 2.89E-10 | 1.061224283 | NA |  |
| 206789_s_at | 22.49 | 2.89E-10 | 1.271480389 | NM_002697| | POU2F1,POU domain, class 2, transcription factor 1 |
| 208626_s_at | 22.48 | 2.91E-10 | 1.223364323 | NM_006373| | VAT1,vesicle amine transport protein 1 |
| 206555_s_at | 22.46 | 2.94E-10 | 1.089221376 | NM_017736| | THUMPD1,THUMP domain containing 1 |
| 223038_s_at | 22.41 | 3.01E-10 | 1.20754514 | NM_021238| | C12orf14,chromosome 12 open reading frame 14 |
| 212053_at | 22.41 | 3.02E-10 | 1.118524129 | NM_015027| | KIAA0251,KIAA0251 protein |
| 203896_s_at | 22.4 | 3.02E-10 | 1.252125383 | NM_000933| | PLCB4,phospholipase C beta 4 isoform a |
| 1553411_s_at | 22.36 | 3.09E-10 | 1.273645718 | NM_171999| | SALL3,sal-like 3 |
| 210674_s_at | 22.36 | 3.08E-10 | 2.614509432 | NM_014005| | PCDHA9,protocadherin alpha 9 isoform 2 precursor |
| 203752_s_at | 22.35 | 3.11E-10 | 1.081269756 | NM_005354| | JUND,jun D proto-oncogene |
| 218186_at | 22.32 | 3.14E-10 | 2.235289387 | NM_020387| | RAB25,RAB25 |
| 218499_at | 22.31 | 3.16E-10 | 1.291237268 | NM_001042452| | NA |
| 208926_at | 22.3 | 3.16E-10 | 1.139583162 | NM_000434| | NEU1,neuraminidase precursor |
| 200977_s_at | 22.29 | 3.19E-10 | 1.117134628 | NM_001079864| | NA |
| 244406_at | 22.28 | 3.20E-10 | 1.183485951 | NM_021143| | ZNF20,zinc finger protein 20 (KOX 13) |
| 231896_s_at | 22.28 | 3.20E-10 | 1.037034065 | NM_003677| | DENR,density-regulated protein |
| 232382_s_at | 22.27 | 3.21E-10 | 1.271015892 | NM_052937| | LOC115294,similar to hypothetical protein FLJ10883 |
| 228598_at | 22.27 | 3.21E-10 | 2.546116468 | NM_001004360| | DPP10,dipeptidylpeptidase 10 isoform 2 |
| 231945_at | 22.27 | 3.20E-10 | 1.788341142 | NM_015687| | FILIP1,filamin A interacting protein 1 |
| 206710_s_at | 22.27 | 3.20E-10 | 2.10311803 | NM_012307| | EPB41L3,erythrocyte membrane protein band 4.1-like 3 |
| 232111_at | 22.26 | 3.21E-10 | 2.157100528 | NA |  |
| 227131_at | 22.26 | 3.22E-10 | 1.134716353 | NM_002401| | MAP3K3,mitogen-activated protein kinase kinase kinase 3 |
| 217967_s_at | 22.24 | 3.24E-10 | 2.47279861 | NM_052966| | C1orf24,niban protein |
| 244050_at | 22.18 | 3.34E-10 | 1.714713113 | NM_001010915| | LOC401494,similar to RIKEN 4933428I03 |
| 1559957_a_at | 22.18 | 3.34E-10 | 1.207935362 | NA |  |
| 237911_at | 22.16 | 3.38E-10 | 3.326253927 | NA |  |
| 222946_s_at | 22.15 | 3.39E-10 | 1.163669832 | NM_024037| | MGC2603,hypothetical protein MGC2603 |
| 209678_s_at | 22.15 | 3.39E-10 | 1.145062339 | NM_002740| | PRKCI,protein kinase C, iota |
| 221832_s_at | 22.13 | 3.42E-10 | 1.159091046 | NM_033631| | LUZP1,leucine zipper protein 1 |
| 220387_s_at | 22.07 | 3.53E-10 | 1.352316452 | NM_001031693| | NA |
| 230195_at | 22.06 | 3.54E-10 | 3.237075629 | NA |  |
| 1554539_a_at | 22.04 | 3.57E-10 | 1.366836469 | NM_019034| | RHOF,ras homolog gene family, member F |
| 225177_at | 22.04 | 3.56E-10 | 1.365055215 | NM_001002233| | RAB11FIP1,Rab coupling protein isoform 2 |
| 226161_at | 22.03 | 3.58E-10 | 1.106927776 | NM_017964| | SLC30A6,solute carrier family 30 (zinc transporter), |
| 209337_at | 22.03 | 3.58E-10 | 1.105500991 | NM_021144| | PSIP1,PC4 and SFRS1 interacting protein 1 |
| 204416_x_at | 22.01 | 3.61E-10 | 1.719475143 | NM_001645| | APOC1,apolipoprotein C-I precursor |
| 240616_at | 22.01 | 3.61E-10 | 1.198040586 | NA |  |
| 201897_s_at | 22 | 3.62E-10 | 1.047826698 | NM_001826| | CKS1B,CDC28 protein kinase 1B |
| 202889_x_at | 21.99 | 3.64E-10 | 1.866040965 | NM_003980| | MAP7,microtubule-associated protein 7 |
| 212647_at | 21.98 | 3.67E-10 | 1.219952786 | NM_006270| | RRAS,related RAS viral (r-ras) oncogene homolog |
| 227063_at | 21.97 | 3.69E-10 | 1.116718229 | NM_152766| | MGC40107,hypothetical protein MGC40107 |
| 210260_s_at | 21.94 | 3.74E-10 | 1.457641445 | NM_001077654| | NA |
| 222033_s_at | 21.93 | 3.75E-10 | 2.111546048 | NA |  |
| 229429_x_at | 21.93 | 3.75E-10 | 1.342143492 | NA |  |
| 219301_s_at | 21.91 | 3.79E-10 | 1.880801923 | NM_014141| | CNTNAP2,cell recognition molecule Caspr2 precursor |
| 228952_at | 21.91 | 3.80E-10 | 1.417548791 | NM_006208| | ENPP1,ectonucleotide pyrophosphatase/phosphodiesterase |
| 214239_x_at | 21.9 | 3.80E-10 | 1.179387903 | NM_007144| | PCGF2,ring finger protein 110 |
| 206891_at | 21.87 | 3.86E-10 | 1.793842173 | NM_001104| | ACTN3,skeletal muscle specific actinin, alpha 3 |
| 220474_at | 21.83 | 3.95E-10 | 1.942720728 | NM_030631| | SLC25A21,solute carrier family 25 (mitochondrial |
| 203233_at | 21.82 | 3.96E-10 | 1.507027442 | NM_000418| | IL4R,interleukin 4 receptor alpha chain isoform a |
| 229139_at | 21.82 | 3.96E-10 | 1.176204302 | NM_020647| | JPH1,junctophilin 1 |
| 204853_at | 21.81 | 3.98E-10 | 1.142391827 | NM_006190| | ORC2L,origin recognition complex, subunit 2 |
| 203984_s_at | 21.81 | 3.98E-10 | 1.207851824 | NM_001229| | CASP9,caspase 9 isoform alpha preproprotein |
| 242328_at | 21.79 | 4.01E-10 | 1.54045819 | NM_138453| | RAB3C,RAB3C, member RAS oncogene family |
| 235141_at | 21.79 | 4.01E-10 | 1.621292838 | NM_001038603| | NA |
| 228340_at | 21.79 | 4.00E-10 | 1.272841337 | NM_001105192| | NA |
| 201605_x_at | 21.77 | 4.04E-10 | 1.294225907 | NM_004368| | CNN2,calponin 2 isoform a |
| 203139_at | 21.77 | 4.04E-10 | 1.129130841 | NM_004938| | DAPK1,death-associated protein kinase 1 |
| 202621_at | 21.76 | 4.05E-10 | 1.100946383 | NM_001571| | IRF3,interferon regulatory factor 3 |
| 202626_s_at | 21.73 | 4.12E-10 | 1.463956712 | NM_001111097| | NA |
| 203650_at | 21.73 | 4.12E-10 | 2.094937362 | NM_006404| | PROCR,endothelial protein C receptor precursor |
| 231079_at | 21.73 | 4.13E-10 | 2.224806897 | NA |  |
| 218694_at | 21.71 | 4.17E-10 | 1.28505282 | NM_016608| | ARMCX1,armadillo repeat containing, X-linked 1 |
| 206472_s_at | 21.69 | 4.20E-10 | 1.272489299 | NM_001105192| | NA |
| 205980_s_at | 21.69 | 4.20E-10 | 1.896535042 | NM_001017526| | NA |
| 202956_at | 21.69 | 4.20E-10 | 1.162266385 | NM_006421| | ARFGEF1,brefeldin A-inhibited guanine |
| 219155_at | 21.68 | 4.22E-10 | 1.182871248 | NM_012417| | PITPNC1,phosphatidylinositol transfer protein, |
| 224439_x_at | 21.67 | 4.24E-10 | 1.081314644 | NM_014245| | RNF7,ring finger protein 7 isoform 1 |
| 225195_at | 21.67 | 4.24E-10 | 1.297238354 | NM_001047434| | NA |
| 236163_at | 21.67 | 4.23E-10 | 1.551166492 | NM_153234| | LIX1,limb expression 1 |
| 207966_s_at | 21.64 | 4.30E-10 | 1.039182314 | NM_012201| | GLG1,golgi apparatus protein 1 |
| 224721_at | 21.63 | 4.33E-10 | 1.054854477 | NM_032168| | FLJ12519,hypothetical protein FLJ12519 |
| 205373_at | 21.63 | 4.32E-10 | 1.788626025 | NM_004389| | CTNNA2,catenin, alpha 2 |
| 227645_at | 21.61 | 4.37E-10 | 1.430128271 | NM_014308| | PIK3R5,phosphoinositide-3-kinase, regulatory subunit 5, |
| 222641_s_at | 21.61 | 4.37E-10 | 1.10923912 | NM_001077498| | NA |
| 35617_at | 21.6 | 4.39E-10 | 1.204476143 | NM_002749| | MAPK7,mitogen-activated protein kinase 7 isoform 1 |
| 201926_s_at | 21.6 | 4.39E-10 | 1.916711117 | NM_000574| | DAF,decay accelerating factor for complement (CD55, |
| 208886_at | 21.59 | 4.40E-10 | 1.420197938 | NM_005318| | H1F0,H1 histone family, member 0 |
| 201548_s_at | 21.56 | 4.47E-10 | 1.212202753 | NM_006618| | JARID1B,Jumonji, AT rich interactive domain 1B |
| 229724_at | 21.56 | 4.47E-10 | 2.303981058 | NM_000814| | GABRB3,gamma-aminobutyric acid (GABA) A receptor, beta |
| 239231_at | 21.54 | 4.51E-10 | 1.327536144 | NA |  |
| 218574_s_at | 21.54 | 4.52E-10 | 1.449865652 | NM_014583| | LMCD1,LIM and cysteine-rich domains 1 |
| 239202_at | 21.53 | 4.53E-10 | 1.524637452 | NA |  |
| 200986_at | 21.53 | 4.53E-10 | 1.761860736 | NM_000062| | SERPING1,complement component 1 inhibitor precursor |
| 229427_at | 21.53 | 4.53E-10 | 1.544151518 | NM_003966| | SEMA5A,semaphorin 5A |
| 223734_at | 21.47 | 4.65E-10 | 1.380307022 | NM_032623| | OSAP,ovary-specific acidic protein |
| 53720_at | 21.47 | 4.66E-10 | 1.266989478 | NM_018381| | FLJ11286,hypothetical protein FLJ11286 |
| 202352_s_at | 21.47 | 4.65E-10 | 1.087547716 | NM_002816| | PSMD12,proteasome 26S non-ATPase subunit 12 isoform 1 |
| 203616_at | 21.44 | 4.73E-10 | 1.112459877 | NM_002690| | POLB,polymerase (DNA directed), beta |
| 230896_at | 21.44 | 4.73E-10 | 2.448350657 | NM_207406| | FLJ43965,FLJ43965 protein |
| 209685_s_at | 21.43 | 4.76E-10 | 2.273735612 | NM_002738| | PRKCB1,protein kinase C, beta isoform 2 |
| 214442_s_at | 21.42 | 4.77E-10 | 1.137568142 | NM_004671| | PIAS2,protein inhibitor of activated STAT X isoform |
| 221666_s_at | 21.42 | 4.77E-10 | 2.079803257 | NM_013258| | PYCARD,PYD and CARD domain containing isoform a |
| 1552897_a_at | 21.36 | 4.93E-10 | 2.717559425 | NM_133329| | KCNG3,potassium voltage-gated channel, subfamily G, |
| 205483_s_at | 21.35 | 4.95E-10 | 1.357923252 | NM_005101| | G1P2,interferon, alpha-inducible protein (clone |
| 235126_at | 21.35 | 4.95E-10 | 1.248761693 | NA |  |
| 49452_at | 21.35 | 4.95E-10 | 1.330651041 | NM_001093| | ACACB,acetyl-Coenzyme A carboxylase beta |
| 209632_at | 21.33 | 4.99E-10 | 1.187239391 | NM_002718| | PPP2R3A,alpha isoform of regulatory subunit B'', protein |
| 208785_s_at | 21.32 | 5.03E-10 | 1.104812805 | NM_022818| | MAP1LC3B,microtubule-associated proteins 1A/1B light |
| 218133_s_at | 21.3 | 5.07E-10 | 1.043621656 | NM_021824| | NIF3L1,NIF3 NGG1 interacting factor 3-like 1 |
| 226747_at | 21.28 | 5.12E-10 | 1.14688023 | NM_020784| | KIAA1344,KIAA1344 |
| 206588_at | 21.27 | 5.15E-10 | 1.652920689 | NM_001351| | DAZL,deleted in azoospermia-like |
| 218865_at | 21.27 | 5.16E-10 | 1.115899192 | NM_022746| | FLJ22390,hypothetical protein FLJ22390 |
| 1555971_s_at | 21.27 | 5.16E-10 | 1.165933927 | NM_015176| | FBXO28,F-box protein 28 |
| 38290_at | 21.26 | 5.17E-10 | 1.296206319 | NM_006480| | RGS14,regulator of G-protein signalling 14 |
| 223457_at | 21.24 | 5.22E-10 | 1.08742272 | NM_012133| | COPG2,coatomer protein complex, subunit gamma 2 |
| 204466_s_at | 21.24 | 5.23E-10 | 2.374290305 | NM_000345| | SNCA,alpha-synuclein isoform NACP140 |
| 221207_s_at | 21.21 | 5.29E-10 | 1.928234912 | NM_015678| | NBEA,neurobeachin |
| 236124_at | 21.2 | 5.31E-10 | 1.311804502 | NA |  |
| 235810_at | 21.2 | 5.33E-10 | 1.199291624 | NM_001007088| | ZNF21,zinc finger protein 21 isoform 2 |
| 202214_s_at | 21.2 | 5.31E-10 | 1.085571276 | NM_001079872| | NA |
| 211015_s_at | 21.19 | 5.36E-10 | 1.131965437 | NM_002154| | HSPA4,heat shock 70kDa protein 4 isoform a |
| 235947_at | 21.18 | 5.37E-10 | 1.671250599 | NA |  |
| 210776_x_at | 21.15 | 5.45E-10 | 1.055692674 | NM_003200| | TCF3,transcription factor 3 |
| 201831_s_at | 21.15 | 5.46E-10 | 1.188292821 | NM_003715| | VDP,vesicle docking protein p115 |
| 226720_at | 21.12 | 5.55E-10 | 1.308551895 | NM_052927| | NA |
| 236501_at | 21.11 | 5.55E-10 | 2.131971518 | NM_020436| | SALL4,sal-like 4 |
| 228010_at | 21.09 | 5.61E-10 | 1.826207453 | NM_020416| | PPP2R2C,gamma isoform of regulatory subunit B55, protein |
| 217223_s_at | 21.07 | 5.68E-10 | 1.171226024 | NM_004327| | BCR,breakpoint cluster region isoform 1 |
| 202550_s_at | 21.07 | 5.67E-10 | 1.072132203 | NM_004738| | VAPB,VAMP-associated protein B/C |
| 226771_at | 21.07 | 5.68E-10 | 1.169994842 | NM_001005855| | ATP8B2,ATPase, Class I, type 8B, member 2 isoform b |
| 209792_s_at | 21.06 | 5.69E-10 | 1.472084428 | NM_001077500| | NA |
| 227417_at | 21.04 | 5.74E-10 | 1.253658172 | NM_017898| | FLJ20605,hypothetical protein FLJ20605 |
| 1554340_a_at | 21.04 | 5.76E-10 | 1.517457776 | NM_198545| | LOC374946,hypothetical gene supported by AK075558; |
| 222635_s_at | 21.04 | 5.74E-10 | 1.190714065 | NM_025205| | MED28,mediator of RNA polymerase II transcription, |
| 210835_s_at | 21.03 | 5.79E-10 | 1.073818445 | NM_001083914| | NA |
| 204678_s_at | 21.02 | 5.81E-10 | 1.793191585 | NM_002245| | KCNK1,potassium channel, subfamily K, member 1 |
| 209238_at | 21.02 | 5.81E-10 | 2.34744462 | NM_004177| | STX3A,syntaxin 3A |
| 218928_s_at | 21.02 | 5.82E-10 | 1.461299047 | NM_018964| | SLC37A1,solute carrier family 37 member 1 |
| 1555962_at | 21.01 | 5.83E-10 | 2.240994806 | NM_145236| | B3GNT7,UDP-GlcNAc:betaGal |
| 223642_at | 21.01 | 5.84E-10 | 3.573657985 | NM_007129| | ZIC2,zinc finger protein of the cerebellum 2 |
| 219316_s_at | 21 | 5.86E-10 | 1.3416848 | NM_017791| | C14orf58,chromosome 14 open reading frame 58 |
| 235687_at | 20.98 | 5.90E-10 | 1.534865027 | NM_001076675| | NA |
| 227566_at | 20.98 | 5.92E-10 | 2.720737613 | NM_001048209| | NA |
| 226555_at | 20.98 | 5.92E-10 | 1.243284335 | NM_017759| | FLJ20309,hypothetical protein FLJ20309 |
| 223068_at | 20.91 | 6.15E-10 | 1.103158215 | NM_019063| | EML4,echinoderm microtubule associated protein like |
| 224710_at | 20.9 | 6.15E-10 | 1.165340837 | NM_031934| | RAB34,RAB39 |
| 213556_at | 20.88 | 6.23E-10 | 1.667740952 | NA |  |
| 203974_at | 20.85 | 6.34E-10 | 1.160475309 | NM_012080| | HDHD1A,haloacid dehalogenase-like hydrolase domain |
| 207571_x_at | 20.85 | 6.33E-10 | 1.450980227 | NM_001039477| | NA |
| 202894_at | 20.84 | 6.36E-10 | 1.124638827 | NM_004444| | EPHB4,ephrin receptor EphB4 precursor |
| 227684_at | 20.84 | 6.36E-10 | 1.193573572 | NM_004230| | EDG5,endothelial differentiation, sphingolipid |
| 213235_at | 20.82 | 6.43E-10 | 1.088847163 | NM_001012991| | NA |
| 211202_s_at | 20.8 | 6.49E-10 | 1.163644267 | NM_006618| | JARID1B,Jumonji, AT rich interactive domain 1B |
| 206355_at | 20.8 | 6.49E-10 | 1.927771711 | NM_002071| | GNAL,guanine nucleotide binding protein (G protein), |
| 201973_s_at | 20.78 | 6.55E-10 | 1.058238472 | NM_015622| | C7orf28A,chromosome 7 open reading frame 28A |
| 201611_s_at | 20.78 | 6.54E-10 | 1.127380605 | NM_012405| | ICMT,isoprenylcysteine carboxyl methyltransferase |
| 231018_at | 20.77 | 6.56E-10 | 1.57930154 | NA |  |
| 207173_x_at | 20.77 | 6.56E-10 | 1.330516101 | NM_001797| | CDH11,cadherin 11, type 2 isoform 1 preproprotein |
| 205068_s_at | 20.77 | 6.56E-10 | 1.651934191 | NM_015071| | ARHGAP26,GTPase regulator associated with the focal |
| 220367_s_at | 20.76 | 6.59E-10 | 1.071539493 | NM_024545| | SAP130,mSin3A-associated protein 130 |
| 1567107_s_at | 20.75 | 6.63E-10 | 1.167534496 | NM_003290| | TPM4,tropomyosin 4 |
| 218464_s_at | 20.74 | 6.67E-10 | 1.081619421 | NM_001077498| | NA |
| 211921_x_at | 20.74 | 6.64E-10 | 1.036029823 | NM_001099285| | NA |
| 206756_at | 20.73 | 6.69E-10 | 1.363229853 | NM_019886| | CHST7,carbohydrate (N-acetylglucosamine 6-O) |
| 221016_s_at | 20.73 | 6.68E-10 | 1.334196094 | NM_031283| | TCF7L1,HMG-box transcription factor TCF-3 |
| 214843_s_at | 20.72 | 6.70E-10 | 1.111027658 | NM_015017| | USP33,ubiquitin specific protease 33 isoform 1 |
| 229519_at | 20.7 | 6.80E-10 | 1.132674348 | NM_001013438| | NA |
| 208869_s_at | 20.68 | 6.89E-10 | 1.662209721 | NM_031412| | GABARAPL1,GABA(A) receptor-associated protein like 1 |
| 209892_at | 20.67 | 6.91E-10 | 1.547869208 | NM_002033| | FUT4,fucosyltransferase 4 |
| 244261_at | 20.67 | 6.91E-10 | 1.666062467 | NM_170743| | IL28RA,interleukin 28 receptor, alpha isoform 1 |
| 204411_at | 20.66 | 6.93E-10 | 1.503419617 | NM_017596| | NA |
| 236037_at | 20.63 | 7.04E-10 | 1.570111635 | NM_020340| | KIAA1244,KIAA1244 |
| 218780_at | 20.6 | 7.15E-10 | 1.192709427 | NM_001100176| | NA |
| 223427_s_at | 20.58 | 7.25E-10 | 1.619280479 | NM_018424| | EPB41L4B,erythrocyte membrane protein band 4.1 like 4B |
| 227794_at | 20.57 | 7.29E-10 | 1.809706537 | NM_080661| | MGC15937,hypothetical protein MGC15937 similar to |
| 223122_s_at | 20.57 | 7.29E-10 | 2.567374971 | NM_003013| | SFRP2,secreted frizzled-related protein 2 precursor |
| 202006_at | 20.56 | 7.31E-10 | 1.089204814 | NM_002835| | PTPN12,protein tyrosine phosphatase, non-receptor type |
| 215227_x_at | 20.56 | 7.31E-10 | 1.043045111 | NM_001040649| | NA |
| 224817_at | 20.53 | 7.45E-10 | 1.254858733 | NM_014631| | SH3MD1,SH3 multiple domains 1 |
| 206142_at | 20.53 | 7.44E-10 | 1.513223828 | NM_003436| | ZNF135,zinc finger protein 135 (clone pHZ-17) |
| 233297_s_at | 20.52 | 7.46E-10 | 1.66416759 | NA |  |
| 224561_s_at | 20.5 | 7.57E-10 | 1.074659904 | NM_006791| | MORF4L1,MORF-related gene 15 isoform 1 |
| 212445_s_at | 20.48 | 7.63E-10 | 1.209206035 | NM_015277| | NEDD4L,ubiquitin-protein ligase NEDD4-like |
| 227599_at | 20.48 | 7.62E-10 | 1.273050433 | NM_178496| | LOC151963,similar to BcDNA:GH11415 gene product |
| 207644_at | 20.46 | 7.70E-10 | 1.372044184 | NM_003923| | FOXH1,forkhead box H1 |
| 224129_s_at | 20.44 | 7.82E-10 | 1.073424264 | NM_032574| | LOC84661,dpy-30-like protein |
| 208815_x_at | 20.43 | 7.82E-10 | 1.098257409 | NM_002154| | HSPA4,heat shock 70kDa protein 4 isoform a |
| 225944_at | 20.39 | 8.02E-10 | 1.093264938 | NM_020726| | NLN,neurolysin |
| 222610_s_at | 20.36 | 8.12E-10 | 1.174259228 | NM_001017406| | NA |
| 221543_s_at | 20.35 | 8.17E-10 | 1.103234102 | NM_001003790| | SPFH2,SPFH domain family, member 2 isoform 2 |
| 202808_at | 20.35 | 8.17E-10 | 1.106653912 | NM_001083913| | NA |
| 1553858_at | 20.34 | 8.19E-10 | 1.273581781 | NM_024784| | ZBTB3,zinc finger and BTB domain containing 3 |
| 202437_s_at | 20.34 | 8.20E-10 | 2.254550326 | NM_000104| | CYP1B1,cytochrome P450, family 1, subfamily B, |
| 205961_s_at | 20.33 | 8.26E-10 | 1.119286526 | NM_021144| | PSIP1,PC4 and SFRS1 interacting protein 1 |
| 205308_at | 20.33 | 8.25E-10 | 1.174457706 | NM_016010| | CGI-62,CGI-62 protein |
| 226415_at | 20.33 | 8.26E-10 | 3.119972075 | NM_020927| | KIAA1576,KIAA1576 protein |
| 210547_x_at | 20.33 | 8.26E-10 | 1.222761312 | NM_004968| | ICA1,islet cell autoantigen 1 isoform 2 |
| 225648_at | 20.3 | 8.36E-10 | 1.152913137 | NM_080836| | STK35,serine/threonine kinase 35 |
| 239422_at | 20.3 | 8.38E-10 | 1.126796432 | NM_152742| | GPC2,glypican 2 |
| 209033_s_at | 20.29 | 8.43E-10 | 1.064764033 | NM_001396| | DYRK1A,dual-specificity tyrosine-(Y)-phosphorylation |
| 225401_at | 20.29 | 8.43E-10 | 1.478258111 | NM_144580| | MGC31963,kidney predominant protein NCU-G1 |
| 204048_s_at | 20.29 | 8.43E-10 | 1.269784753 | NM_001100164| | NA |
| 226015_at | 20.28 | 8.47E-10 | 1.050066097 | NM_006956| | ZNF12,zinc finger protein 12 (KOX 3) |
| 1552938_at | 20.27 | 8.53E-10 | 1.985219028 | NM_033132| | ZIC5,zinc finger protein of the cerebellum 5 |
| 205704_s_at | 20.26 | 8.56E-10 | 1.128661127 | NM_012463| | ATP6V0A2,ATPase, H+ transporting, lysosomal V0 subunit a |
| 1556047_s_at | 20.26 | 8.56E-10 | 1.313346991 | NM_020932| | MAGEE1,melanoma antigen family E, 1 |
| 222603_at | 20.26 | 8.54E-10 | 1.210013128 | NM_024896| | KIAA1815,KIAA1815 |
| 204483_at | 20.25 | 8.59E-10 | 1.259113706 | NM_001976| | ENO3,enolase 3 |
| 202951_at | 20.24 | 8.67E-10 | 1.082336148 | NM_007271| | STK38,serine/threonine kinase 38 |
| 201427_s_at | 20.23 | 8.71E-10 | 2.115852759 | NM_001085486| | NA |
| 219439_at | 20.22 | 8.74E-10 | 1.368211816 | NM_020156| | C1GALT1,core 1 synthase, |
| 209823_x_at | 20.22 | 8.73E-10 | 1.235621814 | NM_002123| | HLA-DQB1,major histocompatibility complex, class II, DQ |
| 204891_s_at | 20.21 | 8.75E-10 | 2.274260121 | NM_001042771| | NA |
| 235052_at | 20.21 | 8.77E-10 | 1.211870335 | NM_175872| | FLJ38451,FLJ38451 protein |
| 234725_s_at | 20.2 | 8.83E-10 | 1.101888284 | NM_020210| | SEMA4B,semaphorin 4B precursor |
| 203593_at | 20.19 | 8.88E-10 | 1.294068512 | NM_012120| | CD2AP,CD2-associated protein |
| 203160_s_at | 20.17 | 8.92E-10 | 1.126040327 | NM_003958| | RNF8,ring finger protein 8 isoform 1 |
| 238510_at | 20.15 | 9.02E-10 | 1.173835383 | NM_001004300| | LOC124411,hypothetical protein LOC124411 |
| 204579_at | 20.13 | 9.14E-10 | 1.538547554 | NM_002011| | FGFR4,fibroblast growth factor receptor 4 isoform 1 |
| 228189_at | 20.13 | 9.10E-10 | 1.136600797 | NM_004874| | BAG4,BCL2-associated athanogene 4 |
| 214889_at | 20.12 | 9.18E-10 | 1.275619579 | NM_001006655| | DKFZP564J102,DKFZP564J102 protein |
| 226682_at | 20.12 | 9.18E-10 | 1.42661102 | NA |  |
| 214285_at | 20.1 | 9.26E-10 | 1.975219278 | NM_004102| | FABP3,fatty acid binding protein 3 |
| 226863_at | 20.09 | 9.32E-10 | 2.291173785 | NM_001077710| | NA |
| 242890_at | 20.09 | 9.30E-10 | 1.160990541 | NA |  |
| 220408_x_at | 20.09 | 9.32E-10 | 1.07291959 | NM_001014286| | NA |
| 225601_at | 20.08 | 9.37E-10 | 1.132353527 | NM_005342| | HMGB3,high-mobility group box 3 |
| 229649_at | 20.08 | 9.36E-10 | 1.794528717 | NM_001105250| | NA |
| 213256_at | 20.07 | 9.38E-10 | 1.4761562 | NM_178450| | MARCH3,membrane-associated ring finger (C3HC4) 3 |
| 227202_at | 20.06 | 9.45E-10 | 2.069519417 | NM_001843| | CNTN1,contactin 1 isoform 1 precursor |
| 236236_at | 20.06 | 9.43E-10 | 1.19728607 | NA |  |
| 204695_at | 20.04 | 9.57E-10 | 1.103096273 | NM_001789| | CDC25A,cell division cycle 25A isoform a |
| 225739_at | 20.04 | 9.58E-10 | 1.405904746 | NM_032932| | RAB11FIP4,RAB11 family interacting protein 4 (class II) |
| 212699_at | 20.03 | 9.59E-10 | 1.149162472 | NM_138967| | SCAMP5,secretory carrier membrane protein 5 |
| 212842_x_at | 20.03 | 9.58E-10 | 1.073418041 | NM_001123363| | NA |
| 203407_at | 20.03 | 9.60E-10 | 1.638129833 | NM_002705| | PPL,periplakin |
| 238765_at | 20.03 | 9.59E-10 | 1.160450328 | NM_004888| | ATP6V1G1,ATPase, H+ transporting, lysosomal, V1 subunit G |
| 219961_s_at | 20 | 9.75E-10 | 1.287716389 | NM_018474| | C20orf19,uncharacterized hypothalamus protein HT013 |
| 222895_s_at | 19.99 | 9.82E-10 | 1.936322447 | NM_022898| | BCL11B,B-cell CLL/lymphoma 11B isoform 2 |
| 202011_at | 19.98 | 9.85E-10 | 1.09527318 | NM_003257| | TJP1,tight junction protein 1 isoform a |
| 223358_s_at | 19.98 | 9.88E-10 | 1.186668397 | NA |  |
| 1568609_s_at | 19.97 | 9.90E-10 | 1.915005578 | NA |  |
| 1554689_a_at | 19.97 | 9.90E-10 | 1.243704605 | NM_020742| | NLGN4X,X-linked neuroligin 4 |
| 220108_at | 19.97 | 9.89E-10 | 2.13352708 | NM_004297| | GNA14,guanine nucleotide binding protein (G protein), |
| 227911_at | 19.96 | 9.96E-10 | 2.199471014 | NM_001010000| | ARHGAP28,Rho GTPase activating protein 28 isoform a |
| 242629_at | 19.96 | 9.96E-10 | 1.720838173 | NA |  |
| 227247_at | 19.93 | 1.01E-09 | 1.143322391 | NA |  |
| 223556_at | 19.92 | 1.02E-09 | 1.195881721 | NM_018063| | HELLS,helicase, lymphoid-specific |
| 221774_x_at | 19.92 | 1.01E-09 | 1.068977018 | NM_001014286| | NA |
| 210683_at | 19.9 | 1.03E-09 | 1.433213019 | NM_004558| | NRTN,neurturin precursor |
| 216985_s_at | 19.89 | 1.04E-09 | 1.957897882 | NM_004177| | STX3A,syntaxin 3A |
| 213676_at | 19.89 | 1.03E-09 | 1.574647986 | NA |  |
| 213652_at | 19.88 | 1.04E-09 | 1.987409103 | NM_006200| | PCSK5,proprotein convertase subtilisin/kexin type 5 |
| 205405_at | 19.87 | 1.04E-09 | 1.251053313 | NM_003966| | SEMA5A,semaphorin 5A |
| 223786_at | 19.84 | 1.06E-09 | 1.418325475 | NM_021615| | CHST6,carbohydrate (N-acetylglucosamine 6-O) |
| 202303_x_at | 19.84 | 1.06E-09 | 1.084912834 | NM_003601| | SMARCA5,SWI/SNF-related matrix-associated |
| 220992_s_at | 19.84 | 1.06E-09 | 1.24992946 | NM_030934| | C1orf25,N2,N2-dimethylguanosine tRNA |
| 217665_at | 19.83 | 1.07E-09 | 1.412727436 | NA |  |
| 209931_s_at | 19.83 | 1.07E-09 | 1.352868167 | NM_004116| | FKBP1B,FK506-binding protein 1B isoform a |
| 226723_at | 19.82 | 1.07E-09 | 1.180364597 | NM_199342| | LOC374969,hypothetical protein LOC374969 |
| 203350_at | 19.8 | 1.08E-09 | 1.095307259 | NM_001030007| | NA |
| 239752_at | 19.8 | 1.08E-09 | 1.250893586 | NA |  |
| 206506_s_at | 19.79 | 1.09E-09 | 1.222920047 | NM_003599| | SUPT3H,suppressor of Ty 3 homolog |
| 223531_x_at | 19.78 | 1.09E-09 | 1.053614022 | NM_001097612| | NA |
| 216054_x_at | 19.78 | 1.09E-09 | 1.249926576 | NM_001002841| | MYL4,atrial/embryonic alkali myosin light chain |
| 243000_at | 19.78 | 1.09E-09 | 1.288329295 | NM_001259| | CDK6,cyclin-dependent kinase 6 |
| 218441_s_at | 19.76 | 1.10E-09 | 1.103581875 | NM_015540| | RPAP1,RNA polymerase II associated protein 1 |
| 226267_at | 19.75 | 1.11E-09 | 1.717103934 | NM_130469| | JDP2,Jun dimerization protein |
| 228008_at | 19.74 | 1.12E-09 | 2.015804777 | NA |  |
| 208941_s_at | 19.72 | 1.13E-09 | 1.143741846 | NM_012247| | SEPHS1,selenophosphate synthetase |
| 212114_at | 19.71 | 1.13E-09 | 1.070490146 | NA |  |
| 225769_at | 19.71 | 1.14E-09 | 1.083421181 | NM_020751| | COG6,component of oligomeric golgi complex 6 |
| 220615_s_at | 19.71 | 1.14E-09 | 1.429723513 | NM_018099| | MLSTD1,male sterility domain containing 1 |
| 219534_x_at | 19.7 | 1.14E-09 | 1.366857072 | NM_000076| | CDKN1C,cyclin-dependent kinase inhibitor 1C |
| 218457_s_at | 19.69 | 1.14E-09 | 1.153640269 | NM_022552| | DNMT3A,DNA cytosine methyltransferase 3 alpha isoform |
| 203433_at | 19.68 | 1.15E-09 | 1.197935256 | NM_001100879| | NA |
| 230047_at | 19.68 | 1.15E-09 | 1.361008019 | NA |  |
| 201071_x_at | 19.67 | 1.15E-09 | 1.046676806 | NM_001005526| | SF3B1,splicing factor 3b, subunit 1 isoform 2 |
| 201739_at | 19.67 | 1.15E-09 | 1.350644979 | NM_005627| | SGK,serum/glucocorticoid regulated kinase |
| 206572_x_at | 19.66 | 1.16E-09 | 1.170417222 | NM_003429| | ZNF85,zinc finger protein 85 (HPF4, HTF1) |
| 222494_at | 19.66 | 1.16E-09 | 1.16167159 | NM_001085471| | NA |
| 224846_at | 19.66 | 1.16E-09 | 1.152140406 | NM_138392| | SHKBP1,SH3KBP1 binding protein 1 |
| 224445_s_at | 19.62 | 1.18E-09 | 1.114034753 | NM_024071| | ZFYVE21,zinc finger, FYVE domain containing 21 |
| 238147_at | 19.57 | 1.22E-09 | 1.20993962 | NM_025058| | TRIM46,tripartite motif-containing 46 |
| 226597_at | 19.57 | 1.22E-09 | 1.276548581 | NM_138393| | C19orf32,polyposis locus protein 1-like 1 |
| 201603_at | 19.57 | 1.22E-09 | 1.184857696 | NM_002480| | PPP1R12A,protein phosphatase 1, regulatory (inhibitor) |
| 228577_x_at | 19.55 | 1.23E-09 | 1.370258395 | NM_001007022| | KIAA1229,KIAA1229 protein isoform b |
| 228336_at | 19.55 | 1.23E-09 | 1.187426753 | NM_052927| | NA |
| 215783_s_at | 19.55 | 1.23E-09 | 1.803745004 | NM_000478| | ALPL,tissue non-specific alkaline phosphatase |
| 222162_s_at | 19.54 | 1.24E-09 | 2.196647694 | NM_006988| | ADAMTS1,a disintegrin and metalloprotease with |
| 217605_at | 19.54 | 1.24E-09 | 1.215317213 | NA |  |
| 1564083_at | 19.53 | 1.24E-09 | 2.249222084 | NA |  |
| 212371_at | 19.52 | 1.25E-09 | 1.104459616 | NM_016076| | PNAS-4,CGI-146 protein |
| 226048_at | 19.51 | 1.26E-09 | 1.169861727 | NM_002750| | MAPK8,mitogen-activated protein kinase 8 isoform 2 |
| 232165_at | 19.51 | 1.25E-09 | 2.175661893 | NM_031308| | EPPK1,epiplakin 1 |
| 221778_at | 19.49 | 1.27E-09 | 1.263325793 | NM_030647| | NA |
| 212311_at | 19.48 | 1.27E-09 | 1.334499254 | NM_015187| | KIAA0746,KIAA0746 protein |
| 204465_s_at | 19.47 | 1.28E-09 | 1.154732946 | NM_032727| | INA,internexin neuronal intermediate filament |
| 223239_at | 19.47 | 1.28E-09 | 1.159283872 | NM_016472| | C14orf129,chromosome 14 open reading frame 129 |
| 200639_s_at | 19.47 | 1.28E-09 | 1.126040951 | NM_003406| | YWHAZ,tyrosine 3/tryptophan 5 -monooxygenase |
| 211571_s_at | 19.47 | 1.28E-09 | 1.234347606 | NM_001126336| | NA |
| 229432_at | 19.46 | 1.29E-09 | 1.443968585 | NM_153006| | NAGS,N-acetylglutamate synthase |
| 202677_at | 19.46 | 1.29E-09 | 1.096770772 | NM_002890| | RASA1,RAS p21 protein activator 1 isoform 1 |
| 202311_s_at | 19.46 | 1.29E-09 | 2.036587726 | NM_000088| | COL1A1,alpha 1 type I collagen preproprotein |
| 226052_at | 19.46 | 1.29E-09 | 1.099094065 | NM_014299| | BRD4,bromodomain-containing protein 4 isoform short |
| 227386_s_at | 19.46 | 1.29E-09 | 1.488543282 | NM_001003682| | DKFZp434C184,cDNA DKFZp434C184 gene |
| 233252_s_at | 19.45 | 1.29E-09 | 1.204486127 | NM_018387| | STRBP,spermatid perinuclear RNA-binding protein |
| 209545_s_at | 19.45 | 1.29E-09 | 1.074586893 | NM_003821| | RIPK2,receptor-interacting serine-threonine kinase 2 |
| 213136_at | 19.44 | 1.30E-09 | 1.133700228 | NM_002828| | PTPN2,protein tyrosine phosphatase, non-receptor type |
| 208742_s_at | 19.43 | 1.30E-09 | 1.084248146 | NM_005870| | SAP18,sin3 associated polypeptide p18 |
| 219650_at | 19.42 | 1.31E-09 | 1.196071945 | NM_017669| | FLJ20105,FLJ20105 protein isoform a |
| 206214_at | 19.42 | 1.32E-09 | 1.846829739 | NM_005084| | PLA2G7,phospholipase A2, group VII |
| 212660_at | 19.42 | 1.31E-09 | 1.206177634 | NM_015288| | PHF15,PHD finger protein 15 |
| 235343_at | 19.41 | 1.32E-09 | 1.527252201 | NM_024749| | FLJ12505,hypothetical protein FLJ12505 |
| 213629_x_at | 19.39 | 1.33E-09 | 1.364030906 | NM_005949| | MT1F,metallothionein 1F |
| 218472_s_at | 19.36 | 1.36E-09 | 1.127550619 | NM_015946| | PELO,CGI-17 protein |
| 204049_s_at | 19.36 | 1.36E-09 | 1.200732364 | NM_001100164| | NA |
| 204047_s_at | 19.35 | 1.36E-09 | 1.291949088 | NM_001100164| | NA |
| 201311_s_at | 19.35 | 1.37E-09 | 1.197379333 | NM_003022| | SH3BGRL,SH3 domain binding glutamic acid-rich protein |
| 213523_at | 19.34 | 1.37E-09 | 1.149937027 | NM_001238| | CCNE1,cyclin E1 isoform 1 |
| 1552390_a_at | 19.33 | 1.38E-09 | 1.608491151 | NM_173549| | FLJ39553,hypothetical protein FLJ39553 |
| 220028_at | 19.33 | 1.38E-09 | 1.170527873 | NM_001106| | ACVR2B,activin A type IIB receptor precursor |
| 203098_at | 19.33 | 1.38E-09 | 1.077724849 | NM_004824| | CDYL,chromodomain protein, Y chromosome-like isoform |
| 223524_s_at | 19.32 | 1.39E-09 | 1.607976189 | NM_023943| | MGC3040,hypothetical protein MGC3040 |
| 228245_s_at | 19.32 | 1.39E-09 | 1.296489177 | NM_001080502| | NA |
| 206116_s_at | 19.31 | 1.40E-09 | 1.45804855 | NM_000366| | TPM1,tropomyosin 1 (alpha) |
| 222209_s_at | 19.3 | 1.40E-09 | 1.093096784 | NM_022918| | FLJ22104,hypothetical protein FLJ22104 |
| 235723_at | 19.3 | 1.40E-09 | 2.068355153 | NM_017637| | BNC2,basonuclin 2 |
| 200039_s_at | 19.29 | 1.41E-09 | 1.03887939 | NM_002794| | PSMB2,proteasome beta 2 subunit |
| 224595_at | 19.27 | 1.43E-09 | 1.166693135 | NM_080546| | CDW92,CDW92 antigen |
| 204702_s_at | 19.26 | 1.43E-09 | 1.496066763 | NM_004289| | NFE2L3,nuclear factor (erythroid-derived 2)-like 3 |
| 230708_at | 19.26 | 1.43E-09 | 1.920962945 | NM_153026| | PRICKLE1,prickle-like 1 |
| 202670_at | 19.24 | 1.45E-09 | 1.104316585 | NM_002755| | MAP2K1,mitogen-activated protein kinase kinase 1 |
| 230623_x_at | 19.24 | 1.45E-09 | 1.24967669 | NM_020886| | USP28,ubiquitin specific protease 28 |
| 212082_s_at | 19.24 | 1.45E-09 | 1.069395566 | NM_002475| | MLC1SA,myosin alkali light chain 1 slow a |
| 214969_at | 19.24 | 1.45E-09 | 1.192693517 | NM_033141| | MAP3K9,mitogen-activated protein kinase kinase kinase |
| 202886_s_at | 19.23 | 1.46E-09 | 1.103010319 | NM_002716| | PPP2R1B,beta isoform of regulatory subunit A, protein |
| 201058_s_at | 19.23 | 1.46E-09 | 1.79933148 | NM_006097| | MYL9,myosin regulatory light polypeptide 9 isoform a |
| 236656_s_at | 19.22 | 1.47E-09 | 1.316519115 | NA |  |
| 223628_at | 19.22 | 1.47E-09 | 1.48505864 | NA |  |
| 210639_s_at | 19.19 | 1.49E-09 | 1.179842288 | NM_004849| | APG5L,APG5 autophagy 5-like |
| 228537_at | 19.18 | 1.50E-09 | 1.201624913 | NM_005270| | GLI2,GLI-Kruppel family member GLI2 isoform delta |
| 218905_at | 19.14 | 1.53E-09 | 1.050813234 | NM_017864| | FLJ20530,hypothetical protein FLJ20530 |
| 235045_at | 19.14 | 1.53E-09 | 1.187570976 | NM_016090| | RBM7,RNA binding motif protein 7 |
| 202763_at | 19.13 | 1.54E-09 | 1.110175189 | NM_004346| | CASP3,caspase 3 preproprotein |
| 37950_at | 19.13 | 1.54E-09 | 1.08462824 | NM_002726| | PREP,prolyl endopeptidase |
| 202686_s_at | 19.11 | 1.56E-09 | 1.225090213 | NM_001699| | AXL,AXL receptor tyrosine kinase isoform 2 |
| 212737_at | 19.11 | 1.56E-09 | 1.192188794 | NM_000405| | GM2A,GM2 ganglioside activator precursor |
| 227627_at | 19.11 | 1.56E-09 | 1.461024022 | NM_001033578| | NA |
| 219464_at | 19.1 | 1.57E-09 | 1.255251738 | NM_012113| | CA14,carbonic anhydrase XIV precursor |
| 204740_at | 19.1 | 1.56E-09 | 1.238407011 | NM_006314| | CNKSR1,connector enhancer of kinase suppressor of Ras |
| 203574_at | 19.09 | 1.58E-09 | 1.268600308 | NM_005384| | NFIL3,nuclear factor, interleukin 3 regulated |
| 217893_s_at | 19.08 | 1.58E-09 | 1.14727878 | NM_024595| | FLJ12666,hypothetical protein FLJ12666 |
| 213182_x_at | 19.06 | 1.60E-09 | 1.358442956 | NM_000076| | CDKN1C,cyclin-dependent kinase inhibitor 1C |
| 229661_at | 19.05 | 1.61E-09 | 2.068926649 | NM_020436| | SALL4,sal-like 4 |
| 226329_s_at | 19.03 | 1.63E-09 | 1.091211512 | NM_138798| | LOC129531,hypothetical protein BC018453 |
| 227494_at | 19.02 | 1.63E-09 | 1.783655473 | NM_001489| | NR6A1,nuclear receptor subfamily 6, group A, member 1 |
| 91826_at | 19.02 | 1.64E-09 | 1.440649385 | NM_017729| | EPS8L1,epidermal growth factor receptor pathway |
| 206490_at | 19.01 | 1.65E-09 | 1.768876202 | NM_001003809| | DLGAP1,discs large homolog-associated protein 1 isoform |
| 218226_s_at | 19.01 | 1.64E-09 | 1.038868249 | NM_004547| | NDUFB4,NADH dehydrogenase (ubiquinone) 1 beta |
| 209723_at | 19 | 1.65E-09 | 3.207316738 | NM_004155| | SERPINB9,serine (or cysteine) proteinase inhibitor, clade |
| 266_s_at | 19 | 1.65E-09 | 1.249057379 | NM_013230| | CD24,CD24 antigen |
| 213503_x_at | 18.99 | 1.66E-09 | 1.117490986 | NM_001002857| | ANXA2,annexin A2 isoform 2 |
| 220239_at | 18.98 | 1.67E-09 | 1.156316625 | NM_001031710| | NA |
| 200706_s_at | 18.97 | 1.68E-09 | 1.196902822 | NM_004862| | LITAF,LPS-induced TNF-alpha factor |
| 220022_at | 18.97 | 1.68E-09 | 1.457147042 | NM_018102| | ZNF334,zinc finger protein 334 isoform a |
| 204791_at | 18.97 | 1.68E-09 | 1.141094941 | NM_001032287| | NA |
| 228440_at | 18.95 | 1.70E-09 | 2.052402429 | NA |  |
| 229088_at | 18.94 | 1.71E-09 | 1.557573263 | NM_006208| | ENPP1,ectonucleotide pyrophosphatase/phosphodiesterase |
| 204568_at | 18.93 | 1.72E-09 | 1.191273492 | NM_014924| | KIAA0831,KIAA0831 |
| 221916_at | 18.92 | 1.73E-09 | 1.614628253 | NM_006158| | NEFL,neurofilament, light polypeptide 68kDa |
| 205774_at | 18.92 | 1.73E-09 | 1.191705672 | NM_000505| | F12,coagulation factor XII precursor |
| 200960_x_at | 18.89 | 1.75E-09 | 1.037407882 | NM_001076677| | NA |
| 1555950_a_at | 18.89 | 1.75E-09 | 1.712542015 | NM_000574| | DAF,decay accelerating factor for complement (CD55, |
| 225235_at | 18.86 | 1.79E-09 | 1.118564129 | NM_001006616| | TM4SF17,transmembrane 4 superfamily member 17 isoform c |
| 213106_at | 18.86 | 1.79E-09 | 1.7361751 | NM_001105529| | NA |
| 219714_s_at | 18.86 | 1.79E-09 | 1.292931686 | NM_018398| | CACNA2D3,calcium channel, voltage-dependent, alpha |
| 210643_at | 18.85 | 1.80E-09 | 2.572132797 | NM_003701| | TNFSF11,tumor necrosis factor ligand superfamily, member |
| 244227_at | 18.85 | 1.80E-09 | 1.684098151 | NM_205848| | SYT6,synaptotagmin VI |
| 210026_s_at | 18.84 | 1.80E-09 | 1.296830582 | NM_014550| | CARD10,caspase recruitment domain protein 10 |
| 207717_s_at | 18.83 | 1.82E-09 | 2.54631741 | NM_001005242| | PKP2,plakophilin 2 isoform 2a |
| 1564413_at | 18.81 | 1.84E-09 | 1.669732448 | NA |  |
| 226591_at | 18.81 | 1.84E-09 | 1.360528192 | NA |  |
| 224336_s_at | 18.81 | 1.84E-09 | 1.429439663 | NM_030640| | DUSP16,dual specificity phosphatase 16 |
| 205213_at | 18.77 | 1.88E-09 | 1.449882482 | NM_014716| | CENTB1,centaurin beta1 |
| 228624_at | 18.77 | 1.88E-09 | 1.717356103 | NM_018342| | FLJ11155,hypothetical protein FLJ11155 |
| 203301_s_at | 18.76 | 1.89E-09 | 1.079614439 | NM_021145| | DMTF1,cyclin D binding myb-like transcription factor |
| 233002_at | 18.76 | 1.90E-09 | 2.090466828 | NM_020958| | KIAA1622,HEAT-like repeat-containing protein isoform 2 |
| 217750_s_at | 18.76 | 1.90E-09 | 1.100272981 | NM_023079| | FLJ13855,hypothetical protein FLJ13855 |
| 225080_at | 18.75 | 1.90E-09 | 1.150725693 | NM_001080779| | NA |
| 227027_at | 18.75 | 1.90E-09 | 1.358045549 | NA |  |
| 238974_at | 18.75 | 1.91E-09 | 1.093664375 | NM_153689| | FLJ38973,hypothetical protein FLJ38973 |
| 204750_s_at | 18.74 | 1.91E-09 | 2.311426027 | NM_004949| | DSC2,desmocollin 2 isoform Dsc2b preproprotein |
| 35776_at | 18.74 | 1.91E-09 | 1.195503003 | NM_001001132| | ITSN1,intersectin 1 isoform ITSN-s |
| 201906_s_at | 18.73 | 1.92E-09 | 1.152070144 | NM_001008392| | CTDSPL,small CTD phosphatase 3 isoform 1 |
| 223174_at | 18.72 | 1.94E-09 | 1.087403828 | NM_032320| | GMRP-1,K+ channel tetramerization protein |
| 201880_at | 18.71 | 1.95E-09 | 1.087811385 | NM_005744| | ARIH1,ariadne homolog, ubiquitin-conjugating enzyme E2 |
| 214933_at | 18.7 | 1.95E-09 | 1.670924243 | NM_000068| | CACNA1A,calcium channel, alpha 1A subunit isoform 1 |
| 218775_s_at | 18.67 | 1.99E-09 | 1.18473615 | NM_024949| | BOMB,BH3-only member B protein |
| 222936_s_at | 18.67 | 1.99E-09 | 1.219541783 | NM_016076| | PNAS-4,CGI-146 protein |
| 203975_s_at | 18.66 | 2.00E-09 | 1.07139743 | NM_005483| | CHAF1A,chromatin assembly factor 1, subunit A (p150) |
| 219352_at | 18.66 | 1.99E-09 | 1.579046269 | NM_017912| | HERC6,hect domain and RLD 6 |
| 218537_at | 18.65 | 2.01E-09 | 1.259252975 | NM_001002017| | HCFC1R1,host cell factor C1 regulator 1 (XPO1 dependant) |
| 213285_at | 18.64 | 2.02E-09 | 2.176325331 | NM_001017970| | NA |
| 202003_s_at | 18.63 | 2.03E-09 | 1.098767828 | NM_006111| | ACAA2,acetyl-coenzyme A acyltransferase 2 |
| 228974_at | 18.62 | 2.04E-09 | 1.415059021 | NA |  |
| 203628_at | 18.6 | 2.07E-09 | 1.119358355 | NM_000875| | IGF1R,insulin-like growth factor 1 receptor precursor |
| 219622_at | 18.6 | 2.06E-09 | 1.471902973 | NM_017817| | RAB20,RAB20, member RAS oncogene family |
| 210785_s_at | 18.59 | 2.08E-09 | 1.435935151 | NM_001039477| | NA |
| 217739_s_at | 18.59 | 2.07E-09 | 1.141619522 | NM_005746| | PBEF1,pre-B-cell colony enhancing factor 1 isoform a |
| 237193_s_at | 18.58 | 2.09E-09 | 2.703393897 | NA |  |
| 243356_at | 18.57 | 2.09E-09 | 1.506497205 | NA |  |
| 204613_at | 18.57 | 2.10E-09 | 1.218933035 | NM_002661| | PLCG2,phospholipase C, gamma 2 |
| 208943_s_at | 18.56 | 2.11E-09 | 1.064200702 | NM_003262| | TLOC1,translocation protein 1 |
| 224325_at | 18.56 | 2.10E-09 | 1.992478615 | NM_031866| | FZD8,frizzled 8 |
| 226886_at | 18.55 | 2.11E-09 | 1.325659374 | NA |  |
| 239155_at | 18.55 | 2.12E-09 | 1.51731279 | NA |  |
| 214474_at | 18.54 | 2.13E-09 | 1.197529415 | NM_005399| | PRKAB2,AMP-activated protein kinase beta 2 |
| 203713_s_at | 18.53 | 2.14E-09 | 1.579661582 | NM_001015002| | NA |
| 218429_s_at | 18.52 | 2.16E-09 | 1.141229343 | NM_018381| | FLJ11286,hypothetical protein FLJ11286 |
| 235152_at | 18.5 | 2.18E-09 | 1.599856464 | NA |  |
| 227123_at | 18.5 | 2.18E-09 | 1.433464116 | NM_002867| | RAB3B,RAB3B, member RAS oncogene family |
| 201845_s_at | 18.5 | 2.18E-09 | 1.212298331 | NM_012234| | RYBP,RING1 and YY1 binding protein |
| 212745_s_at | 18.5 | 2.18E-09 | 1.122486355 | NM_033028| | BBS4,Bardet-Biedl syndrome 4 |
| 217982_s_at | 18.49 | 2.19E-09 | 1.047856099 | NM_006791| | MORF4L1,MORF-related gene 15 isoform 1 |
| 234974_at | 18.48 | 2.21E-09 | 1.137581655 | NM_138801| | GALM,galactose mutarotase (aldose 1-epimerase) |
| 204620_s_at | 18.47 | 2.22E-09 | 1.21745155 | NM_001126336| | NA |
| 1553972_a_at | 18.47 | 2.22E-09 | 1.098458051 | NM_000071| | CBS,cystathionine-beta-synthase |
| 201263_at | 18.46 | 2.23E-09 | 1.131528094 | NM_152295| | TARS,threonyl-tRNA synthetase |
| 244353_s_at | 18.46 | 2.23E-09 | 1.303931946 | NM_145176| | SLC2A12,solute carrier family 2 (facilitated glucose |
| 229513_at | 18.46 | 2.23E-09 | 1.215539377 | NM_018387| | STRBP,spermatid perinuclear RNA-binding protein |
| 226834_at | 18.45 | 2.24E-09 | 1.86267146 | NA |  |
| 209642_at | 18.45 | 2.25E-09 | 1.122147048 | NM_004336| | BUB1,BUB1 budding uninhibited by benzimidazoles 1 |
| AFFX-M27830_M_at | 18.44 | 2.26E-09 | 1.109288223 | NA |  |
| 202832_at | 18.43 | 2.27E-09 | 1.165201712 | NM_014635| | GCC2,GRIP coiled-coil protein GCC185 isoform b |
| 200604_s_at | 18.43 | 2.27E-09 | 1.134757923 | NM_002734| | PRKAR1A,cAMP-dependent protein kinase, regulatory |
| 208690_s_at | 18.42 | 2.29E-09 | 1.386346893 | NM_020992| | PDLIM1,PDZ and LIM domain 1 (elfin) |
| 205531_s_at | 18.4 | 2.31E-09 | 1.73163115 | NM_013267| | GLS2,glutaminase GA isoform a |
| 211954_s_at | 18.39 | 2.33E-09 | 1.034686027 | NM_002271| | RANBP5,RAN binding protein 5 |
| 218530_at | 18.38 | 2.35E-09 | 1.146984348 | NM_013241| | FHOD1,formin homology 2 domain containing 1 |
| 200704_at | 18.37 | 2.36E-09 | 1.202800904 | NM_004862| | LITAF,LPS-induced TNF-alpha factor |
| 203000_at | 18.37 | 2.36E-09 | 1.88783715 | NM_007029| | STMN2,superiorcervical ganglia, neural specific 10 |
| 32069_at | 18.37 | 2.35E-09 | 1.144255169 | NM_153029| | N4BP1,Nedd4 binding protein 1 |
| 218127_at | 18.36 | 2.37E-09 | 1.08067313 | NM_006166| | NFYB,nuclear transcription factor Y, beta |
| 207268_x_at | 18.36 | 2.36E-09 | 1.066923682 | NM_005759| | ABI2,abl interactor 2 |
| 65517_at | 18.34 | 2.39E-09 | 1.678368883 | NM_005498| | AP1M2,adaptor-related protein complex 1, mu 2 subunit |
| 224367_at | 18.31 | 2.43E-09 | 1.400072077 | NM_032621| | BEX2,brain expressed X-linked 2 |
| 220030_at | 18.3 | 2.44E-09 | 1.455055892 | NM_018423| | STYK1,serine/threonine/tyrosine kinase 1 |
| 212759_s_at | 18.3 | 2.44E-09 | 1.178166451 | NM_030756| | TCF7L2,transcription factor 7-like 2 (T-cell specific, |
| 201015_s_at | 18.3 | 2.44E-09 | 1.526788954 | NM_002230| | JUP,junction plakoglobin |
| 207949_s_at | 18.28 | 2.47E-09 | 1.218226667 | NM_004968| | ICA1,islet cell autoantigen 1 isoform 2 |
| 202022_at | 18.28 | 2.48E-09 | 1.247222611 | NM_005165| | ALDOC,aldolase C, fructose-bisphosphate |
| 35820_at | 18.28 | 2.48E-09 | 1.250094473 | NM_000405| | GM2A,GM2 ganglioside activator precursor |
| 212943_at | 18.27 | 2.49E-09 | 1.076889676 | NM_014802| | KIAA0528,KIAA0528 gene product |
| 244370_at | 18.27 | 2.49E-09 | 1.581479358 | NM_001008537| | KIAA2022,KIAA2022 protein |
| 206495_s_at | 18.27 | 2.48E-09 | 1.083439221 | NM_015517| | MIZF,MBD2 (methyl-CpG-binding protein)-interacting |
| 206818_s_at | 18.27 | 2.48E-09 | 1.196901008 | NM_017649| | CNNM2,cyclin M2 isoform 1 |
| 203594_at | 18.25 | 2.52E-09 | 1.134762525 | NM_003729| | RTCD1,RNA terminal phosphate cyclase domain 1 |
| 221731_x_at | 18.24 | 2.52E-09 | 1.216206342 | NM_001126336| | NA |
| 202388_at | 18.24 | 2.53E-09 | 1.269087535 | NM_002923| | RGS2,regulator of G-protein signalling 2, 24kDa |
| 202822_at | 18.24 | 2.52E-09 | 1.174546132 | NM_005578| | LPP,LIM domain containing preferred translocation |
| 207992_s_at | 18.23 | 2.54E-09 | 1.585077363 | NM_000480| | AMPD3,adenosine monophosphate deaminase (isoform E) |
| 219992_at | 18.23 | 2.54E-09 | 1.510880551 | NM_001006667| | TAC3,tachykinin 3 |
| 225093_at | 18.21 | 2.57E-09 | 1.269052282 | NM_007124| | UTRN,utrophin |
| 200797_s_at | 18.21 | 2.58E-09 | 1.051199831 | NM_021960| | MCL1,myeloid cell leukemia sequence 1 isoform 1 |
| 217768_at | 18.21 | 2.58E-09 | 1.030493621 | NM_016039| | C14orf166,chromosome 14 open reading frame 166 |
| 32137_at | 18.2 | 2.59E-09 | 1.257135753 | NM_002226| | JAG2,jagged 2 isoform a precursor |
| 213301_x_at | 18.2 | 2.59E-09 | 1.181470774 | NM_003852| | TIF1,transcriptional intermediary factor 1 alpha |
| 204589_at | 18.2 | 2.58E-09 | 1.265118049 | NM_014840| | ARK5,AMPK-related protein kinase 5 |
| 222606_at | 18.18 | 2.62E-09 | 1.073376964 | NM_017975| | FLJ10036,Zwilch |
| 1553979_at | 18.16 | 2.65E-09 | 1.069268099 | NA |  |
| 208724_s_at | 18.16 | 2.65E-09 | 1.038508127 | NM_004161| | RAB1A,RAB1A, member RAS oncogene family |
| 225274_at | 18.15 | 2.67E-09 | 1.142121281 | NM_016297| | PCYOX1,prenylcysteine oxidase 1 |
| 212816_s_at | 18.15 | 2.66E-09 | 1.074685407 | NM_000071| | CBS,cystathionine-beta-synthase |
| 204866_at | 18.14 | 2.67E-09 | 1.216078454 | NM_001077445| | NA |
| 210395_x_at | 18.13 | 2.68E-09 | 1.274948275 | NM_001002841| | MYL4,atrial/embryonic alkali myosin light chain |
| 202027_at | 18.13 | 2.69E-09 | 1.090121073 | NM_012264| | C22orf5,chromosome 22 open reading frame 5 |
| 235333_at | 18.1 | 2.74E-09 | 1.236902897 | NM_004775| | B4GALT6,UDP-Gal:betaGlcNAc beta 1,4- |
| 222071_s_at | 18.09 | 2.74E-09 | 2.082074618 | NM_180991| | SLCO4C1,solute carrier organic anion transporter family, |
| 222234_s_at | 18.09 | 2.74E-09 | 1.136644091 | NM_001042610| | NA |
| 202274_at | 18.08 | 2.77E-09 | 1.712491594 | NM_001615| | ACTG2,actin, gamma 2 propeptide |
| 203981_s_at | 18.07 | 2.78E-09 | 1.056659231 | NM_005050| | ABCD4,ATP-binding cassette, sub-family D, member 4 |
| 201881_s_at | 18.07 | 2.78E-09 | 1.072919768 | NM_005744| | ARIH1,ariadne homolog, ubiquitin-conjugating enzyme E2 |
| 209529_at | 18.07 | 2.78E-09 | 1.425072596 | NM_003712| | PPAP2C,phosphatidic acid phosphatase type 2C isoform 1 |
| 214047_s_at | 18.06 | 2.79E-09 | 1.076818938 | NM_003925| | MBD4,methyl-CpG binding domain protein 4 |
| 209781_s_at | 18.05 | 2.80E-09 | 1.178274337 | NM_006558| | KHDRBS3,KH domain containing, RNA binding, signal |
| 205478_at | 18.05 | 2.82E-09 | 2.014867872 | NM_006741| | PPP1R1A,protein phosphatase 1, regulatory (inhibitor) |
| 232282_at | 18.03 | 2.84E-09 | 1.247414119 | NM_001002838| | WNK3,WNK lysine deficient protein kinase 3 isoform 2 |
| 206296_x_at | 18.03 | 2.85E-09 | 1.589868802 | NM_001042600| | NA |
| 215603_x_at | 18.02 | 2.86E-09 | 1.170462218 | NM_001032364| | NA |
| 212012_at | 18.02 | 2.86E-09 | 1.117107559 | NM_012293| | NA |
| 232172_at | 17.99 | 2.92E-09 | 1.235012865 | NA |  |
| 208771_s_at | 17.99 | 2.92E-09 | 1.096254818 | NM_000895| | LTA4H,leukotriene A4 hydrolase |
| 226602_s_at | 17.98 | 2.93E-09 | 1.096520997 | NM_004327| | BCR,breakpoint cluster region isoform 1 |
| 1566766_a_at | 17.97 | 2.94E-09 | 1.410732645 | NM_182762| | 7A5,putative binding protein 7a5 |
| 212681_at | 17.97 | 2.95E-09 | 2.184348872 | NM_012307| | EPB41L3,erythrocyte membrane protein band 4.1-like 3 |
| 211417_x_at | 17.97 | 2.95E-09 | 1.155368131 | NM_001032364| | NA |
| 225822_at | 17.96 | 2.96E-09 | 1.720294835 | NM_144626| | MGC17299,hypothetical protein MGC17299 |
| 204879_at | 17.96 | 2.98E-09 | 1.421844542 | NM_001006624| | T1A-2,lung type-I cell membrane-associated |
| 228223_at | 17.95 | 2.98E-09 | 1.172874631 | NM_080752| | ZSWIM3,zinc finger, SWIM domain containing 3 |
| 204479_at | 17.94 | 3.00E-09 | 1.147357099 | NM_012383| | OSTF1,osteoclast stimulating factor 1 |
| 228561_at | 17.94 | 3.01E-09 | 1.157284682 | NM_017913| | CDC37L1,cell division cycle 37 homolog (S. |
| 212457_at | 17.94 | 3.00E-09 | 1.064718691 | NM_006521| | TFE3,transcription factor binding to IGHM enhancer 3 |
| 227111_at | 17.93 | 3.02E-09 | 1.226789315 | NM_001099270| | NA |
| 203288_at | 17.93 | 3.02E-09 | 1.065821898 | NM_014686| | KIAA0355,KIAA0355 |
| 219376_at | 17.92 | 3.04E-09 | 1.180219312 | NM_199005| | ZNF322B,zinc finger protein 322B |
| 212727_at | 17.92 | 3.03E-09 | 1.195863193 | NM_020730| | NA |
| 201602_s_at | 17.92 | 3.03E-09 | 1.191193896 | NM_002480| | PPP1R12A,protein phosphatase 1, regulatory (inhibitor) |
| 227827_at | 17.91 | 3.06E-09 | 2.40310721 | NA |  |
| 204164_at | 17.91 | 3.06E-09 | 1.188643106 | NM_006747| | SIPA1,signal-induced proliferation-associated protein |
| 225919_s_at | 17.91 | 3.05E-09 | 1.129459275 | NM_018325| | C9orf72,hypothetical protein MGC23980 isoform a |
| 209082_s_at | 17.91 | 3.05E-09 | 1.39844074 | NM_030582| | COL18A1,alpha 1 type XVIII collagen isoform 1 precursor |
| 201418_s_at | 17.91 | 3.05E-09 | 1.09064899 | NM_003107| | SOX4,SRY (sex determining region Y)-box 4 |
| 205416_s_at | 17.9 | 3.07E-09 | 1.209321805 | NM_004993| | ATXN3,ataxin 3 isoform 1 |
| 216905_s_at | 17.9 | 3.07E-09 | 1.65187704 | NM_021978| | ST14,matriptase |
| 226119_at | 17.89 | 3.09E-09 | 1.244935854 | NM_052937| | LOC115294,similar to hypothetical protein FLJ10883 |
| 237289_at | 17.89 | 3.09E-09 | 1.125428609 | NM_004379| | CREB1,cAMP responsive element binding protein 1 |
| 203342_at | 17.88 | 3.10E-09 | 1.084114835 | NM_005834| | TIMM17B,translocase of inner mitochondrial membrane 17 |
| 209344_at | 17.88 | 3.10E-09 | 1.130803526 | NM_003290| | TPM4,tropomyosin 4 |
| 205372_at | 17.87 | 3.13E-09 | 2.449166578 | NM_001114634| | NA |
| 210664_s_at | 17.86 | 3.14E-09 | 2.425361189 | NM_001032281| | NA |
| 209262_s_at | 17.86 | 3.14E-09 | 1.122782769 | NM_005234| | NR2F6,nuclear receptor subfamily 2, group F, member 6 |
| 238778_at | 17.83 | 3.21E-09 | 2.474920889 | NM_173496| | MPP7,palmitoylated membrane protein 7 |
| 202385_s_at | 17.82 | 3.21E-09 | 1.106332745 | NM_000356| | TCOF1,Treacher Collins-Franceschetti syndrome 1 |
| 228497_at | 17.82 | 3.21E-09 | 1.596296408 | NM_018420| | SLC22A15,solute carrier family 22 (organic cation |
| 221065_s_at | 17.82 | 3.22E-09 | 1.270504439 | NM_022467| | CHST8,carbohydrate (N-acetylgalactosamine 4-0) |
| 226538_at | 17.8 | 3.25E-09 | 1.17110831 | NM_002372| | MAN2A1,mannosidase, alpha, class 2A, member 1 |
| 202638_s_at | 17.78 | 3.31E-09 | 1.526095151 | NM_000201| | ICAM1,intercellular adhesion molecule 1 precursor |
| 212436_at | 17.76 | 3.35E-09 | 1.134476611 | NM_015906| | TRIM33,tripartite motif-containing 33 protein |
| 225309_at | 17.75 | 3.36E-09 | 1.071961938 | NM_032758| | PHF5A,PHD-finger 5A |
| 215017_s_at | 17.75 | 3.36E-09 | 1.078523128 | NM_001024948| | NA |
| 203585_at | 17.75 | 3.36E-09 | 1.331337503 | NM_007150| | ZNF185,zinc finger protein 185 (LIM domain) |
| 201522_x_at | 17.73 | 3.41E-09 | 1.172279918 | NM_003097| | SNRPN,small nuclear ribonucleoprotein polypeptide N |
| 226503_at | 17.72 | 3.43E-09 | 1.117034522 | NM_018151| | RIF1,RAP1 interacting factor 1 |
| 228961_at | 17.72 | 3.42E-09 | 1.094812491 | NM_152622| | FLJ35954,hypothetical protein FLJ35954 |
| 244246_at | 17.72 | 3.43E-09 | 1.370244967 | NM_138731| | MIPOL1,mirror-image polydactyly 1 |
| 226869_at | 17.7 | 3.47E-09 | 1.781313424 | NM_001409| | EGFL3,EGF-like-domain, multiple 3 |
| 222609_s_at | 17.69 | 3.49E-09 | 1.096395213 | NM_016046| | EXOSC1,exosomal core protein CSL4 |
| 203918_at | 17.69 | 3.49E-09 | 1.418898343 | NM_002587| | PCDH1,protocadherin 1 isoform 1 precursor |
| 210540_s_at | 17.68 | 3.52E-09 | 1.343395975 | NM_003778| | B4GALT4,UDP-Gal:betaGlcNAc beta 1,4- |
| 202575_at | 17.68 | 3.50E-09 | 1.555896413 | NM_001878| | CRABP2,cellular retinoic acid binding protein 2 |
| 215195_at | 17.67 | 3.53E-09 | 1.187598619 | NM_002737| | PRKCA,protein kinase C, alpha |
| 223331_s_at | 17.66 | 3.55E-09 | 1.042228829 | NM_007204| | DDX20,DEAD (Asp-Glu-Ala-Asp) box polypeptide 20 |
| 224395_s_at | 17.66 | 3.54E-09 | 1.090356729 | NM_014245| | RNF7,ring finger protein 7 isoform 1 |
| 204023_at | 17.66 | 3.54E-09 | 1.05681981 | NM_002916| | RFC4,replication factor C 4 |
| 228647_at | 17.64 | 3.58E-09 | 1.44870172 | NA |  |
| 222870_s_at | 17.62 | 3.63E-09 | 1.491362478 | NM_006577| | B3GNT1,beta-1,3-N-acetylglucosaminyltransferase bGnT-1 |
| 204199_at | 17.6 | 3.68E-09 | 1.519583673 | NM_014636| | RALGPS1,Ral GEF with PH domain and SH3 binding motif 1 |
| 219036_at | 17.6 | 3.68E-09 | 1.163043468 | NM_024491| | Cep70,centrosomal protein 70 kDa |
| 225439_at | 17.6 | 3.67E-09 | 1.064672437 | NM_032869| | NUDCD1,NudC domain containing 1 |
| 227070_at | 17.6 | 3.69E-09 | 2.475678155 | NM_031302| | GLT8D2,glycosyltransferase 8 domain containing 2 |
| 1568604_a_at | 17.59 | 3.70E-09 | 1.661783981 | NM_003716| | CADPS,Ca2+-dependent secretion activator isoform 1 |
| 219410_at | 17.59 | 3.70E-09 | 2.867901175 | NM_018004| | TMEM45A,transmembrane protein 45A |
| 213039_at | 17.58 | 3.71E-09 | 1.064404086 | NM_015318| | ARHGEF18,Rho-specific guanine nucleotide exchange factor |
| 202998_s_at | 17.57 | 3.74E-09 | 1.71174397 | NM_002318| | LOXL2,lysyl oxidase-like 2 |
| 227862_at | 17.57 | 3.74E-09 | 1.178743485 | NM_001013642| | NA |
| 225463_x_at | 17.55 | 3.79E-09 | 1.055457895 | NM_001097612| | NA |
| 201364_s_at | 17.55 | 3.79E-09 | 1.179192184 | NM_002537| | OAZ2,ornithine decarboxylase antizyme 2 |
| 203091_at | 17.54 | 3.81E-09 | 1.062329314 | NM_003902| | FUBP1,far upstream element-binding protein |
| 205626_s_at | 17.53 | 3.83E-09 | 2.523350642 | NM_004929| | CALB1,calbindin 1 |
| 227297_at | 17.53 | 3.84E-09 | 1.797781686 | NM_002207| | ITGA9,integrin, alpha 9 precursor |
| 210426_x_at | 17.53 | 3.83E-09 | 1.711855602 | NM_002943| | RORA,RAR-related orphan receptor A isoform c |
| 217794_at | 17.51 | 3.88E-09 | 1.099666277 | NM_001005354| | DKFZp564J157,DKFZp564J157 protein isoform 2 |
| 218129_s_at | 17.51 | 3.88E-09 | 1.181106761 | NM_006166| | NFYB,nuclear transcription factor Y, beta |
| 218878_s_at | 17.5 | 3.91E-09 | 1.342331845 | NM_012238| | SIRT1,sirtuin 1 |
| 208680_at | 17.49 | 3.93E-09 | 1.031790049 | NM_002574| | PRDX1,peroxiredoxin 1 |
| 217966_s_at | 17.48 | 3.95E-09 | 1.958413109 | NM_052966| | C1orf24,niban protein |
| 213217_at | 17.48 | 3.93E-09 | 1.661915306 | NM_020546| | ADCY2,adenylate cyclase 2 |
| 201867_s_at | 17.46 | 4.00E-09 | 1.15631122 | NM_005647| | TBL1X,transducin beta-like 1X |
| 228194_s_at | 17.46 | 3.98E-09 | 1.553492025 | NM_001013031| | NA |
| 225231_at | 17.45 | 4.03E-09 | 1.131292082 | NM_005188| | CBL,Cas-Br-M (murine) ecotropic retroviral |
| 235874_at | 17.44 | 4.05E-09 | 1.888189172 | NM_153362| | PRSS35,protease, serine, 35 |
| 201604_s_at | 17.44 | 4.04E-09 | 1.201782826 | NM_002480| | PPP1R12A,protein phosphatase 1, regulatory (inhibitor) |
| 202037_s_at | 17.43 | 4.07E-09 | 1.374306191 | NM_003012| | SFRP1,secreted frizzled-related protein 1 |
| 222573_s_at | 17.41 | 4.12E-09 | 1.124684275 | NM_021818| | SAV1,WW45 protein |
| 226878_at | 17.41 | 4.12E-09 | 1.969123803 | NM_002119| | HLA-DOA,major histocompatibility complex, class II, DO |
| 231118_at | 17.41 | 4.12E-09 | 1.530140068 | NM_144698| | ANKRD35,ankyrin repeat domain 35 |
| 225806_at | 17.4 | 4.15E-09 | 1.359995779 | NM_032876| | JUB,jub, ajuba homolog isoform 1 |
| 209366_x_at | 17.4 | 4.14E-09 | 1.072953144 | NM_001914| | CYB5,cytochrome b-5 isoform 2 |
| 218651_s_at | 17.4 | 4.13E-09 | 1.159441233 | NM_018357| | FLJ11196,acheron isoform 1 |
| 219343_at | 17.39 | 4.16E-09 | 1.222415312 | NM_017913| | CDC37L1,cell division cycle 37 homolog (S. |
| 206712_at | 17.39 | 4.15E-09 | 1.498314386 | NM_024719| | GRTP1,growth hormone regulated TBC protein 1 |
| 212154_at | 17.38 | 4.17E-09 | 1.261760563 | NM_002998| | SDC2,syndecan 2 precursor |
| 211421_s_at | 17.38 | 4.19E-09 | 2.057629285 | NM_020630| | RET,ret proto-oncogene isoform c |
| 1553764_a_at | 17.35 | 4.25E-09 | 1.429750567 | NM_032876| | JUB,jub, ajuba homolog isoform 1 |
| 232136_s_at | 17.35 | 4.25E-09 | 1.697295174 | NM_033427| | CTTNBP2,cortactin binding protein 2 |
| 239292_at | 17.34 | 4.29E-09 | 1.779360047 | NA |  |
| 228909_at | 17.33 | 4.31E-09 | 1.158355316 | NA |  |
| 202558_s_at | 17.33 | 4.32E-09 | 1.186688622 | NM_006948| | STCH,stress 70 protein chaperone, |
| 213172_at | 17.33 | 4.31E-09 | 2.513623601 | NM_015351| | NA |
| 231296_at | 17.33 | 4.32E-09 | 1.150737662 | NA |  |
| 204832_s_at | 17.3 | 4.41E-09 | 1.132750156 | NM_004329| | BMPR1A,bone morphogenetic protein receptor, type IA |
| 227917_at | 17.3 | 4.40E-09 | 1.222684248 | NA |  |
| 56197_at | 17.28 | 4.45E-09 | 1.104234625 | NM_020360| | PLSCR3,phospholipid scramblase 3 |
| 203666_at | 17.27 | 4.49E-09 | 1.495981734 | NM_000609| | CXCL12,chemokine (C-X-C motif) ligand 12 (stromal |
| 219298_at | 17.27 | 4.47E-09 | 1.289703592 | NM_024693| | ECHDC3,enoyl Coenzyme A hydratase domain containing 3 |
| 231271_x_at | 17.27 | 4.47E-09 | 1.190084056 | NM_020677| | HSCARG,HSCARG protein |
| 223404_s_at | 17.26 | 4.52E-09 | 1.264576618 | NM_030934| | C1orf25,N2,N2-dimethylguanosine tRNA |
| 201590_x_at | 17.26 | 4.50E-09 | 1.116325014 | NM_001002857| | ANXA2,annexin A2 isoform 2 |
| 209421_at | 17.26 | 4.52E-09 | 1.140951251 | NM_000251| | MSH2,mutS homolog 2 |
| 231897_at | 17.24 | 4.57E-09 | 1.072634415 | NM_012212| | LTB4DH,NADP-dependent leukotriene B4 |
| 240382_at | 17.24 | 4.57E-09 | 1.793345958 | NA |  |
| 225925_s_at | 17.24 | 4.57E-09 | 1.105434514 | NM_001032730| | NA |
| 219000_s_at | 17.23 | 4.60E-09 | 1.116085029 | NM_024094| | DCC1,hypothetical protein MGC5528 |
| 203179_at | 17.23 | 4.59E-09 | 1.401110829 | NM_000155| | GALT,galactose-1-phosphate uridylyltransferase |
| 204749_at | 17.21 | 4.66E-09 | 1.479293247 | NM_004538| | NAP1L3,nucleosome assembly protein 1-like 3 |
| 1552736_a_at | 17.21 | 4.66E-09 | 1.733238102 | NM_138966| | NETO1,neuropilin- and tolloid-like protein 1 isoform 3 |
| 226899_at | 17.21 | 4.66E-09 | 1.204712068 | NM_170744| | UNC5B,unc-5 homolog B |
| 235763_at | 17.2 | 4.70E-09 | 1.657596949 | NM_152697| | MGC34032,hypothetical protein MGC34032 |
| 211776_s_at | 17.19 | 4.70E-09 | 1.769417421 | NM_012307| | EPB41L3,erythrocyte membrane protein band 4.1-like 3 |
| 224509_s_at | 17.19 | 4.70E-09 | 1.113521853 | NM_032730| | RTN4IP1,reticulon 4 interacting protein 1 |
| 218764_at | 17.19 | 4.72E-09 | 1.48568209 | NM_006255| | PRKCH,protein kinase C, eta |
| 202981_x_at | 17.19 | 4.70E-09 | 1.054903958 | NM_001006610| | SIAH1,seven in absentia homolog 1 isoform b |
| 203728_at | 17.19 | 4.70E-09 | 1.083896386 | NM_001188| | BAK1,BCL2-antagonist/killer 1 |
| 232636_at | 17.17 | 4.76E-09 | 2.193716004 | NM_173078| | SLITRK4,slit and trk like 4 protein |
| 233841_s_at | 17.15 | 4.83E-09 | 1.152044065 | NM_022491| | SDS3,hypothetical protein FLJ00052 |
| 209682_at | 17.14 | 4.86E-09 | 1.182016027 | NM_170662| | CBLB,Cas-Br-M (murine) ecotropic retroviral |
| 235355_at | 17.14 | 4.86E-09 | 1.696341663 | NA |  |
| 231039_at | 17.14 | 4.86E-09 | 1.263926019 | NA |  |
| 204984_at | 17.13 | 4.87E-09 | 1.464592544 | NM_001448| | GPC4,glypican 4 |
| 202117_at | 17.12 | 4.92E-09 | 1.03429623 | NM_004308| | ARHGAP1,Rho GTPase activating protein 1 |
| 1553957_at | 17.1 | 4.97E-09 | 1.1669649 | NM_144976| | ZNF564,zinc finger protein 564 |
| 205138_s_at | 17.09 | 5.01E-09 | 1.352287724 | NM_005715| | UST,uronyl-2-sulfotransferase |
| 203680_at | 17.09 | 4.99E-09 | 1.125390323 | NM_002736| | PRKAR2B,cAMP-dependent protein kinase, regulatory |
| 232687_at | 17.08 | 5.04E-09 | 1.505310951 | NA |  |
| 226107_at | 17.08 | 5.04E-09 | 1.191531648 | NA |  |
| 209533_s_at | 17.07 | 5.06E-09 | 1.08196678 | NM_001031689| | NA |
| 219505_at | 17.07 | 5.07E-09 | 1.48371491 | NM_017424| | CECR1,cat eye syndrome critical region protein 1 |
| 202455_at | 17.06 | 5.10E-09 | 1.215907896 | NM_001015053| | NA |
| 225232_at | 17.06 | 5.11E-09 | 1.136931913 | NM_001040446| | NA |
| 202435_s_at | 17.06 | 5.09E-09 | 1.441827104 | NM_000104| | CYP1B1,cytochrome P450, family 1, subfamily B, |
| 241353_s_at | 17.04 | 5.15E-09 | 1.263449795 | NA |  |
| 219704_at | 17.02 | 5.23E-09 | 1.298259969 | NM_015982| | YBX2,germ cell specific Y-box binding protein |
| 208636_at | 17.01 | 5.26E-09 | 1.056156488 | NM_001102| | ACTN1,actinin, alpha 1 |
| 203491_s_at | 17.01 | 5.26E-09 | 1.124333629 | NM_014679| | PIG8,translokin |
| 203935_at | 17.01 | 5.25E-09 | 1.465553067 | NM_001105| | ACVR1,activin A type I receptor precursor |
| 201649_at | 17 | 5.28E-09 | 1.107808373 | NM_004223| | UBE2L6,ubiquitin-conjugating enzyme E2L 6 isoform 1 |
| 227878_s_at | 16.99 | 5.30E-09 | 1.155281198 | NM_032306| | SPATA11,spermatogenesis associated 11 |
| 214909_s_at | 16.99 | 5.30E-09 | 1.126628817 | NM_013974| | DDAH2,dimethylarginine dimethylaminohydrolase 2 |
| 223710_at | 16.99 | 5.31E-09 | 1.964932596 | NM_006072| | CCL26,chemokine (C-C motif) ligand 26 precursor |
| 203001_s_at | 16.99 | 5.32E-09 | 2.329281529 | NM_007029| | STMN2,superiorcervical ganglia, neural specific 10 |
| 206042_x_at | 16.97 | 5.36E-09 | 1.210988135 | NM_003097| | SNRPN,small nuclear ribonucleoprotein polypeptide N |
| 204565_at | 16.96 | 5.41E-09 | 1.194827589 | NM_018473| | THEM2,thioesterase superfamily member 2 |
| 220289_s_at | 16.96 | 5.40E-09 | 1.790114472 | NM_001039775| | NA |
| 207843_x_at | 16.96 | 5.40E-09 | 1.07260124 | NM_001914| | CYB5,cytochrome b-5 isoform 2 |
| 205253_at | 16.94 | 5.48E-09 | 1.712300581 | NM_002585| | PBX1,pre-B-cell leukemia transcription factor 1 |
| 205577_at | 16.94 | 5.47E-09 | 1.438032046 | NM_005609| | PYGM,glycogen phosphorylase |
| 230793_at | 16.93 | 5.53E-09 | 1.385351189 | NM_017640| | LRRC16,leucine rich repeat containing 16 |
| 227909_at | 16.93 | 5.53E-09 | 1.609177708 | NM_001031705| | NA |
| 222981_s_at | 16.9 | 5.62E-09 | 1.098045543 | NM_016131| | RAB10,ras-related GTP-binding protein RAB10 |
| 241749_at | 16.9 | 5.62E-09 | 1.539326668 | NM_001018116| | NA |
| 218180_s_at | 16.89 | 5.67E-09 | 1.583097425 | NM_022772| | EPS8L2,epidermal growth factor receptor pathway |
| 236297_at | 16.89 | 5.64E-09 | 1.714024953 | NA |  |
| 209620_s_at | 16.86 | 5.77E-09 | 1.117350287 | NM_004299| | ABCB7,ATP-binding cassette, sub-family B, member 7 |
| 226065_at | 16.85 | 5.79E-09 | 2.227873059 | NM_153026| | PRICKLE1,prickle-like 1 |
| 231310_at | 16.85 | 5.80E-09 | 3.11574351 | NA |  |
| 230597_at | 16.85 | 5.79E-09 | 2.481686679 | NM_001048164| | NA |
| 201329_s_at | 16.85 | 5.80E-09 | 1.188691811 | NM_005239| | ETS2,v-ets erythroblastosis virus E26 oncogene |
| 233911_s_at | 16.84 | 5.82E-09 | 1.382145008 | NM_020700| | NA |
| 203954_x_at | 16.84 | 5.84E-09 | 1.315138843 | NM_001306| | CLDN3,claudin 3 |
| 226148_at | 16.82 | 5.91E-09 | 1.086428926 | NM_014155| | HSPC063,HSPC063 protein |
| 218047_at | 16.82 | 5.92E-09 | 1.100403363 | NM_024586| | OSBPL9,oxysterol-binding protein-like protein 9 isoform |
| 229830_at | 16.8 | 5.98E-09 | 1.193022695 | NA |  |
| 212481_s_at | 16.8 | 5.97E-09 | 1.140624018 | NM_003290| | TPM4,tropomyosin 4 |
| 206117_at | 16.8 | 5.98E-09 | 1.634752935 | NM_000366| | TPM1,tropomyosin 1 (alpha) |
| 1556242_a_at | 16.8 | 5.99E-09 | 1.248779406 | NA |  |
| 204944_at | 16.79 | 6.02E-09 | 1.173697909 | NM_002841| | PTPRG,protein tyrosine phosphatase, receptor type, G |
| 239761_at | 16.78 | 6.04E-09 | 1.919123871 | NM_001097633| | NA |
| 230075_at | 16.77 | 6.11E-09 | 1.164331026 | NM_171998| | RAB39B,RAB39B, member RAS oncogene family |
| 244565_at | 16.76 | 6.12E-09 | 1.583658508 | NM_005519| | NA |
| 232151_at | 16.76 | 6.12E-09 | 1.783348679 | NM_182762| | 7A5,putative binding protein 7a5 |
| 202035_s_at | 16.76 | 6.12E-09 | 1.383794057 | NM_003012| | SFRP1,secreted frizzled-related protein 1 |
| 205541_s_at | 16.75 | 6.16E-09 | 1.15550129 | NM_018094| | GSPT2,peptide chain release factor 3 |
| 225111_s_at | 16.75 | 6.16E-09 | 1.107160573 | NM_022080| | NAPB,N-ethylmaleimide-sensitive factor attachment |
| 221763_at | 16.75 | 6.16E-09 | 1.154604426 | NM_004241| | JMJD1C,jumonji domain containing 1C |
| 240181_at | 16.75 | 6.17E-09 | 1.342758861 | NA |  |
| 203204_s_at | 16.75 | 6.16E-09 | 1.138477216 | NM_014663| | JMJD2A,jumonji domain containing 2A |
| 218917_s_at | 16.73 | 6.26E-09 | 1.072763313 | NM_006015| | ARID1A,AT rich interactive domain 1A (SWI- like) |
| 206981_at | 16.73 | 6.24E-09 | 1.136206158 | NM_000334| | SCN4A,sodium channel, voltage-gated, type IV, alpha |
| 201904_s_at | 16.73 | 6.25E-09 | 1.146095551 | NM_001008392| | CTDSPL,small CTD phosphatase 3 isoform 1 |
| 243174_at | 16.72 | 6.30E-09 | 1.414295504 | NA |  |
| 211999_at | 16.71 | 6.32E-09 | 1.045887457 | NM_002107| | H3F3A,H3 histone, family 3A |
| 219987_at | 16.71 | 6.33E-09 | 1.822260697 | NA |  |
| 218319_at | 16.69 | 6.39E-09 | 1.153191837 | NM_020651| | PELI1,pellino protein |
| 203830_at | 16.69 | 6.41E-09 | 1.132983791 | NM_022344| | NJMU-R1,protein kinase Njmu-R1 |
| 223311_s_at | 16.68 | 6.46E-09 | 1.453409915 | NM_020744| | MTA3,metastasis associated 1 family, member 3 |
| 223589_at | 16.68 | 6.46E-09 | 1.076919424 | NM_017879| | ZNF416,zinc finger protein 416 |
| 220297_at | 16.68 | 6.46E-09 | 1.258736005 | NM_001002860| | BTBD7,BTB (POZ) domain containing 7 isoform 1 |
| 225517_at | 16.67 | 6.50E-09 | 1.135423124 | NM_014106| | FLJ20582,hypothetical protein FLJ20582 |
| 220588_at | 16.67 | 6.50E-09 | 1.341744055 | NM_001010974| | BCAS4,breast carcinoma amplified sequence 4 isoform c |
| 1552524_at | 16.65 | 6.57E-09 | 1.219595465 | NM_001079536| | NA |
| 210069_at | 16.65 | 6.56E-09 | 1.498928797 | NM_004377| | CPT1B,carnitine palmitoyltransferase 1B isoform a |
| 211203_s_at | 16.65 | 6.56E-09 | 1.369040619 | NM_001843| | CNTN1,contactin 1 isoform 1 precursor |
| 225579_at | 16.65 | 6.56E-09 | 1.186728044 | NM_152391| | C2orf22,hypothetical protein MGC33602 |
| 222271_at | 16.63 | 6.63E-09 | 1.540995084 | NA |  |
| 222590_s_at | 16.63 | 6.64E-09 | 1.164433935 | NM_016231| | NLK,nemo like kinase |
| 222909_s_at | 16.62 | 6.68E-09 | 1.159050045 | NM_004874| | BAG4,BCL2-associated athanogene 4 |
| 213927_at | 16.62 | 6.68E-09 | 1.974100367 | NM_033141| | MAP3K9,mitogen-activated protein kinase kinase kinase |
| 210754_s_at | 16.62 | 6.67E-09 | 1.375055232 | NM_001111097| | NA |
| 234085_at | 16.6 | 6.75E-09 | 1.654882688 | NA |  |
| 209962_at | 16.6 | 6.74E-09 | 1.360068123 | NM_000121| | EPOR,erythropoietin receptor precursor |
| 201502_s_at | 16.6 | 6.77E-09 | 1.18913753 | NM_020529| | NFKBIA,nuclear factor of kappa light polypeptide gene |
| 228810_at | 16.6 | 6.76E-09 | 1.174374259 | NM_152523| | FLJ40432,hypothetical protein FLJ40432 |
| 200978_at | 16.59 | 6.79E-09 | 1.068416577 | NM_005917| | MDH1,cytosolic malate dehydrogenase |
| 226129_at | 16.59 | 6.81E-09 | 1.938290947 | NM_198488| | FLJ46072,FLJ46072 protein |
| 227290_at | 16.59 | 6.80E-09 | 1.376091035 | NA |  |
| 220520_s_at | 16.58 | 6.83E-09 | 1.717008243 | NM_017681| | FLJ20130,hypothetical protein FLJ20130 |
| 244071_at | 16.57 | 6.87E-09 | 1.161456376 | NA |  |
| 212805_at | 16.57 | 6.87E-09 | 1.405649324 | NM_015225| | NA |
| 238751_at | 16.57 | 6.90E-09 | 2.077018083 | NA |  |
| 202310_s_at | 16.54 | 7.01E-09 | 2.062930958 | NM_000088| | COL1A1,alpha 1 type I collagen preproprotein |
| 223402_at | 16.53 | 7.05E-09 | 1.128660985 | NM_017823| | DUSP23,dual specificity phosphatase 23 |
| 230519_at | 16.53 | 7.06E-09 | 1.435569548 | NM_145019| | FLJ30707,hypothetical protein FLJ30707 |
| 205368_at | 16.53 | 7.06E-09 | 1.351627938 | NM_001031690| | NA |
| 213926_s_at | 16.52 | 7.09E-09 | 1.167773748 | NM_004504| | HRB,HIV-1 Rev binding protein |
| 229355_at | 16.51 | 7.15E-09 | 1.13950647 | NA |  |
| 207695_s_at | 16.5 | 7.19E-09 | 1.963297421 | NM_001555| | IGSF1,immunoglobulin superfamily, member 1 isoform 1 |
| 209357_at | 16.5 | 7.19E-09 | 1.207933005 | NM_006079| | CITED2,Cbp/p300-interacting transactivator, with |
| 222810_s_at | 16.5 | 7.23E-09 | 1.24041371 | NM_004841| | RASAL2,RAS protein activator like 2 isoform 1 |
| 205105_at | 16.5 | 7.20E-09 | 1.150916133 | NM_002372| | MAN2A1,mannosidase, alpha, class 2A, member 1 |
| 224049_at | 16.49 | 7.25E-09 | 1.284900073 | NM_031460| | KCNK17,potassium channel, subfamily K, member 17 |
| 213725_x_at | 16.49 | 7.27E-09 | 2.7354613 | NM_022166| | XYLT1,xylosyltransferase I |
| 235515_at | 16.48 | 7.27E-09 | 1.725961005 | NM_001039876| | NA |
| 213671_s_at | 16.48 | 7.31E-09 | 1.078683346 | NM_004990| | MARS,methionine-tRNA synthetase |
| 222482_at | 16.48 | 7.30E-09 | 1.168251154 | NM_001009955| | SSBP3,single stranded DNA binding protein 3 isoform c |
| 242515_x_at | 16.48 | 7.30E-09 | 1.076303808 | NM_020642| | C11orf17,chromosome 11 open reading frame 17 |
| 234665_x_at | 16.46 | 7.37E-09 | 1.216667177 | NM_001031693| | NA |
| 210381_s_at | 16.45 | 7.45E-09 | 1.554587019 | NM_176875| | CCKBR,cholecystokinin B receptor |
| 205505_at | 16.45 | 7.45E-09 | 2.053127126 | NM_001097633| | NA |
| 211700_s_at | 16.43 | 7.55E-09 | 1.140745755 | NM_001039705| | NA |
| 209904_at | 16.42 | 7.58E-09 | 1.529361049 | NM_003280| | TNNC1,troponin C, slow |
| 221584_s_at | 16.41 | 7.61E-09 | 1.451980362 | NM_001014797| | NA |
| 232780_s_at | 16.41 | 7.62E-09 | 1.123754681 | NM_015911| | LOC51058,hypothetical protein LOC51058 |
| 218943_s_at | 16.41 | 7.62E-09 | 1.599577368 | NM_014314| | DDX58,DEAD/H (Asp-Glu-Ala-Asp/His) box polypeptide |
| 223383_at | 16.39 | 7.73E-09 | 1.098976897 | NM_032268| | ZNRF1,zinc and ring finger protein 1 |
| 226312_at | 16.39 | 7.72E-09 | 1.13337074 | NM_152756| | AVO3,rapamycin-insensitive companion of mTOR |
| 201216_at | 16.39 | 7.73E-09 | 1.107978458 | NM_001034025| | NA |
| 207749_s_at | 16.38 | 7.75E-09 | 1.246416156 | NM_002718| | PPP2R3A,alpha isoform of regulatory subunit B'', protein |
| 200772_x_at | 16.38 | 7.79E-09 | 1.03019597 | NM_001099285| | NA |
| 212658_at | 16.38 | 7.78E-09 | 1.163075921 | NM_005779| | LHFPL2,lipoma HMGIC fusion partner-like 2 |
| 241897_at | 16.37 | 7.84E-09 | 1.345603675 | NA |  |
| 239302_s_at | 16.36 | 7.90E-09 | 1.442402147 | NA |  |
| 224702_at | 16.35 | 7.94E-09 | 1.173115523 | NM_174909| | MGC23909,hypothetical protein MGC23909 |
| 223160_s_at | 16.35 | 7.92E-09 | 1.096813254 | NM_032560| | KIAA2010,KIAA2010 isoform 1 |
| 201034_at | 16.35 | 7.94E-09 | 1.088811826 | NM_001121| | NA |
| 219793_at | 16.34 | 8.00E-09 | 1.538542176 | NM_022133| | SNX16,sorting nexin 16 isoform a |
| 227139_s_at | 16.34 | 7.97E-09 | 1.16201056 | NM_032383| | HPS3,Hermansky-Pudlak syndrome 3 protein |
| 231609_at | 16.33 | 8.01E-09 | 1.626202395 | NM_144661| | C10orf82,chromosome 10 open reading frame 82 |
| 219823_at | 16.33 | 8.01E-09 | 3.476757489 | NM_024674| | LIN28,lin-28 homolog |
| 220275_at | 16.32 | 8.10E-09 | 2.325150998 | NM_022034| | CUZD1,CUB and zona pellucida-like domains 1 |
| 230359_at | 16.32 | 8.09E-09 | 1.366964825 | NM_152643| | KNDC1,kinase non-catalytic C-lobe domain (KIND) |
| 228970_at | 16.31 | 8.12E-09 | 1.120558572 | NM_178547| | ARCH,archease |
| 203322_at | 16.31 | 8.14E-09 | 1.057812531 | NM_014913| | KIAA0863,KIAA0863 protein |
| 203266_s_at | 16.3 | 8.19E-09 | 1.064465141 | NM_003010| | MAP2K4,mitogen-activated protein kinase kinase 4 |
| 203120_at | 16.29 | 8.21E-09 | 1.038906527 | NM_001031685| | NA |
| 210657_s_at | 16.29 | 8.21E-09 | 1.419793802 | NM_004574| | SEPT4,septin 4 isoform 1 |
| 222395_s_at | 16.29 | 8.21E-09 | 1.092846328 | NM_023079| | FLJ13855,hypothetical protein FLJ13855 |
| 210315_at | 16.29 | 8.23E-09 | 1.284207949 | NM_003178| | SYN2,synapsin II isoform IIb |
| 210275_s_at | 16.28 | 8.24E-09 | 1.041853717 | NM_001102420| | NA |
| 228145_s_at | 16.28 | 8.28E-09 | 1.141881921 | NM_020781| | ZNF398,zinc finger 398 isoform b |
| 229080_at | 16.27 | 8.31E-09 | 1.546287701 | NM_133457| | EMID2,putative emu2 |
| 203635_at | 16.26 | 8.39E-09 | 1.06650687 | NM_006052| | DSCR3,Down syndrome critical region protein 3 |
| 206356_s_at | 16.25 | 8.44E-09 | 1.751150366 | NM_002071| | GNAL,guanine nucleotide binding protein (G protein), |
| 37012_at | 16.25 | 8.43E-09 | 1.095628652 | NM_004930| | CAPZB,F-actin capping protein beta subunit |
| 203434_s_at | 16.25 | 8.43E-09 | 2.060091087 | NM_000902| | MME,membrane metallo-endopeptidase |
| 211240_x_at | 16.24 | 8.49E-09 | 1.133780523 | NM_001085458| | NA |
| 212295_s_at | 16.24 | 8.50E-09 | 1.049146343 | NM_003045| | SLC7A1,solute carrier family 7 (cationic amino acid |
| 202719_s_at | 16.24 | 8.48E-09 | 1.29061529 | NM_015641| | TES,testin isoform 1 |
| 221900_at | 16.23 | 8.52E-09 | 1.30068993 | NM_005202| | COL8A2,collagen, type VIII, alpha 2 |
| 226541_at | 16.23 | 8.54E-09 | 1.098345362 | NM_032145| | FBXO30,F-box only protein 30 |
| 223318_s_at | 16.22 | 8.58E-09 | 1.132083825 | NM_032306| | SPATA11,spermatogenesis associated 11 |
| 204309_at | 16.22 | 8.57E-09 | 1.296493893 | NM_000781| | CYP11A1,cytochrome P450, subfamily XIA precursor |
| 224451_x_at | 16.22 | 8.56E-09 | 1.428480204 | NM_001080156| | NA |
| 238983_at | 16.21 | 8.61E-09 | 2.325498343 | NM_024677| | FLJ14001,hypothetical protein FLJ14001 |
| 222839_s_at | 16.2 | 8.70E-09 | 1.163816702 | NM_022894| | PAPOLG,poly(A) polymerase gamma |
| 218945_at | 16.2 | 8.67E-09 | 1.111658338 | NM_024109| | MGC2654,hypothetical protein MGC2654 |
| 222466_s_at | 16.19 | 8.75E-09 | 1.059483776 | NM_014050| | MRPL42,mitochondrial ribosomal protein L42 isoform a |
| 235216_at | 16.18 | 8.81E-09 | 1.237330415 | NM_052911| | ESCO1,establishment of cohesion 1 homolog 1 |
| 219360_s_at | 16.16 | 8.96E-09 | 1.443624667 | NM_017636| | TRPM4,transient receptor potential cation channel, |
| 202625_at | 16.15 | 8.97E-09 | 1.590923145 | NM_001111097| | NA |
| 201970_s_at | 16.14 | 9.06E-09 | 1.060110927 | NM_002482| | NASP,nuclear autoantigenic sperm protein isoform 2 |
| 223568_s_at | 16.14 | 9.08E-09 | 1.08881031 | NM_001102559| | NA |
| 228430_at | 16.14 | 9.02E-09 | 1.103718952 | NA |  |
| 202481_at | 16.13 | 9.09E-09 | 1.775806006 | NM_004753| | DHRS3,dehydrogenase/reductase (SDR family) member 3 |
| 221555_x_at | 16.13 | 9.11E-09 | 1.154868348 | NM_001077181| | NA |
| 212744_at | 16.12 | 9.16E-09 | 1.132260945 | NM_033028| | BBS4,Bardet-Biedl syndrome 4 |
| 201549_x_at | 16.11 | 9.25E-09 | 1.17375014 | NM_006618| | JARID1B,Jumonji, AT rich interactive domain 1B |
| 240681_at | 16.11 | 9.26E-09 | 3.192365608 | NA |  |
| 47550_at | 16.1 | 9.27E-09 | 1.715733105 | NM_021020| | LZTS1,leucine zipper, putative tumor suppressor 1 |
| 201981_at | 16.1 | 9.30E-09 | 1.235848719 | NM_002581| | PAPPA,pregnancy-associated plasma protein A |
| 215758_x_at | 16.08 | 9.40E-09 | 1.27155556 | NM_031218| | ZNF505,zinc finger protein 505 isoform a |
| 236826_at | 16.08 | 9.39E-09 | 1.317879054 | NM_152574| | C9orf52,hypothetical protein FLJ33868 |
| 223705_s_at | 16.08 | 9.40E-09 | 1.076011483 | NM_001127235| | NA |
| 204759_at | 16.08 | 9.40E-09 | 1.249683957 | NM_001268| | CHC1L,RCC1-like G exchanging factor RLG |
| 223737_x_at | 16.08 | 9.42E-09 | 2.129027292 | NM_031422| | CHST9,GalNAc-4-sulfotransferase 2 |
| 1560853_x_at | 16.08 | 9.44E-09 | 1.896300628 | NM_001039884| | NA |
| 1569039_s_at | 16.07 | 9.44E-09 | 1.546383433 | NM_182609| | MGC48625,hypothetical protein MGC48625 |
| 1553991_s_at | 16.07 | 9.46E-09 | 1.251532258 | NM_019086| | FLJ20674,hypothetical protein FLJ20674 |
| 220372_at | 16.05 | 9.59E-09 | 1.421468786 | NM_001040192| | NA |
| 206456_at | 16.04 | 9.63E-09 | 1.68321562 | NM_000810| | GABRA5,gamma-aminobutyric acid (GABA) A receptor, alpha |
| 228799_at | 16.04 | 9.67E-09 | 1.164601889 | NA |  |
| 226397_s_at | 16.03 | 9.69E-09 | 1.767613958 | NA |  |
| 228407_at | 16.03 | 9.69E-09 | 1.280884783 | NM_152753| | SCUBE3,signal peptide, CUB domain, EGF-like 3 |
| 227468_at | 16.02 | 9.78E-09 | 1.275738903 | NM_152359| | CPT1C,carnitine palmitoyltransferase 1C |
| 214339_s_at | 16.02 | 9.79E-09 | 1.608735737 | NM_001042600| | NA |
| 202053_s_at | 16.01 | 9.85E-09 | 1.104513479 | NM_000382| | ALDH3A2,aldehyde dehydrogenase 3A2 |
| 223625_at | 16 | 9.89E-09 | 1.199087318 | NM_032581| | DRCTNNB1A,down-regulated by Ctnnb1, a |
| 219300_s_at | 16 | 9.94E-09 | 2.284795441 | NM_014141| | CNTNAP2,cell recognition molecule Caspr2 precursor |
| 215084_s_at | 16 | 9.91E-09 | 1.154194227 | NM_052940| | MGC8974,hypothetical protein MGC8974 |
| 1555579_s_at | 15.99 | 9.99E-09 | 1.587929526 | NM_001105244| | NA |
| 242923_at | 15.99 | 9.96E-09 | 1.135513132 | NM_178549| | MGC42493,hypothetical protein MGC42493 |
| 218092_s_at | 15.98 | 1.00E-08 | 1.156833626 | NM_004504| | HRB,HIV-1 Rev binding protein |
| 205022_s_at | 15.98 | 1.01E-08 | 1.151248076 | NM_001085471| | NA |
| 216563_at | 15.98 | 1.01E-08 | 1.116846528 | NM_001083625| | NA |
| 218906_x_at | 15.97 | 1.01E-08 | 1.064201483 | NM_022822| | KLC2,likely ortholog of kinesin light chain 2 |
| 224895_at | 15.97 | 1.01E-08 | 1.142442942 | NM_006106| | YAP1,Yes-associated protein 1, 65 kD |
| 227262_at | 15.97 | 1.01E-08 | 1.196819255 | NM_178232| | HAPLN3,hyaluronan and proteoglycan link protein 3 |
| 1552754_a_at | 15.97 | 1.01E-08 | 1.696776869 | NM_153184| | IGSF4D,immunoglobulin superfamily, member 4D |
| 222459_at | 15.96 | 1.02E-08 | 1.191671921 | NM_024595| | FLJ12666,hypothetical protein FLJ12666 |
| 204359_at | 15.95 | 1.02E-08 | 1.922583253 | NM_013231| | FLRT2,fibronectin leucine rich transmembrane protein |
| 1563022_at | 15.95 | 1.02E-08 | 1.692774524 | NM_001101357| | NA |
| 1555845_at | 15.95 | 1.02E-08 | 1.191205683 | NA |  |
| 205083_at | 15.95 | 1.02E-08 | 1.605902955 | NM_001159| | AOX1,aldehyde oxidase 1 |
| 210758_at | 15.94 | 1.03E-08 | 1.137642897 | NM_021144| | PSIP1,PC4 and SFRS1 interacting protein 1 |
| 203058_s_at | 15.93 | 1.04E-08 | 1.634733042 | NM_001015880| | NA |
| 235165_at | 15.93 | 1.03E-08 | 1.492860693 | NM_032521| | NA |
| 213187_x_at | 15.93 | 1.04E-08 | 1.051055821 | NM_000146| | FTL,ferritin, light polypeptide |
| 226330_s_at | 15.92 | 1.04E-08 | 1.066023332 | NM_001014286| | NA |
| 238752_at | 15.89 | 1.07E-08 | 1.263558596 | NA |  |
| 228782_at | 15.89 | 1.07E-08 | 1.662684135 | NM_054023| | SCGB3A2,secretoglobin, family 3A, member 2 |
| 221217_s_at | 15.89 | 1.06E-08 | 1.393309209 | NM_018723| | A2BP1,ataxin 2-binding protein 1 isoform 4 |
| 214890_s_at | 15.88 | 1.07E-08 | 1.146485909 | NM_001006655| | DKFZP564J102,DKFZP564J102 protein |
| 203476_at | 15.88 | 1.07E-08 | 1.21121725 | NM_006670| | TPBG,5T4 oncofetal trophoblast glycoprotein |
| 218535_s_at | 15.88 | 1.07E-08 | 1.076815744 | NM_018343| | RIOK2,RIO kinase 2 |
| 1555355_a_at | 15.87 | 1.08E-08 | 1.500656429 | NM_005238| | ETS1,v-ets erythroblastosis virus E26 oncogene |
| 225809_at | 15.87 | 1.08E-08 | 1.667699153 | NM_015393| | DKFZP564O0823,DKFZP564O0823 protein |
| 213849_s_at | 15.87 | 1.08E-08 | 2.012128606 | NM_004576| | PPP2R2B,beta isoform of regulatory subunit B55, protein |
| 221556_at | 15.86 | 1.09E-08 | 1.16268241 | NM_001077181| | NA |
| 243439_at | 15.85 | 1.09E-08 | 1.235407321 | NM_133460| | ZNF418,zinc finger protein 418 |
| 229039_at | 15.85 | 1.09E-08 | 1.262649079 | NM_003178| | SYN2,synapsin II isoform IIb |
| 202363_at | 15.85 | 1.09E-08 | 1.441458064 | NM_004598| | SPOCK,sparc/osteonectin, cwcv and kazal-like domains |
| 228771_at | 15.84 | 1.10E-08 | 1.852495518 | NM_005160| | ADRBK2,beta adrenergic receptor kinase 2 |
| 218379_at | 15.83 | 1.10E-08 | 1.168715315 | NM_016090| | RBM7,RNA binding motif protein 7 |
| 214697_s_at | 15.82 | 1.11E-08 | 1.238417929 | NM_005156| | ROD1,ROD1 regulator of differentiation 1 |
| 212464_s_at | 15.82 | 1.11E-08 | 1.741589503 | NM_002026| | FN1,fibronectin 1 isoform 3 preproprotein |
| 219420_s_at | 15.8 | 1.13E-08 | 1.084957035 | NM_023077| | FLJ12439,hypothetical protein FLJ12439 |
| 227172_at | 15.8 | 1.13E-08 | 1.152219226 | NM_138341| | LOC89894,hypothetical protein BC000282 |
| 219697_at | 15.79 | 1.14E-08 | 1.404681919 | NM_006043| | HS3ST2,heparan sulfate D-glucosaminyl |
| 227220_at | 15.79 | 1.14E-08 | 1.205713826 | NM_152995| | HOZFP,ovarian zinc finger protein |
| 216442_x_at | 15.78 | 1.15E-08 | 1.693390687 | NM_002026| | FN1,fibronectin 1 isoform 3 preproprotein |
| 200976_s_at | 15.78 | 1.14E-08 | 1.075994278 | NM_001079864| | NA |
| 227530_at | 15.77 | 1.16E-08 | 1.179331907 | NM_005100| | AKAP12,A-kinase anchor protein 12 isoform 1 |
| 201798_s_at | 15.76 | 1.17E-08 | 1.843848789 | NM_013451| | FER1L3,myoferlin isoform a |
| 202724_s_at | 15.76 | 1.17E-08 | 1.21026025 | NM_002015| | FOXO1A,forkhead box O1A |
| 236454_at | 15.76 | 1.16E-08 | 1.118060081 | NM_194439| | LOC285498,hypothetical protein LOC285498 |
| 211267_at | 15.76 | 1.17E-08 | 1.51854859 | NM_003865| | HESX1,homeo box (expressed in ES cells) 1 |
| 206091_at | 15.75 | 1.17E-08 | 1.830679154 | NM_002381| | MATN3,matrilin 3 precursor |
| 220195_at | 15.75 | 1.17E-08 | 1.159031346 | NM_018328| | MBD5,methyl-CpG binding domain protein 5 |
| 202919_at | 15.73 | 1.19E-08 | 1.091133008 | NM_001100819| | NA |
| 41512_at | 15.73 | 1.19E-08 | 1.073460574 | NA |  |
| 229264_at | 15.73 | 1.19E-08 | 1.473477292 | NA |  |
| 228750_at | 15.72 | 1.19E-08 | 1.637977463 | NA |  |
| 219685_at | 15.72 | 1.20E-08 | 1.3183029 | NM_021637| | TMEM35,transmembrane protein 35 |
| 225959_s_at | 15.72 | 1.20E-08 | 1.05575345 | NM_032268| | ZNRF1,zinc and ring finger protein 1 |
| 212318_at | 15.71 | 1.20E-08 | 1.083896778 | NM_012470| | TNPO3,transportin 3 |
| 219033_at | 15.71 | 1.20E-08 | 2.021645448 | NM_024615| | PARP8,poly (ADP-ribose) polymerase family, member 8 |
| 221648_s_at | 15.7 | 1.21E-08 | 1.571587521 | NA |  |
| 227004_at | 15.7 | 1.21E-08 | 2.215491384 | NA |  |
| 218615_s_at | 15.7 | 1.21E-08 | 1.201580782 | NM_018266| | TMEM39A,transmembrane protein 39A |
| 203488_at | 15.7 | 1.21E-08 | 1.101757366 | NM_001008701| | LPHN1,latrophilin 1 isoform 1 precursor |
| 212864_at | 15.69 | 1.22E-08 | 1.221294494 | NM_003818| | CDS2,phosphatidate cytidylyltransferase 2 |
| 222674_at | 15.69 | 1.21E-08 | 1.071440965 | NM_016390| | C9orf114,chromosome 9 open reading frame 114 |
| 204114_at | 15.68 | 1.22E-08 | 2.333932754 | NM_007361| | NID2,nidogen 2 |
| 220334_at | 15.68 | 1.23E-08 | 1.142662673 | NM_012419| | RGS17,regulator of G-protein signalling 17 |
| 224940_s_at | 15.68 | 1.23E-08 | 1.648262742 | NM_002581| | PAPPA,pregnancy-associated plasma protein A |
| 201110_s_at | 15.67 | 1.24E-08 | 2.613386646 | NM_003246| | THBS1,thrombospondin 1 precursor |
| 204068_at | 15.67 | 1.23E-08 | 1.12578337 | NM_006281| | STK3,serine/threonine kinase 3 (STE20 homolog, |
| 227271_at | 15.67 | 1.23E-08 | 1.232117 | NM_004112| | FGF11,fibroblast growth factor 11 |
| 232306_at | 15.66 | 1.24E-08 | 1.372504955 | NM_021810| | CDH26,cadherin-like 26 isoform b |
| 202842_s_at | 15.66 | 1.24E-08 | 1.067645421 | NM_012328| | DNAJB9,DnaJ (Hsp40) homolog, subfamily B, member 9 |
| 206044_s_at | 15.66 | 1.24E-08 | 1.394647097 | NM_004333| | BRAF,v-raf murine sarcoma viral oncogene homolog B1 |
| 219578_s_at | 15.64 | 1.26E-08 | 1.33157061 | NM_001079533| | NA |
| 239007_at | 15.64 | 1.26E-08 | 1.268037743 | NM_178523| | ZNF616,zinc finger protein 616 |
| 1555137_a_at | 15.64 | 1.26E-08 | 1.803657552 | NM_018351| | FGD6,FYVE, RhoGEF and PH domain containing 6 |
| 201924_at | 15.64 | 1.26E-08 | 1.215838315 | NM_005935| | MLLT2,myeloid/lymphoid or mixed-lineage leukemia |
| 219123_at | 15.64 | 1.26E-08 | 1.165470076 | NM_014519| | ZNF232,zinc finger protein 232 |
| 210038_at | 15.63 | 1.27E-08 | 1.503066331 | NM_006257| | PRKCQ,protein kinase C, theta |
| 1557014_a_at | 15.63 | 1.27E-08 | 1.686946514 | NA |  |
| 210058_at | 15.63 | 1.27E-08 | 1.365159015 | NM_002754| | MAPK13,mitogen-activated protein kinase 13 |
| 210427_x_at | 15.63 | 1.27E-08 | 1.111862385 | NM_001002857| | ANXA2,annexin A2 isoform 2 |
| 205850_s_at | 15.63 | 1.27E-08 | 2.321509179 | NM_000814| | GABRB3,gamma-aminobutyric acid (GABA) A receptor, beta |
| 202637_s_at | 15.63 | 1.27E-08 | 1.219918554 | NM_000201| | ICAM1,intercellular adhesion molecule 1 precursor |
| 210880_s_at | 15.62 | 1.27E-08 | 1.315130445 | NM_005864| | EFS,embryonal Fyn-associated substrate isoform 1 |
| 217478_s_at | 15.61 | 1.28E-08 | 1.741267346 | NM_006120| | HLA-DMA,major histocompatibility complex, class II, DM |
| 203872_at | 15.6 | 1.29E-08 | 2.120261036 | NM_001100| | ACTA1,alpha 1 actin precursor |
| 231470_at | 15.6 | 1.29E-08 | 2.22690039 | NA |  |
| 202610_s_at | 15.6 | 1.29E-08 | 1.125786291 | NM_004229| | CRSP2,cofactor required for Sp1 transcriptional |
| 222690_s_at | 15.6 | 1.29E-08 | 1.183160564 | NM_018266| | TMEM39A,transmembrane protein 39A |
| 227088_at | 15.59 | 1.30E-08 | 2.436237702 | NM_001083| | PDE5A,phosphodiesterase 5A isoform 1 |
| 222636_at | 15.59 | 1.30E-08 | 1.246464407 | NM_025205| | MED28,mediator of RNA polymerase II transcription, |
| 221522_at | 15.58 | 1.30E-08 | 1.085773788 | NM_032139| | ANKRD27,ankyrin repeat domain 27 (VPS9 domain) |
| 1558212_at | 15.58 | 1.30E-08 | 1.680753683 | NA |  |
| 214597_at | 15.57 | 1.32E-08 | 1.580415864 | NM_001050| | SSTR2,somatostatin receptor 2 |
| 213201_s_at | 15.56 | 1.32E-08 | 2.1095811 | NM_001126132| | NA |
| 218622_at | 15.56 | 1.33E-08 | 1.043524561 | NM_024057| | NUP37,nucleoporin 37kDa |
| 228151_at | 15.54 | 1.34E-08 | 1.096731094 | NA |  |
| 229559_at | 15.54 | 1.34E-08 | 1.545968887 | NM_001080401| | NA |
| 1557369_a_at | 15.54 | 1.34E-08 | 1.879427382 | NA |  |
| 204391_x_at | 15.54 | 1.35E-08 | 1.182710823 | NM_003852| | TIF1,transcriptional intermediary factor 1 alpha |
| 212465_at | 15.54 | 1.34E-08 | 1.0934163 | NM_032233| | C14orf154,chromosome 14 open reading frame 154 isoform a |
| 214761_at | 15.53 | 1.35E-08 | 1.189284343 | NM_015069| | ZNF423,zinc finger protein 423 |
| 219004_s_at | 15.52 | 1.36E-08 | 1.063798474 | NM_018944| | C21orf45,chromosome 21 open reading frame 45 |
| 204043_at | 15.52 | 1.36E-08 | 1.346700389 | NM_000355| | TCN2,transcobalamin II precursor |
| 219185_at | 15.52 | 1.35E-08 | 1.107912532 | NM_012241| | SIRT5,sirtuin 5 isoform 1 |
| 206460_at | 15.52 | 1.35E-08 | 1.86137533 | NM_001042478| | NA |
| 218228_s_at | 15.51 | 1.37E-08 | 1.084086769 | NM_025235| | TNKS2,tankyrase, TRF1-interacting ankyrin-related |
| 228859_at | 15.51 | 1.37E-08 | 1.158191173 | NM_001099776| | NA |
| 244264_at | 15.51 | 1.37E-08 | 1.623817698 | NM_198508| | FLJ44186,FLJ44186 protein |
| 213349_at | 15.49 | 1.38E-08 | 1.126754121 | NM_001017395| | NA |
| 219335_at | 15.49 | 1.38E-08 | 1.129481139 | NM_022838| | ARMCX5,armadillo repeat containing, X-linked 5 |
| 240293_at | 15.48 | 1.40E-08 | 1.324479679 | NM_001033658| | NA |
| 225694_at | 15.47 | 1.41E-08 | 1.047932499 | NM_015083| | NA |
| 209925_at | 15.47 | 1.41E-08 | 1.894128892 | NM_002538| | OCLN,occludin |
| 219928_s_at | 15.47 | 1.41E-08 | 1.157647614 | NM_012189| | CABYR,calcium-binding tyrosine |
| 227209_at | 15.46 | 1.42E-08 | 2.178103007 | NM_001843| | CNTN1,contactin 1 isoform 1 precursor |
| 205037_at | 15.45 | 1.42E-08 | 1.179567663 | NM_006860| | RABL4,RAB, member of RAS oncogene family-like 4 |
| 203588_s_at | 15.44 | 1.43E-08 | 1.181065265 | NM_006286| | TFDP2,transcription factor Dp-2 (E2F dimerization |
| 228944_at | 15.43 | 1.45E-08 | 1.221293661 | NA |  |
| 213982_s_at | 15.43 | 1.44E-08 | 1.876818316 | NM_001035230| | NA |
| 231530_s_at | 15.43 | 1.45E-08 | 1.144252617 | NM_022761| | C11orf1,hypothetical protein FLJ23499 |
| 205789_at | 15.43 | 1.44E-08 | 1.925370189 | NM_001766| | CD1D,CD1D antigen, d polypeptide |
| 240152_at | 15.42 | 1.46E-08 | 1.368253797 | NA |  |
| 230441_at | 15.41 | 1.47E-08 | 1.350416245 | NM_052909| | KIAA1909,KIAA1909 protein |
| 217234_s_at | 15.41 | 1.47E-08 | 1.211808054 | NM_001111077| | NA |
| 227529_s_at | 15.4 | 1.47E-08 | 1.231281203 | NM_005100| | AKAP12,A-kinase anchor protein 12 isoform 1 |
| 201331_s_at | 15.39 | 1.49E-08 | 1.510200887 | NM_003153| | STAT6,signal transducer and activator of transcription |
| 223665_at | 15.39 | 1.48E-08 | 1.6530211 | NM_032487| | ARPM1,actin related protein M1 |
| 217574_at | 15.38 | 1.49E-08 | 1.773354044 | NM_001796| | CDH8,cadherin 8, type 2 preproprotein |
| 32502_at | 15.38 | 1.49E-08 | 1.073995329 | NM_030792| | PP1665,hypothetical protein PP1665 |
| 229782_at | 15.37 | 1.51E-08 | 1.246006083 | NA |  |
| 209615_s_at | 15.37 | 1.51E-08 | 2.073599215 | NM_002576| | PAK1,p21-activated kinase 1 |
| 213668_s_at | 15.37 | 1.51E-08 | 1.129249216 | NM_003107| | SOX4,SRY (sex determining region Y)-box 4 |
| 213186_at | 15.35 | 1.53E-08 | 1.145705005 | NM_014648| | DZIP3,zinc finger DAZ interacting protein 3 |
| 212483_at | 15.35 | 1.53E-08 | 1.057422286 | NM_015384| | NIPBL,delangin isoform B |
| 223235_s_at | 15.34 | 1.54E-08 | 2.240182357 | NM_022138| | SMOC2,secreted modular calcium-binding protein 2 |
| 211538_s_at | 15.33 | 1.55E-08 | 1.431013482 | NM_021979| | HSPA2,heat shock 70kDa protein 2 |
| 225816_at | 15.32 | 1.56E-08 | 1.196394618 | NM_024900| | PHF17,Jade1 protein short isoform |
| 205122_at | 15.32 | 1.56E-08 | 1.308741864 | NM_003692| | TMEFF1,transmembrane protein with EGF-like and two |
| 228337_at | 15.31 | 1.57E-08 | 1.197781152 | NM_052927| | NA |
| 227651_at | 15.31 | 1.58E-08 | 1.134408407 | NM_052876| | BTBD14B,transcriptional repressor NAC1 |
| 230900_at | 15.31 | 1.57E-08 | 1.60791714 | NM_152775| | KM-HN-1,KM-HN-1 protein |
| 233543_s_at | 15.3 | 1.59E-08 | 1.127800562 | NM_139076| | FLJ13614,hypothetical protein FLJ13614 |
| 207076_s_at | 15.3 | 1.59E-08 | 1.240861846 | NM_000050| | ASS,argininosuccinate synthetase |
| 209580_s_at | 15.3 | 1.58E-08 | 1.084875843 | NM_003925| | MBD4,methyl-CpG binding domain protein 4 |
| 230466_s_at | 15.3 | 1.58E-08 | 1.270702435 | NA |  |
| 38269_at | 15.29 | 1.60E-08 | 1.111662644 | NM_001079880| | NA |
| 208847_s_at | 15.29 | 1.60E-08 | 1.066238564 | NM_000671| | ADH5,class III alcohol dehydrogenase 5 chi subunit |
| 204715_at | 15.27 | 1.62E-08 | 1.144201865 | NM_015368| | PANX1,pannexin 1 |
| 220011_at | 15.27 | 1.62E-08 | 1.142434206 | NM_024037| | MGC2603,hypothetical protein MGC2603 |
| 226562_at | 15.27 | 1.62E-08 | 1.062960616 | NM_152455| | FLJ35867,hypothetical protein FLJ35867 |
| 205485_at | 15.27 | 1.62E-08 | 1.31151901 | NM_000540| | RYR1,ryanodine receptor 1 (skeletal) |
| 201854_s_at | 15.26 | 1.63E-08 | 1.082436963 | NM_015251| | KIAA0431,KIAA0431 protein |
| 206552_s_at | 15.26 | 1.63E-08 | 2.599643472 | NM_003182| | TAC1,tachykinin 1 isoform beta precursor |
| 230423_at | 15.25 | 1.64E-08 | 1.992335158 | NA |  |
| 205474_at | 15.25 | 1.64E-08 | 1.095494676 | NM_015986| | CRLF3,cytokine receptor-like factor 3 |
| 205132_at | 15.25 | 1.64E-08 | 1.68101044 | NM_005159| | ACTC,cardiac muscle alpha actin proprotein |
| 216945_x_at | 15.24 | 1.65E-08 | 1.077362527 | NM_015148| | PASK,PAS domain containing serine/threonine kinase |
| 217972_at | 15.23 | 1.66E-08 | 1.040412307 | NM_017812| | CHCHD3,coiled-coil-helix-coiled-coil-helix domain |
| 206723_s_at | 15.23 | 1.66E-08 | 1.47921232 | NM_004720| | EDG4,endothelial differentiation, lysophosphatidic |
| 229854_at | 15.22 | 1.68E-08 | 1.312638857 | NM_001098623| | NA |
| 223794_at | 15.21 | 1.69E-08 | 1.295897121 | NM_018076| | ARMC4,armadillo repeat containing 4 |
| 230588_s_at | 15.21 | 1.69E-08 | 1.114157585 | NA |  |
| 219196_at | 15.21 | 1.69E-08 | 2.370283807 | NM_013243| | SCG3,secretogranin III |
| 212749_s_at | 15.21 | 1.69E-08 | 1.075018801 | NM_001008925| | RCHY1,ring finger and CHY zinc finger domain |
| 224467_s_at | 15.2 | 1.69E-08 | 1.105291923 | NM_032346| | MGC13096,hypothetical protein MGC13096 |
| 204905_s_at | 15.2 | 1.69E-08 | 1.062033712 | NM_004280| | EEF1E1,eukaryotic translation elongation factor 1 |
| 244650_at | 15.2 | 1.70E-08 | 1.219131103 | NA |  |
| 231698_at | 15.2 | 1.70E-08 | 2.18076834 | NA |  |
| 224845_s_at | 15.19 | 1.72E-08 | 1.136669608 | NM_020846| | NA |
| 219114_at | 15.18 | 1.72E-08 | 1.151535691 | NM_016210| | C3orf18,chromosome 3 open reading frame 18 |
| 64486_at | 15.18 | 1.72E-08 | 1.104804837 | NM_001018070| | NA |
| 227190_at | 15.18 | 1.72E-08 | 1.523550416 | NM_183240| | TMEM37,transmembrane protein 37 |
| 225010_at | 15.17 | 1.73E-08 | 1.059679194 | NM_005436| | CCDC6,coiled-coil domain containing 6 |
| 221613_s_at | 15.17 | 1.74E-08 | 1.11131717 | NM_019006| | ZA20D3,zinc finger, A20 domain containing 3 |
| 202193_at | 15.17 | 1.73E-08 | 1.189558274 | NM_001031801| | NA |
| 227569_at | 15.17 | 1.73E-08 | 1.220391856 | NM_153371| | LNX2,PDZ domain containing ring finger 1 |
| 227399_at | 15.17 | 1.73E-08 | 2.141052276 | NM_016206| | VGL-3,colon carcinoma related protein |
| 222730_s_at | 15.17 | 1.73E-08 | 1.086743921 | NM_016353| | ZDHHC2,rec |
| 218517_at | 15.16 | 1.75E-08 | 1.195567306 | NM_024900| | PHF17,Jade1 protein short isoform |
| 1553252_a_at | 15.15 | 1.76E-08 | 1.176269871 | NM_153252| | BRWD3,bromo domain-containing protein disrupted in |
| 218683_at | 15.15 | 1.75E-08 | 1.204189218 | NM_021190| | PTBP2,polypyrimidine tract binding protein 2 |
| 208310_s_at | 15.14 | 1.76E-08 | 1.088998254 | NM_015622| | C7orf28A,chromosome 7 open reading frame 28A |
| 223784_at | 15.13 | 1.78E-08 | 1.493355997 | NM_020665| | TMEM27,transmembrane protein 27 |
| 204354_at | 15.13 | 1.78E-08 | 1.098910873 | NM_001042594| | NA |
| 225234_at | 15.12 | 1.79E-08 | 1.106121583 | NM_005188| | CBL,Cas-Br-M (murine) ecotropic retroviral |
| 212873_at | 15.12 | 1.79E-08 | 1.511126149 | NM_012292| | HA-1,minor histocompatibility antigen HA-1 |
| 230166_at | 15.12 | 1.80E-08 | 1.143133408 | NM_133465| | KIAA1958,KIAA1958 |
| 202923_s_at | 15.11 | 1.81E-08 | 1.12000733 | NM_001498| | GCLC,glutamate-cysteine ligase, catalytic subunit |
| 1560537_at | 15.1 | 1.82E-08 | 1.341259961 | NA |  |
| 205053_at | 15.1 | 1.82E-08 | 1.106087702 | NM_000946| | PRIM1,DNA primase small subunit, 49kDa |
| 209426_s_at | 15.09 | 1.83E-08 | 1.191554012 | NM_014324| | AMACR,alpha-methylacyl-CoA racemase isoform 1 |
| 202891_at | 15.09 | 1.83E-08 | 1.100852565 | NM_005600| | NIT1,nitrilase 1 |
| 204423_at | 15.08 | 1.84E-08 | 1.205719542 | NM_013255| | MKLN1,muskelin 1, intracellular mediator containing |
| 224792_at | 15.08 | 1.84E-08 | 1.193194554 | NM_033396| | TNKS1BP1,tankyrase 1-binding protein of 182 kDa |
| 37802_r_at | 15.08 | 1.85E-08 | 1.124910353 | NM_001040450| | NA |
| 226553_at | 15.07 | 1.86E-08 | 1.262921174 | NM_005656| | TMPRSS2,transmembrane protease, serine 2 |
| 201706_s_at | 15.07 | 1.86E-08 | 1.092812938 | NM_002857| | PEX19,peroxisomal biogenesis factor 19 |
| 223557_s_at | 15.06 | 1.87E-08 | 1.538326688 | NM_016192| | TMEFF2,transmembrane protein with EGF-like and two |
| 203166_at | 15.06 | 1.87E-08 | 1.071147774 | NM_006324| | CFDP1,craniofacial development protein 1 |
| 202085_at | 15.06 | 1.87E-08 | 1.148245715 | NM_004817| | TJP2,tight junction protein 2 (zona occludens 2) |
| 213061_s_at | 15.05 | 1.88E-08 | 1.065891746 | NM_173474| | NTAN1,N-terminal Asn amidase |
| 211950_at | 15.05 | 1.88E-08 | 1.080947801 | NM_020765| | RBAF600,retinoblastoma-associated factor 600 |
| 202517_at | 15.04 | 1.90E-08 | 1.137133018 | NM_001014809| | NA |
| 236385_at | 15.04 | 1.90E-08 | 1.323809675 | NA |  |
| 202697_at | 15.04 | 1.90E-08 | 1.201607746 | NM_007006| | CPSF5,cleavage and polyadenylation specific factor 5 |
| 203786_s_at | 15.04 | 1.90E-08 | 1.443456453 | NM_001003395| | TPD52L1,tumor protein D52-like 1 isoform 2 |
| 204948_s_at | 15.04 | 1.90E-08 | 1.60977257 | NM_006350| | FST,follistatin isoform FST317 precursor |
| 210389_x_at | 15.03 | 1.90E-08 | 1.083408661 | NM_016261| | TUBD1,delta-tubulin |
| 205011_at | 15.03 | 1.91E-08 | 1.135665987 | NM_014622| | LOH11CR2A,BCSC-1 isoform 1 |
| 210587_at | 15.02 | 1.93E-08 | 2.422564954 | NM_031479| | INHBE,activin beta E |
| 206729_at | 15.01 | 1.93E-08 | 1.584981898 | NM_001243| | TNFRSF8,tumor necrosis factor receptor superfamily, |
| 227496_at | 15.01 | 1.94E-08 | 1.819787392 | NM_001489| | NR6A1,nuclear receptor subfamily 6, group A, member 1 |
| 227444_at | 15 | 1.95E-08 | 1.176207687 | NM_152583| | ARMCX4,armadillo repeat containing, X-linked 4 |
| 228082_at | 14.99 | 1.97E-08 | 1.658056303 | NM_024769| | ASAM,adipocyte-specific adhesion molecule |
| 204453_at | 14.97 | 1.99E-08 | 1.109547735 | NM_003428| | ZNF84,zinc finger protein 84 (HPF2) |
| 232265_at | 14.96 | 2.00E-08 | 1.767123681 | NM_020725| | NA |
| 209582_s_at | 14.96 | 2.01E-08 | 1.130717635 | NM_001004196| | CD200,CD200 antigen isoform b |
| 203638_s_at | 14.96 | 2.01E-08 | 1.249616872 | NM_000141| | FGFR2,fibroblast growth factor receptor 2 isoform 1 |
| 218951_s_at | 14.95 | 2.02E-08 | 1.199446191 | NM_018390| | PLCXD1,phosphatidylinositol-specific phospholipase C, X |
| 219786_at | 14.95 | 2.02E-08 | 1.875952346 | NM_001039656| | NA |
| 229687_s_at | 14.95 | 2.02E-08 | 1.193547209 | NA |  |
| 210123_s_at | 14.94 | 2.03E-08 | 1.28558776 | NM_000746| | CHRNA7,cholinergic receptor, nicotinic, alpha |
| 213280_at | 14.94 | 2.02E-08 | 1.852622037 | NM_001100398| | NA |
| 204712_at | 14.94 | 2.03E-08 | 2.515145578 | NM_007191| | WIF1,Wnt inhibitory factor-1 precursor |
| 228266_s_at | 14.93 | 2.04E-08 | 1.083927192 | NM_016073| | HDGFRP3,hepatoma-derived growth factor, related protein |
| 228596_at | 14.93 | 2.05E-08 | 1.967377231 | NA |  |
| 205060_at | 14.92 | 2.06E-08 | 1.071605623 | NM_003631| | PARG,poly (ADP-ribose) glycohydrolase |
| 201637_s_at | 14.92 | 2.06E-08 | 1.080708437 | NM_001013438| | NA |
| 229234_at | 14.9 | 2.10E-08 | 1.372693747 | NM_001010888| | CXorf32,chromosome X open reading frame 32 |
| 226099_at | 14.88 | 2.12E-08 | 1.750317578 | NM_012081| | ELL2,elongation factor, RNA polymerase II, 2 |
| 226513_at | 14.87 | 2.13E-08 | 1.088561577 | NA |  |
| 231233_at | 14.87 | 2.14E-08 | 1.567176662 | NA |  |
| 210582_s_at | 14.86 | 2.15E-08 | 1.141409591 | NM_001031801| | NA |
| 242517_at | 14.86 | 2.14E-08 | 2.379393075 | NM_032551| | GPR54,G protein-coupled receptor 54 |
| 201348_at | 14.86 | 2.15E-08 | 1.316983348 | NM_002084| | GPX3,plasma glutathione peroxidase 3 precursor |
| 211656_x_at | 14.86 | 2.15E-08 | 1.19638978 | NM_002123| | HLA-DQB1,major histocompatibility complex, class II, DQ |
| 244758_at | 14.84 | 2.19E-08 | 1.563584992 | NM_052923| | ZNF452,zinc finger protein 452 |
| 222900_at | 14.84 | 2.19E-08 | 1.29091763 | NA |  |
| 231381_at | 14.84 | 2.18E-08 | 3.618519565 | NA |  |
| 236901_at | 14.84 | 2.19E-08 | 1.517164399 | NA |  |
| 212591_at | 14.83 | 2.20E-08 | 1.044215261 | NM_015014| | KIAA0117,KIAA0117 protein |
| 205414_s_at | 14.82 | 2.22E-08 | 1.663024566 | NM_014859| | KIAA0672,KIAA0672 gene product |
| 210775_x_at | 14.82 | 2.22E-08 | 1.167679835 | NM_001229| | CASP9,caspase 9 isoform alpha preproprotein |
| 238819_at | 14.82 | 2.22E-08 | 1.27637268 | NM_032584| | ZNF347,zinc finger protein 347 |
| 225784_s_at | 14.82 | 2.22E-08 | 1.189929429 | NM_018684| | KIAA1166,KIAA1166 |
| 217990_at | 14.81 | 2.23E-08 | 1.102645947 | NM_001002000| | GMPR2,guanosine monophosphate reductase 2 isoform 2 |
| 225183_at | 14.81 | 2.24E-08 | 1.117842325 | NM_014117| | PRO0149,PRO0149 protein |
| 218967_s_at | 14.81 | 2.23E-08 | 1.156660133 | NM_001001484| | PTER,phosphotriesterase related |
| 225005_at | 14.8 | 2.24E-08 | 1.084797183 | NM_153812| | PHF13,PHD finger protein 13 |
| 213938_at | 14.79 | 2.26E-08 | 1.275335978 | NM_015576| | CAST,cytomatrix protein p110 |
| 206662_at | 14.79 | 2.26E-08 | 1.324726147 | NM_001118890| | NA |
| 201328_at | 14.79 | 2.27E-08 | 1.321257586 | NM_005239| | ETS2,v-ets erythroblastosis virus E26 oncogene |
| 208091_s_at | 14.78 | 2.27E-08 | 1.08465768 | NM_030796| | DKFZP564K0822,hypothetical protein DKFZp564K0822 |
| 222821_s_at | 14.78 | 2.28E-08 | 1.100005442 | NM_001007269| | GEMIN7,gemin 7 |
| 206055_s_at | 14.78 | 2.27E-08 | 1.042163994 | NM_003090| | SNRPA1,small nuclear ribonucleoprotein polypeptide A' |
| 232087_at | 14.78 | 2.28E-08 | 1.200619124 | NM_198279| | CXorf23,chromosome X open reading frame 23 |
| 238478_at | 14.78 | 2.28E-08 | 1.593022993 | NM_017637| | BNC2,basonuclin 2 |
| 214116_at | 14.77 | 2.30E-08 | 1.328076326 | NM_000060| | BTD,biotinidase precursor |
| 202918_s_at | 14.77 | 2.29E-08 | 1.119250211 | NM_001100819| | NA |
| 214057_at | 14.77 | 2.30E-08 | 1.088022478 | NM_021960| | MCL1,myeloid cell leukemia sequence 1 isoform 1 |
| 214414_x_at | 14.76 | 2.31E-08 | 1.350425165 | NM_000517| | HBA2,alpha 2 globin |
| 221245_s_at | 14.76 | 2.31E-08 | 1.507777838 | NM_003468| | FZD5,frizzled 5 |
| 218111_s_at | 14.76 | 2.30E-08 | 1.153174982 | NM_018686| | CMAS,cytidine 5'-monophosphate N-acetylneuraminic |
| 1562848_at | 14.75 | 2.32E-08 | 1.150812456 | NA |  |
| 205190_at | 14.74 | 2.35E-08 | 1.238204175 | NM_002670| | PLS1,plastin 1 |
| 203060_s_at | 14.74 | 2.34E-08 | 1.512932511 | NM_001015880| | NA |
| 223294_at | 14.74 | 2.35E-08 | 1.074548688 | NM_016500| | CXorf26,chromosome X open reading frame 26 |
| 235269_at | 14.73 | 2.36E-08 | 1.560449334 | NM_138435| | LOC113828,hypothetical protein BC011204 |
| 222351_at | 14.73 | 2.36E-08 | 1.283651052 | NM_002716| | PPP2R1B,beta isoform of regulatory subunit A, protein |
| 217925_s_at | 14.72 | 2.38E-08 | 1.160140862 | NM_022758| | C6orf106,chromosome 6 open reading frame 106 isoform b |
| 220085_at | 14.71 | 2.40E-08 | 1.149928101 | NM_018063| | HELLS,helicase, lymphoid-specific |
| 34408_at | 14.71 | 2.38E-08 | 1.323668442 | NM_005619| | RTN2,reticulon 2 isoform A |
| 220040_x_at | 14.7 | 2.41E-08 | 1.150371919 | NM_018684| | KIAA1166,KIAA1166 |
| 244497_at | 14.69 | 2.42E-08 | 1.267174576 | NA |  |
| 208758_at | 14.68 | 2.45E-08 | 1.063146353 | NM_004044| | ATIC,5-aminoimidazole-4-carboxamide ribonucleotide |
| 206494_s_at | 14.68 | 2.44E-08 | 1.258279762 | NM_000419| | ITGA2B,integrin alpha 2b precursor |
| 213859_x_at | 14.68 | 2.45E-08 | 1.085945352 | NM_003601| | SMARCA5,SWI/SNF-related matrix-associated |
| 224562_at | 14.68 | 2.45E-08 | 1.05423418 | NM_006990| | WASF2,WAS protein family, member 2 |
| 210457_x_at | 14.67 | 2.46E-08 | 1.247060913 | NM_002131| | HMGA1,high mobility group AT-hook 1 isoform b |
| 205670_at | 14.67 | 2.46E-08 | 1.361513874 | NM_004861| | GAL3ST1,galactose-3-O-sulfotransferase 1 |
| 229978_at | 14.66 | 2.48E-08 | 1.510920923 | NA |  |
| 208096_s_at | 14.65 | 2.50E-08 | 1.361188094 | NM_030820| | COL21A1,alpha 1 type XXI collagen precursor |
| 204005_s_at | 14.64 | 2.52E-08 | 1.194503536 | NM_002583| | PAWR,PRKC, apoptosis, WT1, regulator |
| 203765_at | 14.64 | 2.52E-08 | 1.55979147 | NM_012198| | GCA,grancalcin, EF-hand calcium binding protein |
| 200949_x_at | 14.64 | 2.52E-08 | 1.019216071 | NM_001023| | RPS20,ribosomal protein S20 |
| 204257_at | 14.64 | 2.51E-08 | 1.13260446 | NM_021727| | FADS3,fatty acid desaturase 3 |
| 201385_at | 14.63 | 2.52E-08 | 1.032168344 | NM_001358| | DHX15,DEAH (Asp-Glu-Ala-His) box polypeptide 15 |
| 222158_s_at | 14.63 | 2.54E-08 | 1.112452126 | NM_016076| | PNAS-4,CGI-146 protein |
| 222785_x_at | 14.62 | 2.56E-08 | 1.165135852 | NM_022761| | C11orf1,hypothetical protein FLJ23499 |
| 1552389_at | 14.61 | 2.57E-08 | 1.442514607 | NM_173549| | FLJ39553,hypothetical protein FLJ39553 |
| 225900_at | 14.6 | 2.59E-08 | 1.195702922 | NM_015189| | NA |
| 219572_at | 14.59 | 2.60E-08 | 1.179552227 | NM_001009571| | CADPS2,Ca2+-dependent activator protein for secretion 2 |
| 226485_at | 14.57 | 2.64E-08 | 1.175208802 | NA |  |
| 219894_at | 14.56 | 2.66E-08 | 1.454128132 | NM_019066| | MAGEL2,MAGE-like protein 2 |
| 229582_at | 14.56 | 2.68E-08 | 1.062533537 | NM_001098817| | NA |
| 222696_at | 14.56 | 2.66E-08 | 1.479456961 | NM_004655| | AXIN2,axin 2 |
| 201930_at | 14.55 | 2.68E-08 | 1.054838157 | NM_005915| | MCM6,minichromosome maintenance protein 6 |
| 241834_at | 14.55 | 2.69E-08 | 1.385291724 | NA |  |
| 227296_at | 14.54 | 2.71E-08 | 1.211459854 | NM_138431| | LOC113655,hypothetical protein BC011982 |
| 208549_x_at | 14.54 | 2.71E-08 | 1.059747166 | NA |  |
| 242800_at | 14.54 | 2.71E-08 | 1.22090828 | NM_198270| | NHS,Nance-Horan syndrome protein |
| 224212_s_at | 14.54 | 2.70E-08 | 1.812835256 | NM_014005| | PCDHA9,protocadherin alpha 9 isoform 2 precursor |
| 212731_at | 14.53 | 2.73E-08 | 1.155435331 | NM_198401| | LOC157567,hypothetical protein LOC157567 |
| 222991_s_at | 14.53 | 2.72E-08 | 1.102559325 | NM_013438| | UBQLN1,ubiquilin 1 isoform 1 |
| 212013_at | 14.52 | 2.74E-08 | 1.120226601 | NM_012293| | NA |
| 202543_s_at | 14.52 | 2.76E-08 | 1.143775373 | NM_004124| | GMFB,glia maturation factor, beta |
| 204117_at | 14.52 | 2.76E-08 | 1.072018477 | NM_002726| | PREP,prolyl endopeptidase |
| 226354_at | 14.51 | 2.77E-08 | 1.238353771 | NM_032857| | LACTB,lactamase, beta isoform a |
| 1569287_at | 14.51 | 2.77E-08 | 1.18244345 | NA |  |
| 214352_s_at | 14.5 | 2.78E-08 | 1.127319437 | NM_004985| | KRAS,c-K-ras2 protein isoform b |
| 205007_s_at | 14.5 | 2.78E-08 | 1.222315562 | NM_006383| | CIB2,DNA-dependent protein kinase catalytic |
| 210028_s_at | 14.49 | 2.80E-08 | 1.083237539 | NM_012381| | ORC3L,origin recognition complex, subunit 3 isoform 2 |
| 242565_x_at | 14.49 | 2.81E-08 | 1.168770882 | NM_001006114| | C21orf57,chromosome 21 open reading frame 57 isoform 2 |
| 223060_at | 14.49 | 2.80E-08 | 1.093849654 | NM_017924| | C14orf119,chromosome 14 open reading frame 119 |
| 208959_s_at | 14.49 | 2.80E-08 | 1.076581935 | NM_015051| | TXNDC4,thioredoxin domain containing 4 (endoplasmic |
| 225871_at | 14.49 | 2.80E-08 | 1.469928095 | NM_001040665| | NA |
| 218098_at | 14.48 | 2.82E-08 | 1.067988774 | NM_006420| | ARFGEF2,ADP-ribosylation factor guanine |
| 1570153_at | 14.48 | 2.83E-08 | 1.399062444 | NM_017826| | FLJ20449,hypothetical protein FLJ20449 |
| 226982_at | 14.48 | 2.84E-08 | 1.360772733 | NM_012081| | ELL2,elongation factor, RNA polymerase II, 2 |
| 213268_at | 14.48 | 2.83E-08 | 1.627065744 | NM_015215| | CAMTA1,calmodulin-binding transcription activator 1 |
| 225824_at | 14.47 | 2.84E-08 | 1.05947442 | NM_001099402| | NA |
| 41037_at | 14.46 | 2.87E-08 | 1.099288629 | NM_003213| | TEAD4,TEA domain family member 4 isoform 1 |
| 217165_x_at | 14.45 | 2.88E-08 | 1.192079459 | NM_005949| | MT1F,metallothionein 1F |
| 213698_at | 14.45 | 2.88E-08 | 1.147842507 | NM_007167| | ZNF258,zinc finger protein 258 |
| 225407_at | 14.44 | 2.90E-08 | 1.603605785 | NM_001025081| | NA |
| 205882_x_at | 14.44 | 2.90E-08 | 1.111085046 | NM_001121| | NA |
| 224279_s_at | 14.44 | 2.91E-08 | 1.206210466 | NM_012189| | CABYR,calcium-binding tyrosine |
| 210495_x_at | 14.44 | 2.91E-08 | 1.749126963 | NM_002026| | FN1,fibronectin 1 isoform 3 preproprotein |
| 235199_at | 14.43 | 2.93E-08 | 1.601747846 | NM_017831| | RNF125,ring finger protein 125 |
| 207183_at | 14.41 | 2.96E-08 | 1.13548886 | NM_006143| | GPR19,G protein-coupled receptor 19 |
| 213793_s_at | 14.41 | 2.96E-08 | 1.07227369 | NM_004272| | HOMER1,homer 1 |
| 203389_at | 14.41 | 2.98E-08 | 1.168134079 | NM_002254| | KIF3C,kinesin family member 3C |
| 220391_at | 14.41 | 2.98E-08 | 1.372555541 | NM_024784| | ZBTB3,zinc finger and BTB domain containing 3 |
| 225351_at | 14.4 | 2.98E-08 | 1.147450154 | NM_018472| | FAM45B,family with sequence similarity 45, member B |
| 205591_at | 14.4 | 2.99E-08 | 1.889777316 | NM_006334| | OLFM1,olfactomedin related ER localized protein |
| 207819_s_at | 14.4 | 3.00E-08 | 1.205316133 | NM_000443| | ABCB4,ATP-binding cassette, subfamily B, member 4 |
| 203263_s_at | 14.4 | 2.98E-08 | 1.237356073 | NM_015185| | ARHGEF9,Cdc42 guanine exchange factor 9 |
| 224492_s_at | 14.39 | 3.02E-08 | 1.089066347 | NM_145295| | ZNF627,zinc finger protein 627 |
| 242283_at | 14.38 | 3.03E-08 | 1.230414195 | NM_144989| | NA |
| 225494_at | 14.37 | 3.06E-08 | 1.0535488 | NA |  |
| 220677_s_at | 14.37 | 3.07E-08 | 1.439311909 | NM_007037| | ADAMTS8,a disintegrin and metalloprotease with |
| 1556469_s_at | 14.37 | 3.05E-08 | 1.780092694 | NA |  |
| 200726_at | 14.37 | 3.06E-08 | 1.036920248 | NM_002710| | PPP1CC,protein phosphatase 1, catalytic subunit, gamma |
| 208490_x_at | 14.36 | 3.09E-08 | 1.091939098 | NM_003518| | HIST1H2BG,H2B histone family, member A |
| 223729_at | 14.35 | 3.11E-08 | 1.302739019 | NM_031413| | NA |
| 209735_at | 14.34 | 3.14E-08 | 1.481935941 | NM_004827| | ABCG2,ATP-binding cassette, sub-family G, member 2 |
| 218084_x_at | 14.34 | 3.13E-08 | 1.492198513 | NM_014164| | FXYD5,FXYD domain-containing ion transport regulator |
| 242957_at | 14.34 | 3.12E-08 | 1.219395504 | NM_152718| | FLJ32009,hypothetical protein FLJ32009 |
| 205016_at | 14.33 | 3.15E-08 | 1.555585707 | NM_001099691| | NA |
| 228338_at | 14.33 | 3.15E-08 | 1.594939189 | NA |  |
| 236045_x_at | 14.33 | 3.14E-08 | 1.437187323 | NA |  |
| 215193_x_at | 14.33 | 3.16E-08 | 1.748742573 | NM_001023561| | NA |
| 209035_at | 14.32 | 3.17E-08 | 1.178942089 | NM_001012333| | MDK,midkine |
| 223140_s_at | 14.31 | 3.20E-08 | 1.076113037 | NM_001114397| | NA |
| 212168_at | 14.31 | 3.19E-08 | 1.042516596 | NM_006047| | RBM12,RNA binding motif protein 12 |
| 213954_at | 14.31 | 3.20E-08 | 1.34771311 | NM_015566| | NA |
| 221880_s_at | 14.31 | 3.19E-08 | 1.288683571 | NM_207446| | LOC400451,hypothetical gene supported by AK075564; |
| 235241_at | 14.31 | 3.20E-08 | 1.248313695 | NM_173514| | FLJ90709,hypothetical protein FLJ90709 |
| 40359_at | 14.3 | 3.22E-08 | 1.095188526 | NM_003475| | C11orf13,HRAS1-related cluster-1 |
| 219418_at | 14.3 | 3.23E-08 | 1.069355228 | NM_024782| | FLJ12610,similar to mouse 1700029B21Rik protein |
| 212728_at | 14.3 | 3.22E-08 | 1.267791334 | NM_020730| | NA |
| 220215_at | 14.29 | 3.24E-08 | 1.11096466 | NM_024804| | FLJ12606,hypothetical protein FLJ12606 |
| 218885_s_at | 14.29 | 3.23E-08 | 1.349864722 | NM_024642| | GALNT12,UDP-N-acetyl-alpha-D-galactosamine:polypeptide |
| 202372_at | 14.29 | 3.24E-08 | 1.042384806 | NM_012414| | RAB3-GAP150,rab3 GTPase-activating protein, non-catalytic |
| 202793_at | 14.29 | 3.24E-08 | 1.106211938 | NM_005768| | C3F,gene rich cluster, C3f gene |
| 220230_s_at | 14.28 | 3.26E-08 | 1.817855469 | NM_016229| | CYB5R2,cytochrome b5 reductase b5R.2 isoform 1 |
| 232549_at | 14.28 | 3.26E-08 | 1.591155558 | NM_144770| | RBM11,RNA binding motif protein 11 |
| 209659_s_at | 14.28 | 3.26E-08 | 1.052643361 | NM_001078645| | NA |
| 215354_s_at | 14.27 | 3.29E-08 | 1.106308646 | NM_014389| | PELP1,proline-, glutamic acid-, leucine-rich protein |
| 212761_at | 14.27 | 3.29E-08 | 1.104615404 | NM_030756| | TCF7L2,transcription factor 7-like 2 (T-cell specific, |
| 207318_s_at | 14.26 | 3.31E-08 | 1.058619466 | NM_003718| | CDC2L5,cell division cycle 2-like 5 isoform 1 |
| 226963_at | 14.26 | 3.31E-08 | 1.066239352 | NM_152265| | MGC23908,similar to transcription factor BTF3 |
| 212876_at | 14.26 | 3.31E-08 | 1.343819445 | NM_003778| | B4GALT4,UDP-Gal:betaGlcNAc beta 1,4- |
| 1555281_x_at | 14.26 | 3.31E-08 | 1.121781377 | NM_014154| | ARMC8,armadillo repeat containing 8 |
| 218394_at | 14.26 | 3.31E-08 | 1.191226859 | NM_024589| | FLJ22386,leucine zipper domain protein |
| 224990_at | 14.26 | 3.30E-08 | 1.308011371 | NM_174921| | LOC201895,hypothetical protein LOC201895 |
| 202205_at | 14.26 | 3.31E-08 | 1.228578038 | NM_003370| | VASP,vasodilator-stimulated phosphoprotein isoform 1 |
| 221881_s_at | 14.25 | 3.33E-08 | 1.395892816 | NM_013943| | CLIC4,chloride intracellular channel 4 |
| 227694_at | 14.25 | 3.34E-08 | 1.2016512 | NM_178122| | LOC90529,hypothetical protein LOC90529 |
| 210110_x_at | 14.25 | 3.33E-08 | 1.036162123 | NM_012207| | HNRPH3,heterogeneous nuclear ribonucleoprotein H3 |
| 228345_at | 14.24 | 3.36E-08 | 1.264768928 | NM_001039840| | NA |
| 201417_at | 14.24 | 3.35E-08 | 1.071166293 | NM_003107| | SOX4,SRY (sex determining region Y)-box 4 |
| 203966_s_at | 14.24 | 3.36E-08 | 1.105612035 | NM_021003| | PPM1A,protein phosphatase 1A isoform 1 |
| 218828_at | 14.23 | 3.38E-08 | 1.133833251 | NM_020360| | PLSCR3,phospholipid scramblase 3 |
| 215537_x_at | 14.23 | 3.38E-08 | 1.1170595 | NM_013974| | DDAH2,dimethylarginine dimethylaminohydrolase 2 |
| 214550_s_at | 14.22 | 3.41E-08 | 1.077509908 | NM_012470| | TNPO3,transportin 3 |
| 219405_at | 14.22 | 3.41E-08 | 1.108416639 | NM_018073| | TRIM68,ring finger protein 137 |
| 221974_at | 14.21 | 3.42E-08 | 1.325934899 | NM_003097| | SNRPN,small nuclear ribonucleoprotein polypeptide N |
| 207981_s_at | 14.21 | 3.43E-08 | 1.601327754 | NM_001438| | ESRRG,estrogen-related receptor gamma isoform 1 |
| 240261_at | 14.21 | 3.42E-08 | 1.196562162 | NM_005486| | TOM1L1,target of myb1-like 1 |
| 226276_at | 14.21 | 3.42E-08 | 1.077786309 | NM_174909| | MGC23909,hypothetical protein MGC23909 |
| 225378_at | 14.2 | 3.45E-08 | 1.147553398 | NM_152415| | FLJ32642,hypothetical protein FLJ32642 |
| 212132_at | 14.2 | 3.46E-08 | 1.04908822 | NM_001114093| | NA |
| 200011_s_at | 14.2 | 3.45E-08 | 1.052638395 | NM_001659| | ARF3,ADP-ribosylation factor 3 |
| 202087_s_at | 14.19 | 3.47E-08 | 1.126588935 | NM_001912| | CTSL,cathepsin L preproprotein |
| 213234_at | 14.19 | 3.47E-08 | 1.148212982 | NM_020853| | NA |
| 205209_at | 14.19 | 3.48E-08 | 1.071185396 | NM_004302| | ACVR1B,activin A type IB receptor isoform a precursor |
| 208783_s_at | 14.19 | 3.49E-08 | 1.104906061 | NM_002389| | MCP,membrane cofactor protein isoform 1 precursor |
| 1558404_at | 14.18 | 3.49E-08 | 1.758793408 | NA |  |
| 227702_at | 14.18 | 3.49E-08 | 1.878208221 | NM_178033| | CYP4X1,cytochrome P450, family 4, subfamily X, |
| 241772_at | 14.17 | 3.52E-08 | 2.257559511 | NA |  |
| 213916_at | 14.16 | 3.54E-08 | 1.164020937 | NM_021143| | ZNF20,zinc finger protein 20 (KOX 13) |
| 1554609_at | 14.16 | 3.55E-08 | 1.332656206 | NM_006591| | POLD3,polymerase (DNA directed), delta 3 |
| 218261_at | 14.16 | 3.55E-08 | 1.534891532 | NM_005498| | AP1M2,adaptor-related protein complex 1, mu 2 subunit |
| 212158_at | 14.16 | 3.55E-08 | 1.238426296 | NM_002998| | SDC2,syndecan 2 precursor |
| 225164_s_at | 14.16 | 3.57E-08 | 1.118259836 | NM_001013703| | NA |
| 1569108_a_at | 14.15 | 3.57E-08 | 1.301403655 | NM_016089| | ZNF589,zinc finger protein 589 |
| 201482_at | 14.14 | 3.61E-08 | 1.145596206 | NM_001004128| | QSCN6,quiescin Q6 isoform b |
| 221475_s_at | 14.13 | 3.64E-08 | 1.015194919 | NM_002948| | RPL15,ribosomal protein L15 |
| 230067_at | 14.13 | 3.64E-08 | 1.616175361 | NM_145019| | FLJ30707,hypothetical protein FLJ30707 |
| 221957_at | 14.12 | 3.68E-08 | 1.32830567 | NM_005391| | PDK3,pyruvate dehydrogenase kinase, isoenzyme 3 |
| 219025_at | 14.12 | 3.66E-08 | 1.120761038 | NM_020404| | CD248,tumor endothelial marker 1 precursor |
| 230201_at | 14.12 | 3.67E-08 | 1.140504816 | NA |  |
| 212455_at | 14.12 | 3.66E-08 | 1.074485675 | NM_001031732| | NA |
| 213533_at | 14.11 | 3.68E-08 | 1.400005244 | NM_001040101| | NA |
| 219470_x_at | 14.1 | 3.72E-08 | 1.082720103 | NM_019084| | CCNJ,cyclin J |
| 208939_at | 14.1 | 3.71E-08 | 1.175686572 | NM_012247| | SEPHS1,selenophosphate synthetase |
| 229168_at | 14.09 | 3.75E-08 | 1.44405048 | NM_173465| | COL23A1,collagen, type XXIII, alpha 1 |
| 231035_s_at | 14.08 | 3.79E-08 | 1.289539116 | NA |  |
| 223689_at | 14.07 | 3.81E-08 | 1.705740626 | NM_006546| | IMP-1,IGF-II mRNA-binding protein 1 |
| 239835_at | 14.07 | 3.80E-08 | 1.364701603 | NM_032505| | TA-KRP,T-cell activation kelch repeat protein |
| 201181_at | 14.07 | 3.80E-08 | 1.056436536 | NM_006496| | GNAI3,guanine nucleotide binding protein (G protein), |
| 235149_at | 14.06 | 3.82E-08 | 1.655942839 | NM_173582| | PGM2L1,phosphoglucomutase 2-like 1 |
| 221664_s_at | 14.06 | 3.83E-08 | 2.163585369 | NM_016946| | F11R,F11 receptor isoform a precursor |
| 205489_at | 14.06 | 3.83E-08 | 1.130629047 | NM_001014444| | NA |
| 1556643_at | 14.05 | 3.85E-08 | 1.247978119 | NA |  |
| 202201_at | 14.05 | 3.85E-08 | 1.354282056 | NM_000713| | BLVRB,biliverdin reductase B (flavin reductase |
| 222361_at | 14.05 | 3.86E-08 | 1.680181778 | NA |  |
| 1553103_at | 14.05 | 3.85E-08 | 1.122151476 | NM_002504| | NFX1,nuclear transcription factor, X-box binding 1 |
| 223254_s_at | 14.04 | 3.89E-08 | 1.088437223 | NM_017769| | KIAA1333,KIAA1333 |
| 238477_at | 14.03 | 3.93E-08 | 1.140149758 | NA |  |
| 211719_x_at | 14.03 | 3.91E-08 | 1.781389117 | NM_002026| | FN1,fibronectin 1 isoform 3 preproprotein |
| 219167_at | 14.02 | 3.96E-08 | 1.455130219 | NM_016563| | RASL12,RAS-like, family 12 protein |
| 230406_at | 14.02 | 3.96E-08 | 1.352116008 | NA |  |
| 218935_at | 14.01 | 3.97E-08 | 1.14703813 | NM_014600| | EHD3,EH-domain containing 3 |
| 241416_at | 14.01 | 3.97E-08 | 1.299222668 | NA |  |
| 220382_s_at | 14.01 | 3.98E-08 | 1.482617385 | NM_001010000| | ARHGAP28,Rho GTPase activating protein 28 isoform a |
| 218368_s_at | 14 | 4.02E-08 | 1.101317765 | NM_016639| | TNFRSF12A,type I transmembrane protein Fn14 |
| 244362_at | 14 | 4.02E-08 | 1.34966024 | NA |  |
| 214636_at | 14 | 4.01E-08 | 1.791055221 | NM_000728| | CALCB,calcitonin-related polypeptide, beta |
| 212865_s_at | 13.99 | 4.05E-08 | 2.011253592 | NM_021110| | COL14A1,collagen, type XIV, alpha 1 |
| 217938_s_at | 13.98 | 4.06E-08 | 1.160421417 | NM_020122| | KCMF1,potassium channel modulatory factor 1 |
| 220319_s_at | 13.98 | 4.06E-08 | 1.362612167 | NM_013262| | MYLIP,myosin regulatory light chain interacting |
| 208652_at | 13.98 | 4.05E-08 | 1.031375161 | NM_002715| | PPP2CA,protein phosphatase 2, catalytic subunit, alpha |
| 205141_at | 13.98 | 4.06E-08 | 1.209484066 | NM_001097577| | NA |
| 206231_at | 13.97 | 4.08E-08 | 1.116141174 | NM_002248| | KCNN1,potassium intermediate/small conductance |
| 219270_at | 13.97 | 4.10E-08 | 1.50522384 | NM_024111| | MGC4504,hypothetical protein MGC4504 |
| 210101_x_at | 13.97 | 4.08E-08 | 1.056582442 | NM_016009| | SH3GLB1,SH3-containing protein SH3GLB1 |
| 222857_s_at | 13.96 | 4.12E-08 | 1.715772886 | NM_014505| | KCNMB4,calcium-activated potassium channel beta 4 |
| 204281_at | 13.96 | 4.11E-08 | 1.072453298 | NM_003213| | TEAD4,TEA domain family member 4 isoform 1 |
| 41577_at | 13.96 | 4.11E-08 | 1.51919723 | NM_015568| | PPP1R16B,protein phosphatase 1 regulatory inhibitor |
| 219084_at | 13.95 | 4.14E-08 | 1.199772331 | NM_022455| | NSD1,nuclear receptor binding SET domain protein 1 |
| 46665_at | 13.94 | 4.20E-08 | 1.127468834 | NM_017789| | SEMA4C,semaphorin 4C |
| 231597_x_at | 13.94 | 4.18E-08 | 1.542411415 | NA |  |
| 204328_at | 13.94 | 4.18E-08 | 1.377768943 | NM_001127198| | NA |
| 208726_s_at | 13.94 | 4.19E-08 | 1.035117043 | NM_003908| | EIF2S2,eukaryotic translation initiation factor 2 beta |
| 202338_at | 13.93 | 4.23E-08 | 1.091404282 | NM_003258| | TK1,thymidine kinase 1, soluble |
| 227432_s_at | 13.92 | 4.25E-08 | 1.236308058 | NA |  |
| 242463_x_at | 13.92 | 4.25E-08 | 1.489873044 | NM_198457| | ZNF600,zinc finger protein 600 |
| 210216_x_at | 13.92 | 4.25E-08 | 1.038978357 | NM_002853| | RAD1,RAD1 homolog isoform 1 |
| 221260_s_at | 13.92 | 4.26E-08 | 1.111236174 | NM_030809| | C12orf22,TGF-beta induced apotosis protein 12 |
| 227920_at | 13.91 | 4.30E-08 | 1.134632273 | NM_001080450| | NA |
| 203677_s_at | 13.9 | 4.34E-08 | 1.065034261 | NM_004178| | TARBP2,TAR RNA binding protein 2 isoform c |
| 200899_s_at | 13.9 | 4.34E-08 | 1.107323827 | NM_012215| | MGEA5,meningioma expressed antigen 5 (hyaluronidase) |
| 212632_at | 13.9 | 4.31E-08 | 1.148438641 | NM_003569| | STX7,syntaxin 7 |
| 218195_at | 13.9 | 4.34E-08 | 1.212694279 | NM_024573| | C6orf211,chromosome 6 open reading frame 211 |
| 212367_at | 13.89 | 4.36E-08 | 1.08853235 | NM_015322| | FEM1B,fem-1 homolog b |
| 216996_s_at | 13.89 | 4.34E-08 | 1.042865107 | NM_014929| | KIAA0971,KIAA0971 |
| 242462_at | 13.89 | 4.37E-08 | 1.230401211 | NA |  |
| 1555742_at | 13.89 | 4.37E-08 | 1.379697489 | NA |  |
| 218302_at | 13.88 | 4.39E-08 | 1.068442377 | NM_172341| | PSENEN,presenilin enhancer 2 |
| 209893_s_at | 13.88 | 4.38E-08 | 1.433382778 | NM_002033| | FUT4,fucosyltransferase 4 |
| 205542_at | 13.88 | 4.38E-08 | 1.931483926 | NM_012449| | STEAP,six transmembrane epithelial antigen of the |
| 228252_at | 13.87 | 4.40E-08 | 1.084541668 | NM_025049| | C15orf20,DNA helicase homolog PIF1 |
| 217952_x_at | 13.86 | 4.44E-08 | 1.063367358 | NM_015153| | PHF3,PHD finger protein 3 |
| 217798_at | 13.85 | 4.50E-08 | 1.053199572 | NM_014515| | CNOT2,CCR4-NOT transcription complex, subunit 2 |
| 209569_x_at | 13.85 | 4.49E-08 | 1.903369061 | NM_001040101| | NA |
| 219588_s_at | 13.85 | 4.49E-08 | 1.042246261 | NM_017760| | MTB,more than blood homolog |
| 218986_s_at | 13.84 | 4.52E-08 | 1.189565364 | NM_017631| | FLJ20035,hypothetical protein FLJ20035 |
| 224725_at | 13.83 | 4.56E-08 | 1.104778765 | NM_020774| | MIB1,mindbomb homolog 1 |
| 213229_at | 13.82 | 4.59E-08 | 1.141615633 | NM_030621| | DICER1,dicer1 |
| 210981_s_at | 13.82 | 4.60E-08 | 1.115078604 | NM_001004105| | GRK6,G protein-coupled receptor kinase 6 isoform C |
| 210813_s_at | 13.81 | 4.62E-08 | 1.12654683 | NM_003401| | XRCC4,X-ray repair cross complementing protein 4 |
| 202036_s_at | 13.81 | 4.62E-08 | 1.439313754 | NM_003012| | SFRP1,secreted frizzled-related protein 1 |
| 233080_s_at | 13.81 | 4.63E-08 | 1.042947478 | NM_017892| | NA |
| 209576_at | 13.81 | 4.63E-08 | 1.312781451 | NM_002069| | GNAI1,guanine nucleotide binding protein (G protein), |
| 207513_s_at | 13.8 | 4.65E-08 | 1.077927525 | NM_003452| | ZNF189,zinc finger protein 189 |
| 224984_at | 13.8 | 4.66E-08 | 1.091134561 | NM_001113178| | NA |
| 1553874_a_at | 13.79 | 4.69E-08 | 1.871638109 | NM_032805| | ZNF206,zinc finger protein 206 |
| 218642_s_at | 13.79 | 4.70E-08 | 1.297713559 | NM_001011667| | CHCHD7,coiled-coil-helix-coiled-coil-helix domain |
| 212666_at | 13.79 | 4.70E-08 | 1.093060753 | NM_020429| | SMURF1,Smad ubiquitination regulatory factor 1 isoform |
| 206924_at | 13.78 | 4.71E-08 | 1.415290806 | NM_000641| | IL11,interleukin 11 precursor |
| 234192_s_at | 13.78 | 4.71E-08 | 1.134182369 | NM_025211| | GKAP1,G kinase anchoring protein 1 |
| 228868_x_at | 13.78 | 4.71E-08 | 1.082835575 | NM_030928| | CDT1,DNA replication factor |
| 225664_at | 13.77 | 4.78E-08 | 1.390722594 | NM_004370| | COL12A1,alpha 1 type XII collagen long isoform |
| 64942_at | 13.77 | 4.76E-08 | 1.07462476 | NM_207370| | GPR153,G protein-coupled receptor 153 |
| 226986_at | 13.76 | 4.82E-08 | 1.172889639 | NM_001033518| | NA |
| 205560_at | 13.74 | 4.90E-08 | 1.753857312 | NM_006200| | PCSK5,proprotein convertase subtilisin/kexin type 5 |
| 208651_x_at | 13.74 | 4.90E-08 | 1.277769017 | NM_013230| | CD24,CD24 antigen |
| 209130_at | 13.74 | 4.90E-08 | 1.151174433 | NM_003825| | SNAP23,synaptosomal-associated protein 23 isoform |
| 213558_at | 13.74 | 4.90E-08 | 1.582685258 | NM_014510| | NA |
| 241417_at | 13.73 | 4.91E-08 | 1.279255483 | NA |  |
| 225820_at | 13.73 | 4.90E-08 | 1.191242407 | NM_024900| | PHF17,Jade1 protein short isoform |
| 208747_s_at | 13.72 | 4.98E-08 | 1.387882804 | NM_001734| | C1S,complement component 1, s subcomponent |
| 204681_s_at | 13.72 | 4.96E-08 | 1.250269244 | NM_012294| | RAPGEF5,Rap guanine nucleotide exchange factor (GEF) 5 |
| 221805_at | 13.71 | 5.01E-08 | 1.660966448 | NM_006158| | NEFL,neurofilament, light polypeptide 68kDa |
| 222955_s_at | 13.71 | 5.01E-08 | 1.128060524 | NM_018472| | FAM45B,family with sequence similarity 45, member B |
| 222411_s_at | 13.71 | 5.01E-08 | 1.045032294 | NM_007107| | SSR3,signal sequence receptor gamma subunit |
| 218283_at | 13.71 | 4.99E-08 | 1.078390162 | NM_016305| | SS18L2,synovial sarcoma translocation gene on |
| 221802_s_at | 13.7 | 5.04E-08 | 1.176876986 | NM_001127211| | NA |
| 204811_s_at | 13.7 | 5.05E-08 | 1.565706866 | NM_001005505| | CACNA2D2,calcium channel, voltage-dependent, alpha |
| 212372_at | 13.69 | 5.07E-08 | 1.073754688 | NM_005964| | MYH10,myosin, heavy polypeptide 10, non-muscle |
| 234973_at | 13.68 | 5.12E-08 | 1.722536596 | NM_033518| | SLC38A5,amino acid transport system N2 |
| 223380_s_at | 13.68 | 5.11E-08 | 1.16895713 | NM_014572| | LATS2,LATS, large tumor suppressor, homolog 2 |
| 228109_at | 13.68 | 5.12E-08 | 1.289919092 | NM_006909| | RASGRF2,Ras protein-specific guanine |
| 223650_s_at | 13.68 | 5.10E-08 | 1.098171285 | NM_030759| | NRBF2,nuclear receptor binding factor 2 |
| 227701_at | 13.67 | 5.17E-08 | 1.403697556 | NM_018017| | C10orf118,CTCL tumor antigen L14-2 |
| 201165_s_at | 13.67 | 5.15E-08 | 1.037657977 | NM_001020658| | NA |
| 230619_at | 13.66 | 5.18E-08 | 1.146488679 | NM_001668| | ARNT,aryl hydrocarbon receptor nuclear translocator |
| 222986_s_at | 13.66 | 5.20E-08 | 1.067854019 | NM_016479| | SCOTIN,scotin |
| 1557223_at | 13.66 | 5.18E-08 | 1.225695052 | NA |  |
| 235957_at | 13.65 | 5.22E-08 | 1.664356381 | NA |  |
| 209348_s_at | 13.65 | 5.21E-08 | 1.522088059 | NM_001031804| | NA |
| 227647_at | 13.64 | 5.27E-08 | 1.582196355 | NM_005472| | KCNE3,potassium voltage-gated channel, Isk-related |
| 205992_s_at | 13.64 | 5.28E-08 | 1.297311636 | NM_000585| | IL15,interleukin 15 isoform 1 precursor |
| 219232_s_at | 13.64 | 5.28E-08 | 1.626412832 | NM_022073| | EGLN3,egl nine homolog 3 |
| 203135_at | 13.64 | 5.26E-08 | 1.045882297 | NM_003194| | TBP,TATA box binding protein |
| 237203_at | 13.63 | 5.32E-08 | 1.747259616 | NA |  |
| 223337_at | 13.63 | 5.32E-08 | 1.107603269 | NM_005869| | SDCCAG10,serologically defined colon cancer antigen 10 |
| 226344_at | 13.63 | 5.31E-08 | 1.291747659 | NM_001011657| | ZMAT1,zinc finger, matrin type 1 isoform 1 |
| 1555851_s_at | 13.63 | 5.32E-08 | 1.052935954 | NM_003009| | SEPW1,selenoprotein W, 1 |
| 1562988_at | 13.63 | 5.32E-08 | 1.392541481 | NM_001112734| | NA |
| 201416_at | 13.62 | 5.35E-08 | 1.083142755 | NM_003107| | SOX4,SRY (sex determining region Y)-box 4 |
| 218274_s_at | 13.62 | 5.34E-08 | 1.108630749 | NM_001042410| | NA |
| 237040_at | 13.62 | 5.33E-08 | 1.163150503 | NM_152434| | CWF19L2,CWF19-like 2, cell cycle control |
| 228141_at | 13.62 | 5.34E-08 | 1.506647186 | NM_001008397| | LOC493869,similar to 2310016C16Rik protein |
| 213271_s_at | 13.61 | 5.37E-08 | 1.078937702 | NM_015018| | KIAA1117,KIAA1117 protein |
| 235017_s_at | 13.61 | 5.37E-08 | 1.580894196 | NA |  |
| 209647_s_at | 13.6 | 5.40E-08 | 1.137829657 | NM_014011| | SOCS5,suppressor of cytokine signaling 5 |
| 213384_x_at | 13.6 | 5.41E-08 | 1.065375047 | NM_000932| | PLCB3,phospholipase C, beta 3 |
| 236798_at | 13.59 | 5.48E-08 | 1.252780746 | NA |  |
| 201276_at | 13.59 | 5.46E-08 | 1.086337262 | NM_002868| | RAB5B,RAB5B, member RAS oncogene family |
| 234103_at | 13.58 | 5.52E-08 | 1.259236603 | NM_198503| | SLICK,sodium- and chloride-activated ATP-sensitive |
| 226666_at | 13.58 | 5.49E-08 | 1.064815826 | NM_014992| | DAAM1,dishevelled-associated activator of |
| 43427_at | 13.58 | 5.50E-08 | 1.324505412 | NM_001093| | ACACB,acetyl-Coenzyme A carboxylase beta |
| 218178_s_at | 13.58 | 5.49E-08 | 1.080754231 | NM_020412| | CHMP1.5,CHMP1.5 protein |
| 240117_at | 13.58 | 5.50E-08 | 1.582889793 | NM_032447| | FBN3,fibrillin 3 precursor |
| 226657_at | 13.58 | 5.52E-08 | 1.253404334 | NM_152914| | MGC33894,transcript expressed during hematopoiesis 2 |
| 227109_at | 13.58 | 5.49E-08 | 1.227256361 | NM_024514| | CYP2R1,cytochrome P450, family 2, subfamily R, |
| 203764_at | 13.57 | 5.54E-08 | 1.073447066 | NM_014750| | DLG7,discs large homolog 7 |
| 224329_s_at | 13.57 | 5.56E-08 | 1.580440761 | NM_032488| | CNFN,cornifelin |
| 204595_s_at | 13.56 | 5.57E-08 | 1.727737127 | NM_003155| | STC1,stanniocalcin 1 |
| 227506_at | 13.55 | 5.63E-08 | 1.14848005 | NM_194298| | SLC16A9,solute carrier family 16 (monocarboxylic acid |
| 226875_at | 13.55 | 5.64E-08 | 1.185808621 | NM_144658| | DOCK11,dedicator of cytokinesis 11 |
| 213322_at | 13.54 | 5.68E-08 | 1.064477079 | NM_145063| | C6orf130,chromosome 6 open reading frame 130 |
| 224407_s_at | 13.54 | 5.68E-08 | 1.473463435 | NM_001042452| | NA |
| 204194_at | 13.53 | 5.70E-08 | 1.096712582 | NM_001011545| | BACH1,BTB and CNC homology 1 isoform b |
| 211385_x_at | 13.53 | 5.72E-08 | 1.075072234 | NM_001054| | SULT1A2,sulfotransferase family, cytosolic, 1A, |
| 221933_at | 13.53 | 5.74E-08 | 1.233784809 | NM_020742| | NLGN4X,X-linked neuroligin 4 |
| 216202_s_at | 13.52 | 5.78E-08 | 1.214698907 | NM_004863| | SPTLC2,serine palmitoyltransferase, long chain base |
| 226231_at | 13.52 | 5.76E-08 | 1.255380737 | NA |  |
| 226803_at | 13.5 | 5.88E-08 | 1.784451843 | NM_152284| | Shax3,Snf7 homologue associated with Alix 3 |
| 219527_at | 13.5 | 5.89E-08 | 1.218892875 | NM_017898| | FLJ20605,hypothetical protein FLJ20605 |
| 203791_at | 13.5 | 5.84E-08 | 1.162711109 | NM_005509| | DMXL1,Dmx-like 1 |
| 204937_s_at | 13.49 | 5.93E-08 | 1.129210153 | NM_016324| | ZNF274,zinc finger protein 274 isoform b |
| 201206_s_at | 13.49 | 5.91E-08 | 1.165563194 | NM_001042576| | NA |
| 219080_s_at | 13.49 | 5.91E-08 | 1.258282053 | NM_019857| | CTPS2,cytidine triphosphate synthase II |
| 228422_at | 13.49 | 5.90E-08 | 1.509491282 | NM_198560| | LOC375323,lipoma HMGIC fusion partner-like protein 4 |
| 224857_s_at | 13.48 | 5.97E-08 | 1.07916319 | NM_015972| | POLR1D,RNA polymerase I 16 kDa subunit |
| 205632_s_at | 13.47 | 5.99E-08 | 2.073252472 | NM_003558| | PIP5K1B,phosphatidylinositol-4-phosphate 5-kinase, type |
| 221677_s_at | 13.47 | 6.00E-08 | 1.08343974 | NM_017613| | DONSON,downstream neighbor of SON isoform a |
| 201194_at | 13.47 | 5.99E-08 | 1.063707184 | NM_003009| | SEPW1,selenoprotein W, 1 |
| 229974_at | 13.47 | 6.02E-08 | 1.73568226 | NM_147127| | EVC2,limbin |
| 201949_x_at | 13.46 | 6.04E-08 | 1.074322269 | NM_004930| | CAPZB,F-actin capping protein beta subunit |
| 1554795_a_at | 13.46 | 6.06E-08 | 1.40363045 | NM_001024215| | NA |
| 213627_at | 13.46 | 6.04E-08 | 1.11557682 | NM_014599| | MAGED2,melanoma antigen family D, 2 |
| 237192_at | 13.46 | 6.06E-08 | 2.183225204 | NA |  |
| 213358_at | 13.46 | 6.06E-08 | 1.128529107 | NM_015210| | KIAA0802,KIAA0802 |
| 213467_at | 13.45 | 6.07E-08 | 1.18491451 | NM_005440| | RND2,GTP-binding protein Rho7 |
| 202431_s_at | 13.45 | 6.09E-08 | 2.052320519 | NM_002467| | MYC,v-myc myelocytomatosis viral oncogene homolog |
| 223426_s_at | 13.45 | 6.08E-08 | 1.15914756 | NM_018424| | EPB41L4B,erythrocyte membrane protein band 4.1 like 4B |
| 204342_at | 13.45 | 6.11E-08 | 1.192477304 | NM_013386| | SLC25A24,solute carrier family 25 member 24 isoform 1 |
| 203860_at | 13.44 | 6.15E-08 | 1.159607386 | NM_000282| | PCCA,propionyl-Coenzyme A carboxylase, alpha |
| 208841_s_at | 13.43 | 6.19E-08 | 1.066344128 | NM_012297| | G3BP2,Ras-GTPase activating protein SH3 domain-binding |
| 212653_s_at | 13.43 | 6.17E-08 | 1.09831117 | NM_015252| | EHBP1,EH domain binding protein 1 |
| 212264_s_at | 13.43 | 6.21E-08 | 1.12046088 | NM_015045| | KIAA0261,KIAA0261 |
| 235648_at | 13.43 | 6.18E-08 | 1.090399487 | NM_152603| | ZNF567,zinc finger protein 567 |
| 212345_s_at | 13.42 | 6.24E-08 | 1.130664762 | NM_194071| | CREB3L2,cAMP responsive element binding protein 3-like |
| 235955_at | 13.42 | 6.21E-08 | 1.907912132 | NM_001038603| | NA |
| 205052_at | 13.42 | 6.24E-08 | 1.130676555 | NM_001698| | AUH,AU RNA-binding protein/enoyl-Coenzyme A |
| 204890_s_at | 13.41 | 6.30E-08 | 1.927552162 | NM_001042771| | NA |
| 230839_at | 13.41 | 6.30E-08 | 1.17955028 | NM_019854| | HRMT1L4,protein arginine N-methyltransferase 4 |
| 243580_at | 13.41 | 6.30E-08 | 1.076887378 | NM_004297| | GNA14,guanine nucleotide binding protein (G protein), |
| 217127_at | 13.39 | 6.37E-08 | 1.256530778 | NM_001902| | CTH,cystathionase isoform 1 |
| 218909_at | 13.39 | 6.41E-08 | 1.088290317 | NM_012424| | RPS6KC1,ribosomal protein S6 kinase, 52kDa, polypeptide |
| 226870_at | 13.39 | 6.37E-08 | 1.236008596 | NM_144589| | COMTD1,catechol-O-methyltransferase domain containing |
| 235507_at | 13.39 | 6.38E-08 | 1.139886827 | NM_052937| | LOC115294,similar to hypothetical protein FLJ10883 |
| 203035_s_at | 13.39 | 6.37E-08 | 1.07567492 | NM_006099| | PIAS3,protein inhibitor of activated STAT, 3 |
| 228373_at | 13.38 | 6.46E-08 | 1.078742638 | NM_014117| | PRO0149,PRO0149 protein |
| 229065_at | 13.38 | 6.44E-08 | 1.29983386 | NM_173508| | SLC35F3,solute carrier family 35, member F3 |
| 216548_x_at | 13.38 | 6.42E-08 | 1.048260728 | NA |  |
| 201345_s_at | 13.38 | 6.42E-08 | 1.064656988 | NM_003339| | UBE2D2,ubiquitin-conjugating enzyme E2D 2 isoform 1 |
| 201841_s_at | 13.38 | 6.42E-08 | 1.061602539 | NM_001540| | HSPB1,heat shock 27kDa protein 1 |
| 228856_at | 13.37 | 6.47E-08 | 1.62763577 | NM_023931| | MGC2474,hypothetical protein MGC2474 |
| 211997_x_at | 13.37 | 6.50E-08 | 1.042487153 | NM_002107| | H3F3A,H3 histone, family 3A |
| 219465_at | 13.36 | 6.54E-08 | 1.657284869 | NM_001643| | APOA2,apolipoprotein A-II precursor |
| 210621_s_at | 13.36 | 6.51E-08 | 1.127253495 | NM_002890| | RASA1,RAS p21 protein activator 1 isoform 1 |
| 1562701_at | 13.36 | 6.53E-08 | 1.384883574 | NA |  |
| 227628_at | 13.35 | 6.61E-08 | 1.157093997 | NM_001008397| | LOC493869,similar to 2310016C16Rik protein |
| 202525_at | 13.35 | 6.60E-08 | 1.886391686 | NM_002773| | PRSS8,prostasin preproprotein |
| 218491_s_at | 13.35 | 6.57E-08 | 1.058320867 | NM_001037304| | NA |
| 215594_at | 13.34 | 6.63E-08 | 1.647762104 | NA |  |
| 209436_at | 13.34 | 6.62E-08 | 1.295612621 | NM_006108| | SPON1,spondin 1, extracellular matrix protein |
| 37986_at | 13.34 | 6.64E-08 | 1.233274288 | NM_000121| | EPOR,erythropoietin receptor precursor |
| 202795_x_at | 13.34 | 6.64E-08 | 1.093382188 | NM_001039141| | NA |
| 217765_at | 13.33 | 6.68E-08 | 1.06601858 | NM_013392| | NRBP,nuclear receptor binding protein |
| 225577_at | 13.33 | 6.67E-08 | 1.107161222 | NA |  |
| 238654_at | 13.33 | 6.67E-08 | 1.432124014 | NA |  |
| 228912_at | 13.33 | 6.70E-08 | 1.469056925 | NM_007127| | VIL1,villin 1 |
| 210554_s_at | 13.33 | 6.69E-08 | 1.070878262 | NM_001083914| | NA |
| 228100_at | 13.33 | 6.66E-08 | 1.411729708 | NM_181643| | LOC128344,hypothetical protein LOC128344 |
| 1556069_s_at | 13.32 | 6.76E-08 | 1.646501378 | NM_022462| | HIF3A,hypoxia-inducible factor-3 alpha isoform b |
| 203656_at | 13.31 | 6.80E-08 | 1.138726892 | NM_014845| | KIAA0274,Sac domain-containing inositol phosphatase 3 |
| 204597_x_at | 13.31 | 6.81E-08 | 2.00921847 | NM_003155| | STC1,stanniocalcin 1 |
| 228398_at | 13.31 | 6.78E-08 | 1.10782352 | NM_145204| | SENP8,SUMO/sentrin specific protease family member 8 |
| 207522_s_at | 13.31 | 6.79E-08 | 1.471111678 | NM_005173| | ATP2A3,sarco/endoplasmic reticulum Ca2+ -ATPase isoform |
| 229016_s_at | 13.3 | 6.85E-08 | 1.396313438 | NM_033502| | TRERF1,transcriptional regulating factor 1 isoform 1 |
| 213567_at | 13.3 | 6.85E-08 | 1.11266885 | NA |  |
| 210907_s_at | 13.3 | 6.86E-08 | 1.058726574 | NM_007217| | PDCD10,programmed cell death 10 |
| 229752_at | 13.3 | 6.84E-08 | 1.106254148 | NM_022465| | ZNFN1A4,zinc finger protein, subfamily 1A, 4 |
| 1559827_at | 13.3 | 6.86E-08 | 1.780106693 | NA |  |
| 236266_at | 13.29 | 6.90E-08 | 1.48131629 | NA |  |
| 201100_s_at | 13.29 | 6.88E-08 | 1.197131342 | NM_001039590| | NA |
| 206696_at | 13.29 | 6.88E-08 | 1.678471306 | NM_000273| | GPR143,G protein-coupled receptor 143 |
| 202752_x_at | 13.28 | 6.94E-08 | 1.556387126 | NM_012244| | SLC7A8,solute carrier family 7 (cationic amino acid |
| 229012_at | 13.28 | 6.95E-08 | 1.239567156 | NM_032596| | C9orf24,testes development-related NYD-SP22 isoform 1 |
| 213572_s_at | 13.26 | 7.07E-08 | 1.379171645 | NM_030666| | SERPINB1,serine (or cysteine) proteinase inhibitor, clade |
| 204590_x_at | 13.26 | 7.09E-08 | 1.141218468 | NM_022916| | VPS33A,vacuolar protein sorting 33A |
| 203494_s_at | 13.26 | 7.05E-08 | 1.102896156 | NM_014679| | PIG8,translokin |
| 229693_at | 13.26 | 7.08E-08 | 1.518484708 | NM_001004313| | LOC388335,similar to RIKEN cDNA A730055C05 gene |
| 218217_at | 13.25 | 7.13E-08 | 1.154357598 | NM_021626| | SCPEP1,serine carboxypeptidase 1 precursor protein |
| 228994_at | 13.25 | 7.13E-08 | 1.186154032 | NM_152499| | MGC45441,hypothetical protein MGC45441 |
| 202723_s_at | 13.25 | 7.14E-08 | 1.163254476 | NM_002015| | FOXO1A,forkhead box O1A |
| 209900_s_at | 13.24 | 7.20E-08 | 1.123351111 | NM_003051| | SLC16A1,solute carrier family 16, member 1 |
| 220199_s_at | 13.24 | 7.18E-08 | 1.110525429 | NM_022831| | FLJ12806,hypothetical protein FLJ12806 |
| 224625_x_at | 13.24 | 7.18E-08 | 1.030125998 | NM_001018108| | NA |
| 225917_at | 13.23 | 7.23E-08 | 1.079690588 | NA |  |
| 225175_s_at | 13.23 | 7.22E-08 | 1.15967856 | NM_020428| | CTL2,CTL2 gene |
| 218258_at | 13.23 | 7.22E-08 | 1.06043968 | NM_015972| | POLR1D,RNA polymerase I 16 kDa subunit |
| 241879_at | 13.22 | 7.27E-08 | 1.246756318 | NA |  |
| 222835_at | 13.22 | 7.27E-08 | 1.464397085 | NM_024817| | FLJ13710,hypothetical protein FLJ13710 |
| 203936_s_at | 13.21 | 7.35E-08 | 1.237684431 | NM_004994| | MMP9,matrix metalloproteinase 9 preproprotein |
| 204601_at | 13.21 | 7.33E-08 | 1.131228358 | NM_153029| | N4BP1,Nedd4 binding protein 1 |
| 211926_s_at | 13.21 | 7.36E-08 | 1.07119381 | NM_002473| | MYH9,myosin, heavy polypeptide 9, non-muscle |
| 204783_at | 13.21 | 7.38E-08 | 1.160184236 | NM_022443| | MLF1,myeloid leukemia factor 1 |
| 223199_at | 13.2 | 7.38E-08 | 1.12024723 | NM_017572| | MKNK2,MAP kinase-interacting serine/threonine kinase |
| 226985_at | 13.2 | 7.44E-08 | 1.710150532 | NM_152536| | FGD5,FYVE, RhoGEF and PH domain containing 5 |
| 210287_s_at | 13.2 | 7.42E-08 | 1.826810978 | NM_002019| | FLT1,fms-related tyrosine kinase 1 (vascular |
| 223211_at | 13.19 | 7.49E-08 | 1.123299342 | NM_012260| | HPCL2,2-hydroxyphytanoyl-CoA lyase |
| 219382_at | 13.19 | 7.47E-08 | 1.253928794 | NM_013368| | SERTAD3,RPA-binding trans-activator |
| 201573_s_at | 13.19 | 7.50E-08 | 1.053622996 | NM_004730| | ETF1,eukaryotic translation termination factor 1 |
| 203090_at | 13.18 | 7.51E-08 | 1.054647606 | NM_006923| | SDF2,stromal cell-derived factor 2 precursor |
| 227826_s_at | 13.18 | 7.51E-08 | 2.326128925 | NA |  |
| 220642_x_at | 13.18 | 7.53E-08 | 1.054618594 | NM_001097612| | NA |
| 216511_s_at | 13.18 | 7.53E-08 | 1.12356687 | NM_030756| | TCF7L2,transcription factor 7-like 2 (T-cell specific, |
| 208621_s_at | 13.18 | 7.54E-08 | 1.209728255 | NM_001111077| | NA |
| 226918_at | 13.18 | 7.53E-08 | 1.74129382 | NM_032452| | JPH4,junctophilin 4 |
| 226521_s_at | 13.17 | 7.59E-08 | 1.120532734 | NM_139076| | FLJ13614,hypothetical protein FLJ13614 |
| 239132_at | 13.17 | 7.56E-08 | 1.458813101 | NM_000620| | NOS1,nitric oxide synthase 1 (neuronal) |
| 1557170_at | 13.17 | 7.59E-08 | 1.092364634 | NM_178170| | NEK8,NIMA-related kinase 8 |
| 209472_at | 13.17 | 7.57E-08 | 1.108283672 | NM_001008661| | KAT3,kynurenine aminotransferase III isoform 1 |
| 209560_s_at | 13.17 | 7.57E-08 | 2.291140265 | NM_003836| | DLK1,delta-like homolog |
| 229280_s_at | 13.16 | 7.65E-08 | 1.294186545 | NA |  |
| 225890_at | 13.15 | 7.71E-08 | 1.080190862 | NM_052865| | C20orf72,chromosome 20 open reading frame 72 |
| 227551_at | 13.14 | 7.77E-08 | 1.103420809 | NM_001025780| | NA |
| 204280_at | 13.14 | 7.78E-08 | 1.18574145 | NM_006480| | RGS14,regulator of G-protein signalling 14 |
| 232353_s_at | 13.14 | 7.76E-08 | 1.11047868 | NM_016086| | DUSP24,map kinase phosphatase-like protein MK-STYX |
| 232615_at | 13.13 | 7.86E-08 | 1.617951151 | NA |  |
| 210946_at | 13.13 | 7.86E-08 | 1.491345619 | NM_003711| | PPAP2A,phosphatidic acid phosphatase type 2A isoform 1 |
| 213553_x_at | 13.12 | 7.91E-08 | 1.180258304 | NM_001645| | APOC1,apolipoprotein C-I precursor |
| 238205_at | 13.12 | 7.89E-08 | 1.673373866 | NM_178470| | WDR40B,WD repeat domain 40B |
| 226955_at | 13.12 | 7.89E-08 | 1.34035288 | NM_152406| | FLJ36748,hypothetical protein FLJ36748 |
| 212945_s_at | 13.11 | 7.98E-08 | 1.069281591 | NM_001080541| | NA |
| 220137_at | 13.11 | 7.96E-08 | 1.24190467 | NM_019086| | FLJ20674,hypothetical protein FLJ20674 |
| 230633_at | 13.11 | 7.97E-08 | 1.199635775 | NM_178518| | FLJ36878,hypothetical protein FLJ36878 |
| 209276_s_at | 13.11 | 7.97E-08 | 1.397774172 | NM_001118890| | NA |
| 209102_s_at | 13.1 | 8.03E-08 | 1.09779318 | NM_012257| | HBP1,HMG-box transcription factor 1 |
| 231022_at | 13.1 | 8.04E-08 | 1.555650826 | NA |  |
| 209392_at | 13.1 | 8.01E-08 | 1.390829763 | NM_001040092| | NA |
| 226638_at | 13.09 | 8.06E-08 | 1.191151601 | NA |  |
| 215148_s_at | 13.09 | 8.06E-08 | 1.083337836 | NM_004886| | APBA3,amyloid beta (A4) precursor protein-binding, |
| 229278_at | 13.09 | 8.10E-08 | 1.356220943 | NA |  |
| 218757_s_at | 13.09 | 8.11E-08 | 1.105243705 | NM_023010| | UPF3B,UPF3 regulator of nonsense transcripts homolog B |
| 223249_at | 13.08 | 8.18E-08 | 1.147667374 | NM_012129| | CLDN12,claudin 12 |
| 201315_x_at | 13.08 | 8.18E-08 | 1.25745912 | NM_006435| | IFITM2,interferon induced transmembrane protein 2 |
| 44822_s_at | 13.08 | 8.16E-08 | 1.14140771 | NM_017550| | KIAA1193,KIAA1193 |
| 218847_at | 13.07 | 8.19E-08 | 1.105075069 | NM_001007225| | IMP-2,IGF-II mRNA-binding protein 2 isoform b |
| 244052_at | 13.07 | 8.19E-08 | 1.138868295 | NM_032783| | CBR4,carbonic reductase 4 |
| 225519_at | 13.07 | 8.22E-08 | 1.102934044 | NM_174907| | PPP4R2,protein phosphatase 4, regulatory subunit 2 |
| 216037_x_at | 13.07 | 8.22E-08 | 1.13155569 | NM_030756| | TCF7L2,transcription factor 7-like 2 (T-cell specific, |
| 203857_s_at | 13.07 | 8.23E-08 | 1.091232102 | NM_006810| | PDIR,for protein disulfide isomerase-related |
| 209578_s_at | 13.07 | 8.23E-08 | 1.085550737 | NM_015227| | POFUT2,protein O-fucosyltransferase 2 isoform A |
| 1569191_at | 13.06 | 8.25E-08 | 2.290842001 | NM_001039884| | NA |
| 213018_at | 13.06 | 8.27E-08 | 1.069226244 | NM_021167| | ODAG,ocular development-associated gene |
| 201659_s_at | 13.06 | 8.29E-08 | 1.064432106 | NM_001177| | ARL1,ADP-ribosylation factor-like 1 |
| 1555864_s_at | 13.05 | 8.34E-08 | 1.043901277 | NM_000284| | PDHA1,pyruvate dehydrogenase (lipoamide) alpha 1 |
| 225660_at | 13.04 | 8.44E-08 | 1.171857203 | NM_020796| | SEMA6A,semaphorin 6A1 |
| 201369_s_at | 13.04 | 8.38E-08 | 1.224389155 | NM_006887| | ZFP36L2,butyrate response factor 2 |
| 202511_s_at | 13.03 | 8.46E-08 | 1.103908489 | NM_004849| | APG5L,APG5 autophagy 5-like |
| 235177_at | 13.03 | 8.46E-08 | 1.096929848 | NM_145280| | LOC151194,hepatocellular carcinoma-associated antigen |
| 203718_at | 13.03 | 8.47E-08 | 1.114747622 | NM_006702| | NTE,neuropathy target esterase |
| 244133_at | 13.03 | 8.51E-08 | 1.144754366 | NM_018561| | USP49,ubiquitin specific protease 49 |
| 242064_at | 13.02 | 8.56E-08 | 1.167950636 | NM_019064| | SDK2,sidekick 2 |
| 203798_s_at | 13.01 | 8.65E-08 | 2.502819306 | NM_003385| | VSNL1,visinin-like 1 |
| 226809_at | 13.01 | 8.61E-08 | 1.597313913 | NA |  |
| 201220_x_at | 13 | 8.67E-08 | 1.064928712 | NM_001083914| | NA |
| 223424_s_at | 12.99 | 8.75E-08 | 1.111082774 | NM_145914| | ZNF38,zinc finger protein 38 |
| 219403_s_at | 12.99 | 8.74E-08 | 1.548845116 | NM_001098540| | NA |
| 1563327_a_at | 12.99 | 8.79E-08 | 1.347882283 | NA |  |
| 203087_s_at | 12.98 | 8.80E-08 | 1.082505745 | NM_001098511| | NA |
| 223807_at | 12.98 | 8.80E-08 | 1.582130405 | NM_001555| | IGSF1,immunoglobulin superfamily, member 1 isoform 1 |
| 203374_s_at | 12.98 | 8.82E-08 | 1.078447273 | NM_003291| | TPP2,tripeptidyl peptidase II |
| 206747_at | 12.98 | 8.82E-08 | 1.22714571 | NM_014696| | KIAA0514,KIAA0514 |
| 201925_s_at | 12.97 | 8.93E-08 | 1.871285642 | NM_000574| | DAF,decay accelerating factor for complement (CD55, |
| 204776_at | 12.97 | 8.91E-08 | 1.13445591 | NM_003248| | THBS4,thrombospondin 4 precursor |
| 228205_at | 12.96 | 9.00E-08 | 1.211056293 | NM_001064| | TKT,transketolase |
| 229085_at | 12.96 | 9.00E-08 | 1.259504398 | NM_052953| | LRRC3B,leucine rich repeat containing 3B |
| 203100_s_at | 12.96 | 8.95E-08 | 1.079744387 | NM_004824| | CDYL,chromodomain protein, Y chromosome-like isoform |
| 205719_s_at | 12.96 | 8.95E-08 | 1.804343786 | NM_000277| | PAH,phenylalanine hydroxylase |
| 213661_at | 12.96 | 8.95E-08 | 1.454442655 | NM_001001991| | DKFZP586H2123,regeneration associated muscle protease isoform |
| 223264_at | 12.95 | 9.04E-08 | 1.100565293 | NM_022566| | MESDC1,mesoderm development candidate 1 |
| 218922_s_at | 12.94 | 9.10E-08 | 1.161181026 | NM_024552| | LASS4,LAG1 longevity assurance homolog 4 |
| 230951_at | 12.94 | 9.14E-08 | 2.052026728 | NA |  |
| 205401_at | 12.94 | 9.13E-08 | 1.186540926 | NM_003659| | AGPS,alkylglycerone phosphate synthase precursor |
| 209016_s_at | 12.93 | 9.19E-08 | 1.754278316 | NM_005556| | KRT7,keratin 7 |
| 208711_s_at | 12.93 | 9.19E-08 | 1.054965902 | NM_053056| | CCND1,cyclin D1 |
| 210511_s_at | 12.93 | 9.23E-08 | 1.607303535 | NM_002192| | INHBA,inhibin beta A subunit precursor |
| 209619_at | 12.92 | 9.30E-08 | 1.414287163 | NM_001025158| | NA |
| 225755_at | 12.92 | 9.27E-08 | 1.070342491 | NM_173546| | MGC35097,hypothetical protein MGC35097 |
| 204285_s_at | 12.92 | 9.28E-08 | 1.272907472 | NM_021127| | PMAIP1,phorbol-12-myristate-13-acetate-induced protein |
| 242070_at | 12.91 | 9.34E-08 | 1.611256615 | NA |  |
| 232847_at | 12.91 | 9.32E-08 | 1.264110636 | NM_171999| | SALL3,sal-like 3 |
| 202537_s_at | 12.91 | 9.38E-08 | 1.14121582 | NM_014043| | DKFZP564O123,DKFZP564O123 protein |
| 1560250_s_at | 12.9 | 9.44E-08 | 1.409194442 | NA |  |
| 235144_at | 12.9 | 9.40E-08 | 1.615425397 | NA |  |
| 204804_at | 12.9 | 9.40E-08 | 1.209170455 | NM_003141| | TRIM21,52kD Ro/SSA autoantigen |
| 205139_s_at | 12.89 | 9.51E-08 | 1.236365346 | NM_005715| | UST,uronyl-2-sulfotransferase |
| 204684_at | 12.89 | 9.47E-08 | 2.290048298 | NM_002522| | NPTX1,neuronal pentraxin I precursor |
| 209633_at | 12.89 | 9.48E-08 | 1.177658681 | NM_002718| | PPP2R3A,alpha isoform of regulatory subunit B'', protein |
| 242871_at | 12.89 | 9.48E-08 | 1.887347464 | NM_001104554| | NA |
| 229933_at | 12.88 | 9.55E-08 | 1.252339592 | NM_152485| | FLJ25078,hypothetical protein FLJ25078 |
| 235358_at | 12.87 | 9.65E-08 | 1.284476311 | NA |  |
| 1568780_at | 12.86 | 9.72E-08 | 1.596551851 | NA |  |
| 212770_at | 12.86 | 9.76E-08 | 1.150727291 | NM_001105192| | NA |
| 215726_s_at | 12.85 | 9.82E-08 | 1.085246481 | NM_001914| | CYB5,cytochrome b-5 isoform 2 |
| 221794_at | 12.85 | 9.79E-08 | 1.072620235 | NM_020812| | DOCK6,dedicator of cytokinesis 6 |
| 217916_s_at | 12.85 | 9.85E-08 | 1.070486614 | NM_016623| | FAM49B,family with sequence similarity 49, member B |
| 225330_at | 12.85 | 9.79E-08 | 1.07893636 | NM_000875| | IGF1R,insulin-like growth factor 1 receptor precursor |
| 212920_at | 12.85 | 9.79E-08 | 1.191221112 | NA |  |
| 223373_s_at | 12.84 | 9.93E-08 | 1.153034943 | NM_030821| | PLA2G12A,phospholipase A2, group XIIA |
| 207223_s_at | 12.84 | 9.87E-08 | 1.277000891 | NM_005156| | ROD1,ROD1 regulator of differentiation 1 |
| 221831_at | 12.83 | 9.99E-08 | 1.144538742 | NM_033631| | LUZP1,leucine zipper protein 1 |
| 229545_at | 12.83 | 9.98E-08 | 1.188039159 | NM_017671| | C20orf42,chromosome 20 open reading frame 42 |
| 219675_s_at | 12.83 | 9.94E-08 | 1.102893694 | NM_025076| | UXS1,UDP-glucuronate decarboxylase 1 |
| 56256_at | 12.82 | 1.01E-07 | 1.073722673 | NM_001040455| | NA |
| 204619_s_at | 12.82 | 1.00E-07 | 1.341399325 | NM_001126336| | NA |
| 211153_s_at | 12.81 | 1.02E-07 | 1.312217252 | NM_003701| | TNFSF11,tumor necrosis factor ligand superfamily, member |
| 203988_s_at | 12.81 | 1.02E-07 | 1.082709995 | NM_004480| | FUT8,fucosyltransferase 8 isoform b |
| 201850_at | 12.81 | 1.01E-07 | 1.486750364 | NM_001747| | CAPG,capping protein (actin filament), gelsolin-like |
| 201712_s_at | 12.81 | 1.01E-07 | 1.140213391 | NM_006267| | RANBP2,RAN binding protein 2 |
| 214749_s_at | 12.8 | 1.03E-07 | 1.065794903 | NM_001009584| | ARMCX6,armadillo repeat containing, X-linked 6 |
| 225278_at | 12.8 | 1.02E-07 | 1.135628807 | NM_005399| | PRKAB2,AMP-activated protein kinase beta 2 |
| 228041_at | 12.8 | 1.02E-07 | 1.056272222 | NM_181806| | NRPS998,2-aminoadipic 6-semialdehyde dehydrogenase |
| 224150_s_at | 12.79 | 1.03E-07 | 1.173034756 | NM_024491| | Cep70,centrosomal protein 70 kDa |
| 218221_at | 12.79 | 1.03E-07 | 1.077004687 | NM_001668| | ARNT,aryl hydrocarbon receptor nuclear translocator |
| 242127_at | 12.79 | 1.03E-07 | 2.073658015 | NA |  |
| 202721_s_at | 12.79 | 1.03E-07 | 1.282864403 | NM_002056| | GFPT1,glucosamine-fructose-6-phosphate |
| 214829_at | 12.79 | 1.03E-07 | 1.280899762 | NM_005763| | AASS,aminoadipate-semialdehyde synthase |
| 208855_s_at | 12.78 | 1.04E-07 | 1.060252226 | NM_001032296| | NA |
| 221378_at | 12.78 | 1.04E-07 | 1.910979922 | NM_005454| | CER1,cerberus 1 |
| 215646_s_at | 12.78 | 1.04E-07 | 1.262513703 | NM_001126336| | NA |
| 1568617_a_at | 12.77 | 1.04E-07 | 1.666414374 | NM_001080429| | NA |
| 214820_at | 12.77 | 1.04E-07 | 1.126760776 | NM_001007246| | WDR9,WD repeat domain 9 isoform C |
| 220157_x_at | 12.77 | 1.05E-07 | 1.155944804 | NM_015899| | PLEKHA9,pleckstrin homology domain containing, family A |
| 227388_at | 12.76 | 1.06E-07 | 1.272457005 | NM_001004125| | TUSC1,tumor suppressor candidate 1 |
| 1558368_s_at | 12.76 | 1.06E-07 | 1.267906461 | NM_198545| | LOC374946,hypothetical gene supported by AK075558; |
| 236578_at | 12.75 | 1.07E-07 | 1.269791474 | NA |  |
| 220760_x_at | 12.75 | 1.06E-07 | 1.130629285 | NM_024733| | FLJ14345,hypothetical protein FLJ14345 |
| 224819_at | 12.75 | 1.06E-07 | 1.215237583 | NM_001006684| | TCEAL8,transcription elongation factor A (SII)-like 8 |
| 227662_at | 12.74 | 1.08E-07 | 1.938138154 | NM_133477| | SYNPO2,synaptopodin 2 |
| 1559072_a_at | 12.74 | 1.08E-07 | 1.10033794 | NM_052906| | KIAA1904,KIAA1904 protein |
| 206541_at | 12.73 | 1.08E-07 | 1.494760135 | NM_000892| | KLKB1,plasma kallikrein B1 precursor |
| 221047_s_at | 12.72 | 1.09E-07 | 1.116514601 | NM_018650| | MARK1,MAP/microtubule affinity-regulating kinase 1 |
| 1563658_a_at | 12.71 | 1.10E-07 | 1.175010812 | NM_175733| | SYT9,synaptotagmin IX |
| 211668_s_at | 12.71 | 1.10E-07 | 1.522583457 | NM_002658| | PLAU,urokinase plasminogen activator preproprotein |
| 227985_at | 12.71 | 1.10E-07 | 1.834323771 | NA |  |
| 203714_s_at | 12.71 | 1.10E-07 | 1.103021102 | NM_001079515| | NA |
| 212860_at | 12.7 | 1.11E-07 | 1.082549316 | NM_032283| | ZDHHC18,zinc finger, DHHC domain containing 18 |
| 217149_x_at | 12.69 | 1.12E-07 | 1.137537102 | NM_003985| | TNK1,tyrosine kinase, non-receptor, 1 |
| 226659_at | 12.69 | 1.12E-07 | 1.460012601 | NM_022047| | DEF6,differentially expressed in FDCP 6 homolog |
| 209174_s_at | 12.69 | 1.12E-07 | 1.048040358 | NM_017730| | FLJ20259,FLJ20259 protein |
| 224832_at | 12.68 | 1.13E-07 | 1.213694817 | NM_030640| | DUSP16,dual specificity phosphatase 16 |
| 218318_s_at | 12.68 | 1.13E-07 | 1.1639833 | NM_016231| | NLK,nemo like kinase |
| 206374_at | 12.68 | 1.13E-07 | 1.317367479 | NM_004420| | DUSP8,dual specificity phosphatase 8 |
| 228256_s_at | 12.68 | 1.13E-07 | 1.727939478 | NM_022140| | EPB41L4A,erythrocyte protein band 4.1-like 4 |
| 214714_at | 12.68 | 1.13E-07 | 1.087309429 | NM_032164| | ZNF394,zinc finger protein 99 |
| 206348_s_at | 12.67 | 1.14E-07 | 1.381456573 | NM_005391| | PDK3,pyruvate dehydrogenase kinase, isoenzyme 3 |
| 209204_at | 12.67 | 1.14E-07 | 1.097213124 | NM_006769| | LMO4,LIM domain only 4 |
| 202054_s_at | 12.67 | 1.14E-07 | 1.11361847 | NM_000382| | ALDH3A2,aldehyde dehydrogenase 3A2 |
| 238600_at | 12.66 | 1.15E-07 | 1.422802941 | NM_001099433| | NA |
| 201518_at | 12.66 | 1.14E-07 | 1.0775647 | NM_006807| | CBX1,chromobox homolog 1 (HP1 beta homolog Drosophila |
| 241574_s_at | 12.66 | 1.15E-07 | 1.666349148 | NM_006546| | IMP-1,IGF-II mRNA-binding protein 1 |
| 1558689_a_at | 12.66 | 1.15E-07 | 1.234270122 | NA |  |
| 225444_at | 12.65 | 1.16E-07 | 1.106541637 | NM_173569| | NA |
| 206424_at | 12.65 | 1.15E-07 | 2.444386257 | NM_000783| | CYP26A1,cytochrome P450, family 26, subfamily A, |
| 210839_s_at | 12.64 | 1.17E-07 | 1.361141957 | NM_001040092| | NA |
| 217887_s_at | 12.63 | 1.18E-07 | 1.115491945 | NM_001981| | EPS15,epidermal growth factor receptor pathway |
| 1556308_at | 12.63 | 1.18E-07 | 1.143211318 | NM_207351| | FLJ33674,hypothetical protein FLJ33674 |
| 204526_s_at | 12.63 | 1.18E-07 | 1.097950112 | NM_001102426| | NA |
| 224400_s_at | 12.62 | 1.19E-07 | 2.427865294 | NM_031422| | CHST9,GalNAc-4-sulfotransferase 2 |
| 209992_at | 12.62 | 1.19E-07 | 1.382031757 | NM_001018053| | NA |
| 212931_at | 12.61 | 1.20E-07 | 1.098165237 | NM_005650| | TCF20,transcription factor 20 isoform 1 |
| 228437_at | 12.61 | 1.19E-07 | 1.223285913 | NM_014184| | HSPC163,HSPC163 protein |
| 203436_at | 12.61 | 1.20E-07 | 1.075274436 | NM_001104546| | NA |
| 207307_at | 12.6 | 1.20E-07 | 1.553379416 | NM_000868| | HTR2C,5-hydroxytryptamine (serotonin) receptor 2C |
| 213225_at | 12.6 | 1.20E-07 | 1.319247269 | NM_001033556| | NA |
| 218845_at | 12.6 | 1.21E-07 | 1.073319923 | NM_020185| | DUSP22,dual specificity phosphatase 22 |
| 218676_s_at | 12.6 | 1.21E-07 | 1.364484996 | NM_001102402| | NA |
| 229700_at | 12.59 | 1.21E-07 | 1.163049878 | NA |  |
| 201316_at | 12.59 | 1.21E-07 | 1.07316715 | NM_002787| | PSMA2,proteasome alpha 2 subunit |
| 225969_at | 12.59 | 1.22E-07 | 1.123335677 | NM_001039876| | NA |
| 226873_at | 12.59 | 1.22E-07 | 1.176882282 | NA |  |
| 222203_s_at | 12.57 | 1.24E-07 | 1.097082832 | NM_001002006| | NT5C1B,5' nucleotidase, cytosolic IB isoform 1 |
| 201842_s_at | 12.57 | 1.23E-07 | 1.968634247 | NM_001039348| | NA |
| 212025_s_at | 12.56 | 1.25E-07 | 1.127826173 | NM_002018| | FLII,flightless I homolog |
| 201998_at | 12.56 | 1.25E-07 | 1.163712234 | NM_003032| | ST6GAL1,sialyltransferase 1 isoform a |
| 205441_at | 12.55 | 1.26E-07 | 1.19012587 | NM_024578| | FLJ22709,hypothetical protein FLJ22709 |
| 202777_at | 12.55 | 1.26E-07 | 1.027210718 | NM_007373| | SHOC2,soc-2 suppressor of clear homolog |
| 207153_s_at | 12.54 | 1.27E-07 | 1.161810866 | NM_053274| | GLMN,glomulin isoform FAP68 |
| 202074_s_at | 12.54 | 1.27E-07 | 1.468488289 | NM_001008211| | OPTN,optineurin |
| 208752_x_at | 12.54 | 1.27E-07 | 1.014532592 | NM_004537| | NAP1L1,nucleosome assembly protein 1-like 1 |
| 1552477_a_at | 12.53 | 1.28E-07 | 1.583210257 | NM_006147| | IRF6,interferon regulatory factor 6 |
| 209422_at | 12.53 | 1.29E-07 | 1.086391029 | NM_016436| | PHF20,PHD finger protein 20 |
| 221801_x_at | 12.53 | 1.28E-07 | 1.835194216 | NM_006158| | NEFL,neurofilament, light polypeptide 68kDa |
| 209696_at | 12.53 | 1.28E-07 | 1.17323365 | NM_000507| | FBP1,fructose-1,6-bisphosphatase 1 |
| 235969_at | 12.52 | 1.30E-07 | 1.203660766 | NA |  |
| 201099_at | 12.51 | 1.31E-07 | 1.228486286 | NM_001039590| | NA |
| 242592_at | 12.51 | 1.31E-07 | 1.711727481 | NM_001099652| | NA |
| 235904_at | 12.5 | 1.31E-07 | 1.888186023 | NM_152404| | FLJ34658,hypothetical protein FLJ34658 |
| 208816_x_at | 12.5 | 1.32E-07 | 1.138498149 | NA |  |
| 232263_at | 12.49 | 1.32E-07 | 1.514351128 | NM_018057| | SLC6A15,solute carrier family 6, member 15 isoform 2 |
| 204327_s_at | 12.49 | 1.33E-07 | 1.09695829 | NM_003455| | ZNF202,zinc finger protein 202 |
| 203274_at | 12.48 | 1.34E-07 | 1.050742431 | NM_001007523| | F8A2,coagulation factor VIII-associated (intronic |
| 219488_at | 12.48 | 1.34E-07 | 1.194507908 | NM_017436| | A4GALT,alpha 1,4-galactosyltransferase |
| 209526_s_at | 12.48 | 1.34E-07 | 1.075161164 | NM_016073| | HDGFRP3,hepatoma-derived growth factor, related protein |
| 214106_s_at | 12.48 | 1.34E-07 | 1.089674305 | NM_001500| | GMDS,GDP-mannose 4,6-dehydratase |
| 209154_at | 12.48 | 1.34E-07 | 1.069342122 | NM_014604| | TAX1BP3,Tax1 (human T-cell leukemia virus type I) |
| 220144_s_at | 12.47 | 1.34E-07 | 1.326325523 | NM_022096| | ANKRD5,ankyrin repeat domain protein 5 |
| 235423_at | 12.47 | 1.34E-07 | 1.127853608 | NA |  |
| 203797_at | 12.46 | 1.36E-07 | 2.383995313 | NM_003385| | VSNL1,visinin-like 1 |
| 1564383_s_at | 12.46 | 1.36E-07 | 1.558567289 | NA |  |
| 218470_at | 12.45 | 1.38E-07 | 1.060192297 | NM_001040436| | NA |
| 201868_s_at | 12.45 | 1.37E-07 | 1.107062458 | NM_005647| | TBL1X,transducin beta-like 1X |
| 212350_at | 12.45 | 1.38E-07 | 1.107034948 | NM_015173| | TBC1D1,TBC1 (tre-2/USP6, BUB2, cdc16) domain family, |
| 218946_at | 12.45 | 1.37E-07 | 1.099297179 | NM_001002755| | HIRIP5,HIRA interacting protein 5 isoform 2 |
| 208398_s_at | 12.45 | 1.37E-07 | 1.142140687 | NM_004865| | TBPL1,TBP-like 1 |
| 221667_s_at | 12.45 | 1.38E-07 | 1.549803577 | NM_014365| | HSPB8,heat shock 27kDa protein 8 |
| 219736_at | 12.45 | 1.37E-07 | 1.278762083 | NM_001017397| | NA |
| 218342_s_at | 12.44 | 1.38E-07 | 1.156782176 | NM_024896| | KIAA1815,KIAA1815 |
| 218041_x_at | 12.44 | 1.38E-07 | 1.088553083 | NM_018976| | SLC38A2,solute carrier family 38, member 2 |
| 209874_x_at | 12.44 | 1.39E-07 | 1.202840835 | NM_017649| | CNNM2,cyclin M2 isoform 1 |
| 219319_at | 12.44 | 1.38E-07 | 1.289737589 | NM_022462| | HIF3A,hypoxia-inducible factor-3 alpha isoform b |
| 212242_at | 12.44 | 1.38E-07 | 1.286449267 | NM_006000| | TUBA1,tubulin, alpha 1 |
| 207305_s_at | 12.44 | 1.39E-07 | 1.115811114 | NM_014939| | KIAA1012,KIAA1012 |
| 222580_at | 12.43 | 1.40E-07 | 1.095585269 | NM_016620| | ZNF644,zinc finger protein 644 isoform 2 |
| 218522_s_at | 12.43 | 1.39E-07 | 1.116547107 | NM_018174| | BPY2IP1,BPY2 interacting protein 1 |
| 219494_at | 12.42 | 1.40E-07 | 1.07715679 | NM_012415| | RAD54B,RAD54 homolog B isoform 1 |
| 222589_at | 12.42 | 1.40E-07 | 1.193061102 | NM_016231| | NLK,nemo like kinase |
| 202073_at | 12.42 | 1.41E-07 | 1.603277937 | NM_001008211| | OPTN,optineurin |
| 226272_at | 12.42 | 1.40E-07 | 1.271944056 | NM_013441| | DSCR1L2,Down syndrome critical region gene 1-like 2 |
| 232146_at | 12.41 | 1.41E-07 | 1.133118364 | NM_002494| | NDUFC1,NADH dehydrogenase (ubiquinone) 1, subcomplex |
| 215693_x_at | 12.41 | 1.42E-07 | 1.059055765 | NM_017895| | DDX27,DEAD (Asp-Glu-Ala-Asp) box polypeptide 27 |
| 204500_s_at | 12.4 | 1.43E-07 | 1.189707907 | NM_015239| | AGTPBP1,ATP/GTP binding protein 1 |
| 1554747_a_at | 12.4 | 1.42E-07 | 1.142043897 | NM_001008491| | SEPT2,septin 2 |
| 204814_at | 12.39 | 1.45E-07 | 1.178809218 | NM_003716| | CADPS,Ca2+-dependent secretion activator isoform 1 |
| 228018_at | 12.39 | 1.45E-07 | 1.178245869 | NM_152864| | C20orf58,chromosome 20 open reading frame 58 |
| 212104_s_at | 12.38 | 1.46E-07 | 1.092690277 | NM_001031695| | NA |
| 219924_s_at | 12.38 | 1.45E-07 | 1.11723484 | NM_007167| | ZNF258,zinc finger protein 258 |
| 202925_s_at | 12.37 | 1.47E-07 | 1.138526637 | NM_002657| | PLAGL2,pleiomorphic adenoma gene-like 2 |
| 204901_at | 12.37 | 1.47E-07 | 1.105641005 | NM_003939| | BTRC,beta-transducin repeat containing protein |
| 235129_at | 12.37 | 1.47E-07 | 1.549397159 | NM_006741| | PPP1R1A,protein phosphatase 1, regulatory (inhibitor) |
| 218977_s_at | 12.36 | 1.48E-07 | 1.112697012 | NM_017846| | SECP43,tRNA selenocysteine associated protein |
| 204421_s_at | 12.36 | 1.48E-07 | 1.468809191 | NM_002006| | FGF2,fibroblast growth factor 2 |
| 217591_at | 12.36 | 1.47E-07 | 1.397002558 | NA |  |
| 222052_at | 12.35 | 1.50E-07 | 1.147787006 | NM_198476| | FLJ41131,FLJ41131 protein |
| 224665_at | 12.34 | 1.50E-07 | 1.046303452 | NM_173473| | C10orf104,chromosome 10 open reading frame 104 |
| 230083_at | 12.34 | 1.51E-07 | 1.510995913 | NM_019050| | USP53,ubiquitin specific protease 53 |
| 219327_s_at | 12.33 | 1.52E-07 | 1.450630494 | NM_018653| | GPRC5C,G protein-coupled receptor family C, group 5, |
| 208679_s_at | 12.33 | 1.51E-07 | 1.027188131 | NM_005731| | ARPC2,actin related protein 2/3 complex subunit 2 |
| 221486_at | 12.33 | 1.52E-07 | 1.067960731 | NM_004436| | ENSA,endosulfine alpha isoform 3 |
| 221025_x_at | 12.33 | 1.52E-07 | 1.159603756 | NM_001098614| | NA |
| 235176_at | 12.32 | 1.53E-07 | 1.373196205 | NM_133466| | ZNF545,zinc finger protein 545 |
| 1552643_at | 12.32 | 1.54E-07 | 1.364619434 | NM_001076675| | NA |
| 1568603_at | 12.32 | 1.54E-07 | 1.634141292 | NM_003716| | CADPS,Ca2+-dependent secretion activator isoform 1 |
| 229298_at | 12.32 | 1.53E-07 | 1.111587635 | NM_032138| | KBTBD7,kelch repeat and BTB (POZ) domain containing 7 |
| 203702_s_at | 12.32 | 1.53E-07 | 1.099201179 | NM_014640| | TTLL4,tubulin tyrosine ligase-like family, member 4 |
| 230185_at | 12.32 | 1.53E-07 | 1.226398375 | NM_024672| | THAP9,THAP domain containing 9 |
| 204234_s_at | 12.31 | 1.55E-07 | 1.073182809 | NM_007152| | ZNF195,zinc finger protein 195 |
| 209069_s_at | 12.31 | 1.55E-07 | 1.044164051 | NM_002107| | H3F3A,H3 histone, family 3A |
| 204927_at | 12.3 | 1.56E-07 | 1.250400643 | NM_003475| | C11orf13,HRAS1-related cluster-1 |
| 227058_at | 12.3 | 1.56E-07 | 1.440251567 | NM_032849| | FLJ14834,hypothetical protein FLJ14834 |
| 228787_s_at | 12.29 | 1.58E-07 | 1.271496346 | NM_001010974| | BCAS4,breast carcinoma amplified sequence 4 isoform c |
| 212488_at | 12.29 | 1.58E-07 | 1.634126915 | NM_000093| | COL5A1,alpha 1 type V collagen preproprotein |
| 224416_s_at | 12.28 | 1.59E-07 | 1.176471364 | NM_025205| | MED28,mediator of RNA polymerase II transcription, |
| 239670_at | 12.28 | 1.58E-07 | 1.176804813 | NA |  |
| 213401_s_at | 12.27 | 1.60E-07 | 1.187576659 | NM_005647| | TBL1X,transducin beta-like 1X |
| 221215_s_at | 12.27 | 1.60E-07 | 1.383810282 | NM_020639| | RIPK4,ankyrin repeat domain 3 |
| 212224_at | 12.27 | 1.60E-07 | 1.200286837 | NM_000689| | ALDH1A1,aldehyde dehydrogenase 1A1 |
| 238576_at | 12.26 | 1.62E-07 | 1.363388266 | NA |  |
| 218002_s_at | 12.26 | 1.61E-07 | 1.525752158 | NM_004887| | CXCL14,small inducible cytokine B14 precursor |
| 218942_at | 12.26 | 1.61E-07 | 1.098794975 | NM_024779| | PIP5K2C,phosphatidylinositol-4-phosphate 5-kinase, type |
| 203653_s_at | 12.26 | 1.62E-07 | 1.074503301 | NM_004645| | COIL,coilin |
| 1566764_at | 12.26 | 1.62E-07 | 1.395985059 | NM_182762| | 7A5,putative binding protein 7a5 |
| 203990_s_at | 12.26 | 1.61E-07 | 1.443482239 | NM_021140| | UTX,ubiquitously transcribed tetratricopeptide |
| 212626_x_at | 12.25 | 1.63E-07 | 1.040635629 | NM_001077442| | NA |
| 201829_at | 12.25 | 1.63E-07 | 1.158635187 | NM_001047160| | NA |
| 219988_s_at | 12.24 | 1.64E-07 | 1.065878941 | NM_018150| | FLJ10597,hypothetical protein FLJ10597 |
| 224618_at | 12.24 | 1.64E-07 | 1.226261113 | NM_005156| | ROD1,ROD1 regulator of differentiation 1 |
| 228346_at | 12.23 | 1.65E-07 | 1.189276497 | NA |  |
| 219326_s_at | 12.23 | 1.65E-07 | 1.588845115 | NM_006577| | B3GNT1,beta-1,3-N-acetylglucosaminyltransferase bGnT-1 |
| 227601_at | 12.23 | 1.66E-07 | 1.083467222 | NM_020961| | KIAA1627,KIAA1627 protein |
| 206290_s_at | 12.23 | 1.66E-07 | 1.357354586 | NM_002924| | RGS7,regulator of G-protein signalling 7 |
| 1555480_a_at | 12.22 | 1.67E-07 | 1.202866707 | NM_001024215| | NA |
| 223523_at | 12.22 | 1.66E-07 | 1.600231017 | NM_023943| | MGC3040,hypothetical protein MGC3040 |
| 220987_s_at | 12.22 | 1.66E-07 | 1.454533413 | NM_020642| | C11orf17,chromosome 11 open reading frame 17 |
| 228567_at | 12.21 | 1.68E-07 | 1.074517673 | NA |  |
| 203088_at | 12.21 | 1.68E-07 | 1.262167889 | NM_006329| | FBLN5,fibulin 5 precursor |
| 234924_s_at | 12.21 | 1.69E-07 | 1.127450767 | NM_020832| | KIAA1441,KIAA1441 protein |
| 204108_at | 12.21 | 1.68E-07 | 1.066943378 | NM_002505| | NFYA,nuclear transcription factor Y, alpha isoform 1 |
| 228360_at | 12.21 | 1.69E-07 | 1.369592599 | NM_177964| | LOC130576,hypothetical protein LOC130576 |
| 225165_at | 12.21 | 1.68E-07 | 1.464663237 | NM_032192| | PPP1R1B,protein phosphatase 1, regulatory (inhibitor) |
| 214426_x_at | 12.21 | 1.69E-07 | 1.049853216 | NM_005483| | CHAF1A,chromatin assembly factor 1, subunit A (p150) |
| 219773_at | 12.2 | 1.70E-07 | 1.448871843 | NM_016931| | NOX4,NADPH oxidase 4 |
| 212762_s_at | 12.2 | 1.70E-07 | 1.1648257 | NM_030756| | TCF7L2,transcription factor 7-like 2 (T-cell specific, |
| 201351_s_at | 12.2 | 1.69E-07 | 1.038590934 | NM_014263| | YME1L1,YME1-like 1 isoform 3 |
| 242387_at | 12.2 | 1.69E-07 | 1.479085308 | NM_175075| | INM01,hypothetical protein INM01 |
| 212914_at | 12.2 | 1.70E-07 | 1.316260504 | NM_175709| | CBX7,chromobox homolog 7 |
| 201204_s_at | 12.19 | 1.71E-07 | 1.179453497 | NM_001042576| | NA |
| 223458_at | 12.19 | 1.72E-07 | 1.33463062 | NM_001114099| | NA |
| 221637_s_at | 12.19 | 1.70E-07 | 1.053642448 | NM_024099| | MGC2477,hypothetical protein MGC2477 |
| 228027_at | 12.19 | 1.71E-07 | 1.11432169 | NM_001004051| | GPRASP2,G protein-coupled receptor associated sorting |
| 203006_at | 12.18 | 1.72E-07 | 1.079152269 | NM_005539| | INPP5A,inositol polyphosphate-5-phosphatase A |
| 234734_s_at | 12.17 | 1.75E-07 | 1.053959534 | NM_014494| | TNRC6A,trinucleotide repeat containing 6A |
| 244334_at | 12.16 | 1.76E-07 | 1.14376651 | NM_152402| | TRAM1L1,translocation associated membrane protein 1-like |
| 203793_x_at | 12.16 | 1.76E-07 | 1.095023915 | NM_007144| | PCGF2,ring finger protein 110 |
| 226305_at | 12.15 | 1.78E-07 | 1.195585946 | NM_023946| | LYNX1,Ly-6 neurotoxin-like protein 1 isoform a |
| 205865_at | 12.15 | 1.78E-07 | 1.714922199 | NM_005224| | ARID3A,AT rich interactive domain 3A (BRIGHT- like) |
| 225490_at | 12.15 | 1.77E-07 | 1.070019869 | NM_152641| | ARID2,AT rich interactive domain 2 (ARID, RFX-like) |
| 205768_s_at | 12.15 | 1.78E-07 | 1.429111549 | NM_003645| | SLC27A2,solute carrier family 27 (fatty acid |
| 227829_at | 12.15 | 1.78E-07 | 1.448089865 | NM_152312| | GYLTL1B,glycosyltransferase-like 1B |
| 201109_s_at | 12.15 | 1.78E-07 | 1.931363978 | NM_003246| | THBS1,thrombospondin 1 precursor |
| 52940_at | 12.14 | 1.79E-07 | 1.199476826 | NM_021805| | SIGIRR,single Ig IL-1R-related molecule |
| 229751_s_at | 12.14 | 1.78E-07 | 1.182460466 | NM_001098614| | NA |
| 203234_at | 12.14 | 1.79E-07 | 1.257424448 | NM_003364| | UPP1,uridine phosphorylase 1 |
| 231514_at | 12.13 | 1.80E-07 | 1.413220291 | NM_032884| | MGC15882,hypothetical protein MGC15882 |
| 221760_at | 12.13 | 1.81E-07 | 1.768818718 | NM_005907| | MAN1A1,mannosidase, alpha, class 1A, member 1 |
| 209945_s_at | 12.13 | 1.81E-07 | 1.098766882 | NM_002093| | GSK3B,glycogen synthase kinase 3 beta |
| 204182_s_at | 12.12 | 1.82E-07 | 1.060095723 | NM_014007| | ZNF297B,zinc finger protein 297B |
| 201963_at | 12.12 | 1.81E-07 | 1.710624176 | NM_001995| | ACSL1,acyl-CoA synthetase long-chain family member 1 |
| 244640_at | 12.12 | 1.82E-07 | 1.377100953 | NA |  |
| 212461_at | 12.12 | 1.82E-07 | 1.116215271 | NM_015878| | OAZIN,ornithine decarboxylase antizyme inhibitor |
| 228959_at | 12.11 | 1.83E-07 | 1.109704524 | NA |  |
| 229177_at | 12.11 | 1.83E-07 | 1.581617694 | NM_001098514| | NA |
| 203530_s_at | 12.11 | 1.83E-07 | 1.071491423 | NM_004604| | STX4A,syntaxin 4A (placental) |
| 220065_at | 12.11 | 1.83E-07 | 1.742934733 | NM_022144| | TNMD,tenomodulin |
| 208913_at | 12.1 | 1.86E-07 | 1.051574558 | NM_015044| | GGA2,ADP-ribosylation factor binding protein 2 |
| 205340_at | 12.1 | 1.85E-07 | 1.079286381 | NM_014797| | ZBTB24,zinc finger and BTB domain containing 24 |
| 214853_s_at | 12.1 | 1.84E-07 | 1.067750451 | NM_003029| | SHC1,SHC (Src homology 2 domain containing) |
| 226434_at | 12.09 | 1.87E-07 | 1.076892773 | NM_145030| | MGC22793,hypothetical protein MGC22793 |
| 202404_s_at | 12.09 | 1.87E-07 | 2.303356319 | NM_000089| | COL1A2,alpha 2 type I collagen |
| 213900_at | 12.08 | 1.88E-07 | 1.397115357 | NM_004816| | C9orf61,chromosome 9 open reading frame 61 |
| 227046_at | 12.08 | 1.88E-07 | 1.07076329 | NM_139177| | SLC39A11,solute carrier family 39 (metal ion |
| 221218_s_at | 12.08 | 1.89E-07 | 1.466630562 | NM_001042482| | NA |
| 1555279_at | 12.08 | 1.88E-07 | 1.125295889 | NM_014154| | ARMC8,armadillo repeat containing 8 |
| 230464_at | 12.07 | 1.90E-07 | 1.329726752 | NM_030760| | EDG8,endothelial differentiation, sphingolipid |
| 224675_at | 12.07 | 1.91E-07 | 1.035141548 | NM_015154| | NA |
| 227166_at | 12.06 | 1.92E-07 | 1.104237765 | NM_152686| | MGC29463,hypothetical protein MGC29463 |
| 226291_at | 12.06 | 1.93E-07 | 1.079496149 | NM_020919| | ALS2,alsin |
| 225976_at | 12.05 | 1.93E-07 | 1.038770645 | NM_152265| | MGC23908,similar to transcription factor BTF3 |
| 201436_at | 12.05 | 1.93E-07 | 1.120696806 | NM_001968| | EIF4E,eukaryotic translation initiation factor 4E |
| 212620_at | 12.04 | 1.96E-07 | 1.056921749 | NM_015042| | NA |
| 218341_at | 12.04 | 1.95E-07 | 1.412266588 | NM_001077447| | NA |
| 226789_at | 12.03 | 1.98E-07 | 2.202642741 | NA |  |
| 227947_at | 12.03 | 1.97E-07 | 1.257238399 | NM_001100164| | NA |
| 201298_s_at | 12.03 | 1.98E-07 | 1.059893096 | NM_018221| | MOBK1B,Mob4B protein |
| 226187_at | 12.02 | 1.99E-07 | 1.418441003 | NA |  |
| 226282_at | 12.02 | 1.99E-07 | 1.136657389 | NA |  |
| 225447_at | 12.02 | 1.99E-07 | 1.154732693 | NM_000408| | GPD2,glycerol-3-phosphate dehydrogenase 2 |
| 239350_at | 12.02 | 1.98E-07 | 1.737760788 | NM_001017967| | NA |
| 239648_at | 12.02 | 1.99E-07 | 1.193024668 | NM_173475| | MGC48972,hypothetical protein MGC48972 |
| 221606_s_at | 12.01 | 2.01E-07 | 1.553426456 | NM_030763| | NSBP1,nucleosomal binding protein 1 |
| 209147_s_at | 12.01 | 2.01E-07 | 1.483643606 | NM_003711| | PPAP2A,phosphatidic acid phosphatase type 2A isoform 1 |
| 204588_s_at | 12.01 | 2.00E-07 | 1.412064474 | NM_001126105| | NA |
| 224648_at | 12.01 | 2.01E-07 | 1.041624424 | NM_001127235| | NA |
| 228249_at | 12.01 | 2.00E-07 | 1.112050404 | NM_138787| | LOC119710,hypothetical protein BC009561 |
| 205171_at | 12.01 | 2.01E-07 | 1.17857443 | NM_002830| | PTPN4,protein tyrosine phosphatase, non-receptor type |
| 235226_at | 12 | 2.02E-07 | 1.194403975 | NM_015076| | CDC2L6,cyclin-dependent kinase (CDC2-like) 11 |
| 223495_at | 12 | 2.01E-07 | 1.139927596 | NM_032040| | CCDC8,coiled-coil domain containing 8 |
| 225735_at | 12 | 2.01E-07 | 1.108156883 | NM_020337| | NA |
| 231822_at | 12 | 2.01E-07 | 1.215099499 | NM_018704| | DKFZp547A023,hypothetical protein DKFZp547A023 |
| 222994_at | 11.99 | 2.03E-07 | 1.066527629 | NM_012094| | PRDX5,peroxiredoxin 5 precursor, isoform a |
| 219520_s_at | 11.99 | 2.05E-07 | 1.063426201 | NM_015691| | KIAA1280,KIAA1280 protein |
| 236451_at | 11.99 | 2.04E-07 | 1.555055333 | NA |  |
| 203352_at | 11.98 | 2.06E-07 | 1.153911428 | NM_002552| | ORC4L,origin recognition complex subunit 4 |
| 225606_at | 11.98 | 2.05E-07 | 1.068186794 | NM_006538| | BCL2L11,BCL2-like 11 isoform 6 |
| 224679_at | 11.98 | 2.05E-07 | 1.059704788 | NM_015154| | NA |
| 1557918_s_at | 11.98 | 2.05E-07 | 1.138243058 | NM_003051| | SLC16A1,solute carrier family 16, member 1 |
| 209790_s_at | 11.97 | 2.08E-07 | 1.18412333 | NM_001226| | CASP6,caspase 6 isoform alpha preproprotein |
| 202005_at | 11.97 | 2.07E-07 | 1.245771622 | NM_021978| | ST14,matriptase |
| 201972_at | 11.97 | 2.07E-07 | 1.08123393 | NM_001690| | ATP6V1A,ATPase, H+ transporting, lysosomal 70kD, V1 |
| 203457_at | 11.97 | 2.07E-07 | 1.08751141 | NM_003569| | STX7,syntaxin 7 |
| 225418_at | 11.96 | 2.10E-07 | 1.1869559 | NM_001042724| | NA |
| 202013_s_at | 11.95 | 2.10E-07 | 1.070249608 | NM_000401| | EXT2,exostosin 2 |
| 225480_at | 11.95 | 2.10E-07 | 1.038665711 | NM_198446| | FLJ45459,FLJ45459 protein |
| 228248_at | 11.95 | 2.11E-07 | 1.149333007 | NM_152756| | AVO3,rapamycin-insensitive companion of mTOR |
| 239344_at | 11.94 | 2.13E-07 | 1.245843435 | NA |  |
| 218451_at | 11.94 | 2.14E-07 | 1.364869444 | NM_022842| | CDCP1,CUB domain-containing protein 1 isoform 1 |
| 206600_s_at | 11.94 | 2.14E-07 | 1.811276332 | NM_004695| | SLC16A5,solute carrier family 16, member 5 |
| 225007_at | 11.93 | 2.14E-07 | 1.122042602 | NM_005754| | G3BP,Ras-GTPase-activating protein SH3-domain-binding |
| 224250_s_at | 11.93 | 2.14E-07 | 1.062554256 | NM_024077| | SECISBP2,SECIS binding protein 2 |
| 220638_s_at | 11.93 | 2.15E-07 | 1.687639446 | NM_012116| | CBLC,Cas-Br-M (murine) ecotropic retroviral |
| 1554489_a_at | 11.93 | 2.15E-07 | 1.19629926 | NM_024491| | Cep70,centrosomal protein 70 kDa |
| 244694_at | 11.93 | 2.15E-07 | 1.702839746 | NM_001101372| | NA |
| 219349_s_at | 11.93 | 2.15E-07 | 1.169063362 | NM_018303| | SEC5L1,Sec5 protein |
| 219517_at | 11.93 | 2.14E-07 | 1.183582857 | NM_025165| | ELL3,elongation factor RNA polymerase II-like 3 |
| 200616_s_at | 11.92 | 2.15E-07 | 1.047864053 | NM_014730| | KIAA0152,KIAA0152 gene product |
| 1552580_at | 11.91 | 2.18E-07 | 1.606074911 | NM_173553| | FLJ25801,hypothetical protein FLJ25801 |
| 218486_at | 11.91 | 2.17E-07 | 1.136300251 | NM_003597| | KLF11,Kruppel-like factor 11 |
| 231260_at | 11.9 | 2.20E-07 | 1.280311765 | NA |  |
| 202016_at | 11.9 | 2.21E-07 | 1.053105655 | NM_002402| | MEST,mesoderm specific transcript isoform a |
| 207071_s_at | 11.9 | 2.21E-07 | 1.107824518 | NM_002197| | ACO1,aconitase 1 |
| 204618_s_at | 11.9 | 2.21E-07 | 1.060541327 | NM_002041| | GABPB2,GA binding protein transcription factor, beta |
| 222471_s_at | 11.89 | 2.21E-07 | 1.1127168 | NM_020122| | KCMF1,potassium channel modulatory factor 1 |
| 208739_x_at | 11.89 | 2.22E-07 | 1.037269445 | NM_001005849| | SUMO2,small ubiquitin-like modifier 2 isoform b |
| 226512_at | 11.88 | 2.24E-07 | 1.191298956 | NM_003453| | ZNF198,zinc finger protein 198 |
| 219438_at | 11.88 | 2.25E-07 | 1.12949504 | NM_024522| | FLJ12650,hypothetical protein FLJ12650 |
| 207109_at | 11.87 | 2.26E-07 | 1.314287968 | NM_014352| | POU2F3,POU transcription factor |
| 220419_s_at | 11.87 | 2.26E-07 | 1.20151181 | NM_013396| | USP25,ubiquitin specific protease 25 |
| 1565836_at | 11.86 | 2.28E-07 | 1.080080132 | NA |  |
| 205165_at | 11.86 | 2.28E-07 | 1.087642832 | NM_001040454| | NA |
| 201014_s_at | 11.86 | 2.29E-07 | 1.044557432 | NM_001079524| | NA |
| 223796_at | 11.86 | 2.28E-07 | 1.556662259 | NM_033655| | CNTNAP3,cell recognition molecule CASPR3 |
| 206673_at | 11.86 | 2.29E-07 | 1.484559192 | NM_007223| | GPR,putative G protein coupled receptor |
| 205627_at | 11.85 | 2.29E-07 | 1.791171924 | NM_001785| | CDA,cytidine deaminase |
| 201425_at | 11.85 | 2.29E-07 | 1.161928106 | NM_000690| | ALDH2,mitochondrial aldehyde dehydrogenase 2 |
| 220381_at | 11.85 | 2.30E-07 | 1.270051072 | NM_001010000| | ARHGAP28,Rho GTPase activating protein 28 isoform a |
| 225082_at | 11.85 | 2.29E-07 | 1.035158396 | NM_016207| | CPSF3,cleavage and polyadenylation specific factor 3, |
| 204076_at | 11.85 | 2.30E-07 | 1.10008372 | NM_004901| | ENTPD4,ectonucleoside triphosphate diphosphohydrolase |
| 212978_at | 11.84 | 2.33E-07 | 1.076787527 | NM_015350| | TA-LRRP,T-cell activation leucine repeat-rich protein |
| 207714_s_at | 11.84 | 2.32E-07 | 1.12592624 | NM_001235| | SERPINH1,serine (or cysteine) proteinase inhibitor, clade |
| 220073_s_at | 11.84 | 2.31E-07 | 1.296931081 | NM_018173| | FLJ10665,hypothetical protein FLJ10665 |
| 227015_at | 11.83 | 2.35E-07 | 1.269061016 | NM_020437| | LOC57168,similar to aspartate beta hydroxylase (ASPH) |
| 206383_s_at | 11.83 | 2.35E-07 | 1.120922216 | NM_012297| | G3BP2,Ras-GTPase activating protein SH3 domain-binding |
| 214519_s_at | 11.83 | 2.34E-07 | 2.074462616 | NM_005059| | RLN2,relaxin 2 isoform 2 |
| 227208_at | 11.82 | 2.36E-07 | 1.070032762 | NM_198489| | DLNB14,similar to DLNB14 |
| 235088_at | 11.82 | 2.37E-07 | 1.221773048 | NM_001008393| | LOC201725,hypothetical protein LOC201725 |
| 204009_s_at | 11.82 | 2.35E-07 | 1.081681151 | NM_004985| | KRAS,c-K-ras2 protein isoform b |
| 243070_at | 11.81 | 2.39E-07 | 1.123526967 | NM_017759| | FLJ20309,hypothetical protein FLJ20309 |
| 201773_at | 11.81 | 2.39E-07 | 1.036629882 | NM_015339| | ADNP,activity-dependent neuroprotector |
| 207037_at | 11.81 | 2.39E-07 | 1.636017584 | NM_003839| | TNFRSF11A,tumor necrosis factor receptor superfamily, |
| 236113_at | 11.81 | 2.40E-07 | 1.311428982 | NA |  |
| 210319_x_at | 11.81 | 2.39E-07 | 1.484002875 | NM_002449| | MSX2,msh homeo box homolog 2 |
| 239468_at | 11.8 | 2.42E-07 | 1.318237602 | NM_173576| | C10orf48,chromosome 10 open reading frame 48 |
| 223559_s_at | 11.8 | 2.41E-07 | 1.064441582 | NM_021218| | C9orf80,chromosome 9 open reading frame 80 |
| 224812_at | 11.8 | 2.42E-07 | 1.077135991 | NM_152740| | HIBADH,3-hydroxyisobutyrate dehydrogenase |
| 212888_at | 11.79 | 2.43E-07 | 1.147973443 | NM_030621| | DICER1,dicer1 |
| 226105_at | 11.79 | 2.43E-07 | 1.235671353 | NA |  |
| 225583_at | 11.79 | 2.42E-07 | 1.087166503 | NM_025076| | UXS1,UDP-glucuronate decarboxylase 1 |
| 224671_at | 11.79 | 2.43E-07 | 1.067941826 | NM_145255| | MRPL10,mitochondrial ribosomal protein L10 isoform a |
| 213258_at | 11.79 | 2.44E-07 | 1.992004639 | NM_001032281| | NA |
| 33814_at | 11.78 | 2.46E-07 | 1.083131257 | NM_001014831| | NA |
| 227112_at | 11.78 | 2.46E-07 | 1.072646184 | NM_001017395| | NA |
| 226027_at | 11.78 | 2.46E-07 | 1.071993592 | NM_001040011| | NA |
| 231963_at | 11.78 | 2.46E-07 | 1.443417807 | NA |  |
| 206085_s_at | 11.77 | 2.48E-07 | 1.253989874 | NM_001902| | CTH,cystathionase isoform 1 |
| 235552_at | 11.77 | 2.48E-07 | 1.082785177 | NM_020961| | KIAA1627,KIAA1627 protein |
| 203304_at | 11.76 | 2.49E-07 | 1.222871036 | NM_012342| | BAMBI,BMP and activin membrane-bound inhibitor |
| 213067_at | 11.76 | 2.49E-07 | 1.107843258 | NM_005964| | MYH10,myosin, heavy polypeptide 10, non-muscle |
| 235366_at | 11.76 | 2.49E-07 | 1.096186353 | NM_015394| | ZNF10,zinc finger protein 10 |
| 235533_at | 11.76 | 2.50E-07 | 1.209836005 | NM_001031617| | NA |
| 204584_at | 11.76 | 2.50E-07 | 1.510011246 | NM_000425| | L1CAM,L1 cell adhesion molecule isoform 1 precursor |
| 223255_at | 11.75 | 2.52E-07 | 1.082440786 | NM_017769| | KIAA1333,KIAA1333 |
| 203397_s_at | 11.75 | 2.51E-07 | 3.083681328 | NM_004482| | GALNT3,polypeptide N-acetylgalactosaminyltransferase 3 |
| 208620_at | 11.74 | 2.55E-07 | 1.043543935 | NM_006196| | PCBP1,poly(rC) binding protein 1 |
| 213729_at | 11.74 | 2.54E-07 | 1.049089637 | NM_017892| | NA |
| 243295_at | 11.74 | 2.54E-07 | 1.080381002 | NM_018989| | NA |
| 216550_x_at | 11.73 | 2.57E-07 | 1.111166489 | NM_001083625| | NA |
| 204286_s_at | 11.73 | 2.56E-07 | 1.335317931 | NM_021127| | PMAIP1,phorbol-12-myristate-13-acetate-induced protein |
| 224920_x_at | 11.73 | 2.56E-07 | 1.171074965 | NM_001020818| | NA |
| 1558942_at | 11.73 | 2.56E-07 | 1.083500797 | NM_001040185| | NA |
| 201512_s_at | 11.73 | 2.57E-07 | 1.063638012 | NM_014820| | TOMM70A,translocase of outer mitochondrial membrane 70 |
| 226588_at | 11.73 | 2.57E-07 | 1.12558903 | NM_020943| | KIAA1604,KIAA1604 protein |
| 212268_at | 11.72 | 2.58E-07 | 1.278403631 | NM_030666| | SERPINB1,serine (or cysteine) proteinase inhibitor, clade |
| 221261_x_at | 11.72 | 2.59E-07 | 1.096475193 | NM_001098800| | NA |
| 216468_s_at | 11.7 | 2.64E-07 | 1.316540293 | NM_001077349| | NA |
| 215239_x_at | 11.7 | 2.64E-07 | 1.188221412 | NM_021148| | ZNF273,zinc finger protein 273 |
| 235245_at | 11.7 | 2.64E-07 | 1.518115372 | NM_153229| | FLJ33318,hypothetical protein FLJ33318 |
| 1554980_a_at | 11.7 | 2.63E-07 | 1.196800398 | NM_001030287| | NA |
| 201475_x_at | 11.69 | 2.65E-07 | 1.084385481 | NM_004990| | MARS,methionine-tRNA synthetase |
| 200069_at | 11.69 | 2.65E-07 | 1.043674448 | NM_014706| | SART3,squamous cell carcinoma antigen recognized by T |
| 215076_s_at | 11.68 | 2.69E-07 | 2.18609668 | NM_000090| | COL3A1,alpha 1 type III collagen |
| 219342_at | 11.68 | 2.69E-07 | 1.149079128 | NM_022900| | CAS1,O-acetyltransferase |
| 203715_at | 11.68 | 2.69E-07 | 1.137812857 | NM_001079515| | NA |
| 209278_s_at | 11.68 | 2.68E-07 | 1.668178529 | NM_006528| | TFPI2,tissue factor pathway inhibitor 2 |
| 235527_at | 11.68 | 2.68E-07 | 2.209034688 | NM_001003809| | DLGAP1,discs large homolog-associated protein 1 isoform |
| 227174_at | 11.68 | 2.69E-07 | 2.229733492 | NM_182758| | FLJ38736,hypothetical protein FLJ38736 |
| 213457_at | 11.67 | 2.71E-07 | 1.124133425 | NM_004225| | MFHAS1,malignant fibrous histiocytoma amplified |
| 236759_at | 11.67 | 2.72E-07 | 1.17465627 | NA |  |
| 228206_at | 11.67 | 2.69E-07 | 1.524819781 | NM_006040| | HS3ST4,heparan sulfate D-glucosaminyl |
| 203953_s_at | 11.67 | 2.71E-07 | 1.721690005 | NM_001306| | CLDN3,claudin 3 |
| 219875_s_at | 11.67 | 2.70E-07 | 1.142906482 | NM_016076| | PNAS-4,CGI-146 protein |
| 210115_at | 11.66 | 2.74E-07 | 1.122405609 | NM_052969| | RPL39L,ribosomal protein L39-like protein |
| 213792_s_at | 11.66 | 2.73E-07 | 1.22038182 | NM_000208| | INSR,insulin receptor |
| 227408_s_at | 11.66 | 2.73E-07 | 1.196553567 | NM_031953| | SNX25,sorting nexin 25 |
| 212886_at | 11.65 | 2.76E-07 | 1.734053355 | NM_015621| | DKFZP434C171,DKFZP434C171 protein |
| 201786_s_at | 11.65 | 2.76E-07 | 1.054070278 | NM_001025107| | NA |
| 230972_at | 11.65 | 2.76E-07 | 1.064438137 | NM_152326| | ANKRD9,ankyrin repeat domain 9 |
| 211097_s_at | 11.64 | 2.77E-07 | 1.259914765 | NM_002586| | PBX2,pre-B-cell leukemia transcription factor 2 |
| 212157_at | 11.63 | 2.80E-07 | 1.299237883 | NM_002998| | SDC2,syndecan 2 precursor |
| 222939_s_at | 11.62 | 2.84E-07 | 1.217402459 | NM_018593| | SLC16A10,solute carrier family 16, member 10 |
| 230063_at | 11.62 | 2.84E-07 | 1.112040006 | NM_003417| | ZNF264,zinc finger protein 264 |
| 218640_s_at | 11.61 | 2.85E-07 | 1.169587119 | NM_024613| | PLEKHF2,phafin 2 |
| 205342_s_at | 11.61 | 2.86E-07 | 1.218688417 | NM_001056| | SULT1C1,sulfotransferase family, cytosolic, 1C, member 1 |
| 204417_at | 11.61 | 2.87E-07 | 1.137558816 | NM_000153| | GALC,galactosylceramidase precursor |
| 203435_s_at | 11.61 | 2.86E-07 | 1.407775325 | NM_000902| | MME,membrane metallo-endopeptidase |
| 202147_s_at | 11.6 | 2.88E-07 | 1.158230733 | NM_001007245| | IFRD1,interferon-related developmental regulator 1 |
| 218321_x_at | 11.6 | 2.87E-07 | 1.11390351 | NM_016086| | DUSP24,map kinase phosphatase-like protein MK-STYX |
| 228226_s_at | 11.6 | 2.89E-07 | 1.165479497 | NM_173680| | MGC33584,hypothetical protein MGC33584 |
| 215695_s_at | 11.6 | 2.88E-07 | 1.17411692 | NM_001079855| | NA |
| 225438_at | 11.6 | 2.89E-07 | 1.171367967 | NM_032869| | NUDCD1,NudC domain containing 1 |
| 214698_at | 11.59 | 2.92E-07 | 1.187724432 | NM_005156| | ROD1,ROD1 regulator of differentiation 1 |
| 210652_s_at | 11.59 | 2.90E-07 | 1.709603732 | NM_001080494| | NA |
| 201434_at | 11.59 | 2.90E-07 | 1.070600794 | NM_003314| | TTC1,tetratricopeptide repeat domain 1 |
| 218152_at | 11.58 | 2.95E-07 | 1.044156995 | NM_018200| | HMG20A,high-mobility group 20A |
| 223108_s_at | 11.58 | 2.93E-07 | 1.061495905 | NM_016505| | PS1D,putative S1 RNA binding domain protein |
| 225887_at | 11.58 | 2.94E-07 | 1.051769416 | NM_025138| | C13orf23,hypothetical protein FLJ12661 |
| 1555241_at | 11.58 | 2.94E-07 | 1.134609997 | NM_001099670| | NA |
| 228855_at | 11.58 | 2.93E-07 | 1.306222721 | NM_001105663| | NA |
| 224486_s_at | 11.57 | 2.95E-07 | 1.201390958 | NM_032499| | HH114,hypothetical protein HH114 |
| 211809_x_at | 11.57 | 2.97E-07 | 1.111099791 | NM_005203| | COL13A1,alpha 1 type XIII collagen isoform 1 |
| 225750_at | 11.57 | 2.97E-07 | 1.099172162 | NA |  |
| 224452_s_at | 11.57 | 2.96E-07 | 1.111854294 | NM_001037163| | NA |
| 211137_s_at | 11.57 | 2.96E-07 | 1.118814876 | NM_001001485| | ATP2C1,calcium-transporting ATPase 2C1 isoform 1c |
| 222862_s_at | 11.57 | 2.96E-07 | 1.587526277 | NM_012093| | AK5,adenylate kinase 5 isoform 2 |
| 226086_at | 11.57 | 2.95E-07 | 2.016769923 | NM_020826| | SYT13,synaptotagmin XIII |
| 202403_s_at | 11.57 | 2.97E-07 | 2.019853138 | NM_000089| | COL1A2,alpha 2 type I collagen |
| 235191_at | 11.56 | 2.98E-07 | 1.068131286 | NA |  |
| 229351_at | 11.56 | 2.98E-07 | 1.132238494 | NM_018561| | USP49,ubiquitin specific protease 49 |
| 1560741_at | 11.56 | 2.98E-07 | 1.263212282 | NM_003097| | SNRPN,small nuclear ribonucleoprotein polypeptide N |
| 231067_s_at | 11.55 | 3.01E-07 | 1.332946587 | NA |  |
| 228713_s_at | 11.55 | 3.01E-07 | 1.298593763 | NM_016246| | DHRS10,dehydrogenase/reductase (SDR family) member 10 |
| 217813_s_at | 11.55 | 3.01E-07 | 1.130817349 | NM_006717| | SPIN,spindlin |
| 213370_s_at | 11.55 | 3.02E-07 | 1.098778332 | NM_001005158| | SFMBT1,Scm-like with four mbt domains 1 |
| 219765_at | 11.55 | 3.02E-07 | 1.240686925 | NM_024620| | ZNF329,zinc finger protein 329 |
| 1569796_s_at | 11.54 | 3.03E-07 | 1.446581608 | NM_207303| | ATRNL1,attractin-like 1 |
| 203474_at | 11.54 | 3.03E-07 | 1.313832813 | NM_006633| | IQGAP2,IQ motif containing GTPase activating protein 2 |
| 222662_at | 11.53 | 3.07E-07 | 1.248099164 | NM_024607| | PPP1R3B,protein phosphatase 1, regulatory (inhibitor) |
| 44696_at | 11.53 | 3.08E-07 | 1.073610555 | NM_018201| | TBC1D13,TBC1 domain family, member 13 |
| 223062_s_at | 11.52 | 3.09E-07 | 1.070687522 | NM_021154| | PSAT1,phosphoserine aminotransferase isoform 2 |
| 223993_s_at | 11.52 | 3.09E-07 | 1.058525204 | NM_014184| | HSPC163,HSPC163 protein |
| 212841_s_at | 11.52 | 3.10E-07 | 1.334371973 | NM_003621| | PPFIBP2,PTPRF interacting protein, binding protein 2 |
| 200963_x_at | 11.51 | 3.13E-07 | 1.01636619 | NM_000993| | RPL31,ribosomal protein L31 |
| 210372_s_at | 11.51 | 3.12E-07 | 1.689509639 | NM_001003395| | TPD52L1,tumor protein D52-like 1 isoform 2 |
| 201569_s_at | 11.51 | 3.12E-07 | 1.034999007 | NM_015380| | CGI-51,CGI-51 protein |
| 227226_at | 11.5 | 3.15E-07 | 1.20030129 | NM_138409| | C6orf117,chromosome 6 open reading frame 117 |
| 227405_s_at | 11.5 | 3.15E-07 | 1.727533783 | NM_031866| | FZD8,frizzled 8 |
| 55065_at | 11.49 | 3.18E-07 | 1.116401492 | NM_031417| | MARK4,MAP/microtubule affinity-regulating kinase 4 |
| 209583_s_at | 11.49 | 3.17E-07 | 1.138333998 | NM_001004196| | CD200,CD200 antigen isoform b |
| 217988_at | 11.49 | 3.18E-07 | 1.075789753 | NM_021178| | CCNB1IP1,cyclin B1 interacting protein 1 isoform a |
| 226773_at | 11.48 | 3.20E-07 | 1.165497496 | NA |  |
| 200614_at | 11.48 | 3.20E-07 | 1.022134742 | NM_004859| | CLTC,clathrin heavy chain 1 |
| 228233_at | 11.47 | 3.25E-07 | 1.263999685 | NM_144966| | FREM1,FRAS1 related extracellular matrix 1 |
| 215464_s_at | 11.47 | 3.24E-07 | 1.067551374 | NM_014604| | TAX1BP3,Tax1 (human T-cell leukemia virus type I) |
| 243672_at | 11.47 | 3.23E-07 | 1.230023633 | NM_171999| | SALL3,sal-like 3 |
| 214150_x_at | 11.47 | 3.23E-07 | 1.063425437 | NM_003945| | ATP6V0E,ATPase, H+ transporting, lysosomal, V0 subunit |
| 202932_at | 11.46 | 3.29E-07 | 1.061609932 | NM_005433| | YES1,viral oncogene yes-1 homolog 1 |
| 213205_s_at | 11.45 | 3.32E-07 | 1.063361535 | NM_015106| | SRISNF2L,KIAA0809 protein |
| 208427_s_at | 11.44 | 3.34E-07 | 1.705037229 | NM_004432| | ELAVL2,ELAV (embryonic lethal, abnormal vision, |
| 217738_at | 11.44 | 3.35E-07 | 1.124969638 | NM_005746| | PBEF1,pre-B-cell colony enhancing factor 1 isoform a |
| 42361_g_at | 11.44 | 3.34E-07 | 1.124607487 | NM_001105563| | NA |
| 33322_i_at | 11.44 | 3.33E-07 | 1.110823819 | NM_006142| | SFN,stratifin |
| 224833_at | 11.43 | 3.36E-07 | 1.591125768 | NM_005238| | ETS1,v-ets erythroblastosis virus E26 oncogene |
| 37512_at | 11.43 | 3.38E-07 | 1.289346936 | NM_003725| | RODH,3-hydroxysteroid epimerase |
| 228875_at | 11.43 | 3.36E-07 | 1.924645538 | NM_001085480| | NA |
| 233354_at | 11.42 | 3.39E-07 | 1.111719859 | NA |  |
| 206363_at | 11.42 | 3.39E-07 | 1.507743862 | NM_001031804| | NA |
| 222458_s_at | 11.42 | 3.39E-07 | 1.172900087 | NM_024595| | FLJ12666,hypothetical protein FLJ12666 |
| 218779_x_at | 11.42 | 3.41E-07 | 1.366463385 | NM_017729| | EPS8L1,epidermal growth factor receptor pathway |
| 223595_at | 11.42 | 3.39E-07 | 1.209355526 | NM_032021| | AD031,AD031 protein |
| 233403_x_at | 11.41 | 3.43E-07 | 1.090865538 | NM_001001524| | TM6SF2,transmembrane 6 superfamily member 2 isoform 1 |
| 211712_s_at | 11.41 | 3.42E-07 | 1.339093792 | NM_003568| | ANXA9,annexin A9 |
| 221381_s_at | 11.41 | 3.44E-07 | 1.105478736 | NM_006791| | MORF4L1,MORF-related gene 15 isoform 1 |
| 200631_s_at | 11.41 | 3.44E-07 | 1.021287453 | NM_001122821| | NA |
| 209681_at | 11.4 | 3.48E-07 | 1.137497487 | NM_006996| | SLC19A2,solute carrier family 19, member 2 |
| 204422_s_at | 11.4 | 3.44E-07 | 1.297286937 | NM_002006| | FGF2,fibroblast growth factor 2 |
| 221515_s_at | 11.39 | 3.49E-07 | 1.113728425 | NM_001032391| | NA |
| 236835_at | 11.39 | 3.48E-07 | 1.321470991 | NA |  |
| 239108_at | 11.39 | 3.48E-07 | 1.342865997 | NM_018099| | MLSTD1,male sterility domain containing 1 |
| 217789_at | 11.38 | 3.52E-07 | 1.113867156 | NM_021249| | SNX6,sorting nexin 6 isoform a |
| 225807_at | 11.38 | 3.52E-07 | 1.44372274 | NM_032876| | JUB,jub, ajuba homolog isoform 1 |
| 229573_at | 11.38 | 3.54E-07 | 1.154113551 | NM_001039590| | NA |
| 225548_at | 11.38 | 3.53E-07 | 1.088159314 | NM_020859| | ShrmL,Shroom-related protein |
| 222433_at | 11.38 | 3.52E-07 | 1.060763944 | NM_001008493| | ENAH,enabled homolog isoform a |
| 1553288_a_at | 11.37 | 3.54E-07 | 1.302696248 | NM_173564| | FLJ37538,hypothetical protein FLJ37538 |
| 211404_s_at | 11.37 | 3.55E-07 | 1.054540851 | NM_001642| | APLP2,amyloid beta (A4) precursor-like protein 2 |
| 235435_at | 11.37 | 3.55E-07 | 1.060458619 | NM_181806| | NRPS998,2-aminoadipic 6-semialdehyde dehydrogenase |
| 205235_s_at | 11.36 | 3.60E-07 | 1.070522496 | NM_016195| | MPHOSPH1,M-phase phosphoprotein 1 |
| 226440_at | 11.36 | 3.58E-07 | 1.121115133 | NM_020185| | DUSP22,dual specificity phosphatase 22 |
| 201312_s_at | 11.36 | 3.58E-07 | 1.174557764 | NM_003022| | SH3BGRL,SH3 domain binding glutamic acid-rich protein |
| 207558_s_at | 11.36 | 3.59E-07 | 1.857809717 | NM_000325| | PITX2,paired-like homeodomain transcription factor 2 |
| 208812_x_at | 11.35 | 3.62E-07 | 1.067248815 | NM_002117| | HLA-C,major histocompatibility complex, class I, C |
| 202778_s_at | 11.35 | 3.63E-07 | 1.081672663 | NM_003453| | ZNF198,zinc finger protein 198 |
| 202454_s_at | 11.35 | 3.63E-07 | 1.13254297 | NM_001005915| | ERBB3,erbB-3 isoform s precursor |
| 242481_at | 11.34 | 3.64E-07 | 1.547157786 | NA |  |
| 203875_at | 11.34 | 3.66E-07 | 1.092422948 | NM_003069| | SMARCA1,SWI/SNF-related matrix-associated |
| 1556344_at | 11.34 | 3.65E-07 | 1.223198419 | NA |  |
| 209524_at | 11.33 | 3.69E-07 | 1.069356242 | NM_016073| | HDGFRP3,hepatoma-derived growth factor, related protein |
| 228003_at | 11.33 | 3.70E-07 | 1.2323135 | NM_014488| | RAB30,RAB30, member RAS oncogene family |
| 204837_at | 11.33 | 3.70E-07 | 1.069639508 | NM_015458| | MTMR9,myotubularin-related protein 9 |
| 239647_at | 11.33 | 3.70E-07 | 1.395493157 | NM_152889| | CHST13,carbohydrate (chondroitin 4) sulfotransferase |
| 1556012_at | 11.32 | 3.71E-07 | 1.643834245 | NM_152375| | FLJ38753,hypothetical protein FLJ38753 |
| 219108_x_at | 11.32 | 3.71E-07 | 1.057602281 | NM_017895| | DDX27,DEAD (Asp-Glu-Ala-Asp) box polypeptide 27 |
| 225584_at | 11.32 | 3.71E-07 | 1.076941273 | NA |  |
| 228482_at | 11.32 | 3.72E-07 | 1.683029525 | NM_145301| | LOC201158,similar to CGI-148 protein |
| 237988_at | 11.32 | 3.71E-07 | 1.128419033 | NM_005875| | GC20,translation factor sui1 homolog |
| 224969_at | 11.31 | 3.77E-07 | 1.067532371 | NM_001098833| | NA |
| 219227_at | 11.31 | 3.75E-07 | 1.242898496 | NM_024565| | FLJ14166,hypothetical protein FLJ14166 |
| 214109_at | 11.31 | 3.76E-07 | 1.189186182 | NM_006726| | LRBA,LPS-responsive vesicle trafficking, beach and |
| 215157_x_at | 11.31 | 3.75E-07 | 1.008692485 | NM_002568| | PABPC1,poly(A) binding protein, cytoplasmic 1 |
| 225475_at | 11.31 | 3.76E-07 | 1.126833541 | NM_001077700| | NA |
| 209652_s_at | 11.31 | 3.75E-07 | 1.275114866 | NM_002632| | PGF,placental growth factor, vascular endothelial |
| 225649_s_at | 11.3 | 3.79E-07 | 1.111507708 | NM_080836| | STK35,serine/threonine kinase 35 |
| 201289_at | 11.3 | 3.78E-07 | 1.266563387 | NM_001554| | CYR61,cysteine-rich, angiogenic inducer, 61 |
| 220911_s_at | 11.3 | 3.79E-07 | 1.320701862 | NM_025081| | NA |
| 216615_s_at | 11.29 | 3.82E-07 | 1.572518336 | NM_000869| | HTR3A,5-hydroxytryptamine (serotonin) receptor 3A |
| 200815_s_at | 11.29 | 3.82E-07 | 1.117904776 | NM_000430| | PAFAH1B1,platelet-activating factor acetylhydrolase, |
| 230746_s_at | 11.29 | 3.82E-07 | 2.350961724 | NM_003155| | STC1,stanniocalcin 1 |
| 226068_at | 11.29 | 3.83E-07 | 1.264776266 | NM_003177| | SYK,spleen tyrosine kinase |
| 217766_s_at | 11.28 | 3.87E-07 | 1.092736255 | NM_014313| | SMP1,small membrane protein 1 |
| 201622_at | 11.27 | 3.89E-07 | 1.035894856 | NM_014390| | SND1,staphylococcal nuclease domain containing 1 |
| 206858_s_at | 11.27 | 3.89E-07 | 2.151244917 | NM_004503| | HOXC6,homeo box C6 isoform 1 |
| 226213_at | 11.27 | 3.90E-07 | 1.173300926 | NM_001005915| | ERBB3,erbB-3 isoform s precursor |
| 201332_s_at | 11.27 | 3.89E-07 | 1.227843099 | NM_003153| | STAT6,signal transducer and activator of transcription |
| 206949_s_at | 11.27 | 3.90E-07 | 1.101845741 | NM_001105203| | NA |
| 219559_at | 11.26 | 3.92E-07 | 1.166639081 | NM_022082| | C20orf59,chromosome 20 open reading frame 59 |
| 222982_x_at | 11.26 | 3.94E-07 | 1.068317958 | NM_018976| | SLC38A2,solute carrier family 38, member 2 |
| 226524_at | 11.26 | 3.92E-07 | 1.091892925 | NM_173824| | MGC26717,hypothetical protein MGC26717 |
| 214253_s_at | 11.26 | 3.93E-07 | 1.117942694 | NM_021907| | DTNB,dystrobrevin, beta isoform 1 |
| 208727_s_at | 11.25 | 3.98E-07 | 1.067558124 | NM_001039802| | NA |
| 219014_at | 11.25 | 3.99E-07 | 1.421461786 | NM_016619| | PLAC8,placenta-specific 8 |
| 223901_at | 11.25 | 3.99E-07 | 1.274253675 | NM_032298| | SYT3,synaptotagmin 3 |
| 224775_at | 11.24 | 4.01E-07 | 1.160462561 | NM_017969| | FLJ10006,hypothetical protein FLJ10006 |
| 228580_at | 11.24 | 4.02E-07 | 1.155179667 | NM_053044| | HTRA3,HtrA serine peptidase 3 |
| 238653_at | 11.24 | 4.01E-07 | 1.13712759 | NM_014813| | LRIG2,leucine-rich repeats and immunoglobulin-like |
| 213846_at | 11.24 | 4.02E-07 | 1.074634466 | NM_001867| | COX7C,cytochrome c oxidase subunit VIIc precursor |
| 202066_at | 11.24 | 4.02E-07 | 1.115752321 | NM_003626| | PPFIA1,PTPRF interacting protein alpha 1 isoform b |
| 229849_at | 11.24 | 3.99E-07 | 1.55244764 | NA |  |
| 203968_s_at | 11.23 | 4.05E-07 | 1.057036254 | NM_001254| | CDC6,CDC6 homolog |
| 219061_s_at | 11.23 | 4.05E-07 | 1.102742789 | NM_006014| | DXS9879E,ESO3 protein |
| 235018_at | 11.22 | 4.10E-07 | 1.429500538 | NA |  |
| 225084_at | 11.22 | 4.10E-07 | 1.072181633 | NM_006544| | SEC10L1,brain secretory protein SEC10P |
| 1563770_at | 11.21 | 4.11E-07 | 1.299527518 | NA |  |
| 225991_at | 11.21 | 4.11E-07 | 1.103309835 | NM_080652| | TMEM41A,transmembrane protein 41A |
| 226468_at | 11.21 | 4.12E-07 | 1.053012431 | NA |  |
| 215548_s_at | 11.21 | 4.11E-07 | 1.09250676 | NM_016106| | SCFD1,vesicle transport-related protein isoform a |
| 225359_at | 11.21 | 4.14E-07 | 1.047387849 | NM_145261| | TIM14,homolog of yeast TIM14 isoform a |
| 202838_at | 11.2 | 4.17E-07 | 1.22284373 | NM_000147| | FUCA1,fucosidase, alpha-L- 1, tissue |
| 228705_at | 11.19 | 4.19E-07 | 1.386318166 | NM_144691| | CAPN12,calpain 12 |
| 236391_at | 11.19 | 4.20E-07 | 1.206481845 | NA |  |
| 227040_at | 11.19 | 4.21E-07 | 1.23724897 | NM_001012754| | NA |
| 1555679_a_at | 11.19 | 4.20E-07 | 1.099529002 | NM_032730| | RTN4IP1,reticulon 4 interacting protein 1 |
| 204173_at | 11.19 | 4.20E-07 | 1.067628678 | NM_002475| | MLC1SA,myosin alkali light chain 1 slow a |
| 1555443_at | 11.19 | 4.20E-07 | 1.47565809 | NM_198515| | NA |
| 213183_s_at | 11.18 | 4.25E-07 | 1.37725008 | NM_000076| | CDKN1C,cyclin-dependent kinase inhibitor 1C |
| 243252_at | 11.18 | 4.22E-07 | 1.45442298 | NA |  |
| 224369_s_at | 11.18 | 4.22E-07 | 1.064138317 | NM_030793| | FBXO38,F-box protein 38 isoform a |
| 203985_at | 11.18 | 4.22E-07 | 1.054072704 | NM_012256| | ZNF212,zinc finger protein 212 |
| 203962_s_at | 11.17 | 4.27E-07 | 1.862239456 | NM_006393| | NEBL,nebulette sarcomeric isoform |
| 221027_s_at | 11.16 | 4.30E-07 | 1.252334165 | NM_030821| | PLA2G12A,phospholipase A2, group XIIA |
| 204668_at | 11.16 | 4.33E-07 | 1.182590886 | NM_007219| | RNF24,ring finger protein 24 |
| 227850_x_at | 11.16 | 4.33E-07 | 2.045309912 | NM_145057| | CDC42EP5,CDC42 effector protein 5 |
| 224808_s_at | 11.16 | 4.33E-07 | 1.049228587 | NM_015949| | C7orf20,chromosome 7 open reading frame 20 |
| 214143_x_at | 11.15 | 4.35E-07 | 1.011410305 | NM_000986| | RPL24,ribosomal protein L24 |
| 1570523_s_at | 11.15 | 4.34E-07 | 1.128391207 | NM_031482| | APG10L,APG10 autophagy 10-like |
| 202164_s_at | 11.15 | 4.35E-07 | 1.046457231 | NM_004779| | CNOT8,CCR4-NOT transcription complex, subunit 8 |
| 219615_s_at | 11.14 | 4.39E-07 | 1.696303585 | NM_003740| | KCNK5,potassium channel, subfamily K, member 5 |
| 203920_at | 11.14 | 4.40E-07 | 1.115398715 | NM_005693| | NR1H3,nuclear receptor subfamily 1, group H, member 3 |
| 200629_at | 11.14 | 4.37E-07 | 1.066772292 | NM_004184| | WARS,tryptophanyl-tRNA synthetase isoform a |
| 1568954_s_at | 11.14 | 4.39E-07 | 1.105143521 | NM_014117| | PRO0149,PRO0149 protein |
| 202804_at | 11.13 | 4.43E-07 | 1.080730471 | NM_004996| | ABCC1,ATP-binding cassette, sub-family C, member 1 |
| 201343_at | 11.12 | 4.48E-07 | 1.073064859 | NM_003339| | UBE2D2,ubiquitin-conjugating enzyme E2D 2 isoform 1 |
| 230467_at | 11.12 | 4.47E-07 | 1.155461694 | NM_178545| | LOC339456,hypothetical protein LOC339456 |
| 35148_at | 11.12 | 4.47E-07 | 1.609736335 | NM_014428| | TJP3,tight junction protein 3 (zona occludens 3) |
| 227429_at | 11.12 | 4.47E-07 | 1.361112489 | NM_173584| | MGC45840,hypothetical protein MGC45840 |
| 229874_x_at | 11.11 | 4.51E-07 | 1.2287529 | NA |  |
| 200751_s_at | 11.11 | 4.54E-07 | 1.03957345 | NM_001077442| | NA |
| 214834_at | 11.11 | 4.53E-07 | 1.474339562 | NA |  |
| 204718_at | 11.1 | 4.54E-07 | 1.411280861 | NM_004445| | EPHB6,ephrin receptor EphB6 precursor |
| 223592_s_at | 11.1 | 4.56E-07 | 1.366155056 | NM_032322| | RNF135,ring finger protein 135 isoform 1 |
| 208712_at | 11.1 | 4.54E-07 | 1.05932407 | NM_053056| | CCND1,cyclin D1 |
| 208998_at | 11.1 | 4.57E-07 | 1.184459022 | NM_003355| | UCP2,uncoupling protein 2 |
| 235181_at | 11.1 | 4.55E-07 | 1.16935474 | NM_001039693| | NA |
| 223635_s_at | 11.1 | 4.57E-07 | 1.190543311 | NM_001009955| | SSBP3,single stranded DNA binding protein 3 isoform c |
| 220750_s_at | 11.09 | 4.60E-07 | 1.141562335 | NM_022356| | LEPRE1,leucine proline-enriched proteoglycan (leprecan) |
| 210713_at | 11.08 | 4.66E-07 | 1.155143764 | NM_001001132| | ITSN1,intersectin 1 isoform ITSN-s |
| 217954_s_at | 11.08 | 4.65E-07 | 1.070259061 | NM_015153| | PHF3,PHD finger protein 3 |
| 204773_at | 11.08 | 4.63E-07 | 1.106305687 | NM_004512| | IL11RA,interleukin 11 receptor, alpha isoform 1 |
| 200971_s_at | 11.08 | 4.63E-07 | 1.042788302 | NM_014445| | SERP1,stress-associated endoplasmic reticulum protein |
| 221035_s_at | 11.08 | 4.65E-07 | 1.725023105 | NM_031272| | TEX14,testis expressed sequence 14 isoform b |
| 1558692_at | 11.07 | 4.71E-07 | 1.785920123 | NM_144580| | MGC31963,kidney predominant protein NCU-G1 |
| 228773_at | 11.07 | 4.67E-07 | 1.318399167 | NA |  |
| 214606_at | 11.06 | 4.72E-07 | 1.278319795 | NM_005725| | TSPAN2,tetraspan 2 |
| 213413_at | 11.06 | 4.72E-07 | 1.211038619 | NM_006873| | SBLF,stoned B-like factor |
| 221781_s_at | 11.06 | 4.73E-07 | 1.085974451 | NM_018981| | DNAJC10,DnaJ (Hsp40) homolog, subfamily C, member 10 |
| 213025_at | 11.06 | 4.75E-07 | 1.095138786 | NM_017736| | THUMPD1,THUMP domain containing 1 |
| 209073_s_at | 11.06 | 4.72E-07 | 1.155661481 | NM_001005743| | NUMB,numb homolog isoform 1 |
| 223064_at | 11.05 | 4.80E-07 | 1.076809057 | NM_016494| | LOC51255,hypothetical protein LOC51255 |
| 213230_at | 11.04 | 4.82E-07 | 1.063684183 | NM_014603| | HUMPPA,paraneoplastic antigen |
| 218722_s_at | 11.04 | 4.84E-07 | 1.055509942 | NM_024661| | FLJ12436,hypothetical protein FLJ12436 |
| 223349_s_at | 11.04 | 4.80E-07 | 1.254492217 | NM_032515| | BOK,BCL2-related ovarian killer |
| 206310_at | 11.04 | 4.83E-07 | 1.571274682 | NM_021114| | SPINK2,serine protease inhibitor, Kazal type 2 |
| 201615_x_at | 11.04 | 4.82E-07 | 1.102984172 | NM_004342| | CALD1,caldesmon 1 isoform 2 |
| 224974_at | 11.03 | 4.86E-07 | 1.183602673 | NM_022491| | SDS3,hypothetical protein FLJ00052 |
| 51158_at | 11.03 | 4.88E-07 | 1.474231746 | NM_207446| | LOC400451,hypothetical gene supported by AK075564; |
| 201755_at | 11.03 | 4.86E-07 | 1.04864505 | NM_006739| | MCM5,minichromosome maintenance deficient protein 5 |
| 210062_s_at | 11.03 | 4.85E-07 | 1.188899451 | NM_016089| | ZNF589,zinc finger protein 589 |
| 242522_at | 11.02 | 4.90E-07 | 1.57609001 | NA |  |
| 213440_at | 11.02 | 4.90E-07 | 1.042331629 | NM_004161| | RAB1A,RAB1A, member RAS oncogene family |
| 239710_at | 11.02 | 4.92E-07 | 1.189458435 | NM_018086| | FIGN,fidgetin |
| 239457_at | 11.02 | 4.93E-07 | 1.508258397 | NM_138813| | ATP8B3,ATPase, Class I, type 8B, member 3 |
| 204446_s_at | 11.02 | 4.90E-07 | 1.969135609 | NM_000698| | ALOX5,arachidonate 5-lipoxygenase |
| 235720_at | 11.01 | 4.96E-07 | 1.277193719 | NM_206922| | CRIP3,cysteine-rich protein 3 |
| 212184_s_at | 11.01 | 4.94E-07 | 1.084978101 | NM_015093| | MAP3K7IP2,mitogen-activated protein kinase kinase kinase 7 |
| 218370_s_at | 11.01 | 4.96E-07 | 1.133552877 | NM_001017406| | NA |
| 229202_at | 11.01 | 4.96E-07 | 1.237087004 | NA |  |
| 209210_s_at | 11 | 5.00E-07 | 1.082267563 | NM_006832| | PLEKHC1,pleckstrin homology domain containing, family C |
| 204050_s_at | 11 | 4.99E-07 | 1.042065704 | NM_001076677| | NA |
| 217951_s_at | 11 | 5.02E-07 | 1.075068145 | NM_015153| | PHF3,PHD finger protein 3 |
| 223072_s_at | 11 | 4.99E-07 | 1.066242643 | NM_012477| | WBP1,WW domain binding protein 1 |
| 229534_at | 10.99 | 5.07E-07 | 1.520655388 | NM_152331| | PTE2B,peroxisomal acyl-CoA thioesterase 2B |
| 1555491_a_at | 10.99 | 5.06E-07 | 1.175512059 | NM_018381| | FLJ11286,hypothetical protein FLJ11286 |
| 238716_at | 10.98 | 5.12E-07 | 1.269956291 | NA |  |
| 222793_at | 10.98 | 5.12E-07 | 1.29956464 | NM_014314| | DDX58,DEAD/H (Asp-Glu-Ala-Asp/His) box polypeptide |
| 217707_x_at | 10.98 | 5.08E-07 | 1.773580324 | NM_003070| | SMARCA2,SWI/SNF-related matrix-associated |
| 211020_at | 10.98 | 5.08E-07 | 1.256729264 | NM_001491| | GCNT2,glucosaminyl (N-acetyl) transferase 2 isoform B |
| 204823_at | 10.98 | 5.08E-07 | 1.363402679 | NM_014903| | NAV3,neuron navigator 3 |
| 205229_s_at | 10.97 | 5.16E-07 | 1.229381385 | NM_004086| | COCH,coagulation factor C homolog, cochlin precursor |
| 224991_at | 10.97 | 5.14E-07 | 1.128206156 | NM_030629| | CMIP,c-Maf-inducing protein Tc-mip isoform |
| 235649_at | 10.97 | 5.14E-07 | 1.648144029 | NM_007037| | ADAMTS8,a disintegrin and metalloprotease with |
| 223279_s_at | 10.96 | 5.21E-07 | 1.168551243 | NM_001008224| | UACA,uveal autoantigen with coiled-coil domains and |
| 205486_at | 10.96 | 5.21E-07 | 1.298033178 | NM_007170| | TESK2,testis-specific protein kinase 2 |
| 239355_at | 10.96 | 5.21E-07 | 1.060271959 | NA |  |
| 203851_at | 10.96 | 5.21E-07 | 1.463617296 | NM_002178| | IGFBP6,insulin-like growth factor binding protein 6 |
| 207785_s_at | 10.95 | 5.25E-07 | 1.109660026 | NM_005349| | RBPSUH,recombining binding protein suppressor of |
| 232028_at | 10.95 | 5.24E-07 | 1.086257791 | NM_178549| | MGC42493,hypothetical protein MGC42493 |
| 1555167_s_at | 10.95 | 5.24E-07 | 1.118373093 | NM_005746| | PBEF1,pre-B-cell colony enhancing factor 1 isoform a |
| 228662_at | 10.95 | 5.25E-07 | 1.050244549 | NM_014598| | SOCS7,suppressor of cytokine signaling 7 |
| 201599_at | 10.94 | 5.29E-07 | 1.077119158 | NM_000274| | OAT,ornithine aminotransferase precursor |
| 204680_s_at | 10.94 | 5.30E-07 | 1.170652631 | NM_012294| | RAPGEF5,Rap guanine nucleotide exchange factor (GEF) 5 |
| 238742_x_at | 10.94 | 5.27E-07 | 1.302743322 | NA |  |
| 200860_s_at | 10.93 | 5.36E-07 | 1.055718866 | NM_016284| | CNOT1,CCR4-NOT transcription complex, subunit 1 |
| 218816_at | 10.93 | 5.35E-07 | 1.184060016 | NM_018214| | LRRC1,leucine rich repeat containing 1 |
| 220924_s_at | 10.93 | 5.35E-07 | 1.087150638 | NM_018976| | SLC38A2,solute carrier family 38, member 2 |
| 207267_s_at | 10.93 | 5.35E-07 | 1.314996597 | NM_018962| | DSCR6,Down syndrome critical region protein 6 |
| 204670_x_at | 10.93 | 5.36E-07 | 1.325588559 | NM_001023561| | NA |
| 225525_at | 10.93 | 5.35E-07 | 1.06012164 | NA |  |
| 40020_at | 10.93 | 5.33E-07 | 1.092238556 | NM_001040454| | NA |
| 228908_s_at | 10.92 | 5.38E-07 | 1.171006739 | NA |  |
| 230136_at | 10.92 | 5.41E-07 | 1.155796554 | NA |  |
| 212529_at | 10.92 | 5.39E-07 | 1.052232754 | NM_152344| | FLJ30656,hypothetical protein FLJ30656 |
| 203964_at | 10.91 | 5.46E-07 | 1.283117576 | NM_004688| | NMI,N-myc and STAT interactor |
| 240419_at | 10.91 | 5.43E-07 | 1.378376696 | NM_018057| | SLC6A15,solute carrier family 6, member 15 isoform 2 |
| 208813_at | 10.91 | 5.47E-07 | 1.107765147 | NM_002079| | GOT1,aspartate aminotransferase 1 |
| 211702_s_at | 10.91 | 5.43E-07 | 1.194194081 | NM_032582| | USP32,ubiquitin specific protease 32 |
| 229730_at | 10.91 | 5.43E-07 | 1.74124339 | NM_001114974| | NA |
| 222549_at | 10.91 | 5.44E-07 | 1.70337959 | NM_021101| | CLDN1,claudin 1 |
| 238175_at | 10.91 | 5.43E-07 | 1.605989298 | NA |  |
| 225478_at | 10.9 | 5.48E-07 | 1.126283255 | NM_004225| | MFHAS1,malignant fibrous histiocytoma amplified |
| 225864_at | 10.9 | 5.48E-07 | 1.166208841 | NM_174911| | NSE2,breast cancer membrane protein 101 |
| 224746_at | 10.9 | 5.48E-07 | 1.512796692 | NM_020888| | NA |
| 211518_s_at | 10.9 | 5.48E-07 | 1.492555091 | NM_001202| | BMP4,bone morphogenetic protein 4 preproprotein |
| 224813_at | 10.9 | 5.48E-07 | 1.074769828 | NM_003941| | WASL,Wiskott-Aldrich syndrome gene-like protein |
| 226994_at | 10.89 | 5.57E-07 | 1.141285947 | NM_005880| | DNAJA2,DnaJ subfamily A member 2 |
| 225776_at | 10.88 | 5.61E-07 | 1.106838176 | NA |  |
| 201203_s_at | 10.88 | 5.63E-07 | 1.151122334 | NM_001042576| | NA |
| 219557_s_at | 10.88 | 5.59E-07 | 1.366308051 | NM_020645| | NRIP3,nuclear receptor interacting protein 3 |
| 211707_s_at | 10.88 | 5.58E-07 | 1.093422134 | NM_001023570| | NA |
| 203665_at | 10.88 | 5.60E-07 | 1.325627678 | NM_002133| | HMOX1,heme oxygenase (decyclizing) 1 |
| 202267_at | 10.88 | 5.60E-07 | 1.334535233 | NM_005562| | LAMC2,laminin, gamma 2 isoform a precursor |
| 229612_at | 10.88 | 5.58E-07 | 1.164485076 | NA |  |
| 204608_at | 10.87 | 5.67E-07 | 1.070890915 | NM_000048| | ASL,argininosuccinate lyase |
| 207194_s_at | 10.87 | 5.68E-07 | 1.441681697 | NM_001039132| | NA |
| 31845_at | 10.87 | 5.67E-07 | 1.150593403 | NM_001127197| | NA |
| 226038_at | 10.87 | 5.67E-07 | 1.065852751 | NM_152271| | FLJ23749,hypothetical protein FLJ23749 |
| 224578_at | 10.87 | 5.67E-07 | 1.039134923 | NM_018715| | TD-60,RCC1-like |
| 201883_s_at | 10.87 | 5.67E-07 | 1.120229916 | NM_001497| | B4GALT1,UDP-Gal:betaGlcNAc beta 1,4- |
| 225455_at | 10.86 | 5.72E-07 | 1.094436431 | NM_053053| | STAF42,SPT3-associated factor 42 |
| 209071_s_at | 10.86 | 5.70E-07 | 1.896283289 | NM_003617| | RGS5,regulator of G-protein signalling 5 |
| 219778_at | 10.86 | 5.74E-07 | 1.358299281 | NM_012082| | ZFPM2,zinc finger protein, multitype 2 |
| 206900_x_at | 10.86 | 5.73E-07 | 1.304504388 | NM_021047| | ZNF253,DNA-binding protein |
| 209297_at | 10.86 | 5.74E-07 | 1.172199874 | NM_001001132| | ITSN1,intersectin 1 isoform ITSN-s |
| 234000_s_at | 10.85 | 5.75E-07 | 1.021480268 | NM_016395| | HSPC121,butyrate-induced transcript 1 |
| 232112_at | 10.84 | 5.84E-07 | 1.377660989 | NM_018037| | RALGPS2,Ral GEF with PH domain and SH3 binding motif 2 |
| 230942_at | 10.84 | 5.83E-07 | 1.122007562 | NM_001037288| | NA |
| 223129_x_at | 10.84 | 5.85E-07 | 1.394246144 | NM_013262| | MYLIP,myosin regulatory light chain interacting |
| 244065_at | 10.84 | 5.83E-07 | 1.365314624 | NA |  |
| 203544_s_at | 10.84 | 5.81E-07 | 1.070045735 | NM_003473| | STAM,signal transducing adaptor molecule 1 |
| 212103_at | 10.84 | 5.84E-07 | 1.047041505 | NM_012316| | KPNA6,karyopherin alpha 6 |
| 226150_at | 10.83 | 5.90E-07 | 1.115657558 | NM_001102559| | NA |
| 206960_at | 10.83 | 5.91E-07 | 1.49936867 | NM_005296| | GPR23,G protein-coupled receptor 23 |
| 220127_s_at | 10.82 | 5.97E-07 | 1.0905865 | NM_017703| | FBXL12,F-box and leucine-rich repeat protein 12 |
| 1560577_at | 10.82 | 5.94E-07 | 1.083014612 | NA |  |
| 208614_s_at | 10.82 | 5.92E-07 | 1.143963979 | NM_001457| | FLNB,filamin B, beta (actin binding protein 278) |
| 227727_at | 10.82 | 5.94E-07 | 1.593083185 | NM_001098515| | NA |
| 201738_at | 10.82 | 5.93E-07 | 1.103134649 | NM_005875| | GC20,translation factor sui1 homolog |
| 221729_at | 10.81 | 5.99E-07 | 1.764188628 | NM_000393| | COL5A2,alpha 2 type V collagen preproprotein |
| 221765_at | 10.81 | 6.02E-07 | 1.114319507 | NM_003358| | UGCG,ceramide glucosyltransferase |
| 201000_at | 10.81 | 5.97E-07 | 1.090016256 | NM_001605| | AARS,alanyl-tRNA synthetase |
| 222492_at | 10.8 | 6.08E-07 | 1.106444798 | NM_003681| | PDXK,pyridoxal kinase |
| 234341_x_at | 10.8 | 6.08E-07 | 1.113088696 | NA |  |
| 219268_at | 10.8 | 6.05E-07 | 1.166219964 | NM_018208| | ETNK2,ethanolamine kinase 2 |
| 201656_at | 10.8 | 6.04E-07 | 1.109243731 | NM_000210| | ITGA6,integrin alpha chain, alpha 6 |
| 201833_at | 10.8 | 6.07E-07 | 1.058097983 | NM_001527| | HDAC2,histone deacetylase 2 |
| 222354_at | 10.8 | 6.05E-07 | 1.592012992 | NM_016946| | F11R,F11 receptor isoform a precursor |
| 212320_at | 10.79 | 6.11E-07 | 1.0347233 | NM_178014| | TUBB,tubulin, beta polypeptide |
| 202720_at | 10.79 | 6.10E-07 | 1.296937007 | NM_015641| | TES,testin isoform 1 |
| 215789_s_at | 10.79 | 6.10E-07 | 1.33433031 | NM_001042478| | NA |
| 218398_at | 10.78 | 6.19E-07 | 1.044948171 | NM_016640| | MRPS30,mitochondrial ribosomal protein S30 |
| 203167_at | 10.78 | 6.18E-07 | 1.165020457 | NM_003255| | TIMP2,tissue inhibitor of metalloproteinase 2 |
| 224629_at | 10.78 | 6.16E-07 | 1.032709346 | NM_005570| | LMAN1,lectin, mannose-binding, 1 precursor |
| 204167_at | 10.78 | 6.18E-07 | 1.182267275 | NM_000060| | BTD,biotinidase precursor |
| 218128_at | 10.78 | 6.14E-07 | 1.114257871 | NM_006166| | NFYB,nuclear transcription factor Y, beta |
| 219874_at | 10.77 | 6.23E-07 | 1.34613085 | NM_024628| | SLC12A8,solute carrier family 12, member 8 |
| 237451_x_at | 10.77 | 6.21E-07 | 1.164970276 | NA |  |
| 205664_at | 10.77 | 6.25E-07 | 1.112193458 | NM_012311| | KIN,HsKin17 protein |
| 209999_x_at | 10.77 | 6.20E-07 | 1.383765091 | NM_003745| | SOCS1,suppressor of cytokine signaling 1 |
| 209263_x_at | 10.76 | 6.31E-07 | 1.099072768 | NM_001025234| | NA |
| 208682_s_at | 10.76 | 6.30E-07 | 1.107567194 | NM_014599| | MAGED2,melanoma antigen family D, 2 |
| 226599_at | 10.76 | 6.27E-07 | 1.504773674 | NM_033393| | KIAA1727,KIAA1727 protein |
| 226199_at | 10.76 | 6.31E-07 | 1.218693582 | NM_145052| | MGC23937,hypothetical protein MGC23937 similar to CG4798 |
| 206461_x_at | 10.75 | 6.38E-07 | 1.123537256 | NM_005951| | MT1H,metallothionein 1H |
| 210601_at | 10.75 | 6.32E-07 | 1.428364704 | NM_004932| | CDH6,cadherin 6, type 2 preproprotein |
| 221019_s_at | 10.74 | 6.41E-07 | 1.954132148 | NM_130386| | COLEC12,collectin sub-family member 12 isoform I |
| 201218_at | 10.74 | 6.39E-07 | 1.074644337 | NM_001083914| | NA |
| 201310_s_at | 10.74 | 6.41E-07 | 1.095058222 | NM_004772| | C5orf13,neuronal protein 3.1 |
| 203672_x_at | 10.74 | 6.43E-07 | 1.148634096 | NM_000367| | TPMT,thiopurine S-methyltransferase |
| 214117_s_at | 10.74 | 6.43E-07 | 1.296733108 | NM_000060| | BTD,biotinidase precursor |
| 218710_at | 10.74 | 6.43E-07 | 1.076908542 | NM_017735| | FLJ20272,hypothetical protein FLJ20272 |
| 204535_s_at | 10.73 | 6.44E-07 | 1.137046186 | NM_005612| | REST,RE1-silencing transcription factor |
| 222738_at | 10.73 | 6.48E-07 | 1.272372122 | NM_024949| | BOMB,BH3-only member B protein |
| 218448_at | 10.73 | 6.44E-07 | 1.116572908 | NM_017896| | C20orf11,chromosome 20 open reading frame 11 |
| 218973_at | 10.73 | 6.50E-07 | 1.104686219 | NM_001040610| | NA |
| 244811_at | 10.73 | 6.47E-07 | 1.136169982 | NA |  |
| 224482_s_at | 10.72 | 6.52E-07 | 1.324379024 | NM_032932| | RAB11FIP4,RAB11 family interacting protein 4 (class II) |
| 218412_s_at | 10.72 | 6.55E-07 | 1.129804868 | NM_005685| | GTF2IRD1,GTF2I repeat domain containing 1 isoform 2 |
| 230263_s_at | 10.72 | 6.55E-07 | 1.121641227 | NM_024940| | DOCK5,dedicator of cytokinesis 5 |
| 218551_at | 10.72 | 6.54E-07 | 1.10983212 | NM_021933| | FLJ12438,IGFBP-2-Binding Protein, IIp45 |
| 1555716_a_at | 10.72 | 6.54E-07 | 1.295964463 | NM_001338| | CXADR,coxsackie virus and adenovirus receptor |
| 205942_s_at | 10.72 | 6.54E-07 | 1.640942181 | NM_005622| | SAH,SA hypertension-associated homolog isoform 1 |
| 224704_at | 10.72 | 6.55E-07 | 1.068850398 | NM_014494| | TNRC6A,trinucleotide repeat containing 6A |
| 55705_at | 10.72 | 6.52E-07 | 1.057504063 | NM_138774| | C19orf22,chromosome 19 open reading frame 22 |
| 1568817_at | 10.71 | 6.58E-07 | 1.290937458 | NA |  |
| 223385_at | 10.71 | 6.59E-07 | 1.94277376 | NM_030622| | CYP2S1,cytochrome P450, family 2, subfamily S, |
| 235256_s_at | 10.71 | 6.57E-07 | 1.095460549 | NM_138801| | GALM,galactose mutarotase (aldose 1-epimerase) |
| 204130_at | 10.71 | 6.58E-07 | 1.67418631 | NM_000196| | HSD11B2,hydroxysteroid (11-beta) dehydrogenase 2 |
| 215346_at | 10.71 | 6.58E-07 | 1.5067527 | NM_001250| | CD40,CD40 antigen isoform 1 precursor |
| 202874_s_at | 10.71 | 6.59E-07 | 1.079550086 | NM_001695| | ATP6V1C1,ATPase, H+ transporting, lysosomal 42kDa, V1 |
| 223382_s_at | 10.7 | 6.66E-07 | 1.096829755 | NM_032268| | ZNRF1,zinc and ring finger protein 1 |
| 218762_at | 10.7 | 6.63E-07 | 1.101582289 | NM_022752| | ZNF574,zinc finger protein 574 |
| 214111_at | 10.7 | 6.67E-07 | 1.495644836 | NM_001012393| | OPCML,opioid binding protein/cell adhesion |
| 212664_at | 10.7 | 6.64E-07 | 1.089808612 | NM_006087| | TUBB4,tubulin, beta 4 |
| 205544_s_at | 10.7 | 6.68E-07 | 1.474856859 | NM_001006658| | CR2,complement component (3d/Epstein Barr virus) |
| 217687_at | 10.69 | 6.72E-07 | 1.281674546 | NM_020546| | ADCY2,adenylate cyclase 2 |
| 229797_at | 10.69 | 6.73E-07 | 1.44486868 | NM_018298| | MCOLN3,mucolipin 3 |
| 201506_at | 10.69 | 6.74E-07 | 1.56871865 | NM_000358| | TGFBI,transforming growth factor, beta-induced, 68kDa |
| 244407_at | 10.69 | 6.70E-07 | 1.361752162 | NM_016593| | CYP39A1,cytochrome P450, family 39, subfamily A, |
| 228937_at | 10.69 | 6.70E-07 | 1.463377399 | NM_153218| | FLJ38725,hypothetical protein FLJ38725 |
| 202844_s_at | 10.69 | 6.73E-07 | 1.064335521 | NM_006788| | RALBP1,ralA binding protein 1 |
| 225472_at | 10.69 | 6.73E-07 | 1.039826204 | NM_033177| | BAT4,HLA-B associated transcript 4 |
| 223389_s_at | 10.68 | 6.78E-07 | 1.1285429 | NM_016535| | ZNF581,zinc finger protein 581 |
| 200681_at | 10.68 | 6.79E-07 | 1.05014364 | NM_006708| | GLO1,glyoxalase I |
| 219248_at | 10.68 | 6.81E-07 | 1.086380649 | NM_025264| | THUMPD2,THUMP domain containing 2 |
| 221447_s_at | 10.68 | 6.75E-07 | 1.640763631 | NM_031302| | GLT8D2,glycosyltransferase 8 domain containing 2 |
| 200009_at | 10.68 | 6.78E-07 | 1.036290206 | NM_001115156| | NA |
| 214819_at | 10.68 | 6.78E-07 | 1.213391652 | NM_001111125| | NA |
| 213534_s_at | 10.67 | 6.82E-07 | 1.06667819 | NM_015148| | PASK,PAS domain containing serine/threonine kinase |
| 200800_s_at | 10.67 | 6.84E-07 | 1.070602016 | NM_005345| | HSPA1A,heat shock 70kDa protein 1A |
| 226600_at | 10.67 | 6.82E-07 | 1.128594091 | NM_181783| | SMILE,SMILE protein |
| 216693_x_at | 10.67 | 6.86E-07 | 1.079296577 | NM_016073| | HDGFRP3,hepatoma-derived growth factor, related protein |
| 223711_s_at | 10.66 | 6.92E-07 | 1.082166155 | NM_001037304| | NA |
| 209885_at | 10.66 | 6.94E-07 | 1.264710829 | NM_014578| | RHOD,ras homolog D |
| 55583_at | 10.66 | 6.92E-07 | 1.068760669 | NM_020812| | DOCK6,dedicator of cytokinesis 6 |
| 213578_at | 10.65 | 7.01E-07 | 1.10861051 | NM_004329| | BMPR1A,bone morphogenetic protein receptor, type IA |
| 227294_at | 10.65 | 6.99E-07 | 1.095018354 | NM_138447| | LOC115509,hypothetical protein BC014000 |
| 201362_at | 10.65 | 6.97E-07 | 1.075045076 | NM_006469| | IVNS1ABP,influenza virus NS1A binding protein isoform a |
| 233982_x_at | 10.65 | 6.95E-07 | 1.116504462 | NM_016086| | DUSP24,map kinase phosphatase-like protein MK-STYX |
| 219142_at | 10.65 | 6.99E-07 | 1.533410847 | NM_023940| | RASL11B,RAS-like family 11 member B |
| 220160_s_at | 10.65 | 6.96E-07 | 1.155562335 | NM_007059| | KPTN,kaptin (actin binding protein) |
| 203522_at | 10.64 | 7.03E-07 | 1.129360614 | NM_005125| | CCS,copper chaperone for superoxide dismutase |
| 227621_at | 10.64 | 7.03E-07 | 1.121941178 | NA |  |
| 215925_s_at | 10.64 | 7.05E-07 | 1.490239617 | NM_001782| | CD72,CD72 antigen |
| 209579_s_at | 10.64 | 7.04E-07 | 1.059443925 | NM_003925| | MBD4,methyl-CpG binding domain protein 4 |
| 208899_x_at | 10.64 | 7.05E-07 | 1.061885367 | NM_015994| | ATP6V1D,ATPase, H+ transporting, lysosomal 34kD, V1 |
| 227732_at | 10.64 | 7.05E-07 | 1.413280126 | NM_020725| | NA |
| 1554266_at | 10.63 | 7.11E-07 | 1.294557913 | NA |  |
| 213254_at | 10.63 | 7.12E-07 | 1.044962307 | NM_001024843| | NA |
| 205881_at | 10.63 | 7.09E-07 | 1.104003282 | NM_003426| | ZNF74,zinc finger protein 74 (Cos52) |
| 226140_s_at | 10.63 | 7.09E-07 | 1.207739971 | NA |  |
| 200013_at | 10.62 | 7.16E-07 | 1.010479773 | NM_000986| | RPL24,ribosomal protein L24 |
| 225679_at | 10.62 | 7.17E-07 | 1.054080194 | NM_001011713| | C14orf35,chromosome 14 open reading frame 35 |
| 209157_at | 10.62 | 7.17E-07 | 1.037893661 | NM_005880| | DNAJA2,DnaJ subfamily A member 2 |
| 220089_at | 10.62 | 7.19E-07 | 1.110636855 | NM_024884| | C14orf160,chromosome 14 open reading frame 160 |
| 201120_s_at | 10.62 | 7.19E-07 | 1.148873153 | NM_006667| | PGRMC1,progesterone receptor membrane component 1 |
| 1554241_at | 10.62 | 7.20E-07 | 1.098245688 | NM_004086| | COCH,coagulation factor C homolog, cochlin precursor |
| 210608_s_at | 10.62 | 7.21E-07 | 1.452253705 | NM_000511| | FUT2,fucosyltransferase 2 (secretor status included) |
| 237060_at | 10.61 | 7.27E-07 | 1.258169985 | NA |  |
| 208613_s_at | 10.61 | 7.23E-07 | 1.247762114 | NM_001457| | FLNB,filamin B, beta (actin binding protein 278) |
| 219506_at | 10.61 | 7.26E-07 | 1.16722426 | NM_024579| | FLJ23221,hypothetical protein FLJ23221 |
| 205297_s_at | 10.6 | 7.32E-07 | 1.146836244 | NM_000626| | CD79B,CD79B antigen isoform 1 precursor |
| 219942_at | 10.6 | 7.32E-07 | 1.410417977 | NM_021223| | MYL7,myosin light chain 2a |
| 209984_at | 10.6 | 7.29E-07 | 1.109238816 | NM_015061| | JMJD2C,jumonji domain containing 2C |
| 227583_at | 10.6 | 7.30E-07 | 1.1198133 | NM_006627| | POP4,POP4 (processing of precursor , S. cerevisiae) |
| 235285_at | 10.6 | 7.32E-07 | 1.445452078 | NA |  |
| 202760_s_at | 10.59 | 7.36E-07 | 1.110529447 | NM_007203| | PALM2-AKAP2,PALM2-AKAP2 protein isoform 1 |
| 220047_at | 10.59 | 7.39E-07 | 1.25988156 | NM_012240| | SIRT4,sirtuin 4 |
| 236351_at | 10.58 | 7.45E-07 | 1.770033722 | NA |  |
| 230534_at | 10.57 | 7.49E-07 | 1.206824156 | NA |  |
| 38918_at | 10.56 | 7.58E-07 | 1.283582655 | NM_005686| | SOX13,SRY-box 13 |
| 229289_at | 10.56 | 7.62E-07 | 1.41591272 | NM_138411| | NA |
| 208116_s_at | 10.56 | 7.60E-07 | 1.428011125 | NM_005907| | MAN1A1,mannosidase, alpha, class 1A, member 1 |
| 209142_s_at | 10.55 | 7.65E-07 | 1.100641251 | NM_003342| | UBE2G1,ubiquitin-conjugating enzyme E2G 1 isoform 1 |
| 202236_s_at | 10.55 | 7.68E-07 | 1.080173351 | NM_003051| | SLC16A1,solute carrier family 16, member 1 |
| 202912_at | 10.54 | 7.74E-07 | 1.270245821 | NM_001124| | ADM,adrenomedullin |
| 231946_at | 10.54 | 7.77E-07 | 1.457740401 | NA |  |
| 201141_at | 10.54 | 7.78E-07 | 1.270231446 | NM_001005340| | GPNMB,glycoprotein (transmembrane) nmb isoform a |
| 227493_s_at | 10.53 | 7.83E-07 | 1.079665015 | NM_020696| | KIAA1143,KIAA1143 protein |
| 33323_r_at | 10.53 | 7.82E-07 | 1.351189972 | NM_006142| | SFN,stratifin |
| 1556029_s_at | 10.52 | 7.91E-07 | 1.374051505 | NM_015039| | NMNAT2,nicotinamide mononucleotide adenylyltransferase |
| 209911_x_at | 10.52 | 7.90E-07 | 1.243820375 | NM_021063| | HIST1H2BD,H2B histone family, member B |
| 203376_at | 10.52 | 7.92E-07 | 1.044339528 | NM_015891| | CDC40,pre-mRNA splicing factor 17 |
| 1552740_at | 10.52 | 7.89E-07 | 1.359036228 | NM_144706| | C2orf15,chromosome 2 open reading frame 15 |
| 207316_at | 10.52 | 7.91E-07 | 1.138550519 | NM_001523| | HAS1,hyaluronan synthase 1 |
| 217491_x_at | 10.52 | 7.94E-07 | 1.0463729 | NM_001867| | COX7C,cytochrome c oxidase subunit VIIc precursor |
| 238533_at | 10.51 | 8.00E-07 | 1.557354268 | NM_004440| | EPHA7,ephrin receptor EphA7 |
| 220338_at | 10.51 | 7.95E-07 | 1.196067515 | NM_018037| | RALGPS2,Ral GEF with PH domain and SH3 binding motif 2 |
| 232720_at | 10.51 | 8.02E-07 | 1.647372517 | NM_152570| | FLJ31810,hypothetical protein FLJ31810 |
| 208393_s_at | 10.5 | 8.06E-07 | 1.138179185 | NM_005732| | RAD50,RAD50 homolog isoform 1 |
| 1554805_at | 10.5 | 8.06E-07 | 1.071055866 | NM_001123395| | NA |
| 219296_at | 10.5 | 8.06E-07 | 1.086586392 | NM_001001483| | ZDHHC13,zinc finger, DHHC domain containing 13 isoform |
| 1553741_at | 10.5 | 8.09E-07 | 1.523286132 | NA |  |
| 200630_x_at | 10.5 | 8.06E-07 | 1.028683748 | NM_001122821| | NA |
| 228211_at | 10.5 | 8.07E-07 | 1.11808063 | NM_001010895| | LOC375748,RAD26L hypothetical protein |
| 1557385_at | 10.49 | 8.16E-07 | 1.073564609 | NM_032180| | NA |
| 222420_s_at | 10.49 | 8.16E-07 | 1.070747216 | NM_003344| | UBE2H,ubiquitin-conjugating enzyme E2H isoform 1 |
| 222408_s_at | 10.49 | 8.15E-07 | 1.089489855 | NM_016061| | YPEL5,yippee-like 5 |
| 223234_at | 10.48 | 8.23E-07 | 1.163041476 | NM_006341| | MAD2L2,MAD2 homolog |
| 234976_x_at | 10.48 | 8.25E-07 | 1.174987391 | NM_021196| | SLC4A5,sodium bicarbonate transporter 4 isoform a |
| 223008_s_at | 10.48 | 8.25E-07 | 1.039363725 | NM_001099734| | NA |
| 204435_at | 10.48 | 8.19E-07 | 1.1384453 | NM_001008564| | NUPL1,nucleoporin like 1 isoform b |
| 228742_at | 10.48 | 8.22E-07 | 1.385773542 | NA |  |
| 220740_s_at | 10.48 | 8.24E-07 | 1.242421781 | NM_001042494| | NA |
| 201709_s_at | 10.48 | 8.22E-07 | 1.059402763 | NM_003634| | NIPSNAP1,nipsnap homolog 1 |
| 1552486_s_at | 10.48 | 8.23E-07 | 1.261713758 | NM_032857| | LACTB,lactamase, beta isoform a |
| 209163_at | 10.47 | 8.26E-07 | 1.057548191 | NM_001017916| | NA |
| 217767_at | 10.47 | 8.28E-07 | 1.269906531 | NM_000064| | C3,complement component 3 precursor |
| 216565_x_at | 10.47 | 8.33E-07 | 1.274880573 | NA |  |
| 214700_x_at | 10.47 | 8.26E-07 | 1.087857983 | NM_018151| | RIF1,RAP1 interacting factor 1 |
| 208709_s_at | 10.47 | 8.29E-07 | 1.036269268 | NM_001101662| | NA |
| 218815_s_at | 10.46 | 8.39E-07 | 1.09713083 | NM_018022| | FLJ10199,hypothetical protein FLJ10199 |
| 1556619_at | 10.46 | 8.36E-07 | 1.2507701 | NA |  |
| 201234_at | 10.46 | 8.38E-07 | 1.11455479 | NM_001014794| | NA |
| 200912_s_at | 10.46 | 8.36E-07 | 1.065918534 | NM_001967| | EIF4A2,eukaryotic translation initiation factor 4A, |
| 221636_s_at | 10.46 | 8.36E-07 | 1.232612579 | NM_017898| | FLJ20605,hypothetical protein FLJ20605 |
| 242136_x_at | 10.46 | 8.39E-07 | 1.15526484 | NA |  |
| 236465_at | 10.46 | 8.36E-07 | 1.265798548 | NM_173662| | RNF175,ring finger protein 175 |
| 202542_s_at | 10.45 | 8.50E-07 | 1.049636114 | NM_004757| | SCYE1,small inducible cytokine subfamily E, member 1 |
| 206233_at | 10.45 | 8.44E-07 | 1.280674934 | NM_004775| | B4GALT6,UDP-Gal:betaGlcNAc beta 1,4- |
| 216092_s_at | 10.45 | 8.42E-07 | 1.561510761 | NM_012244| | SLC7A8,solute carrier family 7 (cationic amino acid |
| 47608_at | 10.45 | 8.46E-07 | 1.025571881 | NM_080604| | TJP4,tight junction protein 4 (peripheral) |
| 217150_s_at | 10.44 | 8.58E-07 | 1.329440981 | NM_000268| | NF2,neurofibromin 2 isoform 1 |
| 1556678_a_at | 10.43 | 8.65E-07 | 1.309323886 | NA |  |
| 236214_at | 10.43 | 8.64E-07 | 1.496065033 | NM_032599| | NYD-SP18,testes development-related NYD-SP18 |
| 219147_s_at | 10.43 | 8.63E-07 | 1.304048584 | NM_017881| | C9orf95,nicotinamide riboside kinase 1 |
| 209919_x_at | 10.43 | 8.62E-07 | 1.246750338 | NM_001032364| | NA |
| 213446_s_at | 10.43 | 8.62E-07 | 1.06523402 | NM_003870| | IQGAP1,IQ motif containing GTPase activating protein 1 |
| 204485_s_at | 10.42 | 8.68E-07 | 1.137798079 | NM_005486| | TOM1L1,target of myb1-like 1 |
| 221775_x_at | 10.42 | 8.70E-07 | 1.013191938 | NM_000983| | RPL22,ribosomal protein L22 proprotein |
| 220274_at | 10.42 | 8.74E-07 | 1.300676858 | NM_024726| | IQCA,IQ motif containing with AAA domain |
| 224169_at | 10.41 | 8.84E-07 | 1.644534667 | NM_004885| | GPR74,G protein-coupled receptor 74 |
| 208009_s_at | 10.41 | 8.77E-07 | 1.114216447 | NM_014448| | ARHGEF16,Rho guanine exchange factor 16 |
| 206149_at | 10.41 | 8.84E-07 | 1.37689235 | NM_022097| | LOC63928,hepatocellular carcinoma antigen gene 520 |
| 238708_at | 10.41 | 8.81E-07 | 1.115334296 | NA |  |
| 225639_at | 10.41 | 8.77E-07 | 1.799491495 | NM_003930| | SCAP2,src family associated phosphoprotein 2 |
| 225636_at | 10.4 | 8.93E-07 | 1.112643454 | NM_005419| | STAT2,signal transducer and activator of transcription |
| 232180_at | 10.4 | 8.85E-07 | 1.602100929 | NM_001001521| | UGP2,UDP-glucose pyrophosphorylase 2 isoform b |
| 204450_x_at | 10.4 | 8.90E-07 | 1.332941016 | NM_000039| | APOA1,apolipoprotein A-I precursor |
| 223526_at | 10.4 | 8.91E-07 | 1.042017165 | NM_031446| | C18orf21,chromosome 18 open reading frame 21 |
| 202654_x_at | 10.4 | 8.92E-07 | 1.096802309 | NM_022826| | MARCH7,axotrophin |
| 201900_s_at | 10.4 | 8.91E-07 | 1.07071184 | NM_006066| | AKR1A1,aldo-keto reductase family 1, member A1 |
| 241754_at | 10.39 | 8.96E-07 | 1.450398428 | NM_173690| | C9orf126,chromosome 9 open reading frame 126 |
| 218556_at | 10.39 | 9.02E-07 | 1.05323156 | NM_014182| | ORMDL2,ORMDL2 |
| 205506_at | 10.39 | 9.02E-07 | 1.57251361 | NM_007127| | VIL1,villin 1 |
| 202114_at | 10.39 | 8.95E-07 | 1.044052223 | NM_003100| | SNX2,sorting nexin 2 |
| 207801_s_at | 10.39 | 9.02E-07 | 1.026406218 | NM_014868| | RNF10,ring finger protein 10 |
| 201397_at | 10.38 | 9.03E-07 | 1.07752919 | NM_006623| | PHGDH,phosphoglycerate dehydrogenase |
| 1552634_a_at | 10.38 | 9.10E-07 | 1.315442657 | NM_033204| | ZNF101,zinc finger protein 101 |
| 235618_at | 10.38 | 9.10E-07 | 1.124435937 | NM_014910| | ZNF507,zinc finger protein 507 |
| 48659_at | 10.38 | 9.10E-07 | 1.110680636 | NM_021933| | FLJ12438,IGFBP-2-Binding Protein, IIp45 |
| 202733_at | 10.38 | 9.03E-07 | 1.219335249 | NM_001017973| | NA |
| 219096_at | 10.38 | 9.06E-07 | 1.055643751 | NM_024585| | ARMC7,armadillo repeat containing 7 |
| 204454_at | 10.37 | 9.15E-07 | 1.125849014 | NM_012317| | LDOC1,leucine zipper, down-regulated in cancer 1 |
| 210991_s_at | 10.37 | 9.15E-07 | 1.165919724 | NM_014747| | RIMS3,regulating synaptic membrane exocytosis 3 |
| 222773_s_at | 10.37 | 9.12E-07 | 1.413813577 | NM_024642| | GALNT12,UDP-N-acetyl-alpha-D-galactosamine:polypeptide |
| 211033_s_at | 10.36 | 9.24E-07 | 1.048776412 | NM_000288| | PEX7,peroxisomal biogenesis factor 7 |
| 223217_s_at | 10.36 | 9.23E-07 | 1.327479058 | NM_001005474| | NFKBIZ,nuclear factor of kappa light polypeptide gene |
| 212338_at | 10.36 | 9.28E-07 | 1.803773357 | NM_015194| | MYO1D,myosin ID |
| 229110_at | 10.36 | 9.26E-07 | 1.306205736 | NA |  |
| 213647_at | 10.35 | 9.38E-07 | 1.11670476 | NM_001080449| | NA |
| 219229_at | 10.35 | 9.32E-07 | 1.211377957 | NM_013272| | SLCO3A1,solute carrier organic anion transporter family, |
| 238053_at | 10.35 | 9.31E-07 | 1.095648259 | NM_145177| | DHRSX,dehydrogenase/reductase (SDR family) X-linked |
| 223167_s_at | 10.35 | 9.30E-07 | 1.249687122 | NM_013396| | USP25,ubiquitin specific protease 25 |
| 1554704_at | 10.34 | 9.39E-07 | 1.194845363 | NM_138813| | ATP8B3,ATPase, Class I, type 8B, member 3 |
| 205027_s_at | 10.33 | 9.51E-07 | 1.235749209 | NM_005204| | MAP3K8,mitogen-activated protein kinase kinase kinase |
| 218248_at | 10.33 | 9.58E-07 | 1.273587955 | NM_022074| | FLJ22794,FLJ22794 protein |
| 208994_s_at | 10.33 | 9.51E-07 | 1.042215847 | NM_004792| | PPIG,peptidyl-prolyl isomerase G (cyclophilin G) |
| 224427_s_at | 10.33 | 9.49E-07 | 1.184437778 | NM_022894| | PAPOLG,poly(A) polymerase gamma |
| 231090_s_at | 10.32 | 9.63E-07 | 1.140669793 | NM_152641| | ARID2,AT rich interactive domain 2 (ARID, RFX-like) |
| 222103_at | 10.32 | 9.59E-07 | 1.152678621 | NM_005171| | ATF1,activating transcription factor 1 |
| 208898_at | 10.32 | 9.65E-07 | 1.058628027 | NM_015994| | ATP6V1D,ATPase, H+ transporting, lysosomal 34kD, V1 |
| 236657_at | 10.32 | 9.65E-07 | 1.317062346 | NA |  |
| 202996_at | 10.32 | 9.63E-07 | 1.362090177 | NM_021173| | POLD4,polymerase (DNA-directed), delta 4 |
| 225210_s_at | 10.32 | 9.62E-07 | 1.116166888 | NM_031452| | MGC2560,hypothetical LOC83640 |
| 218304_s_at | 10.31 | 9.70E-07 | 1.119031015 | NM_022776| | OSBPL11,oxysterol-binding protein-like protein 11 |
| 215022_x_at | 10.31 | 9.71E-07 | 1.2076162 | NM_006955| | ZNF11B,zinc finger protein 11b (KOX 2) |
| 221730_at | 10.3 | 9.80E-07 | 1.97900358 | NM_000393| | COL5A2,alpha 2 type V collagen preproprotein |
| 224894_at | 10.3 | 9.83E-07 | 1.115228252 | NM_006106| | YAP1,Yes-associated protein 1, 65 kD |
| 219305_x_at | 10.3 | 9.87E-07 | 1.850164796 | NM_012168| | FBXO2,F-box only protein 2 |
| 232713_at | 10.29 | 9.89E-07 | 1.172013239 | NA |  |
| 219286_s_at | 10.29 | 9.96E-07 | 1.038878463 | NM_022768| | RBM15,RNA binding motif protein 15 |
| 208693_s_at | 10.29 | 9.90E-07 | 1.055690783 | NM_002047| | GARS,glycyl-tRNA synthetase |
| 227133_at | 10.29 | 9.90E-07 | 1.113123092 | NM_207318| | CXorf39,chromosome X open reading frame 39 |
| 218443_s_at | 10.29 | 9.96E-07 | 1.089223167 | NM_018959| | DAZAP1,DAZ associated protein 1 isoform b |
| 231914_at | 10.28 | 1.00E-06 | 1.334121887 | NM_177533| | NUDT14,nudix -type motif 14 |
| 211974_x_at | 10.28 | 1.00E-06 | 1.06641943 | NM_005349| | RBPSUH,recombining binding protein suppressor of |
| 226046_at | 10.28 | 1.00E-06 | 1.109992471 | NM_002750| | MAPK8,mitogen-activated protein kinase 8 isoform 2 |
| 231772_x_at | 10.28 | 1.00E-06 | 1.089316686 | NM_022909| | CENPH,centromere protein H |
| 208093_s_at | 10.28 | 1.00E-06 | 1.069790831 | NM_001025579| | NA |
| 37425_g_at | 10.28 | 1.00E-06 | 1.094453437 | NM_001105563| | NA |
| 222016_s_at | 10.27 | 1.01E-06 | 1.128223943 | NM_030899| | ZNF323,zinc finger protein 323 |
| 217862_at | 10.27 | 1.01E-06 | 1.041355798 | NM_016166| | PIAS1,protein inhibitor of activated STAT, 1 |
| 226625_at | 10.27 | 1.01E-06 | 1.205764144 | NM_003243| | TGFBR3,transforming growth factor, beta receptor III |
| 220053_at | 10.27 | 1.01E-06 | 1.554853167 | NM_020634| | GDF3,growth differentiation factor 3 precursor |
| 221540_x_at | 10.27 | 1.01E-06 | 1.079777629 | NM_001042490| | NA |
| 218631_at | 10.27 | 1.01E-06 | 1.107107968 | NM_021732| | AVPI1,vasopressin-induced protein, 32kDa |
| 209141_at | 10.27 | 1.01E-06 | 1.106034527 | NM_003342| | UBE2G1,ubiquitin-conjugating enzyme E2G 1 isoform 1 |
| 214305_s_at | 10.27 | 1.01E-06 | 1.0675995 | NM_001005526| | SF3B1,splicing factor 3b, subunit 1 isoform 2 |
| 1563392_at | 10.26 | 1.02E-06 | 1.140919523 | NA |  |
| 205388_at | 10.26 | 1.02E-06 | 1.443004812 | NM_003279| | TNNC2,fast skeletal muscle troponin C |
| 200615_s_at | 10.26 | 1.02E-06 | 1.12204487 | NM_001030006| | NA |
| 204512_at | 10.26 | 1.02E-06 | 1.178292805 | NM_002114| | HIVEP1,human immunodeficiency virus type I enhancer |
| 228242_at | 10.26 | 1.03E-06 | 1.26522926 | NM_018177| | N4BP2,Nedd4 binding protein 2 |
| 203271_s_at | 10.26 | 1.02E-06 | 1.153803113 | NM_005148| | UNC119,unc119 (C.elegans) homolog isoform a |
| 231975_s_at | 10.26 | 1.02E-06 | 1.104070324 | NM_152622| | FLJ35954,hypothetical protein FLJ35954 |
| 213036_x_at | 10.25 | 1.03E-06 | 1.300423499 | NM_005173| | ATP2A3,sarco/endoplasmic reticulum Ca2+ -ATPase isoform |
| 208836_at | 10.25 | 1.03E-06 | 1.039364804 | NM_001679| | ATP1B3,Na+/K+ -ATPase beta 3 subunit |
| 222798_at | 10.25 | 1.03E-06 | 1.202758806 | NM_001001484| | PTER,phosphotriesterase related |
| 228396_at | 10.25 | 1.03E-06 | 1.295041875 | NM_001098512| | NA |
| 209205_s_at | 10.25 | 1.03E-06 | 1.040082837 | NM_006769| | LMO4,LIM domain only 4 |
| 1554408_a_at | 10.24 | 1.04E-06 | 1.066370614 | NM_003258| | TK1,thymidine kinase 1, soluble |
| 228057_at | 10.24 | 1.05E-06 | 1.545733159 | NM_145244| | DDIT4L,DNA-damage-inducible transcript 4-like |
| 231122_x_at | 10.24 | 1.04E-06 | 1.206757143 | NM_001039617| | NA |
| 213278_at | 10.24 | 1.04E-06 | 1.101390853 | NM_015458| | MTMR9,myotubularin-related protein 9 |
| 201070_x_at | 10.24 | 1.04E-06 | 1.074150583 | NM_001005526| | SF3B1,splicing factor 3b, subunit 1 isoform 2 |
| 1557348_at | 10.23 | 1.05E-06 | 1.456495703 | NA |  |
| 210231_x_at | 10.23 | 1.05E-06 | 1.03512509 | NM_001122821| | NA |
| 206557_at | 10.23 | 1.05E-06 | 1.582626413 | NA |  |
| 236471_at | 10.23 | 1.05E-06 | 1.420428599 | NM_004289| | NFE2L3,nuclear factor (erythroid-derived 2)-like 3 |
| 214656_x_at | 10.23 | 1.06E-06 | 1.073792767 | NM_001080779| | NA |
| 224911_s_at | 10.23 | 1.05E-06 | 1.085530783 | NM_080927| | DCBLD2,discoidin, CUB and LCCL domain containing 2 |
| 203605_at | 10.23 | 1.05E-06 | 1.086382106 | NM_003136| | SRP54,signal recognition particle 54kDa |
| 243154_at | 10.23 | 1.05E-06 | 1.502616745 | NA |  |
| 229373_at | 10.22 | 1.06E-06 | 1.320985428 | NA |  |
| 227152_at | 10.22 | 1.06E-06 | 1.3735879 | NM_018169| | FLJ10652,hypothetical protein FLJ10652 |
| 218921_at | 10.22 | 1.06E-06 | 1.227618194 | NM_021805| | SIGIRR,single Ig IL-1R-related molecule |
| 226103_at | 10.22 | 1.06E-06 | 1.737017536 | NM_144573| | NEXN,nexilin (F actin binding protein) |
| 205691_at | 10.22 | 1.07E-06 | 1.191610906 | NM_004209| | SYNGR3,synaptogyrin 3 |
| 202970_at | 10.21 | 1.07E-06 | 1.100442267 | NM_003583| | DYRK2,dual-specificity tyrosine-(Y)-phosphorylation |
| 213610_s_at | 10.21 | 1.07E-06 | 1.053421441 | NM_144711| | MGC2610,hypothetical protein MGC2610 |
| 220742_s_at | 10.21 | 1.08E-06 | 1.052237566 | NM_018297| | NGLY1,N-glycanase 1 |
| 219217_at | 10.21 | 1.07E-06 | 1.057467616 | NM_024678| | FLJ23441,hypothetical protein FLJ23441 |
| 212750_at | 10.21 | 1.07E-06 | 1.578997492 | NM_015568| | PPP1R16B,protein phosphatase 1 regulatory inhibitor |
| 222449_at | 10.21 | 1.08E-06 | 1.1447562 | NM_020182| | TMEPAI,transmembrane prostate androgen-induced protein |
| 203075_at | 10.21 | 1.07E-06 | 1.090169465 | NM_001003652| | SMAD2,Sma- and Mad-related protein 2 |
| 226384_at | 10.2 | 1.08E-06 | 1.085279481 | NM_001102559| | NA |
| 239525_at | 10.2 | 1.09E-06 | 1.183729296 | NM_018704| | DKFZp547A023,hypothetical protein DKFZp547A023 |
| 239482_x_at | 10.2 | 1.09E-06 | 1.176255436 | NM_021269| | ZNF15L1,zinc finger protein 15-like 1 (KOX 8) |
| 203912_s_at | 10.19 | 1.10E-06 | 1.204131842 | NM_001009932| | DNASE1L1,deoxyribonuclease I-like 1 precursor |
| 228135_at | 10.19 | 1.09E-06 | 1.032143295 | NM_198077| | C1orf52,chromosome 1 open reading frame 52 |
| 205244_s_at | 10.19 | 1.10E-06 | 1.304678077 | NM_001011554| | SLC13A3,solute carrier family 13 member 3 isoform b |
| 210039_s_at | 10.19 | 1.09E-06 | 1.462370283 | NM_006257| | PRKCQ,protein kinase C, theta |
| 222812_s_at | 10.19 | 1.09E-06 | 1.313147553 | NM_019034| | RHOF,ras homolog gene family, member F |
| 210882_s_at | 10.18 | 1.11E-06 | 1.251857856 | NM_001039705| | NA |
| 232080_at | 10.18 | 1.10E-06 | 1.259525053 | NM_020760| | HECW2,HECT, C2 and WW domain containing E3 ubiquitin |
| 213669_at | 10.18 | 1.11E-06 | 1.179877213 | NM_015122| | FCHO1,FCH domain only 1 |
| 243924_at | 10.17 | 1.12E-06 | 1.142444864 | NA |  |
| 224985_at | 10.17 | 1.12E-06 | 1.041141241 | NM_002524| | NRAS,neuroblastoma RAS viral (v-ras) oncogene |
| 214091_s_at | 10.17 | 1.11E-06 | 1.310432443 | NM_002084| | GPX3,plasma glutathione peroxidase 3 precursor |
| 1555841_at | 10.17 | 1.12E-06 | 1.097513937 | NM_003692| | TMEFF1,transmembrane protein with EGF-like and two |
| 228221_at | 10.17 | 1.12E-06 | 1.471927645 | NM_001114106| | NA |
| 235099_at | 10.17 | 1.12E-06 | 1.162597955 | NM_178868| | CKLFSF8,chemokine-like factor superfamily 8 |
| 227383_at | 10.16 | 1.13E-06 | 1.505032096 | NA |  |
| 220345_at | 10.16 | 1.13E-06 | 1.794664593 | NM_024993| | LRRTM4,leucine rich repeat transmembrane neuronal 4 |
| 201172_x_at | 10.16 | 1.13E-06 | 1.045138631 | NM_003945| | ATP6V0E,ATPase, H+ transporting, lysosomal, V0 subunit |
| 221622_s_at | 10.16 | 1.13E-06 | 1.042180192 | NM_018480| | HT007,uncharacterized hypothalamus protein HT007 |
| 226274_at | 10.16 | 1.13E-06 | 1.099667402 | NM_000084| | CLCN5,chloride channel 5 |
| 205700_at | 10.16 | 1.12E-06 | 1.213297077 | NM_003725| | RODH,3-hydroxysteroid epimerase |
| 237275_at | 10.15 | 1.13E-06 | 1.776945325 | NA |  |
| 228153_at | 10.15 | 1.15E-06 | 1.27805174 | NM_182757| | IBRDC2,IBR domain containing 2 |
| 207890_s_at | 10.15 | 1.14E-06 | 1.1901466 | NM_022468| | MMP25,matrix metalloproteinase 25 |
| 228827_at | 10.15 | 1.14E-06 | 1.111182279 | NA |  |
| 216274_s_at | 10.15 | 1.14E-06 | 1.034588369 | NM_014300| | SEC11L1,SEC11-like 1 |
| 226914_at | 10.15 | 1.14E-06 | 1.045863242 | NM_030978| | ARPC5L,actin related protein 2/3 complex, subunit |
| 206593_s_at | 10.15 | 1.14E-06 | 1.09167097 | NM_133640| | SURF5,surfeit 5 isoform b |
| 233385_x_at | 10.14 | 1.15E-06 | 1.067349515 | NM_016154| | RAB4B,ras-related GTP-binding protein 4b |
| 227470_at | 10.14 | 1.15E-06 | 1.062558769 | NM_152652| | ZNF553,zinc finger protein 553 |
| 208840_s_at | 10.14 | 1.15E-06 | 1.08739517 | NM_012297| | G3BP2,Ras-GTPase activating protein SH3 domain-binding |
| 209982_s_at | 10.14 | 1.15E-06 | 1.459525957 | NM_015080| | NRXN2,neurexin 2 isoform alpha-1 precursor |
| 228861_at | 10.14 | 1.15E-06 | 1.099574304 | NA |  |
| 227817_at | 10.13 | 1.16E-06 | 1.522326982 | NM_002738| | PRKCB1,protein kinase C, beta isoform 2 |
| 242727_at | 10.13 | 1.16E-06 | 1.397798828 | NM_178815| | ARL8,ADP-ribosylation factor-like 8 |
| 226033_at | 10.13 | 1.16E-06 | 1.137358007 | NM_020718| | USP31,ubiquitin specific protease 31 |
| 206429_at | 10.13 | 1.16E-06 | 1.514662421 | NM_005242| | F2RL1,coagulation factor II (thrombin) receptor-like 1 |
| 239083_at | 10.12 | 1.17E-06 | 1.116490882 | NM_152411| | DKFZp762I137,hypothetical protein DKFZp762I137 |
| 222700_at | 10.12 | 1.17E-06 | 1.132342552 | NM_022374| | ARL6IP2,ADP-ribosylation factor-like 6 interacting |
| 228176_at | 10.12 | 1.17E-06 | 1.221521676 | NM_005226| | EDG3,endothelial differentiation, sphingolipid |
| 225845_at | 10.12 | 1.18E-06 | 1.088163946 | NM_014155| | HSPC063,HSPC063 protein |
| 201955_at | 10.11 | 1.19E-06 | 1.0586341 | NM_001013399| | NA |
| 232416_at | 10.11 | 1.19E-06 | 1.422242586 | NM_021938| | BRUNOL5,bruno-like 5, RNA binding protein |
| 213097_s_at | 10.11 | 1.19E-06 | 1.031530464 | NM_014377| | NA |
| 211535_s_at | 10.11 | 1.19E-06 | 1.067298488 | NM_015850| | FGFR1,fibroblast growth factor receptor 1 isoform 2 |
| 204353_s_at | 10.11 | 1.19E-06 | 1.121999576 | NM_001042594| | NA |
| 209966_x_at | 10.11 | 1.19E-06 | 1.37628947 | NM_001438| | ESRRG,estrogen-related receptor gamma isoform 1 |
| 213044_at | 10.1 | 1.20E-06 | 1.069464033 | NM_005406| | ROCK1,Rho-associated, coiled-coil containing protein |
| 209679_s_at | 10.1 | 1.20E-06 | 1.278541013 | NM_001031628| | NA |
| 1555751_a_at | 10.1 | 1.20E-06 | 1.085733335 | NM_001007269| | GEMIN7,gemin 7 |
| 206631_at | 10.1 | 1.20E-06 | 1.263292465 | NM_000956| | PTGER2,prostaglandin E receptor 2 (subtype EP2), 53kDa |
| 226884_at | 10.09 | 1.21E-06 | 1.118577826 | NM_020873| | LRRN1,leucine rich repeat neuronal 1 |
| 231837_at | 10.08 | 1.23E-06 | 1.236956456 | NM_020886| | USP28,ubiquitin specific protease 28 |
| 205953_at | 10.08 | 1.22E-06 | 1.29603517 | NM_014813| | LRIG2,leucine-rich repeats and immunoglobulin-like |
| 206013_s_at | 10.08 | 1.23E-06 | 1.42341785 | NM_016188| | ACTL6B,actin-like 6B |
| 208535_x_at | 10.08 | 1.23E-06 | 1.090215133 | NM_005203| | COL13A1,alpha 1 type XIII collagen isoform 1 |
| 1558643_s_at | 10.08 | 1.22E-06 | 1.342813889 | NM_005711| | EDIL3,EGF-like repeats and discoidin I-like |
| 201811_x_at | 10.07 | 1.23E-06 | 1.111121185 | NM_001018009| | NA |
| 210056_at | 10.07 | 1.24E-06 | 1.240996752 | NM_014470| | RND1,GTP-binding protein RHO6 |
| 202302_s_at | 10.07 | 1.24E-06 | 1.039578328 | NM_023012| | FLJ11021,similar to splicing factor, arginine/serine-rich |
| 1553575_at | 10.07 | 1.24E-06 | 1.066650092 | NA |  |
| 200741_s_at | 10.07 | 1.24E-06 | 1.012928026 | NM_001030| | RPS27,ribosomal protein S27 |
| 211063_s_at | 10.06 | 1.25E-06 | 1.054741754 | NM_006153| | NCK1,NCK adaptor protein 1 |
| 201368_at | 10.06 | 1.25E-06 | 1.11061082 | NM_006887| | ZFP36L2,butyrate response factor 2 |
| 201317_s_at | 10.06 | 1.25E-06 | 1.035463093 | NM_002787| | PSMA2,proteasome alpha 2 subunit |
| 226730_s_at | 10.05 | 1.26E-06 | 1.083168 | NM_020935| | USP37,ubiquitin specific protease 37 |
| 208622_s_at | 10.05 | 1.27E-06 | 1.185862278 | NM_001111077| | NA |
| 228658_at | 10.05 | 1.26E-06 | 1.23955547 | NA |  |
| 1557924_s_at | 10.05 | 1.26E-06 | 1.189296207 | NM_000478| | ALPL,tissue non-specific alkaline phosphatase |
| 223327_x_at | 10.05 | 1.26E-06 | 1.090805143 | NA |  |
| 40093_at | 10.05 | 1.27E-06 | 1.134891202 | NM_001013257| | NA |
| 226984_at | 10.04 | 1.28E-06 | 1.107973584 | NM_152536| | FGD5,FYVE, RhoGEF and PH domain containing 5 |
| 220188_at | 10.03 | 1.29E-06 | 1.266088838 | NM_020655| | JPH3,junctophilin 3 |
| 222657_s_at | 10.02 | 1.31E-06 | 1.05794322 | NM_001001481| | FLJ11011,hypothetical protein FLJ11011 isoform 1 |
| 202235_at | 10.02 | 1.30E-06 | 1.182345894 | NM_003051| | SLC16A1,solute carrier family 16, member 1 |
| 223138_s_at | 10.02 | 1.30E-06 | 1.045444141 | NM_001114397| | NA |
| 204298_s_at | 10.02 | 1.30E-06 | 1.471876702 | NM_002317| | LOX,lysyl oxidase preproprotein |
| 228277_at | 10.01 | 1.32E-06 | 1.079263694 | NM_001099784| | NA |
| 1569241_a_at | 10.01 | 1.32E-06 | 1.652535137 | NM_031218| | ZNF505,zinc finger protein 505 isoform a |
| 209454_s_at | 10.01 | 1.31E-06 | 1.333484773 | NM_003214| | TEAD3,TEA domain family member 3 |
| 236026_at | 10 | 1.33E-06 | 1.149530393 | NM_018040| | GPATC2,G patch domain containing 2 |
| 225114_at | 10 | 1.33E-06 | 1.165488913 | NM_003659| | AGPS,alkylglycerone phosphate synthase precursor |
| AFFX-HUMISGF3A/M97935_3_at | 10 | 1.33E-06 | 1.082660396 | NM_007315| | STAT1,signal transducer and activator of transcription |
| 226997_at | 10 | 1.33E-06 | 1.321535079 | NM_030955| | ADAMTS12,a disintegrin-like and metalloprotease with |
| 205310_at | 9.99 | 1.34E-06 | 1.048815412 | NM_001080469| | NA |
| 204366_s_at | 9.99 | 1.34E-06 | 1.03624251 | NM_001035521| | NA |
| 218792_s_at | 9.99 | 1.35E-06 | 1.43645467 | NM_017688| | BSPRY,B-box and SPRY domain containing |
| 221583_s_at | 9.99 | 1.35E-06 | 1.352532382 | NM_001014797| | NA |
| 220255_at | 9.98 | 1.36E-06 | 1.109381919 | NM_021922| | FANCE,Fanconi anemia, complementation group E |
| 212906_at | 9.98 | 1.35E-06 | 1.129034296 | NM_020716| | NA |
| 204906_at | 9.98 | 1.36E-06 | 1.126939408 | NM_001006932| | RPS6KA2,ribosomal protein S6 kinase, 90kDa, polypeptide |
| 204696_s_at | 9.97 | 1.37E-06 | 1.070618687 | NM_001789| | CDC25A,cell division cycle 25A isoform a |
| 227182_at | 9.97 | 1.37E-06 | 1.500610254 | NM_145006| | SUSD3,sushi domain containing 3 |
| 233695_s_at | 9.97 | 1.37E-06 | 1.252191111 | NM_031413| | NA |
| 229286_at | 9.97 | 1.37E-06 | 1.221688 | NM_020932| | MAGEE1,melanoma antigen family E, 1 |
| 227618_at | 9.97 | 1.37E-06 | 1.217414658 | NA |  |
| 1553640_at | 9.97 | 1.37E-06 | 1.063545087 | NM_173683| | C8orf21,XK-related protein 6 isoform b |
| 218231_at | 9.96 | 1.38E-06 | 1.078801361 | NM_017567| | NAGK,N-Acetylglucosamine kinase |
| 206824_at | 9.96 | 1.39E-06 | 1.096917844 | NA |  |
| 218564_at | 9.95 | 1.40E-06 | 1.066467823 | NM_018124| | NA |
| 1554469_at | 9.95 | 1.40E-06 | 1.307152068 | NM_014155| | HSPC063,HSPC063 protein |
| 204838_s_at | 9.95 | 1.40E-06 | 1.132295773 | NM_001040108| | NA |
| 204875_s_at | 9.95 | 1.40E-06 | 1.113351546 | NM_001500| | GMDS,GDP-mannose 4,6-dehydratase |
| 205290_s_at | 9.95 | 1.40E-06 | 1.092068535 | NM_001200| | BMP2,bone morphogenetic protein 2 precursor |
| 221985_at | 9.94 | 1.41E-06 | 1.193822207 | NM_017644| | DRE1,DRE1 protein |
| 242348_at | 9.94 | 1.42E-06 | 1.650644768 | NM_001005527| | FAM19A4,family with sequence similarity 19 (chemokine |
| 219870_at | 9.94 | 1.41E-06 | 1.0823599 | NM_024997| | ATF7IP2,activating transcription factor 7 interacting |
| 203951_at | 9.94 | 1.42E-06 | 1.612055834 | NM_001299| | CNN1,calponin 1, basic, smooth muscle |
| 228927_at | 9.94 | 1.41E-06 | 1.119786905 | NM_032347| | ZNF397,zinc finger protein 397 |
| 239147_at | 9.93 | 1.42E-06 | 1.11329954 | NM_198150| | DKFZp313G1735,hypothetical protein DKFZp313G1735 |
| 235258_at | 9.93 | 1.43E-06 | 1.143718681 | NM_152624| | DCP2,DCP2 decapping enzyme |
| 223304_at | 9.93 | 1.43E-06 | 1.08208581 | NM_032295| | SLC37A3,solute carrier family 37 (glycerol-3-phosphate |
| 220150_s_at | 9.93 | 1.42E-06 | 1.127995839 | NM_001100411| | NA |
| 223478_at | 9.93 | 1.42E-06 | 1.052484967 | NM_012459| | TIMM8B,translocase of inner mitochondrial membrane 8 |
| 202375_at | 9.93 | 1.43E-06 | 1.431863534 | NM_014822| | SEC24D,Sec24-related protein D |
| 203347_s_at | 9.93 | 1.43E-06 | 1.253983803 | NM_007358| | M96,putative DNA binding protein |
| 228487_s_at | 9.93 | 1.43E-06 | 1.171911645 | NA |  |
| 226139_at | 9.92 | 1.44E-06 | 1.178972468 | NA |  |
| 212904_at | 9.92 | 1.44E-06 | 1.053192603 | NM_020710| | KIAA1185,KIAA1185 protein |
| 211528_x_at | 9.92 | 1.45E-06 | 1.079461562 | NM_002127| | HLA-G,major histocompatibility complex, class I, G |
| 209862_s_at | 9.92 | 1.45E-06 | 1.106457927 | NM_014679| | PIG8,translokin |
| 219311_at | 9.92 | 1.45E-06 | 1.060943769 | NM_024899| | C18orf9,chromosome 18 open reading frame 9 |
| 201761_at | 9.91 | 1.46E-06 | 1.062731216 | NM_001040409| | NA |
| 217783_s_at | 9.91 | 1.47E-06 | 1.105345535 | NM_016061| | YPEL5,yippee-like 5 |
| 222062_at | 9.91 | 1.46E-06 | 1.12772086 | NM_004843| | IL27RA,class I cytokine receptor |
| 227423_at | 9.91 | 1.45E-06 | 1.063653024 | NM_144598| | LRRC28,leucine rich repeat containing 28 |
| 213811_x_at | 9.91 | 1.46E-06 | 1.050215975 | NM_003200| | TCF3,transcription factor 3 |
| 205899_at | 9.91 | 1.45E-06 | 1.416960113 | NM_001111045| | NA |
| 206046_at | 9.91 | 1.46E-06 | 1.07375519 | NM_003812| | ADAM23,a disintegrin and metalloproteinase domain 23 |
| 218716_x_at | 9.91 | 1.45E-06 | 1.053857423 | NM_001123226| | NA |
| 205081_at | 9.9 | 1.48E-06 | 1.308702426 | NM_001311| | CRIP1,cysteine-rich protein 1 (intestinal) |
| 1563321_s_at | 9.9 | 1.47E-06 | 1.13397889 | NM_001009569| | MLLT10,myeloid/lymphoid or mixed-lineage leukemia |
| 217975_at | 9.9 | 1.47E-06 | 1.08027784 | NM_001006612| | WBP5,WW domain binding protein 5 |
| 1553138_a_at | 9.9 | 1.48E-06 | 1.535295497 | NM_152363| | FLJ39369,hypothetical protein FLJ39369 |
| 203510_at | 9.9 | 1.48E-06 | 1.597246913 | NM_000245| | MET,met proto-oncogene precursor |
| 219822_at | 9.89 | 1.48E-06 | 1.090009123 | NM_004294| | MTRF1,mitochondrial translational release factor 1 |
| 219616_at | 9.89 | 1.49E-06 | 1.084292562 | NM_024560| | FLJ21963,FLJ21963 protein |
| 212244_at | 9.89 | 1.49E-06 | 1.100422812 | NM_001018090| | NA |
| 1557411_s_at | 9.89 | 1.49E-06 | 2.245642033 | NM_145305| | LOC203427,mitochondrial solute carrier protein |
| 219317_at | 9.89 | 1.49E-06 | 1.177085119 | NM_007195| | POLI,polymerase (DNA directed) iota |
| 203874_s_at | 9.89 | 1.49E-06 | 1.13522863 | NM_003069| | SMARCA1,SWI/SNF-related matrix-associated |
| 241968_at | 9.88 | 1.50E-06 | 1.164882008 | NA |  |
| 210964_s_at | 9.88 | 1.51E-06 | 1.17156186 | NM_001079855| | NA |
| 226586_at | 9.88 | 1.51E-06 | 1.075383801 | NM_173551| | SAMD6,sterile alpha motif domain containing 6 |
| 229172_at | 9.88 | 1.50E-06 | 1.454383128 | NM_052970| | HSPA12B,heat shock 70kD protein 12B |
| 217980_s_at | 9.88 | 1.50E-06 | 1.038703824 | NM_017840| | MRPL16,mitochondrial ribosomal protein L16 |
| 235110_at | 9.88 | 1.50E-06 | 1.169438109 | NM_007069| | HRASLS3,HRAS-like suppressor 3 |
| 216080_s_at | 9.87 | 1.52E-06 | 1.136871647 | NM_021727| | FADS3,fatty acid desaturase 3 |
| 243764_at | 9.87 | 1.53E-06 | 1.384799667 | NM_182607| | VSIG1,V-set and immunoglobulin domain containing 1 |
| 207732_s_at | 9.87 | 1.52E-06 | 1.263313833 | NM_020730| | NA |
| 224374_s_at | 9.86 | 1.53E-06 | 1.265121476 | NM_032048| | EMILIN2,elastin microfibril interfacer 2 |
| 220956_s_at | 9.86 | 1.54E-06 | 1.071530639 | NM_053046| | EGLN2,EGL nine (C.elegans) homolog 2 isoform 1 |
| 220798_x_at | 9.86 | 1.54E-06 | 1.441149322 | NM_024888| | FLJ11535,hypothetical protein FLJ11535 |
| 226857_at | 9.86 | 1.53E-06 | 1.284520563 | NM_153213| | ARHGEF19,Rho guanine nucleotide exchange factor (GEF) 19 |
| 201130_s_at | 9.86 | 1.54E-06 | 1.801994857 | NM_004360| | CDH1,cadherin 1, type 1 preproprotein |
| 208763_s_at | 9.86 | 1.54E-06 | 1.440033665 | NM_001015881| | NA |
| 225537_at | 9.85 | 1.55E-06 | 1.112309577 | NM_001079537| | NA |
| 213484_at | 9.84 | 1.57E-06 | 1.431612305 | NA |  |
| 36553_at | 9.84 | 1.57E-06 | 1.113145245 | NM_004192| | ASMTL,acetylserotonin O-methyltransferase-like |
| 235953_at | 9.84 | 1.56E-06 | 1.325270293 | NM_173530| | ZNF610,zinc finger protein 610 |
| 205795_at | 9.84 | 1.57E-06 | 1.376115265 | NM_001105250| | NA |
| 37424_at | 9.83 | 1.59E-06 | 1.095620612 | NM_001105563| | NA |
| 37254_at | 9.83 | 1.59E-06 | 1.068705776 | NM_001083330| | NA |
| 40189_at | 9.83 | 1.59E-06 | 1.035858677 | NM_001122821| | NA |
| 241808_at | 9.83 | 1.59E-06 | 1.302898515 | NA |  |
| 219826_at | 9.82 | 1.60E-06 | 1.099540459 | NM_001098491| | NA |
| 208306_x_at | 9.82 | 1.61E-06 | 1.262725119 | NM_002124| | HLA-DRB1,major histocompatibility complex, class II, DR |
| 226265_at | 9.82 | 1.60E-06 | 1.033183035 | NM_001076786| | NA |
| 227200_at | 9.81 | 1.63E-06 | 1.116178607 | NA |  |
| 204928_s_at | 9.81 | 1.63E-06 | 1.087292354 | NM_019848| | SLC10A3,solute carrier family 10, member 3 |
| 236369_at | 9.81 | 1.62E-06 | 1.126466676 | NA |  |
| 225675_at | 9.8 | 1.64E-06 | 1.090214865 | NM_017799| | C14orf101,chromosome 14 open reading frame 101 |
| 221741_s_at | 9.8 | 1.64E-06 | 1.038142926 | NM_017798| | YTHDF1,YTH domain family, member 1 |
| 218263_s_at | 9.8 | 1.63E-06 | 1.037394596 | NA |  |
| 241829_at | 9.8 | 1.64E-06 | 1.80505895 | NM_145019| | FLJ30707,hypothetical protein FLJ30707 |
| 206239_s_at | 9.8 | 1.64E-06 | 1.417869419 | NM_003122| | SPINK1,serine protease inhibitor, Kazal type 1 |
| 219620_x_at | 9.79 | 1.65E-06 | 1.31923853 | NM_017723| | FLJ20245,hypothetical protein FLJ20245 |
| 220624_s_at | 9.79 | 1.65E-06 | 1.096199885 | NM_001422| | ELF5,E74-like factor 5 ESE-2b |
| 1552678_a_at | 9.79 | 1.65E-06 | 1.306851559 | NM_020886| | USP28,ubiquitin specific protease 28 |
| 212871_at | 9.78 | 1.68E-06 | 1.050002969 | NM_003668| | MAPKAPK5,mitogen-activated protein kinase-activated |
| 227279_at | 9.78 | 1.68E-06 | 1.05603308 | NM_001006933| | TCEAL3,transcription elongation factor A (SII)-like 3 |
| 210059_s_at | 9.78 | 1.67E-06 | 1.373677224 | NM_002754| | MAPK13,mitogen-activated protein kinase 13 |
| 211654_x_at | 9.77 | 1.68E-06 | 1.548060883 | NM_002123| | HLA-DQB1,major histocompatibility complex, class II, DQ |
| 209209_s_at | 9.77 | 1.68E-06 | 1.155476455 | NM_006832| | PLEKHC1,pleckstrin homology domain containing, family C |
| 201769_at | 9.77 | 1.68E-06 | 1.06763601 | NM_014666| | ENTH,enthoprotin |
| 201112_s_at | 9.77 | 1.69E-06 | 1.029989735 | NM_001316| | CSE1L,CSE1 chromosome segregation 1-like protein |
| 221962_s_at | 9.77 | 1.69E-06 | 1.129777695 | NM_003344| | UBE2H,ubiquitin-conjugating enzyme E2H isoform 1 |
| 214435_x_at | 9.77 | 1.69E-06 | 1.109564118 | NM_005402| | RALA,ras related v-ral simian leukemia viral oncogene |
| 209739_s_at | 9.77 | 1.69E-06 | 1.887390488 | NM_004650| | PNPLA4,GS2 gene |
| 201635_s_at | 9.77 | 1.68E-06 | 1.101019209 | NM_001013438| | NA |
| 222718_at | 9.77 | 1.68E-06 | 1.105147692 | NM_021259| | TMEM8,transmembrane protein 8 (five membrane-spanning |
| 211971_s_at | 9.76 | 1.70E-06 | 1.032312157 | NM_133259| | LRPPRC,leucine-rich PPR motif-containing protein |
| 231835_at | 9.76 | 1.71E-06 | 1.062038676 | NM_152371| | MGC26818,hypothetical protein MGC26818 |
| 238992_at | 9.76 | 1.71E-06 | 1.090549831 | NM_007195| | POLI,polymerase (DNA directed) iota |
| 235474_at | 9.76 | 1.70E-06 | 1.079028482 | NA |  |
| 212908_at | 9.76 | 1.71E-06 | 1.036216913 | NM_015291| | KIAA0962,KIAA0962 protein |
| 228570_at | 9.75 | 1.73E-06 | 1.380850965 | NM_001017523| | NA |
| 210751_s_at | 9.75 | 1.72E-06 | 1.359745723 | NM_004683| | RGN,regucalcin |
| 227655_at | 9.75 | 1.72E-06 | 1.372561935 | NA |  |
| 221928_at | 9.75 | 1.72E-06 | 1.150499052 | NM_001093| | ACACB,acetyl-Coenzyme A carboxylase beta |
| 204136_at | 9.75 | 1.73E-06 | 1.246423449 | NM_000094| | COL7A1,alpha 1 type VII collagen precursor |
| 214212_x_at | 9.75 | 1.73E-06 | 1.131814658 | NM_006832| | PLEKHC1,pleckstrin homology domain containing, family C |
| 222116_s_at | 9.74 | 1.74E-06 | 1.058794408 | NM_019020| | TBC1D16,TBC1 domain family, member 16 |
| 209662_at | 9.74 | 1.75E-06 | 1.06485081 | NM_004365| | CETN3,centrin 3 |
| 200961_at | 9.74 | 1.74E-06 | 1.063035457 | NM_012248| | SEPHS2,selenophosphate synthetase 2 |
| 228036_s_at | 9.74 | 1.74E-06 | 1.123757029 | NM_012168| | FBXO2,F-box only protein 2 |
| 225506_at | 9.74 | 1.73E-06 | 1.07909717 | NM_020854| | KIAA1468,KIAA1468 |
| 226648_at | 9.74 | 1.73E-06 | 1.065684991 | NM_017902| | HIF1AN,hypoxia-inducible factor 1, alpha subunit |
| 206291_at | 9.74 | 1.74E-06 | 2.768811179 | NM_006183| | NTS,neurotensin/neuromedin N preproprotein |
| 226345_at | 9.73 | 1.75E-06 | 1.062824116 | NA |  |
| 201020_at | 9.73 | 1.76E-06 | 1.118719425 | NM_003405| | YWHAH,tyrosine 3/tryptophan 5 -monooxygenase |
| 238805_at | 9.73 | 1.77E-06 | 1.199439657 | NM_080659| | MGC14839,hypothetical protein MGC14839 |
| 223611_s_at | 9.72 | 1.78E-06 | 1.648643819 | NM_001126328| | NA |
| 209333_at | 9.72 | 1.79E-06 | 1.128551504 | NM_003565| | ULK1,unc-51-like kinase 1 |
| 220484_at | 9.72 | 1.79E-06 | 1.255078224 | NM_018298| | MCOLN3,mucolipin 3 |
| 216237_s_at | 9.72 | 1.79E-06 | 1.05306859 | NM_006739| | MCM5,minichromosome maintenance deficient protein 5 |
| 212564_at | 9.72 | 1.78E-06 | 1.117857863 | NM_015353| | KCTD2,potassium channel tetramerisation domain |
| 226206_at | 9.72 | 1.77E-06 | 1.172025933 | NM_002360| | MAFK,v-maf musculoaponeurotic fibrosarcoma oncogene |
| 217546_at | 9.71 | 1.80E-06 | 1.629045474 | NM_176870| | MT1K,metallothionein 1K |
| 228468_at | 9.71 | 1.80E-06 | 1.1495038 | NM_032844| | MASTL,microtubule associated serine/threonine |
| 220151_at | 9.71 | 1.80E-06 | 1.072975 | NM_018111| | FLJ10490,hypothetical protein FLJ10490 |
| 204362_at | 9.71 | 1.80E-06 | 1.730582307 | NM_003930| | SCAP2,src family associated phosphoprotein 2 |
| 200851_s_at | 9.71 | 1.80E-06 | 1.056245949 | NM_014761| | KIAA0174,KIAA0174 gene product |
| 207837_at | 9.7 | 1.82E-06 | 1.217769268 | NM_001008710| | RBPMS,RNA-binding protein with multiple splicing |
| 203315_at | 9.7 | 1.82E-06 | 1.073195399 | NM_001004720| | NCK2,NCK adaptor protein 2 isoform A |
| 228004_at | 9.7 | 1.82E-06 | 1.273096736 | NA |  |
| 241455_at | 9.7 | 1.81E-06 | 2.240091405 | NA |  |
| 225568_at | 9.7 | 1.82E-06 | 1.124923077 | NM_032928| | MGC14141,hypothetical protein MGC14141 |
| 242560_at | 9.69 | 1.83E-06 | 1.048938799 | NM_001018115| | NA |
| 238183_at | 9.69 | 1.83E-06 | 1.378471566 | NA |  |
| 221735_at | 9.69 | 1.84E-06 | 1.089898891 | NM_020839| | WDR48,WD repeat domain 48 |
| 202922_at | 9.69 | 1.84E-06 | 1.076373443 | NM_001498| | GCLC,glutamate-cysteine ligase, catalytic subunit |
| 230917_at | 9.69 | 1.84E-06 | 1.388716455 | NA |  |
| 1552575_a_at | 9.68 | 1.86E-06 | 1.449015847 | NA |  |
| 223051_at | 9.68 | 1.86E-06 | 1.062813896 | NM_014188| | HSPC182,HSPC182 protein |
| 222008_at | 9.67 | 1.87E-06 | 1.189648691 | NM_001851| | COL9A1,alpha 1 type IX collagen isoform 1 precursor |
| 218804_at | 9.67 | 1.87E-06 | 1.640722763 | NM_018043| | TMEM16A,transmembrane protein 16A |
| 223963_s_at | 9.67 | 1.87E-06 | 1.147858452 | NM_001007225| | IMP-2,IGF-II mRNA-binding protein 2 isoform b |
| 209698_at | 9.66 | 1.89E-06 | 1.096776299 | NM_001105563| | NA |
| 232322_x_at | 9.66 | 1.90E-06 | 1.228200483 | NM_006645| | STARD10,START domain containing 10 |
| 203991_s_at | 9.65 | 1.92E-06 | 1.446517445 | NM_021140| | UTX,ubiquitously transcribed tetratricopeptide |
| 230425_at | 9.65 | 1.91E-06 | 1.333763284 | NM_004441| | EPHB1,ephrin receptor EphB1 precursor |
| 200921_s_at | 9.65 | 1.92E-06 | 1.129712086 | NM_001731| | BTG1,B-cell translocation protein 1 |
| 223591_at | 9.65 | 1.92E-06 | 1.411506899 | NM_032322| | RNF135,ring finger protein 135 isoform 1 |
| 211456_x_at | 9.65 | 1.91E-06 | 1.104536758 | NA |  |
| 213921_at | 9.65 | 1.92E-06 | 1.214244027 | NM_001048| | SST,somatostatin |
| 206542_s_at | 9.64 | 1.94E-06 | 1.891865412 | NM_003070| | SMARCA2,SWI/SNF-related matrix-associated |
| 210692_s_at | 9.64 | 1.93E-06 | 1.278626511 | NM_014096| | SLC43A3,solute carrier family 43, member 3 |
| 229664_at | 9.64 | 1.94E-06 | 1.125610926 | NM_002750| | MAPK8,mitogen-activated protein kinase 8 isoform 2 |
| 202102_s_at | 9.64 | 1.94E-06 | 1.068570213 | NM_014299| | BRD4,bromodomain-containing protein 4 isoform short |
| 209930_s_at | 9.64 | 1.94E-06 | 1.33479137 | NM_006163| | NFE2,nuclear factor (erythroid-derived 2), 45kDa |
| 225582_at | 9.64 | 1.93E-06 | 1.214156871 | NM_033397| | KIAA1754,KIAA1754 |
| 201422_at | 9.64 | 1.94E-06 | 1.311160553 | NM_005027| | PIK3R2,phosphoinositide-3-kinase, regulatory subunit 2 |
| 210770_s_at | 9.63 | 1.96E-06 | 1.34411631 | NM_000068| | CACNA1A,calcium channel, alpha 1A subunit isoform 1 |
| 203321_s_at | 9.63 | 1.95E-06 | 1.044037355 | NM_014913| | KIAA0863,KIAA0863 protein |
| 221873_at | 9.63 | 1.96E-06 | 1.066076029 | NM_003442| | ZNF143,zinc finger protein 143 (clone pHZ-1) |
| 219066_at | 9.63 | 1.95E-06 | 1.046390471 | NM_021823| | MDS018,hypothetical protein MDS018 |
| 210845_s_at | 9.63 | 1.97E-06 | 1.251842762 | NM_001005376| | PLAUR,plasminogen activator, urokinase receptor |
| 203853_s_at | 9.63 | 1.96E-06 | 1.154300646 | NM_012296| | GAB2,GRB2-associated binding protein 2 isoform b |
| 209193_at | 9.63 | 1.96E-06 | 1.367789955 | NM_002648| | PIM1,pim-1 oncogene |
| 235072_s_at | 9.62 | 1.97E-06 | 1.066805401 | NA |  |
| 223248_at | 9.62 | 1.97E-06 | 1.096613496 | NM_031463| | HSDL1,hydroxysteroid dehydrogenase like 1 |
| 205024_s_at | 9.62 | 1.97E-06 | 1.059704834 | NM_002875| | RAD51,RAD51 homolog protein isoform 1 |
| 244024_at | 9.62 | 1.98E-06 | 1.113137329 | NM_001007088| | ZNF21,zinc finger protein 21 isoform 2 |
| 1556826_s_at | 9.62 | 1.98E-06 | 1.266801744 | NM_198545| | LOC374946,hypothetical gene supported by AK075558; |
| 226759_at | 9.61 | 2.00E-06 | 1.151037775 | NM_022465| | ZNFN1A4,zinc finger protein, subfamily 1A, 4 |
| 229569_at | 9.61 | 1.99E-06 | 1.382819934 | NA |  |
| 227729_at | 9.61 | 1.99E-06 | 1.104949764 | NA |  |
| 207524_at | 9.61 | 1.99E-06 | 1.126074041 | NM_018412| | ST7,suppression of tumorigenicity 7 isoform a |
| 242887_at | 9.61 | 1.99E-06 | 1.23665928 | NM_020122| | KCMF1,potassium channel modulatory factor 1 |
| 206544_x_at | 9.6 | 2.03E-06 | 1.433573256 | NM_003070| | SMARCA2,SWI/SNF-related matrix-associated |
| 1557816_a_at | 9.6 | 2.02E-06 | 1.195561212 | NA |  |
| 220360_at | 9.6 | 2.02E-06 | 1.285247419 | NM_024672| | THAP9,THAP domain containing 9 |
| 201256_at | 9.6 | 2.03E-06 | 1.043437917 | NM_004718| | COX7A2L,cytochrome c oxidase subunit VIIa polypeptide 2 |
| 200096_s_at | 9.6 | 2.01E-06 | 1.045758697 | NM_003945| | ATP6V0E,ATPase, H+ transporting, lysosomal, V0 subunit |
| 238455_at | 9.6 | 2.01E-06 | 1.568491069 | NA |  |
| 220291_at | 9.6 | 2.02E-06 | 1.190884885 | NM_017711| | GDPD2,osteoblast differentiation promoting factor |
| 217778_at | 9.59 | 2.05E-06 | 1.121307384 | NM_014437| | SLC39A1,solute carrier family 39 (zinc transporter), |
| 219304_s_at | 9.59 | 2.04E-06 | 1.57450694 | NM_025208| | PDGFD,platelet derived growth factor D isoform 1 |
| 225222_at | 9.59 | 2.04E-06 | 1.044138232 | NM_033055| | HIAT1,hippocampus abundant transcript 1 |
| 223677_at | 9.59 | 2.05E-06 | 1.159629715 | NM_031482| | APG10L,APG10 autophagy 10-like |
| 207791_s_at | 9.58 | 2.05E-06 | 1.080634569 | NM_004161| | RAB1A,RAB1A, member RAS oncogene family |
| 214355_x_at | 9.58 | 2.05E-06 | 1.164484927 | NM_001008747| | LOC441294,similar to CTAGE family, member 6 |
| 205925_s_at | 9.58 | 2.06E-06 | 1.352577311 | NM_002867| | RAB3B,RAB3B, member RAS oncogene family |
| 34858_at | 9.57 | 2.08E-06 | 1.094207895 | NM_015353| | KCTD2,potassium channel tetramerisation domain |
| 217730_at | 9.57 | 2.07E-06 | 1.303270365 | NM_022152| | PP1201,PP1201 protein |
| 213047_x_at | 9.57 | 2.08E-06 | 1.035356842 | NM_001122821| | NA |
| 218313_s_at | 9.57 | 2.08E-06 | 1.064313169 | NM_017423| | GALNT7,polypeptide N-acetylgalactosaminyltransferase 7 |
| 210416_s_at | 9.57 | 2.08E-06 | 1.146621151 | NM_001005735| | CHEK2,protein kinase CHK2 isoform c |
| 213843_x_at | 9.57 | 2.08E-06 | 1.061581625 | NM_005629| | SLC6A8,solute carrier family 6 (neurotransmitter |
| 235840_at | 9.56 | 2.11E-06 | 1.109902729 | NM_144597| | MGC29937,hypothetical protein MGC29937 |
| 222583_s_at | 9.56 | 2.10E-06 | 1.121985559 | NM_007172| | NUP50,nucleoporin 50kDa isoform b |
| 203755_at | 9.56 | 2.10E-06 | 1.045597287 | NM_001211| | BUB1B,BUB1 budding uninhibited by benzimidazoles 1 |
| 223541_at | 9.56 | 2.11E-06 | 1.387742746 | NM_005329| | HAS3,hyaluronan synthase 3 isoform a |
| 217553_at | 9.56 | 2.10E-06 | 1.571305902 | NA |  |
| 232811_x_at | 9.55 | 2.14E-06 | 1.189646734 | NM_153026| | PRICKLE1,prickle-like 1 |
| 225068_at | 9.55 | 2.13E-06 | 1.080618339 | NM_021633| | KLHL12,kelch-like 12 |
| 203827_at | 9.55 | 2.14E-06 | 1.241941903 | NM_017983| | WIPI49,hypothetical protein FLJ10055 |
| 241341_at | 9.54 | 2.15E-06 | 1.222965225 | NA |  |
| 224664_at | 9.54 | 2.15E-06 | 1.033986753 | NM_173473| | C10orf104,chromosome 10 open reading frame 104 |
| 218209_s_at | 9.54 | 2.16E-06 | 1.088481852 | NM_018170| | P15RS,hypothetical protein FLJ10656 |
| 203278_s_at | 9.54 | 2.14E-06 | 1.035483536 | NM_001101802| | NA |
| 211962_s_at | 9.54 | 2.16E-06 | 1.108537397 | NM_004926| | ZFP36L1,butyrate response factor 1 |
| 209581_at | 9.53 | 2.17E-06 | 1.462443062 | NM_007069| | HRASLS3,HRAS-like suppressor 3 |
| 225095_at | 9.53 | 2.18E-06 | 1.050341518 | NM_004863| | SPTLC2,serine palmitoyltransferase, long chain base |
| 220448_at | 9.53 | 2.18E-06 | 1.723295899 | NM_022055| | KCNK12,potassium channel, subfamily K, member 12 |
| 224177_s_at | 9.53 | 2.17E-06 | 1.075597113 | NM_016500| | CXorf26,chromosome X open reading frame 26 |
| 212965_at | 9.52 | 2.20E-06 | 1.314452304 | NM_015094| | HIC2,hypermethylated in cancer 2 |
| 209040_s_at | 9.52 | 2.21E-06 | 1.083617877 | NM_004159| | PSMB8,proteasome beta 8 subunit isoform E1 proprotein |
| 224811_at | 9.51 | 2.22E-06 | 1.170685536 | NA |  |
| 225208_s_at | 9.51 | 2.22E-06 | 1.102338762 | NM_031452| | MGC2560,hypothetical LOC83640 |
| 229766_at | 9.51 | 2.23E-06 | 1.047188969 | NM_181489| | ZNF445,zinc finger protein 445 |
| 226612_at | 9.51 | 2.23E-06 | 1.336140138 | NA |  |
| 204873_at | 9.51 | 2.21E-06 | 1.17594119 | NM_000466| | PEX1,peroxisome biogenesis factor 1 |
| 243722_at | 9.51 | 2.22E-06 | 1.430940261 | NM_152901| | PYC1,pyrin-domain containing protein 1 |
| 1555399_a_at | 9.51 | 2.23E-06 | 1.166851959 | NM_030640| | DUSP16,dual specificity phosphatase 16 |
| 208884_s_at | 9.5 | 2.24E-06 | 1.050526635 | NM_015902| | EDD,progestin-induced protein |
| 205616_at | 9.5 | 2.26E-06 | 1.221736875 | NA |  |
| 203038_at | 9.5 | 2.24E-06 | 1.061407347 | NM_002844| | PTPRK,protein tyrosine phosphatase, receptor type, K |
| 222989_s_at | 9.5 | 2.25E-06 | 1.098871739 | NM_013438| | UBQLN1,ubiquilin 1 isoform 1 |
| 227239_at | 9.5 | 2.24E-06 | 1.128911192 | NM_032581| | DRCTNNB1A,down-regulated by Ctnnb1, a |
| 220454_s_at | 9.49 | 2.26E-06 | 1.252471633 | NM_020796| | SEMA6A,semaphorin 6A1 |
| 228113_at | 9.49 | 2.27E-06 | 1.496417027 | NM_001006638| | RAB37,RAB37, member RAS oncogene family |
| 225502_at | 9.49 | 2.28E-06 | 1.242600941 | NM_203447| | DOCK8,dedicator of cytokinesis 8 |
| 207232_s_at | 9.49 | 2.27E-06 | 1.102204734 | NM_014648| | DZIP3,zinc finger DAZ interacting protein 3 |
| 207292_s_at | 9.49 | 2.28E-06 | 1.137386159 | NM_002749| | MAPK7,mitogen-activated protein kinase 7 isoform 1 |
| 200611_s_at | 9.48 | 2.29E-06 | 1.08899811 | NM_005112| | WDR1,WD repeat-containing protein 1 isoform 2 |
| 209869_at | 9.48 | 2.29E-06 | 1.884147363 | NM_000681| | ADRA2A,alpha-2A-adrenergic receptor |
| 204880_at | 9.48 | 2.28E-06 | 1.15934178 | NM_002412| | MGMT,O-6-methylguanine-DNA methyltransferase |
| 238135_at | 9.48 | 2.29E-06 | 1.129391335 | NM_001040194| | NA |
| 203011_at | 9.47 | 2.31E-06 | 1.061678783 | NM_005536| | IMPA1,inositol(myo)-1(or 4)-monophosphatase 1 |
| 224947_at | 9.47 | 2.32E-06 | 1.069025871 | NM_032015| | RNF26,ring finger protein 26 |
| 221764_at | 9.47 | 2.32E-06 | 1.074064256 | NM_138774| | C19orf22,chromosome 19 open reading frame 22 |
| 209363_s_at | 9.47 | 2.33E-06 | 1.032290591 | NM_004264| | SURB7,SRB7 suppressor of RNA polymerase B homolog |
| 230889_at | 9.47 | 2.32E-06 | 1.288346182 | NA |  |
| 242292_at | 9.46 | 2.36E-06 | 1.176214795 | NA |  |
| 203738_at | 9.46 | 2.35E-06 | 1.067580761 | NM_018356| | FLJ11193,hypothetical protein FLJ11193 |
| 201055_s_at | 9.46 | 2.34E-06 | 1.067207242 | NM_006805| | HNRPA0,heterogeneous nuclear ribonucleoprotein A0 |
| 210247_at | 9.45 | 2.37E-06 | 1.315895772 | NM_003178| | SYN2,synapsin II isoform IIb |
| 203819_s_at | 9.45 | 2.36E-06 | 1.07262874 | NM_006547| | IMP-3,IGF-II mRNA-binding protein 3 |
| 238346_s_at | 9.45 | 2.37E-06 | 1.06843268 | NM_024831| | NCOA6IP,PRIP-interacting protein PIPMT |
| 227333_at | 9.45 | 2.36E-06 | 1.102724991 | NA |  |
| 230003_at | 9.44 | 2.40E-06 | 1.332088651 | NA |  |
| 225117_at | 9.44 | 2.38E-06 | 1.058327432 | NM_015443| | LOC284058,hypothetical protein LOC284058 |
| 209258_s_at | 9.44 | 2.40E-06 | 1.067711666 | NM_005445| | CSPG6,chondroitin sulfate proteoglycan 6 (bamacan) |
| 39650_s_at | 9.44 | 2.40E-06 | 1.246828348 | NM_014801| | PCNXL2,pecanex-like 2 |
| 207988_s_at | 9.44 | 2.40E-06 | 1.039475294 | NM_005731| | ARPC2,actin related protein 2/3 complex subunit 2 |
| 217963_s_at | 9.44 | 2.41E-06 | 1.036987069 | NM_014380| | NGFRAP1,nerve growth factor receptor (TNFRSF16) |
| 219877_at | 9.44 | 2.40E-06 | 1.574720341 | NM_024645| | FLJ13842,hypothetical protein FLJ13842 |
| 204936_at | 9.44 | 2.38E-06 | 1.101015704 | NM_004579| | MAP4K2,mitogen-activated protein kinase kinase kinase |
| 211529_x_at | 9.43 | 2.42E-06 | 1.052191565 | NM_002127| | HLA-G,major histocompatibility complex, class I, G |
| 215891_s_at | 9.43 | 2.42E-06 | 1.210248878 | NM_000405| | GM2A,GM2 ganglioside activator precursor |
| 211185_s_at | 9.43 | 2.41E-06 | 1.050430134 | NM_001005526| | SF3B1,splicing factor 3b, subunit 1 isoform 2 |
| 238695_s_at | 9.43 | 2.43E-06 | 1.136685548 | NM_171998| | RAB39B,RAB39B, member RAS oncogene family |
| 1563638_at | 9.42 | 2.45E-06 | 1.189355089 | NM_001079512| | NA |
| 203335_at | 9.42 | 2.44E-06 | 1.047572432 | NM_001037537| | NA |
| 209349_at | 9.42 | 2.44E-06 | 1.078375462 | NM_005732| | RAD50,RAD50 homolog isoform 1 |
| 213801_x_at | 9.41 | 2.46E-06 | 1.015539381 | NM_001005472| | LOC388524,similar to Laminin receptor 1 |
| 220193_at | 9.41 | 2.48E-06 | 1.13785558 | NM_024676| | FLJ22938,hypothetical protein FLJ22938 |
| 214219_x_at | 9.41 | 2.48E-06 | 1.637856889 | NM_001042600| | NA |
| 213270_at | 9.41 | 2.48E-06 | 1.153493498 | NM_005374| | MPP2,palmitoylated membrane protein 2 |
| 203307_at | 9.41 | 2.47E-06 | 1.060622112 | NM_005275| | GNL1,guanine nucleotide binding protein-like 1 |
| 212512_s_at | 9.41 | 2.48E-06 | 1.091364049 | NM_199141| | CARM1,coactivator-associated arginine |
| 225268_at | 9.41 | 2.47E-06 | 1.043103084 | NM_002268| | KPNA4,karyopherin alpha 4 |
| 218006_s_at | 9.41 | 2.46E-06 | 1.048793753 | NM_006963| | ZNF22,zinc finger protein 22 (KOX 15) |
| 206336_at | 9.41 | 2.46E-06 | 2.664754402 | NM_002993| | CXCL6,chemokine (C-X-C motif) ligand 6 (granulocyte |
| 201354_s_at | 9.41 | 2.47E-06 | 1.084194554 | NM_013449| | BAZ2A,bromodomain adjacent to zinc finger domain, 2A |
| 45828_at | 9.4 | 2.48E-06 | 1.046389229 | NM_018035| | FLJ10241,hypothetical protein FLJ10241 |
| 215356_at | 9.4 | 2.50E-06 | 1.294249955 | NM_001110822| | NA |
| 235704_at | 9.4 | 2.51E-06 | 1.407644707 | NM_014764| | DAZAP2,DAZ associated protein 2 |
| 235529_x_at | 9.4 | 2.49E-06 | 1.188880431 | NA |  |
| 208868_s_at | 9.4 | 2.49E-06 | 1.371090617 | NM_031412| | GABARAPL1,GABA(A) receptor-associated protein like 1 |
| 1556421_at | 9.4 | 2.50E-06 | 1.374816364 | NA |  |
| 200627_at | 9.4 | 2.51E-06 | 1.027634392 | NM_006601| | TEBP,unactive progesterone receptor, 23 kD |
| 225014_at | 9.4 | 2.49E-06 | 1.063003705 | NA |  |
| 206082_at | 9.4 | 2.50E-06 | 1.32711713 | NM_006674| | HCP5,HLA complex P5 |
| 224761_at | 9.4 | 2.50E-06 | 1.076867479 | NM_006572| | GNA13,guanine nucleotide binding protein (G protein), |
| 221790_s_at | 9.39 | 2.53E-06 | 1.199787521 | NM_015627| | ARH,LDL receptor adaptor protein |
| 206191_at | 9.38 | 2.54E-06 | 1.191637359 | NM_001248| | ENTPD3,ectonucleoside triphosphate diphosphohydrolase |
| 209308_s_at | 9.38 | 2.55E-06 | 1.109648316 | NM_004330| | BNIP2,BCL2/adenovirus E1B 19kD interacting protein 2 |
| 203136_at | 9.38 | 2.56E-06 | 1.057370551 | NM_006423| | RABAC1,Rab acceptor 1 |
| 1552388_at | 9.38 | 2.56E-06 | 1.187052221 | NA |  |
| 210292_s_at | 9.37 | 2.58E-06 | 1.839306886 | NM_014522| | PCDH11X,protocadherin 11 X-linked isoform a precursor |
| 225326_at | 9.37 | 2.58E-06 | 1.072754593 | NM_018989| | NA |
| 227306_at | 9.37 | 2.58E-06 | 1.297355347 | NA |  |
| 220054_at | 9.37 | 2.58E-06 | 1.204900586 | NM_016584| | IL23A,interleukin 23, alpha subunit p19 precursor |
| 201597_at | 9.37 | 2.58E-06 | 1.018779474 | NM_001865| | COX7A2,cytochrome c oxidase subunit VIIa polypeptide 2 |
| 215718_s_at | 9.37 | 2.57E-06 | 1.090901714 | NM_015153| | PHF3,PHD finger protein 3 |
| 206217_at | 9.36 | 2.60E-06 | 1.336413956 | NM_001005609| | EDA,ectodysplasin A isoform EDA-A2 |
| 219689_at | 9.36 | 2.60E-06 | 1.37946278 | NM_020163| | LOC56920,semaphorin sem2 |
| 227711_at | 9.36 | 2.59E-06 | 1.18636821 | NM_144594| | FLJ32942,hypothetical protein FLJ32942 |
| 223465_at | 9.36 | 2.59E-06 | 1.193663859 | NM_005713| | COL4A3BP,alpha 3 type IV collagen binding protein isoform |
| 205894_at | 9.36 | 2.60E-06 | 1.259351285 | NM_000047| | ARSE,arylsulfatase E precursor |
| 230916_at | 9.36 | 2.61E-06 | 1.456293448 | NM_018055| | NODAL,nodal-related protein |
| 222815_at | 9.35 | 2.64E-06 | 1.117259457 | NM_016120| | RNF12,ring finger protein 12 |
| 227679_at | 9.35 | 2.64E-06 | 1.103903581 | NA |  |
| 218796_at | 9.35 | 2.64E-06 | 1.209505539 | NM_017671| | C20orf42,chromosome 20 open reading frame 42 |
| 205995_x_at | 9.35 | 2.63E-06 | 1.090053007 | NM_001023570| | NA |
| 1560782_at | 9.35 | 2.63E-06 | 1.167003687 | NA |  |
| 215471_s_at | 9.35 | 2.64E-06 | 1.765456696 | NM_003980| | MAP7,microtubule-associated protein 7 |
| 209434_s_at | 9.34 | 2.65E-06 | 1.060185854 | NM_002703| | PPAT,phosphoribosyl pyrophosphate amidotransferase |
| 236620_at | 9.34 | 2.65E-06 | 1.038725245 | NM_018151| | RIF1,RAP1 interacting factor 1 |
| 227018_at | 9.34 | 2.65E-06 | 1.095992278 | NM_017743| | DPP8,dipeptidyl peptidase 8 isoform 2 |
| 1553286_at | 9.33 | 2.70E-06 | 1.155781431 | NM_152791| | ZNF555,zinc finger protein 555 |
| 219983_at | 9.33 | 2.67E-06 | 1.180427654 | NM_020386| | HRASLS,HRAS-like suppressor |
| 220714_at | 9.33 | 2.68E-06 | 1.675676066 | NM_024504| | PRDM14,PR domain containing 14 |
| 205361_s_at | 9.33 | 2.70E-06 | 1.110029342 | NM_002623| | PFDN4,prefoldin 4 |
| 216319_at | 9.33 | 2.68E-06 | 1.805341962 | NA |  |
| 210619_s_at | 9.32 | 2.72E-06 | 1.261300125 | NM_007312| | HYAL1,hyaluronoglucosaminidase 1 isoform 1 |
| 202113_s_at | 9.32 | 2.72E-06 | 1.081287497 | NM_003100| | SNX2,sorting nexin 2 |
| 207735_at | 9.32 | 2.71E-06 | 1.411173836 | NM_017831| | RNF125,ring finger protein 125 |
| 223616_at | 9.32 | 2.73E-06 | 1.266989102 | NM_023074| | FLJ12644,hypothetical protein FLJ12644 |
| 223168_at | 9.32 | 2.70E-06 | 1.420436869 | NM_021205| | RHOU,ras homolog gene family, member U |
| 239636_at | 9.32 | 2.70E-06 | 1.425180118 | NM_001112732| | NA |
| 223753_s_at | 9.31 | 2.75E-06 | 1.72352588 | NM_001079530| | NA |
| 203822_s_at | 9.31 | 2.73E-06 | 1.090955613 | NM_006874| | ELF2,E74-like factor 2 (ets domain transcription |
| 205528_s_at | 9.31 | 2.75E-06 | 1.133998699 | NM_004349| | RUNX1T1,acute myelogenous leukemia 1 translocation 1 |
| 210242_x_at | 9.31 | 2.74E-06 | 1.14444404 | NM_001100879| | NA |
| 231785_at | 9.31 | 2.75E-06 | 1.366821749 | NM_006179| | NTF5,neurotrophin 5 preproprotein |
| 210740_s_at | 9.31 | 2.75E-06 | 1.079565818 | NM_014216| | ITPK1,inositol 1,3,4-triphosphate 5/6 kinase |
| 244563_at | 9.31 | 2.74E-06 | 1.250853877 | NM_001076786| | NA |
| 214044_at | 9.31 | 2.75E-06 | 1.172959239 | NM_001035| | RYR2,ryanodine receptor 2 |
| 200047_s_at | 9.31 | 2.73E-06 | 1.058271331 | NM_003403| | YY1,YY1 transcription factor |
| 229360_at | 9.3 | 2.76E-06 | 1.197785274 | NM_080764| | SUHW2,suppressor of hairy wing homolog 2 |
| 205236_x_at | 9.3 | 2.78E-06 | 1.088943367 | NM_003102| | SOD3,superoxide dismutase 3, extracellular |
| 227052_at | 9.3 | 2.77E-06 | 1.152131625 | NA |  |
| 231515_at | 9.3 | 2.77E-06 | 1.24897804 | NA |  |
| 219299_at | 9.3 | 2.78E-06 | 1.05176161 | NM_017956| | FLJ20772,hypothetical protein FLJ20772 |
| 1555168_a_at | 9.3 | 2.76E-06 | 1.444340148 | NM_001017440| | NA |
| 33304_at | 9.3 | 2.77E-06 | 1.198709976 | NM_002201| | ISG20,interferon stimulated gene 20kDa |
| 209101_at | 9.3 | 2.78E-06 | 1.388982156 | NM_001901| | CTGF,connective tissue growth factor |
| 209650_s_at | 9.29 | 2.81E-06 | 1.041580618 | NM_014346| | TBC1D22A,TBC1 domain family, member 22A |
| 242056_at | 9.29 | 2.79E-06 | 1.078131122 | NM_025188| | TRIM45,tripartite motif-containing 45 |
| 212989_at | 9.29 | 2.79E-06 | 1.180202979 | NM_147156| | TMEM23,phosphatidylcholine:ceramide |
| 235812_at | 9.29 | 2.79E-06 | 1.096059222 | NM_153261| | FLJ38101,hypothetical protein FLJ38101 |
| 223103_at | 9.29 | 2.81E-06 | 1.229599045 | NM_006645| | STARD10,START domain containing 10 |
| 207057_at | 9.29 | 2.81E-06 | 1.345290517 | NM_004731| | SLC16A7,solute carrier family 16, member 7 |
| 221738_at | 9.28 | 2.84E-06 | 1.081586811 | NM_020336| | KIAA1219,KIAA1219 protein |
| 218489_s_at | 9.28 | 2.83E-06 | 1.118077844 | NM_000031| | ALAD,delta-aminolevulinic acid dehydratase isoform b |
| 227285_at | 9.28 | 2.82E-06 | 1.13165887 | NM_144697| | C1orf51,chromosome 1 open reading frame 51 |
| 201837_s_at | 9.28 | 2.84E-06 | 1.047226813 | NM_014860| | STAF65(gamma),SPTF-associated factor 65 gamma |
| 238504_at | 9.28 | 2.84E-06 | 1.126537009 | NM_145267| | C6orf57,chromosome 6 open reading frame 57 |
| 206757_at | 9.28 | 2.84E-06 | 1.471594252 | NM_001083| | PDE5A,phosphodiesterase 5A isoform 1 |
| 225627_s_at | 9.28 | 2.82E-06 | 1.107098261 | NM_020925| | KIAA1573,KIAA1573 protein |
| 235657_at | 9.27 | 2.85E-06 | 1.460801218 | NA |  |
| 214974_x_at | 9.27 | 2.87E-06 | 2.807632382 | NM_002994| | CXCL5,chemokine (C-X-C motif) ligand 5 precursor |
| 220487_at | 9.27 | 2.87E-06 | 1.465420688 | NM_018968| | SNTG2,syntrophin, gamma 2 |
| 1554043_a_at | 9.27 | 2.86E-06 | 1.237104604 | NA |  |
| 203803_at | 9.27 | 2.85E-06 | 1.136273658 | NM_016297| | PCYOX1,prenylcysteine oxidase 1 |
| 207405_s_at | 9.27 | 2.85E-06 | 1.099603771 | NM_002873| | RAD17,RAD17 homolog isoform 1 |
| 208496_x_at | 9.27 | 2.87E-06 | 1.130807047 | NM_003520| | HIST1H2BN,H2B histone family, member D |
| 208858_s_at | 9.26 | 2.89E-06 | 1.115214833 | NM_015292| | MBC2,KIAA0747 protein |
| 209914_s_at | 9.26 | 2.90E-06 | 1.482675773 | NM_004801| | NRXN1,neurexin 1 isoform alpha precursor |
| 227475_at | 9.26 | 2.88E-06 | 1.578113821 | NM_033260| | FOXQ1,forkhead box Q1 |
| 1558097_at | 9.26 | 2.91E-06 | 1.099477489 | NM_173566| | MGC50372,hypothetical protein MGC50372 |
| 213743_at | 9.25 | 2.93E-06 | 1.128978518 | NM_001241| | CCNT2,cyclin T2 isoform a |
| 204727_at | 9.25 | 2.93E-06 | 1.276000123 | NM_001008396| | WDHD1,WD repeat and HMG-box DNA binding protein 1 |
| 233589_x_at | 9.25 | 2.93E-06 | 1.332244355 | NM_017723| | FLJ20245,hypothetical protein FLJ20245 |
| 204352_at | 9.25 | 2.91E-06 | 1.160518646 | NM_001033910| | NA |
| 221014_s_at | 9.25 | 2.93E-06 | 1.221701131 | NM_031296| | RAB33B,RAB33B, member RAS oncogene family |
| 237227_at | 9.25 | 2.91E-06 | 1.077174042 | NM_001031741| | NA |
| 218645_at | 9.25 | 2.93E-06 | 1.079121559 | NM_021994| | ZNF277,zinc finger protein (C2H2 type) 277 |
| 219580_s_at | 9.24 | 2.94E-06 | 1.288304159 | NM_001105248| | NA |
| 226216_at | 9.24 | 2.94E-06 | 1.176394472 | NM_000208| | INSR,insulin receptor |
| 206550_s_at | 9.24 | 2.94E-06 | 1.036910868 | NM_004298| | NUP155,nucleoporin 155kDa isoform 2 |
| 237054_at | 9.24 | 2.95E-06 | 1.60183437 | NM_021572| | ENPP5,ectonucleotide pyrophosphatase/phosphodiesterase |
| 213122_at | 9.24 | 2.95E-06 | 1.237236342 | NM_033512| | TSPYL5,TSPY-like 5 |
| 230789_at | 9.23 | 2.98E-06 | 1.233695948 | NM_080764| | SUHW2,suppressor of hairy wing homolog 2 |
| 201074_at | 9.22 | 3.02E-06 | 1.038141924 | NM_003074| | SMARCC1,SWI/SNF-related matrix-associated |
| 218728_s_at | 9.22 | 3.04E-06 | 1.075977443 | NM_014184| | HSPC163,HSPC163 protein |
| 1564662_at | 9.22 | 3.02E-06 | 1.173033906 | NA |  |
| 202965_s_at | 9.21 | 3.04E-06 | 1.712014838 | NM_014289| | CAPN6,calpain 6 |
| 212203_x_at | 9.21 | 3.05E-06 | 1.156179275 | NM_021034| | IFITM3,interferon-induced transmembrane protein 3 |
| 205593_s_at | 9.21 | 3.05E-06 | 1.244585316 | NM_001001567| | PDE9A,phosphodiesterase 9A isoform b |
| 1556911_at | 9.21 | 3.07E-06 | 1.454564942 | NA |  |
| 243790_at | 9.21 | 3.04E-06 | 1.172582679 | NM_152655| | ZNF585A,zinc finger protein 585A |
| 220792_at | 9.21 | 3.06E-06 | 1.203930135 | NM_018699| | PRDM5,PR domain containing 5 |
| 223491_at | 9.21 | 3.07E-06 | 1.071087339 | NM_016094| | COMMD2,COMM domain containing 2 |
| 1552684_a_at | 9.2 | 3.10E-06 | 1.125341902 | NM_145204| | SENP8,SUMO/sentrin specific protease family member 8 |
| 228956_at | 9.2 | 3.07E-06 | 1.965014818 | NM_003360| | UGT8,UDP glycosyltransferase 8 (UDP-galactose |
| 213579_s_at | 9.2 | 3.09E-06 | 1.10092932 | NM_001429| | EP300,E1A binding protein p300 |
| 220079_s_at | 9.2 | 3.09E-06 | 1.052101264 | NM_001032730| | NA |
| 223548_at | 9.2 | 3.09E-06 | 1.122151218 | NM_001105518| | NA |
| 216228_s_at | 9.2 | 3.09E-06 | 1.200918759 | NM_001008396| | WDHD1,WD repeat and HMG-box DNA binding protein 1 |
| 204502_at | 9.19 | 3.12E-06 | 1.21061037 | NM_015474| | SAMHD1,SAM domain- and HD domain-containing protein 1 |
| 218503_at | 9.19 | 3.13E-06 | 1.069158475 | NM_017794| | KIAA1797,KIAA1797 |
| 235791_x_at | 9.19 | 3.10E-06 | 1.065816658 | NM_001270| | CHD1,chromodomain helicase DNA binding protein 1 |
| 223440_at | 9.19 | 3.12E-06 | 1.094998696 | NM_025187| | Lin10,lin-10 |
| 204793_at | 9.19 | 3.12E-06 | 1.19634785 | NM_001099410| | NA |
| 209076_s_at | 9.18 | 3.15E-06 | 1.025525629 | NM_019613| | WDR45L,WDR45-like |
| 204922_at | 9.18 | 3.15E-06 | 1.211938101 | NM_024650| | FLJ22531,hypothetical protein FLJ22531 |
| 239492_at | 9.18 | 3.16E-06 | 1.52543744 | NM_174977| | SEC14L4,SEC14p-like protein TAP3 |
| 223574_x_at | 9.18 | 3.15E-06 | 1.161370456 | NM_020416| | PPP2R2C,gamma isoform of regulatory subunit B55, protein |
| 203654_s_at | 9.18 | 3.15E-06 | 1.036836216 | NM_004645| | COIL,coilin |
| 223218_s_at | 9.18 | 3.16E-06 | 1.42185702 | NM_001005474| | NFKBIZ,nuclear factor of kappa light polypeptide gene |
| 215425_at | 9.18 | 3.15E-06 | 1.401787732 | NM_006806| | BTG3,B-cell translocation gene 3 |
| 218251_at | 9.17 | 3.19E-06 | 1.061707143 | NM_001098790| | NA |
| 229750_at | 9.17 | 3.18E-06 | 1.113668974 | NM_002698| | POU2F2,POU domain, class 2, transcription factor 2 |
| 215706_x_at | 9.17 | 3.18E-06 | 1.068752019 | NM_001010972| | ZYX,zyxin |
| 239208_s_at | 9.17 | 3.18E-06 | 1.185949469 | NM_001006114| | C21orf57,chromosome 21 open reading frame 57 isoform 2 |
| 1555229_a_at | 9.17 | 3.20E-06 | 1.146132859 | NM_001734| | C1S,complement component 1, s subcomponent |
| 208951_at | 9.17 | 3.18E-06 | 1.049225412 | NM_001182| | ALDH7A1,antiquitin |
| 223084_s_at | 9.17 | 3.18E-06 | 1.106617469 | NM_012142| | CCNDBP1,cyclin D-type binding-protein 1 |
| 210702_s_at | 9.17 | 3.18E-06 | 1.351849783 | NM_000961| | PTGIS,prostaglandin I2 (prostacyclin) synthase |
| 210132_at | 9.16 | 3.21E-06 | 1.134469359 | NM_004952| | EFNA3,ephrin A3 |
| 226088_at | 9.16 | 3.23E-06 | 1.094895809 | NM_032799| | ZDHHC12,zinc finger, DHHC domain containing 12 |
| 1564381_s_at | 9.16 | 3.22E-06 | 1.146918853 | NA |  |
| 235024_at | 9.16 | 3.23E-06 | 1.195897441 | NM_024900| | PHF17,Jade1 protein short isoform |
| 225766_s_at | 9.16 | 3.22E-06 | 1.075059059 | NM_002270| | TNPO1,transportin 1 |
| 1552370_at | 9.16 | 3.24E-06 | 1.239397298 | NM_001099783| | NA |
| 232254_at | 9.16 | 3.23E-06 | 1.260022722 | NA |  |
| 208321_s_at | 9.15 | 3.27E-06 | 1.190391716 | NM_001033677| | NA |
| 227790_at | 9.15 | 3.25E-06 | 1.111357936 | NM_198920| | C6orf157,chromosome 6 open reading frame 157 |
| 203406_at | 9.15 | 3.26E-06 | 1.040714744 | NM_005926| | MFAP1,microfibrillar-associated protein 1 |
| 218689_at | 9.15 | 3.26E-06 | 1.173195529 | NM_022725| | FANCF,Fanconi anemia, complementation group F |
| 205689_at | 9.15 | 3.25E-06 | 1.208385702 | NM_014801| | PCNXL2,pecanex-like 2 |
| 227781_x_at | 9.15 | 3.26E-06 | 1.128010242 | NM_031478| | DKFZP434I2117,hypothetical protein DKFZp434I2117 |
| 201406_at | 9.15 | 3.25E-06 | 1.015101228 | NM_021029| | RPL36A,ribosomal protein L36a |
| 226450_at | 9.14 | 3.30E-06 | 1.224973492 | NM_000208| | INSR,insulin receptor |
| 204494_s_at | 9.14 | 3.29E-06 | 1.100229855 | NM_015492| | DKFZP434H132,DKFZP434H132 protein |
| 214382_at | 9.14 | 3.30E-06 | 1.150007027 | NM_018974| | UNC93A,unc-93 homolog A |
| 209368_at | 9.14 | 3.28E-06 | 1.526853777 | NM_001979| | EPHX2,epoxide hydrolase 2, cytoplasmic |
| 225705_at | 9.14 | 3.29E-06 | 1.041552371 | NM_138363| | LOC90799,hypothetical protein BC009518 |
| 225853_at | 9.14 | 3.30E-06 | 1.063933397 | NM_198066| | GNPNAT1,glucosamine-phosphate N-acetyltransferase 1 |
| 205621_at | 9.14 | 3.29E-06 | 1.067624541 | NM_006020| | ALKBH,alkB, alkylation repair homolog |
| 1559059_s_at | 9.13 | 3.34E-06 | 1.113013028 | NM_030972| | ZNF611,zinc finger protein 611 |
| 212209_at | 9.13 | 3.32E-06 | 1.109852816 | NM_015335| | THRAP2,thyroid hormone receptor associated protein 2 |
| 204716_at | 9.13 | 3.33E-06 | 1.102636821 | NM_005436| | CCDC6,coiled-coil domain containing 6 |
| 1557522_x_at | 9.13 | 3.31E-06 | 1.273203033 | NA |  |
| 227140_at | 9.13 | 3.34E-06 | 1.915506804 | NA |  |
| 201319_at | 9.12 | 3.36E-06 | 1.078535672 | NM_006471| | MRCL3,myosin regulatory light chain MRCL3 |
| 211464_x_at | 9.12 | 3.36E-06 | 1.155484075 | NM_001226| | CASP6,caspase 6 isoform alpha preproprotein |
| 225135_at | 9.12 | 3.35E-06 | 1.11310146 | NM_015477| | SIN3A,transcriptional co-repressor Sin3A |
| 208546_x_at | 9.12 | 3.36E-06 | 1.122686685 | NM_003524| | HIST1H2BH,H2B histone family, member J |
| 204945_at | 9.11 | 3.41E-06 | 1.142387482 | NM_002846| | PTPRN,protein tyrosine phosphatase, receptor type, N |
| 207827_x_at | 9.1 | 3.44E-06 | 1.309718565 | NM_000345| | SNCA,alpha-synuclein isoform NACP140 |
| 226423_at | 9.1 | 3.44E-06 | 1.104775388 | NM_133367| | C6orf33,chromosome 6 open reading frame 33 |
| 226421_at | 9.09 | 3.48E-06 | 1.098425886 | NM_001025580| | NA |
| 218638_s_at | 9.09 | 3.48E-06 | 1.158303483 | NM_012445| | SPON2,spondin 2, extracellular matrix protein |
| 235037_at | 9.09 | 3.49E-06 | 1.073960174 | NM_080652| | TMEM41A,transmembrane protein 41A |
| 205747_at | 9.09 | 3.49E-06 | 1.401423363 | NM_004352| | CBLN1,cerebellin |
| 210588_x_at | 9.09 | 3.47E-06 | 1.03681774 | NM_012207| | HNRPH3,heterogeneous nuclear ribonucleoprotein H3 |
| 243729_at | 9.09 | 3.48E-06 | 1.644493477 | NA |  |
| 231919_at | 9.09 | 3.48E-06 | 1.106746133 | NM_001918| | DBT,dihydrolipoamide branched chain transacylase |
| 218479_s_at | 9.09 | 3.50E-06 | 1.082896571 | NM_022459| | XPO4,exportin 4 |
| 201950_x_at | 9.08 | 3.51E-06 | 1.099461178 | NM_004930| | CAPZB,F-actin capping protein beta subunit |
| 212247_at | 9.08 | 3.52E-06 | 1.064327971 | NM_015135| | NUP205,nucleoporin 205kDa |
| 227948_at | 9.08 | 3.52E-06 | 1.100024545 | NM_139241| | FGD4,FYVE, RhoGEF and PH domain containing 4 |
| 244533_at | 9.08 | 3.53E-06 | 1.167493928 | NA |  |
| 35436_at | 9.08 | 3.52E-06 | 1.074918913 | NM_004486| | GOLGA2,Golgi autoantigen, golgin subfamily a, 2 |
| 218665_at | 9.08 | 3.53E-06 | 1.116405922 | NM_012193| | FZD4,frizzled 4 |
| 206182_at | 9.08 | 3.53E-06 | 1.148684159 | NM_003435| | ZNF134,zinc finger protein 134 |
| 209856_x_at | 9.07 | 3.56E-06 | 1.082900439 | NM_005759| | ABI2,abl interactor 2 |
| 203636_at | 9.07 | 3.54E-06 | 1.078399217 | NM_000381| | MID1,midline 1 isoform alpha |
| 215812_s_at | 9.07 | 3.55E-06 | 1.05141108 | NM_005629| | SLC6A8,solute carrier family 6 (neurotransmitter |
| 1554300_a_at | 9.07 | 3.55E-06 | 1.499597907 | NM_174959| | LOC136306,hypothetical protein LOC136306 |
| 226925_at | 9.07 | 3.55E-06 | 1.103760323 | NM_001037172| | NA |
| 204370_at | 9.07 | 3.58E-06 | 1.05956994 | NM_006831| | HEAB,ATP/GTP-binding protein |
| 208848_at | 9.07 | 3.55E-06 | 1.081216139 | NM_000671| | ADH5,class III alcohol dehydrogenase 5 chi subunit |
| 201508_at | 9.06 | 3.59E-06 | 1.303982247 | NM_001552| | IGFBP4,insulin-like growth factor binding protein 4 |
| 219662_at | 9.06 | 3.58E-06 | 1.110836595 | NM_024093| | MGC5509,hypothetical protein MGC5509 |
| 218456_at | 9.06 | 3.58E-06 | 1.110234948 | NM_001002259| | C1QDC1,C1q domain containing 1 isoform 1 |
| 237790_at | 9.06 | 3.61E-06 | 1.322429752 | NA |  |
| 210721_s_at | 9.06 | 3.60E-06 | 1.42278192 | NM_020341| | PAK7,p21-activated kinase 7 |
| 219330_at | 9.06 | 3.60E-06 | 1.353571983 | NM_138959| | VANGL1,vang-like 1 |
| 223315_at | 9.05 | 3.64E-06 | 1.366320676 | NM_021229| | NTN4,netrin 4 |
| 219929_s_at | 9.05 | 3.66E-06 | 1.130058056 | NM_024071| | ZFYVE21,zinc finger, FYVE domain containing 21 |
| 225476_at | 9.05 | 3.64E-06 | 1.055530469 | NM_033177| | BAT4,HLA-B associated transcript 4 |
| 218931_at | 9.05 | 3.66E-06 | 1.274653442 | NM_022449| | RAB17,RAB17, member RAS oncogene family |
| 1565939_at | 9.04 | 3.70E-06 | 1.158646792 | NM_018356| | FLJ11193,hypothetical protein FLJ11193 |
| 40284_at | 9.04 | 3.70E-06 | 1.389243456 | NM_021784| | FOXA2,forkhead box A2 |
| 206176_at | 9.04 | 3.70E-06 | 1.203075524 | NM_001718| | BMP6,bone morphogenetic protein 6 precursor |
| 225834_at | 9.03 | 3.73E-06 | 1.062581726 | NM_001100910| | NA |
| 226746_s_at | 9.03 | 3.73E-06 | 1.093110823 | NM_001105562| | NA |
| 218818_at | 9.03 | 3.71E-06 | 1.166045911 | NM_004468| | FHL3,four and a half LIM domains 3 |
| 213943_at | 9.03 | 3.72E-06 | 1.469879233 | NM_000474| | TWIST1,twist |
| 219844_at | 9.02 | 3.78E-06 | 1.285603261 | NM_018017| | C10orf118,CTCL tumor antigen L14-2 |
| 204291_at | 9.02 | 3.78E-06 | 1.248307824 | NM_014803| | ZNF518,zinc finger protein 518 |
| 227307_at | 9.02 | 3.78E-06 | 1.64051252 | NM_001031730| | NA |
| 241399_at | 9.02 | 3.77E-06 | 1.226984208 | NM_178539| | FAM19A2,family with sequence similarity 19 (chemokine |
| 201995_at | 9.02 | 3.78E-06 | 1.062881458 | NM_000127| | EXT1,exostosin 1 |
| 226358_at | 9.01 | 3.82E-06 | 1.161988997 | NA |  |
| 213198_at | 9.01 | 3.79E-06 | 1.09695059 | NM_004302| | ACVR1B,activin A type IB receptor isoform a precursor |
| 202266_at | 9.01 | 3.83E-06 | 1.062235674 | NM_016614| | TTRAP,TRAF and TNF receptor-associated protein |
| 209518_at | 9.01 | 3.80E-06 | 1.037617472 | NM_003076| | SMARCD1,SWI/SNF-related matrix-associated |
| 219113_x_at | 9 | 3.87E-06 | 1.256234509 | NM_016246| | DHRS10,dehydrogenase/reductase (SDR family) member 10 |
| 1553071_a_at | 9 | 3.83E-06 | 1.271481906 | NM_001122853| | NA |
| 235253_at | 9 | 3.83E-06 | 1.091586541 | NM_002853| | RAD1,RAD1 homolog isoform 1 |
| 218241_at | 9 | 3.86E-06 | 1.059176362 | NM_005113| | GOLGA5,Golgi autoantigen, golgin subfamily a, 5 |
| 206779_s_at | 9 | 3.86E-06 | 1.269494231 | NM_004043| | ASMT,acetylserotonin O-methyltransferase |
| 219069_at | 9 | 3.84E-06 | 1.0422397 | NM_017704| | FGIF,fetal globin inducing factor |
| 227358_at | 9 | 3.85E-06 | 1.392971689 | NM_025224| | BTBD4,BTB (POZ) domain containing 4 |
| 1557135_at | 9 | 3.85E-06 | 1.302832233 | NA |  |
| 209724_s_at | 8.99 | 3.91E-06 | 1.091240649 | NM_003409| | ZFP161,zinc finger protein 161 homolog |
| 205429_s_at | 8.99 | 3.90E-06 | 1.154050314 | NM_016447| | MPP6,membrane protein, palmitoylated 6 |
| 227609_at | 8.99 | 3.89E-06 | 1.472998283 | NM_001002264| | EPSTI1,epithelial stromal interaction 1 |
| 235399_at | 8.98 | 3.96E-06 | 1.14272479 | NA |  |
| 1554246_at | 8.98 | 3.94E-06 | 1.462816141 | NM_182517| | MGC52423,hypothetical protein MGC52423 |
| 237247_at | 8.98 | 3.94E-06 | 1.504274956 | NM_201286| | USP51,ubiquitin specific protease 51 |
| 222161_at | 8.98 | 3.94E-06 | 1.759657361 | NM_005467| | NAALAD2,N-acetylated alpha-linked acidic dipeptidase 2 |
| 209307_at | 8.97 | 3.96E-06 | 1.092687423 | NM_015055| | SWAP70,SWAP-70 protein |
| 200624_s_at | 8.97 | 3.98E-06 | 1.044667759 | NM_018834| | MATR3,matrin 3 |
| 204260_at | 8.97 | 3.99E-06 | 1.309785037 | NM_001819| | CHGB,chromogranin B precursor |
| 222020_s_at | 8.97 | 4.00E-06 | 1.675318998 | NM_001048209| | NA |
| 205347_s_at | 8.97 | 4.00E-06 | 1.146519139 | NM_021992| | TMSL8,thymosin-like 8 |
| 208734_x_at | 8.97 | 3.98E-06 | 1.044579666 | NM_002865| | RAB2,RAB2, member RAS oncogene family |
| 222514_at | 8.97 | 3.99E-06 | 1.103478669 | NM_022157| | RRAGC,Ras-related GTP binding C |
| 204212_at | 8.97 | 3.97E-06 | 1.099110354 | NM_005469| | PTE1,peroxisomal acyl-CoA thioesterase isoform a |
| 239031_at | 8.97 | 3.99E-06 | 1.344638284 | NM_001050| | SSTR2,somatostatin receptor 2 |
| 232989_s_at | 8.97 | 3.98E-06 | 1.203758567 | NM_022742| | NAG6,hypothetical protein DKFZp434G156 |
| 232000_at | 8.96 | 4.05E-06 | 1.737220011 | NM_152574| | C9orf52,hypothetical protein FLJ33868 |
| 1554646_at | 8.96 | 4.03E-06 | 1.215905906 | NM_018030| | OSBPL1A,oxysterol-binding protein-like 1A isoform A |
| 236265_at | 8.96 | 4.03E-06 | 1.080147866 | NM_003112| | SP4,Sp4 transcription factor |
| 1558943_x_at | 8.96 | 4.03E-06 | 1.100736063 | NM_001040185| | NA |
| 226575_at | 8.95 | 4.06E-06 | 1.047939375 | NM_021224| | ZNF462,zinc finger protein 462 |
| 230287_at | 8.95 | 4.07E-06 | 1.479378553 | NM_001039948| | NA |
| 227384_s_at | 8.95 | 4.06E-06 | 1.354341891 | NA |  |
| 207219_at | 8.95 | 4.09E-06 | 1.168057896 | NM_023070| | ZNF643,zinc finger protein 643 |
| 209380_s_at | 8.95 | 4.07E-06 | 1.097468352 | NM_001023587| | NA |
| 201189_s_at | 8.95 | 4.09E-06 | 1.533047218 | NM_002224| | ITPR3,inositol 1,4,5-triphosphate receptor, type 3 |
| 223803_s_at | 8.94 | 4.13E-06 | 1.108339561 | NM_017665| | ZCCHC10,zinc finger, CCHC domain containing 10 |
| 225021_at | 8.94 | 4.14E-06 | 1.082865596 | NM_018181| | ZNF532,zinc finger protein 532 |
| 1558890_at | 8.94 | 4.13E-06 | 1.250190406 | NA |  |
| 230574_at | 8.94 | 4.12E-06 | 1.236938535 | NA |  |
| 220238_s_at | 8.93 | 4.16E-06 | 1.240392429 | NM_001031710| | NA |
| 201297_s_at | 8.93 | 4.17E-06 | 1.078617399 | NM_018221| | MOBK1B,Mob4B protein |
| 225305_at | 8.93 | 4.16E-06 | 1.079583205 | NM_001039355| | NA |
| 1554441_a_at | 8.93 | 4.18E-06 | 1.127162205 | NM_015045| | KIAA0261,KIAA0261 |
| 215485_s_at | 8.93 | 4.18E-06 | 1.102001683 | NM_000201| | ICAM1,intercellular adhesion molecule 1 precursor |
| 231511_at | 8.93 | 4.18E-06 | 1.332096774 | NM_025074| | FRAS1,Fraser syndrome 1 isoform 1 |
| 221542_s_at | 8.92 | 4.24E-06 | 1.079450594 | NM_001003790| | SPFH2,SPFH domain family, member 2 isoform 2 |
| 230876_at | 8.92 | 4.21E-06 | 1.319330711 | NM_001101338| | NA |
| 236305_at | 8.92 | 4.22E-06 | 1.472834072 | NM_173362| | LOC317671,LOC317671 |
| 202421_at | 8.92 | 4.21E-06 | 1.083765172 | NM_001007237| | IGSF3,immunoglobulin superfamily, member 3 isoform 2 |
| 202228_s_at | 8.92 | 4.23E-06 | 1.076990622 | NM_012428| | SDFR1,stromal cell derived factor receptor 1 isoform |
| 231644_at | 8.92 | 4.24E-06 | 1.395701731 | NA |  |
| 207413_s_at | 8.91 | 4.28E-06 | 1.286608045 | NM_000335| | SCN5A,voltage-gated sodium channel type V alpha |
| 233665_x_at | 8.91 | 4.29E-06 | 1.048140419 | NM_001123226| | NA |
| 223811_s_at | 8.91 | 4.29E-06 | 1.073049454 | NM_015949| | C7orf20,chromosome 7 open reading frame 20 |
| 211320_s_at | 8.91 | 4.28E-06 | 1.213509838 | NM_005704| | PTPRU,protein tyrosine phosphatase, receptor type, U |
| 205043_at | 8.91 | 4.26E-06 | 1.19433278 | NM_000492| | CFTR,cystic fibrosis transmembrane conductance |
| 1565269_s_at | 8.91 | 4.25E-06 | 1.098260456 | NM_005171| | ATF1,activating transcription factor 1 |
| 1561912_at | 8.91 | 4.26E-06 | 1.095552718 | NA |  |
| 219937_at | 8.9 | 4.33E-06 | 1.425039186 | NM_013381| | TRHDE,thyrotropin-releasing hormone degrading |
| 243489_at | 8.9 | 4.33E-06 | 1.298355532 | NA |  |
| 60474_at | 8.9 | 4.31E-06 | 1.248295193 | NM_017671| | C20orf42,chromosome 20 open reading frame 42 |
| 229353_s_at | 8.9 | 4.29E-06 | 1.024952741 | NM_022731| | NUCKS,nuclear ubiquitous casein kinase and |
| 223396_at | 8.9 | 4.34E-06 | 1.05669654 | NM_032936| | C7orf35,chromosome 7 open reading frame 35 |
| 217877_s_at | 8.9 | 4.30E-06 | 1.042785021 | NM_021639| | SP192,hypothetical protein SP192 |
| 202213_s_at | 8.9 | 4.32E-06 | 1.137266252 | NM_001079872| | NA |
| 223441_at | 8.9 | 4.30E-06 | 1.169461248 | NM_012434| | SLC17A5,solute carrier family 17 (anion/sugar |
| 218765_at | 8.9 | 4.30E-06 | 1.072068421 | NM_001040455| | NA |
| 206702_at | 8.89 | 4.34E-06 | 1.589021322 | NM_000459| | TEK,TEK tyrosine kinase, endothelial |
| 206314_at | 8.89 | 4.38E-06 | 1.340086671 | NM_018651| | ZNF167,zinc finger protein ZFP isoform 1 |
| 238815_at | 8.88 | 4.41E-06 | 1.20350468 | NM_178839| | LRRTM1,leucine rich repeat transmembrane neuronal 1 |
| 221619_s_at | 8.88 | 4.41E-06 | 1.01873524 | NM_014341| | MTCH1,mitochondrial carrier homolog 1 |
| 1561341_at | 8.88 | 4.43E-06 | 1.211298507 | NA |  |
| 211762_s_at | 8.88 | 4.40E-06 | 1.020990158 | NM_002266| | KPNA2,karyopherin alpha 2 |
| 212959_s_at | 8.88 | 4.40E-06 | 1.275785238 | NM_024312| | MGC4170,MGC4170 protein |
| 213836_s_at | 8.87 | 4.45E-06 | 1.183960227 | NM_017983| | WIPI49,hypothetical protein FLJ10055 |
| 227352_at | 8.87 | 4.45E-06 | 1.14278806 | NM_175871| | FLJ35119,hypothetical protein FLJ35119 |
| 231853_at | 8.87 | 4.44E-06 | 1.12207184 | NM_016261| | TUBD1,delta-tubulin |
| 214775_at | 8.87 | 4.48E-06 | 1.322932751 | NM_015111| | NA |
| 219277_s_at | 8.87 | 4.47E-06 | 1.187450835 | NM_018245| | OGDHL,oxoglutarate dehydrogenase-like |
| 224858_at | 8.87 | 4.45E-06 | 1.04286627 | NM_015457| | ZDHHC5,zinc finger, DHHC domain containing 5 |
| 225659_at | 8.87 | 4.49E-06 | 1.159642941 | NM_001001664| | LOC339745,hypothetical protein LOC339745 |
| 219035_s_at | 8.87 | 4.44E-06 | 1.038982065 | NM_025126| | RNF34,ring finger protein 34 isoform 2 |
| 1553696_s_at | 8.86 | 4.52E-06 | 1.122208694 | NM_152484| | ZNF569,zinc finger protein 569 |
| 214614_at | 8.86 | 4.52E-06 | 1.171663247 | NM_005515| | HLXB9,homeo box HB9 |
| 227718_at | 8.86 | 4.50E-06 | 1.12418069 | NM_033224| | PURB,purine-rich element binding protein B |
| 228330_at | 8.86 | 4.53E-06 | 1.098332611 | NM_145062| | C6orf113,chromosome 6 open reading frame 113 |
| 233634_at | 8.86 | 4.50E-06 | 1.506049105 | NM_001017967| | NA |
| 213307_at | 8.86 | 4.50E-06 | 1.425848969 | NM_012309| | SHANK2,SH3 and multiple ankyrin repeat domains 2 |
| 219513_s_at | 8.86 | 4.53E-06 | 1.644922137 | NM_005490| | SH2D3A,SH2 domain containing 3A |
| 235964_x_at | 8.86 | 4.52E-06 | 1.199743566 | NA |  |
| 218325_s_at | 8.86 | 4.49E-06 | 1.208874319 | NM_022105| | DATF1,death associated transcription factor 1 isoform |
| 203186_s_at | 8.85 | 4.58E-06 | 1.219485345 | NM_002961| | S100A4,S100 calcium-binding protein A4 |
| 210394_x_at | 8.85 | 4.55E-06 | 1.206959231 | NM_001034832| | NA |
| 222807_at | 8.85 | 4.57E-06 | 1.066061528 | NM_020193| | C11orf30,EMSY protein |
| 225142_at | 8.84 | 4.64E-06 | 1.511567239 | NM_030647| | NA |
| 219231_at | 8.84 | 4.60E-06 | 1.090834802 | NM_024831| | NCOA6IP,PRIP-interacting protein PIPMT |
| 221780_s_at | 8.84 | 4.63E-06 | 1.050761996 | NM_017895| | DDX27,DEAD (Asp-Glu-Ala-Asp) box polypeptide 27 |
| 226972_s_at | 8.84 | 4.61E-06 | 1.196190262 | NM_022742| | NAG6,hypothetical protein DKFZp434G156 |
| 238860_at | 8.84 | 4.62E-06 | 1.101219311 | NM_145063| | C6orf130,chromosome 6 open reading frame 130 |
| 237322_at | 8.84 | 4.59E-06 | 1.236923163 | NA |  |
| 242045_at | 8.84 | 4.59E-06 | 1.256731459 | NA |  |
| 201555_at | 8.83 | 4.67E-06 | 1.052035175 | NM_002388| | MCM3,minichromosome maintenance protein 3 |
| 213251_at | 8.83 | 4.66E-06 | 1.088796975 | NM_003601| | SMARCA5,SWI/SNF-related matrix-associated |
| 209298_s_at | 8.83 | 4.66E-06 | 1.252787144 | NM_001001132| | ITSN1,intersectin 1 isoform ITSN-s |
| 1598_g_at | 8.83 | 4.65E-06 | 1.073988403 | NM_000820| | GAS6,growth arrest-specific 6 |
| 203175_at | 8.83 | 4.70E-06 | 1.062484843 | NM_001665| | RHOG,ras homolog gene family, member G |
| 224366_s_at | 8.83 | 4.67E-06 | 1.075067867 | NM_031922| | REPS1,RALBP1 associated Eps domain containing 1 |
| 207869_s_at | 8.83 | 4.66E-06 | 1.230560895 | NM_018896| | CACNA1G,voltage-dependent calcium channel alpha 1G |
| 203238_s_at | 8.82 | 4.70E-06 | 1.110567321 | NM_000435| | NOTCH3,Notch homolog 3 |
| 218900_at | 8.82 | 4.70E-06 | 1.116653757 | NM_020184| | CNNM4,cyclin M4 |
| 201176_s_at | 8.82 | 4.72E-06 | 1.071701485 | NM_001655| | ARCN1,archain |
| 201810_s_at | 8.82 | 4.74E-06 | 1.096689059 | NM_001018009| | NA |
| 1552400_a_at | 8.82 | 4.74E-06 | 1.393504411 | NM_152335| | C15orf27,chromosome 15 open reading frame 27 |
| 1557143_at | 8.82 | 4.70E-06 | 1.366856557 | NM_052896| | CSMD2,CUB and Sushi multiple domains 2 |
| 228991_at | 8.82 | 4.75E-06 | 1.145138042 | NA |  |
| 1556236_at | 8.81 | 4.78E-06 | 1.270218013 | NA |  |
| 203325_s_at | 8.81 | 4.78E-06 | 1.672194131 | NM_000093| | COL5A1,alpha 1 type V collagen preproprotein |
| 215722_s_at | 8.81 | 4.80E-06 | 1.046710864 | NM_003090| | SNRPA1,small nuclear ribonucleoprotein polypeptide A' |
| 211689_s_at | 8.8 | 4.81E-06 | 1.329847167 | NM_005656| | TMPRSS2,transmembrane protease, serine 2 |
| 202856_s_at | 8.8 | 4.83E-06 | 1.856793084 | NM_001042422| | NA |
| 224574_at | 8.8 | 4.83E-06 | 1.045243462 | NM_001004333| | MGC71993,similar to DNA segment, Chr 11, Brigham & Womens |
| 227616_at | 8.8 | 4.81E-06 | 1.199856546 | NM_182557| | BCL9L,B-cell CLL/lymphoma 9-like |
| 221435_x_at | 8.8 | 4.83E-06 | 1.07876686 | NM_031207| | HT036,hypothetical protein HT036 |
| 225263_at | 8.8 | 4.84E-06 | 1.099515971 | NM_004807| | HS6ST1,heparan sulfate 6-O-sulfotransferase |
| 222404_x_at | 8.8 | 4.81E-06 | 1.016323906 | NM_016395| | HSPC121,butyrate-induced transcript 1 |
| 212358_at | 8.8 | 4.82E-06 | 1.086082273 | NM_015526| | CLIPR-59,CLIP-170-related protein |
| 228976_at | 8.8 | 4.83E-06 | 1.154197084 | NM_015259| | ICOSLG,inducible T-cell co-stimulator ligand |
| 239278_at | 8.8 | 4.85E-06 | 1.207133537 | NA |  |
| 213159_at | 8.8 | 4.81E-06 | 1.165790308 | NM_014982| | PCNX,pecanex homolog |
| 219540_at | 8.8 | 4.81E-06 | 1.099243539 | NM_003414| | ZNF267,zinc finger protein 267 |
| 225538_at | 8.79 | 4.89E-06 | 1.084397558 | NM_032280| | ZCCHC9,zinc finger, CCHC domain containing 9 |
| 242998_at | 8.79 | 4.89E-06 | 1.453688375 | NM_152443| | RDH12,retinol dehydrogenase 12 (all-trans and 9-cis) |
| 222531_s_at | 8.79 | 4.90E-06 | 1.079551209 | NM_018229| | C14orf108,chromosome 14 open reading frame 108 |
| 212902_at | 8.79 | 4.91E-06 | 1.16451926 | NM_021982| | NA |
| 225260_s_at | 8.79 | 4.86E-06 | 1.04507421 | NM_031903| | MRPL32,mitochondrial ribosomal protein L32 |
| 227151_at | 8.79 | 4.89E-06 | 1.08199661 | NM_153271| | MGC32065,hypothetical protein MGC32065 |
| 230370_x_at | 8.78 | 4.96E-06 | 1.098716189 | NM_016086| | DUSP24,map kinase phosphatase-like protein MK-STYX |
| 213879_at | 8.78 | 4.96E-06 | 1.04146802 | NA |  |
| 214246_x_at | 8.78 | 4.93E-06 | 1.064012398 | NM_001024937| | NA |
| 213048_s_at | 8.78 | 4.95E-06 | 1.035645064 | NA |  |
| 219528_s_at | 8.78 | 4.94E-06 | 1.412009904 | NM_022898| | BCL11B,B-cell CLL/lymphoma 11B isoform 2 |
| 1555250_a_at | 8.78 | 4.96E-06 | 1.083305242 | NM_014912| | CPEB3,cytoplasmic polyadenylation element binding |
| 221326_s_at | 8.78 | 4.95E-06 | 1.089303978 | NM_016261| | TUBD1,delta-tubulin |
| 238426_at | 8.77 | 5.00E-06 | 1.311038932 | NM_152913| | DKFZp761L1417,hypothetical protein DKFZp761L1417 |
| 210205_at | 8.77 | 4.98E-06 | 1.121687614 | NM_003782| | B3GALT4,UDP-Gal:betaGlcNAc beta |
| 1552727_s_at | 8.77 | 4.99E-06 | 1.441796576 | NM_139057| | ADAMTS17,a disintegrin-like and metalloprotease |
| 217349_s_at | 8.76 | 5.06E-06 | 1.144358524 | NM_006150| | LMO6,LIM domain only 6 |
| 205041_s_at | 8.76 | 5.04E-06 | 1.604710901 | NM_000607| | ORM1,orosomucoid 1 precursor |
| 238709_at | 8.76 | 5.03E-06 | 1.028917681 | NA |  |
| 225673_at | 8.76 | 5.04E-06 | 1.077212893 | NM_001020818| | NA |
| 201188_s_at | 8.76 | 5.03E-06 | 1.375584988 | NM_002224| | ITPR3,inositol 1,4,5-triphosphate receptor, type 3 |
| 203529_at | 8.76 | 5.06E-06 | 1.04656072 | NM_001123355| | NA |
| 225693_s_at | 8.76 | 5.08E-06 | 1.032779562 | NM_015215| | CAMTA1,calmodulin-binding transcription activator 1 |
| 226961_at | 8.76 | 5.03E-06 | 1.805143945 | NM_175887| | LOC222171,hypothetical protein LOC222171 |
| 244738_at | 8.76 | 5.08E-06 | 1.198945235 | NM_153252| | BRWD3,bromo domain-containing protein disrupted in |
| 219007_at | 8.75 | 5.13E-06 | 1.05606753 | NM_198887| | NUP43,nucleoporin 43kDa |
| 220721_at | 8.75 | 5.10E-06 | 1.218653344 | NM_014650| | ZNF432,zinc finger protein 432 |
| 226247_at | 8.75 | 5.13E-06 | 1.051594956 | NM_001001974| | PLEKHA1,pleckstrin homology domain containing, family A |
| 215823_x_at | 8.75 | 5.10E-06 | 1.024139607 | NM_002568| | PABPC1,poly(A) binding protein, cytoplasmic 1 |
| 241985_at | 8.75 | 5.09E-06 | 1.143562354 | NM_152405| | JMY,junction-mediating and regulatory protein |
| 230835_at | 8.75 | 5.11E-06 | 1.334273611 | NM_207392| | UNQ467,KIPV467 |
| 208079_s_at | 8.75 | 5.08E-06 | 1.034208514 | NM_003600| | STK6,serine/threonine protein kinase 6 |
| 210061_at | 8.75 | 5.09E-06 | 1.190657946 | NM_016089| | ZNF589,zinc finger protein 589 |
| 223680_at | 8.75 | 5.09E-06 | 1.134219527 | NM_032689| | ZNF607,zinc finger protein 607 |
| 227653_at | 8.74 | 5.19E-06 | 1.065352027 | NM_020810| | KIAA1393,tRNA-(N1G37) methyltransferase |
| 221009_s_at | 8.74 | 5.19E-06 | 1.170969943 | NM_001039667| | NA |
| 219179_at | 8.73 | 5.23E-06 | 1.418788686 | NM_001079520| | NA |
| 224172_at | 8.73 | 5.25E-06 | 1.225945468 | NA |  |
| 215313_x_at | 8.73 | 5.24E-06 | 1.031722348 | NM_002116| | HLA-A,major histocompatibility complex, class I, A |
| 202262_x_at | 8.73 | 5.22E-06 | 1.123301648 | NM_013974| | DDAH2,dimethylarginine dimethylaminohydrolase 2 |
| 215491_at | 8.73 | 5.22E-06 | 1.205854747 | NM_001033081| | NA |
| 1558027_s_at | 8.73 | 5.22E-06 | 1.153045729 | NM_005399| | PRKAB2,AMP-activated protein kinase beta 2 |
| 204168_at | 8.72 | 5.27E-06 | 1.121308211 | NM_002413| | MGST2,microsomal glutathione S-transferase 2 |
| 205463_s_at | 8.72 | 5.27E-06 | 1.136622026 | NM_002607| | NA |
| 219741_x_at | 8.72 | 5.30E-06 | 1.082938129 | NM_024762| | ZNF552,zinc finger protein 552 |
| 223313_s_at | 8.72 | 5.28E-06 | 1.098246946 | NM_001098800| | NA |
| 228029_at | 8.72 | 5.30E-06 | 1.172102152 | NM_133474| | KIAA1982,KIAA1982 protein |
| 226915_s_at | 8.72 | 5.29E-06 | 1.058853576 | NM_030978| | ARPC5L,actin related protein 2/3 complex, subunit |
| 224934_at | 8.72 | 5.27E-06 | 1.054702561 | NM_001024947| | NA |
| 225039_at | 8.71 | 5.36E-06 | 1.061194219 | NM_006916| | RPE,ribulose-5-phosphate-3-epimerase isoform 2 |
| 226431_at | 8.71 | 5.34E-06 | 1.074691373 | NM_173511| | ALS2CR13,amyotrophic lateral sclerosis 2 (juvenile) |
| 219551_at | 8.71 | 5.35E-06 | 1.166870745 | NM_018456| | EAF2,ELL associated factor 2 |
| 230006_s_at | 8.71 | 5.34E-06 | 1.323400652 | NM_148893| | NA |
| 208206_s_at | 8.71 | 5.36E-06 | 1.342997945 | NM_001098670| | NA |
| 209257_s_at | 8.71 | 5.36E-06 | 1.045660031 | NM_005445| | CSPG6,chondroitin sulfate proteoglycan 6 (bamacan) |
| 206174_s_at | 8.7 | 5.38E-06 | 1.030431951 | NM_001123355| | NA |
| 210002_at | 8.7 | 5.40E-06 | 2.207983254 | NM_005257| | GATA6,GATA binding protein 6 |
| 201438_at | 8.7 | 5.40E-06 | 2.053494894 | NM_004369| | COL6A3,alpha 3 type VI collagen isoform 1 precursor |
| 205071_x_at | 8.7 | 5.38E-06 | 1.094147651 | NM_003401| | XRCC4,X-ray repair cross complementing protein 4 |
| 238513_at | 8.7 | 5.40E-06 | 1.586934018 | NM_024081| | PRRG4,proline rich Gla (G-carboxyglutamic acid) 4 |
| 201245_s_at | 8.7 | 5.38E-06 | 1.034526261 | NM_017670| | OTUB1,OTU domain, ubiquitin aldehyde binding 1 |
| 244170_at | 8.69 | 5.44E-06 | 1.503787718 | NA |  |
| 221269_s_at | 8.69 | 5.49E-06 | 1.084282336 | NM_031286| | SH3BGRL3,SH3 domain binding glutamic acid-rich protein |
| 230087_at | 8.69 | 5.46E-06 | 1.119275763 | NM_178013| | PRIMA1,proline rich membrane anchor 1 |
| 227273_at | 8.69 | 5.45E-06 | 1.048590232 | NA |  |
| 228408_s_at | 8.69 | 5.44E-06 | 1.039293235 | NM_018115| | SDAD1,SDA1 domain containing 1 |
| 217822_at | 8.69 | 5.45E-06 | 1.034242557 | NM_016312| | WBP11,WW domain binding protein 11 |
| 206061_s_at | 8.69 | 5.44E-06 | 1.14452157 | NM_030621| | DICER1,dicer1 |
| 220960_x_at | 8.68 | 5.52E-06 | 1.011040964 | NM_000983| | RPL22,ribosomal protein L22 proprotein |
| 1560228_at | 8.68 | 5.53E-06 | 1.329098769 | NM_178310| | NA |
| 209963_s_at | 8.68 | 5.53E-06 | 1.161598483 | NM_000121| | EPOR,erythropoietin receptor precursor |
| 202644_s_at | 8.68 | 5.53E-06 | 1.265156647 | NM_006290| | TNFAIP3,tumor necrosis factor, alpha-induced protein 3 |
| 202790_at | 8.68 | 5.51E-06 | 1.716946504 | NM_001307| | CLDN7,claudin 7 |
| 224918_x_at | 8.68 | 5.55E-06 | 1.055529126 | NM_020300| | MGST1,microsomal glutathione S-transferase 1 |
| 244038_at | 8.68 | 5.53E-06 | 1.065925652 | NM_001008726| | C14orf150,chromosome 14 open reading frame 150 |
| 218349_s_at | 8.67 | 5.57E-06 | 1.055746138 | NM_017975| | FLJ10036,Zwilch |
| 229312_s_at | 8.67 | 5.58E-06 | 1.077470752 | NM_025211| | GKAP1,G kinase anchoring protein 1 |
| 225547_at | 8.67 | 5.58E-06 | 1.042100197 | NA |  |
| 206128_at | 8.67 | 5.62E-06 | 1.241854195 | NM_000683| | ADRA2C,alpha-2C-adrenergic receptor |
| 1555272_at | 8.66 | 5.68E-06 | 1.095602143 | NM_001099697| | NA |
| 202234_s_at | 8.66 | 5.67E-06 | 1.167279243 | NM_003051| | SLC16A1,solute carrier family 16, member 1 |
| 231382_at | 8.66 | 5.65E-06 | 1.449079646 | NM_003862| | FGF18,fibroblast growth factor 18 precursor |
| 235466_s_at | 8.66 | 5.66E-06 | 1.120419313 | NM_032890| | DISP1,dispatched A |
| 241372_at | 8.65 | 5.73E-06 | 1.169534992 | NM_198581| | ZC3HDC6,zinc finger CCCH type domain containing 6 |
| 204061_at | 8.65 | 5.73E-06 | 1.05963618 | NM_005044| | PRKX,protein kinase, X-linked |
| 207610_s_at | 8.65 | 5.69E-06 | 1.29822446 | NM_013447| | EMR2,egf-like module containing, mucin-like, hormone |
| 226797_at | 8.65 | 5.71E-06 | 1.08356847 | NM_017643| | MBTD1,mbt domain containing 1 |
| 200055_at | 8.65 | 5.73E-06 | 1.034878968 | NM_006284| | TAF10,TBP-related factor 10 |
| 225954_s_at | 8.65 | 5.71E-06 | 1.073812589 | NM_177401| | MIDN,midnolin |
| 222723_at | 8.64 | 5.78E-06 | 1.281837632 | NA |  |
| 208536_s_at | 8.64 | 5.80E-06 | 1.36532264 | NM_006538| | BCL2L11,BCL2-like 11 isoform 6 |
| 210551_s_at | 8.64 | 5.75E-06 | 1.258480303 | NM_004043| | ASMT,acetylserotonin O-methyltransferase |
| 228186_s_at | 8.64 | 5.79E-06 | 1.84263384 | NM_032784| | THSD2,thrombospondin, type I, domain containing 2 |
| 213483_at | 8.64 | 5.76E-06 | 1.069295142 | NM_015342| | KIAA0073,KIAA0073 protein |
| 202469_s_at | 8.63 | 5.86E-06 | 1.036441916 | NM_007007| | CPSF6,cleavage and polyadenylation specific factor 6, |
| 231408_at | 8.63 | 5.85E-06 | 1.304031623 | NM_153229| | FLJ33318,hypothetical protein FLJ33318 |
| 236513_at | 8.63 | 5.87E-06 | 1.28592478 | NA |  |
| 214737_x_at | 8.63 | 5.86E-06 | 1.039265186 | NM_001077442| | NA |
| 237301_at | 8.63 | 5.87E-06 | 1.283225952 | NA |  |
| 207980_s_at | 8.62 | 5.92E-06 | 1.177834308 | NM_006079| | CITED2,Cbp/p300-interacting transactivator, with |
| 210840_s_at | 8.62 | 5.92E-06 | 1.079544101 | NM_003870| | IQGAP1,IQ motif containing GTPase activating protein 1 |
| 230085_at | 8.62 | 5.90E-06 | 1.237948069 | NA |  |
| 226917_s_at | 8.62 | 5.94E-06 | 1.043909097 | NM_013367| | ANAPC4,anaphase-promoting complex subunit 4 |
| 217249_x_at | 8.62 | 5.95E-06 | 1.026671159 | NM_001865| | COX7A2,cytochrome c oxidase subunit VIIa polypeptide 2 |
| 202337_at | 8.62 | 5.95E-06 | 1.100790906 | NM_007221| | PMF1,polyamine-modulated factor 1 |
| 217746_s_at | 8.62 | 5.95E-06 | 1.0251631 | NM_013374| | PDCD6IP,programmed cell death 6 interacting protein |
| 211796_s_at | 8.62 | 5.92E-06 | 1.749179475 | NA |  |
| 231817_at | 8.61 | 5.98E-06 | 1.509660705 | NM_019050| | USP53,ubiquitin specific protease 53 |
| 214430_at | 8.61 | 5.98E-06 | 1.080066077 | NM_000169| | GLA,galactosidase, alpha |
| 239403_at | 8.61 | 5.98E-06 | 1.151303927 | NM_033626| | JM11,JM11 protein |
| 204036_at | 8.61 | 5.98E-06 | 1.154570519 | NM_001401| | EDG2,endothelial differentiation, lysophosphatidic |
| 210524_x_at | 8.61 | 5.98E-06 | 1.26611105 | NA |  |
| 210766_s_at | 8.61 | 5.98E-06 | 1.04080625 | NM_001316| | CSE1L,CSE1 chromosome segregation 1-like protein |
| 233305_at | 8.6 | 6.04E-06 | 1.570893088 | NM_022351| | EFCBP1,EF hand calcium binding protein 1 |
| 205380_at | 8.6 | 6.05E-06 | 1.459598102 | NM_002614| | PDZK1,PDZ domain containing 1 |
| 204094_s_at | 8.6 | 6.05E-06 | 1.097002949 | NM_014779| | KIAA0669,KIAA0669 gene product |
| 212823_s_at | 8.59 | 6.11E-06 | 1.140921272 | NM_015549| | PLEKHG3,pleckstrin homology domain containing, family G, |
| 214898_x_at | 8.59 | 6.11E-06 | 1.376831929 | NA |  |
| 1559266_s_at | 8.59 | 6.13E-06 | 1.180400303 | NM_207371| | FLJ45187,FLJ45187 protein |
| 242452_at | 8.59 | 6.10E-06 | 1.098107391 | NA |  |
| 1553686_at | 8.59 | 6.10E-06 | 1.148063097 | NM_001008239| | C18orf25,chromosome 18 open reading frame 25 isoform b |
| 225230_at | 8.58 | 6.20E-06 | 1.07900209 | NM_178454| | MGC54289,hypothetical protein MGC54289 |
| 205944_s_at | 8.58 | 6.20E-06 | 1.160339085 | NM_007098| | CLTCL1,clathrin, heavy polypeptide-like 1 isoform b |
| 235390_at | 8.58 | 6.19E-06 | 1.101528621 | NM_173829| | FLJ36754,hypothetical protein FLJ36754 |
| 225841_at | 8.58 | 6.22E-06 | 1.408732784 | NM_001102592| | NA |
| 205578_at | 8.58 | 6.22E-06 | 1.518643999 | NM_004560| | ROR2,receptor tyrosine kinase-like orphan receptor 2 |
| 239067_s_at | 8.58 | 6.17E-06 | 1.21821717 | NM_052839| | PANX2,pannexin 2 |
| 226392_at | 8.58 | 6.18E-06 | 1.248144357 | NA |  |
| 205529_s_at | 8.58 | 6.19E-06 | 1.170007037 | NM_004349| | RUNX1T1,acute myelogenous leukemia 1 translocation 1 |
| 1554868_s_at | 8.57 | 6.27E-06 | 1.078291127 | NM_020357| | PCNP,PEST-containing nuclear protein |
| 219617_at | 8.57 | 6.24E-06 | 1.136868169 | NM_024766| | FLJ23451,hypothetical protein FLJ23451 |
| 221010_s_at | 8.57 | 6.29E-06 | 1.095279613 | NM_012241| | SIRT5,sirtuin 5 isoform 1 |
| 218424_s_at | 8.57 | 6.27E-06 | 1.058919203 | NM_001008410| | TSAP6,dudulin 2 isoform b |
| 218404_at | 8.57 | 6.27E-06 | 1.125785022 | NM_013322| | SNX10,sorting nexin 10 |
| 218836_at | 8.57 | 6.24E-06 | 1.05808247 | NM_024839| | RPP21,ribonuclease P 21kDa subunit |
| 218606_at | 8.57 | 6.24E-06 | 1.039357555 | NM_017740| | ZDHHC7,zinc finger, DHHC domain containing 7 |
| 226349_at | 8.57 | 6.26E-06 | 1.067458918 | NM_152318| | MGC40397,hypothetical protein MGC40397 |
| 212584_at | 8.57 | 6.24E-06 | 1.083255684 | NM_014691| | AQR,aquarius |
| 235467_s_at | 8.56 | 6.31E-06 | 1.216717697 | NM_001039574| | NA |
| 204819_at | 8.56 | 6.33E-06 | 1.078151289 | NM_004463| | FGD1,faciogenital dysplasia protein |
| 214993_at | 8.56 | 6.37E-06 | 1.141183849 | NM_181718| | LOC253982,hypothetical protein LOC253982 |
| 219518_s_at | 8.56 | 6.36E-06 | 1.221168073 | NM_025165| | ELL3,elongation factor RNA polymerase II-like 3 |
| 238122_at | 8.56 | 6.31E-06 | 1.114923961 | NM_203390| | LOC389677,similar to RIKEN cDNA 3000004N20 |
| 216304_x_at | 8.56 | 6.37E-06 | 1.032220702 | NM_014263| | YME1L1,YME1-like 1 isoform 3 |
| 208382_s_at | 8.56 | 6.31E-06 | 1.376865218 | NM_007068| | DMC1,DMC1 dosage suppressor of mck1 homolog |
| 222027_at | 8.56 | 6.35E-06 | 1.072605758 | NM_022731| | NUCKS,nuclear ubiquitous casein kinase and |
| 204071_s_at | 8.55 | 6.43E-06 | 1.091151247 | NM_005802| | TOPORS,topoisomerase I binding, arginine/serine-rich |
| 241704_x_at | 8.55 | 6.38E-06 | 1.221095914 | NM_207333| | NA |
| 205769_at | 8.55 | 6.43E-06 | 1.354969276 | NM_003645| | SLC27A2,solute carrier family 27 (fatty acid |
| 213386_at | 8.55 | 6.43E-06 | 1.717013106 | NM_032342| | C9orf125,chromosome 9 open reading frame 125 |
| 227445_at | 8.55 | 6.43E-06 | 1.062230435 | NM_138447| | LOC115509,hypothetical protein BC014000 |
| 222212_s_at | 8.55 | 6.39E-06 | 1.04575698 | NM_022075| | LASS2,LAG1 longevity assurance homolog 2 isoform 1 |
| 213074_at | 8.55 | 6.43E-06 | 1.050207414 | NA |  |
| 223543_at | 8.55 | 6.45E-06 | 1.258215821 | NM_032512| | PDZK4,PDZ domain containing 4 |
| 216641_s_at | 8.55 | 6.38E-06 | 1.193578871 | NM_005558| | LAD1,ladinin 1 |
| 225637_at | 8.55 | 6.44E-06 | 1.059955607 | NM_017702| | FLJ20186,differentially expressed in FDCP 8 isoform 2 |
| 203153_at | 8.55 | 6.44E-06 | 1.41461483 | NM_001548| | IFIT1,interferon-induced protein with |
| 206562_s_at | 8.54 | 6.48E-06 | 1.045396524 | NM_001025105| | NA |
| 205890_s_at | 8.54 | 6.51E-06 | 1.256941776 | NM_001470| | GABBR1,gamma-aminobutyric acid (GABA) B receptor 1 |
| 203909_at | 8.54 | 6.47E-06 | 1.044001427 | NM_001042537| | NA |
| 1553528_a_at | 8.54 | 6.46E-06 | 1.071305488 | NM_006951| | TAF5,TBP-associated factor 5 |
| 225306_s_at | 8.54 | 6.46E-06 | 1.075593719 | NM_001039355| | NA |
| 214901_at | 8.54 | 6.46E-06 | 1.09340522 | NM_021089| | ZNF8,zinc finger protein 8 |
| 220335_x_at | 8.54 | 6.45E-06 | 1.231440445 | NM_024922| | FLJ21736,esterase 31 |
| 223845_at | 8.53 | 6.55E-06 | 1.12732483 | NA |  |
| 220129_at | 8.53 | 6.58E-06 | 1.318031073 | NM_017826| | FLJ20449,hypothetical protein FLJ20449 |
| 235781_at | 8.53 | 6.56E-06 | 1.397846427 | NM_000718| | CACNA1B,calcium channel, voltage-dependent, L type, |
| 205256_at | 8.53 | 6.60E-06 | 1.14857592 | NM_014830| | KIAA0352,KIAA0352 gene product |
| 226880_at | 8.52 | 6.64E-06 | 1.033940399 | NM_022731| | NUCKS,nuclear ubiquitous casein kinase and |
| 201657_at | 8.52 | 6.65E-06 | 1.116897008 | NM_001177| | ARL1,ADP-ribosylation factor-like 1 |
| 228916_at | 8.52 | 6.68E-06 | 1.192731049 | NM_152434| | CWF19L2,CWF19-like 2, cell cycle control |
| 208702_x_at | 8.52 | 6.68E-06 | 1.051703313 | NM_001642| | APLP2,amyloid beta (A4) precursor-like protein 2 |
| 230296_at | 8.52 | 6.67E-06 | 1.045622828 | NM_173501| | LOC146174,hypothetical protein LOC146174 |
| 212415_at | 8.51 | 6.74E-06 | 1.689367078 | NM_015129| | SEPT6,septin 6 isoform B |
| 212373_at | 8.51 | 6.72E-06 | 1.193542129 | NM_015322| | FEM1B,fem-1 homolog b |
| 229901_at | 8.51 | 6.70E-06 | 1.331493883 | NM_153034| | ZNF488,zinc finger protein 488 |
| 238740_at | 8.51 | 6.71E-06 | 1.369885426 | NM_025267| | MGC2744,hypothetical protein MGC2744 |
| 227804_at | 8.51 | 6.75E-06 | 1.111360707 | NM_138463| | LOC116238,hypothetical protein BC014072 |
| 205825_at | 8.51 | 6.69E-06 | 1.452343074 | NM_000439| | PCSK1,proprotein convertase subtilisin/kexin type 1 |
| 205467_at | 8.51 | 6.69E-06 | 1.098361111 | NM_001230| | CASP10,Caspase 10 isoform a preproprotein |
| 238174_at | 8.51 | 6.73E-06 | 1.135150238 | NA |  |
| 202430_s_at | 8.51 | 6.76E-06 | 1.26769178 | NM_021105| | PLSCR1,phospholipid scramblase 1 |
| 217756_x_at | 8.5 | 6.78E-06 | 1.026811564 | NM_001018108| | NA |
| 231808_at | 8.5 | 6.78E-06 | 1.106605105 | NM_033061| | KRTAP4-7,keratin associated protein 4-7 |
| 241689_at | 8.5 | 6.80E-06 | 1.120880007 | NA |  |
| 218117_at | 8.5 | 6.76E-06 | 1.027698186 | NM_014248| | RBX1,ring-box 1 |
| 232667_at | 8.5 | 6.79E-06 | 1.198938539 | NA |  |
| 227053_at | 8.5 | 6.82E-06 | 1.633780401 | NM_020804| | PACSIN1,protein kinase C and casein kinase substrate in |
| 205295_at | 8.5 | 6.78E-06 | 1.131798942 | NM_001099735| | NA |
| 244687_at | 8.49 | 6.86E-06 | 1.046825608 | NM_001918| | DBT,dihydrolipoamide branched chain transacylase |
| 238650_x_at | 8.49 | 6.87E-06 | 1.061506414 | NM_001008726| | C14orf150,chromosome 14 open reading frame 150 |
| 213351_s_at | 8.49 | 6.85E-06 | 1.064476821 | NM_001017395| | NA |
| 210753_s_at | 8.49 | 6.90E-06 | 1.179685124 | NM_004441| | EPHB1,ephrin receptor EphB1 precursor |
| 235759_at | 8.49 | 6.91E-06 | 1.178010315 | NA |  |
| 235049_at | 8.49 | 6.87E-06 | 1.0815372 | NM_021116| | ADCY1,brain adenylate cyclase 1 |
| 224539_s_at | 8.49 | 6.91E-06 | 1.221403224 | NM_018898| | PCDHAC1,protocadherin alpha subfamily C, 1 isoform 1 |
| 204208_at | 8.49 | 6.84E-06 | 1.081553038 | NM_003800| | RNGTT,RNA guanylyltransferase and 5'-phosphatase |
| 201753_s_at | 8.48 | 6.96E-06 | 1.095948507 | NM_001121| | NA |
| 231385_at | 8.48 | 6.97E-06 | 2.21304832 | NM_199286| | DPPA3,stella |
| 220317_at | 8.48 | 6.98E-06 | 1.77948101 | NM_004744| | LRAT,lecithin retinol acyltransferase |
| 228733_at | 8.48 | 6.97E-06 | 1.060964742 | NM_153339| | PUSL1,pseudouridylate synthase-like 1 |
| 212542_s_at | 8.48 | 6.96E-06 | 1.047896855 | NM_017934| | PHIP,pleckstrin homology domain interacting protein |
| 235121_at | 8.48 | 6.98E-06 | 1.2329018 | NA |  |
| 222883_at | 8.48 | 6.94E-06 | 1.073640868 | NM_023077| | FLJ12439,hypothetical protein FLJ12439 |
| 215075_s_at | 8.48 | 6.92E-06 | 1.040814812 | NM_002086| | GRB2,growth factor receptor-bound protein 2 isoform |
| 239330_at | 8.48 | 6.93E-06 | 1.500677549 | NA |  |
| 220426_at | 8.48 | 6.95E-06 | 1.222343108 | NM_024059| | MGC5356,hypothetical protein MGC5356 |
| 230769_at | 8.48 | 6.98E-06 | 1.683540689 | NM_198459| | FLJ37099,similar to mouse A930010I20 protein |
| 239177_at | 8.47 | 7.04E-06 | 1.126081934 | NA |  |
| 1559360_at | 8.47 | 7.05E-06 | 1.146190901 | NA |  |
| 232523_at | 8.47 | 7.05E-06 | 1.107853165 | NM_032446| | MEGF10,MEGF10 protein |
| 218469_at | 8.47 | 7.00E-06 | 1.367735884 | NM_013372| | GREM1,cysteine knot superfamily 1, BMP antagonist 1 |
| 227036_at | 8.47 | 7.04E-06 | 1.238754202 | NA |  |
| 226004_at | 8.46 | 7.13E-06 | 1.086558526 | NM_031215| | CABLES2,Cdk5 and Abl enzyme substrate 2 |
| 223886_s_at | 8.46 | 7.14E-06 | 1.02919733 | NM_030963| | RNF146,ring finger protein 146 |
| 203002_at | 8.46 | 7.15E-06 | 1.293022596 | NM_016201| | AMOTL2,angiomotin like 2 |
| 204694_at | 8.46 | 7.11E-06 | 1.503143381 | NM_001134| | AFP,alpha-fetoprotein precursor |
| 238028_at | 8.46 | 7.13E-06 | 1.981166431 | NA |  |
| 221721_s_at | 8.46 | 7.15E-06 | 1.111110129 | NM_021020| | LZTS1,leucine zipper, putative tumor suppressor 1 |
| 210280_at | 8.46 | 7.12E-06 | 1.209550642 | NM_000530| | MPZ,myelin protein zero |
| 211450_s_at | 8.45 | 7.22E-06 | 1.118628925 | NM_000179| | MSH6,mutS homolog 6 |
| 220603_s_at | 8.45 | 7.17E-06 | 1.187586368 | NM_018349| | MCTP2,multiple C2-domains with two transmembrane |
| 218538_s_at | 8.45 | 7.19E-06 | 1.297115621 | NM_020662| | MRS2L,MRS2-like, magnesium homeostasis factor |
| 226550_at | 8.45 | 7.21E-06 | 1.211233506 | NA |  |
| 223989_s_at | 8.45 | 7.19E-06 | 1.03719311 | NM_015523| | DKFZP566E144,small fragment nuclease |
| 1556409_a_at | 8.45 | 7.22E-06 | 1.331723249 | NA |  |
| 221496_s_at | 8.45 | 7.16E-06 | 1.191821092 | NM_016272| | TOB2,transducer of ERBB2, 2 |
| 1569923_s_at | 8.45 | 7.19E-06 | 1.372158866 | NA |  |
| 1555923_a_at | 8.45 | 7.16E-06 | 1.381631021 | NM_001010911| | C10orf114,chromosome 10 open reading frame 114 |
| 1555743_s_at | 8.44 | 7.31E-06 | 1.191758634 | NA |  |
| 207700_s_at | 8.44 | 7.29E-06 | 1.121248343 | NM_006534| | NCOA3,nuclear receptor coactivator 3 isoform b |
| 207993_s_at | 8.44 | 7.28E-06 | 1.065932683 | NM_007236| | CHP,calcium binding protein P22 |
| 242602_x_at | 8.44 | 7.28E-06 | 1.090315424 | NM_203282| | ZNF539,zinc finger protein 539 |
| 229066_at | 8.44 | 7.27E-06 | 1.118308367 | NA |  |
| 233767_at | 8.43 | 7.36E-06 | 1.296597801 | NA |  |
| 223769_x_at | 8.43 | 7.33E-06 | 1.092108492 | NM_031207| | HT036,hypothetical protein HT036 |
| 229927_at | 8.43 | 7.32E-06 | 1.148654814 | NM_001001552| | LEMD1,LEM domain containing 1 |
| 215044_s_at | 8.43 | 7.33E-06 | 1.145404453 | NM_005843| | STAM2,signal transducing adaptor molecule 2 |
| 217978_s_at | 8.42 | 7.45E-06 | 1.016544851 | NM_017582| | UBE2Q,ubiquitin-conjugating enzyme E2Q |
| 204243_at | 8.42 | 7.44E-06 | 1.078988996 | NM_012421| | RLF,rearranged L-myc fusion sequence |
| 218330_s_at | 8.42 | 7.49E-06 | 1.068698303 | NM_001111018| | NA |
| 213551_x_at | 8.42 | 7.46E-06 | 1.151562008 | NM_007144| | PCGF2,ring finger protein 110 |
| 222512_at | 8.42 | 7.44E-06 | 1.050379292 | NM_016118| | NYREN18,NEDD8 ultimate buster-1 |
| 209306_s_at | 8.42 | 7.44E-06 | 1.145647475 | NM_015055| | SWAP70,SWAP-70 protein |
| 215359_x_at | 8.42 | 7.45E-06 | 1.107087322 | NM_016264| | ZNF44,zinc finger protein 44 |
| 239250_at | 8.42 | 7.44E-06 | 1.289901794 | NA |  |
| 225155_at | 8.42 | 7.46E-06 | 1.027862841 | NA |  |
| 209061_at | 8.41 | 7.55E-06 | 1.176385245 | NM_006534| | NCOA3,nuclear receptor coactivator 3 isoform b |
| 211778_s_at | 8.41 | 7.58E-06 | 1.502550621 | NM_021220| | ZNF339,zinc finger protein 339 |
| 219081_at | 8.41 | 7.50E-06 | 1.10472789 | NM_003732| | EIF4EBP3,eukaryotic translation initiation factor 4E |
| 232909_s_at | 8.4 | 7.63E-06 | 1.050736468 | NM_004459| | FALZ,fetal Alzheimer antigen isoform 2 |
| 213010_at | 8.4 | 7.63E-06 | 2.040870337 | NM_145040| | PRKCDBP,protein kinase C, delta binding protein |
| 225040_s_at | 8.4 | 7.62E-06 | 1.043033976 | NM_006916| | RPE,ribulose-5-phosphate-3-epimerase isoform 2 |
| 203775_at | 8.4 | 7.63E-06 | 1.087182734 | NM_014251| | SLC25A13,solute carrier family 25, member 13 (citrin) |
| 219310_at | 8.4 | 7.66E-06 | 1.351833425 | NM_024893| | C20orf39,chromosome 20 open reading frame 39 |
| 202717_s_at | 8.4 | 7.63E-06 | 1.046160281 | NM_001078645| | NA |
| 1565162_s_at | 8.4 | 7.66E-06 | 1.07018048 | NM_020300| | MGST1,microsomal glutathione S-transferase 1 |
| 202313_at | 8.4 | 7.60E-06 | 1.038309824 | NM_002717| | PPP2R2A,alpha isoform of regulatory subunit B55, protein |
| 203341_at | 8.39 | 7.68E-06 | 1.076383407 | NM_005760| | CEBPZ,CCAAT/enhancer binding protein zeta |
| 242915_at | 8.39 | 7.69E-06 | 1.196108375 | NM_001077349| | NA |
| 226631_at | 8.39 | 7.75E-06 | 1.092848946 | NM_212554| | LOC399818,LOC399818 protein |
| 210982_s_at | 8.39 | 7.71E-06 | 1.126682343 | NM_019111| | HLA-DRA,major histocompatibility complex, class II, DR |
| 1554544_a_at | 8.39 | 7.71E-06 | 1.183205897 | NM_001025081| | NA |
| 212496_s_at | 8.38 | 7.82E-06 | 1.082939225 | NM_015015| | JMJD2B,jumonji domain containing 2B |
| 222660_s_at | 8.38 | 7.81E-06 | 1.109486866 | NM_022781| | RNF38,ring finger protein 38 isoform 1 |
| 202761_s_at | 8.38 | 7.85E-06 | 1.105093419 | NM_015180| | SYNE2,synaptic nuclei expressed gene 2 isoform a |
| 218031_s_at | 8.38 | 7.81E-06 | 1.142250749 | NM_001085471| | NA |
| 1560119_at | 8.38 | 7.80E-06 | 1.131190559 | NA |  |
| 212101_at | 8.38 | 7.84E-06 | 1.065706112 | NM_012316| | KPNA6,karyopherin alpha 6 |
| 208914_at | 8.38 | 7.81E-06 | 1.061658842 | NM_015044| | GGA2,ADP-ribosylation factor binding protein 2 |
| 1558815_at | 8.37 | 7.92E-06 | 1.249704352 | NM_003603| | ARGBP2,Arg/Abl-interacting protein 2 isoform 1 |
| 231909_x_at | 8.37 | 7.94E-06 | 1.127038065 | NM_001007022| | KIAA1229,KIAA1229 protein isoform b |
| 238566_at | 8.37 | 7.93E-06 | 1.308641662 | NA |  |
| 213582_at | 8.37 | 7.90E-06 | 1.086944676 | NM_015205| | ATP11A,ATPase, Class VI, type 11A isoform a |
| 229118_at | 8.37 | 7.88E-06 | 1.618920473 | NA |  |
| 233979_s_at | 8.37 | 7.92E-06 | 1.148347272 | NM_031475| | ESPN,espin |
| 59999_at | 8.37 | 7.90E-06 | 1.075813395 | NM_017902| | HIF1AN,hypoxia-inducible factor 1, alpha subunit |
| 230551_at | 8.36 | 8.04E-06 | 1.478797398 | NM_173598| | KSR2,kinase suppressor of ras 2 |
| 235857_at | 8.36 | 8.00E-06 | 1.140849426 | NM_001002914| | KCTD11,potassium channel tetramerisation domain |
| 200007_at | 8.36 | 7.95E-06 | 1.046039554 | NM_003134| | SRP14,signal recognition particle 14kDa (homologous |
| 210613_s_at | 8.36 | 8.03E-06 | 1.228489543 | NM_004711| | SYNGR1,synaptogyrin 1 isoform 1a |
| 213174_at | 8.36 | 7.97E-06 | 1.575433091 | NM_015351| | NA |
| 241925_x_at | 8.36 | 7.97E-06 | 1.191326984 | NA |  |
| 226683_at | 8.36 | 8.02E-06 | 1.080065902 | NM_001102575| | NA |
| 209707_at | 8.36 | 7.98E-06 | 1.115810356 | NM_005482| | PIGK,phosphatidylinositol glycan, class K precursor |
| 213324_at | 8.36 | 7.96E-06 | 1.069472312 | NM_005417| | SRC,proto-oncogene tyrosine-protein kinase SRC |
| 201309_x_at | 8.35 | 8.06E-06 | 1.108273591 | NM_004772| | C5orf13,neuronal protein 3.1 |
[truncated: 136,318 more chars]
