# Supplementary material for: Transcriptional Signature and Memory Retention of Human-Induced Pluripotent Stem Cells
Source: PLoS One. 2009 Sep 18;4(9):e7076. doi: 10.1371/journal.pone.0007076 (PMC2741600; doi:10.1371/journal.pone.0007076)
Supplement: Table S8 — NSC-enriched probes in IPSC, ES versus NSC. Probesets enriched in group-wise comparisons: Column headings are probeset identifiers, T-statistic, P-value, Fold-Change (log2), Refseq identifier and Description of the gene. (NA indicates no Refseq annotation). (12.08 MB DOC) [file pone.0007076.s012.doc]

| Probeset | T-statistic | P-value | Fold-Change (log2) | Refseq | Description |
| --- | --- | --- | --- | --- | --- |
| 238877_at | 144.11 | 1.78E-17 | 3.745350458 | NM_004100| | EYA4,eyes absent 4 isoform a |
| 207327_at | 122.66 | 7.22E-17 | 3.134173531 | NM_004100| | EYA4,eyes absent 4 isoform a |
| 230272_at | 111.13 | 8.68E-17 | 3.215447436 | NA |  |
| 232195_at | 107.98 | 1.08E-16 | 2.6731112 | NM_020752| | GPR158,G protein-coupled receptor 158 |
| 227666_at | 106.56 | 1.12E-16 | 2.206708978 | NM_001040260| | NA |
| 211467_s_at | 100.21 | 1.81E-16 | 2.165885575 | NM_005596| | NFIB,nuclear factor I/B |
| 202132_at | 94.44 | 3.12E-16 | 1.345714983 | NM_015472| | WWTR1,WW domain containing transcription regulator 1 |
| 238058_at | 90.82 | 4.36E-16 | 2.93240594 | NA |  |
| 203540_at | 89.67 | 4.69E-16 | 2.976715769 | NM_002055| | GFAP,glial fibrillary acidic protein |
| 235977_at | 86.93 | 6.08E-16 | 2.126941308 | NM_198461| | FLJ45273,FLJ45273 protein |
| 236761_at | 86.85 | 6.08E-16 | 2.699049545 | NM_199000| | LHFPL3,lipoma HMGIC fusion partner-like 3 |
| 217870_s_at | 83.35 | 9.47E-16 | 1.192536605 | NM_016308| | UMP-CMPK,UMP-CMP kinase |
| 210138_at | 81.17 | 1.20E-15 | 2.138081951 | NM_003702| | RGS20,regulator of G-protein signalling 20 |
| 202910_s_at | 81.02 | 1.20E-15 | 1.892245724 | NM_001025160| | NA |
| 220316_at | 78.81 | 1.55E-15 | 2.192004824 | NM_022123| | NPAS3,neuronal PAS domain protein 3 |
| 213029_at | 77.87 | 1.75E-15 | 1.837723949 | NM_005596| | NFIB,nuclear factor I/B |
| 238850_at | 77.36 | 1.81E-15 | 2.551507867 | NA |  |
| 204069_at | 77.32 | 1.81E-15 | 2.015373758 | NM_002398| | MEIS1,Meis1 homolog |
| 225182_at | 75.92 | 2.03E-15 | 1.391961532 | NM_006134| | C21orf4,HCV p7-transregulated protein 3 |
| 225325_at | 75.49 | 2.13E-15 | 1.387816941 | NM_017694| | FLJ20160,FLJ20160 protein |
| 213158_at | 74.58 | 2.41E-15 | 2.666586628 | NA |  |
| 219415_at | 74.41 | 2.41E-15 | 1.423386597 | NM_001005367| | TTYH1,tweety 1 isoform 2 |
| 213395_at | 74.33 | 2.41E-15 | 2.630625391 | NM_015166| | MLC1,megalencephalic leukoencephalopathy with |
| 228708_at | 72.71 | 2.92E-15 | 2.49785949 | NM_004163| | RAB27B,RAB27B, member RAS oncogene family |
| 244797_at | 71.24 | 3.48E-15 | 1.645875499 | NA |  |
| 206765_at | 70.11 | 4.11E-15 | 1.814549109 | NM_000891| | KCNJ2,potassium inwardly-rectifying channel J2 |
| 223184_s_at | 69.42 | 4.50E-15 | 1.721522252 | NM_001037553| | NA |
| 217143_s_at | 69.09 | 4.69E-15 | 2.334403656 | NA |  |
| 219148_at | 68.2 | 5.35E-15 | 1.382196523 | NM_018492| | PBK,T-LAK cell-originated protein kinase |
| 205112_at | 67.79 | 5.67E-15 | 2.23224011 | NM_016341| | PLCE1,pancreas-enriched phospholipase C |
| 207017_at | 67.61 | 5.67E-15 | 1.684611478 | NM_004163| | RAB27B,RAB27B, member RAS oncogene family |
| 37408_at | 67.11 | 6.07E-15 | 1.248517425 | NM_006039| | MRC2,mannose receptor, C type 2 |
| 226377_at | 66.01 | 7.26E-15 | 2.159769327 | NA |  |
| 1559477_s_at | 66 | 7.26E-15 | 2.406130279 | NM_002398| | MEIS1,Meis1 homolog |
| 1559966_a_at | 65.61 | 7.71E-15 | 2.262228517 | NA |  |
| 205103_at | 64.99 | 8.27E-15 | 2.012972658 | NM_006365| | CROC4,transcriptional activator of the c-fos promoter |
| 1557852_at | 64.63 | 8.69E-15 | 1.61160386 | NA |  |
| 224520_s_at | 64.33 | 8.75E-15 | 1.902900759 | NM_032735| | MGC13168,hypothetical protein MGC13168 |
| 225533_at | 64.25 | 8.75E-15 | 1.22585492 | NM_001009936| | PHF19,PHD finger protein 19 isoform b |
| 231430_at | 64.17 | 8.75E-15 | 1.85143726 | NM_175885| | MGC33846,hypothetical protein MGC33846 |
| 244403_at | 63.84 | 9.24E-15 | 2.595126683 | NM_201253| | CRB1,crumbs homolog 1 isoform II precursor |
| 230864_at | 63.38 | 9.75E-15 | 2.297046442 | NM_153361| | MGC42105,hypothetical protein MGC42105 |
| 227082_at | 62.96 | 1.05E-14 | 2.804181379 | NA |  |
| 215290_at | 62.24 | 1.17E-14 | 2.251599723 | NA |  |
| 204457_s_at | 61.64 | 1.28E-14 | 1.447686411 | NM_002048| | GAS1,growth arrest-specific 1 |
| 201142_at | 61.6 | 1.28E-14 | 1.233773936 | NM_004094| | EIF2S1,eukaryotic translation initiation factor 2, |
| 235829_at | 61.56 | 1.28E-14 | 1.611055099 | NA |  |
| 226250_at | 61.55 | 1.28E-14 | 1.94341886 | NA |  |
| 223182_s_at | 60.84 | 1.45E-14 | 1.846552356 | NM_001037553| | NA |
| 32094_at | 59.99 | 1.72E-14 | 1.293853679 | NM_004273| | CHST3,carbohydrate (chondroitin 6) sulfotransferase 3 |
| 202484_s_at | 59.63 | 1.80E-14 | 2.814836446 | NM_003927| | MBD2,methyl-CpG binding domain protein 2 isoform 1 |
| 226806_s_at | 59.47 | 1.85E-14 | 2.482862935 | NA |  |
| 225381_at | 59.3 | 1.90E-14 | 2.743392282 | NA |  |
| 203232_s_at | 57.76 | 2.48E-14 | 1.529414645 | NM_000332| | ATXN1,ataxin 1 |
| 227121_at | 57.76 | 2.48E-14 | 2.363212221 | NA |  |
| 206071_s_at | 57.44 | 2.57E-14 | 2.12560489 | NM_005233| | EPHA3,ephrin receptor EphA3 isoform a precursor |
| 210393_at | 57.29 | 2.63E-14 | 1.774845623 | NM_003667| | LGR5,leucine-rich repeat-containing G protein-coupled |
| 219511_s_at | 57.05 | 2.68E-14 | 1.459876763 | NM_005460| | SNCAIP,synuclein alpha interacting protein |
| 242837_at | 56.75 | 2.84E-14 | 1.352059239 | NM_005626| | SFRS4,splicing factor, arginine/serine-rich 4 |
| 201983_s_at | 56.2 | 3.14E-14 | 1.654885492 | NM_005228| | EGFR,epidermal growth factor receptor isoform a |
| 201143_s_at | 56.14 | 3.16E-14 | 1.27531492 | NM_004094| | EIF2S1,eukaryotic translation initiation factor 2, |
| 203661_s_at | 56.03 | 3.20E-14 | 1.50510304 | NM_003275| | TMOD1,tropomodulin 1 |
| 238906_s_at | 56 | 3.20E-14 | 2.42228276 | NM_020663| | RHOJ,TC10-like Rho GTPase |
| 203231_s_at | 55.96 | 3.21E-14 | 1.674410642 | NM_000332| | ATXN1,ataxin 1 |
| 219051_x_at | 55.38 | 3.61E-14 | 1.477105274 | NM_024042| | METRN,meteorin, glial cell differentiation regulator |
| 242985_x_at | 55.21 | 3.66E-14 | 1.851822058 | NM_001113561| | NA |
| 229975_at | 55.06 | 3.77E-14 | 1.998336777 | NM_001203| | BMPR1B,bone morphogenetic protein receptor, type IB |
| 215014_at | 55 | 3.78E-14 | 2.914918543 | NM_004980| | KCND3,potassium voltage-gated channel, Shal-related |
| 227124_at | 54.78 | 3.93E-14 | 1.246161142 | NA |  |
| 224976_at | 54.47 | 4.14E-14 | 2.329019731 | NM_005595| | NFIA,nuclear factor I/A |
| 212357_at | 54.42 | 4.15E-14 | 1.403575733 | NM_015159| | NA |
| 204364_s_at | 54.35 | 4.18E-14 | 1.642257342 | NM_022912| | C2orf23,receptor expression enhancing protein 1 |
| 216331_at | 54.17 | 4.31E-14 | 1.290851744 | NM_002206| | ITGA7,integrin alpha 7 precursor |
| 203662_s_at | 53.86 | 4.57E-14 | 1.604986064 | NM_003275| | TMOD1,tropomodulin 1 |
| 223035_s_at | 53.8 | 4.61E-14 | 1.126658928 | NM_005687| | FARSLB,phenylalanine-tRNA synthetase-like, beta |
| 226372_at | 53.58 | 4.82E-14 | 1.526050753 | NM_018413| | CHST11,carbohydrate (chondroitin 4) sulfotransferase |
| 231128_at | 53.44 | 4.92E-14 | 1.883065225 | NM_175885| | MGC33846,hypothetical protein MGC33846 |
| 204341_at | 53.36 | 4.98E-14 | 1.317245824 | NM_006470| | TRIM16,tripartite motif-containing 16 |
| 53991_at | 53.33 | 4.98E-14 | 1.362565405 | NM_015689| | KIAA1277,KIAA1277 protein |
| 31874_at | 53.3 | 4.98E-14 | 1.279861852 | NM_006478| | GAS2L1,growth arrest-specific 2 like 1 isoform a |
| 212294_at | 52.91 | 5.34E-14 | 1.253960696 | NM_018841| | GNG12,G-protein gamma-12 subunit |
| 234985_at | 52.88 | 5.34E-14 | 1.234415046 | NM_174902| | LOC143458,hypothetical protein LOC143458 |
| 235990_at | 52.88 | 5.34E-14 | 1.505835714 | NA |  |
| 213768_s_at | 52.71 | 5.53E-14 | 1.872213266 | NM_004316| | ASCL1,achaete-scute complex homolog-like 1 |
| 232125_at | 52.48 | 5.77E-14 | 2.894497499 | NA |  |
| 213832_at | 52.41 | 5.79E-14 | 2.45342376 | NM_004980| | KCND3,potassium voltage-gated channel, Shal-related |
| 205996_s_at | 52.36 | 5.83E-14 | 1.537788878 | NM_001625| | AK2,adenylate kinase 2 isoform a |
| 227326_at | 52.18 | 6.04E-14 | 1.266215857 | NM_001008528| | TMAP1,transmembrane anchor protein 1 isoform 1 |
| 205383_s_at | 52.15 | 6.04E-14 | 1.89627347 | NM_015642| | ZBTB20,zinc finger and BTB domain containing 20 |
| 223283_s_at | 51.86 | 6.35E-14 | 2.36631915 | NM_005786| | SDCCAG33,serologically defined colon cancer antigen 33 |
| 200794_x_at | 51.7 | 6.53E-14 | 1.251258933 | NM_014764| | DAZAP2,DAZ associated protein 2 |
| 205638_at | 51.51 | 6.76E-14 | 2.310931742 | NM_001704| | BAI3,brain-specific angiogenesis inhibitor 3 |
| 205143_at | 51.32 | 7.05E-14 | 1.990995059 | NM_004386| | CSPG3,chondroitin sulfate proteoglycan 3 (neurocan) |
| 201185_at | 51.03 | 7.55E-14 | 1.395271332 | NM_002775| | PRSS11,protease, serine, 11 |
| 210869_s_at | 50.96 | 7.64E-14 | 1.265983925 | NM_006500| | MCAM,melanoma cell adhesion molecule |
| 227452_at | 50.75 | 7.95E-14 | 2.028449691 | NA |  |
| 200916_at | 50.7 | 7.95E-14 | 1.181806851 | NM_003564| | TAGLN2,transgelin 2 |
| 232113_at | 50.7 | 7.95E-14 | 2.242141513 | NA |  |
| 214175_x_at | 50.62 | 7.99E-14 | 1.362368842 | NM_003687| | PDLIM4,PDZ and LIM domain 4 |
| 209834_at | 50.61 | 7.99E-14 | 1.335250122 | NM_004273| | CHST3,carbohydrate (chondroitin 6) sulfotransferase 3 |
| 212991_at | 50.58 | 7.99E-14 | 1.787606097 | NM_012347| | FBXO9,F-box only protein 9 isoform 1 |
| 239952_at | 50.58 | 7.99E-14 | 1.92205922 | NM_030751| | TCF8,transcription factor 8 (represses interleukin 2 |
| 229435_at | 50.08 | 8.83E-14 | 2.463928032 | NM_001042413| | NA |
| 235308_at | 49.89 | 9.12E-14 | 1.90563953 | NM_015642| | ZBTB20,zinc finger and BTB domain containing 20 |
| 222871_at | 49.18 | 1.08E-13 | 1.810051803 | NM_018203| | FLJ10748,hypothetical protein FLJ10748 |
| 200884_at | 48.97 | 1.12E-13 | 1.159595585 | NM_001823| | CKB,brain creatine kinase |
| 222217_s_at | 48.82 | 1.16E-13 | 1.330787389 | NM_024330| | SLC27A3,solute carrier family 27 member 3 |
| 223419_at | 48.67 | 1.20E-13 | 1.217018508 | NM_032301| | FBXW9,F-box and WD-40 domain protein 9 |
| 206701_x_at | 48.54 | 1.22E-13 | 1.267550462 | NM_000115| | EDNRB,endothelin receptor type B isoform 1 |
| 232269_x_at | 48.19 | 1.31E-13 | 1.444316246 | NM_024042| | METRN,meteorin, glial cell differentiation regulator |
| 222301_at | 48.17 | 1.31E-13 | 2.048198547 | NM_006365| | CROC4,transcriptional activator of the c-fos promoter |
| 229656_s_at | 48.14 | 1.31E-13 | 2.035983969 | NA |  |
| 219107_at | 48.09 | 1.31E-13 | 1.578921213 | NM_021948| | BCAN,brevican isoform 1 |
| 204685_s_at | 48 | 1.33E-13 | 1.549126766 | NM_001001331| | ATP2B2,plasma membrane calcium ATPase 2 isoform a |
| 222231_s_at | 47.77 | 1.40E-13 | 1.103412534 | NM_018509| | PRO1855,hypothetical protein PRO1855 |
| 204099_at | 47.63 | 1.45E-13 | 1.397879389 | NM_001003801| | SMARCD3,SWI/SNF related, matrix associated, actin |
| 202136_at | 47.27 | 1.56E-13 | 1.166351296 | NM_006624| | ZMYND11,zinc finger, MYND domain containing 11 isoform |
| 217529_at | 47.2 | 1.58E-13 | 1.369054874 | NA |  |
| 238041_at | 47.18 | 1.58E-13 | 2.045998834 | NA |  |
| 226779_at | 47.05 | 1.64E-13 | 1.159229962 | NA |  |
| 208221_s_at | 46.58 | 1.83E-13 | 1.722356711 | NM_003061| | SLIT1,slit homolog 1 |
| 1554251_at | 46.41 | 1.90E-13 | 1.337760901 | NM_016287| | HP1-BP74,HP1-BP74 |
| 201941_at | 46.27 | 1.95E-13 | 1.120412783 | NM_001304| | CPD,carboxypeptidase D precursor |
| 212510_at | 46.23 | 1.97E-13 | 1.548445805 | NM_015141| | GPD1L,glycerol-3-phosphate dehydrogenase 1-like |
| 236834_at | 46.19 | 1.97E-13 | 1.510352619 | NM_152540| | SCFD2,sec1 family domain containing 2 |
| 221216_s_at | 45.92 | 2.11E-13 | 1.261522618 | NM_001031694| | NA |
| 227498_at | 45.86 | 2.13E-13 | 1.960026918 | NA |  |
| 230412_at | 45.81 | 2.13E-13 | 3.053143238 | NM_022123| | NPAS3,neuronal PAS domain protein 3 |
| 230291_s_at | 45.74 | 2.16E-13 | 2.389411864 | NM_005596| | NFIB,nuclear factor I/B |
| 242138_at | 45.68 | 2.19E-13 | 2.610500255 | NM_001038493| | NA |
| 225996_at | 45.62 | 2.20E-13 | 2.819611859 | NM_198461| | FLJ45273,FLJ45273 protein |
| 1568598_at | 45.2 | 2.44E-13 | 1.54827542 | NM_030929| | KAZALD1,Kazal-type serine protease inhibitor domain 1 |
| 201464_x_at | 45.06 | 2.52E-13 | 1.200707276 | NM_002228| | JUN,v-jun avian sarcoma virus 17 oncogene homolog |
| 239432_at | 44.8 | 2.67E-13 | 1.524025638 | NA |  |
| 204916_at | 44.69 | 2.75E-13 | 1.491058444 | NM_005855| | RAMP1,receptor (calcitonin) activity modifying protein |
| 227605_at | 44.68 | 2.75E-13 | 1.144597909 | NM_004757| | SCYE1,small inducible cytokine subfamily E, member 1 |
| 205363_at | 44.63 | 2.77E-13 | 2.053217238 | NM_003986| | BBOX1,gamma-butyrobetaine hydroxylase |
| 240122_at | 44.3 | 3.01E-13 | 2.378560241 | NM_017594| | DIRAS2,Di-Ras2 |
| 201152_s_at | 44.07 | 3.17E-13 | 1.44709815 | NM_021038| | MBNL1,muscleblind-like 1 isoform a |
| 204035_at | 44.06 | 3.17E-13 | 2.316927957 | NM_003469| | SCG2,secretogranin II precursor |
| 204011_at | 44.05 | 3.17E-13 | 1.262644791 | NM_005842| | SPRY2,sprouty 2 |
| 229126_at | 43.92 | 3.29E-13 | 1.315234973 | NM_018279| | TMEM19,transmembrane protein 19 |
| 224621_at | 43.81 | 3.37E-13 | 1.073879896 | NM_002745| | MAPK1,mitogen-activated protein kinase 1 |
| 205111_s_at | 43.8 | 3.38E-13 | 2.186738134 | NM_016341| | PLCE1,pancreas-enriched phospholipase C |
| 213830_at | 43.72 | 3.44E-13 | 1.923686583 | NA |  |
| 239999_at | 43.62 | 3.52E-13 | 1.701699144 | NM_001005732| | C21orf34,chromosome 21 open reading frame 34 isoform a |
| 226016_at | 43.37 | 3.74E-13 | 1.38305541 | NM_001025079| | NA |
| 225127_at | 43.36 | 3.74E-13 | 1.17866706 | NM_020823| | NA |
| 214722_at | 42.97 | 4.16E-13 | 1.195586001 | NM_203458| | NOTCH2NL,Notch homolog 2 N-terminal like protein |
| 202202_s_at | 42.86 | 4.24E-13 | 2.477070442 | NM_001105206| | NA |
| 235743_at | 42.84 | 4.26E-13 | 2.097558443 | NA |  |
| 229259_at | 42.69 | 4.42E-13 | 2.007421151 | NM_002055| | GFAP,glial fibrillary acidic protein |
| 229512_at | 42.65 | 4.46E-13 | 1.398884072 | NM_017848| | CXorf17,chromosome X open reading frame 17 |
| 226713_at | 42.56 | 4.53E-13 | 1.605128745 | NM_174908| | C3orf6,Ymer protein short isoform |
| 201028_s_at | 42.54 | 4.54E-13 | 1.301458401 | NM_001122898| | NA |
| 226623_at | 42.54 | 4.54E-13 | 1.965125561 | NM_032439| | PHYHIPL,phytanoyl-CoA hydroxylase interacting |
| 233364_s_at | 42.36 | 4.71E-13 | 2.260142293 | NA |  |
| 203354_s_at | 42.34 | 4.71E-13 | 1.253483302 | NM_015310| | PSD3,ADP-ribosylation factor guanine nucleotide |
| 228461_at | 42.17 | 4.94E-13 | 1.778013814 | NM_001099289| | NA |
| 218055_s_at | 42.12 | 4.99E-13 | 1.116511122 | NM_018268| | WDR41,WD repeat domain 41 |
| 229266_at | 42.04 | 5.07E-13 | 1.77351709 | NA |  |
| 212830_at | 41.97 | 5.17E-13 | 1.881615843 | NM_001080497| | NA |
| 239221_at | 41.87 | 5.30E-13 | 1.535447058 | NM_001083909| | NA |
| 203908_at | 41.72 | 5.50E-13 | 1.954455828 | NM_001098484| | NA |
| 212361_s_at | 41.6 | 5.69E-13 | 1.161883441 | NM_001681| | ATP2A2,ATPase, Ca++ transporting, cardiac muscle, slow |
| 209644_x_at | 41.52 | 5.82E-13 | 1.437514387 | NM_000077| | CDKN2A,cyclin-dependent kinase inhibitor 2A isoform 1 |
| 223093_at | 41.51 | 5.82E-13 | 1.630346681 | NM_054027| | ANKH,ankylosis, progressive homolog |
| 226895_at | 41.33 | 6.12E-13 | 1.747212672 | NM_005597| | NFIC,nuclear factor I/C isoform 1 |
| 233562_at | 41.28 | 6.19E-13 | 1.917023791 | NA |  |
| 214541_s_at | 41.12 | 6.43E-13 | 1.697092259 | NM_006775| | QKI,quaking homolog, KH domain RNA binding isoform |
| 203845_at | 41.12 | 6.43E-13 | 2.172712373 | NM_003884| | PCAF,p300/CBP-associated factor |
| 227049_at | 41.02 | 6.55E-13 | 1.774870758 | NM_175907| | ZADH2,zinc binding alcohol dehydrogenase, domain |
| 223214_s_at | 40.88 | 6.80E-13 | 1.742996768 | NM_001017926| | NA |
| 204271_s_at | 40.82 | 6.89E-13 | 1.200252725 | NM_000115| | EDNRB,endothelin receptor type B isoform 1 |
| 213156_at | 40.52 | 7.51E-13 | 2.549077871 | NA |  |
| 221885_at | 40.48 | 7.57E-13 | 1.374268022 | NM_015689| | KIAA1277,KIAA1277 protein |
| 229715_at | 40.26 | 8.09E-13 | 2.042385723 | NA |  |
| 202133_at | 40.22 | 8.18E-13 | 1.294947831 | NM_015472| | WWTR1,WW domain containing transcription regulator 1 |
| 209121_x_at | 39.92 | 8.85E-13 | 1.457821197 | NM_021005| | NR2F2,nuclear receptor subfamily 2, group F, member 2 |
| 224970_at | 39.92 | 8.85E-13 | 1.919312184 | NM_005595| | NFIA,nuclear factor I/A |
| 216191_s_at | 39.87 | 8.97E-13 | 2.97247817 | NA |  |
| 203217_s_at | 39.85 | 8.98E-13 | 1.387900856 | NM_001042437| | NA |
| 204646_at | 39.77 | 9.18E-13 | 3.012530982 | NM_000110| | DPYD,dihydropyrimidine dehydrogenase |
| 217867_x_at | 39.76 | 9.18E-13 | 1.367864353 | NM_012105| | BACE2,beta-site APP-cleaving enzyme 2 isoform A |
| 226368_at | 39.7 | 9.25E-13 | 1.554895945 | NM_018413| | CHST11,carbohydrate (chondroitin 4) sulfotransferase |
| 213601_at | 39.67 | 9.27E-13 | 1.839119128 | NM_003061| | SLIT1,slit homolog 1 |
| 238008_at | 39.63 | 9.34E-13 | 1.451521266 | NM_175922| | MGC35308,hypothetical protein MGC35308 |
| 209199_s_at | 39.59 | 9.46E-13 | 1.834005765 | NM_002397| | MEF2C,MADS box transcription enhancer factor 2, |
| 221571_at | 39.44 | 9.81E-13 | 1.302984626 | NM_003300| | TRAF3,TNF receptor-associated factor 3 isoform 1 |
| 233167_at | 39.36 | 1.01E-12 | 1.233036353 | NM_031454| | SELO,selenoprotein O |
| 57163_at | 39.35 | 1.01E-12 | 1.14073243 | NM_022821| | ELOVL1,elongation of very long chain fatty acids |
| 200774_at | 39.21 | 1.05E-12 | 1.161713942 | NM_014612| | C9orf10,C9orf10 protein |
| 230258_at | 39.13 | 1.07E-12 | 2.091093855 | NM_001042413| | NA |
| 211564_s_at | 39.02 | 1.11E-12 | 1.532938691 | NM_003687| | PDLIM4,PDZ and LIM domain 4 |
| 228278_at | 38.92 | 1.13E-12 | 1.641815666 | NM_002501| | NFIX,nuclear factor I/X (CCAAT-binding transcription |
| 237833_s_at | 38.82 | 1.17E-12 | 1.590844452 | NM_005460| | SNCAIP,synuclein alpha interacting protein |
| 209663_s_at | 38.79 | 1.17E-12 | 1.367243866 | NM_002206| | ITGA7,integrin alpha 7 precursor |
| 202042_at | 38.79 | 1.17E-12 | 1.093492346 | NM_002109| | HARS,histidyl-tRNA synthetase |
| 212613_at | 38.77 | 1.17E-12 | 1.256099782 | NM_007047| | BTN3A2,butyrophilin, subfamily 3, member A2 |
| 209290_s_at | 38.71 | 1.19E-12 | 1.835311026 | NM_005596| | NFIB,nuclear factor I/B |
| 222503_s_at | 38.58 | 1.23E-12 | 1.128867889 | NM_018268| | WDR41,WD repeat domain 41 |
| 213618_at | 38.49 | 1.26E-12 | 2.216597545 | NM_015230| | CENTD1,centaurin delta 1 isoform a |
| 209686_at | 38.47 | 1.26E-12 | 2.410737661 | NM_006272| | S100B,S100 calcium-binding protein, beta |
| 238472_at | 38.47 | 1.26E-12 | 1.847340279 | NM_012347| | FBXO9,F-box only protein 9 isoform 1 |
| 238736_at | 38.39 | 1.29E-12 | 1.923153013 | NM_002912| | REV3L,REV3-like, catalytic subunit of DNA polymerase |
| 236207_at | 38.32 | 1.31E-12 | 1.861187246 | NM_006751| | SSFA2,sperm specific antigen 2 |
| 203758_at | 38.26 | 1.32E-12 | 1.388419066 | NM_001334| | CTSO,cathepsin O preproprotein |
| 218508_at | 38.24 | 1.33E-12 | 1.091821644 | NM_018403| | DCP1A,decapping enzyme |
| 201029_s_at | 38.22 | 1.33E-12 | 1.232654858 | NM_001122898| | NA |
| 220559_at | 38.2 | 1.33E-12 | 2.588343492 | NM_001426| | EN1,engrailed homolog 1 |
| 1555233_at | 38.16 | 1.35E-12 | 2.158230716 | NM_020663| | RHOJ,TC10-like Rho GTPase |
| 224999_at | 38.04 | 1.39E-12 | 2.090758784 | NA |  |
| 206022_at | 37.96 | 1.41E-12 | 2.473122639 | NM_000266| | NDP,Norrie disease protein |
| 230023_at | 37.87 | 1.45E-12 | 1.213047914 | NM_199044| | NSUN4,NOL1/NOP2/Sun domain family 4 protein |
| 226808_at | 37.85 | 1.46E-12 | 1.281941587 | NM_001099220| | NA |
| 224928_at | 37.65 | 1.54E-12 | 1.434610454 | NM_030648| | SET7,SET domain-containing protein 7 |
| 212022_s_at | 37.58 | 1.57E-12 | 1.167063527 | NM_002417| | MKI67,antigen identified by monoclonal antibody Ki-67 |
| 228679_at | 37.56 | 1.58E-12 | 2.366924977 | NA |  |
| 209301_at | 37.54 | 1.59E-12 | 1.234713614 | NM_000067| | CA2,carbonic anhydrase II |
| 234977_at | 37.51 | 1.60E-12 | 1.753739161 | NM_175907| | ZADH2,zinc binding alcohol dehydrogenase, domain |
| 227178_at | 37.42 | 1.65E-12 | 1.310437374 | NM_001025076| | NA |
| 213587_s_at | 37 | 1.86E-12 | 1.362163345 | NM_001100592| | NA |
| 1559965_at | 36.87 | 1.94E-12 | 3.023655506 | NA |  |
| 229281_at | 36.82 | 1.97E-12 | 2.714387957 | NM_022123| | NPAS3,neuronal PAS domain protein 3 |
| 215259_s_at | 36.79 | 1.98E-12 | 1.580282422 | NM_145296| | IGSF4C,immunoglobulin superfamily, member 4C |
| 202386_s_at | 36.73 | 2.01E-12 | 1.14011803 | NM_014647| | LKAP,limkain b1 isoform 1 |
| 201826_s_at | 36.73 | 2.01E-12 | 1.224666557 | NM_016002| | CGI-49,CGI-49 protein |
| 37384_at | 36.71 | 2.02E-12 | 1.16481723 | NM_014634| | PPM1F,protein phosphatase 1F |
| 213714_at | 36.7 | 2.02E-12 | 1.568508356 | NM_000724| | CACNB2,calcium channel, voltage-dependent, beta 2 |
| 205741_s_at | 36.56 | 2.10E-12 | 1.277117843 | NM_001390| | DTNA,dystrobrevin alpha isoform 1 |
| 224738_x_at | 36.52 | 2.13E-12 | 1.079570746 | NM_198486| | RPL7L1,ribosomal protein L7-like 1 |
| 238898_at | 36.5 | 2.13E-12 | 1.828943316 | NA |  |
| 213500_at | 36.47 | 2.15E-12 | 1.356485419 | NA |  |
| 200767_s_at | 36.43 | 2.17E-12 | 1.269698323 | NM_014612| | C9orf10,C9orf10 protein |
| 225562_at | 36.18 | 2.35E-12 | 1.184904993 | NM_007368| | RASA3,RAS p21 protein activator 3 |
| 228480_at | 36.15 | 2.37E-12 | 1.805705715 | NM_003574| | VAPA,vesicle-associated membrane protein-associated |
| 213628_at | 36.05 | 2.44E-12 | 1.178861135 | NM_001048210| | NA |
| 217819_at | 36.04 | 2.45E-12 | 1.067756041 | NM_001002296| | GOLGA7,golgi autoantigen, golgin subfamily a, 7 |
| 228375_at | 35.93 | 2.52E-12 | 1.711022123 | NM_001015887| | NA |
| 222784_at | 35.81 | 2.61E-12 | 1.441233344 | NM_001034852| | NA |
| 227998_at | 35.79 | 2.63E-12 | 1.319227426 | NM_080388| | S100A16,S100 calcium binding protein A16 |
| 218187_s_at | 35.74 | 2.67E-12 | 1.137308994 | NM_023080| | FLJ20989,hypothetical protein FLJ20989 |
| 212360_at | 35.67 | 2.72E-12 | 1.179319199 | NM_004037| | AMPD2,adenosine monophosphate deaminase 2 (isoform L) |
| 210875_s_at | 35.64 | 2.75E-12 | 2.291260519 | NM_030751| | TCF8,transcription factor 8 (represses interleukin 2 |
| 214334_x_at | 35.62 | 2.76E-12 | 1.205498891 | NM_014764| | DAZAP2,DAZ associated protein 2 |
| 219038_at | 35.6 | 2.77E-12 | 1.574212985 | NM_001085354| | NA |
| 213939_s_at | 35.6 | 2.77E-12 | 1.316487763 | NM_001037442| | NA |
| 217077_s_at | 35.37 | 2.96E-12 | 1.621160458 | NM_005458| | GPR51,G protein-coupled receptor 51 |
| 222446_s_at | 35.32 | 3.01E-12 | 1.32082658 | NM_012105| | BACE2,beta-site APP-cleaving enzyme 2 isoform A |
| 212509_s_at | 35.31 | 3.01E-12 | 1.182352643 | NM_001008528| | TMAP1,transmembrane anchor protein 1 isoform 1 |
| 236261_at | 35.25 | 3.05E-12 | 1.547092936 | NM_032523| | OSBPL6,oxysterol-binding protein-like protein 6 isoform |
| 226923_at | 35.25 | 3.05E-12 | 1.280850155 | NM_152540| | SCFD2,sec1 family domain containing 2 |
| 236154_at | 35.25 | 3.05E-12 | 2.237995177 | NM_006775| | QKI,quaking homolog, KH domain RNA binding isoform |
| 227977_at | 35.19 | 3.11E-12 | 1.658767176 | NM_175907| | ZADH2,zinc binding alcohol dehydrogenase, domain |
| 201174_s_at | 35.16 | 3.14E-12 | 1.168044723 | NM_018975| | TERF2IP,TRF2-interacting telomeric RAP1 protein |
| 228581_at | 35.14 | 3.16E-12 | 1.994359377 | NM_002241| | KCNJ10,potassium inwardly-rectifying channel J10 |
| 219619_at | 35.12 | 3.16E-12 | 2.452373172 | NM_017594| | DIRAS2,Di-Ras2 |
| 208978_at | 35.05 | 3.24E-12 | 1.578241467 | NM_001312| | CRIP2,cysteine-rich protein 2 |
| 203799_at | 35.03 | 3.26E-12 | 1.757947242 | NM_014880| | CD302,CD302 antigen |
| 203723_at | 34.95 | 3.33E-12 | 1.442807225 | NM_002221| | ITPKB,1D-myo-inositol-trisphosphate 3-kinase B |
| 235289_at | 34.91 | 3.37E-12 | 1.708125951 | NM_020390| | EIF5A2,eIF-5A2 protein |
| 228738_at | 34.79 | 3.49E-12 | 1.202850662 | NM_152783| | MGC25181,hypothetical protein MGC25181 |
| 239229_at | 34.74 | 3.54E-12 | 1.846230295 | NA |  |
| 228555_at | 34.64 | 3.65E-12 | 1.704689138 | NM_001221| | CAMK2D,calcium/calmodulin-dependent protein kinase II |
| 225288_at | 34.52 | 3.80E-12 | 1.331386064 | NM_032888| | COL27A1,collagen, type XXVII, alpha 1 |
| 212774_at | 34.52 | 3.81E-12 | 1.205476059 | NM_006352| | ZNF238,zinc finger protein 238 isoform 2 |
| 209985_s_at | 34.49 | 3.82E-12 | 1.893266268 | NM_004316| | ASCL1,achaete-scute complex homolog-like 1 |
| 226018_at | 34.45 | 3.87E-12 | 1.50391118 | NM_152793| | Ells1,hypothetical protein Ells1 |
| 213194_at | 34.44 | 3.88E-12 | 1.127017792 | NM_002941| | ROBO1,roundabout 1 isoform a |
| 238711_s_at | 34.44 | 3.88E-12 | 1.254075411 | NM_021964| | ZNF148,zinc finger protein 148 (pHZ-52) |
| 204030_s_at | 34.37 | 3.94E-12 | 1.235410753 | NM_014575| | SCHIP1,schwannomin interacting protein 1 |
| 218208_at | 34.36 | 3.94E-12 | 1.173321444 | NM_025078| | PQLC1,PQ loop repeat containing 1 |
| 220576_at | 34.34 | 3.96E-12 | 1.458852482 | NM_024989| | PGAP1,GPI deacylase |
| 232015_at | 34.32 | 3.96E-12 | 1.34436887 | NA |  |
| 225383_at | 34.32 | 3.96E-12 | 1.162375275 | NM_001080485| | NA |
| 218692_at | 34.27 | 4.04E-12 | 2.034642399 | NM_001099743| | NA |
| 220298_s_at | 34.2 | 4.13E-12 | 1.402149755 | NM_019073| | SPATA6,spermatogenesis associated 6 |
| 222778_s_at | 34.17 | 4.17E-12 | 1.1881154 | NM_001042424| | NA |
| 238532_at | 34.14 | 4.21E-12 | 2.214268273 | NM_012074| | DPF3,cer-d4 (mouse) homolog |
| 226072_at | 34.12 | 4.22E-12 | 1.208655908 | NM_145059| | FUK,fucokinase |
| 213032_at | 34.1 | 4.25E-12 | 2.154039061 | NM_005596| | NFIB,nuclear factor I/B |
| 228720_at | 34.02 | 4.37E-12 | 1.488227194 | NM_020777| | SORCS2,VPS10 domain receptor protein SORCS 2 |
| 220265_at | 33.9 | 4.56E-12 | 1.562304294 | NM_020960| | GPR107,G protein-coupled receptor 107 |
| 222834_s_at | 33.86 | 4.60E-12 | 1.336399916 | NM_018841| | GNG12,G-protein gamma-12 subunit |
| 1554239_s_at | 33.82 | 4.65E-12 | 1.591796782 | NM_175907| | ZADH2,zinc binding alcohol dehydrogenase, domain |
| 1553108_at | 33.8 | 4.69E-12 | 1.482812171 | NM_152409| | FLJ37562,hypothetical protein FLJ37562 |
| 204105_s_at | 33.79 | 4.69E-12 | 1.591365194 | NM_001037132| | NA |
| 202554_s_at | 33.77 | 4.72E-12 | 1.445900836 | NM_000849| | GSTM3,glutathione S-transferase M3 |
| 219779_at | 33.65 | 4.88E-12 | 1.388860309 | NM_024721| | ZFHX4,zinc finger homeodomain 4 |
| 242263_at | 33.63 | 4.92E-12 | 1.881694657 | NM_016040| | TMED5,transmembrane emp24 protein transport domain |
| 227805_at | 33.63 | 4.92E-12 | 1.343869522 | NA |  |
| 224472_x_at | 33.58 | 4.99E-12 | 1.094806557 | NM_016176| | Cab45,calcium binding protein Cab45 precursor |
| 209587_at | 33.56 | 5.02E-12 | 1.312837665 | NM_002653| | PITX1,paired-like homeodomain transcription factor 1 |
| 1559402_a_at | 33.49 | 5.13E-12 | 1.637389638 | NM_006365| | CROC4,transcriptional activator of the c-fos promoter |
| 225723_at | 33.49 | 5.14E-12 | 1.18528875 | NM_138493| | NA |
| 235164_at | 33.41 | 5.28E-12 | 1.807038375 | NM_145011| | ZNF25,zinc finger protein 25 |
| 201053_s_at | 33.35 | 5.38E-12 | 1.2054057 | NM_006814| | PSMF1,proteasome inhibitor subunit 1 isoform 1 |
| 224994_at | 33.3 | 5.48E-12 | 1.491887826 | NM_001221| | CAMK2D,calcium/calmodulin-dependent protein kinase II |
| 227954_at | 33.26 | 5.56E-12 | 1.385056547 | NM_001034841| | NA |
| 224818_at | 33.26 | 5.56E-12 | 1.136747076 | NM_002959| | SORT1,sortilin 1 preproprotein |
| 202096_s_at | 33.25 | 5.56E-12 | 1.373362775 | NM_000714| | BZRP,peripheral benzodiazapine receptor isoform PBR |
| 226252_at | 33.23 | 5.60E-12 | 2.248092296 | NA |  |
| 213103_at | 33.21 | 5.62E-12 | 1.582875478 | NM_052851| | STARD13,START domain containing 13 isoform gamma |
| 219737_s_at | 33.17 | 5.71E-12 | 2.008944128 | NM_020403| | PCDH9,protocadherin 9 isoform 2 precursor |
| 230577_at | 33.02 | 5.98E-12 | 2.558126668 | NA |  |
| 219600_s_at | 33 | 6.01E-12 | 1.115849825 | NM_006134| | C21orf4,HCV p7-transregulated protein 3 |
| 202695_s_at | 32.96 | 6.09E-12 | 1.682530673 | NM_004760| | STK17A,serine/threonine kinase 17a |
| 218589_at | 32.88 | 6.26E-12 | 1.678767446 | NM_005767| | P2RY5,G-protein coupled purinergic receptor P2Y5 |
| 207776_s_at | 32.84 | 6.34E-12 | 1.433239975 | NM_000724| | CACNB2,calcium channel, voltage-dependent, beta 2 |
| 1555945_s_at | 32.81 | 6.41E-12 | 1.208411521 | NM_014612| | C9orf10,C9orf10 protein |
| 220029_at | 32.78 | 6.46E-12 | 1.920519609 | NM_017770| | ELOVL2,elongation of very long chain fatty acids |
| 224636_at | 32.78 | 6.46E-12 | 1.114077219 | NM_053023| | ZFP91,zinc finger protein 91 isoform 1 |
| 226114_at | 32.69 | 6.67E-12 | 1.801638972 | NM_001077195| | NA |
| 213132_s_at | 32.69 | 6.66E-12 | 1.232423619 | NM_014507| | MT,mitochondrial malonyltransferase isoform b |
| 226691_at | 32.64 | 6.79E-12 | 1.43207856 | NM_001013722| | NA |
| 218599_at | 32.61 | 6.84E-12 | 1.206809108 | NM_001048205| | NA |
| 235489_at | 32.59 | 6.86E-12 | 2.547287195 | NM_020663| | RHOJ,TC10-like Rho GTPase |
| 227249_at | 32.51 | 7.05E-12 | 1.163739083 | NM_017668| | NDE1,nuclear distribution gene E homolog 1 |
| 218834_s_at | 32.48 | 7.13E-12 | 1.252048082 | NM_017870| | HSPA5BP1,GBP protein isoform a |
| 44790_s_at | 32.47 | 7.13E-12 | 1.760457528 | NM_025113| | C13orf18,chromosome 13 open reading frame 18 |
| 208923_at | 32.4 | 7.29E-12 | 1.128603111 | NM_001033028| | NA |
| 227665_at | 32.39 | 7.29E-12 | 1.216082305 | NA |  |
| 209539_at | 32.34 | 7.39E-12 | 1.424730194 | NM_004840| | ARHGEF6,Rac/Cdc42 guanine nucleotide exchange factor 6 |
| 211066_x_at | 32.22 | 7.70E-12 | 1.207693801 | NM_002588| | PCDHGC3,protocadherin gamma subfamily C, 3 isoform 1 |
| 225214_at | 32.22 | 7.70E-12 | 1.27350515 | NM_002799| | PSMB7,proteasome beta 7 subunit proprotein |
| 209794_at | 32.14 | 7.92E-12 | 1.494843353 | NM_001033117| | NA |
| 205240_at | 32.13 | 7.92E-12 | 1.271232493 | NM_013296| | GPSM2,G-protein signalling modulator 2 (AGS3-like, C. |
| 212387_at | 32.12 | 7.95E-12 | 1.185994524 | NM_001083962| | NA |
| 218691_s_at | 32.11 | 7.98E-12 | 2.027271931 | NM_003687| | PDLIM4,PDZ and LIM domain 4 |
| 224184_s_at | 32.09 | 8.00E-12 | 1.416510673 | NM_033254| | BOC,brother of CDO |
| 1556601_a_at | 32.04 | 8.10E-12 | 1.231776709 | NM_153023| | SPATA13,spermatogenesis associated 13 |
| 204273_at | 32.03 | 8.10E-12 | 1.817533728 | NM_000115| | EDNRB,endothelin receptor type B isoform 1 |
| 37590_g_at | 32.03 | 8.10E-12 | 1.341209516 | NA |  |
| 1568597_at | 31.98 | 8.23E-12 | 1.207724889 | NA |  |
| 239425_at | 31.98 | 8.24E-12 | 1.475472223 | NA |  |
| 204159_at | 31.97 | 8.24E-12 | 2.465939396 | NM_001262| | CDKN2C,cyclin-dependent kinase inhibitor 2C |
| 226844_at | 31.96 | 8.25E-12 | 1.119427154 | NM_024761| | MOBKL2B,MOB1, Mps One Binder kinase activator-like 2B |
| 206083_at | 31.93 | 8.32E-12 | 1.412269145 | NM_001702| | BAI1,brain-specific angiogenesis inhibitor 1 |
| 1569854_at | 31.9 | 8.40E-12 | 2.089850255 | NA |  |
| 208912_s_at | 31.87 | 8.49E-12 | 1.119970017 | NM_033133| | CNP,2',3'-cyclic nucleotide 3' phosphodiesterase |
| 223092_at | 31.86 | 8.49E-12 | 1.385948981 | NM_054027| | ANKH,ankylosis, progressive homolog |
| 227448_at | 31.83 | 8.58E-12 | 1.460820803 | NM_018011| | FLJ10154,hypothetical protein FLJ10154 |
| 219331_s_at | 31.81 | 8.62E-12 | 1.899914047 | NM_018203| | FLJ10748,hypothetical protein FLJ10748 |
| 204401_at | 31.76 | 8.75E-12 | 1.911277845 | NM_002250| | KCNN4,intermediate conductance calcium-activated |
| 224882_at | 31.73 | 8.85E-12 | 1.612215321 | NM_032501| | ACAS2L,acetyl-CoA synthetase 2-like |
| 226726_at | 31.7 | 8.94E-12 | 1.195296127 | NM_138799| | OACT2,O-acyltransferase (membrane bound) domain |
| 227138_at | 31.68 | 9.01E-12 | 1.383827039 | NM_006371| | CRTAP,cartilage associated protein precursor |
| 205475_at | 31.65 | 9.08E-12 | 2.565602016 | NM_007281| | SCRG1,scrapie responsive protein 1 |
| 207039_at | 31.64 | 9.09E-12 | 1.685823822 | NM_000077| | CDKN2A,cyclin-dependent kinase inhibitor 2A isoform 1 |
| 218009_s_at | 31.59 | 9.21E-12 | 1.139293601 | NM_003981| | PRC1,protein regulator of cytokinesis 1 isoform 1 |
| 241701_at | 31.49 | 9.44E-12 | 1.488310043 | NM_020824| | ARHGAP21,Rho GTPase activating protein 21 |
| 205278_at | 31.47 | 9.49E-12 | 1.56628139 | NM_000817| | GAD1,glutamate decarboxylase 1 isoform GAD67 |
| 202048_s_at | 31.44 | 9.56E-12 | 1.169613817 | NM_014292| | CBX6,chromobox homolog 6 |
| 211814_s_at | 31.35 | 9.89E-12 | 1.752287244 | NM_057749| | CCNE2,cyclin E2 isoform 1 |
| 213712_at | 31.29 | 1.01E-11 | 2.129303863 | NM_017770| | ELOVL2,elongation of very long chain fatty acids |
| 229176_at | 31.27 | 1.01E-11 | 1.424852997 | NM_054027| | ANKH,ankylosis, progressive homolog |
| 221623_at | 31.14 | 1.06E-11 | 1.541968078 | NM_021948| | BCAN,brevican isoform 1 |
| 217922_at | 31.07 | 1.09E-11 | 1.171127667 | NA |  |
| 231042_s_at | 31.03 | 1.11E-11 | 1.685308244 | NA |  |
| 210830_s_at | 31.03 | 1.11E-11 | 1.208169836 | NM_000305| | PON2,paraoxonase 2 |
| 207233_s_at | 30.91 | 1.15E-11 | 1.426757554 | NM_000248| | MITF,microphthalmia-associated transcription factor |
| 215000_s_at | 30.87 | 1.16E-11 | 1.111059636 | NM_001042548| | NA |
| 212646_at | 30.86 | 1.17E-11 | 1.262391299 | NM_015150| | RAFTLIN,raft-linking protein |
| 201007_at | 30.78 | 1.20E-11 | 1.07226893 | NM_000183| | HADHB,hydroxyacyl dehydrogenase, subunit B |
| 218048_at | 30.75 | 1.21E-11 | 1.400260812 | NM_012071| | COMMD3,COMM domain containing 3 |
| 1552621_at | 30.72 | 1.22E-11 | 1.3115928 | NM_032959| | POLR2J2,DNA directed RNA polymerase II polypeptide |
| 202089_s_at | 30.68 | 1.23E-11 | 1.134188627 | NM_001099406| | NA |
| 209094_at | 30.67 | 1.24E-11 | 1.116458411 | NM_012137| | DDAH1,dimethylarginine dimethylaminohydrolase 1 |
| 241700_at | 30.66 | 1.24E-11 | 1.519936977 | NM_024721| | ZFHX4,zinc finger homeodomain 4 |
| 1438_at | 30.66 | 1.24E-11 | 1.415615322 | NM_004443| | EPHB3,ephrin receptor EphB3 precursor |
| 235192_at | 30.63 | 1.25E-11 | 1.244741483 | NM_033550| | TP53RK,p53-related protein kinase |
| 217792_at | 30.62 | 1.25E-11 | 1.069219297 | NM_014426| | SNX5,sorting nexin 5 |
| 244260_at | 30.6 | 1.26E-11 | 1.378789819 | NA |  |
| 218132_s_at | 30.55 | 1.29E-11 | 1.132705945 | NM_001077446| | NA |
| 225546_at | 30.53 | 1.29E-11 | 1.266273095 | NM_013302| | EEF2K,elongation factor-2 kinase |
| 218633_x_at | 30.51 | 1.30E-11 | 1.185014998 | NM_018394| | ABHD10,abhydrolase domain containing 10 |
| 205152_at | 30.5 | 1.30E-11 | 2.508178911 | NM_003042| | SLC6A1,solute carrier family 6 (neurotransmitter |
| 225921_at | 30.49 | 1.30E-11 | 1.379157933 | NM_016350| | NIN,ninein isoform 4 |
| 212070_at | 30.45 | 1.32E-11 | 1.941723333 | NM_005682| | GPR56,G protein-coupled receptor 56 isoform a |
| 209104_s_at | 30.43 | 1.33E-11 | 1.063409718 | NM_001034833| | NA |
| 209460_at | 30.43 | 1.33E-11 | 1.252389361 | NM_000663| | ABAT,4-aminobutyrate aminotransferase precursor |
| 202370_s_at | 30.42 | 1.33E-11 | 1.224364541 | NM_001755| | CBFB,core-binding factor, beta subunit isoform 2 |
| 230958_s_at | 30.41 | 1.33E-11 | 2.011199642 | NA |  |
| 210345_s_at | 30.4 | 1.33E-11 | 1.421251841 | NM_001372| | DNAH9,dynein, axonemal, heavy polypeptide 9 isoform 2 |
| 212895_s_at | 30.3 | 1.38E-11 | 1.190371121 | NM_001092| | ABR,active breakpoint cluster region-related |
| 205005_s_at | 30.29 | 1.39E-11 | 1.142034019 | NM_004808| | NMT2,glycylpeptide N-tetradecanoyltransferase 2 |
| 220940_at | 30.26 | 1.39E-11 | 1.441539391 | NM_025190| | NA |
| 200760_s_at | 30.26 | 1.39E-11 | 1.127089928 | NM_006407| | ARL6IP5,ADP-ribosylation-like factor 6 interacting |
| 229487_at | 30.23 | 1.41E-11 | 1.67846062 | NM_024007| | EBF,early B-cell factor |
| 213713_s_at | 30.23 | 1.41E-11 | 1.21179333 | NM_138342| | LOC89944,hypothetical protein BC008326 |
| 204476_s_at | 30.19 | 1.42E-11 | 1.22699471 | NM_000920| | PC,pyruvate carboxylase precursor |
| 205737_at | 30.19 | 1.42E-11 | 1.378331308 | NM_004518| | KCNQ2,potassium voltage-gated channel KQT-like protein |
| 223594_at | 30.18 | 1.43E-11 | 1.684934956 | NM_032256| | DKFZp434K2435,hypothetical protein DKFZp434K2435 |
| 208903_at | 30.13 | 1.45E-11 | 1.266780174 | NM_001031| | RPS28,ribosomal protein S28 |
| 232377_at | 30.09 | 1.47E-11 | 2.512188118 | NM_152745| | NXPH1,neurexophilin 1 |
| 204482_at | 30.07 | 1.48E-11 | 1.445815221 | NM_003277| | CLDN5,claudin 5 |
| 232421_at | 29.97 | 1.54E-11 | 1.704744905 | NM_001082959| | NA |
| 225460_at | 29.95 | 1.55E-11 | 1.061676478 | NM_004206| | SEC22L3,vesicle trafficking protein isoform b |
| 204202_at | 29.9 | 1.57E-11 | 1.301052319 | NM_001100390| | NA |
| 242033_at | 29.78 | 1.65E-11 | 2.362186121 | NM_001113561| | NA |
| 220299_at | 29.75 | 1.67E-11 | 1.348235487 | NM_019073| | SPATA6,spermatogenesis associated 6 |
| 206580_s_at | 29.66 | 1.72E-11 | 1.223497507 | NM_016938| | EFEMP2,EGF-containing fibulin-like extracellular matrix |
| 206670_s_at | 29.65 | 1.72E-11 | 1.520560612 | NM_000817| | GAD1,glutamate decarboxylase 1 isoform GAD67 |
| 224814_at | 29.65 | 1.73E-11 | 1.534435502 | NM_013379| | DPP7,dipeptidyl peptidase 7 preproprotein |
| 210415_s_at | 29.64 | 1.73E-11 | 1.390053525 | NM_002540| | ODF2,outer dense fiber of sperm tails 2 isoform 1 |
| 210749_x_at | 29.63 | 1.73E-11 | 1.139661873 | NM_001954| | DDR1,discoidin receptor tyrosine kinase isoform b |
| 204363_at | 29.63 | 1.73E-11 | 1.319377548 | NM_001993| | F3,coagulation factor III precursor |
| 200699_at | 29.61 | 1.74E-11 | 1.134225792 | NM_001100603| | NA |
| 210089_s_at | 29.57 | 1.77E-11 | 1.965037057 | NM_001105206| | NA |
| 208683_at | 29.55 | 1.78E-11 | 1.177661585 | NM_001748| | CAPN2,calpain 2, large subunit |
| 209079_x_at | 29.54 | 1.78E-11 | 1.205658456 | NM_002588| | PCDHGC3,protocadherin gamma subfamily C, 3 isoform 1 |
| 235106_at | 29.54 | 1.78E-11 | 1.550376784 | NM_032427| | MAML2,mastermind-like 2 |
| 202191_s_at | 29.46 | 1.84E-11 | 1.291109396 | NM_003644| | GAS7,growth arrest-specific 7 isoform a |
| 1556194_a_at | 29.3 | 1.95E-11 | 1.930174998 | NA |  |
| 226336_at | 29.24 | 1.99E-11 | 1.069240171 | NM_021130| | PPIA,peptidylprolyl isomerase A isoform 1 |
| 209292_at | 29.19 | 2.03E-11 | 1.6353861 | NM_001546| | ID4,inhibitor of DNA binding 4, dominant negative |
| 217874_at | 29.17 | 2.05E-11 | 1.130298994 | NM_003849| | SUCLG1,succinate-CoA ligase, GDP-forming, alpha |
| 218613_at | 29.17 | 2.05E-11 | 1.289567477 | NM_015310| | PSD3,ADP-ribosylation factor guanine nucleotide |
| 222752_s_at | 29.16 | 2.05E-11 | 1.098794892 | NM_018252| | FLJ10874,hypothetical protein FLJ10874 |
| 201104_x_at | 29.15 | 2.05E-11 | 1.226148909 | NM_001037501| | NA |
| 202693_s_at | 29.12 | 2.07E-11 | 1.356752414 | NM_004760| | STK17A,serine/threonine kinase 17a |
| 202092_s_at | 29.1 | 2.08E-11 | 1.289279811 | NM_012106| | ARL2BP,binder of Arl Two |
| 213574_s_at | 29.07 | 2.11E-11 | 1.11978423 | NA |  |
| 212909_at | 29.02 | 2.15E-11 | 1.52455377 | NM_001077427| | NA |
| 206692_at | 28.96 | 2.20E-11 | 1.483180191 | NM_002241| | KCNJ10,potassium inwardly-rectifying channel J10 |
| 1568763_s_at | 28.9 | 2.24E-11 | 1.072668975 | NM_013232| | PDCD6,programmed cell death 6 |
| 213298_at | 28.89 | 2.25E-11 | 1.581041256 | NM_005597| | NFIC,nuclear factor I/C isoform 1 |
| 241505_at | 28.86 | 2.27E-11 | 1.371338627 | NA |  |
| 243579_at | 28.86 | 2.27E-11 | 1.261303231 | NM_138962| | MSI2,musashi 2 isoform a |
| 1552474_a_at | 28.83 | 2.30E-11 | 1.214328242 | NM_000156| | GAMT,guanidinoacetate N-methyltransferase isoform a |
| 225019_at | 28.82 | 2.30E-11 | 1.302367263 | NM_001221| | CAMK2D,calcium/calmodulin-dependent protein kinase II |
| 205354_at | 28.76 | 2.35E-11 | 1.257449296 | NM_000156| | GAMT,guanidinoacetate N-methyltransferase isoform a |
| 203063_at | 28.75 | 2.37E-11 | 1.18164201 | NM_014634| | PPM1F,protein phosphatase 1F |
| 202986_at | 28.74 | 2.37E-11 | 1.369497345 | NM_014862| | ARNT2,aryl-hydrocarbon receptor nuclear translocator |
| 211763_s_at | 28.74 | 2.37E-11 | 1.190317214 | NM_003337| | UBE2B,ubiquitin-conjugating enzyme E2B |
| 242488_at | 28.7 | 2.40E-11 | 1.773824294 | NA |  |
| 217825_s_at | 28.7 | 2.40E-11 | 1.339981631 | NM_016021| | UBE2J1,ubiquitin-conjugating enzyme E2, J1 |
| 204610_s_at | 28.67 | 2.43E-11 | 1.188802939 | NM_006848| | DIPA,hepatitis delta antigen-interacting protein A |
| 229535_at | 28.62 | 2.47E-11 | 1.409266493 | NM_175732| | NA |
| 205672_at | 28.61 | 2.48E-11 | 1.341711822 | NM_000380| | XPA,xeroderma pigmentosum, complementation group A |
| 1007_s_at | 28.6 | 2.49E-11 | 1.114622222 | NM_001954| | DDR1,discoidin receptor tyrosine kinase isoform b |
| 239780_at | 28.59 | 2.50E-11 | 1.522118181 | NA |  |
| 206154_at | 28.58 | 2.50E-11 | 1.372595342 | NM_000326| | RLBP1,retinaldehyde binding protein 1 |
| 218718_at | 28.57 | 2.51E-11 | 1.456799559 | NM_016205| | PDGFC,platelet-derived growth factor C precursor |
| 223071_at | 28.57 | 2.51E-11 | 1.09843019 | NM_016097| | IER3IP1,immediate early response 3 interacting protein |
| 209589_s_at | 28.54 | 2.54E-11 | 1.207374299 | NM_004442| | EPHB2,ephrin receptor EphB2 isoform 2 precursor |
| 229430_at | 28.53 | 2.55E-11 | 1.678942235 | NM_152765| | MGC33510,hypothetical protein MGC33510 |
| 212251_at | 28.5 | 2.58E-11 | 1.115036631 | NM_178812| | LYRIC,LYRIC/3D3 |
| 223741_s_at | 28.45 | 2.63E-11 | 1.332591862 | NM_032646| | TTYH2,tweety 2 isoform 1 |
| 204736_s_at | 28.43 | 2.64E-11 | 1.527309467 | NM_001897| | CSPG4,melanoma-associated chondroitin sulfate |
| 227035_x_at | 28.4 | 2.67E-11 | 1.140865098 | NA |  |
| 238022_at | 28.37 | 2.70E-11 | 1.182910678 | NA |  |
| 228259_s_at | 28.36 | 2.72E-11 | 1.358148414 | NM_022140| | EPB41L4A,erythrocyte protein band 4.1-like 4 |
| 238009_at | 28.32 | 2.75E-11 | 1.645302161 | NA |  |
| 236291_at | 28.32 | 2.75E-11 | 1.26487621 | NM_002905| | RDH5,retinol dehydrogenase 5 (11-cis and 9-cis) |
| 36612_at | 28.32 | 2.75E-11 | 1.345905827 | NM_015159| | NA |
| 228067_at | 28.31 | 2.75E-11 | 1.529645209 | NM_207362| | MGC42367,similar to 2010300C02Rik protein |
| 203336_s_at | 28.31 | 2.75E-11 | 1.190270187 | NM_004763| | ITGB1BP1,integrin cytoplasmic domain-associated protein 1 |
| 242826_at | 28.21 | 2.86E-11 | 1.2127209 | NA |  |
| 236120_at | 28.17 | 2.90E-11 | 1.747102972 | NA |  |
| 210508_s_at | 28.15 | 2.92E-11 | 1.251716247 | NM_004518| | KCNQ2,potassium voltage-gated channel KQT-like protein |
| 227051_at | 28.13 | 2.95E-11 | 1.885441619 | NA |  |
| 213296_at | 28.09 | 3.00E-11 | 1.117587845 | NM_007033| | RER1,RER1 retention in endoplasmic reticulum 1 |
| 222780_s_at | 28.07 | 3.01E-11 | 1.875231838 | NM_001024372| | NA |
| 218548_x_at | 27.91 | 3.22E-11 | 1.223244622 | NM_015926| | TEX264,testis expressed sequence 264 |
| 209200_at | 27.89 | 3.24E-11 | 1.808780376 | NM_002397| | MEF2C,MADS box transcription enhancer factor 2, |
| 217904_s_at | 27.88 | 3.25E-11 | 1.485790654 | NM_012104| | BACE1,beta-site APP-cleaving enzyme 1 isoform A |
| 212121_at | 27.87 | 3.27E-11 | 1.088445428 | NM_015631| | C10orf61,chromosome 10 open reading frame 61 |
| 211520_s_at | 27.86 | 3.29E-11 | 1.967827912 | NM_000827| | GRIA1,glutamate receptor, ionotropic, AMPA 1 |
| 209793_at | 27.85 | 3.29E-11 | 2.332158118 | NM_000827| | GRIA1,glutamate receptor, ionotropic, AMPA 1 |
| 226395_at | 27.84 | 3.30E-11 | 1.206090897 | NM_032410| | HOOK3,golgi-associated microtubule-binding protein |
| 232231_at | 27.83 | 3.30E-11 | 2.050409174 | NM_001015051| | NA |
| 209291_at | 27.83 | 3.30E-11 | 1.409543681 | NM_001546| | ID4,inhibitor of DNA binding 4, dominant negative |
| 204820_s_at | 27.83 | 3.30E-11 | 1.230554641 | NM_006994| | BTN3A3,butyrophilin, subfamily 3, member A3 isoform a |
| 205717_x_at | 27.71 | 3.47E-11 | 1.308869787 | NM_002588| | PCDHGC3,protocadherin gamma subfamily C, 3 isoform 1 |
| 223472_at | 27.7 | 3.48E-11 | 1.402984313 | NM_001042424| | NA |
| 214164_x_at | 27.68 | 3.50E-11 | 1.636176972 | NM_001218| | CA12,carbonic anhydrase XII isoform 1 precursor |
| 202897_at | 27.67 | 3.52E-11 | 1.203344967 | NM_001040022| | NA |
| 208902_s_at | 27.64 | 3.55E-11 | 1.336474641 | NM_001031| | RPS28,ribosomal protein S28 |
| 204967_at | 27.64 | 3.55E-11 | 1.163229366 | NM_001649| | APXL,apical protein of Xenopus-like |
| 202694_at | 27.62 | 3.58E-11 | 1.937088494 | NM_004760| | STK17A,serine/threonine kinase 17a |
| 235111_at | 27.58 | 3.63E-11 | 1.790556802 | NA |  |
| 212993_at | 27.56 | 3.65E-11 | 1.217926805 | NM_144653| | BTBD14A,BTB (POZ) domain containing 14A |
| 226740_x_at | 27.56 | 3.65E-11 | 1.162763668 | NM_001037501| | NA |
| 202305_s_at | 27.56 | 3.65E-11 | 1.104676128 | NM_001042548| | NA |
| 224690_at | 27.55 | 3.66E-11 | 1.249704511 | NM_080821| | C20orf108,chromosome 20 open reading frame 108 |
| 204836_at | 27.53 | 3.69E-11 | 1.057587469 | NM_000170| | GLDC,glycine dehydrogenase (decarboxylating; glycine |
| 219182_at | 27.48 | 3.76E-11 | 1.341477025 | NM_001077416| | NA |
| 238753_at | 27.47 | 3.77E-11 | 1.160717652 | NM_014286| | FREQ,frequenin homolog |
| 209990_s_at | 27.43 | 3.84E-11 | 1.648095915 | NM_005458| | GPR51,G protein-coupled receptor 51 |
| 227514_at | 27.41 | 3.85E-11 | 1.461501727 | NM_001034841| | NA |
| 223203_at | 27.39 | 3.89E-11 | 1.126001461 | NM_001099684| | NA |
| 228302_x_at | 27.38 | 3.90E-11 | 1.562914856 | NM_018584| | CaMKIINalpha,calcium/calmodulin-dependent protein kinase II |
| 228831_s_at | 27.36 | 3.93E-11 | 1.313594183 | NM_052847| | GNG7,guanine nucleotide binding protein (G protein), |
| 200663_at | 27.35 | 3.94E-11 | 1.053500659 | NM_001040034| | NA |
| 227197_at | 27.35 | 3.94E-11 | 1.168302929 | NM_015595| | SGEF,DKFZP434D146 protein |
| 231319_x_at | 27.33 | 3.97E-11 | 1.33184793 | NM_022342| | KIF9,kinesin family member 9 |
| 222914_s_at | 27.3 | 4.02E-11 | 1.481042216 | NM_025268| | MGC4659,hole protein |
| 218473_s_at | 27.27 | 4.07E-11 | 1.122620175 | NM_024656| | GLT25D1,glycosyltransferase 25 domain containing 1 |
| 244690_at | 27.26 | 4.09E-11 | 1.394888571 | NA |  |
| 224666_at | 27.26 | 4.08E-11 | 1.092688391 | NM_145080| | NSMCE1,non-SMC element 1 homolog |
| 222532_at | 27.23 | 4.14E-11 | 1.137718959 | NM_021203| | SRPRB,signal recognition particle receptor, beta |
| 205088_at | 27.21 | 4.17E-11 | 1.6146899 | NM_005491| | CXorf6,chromosome X open reading frame 6 |
| 235635_at | 27.18 | 4.21E-11 | 1.346994805 | NM_001030055| | NA |
| 232590_at | 27.16 | 4.24E-11 | 1.460629058 | NA |  |
| 239118_at | 27.16 | 4.24E-11 | 2.409411841 | NM_004974| | KCNA2,potassium voltage-gated channel, shaker-related |
| 227400_at | 27.16 | 4.24E-11 | 1.847491328 | NM_002501| | NFIX,nuclear factor I/X (CCAAT-binding transcription |
| 222719_s_at | 27.14 | 4.28E-11 | 1.871626806 | NM_016205| | PDGFC,platelet-derived growth factor C precursor |
| 209012_at | 27.13 | 4.30E-11 | 1.144408811 | NM_007118| | TRIO,triple functional domain (PTPRF interacting) |
| 210410_s_at | 27.12 | 4.31E-11 | 1.340343617 | NM_001039651| | NA |
| 215867_x_at | 27.1 | 4.34E-11 | 1.591119558 | NM_001218| | CA12,carbonic anhydrase XII isoform 1 precursor |
| 219581_at | 27.09 | 4.34E-11 | 1.142308488 | NM_025265| | SEN2L,hypothetical protein MGC2776 |
| 201162_at | 27.09 | 4.34E-11 | 1.289204626 | NM_001553| | IGFBP7,insulin-like growth factor binding protein 7 |
| 210006_at | 27.05 | 4.41E-11 | 1.303528701 | NM_015407| | DKFZP564O243,DKFZP564O243 protein |
| 226748_at | 27.04 | 4.41E-11 | 1.324110433 | NM_153374| | MGC35274,hypothetical protein MGC35274 |
| 205280_at | 27 | 4.50E-11 | 2.226739319 | NM_000824| | GLRB,glycine receptor, beta |
| 1553111_a_at | 26.99 | 4.51E-11 | 1.294998035 | NM_152903| | KBTBD6,kelch repeat and BTB (POZ) domain-containing 6 |
| 223805_at | 26.97 | 4.54E-11 | 1.243183564 | NM_032523| | OSBPL6,oxysterol-binding protein-like protein 6 isoform |
| 225293_at | 26.97 | 4.54E-11 | 1.270956886 | NM_032888| | COL27A1,collagen, type XXVII, alpha 1 |
| 224592_x_at | 26.95 | 4.56E-11 | 1.184430237 | NM_016287| | HP1-BP74,HP1-BP74 |
| 1559419_at | 26.94 | 4.60E-11 | 1.8606281 | NM_000724| | CACNB2,calcium channel, voltage-dependent, beta 2 |
| 235350_at | 26.92 | 4.62E-11 | 1.781920251 | NM_001104629| | NA |
| 213451_x_at | 26.88 | 4.69E-11 | 1.146761786 | NM_019105| | TNXB,tenascin XB isoform 1 |
| 243619_at | 26.81 | 4.85E-11 | 1.370882979 | NM_015633| | FGFR1OP2,FGFR1 oncogene partner 2 |
| 1558044_s_at | 26.79 | 4.88E-11 | 1.146216345 | NM_058219| | EXOSC6,homolog of yeast mRNA transport regulator 3 |
| 225842_at | 26.78 | 4.90E-11 | 1.162953357 | NM_007350| | PHLDA1,pleckstrin homology-like domain, family A, |
| 228395_at | 26.77 | 4.92E-11 | 1.369581667 | NM_001010983| | GLT8D1,glycosyltransferase 8 domain containing 1 |
| 213288_at | 26.73 | 5.00E-11 | 1.406698438 | NM_138799| | OACT2,O-acyltransferase (membrane bound) domain |
| 212636_at | 26.73 | 5.00E-11 | 1.40144087 | NM_006775| | QKI,quaking homolog, KH domain RNA binding isoform |
| 219279_at | 26.71 | 5.02E-11 | 2.41058449 | NM_014689| | DOCK10,dedicator of cytokinesis 10 |
| 238003_at | 26.71 | 5.02E-11 | 1.414645361 | NM_152722| | FLJ25530,hypothetical protein FLJ25530 |
| 235775_at | 26.69 | 5.06E-11 | 1.458254663 | NM_152588| | DKFZp762A217,hypothetical protein DKFZp762A217 |
| 224801_at | 26.69 | 5.07E-11 | 1.388257234 | NM_019080| | NDFIP2,Nedd4 family interacting protein 2 |
| 218140_x_at | 26.68 | 5.08E-11 | 1.160694008 | NM_021203| | SRPRB,signal recognition particle receptor, beta |
| 201067_at | 26.67 | 5.10E-11 | 1.18560796 | NM_002803| | PSMC2,proteasome 26S ATPase subunit 2 |
| 200923_at | 26.66 | 5.11E-11 | 1.312970339 | NM_005567| | LGALS3BP,galectin 3 binding protein |
| 221766_s_at | 26.65 | 5.13E-11 | 1.39908205 | NM_017633| | FAM46A,family with sequence similarity 46, member A |
| 224641_at | 26.64 | 5.16E-11 | 1.216567233 | NM_001011537| | FYTTD1,forty-two-three domain containing 1 isoform 2 |
| 236766_at | 26.63 | 5.16E-11 | 1.560183705 | NA |  |
| 1557953_at | 26.6 | 5.22E-11 | 1.214653296 | NM_003439| | ZKSCAN1,zinc finger protein 36 |
| 228574_at | 26.6 | 5.22E-11 | 1.391603854 | NM_152588| | DKFZp762A217,hypothetical protein DKFZp762A217 |
| 201466_s_at | 26.58 | 5.25E-11 | 1.357927633 | NM_002228| | JUN,v-jun avian sarcoma virus 17 oncogene homolog |
| 228131_at | 26.56 | 5.30E-11 | 1.426530504 | NM_001983| | ERCC1,excision repair cross-complementing 1 isofrom 2 |
| 52975_at | 26.56 | 5.29E-11 | 1.192815123 | NM_001011703| | C9orf28,chromosome 9 open reading frame 28 isoform 2 |
| 201360_at | 26.55 | 5.30E-11 | 1.298495507 | NM_000099| | CST3,cystatin C precursor |
| 203526_s_at | 26.55 | 5.30E-11 | 1.385609238 | NM_000038| | APC,adenomatosis polyposis coli |
| 225157_at | 26.53 | 5.33E-11 | 1.122213797 | NA |  |
| 231866_at | 26.49 | 5.42E-11 | 1.249122902 | NM_005575| | LNPEP,leucyl/cystinyl aminopeptidase |
| 227610_at | 26.49 | 5.43E-11 | 1.53144366 | NA |  |
| 205931_s_at | 26.47 | 5.46E-11 | 1.754420465 | NM_001011666| | CREB5,cAMP responsive element binding protein 5 |
| 226751_at | 26.45 | 5.50E-11 | 1.309345447 | NM_001111101| | NA |
| 209289_at | 26.44 | 5.51E-11 | 1.880665139 | NM_005596| | NFIB,nuclear factor I/B |
| 224693_at | 26.42 | 5.57E-11 | 1.299534324 | NM_080821| | C20orf108,chromosome 20 open reading frame 108 |
| 229382_at | 26.36 | 5.70E-11 | 1.296329928 | NM_019099| | LOC55924,hypothetical protein LOC55924 isoform 1 |
| 202830_s_at | 26.36 | 5.71E-11 | 1.087242139 | NM_001467| | SLC37A4,solute carrier family 37 (glycerol-6-phosphate |
| 203285_s_at | 26.35 | 5.72E-11 | 1.120944573 | NM_012262| | HS2ST1,heparan sulfate 2-O-sulfotransferase 1 |
| 226902_at | 26.35 | 5.73E-11 | 1.138129749 | NA |  |
| 212411_at | 26.33 | 5.75E-11 | 1.129520046 | NM_033416| | IMP4,IMP4, U3 small nucleolar ribonucleoprotein, |
| 225483_at | 26.33 | 5.75E-11 | 1.173227914 | NM_052875| | MGC10485,hypothetical protein MGC10485 |
| 203724_s_at | 26.31 | 5.79E-11 | 1.434440589 | NM_001037442| | NA |
| 200761_s_at | 26.29 | 5.85E-11 | 1.101052725 | NM_006407| | ARL6IP5,ADP-ribosylation-like factor 6 interacting |
| 205251_at | 26.26 | 5.93E-11 | 1.25995503 | NM_022817| | PER2,period 2 isoform 1 |
| 229674_at | 26.24 | 5.96E-11 | 1.237049059 | NM_019605| | SERTAD4,SERTA domain containing 4 |
| 209729_at | 26.24 | 5.96E-11 | 1.228682852 | NM_006478| | GAS2L1,growth arrest-specific 2 like 1 isoform a |
| 201148_s_at | 26.21 | 6.04E-11 | 1.242826474 | NM_000362| | TIMP3,tissue inhibitor of metalloproteinase 3 |
| 200787_s_at | 26.21 | 6.04E-11 | 1.24802593 | NM_003768| | PEA15,phosphoprotein enriched in astrocytes 15 |
| 229454_at | 26.18 | 6.10E-11 | 1.362407888 | NM_001077440| | NA |
| 224869_s_at | 26.18 | 6.11E-11 | 1.095562869 | NM_022497| | MRPS25,mitochondrial ribosomal protein S25 |
| 1557384_at | 26.12 | 6.27E-11 | 1.264286775 | NM_003432| | NA |
| 203608_at | 26.05 | 6.47E-11 | 1.478656327 | NM_001080| | ALDH5A1,aldehyde dehydrogenase 5A1 precursor, isoform 2 |
| 221858_at | 26.04 | 6.50E-11 | 1.332248389 | NM_015188| | NA |
| 224784_at | 26.02 | 6.54E-11 | 1.594783456 | NM_005937| | MLLT6,myeloid/lymphoid or mixed-lineage leukemia |
| 227467_at | 25.97 | 6.69E-11 | 1.471896112 | NM_172037| | RDH10,retinol dehydrogenase 10 |
| 217848_s_at | 25.96 | 6.71E-11 | 1.071520723 | NM_021129| | PP,inorganic pyrophosphatase |
| 202157_s_at | 25.92 | 6.82E-11 | 1.110921097 | NM_001025076| | NA |
| 211976_at | 25.91 | 6.82E-11 | 1.13786842 | NA |  |
| 235405_at | 25.89 | 6.90E-11 | 1.27965153 | NM_001512| | GSTA4,glutathione S-transferase A4 |
| 209029_at | 25.89 | 6.89E-11 | 1.09658384 | NM_016319| | COPS7A,COP9 complex subunit 7a |
| 204647_at | 25.87 | 6.96E-11 | 1.172194505 | NM_004838| | HOMER3,Homer, neuronal immediate early gene, 3 |
| 212005_at | 25.87 | 6.96E-11 | 1.134333203 | NM_001114600| | NA |
| 225098_at | 25.84 | 7.04E-11 | 1.082241484 | NM_005759| | ABI2,abl interactor 2 |
| 212831_at | 25.84 | 7.04E-11 | 1.852341337 | NM_001080497| | NA |
| 227903_x_at | 25.76 | 7.27E-11 | 1.175116537 | NM_033513| | C19orf20,chromosome 19 open reading frame 20 |
| 227061_at | 25.75 | 7.30E-11 | 1.800701501 | NA |  |
| 220773_s_at | 25.74 | 7.33E-11 | 1.270416636 | NM_001024218| | NA |
| 221264_s_at | 25.7 | 7.45E-11 | 1.182257981 | NM_007375| | TARDBP,TAR DNA binding protein |
| 225129_at | 25.69 | 7.46E-11 | 1.276388186 | NM_152727| | CPNE2,copine II |
| 204249_s_at | 25.66 | 7.57E-11 | 1.516226414 | NM_005574| | LMO2,LIM domain only 2 |
| 201149_s_at | 25.66 | 7.57E-11 | 1.381346303 | NM_000362| | TIMP3,tissue inhibitor of metalloproteinase 3 |
| 220939_s_at | 25.66 | 7.57E-11 | 1.079945536 | NM_017743| | DPP8,dipeptidyl peptidase 8 isoform 2 |
| 202566_s_at | 25.65 | 7.58E-11 | 1.284546598 | NM_003174| | SVIL,supervillin isoform 1 |
| 201115_at | 25.65 | 7.57E-11 | 1.100303328 | NM_001127218| | NA |
| 219076_s_at | 25.64 | 7.62E-11 | 1.344657108 | NM_018663| | PXMP2,peroxisomal membrane protein 2, 22kDa |
| 228548_at | 25.56 | 7.88E-11 | 1.330280725 | NA |  |
| 206052_s_at | 25.55 | 7.88E-11 | 1.079557816 | NM_006527| | SLBP,histone stem-loop binding protein |
| 237314_at | 25.54 | 7.91E-11 | 1.94281512 | NM_145010| | C10orf63,enkurin |
| 238034_at | 25.54 | 7.91E-11 | 1.167257478 | NM_001024649| | NA |
| 208660_at | 25.47 | 8.18E-11 | 1.064644121 | NM_004077| | CS,citrate synthase precursor, isoform a |
| 205279_s_at | 25.41 | 8.36E-11 | 1.905515797 | NM_000824| | GLRB,glycine receptor, beta |
| 202170_s_at | 25.4 | 8.40E-11 | 1.079194817 | NM_015423| | AASDHPPT,aminoadipate-semialdehyde |
| 202584_at | 25.4 | 8.40E-11 | 1.300642554 | NM_002504| | NFX1,nuclear transcription factor, X-box binding 1 |
| 207336_at | 25.39 | 8.43E-11 | 1.670083101 | NM_006940| | SOX5,SRY (sex determining region Y)-box 5 isoform a |
| 236664_at | 25.37 | 8.52E-11 | 1.427571849 | NM_001626| | AKT2,v-akt murine thymoma viral oncogene homolog 2 |
| 213257_at | 25.35 | 8.57E-11 | 1.530061483 | NM_015077| | SARM1,sterile alpha and TIR motif containing 1 |
| 209617_s_at | 25.33 | 8.62E-11 | 1.337641622 | NM_001332| | CTNND2,catenin (cadherin-associated protein), delta 2 |
| 210735_s_at | 25.31 | 8.73E-11 | 1.747263621 | NM_001218| | CA12,carbonic anhydrase XII isoform 1 precursor |
| 217933_s_at | 25.29 | 8.81E-11 | 1.110924509 | NM_015907| | LAP3,leucine aminopeptidase |
| 232204_at | 25.26 | 8.90E-11 | 1.784927097 | NM_024007| | EBF,early B-cell factor |
| 213392_at | 25.25 | 8.91E-11 | 1.21188469 | NM_153208| | MGC35048,hypothetical protein MGC35048 |
| 201666_at | 25.23 | 8.97E-11 | 1.145580061 | NM_003254| | TIMP1,tissue inhibitor of metalloproteinase 1 |
| 228429_x_at | 25.23 | 8.97E-11 | 1.339463442 | NM_022342| | KIF9,kinesin family member 9 |
| 203759_at | 25.23 | 8.97E-11 | 1.26174403 | NM_006278| | ST3GAL4,ST3 beta-galactoside alpha-2,3-sialyltransferase |
| 210978_s_at | 25.23 | 8.97E-11 | 1.200467781 | NM_003564| | TAGLN2,transgelin 2 |
| 204456_s_at | 25.2 | 9.08E-11 | 1.573204338 | NM_002048| | GAS1,growth arrest-specific 1 |
| 238735_at | 25.17 | 9.19E-11 | 2.138890296 | NA |  |
| 212206_s_at | 25.17 | 9.19E-11 | 1.188763181 | NM_012412| | H2AFV,H2A histone family, member V isoform 1 |
| 206408_at | 25.08 | 9.57E-11 | 1.786357552 | NM_015564| | LRRTM2,leucine rich repeat transmembrane neuronal 2 |
| 201207_at | 25.08 | 9.57E-11 | 1.119104526 | NM_021137| | TNFAIP1,tumor necrosis factor, alpha-induced protein 1 |
| 213411_at | 25.06 | 9.61E-11 | 1.508351442 | NM_004194| | ADAM22,a disintegrin and metalloproteinase domain 22 |
| 243071_at | 25.02 | 9.79E-11 | 1.411121435 | NA |  |
| 1558034_s_at | 25.02 | 9.79E-11 | 1.913652957 | NM_000096| | CP,ceruloplasmin (ferroxidase) |
| 240339_at | 24.99 | 9.93E-11 | 1.263645293 | NA |  |
| 215836_s_at | 24.99 | 9.94E-11 | 1.212423037 | NM_002588| | PCDHGC3,protocadherin gamma subfamily C, 3 isoform 1 |
| 227407_at | 24.98 | 9.94E-11 | 1.151615031 | NM_153365| | FLJ90013,hypothetical protein FLJ90013 |
| 232793_at | 24.97 | 1.00E-10 | 1.611459255 | NA |  |
| 201734_at | 24.95 | 1.01E-10 | 1.096969361 | NM_001829| | CLCN3,chloride channel 3 isoform b |
| 226805_at | 24.95 | 1.01E-10 | 1.315129451 | NM_001080472| | NA |
| 222995_s_at | 24.89 | 1.03E-10 | 1.179053701 | NM_001040456| | NA |
| 223571_at | 24.89 | 1.03E-10 | 1.185526047 | NM_031910| | C1QTNF6,C1q and tumor necrosis factor related protein 6 |
| 203683_s_at | 24.85 | 1.06E-10 | 1.137413361 | NM_003377| | VEGFB,vascular endothelial growth factor B |
| 1555240_s_at | 24.84 | 1.06E-10 | 1.384455077 | NM_018841| | GNG12,G-protein gamma-12 subunit |
| 236350_at | 24.84 | 1.06E-10 | 1.471219616 | NA |  |
| 227667_at | 24.82 | 1.07E-10 | 1.312862526 | NM_017949| | CUEDC1,CUE domain-containing 1 |
| 229228_at | 24.82 | 1.07E-10 | 1.793672008 | NM_001011666| | CREB5,cAMP responsive element binding protein 5 |
| 221840_at | 24.79 | 1.08E-10 | 1.58191929 | NM_006504| | PTPRE,protein tyrosine phosphatase, receptor type, E |
| 217806_s_at | 24.78 | 1.08E-10 | 1.06368748 | NM_015584| | POLDIP2,DNA polymerase delta interacting protein 2 |
| 241464_s_at | 24.75 | 1.10E-10 | 1.546510434 | NA |  |
| 230212_at | 24.75 | 1.10E-10 | 1.344788463 | NA |  |
| 219525_at | 24.72 | 1.11E-10 | 1.322061805 | NM_018242| | FLJ10847,hypothetical protein FLJ10847 |
| 220688_s_at | 24.72 | 1.11E-10 | 1.116130006 | NM_016183| | C1orf33,ribosomal protein P0-like protein |
| 226255_at | 24.69 | 1.12E-10 | 1.123848025 | NM_006777| | ZBTB33,kaiso |
| 226446_at | 24.68 | 1.13E-10 | 1.3768758 | NM_018645| | HES6,hairy and enhancer of split 6 |
| 203897_at | 24.66 | 1.14E-10 | 1.125464026 | NM_020424| | LOC57149,hypothetical protein A-211C6.1 |
| 221942_s_at | 24.66 | 1.14E-10 | 1.559018226 | NM_000856| | GUCY1A3,guanylate cyclase 1, soluble, alpha 3 |
| 232921_at | 24.64 | 1.15E-10 | 1.340138915 | NM_020910| | NA |
| 1557433_at | 24.62 | 1.16E-10 | 1.608116606 | NA |  |
| 218648_at | 24.6 | 1.17E-10 | 1.182437669 | NM_001042574| | NA |
| 222163_s_at | 24.59 | 1.17E-10 | 1.073458383 | NM_024063| | SPATA5L1,spermatogenesis associated 5-like 1 |
| 235433_at | 24.58 | 1.17E-10 | 1.201342662 | NM_198450| | CXorf33,chromosome X open reading frame 33 |
| 209086_x_at | 24.58 | 1.17E-10 | 1.240322934 | NM_006500| | MCAM,melanoma cell adhesion molecule |
| 212276_at | 24.57 | 1.18E-10 | 1.223900937 | NM_145693| | LPIN1,lipin 1 |
| 228060_at | 24.5 | 1.21E-10 | 1.281468942 | NM_001029858| | NA |
| 204126_s_at | 24.49 | 1.22E-10 | 1.154661721 | NM_003504| | CDC45L,CDC45-like |
| 214805_at | 24.48 | 1.22E-10 | 1.193778037 | NM_001416| | EIF4A1,eukaryotic translation initiation factor 4A, |
| 219973_at | 24.48 | 1.22E-10 | 1.723735036 | NM_024590| | ARSJ,arylsulfatase J |
| 1569303_s_at | 24.47 | 1.23E-10 | 1.272592201 | NM_003702| | RGS20,regulator of G-protein signalling 20 |
| 242579_at | 24.47 | 1.23E-10 | 1.897063413 | NM_001203| | BMPR1B,bone morphogenetic protein receptor, type IB |
| 216120_s_at | 24.44 | 1.24E-10 | 1.599905469 | NM_001001331| | ATP2B2,plasma membrane calcium ATPase 2 isoform a |
| 31799_at | 24.44 | 1.24E-10 | 1.406740775 | NA |  |
| 214297_at | 24.44 | 1.24E-10 | 1.706652336 | NM_001897| | CSPG4,melanoma-associated chondroitin sulfate |
| 224642_at | 24.43 | 1.24E-10 | 1.28565993 | NM_001011537| | FYTTD1,forty-two-three domain containing 1 isoform 2 |
| 238469_at | 24.42 | 1.25E-10 | 2.017101111 | NM_024576| | OGFRL1,opioid growth factor receptor-like 1 |
| 224764_at | 24.41 | 1.26E-10 | 1.257448474 | NM_020824| | ARHGAP21,Rho GTPase activating protein 21 |
| 225246_at | 24.4 | 1.26E-10 | 1.287026572 | NM_020860| | STIM2,stromal interaction molecule 2 |
| 211471_s_at | 24.39 | 1.26E-10 | 1.475670938 | NM_004914| | RAB36,RAB36, member RAS oncogene family |
| 227135_at | 24.38 | 1.27E-10 | 1.411036173 | NM_001042402| | NA |
| 229111_at | 24.38 | 1.27E-10 | 1.325319697 | NA |  |
| 204276_at | 24.37 | 1.28E-10 | 1.342039698 | NM_004614| | TK2,thymidine kinase 2, mitochondrial |
| 222409_at | 24.37 | 1.27E-10 | 1.103023245 | NM_014325| | CORO1C,coronin, actin binding protein, 1C |
| 230782_at | 24.33 | 1.30E-10 | 1.482773471 | NM_003104| | SORD,sorbitol dehydrogenase |
| 213353_at | 24.24 | 1.36E-10 | 1.523440798 | NM_018672| | ABCA5,ATP-binding cassette, sub-family A , member 5 |
| 202920_at | 24.24 | 1.35E-10 | 1.372565663 | NM_001148| | ANK2,ankyrin 2 isoform 1 |
| 212758_s_at | 24.22 | 1.36E-10 | 1.705097572 | NM_030751| | TCF8,transcription factor 8 (represses interleukin 2 |
| 220443_s_at | 24.22 | 1.36E-10 | 1.604418483 | NM_012476| | VAX2,ventral anterior homeobox 2 |
| 35626_at | 24.19 | 1.38E-10 | 1.120617524 | NM_000199| | SGSH,N-sulfoglucosamine sulfohydrolase (sulfamidase) |
| 225823_at | 24.16 | 1.40E-10 | 1.176117883 | NM_205767| | QIL1,QIL1 protein |
| 209341_s_at | 24.16 | 1.41E-10 | 1.164452717 | NM_001556| | IKBKB,inhibitor of kappa light polypeptide gene |
| 229269_x_at | 24.15 | 1.41E-10 | 1.221963603 | NM_001009998| | SSBP4,single stranded DNA binding protein 4 isoform b |
| 204541_at | 24.15 | 1.41E-10 | 1.470715773 | NM_012429| | SEC14L2,SEC14-like 2 |
| 230596_at | 24.1 | 1.44E-10 | 1.375517526 | NA |  |
| 222651_s_at | 24.1 | 1.44E-10 | 1.471141759 | NM_014112| | TRPS1,zinc finger transcription factor TRPS1 |
| 229608_at | 24.08 | 1.45E-10 | 1.282915454 | NM_019099| | LOC55924,hypothetical protein LOC55924 isoform 1 |
| 212386_at | 24.07 | 1.46E-10 | 1.126568669 | NM_001083962| | NA |
| 1554774_at | 24.05 | 1.47E-10 | 1.44221721 | NM_001042533| | NA |
| 232295_at | 24.05 | 1.47E-10 | 1.457615115 | NM_024996| | GFM1,G elongation factor, mitochondrial 1 |
| 202834_at | 24.04 | 1.48E-10 | 1.530643292 | NM_000029| | AGT,angiotensinogen precursor |
| 204556_s_at | 24.03 | 1.48E-10 | 1.190488598 | NM_014934| | DZIP1,DAZ interacting protein 1 |
| 233255_s_at | 24.02 | 1.49E-10 | 1.396575774 | NM_017693| | BIVM,basic, immunoglobulin-like variable motif |
| 227646_at | 24.02 | 1.49E-10 | 1.717789076 | NM_024007| | EBF,early B-cell factor |
| 207147_at | 24.02 | 1.49E-10 | 2.049186041 | NM_004405| | DLX2,distal-less homeo box 2 |
| 204033_at | 24.02 | 1.49E-10 | 1.102980202 | NM_004237| | TRIP13,thyroid hormone receptor interactor 13 |
| 218502_s_at | 24.01 | 1.49E-10 | 1.505583311 | NM_014112| | TRPS1,zinc finger transcription factor TRPS1 |
| 214773_x_at | 23.99 | 1.51E-10 | 1.104712024 | NM_001031800| | NA |
| 212362_at | 23.98 | 1.51E-10 | 1.247952268 | NM_001681| | ATP2A2,ATPase, Ca++ transporting, cardiac muscle, slow |
| 226774_at | 23.96 | 1.51E-10 | 1.251526946 | NM_032448| | KIAA1838,KIAA1838 |
| 205543_at | 23.96 | 1.51E-10 | 1.364004225 | NM_014278| | HSPA4L,heat shock 70kDa protein 4-like |
| 218584_at | 23.96 | 1.51E-10 | 1.317599159 | NM_001082537| | NA |
| 1555594_a_at | 23.96 | 1.51E-10 | 1.27547663 | NM_021038| | MBNL1,muscleblind-like 1 isoform a |
| 226656_at | 23.95 | 1.51E-10 | 1.301913909 | NM_006371| | CRTAP,cartilage associated protein precursor |
| 205481_at | 23.94 | 1.53E-10 | 1.497847359 | NM_000674| | ADORA1,adenosine A1 receptor |
| 1560792_at | 23.93 | 1.53E-10 | 1.830272205 | NA |  |
| 1552622_s_at | 23.93 | 1.53E-10 | 1.381601846 | NM_032959| | POLR2J2,DNA directed RNA polymerase II polypeptide |
| 241938_at | 23.93 | 1.53E-10 | 1.498521437 | NM_006775| | QKI,quaking homolog, KH domain RNA binding isoform |
| 200911_s_at | 23.92 | 1.53E-10 | 1.231545016 | NM_001122824| | NA |
| 231406_at | 23.91 | 1.54E-10 | 1.325222754 | NA |  |
| 233648_at | 23.9 | 1.55E-10 | 1.320603305 | NA |  |
| 241840_at | 23.89 | 1.55E-10 | 1.256454403 | NA |  |
| 233168_s_at | 23.88 | 1.55E-10 | 1.153709249 | NM_031454| | SELO,selenoprotein O |
| 204032_at | 23.88 | 1.55E-10 | 1.117823375 | NM_003567| | BCAR3,breast cancer antiestrogen resistance 3 |
| 224517_at | 23.86 | 1.57E-10 | 1.177852901 | NA |  |
| 216959_x_at | 23.8 | 1.62E-10 | 1.366798889 | NM_001037132| | NA |
| 238963_at | 23.78 | 1.63E-10 | 1.332887202 | NA |  |
| 205283_at | 23.77 | 1.64E-10 | 1.236730808 | NM_001079802| | NA |
| 218557_at | 23.76 | 1.64E-10 | 1.089099245 | NM_020202| | NIT2,nitrilase family, member 2 |
| 212017_at | 23.74 | 1.66E-10 | 1.099211751 | NM_001009993| | LOC130074,hypothetical protein LOC130074 |
| 232884_s_at | 23.74 | 1.66E-10 | 1.312437312 | NA |  |
| 238670_at | 23.74 | 1.66E-10 | 1.336050537 | NA |  |
| 208920_at | 23.73 | 1.66E-10 | 1.567591607 | NM_003130| | SRI,sorcin isoform a |
| 1559603_at | 23.72 | 1.67E-10 | 1.649908497 | NA |  |
| 209032_s_at | 23.72 | 1.67E-10 | 1.232492329 | NM_001098517| | NA |
| 224591_at | 23.72 | 1.67E-10 | 1.151209498 | NM_016287| | HP1-BP74,HP1-BP74 |
| 225371_at | 23.7 | 1.68E-10 | 1.133930758 | NM_001003722| | GLE1L,GLE1-like, RNA export mediator isoform 1 |
| 218862_at | 23.65 | 1.73E-10 | 1.193807225 | NM_024701| | ASB13,ankyrin repeat and SOCS box-containing protein |
| 221910_at | 23.64 | 1.73E-10 | 1.451417364 | NM_004956| | ETV1,ets variant gene 1 |
| 229335_at | 23.64 | 1.73E-10 | 1.400965845 | NM_145296| | IGSF4C,immunoglobulin superfamily, member 4C |
| 231403_at | 23.64 | 1.74E-10 | 1.740865352 | NM_007118| | TRIO,triple functional domain (PTPRF interacting) |
| 225626_at | 23.63 | 1.74E-10 | 1.885112769 | NM_018440| | PAG,phosphoprotein associated with |
| 202985_s_at | 23.63 | 1.74E-10 | 1.11363153 | NM_001015048| | NA |
| 203355_s_at | 23.61 | 1.76E-10 | 1.23678794 | NM_015310| | PSD3,ADP-ribosylation factor guanine nucleotide |
| 200046_at | 23.61 | 1.76E-10 | 1.043879982 | NM_001344| | DAD1,defender against cell death 1 |
| 224975_at | 23.6 | 1.76E-10 | 2.09873371 | NM_005595| | NFIA,nuclear factor I/A |
| 206070_s_at | 23.59 | 1.77E-10 | 3.125221236 | NM_005233| | EPHA3,ephrin receptor EphA3 isoform a precursor |
| 202921_s_at | 23.58 | 1.78E-10 | 1.40111957 | NM_001148| | ANK2,ankyrin 2 isoform 1 |
| 212174_at | 23.57 | 1.78E-10 | 1.642528413 | NM_001625| | AK2,adenylate kinase 2 isoform a |
| 226840_at | 23.57 | 1.78E-10 | 1.45627087 | NM_001040158| | NA |
| 200048_s_at | 23.55 | 1.80E-10 | 1.063178061 | NM_006694| | JTB,jumping translocation breakpoint |
| 219471_at | 23.52 | 1.83E-10 | 1.526921256 | NM_025113| | C13orf18,chromosome 13 open reading frame 18 |
| 228930_at | 23.49 | 1.85E-10 | 1.389319292 | NA |  |
| 225615_at | 23.47 | 1.87E-10 | 1.191550263 | NA |  |
| 222761_at | 23.45 | 1.89E-10 | 1.353590283 | NM_017693| | BIVM,basic, immunoglobulin-like variable motif |
| 204317_at | 23.43 | 1.90E-10 | 1.225987524 | NM_016426| | GTSE1,G-2 and S-phase expressed 1 |
| 212021_s_at | 23.42 | 1.91E-10 | 1.220232253 | NM_002417| | MKI67,antigen identified by monoclonal antibody Ki-67 |
| 225000_at | 23.41 | 1.92E-10 | 1.081039538 | NM_004157| | PRKAR2A,cAMP-dependent protein kinase, regulatory |
| 203318_s_at | 23.4 | 1.93E-10 | 1.140252865 | NM_021964| | ZNF148,zinc finger protein 148 (pHZ-52) |
| 233350_s_at | 23.39 | 1.94E-10 | 1.270500981 | NM_015926| | TEX264,testis expressed sequence 264 |
| 213429_at | 23.39 | 1.94E-10 | 2.082246958 | NM_001080512| | NA |
| 211302_s_at | 23.39 | 1.93E-10 | 1.68633854 | NM_001037339| | NA |
| 221908_at | 23.37 | 1.96E-10 | 1.164387495 | NM_001109903| | NA |
| 206833_s_at | 23.36 | 1.96E-10 | 1.264927787 | NM_138448| | ACYP2,muscle-type acylphosphatase 2 |
| 244788_at | 23.36 | 1.96E-10 | 1.507180378 | NA |  |
| 212625_at | 23.34 | 1.98E-10 | 1.154449045 | NM_003765| | STX10,syntaxin 10 |
| 208664_s_at | 23.33 | 1.99E-10 | 1.313731701 | NM_001001894| | TTC3,tetratricopeptide repeat domain 3 |
| 230071_at | 23.31 | 2.01E-10 | 1.871492644 | NM_018243| | SEPT11,septin 11 |
| 212913_at | 23.3 | 2.02E-10 | 1.353615659 | NM_001039651| | NA |
| 1555091_at | 23.29 | 2.02E-10 | 1.525690568 | NM_014634| | PPM1F,protein phosphatase 1F |
| 204279_at | 23.29 | 2.02E-10 | 1.174045057 | NM_002800| | PSMB9,proteasome beta 9 subunit isoform 1 proprotein |
| 209087_x_at | 23.29 | 2.02E-10 | 1.280064841 | NM_006500| | MCAM,melanoma cell adhesion molecule |
| 242086_at | 23.28 | 2.03E-10 | 1.383997367 | NA |  |
| 228391_at | 23.26 | 2.05E-10 | 2.023932818 | NM_207352| | CYP4V2,cytochrome P450, family 4, subfamily v, |
| 213803_at | 23.26 | 2.05E-10 | 1.141755993 | NA |  |
| 209435_s_at | 23.24 | 2.07E-10 | 1.120021638 | NM_004723| | ARHGEF2,rho/rac guanine nucleotide exchange factor 2 |
| 226324_s_at | 23.22 | 2.09E-10 | 1.228724865 | NM_015662| | SLB,selective LIM binding factor, rat homolog |
| 225725_at | 23.21 | 2.09E-10 | 1.44427833 | NA |  |
| 206718_at | 23.2 | 2.10E-10 | 1.389465352 | NM_002315| | LMO1,LIM domain only 1 |
| 227625_s_at | 23.2 | 2.10E-10 | 1.080942527 | NM_005861| | STUB1,STIP1 homology and U-box containing protein 1 |
| 228606_at | 23.19 | 2.11E-10 | 1.109409897 | NM_138461| | LOC116211,hypothetical protein BC013113 |
| 227699_at | 23.19 | 2.11E-10 | 1.512107311 | NM_144581| | C14orf149,chromosome 14 open reading frame 149 |
| 208072_s_at | 23.19 | 2.11E-10 | 1.109951155 | NM_003648| | DGKD,diacylglycerol kinase, delta 130kDa isoform 1 |
| 226043_at | 23.16 | 2.13E-10 | 1.1939101 | NM_015597| | GPSM1,G-protein signalling modulator 1 (AGS3-like, C. |
| 217764_s_at | 23.16 | 2.13E-10 | 1.240415882 | NM_006868| | RAB31,RAB31, member RAS oncogene family |
| 241710_at | 23.15 | 2.14E-10 | 1.468627593 | NM_001101330| | NA |
| 226810_at | 23.15 | 2.14E-10 | 1.641607039 | NM_024576| | OGFRL1,opioid growth factor receptor-like 1 |
| 225704_at | 23.12 | 2.17E-10 | 1.353793323 | NA |  |
| 241434_at | 23.12 | 2.17E-10 | 1.261360645 | NA |  |
| 219944_at | 23.08 | 2.21E-10 | 1.476315986 | NM_024692| | RSNL2,restin-like 2 |
| 224826_at | 23.08 | 2.21E-10 | 1.335797205 | NM_019593| | KIAA1434,hypothetical protein KIAA1434 |
| 208779_x_at | 23.07 | 2.21E-10 | 1.133420674 | NM_001954| | DDR1,discoidin receptor tyrosine kinase isoform b |
| 203275_at | 23.05 | 2.24E-10 | 1.122465106 | NM_002199| | IRF2,interferon regulatory factor 2 |
| 219634_at | 23.04 | 2.25E-10 | 1.194181054 | NM_018413| | CHST11,carbohydrate (chondroitin 4) sulfotransferase |
| 226425_at | 23.03 | 2.26E-10 | 1.978021834 | NM_024692| | RSNL2,restin-like 2 |
| 201105_at | 23.02 | 2.27E-10 | 1.270738017 | NM_002305| | LGALS1,beta-galactosidase binding lectin precursor |
| 204254_s_at | 23.02 | 2.27E-10 | 1.41992288 | NM_000376| | VDR,vitamin D (1,25- dihydroxyvitamin D3) receptor |
| 227354_at | 23.02 | 2.27E-10 | 2.075483854 | NM_018440| | PAG,phosphoprotein associated with |
| 209987_s_at | 23.01 | 2.27E-10 | 2.246904667 | NM_004316| | ASCL1,achaete-scute complex homolog-like 1 |
| 243432_at | 23 | 2.28E-10 | 1.27167737 | NA |  |
| 212534_at | 23 | 2.28E-10 | 1.093826799 | NA |  |
| 231166_at | 22.99 | 2.29E-10 | 2.018261057 | NM_001033045| | NA |
| 1557137_at | 22.98 | 2.30E-10 | 1.222307464 | NM_198276| | TMEM17,transmembrane protein 17 |
| 225669_at | 22.96 | 2.32E-10 | 1.42835368 | NM_000629| | IFNAR1,interferon-alpha receptor 1 precursor |
| 213134_x_at | 22.96 | 2.32E-10 | 1.134190634 | NM_006806| | BTG3,B-cell translocation gene 3 |
| 209000_s_at | 22.95 | 2.33E-10 | 1.262963955 | NM_001098811| | NA |
| 212419_at | 22.95 | 2.34E-10 | 1.292900141 | NM_153367| | C10orf56,chromosome 10 open reading frame 56 |
| 219416_at | 22.93 | 2.35E-10 | 1.212553688 | NM_016240| | SCARA3,scavenger receptor class A, member 3 isoform 1 |
| 212020_s_at | 22.91 | 2.37E-10 | 1.233696646 | NM_002417| | MKI67,antigen identified by monoclonal antibody Ki-67 |
| 1568629_s_at | 22.9 | 2.38E-10 | 1.147993197 | NM_005027| | PIK3R2,phosphoinositide-3-kinase, regulatory subunit 2 |
| 201136_at | 22.89 | 2.39E-10 | 1.350454906 | NM_002668| | PLP2,proteolipid protein 2 (colonic |
| 203235_at | 22.88 | 2.40E-10 | 1.111411773 | NM_003249| | THOP1,thimet oligopeptidase 1 |
| 212552_at | 22.88 | 2.40E-10 | 1.220611888 | NM_002149| | HPCAL1,hippocalcin-like 1 |
| 208804_s_at | 22.88 | 2.40E-10 | 1.127805483 | NM_006275| | SFRS6,arginine/serine-rich splicing factor 6 |
| 221567_at | 22.87 | 2.41E-10 | 1.219466997 | NM_003946| | NOL3,nucleolar protein 3 |
| 216933_x_at | 22.87 | 2.41E-10 | 1.475095924 | NM_000038| | APC,adenomatosis polyposis coli |
| 235954_at | 22.85 | 2.44E-10 | 1.213287865 | NA |  |
| 212385_at | 22.85 | 2.44E-10 | 1.246704769 | NM_001083962| | NA |
| 212708_at | 22.8 | 2.49E-10 | 1.09423958 | NM_001012241| | NA |
| 213891_s_at | 22.78 | 2.52E-10 | 1.13727258 | NM_001083962| | NA |
| 228953_at | 22.77 | 2.53E-10 | 1.330476864 | NM_001080435| | NA |
| 229354_at | 22.74 | 2.57E-10 | 1.662969277 | NM_013232| | PDCD6,programmed cell death 6 |
| 219825_at | 22.73 | 2.58E-10 | 1.473263674 | NM_019885| | CYP26B1,cytochrome P450, family 26, subfamily b, |
| 228545_at | 22.72 | 2.60E-10 | 1.205790073 | NM_021964| | ZNF148,zinc finger protein 148 (pHZ-52) |
| 228028_at | 22.71 | 2.60E-10 | 1.231064283 | NA |  |
| 222423_at | 22.68 | 2.64E-10 | 1.189431824 | NM_030571| | NDFIP1,Nedd4 family interacting protein 1 |
| 224716_at | 22.6 | 2.75E-10 | 1.179227076 | NM_178148| | SLC35B2,solute carrier family 35, member B2 |
| 209883_at | 22.6 | 2.75E-10 | 1.634613378 | NM_015101| | GLT25D2,glycosyltransferase 25 domain containing 2 |
| 1554724_at | 22.59 | 2.76E-10 | 2.245656553 | NM_014229| | SLC6A11,solute carrier family 6 (neurotransmitter |
| 227055_at | 22.57 | 2.79E-10 | 1.515413626 | NM_152637| | MGC17301,hypothetical protein MGC17301 |
| 228540_at | 22.56 | 2.80E-10 | 1.409804209 | NM_006775| | QKI,quaking homolog, KH domain RNA binding isoform |
| 226889_at | 22.56 | 2.81E-10 | 1.197161923 | NM_001006657| | WDR35,WD repeat domain 35 isoform 1 |
| 217737_x_at | 22.55 | 2.82E-10 | 1.063834221 | NM_016407| | C20orf43,chromosome 20 open reading frame 43 |
| 213157_s_at | 22.52 | 2.86E-10 | 1.211919733 | NM_015253| | KIAA0523,KIAA0523 protein |
| 1552439_s_at | 22.51 | 2.87E-10 | 1.836344117 | NM_032445| | MEGF11,MEGF11 protein |
| 225661_at | 22.51 | 2.87E-10 | 1.48887697 | NM_000629| | IFNAR1,interferon-alpha receptor 1 precursor |
| 202762_at | 22.5 | 2.88E-10 | 1.184178297 | NM_004850| | ROCK2,Rho-associated, coiled-coil containing protein |
| 222437_s_at | 22.49 | 2.90E-10 | 1.265263964 | NM_001005753| | VPS24,vacuolar protein sorting 24 isoform 2 |
| 212458_at | 22.47 | 2.92E-10 | 1.188835506 | NM_181784| | SPRED2,sprouty-related protein with EVH-1 domain 2 |
| 224993_at | 22.47 | 2.92E-10 | 1.126678636 | NM_005934| | MLLT1,myeloid/lymphoid or mixed-lineage leukemia |
| 233257_at | 22.46 | 2.93E-10 | 1.880120868 | NA |  |
| 219663_s_at | 22.42 | 3.00E-10 | 1.418249297 | NM_025268| | MGC4659,hole protein |
| 204139_x_at | 22.42 | 3.00E-10 | 1.108751277 | NM_003422| | ZNF42,zinc finger protein 42 isoform 1 |
| 226745_at | 22.4 | 3.03E-10 | 1.663007326 | NM_207352| | CYP4V2,cytochrome P450, family 4, subfamily v, |
| 227617_at | 22.36 | 3.09E-10 | 1.386993024 | NM_001010866| | RP13-15M17.2,novel protein |
| 205885_s_at | 22.36 | 3.09E-10 | 1.517103322 | NM_000885| | ITGA4,integrin alpha 4 precursor |
| 218199_s_at | 22.34 | 3.12E-10 | 1.09785356 | NM_022917| | NOL6,nucleolar RNA-associated protein alpha isoform |
| 204377_s_at | 22.33 | 3.13E-10 | 1.19514598 | NM_014703| | VprBP,Vpr-binding protein |
| 31807_at | 22.32 | 3.14E-10 | 1.096520776 | NM_019070| | DDX49,DEAD (Asp-Glu-Ala-Asp) box polypeptide 49 |
| 227229_at | 22.32 | 3.15E-10 | 1.057357305 | NA |  |
| 212629_s_at | 22.32 | 3.14E-10 | 1.068985896 | NM_006256| | PKN2,protein kinase N2 |
| 211596_s_at | 22.31 | 3.16E-10 | 1.049713628 | NM_015541| | LRIG1,leucine-rich repeats and immunoglobulin-like |
| 204808_s_at | 22.3 | 3.17E-10 | 1.161685557 | NM_014254| | TMEM5,transmembrane protein 5 |
| 208890_s_at | 22.3 | 3.16E-10 | 1.136181436 | NM_012401| | NA |
| 244509_at | 22.3 | 3.16E-10 | 1.408345136 | NM_001033045| | NA |
| 203384_s_at | 22.29 | 3.19E-10 | 1.368350229 | NM_002077| | GOLGA1,golgin 97 |
| 214558_at | 22.28 | 3.20E-10 | 1.80250552 | NM_005288| | GPR12,G protein-coupled receptor 12 |
| 212456_at | 22.27 | 3.20E-10 | 1.152104886 | NM_015229| | KIAA0664,KIAA0664 protein |
| 244411_at | 22.26 | 3.21E-10 | 1.269057901 | NA |  |
| 209506_s_at | 22.24 | 3.25E-10 | 1.453121652 | NM_005654| | NR2F1,nuclear receptor subfamily 2, group F, member 1 |
| 217911_s_at | 22.24 | 3.25E-10 | 1.178262956 | NM_004281| | BAG3,BCL2-associated athanogene 3 |
| 235471_at | 22.22 | 3.28E-10 | 1.361259202 | NM_001031746| | NA |
| 218065_s_at | 22.22 | 3.28E-10 | 1.138560514 | NM_020644| | C11orf15,chromosome 11 open reading frame 15 |
| 241355_at | 22.21 | 3.30E-10 | 1.462172642 | NM_005144| | HR,hairless protein isoform a |
| 223099_s_at | 22.2 | 3.31E-10 | 1.132618952 | NM_031490| | LONP,peroxisomal lon protease |
| 229590_at | 22.19 | 3.32E-10 | 1.107774618 | NM_000977| | RPL13,ribosomal protein L13 |
| 226484_at | 22.18 | 3.34E-10 | 1.398552887 | NM_145166| | ZNF651,zinc finger protein 651 |
| 218028_at | 22.17 | 3.36E-10 | 1.144505841 | NM_022821| | ELOVL1,elongation of very long chain fatty acids |
| 218653_at | 22.15 | 3.39E-10 | 1.125002461 | NM_014252| | SLC25A15,solute carrier family 25 (mitochondrial carrier; |
| 236024_at | 22.15 | 3.39E-10 | 1.661919702 | NM_005277| | GPM6A,glycoprotein M6A isoform 1 |
| 204908_s_at | 22.13 | 3.42E-10 | 1.303376848 | NM_005178| | BCL3,B-cell CLL/lymphoma 3 |
| 225729_at | 22.13 | 3.42E-10 | 1.235384001 | NM_152734| | C6orf89,hypothetical protein FLJ25357 |
| 226412_at | 22.11 | 3.47E-10 | 1.194802242 | NM_015491| | NA |
| 241198_s_at | 22.1 | 3.48E-10 | 1.397133856 | NM_032930| | MGC13040,hypothetical protein MGC13040 |
| 223500_at | 22.1 | 3.48E-10 | 1.609926761 | NM_006651| | CPLX1,complexin 1 |
| 236208_at | 22.1 | 3.47E-10 | 1.651611761 | NM_004531| | MOCS2,molybdopterin synthase large subunit MOCS2B |
| 221965_at | 22.08 | 3.52E-10 | 1.175625885 | NM_022782| | MPHOSPH9,M-phase phosphoprotein 9 |
| 225553_at | 22.08 | 3.50E-10 | 1.173592859 | NA |  |
| 1554807_a_at | 22.07 | 3.53E-10 | 1.344964192 | NM_020148| | SPIRE1,spire homolog 1 |
| 215091_s_at | 22.07 | 3.52E-10 | 1.12518184 | NM_002097| | GTF3A,general transcription factor IIIA |
| 218714_at | 22.06 | 3.53E-10 | 1.227950572 | NM_024031| | MGC3121,hypothetical protein MGC3121 |
| 203188_at | 22.06 | 3.53E-10 | 1.133057928 | NM_006876| | B3GNT6,beta-1,3-N-acetylglucosaminyltransferase bGnT-6 |
| 201584_s_at | 22.05 | 3.55E-10 | 1.053435814 | NM_005804| | DDX39,DEAD (Asp-Glu-Ala-Asp) box polypeptide 39 |
| 237400_at | 22.05 | 3.56E-10 | 1.46695831 | NM_001003803| | ATP5S,ATP synthase, H+ transporting, mitochondrial F0 |
| 201825_s_at | 22.04 | 3.57E-10 | 1.1324862 | NM_016002| | CGI-49,CGI-49 protein |
| 212277_at | 22.04 | 3.56E-10 | 1.144942245 | NM_004687| | MTMR4,myotubularin related protein 4 |
| 239053_at | 22.03 | 3.58E-10 | 1.095603417 | NM_004804| | WDR39,WD repeat domain 39 |
| 219569_s_at | 22.02 | 3.59E-10 | 1.277881324 | NM_001097599| | NA |
| 225421_at | 22.02 | 3.59E-10 | 1.301050505 | NM_001010853| | ACY1L2,aminoacylase 1-like 2 |
| 207163_s_at | 22.02 | 3.59E-10 | 1.19370048 | NM_001014431| | NA |
| 222476_at | 21.99 | 3.65E-10 | 1.092126169 | NM_015455| | CNOT6,CCR4-NOT transcription complex, subunit 6 |
| 227193_at | 21.99 | 3.64E-10 | 1.438747729 | NA |  |
| 225440_at | 21.98 | 3.67E-10 | 1.317480896 | NM_001037553| | NA |
| 222463_s_at | 21.97 | 3.69E-10 | 1.156173083 | NM_012104| | BACE1,beta-site APP-cleaving enzyme 1 isoform A |
| 221739_at | 21.95 | 3.71E-10 | 1.1189732 | NM_019107| | C19orf10,chromosome 19 open reading frame 10 |
| 208979_at | 21.92 | 3.77E-10 | 1.1838553 | NM_014071| | NCOA6,nuclear receptor coactivator 6 |
| 204724_s_at | 21.91 | 3.80E-10 | 1.118464537 | NM_001853| | COL9A3,alpha 3 type IX collagen |
| 225612_s_at | 21.91 | 3.80E-10 | 1.210487732 | NM_032047| | B3GNT5,beta-1,3-N-acetylglucosaminyltransferase bGnT-5 |
| 228273_at | 21.9 | 3.81E-10 | 1.126009227 | NA |  |
| 227272_at | 21.9 | 3.81E-10 | 1.568931296 | NM_207380| | FLJ43339,FLJ43339 protein |
| 228486_at | 21.89 | 3.82E-10 | 1.511655084 | NM_080546| | CDW92,CDW92 antigen |
| 221039_s_at | 21.88 | 3.85E-10 | 1.191922651 | NM_018482| | DDEF1,development and differentiation enhancing factor |
| 218071_s_at | 21.88 | 3.85E-10 | 1.158432867 | NM_014160| | MKRN2,makorin, ring finger protein, 2 |
| 209846_s_at | 21.85 | 3.89E-10 | 1.259000179 | NM_007047| | BTN3A2,butyrophilin, subfamily 3, member A2 |
| 204376_at | 21.84 | 3.93E-10 | 1.4113072 | NM_014703| | VprBP,Vpr-binding protein |
| 204311_at | 21.83 | 3.95E-10 | 1.261290426 | NM_001678| | ATP1B2,Na+/K+ -ATPase beta 2 subunit |
| 226156_at | 21.82 | 3.96E-10 | 1.278146797 | NM_001626| | AKT2,v-akt murine thymoma viral oncogene homolog 2 |
| 217771_at | 21.82 | 3.96E-10 | 1.198331984 | NM_016548| | GOLPH2,golgi phosphoprotein 2 |
| 205471_s_at | 21.82 | 3.96E-10 | 1.958477111 | NM_004392| | DACH1,dachshund homolog 1 isoform c |
| 237315_at | 21.82 | 3.96E-10 | 1.377671583 | NA |  |
| 202636_at | 21.81 | 3.97E-10 | 1.165023543 | NM_005667| | RNF103,ring finger protein 103 |
| 241774_at | 21.8 | 3.98E-10 | 1.627652602 | NA |  |
| 204519_s_at | 21.79 | 4.01E-10 | 1.307157236 | NM_015993| | TM4SF11,plasmolipin |
| 1556606_at | 21.77 | 4.05E-10 | 1.814756481 | NM_001111018| | NA |
| 208795_s_at | 21.77 | 4.05E-10 | 1.062061209 | NM_005916| | MCM7,minichromosome maintenance protein 7 isoform 1 |
| 217485_x_at | 21.76 | 4.06E-10 | 1.108525259 | NA |  |
| 214743_at | 21.76 | 4.05E-10 | 1.07390047 | NM_001913| | CUTL1,CCAAT displacement protein isoform b |
| 223056_s_at | 21.71 | 4.17E-10 | 1.078383763 | NM_020750| | XPO5,exportin 5 |
| 217837_s_at | 21.7 | 4.18E-10 | 1.215644181 | NM_001005753| | VPS24,vacuolar protein sorting 24 isoform 2 |
| 225002_s_at | 21.69 | 4.20E-10 | 1.097543698 | NM_001042468| | NA |
| 204824_at | 21.68 | 4.22E-10 | 1.16326386 | NM_004435| | ENDOG,endonuclease G precursor |
| 225530_at | 21.68 | 4.23E-10 | 1.276327138 | NM_130807| | MOBKL2A,MOB-LAK |
| 225990_at | 21.66 | 4.25E-10 | 1.378743585 | NM_033254| | BOC,brother of CDO |
| 213701_at | 21.66 | 4.25E-10 | 1.188547681 | NM_001009894| | DKFZp434N2030,hypothetical protein DKFZp434N2030 |
| 213658_at | 21.62 | 4.34E-10 | 1.422251169 | NA |  |
| 223158_s_at | 21.62 | 4.35E-10 | 1.251611479 | NM_014397| | NEK6,putative serine-threonine protein kinase |
| 1552287_s_at | 21.6 | 4.39E-10 | 1.22678227 | NA |  |
| 219537_x_at | 21.6 | 4.38E-10 | 1.20168464 | NM_016941| | DLL3,delta-like 3 protein isoform 1 precursor |
| 221214_s_at | 21.59 | 4.40E-10 | 1.180740336 | NM_015537| | NELF,nasal embryonic LHRH factor |
| 202095_s_at | 21.58 | 4.42E-10 | 1.052437023 | NM_001012270| | BIRC5,baculoviral IAP repeat-containing protein 5 |
| 212546_s_at | 21.57 | 4.44E-10 | 1.173682389 | NM_015030| | NA |
| 203292_s_at | 21.55 | 4.50E-10 | 1.086898099 | NM_021729| | VPS11,vacuolar protein sorting 11 (yeast homolog) |
| 239632_at | 21.54 | 4.52E-10 | 1.500983688 | NA |  |
| 238459_x_at | 21.54 | 4.51E-10 | 1.627390698 | NM_019073| | SPATA6,spermatogenesis associated 6 |
| 205972_at | 21.53 | 4.53E-10 | 1.242768035 | NM_006841| | SLC38A3,solute carrier family 38, member 3 |
| 224977_at | 21.53 | 4.53E-10 | 1.286421505 | NM_152734| | C6orf89,hypothetical protein FLJ25357 |
| 212558_at | 21.52 | 4.56E-10 | 1.241252805 | NM_005841| | SPRY1,sprouty homolog 1, antagonist of FGF signaling |
| 207672_at | 21.52 | 4.56E-10 | 1.965494212 | NA |  |
| 202701_at | 21.51 | 4.56E-10 | 1.196550246 | NM_001199| | BMP1,bone morphogenetic protein 1 isoform 1, |
| 222435_s_at | 21.51 | 4.56E-10 | 1.310205061 | NM_016021| | UBE2J1,ubiquitin-conjugating enzyme E2, J1 |
| 227153_at | 21.5 | 4.60E-10 | 1.132924917 | NM_032549| | IMMP2L,IMP2 inner mitochondrial membrane protease-like |
| 241470_x_at | 21.5 | 4.59E-10 | 1.759320331 | NA |  |
| 208817_at | 21.49 | 4.62E-10 | 1.233335881 | NM_000754| | COMT,catechol-O-methyltransferase isoform MB-COMT |
| 213224_s_at | 21.49 | 4.61E-10 | 1.176086557 | NA |  |
| 213708_s_at | 21.49 | 4.62E-10 | 1.067359707 | NM_170607| | MLX,transcription factor-like protein 4 isoform |
| 220807_at | 21.49 | 4.62E-10 | 1.231067329 | NM_005331| | HBQ1,theta 1 globin |
| 201103_x_at | 21.48 | 4.63E-10 | 1.160993403 | NM_001037501| | NA |
| 236640_at | 21.47 | 4.66E-10 | 1.567342289 | NA |  |
| 219549_s_at | 21.45 | 4.71E-10 | 1.063656986 | NM_006054| | RTN3,reticulon 3 isoform a |
| 236442_at | 21.42 | 4.77E-10 | 1.898402553 | NM_012074| | DPF3,cer-d4 (mouse) homolog |
| 202265_at | 21.42 | 4.77E-10 | 1.25369912 | NM_005180| | PCGF4,polycomb group ring finger 4 |
| 212169_at | 21.41 | 4.78E-10 | 1.345989247 | NM_007270| | FKBP9,FK506 binding protein 9 |
| 212899_at | 21.41 | 4.78E-10 | 1.129567637 | NM_015076| | CDC2L6,cyclin-dependent kinase (CDC2-like) 11 |
| 1554317_s_at | 21.38 | 4.87E-10 | 1.164356245 | NM_152307| | C14orf172,chromosome 14 open reading frame 172 |
| 213657_s_at | 21.37 | 4.90E-10 | 1.320720424 | NA |  |
| 203185_at | 21.36 | 4.92E-10 | 1.500692142 | NM_014737| | RASSF2,Ras association domain family 2 isoform 1 |
| 227543_at | 21.36 | 4.92E-10 | 1.33802788 | NM_032193| | AYP1,AYP1 protein |
| 229549_at | 21.36 | 4.92E-10 | 1.190246051 | NA |  |
| 238012_at | 21.33 | 5.00E-10 | 1.366984584 | NM_013379| | DPP7,dipeptidyl peptidase 7 preproprotein |
| 232990_at | 21.32 | 5.03E-10 | 1.468906005 | NM_138362| | CXorf44,chromosome X open reading frame 44 |
| 223398_at | 21.28 | 5.14E-10 | 1.172100435 | NM_032310| | C9orf89,chromosome 9 open reading frame 89 |
| 239283_at | 21.26 | 5.17E-10 | 1.380146798 | NM_016040| | TMED5,transmembrane emp24 protein transport domain |
| 239654_at | 21.26 | 5.19E-10 | 1.370383777 | NM_025134| | CHD9,chromodomain helicase DNA binding protein 9 |
| 225458_at | 21.25 | 5.20E-10 | 1.19993311 | NA |  |
| 227497_at | 21.25 | 5.20E-10 | 1.603073348 | NA |  |
| 218024_at | 21.24 | 5.23E-10 | 1.303977653 | NM_016098| | BRP44L,brain protein 44-like |
| 223593_at | 21.23 | 5.24E-10 | 1.199416749 | NM_016228| | AADAT,alpha-aminoadipate aminotransferase |
| 226197_at | 21.23 | 5.24E-10 | 1.180819354 | NA |  |
| 213155_at | 21.23 | 5.26E-10 | 1.19115586 | NM_015253| | KIAA0523,KIAA0523 protein |
| 207959_s_at | 21.22 | 5.26E-10 | 1.718634353 | NM_001372| | DNAH9,dynein, axonemal, heavy polypeptide 9 isoform 2 |
| 202010_s_at | 21.21 | 5.31E-10 | 1.117555137 | NM_021188| | ZNF410,clones 23667 and 23775 zinc finger protein |
| 223020_at | 21.2 | 5.33E-10 | 1.10616594 | NM_030782| | CRR9,cisplatin resistance related protein CRR9p |
| 223025_s_at | 21.19 | 5.33E-10 | 1.174225598 | NM_032493| | AP1M1,adaptor-related protein complex 1, mu 1 subunit |
| 239297_at | 21.19 | 5.36E-10 | 1.262346411 | NM_001099677| | NA |
| 214964_at | 21.17 | 5.42E-10 | 1.340987476 | NA |  |
| 212733_at | 21.16 | 5.42E-10 | 1.16930741 | NM_014687| | NA |
| 202084_s_at | 21.16 | 5.43E-10 | 1.119587757 | NM_001039573| | NA |
| 204639_at | 21.16 | 5.43E-10 | 1.093071433 | NM_000022| | ADA,adenosine deaminase |
| 212172_at | 21.16 | 5.43E-10 | 1.282391461 | NM_001625| | AK2,adenylate kinase 2 isoform a |
| 225897_at | 21.16 | 5.43E-10 | 1.18206298 | NM_002356| | MARCKS,myristoylated alanine-rich protein kinase C |
| 224160_s_at | 21.15 | 5.44E-10 | 1.127732832 | NM_014049| | ACAD9,acyl-Coenzyme A dehydrogenase family, member 9 |
| 211852_s_at | 21.13 | 5.52E-10 | 1.198893965 | NM_139321| | ATRN,attractin isoform 1 |
| 219477_s_at | 21.12 | 5.55E-10 | 1.440868688 | NM_018676| | THSD1,thrombospondin type I domain-containing 1 |
| 220441_at | 21.11 | 5.55E-10 | 1.456487531 | NM_024902| | FLJ13236,hypothetical protein FLJ13236 |
| 231716_at | 21.1 | 5.58E-10 | 1.090970346 | NM_001100588| | NA |
| 202070_s_at | 21.1 | 5.58E-10 | 1.196187153 | NM_005530| | IDH3A,isocitrate dehydrogenase 3 (NAD+) alpha |
| 201795_at | 21.1 | 5.58E-10 | 1.214423601 | NM_002296| | LBR,lamin B receptor |
| 213448_at | 21.09 | 5.61E-10 | 1.159109078 | NA |  |
| 222462_s_at | 21.09 | 5.60E-10 | 1.155063044 | NM_012104| | BACE1,beta-site APP-cleaving enzyme 1 isoform A |
| 243664_at | 21.08 | 5.65E-10 | 1.304686509 | NM_004786| | TXNL1,thioredoxin-like 1 |
| 230150_at | 21.07 | 5.68E-10 | 1.472948404 | NM_001008405| | BCAP29,B-cell receptor-associated protein BAP29 isoform |
| 203411_s_at | 21.07 | 5.67E-10 | 1.261677399 | NM_005572| | LMNA,lamin A/C isoform 2 |
| 216870_x_at | 21.05 | 5.71E-10 | 1.413346317 | NA |  |
| 203719_at | 21.02 | 5.82E-10 | 1.080826912 | NM_001983| | ERCC1,excision repair cross-complementing 1 isofrom 2 |
| 203132_at | 21.01 | 5.82E-10 | 1.256596205 | NM_000321| | RB1,retinoblastoma 1 |
| 217226_s_at | 21 | 5.85E-10 | 1.753987811 | NM_030971| | SFXN3,sideroflexin 3 |
| 208972_s_at | 21 | 5.86E-10 | 1.116784648 | NM_001002027| | ATP5G1,ATP synthase, H+ transporting, mitochondrial F0 |
| 225652_at | 21 | 5.85E-10 | 1.141243089 | NA |  |
| 240128_at | 21 | 5.87E-10 | 1.307373873 | NA |  |
| 203150_at | 20.98 | 5.92E-10 | 1.114159229 | NM_005833| | RAB9P40,Rab9 effector p40 |
| 215489_x_at | 20.97 | 5.95E-10 | 1.221479266 | NM_004838| | HOMER3,Homer, neuronal immediate early gene, 3 |
| 226712_at | 20.96 | 5.96E-10 | 1.169017103 | NM_003144| | SSR1,signal sequence receptor, alpha |
| 201153_s_at | 20.96 | 5.96E-10 | 1.451246069 | NM_021038| | MBNL1,muscleblind-like 1 isoform a |
| 201580_s_at | 20.95 | 6.01E-10 | 1.204482051 | NM_021156| | DJ971N18.2,hypothetical protein DJ971N18.2 |
| 244659_at | 20.95 | 5.99E-10 | 1.326681854 | NA |  |
| 218678_at | 20.92 | 6.09E-10 | 1.107609842 | NM_006617| | NES,nestin |
| 203885_at | 20.91 | 6.13E-10 | 1.075350078 | NM_014999| | RAB21,RAB21, member RAS oncogene family |
| 226021_at | 20.91 | 6.14E-10 | 1.377199914 | NM_172037| | RDH10,retinol dehydrogenase 10 |
| 226192_at | 20.89 | 6.20E-10 | 1.219396591 | NA |  |
| 201338_x_at | 20.89 | 6.20E-10 | 1.123227458 | NM_002097| | GTF3A,general transcription factor IIIA |
| 217124_at | 20.88 | 6.23E-10 | 1.37697708 | NM_001100390| | NA |
| 200972_at | 20.88 | 6.23E-10 | 1.117351667 | NM_005724| | TM4SF8,transmembrane 4 superfamily member 8 isoform 1 |
| 226946_at | 20.87 | 6.24E-10 | 1.252136393 | NM_001085411| | NA |
| 226968_at | 20.86 | 6.28E-10 | 1.077163099 | NM_015074| | KIF1B,kinesin family member 1B isoform b |
| 238587_at | 20.85 | 6.33E-10 | 1.574347132 | NM_032873| | STS-1,Cbl-interacting protein Sts-1 |
| 228049_x_at | 20.83 | 6.38E-10 | 1.22125682 | NA |  |
| 1555326_a_at | 20.83 | 6.38E-10 | 1.742717573 | NM_001005845| | ADAM9,a disintegrin and metalloproteinase domain 9 |
| 202069_s_at | 20.82 | 6.43E-10 | 1.10831228 | NM_005530| | IDH3A,isocitrate dehydrogenase 3 (NAD+) alpha |
| 211110_s_at | 20.81 | 6.44E-10 | 1.242544628 | NM_000044| | AR,androgen receptor isoform 1 |
| 213405_at | 20.81 | 6.44E-10 | 1.214426121 | NM_020673| | RAB22A,RAS-related protein RAB-22A |
| 226781_at | 20.81 | 6.44E-10 | 1.221453562 | NM_197964| | HSPC268,hypothetical protein HSPC268 |
| 227250_at | 20.8 | 6.49E-10 | 1.388897223 | NM_001039570| | NA |
| 211466_at | 20.79 | 6.53E-10 | 1.518173661 | NM_005596| | NFIB,nuclear factor I/B |
| 217855_x_at | 20.79 | 6.51E-10 | 1.093280654 | NM_016176| | Cab45,calcium binding protein Cab45 precursor |
| 208663_s_at | 20.79 | 6.50E-10 | 1.252422577 | NM_001001894| | TTC3,tetratricopeptide repeat domain 3 |
| 230002_at | 20.78 | 6.55E-10 | 1.399839517 | NM_001048210| | NA |
| 201751_at | 20.78 | 6.55E-10 | 1.113821956 | NM_014876| | KIAA0063,KIAA0063 gene product |
| 235428_at | 20.77 | 6.56E-10 | 1.474821147 | NA |  |
| 210434_x_at | 20.77 | 6.57E-10 | 1.072954394 | NM_006694| | JTB,jumping translocation breakpoint |
| 206235_at | 20.76 | 6.60E-10 | 1.316478693 | NM_001098268| | NA |
| 225619_at | 20.76 | 6.59E-10 | 1.283250487 | NM_001040153| | NA |
| 204591_at | 20.74 | 6.64E-10 | 2.931435229 | NM_006614| | CHL1,cell adhesion molecule with homology to L1CAM |
| 228752_at | 20.74 | 6.66E-10 | 1.531183005 | NA |  |
| 210239_at | 20.73 | 6.68E-10 | 1.464027527 | NM_005853| | IRX5,iroquois homeobox protein 5 |
| 226760_at | 20.72 | 6.71E-10 | 1.151082736 | NA |  |
| 207616_s_at | 20.68 | 6.86E-10 | 1.100200345 | NM_004180| | TANK,TRAF interacting protein TANK isoform a |
| 242476_at | 20.67 | 6.90E-10 | 2.012159231 | NA |  |
| 224966_s_at | 20.66 | 6.92E-10 | 1.206163912 | NM_020175| | LOC56931,hypothetical protein from EUROIMAGE 1967720 |
| 205548_s_at | 20.66 | 6.94E-10 | 1.134188296 | NM_006806| | BTG3,B-cell translocation gene 3 |
| 1558189_a_at | 20.65 | 6.97E-10 | 1.513608216 | NA |  |
| 201908_at | 20.64 | 7.01E-10 | 1.109211072 | NM_004423| | DVL3,dishevelled 3 |
| 208921_s_at | 20.63 | 7.04E-10 | 1.100354465 | NM_003130| | SRI,sorcin isoform a |
| 213897_s_at | 20.62 | 7.09E-10 | 1.081410167 | NM_021134| | MRPL23,mitochondrial ribosomal protein L23 |
| 209991_x_at | 20.61 | 7.12E-10 | 1.56685627 | NM_005458| | GPR51,G protein-coupled receptor 51 |
| 201896_s_at | 20.61 | 7.12E-10 | 1.131881412 | NM_001005290| | DDA3,p53-regulated DDA3 isoform b |
| 49329_at | 20.6 | 7.18E-10 | 1.138736718 | NM_032775| | KELCHL,kelch-like |
| 221909_at | 20.59 | 7.20E-10 | 1.088633691 | NM_001109903| | NA |
| 203323_at | 20.58 | 7.25E-10 | 1.724933686 | NM_001233| | CAV2,caveolin 2 isoform a and b |
| 232032_x_at | 20.56 | 7.32E-10 | 1.096161592 | NM_016176| | Cab45,calcium binding protein Cab45 precursor |
| 217762_s_at | 20.56 | 7.32E-10 | 1.256878294 | NM_006868| | RAB31,RAB31, member RAS oncogene family |
| 235036_at | 20.55 | 7.37E-10 | 1.582132588 | NM_153713| | LIX1L,Lix1 homolog (mouse) like |
| 226508_at | 20.54 | 7.37E-10 | 1.076112213 | NM_024947| | PHC3,polyhomeotic like 3 |
| 227737_at | 20.5 | 7.55E-10 | 1.18410936 | NM_021203| | SRPRB,signal recognition particle receptor, beta |
| 235260_s_at | 20.49 | 7.59E-10 | 1.210378495 | NM_145048| | MGC29898,hypothetical protein MGC29898 |
| 213369_at | 20.48 | 7.65E-10 | 1.200932887 | NM_033100| | PCDH21,protocadherin 21 precursor |
| 231650_s_at | 20.48 | 7.65E-10 | 1.508469275 | NM_021115| | SEZ6L,seizure related 6 homolog (mouse)-like |
| 238810_at | 20.46 | 7.70E-10 | 1.480527213 | NM_002919| | RFX3,regulatory factor X3 isoform a |
| 218812_s_at | 20.45 | 7.75E-10 | 1.302270256 | NM_001126340| | NA |
| 224900_at | 20.45 | 7.75E-10 | 1.061478747 | NM_016376| | ANKFY1,ankyrin repeat and FYVE domain containing 1 |
| 202461_at | 20.43 | 7.82E-10 | 1.057581885 | NM_014239| | EIF2B2,eukaryotic translation initiation factor 2B, |
| 242307_at | 20.43 | 7.85E-10 | 1.155625318 | NM_001013258| | NA |
| 226113_at | 20.43 | 7.84E-10 | 1.916433599 | NM_001077195| | NA |
| 202125_s_at | 20.42 | 7.86E-10 | 1.192949995 | NM_015049| | ALS2CR3,amyotrophic lateral sclerosis 2 (juvenile) |
| 227119_at | 20.42 | 7.87E-10 | 1.310496806 | NM_144571| | CNOT6L,CCR4-NOT transcription complex, subunit 6-like |
| 231793_s_at | 20.41 | 7.91E-10 | 1.62514868 | NM_001221| | CAMK2D,calcium/calmodulin-dependent protein kinase II |
| 230477_at | 20.41 | 7.91E-10 | 1.209859887 | NA |  |
| 218723_s_at | 20.41 | 7.90E-10 | 1.668534074 | NM_014059| | RGC32,response gene to complement 32 |
| 243481_at | 20.4 | 7.98E-10 | 2.031456981 | NM_020663| | RHOJ,TC10-like Rho GTPase |
| 213489_at | 20.39 | 8.01E-10 | 1.100412025 | NM_014268| | MAPRE2,microtubule-associated protein, RP/EB family, |
| 204795_at | 20.37 | 8.09E-10 | 1.233720527 | NM_001077497| | NA |
| 91920_at | 20.36 | 8.15E-10 | 1.228881424 | NM_021948| | BCAN,brevican isoform 1 |
| 224973_at | 20.36 | 8.14E-10 | 1.666508278 | NM_017633| | FAM46A,family with sequence similarity 46, member A |
| 202443_x_at | 20.36 | 8.15E-10 | 1.110724496 | NM_024408| | NOTCH2,notch 2 preproprotein |
| 219468_s_at | 20.35 | 8.16E-10 | 1.362372726 | NM_017949| | CUEDC1,CUE domain-containing 1 |
| 234140_s_at | 20.35 | 8.15E-10 | 1.265861691 | NM_020860| | STIM2,stromal interaction molecule 2 |
| 204640_s_at | 20.34 | 8.20E-10 | 1.15092672 | NM_001007226| | SPOP,speckle-type POZ protein |
| 203333_at | 20.31 | 8.34E-10 | 1.167441131 | NM_014970| | KIFAP3,kinesin-associated protein 3 |
| 235051_at | 20.31 | 8.33E-10 | 1.287783637 | NM_174908| | C3orf6,Ymer protein short isoform |
| 212321_at | 20.3 | 8.38E-10 | 1.057305636 | NM_003901| | SGPL1,sphingosine-1-phosphate lyase 1 |
| 222146_s_at | 20.27 | 8.53E-10 | 1.19535411 | NM_001083962| | NA |
| 202381_at | 20.26 | 8.53E-10 | 1.320427637 | NM_001005845| | ADAM9,a disintegrin and metalloproteinase domain 9 |
| 206929_s_at | 20.23 | 8.67E-10 | 1.489877261 | NM_005597| | NFIC,nuclear factor I/C isoform 1 |
| 204642_at | 20.22 | 8.73E-10 | 1.415881977 | NM_001400| | EDG1,endothelial differentiation, sphingolipid |
| 235935_at | 20.22 | 8.74E-10 | 1.215040361 | NM_001012974| | NA |
| 225259_at | 20.21 | 8.76E-10 | 1.112242618 | NM_016577| | RAB6B,RAB6B, member RAS oncogene family |
| 239741_at | 20.2 | 8.81E-10 | 1.824525128 | NA |  |
| 223041_at | 20.19 | 8.87E-10 | 1.287925045 | NM_031462| | CD99L2,CD99 antigen-like 2 |
| 208828_at | 20.19 | 8.87E-10 | 1.134733657 | NM_017443| | POLE3,DNA polymerase epsilon subunit 3 |
| 224688_at | 20.19 | 8.88E-10 | 1.130034028 | NM_017994| | FLJ10099,hypothetical protein FLJ10099 |
| 231898_x_at | 20.18 | 8.88E-10 | 2.159320771 | NA |  |
| 221495_s_at | 20.18 | 8.90E-10 | 1.178597402 | NM_014972| | KIAA1049,KIAA1049 protein |
| 227816_at | 20.18 | 8.88E-10 | 1.421578269 | NM_004822| | NTN1,netrin 1 |
| 210270_at | 20.18 | 8.91E-10 | 1.318297763 | NM_004296| | RGS6,regulator of G-protein signalling 6 |
| 221335_x_at | 20.18 | 8.88E-10 | 1.172483423 | NM_019108| | FLJ12886,hypothetical protein FLJ12886 |
| 212237_at | 20.18 | 8.88E-10 | 1.233460911 | NM_015338| | ASXL1,additional sex combs like 1 |
| 225018_at | 20.17 | 8.92E-10 | 1.253301429 | NM_020148| | SPIRE1,spire homolog 1 |
| 221834_at | 20.16 | 9.00E-10 | 1.554625156 | NM_031490| | LONP,peroxisomal lon protease |
| 224952_at | 20.16 | 8.97E-10 | 1.319055048 | NM_025185| | NA |
| 218017_s_at | 20.15 | 9.01E-10 | 1.22550169 | NM_152419| | NA |
| 226501_at | 20.15 | 9.04E-10 | 1.142658816 | NM_022098| | LOC63929,hypothetical protein LOC63929 |
| 235676_at | 20.14 | 9.06E-10 | 1.582527383 | NA |  |
| 209030_s_at | 20.12 | 9.19E-10 | 1.240793648 | NM_001098517| | NA |
| 205970_at | 20.1 | 9.28E-10 | 1.31388949 | NM_005954| | MT3,metallothionein 3 |
| 1554600_s_at | 20.1 | 9.28E-10 | 1.345494144 | NM_005572| | LMNA,lamin A/C isoform 2 |
| 221069_s_at | 20.1 | 9.26E-10 | 1.07798553 | NM_016360| | LOC51204,clone HQ0477 PRO0477p |
| 223230_at | 20.09 | 9.32E-10 | 1.143944732 | NM_032864| | FLJ14936,hypothetical protein FLJ14936 |
| 227212_s_at | 20.09 | 9.31E-10 | 1.211847119 | NM_001009936| | PHF19,PHD finger protein 19 isoform b |
| 209050_s_at | 20.08 | 9.34E-10 | 1.20450506 | NM_001042368| | NA |
| 235494_at | 20.07 | 9.39E-10 | 1.884904867 | NA |  |
| 204084_s_at | 20.03 | 9.62E-10 | 1.286839674 | NM_006493| | CLN5,ceroid-lipofuscinosis, neuronal 5 |
| 226742_at | 20.02 | 9.68E-10 | 1.215876792 | NA |  |
| 218529_at | 20.01 | 9.69E-10 | 1.169552874 | NM_016579| | CD320,8D6 antigen |
| 205304_s_at | 20 | 9.74E-10 | 1.622274509 | NM_004982| | KCNJ8,potassium inwardly-rectifying channel J8 |
| 201876_at | 20 | 9.77E-10 | 1.196732179 | NM_000305| | PON2,paraoxonase 2 |
| 209618_at | 20 | 9.77E-10 | 1.358099565 | NM_001332| | CTNND2,catenin (cadherin-associated protein), delta 2 |
| 1558111_at | 19.97 | 9.89E-10 | 1.6268003 | NM_021038| | MBNL1,muscleblind-like 1 isoform a |
| 205379_at | 19.96 | 9.96E-10 | 1.252935833 | NM_001236| | CBR3,carbonyl reductase 3 |
| 238613_at | 19.96 | 9.96E-10 | 1.428517694 | NM_016653| | ZAK,sterile-alpha motif and leucine zipper |
| 202661_at | 19.96 | 9.96E-10 | 1.769896726 | NM_002223| | ITPR2,inositol 1,4,5-triphosphate receptor, type 2 |
| 212970_at | 19.96 | 9.96E-10 | 1.187704874 | NA |  |
| 217896_s_at | 19.94 | 1.00E-09 | 1.138198656 | NM_024946| | NIP30,NEFA-interacting nuclear protein NIP30 |
| 230079_at | 19.93 | 1.01E-09 | 1.26933243 | NM_017744| | ST7L,suppression of tumorigenicity 7-like isoform 1 |
| 211219_s_at | 19.92 | 1.02E-09 | 1.75942649 | NM_004789| | LHX2,LIM homeobox protein 2 |
| 1559283_a_at | 19.91 | 1.02E-09 | 1.796422204 | NM_001103176| | NA |
| 219628_at | 19.91 | 1.02E-09 | 1.417695229 | NM_022470| | WIG1,p53 target zinc finger protein isoform 1 |
| 202088_at | 19.9 | 1.03E-09 | 1.108609605 | NM_001099406| | NA |
| 232095_at | 19.9 | 1.03E-09 | 1.273749452 | NA |  |
| 216602_s_at | 19.87 | 1.04E-09 | 1.105560592 | NM_004461| | FARSLA,phenylalanine-tRNA synthetase-like protein |
| 210383_at | 19.86 | 1.05E-09 | 1.96849156 | NM_006920| | SCN1A,sodium channel, voltage-gated, type I, alpha |
| 219709_x_at | 19.86 | 1.05E-09 | 1.137061804 | NM_023933| | MGC2494,hypothetical protein MGC2494 |
| 222554_s_at | 19.86 | 1.05E-09 | 1.105454439 | NM_022917| | NOL6,nucleolar RNA-associated protein alpha isoform |
| 233049_x_at | 19.85 | 1.05E-09 | 1.092913312 | NM_005861| | STUB1,STIP1 homology and U-box containing protein 1 |
| 218611_at | 19.84 | 1.06E-09 | 1.228629622 | NM_016545| | IER5,immediate early response 5 |
| 222574_s_at | 19.84 | 1.06E-09 | 1.158851685 | NM_024612| | DHX40,DEAH (Asp-Glu-Ala-His) box polypeptide 40 |
| 225238_at | 19.82 | 1.07E-09 | 1.272855107 | NM_138962| | MSI2,musashi 2 isoform a |
| 222824_at | 19.82 | 1.07E-09 | 1.202237956 | NA |  |
| 205675_at | 19.81 | 1.08E-09 | 1.918545281 | NM_000253| | MTP,microsomal triglyceride transfer protein large |
| 222797_at | 19.81 | 1.08E-09 | 1.37096966 | NM_020134| | DPYSL5,dihydropyrimidinase-like 5 |
| 231899_at | 19.81 | 1.08E-09 | 1.281191469 | NM_033390| | NA |
| 218952_at | 19.8 | 1.09E-09 | 1.341674085 | NM_013271| | PCSK1N,proprotein convertase subtilisin/kexin type 1 |
| 229000_at | 19.78 | 1.10E-09 | 1.109829759 | NM_021217| | ZNF77,zinc finger protein 77 |
| 215436_at | 19.78 | 1.09E-09 | 1.481321446 | NM_032303| | HSDL2,hydroxysteroid dehydrogenase like 2 |
| 202185_at | 19.77 | 1.10E-09 | 1.163766866 | NM_001084| | PLOD3,procollagen-lysine, 2-oxoglutarate 5-dioxygenase |
| 222669_s_at | 19.77 | 1.10E-09 | 1.113420121 | NM_016038| | SBDS,Shwachman-Bodian-Diamond syndrome protein |
| 1554690_a_at | 19.76 | 1.11E-09 | 1.24690212 | NM_001122824| | NA |
| 230304_at | 19.76 | 1.10E-09 | 1.329593192 | NA |  |
| 215429_s_at | 19.76 | 1.11E-09 | 1.258330474 | NM_182498| | MGC51082,hypothetical protein MGC51082 |
| 219098_at | 19.75 | 1.11E-09 | 1.090250576 | NM_001105538| | NA |
| 226780_s_at | 19.74 | 1.12E-09 | 1.175667774 | NM_197964| | HSPC268,hypothetical protein HSPC268 |
| 202936_s_at | 19.73 | 1.12E-09 | 1.523351704 | NM_000346| | SOX9,transcription factor SOX9 |
| 222403_at | 19.73 | 1.12E-09 | 1.342979201 | NM_014342| | MTCH2,mitochondrial carrier homolog 2 |
| 238919_at | 19.71 | 1.13E-09 | 2.755168379 | NA |  |
| 1559633_a_at | 19.7 | 1.14E-09 | 1.605488999 | NM_000740| | CHRM3,cholinergic receptor, muscarinic 3 |
| 225471_s_at | 19.7 | 1.14E-09 | 1.141275287 | NM_001626| | AKT2,v-akt murine thymoma viral oncogene homolog 2 |
| 211679_x_at | 19.7 | 1.14E-09 | 1.434417091 | NM_005458| | GPR51,G protein-coupled receptor 51 |
| 238929_at | 19.7 | 1.14E-09 | 1.195368282 | NM_032102| | SRP46,Splicing factor, arginine/serine-rich, 46kD |
| 202616_s_at | 19.69 | 1.14E-09 | 1.215903717 | NM_001110792| | NA |
| 230401_at | 19.69 | 1.14E-09 | 1.2374735 | NA |  |
| 230728_at | 19.68 | 1.15E-09 | 1.180292167 | NA |  |
| 225368_at | 19.67 | 1.15E-09 | 1.095104894 | NM_001113239| | NA |
| 208756_at | 19.67 | 1.16E-09 | 1.049390594 | NM_003757| | EIF3S2,eukaryotic translation initiation factor 3, |
| 225617_at | 19.66 | 1.16E-09 | 1.369175839 | NM_002540| | ODF2,outer dense fiber of sperm tails 2 isoform 1 |
| 213763_at | 19.65 | 1.17E-09 | 1.163024401 | NM_001113239| | NA |
| 233289_at | 19.65 | 1.16E-09 | 1.658308222 | NA |  |
| 227259_at | 19.65 | 1.17E-09 | 1.502933927 | NM_001025079| | NA |
| 205590_at | 19.64 | 1.17E-09 | 1.427108109 | NM_005739| | RASGRP1,RAS guanyl releasing protein 1 |
| 204600_at | 19.64 | 1.17E-09 | 1.429802128 | NM_004443| | EPHB3,ephrin receptor EphB3 precursor |
| 241741_at | 19.64 | 1.17E-09 | 1.426782716 | NM_019095| | C20orf155,chromosome 20 open reading frame 155 |
| 203527_s_at | 19.64 | 1.17E-09 | 1.470542198 | NM_000038| | APC,adenomatosis polyposis coli |
| 218624_s_at | 19.64 | 1.17E-09 | 1.158498005 | NA |  |
| 228897_at | 19.63 | 1.18E-09 | 1.418959244 | NM_001002862| | DERL3,derlin-3 protein isoform b |
| 242065_x_at | 19.63 | 1.18E-09 | 1.13410237 | NM_004508| | IDI1,isopentenyl-diphosphate delta isomerase |
| 219001_s_at | 19.63 | 1.18E-09 | 1.121929032 | NM_024345| | MGC10765,hypothetical protein MGC10765 |
| 208070_s_at | 19.62 | 1.18E-09 | 1.391079434 | NM_002912| | REV3L,REV3-like, catalytic subunit of DNA polymerase |
| 238462_at | 19.62 | 1.18E-09 | 2.104828295 | NM_032873| | STS-1,Cbl-interacting protein Sts-1 |
| 208707_at | 19.61 | 1.19E-09 | 1.334859442 | NM_001969| | EIF5,eukaryotic translation initiation factor 5 |
| 223707_at | 19.6 | 1.20E-09 | 1.538288653 | NM_000990| | RPL27A,ribosomal protein L27a |
| 219056_at | 19.6 | 1.20E-09 | 1.318601763 | NM_024570| | FLJ11712,hypothetical protein FLJ11712 |
| 235131_at | 19.56 | 1.22E-09 | 1.436731558 | NM_020663| | RHOJ,TC10-like Rho GTPase |
| 225144_at | 19.56 | 1.23E-09 | 1.350493286 | NM_001204| | BMPR2,bone morphogenetic protein receptor, type II |
| 225240_s_at | 19.55 | 1.23E-09 | 1.191008692 | NM_138962| | MSI2,musashi 2 isoform a |
| 223282_at | 19.55 | 1.23E-09 | 2.125430275 | NM_005786| | SDCCAG33,serologically defined colon cancer antigen 33 |
| 228516_at | 19.54 | 1.23E-09 | 1.11088628 | NM_138477| | CDAN1,codanin 1 |
| 213239_at | 19.54 | 1.23E-09 | 1.192731225 | NM_006346| | C13orf24,chromosome 13 open reading frame 24 |
| 227558_at | 19.53 | 1.24E-09 | 1.271536883 | NM_003655| | CBX4,chromobox homolog 4 |
| 212528_at | 19.53 | 1.24E-09 | 1.150575175 | NA |  |
| 235457_at | 19.53 | 1.24E-09 | 1.387021248 | NM_032427| | MAML2,mastermind-like 2 |
| 221675_s_at | 19.53 | 1.24E-09 | 1.095609097 | NM_020244| | CHPT1,choline phosphotransferase 1 |
| 203261_at | 19.52 | 1.25E-09 | 1.082824967 | NM_006571| | DCTN6,dynactin 6 |
| 204508_s_at | 19.51 | 1.26E-09 | 1.711259647 | NM_001218| | CA12,carbonic anhydrase XII isoform 1 precursor |
| 203284_s_at | 19.5 | 1.27E-09 | 1.134757632 | NM_012262| | HS2ST1,heparan sulfate 2-O-sulfotransferase 1 |
| 1552378_s_at | 19.5 | 1.26E-09 | 1.448579348 | NM_172037| | RDH10,retinol dehydrogenase 10 |
| 212240_s_at | 19.5 | 1.26E-09 | 1.313447738 | NM_181504| | PIK3R1,phosphoinositide-3-kinase, regulatory subunit, |
| 224218_s_at | 19.49 | 1.27E-09 | 1.535075267 | NM_014112| | TRPS1,zinc finger transcription factor TRPS1 |
| 243718_at | 19.49 | 1.27E-09 | 1.628236654 | NA |  |
| 220155_s_at | 19.49 | 1.27E-09 | 1.087360216 | NM_001009877| | BRD9,bromodomain containing 9 isoform 2 |
| 206811_at | 19.48 | 1.27E-09 | 1.683511137 | NM_001115| | ADCY8,adenylate cyclase 8 |
| 235067_at | 19.47 | 1.28E-09 | 1.138076569 | NM_013255| | MKLN1,muskelin 1, intracellular mediator containing |
| 203206_at | 19.46 | 1.29E-09 | 1.13318281 | NM_014661| | FAM53B,family with sequence similarity 53, member B |
| 227771_at | 19.44 | 1.30E-09 | 1.370174301 | NM_002310| | LIFR, |
| 204577_s_at | 19.44 | 1.30E-09 | 1.581589214 | NM_015041| | CLUAP1,clusterin associated protein 1 |
| 217824_at | 19.43 | 1.30E-09 | 1.426164682 | NM_016021| | UBE2J1,ubiquitin-conjugating enzyme E2, J1 |
| 226343_at | 19.43 | 1.30E-09 | 1.14692111 | NA |  |
| 212423_at | 19.42 | 1.32E-09 | 1.252976987 | NM_153367| | C10orf56,chromosome 10 open reading frame 56 |
| 208661_s_at | 19.41 | 1.32E-09 | 1.253793701 | NM_001001894| | TTC3,tetratricopeptide repeat domain 3 |
| 238070_at | 19.4 | 1.33E-09 | 1.351577981 | NM_004284| | CHD1L,chromodomain helicase DNA binding protein |
| 230741_at | 19.38 | 1.34E-09 | 1.441114159 | NA |  |
| 227188_at | 19.37 | 1.35E-09 | 1.286194069 | NM_058187| | C21orf63,chromosome 21 open reading frame 63 |
| 229447_x_at | 19.37 | 1.35E-09 | 1.165135267 | NM_001037501| | NA |
| 222398_s_at | 19.36 | 1.36E-09 | 1.07117917 | NM_004247| | U5-116KD,U5 snRNP-specific protein, 116 kD |
| 207401_at | 19.34 | 1.37E-09 | 1.375511254 | NM_002763| | PROX1,prospero-related homeobox 1 |
| 218175_at | 19.34 | 1.37E-09 | 1.539108318 | NM_025140| | FLJ22471,limkain beta 2 |
| 213031_s_at | 19.34 | 1.37E-09 | 1.114514046 | NM_032856| | FLJ14888,hypothetical protein FLJ14888 |
| 1555948_s_at | 19.33 | 1.38E-09 | 1.233659051 | NM_014612| | C9orf10,C9orf10 protein |
| 204764_at | 19.32 | 1.39E-09 | 1.249290941 | NM_002028| | FNTB,farnesyltransferase, CAAX box, beta |
| 219746_at | 19.31 | 1.39E-09 | 1.349389772 | NM_012074| | DPF3,cer-d4 (mouse) homolog |
| 207169_x_at | 19.27 | 1.43E-09 | 1.137406002 | NM_001954| | DDR1,discoidin receptor tyrosine kinase isoform b |
| 227812_at | 19.27 | 1.43E-09 | 1.208489317 | NM_018647| | TNFRSF19,tumor necrosis factor receptor superfamily, |
| 212023_s_at | 19.27 | 1.42E-09 | 1.249282104 | NM_002417| | MKI67,antigen identified by monoclonal antibody Ki-67 |
| 225560_at | 19.27 | 1.43E-09 | 1.167675643 | NM_013382| | POMT2,putative protein O-mannosyltransferase |
| 238161_at | 19.26 | 1.43E-09 | 1.223452804 | NA |  |
| 228299_at | 19.24 | 1.45E-09 | 1.162624738 | NM_173562| | C6orf69,hypothetical protein MGC14254 |
| 209339_at | 19.23 | 1.45E-09 | 1.141989385 | NM_005067| | SIAH2,seven in absentia homolog 2 |
| 218915_at | 19.23 | 1.45E-09 | 1.084499788 | NM_000268| | NF2,neurofibromin 2 isoform 1 |
| 215258_at | 19.21 | 1.47E-09 | 1.341711049 | NM_145296| | IGSF4C,immunoglobulin superfamily, member 4C |
| 207629_s_at | 19.21 | 1.47E-09 | 1.169708871 | NM_004723| | ARHGEF2,rho/rac guanine nucleotide exchange factor 2 |
| 203580_s_at | 19.21 | 1.47E-09 | 1.140692158 | NM_001076785| | NA |
| 209120_at | 19.2 | 1.48E-09 | 1.485830757 | NM_021005| | NR2F2,nuclear receptor subfamily 2, group F, member 2 |
| 1564746_at | 19.19 | 1.49E-09 | 1.519707173 | NM_178833| | LOC133308,hypothetical protein BC009732 |
| 228315_at | 19.18 | 1.50E-09 | 1.332886826 | NA |  |
| 223183_at | 19.18 | 1.50E-09 | 1.732146269 | NM_001037553| | NA |
| 204248_at | 19.17 | 1.51E-09 | 1.166818889 | NM_002067| | GNA11,guanine nucleotide binding protein (G protein), |
| 218309_at | 19.16 | 1.52E-09 | 1.43255263 | NM_018584| | CaMKIINalpha,calcium/calmodulin-dependent protein kinase II |
| 205875_s_at | 19.15 | 1.52E-09 | 1.381482713 | NM_016381| | TREX1,three prime repair exonuclease 1 isoform a |
| 1554640_at | 19.15 | 1.52E-09 | 1.463295069 | NM_001037293| | NA |
| 225221_at | 19.15 | 1.52E-09 | 1.155021627 | NA |  |
| 217727_x_at | 19.15 | 1.53E-09 | 1.048849858 | NM_018206| | VPS35,vacuolar protein sorting 35 |
| 209459_s_at | 19.14 | 1.53E-09 | 1.273393541 | NM_000663| | ABAT,4-aminobutyrate aminotransferase precursor |
| 227624_at | 19.14 | 1.53E-09 | 1.416565599 | NM_001127208| | NA |
| 201700_at | 19.14 | 1.53E-09 | 1.124498044 | NM_001760| | CCND3,cyclin D3 |
| 243030_at | 19.1 | 1.56E-09 | 1.333166755 | NA |  |
| 218270_at | 19.1 | 1.56E-09 | 1.187956196 | NM_024540| | MRPL24,mitochondrial ribosomal protein L24 |
| 227792_at | 19.09 | 1.58E-09 | 1.28476416 | NM_001034841| | NA |
| 236882_at | 19.08 | 1.59E-09 | 1.446891209 | NA |  |
| 221973_at | 19.08 | 1.58E-09 | 1.464243514 | NA |  |
| 226066_at | 19.07 | 1.59E-09 | 1.485029763 | NM_000248| | MITF,microphthalmia-associated transcription factor |
| 212609_s_at | 19.07 | 1.59E-09 | 1.212308925 | NM_005465| | AKT3,v-akt murine thymoma viral oncogene homolog 3 |
| 203456_at | 19.06 | 1.60E-09 | 1.193433963 | NM_007213| | PRAF2,JM4 protein |
| 215185_at | 19.05 | 1.61E-09 | 1.326291531 | NA |  |
| 204041_at | 19.05 | 1.61E-09 | 1.224642479 | NM_000898| | MAOB,amine oxidase (flavin-containing) |
| 1552256_a_at | 19.05 | 1.61E-09 | 1.2371189 | NM_001082959| | NA |
| 202784_s_at | 19.05 | 1.61E-09 | 1.292071027 | NM_012343| | NNT,nicotinamide nucleotide transhydrogenase |
| 215073_s_at | 19.03 | 1.63E-09 | 2.071104116 | NM_021005| | NR2F2,nuclear receptor subfamily 2, group F, member 2 |
| 209042_s_at | 19.03 | 1.63E-09 | 1.086686879 | NM_003343| | UBE2G2,ubiquitin-conjugating enzyme E2G 2 isoform 1 |
| 233587_s_at | 19.03 | 1.63E-09 | 1.253161488 | NM_020808| | SIPA1L2,signal-induced proliferation-associated 1 like |
| 209598_at | 19.02 | 1.64E-09 | 1.389309073 | NM_007257| | NA |
| 206669_at | 19.02 | 1.64E-09 | 1.349608433 | NM_000817| | GAD1,glutamate decarboxylase 1 isoform GAD67 |
| 209716_at | 19.01 | 1.64E-09 | 1.141501532 | NM_000757| | CSF1,colony stimulating factor 1 isoform a precursor |
| 237008_at | 19.01 | 1.64E-09 | 1.532882903 | NA |  |
| 230494_at | 19 | 1.65E-09 | 1.387156929 | NM_005415| | SLC20A1,solute carrier family 20 (phosphate |
| 215169_at | 18.97 | 1.68E-09 | 1.191277893 | NM_001110781| | NA |
| 202730_s_at | 18.96 | 1.70E-09 | 1.233332902 | NM_014456| | PDCD4,programmed cell death 4 isoform 1 |
| 228977_at | 18.95 | 1.70E-09 | 1.297585918 | NA |  |
| 1558796_a_at | 18.94 | 1.71E-09 | 1.557960125 | NA |  |
| 212753_at | 18.94 | 1.72E-09 | 1.133268999 | NM_006315| | PCGF3,ring finger protein 3 |
| 207160_at | 18.93 | 1.72E-09 | 1.2734183 | NM_000882| | IL12A,interleukin 12A precursor |
| 225514_at | 18.93 | 1.72E-09 | 1.119248579 | NM_174913| | C14orf21,chromosome 14 open reading frame 21 |
| 1560371_at | 18.93 | 1.72E-09 | 1.440254017 | NA |  |
| 212256_at | 18.92 | 1.73E-09 | 1.172668721 | NM_017540| | GALNT10,GalNAc transferase 10 isoform b |
| 238575_at | 18.92 | 1.73E-09 | 1.246037035 | NM_032523| | OSBPL6,oxysterol-binding protein-like protein 6 isoform |
| 234583_at | 18.92 | 1.73E-09 | 1.544280757 | NM_006614| | CHL1,cell adhesion molecule with homology to L1CAM |
| 235989_at | 18.92 | 1.73E-09 | 1.291672521 | NA |  |
| 226594_at | 18.91 | 1.74E-09 | 1.180077021 | NA |  |
| 207144_s_at | 18.89 | 1.75E-09 | 1.511472964 | NM_004143| | CITED1,Cbp/p300-interacting transactivator, with |
| 205344_at | 18.89 | 1.75E-09 | 1.452410322 | NM_006574| | CSPG5,chondroitin sulfate proteoglycan 5 (neuroglycan |
| 228419_at | 18.88 | 1.77E-09 | 1.359224386 | NM_024042| | METRN,meteorin, glial cell differentiation regulator |
| 214252_s_at | 18.87 | 1.77E-09 | 1.383431084 | NM_006493| | CLN5,ceroid-lipofuscinosis, neuronal 5 |
| 218277_s_at | 18.87 | 1.77E-09 | 1.180379745 | NM_024612| | DHX40,DEAH (Asp-Glu-Ala-His) box polypeptide 40 |
| 1554260_a_at | 18.86 | 1.79E-09 | 1.225169863 | NM_015030| | NA |
| 233825_s_at | 18.86 | 1.79E-09 | 1.332117928 | NM_031462| | CD99L2,CD99 antigen-like 2 |
| 207360_s_at | 18.86 | 1.79E-09 | 1.205822972 | NM_002531| | NTSR1,neurotensin receptor 1 |
| 228904_at | 18.85 | 1.80E-09 | 2.069284595 | NM_002146| | HOXB3,homeo box B3 |
| 212097_at | 18.84 | 1.80E-09 | 1.349360502 | NM_001753| | CAV1,caveolin 1 |
| 223012_at | 18.84 | 1.81E-09 | 1.145923121 | NM_025241| | UBXD1,UBX domain containing 1 |
| 225340_s_at | 18.84 | 1.81E-09 | 1.045738736 | NM_005898| | M11S1,membrane component, chromosome 11, surface |
| 203257_s_at | 18.83 | 1.82E-09 | 1.102839973 | NM_001003676| | MGC4707,MGC4707 protein isoform 1 |
| 203725_at | 18.83 | 1.82E-09 | 1.166222537 | NM_001924| | GADD45A,growth arrest and DNA-damage-inducible, alpha |
| 229572_at | 18.8 | 1.85E-09 | 1.329502938 | NA |  |
| 242825_at | 18.79 | 1.86E-09 | 1.353562454 | NM_001010861| | NA |
| 236322_at | 18.78 | 1.87E-09 | 1.598522921 | NA |  |
| 217895_at | 18.77 | 1.88E-09 | 1.079790544 | NM_017952| | FLJ20758,FLJ20758 protein |
| 221566_s_at | 18.76 | 1.90E-09 | 1.284429398 | NM_003946| | NOL3,nucleolar protein 3 |
| 220495_s_at | 18.74 | 1.91E-09 | 1.081276668 | NM_024715| | C5orf14,disulfide isomerase |
| 226848_at | 18.74 | 1.91E-09 | 1.266593438 | NA |  |
| 201012_at | 18.73 | 1.92E-09 | 1.272198941 | NM_000700| | ANXA1,annexin I |
| 236958_at | 18.73 | 1.92E-09 | 1.160592606 | NA |  |
| 201729_s_at | 18.72 | 1.93E-09 | 1.105888675 | NM_014680| | KIAA0100,antigen MLAA-22 |
| 203068_at | 18.72 | 1.93E-09 | 1.121386994 | NM_014851| | KLHL21,kelch-like 21 |
| 224835_at | 18.72 | 1.93E-09 | 1.23243784 | NM_019593| | KIAA1434,hypothetical protein KIAA1434 |
| 1557996_at | 18.72 | 1.93E-09 | 1.229317993 | NA |  |
| 212498_at | 18.71 | 1.94E-09 | 1.057215839 | NA |  |
| 209637_s_at | 18.71 | 1.94E-09 | 1.457951111 | NM_002926| | RGS12,regulator of G-protein signalling 12 isoform 2 |
| 218496_at | 18.71 | 1.95E-09 | 1.09801182 | NM_002936| | RNASEH1,ribonuclease H1 |
| 823_at | 18.71 | 1.95E-09 | 1.370277532 | NM_002996| | CX3CL1,chemokine (C-X3-C motif) ligand 1 |
| 201581_at | 18.7 | 1.96E-09 | 1.25136185 | NM_021156| | DJ971N18.2,hypothetical protein DJ971N18.2 |
| 213033_s_at | 18.69 | 1.96E-09 | 1.967437503 | NM_005596| | NFIB,nuclear factor I/B |
| 222445_at | 18.69 | 1.96E-09 | 1.089498244 | NM_018375| | SLC39A9,solute carrier family 39 (zinc transporter), |
| 223296_at | 18.67 | 1.99E-09 | 1.072774935 | NM_032315| | MGC4399,mitochondrial carrier protein MGC4399 |
| 209859_at | 18.66 | 1.99E-09 | 2.115117976 | NM_015163| | TRIM9,tripartite motif protein 9 isoform 1 |
| 218899_s_at | 18.66 | 1.99E-09 | 1.863864147 | NM_001024372| | NA |
| 215667_x_at | 18.66 | 2.00E-09 | 1.110808924 | NA |  |
| 225276_at | 18.66 | 1.99E-09 | 1.139133862 | NA |  |
| 233540_s_at | 18.64 | 2.02E-09 | 1.126080959 | NM_001011649| | CDK5RAP2,CDK5 regulatory subunit associated protein 2 |
| 222617_s_at | 18.64 | 2.02E-09 | 1.105888234 | NM_022063| | C10orf84,chromosome 10 open reading frame 84 |
| 220050_at | 18.64 | 2.02E-09 | 1.34056065 | NM_018956| | C9orf9,chromosome 9 open reading frame 9 |
| 202935_s_at | 18.63 | 2.03E-09 | 1.590097671 | NM_000346| | SOX9,transcription factor SOX9 |
| 218086_at | 18.63 | 2.03E-09 | 1.245506271 | NM_015392| | NPDC1,neural proliferation, differentiation and |
| 235709_at | 18.63 | 2.03E-09 | 1.306716055 | NM_174942| | GAS2L3,growth arrest-specific 2 like 3 |
| 204131_s_at | 18.62 | 2.04E-09 | 1.075007897 | NM_001455| | FOXO3A,forkhead box O3A |
| 235985_at | 18.62 | 2.04E-09 | 1.43844372 | NA |  |
| 1559822_s_at | 18.62 | 2.04E-09 | 1.301386898 | NA |  |
| 238853_at | 18.61 | 2.06E-09 | 1.443845927 | NM_001024647| | NA |
| 228661_s_at | 18.6 | 2.06E-09 | 1.197440787 | NA |  |
| 225799_at | 18.6 | 2.06E-09 | 1.237774402 | NA |  |
| 213724_s_at | 18.6 | 2.06E-09 | 1.154025247 | NM_002611| | PDK2,pyruvate dehydrogenase kinase, isoenzyme 2 |
| 226933_s_at | 18.6 | 2.06E-09 | 1.595052257 | NM_001546| | ID4,inhibitor of DNA binding 4, dominant negative |
| 238905_at | 18.6 | 2.07E-09 | 1.687591531 | NM_020663| | RHOJ,TC10-like Rho GTPase |
| 230655_at | 18.6 | 2.07E-09 | 1.219527 | NA |  |
| 1560029_a_at | 18.59 | 2.07E-09 | 1.414738378 | NM_001082969| | NA |
| 201490_s_at | 18.59 | 2.08E-09 | 1.148749695 | NM_005729| | PPIF,peptidylprolyl isomerase F precursor |
| 213164_at | 18.58 | 2.08E-09 | 1.123559894 | NM_006933| | SLC5A3,solute carrier family 5 (inositol transporters), |
| 244623_at | 18.57 | 2.10E-09 | 2.080958392 | NM_019842| | KCNQ5,potassium voltage-gated channel, KQT-like |
| 213493_at | 18.57 | 2.10E-09 | 1.78267952 | NM_001080437| | NA |
| 40562_at | 18.57 | 2.10E-09 | 1.183184953 | NM_002067| | GNA11,guanine nucleotide binding protein (G protein), |
| 207048_at | 18.57 | 2.10E-09 | 1.295423916 | NM_014229| | SLC6A11,solute carrier family 6 (neurotransmitter |
| 230088_at | 18.56 | 2.10E-09 | 1.528540824 | NA |  |
| 200788_s_at | 18.56 | 2.10E-09 | 1.180696553 | NM_003768| | PEA15,phosphoprotein enriched in astrocytes 15 |
| 239810_at | 18.54 | 2.13E-09 | 1.261854708 | NM_014909| | KIAA1036,KIAA1036 |
| 203212_s_at | 18.52 | 2.16E-09 | 1.096354566 | NM_016156| | MTMR2,myotubularin-related protein 2 isoform 1 |
| 221951_at | 18.51 | 2.16E-09 | 1.203961002 | NM_001042463| | NA |
| 222266_at | 18.5 | 2.18E-09 | 1.222528122 | NM_003796| | C19orf2,RPB5-mediating protein isoform a |
| 227081_at | 18.48 | 2.20E-09 | 1.217254355 | NM_003462| | DNALI1,axonemal dynein light chain |
| 215707_s_at | 18.47 | 2.22E-09 | 1.257047219 | NM_000311| | PRNP,prion protein preproprotein |
| 222774_s_at | 18.46 | 2.23E-09 | 1.270090389 | NM_018092| | NETO2,neuropilin- and tolloid-like protein 2 |
| 228915_at | 18.46 | 2.23E-09 | 1.588702415 | NM_004392| | DACH1,dachshund homolog 1 isoform c |
| 214670_at | 18.45 | 2.25E-09 | 1.223040864 | NM_003439| | ZKSCAN1,zinc finger protein 36 |
| 1554085_at | 18.44 | 2.26E-09 | 1.170447788 | NM_175066| | DDX51,DEAD (Asp-Glu-Ala-Asp) box polypeptide 51 |
| 212850_s_at | 18.4 | 2.32E-09 | 1.208450296 | NM_002334| | NA |
| 214671_s_at | 18.4 | 2.31E-09 | 1.279191513 | NM_001092| | ABR,active breakpoint cluster region-related |
| 227604_at | 18.39 | 2.33E-09 | 1.18857507 | NA |  |
| 242051_at | 18.39 | 2.33E-09 | 1.825836225 | NA |  |
| 226684_at | 18.38 | 2.35E-09 | 1.065731472 | NM_018036| | C14orf103,chromosome 14 open reading frame 103 |
| 210927_x_at | 18.37 | 2.35E-09 | 1.074694679 | NM_006694| | JTB,jumping translocation breakpoint |
| 208662_s_at | 18.37 | 2.35E-09 | 1.261838509 | NM_001001894| | TTC3,tetratricopeptide repeat domain 3 |
| 1558724_at | 18.36 | 2.36E-09 | 1.809968345 | NA |  |
| 231214_at | 18.36 | 2.37E-09 | 1.467298417 | NA |  |
| 222222_s_at | 18.36 | 2.37E-09 | 1.207402136 | NM_004838| | HOMER3,Homer, neuronal immediate early gene, 3 |
| 202283_at | 18.36 | 2.36E-09 | 1.137703178 | NM_002615| | SERPINF1,serine (or cysteine) proteinase inhibitor, clade |
| 212719_at | 18.35 | 2.38E-09 | 1.101648077 | NM_194449| | PLEKHE1,suprachiasmatic nucleus circadian oscillatory |
| 1554089_s_at | 18.34 | 2.39E-09 | 1.099025026 | NM_016038| | SBDS,Shwachman-Bodian-Diamond syndrome protein |
| 201259_s_at | 18.34 | 2.39E-09 | 1.109147258 | NM_006754| | SYPL,synaptophysin-like protein isoform a |
| 227067_x_at | 18.34 | 2.40E-09 | 1.251271654 | NM_203458| | NOTCH2NL,Notch homolog 2 N-terminal like protein |
| 239219_at | 18.33 | 2.40E-09 | 1.155163158 | NM_004217| | AURKB,aurora kinase B |
| 1563014_at | 18.33 | 2.40E-09 | 1.248043568 | NM_001018| | RPS15,ribosomal protein S15 |
| 1552822_at | 18.32 | 2.43E-09 | 1.654838602 | NM_019022| | TXNDC10,thioredoxin domain containing 10 |
| 203557_s_at | 18.32 | 2.43E-09 | 1.113081491 | NM_000281| | PCBD1,pterin-4 alpha-carbinolamine dehydratase isoform |
| 225492_at | 18.31 | 2.43E-09 | 1.066859834 | NM_018126| | TMEM33,transmembrane protein 33 |
| 222916_s_at | 18.31 | 2.44E-09 | 1.231625457 | NM_005336| | HDLBP,high density lipoprotein binding protein |
| 212217_at | 18.31 | 2.43E-09 | 1.102554978 | NM_001042385| | NA |
| 236511_at | 18.3 | 2.44E-09 | 1.547136239 | NA |  |
| 239483_at | 18.3 | 2.44E-09 | 1.21245327 | NA |  |
| 218146_at | 18.3 | 2.44E-09 | 1.075848224 | NM_001010983| | GLT8D1,glycosyltransferase 8 domain containing 1 |
| 212274_at | 18.29 | 2.46E-09 | 1.24785949 | NM_145693| | LPIN1,lipin 1 |
| 201334_s_at | 18.28 | 2.48E-09 | 1.195573766 | NM_015313| | ARHGEF12,Rho guanine nucleotide exchange factor (GEF) 12 |
| 229537_at | 18.28 | 2.48E-09 | 1.399473254 | NA |  |
| 212063_at | 18.28 | 2.47E-09 | 1.799480345 | NM_000610| | CD44,CD44 antigen isoform 1 precursor |
| 229132_at | 18.26 | 2.50E-09 | 1.39164513 | NM_001042533| | NA |
| 208073_x_at | 18.26 | 2.50E-09 | 1.245215435 | NM_001001894| | TTC3,tetratricopeptide repeat domain 3 |
| 205472_s_at | 18.26 | 2.51E-09 | 1.810047782 | NM_004392| | DACH1,dachshund homolog 1 isoform c |
| 219377_at | 18.25 | 2.52E-09 | 1.132963358 | NM_022751| | C18orf11,chromosome 18 open reading frame 11 |
| 230119_at | 18.23 | 2.55E-09 | 1.292667284 | NA |  |
| 1569243_at | 18.22 | 2.56E-09 | 1.324029702 | NA |  |
| 205710_at | 18.22 | 2.56E-09 | 1.357167954 | NM_004525| | LRP2,low density lipoprotein-related protein 2 |
| 209230_s_at | 18.2 | 2.58E-09 | 1.263256694 | NM_001042483| | NA |
| 1557543_at | 18.2 | 2.59E-09 | 1.551185287 | NA |  |
| 227415_at | 18.19 | 2.61E-09 | 1.157551474 | NA |  |
| 220739_s_at | 18.19 | 2.60E-09 | 1.119807407 | NM_017623| | CNNM3,cyclin M3 isoform 1 |
| 235309_at | 18.18 | 2.61E-09 | 1.147024938 | NM_001019| | RPS15A,ribosomal protein S15a |
| 203089_s_at | 18.17 | 2.63E-09 | 1.122528309 | NM_013247| | PRSS25,protease, serine, 25 isoform 1 preproprotein |
| 225055_at | 18.16 | 2.65E-09 | 1.231430687 | NA |  |
| 221922_at | 18.16 | 2.65E-09 | 1.275697047 | NM_013296| | GPSM2,G-protein signalling modulator 2 (AGS3-like, C. |
| 205109_s_at | 18.16 | 2.65E-09 | 1.245576504 | NM_015320| | ARHGEF4,Rho guanine nucleotide exchange factor 4 isoform |
| 221879_at | 18.15 | 2.66E-09 | 1.083931582 | NM_001031733| | NA |
| 228645_at | 18.15 | 2.67E-09 | 1.361935276 | NA |  |
| 227101_at | 18.15 | 2.67E-09 | 1.45740043 | NM_176814| | LOC168850,hypothetical protein LOC168850 |
| 221972_s_at | 18.15 | 2.67E-09 | 1.134788902 | NM_016176| | Cab45,calcium binding protein Cab45 precursor |
| 230667_at | 18.15 | 2.67E-09 | 1.242519119 | NA |  |
| 230791_at | 18.14 | 2.67E-09 | 2.237361316 | NA |  |
| 242186_x_at | 18.14 | 2.68E-09 | 2.008505269 | NM_015236| | LPHN3,latrophilin 3 precursor |
| 204187_at | 18.13 | 2.68E-09 | 1.496009141 | NM_006877| | GMPR,guanosine monophosphate reductase |
| 213838_at | 18.13 | 2.69E-09 | 1.303819382 | NM_016167| | NOL7,nucleolar protein 7, 27kDa |
| 208502_s_at | 18.13 | 2.70E-09 | 1.694950796 | NM_002653| | PITX1,paired-like homeodomain transcription factor 1 |
| 206106_at | 18.13 | 2.69E-09 | 1.323500612 | NM_002969| | MAPK12,mitogen-activated protein kinase 12 |
| 219281_at | 18.12 | 2.71E-09 | 1.222242641 | NM_012331| | MSRA,methionine sulfoxide reductase A |
| 202771_at | 18.12 | 2.70E-09 | 1.162730039 | NA |  |
| 206397_x_at | 18.12 | 2.71E-09 | 1.291518453 | NM_001492| | GDF1,growth differentiation factor 1 |
| 1554102_a_at | 18.1 | 2.74E-09 | 1.257275556 | NM_001079669| | NA |
| 201953_at | 18.1 | 2.74E-09 | 1.134669845 | NM_006384| | CIB1,calcium and integrin binding 1 (calmyrin) |
| 221838_at | 18.1 | 2.74E-09 | 1.221639396 | NM_032775| | KELCHL,kelch-like |
| 222747_s_at | 18.1 | 2.74E-09 | 1.514819671 | NM_001037535| | NA |
| 201346_at | 18.09 | 2.76E-09 | 1.09148173 | NM_024551| | ADIPOR2,adiponectin receptor 2 |
| 216483_s_at | 18.09 | 2.75E-09 | 1.114862666 | NM_019107| | C19orf10,chromosome 19 open reading frame 10 |
| 1558388_a_at | 18.09 | 2.75E-09 | 1.755600198 | NA |  |
| 1552767_a_at | 18.09 | 2.75E-09 | 1.244880822 | NM_001077188| | NA |
| 225491_at | 18.08 | 2.77E-09 | 1.757328737 | NM_004171| | SLC1A2,solute carrier family 1, member 2 |
| 201819_at | 18.08 | 2.77E-09 | 1.280200438 | NM_001082959| | NA |
| 244214_at | 18.08 | 2.77E-09 | 1.156049402 | NM_001098510| | NA |
| 203973_s_at | 18.07 | 2.77E-09 | 1.138032746 | NM_005195| | CEBPD,CCAAT/enhancer binding protein delta |
| 1561660_at | 18.07 | 2.77E-09 | 1.527712641 | NM_001012755| | NA |
| 205496_at | 18.06 | 2.79E-09 | 1.435225069 | NM_014702| | KIAA0408,KIAA0408 protein |
| 226579_at | 18.06 | 2.79E-09 | 1.289268957 | NA |  |
| 201516_at | 18.05 | 2.81E-09 | 1.114231149 | NM_003132| | SRM,spermidine synthase |
| 228495_at | 18.04 | 2.82E-09 | 1.240274523 | NM_174931| | FLJ38348,hypothetical protein FLJ38348 |
| 229157_at | 18.04 | 2.83E-09 | 1.360314129 | NA |  |
| 1555009_a_at | 18.04 | 2.82E-09 | 1.359523898 | NM_003898| | SYNJ2,synaptojanin 2 |
| 564_at | 18.03 | 2.85E-09 | 1.184460588 | NM_002067| | GNA11,guanine nucleotide binding protein (G protein), |
| 221886_at | 18.02 | 2.86E-09 | 1.731914806 | NM_015689| | KIAA1277,KIAA1277 protein |
| 212351_at | 18.02 | 2.86E-09 | 1.09454567 | NM_003907| | EIF2B5,eukaryotic translation initiation factor 2B, |
| 222664_at | 18.01 | 2.88E-09 | 1.1352307 | NM_024076| | KCTD15,potassium channel tetramerisation domain |
| 1773_at | 17.98 | 2.93E-09 | 1.194937981 | NM_002028| | FNTB,farnesyltransferase, CAAX box, beta |
| 205265_s_at | 17.95 | 2.99E-09 | 1.205689957 | NM_005876| | APEG1,aortic preferentially expressed gene 1 |
| 201391_at | 17.95 | 2.98E-09 | 1.10667206 | NM_016292| | TRAP1,TNF receptor-associated protein 1 |
| 233575_s_at | 17.94 | 3.00E-09 | 1.501875374 | NM_007005| | TLE4,transducin-like enhancer protein 4 |
| 227580_s_at | 17.94 | 3.01E-09 | 1.178389453 | NM_015395| | DKFZP434B0335,DKFZP434B0335 protein |
| 212786_at | 17.93 | 3.02E-09 | 1.139899824 | NM_015226| | KIAA0350,KIAA0350 protein |
| 222668_at | 17.93 | 3.03E-09 | 1.116438725 | NM_024076| | KCTD15,potassium channel tetramerisation domain |
| 202345_s_at | 17.93 | 3.02E-09 | 1.066040645 | NM_001444| | FABP5,fatty acid binding protein 5 |
| 226445_s_at | 17.92 | 3.04E-09 | 1.078438712 | NM_033549| | TRIM41,tripartite motif-containing 41 isform 1 |
| 1562062_at | 17.91 | 3.06E-09 | 1.108108941 | NM_001037501| | NA |
| 228185_at | 17.91 | 3.05E-09 | 1.674219067 | NM_145011| | ZNF25,zinc finger protein 25 |
| 202508_s_at | 17.9 | 3.07E-09 | 1.717522256 | NM_003081| | SNAP25,synaptosomal-associated protein 25 isoform |
| 1553959_a_at | 17.88 | 3.10E-09 | 1.105255915 | NM_080605| | B3GALT6,UDP-Gal:betaGal beta 1,3-galactosyltransferase |
| 211569_s_at | 17.88 | 3.10E-09 | 1.120897187 | NM_005327| | HADHSC,L-3-hydroxyacyl-Coenzyme A dehydrogenase, short |
| 226189_at | 17.87 | 3.11E-09 | 1.485870535 | NM_002214| | ITGB8,integrin, beta 8 |
| 202159_at | 17.86 | 3.15E-09 | 1.088437429 | NM_004461| | FARSLA,phenylalanine-tRNA synthetase-like protein |
| 235316_at | 17.86 | 3.13E-09 | 1.249376097 | NM_178557| | FLJ37478,hypothetical protein FLJ37478 |
| 227401_at | 17.84 | 3.19E-09 | 1.59760662 | NM_138284| | IL17D,interleukin 17D precursor |
| 217511_at | 17.84 | 3.18E-09 | 1.22335721 | NM_030929| | KAZALD1,Kazal-type serine protease inhibitor domain 1 |
| 218447_at | 17.83 | 3.21E-09 | 1.059906674 | NM_020188| | DC13,DC13 protein |
| 223312_at | 17.83 | 3.20E-09 | 1.120223679 | NM_032319| | C2orf7,chromosome 2 open reading frame 7 |
| 225415_at | 17.83 | 3.21E-09 | 1.175235391 | NM_138287| | DTX3L,deltex 3-like |
| 241722_x_at | 17.83 | 3.21E-09 | 1.196977429 | NA |  |
| 225250_at | 17.82 | 3.22E-09 | 1.546552626 | NM_020860| | STIM2,stromal interaction molecule 2 |
| 211488_s_at | 17.81 | 3.25E-09 | 1.466869677 | NM_002214| | ITGB8,integrin, beta 8 |
| 231171_at | 17.8 | 3.26E-09 | 1.247658692 | NA |  |
| 208563_x_at | 17.8 | 3.26E-09 | 1.373269597 | NM_006236| | POU3F3,POU domain, class 3, transcription factor 3 |
| 229754_at | 17.79 | 3.27E-09 | 1.163307253 | NA |  |
| 238885_at | 17.79 | 3.27E-09 | 1.307453755 | NM_020910| | NA |
| 217998_at | 17.78 | 3.29E-09 | 1.198058485 | NM_007350| | PHLDA1,pleckstrin homology-like domain, family A, |
| 229876_at | 17.78 | 3.29E-09 | 1.474495821 | NM_001122670| | NA |
| 230286_at | 17.77 | 3.32E-09 | 1.663839669 | NA |  |
| 203926_x_at | 17.76 | 3.34E-09 | 1.071803655 | NM_001001975| | ATP5D,ATP synthase, H+ transporting, mitochondrial F1 |
| 201716_at | 17.73 | 3.40E-09 | 1.121005661 | NM_003099| | SNX1,sorting nexin 1 isoform a |
| 209123_at | 17.72 | 3.43E-09 | 1.117393029 | NM_000320| | QDPR,quinoid dihydropteridine reductase |
| 212234_at | 17.72 | 3.42E-09 | 1.242015097 | NM_015338| | ASXL1,additional sex combs like 1 |
| 230030_at | 17.72 | 3.43E-09 | 1.264368423 | NM_001077188| | NA |
| 227983_at | 17.71 | 3.45E-09 | 1.135407237 | NM_145058| | MGC7036,hypothetical protein MGC7036 |
| 226728_at | 17.7 | 3.47E-09 | 1.202094581 | NM_198580| | SLC27A1,solute carrier family 27 (fatty acid |
| 205303_at | 17.7 | 3.46E-09 | 1.477358471 | NM_004982| | KCNJ8,potassium inwardly-rectifying channel J8 |
| 208030_s_at | 17.69 | 3.48E-09 | 1.08330237 | NM_001119| | ADD1,adducin 1 (alpha) isoform a |
| 218408_at | 17.69 | 3.49E-09 | 1.137151754 | NM_012456| | TIMM10,translocase of inner mitochondrial membrane 10 |
| 236723_at | 17.69 | 3.49E-09 | 1.192435513 | NA |  |
| 204489_s_at | 17.68 | 3.50E-09 | 1.254644666 | NM_000610| | CD44,CD44 antigen isoform 1 precursor |
| 214274_s_at | 17.67 | 3.53E-09 | 1.106184794 | NM_001607| | ACAA1,acetyl-Coenzyme A acyltransferase 1 |
| 238932_at | 17.67 | 3.52E-09 | 1.201198951 | NA |  |
| 238861_at | 17.67 | 3.53E-09 | 1.368244809 | NA |  |
| 229860_x_at | 17.66 | 3.55E-09 | 1.16846978 | NA |  |
| 1568594_s_at | 17.65 | 3.58E-09 | 1.188833306 | NM_032765| | TRIM52,tripartite motif-containing 52 |
| 64900_at | 17.65 | 3.56E-09 | 1.381507867 | NM_001077416| | NA |
| 238948_at | 17.65 | 3.56E-09 | 1.139882725 | NM_001014842| | NA |
| 242673_at | 17.65 | 3.56E-09 | 1.327408538 | NA |  |
| 244354_at | 17.64 | 3.58E-09 | 1.576035143 | NA |  |
| 218039_at | 17.63 | 3.60E-09 | 1.048670797 | NM_016359| | NUSAP1,nucleolar and spindle associated protein 1 |
| 228605_at | 17.63 | 3.61E-09 | 1.107510639 | NA |  |
| 230998_at | 17.62 | 3.63E-09 | 1.275203099 | NA |  |
| 228032_s_at | 17.62 | 3.64E-09 | 1.217643606 | NA |  |
| 244801_at | 17.62 | 3.64E-09 | 1.338946081 | NM_002799| | PSMB7,proteasome beta 7 subunit proprotein |
| 219564_at | 17.61 | 3.65E-09 | 1.855245423 | NM_018658| | KCNJ16,potassium inwardly-rectifying channel J16 |
| 225793_at | 17.61 | 3.66E-09 | 1.360680992 | NM_153713| | LIX1L,Lix1 homolog (mouse) like |
| 220476_s_at | 17.6 | 3.68E-09 | 1.3034481 | NM_019099| | LOC55924,hypothetical protein LOC55924 isoform 1 |
| 212522_at | 17.59 | 3.70E-09 | 1.317204344 | NM_002605| | PDE8A,phosphodiesterase 8A isoform 1 |
| 224901_at | 17.59 | 3.71E-09 | 1.831163134 | NM_001037582| | NA |
| 232478_at | 17.58 | 3.71E-09 | 1.347307436 | NA |  |
| 203202_at | 17.58 | 3.72E-09 | 1.072981151 | NM_007043| | HRB2,HIV-1 rev binding protein 2 |
| 213645_at | 17.57 | 3.74E-09 | 1.232880536 | NM_001126123| | NA |
| 235008_at | 17.56 | 3.75E-09 | 1.159501367 | NA |  |
| 201764_at | 17.56 | 3.77E-09 | 1.327581288 | NM_024056| | MGC5576,hypothetical protein MGC5576 |
| 239515_at | 17.55 | 3.79E-09 | 1.442583563 | NA |  |
| 230776_at | 17.55 | 3.79E-09 | 1.370851797 | NM_052916| | RNF157,ring finger protein 157 |
| 232226_at | 17.54 | 3.81E-09 | 1.637466939 | NM_020929| | NGL-1,netrin-G1 ligand |
| 225050_at | 17.53 | 3.84E-09 | 1.071680276 | NM_032434| | ZNF512,zinc finger protein 512 |
| 207809_s_at | 17.53 | 3.83E-09 | 1.083873993 | NM_001183| | ATP6AP1,ATPase, H+ transporting, lysosomal accessory |
| 212593_s_at | 17.52 | 3.86E-09 | 1.157520352 | NM_014456| | PDCD4,programmed cell death 4 isoform 1 |
| 213726_x_at | 17.51 | 3.88E-09 | 1.049557278 | NM_006088| | TUBB2,tubulin, beta, 2 |
| 203518_at | 17.5 | 3.91E-09 | 1.642490721 | NM_000081| | LYST,lysosomal trafficking regulator isoform 1 |
| 216997_x_at | 17.5 | 3.90E-09 | 1.40564808 | NM_007005| | TLE4,transducin-like enhancer protein 4 |
| 1554355_a_at | 17.5 | 3.90E-09 | 1.822335782 | NM_170601| | CSE-C,cytosolic sialic acid 9-O-acetylesterase |
| 233341_s_at | 17.5 | 3.91E-09 | 1.091577762 | NM_019014| | POLR1B,RNA polymerase I polypeptide B |
| 209046_s_at | 17.5 | 3.90E-09 | 1.107453481 | NM_007285| | GABARAPL2,GABA(A) receptor-associated protein-like 2 |
| 239273_s_at | 17.49 | 3.93E-09 | 1.620905438 | NM_001032278| | NA |
| 201778_s_at | 17.49 | 3.93E-09 | 1.055320721 | NM_014774| | KIAA0494,KIAA0494 gene product |
| 220525_s_at | 17.49 | 3.93E-09 | 1.080757659 | NM_181575| | AUP1,ancient ubiquitous protein 1 isoform 2 |
| 241989_at | 17.47 | 3.96E-09 | 1.260910156 | NM_001004056| | GRK4,G protein-coupled receptor kinase 4 isoform |
| 201335_s_at | 17.47 | 3.98E-09 | 1.327751826 | NM_015313| | ARHGEF12,Rho guanine nucleotide exchange factor (GEF) 12 |
| 212608_s_at | 17.47 | 3.96E-09 | 1.142706974 | NA |  |
| 231967_at | 17.47 | 3.96E-09 | 1.498564978 | NM_016018| | PHF20L1,PHD finger protein 20-like 1 isoform 1 |
| 222006_at | 17.46 | 3.98E-09 | 1.123515075 | NM_012318| | LETM1,leucine zipper-EF-hand containing transmembrane |
| 212563_at | 17.46 | 3.98E-09 | 1.134615609 | NM_015201| | BOP1,block of proliferation 1 |
| 213281_at | 17.46 | 4.00E-09 | 1.297096437 | NM_002228| | JUN,v-jun avian sarcoma virus 17 oncogene homolog |
| 226907_at | 17.45 | 4.03E-09 | 1.74003678 | NM_030949| | PPP1R14C,protein phosphatase 1, regulatory (inhibitor) |
| 224631_at | 17.45 | 4.03E-09 | 1.1398195 | NM_053023| | ZFP91,zinc finger protein 91 isoform 1 |
| 219254_at | 17.45 | 4.01E-09 | 1.25045256 | NM_024648| | FLJ22222,hypothetical protein FLJ22222 |
| 235488_at | 17.43 | 4.07E-09 | 1.061589906 | NM_033315| | RASL10B,RAS-like, family 10, member B |
| 219152_at | 17.43 | 4.07E-09 | 1.276535831 | NM_015720| | PODXL2,endoglycan |
| 1556473_at | 17.43 | 4.06E-09 | 1.474683732 | NA |  |
| 214331_at | 17.41 | 4.12E-09 | 1.311838682 | NM_005726| | TSFM,Ts translation elongation factor, mitochondrial |
| 209902_at | 17.41 | 4.11E-09 | 1.104587997 | NM_001184| | ATR,ataxia telangiectasia and Rad3 related protein |
| 210651_s_at | 17.41 | 4.12E-09 | 1.18244188 | NM_004442| | EPHB2,ephrin receptor EphB2 isoform 2 precursor |
| 212397_at | 17.41 | 4.11E-09 | 1.046718783 | NM_002906| | RDX,radixin |
| 208370_s_at | 17.4 | 4.15E-09 | 1.323315508 | NM_004414| | DSCR1,calcipressin 1 isoform a |
| 220460_at | 17.4 | 4.15E-09 | 1.808105638 | NM_017435| | SLCO1C1,solute carrier organic anion transporter family, |
| 235468_at | 17.4 | 4.14E-09 | 1.144650652 | NM_001082575| | NA |
| 211930_at | 17.39 | 4.17E-09 | 1.134900562 | NM_194247| | HNRPA3,heterogeneous nuclear ribonucleoprotein A3 |
| 229803_s_at | 17.38 | 4.20E-09 | 1.112780195 | NA |  |
| 204962_s_at | 17.38 | 4.17E-09 | 1.095139563 | NM_001042426| | NA |
| 225237_s_at | 17.37 | 4.22E-09 | 1.235415621 | NM_138962| | MSI2,musashi 2 isoform a |
| 225096_at | 17.37 | 4.20E-09 | 1.089960105 | NM_018405| | HSA272196,hypothetical protein, clone 2746033 |
| 210993_s_at | 17.37 | 4.20E-09 | 1.141549909 | NM_001003688| | SMAD1,Sma- and Mad-related protein 1 |
| 205130_at | 17.37 | 4.22E-09 | 1.366506332 | NM_014226| | RAGE,MAPK/MAK/MRK overlapping kinase |
| 209317_at | 17.36 | 4.24E-09 | 1.094467535 | NM_004875| | POLR1C,RNA polymerase I subunit isoform 2 |
| 229653_at | 17.36 | 4.23E-09 | 1.263990573 | NA |  |
| 1556476_at | 17.35 | 4.25E-09 | 1.49624421 | NA |  |
| 213289_at | 17.35 | 4.26E-09 | 1.188077399 | NM_198450| | CXorf33,chromosome X open reading frame 33 |
| 1555953_at | 17.34 | 4.29E-09 | 1.293776696 | NM_030582| | COL18A1,alpha 1 type XVIII collagen isoform 1 precursor |
| 202585_s_at | 17.33 | 4.31E-09 | 1.187670557 | NM_002504| | NFX1,nuclear transcription factor, X-box binding 1 |
| 225682_s_at | 17.33 | 4.32E-09 | 1.154725186 | NM_001018050| | NA |
| 215434_x_at | 17.33 | 4.31E-09 | 1.188826511 | NM_001037501| | NA |
| 205412_at | 17.32 | 4.35E-09 | 1.120317883 | NM_000019| | ACAT1,acetyl-Coenzyme A acetyltransferase 1 precursor |
| 202156_s_at | 17.3 | 4.39E-09 | 1.119686942 | NM_001025076| | NA |
| 217763_s_at | 17.3 | 4.41E-09 | 1.24288764 | NM_006868| | RAB31,RAB31, member RAS oncogene family |
| 225255_at | 17.29 | 4.44E-09 | 1.223421796 | NM_016622| | MRPL35,mitochondrial ribosomal protein L35 isoform a |
| 209168_at | 17.28 | 4.46E-09 | 1.152928458 | NM_001001994| | GPM6B,glycoprotein M6B isoform 4 |
| 224391_s_at | 17.27 | 4.48E-09 | 1.411073319 | NM_170601| | CSE-C,cytosolic sialic acid 9-O-acetylesterase |
| 209866_s_at | 17.27 | 4.47E-09 | 1.227940375 | NM_015236| | LPHN3,latrophilin 3 precursor |
| 222138_s_at | 17.26 | 4.52E-09 | 1.183647734 | NM_017883| | WDR13,WD repeat domain 13 protein |
| 1558795_at | 17.26 | 4.52E-09 | 1.761670584 | NA |  |
| 225242_s_at | 17.24 | 4.57E-09 | 1.417186597 | NM_199511| | URB,steroid-sensitive protein 1 |
| 209444_at | 17.24 | 4.57E-09 | 1.082767459 | NM_001100426| | NA |
| 211152_s_at | 17.22 | 4.61E-09 | 1.120686789 | NM_013247| | PRSS25,protease, serine, 25 isoform 1 preproprotein |
| 201339_s_at | 17.22 | 4.61E-09 | 1.056806096 | NM_001007098| | SCP2,sterol carrier protein X isoform 2 |
| 223307_at | 17.22 | 4.62E-09 | 1.083476567 | NM_031299| | CDCA3,trigger of mitotic entry 1 |
| 1554789_a_at | 17.19 | 4.70E-09 | 1.614376146 | NM_001029851| | NA |
| 210251_s_at | 17.19 | 4.71E-09 | 1.055890085 | NM_001037442| | NA |
| 236250_at | 17.19 | 4.72E-09 | 1.339796 | NA |  |
| 64438_at | 17.18 | 4.72E-09 | 1.09891242 | NM_024648| | FLJ22222,hypothetical protein FLJ22222 |
| 240111_at | 17.17 | 4.77E-09 | 1.361448746 | NM_014899| | RHOBTB3,rho-related BTB domain containing 3 |
| 225575_at | 17.17 | 4.77E-09 | 1.316863007 | NM_002310| | LIFR, |
| 38241_at | 17.16 | 4.78E-09 | 1.529717517 | NM_006994| | BTN3A3,butyrophilin, subfamily 3, member A3 isoform a |
| 241950_at | 17.16 | 4.80E-09 | 1.386820967 | NA |  |
| 220183_s_at | 17.16 | 4.81E-09 | 1.162229951 | NM_007083| | NUDT6,nudix-type motif 6 isoform a |
| 211611_s_at | 17.16 | 4.80E-09 | 1.240536577 | NM_004381| | CREBL1,cAMP responsive element binding protein-like 1 |
| 1557049_at | 17.16 | 4.80E-09 | 1.348641454 | NA |  |
| 229434_at | 17.15 | 4.83E-09 | 1.214935434 | NA |  |
| 236404_at | 17.15 | 4.81E-09 | 1.33522158 | NA |  |
| 204142_at | 17.14 | 4.84E-09 | 1.207433154 | NM_001126123| | NA |
| 225136_at | 17.14 | 4.85E-09 | 1.299187627 | NM_021623| | NA |
| 201535_at | 17.14 | 4.86E-09 | 1.188097261 | NM_007106| | UBL3,ubiquitin-like 3 |
| 217910_x_at | 17.13 | 4.87E-09 | 1.064166998 | NM_170607| | MLX,transcription factor-like protein 4 isoform |
| 1553984_s_at | 17.13 | 4.88E-09 | 1.116385396 | NM_012145| | DTYMK,deoxythymidylate kinase (thymidylate kinase) |
| 226237_at | 17.13 | 4.86E-09 | 1.591214024 | NA |  |
| 216231_s_at | 17.12 | 4.90E-09 | 1.11591844 | NM_004048| | B2M,beta-2-microglobulin precursor |
| 225143_at | 17.12 | 4.92E-09 | 1.195363451 | NM_213649| | SFXN4,sideroflexin 4 isoform 1 |
| 203065_s_at | 17.12 | 4.92E-09 | 1.311009478 | NM_001753| | CAV1,caveolin 1 |
| 228479_at | 17.12 | 4.90E-09 | 1.418559555 | NA |  |
| 225794_s_at | 17.1 | 4.97E-09 | 1.08694559 | NM_033318| | LOC91689,hypothetical protein supported by AL449243 |
| 242260_at | 17.1 | 4.99E-09 | 1.173432942 | NM_018834| | MATR3,matrin 3 |
| 238756_at | 17.08 | 5.03E-09 | 1.232843496 | NM_174942| | GAS2L3,growth arrest-specific 2 like 3 |
| 211165_x_at | 17.08 | 5.04E-09 | 1.261378126 | NM_004442| | EPHB2,ephrin receptor EphB2 isoform 2 precursor |
| 244826_at | 17.06 | 5.09E-09 | 1.300951661 | NA |  |
| 202358_s_at | 17.06 | 5.09E-09 | 1.084819768 | NM_014758| | NA |
| 236609_at | 17.06 | 5.09E-09 | 1.314753474 | NA |  |
| 221850_x_at | 17.06 | 5.11E-09 | 1.074937059 | NM_001077665| | NA |
| 225384_at | 17.06 | 5.11E-09 | 1.137395132 | NM_033407| | DOCK7,dedicator of cytokinesis 7 |
| 236961_at | 17.05 | 5.12E-09 | 1.215192243 | NA |  |
| 203441_s_at | 17.05 | 5.12E-09 | 1.28688036 | NM_001792| | CDH2,cadherin 2, type 1 preproprotein |
| 225590_at | 17.05 | 5.11E-09 | 1.160729007 | NM_020870| | SH3MD2,SH3 multiple domains 2 |
| 239581_at | 17.05 | 5.12E-09 | 1.226364237 | NA |  |
| 221892_at | 17.04 | 5.15E-09 | 1.205620039 | NM_004285| | H6PD,hexose-6-phosphate dehydrogenase precursor |
| 225327_at | 17.04 | 5.16E-09 | 1.223012675 | NM_019600| | FLJ10980,hypothetical protein FLJ10980 |
| 228148_at | 17.03 | 5.20E-09 | 1.24547453 | NM_173548| | ZNF584,zinc finger protein 584 |
| 209281_s_at | 17.03 | 5.20E-09 | 1.192882742 | NM_001001323| | ATP2B1,plasma membrane calcium ATPase 1 isoform 1a |
| 225479_at | 17.03 | 5.18E-09 | 1.204837896 | NM_001099678| | NA |
| 225578_at | 17.02 | 5.23E-09 | 1.200774679 | NM_001071775| | NA |
| 239082_at | 17.02 | 5.21E-09 | 1.185215296 | NA |  |
| 208050_s_at | 17.01 | 5.26E-09 | 1.150862874 | NM_032982| | CASP2,caspase 2 isoform 1 preproprotein |
| 205525_at | 17.01 | 5.24E-09 | 1.190323974 | NM_004342| | CALD1,caldesmon 1 isoform 2 |
| 209171_at | 17.01 | 5.24E-09 | 1.11258673 | NM_033453| | ITPA,inosine triphosphatase isoform a |
| 1561691_at | 17 | 5.27E-09 | 1.658377879 | NA |  |
| 219450_at | 17 | 5.27E-09 | 1.672035648 | NM_001104629| | NA |
| 209177_at | 17 | 5.29E-09 | 1.101472098 | NM_199069| | DKFZP564J0123,nuclear protein E3-3 isoform a |
| 1558369_at | 17 | 5.28E-09 | 1.132815681 | NM_022782| | MPHOSPH9,M-phase phosphoprotein 9 |
| 204238_s_at | 16.99 | 5.30E-09 | 1.135382364 | NM_006443| | C6orf108,putative c-Myc-responsive isoform 1 |
| 201133_s_at | 16.99 | 5.31E-09 | 1.115074378 | NM_014819| | PJA2,praja 2, RING-H2 motif containing |
| 232541_at | 16.99 | 5.31E-09 | 1.882936353 | NA |  |
| 229086_at | 16.99 | 5.30E-09 | 1.330826857 | NM_001008896| | LOC148898,hypothetical protein BC007899 isoform 2 |
| 227581_at | 16.99 | 5.31E-09 | 1.119196758 | NM_015395| | DKFZP434B0335,DKFZP434B0335 protein |
| 200810_s_at | 16.97 | 5.36E-09 | 1.075038792 | NM_001280| | CIRBP,cold inducible RNA binding protein |
| 230652_at | 16.97 | 5.38E-09 | 1.360216971 | NM_001654| | ARAF,v-raf murine sarcoma 3611 viral oncogene |
| 212594_at | 16.96 | 5.41E-09 | 1.214822923 | NM_014456| | PDCD4,programmed cell death 4 isoform 1 |
| 207618_s_at | 16.96 | 5.42E-09 | 1.145795194 | NM_001079866| | NA |
| 212377_s_at | 16.96 | 5.40E-09 | 1.118062665 | NM_024408| | NOTCH2,notch 2 preproprotein |
| 226394_at | 16.95 | 5.46E-09 | 1.142578108 | NM_017824| | MARCH5,ring finger protein 153 |
| 200006_at | 16.95 | 5.46E-09 | 1.043477813 | NM_001123377| | NA |
| 239848_at | 16.95 | 5.44E-09 | 1.89517566 | NA |  |
| 201465_s_at | 16.93 | 5.52E-09 | 1.214648959 | NM_002228| | JUN,v-jun avian sarcoma virus 17 oncogene homolog |
| 221307_at | 16.92 | 5.57E-09 | 1.793787652 | NM_001034837| | NA |
| 209789_at | 16.91 | 5.59E-09 | 1.804120893 | NM_006091| | CORO2B,coronin, actin binding protein, 2B |
| 201306_s_at | 16.9 | 5.62E-09 | 1.085850474 | NM_006401| | ANP32B,acidic (leucine-rich) nuclear phosphoprotein 32 |
| 235606_at | 16.9 | 5.62E-09 | 1.359946695 | NA |  |
| 218966_at | 16.89 | 5.65E-09 | 1.325078685 | NM_018728| | MYO5C,myosin VC |
| 221506_s_at | 16.89 | 5.65E-09 | 1.081939472 | NM_013433| | TNPO2,transportin 2 (importin 3, karyopherin beta 2b) |
| 201494_at | 16.89 | 5.66E-09 | 1.04631588 | NM_005040| | PRCP,prolylcarboxypeptidase isoform 1 preproprotein |
| 206342_x_at | 16.89 | 5.64E-09 | 1.193999016 | NM_000202| | IDS,iduronate-2-sulfatase isoform a precursor |
| 213381_at | 16.88 | 5.69E-09 | 1.245703675 | NM_001031746| | NA |
| 231779_at | 16.88 | 5.68E-09 | 1.376466962 | NM_001570| | IRAK2,interleukin-1 receptor-associated kinase 2 |
| 205078_at | 16.88 | 5.68E-09 | 1.056391146 | NM_002643| | PIGF,phosphatidylinositol glycan, class F isoform 1 |
| 244680_at | 16.88 | 5.68E-09 | 1.459993457 | NM_000824| | GLRB,glycine receptor, beta |
| 238021_s_at | 16.87 | 5.73E-09 | 1.149180893 | NA |  |
| 223213_s_at | 16.87 | 5.74E-09 | 1.703952494 | NM_001017926| | NA |
| 1555595_at | 16.87 | 5.72E-09 | 1.21281898 | NM_024583| | SCRN3,secernin 3 |
| 226627_at | 16.87 | 5.72E-09 | 1.656258772 | NM_001098811| | NA |
| 242989_at | 16.87 | 5.72E-09 | 1.628975046 | NA |  |
| 225656_at | 16.87 | 5.74E-09 | 1.238533501 | NM_018100| | EFHC1,EF-hand domain (C-terminal) containing 1 |
| 224715_at | 16.86 | 5.74E-09 | 1.214260482 | NM_052844| | WDR34,WD repeat domain 34 |
| 239266_at | 16.86 | 5.76E-09 | 1.13885776 | NA |  |
| 208977_x_at | 16.86 | 5.75E-09 | 1.045011752 | NM_006088| | TUBB2,tubulin, beta, 2 |
| 225056_at | 16.86 | 5.74E-09 | 1.218474205 | NM_020808| | SIPA1L2,signal-induced proliferation-associated 1 like |
| 225473_at | 16.85 | 5.80E-09 | 1.261521546 | NM_199181| | FLJ44670,FLJ44670 protein |
| 236229_at | 16.84 | 5.82E-09 | 1.528467477 | NA |  |
| 204027_s_at | 16.83 | 5.84E-09 | 1.111592509 | NM_005371| | METTL1,methyltransferase-like protein 1 isoform a |
| 218198_at | 16.82 | 5.91E-09 | 1.090016694 | NM_018180| | DHX32,DEAD/H (Asp-Glu-Ala-Asp/His) box polypeptide 32 |
| 230184_at | 16.82 | 5.92E-09 | 1.755792807 | NA |  |
| 218135_at | 16.81 | 5.92E-09 | 1.047619635 | NM_016570| | PTX1,CDA14 |
| 209236_at | 16.8 | 5.98E-09 | 1.156909768 | NM_005116| | SLC23A2,solute carrier family 23 (nucleobase |
| 226134_s_at | 16.8 | 5.98E-09 | 1.235399984 | NA |  |
| 218616_at | 16.8 | 5.98E-09 | 1.12883366 | NM_020395| | LOC57117,hypothetical nuclear factor SBBI22 |
| 216044_x_at | 16.79 | 6.02E-09 | 1.12671343 | NM_001006605| | LOC388650,hypothetical LOC388650 |
| 222618_at | 16.78 | 6.04E-09 | 1.055912955 | NM_018225| | SMU1,smu-1 suppressor of mec-8 and unc-52 homolog |
| 201190_s_at | 16.78 | 6.07E-09 | 1.104219287 | NM_006224| | PITPNA,phosphatidylinositol transfer protein, alpha |
| 229800_at | 16.77 | 6.09E-09 | 1.142280871 | NM_004734| | DCAMKL1,doublecortin and CaM kinase-like 1 |
| 222783_s_at | 16.76 | 6.15E-09 | 1.490602114 | NM_001034852| | NA |
| 224743_at | 16.75 | 6.16E-09 | 1.133457113 | NM_017813| | IMPA3,myo-inositol monophosphatase A3 |
| 223370_at | 16.75 | 6.16E-09 | 1.094519461 | NM_019091| | PLEKHA3,pleckstrin homology domain containing, family A |
| 243417_at | 16.75 | 6.16E-09 | 1.838381302 | NA |  |
| 204143_s_at | 16.75 | 6.16E-09 | 1.253177957 | NM_001126123| | NA |
| 53071_s_at | 16.74 | 6.22E-09 | 1.235251558 | NM_024648| | FLJ22222,hypothetical protein FLJ22222 |
| 236146_at | 16.74 | 6.18E-09 | 1.216093471 | NM_006372| | SYNCRIP,synaptotagmin binding, cytoplasmic RNA |
| 202254_at | 16.73 | 6.23E-09 | 1.181068985 | NM_015556| | SIPA1L1,signal-induced proliferation-associated 1 like |
| 226487_at | 16.73 | 6.23E-09 | 1.342970048 | NM_032829| | FLJ14721,hypothetical protein FLJ14721 |
| 205227_at | 16.73 | 6.23E-09 | 1.266987163 | NM_002182| | IL1RAP,interleukin 1 receptor accessory protein isoform |
| 225795_at | 16.71 | 6.30E-09 | 1.088123541 | NM_033318| | LOC91689,hypothetical protein supported by AL449243 |
| 211966_at | 16.71 | 6.33E-09 | 1.235102771 | NM_001846| | COL4A2,alpha 2 type IV collagen preproprotein |
| 1559901_s_at | 16.69 | 6.41E-09 | 1.758636581 | NM_001005732| | C21orf34,chromosome 21 open reading frame 34 isoform a |
| 217367_s_at | 16.69 | 6.40E-09 | 1.339856453 | NM_015035| | ZHX3,zinc fingers and homeoboxes 3 protein |
| 218153_at | 16.68 | 6.46E-09 | 1.087059506 | NM_024537| | FLJ12118,hypothetical protein FLJ12118 |
| 206501_x_at | 16.68 | 6.45E-09 | 1.181739488 | NM_004956| | ETV1,ets variant gene 1 |
| 227524_at | 16.68 | 6.43E-09 | 1.25786621 | NA |  |
| 225545_at | 16.67 | 6.47E-09 | 1.075803237 | NM_013302| | EEF2K,elongation factor-2 kinase |
| 204246_s_at | 16.67 | 6.50E-09 | 1.199774976 | NM_007234| | DCTN3,dynactin 3 isoform 1 |
| 1562012_at | 16.66 | 6.54E-09 | 1.487361296 | NA |  |
| 214693_x_at | 16.66 | 6.54E-09 | 1.208035599 | NM_001037501| | NA |
| 203963_at | 16.65 | 6.55E-09 | 1.746839699 | NM_001218| | CA12,carbonic anhydrase XII isoform 1 precursor |
| 201266_at | 16.65 | 6.57E-09 | 1.076169012 | NM_001093771| | NA |
| 218494_s_at | 16.65 | 6.56E-09 | 1.110105327 | NM_020062| | SLC2A4RG,SLC2A4 regulator |
| 239978_at | 16.64 | 6.60E-09 | 1.479370674 | NA |  |
| 214526_x_at | 16.64 | 6.60E-09 | 1.120604243 | NA |  |
| 213508_at | 16.64 | 6.60E-09 | 1.119752258 | NM_138288| | C14orf147,chromosome 14 open reading frame 147 |
| 203349_s_at | 16.63 | 6.64E-09 | 1.297223664 | NM_004454| | ETV5,ets variant gene 5 (ets-related molecule) |
| 235754_at | 16.63 | 6.65E-09 | 1.305887348 | NM_000410| | HFE,hemochromatosis protein isoform 1 precursor |
| 227100_at | 16.63 | 6.67E-09 | 1.240151658 | NM_194318| | B3GTL,beta 3-glycosyltransferase-like |
| 1556082_a_at | 16.62 | 6.67E-09 | 1.624132219 | NA |  |
| 205182_s_at | 16.62 | 6.68E-09 | 1.139817442 | NM_014347| | ZNF324,zinc finger protein 324 |
| 203994_s_at | 16.62 | 6.70E-09 | 1.145399909 | NM_004928| | C21orf2,chromosome 21 open reading frame 2 |
| 208609_s_at | 16.62 | 6.68E-09 | 1.172118397 | NM_019105| | TNXB,tenascin XB isoform 1 |
| 227557_at | 16.62 | 6.67E-09 | 1.204896957 | NM_153334| | SCARF2,scavenger receptor class F, member 2 isoform 1 |
| 203039_s_at | 16.62 | 6.67E-09 | 1.030951507 | NM_005006| | NDUFS1,NADH dehydrogenase (ubiquinone) Fe-S protein 1, |
| 223226_x_at | 16.61 | 6.70E-09 | 1.179309537 | NM_001009998| | SSBP4,single stranded DNA binding protein 4 isoform b |
| 225363_at | 16.61 | 6.73E-09 | 1.091562188 | NM_000314| | PTEN,phosphatase and tensin homolog |
| 211792_s_at | 16.6 | 6.76E-09 | 1.137162445 | NM_001262| | CDKN2C,cyclin-dependent kinase inhibitor 2C |
| 231034_s_at | 16.6 | 6.75E-09 | 1.21835065 | NA |  |
| 225355_at | 16.59 | 6.79E-09 | 1.175307938 | NA |  |
| 224593_at | 16.58 | 6.85E-09 | 1.16974493 | NM_152437| | ZFOC1,zinc finger protein ZFOC1 |
| 224417_at | 16.58 | 6.86E-09 | 1.443793672 | NA |  |
| 225469_at | 16.57 | 6.87E-09 | 1.236488482 | NM_001001660| | LOC144363,hypothetical protein LOC144363 |
| 206448_at | 16.57 | 6.89E-09 | 1.443612506 | NM_014951| | NA |
| 229593_at | 16.56 | 6.95E-09 | 1.333211875 | NA |  |
| 214361_s_at | 16.55 | 6.96E-09 | 1.228137513 | NM_002926| | RGS12,regulator of G-protein signalling 12 isoform 2 |
| 223468_s_at | 16.55 | 6.96E-09 | 1.166784431 | NM_020211| | RGMA,RGM domain family, member A |
| 217944_at | 16.55 | 6.96E-09 | 1.105993675 | NM_017739| | FLJ20277,O-linked mannose |
| 218109_s_at | 16.54 | 7.03E-09 | 1.187528382 | NM_022736| | MFSD1,major facilitator superfamily domain containing |
| 231117_at | 16.54 | 7.04E-09 | 1.494000552 | NM_138344| | C14orf152,chromosome 14 open reading frame 152 |
| 209505_at | 16.54 | 7.05E-09 | 1.671551569 | NM_005654| | NR2F1,nuclear receptor subfamily 2, group F, member 1 |
| 225939_at | 16.53 | 7.05E-09 | 1.235817643 | NM_173359| | NA |
| 45687_at | 16.53 | 7.06E-09 | 1.134598676 | NM_024031| | MGC3121,hypothetical protein MGC3121 |
| 211337_s_at | 16.52 | 7.09E-09 | 1.133813479 | NM_014444| | 76P,gamma tubulin ring complex protein (76p gene) |
| 202945_at | 16.52 | 7.09E-09 | 1.045624717 | NM_001018078| | NA |
| 39966_at | 16.51 | 7.14E-09 | 1.360267484 | NM_006574| | CSPG5,chondroitin sulfate proteoglycan 5 (neuroglycan |
| 226490_at | 16.51 | 7.14E-09 | 1.25944989 | NA |  |
| 204576_s_at | 16.5 | 7.20E-09 | 1.113767392 | NM_015041| | CLUAP1,clusterin associated protein 1 |
| 204649_at | 16.5 | 7.19E-09 | 1.079940122 | NM_001100620| | NA |
| 225718_at | 16.49 | 7.24E-09 | 1.225197297 | NM_030650| | KIAA1715,Lunapark |
| 218407_x_at | 16.49 | 7.24E-09 | 1.089973507 | NM_013349| | SCIRP10,SCIRP10-related protein |
| 203151_at | 16.48 | 7.28E-09 | 1.261535676 | NM_002373| | MAP1A,microtubule-associated protein 1A |
| 208949_s_at | 16.48 | 7.32E-09 | 1.223134874 | NM_002306| | LGALS3,galectin-3 |
| 209513_s_at | 16.47 | 7.33E-09 | 1.121001309 | NM_032303| | HSDL2,hydroxysteroid dehydrogenase like 2 |
| 1552790_a_at | 16.47 | 7.33E-09 | 1.245809582 | NM_003262| | TLOC1,translocation protein 1 |
| 210448_s_at | 16.47 | 7.34E-09 | 1.284017424 | NM_002561| | P2RX5,purinergic receptor P2X5 isoform A |
| 205260_s_at | 16.47 | 7.32E-09 | 1.103969578 | NM_001107| | ACYP1,erythrocyte acylphosphatase 1 isoform a |
| 201178_at | 16.46 | 7.40E-09 | 1.035311965 | NM_001033024| | NA |
| 202115_s_at | 16.46 | 7.36E-09 | 1.068163215 | NM_015658| | DKFZP564C186,DKFZP564C186 protein |
| 231767_at | 16.46 | 7.37E-09 | 1.275644996 | NM_024015| | HOXB4,homeo box B4 |
| 212656_at | 16.46 | 7.37E-09 | 1.065486255 | NM_005726| | TSFM,Ts translation elongation factor, mitochondrial |
| 229380_at | 16.46 | 7.37E-09 | 1.699987576 | NA |  |
| 212504_at | 16.45 | 7.43E-09 | 1.144343092 | NM_014974| | KIAA0934,KIAA0934 |
| 239272_at | 16.44 | 7.47E-09 | 1.817528825 | NM_001032278| | NA |
| 223945_x_at | 16.43 | 7.51E-09 | 1.184105759 | NA |  |
| 206788_s_at | 16.43 | 7.51E-09 | 1.18831301 | NM_001755| | CBFB,core-binding factor, beta subunit isoform 2 |
| 208451_s_at | 16.42 | 7.58E-09 | 1.182385391 | NM_000592| | C4B,complement component 4B preproprotein |
| 204365_s_at | 16.42 | 7.58E-09 | 1.367324777 | NM_022912| | C2orf23,receptor expression enhancing protein 1 |
| 211060_x_at | 16.42 | 7.56E-09 | 1.12037892 | NM_003801| | GPAA1,anchor attachment protein 1 |
| 1563111_a_at | 16.41 | 7.62E-09 | 1.186388445 | NM_017861| | PIGX,GPI-mannosyltransferase subunit |
| 230257_s_at | 16.41 | 7.61E-09 | 1.114163621 | NM_052965| | C1orf19,chromosome 1 open reading frame 19 |
| 236821_at | 16.41 | 7.61E-09 | 1.256603219 | NA |  |
| 235652_at | 16.4 | 7.70E-09 | 1.735461412 | NA |  |
| 229531_at | 16.39 | 7.71E-09 | 1.388437854 | NA |  |
| 219154_at | 16.39 | 7.74E-09 | 1.196729733 | NM_019034| | RHOF,ras homolog gene family, member F |
| 202799_at | 16.39 | 7.72E-09 | 1.059725757 | NM_006012| | CLPP,endopeptidase Clp precursor |
| 206879_s_at | 16.38 | 7.79E-09 | 1.167577709 | NM_004883| | NRG2,neuregulin 2 isoform 1 |
| 217897_at | 16.38 | 7.75E-09 | 1.164153346 | NM_022003| | FXYD6,FXYD domain-containing ion transport regulator |
| 201125_s_at | 16.37 | 7.79E-09 | 1.067886328 | NM_002213| | ITGB5,integrin, beta 5 |
| 229645_at | 16.36 | 7.89E-09 | 1.483534099 | NM_001044369| | NA |
| 208825_x_at | 16.36 | 7.90E-09 | 1.01023814 | NM_000984| | RPL23A,ribosomal protein L23a |
| 213052_at | 16.35 | 7.93E-09 | 1.095017368 | NM_004157| | PRKAR2A,cAMP-dependent protein kinase, regulatory |
| 221507_at | 16.35 | 7.93E-09 | 1.092463881 | NM_013433| | TNPO2,transportin 2 (importin 3, karyopherin beta 2b) |
| 203687_at | 16.35 | 7.93E-09 | 1.208741707 | NM_002996| | CX3CL1,chemokine (C-X3-C motif) ligand 1 |
| 1557353_at | 16.34 | 7.95E-09 | 1.450853726 | NA |  |
| 238164_at | 16.33 | 8.00E-09 | 1.415881426 | NM_001080491| | NA |
| 205034_at | 16.33 | 8.02E-09 | 1.43554118 | NM_057749| | CCNE2,cyclin E2 isoform 1 |
| 201146_at | 16.33 | 8.01E-09 | 1.083085628 | NM_006164| | NFE2L2,nuclear factor (erythroid-derived 2)-like 2 |
| 230026_at | 16.32 | 8.08E-09 | 1.297049244 | NM_032112| | MRPL43,mitochondrial ribosomal protein L43 isoform a |
| 226831_at | 16.32 | 8.08E-09 | 1.333838369 | NM_138773| | LOC91137,hypothetical protein BC017169 |
| 224820_at | 16.32 | 8.08E-09 | 1.204768211 | NM_198076| | FAM36A,family with sequence similarity 36, member A |
| 203314_at | 16.31 | 8.14E-09 | 1.113533248 | NM_012227| | GTPBP6,pseudoautosomal GTP-binding protein-like |
| 210645_s_at | 16.31 | 8.14E-09 | 1.250372883 | NM_001001894| | TTC3,tetratricopeptide repeat domain 3 |
| 209470_s_at | 16.31 | 8.14E-09 | 2.130378756 | NM_005277| | GPM6A,glycoprotein M6A isoform 1 |
| 205449_at | 16.31 | 8.14E-09 | 1.115170306 | NM_013299| | SHD1,Sac3 homology domain 1 |
| 232064_at | 16.31 | 8.14E-09 | 1.408334782 | NA |  |
| 211621_at | 16.3 | 8.19E-09 | 1.277922462 | NM_000044| | AR,androgen receptor isoform 1 |
| 239024_at | 16.3 | 8.19E-09 | 1.255372512 | NM_021964| | ZNF148,zinc finger protein 148 (pHZ-52) |
| 203152_at | 16.3 | 8.19E-09 | 1.142491154 | NM_003776| | MRPL40,mitochondrial ribosomal protein L40 |
| 235213_at | 16.3 | 8.19E-09 | 1.644944546 | NM_002221| | ITPKB,1D-myo-inositol-trisphosphate 3-kinase B |
| 226941_at | 16.29 | 8.21E-09 | 1.147020062 | NA |  |
| 223040_at | 16.29 | 8.21E-09 | 1.096943547 | NM_016100| | NAT5,N-acetyltransferase 5 isoform a |
| 202119_s_at | 16.29 | 8.20E-09 | 1.06742487 | NM_003909| | CPNE3,copine III |
| 37860_at | 16.28 | 8.25E-09 | 1.114612728 | NM_015655| | ZNF337,zinc finger protein 337 |
| 218329_at | 16.27 | 8.30E-09 | 1.071441988 | NM_012406| | PRDM4,PR domain containing 4 |
| 1568795_at | 16.27 | 8.32E-09 | 1.730181606 | NA |  |
| 228615_at | 16.27 | 8.30E-09 | 1.216985833 | NA |  |
| 201151_s_at | 16.26 | 8.35E-09 | 1.634723315 | NM_021038| | MBNL1,muscleblind-like 1 isoform a |
| 212954_at | 16.26 | 8.40E-09 | 1.194132182 | NM_003845| | DYRK4,dual-specificity tyrosine-(Y)-phosphorylation |
| 225948_at | 16.26 | 8.35E-09 | 1.048029408 | NM_032374| | C14orf153,chromosome 14 open reading frame 153 |
| 209247_s_at | 16.25 | 8.43E-09 | 1.140387638 | NM_005692| | ABCF2,ATP-binding cassette, sub-family F, member 2 |
| 224783_at | 16.24 | 8.48E-09 | 1.132229877 | NM_182565| | MGC29814,hypothetical protein MGC29814 |
| 202506_at | 16.24 | 8.50E-09 | 1.593369049 | NM_006751| | SSFA2,sperm specific antigen 2 |
| 1553348_a_at | 16.24 | 8.48E-09 | 1.226972406 | NM_002504| | NFX1,nuclear transcription factor, X-box binding 1 |
| 223429_x_at | 16.24 | 8.50E-09 | 1.085387978 | NM_020701| | KIAA1160,KIAA1160 protein |
| 230465_at | 16.23 | 8.55E-09 | 1.810006957 | NM_012262| | HS2ST1,heparan sulfate 2-O-sulfotransferase 1 |
| 233224_at | 16.23 | 8.52E-09 | 1.388337274 | NA |  |
| 208967_s_at | 16.23 | 8.51E-09 | 1.067708758 | NM_001625| | AK2,adenylate kinase 2 isoform a |
| 1555471_a_at | 16.22 | 8.56E-09 | 1.68782769 | NM_020066| | FMN2,formin 2 |
| 219544_at | 16.22 | 8.57E-09 | 1.115412612 | NM_024808| | FLJ22624,FLJ22624 protein |
| 224512_s_at | 16.22 | 8.57E-09 | 1.162997543 | NM_032356| | MGC14151,hypothetical protein MGC14151 |
| 200698_at | 16.21 | 8.65E-09 | 1.105912955 | NM_001100603| | NA |
| 1559132_at | 16.2 | 8.70E-09 | 1.168452735 | NM_001042463| | NA |
| 211052_s_at | 16.19 | 8.78E-09 | 1.061889355 | NM_005993| | TBCD,beta-tubulin cofactor D |
| 212854_x_at | 16.19 | 8.78E-09 | 1.121686627 | NM_001039703| | NA |
| 232244_at | 16.19 | 8.73E-09 | 1.342044427 | NM_020702| | KIAA1161,KIAA1161 |
| 212049_at | 16.19 | 8.76E-09 | 1.093596507 | NM_133264| | WIRE,WIRE protein |
| 222760_at | 16.18 | 8.80E-09 | 1.319418172 | NM_025069| | FLJ14299,hypothetical protein FLJ14299 |
| 206453_s_at | 16.18 | 8.83E-09 | 1.224325328 | NM_016250| | NDRG2,N-myc downstream-regulated gene 2 isoform b |
| 222517_at | 16.17 | 8.89E-09 | 1.324536728 | NM_012095| | AP3M1,adaptor-related protein complex 3, mu 1 subunit |
| 210243_s_at | 16.16 | 8.94E-09 | 1.065707762 | NM_003779| | B4GALT3,UDP-Gal:betaGlcNAc beta 1,4- |
| 218898_at | 16.16 | 8.97E-09 | 1.116513652 | NM_024792| | CT120,membrane protein expressed in epithelial-like |
| 210157_at | 16.15 | 8.99E-09 | 1.109721212 | NM_003796| | C19orf2,RPB5-mediating protein isoform a |
| 211715_s_at | 16.15 | 9.01E-09 | 1.124347046 | NM_004051| | BDH,3-hydroxybutyrate dehydrogenase precursor |
| 202565_s_at | 16.15 | 8.99E-09 | 1.234126336 | NM_003174| | SVIL,supervillin isoform 1 |
| 224796_at | 16.15 | 8.97E-09 | 1.131555898 | NM_018482| | DDEF1,development and differentiation enhancing factor |
| 240066_at | 16.15 | 8.98E-09 | 1.746253006 | NA |  |
| 201408_at | 16.14 | 9.03E-09 | 1.200543245 | NM_002709| | PPP1CB,protein phosphatase 1, catalytic subunit, beta |
| 212111_at | 16.14 | 9.05E-09 | 1.149637809 | NM_177424| | STX12,syntaxin 12 |
| 229058_at | 16.13 | 9.09E-09 | 1.134208773 | NM_001009941| | ANKRD16,ankyrin repeat domain 16 isoform a |
| 228515_at | 16.11 | 9.23E-09 | 1.19097542 | NA |  |
| 203795_s_at | 16.11 | 9.23E-09 | 1.165917254 | NM_001024808| | NA |
| 36499_at | 16.11 | 9.21E-09 | 1.115313802 | NM_001408| | CELSR2,cadherin EGF LAG seven-pass G-type receptor 2 |
| 243591_at | 16.11 | 9.24E-09 | 1.447154125 | NA |  |
| 235760_at | 16.11 | 9.24E-09 | 1.384948408 | NM_022455| | NSD1,nuclear receptor binding SET domain protein 1 |
| 228932_at | 16.1 | 9.27E-09 | 1.268979913 | NA |  |
| 226935_s_at | 16.09 | 9.39E-09 | 1.094013581 | NM_030782| | CRR9,cisplatin resistance related protein CRR9p |
| 218126_at | 16.09 | 9.33E-09 | 1.104626131 | NM_018145| | FLJ10579,hypothetical protein FLJ10579 |
| 203270_at | 16.08 | 9.40E-09 | 1.135777732 | NM_012145| | DTYMK,deoxythymidylate kinase (thymidylate kinase) |
| 212726_at | 16.08 | 9.40E-09 | 1.219782161 | NM_005392| | PHF2,PHD finger protein 2 isoform a |
| 209469_at | 16.08 | 9.40E-09 | 1.945002217 | NM_005277| | GPM6A,glycoprotein M6A isoform 1 |
| 226883_at | 16.07 | 9.46E-09 | 1.107458942 | NA |  |
| 212238_at | 16.06 | 9.53E-09 | 1.174372313 | NM_015338| | ASXL1,additional sex combs like 1 |
| 235688_s_at | 16.05 | 9.58E-09 | 1.245556378 | NM_004295| | TRAF4,TNF receptor-associated factor 4 isoform 1 |
| 209944_at | 16.05 | 9.61E-09 | 1.126701236 | NM_021188| | ZNF410,clones 23667 and 23775 zinc finger protein |
| 1558750_a_at | 16.05 | 9.59E-09 | 1.274770172 | NA |  |
| 208969_at | 16.05 | 9.61E-09 | 1.040392122 | NM_005002| | NDUFA9,NADH dehydrogenase (ubiquinone) 1 alpha |
| 201645_at | 16.05 | 9.59E-09 | 1.66280384 | NM_002160| | TNC,tenascin C (hexabrachion) |
| 212388_at | 16.04 | 9.68E-09 | 1.076626475 | NM_015306| | NA |
| 222875_at | 16.04 | 9.65E-09 | 1.05122875 | NM_020162| | DHX33,DEAH (Asp-Glu-Ala-His) box polypeptide 33 |
| 219373_at | 16.04 | 9.63E-09 | 1.123989399 | NM_018973| | DPM3,dolichyl-phosphate mannosyltransferase |
| 224694_at | 16.03 | 9.73E-09 | 1.221159445 | NM_018153| | ANTXR1,tumor endothelial marker 8 isoform 3 precursor |
| 222551_s_at | 16.03 | 9.75E-09 | 1.133290644 | NM_023080| | FLJ20989,hypothetical protein FLJ20989 |
| 202171_at | 16.02 | 9.79E-09 | 1.077569931 | NM_007146| | ZNF161,zinc finger protein 161 |
| 217934_x_at | 16.02 | 9.79E-09 | 1.092200058 | NM_005861| | STUB1,STIP1 homology and U-box containing protein 1 |
| 214073_at | 16.01 | 9.84E-09 | 1.206063283 | NM_005231| | CTTN,cortactin isoform a |
| 225804_at | 16.01 | 9.84E-09 | 1.174322039 | NM_144611| | MGC32124,hypothetical protein MGC32124 |
| 227584_at | 16.01 | 9.84E-09 | 1.088224233 | NM_020443| | NAV1,neuron navigator 1 |
| 235803_at | 16 | 9.90E-09 | 1.796065584 | NA |  |
| 203353_s_at | 15.99 | 9.95E-09 | 1.06602486 | NM_002384| | MBD1,methyl-CpG binding domain protein 1 isoform 4 |
| 229366_at | 15.99 | 9.98E-09 | 1.205546611 | NA |  |
| 221406_s_at | 15.96 | 1.02E-08 | 1.331176234 | NM_001039651| | NA |
| 239084_at | 15.96 | 1.02E-08 | 1.309032683 | NA |  |
| 1555952_at | 15.96 | 1.02E-08 | 1.196878957 | NM_194255| | SLC19A1,solute carrier family 19 member 1 isoform a |
| 223095_at | 15.96 | 1.02E-08 | 1.191733871 | NA |  |
| 204029_at | 15.95 | 1.03E-08 | 1.136467502 | NM_001408| | CELSR2,cadherin EGF LAG seven-pass G-type receptor 2 |
| 233304_at | 15.95 | 1.02E-08 | 1.813092844 | NM_005596| | NFIB,nuclear factor I/B |
| 217826_s_at | 15.95 | 1.03E-08 | 1.339096084 | NM_016021| | UBE2J1,ubiquitin-conjugating enzyme E2, J1 |
| 228530_at | 15.95 | 1.02E-08 | 1.283197243 | NM_001071775| | NA |
| 225441_x_at | 15.95 | 1.02E-08 | 1.16115738 | NM_032356| | MGC14151,hypothetical protein MGC14151 |
| 205521_at | 15.95 | 1.02E-08 | 1.197026451 | NM_005107| | ENDOGL1,endonuclease G-like 1 |
| 65884_at | 15.94 | 1.03E-08 | 1.098512575 | NM_016219| | MAN1B1,alpha 1,2-mannosidase |
| 202867_s_at | 15.94 | 1.03E-08 | 1.120508585 | NM_001002762| | DNAJB12,DnaJ (Hsp40) homolog, subfamily B, member 12 |
| 206502_s_at | 15.94 | 1.03E-08 | 1.635426022 | NM_002196| | INSM1,insulinoma-associated 1 |
| 209090_s_at | 15.94 | 1.03E-08 | 1.112631981 | NM_016009| | SH3GLB1,SH3-containing protein SH3GLB1 |
| 219999_at | 15.93 | 1.04E-08 | 1.31582895 | NM_006122| | MAN2A2,mannosidase, alpha, class 2A, member 2 |
| 205880_at | 15.93 | 1.04E-08 | 1.556598199 | NM_002742| | PRKD1,protein kinase D1 |
| 224755_at | 15.92 | 1.04E-08 | 1.22209808 | NA |  |
| 203543_s_at | 15.92 | 1.04E-08 | 1.94175164 | NM_001206| | KLF9,Kruppel-like factor 9 |
| 235079_at | 15.91 | 1.05E-08 | 1.484108508 | NA |  |
| 236310_at | 15.91 | 1.05E-08 | 1.389208479 | NA |  |
| 1553954_at | 15.91 | 1.05E-08 | 1.313816867 | NM_144988| | MGC19780,hypothetical protein MGC19780 |
| 216005_at | 15.91 | 1.05E-08 | 1.56524705 | NM_002160| | TNC,tenascin C (hexabrachion) |
| 211499_s_at | 15.89 | 1.07E-08 | 1.301045351 | NM_002751| | MAPK11,mitogen-activated protein kinase 11 |
| 228333_at | 15.89 | 1.07E-08 | 1.464187607 | NA |  |
| 211985_s_at | 15.88 | 1.07E-08 | 1.160199611 | NM_001743| | CALM2,calmodulin 2 |
| 209447_at | 15.88 | 1.07E-08 | 1.439932027 | NM_015293| | SYNE1,nesprin 1 isoform beta |
| 205453_at | 15.88 | 1.07E-08 | 1.491279531 | NM_002145| | HOXB2,homeo box B2 |
| 234321_x_at | 15.87 | 1.08E-08 | 1.20815171 | NA |  |
| 212797_at | 15.87 | 1.08E-08 | 1.155128134 | NM_002959| | SORT1,sortilin 1 preproprotein |
| 222697_s_at | 15.86 | 1.08E-08 | 1.16135422 | NM_018394| | ABHD10,abhydrolase domain containing 10 |
| 222507_s_at | 15.86 | 1.09E-08 | 1.163421964 | NM_020644| | C11orf15,chromosome 11 open reading frame 15 |
| 1557133_at | 15.86 | 1.09E-08 | 1.235595169 | NM_001126492| | NA |
| 225132_at | 15.85 | 1.09E-08 | 1.25210784 | NM_012158| | FBXL3,F-box and leucine-rich repeat protein 3 |
| 239585_at | 15.85 | 1.09E-08 | 1.440610435 | NM_003884| | PCAF,p300/CBP-associated factor |
| 213008_at | 15.85 | 1.10E-08 | 1.099056802 | NM_001113378| | NA |
| 235543_at | 15.85 | 1.10E-08 | 1.290386113 | NA |  |
| 213598_at | 15.84 | 1.10E-08 | 1.098275182 | NM_014473| | HSA9761,putative dimethyladenosine transferase |
| 243768_at | 15.83 | 1.11E-08 | 1.143319114 | NA |  |
| 1553974_at | 15.83 | 1.11E-08 | 1.120902951 | NM_173793| | LOC128977,hypothetical protein LOC128977 |
| 203916_at | 15.82 | 1.12E-08 | 1.067993634 | NM_003635| | NDST2,N-deacetylase/N-sulfotransferase (heparan |
| 213509_x_at | 15.82 | 1.11E-08 | 1.12096972 | NM_003869| | CES2,carboxylesterase 2 isoform 1 |
| 200015_s_at | 15.82 | 1.12E-08 | 1.044250518 | NM_001008491| | SEPT2,septin 2 |
| 227158_at | 15.82 | 1.11E-08 | 1.10285207 | NM_080664| | C14orf126,hypothetical protein MGC9912 |
| 227746_at | 15.81 | 1.12E-08 | 1.37977649 | NM_001419| | ELAVL1,ELAV-like 1 |
| 201853_s_at | 15.81 | 1.12E-08 | 1.159690153 | NM_004358| | CDC25B,cell division cycle 25B isoform 1 |
| 230069_at | 15.81 | 1.12E-08 | 1.145936923 | NM_022754| | SFXN1,sideroflexin 1 |
| 224335_s_at | 15.8 | 1.13E-08 | 1.229125641 | NM_012104| | BACE1,beta-site APP-cleaving enzyme 1 isoform A |
| 37965_at | 15.8 | 1.13E-08 | 1.189262513 | NM_001003828| | PARVB,parvin, beta isoform a |
| 226919_at | 15.77 | 1.16E-08 | 1.284814949 | NM_020466| | DJ122O8.2,hypothetical protein dJ122O8.2 |
| 1558088_a_at | 15.76 | 1.16E-08 | 1.182977754 | NM_003345| | UBE2I,ubiquitin-conjugating enzyme E2I |
| 201870_at | 15.75 | 1.17E-08 | 1.112991177 | NM_006809| | TOMM34,translocase of outer mitochondrial membrane 34 |
| 229666_s_at | 15.75 | 1.17E-08 | 1.134940102 | NM_001033505| | NA |
| 223712_at | 15.73 | 1.19E-08 | 1.185479218 | NM_032151| | PCBD2,dimerization cofactor of hepatocyte nuclear |
| 217721_at | 15.73 | 1.19E-08 | 1.316557702 | NA |  |
| 216033_s_at | 15.73 | 1.19E-08 | 1.154596326 | NM_002037| | FYN,protein-tyrosine kinase fyn isoform a |
| 227143_s_at | 15.73 | 1.19E-08 | 1.245244375 | NM_001196| | BID,BH3 interacting domain death agonist isoform 2 |
| 242492_at | 15.73 | 1.18E-08 | 1.260861174 | NA |  |
| 242939_at | 15.71 | 1.20E-08 | 1.497959494 | NM_007111| | TFDP1,transcription factor Dp-1 |
| 225504_at | 15.71 | 1.20E-08 | 1.129067604 | NA |  |
| 230904_at | 15.71 | 1.20E-08 | 1.421793184 | NM_031919| | CCDC10,cystatin and DUF19 domain-containing protein 1 |
| 228108_at | 15.7 | 1.21E-08 | 1.204247909 | NA |  |
| 233924_s_at | 15.7 | 1.21E-08 | 1.214340884 | NM_001013848| | NA |
| 235925_at | 15.68 | 1.23E-08 | 1.441427231 | NM_003205| | TCF12,transcription factor 12 isoform b |
| 222453_at | 15.67 | 1.23E-08 | 1.293195304 | NM_024843| | CYBRD1,cytochrome b reductase 1 |
| 201704_at | 15.67 | 1.24E-08 | 1.119371877 | NM_001114089| | NA |
| 212601_at | 15.67 | 1.23E-08 | 1.124762699 | NM_015113| | ZZEF1,zinc finger, ZZ-type with EF hand domain 1 |
| 204306_s_at | 15.67 | 1.23E-08 | 1.191452131 | NM_001039490| | NA |
| 227476_at | 15.66 | 1.25E-08 | 1.451026121 | NA |  |
| 232473_at | 15.66 | 1.24E-08 | 1.603528799 | NM_003675| | PRPF18,PRP18 pre-mRNA processing factor 18 homolog |
| 215517_at | 15.65 | 1.25E-08 | 1.450479957 | NM_015617| | PYGO1,pygopus homolog 1 |
| 230306_at | 15.65 | 1.25E-08 | 1.162166447 | NM_052875| | MGC10485,hypothetical protein MGC10485 |
| 229994_at | 15.64 | 1.26E-08 | 1.444691377 | NA |  |
| 201350_at | 15.64 | 1.26E-08 | 1.072276939 | NM_004475| | FLOT2,flotillin 2 |
| 227894_at | 15.63 | 1.27E-08 | 1.242440594 | NM_145294| | LOC197336,similar to RIKEN cDNA 3230401M21 [Mus musculus] |
| 227189_at | 15.63 | 1.27E-08 | 1.426530038 | NM_020939| | CPNE5,copine V |
| 228310_at | 15.62 | 1.27E-08 | 1.207511746 | NM_001008493| | ENAH,enabled homolog isoform a |
| 204504_s_at | 15.61 | 1.28E-08 | 1.082982717 | NM_003609| | HIRIP3,HIRA interacting protein 3 |
| 220173_at | 15.61 | 1.28E-08 | 1.47184034 | NM_025057| | C14orf45,chromosome 14 open reading frame 45 |
| 218284_at | 15.61 | 1.28E-08 | 1.140959463 | NM_005902| | SMAD3,MAD, mothers against decapentaplegic homolog 3 |
| 66053_at | 15.61 | 1.28E-08 | 1.139999819 | NM_001079559| | NA |
| 221704_s_at | 15.61 | 1.28E-08 | 1.092644241 | NM_024667| | FLJ12750,hypothetical protein FLJ12750 |
| 209605_at | 15.61 | 1.28E-08 | 1.081069335 | NM_003312| | TST,thiosulfate sulfurtransferase |
| 213005_s_at | 15.61 | 1.28E-08 | 1.122361236 | NM_015158| | ANKRD15,ankyrin repeat domain protein 15 |
| 1557286_at | 15.61 | 1.28E-08 | 1.428800413 | NA |  |
| 219135_s_at | 15.6 | 1.29E-08 | 1.263475986 | NM_022773| | FLJ12681,hypothetical protein FLJ12681 |
| 205651_x_at | 15.6 | 1.29E-08 | 1.747574171 | NM_001100397| | NA |
| 225772_s_at | 15.59 | 1.30E-08 | 1.076989674 | NM_032901| | MGC14288,hypothetical protein MGC14288 |
| 205320_at | 15.59 | 1.30E-08 | 1.28087134 | NM_005883| | APC2,adenomatosis polyposis coli 2 |
| 49485_at | 15.59 | 1.30E-08 | 1.084594635 | NM_012406| | PRDM4,PR domain containing 4 |
| 218776_s_at | 15.59 | 1.30E-08 | 1.381947192 | NM_024956| | FLJ23375,hypothetical protein FLJ23375 |
| 1556328_at | 15.59 | 1.30E-08 | 1.964482977 | NA |  |
| 204843_s_at | 15.59 | 1.30E-08 | 1.171046409 | NM_004157| | PRKAR2A,cAMP-dependent protein kinase, regulatory |
| 241699_at | 15.59 | 1.30E-08 | 1.60512441 | NA |  |
| 209385_s_at | 15.59 | 1.30E-08 | 1.110449612 | NM_007198| | PROSC,proline synthetase co-transcribed homolog |
| 218839_at | 15.58 | 1.31E-08 | 1.408857743 | NM_001040708| | NA |
| 203037_s_at | 15.57 | 1.31E-08 | 1.292921156 | NM_014751| | MTSS1,metastasis suppressor 1 |
| 212605_s_at | 15.57 | 1.32E-08 | 1.154841287 | NA |  |
| 231877_at | 15.56 | 1.32E-08 | 1.275129347 | NM_152292| | RG9MTD2,RNA (guanine-9-) methyltransferase domain |
| 236010_at | 15.56 | 1.33E-08 | 1.192393498 | NA |  |
| 204119_s_at | 15.56 | 1.33E-08 | 1.124177259 | NM_001123| | ADK,adenosine kinase isoform a |
| 223407_at | 15.56 | 1.32E-08 | 1.26127507 | NM_032140| | C16orf48,chromosome 16 open reading frame 48 |
| 235031_at | 15.56 | 1.33E-08 | 1.734351757 | NA |  |
| 215164_at | 15.54 | 1.34E-08 | 1.301964049 | NA |  |
| 231847_at | 15.54 | 1.34E-08 | 1.20565542 | NM_138797| | LOC129138,hypothetical protein BC014641 |
| 235626_at | 15.54 | 1.34E-08 | 1.089796677 | NM_020397| | CAMK1D,calcium/calmodulin-dependent protein kinase ID |
| 232890_at | 15.54 | 1.34E-08 | 1.254621045 | NA |  |
| 216705_s_at | 15.54 | 1.35E-08 | 1.108431783 | NM_000022| | ADA,adenosine deaminase |
| 225115_at | 15.53 | 1.35E-08 | 1.157079042 | NM_001113239| | NA |
| 230777_s_at | 15.53 | 1.35E-08 | 1.109353076 | NM_001040424| | NA |
| 231423_s_at | 15.53 | 1.35E-08 | 1.132713126 | NM_001009941| | ANKRD16,ankyrin repeat domain 16 isoform a |
| 219137_s_at | 15.53 | 1.35E-08 | 1.081176904 | NM_020194| | C2orf33,chromosome 2 open reading frame 33 |
| 220964_s_at | 15.53 | 1.35E-08 | 1.053559014 | NM_030981| | RAB1B,RAB1B, member RAS oncogene family |
| 209361_s_at | 15.52 | 1.35E-08 | 1.216429787 | NM_020418| | PCBP4,poly(rC) binding protein 4 isoform a |
| 223743_s_at | 15.52 | 1.36E-08 | 1.139533076 | NM_015956| | MRPL4,mitochondrial ribosomal protein L4 isoform a |
| 225446_at | 15.52 | 1.36E-08 | 1.104811156 | NM_001007246| | WDR9,WD repeat domain 9 isoform C |
| 1554101_a_at | 15.52 | 1.35E-08 | 1.250519088 | NM_001079669| | NA |
| 1553677_a_at | 15.52 | 1.36E-08 | 1.094257222 | NM_001031800| | NA |
| 40569_at | 15.51 | 1.36E-08 | 1.110364207 | NM_003422| | ZNF42,zinc finger protein 42 isoform 1 |
| 219249_s_at | 15.5 | 1.37E-08 | 1.131393699 | NM_021939| | FKBP10,FK506 binding protein 10, 65 kDa |
| 204514_at | 15.5 | 1.37E-08 | 1.132395392 | NM_001039589| | NA |
| 230653_at | 15.48 | 1.40E-08 | 1.470936692 | NA |  |
| 202650_s_at | 15.48 | 1.40E-08 | 1.098692988 | NM_014738| | KIAA0195,KIAA0195 gene product |
| 215634_at | 15.47 | 1.41E-08 | 1.861893021 | NA |  |
| 221727_at | 15.47 | 1.41E-08 | 1.208415702 | NM_006713| | PC4,activated RNA polymerase II transcription |
| 201864_at | 15.47 | 1.40E-08 | 1.098849597 | NM_001493| | GDI1,GDP dissociation inhibitor 1 |
| 203911_at | 15.46 | 1.42E-08 | 1.116863855 | NM_002885| | RAP1GA1,RAP1, GTPase activating protein 1 |
| 239238_at | 15.46 | 1.41E-08 | 1.286091272 | NA |  |
| 235174_s_at | 15.46 | 1.42E-08 | 1.730132864 | NA |  |
| 213758_at | 15.45 | 1.43E-08 | 1.180458489 | NM_001861| | COX4I1,cytochrome c oxidase subunit IV isoform 1 |
| 238431_at | 15.44 | 1.43E-08 | 1.237733572 | NA |  |
| 206499_s_at | 15.44 | 1.43E-08 | 1.078161653 | NM_001048194| | NA |
| 223641_at | 15.43 | 1.44E-08 | 1.261675846 | NA |  |
| 225594_at | 15.42 | 1.46E-08 | 1.098432642 | NM_001039618| | NA |
| 233842_x_at | 15.41 | 1.47E-08 | 1.064705629 | NM_016407| | C20orf43,chromosome 20 open reading frame 43 |
| 208999_at | 15.41 | 1.47E-08 | 1.11024824 | NM_001098811| | NA |
| 214268_s_at | 15.4 | 1.47E-08 | 1.124564694 | NM_004687| | MTMR4,myotubularin related protein 4 |
| 224534_at | 15.4 | 1.48E-08 | 1.593474758 | NM_001039570| | NA |
| 217526_at | 15.4 | 1.47E-08 | 1.091519477 | NM_032815| | NFATC2IP,nuclear factor of activated T-cells, |
| 202967_at | 15.4 | 1.48E-08 | 1.076205991 | NM_001512| | GSTA4,glutathione S-transferase A4 |
| 235587_at | 15.4 | 1.48E-08 | 1.154918338 | NA |  |
| 209867_s_at | 15.39 | 1.49E-08 | 1.15085645 | NM_015236| | LPHN3,latrophilin 3 precursor |
| 223080_at | 15.39 | 1.48E-08 | 1.260718499 | NM_014905| | GLS,glutaminase C |
| 214545_s_at | 15.39 | 1.48E-08 | 1.139533351 | NM_007198| | PROSC,proline synthetase co-transcribed homolog |
| 201959_s_at | 15.38 | 1.50E-08 | 1.178757456 | NM_015057| | MYCBP2,MYC binding protein 2 |
| 222777_s_at | 15.37 | 1.51E-08 | 1.281654059 | NM_001042424| | NA |
| 227750_at | 15.37 | 1.51E-08 | 1.129241769 | NM_001024660| | NA |
| 240312_at | 15.37 | 1.51E-08 | 1.375981471 | NA |  |
| 200991_s_at | 15.36 | 1.52E-08 | 1.076178459 | NM_014748| | SNX17,sorting nexin 17 |
| 1555460_a_at | 15.35 | 1.53E-08 | 1.187116645 | NM_001099406| | NA |
| 212573_at | 15.35 | 1.53E-08 | 1.283748166 | NM_015036| | NA |
| 224303_x_at | 15.34 | 1.54E-08 | 1.126912404 | NM_016350| | NIN,ninein isoform 4 |
| 224909_s_at | 15.34 | 1.54E-08 | 1.358793465 | NM_020820| | PREX1,PREX1 protein |
| 217927_at | 15.34 | 1.54E-08 | 1.041276239 | NM_014041| | SPCS1,signal peptidase complex subunit 1 homolog |
| 220632_s_at | 15.34 | 1.54E-08 | 1.079807597 | NM_013382| | POMT2,putative protein O-mannosyltransferase |
| 205646_s_at | 15.34 | 1.54E-08 | 1.568653633 | NM_000280| | PAX6,paired box gene 6 isoform a |
| 239533_at | 15.33 | 1.55E-08 | 1.997653062 | NM_001033045| | NA |
| 225118_at | 15.33 | 1.55E-08 | 1.238133014 | NM_020382| | SET8,SET domain-containing protein 8 |
| 229459_at | 15.32 | 1.56E-08 | 1.138506734 | NM_001082967| | NA |
| 227021_at | 15.32 | 1.56E-08 | 1.385852882 | NM_153042| | NA |
| 204846_at | 15.32 | 1.56E-08 | 1.311899038 | NM_000096| | CP,ceruloplasmin (ferroxidase) |
| 239512_at | 15.31 | 1.57E-08 | 1.412044583 | NM_005626| | SFRS4,splicing factor, arginine/serine-rich 4 |
| 218841_at | 15.3 | 1.58E-08 | 1.264044569 | NM_024095| | ASB8,ankyrin repeat and SOCS box-containing 8 |
| 236168_at | 15.29 | 1.60E-08 | 1.351926169 | NA |  |
| 1556054_at | 15.28 | 1.61E-08 | 1.372605811 | NA |  |
| 230210_at | 15.28 | 1.61E-08 | 1.397441994 | NM_025154| | UNC84A,unc-84 homolog A |
| 226120_at | 15.27 | 1.62E-08 | 1.126812707 | NM_144596| | TTC8,tetratricopeptide repeat domain 8 isoform A |
| 232527_at | 15.27 | 1.62E-08 | 1.135552873 | NA |  |
| 209356_x_at | 15.27 | 1.62E-08 | 1.220049514 | NM_016938| | EFEMP2,EGF-containing fibulin-like extracellular matrix |
| 237482_s_at | 15.26 | 1.63E-08 | 1.251071561 | NM_025247| | ACAD10,acyl-Coenzyme A dehydrogenase family, member 10 |
| 219843_at | 15.26 | 1.63E-08 | 1.237363643 | NM_005897| | IPP,intracisternal A particle-promoted polypeptide |
| 1559496_at | 15.25 | 1.64E-08 | 1.453601671 | NM_001034191| | NA |
| 223104_at | 15.24 | 1.65E-08 | 1.101520027 | NM_032492| | JAGN1,jagunal homolog 1 |
| 231954_at | 15.23 | 1.66E-08 | 1.206357897 | NA |  |
| 207839_s_at | 15.23 | 1.67E-08 | 1.249649293 | NM_001042589| | NA |
| 228693_at | 15.22 | 1.68E-08 | 1.523690563 | NM_174908| | C3orf6,Ymer protein short isoform |
| 203596_s_at | 15.22 | 1.68E-08 | 1.362606807 | NM_012420| | IFIT5,interferon-induced protein with |
| 1555476_at | 15.21 | 1.69E-08 | 1.261815694 | NM_004136| | IREB2,iron-responsive element binding protein 2 |
| 228180_at | 15.21 | 1.68E-08 | 1.342847417 | NA |  |
| 232632_at | 15.2 | 1.70E-08 | 1.545415363 | NA |  |
| 215411_s_at | 15.2 | 1.69E-08 | 1.183905663 | NM_147200| | C6orf4,chromosome 6 open reading frame 4 isoform 1 |
| 208919_s_at | 15.2 | 1.69E-08 | 1.078209857 | NM_023018| | FLJ13052,NAD kinase |
| 1564339_a_at | 15.2 | 1.69E-08 | 1.263750102 | NM_000740| | CHRM3,cholinergic receptor, muscarinic 3 |
| 226143_at | 15.19 | 1.71E-08 | 1.259896704 | NM_030665| | RAI1,retinoic acid induced 1 |
| 228844_at | 15.18 | 1.72E-08 | 1.456499284 | NM_177550| | SLC13A5,solute carrier family 13 (sodium-dependent |
| 223499_at | 15.18 | 1.72E-08 | 1.202104087 | NM_015645| | C1QTNF5,C1q and tumor necrosis factor related protein 5 |
| 1556341_s_at | 15.18 | 1.72E-08 | 1.201370774 | NM_002969| | MAPK12,mitogen-activated protein kinase 12 |
| 228664_at | 15.17 | 1.73E-08 | 1.109748462 | NA |  |
| 227148_at | 15.16 | 1.75E-08 | 1.328751857 | NM_172069| | PLEKHH2,pleckstrin homology domain containing, family H |
| 235461_at | 15.15 | 1.76E-08 | 1.290047874 | NM_001127208| | NA |
| 225722_at | 15.14 | 1.76E-08 | 1.096003316 | NA |  |
| 222172_at | 15.14 | 1.77E-08 | 1.271360075 | NM_022123| | NPAS3,neuronal PAS domain protein 3 |
| 209692_at | 15.14 | 1.77E-08 | 1.245074707 | NM_005244| | EYA2,eyes absent 2 isoform a |
| 209988_s_at | 15.13 | 1.79E-08 | 2.271374913 | NM_004316| | ASCL1,achaete-scute complex homolog-like 1 |
| 207107_at | 15.13 | 1.79E-08 | 2.283976864 | NM_000329| | RPE65,retinal pigment epithelium-specific protein |
| 228349_at | 15.12 | 1.80E-08 | 1.19186497 | NA |  |
| 221231_s_at | 15.11 | 1.81E-08 | 1.214270998 | NM_017970| | C14orf102,chromosome 14 open reading frame 102 isoform 1 |
| 230712_at | 15.11 | 1.80E-08 | 1.164087751 | NM_001037501| | NA |
| 225571_at | 15.1 | 1.82E-08 | 1.428147667 | NM_002310| | LIFR, |
| 225633_at | 15.1 | 1.82E-08 | 1.10048287 | NM_207325| | LOC147991,hypothetical protein LOC147991 |
| 228656_at | 15.09 | 1.84E-08 | 1.47136089 | NM_002763| | PROX1,prospero-related homeobox 1 |
| 238452_at | 15.09 | 1.83E-08 | 1.495236068 | NM_001002901| | FCRL2,hypothetical protein FLJ31052 isoform a |
| 203245_s_at | 15.09 | 1.83E-08 | 1.111524292 | NA |  |
| 229989_at | 15.09 | 1.83E-08 | 1.250340934 | NM_138378| | NA |
| 210046_s_at | 15.09 | 1.83E-08 | 1.172179975 | NM_002168| | IDH2,isocitrate dehydrogenase 2 (NADP+), |
| 226913_s_at | 15.09 | 1.83E-08 | 1.381534682 | NM_014587| | SOX8,SRY (sex determining region Y)-box 8 |
| 201410_at | 15.08 | 1.85E-08 | 1.039916831 | NM_001100623| | NA |
| 209293_x_at | 15.08 | 1.84E-08 | 1.432384727 | NM_001546| | ID4,inhibitor of DNA binding 4, dominant negative |
| 226692_at | 15.08 | 1.84E-08 | 1.144609594 | NM_001018108| | NA |
| 231883_at | 15.07 | 1.87E-08 | 1.235304538 | NM_012174| | FBXW8,F-box and WD-40 domain protein 8 isoform 2 |
| 1553106_at | 15.07 | 1.86E-08 | 1.355152018 | NM_152409| | FLJ37562,hypothetical protein FLJ37562 |
| 242473_at | 15.06 | 1.87E-08 | 1.309041773 | NM_004295| | TRAF4,TNF receptor-associated factor 4 isoform 1 |
| 235890_at | 15.06 | 1.87E-08 | 1.257914333 | NA |  |
| 1555216_a_at | 15.06 | 1.87E-08 | 2.006538123 | NA |  |
| 230948_at | 15.06 | 1.87E-08 | 1.321695811 | NA |  |
| 201489_at | 15.06 | 1.87E-08 | 1.120852381 | NM_005729| | PPIF,peptidylprolyl isomerase F precursor |
| 218328_at | 15.06 | 1.87E-08 | 1.092105225 | NM_016035| | COQ4,CGI-92 protein |
| 235736_at | 15.05 | 1.89E-08 | 1.525271906 | NA |  |
| 226897_s_at | 15.05 | 1.88E-08 | 1.070641911 | NM_014153| | ZC3HDC7,zinc finger CCCH type domain containing 7 |
| 212678_at | 15.04 | 1.90E-08 | 1.145021554 | NM_000267| | NF1,neurofibromin |
| 214723_x_at | 15.04 | 1.89E-08 | 1.297297748 | NM_025190| | NA |
| 218481_at | 15.04 | 1.90E-08 | 1.056760748 | NM_020158| | EXOSC5,exosome component Rrp46 |
| 226458_at | 15.04 | 1.90E-08 | 1.153256444 | NA |  |
| 222277_at | 15.03 | 1.91E-08 | 1.16256909 | NM_001014442| | NA |
| 218874_s_at | 15.03 | 1.91E-08 | 1.302387013 | NM_001031722| | NA |
| 212382_at | 15.02 | 1.92E-08 | 1.177127146 | NM_001083962| | NA |
| 223159_s_at | 15.02 | 1.92E-08 | 1.46730327 | NM_014397| | NEK6,putative serine-threonine protein kinase |
| 39817_s_at | 15.01 | 1.93E-08 | 1.108391307 | NM_006443| | C6orf108,putative c-Myc-responsive isoform 1 |
| 209275_s_at | 15.01 | 1.94E-08 | 1.09967945 | NM_000086| | CLN3,CLN3 protein |
| 234049_at | 15 | 1.95E-08 | 1.259938162 | NA |  |
| 201426_s_at | 15 | 1.95E-08 | 1.117051685 | NM_003380| | VIM,vimentin |
| 214756_x_at | 15 | 1.95E-08 | 1.120910567 | NA |  |
| 215287_at | 15 | 1.95E-08 | 1.679588386 | NA |  |
| 226013_at | 15 | 1.94E-08 | 1.237078769 | NM_001042646| | NA |
| 230651_at | 14.99 | 1.96E-08 | 1.362760913 | NA |  |
| 224691_at | 14.98 | 1.98E-08 | 1.098585698 | NA |  |
| 214194_at | 14.98 | 1.98E-08 | 1.267922184 | NM_014953| | KIAA1008,KIAA1008 |
| 200973_s_at | 14.98 | 1.97E-08 | 1.15845496 | NM_005724| | TM4SF8,transmembrane 4 superfamily member 8 isoform 1 |
| 221676_s_at | 14.97 | 2.00E-08 | 1.080795289 | NM_014325| | CORO1C,coronin, actin binding protein, 1C |
| 209597_s_at | 14.97 | 1.99E-08 | 1.240710897 | NM_007257| | NA |
| 220522_at | 14.97 | 2.00E-08 | 1.449522717 | NM_201253| | CRB1,crumbs homolog 1 isoform II precursor |
| 223302_s_at | 14.97 | 2.00E-08 | 1.227314296 | NM_001009958| | ZNF655,zinc finger protein 655 isoform c |
| 242611_at | 14.96 | 2.01E-08 | 1.234494492 | NA |  |
| 227840_at | 14.96 | 2.01E-08 | 1.144855064 | NM_001017927| | NA |
| 1555609_a_at | 14.95 | 2.02E-08 | 1.597815048 | NM_022470| | WIG1,p53 target zinc finger protein isoform 1 |
| 228379_at | 14.95 | 2.02E-08 | 1.224630587 | NM_005796| | NUTF2,nuclear transport factor 2 |
| 211474_s_at | 14.95 | 2.02E-08 | 1.127307872 | NM_004568| | SERPINB6,serine (or cysteine) proteinase inhibitor, clade |
| 204065_at | 14.95 | 2.01E-08 | 1.082301832 | NM_004854| | CHST10,HNK-1 sulfotransferase |
| 203668_at | 14.95 | 2.02E-08 | 1.156855074 | NM_006715| | MAN2C1,mannosidase, alpha, class 2C, member 1 |
| 229448_at | 14.95 | 2.02E-08 | 1.220939059 | NM_021267| | LASS1,longevity assurance gene 1 isoform 1 |
| 219742_at | 14.95 | 2.02E-08 | 1.127957234 | NM_030567| | PRR7,proline rich 7 (synaptic) |
| 209812_x_at | 14.95 | 2.02E-08 | 1.13260493 | NM_032982| | CASP2,caspase 2 isoform 1 preproprotein |
| 203148_s_at | 14.94 | 2.03E-08 | 1.088282082 | NM_014788| | TRIM14,tripartite motif protein TRIM14 isoform alpha |
| 218590_at | 14.94 | 2.03E-08 | 1.080240177 | NM_021830| | PEO1,twinkle |
| 208630_at | 14.94 | 2.03E-08 | 1.045085106 | NM_000182| | HADHA,hydroxyacyl dehydrogenase, subunit A |
| 220354_at | 14.93 | 2.04E-08 | 1.240008555 | NA |  |
| 204497_at | 14.93 | 2.04E-08 | 1.121582121 | NM_001116| | ADCY9,adenylate cyclase 9 |
| 232480_at | 14.93 | 2.05E-08 | 1.477387629 | NA |  |
| 229981_at | 14.92 | 2.05E-08 | 1.245967555 | NM_014426| | SNX5,sorting nexin 5 |
| 202408_s_at | 14.92 | 2.06E-08 | 1.089831367 | NM_015629| | PRPF31,pre-mRNA processing factor 31 homolog |
| 213785_at | 14.92 | 2.07E-08 | 1.222066271 | NM_018085| | IPO9,importin 9 |
| 225139_at | 14.91 | 2.08E-08 | 1.118455172 | NA |  |
| 212326_at | 14.91 | 2.08E-08 | 1.128209288 | NM_015378| | VPS13D,vacuolar protein sorting 13D isoform 1 |
| 226416_at | 14.91 | 2.07E-08 | 1.115815172 | NM_153332| | 3'HEXO,3' exoribonuclease |
| 216375_s_at | 14.91 | 2.08E-08 | 1.355117471 | NM_004454| | ETV5,ets variant gene 5 (ets-related molecule) |
| 213606_s_at | 14.89 | 2.11E-08 | 1.320919259 | NM_004309| | ARHGDIA,Rho GDP dissociation inhibitor (GDI) alpha |
| 217882_at | 14.89 | 2.11E-08 | 1.062778079 | NM_018447| | LOC55831,30 kDa protein |
| 222880_at | 14.88 | 2.12E-08 | 1.376584315 | NM_005465| | AKT3,v-akt murine thymoma viral oncogene homolog 3 |
| 221270_s_at | 14.88 | 2.12E-08 | 1.136206641 | NM_031209| | QTRT1,queuine tRNA-ribosyltransferase 1 (tRNA-guanine |
| 208958_at | 14.87 | 2.14E-08 | 1.477390957 | NM_015051| | TXNDC4,thioredoxin domain containing 4 (endoplasmic |
| 1559993_at | 14.87 | 2.14E-08 | 1.567454277 | NM_030971| | SFXN3,sideroflexin 3 |
| 212995_x_at | 14.87 | 2.14E-08 | 1.05539315 | NM_001085365| | NA |
| 203836_s_at | 14.87 | 2.14E-08 | 1.138358569 | NM_005923| | MAP3K5,mitogen-activated protein kinase kinase kinase |
| 210268_at | 14.87 | 2.14E-08 | 1.259388169 | NM_002504| | NFX1,nuclear transcription factor, X-box binding 1 |
| 243707_at | 14.86 | 2.16E-08 | 1.235086 | NA |  |
| 216724_at | 14.85 | 2.17E-08 | 1.259848575 | NM_001040260| | NA |
| 225336_at | 14.85 | 2.17E-08 | 1.084888276 | NM_004719| | SFRS2IP,splicing factor, arginine/serine-rich 2, |
| 202850_at | 14.83 | 2.21E-08 | 1.156481511 | NM_001122674| | NA |
| 240258_at | 14.83 | 2.20E-08 | 1.326116857 | NM_001428| | ENO1,enolase 1 |
| 203469_s_at | 14.83 | 2.20E-08 | 1.121247435 | NM_001098533| | NA |
| 219175_s_at | 14.83 | 2.21E-08 | 1.090219238 | NM_001008485| | SLC41A3,solute carrier family 41, member 3 isoform 1 |
| 220669_at | 14.83 | 2.20E-08 | 1.366340665 | NM_001102653| | NA |
| 243931_at | 14.82 | 2.22E-08 | 1.402738068 | NA |  |
| 203570_at | 14.82 | 2.22E-08 | 1.206023689 | NM_005576| | LOXL1,lysyl oxidase-like 1 |
| 209371_s_at | 14.82 | 2.22E-08 | 1.367682333 | NM_001122681| | NA |
| 213436_at | 14.8 | 2.25E-08 | 1.656973822 | NM_016083| | CNR1,central cannabinoid receptor isoform a |
| 208873_s_at | 14.8 | 2.24E-08 | 1.142031119 | NM_005669| | C5orf18,deleted in polyposis 1 |
| 212002_at | 14.8 | 2.24E-08 | 1.135672942 | NM_001114600| | NA |
| 223066_at | 14.8 | 2.25E-08 | 1.112422736 | NM_012437| | SNAPAP,SNAP-associated protein |
| 212062_at | 14.8 | 2.25E-08 | 1.161291562 | NM_006045| | NA |
| 229949_at | 14.8 | 2.24E-08 | 1.150680457 | NA |  |
| 202239_at | 14.8 | 2.24E-08 | 1.039899104 | NM_006437| | PARP4,poly (ADP-ribose) polymerase family, member 4 |
| 219354_at | 14.79 | 2.26E-08 | 1.180033041 | NM_018316| | FLJ11078,hypothetical protein FLJ11078 |
| 233442_at | 14.78 | 2.28E-08 | 1.425933821 | NA |  |
| 203031_s_at | 14.78 | 2.28E-08 | 1.191740088 | NM_000375| | UROS,uroporphyrinogen III synthase |
| 209184_s_at | 14.78 | 2.28E-08 | 1.118999381 | NM_003749| | IRS2,insulin receptor substrate 2 |
| 226554_at | 14.78 | 2.27E-08 | 1.064904207 | NM_015898| | ZBTB7,zinc finger and BTB domain-containing 7 |
| 212667_at | 14.77 | 2.29E-08 | 1.178921528 | NM_003118| | SPARC,secreted protein, acidic, cysteine-rich |
| 225812_at | 14.76 | 2.31E-08 | 1.06770608 | NM_001033564| | NA |
| 225094_at | 14.76 | 2.31E-08 | 1.173945957 | NM_020382| | SET8,SET domain-containing protein 8 |
| 225291_at | 14.76 | 2.31E-08 | 1.041670383 | NM_033109| | PNPT1,polyribonucleotide nucleotidyltransferase 1 |
| 202731_at | 14.75 | 2.33E-08 | 1.239297022 | NM_014456| | PDCD4,programmed cell death 4 isoform 1 |
| 1559960_x_at | 14.74 | 2.35E-08 | 1.174441986 | NA |  |
| 203452_at | 14.74 | 2.35E-08 | 1.173549576 | NM_012200| | B3GAT3,beta-1,3-glucuronyltransferase 3 |
| 202896_s_at | 14.74 | 2.34E-08 | 1.172462569 | NM_001040022| | NA |
| 1556346_at | 14.74 | 2.34E-08 | 1.285837487 | NA |  |
| 224331_s_at | 14.73 | 2.36E-08 | 1.084187644 | NM_032479| | MRPL36,mitochondrial ribosomal protein L36 |
| 238880_at | 14.73 | 2.35E-08 | 1.222111449 | NM_002097| | GTF3A,general transcription factor IIIA |
| 219706_at | 14.73 | 2.36E-08 | 1.226855162 | NM_018347| | C20orf29,chromosome 20 open reading frame 29 |
| 235201_at | 14.73 | 2.35E-08 | 1.64837898 | NM_014491| | FOXP2,forkhead box P2 isoform I |
| 208640_at | 14.73 | 2.36E-08 | 1.025020244 | NM_006908| | RAC1,ras-related C3 botulinum toxin substrate 1 |
| 223847_s_at | 14.72 | 2.37E-08 | 1.119896269 | NM_001031711| | NA |
| 222478_at | 14.72 | 2.37E-08 | 1.264907425 | NM_016075| | C13orf9,CGI-145 protein |
| 230404_at | 14.72 | 2.38E-08 | 1.389864815 | NA |  |
| 230403_at | 14.72 | 2.37E-08 | 1.345039617 | NA |  |
| 224860_at | 14.72 | 2.37E-08 | 1.240601579 | NM_033428| | NA |
| 222826_at | 14.72 | 2.38E-08 | 1.067359598 | NM_012388| | PLDN,pallidin |
| 219041_s_at | 14.71 | 2.39E-08 | 1.093944091 | NM_001099695| | NA |
| 204227_s_at | 14.7 | 2.42E-08 | 1.174560866 | NM_004614| | TK2,thymidine kinase 2, mitochondrial |
| 238146_at | 14.7 | 2.40E-08 | 1.441057595 | NA |  |
| 229092_at | 14.69 | 2.42E-08 | 1.593589856 | NM_021005| | NR2F2,nuclear receptor subfamily 2, group F, member 2 |
| 1553107_s_at | 14.69 | 2.44E-08 | 1.158667687 | NM_152409| | FLJ37562,hypothetical protein FLJ37562 |
| 1556950_s_at | 14.68 | 2.45E-08 | 1.383682164 | NM_004568| | SERPINB6,serine (or cysteine) proteinase inhibitor, clade |
| 218742_at | 14.68 | 2.44E-08 | 1.073834921 | NM_022493| | NARFL,nuclear prelamin A recognition factor-like |
| 205729_at | 14.67 | 2.46E-08 | 1.481577668 | NM_003999| | OSMR,oncostatin M receptor |
| 214279_s_at | 14.67 | 2.46E-08 | 1.369560339 | NM_016250| | NDRG2,N-myc downstream-regulated gene 2 isoform b |
| 218124_at | 14.67 | 2.46E-08 | 1.184707801 | NM_017750| | RetSat,all-trans-13,14-dihydroretinol saturase |
| 219097_x_at | 14.66 | 2.48E-08 | 1.057574532 | NM_024104| | MGC2747,hypothetical protein MGC2747 |
| 244015_at | 14.66 | 2.47E-08 | 1.238180291 | NA |  |
| 207515_s_at | 14.66 | 2.48E-08 | 1.110925135 | NM_004875| | POLR1C,RNA polymerase I subunit isoform 2 |
| 213416_at | 14.66 | 2.48E-08 | 1.615998441 | NM_000885| | ITGA4,integrin alpha 4 precursor |
| 204955_at | 14.66 | 2.48E-08 | 1.120323054 | NM_006307| | SRPX,sushi-repeat-containing protein, X-linked |
| 227403_at | 14.66 | 2.48E-08 | 1.297693364 | NM_017861| | PIGX,GPI-mannosyltransferase subunit |
| 215171_s_at | 14.66 | 2.48E-08 | 1.044882739 | NM_006335| | TIMM17A,translocase of inner mitochondrial membrane 17 |
| 226436_at | 14.64 | 2.51E-08 | 1.104140821 | NM_032023| | RASSF4,Ras association domain family 4 isoform a |
| 211914_x_at | 14.64 | 2.52E-08 | 1.105840056 | NM_000267| | NF1,neurofibromin |
| 227696_at | 14.64 | 2.51E-08 | 1.122837898 | NM_058219| | EXOSC6,homolog of yeast mRNA transport regulator 3 |
| 232053_x_at | 14.63 | 2.54E-08 | 1.240452023 | NM_001040456| | NA |
| 209068_at | 14.63 | 2.54E-08 | 1.081049676 | NM_031372| | HNRPDL,heterogeneous nuclear ribonucleoprotein D-like |
| 223220_s_at | 14.63 | 2.54E-08 | 1.26229096 | NM_031458| | PARP9,B aggressive lymphoma gene |
| 215649_s_at | 14.62 | 2.55E-08 | 1.229370906 | NM_000431| | MVK,mevalonate kinase |
| 227424_x_at | 14.62 | 2.54E-08 | 1.216346393 | NA |  |
| 203410_at | 14.61 | 2.57E-08 | 1.09657466 | NM_006803| | AP3M2,adaptor-related protein complex 3, mu 2 subunit |
| 228769_at | 14.6 | 2.59E-08 | 1.154447466 | NM_181846| | HKR2,GLI-Kruppel family member HKR2 |
| 212228_s_at | 14.6 | 2.59E-08 | 1.149527151 | NM_020312| | DKFZP434K046,hypothetical protein DKFZp434K046 |
| 228849_at | 14.6 | 2.59E-08 | 1.106701872 | NM_001007156| | NTRK3,neurotrophic tyrosine kinase, receptor, type 3 |
| 225695_at | 14.6 | 2.59E-08 | 1.139184113 | NM_017877| | C2orf18,chromosome 2 open reading frame 18 |
| 216913_s_at | 14.59 | 2.61E-08 | 1.098382176 | NM_015179| | KIAA0690,KIAA0690 |
| 209360_s_at | 14.58 | 2.63E-08 | 1.685424944 | NM_001001890| | RUNX1,runt-related transcription factor 1 isoform b |
| 200927_s_at | 14.58 | 2.64E-08 | 1.085687348 | NM_016322| | RAB14,GTPase Rab14 |
| 215248_at | 14.57 | 2.65E-08 | 1.287734611 | NM_001001549| | GRB10,growth factor receptor-bound protein 10 isoform |
| 231931_at | 14.57 | 2.66E-08 | 1.173406285 | NM_001040424| | NA |
| 207711_at | 14.57 | 2.66E-08 | 1.359190408 | NM_199181| | FLJ44670,FLJ44670 protein |
| 227022_at | 14.56 | 2.66E-08 | 1.264510744 | NM_138335| | GNPDA2,glucosamine-6-phosphate deaminase 2 |
| 218252_at | 14.56 | 2.66E-08 | 1.148400593 | NM_001098525| | NA |
| 214608_s_at | 14.56 | 2.66E-08 | 1.624512784 | NM_000503| | EYA1,eyes absent 1 isoform b |
| 200919_at | 14.56 | 2.66E-08 | 1.251797198 | NM_004427| | PHC2,polyhomeotic 2-like isoform b |
| 218437_s_at | 14.55 | 2.68E-08 | 1.291880578 | NM_020347| | LZTFL1,leucine zipper transcription factor-like 1 |
| 218440_at | 14.55 | 2.70E-08 | 1.104630065 | NM_020166| | MCCC1,methylcrotonoyl-Coenzyme A carboxylase 1 |
| 220500_s_at | 14.55 | 2.69E-08 | 1.287412827 | NM_001003789| | RABL2B,RAB, member of RAS oncogene family-like 2B |
| 201175_at | 14.55 | 2.68E-08 | 1.057875543 | NM_015959| | TMX2,thioredoxin-related transmembrane protein 2 |
| 213703_at | 14.54 | 2.71E-08 | 1.483830176 | NA |  |
| 242172_at | 14.54 | 2.71E-08 | 1.961297223 | NM_002398| | MEIS1,Meis1 homolog |
| 225810_at | 14.54 | 2.71E-08 | 1.122206687 | NM_017762| | FLJ20313,hypothetical protein FLJ20313 |
| 217786_at | 14.53 | 2.72E-08 | 1.067783072 | NM_001039619| | NA |
| 223176_at | 14.53 | 2.73E-08 | 1.213755268 | NM_173562| | C6orf69,hypothetical protein MGC14254 |
| 227325_at | 14.53 | 2.72E-08 | 1.118636495 | NA |  |
| 201816_s_at | 14.52 | 2.75E-08 | 1.114826994 | NM_001483| | GBAS,nipsnap homolog 2 |
| 227656_at | 14.52 | 2.74E-08 | 1.117178638 | NM_018341| | NA |
| 234299_s_at | 14.51 | 2.77E-08 | 1.448730611 | NM_016350| | NIN,ninein isoform 4 |
| 224282_s_at | 14.51 | 2.77E-08 | 1.199109518 | NM_001037553| | NA |
| 1569788_at | 14.5 | 2.79E-08 | 1.703525706 | NM_003034| | ST8SIA1,ST8 alpha-N-acetyl-neuraminide |
| 242068_at | 14.5 | 2.78E-08 | 1.266813401 | NA |  |
| 204206_at | 14.5 | 2.80E-08 | 1.084350526 | NM_020310| | MNT,MAX binding protein |
| 212676_at | 14.49 | 2.81E-08 | 1.083599728 | NM_000267| | NF1,neurofibromin |
| 244196_at | 14.48 | 2.82E-08 | 1.185701089 | NA |  |
| 229884_s_at | 14.48 | 2.83E-08 | 1.176807836 | NM_015950| | MRPL2,mitochondrial ribosomal protein L2 |
| 238672_at | 14.48 | 2.82E-08 | 1.223145016 | NA |  |
| 232330_at | 14.48 | 2.83E-08 | 1.8529488 | NM_018224| | FLJ10803,hypothetical protein FLJ10803 |
| 232395_x_at | 14.48 | 2.83E-08 | 1.649649837 | NA |  |
| 223319_at | 14.47 | 2.85E-08 | 1.19444248 | NM_001024218| | NA |
| 219325_s_at | 14.47 | 2.85E-08 | 1.516460234 | NM_018696| | ELAC1,elaC homolog 1 |
| 206144_at | 14.47 | 2.84E-08 | 1.264430642 | NM_001033057| | NA |
| 211981_at | 14.46 | 2.87E-08 | 1.139545629 | NM_001845| | COL4A1,alpha 1 type IV collagen preproprotein |
| 224950_at | 14.46 | 2.88E-08 | 1.209465152 | NM_020440| | PTGFRN,prostaglandin F2 receptor negative regulator |
| 219683_at | 14.46 | 2.86E-08 | 1.192147513 | NM_017412| | FZD3,frizzled 3 |
| 236903_at | 14.46 | 2.86E-08 | 1.184252679 | NA |  |
| 213434_at | 14.46 | 2.87E-08 | 1.082285928 | NM_001980| | EPIM,epimorphin isoform 1 |
| 209239_at | 14.45 | 2.89E-08 | 1.2390576 | NM_003998| | NFKB1,nuclear factor kappa-B, subunit 1 |
| 229319_at | 14.45 | 2.89E-08 | 1.197628829 | NA |  |
| 209031_at | 14.45 | 2.89E-08 | 1.248239403 | NM_001098517| | NA |
| 201409_s_at | 14.44 | 2.91E-08 | 1.119349131 | NM_002709| | PPP1CB,protein phosphatase 1, catalytic subunit, beta |
| 228890_at | 14.44 | 2.91E-08 | 1.311805235 | NM_032827| | ATOH8,atonal homolog 8 |
| 227221_at | 14.44 | 2.92E-08 | 1.307789301 | NA |  |
| 212503_s_at | 14.44 | 2.91E-08 | 1.124804188 | NM_014974| | KIAA0934,KIAA0934 |
| 224889_at | 14.43 | 2.92E-08 | 1.058877379 | NM_001455| | FOXO3A,forkhead box O3A |
| 225282_at | 14.43 | 2.92E-08 | 1.307898385 | NM_022733| | LOC64744,hypothetical protein AL133206 |
| 202196_s_at | 14.42 | 2.94E-08 | 1.226253646 | NM_001018057| | NA |
| 214596_at | 14.42 | 2.96E-08 | 1.329867219 | NM_000740| | CHRM3,cholinergic receptor, muscarinic 3 |
| 214053_at | 14.41 | 2.98E-08 | 1.603710221 | NM_001042599| | NA |
| 219957_at | 14.41 | 2.96E-08 | 1.198901148 | NM_001042417| | NA |
| 224525_s_at | 14.41 | 2.96E-08 | 1.442350734 | NM_001011708| | PTD004,GTP-binding protein PTD004 isoform 2 |
| 209575_at | 14.41 | 2.96E-08 | 1.39742209 | NM_000628| | IL10RB,interleukin 10 receptor, beta precursor |
| 202866_at | 14.41 | 2.98E-08 | 1.154299788 | NM_001002762| | DNAJB12,DnaJ (Hsp40) homolog, subfamily B, member 12 |
| 224806_at | 14.4 | 3.00E-08 | 1.100075766 | NM_005082| | TRIM25,tripartite motif-containing 25 |
| 1556551_s_at | 14.4 | 2.99E-08 | 1.178354372 | NM_001099406| | NA |
| 231055_at | 14.4 | 3.00E-08 | 1.475350076 | NA |  |
| 232800_at | 14.39 | 3.02E-08 | 1.577958886 | NA |  |
| 218826_at | 14.39 | 3.01E-08 | 1.084424078 | NM_017515| | SLC35F2,solute carrier family 35, member F2 |
| 223228_at | 14.39 | 3.01E-08 | 1.141520512 | NM_032287| | LDOC1L,leucine zipper, down-regulated in cancer 1-like |
| 228950_s_at | 14.38 | 3.04E-08 | 1.304140151 | NM_001002292| | FLJ23091,putative NFkB activating protein 373 isoform 2 |
| 211973_at | 14.38 | 3.03E-08 | 1.184217059 | NA |  |
| 224790_at | 14.37 | 3.06E-08 | 1.147531043 | NM_018482| | DDEF1,development and differentiation enhancing factor |
| 227359_at | 14.37 | 3.06E-08 | 1.366259989 | NM_145047| | NOR1,oxidored-nitro domain-containing protein isoform |
| 205273_s_at | 14.37 | 3.07E-08 | 1.195825553 | NM_014889| | PITRM1,metalloprotease 1 |
| 214202_at | 14.37 | 3.06E-08 | 1.293004146 | NA |  |
| 201186_at | 14.36 | 3.07E-08 | 1.124169057 | NM_002337| | LRPAP1,low density lipoprotein receptor-related protein |
| 201095_at | 14.36 | 3.07E-08 | 1.090425494 | NM_004394| | DAP,death-associated protein |
| 201923_at | 14.35 | 3.09E-08 | 1.0595671 | NM_006406| | PRDX4,thioredoxin peroxidase |
| 200746_s_at | 14.34 | 3.14E-08 | 1.041161452 | NM_002074| | GNB1,guanine nucleotide-binding protein, beta-1 |
| 211340_s_at | 14.34 | 3.13E-08 | 1.227289515 | NM_006500| | MCAM,melanoma cell adhesion molecule |
| 1558101_at | 14.34 | 3.12E-08 | 1.78395851 | NA |  |
| 224730_at | 14.33 | 3.15E-08 | 1.097876571 | NM_005828| | HAN11,WD-repeat protein |
| 231714_s_at | 14.33 | 3.16E-08 | 1.168350301 | NM_006594| | AP4B1,adaptor-related protein complex 4, beta 1 |
| 208212_s_at | 14.33 | 3.16E-08 | 1.431266388 | NM_004304| | ALK,anaplastic lymphoma kinase Ki-1 |
| 238190_at | 14.33 | 3.14E-08 | 1.212309349 | NM_003321| | TUFM,Tu translation elongation factor, mitochondrial |
| 236264_at | 14.32 | 3.18E-08 | 1.818514891 | NM_015236| | LPHN3,latrophilin 3 precursor |
| 218244_at | 14.32 | 3.17E-08 | 1.117884201 | NM_017948| | NOL8,nucleolar protein 8 |
| 209053_s_at | 14.32 | 3.18E-08 | 1.057920629 | NM_001042424| | NA |
| 226410_at | 14.32 | 3.18E-08 | 1.128892124 | NM_001012759| | NA |
| 204688_at | 14.31 | 3.20E-08 | 1.067322429 | NM_001099400| | NA |
| 204251_s_at | 14.31 | 3.20E-08 | 1.148420846 | NM_014956| | Cep164,KIAA1052 protein |
| 1556694_a_at | 14.31 | 3.21E-08 | 1.293602753 | NA |  |
| 201150_s_at | 14.31 | 3.19E-08 | 1.641425266 | NM_000362| | TIMP3,tissue inhibitor of metalloproteinase 3 |
| 217780_at | 14.3 | 3.22E-08 | 1.0595709 | NM_016145| | PTD008,PTD008 protein |
| 213609_s_at | 14.3 | 3.22E-08 | 1.553625789 | NM_021115| | SEZ6L,seizure related 6 homolog (mouse)-like |
| 224766_at | 14.3 | 3.22E-08 | 1.423034819 | NM_000997| | RPL37,ribosomal protein L37 |
| 212250_at | 14.29 | 3.24E-08 | 1.11367478 | NM_178812| | LYRIC,LYRIC/3D3 |
| 220346_at | 14.29 | 3.23E-08 | 1.446807644 | NM_001004346| | MTHFD2L,methylenetetrahydrofolate dehydrogenase (NADP+ |
| 214661_s_at | 14.29 | 3.23E-08 | 1.107051359 | NM_003703| | C4orf9,gene near HD on 4p16.3 with homology to |
| 1558568_a_at | 14.29 | 3.23E-08 | 1.311656074 | NA |  |
| 209321_s_at | 14.28 | 3.26E-08 | 1.15432859 | NM_004036| | ADCY3,adenylate cyclase 3 |
| 223243_s_at | 14.28 | 3.25E-08 | 1.130290782 | NM_025191| | C1orf22,chromosome 1 open reading frame 22 |
| 202182_at | 14.27 | 3.29E-08 | 1.106388334 | NM_021078| | GCN5L2,GCN5 general control of amino-acid synthesis |
| 235673_at | 14.27 | 3.29E-08 | 1.243524657 | NA |  |
| 230831_at | 14.26 | 3.31E-08 | 1.107189497 | NA |  |
| 207828_s_at | 14.26 | 3.31E-08 | 1.092797102 | NM_016343| | CENPF,centromere protein F (350/400kD) |
| 232528_at | 14.26 | 3.31E-08 | 1.453481948 | NA |  |
| 217823_s_at | 14.26 | 3.32E-08 | 1.298286932 | NM_016021| | UBE2J1,ubiquitin-conjugating enzyme E2, J1 |
| 217832_at | 14.26 | 3.31E-08 | 1.067011087 | NM_006372| | SYNCRIP,synaptotagmin binding, cytoplasmic RNA |
| 225198_at | 14.26 | 3.31E-08 | 1.089053127 | NM_003574| | VAPA,vesicle-associated membrane protein-associated |
| 1555764_s_at | 14.25 | 3.32E-08 | 1.107033133 | NM_012456| | TIMM10,translocase of inner mitochondrial membrane 10 |
| 201940_at | 14.25 | 3.32E-08 | 1.104116097 | NM_001304| | CPD,carboxypeptidase D precursor |
| 207494_s_at | 14.25 | 3.34E-08 | 1.104296181 | NM_003427| | ZNF76,zinc finger protein 76 (expressed in testis) |
| 204085_s_at | 14.25 | 3.34E-08 | 1.475540282 | NM_006493| | CLN5,ceroid-lipofuscinosis, neuronal 5 |
| 225777_at | 14.25 | 3.32E-08 | 1.171346493 | NM_178448| | C9orf140,chromosome 9 open reading frame 140 |
| 229744_at | 14.23 | 3.38E-08 | 1.615613898 | NM_006751| | SSFA2,sperm specific antigen 2 |
| 1553909_x_at | 14.23 | 3.38E-08 | 1.125902065 | NM_018121| | C10orf6,chromosome 10 open reading frame 6 |
| 228739_at | 14.23 | 3.37E-08 | 1.342919244 | NM_001037160| | NA |
| 229870_at | 14.23 | 3.38E-08 | 1.17603231 | NA |  |
| 204241_at | 14.23 | 3.39E-08 | 1.180447248 | NM_001101667| | NA |
| 226180_at | 14.22 | 3.41E-08 | 1.080325701 | NM_139281| | WDR36,WD repeat domain 36 |
| 225112_at | 14.22 | 3.41E-08 | 1.080570248 | NM_005759| | ABI2,abl interactor 2 |
| 244058_at | 14.22 | 3.40E-08 | 1.187061939 | NM_001031746| | NA |
| 219911_s_at | 14.21 | 3.42E-08 | 1.282238623 | NM_016354| | SLCO4A1,solute carrier organic anion transporter family |
| 208877_at | 14.21 | 3.42E-08 | 1.124478557 | NM_002577| | PAK2,p21-activated kinase 2 |
| 203921_at | 14.2 | 3.46E-08 | 1.174730362 | NM_004267| | CHST2,carbohydrate (N-acetylglucosamine-6-O) |
| 200078_s_at | 14.2 | 3.46E-08 | 1.089960187 | NM_001039457| | NA |
| 1552651_a_at | 14.2 | 3.45E-08 | 1.09863178 | NM_001017368| | NA |
| 230729_at | 14.2 | 3.46E-08 | 1.444280498 | NA |  |
| 206140_at | 14.19 | 3.47E-08 | 1.944437259 | NM_004789| | LHX2,LIM homeobox protein 2 |
| 219122_s_at | 14.18 | 3.49E-08 | 1.13421615 | NM_017872| | ICF45,interphase cyctoplasmic foci protein 45 |
| 219072_at | 14.18 | 3.51E-08 | 1.133355137 | NM_004765| | BCL7C,B-cell CLL/lymphoma 7C |
| 228239_at | 14.18 | 3.51E-08 | 1.280700629 | NM_058182| | C21orf51,chromosome 21 open reading frame 51 |
| 203327_at | 14.18 | 3.49E-08 | 1.087198691 | NM_004969| | IDE,insulysin |
| 229194_at | 14.18 | 3.51E-08 | 1.288750814 | NM_032373| | PCGF5,polycomb group ring finger 5 |
| 240575_at | 14.17 | 3.52E-08 | 1.242868094 | NA |  |
| 238824_at | 14.17 | 3.52E-08 | 1.161187625 | NA |  |
| 213259_s_at | 14.17 | 3.52E-08 | 1.318752349 | NM_015077| | SARM1,sterile alpha and TIR motif containing 1 |
| 1569181_x_at | 14.17 | 3.54E-08 | 1.305926141 | NA |  |
| 63825_at | 14.16 | 3.55E-08 | 1.157454191 | NM_007011| | ABHD2,alpha/beta hydrolase domain containing protein |
| 225103_at | 14.16 | 3.55E-08 | 1.121854432 | NM_032478| | MRPL38,mitochondrial ribosomal protein L38 |
| 202447_at | 14.16 | 3.55E-08 | 1.095131523 | NM_001359| | DECR1,2,4-dienoyl CoA reductase 1 precursor |
| 203525_s_at | 14.16 | 3.56E-08 | 1.220625199 | NM_000038| | APC,adenomatosis polyposis coli |
| 209413_at | 14.15 | 3.57E-08 | 1.079631824 | NM_001005417| | B4GALT2,UDP-Gal:betaGlcNAc beta 1,4- |
| 229163_at | 14.15 | 3.58E-08 | 1.504468772 | NM_018584| | CaMKIINalpha,calcium/calmodulin-dependent protein kinase II |
| 217912_at | 14.14 | 3.61E-08 | 1.067284582 | NM_022156| | PP3111,PP3111 protein |
| 235593_at | 14.14 | 3.60E-08 | 1.284166656 | NM_014795| | ZFHX1B,zinc finger homeobox 1b |
| 212936_at | 14.14 | 3.61E-08 | 1.230318818 | NM_032042| | DKFZP564D172,hypothetical protein DKFZp564D172 |
| 223456_s_at | 14.14 | 3.61E-08 | 1.380202735 | NM_032300| | MGC10854,hypothetical protein MGC10854 |
| 1555848_at | 14.13 | 3.64E-08 | 1.263985776 | NA |  |
| 242389_at | 14.13 | 3.64E-08 | 1.263149404 | NA |  |
| 214960_at | 14.12 | 3.67E-08 | 1.23814454 | NM_006595| | API5,apoptosis inhibitor 5 |
| 201986_at | 14.12 | 3.66E-08 | 1.071037023 | NM_005121| | THRAP1,thyroid hormone receptor associated protein 1 |
| 212098_at | 14.11 | 3.70E-08 | 1.102626432 | NA |  |
| 202169_s_at | 14.11 | 3.70E-08 | 1.101066358 | NM_015423| | AASDHPPT,aminoadipate-semialdehyde |
| 1553218_a_at | 14.11 | 3.70E-08 | 1.104754811 | NM_032434| | ZNF512,zinc finger protein 512 |
| 202479_s_at | 14.11 | 3.69E-08 | 1.382558149 | NM_021643| | TRIB2,tribbles homolog 2 |
| 39549_at | 14.1 | 3.72E-08 | 1.296186862 | NM_002518| | NPAS2,neuronal PAS domain protein 2 |
| 48580_at | 14.1 | 3.72E-08 | 1.036325191 | NM_001101654| | NA |
| 232910_at | 14.1 | 3.72E-08 | 1.217949577 | NA |  |
| 52169_at | 14.09 | 3.76E-08 | 1.065820686 | NM_001003786| | LYK5,protein kinase LYK5 isoform 2 |
| 226091_s_at | 14.09 | 3.75E-08 | 1.02462455 | NM_033296| | PGR1,protein associated with MRG, 14 kDa |
| 200814_at | 14.09 | 3.74E-08 | 1.067628011 | NM_006263| | PSME1,proteasome activator subunit 1 isoform 1 |
| 228270_at | 14.08 | 3.77E-08 | 1.143356992 | NA |  |
| 1558426_x_at | 14.08 | 3.77E-08 | 1.18819554 | NM_001126340| | NA |
| 222570_at | 14.08 | 3.78E-08 | 1.143338337 | NM_014286| | FREQ,frequenin homolog |
| 218801_at | 14.08 | 3.76E-08 | 1.333800143 | NM_020121| | UGCGL2,UDP-glucose:glycoprotein glucosyltransferase 2 |
| 201411_s_at | 14.08 | 3.77E-08 | 1.066096829 | NM_001100623| | NA |
| 222131_x_at | 14.08 | 3.77E-08 | 1.036718759 | NM_138769| | RHOT2,ras homolog gene family, member T2 |
| 211755_s_at | 14.07 | 3.80E-08 | 1.031323425 | NM_001688| | ATP5F1,ATP synthase, H+ transporting, mitochondrial F0 |
| 203424_s_at | 14.06 | 3.82E-08 | 1.321497824 | NM_000599| | IGFBP5,insulin-like growth factor binding protein 5 |
| 202423_at | 14.06 | 3.82E-08 | 1.120383328 | NM_001099412| | NA |
| 226009_at | 14.06 | 3.84E-08 | 1.132334415 | NM_015448| | DPCD,DPCD protein |
| 238617_at | 14.06 | 3.83E-08 | 1.527277815 | NA |  |
| 1558802_at | 14.05 | 3.86E-08 | 1.337522692 | NA |  |
| 213088_s_at | 14.05 | 3.87E-08 | 1.08267945 | NM_015190| | DNAJC9,DnaJ homolog, subfamily C, member 9 |
| 225926_at | 14.04 | 3.89E-08 | 1.156922211 | NM_006370| | VTI1B,vesicle transport through interaction with |
| 208986_at | 14.04 | 3.89E-08 | 1.207105356 | NM_003205| | TCF12,transcription factor 12 isoform b |
| 1556110_at | 14.03 | 3.92E-08 | 1.156847799 | NA |  |
| 232256_s_at | 14.03 | 3.93E-08 | 1.477325962 | NA |  |
| 219392_x_at | 14.03 | 3.91E-08 | 1.068122377 | NM_018304| | FLJ11029,hypothetical protein FLJ11029 |
| 202813_at | 14.03 | 3.91E-08 | 1.100855744 | NM_005646| | TARBP1,TAR RNA binding protein 1 |
| 228837_at | 14.02 | 3.96E-08 | 1.408315776 | NM_001083962| | NA |
| 213241_at | 14.02 | 3.95E-08 | 1.316508777 | NM_005761| | PLXNC1,plexin C1 |
| 203907_s_at | 14.02 | 3.96E-08 | 1.081375833 | NM_014869| | IQSEC1,IQ motif and Sec7 domain 1 |
| 225233_at | 14.01 | 3.97E-08 | 1.35748198 | NM_138962| | MSI2,musashi 2 isoform a |
| 226479_at | 14.01 | 3.97E-08 | 1.16201089 | NM_152903| | KBTBD6,kelch repeat and BTB (POZ) domain-containing 6 |
| 244414_at | 14.01 | 3.99E-08 | 1.585520823 | NA |  |
| 213262_at | 14 | 4.01E-08 | 1.165276911 | NM_014363| | SACS,sacsin |
| 237262_at | 14 | 4.01E-08 | 1.181910058 | NA |  |
| 216048_s_at | 14 | 4.01E-08 | 1.418104305 | NM_014899| | RHOBTB3,rho-related BTB domain containing 3 |
| 221792_at | 14 | 4.02E-08 | 1.232473308 | NM_016577| | RAB6B,RAB6B, member RAS oncogene family |
| 243868_at | 14 | 4.01E-08 | 1.240865044 | NA |  |
| 212083_at | 13.99 | 4.04E-08 | 1.301697252 | NM_144582| | TEX261,testis expressed gene 261 |
| 202194_at | 13.99 | 4.04E-08 | 1.092633349 | NM_016040| | TMED5,transmembrane emp24 protein transport domain |
| 239775_at | 13.98 | 4.05E-08 | 1.289734846 | NA |  |
| 232276_at | 13.98 | 4.06E-08 | 1.402167672 | NM_153456| | HS6ST3,heparan sulfate 6-O-sulfotransferase 3 |
| 1555234_a_at | 13.98 | 4.05E-08 | 1.281472154 | NM_020663| | RHOJ,TC10-like Rho GTPase |
| 205450_at | 13.98 | 4.06E-08 | 1.190381067 | NM_001122670| | NA |
| 225443_at | 13.98 | 4.08E-08 | 1.091279065 | NM_018403| | DCP1A,decapping enzyme |
| 205012_s_at | 13.98 | 4.07E-08 | 1.12363895 | NM_001040427| | NA |
| 213334_x_at | 13.97 | 4.10E-08 | 1.062592143 | NM_017518| | NA |
| 241407_at | 13.97 | 4.09E-08 | 1.198559145 | NA |  |
| 225137_at | 13.97 | 4.08E-08 | 1.129483182 | NA |  |
| 220543_at | 13.96 | 4.12E-08 | 1.465845842 | NM_019596| | C21orf62,chromosome 21 open reading frame 62 |
| 213931_at | 13.96 | 4.14E-08 | 1.178424172 | NM_002166| | ID2,inhibitor of DNA binding 2 |
| 228857_at | 13.95 | 4.15E-08 | 1.225445148 | NM_005275| | GNL1,guanine nucleotide binding protein-like 1 |
| 218526_s_at | 13.95 | 4.17E-08 | 1.039988113 | NM_016492| | RANGNRF,RAN guanine nucleotide release factor |
| 212595_s_at | 13.93 | 4.22E-08 | 1.300171846 | NM_014764| | DAZAP2,DAZ associated protein 2 |
| 209268_at | 13.93 | 4.21E-08 | 1.107654576 | NM_007259| | VPS45A,vacuolar protein sorting 45A |
| 212813_at | 13.93 | 4.21E-08 | 1.117873673 | NM_032801| | JAM3,junctional adhesion molecule 3 precursor |
| 202868_s_at | 13.91 | 4.31E-08 | 1.082016701 | NM_006627| | POP4,POP4 (processing of precursor , S. cerevisiae) |
| 203524_s_at | 13.91 | 4.29E-08 | 1.071366785 | NM_001013436| | NA |
| 229404_at | 13.91 | 4.29E-08 | 1.226692111 | NM_057179| | TWIST2,twist homolog 2 |
| 228603_at | 13.91 | 4.29E-08 | 1.159943148 | NA |  |
| 235376_at | 13.91 | 4.28E-08 | 1.348192294 | NA |  |
| 225334_at | 13.9 | 4.31E-08 | 1.338286321 | NM_144591| | C10orf32,hypothetical protein MGC27171 |
| 200947_s_at | 13.9 | 4.31E-08 | 1.072366081 | NM_005271| | GLUD1,glutamate dehydrogenase 1 |
| 224463_s_at | 13.9 | 4.31E-08 | 1.452666439 | NM_032930| | MGC13040,hypothetical protein MGC13040 |
| 201113_at | 13.89 | 4.34E-08 | 1.045914895 | NM_003321| | TUFM,Tu translation elongation factor, mitochondrial |
| 202743_at | 13.89 | 4.35E-08 | 1.211597364 | NM_001114172| | NA |
| 232589_at | 13.89 | 4.37E-08 | 1.212506974 | NA |  |
| 214736_s_at | 13.89 | 4.37E-08 | 1.108780994 | NM_001119| | ADD1,adducin 1 (alpha) isoform a |
| 217061_s_at | 13.89 | 4.37E-08 | 1.178603545 | NM_004956| | ETV1,ets variant gene 1 |
| 1559227_s_at | 13.89 | 4.35E-08 | 1.107315599 | NM_000551| | VHL,von Hippel-Lindau tumor suppressor isoform 1 |
| 241425_at | 13.88 | 4.39E-08 | 1.317281644 | NM_001008564| | NUPL1,nucleoporin like 1 isoform b |
| 232659_at | 13.88 | 4.37E-08 | 1.239553542 | NA |  |
| 232420_x_at | 13.88 | 4.39E-08 | 1.079141027 | NA |  |
| 201618_x_at | 13.88 | 4.38E-08 | 1.120194037 | NM_003801| | GPAA1,anchor attachment protein 1 |
| 234005_x_at | 13.88 | 4.38E-08 | 1.124394408 | NM_015690| | STK36,serine/threonine kinase 36 (fused homolog, |
| 217597_x_at | 13.88 | 4.38E-08 | 1.111753252 | NM_006822| | RAB40B,RAB40B, member RAS oncogene family |
| 213244_at | 13.88 | 4.37E-08 | 1.110500541 | NM_079834| | SCAMP4,secretory carrier membrane protein 4 |
| 230999_at | 13.87 | 4.43E-08 | 1.120544461 | NA |  |
| 222021_x_at | 13.87 | 4.42E-08 | 1.091924286 | NA |  |
| 231930_at | 13.87 | 4.43E-08 | 1.66839189 | NM_018712| | ELMOD1,ELMO domain containing 1 |
| 226500_at | 13.87 | 4.42E-08 | 1.172482426 | NM_145166| | ZNF651,zinc finger protein 651 |
| 238438_at | 13.86 | 4.46E-08 | 1.350244655 | NM_144571| | CNOT6L,CCR4-NOT transcription complex, subunit 6-like |
| 37966_at | 13.86 | 4.46E-08 | 1.234516669 | NM_001003828| | PARVB,parvin, beta isoform a |
| 217538_at | 13.86 | 4.43E-08 | 1.307276213 | NM_001098509| | NA |
| 201999_s_at | 13.85 | 4.48E-08 | 1.039469058 | NM_006519| | TCTEL1,t-complex-associated-testis-expressed 1-like 1 |
| 227084_at | 13.85 | 4.49E-08 | 1.184188746 | NM_001390| | DTNA,dystrobrevin alpha isoform 1 |
| 218074_at | 13.85 | 4.49E-08 | 1.067690575 | NM_016062| | CGI-128,CGI-128 protein |
| 237834_at | 13.85 | 4.48E-08 | 1.369435392 | NM_005460| | SNCAIP,synuclein alpha interacting protein |
| 202041_s_at | 13.83 | 4.55E-08 | 1.054557211 | NM_004214| | FIBP,FGF intracellular binding protein isoform b |
| 228162_at | 13.83 | 4.55E-08 | 1.243490099 | NM_001984| | ESD,esterase D/formylglutathione hydrolase |
| 216242_x_at | 13.83 | 4.55E-08 | 1.108192734 | NM_001097615| | NA |
| 217992_s_at | 13.82 | 4.60E-08 | 1.074378 | NM_024329| | EFHD2,EF hand domain family, member D2 |
| 209871_s_at | 13.82 | 4.60E-08 | 1.286541187 | NM_005503| | APBA2,amyloid beta A4 precursor protein-binding, |
| 207396_s_at | 13.82 | 4.57E-08 | 1.069124627 | NM_005787| | ALG3,alpha-1,3-mannosyltransferase ALG3 isoform a |
| 217844_at | 13.81 | 4.61E-08 | 1.104761171 | NM_021198| | CTDSP1,CTD (carboxy-terminal domain, RNA polymerase II, |
| 227056_at | 13.81 | 4.63E-08 | 1.166894064 | NM_014773| | KIAA0141,KIAA0141 |
| 243531_at | 13.81 | 4.61E-08 | 1.465578928 | NM_153451| | ORAOV1,oral cancer overexpressed 1 |
| 225875_s_at | 13.8 | 4.68E-08 | 1.166409387 | NM_020448| | DJ462O23.2,hypothetical protein dJ462O23.2 |
| 228314_at | 13.8 | 4.68E-08 | 1.222362227 | NA |  |
| 212586_at | 13.8 | 4.68E-08 | 1.174848563 | NM_001042440| | NA |
| 1552680_a_at | 13.79 | 4.68E-08 | 1.120804385 | NM_144508| | AF15Q14,AF15q14 protein isoform 2 |
| 1553976_a_at | 13.79 | 4.69E-08 | 1.151654511 | NM_015448| | DPCD,DPCD protein |
| 1555974_a_at | 13.79 | 4.71E-08 | 1.17231245 | NA |  |
| 204966_at | 13.78 | 4.72E-08 | 1.349878085 | NM_001703| | BAI2,brain-specific angiogenesis inhibitor 2 |
| 214765_s_at | 13.78 | 4.73E-08 | 1.277398531 | NM_001042402| | NA |
| 240153_at | 13.78 | 4.72E-08 | 1.346592696 | NA |  |
| 235628_x_at | 13.77 | 4.76E-08 | 1.24170171 | NA |  |
| 235798_at | 13.77 | 4.75E-08 | 1.634490578 | NM_001100829| | NA |
| 213512_at | 13.77 | 4.75E-08 | 1.177114414 | NM_174891| | C14orf79,chromosome 14 open reading frame 79 |
| 221959_at | 13.76 | 4.82E-08 | 1.182896463 | NM_147189| | MGC39325,hypothetical protein MGC39325 |
| 1559946_s_at | 13.75 | 4.86E-08 | 1.04404535 | NM_006666| | RUVBL2,RuvB-like 2 |
| 201668_x_at | 13.75 | 4.86E-08 | 1.216639746 | NM_002356| | MARCKS,myristoylated alanine-rich protein kinase C |
| 213488_at | 13.74 | 4.87E-08 | 1.486137037 | NM_001080437| | NA |
| 218736_s_at | 13.74 | 4.88E-08 | 1.725035712 | NM_017734| | PALMD,palmdelphin |
| 222121_at | 13.74 | 4.90E-08 | 1.254260233 | NM_015595| | SGEF,DKFZP434D146 protein |
| 219481_at | 13.74 | 4.87E-08 | 1.212130741 | NM_001122835| | NA |
| 242208_at | 13.74 | 4.90E-08 | 1.580028477 | NA |  |
| 203057_s_at | 13.74 | 4.90E-08 | 1.078758688 | NM_001007257| | PRDM2,retinoblastoma protein-binding zinc finger |
| 212607_at | 13.73 | 4.90E-08 | 1.071079248 | NM_005465| | AKT3,v-akt murine thymoma viral oncogene homolog 3 |
| 218168_s_at | 13.73 | 4.92E-08 | 1.097129142 | NM_020247| | CABC1,chaperone, ABC1 activity of bc1 complex like |
| 213861_s_at | 13.72 | 4.97E-08 | 1.178244751 | NM_015433| | DKFZP586D0919,hepatocellularcarcinoma-associated antigen |
| 218099_at | 13.72 | 4.95E-08 | 1.155202387 | NM_018469| | HT008,uncharacterized hypothalamus protein HT008 |
| 213130_at | 13.71 | 4.98E-08 | 1.087794293 | NM_001006656| | ZNF473,zinc finger protein 473 |
| 226418_at | 13.71 | 4.99E-08 | 1.212747213 | NM_016570| | PTX1,CDA14 |
| 238836_at | 13.71 | 4.99E-08 | 1.139114098 | NA |  |
| 235407_at | 13.71 | 4.99E-08 | 1.166065541 | NA |  |
| 220757_s_at | 13.71 | 4.99E-08 | 1.162990256 | NM_025241| | UBXD1,UBX domain containing 1 |
| 229201_at | 13.71 | 4.99E-08 | 1.193804228 | NA |  |
| 239619_at | 13.71 | 5.00E-08 | 1.244751568 | NA |  |
| 213196_at | 13.7 | 5.05E-08 | 1.081180289 | NM_001080417| | NA |
| 225625_at | 13.7 | 5.05E-08 | 1.146216907 | NM_001001655| | MGC90512,similar to hypothetical protein 9530023G02 |
| 221436_s_at | 13.7 | 5.03E-08 | 1.100011562 | NM_031299| | CDCA3,trigger of mitotic entry 1 |
| 212775_at | 13.69 | 5.07E-08 | 1.12555377 | NM_015311| | NA |
| 235795_at | 13.69 | 5.08E-08 | 1.840476613 | NM_000280| | PAX6,paired box gene 6 isoform a |
| 201632_at | 13.69 | 5.06E-08 | 1.059951395 | NM_001414| | EIF2B1,eukaryotic translation initiation factor 2B, |
| 218837_s_at | 13.68 | 5.11E-08 | 1.189089157 | NM_015983| | UBE2D4,ubiquitin-conjugating enzyme E2D 4 (putative) |
| 244766_at | 13.68 | 5.11E-08 | 1.185217487 | NM_015092| | SMG1,PI-3-kinase-related kinase SMG-1 isoform 1 |
| 201433_s_at | 13.68 | 5.10E-08 | 1.093688206 | NM_014754| | PTDSS1,phosphatidylserine synthase 1 |
| 1562699_at | 13.68 | 5.10E-08 | 1.424038047 | NA |  |
| 229287_at | 13.68 | 5.09E-08 | 1.180610865 | NM_014982| | PCNX,pecanex homolog |
| 235010_at | 13.67 | 5.14E-08 | 1.183116127 | NA |  |
| 223742_at | 13.67 | 5.16E-08 | 1.252952313 | NM_015956| | MRPL4,mitochondrial ribosomal protein L4 isoform a |
| 244114_x_at | 13.66 | 5.20E-08 | 1.328066313 | NA |  |
| 244457_at | 13.66 | 5.17E-08 | 1.301352523 | NA |  |
| 228369_at | 13.66 | 5.18E-08 | 1.39958564 | NM_006586| | TNRC5,trinucleotide repeat containing 5 |
| 227640_s_at | 13.66 | 5.18E-08 | 1.160673961 | NM_203288| | RP9,retinitis pigmentosa 9 protein |
| 227146_at | 13.65 | 5.23E-08 | 1.095738834 | NM_181701| | QSCN6L1,quiescin Q6-like 1 |
| 1553705_a_at | 13.65 | 5.25E-08 | 1.363147023 | NM_000740| | CHRM3,cholinergic receptor, muscarinic 3 |
| 225430_at | 13.65 | 5.24E-08 | 1.171460431 | NM_176818| | 15E1.2,hypothetical protein 15E1.2 |
| 212556_at | 13.65 | 5.24E-08 | 1.100126828 | NM_015356| | SCRIB,scribble isoform b |
| 215731_s_at | 13.65 | 5.25E-08 | 1.169471557 | NM_022782| | MPHOSPH9,M-phase phosphoprotein 9 |
| 212715_s_at | 13.65 | 5.23E-08 | 1.181167079 | NM_001122731| | NA |
| 241631_at | 13.64 | 5.28E-08 | 1.220647635 | NA |  |
| 227907_at | 13.64 | 5.27E-08 | 1.213464819 | NA |  |
| 229743_at | 13.64 | 5.27E-08 | 1.16674243 | NM_182755| | LOC220929,hypothetical protein LOC220929 |
| 228628_at | 13.64 | 5.26E-08 | 1.230202576 | NA |  |
| 215099_s_at | 13.64 | 5.27E-08 | 1.2996712 | NM_021976| | RXRB,retinoid X receptor, beta |
| 203319_s_at | 13.62 | 5.33E-08 | 1.201894673 | NM_021964| | ZNF148,zinc finger protein 148 (pHZ-52) |
| 202047_s_at | 13.62 | 5.34E-08 | 1.215326227 | NM_014292| | CBX6,chromobox homolog 6 |
| 212249_at | 13.62 | 5.36E-08 | 1.615926085 | NM_181504| | PIK3R1,phosphoinositide-3-kinase, regulatory subunit, |
| 1560082_at | 13.62 | 5.33E-08 | 1.329185964 | NA |  |
| 204381_at | 13.62 | 5.33E-08 | 1.129910977 | NM_002333| | LRP3,low density lipoprotein receptor-related protein |
| 209409_at | 13.62 | 5.33E-08 | 1.096559363 | NM_001001549| | GRB10,growth factor receptor-bound protein 10 isoform |
| 1566513_a_at | 13.61 | 5.38E-08 | 1.437208125 | NM_001098721| | NA |
| 204511_at | 13.61 | 5.38E-08 | 1.119298485 | NM_014808| | FARP2,FERM, RhoGEF and pleckstrin domain protein 2 |
| 223109_at | 13.61 | 5.36E-08 | 1.076831942 | NM_015679| | TRUB2,TruB pseudouridine (psi) synthase homolog 2 |
| 225703_at | 13.61 | 5.38E-08 | 1.082494928 | NA |  |
| 1569872_a_at | 13.6 | 5.42E-08 | 1.321146303 | NA |  |
| 1555905_a_at | 13.6 | 5.41E-08 | 1.258472428 | NM_001029839| | NA |
| 227964_at | 13.59 | 5.46E-08 | 1.095789271 | NM_031904| | FKSG44,FKSG44 protein |
| 212395_s_at | 13.59 | 5.46E-08 | 1.088790078 | NM_015047| | KIAA0090,KIAA0090 protein |
| 221952_x_at | 13.59 | 5.48E-08 | 1.068054177 | NM_020810| | KIAA1393,tRNA-(N1G37) methyltransferase |
| 235224_s_at | 13.59 | 5.46E-08 | 1.545404302 | NA |  |
| 231964_at | 13.59 | 5.48E-08 | 1.211197884 | NA |  |
| 220232_at | 13.59 | 5.48E-08 | 1.143295713 | NM_001037582| | NA |
| 226186_at | 13.58 | 5.50E-08 | 1.512305985 | NA |  |
| 228790_at | 13.58 | 5.51E-08 | 1.29719358 | NM_147189| | MGC39325,hypothetical protein MGC39325 |
| 231056_at | 13.58 | 5.51E-08 | 1.113728343 | NA |  |
| 223207_x_at | 13.58 | 5.49E-08 | 1.064842296 | NM_014172| | PHPT1,phosphohistidine phosphatase 1 |
| 210756_s_at | 13.58 | 5.52E-08 | 1.187671175 | NM_024408| | NOTCH2,notch 2 preproprotein |
| 215574_at | 13.57 | 5.53E-08 | 1.215176451 | NA |  |
| 212923_s_at | 13.57 | 5.54E-08 | 1.263041631 | NM_183373| | C6orf145,chromosome 6 open reading frame 145 |
| 212144_at | 13.57 | 5.56E-08 | 1.183702058 | NM_015374| | UNC84B,unc-84 homolog B |
| 239280_at | 13.57 | 5.56E-08 | 1.33089935 | NA |  |
| 218786_at | 13.56 | 5.59E-08 | 1.115856523 | NM_001031701| | NA |
| 223261_at | 13.56 | 5.59E-08 | 1.141678238 | NM_016218| | POLK,polymerase (DNA directed) kappa |
| 207000_s_at | 13.55 | 5.61E-08 | 1.178544764 | NM_005605| | PPP3CC,protein phosphatase 3 (formerly 2B), catalytic |
| 41160_at | 13.55 | 5.65E-08 | 1.09667907 | NM_003926| | MBD3,methyl-CpG binding domain protein 3 |
| 235793_at | 13.54 | 5.69E-08 | 1.191496689 | NA |  |
| 213013_at | 13.54 | 5.69E-08 | 1.116005534 | NM_005456| | MAPK8IP1,mitogen-activated protein kinase 8 interacting |
| 222889_at | 13.54 | 5.69E-08 | 1.117957975 | NM_022836| | DCLRE1B,DNA cross-link repair 1B (PSO2 homolog, S. |
| 230724_s_at | 13.54 | 5.66E-08 | 1.194346747 | NM_001082969| | NA |
| 202942_at | 13.54 | 5.69E-08 | 1.084700651 | NM_001014763| | NA |
| 231720_s_at | 13.53 | 5.72E-08 | 1.165269419 | NM_032801| | JAM3,junctional adhesion molecule 3 precursor |
| 218233_s_at | 13.53 | 5.73E-08 | 1.027181453 | NM_013397| | C6orf49,over-expressed breast tumor protein |
| 217791_s_at | 13.52 | 5.77E-08 | 1.102171173 | NM_001017423| | NA |
| 209208_at | 13.52 | 5.76E-08 | 1.085898631 | NM_004870| | MPDU1,mannose-P-dolichol utilization defect 1 |
| 1554783_s_at | 13.52 | 5.76E-08 | 1.196847355 | NM_004723| | ARHGEF2,rho/rac guanine nucleotide exchange factor 2 |
| 201236_s_at | 13.52 | 5.76E-08 | 1.340357079 | NM_006763| | BTG2,B-cell translocation gene 2 |
| 222030_at | 13.52 | 5.78E-08 | 1.16734285 | NM_006427| | SIVA,CD27-binding (Siva) protein isoform 1 |
| 225665_at | 13.5 | 5.88E-08 | 1.257788986 | NM_016653| | ZAK,sterile-alpha motif and leucine zipper |
| 221267_s_at | 13.49 | 5.92E-08 | 1.110586399 | NM_031213| | C19orf27,chromosome 19 open reading frame 27 |
| 236038_at | 13.49 | 5.90E-08 | 1.204368407 | NA |  |
| 227255_at | 13.48 | 5.97E-08 | 1.336976222 | NM_152835| | PDIK1L,PDLIM1 interacting kinase 1 like |
| 202940_at | 13.48 | 5.94E-08 | 1.194553442 | NM_018979| | WNK1,WNK lysine deficient protein kinase 1 |
| 220914_at | 13.48 | 5.97E-08 | 1.209852931 | NA |  |
| 231106_at | 13.47 | 6.02E-08 | 1.176789385 | NA |  |
| 210137_s_at | 13.47 | 6.02E-08 | 1.073195709 | NM_001012732| | NA |
| 226861_at | 13.47 | 5.98E-08 | 1.218813995 | NM_024095| | ASB8,ankyrin repeat and SOCS box-containing 8 |
| 1552925_at | 13.47 | 6.02E-08 | 1.483886487 | NM_020815| | PCDH10,protocadherin 10 isoform 2 precursor |
| 225120_at | 13.46 | 6.05E-08 | 1.118743499 | NM_033224| | PURB,purine-rich element binding protein B |
| 205794_s_at | 13.46 | 6.05E-08 | 1.549610278 | NM_002515| | NOVA1,neuro-oncological ventral antigen 1 isoform 1 |
| 204821_at | 13.46 | 6.04E-08 | 1.563604269 | NM_006994| | BTN3A3,butyrophilin, subfamily 3, member A3 isoform a |
| 213750_at | 13.46 | 6.04E-08 | 1.179496357 | NM_015659| | RSL1D1,ribosomal L1 domain containing 1 |
| 227406_at | 13.46 | 6.06E-08 | 1.088882944 | NM_002041| | GABPB2,GA binding protein transcription factor, beta |
| 225236_at | 13.46 | 6.06E-08 | 1.101584876 | NM_033117| | RBM18,RNA binding motif protein 18 |
| 1566482_at | 13.46 | 6.06E-08 | 1.24445619 | NA |  |
| 212730_at | 13.45 | 6.08E-08 | 1.30143244 | NM_015286| | DMN,desmuslin isoform B |
| 229530_at | 13.45 | 6.10E-08 | 1.743069327 | NA |  |
| 224668_at | 13.45 | 6.08E-08 | 1.064617905 | NM_033542| | C20orf35,uncharacterized hypothalamus protein HSMNP1 |
| 212828_at | 13.45 | 6.08E-08 | 1.300486032 | NM_003898| | SYNJ2,synaptojanin 2 |
| 1554547_at | 13.44 | 6.13E-08 | 1.816067626 | NM_001001971| | FAM13C1,family with sequence similarity 13, member C1 |
| 221235_s_at | 13.44 | 6.15E-08 | 1.12612654 | NA |  |
| 203542_s_at | 13.43 | 6.17E-08 | 1.593255405 | NM_001206| | KLF9,Kruppel-like factor 9 |
| 202246_s_at | 13.43 | 6.18E-08 | 1.066393217 | NM_000075| | CDK4,cyclin-dependent kinase 4 |
| 1553947_at | 13.43 | 6.19E-08 | 1.142500831 | NM_058219| | EXOSC6,homolog of yeast mRNA transport regulator 3 |
| 1554906_a_at | 13.42 | 6.25E-08 | 1.630306685 | NM_005792| | MPHOSPH6,M-phase phosphoprotein 6 |
| 228093_at | 13.42 | 6.24E-08 | 1.231631183 | NM_001007248| | ZNF599,zinc finger protein 599 isoform a |
| 206752_s_at | 13.41 | 6.26E-08 | 1.140599309 | NM_004402| | DFFB,DNA fragmentation factor, 40 kD, beta |
| 220741_s_at | 13.41 | 6.30E-08 | 1.048849341 | NM_001034191| | NA |
| 204653_at | 13.41 | 6.30E-08 | 1.413796395 | NM_001032280| | NA |
| 227620_at | 13.4 | 6.35E-08 | 1.253137291 | NM_080546| | CDW92,CDW92 antigen |
| 228847_at | 13.4 | 6.31E-08 | 1.242462087 | NM_007277| | SEC6L1,Sec6 protein |
| 1559139_at | 13.4 | 6.32E-08 | 1.259398063 | NM_015658| | DKFZP564C186,DKFZP564C186 protein |
| 201792_at | 13.4 | 6.36E-08 | 1.094452476 | NM_001129| | AEBP1,adipocyte enhancer binding protein 1 precursor |
| 202630_at | 13.37 | 6.50E-08 | 1.263314072 | NM_006380| | APPBP2,amyloid beta precursor protein-binding protein |
| 224068_x_at | 13.37 | 6.50E-08 | 1.082080196 | NM_018047| | RBM22,RNA binding motif protein 22 |
| 227251_at | 13.37 | 6.47E-08 | 1.155601126 | NM_003861| | WDR22,Breakpoint cluster region protein, uterine |
| 206874_s_at | 13.37 | 6.48E-08 | 1.083219195 | NA |  |
| 237270_at | 13.37 | 6.50E-08 | 1.318523955 | NA |  |
| 229192_s_at | 13.37 | 6.47E-08 | 1.13592992 | NM_005993| | TBCD,beta-tubulin cofactor D |
| 213573_at | 13.36 | 6.55E-08 | 1.120692014 | NA |  |
| 230872_s_at | 13.36 | 6.54E-08 | 1.174322627 | NM_001025930| | NA |
| 224937_at | 13.36 | 6.53E-08 | 1.157652298 | NM_020440| | PTGFRN,prostaglandin F2 receptor negative regulator |
| 222830_at | 13.36 | 6.53E-08 | 1.240416576 | NM_014552| | TFCP2L2,leader-binding protein 32 isoform 1 |
| 228218_at | 13.36 | 6.54E-08 | 1.58553503 | NA |  |
| 232668_at | 13.36 | 6.55E-08 | 1.305253552 | NA |  |
| 1557123_a_at | 13.35 | 6.59E-08 | 1.167355209 | NA |  |
| 212996_s_at | 13.35 | 6.61E-08 | 1.121720086 | NA |  |
| 242337_at | 13.35 | 6.60E-08 | 1.229378426 | NA |  |
| 1564907_s_at | 13.34 | 6.64E-08 | 1.109064368 | NM_018834| | MATR3,matrin 3 |
| 235980_at | 13.34 | 6.63E-08 | 1.247745109 | NM_006218| | PIK3CA,phosphoinositide-3-kinase, catalytic, alpha |
| 1565681_s_at | 13.34 | 6.64E-08 | 1.224233415 | NM_014974| | KIAA0934,KIAA0934 |
| 1557270_at | 13.34 | 6.64E-08 | 1.224989508 | NA |  |
| 224759_s_at | 13.34 | 6.64E-08 | 1.072321231 | NM_152261| | MGC17943,hypothetical protein MGC17943 |
| 231513_at | 13.34 | 6.62E-08 | 1.655843825 | NA |  |
| 201481_s_at | 13.33 | 6.69E-08 | 1.073026945 | NM_002862| | PYGB,brain glycogen phosphorylase |
| 220607_x_at | 13.33 | 6.68E-08 | 1.200661207 | NM_198976| | TH1L,TH1-like protein |
| 213604_at | 13.32 | 6.76E-08 | 1.062573075 | NA |  |
| 207018_s_at | 13.32 | 6.73E-08 | 2.271670664 | NM_004163| | RAB27B,RAB27B, member RAS oncogene family |
| 218386_x_at | 13.31 | 6.79E-08 | 1.109582391 | NM_001001992| | USP16,ubiquitin specific protease 16 isoform b |
| 203168_at | 13.31 | 6.80E-08 | 1.140694988 | NM_004381| | CREBL1,cAMP responsive element binding protein-like 1 |
| 223791_at | 13.31 | 6.81E-08 | 1.244272813 | NA |  |
| 213947_s_at | 13.31 | 6.81E-08 | 1.075794271 | NM_024923| | NUP210,nucleoporin 210 |
| 209588_at | 13.31 | 6.79E-08 | 1.142529182 | NM_004442| | EPHB2,ephrin receptor EphB2 isoform 2 precursor |
| 211685_s_at | 13.3 | 6.85E-08 | 1.267734356 | NM_001040624| | NA |
| 221713_s_at | 13.3 | 6.86E-08 | 1.197536016 | NM_024871| | FLJ12748,hypothetical protein FLJ12748 |
| 218974_at | 13.3 | 6.85E-08 | 1.233256784 | NM_018013| | NA |
| 220702_at | 13.29 | 6.87E-08 | 1.442793905 | NM_018616| | NA |
| 239151_at | 13.29 | 6.91E-08 | 1.255690462 | NA |  |
| 206935_at | 13.29 | 6.91E-08 | 1.822713002 | NM_002590| | PCDH8,protocadherin 8 isoform 1 precursor |
| 209100_at | 13.29 | 6.87E-08 | 1.078399854 | NM_006764| | IFRD2,interferon-related developmental regulator 2 |
| 224848_at | 13.29 | 6.91E-08 | 1.297174995 | NM_001259| | CDK6,cyclin-dependent kinase 6 |
| 218463_s_at | 13.28 | 6.97E-08 | 1.054429367 | NM_025128| | MUS81,MUS81 endonuclease homolog |
| 244184_at | 13.28 | 6.94E-08 | 1.273165268 | NA |  |
| 1559214_at | 13.28 | 6.94E-08 | 1.471526473 | NA |  |
| 243016_at | 13.26 | 7.09E-08 | 1.716801754 | NA |  |
| 230449_x_at | 13.26 | 7.08E-08 | 1.208451231 | NA |  |
| 235391_at | 13.26 | 7.09E-08 | 1.117466158 | NM_145269| | LOC137392,similar to CG6405 gene product |
| 220633_s_at | 13.26 | 7.07E-08 | 1.174862231 | NM_016287| | HP1-BP74,HP1-BP74 |
| 237094_at | 13.25 | 7.10E-08 | 1.125128503 | NM_001082967| | NA |
| 212427_at | 13.25 | 7.13E-08 | 1.33014087 | NM_001080398| | NA |
| 1556761_at | 13.24 | 7.20E-08 | 1.321161853 | NA |  |
| 218554_s_at | 13.24 | 7.18E-08 | 1.22946756 | NM_018489| | ASH1L,ash1 (absent, small, or homeotic)-like |
| 227925_at | 13.24 | 7.20E-08 | 1.278868767 | NA |  |
| 222077_s_at | 13.23 | 7.22E-08 | 1.095970789 | NM_001126103| | NA |
| 225523_at | 13.23 | 7.24E-08 | 1.072391473 | NM_053050| | MRPL53,mitochondrial ribosomal protein L53 |
| 220010_at | 13.23 | 7.26E-08 | 1.331843959 | NM_012282| | KCNE1L,potassium voltage-gated channel, Isk-related |
| 213043_s_at | 13.22 | 7.29E-08 | 1.062448301 | NM_001079518| | NA |
| 225980_at | 13.22 | 7.28E-08 | 1.164720296 | NM_001043318| | NA |
| 209745_at | 13.21 | 7.33E-08 | 1.09979972 | NM_016138| | COQ7,COQ7 protein |
| 202632_at | 13.21 | 7.36E-08 | 1.105804436 | NM_001383| | DPH2L1,diptheria toxin resistance protein required for |
| 1559739_at | 13.2 | 7.40E-08 | 1.444150463 | NM_020244| | CHPT1,choline phosphotransferase 1 |
| 225877_at | 13.2 | 7.38E-08 | 1.070850815 | NM_001040273| | NA |
| 226807_at | 13.19 | 7.48E-08 | 1.103070222 | NM_153688| | ZFP1,zinc finger protein 1 homolog |
| 209407_s_at | 13.19 | 7.45E-08 | 1.149062719 | NM_021008| | DEAF1,suppressin |
| 227722_at | 13.19 | 7.49E-08 | 1.245663591 | NM_001025| | RPS23,ribosomal protein S23 |
| 205241_at | 13.19 | 7.50E-08 | 1.093723995 | NM_005138| | SCO2,cytochrome oxidase deficient homolog 2 |
| 235427_at | 13.19 | 7.50E-08 | 1.39149202 | NA |  |
| 226459_at | 13.19 | 7.46E-08 | 1.299752156 | NM_152309| | PIK3AP1,phosphoinositide-3-kinase adaptor protein 1 |
| 222709_at | 13.18 | 7.55E-08 | 1.127858749 | NM_006395| | APG7L,APG7 autophagy 7-like |
| 243092_at | 13.18 | 7.50E-08 | 1.251482209 | NA |  |
| 224988_at | 13.18 | 7.52E-08 | 1.175974072 | NM_152734| | C6orf89,hypothetical protein FLJ25357 |
| 214007_s_at | 13.17 | 7.58E-08 | 1.264460221 | NM_002822| | PTK9,twinfilin isoform 1 |
| 204087_s_at | 13.17 | 7.56E-08 | 1.046109869 | NM_021095| | SLC5A6,solute carrier family 5 (sodium-dependent |
| 1563808_at | 13.17 | 7.59E-08 | 1.160936051 | NM_001112732| | NA |
| 208798_x_at | 13.17 | 7.56E-08 | 1.170122977 | NM_181077| | GOLGIN-67,golgin-67 isoform c |
| 230039_at | 13.16 | 7.63E-08 | 1.163838696 | NA |  |
| 226874_at | 13.16 | 7.65E-08 | 1.215899984 | NM_020803| | KLHL8,kelch-like 8 |
| 202783_at | 13.16 | 7.62E-08 | 1.236619728 | NM_012343| | NNT,nicotinamide nucleotide transhydrogenase |
| 225012_at | 13.15 | 7.69E-08 | 1.153419775 | NM_005336| | HDLBP,high density lipoprotein binding protein |
| 208668_x_at | 13.15 | 7.71E-08 | 1.046423322 | NM_005517| | HMGN2,high-mobility group nucleosomal binding domain |
| 242191_at | 13.14 | 7.75E-08 | 1.6434613 | NM_001039703| | NA |
| 235264_at | 13.14 | 7.75E-08 | 1.259850322 | NM_013320| | HCFC2,host cell factor C2 |
| 238659_at | 13.13 | 7.86E-08 | 1.315660048 | NA |  |
| 208722_s_at | 13.13 | 7.86E-08 | 1.063632615 | NM_016237| | ANAPC5,anaphase-promoting complex subunit 5 |
| 204127_at | 13.13 | 7.82E-08 | 1.045663864 | NM_002915| | RFC3,replication factor C 3 isoform 1 |
| 203796_s_at | 13.13 | 7.83E-08 | 1.170340833 | NM_001024808| | NA |
| 218597_s_at | 13.12 | 7.90E-08 | 1.052488167 | NM_018464| | C10orf70,chromosome 10 open reading frame 70 |
| 230482_at | 13.12 | 7.90E-08 | 1.257255878 | NM_030965| | ST6GALNAC5,sialyltransferase 7E |
| 209167_at | 13.12 | 7.88E-08 | 1.302109686 | NM_001001994| | GPM6B,glycoprotein M6B isoform 4 |
| 203247_s_at | 13.12 | 7.91E-08 | 1.098259937 | NM_006965| | ZNF24,zinc finger protein 24 (KOX 17) |
| 223842_s_at | 13.12 | 7.89E-08 | 1.334849758 | NM_016240| | SCARA3,scavenger receptor class A, member 3 isoform 1 |
| 238603_at | 13.12 | 7.90E-08 | 1.349021345 | NA |  |
| 203303_at | 13.11 | 7.93E-08 | 1.195370988 | NM_006520| | TCTE1L,t-complex-associated-testis-expressed 1-like |
| 1553510_s_at | 13.11 | 7.96E-08 | 1.102999585 | NM_005444| | RQCD1,RCD1 required for cell differentiation1 homolog |
| 239329_at | 13.11 | 7.96E-08 | 1.109679694 | NA |  |
| 201783_s_at | 13.09 | 8.09E-08 | 1.158168259 | NM_021975| | RELA,v-rel reticuloendotheliosis viral oncogene |
| 218901_at | 13.09 | 8.07E-08 | 1.353453164 | NM_020353| | PLSCR4,phospholipid scramblase 4 |
| 230144_at | 13.09 | 8.06E-08 | 1.810265403 | NM_000828| | GRIA3,glutamate receptor 3 isoform flop precursor |
| 226179_at | 13.08 | 8.15E-08 | 1.093742516 | NM_016612| | MSCP,mitochondrial solute carrier protein |
| 222725_s_at | 13.08 | 8.15E-08 | 1.447922805 | NM_017734| | PALMD,palmdelphin |
| 217942_at | 13.08 | 8.15E-08 | 1.029935195 | NM_021821| | MRPS35,mitochondrial ribosomal protein S35 |
| 239392_s_at | 13.08 | 8.12E-08 | 1.194364545 | NM_017542| | POGK,pogo transposable element with KRAB domain |
| 220076_at | 13.08 | 8.18E-08 | 1.889561693 | NM_054027| | ANKH,ankylosis, progressive homolog |
| 224478_s_at | 13.08 | 8.13E-08 | 1.132005506 | NM_032350| | MGC11257,hypothetical protein MGC11257 |
| 1556144_at | 13.08 | 8.16E-08 | 1.187934777 | NM_014966| | DHX30,DEAH (Asp-Glu-Ala-His) box polypeptide 30 |
| 221206_at | 13.08 | 8.16E-08 | 1.094072223 | NM_000535| | PMS2,PMS2 |
| 242134_at | 13.07 | 8.19E-08 | 1.303149609 | NA |  |
| 236381_s_at | 13.07 | 8.19E-08 | 1.094013625 | NM_017818| | WDR8,WD repeat domain 8 protein |
| 205745_x_at | 13.07 | 8.23E-08 | 1.047171252 | NM_003183| | ADAM17,a disintegrin and metalloproteinase domain 17 |
| 242785_at | 13.06 | 8.26E-08 | 1.268873277 | NA |  |
| 1553588_at | 13.06 | 8.27E-08 | 1.020155806 | NM_001024666| | NA |
| 234032_at | 13.06 | 8.27E-08 | 1.392818631 | NA |  |
| 209170_s_at | 13.06 | 8.25E-08 | 1.276054275 | NM_001001994| | GPM6B,glycoprotein M6B isoform 4 |
| 212674_s_at | 13.05 | 8.34E-08 | 1.058811217 | NM_014966| | DHX30,DEAH (Asp-Glu-Ala-His) box polypeptide 30 |
| 243278_at | 13.05 | 8.32E-08 | 1.776721495 | NM_014491| | FOXP2,forkhead box P2 isoform I |
| 230571_at | 13.05 | 8.37E-08 | 1.280010388 | NA |  |
| 212019_at | 13.04 | 8.40E-08 | 1.233904686 | NM_015659| | RSL1D1,ribosomal L1 domain containing 1 |
| 230591_at | 13.04 | 8.44E-08 | 1.329107327 | NA |  |
| 225209_s_at | 13.04 | 8.44E-08 | 1.037558384 | NM_058167| | UBE2J2,ubiquitin conjugating enzyme E2, J2 isoform 2 |
| 36545_s_at | 13.03 | 8.50E-08 | 1.158484348 | NM_001007467| | SFI1,spindle assembly associated Sfi1 homolog isoform |
| 234926_s_at | 13.03 | 8.46E-08 | 1.06450566 | NM_016407| | C20orf43,chromosome 20 open reading frame 43 |
| 227170_at | 13.03 | 8.47E-08 | 1.118697258 | NA |  |
| 204863_s_at | 13.02 | 8.52E-08 | 1.606687599 | NM_002184| | IL6ST,interleukin 6 signal transducer isoform 1 |
| 227932_at | 13.02 | 8.55E-08 | 1.201586967 | NM_006321| | ARIH2,ariadne homolog 2 |
| 232605_s_at | 13.02 | 8.57E-08 | 1.288200964 | NA |  |
| 219762_s_at | 13.02 | 8.52E-08 | 1.026432724 | NM_015414| | RPL36,ribosomal protein L36 |
| 226166_x_at | 13.02 | 8.56E-08 | 1.125429599 | NM_015690| | STK36,serine/threonine kinase 36 (fused homolog, |
| 1557804_at | 13.02 | 8.56E-08 | 1.3308784 | NA |  |
| 223165_s_at | 13.02 | 8.57E-08 | 1.057162123 | NM_001005909| | IHPK2,inositol hexaphosphate kinase 2 isoform a |
| 221139_s_at | 13.01 | 8.59E-08 | 1.3060899 | NM_015989| | CSAD,cysteine sulfinic acid decarboxylase-related |
| 224669_at | 13.01 | 8.60E-08 | 1.052205304 | NM_033542| | C20orf35,uncharacterized hypothalamus protein HSMNP1 |
| 204754_at | 13 | 8.66E-08 | 1.222543424 | NM_002126| | HLF,hepatic leukemia factor |
| 224018_s_at | 13 | 8.70E-08 | 1.280955491 | NM_001037582| | NA |
| 201910_at | 13 | 8.70E-08 | 1.077516251 | NM_001001715| | FARP1,FERM, RhoGEF, and pleckstrin domain protein 1 |
| 205356_at | 13 | 8.70E-08 | 1.075454135 | NM_003940| | USP13,ubiquitin specific protease 13 (isopeptidase |
| 231989_s_at | 13 | 8.66E-08 | 1.20337018 | NA |  |
| 201421_s_at | 12.99 | 8.78E-08 | 1.096012966 | NM_024102| | MEP50,methylosome protein 50 |
| 206469_x_at | 12.99 | 8.73E-08 | 1.13292227 | NM_012067| | AKR7A3,aldo-keto reductase family 7, member A3 |
| 207551_s_at | 12.99 | 8.75E-08 | 1.146226895 | NM_006800| | MSL3L1,male-specific lethal 3-like 1 isoform c |
| 202212_at | 12.99 | 8.78E-08 | 1.094958077 | NM_014303| | PES1,pescadillo homolog 1, containing BRCT domain |
| 235327_x_at | 12.98 | 8.83E-08 | 1.034181538 | NM_181713| | UBXD4,UBX domain containing 4 |
| 203855_at | 12.98 | 8.83E-08 | 1.181500594 | NM_014969| | WDR47,WD repeat domain 47 |
| 226839_at | 12.98 | 8.80E-08 | 1.166234645 | NM_176880| | TRA16,TR4 orphan receptor associated protein TRA16 |
| 224662_at | 12.98 | 8.84E-08 | 1.100164512 | NM_004521| | KIF5B,kinesin family member 5B |
| 235211_at | 12.97 | 8.87E-08 | 1.245566007 | NA |  |
| 226017_at | 12.97 | 8.94E-08 | 1.058135477 | NM_138410| | CKLFSF7,chemokine-like factor superfamily 7 isoform a |
| 202032_s_at | 12.97 | 8.87E-08 | 1.209497454 | NM_006122| | MAN2A2,mannosidase, alpha, class 2A, member 2 |
| 215310_at | 12.96 | 8.96E-08 | 1.441711398 | NM_000038| | APC,adenomatosis polyposis coli |
| 225292_at | 12.96 | 8.96E-08 | 1.241244562 | NM_032888| | COL27A1,collagen, type XXVII, alpha 1 |
| 221000_s_at | 12.96 | 8.98E-08 | 1.231767871 | NM_030929| | KAZALD1,Kazal-type serine protease inhibitor domain 1 |
| 242480_at | 12.96 | 8.99E-08 | 1.194429216 | NA |  |
| 214744_s_at | 12.96 | 8.94E-08 | 1.222952768 | NM_000978| | RPL23,ribosomal protein L23 |
| 213970_at | 12.95 | 9.03E-08 | 1.09420839 | NM_173825| | RABL3,RAB, member of RAS oncogene family-like 3 |
| 228780_at | 12.95 | 9.06E-08 | 2.171732177 | NA |  |
| 218793_s_at | 12.95 | 9.04E-08 | 1.718290832 | NM_001037535| | NA |
| 220935_s_at | 12.95 | 9.08E-08 | 1.11434247 | NM_001011649| | CDK5RAP2,CDK5 regulatory subunit associated protein 2 |
| 226433_at | 12.94 | 9.13E-08 | 1.130064248 | NM_052916| | RNF157,ring finger protein 157 |
| 244519_at | 12.94 | 9.13E-08 | 1.290310995 | NM_015338| | ASXL1,additional sex combs like 1 |
| 242725_at | 12.94 | 9.10E-08 | 1.236778939 | NA |  |
| 1569472_s_at | 12.94 | 9.10E-08 | 1.72509844 | NM_001001894| | TTC3,tetratricopeptide repeat domain 3 |
| 224215_s_at | 12.94 | 9.09E-08 | 1.312160058 | NM_005618| | DLL1,delta-like 1 |
| 233803_s_at | 12.93 | 9.17E-08 | 1.142249289 | NM_001105538| | NA |
| 221853_s_at | 12.93 | 9.23E-08 | 1.06225017 | NM_001004060| | NOMO2,nodal modulator 2 isoform 1 |
| 221532_s_at | 12.92 | 9.27E-08 | 1.058481986 | NM_025234| | REC14,recombination protein REC14 |
| 236976_at | 12.92 | 9.30E-08 | 1.29155156 | NM_000135| | FANCA,Fanconi anemia, complementation group A |
| 217909_s_at | 12.92 | 9.24E-08 | 1.099879024 | NM_170607| | MLX,transcription factor-like protein 4 isoform |
| 209522_s_at | 12.92 | 9.26E-08 | 1.14909673 | NM_000755| | CRAT,carnitine acetyltransferase isoform 1 precursor |
| 218032_at | 12.91 | 9.34E-08 | 1.097944825 | NM_003498| | SNN,Stannin |
| 1559121_s_at | 12.91 | 9.33E-08 | 1.373530721 | NM_006321| | ARIH2,ariadne homolog 2 |
| 216192_at | 12.91 | 9.33E-08 | 1.612964707 | NM_001446| | FABP7,fatty acid binding protein 7, brain |
| 235876_at | 12.9 | 9.40E-08 | 1.415418758 | NA |  |
| 230095_at | 12.9 | 9.40E-08 | 1.37200049 | NA |  |
| 229395_at | 12.9 | 9.43E-08 | 1.157923885 | NM_004604| | STX4A,syntaxin 4A (placental) |
| 1555870_at | 12.9 | 9.45E-08 | 1.329337479 | NM_207396| | FLJ46380,FLJ46380 protein |
| 226242_at | 12.9 | 9.38E-08 | 1.113122979 | NM_152379| | DKFZp547B1713,hypothetical protein DKFZp547B1713 |
| 235785_at | 12.89 | 9.52E-08 | 1.184213607 | NA |  |
| 218362_s_at | 12.89 | 9.51E-08 | 1.609529588 | NM_014953| | KIAA1008,KIAA1008 |
| 221926_s_at | 12.89 | 9.52E-08 | 1.144949658 | NM_032732| | IL17RC,interleukin 17 receptor C isoform 3 precursor |
| 225896_at | 12.89 | 9.45E-08 | 1.162999615 | NA |  |
| 220748_s_at | 12.89 | 9.52E-08 | 1.121436278 | NM_016202| | ZNF580,zinc finger protein 580 |
| 218888_s_at | 12.88 | 9.52E-08 | 1.334093671 | NM_018092| | NETO2,neuropilin- and tolloid-like protein 2 |
| 229774_at | 12.88 | 9.56E-08 | 1.520759987 | NM_025212| | CXXC4,CXXC finger 4 |
| 209197_at | 12.88 | 9.57E-08 | 1.226809994 | NM_152280| | SYT11,synaptotagmin 12 |
| 213689_x_at | 12.87 | 9.63E-08 | 1.099476457 | NM_001006605| | LOC388650,hypothetical LOC388650 |
| 229120_s_at | 12.87 | 9.63E-08 | 1.071182485 | NM_001038707| | NA |
| 201415_at | 12.87 | 9.63E-08 | 1.099430261 | NM_000178| | GSS,glutathione synthetase |
| 40149_at | 12.87 | 9.64E-08 | 1.08431308 | NM_015503| | SH2B,SH2-B homolog |
| 230329_s_at | 12.86 | 9.74E-08 | 1.165418643 | NM_007083| | NUDT6,nudix-type motif 6 isoform a |
| 233852_at | 12.86 | 9.69E-08 | 1.344499816 | NM_006502| | POLH,polymerase (DNA directed), eta |
| 201568_at | 12.85 | 9.85E-08 | 1.08404419 | NM_014402| | QP-C,low molecular mass ubiquinone-binding protein |
| 212439_at | 12.84 | 9.86E-08 | 1.10476564 | NM_001006115| | IHPK1,inositol hexaphosphate kinase 1 isoform 2 |
| 228214_at | 12.84 | 9.91E-08 | 1.323146718 | NA |  |
| 223094_s_at | 12.84 | 9.90E-08 | 1.287919052 | NM_054027| | ANKH,ankylosis, progressive homolog |
| 219733_s_at | 12.84 | 9.92E-08 | 1.124517104 | NM_012254| | SLC27A5,solute carrier family 27 (fatty acid |
| 51176_at | 12.84 | 9.90E-08 | 1.065631678 | NM_004269| | CRSP8,cofactor required for Sp1 transcriptional |
| 214247_s_at | 12.83 | 9.98E-08 | 1.168820423 | NM_001018057| | NA |
| 202407_s_at | 12.83 | 9.99E-08 | 1.123833078 | NM_015629| | PRPF31,pre-mRNA processing factor 31 homolog |
| 230408_at | 12.83 | 9.96E-08 | 1.082214644 | NA |  |
| 202800_at | 12.82 | 1.01E-07 | 1.580343436 | NM_004172| | SLC1A3,solute carrier family 1 (glial high affinity |
| 213084_x_at | 12.81 | 1.02E-07 | 1.010445152 | NM_000984| | RPL23A,ribosomal protein L23a |
| 1557370_s_at | 12.81 | 1.02E-07 | 1.225445035 | NM_015057| | MYCBP2,MYC binding protein 2 |
| 210045_at | 12.81 | 1.02E-07 | 1.337893009 | NM_002168| | IDH2,isocitrate dehydrogenase 2 (NADP+), |
| 224772_at | 12.81 | 1.01E-07 | 1.070419295 | NM_020443| | NAV1,neuron navigator 1 |
| 1556329_a_at | 12.81 | 1.02E-07 | 2.069373288 | NA |  |
| 227150_at | 12.81 | 1.01E-07 | 1.108958482 | NM_005955| | MTF1,metal-regulatory transcription factor 1 |
| 212416_at | 12.81 | 1.01E-07 | 1.05051028 | NM_004866| | SCAMP1,secretory carrier membrane protein 1 isoform 1 |
| 1554696_s_at | 12.81 | 1.01E-07 | 1.155117595 | NM_001071| | TYMS,thymidylate synthetase |
| 210896_s_at | 12.8 | 1.02E-07 | 1.179334562 | NM_004318| | ASPH,aspartate beta-hydroxylase isoform a |
| 224948_at | 12.8 | 1.02E-07 | 1.056090251 | NM_032014| | MRPS24,mitochondrial ribosomal protein S24 |
| 212347_x_at | 12.8 | 1.02E-07 | 1.122125295 | NM_006454| | MXD4,MAD4 |
| 212973_at | 12.8 | 1.02E-07 | 1.120062906 | NM_144563| | RPIA,ribose 5-phosphate isomerase A (ribose |
| 1554159_a_at | 12.8 | 1.02E-07 | 1.187417543 | NM_006624| | ZMYND11,zinc finger, MYND domain containing 11 isoform |
| 227125_at | 12.79 | 1.03E-07 | 1.196311724 | NA |  |
| 215364_s_at | 12.79 | 1.03E-07 | 1.172594257 | NM_015284| | KIAA0467,KIAA0467 protein |
| 219543_at | 12.79 | 1.03E-07 | 1.270965054 | NM_001033083| | NA |
| 208803_s_at | 12.78 | 1.03E-07 | 1.036097274 | NM_006947| | SRP72,signal recognition particle 72kDa |
| 225526_at | 12.78 | 1.04E-07 | 1.095491197 | NM_013255| | MKLN1,muskelin 1, intracellular mediator containing |
| 221745_at | 12.78 | 1.04E-07 | 1.097181264 | NM_005828| | HAN11,WD-repeat protein |
| 230761_at | 12.78 | 1.04E-07 | 1.157326804 | NA |  |
| 237675_at | 12.77 | 1.04E-07 | 1.522524563 | NA |  |
| 228274_at | 12.76 | 1.05E-07 | 1.448870257 | NM_138432| | SDSL,serine dehydratase-like |
| 228702_at | 12.76 | 1.06E-07 | 1.475486597 | NA |  |
| 216858_x_at | 12.76 | 1.06E-07 | 1.062043963 | NA |  |
| 203171_s_at | 12.76 | 1.06E-07 | 1.079771723 | NM_015324| | KIAA0409,KIAA0409 protein |
| 226031_at | 12.75 | 1.06E-07 | 1.082510867 | NM_017667| | NA |
| 205010_at | 12.75 | 1.06E-07 | 1.079680239 | NM_019067| | GNL3L,guanine nucleotide binding protein-like 3 |
| 221366_at | 12.75 | 1.07E-07 | 1.510429951 | NM_006168| | NKX6-1,NK6 transcription factor related, locus 1 |
| 223366_at | 12.75 | 1.06E-07 | 1.33581976 | NA |  |
| 211984_at | 12.75 | 1.06E-07 | 1.147303097 | NM_001743| | CALM2,calmodulin 2 |
| 214788_x_at | 12.75 | 1.06E-07 | 1.232111659 | NM_015086| | NA |
| 203801_at | 12.74 | 1.07E-07 | 1.246531319 | NM_022100| | MRPS14,mitochondrial ribosomal protein S14 |
| 213612_x_at | 12.74 | 1.08E-07 | 1.146643552 | NM_001037501| | NA |
| 224523_s_at | 12.74 | 1.08E-07 | 1.064910181 | NM_032359| | MGC4308,hypothetical protein MGC4308 |
| 235193_at | 12.73 | 1.08E-07 | 1.713804155 | NA |  |
| 1558714_at | 12.73 | 1.08E-07 | 1.401143952 | NA |  |
| 1557165_s_at | 12.73 | 1.08E-07 | 1.140151964 | NM_025010| | KLHL18,kelch-like 18 |
| 230605_at | 12.72 | 1.09E-07 | 1.176193531 | NA |  |
| 225311_at | 12.72 | 1.09E-07 | 1.084377895 | NM_002225| | IVD,isovaleryl Coenzyme A dehydrogenase |
| 212946_at | 12.71 | 1.10E-07 | 1.107107686 | NM_001009814| | KIAA0564,KIAA0564 protein isoform b |
| 1564787_at | 12.71 | 1.10E-07 | 1.113125263 | NM_001039772| | NA |
| 213944_x_at | 12.7 | 1.11E-07 | 1.140650374 | NM_002067| | GNA11,guanine nucleotide binding protein (G protein), |
| 204175_at | 12.7 | 1.11E-07 | 1.07364195 | NM_015871| | ZNF593,zinc finger protein LOC51042 |
| 244165_at | 12.7 | 1.11E-07 | 1.333207573 | NM_017782| | NA |
| 212577_at | 12.7 | 1.11E-07 | 1.108439187 | NM_015295| | NA |
| 236247_at | 12.7 | 1.11E-07 | 1.154822433 | NM_199044| | NSUN4,NOL1/NOP2/Sun domain family 4 protein |
| 225310_at | 12.7 | 1.11E-07 | 1.068569426 | NM_002139| | RBMX,RNA binding motif protein, X-linked |
| 220609_at | 12.69 | 1.12E-07 | 1.155985084 | NA |  |
| 240592_at | 12.68 | 1.13E-07 | 1.151532723 | NM_153686| | MLR1,transcription factor MLR1 |
| 235058_at | 12.67 | 1.14E-07 | 1.125106294 | NA |  |
| 202551_s_at | 12.67 | 1.14E-07 | 1.128446815 | NM_016441| | CRIM1,cysteine-rich motor neuron 1 |
| 227720_at | 12.66 | 1.15E-07 | 1.12451829 | NM_152345| | FLJ25555,hypothetical protein FLJ25555 |
| 228791_at | 12.66 | 1.15E-07 | 1.233994341 | NA |  |
| 229384_at | 12.66 | 1.14E-07 | 1.132228288 | NA |  |
| 236004_at | 12.66 | 1.15E-07 | 1.189454924 | NA |  |
| 202629_at | 12.65 | 1.15E-07 | 1.107958591 | NM_006380| | APPBP2,amyloid beta precursor protein-binding protein |
| 1554010_at | 12.65 | 1.15E-07 | 1.191154845 | NM_001543| | NDST1,N-deacetylase/N-sulfotransferase (heparan |
| 227585_at | 12.65 | 1.16E-07 | 1.085000836 | NA |  |
| 202082_s_at | 12.65 | 1.16E-07 | 1.127655743 | NM_001039573| | NA |
| 239476_at | 12.65 | 1.15E-07 | 1.2514907 | NA |  |
| 236916_at | 12.65 | 1.16E-07 | 1.190860575 | NA |  |
| 244391_at | 12.65 | 1.15E-07 | 1.151667627 | NM_025265| | SEN2L,hypothetical protein MGC2776 |
| 239611_at | 12.64 | 1.17E-07 | 1.582727954 | NA |  |
| 230528_s_at | 12.64 | 1.17E-07 | 1.17255538 | NA |  |
| 210811_s_at | 12.64 | 1.16E-07 | 1.138100378 | NM_019070| | DDX49,DEAD (Asp-Glu-Ala-Asp) box polypeptide 49 |
| 219213_at | 12.64 | 1.17E-07 | 1.195431013 | NM_021219| | JAM2,junctional adhesion molecule 2 precursor |
| 214869_x_at | 12.63 | 1.18E-07 | 1.080371411 | NM_015635| | DKFZP434C212,DKFZP434C212 protein |
| 209093_s_at | 12.63 | 1.18E-07 | 1.155661588 | NM_000157| | GBA,glucocerebrosidase precursor |
| 232951_at | 12.63 | 1.18E-07 | 1.106818936 | NA |  |
| 223516_s_at | 12.63 | 1.18E-07 | 1.026279958 | NM_013397| | C6orf49,over-expressed breast tumor protein |
| 200769_s_at | 12.62 | 1.19E-07 | 1.180785281 | NM_005911| | MAT2A,methionine adenosyltransferase II, alpha |
| 216135_at | 12.62 | 1.19E-07 | 1.333753325 | NM_153208| | MGC35048,hypothetical protein MGC35048 |
| 217543_s_at | 12.62 | 1.19E-07 | 1.141353103 | NM_003791| | MBTPS1,membrane-bound transcription factor site-1 |
| 228630_at | 12.62 | 1.19E-07 | 1.263022166 | NM_003428| | ZNF84,zinc finger protein 84 (HPF2) |
| 218097_s_at | 12.62 | 1.18E-07 | 1.099740593 | NM_024040| | CUEDC2,CUE domain containing 2 |
| 228402_at | 12.61 | 1.19E-07 | 1.153418929 | NM_032367| | ZBED3,zinc finger, BED domain containing 3 |
| 236196_at | 12.61 | 1.19E-07 | 1.226451368 | NA |  |
| 221828_s_at | 12.61 | 1.20E-07 | 1.145244584 | NM_001011703| | C9orf28,chromosome 9 open reading frame 28 isoform 2 |
| 213893_x_at | 12.61 | 1.19E-07 | 1.090016781 | NM_174930| | PMS2L5,postmeiotic segregation increased 2-like 5 |
| 212863_x_at | 12.6 | 1.21E-07 | 1.057559522 | NM_001012614| | NA |
| 213152_s_at | 12.6 | 1.21E-07 | 1.112430133 | NM_032102| | SRP46,Splicing factor, arginine/serine-rich, 46kD |
| 238207_at | 12.59 | 1.22E-07 | 1.233569309 | NM_001124767| | NA |
| 32541_at | 12.59 | 1.21E-07 | 1.242903184 | NM_005605| | PPP3CC,protein phosphatase 3 (formerly 2B), catalytic |
| 234971_x_at | 12.59 | 1.22E-07 | 1.100066319 | NM_133373| | PLCD3,phospholipase C delta 3 |
| 223061_at | 12.59 | 1.22E-07 | 1.095478673 | NM_023947| | MGC3234,hypothetical protein MGC3234 |
| 243864_at | 12.58 | 1.23E-07 | 1.293033269 | NM_199511| | URB,steroid-sensitive protein 1 |
| 203669_s_at | 12.58 | 1.23E-07 | 1.09810882 | NM_012079| | DGAT1,diacylglycerol O-acyltransferase 1 |
| 220202_s_at | 12.58 | 1.23E-07 | 1.114633435 | NM_001100588| | NA |
| 229211_at | 12.57 | 1.23E-07 | 1.222143534 | NM_001033575| | NA |
| 201308_s_at | 12.57 | 1.23E-07 | 1.203280702 | NM_018243| | SEPT11,septin 11 |
| 221833_at | 12.57 | 1.23E-07 | 1.662765713 | NM_031490| | LONP,peroxisomal lon protease |
| 225176_at | 12.57 | 1.24E-07 | 1.205387599 | NA |  |
| 205806_at | 12.57 | 1.24E-07 | 1.115196799 | NM_000327| | ROM1,retinal outer segment membrane protein 1 |
| 205077_s_at | 12.57 | 1.23E-07 | 1.039823767 | NM_002643| | PIGF,phosphatidylinositol glycan, class F isoform 1 |
| 221878_at | 12.57 | 1.23E-07 | 1.091787046 | NM_001013649| | NA |
| 218380_at | 12.56 | 1.25E-07 | 1.126742629 | NM_001033053| | NA |
| 200665_s_at | 12.56 | 1.25E-07 | 1.102049984 | NM_003118| | SPARC,secreted protein, acidic, cysteine-rich |
| 209234_at | 12.56 | 1.25E-07 | 1.085608656 | NM_015074| | KIF1B,kinesin family member 1B isoform b |
| 215114_at | 12.56 | 1.25E-07 | 1.167100823 | NM_015670| | SENP3,SUMO1/sentrin/SMT3 specific protease 3 |
| 212548_s_at | 12.56 | 1.24E-07 | 1.12799956 | NM_015030| | NA |
| 35179_at | 12.56 | 1.25E-07 | 1.166462731 | NM_012200| | B3GAT3,beta-1,3-glucuronyltransferase 3 |
| 201598_s_at | 12.55 | 1.26E-07 | 1.122062917 | NM_001567| | INPPL1,inositol polyphosphate phosphatase-like 1 |
| 205160_at | 12.54 | 1.27E-07 | 1.152710401 | NM_003847| | PEX11A,peroxisomal biogenesis factor 11A |
| 226762_at | 12.53 | 1.28E-07 | 1.139532316 | NM_033224| | PURB,purine-rich element binding protein B |
| 1557527_at | 12.53 | 1.28E-07 | 1.358796367 | NA |  |
| 231810_at | 12.53 | 1.28E-07 | 1.293831955 | NM_080626| | BRI3BP,BRI3-binding protein |
| 219383_at | 12.53 | 1.28E-07 | 1.477781682 | NM_024841| | FLJ14213,hypothetical protein FLJ14213 |
| 224896_s_at | 12.52 | 1.29E-07 | 1.06417945 | NM_153712| | TTL,tubulin tyrosine ligase |
| 226821_at | 12.52 | 1.29E-07 | 1.108687735 | NA |  |
| 32209_at | 12.52 | 1.29E-07 | 1.147429164 | NM_001098784| | NA |
| 1555486_a_at | 12.51 | 1.31E-07 | 1.215294754 | NM_024841| | FLJ14213,hypothetical protein FLJ14213 |
| 203340_s_at | 12.51 | 1.31E-07 | 1.125353501 | NM_003705| | SLC25A12,solute carrier family 25 (mitochondrial carrier, |
| 1569110_x_at | 12.51 | 1.30E-07 | 1.065477117 | NA |  |
| 225797_at | 12.51 | 1.31E-07 | 1.071120724 | NM_172251| | MRPL54,mitochondrial ribosomal protein L54 |
| 218483_s_at | 12.51 | 1.30E-07 | 1.085051424 | NM_020153| | FLJ21827,hypothetical protein FLJ21827 |
| 218355_at | 12.5 | 1.32E-07 | 1.05900984 | NM_012310| | KIF4A,kinesin family member 4 |
| 222644_s_at | 12.5 | 1.32E-07 | 1.252928797 | NM_024656| | GLT25D1,glycosyltransferase 25 domain containing 1 |
| 238099_at | 12.5 | 1.32E-07 | 1.157557824 | NA |  |
| 204521_at | 12.48 | 1.34E-07 | 1.123848135 | NM_013300| | HSU79274,protein predicted by clone 23733 |
| 236591_at | 12.48 | 1.34E-07 | 1.681101941 | NA |  |
| 1560028_at | 12.47 | 1.35E-07 | 1.204598869 | NM_001082969| | NA |
| 207628_s_at | 12.47 | 1.35E-07 | 1.074632998 | NM_017528| | WBSCR22,Williams Beuren syndrome chromosome region 22 |
| 223706_at | 12.46 | 1.36E-07 | 1.211768258 | NM_032561| | C22orf23,EVG1 protein |
| 237169_at | 12.46 | 1.36E-07 | 1.89998329 | NA |  |
| 219639_x_at | 12.46 | 1.36E-07 | 1.109656521 | NM_020214| | PARP6,poly (ADP-ribose) polymerase family, member 6 |
| 227155_at | 12.45 | 1.38E-07 | 1.2825647 | NM_006769| | LMO4,LIM domain only 4 |
| 212202_s_at | 12.45 | 1.38E-07 | 1.038697148 | NM_001110503| | NA |
| 208688_x_at | 12.44 | 1.39E-07 | 1.028203382 | NM_001037283| | NA |
| 228584_at | 12.44 | 1.38E-07 | 1.153808791 | NM_000232| | SGCB,sarcoglycan, beta (43kDa dystrophin-associated |
| 230656_s_at | 12.43 | 1.39E-07 | 1.05572009 | NM_032830| | CIRH1A,cirhin |
| 219785_s_at | 12.43 | 1.39E-07 | 1.138512505 | NM_024735| | FBXO31,F-box protein 31 |
| 213880_at | 12.43 | 1.39E-07 | 2.144178171 | NM_003667| | LGR5,leucine-rich repeat-containing G protein-coupled |
| 203030_s_at | 12.43 | 1.39E-07 | 1.335923625 | NM_002847| | PTPRN2,protein tyrosine phosphatase, receptor type, N |
| 232086_at | 12.42 | 1.40E-07 | 1.36275802 | NA |  |
| 204594_s_at | 12.42 | 1.40E-07 | 1.070662979 | NM_019008| | FLJ20232,hypothetical protein FLJ20232 |
| 225241_at | 12.42 | 1.40E-07 | 1.371796621 | NM_199511| | URB,steroid-sensitive protein 1 |
| 226034_at | 12.41 | 1.42E-07 | 1.213860704 | NA |  |
| 50314_i_at | 12.41 | 1.41E-07 | 1.065280718 | NM_001039140| | NA |
| 208832_at | 12.41 | 1.42E-07 | 1.088263074 | NM_013236| | ATXN10,ataxin 10 |
| 201046_s_at | 12.41 | 1.42E-07 | 1.095274406 | NM_005053| | RAD23A,UV excision repair protein RAD23 homolog A |
| 202204_s_at | 12.41 | 1.42E-07 | 1.175157133 | NM_001144| | AMFR,autocrine motility factor receptor isoform a |
| 230418_s_at | 12.41 | 1.42E-07 | 1.242880441 | NM_020692| | NA |
| 1563467_at | 12.4 | 1.43E-07 | 1.365573049 | NA |  |
| 244425_at | 12.4 | 1.43E-07 | 1.329951713 | NA |  |
| 200768_s_at | 12.4 | 1.43E-07 | 1.100868095 | NM_005911| | MAT2A,methionine adenosyltransferase II, alpha |
| 1569519_at | 12.4 | 1.43E-07 | 1.26291353 | NM_001037501| | NA |
| 212639_x_at | 12.4 | 1.43E-07 | 1.03084684 | NM_006082| | K-ALPHA-1,tubulin, alpha, ubiquitous |
| 222803_at | 12.4 | 1.43E-07 | 1.372575919 | NM_020200| | PRTFDC1,phosphoribosyl transferase domain containing 1 |
| 243366_s_at | 12.4 | 1.43E-07 | 1.633255775 | NA |  |
| 228843_at | 12.39 | 1.44E-07 | 1.256749917 | NA |  |
| 1553666_at | 12.38 | 1.45E-07 | 1.893489232 | NM_030771| | NA |
| 1552689_at | 12.38 | 1.45E-07 | 1.094516819 | NM_020764| | CASKIN1,CASK interacting protein 1 |
| 240451_at | 12.38 | 1.45E-07 | 1.259396311 | NA |  |
| 243149_at | 12.38 | 1.46E-07 | 1.256735456 | NA |  |
| 236294_at | 12.38 | 1.46E-07 | 1.194619403 | NM_031407| | NA |
| 219337_at | 12.38 | 1.46E-07 | 1.141320129 | NM_001114103| | NA |
| 211891_s_at | 12.38 | 1.45E-07 | 1.32203537 | NM_015320| | ARHGEF4,Rho guanine nucleotide exchange factor 4 isoform |
| 200795_at | 12.37 | 1.47E-07 | 2.095980653 | NM_004684| | SPARCL1,SPARC-like 1 |
| 228698_at | 12.37 | 1.46E-07 | 1.416291634 | NM_031439| | SOX7,SRY-box 7 |
| 201320_at | 12.36 | 1.48E-07 | 1.143629084 | NM_003075| | SMARCC2,SWI/SNF-related matrix-associated |
| 211067_s_at | 12.36 | 1.48E-07 | 1.236374003 | NM_003644| | GAS7,growth arrest-specific 7 isoform a |
| 209195_s_at | 12.35 | 1.49E-07 | 1.094455946 | NM_015270| | ADCY6,adenylate cyclase 6 isoform a |
| 223320_s_at | 12.35 | 1.49E-07 | 1.263023148 | NM_012089| | ABCB10,ATP-binding cassette, sub-family B, member 10 |
| 215360_at | 12.35 | 1.50E-07 | 1.1321167 | NA |  |
| 203415_at | 12.35 | 1.49E-07 | 1.047431792 | NM_013232| | PDCD6,programmed cell death 6 |
| 227567_at | 12.34 | 1.50E-07 | 1.186126507 | NM_001033569| | NA |
| 219858_s_at | 12.34 | 1.50E-07 | 1.509996404 | NM_017694| | FLJ20160,FLJ20160 protein |
| 226621_at | 12.34 | 1.50E-07 | 1.532295209 | NA |  |
| 215069_at | 12.34 | 1.51E-07 | 1.370171112 | NM_004808| | NMT2,glycylpeptide N-tetradecanoyltransferase 2 |
| 235878_at | 12.34 | 1.51E-07 | 1.099519768 | NM_005680| | TAF1B,TBP-associated factor 1B |
| 242405_at | 12.33 | 1.52E-07 | 1.301129302 | NA |  |
| 203367_at | 12.33 | 1.52E-07 | 1.109940525 | NM_007026| | DUSP14,dual specificity phosphatase 14 |
| 228841_at | 12.33 | 1.52E-07 | 1.147813581 | NM_181705| | LOC90624,hypothetical protein LOC90624 |
| 240458_at | 12.32 | 1.53E-07 | 1.476998797 | NA |  |
| 201066_at | 12.32 | 1.54E-07 | 1.073018713 | NM_001916| | CYC1,cytochrome c-1 |
| 230142_s_at | 12.32 | 1.53E-07 | 1.216181719 | NM_001280| | CIRBP,cold inducible RNA binding protein |
| 203997_at | 12.32 | 1.53E-07 | 1.259858508 | NM_002829| | PTPN3,protein tyrosine phosphatase, non-receptor type |
| 212173_at | 12.31 | 1.55E-07 | 1.256781622 | NM_001625| | AK2,adenylate kinase 2 isoform a |
| 201797_s_at | 12.31 | 1.54E-07 | 1.100411648 | NM_006295| | VARS2,valyl-tRNA synthetase 2 |
| 223227_at | 12.31 | 1.55E-07 | 1.155003036 | NM_031885| | BBS2,Bardet-Biedl syndrome 2 protein |
| 205076_s_at | 12.31 | 1.54E-07 | 1.254143432 | NM_181873| | CRA,cisplatin resistance associated |
| 213365_at | 12.31 | 1.55E-07 | 1.151663532 | NM_080663| | MGC16943,hypothetical protein MGC16943 |
| 225356_at | 12.3 | 1.56E-07 | 1.12347025 | NA |  |
| 214259_s_at | 12.29 | 1.57E-07 | 1.081907323 | NM_003689| | AKR7A2,aldo-keto reductase family 7, member A2 |
| 1557238_s_at | 12.28 | 1.58E-07 | 1.310069252 | NA |  |
| 226208_at | 12.28 | 1.59E-07 | 1.068395374 | NA |  |
| 223598_at | 12.28 | 1.59E-07 | 1.027496319 | NM_002874| | RAD23B,UV excision repair protein RAD23 homolog B |
| 1552766_at | 12.27 | 1.60E-07 | 1.67082772 | NM_001077188| | NA |
| 218105_s_at | 12.27 | 1.60E-07 | 1.088275696 | NM_015956| | MRPL4,mitochondrial ribosomal protein L4 isoform a |
| 231952_at | 12.27 | 1.60E-07 | 1.227820215 | NA |  |
| 229127_at | 12.26 | 1.61E-07 | 1.56702027 | NM_021219| | JAM2,junctional adhesion molecule 2 precursor |
| 203264_s_at | 12.26 | 1.61E-07 | 1.175415946 | NM_015185| | ARHGEF9,Cdc42 guanine exchange factor 9 |
| 212764_at | 12.26 | 1.62E-07 | 1.904314931 | NM_030751| | TCF8,transcription factor 8 (represses interleukin 2 |
| 214531_s_at | 12.25 | 1.63E-07 | 1.135750106 | NM_003099| | SNX1,sorting nexin 1 isoform a |
| 231806_s_at | 12.25 | 1.63E-07 | 1.132919825 | NM_015690| | STK36,serine/threonine kinase 36 (fused homolog, |
| 1558365_at | 12.25 | 1.63E-07 | 1.519564372 | NA |  |
| 212014_x_at | 12.24 | 1.64E-07 | 1.154563378 | NM_000610| | CD44,CD44 antigen isoform 1 precursor |
| 238597_at | 12.24 | 1.64E-07 | 1.257305278 | NM_030816| | DKFZP566D1346,hypothetical protein DKFZp566D1346 |
| 201779_s_at | 12.24 | 1.65E-07 | 1.074380409 | NM_007282| | RNF13,ring finger protein 13 isoform 1 |
| 202651_at | 12.24 | 1.65E-07 | 1.194234266 | NM_014873| | LPGAT1,lysophosphatidylglycerol acyltransferase 1 |
| 231852_at | 12.24 | 1.65E-07 | 1.22694745 | NM_153332| | 3'HEXO,3' exoribonuclease |
| 213850_s_at | 12.24 | 1.64E-07 | 1.078475172 | NM_004719| | SFRS2IP,splicing factor, arginine/serine-rich 2, |
| 222671_s_at | 12.23 | 1.66E-07 | 1.155975696 | NM_023007| | FLJ12517,hypothetical protein FLJ12517 |
| 225878_at | 12.23 | 1.66E-07 | 1.061272924 | NM_015074| | KIF1B,kinesin family member 1B isoform b |
| 206016_at | 12.23 | 1.66E-07 | 1.135240554 | NM_014008| | CXorf37,chromosome X open reading frame 37 |
| 203045_at | 12.23 | 1.65E-07 | 1.190433569 | NM_004148| | NINJ1,ninjurin 1 |
| 215807_s_at | 12.23 | 1.65E-07 | 1.109334439 | NM_002673| | PLXNB1,plexin B1 |
| 1555920_at | 12.22 | 1.67E-07 | 1.223101913 | NM_007276| | CBX3,chromobox homolog 3 |
| 239692_at | 12.22 | 1.66E-07 | 1.21200548 | NA |  |
| 204133_at | 12.22 | 1.67E-07 | 1.113939979 | NM_004704| | RNU3IP2,RNA, U3 small nucleolar interacting protein 2 |
| 236353_at | 12.22 | 1.66E-07 | 1.17187305 | NA |  |
| 1556696_s_at | 12.22 | 1.67E-07 | 1.405062325 | NA |  |
| 220264_s_at | 12.21 | 1.69E-07 | 1.143721593 | NM_020960| | GPR107,G protein-coupled receptor 107 |
| 203508_at | 12.21 | 1.68E-07 | 1.428513213 | NM_001066| | TNFRSF1B,tumor necrosis factor receptor 2 precursor |
| 235421_at | 12.21 | 1.68E-07 | 1.254006514 | NM_005204| | MAP3K8,mitogen-activated protein kinase kinase kinase |
| 218500_at | 12.21 | 1.69E-07 | 1.376313227 | NM_016647| | LOC51337,mesenchymal stem cell protein DSCD75 |
| 228886_at | 12.21 | 1.69E-07 | 1.295660971 | NM_030626| | LRRC27,leucine rich repeat containing 27 |
| 223034_s_at | 12.21 | 1.68E-07 | 1.0567794 | NM_001098616| | NA |
| 228967_at | 12.2 | 1.70E-07 | 1.150380271 | NM_005801| | SUI1,putative translation initiation factor |
| 202451_at | 12.2 | 1.69E-07 | 1.12434146 | NM_005316| | GTF2H1,general transcription factor IIH, polypeptide 1 |
| 231300_at | 12.2 | 1.70E-07 | 1.382512972 | NM_001014979| | NA |
| 200790_at | 12.2 | 1.69E-07 | 1.141204405 | NM_002539| | ODC1,ornithine decarboxylase 1 |
| 1557507_at | 12.2 | 1.69E-07 | 1.263114467 | NA |  |
| 212272_at | 12.19 | 1.72E-07 | 1.541185583 | NM_145693| | LPIN1,lipin 1 |
| 224683_at | 12.19 | 1.71E-07 | 1.078902216 | NM_032807| | FBXO18,F-box only protein, helicase, 18 isoform 1 |
| 200896_x_at | 12.19 | 1.71E-07 | 1.023822039 | NM_001126050| | NA |
| 225300_at | 12.19 | 1.72E-07 | 1.049126592 | NM_033286| | C15orf23,chromosome 15 open reading frame 23 |
| 221796_at | 12.18 | 1.73E-07 | 1.805901363 | NM_001007097| | NTRK2,neurotrophic tyrosine kinase, receptor, type 2 |
| 241100_at | 12.17 | 1.74E-07 | 1.217014757 | NA |  |
| 224925_at | 12.17 | 1.74E-07 | 1.314807462 | NM_020820| | PREX1,PREX1 protein |
| 200028_s_at | 12.17 | 1.74E-07 | 1.03758411 | NM_020151| | STARD7,START domain containing 7 |
| 202459_s_at | 12.16 | 1.76E-07 | 1.090581388 | NM_014646| | LPIN2,lipin 2 |
| 229034_at | 12.15 | 1.77E-07 | 1.301517754 | NM_018013| | NA |
| 226854_at | 12.15 | 1.77E-07 | 1.109675199 | NA |  |
| 1556656_at | 12.15 | 1.77E-07 | 1.272444395 | NA |  |
| 201774_s_at | 12.14 | 1.79E-07 | 1.076260804 | NM_014865| | CNAP1,chromosome condensation-related SMC-associated |
| 222694_at | 12.14 | 1.78E-07 | 1.157959474 | NA |  |
| 202128_at | 12.14 | 1.79E-07 | 1.077007033 | NM_001039479| | NA |
| 1554080_at | 12.14 | 1.79E-07 | 1.193961396 | NM_005444| | RQCD1,RCD1 required for cell differentiation1 homolog |
| 221815_at | 12.14 | 1.78E-07 | 1.185007764 | NM_007011| | ABHD2,alpha/beta hydrolase domain containing protein |
| 1560100_at | 12.14 | 1.79E-07 | 1.298696986 | NM_001038493| | NA |
| 209150_s_at | 12.13 | 1.81E-07 | 1.05516578 | NM_001014842| | NA |
| 230460_at | 12.13 | 1.81E-07 | 1.306396177 | NA |  |
| 214786_at | 12.13 | 1.81E-07 | 1.449424691 | NM_005921| | NA |
| 202976_s_at | 12.12 | 1.82E-07 | 1.2889026 | NM_014899| | RHOBTB3,rho-related BTB domain containing 3 |
| 228743_at | 12.12 | 1.81E-07 | 1.340574161 | NM_032731| | TXNL5,thioredoxin-like 5 |
| 227009_at | 12.12 | 1.82E-07 | 1.198942027 | NA |  |
| 229236_s_at | 12.12 | 1.82E-07 | 1.185371777 | NM_213649| | SFXN4,sideroflexin 4 isoform 1 |
| 208425_s_at | 12.12 | 1.82E-07 | 1.342633877 | NM_025185| | NA |
| 227293_at | 12.12 | 1.82E-07 | 1.085032273 | NA |  |
| 214703_s_at | 12.11 | 1.84E-07 | 1.141654764 | NM_015274| | MAN2B2,mannosidase, alpha, class 2B, member 2 |
| 1556558_s_at | 12.11 | 1.83E-07 | 1.112875294 | NA |  |
| 204839_at | 12.11 | 1.84E-07 | 1.042360861 | NM_015918| | POP5,processing of precursor 5, ribonuclease P/MRP |
| 213362_at | 12.11 | 1.84E-07 | 1.14172471 | NM_001040712| | NA |
| 203115_at | 12.1 | 1.84E-07 | 1.155747126 | NM_000140| | FECH,ferrochelatase isoform b precursor |
| 214924_s_at | 12.1 | 1.86E-07 | 1.110018861 | NM_001042646| | NA |
| 238844_s_at | 12.1 | 1.86E-07 | 1.25376078 | NM_000272| | NPHP1,nephrocystin isoform 1 |
| 214043_at | 12.1 | 1.84E-07 | 1.099084706 | NM_001040712| | NA |
| 52741_at | 12.09 | 1.87E-07 | 1.06065863 | NM_152307| | C14orf172,chromosome 14 open reading frame 172 |
| 204743_at | 12.09 | 1.87E-07 | 1.386918817 | NM_001008272| | TAGLN3,transgelin 3 |
| 227776_at | 12.08 | 1.89E-07 | 1.120092211 | NM_018367| | PHCA,phytoceramidase, alkaline |
| 235595_at | 12.08 | 1.89E-07 | 1.144534819 | NM_004723| | ARHGEF2,rho/rac guanine nucleotide exchange factor 2 |
| 241867_at | 12.08 | 1.89E-07 | 1.303610532 | NA |  |
| 209763_at | 12.08 | 1.88E-07 | 1.384610608 | NM_145234| | CHRDL1,chordin-like 1 |
| 239091_at | 12.07 | 1.90E-07 | 1.463715923 | NA |  |
| 1556186_s_at | 12.07 | 1.91E-07 | 1.114959388 | NM_015047| | KIAA0090,KIAA0090 protein |
| 221869_at | 12.06 | 1.92E-07 | 1.095525132 | NM_020713| | GM632,KIAA1196 protein |
| 229007_at | 12.06 | 1.91E-07 | 1.868896653 | NA |  |
| 244660_at | 12.06 | 1.93E-07 | 1.316964065 | NM_001419| | ELAVL1,ELAV-like 1 |
| 214473_x_at | 12.06 | 1.91E-07 | 1.105339155 | NM_001003686| | PMS2L3,postmeiotic segregation increased 2-like 3 |
| 219034_at | 12.05 | 1.94E-07 | 1.065506936 | NM_017851| | PARP16,poly (ADP-ribose) polymerase family, member 16 |
| 219447_s_at | 12.05 | 1.93E-07 | 1.09911089 | NM_015945| | SLC35C2,ovarian cancer overexpressed 1 isoform a |
| 200098_s_at | 12.05 | 1.94E-07 | 1.053666173 | NM_016237| | ANAPC5,anaphase-promoting complex subunit 5 |
| 209286_at | 12.04 | 1.95E-07 | 1.42278744 | NM_006449| | CDC42EP3,Cdc42 effector protein 3 |
| 222364_at | 12.04 | 1.95E-07 | 1.287367255 | NM_080546| | CDW92,CDW92 antigen |
| 1554740_a_at | 12.04 | 1.95E-07 | 1.215618146 | NM_005897| | IPP,intracisternal A particle-promoted polypeptide |
| 209777_s_at | 12.04 | 1.95E-07 | 1.147227022 | NM_194255| | SLC19A1,solute carrier family 19 member 1 isoform a |
| 203324_s_at | 12.04 | 1.96E-07 | 1.385525474 | NM_001233| | CAV2,caveolin 2 isoform a and b |
| 209067_s_at | 12.03 | 1.97E-07 | 1.031622804 | NM_031372| | HNRPDL,heterogeneous nuclear ribonucleoprotein D-like |
| 210672_s_at | 12.03 | 1.97E-07 | 1.037209353 | NM_001039476| | NA |
| 203753_at | 12.03 | 1.97E-07 | 1.122800408 | NM_001083962| | NA |
| 201857_at | 12.02 | 2.00E-07 | 1.051382925 | NM_016107| | ZFR,zinc finger RNA binding protein |
| 202478_at | 12.02 | 1.98E-07 | 1.294512436 | NM_021643| | TRIB2,tribbles homolog 2 |
| 221098_x_at | 12.02 | 2.00E-07 | 1.180321657 | NM_006649| | UTP14A,UTP14, U3 small nucleolar ribonucleoprotein, |
| 215855_s_at | 12.01 | 2.00E-07 | 1.108719481 | NM_007114| | TMF1,TATA element modulatory factor 1 |
| 240467_at | 12.01 | 2.00E-07 | 1.516423099 | NA |  |
| 233379_at | 12.01 | 2.01E-07 | 1.141891155 | NM_024841| | FLJ14213,hypothetical protein FLJ14213 |
| 229210_at | 12.01 | 2.01E-07 | 1.217687759 | NM_024570| | FLJ11712,hypothetical protein FLJ11712 |
| 231348_s_at | 12.01 | 2.00E-07 | 1.321110184 | NM_001001395| | LMO3,LIM domain only 3 |
| 223519_at | 12.01 | 2.00E-07 | 1.40019716 | NM_016653| | ZAK,sterile-alpha motif and leucine zipper |
| 228579_at | 12 | 2.03E-07 | 1.192916777 | NA |  |
| 217053_x_at | 12 | 2.03E-07 | 1.180411555 | NM_004956| | ETV1,ets variant gene 1 |
| 206103_at | 12 | 2.02E-07 | 1.081072704 | NM_005052| | RAC3,ras-related C3 botulinum toxin substrate 3 (rho |
| 201324_at | 12 | 2.01E-07 | 1.75345578 | NM_001423| | EMP1,epithelial membrane protein 1 |
| 225089_at | 12 | 2.01E-07 | 1.146319556 | NM_018218| | USP40,ubiquitin specific protease 40 |
| 204081_at | 12 | 2.01E-07 | 1.218325739 | NM_001126181| | NA |
| 235173_at | 11.99 | 2.04E-07 | 1.152210348 | NA |  |
| 215690_x_at | 11.99 | 2.05E-07 | 1.111461805 | NM_003801| | GPAA1,anchor attachment protein 1 |
| 202984_s_at | 11.99 | 2.05E-07 | 1.139226447 | NM_001015048| | NA |
| 1564911_at | 11.99 | 2.04E-07 | 1.32282565 | NA |  |
| 214434_at | 11.98 | 2.05E-07 | 1.414455794 | NM_025015| | NA |
| 226112_at | 11.98 | 2.06E-07 | 1.092459241 | NM_000232| | SGCB,sarcoglycan, beta (43kDa dystrophin-associated |
| 202138_x_at | 11.98 | 2.05E-07 | 1.037907869 | NM_006303| | JTV1,JTV1 |
| 1556821_x_at | 11.98 | 2.05E-07 | 1.980577277 | NA |  |
| 223036_at | 11.98 | 2.05E-07 | 1.548793144 | NM_005687| | FARSLB,phenylalanine-tRNA synthetase-like, beta |
| 213646_x_at | 11.98 | 2.05E-07 | 1.033478321 | NM_006082| | K-ALPHA-1,tubulin, alpha, ubiquitous |
| 213211_s_at | 11.98 | 2.05E-07 | 1.241747476 | NM_006473| | TAF6L,TAF6-like RNA polymerase II |
| 217907_at | 11.98 | 2.05E-07 | 1.055283689 | NM_014161| | MRPL18,mitochondrial ribosomal protein L18 |
| 227085_at | 11.98 | 2.05E-07 | 1.0758884 | NM_012412| | H2AFV,H2A histone family, member V isoform 1 |
| 225905_s_at | 11.97 | 2.08E-07 | 1.112448367 | NM_006279| | ST3GAL3,sialyltransferase 6 isoform j |
| 209776_s_at | 11.97 | 2.07E-07 | 1.175478849 | NM_194255| | SLC19A1,solute carrier family 19 member 1 isoform a |
| 228655_at | 11.96 | 2.09E-07 | 1.223982848 | NA |  |
| 204161_s_at | 11.96 | 2.10E-07 | 1.146946202 | NM_014936| | ENPP4,ectonucleotide pyrophosphatase/phosphodiesterase |
| 208297_s_at | 11.95 | 2.11E-07 | 1.179547843 | NM_005665| | EVI5,ecotropic viral integration site 5 |
| 219522_at | 11.95 | 2.11E-07 | 1.17065014 | NM_014344| | FJX1,four jointed box 1 |
| 1557350_at | 11.95 | 2.12E-07 | 1.191339401 | NM_005754| | G3BP,Ras-GTPase-activating protein SH3-domain-binding |
| 230280_at | 11.94 | 2.13E-07 | 1.528583554 | NM_015163| | TRIM9,tripartite motif protein 9 isoform 1 |
| 218760_at | 11.94 | 2.13E-07 | 1.133935607 | NM_182476| | COQ6,CGI-10 protein isoform a |
| 236627_at | 11.94 | 2.13E-07 | 1.600098427 | NA |  |
| 226428_at | 11.93 | 2.15E-07 | 1.081577521 | NM_013433| | TNPO2,transportin 2 (importin 3, karyopherin beta 2b) |
| 201771_at | 11.93 | 2.14E-07 | 1.098135326 | NM_005698| | SCAMP3,secretory carrier membrane protein 3 isoform 1 |
| 212263_at | 11.93 | 2.15E-07 | 1.068841891 | NM_006775| | QKI,quaking homolog, KH domain RNA binding isoform |
| 212088_at | 11.93 | 2.15E-07 | 1.081959191 | NM_015160| | PMPCA,mitochondrial matrix processing protease, alpha |
| 209939_x_at | 11.93 | 2.15E-07 | 1.083807829 | NM_001127183| | NA |
| 216799_at | 11.93 | 2.15E-07 | 1.385346008 | NA |  |
| 221272_s_at | 11.93 | 2.15E-07 | 1.177618352 | NM_030806| | C1orf21,chromosome 1 open reading frame 21 |
| 216028_at | 11.93 | 2.15E-07 | 1.168626369 | NA |  |
| 213435_at | 11.93 | 2.15E-07 | 1.380007009 | NM_015265| | SATB2,SATB family member 2 |
| 212858_at | 11.93 | 2.14E-07 | 1.110909037 | NM_152341| | PAQR4,progestin and adipoQ receptor family member IV |
| 202975_s_at | 11.93 | 2.14E-07 | 1.287351038 | NM_014899| | RHOBTB3,rho-related BTB domain containing 3 |
| 1555912_at | 11.93 | 2.15E-07 | 1.327026409 | NA |  |
| 236536_at | 11.92 | 2.15E-07 | 1.375959453 | NM_052917| | GALNT13,UDP-N-acetyl-alpha-D-galactosamine:polypeptide |
| 222613_at | 11.92 | 2.15E-07 | 1.161824275 | NM_020374| | C12orf4,chromosome 12 open reading frame 4 |
| 204547_at | 11.92 | 2.16E-07 | 1.192003759 | NM_006822| | RAB40B,RAB40B, member RAS oncogene family |
| 203720_s_at | 11.92 | 2.16E-07 | 1.091268309 | NM_001983| | ERCC1,excision repair cross-complementing 1 isofrom 2 |
| 1558215_s_at | 11.91 | 2.18E-07 | 1.114514897 | NM_001076683| | NA |
| 214188_at | 11.91 | 2.19E-07 | 1.24582722 | NM_006460| | HIS1,HMBA-inducible |
| 1554132_a_at | 11.91 | 2.18E-07 | 1.163313496 | NM_018999| | KIAA1128,KIAA1128 |
| 206238_s_at | 11.91 | 2.19E-07 | 1.468417751 | NM_005748| | YAF2,YY1 associated factor 2 isoform a |
| 223923_at | 11.91 | 2.18E-07 | 1.232235363 | NM_019596| | C21orf62,chromosome 21 open reading frame 62 |
| 222193_at | 11.91 | 2.18E-07 | 1.092368401 | NM_021925| | FLJ21820,hypothetical protein FLJ21820 |
| 218785_s_at | 11.9 | 2.21E-07 | 1.202739883 | NM_022777| | RABL5,RAB, member RAS oncogene family-like 5 |
| 235206_at | 11.9 | 2.20E-07 | 1.197509931 | NM_016558| | SCAND1,SCAN domain containing protein 1 |
| 202134_s_at | 11.9 | 2.21E-07 | 1.128418838 | NM_015472| | WWTR1,WW domain containing transcription regulator 1 |
| 204331_s_at | 11.89 | 2.21E-07 | 1.053054183 | NM_021107| | MRPS12,mitochondrial ribosomal protein S12 precursor |
| 225628_s_at | 11.89 | 2.21E-07 | 1.243078815 | NM_005937| | MLLT6,myeloid/lymphoid or mixed-lineage leukemia |
| 210612_s_at | 11.89 | 2.21E-07 | 1.341968436 | NM_003898| | SYNJ2,synaptojanin 2 |
| 210139_s_at | 11.89 | 2.22E-07 | 1.394857092 | NM_000304| | PMP22,peripheral myelin protein 22 |
| 212319_at | 11.89 | 2.21E-07 | 1.174526276 | NM_001098509| | NA |
| 203383_s_at | 11.89 | 2.22E-07 | 1.112216187 | NM_002077| | GOLGA1,golgin 97 |
| 1566257_at | 11.89 | 2.22E-07 | 1.316762388 | NA |  |
| 206352_s_at | 11.89 | 2.23E-07 | 1.061053471 | NM_002617| | PEX10,peroxisome biogenesis factor 10 isoform 2 |
| 238119_at | 11.88 | 2.24E-07 | 1.205015323 | NA |  |
| 204964_s_at | 11.88 | 2.24E-07 | 1.254234993 | NM_005086| | SSPN,sarcospan |
| 231845_at | 11.87 | 2.27E-07 | 1.039872485 | NM_020745| | AARSL,alanyl-tRNA synthetase like |
| 229610_at | 11.87 | 2.26E-07 | 1.074101012 | NM_152515| | FLJ40629,hypothetical protein FLJ40629 |
| 227037_at | 11.87 | 2.26E-07 | 1.325897239 | NM_178836| | LOC201164,similar to CG12314 gene product |
| 225375_at | 11.87 | 2.27E-07 | 1.073838968 | NM_152464| | C17orf32,hypothetical protein MGC45714 |
| 202192_s_at | 11.86 | 2.28E-07 | 1.224950861 | NM_003644| | GAS7,growth arrest-specific 7 isoform a |
| 201491_at | 11.86 | 2.29E-07 | 1.042813524 | NM_012111| | AHSA1,AHA1, activator of heat shock 90kDa protein |
| 202139_at | 11.85 | 2.29E-07 | 1.108426239 | NM_003689| | AKR7A2,aldo-keto reductase family 7, member A2 |
| 244291_x_at | 11.85 | 2.29E-07 | 1.28116502 | NA |  |
| 1554228_a_at | 11.85 | 2.30E-07 | 1.200888383 | NA |  |
| 202617_s_at | 11.84 | 2.33E-07 | 1.211067924 | NM_001110792| | NA |
| 203611_at | 11.84 | 2.33E-07 | 1.088604941 | NM_005652| | TERF2,telomeric repeat binding factor 2 |
| 204860_s_at | 11.84 | 2.32E-07 | 1.449501949 | NM_004536| | BIRC1,baculoviral IAP repeat-containing 1 |
| 222805_at | 11.84 | 2.32E-07 | 1.273777382 | NM_024641| | MANEA,mannosidase, endo-alpha |
| 207084_at | 11.84 | 2.31E-07 | 1.803949399 | NM_005604| | POU3F2,POU domain, class 3, transcription factor 2 |
| 227890_at | 11.83 | 2.33E-07 | 1.213167484 | NM_001005209| | MGC99813,similar to RIKEN cDNA A230078I05 gene |
| 202172_at | 11.83 | 2.35E-07 | 1.129435029 | NM_007146| | ZNF161,zinc finger protein 161 |
| 205094_at | 11.83 | 2.35E-07 | 1.082216867 | NM_000286| | PEX12,peroxisomal biogenesis factor 12 |
| 232758_s_at | 11.82 | 2.35E-07 | 1.197229209 | NA |  |
| 201191_at | 11.81 | 2.39E-07 | 1.119507347 | NM_006224| | PITPNA,phosphatidylinositol transfer protein, alpha |
| 224847_at | 11.81 | 2.39E-07 | 1.278999508 | NM_001259| | CDK6,cyclin-dependent kinase 6 |
| 212222_at | 11.81 | 2.39E-07 | 1.095137202 | NM_014614| | PSME4,proteasome (prosome, macropain) activator |
| 217527_s_at | 11.8 | 2.41E-07 | 1.073212557 | NM_032815| | NFATC2IP,nuclear factor of activated T-cells, |
| 214108_at | 11.8 | 2.41E-07 | 1.147487642 | NM_002382| | MAX,MAX protein isoform a |
| 239657_x_at | 11.79 | 2.42E-07 | 1.116194383 | NA |  |
| 204490_s_at | 11.79 | 2.42E-07 | 1.153164784 | NM_000610| | CD44,CD44 antigen isoform 1 precursor |
| 228170_at | 11.79 | 2.42E-07 | 1.893500553 | NM_138983| | OLIG1,oligodendrocyte transcription factor 1 |
| 214538_x_at | 11.78 | 2.44E-07 | 1.276382337 | NM_004296| | RGS6,regulator of G-protein signalling 6 |
| 223917_s_at | 11.78 | 2.45E-07 | 1.092383761 | NM_144564| | SLC39A3,solute carrier family 39 (zinc transporter), |
| 44783_s_at | 11.78 | 2.45E-07 | 1.388772501 | NM_001040708| | NA |
| 204247_s_at | 11.78 | 2.45E-07 | 1.088587285 | NM_004935| | CDK5,cyclin-dependent kinase 5 |
| 219236_at | 11.77 | 2.47E-07 | 1.272770246 | NM_024897| | PAQR6,progestin and adipoQ receptor family member VI |
| 212054_x_at | 11.77 | 2.46E-07 | 1.061657095 | NM_015043| | KIAA0676,KIAA0676 protein isoform b |
| 228931_at | 11.76 | 2.49E-07 | 1.125626221 | NM_016035| | COQ4,CGI-92 protein |
| 233518_at | 11.76 | 2.49E-07 | 1.558294515 | NA |  |
| 217800_s_at | 11.76 | 2.50E-07 | 1.174468918 | NM_030571| | NDFIP1,Nedd4 family interacting protein 1 |
| 202289_s_at | 11.76 | 2.50E-07 | 1.063997596 | NM_006997| | TACC2,transforming, acidic coiled-coil containing |
| 225436_at | 11.75 | 2.52E-07 | 1.04431355 | NM_021214| | NA |
| 202749_at | 11.75 | 2.53E-07 | 1.057328331 | NM_004627| | WRB,tryptophan rich basic protein |
| 201076_at | 11.75 | 2.51E-07 | 1.055231714 | NM_001003796| | NHP2L1,NHP2 non-histone chromosome protein 2-like 1 |
| 222682_s_at | 11.75 | 2.53E-07 | 1.153446942 | NM_018691| | C5orf3,chromosome 5 open reading frame 3 |
| 226447_at | 11.75 | 2.51E-07 | 1.121170932 | NM_018489| | ASH1L,ash1 (absent, small, or homeotic)-like |
| 229029_at | 11.75 | 2.51E-07 | 1.354124661 | NA |  |
| 204066_s_at | 11.74 | 2.53E-07 | 1.091100432 | NM_001037131| | NA |
| 208818_s_at | 11.74 | 2.54E-07 | 1.238419884 | NM_000754| | COMT,catechol-O-methyltransferase isoform MB-COMT |
| 217437_s_at | 11.73 | 2.55E-07 | 1.274136492 | NM_001122824| | NA |
| 235053_at | 11.73 | 2.55E-07 | 1.168817269 | NA |  |
| 205234_at | 11.73 | 2.57E-07 | 1.696185003 | NM_004696| | SLC16A4,solute carrier family 16, member 4 |
| 218358_at | 11.73 | 2.56E-07 | 1.1114755 | NM_024324| | MGC11256,hypothetical protein MGC11256 |
| 208430_s_at | 11.72 | 2.58E-07 | 1.307072856 | NM_001390| | DTNA,dystrobrevin alpha isoform 1 |
| 221569_at | 11.72 | 2.58E-07 | 1.217652063 | NM_017651| | AHI1,jouberin |
| 228240_at | 11.72 | 2.58E-07 | 1.05212225 | NA |  |
| 220753_s_at | 11.71 | 2.62E-07 | 1.308048432 | NM_015974| | CRYL1,lambda-crystallin |
| 204300_at | 11.71 | 2.62E-07 | 1.097299778 | NM_004564| | PET112L,PET112-like |
| 204993_at | 11.71 | 2.62E-07 | 1.149257102 | NM_002073| | GNAZ,guanine nucleotide binding protein, alpha z |
| 225085_at | 11.71 | 2.60E-07 | 1.19069928 | NM_018218| | USP40,ubiquitin specific protease 40 |
| 228817_at | 11.71 | 2.61E-07 | 1.091726425 | NM_001077690| | NA |
| 227935_s_at | 11.71 | 2.62E-07 | 1.210687404 | NM_032373| | PCGF5,polycomb group ring finger 5 |
| 202471_s_at | 11.7 | 2.64E-07 | 1.085960046 | NM_004135| | IDH3G,isocitrate dehydrogenase 3 (NAD+) gamma isoform |
| 205918_at | 11.69 | 2.66E-07 | 1.213917219 | NM_005070| | SLC4A3,solute carrier family 4, anion exchanger, member |
| 220666_at | 11.69 | 2.67E-07 | 1.148017974 | NA |  |
| 217754_at | 11.68 | 2.68E-07 | 1.070411181 | NM_019082| | DDX56,DEAD (Asp-Glu-Ala-Asp) box polypeptide 56 |
| 243303_at | 11.68 | 2.68E-07 | 1.504385531 | NA |  |
| 1556021_at | 11.68 | 2.69E-07 | 1.475239252 | NA |  |
| 202662_s_at | 11.68 | 2.69E-07 | 1.535141964 | NM_002223| | ITPR2,inositol 1,4,5-triphosphate receptor, type 2 |
| 203823_at | 11.68 | 2.67E-07 | 1.158621 | NM_017790| | RGS3,regulator of G-protein signalling 3 isoform 3 |
| 227756_at | 11.68 | 2.67E-07 | 1.382897854 | NM_152450| | MGC26690,hypothetical protein MGC26690 |
| 209870_s_at | 11.67 | 2.70E-07 | 1.146035826 | NM_005503| | APBA2,amyloid beta A4 precursor protein-binding, |
| 226198_at | 11.67 | 2.71E-07 | 1.11568707 | NM_001033551| | NA |
| 242888_at | 11.67 | 2.70E-07 | 1.482474215 | NA |  |
| 213433_at | 11.67 | 2.71E-07 | 1.212997268 | NM_004311| | ARL3,ADP-ribosylation factor-like 3 |
| 232759_at | 11.67 | 2.70E-07 | 1.165160317 | NA |  |
| 202463_s_at | 11.66 | 2.73E-07 | 1.114006012 | NM_003926| | MBD3,methyl-CpG binding domain protein 3 |
| 209968_s_at | 11.66 | 2.73E-07 | 1.119145156 | NM_000615| | NCAM1,neural cell adhesion molecule 1 |
| 228173_at | 11.66 | 2.73E-07 | 1.173921874 | NM_000516| | GNAS,guanine nucleotide binding protein, alpha |
| 1555906_s_at | 11.66 | 2.73E-07 | 1.115542119 | NM_001029839| | NA |
| 228443_s_at | 11.65 | 2.76E-07 | 1.190814476 | NA |  |
| 222941_at | 11.65 | 2.76E-07 | 1.145649043 | NA |  |
| 203450_at | 11.65 | 2.76E-07 | 1.085446443 | NM_001002880| | PGEA1,PKD2 interactor, golgi and endoplasmic reticulum |
| 227563_at | 11.65 | 2.76E-07 | 1.253855665 | NM_175923| | MGC42630,hypothetical protein MGC42630 |
| 218856_at | 11.65 | 2.76E-07 | 1.025921832 | NM_014452| | TNFRSF21,tumor necrosis factor receptor superfamily, |
| 221516_s_at | 11.64 | 2.78E-07 | 1.073085792 | NM_019008| | FLJ20232,hypothetical protein FLJ20232 |
| 212468_at | 11.64 | 2.78E-07 | 1.139525938 | NM_003971| | SPAG9,sperm associated antigen 9 isoform 1 |
| 218827_s_at | 11.63 | 2.82E-07 | 1.082338525 | NM_032142| | Cep192,centrosomal protein 192 kDa isoform 1 |
| 216583_x_at | 11.63 | 2.80E-07 | 1.093324172 | NM_001034833| | NA |
| 1555573_at | 11.63 | 2.80E-07 | 1.407814408 | NM_173572| | C10orf93,chromosome 10 open reading frame 93 |
| 227462_at | 11.63 | 2.80E-07 | 1.452178856 | NA |  |
| 229905_at | 11.63 | 2.80E-07 | 1.282817165 | NM_001100426| | NA |
| 208985_s_at | 11.63 | 2.80E-07 | 1.076576173 | NM_003758| | EIF3S1,eukaryotic translation initiation factor 3, |
| 1557360_at | 11.62 | 2.83E-07 | 1.119178573 | NM_133259| | LRPPRC,leucine-rich PPR motif-containing protein |
| 227982_at | 11.62 | 2.84E-07 | 1.133666814 | NM_016955| | SLA/LP,soluble liver antigen/liver pancreas antigen |
| 226373_at | 11.62 | 2.84E-07 | 1.156866302 | NM_144579| | SFXN5,sideroflexin 5 |
| 226070_at | 11.62 | 2.83E-07 | 1.140341683 | NM_183241| | LOC286257,hypothetical protein LOC286257 |
| 210473_s_at | 11.61 | 2.86E-07 | 1.055568529 | NM_145290| | GPR125,G protein-coupled receptor 125 |
| 231912_s_at | 11.61 | 2.86E-07 | 1.173087307 | NM_015395| | DKFZP434B0335,DKFZP434B0335 protein |
| 215349_at | 11.61 | 2.85E-07 | 1.259102145 | NA |  |
| 218337_at | 11.61 | 2.87E-07 | 1.093535536 | NM_022749| | RAI16,retinoic acid induced 16 |
| 202756_s_at | 11.6 | 2.88E-07 | 1.135758451 | NM_002081| | GPC1,glypican 1 precursor |
| 1556111_s_at | 11.6 | 2.88E-07 | 1.12363358 | NA |  |
| 204078_at | 11.6 | 2.89E-07 | 1.12347963 | NM_006455| | SC65,synaptonemal complex protein SC65 |
| 220605_s_at | 11.58 | 2.94E-07 | 1.13507284 | NM_012237| | SIRT2,sirtuin 2 isoform 1 |
| 203427_at | 11.57 | 2.95E-07 | 1.097839074 | NM_014034| | ASF1A,ASF1 anti-silencing function 1 homolog A |
| 244532_x_at | 11.57 | 2.96E-07 | 1.504835553 | NA |  |
| 206465_at | 11.57 | 2.97E-07 | 1.527174269 | NM_015162| | BG1,lipidosin |
| 221911_at | 11.57 | 2.97E-07 | 1.165180296 | NM_004956| | ETV1,ets variant gene 1 |
| 224872_at | 11.56 | 2.98E-07 | 1.130426795 | NM_173602| | KIAA1463,KIAA1463 protein |
| 209370_s_at | 11.56 | 2.98E-07 | 1.197400047 | NM_001122681| | NA |
| 200621_at | 11.56 | 2.99E-07 | 1.403955383 | NM_004078| | CSRP1,cysteine and glycine-rich protein 1 |
| 1557053_s_at | 11.55 | 3.02E-07 | 1.065121836 | NM_003343| | UBE2G2,ubiquitin-conjugating enzyme E2G 2 isoform 1 |
| 217890_s_at | 11.55 | 3.02E-07 | 1.235832389 | NM_018222| | PARVA,parvin, alpha |
| 229632_s_at | 11.55 | 3.00E-07 | 1.07899479 | NM_018142| | FLJ10569,hypothetical protein FLJ10569 |
| 1556285_s_at | 11.55 | 3.03E-07 | 1.055066668 | NM_001034191| | NA |
| 213581_at | 11.55 | 3.01E-07 | 1.08499143 | NM_002598| | PDCD2,programmed cell death 2 isoform 1 |
| 1554878_a_at | 11.55 | 3.02E-07 | 1.475624552 | NM_001122674| | NA |
| 46142_at | 11.55 | 3.02E-07 | 1.330653413 | NM_022773| | FLJ12681,hypothetical protein FLJ12681 |
| 226872_at | 11.55 | 3.01E-07 | 1.279215865 | NM_000635| | RFX2,regulatory factor X2 isoform a |
| 221767_x_at | 11.55 | 3.03E-07 | 1.044988389 | NM_005336| | HDLBP,high density lipoprotein binding protein |
| 221748_s_at | 11.55 | 3.01E-07 | 1.324667333 | NM_022648| | TNS,tensin |
| 218939_at | 11.53 | 3.09E-07 | 1.119631689 | NM_012318| | LETM1,leucine zipper-EF-hand containing transmembrane |
| 228323_at | 11.53 | 3.08E-07 | 1.059669172 | NM_144508| | AF15Q14,AF15q14 protein isoform 2 |
| 232947_at | 11.53 | 3.08E-07 | 1.274619327 | NA |  |
| 227499_at | 11.53 | 3.07E-07 | 1.21991284 | NA |  |
| 203933_at | 11.52 | 3.10E-07 | 1.101968343 | NM_014700| | RAB11FIP3,rab11-family interacting protein 3 |
| 212763_at | 11.52 | 3.11E-07 | 1.092966339 | NM_203459| | KIAA1078,KIAA1078 protein |
| 217985_s_at | 11.52 | 3.11E-07 | 1.240269648 | NM_013448| | BAZ1A,bromodomain adjacent to zinc finger domain, 1A |
| 215128_at | 11.52 | 3.09E-07 | 1.114563877 | NA |  |
| 239311_at | 11.52 | 3.10E-07 | 1.262183564 | NA |  |
| 230863_at | 11.52 | 3.11E-07 | 1.347418674 | NM_004525| | LRP2,low density lipoprotein-related protein 2 |
| 242790_at | 11.51 | 3.14E-07 | 1.198235939 | NM_007241| | EAP30,EAP30 subunit of ELL complex |
| 229733_s_at | 11.51 | 3.13E-07 | 1.288095164 | NA |  |
| 224576_at | 11.51 | 3.12E-07 | 1.079180612 | NM_001031711| | NA |
| 223584_s_at | 11.5 | 3.16E-07 | 1.163046315 | NM_015483| | KBTBD2,kelch repeat and BTB (POZ) domain containing 2 |
| 210175_at | 11.5 | 3.15E-07 | 1.284861562 | NM_003203| | C2orf3,chromosome 2 open reading frame 3 |
| 226101_at | 11.5 | 3.17E-07 | 1.278729408 | NM_005400| | PRKCE,protein kinase C, epsilon |
| 222705_s_at | 11.5 | 3.15E-07 | 1.190828246 | NM_014252| | SLC25A15,solute carrier family 25 (mitochondrial carrier; |
| 213222_at | 11.5 | 3.14E-07 | 1.261180935 | NM_015192| | PLCB1,phosphoinositide-specific phospholipase C beta 1 |
| 224931_at | 11.5 | 3.15E-07 | 1.096325059 | NM_001008485| | SLC41A3,solute carrier family 41, member 3 isoform 1 |
| 210105_s_at | 11.5 | 3.14E-07 | 1.087837984 | NM_002037| | FYN,protein-tyrosine kinase fyn isoform a |
| 208853_s_at | 11.5 | 3.15E-07 | 1.095833056 | NM_001024649| | NA |
| 223852_s_at | 11.49 | 3.18E-07 | 1.261178797 | NM_032017| | MGC4796,SINK-homologous serine/threonine kinase |
| 233530_at | 11.49 | 3.20E-07 | 1.228968579 | NA |  |
| 212162_at | 11.49 | 3.18E-07 | 1.062737976 | NM_020738| | KIDINS220,kinase D-interacting substance of 220 kDa |
| 219205_at | 11.49 | 3.19E-07 | 1.207756512 | NM_021947| | SRR,serine racemase |
| 217986_s_at | 11.48 | 3.23E-07 | 1.303259672 | NM_013448| | BAZ1A,bromodomain adjacent to zinc finger domain, 1A |
| 204191_at | 11.47 | 3.24E-07 | 1.183362695 | NM_000629| | IFNAR1,interferon-alpha receptor 1 precursor |
| 240529_at | 11.46 | 3.28E-07 | 1.159637158 | NA |  |
| 228724_at | 11.46 | 3.27E-07 | 1.395888849 | NA |  |
| 224789_at | 11.46 | 3.27E-07 | 1.107502523 | NM_015397| | WDR40A,WD repeat domain 40A |
| 212315_s_at | 11.46 | 3.27E-07 | 1.103239658 | NM_024923| | NUP210,nucleoporin 210 |
| 212710_at | 11.45 | 3.31E-07 | 1.054402998 | NM_015447| | CAMSAP1,calmodulin regulated spectrin-associated protein |
| 204484_at | 11.45 | 3.31E-07 | 1.09037036 | NM_002646| | PIK3C2B,phosphoinositide-3-kinase, class 2, beta |
| 211964_at | 11.45 | 3.32E-07 | 1.156480645 | NM_001846| | COL4A2,alpha 2 type IV collagen preproprotein |
| 230300_at | 11.45 | 3.30E-07 | 1.633043227 | NA |  |
| 202836_s_at | 11.45 | 3.31E-07 | 1.062281301 | NM_006701| | TXNL4A,thioredoxin-like 4A |
| 223614_at | 11.45 | 3.30E-07 | 1.224484595 | NA |  |
| 212792_at | 11.45 | 3.32E-07 | 1.125343412 | NM_015283| | NA |
| 209332_s_at | 11.44 | 3.33E-07 | 1.084539837 | NM_002382| | MAX,MAX protein isoform a |
| 219717_at | 11.44 | 3.35E-07 | 1.052285595 | NM_017741| | FLJ20280,hypothetical protein FLJ20280 |
| 212972_x_at | 11.44 | 3.34E-07 | 1.123601715 | NA |  |
| 229197_at | 11.44 | 3.35E-07 | 1.216224086 | NM_032329| | ING5,inhibitor of growth family, member 5 |
| 203817_at | 11.43 | 3.38E-07 | 1.254269484 | NM_000857| | GUCY1B3,guanylate cyclase 1, soluble, beta 3 |
| 214332_s_at | 11.43 | 3.36E-07 | 1.236015315 | NM_005726| | TSFM,Ts translation elongation factor, mitochondrial |
| 1554520_at | 11.43 | 3.37E-07 | 1.180932177 | NA |  |
| 227634_at | 11.43 | 3.36E-07 | 1.171105078 | NM_173575| | STK32C,serine/threonine kinase 32C |
| 211876_x_at | 11.43 | 3.37E-07 | 1.278257704 | NM_003735| | PCDHGA12,protocadherin gamma subfamily A, 12 isoform 1 |
| 224865_at | 11.43 | 3.38E-07 | 1.188476322 | NM_032228| | MLSTD2,male sterility domain containing 2 |
| 226060_at | 11.43 | 3.37E-07 | 1.070365537 | NM_052859| | RFT1,RFT1 homolog |
| 210425_x_at | 11.42 | 3.39E-07 | 1.173715207 | NM_001023567| | NA |
| 1557950_at | 11.42 | 3.39E-07 | 1.043440146 | NM_004713| | SDCCAG1,serologically defined colon cancer antigen 1 |
| 203196_at | 11.42 | 3.39E-07 | 1.136960652 | NM_001105515| | NA |
| 239572_at | 11.42 | 3.39E-07 | 1.435367407 | NM_021954| | GJA3,gap junction protein, alpha 3, 46kDa (connexin |
| 231894_at | 11.41 | 3.43E-07 | 1.218270649 | NA |  |
| 227579_at | 11.41 | 3.43E-07 | 1.246151699 | NA |  |
| 235882_at | 11.41 | 3.42E-07 | 1.608255091 | NA |  |
| 1560689_s_at | 11.41 | 3.43E-07 | 1.271745109 | NM_001626| | AKT2,v-akt murine thymoma viral oncogene homolog 2 |
| 220002_at | 11.41 | 3.42E-07 | 1.181901159 | NM_018012| | NA |
| 224851_at | 11.41 | 3.44E-07 | 1.314621161 | NM_001259| | CDK6,cyclin-dependent kinase 6 |
| 235015_at | 11.41 | 3.43E-07 | 1.244124978 | NA |  |
| 230868_at | 11.41 | 3.43E-07 | 1.294017695 | NA |  |
| 1558613_at | 11.41 | 3.43E-07 | 1.500383417 | NA |  |
| 226660_at | 11.41 | 3.42E-07 | 1.081696408 | NM_003161| | RPS6KB1,ribosomal protein S6 kinase, 70kDa, polypeptide |
| 1556081_at | 11.4 | 3.45E-07 | 1.574437481 | NA |  |
| 229491_at | 11.4 | 3.47E-07 | 1.373946896 | NM_178833| | LOC133308,hypothetical protein BC009732 |
| 230220_at | 11.4 | 3.47E-07 | 1.204799347 | NA |  |
| 211299_s_at | 11.39 | 3.50E-07 | 1.183727234 | NM_004475| | FLOT2,flotillin 2 |
| 217914_at | 11.39 | 3.48E-07 | 1.08535098 | NM_017901| | TPCN1,two pore segment channel 1 |
| 208965_s_at | 11.39 | 3.49E-07 | 1.558323624 | NM_005531| | IFI16,interferon, gamma-inducible protein 16 |
| 224359_s_at | 11.39 | 3.48E-07 | 1.209031675 | NM_032410| | HOOK3,golgi-associated microtubule-binding protein |
| 229697_at | 11.39 | 3.48E-07 | 1.167999255 | NM_003609| | HIRIP3,HIRA interacting protein 3 |
| 225865_x_at | 11.39 | 3.49E-07 | 1.188599733 | NM_198976| | TH1L,TH1-like protein |
| 213424_at | 11.38 | 3.53E-07 | 1.598360176 | NM_001100425| | NA |
| 209192_x_at | 11.38 | 3.52E-07 | 1.093234932 | NM_006388| | HTATIP,HIV-1 Tat interactive protein, 60kDa isoform 2 |
| 225685_at | 11.38 | 3.53E-07 | 1.353210236 | NA |  |
| 242705_x_at | 11.37 | 3.55E-07 | 1.141183931 | NA |  |
| 210627_s_at | 11.37 | 3.57E-07 | 1.053268136 | NM_006302| | GCS1,mannosyl-oligosaccharide glucosidase |
| 225835_at | 11.37 | 3.57E-07 | 1.199833158 | NM_001046| | SLC12A2,solute carrier family 12 |
| 233401_at | 11.37 | 3.57E-07 | 1.819803531 | NA |  |
| 243224_at | 11.36 | 3.58E-07 | 1.163575713 | NA |  |
| 223605_at | 11.36 | 3.60E-07 | 1.192472871 | NM_031481| | SLC25A18,solute carrier |
| 209651_at | 11.36 | 3.59E-07 | 1.152975256 | NM_001042454| | NA |
| 1558740_s_at | 11.36 | 3.59E-07 | 1.384229836 | NA |  |
| 220974_x_at | 11.36 | 3.59E-07 | 1.613744944 | NM_030971| | SFXN3,sideroflexin 3 |
| 227010_at | 11.36 | 3.57E-07 | 1.093560784 | NA |  |
| 217945_at | 11.36 | 3.58E-07 | 1.063127568 | NM_001011885| | BTBD1,BTB (POZ) domain containing 1 isoform 2 |
| 212565_at | 11.36 | 3.60E-07 | 1.119013036 | NM_015000| | STK38L,serine/threonine kinase 38 like |
| 214760_at | 11.35 | 3.63E-07 | 1.140930798 | NM_015655| | ZNF337,zinc finger protein 337 |
| 201534_s_at | 11.35 | 3.61E-07 | 1.156048634 | NM_007106| | UBL3,ubiquitin-like 3 |
| 214460_at | 11.35 | 3.61E-07 | 1.322570092 | NM_002338| | LSAMP,limbic system-associated membrane protein |
| 211750_x_at | 11.35 | 3.62E-07 | 1.024384615 | NM_032704| | TUBA6,tubulin alpha 6 |
| 211576_s_at | 11.35 | 3.61E-07 | 1.086966718 | NM_194255| | SLC19A1,solute carrier family 19 member 1 isoform a |
| 203931_s_at | 11.35 | 3.61E-07 | 1.044151681 | NM_002949| | MRPL12,mitochondrial ribosomal protein L12 |
| 236585_at | 11.35 | 3.61E-07 | 1.337995515 | NA |  |
| 235255_at | 11.34 | 3.64E-07 | 1.193674544 | NM_012463| | ATP6V0A2,ATPase, H+ transporting, lysosomal V0 subunit a |
| 210752_s_at | 11.34 | 3.64E-07 | 1.162988133 | NM_170607| | MLX,transcription factor-like protein 4 isoform |
| 212852_s_at | 11.34 | 3.64E-07 | 1.059403392 | NM_001042369| | NA |
| 1559517_a_at | 11.34 | 3.66E-07 | 1.402621803 | NM_020148| | SPIRE1,spire homolog 1 |
| 206993_at | 11.34 | 3.64E-07 | 1.210610356 | NM_001003803| | ATP5S,ATP synthase, H+ transporting, mitochondrial F0 |
| 240440_at | 11.34 | 3.64E-07 | 1.200942513 | NM_030769| | NPL,N-acetylneuraminate pyruvate lyase |
| 211534_x_at | 11.33 | 3.68E-07 | 1.132198966 | NM_002847| | PTPRN2,protein tyrosine phosphatase, receptor type, N |
| 223284_at | 11.32 | 3.72E-07 | 1.092257732 | NM_020378| | KLP1,K562 cell-derived leucine-zipper-like protein 1 |
| 225749_at | 11.32 | 3.73E-07 | 1.089310951 | NM_001010878| | LOC283951,hypothetical protein LOC283951 |
| 211022_s_at | 11.32 | 3.73E-07 | 1.155592891 | NM_000489| | ATRX,transcriptional regulator ATRX isoform 1 |
| 213790_at | 11.32 | 3.71E-07 | 1.439854355 | NA |  |
| 208676_s_at | 11.31 | 3.76E-07 | 1.075475874 | NM_006191| | PA2G4,proliferation-associated 2G4, 38kDa |
| 1558021_at | 11.31 | 3.75E-07 | 1.5565531 | NM_005833| | RAB9P40,Rab9 effector p40 |
| 203509_at | 11.31 | 3.76E-07 | 1.061654029 | NM_003105| | SORL1,sortilin-related receptor containing LDLR class |
| 229638_at | 11.31 | 3.74E-07 | 1.15727374 | NM_024336| | IRX3,iroquois homeobox protein 3 |
| 208907_s_at | 11.31 | 3.76E-07 | 1.073852597 | NM_014046| | MRPS18B,mitochondrial ribosomal protein S18B |
| 204947_at | 11.31 | 3.76E-07 | 1.095224057 | NM_005225| | E2F1,E2F transcription factor 1 |
| 209379_s_at | 11.3 | 3.78E-07 | 1.189498731 | NM_018999| | KIAA1128,KIAA1128 |
| 211000_s_at | 11.3 | 3.78E-07 | 1.548598452 | NM_002184| | IL6ST,interleukin 6 signal transducer isoform 1 |
| 204315_s_at | 11.3 | 3.79E-07 | 1.078752395 | NM_016426| | GTSE1,G-2 and S-phase expressed 1 |
| 1555004_a_at | 11.29 | 3.84E-07 | 1.254968215 | NM_002895| | RBL1,retinoblastoma-like protein 1 isoform a |
| 218997_at | 11.28 | 3.85E-07 | 1.082622627 | NM_022490| | PAF53,RNA polymerase I associated factor 53 |
| 212711_at | 11.28 | 3.86E-07 | 1.05838976 | NM_015447| | CAMSAP1,calmodulin regulated spectrin-associated protein |
| 215143_at | 11.28 | 3.87E-07 | 1.629390797 | NA |  |
| 41047_at | 11.28 | 3.87E-07 | 1.051847431 | NM_024112| | C9orf16,chromosome 9 open reading frame 16 |
| 1561578_s_at | 11.28 | 3.85E-07 | 1.358601609 | NM_001012755| | NA |
| 219324_at | 11.27 | 3.89E-07 | 1.161089992 | NM_001039141| | NA |
| 236109_at | 11.27 | 3.90E-07 | 1.319709511 | NA |  |
| 220200_s_at | 11.27 | 3.89E-07 | 1.220225917 | NM_020382| | SET8,SET domain-containing protein 8 |
| 202395_at | 11.27 | 3.90E-07 | 1.105319414 | NM_006178| | NSF,N-ethylmaleimide-sensitive factor |
| 220494_s_at | 11.27 | 3.91E-07 | 1.173264841 | NA |  |
| 244356_at | 11.27 | 3.89E-07 | 1.123396622 | NA |  |
| 219538_at | 11.26 | 3.94E-07 | 1.234883598 | NM_019069| | WDR5B,WD repeat domain 5B |
| 239801_at | 11.26 | 3.92E-07 | 1.141996649 | NA |  |
| 211851_x_at | 11.25 | 3.97E-07 | 1.080242592 | NM_007294| | BRCA1,breast cancer 1, early onset |
| 209567_at | 11.25 | 3.96E-07 | 1.030861115 | NM_015169| | RRS1,homolog of yeast ribosome biogenesis regulatory |
| 227968_at | 11.24 | 4.00E-07 | 1.112086987 | NM_182612| | FLJ34283,hypothetical protein FLJ34283 |
| 218544_s_at | 11.24 | 4.00E-07 | 1.046781979 | NM_005772| | RCL1,RNA cyclase homolog |
| 220212_s_at | 11.24 | 4.02E-07 | 1.08726226 | NM_001083953| | NA |
| 233058_at | 11.24 | 4.00E-07 | 1.318518617 | NA |  |
| 236462_at | 11.23 | 4.04E-07 | 1.430178652 | NA |  |
| 217080_s_at | 11.23 | 4.03E-07 | 1.155868279 | NM_004839| | HOMER2,homer 2 isoform 1 |
| 200901_s_at | 11.23 | 4.03E-07 | 1.037200765 | NM_002355| | M6PR,cation-dependent mannose-6-phosphate receptor |
| 202438_x_at | 11.23 | 4.03E-07 | 1.362960141 | NM_000202| | IDS,iduronate-2-sulfatase isoform a precursor |
| 230755_at | 11.22 | 4.08E-07 | 1.145651971 | NM_138328| | RHBDL4,rhomboid, veinlet-like 4 |
| 217598_at | 11.22 | 4.08E-07 | 1.094856152 | NA |  |
| 204343_at | 11.22 | 4.08E-07 | 1.272713145 | NM_001089| | ABCA3,ATP-binding cassette, sub-family A member 3 |
| 215469_at | 11.22 | 4.07E-07 | 1.330537537 | NA |  |
| 232915_at | 11.22 | 4.10E-07 | 1.137296175 | NM_019070| | DDX49,DEAD (Asp-Glu-Ala-Asp) box polypeptide 49 |
| 223288_at | 11.21 | 4.12E-07 | 1.076083568 | NM_032557| | USP38,ubiquitin specific protease 38 |
| 215211_at | 11.21 | 4.12E-07 | 1.278626596 | NM_145237| | LOC94431,similar to RNA polymerase I transcription factor |
| 201745_at | 11.2 | 4.18E-07 | 1.146010066 | NM_002822| | PTK9,twinfilin isoform 1 |
| 203729_at | 11.2 | 4.17E-07 | 1.275530682 | NM_001425| | EMP3,epithelial membrane protein 3 |
| 212239_at | 11.2 | 4.15E-07 | 1.299184301 | NM_181504| | PIK3R1,phosphoinositide-3-kinase, regulatory subunit, |
| 201805_at | 11.2 | 4.18E-07 | 1.068861865 | NM_002733| | PRKAG1,AMP-activated protein kinase, noncatalytic |
| 227564_at | 11.2 | 4.16E-07 | 1.161289337 | NM_152419| | NA |
| 220113_x_at | 11.2 | 4.18E-07 | 1.085225157 | NM_019014| | POLR1B,RNA polymerase I polypeptide B |
| 220954_s_at | 11.2 | 4.17E-07 | 1.150878386 | NM_013440| | PILRB,paired immunoglobulin-like type 2 receptor beta |
| 229881_at | 11.2 | 4.16E-07 | 1.285076589 | NM_007249| | KLF12,Kruppel-like factor 12 isoform a |
| 203224_at | 11.2 | 4.18E-07 | 1.173519207 | NM_018339| | RFK,riboflavin kinase |
| 217604_at | 11.19 | 4.19E-07 | 1.262424794 | NA |  |
| 206320_s_at | 11.19 | 4.21E-07 | 1.179484982 | NM_001127217| | NA |
| 239544_at | 11.19 | 4.21E-07 | 1.135693409 | NA |  |
| 241798_at | 11.19 | 4.20E-07 | 1.195932208 | NA |  |
| 225302_at | 11.19 | 4.19E-07 | 1.178854591 | NM_019022| | TXNDC10,thioredoxin domain containing 10 |
| 202203_s_at | 11.18 | 4.22E-07 | 1.248678765 | NM_001144| | AMFR,autocrine motility factor receptor isoform a |
| 240733_at | 11.18 | 4.23E-07 | 1.250532302 | NA |  |
| 221845_s_at | 11.18 | 4.23E-07 | 1.181908336 | NM_030813| | SKD3,suppressor of potassium transport defect 3 |
| 201991_s_at | 11.18 | 4.24E-07 | 1.067967209 | NM_004521| | KIF5B,kinesin family member 5B |
| 219978_s_at | 11.18 | 4.22E-07 | 1.041401185 | NM_016359| | NUSAP1,nucleolar and spindle associated protein 1 |
| 212123_at | 11.18 | 4.22E-07 | 1.073860364 | NM_015631| | C10orf61,chromosome 10 open reading frame 61 |
| 225100_at | 11.18 | 4.22E-07 | 1.061336323 | NM_001105573| | NA |
| 209169_at | 11.17 | 4.27E-07 | 1.125699339 | NM_001001994| | GPM6B,glycoprotein M6B isoform 4 |
| 203209_at | 11.17 | 4.28E-07 | 1.070522665 | NM_007370| | RFC5,replication factor C 5 isoform 1 |
| 203595_s_at | 11.17 | 4.26E-07 | 1.390637391 | NM_012420| | IFIT5,interferon-induced protein with |
| 225006_x_at | 11.17 | 4.27E-07 | 1.1944726 | NM_198976| | TH1L,TH1-like protein |
| 202020_s_at | 11.17 | 4.26E-07 | 1.05496301 | NM_006055| | LANCL1,lanthionine synthetase C-like protein 1 |
| 231863_at | 11.17 | 4.26E-07 | 1.510881705 | NM_019071| | ING3,inhibitor of growth family, member 3 isoform 1 |
| 227712_at | 11.17 | 4.26E-07 | 1.208187768 | NM_020466| | DJ122O8.2,hypothetical protein dJ122O8.2 |
| 238704_at | 11.17 | 4.27E-07 | 1.174540033 | NA |  |
| 212808_at | 11.17 | 4.27E-07 | 1.157050903 | NM_032815| | NFATC2IP,nuclear factor of activated T-cells, |
| 223455_at | 11.16 | 4.31E-07 | 1.326487063 | NM_032300| | MGC10854,hypothetical protein MGC10854 |
| 230376_at | 11.16 | 4.31E-07 | 1.143601588 | NA |  |
| 238890_at | 11.16 | 4.32E-07 | 1.350505455 | NM_003720| | DSCR2,Down syndrome critical region protein 2 isoform |
| 241044_x_at | 11.16 | 4.33E-07 | 1.228442858 | NA |  |
| 231957_s_at | 11.16 | 4.30E-07 | 1.167771388 | NM_139159| | DPP9,dipeptidylpeptidase 9 |
| 206902_s_at | 11.15 | 4.36E-07 | 1.342264078 | NM_005107| | ENDOGL1,endonuclease G-like 1 |
| 225170_at | 11.15 | 4.34E-07 | 1.082232208 | NM_017588| | WDR5,WD repeat domain 5 protein |
| 228993_s_at | 11.15 | 4.34E-07 | 1.168054913 | NA |  |
| 225032_at | 11.14 | 4.40E-07 | 1.035225273 | NM_022763| | FNDC3B,fibronectin type III domain containing 3B |
| 229346_at | 11.14 | 4.41E-07 | 1.131824511 | NM_006617| | NES,nestin |
| 211136_s_at | 11.14 | 4.39E-07 | 1.131645752 | NM_001294| | CLPTM1,cleft lip and palate associated transmembrane |
| 203482_at | 11.14 | 4.37E-07 | 1.092520815 | NM_018121| | C10orf6,chromosome 10 open reading frame 6 |
| 212195_at | 11.14 | 4.38E-07 | 1.365960406 | NM_002184| | IL6ST,interleukin 6 signal transducer isoform 1 |
| 225716_at | 11.13 | 4.45E-07 | 1.245306921 | NA |  |
| 209036_s_at | 11.13 | 4.42E-07 | 1.027568759 | NM_005918| | MDH2,mitochondrial malate dehydrogenase precursor |
| 208015_at | 11.13 | 4.46E-07 | 1.338851605 | NM_001003688| | SMAD1,Sma- and Mad-related protein 1 |
| 225733_at | 11.13 | 4.45E-07 | 1.19144257 | NM_080605| | B3GALT6,UDP-Gal:betaGal beta 1,3-galactosyltransferase |
| 209051_s_at | 11.13 | 4.44E-07 | 1.200051281 | NM_001042368| | NA |
| 213656_s_at | 11.13 | 4.42E-07 | 1.054541265 | NM_005552| | KNS2,kinesin 2 60/70kDa |
| 202609_at | 11.12 | 4.48E-07 | 1.170279412 | NM_004447| | EPS8,epidermal growth factor receptor pathway |
| 206284_x_at | 11.12 | 4.49E-07 | 1.056676914 | NM_001834| | CLTB,clathrin, light polypeptide isoform a |
| 222441_x_at | 11.12 | 4.47E-07 | 1.048682346 | NM_016045| | C20orf45,CGI-107 protein |
| 225410_at | 11.11 | 4.52E-07 | 1.040207378 | NM_001008215| | MGC52110,hypothetical protein MGC52110 |
| 225484_at | 11.11 | 4.53E-07 | 1.226731719 | NM_018718| | TSGA14,testis specific, 14 |
| 205760_s_at | 11.11 | 4.52E-07 | 1.166705399 | NM_002542| | OGG1,8-oxoguanine DNA glycosylase isoform 1a |
| 238691_at | 11.11 | 4.52E-07 | 1.555273458 | NA |  |
| 236521_at | 11.11 | 4.52E-07 | 1.305698024 | NA |  |
| 212486_s_at | 11.11 | 4.51E-07 | 1.309471663 | NM_002037| | FYN,protein-tyrosine kinase fyn isoform a |
| 204933_s_at | 11.11 | 4.53E-07 | 1.3661725 | NM_002546| | TNFRSF11B,osteoprotegerin precursor |
| 231397_at | 11.11 | 4.51E-07 | 1.795132365 | NM_001010861| | NA |
| 1554306_at | 11.11 | 4.53E-07 | 1.261054516 | NM_002221| | ITPKB,1D-myo-inositol-trisphosphate 3-kinase B |
| 225261_x_at | 11.11 | 4.51E-07 | 1.193578127 | NM_198976| | TH1L,TH1-like protein |
| 212432_at | 11.1 | 4.55E-07 | 1.088494891 | NM_025196| | GRPEL1,GrpE-like 1, mitochondrial |
| 223289_s_at | 11.1 | 4.58E-07 | 1.100387804 | NM_032557| | USP38,ubiquitin specific protease 38 |
| 224876_at | 11.1 | 4.55E-07 | 1.176117408 | NM_152409| | FLJ37562,hypothetical protein FLJ37562 |
| 212434_at | 11.1 | 4.55E-07 | 1.091796087 | NM_025196| | GRPEL1,GrpE-like 1, mitochondrial |
| 206138_s_at | 11.1 | 4.57E-07 | 1.063928204 | NM_002651| | PIK4CB,phosphatidylinositol 4-kinase, catalytic, beta |
| 221931_s_at | 11.09 | 4.61E-07 | 1.074165402 | NM_001013437| | NA |
| 219759_at | 11.09 | 4.61E-07 | 1.535684134 | NM_022350| | LRAP,leukocyte-derived arginine aminopeptidase |
| 239668_at | 11.08 | 4.65E-07 | 1.243383155 | NA |  |
| 208499_s_at | 11.08 | 4.65E-07 | 1.124677383 | NM_006260| | DNAJC3,DnaJ (Hsp40) homolog, subfamily C, member 3 |
| 213333_at | 11.08 | 4.66E-07 | 1.061204847 | NM_005918| | MDH2,mitochondrial malate dehydrogenase precursor |
| 201943_s_at | 11.08 | 4.64E-07 | 1.11104733 | NM_001304| | CPD,carboxypeptidase D precursor |
| 232500_at | 11.08 | 4.66E-07 | 1.240742199 | NM_020343| | NA |
| 222633_at | 11.08 | 4.65E-07 | 1.058408585 | NM_024665| | TBL1XR1,nuclear receptor co-repressor/HDAC3 complex |
| 202118_s_at | 11.07 | 4.70E-07 | 1.139360236 | NM_003909| | CPNE3,copine III |
| 218157_x_at | 11.07 | 4.71E-07 | 1.070689081 | NM_001038707| | NA |
| 239236_at | 11.07 | 4.68E-07 | 1.337359378 | NA |  |
| 223981_at | 11.07 | 4.68E-07 | 1.396204332 | NM_016350| | NIN,ninein isoform 4 |
| 244433_at | 11.07 | 4.70E-07 | 1.501494347 | NA |  |
| 209408_at | 11.07 | 4.68E-07 | 1.034746257 | NM_006845| | KIF2C,kinesin family member 2C |
| 224577_at | 11.06 | 4.72E-07 | 1.100680609 | NM_001031711| | NA |
| 218510_x_at | 11.06 | 4.71E-07 | 1.256719626 | NM_001034850| | NA |
| 243236_at | 11.06 | 4.75E-07 | 1.281842147 | NA |  |
| 212412_at | 11.06 | 4.75E-07 | 1.094415215 | NM_001011513| | PDLIM5,PDZ and LIM domain 5 isoform b |
| 218953_s_at | 11.06 | 4.73E-07 | 1.099933899 | NM_024028| | MGC3265,hypothetical protein MGC3265 |
| 204842_x_at | 11.06 | 4.74E-07 | 1.061234192 | NM_004157| | PRKAR2A,cAMP-dependent protein kinase, regulatory |
| 205712_at | 11.06 | 4.74E-07 | 1.236311051 | NM_001040712| | NA |
| 201305_x_at | 11.05 | 4.78E-07 | 1.070947773 | NM_006401| | ANP32B,acidic (leucine-rich) nuclear phosphoprotein 32 |
| 235683_at | 11.05 | 4.80E-07 | 1.131917227 | NM_144665| | SESN3,sestrin 3 |
| 1553211_at | 11.05 | 4.78E-07 | 1.212163524 | NM_153228| | FLJ38335,hypothetical protein FLJ38335 |
| 1557812_a_at | 11.05 | 4.76E-07 | 1.328276119 | NA |  |
| 202090_s_at | 11.04 | 4.83E-07 | 1.041944916 | NM_006830| | UQCR,ubiquinol-cytochrome c reductase, 6.4kDa |
| 230763_at | 11.04 | 4.83E-07 | 1.543267486 | NM_138796| | LOC128153,hypothetical protein BC014608 |
| 227766_at | 11.03 | 4.86E-07 | 1.22720068 | NM_001098268| | NA |
| 211330_s_at | 11.03 | 4.88E-07 | 1.262884081 | NM_000410| | HFE,hemochromatosis protein isoform 1 precursor |
| 243984_at | 11.03 | 4.88E-07 | 1.502229758 | NA |  |
| 215204_at | 11.03 | 4.86E-07 | 1.168937 | NA |  |
| 223368_s_at | 11.03 | 4.89E-07 | 1.044457827 | NM_014064| | AD-003,AD-003 protein |
| 224748_at | 11.03 | 4.87E-07 | 1.068200152 | NM_005828| | HAN11,WD-repeat protein |
| 202606_s_at | 11.03 | 4.86E-07 | 1.040934454 | NM_012290| | TLK1,tousled-like kinase 1 |
| 225361_x_at | 11.02 | 4.94E-07 | 1.185198286 | NM_145284| | LOC159090,similar to hypothetical protein MGC17347 |
| 236436_at | 11.02 | 4.92E-07 | 1.310014001 | NM_001077241| | NA |
| 59625_at | 11.02 | 4.92E-07 | 1.256630127 | NM_003946| | NOL3,nucleolar protein 3 |
| 205164_at | 11.02 | 4.90E-07 | 1.163497545 | NM_014291| | GCAT,glycine C-acetyltransferase precursor |
| 243501_at | 11.01 | 4.95E-07 | 1.319552366 | NA |  |
| 238705_at | 11.01 | 4.96E-07 | 1.215286327 | NA |  |
| 207181_s_at | 11.01 | 4.97E-07 | 1.184034375 | NM_001227| | CASP7,caspase 7 isoform alpha precursor |
| 228305_at | 11.01 | 4.97E-07 | 1.129558106 | NM_001042474| | NA |
| 221413_at | 11 | 5.00E-07 | 1.260937159 | NM_004732| | KCNAB3,potassium voltage-gated channel, shaker-related |
| 225352_at | 11 | 5.02E-07 | 1.072366051 | NM_003262| | TLOC1,translocation protein 1 |
| 232004_at | 11 | 4.99E-07 | 1.163798808 | NM_001102397| | NA |
| 207081_s_at | 11 | 5.03E-07 | 1.097781004 | NM_002650| | PIK4CA,phosphatidylinositol 4-kinase, catalytic, alpha |
| 1562056_at | 11 | 5.01E-07 | 1.225310818 | NA |  |
| 202898_at | 11 | 5.02E-07 | 1.158888437 | NM_014654| | SDC3,syndecan 3 |
| 209577_at | 11 | 5.02E-07 | 1.091548246 | NM_002861| | PCYT2,phosphate cytidylyltransferase 2, ethanolamine |
| 1555867_at | 10.99 | 5.05E-07 | 1.450071212 | NM_001098721| | NA |
| 1569652_at | 10.99 | 5.05E-07 | 1.275033855 | NM_004529| | MLLT3,myeloid/lymphoid or mixed-lineage leukemia |
| 201167_x_at | 10.99 | 5.04E-07 | 1.181708004 | NM_004309| | ARHGDIA,Rho GDP dissociation inhibitor (GDI) alpha |
| 236298_at | 10.99 | 5.05E-07 | 1.112509165 | NM_014317| | TPRT,trans-prenyltransferase |
| 235532_at | 10.99 | 5.07E-07 | 1.35869734 | NM_145167| | PIGM,PIG-M mannosyltransferase |
| 1569001_at | 10.99 | 5.04E-07 | 1.119327416 | NM_001199| | BMP1,bone morphogenetic protein 1 isoform 1, |
| 203348_s_at | 10.99 | 5.06E-07 | 1.29922165 | NM_004454| | ETV5,ets variant gene 5 (ets-related molecule) |
| 225666_at | 10.99 | 5.05E-07 | 1.195446593 | NM_001079669| | NA |
| 206066_s_at | 10.99 | 5.07E-07 | 1.207050541 | NM_002876| | RAD51C,RAD51 homolog C isoform 2 |
| 227549_x_at | 10.98 | 5.12E-07 | 1.096183065 | NM_207340| | LOC254359,hypothetical protein LOC254359 |
| 203116_s_at | 10.98 | 5.11E-07 | 1.060791442 | NM_000140| | FECH,ferrochelatase isoform b precursor |
| 236780_at | 10.98 | 5.10E-07 | 1.190415376 | NA |  |
| 204008_at | 10.98 | 5.10E-07 | 1.050641118 | NM_005740| | DNAL4,dynein light chain 4, axonemal |
| 223618_at | 10.97 | 5.17E-07 | 1.523671571 | NM_020066| | FMN2,formin 2 |
| 235065_at | 10.97 | 5.14E-07 | 1.172938835 | NA |  |
| 210990_s_at | 10.97 | 5.16E-07 | 1.342502745 | NM_001105206| | NA |
| 211043_s_at | 10.97 | 5.16E-07 | 1.05924326 | NM_001834| | CLTB,clathrin, light polypeptide isoform a |
| 224883_at | 10.97 | 5.13E-07 | 1.065253686 | NM_012388| | PLDN,pallidin |
| 225328_at | 10.97 | 5.17E-07 | 1.289759803 | NA |  |
| 218550_s_at | 10.97 | 5.16E-07 | 1.104688607 | NM_018205| | LRRC20,leucine rich repeat containing 20 isoform 3 |
| 214046_at | 10.96 | 5.21E-07 | 1.321929394 | NM_006581| | FUT9,fucosyltransferase 9 (alpha (1,3) |
| 227395_at | 10.96 | 5.22E-07 | 1.109850997 | NA |  |
| 204420_at | 10.95 | 5.25E-07 | 1.300403559 | NM_005438| | FOSL1,FOS-like antigen 1 |
| 201477_s_at | 10.95 | 5.25E-07 | 1.061220525 | NM_001033| | RRM1,ribonucleoside-diphosphate reductase M1 chain |
| 47083_at | 10.95 | 5.24E-07 | 1.036962821 | NM_024067| | C7orf26,chromosome 7 open reading frame 26 |
| 224663_s_at | 10.95 | 5.24E-07 | 1.204150464 | NM_021914| | CFL2,cofilin 2 |
| 203762_s_at | 10.95 | 5.24E-07 | 1.080267286 | NM_001012665| | NA |
| 229544_at | 10.95 | 5.25E-07 | 1.128423431 | NA |  |
| 1568815_a_at | 10.95 | 5.24E-07 | 1.301834748 | NM_024045| | DDX50,nucleolar protein GU2 |
| 1552476_s_at | 10.95 | 5.25E-07 | 1.169258229 | NM_133373| | PLCD3,phospholipase C delta 3 |
| 1563724_at | 10.94 | 5.29E-07 | 1.380047839 | NA |  |
| 1558467_a_at | 10.94 | 5.28E-07 | 1.235252 | NA |  |
| 202780_at | 10.94 | 5.30E-07 | 1.114489686 | NM_000436| | OXCT1,3-oxoacid CoA transferase 1 precursor |
| 240078_at | 10.94 | 5.30E-07 | 1.198811108 | NM_004592| | SFRS8,splicing factor, arginine/serine-rich 8 isoform |
| 204242_s_at | 10.93 | 5.34E-07 | 1.167389594 | NM_001101667| | NA |
| 218794_s_at | 10.93 | 5.35E-07 | 1.157861821 | NM_017853| | TXNL4B,thioredoxin-like 4B |
| 200812_at | 10.93 | 5.35E-07 | 1.033238742 | NM_001009570| | CCT7,chaperonin containing TCP1, subunit 7 isoform b |
| 202183_s_at | 10.93 | 5.35E-07 | 1.033992799 | NM_007317| | KIF22,kinesin family member 22 |
| 1554986_a_at | 10.93 | 5.35E-07 | 1.167522931 | NM_014758| | NA |
| 209708_at | 10.93 | 5.36E-07 | 1.207298588 | NM_015529| | MOXD1,monooxygenase, DBH-like 1 |
| 228143_at | 10.92 | 5.39E-07 | 1.534956942 | NM_000096| | CP,ceruloplasmin (ferroxidase) |
| 221744_at | 10.92 | 5.38E-07 | 1.080632616 | NM_005828| | HAN11,WD-repeat protein |
| 236114_at | 10.92 | 5.40E-07 | 1.390409128 | NA |  |
| 208405_s_at | 10.91 | 5.47E-07 | 1.074699092 | NM_006016| | CD164,CD164 antigen, sialomucin |
| 221617_at | 10.9 | 5.49E-07 | 1.104197754 | NM_015975| | TAF9L,TBP-associated factor 9L |
| 216958_s_at | 10.9 | 5.48E-07 | 1.104932558 | NM_002225| | IVD,isovaleryl Coenzyme A dehydrogenase |
| 213499_at | 10.9 | 5.48E-07 | 1.141049556 | NM_004366| | CLCN2,chloride channel 2 |
| 203048_s_at | 10.9 | 5.53E-07 | 1.061985445 | NM_014639| | KIAA0372,KIAA0372 |
| 202181_at | 10.89 | 5.53E-07 | 1.21340272 | NM_014734| | KIAA0247,KIAA0247 |
| 226230_at | 10.89 | 5.54E-07 | 1.11476232 | NM_001122964| | NA |
| 220720_x_at | 10.89 | 5.55E-07 | 1.053422017 | NM_025029| | FLJ14346,hypothetical protein FLJ14346 |
| 36129_at | 10.89 | 5.53E-07 | 1.118980789 | NM_001098509| | NA |
| 238768_at | 10.88 | 5.59E-07 | 1.135364839 | NM_001013649| | NA |
| 233490_at | 10.88 | 5.63E-07 | 1.25587361 | NM_016221| | DCTN4,dynactin 4 (p62) |
| 1555803_a_at | 10.88 | 5.59E-07 | 1.110954759 | NM_001082969| | NA |
| 224856_at | 10.87 | 5.66E-07 | 1.098034022 | NM_004117| | FKBP5,FK506 binding protein 5 |
| 210417_s_at | 10.87 | 5.68E-07 | 1.141927633 | NM_002651| | PIK4CB,phosphatidylinositol 4-kinase, catalytic, beta |
| 204225_at | 10.87 | 5.66E-07 | 1.14736407 | NM_006037| | HDAC4,histone deacetylase 4 |
| 224513_s_at | 10.87 | 5.64E-07 | 1.042362048 | NM_020131| | UBQLN4,ataxin-1 ubiquitin-like interacting protein |
| 226128_at | 10.86 | 5.70E-07 | 1.071245421 | NA |  |
| 208981_at | 10.86 | 5.69E-07 | 1.1343174 | NM_000442| | PECAM1,platelet/endothelial cell adhesion molecule |
| 204303_s_at | 10.86 | 5.71E-07 | 1.150129743 | NM_014772| | KIAA0427,KIAA0427 |
| 230879_at | 10.86 | 5.70E-07 | 1.180254499 | NM_004282| | BAG2,BCL2-associated athanogene 2 |
| 211070_x_at | 10.85 | 5.75E-07 | 1.097585701 | NM_001079862| | NA |
| 239230_at | 10.85 | 5.77E-07 | 1.274861582 | NM_001010926| | HES5,hairy and enhancer of split 5 |
| 1554352_s_at | 10.85 | 5.77E-07 | 1.239559304 | NM_005848| | MYCPBP,c-myc promoter binding protein |
| 203676_at | 10.85 | 5.77E-07 | 1.16722765 | NM_002076| | GNS,glucosamine (N-acetyl)-6-sulfatase precursor |
| 239683_at | 10.84 | 5.81E-07 | 1.173126743 | NM_206808| | CLYBL,citrate lyase beta like |
| 243861_at | 10.84 | 5.83E-07 | 1.40812966 | NA |  |
| 217506_at | 10.84 | 5.84E-07 | 1.265191225 | NA |  |
| 210086_at | 10.83 | 5.89E-07 | 1.24902351 | NM_005144| | HR,hairless protein isoform a |
| 225219_at | 10.83 | 5.87E-07 | 1.126884796 | NM_001001419| | SMAD5,SMAD, mothers against DPP homolog 5 |
| 226201_at | 10.83 | 5.90E-07 | 1.085369394 | NM_032482| | DOT1L,DOT1-like, histone H3 methyltransferase |
| 1552931_a_at | 10.82 | 5.93E-07 | 1.396628689 | NM_002605| | PDE8A,phosphodiesterase 8A isoform 1 |
| 230902_at | 10.82 | 5.94E-07 | 1.374715996 | NA |  |
| 213752_at | 10.82 | 5.94E-07 | 1.169577828 | NM_001017999| | NA |
| 1562013_a_at | 10.82 | 5.96E-07 | 1.312903199 | NA |  |
| 210036_s_at | 10.82 | 5.91E-07 | 1.150571721 | NM_000238| | KCNH2,voltage-gated potassium channel, subfamily H, |
| 212188_at | 10.82 | 5.93E-07 | 1.284598508 | NM_138444| | KCTD12,potassium channel tetramerisation domain |
| 202433_at | 10.81 | 5.97E-07 | 1.037085674 | NM_005827| | SLC35B1,solute carrier family 35, member B1 |
| 236895_at | 10.81 | 5.98E-07 | 1.071408353 | NA |  |
| 201740_at | 10.81 | 5.98E-07 | 1.050517588 | NM_004551| | NDUFS3,NADH dehydrogenase (ubiquinone) Fe-S protein 3, |
| 240344_x_at | 10.81 | 5.97E-07 | 1.151307822 | NM_181705| | LOC90624,hypothetical protein LOC90624 |
| 1556474_a_at | 10.8 | 6.08E-07 | 1.727083604 | NA |  |
| 213041_s_at | 10.8 | 6.04E-07 | 1.062254597 | NM_001001975| | ATP5D,ATP synthase, H+ transporting, mitochondrial F1 |
| 242604_at | 10.8 | 6.05E-07 | 1.150771 | NA |  |
| 212680_x_at | 10.8 | 6.08E-07 | 1.047367424 | NM_138689| | NA |
| 202414_at | 10.8 | 6.06E-07 | 1.093644688 | NM_000123| | ERCC5,XPG-complementing protein |
| 201960_s_at | 10.8 | 6.04E-07 | 1.12289303 | NM_015057| | MYCBP2,MYC binding protein 2 |
| 203420_at | 10.8 | 6.07E-07 | 1.112008926 | NM_016255| | FAM8A1,Autosomal Highly Conserved Protein |
| 204015_s_at | 10.79 | 6.09E-07 | 1.121165381 | NM_001394| | DUSP4,dual specificity phosphatase 4 isoform 1 |
| 209528_s_at | 10.79 | 6.12E-07 | 1.094930875 | NM_016111| | KIAA0683,KIAA0683 gene product |
| 237936_at | 10.79 | 6.11E-07 | 1.669325602 | NA |  |
| 228129_at | 10.79 | 6.12E-07 | 1.054254423 | NM_001018067| | NA |
| 236268_at | 10.79 | 6.09E-07 | 1.173936 | NM_004206| | SEC22L3,vesicle trafficking protein isoform b |
| 1555894_s_at | 10.79 | 6.13E-07 | 1.285083258 | NM_138383| | LOC92154,hypothetical protein BC002770 |
| 203248_at | 10.79 | 6.13E-07 | 1.202499569 | NM_006965| | ZNF24,zinc finger protein 24 (KOX 17) |
| 218351_at | 10.79 | 6.13E-07 | 1.109587143 | NM_017845| | COMMD8,COMM domain containing 8 |
| 208927_at | 10.78 | 6.16E-07 | 1.158121619 | NM_001007226| | SPOP,speckle-type POZ protein |
| 210438_x_at | 10.78 | 6.15E-07 | 1.078409803 | NM_001042369| | NA |
| 201093_x_at | 10.78 | 6.18E-07 | 1.103581037 | NM_004168| | SDHA,succinate dehydrogenase complex, subunit A, |
| 222040_at | 10.78 | 6.17E-07 | 1.136124717 | NM_002136| | HNRPA1,heterogeneous nuclear ribonucleoprotein A1 |
| 229135_at | 10.78 | 6.16E-07 | 1.150849838 | NA |  |
| 212205_at | 10.77 | 6.20E-07 | 1.129839633 | NM_012412| | H2AFV,H2A histone family, member V isoform 1 |
| 212118_at | 10.77 | 6.24E-07 | 1.087952695 | NM_006510| | RFP,ret finger protein isoform alpha |
| 235296_at | 10.77 | 6.24E-07 | 1.221373252 | NM_020390| | EIF5A2,eIF-5A2 protein |
| 213304_at | 10.77 | 6.20E-07 | 1.089057892 | NM_015091| | KIAA0423,KIAA0423 |
| 226183_at | 10.77 | 6.20E-07 | 1.20409465 | NA |  |
| 243367_at | 10.76 | 6.31E-07 | 1.441526113 | NA |  |
| 211360_s_at | 10.76 | 6.27E-07 | 1.187320538 | NM_002223| | ITPR2,inositol 1,4,5-triphosphate receptor, type 2 |
| 201155_s_at | 10.76 | 6.30E-07 | 1.059446132 | NM_014874| | MFN2,mitofusin 2 |
| 1552310_at | 10.76 | 6.29E-07 | 1.137808185 | NM_144597| | MGC29937,hypothetical protein MGC29937 |
| 213383_at | 10.76 | 6.31E-07 | 1.17644266 | NA |  |
| 219015_s_at | 10.75 | 6.37E-07 | 1.182670734 | NM_018466| | GLT28D1,glycosyltransferase 28 domain containing 1 |
| 226229_s_at | 10.75 | 6.36E-07 | 1.225662081 | NM_014188| | HSPC182,HSPC182 protein |
| 200946_x_at | 10.74 | 6.39E-07 | 1.105964688 | NM_005271| | GLUD1,glutamate dehydrogenase 1 |
| 1553569_at | 10.74 | 6.43E-07 | 1.018975542 | NM_022340| | ZFYVE20,FYVE-finger-containing Rab5 effector protein |
| 1556306_at | 10.74 | 6.39E-07 | 1.47244396 | NA |  |
| 236000_s_at | 10.74 | 6.41E-07 | 1.29204026 | NA |  |
| 211075_s_at | 10.74 | 6.43E-07 | 1.155209821 | NM_001025079| | NA |
| 203459_s_at | 10.74 | 6.43E-07 | 1.069303005 | NM_022575| | VPS16,vacuolar protein sorting 16 isoform 1 |
| 212802_s_at | 10.73 | 6.49E-07 | 1.071965865 | NM_015635| | DKFZP434C212,DKFZP434C212 protein |
| 202791_s_at | 10.73 | 6.48E-07 | 1.058341483 | NM_014678| | KIAA0685,KIAA0685 gene product |
| 218811_at | 10.73 | 6.50E-07 | 1.235204929 | NM_001126340| | NA |
| 219458_s_at | 10.73 | 6.50E-07 | 1.070772952 | NM_022072| | NSUN3,NOL1/NOP2/Sun domain family, member 3 |
| 1556447_at | 10.73 | 6.49E-07 | 1.642994537 | NA |  |
| 235889_at | 10.72 | 6.55E-07 | 1.201237247 | NA |  |
| 203989_x_at | 10.72 | 6.53E-07 | 1.226681963 | NM_001992| | F2R,coagulation factor II receptor precursor |
| 213190_at | 10.72 | 6.55E-07 | 1.072600281 | NM_153603| | COG7,component of oligomeric golgi complex 7 |
| 217289_s_at | 10.72 | 6.56E-07 | 1.159243609 | NM_001467| | SLC37A4,solute carrier family 37 (glycerol-6-phosphate |
| 226581_at | 10.72 | 6.51E-07 | 1.084729499 | NM_022340| | ZFYVE20,FYVE-finger-containing Rab5 effector protein |
| 226224_at | 10.72 | 6.53E-07 | 1.080254519 | NM_004514| | FOXK2,forkhead box K2 isoform 1 |
| 233360_at | 10.71 | 6.61E-07 | 1.118119235 | NM_003345| | UBE2I,ubiquitin-conjugating enzyme E2I |
| 239466_at | 10.71 | 6.58E-07 | 1.324686551 | NA |  |
| 225097_at | 10.71 | 6.60E-07 | 1.108580324 | NM_001113239| | NA |
| 1554635_a_at | 10.71 | 6.57E-07 | 1.385668805 | NM_022123| | NPAS3,neuronal PAS domain protein 3 |
| 213220_at | 10.71 | 6.60E-07 | 1.044001465 | NA |  |
| 201163_s_at | 10.71 | 6.57E-07 | 1.884515534 | NM_001553| | IGFBP7,insulin-like growth factor binding protein 7 |
| 1555784_s_at | 10.71 | 6.61E-07 | 1.168520099 | NM_001025242| | NA |
| 1560425_s_at | 10.7 | 6.68E-07 | 1.599512033 | NA |  |
| 238446_at | 10.7 | 6.63E-07 | 1.31710647 | NA |  |
| 1558748_at | 10.7 | 6.68E-07 | 1.313493268 | NA |  |
| 212399_s_at | 10.69 | 6.72E-07 | 1.125443169 | NM_014667| | VGLL4,vestigial like 4 |
| 217838_s_at | 10.69 | 6.73E-07 | 1.096928703 | NM_016337| | EVL,Enah/Vasp-like |
| 225623_at | 10.68 | 6.80E-07 | 1.069681202 | NM_033426| | KIAA1737,KIAA1737 protein |
| 1559412_at | 10.68 | 6.75E-07 | 1.582746556 | NM_001005732| | C21orf34,chromosome 21 open reading frame 34 isoform a |
| 236524_at | 10.68 | 6.77E-07 | 1.349803806 | NA |  |
| 239793_at | 10.68 | 6.79E-07 | 1.541488868 | NA |  |
| 233019_at | 10.68 | 6.78E-07 | 1.266137705 | NM_013354| | CNOT7,CCR4-NOT transcription complex, subunit 7 |
| 225489_at | 10.68 | 6.77E-07 | 1.038121903 | NM_152834| | TMEM18,transmembrane protein 18 |
| 219781_s_at | 10.67 | 6.86E-07 | 1.073330287 | NM_016643| | LOC51333,mesenchymal stem cell protein DSC43 |
| 223148_at | 10.67 | 6.84E-07 | 1.070898376 | NM_033198| | PIGS,phosphatidylinositol glycan class S |
| 233249_at | 10.67 | 6.87E-07 | 1.437835462 | NA |  |
| 238988_at | 10.67 | 6.84E-07 | 1.28214946 | NA |  |
| 213069_at | 10.67 | 6.82E-07 | 1.172713672 | NM_020733| | NA |
| 236531_at | 10.67 | 6.82E-07 | 1.32422495 | NA |  |
| 221844_x_at | 10.67 | 6.84E-07 | 1.092810468 | NA |  |
| 1555847_a_at | 10.67 | 6.87E-07 | 1.152399656 | NA |  |
| 232940_s_at | 10.66 | 6.91E-07 | 1.067900142 | NM_170606| | MLL3,myeloid/lymphoid or mixed-lineage leukemia 3 |
| 231873_at | 10.66 | 6.89E-07 | 1.34436052 | NM_001204| | BMPR2,bone morphogenetic protein receptor, type II |
| 204318_s_at | 10.65 | 6.97E-07 | 1.090774124 | NM_016426| | GTSE1,G-2 and S-phase expressed 1 |
| 229014_at | 10.65 | 7.01E-07 | 1.267438656 | NA |  |
| 242214_at | 10.65 | 6.99E-07 | 1.143984127 | NM_002954| | RPS27A,ubiquitin and ribosomal protein S27a precursor |
| 228411_at | 10.65 | 6.95E-07 | 1.176402015 | NM_057177| | ALS2CR19,amyotrophic lateral sclerosis 2 (juvenile) |
| 204100_at | 10.65 | 6.97E-07 | 1.169700056 | NM_003250| | THRA,thyroid hormone receptor, alpha isoform 2 |
| 213650_at | 10.65 | 6.98E-07 | 1.178655057 | NM_001023567| | NA |
| 239069_s_at | 10.64 | 7.05E-07 | 1.426221854 | NA |  |
| 209376_x_at | 10.64 | 7.05E-07 | 1.087505474 | NM_004719| | SFRS2IP,splicing factor, arginine/serine-rich 2, |
| 209468_at | 10.64 | 7.01E-07 | 1.143143999 | NM_002335| | LRP5,low density lipoprotein receptor-related protein |
| 209971_x_at | 10.64 | 7.07E-07 | 1.036020583 | NM_006303| | JTV1,JTV1 |
| 243118_at | 10.64 | 7.03E-07 | 1.389232967 | NM_001083535| | NA |
| 1556060_a_at | 10.64 | 7.05E-07 | 1.158585885 | NA |  |
| 239144_at | 10.63 | 7.11E-07 | 1.614161365 | NM_080742| | B3GAT2,beta-1,3-glucuronyltransferase 2 |
| 212956_at | 10.63 | 7.12E-07 | 1.250290493 | NM_015130| | KIAA0882,KIAA0882 protein |
| 220371_s_at | 10.63 | 7.09E-07 | 1.036788875 | NM_020246| | SLC12A9,solute carrier family 12 (potassium/chloride |
| 243010_at | 10.63 | 7.12E-07 | 1.173881266 | NM_138962| | MSI2,musashi 2 isoform a |
| 244372_at | 10.63 | 7.11E-07 | 1.158922468 | NA |  |
| 211929_at | 10.63 | 7.10E-07 | 1.080477052 | NM_194247| | HNRPA3,heterogeneous nuclear ribonucleoprotein A3 |
| 1554743_x_at | 10.63 | 7.11E-07 | 1.533243422 | NM_000534| | PMS1,postmeiotic segregation 1 |
| 213311_s_at | 10.63 | 7.14E-07 | 1.126193131 | NM_014972| | KIAA1049,KIAA1049 protein |
| 230235_at | 10.62 | 7.16E-07 | 1.169878432 | NA |  |
| 203943_at | 10.62 | 7.20E-07 | 1.12223732 | NM_004798| | KIF3B,kinesin family member 3B |
| 219262_at | 10.62 | 7.21E-07 | 1.335344315 | NM_024670| | SUV39H2,suppressor of variegation 3-9 homolog 2 |
| 232209_x_at | 10.62 | 7.20E-07 | 1.092666675 | NM_030789| | HM13,minor histocompatibility antigen 13 isoform 1 |
| 218281_at | 10.61 | 7.23E-07 | 1.058537002 | NM_016055| | MRPL48,mitochondrial ribosomal protein L48 |
| 233480_at | 10.61 | 7.23E-07 | 1.514664557 | NM_024334| | TMEM43,transmembrane protein 43 |
| 1558787_a_at | 10.61 | 7.23E-07 | 1.265422231 | NA |  |
| 216397_s_at | 10.61 | 7.25E-07 | 1.1628204 | NM_015201| | BOP1,block of proliferation 1 |
| 224908_s_at | 10.61 | 7.27E-07 | 1.093145871 | NM_153712| | TTL,tubulin tyrosine ligase |
| 209325_s_at | 10.61 | 7.21E-07 | 1.212202276 | NM_002928| | RGS16,regulator of G-protein signalling 16 |
| 212847_at | 10.61 | 7.25E-07 | 1.209229985 | NM_003902| | FUBP1,far upstream element-binding protein |
| 212508_at | 10.61 | 7.24E-07 | 1.060648353 | NM_022151| | MOAP1,modulator of apoptosis 1 |
| 229413_s_at | 10.61 | 7.25E-07 | 1.280827849 | NA |  |
| 214255_at | 10.61 | 7.27E-07 | 1.183452267 | NM_024490| | ATP10A,ATPase, Class V, type 10A |
| 213857_s_at | 10.61 | 7.24E-07 | 1.109481238 | NM_001025079| | NA |
| 230102_at | 10.6 | 7.29E-07 | 1.29178636 | NM_004454| | ETV5,ets variant gene 5 (ets-related molecule) |
| 240499_at | 10.6 | 7.29E-07 | 1.434190398 | NA |  |
| 240165_at | 10.6 | 7.32E-07 | 1.615202092 | NA |  |
| 227203_at | 10.6 | 7.28E-07 | 1.378753084 | NM_022824| | FBXL17,F-box and leucine-rich repeat protein 17 |
| 214258_x_at | 10.6 | 7.33E-07 | 1.098935194 | NM_006388| | HTATIP,HIV-1 Tat interactive protein, 60kDa isoform 2 |
| 228822_s_at | 10.6 | 7.28E-07 | 1.106599781 | NM_001001992| | USP16,ubiquitin specific protease 16 isoform b |
| 224891_at | 10.6 | 7.29E-07 | 1.040898123 | NM_001455| | FOXO3A,forkhead box O3A |
| 217645_at | 10.6 | 7.28E-07 | 1.123971102 | NM_016468| | C14orf112,chromosome 14 open reading frame 112 |
| 213336_at | 10.59 | 7.40E-07 | 1.042857042 | NM_032408| | BAZ1B,bromodomain adjacent to zinc finger domain, 1B |
| 235147_at | 10.59 | 7.37E-07 | 1.79293141 | NA |  |
| 1563629_a_at | 10.59 | 7.38E-07 | 1.312943248 | NA |  |
| 203012_x_at | 10.59 | 7.40E-07 | 1.010202603 | NM_000984| | RPL23A,ribosomal protein L23a |
| 225662_at | 10.59 | 7.37E-07 | 1.283875129 | NM_016653| | ZAK,sterile-alpha motif and leucine zipper |
| 206197_at | 10.59 | 7.41E-07 | 1.317391636 | NM_003551| | NME5,non-metastatic cells 5, protein expressed in |
| 200804_at | 10.58 | 7.43E-07 | 1.031220456 | NM_001098576| | NA |
| 231840_x_at | 10.58 | 7.42E-07 | 1.203163553 | NM_181705| | LOC90624,hypothetical protein LOC90624 |
| 208433_s_at | 10.58 | 7.49E-07 | 1.083224839 | NM_001018054| | NA |
| 209702_at | 10.58 | 7.42E-07 | 1.05410312 | NM_001080432| | NA |
| 202792_s_at | 10.58 | 7.43E-07 | 1.24953591 | NM_014678| | KIAA0685,KIAA0685 gene product |
| 218587_s_at | 10.58 | 7.44E-07 | 1.111146872 | NM_020231| | MDS010,x 010 protein |
| 225712_at | 10.58 | 7.45E-07 | 1.050577803 | NM_015465| | GEMIN5,gemin 5 |
| 231199_at | 10.58 | 7.43E-07 | 1.398767686 | NA |  |
| 226326_at | 10.58 | 7.49E-07 | 1.866451754 | NM_032373| | PCGF5,polycomb group ring finger 5 |
| 1558164_s_at | 10.58 | 7.46E-07 | 1.153950459 | NM_002618| | PEX13,peroxisome biogenesis factor 13 |
| 225510_at | 10.58 | 7.43E-07 | 1.167522322 | NM_178507| | NS5ATP13TP2,NS5ATP13TP2 protein |
| 215032_at | 10.57 | 7.53E-07 | 1.221940164 | NM_001003698| | RREB1,ras responsive element binding protein 1 isoform |
| 206050_s_at | 10.57 | 7.54E-07 | 1.140841339 | NM_002939| | RNH,ribonuclease/angiogenin inhibitor |
| 224522_s_at | 10.57 | 7.53E-07 | 1.159132035 | NM_024819| | FLJ22955,hypothetical protein FLJ22955 |
| 230274_s_at | 10.57 | 7.49E-07 | 1.186203203 | NM_002532| | NUP88,nucleoporin 88kDa |
| 221621_at | 10.57 | 7.55E-07 | 1.436033242 | NA |  |
| 237157_at | 10.56 | 7.61E-07 | 1.279326839 | NA |  |
| 209558_s_at | 10.56 | 7.60E-07 | 1.203878632 | NM_003959| | HIP1R,huntingtin interacting protein-1-related |
| 230343_at | 10.56 | 7.61E-07 | 1.418444803 | NA |  |
| 204132_s_at | 10.56 | 7.58E-07 | 1.086613428 | NM_001455| | FOXO3A,forkhead box O3A |
| 34689_at | 10.56 | 7.57E-07 | 1.135679501 | NM_016381| | TREX1,three prime repair exonuclease 1 isoform a |
| 202282_at | 10.56 | 7.58E-07 | 1.051011101 | NM_001037811| | NA |
| 212776_s_at | 10.56 | 7.59E-07 | 1.134143118 | NM_015311| | NA |
| 222740_at | 10.56 | 7.57E-07 | 1.15495382 | NM_014109| | ATAD2,two AAA domain containing protein |
| 40560_at | 10.55 | 7.65E-07 | 1.329010641 | NM_005994| | TBX2,T-box 2 |
| 212375_at | 10.55 | 7.71E-07 | 1.048586103 | NM_015409| | EP400,E1A binding protein p400 |
| 218052_s_at | 10.55 | 7.68E-07 | 1.091745646 | NM_020410| | ATP13A1,ATPase type 13A1 |
| 218203_at | 10.54 | 7.75E-07 | 1.104169975 | NM_013338| | ALG5,dolichyl phosphate glucosyltransferase |
| 225521_at | 10.54 | 7.72E-07 | 1.462668848 | NM_016238| | ANAPC7,anaphase-promoting complex subunit 7 |
| 217729_s_at | 10.53 | 7.82E-07 | 1.05258915 | NM_001130| | AES,amino-terminal enhancer of split isoform b |
| 232777_s_at | 10.53 | 7.81E-07 | 1.539733505 | NM_144980| | C6orf118,chromosome 6 open reading frame 118 |
| 209389_x_at | 10.53 | 7.82E-07 | 1.102386595 | NM_001079862| | NA |
| 241529_at | 10.53 | 7.84E-07 | 1.441902915 | NA |  |
| 212112_s_at | 10.52 | 7.91E-07 | 1.171880078 | NM_177424| | STX12,syntaxin 12 |
| 203062_s_at | 10.52 | 7.90E-07 | 1.033941432 | NM_014641| | MDC1,mediator of DNA damage checkpoint 1 |
| 218083_at | 10.52 | 7.90E-07 | 1.085224441 | NM_025072| | PTGES2,prostaglandin E synthase 2 isoform 1 |
| 213463_s_at | 10.52 | 7.88E-07 | 1.136022923 | NA |  |
| 208834_x_at | 10.52 | 7.91E-07 | 1.009218012 | NM_000984| | RPL23A,ribosomal protein L23a |
| 200811_at | 10.52 | 7.90E-07 | 1.101819381 | NM_001280| | CIRBP,cold inducible RNA binding protein |
| 235266_at | 10.52 | 7.91E-07 | 1.16847976 | NM_014109| | ATAD2,two AAA domain containing protein |
| 212182_at | 10.52 | 7.94E-07 | 1.281644672 | NM_019094| | NUDT4,nudix (nucleoside diphosphate linked moiety |
| 211555_s_at | 10.52 | 7.88E-07 | 1.34611152 | NM_000857| | GUCY1B3,guanylate cyclase 1, soluble, beta 3 |
| 234193_at | 10.52 | 7.93E-07 | 1.254265494 | NA |  |
| 209497_s_at | 10.52 | 7.88E-07 | 1.175646486 | NM_031492| | RBM30,RNA binding motif protein 30 |
| 228045_at | 10.52 | 7.94E-07 | 1.158328066 | NA |  |
| 201536_at | 10.52 | 7.89E-07 | 1.043497382 | NM_004090| | DUSP3,dual specificity phosphatase 3 |
| 1554078_s_at | 10.52 | 7.89E-07 | 1.070767695 | NM_005147| | DNAJA3,DnaJ (Hsp40) homolog, subfamily A, member 3 |
| 214991_s_at | 10.51 | 7.99E-07 | 1.086767768 | NM_032634| | PIGO,phosphatidylinositol glycan, class O isoform 1 |
| 212092_at | 10.51 | 7.94E-07 | 1.194887673 | NM_001040152| | NA |
| 222816_s_at | 10.5 | 8.03E-07 | 1.061417225 | NM_017742| | ZCCHC2,zinc finger, CCHC domain containing 2 |
| 225677_at | 10.5 | 8.08E-07 | 1.096877669 | NM_001008405| | BCAP29,B-cell receptor-associated protein BAP29 isoform |
| 220917_s_at | 10.5 | 8.04E-07 | 1.086987008 | NM_025132| | WDR19,WD repeat domain 19 |
| 237449_at | 10.5 | 8.06E-07 | 1.45977257 | NM_182700| | SP8,Sp8 transcription factor isoform 1 |
| 224733_at | 10.5 | 8.09E-07 | 1.119945169 | NM_001048251| | NA |
| 223673_at | 10.5 | 8.06E-07 | 2.11105728 | NM_002920| | RFX4,regulatory factor X4 isoform b |
| 234975_at | 10.49 | 8.14E-07 | 1.40689952 | NA |  |
| 214184_at | 10.49 | 8.18E-07 | 1.197614901 | NM_003717| | NPFF,FMFRamide-related protein precursor |
| 208829_at | 10.49 | 8.10E-07 | 1.069732378 | NM_003190| | TAPBP,tapasin isoform 1 precursor |
| 200661_at | 10.49 | 8.17E-07 | 1.09923177 | NM_000308| | PPGB,protective protein for beta-galactosidase |
| 211019_s_at | 10.49 | 8.17E-07 | 1.18811799 | NM_001001438| | LSS,lanosterol synthase |
| 212352_s_at | 10.49 | 8.16E-07 | 1.04700416 | NM_006827| | TMP21,transmembrane trafficking protein |
| 212551_at | 10.49 | 8.16E-07 | 1.110024342 | NM_006366| | CAP2,adenylyl cyclase-associated protein 2 |
| 203660_s_at | 10.48 | 8.20E-07 | 1.065545134 | NM_006031| | PCNT2,pericentrin B |
| 201018_at | 10.48 | 8.24E-07 | 1.062175421 | NM_001412| | EIF1AX,X-linked eukaryotic translation initiation |
| 230256_at | 10.48 | 8.23E-07 | 1.139119605 | NM_001039517| | NA |
| 201680_x_at | 10.48 | 8.19E-07 | 1.035374209 | NM_015908| | ARS2,arsenate resistance protein ARS2 isoform a |
| 244764_at | 10.48 | 8.20E-07 | 1.540023558 | NM_024503| | HIVEP3,human immunodeficiency virus type I enhancer |
| 236644_at | 10.48 | 8.19E-07 | 1.953645645 | NM_001113561| | NA |
| 213209_at | 10.48 | 8.20E-07 | 1.179549201 | NM_006473| | TAF6L,TAF6-like RNA polymerase II |
| 1562940_at | 10.47 | 8.31E-07 | 1.191039637 | NA |  |
| 242182_x_at | 10.47 | 8.26E-07 | 1.14583878 | NA |  |
| 238782_at | 10.47 | 8.26E-07 | 1.497083622 | NA |  |
| 225516_at | 10.47 | 8.29E-07 | 1.124193824 | NM_001008539| | SLC7A2,solute carrier family 7, member 2 isoform 1 |
| 239731_at | 10.47 | 8.33E-07 | 1.264703418 | NA |  |
| 236330_at | 10.46 | 8.36E-07 | 1.328603681 | NA |  |
| 211275_s_at | 10.46 | 8.38E-07 | 1.07773147 | NM_004130| | GYG,glycogenin |
| 223306_at | 10.46 | 8.39E-07 | 1.06248506 | NM_032565| | EBPL,emopamil binding related protein, delta8-delta7 |
| 215307_at | 10.46 | 8.40E-07 | 1.171547252 | NM_020951| | ZNF529,zinc finger protein 529 |
| 226056_at | 10.46 | 8.41E-07 | 1.460233398 | NM_020754| | CDGAP,Cdc42 GTPase-activating protein |
| 218791_s_at | 10.46 | 8.36E-07 | 1.166168289 | NM_024713| | C15orf29,chromosome 15 open reading frame 29 |
| 218679_s_at | 10.46 | 8.36E-07 | 1.086762238 | NM_016208| | VPS28,vacuolar protein sorting 28 isoform 1 |
| 229299_at | 10.45 | 8.49E-07 | 1.312652487 | NM_001085411| | NA |
| 223013_at | 10.45 | 8.47E-07 | 1.060403836 | NM_024665| | TBL1XR1,nuclear receptor co-repressor/HDAC3 complex |
| 226717_at | 10.45 | 8.42E-07 | 1.137208878 | NM_145309| | LOC220074,Hypothetical 55.1 kDa protein F09G8.5 in |
| 219261_at | 10.44 | 8.56E-07 | 1.082674747 | NM_024067| | C7orf26,chromosome 7 open reading frame 26 |
| 216644_at | 10.44 | 8.55E-07 | 1.223412647 | NA |  |
| 212221_x_at | 10.44 | 8.51E-07 | 1.122353057 | NM_000202| | IDS,iduronate-2-sulfatase isoform a precursor |
| 210263_at | 10.44 | 8.52E-07 | 1.292435192 | NM_002236| | KCNF1,potassium voltage-gated channel, subfamily F, |
| 208966_x_at | 10.43 | 8.60E-07 | 1.510223757 | NM_005531| | IFI16,interferon, gamma-inducible protein 16 |
| 229244_at | 10.43 | 8.65E-07 | 1.334048041 | NA |  |
| 236836_at | 10.43 | 8.64E-07 | 1.180928235 | NA |  |
| 231721_at | 10.43 | 8.65E-07 | 1.267712072 | NM_032801| | JAM3,junctional adhesion molecule 3 precursor |
| 209414_at | 10.43 | 8.67E-07 | 1.174200994 | NM_016263| | FZR1,Fzr1 protein |
| 225420_at | 10.43 | 8.60E-07 | 1.081381115 | NM_020918| | GPAM,mitochondrial glycerol 3-phosphate |
| 238590_x_at | 10.43 | 8.67E-07 | 1.081611762 | NM_032354| | MGC10744,hypothetical protein MGC10744 isoform 1 |
| 237452_at | 10.42 | 8.72E-07 | 1.289672581 | NA |  |
| 216220_s_at | 10.42 | 8.68E-07 | 1.155612279 | NM_000674| | ADORA1,adenosine A1 receptor |
| 218560_s_at | 10.42 | 8.72E-07 | 1.152679035 | NM_023007| | FLJ12517,hypothetical protein FLJ12517 |
| 224280_s_at | 10.41 | 8.81E-07 | 1.098135409 | NM_001099625| | NA |
| 1554038_at | 10.4 | 8.91E-07 | 1.121404529 | NM_018078| | FLJ10378,FLJ10378 protein isoform 1 |
| 223260_s_at | 10.4 | 8.93E-07 | 1.173702504 | NM_016218| | POLK,polymerase (DNA directed) kappa |
| 1556126_s_at | 10.4 | 8.91E-07 | 1.270374481 | NM_018040| | GPATC2,G patch domain containing 2 |
| 238530_at | 10.4 | 8.88E-07 | 1.682354514 | NM_012343| | NNT,nicotinamide nucleotide transhydrogenase |
| 226193_x_at | 10.39 | 9.02E-07 | 1.072585264 | NM_001024916| | NA |
| 222425_s_at | 10.39 | 9.01E-07 | 1.101653307 | NM_015584| | POLDIP2,DNA polymerase delta interacting protein 2 |
| 235112_at | 10.39 | 8.95E-07 | 1.116751815 | NA |  |
| 65630_at | 10.39 | 9.00E-07 | 1.259674484 | NM_001042463| | NA |
| 219322_s_at | 10.39 | 8.95E-07 | 1.06745071 | NM_017818| | WDR8,WD repeat domain 8 protein |
| 208564_at | 10.39 | 8.96E-07 | 1.167797303 | NM_004974| | KCNA2,potassium voltage-gated channel, shaker-related |
| 241792_x_at | 10.39 | 8.96E-07 | 1.170753068 | NA |  |
| 201620_at | 10.39 | 8.99E-07 | 1.092397715 | NM_003791| | MBTPS1,membrane-bound transcription factor site-1 |
| 221779_at | 10.38 | 9.08E-07 | 1.074083583 | NM_033386| | MICAL-L1,molecule interacting with Rab13 |
| 1557799_at | 10.38 | 9.08E-07 | 1.163001313 | NM_170746| | C11orf31,selenoprotein H |
| 219708_at | 10.38 | 9.10E-07 | 1.08922921 | NM_020201| | NT5M,5',3'-nucleotidase, mitochondrial precursor |
| 205195_at | 10.38 | 9.08E-07 | 1.077749943 | NM_001283| | AP1S1,adaptor-related protein complex 1, sigma 1 |
| 230748_at | 10.38 | 9.10E-07 | 1.520750281 | NM_004694| | SLC16A6,solute carrier family 16, member 6 |
| 214102_at | 10.37 | 9.15E-07 | 1.171453949 | NM_015230| | CENTD1,centaurin delta 1 isoform a |
| 213449_at | 10.37 | 9.15E-07 | 1.1610786 | NM_015029| | POP1,processing of precursor 1, ribonuclease P/MRP |
| 229375_at | 10.37 | 9.13E-07 | 1.084506305 | NA |  |
| 203622_s_at | 10.37 | 9.17E-07 | 1.057716113 | NM_020143| | LOC56902,putatative 28 kDa protein |
| 225389_at | 10.37 | 9.19E-07 | 1.165899566 | NM_033271| | BTBD6,BTB domain protein BDPL |
| 236270_at | 10.36 | 9.23E-07 | 1.130106816 | NM_004554| | NFATC4,cytoplasmic nuclear factor of activated T-cells |
| 211250_s_at | 10.36 | 9.24E-07 | 1.115754121 | NM_001122681| | NA |
| 218188_s_at | 10.36 | 9.23E-07 | 1.041121925 | NM_012458| | TIMM13,translocase of inner mitochondrial membrane 13 |
| 230463_at | 10.36 | 9.24E-07 | 1.306813927 | NA |  |
| 220251_at | 10.36 | 9.28E-07 | 1.27987807 | NM_014388| | MGC29875,hypothetical protein MGC29875 |
| 221958_s_at | 10.36 | 9.28E-07 | 1.202422449 | NM_001002292| | FLJ23091,putative NFkB activating protein 373 isoform 2 |
| 227927_at | 10.36 | 9.24E-07 | 1.187391145 | NA |  |
| 1554423_a_at | 10.36 | 9.27E-07 | 1.067754595 | NM_001033024| | NA |
| 224468_s_at | 10.35 | 9.32E-07 | 1.034706827 | NM_199249| | MGC13170,multidrug resistance-related protein |
| 230811_at | 10.35 | 9.32E-07 | 1.188447363 | NM_153025| | FLJ31606,hypothetical protein FLJ31606 |
| 224674_at | 10.35 | 9.37E-07 | 1.090911048 | NM_025250| | TTYH3,tweety 3 |
| 202445_s_at | 10.35 | 9.34E-07 | 1.130562203 | NM_024408| | NOTCH2,notch 2 preproprotein |
| 210622_x_at | 10.35 | 9.37E-07 | 1.126413343 | NM_001098533| | NA |
| 213778_x_at | 10.35 | 9.32E-07 | 1.208084901 | NM_001113525| | NA |
| 231848_x_at | 10.34 | 9.44E-07 | 1.1744705 | NM_001032293| | NA |
| 209610_s_at | 10.34 | 9.43E-07 | 1.324470414 | NM_003038| | SLC1A4,solute carrier family 1, member 4 |
| 235968_at | 10.34 | 9.48E-07 | 1.088509106 | NM_001037131| | NA |
| 215747_s_at | 10.33 | 9.58E-07 | 1.105455265 | NM_001048194| | NA |
| 211058_x_at | 10.33 | 9.49E-07 | 1.023596698 | NM_006082| | K-ALPHA-1,tubulin, alpha, ubiquitous |
| 229053_at | 10.33 | 9.49E-07 | 1.112326303 | NM_016524| | LOC51760,B/K protein |
| 239863_at | 10.33 | 9.55E-07 | 1.288609863 | NA |  |
| 243189_at | 10.33 | 9.51E-07 | 1.215865643 | NA |  |
| 224318_s_at | 10.33 | 9.51E-07 | 1.054405445 | NM_001115016| | NA |
| 228043_at | 10.33 | 9.51E-07 | 1.224152839 | NM_032175| | FLJ12787,Src-associated protein SAW |
| 226850_at | 10.33 | 9.54E-07 | 1.056924629 | NM_182760| | SUMF1,sulfatase modifying factor 1 |
| 233303_at | 10.32 | 9.61E-07 | 1.356017075 | NA |  |
| 201459_at | 10.32 | 9.65E-07 | 1.050918058 | NM_006666| | RUVBL2,RuvB-like 2 |
| 205963_s_at | 10.32 | 9.58E-07 | 1.079421506 | NM_005147| | DNAJA3,DnaJ (Hsp40) homolog, subfamily A, member 3 |
| 233678_at | 10.32 | 9.59E-07 | 1.252401699 | NA |  |
| 242303_at | 10.32 | 9.65E-07 | 1.145606896 | NA |  |
| 209256_s_at | 10.31 | 9.73E-07 | 1.065814152 | NM_014997| | KIAA0265,KIAA0265 protein |
| 210983_s_at | 10.31 | 9.76E-07 | 1.076338762 | NM_005916| | MCM7,minichromosome maintenance protein 7 isoform 1 |
| 203337_x_at | 10.31 | 9.75E-07 | 1.068579416 | NM_004763| | ITGB1BP1,integrin cytoplasmic domain-associated protein 1 |
| 233638_s_at | 10.3 | 9.82E-07 | 1.237585522 | NM_017739| | FLJ20277,O-linked mannose |
| 225341_at | 10.3 | 9.87E-07 | 1.161798829 | NM_001033050| | NA |
| 234577_at | 10.3 | 9.86E-07 | 1.142427199 | NA |  |
| 238109_at | 10.3 | 9.83E-07 | 1.12719529 | NA |  |
| 214881_s_at | 10.3 | 9.87E-07 | 1.119436968 | NM_001076683| | NA |
| 203117_s_at | 10.29 | 9.90E-07 | 1.130827479 | NM_014871| | USP52,ubiquitin specific protease 52 |
| 226783_at | 10.29 | 9.89E-07 | 1.13641835 | NM_153373| | MGC15875,hypothetical protein MGC15875 |
| 241391_at | 10.29 | 9.88E-07 | 1.27836575 | NA |  |
| 206093_x_at | 10.29 | 9.90E-07 | 1.119380892 | NM_019105| | TNXB,tenascin XB isoform 1 |
| 241376_at | 10.29 | 9.92E-07 | 1.326605319 | NA |  |
| 204864_s_at | 10.28 | 1.00E-06 | 1.2849724 | NM_002184| | IL6ST,interleukin 6 signal transducer isoform 1 |
| 217624_at | 10.28 | 1.00E-06 | 1.358499177 | NM_014891| | PDAP1,PDGFA associated protein 1 |
| 235694_at | 10.28 | 1.01E-06 | 1.10701862 | NM_006602| | TCFL5,transcription factor-like 5 protein |
| 210015_s_at | 10.27 | 1.01E-06 | 1.49851295 | NM_001039538| | NA |
| 227944_at | 10.27 | 1.01E-06 | 1.232561172 | NM_002829| | PTPN3,protein tyrosine phosphatase, non-receptor type |
| 1565149_at | 10.27 | 1.01E-06 | 1.351356898 | NM_001080463| | NA |
| 1570135_at | 10.27 | 1.01E-06 | 1.112137563 | NM_006300| | ZNF230,zinc finger protein 230 |
| 218227_at | 10.27 | 1.01E-06 | 1.063716354 | NM_012225| | NUBP2,nucleotide binding protein 2 (MinD homolog, E. |
| 210424_s_at | 10.27 | 1.02E-06 | 1.158250888 | NM_001023567| | NA |
| 238683_at | 10.27 | 1.01E-06 | 1.108517898 | NM_153219| | ZNF524,zinc finger protein 524 |
| 203883_s_at | 10.26 | 1.02E-06 | 1.045059082 | NM_014904| | RAB11FIP2,RAB11 family interacting protein 2 (class I) |
| 225876_at | 10.26 | 1.02E-06 | 1.131730759 | NM_020448| | DJ462O23.2,hypothetical protein dJ462O23.2 |
| 1552274_at | 10.26 | 1.02E-06 | 1.217007784 | NM_017771| | PXK,PX domain containing serine/threonine kinase |
| 227318_at | 10.26 | 1.03E-06 | 1.145035272 | NA |  |
| 1557905_s_at | 10.26 | 1.03E-06 | 1.195237201 | NM_000610| | CD44,CD44 antigen isoform 1 precursor |
| 225826_at | 10.25 | 1.03E-06 | 1.29422194 | NM_052845| | MMAB,cob(I)alamin adenosyltransferase |
| 1565833_at | 10.25 | 1.03E-06 | 1.231623287 | NA |  |
| 209712_at | 10.25 | 1.03E-06 | 1.196083952 | NM_015139| | SLC35D1,solute carrier family 35 (UDP-glucuronic |
| 212987_at | 10.25 | 1.03E-06 | 1.161423396 | NM_012347| | FBXO9,F-box only protein 9 isoform 1 |
| 204093_at | 10.25 | 1.04E-06 | 1.044367763 | NM_001239| | CCNH,cyclin H |
| 238633_at | 10.24 | 1.04E-06 | 1.311759402 | NM_025209| | EPC1,enhancer of polycomb 1 |
| 218427_at | 10.24 | 1.04E-06 | 1.075334624 | NM_001039707| | NA |
| 224983_at | 10.24 | 1.05E-06 | 1.077809199 | NM_005506| | SCARB2,scavenger receptor class B, member 2 |
| 208445_s_at | 10.24 | 1.05E-06 | 1.028158791 | NM_032408| | BAZ1B,bromodomain adjacent to zinc finger domain, 1B |
| 201341_at | 10.24 | 1.04E-06 | 1.229141259 | NM_003633| | ENC1,ectodermal-neural cortex (with BTB-like domain) |
| 211568_at | 10.24 | 1.04E-06 | 1.191857057 | NM_001704| | BAI3,brain-specific angiogenesis inhibitor 3 |
| 227211_at | 10.24 | 1.04E-06 | 1.17162748 | NM_001009936| | PHF19,PHD finger protein 19 isoform b |
| 224013_s_at | 10.23 | 1.05E-06 | 1.432095889 | NM_031439| | SOX7,SRY-box 7 |
| 205322_s_at | 10.23 | 1.05E-06 | 1.068620908 | NM_005955| | MTF1,metal-regulatory transcription factor 1 |
| 212262_at | 10.23 | 1.06E-06 | 1.07521048 | NM_006775| | QKI,quaking homolog, KH domain RNA binding isoform |
| 225531_at | 10.23 | 1.05E-06 | 1.185007806 | NM_001100619| | NA |
| 234107_s_at | 10.23 | 1.06E-06 | 1.047381586 | NM_080820| | HARS2,histidyl-tRNA synthetase 2 |
| 212186_at | 10.22 | 1.06E-06 | 1.093258151 | NM_198834| | ACACA,acetyl-Coenzyme A carboxylase alpha isoform 1 |
| 230427_s_at | 10.22 | 1.06E-06 | 1.124196815 | NM_001015048| | NA |
| 214500_at | 10.22 | 1.06E-06 | 1.437318262 | NM_001040158| | NA |
| 210555_s_at | 10.22 | 1.06E-06 | 1.124249796 | NM_004555| | NFATC3,cytoplasmic nuclear factor of activated T-cells |
| 235259_at | 10.21 | 1.08E-06 | 1.314227335 | NM_145048| | MGC29898,hypothetical protein MGC29898 |
| 241091_at | 10.21 | 1.08E-06 | 1.286641996 | NA |  |
| 235046_at | 10.21 | 1.08E-06 | 1.882560405 | NA |  |
| 215710_at | 10.21 | 1.08E-06 | 1.173054783 | NM_006278| | ST3GAL4,ST3 beta-galactoside alpha-2,3-sialyltransferase |
| 202135_s_at | 10.21 | 1.07E-06 | 1.052019139 | NM_005735| | ACTR1B,ARP1 actin-related protein 1 homolog B, |
| 202607_at | 10.21 | 1.07E-06 | 1.051137892 | NM_001543| | NDST1,N-deacetylase/N-sulfotransferase (heparan |
| 221103_s_at | 10.21 | 1.08E-06 | 1.283566605 | NM_018338| | WDR52,WD repeat domain 52 |
| 235248_at | 10.2 | 1.09E-06 | 1.090732739 | NM_001099272| | NA |
| 216902_s_at | 10.2 | 1.09E-06 | 1.14583745 | NM_018427| | RRN3,RRN3 RNA polymerase I transcription factor |
| 210336_x_at | 10.19 | 1.10E-06 | 1.080059096 | NM_003422| | ZNF42,zinc finger protein 42 isoform 1 |
| 235393_at | 10.19 | 1.10E-06 | 1.183530975 | NA |  |
| 1554822_at | 10.19 | 1.10E-06 | 1.235600885 | NM_020432| | PHTF2,putative homeodomain transcription factor 2 |
| 239892_at | 10.19 | 1.10E-06 | 1.334713622 | NA |  |
| 211725_s_at | 10.19 | 1.10E-06 | 1.129040912 | NM_001196| | BID,BH3 interacting domain death agonist isoform 2 |
| 222104_x_at | 10.19 | 1.10E-06 | 1.061235137 | NM_001516| | GTF2H3,general transcription factor IIH, polypeptide 3, |
| 230328_at | 10.18 | 1.10E-06 | 1.139106292 | NM_145237| | LOC94431,similar to RNA polymerase I transcription factor |
| 233496_s_at | 10.18 | 1.11E-06 | 1.220460572 | NM_021914| | CFL2,cofilin 2 |
| 213937_s_at | 10.18 | 1.11E-06 | 1.073959084 | NM_012280| | FTSJ1,FtsJ homolog 1 isoform a |
| 228006_at | 10.18 | 1.10E-06 | 1.235976736 | NA |  |
| 221795_at | 10.18 | 1.10E-06 | 1.381910847 | NM_001007097| | NTRK2,neurotrophic tyrosine kinase, receptor, type 2 |
| 242059_at | 10.17 | 1.12E-06 | 1.240504352 | NA |  |
| 237756_at | 10.17 | 1.12E-06 | 1.238425053 | NM_144711| | MGC2610,hypothetical protein MGC2610 |
| 225498_at | 10.17 | 1.12E-06 | 1.055766675 | NM_176812| | C20orf178,Snf7 homologue associated with Alix 1 |
| 226335_at | 10.17 | 1.12E-06 | 1.082809383 | NM_004586| | RPS6KA3,ribosomal protein S6 kinase, 90kDa, polypeptide |
| 206531_at | 10.17 | 1.12E-06 | 1.091961606 | NM_004647| | DPF1,Neuro-d4 (rat) homolog |
| 237623_at | 10.16 | 1.12E-06 | 1.233525634 | NM_000099| | CST3,cystatin C precursor |
| 210560_at | 10.16 | 1.13E-06 | 1.477926229 | NM_001485| | GBX2,gastrulation brain homeo box 2 |
| 230029_x_at | 10.16 | 1.13E-06 | 1.124740356 | NM_172070| | ZNF650,zinc finger protein 650 |
| 219802_at | 10.15 | 1.14E-06 | 1.191293401 | NM_024854| | FLJ22028,hypothetical protein FLJ22028 |
| 228695_at | 10.15 | 1.15E-06 | 1.38996408 | NM_152765| | MGC33510,hypothetical protein MGC33510 |
| 236866_at | 10.15 | 1.14E-06 | 1.146300028 | NM_000512| | GALNS,N-acetylgalactosamine-6-sulfatase precursor |
| 211975_at | 10.15 | 1.14E-06 | 1.069731607 | NM_032389| | ZNF289,zinc finger protein 289, ID1 regulated |
| 242648_at | 10.15 | 1.14E-06 | 1.163301994 | NM_020803| | KLHL8,kelch-like 8 |
| 224615_x_at | 10.14 | 1.15E-06 | 1.080496396 | NM_030789| | HM13,minor histocompatibility antigen 13 isoform 1 |
| 225543_at | 10.14 | 1.15E-06 | 1.06676517 | NA |  |
| 225574_at | 10.14 | 1.15E-06 | 1.062205656 | NM_152682| | MGC10198,hypothetical protein MGC10198 |
| 212355_at | 10.14 | 1.16E-06 | 1.124221483 | NM_015299| | KIAA0323,KIAA0323 |
| 235962_at | 10.14 | 1.15E-06 | 1.240602307 | NA |  |
| 215684_s_at | 10.14 | 1.16E-06 | 1.070140376 | NM_032204| | ASCC2,activating signal cointegrator 1 complex subunit |
| 226965_at | 10.14 | 1.15E-06 | 1.132373912 | NM_152678| | FLJ34969,hypothetical protein FLJ34969 |
| 230821_at | 10.14 | 1.16E-06 | 1.239897974 | NM_021964| | ZNF148,zinc finger protein 148 (pHZ-52) |
| 232683_s_at | 10.13 | 1.17E-06 | 1.122108117 | NM_020214| | PARP6,poly (ADP-ribose) polymerase family, member 6 |
| 222310_at | 10.13 | 1.16E-06 | 1.253066528 | NM_020706| | SFRS15,splicing factor, arginine/serine-rich 15 |
| 239174_at | 10.13 | 1.17E-06 | 1.235526643 | NA |  |
| 222844_s_at | 10.13 | 1.17E-06 | 1.198052124 | NM_021947| | SRR,serine racemase |
| 221250_s_at | 10.13 | 1.16E-06 | 1.206619665 | NM_031300| | MXD3,MAX dimerization protein 3 |
| 225782_at | 10.12 | 1.17E-06 | 1.462232304 | NM_001031679| | NA |
| 201942_s_at | 10.12 | 1.17E-06 | 1.181100959 | NM_001304| | CPD,carboxypeptidase D precursor |
| 223364_s_at | 10.12 | 1.18E-06 | 1.077313861 | NM_032656| | DHX37,DEAH (Asp-Glu-Ala-His) box polypeptide 37 |
| 202519_at | 10.12 | 1.18E-06 | 1.101078412 | NM_014938| | MONDOA,MondoA |
| 238935_at | 10.12 | 1.18E-06 | 1.36813625 | NM_015920| | RPS27L,ribosomal protein S27-like protein |
| 213471_at | 10.11 | 1.18E-06 | 1.135133855 | NM_015102| | NPHP4,nephroretinin |
| 209478_at | 10.11 | 1.19E-06 | 1.053111575 | NM_144998| | STRA13,stimulated by retinoic acid 13 homolog |
| 244092_at | 10.11 | 1.19E-06 | 1.173672528 | NM_032143| | ZRANB3,zinc finger, RAN-binding domain containing 3 |
| 228361_at | 10.1 | 1.20E-06 | 1.211971318 | NM_004091| | E2F2,E2F transcription factor 2 |
| 239212_at | 10.1 | 1.20E-06 | 1.507978812 | NM_032860| | C6orf93,hypothetical protein FLJ14909 |
| 201814_at | 10.1 | 1.20E-06 | 1.055241108 | NM_014744| | TBC1D5,TBC1 domain family, member 5 |
| 241937_s_at | 10.1 | 1.19E-06 | 1.090642754 | NM_018669| | WDR4,WD repeat domain 4 protein |
| 229425_at | 10.1 | 1.20E-06 | 1.127204716 | NA |  |
| 208094_s_at | 10.09 | 1.21E-06 | 1.108420646 | NM_030818| | MGC10471,hypothetical protein MGC10471 |
| 214004_s_at | 10.09 | 1.21E-06 | 1.128393684 | NM_014667| | VGLL4,vestigial like 4 |
| 205823_at | 10.09 | 1.21E-06 | 1.150513086 | NM_002926| | RGS12,regulator of G-protein signalling 12 isoform 2 |
| 238712_at | 10.08 | 1.23E-06 | 1.25023141 | NA |  |
| 217408_at | 10.07 | 1.24E-06 | 1.047797273 | NM_014046| | MRPS18B,mitochondrial ribosomal protein S18B |
| 236267_at | 10.07 | 1.24E-06 | 1.166796186 | NM_012279| | ZNF346,zinc finger protein 346 |
| 227264_at | 10.07 | 1.23E-06 | 1.214863208 | NA |  |
| 225674_at | 10.07 | 1.24E-06 | 1.096557202 | NM_001008405| | BCAP29,B-cell receptor-associated protein BAP29 isoform |
| 227745_at | 10.06 | 1.26E-06 | 1.186746513 | NA |  |
| 220450_at | 10.06 | 1.25E-06 | 1.395020146 | NA |  |
| 235311_at | 10.06 | 1.25E-06 | 1.32569892 | NM_017946| | FKBP14,FK506 binding protein 14, 22 kDa |
| 226381_at | 10.05 | 1.26E-06 | 1.146177628 | NA |  |
| 1556039_s_at | 10.05 | 1.26E-06 | 1.097658553 | NM_018969| | GPR173,G-protein coupled receptor 173 |
| 223482_at | 10.05 | 1.27E-06 | 1.076133615 | NM_031925| | TMPIT,transmembrane protein induced by tumor necrosis |
| 201554_x_at | 10.05 | 1.27E-06 | 1.110166175 | NM_004130| | GYG,glycogenin |
| 1552789_at | 10.05 | 1.26E-06 | 1.289282334 | NM_003262| | TLOC1,translocation protein 1 |
| 211595_s_at | 10.04 | 1.27E-06 | 1.077717552 | NM_022839| | MRPS11,mitochondrial ribosomal protein S11 isoform a |
| 226121_at | 10.04 | 1.27E-06 | 1.116647346 | NM_144683| | MGC23280,hypothetical protein MGC23280 |
| 217957_at | 10.04 | 1.28E-06 | 1.025078286 | NM_013242| | GTL3,transcription factor IIB |
| 240892_at | 10.04 | 1.28E-06 | 1.36277207 | NA |  |
| 200652_at | 10.04 | 1.28E-06 | 1.025808864 | NM_003145| | SSR2,signal sequence receptor, beta precursor |
| 242523_at | 10.04 | 1.28E-06 | 1.350802612 | NA |  |
| 207622_s_at | 10.04 | 1.27E-06 | 1.149775249 | NM_005692| | ABCF2,ATP-binding cassette, sub-family F, member 2 |
| 218367_x_at | 10.04 | 1.27E-06 | 1.07171574 | NM_001014443| | NA |
| 212856_at | 10.04 | 1.27E-06 | 1.162977193 | NM_015124| | NA |
| 244439_at | 10.04 | 1.27E-06 | 1.194926004 | NM_152594| | SPRED1,sprouty-related protein with EVH-1 domain 1 |
| 219646_at | 10.04 | 1.27E-06 | 1.151317446 | NM_017702| | FLJ20186,differentially expressed in FDCP 8 isoform 2 |
| 226383_at | 10.04 | 1.28E-06 | 1.06793389 | NM_152316| | FLJ38968,hypothetical protein FLJ38968 |
| 225202_at | 10.03 | 1.30E-06 | 1.24091127 | NM_014899| | RHOBTB3,rho-related BTB domain containing 3 |
| 236219_at | 10.03 | 1.29E-06 | 1.456243489 | NA |  |
| 205324_s_at | 10.03 | 1.29E-06 | 1.053980385 | NM_012280| | FTSJ1,FtsJ homolog 1 isoform a |
| 214462_at | 10.03 | 1.30E-06 | 1.289949222 | NM_004232| | SOCS6,suppressor of cytokine signaling 6 |
| 38340_at | 10.03 | 1.30E-06 | 1.116924282 | NM_003959| | HIP1R,huntingtin interacting protein-1-related |
| 218699_at | 10.03 | 1.29E-06 | 1.119854476 | NM_003929| | RAB7L1,RAB7, member RAS oncogene family-like 1 |
| 214543_x_at | 10.03 | 1.30E-06 | 1.109683816 | NM_006775| | QKI,quaking homolog, KH domain RNA binding isoform |
| 236007_at | 10.02 | 1.30E-06 | 1.182427248 | NM_007202| | AKAP10,A-kinase anchor protein 10 precursor |
| 1558410_s_at | 10.02 | 1.31E-06 | 1.14165644 | NA |  |
| 203466_at | 10.02 | 1.30E-06 | 1.066676224 | NM_002437| | MPV17,MpV17 transgene, murine homolog, |
| 241865_at | 10.02 | 1.30E-06 | 1.152688701 | NA |  |
| 241998_at | 10.02 | 1.30E-06 | 1.971428407 | NM_001099334| | NA |
| 203225_s_at | 10.02 | 1.31E-06 | 1.175299352 | NM_018339| | RFK,riboflavin kinase |
| 237885_at | 10.02 | 1.30E-06 | 1.499162367 | NA |  |
| 203776_at | 10.01 | 1.32E-06 | 1.06997232 | NM_015698| | GPKOW,G patch domain and KOW motifs |
| 212782_x_at | 10.01 | 1.32E-06 | 1.058967904 | NM_006234| | POLR2J,DNA directed RNA polymerase II polypeptide J |
| 226959_at | 10.01 | 1.32E-06 | 1.105806154 | NA |  |
| 242110_at | 10.01 | 1.32E-06 | 1.232136253 | NA |  |
| 202528_at | 10.01 | 1.32E-06 | 1.074963269 | NM_000403| | GALE,UDP-galactose-4-epimerase |
| 222407_s_at | 10.01 | 1.32E-06 | 1.038867039 | NM_022473| | ZFP106,zinc finger protein 106 homolog |
| 213055_at | 10.01 | 1.32E-06 | 1.31254729 | NM_001025079| | NA |
| 1557246_at | 10.01 | 1.32E-06 | 1.274129724 | NM_020738| | KIDINS220,kinase D-interacting substance of 220 kDa |
| 209903_s_at | 10.01 | 1.31E-06 | 1.083929558 | NM_001184| | ATR,ataxia telangiectasia and Rad3 related protein |
| 239562_at | 10.01 | 1.31E-06 | 1.42640012 | NM_001004346| | MTHFD2L,methylenetetrahydrofolate dehydrogenase (NADP+ |
| 212248_at | 10.01 | 1.32E-06 | 1.237127877 | NM_178812| | LYRIC,LYRIC/3D3 |
| 224887_at | 10.01 | 1.32E-06 | 1.113880118 | NM_032520| | GNPTG,N-acetylglucosamine-1-phosphotransferase, gamma |
| 244627_at | 10 | 1.33E-06 | 1.156061643 | NM_015533| | DKFZP586B1621,DKFZP586B1621 protein |
| 229406_at | 10 | 1.33E-06 | 1.11092712 | NM_001082575| | NA |
| 224584_at | 10 | 1.33E-06 | 1.033191445 | NM_001009923| | C20orf30,chromosome 20 open reading frame 30 isoform 1 |
| 242108_at | 10 | 1.33E-06 | 1.131760571 | NA |  |
| 225868_at | 10 | 1.33E-06 | 1.233241496 | NM_033452| | TRIM47,gene overexpressed in astrocytoma |
| 244512_at | 10 | 1.33E-06 | 1.27074486 | NA |  |
| 232983_s_at | 9.99 | 1.35E-06 | 1.085036396 | NM_012139| | DELGEF,deafness locus associated putative guanine |
| 243050_at | 9.99 | 1.35E-06 | 1.257418695 | NA |  |
| 227331_at | 9.99 | 1.34E-06 | 1.03264873 | NM_001004304| | LOC283337,hypothetical protein LOC283337 |
| 229515_at | 9.98 | 1.35E-06 | 1.138043735 | NM_002583| | PAWR,PRKC, apoptosis, WT1, regulator |
| 213644_at | 9.98 | 1.36E-06 | 1.250056407 | NM_001037325| | NA |
| 211958_at | 9.98 | 1.35E-06 | 1.282471226 | NM_000599| | IGFBP5,insulin-like growth factor binding protein 5 |
| 232052_at | 9.98 | 1.36E-06 | 1.081063653 | NA |  |
| 236358_at | 9.98 | 1.36E-06 | 1.342310789 | NA |  |
| 228760_at | 9.98 | 1.35E-06 | 1.102027632 | NM_032102| | SRP46,Splicing factor, arginine/serine-rich, 46kD |
| 1558604_a_at | 9.97 | 1.37E-06 | 1.304315618 | NA |  |
| 221127_s_at | 9.97 | 1.37E-06 | 1.326521981 | NA |  |
| 201168_x_at | 9.97 | 1.38E-06 | 1.168166373 | NM_004309| | ARHGDIA,Rho GDP dissociation inhibitor (GDI) alpha |
| 203442_x_at | 9.97 | 1.38E-06 | 1.06276698 | NM_153265| | FLJ35827,hypothetical protein FLJ35827 |
| 1555824_a_at | 9.97 | 1.37E-06 | 1.058338359 | NM_001100913| | NA |
| 212985_at | 9.97 | 1.37E-06 | 1.132309476 | NA |  |
| 204963_at | 9.97 | 1.38E-06 | 1.348388483 | NM_005086| | SSPN,sarcospan |
| 227562_at | 9.97 | 1.37E-06 | 1.202505119 | NA |  |
| 224162_s_at | 9.96 | 1.39E-06 | 1.080697035 | NM_024735| | FBXO31,F-box protein 31 |
| 227649_s_at | 9.96 | 1.38E-06 | 1.248277543 | NM_001042758| | NA |
| 242877_at | 9.96 | 1.39E-06 | 1.137096479 | NA |  |
| 203805_s_at | 9.95 | 1.40E-06 | 1.109322368 | NM_000135| | FANCA,Fanconi anemia, complementation group A |
| 235762_at | 9.95 | 1.40E-06 | 1.362702606 | NM_023922| | TAS2R14,taste receptor, type 2, member 14 |
| 222942_s_at | 9.95 | 1.39E-06 | 1.085769789 | NM_001010927| | TIAM2,T-cell lymphoma invasion and metastasis 2 |
| 232744_x_at | 9.95 | 1.40E-06 | 1.176382998 | NA |  |
| 228686_at | 9.94 | 1.41E-06 | 1.227993535 | NA |  |
| 226932_at | 9.94 | 1.42E-06 | 1.352828605 | NA |  |
| 223871_x_at | 9.94 | 1.42E-06 | 1.158502366 | NM_032329| | ING5,inhibitor of growth family, member 5 |
| 202329_at | 9.94 | 1.41E-06 | 1.07431594 | NM_001127190| | NA |
| 217795_s_at | 9.93 | 1.43E-06 | 1.063143917 | NM_024334| | TMEM43,transmembrane protein 43 |
| 239637_at | 9.93 | 1.43E-06 | 1.251375473 | NA |  |
| 227984_at | 9.93 | 1.44E-06 | 1.392271103 | NA |  |
| 202522_at | 9.93 | 1.42E-06 | 1.041603477 | NM_012399| | PITPNB,phosphatidylinositol transfer protein, beta |
| 244287_at | 9.93 | 1.43E-06 | 1.076465185 | NM_001077199| | NA |
| 227327_at | 9.93 | 1.43E-06 | 1.047851006 | NM_001410| | EGFL4,EGF-like-domain, multiple 4 |
| 226652_at | 9.93 | 1.43E-06 | 1.427228677 | NM_006537| | USP3,ubiquitin specific protease 3 |
| 226365_at | 9.93 | 1.43E-06 | 1.210357087 | NA |  |
| 218628_at | 9.92 | 1.44E-06 | 1.091892608 | NM_016053| | CGI-116,CGI-116 protein |
| 230568_x_at | 9.92 | 1.44E-06 | 1.07449594 | NM_016941| | DLL3,delta-like 3 protein isoform 1 precursor |
| 213398_s_at | 9.91 | 1.46E-06 | 1.025531831 | NM_020195| | C14orf124,HCDI protein |
| 217106_x_at | 9.91 | 1.46E-06 | 1.062883676 | NM_014473| | HSA9761,putative dimethyladenosine transferase |
| 225331_at | 9.91 | 1.46E-06 | 1.049070321 | NM_174908| | C3orf6,Ymer protein short isoform |
| 212223_at | 9.9 | 1.47E-06 | 1.177770711 | NM_000202| | IDS,iduronate-2-sulfatase isoform a precursor |
| 212310_at | 9.9 | 1.47E-06 | 1.115850733 | NM_198551| | NA |
| 229193_at | 9.9 | 1.47E-06 | 1.389252941 | NA |  |
| 228384_s_at | 9.9 | 1.48E-06 | 1.267548149 | NM_032709| | C10orf33,chromosome 10 open reading frame 33 |
| 209251_x_at | 9.9 | 1.48E-06 | 1.023926528 | NM_032704| | TUBA6,tubulin alpha 6 |
| 214789_x_at | 9.9 | 1.47E-06 | 1.073038214 | NM_032102| | SRP46,Splicing factor, arginine/serine-rich, 46kD |
| 232755_at | 9.9 | 1.47E-06 | 1.305846455 | NM_003343| | UBE2G2,ubiquitin-conjugating enzyme E2G 2 isoform 1 |
| 229376_at | 9.9 | 1.48E-06 | 1.440004204 | NM_002763| | PROX1,prospero-related homeobox 1 |
| 1557383_a_at | 9.9 | 1.47E-06 | 1.313045965 | NA |  |
| 201321_s_at | 9.89 | 1.49E-06 | 1.119264942 | NM_003075| | SMARCC2,SWI/SNF-related matrix-associated |
| 222125_s_at | 9.89 | 1.49E-06 | 1.159440552 | NM_177938| | PH-4,hypoxia-inducible factor prolyl 4-hydroxylase |
| 243484_x_at | 9.89 | 1.48E-06 | 1.249326473 | NA |  |
| 217550_at | 9.89 | 1.50E-06 | 1.12584748 | NM_007348| | ATF6,activating transcription factor 6 |
| 211323_s_at | 9.88 | 1.50E-06 | 1.094265377 | NM_001099952| | NA |
| 1562904_s_at | 9.88 | 1.50E-06 | 1.268978222 | NA |  |
| 238045_at | 9.88 | 1.50E-06 | 1.169820972 | NM_194291| | LOC157378,hypothetical protein BC017881 |
| 209878_s_at | 9.88 | 1.50E-06 | 1.159071532 | NM_021975| | RELA,v-rel reticuloendotheliosis viral oncogene |
| 230031_at | 9.88 | 1.50E-06 | 1.184392292 | NM_005347| | HSPA5,heat shock 70kDa protein 5 (glucose-regulated |
| 221512_at | 9.88 | 1.50E-06 | 1.113002038 | NM_032125| | DKFZP564D0478,hypothetical protein DKFZp564D0478 |
| 230168_at | 9.88 | 1.51E-06 | 1.194262839 | NA |  |
| 213321_at | 9.88 | 1.50E-06 | 1.136310274 | NM_000056| | BCKDHB,branched chain keto acid dehydrogenase E1, beta |
| 208336_s_at | 9.88 | 1.51E-06 | 1.038258602 | NM_138501| | GPSN2,glycoprotein, synaptic 2 |
| 201404_x_at | 9.88 | 1.50E-06 | 1.059884387 | NM_002794| | PSMB2,proteasome beta 2 subunit |
| 226217_at | 9.88 | 1.51E-06 | 1.097163225 | NM_133496| | SLC30A7,zinc transporter like 2 |
| 239022_at | 9.87 | 1.51E-06 | 1.192870543 | NA |  |
| 228820_at | 9.87 | 1.53E-06 | 1.084284532 | NM_022098| | LOC63929,hypothetical protein LOC63929 |
| 218708_at | 9.87 | 1.51E-06 | 1.113197019 | NM_013248| | NXT1,NTF2-like export factor 1 |
| 219484_at | 9.86 | 1.53E-06 | 1.214774818 | NM_013320| | HCFC2,host cell factor C2 |
| 221693_s_at | 9.85 | 1.55E-06 | 1.027868717 | NM_018135| | MRPS18A,mitochondrial ribosomal protein S18A |
| 227986_at | 9.85 | 1.55E-06 | 1.194991489 | NM_024325| | ZNF343,zinc finger protein 343 |
| 236370_at | 9.85 | 1.55E-06 | 1.232300252 | NA |  |
| 205029_s_at | 9.85 | 1.55E-06 | 1.786213688 | NM_001446| | FABP7,fatty acid binding protein 7, brain |
| 37793_r_at | 9.85 | 1.54E-06 | 1.146670057 | NM_002878| | RAD51L3,RAD51-like 3 isoform 1 |
| 213320_at | 9.85 | 1.55E-06 | 1.091147021 | NM_005788| | HRMT1L3,HMT1 hnRNP methyltransferase-like 3 |
| 218565_at | 9.84 | 1.57E-06 | 1.109087578 | NM_016390| | C9orf114,chromosome 9 open reading frame 114 |
| 237750_at | 9.84 | 1.57E-06 | 1.214399306 | NM_022098| | LOC63929,hypothetical protein LOC63929 |
| 213286_at | 9.83 | 1.59E-06 | 1.066181821 | NM_016107| | ZFR,zinc finger RNA binding protein |
| 204662_at | 9.83 | 1.59E-06 | 1.12425816 | NM_014711| | CP110,CP110 protein |
| 225163_at | 9.83 | 1.58E-06 | 1.301461081 | NM_018027| | FRMD4A,FERM domain containing 4A |
| 1552641_s_at | 9.83 | 1.59E-06 | 1.116972731 | NM_001039211| | NA |
| 243364_at | 9.83 | 1.59E-06 | 1.259540289 | NM_001127231| | NA |
| 205055_at | 9.82 | 1.61E-06 | 1.097213273 | NM_002208| | ITGAE,integrin, alpha E (antigen CD103, human mucosal |
| 230143_at | 9.82 | 1.60E-06 | 1.326810914 | NM_152470| | C18orf23,chromosome 18 open reading frame 23 |
| 1558507_at | 9.82 | 1.60E-06 | 1.194239839 | NM_001024594| | NA |
| 202541_at | 9.82 | 1.60E-06 | 1.114634586 | NM_004757| | SCYE1,small inducible cytokine subfamily E, member 1 |
| 203785_s_at | 9.82 | 1.61E-06 | 1.073501444 | NM_018380| | DDX28,DEAD (Asp-Glu-Ala-Asp) box polypeptide 28 |
| 230495_at | 9.82 | 1.61E-06 | 1.479026262 | NA |  |
| 242080_at | 9.82 | 1.60E-06 | 1.193414122 | NA |  |
| 228999_at | 9.82 | 1.60E-06 | 1.115067277 | NM_001042572| | NA |
| 225382_at | 9.82 | 1.61E-06 | 1.199750275 | NM_001080485| | NA |
| 227282_at | 9.81 | 1.61E-06 | 1.144598699 | NM_001105243| | NA |
| 201600_at | 9.81 | 1.61E-06 | 1.029527515 | NM_007273| | PHB2,prohibitin 2 |
| 1555288_s_at | 9.81 | 1.62E-06 | 1.072669458 | NM_001080542| | NA |
| 209826_at | 9.81 | 1.62E-06 | 1.113195116 | NM_005155| | PPT2,palmitoyl-protein thioesterase 2 isoform a |
| 226882_x_at | 9.81 | 1.61E-06 | 1.172444499 | NM_018669| | WDR4,WD repeat domain 4 protein |
| 224233_s_at | 9.8 | 1.64E-06 | 1.11286814 | NM_018116| | FLJ10504,misato |
| 1557055_s_at | 9.8 | 1.64E-06 | 1.119341425 | NA |  |
| 230417_at | 9.8 | 1.64E-06 | 1.282369147 | NM_020692| | NA |
| 222054_at | 9.8 | 1.63E-06 | 1.149585722 | NA |  |
| 238273_at | 9.8 | 1.63E-06 | 1.196222957 | NM_012450| | SLC13A4,solute carrier family 13 (sodium/sulfate |
| 226704_at | 9.8 | 1.64E-06 | 1.108961024 | NM_058167| | UBE2J2,ubiquitin conjugating enzyme E2, J2 isoform 2 |
| 204147_s_at | 9.79 | 1.65E-06 | 1.187123365 | NM_007111| | TFDP1,transcription factor Dp-1 |
| 217999_s_at | 9.79 | 1.66E-06 | 1.194884027 | NM_007350| | PHLDA1,pleckstrin homology-like domain, family A, |
| 223298_s_at | 9.79 | 1.66E-06 | 1.072028762 | NM_001002009| | NA |
| 208968_s_at | 9.79 | 1.66E-06 | 1.034460631 | NM_020313| | CIAPIN1,cytokine induced apoptosis inhibitor 1 |
| 232918_at | 9.78 | 1.67E-06 | 1.188849459 | NA |  |
| 221899_at | 9.78 | 1.66E-06 | 1.141073217 | NM_014887| | PFAAP5,phosphonoformate immuno-associated protein 5 |
| 243879_at | 9.78 | 1.68E-06 | 1.29152837 | NA |  |
| 230123_at | 9.78 | 1.67E-06 | 1.175544124 | NM_018090| | FLJ10420,hypothetical protein FLJ10420 |
| 1557315_a_at | 9.78 | 1.67E-06 | 1.170125305 | NA |  |
| 1552257_a_at | 9.78 | 1.68E-06 | 1.054172607 | NM_015140| | KIAA0153,KIAA0153 protein |
| 242723_at | 9.78 | 1.68E-06 | 1.175925133 | NA |  |
| 239672_at | 9.78 | 1.67E-06 | 1.218771612 | NA |  |
| 1554084_a_at | 9.77 | 1.69E-06 | 1.039596246 | NM_024654| | FLJ23323,hypothetical protein FLJ23323 |
| 214063_s_at | 9.77 | 1.69E-06 | 1.250100387 | NM_001063| | TF,transferrin |
| 219558_at | 9.77 | 1.69E-06 | 1.11072585 | NM_024524| | NA |
| 236649_at | 9.77 | 1.69E-06 | 1.171451626 | NM_020234| | MDS009,x 009 protein |
| 230405_at | 9.77 | 1.68E-06 | 1.181577503 | NM_001013717| | NA |
| 228217_s_at | 9.77 | 1.70E-06 | 1.069672047 | NA |  |
| 212365_at | 9.77 | 1.69E-06 | 1.081246163 | NM_012223| | MYO1B,myosin IB |
| 203987_at | 9.77 | 1.69E-06 | 1.079334997 | NM_003506| | FZD6,frizzled 6 |
| 203029_s_at | 9.76 | 1.71E-06 | 1.329483281 | NM_002847| | PTPRN2,protein tyrosine phosphatase, receptor type, N |
| 230057_at | 9.76 | 1.71E-06 | 1.363663641 | NA |  |
| 1553743_at | 9.76 | 1.70E-06 | 1.281009865 | NM_145280| | LOC151194,hepatocellular carcinoma-associated antigen |
| 233271_at | 9.76 | 1.70E-06 | 1.160008213 | NA |  |
| 1556051_a_at | 9.75 | 1.72E-06 | 1.179677718 | NM_001003398| | BICD1,bicaudal D homolog 1 isoform 2 |
| 231316_at | 9.75 | 1.72E-06 | 1.163892893 | NA |  |
| 242932_at | 9.75 | 1.72E-06 | 1.440620126 | NA |  |
| 219766_at | 9.75 | 1.73E-06 | 1.15752416 | NM_030578| | MGC4093,hypothetical protein MGC4093 |
| 202428_x_at | 9.75 | 1.72E-06 | 1.085431648 | NM_001079862| | NA |
| 205030_at | 9.75 | 1.72E-06 | 1.593012564 | NM_001446| | FABP7,fatty acid binding protein 7, brain |
| 211597_s_at | 9.75 | 1.73E-06 | 1.541275484 | NM_032495| | HOP,homeodomain-only protein |
| 233369_at | 9.75 | 1.72E-06 | 1.158696402 | NA |  |
| 219269_at | 9.75 | 1.73E-06 | 1.117925126 | NM_024567| | FLJ21616,hypothetical protein FLJ21616 |
| 222528_s_at | 9.75 | 1.72E-06 | 1.111903753 | NM_016612| | MSCP,mitochondrial solute carrier protein |
| 233512_at | 9.74 | 1.75E-06 | 1.158548243 | NA |  |
| 1559491_at | 9.74 | 1.75E-06 | 1.090461209 | NA |  |
| 209342_s_at | 9.74 | 1.74E-06 | 1.130578772 | NM_001556| | IKBKB,inhibitor of kappa light polypeptide gene |
| 209288_s_at | 9.74 | 1.75E-06 | 1.174872728 | NM_006449| | CDC42EP3,Cdc42 effector protein 3 |
| 1565689_at | 9.73 | 1.76E-06 | 1.292704074 | NA |  |
| 1552719_at | 9.73 | 1.75E-06 | 1.18554342 | NM_138423| | H63,H63 breast cancer expressed gene isoform a |
| 215301_at | 9.73 | 1.77E-06 | 1.15534094 | NA |  |
| 217501_at | 9.73 | 1.77E-06 | 1.073700662 | NM_004804| | WDR39,WD repeat domain 39 |
| 211630_s_at | 9.73 | 1.77E-06 | 1.100671745 | NM_000178| | GSS,glutathione synthetase |
| 205079_s_at | 9.73 | 1.75E-06 | 1.067410303 | NM_003829| | MPDZ,multiple PDZ domain protein |
| 226363_at | 9.73 | 1.75E-06 | 1.222693117 | NM_001023587| | NA |
| 222270_at | 9.73 | 1.76E-06 | 1.50350144 | NM_001122964| | NA |
| 237954_x_at | 9.73 | 1.76E-06 | 1.186797327 | NA |  |
| 243704_at | 9.73 | 1.76E-06 | 1.300461622 | NA |  |
| 208714_at | 9.72 | 1.78E-06 | 1.076671721 | NM_007103| | NDUFV1,NADH dehydrogenase (ubiquinone) flavoprotein 1, |
| 204056_s_at | 9.72 | 1.77E-06 | 1.088490808 | NM_000431| | MVK,mevalonate kinase |
| 224626_at | 9.72 | 1.78E-06 | 1.069988355 | NM_080670| | SLC35A4,solute carrier family 35, member A4 |
| 209198_s_at | 9.71 | 1.80E-06 | 1.286328723 | NM_152280| | SYT11,synaptotagmin 12 |
| 205316_at | 9.71 | 1.79E-06 | 1.40519107 | NA |  |
| 235534_at | 9.71 | 1.80E-06 | 1.240779198 | NA |  |
| 228900_at | 9.71 | 1.80E-06 | 1.179682881 | NM_001033553| | NA |
| 228635_at | 9.71 | 1.79E-06 | 1.547940398 | NM_020815| | PCDH10,protocadherin 10 isoform 2 precursor |
| 209625_at | 9.71 | 1.79E-06 | 1.364781632 | NM_004569| | PIGH,phosphatidylinositol glycan, class H |
| 228880_at | 9.7 | 1.82E-06 | 1.189099395 | NA |  |
| 215994_x_at | 9.7 | 1.81E-06 | 1.072812737 | NM_015043| | KIAA0676,KIAA0676 protein isoform b |
| 235551_at | 9.7 | 1.82E-06 | 1.255364736 | NM_018669| | WDR4,WD repeat domain 4 protein |
| 213094_at | 9.69 | 1.83E-06 | 1.450986231 | NM_001032394| | NA |
| 202656_s_at | 9.69 | 1.83E-06 | 1.089638441 | NM_014755| | SERTAD2,SERTA domain containing 2 |
| 229699_at | 9.69 | 1.84E-06 | 1.193849604 | NM_001123328| | NA |
| 223188_at | 9.69 | 1.83E-06 | 1.145940916 | NM_019108| | FLJ12886,hypothetical protein FLJ12886 |
| 206689_x_at | 9.69 | 1.84E-06 | 1.089604365 | NM_006388| | HTATIP,HIV-1 Tat interactive protein, 60kDa isoform 2 |
| 1558854_a_at | 9.69 | 1.83E-06 | 1.214322776 | NA |  |
| 218758_s_at | 9.69 | 1.83E-06 | 1.077253124 | NM_003683| | D21S2056E,nucleolar protein NOP52 |
| 229570_at | 9.69 | 1.84E-06 | 1.114135691 | NM_005560| | LAMA5,laminin alpha 5 |
| 225528_at | 9.68 | 1.85E-06 | 1.033013724 | NM_006390| | IPO8,importin 8 |
| 242455_at | 9.68 | 1.86E-06 | 1.959805827 | NM_005604| | POU3F2,POU domain, class 3, transcription factor 2 |
| 209711_at | 9.68 | 1.86E-06 | 1.267352227 | NM_015139| | SLC35D1,solute carrier family 35 (UDP-glucuronic |
| 218474_s_at | 9.68 | 1.85E-06 | 1.058237096 | NM_018992| | KCTD5,potassium channel tetramerisation domain |
| 205462_s_at | 9.68 | 1.86E-06 | 1.248903583 | NM_002149| | HPCAL1,hippocalcin-like 1 |
| 238485_at | 9.67 | 1.87E-06 | 1.267714841 | NA |  |
| 1556820_a_at | 9.67 | 1.88E-06 | 1.505520006 | NA |  |
| 202705_at | 9.67 | 1.88E-06 | 1.028834878 | NM_004701| | CCNB2,cyclin B2 |
| 200839_s_at | 9.67 | 1.87E-06 | 1.097479012 | NM_001908| | CTSB,cathepsin B preproprotein |
| 209770_at | 9.67 | 1.86E-06 | 1.082837055 | NM_007048| | BTN3A1,butyrophilin, subfamily 3, member A1 isoform a |
| 1557244_a_at | 9.67 | 1.88E-06 | 1.185872875 | NA |  |
| 214778_at | 9.66 | 1.90E-06 | 1.090580804 | NM_001410| | EGFL4,EGF-like-domain, multiple 4 |
| 225339_at | 9.66 | 1.89E-06 | 1.104728363 | NM_003971| | SPAG9,sperm associated antigen 9 isoform 1 |
| 204480_s_at | 9.66 | 1.89E-06 | 1.077581381 | NM_024112| | C9orf16,chromosome 9 open reading frame 16 |
| 1563796_s_at | 9.66 | 1.90E-06 | 1.081849871 | NM_001083614| | NA |
| 204826_at | 9.66 | 1.90E-06 | 1.039853007 | NM_001761| | CCNF,cyclin F |
| 222129_at | 9.66 | 1.89E-06 | 1.424881013 | NM_024293| | C2orf17,chromosome 2 open reading frame 17 |
| 225298_at | 9.66 | 1.90E-06 | 1.080830401 | NM_001077399| | NA |
| 235109_at | 9.65 | 1.92E-06 | 1.16989257 | NA |  |
| 201147_s_at | 9.65 | 1.92E-06 | 1.353684957 | NM_000362| | TIMP3,tissue inhibitor of metalloproteinase 3 |
| 228917_at | 9.65 | 1.91E-06 | 1.118838893 | NA |  |
| 218882_s_at | 9.65 | 1.91E-06 | 1.036194712 | NM_006784| | WDR3,WD repeat-containing protein 3 |
| 221090_s_at | 9.65 | 1.91E-06 | 1.04168017 | NM_018233| | FLJ10826,hypothetical protein FLJ10826 |
| 213058_at | 9.65 | 1.91E-06 | 1.237033674 | NA |  |
| 242569_at | 9.64 | 1.94E-06 | 1.195139424 | NM_005843| | STAM2,signal transducing adaptor molecule 2 |
| 226319_s_at | 9.64 | 1.94E-06 | 1.064946031 | NM_005782| | THOC4,THO complex 4 |
| 222748_s_at | 9.64 | 1.93E-06 | 1.25847214 | NM_017853| | TXNL4B,thioredoxin-like 4B |
| 227730_at | 9.64 | 1.93E-06 | 1.118169302 | NA |  |
| 224100_s_at | 9.64 | 1.94E-06 | 1.240439861 | NM_020134| | DPYSL5,dihydropyrimidinase-like 5 |
| 211382_s_at | 9.64 | 1.93E-06 | 1.061821659 | NM_006997| | TACC2,transforming, acidic coiled-coil containing |
| 242034_at | 9.64 | 1.94E-06 | 1.338486456 | NM_022824| | FBXL17,F-box and leucine-rich repeat protein 17 |
| 232441_at | 9.64 | 1.94E-06 | 1.195048931 | NM_007043| | HRB2,HIV-1 rev binding protein 2 |
| 221746_at | 9.63 | 1.94E-06 | 1.040398258 | NM_014235| | UBL4,ubiquitin-like 4 |
| 223299_at | 9.63 | 1.96E-06 | 1.134015107 | NM_033280| | SEC11L3,SEC11-like 3 |
| 221800_s_at | 9.62 | 1.98E-06 | 1.121678555 | NM_001109760| | NA |
| 212561_at | 9.62 | 1.98E-06 | 1.059099722 | NM_015213| | RAB6IP1,RAB6 interacting protein 1 |
| 64371_at | 9.62 | 1.98E-06 | 1.123774611 | NM_001017392| | NA |
| 209206_at | 9.62 | 1.98E-06 | 1.11589531 | NM_004892| | SEC22L1,vesicle trafficking protein sec22b |
| 209511_at | 9.61 | 2.00E-06 | 1.084953864 | NM_021974| | POLR2F,DNA directed RNA polymerase II polypeptide F |
| 223575_at | 9.61 | 1.99E-06 | 1.059832196 | NM_020910| | NA |
| 225017_at | 9.6 | 2.02E-06 | 1.05245458 | NM_022757| | CCDC14,coiled-coil domain containing 14 |
| 218592_s_at | 9.6 | 2.02E-06 | 1.055482027 | NM_017829| | CECR5,cat eye syndrome chromosome region, candidate 5 |
| 215515_at | 9.6 | 2.02E-06 | 1.116204024 | NA |  |
| 209322_s_at | 9.6 | 2.02E-06 | 1.051977356 | NM_015503| | SH2B,SH2-B homolog |
| 222026_at | 9.6 | 2.02E-06 | 1.151408282 | NM_006743| | RBM3,RNA binding motif (RNP1, RRM) protein 3 |
| 203440_at | 9.6 | 2.02E-06 | 1.156430202 | NM_001792| | CDH2,cadherin 2, type 1 preproprotein |
| 238506_at | 9.6 | 2.03E-06 | 1.618955389 | NM_001099678| | NA |
| 212108_at | 9.6 | 2.01E-06 | 1.032720477 | NM_014613| | ETEA,protein expressed in T-cells and eosinophils in |
| 201977_s_at | 9.59 | 2.04E-06 | 1.1332204 | NM_014773| | KIAA0141,KIAA0141 |
| 203839_s_at | 9.59 | 2.04E-06 | 1.072985989 | NM_001010938| | TNK2,tyrosine kinase, non-receptor, 2 isoform 2 |
| 220915_s_at | 9.59 | 2.04E-06 | 1.423722763 | NA |  |
| 242671_at | 9.59 | 2.04E-06 | 1.114031633 | NA |  |
| 205634_x_at | 9.59 | 2.04E-06 | 1.133901975 | NM_207340| | LOC254359,hypothetical protein LOC254359 |
| 1556809_a_at | 9.59 | 2.05E-06 | 1.282022022 | NA |  |
| 244496_at | 9.59 | 2.04E-06 | 1.13560335 | NA |  |
| 1558673_s_at | 9.59 | 2.04E-06 | 1.174277981 | NM_021217| | ZNF77,zinc finger protein 77 |
| 1558002_at | 9.59 | 2.04E-06 | 1.154818716 | NM_007178| | STRAP,serine/threonine kinase receptor associated |
| 234317_s_at | 9.59 | 2.03E-06 | 1.100379755 | NM_020225| | DKFZp762K222,hypothetical protein DKFZp762K222 |
| 217457_s_at | 9.58 | 2.05E-06 | 1.087467321 | NM_001100426| | NA |
| 231115_at | 9.58 | 2.07E-06 | 1.434514061 | NM_006502| | POLH,polymerase (DNA directed), eta |
| 226309_at | 9.58 | 2.05E-06 | 1.118880787 | NM_031427| | C14orf168,chromosome 14 open reading frame 168 |
| 239718_at | 9.58 | 2.05E-06 | 1.218244604 | NA |  |
| 212807_s_at | 9.58 | 2.06E-06 | 1.048804704 | NM_002959| | SORT1,sortilin 1 preproprotein |
| 217941_s_at | 9.58 | 2.05E-06 | 1.09778486 | NM_001006600| | ERBB2IP,ERBB2 interacting protein isoform 7 |
| 1554670_at | 9.58 | 2.05E-06 | 1.240708824 | NM_001001560| | GGA1,golgi associated, gamma adaptin ear containing, |
| 239222_at | 9.58 | 2.07E-06 | 1.156038608 | NM_018956| | C9orf9,chromosome 9 open reading frame 9 |
| 220260_at | 9.57 | 2.08E-06 | 1.084765151 | NM_018317| | TBC1D19,TBC1 domain family, member 19 |
| 223792_at | 9.57 | 2.09E-06 | 1.104681654 | NM_001017396| | NA |
| 223464_at | 9.57 | 2.08E-06 | 1.172273581 | NM_020896| | OSBPL5,oxysterol-binding protein-like protein 5 isoform |
| 235656_s_at | 9.57 | 2.08E-06 | 1.26052607 | NA |  |
| 218115_at | 9.56 | 2.10E-06 | 1.081400024 | NM_018154| | ASF1B,ASF1 anti-silencing function 1 homolog B |
| 231392_at | 9.56 | 2.10E-06 | 1.13739578 | NA |  |
| 225758_s_at | 9.56 | 2.10E-06 | 1.138365854 | NM_020461| | TUBGCP6,tubulin, gamma complex associated protein 6 |
| 243176_at | 9.55 | 2.13E-06 | 1.175954106 | NA |  |
| 214006_s_at | 9.55 | 2.12E-06 | 1.106637267 | NM_000821| | GGCX,gamma-glutamyl carboxylase |
| 237305_at | 9.54 | 2.15E-06 | 1.362110022 | NA |  |
| 233746_x_at | 9.54 | 2.15E-06 | 1.067648992 | NM_016400| | HYPK,Huntingtin interacting protein K |
| 233035_at | 9.54 | 2.16E-06 | 1.239833473 | NA |  |
| 221246_x_at | 9.54 | 2.16E-06 | 1.141502261 | NM_022648| | TNS,tensin |
| 218680_x_at | 9.53 | 2.19E-06 | 1.063184545 | NM_016400| | HYPK,Huntingtin interacting protein K |
| 219469_at | 9.53 | 2.17E-06 | 1.196939366 | NM_001080463| | NA |
| 203283_s_at | 9.53 | 2.17E-06 | 1.160434838 | NM_012262| | HS2ST1,heparan sulfate 2-O-sulfotransferase 1 |
| 206543_at | 9.53 | 2.17E-06 | 1.424716892 | NM_003070| | SMARCA2,SWI/SNF-related matrix-associated |
| 217940_s_at | 9.53 | 2.18E-06 | 1.089114003 | NM_018210| | FLJ10769,hypothetical protein FLJ10769 |
| 243740_at | 9.53 | 2.18E-06 | 1.275540835 | NA |  |
| 230134_s_at | 9.53 | 2.18E-06 | 1.083142768 | NM_001100588| | NA |
| 228722_at | 9.53 | 2.17E-06 | 1.152593324 | NM_001535| | HRMT1L1,HMT1 hnRNP methyltransferase-like 1 |
| 231844_at | 9.53 | 2.18E-06 | 1.091204723 | NA |  |
| 1556580_a_at | 9.52 | 2.20E-06 | 1.478163431 | NA |  |
| 201140_s_at | 9.52 | 2.19E-06 | 1.060639537 | NM_004583| | RAB5C,RAB5C, member RAS oncogene family isoform b |
| 230392_at | 9.52 | 2.21E-06 | 1.150428404 | NA |  |
| 1554193_s_at | 9.52 | 2.19E-06 | 1.220002706 | NM_024641| | MANEA,mannosidase, endo-alpha |
| 227873_at | 9.52 | 2.20E-06 | 1.067721329 | NM_024715| | C5orf14,disulfide isomerase |
| 225048_at | 9.52 | 2.21E-06 | 1.167690903 | NM_018288| | PHF10,PHD finger protein 10 isoform a |
| 213720_s_at | 9.52 | 2.21E-06 | 1.028826241 | NM_003072| | SMARCA4,SWI/SNF-related matrix-associated |
| 210296_s_at | 9.52 | 2.20E-06 | 1.060100258 | NM_000318| | PXMP3,peroxisomal membrane protein 3 |
| 218876_at | 9.51 | 2.23E-06 | 1.245966819 | NM_015964| | CGI-38,CGI-38 protein |
| 223024_at | 9.51 | 2.21E-06 | 1.155006293 | NM_032493| | AP1M1,adaptor-related protein complex 1, mu 1 subunit |
| 226328_at | 9.51 | 2.23E-06 | 1.087420586 | NM_031918| | KLF16,BTE-binding protein 4 |
| 241396_at | 9.51 | 2.22E-06 | 1.137842571 | NM_015277| | NEDD4L,ubiquitin-protein ligase NEDD4-like |
| 222429_at | 9.51 | 2.23E-06 | 1.086153471 | NM_001014436| | NA |
| 1554127_s_at | 9.51 | 2.23E-06 | 1.254374636 | NM_001031679| | NA |
| 226382_at | 9.51 | 2.23E-06 | 1.103039684 | NA |  |
| 235984_at | 9.51 | 2.23E-06 | 1.17972721 | NA |  |
| 218287_s_at | 9.51 | 2.22E-06 | 1.087817751 | NM_012199| | EIF2C1,eukaryotic translation initiation factor 2C, 1 |
| 209466_x_at | 9.5 | 2.24E-06 | 1.334926477 | NM_002825| | PTN,pleiotrophin |
| 223253_at | 9.5 | 2.26E-06 | 1.132969892 | NM_017549| | EPDR1,upregulated in colorectal cancer gene 1 protein |
| 236359_at | 9.5 | 2.25E-06 | 1.14686018 | NM_174934| | SCN4B,sodium channel, voltage-gated, type IV, beta |
| 217923_at | 9.49 | 2.28E-06 | 1.102571648 | NM_012392| | PEF,PEF protein with a long N-terminal hydrophobic |
| 214501_s_at | 9.49 | 2.27E-06 | 1.221307785 | NM_001040158| | NA |
| 236072_at | 9.49 | 2.28E-06 | 1.534087857 | NA |  |
| 227770_at | 9.49 | 2.28E-06 | 1.050540609 | NA |  |
| 203033_x_at | 9.49 | 2.28E-06 | 1.042669163 | NM_000143| | FH,fumarate hydratase precursor |
| 224352_s_at | 9.49 | 2.27E-06 | 1.186856707 | NM_021914| | CFL2,cofilin 2 |
| 212394_at | 9.49 | 2.28E-06 | 1.09297006 | NM_015047| | KIAA0090,KIAA0090 protein |
| 228399_at | 9.49 | 2.28E-06 | 1.187700391 | NM_145260| | OSR1,odd-skipped related 1 |
| 213145_at | 9.49 | 2.28E-06 | 1.167455554 | NM_152441| | FBXL14,F-box and leucine-rich repeat protein 14 |
| 239049_at | 9.49 | 2.28E-06 | 1.08349244 | NA |  |
| 212536_at | 9.48 | 2.28E-06 | 1.151373453 | NM_014616| | NA |
| 240105_at | 9.48 | 2.30E-06 | 1.181369238 | NA |  |
| 213127_s_at | 9.48 | 2.30E-06 | 1.071895181 | NM_052877| | MED8,mediator of RNA polymerase II transcription |
| 217880_at | 9.48 | 2.29E-06 | 1.079990017 | NM_001114091| | NA |
| 232309_at | 9.48 | 2.31E-06 | 1.170075057 | NA |  |
| 240452_at | 9.48 | 2.30E-06 | 1.305727088 | NM_002094| | GSPT1,G1 to S phase transition 1 |
| 224304_x_at | 9.47 | 2.32E-06 | 1.078188209 | NM_016350| | NIN,ninein isoform 4 |
| 210111_s_at | 9.47 | 2.33E-06 | 1.049775401 | NM_014997| | KIAA0265,KIAA0265 protein |
| 221311_x_at | 9.47 | 2.33E-06 | 1.189240122 | NM_020466| | DJ122O8.2,hypothetical protein dJ122O8.2 |
| 223222_at | 9.47 | 2.33E-06 | 1.117355449 | NM_001126121| | NA |
| 204807_at | 9.47 | 2.32E-06 | 1.54701345 | NM_014254| | TMEM5,transmembrane protein 5 |
| 221897_at | 9.47 | 2.33E-06 | 1.157334676 | NM_032765| | TRIM52,tripartite motif-containing 52 |
| 1555973_at | 9.47 | 2.31E-06 | 1.196930833 | NA |  |
| 215942_s_at | 9.47 | 2.32E-06 | 1.127987731 | NM_016426| | GTSE1,G-2 and S-phase expressed 1 |
| 201836_s_at | 9.47 | 2.32E-06 | 1.166100093 | NM_014860| | STAF65(gamma),SPTF-associated factor 65 gamma |
| 230243_at | 9.46 | 2.33E-06 | 1.217022043 | NM_152292| | RG9MTD2,RNA (guanine-9-) methyltransferase domain |
| 241922_at | 9.46 | 2.34E-06 | 1.276787354 | NA |  |
| 222898_s_at | 9.46 | 2.33E-06 | 1.337911311 | NM_016941| | DLL3,delta-like 3 protein isoform 1 precursor |
| 225435_at | 9.46 | 2.34E-06 | 1.16281833 | NM_003144| | SSR1,signal sequence receptor, alpha |
| 216247_at | 9.46 | 2.35E-06 | 1.381272429 | NM_001023| | RPS20,ribosomal protein S20 |
| 239451_at | 9.46 | 2.33E-06 | 1.218011841 | NA |  |
| 225967_s_at | 9.46 | 2.36E-06 | 1.107491387 | NM_001086521| | NA |
| 242829_x_at | 9.45 | 2.37E-06 | 1.186810259 | NM_012158| | FBXL3,F-box and leucine-rich repeat protein 3 |
| 233005_at | 9.45 | 2.36E-06 | 1.262368517 | NA |  |
| 202758_s_at | 9.45 | 2.36E-06 | 1.092321437 | NM_003721| | RFXANK,regulatory factor X-associated |
| 219596_at | 9.45 | 2.36E-06 | 1.128638441 | NM_020147| | THAP10,THAP domain containing 10 |
| 236202_at | 9.45 | 2.38E-06 | 1.343337211 | NA |  |
| 226003_at | 9.44 | 2.38E-06 | 1.144752794 | NM_017641| | KIF21A,kinesin family member 21A |
| 201992_s_at | 9.44 | 2.40E-06 | 1.111988528 | NM_004521| | KIF5B,kinesin family member 5B |
| 209420_s_at | 9.44 | 2.41E-06 | 1.127189686 | NM_000543| | SMPD1,sphingomyelin phosphodiesterase 1, acid |
| 226777_at | 9.44 | 2.39E-06 | 1.517508277 | NA |  |
| 203708_at | 9.44 | 2.39E-06 | 1.481983601 | NM_001037339| | NA |
| 1559780_at | 9.44 | 2.39E-06 | 1.141548617 | NA |  |
| 234645_at | 9.44 | 2.40E-06 | 1.267309564 | NA |  |
| 208492_at | 9.43 | 2.42E-06 | 1.178782973 | NM_000538| | RFXAP,regulatory factor X-associated protein |
| 225971_at | 9.43 | 2.43E-06 | 1.122461609 | NM_030637| | DDHD1,DDHD domain containing 1 |
| 1560659_at | 9.43 | 2.43E-06 | 1.183334383 | NA |  |
| 215443_at | 9.43 | 2.43E-06 | 1.243709218 | NM_000369| | TSHR,thyroid stimulating hormone receptor |
| 210778_s_at | 9.43 | 2.41E-06 | 1.154350993 | NM_006454| | MXD4,MAD4 |
| 1564190_x_at | 9.43 | 2.43E-06 | 1.11318995 | NM_145287| | ZNF519,zinc finger protein 519 |
| 227502_at | 9.43 | 2.41E-06 | 1.096904119 | NM_001080392| | NA |
| 1560339_s_at | 9.43 | 2.41E-06 | 1.062965427 | NM_005969| | NAP1L4,nucleosome assembly protein 1-like 4 |
| 211998_at | 9.42 | 2.45E-06 | 1.057719079 | NM_002107| | H3F3A,H3 histone, family 3A |
| 220744_s_at | 9.42 | 2.44E-06 | 1.096029756 | NM_018262| | WDR10,WD repeat domain 10 isoform 3 |
| 236153_at | 9.42 | 2.45E-06 | 1.371595308 | NA |  |
| 214754_at | 9.42 | 2.45E-06 | 1.119637506 | NM_144993| | NA |
| 214126_at | 9.42 | 2.44E-06 | 1.118668995 | NA |  |
| 200854_at | 9.42 | 2.45E-06 | 1.041670943 | NM_006311| | NCOR1,nuclear receptor co-repressor 1 |
| 223223_at | 9.42 | 2.44E-06 | 1.124487305 | NM_022786| | ARV1,ARV1 homolog |
| 240172_at | 9.42 | 2.45E-06 | 1.338345532 | NM_016570| | PTX1,CDA14 |
| 205086_s_at | 9.42 | 2.44E-06 | 1.09174826 | NM_014551| | 384D8-2,hypothetical protein MGC18000 |
| 212930_at | 9.42 | 2.44E-06 | 1.186165798 | NM_001001323| | ATP2B1,plasma membrane calcium ATPase 1 isoform 1a |
| 203156_at | 9.41 | 2.47E-06 | 1.100803643 | NM_016248| | AKAP11,A-kinase anchor protein 11 isoform 1 |
| 235960_at | 9.41 | 2.48E-06 | 1.32317484 | NA |  |
| 201571_s_at | 9.41 | 2.47E-06 | 1.072235687 | NM_001012732| | NA |
| 203867_s_at | 9.41 | 2.48E-06 | 1.065113071 | NM_001014445| | NA |
| 226532_at | 9.41 | 2.46E-06 | 1.083567324 | NA |  |
| 229204_at | 9.41 | 2.46E-06 | 1.253929555 | NM_016287| | HP1-BP74,HP1-BP74 |
| 219204_s_at | 9.41 | 2.48E-06 | 1.136076032 | NM_021947| | SRR,serine racemase |
| 235783_at | 9.41 | 2.48E-06 | 1.180813999 | NM_016183| | C1orf33,ribosomal protein P0-like protein |
| 242686_at | 9.41 | 2.48E-06 | 1.190415335 | NM_052851| | STARD13,START domain containing 13 isoform gamma |
| 1555516_at | 9.4 | 2.50E-06 | 1.327865612 | NM_014491| | FOXP2,forkhead box P2 isoform I |
| 208806_at | 9.4 | 2.49E-06 | 1.094183674 | NM_001005271| | CHD3,chromodomain helicase DNA binding protein 3 |
| 203162_s_at | 9.4 | 2.50E-06 | 1.094845067 | NM_005886| | KATNB1,katanin p80 subunit B 1 |
| 205406_s_at | 9.4 | 2.48E-06 | 1.156779824 | NM_017425| | SPA17,sperm autoantigenic protein 17 |
| 214607_at | 9.4 | 2.49E-06 | 1.078297816 | NM_002578| | PAK3,p21-activated kinase 3 |
| 216895_at | 9.4 | 2.50E-06 | 1.177379408 | NM_033223| | GABRG3,gamma-aminobutyric acid (GABA) A receptor, gamma |
| 214855_s_at | 9.39 | 2.53E-06 | 1.084160223 | NM_014990| | GARNL1,GTPase activating Rap/RanGAP domain-like 1 |
| 209635_at | 9.39 | 2.51E-06 | 1.053051054 | NM_001283| | AP1S1,adaptor-related protein complex 1, sigma 1 |
| 214170_x_at | 9.39 | 2.51E-06 | 1.048655861 | NM_000143| | FH,fumarate hydratase precursor |
| 226475_at | 9.39 | 2.52E-06 | 1.051884569 | NM_001104595| | NA |
| 240603_s_at | 9.39 | 2.51E-06 | 1.218768808 | NM_080663| | MGC16943,hypothetical protein MGC16943 |
| 241946_at | 9.39 | 2.51E-06 | 1.357974667 | NM_178566| | ZDHHC21,zinc finger, DHHC domain containing 21 |
| 208922_s_at | 9.39 | 2.52E-06 | 1.060318703 | NM_001081491| | NA |
| 224560_at | 9.39 | 2.53E-06 | 1.13200778 | NM_003255| | TIMP2,tissue inhibitor of metalloproteinase 2 |
| 235554_x_at | 9.39 | 2.53E-06 | 1.241068516 | NM_145048| | MGC29898,hypothetical protein MGC29898 |
| 233262_at | 9.38 | 2.54E-06 | 1.155546848 | NA |  |
| 214484_s_at | 9.38 | 2.54E-06 | 1.049253137 | NM_005866| | OPRS1,opioid receptor, sigma 1 isoform 1 |
| 212073_at | 9.38 | 2.55E-06 | 1.046754906 | NM_001895| | CSNK2A1,casein kinase II alpha 1 subunit isoform a |
| 223740_at | 9.38 | 2.55E-06 | 1.120540974 | NM_001009994| | C6orf159,chromosome 6 open reading frame 159 |
| 217932_at | 9.38 | 2.54E-06 | 1.044386746 | NM_015971| | MRPS7,mitochondrial ribosomal protein S7 |
| 238936_at | 9.38 | 2.56E-06 | 1.129124472 | NA |  |
| 241737_x_at | 9.38 | 2.54E-06 | 1.280978216 | NA |  |
| 229843_at | 9.38 | 2.55E-06 | 1.216100286 | NM_016033| | CGI-90,CGI-90 protein |
| 233038_at | 9.38 | 2.54E-06 | 1.359683572 | NA |  |
| 213980_s_at | 9.38 | 2.55E-06 | 1.047026411 | NM_001012614| | NA |
| 240332_at | 9.37 | 2.57E-06 | 1.520238333 | NA |  |
| 218664_at | 9.37 | 2.56E-06 | 1.085110635 | NM_001024732| | NA |
| 226690_at | 9.37 | 2.57E-06 | 1.14495171 | NM_001118| | ADCYAP1R1,type I adenylate cyclase activating polypeptide |
| 229742_at | 9.37 | 2.58E-06 | 1.183479048 | NA |  |
| 222275_at | 9.37 | 2.58E-06 | 1.05817148 | NA |  |
| 208797_s_at | 9.37 | 2.57E-06 | 1.15188909 | NM_181077| | GOLGIN-67,golgin-67 isoform c |
| 200957_s_at | 9.37 | 2.57E-06 | 1.04726993 | NM_003146| | SSRP1,structure specific recognition protein 1 |
| 222527_s_at | 9.37 | 2.58E-06 | 1.066509594 | NM_018047| | RBM22,RNA binding motif protein 22 |
| 228619_x_at | 9.37 | 2.57E-06 | 1.112249024 | NM_001031800| | NA |
| 203617_x_at | 9.37 | 2.58E-06 | 1.02208469 | NM_001114123| | NA |
| 208935_s_at | 9.36 | 2.61E-06 | 1.193986384 | NM_006499| | LGALS8,galectin 8 isoform a |
| 204404_at | 9.36 | 2.60E-06 | 1.186889324 | NM_001046| | SLC12A2,solute carrier family 12 |
| 235167_at | 9.36 | 2.60E-06 | 1.268169958 | NA |  |
| 208625_s_at | 9.36 | 2.60E-06 | 1.043588015 | NM_004953| | EIF4G1,eukaryotic translation initiation factor 4 |
| 209149_s_at | 9.36 | 2.61E-06 | 1.11633448 | NM_001014842| | NA |
| 227301_at | 9.36 | 2.61E-06 | 1.106827918 | NA |  |
| 220227_at | 9.36 | 2.59E-06 | 1.233294381 | NM_001794| | CDH4,cadherin 4, type 1 preproprotein |
| 209667_at | 9.36 | 2.61E-06 | 1.142857342 | NM_003869| | CES2,carboxylesterase 2 isoform 1 |
| 239794_at | 9.36 | 2.60E-06 | 1.2642713 | NA |  |
| 220353_at | 9.35 | 2.62E-06 | 1.126286507 | NM_001099653| | NA |
| 213346_at | 9.35 | 2.64E-06 | 1.134203746 | NM_138779| | LOC93081,hypothetical protein BC015148 |
| 200723_s_at | 9.34 | 2.65E-06 | 1.028309139 | NM_005898| | M11S1,membrane component, chromosome 11, surface |
| 226235_at | 9.34 | 2.65E-06 | 1.05929094 | NA |  |
| 200820_at | 9.34 | 2.66E-06 | 1.032354371 | NM_002812| | PSMD8,proteasome 26S non-ATPase subunit 8 |
| 213981_at | 9.34 | 2.65E-06 | 1.114797673 | NM_000754| | COMT,catechol-O-methyltransferase isoform MB-COMT |
| 219985_at | 9.34 | 2.65E-06 | 1.180414431 | NM_006042| | HS3ST3A1,heparan sulfate D-glucosaminyl |
| 225321_s_at | 9.34 | 2.66E-06 | 1.09970955 | NM_013440| | PILRB,paired immunoglobulin-like type 2 receptor beta |
| 218830_at | 9.34 | 2.67E-06 | 1.048193299 | NM_016093| | RPL26L1,ribosomal protein L26-like 1 |
| 243550_at | 9.34 | 2.67E-06 | 1.272667977 | NM_178566| | ZDHHC21,zinc finger, DHHC domain containing 21 |
| 237705_at | 9.34 | 2.66E-06 | 1.144134165 | NA |  |
| 223532_at | 9.34 | 2.65E-06 | 1.119240439 | NM_016466| | MGC41816,hypothetical protein MGC41816 |
| 236726_at | 9.34 | 2.65E-06 | 1.38336576 | NM_004296| | RGS6,regulator of G-protein signalling 6 |
| 243088_at | 9.33 | 2.70E-06 | 1.231325411 | NA |  |
| 217884_at | 9.33 | 2.69E-06 | 1.043690851 | NM_024662| | FLJ10774,hypothetical protein FLJ10774 |
| 217913_at | 9.33 | 2.68E-06 | 1.057311187 | NM_013245| | VPS4A,vacuolar protein sorting factor 4A |
| 227970_at | 9.33 | 2.70E-06 | 1.142321379 | NM_024980| | GPR157,G protein-coupled receptor 157 |
| 212818_s_at | 9.33 | 2.67E-06 | 1.069143288 | NM_001040445| | NA |
| 230270_at | 9.33 | 2.68E-06 | 1.193022195 | NM_018061| | FLJ10330,sarcoma antigen NY-SAR-27 |
| 1553570_x_at | 9.33 | 2.68E-06 | 1.019690226 | NM_022340| | ZFYVE20,FYVE-finger-containing Rab5 effector protein |
| 207346_at | 9.33 | 2.68E-06 | 1.061939796 | NM_001980| | EPIM,epimorphin isoform 1 |
| 217908_s_at | 9.33 | 2.68E-06 | 1.12700077 | NM_001017977| | NA |
| 228066_at | 9.33 | 2.69E-06 | 1.131438625 | NA |  |
| 222629_at | 9.33 | 2.67E-06 | 1.259640378 | NM_001037872| | NA |
| 1557675_at | 9.33 | 2.69E-06 | 1.141508376 | NM_002880| | RAF1,v-raf-1 murine leukemia viral oncogene homolog |
| 218296_x_at | 9.33 | 2.69E-06 | 1.092402093 | NM_018116| | FLJ10504,misato |
| 232710_at | 9.32 | 2.72E-06 | 1.166742195 | NA |  |
| 217891_at | 9.32 | 2.72E-06 | 1.085588598 | NM_022744| | FLJ13868,hypothetical protein FLJ13868 |
| 242440_at | 9.32 | 2.73E-06 | 1.296192502 | NA |  |
| 242356_at | 9.32 | 2.72E-06 | 1.088580641 | NM_145206| | VTI1A,SNARE Vti1a-beta protein |
| 231532_at | 9.32 | 2.71E-06 | 1.216642865 | NM_000615| | NCAM1,neural cell adhesion molecule 1 |
| 240857_at | 9.32 | 2.71E-06 | 1.273329953 | NM_001372| | DNAH9,dynein, axonemal, heavy polypeptide 9 isoform 2 |
| 202993_at | 9.32 | 2.73E-06 | 1.07287126 | NM_006844| | ILVBL,ilvB (bacterial acetolactate synthase)-like |
| 200837_at | 9.32 | 2.73E-06 | 1.050020155 | NM_005745| | BCAP31,B-cell receptor-associated protein 31 |
| 210337_s_at | 9.32 | 2.72E-06 | 1.060041596 | NM_001096| | ACLY,ATP citrate lyase isoform 1 |
| 232453_at | 9.31 | 2.73E-06 | 1.227671322 | NA |  |
| 237158_s_at | 9.31 | 2.73E-06 | 1.143280126 | NM_022782| | MPHOSPH9,M-phase phosphoprotein 9 |
| 202207_at | 9.31 | 2.73E-06 | 1.12300059 | NM_005737| | ARL7,ADP-ribosylation factor-like 7 |
| 217164_at | 9.31 | 2.73E-06 | 1.117015176 | NA |  |
| 230570_at | 9.31 | 2.74E-06 | 1.205862139 | NA |  |
| 205271_s_at | 9.3 | 2.77E-06 | 1.214026722 | NM_001039803| | NA |
| 206574_s_at | 9.3 | 2.78E-06 | 1.105701755 | NM_007079| | PTP4A3,protein tyrosine phosphatase type IVA, member 3 |
| 1570451_at | 9.3 | 2.78E-06 | 1.280582994 | NM_006365| | CROC4,transcriptional activator of the c-fos promoter |
| 243953_at | 9.3 | 2.78E-06 | 1.235699258 | NM_004265| | FADS2,fatty acid desaturase 2 |
| 205439_at | 9.3 | 2.76E-06 | 1.214803309 | NM_000854| | GSTT2,glutathione S-transferase theta 2 |
| 220055_at | 9.29 | 2.79E-06 | 1.329253425 | NM_020653| | ZNF287,zinc finger protein 287 |
| 201135_at | 9.29 | 2.79E-06 | 1.037687888 | NM_004092| | ECHS1,mitochondrial short-chain enoyl-coenzyme A |
| 208933_s_at | 9.29 | 2.79E-06 | 1.288202413 | NM_006499| | LGALS8,galectin 8 isoform a |
| 237521_x_at | 9.29 | 2.79E-06 | 1.089523368 | NA |  |
| 211732_x_at | 9.29 | 2.79E-06 | 1.266559111 | NM_001024074| | NA |
| 209475_at | 9.28 | 2.83E-06 | 1.08476037 | NM_006313| | USP15,ubiquitin specific protease 15 |
| 235967_at | 9.28 | 2.82E-06 | 1.171349036 | NA |  |
| 212804_s_at | 9.28 | 2.84E-06 | 1.05981291 | NM_015635| | DKFZP434C212,DKFZP434C212 protein |
| 209124_at | 9.28 | 2.83E-06 | 1.059287679 | NM_002468| | MYD88,myeloid differentiation primary response gene |
| 213877_x_at | 9.28 | 2.82E-06 | 1.070783343 | NM_007108| | TCEB2,elongin B isoform a |
| 242133_s_at | 9.28 | 2.82E-06 | 1.360609384 | NA |  |
| 225576_at | 9.28 | 2.83E-06 | 1.069200543 | NM_138785| | C6orf72,chromosome 6 open reading frame 72 |
| 218430_s_at | 9.28 | 2.83E-06 | 1.086054918 | NM_022841| | FLJ12994,hypothetical protein FLJ12994 |
| 216381_x_at | 9.28 | 2.82E-06 | 1.112050436 | NM_012067| | AKR7A3,aldo-keto reductase family 7, member A3 |
| 1560297_at | 9.28 | 2.82E-06 | 1.217580113 | NA |  |
| 232135_at | 9.27 | 2.85E-06 | 1.172139245 | NA |  |
| 202664_at | 9.27 | 2.87E-06 | 1.262025665 | NM_001077269| | NA |
| 211373_s_at | 9.27 | 2.85E-06 | 1.06312756 | NM_000447| | PSEN2,presenilin 2 isoform 1 |
| 242782_x_at | 9.27 | 2.87E-06 | 1.217321412 | NM_001005209| | MGC99813,similar to RIKEN cDNA A230078I05 gene |
| 209250_at | 9.27 | 2.87E-06 | 1.054399814 | NM_003676| | DEGS1,degenerative spermatocyte homolog 1, lipid |
| 201214_s_at | 9.27 | 2.85E-06 | 1.056930343 | NM_002712| | PPP1R7,protein phosphatase 1, regulatory subunit 7 |
| 209693_at | 9.27 | 2.87E-06 | 1.049746376 | NM_014010| | ASTN2,astrotactin 2 isoform a |
| 228238_at | 9.27 | 2.85E-06 | 1.070677503 | NA |  |
| 226419_s_at | 9.27 | 2.87E-06 | 1.177749006 | NA |  |
| 206845_s_at | 9.27 | 2.86E-06 | 1.110794583 | NM_014771| | RNF40,ring finger protein 40 isoform 1 |
| 1552291_at | 9.27 | 2.87E-06 | 1.187843219 | NM_017861| | PIGX,GPI-mannosyltransferase subunit |
| 91682_at | 9.26 | 2.88E-06 | 1.11109022 | NA |  |
| 242688_at | 9.26 | 2.90E-06 | 1.324075702 | NA |  |
| 238252_at | 9.26 | 2.88E-06 | 1.136673533 | NA |  |
| 59437_at | 9.26 | 2.89E-06 | 1.177942219 | NM_001048265| | NA |
| 227926_s_at | 9.26 | 2.88E-06 | 1.21660331 | NM_001037501| | NA |
| 201935_s_at | 9.26 | 2.90E-06 | 1.037912876 | NM_003760| | EIF4G3,eukaryotic translation initiation factor 4 |
| 212038_s_at | 9.26 | 2.89E-06 | 1.069538969 | NM_003374| | VDAC1,voltage-dependent anion channel 1 |
| 205540_s_at | 9.25 | 2.93E-06 | 1.117904926 | NM_006064| | RRAGB,Ras-related GTP binding B short isoform |
| 224987_at | 9.25 | 2.92E-06 | 1.149964684 | NM_152734| | C6orf89,hypothetical protein FLJ25357 |
| 229297_at | 9.25 | 2.92E-06 | 1.066367319 | NA |  |
| 225159_s_at | 9.25 | 2.92E-06 | 1.106642588 | NA |  |
| 206020_at | 9.25 | 2.92E-06 | 1.252275655 | NM_004232| | SOCS6,suppressor of cytokine signaling 6 |
| 228573_at | 9.25 | 2.92E-06 | 1.388475963 | NA |  |
| 239539_at | 9.25 | 2.92E-06 | 1.171780627 | NA |  |
| 217099_s_at | 9.25 | 2.91E-06 | 1.073204629 | NM_015721| | GEMIN4,gemin4 |
| 201419_at | 9.24 | 2.96E-06 | 1.047980561 | NM_004656| | BAP1,BRCA1 associated protein-1 |
[truncated: 211,509 more chars]
